# Disentangling the History of Deep Ocean Disposal for DDT and Other Industrial Waste Off Southern California

**DOI:** 10.1021/acs.est.3c08575

**Published:** 2024-02-21

**Authors:** Jacob T. Schmidt, Mong Sin Christine Wu, Hailie E. Kittner, J. Samuel Arey, Douglas E. Hammond, Earth 182A Group, David L. Valentine

**Affiliations:** † Interdepartmental Graduate Program in Marine Science, University of California, Santa Barbara, California 93106, United States; ‡ Department of Earth Science, University of California, Santa Barbara, California 93106, United States; § Oleolytics, LLC, State College, Pennsylvania 16801, United States; ∥ Department of Earth Sciences, University of Southern California, Los Angeles, California 90089, United States; ⊥ Marine Science Institute, University of California, Santa Barbara, California 93106, United States

**Keywords:** chlorinated petrochemicals, ocean dumping, legacy pollution, radioactive waste, pesticide use, DDT

## Abstract

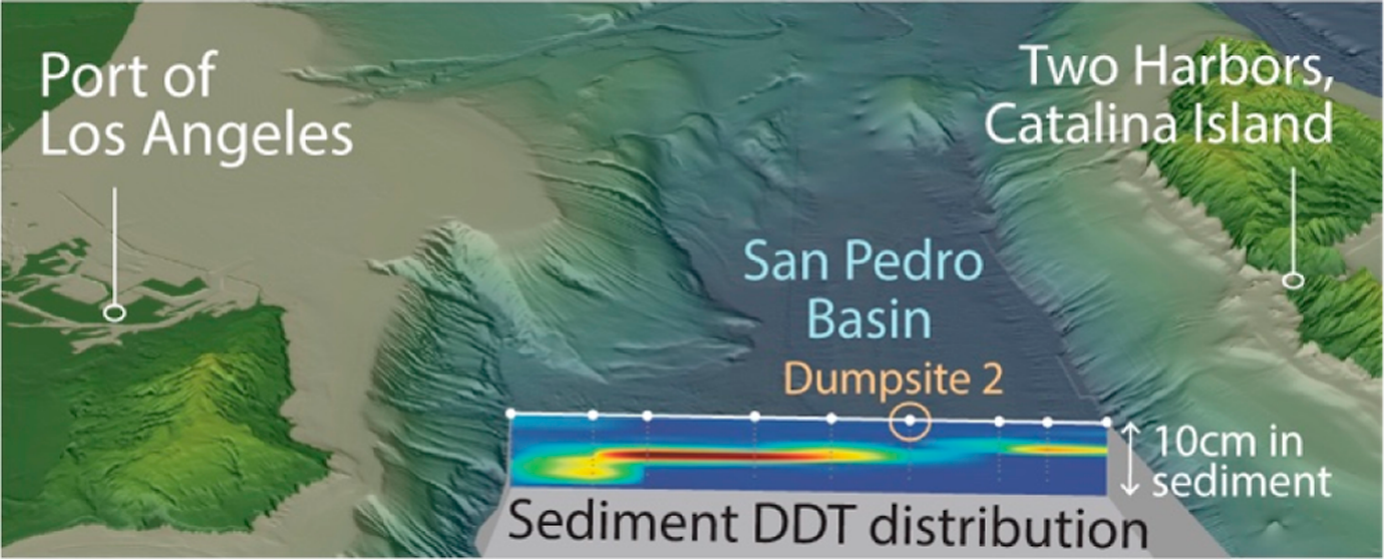

Ocean disposal of industrial waste from technical DDT [mainly 1,1′-(2,2,2-trichloroethane-1,1-diyl)bis(4-chlorobenzene), or 4,4′-DDT] manufacture occurred historically in the Southern California Bight. However, the paucity of historical records highlights uncertainties as to the mode, location, and timing of disposal or ongoing ecological effects of these wastes. This study combines sampling, chemical analysis, and numerical modeling of deep San Pedro Basin sediments revealing substantial DDT contamination that extends at least 25 km from the mainland. These findings narrate bulk DDT waste disposal to the offshore that peaked in the 1950s, prior to the onset of formal regulations; was agnostic to later-designated disposal sites; and has experienced sluggish transformation. Our findings further indicate an attenuating secondary source for the DDT daughter product, 1-chloro-4-[2,2-dichloro-1-(4-chlorophenyl)ethenyl]benzene (4,4′-DDE), which still deposits into deep San Pedro Basin sediments. While demonstrating the severity of DDT contamination to the region, these findings further define the burial potential of DDT wastes and inform the past, present, and future contamination potential that is needed to understand and predict ecological consequences. This work also points firmly to bulk, not containerized, disposal of DDT waste and to potential alternative contents of collocated waste.

## Abstract 2

This work unveils the broad extent of bulk DDT waste disposal to Southern California’s ocean waters and informs the transport and fate of these materials in deep ocean sediments.

## 1 Introduction

Ocean waste disposal was prevalent offshore Southern California during the early to mid 1900s with 15 offshore dump sites identified by the United States Environmental Protection Agency (EPA).^1^ Numerous types of waste were reportedly dumped at these locations including radioactive wastes, refinery and oil drilling wastes, chemical wastes, military munitions, filter cakes, and refuse.^2−4^ One problematic waste stream was derived from the manufacture of technical DDT [mainly 1,1′-(2,2,2-trichloroethane-1,1-diyl)bis(4-chlorobenzene), or 4,4′-DDT], a hydrophobic, persistent, and toxic pesticide, by Montrose Chemical Corporation of California (Montrose). From ca. 1948 until at least 1961, Montrose generated concentrated (75–85 vol %) sulfuric acid waste from the condensation reaction between trichloroacetaldehyde and chlorobenzene (Documents S1 and S2), which contained ∼0.5–2 wt % technical DDT.^2,5^ To dispose of this waste, Montrose contracted a disposal service, California Salvage Company (Cal Salvage), to barge the strong acid waste offshore and discharge it into the ocean.

**Figure 1:**
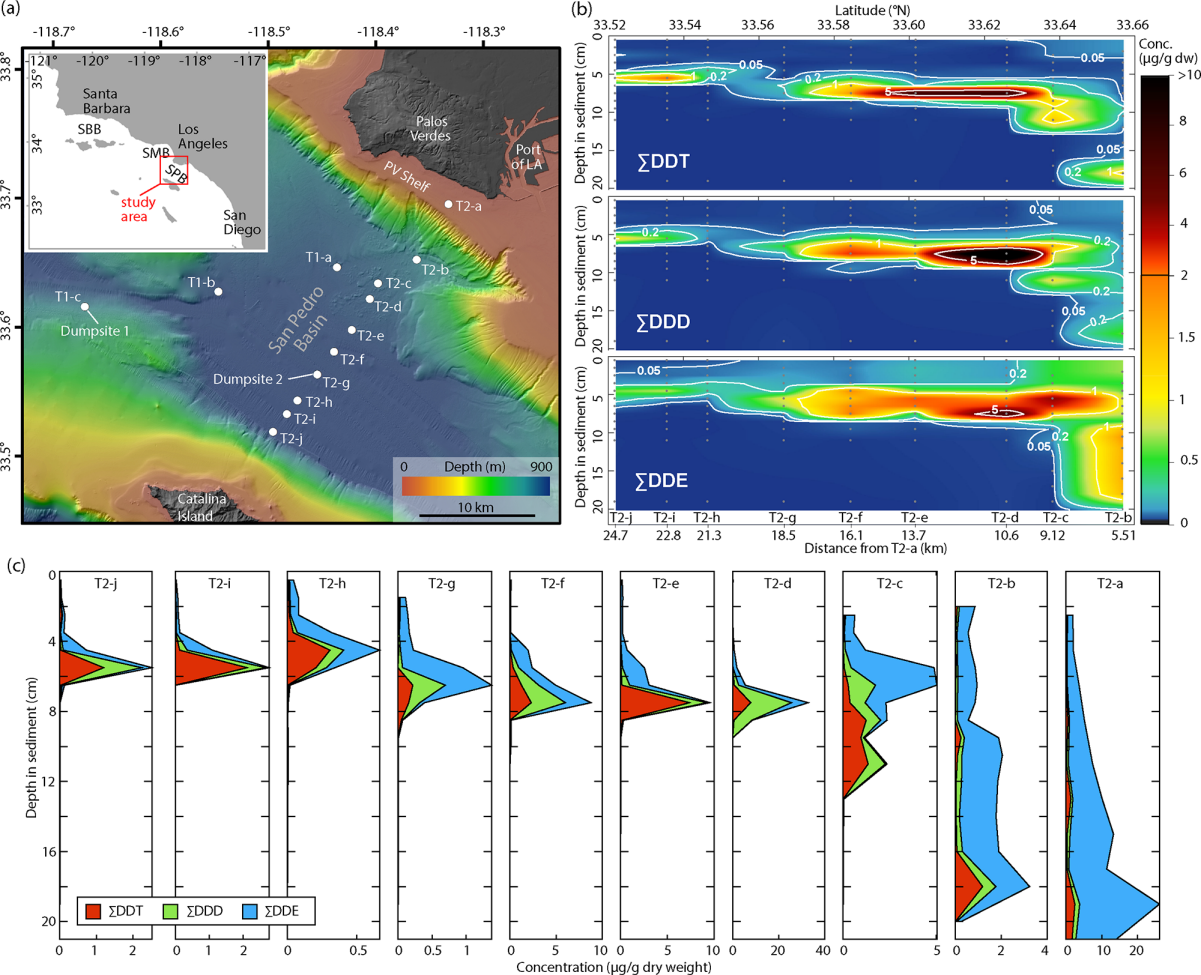
DDX signature across the San Pedro Basin. (a) Sampling stations and bathymetry map of the study area in SP Basin (SPB). Inset shows the location of SP Basin and adjacent basins (Santa Barbara Basin: SBB, Santa Monica Basin: SMB) along the Southern California coast. Dumpsite 1 is centered at station T1-c, and dumpsite 2 is centered at station T2-g. (b) Heat maps of ΣDDT, ΣDDD, and ΣDDE concentrations (μg/g dry weight) in sediments across the deep basin along transect 2. Gray dots indicate where data are present, with values in between estimated by linear interpolation. (c) Depth profiles of stacked concentrations of ΣDDT, ΣDDD, and ΣDDE along transect 2 including deep basin stations and the PV Shelf station T2-a. Note the variable *x*-axes. Data in (b,c) are plotted at mid depths of the sample intervals. See Figure S1 for details.

Located immediately offshore from the Ports of Los Angeles and Long Beach, the San Pedro Basin (SP Basin, Figure 1a) received substantial input of these wastes leading to high DDT concentrations recorded in select sediment samples.^5−7^ Offshore disposal of Montrose’s concentrated sulfuric acid wastes by Cal Salvage began ca. 1948 and declined following the construction of an acid recycling plant on the Montrose property. There was no record of ocean disposal of Montrose sulfuric acid wastes after 1961,^3^ the same year Cal Salvage’s operation became regulated by a regional water quality board (Document S3) and a formal dumpsite was assigned to them (dumpsite 1, Figure 1a). Nonetheless, Cal Salvage continued its industrial waste disposal activity with other clients, reportedly disposing of more than 1.5 million gallons of other industrial waste offshore from 1965 to 1972 with a persistent record of “short dumping” at unpermitted locations including the area known as dumpsite 2 (Figure 1a).^3^ The lack of historical records regarding offshore disposal by Cal Salvage raises important questions that frame this study. How much DDT waste was disposed offshore? When and where did the disposal occur? Was the DDT waste containerized as once suggested^3^ or bulk dumped as indicated more recently by the EPA?^8^ Have these wastes persisted in a manner that can lead to ongoing ecological effects? What other wastes are collocated with DDT waste?

Montrose also discharged a second problematic waste stream from a subsequent step in the same manufacturing process in the form of dilute acid filtered from technical DDT product that was neutralized and disposed of through the local sewage system.^2^ The neutralized waste resulted in concentrated pollution on the Palos Verdes Shelf (PV Shelf, Figure 1a) from sewage outfall pipes located there. Originating from the same synthesis reaction, the neutralized waste is assumed to have had a similar chemical composition to the concentrated sulfuric acid waste that was disposed of offshore. The discharge to the PV Shelf was included in the 2000 lawsuit *United States of America and State of California v. Montrose Chemical Corp. of California et al.*, leading to a settlement of ∼$140 million toward environmental restoration of the PV Shelf along with areas including the Montrose Property in Torrance, CA; these areas have been declared Superfund sites by the EPA under the Comprehensive Environmental Response, Compensation, and Liability Act.^9^

Numerous studies have focused on the transport, fate, and effects of DDT waste on the PV Shelf, which was discharged through the outfall pipes and settled to the seafloor there mainly in the form of 4,4′-DDT. The majority of this DDT waste was transformed into a primary daughter product 1-chloro-4-[2,2-dichloro-1-(4-chlorophenyl)ethenyl]benzene (4,4′-DDE) and its 2,4′-isomer (collectively DDE) which remain abundant in modern PV Shelf sediment.^10^ A second primary transformation product, 1-chloro-4-[2,2-dichloro-1-(4-chlorophenyl)ethyl]benzene (4,4′-DDD) and its 2,4′-isomer (collectively DDD), has also been found on PV Shelf, but at substantially lower (<10% of DDE) concentrations.^10^ The balance of DDE and DDD is interpreted to indicate competing pathways of dehalogenation which depend on environmental conditions; abiotic dehydrochlorination via pH-dependent hydrolysis^11^ and oxidative dehalogenation by microbes to DDE are favored for oxic conditions with low sorption to solids (e.g., in the water column), whereas microbially mediated reductive dechlorination to DDD is favored in anoxic or reducing conditions and high sorption to solids (e.g., in buried sediments).^10,12^ DDD and DDE inventories on the shelf are expected to vary in time as they are produced from DDT and subsequently degraded.^13−15^ However, variability between studies and observed inhomogeneity in PV Shelf sediments^16^ have obscured temporal trends and have led to some confusion within the public arena^17^ over DDT contamination. What remains obvious, however, are characteristically high DDE concentrations in PV Shelf sediments, especially proximate to outfall pipes. Waste exiting sewage outfall pipes was likely also transported off the shelf, with characteristically high DDE concentrations observed in some near-shelf sediments in SP Basin.^7^ The discharge of DDT and accumulation of DDE to the PV Shelf has been linked to bioaccumulation up the trophic food web affecting megafauna that include California Condors,^18,19^ California Sea Lions,^20^ several dolphin species,^21−24^ and local fish including white croaker^25^ and flatfish.^26^

The occurrence of offshore DDT waste disposal in the SP Basin has long been assumed but obscured by poor historical records, relative inaccessibility of the deep basin seafloor (700–950 m), and attention to the litigation surrounding discharge to the PV Shelf. While details surrounding offshore industrial waste disposal practices have been elusive, this issue recently recaptured public interest following the public release of data and imagery which disclosed the disposal of materials at dumpsite 2 in the SP Basin.^5,27−29,55^ Subsequent interest in this issue has been sustained through work linking DDT in the coastal environment to ecosystem effects that include cancer in sea lions and bioaccumulation in endangered California Condors,^18−20,30,31^ as well as the identification of thousands of debris targets throughout the SP Basin that include military munitions, drums, and whale falls.^29,32,33^ Furthermore, human health studies have shown generational health effects from maternal DDT exposure.^34,35^ This renewed interest has further triggered actions at the local, state, and federal levels that include proposed legislation, research support, and proposed mandates for the involvement of state and federal agencies. Given the great uncertainties surrounding Montrose’s offshore disposal activities and its potential effects, we designed a sediment study based on the analysis of a transect of cores to inform the mode, location, and timing of offshore DDT waste disposal activities while simultaneously informing the potential for the transport of DDT and its degradation products from the PV Shelf to the deep SP Basin and the extent to which it has experienced transformation. Our results inform each of these issues and further provide the basis for the development of a numerical model to describe the physical, chemical, and biological processes that affect DDT waste in this setting and to predict its long-term fate. In the course of this work, we further found historical evidence regarding potential low-level radioactive (non-DDT) containerized waste disposed of at dumpsite 2.

## 2 Materials and Methods

Sediment from SP Basin, California, was collected aboard *R/V Yellowfin* on November 07, 2022, and December 15, 2022. A multicorer was used to collect sediment cores of 10 cm diameter from sampling stations along two transects through SP Basin intersecting regulated disposal sites: dumpsite 1 and dumpsite 2 (Figure 1a). Sediment cores were processed shipboard for chemical analysis.

Sediment cores were loaded onto an extruder and sectioned at 1 cm intervals for the top 10 cm of the core, followed by 2 cm intervals for depths below 10 cm. Between each core interval, sectioning equipment was cleaned of sediment particles, rinsed with surface seawater, and blotted dry. For stations visited on December 15, 2022, an isopropyl alcohol (91% v/v) rinse was included prior to drying. Core sections were placed in 125 mL glass jars with PTFE-lined closures and stored at −20 °C until used for chemical analyses.

Sectioned cores were analyzed for pesticide content (including 4,4′-DDT, 2,4′-DDT, 4,4′-DDE, 2,4′-DDE, 4,4′-DDD, and 2,4′-DDD) by gas chromatography with electron capture detection at Alpha Analytical, Mansfield, Massachusetts, by EPA Method 8081, following solvent extraction by EPA Method 3570. Additional QA/QC information for analyses at Alpha Analytical can be found in the Supporting Information. Total organic carbon (TOC) and total nitrogen (TN) content of sediment sections were quantified by a model CEC 440HA elemental analyzer at the Marine Science Institute Analytical Laboratory at University of California, Santa Barbara, California, following carbonate removal. δ^13^C-TOC and δ^15^N-TN content of sediment sections were quantified by a Thermo-Finnigan MAT Delta Plus Advantage isotope ratio mass spectrometer using an elemental analyzer dual method at the Marine Science Institute Analytical Laboratory at University of California, Santa Barbara, California, following carbonate removal. Δ^14^C of TOC was quantified at the Keck Accelerator Mass Spectroscopy facility at the University of California, Irvine, California, following their standard procedures.^36^ Δ^14^C of barrel-associated carbonate was analyzed at the Center for Accelerator Mass Spectrometry at Lawrence Livermore National Laboratory, Livermore, California.^37^^137^Cs activity and ^210^Pb excess was measured by γ-ray spectroscopy at University of Southern California, Los Angeles, California, as described previously.^38^ Additional QA/QC information for these analyses can be found in the Supporting Information.

A 1-D numerical model^39^ was developed to aid our understanding of dynamical transport and degradation processes for individual 4,4′-DDX compounds within the deep SP Basin. The 1-D model accounts for coupled transport processes within and between sediments and the lower water column, and it also accounts for selected degradation pathways in the deep basin. The model is organized as two modules: a Resuspension and Redeposition (RR) module and a Diffusive Exchange, Burial, and Aqueous Export (DEBAE) module. The RR module is a compartment model that simulates initial DDX deposition due to dumping; sediment–water exchange by resuspension of DDX-laden sediments; redeposition of the resuspended DDX; degradation of DDT to DDE within the water column (*k*_water deg. DDT/DDE_); and sedimentation, defined as the transfer of DDX in labile sediments considered available to resuspension to layering sediments considered unavailable to resuspension. The DEBAE module is a partial-differential-equation model that simulates burial, diffusive transport, and degradation within sediments (*k*_sed. biodeg. DDT/DDD_ and *k*_sed. deg. DDT/DDE_); diffusive exchange at the sediment–water interface; and export of DDX from the deep SP Basin by upward transport through the lower water column. The two modules are linked through the sedimentation flux simulated by the RR module. The scientific methodology of the 1-D model of DDX transport and degradation is described in the Supporting Information. The model software is proprietary to Oleolytics LLC and can be made available according to the journal requirements.

## 3 Results and Discussion

### 3.1 Disposition, Chronology, and Depositional Dynamics of DDT Waste

Stations T2-a–T2-j comprising the transect from the PV Shelf through dumpsite 2 (transect 2, Figure 1) exhibit highly elevated concentrations above background of DDT-family compounds (collectively referred to as DDX, see Table S1) with pronounced subsurface maxima that range from 665–32,800 μg/kg DDX (Figures 1 and S1). The seven furthest offshore stations in transect 2 (T2-d–T2-j) exhibit similar depth distributions with peak DDX located consistently at 4–8 cm below seafloor, indicating that alarming concentrations of DDT, DDD and DDE span the entire swath of the SP Basin. The two stations in transect 2 located in the deep basin closest to the PV Shelf (T2-b and T2-c) exhibit broad maxima extending to greater sediment depths, with notable concentrations of DDT and DDD at depth, overlain by a broad maximum composed of mainly DDE. Heat plots of DDT and DDE for the nine deep basin stations in transect 2 (Figure 1b) exhibit differentiated patterns of deposition and preservation and are interpreted here as an offshore depositional event followed by transport of DDE to the basin’s northeastern margin from the PV Shelf.

Elevated concentrations above background of DDX were also found for the transect to dumpsite 1 (transect 1, Figures S1 and S2), which comprised three unique stations (T1-a, T1-b, and T1-c) and a tie-in to transect 2 at T2-b. The two unique stations located in the SP Basin (T1-a and T1-b) shared common features with the other SP Basin stations including a subsurface DDX maximum located 4–8 cm beneath seafloor and exceeding 1000 μg/kg. The more distal of these stations from the PV Shelf, T1-b, exhibited high relative abundance of DDE in the subsurface maximum, distinguishing this location from other stations in the deep SP Basin. Station T1-c located outside of the SP Basin at the center of dumpsite 1 exhibited elevated concentrations of DDX to depths exceeding 12 cm, but with no distinctive subsurface maximum and with peak DDX concentrations notably less (<90 μg/kg) than in the SP Basin. Heat maps along transect 1 for various DDX components are shown in Figure S2.

**Figure 2:**
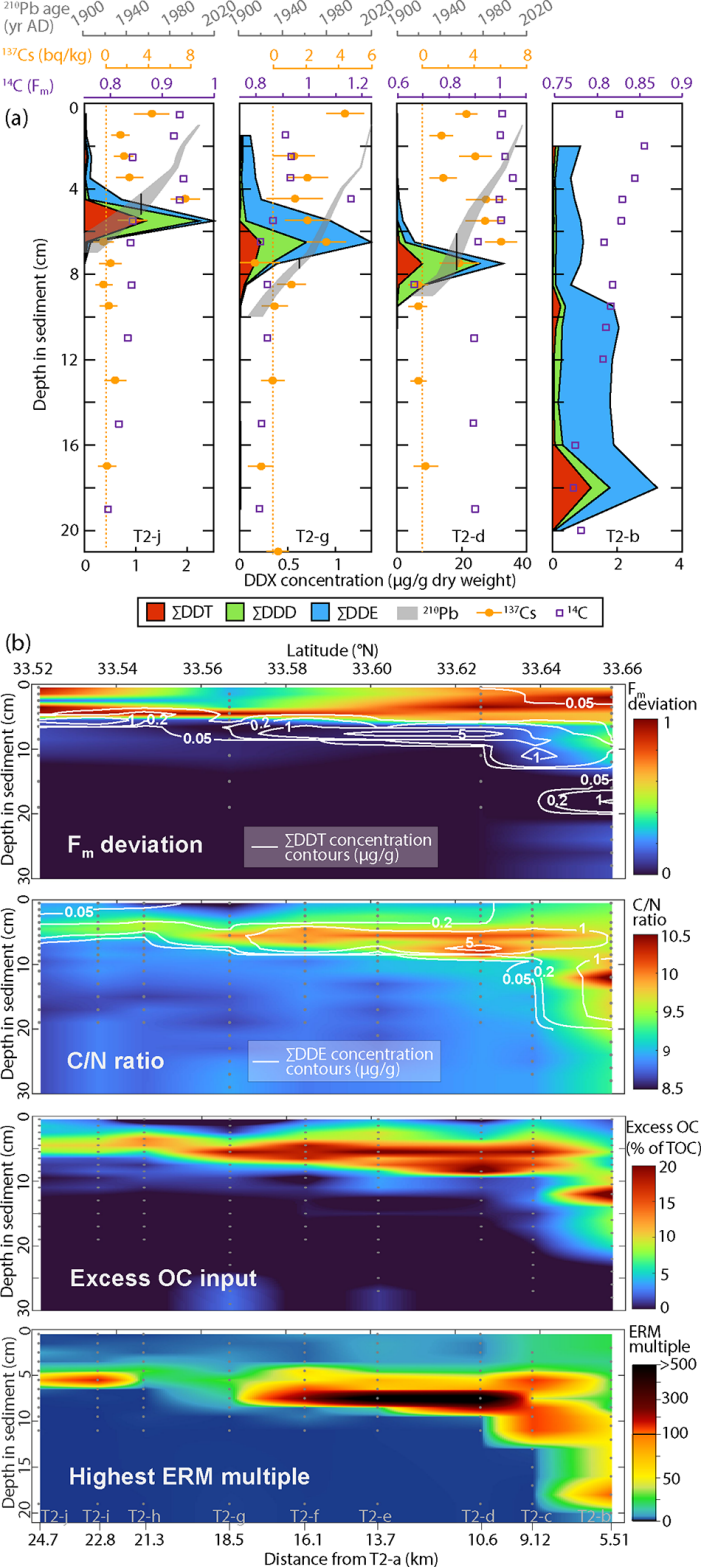
Sediment chronology and geochemistry. (a) Comparison between DDX depth profiles and sediment chronology, including ^210^Pb-derived age model (gray shading shows 1σ uncertainties, black vertical line indicates the year 1955; see Figure S4 for details), as well as radiocarbon fraction modern (*F*_m_) and ^137^Cs concentration (with 1σ uncertainties). Note that zero for ^137^Cs is offset from the scale for DDX. (b) Heat maps showing sediment geochemical properties along transect 2. Figures include (from top to bottom): radiocarbon fraction modern (*F*_m_) deviation (see the Supporting Information and Figure S3 for details) overlaid by ΣDDT concentration contours; organic carbon-to-nitrogen ratio (C/N ratio) overlaid by ΣDDE concentration contours; estimate of excess organic carbon (OC) attributed to anthropogenic inputs; and highest effects range median^43^ multiples among 4,4′-DDT, 4,4′-DDD, and 4,4′-DDE (see Figure S5 for details). Gray dots indicate where data is present, with values in between estimated by linear interpolation.

In order to contextualize the observed depth distributions of DDX, we investigated cross-basin sediment chronology using multiple radioisotope proxies: ^137^Cs, radiocarbon (^14^C) and ^210^Pb (Figure 2a). From depth distributions at T2-d, T2-g, and T2-j, we find the first appearance of ^137^Cs in the same depth interval as the subsurface 4,4′-DDT maximum, which lies at or immediately below the interval of peak concentration for ^137^Cs. The initial appearance of ^137^Cs in sediment cores is typically attributed to nuclear weapons testing in 1955 whereas the peak in ^137^Cs is typically attributed to 1963.^40^ Based on the accumulation rates for these cores, this eight-year time span may be fully incorporated in a single 1 cm sample interval or may be split between adjacent intervals, both of which are observed in our data. Radiocarbon profiles of bulk organic carbon in the cores exhibit a positive shift due to the incorporation of radiocarbon from nuclear weapons testing, which peaked in surface waters of this region in the 1970s (Figures 2 and S3);^41,42^ this feature overlies peak 4,4′-DDT in each core (Figure 2), consistent with the results from ^137^Cs. Results based on excess ^210^Pb are further consistent with the ^137^Cs chronology, with some variability arising based on the assumptions for the age model used in the calculation (Figure S4). These results indicate intense deposition of DDT mainly in the 1950s, followed by partial transformation of emplaced DDT. This chronology is consistent with historical records of DDT production with offshore waste disposal increasing commensurate with Montrose’s production from 1947 to 1961, and then decreasing following the construction of an internal acid recycling facility in 1961. Importantly, this timing places the bulk of the DDT waste disposal activities prior to the onset of regulations governing disposal activities in SP Basin.

**Figure 3:**
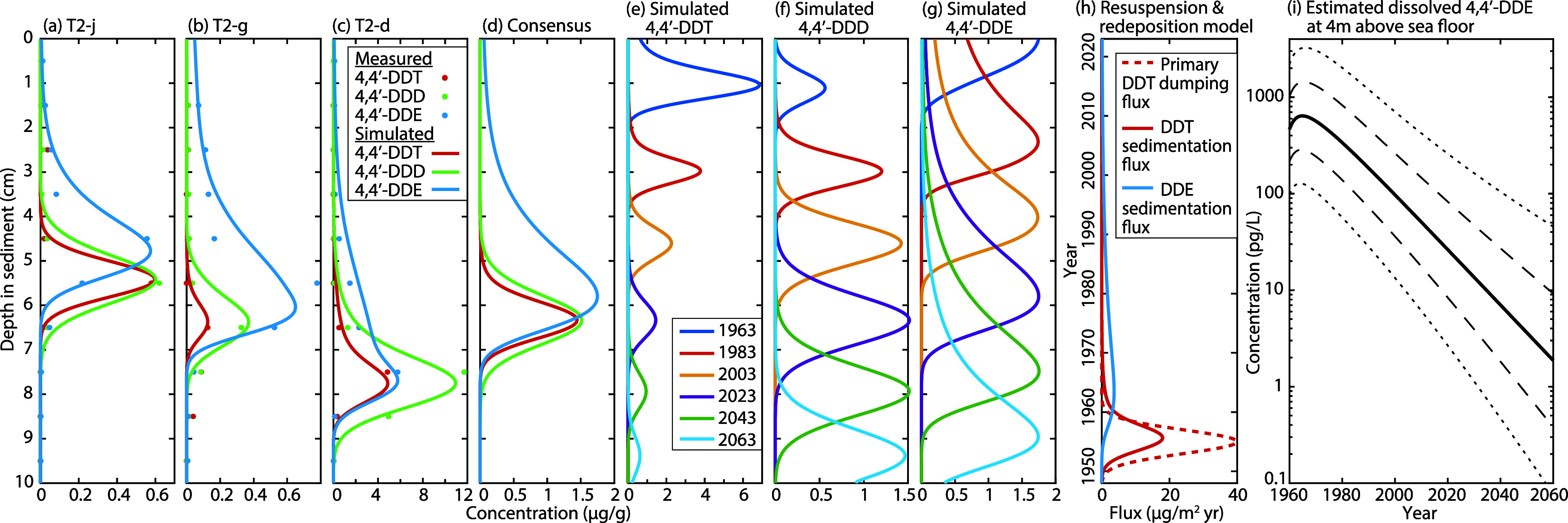
Results from a 1-D model of 4,4′-DDX transport and degradation processes in the deep SP Basin. Measured concentrations in sediment profiles are compared to simulation results for stations (a) T2-j, (b) T2-g, and (c) T2-d, based on a best fit of adjustable model parameters separately for each station. (d) Simulated concentration profiles of 4,4′-DDX compounds in sediments according to a consensus model which employs the midpoint between average and median values of adjustable model parameters fitted to stations T2-d, T2-e, T2-f, T2-g, T2-h, T2-i, and T2-j. Simulated concentration profiles of the consensus model are displayed at past, present, and future time points for (e) 4,4′-DDT, (f) 4,4′-DDD, and (g) 4,4′-DDE. (h) Historical time course for fluxes into layering sediments derived from the consensus model. (i) Time trajectory of estimated dissolved 4,4′-DDE concentrations at 4 m above seafloor, based on the fitted models to stations T2-d, T2-e, T2-f, T2-g, T2-h, T2-i, and T2-j, showing the median prediction (solid line), 1σ estimate (dashed lines), and 2σ estimate (dotted lines).

We sought to interpret the observed depth profiles of 4,4′-DDT, 4,4′-DDD, and 4,4′-DDE by the development of a 1-D model of coupled transport and degradation processes in the deep SP Basin (Figures 3a–c and S6). The model provides a quantitative framework to deepen our understanding of the physical, chemical, and biological processes acting on these compounds in a deep basin environment. The model was fitted to observed 4,4′-DDX distributions for each deep basin station along transect 2, by tuning station-specific parameters which represent certain physical and chemical processes: the maximum flux of a unimodal dumping input of 4,4′-DDT which is centered on the year 1955; the solids concentration in the deep water column; a sediment resuspension rate constant; a redeposition rate constant; a sedimentation rate constant which describes input to layering sediments; and first-order degradation kinetics constants which represent the decomposition of 4,4′-DDT by distinct pathways into 4,4′-DDD and 4,4′-DDE. A conceptual schematic of these parameters can be found in the Supporting Information (Figure S12).

The model explains the observed vertical thickness and depth of 4,4′-DDT profiles predominantly by diffusive transport coupled with burial and compaction by sedimentation. The model interprets trends in 4,4′-DDD profiles predominantly as within-sediment biodegradation of 4,4′-DDT further modulated by diffusion and burial processes. However, a secondary DDX input is needed to explain two notable asymmetries: first, 20 of 27 observed vertical distributions of the 4,4′-DDX compounds are skewed toward the sediment–water interface (Table S2); and second, the centroids of 4,4′-DDE abundance are vertically dislocated, lying ∼1 cm (more at stations near the PV Shelf) above the centroids of 4,4′-DDT and 4,4′-DDD (Table S2). The model explains these features by a secondary input to sediments that arises from dynamical interactions among resuspension, redeposition, sedimentation, and degradation processes. In the model, these processes prolong the deposition and burial of 4,4′-DDT, and they also produce a 4,4′-DDE input to sediments on a longer time frame (Figure 3h). The 1-D model therefore can explain many features of the observed modern sediment profiles of 4,4′-DDT, 4,4′-DDD, and 4,4′-DDE in terms of dynamical interactions among transport and fate processes within the deep basin. The model excludes inputs that are external to the deep SP basin such as the PV Shelf or wastewater outfalls.

### 3.2 Reactivity, Recalcitrance, and Sources of DDT Pollution

Our survey of DDT waste in the SP Basin reveals widespread contamination spanning from the Superfund site on the PV Shelf to the base of the Catalina Rise, 25 km away (Figures 1, S1, S5, and S7). The spatial pattern of contamination is contextualized by the chronology of the cores and enables the differentiation between direct inputs of DDT waste by offshore disposal versus lingering inputs of mainly DDE that continue to deposit in the sediment at attenuating rates. Each of the 10 stations along transect 2 exhibited peak 4,4′-DDX concentrations between 480 and 22,300 μg/kg, values exceeding NOAA’s sediment quality [effects range median of 4,4′-(DDT + DDD + DDE) = 46.1 μg/kg] guidelines^43^ by 10–484 fold (Figures 2b and S5). That is, the buried sediments of the deep SP Basin are polluted with ocean-dumped DDT waste, stretching from the Catalina Rise to the PV Shelf, with active but attenuating deposition of mainly DDE through the present day.

**Figure 4:**
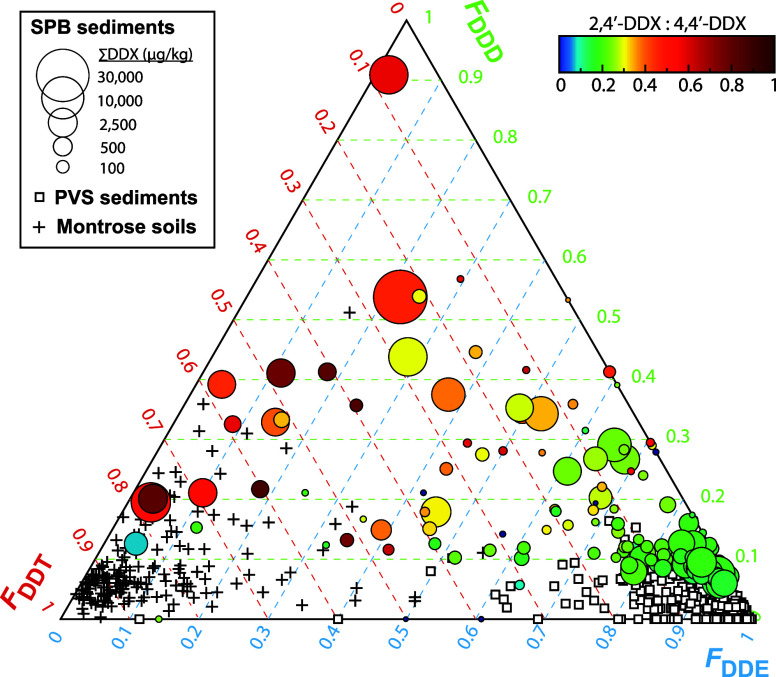
Ternary plot comparison of different sample types. Ternary plot parsing out the relative fractions of DDT, DDD, and DDE in different types of samples, including the deep SP Basin sediments (circles; this study), PV Shelf sediments (white squares),^45^ and soils around Montrose Facility (plus).^2^ For deep SP Basin sediments, symbol color represents the ratio between 2,4′ and 4,4′ DDX isomers and symbol size represents the concentration of ∑DDX.

The relative abundance of DDT compared to its primary degradation products, DDD and DDE, further provides insights into the limited extent of DDT transformation in the deep marine sediments of the SP Basin and also punctuates environmental concerns. In contrast to the PV Shelf where DDE is the primary DDX compound present, the offshore stations exhibit higher but variable proportions of DDT and DDD, especially within the most highly contaminated strata associated with offshore disposal in the 1950s (Figure 4). A high proportion of DDT left behind in these strata, like in soils at Montrose, points to slow degradation rates, as this DDT has gone unaltered since its disposal more than 70 years ago (Figure 4). However, variation in isomer ratios (2,4′- to 4,4′-; Figure 4) leaves open the possibility that additional dechlorination beyond DDE and DDD are at work. For example, several lines of evidence indicate that 4,4′-DDE undergoes reductive dechlorination to 4,4′-DDMU in PV Shelf sediments,^13^ and Kivenson and co-workers reported the pervasive presence of 4,4′-DDMU in deep SP Basin sediments sampled at dumpsite 2.^5^ The commonly used ERM sediment quality guideline^43^ for 4,4′-DDT in marine sediment sets a lower concentration threshold than for other DDX compounds (Table S1), and the peak observed 4,4′-DDT concentration is 690-fold greater than published guidelines (Figures 2b and S5). The high relative abundance of 4,4′-DDD compared to 4,4′-DDE for some stations is further interpreted as evidence for an anaerobic degradation pathway, consistent with slow degradation and the anaerobic biogeochemistry of the deep SP Basin sediment.^44^ Using the 1-D model to constrain these degradation processes, we obtained fitted rate constant values of *k*_sed. biodeg. DDT/DDD_ = (1.2 ± 0.5) × 10^–2^ year^–1^ for 4,4′-DDT → 4,4′-DDD in buried sediments [half-life = (5.8 ± 2.0) × 10^1^ years], *k*_sed. deg. DDT/DDE_ = (4.2 ± 3.5) × 10^–3^ year^–1^ for 4,4′-DDT → 4,4′-DDE in buried sediments [half-life = (1.7 ± 0.7) × 10^2^ years], and *k*_water deg. DDT/DDE_ = (2.4 ± 1.5) × 10^–1^ year^–1^ for 4,4′-DDT → 4,4′-DDE (half-life = 2.9 ± 1.0 years) in suspended particles and surface sediments (Table S3). The model-fitted value of the rate constant for 4,4′-DDT transformation to 4,4′-DDE (*k*_water deg. DDT/DDE_) is likely conservative because 4,4′-DDE degradation processes are neglected by the 1-D model. This parameter estimate is slightly lower than the rate constant value of ∼5 × 10^–1^ year^–1^ for hydrolytic dehydrochlorination of 4,4′-DDT to 4,4′-DDE in the PV Shelf water column that was calculated by Eganhouse and co-workers^10^ based on earlier measurements of this pH- and sorption-dependent abiotic reaction.^11^

Sediment strata overlying the offshore disposal peak all exhibit high proportions of 4,4′-DDE similar to the PV Shelf, consistent with limited prior observations.^5,7^ The results of our modeling effort indicate that upward flow of porewater-accommodated 4,4′-DDX coupled to reflux of particle associated 4,4′-DDX to the seafloor is unlikely to account for this feature, pointing to a secondary input of 4,4′-DDE-laden sediment over longer time frames. The PV Shelf is a likely contributor to the observed 4,4′-DDE-rich strata in deep basin sediments, especially in the vicinity of the slope base. In addition, protracted secondary inputs may arise from local resuspension–redeposition dynamics driven by bottom boundary layer turbulence or exhumation from burrowing faunae in deep basin sediments. Burrowing activity is consistent with rough seafloor texturing as has been reported in portions of the San Pedro Basin,^32^ and it may explain secondary 4,4′-DDE maxima in some cores especially impacted by burrowing, such as at stations T2-b, T2-c, and T1-b.

### 3.3 Historical Disposal Practices and Exposure Predictions

In assessing potential ongoing sources of DDE to the SP Basin, we found a spatial and temporal association of 4,4′-DDE with the carbon to nitrogen ratio (C/N) of sediment organic material (Figures 2b and S8–S10) that points to the PV Shelf and the Los Angeles County Sanitation Districts’ (LACSD) Joint Wastewater Treatment Plant outflow pipes as a substantial source. Sediment distributions with subsurface C/N maxima are consistent with both a high C/N source of sediment particles from sewage outflow^46,47^ and the history of suspended solid discharge in effluent from the LACSD outflow pipes which increased until the early 1970s and then decreased commensurate with enhanced treatment as regulated under the Clean Water Act.^48^ Solids suspended in the sewage effluent, along with DDX, are known to have been transported toward the northwest laterally along PV Shelf, but models also show a cross-shelf trajectory in average annual horizontal sediment transport rates;^49^ cross-basin transport may also have been enhanced under strong eddy regimes, a phenomenon modeled in nearby San Pedro and Santa Monica Bays,^50^ or through sediment transport off the shelf, e.g., into Redondo Canyon. Using the maximum C/N values from station T2-a on the PV Shelf, similar to those observed previously,^46,47^ we calculate a potential contribution of 10–25% toward total organic matter deposition across the SP Basin, spanning approximately two or more decades—the 1960s and 1970s (Figure 2b). The loading of nitrogen-depleted carbon sourced from the outflow pipes into the deep sediments of the SP Basin is consistent with observed profiles as the outflow pipes released abundant DDX during this same time frame.

The 1-D model of transport and degradation constrains the exposure potential caused by DDX compounds in the deep SP Basin, including predictions for past and future conditions. After calibration to 4,4′-DDX profiles in sediments at seven stations, the model estimates that dissolved concentrations of 4,4′-DDE in seawater today may range from 2 to 2 × 10^2^ pg L^–1^ within the bottom boundary layer which spans a height of several meters above the basin floor (Figure 3i). The model further predicts that the DDX burden in the deep water column has decreased over time and will continue to decline as sedimentation continues to bury the primary deposits deeper into the seafloor (Figure 3). The extent to which 4,4′-DDE is expected to linger in the deep SP Basin and further deposit to the sediments is intimately linked to the transport processes that appear to have been active for more than 70 years, such as resuspension processes including exhumation of buried sediments. The 1-D model excludes inputs from the PV Shelf or from wastewater outflow and neglects degradation processes affecting 4,4′-DDD or 4,4′-DDE, but the optimized model may proxy these processes through parameters which are fitted to 4,4′-DDX profiles at coring stations (Figure 3 and Table S3). Parsing these potential sources of DDE to the deep basin will be important for understanding modern and future exposure potential.

In planning our survey, we anticipated the highest concentrations of DDX would be found in the immediate vicinity of Cal Salvage’s preferred dump site, dumpsite 2, or perhaps at their originally assigned dump site, dumpsite 1. In contrast to expectation, the observed pattern showed a highly DDX-contaminated area to the east of dumpsite 2 and a second notable area located west of dumpsite 2 and southeast of dumpsite 1 (Figure 1). We interpret these more highly contaminated locations as areas where substantial amounts of historical dumping occurred, and in turn, we suggest the easternmost area may have been commonly used by Cal Salvage for dumping, prior to the onset of regulations.^3^ The explanation for elevated DDX at the westernmost reaches of the SP Basin remains uncertain but could be related to disposal in that area or to physical transport that affects deposition patterns. Either way, the occurrence of DDX at this location is notable because it is just 6.7 km from Catalina Island and may inform historical observations by providing a contributory mechanism as to how Catalina Island’s Bald Eagle population was completely lost in the 1950s.^51−54^

### 3.4 Disposal of Bulk versus Containerized Waste

**Figure 5:**
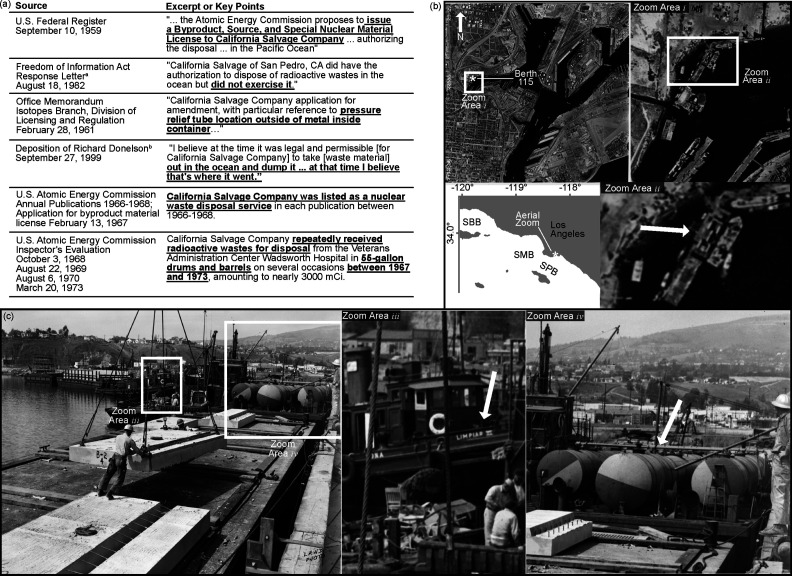
Evidence for the bulk disposal of DDT wastes and low-level radioactive wastes by Cal Salvage. (a) Tabulated excerpts or key points from documents acquired through the Freedom of Information Act. ^a^the Freedom of Information Act Response Letter was received through the Division of Rules and Records, Office of Administration, U.S. Nuclear Regulatory Commission; ^b^Richard Donelson’s September 27, 1999, deposition was from the case *Joseph A. Thomson and Virginia Thomson v. ICN Pharmaceuticals, Inc., a Delaware Corporation; NUCOR Corporation, a Delaware Corporation; and Rhone-Poulenc, Inc., a New York Corporation*. (b) An aerial image of Port of Los Angeles including Berth 115 (star, ca. 33.75° N, 118.29° W) used by Cal Salvage to load industrial wastes for ocean disposal. Zoom area *i* shows the greater Berth 115 area, and zoom area *ii* includes a barge containing large tanks that may have stored wastes for ocean disposal. Image from Flight C-22555, Frame 29–27, July 01, 1956. Geospatial Collection. Department of Special Research Collections, UC Santa Barbara Library, University of California, Santa Barbara. (c) An oblique image taken on April 12, 1961, at Berth 115 in the Port of Los Angeles facing west. Zoom panel *iii* and *iv*, respectively, highlight *Limpiar VI* of Cal Salvage’s fleet used for ocean disposal activities and large tanks aboard a barge that may have been used for ocean disposal activities. Image from the Los Angeles Harbor Department—Reuse restrictions apply.

High concentrations and sediment depth distributions of DDT family compounds observed in this work point to the bulk disposal of DDT waste in the SP Basin. This interpretation is consistent with recent claims made by the EPA^8^ but stands in contrast to historical interpretations^3^ likely because those historical interpretations conflated physical barrels with “barrels” as the volumetric unit of measure. To further assess our interpretation of bulk disposal, we searched historical photographic archives including images from the Ports of Los Angeles and Long Beach as well as aerial photo archives. A photo of the Cal Salvage dock facility (Berth 115) is shown in Figures 5b,c and S11, highlighting a tank barge docked there, and hosting various tank configurations between 1947 and 1968. Given the presence of this barge at the Cal Salvage dock facility during the era of active DDT waste disposal, we suggest that this could be the barge used to transport DDT waste (and other wastes) for bulk offshore disposal.

The realization that DDT waste was disposed of in bulk, and not containerized in barrels, raises another important question as to what material is contained within the barrels and drums observed previously in the SP Basin^5,32^ and that have drawn substantial public interest.^27−29,55^ Toward addressing this question, we searched through historical archives and materials provided through the US Freedom of Information Act for insight about containerized waste disposal by Cal Salvage during this era. We identified seven independent lines of evidence that collectively point to the possibility of clandestine disposal of containerized low-level radioactive waste by Cal Salvage. The 11 documents supporting these seven lines of evidence are provided in full in the Supporting Information, with key excerpts provided in Figure 5a. Two key revelations frame our analysis. First, according to the US Federal Register (Document S4), in 1959, Cal Salvage applied for and received a permit for the disposal of containerized radioactive waste at a location of 32.0° N, 121.5° W at depth greater than 1000 fathoms, roughly ∼358 km southwest of the Port of Los Angeles and Long Beach (US Atomic Energy Commission permit 04-05479-01). Second, according to a Freedom of Information Act response letter dated August 18, 1982, from the US Nuclear Regulatory Commission, Cal Salvage never activated their permit with the US Atomic Energy Commission (the predecessor agency to the Nuclear Regulatory Commission) and never (legally) disposed of radioactive waste (Document S5), a contention supported with testimony by representatives of the Atomic Energy Commission for the April 6, 1971, congressional hearing on Ocean dumping of waste material (Document S6). However, five lines of evidence collectively point to sustained radioactive waste disposal activity by Cal Salvage: (1) according to an internal February 28, 1961, memo from the US Atomic Energy Commission (Document S7), Cal Salvage applied for an amendment, with particular reference to the design of a pressure relief tube, indicating a sustained business interest; (2) according to a 1999 legal deposition, Cal Salvage accepted radioactive waste material from the ca. 1961 decommissioning of a radioisotope facility in Burbank, California, with the explicit purpose of offshore disposal (Document S8); (3) from 1966-68, Cal Salvage passively advertised their radioactive waste disposal services in an annual publication of the US Atomic Energy Commission (Documents S9–S11); (4) in 1967, Cal Salvage was listed as the intended commercial waste disposal service provider (Document S12) for a Byproduct Material License Application to the Atomic Energy Commission; and (5) according to US federal records from the Atomic Energy Commission, Cal Salvage was reported to have accepted radioactive waste material quarterly from 1968-73, from a regional Veterans Administration hospital facility (Documents S13–S16).

To our knowledge, no samples have been collected of the interior contents of barrels disposed in the SP Basin that would inform the issue of radioactive waste disposal, but we did previously collect samples from mineral growth on the exterior of one such barrel. Based on the recorded occurrence of radiocarbon in containerized waste accepted by Cal Salvage (Documents S13–S16), we applied accelerator mass spectrometry to the carbonate fraction of this feature, finding no anomaly (*F*_m_ = 0.89 ± 0.04; *N* = 10). Nonetheless, the historical record points to a scenario in which Cal Salvage was potentially able to openly operate as an offshore radioactive waste disposal company without triggering oversight, regulatory compliance required by the permitting process, or even activating their permit. A final piece of (circumstantial) evidence further informs this issue: recently identified debris trails of mainly military munitions extrapolate from the Port of Los Angeles and Long Beach, through the SP Basin,^32^ toward the dumpsite location provided to Cal Salvage by the US Atomic Energy Commission. Disposal along such a track might have provided Cal Salvage some mitigation or plausible deniability had their activities come to the notice of regulators.

### 3.5 San Pedro Basin Was an Ocean Dump

In summary, the results from our seabed analysis campaign point to bulk ocean disposal of DDT manufacturing wastes over an area that extends to a distance of at least 25 km from the mainland of Southern California, is focused outside of designated disposal areas, and began prior to the onset of regulation. Substantial amounts of DDT remain in these sediments, which are largely unaltered after more than 70 years. Evidence points to long-term burial overprinted by a secondary source of DDE with targeted studies needed to capture the full disposition of these wastes and the extent to which they have degraded and to provide predictions of ongoing deposition and interaction with the water column and biota. Circumstantial historical evidence further points to concurrent or subsequent disposal of containerized low-level radioactive wastes by the same disposal company responsible for DDT waste. Together, these findings point to a pervasive industrial waste disposal campaign that took place off the coast of Southern California, with environmental effects that still linger today.

## Data Availability

The data underlying this study are available in the published article and its Supporting Information. All study data are also publicly available to readers in the NOAA NCEI database at https://www.ncei.noaa.gov/.

## Supporting Information Available

Detailed QA/QC of certain analyses; definition of radiocarbon fraction modern deviation; in-depth description of a 1-D model; additional DDX and other sediment chemical profiles; tables of abbreviations, statistics, and model parameters; and documents procured under a Freedom of Information Act request for the discussion of disposal of bulk versus containerized waste into San Pedro Basin (PDF)
Complete data set for all sample sites referenced in this work (XLSX)
