# Correction to “Disentangling the History of Deep Ocean Disposal for DDT and Other Industrial Waste Off Southern California”

**DOI:** 10.1021/acs.est.4c03637

**Published:** 2024-04-23

**Authors:** Jacob T. Schmidt, Mong Sin Christine Wu, Hailie E. Kittner, J. Samuel Arey, Douglas E. Hammond, Earth 182A Group, David L. Valentine

The Acknowledgment section has been updated and revised to reflect the contribution of each of the members of the Earth 182A Group more appropriately. This correction does not affect any other results or conclusions in our work.

