# Supplementary material for: Disentangling the History of Deep Ocean Disposal for DDT and Other Industrial Waste Off Southern California
Source: Environ Sci Technol. 2024 Feb 21;58(9):4346–56. doi: 10.1021/acs.est.3c08575 (PMC10919092; doi:10.1021/acs.est.3c08575)
Supplement: Supplementary file 1 — es3c08575_si_001.pdf [file es3c08575_si_001.pdf]

Supporting Information for

Disentangling the History of Deep Ocean Disposal for DDT and Other Industrial Waste Off Southern California.

Jacob T. Schmidt[a], *Mong Sin Christine Wu*[b], *Hailie E. Kittner*[b], *J. Samuel Arey*[c], *Douglas E. Hammond*[d], *Earth 182A Group*[b], **David L. Valentine*[b,e]

[a]Interdepartmental Graduate Program in Marine Science, University of California, Santa Barbara, California, 93106 United States.

[b]Department of Earth Science, University of California, Santa Barbara, California, 93106 United States.

[c]Oleolytics, LLC, State College, Pennsylvania, 16801 United States.

[d]Department of Earth Sciences, University of Southern California, Los Angeles, California, 90089 United States.

[e]Marine Science Institute, University of California, Santa Barbara, California, 93106 United States.

*Corresponding author: David L. Valentine
Email: valentine@ucsb.edu

Summary: 1741 pages, 12 figures, 4 tables

Table of Contents

**Supporting Information Text**	**S3**
SI 1	S3
SI 2	S4
SI 3	S4
**Figures**	**S17**
Figure S1	S17
Figure S2	S18
Figure S3	S19
Figure S4	S20
Figure S5	S21
Figure S6	S22
Figure S7	S23
Figure S8	S24
Figure S9	S25
Figure S10	S26
Figure S11	S27
Figure S12	S28
**Tables**	**S29**
Table S1	S29
Table S2	S30
Table S3	S31
Table S4	S32
**Document Descriptions**	**S33**
**References**	**S37**
**Documents**	**S39**
Document S1	S39
Document S2	S162
Document S3	S232
Document S4	S242
Document S5	S282
Document S6	S283
Document S7	S844
Document S8	S848
Document S9	S988
Document S10	S1172
Document S11	S1369
Document S12	S1642
Document S13	S1668
Document S14	S1679
Document S15	S1691
Document S16	S1709

Supporting Information

SI 1. Relevant QA/QC details for select analyses.

EPA methods were used for analysis at Alpha Analytical, Mansfield, Massachusetts, including extraction (EPA 3570), cleanup (EPA 3630) and quantitation (EPA 8081B), each with their own high degree of QA/QC. Alpha Analytical retains the following certifications & approvals: MA (M-MA030), NH NELAP (2062), CT (PH-0825), DoD (L2474), FL (E87814), IL (200081), IN (C-MA-04), KY (KY98046), LA (85084), ME (MA00030), MD (350), MI (99110) , NJ (MA015), NY (11627), NC (685), OH (CL106), OR (MA-0262), PA (68-02089), RI (LAO00299), TX (T104704419), VT (VT-0015), VA (460194), WA (C954), US Army Corps of Engineers, USDA (Permit #525-23-107-88708), USFWS (Permit #206964). The Pesticides Analysis method includes recovery standards and internal standards, and the analysis relies on a dual-column, Dual ECD. The analytes quantified by this method include: alpha-BHC, Hexachlorobenzene, beta-BHC, gamma-BHC, delta-BHC, Heptachlor, Aldrin, Heptachlor epoxide (B), Oxychlordane, gamma-Chlordane, 2,4'-DDE, Endosulfan I, alpha-Chlordane, trans-Nonachlor, 4,4'-DDE, Dieldrin, 2,4'-DDD, Endrin, Endosulfan II, 4,4'-DDD, 2,4'-DDT, cis-Nonachlor, Endrin aldehyde, Endosulfan sulfate, 4,4'-DDT, Endrin ketone, Methoxychlor and Mirex (note that only 2,4'-DDE, 4,4'-DDE, 2,4'-DDD, 4,4'-DDD, 2,4'-DDT, and 4,4'-DDT exceeded the Detection Limit).

^{137}Cs activity and ^{210}Pb excess was measured at University of Southern California. ^{210}Pb activity was calibrated using NIST SRM 4337 with corrections made for self-adsorption in similar sediments. The ^{226}Ra parent activity was calibrated using SRM-DP2 diluted pitchblende supplied by EPA, with corrections made for a 10% ^{222}Rn loss measured from

similar sediments. Secular equilibrium for ^{210}Pb and ^{226}Ra at depth was observed when using these corrections. ^{137}Cs activity was calibrated with IAEA marine sediment standard 385, and a standard solution supplied by EPA. Error bars reflect ±1 sigma, based on counting statistics.

SI 2. Definition and calculation of radiocarbon F_m deviation.

To aid the comparison of radiocarbon (fraction modern, F_m) profiles for bulk sediment organic carbon at stations across the deep SP Basin with different radiocarbon backgrounds (T2-b, T2-d, T2-g, T2-j; from PV Shelf to Catalina Rise), we defined a 'F_m deviation' variable, which is calculated as the deviation of downcore F_m values normalized between the 'baseline' value (defined as 0) and the peak value (defined as 1) in each individual core. The 'baseline' F_m value in each core is calculated as the average of data points prior to the abrupt positive shift in F_m attributed to the bomb spike (Figure S3a-d). One outlier at 8.5 cm depth at station T2-d has been removed from the calculation of average baseline value as it has highly depleted radiocarbon abundance ($F_m = 0.565$) not consistent with the other pre-bomb spike values (average $F_m = 0.802$). The normalized downcore F_m deviation trends (Figure S3e) allow better comparison and visualization of the bomb-spike depths along Transect 2 (Figure 2).

SI 3. 1-D model of dynamical transport and degradation processes of DDX compounds in the deep San Pedro Basin.[1]

Overview.

To interpret the observed profiles of 4,4'-DDX in sediment cores, we developed a 1-D model which simulates dynamical transport and degradation processes of individual 4,4'-DDX compounds in the deep SP Basin. The 1-D model accounts for coupled transport processes within and between sediments and the lower water column, and it also accounts

for selected degradation pathways in the deep basin. We adapted the 1-D model to seven individual sediment coring stations by use of fitted parameters. Each of these seven individually optimized models provide an independent estimate of transport and degradation processes in the deep basin system. Collectively as a set, the individually optimized models are used to derive a consensus model, which is viewed as a best estimate of processes affecting 4,4'-DDX in the deep SP Basin (excluding representation from stations on or immediately proximate to the PV Shelf), within the assumptions and limitations of the model framework.

The 1-D model simulates transport processes along a vertical axis, and it assumes that lateral gradients are negligible. In other words, the model assumes that lateral migration of 4,4'-DDX can be averaged, and this assumption is inappropriate for describing certain processes. However, the approach demonstrates that many features of the observed 4,4'-DDX profiles in sediments can be interpreted through vertical transport and coupled degradation processes. The model provides a quantitative framework to deepen our understanding of the interactions between certain physical, chemical, and biological processes, and it does not evaluate all plausible mechanisms or hypotheses that may explain contemporary 4,4'-DDX observations in sediments of the deep SP Basin.

The 1-D model is dynamical in the sense that the simulated 4,4'-DDX concentrations vary both temporally and vertically in space. The transport and degradation processes are represented and solved as coupled systems of time-dependent differential equations. Consequently, the 1-D model provides an interpreted dynamical history of 4,4'-DDX concentrations, transport, and degradation within the deep SP Basin environment, as well as future predictions.

Parameter fitting and the consensus model.

For the purpose of developing models that are individually optimized to station-specific data, two types of parameters are distinguished: assigned parameters and fitted parameters. Assigned parameters are prescribed or estimated by the authors based on measured environmental data for the SP Basin and/or previous literature. Some assigned parameters are given station-specific values or distributions that vary from one coring station to another, and other assigned parameters are prescribed a single value or distribution which represents conditions at all coring stations, depending on the availability of environmental data.

Unlike assigned parameters, fitted parameters are optimized separately for individual stations. Up to eight model parameters were fitted for each station (Table S3). Fitted parameters were determined by minimizing the weighted sum of squared deviations between observed and simulated 4,4'-DDX concentrations within the top 10 cm, with manual parameter adjustment in some cases. These parameter optimizations applied greater weight to 4,4'-DDX measurements at <3 cm depth relative to those at >3 cm depth. This weighting scheme was justified by two considerations. Firstly, the observed 4,4'-DDX concentrations at <3 cm depth were consistently lower than the highest 4,4'-DDX concentrations occurring in deeper sediment layers, and thus the optimization may lead to larger relative errors for shallower (<3 cm depth) sediment data unless they are weighted. Secondly, the 4,4'-DDX concentrations in near-surface sediments (i.e., <3 cm depth) are relevant to contemporary sediment-water exchanges of 4,4'-DDX and related exposures of marine biota in the lower water column, and we aimed to capture these ongoing processes accurately. The parameter fitting procedure tests the model's ability to reproduce observed

4,4’-DDX profiles in sediments at individual stations in the deep SP Basin (Figure 3), and it also provides a systematic basis to compare the magnitudes of simulated processes among the different stations.

The model fitting procedure additionally provided the basis to derive a consensus model, which is interpreted as a best estimate of the modeled processes in the deep SP Basin environment based on seven stations (T2-d, T2-e, T2-f, T2-g, T2-h, T2-i, T2-j), excluding representation from stations (T2-a, T2-b, and T2-c) on or immediately proximate to the PV Shelf. The consensus model is defined as a model simulation which employs the mid-point between the average and median values of the station-specific parameters for the seven sediment stations (Table S3).

The two modules of the 1-D model.

The 1-D model is organized as two modules, referred to as (a) the Resuspension & Redeposition (RR) module and (b) the Diffusive Exchange, Burial, and Aqueous Export (DEBAE) module. The RR module is a compartment model that simulates the processes of initial DDX deposition due to dumping; sediment-water exchange by resuspension of DDX-laden sediments; redeposition of the resuspended DDX; degradation of DDX within the water column; and finally, sedimentation of DDX, defined as the transfer of DDX from labile sediments (considered available to resuspension) to non-labile sediments (considered unavailable to resuspension). The DEBAE module is a partial-differential-equation model that simulates the processes of burial, diffusive transport, and degradation within sediments; diffusive exchange at the sediment-water interface; and export of DDX from the deep SP Basin by upward transport through the lower water column. The two modules are linked through the sedimentation flux simulated by the RR module, which represents

the flux of DDX transfer from labile sediments (which undergo resuspension) to non-labile sediments (which are committed to layering). The sedimentation flux simulated by the RR module provides the flux of DDX into the surface sediment in the DEBAE module. The DEBAE module omits resuspension processes. Each module is described below in further detail.

Resuspension & Redeposition (RR) module.

The RR module is a compartment model that handles the time-dependent processes of initial dumping of 4,4'-DDT to sediments, assumed to occur historically from 1948 to 1961; transfer of 4,4'-DDT and 4,4'-DDE from sediments into the lower water column due to sediment resuspension; degradation of 4,4'-DDT into 4,4'-DDE within the lower water column; redeposition of 4,4'-DDT and 4,4'-DDE originating from the resuspended sediments; and finally sedimentation, defined as transfer of 4,4'-DDT and 4,4'-DDE from sediments which are labile to resuspension into sediments that are committed to layering (unavailable to resuspension). The sedimentation process, which incorporates labile sediment layers into non-labile sediment layers, represents a time-dependent input of 4,4'-DDX to the surface sediment layer in the DEBAE module. The DEBAE module excludes resuspension processes.

The RR module describes the initial flux of 4,4'-DDT dumping to surface sediments by a time-dependent Gaussian function centered at the year 1955 with an assigned standard deviation of 2 yr. The 4,4'-DDT dumping flux is annotated as $g(t)$ in Figure S12. Although the exact composition of the dumped waste is unknown, the source comes from the same batch synthesis as wastes at the Montrose facility and from the PV Shelf, which is predominantly 4,4'-DDT (with some 2,4'-DDT). By assuming a dumping source of (only)

4,4'-DDT, we implement a parsimonious model parameterization which allows the appearance of other 4,4'-DDX products to be straightforwardly interpreted in terms of the simulated environmental processes. This initial dumping input is described as a time-varying process because the SP Basin was affected by numerous dumping events through time, rather than an instantaneous dumping event. The maximum flux value of *g(t)* is a fitted parameter that is optimized separately for each coring station (Table S3). The dumping input, *g(t)*, transfers 4,4'-DDT into an environmental compartment interpreted as sediments that are labile to resuspension (Figure S12).

From the labile sediments in the RR module, the 4,4'-DDT can undergo resuspension into the lower water column, according to a first-order process with rate constant $k_{resuspension}$, which is a fitted parameter optimized separately for each coring station (Table S3). The 4,4'-DDT in the labile sediments also undergoes sedimentation, defined as transfer to layering sediments considered unavailable to resuspension (non-labile), according to a first-order process with rate constant $k_{sedimentation}$ (year^{-1}). This rate constant is a fitted parameter optimized separately for each station (Table S3). Once 4,4'-DDT has reached the lower water column compartment, it can undergo redeposition back to the labile sediments compartment, according to a first-order rate constant $k_{redeposition}$. The value of $k_{redeposition}$ is a fitted parameter optimized separately for each station (Table S3). From the lower water column compartment, 4,4'-DDT can undergo degradation to 4,4'-DDE according to a first-order rate constant which is fitted parameter, optimized separately for each coring station (Table S3). The resulting 4,4'-DDE can undergo the same transport processes that affect 4,4'-DDT (Figure S12).

Numerical implementation of the RR module.

The RR module was implemented as a system of ordinary differential equations (ODEs). To robustly determine the time-dependent solution of this model, we employed an implicit ODE solver, ode15s, implemented in Matlab,[2] with a user-supplied Jacobian matrix.

The RR module simulation produces time-dependent sedimentation fluxes of both 4,4'-DDT and 4,4'-DDE, defined as the flux of these compounds to layering sediments which are unavailable for further resuspension processes (Figure S12). The sedimentation flux from the RR module provides the 4,4'-DDX input flux to surface sediments in the DEBAE module. The DEBAE module omits resuspension processes.

Sediment-Water Exchange, Burial, & Aqueous Export (DEBAE) module.

The DEBAE module is a partial-differential-equation model that simulates the time-dependent processes of transformation of 4,4'-DDT into 4,4'-DDE and 4,4'-DDD within buried sediments; burial of 4,4'-DDX through deeper sediment layers due to sedimentation; vertical migration of 4,4'-DDX within sediments due to desorption from sediments and diffusion through porewater; diffusive exchange of dissolved 4,4'-DDX at the sediment-water interface; and vertical migration of 4,4'-DDX within the lower water column through dispersive mixing by turbulent eddies and settling of the particle-associated fraction. Potential transformation pathways affecting 4,4'-DDE are assumed negligible in the present model implementation, because we did not measure for relevant metabolites (e.g., 4,4'-DDMU) in SP Basin sediments. However, future model implementations can include such transformation pathways in tandem with efforts to expand the measurement of DDX analytes in the deep SP Basin. Field evidence from the PV Shelf suggests that transformation rates of 4,4'-DDD are negligible or very slow

relative to other considered processes.[3,4] The DEBAE module receives 4,4'-DDX compounds as a time-dependent sedimentation flux from the RR module.

Within layered sediments, transport and fate of 4,4'-DDX is simulated with an advection, diffusion, and reaction equation in one dimension:

$$\frac{\partial C_i}{\partial t}_{sediments} = f_{i,mobile}\, D_{i,diffusion} \frac{\partial^2 C_i}{\partial z^2} - v_{burial} \frac{\partial C_i}{\partial z} - \Sigma k_{i,\text{sed}.deg.removal}\, C_i + k_{i,\text{sed}.deg.source}\, C_i \qquad \text{(S1)}$$

where C_i represents the concentration of a solute *i* in bulk sediments (sorbed plus dissolved phases); *t* represents time; *z* represents depth within the sediment column; $f_{i,mobile}$ represents the mass fraction of solute *i* that is mobile and can travel through porewater; $D_{i,diffusion}$ is the molecular diffusion coefficient of solute *i* in water; v_{burial} is the downward "velocity" of sediments through the sediment column, relative to the sediment-water interface, due to sedimentation processes; $k_{i,sed.deg.removal}$ represents a first-order rate constant of degradation that removes solute *i* within the sediment column; and $k_{i,sed.deg.source}$ represents a first-order rate constant of degradation that produces solute *i* within the sediment column. In equation S1, the mobile fraction, $f_{i,mobile}$, is estimated as the fraction that is dissolved in porewater plus the fraction that is sorbed to dissolved organic carbon within the sediment layer.[5] To determine $f_{i,mobile}$, we estimated the solids-water partition coefficient, K_d, by $K_d = f_{oc} \times K_{oc}$, where f_{oc} represents the fraction of organic carbon in suspended solids, assigned based on measured organic carbon abundances in the studied sediment cores (Table S3), and K_{oc} is the organic carbon-water partition coefficient. Published estimates of K_{oc} for DDX compounds vary substantially,[6] indicative of the difficulty of constraining this chemical property. We estimated K_{oc} values of 10^7, $10^{6.5}$, and 10^7 L kg^{-1} for 4,4'-DDT, 4,4'-DDD, and 4,4'-DDE, respectively, based on available literature data[6] and consideration of related partitioning property values such as the octanol-water partition coefficient.[5] To determine

$f_{i,\mathrm{mobile}}$, we estimated the dissolved organic carbon-water partition coefficients, K_{doc}, as 10^5, 10^4, and 10^5 L kg^{-1} for 4,4'-DDT, 4,4'-DDD, and 4,4'-DDE, respectively, based on available measurement data[6] from sediment cores in the PV Shelf. We estimated $D_{i,diffusion}$ values for 4,4'-DDX compounds based on empirical correlation to molecular weight.[5] For the purposes of calculating $f_{i,mobile}$, we further assumed a solids density of 2.65 kg L^{-1} (i.e., representative of quartz) and a dissolved organic carbon concentration of 10 mg L^{-1} in sediment porewater. We assigned a depth-dependent solids-to-water ratio profile separately for each coring station based on the measured values in cores at 1 cm intervals. The solids-to-water ratio affects $f_{i,mobile}$ and also v_{burial}, hence both of these model parameters vary with depth in the modeled sediment column. The degradation rate constants in equation S1 were fitted by optimization of the model to individual coring stations (Table S3).

Within the lower water column, transport and fate of 4,4'-DDX is simulated with an advection and dispersion equation in one dimension:

$$\frac{\partial C_i}{\partial t}_{water} = D_{eddy}\frac{\partial^2 C_i}{\partial z^2} - v_{i,settling}\frac{\partial C_i}{\partial z} \qquad \text{(S2)}$$

where $v_{i,settling}$ is an effective settling velocity of particle-associated 4,4'-DDX in the water column, and D_{eddy} represents the dispersion coefficient of vertical mixing by turbulent eddies. The value of $v_{settling}$ in equation S2 is estimated from the particle settling velocity (assigned as 30 m day^{-1}); the solids concentration in the water column, which is a fitted parameter optimized separately for each coring station (Table S3); and the solids-water partition coefficient, K_d.[5] The solids-water partition coefficient is estimated by $K_d = f_{oc} \times K_{oc}$, where f_{oc} represents the fraction of organic carbon in suspended solids, assigned based on measured organic carbon abundances in sediment cores (Table S3), and K_{oc} is the

organic carbon-water partition coefficient. We assigned K_{oc} values of 10^7 for both 4,4'-DDT and 4,4'-DDE (*vide supra*). In the DEBAE module, the $v_{i,settling}$ parameter is assumed constant with respect to water depth, whereas the D_{eddy} parameter is assumed to vary with height above the seafloor. To estimate D_{eddy}, we applied a simple K-Profile model for oceanic boundary layer mixing.[7] To apply the K-Profile model to the deep SP Basin, we assumed a bottom boundary layer thickness of 30 m, a friction velocity of 0.5 cm s^{-1}, a von Karman constant value of 0.4, and a D_{eddy} value for interior waters (>30 m height) of 10^{-3} m^2 s^{-1}. The D_{eddy} value[8] of 10^{-3} m^2 s^{-1} is viewed as representative of the vertical mixing by turbulent eddies in the quiescent deep interior of the SP Basin.

At the sediment-water interface of the DEBAE module, deposition of settling particles transfers 4,4'-DDX from the adjacent water layer to the sediment surface layer. In addition, the DEBAE module simulates diffusive exchange of 4,4'-DDX between the surface sediment layer and the adjacent water layer as a first-order mass transfer process. The diffusive flux across the interface is calculated by assuming molecular diffusion through an aqueous boundary layer having a thickness of $\delta = 0.02$ cm, based on the simulated time-dependent dissolved concentration gradient across the interface. This value of δ is the same as that employed by Fernandez and coworkers in their model of DDX sediment-water fluxes of the PV Shelf,[9] who also assumed a friction velocity of 0.5 cm s^{-1}. By comparison, Schwarzenbach, Gschwend, and Imboden assumed a value of $\delta = 0.01$ cm for sediment-water fluxes of PCBs in Boston Harbor.[5]

Boundary conditions of the DEBAE module.

The sediment column boundary conditions of the DEBAE module were handled as follows. The deepest layer of the sediment column in the model (typically ~10 cm sediment

depth) employs a downstream Dankwerts' boundary as implemented in equation 6.127 of Beers.[10] At the sediment-water interface, the sediment boundary employs an upstream Dankwerts' boundary as implemented by equation 6.126 in Beers.[10] The Dankwerts' boundary conditions are appropriate for handling boundaries with coupled advection and diffusion/dispersion processes, such as the expressions in equations S1 and S2.

The water column boundary conditions of the DEBAE module were handled as follows. The uppermost boundary of the water column in the model enforces a zero concentration at the boundary (Dirichlet-type boundary). This water column boundary was set at a height of 300 m above the seafloor, based on the estimated depth at which water layers in the SP Basin exhibit lateral transfer to neighboring basins.[11] In other words, the model assumes that the deepest 300 m of water in the SP Basin undergoes relatively slow transfer to lateral basins, which allows DDX to transport vertically through this span of water column until it reaches an altitude of 300 m above the seafloor, where it undergoes (immediate) lateral export by flushing of the SP Basin. Simulated 4,4'-DDX mass reaching this boundary exits the DEBAE module, interpreted as exported from the deep SP Basin (e.g., by lateral transport to other basins). The deepest layer of the water column, at the sediment-water interface, employs a downstream Dankwerts' boundary as implemented in equation 6.127 of Beers.[10]

Numerical implementation of the DEBAE module.

We converted equations S1, S2, and coupled expressions into a system of coupled ordinary differential equations by implementing an upwind finite difference approximation. The DEBAE module features v_{burial}, f_{mobile}, and D_{eddy} parameters that vary with z (depth), according to complicated expressions and numerical datasets. Therefore,

the DEBAE implements a spatial discretization of sediment layers and water layers such that all nodes experience an approximately equivalent local Peclet number within each environmental compartment (sediment or water). By ensuring a near-invariant local Peclet number, the implementation dramatically decreases artifactual mass leakage errors that can arise from the finite difference approximation.

To robustly solve the finite difference model of the DEBAE module, we implemented the right-hand-side of the system of coupled ordinary differential equations as a near-diagonal matrix of constant coefficients multiplied by the vector of system variables (concentrations in each model layer), plus a vector of constants (source terms) at each time point. This linearized ODE formulation of the finite difference model, which requires the repeated inversion of a near-diagonal matrix, can be computed very efficiently using modern numerical methods. We solved the stiff ODE system with an implicit solver, ode15s, implemented in Matlab,[2] employing a user-supplied Jacobian matrix which further facilitated computational accuracy and efficiency. To further ensure the stability and accuracy of the ODE solver, we employed a spatial discretization which enforced a local Peclet number $\leq 10^{-1}$ for sediment layers and a local Peclet number $\leq 10^{-3}$ for water layers. For a typical DEBAE model run which employed three species (4,4'-DDT, 4,4'-DDD, and 4,4'-DDE), a 10 cm sediment column, and a 300 m water column, we simulated ~39,000 model variables (i.e., 3 species × ~13,000 discretization layers) through a model time period of 70+ years.

Relevant software language, version, and operating system information.

The model software was implemented using Matlab version 9.13.0 (R2022b).[2] Model simulations were conducted on a Windows 10 platform with an Intel® Core™ i9-10900K (3.70 GHz) 10-core processor.

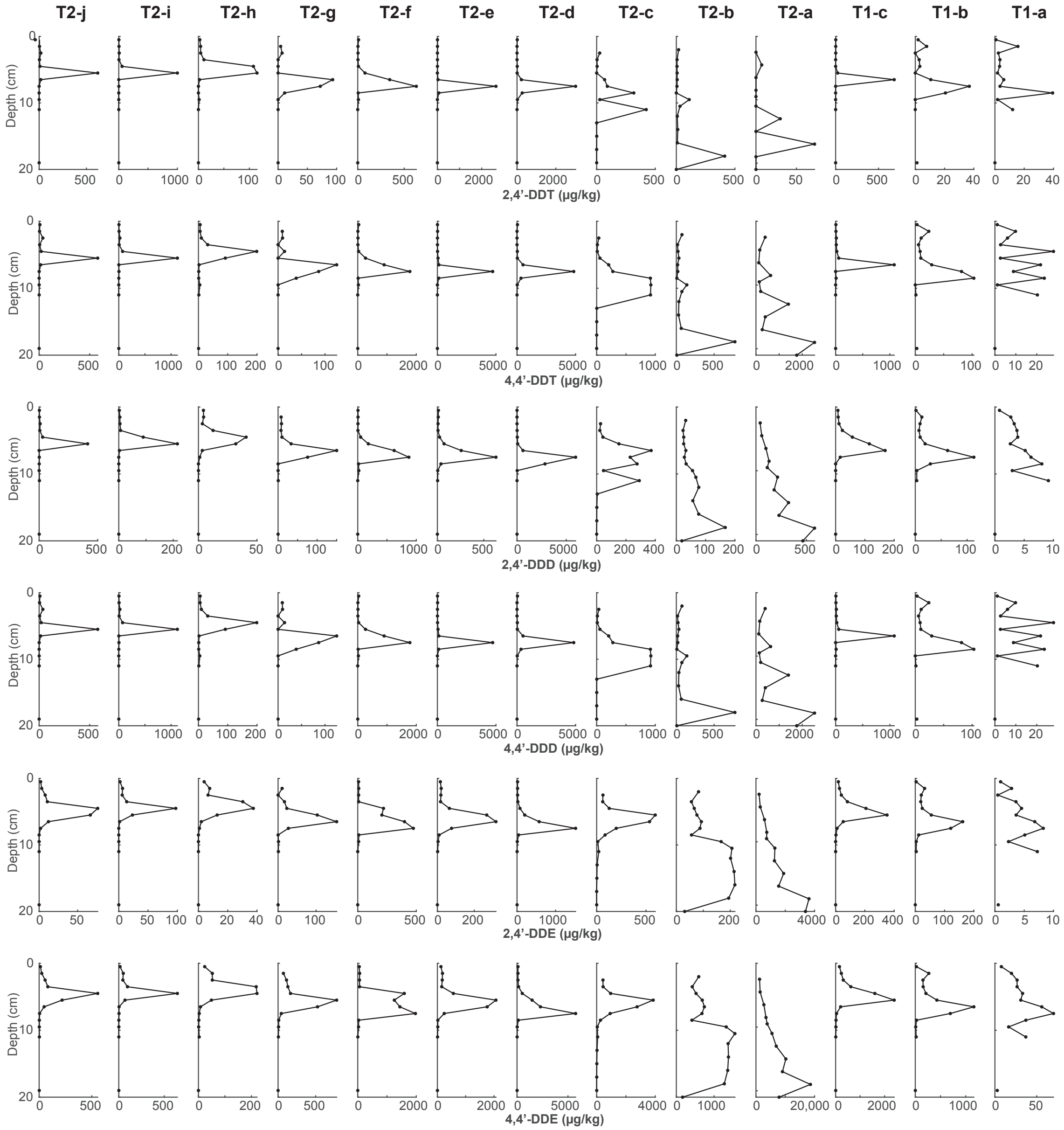

Figure S1. Depth profiles of 2,4’-DDT, 4,4’-DDT, 2,4’-DDD, 4,4’-DDD, 2,4’-DDE, and 4,4’-DDE concentrations (µg/g dry weight) in sediments at each sample station.

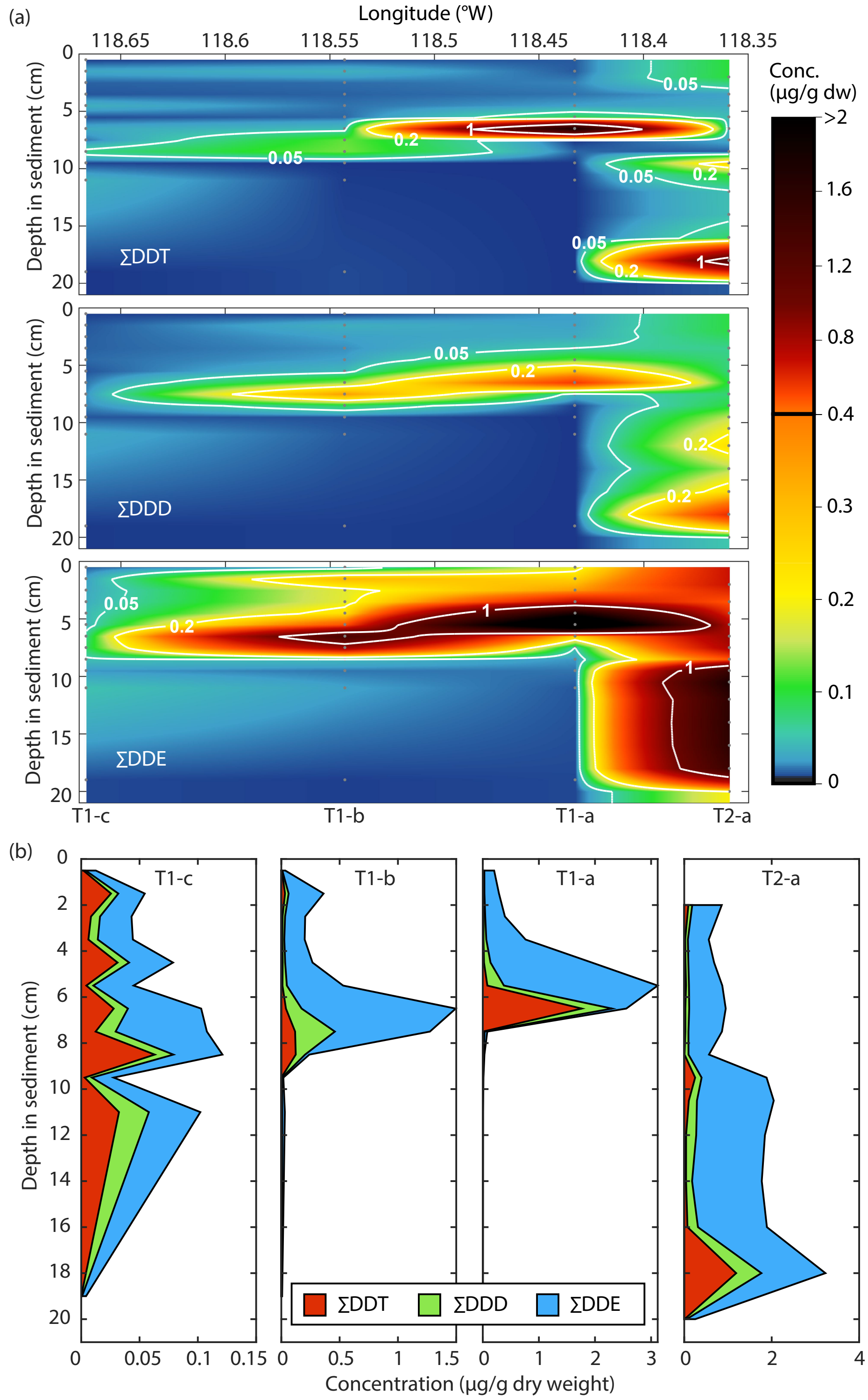

Figure S2. Heat maps (a) and stacked depth profiles (b) of ΣDDT, ΣDDD, and ΣDDE concentrations (µg/kg dry weight) in sediments along transect 1. Gray dots indicate where data is present, with values in between estimated by linear interpolation. Data is plotted at mid depths of sample intervals.

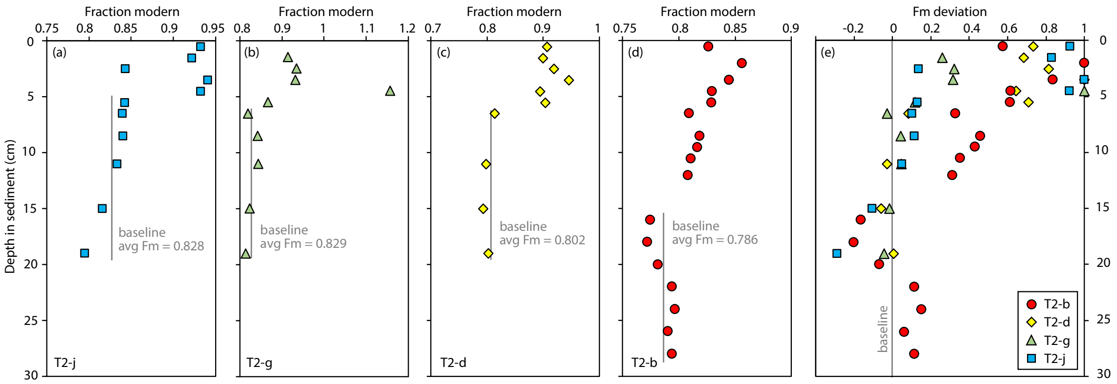

Figure S3. (a-d) Downcore profiles of radiocarbon F_m in four stations along transect 2, with the calculated 'baseline' average F_m values shown. (e) Comparison of F_m deviation profiles in the four stations. The bomb spike at station T2-b appears to be at greater depth than stations T2-d, T2-g, and T2-j.

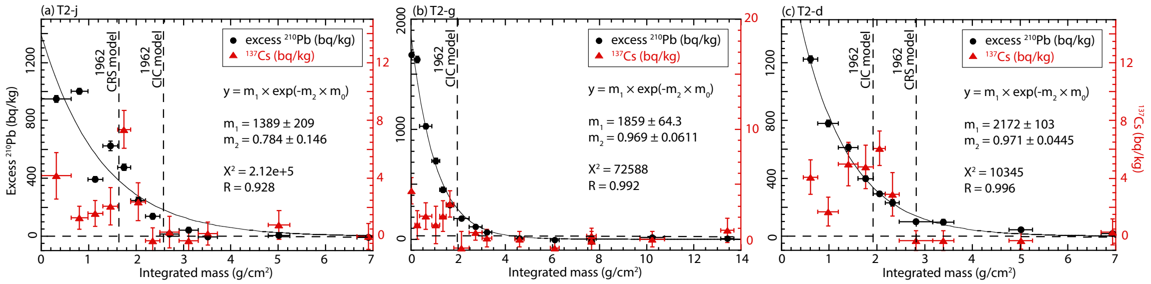

Figure S4. Profiles of excess ^{210}Pb and ^{137}Cs from stations T2-d, T2-g, and T2-j. Best-fit curves of the ^{210}Pb profiles and their statistics are shown. Dashed vertical lines indicate the calendar year 1962 estimated from the Constant Rate of Supply (CRS) and Constant Initial Concentration (CIC) models of ^{210}Pb chronology. By comparing that with the timing of the ^{137}Cs bomb spike, we decided to adopt the CRS model at station T2-j and CIC model at stations T2-d and T2-g, the model that provides age estimates that better fit the ^{137}Cs profile at each station.

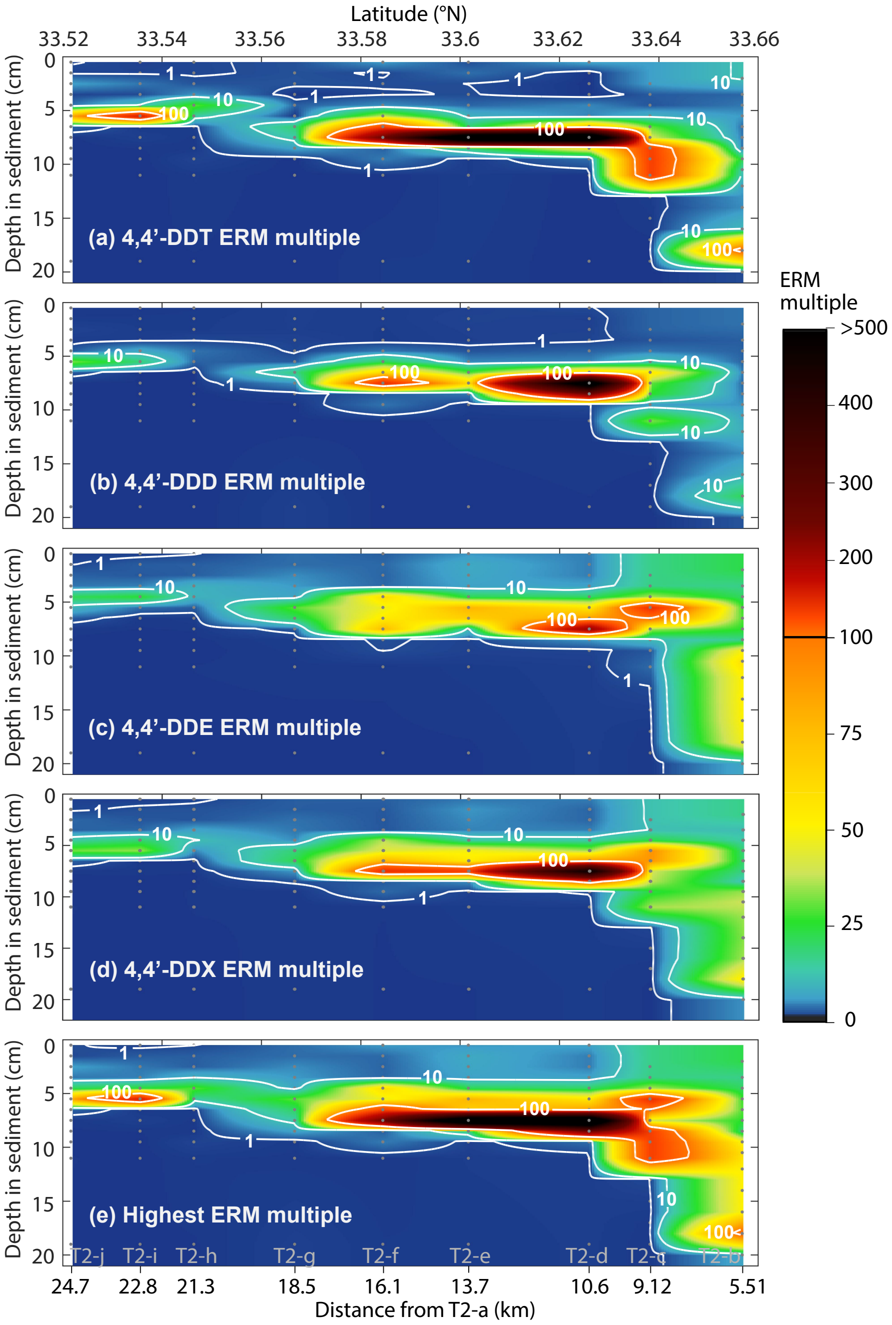

Figure S5. Heat maps showing the concentration of DDX relative to the effects range median (ERM)[12] values in NOAA sediment quality guidelines along transect 2. 'ERM multiple' was calculated for each individual compound of (a) 4,4'-DDT, (b) 4,4'-DDD, (c) 4,4'-DDE, and (d) for the sum of 4,4'-DDX, by dividing their concentrations with their respective ERM values (7 ng/g for 4,4'-DDT, 20 ng/g for 4,4'-DDD, 27 ng/g for 4,4'-DDE, and 46.1 ng/g for 4,4'-DDX, Table S1). The highest ERM multiple among 4,4'-DDT, 4,4'-DDD, and 4,4'-DDE is shown in (e).

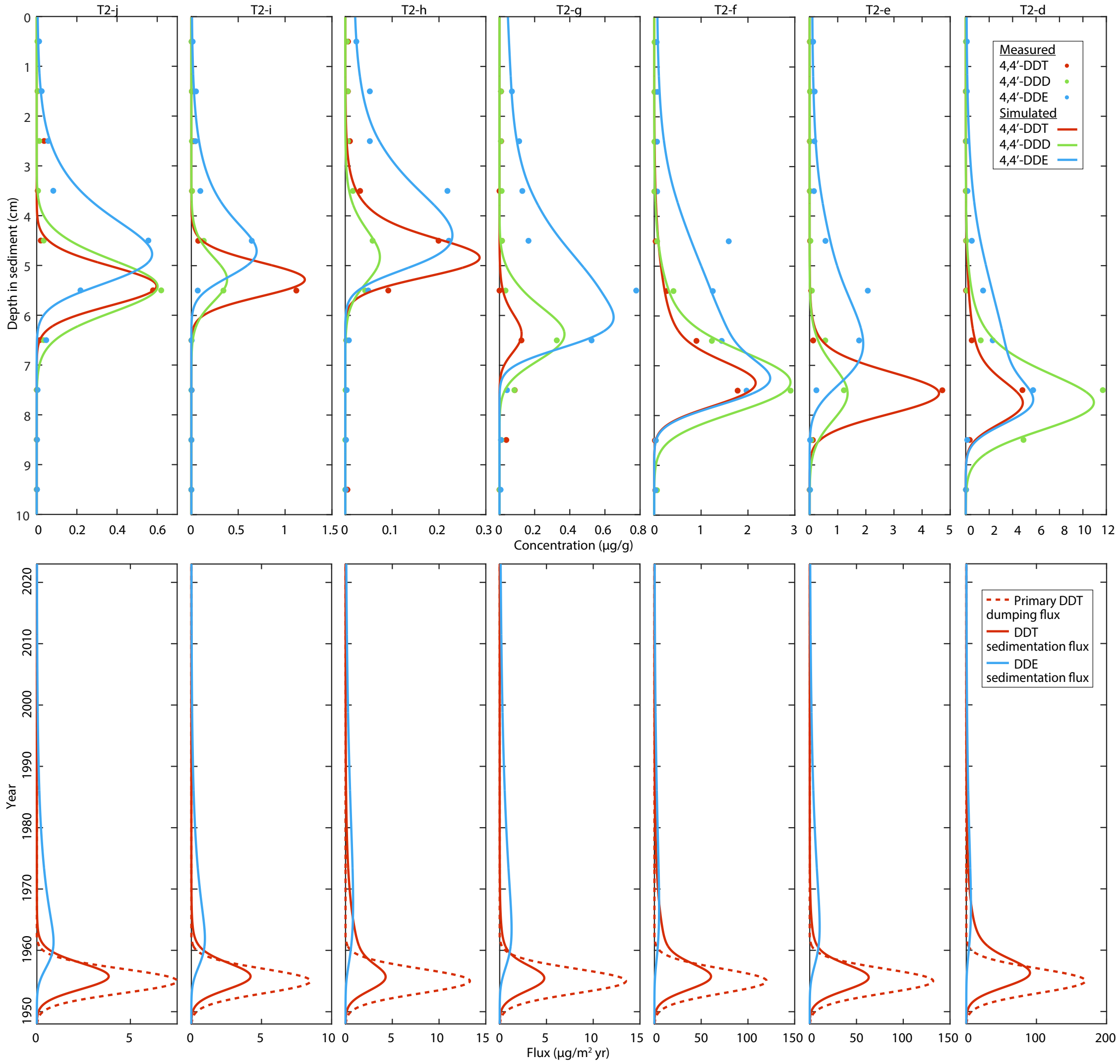

Figure S6. Simulated 4,4'-DDX concentrations (upper panels) and sedimentation fluxes (lower panels) from a 1-D model of transport and degradation for seven stations along transect 2 from station T2-d to T2-j.

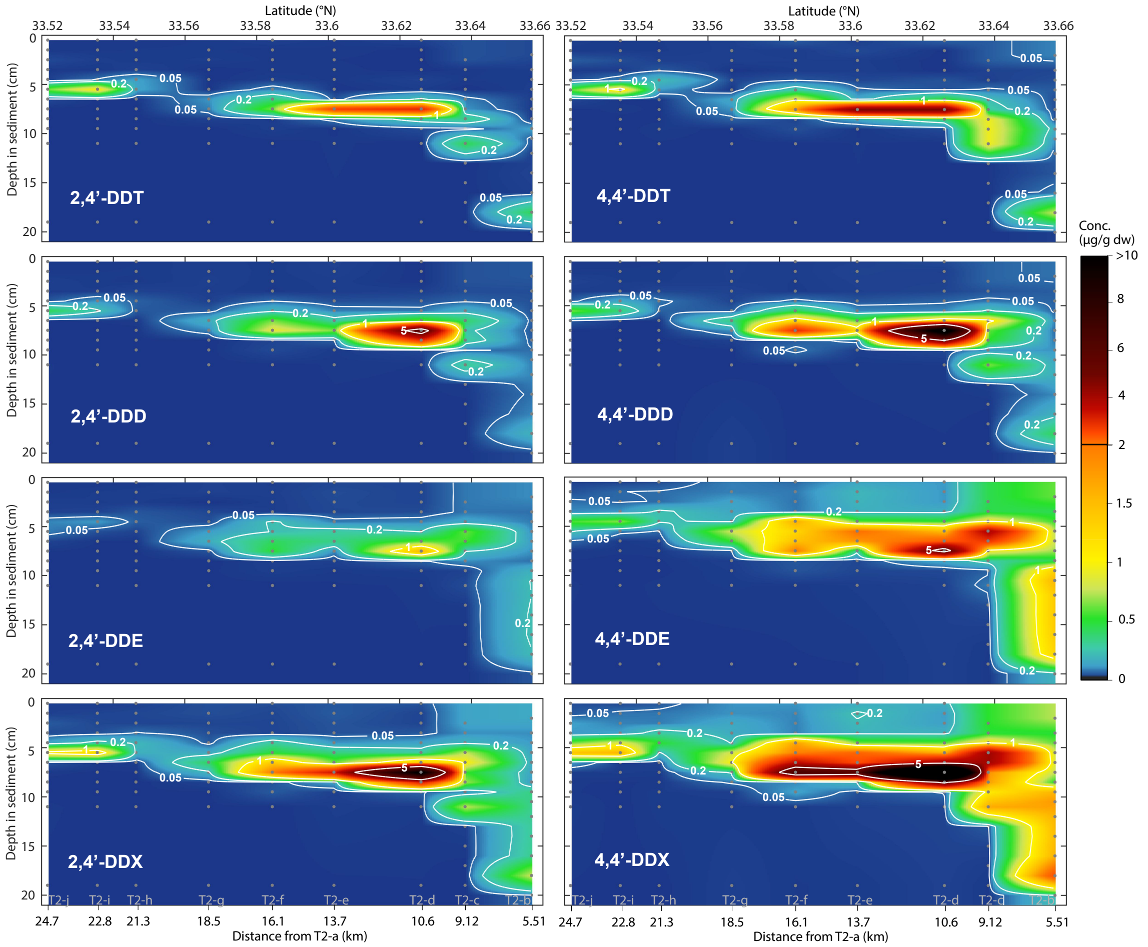

Figure S7. Heat maps of the 2,4'- and 4,4'- isomers of DDT, DDD, DDE, and DDX along transect 2.

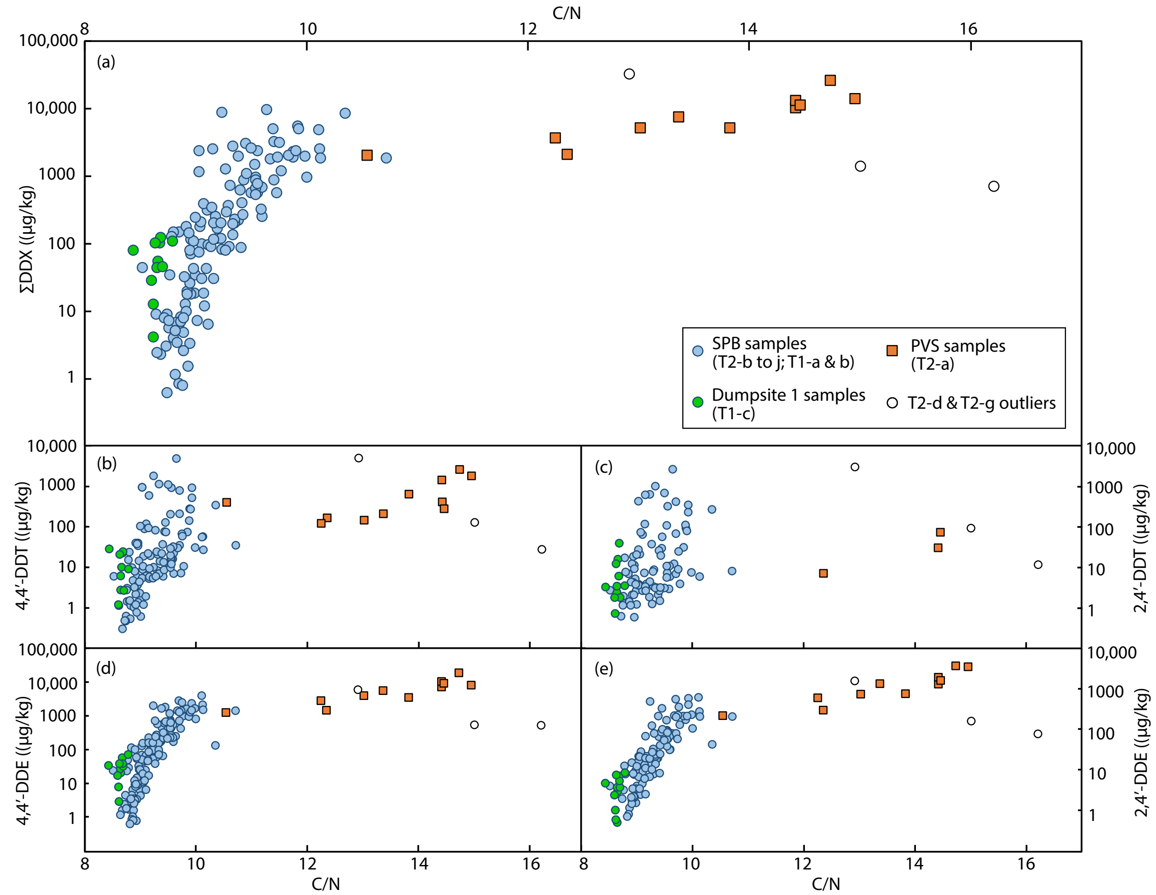

Figure S8. Correlation between C/N ratio and (a) ΣDDX, (b) 4,4'-DDT, (c) 2,4'-DDT, (d) 4,4'-DDE, and (e) 2,4'-DDE. Data are from SP Basin stations (T2-e – T2-j; T1-a and T1-b, blue circles), the dumpsite 1 station (T1-c; green circles), and the PV Shelf station (T2-a, orange squares). White circles indicate the three outliers from stations T2-d and T2-g with elevated C/N ratio. Elevated C/N ratios from station T2-d are attributed to contamination from tar that was visible during field sampling. Note the y-axis is plotted in log scale.

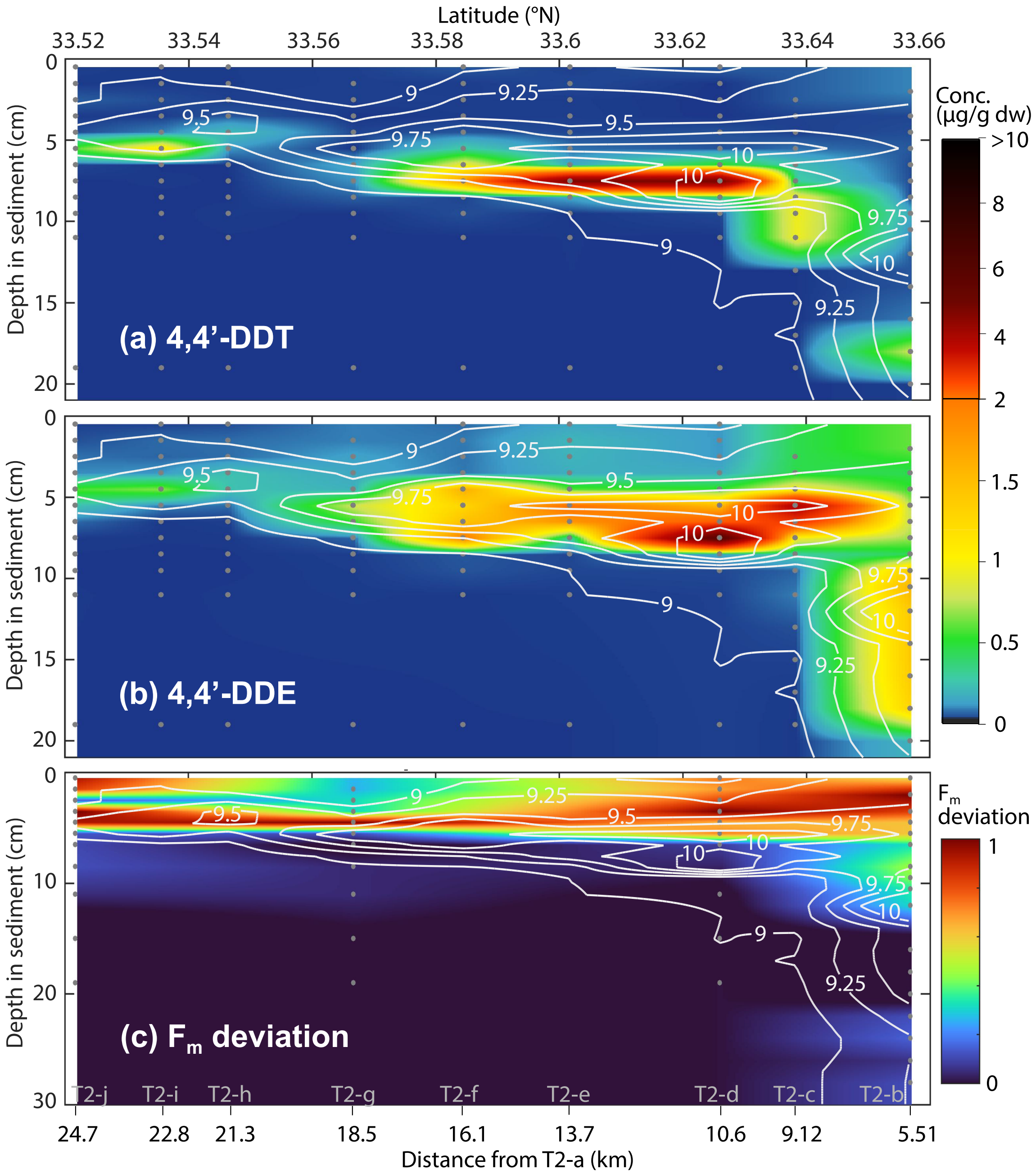

Figure S9. Heat maps of (a) 4,4'-DDT, (b) 4,4'-DDE, and (c) F_m deviation, overlaid by contours of C/N ratio for comparison, along transect 2.

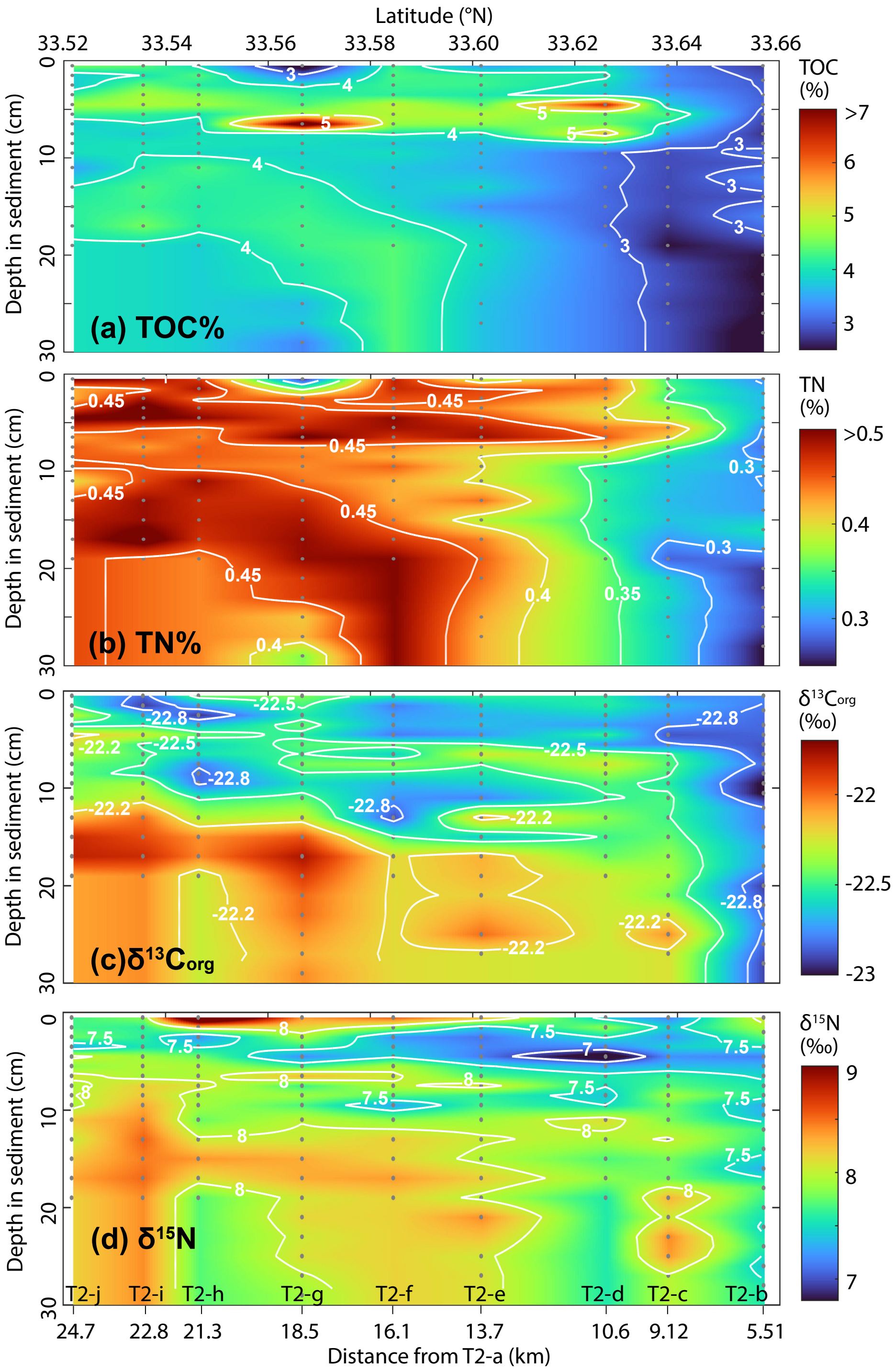

Figure S10. Heat maps of total organic carbon (TOC%), total nitrogen (TN%), organic carbon isotopic composition ($\delta^{13}C_{org}$), and nitrogen isotopic composition ($\delta^{15}N$) along transect 2.

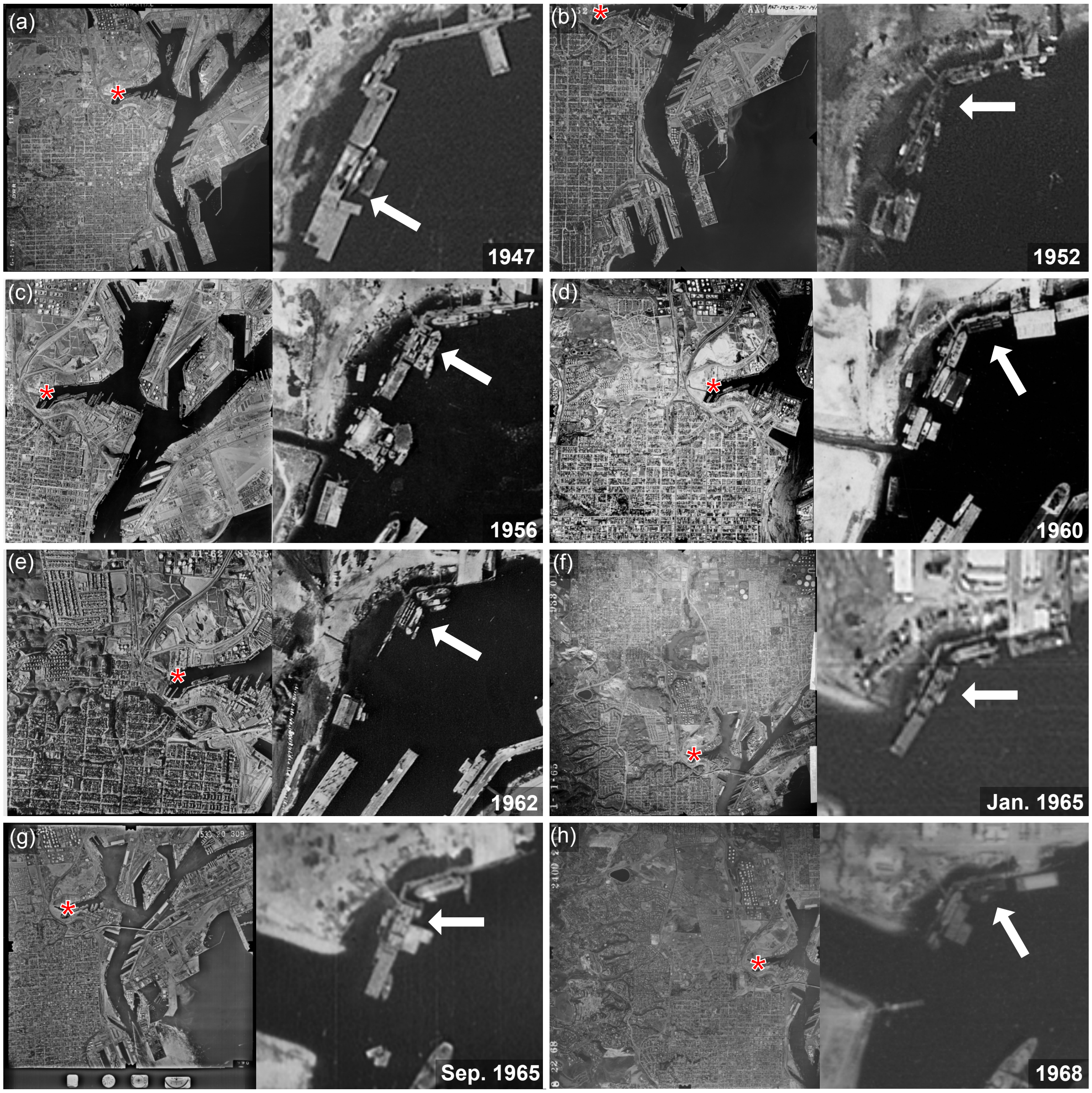

Figure S11. Eight panels (a-h) of Port of Los Angeles aerial images including Berth 115 (star, ca. 33.75° N, 118.29° W) used by Cal Salvage to load industrial wastes for ocean disposal (left). Zoom area (right) shows Berth 115 including vessels with large tanks that may have stored wastes for ocean disposal, indicated with an arrow. Each panel is dated for when the image was taken. (a) Image from Flight C-11351, Frame 7-20, 01 May 1947. (b) Image from Flight AXJ-1952, Frame 7k-141, 03 November 1952. (c) Image from Flight C-22555, Frame 29-27, 01 July 1956. (d) Image from Flight C-23870, Frame 509, 01 May 1960. (e) Image from Flight C-24400, Frame 8-215, 01 October 1962. (f) Image from Flight PAI_LA_Basin-65, Frame 1933_01_17, 01 January 1965. (g) Image from Flight C-25019, Frame 309, 22 September 1965. (h) Image from Flight TG-2400, Frame 2-101, 01 March 1968. All images from the Geospatial Collection. Department of Special Research Collections, UC Santa Barbara Library, University of California, Santa Barbara.

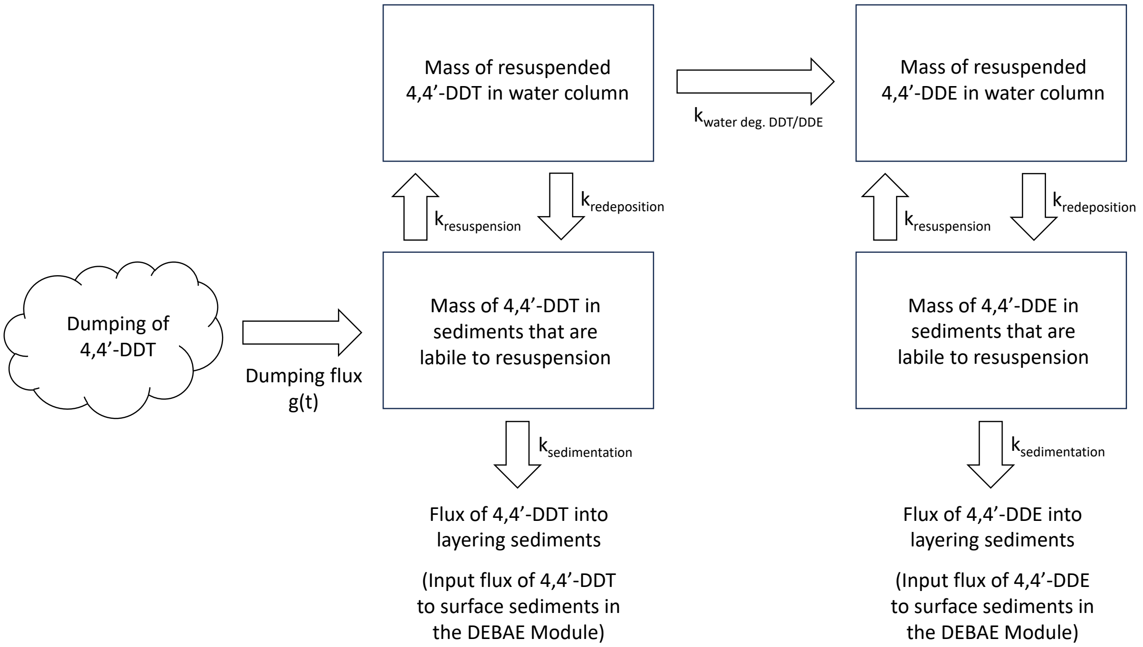

Figure S12. Conceptual schematic of the RR module in the 1-D model of transport and degradation processes affecting DDX compounds in the deep SP Basin.

Table S1. DDT family compounds quantified in this study.

IUPAC Name	Common Names	Sediment ERM*
1-chloro-4-[2,2,2-trichloro-1-(4-chlorophenyl)ethyl]benzene	4,4'-DDT / p,p'-DDT	7 ng/g
1-chloro-2-[2,2,2-trichloro-1-(4-chlorophenyl)ethyl]benzene	2,4'-DDT / o,p'-DDT	NA
1-chloro-4-[2,2-dichloro-1-(4-chlorophenyl)ethenyl]benzene	4,4'-DDE / p,p'-DDE	27 ng/g
1-chloro-2-[2,2-dichloro-1-(4-chlorophenyl)ethenyl]benzene	2,4'-DDE / o,p'-DDE	NA
1-chloro-4-[2,2-dichloro-1-(4-chlorophenyl)ethyl]benzene	4,4'-DDD / p,p'-DDD	20 ng/g
1-chloro-2-[2,2-dichloro-1-(4-chlorophenyl)ethyl]benzene	2,4'-DDD / o,p'-DDD	NA
(Sum of all 4,4'- isomers)	4,4'-DDX**	46.1 ng/g
(Sum of all 6 compounds)	DDX	NA

* Effect range median (ERM) values[12] for sediment quality guidelines for marine sediments as published by the National Oceanic and Atmospheric Administration.

** DDX is defined here as the sum concentration of each compound listed in this Table. Similarly, 4,4'-DDX and 2,4'-DDX are defined here as the sum values DDT + DDE + DDD for the respective 4,4'- and 2,4'- isomers.

Table S2. Statistics of the concentration depth profiles of 4,4’ DDX compounds.

	Offshore stations along transect 2 between T2-d – T2-j									Other stations				
	**T2-j**	**T2-i**	**T2-h**	**T2-g**	**T2-f**	**T2-e**	**T2-d**	**mean**	**σ**	**T2-c**	**T2-b**	**T1-c**	**T1-b**	**T1-a**
Weighted Mean Depth (cm)														
4,4'-DDE	4.55	4.19	3.76	5.30	5.99	5.44	6.67			5.82	10.80	6.41	5.70	4.60
4,4'-DDD	5.42	5.12	4.46	6.60	7.03	7.03	7.67			7.79	12.66	7.20	6.85	5.68
4,4'-DDT	5.23	5.36	4.65	6.69	6.92	7.42	7.41			9.12	14.35	6.53	6.87	6.31
Dislocation between 4,4'-DDE and 4,4'-DDT	0.68	1.17	0.89	1.38	0.93	1.99	0.73	1.11	0.46	3.29	3.55	0.12	1.17	1.71
Dislocation between 4,4'-DDE and 4,4'-DDD	0.86	0.93	0.71	1.29	1.04	1.59	1.00	1.06	0.29	1.96	1.86	0.79	1.15	1.08
Mode Depth (cm)														
4,4'-DDE	4.5	4.5	4.5	5.5	7.5	5.5	7.5			5.5	10.5	7.5	6.5	5.5
4,4'-DDD	5.5	5.5	4.5	6.5	7.5	7.5	7.5			6.5	18	11	7.5	6.5
4,4'-DDT	5.5	5.5	4.5	6.5	7.5	7.5	7.5			9.5	18	4.5	8.5	6.5
Standard Deviation (cm)														
4,4'-DDE	1.25	1.12	1.38	1.43	1.44	1.53	1.37			1.45	4.63	2.94	1.96	1.43
4,4'-DDD	0.88	0.74	1.32	2.03	0.87	1.08	0.68			1.99	5.07	3.30	1.93	1.36
4,4'-DDT	0.97	0.67	1.34	1.53	1.09	0.66	0.73			1.63	5.20	2.88	2.59	0.81
*Pearson Mode Skewness**														
4,4'-DDE	0.04	-0.28	-0.54	-0.14	-1.05	-0.04	-0.60	-0.37	0.38	0.22	0.07	-0.37	-0.41	-0.63
4,4'-DDD	-0.09	-0.51	-0.03	0.05	-0.53	-0.44	0.25	-0.19	0.31	0.65	-1.05	-1.15	-0.34	-0.60
4,4'-DDT	-0.27	-0.21	0.11	0.12	-0.53	-0.11	-0.13	-0.15	0.23	-0.24	-0.70	0.71	-0.63	-0.24

* Pearson mode skewness is calculated as (weighted mean depth - mode depth) / standard deviation.

Table S3. Station-specific parameters used in the 1-D model of coupled transport and degradation processes affecting 4,4'-DDX compounds in the deep SP Basin.

Parameter	T2-j	T2-i	T2-h	T2-g	T2-f	T2-e	T2-d	Consensus[a]	Units
Maximum DDT dumping flux[b]	7.5	8.5	13.3	13.5	120	132	170	40	$\mu g\ kg^{-1}\ m^{-2}\ yr^{-1}$
$k_{resuspension}$[c]	0.70	0.79	3.6	2.5	1.0	1.25	0.29	1.22	yr^{-1}
$k_{water\ deg.\ DDT/DDE}$[d]	0.60	0.41	0.076	0.28	0.064	0.23	0.085	0.24	yr^{-1}
$k_{redeposition}$[e]	0.18	0.19	0.22	0.15	0.15	0.13	0.15	0.16	yr^{-1}
$k_{sedimentation}$[f]	0.70	0.70	1.15	1.10	0.84	0.99	0.40	0.84	yr^{-1}
Fraction of organic carbon[g]	0.042	0.044	0.043	0.049	0.045	0.043	0.046	0.044	-
Solids concentration in the lower water column[h]	2.8	2.7	1.8	2.2	2.6	3.0	3.3	2.7	$10^{-5}\ kg\ L^{-1}$
$k_{sed.\ biodeg.\ DDT/DDD}$[i]	0.013	0.005	0.004	0.019	0.013	0.005	0.018	0.012	yr^{-1}
$k_{sed.\ deg.\ DDT/DDE}$[j]	0	0	0.004	0.012	0.007	0	0.006	0.004	yr^{-1}

[a] For each type of station-specific model parameter, a "consensus value" is calculated as the mid-point between the average and median of the set of 7 station-specific values. The consensus model is interpreted as a best estimate of 4,4'-DDX transport and degradation processes in the deep SP Basin, excluding representation from stations T2-a – T2-c which are on or proximate to the PV Shelf.

[b] Maximum flux of 4,4'-DDT input due to dumping during the period 1948 to 1961, fitted to match observed 4,4'-DDX profiles for individual cores. The dumping flux was assumed to follow a Gaussian distribution with a sigma value of 2 years, centered on the year 1955.

[c] Resuspension rate constant, fitted to match observed 4,4'-DDX profiles for individual cores.

[d] First-order rate constant for degradation of 4,4'-DDT to 4,4'-DDE in the water column, fitted to match 4,4'-DDX profiles for individual cores.

[e] Redeposition rate constant, fitted to match observed 4,4'-DDX profiles for individual cores.

[f] Sedimentation rate constant of 4,4'-DDX in labile sediments, fitted to match observed 4,4'-DDX profiles for individual cores. The sedimentation rate describes the flux of 4,4'-DDX in labile sediments (available to resuspension processes) to layering sediments (unavailable to resuspension).

[g] Fraction of organic carbon in particles and sediments, estimated from measurement data of individual cores.

[h] Solids concentration produced by suspended particles in the water column, fitted to match observed 4,4'-DDX profiles for individual cores. This parameter affects the burial velocity of sediments which differs for each sediment core, and it affects the downward transport of particle-associated DDX compounds in the water column.

[i] First-order rate constant for biodegradation of 4,4'-DDT to 4,4'-DDD in the sediment, fitted to match 4,4'-DDX profiles for individual cores.

[j] First-order rate constant for degradation of 4,4'-DDT to 4,4'-DDE in the sediment, fitted to match 4,4'-DDX profiles for individual cores

Table S4. Key for original station numbers in existing datasets to new consecutive station numbers used in this manuscript.

Manuscript Station Numbers	Original Station Numbers
T1-a	S13
T1-b	S12
T1-c	S11
T2-a	S1
T2-b	S6
T2-c	S5
T2-d	S4
T2-e	S2
T2-f	S7
T2-g	S3
T2-h	S8
T2-i	S9
T2-j	S10

Document Descriptions

All supporting information documents referenced in this section and made publicly available in the published supplement due to their limited accessibility and can be found compiled in this Supporting Information document starting on page S39.

Document S1 (separate file). Volume 1 of the deposition of Ferdinand Suhrer from July 30, 1996, in the case *United States of America and State of California v. Montrose Chemical Corp. of California, et al.,* that contains testimony about Montrose waste disposal and waste content.

Document S2 (separate file). Volume 1 of the deposition of Bernard Bratter from March 20, 1989, in the case *United States of America and State of California v. Montrose Chemical Corp. of California, et al.*, that contains testimony about Montrose waste disposal and waste content.

Document S3 (separate file). Los Angeles Regional Water Pollution Control Board resolution number 61-43 from 1961 that describes requirements to California Salvage Company for the regulated disposal of industrial waste.

Document S4 (separate file). A docket (No. 27-3) from the Thursday September 10, 1959, issue of the United States Federal Register stating that the Atomic Energy Commission had proposed to issue California Salvage Company a Byproduct, Source and Special Nuclear Material License that would allow for deep ocean waste disposal.

Document S5 (separate file). A letter dated August 18, 1982, from J. M. Felton – the Director of the Division of Rules and Records at the Office of Administration, United States Nuclear Regulatory Commission – to Mr. John Sullivan in response to a Freedom of Information Act Request (82-349).

Document S6 (separate file). Hearings in the House of Representatives on Ocean Dumping of Waste Materials at the Ninety-Second Congress of the United States of America from April 5-7, 1971. The document includes a statement by Hon. James T. Ramey, Member, Joseph F. Hennessey, General Counsel, and Harold L. Price, Director of Regulations, Atomic Energy Commission on April 6, 1971, that describes California Salvage Company as not disposing of radioactive wastes under its permit.

Document S7 (separate file). A United States Government Office Memorandum dated February 28, 1961, from W. S. Cool, Isotopes Branch in the Division of Licensing and Regulation to James R. Mason, Chief, Isotopes Branch in the Division of Licensing and Regulation. The office memorandum specifically discusses an application to amend the California Salvage Company Byproduct, Source and Special Nuclear Material License.

Document S8 (separate file). Deposition of Richard Donelson on September 27, 1999, in Los Angeles, California, in the case *Joseph A. Thomson and Virginia Thomson v. ICN Pharmaceuticals, Inc., a Delaware Corporation; NUCOR Corporation, a Delaware Corporation; Rhone-Poulenc Inc., a New York Corporation*. Richard Donelson describes wastes from radioactive decontamination being sent to California Salvage Company for disposal.

Document S9 (separate file). Report on The Nuclear Industry by the U.S. Atomic Energy Commission, Division of Industrial Participation, 1966. California Salvage Company is advertised as licensed for disposal of radioactive wastes on page 153.

Document S10 (separate file). Report on The Nuclear Industry by the U.S. Atomic Energy Commission, Division of Industrial Participation, 1967. California Salvage Company is advertised as licensed for disposal of radioactive wastes on page 161.

Document S11 (separate file). Report on The Nuclear Industry by the U.S. Atomic Energy Commission, Division of Industrial Participation, 1968. California Salvage Company is advertised as licensed for disposal of radioactive wastes on page 201.

Document S12 (separate file). An application for Byproduct Material License through the United States Atomic Energy Commission for Philco-Ford Corporation, Aeronutronic Division & Space and Re-entry Systems Division dated January 31, 1967. The application lists California Salvage Company employed as a commercial waste disposal service on page 2.

Document S13 (separate file). A United States Atomic Energy Commission Division of Compliance Radiation Specialist Inspector's Evaluation Report of the Veterans Administration Center, Wadsworth Hospital, Los Angeles, California, dated October 3, 1968. In section 21 of the report, California Salvage Company is described as being transferred radioactive wastes.

Document S14 (separate file). A United States Atomic Energy Commission Division of Compliance Radiation Specialist Inspector's Evaluation Report of the Veterans Administration Center, Wadsworth Hospital, Los Angeles, California, dated August 22, 1969. In section 16 of the report, California Salvage Company is described as being transferred radioactive wastes.

Document S15 (separate file). A United States Atomic Energy Commission Division of Compliance Radiation Specialist Inspector's Evaluation Report of the Veterans Administration Center, Wadsworth Hospital, Los Angeles, California, dated August 6, 1970. In sections 41 and 42 of the report, California Salvage Company is described as being transferred radioactive wastes.

Document S16 (separate file). A United States Atomic Energy Commission Division of Compliance Radiation Specialist Inspector's Evaluation Report of the Veterans Administration Center, Wadsworth Hospital, Los Angeles, California, dated September 28, 1973. In section 21 of the report, California Salvage Company is described as being transferred radioactive wastes.

References

(1) Arey, J. Samuel. *1-D model of transport and degradation processes affecting hydrophobic compounds in sediments and water of a deep basin*, Version 1.0, Oleolytics LLC: State College, PA, 2023. https://oleolytics.com.

(2) The MathWorks Inc. (2022). MATLAB version: 9.13.0 (R2022b), Natick, Massachusetts: The MathWorks Inc. https://www.mathworks.com.

(3) Eganhouse, R. P.; Pontolillo, J.; Leiker, T. J. Diagenetic Fate of Organic Contaminants on the Palos Verdes Shelf, California. *Marine Chemistry* **2000**, *70* (4), 289–315. https://doi.org/10.1016/S0304-4203(00)00034-7.

(4) Eganhouse, R. P.; Pontolillo, J. DDE in Sediments of the Palos Verdes Shelf, California: In Situ Transformation Rates and Geochemical Fate. *Environ. Sci. Technol.* **2008**, *42* (17), 6392–6398. https://doi.org/10.1021/es7029619.

(5) Schwarzenbach, R. P.; Gschwend, P. M.; Imboden, D. M. *Environmental Organic Chemistry*, 3rd ed.; Wiley, 2016.

(6) Eganhouse, R. P.; DiFilippo, E.; Pontolillo, J.; Orem, W.; Hackley, P.; Edwards, B. D. DDT and Related Compounds in Pore Water of Shallow Sediments on the Palos Verdes Shelf, California, USA. *Mar. Chem.* 2018, *203*, 78–90. https://doi.org/10.1016/j.marchem.2018.05.003.

(7) Large, W. G.; McWilliams, J. C.; Doney, S. C. Oceanic Vertical Mixing: A Review and a Model with a Nonlocal Boundary Layer Parameterization. *Rev. Geophys.* 1994, *32* (4), 363. https://doi.org/10.1029/94RG01872.

(8) Ledwell, J. R.; Bratkovich, A. A Tracer Study of Mixing in the Santa Cruz Basin. *J. Geophys. Res.* 1995, *100* (C10), 20681. https://doi.org/10.1029/95JC02164.

(9) Fernandez, L. A.; Lao, W.; Maruya, K. A.; Burgess, R. M. Calculating the Diffusive Flux of Persistent Organic Pollutants between Sediments and the Water Column on the Palos Verdes Shelf Superfund Site Using Polymeric Passive Samplers. *Environ. Sci. Technol.* 2014, *48* (7), 3925–3934. https://doi.org/10.1021/es404475c.

(10) Beers, K. J. *Numerical Methods for Chemical Engineering: Applications in MATLAB*, 1st ed.; Cambridge University Press, 2006.

(11) Heintz, M. B.; Mau, S.; Valentine, D. L. Physical Control on Methanotrophic Potential in Waters of the Santa Monica Basin, Southern California. *Limnol. Oceanogr.* 2012, *57* (2), 420–432. https://doi.org/10.4319/lo.2012.57.2.0420.

(12) National Oceanic and Atmospheric Administration. *Screening Quick Reference Tables*. https://response.restoration.noaa.gov/environmental-restoration/environmental-assessment-tools/squirt-cards.html (accessed 2023-08-09).

UNITED STATES DISTRICT COURT
CENTRAL DISTRICT OF CALIFORNIA
---o0o---

UNITED STATES OF AMERICA, et al.
Plaintiffs,
vs. NO. CV 90-3122-AAH
MONTROSE CHEMICAL CORPORATION OF
CALIFORNIA, et al.,
Defendants.
--------------------------------------//
AND RELATED ACTIONS.
--------------------------------------//
Pages 1 through 197, inclusive.

DEPOSITION OF FERDINAND SUHRER
July 30, 1996
VOLUME I

REPORTED BY:
JILL N. HORAN, CSR NO. 10306

I N D E X

DEPOSITION OF FERDINAND SUHRER
VOLUME I

EXAMINATION BY:	PAGE
MR. O'ROURKE	6, 184
MR. KUSHNER	183
AFTERNOON SESSION	101

E X H I B I T S

Plaintiffs' Exhibits:
1 - Photograph of Montrose plant at Torrance 48

2 - Drawing of Oliver filter 147

---o0o---

A P P E A R A N C E S

FOR THE PLAINTIFFS:
UNITED STATES DEPARTMENT OF JUSTICE
ENVIRONMENTAL ENFORCEMENT SECTION
BY: STEVEN O'ROURKE, ATTORNEY AT LAW
ADAM KUSHNER, ATTORNEY AT LAW
1425 New York Avenue, NW
Washington, DC 20005

STATE OF CALIFORNIA
DEPARTMENT OF JUSTICE
BY: JOHN A. SAURENMAN, DEPUTY ATTORNEY GENERAL
300 S. Spring Street, Suite 500
Los Angeles, California 90013

FOR DEFENDANT MONTROSE CHEMICAL CORPORATION:
LATHAM & WATKINS
BY: KARL S. LYTZ, ATTORNEY AT LAW
505 Montgomery Street, Suite 1900
San Francisco, California 94111-2562

LATHAM & WATKINS
BY: KIMBERLY M. MCCORMICK, ATTORNEY AT LAW
701 B Street, Suite 2100
San Diego, California 92101

Appearances, (continued).

FOR DEFENDANT CHRIS-CRAFT INDUSTRIES, INC.:
SKADDEN, ARPS, SLATE, MEAGHER & FLOM

BY: PETER SIMSHAUSER, ATTORNEY AT LAW
300 South Grand Avenue
Los Angeles, California 90071-3144

FOR DEFENDANTS ZENECA HOLDINGS, INC., STAUFFER MANAGEMENT, CO., ATKEMIX 37, AND RHONO-POALENC, INC.:
ROPES & GRAY
BY: DAVID A. BROWN, ATTORNEY AT LAW
One International Place
Boston, Massachusetts 02110-2624

ALSO PRESENT:
Bernard I. Bratter, Bernard I. Bratter, Inc.
Jefferey Dhont, EPA, San Francisco
John Lyons, EPA, San Francisco

TAKEN AT:
DEPARTMENT OF JUSTICE, USEPA OFFICE
75 Hawthorne Street
San Francisco, California 94103

BE IT REMEMBERED that, pursuant to Notice of Taking Deposition, and on, July 30, 1996, commencing at the hour of 9:16 a.m., before me, JILL N. HORAN, CSR NO. 10306, a Certified Shorthand Reporter, in and for the State of California, there personally appeared

FERDINAND SUHRER,

called as a witness by the Plaintiffs, who having been first duly sworn, was examined and testified as hereinafter set forth.

---o0o---

page 5

Deposition of Suhrer, Ferdinand
page 6

(Reporter reads case caption into record and
identifies all present).

FERDINAND SUHRER

having been duly sworn, testified as follows:

EXAMINATION BY MR. O'ROURKE

MR. SIMSHAUSER: Just one correction for
the record. I'm Peter Simshauser from Skadden Arps
representing Chris-Craft.
MR. LYTZ: Mr. O'Rourke, before we begin
this morning, may we enter a stipulation concerning
the entry of objections. I would propose, if all 09:17
agree, that we agree, collectively, that an objection
made by any party as deemed to be an objection made
by all, unless otherwise stipulated.
MR. O'ROURKE: Objections made by any
defendant is an objection made by all defendants. We 09:17
agree to that.
Q. Good morning Mr. Suhrer.
A. Good morning.
Q. Have you ever been deposed before?
A. Never. 09:17

page 6

Deposition of Suhrer, Ferdinand
page 7

Q. I'll explain a bit of the purpose. This
deposition is being taken pursuant to Federal Rules
of Civil Procedure. I'm going to ask you a series of
questions, and you answer the questions; it's that
simple. I ask you to answer the questions verbally. 09:17
Even though you are being filmed, the court reporter
also has to take down your answers. She can't take
down a nod of the head. So I'll ask you to say "Yes"
or "No," in addition.
If I ask you any questions you don't 09:18
understand, you can tell me that and ask me to

clarify, and I'll attempt to do so. Attorneys for the defendants are present, they may object to some of my questions. They may reformulate the questions or ask you to answer over the objections. But you 09:18
will still be answering the questions ultimately. It's not like television where there's an objection sustained or overruled, it's just going to go on.
And we'll take breaks occasionally today. Probably every hour, more or less. If you need a 09:18
break sooner, let me know, then we can stop.
So to begin, could you state your name for the record?
A. Ferdinand Charles Suhrer.
Q. And are you currently employed? 09:18

A. No.
Q. Retired?
A. I'm retired, yes.
Q. And how long have you been retired?
A. Since 1985. 09:18
Q. What have you done in the past ten years?
A. Enjoyed retirement. I've traveled some. I do some volunteer work.
Q. What kind of volunteer work do you do?
A. I tutor math to illiterate adults. And I do 09:19
-- in tax season, I prepare income tax for low-income seniors.
Q. I'm going to back and trace some of your life history, now. Did you go to college?
A. Yes. 09:19
Q. When was that?
A. The first time at the University of Florida, 1936 to 1940.
Q. Uh-huh. Did you take a degree there?
A. No degree. 09:19
Q. You said the first time. Did you go to college a second time?
A. Yes, I was in the Navy. I was a naval pilot for 1941 to 1945. 1945 I entered Cal Tech and took a degree there in applied chemistry. 09:20

Q. And this was a Bachelor of Science?
A. That's correct.
Q. Uh-huh. When did you enroll in the Navy?
A. In 1941.
Q. Uh-huh. 09:20
A. January, 1941.
Q. And where did you do your duty?
A. I served in the South Pacific.
Q. For four years that was?
A. Off and on, yes. 09:20
Q. Did you see any action?
A. Oh, yes.
Q. Uh-huh. And how old were you when you were enrolled in the Navy?
A. 21. 09:20
Q. And had you -- you had gone to Florida State before that?
A. Let me correct that. I was -- yeah, 21 is right. I beg your pardon?
Q. Had you already been to your first college in Florida before that? 09:20
A. Yes.
Q. And pardon me, I forget what college you went to, Cal Tech?
A. Cal Tech. And in 1953-- excuse me, 1972, 09:20

the job I was in, the company discontinued the plant. The plant stopped producing. And I was lucky enough to get an EPA study grant. And took a Master's Degree at Vanderbilt in Environmental Engineering. 09:21
Q. Did you receive any further formalized education after your Master's Degree?
A. No.
Q. So you went to Cal Tech in 1945?
A. Yes, '45 through '48. 09:21
MR. SIMSHAUSER: I missed the last part of the answer. '45 through when, madam reporter?
(Record read).
MR. SIMSHAUSER: Thank you.
MR. O'ROURKE: Q. For your Master's Degree did you have to write a thesis? 09:21
A. Yes.
Q. What was that in?

A. It was rapid evaluation of BOD.
Q. And what's BOD? 09:22
A. Biological oxygen demand.
Q. Was your thesis published?
A. No.
Q. Have you ever had any publications?
A. There was a -- back when I was at the 09:22

University of Florida there was a short, I don't know
what you would call it, a few paragraphs on
laboratory preparation of hydrogen sulfite.
Q. Uh-huh. Where was that published?
A. It was -- I really couldn't tell you. I 09:22
don't know. I've seen it, but I don't know what the
publication was.
Q. Do you have any professional licenses or
certifications?
A. When I was an active engineer, I was a 09:22
licensed professional engineer in California, in
Tennessee, and in Nevada.
Q. What were the years you were licensed in
California?
A. Let's see, that would have been about 1975 09:22
to '85.
Q. What years were you licensed in Tennessee?
A. That would have been probably '68 to '72.
Q. What was the third state?
A. Nevada. 09:23
Q. When was that?
A. That was between about '62 to '68.
Q. Uh-huh. So you took your degree from Cal
Tech in 1948?
A. Right. 09:23

Q. What was the first job you had after that?
A. I worked as a process engineer at the Texaco
refinery in Wilmington, California.
Q. Did you start that in 1948?
A. '48. 09:23
Q. How long did you stay at that job?

A. I left there in 1951.
Q. Uh-huh. What were the nature of your duties at the Texaco plant?
A. Initially, I was doing process work. Mostly keeping records of various processes in the refinery. And some time later, I was transferred to the testing laboratory. 09:24
Q. What did you do in the testing laboratory?
A. Tested customer samples. Customers would return samples of oil and grease which were -- they claimed defective, and I would analyze it to see if it met company specs. 09:24
Q. What was the business of the Texaco plant?
A. It was a gasoline refinery. 09:24
Q. And you stayed there until 1941 -- 1951?
A. '51.
Q. Where did you go after that?
A. Montrose Chemical.
Q. Montrose Chemical of California? 09:24

A. That's correct, at Torrance.
Q. At the Torrance.
(Reporter asks witness to repeat answer).
THE WITNESS: At the Torrance plant.
MR. O'ROURKE: Q. And how long did you stay there? 09:25
A. Until 1961.
Q. What was -- what were your job titles at Montrose?
A. I was Production Supervisor there over an eight-hour shift producing DDT. 09:25
Q. And you had like the same position for the ten years you were there?
A. That's correct.
Q. What did you do after 1961? 09:25
A. I was transferred to Montrose's plant in Henderson, Nevada.
Q. And that's Montrose of California?
A. Montrose of California.
Q. And how long did you stay there? 09:25
A. I stayed there until 1968.
Q. It was during that job that you got your Nevada certification?
A. That's correct.
Q. What was the business of the Henderson 09:25

plant?
A. They -- their main business was preparing
the raw materials for the DDT plant in Torrance.
Q. What was your job function at Henderson?
A. I was the plant engineer. I designed and 09:26
developed new plant improvements. If we had a new
product coming on line, I would design something for
that.
Q. And where did you go after 1968?
A. I went to Chattanooga, Tennessee to Atlas 09:26
Chemical Company.
Q. Why did you leave the Montrose Chemical
Company of California?
A. It was my opinion that DDT was fading out as
a pesticide. I thought it was going to be ruled 09:26
out. And since DDT was the only product that
Montrose had, I thought it was best to seek
elsewhere.
Q. And why did you think DDT was going to be
phased out? 09:27
A. Well, Silent Spring had been published a
number of years back. And I thought I could see
increased regulation of DDT coming up, as
subsequently happened.
Q. And what was the business of the Atlas 09:27

company?
A. They made -- they operated a TNT plant for
the US Army.
Q. And you thought that manufacturing TNT would
just be an economically more stable job? 09:27
A. As it turned out, it was not. But I thought
so at the time.
Q. So the TNT market was unstable?
A. Well, in 1972 you may remember the Southeast
Asia thing quieted down. And there was much less 09:27
demand for TNT after 1972. A number of TNT plants
were mothballed, my plant included. A number of us
were laid off.

Q. So you worked -- how long did you work at the Atlas plant then? 09:28
A. From about '68 to '72.
Q. And what was your job function there?
A. I was -- my job was designing and bringing in a new improved method for making TNT.
Q. It was a new process or -- 09:28
A. New process. The old process was a batch process. The new process was going to be a continuous process.
Q. Why do you say that was an improvement?
A. Economically it was a better -- it was a -- 09:28

the production was better for economic reasons. And you had better control over the quality of TNT.
Q. What did you do after 1972?
A. As I said, I was lucky enough to get an EPA study grant. And I went to Vanderbilt. Graduated 09:29 from Vanderbilt in either '73 or '74 -- '73, probably.
Q. Where did you go after that?
A. I took a job at the EPA's regional office in Atlanta. 09:29
Q. What was your job there?
A. It was overseeing the sum of the grants that it made to sewage treatment plants in Alabama. My job was to go to various plants and see that the Government's money was well spent, and that the 09:30 plants were being well maintained.
Q. Was your job as an engineer or --
A. Yes.
Q. How long did you stay at EPA in Atlanta?
A. I left there in mid 1974. 09:30
Q. To go where?
A. To the regional office in San Francisco.
Q. Office of EPA?
A. Yes.
Q. How long did you stay there? 09:30

A. I retired from the regional office in 1985.

Q. And that was your last job?
A. Last job.
Q. What were your jobs at EPA in San Francisco?
A. In San Francisco, I was initially in the 09:30
solid waste section. After solid waste, I was in the
hazardous waste materials.
Q. What did you do in the solid waste section?
A. I had something to do with a computer
program to computerize the collection of solid waste, 09:31
they called it Colmis. And this was one thing that I
worked on. Another, I went to the various solid
waste plants and -- throughout the state who were
more of an inspection tour than anything. And we did
some licensing of solid waste plants in my section. 09:31
Q. When did you switch from solid waste to the
hazardous waste sections?
A. That would have been -- for four years, in
1946 to 1950, EPA -- I was on loan from EPA to the
State Health Department. And with the State Health 09:32
Department I -- over in Berkeley, I visited the state
licensed hazardous waste dumps throughout the state.
Q. So you were working for the State of
California at that time?
A. I was paid by EPA, but on loan to State 09:32

Health Department.
Q. Uh-huh. How long -- what were the years for
that?
A. 1976 to 1980.
Q. So where were you physically working for 09:32
those four years?
A. Berkeley.
Q. Uh-huh. And why did you come back after
1980?
A. These tours of duty only lasted four years. 09:32
So I returned to the regional office in 1980.
Q. You returned to the hazardous waste section?
A. That's correct.
Q. What were your duties there?
A. Let's see. Developing regulations for 09:33
hazardous waste. And visiting hazardous waste
sites. Lecturing at some localities that wanted some
instruction on hazardous waste.
Q. What kind of places would that be?
A. Well, one that comes to mind was in Eureka, 09:33

I think it was College of the Redwoods. And I lectured some workers there about hazardous waste and what to watch out for. Safety measures.
     Q. You mentioned you were involved in developing regulations. What kind of regulations 09:33

were they?
     A. It was a minor involvement, but they would be: Hazardous waste regulations. And I did some review on some state -- some state proposed regulations -- legislation on hazardous waste. 09:34
     Q. Why did you leave EPA eventually?
     A. To retire.
     Q. What age were you then?
     A. 66.
     Q. And how old are you now? 09:34
     A. I'm 76.
     Q. When you were at the Montrose Torrance plant, what were the nature of your duties there?
     A. I was responsible for an eight-hour shift production of DDT. I had a crew under me, it varied, 09:35 usually it was ten or 12 people.
     Q. What shift did you work?
     A. We rotated shifts.
     Q. How many days a week did you work?
     A. It averaged out 40 hours a week, it depended 09:35 on the particular shift. On graveyard shift it was either more or less, depending on how it worked out. But you worked five eight-hour shifts a week.
     Q. How many shifts per day ran at the Torrance plant? 09:35

     A. They ran 'round the clock, three shifts a day.
     Q. And did the Torrance plant operate three shifts a day the entire time you were there?
     A. Yes. 09:35
     Q. When you worked a shift, did you have the same people working under you, or did it change?
     A. That's correct.

Q. The same?
A. Same people. 09:36
Q. Who were the other shift supervisors?
A. Well, let's see: A Shift was Guy A.
DeMichelle; B Shift was Anitol Nicholaus Leonovich; C
Shift was -- excuse me, A Shift was Lane Hodges;
DeMichelle was C Shift; I was D. 09:36
Q. You said there were three shifts per day.
But there were four --
A. Right. One shift is off.
Q. And what would be the title of the people
that worked for you? 09:37
A. Well, there would be a chemical operator, a
chemical operator helper, a flaker operator. There
would be several laborers. There was also a
stationary engineer who operated the plant steam
boiler. 09:37

Q. What did the chemical operator do?
A. His responsibility was preparing the acid
strength. And charging the condensators with the
ingredients for DDT. And for seeing that the process
went properly. This was instrumented, and he had to 09:37
follow his instruments.
Q. What did the flaker operator do?
A. He operated the machines that transform
liquid molten DDT into a dry flake for packaging.
Q. What did you do on a day-to-day basis? 09:38
A. I kept records. I would analyze the
shipments of raw materials that came in. Generally
to see that things went well. If any troubles came
up anywhere, I was supposed to take care of that,
correct them. 09:38
Q. Were you responsible for overseeing the
production of any materials other than DDT?
A. No.
Q. Just DDT?
A. Just DDT. That was our only product. 09:38
Q. So you had -- did you have the same position
for the whole ten years you worked at Montrose?
A. That's correct.
Q. Every day you worked there you had hands-on
dealing with the production of DDT? 09:39

A. Yes, indeed.
Q. Do you know when the Torrance plant was constructed?
MR. SIMSHAUSER: Objection, calls for speculation. 09:39
THE WITNESS: No, I don't know.
MR. O'ROURKE: Q. Do you know if Montrose Chemical of California had any plants other than Torrance and Henderson plants?
A. No, I'm not aware of anything else that they 09:39 had. There was Montrose of New Jersey was a parent company, and they had operations back East, which I know nothing. Montrose in 1949 -- excuse me, 1959, Montrose designed and built a plant in Mexico. And I'm not sure just what the relationship of Montrose 09:40 of California had with the Mexico plant.
Q. Was the Mexican plant designed by Montrose of California?
A. That's correct.
Q. What city was that in, do you know? 09:40
A. It was in Salamanca, Mexico.
Q. Do you know what the business of the Mexican plant was?
A. Only DDT.
Q. Manufacturing DDT? 09:40

A. Yes.
Q. Who were Montrose's competitors in the DDT market, when you started working there?
A. Oh, there were a number of them. There was Olin. Pen Salt, was at one time manufacturing DDT. 09:41 I think Monsanto. I had heard that Diamond Shamrock was also a manufacturer. I'm not aware of any others.
Q. Were any of those plants located in Southern California?
A. No.
Q. And how successful was Montrose compared to those other companies?
A. Very successful. We could out produce at lower cost than anybody else. So all the others 09:41

finally dropped out of the business.

Q. Why was Montrose so much more successful?

A. They had the advantage that they did not have to use liquid chlorine. DDT is, by weight, one half chlorine, which is a major ingredient. And 09:42 Montrose could use sill gas chlorine at Henderson, rather than have to liquefy it and transport it to where it was going to be used. It was manufactured right at Henderson. So there was only a pipeline between the Stauffer plant that manufactured the 09:42

chlorine and the Montrose plant that used it.

Q. The Stauffer plant was located near the Henderson plant?

A. Right in the same series of buildings.

Q. So which company was manufacturing the 09:42 chlorine?

A. Stauffer.

Q. And did Henderson buy it from Stauffer, or what was the arrangement?

A. I assume it was bought. But I'm not 09:42 familiar with the arrangements.

MR. SIMSHAUSER: Move to strike.

MR. O'ROURKE: Q. Do you know the price -- you don't know the price?

A. I have no idea what the price of chlorine 09:43 was.

Q. Did the Henderson plant have to further process the chlorine to prepare it for the Torrance plant?

A. Well, the Henderson plant used the chlorine 09:43 to manufacture chloral and monochloral benzene, which are the ingredients for DDT.

Q. Do you know if that -- if Stauffer continued to make chlorine for Henderson the entire time you worked there? 09:43

A. Yes.

Q. Now you mentioned four other shift supervisors. How many people --

A. Three other shift supervisors.
Q. How many people worked in those other shifts? 09:44
A. Probably the same as mine. We were all about the same size.
Q. Uh-huh. Who was your direct supervisor at the Torrance plant? 09:44
A. Max Sobelman.
MR. LYTZ: Excuse me, could you read back the last question please?
(Record read).
THE WITNESS: Max Sobelman, S-o-b-e-l-m-a-n. 09:44
MR. O'ROURKE: Q. What was his title?
A. Let's see. Production Superintendent, I believe.
Q. Was he the only production superintendent at the plant? 09:44
A. That's correct.
Q. Was he there the whole time you worked there?
A. Yes. 09:45

Q. Was he your boss the whole time you worked there?
A. Yes.
Q. And who was his boss?
A. Albert Ross Wilcox. 09:45
Q. What was his title?
A. I believe he was Plant Manager.
Q. Was there a plant engineer?
A. Yes.
Q. Who was that? 09:45
A. Bernard I. Bratter, I believe you're present.
Q. Was Mr. Wilcox the plant manager the entire time you worked there?
A. That's correct. 09:45
Q. He was the only manager?
A. That's correct.
Q. Was Mr. Bratter the plant engineer the entire time you worked there?
A. I believe so. 09:45
Q. Was he the only plant engineer?
A. I believe so, yes.

Q. Are there any other supervisors or managers, that you have not mentioned yet, that worked at the plant, above you? 09:46

page 26

A. Directly above me? No. John Kalock was the engineering and maintenance superintendent.
Q. And did he -- was he in charge of a different part of the plant than you worked in?
A. He was in charge of the maintenance crew. 09:47
And he was responsible for the engineering, I believe.
Q. Do you know what the difference in jobs between Mr. Bratter as plant engineer and Mr. Kalock as -- 09:47
A. I would think that Kalock was Bratter's boss.
Q. Uh-huh. So when the plant was in full operation, how many people would be at the plant?
A. You mean production people? 09:47
Q. People in total.
A. I'd have to add that up. I would guess probably 50 people in maintenance. Probably 45 or 50 in production. There would be probably maybe 15 in grinding and shipping. And office staff would be 09:48
another dozen people.
MR. SIMSHAUSER: Move to strike, nonresponsive.
MR. O'ROURKE: Q. Is that the total number of people that were employed by the plant? 09:48

page 27

A. As I remember, yes.
Q. On any given afternoon, how many people would be working at the plant?
MR. LYTZ: Objection, asked and answered.
THE WITNESS: Counting office personnel, 09:48
maybe 70, 75. That would be the maintenance crew plus the production people that were on board, plus the grinding and shipping people, and office staff, that's a guess.
MR. O'ROURKE: It's coming up on 10:00. 09:49

Why don't we take our first break.
THE VIDEOGRAPHER: We're going off the record now at 9:49.
(Recess taken).
THE VIDEOGRAPHER: We're back on the record now at 10:01. 10:01
MR. O'ROURKE: Q. When you worked at the Torrance plant, did you ever have occasion to go to the Henderson plant?
A. No, I was never there. 10:02
Q. When you worked at the Henderson plant, did you ever go back to the Torrance plant?
A. Yes.
Q. When was that?
A. There was a strike in -- and -- it's 10:02

about '65. And a number of management people from the Henderson plant came back to help operate the Torrance plant during the strike.
Q. Was the strike in the Henderson plant as well? 10:02
A. No.
Q. How long was that for that period when you went back to Torrance?
A. It could not have lasted more than four or five days. 10:02
Q. Did you ever have occasion to go to the Mexican plant?
A. Oh, yes. I was down -- I went to the Mexican plant to help with the start up, when they first came on line. 10:02
Q. When was that?
A. This was in 1959 -- '58 or '59. And the -- they needed help to start the plant up and keep it running, and train the Mexican workers on how to operate the plant. 10:03
Q. Were you working at the Torrance plant at that time?
A. Yes.
Q. How long did you spend at the Mexican plant?
A. It was about four or five months. 10:03

Q. What were the nature of your duties at the plant in Mexico.
A. Teach people how to operate the plant, which meant I did a lot of the operating myself.
Q. Was it similar to your duties at Torrance? 10:03
A. Close, except that I was more of an advisor. I was not directly in charge of the people that were operating the plant. But actually I did a lot of the operating myself. And I was training the foremen and training the operators, as well. 10:03
Q. How many other people went from the Torrance plant to the Mexican plant?
A. I remember Bernie Bratter went. Max Sobelman was there for a while. I can't recall anybody else. The plant manager was sent down from 10:04 the Henderson plant, his name is Hal Wurzer.
Q. Was he the manager when you were at Henderson, as well?
A. When he came back from Mexico, he was the manager there. 10:04
Q. Do you know if the Montrose plant in Mexico still exists?
A. I asked Mr. Bratter that a few minutes ago, and he thought it might be still running.
Q. Now, when you finished in Mexico, did you go 10:04

back to Torrance?
A. That's correct.
Q. How long were you there before you went over to Henderson?
A. Probably a year, year and a half. 10:05
Q. Who signed your pay checks when you worked at the Torrance plant?
A. The pay checks came out of -- as I recall, they came out of the Stauffer office. I think they were -- Stauffer handled the payroll. I don't know 10:05 who signed them.
Q. Do you know where the Stauffer office was?
A. They had a number of offices around. I think the main office was in Vernon, California.
Q. Who pays your pension? 10:05
A. I have a small pension that comes from the -- originated at the Henderson plant, Montrose. And

I have a civil service pension from my EPA tour.
Q. When you were at Henderson, where did your pay checks come from? 10:06
A. I believe still Stauffer. But it would have been the Stauffer Henderson operation, rather than the California operation.
Q. Where did your -- from Henderson you said you received a pension, where were those checks 10:06

coming from?
A. Now they come from some place in Florida -- Boca Raton, I think, Florida. Stauffer has changed hands a number of times since I left them. And the pension administration changes hands at the same 10:06 time. I think there's been at least four or five different offices that the pensions have come from.
Q. Do you remember where the pension was coming from when you first started to receive it?
A. No. 10:06
Q. Did you receive any bonuses when you worked at the Torrance plant?
A. The owner of Montrose had a policy, when I first got there, of every year giving a month's pay as a bonus if we were producing well, which we were 10:07 those days. And that continued for three or four years, and then it stopped.
Q. Who was the owner of Montrose?
A. Pinkers Rothberg.
Q. Do you know where he lived? 10:07
A. At the time I think he lived in Miami.
Q. Was he involved with Montrose of New Jersey?
A. Probably, I don't know for sure.
Q. Did you ever meet him?
A. Oh, yes. 10:07

Q. How often?
A. During the first few years, Dr. Rothberg would come out to the plant, oh, two or three times a year.
Q. What did he do during his visits? 10:07

A. Usually took us all out to a steak dinner.
Q. Where?
A. I can remember that clearly. It was the House of Murphy in -- down near La Cienega in LA.
Q. Did he take you to dinner just because he was in town? 10:08
A. Because he felt good about production. And he was a good guy to work for.
Q. How many years did that go on that he used to come to the plant? 10:08
A. I don't think he came down after about 1955. He was getting on in years. And his wife was in poor health, which may have accounted for him not visiting as much.
Q. Did -- 10:08
A. His son Benjamin Rothberg was gradually taking over some of these chores, and we would see Ben Rothberg quite frequently.
Q. How often did he come up to the Torrance plant? 10:08

A. Oh, couple of times a year, I would guess.
Q. Did that continue the entire time you worked at Torrance?
A. I think it tailed off a little bit at the end. I don't think he was out to the plant nearly as 10:08 much in the later 50's. I did see Ben at the Mexican plant, though.
Q. Did he continue the tradition of taking you all to dinner?
A. Not so much. 10:09
Q. Who made the hiring and firing decisions at the Torrance plant?
A. Wilcox and Sobelman. And I'm sure for the maintenance people, John Kalock.
Q. Did any officers from the Stauffer Chemical 10:09 Corporation have offices at the Torrance plant?
A. Early on, in the early 50's, there was a -- one of the Stauffer offices did have an office, not in our building, within our belated building further away. I don't remember what his name was. 10:10
Q. Where was this other building?
A. There was a -- Stauffer had a small production facility that made benzene hudson chloride, and it was, oh, 50 yards away from the

Montrose offices. And I think that probably this   10:10

page 34

Deposition of Suhrer, Ferdinand
page 35

Stauffer official had an office in that building.
     Q. Was this Stauffer facility located on the
same property as the Montrose Torrance facility?
     A. Yes.
     Q. Was benzene hudson chloride the only product 10:10
they produced at that facility -- Stauffer produced
at that facility?
     A. As far as I know. I'm really not familiar
with what they did. But I know they made BHC.
     Q. How long did that plant operate?   10:11
     A. Not very long. I think they closed down
probably after -- I think they closed down maybe 1953
or '54.
     Q. Do you know why it closed down?
     A. I have no idea.   10:11
     Q. Do you know if that Stauffer plant ever
received or stored chlorine?
     A. I'm sure they must have, if they were making
BHC.
     Q. Did you ever manufacture BHC yourself?   10:11
     A. No.
     Q. Do you know if the Stauffer Chemical Company
ever provided personnel to the Montrose Torrance
plant?
     A. Would you repeat that?   10:11

page 35

Deposition of Suhrer, Ferdinand
page 36

     Q. Do you know if Stauffer Chemical Company
ever provided personnel to the Montrose Torrance
plant?
     A. No, I'm not aware that any people came. You
mean on a long term basis?   10:12
     Q. Yes.
     A. I'm not aware of any like that.
     Q. Do you know who selected Mr. Wilcox to be
the plant manager?
     A. Probably Pinkers Rothberg.   10:12
         MR. SIMSHAUSER: Move to strike.
         THE WITNESS: Although this was before my

time. I would be guessing there.

MR. SIMSHAUSER: Move to strike -- that is, I move to strike the previous answer, based on the 10:12 answer that was just given. Could you read back the previous question and answer, please?

(Record read).

MR. O'ROURKE: Q. Do you know who selected Mr. Wilcox to be plant manager? 10:12

MR. SIMSHAUSER: Objection, asked and answered. I'm sorry, you need to let me finish my objection, sir, before you interpose an answer.

THE WITNESS: Excuse me.

MR. SIMSHAUSER: And I object to the 10:13

question and move to strike the answer.

MR. O'ROURKE: Q. What were the raw materials used to make DDT at the Torrance plant?

A. Chloral and monochloral benzene, which we refer to as MCB. 10:13

Q. Did you have a nickname for chloral?

A. No.

Q. What was the basic chemical reaction by which you manufactured DDT from those two compounds?

A. The monochloral benzene and the chloral 10:13 condensed to form DDT, in the presence of sulfuric acid -- strong sulfuric acid.

Q. Are there any other products or by-products of the reaction?

A. There are none that you would like to have. 10:14 There are -- some of the MCB is sulfonated by the acid, which is an undesirable reaction because it wastes MCB.

Q. What's the product of that?

A. Monochloral benzene sulfonic acid, MCBS. 10:14

Q. Are there different isomers of DDT?

A. Yes.

Q. Was one of those isomers more desirable in the final product at Torrance?

A. The para-para isomer is the desired form. 10:14

The others have very little insecticidal value.
Q. Do you know what the composition of the final product of the Torrance plant was?
A. I don't know what the percentage of para-para was. The government had a spec of an 89 set point. The setting point of pure para-para is up in the mid 90's. So it's a test of the purity of the DDT. The higher the set point, the more the desired isomers present. And the government had a spec of 89 degrees centigrade for the setting point. We in the plant kept a plant spec of 90 degrees. We wouldn't ship anything less than 90 degrees set point. 10:15 10:15
Q. Do you know what governmental agency set the set point at 89?
A. I don't know. 10:15
Q. So was the final product from Torrance a combination of more than one isomer?
A. It was probably chiefly para-para, some ortho para isomers.
Q. And would that -- was that the product that was produced the entire time you worked there? 10:16
A. Yes.
Q. Do you know what technical grade DDT is?
A. It is DDT with a setting point above 89 degrees centigrade. 10:16

Q. That's the product that you all manufactured?
A. That's the product.
Q. Could you very briefly describe the steps in the process that was used to convert the raw materials to the finer product at the Torrance plant? 10:16
A. The chloral came into the plant in a mixture with monochloral benzene, which was chloral rich, richer than we would want in the process. So when we charged the condensators with the chloral MCB mix, we would have to add additional monochloral benzene to bring it up to proper proportions. 10:17
When you charge a condensator with the chloral and MCB, then you bleed in slowly with strong sulfuric acid, almost 100 percent sulfuric acid, and this is done under control, because the temperature tends to rise. If the temperature gets too warm, you will start decomposing the monochloral benzene, it will start sulfonating, giving you an undesirable 10:17

product. 10:18
So the temperature was controlled at a
fairly low temperature by means of brine coils in the
condensator. Cold brine would circulate through
there to keep the reaction cold. And acid would be
bled in with an instrument, just keeping the 10:18

temperature where it was. A little acid, all the
time, until the temperature was well under control,
then you could add a little more acid.
Q. Uh-huh.
A. And this continued until the condensator was 10:18
full.
Q. Then what happened?
A. By that time you had enough acid, and enough
mix and enough monochloral benzene in there to make
DDT. And the DDT would start to form about midway 10:18
through the process. It would form -- it looked
exactly like beans. And we referred to them as
beans. Looked like pork and beans. Red acid and
stirred up in this mixture, all kept cold.
Q. And -- 10:18
A. At the end of the process, maybe six hours
later, depending on what -- when I got there first,
we would send that material to a -- let's see, how
this would work. We try to pump off as much acid as
we could, and add monochloral benzene to dissolve the 10:19
DDT. Pump this to a stripper, where the solvent MCB
would be stripped off by steam, leaving molten DDT in
the bottom of the stripper. This would then be
poured out into long pans, about not too much
different than the size of this table. And poured to 10:19

a depth of maybe five or six inches, and allowed to
harden, and then chopped up to put into bags. This
was the initial process.
Later on, we developed a -- more of a
continuous process. When the beans, when they were 10:20
formed, were pumped to a postreactor where additional
reaction took place to finish off the DDT. Then this

slurry of DDT and acid was sent to an Oliver filter
that sucked out the beans and dropped them on a
conveyer belt. The acid remained behind, and was 10:20
sent back to be refortified for the next batch.
Q. And what happened to the DDT that went on
the conveyer belt?
A. The DDT on the conveyer belt was taken up to
a small tank where steam was brought in and melted 10:20
the DDT. The DDT was then fed into a separator where
the residual acid was drained off and the molten DDT
was sent to a caustic scrubber to neutralize the
acid, then to a water scrubber to take out any
caustic, or any acid that could be left. Then it was 10:21
sent to a holding tank. Molten DDT went into a
holding tank and was pumped either to the flakers, or
to a chip table, or to wherever you wanted --
whatever product you wanted the final form to be.
Q. When did that process change take place? 10:21

page 41

Deposition of Suhrer, Ferdinand
page 42

A. I'm guessing on this, but I would say about
1955 -- '54 or '55.
Q. And the second process, which you said was
more continuous, was that the process that was used
for the rest of your time at the Torrance plant? 10:22
A. That's correct.
Q. Do you know if that process continued to be
used after you left the Torrance plant?
MR. LYTZ: Objection, calls for
speculation. 10:22
MR. O'ROURKE: Q. Do you know?
A. I don't know. Well, I do know, too.
Because when I went back during the strike, it was
still in use then.
Q. And that was in -- 10:22
A. That was about '64 or '65.
Q. Where did the Torrance plant get the chloral
to be used to manufacture the DDT?
A. The chloral and MCB were manufactured at the
Henderson plant -- Henderson, Nevada. 10:22
Q. The Henderson Montrose plant?
A. Right.
Q. How did they get from the Henderson plant to
the Torrance plant?
A. It was sent down in tank trucks by Post 10:22

page 42

Deposition of Suhrer, Ferdinand
page 43

Transportation Company.
     Q. Do you know the volume of those tank trucks?
     A. Probably the truck and trailer together would be about 5,000 gallons.
     Q. How many shipments per day did the Torrance plant receive from Henderson? 10:23
          MR. LYTZ: Objection, assumes a fact not in evidence.
          THE WITNESS: It would depend on the production rate. If you were making a lot of DDT, you would maybe need two or three trucks a day. It just depended on how -- what your production schedule was. 10:23
          MR. O'ROURKE: Q.   How did the production schedule vary over time? 10:23
     A. This I really could not say. It was fairly constant for most of the time. Occasionally we'd be in a bind where we would have lots of orders for DDT, and we would step it up. We would step it up by shortening the cycle of DDT in the condensators. If you shorten the cycle, you get more batches out per day, and make more DDT. You may take a slight loss in quality and in yield of DDT by doing that. So if you had time, you'd rather take a long cycle. I don't think there was much variation in production. 10:24 10:24

page 43

Deposition of Suhrer, Ferdinand
page 44

     Q. You mentioned that MCB and chloral were mixed. Were they mixed when they arrived at the plant?
     A. Yes, they were mixed at the Henderson plant for shipment down to Torrance. 10:24
     Q. Do you know what the ratio of the two compounds in the mix was?
     A. I don't remember that. It's easy to calculate, but I don't remember that.
     Q. And how come those two compounds did not react in the truck on the way over? 10:25
     A. Because there was no acid present. The sulfuric acid has to be added to promote the reaction.

Q. What's the function of the sulfuric action? 10:25
A. To make the reaction go. And to pick up -- see, a molecule of water is formed for every molecule of DDT. And the sulfuric acid picks up that water. It's a strong dehydrating agent, sulfuric acid.
Q. Is sulfuric acid incorporated into the final 10:25 product?
A. No.
Q. Does that mean it's a catalyst?
A. Yes, it's a catalyst. But it does enter into the reaction by picking up the water. A true 10:25

catalyst does not enter into the reaction at all.
Q. Were chloral and MCB also shipped separately from the Henderson plant to the Torrance plant?
A. MCB was shipped separately to make up the correct proportions. But the chloral was always 10:26 shipped down in mixture with MCB.
Q. Why was that?
A. Chloral tends to hydrate. And the hydrate does not do any good for the reaction, is not used in the reaction. It has to be anhydrous chloral. And 10:26 the mono in the mixture tended to repress this hydration.
Q. What was the purity of the MCB that was received from Henderson to Torrance?
A. I don't know. 10:27
Q. Do you know if there were any contaminants or any non MCB?
A. If there was a contaminant, it could have been benzene. See, MCB is made from benzene. And if there were any unreacted benzene left in the MCB, 10:27 this would be not good, because benzene does not promote the reaction.
MR. SIMSHAUSER: Move to strike the testimony. You elicited an I don't know answer, and you asked him a question which called for 10:27

speculation.
MR. O'ROURKE: No, I asked him if he knew

the purity. And he said he didn't know the
proportions, but he knows what contaminants might
have been in it.
MR. SIMSHAUSER: The record will speak for
itself.
MR. O'ROURKE: Q. Where did the Torrance
plant get the sulfuric acid from?
A. We bought the sulfuric acid from Stauffer. 10:28
Q. Which Stauffer plant?
A. I think Stauffer had a large acid plant in
Vernon and another acid plant in Dominguez, both in
California, and I think both in LA County. Where it
came from, I don't know. Which one of the plants, I 10:28
don't know.
Q. Do you know what was the purity of the
sulfuric acid you were getting?
A. We would get sulfuric acid in the form
called oleum. 10:28
Q. What's that?
A. That is a super-concentrated sulfuric acid.
It's highly corrosive to your skin. It'll just tear
holes in you quickly. Oleum is very, very
corrosive. But it's peculiar in that you can store 10:29

it in an iron tank. Weaker sulfuric acid, you
cannot. But oleum, you can store.
Q. Did the Torrance plant use an iron tank?
A. Yes.
Q. Do you know what the concentration of oleum 10:29
was?
A. There were two concentrations. They
reported the concentration in two different forms:
One of the concentrations that was formed was
reported was in percent of S03 of sulfur trioxide, 10:29
which was added to the sulfuric acid. And I think
that was around 30 percent. There was another way
that you could give the same concentration, which
was, I think, 105 percent. I have no idea what that
meant -- exactly what that meant. But oleum is a -- 10:30
is sulfur trioxide dissolved in sulfuric acid.
Q. And when you received oleum from the
Stauffer plant, it was oleum, you did not have to add
the sulfur trioxide yourself?
A. No, we received it as oleum. As oleum it 10:30
was not useful in our plant. So we use that to

fortify the acid that has been used already. The spent oleum was fortified with oleum to bring it up to just under 100 percent.
Q. What was the percentage of the oleum when it 10:31

was used in the reaction?
A. Just under 100 percent. It was not oleum then, it was what we call soo. Spent oleum, oleum, s-o-o, and the nickname in the plant was soo.
Q. Uh-huh. Where did the spent oleum come from? 10:31
A. It came from the -- when we are operating the Oliver filter, it came -- it was the oleum -- the acid that was drained off the filter.
MR. O'ROURKE: Off the record for a minute. 10:31
THE VIDEOGRAPHER: We're going off the record now at 10:31.
(Recess taken).
THE VIDEOGRAPHER: We're going back on the record now at 10:51. 10:51
MR. O'ROURKE: Q. All right. We've put up a photograph which is Exhibit 1. Do you want to mark it right now?
(Document referred to herein marked Plaintiff's Exhibit 1).
MR. O'ROURKE: Q. Mr. Suhrer, do you recognize the contents of this photograph?
A. Yes, I do.
Q. What is it?
A. It's the Montrose plant at Torrance. 10:51

Q. This is the plant that you worked at?
A. Right.
Q. Does it -- this photograph -- does the plant in the photograph look like the plant when you worked there? 10:51
A. Yes.
Q. Are there any substantial differences between the way you remember it and the way this photograph depicts it?

A. No, I don't right now see any differences. I recognize the various buildings. 10:51
MR. SIMSHAUSER: Steve, are you going to try to lay a time?
MR. O'ROURKE: Q. I was just about to ask. Can you tell by looking at this photograph approximately when it would have been taken? 10:52
A. One thing I notice, there was a change house right here at the end of -- and I think that went in, oh, maybe 1956, that would be a guess. I don't see the change house there now. But it should be right in here. 10:52
(Witness indicates).
MR. O'ROURKE: Q. Does that imply to you that the photograph is from before 1956?
A. This I would guess, yes. 10:52

Q. Before the break, we were discussing the MCB and chloral, where were they stored on site?
A. Let's see. Tanks here and here.
(Witness indicates).
THE WITNESS: These were for chloral and mono. 10:53
MR. O'ROURKE: Q. And did those tanks contain mix or --
A. Yes, mix -- mix and mono, excuse me.
Q. Do you remember which one was the mix tank? 10:53
A. No, I don't. I believe -- well, let me say I believe the tank in the corner here was mix. And perhaps this one, too, this one was probably mono.
Q. And when you say "mono," you mean MCB?
A. Monochloral benzene, yes. 10:53
Q. And what is the building that's next to the tanks you were just pointing at?
A. This building?
Q. Yes, sir.
A. This is the process building. 10:54
Q. Is that all one building?
A. Yes.
Q. It appears that the roofs are at different angles?
A. Yes. 10:54

Q. It's still one?
A. One building.
Q. Okay. Do you know which direction is north on this photograph?
A. That's north. 10:54
(Witness indicates).
MR. O'ROURKE: Q. Okay. And --
THE VIDEOGRAPHER: Do you want to point to that again?
THE WITNESS: North is the top of the 10:54 photograph.
MR. O'ROURKE: Q. What street is that on the right side?
A. This over here?
Q. Yes, sir. 10:54
A. That would be Normandie.
Q. Where was the pure oleum stored that was shipped from Henderson -- pardon me. Where did the oleum come from?
A. The oleum came from several Stauffer plants 10:55 in LA County.
Q. Stauffer Chemical Company?
A. Stauffer Chemical at Dominguez, at Vernon. I don't know where else.
Q. Uh-huh. 10:55

A. The storage there was in a horizontal tank right here.
(Witness indicates).
MR. O'ROURKE: Q. And can you tell me where that tank is in relation to the process 10:55 building?
A. It's alongside the process building. Just to the east of the process building.
Q. Do you know the capacity of that oleum storage tank? 10:55
A. As I remember, 5,000 gallons.
Q. And I forgot to ask: Do you know the capacity of the mix storage tanks?
A. Mix storage tanks and the mono storage tanks were 10,000 gallons. 10:55
Q. Were those -- are those vertical tanks?

A. The mix and the mono were vertical tanks. The oleum storage was a horizontal tank.
Q. Was there a separate soo storage tank?
A. Yes. 10:56
Q. Where was that?
A. That was right alongside the oleum tank, another horizontal tank.
Q. They're parallel next to each other?
A. Yes. 10:56

Q. And do you know the capacity of the soo storage tank?
A. It was the same size as the oleum, I'd say 5,000.
Q. Was there a spent oleum tank? 10:56
A. Yes. And that was right alongside here. One of these tanks. I don't recall just where that was. One of these here was the spent oleum.
(Witness indicates).
THE WITNESS: So we have spent oleum, soo, 10:56 and oleum.
MR. O'ROURKE: Q. And was there some kind of mixing system for the oleum and spent oleum?
A. You would agitate and circulate. Circulate the tank, just pump from the bottom to the top of the 10:57 tank, and keep it going around like that, which would mix it. And also some of the tanks had agitators.
Q. Were there agitators like that in the mono tank?
A. No. 10:57
Q. In the mix tank?
A. No.
Q. But in all three of the oleum?
A. Yes.
Q. And was there a system to mix the spent 10:57

oleum with the fresh oleum?
A. Yes. You mix those in a tank. You mix those in an agitated tank. The operators got pretty good at knowing just how much to put in. How much

oleum and how much spent oleum, what the proportions 10:57
were. The operators were pretty good at that. So
they would know in advance how much to put in. And
it would always be tested before we used it.
Q. And who were the -- what was the title of
the person who would operate the mixing system for 10:57
the oleum?
A. This would be the chemical operator. And
sometimes he would have his helper -- chemical
operator helper do the work.
Q. Were you the supervisor of the chemical 10:58
operator?
A. Yes.
Q. Did you oversee the oleum mixing system --
A. Yes.
Q. -- operations -- 10:58
A. Yes.
Q. -- on a day-to-day basis?
A. Yes.
Q. What was the concentration of the spent
oleum when it came out of the process? 10:58

A. From memory, I would say probably 95
percent.
Q. Did the spent oleum ever get used up to the
extent that it could not be refreshed?
A. No. It would be used, as long as you had it 10:58
there. You needed spent oleum for the soo. Now what
acid that was not used, was the separator acid. If
you remember, when coming off the filter, you would
conveyer belt the DDT with some acid still in it up
to a tank where you melted the DDT with steam. This 10:59
produced a weak acid, probably 85 percent acid, which
was not used for soo. That had to be discarded.
Q. Which parts of the plant of the process
generated spent oleum that was generated to soo
again? 10:59
A. Inside the process building, the
condensators.
Q. Condensators.
THE VIDEOGRAPHER: Would you like to point
to that one more time, please? 10:59
THE WITNESS: Inside the process building,
the condensators.
(Witness indicates).

MR. O'ROURKE: Q. How many condensators
were in there? 10:59

page 55

A. Initially there were eight. And later on, oh, after a few years, they added two more somewhat larger condensators outside.
Q. Can you tell me where those were added later on? 11:00
A. They were vertical tanks here and here.
(Witness indicates).
MR. O'ROURKE: Q. Is that in between the mix tanks and the oleum tanks?
A. Yes, between those. 11:00
Q. Were they -- when were they added, do you know?
A. 1956 is a guess.
Q. Now the condensators were the reaction vessel; is that true? 11:00
A. That's correct.
Q. What was first added to the condensator?
A. You would put in -- first you would put in the mix, measured amount of mix. And then a metered amount of MCB to the correct proportion, which would 11:00 be calculated by myself. I would give the operator a chart saying mix so many gallons and mono so many gallons. They would charge the condensator with this, and then start bleeding in the soo.
Q. Do you know what the approximate volumes of 11:01

page 56

the ingredients were that were put in the condensators?
A. Perhaps 500 gallons of mix and mono. And perhaps the same amount, or somewhat more, of the soo. 11:01
Q. Uh-huh. Was the mix a liquid?
A. Yes.
Q. Did it have a smell?
A. Yes, strong.
Q. Could you describe the smell? 11:01
A. Very strong, pungent smell of chloral.

Q. Uh-huh. Does MCB have a smell?
A. It's also an organic smell, with much less pungent than the chloral.
Q. And is MCB viscus, or watery consistency, or -- 11:01
A. It was about the consistency of kerosene.
Q. Was it clear, or opaque, or --
A. Very clear.
Q. And chloral, what was its consistency? 11:01
A. The same. You could not tell them apart by looking at them.
Q. Could you tell them apart by smell?
A. Oh, yes, indeed.
Q. And the oleum, was that a liquid also? 11:02

A. That's a liquid. That's a very heavy liquid. Almost twice the density of water. Very heavy liquid. And fumes, a lot, very irritating to the nose.
Q. Were the fumes visible? 11:02
A. Oh, yes, smoked.
Q. Was it clear, the liquid?
A. Yes.
Q. And once you put in all of the three ingredients into the condensator, did the reaction start immediately? 11:02
A. No. You have the mono and the mix in to start. And the acid is metered in slowly. Because if you put a lot of it in, the temperature would go too high. You could not control the temperature. You bled in acid slowly to keep the temperature under control. 11:03
Q. What was the size of these condensators?
A. Perhaps six feet diameter, maybe eight feet depth. 11:03
Q. Did they have a cover?
A. Yes.
Q. Made out of -- what were they made out of?
A. Made out of steel.
Q. Were all eight condensators about the same 11:03

size?
A. Yes -- well, the original eight were the same size. The two that were added later were larger.
Q. Do you remember the volumes on those, or the sizes of those? 11:03
A. Perhaps 8,000 -- 800 gallons.
Q. Who was in charge of putting the soo into the reaction vessel?
A. The chemical operator did this. 11:04
Q. Did you oversee that --
A. Yes.
Q. -- process?
A. Yes, I did.
Q. Now you mentioned temperature control. What temperature did the reaction occur at? 11:04
A. The reaction would occur -- I'm trying to think. I don't remember. It sticks in my mind that it might be around 25 centigrade.
Q. And what -- 11:04
A. That doesn't sound right. I don't recall.
Q. You mentioned set point earlier, what does that mean?
A. It's the test on molten DDT. You have the molten DDT in a test tube. And it would be up 11:05

probably around 95 centigrade, 98 centigrade. Agitated with a thermometer in there, when the DDT starts to set up, first starts to crystallize, it would take a little jump in temperature, that jump is what you measure. So you start it off at maybe 95, 11:05 96, in a hot water bath, and keep it stirring, and let the temperature come down slowly. When the temperature got down, when it first started to set up, you would get this little rise in temperature, that's what you would measure. Then it would turn 11:05 solid, and you, of course, dump it out.
Q. And who was responsible for doing that test?
A. We had lab technicians for that.
Q. Who took the sample out of the condensator?
A. The -- this would be taken by probably the 11:06 chemical operator helper and run over to the laboratory for tests.
Q. So you oversaw the sampling but not the

analysis?
A. That's right. I spent some time in the laboratory, and saw many analysis run. But it was not part of my particular job to do that. 11:06
Q. How long did it take to get results back from the lab on the set point test?
A. Depending on the technician, maybe if you 11:06

were lucky, ten minutes.
Q. And how long did it take -- what was the longest you remember it taking?
A. To run --
Q. An analysis. 11:07
A. A lot of samples never did get run.
Q. And what would you do if you did not get results back from the lab?
A. I would complain -- if set point were a problem, if we were running close to the 90-degree 11:07 plant specification, I would get on it right away and want to know what it was. If we were having good set points up around 93, 94, it did not matter that much.
Q. How did you know what the set points were if you did not -- 11:07
A. If the previous set points had been perhaps above 92, then the chances are pretty good that the subsequent set points are going to be pretty good, too. They don't just fall apart all at once. It's a gradual decline over a matter of hours. 11:07
Q. How many times did you check the set points during the one batch in the condensator?
A. You would not check it in the condensator. The set point would be checked from molten DDT. And you probably do that, oh, two or three times a 11:08

shift. More if you were in trouble with set points.
Q. What temperature did you try to keep your condensator at during the reaction?
A. This is the temperature that I'm trying hard to remember, and can't. It was -- I don't know. I 11:08 don't remember.

Q. Was it -- was the reaction -- did it need to
be cooled down or heated up?
A. It needed to be cooled down. The reaction
would tend to run away. If you somehow lost the 11:08
brine circulation, where you were not getting
cooling, the temperature would just go off the chart.
Q. And what would be the result then?
A. It would cook the DDT and you would have a
lost batch. 11:08
Q. What do you mean by "cooked"?
A. It would be a wasted batch. The spec --
quality of DDT would be very poor.
Q. What would be the products in a reaction
that would be allowed to run at a higher temperature? 11:08
A. It would be --
MR. LYTZ: Objection, assumes facts not in
evidence.
MR. O'ROURKE: I don't understand the basis
of your objection. 11:09

MR. LYTZ: You're making an assumption that
there were point in times that there were runaway
batches of DDT. You have not established for the
record yet that such an event ever occurred. To
date, Mr. Suhrer has only described that to you in a 11:09
hypothetical context.
MR. O'ROURKE: Q. Do you know what would
happen if DDT were allowed to react at higher
temperatures?
A. If the condensator temperature got beyond 11:09
control?
Q. Yes.
A. Is that what you're asking?
Q. Yes.
A. I know very well what would happen. The 11:09
acid would start working on the funnel, and convert
it all into MCBS. And the reaction would not take
place, or it would be, if it, depending on when this
problem happened, if you got a leak in the brine
coil, then brine would leak into the acid, instead of 11:09
cooling the acid, it would make it very, very hot.
You would say the con would take off and run away.
And when that happened, the only thing you could do
is throw the stuff away, dump it.
Q. Did it ever happen that the reaction 11:10

page 63

Deposition of Suhrer, Ferdinand
page 64

temperature got out of control?
A. The first night I was on duty it happened to
me.
Q. And what was the result that night?
A. Well, there was a leak in brine coil, and 11:10
the temperature got out of control. I didn't, being
the first time I was on, had no idea what to do. But
I thought it was a good idea to dump in more mono,
which was not a particularly good plan, because it
sulfonated more, and we lost more mono. It did tend 11:10
to quench the reaction, because the acid got diluted
in a hurry.
Q. And did it happen at any other times while
you worked at the Torrance plant?
A. I'm sure it did. I'm sure we had runaway 11:10
cons before.
Q. Do you remember it happening?
A. No.
Q. What did you do with the batch that night,
your first night of work? 11:11
A. I put it -- as I recall, I put the mono
solution in a storage tank that I happened to have
empty. And I tried to drain all the acid off. And I
believe the mono went into a storage tank. I thought
possibly we could reuse it. 11:11

page 64

Deposition of Suhrer, Ferdinand
page 65

Q. Where did you drain the acid out to?
A. I don't recall.
Q. You mentioned brine coils, what were they?
A. They were steel coils, circular coils in the
bottom of the condensator, and you circulated brine 11:11
inside the -- cold brine inside the coils.
Q. These coils were inside the vessel, so in
contact --
A. Inside the vessel.
Q. And was there a brine cooling system or -- 11:12
A. Yes. There was a -- in this, over in this
direction here, there was a stationary engineer who
operated the steam boiler, and also operated five or

six large ammonia compressors. And these ammonia
compressors were used to liquefy ammonia to run 11:12
through and cool a brine tank. Circulated brine from
the brine tank to the process, and then back to the
brine tank. The brine was kept very cold.
THE VIDEOGRAPHER: Could you point to that
area one more time, please? 11:12
(Witness indicates).
MR. O'ROURKE: Q. Could you describe
where it is in relation to the process building?
A. This is where the stationary engineer place
was. The boiler is right here. And the ammonia 11:12

compressors were inside the house, and I think there
was one or two outside.
(Witness indicates).
MR. O'ROURKE: Q. Could you state orally
which direction that is? 11:13
A. Yes. That's close to the northwest corner
of the process building.
Q. Is the stationary engineer someone you
oversaw?
A. Yes. He was a licensed stationary engineer. 11:13
Q. Was his sole job to run the cooling and
refrigeration part of the plant?
A. And the boiler.
Q. What was the boiler for?
A. The boiler was for process steam. 11:13
Q. Uh-huh. Did a higher temperature of
reaction have any effect on which isomers of DDT were
produced?
A. I don't know that.
Q. How long did the reaction take in the 11:13
condensator?
A. Depending on what your production schedule
was. Normally they ran -- the reaction was about a
six-hour cycle.
Q. And how did you know when the reaction was 11:14

complete?

A. When the six hours were up, you knew it was -- the reaction, you could see it was demanding less and less brine. When all the acid -- when the condensator was full of acid then, then you knew the 11:14
reaction was finished. And you would look at the temperature chart. If the temperature chart showed no more tendency to rise, that meant your reaction was through. Six hours was always normal, or was always plenty to complete the reaction. 11:14
Q. And you mentioned that during times of higher production, you would make the reaction run shorter?
A. Shorter than that.
Q. What was the shortest length of time you 11:14
would run it?
A. I don't recall that. Perhaps five and a half hours, maybe five hours.
Q. Did you ever run it longer than six hours?
A. Occasionally. 11:15
Q. Why would that be?
A. Well, occasionally when -- if you were having a problem with the Oliver filter, when you could not take the slurry, couldn't accept any more, you would leave it in the condensators, just in 11:15

storage. And also if your production schedule was fairly light, if you did not need the DDT, you would keep it in the condensator to improve the purity and improve the yield.
Q. So a reaction longer than six hours might 11:15
improve the yield?
A. Yes.
Q. And the purity?
A. That's correct.
Q. Did you measure the yield in your reactions? 11:15
A. This was not done by myself. The monthly report would always have a calculation of the overall yield for the month.
Q. But you never measured the yield for any given batch -- 11:16
A. No.
Q. -- in the condensator?
A. No.
Q. Do you know what -- was the yield measured in a percentage? 11:16

A. Yes.
Q. Do you know what percentage was typically -- what percentage yield was typically obtained by the plant?
A. As I recall, I think the chloral yield, 11:16

page 68

Deposition of Suhrer, Ferdinand
page 69

based on the choral used, was around 90 percent. I think the mono yield was somewhat less than that.
Q. Do you know why there was a difference?
A. No.
Q. What are the DDT beans that you mentioned earlier? 11:16
MR. LYTZ: Could you repeat the question, please?
(Record read).
THE WITNESS: They're small lumps of DDT. 11:16
MR. O'ROURKE: Q. About how big?
A. About the size of a bean.
Q. And did all the reactions in the condensators end up forming these beans, or is that typical? 11:17
A. Yes.
Q. Is DDT a solid at ordinary temperatures?
A. Yes, it's a waxy solid.
Q. Were the beans waxy then?
A. Soft and waxy, yes. 11:17
Q. What was the limiting reagent in the reaction that occurred in the condensators?
MR. LYTZ: Excuse me, would you read the question back please?
(Record read).

page 69

Deposition of Suhrer, Ferdinand
page 70

MR. LYTZ: Objection, ambiguous.
MR. O'ROURKE: Let me re-ask it.
THE WITNESS: I think the --
MR. O'ROURKE: Q. What was the limiting reagent in the reaction that occurred in the condensators? 11:18
A. I think the limiting reagent was the chloral. I think there was always included a slight

excess of MCB in the charge.
Q. Did every batch contain some MCBS at the end? 11:18
A. We never measured that. But I would assume there was a trace of MCB remaining.
MR. SIMSHAUSER: Objection, move to strike.
MR. O'ROURKE: Q. You mentioned earlier that there was a competing reaction to sulfonating the MCB to make MCBS? 11:18
A. Right.
Q. Did that happen in every batch?
A. I would assume so. We didn't measure -- we didn't try to determine the presence of MCBS in the DDT, because it was not going to stay in the DDT. And it would be a measure of the inefficiency of the process. We knew that from other reasons, so there was no need to do that. 11:19 11:19

page 70

Deposition of Suhrer, Ferdinand
page 71

Q. We've talked about running the reaction at too high a temperature. What would happen if the reaction was run at too low a temperature?
A. It would take a long time to react.
Q. Did that ever happen at the plant while you worked there? 11:19
A. We never ran it too low, no.
Q. Did you keep records of the temperature of the reaction?
A. There was a monthly report that covered all of this in detail. 11:20
Q. Did you personally --
A. And also, each reaction, each condensator had a temperature chart recorder. And the recorder charts were kept on file. 11:20
Q. That was an automatic recorder?
A. Yes.
Q. Were any other reaction parameters measured, besides temperature, such as pressure or time?
A. No. 11:20
Q. How much DDT was produced in one batch in a condensator?
A. I really don't remember.
Q. Do you how much DDT was produced per month or per year? 11:21

page 71

A. Let's see. I remember the month that we went over three million pounds. I don't know -- I don't remember what you would call a normal month. This varied -- varied on the demand for DDT.
Q. But three million was unusually high? 11:21
A. Three million was a milestone.
Q. Uh-huh. Did you get taken out to a steak dinner for that?
A. Probably.
MR. SIMSHAUSER: Move to strike. 11:21
MR. O'ROURKE: Q. Did the plant ever exceed the three million pounds in one month after that?
MR. LYTZ: Objection, asked and answered.
MR. KUSHNER: Let him answer the question. 11:22
I don't think it was finished. Let him restate it.
MR. LYTZ: Would you read the question back?
MR. O'ROURKE: Q. I'll ask it again. Did the plant ever again exceed that three million pound 11:22
amount that you know of?
A. I'm sure that we did.
MR. LYTZ: Move to strike.
MR. O'ROURKE: Q. How many reactions were run on the condensator per day? 11:23

MR. LYTZ: Objection, calls for speculation on the part of the witness.
MR. O'ROURKE: Q. While you worked at the plant, how many reactions were run on the condensator per day? 11:23
MR. LYTZ: Same objection.
THE WITNESS: During an eight-hour shift, you probably would run between six and eight.
MR. O'ROURKE: Q. Is that per -- that's total for your shift? 11:23
A. Eight-hour shift, yes.
Q. So did some of the condensators go unused during your shifts?
A. No. Not if they were in service. If they were not being maintained, they were in service, they 11:23

were used.
Q. If you were working one shift, did you start a reaction when your shift started?
A. No. You would come in, and the condensators would be in all different stages, all up and down the line. The two operators -- the relieving operator would find out what condensators would be due to come off next, so he'd be watching for that. 11:24
Q. Now where was this sample of DDT taken to be sent to the lab for set point tests? 11:24

A. It was taken -- the sample DDT would be taken in the plant, from the plant over to the laboratory.
Q. What part in the process in the plant was it taken from? 11:25
A. Sometimes from the DDT holding tank. Sometimes from the flaker feed line. Sometimes from -- at first, when we were pouring into these long galvanized pans, it was taken directly from the pan.
Q. So by the time the set point was measured, 11:25 the entire reaction was done and the DDT was made?
A. Oh, yes.
Q. What happened to the DDT that was found to have a set point below the 90-degree spec?
A. It would be set aside for rework. 11:25
Q. And was the rework done at the plant?
A. Yes.
Q. Was it possible to have a set point that was too high?
A. No. 11:26
Q. After the reaction was complete in the condensator, was the DDT removed from the condensator?
A. The DDT was -- let's see. During the time when we ran the Oliver filter, the DDT and the acid 11:26

were stirred up together and pumped as one mass over to a postreactor where the reaction was completed.
Q. What happened in the earlier years you

worked at the plant, there was a separate process,
right? 11:26
A. Yes.
Q. What happened under the original process?
A. Okay. You would, in the original process,
try to settle off the acid, and pump it off. DDT
would tend to float to the top. If you shut the 11:26
agitator off, the DDT would float to the top. You
try to drain off as much of the acid as you could,
then put in mono to dissolve the DDT.
Q. How did you drain out -- how long did it
take for the acid layer to separate from the DDT 11:27
layer?
A. Five minutes.
Q. How did --
A. Ten minutes.
Q. How did you drain off the acid? 11:27
A. Pump this off to spent oleum storage.
Q. Was there a faucet on the bottom of the tank
or something?
A. Valves.
Q. Uh-huh. And how was it connected to the 11:27

spent oleum storage tank?
A. By a pipeline.
Q. And was it pumped, or did it just flow by
gravity?
A. Always pumped. 11:27
Q. Was the -- were the valves operated
manually --
A. Yes.
Q. -- by the people under your supervision?
A. That's right. 11:27
Q. Laborers?
A. Yes. Chemical operator helper usually.
Q. And how did he know -- the condensator had
opaque sides, iron sides?
A. That's right. 11:28
Q. How did he know when to turn off the valve
to prevent DDT from --
A. The chemical operator himself is looking
through a sight glass at the top of the tank. And he
can see when most of the acid has been pumped off, so 11:28
he turns the pump off.
Q. Was there a color difference between the two

layers?
A. Oh, yes. The DDT is white and the acid is
red. 11:28

page 76

Deposition of Suhrer, Ferdinand
page 77

Q. What's in the acid that makes it red?
A. I don't know. It's a peculiar compound
that's found only in DDT manufacture. Normally when
sulfuric acid is diluted, or used, it darkens, it
turns black. But there's some particular dye, and I 11:28
don't know what it is.
Q. And how did the chemical operator assistant
actually go about the logistics of draining the acid
out of the condensator?
A. You shut the agitator off, and let it 11:29
settle. When settled, you turn on the pump. And the
acid by this time is on the bottom layer. Pump off
the acid. By looking through the top, you can tell
about when all the acid is gone. Shut the pump off.
And tell the chemical operator helper down below to 11:29
close the valve.
Q. Was there anything else in the spent acid,
besides spent acid, in this red dye?
A. Probably some MCBS.
Q. And what was the next step, after removing 11:29
the acid from the condensator?
A. You dissolve the mono. Dissolve the DDT
with a charge of mono with agitation.
Q. Do you know how much mono was added?
A. I don't remember. 11:30

page 77

Deposition of Suhrer, Ferdinand
page 78

Q. Why did you have to dissolve it?
A. Because you can't pump a solid. It has to
be liquid to pump. And DDT was solid, the soft beans
would not pump.
Q. How long did it take to dissolve the beans 11:30
into an MCB liquid?
A. Five, ten minutes.
Q. And then what happened to the contents of
the condensator?
A. You would pump -- and this is in the early 11:30

stages of the plant, early stages of the process. You pump the mono DDT solution to a stripper, which is a tank with steam jets in the bottom. Pump all this into a stripper, and turn on the steam.

Q. And this was pumped by pipe directly from 11:30 the condensator to the stripper?

A. Right.

Q. Can you show us on the photograph where the stripper was located?

A. Everything was in the process building. 11:31

Q. Uh-huh. How big was the stripper?

A. About the same size as the condensator. And I would guess, and make a guess of 500 gallons -- on the order of 500 gallons.

Q. What's the shape like? 11:31

A. A cylindrical -- circular cylindrical tank with a cone bottom.

Q. Is a stripper similar to a still or distillation --

A. Not a distillation column. The stripper was 11:31 almost identical in size with the condensator, except it had the steam jets in the bottom so you could bubble steam up through this mixture.

Q. And what happened when you pumped steam into the stripper? 11:32

A. The whole liquid would get hot and boil off the mono. The mono would be stripped out in the steam, accompany the steam to a condenser, where the whole thing would be condensed, where you could recover the mono. The DDT would not be stripped off 11:32 with the steam, and would stay in the stripper.

Q. Why did the two compounds act differently to the steam?

A. DDT is a solid, and mono is a liquid. Mono has a much lower boiling point. And you can strip 11:32 out the mono, the DDT would stay in the bottom.

Q. Where was the condenser for the mono steam?

A. Up above.

Q. Inside the process building?

A. Oh, yes. 11:32

Q. On the second floor or something?
A. Yes.
Q. Do you know what the volume of the condenser
was?
A. I don't know. 11:32
Q. Where did the -- what happened in the
condenser?
A. In the condenser, the condenser would
condense the vapors, the mono vapor and the water
vapor. Condense those into a little holding tank. 11:33
And the water would -- water and mono are not
mutually soluble. The mono would be on the bottom,
would form a bottom layer, and the water would be on
the top layer. You would drain off the mono for use
in the process for reuse. The water would be 11:33
discharged, would be discarded.
Q. Where did the mono go from the tanks?
A. It would go back to a mono holding tank.
Q. Is that different from the MCB holding tank?
A. I believe we had a separate mono holding 11:33
tank inside the building for this recovered mono.
Q. Was the recovered mono treated any
differently from fresh mono?
A. No.
Q. What happened to the water layer from the 11:33

condenser?
A. It was discarded to the sewer.
Q. Which sewer?
A. In those days, in the days of the stripper,
there was a sewer -- it was a ditch that came out of 11:34
the process building right about here, and ran in --
generally in this direction to a pond.
(Witness indicates).
MR. O'ROURKE: Q. Can you see the pond on
that? 11:34
A. The pond would be -- well, in those days,
the pond would have been right here -- right there.
(Witness indicates).
MR. O'ROURKE: Q. Do you know if there
was any MCB in the water layer that came out of the 11:34
condenser?
A. Probably was some residual MCB. You could

always smell MCB in the water.
Q. How long was the steam driven through the stripper, before the stripping -- 11:35
A. How long did it take to strip off the mono?
Q. Yes, sir.
A. Maybe 40 minutes.
Q. And what was left in the stripper after you shut the steam off? 11:35

A. DDT -- DDT with a certain amount of residual water on top of it.
Q. Any residual MCB?
A. No. You tested for that. You tested the vapor coming off. When there was no mono left in the 11:35 DDT, then you assumed it was ready to pour.
Q. And how did you test -- you tested inside the stripper?
A. Yes. You test by sampling the vapor into a condensing coil. When the vapor condenses, you draw 11:36 that off into a test tube. And you can sniff and see if there's any mono -- there's water, most of it's water. But there may be a little residual mono. If there is residual mono, you continue stripping.
Q. Uh-huh. 11:36
A. Because you don't want mono in the DDT, it cuts the set point.
Q. What was done after you shut off the steam?
A. You would then have a layer of molten DDT in the bottom of the stripper. And a layer on top of -- 11:36 maybe two or three feet of water on top of it. You would drain off the molten DDT into these crystallization pans for process, for cooling and chopping up for bags.
Q. How did you drain the DDT out of the 11:36

stripper?
A. Manually. You have a rubber hose there. You run the rubber hose to this pan, open the valve. And you could tell when you reach the water layer, and stop it right there. 11:37

Q. How could you tell when you reached the
water layer?
A. Usually -- well, you get water coming out of
the hose that you were draining. And you had
somebody keep a careful watch on that, because you 11:37
don't want to get a lot of water in the DDT. A
gallon or so, probably no harm, that will just
evaporate off.
Q. And how was the flow of water shut off?
A. By a valve. 11:37
Q. Was the same person on both ends of the
hose?
A. Yes. One person on the end of the hose
pouring into the pan, another person on the valve to
shut it off. 11:37
Q. How did -- did one of them yell to the
other?
A. That's exactly right.
Q. And then what would be left in the stripper
at this point? 11:38

A. Left in the stripper would be water with
traces of mono.
Q. Would there be any traces of DDT in that
water?
A. No. Unless you did not get a clear 11:38
separation. If separation was clear, like if you had
one clear layer, and another clear layer below, then
there was no DDT carried off in the water. If you
got what they call an interfacial cuff, you would
have two or three inches of a soupy mixture of water 11:38
and DDT, and that you had to send out to rework.
MR. O'ROURKE: Okay. I think we have to
change the video cassette. So we'll take a break
right there, just for however long it takes. Off the
record.
THE VIDEOGRAPHER: We're going off the
record now. The time is 11:39. This denotes the end
of Tape No. 1 in the deposition of Ferdinand Suhrer
on February -- I'm sorry, on July 30th, 1996.
(Recess taken). 11:46
MR. O'ROURKE: Q. Thank you. Before we
changed tapes, we were discussing the stripper. You
were telling me there could be two different layers
left in the stripper. What were the two layers?

A. There would always be two layers. There 11:47

would be molten DDT liquid and mono.
Q. And after the DDT was drawn off, what was left in the stripper?
A. Water.
Q. And were you saying, before we changed 11:47 tapes, there could be a cusp layer?
A. Sometimes there would be an interfacial cuff, maybe two or three inches, which was not a clear break between mono -- between DDT and water. It would be both large globules of water mixed in 11:48 with the DDT, and this would have to be drained out for rework.
Q. How was it collected when drained out?
A. I don't recall that.
Q. How often did you get the interfacial cuff? 11:48
A. Oh, perhaps a third of the time.
Q. How many condensators would be emptied into the stripper at one time?
A. Just one.
Q. What happened to the water that was left in 11:48 the stripper, after the other layers were drawn off?
A. It was drained to the sewer.
Q. Is that drained to the same pond and sewer you discussed earlier?
A. Yes. 11:49

Q. Was there any MCB left in that water layer?
A. Probably, yes.
Q. Was there any DDT left in that water layer?
A. Usually, no.
Q. Sometimes was there? 11:49
A. Well, in the case -- if there was an interfacial cuff, some of it would probably go out with the water.
Q. And that DDT would go down to the sewer, as well? 11:49
A. It's possible.
Q. Was there ever MCBS in the water layer that

went to the sewer?
A. Probably. We never tested for that, but I'm sure it was present. 11:49
MR. LYTZ: Move to strike, nonresponsive.
MR. SIMSHAUSER: Also speculation.
MR. O'ROURKE: Q. How many strippers were there in the original process?
A. I know of two. I don't know of any more. 11:50
Q. What happened to the DDT after it was drawn out of the stripper?
A. It was -- would drain by a rubber hose to a crystallization pan.
Q. How big was the pan? 11:50

A. About the size of this table.
Q. Could you estimate that?
A. Oh, I'd say probably five feet by 18 feet.
Q. And its depth?
A. Depth was about a foot. 11:50
Q. What was it made out of?
A. It was galvanized steel.
Q. Where was this pan located?
A. There were probably six or seven of those pans spotted around the floor in the process building. 11:51
Q. Were they connected -- bolted to the floor?
A. No.
Q. And what happened to the DDT after it was laid into those pans? 11:51
A. It was allowed to crystallize, to set up. Then it was chopped up by laborers. Chopped up into lumps and shoveled into burlap bags.
Q. What was the consistency or the properties of the DDT? 11:51
A. Waxy solid -- soft waxy solid.
Q. So, how long did it take to set up?
A. Probably 15 minutes.
Q. And at the end of that, was there one big cake inside the pan? 11:51

A. Yes. Normally to get a better appearance of the product, your people would rake this molten DDT, and it would give it a whiter appearance.
Q. While it was setting up?
A. While it was setting up. 11:52
Q. Who broke up the cake into lumps?
A. Laborers.
Q. Did you ever see that process?
A. Oh, yes.
Q. How big were the lumps that were created? 11:52
A. Various sizes. None -- you would not want lumps any bigger than maybe five inches by five inches.
Q. What was used to break it up?
A. Picks. 11:52
Q. What was the volume of the burlap bag?
A. The burlap bag would have a hundred pounds of DDT.
Q. And how many bags would you get out of one tray full of -- 11:52
A. Probably --
Q. -- DDT?
A. Probably -- I don't remember. But I would guess 20.
Q. And was that the process that was used to 11:53

make lump DDT the whole time you worked there?
A. That's right.
Q. Was lump DDT manufactured the whole time you worked there?
A. No. 11:53
Q. When was that discontinued?
A. While we were making lump DDT, we were also making flake DDT. And about -- I would estimate about 1955 we stopped making lump. We had a better product in chips. 11:53
Q. Was chips being made prior to 1955?
A. I don't think so, no. We didn't have a chip table before that.
Q. You also mentioned flake, what was that?
A. Flake DDT. There were -- the flaker was 11:54 three horizontal cylindrical rolls which operated in different rotations. And you would drop -- they were very close together, the rolls were, and all spinning different speeds. You would drop molten DDT between

the first two rolls. The rolls had water -- cooling water running inside the rolls. They were hollow steel shells. And DDT was set up on these rolls. It was set up on the first roll, transfer itself to the second roll, and then to the third roll. By the time it hit the third roll, it would be cool enough so a 11:54 11:54

page 89

Deposition of Suhrer, Ferdinand
page 90

knife could scrape it off the roll into flakes, like soap flakes.
Q. How fast were the cylinders turning?
A. Probably -- well, the first roll was maybe 60 RPM. The second maybe 80. And the last one maybe 90. 11:55
Q. And how long were the cylinders?
A. Five feet.
Q. And what diameter?
A. About a foot. 11:55
Q. And was the flake being manufactured when you first arrived at the plant?
A. Yes.
Q. And continued to be manufactured until you left the plant? 11:55
A. Yes.
Q. Who scraped the DDT off of the cylinders?
A. There was a knife that did this automatically, no person did it. A knife was set close to the roll. When the DDT build up got thick enough, it would peel this off into flakes. 11:55
Q. Where did the flakes go?
A. Onto a conveyer belt. And from a conveyer belt into a hopper. When it was -- hopper with a bagging machine on the bottom end of the hopper. 11:56

page 90

Deposition of Suhrer, Ferdinand
page 91

Q. Was -- the flaker and hopper, where were they located?
A. In the process building.
Q. And how were the flakes bagged up?
A. In 50-pound bags. 11:56
Q. Burlap bags?
A. No, paper bags.

Q. Were there different markets for the two different products, lump versus the flake?
A. I'm sure some customers like flake, and some like lump, some like chips. I think there was probably a price differential between those. I have no idea whether one was used for a particular source. 11:56
Q. Which one would have been cheaper?
A. The lump would have been the cheaper. 11:56
Q. How big were the flakes that came off the flaker?
A. All sizes. You could get a flake -- there was large flake, would be one inch by two inches. Most of them would be smaller than that. Very thin shavings. 11:57
Q. And were the flakes waxy or soapy?
A. No, they were fairly dry. And they did not have this waxy feel to it. The waxy DDT would tend to stick -- the lumps would tend to stick to each 11:57

other. The flake did not do that so much.
Q. So the lumps were waxy?
A. Yes.
Q. You mentioned that chips were introduced?
A. Yes. 11:57
Q. Was that part of the process change you discussed earlier?
A. No. The chips were a different finished product than the lump and the flakes.
Q. Uh-huh. 11:57
A. For chips, you dump molten DDT on a rotating table -- a circular table.
Q. How big was the table?
A. Probably diameter maybe eight feet.
Q. Flat round table like a record player? 11:58
A. Flat round table with water sprays underneath the surface to cool this off. So you drop molten DDT on the table, it would spread out. And the table would be rotating. So by the time it came around to where the knife was on the far end, the DDT 11:58 was solid and could be picked up -- could be peeled off by the knife.
Q. How thick was the layer of DDT on top of the table?
A. Probably an eighth of an inch. 11:58

Q. And when it hit the knife, what came off?
A. Peeled off onto -- the chips peeled off onto a conveyer belt, which went up to a hopper.
Q. How were the chips bagged up?
A. Paper bag. 11:58
Q. 50 pounds?
A. 50 pounds.
Q. What were the chips -- what were the chips like? What size? What shape?
A. They were maybe average about one inch by 11:59 one inch.
Q. And thin like a flake?
A. About an eighth of an inch thick.
Q. Why did the Torrance plant replace lumps with chips? 11:59
A. It was cheaper to produce lump -- produce the chips and the flake, than the lumps. Although I think they got a better price for the lumps -- for the flake and the chips, than they did lump.
Q. I didn't really understand. 11:59
A. Lump was not a real satisfactory product.
Q. Why was that?
A. It was waxy. It did not grind as well as the others did.
Q. Was the grinding done -- did Montrose do 11:59

grinding at the Torrance plant?
A. Montrose had a grinding room.
Q. Was that there when you arrived?
A. Yes.
Q. Did Montrose grind up lump DDT? 11:59
A. Yes.
Q. Did it grind up flake DDT?
A. Sometimes.
Q. And chip?
A. Sometimes. 12:00
Q. Was the grinding room in operation the entire time you worked there?
A. Yes.
Q. Can you point to the grinding room on the

photograph? 12:00
A. Let's see. This is the grinding building.
(Witness indicates).
MR. O'ROURKE: Q. And can you orally describe where it is?
A. Well, it's due west of the plant offices. 12:00
Q. Can you point to the plant offices?
A. The plant offices are here. This is the grinding room.
(Witness indicates).
MR. O'ROURKE: Q. Can you describe the 12:00

building to us, what it looks like -- describe --
A. The grinding room?
Q. Yes.
A. It was a large building.
Q. What color? 12:00
A. On the inside it was DDT color. Because the DDT powder just was on the walls, and the ceiling in the building, and the windows, and everything. It stuck to everything in there.
Q. Uh-huh. 12:01
MR. LYTZ: Move to strike, nonresponsive.
MR. O'ROURKE: Q. What color was it on the outside?
A. On the outside? I don't remember.
Q. How tall was it? 12:01
A. The building? Probably 30 feet.
Q. One story, one big room?
A. Yes.
Q. Can you describe the building orally, so that we can know where it is in the photograph, in 12:01 addition to pointing to it. What does it look like?
A. It's a large, rectangular building. It has windows along the top half. There is a large door so you could get a forklift in and out. Perhaps -- I would guess -- let's say 70 feet long, 70 feet wide, 12:02

and maybe a hundred long, and perhaps 30 feet high.
Q. What was done in the grinding room?

A. They ground DDT to triple-ground powder.
Q. What was the consistency of the powder?
A. Very fine. 12:02
Q. Like a talcum powder or salt?
A. Yes.
Q. And was there much airborne DDT inside the grinding room?
A. The walls -- inside walls are always covered with DDT dust. 12:02
Q. How thick?
A. I don't know.
Q. Were the windows covered with DDT dust?
A. Yes, I'm sure. 12:03
Q. Was the ceiling covered with DDT dust?
A. Yes.
Q. Was the floor covered with DDT dust?
A. We swept the floor.
Q. What did you do with that DDT off the floor? 12:03
A. That went into bins for rework.
Q. Rework was done on site?
A. Not in the grinding room, no. It was done over by the process building.
Q. Did you work in the grinding room? 12:03

A. No.
Q. Did you ever go in there and see what the operations were like?
A. Often.
Q. What brought you in there? 12:03
A. Well, the graveyard foreman, one of his responsibilities was inventorying the entire plant. So you had to count all the DDT. You counted the warehouse, then you counted the grinding room.
Q. And this happened on every graveyard shift? 12:03
A. Right.
Q. So you personally did that every time you worked the graveyard shift?
A. Exactly.
Q. What did you do with that information? 12:04
A. It went into a report which was submitted to the office by the next morning.
Q. And who did you submit the report to?
A. Max Sobelman.
Q. Did you write these reports up yourself? 12:04
A. No, Max wrote the reports. I sent in the

inventory results, which was incorporated into his report.
     Q. And what did you inventory? Did you inventory any final product? 12:04

     A. I inventoried mix, mono, acid, all grains of acid, rework, DDT in process, and DDT in the warehouse, DDT in the grinding room.
     Q. And this information was conveyed to Mr. Sobelman every night? 12:04
     A. Every morning he got it at 8:00.
     Q. Was there any other information you had to collect on the graveyard shift and submit to Mr. Sobelman?
     A. The incoming shipments of mix, and mono, and 12:04 acid, and caustic.
     Q. What was the process used in the grinding room?
     A. They would grind DDT into fine powder.
     Q. Using what kind of equipment? 12:05
     A. Using a hammer mill. I think there were two hammer mills.
     Q. How big is a hammer mill?
     A. Oh, it's -- maybe ten feet by ten feet, and 15 feet high. 12:05
     Q. How was the DDT powder packaged?
     A. Paper bags.
     Q. 50 pounds?
     A. Hundred pounds.
     Q. Hundred-pound bags. Did Montrose have any 12:05

final products -- you mentioned so far chips, flakes, lump and powder?
     A. Yes.
     Q. Were there any others?
     A. No. 12:06
     Q. Did any DDT dust come out of the grinding room -- out of the grinding room building while you worked there?
          MR. SIMSHAUSER: Objection, you're starting

to lead. Can you start phrasing your questions in a non-leading manner? 12:06

MR. O'ROURKE: Q. During the period of time you were there, did you ever see DDT dust coming out of the grinding room building?

MR. SIMSHAUSER: Objection, leading. 12:06

THE WITNESS: Occasionally.

MR. O'ROURKE: Q. Other than in bags?

A. When we had a strong wind blowing, you could see dust coming up.

Q. Where did it go? 12:06

A. In the air.

Q. Do you remember which way?

A. I'm sure it settled on the property.

Q. Uh-huh. Do you -- did any blow off of the property that you remember? 12:06

A. That's possible.

MR. LYTZ: Move to strike, speculation.

MR. O'ROURKE: Q. Now is that the end of the process to manufacture DDT that was used prior to 1955? Have we discussed all the steps? 12:07

A. I think we have, yes.

MR. O'ROURKE: Uh-huh. Well, it's just after noon, maybe we should take lunch. Does that sound agreeable?

THE VIDEOGRAPHER: We're going off the record now at 12:07. 12:07

(Whereupon, at 12:07 p.m., a lunch recess was taken).

---o0o---

AFTERNOON SESSION 1:15 p.m.
THE VIDEOGRAPHER: Back on the record now
at 1:15.
MR. O'ROURKE: Can you zoom so we have the
photograph in as well? 01:16
THE VIDEOGRAPHER: Oh.
MR. O'ROURKE: Thank you.
Q. Mr. Suhrer, I'm going to ask you to take a
look at the photograph and identify everything on
there, basically. Why don't we start in the upper 01:16
left-hand corner. Do you know what that black, long
flat building is at the top?
A. This building I don't recognize. I think it
was Montrose's carpenter shop, I believe.
Q. Can you first draw a line and show me which 01:16
part of this photograph is the Montrose property?
A. Let's see. It started from over here, down
here. Actually, this is old Stauffer buildings. I
don't know if it was Montrose properties or not. But
Montrose did not have any business over here. This 01:17
is a carpenter shop right here. This is a
maintenance yard, maintenance building. The old
cooling tower here.
(Witness indicates).
MR. O'ROURKE: Q. What was -- 01:17

A. No, I'm mistaken there. This would be the
maintenance building right here. This is the cooling
tower. These were some tanks, I know not what of. I
don't remember.
(Witness indicates). 01:17
MR. O'ROURKE: Q. Before you go any
further to the right --
A. Yes.
Q. So which of those two buildings was the
maintenance building? 01:17
A. This is the maintenance building right here.
(Witness indicates).
THE WITNESS: This one I don't recognize.
This is the maintenance. This was a maintenance yard
for storage of all sorts of stuff. I think this is 01:18
the carpenter shop right here.

(Witness indicates).
MR. O'ROURKE: Q. Okay.
A. Here is our steam boiler. The brine tank. And probably these were ammonia compressors here. 01:18
The boiler. The process building here. Mix and mono tankage. Acid tankage. Kwansit hut (phonetic). The warehouse. Grinding room. Offices. Parking lot. And this would have been, I believe that's the Stauffer BHC plant. 01:18

(Witness indicates).
MR. O'ROURKE: Q. Which buildings in that corner were the BHC plant?
A. Right in here, probably. I never went over there, so I'm not sure. That's where Stauffer was. 01:18
(Witness indicates).
MR. O'ROURKE: Q. What's the collection of machinery next to that?
A. This here?
Q. I'll point. 01:19
A. This? I don't know what this was here.
Q. Do you know if that was part of the Stauffer BHC plant?
A. I'm pretty sure it wasn't part of Montrose. I didn't have anything to supervise over here, so it 01:19
would not have been Montrose.
Q. What's the next thing over to the left?
A. This stuff here?
Q. Yes.
A. This is -- I'm not really sure what that 01:19
was. A pond would have been right in here someplace. I don't know what this is right here.
(Witness indicates).
MR. O'ROURKE: Q. Okay. And what's the next set? 01:19

A. This?
Q. In between that and --
A. Right here?
Q. Yes.

A. This I don't know. This is probably 01:19
Stauffer's property, Stauffer's stuff. This is --
Stauffer had a small acid plant here, sulfuric acid
plant, before I got there. And it was shut down
somewhat before I was there. This was what we call
the burner building. I don't know about this other 01:20
stuff.
Q. The burner building was not in use when you
arrived?
A. No, they were not making sulfuric acid when
I arrived. 01:20
Q. So were all of those buildings in the lower
left --
A. All of this is Stauffer.
(Witness indicates).
MR. O'ROURKE: Q. And none of that was 01:20
being used when you arrived?
A. No.
Q. How long did the BHC plant operate while you
were there?
A. I don't know, a couple of years, maybe. 01:20

That's a guess. I had nothing to do with the
Stauffer plant. I walked by it, that was all.
Q. Is that -- I'm going to take this off and
point to another couple of things and ask you what
they are. What's this right along here, this 01:20
straight line? Is that a road or something?
A. It looks like a roadway, but I don't know
what it is. This is the kwansit hut right here.
(Witness indicates).
MR. O'ROURKE: Q. What's this right here 01:21
next to the kwansit hut?
A. This was a structure that eventually became
the acid recovery plant. But at the time of this, I
don't know what that would have been.
Q. What's right here? 01:21
A. Storage area for something. This was -- I
believe this is where we had our sugar lime pile.
(Reporter asks witness to repeat).
THE WITNESS: Sugar lime pile, it's a waste
product. 01:21
MR. O'ROURKE: Q. And what's this
rectangular --
A. Let's see, I don't know. I don't recognize

it.
Q. And what's in this area right in here? 01:21

page 105

Deposition of Suhrer, Ferdinand
page 106

A. Here we had some acid tankage. The rework area was right about here. These look like tanks, I don't remember what for. There was a dump -- we had a dump for wastepaper and waste objects, it should have been right about here. Cooling tower. 01:22
(Witness indicates).
MR. O'ROURKE: Q. What was the floor made of inside the process area -- process building?
A. Cement.
Q. Was there a drainage system in the process building? 01:22
A. There were drainage ditches running through the process building.
Q. Were they dug out of cement as well?
A. I think so, yes. 01:22
Q. What was the depth of those drainage trenches?
A. Four inches, maybe.
Q. And how wide were they?
A. About six inches. 01:23
Q. Where were they located in the building?
A. Running the length of the building -- excuse me. They ran the length of the building north and south, and east and west.
Q. Where did the drainage trenches go to? 01:23

page 106

Deposition of Suhrer, Ferdinand
page 107

A. Initially they went to the pond which would have been just about here.
(Witness indicates).
MR. O'ROURKE: Q. How did they get from the process building to the pond? 01:23
A. There was a drainage ditch that ran through this way. There was some sump tanks, maybe five feet wide, five feet deep, and maybe five feet in diameter, that were agitated. And drainage water came into those tanks, where we hoped to neutralize them with sugar lime. 01:24

Q. What were the tanks made out of?
A. I don't know. Probably steel.
Q. Were they above ground or --
A. Below ground. 01:24
Q. The surface of the tank surface with the earth?
A. Exactly.
Q. And what was the trench made out of that connected the process building to the tanks? 01:24
A. I believe it was brick lined.
Q. How big was it?
A. Oh, six inches wide, maybe a foot deep.
Q. Was it brick lined the entire time you worked at the plant? 01:24

A. I don't think so. I think it was concrete at first.
Q. And do you know when it was changed to brick?
A. No. 01:24
Q. And what flowed into those sump tanks?
A. Into the sump tanks came any surface run off from this building. Any -- we were constantly washing down things inside. And this washed down water came down here. The drainage water from the 01:25 strippers came down this thing here to here.
(Witness indicates).
THE WITNESS: I don't know where the drainage from underneath the condensators went to. This would have been highly acidic. A lot of acid 01:25 went down from under the condensators. Where that went, I don't know.
MR. O'ROURKE: Q. And it was all liquids that flowed down these trenches to the sump tanks?
A. Yes. 01:25
Q. Did those liquids contain any DDT?
A. Occasionally you would have some DDT flecks, powders, beans, floating in the water.
Q. Did it contain any MCB?
A. Probably at some times, yes. 01:25

Q. And did the liquids that float to the sump tanks contain any acid?
A. Occasionally, yes. This is why we had to neutralize it with sugar lime.
Q. Did those waste waters contain any MCBS? 01:25
A. Probably.
Q. Could you describe the process where you neutralized the waste?
A. Okay. There were two subsurface tanks right here. Agitated. The water -- the drainage water -- 01:26 the acidic water from the plant flowed into those. The agitators were always turning. And we had a man standing by with a hose by this lime pile, trying to flush down sugar lime into these two tanks to neutralize the acid. 01:26
Q. What is sugar lime?
A. It's a waste product from the sugar refineries. It decolorizes the sugar, so it comes out white. And the lime itself is sort of a tan color. 01:26
Q. And it's -- is it --
A. Calcium carbonate, basically -- mostly.
Q. It's an alkaline or a basic substance?
A. It's to neutralize the acid.
Q. How much sugar lime was put into the -- 01:27

pardon me, into these sump tanks?
A. As much as we could get in there. We would flush it out with a hose. Hose this lime down, so it would spill over into the tanks, and is agitated with the acidic water. 01:27
Q. Was the lime in a pile?
A. The lime was in a big pile here. And we would flush it out. Just point the hose at the pile and wash it down.
Q. Was the pile just on the earth next to the 01:27 --
A. Right, exactly.
Q. And where on the map was -- was the pile of sugar lime?
A. The lime pile would have been right about 01:27 here -- right about here. Because here were your underground tanks. And this part in here would have been the pond.

(Witness indicates).
MR. O'ROURKE: Q. Okay. And what happened to the liquids that had been neutralized that were in the sump tanks? 01:28
A. They overflowed the sump tanks. And they were constantly overflowing. They overflowed to the pond. And the solids, if any, would settle out in 01:28

the pond, and the liquid would drain off to the sewer.
Q. What was the -- how big was the pond?
A. Probably a hundred feet by a hundred feet.
Q. And what was it made out of? 01:28
A. Earth.
Q. Was just a hole in the ground?
A. Just a hole scooped out of the ground.
Q. And how come the sump tanks were overflowing? 01:28
A. They were designed to overflow. You put drainage water into one tank, and it overflows to the second tank, which overflows to the pump. All of them agitated.
Q. Do you know how many gallons per day would go through the sump tanks? 01:29
A. I have no idea.
Q. And what happened to the waste water once it was in the settling pond?
A. It would overflow to the sewer. 01:29
Q. Do you know where the sewer connection was?
A. The sewer connection was, oh, somewhere in here. And it ran down to the corner of the burner building, and it went out this way. And I think clear to Westborough, which is way over here some 01:29

place.
(Witness indicates).
MR. O'ROURKE: Q. Was there any process to neutralize or remove DDT before it was overflowed from the sump tanks into the settling pond? 01:29
MR. LYTZ: Objection, assumes facts not in

evidence.
MR. SIMSHAUSER: Leading.
MR. O'ROURKE: Q. You stated earlier that there was DDT in the water that washed down to the --
A. Small amounts, yes.
Q. Was there anything to remove that DDT?
A. No.
Q. Did the DDT wash over into the settling pond? 01:30
A. I'm sure it did, yes.
Q. Was there anything done to remove MCB from the waste water that went into the sump tanks?
A. No.
Q. Did that also -- 01:30
A. We're talking very tiny amounts. And nothing was done to recover either mono or tiny amounts of DDT.
Q. Was there anything done to recover or remove MCBS from that waste water in the sump tank? 01:30

A. No.
Q. Did any areas of the plant, besides the process building, empty down to the sump tanks at the settling pond?
A. The -- this acid storage area would have to be connected to that. I don't know where the connection was, or where the lines ran. But it would have to be connected to it. 01:31
Q. Why do you say that?
A. Because if you have wash down in this area, it has to go somewhere. And as far as I know, this was the only drainage from the plant. 01:31
Q. So wash down water from the entire plant --
A. Wash down water, acid spills, anything like that, rain run off, would have to go down there. 01:31
Q. What equipment did you wash down inside the central process area?
A. Well, you're always washing down pumps and areas around pumps. Tanks got washed down.
Q. Were the condensators washed out after each batch? 01:31
A. No.
Q. Was the stripper washed out after each batch?
A. No, there's no need for that. No need for 01:32

page 113

Deposition of Suhrer, Ferdinand
page 114

either one of them to be washed out.
Q. Was there any sampling done of the water
that went down to the sumps?
A. We had a PH meter on the outflow which
registered the PH of the water. PH-7, of course, is 01:32
neutral. If it was anywhere below five, it was
considered acceptable. So we tried to neutralize it
to at least a five PH.
Q. You said anywhere -- if it was anywhere
below five it was acceptable? 01:32
A. Anywhere -- number above five would be
acceptable.
Q. And you just put in sugar lime until that
number was --
A. Right. There was a PH meter on this 01:32
overflow from the tanks. And there was an alarm
attached to this PH meter. An alarm rang a bell in
the foreman's office whenever the acid got above, oh,
got more acidic than a PH-5.
Q. And that would be your office that had the 01:33
bell in it?
A. Right.
Q. When the bell rang, what did you do?
A. We'd run out there and light a fire under
the man who was hosing around the sugar lime. 01:33

page 114

Deposition of Suhrer, Ferdinand
page 115

Q. Was there any sampling done of the waste
waters in the sump tanks? Sampling for DDT?
A. No.
Q. For MCB?
A. No. 01:33
Q. And inside the process area you said you
were constantly washing down. How often were you
washing down?
A. Oh, we would probably -- every time a pump
needed to be packed, there would be a certain amount 01:34
of spillage of acid and DDT. And pumps were packed
almost every day. And each time you had the
spillage, you'd hose it down. And probably be, oh,

15, 20 times a day you'd have to hose down the pump, or hose down something around the process building. 01:34
Q. How much liquid would come out of the pump when you were repacking it?
A. About a gallon.
Q. How much DDT would be in there?
A. Maybe a cup full. 01:34
Q. How much MCB?
A. I don't know. Probably none, or very, very trace amounts.
Q. And each time that happened, you would wash down the liquids into the trenches? 01:35

A. That's right.
Q. And then those liquids would go down to the sump tanks?
A. They would go into the waste treatment tanks, and then into the pond. 01:35
Q. And the waste treatment tanks, those are the same tanks we've been discussing?
A. Yeah. The agitated tanks where you hose the sugar lime down in.
Q. Who repaired the pumps? 01:35
A. Maintenance crew. The pumps were constantly needing packing. And there was one man assigned to do nothing but pack pumps. And he kept busy an eight-hour shift.
Q. So did he work for John Kalock then? 01:35
A. Yes.
Q. Were the lines redundant so that there were parallel lines if a pump broke down?
A. You mean did we have auxiliary pumps to take over -- I don't understand your question. 01:36
Q. If a pump broke down, did the whole process have to stop, or was there an alternative?
A. If the pump broke down, that particular condensator could not be pumped out. There's a pump for each condensator and a pump for each acid tank. 01:36

And if the pump breaks down, or is being packed, then

you can't use it, can't use that tank until it's all
done.
Q. Did you, or people that worked for you at
the plant, ever clean out the trenches that connected 01:36
the process area to the waste water pond and waste
water sump tanks?
A. Occasionally we did that.
Q. How often?
A. We did it at a time when things were slow in 01:37
the plant. Probably you did three, or four, or five
times a week.
Q. And how did you clean them out?
A. Shovel.
Q. What kind of build up was in these trenches? 01:37
A. You would have -- speaking of DDT, there
would be, oh, DDT maybe an eighth of an inch thick,
beans, not clear down all over the ditch, but in
parts of it. And you'd have a man with a shovel,
shoveling this stuff out. 01:37
Q. Where did he bring it?
A. Rework.
Q. What happened to rain water when it rained
on the plant?
A. It ran off in the ditches. 01:38

page 117

Deposition of Suhrer, Ferdinand
page 118

Q. And did it go down to the settling pond as
well?
A. Yes.
Q. Did the rain water wash DDT into the
settling pond? 01:38
A. Probably.
MR. LYTZ: Move to strike.
THE WITNESS: This whole waste
neutralization thing was a real pain. Because this
bell in the foreman's office was constantly ringing. 01:38
At least every 15, 30 minutes you'd have the damn
thing going off, and you'd have to run out there and
see what's happened to the guy that was supposed to
be hosing lime down, and somehow repair the
situation. 01:39
MR. O'ROURKE: Q. You said earlier that
you saw DDT come out of the grinding room and land on
the property. Did the rain water push that DDT
around anywhere?
A. Well, this would have been -- since the 01:39

prevailing wind here comes from the west --
THE VIDEOGRAPHER: I'm sorry, get that again, please?
MR. KUSHNER: Repeat your response again, sir.

THE WITNESS: Okay. The prevailing wind generally was from the west. And this would blow DDT dust into this area right here, and further over into the parking lot. I don't know where the run off from this area went. It could have gone over toward the 01:39 railroad tracks, or could have gone back in the back, I have no idea.
MR. O'ROURKE: Q. Earlier in the day we were discussing, in the process, the stripper. And you said that there was a water layer taken off to a 01:40 condenser, to condense MCB?
A. Out of the stripper. When you stop stripping, there was a layer of DDT on the bottom and a layer of water on the top. And sometimes this interfacial cuff in between. 01:40
Q. And what happened to the water that was on the top layer?
A. It would be drained out, and eventually come down here and wind up in the pond.
Q. Into that same pond we were just discussing? 01:40
A. Right.
Q. Now, what happened to the water that was in the MCB condenser?
A. This would also be drained, same place.
MR. KUSHNER: Can I ask the videographer 01:41

whether or not we're picking up the speech in the other room? May I suggest a five-minute break, and we'll walk around there to see.
THE VIDEOGRAPHER: We're going off the record now at 1:41. 01:41
(Recess taken).
THE VIDEOGRAPHER: We're back on the record now at 1:45.

MR. O'ROURKE: Q. Mr. Suhrer, a couple of
times today you mentioned rework. Where was the 01:45
rework area in the photograph?
A. Rework area is right off the -- let's see.
That would be on the southwest corner of the process
building right about here.
(Witness indicates). 01:45
MR. O'ROURKE: Q. What materials were
sent to the rework area?
A. Dirty DDT, off spec DDT.
Q. Those are two different things?
A. Really it's the same thing. Dirty DDT was 01:45
off spec. If it was off spec, it did not have to be
dirty.
Q. By "off spec," do you mean it was
manufactured and did not meet --
A. Did not meet the set point, generally. 01:46

Q. Where did dirty DDT come from?
A. Off the floor.
Q. Off the floor in the process building?
A. Yes.
Q. Off the floor inside? 01:46
A. Off the floor in the grinding room. Off the
floor in the process building. Anywhere you could
find DDT that had dirt in it, or anything other than
pure acceptable DDT, was called rework, and was
reworked. 01:46
Q. Whose job was it to sweep that up?
A. Any laborer that you could corral to do it.
Q. Uh-huh. Do you know how much DDT was sent
to the rework process area, say per day?
A. Oh, maybe perhaps 500 pounds. Some days you 01:47
had a lot -- if you were making off spec product in
the plant, you had tons of it. If things were going
smoothly, and you did not have much spillage, there
would be very little.
Q. And what happened in the rework area? 01:47
A. Well initially, in rework you would dump the
rework into a tank, which you'd previously fill with
MCB, agitate this. The DDT would go in the solution
in the MCB. And the dirt, and the straw, and
whatever else, paper, whatever else was contaminating 01:47

the DDT, would just float in the solution. So you'd
filter that. There was a plate and frame filter that
you filter this off. Hopefully you would catch all
the dirt and have pure DDT solution going back into
the tank. That rarely happened, it was almost always 01:48
dirty.
Q. When it finally went back to the tank?
A. When it went back to the tank, it was almost
always off color. We tried using activated carbon to
pull out the color, and that worked sometimes. But 01:48
you really could not depend on a good product coming
out of rework.
Q. Was the MCB and the dirty DDT mixture pumped
through the filter or poured through a filter?
A. It was pumped through the filter. 01:48
Q. Did the filter leak?
A. Yes, it did.
Q. Did the pump leak?
A. Pumps always leak.
Q. Did all the pumps in the process area leak 01:48
in the process building?
A. Yeah. After they've just been packed, you
will get very little leakage, maybe a drip. But
after a few hours of running time, you would get
drips coming out. 01:49

Q. What does it mean to repack a pump?
A. You have to pump -- the motor goes on one
end of the shaft. And on the other end of the shaft
is a propeller, inside of a pump enclosure. And
where the shaft goes into the pump enclosure, there's 01:49
a metal to metal contact, and always space. So what
you do is to pack asbestos and linen and graphite
mixtures, pack that in there to try to keep this pump
from leaking out, keep it from coming out past the
shaft. And with a good packing job, you could 01:49
probably expect several hours of absolute drip free.
But beyond that, you could expect drips to come out.
When drips got to be significant, then you call for a
guy to pack the pump.
Q. What happened to the substances that leaked 01:50

out of the filter and pump in the rework area?
A. Went into the ditch.
Q. Where did the ditch go?
A. Went out to the sewer, and out to the two subsurface tanks, and into the pond. 01:50
Q. So it went to the tanks first, then pond, then the sewer?
A. Right.
Q. Was the ditch between the rework area and tanks, was that lined or unlined? 01:50

A. I believe it was unlined. I think it was concrete -- excuse me. I believe it was a concrete ditch.
Q. Was the rework area outside of the process building? 01:50
A. Yes.
Q. And is it on the dirt or was there a floor there?
A. There was a -- I don't remember if there was a concrete pad underneath that or not. It probably 01:50 was right on the dirt. But you understand the rework tank was set up off ground. It was up in the air. The bottom was maybe four or five feet off the ground.
Q. How big was that tank? 01:51
A. Maybe ten feet high, and five, six foot in diameter.
Q. And were there two tanks, one that did the dissolving and one that was filtered into the second?
A. No. 01:51
Q. Ran into the filter and back into the same tank?
A. Yes.
Q. How long did it take to filter one tank full of rework? 01:51

A. If you got a good papering job on the filter, you could probably get it done in two or three hours. If you got a bad job, then you have

leaks and have to stop and do it over again. And
this whole rework system was a real headache.   01:51
     Q. How so?
     A. It never worked right. It never gave you a
good product DDT. It leaked too much mono. It was
just a bad system.
     Q. And the mono that leaked out, did that go   01:52
down into the tanks in the settling pond?
     A. Right.
     Q. Do you know how much mono was leaking out of
the rework area?
     A. It would vary so much from day to day. An   01:52
average might be, oh, four or five gallons, five
gallons of mono. But it would vary tremendously.
     Q. Sometimes more than that?
     A. Much more than that. Sometimes very
little.   01:52
     Q. When you refer to mono, are you referring to
MCB?
     A. MCB, mono, monochloral benzene, all the same
product.
     Q. Was there any chloral present in the rework   01:52

page 125

Deposition of Suhrer, Ferdinand
page 126

area?
     A. No. The only chloral present would be in
the mix storage tank, and in the condensators at the
start. The chloral would then be made into DDT, and
you'd see no more chloral the rest of the way.   01:53
     Q. Did the Torrance plant ever change the
rework area process?
     A. Yes, it did.
     Q. When was that?
     A. This was -- I can tell you this was about   01:53
'55, because it was my design that did this. We
started dissolving, without going through this filter
press, we would dissolve rework DDT in the rework
tank, dissolve it in mono.
     Q. Is that the same way it used to be done?   01:53
     A. Same way it used to be done. But instead of
filtering, we would take this and pump it directly to
the condensators as mono makeup. Remember you had to
add extra mono to the mix. You would make that up
with rework. And there was concern at first that   01:54
this dark solution of dirty DDT would discolor the
DDT, the final product. And this did not happen to

any appreciable extent. So from then on, we did it
that way. It was a much better process.
     Q. And so the filter step was entirely   01:54

eliminated?
     A. Right.
     Q. And why did you -- you said it was your idea
to change the rework area?
     A. Yes.   01:54
     Q. Why did you think it was good to change the
rework process?
     A. Just seemed to be a good idea. I suggested
it. We were all engineers in the plant. And we were
expected to make suggestions for process   01:54
improvement. This was one.
     Q. Uh-huh. And what made you focus on the
rework area as a place to change?
     A. Because it was always a headache. It was
never, as far as I could see, never a successful way 01:55
to deal with the problem.
     Q. And how much of the reworked MCB could you
charge a condensator with?
     A. You could -- it depended on the strength of
the mix, because that regulated how much extra mono   01:55
you had to put in. If the mix was very rich in
chloral, you would have to add more mono to the
charge. And the make up mono could be from rework or
could be from storage tanks.
     Q. Or a combination?   01:55

     A. Or a combination.
     Q. Were there any batches where you used
entirely rework MCB as the make up MCB?
     A. Yes, I'm sure we did. Particularly if the
rework were a lighter color, we could do that.   01:56
     Q. Uh-huh.
     A. If we were running short of mono in the
storage tank, we would certainly do that.
     Q. Does the color of the DDT product affect its
ability to -- insecticidal properties?   01:56

A. No. But it's part of the spec. DDT is supposed to be snow white. That's the description: Snow white. So if it's an off white, it's, to some extent, an off spec product.
Q. If it came out discolored, would you send it to rework? 01:56
A. If it came out off color, we would send it to rework.
Q. Do you know why the color was part of the spec if it didn't affect -- 01:56
A. I have no idea.
Q. So you reworked the rework process. Was the new process used the entire rest of the time you were at the plant?
A. Yes. 01:57

Q. Do you know if it continued to be used after you left?
A. I don't know. I would assume so.
Q. Do you remember if it was still in use that -- at the time you went back for the strike? 01:57
A. It was in use then.
Q. Was the plant ever shut down for general cleaning or maintenance?
A. Yes. I think we had a shut down every year, generally in the summer time. 01:57
Q. How long does that last?
A. I believe it would go three or four days.
Q. And --
A. We would -- particularly any time we had to do work on the boiler, this meant the whole plant had 01:58 to come down.
Q. Because there was no steam?
A. Because there was no steam. For almost anything else, we could maybe run part time, or do a halfway job of running the plant. Maybe half speed. 01:58 When the boiler came down, the whole plant came down.
Q. Did the boiler ever break down or --
A. It had happened, yes.
Q. Uh-huh. And how long --
A. I remember one time the boiler broke down 01:58

because they -- in the oil storage tank, it was an oil-fired boiler. When the oil got fairly low, they drew it down too low, and drew down a layer of water that was on top of the oil. And this plugged up the burners, and the whole boiler came down immediately. 01:58
Q. Did that shut down the whole plant?
A. Yeah.
Q. For how long?
A. I don't remember that.
Q. Was the boiler repaired every summer when the plant shut down? Or maintained? 01:59
A. I don't know what the schedule of maintenance on the boiler was.
Q. During -- was the summer shut down the same time every year? 01:59
A. Roughly yes, I believe so.
Q. Were you there each time that it happened?
A. Each time, yeah.
Q. For the ten years you worked there?
A. We -- the men were encouraged to take vacation during the shut down time. 01:59
Q. Did you do that?
A. No, I didn't.
Q. Uh-huh. What did you do during shut down periods? 02:00

A. We -- there was always work to do. We came out, and there was plant clean up that we were doing. And when you shut down for any particular reason, such as a boiler, you try to bring everything else up to tip-top shape. So you were constantly 02:00 shutting down this unit or that unit. One of the things that I had to do, was to go, before they worked on any tank, it had to be cleaned out of any gas. So I had to certify that the tank was clean and okay for welding, or okay for a man to go inside. 02:00 Agitators had to be rewelded from time to time. And it was something that the foreman had to do constantly, was check on these tanks to make sure that it was safe to go in the tanks. Safety was a very large part of our job. 02:01
Q. Who cleaned out the tanks before somebody could go in them?

A. Maintenance crew. Well, the production crew
would pump the tank out and probably set hoses.
Maintenance people would take the flanges off and get 02:01
everything ready for the clean up. It was in the
hands of maintenance, then.
Q. When the production people emptied the tank
out, would they hose the tank out?
A. Probably not. They would wait. They would 02:01

have to wait until the tank bottom was -- bottom
flanges were opened up, so then the drain water could
drain. If we were asked to do so, we would certainly
hose the tanks down.
Q. Where did the drain water drain from the 02:01
bottom of the tank?
A. Down to the ditch.
Q. And then down into the settling pump?
A. Exactly.
Q. What tanks were cleaned out like that? 02:02
A. All of them.
Q. The mono tank?
A. Yes.
Q. Were the condensators cleaned out like that?
A. Oh, yes. The mix and mono tank rarely 02:02
needed clean out. But occasionally you needed
welding done, which involves the same thing, it has
to be emptied and cleaned out.
Q. How often does that happen?
A. Not often, once every two or three years. 02:02
Q. Was there any maintenance done to keep the
pipes clean and valves clean?
A. When you're pumping a liquid, which is
partially solid, like liquid acid with DDT floating
solid -- solid DDT floating in it, you're constantly 02:03

getting plugs. So you have to stop and clean them
out. Have to stop and clean out the plug before you
can pump the line again.
Q. What is a plug?
A. A solid chunk of DDT plugging up a line. 02:03

DDT collects in an elbow bend. It would collect
there enough, so eventually it might even completely
plug the line so it could not be pumped. Then you
have to get in -- first thing you try is to put in a
settling torch on the outside of the line to try to 02:03
warm it up, to melt the DDT so you can melt the plug
and pump it out. If that does not work, which was
about half the time, you had to break flanges and
break lines, hose out the DDT and the acid onto the
ground, and bring it back up again. 02:03

Q. And where did the acid and the DDT go?

A. In the ditch.

Q. Down to the settling pond?

A. Yes.

Q. How often did the lines plug up? 02:04

A. Oh, if you got through a shift without a
plugged line, you were pretty lucky.

Q. And half the time the lines plugged you had
to empty DDT and the acid onto the ground?

A. Well, you're talking emptying what's in a 02:04

line, which might be maybe ten gallons. You break a
section off this line, and whatever was in there, you
drain out.

Q. How did you do that? You shut off the
valves -- 02:04

A. Shut the valves on either side of the plug
and break the flange. You unscrew the flange,
they're all screwed couplings there. Unscrew the
flange, wash it out, and then put it back up again
and off you go. 02:04

Q. You said when the boiler went down there was
no steam, and so the whole plant would have to shut
down?

A. Right.

Q. Why was steam so important? 02:05

A. Well the steam is important for this
reason: All of the lines that carried molten DDT
had to be kept hot, otherwise the DDT sets up in the
line and you get a horrendous plug there. So most of
the lines were either traced, meaning you had copper 02:05
tube with steam inside the copper tube that you
wrapped around the pipe that you wanted to trace.
And then you insulated over that. The steam tracing
would keep the line hot enough so DDT would not

freeze up inside. If it were a real serious line,   02:05

you would jack at the line, have one pipe inside
another. And the outer pipe would have steam
pressure in it. The inner pipe would be carrying
molten DDT. But if you did not keep DDT very hot,
you had terrific plugs.   02:06
     Q. What happened -- you told me one time the
boiler broke down?
     A. Yes.
     Q. And there was no steam?
     A. Yes.   02:06
     Q. What happened then, did the DDT set up in
the lines?
     A. As soon as the boiler broke down, you
immediately start draining lines. And you may have
some plugs, but you hope to minimize that.   02:06
     Q. Do you remember whether there were a lot of
plugs that night or that day?
     A. I don't remember, no.
     Q. Now we discussed the settling pond, you said
that was connected to the sewer?   02:07
     A. Yes.
     Q. Did that sewer connection ever back up?
     A. This had happened, yes.
     Q. When?
     A. I don't remember just when. But I know it   02:07

happened. When we were flushing sugar lime down
there, occasionally you would get too much sugar
lime. The sewer line would become partially
restricted, and the flow would be slow. And so the
pond would start to rise. Then you had a real   02:08
problem. You tried to clear the plug as best you
could. You could drop an air pipe down -- drop a
pipe with an air hose on one end of it down in there
and hope you could bubble the plug loose. Sometimes
that worked.   02:08
     Q. How often did it back up like that?
     A. Not too often. Perhaps once a week.

Q. And did it -- did the settling pond overflow because of the back ups?
A. I don't recall that it ever did. 02:08
Q. Did the holding pond ever overflow, in your recollection?
MR. LYTZ: Objection, asked and answered.
MR. KUSHNER: That question was not asked and answered.
THE WITNESS: I don't recall that it ever did.
MR. O'ROURKE: We've been going an hour since lunch. Should we take a break now?
THE VIDEOGRAPHER: We're going off the 02:09

record now at 2:09.
(Recess taken).
THE VIDEOGRAPHER: We're going back on the record now at 2:27.
MR. O'ROURKE: Q. Mr. Suhrer, a few 02:27
minutes ago you mentioned that one time the boiler broke down and you said that process people had to drain all the lines. Where did you drain the lines to?
A. Well, when you drain the lines, the DDT you 02:27
recover. You drain all the DDT you can into pans or buckets, someplace where it can be recovered. The acid is probably going to be fairly diluted, that goes to the floor into the drains.
Q. And then down into the settling pond? 02:28
A. Yes. Any DDT that's in the form of beans is probably going to go on the floor, as well. Those you try to shovel up. And you shovel up, oh, half of it, maybe more. But the rest of it gets washed down. 02:28
Q. And did the same thing happen during the annual summer shut down on the boiler?
A. Where you have a program shut down, then you prepare everything. You drain the lines first. You drain all of these acid lines and blow them clean, 02:28

before you pour the shut down in. So you're prepared
for that. It's not an emergency thing. So you get
very little spillage out of a planned shut down.
     Q. When the lines clogged, you said half the
time you'd have to break them open?   02:29
     A. Yes.
     Q. What were they cleaned out with?
     A. Steam. You blow steam through the line to
melt the DDT plug.
     Q. Did you ever use MCB to dissolve DDT out of   02:29
a line?
     A. I don't believe so, but it's an idea. Steam
usually works faster.
     Q. Is DDT soluble in water?
     A. No.   02:29
     Q. How come the steam dissolves the DDT?
     A. Steam does not dissolve it, it melts it,
makes it liquid.
     Q. So it's really the heat from the steam --
     A. Exactly.   02:29
     Q. -- cleaning out the lines. You mentioned
earlier that there was a process change in the way
the DDT was constructed. You said that around 1954
or 1955 there was a change in the process used?
     A. Yes, about the way we finished the DDT. The 02:30

condensators, that part stayed the same. But about
in the middle 50's, instead of going to dissolve --
being dissolved in MCB and stripped, the DDT slurry,
the acid and the beans were pumped to a large
agitated tank, which was called a postreactor,   02:31
because some final residual DDT was formed in there.
And from there it was pumped to a holding tank which
fed the Oliver filter.
     Q. Now, so the entire reaction of the
condensator was the same?   02:31
     A. Yes.
     Q. Using the same ingredients --
     A. Yes.
     Q. -- for the same tanks?
     A. Yes.   02:31
     Q. And did the reaction time in the condensator
change, or did it still last about --
     A. That depended on the demand for DDT.
     Q. The same as it used to before?

A. Exactly. 02:31
Q. So there was no attempt -- the postreactor wasn't a way to shorten the time of the condensator?
A. The postreactor could have been used to shorten the time. If you needed production, you did it that way, you shortened the time in the 02:31

condensator. If you didn't need production so much, if the orders were coming in fairly slowly, then you slowed it down in the condensators.
Q. How many postreactors were there?
A. One -- one large tank. 02:32
Q. All right. So that's not going to be in this photograph, right?
A. No, it's not going to be there. If it were there, it would be right about here.
(Witness indicates). 02:32
MR. O'ROURKE: Q. If this were a picture from the late 50's, there would be a tank over there?
A. Yeah.
Q. How big was the postreactor?
A. It was a large tank. Maybe 16 feet diameter. It was a horizontal tank. 16 feet diameter, maybe 50 feet long. 02:32
Q. And how many condensators emptied into the postreactor?
A. All of them. 02:32
Q. How did the slurry get from condensator to the postreactor?
A. Through a pump line.
Q. And the entire contents of the condensator was pumped into the postreactor? 02:33

A. Yes.
Q. There was no separation at all?
A. No separation at all.
Q. And what was the purpose of the postreactor?
A. Postreactor was a place where you could store slurry, which was one purpose. Another purpose was to finish off the reaction, to get higher yields. 02:33

If you let the reaction, give it an extra few hours, you could improve the yield by finishing it off.

Q. Was the postreactor kept at a controlled temperature? 02:33

A. Yes.

Q. The same temperature as the condensator?

A. Somewhat warmer, I think.

Q. Was it cooled -- was it cooled by the brine coolers the same way -- 02:33

A. Yes.

Q. -- as the condensators?

A. Brine coils.

Q. Why did the Torrance plant change the process? 02:33

A. Because it's a much more efficient way to do it. The stripping was -- you had unavoidable losses of MCB.

Q. Losses to where? 02:34

A. To the air, to the water.

Q. To what water?

A. To the drain water.

MR. LYTZ: Objection, asked and answered.

MR. O'ROURKE: Q. And how was MCB lost in the air in the old process? 02:34

A. In the old process, every time you pump the tank out, there was some that was lost to the air. It wasn't a sealed tank. The vent came up to the condensers, and you always lose a little bit there. 02:34 You don't condense all of it. So you always had some MCB lost.

Q. From the stripper?

A. Yes, to the atmosphere.

Q. Who at the plant made the process change, the 1954, 1955 process change? 02:34

A. That I don't know. I would guess John Kalock. Kalock and Wilcox. Wilcox was responsible for a lot of the design, he had a piece of everything that went in that plant. And Kalock worked closely 02:35 with him. Probably Mr. Bratter had a good deal to do with that, too.

Q. Did Mr. Wilcox design the plant? Or do you know who designed the plant?

A. I don't know. 02:35

Q. Can you continue to tell me why the second
process was more efficient than the first process?
A. You did not have the MCB loss. It was a
much more efficient way to filtering this material
off in the form of beans in this Oliver filter. Was 02:35
a much better way, much cleaner separation than the
separation way. You got DDT with much less acid in
it than the previous way to do it. So it was a much
more efficient way to handle it.
Q. Did it improve the quality of the final 02:36
product?
A. I'm sure it must have. I don't have any
figures to demonstrate this. I'm sure the yield must
have gone up at least slightly because of the new
process. 02:36
Q. Okay. What happened in the -- nothing
really happened in the postreactor, it just sat
there?
A. There was some residual reacting going on
there. There was some -- you probably had one 02:36
percent of the action, or maybe slightly more of the
reaction, taking place in the postreactor.
Q. And the contents of the postreactor were
what substances?
A. It was the slurry -- bean slurry. The DDT 02:37

beans floating in the red sulfuric acid.
Q. What was the next step after the
postreactor?
A. Goes to a tank which fed the Oliver filter.
Q. Can you describe what an Oliver filter looks 02:37
like?
A. I can draw it for you better.
THE VIDEOGRAPHER: Shall we go off the
record while we're getting organized? Let's go off
the record now at 2:37. 02:37
(Recess taken).
THE VIDEOGRAPHER: Okay. We're going back
on the record at 2:40.
MR. O'ROURKE: Q. Mr. Suhrer, could you,

using this easel, draw me a depiction of the Oliver filter and how it worked? 02:41
(Witness draws picture).
THE WITNESS: Now this Oliver filter is floating in a pan, which is up to about this level with the slurry of DDT and acid. And the filter is 02:41 rotating in this direction, slowly. Maybe one revolution a minute.
MR. O'ROURKE: Q. What's the diameter of this?
A. The diameter is probably five feet. 02:41

Q. And this is a cylinder? We're looking at the side of a cylinder?
A. You're looking at the end of a cylinder.
Q. How long?
A. The length is probably about seven or eight 02:41 feet. Now this thing rotates in this direction, and there is a -- you have a vacuum pump over here someplace which pulls a vacuum on this hub inside. And there are valves in there that says that -- that's the vacuum, pull vacuum. This, this, this, 02:42 and this. But the vacuum is off for these here. And there's a blower that operates when it gets to this section and this section. You see these sections, it goes around like this, and as it gets in, it builds up a layer of DDT, because it's sucking the acid 02:42 inside, and leaving the DDT on the filter medium, which is some sort of a woven plastic.
(Witness indicates).
MR. O'ROURKE: Q. And the plastic was designed to let acid through but not DDT? 02:42
A. Right. So you build up this crust of DDT all the way across here. When you get over here to this side - I have not drawn this particularly well, like so - you have a knife blade that contacts this thing right here, and there is a conveyer belt here 02:43

that takes it on up.
(Witness indicates).

MR. O'ROURKE: Q. And the knife just scrapes the DDT?

A. The knife -- you have the blower that frees up the DDT from the cloth, and the knife scrapes it off and drops it in there. And the blower and the suction goes on all the time. So this is a much more efficient way to separate acid and DDT. Once you get down here, is a soft, waxy DDT mass, with some acid included. But most of the acid is pumped off through this vacuum pump and goes over to storage.

Q. And the acid gets sucked into the cylinder and pumped out the side of the cylinder?

A. The acid gets sucked into here and goes out, pumped off to storage.

Q. It goes to spent oleum storage?

A. Right, spent oleum storage.

Q. Could you label the top of that, just say, "Oliver filter." And put your initials underneath. Okay, I think we're probably done with this picture for now. And I'm going to get the court reporter to mark it. Do you want to do that now?

(Discussion held).

MR. O'ROURKE: Thank you. So this drawing

by Mr. Suhrer is Exhibit 2.

(Document referred to herein marked Plaintiff's Exhibit 2).

MR. O'ROURKE: Q. Now in the postreactor -- was there any excess MCB in the postreactor?

A. I'm sure there must have been.

Q. What happened to that?

A. Well, it's -- you hope it's drawn up into the final reaction. See, you're getting reaction going on there. You're getting some DDT form, which means that the material coming into the postreactor has residual amounts of chloral and residual amounts of MCB. When the reaction completes, you hope you used up all the chloral and almost all the MCB. You probably have.

Q. If any MCB were left over, would it go to this Oliver filter?

A. It would go there. It probably would be sulfonated by the acid.

Q. Uh-huh.

A. It's possible it might -- if there was any DDT -- any MCB left in this, it would have stayed with the DDT probably, because the two are mutually soluble. 02:45

page 147

Deposition of Suhrer, Ferdinand
page 148

Q. What about any MCBS?
A. That would have stayed with the acid.
Q. And been pumped off out of the filter?
A. Yes.
Q. So how many condensers were emptying into the postreactor at one go? 02:46
A. The condensators? Would have been, let's see, eight -- I think when we finally finished, we had 12 condensators, and all 12 of them would have gone into that. 02:46
Q. It had volume to receive all 12?
A. Not at the same time. See, they would come up every hour, whatever your cycle was. But you had condensators being pumped off in sequence. When the cycle was finished, you'd pump it to the postreactor 02:46 and recharge it. And a half hour later, hour later, another one would be ready, and it would go off.
Q. Was the flow from the postreactor to the Oliver filter, was that continuous?
A. That was constant, yes. 02:46
Q. What happened to the -- did the filter on the Oliver filter have to get changed?
A. Yes. We had to change the filter cloth from time to time.
Q. How often do you have to do that? 02:47

page 148

Deposition of Suhrer, Ferdinand
page 149

A. Maybe twice a year.
Q. Uh-huh. And do you know what happened to the old cloth?
A. I'm sure it went out in the trash.
Q. What was the next step after the DDT was taken off the conveyer belt of the Oliver filter? 02:47
A. Goes up this conveyer belt and drops into a tank, a rather small tank we call the melter. And you blew steam in there. And when steam hits this

acid, the acid gets very hot right away. Because the 02:47
dilution effect. And also because it is steam. But
this would melt the DDT. So you've got molten DDT
liquid, and acid -- sulfuric acid. This would go
from the melter, it would overflow into an acid
separator. And you had, again, a tank with two 02:48
layers in it. A DDT layer. I believe the DDT layer
would have been on top, and the acid would have been
on the bottom. You separate that. And you can do
that continuously, if you arrange the drain lines a
certain way. Calculate the heights of drain lines, 02:48
you can dump this mixture in there, and you would
continually get stuff coming off the top, acid going
off the bottom.
Q. Would the melter and the separator, they
were two different tanks? 02:48

A. Two different tanks.
Q. Was it considered one unit, or were there
different functions?
A. Two different tanks doing different things:
One was melting, one was separating. 02:49
Q. What was the size of the melter?
A. Rather small tank. I would say probably
four feet in diameter by maybe six feet.
Q. And the only function was to blow steam in
and -- 02:49
A. Was to melt the DDT.
Q. Okay. And how did the DDT get pumped from
the melter into the separator?
A. It overflowed. It was -- the melter was
higher up. This conveyer belt took it up to a level 02:49
where it would overflow from the melter to the
separator.
Q. And how big was the separator tank?
A. The separator tank was probably, oh, five
feet diameter, six feet long. 02:49
Q. And what happened to the molten DDT that
landed in the separator?
A. The molten DDT there is still -- it may be
free of acid, but it's still very acidic from all of
this contact with sulfuric. So it went into an 02:49

agitated small tank where it was caustic neutralized.
     Q. From the melter, where did the acid go?
     A. The acid would go to separator acid storage, which is different from soo, which is different from spent oleum. Because this has had steam blowing into it. And instead of being 90, 95 so percent acid, it's now down around 85, 86 percent. And that is not -- it's not economical to try to fortify that to use for soo, it takes too much oleum. 02:50
     Q. So what was done with the separator acid?
     A. Some of the separator acid, a small amount of it, was shipped up to the Henderson plant for use in their process.
     Q. Montrose of California, Henderson? 02:50
     A. The Montrose Henderson, California plant. It would have to be fortified with oleum to -- up around 90-something percent, before it could be used. But we fortified it and shipped some of it up there. Most of it got shipped out to sea. 02:51
     Q. Do you know who shipped it out to sea?
     A. We had contracted with California Salvage Company to take care of it.
     Q. Were they the only company that Montrose used during the time you worked there? 02:51

     A. Only company I'm aware of, yes.
     Q. How did the separator acid get from the melter to the ocean?
     A. It went -- from the melter it went to separator acid storage. And when the storage tank got, oh, dangerously high, maybe three quarters full, we would call California Salvage and tell them we needed some trucks. 02:51
     Q. What was the volume of the separator acid tank? 02:51
     A. Gee, I don't remember.
          MR. O'ROURKE: We're going to go off the record for a second.
          THE VIDEOGRAPHER: We're going off the record now at 2:51. 02:51
          (Recess taken).

THE VIDEOGRAPHER: We're going back on the record now at 2:53.
MR. O'ROURKE: Q. We're back to Exhibit 1 again. And could you show us where the Oliver filter 02:53 was located. I know it's not on this picture, but where it was located?
A. The Oliver filter would have been right here.
(Witness indicates). 02:53

page 152

Deposition of Suhrer, Ferdinand
page 153

MR. O'ROURKE: Q. And then the next step was the melter; is that right?
A. The melter would have been a small tank directly above it and off a little bit north and above the filter. 02:53
Q. By "above" you mean higher in the air?
A. Yes.
Q. And then where was the separator?
A. The separator would have been a little bit lower and little bit west of the melter, which puts 02:53 it right alongside this platform right here.
(Witness indicates).
MR. O'ROURKE: Q. So for the record, this whole unit is a little bit south of the process building? 02:53
A. Yes.
Q. And where was the separator acid tank put in?
A. Separator acid storage would have been over in this area here. 02:54
(Witness indicates).
MR. O'ROURKE: Q. Is that pretty close to where the postreactor was?
A. Yeah, right next to it.
Q. Okay. Was that a horizontal tank or 02:54

page 153

Deposition of Suhrer, Ferdinand
page 154

vertical or --
A. As I recall, it's a horizontal tank. Size, I don't recall. Probably 5,000 gallons, but that would be a guess.

Q. How often did the California Salvage people come to empty the separator acid tank? 02:54
A. They probably would average in a week, there would probably be a matter of ten loads a week.
Q. What was a load, one truckload?
A. Yes, about 3,000 gallons. 02:54
Q. What kind of truck was this?
A. Tank truck.
Q. Do you know where California Salvage brought the separator acid?
A. California Salvage took it down to their docks in the harbor, and put it on barges, and towed the barges out to sea. 02:54
Q. The separator acid was created by steam being pumped into the melter separator?
A. Exactly. 02:55
Q. Did the steam also blow off any DDT in the separator acid tank?
A. No. See, the melter is where the steam went in. And from the melter, the flow from the melter was liquid DDT and liquid acid. The separator broke 02:55

page 154

those two liquids. You got separator acid going to separator acid storage, DDT going to caustic wash.
Q. Did the separator acid contain any DDT?
A. Minor amounts probably, yes.
Q. Did it contain any MCB? 02:55
A. Probably not.
Q. Do you know if there was any sampling for DDT done on the separator acid?
A. Not to my knowledge.
Q. And do you know where California Salvage took the separator acid? 02:56
A. They were supposed to take it out to sea, I think beyond the Continental Shelf. But there was a common joke among people that they only took it as far as they needed to, just out of sight, and started dumping right there. This I couldn't say. 02:56
Q. Did you ever --
MR. LYTZ: Move to strike, nonresponsive.
MR. O'ROURKE: Q. Did you ever see the barge or the ship from California Salvage? 02:56
A. I've seen the barges. I've been on fishing trips on the tugs that towed the barges.
Q. When was that?

A. I don't remember. California Salvage liked
to keep us happy, particularly the foremen. Because 02:56

page 155

Deposition of Suhrer, Ferdinand
page 156

we could make things difficult for them if we wanted
to. But they would -- every summer we would have a
fishing trip on a California Salvage tug.
Q. And where did they take you?
A. I don't know. 02:57
Q. Out to the ocean somewhere?
A. No idea. Out in the ocean.
Q. Now, you said it was a common joke that
California Salvage dumped closer to shore. Do you
know how that joke started? 02:57
A. No, I don't know how it started.
Q. Do you know if there was any truth to that
rumor?
A. Well, I don't know. But it was a joke. And
the truck drivers all thought it was funny, too. 02:57
Q. Okay. California Salvage truck drivers?
A. Oh, yeah.
Q. Who else was invited on the fishing trip?
Just the foremen?
A. The foremen, Max, Bernie, Kalock, any of the 02:57
staff. I don't know that all of them went. I
remember Max was aboard one time.
Q. When you refer to Bernie, do you mean Mr.
Bratter?
A. Mr. Bratter.

page 156

Deposition of Suhrer, Ferdinand
page 157

Q. And Max is Max Sobelman?
A. Max Sobelman.
Q. Okay. What happened after the separator
acid was removed from the separator? What happened
to -- what was left in the separator? 02:58
A. The separator stayed at a constant level.
Q. Okay.
A. You were constantly putting material into it
-- putting two liquids into it, and taking two
separated liquids out. One out of the top, and one 02:58
out of the bottom. So it kept a constant level.

Q. And the melter was also a continuous process?
A. Yes. As long as the Oliver filter was running, you had to run the melter. 02:58
Q. What came out of the separator? There was a DDT layer that came out of the separator?
A. Yes.
Q. And where did that go next?
A. That went to a small pot where we washed it with caustic, with agitation and heat. 02:59
Q. What was the name of that pot? Did it have a name?
A. Caustic scrubber, I think we called it.
Q. Where was the caustic scrubber on this 02:59

photograph?
A. Well, this is not on here. It would have been about five feet away from the separator.
Q. Can you point to where that would have been again? 02:59
A. It would have been right about here.
(Witness indicates).
MR. O'ROURKE: Q. What was the size of the caustic scrubber tank?
A. It was a small tank, probably four feet diameter, maybe five or six feet long. 02:59
Q. Was it sitting on the ground or --
A. Vertical tank, in the air. Way up in the air.
Q. What happened in the caustic scrubber? 03:00
A. Caustic scrubber, you brought in caustic with a lot of agitation and a lot of heat to keep this DDT molten.
Q. What is caustic?
A. Sodium hydroxide. 03:00
Q. Where did you get the caustic?
A. This was purchased from -- I don't remember the vendor. It may have been Stauffer.
Q. Stauffer Chemical?
A. Yeah. 03:00

Q. Okay. And what happened after the caustic was agitated in the caustic tank?
A. Okay. It went from there to another separator tank, and this was a large tank.
Q. Actually, I want to stay and talk about the caustic scrubber, there. 03:00
A. Yeah.
Q. What happened in the caustic scrubber?
A. In the caustic scrubber, the caustic neutralized remaining amounts of sulfuric acid. 03:00
Q. Whatever had not been removed by the Oliver filter?
A. Whatever had not been removed through the separator. See the separator was supposed to break liquid DDT out and liquid acid, which it did. But 03:01 there was some residual acid still with the DDT that had to be washed out. First you neutralized it, then you washed it with water.
Q. So was the caustic scrubber a continuous process? 03:01
A. Continuous.
Q. And what came out of the caustic scrubber tank?
A. Out of the caustic scrubber comes the caustic, which is mostly neutralized by now, and 03:01

mostly neutralized DDT.
Q. Two different streams?
A. No, the same stream. They've been agitated, and they just overflow this into a large tank.
Q. Okay. 03:01
A. Now you get another separation. You've got a tank with molten DDT on the bottom, and spent caustic, and some water, acquiesce material, on the top. The acquiesce material flows out to the sewer. The DDT flows out the bottom, where it's into another 03:01 separator.
Q. Uh-huh.
A. Another washer.
Q. Now the caustic solution you said was drawn off into the sewer? 03:02
A. Yes.
Q. Is that the same sewer we were discussing earlier?

A. I think by this time we're using the new
water drainage system. 03:02
Q. Where was that?
A. Which we have -- well, that was a large tank
set underground so the surface was flush with the
ground. And it was right here, just south of the
kwansit hut. It was an agitated tank -- there were 03:02

two agitated tanks there. You flowed into one, until
it was filled, until it was neutral, then you release
that to the sewer, and flow it into the other.
Q. Were the two tanks next to each other?
A. Yes. 03:02
Q. Next to that kwansit hut?
A. Yes.
Q. What was the volume or the size?
A. This I don't know. I could give you a
guess. Probably about maybe 12 feet in diameter, 03:03
maybe 12 feet deep. Large tanks.
Q. And how was -- you said there was a
neutralization. What took place in the tanks?
A. In these tanks here? If there was a PH
meter on the tanks, if further neutralization was 03:03
required, they would bleed in caustic to bring up to
neutral.
Q. I thought you said that caustic from the
caustic --
A. We're talking about two different things 03:03
now.
Q. Okay.
A. We're talking about the water drainage
system, which are these underground tanks. The
caustic scrubber took the acidic DDT and washed all 03:03

the acid out. And that caustic was drained, I think,
to these underground tanks.
Q. Okay. And how long did the liquid stay in
the underground tanks?
A. I think they probably filled up either two 03:04
or three times a shift.

Q. And what happened once they were full?
A. Once they were full, and once they were neutral, they were released to the sewer.
Q. And where was the new sewer connection? 03:04
A. This -- there was a drainage of -- new drainage ditch that ran down the plant this way. And then somehow it cut over, I don't know where the connection was into these tanks, probably underground. This drainage wound up in these tanks 03:04 here.
(Witness indicates).
MR. O'ROURKE: Q. Uh-huh. And where did the contents of those tanks go?
A. To the sewer, which my understanding was it 03:04 was out in this direction toward Normandie.
Q. Let's go back and talk about the caustic scrubber again.
A. Good. All right.
Q. You said the caustic was drawn off into 03:05

those tanks?
A. Yes.
Q. Was there any DDT in that caustic?
A. Probably.
Q. Would that DDT have been discharged into the 03:05 sewer?
A. Yes, probably.
Q. Was there any MCB in that caustic?
A. There would have been none.
Q. Okay. And what was the next step after the 03:05 caustic scrubber?
A. After the caustic scrubber, you have DDT with residual amounts of caustic in it. And you go into another separator tank, which is the same size as the first separator, and this one is -- has hot 03:05 water -- 98 degree hot water boiled up through the bottom.
Q. 98 degree Celsius?
A. Uh-huh. To keep the DDT melted, and to absorb -- pick up any traces of caustic or any traces 03:05 of acid that may have lasted this long. When it got out of that final separator, the DDT should have been perfectly neutral.
Q. What happened to the hot water that was put into -- 03:06

A. Overflowed to the sewer.
Q. Down to those same tanks that the caustic wash went to?
A. Yes.
Q. Was there any DDT in that water? 03:06
A. Yes, there was some. Minor amounts.
Q. And did that DDT also go into the sewer?
A. Yes.
Q. And what happened to the DDT once it was taken out of that final washing separator? 03:06
A. After this final wash, it goes through a jacketed pipe to be kept hot. And it ran into a DDT holding tank inside the process building.
Q. And how big was the holding tank?
A. It was probably five feet diameter and six feet long. 03:06
Q. And was it heated?
A. It had steam coils inside.
Q. How long could the DDT stay in that holding tank? 03:07
A. It could stay in there for a long time. But the difficulty with DDT, when you kept it hot, as you needed to, to keep it liquid, it started to decompose. And decomposed DDT is off spec DDT.
Q. What did it decompose to? 03:07

A. It decomposes to -- I don't remember what compounds. Compounds which would give you a bad set point in any case. So you didn't want to keep it molten any longer than you had to. You tried to make these tanks small, and lines as short as you could. 03:07 Out of that tank you fed the flakers, the chip table, and any other source of DDT, anything else.
Q. So all those final steps in taking the DDT and putting into the bags were all the same?
A. Yes. 03:07
Q. Was there still the lump process being used at this time?
A. No, not at that time.

Q. You mentioned there's two tanks that were
installed for connection to the Normandie sewer? 03:08
A. Yes.
Q. Was the plant drainage directed to these two
tanks?
A. It must have been. Because there was no
other drainage. We stopped using the pond. 03:08
Q. When did they stop using the pond?
A. When they put in these two tanks. This, I
would have to guess, and say maybe 1956 -- '55, '56.
Q. Were the tanks put in the same time as the
new process using the Oliver filter was installed? 03:08

A. Yes.
Q. And about that same time --
A. About that same time, yes.
Q. That's about when you stopped using the
settling pond? 03:09
A. Yes.
Q. What happened to the settling pond?
A. Just stayed there. I think we put in an
acid recovery plant about the same vicinity. Whether
it was actually where the pond used to be or not, I 03:09
don't remember.
Q. You don't remember the pond being filled in
or anything?
A. No.
Q. Did any of the run off and drainage from the 03:09
plant continue to go to the pond?
A. No.
Q. Was it all redirected to the two new tanks?
A. Yes.
Q. So we discussed earlier, drainage from the 03:09
process building going down to the settling pond.
After 1955, was that drainage now going to these two
tanks?
A. Right.
Q. And from there -- 03:09

A. Yes. The entire plant run off would have

gone through those two tanks to be neutralized.
Q. And then would have been discharged into the sewer system?
A. Right. 03:10
Q. So earlier when we discussed pump repacking and spill of DDT, would that have gone to these new tanks?
A. That's right.
Q. And from there to the sewer? 03:10
A. That's right.
Q. And were these two tanks still in use when you went back during the strike?
A. Yes.
Q. Those two tanks were in use the entire time 03:10
from 1955 until the end of your tenure there?
A. Let me back up a little bit. They must have been in use. I don't recall I actually went out and looked at them. But the drainage had to go someplace. I'm sure that they went to those tanks. 03:10
Q. From 1955 until you left the Torrance plant, were those two tanks in use and connected to the sewer?
A. Yes.
Q. Do you know how much DDT was going through 03:10

those tanks into the sewer?
A. No. There would be no way to estimate that. I know some DDT did go. How much, it would be impossible to say. Minor amounts, we hoped. Because DDT was an expensive product. And we wanted to save 03:11
it. But I'm sure some of it got away from us.
Q. There was no sampling ever done on the --
A. No, I never did any sampling of it.
Q. We talked about the separator acid being barged to the sea. And that same separator acid 03:11
product was also sent to Henderson?
A. It was -- after being fortified, it was sent to Henderson.
Q. Was there any sampling done on the acid that was sent to Henderson to check if there was DDT in 03:11
it?
A. Only to check the acid strength.
Q. Okay. California Salvage was the only firm that Torrance used to ship acid?
A. Only firm while I was there, yes. 03:11

MR. LYTZ: I'm going to object, asked and answered.
MR. O'ROURKE: Q. Did the discharges -- I mean, did the separator acid shipments to the ocean continue the entire time you were at the plant? 03:12

A. Yes.
Q. From 1955 to '61?
A. Well, there was an attempt made -- brief attempt made to recover the separator acid. They had hoped that they could heat the separator acid hot 03:12 enough to get the MCB to revert to get MCBS to revert to MCB, and recover the MCB that way. I don't think that was really a successful thing.
Q. Was there a lot of MCBS in the separator acid? 03:12
A. Appreciable amounts, yes.
Q. And that was shipped to the ocean dumping as well?
A. Yes.
Q. Was there MCB that went down through the new 03:13 tanks and into the Normandie sewer?
A. Yes, there were.
Q. Rain water run off, after the new tanks were put in, those went down to the tanks as well?
A. Correct. 03:13
Q. Have we discussed all the major steps in the process, after 1955, or is there anything that we left out?
A. I can't think of anything that's left out.
MR. O'ROURKE: Well, the tape is going to 03:13

end in two minutes. So I think it's time for a break.
THE VIDEOGRAPHER: We're going off the record now at 3:14. This concludes the end of Tape 2 on July 30th, 1996. 03:14
(Recess taken).
MR. O'ROURKE: Q. Thank you. Mr. Suhrer, you pointed to the kwansit hut, what went on there?

A. The kwansit hut was originally the change house for the men. There were a couple of toilets in there, and that was it. There were a few lockers. 03:29
Later on, when the new change room was built over right about here, the kwansit hut was -- I don't know what it was used for, the toilets are still there, but I don't know what else. 03:30
(Witness indicates).
MR. O'ROURKE: Q. Was -- speaking now about the two new tanks that were put in near the kwansit hut.
A. Yeah. 03:30
Q. You said that there was a neutralization done on the waters that float down into that?
A. The contents of the -- see, one tank was filling while the other was draining. When a tank was filling, they monitored the PH of the tank. If 03:30

the PH got too acidic, they were going to caustic to correct that. When it was corrected up to the neutral point, or whatever range they wanted, then it was okay to release. And when it was filled up and neutral, out it would go. 03:30
Q. And what happened to the old sugar lime method?
A. Stopped using it.
Q. About the same time the process changed?
A. Yes. The same time that they started using this, there was no use for sugar lime any more. 03:31
Q. When you say this --
A. The new tank system.
Q. That was also about the time when you put in the Oliver filter and the new parts of the process? 03:31
A. Yes, I believe so.
Q. You mentioned earlier that there was a large concern for worker safety at the plant?
A. Very large concern.
Q. Was there a person whose sole job was to watch out for worker safety? 03:31
A. Sometimes I thought it was me. All the foremen were constantly being nagged about safety. Every shift we had a safety meeting, ten, 15 minutes long, to discuss some aspect of safety and what's 03:31

happening. There were always safety contests going
on to see who had the fewest accidents. Safety was a
big item.
     Q. What were the dangerous parts of the plant?
     A. Sulfuric acid is always a danger. Molten   03:32
DDT. You have hot liquids everywhere. Ammonia can
be a danger. Sulfuric certainly is. Organic
chemicals like chloral and mono are always
hazardous.
          We were required to wear safety glasses   03:32
whenever you were in the plant, safety glasses with
side shields and thick glass unbreakable lenses.
People -- men were disciplined for not wearing those
things. And you had to force them to wear them,
because they would tend to fog up, and they would get 03:32
in the way of your work. But we did so.
          And it was a constant struggle to keep the
people wearing hard hats, and safety glasses, and
gloves. Gloves were not so much a problem, they
found the gloves would protect them against acid   03:32
spills, and against all sorts of things. But
anything else, you really had a problem to make the
men do.
     Q. And what happened if you spilled acid on
your hand?   03:33

     A. You washed it off.
     Q. Okay.
     A. If you spill oleum on your hand, you had a
burn immediately. If you spill sulfuric, soo, or any
other acids, if you got it to water within, oh, ten   03:33
to 15 seconds, no harm done.
     Q. What about if you spilled MCB on yourself?
     A. No problem, it would wash off. If you're in
constant contact with MCB, I'm sure you get
blistered. But an occasional splash, you should wash 03:33
it off, and nothing happens.
     Q. What about handling DDT or touching DDT?
     A. It's not any problem. I saw Mr. Wilcox one
time standing on the edge of one of these
crystallization pans, and he slipped and fell into   03:33

molten DDT, up to his ankles. And he got out, and
shook his feet a little bit, and he had crusted DDT
on there, and he had a little bit of burn on his
foot, but that was all.
Q. Did you all ever have to wear respirators at 03:34
the plant?
A. No.
Q. Boots?
A. No. We were offered safety shoes, safety
toe shoes, which were rubber boots. But I think the 03:34

page 173

Deposition of Suhrer, Ferdinand
page 174

main feature there was the safety toe, rather than
the fact they were acid proof.
Q. Uh-huh.
A. But acid was a constant worry. When you had
a pump pumping acid, if a little bit got on the pump 03:34
shaft, it would sling it out in a spray, and you
could walk into a spray like that without even
knowing it. A lot of times my wife would be greatly
surprised when I come home and she'd wash a pair of
pants and they fall apart, because they had acid 03:34
sprayed on them, and I didn't know it. But it just
disappeared like that.
Q. Did people who worked in the grinding room
ever have to wear respirators?
A. They were supposed to wear respirators. I 03:35
believe they did.
Q. Did you ever see DDT placed directly onto
the dirt?
A. I've seen it fall onto the dirt, yeah.
Q. Did you ever see MCB fall or spill directly 03:35
onto the dirt?
A. Yes, I have.
Q. Did Montrose ever store DDT in piles on the
dirt, or anything?
A. We had rework piles on the dirt, 03:35

page 174

Deposition of Suhrer, Ferdinand
page 175

occasionally. Particularly at the early stages of
the plant.
Q. And how big were those piles at the rework

area?
A. I can remember one that was about six feet 03:35
high and maybe 20 or 30 feet in diameter, conical
shape.
Q. How long would a pile like that sit on the
soil?
A. Until you rework it, which might be a month 03:36
or two.
Q. That conical pile you just described, how
much rework was that? How long did it take to build
up a pile like that?
A. This happened after a prolonged period when 03:36
we had, for no reason we ever figured out, off spec
DDT. And we had to store it someplace. We did not
have enough containers, so we just piled it on the
ground.
Q. It was just some production problem? 03:36
A. Yes. It was a continuing production problem
that lasted for a matter of a week or two. Off spec
DDT. And we never did find out what happened.
Finally the problem went away.
Q. So all of the off spec had to be stored? 03:36

A. Stored someplace. And stored on the ground,
until we worked it off.
Q. Where was the laboratory on the plant? Show
me on Exhibit 1.
A. Right next to the plant office. Right in 03:37
this corner, in the southwest corner of the office
building.
(Witness indicates).
MR. O'ROURKE: Q. Could you describe
verbally where that office building is on this 03:37
picture?
A. The office building is due east of the
grinding room. And let's say between the grinding
room and Normandie Boulevard.
Q. How many people worked in the lab? 03:37
A. During days, there were probably three
people. Around the clock, there was always one man
there.
Q. Uh-huh. And who was in charge of the lab?
A. Mr. Walter Carey. 03:37
Q. How do you spell that?
A. C-a-r-e-y. Red Carey.

Q. Red?
A. Yeah.
Q. Do you know if there was any connection 03:38

page 176

Deposition of Suhrer, Ferdinand
page 177

between the laboratory and the Stauffer BHC plant?
MR. LYTZ: Objection, ambiguous.
MR. KUSHNER: Could you repeat your response, sir?
THE WITNESS: I know of no connection. 03:38
MR. O'ROURKE: You told me the lab did set point checks. What other kind of work was done in the lab?
A. They tested soo to make sure it was proper strength. Really, they were just checking on the 03:38 plant work, because most of the soo checking took place in the process building. But they did soo tests. They did set points. They did some occasional analysis of incoming material. Mono and chloral. They did -- any time there was some 03:39 experimental work going on, and Mr. Wilcox believed in doing lots of experimental work, the laboratory did that.
Q. What kind of experiments would go on?
A. Better ways to make DDT. 03:39
Q. Process improvement?
A. Process improvement, yes.
Q. Who was involved in that kind of research in there?
A. Red Carey did most of that, I believe. 03:39

page 177

Deposition of Suhrer, Ferdinand
page 178

Although possibly -- it was possible that Mr. Bratter or Mr. Sobelman had a finger in that, too.
Q. Do you know if records were kept of the laboratory operations?
A. I'm sure there were, but I don't know. 03:39
Q. Did the Torrance plant ever formulate DDT for commercial use?
A. No, I don't believe we ever did, not while I was there. Most of -- I think all of our customers were formulators, like United Heckthorn up in the Bay 03:40

Area. We did a lot of work for Pen Salt. We packaged DDT in Pen Salt's bags, for their formulation. I don't remember any other. We shipped to Chevron quite a bit.
Q. Where was this Chevron? 03:40
A. Chevron in the Bay Area, Richmond I believe. Ortho Brand pesticides.
Q. Do you know if the Torrance plant ever began to formulate pesticides after you left?
MR. LYTZ: Objection, calls for speculation. 03:40
THE WITNESS: I know that it -- at the Henderson plant, there was a new pesticide that I was doing pilot plant work on when I left.
MR. LYTZ: Move to strike, nonresponsive. 03:41

MR. O'ROURKE: Q. Was there a formulating plant at the Torrance plant when you went back during the strike?
A. No. Well, let me correct that. They were making a product which was unfamiliar to me when we went back, which was a wettable powder. 03:41
Q. Was this a technical grade DDT powder?
A. I believe so.
Q. Was it the same product that had been manufactured when you were there, only in a different form? 03:41
A. In a different form and a different formulation. And being wettable, there was something else added, perhaps a soap, I don't know. I knew nothing of that.
Q. You weren't involved in that when you went back for the strike?
A. No.
Q. Did the production people at the Torrance plant try to keep track of losses of MCB? 03:42
A. We tried to recover all MCB we could.
Q. Was there any record keeping to keep a record of how much MCB was not recovered?
A. No.
Q. You mentioned earlier that John Kalock was 03:42

the head of the maintenance and engineering
department?
A. That's right.
Q. What did his department do?
A. His department repaired and maintained all 03:43
of the plant equipment. Whenever we had a new
process, which involved new construction, generally
-- particularly if it was not a huge thing, Kalock's
group did it.
Q. Did Kalock's group do the construction to 03:43
put in the new process change in 1955?
A. I believe they did.
Q. Do you know what they did with the old tanks
that they had to dispose of?
A. I have no idea. 03:44
Q. Do you know what they did with any dredging
of earth they had to do, where they disposed of the
earth?
A. I have no idea, no.
Q. Did they also put in the new sewer 03:44
connecting tanks?
A. Now that I don't know. I think that
Kalock's group would do the fairly small modification
to the plant. Anything major like that, particularly
to something that would involve a lot of digging, I 03:44

believe Kalock contracted that out.
Q. Uh-huh. So it was Kalock's people who had
to do pump repacking?
A. Oh, yes.
Q. And who would be the people who would break 03:44
open lines to unplug them when that happened?
A. This would be Kalock's group.
Q. Uh-huh.
A. If it happened on the off shift, we had the
option of either -- if it looked a short line and 03:45
easy job, I would do it with my own people. If it
looked like it was going to be an involved thing, I
would call one of the maintenance people in on
overtime.
Q. Uh-huh. Have you ever met Samuel Rothrosin? 03:45
A. I think I did once.
Q. Who was he?

A. Rothrosin was, I think, Pinkers Rothberg's son-in-law. And he was a high official in Montrose. Just what his capacity -- what capacity, I don't know. 03:46
Q. You mention that you get a pension from Montrose from your time at Montrose?
A. Yes.
Q. Do you know what company sends the pension 03:46

to you?
MR. LYTZ: Objection, asked and answered.
MR. O'ROURKE: Q. Excuse me?
A. No, I don't know. It's been different companies. Must have been six different companies in the ten years that I've been retired. 03:46
Q. Have you ever heard of ICI Corporation?
A. Oh, yes.
Q. Are they the ones that give you your pension? 03:46
A. At one time they were the ones. They were the -- somehow the heirs of Stauffer, and my pension came through ICI.
Q. Have you ever heard of Zeneca? Begins with a Z. 03:47
A. I believe that may be the outfit that's sending the pension now.
Q. Have you ever heard of Rhono-Poalenc?
A. Rhono-Poalenc, the French outfit?
Q. Yes, sir. 03:47
A. Yes, I have.
Q. Did they ever send you a pension?
A. I'm not aware of that. The pension amounts to $145 a month, so it's not a real big item.
Q. Do you have any copies of the checks at home 03:47

that would allow you to confirm whether it was Zeneca?
A. Sure.
Q. And when you go home tonight, would you be able to find a copy of that? 03:47

A. I'll see if I can find that, yes.
Q. And you can tell us tomorrow morning.
Unless Adam has any more questions, I think this is probably a good time to break. And maybe break for the day. 03:48
MR. KUSHNER: I just have one clarifying question.
EXAMINATION BY MR. KUSHNER
MR. KUSHNER: Q. What was the name of the plant manager at Henderson that you mentioned had gone down to Mexico at one point? 03:48
A. The Montrose plant manager?
Q. Henderson, right.
A. Hal Wurzer.
Q. How do you spell that? 03:48
A. W-u-r-z-e-r.
Q. Okay.
A. H.J. Wurzer.
MR. O'ROURKE: Does everybody agree it's a good time to stop? 03:48

MR. LYTZ: How much longer are you going to take tomorrow?
THE VIDEOGRAPHER: You want this off the record?
MR. LYTZ: Why don't we go off the record.
THE VIDEOGRAPHER: We're going off the record now at 3:48.
(Discussion held off the record).
THE VIDEOGRAPHER: We're going back on the record now at 3:50. 03:50
FURTHER EXAMINATION BY MR. O'ROURKE
MR. O'ROURKE: Q. When did you move over to the Henderson plant?
A. In 1961. January, 1961.
Q. What job title did you get at Henderson? 03:50
A. I was Plant Engineer.
Q. Did you hold that job the entire time you were at Henderson?
A. Yes.
Q. When did you leave Henderson? 03:50
A. In '68.
Q. What products were manufactured at Henderson?
A. At Henderson, we manufactured monochloral

benzene. We manufactured chloral. And toward the   03:50

end of my tour there, we manufactured a new
pesticide, I think the name of it was
dichlorobenzene. It was a bright, intensely yellow
compound. I did some of the pilot plant work on it
there at Henderson. We were not in full production   03:51
by it, when I left. I understand that they later did
come up to full production.
     Q. What was the reaction used to generate mono
chloral benzene?
     A. You bubble chlorine gas through benzene.   03:51
And this produces mono chloral benzene. You try to
convert just part of it into mono chloral benzene.
Because if you do it too long, you get
dichlorobenzenes, which have very limited sale.
          So the object was to bubble in chlorine for   03:51
a certain length of time, then distill that. The
benzene -- unreactive benzene would come off the top,
and your mono would come off the bottom. And you
would then distill that to get the mono off the top
and the polychlors, dichlors, trichlors, tetrichlors, 03:52
off the bottom. These were in fractionated columns.
     Q. So what was the purity of the final MCB
product?
          MR. LYTZ: Objection, ambiguous.
          THE WITNESS: This I don't know. As pure   03:52

as it had to be to make DDT. I don't know what that
was.
          MR. O'ROURKE: Q.   And how was the chloral
manufactured at Henderson?
     A. Initially chloral was manufactured by   03:52
bubbling chlorine gas into alcohol -- ethyl alcohol.
And when about halfway through my tour of duty at
Henderson, they changed the process to bubble
chlorine gas through acid aldehyde. Which was an
improvement, in that it made -- see, when you bubble   03:52
chlorine in any of these hydrocarbons, one of the
by-products is hydrochloric acid. And hydrochloric

acid in that day had very limited sale. And we had trouble selling it. And I think in some cases, we almost gave the stuff away. If you could sell all of your acid, you were in good shape. If you made less acid, you were in better shape. And that was why the acid aldehyde process was so successful. Because it made something like, oh, 60 percent of the acid that the former process made. 03:53 03:53
Q. Is ethyl alcohol the alcohol that gets you drunk?
A. The ethyl alcohol? Oh, yes. But in this case it was denatured by putting in a very foul smelling oil into the alcohol. 03:53

Q. Do you know if any of the MCB that was sent from Henderson to Torrance contained pure benzene?
A. I'm sure there were trace amounts.
Q. Can you tell me what kind of percentage that would be? 03:54
A. No, I can't. I would expect it would be a very tiny amount -- fraction of a percent.
Q. So those are the only three products that you know that Henderson manufactured: MCB, chloral and dichlorobenzene? 03:54
A. There was -- well, the by-product of muriatic acid, hydrochloric acid, was a product which did have some limited sale. Another product was the dichlorobenzenes have a limited sale. They're -- very limited sale on that. When you found a customer for dichlors, you were very happy about that. 03:54
Q. Was there any market for more richly chlorinated benzenes, tri or tetra?
A. No.
Q. You mention that Henderson received some acids back from Torrance? 03:55
A. Yes.
Q. When did that happen?
A. This happened when they were chlorinating alcohol. What they did there, was bring the acid in 03:55

and mix it with the crude chloral to pick up all the
water and to dehydrate the chloral. I think I said
that you couldn't have water with the chloral,
because it fouled up the process at Torrance. So if
you brought in this acid, which is a great 03:55
dehydrating agent, would pick up all the water from
the process and dehydrate the chloral, so it would be
suitable for DDT manufacturing.
     Q. And what was then done with the hydrated
acid? 03:55
     A. Down the sewer.
     Q. What part of the Torrance process did that
acid come from?
     A. That would come from the separator acid
fortified. For a long time it came from spent acid. 03:56
     Q. Where did spent acid come from?
     A. Spent acid is what comes off the bottom of
the condensators. You fortify the spent acid, with
oleum to make soo. If the spent acid was suitable
strength, so it could be shipped directly to 03:56
Henderson for there purpose.
     Q. And what time periods was the spent acid
being sent to Henderson?
     A. I'd say probably they stopped sending that
up probably around '62, '63. 03:56

     Q. Were they sending that the entire time you
worked at the Torrance plant?
     A. Yes.
     Q. And then they started sending the separator
acid in around 1955 or something? 03:57
     A. Well, they started -- when they started
making separator acid, they started fortifying it for
Henderson use. Before they made separator acid, they
used spent oleum for Henderson use.
     Q. What portion of the spent oleum from the 03:57
condensator went to Henderson, and what part of it
went to the spent oleum tank to be made into soo?
     A. Well, just looking at a material balance, I
would say the volume of oleum that was taken into the
plant, the only place it could leave the plant was 03:57
going out -- was going to Henderson. Unless -- and
they may have shipped some to California Salvage, I'm
not sure about that.
     Q. Uh-huh. And at the Henderson plant, is that

where the MCB and the chloral were mixed? 03:58
A. Yes.
Q. And shipped from there as a mix to Torrance?
A. That's right.
Q. Who calculated -- who decided what ratio of
MCB to chloral to mix together? 03:58

page 189

Deposition of Suhrer, Ferdinand
page 190

A. At Henderson?
Q. Yes.
A. Probably the operator.
Q. Was it always the same ratios?
A. No, different ratios. It varied. Not 03:58
widely, but it varied.
Q. Why did it vary?
A. Because one operator would put in a certain
amount, and another one would maybe put in a little
bit more. They gauged the tanks and dropped in a 03:58
certain amount from the tank into the trucks. And it
was mixed with -- with MCB. And the amounts would
vary from time to time. We always tested when they
got to Torrance. We tested the specific gravity to
find out what the chloral content was. That's how we 03:58
could send up to the chemical operator the charge for
this particular load of mix. It had to be so many
gallons of MCB and so many gallons of mix. And that
varied from load to load.
Q. The amount of make up MCB depended on the 03:59
ratio --
A. Right, depended on the chloral strength.
Q. And was the testing of specific gravity
something you did yourself?
A. Oh, yes. 03:59

page 190

Deposition of Suhrer, Ferdinand
page 191

Q. You did not have to use lab?
A. No.
Q. Did you do the calculations yourself --
A. Yes.
Q. -- from the specific gravity to the -- 03:59
A. Sure.
Q. -- to the number of gallons needed?

A. Yes.
Q. Do you remember how to do that calculation?
A. It's not an involved calculation. I think we may have had a table to work with. 03:59
Q. Uh-huh.
A. We had tables and we had -- it was an easy calculation. I could do it if I was looking at it.
Q. Did the Henderson plant ever receive any DDT shipments from Torrance? 03:59
A. Yes.
Q. When was that?
A. When they were making this new product, this new pesticide, started as a base material with DDT. 04:00
Q. When they were making the dichlorobenzene?
A. Yes.
Q. So how much DDT was shipped from Torrance to Henderson?
A. While I was there, probably, oh, maybe as 04:00

much as six, 8,000 pounds.
Q. And this was when, around 1960 --
A. This would have been '67 and '68 -- '67.
Q. And was the DDT that was shipped to Henderson used for product research, or there was no production in scale? 04:00
A. It was used only for the production of dichlorobenzene. It was the starting point from there.
Q. Was it -- when you were there, was it a pilot plant or was it a full operation production plant? 04:00
A. It was a pilot plant. It was big enough so we could have sold some of the product.
Q. So how long did shipments of DDT from Torrance to Henderson go on? 04:01
A. It was an occasional thing. See, pilot plant, a pallet of DDT contained 2,000 pounds. I think we probably used three or four pallets of DDT, while I was there. You would use four or 500 pounds for a batch of dichlorobenzene. This is strictly experimental, and the pilot plant worked. But I don't think we used any more than three or four pallets of DDT there at all. Once they got into production, they would undoubtedly use a lot more. 04:01

Q. Was -- to your knowledge, was all the DDT incorporated into the final product?
A. Yes.
Q. Did the plant in Mexico use the same process as the Torrance plant? 04:02
A. Same plant, same design, same everything.
Q. When the switch was made from the settling pond to the two new tanks for drainage to the sewer, was there a lot of new trenches dug, or were the trenches redirected to the tanks? Or do you remember 04:02 what happened?
MR. SIMSHAUSER: I'm sorry, what was the last part? You need to speak up.
MR. KUSHNER: You need to drowned out that person.
MR. O'ROURKE: Q. When the switch was made from the settling pond to the two new tanks, were there a lot of new trenches dug on the property?
A. I don't recall. They used the old trenches just as much as they possibly could, obviously. 04:03 There probably had to be some new trenches dug. But couldn't say where or how much there were.
Q. Whose job was it to maintain the trenches?
A. If the production people spilled anything in the trenches, it was their job to clean it out. The 04:03

maintenance of the trenches, repairing broken bricks, and doing work like that, would have been the maintenance group.
Q. Kalock's group?
A. Kalock's bunch. 04:03
Q. And when you say the production people had to clean out the trenches, would that involve hosing the trenches down or --
A. Yeah. Shoveling out what DDT that could get out; hosing down everything else. 04:04
MR. O'ROURKE: Okay. I think that's probably it for today then. We'll go off the record for Monday.
MR. KUSHNER: This is Tuesday.

MR. O'ROURKE: For Tuesday. Thanks for your time and effort, Mr. Suhrer. 04:04

THE WITNESS: Okay.

MR. KUSHNER: And we'll reconvene tomorrow at 9:00 a.m., same place.

THE VIDEOGRAPHER: We're going off the record now, the time is 4:04. This adjourns the deposition of Ferdinand Suhrer on July 30th, 1996, at 4:04. 04:04

(Whereupon, at 4:04 p.m., the deposition was adjourned).

____
FERDINAND SUHRER

---o0o---

TO: FERDINAND SUHRER

c/o STEVEN O'ROURKE, ATTORNEY AT LAW
1425 NEW YORK AVENUE, NW
WASHINGTON, DC 20005

RE: UNITED STATES OF AMERICA, et al.
V.
MONTROSE CHEMICAL CORP. OF CALIFORNIA, et al.

DEPOSITION OF: FERDINAND SUHRER, VOLUME I

DATE OF DEPOSITION: July 30, 1996

Dear Mr. Suhrer:

The transcript of your deposition taken in the above matter is available at this office for your review. If it is more convenient, you may read your attorney's copy.

In the event you have not read, corrected and signed your deposition within thirty (30) days of receipt of this letter, it may be used with the full force and effect as though it has been read, corrected and signed.

If you have any questions regarding this matter, please contact this office at the below telephone number.

(415)777-2111

cc: All Counsel

STATE OF CALIFORNIA)
ss.
CITY AND COUNTY OF SAN FRANCISCO)

I hereby certify that the witness in the foregoing deposition was by me duly sworn to testify to the truth, the whole truth and nothing but the truth, in the within-entitled cause; that said deposition was taken at the time and place herein named; that the deposition is a true record of the witness' testimony as reported to the best of my ability, a duly licensed Certified Shorthand Reporter and disinterested person, and was thereafter transcribed under my direction into typewriting by computer; that the witness was given an opportunity to read, correct and sign the deposition.

I further certify that I am not interested in the outcome of said action, nor connected with, nor related to any of the parties in said action, nor to their respective counsel.

JILL N. HORAN, CSR #10306

Deposition of Bratter, Bernard
page 1

Delivery regular
invoice number / / / / /
a b c d e
job number __________
deposition date 3/20/89
client file number x
court name and number LASC C 594 148
consolidated with c 597 389

plaintiff(s) Montrose Chemical Corporation of california, a corporation,

defendant(s) canadian universal insurance company, inc., a corporation, et al.
____

stauffer chemical company, a corporation

vs.

american motorists insurance company, a corporation, et al.

page 1

Deposition of Bratter, Bernard
page 2

deponent name Bernard I. Bratter
volume one
reporter number and name h.sullivan 6600
reporter class c
delivery rate r
charge rate c
number of pages 110
appearance (only or certificate) x
summary,number of pages x
parking amount $19.00
hourly pay (overtime or waiting) wait 2 hours, 25 mins. or half day per diem
interpreter x

exhibits (number of pages) 1
original instructions to Mr. Conn
shipping (ups/overnight/messenger) x
original and (number of copies) 10
penalty of perjury or any notary POP
pursuant to subpoena

page 2

Deposition of Bratter, Bernard
page 3

firm name	deliver	bill
Gibson dunn crutcher	1cc	0 & 1
a.latham & watkins	original	cc
b. charlston, revich	1cc	1cc
c. ericksen arbuthnot	1cc	1cc
d.orrick, herrington	1cc	1cc
e.boornazian, jensen	1cc	1cc
wilson, kenna	1cc	1cc
buchalter, nemer	1cc	1cc
coudert brothers (5 1/4 ascii disk)	1cc	1cc
o'melveny & myers	1cc	1cc
mindlin, tigerman	1cc	1cc

please send a copy of the transcript and ascii disk to:
Drinker, Biddle & Reath
attn: Patricia A. Gotschalk, attorney at law
901 fifteenth street NW, suite 900
washington, D.C. 20005

any questions call michael holtzman (213) 277-5233
(he said 3" discover format for the disk)

Also send 5 1/4 ascii disk of transcript to coudert brothers.

page 3

Deposition of Bratter, Bernard
page 4

THE DEPOSITION OF Bernard I. Bratter, witness, taken ON BEHALF OF defendants, AT 333 south grand avenue, 48th floor, Los Angeles, CALIFORNIA 90071, AT 10:30 A.M.,

Monday, March 20, 1989, BEFORE heidi sullivan,
A CERTIFIED SHORTHAND REPORTER IN THE STATE OF
CALIFORNIA, LICENSE No. 6600, PURSUANT TO subpoena.

* * *

APPEARANCES OF COUNSEL:

FOR Plaintiff montrose chemical co.:

Latham & Watkins
BY: Richard A. Conn, jr., ESQUIRE
701 "b" street, suite 2100
san diego, California 92101
(619) 236-1234

FOR international insurance:

orrick, herrington & sutcliffe
BY: WIlliam W. Oxley, esquire
333 south hope street, suite 3100
Los angeles, California 90017
(213) 680-7000

FOR centaur insurance co.:

boornazian, jensen & Garthe
BY: Blaise S. Curet, ESQUIRE
300 lakeside drive, 16th floor
P.O. Box 12925
oakland, California 94604
(415) 834-4350

FOR admiral insurance company:

Wilson, Kenna & Borys
BY: andrew L. Finn, esquire
11075 santa monica boulevard, third floor
los angeles, California 90025

(213) 478-4285

page 5

For National Union Fire Insurance:

Buchalter, Nemer, FIelds & Younger
BY: Patricia M. Wilson, attorney at law
700 south flower street
los angeles, california 90017-4183
(213)626-6700

for Travelers insurance company:

Coudert brothers
by: craig H. Missakian, esquire
707 wilshire boulevard, suite 4600
los angeles, california 90017
(213) 688-9088

For INA:

o'melveny & myers
by: H. Douglas galt, esquire
400 south hope street
Los ANgeles, california 90017-2899
(213) 669-6000

page 6

I N D E X

WITNESS	EXAMINATION	PAGE
Bernard I. Bratter	BY Mr. Rezen	7

E X H I B I T S

Defendants'		Page	LIne
1	8/8/67 letter, bratter to Bridges	77	18

QUESTIONS WITNESS INSTRUCTED NOT TO ANSWER

NONE

Bernard I. Bratter,

THE witness HEREIN, HAVING BEEN CALLED BY AND ON BEHALF OF THE DEFENDANTS, WAS SWORN AND TESTIFIED AS FOLLOWS:

EXAMINATION

BY MR. REZEN:

Q Could you state your name for the record.

A Bernard I. Bratter.

Q Could you spell it for the reporter?

A b-e-r-n-a-r-d. Last name is b-r-a-t-t-e-r.

MR. CONN: Counsel, if I may interject, just prior to beginning the deposition you had asked me if I would attempt to make contact with Stauffer counsel to ascertain what their position would be with respect to use of Mr. Bratter's prior depositions in the underlying actions. In addition I took that to be a request for me to ascertain that from my client and I attempted to do that as well. I was not able to reach any of those parties. I took a couple minutes to figure out the phone system, but they were not available and I will certainly make further efforts to do that.

I take it from your request that perhaps the carriers are sensitive to the prospects that through this

deposition facts potentially could come out that would be harmful to their insured and are perhaps seeking ways of avoiding that from happening. We would suggest that if you would like a response to your request that again you posed to me just this morning that we adjourn the deposition to provide me with an opportunity to ascertain what that position would be and to continue the deposition when we have a response for you. I didn't want to take any more than the 20 minutes that I took me this morning, but I would be happy to get back to them if that was the concern of the carrier.

MR. REZEN: No, it wasn't a concern really. It was an attempt by me to not go over a lot of questioning that had taken place in the other depositions so we could save time and save Mr. Bratter some time.

MR. CONN: I would make the same suggestion. You asked me that when I walked in the room this morning. I am happy to ascertain the position of Stauffer and our client. I'm confident that I could do that and reach people; but people, for example, in Washington, where Stauffer's people are located, are out to lunch and might be available shortly. It's up to you, but I'm willing to do and that and see if I can accommodate you.

MR. REZEN: It is my opinion that we can use those transcripts -- And when I say "we", I'm talking about the

defendants in this coverage litigation -- that we can use those in this case regardless of whether or not we get a stipulation from you. It's also my understanding that that's not 100 percent clear because I want to make sure that there's not going to be any dispute in that regard later on in the case. I would feel more comfortable getting a stipulation from you and the attorneys from Stauffer before I go forward relying on my understanding.

With respect to your getting a definitive answer from your client and also from Stauffer in this regard, is it possible that you could leave word, if you haven't already, with both your office and the offices of the attorneys for Stauffer so that they could contact you during the lunch period? And by then if I get a stipulation from you and from Stauffer, the afternoon at least will go more quickly and I'll be able to refrain from asking a lot of questions this afternoon that I would otherwise ask if I get that stipulation at lunch. I'd

rather not adjourn the deposition at this point, but again if we could get a stipulation at lunch, that would save a lot of time this afternoon.
MR. CONN: The choice is yours. Obviously we're here pursuant to subpoena and the court on Friday in a telephonic discussion indicated that on Friday evening, anyway, it was too late to hear any questions with respect

to proceeding with the deposition. Therefore, it's your choice, in my view, as to how you would like to proceed. I am happy to leave messages, ask them to call me here. I don't know how to be reached and which conference room we were in so I told people I would get back. If you like I can leave messages specifically asking to be interrupted here in the conference room. That's up to you.
MR. REZEN: I'd prefer that. Once you've left those messages, we can get moving this morning.
MR. CONN: Your proposal would be we take a few minutes now for me to leave those messages?
MR. REZEN: Right.
MR. CONN: I'll do that.
(recess.)
MR. REZEN: We're on the record now and Mr. Conn has represented to me that he had spoken with the attorneys for Stauffer chemical company and that he on behalf of Montrose and they on behalf of Stauffer are willing to stipulate that the defendants in this litigation can use the transcripts of the deposition testimony of Mr. Bratter in the underlying Kelly, Newman case and in the underlying United States versus Stringfellow case in this litigation as though that testimony were given in this litigation.
Is that accurate, mr. conn?

MR. CONN: It is with a couple provisos, that is, an understanding that it is subject to our right to make any objections to the use of such testimony that would be available to us had in fact the deposition been taken in this case, and that would include but not be limited to the assertions of bad faith that we have ad nauseam raised

with you in previous depositions and we presume have been communicated to your respective clients.
Beyond that it is our understanding that we will discuss further the question as to whether at some point following this deposition we will reach agreement that the carriers would as part of this stipulation agree to provide us with some indication of areas that the carriers will be inquiring into with respect to depositions that have been taken in the underlying actions where the carriers contend that is not complete with respect to certain issues. I understand, however, that is an open point at this stage and that the carriers have by any means not agreed to that.
We have agreed in proceeding here today that the carriers would grant to the policyholders a continuing standing objection on the grounds of asked and answered since we have not reached any type of agreement with respect to the precise scope of the questioning today. That will relieve us from making those objections during

the course of the deposition.
MR. REZEN: We will agree that you have that standing objection but not that it's necessarily a valid objection. You have the right to make that standing objection, but the court will rule on whether or not it's valid.
MR. CONN: Right. All I'm saying, and I think we agree, it is as if I were making the objection as each question.
MR. REZEN: Right.
MR. CONN: And it's clear, again, that this stipulation is in no way a waiver of any other objections that the policyholders have made in this case to date or may make with the respect to the possible usage of this deposition, and without further ado --
MS. ABBOTT: I would like one clarifying question. You refer to bad faith as an objection, and I want to have it clear on the record the nature of that objection. It has to do with the relevance of the questions and the possible prejudice to your client as a result of those questions, does it not?
MR. CONN: I will state that I think it has been stated in part by David mulliken in the deposition of Mr. rotrosen that took place in New York. I think we've articulated it in part in previous depositions as well as

page 13

in certain court pleadings as well as in certain interrogatory requests for admission responses. I put it this way: It is the position of at least Montrose -- and I presume Stauffer would have the same viewpoint although I'm not speaking for them at this moment -- that there are a couple of acts of bad faith that we perceive taking place at this point.
One is indeed the prejudice the development of underlying facts has on the insured in this instance, both Montrose and Stauffer. We believe that makes it more likely that there will be a finding of liability of your insured in the underlying actions and we feel that that is indeed a situation where you're placing your interest ahead of those of the policyholder.
MS. ABBOTT: I mentioned that. I'm not asking you to make a speech about that, but just if there are any other aspects. What I'm concerned about is you're suggesting that we might be in bad faith for questions that someone in the underlying litigation asked. I assume you only mean if we attempt to use that information in this litigation; is that correct?
MR. CONN: Let me make it clear. I'm not trying to make a speech but I want to make sure the record is clear. We also assert the bad faith because we feel you are interfering with the settlement process in the underlying

page 14

actions and this conduct will make it far more difficult for the insured to settle the case and we feel that is in violation of duties to settle under California law. We feel the financial strain you're placing on Montrose -- again, I'm representing only them in terms of this speech. We feel you're breaching your contract to provide a defense by forcing us to pay that cost. That is a bad faith breach as well. This is by no means an exclusive --
MS. ABBOTT: as well as with respect to the use of Mr. Bratter's prior deposition?
MR. CONN: It would be our view that any use of such deposition testimony would indeed constitute an act of bad faith. We don't want to preclude that. The

address of any further development of factual information could indeed become the basis of an assertion of bad faith against each of your respective clients.
MS. ABBOTT: I think you answered the question. Thank you.
Q BY MR. REZEN: Mr. Bratter, my name is Randy Rezen. I'm an attorney in this insurance coverage litigation. I represent one of the insurance companies in this litigation between Montrose chemical corporation and Stauffer chemical company and the various insurance companies. It's my understanding that you have had your deposition taken in two underlying cases, one being the

United States versus Stringfellow case and the other one being the Kelly, Newman litigation; is that correct?
A Yes.
Q Have you had your deposition taken in any other litigations in the past?
A No.
Q I'm sure that your attorneys in those underlying actions to which I just made reference explained to you the deposition process, however, I feel compelled to explain that process to you once again so you have the rules in mind during this deposition.
First of all, you've been given an oath and that is the same oath that you'd be given in the court of law and carries with it the same penalties of perjury so it's important that you tell the truth today. Do you understand that?
A Yes.
Q On my left is a court reporter who is going to be taking down everything we say today, and after your deposition has been completed she will have what has been said prepared in transcript form and the transcript will be sent to you and you will have an opportunity to read the transcript, make any changes that you feel necessary in your testimony, and sign the transcript; however, I caution you that if you do make changes in your testimony

at a later time, those changes could adversely affect your

credibility at the time of trial in this case. It's important that you try to give your best and most accurate testimony today. Do you understand that?
A Yes.
Q Will you do that?
A Yes.
Q If at any time today you don't hear a question I ask, you should tell me that you don't hear it and I will speak more loudly. Will you do that?
A Yes.
Q If at any time today you don't understand a question that I ask, you should tell me that you don't understand and I will rephrase the question until you do understand it. Will you do that?
A Yes.
Q In everyday conversation we have a tendency at times to interrupt one another when we anticipate what the other person is going to say. It's important that we don't do that today because it's important for the court reporter to record what we're saying so I will try to wait until you're finished answering my questions before I ask another question, and I ask that you wait until I'm finished asking my question before you respond. Will you do that?

A I will try to.
Q We in this deposition are entitled to your best recollection with respect to events which have taken place in the past, and during this deposition I will be asking you questions about things that happened in the 1960s and 1970s. I realize you may not have precise recollections of specific dates and times, for example. If you have no recollection with respect to one or more of my questions, I would like you to tell me that. I do not want you to guess or speculate in responding to any of my questions, however, if you do have an estimate with respect to when a particular event took place, for example, I am entitled to your best estimate. Will you give me your best estimate when you have one?
A It's difficult for me to know the difference between an estimate and a speculation. I will try to differentiate but to me they sound very similar.
Q By way of example let me try to explain the difference to you. Let's say I asked you a question with respect to the date on which an event took place, and you

can recall that the event took place at some time during the 1960s, for example, you can recall that an event took place prior to the time Montrose began shipping waste to the Stringfellow site. You can't recall exactly when the event took place. You can't recall which month, for

example, it took place. If you were to tell me that it's your recollection that the event took place sometime prior to when Montrose began shipping waste to the Stringfellow site, that would be an estimate. If you were to tell me the specific month and the specific day on which the event took place that would be a guess. I'd be entitled to the former. I wouldn't be entitled to the latter. Does that explanation that I gave you give you an idea with respect to what I mean when I talk about the difference between an estimate and a guess?

A It gives me a good idea.

Q Okay. Is there any reason mental or physical today why you can't give your best testimony?

A None that I know of.

Q You presently live in the Southern California area; is that correct, Mr. Bratter?

A Yes.

Q How long have you lived in the Southern California area?

A Over 40 years.

Q Do you have relatives living in the Southern California area?

A Yes.

Q Do you have any relatives that live in riverside county?

A Not that I know of.

Q Have you ever had any relatives who live in Riverside county?

A Not on a permanent basis.

Q Have you ever had any relatives who lived there on a temporary basis?

A If Palm Springs is in that area I have a lot of relatives who come out there for a month or two.

Q Other than relatives who go to Palm Springs periodically have you ever had any other relatives that lived in the Riverside area?
A Not that I know of.
Q Do you have any acquaintances who live in the Riverside area?
A I have friends that have moved to Palm Springs.
Q Do you have any acquaintances who have moved to any place in the Riverside area other than Palm Springs?
A Not that I can think of.
Q How many acquaintances do you have who have moved to the Palm Springs area?
A Offhand I'd have trouble listing them. Maybe a dozen or two.
Q Can you remember when your first acquaintance

moved to the Palm Springs area?
A No.
Q Can you remember if it was before 1970?
A I don't think it was.
Q Do you recall if it was before 1980?
A I'm not sure when people moved there. It's nothing that happened at an exact time.
Q Do you recall ever having any conversations with any of these acquaintances regarding the Stringfellow site?
A I can't remember any conversations with them on Stringfellow.
Q Do you recall ever having any conversations with any of your relatives who visit the Palm Springs area regarding the Stringfellow site?
A No, I can't.
Q Can you provide me with the dates during which you were employed by Montrose chemical corporation?
A Yes.
Q What were those dates?
A Started in 1948, and was employed by them until 1983.
Q Have you done any work for Montrose since 1983?
A Yes.

Q On how many occasions have you done work for Montrose since 1983?
MR. CONN: I'd have to object. I think the question is vague in terms of what you mean by work.
You can answer.
Q BY MR. REZEN: Do you understand the question?
A I understand the question, but I don't know how to answer it.
Q What type of work have you done for Montrose since 1983?
A I'm a consulting engineer and I do consulting work for Montrose chemical.
Q You've done that since 1983?
A Yes.
Q Have you done consulting work for anyone other than Montrose chemical since 1983?
A Yes.
MR. CURET: Randy, before we go on can we make sure we all understand that Montrose is Montrose chemical corporation of California?
MR. REZEN: Sure.
Q I've used the term Montrose chemical corporation on several occasions so far. I was referring to montrose chemical corporation of California. Did you

understand that?
A That's my understanding.
Q For which entities other than Montrose chemical corporation have you done consulting work since 1983?
A I've done for a few other companies. I don't have their names Handy.
Q Are you being paid by Montrose for your services today?
MR. CONN: If I may put on the record, Randy, in my review of the different transcripts it struck me that with respect to the arrangements with respect to the consulting agreement that there was some type of confidentiality order in place. Do you recall that section of the transcript I'm referring to where there was a separate

transcription of the section in the Newman case?
MR. REZEN: You know, I really don't.
MR. CONN: I don't have a problem obviously with inquiry about that in terms of what his consulting arrangement is. What I would request is we have an opportunity to at least -- obviously we'll be continuing this tomorrow. I'd like to find out what that confidentiality provision is and be sure we're not violating anything by testifying about that. If you can put that on your checklist tomorrow we'll have a response

to you.
MR. CURET: Richard, let me refer you to Mr. Bratter's deposition, Volume two, taken April 20, 1988, page 235, et seq, which will hopefully shed some light for you.
MR. CONN: 235?
MR. CURET: Pages 235, et seq.
MR. CONN: I will review that and we'll talk to the attorney involved in that, mr. gotto. Thank you.
Q BY MR. REZEN: What was your job capacity when you began working for Montrose chemical corporation in 1948?
A I was a production supervisor.
Q During what period of time did you continue to be a production supervisor?
A The exact dates are vague, but it was around two years.
Q What was your job title after you discontinued being a production supervisor?
A I was a project engineer.
Q For what period of time were you a project engineer?
A 10 or 15 years.
Q Is it your best recollection that you continued to be a project engineer until approximately

1965?
A Somewhere in that range.
Q What was your job title after you

discontinued being a project engineer?

A   Plant superintendent.

Q   Did you continue to be a plant superintendent for Montrose until you retired in 1983?

A   Yes.

Q   Did you have any other job title other than plant superintendent at any time between the mid 1960s and 1983?

A   At Montrose the job title was not clearly defined, and it's difficult to put dates down for each one of these. These jobs evolved over a period of time and responsibilities we changed slowly. It was not a cut and dried thing from day-to-day on the various jobs.

Q   Were you ever told at any time after you became a plant superintendent for Montrose that your title had changed to something else?

A   No.

Q   Did your job duties at some time after you originally became a plant superintendent change?

A   The job duties would vary depending on conditions.

Q   What were your job duties and

responsibilities when you first became a plant superintendent for Montrose in the mid 1960s?

A   Basically it was to run the plant in a proper manner, to make high quality material at an efficient cost.

Q   At any time during the period of time you were a plant superintendent did your job duties in that regard change?

A   There were many occasions where different needs would come up, and I'd do various jobs in addition to what else I was doing.

Q   Can you provide me with a list of the various job duties and responsibilities you had during the time you were plant superintendent for Montrose?

A   At this date it would be impossible to provide you with an accurate list.

Q   you can take some time, if you like, and think about it and provide me with a list of what you can recall in that regard.

A   Well, one thing, I would negotiate contracts with the union. I would do purchasing of certain materials, hire new employees, supervise shipping,

basically anything else that was assigned for me to do.

Q Can you remember any of the other job duties and responsibilities you had? I'd like as complete a list

as you can provide me as you sit here today.

MR. CONN: For the record, you're asking him for everything he's done or everything that he had responsibility for for long periods of time? I'm not sure exactly what types of things you're going for. I'm not sure if the witness -- for example, if he took out a box once, I assume that wouldn't be the thing you're calling for. Are you looking for a course of responsibility?

Q BY MR. REZEN: I think what you've been giving me so far, Mr. Bratter, is responsive to the question I asked you, and to restate my question I'd like you to continue to list for me your job duties and responsibilities while you were a plant superintendent for Montrose.

A What you're asking me to do is turn my mind back to a certain period of time and it's difficult now for me to know if I Did something, whether it was in 1960 or 1975 or 1970 or '75. I'm not sure which jobs were done at what time anymore.

Q To the extent that you have a recollection of having any job duties and responsibilities in addition to those ones you've just listed while you were plant superintendent, I'd like you to provide them to me. If you can't remember any more, then I'll move on.

A I Did a fair amount of design work, too.

It's Design of different pieces of equipment that we use.

Q Can you recall anything else?

A Not offhand.

Q Is it your recollection that you became a plant superintendent for Montrose prior to the time Montrose began sending waste material to the Stringfellow site?

A Yes.

Q Can you recall approximately how long prior to that time you became a plant superintendent?

A I don't quite understand what you mean.
Q I'm trying to get an idea, a little bit more specific idea, with respect to when you became a plant superintendent. You've already testified it's your best recollection it was sometime in the mid 1960s. If you can tell me it was two or three years prior to the time Montrose began shipping waste to the Stringfellow site, that would provide me with a more specific idea with respect to when you became plant superintendent.
A It was in the neighborhood of three years.
Q To whom did you report at Montrose chemical corporation after you became plant superintendent?
A To Max Sobelman.
Q Did you report to anyone other than Max Sobelman?

A Mr. Ross Wilcox.
Q Were there any other individuals to whom you reported other than Max Sobelman and Ross Wilcox?
A Not directly.
Q Were there other individuals to whom you reported indirectly?
A President of Montrose.
Q That was Mr. Rotrosen; correct?
A Correct.
Q Samuel Rotrosen?
A Yes.
Q Did you continue to report to these three individuals during the entire period of time you were plant superintendent for Montrose?
MR. CONN: I'm going to object. I think the question is vague with respect to reporting to three individuals. To the extent you're encompassing both direct and indirect, I'm not sure what we have are respect to the indirect reporting. I think it's vague in that it encompasses direct and indirect.
Q BY MR. REZEN: Did you continue to report to these three individuals either direct or indirect during the entire time you were plant superintendent for montrose?
A No.

Q were there other individuals you reported to as plant superintendent?
A Yes.
Q Who were they?
A John Kallok.
Q Were there any other other than John Kallok?
A Richard Wilson.
Q Were there others?
A None that I can think of.
Q What was Richard Wilson's title?
A I'm not sure what his title was.
Q Did anyone ever tell you what his title was?
A If they Did, I don't remember. It was a short period of time.
Q What was John Kallok's job title?
A What period of time?
Q When you first began reporting to go him.
A He was plant manager.
Q When did you first begin reporting to John Kallok?
A Somewhere in the 1970s.
Q Can you recall if it was before 1975?
A I believe so.
Q Did John Kallok's job title change to something other than plant manager at some time after you

began reporting to him?
A I don't know when his job title changed because we never had official notices put on out on job titles.
Q But it was your understanding that at some point in time his job title Did change?
A I believe so.
Q Do you know to what?
A As I said, he was plant manager.
Q Did he become something else at some point in time other than plant manager?
A When our plant shut down that was his title.
Q When Did the plant shut down?
A In 1982.
Q When did you begin reporting to Richard Wilson?
A In 1981.

MR. REZEN: For the record, I would like conferences between Mr. Bratter and Mr. Conn noted.
(witness conferring with his counsel off the record.)
Q BY MR. REZEN: Did you report directly or indirectly to Mr. Richard Wilson?
A Indirectly.
Q When you use the term indirectly what do you mean?

MR. CONN: Objection. Overbroad.
Q BY MR. REZEN: When I say the term indirectly, I'm talking about in the context of communicating or reporting to these individuals.
MR. CONN: You're asking with respect to Mr. Wilson in particular?
MR. REZEN: Yes.
THE WITNESS: Generally I reported through Mr. Kallok.
Q BY MR. REZEN: Did there come a time when you stopped reporting directly to Richard Wilson?
A Yes.
Q When was that?
A Sometime in 1982.
Q Did there come a time when you stopped reporting to John Kallok?
A Yes.
Q Was that 1982 also?
A No.
Q When was it?
A When I left Montrose.
Q In 1983; correct?
A Correct.
Q Why did you stop reporting indirectly to Richard Wilson in 1982?

A He left Montrose's employment.
Q Did someone take his place at that point?
A No.
Q What was Max Sobelman's title when you reported to him?

A It depends on what period of time. He had various titles.
Q What was his title when you first started reporting to him?
A I'm not sure. He might have been plant manager.
Q Can you recall the titles he held during the period of time you reported to him?
A He later became a vice president of Montrose.
Q Was he ever anything, to your knowledge, other than plant manager or vice president?
A He might have been.
Q Can you recall what else he might have been?
MR. CONN: I'm going to object to that as calling for speculation.
Of course, if you know you should say so, but you shouldn't guess. I know the questioner doesn't want you to guess.
Q BY MR. REZEN: That's right. I don't want you to guess or speculate, but if you have an estimate or

best recollection with respect to other titles he held, I'd like you to tell me what they were.
A I don't have any clear knowledge of exact titles he had.
Q Can you recall when you first began reporting to Mr. Sobelman?
A When I went to work at Montrose in 1948.
Q Can you recall when you stopped reporting to Mr. Sobelman?
A Yes.
Q When was that?
A 1981.
Q Did you report directly to Mr. Sobelman?
A Some of the time.
Q When did you start reporting to Ross Wilcox?
A In 1948 when I went to work for Montrose.
Q When did you stop reporting to Mr. Wilcox?
A When he retired.
Q Can you recall when that was?
A In the early 1970s.
Q Did Ross Wilcox have more than one job title during the period of time you reported to him?
A Yes.
Q Can you recall what job titles he had during

that period of time?

A   I believe when I came there he was plant manager and later he became general manager.
Q   Did he ever have any job titles other than plant manager or general manager during the period of time you reported to him to your knowledge?
A   Not to my knowledge.
Q   During what period of time did you report to Samuel Rotrosen?
MR. CONN: Object to that as vague and uncertain. It also assumes facts not in evidence and is without foundation. I think his testimony was indirect. I don't think the question included that.
Q   BY MR. REZEN: During what period of time did you report on an indirect basis to Mr. Rotrosen?
A   Basically all the time I was there.
Q   From 1948 to 1983?
A   No. From the time I became a plant superintendent.
Q   Okay. Until 1983?
A   Yes.
Q   Did you have individuals who reported to you during the period of time you were a plant superintendent for Montrose?
A   Yes.
Q   Can you recall the names of the individuals

who reported to you when you first became a plant superintendent?
A   I had various production supervisors, and they had many people under them who indirectly reported to me and I couldn't list them all.
Q   What were the job titles of the individuals under them, the production supervisors who reported to you?
A   Chemical operators, operator helpers, various titles of type of equipment they would handle, all the way down to laborers.
Q   Can you give me in list form the job titles

of others who reported indirectly to you other than those you've already provided me?
A Shipping supervisor, and he had his crew under him, laborers, lift truck operators.
Q Can you recall any others?
A Not offhand.
Q Did these individuals -- the production supervisors, the chemical operators, the operator helpers, the laborers, and the shipping supervisors -- continue to report to you during the entire period you were a plant superintendent for Montrose?
A Yes.
Q Can you give me the names of these

individuals who you can recall who reported to you between 1965 and 1970?
A Lane Hodgins.
Q Is his first name spelled l-a-n-e?
A Yes.
Q How is his last name spelled?
A I'm not that good at spelling. I think it's h-o-d-g-i-n-s.
Q Can you list for me the other names you can recall?
A Nick Leonovitch, Bernie Hollander. Any of the others I'm not sure of the dates that they had the job.
Q Are the three that you just mentioned the only three you can recall who definitely reported to you between 1965 and 1970?
A Yes.
MS. ABBOTT: Can I ask for clarification are we talking about directly or indirectly here?
THE WITNESS: These reported directly to me.
MS. ABBOTT: Thank you.
Q BY MR. REZEN: What was lane Hodgins's title?
A At that time he was production supervisor.
Q What was Nick Leonovitch's title?
A The same.

Q What about Bernie Hollander?
A The same.
Q In addition to these individuals you've just identified I take it there were other individuals who reported to you at various times and you cannot recall the specific dates on which they reported to you; is that correct?
A Yes.
Q Can you give me the names of those other individuals?
A Jack Vaga.
Q What was Mr. Vaga's job title?
A Same. And Jerry Tenney.
Q What was Jerry Tenney's job title?
A The same. Frank Sains.
Q How is Mr. Sains's last name spelled?
A s-a-i-n-s, I think.
Q Was Mr. Sains also a production supervisor?
A Yes.
Q Can you recall the names of others who reported to you between 1965 and 1983?
A These are the only names of the supervisors I can think of.
Q And these individuals all reported to you directly; correct?

A Correct.
Q It is the case that the other categories of individuals which you identified earlier reported to you indirectly?
A Yes.
Q Did any of those other categories report to you directly?
A Not normally.
Q At what location did you work when you were a plant superintendent for Montrose?
A I worked at Montrose's Torrance plant.
Q Approximately how many individuals were employed at that plant in the mid 1960s when you became a plant superintendent?
A A payroll of the hourly people varied from, like, 50 to 200; in addition there were salaried people of around 20 to 30.
Q I take it that the individuals who you just identified were salaried people; correct?

A The production supervisors, most of them were salaried.

Q Can you recall any of the production supervisors who were not salaried?

A I believe it was the last three listed started out on the hourly payroll and at some point in

time got promoted to the salaried payroll. I don't have any accurate dates on it.

Q While you were employed by Montrose, Did there ever come a time when you became familiar with any federal, state, or local environmental rules or regulations?

A I don't quite understand the statement because it's so vague.

Q What part of the statement don't you understand?

MR. CONN: Objection. Arguing with the witness. I think, Randy, you invited the witness to tell you if he didn't understand and you would rephrase it during your prefatory remarks. he's just told you that and instead of arguing you could clarify it.

Q BY MR. REZEN: The reason I asked you that is because I want to know what part of the question you don't understand so I can rephrase it.

MR. CONN: Why don't we have the question read back.

(record read.)

MR. CONN: Maybe I can help. It seems to me the question is overbroad. It's also compound. It seems as if you certainly could break that down.

MR. REZEN: That's what I'm trying to do. First of

all I'd like to have the witness give me some feedback so I can start breaking it down.

MR. CONN: It seems to me if he says he doesn't understand it or it's vague, you should do that, go ahead and try to make it clear for him. I'm not going to have him enter into discussion with you about how to phrase your questions.

Q BY MR. REZEN: Were there specific terms in that question that you didn't understand?
MR. CONN: I'm going to object to the question as arguing with the witness.
MR. REZEN: I'll represent to you I'm simply trying to get some information from the witness so I can rephrase the question. It's not my intent to argue.
MR. CONN: Why don't you break it down. It's obviously compound.
MR. REZEN: Because if he tells me what words he doesn't understand, I can use different words.
MR. CONN: His comment is he thought the question was vague. Asking if there was not a word he understands isn't logical. Instead of battling over it, why don't you break it down.
MR. REZEN: It seems entirely logical to me if I determine from him which words he doesn't understand if any I can use different words --

page 41

Deposition of Bratter, Bernard
page 42

MR. CONN: You have my objection. I think you are arguing with the witness. If he can add anything, he's free to do it but it's not up to him to help you construct your question.
THE WITNESS: I'm not sure what you mean by environmental rule. I don't remember any city rules about it. The specific rules that you're referring to, I would like to see them and I can tell you whether I'm acquainted with them.
MR. CONN: That's fine.
Q BY MR. REZEN: Are you familiar with the resource conservation and recovery act?
MR. CONN: I'm going to object to the question to the extent it's vague and ambiguous with respect to the term familiar.
Q BY MR. REZEN: Have you ever heard of the federal resource conservation and recovery act?
MR. CONN: Sitting here today?
MR. REZEN: Yes.
THE WITNESS: I don't remember that.
Q BY MR. REZEN: Have you ever heard of the comprehensive environmental response, compensation and liability act?
MR. CONN: Sitting here today?
MR. REZEN: yes.

page 42

Deposition of Bratter, Bernard
page 43

THE WITNESS: No, I don't remember that one.
Q   BY MR. REZEN: Have you ever heard of the federal water pollution control act?
MR. CONN: Same thing, as of today?
THE WITNESS: I've heard of it.
Q   BY MR. REZEN: Can you recall when you first heard of it?
A   No.
Q   Can you recall how you first heard of it?
A   No.
Q   Can you recall if you heard of it in the context of your job as plant superintendent for Montrose?
MR. CONN: Are you asking if he heard about it back in a certain time frame in the 1970s?
MR. REZEN: Yes. And once he tells me whether or not he Did, I'll try to narrow the time down.
THE WITNESS: I've heard of water pollution acts. I'm not positive of the one you mentioned, whether that's the one I heard about or not.
Q   BY MR. REZEN: Okay. Can you recall when you first heard about a water pollution control act?
A   I think it was in the 1970s.
Q   Can you remember if it was before 1975?
A   I believe it was, but I'm not positive.
Q   Can you recall from whom you heard about the

page 43

Deposition of Bratter, Bernard
page 44

water pollution control act?
A   No.
Q   Can you recall if it was someone at Montrose?
MR. CONN: Object as assuming facts not in evidence, namely that he Did in fact hear about it during that time frame. I think he testified that he's not sure. Your question also assumes facts not in evidence assuming it's an individual who advised him of this. I don't think you established that foundation.
Q   BY MR. REZEN: Can you recall from what source you heard of the water pollution control act?
A   No.
Q   Can you recall if it was someone at Montrose?
A   No.

Q Can you recall if you heard of the water pollution control act in the context of your job duties and responsibilities with Montrose?
A I really don't know how to answer that question.
MR. CONN: If you know, you should say so; if not, you shouldn't speculate or guess.
THE WITNESS: Okay.
MR. CONN: If you don't know, you should say you don't know.
THE WITNESS: I don't know.

Q BY MR. REZEN: Have you ever heard of the toxic substances control act?
MR. CONN: Sitting here today?
MR. REZEN: Yes.
THE WITNESS: Yes.
Q BY MR. REZEN: Can you recall when you first heard of that?
A No.
Q Can you recall if it was before 1975?
A I don't believe it was.
Q Can you recall if it was before 1980?
A I'm not sure whether it was or not.
Q Can you recall from whom you heard of the toxic substances control act?
MR. CONN: I'm going to object again assuming facts not in evidence. Lack of foundation.
MR. REZEN: I'll rephrase the question.
THE WITNESS: Can I have a minute for a conference?
MR. REZEN: Sure.
(witness confers with his counsel outside the room.)
MR. CONN: One problem the witness is having, to call it to your attention, is that he is part of the technical committee in the Stringfellow case, and it is my understanding as well as his that all discussions involved in that are confidential and some of your recent

questioning, Randy, I think, in asking for his knowledge about certain things puts him in a position of perhaps

testifying about things he learned in the course of those committee meetings and he of course cannot testify as to that. There is apparently some type of an agreement in that case and for that reason he wanted to have a discussion about that. I've advised him not to violate any confidentiality agreement that has been reached in connection with his duties on the technical committee.

MR. REZEN: I'm not particularly interested in his activities on the technical committee to the extent that they occurred after 1980, and I think they Did. I'm sure other people will be interested in that and will want to question him on that. I'm sure that this issue is going to come up again.

MR. CONN: So be it.

Q BY MR. REZEN: Mr. Bratter, are you being represented by Mr. Conn today?

A Yes.

Q Is he your attorney?

MR. CONN: I'm going to object as asked and answered. We are representing Mr. Bratter today.

Q BY MR. REZEN: Mr. Conn just stated on the record that you are a member of a technical committee relating to the Stringfellow case or Stringfellow site,

page 46

rather. Is that true that you are a member of such a committee?

A Yes.

Q Does that committee have a title?

A Stringfellow technical committee.

Q When did you become a committee member of that committee?

A 1982 or '83.

Q And are you still a member of that committee today?

A Yes.

Q Later in the deposition I may ask you some further questions regarding your membership in that committee and things that you've learned as a result of being a member of that committee. For the time being, however, you can assume that the questions that I ask regarding knowledge of environmental rules and regulations refer to the time period prior to when you became a member of that Stringfellow technical committee. With that in mind can you recall at any time in the 1970s becoming aware of the toxic substances control act?

A I don't remember whether I Did in the 1970s or not.
Q Can you recall how you became aware of the toxic substances control act?

MR. CONN: I'm going to object as assuming facts not in evidence in that he became aware of it. It's particularly difficult, Randy, given the comment you made that it should be taken without regard to what he may have learned on the committee. I'm not sure how he can piece his way through this. I don't understand what he should be answering here.
Q BY MR. REZEN: You have heard of the toxic substances control act; correct?
A Yes.
Q Do you have a belief with respect to what the toxic substances control act is?
A Only a vague understanding.
Q Can you describe for me what your understanding is?
MR. CONN: I would caution the witness to the extent this does get into areas where you've learned information from your role in that committee that we've discussed in order to answer that we would take a look to see if a court order would be necessary on anything you learned during those meetings.
MR. REZEN: You're really pushing it on his membership in that meeting. I'm not sure of the basis of any objection to questions relating to that membership. At this point I've said that I'm not going to ask specific

questions about that. I may do so later in the deposition, but I do clearly think I'm entitled to know his understanding of what the toxic substances control act is at this point.
MR. CONN: I guess, counsel -- I'm not trying to be difficult. I think there are plenty of things he can answer. I'm only carving out what he has learned in that context. The analogy i provide you with is obviously in an attorney-client context you may have discussions about

a topic and then if a questioner says, "what is your understanding about a subject area?" If that comes from discussions with your attorney, that obviously is privileged. I think what you're asking is the same type of things.

I'm telling him if it comes from his committee work and he learned it in that context, it is my understanding that is confidential and there's an agreement to that effect. I'm saying until such time as we can determine whether that can be waived or breached without court order I'm not going to permit him to answer questions in that regard. I'm not stopping him from giving his understanding independant of what he gained other than at that committee.

MR. REZEN: Why don't we start with that.

Q Could you provide me, Mr. Bratter, with your

understanding of what the toxic substances control act is?

MR. CONN: With that proviso, that you have the understanding without respect to your work on the committee.

THE WITNESS: I've heard of the act, but I'd have to see it in front of me to discuss it with you.

Q BY MR. REZEN: From what source did you hear of the act?

A I don't remember.

Q Can you recall becoming aware of any state rules or regulations regarding water quality while you were a plant superintendent for Montrose?

MR. CONN: Object as overbroad and vague and uncertain.

Q BY MR. REZEN: For your information, Mr. Bratter, Mr. Conn throughout today and probably tomorrow will at times make objections for the record.

MR. CONN: Meritorious objections for the record.

Q BY MR. REZEN: If after he makes an objection you forget the question, please ask the reporter to read it back and she'll be happy to do so. Unless he instructs you not to answer, I would like you to answer and I think he'll tell you that I have the right to your answer unless he instructs you otherwise. With that in mind --

MR. CONN: I agree except to the extent, counsel,

if he does not understand a question or wants it clarified, he is entitled to indicate that to you.
MR. REZEN: Clearly, yes. I've made that clear, I think.
Q Do you have the question in mind?
A What is the question?
(record read.)
THE WITNESS: Right now I don't know which of any regulations were state, city, or federal so I couldn't recall any.
Q BY MR. REZEN: Can you recall becoming aware of any such regulations regardless of whether they were state, city, or federal -- again, regulations relating to water quality?
MR. CONN: Question is vague and uncertain as to time and overbroad.
Q BY MR. REZEN: The time period I'm talking about is the time during which you were plant superintendent for Montrose.
MR. CONN: It's also ambiguous as to aware.
THE WITNESS: I'm not sure what you mean by it.
MR. CONN: Why don't we have the question reread.
(record read.)
MR. CONN: Perhaps you could, since it came out a bit disjointed, you would rephrase that, Counsel. It may

help.
Q BY MR. REZEN: Have you at any time in the past become aware of -- let me strike that and start again.
During the period of time you were plant superintendent for Montrose, were you ever made aware of the existence of any governmental rules or regulations regarding water quality control?
MR. CONN: Same objections. I think it's overbroad. It's vague as to time. I'm not sure exactly what years we are talking about, and it's also ambiguous as to aware.
Q BY MR. REZEN: Do you have the question in mind?
A Well, I'm not sure what you mean by water. There were regulations on various kind of water. I don't

know which ones you mean.
Q Well, which ones do you mean?
A It's your question.
Q You just said there were various regulations.
A Yeah. Drinking water, for example.
Q Can you remember any others?
MR. CONN: What time period are we talking about?
MR. REZEN: I've identified the time period and I'll be more specific when I got a specific answer from

him.
MR. CONN: I think the question is vague and ambiguous.
Q BY MR. REZEN: Mr. Bratter, you've just made reference to drinking water quality control regulations. Can you recall water quality control regulations of any other nature of which you became aware while you were plant superintendent for Montrose?
MR. CONN: Mischaracterizes testimony.
THE WITNESS: There were regulations by the county sanitation district on what material could flow into their sewer lines.
Q BY MR. REZEN: Can you recall any others?
A Not at this time.
Q How did you become aware of the drinking water quality regulations to which you made reference?
A I would read about it in the literature.
Q What literature was that?
A I don't remember.
Q Was it literature you received from Montrose?
A I'm not sure where I got the literature.
Q Can you recall when you first became aware of such regulations?
A No.
Q How did you first become aware of the county

sanitation district's rules regarding materials which could flow into their sewer lines?
MR. CONN: The question is vague and ambiguous as to aware.

But You may answer.
THE WITNESS: At some point in time we used to run tests on samples in our sewer line and report to county sanitation what was in it. It wasn't part of my responsibility but I used to hear about it.
Q BY MR. REZEN: Can you remember when you first started running such tests?
A No.
Q Can you remember if it was before 1970?
A No.
Q From whom did you hear about it?
A Other people at Montrose.
Q What were their names?
A I'm not sure who I heard it from.
Q Can you remember the names of any of those individuals?
A Not with any accuracy.
Q Can you give me a best recollection without speculating or guessing?
A No.
Q Earlier I asked you to provide me with the

names of individuals who reported to you at Montrose and individuals to whom you reported, and you provided me with lists of both. In addition to those individuals you've already identified can you identify all other individuals with whom you communicated while at Montrose, that is, while you were plant superintendent?
MR. CONN: Objection. The question is overbroad and unduly burdensome, calls for a narrative answer. I'd like to hear it again. I'm not sure I want him to answer that one. You're asking for all communications he's had with anyone?
MR. REZEN: No.
MR. CONN: Why don't we have it read back, then.
(record read.)
MR. CONN: I think the question is vague and uncertain. It is unduly burdensome and overbroad. It doesn't sound like you've limited that in any way.
THE WITNESS: The answer is no.
Q BY MR. REZEN: Can you provide me with the names of such individuals you can recall as you sit here today?
MR. CONN: Same objections.
THE WITNESS: I probably communicated with hundreds

of people, and I wouldn't even attempt to try to list them. This was 20 some years ago. It would be impossible

for me to attempt that.
Q BY MR. REZEN: To the extent you can recall any of their names in addition to the names you've given me I'd like you to provide me with those names. I realize you can't recall all of them, but if you can remember some I'd like those names.
MR. CONN: You're asking everyone he spoke with over a 30-year period, is that your question?
MR. REZEN: I believe he testified he was plant superintendent from the mid 1960s to --
MR. CONN: '83.
THE WITNESS: 18.
MR. CONN: You want all conversations over 18 years?
MR. REZEN: I'm not asking conversations. I'm asking individuals with whom he communicated and only those he can remember as he sits here today.
MR. CONN: You have my objection. I'll incorporate them into this question.
If you have anything you want to add, you may do so.
THE WITNESS: I don't have anything to add. It's like trying to empty a river by using a spoon. You never get anywhere.
Q BY MR. REZEN: Can you recall any other

names, Mr. Bratter, other than the ones you've given me?
MR. CONN: I realize I have an asked and answered objection standing here so I won't make it again, but if you have anything to add, say so. If you don't --
THE WITNESS: Not at the moment.
MR. CONN: Perhaps if you're more specific, counsel, he'll be able to help you.
Q BY MR. REZEN: Do you know where Richard Wilson now lives?
A No.
Q When did you last communicate with Mr.

Wilson?
A 1982.
Q Did you know where he lived at that time?
A No.
Q Did you have an understanding with respect to what Mr. Wilson's job duties and responsibilities were when you reported indirectly to him?
MR. CONN: Object as calling for speculation.
If you personally know, you may say so.
THE WITNESS: Well, he was above Mr. Kallok in the line of command.
Q BY MR. REZEN: Do you have any additional understanding of what his job duties and responsibilities were other than being above Mr. Kallok in the line of

command?
A No.
Q Is Mr. Sobelman alive to your knowledge?
A No.
Q What about Ross Wilcox?
A No.
Q Do you know where Lane Hodgins lives?
A Yes.
Q Where does he live?
A In Westchester.
Q When is the last time you communicated with him?
A About a month ago.
Q What about Nick Leonovitch?
A He had a house in Lakewood.
Q When did you last communicate with him?
A Four or five years ago.
Q What about Bernie hollander?
A He lives in Torrance, California.
Q When did you last communicate with him?
A About a month ago.
Q Do you know where Jack Vaga lives?
A No.
Q When did you last communicate with Jack Vaga?
A 1982.

Q Did you know where he lived at that time?
A He lived in Torrance.
Q Do you know where Jerry Tenney lives?
A No.
Q When did you last communicate with Jerry Tenney?
A 1982.
Q Did you know where he lived at that time?
A It was somewhere in the torrance area. I don't remember the exact address.
Q What about Frank Sains? Do you know where he lives?
A I was told that he passed away some time ago.
Q Have you ever heard of an individual by the name of Walter Winans?
A I don't recall the name.
Q Have you ever heard of a name individual by the name of Jim Gallagher?
A I don't remember meeting anyone with that name.
Q Have you ever heard of anyone by the name of Janet Ignatovich?
A No.
Q Did you ever communicate with anyone in Stauffer chemical company's insurance department?

A I don't believe so.
Q Were you at any time before today aware of the fact that Stauffer chemical company had an insurance department?
A Yes.
Q Can you remember when you first became aware of that fact?
A When I was with Montrose chemical we carried medical insurance on all our employees, and it was carried with the same carrier that Stauffer chemical used, and I believe that our medical insurance claims were processed through Stauffer's insurance department.
Q Were you ever told by anyone that Montrose had any insurance through Stauffer other than medical insurance?
A Not that I can remember.
Q Were you at any time while a plant superintendent for Montrose told that Montrose had

liability insurance?
A I don't remember hearing that.
Q Earlier you described your job duties and responsibilities as plant superintendent for Montrose, and one of those job duties and responsibilities was, in your words, to run the plant in a proper manner and to make high quality material at an efficient cost. The question

I have for you is this: What material were you referring to?
MR. CONN: Objection. Overbroad.
Q BY MR. REZEN: Could you answer the question, Mr. Bratter?
MR. CONN: If you can.
THE WITNESS: Well, the main product Montrose produced was DDT.
Q BY MR. REZEN: So was it your job responsibility while plant superintendent at Montrose to make sure that montrose manufactured high quality DDT?
A Yes.
Q Was it your responsibility while a plant superintendent to make sure that Montrose manufactured anything other than DDT?
A Well, DDT was the main product that we produced and sold. We had a by-product that was sold part of the time.
Q What was that by-product?
A It was recovered acid.
Q Were there any other materials which you were responsible for manufacturing?
A No.
Q While a plant superintendent for Montrose, that is?

A No.
Q Did you refer to the recovered acid to which you just made reference as spent sulfuric acid?
A It was also called that.
Q What was the chemical content of spent sulfuric acid manufactured by Montrose?

MR. CONN: Counsel, I would only note that it does seem to me that this area you're in now has been very extensively covered in previous depositions. Again I understand if you want to lay a couple foundational points to get into new areas, but it seems we're in the meat of what was covered previously.

Q BY MR. REZEN: Is it correct that the spent sulfuric acid was made up of sulfuric acid, chlorobenzene, sulfonic acid, and DDT?

A No.

Q What was the chemical content of spent sulfuric acid which was a by-product of what you manufactured at Montrose while being a plant superintendent?

A The main part was sulfuric acid which was roughly 75 to 80 percent. {mainmay not} impurity was chlorobenzene sulfonic acid, roughly 2 to 6 percent. The rest of the composition was water.

Q You would characterize the chlorobenzene

sulfonic acid as the main impurity. What did you mean by that?

A There were traces of other impurities.

Q What did you mean by the word impurity?

A Something other than the ingredients listed.

Q The ingredients listed where?

A The ones I just listed for you.

Q Well, chlorobenzene sulfonic acid was one of the ones you listed.

A Yes.

MR. CONN: Objection. Arguing with the witness.

MR. REZEN: I don't mean to argue.

A Okay.

Q I'm trying to clarify your testimony in my own mind.

You characterized chlorobenzene sulfonic acid as an impurity; correct?

A Correct.

Q Would you characterize sulfuric acid as an impurity?

A That was the main ingredient. No.

Q What other impurities, if any, were contained in this spent sulfuric acid?

A There were traces of DDT and traces of tar.

Q Were there any other impurities in this spent

page 63

Deposition of Bratter, Bernard
page 64

sulfuric acid which we're talking about?
A There was some decomposition products of DDT but I'm not sure what they were.
Q Any others?
A Not that I know of.
Q Can you recall when you first became aware of the materials which were contained in this spent sulfuric acid?
MR. CONN: I'm going to object as vague and uncertain. I'm not sure what you're asking the witness.
If you understand it, you may answer.
Q BY MR. REZEN: Did you understand the question?
A No.
Q I don't want to belabor this point but I would like you, Mr. Bratter, to explain to me, if you can, how the spent sulfuric acid was generated in the process of producing DDT.
MR. CONN: I would simply note again, counsel, I think this all has been extensively covered, and I guess while we were discussing off the record how our stipulation would work it seemed to me one thing we were talking about is a reasonableness standard in terms of permitting you to lay certain foundation but not to be duplicating material. We're only moving forward with Mr.

page 64

Deposition of Bratter, Bernard
page 65

Bratter's deposition under that stipulation As a test case for us. I would ask you not to repeat things that were covered extensively.
MR. REZEN: That's why I prefaced it with the fact I didn't want to belabor it. I will represent to you if you don't continue to make the objections we'll move relatively quickly but if you continue to, its going to take a long time.
MR. CONN: I'm trying to call your attention to the fact that there are major subjects we have covered.
MS. ABBOTT: I understood there was a stipulation with you with a reservation of certain objections. I want to make it clear that I have not stipulated that areas

previously covered are completely off limits or off limits except for foundational reasons. On behalf of Canadian universal I've made no stipulation of any sort. You have your objections.
Q BY MR. REZEN: Do you have the question in mind?
A Would you repeat it, please?
MR. REZEN: I'll have the reporter read it back. Please listen to the question carefully, Mr. Bratter, and provide me with a response.
MR. CONN: Let me clarify one thing. Is it Canadian's position, counsel, that even though we are

permitting, under the stipulation that we've discussed, you to use the deposition under the caveats we laid out earlier, that Canadian nonetheless may repeat all examination contained in those depositions? Is that your position?
MS. ABBOTT: I don't have any expectation that that would occur.
MR. CONN: at this point we're talking about a reasonableness standard. We haven't gotten down to any specific line, but we're talking about reasonableness. Do you find something objectionable in that?
MS. ABBOTT: I don't want to be bound by any stipulation as to what can or cannot be covered in relation to previous testimony. If the issue arises, it will have to be dealt with on a law and motion basis or agreement basis or whatever stipulation basis we can arrive at. At this point I don't want to make any binding stipulation.
MR. REZEN: We've already said we're going to have to deal with this issue after today with respect to future witnesses. For purposes of Mr. Bratter's deposition we're not going to get bogged down with that issue. We're going to have to deal with that later, but I don't think we have to deal with that now. I thought you understood that at the beginning of today's deposition.

MR. CONN: I understand what we agreed to. What

concerns me is when she begins her questioning she can feel she can go through everything that is covered in the underlying deposition transcripts. I don't view that as what we stipulated to. I understand we haven't put a bright line in, but I understand we are trying to act reasonably.

I understand what reasonable is with respect to this deposition is something that could be brought to a court, but I am troubled if it's Canadian's position that our willingness to save time here by providing these underlying depositions is simply going to be ignored by Canadian and you're going to make our witness go through everything again it would seem we gain nothing by the stipulation. I don't think that's fair. Is there a problem that I'm missing?

MS. ABBOTT: It is not my intention to go into detail into the matters discussed in Mr. Bratter's prior deposition or to do anything other than what is reasonable.

MR. CONN: Why don't we proceed then.

MR. REZEN: I'd like to have the question read back, Mr. Bratter. Listen to it carefully and we'll try to move along.

(record read as follows: I don't want to belabor

this point but I would like you, Mr. Bratter, to explain to me, if you can, how the spent sulfuric acid was generated in the process of producing DDT.)

THE WITNESS: In the previous deposition it was covered quite extensively, but briefly DDT is produced in the presence of sulfuric acid. The DDT is filtered out of the acid leaving a spent acid that has 20 to 25 percent MCBS or monochlorobenzene sulfonic acid. This acid is then washed with monochlorobenzene removing most of the MCBS and leaving the recovered acid with the analysis that we stated earlier.

MR. FINN: Can you read back the answer.

(record read.)

MR. CONN: For the record, counsel, it's sort of unstated but I presume that all counsel are aware that the transcripts of these underlying depositions have been made available in connection with the monitoring, if you will, of the underlying actions, and I presume all counsel here are familiar with those transcripts or have had them made available to you.

MR. REZEN: I'm familiar with them. I don't know about the other people. I assume they are.
MR. CONN: Does anyone here not have access to them or need them?
MR. CURET: I can state for the record simply I do

not have a copy of the transcript of Mr. Bratter's deposition in the U.S.A. versus Stringfellow action.
MR. CONN: I guess my thought would be if we are thinking of streamlining this and you would like to ask questions of this witness in light, particularly, of the stipulation, perhaps it would make sense to obtain those from one of the other counsel and review that. I want to be sure we are all operating from the same premises.
I'm sorry to interrupt, counsel.
Q BY MR. REZEN: When you became plant superintendent for Montrose, did your job duties and responsibilities include making decisions with respect to what was done with the spent sulfuric acid?
A Yes.
Q Was there anyone else at Montrose at the time you were plant superintendent who participated in those decisions?
A Yes.
Q Who was that person?
A Max Sobelman and Ross Wilcox.
Q Anyone else?
A Not that I'm aware of.
Q Were you aware of the fact that spent sulfuric acid was generated as a by-product of the DDT manufacturing process before you became plant

superintendent for Montrose?
A Yes.
Q When you became plant superintendent for Montrose did you have conversations with either Max Sobelman or Ross Wilcox regarding what should be done with the spent sulfuric acid?
MR. CONN: I'd caution the witness if you have a recollection give it but not to speculate or guess.

THE WITNESS: You're referring to the time I became plant superintendent?
Q BY MR. REZEN: Yes.
A I don't believe we Did at that time.
Q Do you recall what was being done with the spent sulfuric acid at that time?
A I believe it was being sold to the fertilizer industry.
Q After you became plant superintendent did you communicate with others regarding the selling of spent sulfuric acid to the fertilizer company?
MR. CONN: Objection. the question is vague and uncertain and overbroad.
If you understand it you can answer.
THE WITNESS: My function was to produce the acid, not to sell it.
Q BY MR. REZEN: You Did testify earlier that

it was also your function to decide what to do with it; isn't that correct?
MR. CONN: Object as arguing with the witness.
Q BY MR. REZEN: I'm not trying to argue with you. I'm trying to make sure I have your testimony straight in my own mind so I can ask some follow up questions.
MR. CONN: Why don't we have the question reread and see if it does correctly state your previous testimony.
(record read.)
THE WITNESS: My understanding in your question was that when you asked me if I was to decide what to do with the acid it was at a different period of time from the time I became plant superintendent. Second question you asked, when I became plant superintendent Did I discuss the sale of the acid.
Q BY MR. REZEN: Okay. Was there a period of time when you were plant superintendent when it was your job responsibility to decide what to do with the spent sulfuric acid?
A Yes.
Q During what period of time was that your job responsibility?
A When sales department was not able to sell

all the recovered acid we produced, it became necessary to dispose of the excess, and then I had a hand in deciding where it would go.
Q   Can you recall being told at some time after the time you became plant superintendent that Montrose was unable to sell all of Its spent sulfuric acid?
A   I don't --
MR. CONN: Object as overbroad and vague in terms of time periods, whether it's a constant practice. It's unclear. He can answer it.
THE WITNESS: I don't remember hearing anything said that way.
Q   BY MR. REZEN: Can you recall how you learned that Montrose was unable to sell all of Its spent sulfuric acid?
MR. CONN: Are you asking generally or at any given event?
MR. REZEN: For the first time.
THE WITNESS: I don't remember when the first time was.
Q   BY MR. REZEN: Do you have a recollection of ever being informed that Montrose was unable to sell all of Its spent sulfuric acid?
A   I remember discussions on it. I don't know when and with whom.

Q   What can you recall about those discussions?
A   Montrose produced this recovered acid and we had large storage tanks. When we sold it, it would be shipped out of the storage tanks. When the tanks would start to get too full then there was a concern where the acid should go to. If it wasn't sold it would have to be disposed of.
Q   Can you recall at any time while you were a plant superintendent for Montrose making a decision with respect to what to do with spent sulfuric acid?
A   Can you clarify it by decision? Are you talking about my decision or joint decision?
Q   Let's start with your decision and then we'll go from there.
A   No. I don't recall making the decision

specifically.

Q   Can you recall at any time while you were plant superintendent for Montrose participating in a decision regarding what to do with spent sulfuric acid?

A   I can remember being informed that we should ship some to a disposal site.

MR. REZEN: Could I have that answer read back.

(record read.)

Q   BY MR. REZEN: Who informed you of that?

A   It's hard to say specifically who I heard it

from many years ago.

Q   Is it your best recollection that either Max Sobelman or Ross Wilcox informed you that the spent sulfuric acid should be shipped to a disposal site?

MR. CONN: Again, I caution the witness not to guess.

THE WITNESS: I believe it was one of the two gentlemen.

Q   BY MR. REZEN: Did you have an understanding at that time with respect to what they meant by disposal site?

MR. CONN: I'm going to object at your asking the witness to speculate as to the thoughts of a third party.

Q   BY MR. REZEN: Actually I'm not asking you to tell me what they meant. I'm asking if you had an understanding as to what they meant.

MR. CONN: Same objection.

Q   BY MR. REZEN: Or a belief with respect to what they meant.

MR. CONN: Again, you're calling for him to speculate.

THE WITNESS: It's too vague for me to know what you mean.

Q   BY MR. REZEN: When you were told by either Max Sobelman or Ross Wilcox that the spent sulfuric acid

should be shipped to a disposal site did you have a belief with respect to what was meant by disposal site in that context?

MR. CONN: Assuming facts not in evidence. Are you suggesting that that's a quotation from one of those individuals, the word disposal site? Because I'm not sure there has been any testimony to that effect. Your question premised on those words being used.

MR. REZEN: Mr. Bratter testified that he was told by someone, either Max Sobelman or Ross Wilcox, he believes, that the sulfuric acid should be sent to a disposal site.

MR. CONN: I didn't understand his testimony to be quoting anyone 20 years ago, however, it's your deposition. If you want to leave the record as it is, that's fine.

Q BY MR. REZEN: Is it your general recollection that the words disposal site were used in that context?

A I don't remember if they used those words or not.

Q You do have a recollection, do you not, that you were told the sulfuric acid should be shipped someplace?

MR. CONN: Object as vague and also

mischaracterizes the testimony.

Q BY MR. REZEN: I'm just asking for your best recollection, Mr. Bratter.

A I don't know when we're talking about.

Q Well, earlier you were unable to give me a specific date, but you said you had a general recollection, and so all I can do is work with that general recollection unless you can give me a specific date or a general date for that matter.

MR. CONN: What is the specific question now?

If you don't have the question in mind, why don't you read it back or you can pose one.

MR. REZEN: Could I have it back.

(record read.)

MR. CONN: I think it's overbroad and vague in terms of time.

If you can answer, go ahead.

THE WITNESS: Again, such a broad period of time we're talking about. It might have happened on many occasions, and I don't know what we're referring to specifically.

Q BY MR. REZEN: Well, if you can narrow the

time for me, I'd like you to do that.
MR. CONN: Is there a specific question pending? If you can give him a question, I'll have him answer it.

MR. REZEN: We just read the question and that's the question that is pending.
MR. CONN: He told you he's having difficulty with the question. You're then making statements. I'm asking if you can form a question.
MR. REZEN: Let me explain the problem.
Q I've asked you for dates and you told me you couldn't remember dates but then you told me you could recall generally being told sometime in the past that the sulfuric acid should be shipped to a disposal site and now you're telling me that my follow up questions are difficult for you because I'm not putting a date on them when you've already told me you're having a problem putting a date on them yourself so that's the problem we have.
All I'm trying to do here, Mr. Bratter, is to get your recollection and I'm trying to do the best I can with the recollection you have. If you can't remember anything that happened over the last 20 years we're not going to go anyplace and we'll have to conclude the deposition, but I think you can remember some things and I'd like your best recollection keeping in mind that some of these things happened 10 to 20 years ago.
MR. CONN: I find your comments argumentative and harrassing and I think it's inappropriate for you to

direct such comments towards the witness. If you have questions to ask, that's why we're here. I then put objections on the record and The witness does his best. If he doesn't understand them, he'll tell you. if he finds it vague, you can rephrase them. I don't think you should batter and berate the witness for not having an easier time with your questions. I find them vague. I put it on the record. The witness has told you when he's having difficulty with them. Again, I ask you to pose them to him and he will do his best.

Q BY MR. REZEN: I'm going to hand you a document, Mr. Bratter, which we'll mark as Defendants' Exhibit No. 1.
For the record this is a document which has at the bottom the number LW001245. It appears to be a letter dated August 8, 1967, from Mr. Bratter to a Ms. Marjorie bridges at the Stringfellow quarry company.
(The above-mentioned document was marked for identification by the certified shorthand reporter And attached hereto.)
Q BY MR. REZEN: Do you recognize this letter, Mr. Bratter?
A Yes.
Q This letter was used, I believe, at your previous depositions. Do you have a recollection, a

general recollection, that is, of sending this letter to Ms. bridges on or about Its date, August 8, 1967?
A Yes.
Q Does this letter refresh your recollection with respect to when you were told that spent sulfuric acid should be shipped to a disposal site?
MR. CONN: Could I have that read back, please.
(record read.)
THE WITNESS: No.
Q BY MR. REZEN: Is it your best recollection that you were told at some time prior to the date of this letter that Montrose's spent sulfuric acid should be sent to a disposal site?
MR. CONN: Again, I caution the witness not to speculate.
THE WITNESS: I don't remember any conversation about that at that time.
Q BY MR. REZEN: But you do recall generally sending this letter to Ms. bridges; correct?
A Correct.
Q I'd like you to refer to the first sentence of this letter which reads as follows, quote, we produce a spent sulfuric acid and we may need of a place to dispose of this acid, end of quote. The question I have for you is this: Did you ever consider simply flushing the acid

down the disposal drains at the Montrose Torrance facility?

MR. CONN: Can I have that question read back, please.

(record read.)

MR. CONN: Object as argumentative, calling for speculation. I believe the question is also -- I take it to be hypothetical.

But if you can answer, go ahead.

I'm not certain the relationship between the preface, that is, what has been read into the record, and the question that you posed. I think, therefore, the question is unintelligible and vague.

MR. REZEN: I disagree absolutely with everything you said, but go ahead.

THE WITNESS: I think the question is --

MR. REZEN: Let me say this, Richard, you are really complicating this deposition, and I am trying to get very simple testimony here, very preliminary answers to preliminary questions, and you're making the process very time consuming and difficult. You can continue to do that. We'll continue to depose Mr. Bratter for the next two weeks or whatever if you want to continue to do that, but I view these questions as very clear-cut questions, good faith questions, calculated to simply get answers to

simple foundational questions and you're attempting to prevent me from doing that.

MR. CONN: Counsel, my view is that your comments are self-serving and of course I do disagree with your characterization of what is happening here. It seems to me that your questioning has to date been vague and uncertain as a general matter. My job is to place those objections on the record. I'm doing that. I do not intend to argue with you about those objections. I take it for granted that you will disagree with me with respect to those otherwise you would not be posing your questions. What I suggest is I'll make the objections and you follow along with whatever questions you like. My objection is on the record. The witness may answer.

Q BY MR. REZEN: Do you have the question in mind?

A If the question is Did I ever consider

flushing the acid down the drain, the answer is no.

Q That was the question. Thank you for your answer. Why did you not consider flushing the acid down the drain?

MR. CONN: Object. Calling for speculation. It's hypothetical, unintelligible to ask somebody why they did not consider something.

But go ahead if you can speculate.

Are you asking for his recollection back in 1960 something, some specific date?

MR. REZEN: Yes. I'm asking him for his general recollection.

MR. CONN: At what date?

MR. REZEN: At or about the time he sent this letter on August 8, 1967.

MR. CONN: So it's clear he's asking for your recollection in 1967 not today.

THE WITNESS: By the drain do you mean drain line or sewer line?

Q BY MR. REZEN: Either.

A The answer is no to both.

Q Why didn't you consider disposing of the acid that way?

A It was the regulation by the county sanitation district of any liquid that went to their sewer line had to be a PH of five or above and this acid would be well below it.

Q If it hadn't been for this regulation would you have flushed the acid down the sewer?

MR. CONN: I'm going to object to the hypothetical nature of "the sewer" and also calls for speculation.

he's also calling for your memory at that time. He's asking you to speculate how you would have

speculated in '67.

Q BY MR. REZEN: I'm not asking for speculation. I'm asking for a general recollection. I realize you may not have a specific recollection of what you were thinking at the time.

MR. CONN: I would caution the witness not to speculate. If you have knowledge as to something, you may testify as to that.
MR. HOYT: I quite frankly find your interjection of a speculation objection after every question to be coaching, and I think it's improper. I think it's already out on the table and understood we're not seeking speculation and that's clear. The only purpose for interjecting admonitions about don't speculate after each question is to suggest to the witness not to respond.
MR. CONN: Counsel, I disagree with your characterization. I take it we are going to disagree today on a variety of these points. That last question seems to be clearly calling for speculation. It's hypothetical If you're asking someone to guess about something years in the past. If the witness can do that, he's free to do that. I'm not instructing him not to answer.
MR. REZEN: Again, I disagree with all of your objections. I think it's an entirely proper question.

I'd like to have it read back so Mr. Bratter can have it in mind.
MR. CONN: Why don't we stipulate that we disagree with each other so we don't have this banter each time.
Go ahead.
(record read as follows: If it hadn't been for this regulation would you have flushed the acid down the sewer?)
THE WITNESS: No.
Q BY MR. REZEN: Why not?
MR. CONN: Same objections.
THE WITNESS: It's just something I wouldn't consider doing with a strong acid like this.
Q BY MR. REZEN: Why wouldn't you consider doing that?
MR. CONN: Same objection. Calling for hypothetical and speculative answers.
THE WITNESS: I previously stated that it didn't meet the standards, and my speculation doesn't go any further than that once it doesn't meet standards.
Q BY MR. REZEN: I'm going to ask you about the county regulations in a second. For the time being I'd like to concentrate on the reason why you wouldn't have flushed the acid down the drain even if there hadn't been

a regulation against it.

MR. CONN: If you have anything to add to your answer, you should; if not, you should say no.
THE WITNESS: I have nothing further to answer on that.
Q BY MR. REZEN: You said that you wouldn't have flushed it down the sewer -- or rather you said you wouldn't have flushed strong acid like this down the sewer. Is it correct that the reason you wouldn't have flushed the strong acid down the sewer was that you realize that there was a risk of ground water contamination if you had done so?
MR. CONN: You're asking him today or are you asking him to tell you what his thinking was back in 1967 or '68?
MR. REZEN: I'm asking for a general recollection with respect to why he wouldn't have flushed the acid down the sewer in '68.
MR. CONN: I'll object. It seems you're calling for speculation not recollection.
MR. REZEN: I just said I was.
MR. CONN: It doesn't seem that you are so I'm objecting on the grounds of speculation.
THE WITNESS: There's nothing I can add other than I know it wasn't the right thing to do and I didn't do it.
Q BY MR. REZEN: I'd like you to tell me why

you realized why it wasn't the right thing to do.
A I've told you why.
MR. REZEN: Why don't we take a five or ten minute break and we'll come back and finish up the first day.
(recess.)
MR. CONN: For the record, with respect to Mr. Kallok's deposition, at his request he would like not to be deposed on Wednesday, and he will be available on Thursday. Counsel here have graciously permitted him to be deposed on Thursday with the understanding that of course his deposition to the extent it is not concluded on that date -- and we presume it will not be -- that it will

be rescheduled for a mutually convenient time and it may be double tracked at some later date.

We've also discussed -- although I don't think we've come to any firm terms -- as to what will happen on Wednesday. Mr. Bratter is available until 11 o'clock and we would be willing to start early that morning if counsel so choose; and I believe counsel are going to discuss that specific question at the conclusion of today's deposition and let us know about that perhaps tomorrow.

With that we can continue with this deposition.

Q BY MR. REZEN: Regarding the spent sulfuric

acid that we've been discussing, Mr. Bratter, in 1967 you realized, did you not, that there were certain hazards associated with this material?

MR. CONN: I'm going to object as vague and overbroad and uncertain as to hazards and ambiguous as well.

But you may answer.

THE WITNESS: My understanding of this material is that if it was to be disposed of it should be taken to a Class 1 landfill, which was qualified to take hazardous waste.

MS. ABBOTT: Objection. Nonresponsive.

Q BY MR. REZEN: How did you obtain that understanding?

A Right now I don't remember.

Q How did you become aware of the county regulation limiting the types of materials that could be placed in a sewer line?

MR. CONN: I think that has been asked but you may answer again.

THE WITNESS: Los Angeles County sanitation district informed us of their limits of what could be put into a sewer line.

Q BY MR. REZEN: Did you understand in or about August of 1967 that the spent sulfuric acid to which we've

been making reference could be dangerous --
MR. CONN: Is that the entire question?
MR. REZEN: No.
MR. CONN: Sorry.
Q BY MR. REZEN: -- If an individual came into contact with it?
MR. CONN: Objection. The question is vague and uncertain and ambiguous.
But go ahead.
THE WITNESS: Having used sulfuric acid for many years, if an individual came into contact with it they could get a severe burn on their skin. In that respect it was dangerous.
Q BY MR. REZEN: Did Montrose in 1967 take precautions with respect to limiting worker contact with the spent sulfuric acid?
A Yes.
Q What precautions Did they take?
A Sulfuric acid was handled in equipment that wouldn't corrode. We would prevent the equipment from leaking. Employees were furnished with safety equipment.
Q Were there other safety precautions which Montrose took with respect to the sulfuric acid?
A Yes.
Q Earlier I think I misspoke. I may have made

reference to precautions which Stauffer took and if I Did misspeak I meant to refer to Montrose. Did you understand that?
A I didn't notice it.
Q Could you list for me the other precautions which Montrose took with regard to the handling of this sulfuric acid?
A Well, workers were furnished with safety glasses, with side shields. They had gloves that were resistant to sulfuric acid and hard hats with face shields that could protect splashes on their face. Safety showers were installed so it would be easy to wash any acid that came into contact with the skin. eye fountains were installed to wash eyes out in case any acid got in the eyes. Probably other things I can't think of right now.
MR. CONN: I would interpose an objection on the grounds the question was uncertain as to time.
Q BY MR. REZEN: We were talking about the 1967 time period. Did you understand that?

A We took these precautions from the time the plant started in business.
Q When Did the plant start business?
A 1947.
Q You mentioned the spent sulfuric acid was stored. Was it stored in tanks?

A Yes.
Q I'd like you to make reference to Exhibit No. 1. Can you recall communicating with Ms. Margery bridges in 1967?
A Yes.
Q Can you recall approximately how many times you communicated with her in 1967?
A No.
Q Can you recall if it was more than ten times?
A It might have been once or twice.
Q Did you communicate with her after 1967?
A Yes.
Q did you communicate with her before 1967?
A No.
Q Can you remember approximately how many times you communicated with her after 1967?
A No.
Q Can you recall if it was more than five times?
A I don't know.
Q Do you recall when you last communicated with her?
A No.
Q Do you recall if it was before 1970?
A No, I don't recall.

Q Did you ever have an in-person communication with Ms. bridges?
A What do you mean by in-person?
Q As opposed to by telephone or through written correspondence.
A I have never met miss bridges in person.
Q Did you communicate with her by telephone at

any time in the past?
A The first communication I had with her was by telephone, and if there are any others they are only by telephone.
Q You also communicated to her in writing, though?
A Yes.
Q Can you recall talking to Ms. bridges regarding the chemical content of the spent sulfuric acid?
A Yes.
Q Can you recall when you first spoke to her in that regard?
A It was probably a day or two before this letter was written.
Q Do you have a specific recollection of that conversation?
A Yes.
Q Tell me what she said during that

conversation.
A She asked me what we had to dispose of at their site, and I told her.
Q What did you tell her?
A I told her that we had a spent sulfuric acid that was approximately 76 percent sulfuric acid by titer and that the main impurity was chlorobenzene sulfonic acid approximately 2 percent, that it had traces of tar and DDT in it.
Q Do you have a specific recollection as you sit here of telling her it had tar and DDT in it at that time?
A Yes.
Q Do you have a specific recollection as you sit here today of any of the conversations that you had with Max Sobelman prior to 1970 regarding the disposal of spent sulfuric acid waste?
A I don't remember any specific conversations.
Q Do you have a specific recollection as you sit here today of any of the communications you had with Ross Wilcox prior to 1970 regarding the disposal of Montrose's spent sulfuric acid waste?
A Ross Wilcox sent me a memo requesting or ordering that I check with Stringfellow to see if it was a suitable place to dispose of our acid.

page 92

Deposition of Bratter, Bernard
page 93

Q Do you have a specific recollection of any oral communications you had with him regarding the disposal of the acid waste prior to 1970?
A I don't remember if they were written or oral but we discussed checking on Stringfellow and the results of this checking.
Q So you don't have a specific recollection of any oral communications with Mr. Wilcox regarding waste disposal prior to 1970; is that correct?
A No specific one.
Q Do you have a specific recollection of communications with anyone prior to 1970 regarding the disposal of acid waste, Montrose's acid waste, that is, other than miss bridges?
MR. CONN: Objection. Overbroad.
THE WITNESS: Well, the specific one was not miss bridges but with Stringfellow quarry to check to find out if that was a suitable disposal site.
Q BY MR. REZEN: You spoke with someone else, then, other than miss bridges at the Stringfellow site regarding acid waste disposal; is that correct?
A Yes.
Q With whom did you speak?
A There was a Mr. Nutt who called me after I had spoken or wrote the letter to miss bridges.

page 93

Deposition of Bratter, Bernard
page 94

Q I take it when you make reference to the letter, you're making reference to the Defendants' Exhibit 1; correct?
A Yes.
Q How long after you wrote that letter did you communicate with Mr. Nutt in this regard?
A Well, my recollection is that he communicated with me, and it was very shortly after, a matter of a day or two.
Q What did he say during that communication?
A He was concerned about the traces of tar. He wasn't particularly concerned about the traces of DDT, and the way he explained to me is that they had this Stringfellow quarry which had pits in it and below the

pits was bedrock, and any acid was put into these pits and would percolate down through the rock quarry to the bedrock, and he was concerned if there was any kind of tar in our acid it would plug up the quarry pits and the acid would not be able to percolate down to the rock bed and they wouldn't have enough capacity.
Q At the time you had that communication with Mr. Nutt did you know how long the Stringfellow site had been in operation?
A No.
Q Did you talk to him about that?

A Not that I remember.
Q Did you ever talk to anyone about that?
A Not that I can recall.
Q As you sit here today do you know how long the Stringfellow site was in operation?
A Yes.
Q Do you recall being aware of how long the Stringfellow site had been in operation in the 1960s?
A No.
Q Did you have a belief at the time that you communicated with Mr. Nutt regarding whether or not other waste generators used the Stringfellow site?
A My understanding was that there were many that used it.
Q Did you have a belief at the time you communicated with Mr. Nutt regarding how big the Stringfellow site was?
A No.
Q Did you have a belief at the time you communicated with Mr. Nutt regarding what ultimately happened to the liquid waste which was disposed of at the site?
MR. CONN: I'm going to object to that question as to vague and uncertain as to the term what ultimately happened. I don't understand that. I think it's

ambiguous and there's No Foundation for it.
You can answer.

THE WITNESS: Well, earlier I stated that I was told that the acid went into the pit and percolated down the bedrock where it would be held. That was my whole impression of what happened to it.
Q BY MR. REZEN: Did you ever wonder what would happen after the pit got full?
A No.
Q Did Mr. Nutt tell you about the percolation process?
A Very vaguely.
Q Did anyone else tell you about it prior to the time you had a communication with him?
A No.
Q You've already described one communication you had with Ms. bridges regarding the disposal of Montrose's sulfuric acid at the Stringfellow site. You indicated you had a specific recollection of that conversation. Do you have a specific recollection of any of the other conversations you had with Ms. bridges in the 1960s?
A No.
Q Do you have a general recollection of any of those other communications?

A I don't really remember talking to her again.
Q Do you recall what you said to Mr. Nutt, if anything, during that communication to which you just made reference when he indicated to you he was concerned about the traces of tar in the Montrose sulfuric acid waste?
A I asked him if he'd like to see a sample of our acid and he requested one so I had a sample prepared and shipped it to him.
Q Can you remember anything else that he said during that conversation that we're talking about?
A Well, after he would check the sample he would let me know whether the acid was acceptable for them to dispose of or not.
Q Can you remember anything else he said during that conversation?
A No.
Q Can you remember anything else you said during the conversation?
A No.
Q Can you recall communicating with Mr. Nutt after that occasion?

A At some time in the future we decided to use Stringfellow to dispose of some of our acid and before we Did we communicated with him to make arrangements.
Q How many times did you communicate with him

after that first communication and before you started using the Stringfellow site?
A I don't remember the number of times.
Q Do you have a recollection of any of those communications?
A No.
Q is it your best recollection that Montrose first began using the Stringfellow site in late 1968 for disposal purposes?
A Yes.
Q You used -- and when I say you, I'm referring to Montrose chemical -- used transporters to transport this sulfuric acid to the Stringfellow site; is that correct?
A We used one transporter.
Q That was Post Transportation company?
A Yes.
Q Can you give me the names of the individuals at post who you knew, if any?
A The man I dealt with at Post Transportation was named pete Sands.
Q Is that s-a-n-d-s?
A I believe it is.
Q When is the last time you communicated with Mr. Sands?

A He passed away about ten years ago or so, and it was shortly before that.
Q Did you ever communicate with anyone at post other than Mr. Sands?
A Well, there's Bob kirtsey.
Q How is Mr. kirtsey's last name spelled if you know?
A I'm not sure.
Q Could you give me an approximation?

A   k-i-r-t-s-e-y, I think.
Q   Were there any other individuals at Post Transportation with whom you communicated?
A   Not whose names I can recall.
Q   When is the last time you spoke to Bob Kirtsey?
MR. CONN: Objection. I think you just asked that.
MR. REZEN: No. I asked that about Mr. Sands.
THE WITNESS: It was probably 1982, but I'm not sure of the date.
Q   BY MR. REZEN: Did you know where Mr. Kirtsey lived at that time?
A   No, I didn't.
Q   Do you recall the context of your communication with him in 1982?
A   No.

Q   Do you remember anything about that communication?
A   No.
Q   Do you remember where you were at the time of the communication?
A   I was at Montrose's plant in Torrance.
Q   Do you recall having conversations with Pete Sands regarding the Stringfellow site?
A   Yes.
Q   Can you remember when you first had such a conversation with him in that regard?
A   I believe it was to haul the first load to Stringfellow. Somewhere around 1968.
Q   It's your best recollection that was in the late part of 1968; is that correct?
A   That's correct.
Q   Do you recall calling him on the telephone to ask him to transport the waste?
A   Yes.
Q   Do you remember what he said at that time?
A   My recollection is that he was glad to have the business.
Q   Did he tell you that he was familiar with the Stringfellow site?
A   I don't remember the details.

Q Do you recall generally whether or not he said anything about the Stringfellow site during that first communication?
A I don't remember him expressing any difficulty in handling it.
Q How often did you communicate with Pete Sands after you first communicated with him regarding the Stringfellow site?
A It was on a regular basis because Post Transportation Did other hauling for Montrose in addition to the recovered acid.
Q When you say on a regular basis, are you talking about on a weekly basis or daily basis or monthly basis?
A Probably several times a week.
Q And did you continue to communicate with Mr. Sands several times a week for the entire period of time during which you sent acid waste to the Stringfellow site?
A I believe so.
Q Do you have a general recollection of any of the conversations you had with Pete Sands about the Stringfellow site after that initial conversation?
A Generally it was to inform him of how many truckloads he should haul for any given period of time.
Q Did you ever talk to Mr. Sands about site

overflow problems at the Stringfellow site?
MR. CONN: Objection. Assumes facts not in evidence.
THE WITNESS: The answer is no.
Q BY MR. REZEN: Did you ever talk to Mr. Sands about the possible closure of the Stringfellow site?
MR. CONN: Same objection. No foundation. assumes facts not in evidence.
THE WITNESS: The answer is no.
Q BY MR. REZEN: Did you ever talk to Mr. Sands about operational problems which the Stringfellow site was experiencing?
MR. CONN: Same objection. No foundation. Assumes facts not in evidence.
THE WITNESS: I don't remember any conversations about any problems at Stringfellow with Mr. Sands.

Q BY MR. REZEN: Did you also communicate with Bob Kirtsey on a weekly basis during the period of time Montrose was sending acid waste to the Stringfellow site?
A No.
Q How often did you communicate with Mr. Kirtsey during this period of time?
A Very rarely.
Q Can you give me a more precise estimate of how often those communications took place?

A I believe it was less than once a month.
Q Why would you communicate with Mr. Kirtsey on these occasions?
A It might have been because Mr. Sands wasn't around or might have been information that Mr. Sands didn't have.
MR. CONN: Move to strike only to the extent you're testifying based on speculation. If you have a specific recollection, you should say so.
Q BY MR. REZEN: What was Mr. Sands's job title if you know?
A I think he was dispatcher and maybe general manager.
Q What was Mr. Kirtsey's job title?
A I believe he became president of Post Transportation.
Q Was he president of post at any time during the period of time Montrose was sending acid waste to Stringfellow if you know?
A I don't know.
Q Do you know what his job title was before he became president of Post Transportation?
A No.
Q With respect to the acid waste which was sent to the Stringfellow site by Montrose, where specifically

at the Montrose plant was this waste picked up by the transporter?
A We installed a loading facility next to our storage tanks.

Q And it was picked up at that facility?
A Yes.
Q Was there typically someone from Montrose present at the loading facility when the waste was picked up?
A Yes.
Q Who was that?
A The procedure I set up is that when a truck driver arrived at our plant to pick up a load of recovered acid, he would get ahold of the production supervisor on duty. The production supervisor would take his operator who loaded the trucks out to the truck and show him which tank to pump from to load the truck, and then our operator would load the truck.
Q These production supervisors included Lane Hodgins, Nick Leonovitch, and Bernie Hollander; correct?
A Correct.
Q Can you remember the names of any of the operators who loaded the trucks?
A No.
Q Were you ever present at the time of the pick

up of any of this waste acid?
A Yes.
Q How often would you be present at those times?
MR. CONN: I'm going to object. It's not clear if you're asking whether he was actually at the site or simply at the facility. I think the question is vague in that respect.
Q BY MR. REZEN: I'm asking if you were present at the loading facility at any time when the waste was picked up?
A Yes.
Q How often during the period of time when Montrose was sending waste to Stringfellow were you present at the loading facility at those times?
A Probably I'd see two or three loads a week being loaded.
Q Why would you be present?
A Just I would like to check on all operations of the plant, and that is one of the things I would check on.
Q Were there certain things you wouldn't check on?

MR. CONN: Objection. Vague and overbroad.
Uncertain.

page 105

Deposition of Bratter, Bernard
page 106

THE WITNESS: Not to my knowledge.
Q BY MR. REZEN: Montrose paid the transporter to transport the acid waste; isn't that correct?
A Yes.
Q And Montrose also paid the Stringfellow site for the opportunity to dispose of Its waste at that location?
MR. CONN: I'm going to object to the phraseology. I think it's vague and uncertain as to "opportunity". And vague and uncertain as the Stringfellow site as the payee.
Q BY MR. REZEN: is that correct?
A There was a fee paid for disposal to Stringfellow.
Q At what time was the transporter paid --
MR. CONN: Objection. Vague and uncertain.
Q BY MR. REZEN: -- For Its services?
MR. CONN: Same objection.
THE WITNESS: I didn't pay the bills so I don't know.
Q BY MR. REZEN: In other words, the payment wasn't made at the time of the pick up?
A No.
Q Who Did pay the bills?
A All the bills were sent to our accounting department, and we had a procedure set up at which they

page 106

Deposition of Bratter, Bernard
page 107

would be paid by.
Q Did the accounting department also take care of the payments to the Stringfellow site?
A Yes.
Q Can you remember who was in the accounting department at Montrose at the time during which Montrose was using the Stringfellow site?
A No.
Q Can you remember the names of any of the individuals in the accounting department at that time?
A I'm not sure who was in the accounting

department at that time.
Q When you were present at the loading facility when the Montrose waste was picked up did you typically communicate with the individual from Post Transportation who was driving the truck?
A No.
Q Did you typically communicate with your production supervisor on those occasions?
A Yes.
Q Did you ever communicate with the Post Transportation company truck drivers when they picked up the acid waste at the loading facility at Montrose?
A To the extent of saying hello to them I Did.
Q Can you recall ever having any communications

with any of those individuals which involved something other than saying hello to them?
A It's very vague. I might have said "merry christmas" too. I don't know.
Q Can you remember the names of any of those individuals?
A Not now, no.
Q Can you recall any of those individuals ever saying anything about the Stringfellow site in your presence?
A No. I don't remember any discussions about Stringfellow.
Q When did you first become aware of the existence of the Stringfellow site?
A The same day that I communicated with miss bridges.
Q How did you become aware of the Stringfellow site?
A My recollection is that I received a memo from Mr. Wilcox asking me to check out Stringfellow.
Q Do you remember what Mr. Wilcox said in the memo other than asking you to check out Stringfellow?
A That's what I recall, that he asked me to check it out.
Q Did you then check it out?

A Yes. Immediately.
Q Can you tell me all the steps you took in checking it out?
A The first step I took was to get a list of hazardous waste sites from the California water resources board to get a list of all places that were licensed by the state to accept hazardous waste, and I found Stringfellow quarry is one of them listed. Then I called miss bridges to find out what procedure there was to get our material accepted for disposal there.
Q How did you know to call miss bridges?
A I stated earlier that I received a memo from Mr. Wilcox.
Q In the memo did he make reference to miss bridges?
A I don't remember whether he Did or not.
Q How did you know to contact the California water resources control board?
A California water resources control board was in charge of setting up and licensing landfills. According to the state law, the only landfill that could receive hazardous waste was a Class 1 landfill and they were licensed by the state.
Q Can you remember how you learned the things that you just told me?

A No.
Q Can you remember from whom you learned those things?
A No.
MR. CONN: Objection. Assumes facts not in evidence.
Q BY MR. REZEN: Can you remember with whom you communicated at the California water resources control board when you checked out the Stringfellow site?
A No.
MR. CONN: Objection. Assumes facts not in evidence.
Q BY MR. REZEN: Can you remember the names of any individuals at the water resources control board with whom you communicated at any time in the past?
A You mean in 1967?
Q Then or at any time subsequent to that.
MR. CONN: Through today, counsel?

MR. REZEN: Yeah.
THE WITNESS: I was at the Santa Ana district of the California water resources board sometime in the 1980s, and I met a Jim Anderson there who was in charge of that district.
Q BY MR. REZEN: Had you met Mr. Anderson prior to that time?

A No.
Q Why were you at the board on that occasion when you met Mr. Anderson?
A I was seeking information about Stringfellow.
Q Why were you seeking information about Stringfellow at that time?
A I was asked to.
Q By whom?
A I don't remember who asked me to.
MR. CONN: Counsel, I note it's by my watch 5 o'clock now. I would suggest we resume tomorrow at 10:00 and if you'd like at that point to advise us, as I said, we're willing to start early on Wednesday.
MR. REZEN: Okay.
MR. CONN: And counsel can come to some accord as to what time that would be, 8:30, 9:00. Whatever you would like to do, we'd be happy to do that and we appreciate the courtesy regarding Mr. Kallok.
MR. REZEN: Okay.
///

WITNESS:________________________________

FOR defendants lloyds of london:

gibson, dunn & crutcher
BY: Scott R. Hoyt, ESQUIRE
333 south grand avenue, 48th floor

los angeles, california 90071
(213) 229-7000

FOR home insurance co.:

charlston, revich & williams
BY: Randy Rezen, ESQUIRE
2049 century park east, 42nd floor
los angeles, California 90067
(213) 551-7000

FOR canadian universal insurance company:

ericksen, arbuthnot, brown, kilduff & day
BY: beth abbott, attorney at law
150 west first street, suite 270
claremont, California 91711
(714)621-6862

State of California
REGIONAL WATER POLLUTION CONTROL BOARD NO. 4
Los Angeles Region

RESOLUTION NO. 61-43

PRESCRIBING REQUIREMENTS FOR THE DISPOSAL OF INDUSTRIAL WASTE
CALIFORNIA SALVAGE COMPANY
Los Angeles, California
(File 60-28)

WHEREAS, in compliance with the provisions of Section 13054 of the Water Code of the State of California, CALIFORNIA SALVAGE COMPANY has filed with this Board a Report on Waste Discharge (our Report No. 464) dated February 24, 1960, for its existing operations of the ocean disposal of industrial waste; and

WHEREAS, this Board has caused the following investigations to be made with respect to this proposal:

1. Field investigations were conducted by the staff of the Board.

2. Review was made of Board Resolution No. 54-1 "Adopting Long Range Waste Disposal and Water Quality Objectives for Los Angeles and Long Beach Harbors".

3. This Board has received written comments on this proposal from the State Departments of Fish and Game, and Water Resources; and

WHEREAS, based on the foregoing investigations this Board finds:

1. California Salvage Company receives for discharge at sea a variety of extremely noxious industrial wastes.

2. At the present time the company [illegible] not handle the disposal of radioactive materials.

3. The materials are received at the company's operation's site Berth [illegible], Port of Los Angeles. The materials are loaded on a barge and pulled out to sea where they are disposed of.

CALIFORNIA SALVAGE COMPANY
Los Angeles, California
(File 60-28)

WHEREAS, in compliance with the provisions of Section 13054 of the Water Code of the State of California, CALIFORNIA SALVAGE COMPANY has filed with this Board a Report on Waste Discharge (our Report No. 464) dated February 24, 1960, for its existing operations of the ocean disposal of industrial waste; and

WHEREAS, this Board has caused the following investigations to be made with respect to this proposal:

1. Field investigations were conducted by the staff of the Board.

2. Review was made of Board Resolution No. 54-1 "Adopting Long Range Waste Disposal and Water Quality Objectives for Los Angeles and Long Beach Harbors".

3. This Board has received written comments on this proposal from the State Departments of Fish and Game, and Water Resources; and

WHEREAS, based on the foregoing investigations this Board finds:

1. California Salvage Company receives for discharge at sea a variety of extremely noxious industrial wastes.

2. At the present time the compa[illegible] not handle the disposal of radioactive materials.

3. The materials are received at the company's operation's site Berth 115, Port of Los Angeles. The materials are loaded on a barge and pulled out to sea where they are disposed of.

4. The ocean disposal area is that area shown as "Dumping Area", located northerly and westerly of Santa Catalina Island. This dumping area

is that area within a radius of three nautical miles of Longitude W 118° 40 min., Latitude N 33° 37 min. (This area is depicted on Chart A, attached hereto).

5. The operations are satisfactory provided:
   1. There is no storage of waste materials received from disposal other than in impervious containers; and
   2. Precautions be continued against materials being spilled, such that they can enter into the waters of the Port of Los Angeles.

6. The character of the wastes which will be disposed of by this company, together with the significant import of the waters of the State in this area to fish and aquatic life, and as used in varied recreational pursuits, is such that these wastes must be precluded from being discharged therein. It is therefore found by the Board that the beneficial uses of the waters of this area can be protected only if these wastes are discharged into the "Dumping Area", northerly and westerly of Santa Catalina Island, as depicted on Chart A, attached hereto.

WHEREAS, on October 5, 1961, a letter transmitting a tentative copy of this resolution was submitted to the proponent and copies thereof sent to interested governmental agencies with the advice that objections thereto would be considered by the Board if submitted in writing on or before October 17, 1961; and

WHEREAS, objections and recommendations received by the Board have been reviewed and considered;

NOW, THEREFORE, BE IT RESOLVED, that in order to prevent pollution of the receiving waters, and to prevent creation of a nuisance, this Regional Water Pollution Control Board in accordance with authority granted by Division 7 of the Water Code of the State of California, prescribes the following requirements with respect to the discharge of noxious industrial wastes by California Salvage Company, located at Berth 115, Port of Los Angeles, subject to the provisions of 13054 of the Water Code, which reads in part that requirements may be revised from time to time:

Chart A, attached hereto).

5. The operations are satisfactory provided:
    1. There is no storage of waste materials received from disposal other than in impervious containers; and
    2. Precautions be continued against materials being spilled, such that they can enter into the waters of the Port of Los Angeles.

6. The character of the wastes which will be disposed of by this company, together with the significant import of the waters of the State in this area to fish and aquatic life, and as used in varied recreational pursuits, is such that these wastes must be precluded from being discharged therein. It is therefore found by the Board that the beneficial uses of the waters of this area can be protected only if these wastes are discharged into the "Dumping Area", northerly and westerly of Santa Catalina Island, as depicted on Chart A, attached hereto.

WHEREAS, on October 5, 1961, a letter transmitting a tentative copy of this resolution was submitted to the proponent and copies thereof sent to interested governmental agencies with the advice that objections thereto would be considered by the Board if submitted in writing on or before October 17, 1961; and

WHEREAS, objections and recommendations received by the Board have been reviewed and considered;

NOW, THEREFORE, BE IT RESOLVED, that in order to prevent pollution of the receiving waters, and to prevent creation of a nuisance, this Regional Water Pollution Control Board in accordance with authority granted by Division 7 of the Water Code of the State of California, prescribes the following requirements with respect to the discharge of noxious industrial wastes by California Salvage Company, located at Berth 115, Port of Los Angeles, subject to the provisions of 13054 of the Water Code, which reads in part that requirements may be revised from time to time:

1. Land operations and loading operations shall be such that there are no spillages or droppages of the waste materials into the waters of the Port of Los Angeles.

2. The ocean area into which waste materials accepted for disposal by this company may be discharged is that area shown as "Dumping Area", as defined herein and depicted on Chart A.

3. This discharge shall not cause the presence of visible floating oil nor the appearance of floating or suspended matter.

4. All Federal, State, County and City rules, regulations and ordinances pertinent to this disposal of wastes shall be complied with in these waste disposal operations; and

BE IT FURTHER RESOLVED, that for the purpose of checking compliance with the conditions hereinbefore specified, and pursuant to the authority granted by Section 13055 of the Water Code of the State of California, the following technical records shall be maintained and technical reports shall be filed with this Regional Water Pollution Control Board as indicated below:

1. All wastes received shall be accounted for by adequate records kept by the company, including certifications as to their type, producer and quantity. After such certifications are endorsed by the producer of the wastes, hauler, and operator, they shall be filed by the company once every two months with this Regional Water Pollution Control Board.

2. Logs of all trips to the disposal area shall be maintained. They shall be filed once every two months with this Regional Water Pollution Control Board; and

BE IT FURTHER RESOLVED, that in prescribing these requirements it is the intent of this Board to:

1. Preserve the quality of the receiving waters in the Pacific Ocean for fish and aquatic life, and for recreational use; and

BE IT FURTHER RESOLVED, that California Salvage Company be advised that these requirements do not exempt the operator of this disposal facility from

[illegible] company may be discharged is that area shown as "Dumping Area", as defined herein and depicted on Chart A.

3. This discharge shall not cause the presence of visible floating oil nor the appearance of floating or suspended matter.

4. All Federal, State, County and City rules, regulations and ordinances pertinent to this disposal of wastes shall be complied with in these waste disposal operations; and

BE IT FURTHER RESOLVED, that for the purpose of checking compliance with the conditions hereinbefore specified, and pursuant to the authority granted by Section 13055 of the Water Code of the State of California, the following technical records shall be maintained and technical reports shall be filed with this Regional Water Pollution Control Board as indicated below:

1. All wastes received shall be accounted for by adequate records kept by the company, including certifications as to their type, producer and quantity. After such certifications are endorsed by the producer of the wastes, hauler, and operator, they shall be filed by the company once every two months with this Regional Water Pollution Control Board.

2. Logs of all trips to the disposal area shall be maintained. They shall be filed once every two months with this Regional Water Pollution Control Board; and

BE IT FURTHER RESOLVED, that in prescribing these requirements it is the intent of this Board to:

1. Preserve the quality of the receiving waters in the Pacific Ocean for fish and aquatic life, and for recreational use; and

BE IT FURTHER RESOLVED, that California Salvage Company be advised that these requirements do not exempt the operator of this disposal facility from

Requirements
Calif. Salvage Co.

File 60-28

any other law which may be applicable. These requirements are not a permit; they do not legalize this waste disposal facility and they leave unaffected any further restraints on this disposal of wastes which might be contained in other statutes: and

BE IT FURTHER RESOLVED, that the Executive Officer of this Board be authorized, and he is hereby directed to certify and submit copies of this resolution to California Salvage Company and to such agencies and individuals as may have need therefor, or as may request same.

I, Linne C. Larson, Executive Officer of the Los Angeles Regional Water Pollution Control Board, State of California, do hereby certify that the foregoing is a full, true, and correct copy of a resolution adopted by the Los Angeles Regional Water Pollution Control Board at a Board Meeting held on October 16, 1961.

LINNE C. LARSON
Executive Officer

I, Linne C. Larson, Executive Officer of the Los Angeles Regional Water Pollution Control Board, State of California, do hereby certify that the foregoing is a full, true, and correct copy of a resolution adopted by the Los Angeles Regional Water Pollution Control Board at a Board Meeting held on October 18, 1961.

LINNE C. LARSON
Executive Officer

[illegible]
[illegible]
[illegible]
Revised 10-18-61

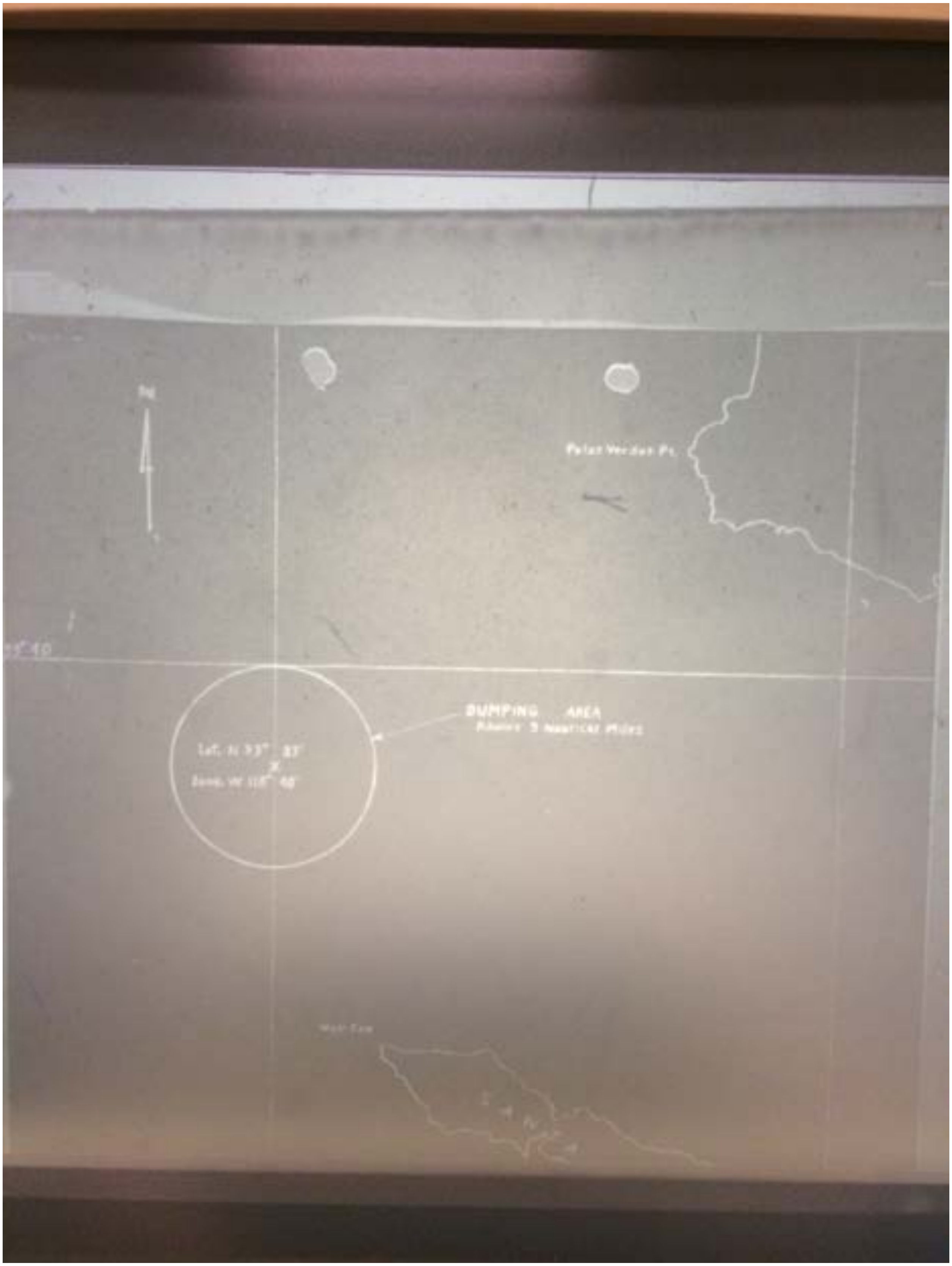
N
DUMPING AREA

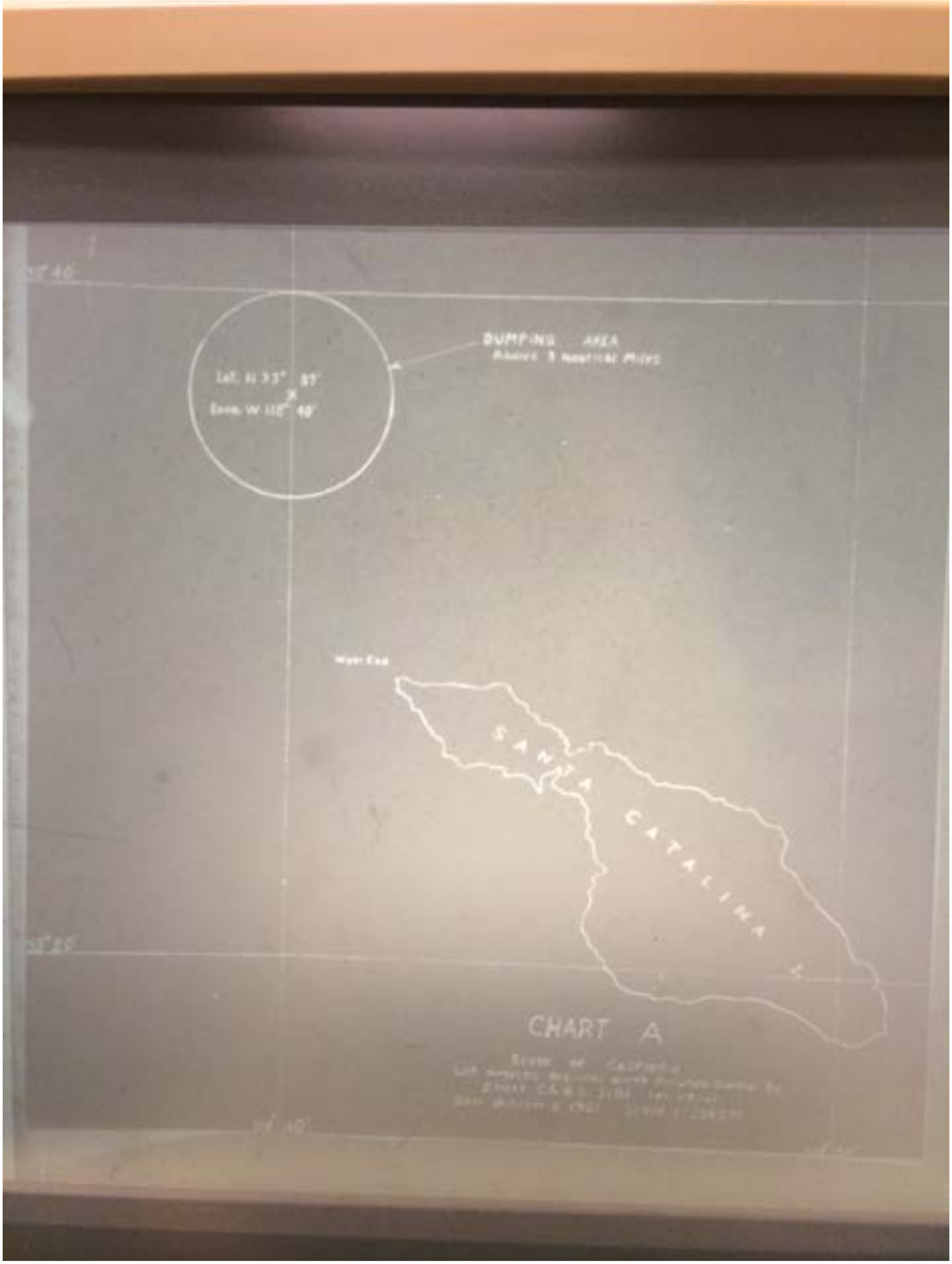
DUMPING AREA
SANTA CATALINA
CHART A
State of California

FEDERAL REGISTER

THE NATIONAL ARCHIVES OF THE UNITED STATES 1934 LITTERA SCRIPTA MANET

VOLUME 24 NUMBER 177

Washington, Thursday, September 10, 1959

Title 3—THE PRESIDENT

EXECUTIVE ORDER 10836

AMENDMENT OF EXECUTIVE ORDER NO. 10530,[1] PROVIDING FOR THE PERFORMANCE OF CERTAIN FUNCTIONS VESTED IN OR SUBJECT TO THE APPROVAL OF THE PRESIDENT

By virtue of the authority vested in me by section 301 of title 3 of the United States Code, and as President of the United States, it is ordered that Executive Order No. 10530 of May 10, 1954, entitled "Providing for the Performance of Certain Functions Vested in or Subject to the Approval of the President", be, and it is hereby, amended as follows:

1. Paragraph (1) of section 1 is amended to read:

"(1) The authority vested in the President by the last sentence of section 11 of the act of June 6, 1924, ch. 270, 43 Stat. 463 (as renumbered by section 2 of, and as amended by, the act of July 19, 1952, ch. 949, 66 Stat. 781, *et seq.*), to approve (1) the designation of lands to be acquired by condemnation, (2) contracts for purchase of lands, and (3) agreements between the National Capital Planning Commission and officials of the States of Maryland and Virginia."

2. The following paragraph (o) is added at the end of section 1:

"(o) The authority vested in the President by section 44(a) of the Alaska Omnibus Act, approved June 25, 1959 (Public Law 86-70; 73 Stat. 141, 151), to make transitional grants to the State of Alaska; and the authority vested in the President by section 44(b) of that act (1) to approve requests of the Governor of Alaska that Federal agencies continue to provide services or facilities in Alaska for an interim period, and (2) to allocate to such agencies the funds necessary to finance the provision of such services or facilities."

DWIGHT D. EISENHOWER

THE WHITE HOUSE,
September 8, 1959.

[F.R. Doc. 59-7578; Filed, Sept. 9, 1959; 10:34 a.m.]

---

[1] 19 F.R. 2709; 3 CFR, 1954 Supp., p. 55.

Title 7—AGRICULTURE

Chapter VIII—Commodity Stabilization Service (Sugar), Department of Agriculture

SUBCHAPTER B—SUGAR REQUIREMENTS AND QUOTAS

[Sugar Reg. 811, Amdt. 2]

PART 811—CONTINENTAL SUGAR REQUIREMENTS AND AREA QUOTAS

Requirements, Quotas, and Quota Deficits for 1959

Basis and purpose. The purpose of Sugar Regulation 811 is to determine, pursuant to section 201 of the Sugar Act of 1948, as amended (hereinafter called the act), the amount of sugar needed to meet the requirements of consumers in the continental United States for the calendar year 1959 and to establish, pursuant to sections 202, 204 and 411 of the act sugar quotas and prorations for the supplying areas in terms of short tons of sugar, raw value, equal to the quantity determined by the Secretary of Agriculture to be needed in 1959. This regulation also establishes pursuant to section 207 of the act the quantity of quota that may be filled by direct-consumption sugar and pursuant to section 208, quotas of liquid sugar which may be entered into the continental United States.

The act requires that the Secretary shall revise the determination of sugar requirements at such times during the calendar year as may be necessary. It now appears that an increase in the estimate of requirements for the calendar year 1959 is necessary. The purpose of this amendment is to make such determination conform to the requirements indicated on the basis of the factors specified in section 201 of the act, as amended, to give effect to the revised determination and to further amend § 811.4 to determine and prorate deficits in the quotas for Hawaii, Puerto Rico and the Virgin Islands for sugar to be marketed in the continental United States in 1959 as established in § 811.2, as amended herein.

Section 204(a) of the act provides that the Secretary shall from time to time determine whether any area will be unable to market its quota and prescribes

(Continued on p. 7271)

CONTENTS

THE PRESIDENT

Executive Order	Page
Amendment of Executive Order No. 10530, providing for the performance of certain functions vested in or subject to the approval of the President	7269

EXECUTIVE AGENCIES

**Agricultural Marketing Service**	
Proposed rule making:	
Potatoes, Irish:	
Imports	7279
Limitation of shipments	7278
Rules and regulations:	
Lemons grown in California and Arizona; limitation of handling	7272
Potatoes, Irish, grown in certain parts of California and Arizona; limitation of shipments	7272
**Agriculture Department**	
See Agricultural Marketing Service; Commodity Stabilization Service.	
**Atomic Energy Commission**	
Notices:	
Hearings, etc.:	
Advance Industrial X-Ray Laboratories et al	7282
California Salvage Co	7282
Electro Chemical Laboratories Corp	7284
**Business and Defense Services Administration**	
Proposed rule making:	
Nonagricultural foreign excess property; importation into U.S	7280
**Civil Service Commission**	
Rules and regulations:	
Competitive service exceptions:	
Defense Department	7272
Treasury Department	7273
**Commerce Department**	
See also Business and Defense Services Administration; Federal Maritime Board; Maritime Administration.	
Notices:	
Statements of changes in financial interests:	
Lewis, Richmond	7282
Sauers, Marguerite M	7282

FEDERAL REGISTER

Published daily, except Sundays, Mondays, and days following official Federal holidays, by the Office of the Federal Register, National Archives and Records Service, General Services Administration, pursuant to the authority contained in the Federal Register Act, approved July 26, 1935 (49 Stat. 500, as amended; 44 U.S.C., ch. 8B), under regulations prescribed by the Administrative Committee of the Federal Register, approved by the President. Distribution is made only by the Superintendent of Documents, Government Printing Office, Washington 25, D.C.

The FEDERAL REGISTER will be furnished by mail to subscribers, free of postage, for $1.50 per month or $15.00 per year, payable in advance. The charge for individual copies (minimum 15 cents) varies in proportion to the size of the issue. Remit check or money order, made payable to the Superintendent of Documents, directly to the Government Printing Office, Washington 25, D.C.

The regulatory material appearing herein is keyed to the CODE OF FEDERAL REGULATIONS, which is published, under 50 titles, pursuant to section 11 of the Federal Register Act, as amended August 5, 1953. The CODE OF FEDERAL REGULATIONS is sold by the Superintendent of Documents. Prices of books and pocket supplements vary.

There are no restrictions on the republication of material appearing in the FEDERAL REGISTER, or the CODE OF FEDERAL REGULATIONS.

SEMIANNUAL CFR SUPPLEMENT
(As of July 1, 1959)

The following semiannual cumulative pocket supplement is now available:

Title 46, Parts 146–149, 1959 Supplement 1 ($1.25)

Order from Superintendent of Documents, Government Printing Office, Washington 25, D. C.

CONTENTS—Continued

	Page
**Commodity Stabilization Service**	
Rules and regulations:	
Sugar; continental requirements and area quotas	7269
**Federal Aviation Agency**	
Rules and regulations:	
Restricted areas; modifications and revocations (3 documents)	7273
**Federal Communications Commission**	
Notices:	
Hearings, etc.:	
Audiocasting of Texas, Inc., and Horace K. Jackson, Sr.	7289
Beacon Broadcasting System, Inc., et al	7289
**Federal Communications Commission—Continued**	
Notices—Continued	
Hearings, etc.—Continued	
Bloom Radio (WHLM)	7289
Granite City Broadcasting Co. et al	7288
Hernreich, George T., and Patteson Brothers	7289
Inter-Cities Broadcasting Co.	7288
Laurino, John, and Capital Broadcasting Co. (WNAV)	7289
WMAX, Inc. (WMAX)	7289
Proposed rule making:	
Television broadcast stations; extension of time for filing comments	7280
Rules and regulations:	
Citizens radio service; licensed radio operators	7276
Radio broadcast services:	
Standards of good engineering practice	7274
Columbus, Ga.; table of assignments	7276
Lubbock, Tex.; table of assignments	7275
**Federal Maritime Board**	
Notices:	
Oranje Lijn (Maatschappij Zeetransport), N.V., et al.; agreement filed for approval	7281
Application of American Export Lines, Inc., for permission to provide service	7281
**Federal Power Commission**	
Notices:	
Hearings, etc.:	
Atlantic Seaboard Corp.	7286
Bevly, W. M., and W. M. Bevly, Jr.	7286
Manufacturers Light and Heat Co.	7284
Monsanto Chemical Co.	7284
Monsanto Chemical Co. et al.	7284
Okmar Oil Co.	7285
Osborn, W. B., Jr., et al. (2 documents)	7287
Pacific Northwest Pipeline Corp	7288
Vincent & Welch, Inc., et al.	7285
**Federal Trade Commission**	
Rules and regulations:	
Cease and desist orders:	
Booth, P. E., Co., Inc.	7273
Lenders Service Corp. et al.	7274
**Fish and Wildlife Service**	
Proposed rule making:	
Little Pend Oreille National Wildlife Refuge, Washington	7277
Loxahatchee National Wildlife Refuge, Florida	7277
Rules and regulations:	
Migratory birds; open seasons, bag limits, and possession	7277
**Health, Education, and Welfare Department**	
See Public Health Service.	
**Interior Department**	
See Fish and Wildlife Service.	
**Interstate Commerce Commission**	
Notices:	
Fourth section applications for relief	7290
Motor carriers:	
Applications	7292
"Grandfather" certificate or permit	7290
Transfer proceedings	7291
**Maritime Administration**	
Notices:	
Trade Route 13; tentative conclusions and determinations regarding modification of essentiality and U.S. Flag requirements	7281
**Public Health Service**	
Rules and regulations:	
Commissioned officers; prescription of numbers in grade	7274
**Tariff Commission**	
Notices:	
Starch; investigation and hearing	7290

CODIFICATION GUIDE

A numerical list of the parts of the Code of Federal Regulations affected by documents published in this issue. Proposed rules, as opposed to final actions, are identified as such.

A Cumulative Codification Guide covering the current month appears at the end of each issue beginning with the second issue of the month.

	Page
**3 CFR**	
Executive orders:	
10530 (amended by EO 10836)	7269
10836	7269
**5 CFR**	
6 (2 documents)	7272, 7273
**7 CFR**	
811	7269
953	7272
959	7272
Proposed rules:	
970	7278
1066	7279
**14 CFR**	
608 (3 documents)	7273
**16 CFR**	
13 (2 documents)	7273, 7274
**42 CFR**	
21	7274
**44 CFR**	
Proposed rules:	
401	7280
**47 CFR**	
3 (3 documents)	7274–7276
19	7276
Proposed rules:	
3	7280
**50 CFR**	
6	7277
Proposed rules:	
31	7277
34	7277

the manner in which any deficit in a quota for a domestic area or Cuba is to be prorated to such other areas able to supply the additional sugar. Such section provides that any deficit in any domestic producing area occurring by reason of inability to market that part of the quota for such area allotted under the provisions of section 202(a)(2) of the act, shall first be prorated to other domestic areas on the basis of the quotas then in effect, and the remainder of such deficit to be prorated to other domestic areas and Cuba on the basis of quotas then in effect.

The act also provides that the quota for any area as established under the provisions of section 202 shall not be reduced by reason of any detemination of a deficit.

In order to afford sellers of sugar in affected areas an adequate opportunity to plan marketings and to market the additional sugar authorized by this amendment, and thereby protect the welfare of consumers, it is essential that this amendment be made effective immediately. Therefore, it is hereby determined and found that compliance with the notice, procedure and effective date requirements of the Administrative Procedure Act is unnecessary, impracticable and contrary to the public interest and the amendment herein shall become effective when published in the FEDERAL REGISTER.

By virtue of the authority vested in the Secretary of Agriculture by the Sugar Act of 1948, as amended (61 Stat. 922, as amended) and the Administrative Procedure Act (60 Stat. 237), §§ 811.1, 811.2, 811.3 and 811.4 of Sugar Regulation 811 (24 F.R. 1; 24 F.R. 6473) are amended to read as hereinafter set forth.

Sections 811.1 to 811.4 are amended to read:

§ 811.1 Sugar requirements, 1959.

The amount of sugar needed to meet the requirements of consumers in the continental United States for the calendar year 1959 is hereby determined to be 9,300,000 short tons, raw value.

§ 811.2 Quotas for domestic areas.

(a) For the calendar year 1959, quotas for consumption in the continental United States from domestic areas are established in column (1) and the amounts of such quotas for offshore areas that may be filled by direct-consumption sugar are established in column (2) as follows:

[Short tons, raw value]

Area	Quota (1)	Direct-consumption limit (2)
Domestic beet sugar	2,021,098	(1)
Mainland cane sugar	621,912	(1)
Hawaii	1,127,970	31,705
Puerto Rico	1,179,437	137,637
Virgin Islands	16,083	0

[1] No limit.

(b) Of the quantity established in paragraph (a) of this section for Puerto Rico which may be filled by direct-consumption sugar, 126,033 short tons, raw value, may be filled only by sugar principally of crystalline structure.

§ 811.3 Quotas for foreign countries.

For the calendar year 1959, quotas for sugar to be imported into the continental United States for consumption therein from foreign countries are established in column (1) and the amount of each such quota that may be filled by direct-consumption sugar is established in column (2), as follows:

[Short tons, raw value]

Country	(1) Quota	(2) Direct-consumption limit
Republic of the Philippines	980,000	59,920
Cuba	3,090,065	375,189
Peru	91,197	8,962
Dominican Republic	76,917	8,889
Mexico	89,709	16,130
Nicaragua	13,473	10,637
Haiti	6,906	6,905
Netherlands	3,662	3,662
China	3,564	3,564
Panama	3,564	3,564
Costa Rica	3,537	3,537
Canada	631	631
United Kingdom	516	516
Belgium	182	182
British Guiana	84	84
Hong Kong	3	3
All other countries	0	0

§ 811.4 Determination and proration of area deficits and adjusted quotas.

(a) *Deficit in quotas established in § 811.2.* It is hereby determined, pursuant to subsection (a) of section 204 of the act, that for the calendar year 1959, Hawaii, Puerto Rico and the Virgin Islands will be unable by 150,000, 209,562 and 3,678 short tons, raw value, of sugar, respectively, to market the quotas established for such areas in § 811.2.

(b) *Quotas in effect upon proration of deficits in parts of quotas established pursuant to section 202(a)(2).* The part of the deficits determined in paragraph (a) of this section applicable to that portion of the quotas in § 811.2 established pursuant to the provisions of section 202(a)(2) of the act, which amounts to 179,085 short tons, raw value, is hereby prorated on the basis of the quotas established in § 811.2 to domestic areas to the extent each such area is able to supply additional quantities. The quotas for such areas in effect upon publication of this paragraph in the FEDERAL REGISTER shall be those established in § 811.2 plus the quantities prorated herein, as follows:

[Short tons, raw value]

Area	Prorated herein	Quotas including prorations herein
Domestic beet sugar	136,965	2,158,064
Mainland cane sugar	42,120	664,051
Hawaii	0	1,127,970
Puerto Rico	0	1,179,437
Virgin Islands	0	16,083

(c) *Quotas in effect upon proration of deficits in part of quotas otherwise established.* Immediately after the quotas established in paragraph (b) of this section become effective, the quantity by which the deficit determined in paragraph (a) of this section exceeds the quantity prorated in paragraph (b) of this section, which amounts to 184,155 short tons, raw value, is hereby prorated on the basis of the quotas in effect pursuant to paragraph (b) of this section for domestic areas and pursuant to § 811.3 for Cuba, to the domestic areas able to supply additional sugar and Cuba. Thereupon, the following quotas shall be in effect, such quotas consisting of those established in paragraph (b) of this section for domestic areas and in § 811.3 for Cuba plus the quantities prorated in this paragraph:

[Short tons, raw value]

Area	Prorated herein	Quotas including prorations herein and in par. (b) of this section
Domestic beet sugar	67,220	2,225,284
Mainland cane sugar	20,684	684,735
Hawaii	0	1,127,970
Puerto Rico	0	1,179,437
Virgin Islands	0	16,083
Cuba	96,231	3,186,319

Statement of bases and considerations. Total sugar quotas for 1959 were established at 9,200,000 tons the 29th of December 1958.

Since May sugar distribution has been running at a record level. The extreme heat which prevailed this summer in many parts of the country no doubt increased the consumption of soft drinks, ice cream and other sugar-containing products. More recently the danger of a waterfront work stoppage, beginning September 30, at eastern and southern ports has been a f[illegible]tor in the market and should continue to be until the issue is settled. Sugar refiners have been buying and scheduling the receipt of raw sugar to meet continued high distribution and to build up inventories.

The tightness of sugar supplies, especially for the Atlantic and Gulf Coasts, is reflected in current sugar prices. Raw sugar prices at New York have ranged from a low of 5.75 cents per pound, duty paid, in early April to 6.50 cents on September 1. Recently increases of 20 cents per 100 pounds have been announced that would raise the price of refined sugar to 9.55 cents per pound, the highest price since 1920.

To meet the increased needs, total quotas are increased to 9,300,000 tons.

Deficits in the quotas for Puerto Rico, Virgin Islands and Hawaii were determined and prorated on July 31, 1959. Partially as a result of the increase in total quotas provided for herein, it is determined that the Hawaiian deficit will approximate 150,000 tons rather than 100,000 tons, previously determined. Accordingly, quota deficits of 209,562 tons for Puerto Rico, 3,678 tons for the Virgin Islands and 150,000 tons for Hawaii are hereby determined and pursuant to section 204(a) of the act 179,085 tons are prorated to domestic areas able to market additional sugar on the basis of the quotas for such areas as established in § 811.2 and 184,155 short

tons, raw value, are prorated to such domestic areas and Cuba on the basis of the quotas in effect after proration of the 179,085 tons.

The quotas established in §§ 811.2 and 811.3 were determined in accordance with the specific procedures provided in section 202 of the act. The amounts of the quotas which may be filled by direct-consumption sugar were established in accordance with section 207 of the act.

(Sec. 403, 61 Stat. 932; 7 U.S.C. 1153. Interprets or applies secs. 202, 204; 61 Stat. 924, 925; 7 U.S.C. 1112, 1114)

Done at Washington, D.C., this 3d day of September, 1959.

TRUE D. MORSE,
Acting Secretary.

[F.R. Doc. 59-7510; Filed, Sept. 9, 1959; 8:47 a.m.]

Chapter IX—Agricultural Marketing Service (Marketing Agreements and Orders), Department of Agriculture

[Lemon Reg. 807, Amdt. 1]

PART 953—LEMONS GROWN IN CALIFORNIA AND ARIZONA

Limitation of Handling

(a) *Findings.* (1) Pursuant to the marketing agreement, as amended, and Order No. 53, as amended (7 CFR Part 953), regulating the handling of lemons grown in California and Arizona, effective under the applicable provisions of the Agricultural Marketing Agreement Act of 1937, as amended (7 U.S.C. 601 et seq.; 68 Stat. 906, 1047), and upon the basis of the recommendation and information submitted by the Lemon Administrative Committee, established under the said amended marketing agreement and order, and upon other available information, it is hereby found that the limitation of handling of such lemons as hereinafter provided will tend to effectuate the declared policy of the act.

(2) It is hereby further found that it is impracticable and contrary to the public interest to give preliminary notice, engage in public rule-making procedure, and postpone the effective date of this amendment until 30 days after publication hereof in the FEDERAL REGISTER (60 Stat. 237; 5 U.S.C. 1001 et seq.) because the time intervening between the date when information upon which this amendment is based became available and the time when this amendment must become effective in order to effectuate the declared policy of the Agricultural Marketing Agreement Act of 1937, as amended, is insufficient, and this amendment relieves restriction on the handling of lemons grown in California and Arizona.

(b) *Order, as amended.* The provisions in paragraph (b) (1) (ii) of § 953.914 (Lemon Regulation 807, 24 F.R. 6994) are hereby amended to read as follows:

(ii) District 2: 348,750 cartons.

(Secs. 1-19, 48 Stat. 31, as amended; 7 U.S.C. 601-674)

Dated: September 4, 1959.

FLOYD F. HEDLUND,
Acting Director, Fruit and Vegetable Division, Agricultural Marketing Service.

[F.R. Doc. 59-7508; Filed, Sept. 9, 1959; 8:47 a.m.]

[959.317, Amdt. 1]

PART 959—IRISH POTATOES GROWN IN MODOC AND SISKIYOU COUNTIES, CALIF., AND IN ALL COUNTIES IN OREGON, EXCEPT MALHEUR COUNTY

Limitation of Shipments

Findings. (a) Pursuant to Marketing Agreement No. 114, as amended, and Order No. 59, as amended (7 CFR Part 959), regulating the handling of Irish potatoes grown in Modoc and Siskiyou Counties, California and in all counties in Oregon except Malheur County, effective under the applicable provisions of the Agricultural Marketing Agreement Act of 1937, as amended (7 U.S.C. 601-674), and upon the basis of recommendations and information submitted by the Oregon-California Potato Committee, established pursuant to the said amended marketing agreement and order, and other available information, it is hereby found that the amendment to the limitation of shipments regulation hereinafter set forth, will tend to effectuate the declared policy of the act.

(b) It is hereby found that it is impracticable and contrary to the public interest to give preliminary notice, and engage in public rule making procedure, and postpone the effective date of this amendment until 30 days after publication in the FEDERAL REGISTER (5 U.S.C. 1001-1011) in that (1) the time intervening between the date when information upon which this amendment is based became available and the time when this amendment must become effective in order to effectuate the declared policy of the act is insufficient, (2) the currently effective limitation of shipments regulation (§ 959.317, 24 F.R. 5599) terminates on September 10, 1959 and as amended herein the period of regulation is extended for an additional thirty (30) days through October 10, 1959, (3) more orderly marketing in the public interest than would otherwise prevail will be promoted by extending the period of regulation the additional thirty (30) days, (4) compliance with the regulation as amended herein will not require any special preparation on the part of handlers which cannot be completed by the effective date hereof, and (5) information regarding the committee's recommendation has been disseminated to producers and handlers in the production area.

Order. The first paragraph and paragraph (b) (1) of the said regulation are hereby amended as set forth below.

§ 959.317 Limitation of shipments.

During the period from July 13, 1959, through October 10, 1959, no person shall handle any lot of potatoes unless such potatoes meet the requirements of paragraphs (a) and (b) of this section or unless such potatoes are handled in accordance with paragraphs (c), (d), (e), and (f) of this section.

* * * * *

(b) *Minimum maturity requirements*—(1) *All varieties.* "Slightly skinned," which means that not more skinned", which means that not more than 10 percent of the potatoes in any lot may have more than one-fourth of the skin missing or "feathered."

* * * * *

(Secs. 1-19, 48 Stat. 31, as amended; 7 U.S.C. 601-674)

Dated: September 4, 1959, to become effective September 10, 1959.

FLOYD F. HEDLUND,
Acting Director, Fruit and Vegetable Division, Agricultural Marketing Service.

[F.R. Doc. 59-7535; Filed, Sept. 9, 1959; 8:51 a.m.]

Title 5—ADMINISTRATIVE PERSONNEL

Chapter I—Civil Service Commission

PART 6—EXCEPTIONS FROM THE COMPETITIVE SERVICE

Department of Defense

Effective upon publication in the FEDERAL REGISTER, subparagraphs (2) and (17) of § 6.304(a) are amended as set out below.

§ 6.304 Department of Defense.

(a) *Office of the Secretary.* * * *

(2) Two Confidential Assistants (Private Secretaries) to the Deputy Secretary of Defense and one Confidential Assistant (Private Secretary) to each of the following: the Director of Defense Research and Engineering; the Assistant Secretary of Defense, Manpower, Personnel and Reserve; the Assistant Secretary of Defense, International Security Affairs; the Chairman of the Joint Chiefs of Staff; the Defense Liaison Officer to the White House; the Assistant Secretary of Defense, Public Affairs; the Assistant Secretary of Defense, Properties and Installations; the Assistant Secretary of Defense, Health and Medical; the Assistant Secretary of Defense, Supply and Logistics; the General Counsel; the U.S. Military Representative, NATO Standing Group; and the Assistant to the Secretary of Defense, Atomic Energy.

* * * * *

(17) One Confidential Secretary to the Special Assistant to the Director of Defense Research and Engineering.

(R.S. 1753, sec. 2, 22 Stat. 403, as amended; 5 U.S.C. 631, 633)

UNITED STATES CIVIL SERVICE COMMISSION,
[SEAL] WM. C. HULL,
Executive Assistant.

[F.R. Doc. 59-7512; Filed, Sept. 9, 1959; 8:48 a.m.]

PART 6—EXCEPTIONS FROM THE COMPETITIVE SERVICE

Treasury Department

Effective upon publication in the FEDERAL REGISTER, subparagraph (10) is added to § 6.303(a) as set out below.

§ 6.303(a) **Treasury Department.**

(a) *Office of the Secretary.* * * *

(10) One Deputy to the Secretary.

(R.S. 1753, sec. 2, 22 Stat. 403, as amended; 5 U.S.C. 631, 633)

UNITED STATES CIVIL SERVICE COMMISSION,
[SEAL] WM. C. HULL,
Executive Assistant.

[F.R. Doc. 59-7533; Filed, Sept. 9, 1959; 8:51 a.m.]

Title 14—AERONAUTICS AND SPACE

Chapter III—Federal Aviation Agency

[Airspace Docket No. 59-LA-24; Amdt. 18]

PART 608—RESTRICTED AREAS

Revocation of Restricted Area

The purpose of this action is to revoke the Sand Hills, California, Restricted Area (R314).

The U.S. Navy no longer has a requirement for this restricted area, and has requested that it be revoked.

Since this amendment eliminates a burden on the public, compliance with the notice, public procedure, and effective date requirements of section 4 of the Administrative Procedure Act is unnecessary.

In consideration of the foregoing, the following action is taken: in § 608.14 the Sand Hills, California, Restricted Area (R-314) (23 F.R. 8578, 24 F.R. 524) is revoked.

This amendment shall become effective upon the date of publication in the FEDERAL REGISTER.

(Secs. 307(a) and 313(a), 72 Stat. 749, 752; 49 U.S.C. 1348, 1354)

Issued in Washington, D.C., on September 2, 1959.

JAMES T. PYLE,
Acting Administrator.

SEPTEMBER 2, 1959.

[F.R. Doc. 59-7491; Filed, Sept. 9, 1959; 8:46 a.m.]

[Airspace Docket No. 59-WA-22; Amdt. 21]

PART 608—RESTRICTED AREAS

Modification of Restricted Area

The purpose of this amendment is to change the designated altitudes of the Camp Pickett, Virginia, Restricted Area (R-44) from "Surface to unlimited" to "Surface to 22,000 feet, MSL".

The U.S. Army no longer has a requirement for the Airspace above 22,000 feet within this restricted area and has requested that an upper altitude limit be designated accordingly.

Since this amendment reduces a burden on the public, compliance with the notice, public procedure, and effective date requirements of section 4 of the Administrative Procedure Act is unnecessary.

In consideration of the foregoing, the following action is taken: In § 608.54, the Camp Pickett, Virginia, Restricted Area (R-44) (23 F.R. 8588) is amended by deleting "Surface to unlimited" and substituting therefor "Surface to 22,000 feet MSL".

This amendment shall become effective upon the date of publication in the FEDERAL REGISTER.

(Secs. 307(a) and 313(a), 72 Stat. 749, 752; 49 U.S.C. 1348, 1354)

Issued in Washington, D.C., on September 2, 1959.

JAMES T. PYLE,
Acting Administrator.

SEPTEMBER 2, 1959.

[F.R. Doc. 59-7489; Filed, Sept. 9, 1959; 8:45 a.m.]

[Airspace Docket No. 59-WA-78; Amdt. 19]

PART 608—RESTRICTED AREAS

Modification of a Restricted Area

The purpose of this amendment is to reduce the upper altitude limit of the Waldron Island, Washington, Restricted Area (R-234) from 10,000 feet to 4,000 feet, MSL.

The U.S. Navy no longer has a requirement for the airspace above 4,000 feet within this restricted area and has requested that the upper altitude limit be lowered accordingly.

Since this amendment reduces a burden on the public, compliance with the notice, public procedure, and effective date requirements of section 4 of the Administrative Procedure Act is unnecessary.

In consideration of the foregoing, the following action is taken: In § 608.55, the Waldron, Washington Restricted Area (R-234) (23 F.R. 8590) is amended by deleting "Surface to 10,000 feet" and substituting therefor "Surface to 4,000 feet MSL".

This amendment shall become effective upon the date of publication in the FEDERAL REGISTER.

(Secs. 307(a) and 313(a), 72 Stat. 749, 752; 49 U.S.C. 1348, 1354)

Issued in Washington, D.C., on September 2, 1959.

JAMES T. PYLE,
Acting Administrator.

SEPTEMBER 2, 1959.

[F.R. Doc. 59-7490; Filed, Sept. 9, 1959; 8:45 a.m.]

Title 16—COMMERCIAL PRACTICES

Chapter I—Federal Trade Commission

[Docket 7438 c.o.]

PART 13—DIGEST OF CEASE AND DESIST ORDERS

F. E. Booth Company, Inc.

Subpart—*Discriminating in price under section 2, Clayton Act, as amended*—Payment or acceptance of commission, brokerage, or other compensation under 2(c): § 13.820 *Direct buyers.*

(Sec. 6, 38 Stat. 721; 15 U.S.C. 46. Interprets or applies sec. 2, 38 Stat. 730, as amended; 15 U.S.C. 13) [Cease and desist order, F. E. Booth Company, Inc., San Francisco, Calif., Docket 7438, Aug. 4, 1959]

This proceeding was heard by a hearing examiner on the complaint of the Commission charging a processor and canner of fish, fruits, and vegetables, in San Francisco, Calif., with violating section 2(c) of the Clayton Act by giving to customers buying directly for their own accounts for resale, discounts or allowances reflecting the usual 2½ percent brokerage fee.

After acceptance of an agreement containing consent order, the hearing examiner made his initial decision and order to cease and desist which became on August 4 the decision of the Commission.

The order to cease and desist is as follows:

It is ordered, That Respondent F. E. Booth Company, Inc., a corporation, its officers, representatives, agents or employees, directly or through any corporate or other device, in connection with the sale and distribution of canned foods, or other food products, in commerce, as "commerce" is defined in the Clayton Act, as amended, do forthwith cease and desist from: Paying, granting or allowing, directly or indirectly, to any buyer, or anyone acting for or in behalf of, or who is subject to, the direct or indirect control of such buyers, anything of value as a commission, brokerage or other compensation or any allowance or discount in lieu thereof upon, or in connection with, any sale to such buyer for its own account.

By "Decision of the Commission," etc., report of compliance was required as follows:

It is ordered, That Respondent F. E. Booth Company, Inc., a corporation, shall, within sixty (60) days after service upon it of this order, file with the Commission a report in writing, setting forth in detail the manner and form in which it has complied with the order to cease and desist.

Issued: August 4, 1959.

By the Commission.

[SEAL] ROBERT M. PARRISH,
Secretary.

[F.R. Doc. 59-7503; Filed, Sept. 9, 1959; 8:47 a.m.]

[Docket 7449 c.o.]

PART 13—DIGEST OF CEASE AND DESIST ORDERS

Lenders Service Corp. et al.

Subpart—*Advertising falsely or misleadingly:* § 13.15 *Business status, advantages, or connections:* Financing activities; institutional connections; § 13.85 *Government approval, action, connection or standards:* Small Business Administration,[1] § 13.185 *Refunds, repairs, and replacements;* § 13.205 *Scientific or other relevant facts;* § 13.225 *Services.* Subpart—*Misrepresenting oneself and goods*—Business status, advantages or connections: § 13.1417 *Financing activities;*[1] § 13.1553 *Services;* [*Misrepresenting oneself and goods*]—Goods: § 13.1740 *Scientific or other relevant facts.*

(Sec. 6, 38 Stat. 721; 15 U.S.C. 46. Interpret or apply sec. 5, 38 Stat. 719, as amended; 15 U.S.C. 45) [Cease and desist order, Lenders Service Corporation et al., Los Angeles, Calif., Docket 7449, August 6, 1959]

In the Matter of Lenders Service Corporation, a Corporation; Ralph L. Sampson, Leonard Miller, Herbert Ruttenberg and Harvey Cova, Individually and as Officers of Said Corporation; U. T. Thompson, Individually and as Executive Vice President of Said Corporation; and William VanPinsker, Charles McCarthy and William Mitchell, Individually

This proceeding was heard by a hearing examiner on the complaint of the Commission charging a lending concern in Los Angeles, Calif., with misrepresenting the services it rendered in helping businessmen to obtain loans by such false misrepresentations as that it was an affiliate or agent of banks and other lending institutions which would make loans to anyone it recommended, that upon payment of a fee it would get clients larger loans than they applied for and would refund the fee if the loan was not obtained, and that it often made loans from its own funds or would get clients loans from the Small Business Administration.

On the basis of a consent agreement, the hearing examiner made his initial decision and order to cease and desist which became on August 6 the decision of the Commission.

The order to cease and desist is as follows:

It is ordered, That Respondents Lenders Service Corporation, a corporation, and its officers; Ralph L. Sampson, Leonard Miller, Herbert Ruttenberg and Harvey Cova, individually and as officers of said corporation; U. T. Thompson, individually and as Executive Vice President of said corporation; and William VanPinsker, Charles McCarthy and William Mitchell, individually, and Respondents' representatives, agents and employees, directly or through any corporate or other device, in connection with the advertising of or offering for sale or sale of their services in obtaining loans or financial assistance for businessmen or others, in commerce, as "commerce" is defined in the Federal Trade Commission Act, do forthwith cease and desist from representing, directly or by implication, that:

1. Respondents will obtain loans within a short period of time; or in any period of time that is not in accordance with the fact;
2. Respondents will refund the fee paid in event they do not obtain a loan, unless such is the fact;
3. Respondents can or will obtain larger loans than the loans applied for;
4. Respondents are affiliated with banks or lending institutions;
5. Banks or other lending institutions will make loans to everyone recommended by Respondents;
6. Respondents are the agents of banks, insurance companies or other lending or financial institutions;
7. Respondents will obtain loans from the Small Business Administration for those who pay Respondents for their service;
8. Respondents will make loans to clients from their own funds.

By "Decision of the Commission," etc., report of compliance was required as follows:

It is ordered, That the respondents named in the caption hereof shall, within sixty (60) days after service upon them of this order, file with the Commission a report in writing, setting forth in detail the manner and form in which they have complied with the order to cease and desist.

Issued: August 6, 1959.

By the Commission.

[SEAL] ROBERT M. PARRISH, *Secretary.*

[F.R. Doc. 59-7503; Filed, Sept. 9, 1959; 8:47 a.m.]

[1] New.

Title 42—PUBLIC HEALTH

Chapter I—Public Health Service, Department of Health, Education, and Welfare

PART 21—COMMISSIONED OFFICERS

Prescription of Numbers in Grade

Section 21.111 of Subpart G is amended to read as follows:

§ 21.111 Prescription of numbers in grade.

The following maximum number of officers is authorized to be on active duty in the Regular Corps in each of the grades from the junior assistant grade to the director grade, inclusive, during the fiscal year beginning July 1, 1959, and ending June 30, 1960:

Director Grade	480
Senior Grade	590
Full Grade	435
Senior Assistant Grade	335
Assistant Grade	60
Junior Assistant Grade	0

(Sec. 206, 58 Stat. 694, as amended; 42 U.S.C. and Sup., 207)

This amendment shall be effective as of July 1, 1959.

Dated: August 20, 1959.

[SEAL] L. E. BURNEY, *Surgeon General.*

Approved: September 3, 1959.

ARTHUR S. FLEMMING, *Secretary.*

[F.R. Doc. 59-7517; Filed, Sept. 9, 1959; 8:49 a.m.]

Title 47—TELECOMMUNICATION

Chapter I—Federal Communications Commission

[Docket No. 11233; FCC 59-908]

PART 3—RADIO BROADCAST SERVICES

Standards of Good Engineering Practice

In the matter of Amendment of Part 3 of the Commission's rules and regulations and the Standards of Good Engineering Practice Concerning Bandwidth and Spurious Emissions of AM and FM Stations; Docket No. 11233.

1. On December 9, 1954 the Commission released a Notice of Proposed Rule Making (FCC 54-1518) in the above-entitled matter. The Notice of Proposed Rule Making was published in the FEDERAL REGISTER December 14, 1954. The date for filing comments was specified as March 7, 1955 and was subsequently extended to June 6, 1955. The purpose of the proposed rules is to establish definitions of bandwidth and specifications for suppression of spurious emissions of AM and FM broadcast stations.

2. Comments were received from licensees of AM and FM broadcast stations,[1] the National Association of Radio and Television Broadcasters (NARTB), the Radio-Electronics-Television Manufacturers Association (RETMA) and the General Electric Company. A review of the comments shows that they may be divided into two categories: Those concerning transmitters presently in use, and those applying to transmitters to be installed in the future.

3. The comments generally favored the adoption of the proposed performance standards with certain modifications, but expressed the opinion that existing transmitting equipment should be exempted from the proposed requirements until a further study could be made.

[1] The following stations filed comments: The Good Music Station, Inc. (WOMS); The Montana Network (KOOK); Tri-City Radio Corp. (WLBC, WMUN); Chanticleer Broadcasting Co. (WCTC); Indian River Broadcasting Co. (WIRA); General Teleradio, Inc.; Havens and Martin, Inc. (WMBG, WOOD); Albuquerque Broadcasting Co. (KOB); KOMA, Inc. (KOMA); Crosley Broadcasting Corp.; E. Harold Munn, Jr. (WTVV); American Broadcasting Co.; Queen City Broadcasting Co. (KIRO); The Philadelphia Inquirer Stations (WFIL); WRAK, Inc. (WRAK); Owensboro Publishing Co. (WOMI); Alvin E. O'Konski (WOSA, WLIN); and Corn Belt Publishers, Inc. (WAAF).

Such a survey was conducted by NAB (formerly NARTB) and the results submitted to the Commission on November 16, 1956. These results showed that although many existing stations were achieving suppression in excess of that proposed to be required, it would impose an extreme hardship on some to make the necessary modifications in existing equipment to comply strictly with the proposed standards.

4. Evidence was also submitted indicating that the Commission's original proposal would require an abrupt transition from the amplitude of the emissions required for the proper performance of the broadcasting system and the so-called out-of-band or spurious emissions in the region of the spectrum immediately adjacent to the channel occupied by the broadcast station. Such a transition not only imposes a severe requirement on the equipment design but also may result in other undesirable effects which could lead to other spurious emissions. The comments also point out that the degree of suppression under the formula proposed in the initial Notice would require attenuation of some out-of-band emissions by more than 90 decibels.

5. On the basis of the comments submitted and the record in the proceeding in Docket No. 11654,[1] the Commission has concluded that its rules should be amended as set forth below. It will be noted that the maximum suppression required as a matter of equipment acceptance and normal operation is 80 decibels. Should such suppression in individual cases be insufficient to prevent the interference to the reception of other radio stations, the Commission may require a licensee to take such further steps as may be necessary to eliminate the interference. It may also be noted that although existing stations are not required to adhere strictly to the performance requirements they are expected to achieve the highest degree of compliance within the capabilities of their present equipment. This exemption does not extend to individual cases where actual interference occurs. In such cases the Commission may require licensees to take further steps to eliminate the intereference. All transmitting apparatus which is type accepted after January 1, 1960, must meet the new requirements.

6. In view of the foregoing considerations and pursuant to the authority contained in sections 4(i), 301 and 303 of the Communications Act of 1934, as amended: *It is ordered*, That, effective January 1, 1960, Part 3 of the Commission's rules is amended as set forth below.

(Sec. 4, 48 Stat. 1066, as amended; 47 U.S.C. 154. Interpret or apply secs. 301, 303, 48 Stat. 1081, 1082; 47 U.S.C. 301, 303)

Adopted: September 2, 1959.

Released: September 4, 1959.

FEDERAL COMMUNICATIONS COMMISSION,
[SEAL] MARY JANE MORRIS,
Secretary.

[1] See Notice of Proposed Rule Making (FCC 56-231) released on March 23, 1956.

1. Section 3.40(a) is amended by adding thereto the following new subparagraphs:

§ 3.40 Transmitter; design, construction, and safety of life requirements.

(a) * * *

(12) Any emission appearing on a frequency removed from the carrier by between 15 kc and 30 kc, inclusive, shall be attenuated at least 25 db below the level of the unmodulated carrier. Compliance with the specification will be deemed to show the occupied bandwidth to be 30 kc or less.

(13) Any emission appearing on a frequency removed from the carrier by more than 30 kc and up to and including 75 kc, inclusive, shall be attenuated at least 35 db below the level of the unmodulated carrier.

(14) Any emission appearing on a frequency removed from the carrier by more than 75 kc shall be attenuated at least 43+ 10 Log_{10} (Power, in watts) decibels below the level of the unmodulated carrier, or 80 decibels, whichever is the lesser attenuation.

2. Section 3.46(c) is amended by deleting the present text and substituting the following:

§ 3.46 Transmitter.

* * * * *

(c) The station equipment shall be so operated, tuned, and adjusted that emissions outside of the authorized channel do not cause harmful interference to the reception of other radio stations. Standard broadcast stations employing radio transmitters type accepted after January 1, 1960, shall maintain the bandwidth occupied by their emissions in accordance with the specifications set forth in § 3.40(a). Stations employing transmitters installed or type accepted prior to January 1, 1960, shall achieve the highest degree of compliance practicable with their existing equipment. In either case, should harmful interference to the reception of other radio stations occur, the licensee may be required to take such further steps as may be necessary to eliminate the interference.

3. Section 3.317 is amended by adding new subparagraphs to paragraph (a) and by substituting new text for paragraph (f)(2), as follows:

§ 3.317 Transmitter and associated equipment.

(a) * * *

(12) Any emission appearing on a frequency removed from the carrier by between 120 kc and 240 kc inclusive shall be attenuated at least 25 decibels below the level of the unmodulated carrier. Compliance with this specification will be deemed to show the occupied bandwith to be 240 kc or less.

(13) Any emission appearing on a frequency removed from the carrier by more than 240 kc and up to and including 600 kc shall be attenuated at least 35 db below the level of the unmodulated carrier.

(14) Any emission appearing on a frequency removed from the carrier by more than 600 kc shall be attenuated at least 43+10 Log_{10} (Power, in watts) decibels below the level of the unmodulated carrier, or 80 decibels, whichever is the lesser attenuation.

* * * * *

(f) * * *

(2) The station equipment shall be so operated, tuned, and adjusted that emissions outside of the authorized channel do not cause harmful interference to the reception of other radio stations. FM broadcast stations employing transmitters type accepted after January 1, 1960, shall maintain the bandwidth occupied by their emissions in accordance with the specifications set forth in paragraph (a) of this section. Stations employing transmitters installed or type accepted prior to January 1, 1960, shall achieve the highest degree of compliance practicable with their existing equipment. In either case, should harmful interference to the reception of other radio stations occur, the licensee may be required to take such further steps as may be necessary to eliminate the interference.

[F.R. Doc. 59-7519; Filed, Sept. 9, 1959; 8:49 a.m.]

[Docket No. 11779; FCC 59-907]

PART 3—RADIO BROADCAST SERVICES

Table of Assignments; Lubbock, Tex.

In the matter of amendment of § 3.606 *Table of assignments*, Television Broadcast Stations (Lubbock, Tex.); Docket No. 11779.

1. The Commission has before it for consideration a Petition filed on November 26, 1956, by Texas Technological College, for reconsideration of the Commission's Report and Order issued in this proceeding on October 25, 1956 (FCC 56-1032), denying the College's request that the educational reservation in Lubbock, Texas, be changed from UHF Channel 20 to VHF Channel 5; an Opposition filed by C. L. Trigg on December 6, 1956; and a Reply filed by Texas Technological College on December 20, 1956.

2. In the above-described Report and Order denying Texas Technological College's petition, we concluded at that time that the public interest would not be served by reserving Channel 5 in Lubbock for exclusive use by a noncommercial educational television station. This conclusion was based in part on the fact that applications had then been filed for a noncommercial station by the College and for a commercial station by another applicant, and on our judgment that in such a case the channel should not be set aside exclusively for noncommercial educational use where the educational interests, as well as the commercial applicant were ready to proceed and the comparative merits of all applicants could be evaluated in a hearing. We are not persuaded by the pleadings before us that this judgment was in error. However, subsequent events in this proceeding, included in the record now before us, and summarized below, render the contentions and arguments advanced

in the above-described petition, opposition, and reply moot.

3. On July 16, 1957, which was subsequent to the date of the Order here under consideration, an evidentiary hearing was held on Texas Technological College's application for a construction permit to operate noncommercially on Channel 5 at Lubbock; on September 17, 1957, an Initial Decision was released, looking toward the grant of the application; on October 24, 1957, the Commission stayed the effect of the Initial Decision, and remanded the case to the Examiner for the taking of additional evidence, and on April 15, 1958, the Examiner's Supplemental Initial Decision, looking toward grant of the application, was released. This decision became effective on May 21, 1958, when Texas Technological College's application to operate noncommercially on Channel 5 at Lubbock was granted. A construction permit was issued on July 21, 1958, and was extended on January 21, 1959, until July 21, 1959. On June 26, 1959, the permit was extended until January 21, 1960. Petitioner continues to propose a noncommercial operation.

4. In view of the foregoing facts, we now find it appropriate to grant petitioner's request that the educational reservation in Lubbock, Texas, be changed from UHF Channel 20 to VHF Channel 5.

5. Authority for the amendments ordered herein is contained in sections 4(i), 301, 303 (c), (d), (f), and (r) and 307(b) of the Communications Act of 1934, as amended.

6. *Accordingly, it is ordered*, Effective October 9, 1959, that the assignments contained in § 3.606 of the Commisison's rules are amended, insofar as the community named is concerned, to read as follows:

City	Channel No.
Lubbock, Texas	*5—, 11, 13—, 20, 28

(Sec. 4, 48 Stat. 1066, as amended; 47 U.S.C. 154. Interpret or apply secs. 301, 303, 307, 48 Stat. 1081, 1082, 1083; 47 U.S.C. 301, 303, 307)

Adopted: September 2, 1959.

Released: September 4, 1959.

FEDERAL COMMUNICATIONS COMMISSION,
[SEAL] MARY JANE MORRIS,
Secretary.

[F.R. Doc. 59-7520; Filed, Sept. 9, 1959; 8:49 a.m.]

[Docket No. 12054; FCC 59-910]

PART 3—RADIO BROADCAST SERVICES

Table of Assignments; Columbus, Ga.

In the matter of Amendment of § 3.606 *Table of assignments*, Television Broadcast Stations (Columbus, Georgia); Docket No. 12054.

At a session of the Federal Communications Commission held at its Offices in Washington, D.C., on the 2d day of September 1959.

The Commission having under consideration a Petition for Reconsideration filed August 17, 1959, by WTVY, Inc., licensee of WTVY, Dothan Alabama, requesting reconsideration of the Report and Order (FCC 59-721) released in this proceeding on July 17, 1959; and

It appearing that the Commission will require additional time for review of the matters raised in the foregoing Petition for Reconsideration;

It further appearing that the Report and Order (FCC 59-721, Paragraph 29), specified August 24, 1959, as the effective date for the amendments to § 3.606 of the Commission's rules and regulations; and

It further appearing that the channels ordered to be reassigned must continue to be occupied by stations licensed to operate on them in Columbus, Georgia, and Dothan, Alabama, until disposition is made of matters raised in the foregoing Petition for Reconsideration;

It is ordered on the Commission's own motion, That the effective date of that portion of the Report and Order released in this proceeding on July 17, 1959 (24 F.R. 5834), which amends § 3.606 of the Commission's rules and regulations, is stayed, until further order of the Commission.

Released: September 4, 1959.

FEDERAL COMMUNICATIONS COMMISSION,
[SEAL] MARY JANE MORRIS,
Secretary.

[F.R. Doc. 59-7521; Filed, Sept. 9, 1959; 8:49 a.m.]

[Docket No. 12902; FCC 59-924]

PART 19—CITIZENS RADIO SERVICE

Licensed Radio Operators

In the matter of amendment of § 19.71 of Part 19, Citizens Radio Service, to specify certain exceptions to the requirements regarding licensed radio operators in connection with Class C and Class D stations in that service; upon certification of the equipment by the manufacturer, Docket No. 12902.

1. On June 10, 1959, the Commission adopted a Notice of Proposed Rule Making in the above entitled matter which was published in the FEDERAL REGISTER of June 18, 1959 (24 F.R. 4969). The time allowed for filing both original and reply comments has now expired.

2. Comments were received from the Citizenship Radio Corporation and the Kaar Engineering Corporation. No reply comments were received. The statement of Citizenship was simply that it had no objections to the proposal. Kaar supports the proposal insofar as it relates to factory-assembled equipment but opposes the exception to the requirement for a licensed operator as it relates to home-assembled kits.

3. The opposition of Kaar appears to be based on a misconception of the restated in the proposed rule and a mistaken idea that the licensee who home-assembles a factory kit is relieved of any responsibility for the proper performance of the equipment. In order to come within the proposed exception, a kit which is to be further home-assembled must comply with all the conditions listed in paragraph (d) of the amended § 19.71, one of which is that the frequency determining elements of the transmitter, including the crystal and all components of the crystal oscillator circuit, shall have been pre-assembled by the manufacturer, pre-tuned to a specific available frequency, and sealed. Thus the proposed rule would not include the complete un-supervised home-construction and activation of a transmitter but only the addition to this sealed unit of certain components, preselected by the manufacturer of the kit, that may reasonably be expected not to result in off-frequency operation, excessive plate input power, over-modulation, excessive harmonics or other spurious emissions. Compliance with § 19.71 (d) does not relieve the licensee of responsibility for the proper performance of the equipment since he may still be cited for violation of the appropriate section of Subpart C of Part 19 if this equipment fails to comply with any of the technical standards contained therein. Compliance with § 19.71(d) simply relieves the licensee of the necessity of determining in advance whether or not certain adjustments of the transmitting equipment may reasonably be expected to result in violation of those technical standards.

4. The Commission is of the opinion that the limited exception to the licensed operator requirements contained in the proposal is in the public interest in that it would facilitate the fullest development and use of Class C and D stations and at the same time contains adequate safeguards to prevent a degradation of the service, particularly with respect to those persons who, for economic or other reasons, wish to use equipment involving some degree of home construction.

5. In view of the foregoing, and pursuant to authority contained in sections 4(c) and 303 of the Communications Act of 1934, as amended, *It is ordered*, That effective November 15, 1959, Section 19.71 of Part 19, Citizens Radio Service, be amended as set forth below.

(Sec. 4, 48 Stat. 1066, as amended; 47 U.S.C. 154. Interprets or applies sec. 303, 48 Stat. 1082, as amended; 47 U.S.C. 303)

Adopted: September 2, 1959.

Released: September 4, 1959.

FEDERAL COMMUNICATIONS COMMISSION,
[SEAL] MARY JANE MORRIS,
Secretary.

In § 19.71, paragraph (c) is amended and new paragraphs (d) and (e) are added, as follows:

§ 19.71 Operator requirements.

* * * * *

(c) Except as provided in paragraph (d) of this section, all transmitter adjustments or tests while radiating energy during or coincident with the construction, installation, servicing, or maintenance of a radio station in this service which may affect the proper operation of

such station, shall be made by or under the immediate supervision and responsibility of a person holding a first- or second-class commercial radio operator license, either radiotelephone or radiotelegraph, as may be appropriate for the type of emission employed, and such person shall be responsible for the proper functioning of the station equipment at the conclusion of such adjustments or tests.

(d) In the case of Class C or Class D stations in this service, no commercial radio operator license is required to be held by the person performing transmitter adjustments or tests during or coincident with the construction, installation, servicing, or maintenance of such stations: *Provided*, That there is compliance with all of the following conditions:

(1) The transmitting equipment shall be crystal-controlled with a crystal capable of maintaining the station frequency within the prescribed tolerance;

(2) The transmitting equipment either shall have been factory-assembled or shall have been provided in kit form by a manufacturer who provided all components together with full and detailed instructions for their assembly by nonfactory personnel;

(3) The frequency determining elements of the transmitter, including the crystal(s) and all other components of the crystal oscillator circuit, shall have been pre-assembled by the manufacturer, pre-tuned to a specific available frequency, and sealed by the manufacturer so that replacement of any component or any adjustment which might cause off-frequency operation cannot be made without breaking such seal and thereby voiding the certification of the manufacturer required by this paragraph;

(4) The transmitting equipment shall have been so designed that none of the transmitter adjustments or tests normally performed during or coincident with the installation, servicing, or maintenance of the station, or during the normal rendition of the service of the station, or during the final assembly of kits or partially pre-assembled units, may reasonably be expected to result in off-frequency operation, excessive plate input power, over-modulation, or excessive harmonics or other spurious emissions; and

(5) The manufacturer of the transmitting equipment or of the kit from which the transmitting equipment is assembled shall have certified in writing to the purchaser of the equipment (and to the Commission upon request) that the equipment has been designed, manufactured and furnished in accordance with the specifications contained in the foregoing subparagraphs of this paragraph:

And provided further, That, notwithstanding the foregoing provisions of this paragraph, whenever the transmitting equipment of a station is found operating contrary to any of the technical regulations contained in Subpart C of this part, all transmitter adjustments or tests while radiating energy during or coincident with the servicing of that equipment for the purpose of restoring compliance with those regulations shall be made by or under the immediate supervision and responsibility of a person holding a first- or second-class commercial radio operator license, either radiotelephone or radiotelegraph, as may be appropriate for the type of emission employed, and such person shall be responsible for the proper functioning of the station equipment at the conclusion of such adjustments or tests.

(e) The manufacturer's certification concerning design and construction features of Class C or Class D station transmitting equipment, as required if the provisions of paragraph (d) of this section are invoked, may be specific as to a particular unit of transmitting equipment or general as to a group or model of such equipment, and may be in any form adequate to assure the purchaser of the equipment or the Commission that the conditions described in that paragraph have been fulfilled.

[F.R. Doc. 59-7522; Filed, Sept. 9, 1959; 8:50 a.m.]

Title 50—WILDLIFE

Chapter I—Fish and Wildlife Service, Department of the Interior

SUBCHAPTER B—HUNTING AND POSSESSION OF WILDLIFE

PART 6—MIGRATORY BIRDS

Open Seasons, Bag Limits, and Possession of Certain Migratory Game Birds

Correction

In F.R. Doc. 59-7315, appearing at page 7079 of the issue of Tuesday, September 1, 1959, the "do" listed for South Carolina in the "Ducks and coots" column of § 6.51(c) should read "Nov. 30-Jan. 8."

PROPOSED RULE MAKING

DEPARTMENT OF THE INTERIOR

Fish and Wildlife Service

[50 CFR Part 31]

LITTLE PEND OREILLE NATIONAL WILDLIFE REFUGE, WASHINGTON

Notice of Proposed Rule Making

Notice is hereby given that pursuant to the authority contained in section 10 of the Migratory Bird Conservation Act of February 18, 1929 (45 Stat. 1224; 16 U.S.C. 715i), and under authority delegated by Commissioner's Order 4 (22 F.R. 8126), it is proposed to revise § 31.177 and to revoke §§ 31.178 and 31.179 of Subpart—Little Pend Oreille National Wildlife Refuge, Washington, Chapter I, Title 50, Code of Federal Regulations, to read as set forth in tentative form below. The purpose is to permit the hunting of deer, bear, and grouse on certain lands of the Little Pend Oreille National Wildlife Refuge in accordance with existing State procedures and regulations.

No. 177—2

Interested persons may submit in duplicate written comments, suggestions, or objections with respect to the proposed revision and revocations to the Director, Bureau of Sport Fisheries and Wildlife, Washington 25, D.C., within thirty days of the date of publication of this notice in the FEDERAL REGISTER.

Dated: September 3, 1959.

A. V. TUNISON,
Acting Director, Bureau of Sport Fisheries and Wildlife.

§ 31.177 Deer, bear, and grouse hunting permitted.

Subject to compliance with the provisions of Parts 18 and 21 of this chapter, hunting of deer, bear, and grouse is permitted on the hereinafter described lands of the Little Pend Oreille National Wildlife Refuge subject to the following conditions, restrictions, and requirements:

(a) *State laws.* Strict compliance with all applicable State laws and regulations is required.

(b) *Entry.* A valid State hunting license, if required under State law, will serve as a Federal permit for hunting on that portion of the refuge opened to hunting.

(c) *Dogs.* Dogs are not permitted on the refuge for use in the hunting of deer, bear, or grouse.

(d) *Hunting period.* The period of hunting will be in compliance with regulations prescribed by the Washington Fish and Game Commission.

(e) *Hunting area.* All of the lands of the Little Pend Oreille National Wildlife Refuge will be opened to the hunting of deer, bear, and grouse.

[F.R. Doc. 59-7504; Filed, Sept. 9, 1959; 8:47 a.m.]

[50 CFR Part 34]

LOXAHATCHEE NATIONAL WILDLIFE REFUGE, FLORIDA

Notice of Proposed Rule Making

Notice is hereby given that pursuant to the authority contained in section 10 of the Migratory Bird Conservation Act of February 18, 1929 (45 Stat. 1224; 16

U.S.C. 715i), and under authority delegated by Commissioner's Order 4 (22 F.R. 8126), it is proposed to revise paragraph (b) of § 34.93 of Subpart—Loxahatchee National Wildlife Refuge, Florida, Title 50, Code of Federal Regulations, to read as set forth in tentative form below. The purpose is to eliminate discrepancies and clarify the regulations governing the use of boats on areas of the Loxahatchee National Wildlife Refuge in accordance with existing State procedures and regulations.

Interested persons may submit in duplicate written comments, suggestions, or objections with respect to the proposed revision to the Director, Bureau of Sport Fisheries and Wildlife, Washington 25, D.C., within thirty days of the date of publication of this notice in the FEDERAL REGISTER.

Dated: September 3, 1959.

A. V. TUNISON,
Acting Director, Bureau of Sport Fisheries and Wildlife.

(b) *Limitation.* Inboard and outboard motor boats may not be used during the open season for the hunting of migratory birds or fishing in areas designated by suitable posting by the Refuge Officer in charge as closed to motor boat operation during such open season.

[F.R. Doc. 59-7503; Filed, Sept. 9, 1959; 8:47 a.m.]

DEPARTMENT OF AGRICULTURE

Agricultural Marketing Service

[7 CFR Part 970]

IRISH POTATOES GROWN IN MAINE

Limitation of Shipments

Notice is hereby given that the Secretary of Agriculture is considering the limitation of shipments as hereinafter set forth, which was recommended by the Maine Potato Marketing Committee pursuant to Marketing Agreement No. 122 and Order No. 70 (7 CFR Part 970) regulating the handling of Irish potatoes grown in Maine, issued under the Agricultural Marketing Agreement Act of 1937, as amended (Secs. 1-19, 48 Stat. 31, as amended; 7 U.S.C. 601-674).

Consideration will be given to any data, views, or arguments pertaining thereto, which are received by the Director, Fruit and Vegetable Division, Agricultural Marketing Service, U.S. Department of Agriculture, Washington 25, D.C., not later than five days following publication of this notice in the FEDERAL REGISTER. The proposals are as follows:

§ 970.306 Limitation of shipments.

During the period from September 21, 1959, through July 16, 1960, no person such potatoes meet the requirements of paragraph (a) of this section or unless such potatoes are handled in accordance with the provisions of paragraphs (b), (c), (d), (e), (f), or (g) of this section.

(a) *Minimum grade, size, and cleanliness requirements*—(1) *Round varieties.* U.S. No. 1, or better, grade, 2¼ inches minimum diameter and 4 inches maximum diameter.

(2) *Long varieties.* U.S. No. 2, or better, grade, size A, 2 inches minimum diameter or 4 ounces minimum weight.

(3) *Cleanliness.* (i) round varieties—at least 90 percent fairly clean. (ii) long varieties—packs of less than fifty pounds, at least generally fairly clean to clean, mostly clean, which means that not less than 55 percent of such potatoes are clean and not more than 10 percent are slightly dirty; packs of fifty pounds or larger may be shipped if at least 90 percent fairly clean.

(b) *Special purpose shipments*—(1) *Modified grade, size, and cleanliness requirements.* In addition to potatoes which meet the requirements of paragraph (a), potatoes may be shipped for the special purposes set forth in this paragraph if such potatoes meet the grade and size requirements specified for the particular purpose and the handler complies with the applicable provisions of paragraph (c) of this section.

(i) *Chipping.* U.S. No. 1, or better, grade (except for damage by shriveling and sprouting), 1½ inches minimum diameter and 4 inches maximum diameter.

(ii) *Processing into potato salad, fish cakes, hash, knishes, or pancakes.* 85 percent U.S. No. 1, or better, quality, 1½ inches minimum diameter and 2½ inches maximum diameter.

(iii) *Export.* U.S. No. 1, or better, grade, 1½ inches minimum diameter.

(2) *Exemptions from grade, size, and cleanliness requirements.* The minimum grade, size, and cleanliness requirements of paragraph (a) of this section shall not apply to shipments of potatoes for the following purposes if the handler complies with the applicable provisions of paragraph (c) of this section.

(i) Seed, prior to June 1, 1960;

(ii) For grading or storing within the production area;

(iii) Dehydration or potato flakes;

(iv) Manufacture or conversion into starch, flour, or alcohol;

(v) Canning or freezing;

(vi) Livestock feed;

(vii) For distribution by the Federal government; and

(viii) Charity.

(c) *Safeguards.* (1) Each handler making shipment of potatoes under the provisions of paragraph (b) of this section for chipping, processing into potato salad, fish cakes, hash, knishes or pancakes, export, dehydration or potato flakes, canning or freezing, livestock feed, or charity shall:

(i) Prior to making shipment, apply for and obtain an approved Certificate of Privilege from the committee pursuant to the provisions of § 970.130;

(ii) Obtain inspection of, and pay assessments on such shipments except shipments for canning or freezing, livestock feed, dehydration, potato flakes, and charity;

(iii) Pay assessments on shipments of seed; and

(iv) Furnish the committee such reports and documents as may be requested, including certification by the buyer or consignee as to the use of such potatoes.

(2) Prior to the issuance of a Certificate of Privilege to ship potatoes for chipping, the applicant shall provide the committee with appropriate evidence that such potatoes have been treated and conditioned (or are in the process of being treated and conditioned) for use for potato chipping and that such potatoes, except for damage resulting from shriveling or sprouting, meet the applicable grade and size requirements set forth in paragraph (b), (1), (i) of this section.

(3) The safeguards set forth in this paragraph shall not apply to shipments of potatoes within the production area of less than 15,000 pounds for canning or freezing, dehydration, potatoe flakes or livestock feed when such potatoes are shipped in bulk, barrels, or in unsewn 100-pound burlap bags.

(d) *State requirements.* The obtaining of a Certificate of Privilege for shipments to specified outlets as provided for in this section does not relieve the handler of the responsibility of complying with the provisions of the Maine Branding Law (Me. Rev. Stat. Ch. 32, sections 295-301 (1954), as amended (Supp. 1957)).

(e) *Minimum quantities.* Each handler may handle not in excess of thirty (30) hundredweight of potatoes per week free from regulation effective pursuant to § 970.45 (assessments) and § 970.65 (Inspection and Certification); if such potatoes meet the requirements of subparagraph (1) or (2) of paragraph (a) and are 90 percent "fairly clean". This exemption shall not apply to any portion of a shipment of over thirty (30) hundredweight of such potatoes.

(f) *Inspection.* No handler shall ship potatoes for which inspection is required unless an appropriate inspection certificate has been issued with respect thereto and the certificate is valid at the time of shipment. Under § 970.65 (c), for administration thereof each inspection certificate shall be valid for only 72 hours following completion of inspection as shown in the certificate.

(g) *Certificates of Exemption.* Each handler making shipments of potatoes under a Certificate of Exemption issued under § 970.122 which are exempted from the grade and size limitations set forth in paragraph (a) of this section shall pack such potatoes in 50-pound, or larger, packs.

(h) *Definitions.* The terms "clean", "fairly clean" and the grades and sizes used in this section shall have the same meaning assigned such terms, grades, and sizes in the United States Standards for Potatoes (§§ 51.1540 to 51.1556 of this title) including the tolerances set forth therein; the term "week" as used in paragraph (e) means a calendar week beginning midnight (12:01 a.m.) Sunday; other terms used in this section shall have the same meaning as when used in

Marketing Agreement No. 122 and Order No. 79.

(Secs. 1-19, 48 Stat. 31, as amended; 7 U.S.C. 601-674)

Dated: September 3, 1959.

S. R. SMITH,
Director, Fruit and Vegetable Division, Agricultural Marketing Service.

[F.R. Doc. 59-7509; Filed, Sept. 9, 1959; 8:47 a.m.]

[7 CFR Part 1066]

IRISH POTATOES

Imports

Notice is hereby given that the Secretary of Agriculture is giving consideration to grade, size, quality and maturity regulations and inspection requirements that are to be made applicable to the importation of Irish potatoes into the United States pursuant to the provisions of section 608e of the Agricultural Marketing Agreement Act of 1937, as amended (7 U.S.C. 601-674), and the applicable General Regulations (Part 1060 of this chapter).

The act requires that whenever two or more marketing orders regulating the same commodity produced in different areas of the United States are concurrently in effect, the importation into the United States of any such commodity shall be prohibited unless it complies with the grade, size, quality and maturity provisions of the order which, as determined by the Secretary, regulate the commodity produced in the area with which the imported commodity is in most direct competition. Shipments of Irish potatoes are regulated under two or more of the marketing orders applicable to this commodity during each of the twelve months throughout the calendar year, and the regulations issued thereunder, based upon experience during the past several seasons, follow a reasonably uniform pattern from one season to the next. Therefore, determinations are set forth in the proposed notice on the portions of the marketing year during which imports of potatoes are in most direct competition with regulations issued or to be issued under specific marketing orders and the attached proposal sets forth the basis on which the grade, size, quality and maturity regulations issued under such marketing orders on the domestic commodity shall apply within the period set forth herein of such imports.

Consideration will be given to any data, views, or arguments pertaining thereto, which are filed with the Director, Fruit and Vegetable Division, Agricultural Marketing Service, United States Department of Agriculture, Washington 25, D.C., not later than 10 days following publication of this notice in the FEDERAL REGISTER. The proposals are as follows:

Sections 1066.2 through 1066.6 (1066.2–1066.6 of this chapter) shall be terminated and, in lieu thereof, the following general regulations applicable to imports of Irish potatoes pursuant to section 608e of the Agricultural Marketing Agreement Act, as amended (7 U.S.C. 601-674), shall be effective.

§ 1066.1 Import regulations.

(a) *Findings and determinations with respect to imports of Irish potatoes.* (1) Pursuant to section 8e of the Agricultural Marketing Agreement Act of 1937, as amended (7 U.S.C. 631-674), it is hereby found that:

(i) Grade, size, quality, and maturity regulations have been issued from time to time pursuant to the following marketing orders: No. 38 (Part 938 of this chapter), No. 57 (Part 957 of this chapter), No. 58 (Part 958 of this chapter), No. 59 (Part 959 of this chapter), No. 70 (Part 97 of this chapter), and No. 92 (Part 992 of this chapter);

(ii) During the past several years grade, size, quality and maturity regulations have been in effect pursuant to two or more of such orders during each month of the year;

(iii) The marketing of Irish potatoes can be reasonably distinguished by two seasonal categories, namely, first, fall or winter potatoes usually marketed during the months of October through the following June, with the great bulk of such marketings being out of storage, and, second, potatoes marketed during July through September, with the great bulk of such marketings being made as the potatoes are harvested;

(iv) Concurrent grade, size, quality, and maturity regulations under two or more of the aforesaid marketing orders are expected in the ensuing and future seasons, as in the past.

(2) Therefore it is hereby determined that:

(i) Imports of round type potatoes during the months of October through the following June are in most direct competition with marketing of the same type potatoes produced in the area covered by Order No. 70;

(ii) Imports of long type potatoes during each month and imports of round type potatoes during the months of July through September are in most direct competition with potatoes of the same type produced in the area covered by Order No. 57.

(b) *Grade, size, quality, and maturity requirements.* On and after October 1, 1959, the importation of Irish potatoes, except certified seed potatoes, shall be prohibited unless they comply with the following requirements:

(1) For the period July 1 through September 30 of each marketing year, the grade, size, quality, and maturity requirements of Marketing Order No. 57 applicable to potatoes of the long or round types shall be the respective grade, size, quality and maturity requirements for imported potatoes of the long or round types.

(2) For the period October 1 through June 30 of each marketing year, the grade, size, quality and maturity requirements of Marketing Order No. 57 applicable to long type potatoes and the grade, size, quality and maturity requirements of Marketing Order No. 70 applicable to round type potatoes shall be the respective grade, size, quality and maturity requirements for imported potatoes of the long or round types.

(3) The grade, size, quality and maturity requirements specified in paragraph (b) shall apply to imports of potatoes, unless otherwise ordered, on and after the effective date of the applicable domestic regulation or amendment thereto, specified in paragraph (b), or three days following its publication in the FEDERAL REGISTER, whichever is later.

(c) *Minimum quantities.* Any importation which in the aggregate, does not exceed 500 pounds may be imported without regard to the provisions of this section.

(d) *Plant quarantine.* No provisions of this section shall supersede the restrictions or prohibitions of potatoes under the Plant Quarantine Act of 1912.

(e) *Certified seed.* Certified seed potatoes shall include only those potatoes which are officially certified and tagged as seed potatoes by the Plant Protection Division, Science Service, Canada Department of Agriculture.

(f) *Designation of Governmental inspection service.* Fruit and Vegetable Inspection Service, Fruit and Vegetable Division, Marketing Service, Canada Department of Agriculture, is hereby designated, pursuant to § 1060.4(a) of this chapter, as a governmental inspection service for the purpose of certifying the grade, size, quality and maturity of Irish potatoes that are imported, or to be imported, from Canada into the United States under the provisions of section 8e of the Act.

(g) *Inspection and official inspection certificates.* (1) Inspection by the Federal or Federal-State Inspection Service, by the Fruit and Vegetable Inspection Service, Fruit and Vegetable Division, Marketing Service, Canada Department of Agriculture, or by such other governmental inspection service as may be designated, or approved, by the administrator with appropriate evidence thereof in the form of an official inspection certificate issued by the respective service and applicable to a particular shipment of potatoes, is required on all imports of potatoes, other than certified seed, pursuant to § 1060.3 of this chapter.

(2) Inspection certificates shall cover only the quantity of potatoes that is being imported at a particular port of entry by particular importers.

(3) The inspections performed, and certificates issued, by the Federal or Federal-State Inspection Service shall be in accordance with the rules and regulations of the Department governing the inspection and certification of fresh fruits, vegetables, and other products (Part 51 of this title). Cost of inspection and certification shall be borne by the applicant therefor.

(4) Each inspection certificate issued with respect to any Irish potatoes to be imported into the United States shall set forth, among other things:

(i) The date and place of inspection;

(ii) The name of the shipper, or applicant;

(iii) The name of the importer (consignee);

(iv) The commodity inspected;

(v) The quantity of the commodity covered by the Certificate;

(vi) The principal identifying marks of the containers;

(vii) The railroad car initials and number, the truck and trailer number, the name of the vessel, or other identification of the shipment; and

(viii) The following statement if the facts warrant: Meets U.S. Import requirements under section 8e of the Agricultural Marketing Agreement Act of 1937.

(h) *Definitions.* (1) For purposes of this part potatoes meeting the requirements of Canada No. 1 grade and Canada No. 2 grade shall be deemed to comply with the requirements of the U.S. No. 1 grade and U.S. No. 2 grade, respectively, and the tolerances for size, as set forth in the United States Standards for Potatoes (§§ 51.1540 to 51.1556, inclusive, of this title), may be used.

(2) All other terms shall have the same meaning as when used in the General Regulations (Part 1060 of this chapter) applicable to the importation of listed commodities.

(Secs. 1-19, 48 Stat. 31, as amended; 7 U.S.C. 601-674)

Dated: September 3, 1959.

S. R. SMITH,
Director,
Fruit and Vegetable Division.

[F.R. Doc. 59-7534; Filed, Sept. 9, 1959; 8:51 a.m.]

DEPARTMENT OF COMMERCE

Business and Defense Services Administration

[44 CFR Part 401]

IMPORTATION INTO THE UNITED STATES OF NONAGRICULTURAL FOREIGN EXCESS PROPERTY

Notice of Proposed Rule Making

Insofar as the Administrative Procedure Act may be applicable herein, notice is hereby given of the proposed amendment of Foreign Excess Property Order No. 1 (Revised), Importation Into the United States of Nonagricultural Foreign Excess Property (24 F. R. 366).

The purpose of the proposed amendments is to accomplish the following changes in Foreign Excess Property Order No. 1 (Revised):

1. To establish that property of lend-lease origin is not regarded as foreign excess property. This change is accomplished by amendments to § 401.2(a) and § 401.4(c)(14) set forth below. Former § 401.4(c)(15) is redesignated as § 401.4(c)(14) by reason of the revocation of § 401.4(c)(14).

2. To provide additional clarification of the respective responsibilities and functions of the Administrator of the Business and Defense Services Administration and of the Foreign Excess Property Officer. This is accomplished by amendment of § 401.3(b) set forth below.

3. To provide for the exemption from requirements of application to and action by the Foreign Excess Property Officer with respect to the importation of foreign excess property in the form of metal scrap which is exempt from import duty or duties or with respect to which all import duties imposed by or pursuant to the Tariff Act of 1930, as amended, are at the time of such importation suspended by law. These amendments are considered necessary and desirable in the interest of improved and simplified administration of the responsibilities of the Secretary of Commerce as set forth in section 402 of the Federal Property and Administrative Services Act of 1949 as delegated to the Administrator of the Business and Defense Services Administration by Commerce Department Order No. 152 (23 F.R. 7951).

It is proposed to make the amendments herein set forth effective upon the date of their publication in the FEDERAL REGISTER which will be not less than 30 days subsequent to the date of publication of this notice.

It is proposed to publish these amendments in substantially the following form:

§ 401.2 [Amendment]

Section 401.2(a) is amended to read as follows:

(a) "Foreign excess property" means any property (except any agricultural commodity, food, or cotton or woolen goods) located outside the continental United States, Hawaii, Alaska, Puerto Rico, and the Virgin Islands, under the control of any Federal agency, which is not required for its needs and the discharge of its responsibilities as determined by the need thereof. It includes any such property after it has been disposed of by such Federal agency, notwithstanding any subsequent change of ownership. The importation of surplus property sold by the Government or any agency thereof in foreign areas before July 1, 1949, is governed by Foreign Liquidation Commissioner's Reg. 8, which delegates to the Secretary of Commerce jurisdiction over some but not all of such property (§ 308.15 of this title). To the extent that such jurisdiction over such property is delegated to the Secretary of Commerce, such property shall be deemed to be foreign excess property, and is governed by the provisions of this part. All persons owning or acquiring property disposed of by the Government in foreign areas before July 1, 1949, are referred to the said Foreign Liquidation Commissioner's Reg. 8, § 308.15 of this title, for the rules applicable to the importation of any such property which is not subject to the jurisdiction of the Secretary of Commerce. Property of land-lease origin is not regarded as foreign excess property, nor is the importation of such property prohibited or otherwise limited by Foreign Liquidation Commissioner's Reg. 8.

§ 401.3 [Amendment]

Section 401.3(b) is amended to read as follows:

(b) Under the general direction and supervision of the Administrator, the FEPO, or, in his absence the Deputy FEPO, is authorized to carry out the functions assigned to him in this part.

§ 401.4 [Revocation and redesignation]

a. Section 401.4(c)(14) is revoked.

b. Section 401.4(c)(15) is redesignated as § 401.4(c)(14).

Section 401.11 is amended to read as follows:

§ 401.11 Metal scrap.

(a) The determination made on August 23, 1950 (15 F.R. 5847, 5849) that no authorization is required for importation of foreign excess property in the form of metal scrap is revoked. Except as provided in paragraphs (b) and (c) of this section, applications for the importation of metal scrap should be made to the FEPO in accordance with the provisions of this part.

(b) No application need be filed with the FEPO for the importation of foreign excess property in the form of metal scrap which is exempt from import duty or duties or with respect to which all import duties imposed by or pursuant to the Tariff Act of 1930, as amended, are, at the time of such importation, suspended by law.

(c) No application need be filed with the FEPO for the importation of foreign excess property in the form of metal scrap delivered to a United States port of entry on or before February 15, 1959.

(d) Every FEP Import Authorization for the importation of metal scrap issued by the FEPO shall require, as a condition precedent to such importation, the importer to furnish an undertaking in a form and an amount to be prescribed by the Treasury Department to insure that none of the property will be diverted from use as metal scrap. Importers of metal scrap pursuant to paragraphs (b) and (c) of this section shall be required to furnish like undertakings.

Interested persons may submit to the Foreign Excess Property Officer, Room 4816, Department of Commerce, Washington 25, D.C., data, views or arguments in writing but not orally relative to the proposed issuance of the amendments herein set forth. All relevant material received within 20 days following the day of publication of this notice will be considered.

Dated: September 3, 1959

BUSINESS AND DEFENSE SERVICES ADMINISTRATION,
H. HERBERT HUGHES,
Acting Administrator.

[F.R. Doc. 59-7532; Filed, Sept. 9, 1959; 8:51 a.m.]

FEDERAL COMMUNICATIONS COMMISSION

[47 CFR Part 3]

[Docket No. 12945]

TABLE OF ASSIGNMENTS; TELEVISION BROADCAST STATIONS

Order Extending Time for Filing Reply Comments

In the matter of amendment of § 3.606 *Table of assignments,* Television Broadcast Stations (Montgomery, Selma,

Tuscaloosa and Birmingham, Alabama; Tupelo, Columbus and State College, Miss.); Docket No. 12945.

1. The Commission has before it for consideration a petition filed on September 2, 1959, by Capitol Broadcasting Co., licensee of Station WCOV-TV, Montgomery, Alabama, requesting that the time for filing reply comments in the above-entitled proceeding be extended from September 8, 1959, to September 18, 1959.

2. Petitioner states that numerous comments have been filed in this proceeding; that they are replete with engineering and economic data; and that they propose various television reallocations affecting all of the communities named in the above caption. It urges that the additional time requested is needed for it to properly analyze all the comments and to file adequate reply comments; that the extension would not prejudice the rights of any party; and that it will permit the filing of better prepared reply comments. Petitioner advises that counsel for Deep South Broadcasting Company and Frank K. Spain have informed it that they join in its request for a ten day extension and that counsel for WTVY, Inc., Edward L. Norton and Thad Holt, the Regents of the University System of Georgia for and on behalf of the University of Georgia, The Washington Post Company and the Association of Maximum Service Telecasters, Inc., American Broadcasting Company and Birmingham Television Corporation, and Birney Imes, Jr., have informed that they will interprose no objection to the granting of the extension.

3. In view of the number of proposals to be considered in this proceeding, the many comments received, and the representations of the petitioner, the Commission believes that the public interest, convenience and necessity would be served by affording the additional time requested for filing replies.

4. *Accordingly, it is ordered*, This 3d day of September 1959, that the above-mentioned request of Capitol Broadcasting Co. for additional time to file reply comments is granted, and that the time for filing reply comments in the above-entitled proceeding is extended from September 8, 1959, to September 18, 1959.

Released: September 3, 1959.

FEDERAL COMMUNICATIONS COMMISSION,
[SEAL] MARY JANE MORRIS,
Secretary.

[F.R. Doc. 59-7523; Filed, Sept. 9, 1959; 8:50 a.m.]

NOTICES

DEPARTMENT OF COMMERCE

Federal Maritime Board

ORANJE LIJN (MAATSCHAPPIJ ZEETRANSPORT) N.V. ET AL.

Notice of Agreements Filed for Approval

Notice is hereby given that the following described agreements have been filed with the Board for approval pursuant to section 15 of the Shipping Act, 1916 (39 Stat. 733, 46 U.S.C. 814):

(1) Agreement No. 8067-2, between Oranje Lijn (Maatschappij Zeetransport) N.V., and the carriers comprising the Fjell Line joint service, modifies approved sailing and pooling arrangement of the parties (Agreement No. 8067, as amended), in the trades between the Great Lakes of the United States and Canada, the St. Lawrence River and Seaway, Newfoundland and the Canadian Maritimes, on the one hand, and the United Kingdom, and ports in the Bordeaux/Hamburg and Scandinavian Ranges, and in the Mediterranean and adjacent seas, on the other hand. The purpose of the modification is to extend the term of Agreement No. 8067, as amended to December 31, 1979, renewable thereafter for additional periods of ten years from said date, unless cancelled by either party. The agreement presently provides that it shall continue for ten years from date of approval (April 13, 1956), renewable for additional periods of ten years, unless cancelled by either party.

(2) Agreement No. 8253-2, between Lamport & Holt Line, Ltd., and Bull Insular Line, Inc., modifies their approved Agreement No. 8253, as amended, covering a through billing arrangement in the trade from Argentina, Brazil, Peru and Uruguay to Puerto Rico, with transhipment at certain designated U.S. Atlantic and Gulf ports. The purpose of the modification is to delete Peru from the scope of the agreement.

Interested parties may inspect these agreements and obtain copies thereof at the Regulation Office, Federal Maritime Board, Washington, D.C., and may submit, within 20 days after publication of this notice in the FEDERAL REGISTER, written statements with reference to either of the agreements and their position as to approval, disapproval, or modification, together with request for hearing should such hearing be desired.

Dated: September 4, 1959.

By order of the Federal Maritime Board.

[SEAL] JAMES L. PIMPER,
Secretary.

[F.R. Doc. 59-7510; Filed, Sept. 9, 1959; 8:49 a.m.]

AMERICAN EXPORT LINES, INC.

Notice of Application

Notice is hereby given that American Export Lines, Inc., seeks permission to provide service between ports in North Carolina, and ports in the Mediterranean on Trade Route No. 13, with combination vessels and freight vessels operating on Trade Route No. 10.

Any person, firm or corporation having any interest in such application and desiring a hearing on issues pertinent to section 605(c) of the Merchant Marine Act, 1936, as amended, 46 U.S.C. 1175, should by the close of business on September 25, 1959, notify the Secretary, Federal Maritime Board in writing in triplicate, and file petition for leave to intervene in accordance with the rules of practice and procedure of the Federal Maritime Board.

If no request for hearing and petition for leave to intervene is received within the specified time, or if the Federal Maritime Board determines that petitions to intervene filed within the specified time do not demonstrate sufficient interest to warrant a hearing, the Federal Maritime Board will take such action as may be deemed appropriate.

Dated: September 8, 1959.

By order of the Federal Maritime Board.

JAMES L. PIMPER,
Secretary.

[F.R. Doc. 59-7576; Filed, Sept. 9, 1959; 9:04 a.m.]

Maritime Administration

TRADE ROUTE NO. 13

Notice of Tentative Conclusions and Determinations Regarding Modification of Essentiality and United States Flag Requirements

Notice is hereby given that on September 8, 1959, the Maritime Administrator, acting pursuant to section 211 of the Merchant Marine Act, 1936, as amended, tentatively found and determined that service between ports in North Carolina and ports in the Mediterranean on Trade Route No. 13 by combination vessels and freight vessels operating on Trade Route No. 10, is essential to the promotion, development, expansion and maintenance of the foreign commerce of the United States.

Any person, firm or corporation having an interest in the foregoing who desires to offer comments and views or request a hearing thereon should submit same in writing to the Chief, Office of Government Aid, Maritime Administration, Department of Commerce, Washington 25, D.C., by the close of business on September 25, 1959. In the event a hearing is requested, a statement must be included giving the reasons therefor. Any hear-

ing thereby afforded will be before an Examiner on an informal basis only. The Maritime Administrator will consider these comments and views and take such action with respect thereto as in his discretion he deems warranted.

Dated: September 8, 1959.

By order of the Maritime Administrator.

JAMES L. PIMPER,
Secretary.

[F.R. Doc. 59-7577; Filed, Sept. 9, 1959; 9:04 a.m.]

Office of the Secretary

RICHMOND LEWIS

Statement of Changes in Financial Interests

In accordance with the requirements of section 710(b)(6) of the Defense Production Act of 1950, as amended, and Executive Order 10647 of November 28, 1955, the following changes have taken place in my financial interests as reported in the FEDERAL REGISTER during the last six months:

A. Deletions: Steep Rock Mines, Westates Petroleum, Stanwell Oil & Gas, Catalin Corporation.
B. Additions: None.

This statement is made as of August 27, 1959.

RICHMOND LEWIS.

AUGUST 31, 1959.

[F.R. Doc. 59-7487; Filed, Sept. 9, 1959; 8:45 a.m.]

MARGUERITE M. SAUERS

Statement of Changes in Financial Interests

In accordance with the requirements of section 710(b)(6) of the Defense Production Act of 1950, as amended, and Executive Order 10647 of November 28, 1955, the following changes have taken place in my financial interests as reported in the FEDERAL REGISTER during the last six months:

A. Deletions: None.
B. Additions: None.

This statement is made as of August 29, 1959.

MARGUERITE M. SAUERS.

AUGUST 31, 1959.

[F.R. Doc. 59-7488; Filed, Sept. 9, 1959; 8:45 a.m.]

ATOMIC ENERGY COMMISSION

[Byproduct Material License No. 41595-2B59]

ADVANCE INDUSTRIAL X-RAY LABORATORIES ET AL.

Notice of Hearing

Notice is hereby given that pursuant to a stipulation as to available dates between counsel, a hearing will be held at 10:00 a.m. on Wednesday, September 16, 1959 in a courtroom of the United States District Courthouse, 312 North Spring Street, Los Angeles, California respecting the above consolidated dockets for a consideration of the issues presented by the application dated April 15, 1959 for a byproduct material license filed by Advance Industrial X-Ray Laboratories, Inc., as well as for a consideration of the issues presented by the Order of this Commission dated July 15, 1959. In the Matter of Advance Industrial X-Ray Laboratories, a Division of Air-Frame Inspection, Inc., including among other properly presented issues, the following:

1. The effect upon the proceeding of the alleged change of the ownership and management of Respondent, Advance Industrial X-Ray Laboratories;

2. Whether or not a license should be issued to Applicant Advance Industrial X-Ray Laboratories, Inc.;

3. Compliance by above designated Respondent and its personnel, including such personnel as may now be connected with above designated applicant, with the terms of the Temporary Order of the Commission dated June 13, 1958, as modified.

The Order to Show Cause and for hearing previously set for September 10, 1959 respecting the above designated Applicant is superseded by this Notice, but respecting date only, and the herein prescribed hearing will also consider the issues set forth in the previously issued Order to Show Cause.

Issued: August 29, 1959, Germantown, Md.

SAMUEL W. JENSCH,
Presiding Officer.

[F.R. Doc. 59-7484; Filed, Sept. 9, 1959; 8:45 a.m.]

[Docket No. 27-3]

CALIFORNIA SALVAGE CO.

Notice of Proposed Issuance of Byproduct, Source, and Special Nuclear Material License

Please take notice that the Atomic Energy Commission proposes to issue a Byproduct, Source and Special Nuclear Material License to California Salvage Company, 709 North Pacific Avenue, San Pedro, California substantially in the following form, authorizing the disposal of waste byproduct, source and special nuclear material in the Pacific Ocean at a minimum depth of 1,000 fathoms unless within fifteen (15) days after filing of this notice with the Federal Register Division a petition to intervene and a request for a formal hearing is filed with the Commission in the manner prescribed by Title 10, Code of Federal Regulations, Chapter 1, Part 2, Rules of Practice. There is also set forth below a memorandum submitted by the Division of Licensing and Regulation which summarizes the principal factors considered in reviewing the application for a license.

For further details see (1) the application submitted by the California Salvage Company and amendments thereto and (2) a copy of Appendix A to the proposed license which contains transportation container specifications substantially similar to those contained in Title 49, Code of Federal Regulations, Part 78 and referenced in Condition No. 6 of the proposed license, both on file at the Commission's Public Document Room, 1717 H Street NW., Washington, D.C. A copy of (2) above may be obtained at the Commission's Public Document Room or by request addressed to the Atomic Energy Commission, Washington 25, D.C., Attention: Director, Division of Licensing and Regulation.

Dated at Germantown, Md., this 2d day of September, 1959.

For the Atomic Energy Commission.

H. L. PRICE,
Director, Licensing and Regulation.

[License No. 4-5479-1 (H61)]

Pursuant to the Atomic Energy Act of 1954, as amended, and 10 CFR Part 30, "Licensing of Byproduct Material", 10 CFR Part 40, "Control of Source Material", and 10 CFR Part 70, "Special Nuclear Material", and in reliance upon the statements and representations contained in the applications dated June 13, 1957 and August 11, 1958, and the amendments thereto dated November 6, 1958, February 13, 1959 and April 23, 1959, hereinafter referred to as "the application", a license is hereby issued to California Salvage Company, 709 North Pacific Avenue, San Pedro, California to receive, possess, package, and dispose of byproduct, source and special nuclear material.

This license shall be deemed to contain the conditions specified in Section 183 of the Atomic Energy Act of 1954, as amended, and is subject to the provisions of 10 CFR Part 20, "Standards for Protection Against Radiation", all other applicable rules, regulations, orders of the Atomic Energy Commission now or hereafter in effect, and to the following conditions:

1. The licensee shall not possess more than 2 curies of byproduct material, 500 pounds of source material and 10 grams of special nuclear material at any one time.

2. Byproduct, source and special nuclear material shall be received, packaged, and disposed of by, or under the direct personal supervision of, Harvey F. Ludwig or Frank Orlando.

3. The licensee shall receive, package, possess and dispose of the byproduct, source and special nuclear material in accordance with the procedures described in the application, except as provided otherwise in this license.

4. A copy of the "Radiological Safety Manual and Operation Instructions", described in the licensee's application dated February 13, 1959, shall be supplied to each employee of the licensee involved in the receipt, packaging and disposal of byproduct, source and special nuclear material.

5. The licensee shall receive only waste materials which have been prepackaged by the licensee's customers in containers in compliance with applicable Interstate Commerce Commission regulations or with Condition 6 of this license. Prepackaged waste containers shall not be opened by the licensee. The licensee shall instruct each customer to package waste material in such a manner as to assure the absence of any significant voids.

6. The transportation of AEC—licensed material to and from the location designated in Condition 7 shall be subject to the applicable regulations of the Interstate Commerce Commission, United States Coast Guard and other agencies of the United

States having appropriate jurisdiction, and where such regulations are not applicable shall be in accordance with the following requirements except as specifically provided by the Atomic Energy Commission:

A. *Outside shipping containers.* (1) The containers shall meet the specifications for sea disposal containers as described in the application or any one of the following specifications described in Appendix A attached hereto:

a. 15A, 15B, 12B, 6A, 6B, 6C, 17C, 17H, 19A, or 19B for the containment of radioactivity in amounts not in excess of 2.7 curies; except polonium 2 curies; or

b. Specification 55 for containment of solid cobalt 60, cesium 137, iridium 192, or gold 198 in amounts not in excess of 300 curies.

(2) There shall be no radioactive contamination on any exterior surface of the container in excess of 500 d/m/100 sq. cm. alpha and 0.1 mrep/hr beta-gamma radiation.

(3) The smallest dimension of the container shall not be less than 4 inches.

(4) The radiation level of any accessible surface of the container shall not exceed 200 mrem/hr.

(5) At one meter from any point on the radioactive source the radiation level shall not exceed 10 mrem/hr.

(6) Containers which contain radioactive material emitting only alpha and/or beta radiation shall contain sufficient shielding to prevent the escape of primary corpuscular radiation to the exterior surface and to reduce the secondary radiation at the surface of the container to at least 10 mrem/24 hours at any time during transportation.

B. *Inside containers.* (1) Solid and gaseous radioactive materials shall be packed in suitable inside containers designed to prevent rupture and leakage under conditions incident to transportation.

(2) Liquid radioactive materials must be packed in sealed glass, earthenware, or other suitable containers. The container must be surrounded on all sides by an absorbent material sufficient to absorb the entire liquid contents and be of such nature that its efficiency will not be impaired by chemical reactions with the contents. Where shielding is required the absorbent material must be placed within the shield. If the inside container meets the Specification 2R in Appendix A the absorbent material is not required.

(3) Materials containing radioisotopes of plutonium, americium, polonium, or curium, or the isotope strontium 90, in quantities in excess of 100 microcuries, must be packed in containers which meet Specification 2R in Appendix A.

C. *Shielding.* Inside containers must be completely surrounded with sufficient shielding to meet the requirements of subparagraphs A(4), A(5), and A(6) of this condition. The shield must be so designed that it will not open or break under normal conditions incident to transportation.

D. *Labeling.* Each outside container label required under § 20.203(f) of 10 CFR Part 20 shall bear the following information:

(1) Total activity in millicuries, or in the case of source and special nuclear material, the total weight;

(2) principal radioisotope;

(3) radiation level at the surface of the container and at one meter from the source; and

(4) the name and address of the licensee.

E. Each vehicle in which licensed material is transported shall be marked or placarded on each side and the rear with the lettering at least 3 inches high as follows: "DANGEROUS—RADIOACTIVE MATERIAL".

F. *Accidents.* In the event of an accident involving any vehicle transporting licensed material, immediate steps shall be taken to prevent radiation exposure of persons and to control contamination.

G. *Exemptions.* Specific approval must be obtained from the Atomic Energy Commission for modification of, or exemption from, the requirements of the license condition. Requests for such approval should be directed to the Chief, Isotopes Branch, Division of Licensing and Regulation, Atomic Energy Commission, and should contain sufficient information to support such a request.

7. The licensee shall store and package byproduct, source and special nuclear material for sea disposal only at California Salvage Company's Yard, Berth 115, San Pedro, California as described in the licensee's application.

8. The licensee shall dispose of byproduct, source and special nuclear material at a minimum depth of 1,000 fathoms in the Pacific Ocean within 5 miles of:

(1) Parallel of 32°00′ north latitude, meridian of 121°30′ west longitude, or

(2) Other locations in the Pacific Ocean when approved by the Commission.

9. Packaged radioactive waste containing special nuclear material shall be transported only aboard vessels of American registry.

10. The licensee shall notify the Chief, Isotopes Branch, Division of Licensing and Regulation, Atomic Energy Commission, at least 20 days prior to each disposal, by letter deposited in the United States mail properly stamped and addressed, of the proposed date for disposal, the total number of containers, the total activity of byproduct material in millicuries, the amount of source material in pounds, the amount of special nuclear material in grams, and the most hazardous radioisotope contained in each container.

11. Waste byproduct, source and special nuclear material shall be disposed of at sea within 21 months from the date on which California Salvage Company first takes possession of such material.

12. The licensee shall not receive any byproduct, source or special nuclear material until and unless the facility for packaging and storing such material has been erected and completed in accordance with the application. Upon completion of the facility the licensee shall notify the Chief, Isotopes Branch, Division of Licensing and Regulation, Atomic Energy Commission, of the fact of such completion.

This license shall be effective on the date issued and shall expire on August 31, 1961.

Date of issuance:

For the Atomic Energy Commission.

MEMORANDUM

By applications dated June 13, 1957 and August 11, 1958, and amendments thereto, California Salvage Company, 709 North Pacific Avenue, San Pedro, California requested a license to receive, possess, package and dispose of byproduct, source and special nuclear material wastes in the Pacific Ocean.

Based on the consideration set forth in this memorandum the Atomic Energy Commission has found that:

(a) The applicant's proposed equipment, facilities and procedures are adequate to protect health and minimize danger of life or property;

(b) The applicant is qualified by training and experience to conduct the proposed waste disposal service for byproduct, source and special nuclear material in such a manner as to protect health and minimize danger to life or property;

(c) The issuance of a byproduct, source and special nuclear material license to California Salvage Company will not be inimical to the health and safety of the public.

Experience of personnel. Mr. Harvey F. Ludwig, President of Engineering-Science Incorporated (consultant to California Salvage Company), and Mr. Frank Orlando, Marine Superintendent, will directly supervise the receipt, packaging and disposal operations. Mr. Ludwig has had about 10 years experience in radiation protection and the handling of radioactive materials. This training and experience included the principles of radiation monitoring and measurement, and the principles and practices of radiation protection. Mr. Orlando has taken a three-month course in radiological monitoring given by the Los Angeles Board of Education which included radiation monitoring and measurement, contamination control, decontamination, shielding and the principles and the practices of radiation protection. Therefore, it appears that the applicant has personnel with sufficient training and experience in the handling of radioactive materials to provide assurance that the waste disposal operation will be conducted in a manner to protect the health and safety of the public and minimize the danger to life and property.

Equipment, facilities and procedures. The waste packaging and storage site is located at San Pedro, California, at Berth 115 on the West Basin, San Pedro Bay. The area is on the waterfront and is chiefly industrial. The proposed site is approximately 50′ x 50′ and will be enclosed with a six-foot high cyclone fence topped with barbed wire. Within this area there will be a storage vault approximately 400 square feet which will be 4 feet below ground level. Access will be through a door on the roof of the vault. The storage and processing areas will be locked to prevent unauthorized entry.

The waste material will be packaged by the applicant's customers to meet container specifications prescribed by the Interstate Commerce Commission or Condition 6 of the proposed license and will not be opened by the applicant. Adequate radiation safety and emergency procedures have been established covering each phase of the waste disposal program. Written instructions on proper radiation protection precautions and procedures will be given to each employee involved in the waste disposal operation. Necessary equipment for packaging the waste and transporting it to the disposal site is available to the applicant.

Transportation of waste material to and from the applicant's proposed site will be conducted in accordance with the regulations of the Interstate Commerce Commission and the U.S. Coast Guard where such regulations apply. Where these regulations do not apply, transportation will be conducted in accordance with Condition 6 of the proposed license which establishes transportation requirements substantially the same as those of the Interstate Commerce Commission regulations.

The facilities, equipment and operating procedures described by the applicant appear adequate to assure that the disposal operations will be conducted in compliance with the Commission's regulations and the conditions of the proposed license.

Containers and disposal site. The byproduct, source, and special nuclear material wastes will be prepackaged for transportation to the applicant's site by the applicant's customers. The applicant will encase the containers received, without opening, in concrete inside of a 55-gallon drum, previously lined with at least 3 inches of concrete, to obtain a density of at least 10 pounds per gallon to assure sinking in the ocean. Each drum will be labeled to indicate the company's name, the date of packaging, the most hazardous radioisotope, and the level of activity. All drums will be checked for outside contamination and proper weight upon completion of packaging.

Disposal will be in the Pacific Ocean where the minimum depth is 1,000 fathoms at the site specified in Condition 8 of the proposed license. The site is about 165 miles from the coast and is beyond the continental shelf. The licensee will be required to maintain a certified true copy of the ship's log to verify disposal at this site.

At least 20 days prior to each sea disposal operation the Commission will be notified of

the proposed date for disposal, total number of containers, total activity of byproduct material in millicuries, total amount of source material in pounds, and special nuclear material in grams, and the most hazardous radioisotope in each container.

The sea disposal containers and disposal location proposed meet the recommendations of the National Committee on Radiation Protection contained in the National Bureau of Standards Handbook 58, "Radioactive Waste Disposal in the Ocean."

The sea disposal of radioactive wastes where the depth is 1,000 fathoms when packaged in accordance with the requirements of the proposed license is considered a safe method of radioactive waste disposal. These small amounts of radioactive material if released into sea water at the specified location would be diluted and dispersed by the ocean and would not result in concentrations of radioactivity of public health significance.

[F.R. Doc. 59-7485; Filed, Sept. 9, 1959; 8:45 a.m.]

[Docket No. 27-21]

ELECTRO CHEMICAL LABORATORIES CORP.

Notice of Postponement and Setting New Date of Hearing and Prehearing Conference

Notice is hereby given that pursuant to stipulation between counsel as to available dates, the hearing previously set for September 15, 1959, by the Notice of Hearing issued by the Commission in the above designated matter is hereby postponed and in lieu thereof notice is hereby given that the hearing concerning the issues prescribed by the Commission will be held at 10:30 a.m. on October 21, 1959, in the Auditorium of the Headquarters of the Atomic Energy Commission, Germantown, Maryland.

Notice is also hereby given that in accordance with a request of the parties, a pre-hearing conference in this proceeding will be held at 10:30 a.m. on October 14, 1959, in the Press Room of the Auditorium of the Headquarters of the Atomic Energy Commission, Germantown, Maryland.

In view of the change in date for hearing in this proceeding, the date on or before which petitions to intervene may be filed is hereby designated to be September 15, 1959, and the date on or before which Electro Chemical Laboratories Corporation shall file an answer to the Notice of Hearing issued by the Commission is hereby designated to be September 21, 1959.

Issued August 31, 1959, Germantown, Md.

SAMUEL W. JENSCH,
Presiding Officer.

[F.R. Doc. 59-7486; Filed, Sept. 9, 1959; 8:45 a.m.]

FEDERAL POWER COMMISSION

[Docket Nos. G-12197, G-16404]

MANUFACTURERS LIGHT AND HEAT CO.

Notice of Date of Hearing

SEPTEMBER 3, 1959.

Take notice that pursuant to the authority conferred upon the Federal Power Commission by sections 4 and 15 of the Natural Gas Act and the Commission's rules of practice and procedure (18 CFR Ch. I) a hearing will be held on September 30, 1959, at 10:00 a.m., e.d.s.t., in a hearing room of the Federal Power Commission, 441 G Street NW., Washingon, D.C., concerning the matters involved and the issues presented in the Order Suspending Proposed Changes In Rates issued on March 14, 1957 in Docket No. G-12197 and the Order For Hearing, Suspending Proposed Tariff Sheets, Allowing Tariff Sheets to Become Effective Upon filing Of Motion and Undertaking to Assure Refund of Excess Charges, and Consolidating Proceedings issued on September 30, 1958 in Docket Nos. G-12197 and G-16404.

Protests or petitions to intervene may be filed with the Federal Power Commission, Washington 25, D.C., in accordance with the rules of practice and procedure (18 CFR 1.8 or 1.10) on or before September 20, 1959.

Notices of the orders issued herein were published in the FEDERAL REGISTER on March 21, 1957 (22 F.R. 1904) and October 7, 1958 (23 F.R. 7748).

MICHAEL J. FARRELL,
Acting Secretary.

[F.R. Doc. 59-7492; Filed, Sept. 9, 1959; 8:46 a.m.]

[Docket No. G-19258]

MONSANTO CHEMICAL CO.

Order for Hearing and Suspending Proposed Changes in Rates

SEPTEMBER 1, 1959.

Monsanto Chemical Company (Monsanto) on August 3, 1959, tendered for filing proposed changes in its presently effective rate schedules[1] for sales of natural gas subject to the jurisdiction of the Commission. The proposed changes, which constitute increased rates and charges, are contained in the following designated filings:

Description: Notices of change, dated June 24, 1959.
Purchaser: Texas Eastern Transmission Corporation.
Rate schedule designation: Supplement No. 10 to Monsanto's FPC Gas Rate Schedule No. 1. Supplement No. 15 to Monsanto's FPC Gas Rate Schedule No. 2.
Effective date: November 1, 1959 (stated effective date is the effective date proposed by Monsanto).

In support of the proposed periodic rate increases, Monsanto cites the contract provisions and states that the contracts were negotiated at arm's length.

The increased rates and charges so proposed have not been shown to be justified, and may be unjust, unreasonable, unduly discriminatory, or preferential, or otherwise unlawful.

The Commission finds: It is necessary and proper in the public interest and to aid in the enforcement of the provisions of the Natural Gas Act that the Commission enter upon a hearing concerning the lawfulness of the said proposed changes, and that Supplement No. 10 to Monsanto's FPC Gas Rate Schedule No. 1, and Supplement No. 15 to Monsanto's FPC Gas Rate Schedule No. 2, be suspended and the use thereof deferred as hereinafter ordered.

The Commission orders:

(A) Pursuant to the authority of the Natural Gas Act, particularly sections 4 and 15 thereof, the Commission's rules of practice and procedure, and the regulations under the Natural Gas Act (18 CFR, Ch. I), a public hearing be held upon a date to be fixed by notice from the Secretary concerning the lawfulness of the proposed increased rates and charges contained in Supplement No. 10 to Monsanto's FPC Gas Rate Schedule No. 1, and Supplement No. 15 to Monsanto's FPC Gas Rate Schedule No. 2.

(B) Pending such hearing and decision thereon, said supplements be and they are each hereby suspended and the use thereof deferred until April 1, 1960, and until such further time as they are made effective in the manner prescribed by the Natural Gas Act.

(C) Neither the supplements hereby suspended nor the rate schedules sought to be altered thereby shall be changed until this proceeding has been disposed of or until the periods of suspension have expired, unless otherwise ordered by the Commission.

(D) Interested State commissions may participate as provided by §§ 1.8 and 1.37 (f) of the Commission's rules of practice and procedure (18 CFR 1.8 and 1.37(f)).

By the Commission.

MICHAEL J. FARRELL,
Acting Secretary.

[F.R. Doc. 59-7493; Filed, Sept. 9, 1959; 8:46 a.m.]

[1] Present rate (contained in Supplement No. 8 to Monsanto's FPC Gas Rate Schedule No. 1) previously suspended and is in effect subject to refund in Docket No. G-16807. Also subject to the Commission's orders in Docket Nos. G-13612, G-11368 and G-9681. Present rate (contained in Supplement No. 13 to Monsanto's FPC Gas Rate Schedule No. 2) previously suspended and is in effect subject to refund in Docket No. G-16807. Also subject to the Commission's orders in Docket Nos. G-13612, G-11369 and G-9682.

[Docket No. G-19259]

MONSANTO CHEMICAL CO. ET AL.

Order for Hearing and Suspending Proposed Change in Rates

SEPTEMBER 1, 1959.

Monsanto Chemical Company (Operator), et al. (Monsanto), on August 3, 1959, tendered for filing a proposed change in its presently effective rate schedule[1] for sales of natural gas subject to the jurisdiction of the Commission. The proposed change, which constitutes an increased rate and charge, is contained in the following designated filing:

Description: Notice of change, dated June 24, 1959.
Purchaser: Texas Eastern Transmission Corporation.

[1] Present rate previously suspended and is in effect subject to refund in Docket No. G-16808. Also subject to the Commission's order in Docket No. G-14728.

Rate schedule designation: Supplement No. 6 to Monsanto's FPC Gas Rate Schedule No. 20.

Effective date: November 1, 1959 (stated effective date is the effective date proposed by Monsanto).

In support of the proposed periodic rate increase, Monsanto cites the contract provisions and states that the contract was negotiated at arm's length.

The increased rate and charge so proposed has not been shown to be justified, and may be unjust, unreasonable, unduly discriminatory, or preferential, or otherwise unlawful.

The Commission finds: It is necessary and proper in the public interest and to aid in the enforcement of the provisions of the Natural Gas Act that the Commission enter upon a hearing concerning the lawfulness of the said proposed change, and that Supplement No. 6 to Monsanto's FPC Gas Rate Schedule No. 20 be suspended and the use thereof deferred as hereinafter ordered.

The Commission orders:

(A) Pursuant to the authority of the Natural Gas Act, particularly sections 4 and 15 thereof, the Commission's rules of practice and procedure, and the regulations under the Natural Gas Act (18 CFR Ch. I), a public hearing be held upon a date to be fixed by notice from the Secretary concerning the lawfulness of the proposed increased rate and charge contained in Supplement No. 6 to Monsanto's FPC Gas Rate Schedule No. 20.

(B) Pending such hearing and decision thereon, said supplement be and it is hereby suspended and the use thereof deferred until April 1, 1960, and until such further time as it is made effective in the manner prescribed by the Natural Gas Act.

(C) Neither the supplement hereby suspended nor the rate schedule sought to be altered thereby shall be changed until this proceeding has been disposed of or until the period of suspension has expired, unless otherwise ordered by the Commission.

(D) Interested State commissions may participate as provided by §§ 1.8 and 1.37(f) of the Commission's rules of practice and procedure (18 CFR 1.8 and 1.37(f)).

By the Commission.

MICHAEL J. FARRELL,
Acting Secretary.

[F.R. Doc. 59-7494; Filed, Sept. 9, 1959; 8:46 a.m.]

[Docket No. G-19260]

OKMAR OIL CO.

Order for Hearing and Suspending Proposed Change in Rates

SEPTEMBER 1, 1959.

Okmar Oil Company (Okmar) on August 3, 1959, tendered for filing a proposed change in its presently effective rate schedule for sales of natural gas subject to the jurisdiction of the Commission. The proposed change, which constitutes an increased rate and charge, is contained in the following designated filing:

Description: Notice of change, undated.

Purchaser: Hope Natural Gas Company.

Rate schedule designation: Supplement No. 8 to Okmar's FPC Gas Rate Schedule No. 1.

Effective date: September 3, 1959 (stated effective date is the first day after expiration of the required thirty days' notice).

In support of the proposed favored-nation rate increase, Okmar cites the contract provisions, states that the contract was negotiated at arm's length and provides for just and reasonable prices. Okmar also states that costs have increased and requests that the higher rate be allowed to become effective at the same time as the 25.0¢ per Mcf initial rates of three other producers[1] so that there will be no discrimination.

The increased rate and charge so proposed has not been shown to be justified, and may be unjust, unreasonable, unduly discriminatory, or preferential, or otherwise unlawful.

The Commission finds: It is necessary and proper in the public interest and to aid in the enforcement of the provisions of the Natural Gas Act that the Commission enter upon a hearing concerning the lawfulness of the said proposed change, and that Supplement No. 8 to Okmar's FPC Gas Rate Schedule No. 1 be suspended and the use thereof deferred as hereinafter ordered.

The Commission orders:

(A) Pursuant to the authority of the Natural Gas Act, particularly sections 4 and 15 thereof, the Commission's rules of practice and procedure, and the regulations under the Natural Gas Act (18 CFR Ch. I), a public hearing be held upon a date to be fixed by notice from the Secretary concerning the lawfulness of the proposed increased rate and charge contained in Supplement No. 8 to Okmar's FPC Gas Rate Schedule No. 1.

(B) Pending such hearing and decision thereon, said supplement be and it is hereby suspended and the use thereof deferred until February 3, 1960, and until such further time as it is made effective in the manner prescribed by the Natural Gas Act.

(C) Neither the supplement hereby suspended nor the rate schedule sought to be altered thereby shall be changed until this proceeding has been disposed of or until the period of suspension has expired, unless otherwise ordered by the Commission.

(D) Interested State commissions may participate as provided by §§ 1.8 and 1.37(f) of the Commission's rules of practice and procedure (18 CFR 1.8 and 1.37(f)).

By the Commission.

MICHAEL J. FARRELL,
Acting Secretary.

[F.R. Doc. 59-7495; Filed, Sept. 9, 1959; 8:46 a.m.]

[1] Pell Oil & Gas Company, Harry Stevens, et al., and Orma Oil & Gas Company.

[Docket No. G-19261]

VINCENT & WELCH, INC., ET AL.

Order for Hearing and Suspending Proposed Change in Rates

SEPTEMBER 1, 1959.

Vincent & Welch, Inc. (Operator), et al., (Vincent & Welch), on August 3, 1959, tendered for filing a proposed change in their presently effective rate schedule for sales of natural gas subject to the jurisdiction of the Commission. The proposed change, which constitutes an increased rate and charge, is contained in the following designated filing:

Description: Notice of change, undated.

Purchaser: Transcontinental Gas Pipe Line Corporation.

Rate schedule designation: Supplement No. 3 to Vincent & Welch's FPC Gas Rate Schedule No. 2.

Effective date: November 1, 1959 (stated effective date is the effective date proposed by Vincent & Welch).

In support of the proposed periodic rate increase, Vincent & Welch state that the contract provisions were arrived at through arm's-length bargaining and that the proposed rate is just and reasonable and is in line with the going price for gas in the area.

The increased rate and charge so proposed has not been shown to be justified, and may be unjust, unreasonable, unduly discriminatory, or preferential, or otherwise unlawful.

The Commission finds: It is necessary and proper in the public interest and to aid in the enforcement of the provisions of the Natural Gas Act that the Commission enter upon a hearing concerning the lawfulness of the said proposed change, and that Supplement No. 3 to Vincent & Welch's FPC Gas Rate Schedule No. 2 be suspended and the use thereof deferred as hereinafter ordered.

The Commission orders:

(A) Pursuant to the authority of the Natural Gas Act, particularly sections 4 and 15 thereof, the Commission's rules of practice and procedure, and the regulations under the Natural Gas Act (18 CFR Ch. I), a public hearing be held upon a date to be fixed by notice from the Secretary concerning the lawfulness of the proposed increased rate and charge contained in Supplement No. 3 to Vincent & Welch's FPC Gas Rate Schedule No. 2.

(B) Pending such hearing and decision thereon, said supplement be and it is hereby suspended and the use thereof deferred until April 1, 1960, and until such further time as it is made effective in the manner prescribed by the Natural Gas Act.

(C) Neither the supplement hereby suspended nor the rate schedule sought to be altered thereby shall be changed until this proceeding has been disposed of or until the period of suspension has expired, unless otherwise ordered by the Commission.

(D) Interested State commissions may participate as provided by §§ 1.8 and 1.37(f) of the Commission's rules of

practice and procedure (18 CFR 1.8 and 1.37(f)).

By the Commission.

MICHAEL J. FARRELL,
Acting Secretary.

[F.R. Doc. 59-7496; Filed, Sept. 9, 1959; 8:46 a.m.]

[Docket No. G-19326]

ATLANTIC SEABOARD CORP.

Order for Hearing, Suspending Proposed Revised Tariff Sheets and Allowing Tariff Sheets To Become Effective Upon Filing of Motion and Undertaking To Assure Refund of Excess Charges

SEPTEMBER 2, 1959.

On August 3, 1959, Atlantic Seaboard Corporation (Atlantic) tendered for filing First Revised Sheets Nos. 10, 15, 16E, 16F, 17E and 17F to its FPC Gas Tariff, Seventh Revised Volume No. 1, proposing to change the basis upon which the heating value adjustments in its billings are made. Atlantic requests an effective date of August 1, 1959.

The present Btu adjustment[1] provides for an increase or decrease in total payment proportional to the deviation in heating value from 1000 Btu of the gas delivered during the current month.

The proposed Btu adjustment retains the present Btu adjustment as to the commodity charge, whereas, with respect to the demand charge, the proposed adjustment is proportional to the deviation in heating value from 1000 Btu of the gas delivered during the month in which the billing demand was established.

Atlantic states that the proposed change is necessery:

* * * to prevent Buyers under the subject rate schedules from being unduly penalized in the Monthly Demand Charge because of the delivery by Seller to its customers during the summer months of gas containing a Btu content higher than the Btu content of gas delivered by Seller during the winter months, during which winter months the Billing Demand for each Buyer is established.

The proposed change tendered by Atlantic has not been shown to be justified and may be unjust, unreasonable, unduly discriminatory, or preferential, or otherwise unlawful.

The Commission finds:

(1) It is necessary and proper in the public interest, and to aid in the enforcement of the provisions of the Natural Gas Act, that the Commission enter upon a hearing concerning the lawfulness of the rates, charges, classifications, and services contained in Atlantic's FPC Gas Tariff Seventh Revised Volume No. 1 as proposed to be amended by First Revised Sheets Nos. 10, 15, 16E, 16F, 17E and 17F as tendered for filing on August 3, 1959, and that said proposed revised tariff sheets and the rates contained therein be suspended and the use thereof deferred as hereinafter provided.

(2) It is appropriate in the public interest and in carrying out the provisions of the Natural Gas Act that Atlantic's proposed tariff sheets be made effective as hereinafter provided and that Atlantic be required to file an undertaking as hereinafter ordered and conditioned.

The Commission orders:

(A) Pursuant to the authority contained in and subject to the jurisdiction conferred upon the Federal Power Commission by sections 4 and 15 of the Natural Gas Act, and the Commission's Regulations under the Natural Gas Act, including rules of practice and procedure (18 CFR Ch. I), a public hearing be held at a time and date to be fixed by notice from the Secretary of this Commission, concerning the lawfulness of the rates, charges, classifications, and services, subject to the jurisdiction of the Commission ,contained in Atlantic's FPC Gas Tariff Seventh Revised Volume No. 1 as proposed to be amended by First Revised Sheets Nos. 10, 15, 16E, 16F, 17E and 17F as tendered for filing on August 3, 1959.

(B) Pending such hearing and decision thereon First Revised Sheets Nos. 10, 15, 16E, 16F, 17E and 17F to Atlantic's FPC Gas Tariff, Seventh Revised Volume No. 1 as tendered for filing on August 3, 1959, are each hereby suspended, and their use deferred until September 4, 1959, and until such further time as they are made effective in the manner hereinafter prescribed.

(C) The rates, charges, classifications, and services set forth in the above-designated filings shall be effective as of September 4, 1959: *Provided, however,* That, within 20 days from the date of this order, Atlantic shall file a motion as required by section 4(e) of the Natural Gas Act and concurrently execute and file with the Secretary of the Commission the agreement and undertaking described in paragraph (E) below.

(D) Atlantic shall refund at such times and in such amounts to persons entitled thereto, and in such manner as may be required by final order of the Commission, the portion of the increased rates and charges found by the Commission in this proceeding not justified, together with interest thereon at the rate of 6 percent per annum from the date of payment to Atlantic until refunded; shall bear all costs of any such refunding, shall keep accurate accounts in detail of all amounts received by reason of the increased rates or charges effective as of September 4, 1959, for each billing period, specifying by whom and in whose behalf such amounts were paid; and shall report (original and one copy), in writing and under oath, to the Commission monthly, for each billing period and for each purchaser, the billing determinants of natural gas sales to such purchasers and the revenues resulting therefrom as computed under the rates in effect immediately prior to July 14, 1957, until Commission determination of the rates and charges in Docket No. G-12196, and in Docket Nos. G-16817 and G-18422, then under each of those rates, and under the rates and charges allowed by this order to become effective, together with the differences in the revenues so computed.

(E) As a condition of this order, within 20 days from the date of issuance hereof, Atlantic shall concurrently execute and file (original and three (3) copies) with the Secretary of the Commission its motion to make rates effective and its written agreement and undertaking to comply with the terms of paragraph (D) hereof, signed by a responsible officer of the corporation, evidenced by proper authority from the Board of Directors, and accompanied by a certificate showing service of copies thereof upon all purchasers under the tariff sheets involved, as follows:

Agreement and Undertaking of Atlantic Seaboard Corporation to Comply with the Terms and Conditions of Paragraph (D) of Federal Power Commission's Order for Hearing, Suspending Proposed Revised Tariff Sheets, and Allowing Revised Tariff Sheets to Become Effective Upon Filing of Motion and Undertaking to Assure Refund of Excess Charges.

In conformity with the requirements of the order issued (Date), in Docket No. G-19326, Atlantic Seaboard Corporation hereby agrees and undertakes to comply with the terms and conditions of paragraph (D) of said order, and has caused this agreement and undertaking to be executed and sealed in its name by its officers, thereupon duly authorized in accordance with the terms of the resolution of its Board of Directors, a certified copy of which is appended hereto this ______ day of ______, 1959.

ATLANTIC SEABOARD CORPORATION,
By ______________________________

Attest:

____
(Secretary)

Unless Atlantic is advised to the contrary within 15 days after the date of filing such agreement and undertaking, the agreement and undertaking shall be deemed to have been accepted.

(F) If Atlantic shall, in conformity with the terms and conditions of paragraph (D) of this order, make the refunds as may be required by order of the Commission, the undertaking shall be discharged, otherwise it shall remain in full force and effect.

(G) Neither the tariff here proposed to be amended nor the revised tariff sheets hereby suspended shall be changed until the period of suspension has expired, unless otherwise ordered by the Commission.

(H) Interested State commissions may participate as provided by §§ 1.8 and 1.37(f) of the Commission's rules of practice and procedure (18 CFR 1.8 and 1.37(f)).

By the Commission.

MICHAEL J. FARRELL,
Acting Secretary.

[F.R. Doc. 59-7497; Filed, Sept. 9, 1959; 8:46 a.m.]

[1] Effective as part of rates in effect subject to refund in Docket No. G-12196.

[Docket No. G-13681]

W. M. BEVLY AND W. M. BEVLY, JR.

Notice of Application and Date of Hearing

SEPTEMBER 3, 1959.

Take notice that W. M. Bevly and W. M. Bevly, Jr. (Applicants), independent

producers of natural gas with their principal place of business in Corpus Christi, Texas, filed, on November 8, 1957, an application in Docket No. G-13681 for a certificate of public convenience and necessity, pursuant to section 7(c) of the Natural Gas Act, authorizing Applicants to sell natural gas as hereinafter described, subject to the jurisdiction of the Commission, and on June 9, 1959, Applicants filed an amendment to the foregoing requesting permission to abandon the afore-mentioned service pursuant to section 7(b) of the Natural Gas Act, all as more fully set forth in the application and amendment which are on file with the Commission and open to public inspection.

Applicants requested authorization to render service to Trunkline Gas Company (Trunkline) from certain acreage in the Howell Field in Jim Wells County, Texas, pursuant to a gas sales contract dated October 1, 1957, between Applicants, as sellers, and Trunkline, as buyer, on file with the Commission as W. M. Bevly, et al., FPC Gas Rate Schedule No. 1. Applicants were authorized to commence service on a temporary basis pursuant to § 157.28 of the regulations under the Natural Gas Act by airmail letter dated December 6, 1957.

In the amendment filed June 9, 1959, Applicants state that the gas well on said acreage has been plugged and abandoned and all rights to the oil and gas leases have been released.

This matter is one that should be disposed of as promptly as possible under the applicable rules and regulations and to that end:

Take further notice that, pursuant to the authority contained in and subject to the jurisdiction conferred upon the Federal Power Commission by sections 7 and 15 of the Natural Gas Act, and the Commission's rules of practice and procedure, a hearing will be held on October 13, 1959 at 9:30 a.m., e.d.s.t., in a Hearing Room of the Federal Power Commission, 441 G Street NW., Washington, D.C., concerning the matters involved in and the issues presented by such applications: *Provided, however*, That the Commission may, after a non-contested hearing, dispose of the proceedings pursuant to the provisions of § 1.30(c) (1) or (2) of the Commission's rules of practice and procedure. Under the procedure herein provided for, unless otherwise advised, it will be unnecessary for Applicants to appear or be represented at the hearing.

Protests or petitions to intervene may be filed with the Federal Power Commission, Washington 25, D.C., in accordance with the rules of practice and procedure (18 CFR 1.8 or 1.10) on or before October 2, 1959. Failure of any party to appear at and participate in the hearing shall be construed as waiver of and concurrence in omission herein of the intermediate decision procedure in cases where a request therefor is made.

MICHAEL J. FARRELL,
Acting Secretary.

[F.R. Doc. 59-7498; Filed, Sept. 9, 1959; 8:46 a.m.]

[Docket No. G-19262, etc.]

W. B. OSBORN, JR., ET AL.

Order for Hearings and Suspending Proposed Changes in Rates [1]

SEPTEMBER 2, 1959.

In the matters of W. B. Osborn, Jr., Executor of the Estate of W. B. Osborn, Sr., Docket No. G-19262; Lee Minton, Docket No. G-19264; Della Minton, Docket No. G-19265; Winnie Lou Jones, Docket No. G-19266; W. B. Osborn, Jr., Docket No. G-19267; Charlotte Osborn Barrett, Docket No. G-19268; The Altex Corporation, Docket No. G-19271; Jewel Osborn, Docket No. G-19272; Betty Osborn Biedenharn, Docket No. G-19274.

The proposed changes hereinafter designated, which constitute increased rates and charges in presently effective rate schedules for sales of natural gas subject to the jurisdiction of the Commission, have been tendered for filing by the above-named Respondents. In each filing, the purchaser is the Tennessee Gas Transmission Company and the Respondents have proposed November 1, 1959, as the effective date of the changes. Each Notice of Change is dated August 3, 1959, and was tendered for filing on August 5, 1959.

The Commission finds: It is necessary and proper in the public interest and to aid in the enforcement of the provisions of the Natural Gas Act that the Commission enter upon hearings concerning the lawfulness of the several proposed changes, and that each of the above-designated supplements be suspended and the use thereof deferred as hereinafter ordered.

The Commission orders:

(A) Pursuant to the authority of the Natural Gas Act, particularly sections 4 and 15 thereof, the Commission's rules of practice and procedure, and the regulations under the Natural Gas Act (18 CFR Ch. I), public hearings be held upon dates to be fixed by notices from the Secretary concerning the lawfulness of the several proposed increased rates and charges contained in the above-designated supplements.

(B) Pending hearings and decisions thereon, Supplement No. 2 to Altex's FPC Gas Rate Schedule No. 1, and Supplement No. 4 to each of the other aforementioned Respondents' FPC Gas Rate Schedule No. 1, are hereby suspended and the use thereof deferred until April 1, 1960; each of the aforementioned supplements shall remain suspended until such further time as they are made effective in the manner prescribed by the Natural Gas Act.

(C) Neither the supplements hereby suspended, nor the rate schedules sought to be altered thereby, shall be changed until these proceedings have been disposed of or until the periods of suspension have expired, unless otherwise ordered by the Commission.

(D) Interested State commissions may participate as provided by §§ 1.8 and 1.37(f) of the Commission's rules of practice and procedure (18 CFR 1.8 and 1.37(f)).

By the Commission (Commissioner Hussey dissenting).

MICHAEL J. FARRELL,
Acting Secretary.

[F.R. Doc. 59-7499; Filed, Sept. 9, 1959; 8:46 a.m.]

[1] This order does not provide for the consolidation for hearing or disposition of the separately docketed matters covered herein, nor should it be so construed.

[Docket No. G-19263, etc.]

W. B. OSBORN, JR., ET AL.

Order for Hearings and Suspending Proposed Changes in Rates [1]

SEPTEMBER 2, 1959.

In the matters of W. B. Osborn, Jr., Executor of the Estate of W. B. Osborn, Sr. (Operator), et al., Docket No. G-19263; Jewel Osborn, Docket No. G-19269; W. B. Osborn, Jr., Docket No. G-19270; Charlotte Osborn Barrett, Docket No. G-19273; Betty Osborn Biedenharn, Docket No. G-19275.

The proposed changes hereinafter designated, which constitute increased rates and charges in presently effective rate schedules for sales of natural gas subject to the jurisdiction of the Commission, have been tendered for filing by the above-named Respondents. In each filing, the purchaser is The Altex Corporation (Altex) and the effective date is November 2, 1959.[2] Each Notice of Change is dated August 3, 1959, and was tendered for filing on August 5, 1959.

Respondent	Rate schedule No.	Supplement No.
W. B. Osborn, Jr., executor of the estate of W. B. Osborn, Sr. (operator), et al.	11	2
Jewel Osborn	11	2
W. B. Osborn, Jr.	9	2
Charlotte Osborn Barrett	9	2
Betty Osborn Biedenharn	9	2

Respondents' proposed revenue-sharing rate increases are based on the buyer's (Altex) proposed rate increased to Tennessee Gas Transmission Company suspended concurrently herewith in Docket No. G-19271 until April 1, 1960, and thereafter until it is made effective in the manner prescribed by the Natural Gas Act. In support of their proposed increases, Respondents state that it will be necessary to install additional compressor facilities due to the decline of bottom hole pressure and depletion of the wells in question and submit a graph depicting a decline in deliverability and well pressure. The cost data submitted by the Respondents in support of their increases is deemed insufficient for the Commission's staff to make an adequate

[1] This order does not provide for the consolidation for hearing or disposition of the separately docketed matters covered herein, nor should it be so construed.

[2] The stated effective date is the date following the effective date of the redetermined rate increase filed by the buyer (Altex) and suspended concurrently herewith in Docket No. G-19271.

cost study to determine the justness and reasonableness of the proposed rates.

The increased rates and charges so proposed have not been shown to be justified, and may be unjust, unreasonable, unduly discriminatory, or preferential, or otherwise unlawful.

The Commission finds: It is necessary and proper in the public interest and to aid in the enforcement of the provisions of the Natural Gas Act that the Commission enter upon hearings concerning the lawfulness of the several proposed changes, and that each of the above-designated supplements be suspended and the use thereof deferred as hereinafter ordered.

The Commission orders:

(A) Pursuant to the authority of the Natural Gas Act, particularly sections 4 and 15 thereof, the Commission's rules of practice and procedure, and the regulations under the Natural Gas Act (18 CFR Ch. I), public hearings be held upon dates to be fixed by notices from the Secretary concerning the lawfulness of the several proposed increased rates and charges contained in the above-designated supplements.

(B) Pending such hearings and decision thereon, Supplements No. 2 to W. B. Osborn, Jr., Executor of the Estate of W. B. Osborn, Sr. (Operator), et al., and Jewel Osborn's FPC Gas Rate Schedules No. 11, respectively, and Supplements No. 2 to W. B. Osborn, Jr., Charlotte Osborn Barrett and Betty Osborn Biedenharn's FPC Gas Rate Schedules No. 9, respectively, are each hereby suspended and the use thereof deferred until April 2, 1960, or, if later, until such date as is one day beyond the date the redetermined rate increase of Altex, suspended in Docket No. G-19271, is made effective in the manner prescribed by the Natural Gas Act, and until such further time as the aforementioned Respondents' supplements are made effective in the manner prescribed by the Natural Gas Act.

(C) Neither the supplements hereby suspended, nor the rate schedules sought to be altered thereby, shall be changed until these proceedings have been disposed of or until the periods of suspension have expired, unless otherwise ordered by the Commission.

(D) Interested State commissions may participate as provided by §§ 1.8 and 1.37(f) of the Commission's rules of practice and procedure (18 CFR 1.8 and 1.37(f)).

By the Commission.

MICHAEL J. FARRELL,
Acting Secretary.

[F.R. Doc. 59-7500; Filed, Sept. 9, 1959; 8:46 a.m.]

[Docket No. G-18762]

PACIFIC NORTHWEST PIPELINE CORP.

Notice of Application and Date of Hearing

SEPTEMBER 3, 1959.

On June 10, 1959, Pacific Northwest Pipeline Corporation (Applicant) filed an application in Docket No. G-18762, pursuant to section 7(c) of the Natural Gas Act, seeking authorization to construct and operate a tap and measuring and regulating station on its 26-inch main transmission line in Washington, approximately one quarter of a mile north of the town of Stevenson, Skamania County, Washington. Applicant also seeks authorization to sell and deliver natural gas to Columbia Gas Company (Columbia)[1] at the meter station for distribution in the town of Stevenson and environs.

The peak day and annual gas requirements of the Stevenson area are estimated as follows:

	1st year	2d year	3d year
Peak day	184	216	408
Annual	49,188	57,878	114,748
Annual revenue @45 cents per Mcf	$22,134	$26,045	$51,636

The cost of Pacific's facilities is estimated at $4,861, which is to be financed from currently available funds.

Applicant proposes to sell and deliver natural gas on a firm basis to Columbia under terms and provisions of Applicant's Rate Schedule DS-1, on file with the Commission.

This matter is one that should be disposed of as promptly as possible under the applicable rules and regulations and to that end:

Take further notice that, pursuant to the authority contained in and subject to the jurisdiction conferred upon the Federal Power Commission by sections 7 and 15 of the Natural Gas Act, and the Commission's rules of practice and procedure, a hearing will be held on October 8, 1959, at 9:30 a.m., e.d.s.t., in a Hearing Room of the Federal Power Commission, 441 G Street NW., Washington, D.C., concerning the matters involved in and the issues presented by such application: *Provided, however,* That the Commission may, after a non-contested hearing, dispose of the proceedings pursuant to the provisions of § 1.30(c) (1) or (2) of the Commission's rules of practice and procedure. Under the procedure herein provided for, unless otherwise advised, it will be unnecessary for Applicant to appear or be represented at the hearing.

Protests or petitions to intervene may be filed with the Federal Power Commission, Washington 25, D.C., in accordance with the rules of practice and procedure (18 CFR 1.8 or 1.10) on or before September 28, 1959. Failure of any party to appear at and participate in the hearing shall be construed as waiver of and concurrence in omission herein of the intermediate decision procedure in cases where a request therefor is made.

MICHAEL J. FARRELL,
Acting Secretary.

[F.R. Doc. 59-7501; Filed, Sept. 9, 1959; 8:46 a.m.]

[1] Formerly Eastern Washington Natural Gas Company.

FEDERAL COMMUNICATIONS COMMISSION

[Docket No. 12742, etc.; FCC 59M-1120]

GRANITE CITY BROADCASTING CO. ET AL.

Order Continuing Hearing

In re applications of Seibert McRae Wood, Clagett "Woody" Wood, Tycho Heckard Wood and Paul Edgar Johnson, d/b as Granite City Broadcasting Company, Mount Airy, North Carolina, Docket No. 12742, File No. BP-11811; Cumberland Publishing Company (WLSI), Pikeville, Kentucky, Docket No. 12743, File No. BP-11997; S. L. Goodman, Bassett, Virginia, Docket No. 12869, File No. BP-12611; for construction permits for standard broadcast stations.

On the Examiner's own motion: *It is ordered,* This 3d day of September 1959, that the hearing in the above-entitled proceeding, presently scheduled for September 9, 1959, is hereby continued to a date to be set by subsequent order.

Released: September 4, 1959.

FEDERAL COMMUNICATIONS COMMISSION,
[SEAL] MARY JANE MORRIS,
Secretary.

[F.R. Doc. 59-7524; Filed, Sept. 9, 1959; 8:50 a.m.]

[Docket No. 12824; FCC 59M-1111]

INTER-CITIES BROADCASTING CO.

Order Continuing Hearing

In re application of Theodore A. Kolasa, Henry J. Kolasa, Mitchell A. Kolasa and Alphonse R. Deresz, d/b as Inter-Cities Broadcasting Company, Livonia, Michigan, Docket No. 12824, File No. BP-10991; for construction permit for a new standard broadcast station.

At a pre-hearing conference held on September 2, 1959, it was agreed by all the parties that the following calendar of events should govern future course of hearing:

September 14, 1959 (10:00 a.m.)—Inter-Cities will exchange with the other parties supplemental engineering showing;

September 18, 1959—Informal engineering conference, the time and place to be arranged by the parties;

September 25, 1959—Peoples Broadcasting Corporation (WGAR) will exchange its supplemental engineering showing, if any;

September 28, 1959 (10:00 a.m.)—Hearing (Cont. from Sept. 8, 1959.)

So ordered.

Released: September 3, 1959.

FEDERAL COMMUNICATIONS COMMISSION,
[SEAL] MARY JANE MORRIS,
Secretary.

[F.R. Doc. 59-7525; Filed, Sept. 9, 1959; 8:50 a.m.]

[Docket Nos. 12833, 12834; FCC 59M-1119]

GEORGE T. HERNREICH AND PATTESON BROTHERS

Order Continuing Hearing

In re applications of George T. Hernreich, Jonesboro, Arkansas, Docket No. 12833, File No. BPCT-2538; Alan G. Patteson, Jr. and Mathew Carter Patteson, d/b as Patteson Brothers, Jonesboro, Arkansas, Docket No. 12834, File No. BPCT-2567; for construction permits for new television broadcast stations (Channel 8).

The Hearing Examiner having under consideration a petition filed on August 28, 1959 by George T. Hernreich, requesting (1) an extension of time from September 9, 1959 to October 15, 1959 within which to exchange direct written cases; (2) a continuance of the date for the commencement of the evidentiary hearing and the introduction into evidence of applicants' written cases from September 16, 1959 to November 3, 1959; and (3) a continuance of the date for presentation of oral testimony, if needed, or if requested, from September 23, 1959 to November 9, 1959;

It appearing that additional time is necessary to prepare for the hearing in the above-entitled proceeding;

It further appearing that counsel for Patteson Brothers and for the Broadcast Bureau have no objection to a grant of the petition for continuance of dates as requested; and good cause has been shown for the grant of the relief requested;

It is ordered, This 3d day of September 1959, that the petition be and it is hereby granted; the date for exchange of direct written cases be and it is hereby continued to October 15, 1959; the date for commencement of the evidentiary hearing and the introduction into evidence of applicants' written cases be and it is hereby continued to November 3, 1959; and the date for presentation of oral testimony, if any is needed or requested, be and it is hereby continued to November 9, 1959.

Released: September 4, 1959.

FEDERAL COMMUNICATIONS COMMISSION,
[SEAL] MARY JANE MORRIS,
Secretary.

[F.R. Doc. 59-7526; Filed, Sept. 9, 1959; 8:50 a.m.]

[Docket Nos. 12876, 12877; FCC 59M-1112]

AUDIOCASTING OF TEXAS, INC. AND HORACE K. JACKSON, SR.

Order Continuing Hearing

In re applications of Audiocasting of Texas, Inc., Waco, Texas, Docket No. 12876, File No. BP-11851; Horace K. Jackson, Sr., Gatesville, Texas, Docket No. 12877, File No. BP-12550; for construction permits for new standard broadcast stations.

The Hearing Examiner has before him a petition filed on August 31, 1959, by Audiocasting of Texas, Inc., to extend the hearing date in the above-entitled proceedings;

It appearing that an additional week will be required by Audiocasting's engineer to complete the work on the additional engineering information requested by the Broadcast Bureau; and

It further appearing that the other parties to the proceeding have consented to the requested continuance;

It is ordered, This 2d day of September 1959, that the petition referred to above is granted; and the hearing now scheduled for September 11, 1959, is continued to 10:00 a.m., September 18, 1959.

Released: September 3, 1959.

FEDERAL COMMUNICATIONS COMMISSION,
[SEAL] MARY JANE MORRIS,
Secretary.

[F.R. Doc. 59-7527; Filed, Sept. 9, 1959; 8:50 a.m.]

[Docket Nos. 12900, 12901; FCC 59M-1114]

JOHN LAURINO AND THE CAPITAL BROADCASTING CO. (WNAV)

Order Continuing Hearing

In re applications of John Laurino, Ashland, Virginia, Docket No. 12900, File No. BP-12112; The Capital Broadcasting Company (WNAX), Annapolis, Maryland, Docket No. 12901, File No. BP-12773; for construction permits for standard broadcast stations.

The Acting Chief Hearing Examiner, on his own motion, having under consideration a request for postponement of hearing in this proceeding filed September 1, 1959 by The Capital Broadcasting Company (WNAV);

It is ordered, This the 3d day of September 1959, that the hearing now scheduled for September 10, 1959 be and the same is hereby continued without date.

Released: September 4, 1959.

FEDERAL COMMUNICATIONS COMMISSION,
[SEAL] MARY JANE MORRIS,
Secretary.

[F.R. Doc. 59-7528; Filed, Sept. 9, 1959; 8:50 a.m.]

[Docket No. 12904; FCC 59M-1110]

WMAX, INC. (WMAX)

Memorandum Opinion and Order Continuing Hearing

In re application of WMAX, Inc. (WMAX) Grand Rapids, Michigan, Docket No. 12904, File No. BP-11744; for construction permit for standard broadcast station.

The Acting Chief Hearing Examiner having under consideration a communication from counsel for WMAX, Inc., dated and filed with the Secretary on August 26, 1959, requesting the change of certain dates, to wit: That the date for exchanging exhibits be changed from August 25 to September 8, 1959, and the hearing now set for September 17, 1959, be continued for a period of two weeks. The communication states that counsel for all parties concur in this request.

Accordingly, it is ordered, This 2d day of September 1959, that the exchange of exhibits in this proceeding will be accomplished on or before September 8, 1959, and the hearing herein now scheduled for September 17, 1959, be, and the same is hereby, rescheduled for October 1, 1959, at 10:00 a.m., in the Commission's Offices in Washington, D.C.

Released: September 3, 1959.

FEDERAL COMMUNICATIONS COMMISSION,
[SEAL] MARY JANE MORRIS,
Secretary.

[F.R. Doc. 59-7529; Filed, Sept. 9, 1959; 8:50 a.m.]

[Docket No. 13086, etc.; FCC 59M-1108]

BEACON BROADCASTING SYSTEM, INC. ET AL.

Order Scheduling Prehearing Conference

In re applications of Beacon Broadcasting System, Inc., Grafton-Cedarburg, Wisconsin, Docket No. 13086, File No. BP-10518; American Broadcasting Stations, Inc. (KWMT), Fort Dodge, Iowa, Docket No. 13087, File No. BP-12201; Suburban Broadcasting Co., Inc., Jackson, Wisconsin, Docket No. 13088, File No. BP-12802; for construction permits.

It is ordered, This 2d day of September 1959, that a prehearing conference, in accordance with § 1.111 of the rules, will be held in the above-entitled matter at 10:00 a.m. on Friday, September 11, 1959, in the offices of the Commission, Washington, D.C.

Released: September 3, 1959.

FEDERAL COMMUNICATIONS COMMISSION,
[SEAL] MARY JANE MORRIS,
Secretary.

[F.R. Doc. 59-7530; Filed, Sept. 9, 1959; 8:50 a.m.]

[Docket No. 13148; FCC 59M-1116]

BLOOM RADIO (WHLM)

Order Scheduling Prehearing Conference

In re application of Harry L. Magee, tr/as Bloom Radio (WHLM) Bloomsburg, Pennsylvania, Docket No. 13148, File No. BP-12002; for construction permit.

It is ordered, This 3d day of September 1959, that a prehearing conference, in accordance with § 1.111 of the rules, will be held in the above-entitled matter at 10:00 a.m. on Thursday, September 17, 1959, in the offices of the Commission, Washington, D.C.

Released: September 4, 1959.

FEDERAL COMMUNICATIONS COMMISSION,
[SEAL] MARY JANE MORRIS,
Secretary.

[F.R. Doc. 59-7531; Filed, Sept. 9, 1959; 8:50 a.m.]

TARIFF COMMISSION

[332-37]

STARCH

Notice of Investigation and Date of Hearing

Pursuant to a resolution adopted September 2, 1959, by the Committee on Finance, United States Senate, the United States Tariff Commission has instituted a general investigation under the authority of section 332 of the Tariff Act of 1930 (19 U.S.C. 1332) of the conditions of competition in the United States between starch produced in the United States and in foreign countries.

The Finance Committee resolution directs the Commission to include in its report of the results of the investigation a summary of the facts obtained in the investigation, including a description of the domestic industry, domestic production, foreign production, comparative costs of domestic and foreign production, including labor costs, imports, consumption, channels and methods of distribution, United States exports, United States customs treatment since 1930, and other factors affecting the competition between domestic and imported starch.

Hearings. Public hearings, at which all interested parties will be given opportunity to appear and to be heard, will be held in connection with the investigation in the Hearing Room of the Tariff Commission, 8th and E Streets NW., Washington, D.C., beginning at 10 a.m., e.s.t., on January 26, 1960.

Requests to appear. Interested parties desiring to appear and to be heard at the hearings should notify the Secretary of the Tariff Commission, in writing, at its offices in Washington, D.C., at least five days in advance of the opening of the hearings.

Issued: September 4, 1959.

DONN N. BENT,
Secretary.

[F.R. Doc. 59-7511; Filed, Sept. 9, 1959; 8:48 a.m.]

INTERSTATE COMMERCE COMMISSION

FOURTH SECTION APPLICATIONS

SEPTEMBER 4, 1959.

Protests to the granting of an application must be prepared in accordance with Rule 40 of the general rules of practice (49 CFR 1.40) and filed within 15 days from the date of publication of this notice in the FEDERAL REGISTER.

LONG-AND-SHORT HAUL

FSA No. 35657: *Cement between points in southern territory and border points.* Filed by O. W. South, Jr., Agent (SFA No. A3835), for interested rail carriers. Rates on cement and related articles, carloads between points in southern territory, also from Hagerstown (Security), Md., Alpha, Marquette, Prospect Hill, St. Louis, Mo., and E. St. Louis, Ill., to points in southern territory.

Grounds for relief: Short-line distance formula, grouping relief line arbitraries, and motor truck competition.

Tariff: Southern Freight Association, Agent, tariff I.C.C. S-61.

FSA No. 35658: *Liquified chlorine gas—Saltville, Va., to Tennessee.* Filed by O. W. South, Jr., Agent (SFA No. A3836), for interested rail carriers. Rates on liquified chlorine gas, tank-car loads from Saltville, Va., to Boyce, Calhoun, Chattanooga, and North Chattanooga, Tenn.

Grounds for relief: Market competition.

Tariff: Supplement 115 to Southern Freight Association, Agent, tariff I.C.C. 1538.

FSA No. 35659: *Window glass—Southwestern points to Florida.* Filed by Southwestern Freight Bureau, Agent (No. B-7629), for interested rail carriers. Rates on window glass (other than plate), carloads from Ft. Smith, Ark., Henryetta, Okmulgee, Okla., and Shreveport, La., to specified points in Florida.

Grounds for relief: Market competition with foreign producers.

Tariff: Supplement No. 55 to Southwestern Freight Bureau tariff I.C.C. 4100.

FSA No. 35660: *Bituminous fine coal to Oak Creek Power Plant, Wis.* Filed by Illinois Freight Association, Agent (No. 74), for interested rail carriers. Rates on bituminous fine coal, carloads from mines in Illinois, Indiana, and western Kentucky to Oak Creek Power Plant, Wis.

Grounds for relief: Rail, lake-vessel competition.

Tariffs: Supplement 31 to Illinois Freight Association, Agent, tariff I.C.C. 898 and other schedules.

FSA No. 35661: *Caustic soda—Wichita, Kans., to western points.* Filed by Western Trunk Line Committee, Agent, (A-2081), for interested rail carriers. Rates on sodium (soda), caustic (sodium hydroxide) liquid, tank-car loads, from Wichita, Kans., to points in Michigan, Minnesota and Wisconsin.

Grounds for relief: Short-line distance formula and market competition.

Tariff: Supplement 215 to Western Trunk Line Committee, Agent, tariff I.C.C. A-3991.

FSA No. 35662: *Petroleum and its products from the southwest to the south.* Filed by Southwestern Freight Bureau, Agent (B-7623), for interested rail carriers. Rates on petroleum, petroleum products, and related articles, carloads from specified points in Kansas, Louisiana, Missouri, Oklahoma, and Texas to points in southern territory.

Grounds for relief: Maintenance of rates revised to restore origin relationships disrupted on account of inclusion of different Ex Parte increases.

Tariffs: Supplement 92 to Southwestern Freight Bureau, Agent, tariff I.C.C. 4172. Southwestern Freight Bureau, Agent, tariff I.C.C. 4330.

FSA No. 35663: *Cement and related articles—East to south.* Filed by O. E. Schultz, Agent (ER No. 2507), for interested rail carriers. Rates on cement and related articles, carloads from points in trunkline territory to points in southern and border territories.

Grounds for relief: Short-line distance formula, short-line arbitraties, grouping and market competition.

Tariffs: Supplement 41 to Baltimore and Ohio Railroad Company's tariff I.C.C. 24338 and other schedules.

FSA No. 35664: *Lowe's Transportation Company—Joint rates.* Filed by O. W. South, Jr., Agent (SFA No. A3837), for interested carriers. Rates on all commodities moving on class and commodity rates as described in the application between Carthage, N.C., on the one hand, and points in the United States and Canada, on the other.

Grounds for relief: Abandonment of railroad service from and to Carthage and establishment of joint service by Lowe with rail and water carriers.

By the Commission.

[SEAL] HAROLD D. MCCOY,
Secretary.

[F.R. Doc. 59-7513; Filed, Sept. 9, 1959; 8:48 a.m.]

[Notice 30]

APPLICATIONS FOR MOTOR CARRIER "GRANDFATHER" CERTIFICATE OR PERMIT

SEPTEMBER 4, 1959.

The following applications and certain other procedural matters relating thereto are filed under the "grandfather" clause of section 7(c) of the Transportation Act of 1958. These matters are governed by special rule § 1.243 published in the FEDERAL REGISTER issue of January 8, 1959, page 205, which provides, among other things, that this publication constitutes the only notice to interested persons of filing that will be given; that appropriate protests to an application (consisting of an original and six copies each) must be filed with the Commission at Washington, D.C., within 30 days from the date of this publication in the FEDERAL REGISTER; that failure to so file seasonably will be construed as a waiver of opposition and participation in such proceeding, regardless of whether or not an oral hearing is held in the matter; and that a copy of the protest also shall be served upon applicant's representative (or applicant, if no practitioner representing him is named in the notice of filing).

These notices reflect the operations described in the applications as filed on or before the statutory date of December 10, 1958.

No. MC 117967 (Sub No. 1), (REPUBLICATION), filed December 4, 1958, published issue of FEDERAL REGISTER of March 18, 1959, at page 2007. Applicant: AIR LINE TRUCKING SERVICE, INC., 275 Spring Street SW., Atlanta, Ga. Applicant's attorney: Paul M. Daniell, 214 Grant Building, Atlanta 3, Ga. Grandfather authority sought under section 7 of the Transportation Act of 1958, to continue to operate as a *common carrier*, by motor vehicle, over irregular routes, transporting: *Frozen fruits, frozen berries*, and *frozen vegetables*, in mixed and in straight loads with *certain exempt commodities*, (1) between points in Geor-

gia, Alabama, North Carolina, South Carolina, Tennessee, Virginia, Mississippi, Florida, and Louisiana, on the one hand, and, on the other, points in Georgia, Alabama, North Carolina, South Carolina, Tennessee, Virginia, Mississippi, Louisiana, Texas, Oklahoma, Arkansas, Wisconsin, Illinois, St. Louis, Mo., Kentucky, Indiana, Michigan, Ohio, District of Columbia, Connecticut, Delaware, Maryland, Pennsylvania, New Jersey, New York and Massachusetts. (2) from points in New York, New Jersey, and Boston, Mass., to points in Ohio, Michigan, and Illinois. *Bananas*, from New Orleans, La., Mobile, Ala., and points in Florida to points in Alabama, Georgia, South Carolina and Tennessee.

NOTE: The purpose of this republication is to reflect service involving the State of Florida substantiated by the representative list of shipments, which was inadvertently omitted from the application.

HEARING: Remains as assigned October 5, 1959, 680 West Peachtree St. NW., Atlanta, Ga., before Examiner Robert A. Joyner.

By the Commission.

[SEAL] HAROLD D. MCCOY, *Secretary*.

[F.R. Doc. 59-7514; Filed, Sept. 9, 1959; 8:48 a.m.]

[Notice 186]

MOTOR CARRIER TRANSFER PROCEEDINGS

SEPTEMBER 4, 1959.

Synopses of orders entered pursuant to section 212(b) of the Interstate Commerce Act, and rules and regulations prescribed thereunder (49 CFR Part 179), appear below:

As provided in the Commission's special rules of practice any interested person may file a petition seeking reconsideration of the following numbered proceedings within 20 days from the date of publication of this notice. Pursuant to section 17(8) of the Interstate Commerce Act, the filing of such a petition will postpone the effective date of the order in that proceeding pending its disposition. The matters relied upon by petitioners must be specified in their petitions with particularity.

No. MC-FC 62336. By order of August 31, 1959, the Transfer Board approved the transfer to Warren C. Kreitz and Robert E. Kreitz, a Partnership, doing business as Morris Kreitz and Sons, Reading, Pennsylvania, of the operating rights in Certificate No. MC 115793, issued February 20, 1956, to Lamon L. Kreitz, Margaret M. Kreitz, Administratrix, Warren C. Kreitz, and Robert E. Kreitz, a Partnership, doing business as Morris Kreitz & Sons, Reading, Pennsylvania, authorizing the transportation, over irregular routes, of such commodities as contractors' equipment, heavy and bulky articles, machinery and machine parts, and articles requiring specialized handling or rigging, between points in Berks County, Pa., on the one hand, and, on the other, points in New York, New Jersey, Massachusetts, Connecticut, Rhode Island, Delaware, Maryland, Ohio, Virginia, West Virginia, and the District of Columbia, and general commodities, excluding household goods, commodities in bulk, and other specified commodities, between Reading, Pa., and points within ten miles thereof. Paul H. Edelman, 614 Washington Street, Reading, Pa., for applicants.

No. MC-FC 62353. By order of August 31, 1959, the Transfer Board approved the transfer to Mainline Transportation System, A Corporation, Whittier, Calif., of Certificate in No. MC 59313, issued April 28, 1941, to Higgins Trucks, Inc., Los Angeles, Calif., authorizing the transportation of: *Canned goods, lubricating oil, battery testers*, and *general commodities* with the usual exceptions including household goods and commodities in bulk, between specified points in California. Wade and Wade, 729 Citizens Nat'l Bank Bldg., 453 S. Spring St., Los Angeles 13, Calif., for applicants.

No. MC-FC 62359. By order of August 31, 1959, the Transfer Board approved the transfer to Martin A. Crowley, doing business as Martin A. Crowley Trucking, Franklin, N.H., of Certificates Nos. 95979 and MC 95979 Sub 1, issued December 19, 1940, and May 16, 1944, respectively, to Earl H. Dallmeyer, doing business as E. H. Dallmeyer, Pittsfield, Mass., authorizing the transportation of: Household goods, between Pittsfield, Mass., and points within 35 miles of Pittsfield, on the one hand, and, on the other, points in Connecticut, Rhode Island, Vermont, New York, New Jersey, and Pennsylvania, fresh fruits and vegetables, between points in Connecticut, New York, New Jersey, and Rhode Island, within 150 miles of Pittsfield, Mass.; lumber and logs, from Hancock, Mass., to Albany, N.Y., Wallingford, Vt., and points in Rensselear County, N.Y.; and machinery, between Pittsfield, Mass., on the one hand, and, on the other, points in Connecticut, New Jersey, New York, Pennsylvania, Rhode Island, and Vermont. Arthur A. Marshall, 145 State Street, Springfield 3, Mass., for applicants.

No. MC-FC 62363. By order of August 31, 1959, the Transfer Board approved the transfer to Eleveld Chicago Furniture Service, Inc., Chicago, Ill., of Certificate in No. MC 3619, issued April 10, 1953, to Bouma Cartage Company, A Corporation, Grand Rapids, Mich., authorizing the transportation of: *New furniture*, from Grand Rapids, Mich., to points in Illinois, Indiana, and Ohio. Eugene L. Cohn, One North La Salle St., Chicago 2, Ill., for applicants.

No. MC-FC 62366. By order of August 31, 1959, the Transfer Board approved the transfer to Sperry Transportation Company, Charles City, Iowa, of Permit No. MC 60465, issued May 28, 1957, to Dick D. Sperry, Charles City, Iowa, and of Certificate No. MC 106053 Sub 7, issued April 21, 1950, to W. R. Cordle, doing business as Cordle Cartage Company, and acquired by Dick D. Sperry pursuant to MC-F 6937. The Permit authorizes the transportation of: Tractors and tractor parts, between Charles City, Iowa, on the one hand, and, on the other, points in a specified portion of Illinois. The Certificate authorizes the transportation of: Household goods, between Nashua, Iowa, and points within 20 miles thereof, on the one hand, and, on the other, points in Illinois, Wisconsin, and Minnesota. Erwin Larson, Ellis Block, Charles City, Iowa, for applicants.

No. MC-FC 62368. By order of August 31, 1959, the Transfer Board approved the transfer to Robertson's Express, Inc., White Plains, N.Y., of Certificate in No. MC 86320, issued October 13, 1958, to Samuel Irving Lightbody, doing business as Chatsworth Moving and Storage Co., Mamaroneck, N.Y., authorizing the transportation of: *Uncrated household goods* and *uncrated store and office furniture or fixtures*, between New York, N.Y., and points in Westchester County, N.Y., on the one hand, and, on the other, points in Connecticut, Massachusetts, New Jersey, New York, Pennsylvania, and Rhode Island. David Brodsky, 1776 Broadway, New York 19, N.Y., for applicants.

No. MC-FC 62380. By order of August 31, 1959, The Transfer Board approved the transfer to Frederick A. Zank, doing business as North Central Bus Lines, 316 Eau Claire Street, Eau Claire, Wis., of Certificate No. MC 81592 Sub 2, issued June 2, 1959, to Henry C. Wenzel, doing business as North Central Bus Lines, 419 Eau Claire Street, Eau Claire, Wis., authorizing the transportation of: Passengers and their baggage, and express and newspapers in the same vehicle with passengers, between Prentice, Wis., and Park Falls, Wis., serving intermediate points on designated highways, restricted against the transportation of passengers between Park Falls and Fifield, Wis., unless destined to or originating at points beyond Fifield; and between Eau Claire, Wis., and Ladysmith, Wis., serving intermediate points on designated highways.

No. MC-FC 62399. By order of September 1, 1959, The Transfer Board approved the transfer to Alexander Moving and Storage of Alliance, Ohio, Inc., of Certificate in No. MC 17233, issued December 13, 1956, to Clyde B. Alexander, doing business as Alexander's Moving and Storage, Alliance, Ohio, authorizing the transportation of: Household goods between Alliance, Ohio, on the one hand, and, on the other, points in Michigan, Pennsylvania, and West Virginia. Nicholas G. Caples, Attorney, 524 Renkert Bldg., Canton, Ohio.

No. MC-FC 62464. By order of August 31, 1959, The Transfer Board approved the transfer to Ray Carter, Inc., Nashville, Tenn., of Certificate No. MC 96176, issued July 3, 1941, to Paul Clancy, doing business as Clancy Heavy Hauling, Memphis, Tenn., authorizing the transportation of: Heavy machinery, contractors' equipment, and parts and accessories, in special equipment, in quantities of not less than 10,000 pounds, between points in Tennessee, Louisiana, Arkansas, Mississippi, Kentucky, and Alabama. James W. Wrape, 2111 Sterick Bldg., Memphis 3, Tenn., for applicants.

No. MC-FC 62467. By order of August 31, 1959, The Transfer Board approved the transfer to William Hawthorne and Mary H. Wiegand, a partnership, doing

business as Hawthorne & Co., Philadelphia, Pennsylvania, of Certificate in No. MC 26871, issued June 19, 1941, to Harvey Braddock, doing business as Braddock Brothers, Wilmington, Delaware, authorizing the transportation of: Machinery, material, and supplies used in the construction, repair and maintenance of telephone and telegraph systems, from Wilmington, Del., to points in Pa., and Md., within 35 miles of Wilmington, and heavy machinery between Wilmington, Del., and points in Del., Pa., and Md., within 35 miles of Wilmington. Jacob Polin, 314 Old Lancaster Road, Merion, Pa.

No. MC-FC 62479. By order of August 31, 1959, The Transfer Board approved the transfer to Mid Columbia Motor Freight, Inc., Portland, Oregon, of Certificate No. MC 113212 issued May 7, 1954, to C. C. Horger, doing business as Oregon Freight Lines, Portland, Oregon, authorizing the transportation of general commodities, excluding household goods, commodities in bulk, and various specified commodities, between Portland, Oreg., and Washougal, Wash., serving the intermediate point of Vancouver, Wash., and intermediate and off-route points within ten miles of Washougal; fruit, over irregular routes, from points in Hood River County, Oreg., and those in Wasco County, Oreg., on and west of Oregon Highway 23, to Vancouver, Wash.; paper products, from Camas, Wash., to points in Hood River County, Oreg., and those in Wasco County, Oreg., located on and west of Oregon Highway 23; box shooks, from Vancouver and Bingen, Wash., to points in Hood River County, Oreg., and those in Wasco, County, Oreg., on and west of Oregon Highway 23; general commodities, excluding commodities in bulk, other commodities, but including household goods, between points in Hood River County, Oreg., on the one hand, and, on the other, points in Klickitat County, Wash.; fruit, from points in Hood River County, Oreg., and those in Klickitat County, Wash., to Portland, Oreg.; agricultural commodities, from points in Wasco, Sherman, Gilliam, Hood River, and Jefferson Counties, Oreg., to Portland, Oreg.; petroleum products, in containers, fuel, merchandise, farm machinery, salt, bags, and twine, from Portland, Oreg., to points in Wasco, Sherman, Gilliam, Hood River and Jefferson Counties, Oreg.; fresh fruits and vegetables, fruit-spraying compounds, machinery and machines used in fruit-packing plants, fruit-packing house and cannery waste and by-products, empty containers, box shook and box tops between points in Wasco and Hood River Counties, Oreg., on the one hand, and, on the other, points in Yakima County, Wash. C. C. Horger, 1419 N. W. Overton St., Portland 9, Oregon, for applicants.

No. MC-FC 62496. By order of August 31, 1959, The Transfer Board approved the transfer to Beverage Transportation Inc., Cleveland, Ohio, of Permits in Nos. MC 109513, MC 109513 Sub 4, MC 109513 Sub 5, and MC 109513 Sub 8, issued October 10, 1952, February 4, 1957, February 25, 1959, and July 14, 1959, respectively, to Charles B. Retzer, doing business as Beverage Transportation Company, Cleveland, Ohio, authorizing the transportation of specified commodities, from, to, and between specified points in Ohio, Illinois, Indiana, New Jersey, New York, Pennsylvania, Wisconsin, Missouri, and Massachusetts. Transferee was also substituted in lieu of transferor as applicant in No. MC 109513 Sub 6. G. H. Dilla, 3350 Superior Avenue, Cleveland 14, Ohio.

No. MC-FC 62509. By order of August 31, 1959, The Transfer Board approved the transfer to Scherr Motor Freight, Inc., Philadelphia, Pennsylvania, of a Certificate in No. MC 44142, issued October 30, 1957, to Irene Scherr, doing business as Scherr Motor Freight, Philadelphia, Pennsylvania, authorizing the transportation of specified commodities, from, to, and between, specified points in Pennsylvania, Delaware, New Jersey, New Hampshire, Vermont, Rhode Island, Connecticut, New York, Maryland, Florida, Georgia, Virginia, Massachusetts, Ohio, Tennessee, Indiana, North Carolina, South Carolina, Michigan, and the District of Columbia, and household goods, as defined by the Commission, between Philadelphia, Pa., on the one hand, and, on the other, points in New Jersey. Raymond A. Thistle, Jr., Shertz, Barnes & Shertz, 811-819 Lewis Tower Bldg., 225 S. 15th St., Philadelphia 2, Pennsylvania.

[SEAL] HAROLD D. MCCOY, *Secretary.*

[F.R. Doc. 59-7516; Filed, Sept. 9, 1959; 8:48 a.m.]

[Notice 286]

MOTOR CARRIER APPLICATIONS

SEPTEMBER 4, 1959.

The following applications are governed by the Interstate Commerce Commission's special rules governing notice of filing of applications by motor carriers of property or passengers or brokers under sections 206, 209 and 211 of the Interstate Commerce Act and certain other proceedings with respect thereto.

All hearings will be called at 9:30 o'clock a.m., U.S.s.t. (or 9:30 o'clock a.m., local d.s.t.), unless otherwise specified.

APPLICATIONS ASSIGNED FOR ORAL HEARING OR PRE-HEARING CONFERENCE

MOTOR CARRIERS OF PROPERTY

No. MC 807 (Sub No. 160), filed August 26, 1959. Applicant: ANCHOR MOTOR FREIGHT, INC., OF DELAWARE, 11700 Shaker Boulevard, Cleveland 20, Ohio. Applicant's attorney: Ewald E. Kundtz, 1050 Union Commerce Building, Cleveland 14, Ohio. Authority sought to operate as a *contract carrier*, by motor vehicle, over irregular routes, transporting: (1) *Automotive vehicles, chassis, bodies, cabs* and *trailers*, new, used, finished, unfinished or wrecked, *automobile parts, tools* and *accessories* moving in connection with the above-specified commodities, chassis, bodies, cabs and trailers, and *show equipment or paraphernalia*, restricted to initial movements, in driveaway or truckaway service, from the plant site or sites of Chevrolet Motor Division of General Motors Corporation located in Baltimore, Md., to points in Florida and Georgia; and (2) *Automotive vehicles, chassis, bodies, cabs* and *trailers*, new, used, finished, unfinished or wrecked, *automobile parts, tools* and *accessories* moving in connection with the above-specified commodities, chassis, bodies, cabs and trailers, and *show equipment or paraphernalia*, restricted to secondary movements, in driveaway and truckaway service, between all points described above. Applicant is authorized to conduct operations in New York, Delaware, the District of Columbia, Maryland, Pennsylvania, Virginia, West Virginia, Ohio, Indiana, Kentucky, New Jersey, North Carolina, Tennessee, South Carolina, Illinois, Michigan, Connecticut, Maine, Massachusetts, New Hampshire, Rhode Island and Vermont.

HEARING: October 15, 1959, at the Offices of the Interstate Commerce Commission, Washington, D.C., before Examiner Abraham J. Essrick.

No. MC 873 (Sub No. 34), filed July 31, 1959. Applicant: SOONER FREIGHT LINES, a Corporation, 3000 West Reno, P.O. Box 2488, Exchange Branch, Oklahoma City, Okla. Authority sought to operate as a *common carrier*, by motor vehicle, over regular and irregular routes, transporting: *All liquid or dry commodities*, in collapsible tanks or bins marketed by the U.S. Rubber Company under the trade names of "Sealdtank" or "Sealdbin" or the equivalent thereof, between all points applicant is presently authorized to serve in the transportation of General commodities, as authorized in Certificate No. MC 873 and sub numbers thereunder. Applicant is authorized to conduct operations in Colorado, Kansas, Oklahoma, and Texas.

NOTE: Applicant states that it seeks authority by the instant application, if it does not already have, authority to transport both liquid and dry commodities throughout its entire scope of operations when such transportation takes place in "Sealdtanks" or "Sealdbins" or other collapsible containers of the same or similar nature and design.

HEARING: October 26, 1959, at the Offices of the Interstate Commerce Commission, Washington, D.C., before Examiner Allan F. Borroughs.

No. MC 906 (Sub No. 43), filed August 27, 1959. Applicant: CONSOLIDATED FORWARDING CO., INC., 1300 North Tenth Street, St. Louis, Mo. Applicant's attorney: Glenn W. Stephens, 121 West Doty Street, Madison 3, Wis. Authority sought to operate as a *common carrier*, by motor vehicle, transporting: *General Commodities*, except those of unusual value, Class A and B explosives, household goods as defined by the Commission, gravel, coal, and lumber and those requiring special equipment, serving the site of the Line Material Industries plant near Sherman, Tex., as an off-route point in connection with applicant's authorized regular route operations. Applicant is authorized to conduct operations in Illinois, Indiana, Kansas, Kentucky, Missouri, Ohio, Oklahoma, Texas, and Wisconsin.

NOTE: Applicant is under common control with Triangle Express & Transfer Company (Certificate No. MC 910 and Permit No. MC

911); therefore common control may be involved.

HEARING: October 22, 1959, at the Baker Hotel, Dallas, Tex., before Joint Board No. 77, or, if the Joint Board waives its right to participate, before Examiner James C. Cheseldine.

No. MC 1313 (Sub No. 8), filed September 2, 1959. Applicant: RIDGELY TRANSPORT, doing business as PIONEER-RIDGELY FREIGHT LINES, a Corporation, 1509 Bent Avenue, Cheyenne, Wyo. Applicant's attorney: Marion F. Jones, 526 Denham Building, Denver 2, Colo. Authority sought to operate as a *common carrier*, by motor vehicle, over irregular routes, transporting: *General commodities*, except those of unusual value, Class A and B explosives, household goods as defined by the Commission, livestock, commodities in bulk and those requiring special equipment, serving intercontinental ballistics missile launching sites located in Wyoming within 70 miles of Cheyenne, Wyo., as off-route points in connection with applicant's authorized regular route operations to and from Cheyenne, Wyo. Applicant is authorized to conduct operations in Colorado, Wyoming and Nebraska.

NOTE: Applicant states it is authorized to transport the involved commodities serving intercontinental ballistics missile launching sites located in Wyoming within 25 miles of Cheyenne; and that, because a considerable number of new missile sites are scheduled for construction which are beyond the 25-mile radius authorized it files the instant application to enlarge said area to serve such additional sites.

HEARING: September 29, 1959, at the New Customs House, Denver, Colo., before Joint Board No. 197.

No. MC 2894 (Sub No. 17), filed June 1, 1959. Applicant: RED STAR TRANSIT COMPANY, INC., 7950 Dix Avenue, Detroit, Mich. Applicant's attorney: Rex Eames, 1800 Buhl Building, Detroit 26, Mich. Authority sought to operate as a *common carrier*, by motor vehicle, over regular routes, transporting: *Liquid commodities*, and *dry commodities*, in containers, *including but not limited to Sealdtanks and Sealdbins* when transported in standard vehicles, over the regular routes and in the territory, including all intermediate and off-route points authorized to be served by applicant in Certificate No. MC 2894 and Sub Numbers thereunder covering the transportation of general commodities, with certain exceptions, in Michigan, Ohio, Indiana, Illinois and Pennsylvania. Applicant is authorized to conduct operations in Illinois, Indiana, Michigan, Ohio, Pennsylvania and West Virginia.

HEARING: October 26, 1959, at the Offices of the Interstate Commerce Commission, Washington, D.C., before Examiner Allan F. Borroughs.

No. MC 2962 (Sub No. 23), filed June 17, 1959. Applicant: A. & H. TRUCK LINE, INC., 1277 Maxwell Avenue, Evansville, Ind. Applicant's attorney: Howell Ellis, 520 Illinois Building, Indianapolis, Ind. Authority sought to operate as a *common carrier*, by motor vehicle, over regular routes, transporting: *Liquid and dry commodities;* in containers, including, but not limited to, Sealdtanks and Sealdbins, when transported in standard vehicles over the regular routes and in the territory, including all off-route and intermediate points, authorized to be served by applicant by virtue of Certificates in No. MC 2962 and Sub Numbers thereunder, authorizing the transportation of general commodities, with certain exceptions, in Arkansas, Illinois, Indiana, Kentucky, Missouri, Ohio, and Tennessee. Applicant is authorized to conduct operations over regular routes in the above-specified States, and over irregular routes in Alabama, Arkansas, Colorado, Florida, Georgia, Illinois, Indiana, Iowa, Kansas, Kentucky, Louisiana, Michigan, Minnesota, Mississippi, Missouri, Nebraska, New York, North Carolina, Ohio, Oklahoma, Pennsylvania, South Carolina, Tennessee, Texas, Virginia, West Virginia, and Wisconsin.

HEARING: October 26, 1959, at the Offices of the Interstate Commerce Commission, Washington, D.C., before Examiner Allan F. Borroughs.

No. MC 3062 (Sub No. 15), filed June 29, 1959. Applicant: L. A. TUCKER LINES, INCORPORATED, 1451 Independence Avenue, Cape Girardeau, Mo. Applicant's attorney: G. M. Rebman, Suite 1230 Boatmen's Bank Building, St. Louis 2, Mo. Authority sought to operate as a *common carrier*, by motor vehicle, over regular and irregular routes, transporting: *Liquid or dry commodities*, in collapsible tanks or bins, or the equivalent thereof, including but not limited to tanks or bins known as Sealdtanks or Sealdbins, whether furnished by shipper or shippers or owned or leased by applicant, over the routes and in the territory, including all off-route and intermediate points authorized to be served by applicant by virtue of Certificate No. MC 3062 and subs thereunder, covering the transportation of general commodities, with certain exceptions in Illinois, Missouri, Indiana, Kentucky, Arkansas, Iowa and Tennessee.

NOTE: Applicant has filed a Motion to Dismiss its application on the grounds that it is presently authorized to transport lading tendered to it in containers, regardless of size of the containers.

HEARING: October 26, 1959, at the Offices of the Interstate Commerce Commission, Washington, D.C., before Examiner Allan F. Borroughs.

No. MC 3341 (Sub No. 20), filed July 1, 1959. Applicant: LAKE MOTOR FREIGHT LINES, INC., 2222 West Sample Street, South Bend, Ind. Applicant's attorney: Ferdinand Born, 1017-19 Chamber of Commerce Building, Indianapolis 4, Ind. Authority sought to operate as a *common carrier*, by motor vehicle, over regular and irregular routes, transporting: *Liquid and dry commodities*, in containers, including but not limited to Sealdtank, Sealdbin, and Nest-a-Bin containers, in or upon ordinary vehicles, over the routes and in the territories including all termini and all off-route and all intermediate points authorized to be served by applicant by virtue of Certificate No. MC 3341 and Subs thereunder. Applicant is authorized to conduct operations in Illinois, Indiana, Michigan, and Ohio.

NOTE: Any duplication with present authority to be eliminated. Common control and dual operations under section 210 may be involved.

HEARING: October 26, 1959, at the Offices of the Interstate Commerce Commission, Washington, D.C., before Examiner Allan F. Borroughs.

No. MC 3419 (Sub No. 8), filed July 30, 1959. Applicant: THE CLEVELAND, COLUMBUS & CINCINNATI HIGHWAY, INC., 215 Euclid Avenue, Cleveland 14, Ohio. Applicant's attorney: John S. Fessenden, 618 Perpetual Building, Washington 4, D.C. Authority sought to operate as a *common carrier*, by motor vehicle, over regular and irregular routes, transporting: *Liquid and dry commodities*, in containers, including but not limited to "Sealdtank" and "Sealdbin" containers, in or upon ordinary vehicular equipment, from, to and between all points applicant is authorized to transport general commodities, including all intermediate and off-route points, as authorized in Certificate No. MC 3419 and sub numbers thereunder. Applicant is authorized to conduct operations in Ohio, Indiana, Michigan and West Virginia.

HEARING: October 26, 1959, at the Offices of the Interstate Commerce Commission, Washington, D.C., before Examiner Allan F. Borroughs.

No. MC 3420 (Sub No. 5), filed July 30, 1959. Applicant: MOTOR EXPRESS, INC., an Ohio Corporation, 410 Lincoln Building, Cleveland 14, Ohio. Applicant's attorney: John S. Fessenden, 618 Perpetual Building, Washington 4, D.C. Authority sought to operate as a *common carrier*, by motor vehicle, over regular and irregular routes, transporting: *Liquid and dry commodities*, in containers, including but not limited to "Sealdtank" and "Sealdbin" containers, in or upon ordinary vehicular equipment, from, to and between all points applicant is authorized to serve in the transportation of general commodities, including all intermediate and off-route points, as authorized in Certificate No. MC 3420 and sub numbers thereunder. Applicant is authorized to conduct operations in Ohio, Pennsylvania, New York and West Virginia.

HEARING: October 26, 1959, at the Offices of the Interstate Commerce Commission, Washington, D.C., before Examiner Allan F. Borroughs.

No. MC 10761 (Sub No. 84), filed June 18, 1959. Applicant: TRANSAMERICAN FREIGHT LINES, INC., 1700 North Waterman Avenue, Detroit 9, Mich. Applicant's attorney: Howell Ellis, 520 Illinois Building, Indianapolis, Ind. Authority sought to operate as a *common carrier*, by motor vehicle, over regular and irregular routes, transporting: *Liquid commodities* and *dry commodities*, in containers, including, but not limited to, Sealdtanks and Sealdbins, in standard motor vehicles, over the routes and in the territory, including all off-route and intermediate points authorized to be served by applicant under Certificate No. MC 10761 and sub numbers thereunder and authority granted in MC-F

5501, MC–F 6136, MC–F 6185 and MC–F 6491, to which as yet no sub numbers have been assigned, covering the transportation of general commodities, with certain exceptions, in Arkansas, Colorado, Connecticut, Delaware, Illinois, Indiana, Iowa, Kansas, Kentucky, Maryland, Massachusetts, Michigan, Missouri, New Jersey, New York, Ohio, Oklahoma, Pennsylvania, Rhode Island, Texas, Virginia, West Virginia, Wisconsin and the District of Columbia.

HEARING: October 26, 1959, at the Offices of the Interstate Commerce Commission, Washington, D.C., before Examiner Allan F. Borroughs.

No. MC 10875 (Sub No. 10), filed August 6, 1959. Applicant: BRANCH MOTOR EXPRESS COMPANY, a Corporation, 300 Maspeth Avenue, Brooklyn 11, N.Y. Authority sought to operate as a *common carrier*, by motor vehicle, over regular and irregular routes, transporting: *General commodities* as presently authorized but *including liquid and dry commodities* in containers such as, but not limited to, sealed tank, sealed bin, nest-a-bin, or tote bin type, in or on ordinary vehicles, between all points over the regular and irregular routes presently authorized in Certificate No. MC 10875 and sub numbers thereunder, in Delaware, Maryland, New Jersey, New York, Pennsylvania, Virginia, and the District of Columbia.

HEARING: October 26, 1959, at the Offices of the Interstate Commerce Commission, Washington, D.C., before Examiner Allan F. Borroughs.

No. MC 10928 (Sub No. 37), filed July 27, 1959. Applicant: SOUTHERN-PLAZA EXPRESS, INC., 2001 Irving Boulevard, P.O. Box 10572, Dallas, Tex. Applicant's attorney: Hugh T. Matthews, 305 Empire Bank Building, Dallas 1, Tex. Authority sought to operate as a *common carrier*, by motor vehicle, over regular and irregular routes, transporting: *Liquid or dry commodities* in collapsible tanks or bins, including but not limited to those known as "Sealdtanks" or "Sealdbins", or the equivalent thereof, over all routes and between all points applicant is presently authorized to transport general commodities, with certain exceptions, as authorized in Certificate No. MC 10928 and sub numbers thereunder. Applicant is authorized to conduct operations in Missouri, Illinois, Tennessee, Texas, Arkansas, Oklahoma, and Kansas.

HEARING: October 26, 1959, at the Offices of the Interstate Commerce Commission, Washington, D.C., before Examiner Allan F. Borroughs.

No. MC 17398 (Sub No. 3), filed May 28, 1959. Applicant: THE H. T. SMITH EXPRESS COMPANY, P.O. Box 1000, Staunton, Va. Applicant's attorney: Francis W. McInerny, Commonwealth Building, 1625 E Street NW., Washington 6, D.C. Authority sought to operate as a *common carrier*, by motor vehicle, over regular and irregular routes, transporting: *Liquid or dry commodities*, in collapsible tanks or bins, or the equivalent thereof, over the regular and irregular routes between all points in Connecticut, Massachusetts, New Jersey and New York applicant is presently authorized to engage in the transportation of general commodities in Certificate No. MC 17398. Applicant is authorized to conduct operations in the above-specified States.

HEARING: October 26, 1959, at the Offices of the Interstate Commerce Commission, Washington, D.C., before Examiner Allan F. Borroughs.

No. MC 24379 (Sub No. 26), filed May 29, 1959. Applicant: LONG TRANSPORTATION COMPANY, a Corporation, 3755 Central Avenue, Detroit, Mich. Applicant's attorney: Rex Eames, 1800 Buhl Building, Detroit 26, Mich. Authority sought to operate as a *common carrier*, by motor vehicle, over regular routes, transporting: *Liquid and dry commodities*, in containers, including but not limited to Sealdtanks and Sealdbins when transported in standard vehicles, from, to and between all points applicant is authorized to transport General Commodities, with certain exceptions, including all intermediate and off-route points, in the States of Michigan, Ohio, Indiana, Illinois, Pennsylvania, New York, and New Jersey, as outlined in Certificate No. MC 24379 and sub numbers thereunder. Applicant is authorized to conduct operations in Illinois, Indiana, Michigan, New Jersey, New York, Ohio, and Pennsylvania.

HEARING: October 26, 1959, at the Offices of the Interstate Commerce Commission, Washington, D.C., before Examiner Allan F. Borroughs.

No. MC 28813 (Sub No. 21), filed July 30, 1959. Applicant: MOTOR EXPRESS, INC. OF INDIANA, 701 Illinois Building Indianapolis 4, Ind. Applicant's attorney: John S. Fessenden, 618 Perpetual Building, Washington 4, D.C. Authority sought to operate as a *common carrier*, by motor vehicle, over regular and irregular routes, transporting: *Liquid and dry commodities*, in containers, including but not limited to "Sealdtank" and "Sealdbin" containers, in or upon ordinary vehicular equipment, from, to and between all points applicant is authorized to serve in the transportation of general commodities, including all intermediate and off-route points, as authorized in Certificate No. MC 28813 and sub numbers thereunder. Applicant is authorized to conduct operations in Indiana, Illinois, Ohio and Wisconsin.

HEARING: October 26, 1959, at the Offices of the Interstate Commerce Commission, Washington, D.C., before Examiner Allan F. Borroughs.

No. MC 29910 (Sub No. 51), filed May 22, 1959. Applicant: ARKANSAS-BEST FREIGHT SYSTEM, INC., 301 South 11th Street, Fort Smith, Ark. Applicant's attorney: Thomas Harper, Kelley Building, P.O. Box 297, Fort Smith, Ark. Authority sought to operate as a *common carrier*, by motor vehicle, over regular and irregular routes, transporting: *Liquid and dry commodities* in collapsible tanks or bins bearing the trade names "Sealdtank", "Sealdbin" or the equivalent thereof, between all points and over all routes applicant is presently authorized to serve in the transportation of general commodities as outlined in Certificate No. MC 29910 and Sub numbers thereunder. Applicant is authorized to conduct operations in Arkansas, Louisiana, Oklahoma, Tennessee, Texas, Kansas, Missouri, Illinois, Indiana and Ohio.

HEARING: October 26, 1959, at the Offices of the Interstate Commerce Commission, Washington, D.C., before Examiner Allan F. Borroughs.

No. MC 35484 (Sub No. 41), filed June 24, 1959. Applicant: VIKING FREIGHT COMPANY, a Corporation, 614 South 6th Street, St. Louis 2, Mo. Applicant's attorney: G. M. Rebman, 1230 Boatmen's Bank Building, St. Louis 2, Mo. Authority sought to operate as a *common carrier*, by motor vehicle, over regular and irregular routes, transporting: *Liquid and dry commodities*, in collapsible tanks or bins, or the equivalent thereof, including but not limited to tanks or bins known as Sealdtanks or Sealdbins, whether furnished by shipper or shippers or owned or leased by applicant, over the regular and irregular routes, between the points, and in the territory, including all intermediate and off-route points, authorized to be served by applicant in Certificate No. MC 35484 and Sub Numbers thereunder, covering the transportation of general commodities, with certain exceptions, in Missouri, Illinois, Tennessee, Ohio, Kentucky, Indiana, Oklahoma, Texas, Mississippi, Louisiana, and Arkansas.

NOTE: This application is accompanied by a Motion to Dismiss on the ground that applicant's general commodity authority has no restriction as to the size of the containers in which a shipment may be tendered; that it is applicant's position that in the absence of such a restriction, applicant can presently lawfully handle lading tendered to it in containers as described in the application.

HEARING: October 26, 1959, at the Offices of the Interstate Commerce Commission, Washington, D.C., before Examiner Allan F. Borroughs.

No. MC 40302 (Sub No. 29), filed June 18, 1959. Applicant: FEDERAL EXPRESS, INC., 4930 North Pennsylvania Street, Indianapolis, Ind. Applicant's attorney: Ferdinand Born, 1019 Chamber of Commerce Building, Indianapolis 4, Ind. Authority sought to operate as a *common carrier*, by motor vehicle, over regular routes, transporting: *Liquid and dry commodities*, in containers, including but not limited to sealdtank, sealdbin, sealdrum, and Nest-a-Bin containers, in or upon ordinary vehicles, over the regular routes and in the territory, including all termini and all intermediate and off-route points, authorized to be served by applicant in Certificates in No. MC 40302 and Sub Numbers thereunder, in the transportation of general commodities, with exceptions, in Illinois, Indiana, Kentucky, Michigan, Missouri and Ohio. Applicant is authorized to conduct operations in the above-specified States.

HEARING: October 26, 1959, at the Offices of the Interstate Commerce Commission, Washington, D.C., before Examiner Allan F. Borroughs.

No. MC 41432 (Sub No. 82), filed July 24, 1959. Applicant: EAST TEXAS MOTOR FREIGHT LINES, INC., 623 North Washington, P.O. Box 7667 (Fair Park Station), Dallas 10, Tex. Appli-

cant's attorney: Hugh T. Matthews, Empire Bank Building, Dallas 1, Tex. Authority sought to operate as a *common carrier*, by motor vehicle, over regular and irregular routes, transporting: *Liquid or dry commodities*, in collapsible tanks or bins, including but not limited to those known as "Sealdtanks" or "Sealdbins", or the equivalent thereof, over the specified regular routes and over the irregular routes, and between the points and in the territory between which applicant is presently authorized to transport general commodities, with certain exceptions, in Certificates in No. MC 41422 and Sub Numbers in Arkansas, Illinois, Lousiana, Missouri, Tennessee and Texas. Applicant is authorized to conduct operations in the above-named States.

HEARING: October 26, 1959, at the Offices of the Interstate Commerce Commission, Washington, D.C., before Examiner Allan F. Borroughs.

No. MC 44290 (Sub No. 13), filed June 29, 1959. Applicant: HUSMANN & ROPER FREIGHT LINES, INC., 1717 North Broadway, St. Louis 6, Mo. Applicant's attorney: G. M. Rebman, 1230 Boatmen's Bank Building, St. Louis 2, Mo. Authority sought to operate as a *common carrier*, by motor vehicle, over regular and irregular routes, transporting: *Liquid or dry commodities*, in collapsible tanks or bins, or the equivalent thereof, including but not limited to tanks or bins known as "Sealdtanks" or "Sealdbins", whether furnished by shipper or shippers, or owned or leased by applicant, over the routes and in the territory, including all off-route and intermediate points, authorized to be served by applicant in Certificate MC 44290 and sub numbers thereunder covering the transportation of general commodities, with certain exceptions, in Missouri, Ohio, Kentucky, Indiana, Illinois and Kansas.

NOTE: Applicant has filed a Motion to Dismiss the above application on the ground that it is presently authorized to transport lading tendered to it in containers, regardless of size, and that it requires no additional authority therefor.

HEARING: October 26, 1959, at the Offices of the Interstate Commerce Commission, Washington, D.C., before Examiner Allan F. Borroughs.

No. MC 45657 (Sub No. 23), filed June 26, 1959. Applicant: PIC-WALSH FREIGHT CO., a Missouri Corporation, 731 Campbell Avenue, St. Louis 15, Mo. Applicant's attorney: G. M. Rebman, Suite 1230 Boatmen's Bank Building, St. Louis 2, Mo. Authority sought to operate as a *common carrier*, by motor vehicle, over regular and irregular routes, transporting: *Liquid and dry commodities*, in collapsible tanks or bins, or the equivalent thereof, including but not limited to tanks or bins known as Sealdtanks or Sealdbins, whether furnished by shipper or shippers or owned or leased by applicant, over the routes and in the territory, including all off-route and intermediate points, authorized to be served by applicant in Certificate No. MC 45657 and sub numbers thereunder, covering the transportation of general commodities, with certain exceptions, in the States of Missouri, Illinois, Ohio, Indiana, Arkansas, and Tennessee.

NOTE: Applicant has filed a Motion to Dismiss the above application on the grounds that it presently holds authority to perform the above transportation.

HEARING: October 26, 1959, at the Offices of the Interstate Commerce Commission, Washington, D.C., before Examiner Allan F. Borroughs.

No. MC 48958 (Sub No. 36) Filed August 17, 1959. Applicant: ILLINOIS-CALIFORNIA EXPRESS, INC., 510 East 51st Avenue, Denver, Colo. Authority sought to operate as a *common carrier*, by motor vehicle, over *regular routes*, transporting: *all liquid or dry commodities*, except Class A and B explosives, in collapsible tanks or bins, or the equivalent thereof, including, but not limited to those known as "sealdtanks" or "sealdbins", whether furnished by shipper or shippers, or owned or leased by applicant, over all routes and between all points, including all intermediate and off-route points, authorized to be served by applicant in Certificate No. MC 48958 and sub Numbers thereunder, throughout its entire scope of operations in Illinois, Iowa, Nebraska, Wyoming, Colorado, New Mexico, Arizona, California, and Nevada.

HEARING: October 26, 1959, at the Offices of the Interstate Commerce Commission, Washington, D.C., before Examiner Allan F. Borroughs.

No. MC 49387 (Sub No. 12), filed August 3, 1959. Applicant: ORSCHELN BROS. TRUCK LINES, INC., 339 North Williams Street, Moberly, Mo. Applicant's attorney: G. M. Rebman, 1230 Boatmen's Bank Building, St. Louis 2, Mo. Authority sought to operate as a *common carrier*, by motor vehicle, over regular and irregular routes, transporting: *Liquid or dry commodities*, in collapsible tanks or bins, or the equivalent thereof, including but not limited to tanks or bins known as Sealdtanks or Sealdbins, whether furnished by shipper or shippers or owned or leased by applicant, over the routes and in the territory, including all off-route and intermediate points authorized to be served by applicant by virtue of Certificate No. MC 49387 and Subs thereunder, covering the transportation of General commodities, with certain exceptions, in the States of Missouri, Illinois, Iowa, and Kansas.

NOTE: Applicant has filed a Motion to Dismiss the instant application on the ground that it presently holds the authority requested above.

HEARING: October 26, 1959, at the Offices of the Interstate Commerce Commission, Washington, D.C., before Examiner Allan F. Borroughs.

No. MC 52657 (Sub No. 573), filed August 24, 1959. Applicant: ARCO AUTO CARRIERS, INC., 7530 S. Western Avenue, Chicago 20, Ill. Applicant's attorney: Glenn W. Stephens, 121 West Doty Street, Madison, Wis. Authority sought to operate as a *common carrier*, by motor vehicle, over irregular routes, transporting: *Trailers*, except those designed to be drawn by passenger automobiles, and *trailer chassis*, in initial truckaway service, from points in Beaver and Cambria Counties, Pa., to points in the United States, including Alaska. Applicant is authorized to conduct operations throughout the United States.

HEARING: October 16, 1959, at the Offices of the Interstate Commerce Commission, Washington, D.C., before Examiner Abraham J. Essrick.

No. MC 52709 (Sub No. 93), filed September 2, 1959. Applicant: RINGSBY TRUCK LINES, INC., 3201 Ringsby Court, Denver 5, Colo. Authority sought to operate as a *common carrier*, by motor vehicle, over irregular routes, transporting: *General commodities, including shipper-owned compressed gas trailers loaded with compressed gas* (other than liquefied petroleum gas), *or empty*, but excluding livestock, commodities of unusual value, household goods as defined by the Commission, and those requiring special equipment, and commodities in bulk, serving ballistic missiles testing and launching sites and supply points therefor (1) within sixty (60) miles of Denver, Colo., as off-route points in connection with applicant's authorized regular route operations to and from Denver and (2) within seventy (70) miles of Cheyenne, Wyo., in connection with applicant's authorized regular route operations to and from Cheyenne.

NOTE: Applicant has authority in MC 52709 (Sub No. 85) to serve missile launching sites within twenty-five miles of Cheyenne; the purpose of this application is to broaden such authority in view of new missile sites scheduled for construction in said area which are beyond the scope presently authorized.

HEARING: September 29, 1959, at the New Customs House, Denver, Colo., before Joint Board No. 50.

No. MC 60319 (Sub No. 7), filed August 6, 1959. Applicant: MURDOCH & HATCH MOTOR TRANSPORT, INC., 300 Maspeth Avenue, Brooklyn 11, N.Y. Authority sought to operate as a *common carrier*, by motor vehicle, over regular and irregular routes, transporting: *General commodities, including liquid and dry commodities*, in containers, including but not limited to sealed tank, sealed bin, nest-a-bin, or tote bin type in or on ordinary vehicles, between all points over the regular and irregular route presently authorized in Certificate No. MC 60319 in Connecticut, Massachusetts, New Jersey, New York, Rhode Island, and the District of Columbia. Applicant is authorized to conduct regular route operations in Connecticut, Massachusetts, New York and Rhode Island, and irregular route operations in Connecticut, Massachusetts, New Jersey, New York, and the District of Columbia.

NOTE: Duplication with present authority to be eliminated.

HEARING: October 26, 1959, at the Offices of the Interstate Commerce Commission, Washington, D.C., before Examiner Allan F. Borroughs.

No. MC 61440 (Sub No. 72), filed July 31, 1959. Applicant: LEE WAY MOTOR FREIGHT, INC., 3000 West Reno, Oklahoma City, Okla. Authority sought to operate as a *common carrier*, by motor vehicle, over regular and irregular routes, transporting: *All liquid or dry commodities*, in collapsible tanks or bins marketed by the U.S. Rubber Company

under the trade names "Sealdtanks" or "Sealdbins", or the equivalent thereof, between points applicant is authorized to serve in the transportation of general commodities (between specified points in Texas, Kansas, Oklahoma, Missouri and Illinois), in Certificate No. MC 61440 and sub numbers thereunder.

NOTE: Applicant states it seeks, if it does not already have, authority to transport both liquid and dry commodities throughout its entire scope of operations when such transportation takes place in "Sealdtanks" or "Sealdbins" or other collapsible containers of the same or similar nature and design.

HEARING: October 26, 1959, at the Offices of the Interstate Commerce Commission, Washington, D.C., before Examiner Allan F. Borroughs.

No. MC 62835 (Sub No. 13), filed June 29, 1959. Applicant: C. E. S. TRUCK LINES, INC., Highway 61-67, Crystal City, Mo. Applicant's attorney: G. M. Rebman, 1230 Boatmen's Bank Building, St. Louis 2, Mo. Authority sought to operate as a *common carrier*, by motor vehicle, over regular and irregular routes, transporting: *Liquid or dry commodities*, in collapsible tanks or bins, or the equivalent thereof, including but not limited to tanks or bins known as "Sealdtanks" or "Sealdbins", whether furnished by shipper or shippers or owned or leased by applicant, over the routes and in the territory, including all off-route and intermediate points authorized to be served by applicant in its Certificate No. MC 62835 and sub numbers thereunder, covering the transportation of general commodities, with certain exceptions, as more fully set forth in the application.

NOTE: Applicant has also filed a Motion to Dismiss the above application on the ground that it is presently authorized to transport lading tendered to it in containers, regardless of size thereof.

HEARING: October 26, 1959, at the Offices of the Interstate Commerce Commission, Washington, D.C., before Examiner Allan F. Borroughs.

No. MC 66990 (Sub No. 7) (REPUBLICATION), filed May 14, 1959. Published FEDERAL REGISTER issue of August 5, 1959. Applicant: DON EATON TRANSFER & STORAGE, INCORPORATED, 119 South Frisco, Tulsa, Okla. Applicant's attorney: W. T. Brunson, Leonhardt Building, Oklahoma City 2, Okla. Authority sought to operate as a *common carrier*, by motor vehicle over irregular routes, transporting: *Household goods* as defined by the Commission in Ex Parte No. MC 19, 176.1 as amended, (1) between points in Alaska, and (2) between points in Alaska, on the one hand, and, on the other, points in Oklahoma, Texas, New Mexico, Colorado, Nebraska, Kansas, Missouri, Arkansas, Louisiana, Iowa, Minnesota, Illinois, Indiana, Kentucky, Tennessee, Mississippi, Alabama, Ohio, Michigan, Pennsylvania, New York, and New Jersey. Applicant is authorized to conduct operations in the above-named States except Alaska.

NOTE: This notice was inadvertently published under the "No Hearing" Procedures.

HEARING: Remains as assigned September 15, 1959, at the Mayo Hotel, Tulsa, Okla., before Examiner Jair S. Kaplan.

No. MC 69275 (Sub No. 32), filed June 24, 1959. Applicant: M & M TRANSPORTATION COMPANY, a Massachusetts Corporation, 250 Mystic Avenue, Somerville, Mass. Applicant's attorney: John S. Fessenden, Suite 618 Perpetual Building, 1111 E Street NW., Washington 4, D.C. Authority sought to operate as a *common carrier*, by motor vehicle, over regular and irregular routes, transporting: *Liquid and dry commodities*, in containers, including but not limited to "Sealdtank" and "Sealdbin" containers, in or upon ordinary vehicular equipment, between all points applicant is presently authorized to serve in the transportation of general commodities, including all intermediate and off-route points, as well as all points at which service is to be authorized by reason of the purchase of rights from Chelsea Contracting and Trucking Co., MC-F 6917. Applicant is authorized to conduct operations in Massachusetts, Pennsylvania, Rhode Island, Connecticut, New Jersey, New York, Maryland, and Delaware.

HEARING: October 26, 1959, at the Offices of the Interstate Commerce Commission, Washington, D.C., before Examiner Allan F. Borroughs.

No. MC 69281 (Sub No. 37), filed June 16, 1959. Applicant: THE DAVIDSON TRANSFER & STORAGE CO., a Corporation, 6301 Pulaski Highway, Baltimore 3, Md. Applicant's attorney: John S. Fessenden, 1111 E Street NW., Washington 4, D.C. Authority sought to operate as a *common carrier*, by motor vehicle, over regular and irregular routes, transporting: *Liquid and dry commodities*, in containers, including but not limited to "Sealdtank" and "Sealdbin" containers, in or upon ordinary vehicular equipment, between all points applicant is authorized to serve in the transportation of general commodities, in Certificate No. MC 69281. Applicant is authorized to transport general commodities, with certain exceptions, between specified points in Maryland, New York, Delaware, New Jersey, Pennsylvania, Virginia and the District of Columbia.

NOTE: Applicant states it seeks, if it does not already have, authority to transport both liquid and dry commodities throughout its entire scope of operations when such transportation takes place in "Sealdtanks" or "Sealdbins" or other collapsible containers of the same or similar nature and design, as more fully described in its proposed application.

HEARING: October 26, 1959, at the Offices of the Interstate Commerce Commission, Washington, D.C., before Examiner Allan F. Borroughs.

No. MC 73262 (Sub No. 13), filed July 6, 1959. Applicant: MERCHANTS FREIGHT SYSTEM, INC., 1401 North 13th Street, Terre Haute, Ind. Applicant's attorney: Howell Ellis, 520 Illinois Building, Indianapolis, Ind. Authority sought to operate as a *common carrier*, by motor vehicle over regular and irregular routes, transporting: *Liquid or dry commodities*, in collapsible tanks or bins, or the equivalent thereof, between all points applicant is presently authorized to serve in the transportation of general commodities as authorized in Certificate No. MC 73262 and sub numbers thereunder, in Michigan, Ohio, Illinois, Indiana, Missouri, and Kentucky.

NOTE: Applicant states it is seeking the above authority, if it does not already hold the same under its present authority, when such transportation takes place in "Sealdtanks" or "Sealdbins" marketed by the U.S. Rubber Company, or other collapsible containers of similar nature and design.

HEARING: October 26, 1959, at the Offices of the Interstate Commerce Commission, Washington, D.C., before Examiner Allan F. Borroughs.

No. MC 75320 (Sub No. 90), filed June 4, 1959. Applicant: CAMPBELL SIXTY-SIX EXPRESS, INC., P.O. Box 390, 2330 East Mill Street, Springfield, Mo. Authority sought to operate as a *common carrier*, by motor vehicle, over regular and irregular routes, transporting: *Liquid or dry commodities*, in collapsible tanks or bins similar or identical with so-called "Sealdtank" or "Sealbin" containers, or containers of a similar nature in design, or containers that are of substantial equivalent thereof, between the points, over the regular and irregular routes, and in the territory, including intermediate and off-route points, authorized to be served by applicant in Certificate No. MC 75320 and Sub Numbers thereunder, in Alabama, Arkansas, Illinois, Indiana, Kansas, Louisiana, Mississippi, Missouri, Oklahoma, Tennessee and Texas.

HEARING: October 26, 1959, at the Offices of the Interstate Commerce Commission, Washington, D.C., before Examiner Allan F. Borroughs.

No. MC 75406 (Sub No. 16), filed June 29, 1959. Applicant: SUPERIOR FORWARDING COMPANY, INC., 2800 South Fourth Street, St. Louis 18, Mo. Applicant's attorney: G. M. Rebman, Suite 1230 Boatmen's Bank Building, St. Louis 2, Mo. Authority sought to operate as a *common carrier*, by motor vehicle, over regular and irregular routes, transporting: *Liquid and dry commodities*, in collapsible tanks or bins, or the equivalent thereof, including but not limited to tanks or bins known as Sealdtanks or Sealdbins, whether furnished by shipper or shippers or owned or leased by applicant, over the routes and in the territory, including all off-route and intermediate points, authorized to be served by applicant in Certificate No. MC 75406 and subnumbers thereunder, covering the transportation of general commodities, with certain exceptions, in the States of Missouri, Illinois and Arkansas.

NOTE: Applicant has filed a Motion to Dismiss the instant application on the grounds that it presently holds the above requested authority.

HEARING: October 26, 1959, at the Offices of the Interstate Commerce Commission, Washington, D.C., before Examiner Allan F. Borroughs.

No. MC 75527 (Sub No. 20), filed August 24, 1959. Applicant: LAHN TRANSPORTATION, a Corporation, P.O. Box 17, Bridgeton, N.J. Applicant's attorney: Frank B. Hand, Jr., Transportation Building, Washington 6, D.C. Authority sought to operate as a *common carrier*, by motor vehicle over irregular routes, transporting: *Glassware*, and *glass containers*, from points in Cumber-

land and Salem Counties, N.J., to points in Maine, New Hampshire, and Vermont, and *rejected, returned* and *damaged glassware and glass containers, used pallets, used platforms,* and *used skids,* on return. Applicant is authorized to conduct operations in New York, New Jersey, New Hampshire, Pennsylvania, Delaware, Maryland, Virginia, North Carolina, Ohio, West Virginia, Connecticut, Rhode Island, Massachusetts, and the District of Columbia.

HEARING: October 9, 1959, at the Offices of the Interstate Commerce Commission, Washington, D.C., before Examiner Donald Sutherland.

No. MC 78712 (Sub No. 7), filed June 11, 1959. Applicant: MILLER TRANSPORTATION, INC., 1200 South Home Avenue, Kokomo, Ind. Applicant's attorney: Howell Ellis, 520 Illinois Avenue, Indianapolis, Ind. Authority sought to operate as a *common carrier,* by motor vehicle, over irregular routes, transporting: *Liquid or dry commodities,* in collapsible tanks or bins, or the equivalent thereof, between all points over the irregular routes applicant is presently authorized to serve in the transportation of general commodities in Certificate No. MC 78712, in Indiana, Kentucky, Missouri, Ohio, Illinois and Michigan.

NOTE: Applicant states it is seeking this authority, if it does not already hold the same under the provisions of its present certificate, when such transportation takes place in sealdtanks or sealdbins, marketed by the U.S. Rubber Company, or other collapsible containers of similar nature and design. Applicant is authorized to conduct operations in Illinois, Indiana, Kentucky, Michigan, Missouri, New York, Ohio, Pennsylvania and Wisconsin.

HEARING: October 26, 1959, at the Offices of the Interstate Commerce Commission, Washington, D.C., before Examiner Allan F. Borroughs.

No. MC 80430 (Sub No. 94) filed August 12, 1959. Applicant: GATEWAY TRANSPORTATION CO., an Illinois Corporation, 2130–2150 South Avenue, LaCrosse, Wis. Applicant's attorney: John C. Bradley, Suite 618, Perpetual Building, 1111 E Street NW., Washington 4, D.C. Authority sought to operate as a *common carrier,* by motor vehicle, over regular and irregular routes, transporting: *Liquid and dry commodities,* in containers, including but not limited to "Sealdtank" and "Sealdbin" containers, in or upon ordinary vehicular equipment, from, to and between all points, including all intermediate and off-route points, applicant is authorized to conduct operations as authorized in Certificate No. MC 80430 and Sub numbers thereunder. Applicant is authorized to conduct operations in Illinois, Indiana, Iowa, Kentucky, Michigan, Minnesota, Missouri, Ohio, Pennsylvania, New York and Wisconsin.

HEARING: October 26, 1959, at the Offices of the Interstate Commerce Commission, Washington, D.C., before Examiner Allan F. Borroughs.

No. MC 82944 (Sub No. 6), filed September 2, 1959. Applicant: FREDERIC A. BETHKE, doing business as BETHKE TRUCK LINES, Gilcrest, Colo. Applicant's attorney: Marion F. Jones, 526 Denham Building, Denver 2, Colo. Authority sought to operate as a *common carrier,* by motor vehicle, over irregular routes, transporting: *General commodities,* except Class A and B explosives, household goods as defined by the Commission, livestock, commodities requiring special equipment, and liquid commodities in bulk, serving ballistic missile testing and launching sites and supply points therefor located within a 60-mile radius of Denver, Colo., as off-route points in connection with applicant's authorized regular route operations to and from Denver, Colo. Applicant is authorized to conduct operations in Colorado.

HEARING: September 29, 1959, at the New Customs House, Denver, Colo., before Joint Board No. 126.

No. MC 83539 (Sub No. 51), filed July 9, 1959. Applicant: C & H TRANSPORTATION CO., INC., 1935 West Commerce Street, P.O. Box 5976, Dallas, Tex. Applicant's attorney: W. T. Brunson, Leonhardt Building, Oklahoma City 2, Okla. Authority sought to operate as a *common carrier,* by motor vehicle, over irregular routes, transporting: *Liquid and dry commodities,* in containers, including but not limited to Sealdtanks, Sealdbins, Nest-a-bin, or tote-bin containers, in or upon ordinary vehicles, between points in Alabama Arizona, Arkansas, Colorado, Florida, Georgia, Illinois, Indiana, Iowa, Kentucky, Kansas, Louisiana, Michigan, Minnesota, Mississippi, Missouri, Montana, Nebraska, Nevada, New Jersey, New Mexico, New York, North Dakota, Ohio, Oklahoma, Oregon, Pennsylvania, South Dakota, Tennessee, Texas, Utah, Washington, Wisconsin, and Wyoming. Applicant is authorized to conduct operations in Arkansas, Colorado, Illinois, Indiana, Iowa, Kansas, Kentucky, Louisiana, Michigan, Minnesota, Mississippi, Missouri, Montana, Nebraska, Nevada, New Jersey, New Mexico, New York, North Dakota, Ohio, Oklahoma, Oregon, Pennsylvania, South Dakota, Tennessee, Texas, Utah, Washington, West Virginia, Wisconsin, and Wyoming.

HEARING: October 26, 1959, at the Offices of the Interstate Commerce Commission, Washington, D.C., before Examiner Allan F. Borroughs.

No. MC 96079 (Sub No. 1) (CLARIFICATION), filed April 24, 1959, published in the August 26, 1959 issue of the FEDERAL REGISTER. Applicant: KELLY AUGUSTA CRAWFORD, doing business as KELLY CRAWFORD TRANSFER, Richlands, Va. Applicant's attorney: R. Roy Rush, Box 614, Boxley Building, Roanoke, Va. Authority sought to operate as a *common carrier,* by motor vehicle, over irregular routes, transporting: *Brick,* from Richlands, Va., to points in West Virginia; to points in Kentucky on and east of a line beginning at the Kentucky-Ohio State line and extending along U.S. Highway 25 to Lexington, Ky., thence along U.S. Highway 27 to junction Kentucky Highway 34 near Bryantsville, Ky., thence along Kentucky Highway 34 to Danville, Ky., and thence along Kentucky Highway 35 to the Kentucky-Tennessee State line; to points in Tennessee on and east of Tennessee Highway 28; to points in North Carolina on and west of U.S. Highway 1; and to points in Virginia on and west of U.S. Highway 15. Applicant is authorized to conduct operations in Virginia and West Virginia.

HEARING: Remains as assigned September 28, 1959, at the U.S. Court Rooms, Roanoke, Va., before Examiner Richard H. Roberts.

No. MC 96339 (Sub No. 6), filed September 2, 1959. Applicant: MONA RIDGELY, doing business as ARROW MOVING & STORAGE CO., 1509 Bent Avenue, Cheyenne, Wyo. Applicant's attorney: Marion F. Jones, 526 Denham Building, Denver 2, Colo. Authority sought to operate as a *common carrier,* by motor vehicle, over irregular routes, transporting: *General commodities,* except those of unusual value, Class A and B explosives, household goods as defined by the Commission, and commodities in bulk, between Cheyenne, Wyo., on the one hand, and, on the other, intercontinental ballistics missile launching sites located in Wyoming within a 70-mile radius of Cheyenne, Wyo. Applicant is authorized to conduct operations in Wyoming, Colorado and Nebraska.

NOTE: Applicant states it is authorized to transport the involved commodities between Cheyenne, Wyo., and intercontinental ballistics missile launching sites in Wyoming located within 25 miles of Cheyenne; that because a number of new missile sites are scheduled for construction which are beyond the 25 mile radius authorized, applicant files the instant application for the enlarged area so as to serve such additional sites.

HEARING: September 29, 1959, at the New Customs House, Denver, Colo., before Joint Board No. 197.

No. MC 103654 (Sub No. 49), filed July 2, 1959. Applicant: SCHIRMER TRANSPORTATION COMPANY, INCORPORATED, 649 Pelham Boulevard, St. Paul, Minn. Applicant's attorney: Donald A. Morken, 1100 First National Soo-Line Building, Minneapolis, Minn. Authority sought to operate as a *common carrier,* by motor vehicle, over irregular routes, transporting: *Flour,* in bulk, or in containers including sealdbins, sealdtanks, shipper-owned containers, from Grand Forks, N. Dak., Superior, Wis., and points in Minnesota, to points in Minnesota, Wisconsin, Iowa, Illinois, North Dakota, South Dakota and the Upper Peninsula of Michigan, and *empty containers and rejected shipments,* on return. Applicant is authorized to conduct operations in Illinois, Indiana, Michigan, Minnesota, North Dakota and Wisconsin.

HEARING: October 26, 1959, at the Offices of the Interstate Commerce Commission, Washington, D.C., before Examiner Allan F. Borroughs.

No. MC 104004 (Sub No. 140), filed July 17, 1959. Applicant: ASSOCIATED TRANSPORT, INCORPORATED, 380 Madison Avenue, New York 17, N.Y. Authority sought to operate as a *common carrier,* by motor vehicle, over regular and irregular routes, transporting: *General commodities,* in collapsible containers known by the manufacturer's trade name of "Sealdtanks" and "Sealdbins" but not restricted to that manufacturers containers, from, to and between all points applicant is authorized

to conduct operations as authorized in Certificate No. MC 104004 and sub numbers thereunder. Applicant is authorized to conduct operations in Connecticut, Delaware, Georgia, Maryland, New Jersey, New York, North Carolina, Ohio, Pennsylvania, South Carolina, Tennessee, Virginia, Massachusetts, Rhode Island, and the District of Columbia.

HEARING: October 26, 1959, at the Offices of the Interstate Commerce Commission, Washington, D.C., before Examiner Allan F. Borroughs.

No. MC 106943 (Sub No. 67), filed May 15, 1959. Applicant: EASTERN EXPRESS, INC., 1450 Wabash Avenue, Terre Haute, Ind. Applicant's attorney: John E. Lesow, 3737 North Meridian Street, Indianapolis 8, Ind. Authority sought to operate as a *common carrier*, by motor vehicle, over regular and irregular routes, transporting: *Liquid and dry commodities* (except Class A and B explosives), in collapsible cargo containers, from, to and between all points applicant is authorized to conduct operations, including all intermediate and off-route points, by virtue of Certificate No. MC 106943 and sub numbers thereunder, in the transportation of General commodities, with certain exceptions, in the States of Delaware, Indiana, Illinois, Iowa, Kentucky, Maryland, Michigan, Missouri, New Jersey, New York, Ohio, Pennsylvania, West Virginia, and the District of Columbia.

NOTE: Applicant has filed a Motion to Dismiss the instant application on the grounds that it presently has the authority to perform the above operations.

HEARING: October 26, 1959, at the Offices of the Interstate Commerce Commission, Washington, D.C., before Examiner Allan F. Borroughs.

No. MC 108158 (Sub No. 49), filed June 16, 1959. Applicant: MID-CONTINENT FREIGHT LINES, INC., 4350 West Roosevelt Road, Chicago 24, Ill. Applicant's attorney: John S. Fessenden, 1111 E Street NW., Washington 4, D.C. Authority sought to operate as a *common carrier*, by motor vehicle, over regular routes, transporting: *Liquid and dry commodities*, in containers, including but not limited to "Sealdtank" and "Sealdbin" containers, in or upon ordinary vehicular equipment, between all points applicant is authorized to serve in the transportation of general commodities, in Certificate MC 108158 and sub numbers thereunder. Applicant is authorized to transport general commodities, with certain exceptions, between specified points in Texas, Oklahoma, Illinois, Missouri, Indiana, Minnesota, Wisconsin, Kansas, and Ohio.

NOTE: Applicant states it seeks, if it does not already have, authority to transport both liquid and dry commodities throughout its entire scope of operations when such transportation takes place in "Sealdtanks" or "Sealdbins" or other containers of the same or similar nature and design, as more fully described in its proposed application.

HEARING: October 26, 1959, at the Offices of the Interstate Commerce Commission, Washington, D.C., before Examiner Allan F. Borroughs.

No. MC 108302 (Sub No. 2) (REPUBLICATION), filed June 30, 1959, published issue FEDERAL REGISTER August 5, 1959. Applicant: G. & S. CITY TRUCKING CO., INC., 345 West 38th Street, New York 18, N.Y. Applicant's representative: Charles H. Trayford, 155 East 40th Street, New York 16, N.Y. Authority sought to operate as a *common carrier*, by motor vehicle, over irregular routes, transporting: *Garments, piece goods*, cut or in the original piece, *trimmings, buttons, notions, fasteners*, between Ossining, N.Y., and points in the New York, N.Y., Commercial Zone, as defined by the Commission, on the one hand, and, on the other, Cleveland, Ohio. Applicant is authorized to conduct operations in New Jersey and New York.

HEARING: Remains as reassigned September 29, 1959, at the Offices of the Interstate Commerce Commission, Washington, D.C., before Examiner Thomas F. Kilroy.

No. MC 109095 (Sub No. 8), filed June 29, 1959. Applicant: ANDERSON MOTOR SERVICE, INC., 1516 North Fourteenth Street, St. Louis 6, Mo. Applicant's attorney: Gregory M. Rebman, 1230 Boatmen's Bank Building, St. Louis 2, Mo. Authority sought to operate as a *common carrier*, by motor vehicle, over regular and irregular routes, transporting: Liquid or dry commodities, in collapsible tanks or bins, or the equivalent thereof, including but not limited to tanks or bins known as Sealdtanks or Sealdbins, whether furnished by shipper or shippers or owned or leased by applicant, over the routes and in the territory including all off-route and intermediate points authorized to be served by applicant by virtue of Certificate No. MC 109095 and Subs thereunder covering the transportation of general commodities, with certain exceptions, in the States of Indiana, Ohio, Illinois, and Missouri.

NOTE: Applicant has filed a Motion to Dismiss the instant application on the ground that it presently holds the authority requested above.

HEARING: October 26, 1959, at the Offices of the Interstate Commerce Commission, Washington, D.C., before Examiner Allan F. Borroughs.

No. MC 110388 (Sub No. 16), filed June 17, 1959. Applicant: UNION PACIFIC MOTOR FREIGHT COMPANY, a Corporation, 1416 Dodge Street, Omaha 2, Nebr. Applicant's attorney: John J. Burchell, 1416 Dodge Street, Omaha 2, Nebr. Authority sought to operate as a *common carrier*, by motor vehicle, over regular routes, transporting: *General commodities*, except those of unusual value, household goods as defined by the Commission, and commodities requiring special equipment, (1) between Pocatello, Idaho, and Butte, Mont.: from Pocatello over U.S. Highway 91 to Butte; (2) between Blackfoot, Idaho, and Mackay, Idaho: from Blackfoot over U.S. Highway 26 to Arco, thence over Alternate U.S. Highway 93 to Mackay; (3) between Idaho Falls, Idaho, and Swan Valley, Idaho: from Idaho Falls over U.S. Highway 26 to Swan Valley; (4) between junction U.S. Highways 20 and 26 near Atomic City, Idaho, and West Yellowstone, Mont.: from junction U.S. Highways 20 and 26 over U.S. Highway 20 to West Yellowstone; (5) between Roberts, Idaho, and Ririe, Idaho: from Roberts over Idaho Highway 48 to Ririe; (6) between Menan, Idaho and Lorenzo, Idaho: from Menan over Idaho Highway 80 to Lorenzo; (7) between Swan Valley, Idaho, and junction Idaho Highway 33 and U.S. Highway 20: from Swan Valley over Idaho Highway 31 to Victor, thence over Idaho Highway 33 to junction U.S. Highway 20, near Sugar City; (8) between junction Idaho Highways 32 and 33, and Ashton, Idaho: from junction Idaho Highways 32 and 33, near Tetonia, over Idaho Highway 32 to Ashton; (9) between American Falls, Idaho, and Blackfoot, Idaho: from American Falls over Idaho Highway 39 to Blackfoot; (10) between Pocatello, Idaho, and Boise, Idaho: from Pocatello over U.S. Highway 30-N to Burley, thence over U.S. Highway 30 to Boise; (11) between Kimama and Bliss, Idaho: from Kimama over Idaho Highway 24 to junction Idaho Highway 25, thence over Idaho Highway 25 to Bliss; (12) between junction U.S. Highway 30 and unnumbered county road, and Oakley, Idaho: from junction U.S. Highway 30 and unnumbered county road (approximately one mile west of Murtaugh), thence over such unnumbered county road to Oakley; (13) between Burley, Idaho, and junction Idaho Highway 77 and U.S. Highway 30-N: from Burley over U.S. Highway 30-S to Declo, thence over Idaho Highway 77 to junction U.S. Highway 30-N (near Rupert); (14) between Paul and Oakley, Idaho: from Paul over Idaho Highway 27 to Oakley; (15) between Twin Falls, Idaho, and junction Idaho Highways 25 and 50: from Twin Falls over Idaho Highway 50 to junction Idaho Highway 25; (16) between Twin Falls, Idaho, and junction Idaho Highway 74 and U.S. Highway 93: from Twin Falls over Idaho Highway 74 to junction U.S. Highway 93; (17) between Ketchum, Idaho, and Wells, Nev.: from Ketchum over U.S. Highway 93 to junction U.S. Highway 40, near Wells, thence over U.S. Highway 40 to Wells; (18) between Wendell, Idaho, and junction unnumbered highway and U.S. Highway 30: from Wendell over unnumbered highway to junction U.S. Highway 30, near Hagerman; (19) between junction Idaho Highway 79 and U.S. Highway 93, and junction Idaho Highway 79 and U.S. Highway 93: from junction Idaho Highway 79 and U.S. Highway 93, over Idaho Highway 79, via Jerome, Idaho, to junction Idaho Highway 79 and U.S. Highway 93; (20) between Shoshone, Idaho, and Dietrich, Idaho: from Shoshone over Idaho Highway 24 to Dietrich; (21) between Bliss, Idaho, and junction Idaho Highway 23 and U.S. Highway 93, near Bellevue, Idaho: from Bliss over U.S. Highway 26 to Carey, Idaho, thence over Idaho Highway 23 to junction U.S. Highway 93; (22) between junction Idaho Highways 25 and 46, south of Wendell, Idaho, and Hill City, Idaho: from junction Idaho Highways 25 and 46 over Idaho Highway 46 to junction Idaho Highway 68, thence over Idaho Highway 68 to Hill City; (23) between junction Idaho Highways 46 and 68 and junction Idaho Highway 68 and U.S. Highway 93: from junction Idaho

Highways 46 and 68, approximately 4 miles east of Fairfield, Idaho, thence over Idaho Highway 68 to junction U.S. Highway 93; and return over the above-described routes, serving all intermediate points on said routes and all off-route points which are stations on the lines of the Union Pacific Railroad Company, subject to the following conditions: (1) The service proposed by applicant shall be limited to service which is auxiliary to or supplemental of the service of Union Pacific Railroad Company; (2) No service shall be rendered to or from any point not a station on the rail lines of Union Pacific Railroad Company; (3) No shipment shall be transported by applicant between any of the following points, or through or to or from more than one of said points: Pocatello, Boise and Gooding, Idaho, Butte, Mont., and Wells, Nev.; (4) All contractual arrangements between the carrier and Union Pacific Railroad Company shall be reported to the Commission and shall be subject to revision by it if and as it may be found necessary in order that such arrangements shall be fair and equitable to the parties; (5) Such further conditions as the Commission, in the future, may find it necessary to impose in order to restrict carrier's operation by motor vehicle to service which is auxiliary to, or supplemental of, the rail service of Union Pacific Railroad Company. Applicant is authorized to conduct operations in Idaho, Utah, Missouri, Kansas, Wyoming, Colorado, Iowa, Oregon, Washington, Nevada and California.

NOTE: Applicant states that highway descriptions between Blackfoot and Mackay have been changed due to recent new construction; and that if the authority here sought is granted, it is willing that its Certificate in No. MC 110388 Sub 1 be canceled in order that duplicate authority may be eliminated and its certificates simplified.

HEARING: October 5, 1959, at the U.S. Court Rooms, Pocatello, Idaho, before Joint Board No. 410, or, if the Joint Board waives its right to participate, before Examiner Lucian A. Jackson.

No. MC 110683 (Sub No. 13), filed May 28, 1959. Applicant: SMITH'S TRANSFER CORPORATION OF STAUNTON, VA., P.O. Box 1000, Staunton, Va. Applicant's attorney: Francis W. McInerny, Commonwealth Building, 1625 K Street NW., Washington 6, D.C. Authority sought to operate as a *common carrier*, by motor vehicle, over regular and irregular routes, transporting: *Liquid or dry commodities*, in collapsible tanks or bins, or the equivalent thereof, over the regular and irregular routes, from, to, and between all points in Delaware, Kentucky, Maryland, New Jersey, New York, North Carolina, Pennsylvania, South Carolina, Virginia, West Virginia, and the District of Columbia, that applicant is presently authorized to serve in the transportation of general commodities in Certificate No. MC 110683 and Sub Numbers thereunder. Applicant is authorized to conduct operations in the above-specified States.

NOTE: Common control may be involved.

HEARING: October 26, 1959, at the Offices of the Interstate Commerce Commission, Washington, D.C., before Examiner Allan F. Borroughs.

No. MC 111320 (Sub No. 39), filed August 28, 1959. Applicant: CURTIS KEAL TRANSPORT COMPANY, INC., East 54th Street and Cleveland Shoreway, Cleveland 14, Ohio. Applicant's representative: G. H. Dilla, 3350 Superior Avenue, Cleveland 14, Ohio. Authority sought to operate as a common carrier, by motor vehicle, over irregular routes, transporting: *Roadbuilding, earth moving, mining and quarrying equipment including articles* named in Appendix 8 of Exparte MC 45 and *parts* of such commodities moving with, or separately, from the above-described commodities, in both driveaway and truckaway methods, between Evansville, Ind., on the one hand, and, on the other, points in the United States except Alaska. Applicant is authorized to conduct operations throughout the United States.

HEARING: September 18, 1959, at the U.S. Court Rooms, Indianapolis, Ind., before Examiner Michael B. Driscoll.

No. MC 111320 (Sub No. 40), filed August 28, 1959. Applicant: CURTIS KEAL TRANSPORT COMPANY, INC., East 54th Street and Cleveland Shoreway, Cleveland 14, Ohio. Applicant's representative: G. H. Dilla, 3350 Superior Avenue, Cleveland 14, Ohio. Authority sought to operate as a *common carrier*, by motor vehicle, over irregular routes, transporting: *Water, oil, blast hole, quarry and mining drills*, self-propelled, or mounted on trailers and *parts* of the above commodities when moving with the above-described commodities, or separately, in driveaway, towaway or truckaway methods, between Wayne County, Ind. on the one hand, and, on the other, points in the United States, except Alaska. Applicant is authorized to conduct operations throughout the United States.

HEARING: September 18, 1959, at the U.S. Court Rooms, Indianapolis, Ind., before Examiner Michael B. Driscoll.

No. MC 111594 (Sub No. 15), filed June 22, 1959. Applicant: CENTRAL WISCONSIN MOTOR TRANSPORT COMPANY, a Corporation, 610 High Street, Wisconsin Rapids, Wis. Applicant's attorney: Hamilton R. Winton, Jr., Harris Trust Building, 111 West Monroe Street, Chicago 3, Ill. Authority sought to operate as a *common carrier*, by motor vehicle, over regular and irregular routes, transporting: *Liquid and dry commodities*, in collapsible tanks and bins marketed by the U.S. Rubber Company under the trade names "Sealdtank" or "Sealdbin" or the equivalent thereof, between all points over the regular and irregular routes, including all intermediate and off-route points, which applicant is presently authorized to serve in the transportation of general commodities, with exceptions, in Certificate No. MC 111594 and Sub Numbers thereunder, in Illinois, Indiana, Minnesota and Wisconsin. Applicant is authorized to conduct operations in the above-specified States.

HEARING: October 26, 1959, at the Offices of the Interstate Commerce Commission, Washington, D.C., before Examiner Allan F. Borroughs.

No. MC 112497 (Sub No. 145) (CLARIFICATION), published in the FEDERAL REGISTER August 26, 1959 at page 6929. Applicant: HEARIN TANK LINES, INC., 6440 Rawlins St., P.O. Box 3096, Baton Rouge, La. Applicant's attorney: Harry C. Ames, Jr., Transportation Building, Washington, D.C. Authority sought to operate as a *common carrier*, by motor vehicle, over irregular routes, transporting: *Petroleum products*, in bulk, in tank vehicles, between Mobile, Ala., on the one hand, and, on the other, Baton Rouge, La. Applicant is authorized to conduct operations in Alabama, Arizona, Arkansas, California, Florida, Georgia, Illinois, Indiana, Iowa, Kansas, Kentucky, Louisiana, Mississippi, Missouri, New Jersey, New Mexico, New York, North Carolina, Ohio, Oklahoma, Pennsylvania, South Carolina, Tennessee and Texas.

HEARING: Remains as assigned November 16, 1959, at the Federal Office Building, 600 South Street, New Orleans, La., before Joint Board No. 165, or, if the Joint Board waives its right to participate, before Examiner Harold P. Boss.

No. MC 113255 (Sub No. 15), CORRECTION, filed June 29, 1959 published in the August 5, 1959 issue of the FEDERAL REGISTER. Applicant: MILK TRANSPORT, INC., P.O. Box 398, New Brighton, Minn. Applicant's attorney: Clay R. Moore, 1100 First National-Soo Line Building, Minneapolis 2, Minn. Authority sought to operate as a *common carrier*, by motor vehicle, over irregular routes, transporting: *Citrus juice*, in bulk in insulated tank vehicles without mechanical refrigeration, from points in Florida to points in Illinois, Wisconsin, Minnesota, and Iowa, and *rejected shipments* of the above-specified commodity on return. Applicant is authorized to conduct operations in Arkansas, Colorado, Florida, Illinois, Louisiana, Massachusetts, Minnesota, Missouri, Nebraska, New Jersey, Ohio, Pennsylvania, New Mexico, New York, Oklahoma, and Texas.

HEARING: Remains as assigned September 21, 1959, at the U.S. Custom House, Room 852, 610 South Canal Street, Chicago, Ill., before Examiner Leo A. Riegel.

No. MC 113524 (Sub. No. 18), filed June 11, 1959. Applicant: JAMES F. BLACK, doing business as PARKVILLE TRUCKING COMPANY, 3618 Pulaski Highway, Baltimore, Md. Applicant's attorney: Dale C. Dillon, 1825 Jefferson Place NW., Washington 6, D.C. Authority sought to operate as a *common carrier*, by motor vehicle, over irregular routes, transporting: *Dry commodities*, in bulk, and *liquid commodities*, other than edible products, in bulk, to be transported in collapsible containers, on van type or flat bed trailers, between Baltimore, Md., on the one hand, and, on the other, points in Delaware, New Jersey, Ohio, points in Virginia on and east of U.S. Highway 1, Batavia, Brockport, Fairport, Leroy and points on Long Island, N.Y., Coatesville, Chester, Marcus Hook, Philadelphia and Warren, Pa., and Washington, D.C. Applicant is authorized to conduct operations in Delaware, Maryland, New Jersey, Pennsylvania, Virginia, the District of Columbia, New York, and Ohio.

HEARING: October 26, 1959, at the Offices of the Interstate Commerce Commission, Washington, D.C., before Examiner Allen F. Borroughs.

No. MC 113832 (Sub No. 13) (CORRECTION), filed July 20, 1959, published issue FEDERAL REGISTER August 19, 1959. Applicant: SCHWERMAN TRUCKING CO., a Corporation, 620 South 29th Street, Milwaukee 46, Wis. Applicant's attorney: James R. Ziperski, 620 South 29th Street, Milwaukee 46, Wis. Authority sought to operate as a *contract carrier*, by motor vehicle, over irregular routes, transporting: *Cement*, in bulk and in packages, (1) from the plant site of Marquette Cement Manufacturing Company, located in or near St. Louis, Mo., to points in Kentucky west of Cumberland, Adair, Green, Larue, Nelson, Bullitt and Jefferson Counties, Ky., points in Arkansas north of Polk, Montgomery, Garland, Hot Spring, Dallas, Cleveland, Drew and Chicot Counties, Ark., and points in Missouri, Illinois, and Indiana; and (2) from the plant site of Marquette Cement Manufacturing Company, located in or near Cape Girardeau, Mo., to points in Illinois south of Mercer, Henry, Bureau, Putnam, LaSalle, Grundy and Will Counties, Ill., points in Arkansas north of Polk, Montgomery, Clark, Dallas, Cleveland, Drew and Chicot Counties, Ark., and points in Missouri, Indiana, Kentucky and Tennessee. Applicant is authorized to conduct operations in Illinois, Indiana and Wisconsin.

NOTE: Applicant indicates the above operations are to be performed under a continuing contract with the Marquette Cement Manufacturing Company, Chicago, Ill.

HEARING: Remains as assigned October 14, 1959, at the U.S. Court House and Custom House, 1114 Market Street, St. Louis, Mo., before Examiner Lacy W. Hinely.

No. MC 115975 (Sub No. 1), filed June 1, 1959. Applicant: C. B. W. TRANSPORT SERVICE, INC., 115 West Lorena Street, Wood River, Ill. Applicant's attorney: Ernest A. Brooks II, 1301 Ambassador Building, St. Louis 1, Mo. Authority sought to operate as a *contract carrier*, by motor vehicle, over irregular routes, transporting: *Oleomargarine, shortening, refined vegetable oils, lard, animal fats, salad dressing, sandwich spread, substitute salad oils*, and *cooking oils*, from St. Louis, Mo., to points in Alabama, Arkansas, Colorado, North Dakota, South Dakota, Georgia, Illinois, Indiana, Iowa, Kansas, Kentucky, Louisiana, Michigan, Minnesota, Missouri, Mississippi, Nebraska, New York, Ohio, Oklahoma, Pennsylvania, Tennessee, Texas, West Virginia, and Wisconsin, and *empty containers or other such incidental facilities* (not specified) used in transporting the above-specified commodities, and *vegetable oils, soybeans, corn, cottonseed, and lard*, on return.

HEARING: October 26, 1959, at the Offices of the Interstate Commerce Commission, Washington, D.C., before Examiner Allan F. Borroughs.

No. MC 117340 (Sub No. 2) (REPUBLICATION) Filed July 13, 1959, published FEDERAL REGISTER issue of August 26, 1959. Applicant: TRIO BUTCHERS TRUCKING CORP., 636 West 131st Street, New York 27, N.Y. Applicant's representative: Charles H. Trayford, 155 East 40th Street, New York 16, N.Y. Authority sought to operate as a *contract carrier*, by motor vehicle, over irregular routes, transporting: *Ice cream*, in insulated vehicles with mechanically refrigerated units, from Elizabeth, N.J., to New York, New Rochelle, Scarsdale, Yorktown Heights, Hewlett, Roslyn, Yonkers, Larchmont, Pelham, Harrison, White Plains, Hartsdale, Mamaroneck, Riverdale, Mount Vernon, Ardsley, Briarcliff Manor, Long Beach, Bronxville, Pelham Manor, Tuckahoe, Cedarhurst, Rye, Spring Valley, Elmsford, Great Neck, Monticello, and Rockville Center, N.Y., Bayonne, Passaic and Hackensack, N.J., and Stamford and Greenwich, Conn., and *rejected, returned or damaged shipments* of Ice cream on return movements. Applicant is authorized to conduct operations in Connecticut, New Jersey, and New York.

NOTE: Any duplication with present authority to be eliminated. The purpose of this republication is to show the correct spelling of applicants name.

HEARING: Remains as assigned October 8, 1959 at the Governor Clinton Hotel, 31st & 7th Ave., New York, N.Y., before Examiner Harry Ross, Jr.

No. MC 118876 (Sub No. 2), filed August 24, 1959. Applicant: HENRY G. GRAVES, CHARLES L. GRAVES AND WM. E. GRAVES, doing business as GRAVES TRANSFER, Georgetown, Del. Applicant's attorney: H. James Company, Jr., Bank of Delaware Building, Wilmington, Del. Authority sought to operate as a *common carrier*, by motor vehicle, over irregular routes, transporting: *Fish oil* and *fish solubles* moving in bulk in tanks, and *fish scrap* and *fish meal* in bulk, from Lewes, Del., to Denton and Hagerstown, Md., Rohrerstown, Allentown and York, Pa., Romney, W. Va., Cartersville, Ga., Akron and Wooster, Ohio, and Lakewood, N.J.

HEARING: October 13, 1959, at the Offices of the Interstate Commerce Commission, Washington, D.C., before Examiner Abraham J. Essrick.

No. MC 119177, filed August 26, 1959. Applicant: ALFOSTER FRANCIS BERRY, 5216 Cloud Place NE., Washington 19, D.C. Authority sought to operate as a *contract carrier*, by motor vehicle, over irregular routes, transporting: (1) *Calf skins*, (green salted), from Washington, D.C. to Wilmington, Del., and refused or rejected shipments of the above-described commodity on return, and (2) *dog food*, from Philadelphia, Pa. to Washington, D.C., and *refused* or *rejected shipments* of dog food, on return.

HEARING: October 9, 1959, at the Offices of the Interstate Commerce Commission, Washington, D.C., before Examiner Leo W. Cunningham.

APPLICATION FOR BROKERAGE LICENSE

MOTOR CARRIER OF PASSENGERS

No. MC 12698 (REPUBLICATION), filed March 13, 1959, published issue FEDERAL REGISTER August 26, 1959. Applicant: CLARENCE E. WIDELL, doing business as VIKING TRAVEL AGENCY, 207 North Broadway, Camden 2, N.J. and 50 Tanner Street, Haddonfield, N.J. Applicant's attorney: Walter S. Anderson, Wilson Building, Broadway at Cooper Street, Camden 2, N.J. The previous publications of the notice of filing in the issues of the FEDERAL REGISTER, dated May 27, 1959 and August 26, 1959, did not clearly describe the proposed operations. At the hearing held June 24, 1959, before Examiner Lucian A. Jackson, the discrepancies in the publication of May 27, 1959 were noted. Correctly stated, and as restrictively amended at the hearing and as now amended, applicant proposes to engage in operations as a broker at Camden and Haddonfield, N.J., in arranging for transportation in interstate or foreign commerce by motor vehicle, of *Passengers and their baggage*, in the same vehicle with passengers, as follows: (1) Groups in all-expense or "package" sightseeing or vacation tours, beginning and ending at points in Camden and Gloucester Counties, N.J., Burlington County, N.J. (except Fort Dix and McGuire Air Base), and Philadelphia County, Pa., and extending to points in the United States, including ports of entry on the International Boundary Lines between the United States and Canada and the United States and Mexico, and (2) individual passengers and their baggage, in the same vehicle with such passengers, in both regular-route and special bus operations by common carrier by motor vehicle.

NOTE: Applicant states that, in respect of the all-expense or "package" sightseeing or vacation tours, he "proposes to assemble persons into groups for excursion and sightseeing trips, to issue all-expense coupons to such passengers, to receive from such passengers their acceptance of an appointment of applicant as agent for the group, collectively, for the purpose of obtaining charter transportation by motor carrier, in accordance with the principle which evolved from the Tauck Tours, Inc. cases."

NOTE: The purpose of this republication is to include the symbol (1) above which was inadvertently omitted, and also to correct the spelling of McGuire Air Base.

CONTINUED HEARING: October 8, 1959, at the Penn Sherwood Hotel, 3900 Chestnut Street, Philadelphia, Pa., before Examiner Herbert L. Hanback.

APPLICATIONS IN WHICH HANDLING WITHOUT ORAL HEARINGS IS REQUESTED

MOTOR CARRIERS OF PROPERTY

No. MC 1942 (Sub No. 5) (AMENDMENT), filed July 13, 1959, published FEDERAL REGISTER of August 5, 1959. Applicant: MARVIN STROBEL AND MERRITT McDONALD, doing business as RICHMOND TRUCK LINE, Richmond, Kans. Authority sought to operate as a *common carrier*, by motor vehicle, over regular routes, transporting: *Feed in bulk*, (1) between Richmond, Kans., and Kansas City, Mo., from Richmond over U.S. Highway 59 to Lawrence, Kans., thence over Kansas Highway 10 to Kansas City, Kans., thence over city streets to Kansas City, Mo., and return over the same route, serving the intermediate and off-route points of North Kansas City, Mo., Kansas City, Kans., and those within 20 miles of Richmond, Kans.; (2)

between junction U.S. Highways 50 and 59, four miles west of Baldwin City, Kans., and junction U.S. Highway 50 and Kansas Highway 10, near Merriam, Kans., over U.S. Highway 50, serving no intermediate points, as an alternate route for operating convenience only, serving the termini for purpose of joinder only.

NOTE: Applicant is authorized to transport General commodities, except those of unusual value, Class A and B explosives, commodities in bulk, and those requiring special equipment, over the above-specified routes. The purpose of this application is to remove the restriction against the transportation of feed in bulk.

No. MC 29591 (Sub No. 1), filed August 27, 1959. Applicant: B & M EXPRESS COMPANY, INCORPORATED, Salem, Mo. Applicant's attorney: Joseph R. Nacy, 117 West High Street, Jefferson City, Mo. Authority sought to operate as a common *carrier*, by motor vehicle, over a regular route, transporting: *General commodities*, including *commodities in bulk*, but excluding articles of unusual value, Class A and B explosives, household goods as defined by the Commission, and commodities requiring special equipment, between the site of the Floyd Bros. Charcoal Co. plant, located approximately ¼ mile north of Howes, Mo., and junction unnumbered county road and Missouri Highway 19 at or near Howes, Mo., over unnumbered county road, serving the intermediate point of the rail siding of St. Louis-San Francisco Railway near Howes, Mo. Applicant is authorized to conduct operations in Illinois and Missouri.

NOTE: Applicant indicates it proposes to tack the above operation to the authority presently held by it in order to render through service between said point and the points now authorized to be served.

No. MC 32430 (Sub No. 3) filed August 31, 1959. Applicant: FERGUSON TRANSFER COMPANY, a Corporation, 320 North Front Street, P.O. Box 337, Coos Bay, Oreg. Applicant's attorney: William B. Adams, Pacific Building, Portland 4, Oreg. Authority sought to operate as a *common carrier*, by motor vehicle, over irregular routes, transporting: *Liquid glues*, in bulk, in tank vehicles, from Coos Bay, Oreg. to Samoa, Calif. and points within 5 miles thereof, and *returned* and *rejected shipments* of liquid glues, on return. Applicant is authorized to conduct operations in Oregon.

No. MC 42405 (Sub No. 12), filed, August 27, 1959. Applicant: MISTLETOE EXPRESS SERVICE, doing business as MISTLETOE EXPRESS, 111 Harrison, Oklahoma City, Okla. Applicant's attorney: Max G. Morgan, 443-54 American National Building, Oklahoma City 2, Okla. Authority sought to operate as a *common carrier*, by motor vehicle over regular routes, transporting: *General commodities*, except Class A and B explosives, moving in express service, between Durant, Okla., and Sherman, Tex., over U.S. Highway 75, serving no intermediate points, but serving Durant as a point of joinder only. Applicant is authorized to conduct operations in Texas and Oklahoma.

No. 177——5

No. MC 66562 (Sub No. 1550), filed August 27, 1959. Applicant: RAILWAY EXPRESS AGENCY, INCORPORATED, 219 East 42d Street, New York 17, N.Y. Applicant's attorney: William H. Marx, 219 East 42d Street, New York 17, N.Y. Authority sought to operate as a *common carrier*, by motor vehicle over regular routes, transporting: *General commodities, including Class A and B explosives*, moving in express service, between Binghamton, N.Y., and Ithaca, N.Y., from Binghamton over New York Highway 17 to the junction of New York Highway 26, thence over New York Highway 26 to the junction of New York Highway 38B, thence over New York Highway 38B to the junction of New York Highway 38, thence over New York Highway 38 to the junction of New York Highway 79, thence over New York Highway 79 to Ithaca, and return over the same route, serving the intermediate points of Newark Valley and Berkshire, N.Y. Applicant is authorized to conduct operations throughout the United States.

No. MC 66562 (Sub No. 1552), filed September 1, 1959. Applicant: RAILWAY EXPRESS AGENCY, INCORPORATED, 219 East 42d Street, New York 17, N.Y. Applicant's attorney: William H. Marx, 219 East 42d Street, New York 17, N.Y. Authority sought to operate as a *common carrier*, by motor vehicle, over regular routes, transporting: *General commodities, including Class A and B explosives*, moving in express service, between Boston, Mass., and Brockton, Mass., from Boston over the Southeast Expressway to Quincy, thence over city streets of Quincy and Braintree to junction of Massachusetts Highway 37, thence over Massachusetts Highway 37 to Brockton, and return over the same route, serving the intermediate point of Quincy. Applicant is authorized to conduct operations throughout the United States.

No. MC 66562 (Sub No. 1553), filed September 1, 1959. Applicant: RAILWAY EXPRESS AGENCY, INCORPORATED, 219 East 42d Street, New York, N.Y. Applicant's attorney: William H. Marx, 219 East 42d Street, New York, N.Y. Authority sought to operate as a *common carrier*, by motor vehicle over regular routes, transporting: *General commodities, including Class A and B explosives*, moving in express service, (1) between Mansfield, Pa., and Troy, Pa., over U.S. Highway 6; (2) between Blossburg, Pa., and Trout Run, Pa., over U.S. Highway 15, serving no intermediate points on the above specified routes. Applicant is authorized to conduct operations throughout the United States.

No. MC 66562 (Sub No. 1554), filed September 1, 1959. Applicant: RAILWAY EXPRESS AGENCY, INCORPORATED, 219 East 42d Street, New York, N.Y. Applicant's attorney: William H. Marx, 219 East 42d Street, New York, N.Y. Authority sought to operate as a *common carrier*, by motor vehicle, over regular routes, transporting: *General commodities, including Class A and B explosives*, moving in express service, (1) between Providence, R.I., and Middleboro, Mass., over U.S. Highway 44, serving the intermediate point of Taunton, Mass.; (2) between Wareham, Mass., and Hyannis, Mass., from Wareham over U.S. Highway 6 to junction Massachusetts Highway 28, thence over Massachusetts Highway 28 to junction Massachusetts Highway 132, thence over Massachusetts Highway 132 to junction unnumbered highway near Hyannis, thence over unnumbered highway to Hyannis, and return over the same route, serving the intermediate or off-route points of West Barnstable and Buzzards Bay, Mass. Applicant is authorized to conduct operations throughout the United States.

No. MC 101915 (Sub No. 2), filed August 26, 1959. Applicant: WILLIAM F. MADDEN, doing business as MADDEN'S TRANSFER & STORAGE, 12 Lake Flower Avenue, Saranac Lake, N.Y. Applicant's attorney: John J. Brady, Jr., 75 State Street, Albany 7, N.Y. Authority sought to operate as a *contract carrier*, by motor vehicle, over irregular routes, transporting: *Fresh meats, packing house products* and *dairy products* as defined in Group A, B, and C of Appendix 1, Descriptions in Motor Carrier Certificates, 61 M.C.C. 209 and 766, from Saranac Lake, N.Y., to points in St. Lawrence County, N.Y., and *rejected and damaged shipments* of the commodities specified in this application on return. Applicant is authorized to conduct contract carrier operations in New York.

NOTE: Applicant holds common carrier authority in Certificates No. MC 94170 and Sub 2. Section 210, dual operations may be involved.

No. MC 107022 (Sub 149), filed August 26, 1959. Applicant: W. M. CHAMBERS TRUCK LINE, INC., 920 Louisiana Blvd.-P.O. Box 547, Kenner, La. Authority sought to operate as a *common carrier*, by motor vehicle, over irregular routes, transporting: *Liquid wax*, in bulk, in tank vehicles, from West Lake Charles, La., to Mobile, Ala., and Pensacola, Fla. Applicant is authorized to conduct operations in Louisiana, Mississippi, Tennessee, Arkansas, Alabama, Kentucky, Florida, Georgia, Texas, North Carolina, South Carolina, Oklahoma, Pennsylvania, Missouri, Connecticut, Illinois, Indiana, Maine, Maryland, Massachusetts, Michigan, Minnesota, New Jersey, New York, Ohio, Kansas, Rhode Island, Virginia, Wisconsin, West Virginia and the District of Columbia.

No. MC 112985 (Sub No. 7), filed August 28, 1959. Applicant: R. E. NEWBROUGH, Wesley, Iowa. Applicant's representative: William A. Landau, 1307 East Walnut Street, Des Moines 16, Iowa. Authority sought to operate as a *common carrier*, by motor vehicle, over irregular routes, transporting: *Malt beverages*, from Milwaukee, Wis., and Omaha, Nebr., to Arnolds Park, Iowa and *empty malt beverage containers* on return. Applicant is authorized to conduct operations in Illinois, Iowa, Minnesota, Nebraska, and Wisconsin.

NOTE: Applicant states that occasionally he will traverse Minnesota in performing service between Milwaukee, Wis., and Arnolds Park, Iowa.

No. MC 114106 (Sub No. 17), filed August 28, 1959. Applicant: MAYBELLE TRANSPORT COMPANY, a Corporation, Box 461, 1820 South Main Street, Lexington, N.C. Applicant's attorney: Dale C. Dillon, 1825 Jefferson Place NW., Washington 6, D.C. Authority sought to operate as a *common carrier*, by motor vehicle, over irregular routes, transporting: *Liquid and invert sugar*, in bulk, in tank vehicles, from Port Wentworth, Ga., to Lexington, N.C. Applicant is authorized to conduct operations in Georgia, North Carolina, South Carolina, Tennessee, and Virginia.

NOTE: Applicant also has contract carrier authority under Permit No. MC 115176, dated May 14, 1956. Dual authority under Section 210 may be involved.

No. MC 114194 (Sub No. 23), filed August 14, 1959. Applicant: KREIDER TRUCK SERVICE, INC., 8003 Collinsville Road, East St. Louis, Ill. Authority sought to operate as a *common carrier*, by motor vehicle, over irregular routes, transporting: *Ethylene dibromide* and *methyl bromide*, ranging from 95 percent ethylene dibromide and 5 percent methyl bromide to 95 percent methyl bromide and 5 percent ethylene dibromide, in bulk in tank vehicles, from Filer City, St. Louis, and Midland, Mich., and Eldorado, Ark., to points in the United States, including ports of entry on the International Boundary line between the United States and Canada and including Alaska and *empty containers or other such incidental facilities* (not specified), used in transporting the above commodities, and *rejected* and *refused shipments* on return. Applicant is authorized to conduct operations in Illinois, Missouri, Tennessee, Indiana, Ohio, Iowa, Virginia, West Virginia, South Carolina, North Carolina, North Dakota, South Dakota, Michigan, Wisconsin, Minnesota, Pennsylvania, Kentucky, Arkansas, the District of Columbia, Georgia, Mississippi, Alabama, Louisiana, Oklahoma, Colorado, Kansas, Texas and Nebraska.

NOTE: Applicant states that the above commodities are Class B poison and an end product, not to be further processed but will be packaged and sold to the general public, and will also be applied directly from bulk containers to grain bins as a fumigant. Applicant further states that based on the Maxwell Case, these commodities are not considered liquid chemicals as the term is used in operating authorities.

No. MC 114194 (Sub No. 24), filed Aug. 28, 1959. Applicant: KREIDER TRUCK SERVICE, INC., 8003 Collinsville Road, East St. Louis, Ill. Authority sought to operate as a *common carrier*, by motor vehicle over irregular routes, transporting: *Lime*, common, hydrated quick, or slaked and *limestone products*, in bulk, from Ste. Genevieve and Mosher, Mo., to points in Madison County, Ill., and *refused and rejected shipments*, on return. Applicant is authorized to conduct operations in Arkansas, Colorado, Illinois, Indiana, Iowa, Kentucky, Kansas, Louisiana, Missouri, Michigan, Minnesota, Nebraska, Ohio, Oklahoma, Pennsylvania, Tennessee, Texas and Wisconsin.

No. MC 116282 (Sub No. 1), filed August 7, 1959. Applicant: ONILE P. FRANCOEUR, doing business as NEIL'S BAKERY PRODUCTS TRANSPORTATION, 246 Broad Street, Auburn, Maine. Applicant's attorney: William D. Pinansky, 403-4-5 Clapp Memorial Building, 443 Congress Street, Portland, Maine. Authority sought to operate as a *contract carrier*, by motor vehicle, over irregular routes, transporting: *Bakery products*, from Boston, Mass., to Augusta, Bangor, Biddeford, Brunswick, Ellsworth, Lewiston, Newport, Portland, Rockland, Rumford, and Waterville, Maine, and from Dover, N.H., to Bangor, Livermore Falls, and Rumford, Maine; and *Containers* used in transporting such commodities on return.

NOTE: The purpose of this application is to obtain authority to enter into contracts with the Continental Baking Company of Rye, N.Y., and with the Berwick Cake Company of Boston, Mass., to transport bakery products from and to the above points. In Permit No. MC 116282 the carrier is specifically limited to three shippers, and this application is filed to obtain this additional authority, although it does not request any additional territory.

MOTOR CARRIERS OF PASSENGERS

No. MC 1501 (Sub No. 169), filed August 25, 1959. Applicant: THE GREYHOUND CORPORATION, 5600 Jarvis Avenue, Chicago 48, Ill. Applicant's attorney: Earl A. Bagby, Western Greyhound Lines (Division of The Greyhound Corporation), Greyhound Building, Market and Fremont Streets, San Francisco 5, Calif. Authority sought to operate as a *common carrier*, by motor vehicle, over regular routes, transporting: *Passengers and their baggage*, and *express* and *newspapers* in the same vehicle with passengers, serving all intermediate points. Interstate routes within the State of California: On Proposed Revised Sheet No. 17. Route 71. Between the Nevada-California State line at Cal-Nava and Tahoe City: From the point where California Highway 28 contacts the Nevada-California State line, over California Highway 28 to junction California Highway 89 (Tahoe City). (Connects with Nevada route 3.) Service is authorized to be conducted in special operations only, except that regular service is authorized to be conducted on occasions of and for the purpose of affording transportation to and from advertised special events to be held in the Squaw Valley area to which the general public is invited. Interstate Routes within the State of Nevada. On Proposed Revised Sheet No. 56. Route 3. Between Reno Hot Springs Junction and Cal-Neva: From the junction of U.S. Highway 395 and Nevada Highway 27 (Reno Hot Springs Junction), over Nevada Highway 27 to junction Nevada Highway 28 (Incline), thence over Nevada Highway 28 to the Nevada-California State line (Cal-Neva). (Connects with California route 71.) Service is authorized to be conducted in special operations only, except that regular service is authorized to be conducted between Incline and Cal-Neva on occasions of and for the purpose of affording transportation to and from advertised special events to be held in the Squaw Valley, Calif., area to which the general public is invited. On Proposed Revised Sheet No. 57. Route 5. Between Incline and Spooners Station: From the junction of Nevada Highway 27 and Nevada Highway 28 (Incline), over Nevada Highway 28 to junction U.S. Highway 50 (Spooners Station). Service is authorized to be conducted in special operations only, except that regular service is authorized to be conducted on occasions of and for the purpose of affording transportation to and from advertised special events to be held in the Squaw Valley, Calif., area to which the general public is invited. The carrier is presently authorized to operate over the above-specified regular routes and no new route or change in route authorized is requested, but it is proposed that the special conditions presently applicable to each of such routes reading: "Service is authorized to be conducted in special operations only" be changed to the special condition shown after each of the above numbered routes. Applicant is authorized to conduct operations throughout the United States.

No. MC 1504 (Sub No. 147), filed August 24, 1959. Applicant: ATLANTIC GREYHOUND CORPORATION, 1100 Kanawha Valley Building, Charleston, W. Va. Applicant's attorney: Raymond H. Warns, Commerce Counsel, The Greyhound Corporation, 5600 Jarvis Avenue, Chicago 48, Ill. Authority sought to operate as a *common carrier*, by motor vehicle, over a regular route, transporting: *Passengers and their baggage, and express, newspapers and mail*, in the same vehicle with passengers, between junction of Interstate Highway 95 and U.S. Highway 1, located approximately one mile south of Petersburg, Va., and Richmond, Va., from the junction of Interstate Highway 95 and U.S. Highway 1 over Interstate Highway 95 to Richmond, and return over the same route, serving all intermediate points. Applicant states it proposes to join or tack this authority, if granted, with its present authority at the junction of Interstate Highway 95 and U.S. Highway 1, Richmond, Va., and at all points intermediate to the termini of the proposed route. Applicant is authorized to conduct operations in Georgia, North Carolina, South Carolina, Tennessee, Ohio, Virginia, West Virginia, and the District of Columbia.

No. MC 39416 (Sub No. 3), filed August 26, 1959. Applicant: THE GRAY LINE COMPANY, an Oregon corporation, 628 NW. Sixth Avenue, Portland, Oreg. Applicant's attorney: William B. Adams, Pacific Building, Portland 4, Oreg. Authority sought to operate as a *common carrier*, by motor vehicle, over irregular routes, transporting: *Passengers and their baggage*, in seasonal operations between June 1 and September 15th of each year, in special operations, sightseeing and pleasure tours, between points in Multnomah and Hood River Counties, Oreg., and points in Clark and Skamania Counties, Wash. Applicant is authorized to conduct operations in Oregon.

NOTE: Applicant states that the total service proposed will in every instance be on a round-trip basis, normally one segment thereof will be a boat trip on the Columbia River. The two vehicles will be coordinated

so that one group of passengers will leave Portland, Oreg. (in every instance the origin and destination) by boat and return by bus, and a second group will leave by bus and return on the boat. On rare occasions an individual may use motor carrier service for the round trip.

APPLICATIONS UNDER SECTIONS 5 AND 210a(b)

The following applications are governed by the Interstate Commerce Commission's special rules governing notice of filing of applications by motor carrier of property or passengers under Section 5(a) and 210a(b) of the Interstate Commerce Act and certain other proceedings with respect thereto. (49 CFR 1.240).

MOTOR CARRIERS OF PROPERTY

No. MC-F 7300. Authority sought for purchase by WHITESCARVER TRANSPORTATION CORP., 46 Oakwood Avenue, Orange, N.J., of the operating rights of TUOHY TRUCKING CORPORATION, 733 State Highway 17, Carlstadt, N.J., and for acquisition by FRANKLIN WHITESCARVER, JR., and PAUL S. WHITESCARVER, SR., all of Orange, of control of such rights through the purchase. Applicants' attorney: Bowes & Millner, 1060 Broad Street, Newark 2, N.J. Operating rights sought to be transferred: *Groceries* and *such merchandise and fixtures* as are used in the operation of chain grocery stores, under special and individual contracts or agreements with persons (as defined in section 203(a) of the Interstate Commerce Act) who operate retail stores, the business of which is the sale of food, as a *contract carrier* over irregular routes, between New York, N.Y., on the one hand, and, on the other, points in Fairfield County, Conn., Orange, Rockland, and Westchester Counties, N.Y., and Bergen and Passaic Counties, N.J.; *groceries and such merchandise and fixtures* as are used in the operation of chain grocery stores, under special and individual contracts or agreements between New York, N.Y., on the one hand, and, on the other, points in Morris, Essex, and Sussex Counties, N.J., and between New York, N.Y., on the one hand, and, on the other, points in Pike County, Pa.; *such merchandise* as is dealt in by wholesale, retail, and chain grocery and food business houses, and in connection therewith, *equipment, materials, and supplies* used in the conduct of such business, under special and individual contracts or agreements with persons (as defined in section 203(a) of the Interstate Commerce Act who operate retail stores, the business of which is the sale of such commodities, between New York, N.Y., on the one hand, and, on the other, points in Warren and Union Counties, N.J., and Sullivan and Ulster Counties, N.Y.; *such merchandise* as is dealt in by wholesale, retail, and chain grocery and food business houses, under individual contracts or agreements with persons (as defined in section 203(a) of the Interstate Commerce Act) who operate wholesale, retail, and chain stores, the business of which is the sale of food, between Carlstadt, N.J., on the one hand, and, on the other, certain points in New York, Connecticut and Pennsylvania, and from Carlstadt, N.J., and New York, N.Y., to points in New Haven and Hartford Counties, Conn.; *soaps, soap powders, soap products, shortenings, edible oils, toilet preparations, glycerine, washing compounds,* and *cleansing compounds,* in containers, and *advertising matter* and *premiums* in connection therewith, from Port Ivory, N.Y., to points in Westchester County, N.Y.; *such commodities* as are dealt in by wholesale, retail, and chain grocery and food business houses, under individual contracts or agreements with persons (as defined in section 203(a) of the Interstate Commerce Act) who operate wholesale or retail stores, the business of which is the sale and distribution of food, between New York, N.Y., on the one hand, and, on the other, points in Middlesex, Monmouth, and Somerset Counties, N.J.; *such merchandise* as is dealt in by wholesale, retail, and chain grocery and food business houses, limited to a transportation service to be performed under a continuing contract, or contracts, with the Grand Union Company, East Paterson, N.J., from New York, N.Y., to points in Burlington and Ocean Counties, N.J., and from Mt. Kisco, N.Y., to certain points in New Jersey, Pennsylvania and New York. Vendee has applied for temporary authority, in Docket No. MC 118956 Sub 1TA, to operate as a *contract carrier* in New Jersey, New York and Pennsylvania. Application has not been filed for temporary authority under section 210a(b).

NOTE: A motion to dismiss the Section 5 application has been filed simultaneously.

No. MC-F 7302. Authority sought for purchase by NATIONAL TRANSPORTATION COMPANY, 251 State Street Extension, Bridgeport, Conn., of the operating rights and property of LEON E. TAYLOR, doing business as TAYLOR TRUCKING COMPANY, 247 Summit Street, Norwich, Conn., and for acquisition by DAVID C. GOLD, RAYMOND PULVER and THEODORE KRAMER, all of Bridgeport, of control of such rights and property through the purchase. Applicants' attorney, Joseloff, Murrett & Ahearn, 410 Asylum Street, Hartford 3, Conn. Operating rights sought to be transferred: *General commodities,* excepting, among others, household goods and commodities in bulk, as a *common carrier* over regular routes between New London, Conn., and Providence, R.I., serving certain intermediate and off-route points. Vendee is authorized to operate as a *common carrier* in Massachusetts, Maryland, Connecticut, New York, Pennsylvania, New Jersey, and Delaware. Application has been filed for temporary authority under section 210a(b).

No. MC-F 7303. Authority sought for control and merger by BRADA CARTAGE COMPANY, 4001 Central Avenue, Detroit 10, Mich., of the operating rights and property of MILLER TRANSPORTATION, INC., 1200 South Home Street, Kokomo, Ind., and for acquisition by C. RUSSELL WAGSTAFF, also of Detroit, of control of such rights and property through the transaction. Applicants' attorneys: Axelrod, Goodman & Steiner, 39 South LaSalle Street, Chicago 3, Ill., and Howell Ellis, 520 Illinois Building, Indianapolis, Ind. Operating rights sought to be controlled and merged: *General commodities,* except articles of unusual value, and except dangerous explosives, household goods as defined by the Commission, and liquid commodities in bulk, as a *common carrier* over irregular routes, between Kokomo, Ind., and points within 50 miles of Kokomo, on the one hand, and, on the other, Louisville, Ky., St. Louis, Mo., points in Ohio and Illinois, and certain points in Michigan; *household goods,* as defined by the Commission, between Kokomo, Ind., on the one hand, and, on the other, certain points in Illinois and Michigan; *ferro alloy (stellite), stellite dies, iron and steel products, power pumps, measuring pumps and parts, stellite scrap, chromium and carbon electrodes (graphite), sugar, fertilizer, dry chemicals in bags, liquid chemicals in carboys, farm machinery and farm implements and parts, beans, sugar, malt beverages,* and *composition building slabs,* loose or individually packaged, from, to or between points and areas, varying with the commodity transported, in Indiana, Illinois, Ohio, Missouri, Kentucky, Michigan, Pennsylvania, New York, and Wisconsin. RESTRICTIONS: (1) The authority granted in Certificate No. MC 78712 is subject to the conditions that such operations shall be conducted separately from said carrier's other activities, that a separate accounting system therefor shall be maintained, and that said carrier shall not transport both as a public and private carrier at the same time in the same vehicle; and (2) the authority granted in Certificate No. MC 78712 Sub 4 in restricted against service at points any part of which is traversed by the indicated portions of the highways referred to in said certificate, and also against service at Detroit, Mich., and points in the Detroit, Mich., Commercial Zone, as defined by the Commission. BRADA CARTAGE COMPANY is authorized to operate as a *common carrier* in Michigan, Ohio, Indiana, Illinois and Kentucky. Application has been filed for temporary authority under section 210a(b).

No. MC-F 7304. Authority sought for purchase by COLONIAL REFRIGERATED TRANSPORTATION, INC. (ALABAMA CORPORATION), 1215 Bankhead Highway, Birmingham, Ala., of the operating rights and property of COLONIAL REFRIGERATED TRANSPORTATION, INC. (DELAWARE CORPORATION), 1215 Bankhead Highway West, P.O. Box 2169, Birmingham, Ala., and for acquisition by C. E. McBRIDE, also of Birmingham, of control of such rights and property through the purchase. Applicants' attorney: Maurice F. Bishop, 327 Frank Nelson Building, Birmingham 3, Ala. Operating rights sought to be transferred: *Frozen fish, fresh fish,* including shellfish and crabmeat, *frozen fruits and vegetables, fresh and frozen poultry, frozen foods, dairy products,* as defined by the Commission, *cheese, frozen dough, frozen pastries, fresh meats, meats, meat products, and meat byproducts,* as defined by the Com-

mission, *fresh fruits and vegetables, grape juice, jams, jellies, preserves, tomato juice, chocolate, chocolate confectionery and the ingredients thereof, prepared foods, bakery products, dessert topping, bakery goods topping, canned goods, frozen fruit juices, articles distributed by meatpacking houses, foodstuffs, confectionery, grape products, frozen vegetable juices,* and *cooked or cured meats,* as a *common carrier* over irregular routes, from, to or between points and areas, varying with the commodity transported, in Maine, Massachusetts, Rhode Island, Alabama, Florida, Georgia, Louisiana, Mississippi, North Carolina, South Carolina, Tennessee, Connecticut, Illinois, Indiana, Kentucky, Maryland, Michigan, New Jersey, New York, Ohio, Pennsylvania, Virginia, West Virginia, Wisconsin, the District of Columbia, Delaware, Arkansas, Oklahoma, Texas, Missouri, New Hampshire, Vermont, California, Oregon, and Washington. Vendee holds no authority from this Commission. However, its controlling stockholder also owns the controlling stock in (1) vendor, a newly-formed corporation, (2) COLONIAL & PACIFIC FRIGIDWAYS, INC., and (3) COLONIAL FAST FREIGHT LINES, INC., which are authorized to operate as *common carriers* in (2) Illinois, Iowa, Wisconsin, California, Washington, Oregon, Tennessee, Alabama, Nebraska, Minnesota, Indiana, Missouri, Kansas, Arizona, Idaho, Utah, Arkansas, Michigan, Pennsylvania, New York, Massachusetts, Connecticut, Rhode Island, and the District of Columbia, and (3) Alabama, Florida, Georgia, Tennessee, Mississippi, and Louisiana, respectively. Application has not been filed for temporary authority under section 210a(b).

No. MC-F 7305. Authority sought for control by WOODS INDUSTRIES, INC., 4900 North Santa Fe, Oklahoma City, Okla., of AUTO TRANSPORTS, INC., UNITED TRANSPORTS, INC., TEXAS AUTO TRANSPORTS, INC., AUTO WAREHOUSES, INC., TEXAS AUTO WAREHOUSES, INC., OKLAHOMA CITY TERMINALS, INC., and WOODS PETROLEUM CORPORATION, all of 4900 North Santa Fe, Oklahoma City, Okla., and for acquistion by ROY G. WOODS, also of Oklahoma City, of control of AUTO TRANSPORTS, INC., UNITED TRANSPORTS, INC., TEXAS AUTO TRANSPORTS, INC., AUTO WAREHOUSES, INC., TEXAS AUTO WAREHOUSES, INC., OKLAHOMA CITY TERMINALS, INC., and WOODS PETROLEUM CORPORATION through the acquisition by WOODS INDUSTRIES, INC. Applicant's attorney: James W. Wrape, Sterick Building, Memphis, Tenn. Operating rights sought to be controlled: (AUTO TRANSPORTS, INC.) *New motor vehicles, vehicle cabs and bodies,* and *automobile show equipment and paraphernalia,* when transported with display vehicles, by driveaway and truckaway methods in initial movements, as a *contract carrier* over irregular routes, from points in Wyandotte County, Kans., to points in Arkansas, Colorado, Illinois, Iowa, Louisiana, Minnesota, Missouri, Montana, Nebraska, New Mexico, Oklahoma, South Dakota, Texas, Utah, and Wyoming; *the above-described commodities,* in secondary movements, between points in Arkansas, Colorado, Illinois, Iowa, Louisiana, Minnesota, Missouri, Montana, Nebraska, New Mexico, Oklahoma, South Dakota, Texas, Utah, Wyoming, and Kansas; *motor vehicles,* in initial movements, by driveaway and truckaway methods, and *vehicle cabs and bodies,* and *automobile show equipment and paraphernalia,* from points in Wyandotte County, Kans., to points in North Dakota, Wisconsin, and Indiana; (UNITED TRANSPORTS, INC.) (A) *new automobiles, new trucks, new bodies, new cabs, new chassis,* and *parts thereof,* in initial movements, in driveaway and truckaway service, as a *common carrier* over irregular routes, from places of manufacture and assembly in St. Louis and Kansas City, Mo., to points in Oklahoma and Texas; (B) *new buses, new chassis,* and *parts thereof,* in driveaway service, from Richmond, Ind., to points in Oklahoma, *new trucks,* and *new chassis,* in initial movements, in driveaway service, from Springfield, Ohio, to Joplin, Mo., and points in Oklahoma, with the right to stop shipments in transit for installation of bodies or parts, on shipments from Springfield to points in Oklahoma, and *new trucks, new bodies, new cabs, new chassis,* and *parts thereof,* in initial movements, in truckaway service, from Springfield, Ohio, to Joplin, Mo.; (C) *automobiles, trucks, chassis,* and *automobile parts,* new, used, or unfinished, in secondary movements, in truckaway service, from St. Louis, Mo., to certain points in Missouri and Kansas; (D) *new trucks, new commercial cars, new chassis,* and *parts thereof,* in initial movements, in driveaway service, from places of manufacture and assembly in Fort Wayne, Ind., to points in Oklahoma, with the right to stop shipments in transit for the installation of bodies or parts; (E) *automobiles, trucks, bodies, cabs, chassis,* and *parts thereof,* new, used, unfinished, and/or wrecked, in secondary movements, in driveaway and truckaway service, between St. Louis, Kansas City, and Joplin, Mo., and points in Texas, Oklahoma, and that part of Kansas on and south of U.S. Highway 66; in addition to the authority described in paragraphs (A), (B), (C), (D), and (E), the carrier is authorized to perform through service (1) under a combination of the initial authority described in paragraph (B) and the secondary authority described in paragraph (E), provided the carrier is authorized in paragraph (B) to transport the shipment to a gateway point from which it is authorized to transport such shipments in paragraph (E), and provided that in each instance the movement is made through such authorized gateway point and (2) under a combination of the secondary authorities described in paragraphs (B), (C), and (E) when under one of such paragraphs the carrier is authorized to transport the shipment to a gateway point from which the shipment is authorized to be transported under the other of such paragraphs and provided in each instance the movement is made through such authorized gateway point; *new vehicles or chassis* that have been rejected by a dealer or distributor, from points in Oklahoma to Fort Wayne, Ind., and Springfield, Ohio; *automobiles, trucks, tractors, chassis, cabs, bodies,* and *parts therefor,* restricted to secondary movements, in truckaway or driveaway service, from St. Louis, Mo., to points in New Mexico and certain points in Kansas, and between points in Kansas, on the one hand, and, on the other, points in Missouri, Oklahoma, and Texas; *automobiles, trucks, tractors, chassis, cabs, bodies,* and *parts therefor,* restricted to initial movements, in driveaway service, from points of manufacture and assembly in Fort Wayne, Ind., and Springfield, Ohio, to points in Arizona; *automobiles, trucks, tractors, trailers, chassis, bodies, cabs,* and *parts thereof (when accompanying the vehicle for which they are intended)* in truckaway service, from Memphis, Tenn., to points in Texas, restricted to shipments which have had a prior or which will have a subsequent movement by barge; *automobiles, trucks, tractors, cabs, chassis, bodies,* and *parts thereof* (when accompanying the vehicle for which they are intended), and *automobile show equipment* (when accompanying the motor vehicle), by the driveaway and truckaway methods, restricted to secondary or subsequent movements, between points in Missouri, on the one hand, and, on the other, points in Arizona, New Mexico, Oklahoma, and Texas, between points in Arizona, on the one hand, and, on the other, points in New Mexico, Oklahoma, and Texas, between points in New Mexico, on the one hand, and, on the other, points in Oklahoma and Texas, and from Memphis, Tenn., to points in Arizona, New Mexico, Oklahoma, and Texas, where the shipment has had a prior movement by water carrier; *trailers,* other than those designed to be drawn by passenger automobile, in truckaway service, in secondary movements, from Memphis, Tenn., to points restricted to an immediately prior movement by water carrier; in Arizona, Oklahoma, New Mexico, and Texas, *automobiles, trucks, tractors,* and *chassis,* in secondary movements, by the driveaway and *truckaway methods, and cabs, bodies,* and *automobile show equipment,* from Memphis, Tenn., to points in Arizona, New Mexico, Oklahoma, and Texas; *motor vehicles* (other than automobiles and trailers) and *chassis,* in initial movements, in driveaway service, from Fort Wayne, Ind., and Springfield, Ohio, to points in Kansas, Oklahoma, Texas, Arizona, and New Mexico; *motor vehicles* (other than automobiles and trailers) and *chassis,* in secondary movements, in driveaway service, between points in Kansas, Oklahoma, Texas, Arizona, and New Mexico, restricted to the transportation of vehicles which have previously been transported in initial movements from Bridgeport, Conn., Fort Wayne, Ind., or Springfield, Ohio; *parts of, or accessories for, such vehicles as are described above,* when transported with vehicles moving under the above-described authority, from Fort Wayne, Ind., and Springfield, Ohio, to points in

Kansas, Oklahoma, Texas, Arizona, and New Mexico, and between points in Kansas, Oklahoma, Texas, Arizona, and New Mexico; *new automobiles*, in initial movements, in truckaway service, from the site of the Cadillac Motor Car Division of General Motor Corporation at Detroit, Mich., to points in Kansas, Missouri, Oklahoma, and Texas; *new automobiles, new trucks*, and *new assembled and partially assembled automobile chassis*, in initial movements, in truckaway and driveaway service, from Evansville, Ind., to San Antonio, San Marcos, Austin, Laredo, and Sequin, Tex.; *new automobiles, new trucks*, and *new assembled and partially assembled automobile chassis*, in initial movements, in driveaway service, from Detroit, Mich., to San Antonio, San Marcos, Austin, Laredo, and Sequin, Tex.; *new automobiles, new trucks*, and *new assembled and partially assembled automobile chassis*, in initial movements, in truckaway service, from Detroit, Mich., and Willow Run in Washtenaw County Mich., to certain points in Texas; *new automobiles, new trucks*, and *new assembled and partially assembled automobile chassis*, in secondary movements, in driveaway and truckaway service, from Memphis, Tenn., and Houston, Tex., and points within five miles of each, to certain points in Texas; the initial-movement authority specified in No. MC 71902 Sub 60 is restricted against tacking with carrier's otherwise authorized secondary movement authority so as to prohibit the transportation of traffic moving from Detroit, Mich., and Evansville, Ind., to points in Missouri, Kansas, Oklahoma, Arizona, and New Mexico (TEXAS AUTO TRANSPORTS, INC.); *motor vehicles, vehicle cabs and bodies*, and *automobile show equipment and paraphernalia*, in initial movements, by driveaway and truckaway methods, as a *contract carrier* over irregular routes, from Arlington, Tex., to points in Texas, Louisiana, Oklahoma, New Mexico, Arizona, Utah, Colorado, Kansas, Arkansas, Wyoming, Nebraska, and Missouri, and to Memphis, Tenn. WOODS INDUSTRIES, INC., holds no authority from this Commission. However, its controlling stockholder is affiliated with (1) UNITED TRANSPORTS, INC., (2) AUTO TRANSPORTS, INC., and (3) TEXAS AUTO TRANSPORTS, INC., which are authorized to operate as (1) *common carrier*, (2) and (3) *contract carriers*. Application has not been filed for temporary authority under section 210a(b).

MOTOR CARRIERS OF PASSENGERS

No. MC-F 7301. Authority sought for control by FERGUSON-BROADWAY BUS LINES, INC., 10100 West Florissant Avenue, Dellwood 36, Mo., of BROWN MOTOR LINES, INC., 225 Wilson Avenue, Alton, Ill., and for acquisition by ROY E. KRUPP, 1310 Highmount, Ferguson 35, Mo., CHARLES CALDWELL, 222 Wesley Avenue, Ferguson 35, Mo., BERT CALDWELL, 3023 West Main, Belleville, Ill., CARL BEHLE, 215 Dellwood Avenue, St. Louis 35, Mo., and WALTER FRANKE, 214 North Floridale, St. Louis 36, Mo., of control of BROWN MOTOR LINES, INC., through the acquisition by FERGUSON-BROADWAY BUS LINES, INC. Applicant's attorney: Joseph R. Nacy, 117 West High Street, Jefferson City, Mo. Operating rights sought to be controlled: *Passengers and their baggage*, and *express* and *newspapers*, in the same vehicle with passengers, as a *common carrier* over regular routes, between Alton, Ill., and St. Louis, Mo., serving all intermediate points; *passengers and their baggage*, between Alton, Ill., and St. Louis, Mo., serving all intermediate points on Illinois Highway 111. FERGUSON-BROADWAY BUS LINES, INC., is authorized to operate as a *common carrier* in Missouri and Illinois. Application has been filed for temporary authority under section 210a(b).

By the Commission.

[SEAL] HAROLD D. McCOY, *Secretary*.

[F.R. Doc. 59-7515; Filed, Sept. 9, 1959; 8:48 a.m.]

CUMULATIVE CODIFICATION GUIDE—SEPTEMBER

A numerical list of the parts of the Code of Federal Regulations affected by documents published to date during September. Proposed rules, as opposed to final actions, are identified as such.

3 CFR Page

Executive orders:

July 2, 1910 7251
5904 7143
9173 7253
10530 7269
10836 7269

5 CFR

6 .... 7087, 7127, 7162, 7272, 7273

6 CFR

331 7089
421 .... 7090, 7092, 7237, 7239
483 .... 7239, 7241
484 7092

7 CFR

48 7127
301 7242
722 .... 7055, 7058
723 7243
725 7243
811 7269
903 7198
905 7198
906 7198
907 7198
908 7198
911 7198
912 7198
913 7198
916 7198
917 7198
918 7198
919 7198
921 7198

7 CFR—Continued Page

922 7198
923 7198
924 7198
925 7198
928 7198
929 7198
930 7198
931 7198
932 7198
933 .... 7199, 7243
935 7198
941 7198
942 7198
943 7198
944 7198
946 7198
948 7198
949 7198
952 7198
953 .... 7059, 7199, 7272
954 7198
956 7198
959 7272
965 7198
966 7198
967 7198
968 .... 7059, 7198
971 7198
972 7198
974 7198
975 7198
976 7198
977 7198
978 7198
980 7198

7 CFR—Continued Page

983 7198
985 7198
986 7198
987 7198
988 7198
991 7198
994 7198
995 7198
998 7198
1000 7198
1002 7198
1004 7198
1005 7198
1008 7198
1009 7198
1011 7198
1012 7198
1013 7198
1014 7198
1016 7198
1018 7198
1023 7198

Proposed rules:

319 7107
351 7108
909 7107
924 7163
958 7107
963 7207
970 7278
1024 7138
1066 7279

9 CFR

73 7244
74 7244

12 CFR
Page

217 7062
219 7062
224 7062
329 7062

13 CFR

128 7063

14 CFR

1 7065
3 7065
4b 7067
6 7072
7 7074
13 7076
40 7253
60 .... 7129, 7253
225 7155
241 7253
298 7155
507 .... 7254, 7255
600 7130–7132
601 7130–7132
608 7273
609 .... 7157, 7158, 7255

Proposed rules:
1 7204
3 7204
4b 7204
5 7204
6 7204
7 7204
8 7204
9 7204
10 7204
13 7204
14 7204
60 7164
221 7163
507 7164
514 7165
600 .... 7081, 7083, 7138, 7165–7167
601 .... 7082, 7083, 7165–7167, 7205, 7206
602 7206

15 CFR

361 7087
371 7200
373 7200
374 7200
379 7200
382 7200
385 7200
399 7201

16 CFR

13 .... 7059–7061, 7098, 7099, 7133, 7273, 7274
1—310 7083

17 CFR

Proposed rules:
230 7259

21 CFR
Page

120 7102
141b 7258
146 7103
146a 7258
146b 7258

Proposed rules:
53 7110
120 7167
121 7168

25 CFR

121 7100

Proposed rules:
221 7163

26 (1954) CFR

Proposed rules:
1 7103

27 CFR

5 7078

30 CFR

Proposed rules:
33 7135

32 CFR

45 7134
83 7134
146 7134
148 7134
211 7134
836 7162
1621 7078

38 CFR

3 7259

39 CFR

22 7162
111 7245
112 7245
122 7245
131 7245
132 7245
161 7245
162 7245
168 7249

42 CFR

21 7274

43 CFR

Public land orders:
82 7200
486 7252
657 7253
1061 7251
1071 7252
1621 7200
1654 7252
1964 7079
1965 7200
1966 7251
1967 7251
1968 7251
1969 7251

43 CFR—Continued
Page

Public land orders—Continued
1970 7252
1971 7252
1972 7252
1973 7253

44 CFR

Proposed rules:
401 7280

46 CFR

12 7177
30 7179
32 7179
33 7179
34 7179
35 7181
43 7181
44 7182
45 7182
51 7183
52 7184
54 7185
55 7187
56 7188
57 7190
61 7190
71 7190
76 7191
91 7191
95 7191
110 7192
111 7192
112 7194
113 7194
161 7195
167 7196
172 7197
176 7197
181 7197
183 7197
308 7193

Proposed rules:
201—360 7138

47 CFR

3 .... 7162, 7274–7276
19 7276

Proposed rules:
3 .... 7110, 7259, 7280
7 7110
8 7110
9 7110
10 7110
11 7110
16 7110
19 7110

49 CFR

95 7103

50 CFR

6 .... 7079, 7277

Proposed rules:
31 .... 7105, 7203, 7204, 7277
32 .... 7106, 7204
34 .... 7106, 7277

PDR-016

UNITED STATES
NUCLEAR REGULATORY COMMISSION
WASHINGTON, D. C. 20555

August 18, 1982

Mr. John Sullivan
33 Chartley Brook Lane
Attleboro, Massachusetts 02703

IN RESPONSE REFER
TO FOIA-82-349

Dear Mr. Sullivan:

This is in response to your letter dated July 28, 1982 in which you requested, pursuant to the Freedom of Information Act, documents regarding California Salvage and U.S. Nuclear Corporation; WASH-734 and a microfiche copy of AEC - TR - 7308.

As explained during an August 11, 1982 telephone conversation with Mr. Stephen Isaacs of my staff, California Salvage of San Pedro, CA did have the authorization to dispose of radioactive waste in the ocean but did not exercise it. U.S. Nuclear Corporation of Burbank, CA was never authorized to dispose of radioactive waste in the ocean. As per that conversation, neither docket was reproduced.

A paper copy of WASH-734, "U.S. Sea Disposal Operations" and a microfiche copy of AEC - TR - 7308, "A Study of Artificial Radioactivity of the Atmosphere and Especially the Clouds" are enclosed.

This completes action on your request.

Sincerely,

J. M. Felton, Director
Division of Rules and Records
Office of Administration

8209150108 820818
PDR FOIA
SULLIVA82-349 PDR

OCEAN DUMPING OF WASTE MATERIALS

HEARINGS

BEFORE THE

SUBCOMMITTEE ON FISHERIES AND WILDLIFE CONSERVATION

AND THE

SUBCOMMITTEE ON OCEANOGRAPHY

OF THE

COMMITTEE ON MERCHANT MARINE AND FISHERIES HOUSE OF REPRESENTATIVES

NINETY-SECOND CONGRESS

FIRST SESSION

ON

H.R. 285, H.R. 336, H.R. 337, H.R. 548, H.R. 549, H.R. 805, H.R. 807, H.R. 808, H.R. 983, H.R. 1095, H.R. 1329, H.R. 1381, H.R. 1382, H.R. 1383, H.R. 1661, H.R. 1674, H.R. 2581, H.R. 3662, H.R. 4217, H.R. 4218, H.R. 4247, H.R. 4359, H.R. 4360, H.R. 4361, H.R. 4584, H.R. 4719, H.R. 4723, H.R. 5049, H.R. 5050, H.R. 5239, H.R. 5268, H.R. 5477, H.R. 5705, H.R. 6305, H.R. 6582, H.R. 6610, H.R. 6771, H.R. 7619, and H.R. 8039

APRIL 5, 6, 7, 1971

Serial No. 92–2

Printed for the use of the Committee on Merchant Marine and Fisheries

U.S. GOVERNMENT PRINTING OFFICE

62-513 O WASHINGTON : 1971

COMMITTEE ON MERCHANT MARINE AND FISHERIES

EDWARD A. GARMATZ, Maryland, *Chairman*

LEONOR K. (MRS. JOHN B.) SULLIVAN, Missouri
FRANK M. CLARK, Pennsylvania
THOMAS L. ASHLEY, Ohio
JOHN D. DINGELL, Michigan
ALTON LENNON, North Carolina
THOMAS N. DOWNING, Virginia
JAMES A. BYRNE, Pennsylvania
PAUL G. ROGERS, Florida
FRANK A. STUBBLEFIELD, Kentucky
JOHN M. MURPHY, New York
JOSEPH E. KARTH, Minnesota
WALTER B. JONES, North Carolina
ROBERT L. LEGGETT, California
SPEEDY O. LONG, Louisiana
MARIO BIAGGI, New York
CHARLES H. GRIFFIN, Mississippi
GLENN M. ANDERSON, California
ELIGIO DE LA GARZA, Texas
PETER N. KYROS, Maine
ROBERT O. TIERNAN, Rhode Island
JAMES V. STANTON, Ohio

THOMAS M. PELLY, Washington
WILLIAM S. MAILLIARD, California
CHARLES A. MOSHER, Ohio
JAMES R. GROVER, Jr., New York
HASTINGS KEITH, Massachusetts
PHILIP E. RUPPE, Michigan
GEORGE A. GOODLING, Pennsylvania
WILLIAM G. BRAY, Indiana
PAUL N. McCLOSKEY, Jr., California
JACK H. McDONALD, Michigan
M. G. (GENE) SNYDER, Kentucky
ROBERT H. STEELE, Connecticut
EDWIN B. FORSYTHE, New Jersey
PIERRE S. (PETE) DUPONT, Delaware

RALPH E. CASEY, *Chief Counsel*
ROBERT J. McELROY, *Chief Clerk*
NED P. EVERETT, *Counsel*
FRANCIS D. HEYWARD, *Counsel*
RICHARD N. SHAROOD, *Minority Counsel*

SUBCOMMITTEE ON FISHERIES AND WILDLIFE CONSERVATION

JOHN D. DINGELL, Michigan, *Chairman*

ALTON LENNON, North Carolina
THOMAS N. DOWNING, Virginia
JOSEPH E. KARTH, Minnesota
PAUL G. ROGERS, Florida
ROBERT L. LEGGETT, California
SPEEDY O. LONG, Louisiana
MARIO BIAGGI, New York
GLENN M. ANDERSON, California
ELIGIO DE LA GARZA, Texas
PETER N. KYROS, Maine
ROBERT O. TIERNAN, Rhode Island

THOMAS M. PELLY, Washington
HASTINGS KEITH, Massachusetts
GEORGE A. GOODLING, Pennsylvania
PAUL N. McCLOSKEY, Jr., California
JACK H. McDONALD, Michigan
WILLIAM S. MAILLIARD, California
PHILIP E. RUPPE, Michigan
EDWIN B. FORSYTHE, New Jersey

SUBCOMMITTEE ON OCEANOGRAPHY

ALTON LENNON, North Carolina, *Chairman*

PAUL G. ROGERS, Florida
THOMAS L. ASHLEY, Ohio
THOMAS N. DOWNING, Virginia
JOSEPH E. KARTH, Minnesota
FRANK M. CLARK, Pennsylvania
WALTER B. JONES, North Carolina
ROBERT L. LEGGETT, California
CHARLES H. GRIFFIN, Mississippi
GLENN M. ANDERSON, California
MARIO BIAGGI, New York
ELIGIO DE LA GARZA, Texas
ROBERT O. TIERNAN, Rhode Island
PETER N. KYROS, Maine

CHARLES A. MOSHER, Ohio
HASTINGS KEITH, Massachusetts
GEORGE A. GOODLING, Pennsylvania
WILLIAM S. MAILLIARD, California
ROBERT H. STEELE, Connecticut
EDWIN B. FORSYTHE, New Jersey
PIERRE S. (PETE) DU PONT, Delaware

CONTENTS

	Page
Hearings held—	
April 5, 1971	1
April 6, 1971	211
April 7, 1971	379
Executive Communication No 434	3
Text of—	
H.R. 285	13
H.R. 336	20
H.R. 337	22
H.R. 548	20
H.R. 549	22
H.R. 805	24
H.R. 807	24
H.R. 808	24
H.R. 983	13
H.R. 1095	29
H.R. 1329	24
H.R. 1381	22
H.R. 1382	20
H.R. 1383	41
H.R. 1661	45
H.R. 1674	20
H.R. 2581	24
H.R. 3662	51
H.R. 4217	61
H.R. 4218	61
H.R. 4247	63
H.R. 4359	78
H.R. 4360	78
H.R. 4361	78
H.R. 4584	88
H.R. 4719	61
H.R. 4723	63
H.R. 5049	45
H.R. 5050	45
H.R. 5239	63
H.R. 5268	63
H.R. 5477	63
H.R. 5705	24
H.R. 6305	20
H.R. 6582	63
H.R. 6610	61
H.R. 6771	63
H.R. 7619	24
H.R. 8039	24
Reports from—	
Agriculture Department	90
Army Department	90
Atomic Energy Commission:	
April 7, 1971	93
April 26, 1971	91
Defense Department	94
Environmental Protection Agency	95
Federal Power Commission	103

Reports from—Continued
Health, Education, and Welfare: — Page
May 10, 1971 — 107
May 21, 1971 — 106
Interior Department — 107
Navy Department — 111
State Department:
April 7, 1971 — 114
April 21, 1971 — 113
April 22, 1971 — 112
Statement of—
Amundsen, Paul, executive director of the American Association of Port Authorities — 335
Barber, Dr. Richard, director, Oceanographic Program, Duke Marine Laboratory — 529
Beggs, James M., Under Secretary, Department of Transportation — 345
Bennett, Hon. Charles E., a Representative in Congress from the State of Florida — 151
Brennan, James, acting assistant general counsel, Department of Commerce — 478
Casey, Hon. Bob, a Representative in Congress from the State of Texas — 147
Cone, O. Lincoln, staff member, American Institute of Merchant Shipping — 357
Conte, Hon. Silvio O., a Representative in Congress from the State of Massachusetts — 155
Coughlin, Hon. Lawrence, a Representative in Congress from the State of Pennsylvania — 158
Crawford, Rear Adm. E. R., U.S. Navy, Assistant Deputy Chief of Naval Operations (Logistics) — 487
Dominick, David, Acting Commissioner, Water Quality Office, EPA — 392
Douglas, Henry T., chief of planning, Maryland Port Authority — 471
Edwards, Hon. Don, a Representative in Congress from the State of California — 226
Fascell, Hon. Dante B., a Representative in Congress from the State of Florida — 137
Ford, Hon. Gerald R., a Representative in Congress from the State of Michigan — 228
Frey, Hon. Louis Jr., a Representative in Congress from the State of Florida — 216
Gallagher, Hon. Cornelius E., a Representative in Congress from the State of New Jersey — 387
Garmatz, Hon. Edward A., a Representative in Congress from the State of Maryland — 143
Giaimo, Hon. Robert N., a Representative in Congress from the State of Connecticut — 149
Gibbons, Hon. Sam, a Representative in Congress from the State of Florida — 222
Groves, Brig. Gen. Richard H., Deputy Director of Civil Works, Office, Chief of Engineers, Department of the Army — 487
Halpern, Hon. Seymour, a Representative in Congress from the State of New York — 150
Hammond, Rear Adm. Robert, Chief, Office of Operations, Coast Guard — 345
Harrington, Hon. Michael J., a Representative in Congress from the State of Massachusetts — 379
Hayes, Brig. Gen. George J., U.S. Army Principal Deputy Assistant Secretary of Defense for Health and Environment — 487
Kemp, Hon. Jack F., a Representative in Congress from the State of New York — 152
Ketchum, Dr. Bostwick H., associate director of the Woods Hole Oceanographic Institution, Woods Hole, Mass — 316
Langlois, Edward, chairman, Committee on Environmental Affairs, American Association of Port Authorities — 335
Linduska, Dr. Joseph P., special director, Bureau of Sport Fisheries and Wildlife, Department of the Interior — 266

Statement of—Continued Page
Loesch, Hon. Harrison, Assistant Secretary for Public Lands, Department of the Interior 266
Mikva, Hon. Abner J., a Representative in Congress from the State of Illinois 156
Murphy, Hon. John M., a Representative in Congress from the State of New York 118
Pepper, Hon. Claude, a Representative in Congress from the State of Florida 141
Pollock, Howard, Deputy Administrator for National Oceanic and Atmospheric Administration, Department of Commerce 478
Price, Harold L., Director of Regulations, Atomic Energy Commission 230
Prokop, John, legislative attorney, American Institute of Merchant Shipping 357
Ramey, Hon. James T., member, Atomic Energy Commission 230
Reynolds, James J., president, American Institute of Merchant Shipping 357
Rhodes, Hon. John J., a Representative in Congress from the State of Arizona 156
Robison, Hon. Howard W., Congressman from the State of New York 315
Ruckelshaus, William D., Administrator, Environmental Protection Agency 392
Sandman, Hon. Charles W., a Representative in Congress from the State of New Jersey 211
Stevenson, John R., legal adviser, Department of State 461
Train, Russell E., chairman, Council on Environmental Quality 164
Wakelin, James H., Jr., Assistant Secretary for Science and Technology, Department of Commerce 478
Williams, Hon. Lawrence G., a Representative in Congress from the State of Pennsylvania 229
Wolff, Hon. Lester L., a Representative in Congress from the State of New York 227
Additional information supplied by—
American Association of Port Authorities, supplementary statement 344
American Petroleum Institute, prepared statement 538
Atomic Energy Commission:
Dumping sites of radioactive wastes 238
Monitoring of radioactive dumping sites 238
Radioactive waste labeling requirements 243
Ramey, Commissioner James T., letter to Congressman P. N. McCloskey, enclosing answers to questions submitted for record 249
Recovery of items dumped in the ocean, listing 239
Rules of practice authorizing ocean dumping 265
Sea disposal of radioactive wastes—historical trends, 1946–70, table 235
Step-by-step process for licensing of power reactors by the AEC 256
Barber, Dr. Richard T.:
Prepared statement 531
Résumé of biographical information 530
Commerce Department:
NOAA laboratories capable of assisting with ocean dumping problems 486
NOAA research vessels 485
Defense Department:
Air Force disposal of photographic wastes 499
Air Force memorandum on pollution abatement dated May 6, 1970 503
Air Force regulation on pollution control dated Sept. 23, 1970 505
Amendatory language re vessel "dumping" 522
Army memorandum on environmental protection and preservation dated March 29, 1971 513

Additional information supplied by—Continued
Defense Department—Continued
Page
Army memorandum on environmental quality dated Sept. 14, 1970 516
Disposal of biological warfare agents 500
Environmental statements 496
Establishment of the issuance of permits program 494
Final report of results of Navy investigation to inquire into the circumstances surrounding the discharge of waste material which occurred off the coast of Jacksonville, Fla., on November 30, 1970 541
Limits of tidal influence along certain rivers 523
Refuse Act permit program 495
Sewage treatment timetable 501
Dingell, Hon. John D.:
Boca Ciega case 271
Ecology, Law, and the "Marine Revolution" by Carleton Ray 542
Papers by Dr. Jerold M. Lowenstein on "Risks of Radioactive Pollution of the Oceans" and "Radioactive Pollution of the Seas" 373
Environmental Protection Agency:
AEC permit and regulatory requirements 457
Budget request for 1972 for solid waste control 404
Burden of proof for proposed dumping permit 450
Conditions of a dumping permit 443
Consultation provisions prior to issuing permits 455
Cost of implementing the Marine Protection Act of 1971 399
Deadline dates for municipal and industrial wastes 431
Dumping material from foreign territories in waters seaward of the United States 440
Emergency dumping without a permit to safeguard human life 443
Environmental Impact Statement from the Department of Defense—May 15, 1970 to April 15, 1971 406
Federal preemption of State control of ocean dumping 422
H.R. 4247 effect on memorandum of understanding 446
Intentions of sections 5(f) and 6(f) 456
Notices and/or hearings prior to the issuing of denial permits 455
Position on discharges of mercury 412
Studies on fish habitats of junked automobiles 414
Table: Fiscal year 1972 program plan for ocean-disposal research 452
Table on 6-year cost on implementation of H.R. 4247 399
"Toxic industrial wastes" definition 448
Toxicologist employed by 411
Interior Department:
Memorandum of Understanding between the Secretary of the Interior and the Secretary of the Army 268
Prepared statement of Harrison Loesch, Assistant Secretary of Interior 312
Ketchum, Dr. Bostwick H.:
Biographical information 320
Memorandum to Hon. John D. Dingell, dated April 16, 1971, on proposed research program 328
Lang, Andrew M., president, Waste Management and Compactions Systems, Inc., prepared statement 538
Lennon, Hon. Alton: Position paper on ocean protection
Manufacturing Chemists Association, prepared statement 535
Maryland Port Authority, prepared statement 313
Murphy, Hon. John M.:
Amendments to H.R. 4723 136
New York Bight 121
Report of the Council on Environmental Quality on Ocean Dumping, H. Doc. 91-399 124

Additional information supplied by—Continued

National Wildlife Federation, prepared statement of Louis S. Clapper, Conservation Director ..... Page 390

Perry, Harold L., wildlife representative, the Humane Society of the United States, prepared statement 538

Reynolds, James J., prepared statement 357

Sierra Club:

Prepared statement of Eugene V. Coan 388

Communications submitted by—

Barber, Dr. Richard T., letter to Ned P. Everett dated April 19, 1971 531

Douglas, Henry T., letter from Department of Natural Resources to Andrew Henbeck Jr., dated February 2, 1971 476

Everett, Ned P., letter from Barry Sullivan to Hon. Alton Lennon dated March 31, 1971 521

Johnson, Hayden B., letter to Hon. Edward A. Garmatz dated April 13, 1971 534

Port of New Orleans, telegram to Hon. Edward A. Garmatz dated April 5, 1971 540

Sierra Club, New England Chapter, letter to Hon. Edward A. Garmatz, dated April 29, 1971 540

Western Oil and Gas Association, telegram to R. J. McElroy dated May 11, 1971 539

Zimmerman, Fern, letter to Hon. Alton Lennon dated April 7, 1971 533

OCEAN DUMPING OF WASTE MATERIALS

MONDAY, APRIL 5, 1971

HOUSE OF REPRESENTATIVES,
JOINT SUBCOMMITTEES ON OCEANOGRAPHY AND FISHERIES
AND WILDLIFE CONSERVATION,
Washington, D.C.

The joint subcommittee met, pursuant to notice, at 10 a.m. in room 1334, Longworth House Office Building, Hon. Alton Lennon (chairman of the Subcommittee on Oceanography) and Hon. John D. Dingell (chairman of the Subcommittee on Fisheries and Wildlife Conservation) presiding.

Mr. LENNON. The Joint Subcommittee on Oceanography and Fisheries and Wildlife Conservation of the Committee on Merchant Marine and Fisheries are now convened.

This morning these two subcommittees will begin joint hearings on the series of bills designed to regulate the dumping of waste material in our coastal and off-shore waters.

It seems that no one knows the volume—and I think that is really an understatement—of wastes that have been dumped in the oceans in the past years. In fact, until recently, the question was scarcely asked and then only by an obscure group of scientists, known as ecologists.

Fortunately, however, in the last few years the entire question of ocean disposal of waste material has been thrust into prominence, and I think appropriately so, by the recently disclosed dumping of nerve gas and oil wastes off the coast of Florida, by the dumping of sewage and other municipal wastes off New York Harbor, and a number of other and similar instances, all of which I am delighted to report were the subject of hearings and investigation by these two subcommittees during the 91st Congress.

In October of 1970, the Council of Environmental Quality, which was created as a result of legislation reported by one of these committees, published an excellent report entitled "Ocean Dumping—A National Policy." That report forms the basis for the hearings we are beginning today, and points up the immediacy and the severity of the problems that we may be creating.

These problems may perhaps best be exemplified by reference to the international conference held on the Island of Malta last summer, dealing with the necessity of and techniques for protecting the oceans from ill-advised actions by man. One scientist, regrettably but not surprisingly from the United Nations International Atomic Energy Agency, put the problem into a certain perspective by asking at one point if perhaps the highest and best use of the oceans might not be to serve as a dump for man's waste.

I must say that I personally find such an attitude distressing, and I am gratified to note that a position paper, adopted by most of the scientists at that Conference, stated that:

> Until better evidence is available that irreversible changes are not taking place, reason demands that we proceed more carefully, with greater concern for the health of the seas.

I will ask at this time unanimous consent that this position paper be included in the record at the conclusion of my remarks.

The most recent statistics from the Food and Agricultural Organization of the United Nations indicates that in 1969, the latest year for which we have complete figures, for the first time in 25 years the total fisheries catch from the world's oceans declined by over 1 million metric t[illegible], as compared to the 1968 high of 64.3 million metric tons. I would hasten to remind all those here today that this happened at a time when our efforts to increase the ocean catch were intensifying and at a time when it is becoming increasingly important to provide needed protein to a hungry, growing world population. None of us are prepared to say that the decline was due solely to America's ocean dumping policies nor to those of other nations, but I do feel that this is certainly a very ominous sign, and increases the incentives upon this committee and the Congress to see that this country does nothing to decrease the perhaps fragile productivity of the world's seas.

Simply stated then, we can no longer afford the illusion that out of sight is out of mind, and that the oceans may safely be treated as a gigantic dump. They are, or rather should be, a resource—not an infinite resource, but one of given magnitude—and they must be treated with care and respect.

The main bill under consideration by the two subcommittees during these hearings is the administration bill, H.R. 4723, introduced by the distinguished chairman of our full committee, Congressman Garmatz, and an identical bill introduced by Congressman Pelly, the ranking minority member of this committee; and identical bills introduced by Congressman Kemp; Congressman Ruppe, a distinguished member of this committee; Congressman Chamberlain; and Congressman Gerald Ford, the minority leader of the House.

There are a number of other bills to be heard during these hearings—in fact, a total of 36 in number—some of which are very similar to the administration bill, some of which contain, however, several provisions of the administration bill, and some of which contain provisions and cover areas not included in the administration bill. We will not take the time this morning to enumerate the bill numbers and authors at this time, but Mr. Dingell and I would like to make it clear that these hearings will cover all bills pending before the committee that have to do with ocean dumping of waste material.

Let the bills and departmental reports appear in the record at this point.

(The bills and departmental reports follow:)

[Executive Communication No. 434]

ENVIRONMENTAL PROTECTION AGENCY,
Washington, D.C., March 18, 1971.

Hon. CARL B. ALBERT,
Speaker, House of Representatives,
Washington, D.C.

DEAR MR. SPEAKER: In accordance with Section 102(2)(C) of the National Environmental Policy Act of 1969, enclosed herewith are the environmental impact statements for the four legislative proposals of the Environmental Protection Agency.

This proposed legislation is part of the President's environmental program as announced in his environmental message to the Congress of February 8, 1971, and was transmitted to you on February 10, 1971. We understand that those legislative proposals to amend the Federal Water Pollution Control Act, as amended, were referred to the Committee on Public Works and that the proposed "Marine Protection Act of 1971" was referred to the Committee on Merchant Marine and Fisheries.

Sincerely yours,

WILLIAM D. RUCKELSHAUS,
Administrator.

Enclosures: Environmental impact statements for EPA legislative proposals

PROPOSED AMENDMENTS TO SECTION OF THE FEDERAL WATER POLLUTION CONTROL ACT, AS AMENDED, RELATING TO STATE AND INTERSTATE PROGRAM GRANTS (PREPARED IN COMPLIANCE WITH SECTION 102(2)(C) OF THE NATIONAL ENVIRONMENTAL POLICY ACT OF 1969 (PL 91–190), FEBRUARY 8, 1971)

1. NATURE OF THE PROPOSED BILL

This proposal, in the nature of an amendment to Section 7 of the Federal Water Pollution Control Act, as amended, would provide the EPA Administrator with flexibility in funding signficant pollution control projects in the State program grant context, would provide for bonuses for State achievement of specific program improvements, and would extend and increase the authorization, for the State program grant authority through Fiscal Year 1975.

Key provisions of this proposal would authorize increased appropriations for an additional four years on a sliding scale from $15 million for FY 1972 to $30 million for FY 1975. Ten million dollars of these sums would continue to be available for the basic State and interstate programs. The proposal would also add five grant bonus categories for an improved program consisting of five components: (1) a permit system; (2) a sewage treatment facilities program; (3) training and development of personnel; (4) State recruitment and personnel system; and (5) a planning capability.

2. ENVIRONMENTAL IMPACT OF PROPOSED BILL

Over the past years, we have identified a need for the EPA to increase its support to the States and interstate agencies to enable them to carry out and accelerate their own programs of water quality standards enforcement and implementation. This need becomes even more acute with the implementation of the recently published construction grant regulations, the accelerated construction of waste treatment facilities, and the Refuse Act permit program. The proposed strengthening of the State program grant authority would have a decidedly beneficial impact upon water quality.

3. ADVERSE ENVIRONMENTAL EFFECTS WHICH CANNOT BE AVOIDED

As this proposal is aimed at upgrading State water quality enhancement programs, we can foresee no unavoidable adverse environment effects growing out of this proposal.

4. ALTERNATIVES TO THE PROPOSED BILL

One obvious alternative to the provision of Federal funds for specific State program elements would be the provision of additional Federal funds for general program development and implementation. The option we have chosen, to direct Federal expenditures toward specific State program achievements, is designed to encourage the development of those aspects of State programs which will make the greatest contribution toward water quality protection and enhancement, thereby making the most of both Federal and State investments.

5. RELATION BETWEEN LOCAL SHORT-TERM USES AND LONG-TERM PRODUCTIVITY OF THE ENVIRONMENT

In both the short and long term, this proposal is expected to protect and enhance the environment.

6. IRREVERSIBLE AND IRRETRIEVABLE COMMITMENTS OF RESOURCES

No natural resources will be committed pursuant to this proposal. The financial resources authorized for this program will be justified by the beneficial environmental effects to be derived from the State program grants.

PROPOSED AMENDMENTS TO SECTION 8 OF THE FEDERAL WATER POLLUTION CONTROL ACT, AS AMENDED, RELATING TO WASTE TREATMENT FACILITY CONSTRUCTION

(Prepared in Compliance With Section 102(2) (C) of the National Environmental Policy Act of 1969 (PL 91–190) February 8, 1971)

1. NATURE OF PROPOSED BILL

The proposal, in the nature of an amendment to section 8 of the Federal Water Pollution Control Act, as amended, seeks to assist States and municipalities in financing the construction of needed waste treatment facilities necessary to comply with water quality requirements of the President's environmental program. Specifically, the proposal would authorize appropriations for grants for treatment works construction in an aggregate amount of $6 billion over a period of three fiscal years beginning with fiscal year 1972. In addition, the bill would revise the allocation formula to allocate funds to States on the basis of relative population, availability of State matching funds, outstanding reimbursables, and water pollution control needs.

The bill would direct the Administrator to encourage grantees to achieve institutional and financial capability to maintain, expand and replace necessary treatment works, and would provide for an increased Federal share of project costs if the grantee has made provision to achieve such institutional and financial capability. The bill would authorize grants for the costs of treating industrial wastes only if the grantee makes provision for full cost recovery of construction costs allowable to industrial wastes.

2. ENVIRONMENTAL IMPACT OF PROPOSED BILL

This proposal is prompted by the over-riding need among States and their political subdivisions for additional financial assistance for needed sewage treatment plant construction. Accelerated plant construction will enable States to meet water quality standards established for their waterways for a variety of beneficial water uses as well as to comply with national planning and treatment requirements. It is the provision of these treatment works which is at the foundation of the Federal effort to maintain a high level of water quality nationwide. The environmental impact on water is expected to be entirely beneficial.

3. ADVERSE ENVIRONMENTAL EFFECTS WHICH CANNOT BE AVOIDED

Although this proposal is directed toward water quality enhancement, we anticipate that the construction of additional treatment facilities may increase the potential for adverse environmental effects associated with the movement of soil as plant sites are readied and facilities are placed into operation. The placement of such facilities could potentially interfere with recreational, residential and aesthetic land use considerations. To identify such conflicts, grantees will be obliged to provide the Administrator with an environmental assessment of the project so that such adverse effects can be eliminated or minimized. Further, through the river basin, regional, and metropolitan plans that would be supported by this proposal, we will have an effective tool whereby any adverse environmental effects associated with a project will be identified and eliminated or minimized. It will be incumbent upon Federal, State, and local environmental protection authorities to monitor the construction and operating plans and activities of each jurisdiction to ensure compliance, to the extent possible, with all environmental protection requirements.

4. ALTERNATIVES TO THE PROPOSED BILL

Financial studies conducted by the Environmental Protection Agency, including the Water Quality Office's *Cost of Clean Water*, point to the need for an additional Federal financial support for waste treatment facility construction. This proposal is thought to be the most workable solution to this problem of financial need, providing for State, local and Federal sharing of costs, and providing for the achievement of water quality standards goals by 1974.

5. RELATION BETWEEN LOCAL SHORT-TERM USES AND LONG-TERM PRODUCTIVITY OF THE ENVIRONMENT

Both in the short and long term, this proposal is directed toward environmental enhancement and protection. Local short-term adverse impact associated with by-passing of existing treatment works during the construction of additions of alterations of the plant will no longer be tolerated. Grantees will be required, pursuant to regulations, to provide for the same level of treatment during construction as that which was obtained prior to the initiation of construction.

6. IRREVERSIBLE AND IRRETRIEVABLE COMMITMENTS OF RESOURCES

Although this proposal involves a large commitment of financial resources, we expect the benefits to be derived from such investment in terms of water quality improvement to be more than commensurate.

PROPOSED AMENDMENTS TO SECTION 10 OF THE FEDERAL WATER POLLUTION CONTROL ACT, AS AMENDED, RELATING TO ENFORCEMENT AND WATER QUALITY STANDARDS (PREPARED IN COMPLIANCE WITH SECTION 102(2) (C) OF THE NATIONAL ENVIRONMENTAL POLICY ACT OF 1969 (PL 91-190), FEBRUARY 8, 1971)

1. NATURE OF PROPOSED BILL

The proposal would amend section 10 of the Federal Water Pollution Control Act to strengthen and clarify the authority of the Administrator in the establishment and enforcement of water qualify standards, and would add new authorities relating to monitoring, surveillance, citizens' suits, and abatement of pollution from hazardous substances.

The Water Quality Improvement Act of 1970 provided important new authorities for the enhancement of water quality. These new authorities will assist in controlling water pollution caused by oil and hazardous substances, in carrying out an important new State-Federal program for the prevention of water pollution from federally licensed or permitted activities, and in other areas. However, strengthening of the Act is now necessary to enable the Environmental Protection Agency to play a more active role in working with State and local governments to prevent and abate pollution of our Nation's waters.

Key provisions of the proposed bill would extend Federal jurisdiction for pollution abatement to include expressly ground waters, tributaries of interstate and navigable waters, pollution of waters of the contiguous zone which adversely affects water quality in the territorial sea, and pollution of the high seas resulting from discharges of matter transported from United States territory. The proposed bill would also more adequately define water quality standards to mean water quality standards established under existing law, and in additional, water use designations, water quality criteria, effluent limitations, and plans of implementation and enforcement established pursuant to new requirements contained in the bill.

The proposal would also require the Administrator to publish regulations establishing specifications for water qua'ity criteria and effluent limitations. In doing so, the uncertainty and confusion which have resulted from a lack of guidance to the States in this respect and the delays caused in the establishment of enforceable standards would be eliminated. Water quality standards under the proposed legislation would include two elements not previously specified: water use designations and effluent limitations. Water quality standards in all States would be require' to be revised to include these new elements. It would provide the Administrat r with clear authority to establish standards in areas of exclusive Federal jurisdiction or where the States do not have jurisdiction, after public hearings. The proposed bill would also eliminate the existing enforcement conference. The Administrator would be authorized to abate pollution and enforce water quality standards through the issuance of orders following notice to violators. Such orders would be legally enforceable. Appeal from and judicial review of such orders would be provided.

The Administrator could also call a fact-finding hearing to be conducted with State participation, and, where he finds a threat to health or welfare, he may initiate the speedy revision of water quality standards. Civil penalties of up to $25,000 per day of violation of a final order would be provided. Further provisions would provide the Administrator with authority to establish effluent limitations for hazardous substances, to compel the attendance and testimony of witnesses, to enter and inspect facilities, and to require dischargers to perform effluent monitoring. Court action by citizens would also be authorized to enforce water quality standards or to compel the Administrator to perform non-discretionary acts.

2. ENVIRONMENTAL IMPACT OF PROPOSED BILL

This proposal would greatly strengthen the regulatory tools at the command of the Environmental Protection Agency for water pollution control and would give private citizens a larger role in the enforcement process. Its impact on the environment is anticipated to be a wholly beneficial one. Specific provisions of the bill are designed to broaden the scope of application of water quality standards, increase water quality requirements, provide for speedy, just, and effective enforcement procedures, and allow for public participation through hearings and citizen suits. In short, the proposal would give the EPA the added authority it needs to more effectively carry out its mission of water quality protection and enhancement. All of these provisions are directed toward water quality protection and enhancement.

3. ADVERSE ENVIRONMENTAL EFFECTS WHICH CANNOT BE AVOIDED

We do not foresee any unavoidable adverse effects to the environment which might result from the enactment of this proposal. Its purpose is to expand and increase presently existing regulatory controls over water pollution situations, and its environmental effects, therefore, are expected to be entirely beneficial with respect to environmental quality.

4. ALTERNATIVES TO THE PROPOSED BILL

This proposal reflects the recommendations of Federal, State, and local enforcement officials in the water pollution control field as to the needs in the upgrading of water quality standards and enforcement procedures. We feel this is the best and most comprehensive proposal in the enforcement field which can be submitted at this time. If enacted, we intend to review the operation of these

new procedures to ascertain their adequacy and with a view toward additional refinement that may be indicated by implementation of these provisions.

5. RELATION BETWEEN LOCAL SHORT-TERM USES AND LONG-TERM PRODUCTIVITY OF THE ENVIRONMENT

Since this proposal is wholly directed toward water quality protection and enhancement both legitimate, local, short-term uses and long-term productivity of the environment will benefit from this proposal.

6. IRREVERSIBLE AND IRRETRIEVABLE COMMITMENTS OF RESOURCES

Since this proposal would strengthen the regulatory authority of the EPA, its effect upon natural resources would be one of enhancement, not exploitation or commitment.

PROPOSED AMENDMENT TO SECTION 23 OF THE FEDERAL WATER POLLUTION CONTROL ACT, AS AMENDED, TO INCLUDE AMERICAN SAMOA AND THE TRUST TERRITORY OF THE PACIFIC ISLANDS WITHIN THE MEANING OF "STATE"

(Prepared in compliance with section 102(2) (c) of the National Environmental Policy Act of 1969 (P.L. 91–190), February 8, 1971)

1. NATURE OF PROPOSED BILL

The proposed bill will amend the definition of "State" in Section 23 of the Federal Water Pollution Control Act, as amended, to include American Samoa and the Trust Territory of the Pacific Islands within the meaning of this definition. These areas were included in the definition of "State" for purposes of the 1970 amendments to that Act, dealing with oil pollution, hazardous polluting substances, vessel sewage, training grants and contracts and scholarships. However, these areas are still unable to benefit from or participate in many important EPA programs, including: (1) water quality standards program; (2) waste treatment construction grant program; (3) research, development, and demonstration grant program; (4) performance standards and other requirements of Executive Order 11507, dealing with the prevention, control, abatement of air and water pollution from Federal facilities. In order to obtain consistency with the Act and to extend the pollution control efforts of the Environmental Protection Agency to all areas of United States responsibility, this proposed legislation is offered.

2. ENVIRONMENTAL IMPACT OF PROPOSED BILL

The Southwest Regional Office of the Water Quality Office, Environmental Protection Agency, has received repeated requests from the Governments of American Samoa and the Pacific Trust Territory that they be made eligible for grants and other programs administered by EPA. Officials of these governments are greatly concerned with the deterioration of the quality of their waters and are anxious to participate in programs which would help them protect and enhance water quality in those jurisdictions. This bill would provide such waterway protection and improvement.

3. ADVERSE ENVIRONMENTAL EFFECTS WHICH CANNOT BE AVOIDED

Since this proposed bill is designed to bring American Samoa and the Pacific Trust Territory entirely within the purview of the Environmental Protection Agency, there would not appear to be any significant unavoidable adverse environmental effects resulting from such action. In particular, the inclusion of these jurisdictions under the water quality standards and other programs would help these areas protect and upgrade the quality of their waterways.

4. ALTERNATIVES TO THE PROPOSED BILL

The most obvious alternative to the proposed bill is to do nothing. This would perpetuate a situation already identified as unsatisfactory for the effective protection of environmental values in these areas of American responsibility.

BEST COPY AVAILABLE

5. RELATION BETWEEN LOCAL SHORT-TERM USES AND LONG-TERM PRODUCTIVITY OF THE ENVIRONMENT

Both in the short and long terms, this proposed legislation would bring jurisdictions within the water quality management and planning process and is intended to provide for the enhancement of water resources in those areas.

6. IRREVERSIBLE AND IRRETRIEVABLE COMMITMENTS OF RESOURCES

The proposed bill makes no commitments of natural resources. Rather than committing resources, the proposal is intended to make high quality water resources available in an area where they are presently suffering degradation.

PROPOSED MARINE PROTECTION ACT OF 1971 PREPARED IN COMPLIANCE WITH SECTION 102(2)(C) OF THE NATIONAL ENVIRONMENTAL POLICY ACT OF 1969 (P.L. 91-190), FEBRUARY 8, 1971

A. NATURE OF THE PROPOSAL

The proposed legislation would regulate the dumping of material into the oceans and coastal and other waters. It would bar the transportation of material from the United States for dumping in the oceans, coastal waters, and the Great Lakes and the actual dumping of material in our territorial waters or in the Contiguous Zone, except as authorized by permits issued by the Administrator of the Environmental Protection Agency (EPA). The Administrator would establish and apply criteria for evaluating dumping applications, and in establishing such criteria would consider specified environmental considerations. Additionally, the Administrator would be empowered to ban ocean dumping of certain materials and to designate recommended safe disposal sites. Transportation for dumping or dumping without a permit or in violation of a permit would be subject to civil and criminal penalties. The Coast Guard would perform surveillance and other appropriate enforcement activity.

B. ENVIRONMENTAL SETTING AND HISTORY OF THE PROPOSAL

The proposed legislation would implement the recommendations of the report "Ocean Dumping—A National Policy." That report, requested by the President in his April 15, 1970, message on waste disposal, was prepared by the Council on Environmental Quality and made public by the President on October 7, 1970. The Council was materially assisted in preparing the report by the members of a Federal Task Force, established to provide guidance in formulating the recommended policy. Helpful assistance was also received from agencies and individuals in State and local government and from scientists and academicians, including the National Academy of Sciences and the National Academy of Engineering.

C. ANALYSIS OF ENVIRONMENTAL CONSEQUENCES

(1) The environmental impact of the proposed legislation

(*a*) The proposed bill would establish for the first time a comprehensive, unified Federal regulatory scheme to meet the serious threat of pollution in the oceans and similar waters. It would permit implementation of an anti-dumping policy which has its focus on prevention of damage, and would allow action to be taken before the problem of ocean dumping becomes acute.

The Council report points out that there is a critical need for a national policy on ocean dumping. Many of the wastes now being dumped are heavily concentrated and contain materials that have a number of adverse effects. Many are toxic to human and marine life, deplete oxygen necessary to maintain the marine ecosystem, reduce populations of fish and other economic resources, and damage aesthetic values. In some areas such as the New York Bight, the environmental conditions created by ocean disposal of wastes are serious.

The Council study indicates that the volume of waste materials dumped in the ocean is growing rapidly. Because the capacity of land-based waste disposal sites is becoming exhausted in some coastal cities, communities are looking to the ocean as a dumping ground for their wastes. Faced with higher water quality standards, industries may also look to the oceans for disposal. The result could be a massive increase in the already growing level of ocean dumping. If this occurs, environmental deterioration will become widespread.

In most cases, feasible and economic land-based disposal methods are available for wastes currently being dumped in the ocean. In fact, many alternatives to ocean dumping, such as land reclamation and recycling to recover valuable waste components, can be applied to obtain positive environmental benefits, such as the maintenance and enhancement of valuable associated living marine resources.

Current regulatory activities and authorities are not adequate to handle the problem of ocean dumping. States do not exercise extensive control over ocean dumping, and generally their authority extends only within the three-mile territorial sea. The greater part of current dumping occurs outside these waters. The Army Corps of Engineers has regulatory authority over ocean dumping but, again, this is largely confined to the territorial sea. The Corps also has responsibility to facilitate navigation, chiefly by dredging navigation channels. As such, it is in the position of regulating activities over which it also has operational responsibility. The Coast Guard enforces several Federal laws regarding pollution but has no direct authority to regulate ocean dumping. The authority of the Environmental Protection Agency does not provide for issuance of permits to control ocean dumping. And, the Atomic Energy Commission has authority only for disposal of radioactive materials. New legislative authority is necessary.

Taken together, present responsibilities are dispersed, and operational agencies exercise responsibility to regulate themselves and entities performing work consistent with their primary mission. It is now necessary that responsibility for ocean dumping be centralized in an agency whose chief role is control of pollution.

(*b*) The proposed bill would enable EPA to regulate the dumping of materials in the oceans and similar waters by not only private persons or entities but also all Federal, State, and in appropriate cases, foreign, governmental organizations employees and agents. Thus, even sister Federal organizations would have to comply with the permit and standard-setting provisions of the proposal.

(*c*) The proposed bill sets out specific considerations to be used by EPA in developing criteria for ocean dumping. These considerations would permit EPA to refine and modify the criteria as additional knowledge respecting the effect of ocean dumping is developed.

(*d*) The proposal would enhance the ability of the Federal government to engage in productive research efforts to understand the effects of materials dumped or spilled into the oceans and to develop means of monitoring and controlling such disposal. In developing the criteria and enforcement programs EPA and the Coast Guard would have the impetus to work not only with each other but also to use their present research authority to develop relevant research programs in conjunction with such other agencies as the National Oceanic and Atmospheric Administration.

(*e*) The authority contained in the proposal and the policy contained in the Council's report which would be implemented by the authority, would have an estimated impact on present dumping practices as follows:

About 48 million tons of wastes were dumped at sea in 1968. These wastes included dredge spoils, industrial wastes, sewage sludge, construction and demolition debris, solid waste, explosives, chemical munitions, radioactive wastes, and miscellaneous other materials, the present degree of regulation for these materials varies considerably.

(*i*) As the following table indicates, dredge spoils accounted for 80 per cent by weight of all ocean dumping:

OCEAN DUMPING: TYPES AND AMOUNTS 1968 (66)

[In tons]

Waste type	Atlantic	Gulf	Pacific	Total	Percent of total
Dredge spoils	15,808,000	15,300,000	7,320,000	38,428,000	80
Industrial wastes	3,013,200	696,000	981,300	4,000,500	10
Sewage sludge	4,477,000	0	0	4,477,000	9
Construction and demolition debris	574,000	0	0	574,000	<1
Solid waste	0	0	26,000	26,000	<1
Explosives	15,200	0	0	15,200	<1
Total	23,887,400	15,966,000	8,327,300	48,210,700	100

62-513 O—71——2

The Corps of Engineers estimates that about 34 per cent (13 million tons) of this material is polluted. Disposal of this material at sea can be a serious source of ocean pollution. Dumping of unpolluted material can also be harmful particularly if it occurs in biologically active areas such as shellfish beds.

Present regulatory control over dredge spoil disposal is vested in the Corps, both as a consequence of its authority to regulate dredging and dumping in navigable waters, and as a result of its self-regulation of the dredge spoil produced by its own activity.

Granting EPA a permit authority over the dumping of dredge spoil, even where it is generated by the Corps' own activities, would allow EPA to phase out the ocean disposal of polluted dredge spoils and to base selection of disposal sites for unpolluted material primarily on environmental factors, with a resulting considerable gain in alleviating marine environmental degradation.

The policy on dumping dredge spoil which the legislative proposal is designed to implement may result in carrying out fewer and smaller dredging operations. Most polluted dredge spoil may be appropriate for disposition at land sites, and development and use of these sites would involve greater costs than ocean or other marine dumping sites. Greater disposal costs may be incurred for even unpolluted dredge spoil. Appropriate dumping sites may not exist in close proximity to the dredging area and considerable costs may be incurred in transporting the spoil to suitable areas.

Reducing the amount of dredging may be of some benefit to the environment, for dredging normally involves increasing the turbidity of the relevant waters and suspending some of the pollutants which are present in material being dredged. This would also have a beneficial effect in reducing dredge spoil that might be dumped on coastal marsh areas which are often unique and productive of waterfowl and other shore birds and often serve as the nutrient base for food chains of valuable estuarine living marine resources.

The proposal would allow implementation of the Council's recommendations to bar as soon as possible the dumping of industrial wastes, especially those which are toxic, and undigested sewage sludge. Dumping of digested or other stabilized sludge would be phased out as environmentally-sound land-based alternatives were developed.

Other land-oriented materials which under the proposal and new anti-dumping policy could not be dumped at sea or in similar waters include high-level radioactive wastes and chemical warfare agents. Dumping of other materials, such as explosive munitions, would be phased out.

(2) *Adverse environmental effects which cannot be avoided should the bill be enacted*

(*a*) Each decision not to permit the dumping of material in the ocean or similar waters would to some degree enhance the quality of those waters. Yet, the waste disposal problem would only shift to another part of the environment if long-term alternatives cannot be developed to increase recycling of waters, to conserve resources, and to manage solid wastes effectively from an economic and environmental standpoint.

Nevertheless, the bill seeks to vest the permit authority with the Administrator of EPA, who, because of his responsibility in the areas of air and water pollution control, radiation standard-setting, and solid-waste management research, is probably better qualified than any other Federal government official to choose between competing disposal modes.

(*b*) Arguably, the assumption of an active regulatory role by the Federal government would lead States and local governments to abandon their current efforts to control dumping. However, State and local regulatory efforts of a comprehensive nature have only begun to be developed, and the bill would explicitly save these nascent efforts from preemption.

In California, for example, the San Francisco Bay Area Water Quality Control Board has passed stringent anti-dumping rules which were effective January 1, 1971. These efforts, and those of a similar nature, could be revised to reflect the newly strengthened Federal authority.

(*c*) The bill would repeal or restrict the scope of several existing Federal statutes and add requirements to others. These actions are not expected to have adverse environmental effects.

In its subsections 11(a) and 11(b), the bill would repeal the Supervisory Harbors Act of 1888, as amended (33 U.S.C. § 441–451b), and the provision of the Rivers and Harbors Act of 1899 (33 U.S.C. § 418) which preserves the Super-

visory Harbors Act from supersession by the 1899 Act. The Supervisory Harbors Act provides a special authority to control transit in and from the harbors of New York, Baltimore, and Hampton Roads, Virginia. This authority has been used to regulate ocean dumping. The proposed Act would replace that authority. A portion of the Act of August 5, 1886 (33 U.S.C. § 407a), which pertains to deposits of debris from mines and stamp works, and which is covered by this bill or the Refuse Act, is also repealed. A provision contained in the Rivers and Harbors Act of 1905 (33 U.S.C. § 419), which has been used to buttress the Corps of Engineers' authority to regulate ocean dumping, is superseded, insofar as it authorizes action that would be regulated by this proposal. Lastly, section 13 of the Rivers and Harbors Act of 1899 (33 U.S.C. § 407), commonly known as the Refuse Act, is superseded, but only insofar as it applies to dumping of material in the waters covered by subsection 4(b) of this proposal.

Another portion of the proposed bill, section 7, deals with the relationship of this proposed legislation to other laws. Generally, except as provided in subsections 7(b) and 7(c), it provides that after the Act's effective date, existing licenses, permits, or authorizations would be terminated to the extent they authorize activity covered by this proposal, and that further licenses, permits, or authorizations of a similar nature could not be issued.

Subsection 7(b) maintains present responsibility and authority contained in the Atomic Energy Act of 1954, and provides that the prohibitory provisions of this proposal do not apply to actions taken under that Act. However, the AEC must consult with the Administrator before issuing a permit to conduct any activity otherwise regulated by this proposal. Moreover, the AEC must comply with the radioactive-material standards set by the Administrator, and the Administrator is directed to consider the policy expressed in this proposal along with the proposed criteria-basing factors in setting such standards for the waters covered by this proposal.

Subsection 7(c) relates to authorities contained in the Rivers and Harbors Act of 1899, respecting dredging, filling, harbor works, and maintenance of navigability. The powers are exercised by the Secretary of the Army and the Chief of Engineers. Except for the limited supersession respecting the Refuse Act discussed earlier, the Rivers and Harbors Act authorities are not negated or abrogated, nor are existing licenses or permits issued under the Act terminated. Rather, in situations where this bill would but for the provisions of 7(a) and (c) also apply to dumping of material in connection with a dredge and fill or other permit issued by the Corps of Engineers, such permit is conditioned upon a certification by the Administrator of EPA that the activity is in conformity with this proposal and any regulations issued under it. The Administrator would not issue a separate permit.

(3) Alternatives to the proposed bill

(a) The Federal government's present, scattered regulatory authority over dumping in the oceans and other similar waters could be retained, but each official responsible for administration of a part of the mosaic of laws could implement the policy reflected in the Council's report. Such an approach would give direction to the presently largely uncoordinated regulatory efforts, but it would mean that serious jurisdictional gaps in Federal authority would remain. Most of the relevant Federal laws are not written to reach activity which occurs beyond United States territorial sea. Yet most dumping takes place beyond these waters, notwithstanding the fact that the dumped material originates within the United States.

(b) Greater responsibility could be placed on the States through development of a regulatory system akin to that found in the Federal Water Pollution Control Act, where States would develop criteria and procedures for dumping permits, and the Federal government would approve suitable State systems. But the interstate character of the oceans and the direct effect upon them by actions taken in such similar areas as estuaries suggests that a unified control is desirable. Also, the authority of the States is limited to their territorial seas.

(c) A complete ban on all dumping in the oceans, Great Lakes, and other similar waters could be declared and enforced. This alternative, however, would have unnecessary and undesirable environmental effects. It would force all disposal to land modes and create undue air and water pollution problems as well as hamper land use of some areas which would have to be used as solid waste and dredge spoil disposal sites. Such action would arbitrarily place the oceans

and similar waters in a preferred environmental position at the expense of other portions of our surroundings.

(*d*) A system of taxes on massive disposal could be used in lieu of a permit system. At present, such a tax approach would be difficult to administer because the relative impact of the different materials dumped is difficult to determine. When the variable of dumping locations is added, as would be necessary, the rate of taxation to be assessed for a dumping action seems almost impossible to determine.

(*e*) The present permit system could be modified to incorporate a requirement that the Administrator of EPA consult with the Secretaries of the Interior (Fish and Wildlife Service) and Commerce (National Oceanic and Atmospheric Administration) respecting each permit application. Such a requirement would ensure that added environmental expertise was brought to bear on the application of criteria as well as in the development and modification of the criteria. However, the added administrative burden of mandatory comment in each permit application could be overly burdensome. The proposal now gives the Administrator discretion to consult when he deems such consultation to be necessary.

(*4*) *Relationship between local short-term uses of man's environmental and the maintenance of long-term productivity*

The proposal provides adequate authority to protect the oceans and similar waters from the degradation resulting from dumping wastes in them. In the short term, adoption of the proposed bill would involve (1) markedly increased dredging costs, particularly for areas where the dredge spoil was polluted, (2) increased expenditures for sewage treatment facilities, especially in the New York area where much undigested sewage sludge is dumped at sea, (3) increased funding demands for both public and private solid waste disposal and research, and (4) development of methods to detoxify or render harmless the nation's outdated chemical and explosive munitions. The oceans would no longer be a least-cost, convenient sink for many wastes. In the short term the proposed bill would increase pressures on land disposal sites and may also contribute to some air and water pollution.

In the long term, advances in recycling and resource development technology should decrease the pressures placed on land-based disposal, except perhaps in the case of polluted dredge spoil. Presently, alternatives to diked land disposition of such spoil appear limited, but research and management efforts are being stepped up, as evidenced by the program contained in Section 123 of the recently-enacted River and Harbor Act of 1970 (P.L. 91–611).

(*5*) *Any irreversible or irretrievable commitments of resources*

The bill would commit Federal funds to the administration of the new regulatory machinery. It would also require increased Federal and private financing of efforts to develop alternative means of waste treatment and disposal. Certain activities such as dredging would become sufficiently expensive in some cases that the dredging either might not be done to the detriment of navigation, or might take a greater show of Federal or private funds than at present. And, it would force industries and municipalities to construct new and environmentally desirable methods of disposing of wastes on land.

D. COORDINATION WITH OTHER AGENCIES

The proposed bill was prepared by the Council on Environmental Quality with the guidance and assistance of an interagency task force including representatives of the Office of Management and Budget.

Enclosure.

[H.R. 285, H.R. 983, 92d Cong., 1st sess.]

A BILL

To amend the Fish and Wildlife Coordination Act to provide additional protection to marine and wildlife ecology by requiring the designation of certain water and submerged lands areas where the depositing of certain waste materials will be permitted, to authorize the establishment of standards with respect to such deposits, and for other purposes.

Be it enacted by the Senate and House of Representatives of the United States of America in Congress assembled, That the Fish and Wildlife Coordination Act (16 U.S.C. 661 et seq.) is amended by inserting immediately following section 5A thereof the following new section:

"SEC. 5B. (a) The Secretary of the Interior, acting through the United States Fish and Wildlife Service, shall

I—O

designate those portions of the navigable waters of the United States and those portions of the waters above the Outer Continental Shelf as defined in the Outer Continental Shelf Lands Act, and those portions of the submerged lands beneath the navigable waters and beneath the waters above the Outer Continental Shelf into and onto which he determines sewage, sludge, spoil, or other waste can be safety discharged. In making such designation he shall consider all ecological and environmental factors, including, but not limited to, the effect of such discharging on the marine and wildlife ecology.

"(b) No designation shall be made by the Secretary of the Interior under authority of subsection (a) of this section for the two-year period beginning on the date of enactment of this section. During such two-year period the Secretary of the Interior, in cooperation with the Secretary of the Army acting through the Chief of Engineers, shall make a full and complete investigation and study of potential water and submerged lands areas for designation and shall identify those areas most suitable for such designation.

"(c) As soon as practicable after the designation of an area under subsection (a) of this section, the Secretary of the Interior shall establish standards which shall be applicable to the discharge of material within such designated area. Such standards shall be for the purpose of insuring that no damage to, or loss of, any wildlife or wildlife resources or pollution of

the navigable waters of the United States will result from any such activity. Such standards shall be applicable to all of the departments, agencies, and instrumentalities of the Federal Government, and, except as otherwise provided in this section, in the case of a designated area containing any submerged lands within the jurisdiction of the States, to the States and their agencies, including any person having any license, permit, or other authorization from such State or agency for any such activity with respect to any of such submerged lands.

"(d) If a State establishes within one year after the date that a Federal standard is established under subsection (c) of this section its own standard with respect to the activity covered by such Federal standard, such standard shall be applicable to such activity within the jursidiction of such State if within such one-year period the Secretary, after public hearing, determines that such State standard is equal to or more stringent than the Federal standard established under this section with respect to such activity and that there are adequate procedures for the State to enforce such standard, then such State standard shall apply to such activity within the State's jurisdiction, and the Federal standard shall not apply. If he determines that such State standard is not as stringent as the Federal standard, then

the Federal standard shall apply to such activity in such State.

"(e) Whenever a State's standard is applicable within the jursidiction of that State it shall continue to be applicable until the Secretary, after public hearing, determines that it is not as stringent as the comparable Federal standard. He shall review all of the standards of each State for this purpose at least once each calendar year.

"(f) The Secretary is authorized to issue new standards and to amend existing standards from time to time as he determines necessary, and such new or amended standards shall be considered as initial standards issued under subsection (c) of this section for the purpose of their application to the States under this section.

"(g) The district courts of the United States shall have jurisdiction to restrain violations of this section. Actions to restrain such violations shall be brought by, and in, the name of the United States. In case of contumacy or refusal to obey a subpena upon any person under this subsection, the district court of the United States for any district in which such person is found or resides or transacts business, upon application by the United States and after notice to such person, shall have jurisdiction to issue an order requiring such person to appear and give testimony or to appear and produce documents, and any failure to obey

such order of the court may be punished by such court as a contempt thereof.

"(h) Every department, agency, and instrumentality of the Federal Government and of the States, and every person applying for a license, permit, or other authorization from the United States or from any State to discharge or otherwise dispose of any material in an area designated under subsection (a) of this section shall establish and maintain such records, make such reports, and provide such information as the Secretary may reasonably require to assist him in establishing standards under this section and in determining whether such department, agency, instrumentality, or person has acted or is acting in compliance with this section and shall, upon request by the Secretary, permit him at reasonable times to have access to and to copy such records. All information reported to, or otherwise obtained by, such Secretary or his representative pursuant to this subsection which contains or relates to a trade secret or other matter referred to in section 1905 of title 18 of the United States Code shall be considered confidential for the purpose of that section, except that such information may be disclosed to other officers or employees concerned with carrying out the provisions of this section.

"(i) (1) Whoever discharges (including, but not limited to, any spilling, leaking, pumping, pouring, emitting,

emptying, or dumping) any sewage, sludge, spoil, or other waste into or upon any waters or submerged lands within the jurisdiction of the United States and not within an area designated under subsection (a) of this section shall be subject to a civil penalty of not more than $10,000 for each offense. Any such civil penalty may be compromised by the Secretary referred to in subsection (k) (1) of this section.

"(2) Whoever violates any standard established under subsection (c) of this section shall be liable to a civil penalty of not more than $10,000 for each such violation. In the case of a continuing violation of such a standard, each day of violation shall be considered a separate offense for the purposes of this subsection. The Secretary of the Interior may assess and may mitigate, remit, or compromise any such penalty. In taking any penalty action for violation of a standard, the gravity of the violation, and the demonstrated good faith of the person charged in attempting to achieve rapid compliance, after notification of a violation, shall be considered by the Secretary of the Interior.

"(j) Upon the designation of waters or submerged lands under subsection (a) of this section, all licenses, permits, or authorizations which have been issued by any officer or employee of the United States under authority of any other provision of law shall be terminated and of no effect to the extent they authorize any activity prohibited by subsection

(i) of this section. Thereafter no license, permit, or authority shall be issued by any officer or employee of the United States which would authorize any activity prohibited by subsection (i) of this section.

"(k) (1) The Secretary of the department in which the Coast Guard is operating, acting through the Coast Guard, shall enforce subsection (i) (1) of this section.

"(2) The Secretary of the Interior shall enforce subsection (i) (2) of this section."

[H.R. 336, H.R. 548, H.R. 1382, H.R. 1674, H.R. 6305, 92d Cong., 1st sess.]

A BILL

To require the Council on Environmental Quality to make a full and complete investigation and study of national policy with respect to the discharging of material into the oceans.

Be it enacted by the Senate and House of Representatives of the United States of America in Congress assembled, That the Council on Environmental Quality established by Public Law 91-190 shall make a full and complete investigation and study of all aspects of existing national policy with respect to the discharge of any material of any kind into the waters of the Atlantic and Pacific Oceans, the Gulf of Mexico, and any other waters within the territorial sea and the contiguous zone of the United States. Upon completion of such investigation and study the Council shall report to

I

the President and Congress the results thereof, its recommendations for a national policy with respect to discharges into such waters including any treaties, agreements, and legislation necessary in connection therewith.

[H.R. 337, H.R. 549, H.R. 1381, 92d Cong., 1st sess.]

A BILL

To prohibit the discharge into any of the navigable waters of the United States or into international waters of any military material without a certification by the Council on Environmental Quality approving such discharge.

Be it enacted by the Senate and House of Representatives of the United States of America in Congress assembled, That after the date of enactment of this Act, no person shall discharge, directly or indirectly, into any of the navigable waters of the United States or into international waters any munition, or any chemical, biological, or radiological warfare agent, or any other military material except in accord-

I

ance with a certificate issued by the Council on Environmental Quality established by Public Law 91–190 permitting such discharge and establishing the terms, conditions, and limitations applicable thereto.

[H.R. 805, H.R. 807, H.R. 808, H.R. 1329, H.R. 2581, H.R. 5705, H.R. 7619, H.R. 8039, 92d Cong., 1st sess.]

A BILL

To amend the Fish and Wildlife Coordination Act to provide additional protection to marine and wildlife ecology by providing for the orderly regulation of dumping in the ocean, coastal, and other waters of the United States.

Be it enacted by the Senate and House of Representatives of the United States of America in Congress assembled, That the Fish and Wildlife Coordination Act (16 U.S.C. 661 et seq.) is amended by inserting immediately following section 5A thereof the following new section:

I

"SEC. 5B. For the purposes of this section:

(1) the term "ocean, coastal, and other waters" means oceans, gulfs, bays, salt-water lagoons, salt-water harbors, other coastal waters where the tide ebbs and flows, the Great Lakes, and all waters in a zone contiguous to the United States extending to a line twelve nautical miles seaward from the baseline of the territorial sea as provided in article 24 of the Convention on the Territorial Sea and the Contiguous Zone.

(2) the term "Admini[illegible]rator" means the Administrator of the Environmental Protection Agency.

"(b) The Administrator and the Secretary of the Interior (acting through the United States Fish and Wildlife Service) in consultation with the Secretary of the Army (acting through the Chief of Engineers of the United States Army Corps of Engineers), shall establish standards which apply to the deposit or discharge into the ocean, coastal, and other waters of the United States of all industrial wastes, sludge, spoil, and all other materials that might be harmful to the wildlife or wildlife resources or to the ecology of these waters. Such standards shall be for the purpose of insuring that no damage to the natural environment and ecology including but not limited to marine and wildlife ecology of the ocean, coastal, and other waters of the United States will result from any such activity. Such standards shall

62-513 O - 71 - 3

require, in part, that any person before depositing or discharging of such materials into the ocean, coastal, and other waters of the United States must present sufficient evidence to sustain a burden of proof that such materials in the location in which they are to be deposited will not endanger the natural environment and ecology of these waters, and to meet such additional requirements as the Administrator may deem necessary for the orderly regulation of such activity.

"(c) Such standards shall be adopted and enforced by any department, agency, or instrumentality of the Federal Government or any State department, agency, or instrumentality that issues any license, permit, or other authorization for any such activity with respect to any of such coastal waters.

"(d) Such standards shall be applicable to all of the departments, agencies, and instrumentalities of the Federal Government, to the States and their agencies, including any person having any license, permit, or other authorization from such State or agency for any such activity with respect to any of such ocean, coastal, and other waters.

"(e) After the date that a Federal standard is established under this section, a State may establish its own standard with respect to the activity covered by such Federal standard, except that the State standard must be more strin-

gent than the Federal standard and must provide adequate procedures for enforcement. Such a State standard shall apply to such activity within the State's jurisdiction and the Federal standard shall not apply. If the Administrator determines that such State standard is not as stringent as the Federal standard, or is not being enforced, then the Federal standard shall apply.

"(f) Every department, agency, and instrumentality of the Federal Government and of the States, and every person applying for a license, permit, or other authorization from the United States or from any State to discharge or otherwise dispose of any material in the coastal waters of the United States shall establish and maintain such records, make such reports, and provide such information as the Administrator may reasonably require to assist him in establishing standards under this section and in determining whether such department, agency, instrumentality, or person has acted or is acting in compliance with this section and shall, upon request by the Administrator, permit him to have access to and copy such records.

"(g) The district courts of the United States shall have jurisdiction to restrain violations of this section. Actions to restrain such violations shall be brought by, and in the name of, the United States. In case of contumacy or refusal to obey a subpena upon any person under this subsection, the district

court of the United States for any district in which such person is found or resides or transacts business, upon application by the United States and after notice to such person, shall have jurisdiction to issue an order requiring such person to appear and give testimony or to appear and produce documents, and any failure to obey such order of the courts may be punished by such court as a contempt thereof.

"(h) Whoever violates any standard established under subsection (b) of this section shall be liable to a civil penalty of not more than $10,000 nor less than $5,000 for each violation. In the case of a continuing violation of such a standard, each day of violation shall be considered a separate offense for the purposes of this section.

"(i) Upon the effective date of this section, all licenses, permits, or authorizations which have been issued by any officers or employee of the United States under authority of any other provision of law shall be terminated.

[H.R. 1095, 92d Cong., 1st sess.]

A BILL

To amend the Fish and Wildlife Coordination Act to provide additional protection to marine and wildlife ecology by requiring the designation of certain water and submerged land areas where the depositing of certain waste materials is prohibited, to require the establishment of standards with respect to such deposits in all other areas, and for other purposes.

Be it enacted by the Senate and House of Representatives of the Uniied States of America in Congress assembled, That the Fish and Wildlife Coordination Act (16 U.S.C. 661

I

et seq.) is amended by inserting immediately following section 5A thereof the following new sections:

"SEC. 5B. (a) The Secretary of the Interior, acting through the United States Fish and Wildlife Service, shall designate those portions of the navigable waters of the United States and those portions of the waters above the Outer Continental Shelf as defined in the Outer Continental Shelf Lands Act, and those portions of the submerged lands beneath the navigable waters of the United States and beneath the waters above the Outer Continental Shelf and those lands beneath international waters into and onto which he determines sewage, sludge, spoil, landfill, heated effluents, or any other waste or substance (solid, liquid, or gas) cannot be safely discharged. Areas designated under this section shall be known as "marine sanctuaries".

"(b) In making such designation the Secretary of the Interior shall—

"(1) consider the overall effect on the marine and wildlife ecological balance which discharging of such materials has had or will have in the area.

"(2) consider all effects of such discharges which he may find to be dangerous to the mating, spawning, and other necessary life processes of species of fish, shellfish, and all other forms of marine animal and plant life,

"(3) consider all other ecological and environmental factors, including, but not limited to, the ecological effect of discharging heated effluents into the area, and

"(4) consult with the appropriate Federal, State, and local agencies and officials, and with public or private organizations, institutions, agencies, and individuals with expertise in the sciences of ecology, marine biology, oceanography, and other related disciplines in the physical and biological sciences.

"(c) No designation shall be made by the Secretary of the Interior under authority of subsection (a) of this section during the one-year period beginning on the date of enactment of this section. During such one-year period the Secretary of the Interior, in cooperation with the Secretary of the Army acting through the Chief of Engineers, shall make a full and complete investigation and study of potential water and submerged land areas for designation and shall identify those areas most suitable for such designation.

"(d) Upon the designation of areas under subsection (a) of this section, all licenses, permits, or authorizations which have been issued by any officer or employee of the United States under authority of any other provision of law shall be terminated and of no effect to the extent they authorize any activity prohibited by subsection (e) of this section.

Thereafter no license, permit, or authority shall be issued by any officer or employee of the United States which would authorize any activity prohibited by subsection (e) of this section.

"(e) Whoever discharges spills, leaks, pours, emits, empties, dumps, or in any other way introduces, any sewage, sludge, spoil, landfill, heated effluents, or any other waste or substance (solid, liquid, or gas) into or upon any of the waters designated under subsection (a) of this section shall be fined not more than $10,000 for each offense.

"(f) The Secretary of the department in which the Coast Guard is operating, acting through the Coast Guard, shall enforce this section.

"SEC. 5C. (a) Within one hundred and eighty days after the designation of areas under subsection (a) of section 5B of this Act, the Secretary of the Interior shall establish standards which, after notice, shall be applicable to the discharge of any sewage, sludge, spoil, landfill, heated effluents, or any other waste or substance (solid, liquid, or gas), including but not limited to pesticide, herbicide, silt, and fertilizer runoff, within any area not designated under subsection (a) of section 5B of this Act. Such standards shall be for the purpose of insuring that no damage to, or loss of, any marine life or wildlife or other resources necessary for the ecological balance of the area or pollution of the navigable waters of

the United States will result from any such activity. Such standards shall require, in part, that any person before depositing or discharging such materials into the navigable and coastal waters of the United States and into any international waters must present sufficient evidence that discharging such materials in the location in which they are to be deposited will not endanger the natural environment and ecology of these waters. Such standards shall further include the following:

"(1) No sewage or industrial waste shall be discharged (directly or indirectly) into any area subject to standards issued under subsection (a) of this section after January 1, 1973, unless such sewage or industrial waste has received "at least" primary treatment "or such other treatment equal to primary treatment" in accordance with standards and regulations established by the Secretary of the Interior.

"(2) No sewage or industrial waste shall be discharged (directly or indirectly) into any area subject to standards issued under subsection (a) of this section after January 1, 1975, unless such sewage or industrial waste has received "at least" primary and secondary treatment "or such other treatment equal to primary and secondary treatment" in accordance with standards and regulations established by the Secretary of the Interior.

"(3) No sewage or industrial waste shall be discharged (directly or indirectly) into any area subject to standards issued under subsection (a) of this section after January 1, 1977, unless such sewage or industrial waste has received "at least" primary, secondary, and tertiary treatment "or such other treatment equal to primary, secondary, and tertiary treatment" in accordance with standards and regulations established by the Secretary of the Interior.

In addition, such person, prior to such discharging, must meet such additional requirements as the Secretary of the Interior may deem necessary for the orderly regulation of such activity. Such standards shall be applicable to all of the departments, agencies, and instrumentalities of the United States Government. Except as otherwise provided in this section, in the case of an area containing any submerged land within the jurisdiction of the States, such standards shall be applicable to the States and their agencies, including any person having any license, permit, or other authorization from such State or agency for any such activity with respect to any of such submerged lands.

"(b) Every department, agency, and instrumentality of the Federal Government and of the States, and every person applying for a license, permit, or other authorization from the United States or from any State to discharge or

otherwise dispose of any material in any area subject to standards issued under subsection (a) of this section shall establish and maintain such records, make such reports, and provide such information as the Secretary of the Interior may reasonably require to assist him in establishing standards under this section and in determining whether such department, agency, instrumentality, or person has acted or is acting in compliance with this section. Upon request, the Secretary of the Interior shall, at reasonable times, have access to examine and copy such records. All information reported to, or otherwise obtained by, the Secretary of the Interior, or his representative, pursuant to this subsection which contains or relates to a trade secret or other matter referred to in section 1905 of title 18 of the United States Code shall be considered confidential for the purpose of that section, except that such information may be disclosed to other officers or employees concerned with carrying out the provisions of this section. Officers or employees duly designated by the Secretary of the Interior, upon presenting appropriate credentials to the department, agency, instrumentality or person in charge, are authorized to enter at reasonable times, for the purpose of inspecting any plant, establishment or other property of such department, agency, instrumentality, or person to determine whether such depart-

ment, agency, instrumentality, or person has acted or is acting in compliance with this section.

"(c) The Secretary of the Interior is authorized to issue new standards and to amend existing standards from time to time as he determines necessary. Such new or amended standards, after notice, shall be considered as initial standards issued under subsection (a) of this section for the purpose of their application to the States under this section.

"(d) If a State, within one year of the date that a Federal standard is established under subsection (a) of this section, establishes its own standard with respect to the activity covered by such Federal standard which the Secretary of the Interior determines, after public hearing, is equal to or more stringent than such Federal standard, and if the Secretary of the Interior determines that there are adequate State enforcement procedures for such State standard, then such State standard shall apply to such activity within the State's jurisdiction, and the Federal standard shall not apply. If the Secretary of the Interior determines that such State standard is not as stringent as the Federal standard, then the Federal standard shall apply to such activity in such State.

"(e) Whenever a State's standard is applicable within the jurisdiction of that State it shall continue to be applicable until the Secretary of the Interior, after public hearing, de-

termines either that it is not as stringent as the comparable Federal standard or that there is not adequate State enforcement of such standard. He shall review all of the standards of each State for this purpose at least once during each calendar year.

"(f) Upon the issuance of standards under subsection (a) of this section applicable to any area, all licenses, permits, or authorizations which have been issued by any officer or employee of the United States under authority of any other provision of law with respect to discharges in an area shall be terminated and of no effect to the extent they authorize any activity prohibited by subsection (g) of this section.

"(g) Whoever discharges any waste or substance in violation of the standards established under subsection (a) of this section shall be subject to a civil penalty of not more than $10,000 for each violation. In the case of a continuing violation, each day of violation shall be considered a separate offense for the purposes of this subsection. The Secretary of the Interior may assess and may mitigate, remit, or compromise any such penalty. In taking any penalty action for violation of a standard, the gravity of the violation, and the demonstrated good faith of the person charged in attempting to achieve rapid compliance, after notification of a violation, shall be considered by the Secretary of the Interior.

"(h) The Secretary of the Interior shall enforce subsection (g) of this section.

"(i) The district courts of the United States shall have jurisdiction to restrain violations of this section and of section 5B of this Act. Actions to restrain such violations shall be brought by, and in, the name of the United States. In case of contumacy or refusal to obey a subpena upon any person under this subsection, the district court of the United States for any district in which such person is found or resides or transacts business, upon application by the United States and after notice to such person, shall have jurisdiction to issue an order requiring such person to appear and give testimony or to appear and produce documents, and any failure to obey such order of the court may be punished by such court as a contempt thereof.

"SEC. 5D. (a) Within one hundred and eighty days after the date of enactment of this section, the Secretary of Defense, acting through the Secretaries of the military departments, shall make a complete inventory of all existing munitions, chemical, biological, and radiological warfare agents, and other military materials, the ultimate disposition of which (other than for the purpose for which acquired) may present a danger to man, the environment, or to fish and wildlife, and with respect to each item on such inventory shall determine the date beyond which such items cannot

be safely retained. The Secretary shall submit to the Secretary of Interior, Council on Environmental Quality, the Environmental Protection Agency, and to the Congress an adequate plan (supported by the technology to carry out such plan), along with the inventory list and disposition dates provided for under the preceding sentence, for the demilitarization, detoxification, or decontamination of each such item in order to ultimately dispose of such items.

"(B) Prior to the acquisition, after the date of enactment of this Act, of any munitions, chemical, biological, or radiological warfare agents, or other military materials, the ultimate disposition of which (other than for the purpose for which acquired) may present a danger to man, the environment, or to fish and wildlife, the Secretary of Defense, acting through the Secretaries of the military departments, shall determine the date beyond which such munition, agent, or material cannot be safely retained, and shall submit to the Secretary of Interior, Council on Environmental Quality, and to the Congress an adequate plan (supported by the technology to carry out such plan) for the demilitarization, detoxification, or decontamination of such munition, agent, or material in order to ultimately dispose of such munition, agent, or material.

"(C) Notwithstanding any other provision of law, after the date of enactment of this Act it shall be unlawful for any

Federal official to discharge or dispose of, or cause to be discharged or disposed of, either directly or indirectly, any munitions, chemical, biological, or radiological warfare agents, or any other military materials, that may present a danger to man, the environment, or to fish and wildlife, into any navigable or coastal waters of the United States or into any international waters."

[H.R. 1383, 92d Cong., 1st sess.]

A BILL

To amend the Fish and Wildlife Coordination Act to provide additional protection to marine and wildlife ecology by providing for the orderly regulation of dumping in the coastal waters of the United States.

Be it enacted by the Senate and House of Representatives of the United States of America in Congress assembled, That the Fish and Wildlife Coordination Act (16 U.S.C. 661 et seq.) is amended by inserting immediately following section 5A thereof the following new section:

"SEC. 5B. (a) The Secretary of the Interior, acting through the United States Fish and Wildlife Service, and in consultation with the Chief of Engineers of the United States Army, shall establish standards which apply to the

I—O

62-513 O - 71 - 4

deposit or discharge into the coastal waters of the United States of all industrial wastes, sludge, spoil, and all other materials that might be harmful to the wildlife or wildlife resources or to the ecology of these waters. Such standards shall be for the purpose of insuring that no damage to the natural environment and ecology including but not limited to marine and wildlife ecology of the navigable waters of the United States will result from any such activity. Such standards shall require, in part, that any person before depositing or discharging of such materials into the coastal waters of the United States must present sufficient evidence to sustain a burden of proof that such materials in the location in which they are to be deposited will not endanger the natural environment and ecology of these waters, and to meet such additional requirements as the Secretary of the Interior may deem necessary for the orderly regulation of such activity.

"(b) Such standards shall be adopted and enforced by any department, agency, or instrumentality of the Federal Government or any State department, agency, or instrumentality that issues any license, permit, or other authorization for any such activity with respect to any of such coastal waters.

"(c) Such standards shall be applicable to all of the departments, agencies, and instrumentalities of the Federal

Government, to the States and their agencies, including any person having any license, permit, or other authorization from such State or agency for any such activity with respect to any of such coastal waters.

"(d) After the date that a Federal standard is established under this section, a State may establish its own standard with respect to the activity covered by such Federal standard, except that the State standard must be more stringent than the Federal standard and must provide adequate procedures for enforcement. Such a State standard shall apply to such activity within the State's jurisdiction and the Federal standard shall not apply. If the Secretary of the Interior determines that such State standard is not as stringent as the Federal standard, or is not being enforced, then the Federal standard shall apply.

"(e) Every department, agency, and instrumentality of the Federal Government and of the States, and every person applying for a license, permit, or other authorization from the United States or from any State to discharge or otherwise dispose of any material in the coastal waters of the United States shall establish and maintain such records, make such reports, and provide such information as the Secretary may reasonably require to assist him in establishing standards under this section and in determining whether such department, agency, instrumentality, or person has acted or

is acting in compliance with this section and shall, upon request by the Secretary, permit him to have access to and copy such records.

"(f) The district courts of the United States shall have jurisdiction to restrain violations of this section. Actions to restrain such violations shall be brought by, and in the name of, the United States. In case of contumacy or refusal to obey a subpena upon any person under this subsection, the district court of the United States for any district in which such person is found or resides or transacts business, upon application by the United States and after notice to such person, shall have jurisdiction to issue an order requiring such person to appear and give testimony or to appear and produce documents, and any failure to obey such order of the courts may be punished by such court as a contempt thereof.

"(g) Whoever violates any standard established under subsection (b) of this section shall be liable to a civil penalty of not more than $10,000 nor less than $5,000 for each violation. In the case of a continuing violation of such a standard, each day of violation shall be considered a separate offense for the purposes of this section.

"(h) Upon the effective date of this section, all licenses, permits, or authorizations which have been issued by any officers or employee of the United States under authority of any other provision of law shall be terminated.

[H.R. 1661, H.R. 5049, H.R. 5050, 92d Cong., 1st sess.]

A BILL

To regulate the discharge of wastes in territorial and international waters.

Be it enacted by the Senate and House of Representatives of the United States of America in Congress assembled, That no owner or master of a vessel may load, or permit the loading of, any waste on such vessel while such vessel is in any port of the United States, if such waste is to be discharged in ocean waters, unless such owner or master first—

(1) obtains a permit from the Administrator of the Environmental Protection Agency (hereafter referred to in this Act as the "Administrator") which authorizes the loading of such waste; and

(2) notifies the Coast Guard of such loading as prescribed in section 3.

SEC. 2. (a) The Administrator shall issue to any owner or master of a vessel a permit authorizing the loading of waste on such vessel if the Administrator finds that the discharge of such waste in any ocean waters will not damage the ecology of the marine environment. In making any such finding, the Administrator shall consider the effect of such discharge on human health and welfare (including possible adverse effects on economic, recreational and esthetic values) and on the marine ecosystem, taking into account the proposed location of such discharge and the concentration and volume of the waste to be discharged.

(b) In no event shall any permit be issued for the discharge of any waste whatever between the Continental Shelf and the coast of the United States.

(c) The Administrator shall have the authority to ban the loading, transporting and dumping of any specific matter deemed damaging to the marine environment or to human health and welfare.

(d) The Administrator shall have the authority to designate ocean dumping sites.

(e) Each permit issued under subsection (a) shall specify—

(1) the amount and type of waste authorized to be loaded and discharged;

(2) the exact coordinates of the location at which such discharge is permitted and a statement of the route to that location;

(3) such provisions as the Administrator deems necessary to insure that such waste will be transported to the discharge site without accidental spillage or leakage; and

(4) such other provisions as the Administrator deems necessary to carry out the purposes of this Act.

SEC. 3. (a) Any owner or master of a vessel who is issued a permit under section 2 must notify the Coast Guard and the Army Corps of Engineers of the exact location where the waste covered by such permit is to be discharged. Such notification must be given to the Coast Guard and the Army Corps of Engineers in such manner as the Administrators of the Department in which the Coast Guard is operating shall prescribe and not later than four hours before the departure of the vessel.

(b) The Administrator of the Department in which the Coast Guard is operating shall conduct surveillance and other appropriate enforcement activity to prevent violations of this Act.

SEC. 4. (a) Any owner or master of a vessel who violates the first section of this Act or who violates any provision of a permit issued under section 2 of this Act shall be liable to a civil penalty of not more than $50,000 for the first violation, and not more than $100,000 for each subsequent violation. No penalty shall be assessed until the person charged shall have been given notice and an opportunity for a public hearing on such charge. Upon failure of an offending party to pay the penalty, the Administrator may request the Attorney General to commence an action in the appropriate district court of the United States for such relief as may be appropriate.

(b) A vessel, other than a vessel owned or bargeboat chartered by the United States, or other property used in a violation shall be liable in rem for any civil penalty assessed under this section and may be proceeded against in any district court of the United States having jurisdiction thereof.

SEC. 5. As used in this Act—

(1) The term "discharge" means to place, release,

discharge, or by any means whatsoever to dispose, of waste in ocean waters.

(2) The term "master" includes any person acting in the capacity of a master.

(3) The term "ocean waters" means any estuarine area, coastal waters, Great Lakes, territorial waters, and the high seas adjacent to the territorial waters.

(4) The term "owner" includes any private individual or corporate owner and any public owner, whether a department, agency, or instrumentality of a State or a political subdivision thereof, of an interstate governmental entity, or of the Federal Government.

(5) The term "United States" means the States, the District of Columbia, the Commonwealth of Puerto Rico, Guam, and American Samoa.

(6) The term "vessel" includes any vessel, scow, or boat, whether or not documented under the laws of the United States, capable of being used to transport waste in ocean waters.

(7) The term "waste" means matter of any kind or description, including, but not limited to, dredge spoil, spoil waste, garbage, sewage sludge, munitions, chemical, biological and radiological warfare agents, radioactive materials, wrecked or discarded equipment, rock, sand, cellar dirt, and industrial wastes.

SEC. 6. This Act shall take effect immediately upon final passage as provided by law.

SEC. 7. On and after the effective date of this Act, any license, permit, or authorization issued by any officer or employee of the United States under the authority of any other provision of law shall be terminated and be of no effect whatsoever to the extent that such license, permit, or authorization authorizes any activity to which this Act applies.

92D CONGRESS
1ST SESSION

H. R. 3662

IN THE HOUSE OF REPRESENTATIVES

FEBRUARY 4, 1971

Mr. ROGERS (for himself, Mr. DINGELL, Mr. PELLY, Mr. McCLOSKEY, Mr. KEITH, Mr. MOSS, and Mr. CONTE) introduced the following bill; which was referred to the Committee on Merchant Marine and Fisheries

A BILL

To amend the Fish and Wildlife Coordination Act in order to protect marine environment by regulating the dumping of wastes in the coastal and ocean waters of the United States.

Be it enacted by the Senate and House of Representatives of the United States of America in Congress assembled, That the Fish and Wildlife Coordination Act (16 U.S.C. 661 et seq.) is amended by inserting immediately following section 5A thereof the following new section:

"SEC. 5B. (a) No person may dump waste material into the ocean waters of the United States, or transport such material through such waters, unless he has first obtained a

permit from the Administrator of the Environmental Protection Agency (hereafter referred to in this section as the 'Administrator') authorizing such dumping.

"(b) (1) Except as provided in paragraph (4) of this subsection, the Administrator may issue a permit to any person authorizing such person to dump waste material into ocean waters of the United States if the Administrator finds that such dumping will not damage the ecology of the marine environment. In making such finding, the Administrator shall take into account such factors as he deems appropriate, including the following:

"(A) The need for the proposed dumping.

"(B) The effect of such dumping on human health and welfare, including economic, esthetic, and recreational values.

"(C) The effect of such dumping on fisheries resources.

"(D) The effect of such dumping on marine ecosystems, particularly with respect to—

"(i) the transfer, concentration, and dispersion of such material and its byproducts through biological, physical, and chemical pathways,

"(ii) potential changes in marine ecosystems diversity and stability, and

"(iii) species and community population dynamics.

"(E) The persistence and permanence of the effects of the dumping.

"(F) The effect of dumping of particular volumes and concentrations of such materials.

"(G) Appropriate locations and methods of disposal, including land-based alternatives.

"(2) The Administrator may establish and issue various categories of permits, including general permits for the discharge of any class of waste material which he determines will have a de minimis impact.

"(3) Each permit issued under the authority of this section shall specify—

"(A) the type and amount of waste material authorized to be dumped;

"(B) the location at which such dumping is permitted;

"(C) the length of time for which the permit is valid and its expiration date;

"(D) any special provisions deemed necessary by the Administrator for the monitoring and surveillance of the dumping; and

"(E) such other provisions as the Administrator deems necessary to carry out the purposes of this section.

"(4) (A) The Administrator may not issue any permit authorizing the dumping of the following waste materials in the ocean waters of the United States:

"(i) Radioactive wastes;

"(ii) Toxic industrial wastes; and

"(iii) Chemical and biological warfare materials.

"(B) After January 1, 1972, no permit shall be issued by the Administrator for the dumping of sewage or industrial waste, unless such sewage or industrial waste has received primary treatment in accordance with standards and regulations established by the Administrator.

"(C) After January 1, 1974, no permit shall be issued by the Administrator for the dumping of sewage or industrial waste, unless such sewage or industrial waste has received primary and secondary treatment in accordance with standards and regulations established by the Administrator.

"(D) After January 1, 1976, no permit shall be issued by the Administrator for the dumping of sewage or industrial waste, unless such sewage or industrial waste has received primary, secondary, and tertiary treatment in accordance with standards and regulations established by the Administrator.

"(5) The Administrator may suspend, revoke, revise, or condition, partially or entirely, any permit issued by him for the dumping of waste material if he finds that such dumping cannot or will not be carried out in conformity with the provisions of such permit.

"(6) The Administrator may, considering the factors set forth in paragraph (1) of this subsection, designate recommended sites for the dumping of materials.

"(c) The Secretary of the department in which the Coast Guard is operating shall conduct surveillance and other appropriate enforcement activities in order to prevent violations of this section.

"(d) (1) Whoever violates any provision of this section, of any regulation promulgated under this section, or of any permit issued under this section, shall be liable to a civil penalty of not more than $50,000 for each violation, to be assessed by the Administrator. No penalty shall be assessed until the person charged shall have been given notice and an opportunity for a public hearing on such charge. In determining the amount of the penalty, or the amount agreed upon in compromise, the gravity of the violation and the demonstrated good faith of the person charged in attempting to achieve rapid compliance after notification of a violation shall be considered by the Administrator. Thereafter, upon failure of the offending party to

pay the penalty, the Administrator may request the Attorney General to commence an action in the appropriate district court of the United States for such relief as may be appropriate.

"(2) In addition to any action which may be brought under paragraph (1) of this subsection, whoever knowingly violates any provision of this section, of any regulation promulgated under this section, or of any permit issued under this section, shall be fined not more than $50,000, or imprisoned for not more than one year, or both.

"(3) For the purpose of imposing civil penalties and criminal fines under this subsection, each day of a periodic violation shall constitute a separate offense, and one-half of the penalties and fines recovered shall be payable to the informer who provides the information resulting in the penalties or fines and who may sue for the same.

"(4) The Attorney General or his delegate may bring actions for equitable relief to redress violations of this section, of any regulation promulgated under this section, or of any permit issued under this section, and the district courts of the United States shall have jurisdiction to grant such relief as the equities of the case may require.

"(5) A vessel, other than a vessel owned or bareboat chartered by the United States, or other property used in a violation shall be liable in rem for any civil penalty assessed

and may be proceeded against in any district court of the United States having jurisdiction thereof; except that no vessel or other property shall be liable unless it shall appear that the owner was at the time of the violation a consenting party or privy thereto.

"(e) (1) On and after the effective date of this section, any license, permit, or authorization issued by any officer or employee of the United States under the authority of any other provision of law shall be terminated and be of no effect whatsoever to the extent that such license, permit, or authorization authorizes any activity to which this section applies. After such effective date, no license, permit, or authorization shall be issued by any officer or employee of the United States other than the Administrator which would authorize any activity to which this section or regulations issued hereunder apply.

"(2) Nothing in this section shall be construed as abrogating or negating any existing responsibility or authority contained in the Rivers and Harbors Act of 1899; except that on and after the effective date of this section, any Federal license or permit proposed to be issued under the authority of such Act of 1899 to conduct any activity otherwise regulated by this section and the regulations issued hereunder, shall be conditioned upon certification by the Administrator

62-513 O - 71 - 5

that the activity proposed to be conducted is in conformity with this section.

"(3) Before issuing any permit under this section, where it appears to the Administrator that the disposition of the waste material to be discharged may affect navigation in the navigable waters of the United States or may create an artificial island on the Outer Continental Shelf, the Administrator shall consult with the Secretary of the Army and no permit shall be issued if the Secretary of the Army determines that navigation will be unreasonably impaired.

"(f) Nothing in this section shall be construed as preempting any State or subdivision thereof from imposing more stringent requirements or liabilities regarding the discharge of any waste material (1) having its origin in such State or subdivision or (2) in any area where such State or subdivision has competent jurisdiction.

"(g) As used in this section—

"(1) The term 'waste material' means all solid and liquid products or byproducts of industrial processes (including tailings, sediment, and like materials resulting from marine mining or dredging activities), industrial waste acids, chemicals, sewage, sludge, garbage, dredge spoils, radioactive materials, construction and demolition debris, military ordnance, explosives, and any other form of discarded material or equipment.

"(2) The term 'ocean waters' means any estuarine area, coastal waters, the Great Lakes, and waters above the Outer Continental Shelf.

"(3) The term 'estuarine area' means an estuary and those transitional areas which are consistently influenced or affected by waters from the estuary such as, but not limited to, salt marshes, coastal and intertidal areas, bays, harbors, lagoons, inshore waters, and channels.

"(4) The term 'estuary' includes all or part of the mouth of a navigable or interstate river or stream or other body of water having an unimpaired natural connection with the open sea and within which the sea water is measurably diluted with fresh water derived from land drainage.

"(5) The term 'coastal waters' means the waters lying seaward of the line of ordinary low water along that portion of the coast which is in direct contact with the open sea and the line marking the seaward limit of inland waters to a distance of three miles from such lines.

"(6) The term 'Outer Continental Shelf' means land extending from the three mile territorial limit out to the 200-meter depth contour.

"(7) The term 'United States' means the States,

the District of Columbia, the Commonwealth of Puerto Rico, Guam, and American Samoa.

"(8) The term 'person' means any individual, corporation, firm, association, or other entity and shall include any officer, department, agency, or instrumentality of any State, interstate, or local unit of government, and any officer, department, agency, or instrumentality of the Federal Government.

"(9) The term 'dumping' means to place, release, discharge, or by any means whatsoever dispose of material into ocean, coastal, or other waters.

"(h) The Administrator shall direct and conduct such investigation and research with respect to the marine ecology as is necessary to carry out the purposes of this section. To support such research and investigation, there is authorized to be appropriated $1,000,000 for the fiscal year beginning July 1, 1971, and a like sum for each fiscal year thereafter."

SEC. 2. (a) Section 6 of the Fish and Wildlife Coordination Act is amended by inserting after "Act" the first time it appears therein the following: "(other than section 5B (h))".

(b) Section 7 of such Act is amended by inserting after "Act" the following: "(other than a rule or regulation promulgated pursuant to section 5B)".

[H.R. 4217, H.R. 4218, H.R. 4719, H.R. 6610, 92d Cong., 1st sess.]

A BILL

To prohibit the discharge into any of the navigable waters of the United States or into international waters of any military material or other refuse without a certification by the Environmental Protection Agency approving such discharge.

Be it enacted by the Senate and House of Representatives of the United States of America in Congress assembled, That after the date of enactment of this Act, no person shall discharge, directly or indirectly, into any of the navigable waters of the United States or into international waters (A) any munition, or any chemical, biological, or radiological warfare agent, or any other military material or (B) any

I

other refuse matter of any kind or description whatever except in accordance with a certificate issued by the Administrator of the Environmental Protection Agency permitting such discharge and establishing the terms, conditions, limitation, and penalties applicable thereto.

[H.R. 4247, H.R. 4723, H.R. 5239, H.R. 5268, H.R. 5477, H.R. 6582, H.R. 6771, 92d Cong., 1st sess.]

A BILL

To regulate the dumping of material in the oceans, coastal, and other waters and for other purposes.

Be it enacted by the Senate and House of Representatives of the United States of America in Congress assembled, That this Act may be cited as the "Marine Protection Act of 1971".

FINDING POLICY AND PURPOSE

SEC. 2. (a) Unregulated dumping of material into the oceans, coastal, and other waters endangers human health, welfare, and amenities, and the marine environment, ecological systems, and economic potentialities.

(b) Congress declares that it is the policy of the United States to regulate the dumping of all types of material in the

I

oceans, coastal, and other waters and to prevent or vigorously limit the dumping into the oceans, coastal, and other waters of any material which could adversely affect human health, welfare, or amenities, or the marine environment, ecological systems, or economic potentialities. To this end, it is the purpose of this Act to regulate the transportation of material from the United States for dumping into the oceans, coastal, and other waters, and the dumping of material by any person from any source if the dumping occurs in waters over which the United States has jurisdiction.

DEFINITIONS

SEC. 3. For the purposes of this Act the term—

(a) "Administrator" means the Administrator of the Environmental Protection Agency.

(b) "Oceans, coastal, and other waters" means oceans, gulfs, bays, salt water lagoons, salt water harbors, other coastal waters where the tide ebbs and flows, and the Great Lakes.

(c) "Material" means matter of any kind or description, including, but not limited to, dredge spoil, solid waste, garbage, sewage, sludge, munitions, chemical, biological, and radiological warfare agents, radioactive materials, wrecked or discarded equipment, rock, sand, cellar dirt, and industrial waste: *Provided*, That it does not mean oil within the meaning of section 11 of the Federal Water Pollution Control Act

or sewage from vessels within the meaning of section 13 of said Act.

(d) "United States" includes the several States, the District of Columbia, the Commonwealth of Puerto Rico, the Canal Zone, the territories and possessions of the United States and the Trust Territory of the Pacific Islands.

(e) "Person" means any private person or entity, any employee, agent, department, agency, or instrumentality of any State or local unit of government, or foreign government, and, except as to the provisions of section 6, any employee, agent, department, agency, or instrumentality of the Federal Government.

(f) "Dumping" means a disposition of material: *Provided,* That it does not mean a disposition of any effluent from any outfall structure, or a routine discharge of effluent incidental to the propulsion of vessels: *And provided further,* That it does not mean the intentional placement of any device in the oceans, coastal, or other waters or on the submerged land beneath such waters, for the purpose of using such device there to produce an effect attributable to other than its mere physical presence.

(g) "District Court of the United States" includes the District Court of Guam, the District Court of the Virgin Islands, the District Court of the Canal Zone, and in the case of American Samoa and the Trust Territory of the

Pacific Islands, the District Court of the United States for the District of Hawaii, which court shall have jurisdiction over actions arising therein.

PROHIBITED ACTS

SEC. 4. Except as such transportation or dumping or both may be authorized in a permit issued by the Administrator:

(a) No person shall transport material from the United States for the purpose of dumping it into the oceans, coastal, and other waters, and

(b) No person shall dump material (1) in that part of the oceans, coastal, and other waters which is within the territorial jurisdiction of the United States, or, (2) in a zone contiguous to the territorial sea of the United States, extending to a line twelve nautical miles seaward from the b[illegible]e line of the territorial sea as provided in Article 24 of the Convention on the Territorial Sea and the Contiguous Zone, to the extent that it may affect the territorial sea or the territory of the United States.

PERMITS

SEC. 5. (a) The Administrator may issue permits to transport material for dumping into the oceans, coastal, and other waters, or to dump material into the waters described in subsection 4(b), or both, where the applicant presents information respecting the proposed activity which in the

judgment of the Administrator indicates that such transportation, or dumping, or both will not unreasonably degrade or unreasonably endanger human health, welfare, or amenities, or the marine environment, ecological systems, or economic potentialities. The Administrator shall establish and apply criteria for reviewing and evaluating such permit applications, and, in establishing or revising such criteria, shall consider, but not be limited in his consideration to, the following—

(1) the likely impact of the proposed dumping on human health, welfare, and amenities, and on the marine environment, ecological systems, and economic potentialities, including an assessment of—

(A) the possible persistence or permanence of the effects of the proposed dumping,

(B) the volume and concentration of materials involved, and

(C) the location proposed for the dumping.

(2) alternative locations and methods of disposal, including land-based alternatives; the probable impact of requiring the use of such locations or methods of disposal on considerations affecting the public interest; and the probable impact of issuing or denying permits on considerations affecting the public interest.

In establishing or revising such criteria, the Administrator

shall consult with the Secretaries of Commerce, Interior, State, Defense, Agriculture, Health, Education, and Welfare, and Transportation, the Atomic Energy Commission, and other appropriate Federal, State, and local officials. With respect to such criteria, as may affect the civil works program of the Department of the Army, the Administrator shall also consult with the Secretary of the Army. In reviewing applications for permits, the Administrator shall make such provision for consultation with interested Federal and State agencies as he deems useful or necessary. No permit shall be issued for a dumping of material which will violate applicable water quality standards.

(b) (1) The Administrator may establish and issue various categories of permits, including the general permits described in subsection (e).

(2) The Administrator may require an applicant for a permit under subsection (a) to provide such information as the Administrator may consider necessary to review and evaluate such an application.

(c) Permits issued under subsection (a) may designate and include (1) the type of material authorized to be transported for dumping or to be dumped; (2) the amount of material authorized to be transported for dumping or to be dumped; (3) the location where such transport for dumping will be terminated or where such dumping will

occur; (4) the length of time for which the permits are valid and their expiration date; and (5) such other matters as the Administrator deems appropriate.

(d) The Administrator may prescribe such processing fees for permits and such reporting requirements for actions taken pursuant to permits issued under subsection (a) as he deems appropriate.

(e) Notwithstanding any other provision of this Act, the Administrator may issue general permits for the transportation for dumping, or dumping, or both, of classes of materials which he determines will have a minimal impact, considering the factors, stated in subsection (a).

(f) The Administrator may limit or deny the issuance of permits, or may alter or revoke partially or entirely the terms of permits issued by him under this Act, for the transportation for dumping, or the dumping, or both, of specified material, where he finds that such material cannot be dumped consistently with the criteria established pursuant to subsection (a). No action shall be taken under this subsection unless the affected person or permittee shall have been given notice and opportunity for hearing on such action as proposed.

(g) The Administrator may, considering the criteria established pursuant to subsection (a), designate recommended sites for the dumping of specified materials.

(h) Nothing in this Act shall prohibit any transportation for dumping or dumping of material where such transportation or dumping is necessary, in an emergency, to safeguard human life. Such transportation or dumping shall be reported to the Administrator within such times and under such conditions as he may prescribe by regulation.

PENALTIES

SEC. 6. (a) A person who violates section 4 of this Act, or regulations promulgated under this Act, or a permit issued under this Act by the Administrator shall be liable to a civil penalty of not more than $50,000 for each violation to be assessed by the Administrator. No penalty shall be assessed until the person charged shall have been given notice and an op[illegible]unity for a hearing on such violation. Any such civil penalty may be compromised by the Administrator. In determining the amount of the penalty, or the amount agreed upon in compromise, the gravity of the violation and the demonstrated good faith of the person charged in attempting to achieve rapid compliance after notification of a violation shall be considered by said Administrator. Upon failure of the offending party to pay the penalty, the Administrator may request the Attorney General to commence an action in the appropriate district court of the United States for such relief as may be appropriate.

(b) In addition to any action which may be brought under subsection (a), a person who knowingly and willfully violates section 4 of this Act, regulations promulgated under this Act, or a permit issued under this Act by the Administrator shall be fined not more than $50,000 or imprisoned for not more than one year, or both.

(c) For the purpose of imposing civil penalties and criminal fines under this section, each day of a continuing violation shall constitute a separate offense.

(d) The Attorney General or his delegate may bring actions for equitable relief to redress a violation by any person of this Act, regulations promulgated under this Act, and permits issued under this Act by the Administrator, and the district courts of the United States shall have jurisdiction to grant such relief as the equities of the case may require.

(e) A vessel, except a public vessel within the meaning of subsection 13(a)(3) of the Federal Water Pollution Control Act or other public property of a similar nature, used in a violation shall be liable in rem for any civil penalty assessed or criminal fine imposed and may be proceeded against in any district court of the United States having jurisdiction thereof: *Provided*, That no vessel shall be liable unless it shall appear that the owner was at the time of the violation a consenting party or privy to such violation.

(f) If the provisions of any permit issued under sub-

section (a) of section 5 are violated, the Administrator may revoke the permit or may suspend the permit for a specified period of time. No permit shall be revoked or suspended unless the permittee shall have been given notice and opportunity for a hearing on such violation and proposed suspension or revocation.

RELATIONSHIP TO OTHER LAWS

SEC. 7. (a) After the effective date of this Act, all licenses, permits, or authorizations which have been issued by any officer or employee of the United States under authority of any other provision of law shall be terminated and of no effect to the extent they authorize any activity regulated by this Act. Thereafter, except as hereafter provided, no license, permit, or authority shall be issued by any officer or employee of the United States other than the Administrator which would authorize any activity regulated by this Act or the regulations issued hereunder.

(b) Nothing in this Act shall abrogate or negate any existing responsibility or authority contained in the Atomic Energy Act of 1954, as amended, and section 4 and subsection 7 (a) of this Act shall not apply to any activity regulated by that Act: *Provided,* The Atomic Energy Commission shall consult with the Administrator prior to issuing a permit to conduct any activity which would otherwise be regulated by this Act. In issuing any such permit, the Atomic

Energy Commission shall comply with standards set by the Administrator respecting limits on radiation exposures or levels, or concentrations or quantities of radioactive material. In setting such standards for application to the oceans, coastal, and other waters, or for specific portions of such waters, the Administrator shall consider the policy expressed in subsection 2 (b) of this Act and the factors stated in subsections 5 (a) (1) and 5 (a) (2) of this Act.

(c) (1) The provisions of subsection (a) shall not apply to actions taken before or after the effective date of this Act under the authority of the Rivers and Harbors Act of 1899 (33 U.S.C. 401 et seq.).

(2) Except as provided in subsection 11 (e), nothing in this Act shall be construed as abrogating or negating any existing responsibility or authority contained in the Rivers and Harbors Act of 1899: *Provided,* That after the effective date of this Act, no Federal license or permit shall be issued under the authority of the Rivers and Harbors Act of 1899 to conduct any activity otherwise regulated by section 4 of this Act and the regulations issued hereunder, unless the Administrator has certified that the activity proposed to be conducted is in conformity with the provisions of this Act and with the regulations issued hereunder.

62-513 O - 71 - 6

(3) Where a license or permit to conduct an activity has been granted under the authority of subsections (c) (1) and (c) (2) of this section and of the Rivers and Harbors Act of 1899, no separate permit to conduct such activity shall be required under this Act.

(d) Prior to issuing any permit under this Act, where it appears to the Administrator that the disposition of the material to be transported for dumping or to be dumped may affect navigation in the navigable waters of the United States or may create an artificial island on the Outer Continental Shelf, the Administrator shall consult with the Secretary of the Army and no permit shall be issued if the Secretary of the Army determines that navigation will be unreasonably impaired.

(e) Nothing in this Act shall be construed as preempting any State, Federal territory or commonwealth, or subdivision thereof from imposing any requirement of liability.

ENFORCEMENT

SEC. 8. (a) The Administrator may, whenever appropriate, utilize by agreement, the personnel, services, and facilities of other Federal departments, agencies, and instrumentalities, or State agencies or instrumentalities, whether on a reimbursable or a nonreimbursable basis.

(b) The Administrator may delegate responsibility and authority for reviewing and evaluating permit applications, including the decision as to whether a permit will be issued, to an officer of the Environmental Protection Agency, or he may delegate, by agreement, such responsibility and authority to the heads of other Federal departments or agencies, whether on a reimbursable or nonreimbursable basis.

(c) The Secretary of the department in which the Coast Guard is operating shall conduct surveillance and other appropriate enforcement activity to prevent unlawful transportation of material for dumping or dumping.

REGULATIONS

SEC. 9. In carrying out the responsibilities and authority conferred by this Act, the Administrator is authorized to issue such regulations as he may deem appropriate.

INTERNATIONAL COOPERATION

SEC. 10. The Secretary of State, in consultation with the Administrator, shall seek effective international action and cooperation to ensure protection of the marine environment, and may for this purpose, formulate, present, or support specific proposals in the United Nations and other competent international organizations for the development of appropriate international rules and regulations in support of the policy of this Act.

REPEAL AND SUPERSESSION

SEC. 11. (a) The second proviso to the last paragraph of section 20 of the Act of March 3, 1899 (30 Stat. 1154), as amended (33 U.S.C. 418), is repealed.

(b) Sections 1, 2, 3, 4, 5, 6, and 7 of the Act of June 29, 1888 (25 Stat. 209), as amended (33 U.S.C. 441–451b), are repealed.

(c) Section 2 of the Act of August 5, 1886 (24 Stat. 329; 33 U.S.C. 407a), is repealed.

(d) To the extent that it authorizes action regulated by this Act, section 4 of the Act of March 3, 1905 (33 Stat. 1147; 33 U.S.C. 419), is superseded.

(e) Section 13 of the Rivers and Harbors Act of 1899 (30 Stat. 1152), as amended (33 U.S.C. 407), is superseded insofar as it applies to dumping, as defined in subsection 3 (f) of this Act, of material in the waters covered by subsection 4 (b) of this Act.

EFFECTIVE DATE AND SAVINGS PROVISION

SEC. 12. (a) This Act shall take effect six months after its enactment.

(b) No legal action begun, or right of action accrued, prior to the effective date of this Act shall be affected by any provision of this Act.

AUTHORIZATION FOR APPROPRIATIONS

SEC. 13. There is hereby authorized to be appropriated, out of any moneys in the Treasury not otherwise appropriated, such sums as may be necessary for the purposes and administration of this Act.

[H.R. 4359, H.R. 4360, H.R. 4361, 92d Cong., 1st sess.]

A BILL

To amend the Act of August 3, 1968 (82 Stat. 625), to protect the ecology of estuarine areas by regulating dumping of waste materials, to authorize the establishment of a system of marine sanctuaries, and for other purposes.

Be it enacted by the Senate and House of Representatives of the United States of America in Congress assembled, That section 1 of the Act of August 3, 1968 (82 Stat. 625), is amended by adding at the end thereof the following:

"Congress further finds and declares that many estuaries of the Nation are being subjected to severe ecological degradation through the unregulated dumping into the oceans and into the coastal waters of the United States of polluted dredge spoils, industrial wastes, sewage, and refuse

I—O

in ever-increasing quantities; that such dumping should not be permitted except when it has been determined that the material cannot adversely affect the ecology of the oceans and of the coastal waters and estuaries of the United States, and that portions of the Nation's tidelands, Outer Continental Shelf, seaward areas and land and waters of the Great Lakes should be preserved as marine sanctuaries where industry development and extraction of the nonliving resources of the seabed and subsoil thereof and dumping of any kind should be prohibited."

SEC. 2. Section 6 of the Act of August 3, 1968 (82 Stat. 628), is amended to read as follows:

"After the effective date of this section, no citizen of the United States or other person shall dispose of waste materials into the oceans, coastal waters, and estuarine areas of the United States or into the Great Lakes unless he shall have first secured a permit from the Administrator of the Environmental Protection Agency under authority of this Act for the transportation and dumping or disposal by whatever means contemplated of such waste material."

SEC. 3. The Act of August 3, 1968 (82 Stat. 625 et seq.), is amended by inserting immediately following section 6 thereof the following new sections:

"SEC. 7. (a) The Administrator of the Environmental Protection Agency may issue permits authorizing the dump

ing or disposal of waste material into the oceans, coastal waters, and estuarine areas of the United States, and into the Great Lakes under such terms and conditions as he determines necessary to insure that such dumping or disposal will not damage the ecology of the marine environment.

"(b) In determining whether any dumping or disposal for which a permit is sought will damage the ecology of the marine environment, the Administrator shall consider among other factors the following:

"(1) Present and future impact on the marine environment, human health, welfare, and amenities;

"(2) Irreversibility of the impact of dumping or disposal;

"(3) Volume and concentration of materials involved;

"(4) Location of disposal, depth, and potential impact of one location relative to others;

"(c) No permit shall be issued by the Administrator for the dumping or disposal of the following materials:

"(1) Radioactive wastes;

"(2) Toxic industrial wastes;

"(3) Chemical and biological warfare materials.

"(d) After January 1, 1972, no permit shall be issued by the Administrator for the dumping or disposal of sewage or industrial waste, unless such sewage or industrial waste

has received primary treatment in accordance with standards and regulations established by the Administrator.

"(e) After January 1, 1974, no permit shall be issued by the Administrator for the dumping or disposal of sewage or industrial waste, unless such sewage or industrial waste has received primary and secondary treatment in accordance with standards and regulations established by the Administrator.

"(f) After January 1, 1976, no permit shall be issued by the Administrator for the dumping or disposal of sewage or industrial waste, unless such sewage or industrial waste has received primary, secondary, and tertiary treatment in accordance with standards and regulations established by the Administrator.

"(g) The Administrator of the Environmental Protection Agency may by regulation prohibit the disposal or dumping of any waste material which he determines may damage the ecology of the marine environment, and in making such determination he may rely upon whatever indicators are currently available to him, regardless of the fact that such indicators may not be conclusive.

"SEC. 8. (a) Whoever violates the provisions of section 6 of this Act shall be fined not less than $2,000 nor more than $10,000 for the first offense, and not less than $10,000 nor more than $25,000 for each succeeding offense.

In the case of a continuous disposal extending over a period of time, each day that such disposal occurs shall be considered a separate offense. Any vessel or barge engaged in the dumping or disposal of waste material in violation of this Act shall be forfeited to the United States.

"(b) The Administrator of the Environmental Protection Agency, the Secretary of the Department in which the Coast Guard is operating acting through the Coast Guard, and the Secretary of the Army acting through the Corps of Engineers shall enforce section 6 of this Act under regulations and operational directives jointly agreed to. The Coast Guard is hereby empowered to stop, search, and detain in the territorial sea or contiguous zone of the United States any vessel or barge which appears to be engaged in dumping operations or which appears to be transporting waste material for the purpose of determining whether such vessel or barge is covered by a permit issued under authority of this Act. There is hereby authorized to be appropriated such sums as may be necessary to carry out the enforcement activities authorized by this subsection.

"(c) The district courts of the United States shall have jurisdiction to restrain violations of section 6 of this Act. Actions to restrain such violations shall be brought by, and in, the name of the United States. In the case of contumacy or

refusal to obey a subpena upon any person, the district court of the United States for any district in which such person is found or resides or transacts business, upon application by the United States, and after notice to such person, shall have jurisdiction to issue an order requiring such person to appear and give testimony or to appear and produce documents, and any failure to obey such order of the court may be punished by such court as a contempt thereof.

"SEC. 9. (a) The Secretary of Commerce acting through the Administrator of the National Oceanic and Atmospheric Administration after consultation with the Secretary of the Interior, the Administrator of the Environmental Protection Agency, and the Council on Environmental Quality shall designate as marine sanctuaries those areas of the Nation's tidelands, Outer Continental Shelf, seaward areas, and land and waters of the Great Lakes which the Secretary determines should be preserved or restored for their recreation, conservation, ecologic, or esthetic values.

"(b) The Secretary of Commerce shall make his initial designation under subsection (a) of this section within two years following the date of enactment of this section. Thereafter, he shall periodically designate such additional areas as he deems appropriate. The Secretary shall submit a report to the President and Congress annually setting forth a com-

prehensive review of his actions under the authority of this section, together with such recommendations for further legislation as he deems appropriate to further the designation and preservation of marine sanctuaries.

"(c) In conducting the studies, the Secretary shall schedule hearings in areas contiguous to the proposed sanctuary sites, for the purposes of receiving views on the establishment of such marine sanctuaries.

"(d) The Secretary of the Interior shall not issue or renew any license, permit, or other authorization for the exploration, development, mining, or removal of any minerals (including gas and oil) from any area designated or under study for possible designation as a marine sanctuary.

"(e) While any area is under study for designation as a marine sanctuary, the Secretary is authorized to cooperate with all affected Federal, State, local, and international organizations in order that, until the completion of such study, a moratorium on the industrial development of any portion of the tidelands, Outer Continental Shelf, seaward areas, and land and waters of the Great Lakes under consideration as a possible marine sanctuary may be agreed upon by such interested parties.

"(f) The Administrator of the Environmental Protection Agency shall not issue or renew any permit for the dumping or disposal of any waste material in any area desig-

nated or under study for possible designation as a marine sanctuary.

"(g) There is authorized to be appropriated not to exceed $5,000,000 for the conduct of such studies as may be necessary to carry out the purposes of this section.

"SEC. 10. The provisions of this Act shall be considered as supplementary to the Fish and Wildlife Coordination Act (16 U.S.C. 661 et seq.) and the National Environmental Policy Act of 1969 (42 U.S.C. 4331 et seq.). All other provisions of law which are in conflict with this Act are hereby repealed.

"SEC. 11. For the purposes of this Act—

"(a) The term 'waste material' means all solid and liquid products or byproducts of the industrial processes (including tailings, sediment, and like materials resul[illegible]ng from marine mining or dredging activities), industrial waste acids, chemicals, sewage, sludge, garbage, dredge spoils, radioactive materials, construction and demolition debris, military ordinance, explosives, and any other form of discarded material or equipment.

"(b) The term 'coastal waters' means the waters lying seaward of the line of ordinary low water along that portion of the coast which is in direct contact with the open sea and the line marking the seaward limit of inland waters to a distance of three miles from such lines. As used with reference

to the Great Lakes, 'coastal waters' means those boundary waters between the United States and Canada lying on the United States side of the International Boundary between the United States and Canada.

"(c) The term 'oceans' means those portions of the high seas as defined in the Convention on the High Seas lying seaward of the outer limits of the coastal waters of the United States.

"(d) The terms 'estuary' and 'estuarine areas' mean an environmental system consisting of an estuary and those transitional areas which are consistently influenced or affected by waters from an estuary such as, but not limited to, salt marshes, coastal and intertidal areas, bays, harbors, lagoons, inshore waters, and channels, and the term 'estuary' shall include all or part of the mouth of a navigable or interstate river or stream or other body of water having an unimpaired natural connection with the open sea and within which the sea water is measurably diluted with fresh water derived from land drainage.

"(e) The term 'citizen of the United States' means officers and employees of the United States, or of any political subdivision thereof, all natural persons who are citizens of the United States, all partnerships or other associations which include in their membership one or more citizens of the United States, and the officers and directors

of all corporations organized under the laws of the United States or of any State of the United States.

"(f) The term 'other person' means the resident officers, directors or managers of foreign partnerships, associations, or corporations doing business in the United States.

"(g) The terms 'dumping' and 'disposal' mean to place, release, or discharge by any means whatsoever.

"(h) The term 'tidelands' means bays, estuaries, land, and waters within the three-mile territorial limit of the United States.

"(i) The term 'Outer Continental Shelf' means land and waters extending from the three-mile territorial limit out to the two-hundred-meter depth contour.

"(j) The term 'seaward areas' means land and waters contiguous to and extending from the two-hundred-meter depth contour."

SEC. 4. The provisions of section 2 of this Act shall become effective one hundred and twenty days after the date of enactment of this Act.

92D CONGRESS
1ST SESSION

H. R. 4584

IN THE HOUSE OF REPRESENTATIVES

FEBRUARY 18, 1971

MR. LENNON introduced the following bill; which was referred to the Committee on Merchant Marine and Fisheries

A BILL

To prohibit the discharge into any of the navigable waters of the United States or into international waters of any military or waste material without a certification by the Environmental Protection Agency and the National Oceanic and Atmospheric Administration approving such discharge.

Be it enacted by the Senate and House of Representatives of the United States of America in Congress assembled, That after the date of enactment of this Act, no person shall discharge, directly or indirectly, into any of the navigable waters of the United States or into international waters any munition, or any chemical, biological, or radiological warfare agent, or any other military material except in accordance with a certificate issued by the Environmental Protection

Agency and the National Oceanic and Atmospheric Administration permitting such discharge and establishing the terms, conditions, limitation, and penalties applicable thereto.

82-513 O - 71 - 7

DEPARTMENT OF AGRICULTURE,
OFFICE OF THE SECRETARY,
Washington, D.C., April 19, 1971.

Hon. EDWARD A. GARMATZ,
Chairman, Committee on Merchant Marine and Fisheries,
House of Representatives, Washington, D.C.

DEAR MR. CHAIRMAN: This is in response to your request for reports on H.R. 4247 and H.R. 4723, bills "To regulate the dumping of material in the oceans, coastal, and other waters and for other purposes."

This Department supports the enactment of H.R. 4247 and H.R. 4723 which carry out the recommendations set forth by the President in his February 8, 1971, message on the environment.

Under these bills, the Administrator of the Environmental Protection Agency would be authorized to issue permits for dumping materials into oceans, coastal, and other waters when, in his judgment, such dumping will not unreasonably endanger human health, welfare, or amenities, or the marine environment, ecological systems, or economic potentialities.

The Administrator, EPA, would be directed to establish criteria for evaluating permit applications on the basis of their likely environmental impact including (1) possible persistence of the effects of the proposed dumping, (2) volume and concentration of materials involved, and (3) the location proposed for dumping.

Of especial interest to this Department is the provision (Sec. 5(a)(2) that the Administrator, EPA, consider "alternate locations and methods of disposal including land-based alternatives. . . ." Since most of the land in the United States is rural land, used for farming or forestry, this Department is concerned with any land-based alternatives which might be considered. The Department of Agriculture has information and expertise relevant to the suitability of various land sites for disposal of solids, either as sanitary landfills or through methods by which many solids may be beneficially incorporated in the soil. We wish to point out that the bills very appropriately provide that, in establishing or revising criteria against which dumping permit applications would be approved or denied, the Administrator, EPA, will consult with this Department, along with several other interested Federal agencies.

The Office of Management and Budget advises that there is no objection to the presentation of this report from the standpoint of the Administration's program.

Sincerely,

J. PHIL CAMPBELL,
Under Secretary.

DEPARTMENT OF THE ARMY,
Washington, D.C., April 7, 1971.

Hon. EDWARD A. GARMATZ,
Chairman, Merchant Marine and Fisheries Committee, House of Representatives.

DEAR MR. CHAIRMAN: Reference is made to your request for the views of the Department of Defense on H.R.'s 285, 336, 337, 548, 549, 805, 983, 1095, 1383, 1661, 3662, 4217, 4584 and 5050, 92nd Congress, bills concerning the discharge of military or other material into international waters or waters of the United States, and the transportation of that material for disposal into internaional waters. The Department of the Army has been assigned responsibility for expressing the views of the Department of Defense on these bills.

The purpose of the bills is to prohibit unregulated dumping into the oceans and other waters. The Department of the Army on behalf of the Department of Defense is deeply concerned about the adverse ecological and environmental effects associated with the discharge of wastes and other materials into the navigable, coastal, and ocean waters of the United States. Each of these bills addresses some facet of this area of concern. We are concerned, however, that certain of these bills could unnecessarily prohibit some important activities not necessarily harmful to the marine environment. We are especially concerned that the prohibitive features of certain of these bills could be construed as an attempt to preclude operation of U.S. nuclear powered warships, including the strategic deterrent Fleet Ballistic Missile Submarine force. Such a result would be untenable to the security of the United States.

THIS PAGE INTENTIONALLY LEFT BLANK

The Department of the Army on behalf of the Department of Defense believes that the Administration's bill, H.R. 4723, introduced by you on February 22, 1971, to the 92nd Congress, realistically and comprehensively provides for the intent expressed in the proposed bills cited in the first paragraph, above, with respect to preventing unregulate dumping of harmful substances into estuarine areas.

This report has been coordinated within the Department of Defense in accordance with procedures prescribed by the Secretary of Defense.

The Office of Management and Budget advises that, from the standpoint of the Administration's program, there is no objection to the presentation of this report for the consideration of the Committee.

Sincerely,

STANLEY R. RESOR,
Secretary of the Army.

U.S. ATOMIC ENERGY COMMISSION,
Washington, D.C., April 26, 1971.

Hon. EDWARD A. GARMATZ,
Chairman, Committee on Merchant Marine and Fisheries,
House of Representatives.

DEAR MR. GARMATZ: The Atomic Energy Commission is pleased to reply to your letter of February 19, 1971, requesting our views on H.R. 336 and H.R. 548, identical bills "[t]o require the Council on Environmental Quality to make a full and complete investigation and study of national policy with respect to the discharging of material into the oceans."

These bills are identical to H.R. 18914, which was introduced in the 91st Congress on August 11, 1970. At your request, our views on that bill were submitted for your Committee's consideration by letter dated October 30, 1970. Consistent with the views we expressed at that time, we feel that the proposed legislation is unnecessary.

On October 7, 1970, the President made public the results of a study conducted by the Council on Environmental Quality with respect to the discharge of materials into the oceans. To implement the policy recommendations contained in the Council's report, the Administration recently sent to Congress a proposed bill which would provide for comprehensive regulation of the discharge of materials into the oceans and coastal waters, as well as the Great Lakes. This proposed legislation was introduced in the House on February 10, 1971, as H.R. 4247. Accordingly, in view of these developments it is apparent that the objectives of H.R. 336 and H.R. 548 have already been realized.

The Office of Management and Budget has advised that there is no objection to the presentation of this report from the standpoint of the Administration's program.

Cordially,

GLENN T. SEABORG, *Chairman.*

H.R. 1661 and H.R. 5050.—These bills, which are identical, would impose a specific prohibition on an owner or master of a vessel, in regard to the loading of any waste on a vessel, while it is in a United States port, if the material is to be dumped in territorial or international waters. An authorizing permit would first have to be obtained from the Administrator of the Environment Protection Agency; such authorization would be based on the Administrator's determination that the discharge would not damage the marine environment or human health and welfare. The Administrator would be precluded from authorizing any discharges of wastes between the Continental Shelf and the coast of the United States. The owner or master of the vessel would also be required to notify the Coast Guard of the exact location where the authorized dumping would be effected.

H.R. 3662 and H.R. 4359.—These similar bills would prohibit any person from dumping waste material into the coastal or ocean waters of the United States, including the Great Lakes and estuarine areas, without first obtaining a permit from the Administrator of EPA. The Administrator could issue the permit if he determined that the discharge would not damage the ecology of the marine

environment; the Administrator would be obliged to take into account a number of factors specified in the bills, including the effect of the dumping on human health and welfare. No permit could be issued for the disposal of certain specified wastes, including "radioactive wastes". Section 9(a) of H.R. 4359 (not contained in H.R. 3662) would require that the Secretary of Commerce designate portions of the waters encompassed by the bill, as well as adjacent land areas, as marine sanctuaries. The Administrator of EPA would be prohibited from issuing or renewing any permit for the disposal of any wastes "in any area designated or under study for possible designation as a marine sanctuary."

H.R. 4247 and H.R. 4723.—These identical bills, which are favored by the Administration, would (1) carefully regulate the transportation of materials from the United States for the purpose of disposal in the oceans and coastal and other waters of the United States, and (2) dumping in waters over which the United States has jurisdiction. The term "dumping" and other key words in these bills are clearly defined. Both transportation and dumping would be prohibited unless the Administrator of EPA issues an authorizing permit. The Administrator may issue such permits "where the applicant presents information respecting the proposed activity which in the judgment of the Administrator indicates that such transportation, or dumping, or both will not unreasonably degrade or unreasonably endanger human health, welfare, or amenities, or the marine environment, ecological systems, or economic potentialities."

In reviewing permit applications the Administrator would be guided by criteria to be established by him in consultation with certain named Federal agencies, including the Atomic Energy Commission, as well as "other appropriate Federal, State, and local officials."

The Administrator would have very broad authority with respect to types and scopes of permits, but no permit could be issued for dumping that would violate applicable water quality standards. The bills provide that transportation or dumping without a permit would be permitted in emergency situations where necessary to safeguard human life; in such excepted instances, reports must be furnished to the Administrator "within such time and under such conditions as he may prescribe by regulation."

Under the caption "Relationship to Other Laws" the bills provide, among other things, that:

"(b) Nothing in this Act shall abrogate or negate any existing responsibility or authority contained in the Atomic Energy Act of 1954, as amended, and section 4 and subsection 7(a) of this Act shall not apply to any activity regulated by that Act: Provided, The Atomic Energy Commission shall consult with the Administrator prior to issuing a permit to conduct any activity which would otherwise be regulated by this Act. In issuing any such permit, the Atomic Energy Commission shall comply with standards set by the Administrator respecting limits on radiation exposures or levels, or concentrations or quantities of radioactive material. In setting such standards for application to the oceans, coastal, and other waters, or for specific portions of such waters, the Administrator shall consider the policy expressed in subsection 2(b) of this Act and the factors stated in subsections 5(a)(1) and 5(a)(2) of this Act."

This provision recognizes that the Atomic Energy Act of 1954, as amended, vests the Atomic Energy Commission with regulatory authority over the construction and operation of nuclear facilities and the possession and use of certain defined nuclear materials, including the disposal of all radioactive materials except radioactive material produced in accelerators and naturally occurring radium and its daughters.

AEC has not permitted ocean disposal of high-level radioactive wastes from fuel reprocessing operations. Although the disposal of low-level liquid wastes from such facilities as nuclear power plants and the dumping of solid, packaged radioactive wastes into the ocean have been permitted, AEC has strictly controlled and limited the quantities and types of wastes disposed in this manner. In fact, AEC itself has made no sea disposals during the past eight years and has not issued any licenses for this purpose since 1960. The four existing licenses have seldom been used.

The discharge of radioactive effluents from AEC licensed facilities is subject to a comprehensive system of Federal regulations and licensing requirements, which are contained in 10 CFR Parts 20 and 50 of the Commission's regulations. These regulations are based upon recommendations which have been made

by the Federal Radiation Council. Pursuant to Reorganization Plan No. 3 of 1970 (effective December 2, 1970) the functions of the FRC were transferred to the Environmental Protection Agency, which now has the responsibility to set standards for the protection of the general environment from radioactive materials. As with the disposal of radioactive wastes, the AEC has exercised its authority over the discharge of radioactive effluents by strictly controlling and limiting such releases. We do not believe that experience has shown any need for an additional system of control over such discharges or disposal.

Unlike the other bills mentioned above, H.R. 4247 and H.R. 4723 avoid the problem of dual regulation in the atomic energy field. Under these bills AEC would be required to consult with the Administrator before issuing a permit for any activity which would otherwise be within the scope of the statute, and would also be required to comply with the standards set by the Administrator respecting limits on radiation exposures or levels, or concentrations or quantities of radioactive material.

In our view, the proposed legislation embodied in H.R. 4247 and H.R. 4723 would provide for more comprehensive and effective regulation of the discharge of materials into the marine environment than would the other bills. Moreover, we feel that enactment of any of the other bills could give rise to serious problems which are avoided in the careful draftsmanship of the proposed legislation of the President.

We recommend that favorable consideration be given to enactment of the text of H.R. 4247 and H.R. 4723. We believe that the other bills, which cover many of the same areas as H.R. 4247 and H.R. 4723, are not as well drawn as those two bills and should not be enacted into law in their present form.

The Office of Management and Budget has advised that there is no objection to the presentation of this report from the standpoint of the Administration's program.

Cordially,

GLENN T. SEABORG, *Chairman.*

U.S. ATOMIC ENERGY COMMISSION,
Washington, D.C., April 7, 1971.

Hon. EDWARD A. GARMATZ,
Chairman, Committee on Merchant Marine and Fisheries, House of Representatives, Washington, D.C.

DEAR MR. GARMATZ: The Atomic Energy Commission is pleased to reply to your requests for our views on H.R. 285, H.R. 337, H.R. 549, H.R. 983, H.R. 1095, H.R. 4217, and H.R. 4584, bills relating to the regulation of discharges of specified materials into the navigable waters of the United States or international waters.

We note that these bills are identical or substantially similar to proposed legislation introduced in the 91st Congress. Our comments on the prior bills were submitted for your Committee's consideration by our letters dated October 30, 1970.

As we then explained, we strongly support effective measures to protect and preserve our environment; however, we did not favor enactment of those bills because they appeared to be unnecessary. Additionally, we believe they would have interfered with the functions of AEC under the Atomic Energy Act, without adding something of substantive benefit.

As noted in our earlier replies, at the President's request the Council on Environmental Quality undertook an intensive study of pollution in the marine environment. The results of CEQ's study were subsequently made public by the President on October 7, 1970. In implementation of the policy recommendations embodied in the Council's report, the Administration recently transmitted a proposed bill to the Congress which would provide for comprehensive regulation of the discharge of materials into the oceans and coastal waters, as well as the Great Lakes. H.R. 4247, and your bill H.R. 4723, introduced in February, set forth the Administration's legislative proposal. In a companion letter today, we have submitted our comments to you on H.R. 4247 and H.R. 4723 and several other bills concerned with dumping.

Respecting navigable waters which may not be covered by H.R. 4247 and H.R. 4723, legislative authority already exists for the regulation and control of discharges into such waters. Under the Federal Water Pollution Control Act, as

amended (33 U.S.C. 1151 et seq.), discharges which violate applicable water quality standards are subject to suit for abatement, and, in addition, discharges from federally licensed activities are generally subject to a requirement for certification that there is reasonable assurance that applicable water quality standards will not be violated. Furthermore, the Refuse Act (33 U.S.C. 407) makes it unlawful to discharge any refuse matter, other than liquid effluents flowing from streets or sewers, into any of the navigable waters of the United States or their tributaries, unless otherwise authorized by the Secretary of the Army upon terms and conditions specified by him. With res, ect to this latter authority, in order to further the objectives of the Refuse Act the President, by Executive Order 11574 (December 25, 1970, 35 F.R. 19627), has directed the Secretary of the Army to establish a permit program in cooperation with the Administrator of EPA "to regulate the discharge of pollutants and other refuse matter into the navigable waters of the L .ited States or their tributaries and the placing of such matter upon their banks." Pursuant to that Order, the Army Corps of Engineers published proposed regulations on December 31, 1970 (35 F.R. 20005).

Accordingly, with respect to the oceans, gulfs, bays, sea-water lagoons, salt-water harbors, other coastal waters where the tide ebbs and flows, and the Great Lakes, we favor enactment of the text of H.R. 4247 and H.R. 4723.

In our opinion, H.R. 285, H.R. 337, H.R. 549, H.R. 983, H.R. 1095, H.R. 4217, and H.R. 4584 should not be enacted into law. To reiterate some of the objections we identified in our letters last fall, several of these bills define waste material so broadly as to encompass radioactive materials and effluents. The AEC has licensing authority over effluent discharges and the disposal of all radioactive waste materials, except radioactive material produced in accelerators and naturally occurring radium and its daughters. This is a highly specialized health and safety field; dual regulation or the diffusion of Federal responsibility in this area would, in our judgment, be ..ighly undesirable.

Several of the other bills, in their severe prohibition on discharging vaguely defined material, could be construed in such a way as to constitute a serious interference with our national defense capability. The normal discharge of a military ship's fire and bilge pump system, or firing of any ordnance by a military vessel or aircraft, could be interpreted as the discharge of "military material" into the waters.

In short, for the reasons mentioned above, and those advanced in our companion letter on H.R. 4247, H.R. 4723 and several other bills, we do not favor enactment of H.R. 285, H.R. 337, H.R. 549, H.R. 983, H.R. 1095, H.R. 4217 and H.R. 4584.

The Office of Management and Budget has advised that there is no objection to the presentation of this report from the standpoint of the Administration's program

Cordially,

GLENN T. SEABORG, *Chairman.*

GENERAL COUNSEL OF THE DEPARTMENT OF DEFENSE,
Washington, D.C., April 9, 1971.

Hon. EDWARD A. GARMATZ,
Chairman, Committee on Merchant Marine and Fisheries, House of Representatives, Washington, D.C.

DEAR MR. CHAIRMAN: Reference is made to your request for the views of the Department of Defense on H.R. 4247 and H.R. 4723, 92d Congress, similar bills "To regulate the dumping of material in the oceans, coastal, and other waters and for other purposes".

The purpose of the bills is stated in their titles. If enacted, the bills would make the Administrator of the Environmental Protection Agency responsible for establishing appropriate regulations for the application of the environmental standards contained in the proposals. Any agency or person would have to obtain a permit from the Administrator before transporting materials for dumping or before dumping materials in the protected areas. There are certain exceptions to this latter requirement for routine operation of vessels and for intentional placement of devices in the waters, if such placement is for a purpose other than disposal.

The bills were introduced as a result of a proposal submitted to the Congress in connection with the President's environmental message of February 8, 1971. The Department of Defense supports the bills and recommends enactment.

The Office of Management and Budget advises that, from the standpoint of the Administration's program, there would be no objection to the presentation of this report for the consideration of the Committee, and that the enactment of H.R. 4247 or H.R. 4723 would be in accord with the program of the President.

Sincerely yours,

J. FRED BUZHARDT.

ENVIRONMENTAL PROTECTION AGENCY,
Washington, D.C., April 6, 1971.

Hon. EDWARD A. GARMATZ,
Chairman, Committee on Merchant Marine and Fisheries, House of Representatives, Washington, D.C.

DEAR MR. CHAIRMAN: As requested, we submit herewith the views of the Environmental Protection Agency on the following legislative proposals, most of which will be the subject of joint legislative hearings to be held by the Subcommittee on Oceanography and the Subcommittee on Fisheries and Wildlife Conservation during the week of April 5, 1971: H.R. 285, 336, 337, 548, 549, 805, 807, 808, 983, 1095, 1329, 1381, 1382, 1383, 1661, 1674, 2581, 3662, 4217, 4218, 4247, 4359, 4360, 4361, 4584, 4719, 4723, 5049, 5050, 5239, 5268, 5477, 5705, and 6862.

H.R. 4723 (also 4247, 5239, 5268, 5477, and 6862)

H.R. 4723, which is the Administration's own ocean dumping proposal, provides that, except as authorized in a permit issued by the Administrator of EPA, no person shall (a) transport "material" from the United States for the purpose of dumping it into "oceans, coastal, and other waters," or (b) dump material in that part of such waters within the territorial juridsdiction of the United States, or in the contiguous zone to the extent that the dumping may affect the territorial sea or the territory of the United States. "Material" is defined to include dredge spoil, solid waste, garbage, sewage sludge, munitions, chemical, biological, and radiological warfare agents, radioactive materials, wrecked or discarded equipment, rock, sand, cellar dirt, and industrial waste, but to exclude oil and vessel sewage, discharges of which are regulated by the Federal Water Pollution Control Act. "Oceans, coastal, and other waters" are defined to mean oceans, gulfs, bays, salt-water lagoons, salt water harbors, other coastal waters where the tide ebbs and flows, and the Great Lakes. The "dumping" to which the bill applies includes any disposition of material other than dispositions of effluent from outfall structures, or routine discharges of effluent incidental to the propulsion of vessels.

The Administrator would be authorized to issue permits to dump materials or to transport them for dumping where in his judgment, based on information supplied by the applicant, such activity will not unreasonably degrade or endanger human health, welfare or amenities, or the marine environment, ecological systems, or economic potentialities. He would be required to establish criteria for evaluating permit applications, taking into account the likely environmental impact of the proposed dumping, alternative locations and methods of disposal, and the impact on the public interest of either issuing or denying a permit or of requiring an alternative disposal method. In establishing or revising criteria, the Administrator would be required to consult with the heads of concerned departments and agencies. He would be precluded from issuing any permit which would result in a violation of water quality standards. He would be authorized to impose restrictions relating to the type and amount of materials to be dumped, the place of dumping, and the duration of the permit. He would be authorized to limit, deny, alter or revoke permits where he finds that materials cannot be dumped consistently with the criteria established for the issuance of permits. Dumping of materials in an emergency to safeguard human life would be exempted from the requirements of the Act, but would be required to be reported to the Administrator.

The Administrator would be authorized to impose civil penalties of up to $50,000 per day for violations of the Act or of any regulations or permit issued thereunder. In addition, knowing or willful violations would invite criminal fines of

up to $50,000 per day, imprisonment for up to one year, or both. The Attorney General would be authorized to bring actions for equitable relief to redress any such violations, and the Administrator would be authorized to revoke or suspend a violator's permit. All of the Act's prohibitions and requirements would be applicable to agencies and employees of the Federal Government, except the remedial provisions described in this paragraph. The bill would require the Coast Guard to conduct surveillance and other appropriate enforcement activity.

The bill has a section which defines its relationship with other laws and with actions taken pursuant to other laws. Generally speaking, existing Federal permits would be terminated upon the Act's effective date to the extent that such permits authorize activity covered by the Act, and further permits of a similar nature could not be issued. However, there would be two exceptions to this general supersession of other laws: (1) the AEC's authorities with respect to radioactive materials under the Atomic Energy Act of 1954 would not be affected (although the AEC would be required to consult with EPA prior to issuing any permit to conduct any activity otherwise regulated by this Act, and to comply with radioactive-material standards set by the Administrator); and (2) except as set forth in the next paragraph, the authorities contained in the Rivers and Harbors Act of 1899, as well as all actions taken pursuant to that Act either before or after the effective date of this proposal, would be preserved. In situations in which this Act and the Act of 1899 both apply to dumping of material in connection with a dredge, fill or other permit issued by the Corps of Engineers, the permit would be issued by the latter only after receiving certification from EPA that the proposed activity is in conformity with this Act.

The bill would supersede the Refuse Act insofar as that Act applies to dumping of materials in waters covered by the bill, and would repeal the Supervisory Harbors Act of 1888, an act which has been used to regulate ocean dumping of materials transported from the harbors of New York, Baltimore, and Hampton Roads, Virginia.

EPA recommends the enactment of H.R. 4723. The bill contains the following major elements, all of which are considered essential to a rational and comprehensive ocean dumping policy:

1. In addition to its application to ocean waters, the bill would apply to the Great Lakes as well as to certain internal waters having characteristics of open ocean waters (salt-water gulfs, bays, lagoons, harbors, etc.).

2. The bill would require permits for two types of activity which are not necessarily related: (a) transportation of materials from the United States for dumping in ocean waters *anywhere*; and (b) dumping of materials—whether transported from the United States or not—in waters covered by the Act which are within the territorial jurisdiction of the United States, or in waters of the contiguous zone where the dumping may affect the territory or territorial sea of the United States. Under this approach, the regulatory authority of the United States is utilized to its fullest extent consistent with established principles of international law.

3. The bill is coordinated with other laws and with water quality management programs carried out pursuant to other laws. The bill would for the most part be inapplicable to internal navigable waterways, which are protected by water quality standards established by the States or by joint Federal-State action pursuant to the Federal Water Pollution Control Act, and by the requirements of the Refuse Act of 1899. In order to rationalize the overlap which does exist between this proposal and either the Federal Water Pollution Control Act or the Refuse Act (on overlap which is limited primarily to the Great Lakes and coastal waters out to the three mile limit), the bill provides: (a) that it does not apply to effluent from outfall structures (which are adequately regulated by the Refuse Act and the Federal Water Pollution Control Act);[1] (b) that the Refuse Act is superseded insofar as it applies to dumping of materials in waters covered by the bill; and (c) that no permit may be issued which would violate water quality standards.

[1] H.R. 5966, an Administration proposal to amend section 10 of the Federal Water Pollution Control Act, would, *inter alia*, authorize the Administrator of EPA to establish water quality standards for the high seas applicable to the discharge of material transported from or originating within the United States. This would enable the Administrator to regulate discharges from ocean outfalls, a category of discharge not covered by H.R. 4723.

4. Control over dumping is consolidated in EPA, an agency which has as its chief purpose the protection of the environment, and which possesses the research and regulatory capability necessary for developing and carrying out a comprehensive ocean dumping policy.

H.R. 3662

This bill provides that no person may dump waste material (comprehensively defined) into the "ocean waters of the United States," or "transport such material through such waters" (presumably for dumping without a permit from the Administrator of EPA. "Ocean waters" is defined to mean estuarine areas, coastal waters (out to the three-mile limit), the Great Lakes, and waters above the Outer Continental Shelf (from the three-mile limit to the 200-meter depth contour). The "dumping" to which the bill applies includes disposal of material by any means whatsoever. The Administrator would be authorized to issue permits for dumping where he determines that it will not damage the ecology of the marine environment, taking into account such factors as land-based alternatives and the effect of the dumping on human health and welfare, fisheries resources, and marine ecosystems. Permits would be required to specify restrictions relative to the type and amount of material authorized to be dumped, the location of dumping, and the duration of the permit. The Administrator would not be allowed to issue permits authorizing the dumping of radioactive wastes, toxic industrial wastes, or chemical or biological warfare materials. In the case of permits for the dumping of sewage or industrial wastes, the Administrator would not be allowed to issue a permit (1) after January 1, 1972, unless such wastes had received primary treatment; (2) after January 1, 1974, unless they had also received tertiary treatment; and (3) after January 1, 1976, unless they had also received tertiary treatment. The Administrator would have authority to suspend, revoke, revise or condition permits. The Coast Guard would be required to conduct surveillance and other appropriate enforcement activities. Civil and criminal penalties would be the same as in H.R. 4723, except that one-half of any penalty or fine would be payable to the informer providing the information resulting in such penalty or fine. Equitable relief to redress violation would be available. The Administrator would be required to conduct the investigation and research with respect to marine ecology necessary to carry out the purposes of the Act; appropriations of $1 million per year would be authorized for this purpose.

EPA is generally favorable to the provisions of H.R. 3662, which are similar or identical in many respects to the provisions of the Administration's proposal set forth in H.R. 4723. However, EPA has the following major comments or reservations about H.R. 3662:

1. The prohibition against transport through "ocean waters" (waters out to the 200-meter depth contour) without a permit is not linked to the place of origin of the transporting vessel. Insofar as this provision is made applicable to vessels which are not leaving United States ports, it may violate the rights of innocent passage and freedom of the seas under international law.

2. The prohibition against dumping between the 12-mile limit and the 200-meter depth contour, regardless of the place of origin of the material to be dumped, may also raise problems under international law.

3. EPA is opposed to the Act's broad definition of "dumping," which would include continuous discharges from outfall structures which are already subject to regulation under the Federal Water Pollution Control Act, and, in the case of industrial wastes, by the Refuse Act as well. The imposition of further Federal controls over such discharges, in addition to those already provided under the Federal Water Pollution Control Act and the Refuse Act, is duplicative and unnecessary. There is no provision in the bill for supersession of existing, overlapping legal authorities.

4. EPA is opposed to the provisions of the bill which would prohibit the Administrator from issuing permits to dump specified categories of wastes. It is agreed that, generally speaking, ocean disposal of radioactive wastes, toxic industrial wastes, and chemical and biological warfare agents is undesirable and should not be allowed. However, there may be the rare exceptional case, *e.g.*, reactor components from nuclear powered vessels, in which ocean disposal will present a lesser threat to human health, welfare or the environment than land-based disposal. We favor the approach taken in H.R. 4723, which would give the Ad-

ministrator flexibility in developing an ocean dumping policy which would take account of such special circumstances.

5. EPA is opposed to the provisions of the bill which would prohibit the Administrator from issuing permits to dump sewage or industrial wastes which have received less than a specified level of treatment. This provision appears to be concerned with effluents from municipal and industrial waste treatment plants—a category of discharge apparently within the Act's definition of "dumping." EPA believes that such continuous discharges should continue to be regulated by the Federal Water Pollution Control Act, rather than by a bill concerned primarily with ocean dumping. Furthermore, a requirement of a specified level of treatment for all discharges by a specified date fails to take into account variations in water use designations, the quality or characteristics of the receiving waters, or other factors which bear on the appropriate level of treatment in a given instance. The provisions of the Federal Water Pollution Control Act governing the establishment of water quality standards provide a more flexible and responsive vehicle for the establishment of base levels of treatment.

6. While subsection (e)(2) of the bill provides that "nothing in this section shall be construed as abrogating or negating any existing responsibility or authority contained in the Rivers and Harbors Act of 1899," any outstanding permits authorizing dumping issued under that Act would apparently not survive the enactment of this proposal, in view of subsection (e)(1) which provides for the termination of such permits.

H.R. 4359 (also 4360, 4361)

This bill provides that no citizen of the United States or "other person" may dispose of waste materials (comprehensively defined) into the oceans, coastal waters, or estuarine waters of the United States or into the Great Lakes without a permit from the Administrator of EPA. "Other person" is defined to mean resident officers, directors or managers of foreign partnerships, associations, or corporations doing business in the United States. The Administrator would be authorized to issue permits under such terms as he determines necessary to insure that the dumping will not damage the ecology of the marine environment. The Administrator would not be authorized to issue permits for the dumping of radioactive wastes, toxic industrial wastes, or chemical or biological warfare agents. In the case of permits for the dumping of sewage or industrial wastes, he would not be authorized to issue a permit (1) after January 1, 1972, unless such wastes had received primary treatment; (2) after January 1, 1974, unless they had also received secondary treatment; or (3) after January 1, 1976, unless they had also received tertiary treatment. The Administrator would be authorized to prohibit by regulation the disposal of any waste material which he determines may damage the ecology of the marine environment. The Act would authorize the imposition of criminal fines as follows: fines of $2,000 to $10,000 per day of violation for first offenses, and fines of $10,000 to $20,000 per day of violation for subsequent offenses. Vessels involved in violations would be forfeited to the United States. The permit provisions of the Act would be enforced by EPA, the Secretary of Transportation (Coast Guard), and the Secretary of the Army (Corps of Engineers) under regulations and operational directives jointly agreed to. The Coast Guard would be empowered to stop, search and detain vessels, and district courts would have jurisdiction to restrain violations.

The Secretary of Commerce, acting through NOAA, after consultation with the Secretary of the Interior, EPA, and CEQ, would be directed to designate as marine sanctuaries those areas of the Nation's tidelands, Outer Continental Shelf, seaward areas, and land and waters of the Great Lakes, which the Secretary determines should be preserved or restored for their recreation, conservation, ecologic, or aesthetic values. The Secretary of the Interior would be precluded from issuing or renewing any license for the exploration, mining or removal of any minerals, including oil and gas, from any area designated or under study for possible designation as a marine sanctuary. EPA would be precluded from issuing or renewing permits for dumping in such areas. $5,000,000 would be authorized to be appropriated for studies in connection with the designation of marine sanctuaries.

EPA is generally favorable to the provisions of this proposal, with the following major reservations:

1. The bill applies only to dumping activities carried out by United States citizens or other persons doing business in the United States. It would not cover dumping in United States territorial waters, or transportation for dumping from United States ports, carried out by persons lacking these connections with the United States. EPA believes that this gap in coverage is both unnecessary and undesirable.

2. The bill contains a broad definition of "dumping" which would include continuous discharges from outfall structures. EPA is opposed to Federal permit requirements applicable to such discharges for reasons discussed above in connection with H.R. 3662.

3. EPA is opposed to the dumping prohibitions affecting sewage, industrial wastes, radioactive wastes, and chemical and biological warfare agents, for reasons discussed above in connection with H.R. 3662.

4. The bill does not define its relationship with other laws dealing with Federal permits for dumping, notably the Rivers and Harbors Act of 1899, which includes the Refuse Act. Presumably the overlapping requirements of the Refuse Act would remain in effect in areas in which both Acts apply. The bill states that "other provisions of law which are in conflict with this Act are hereby repealed," but this provision does not solve the problem of duplicative, overlapping requirements.

5. The bill does not provide for administratively as well as judicially imposed penalties, as both H.R. 4723 and H.R. 3662 do, but only for judicial fines. EPA favors the approach taken in H.R. 4723 and H.R. 3662 since it would foster rapid adjudication of violations by administrative personnel having the necessary expertise to deal with the problem.

6. The establishment of "marine sanctuaries" is beyond the scope of the Administration's bill, which deals entirely with the control of ocean dumping. However, EPA is completely in accord that certain critical marine areas should be protected from dumping, and would have this objective in mind in administering H.R. 4723, which provides ample authority to ban dumping in certain areas. The relationship of the marine sanctuaries proposal to the land use programs proposed by the administration in H.R. 4332 should be examined. Under H.R. 4332, the Secretary of the Interior would be authorized to make grants to States to assist them in developing land use programs which would include State controls over the use and development of "areas of critical environmental concern," defined in the bill to include coastal zones, estuaries, and the Great Lakes.

H.R. 1661 (also 5049, 5050)

This bill provides that no owner or master of a vessel may load or permit the loading of any waste (comprehensively defined) while in any port of the United States, if such waste is to be discharged in "ocean waters," unless such owner or master first obtains a loading permit from the Administrator of EPA and notifies the Coast Guard. "Ocean waters" is defined to mean "any estuarine area, coastal waters, Great Lakes, territorial waters, and the high seas adjacent to the territorial waters." The Administrator would be required to issue loading permits if he determines that dumping of the wastes into ocean waters will not damage the ecology of the marine environment. He would be precluded from issuing any permit for the discharge of any waste between the Continental Shelf and the coast of the United States (meaning, it would appear, within the three-mile territorial sea). The Administrator would have authority to ban loading, transportation and dumping of matter deemed damaging to the marine environment or to human health or welfare. The Coast Guard would be required to conduct surveillance and other appropriate enforcement activity. The bill would authorize administratively imposed civil penalties as follows: up to $50,000 for the first violation, and up to $100,000 for each subsequent violation. Upon failure of an offending party to pay a penalty, the Administrator would be authorized to request the Attorney General to commence a district court action for appropriate relief. Outstanding Federal permits authorizing any activities to which the bill applies would be terminated as of the bill's effective date.

EPA is generally favorable to H.R. 1661, with the following major reservations:

1. It would not apply, as H.R. 4723 would, to dumping of material in the U.S. territorial sea or contiguous zone which is not loaded on vessels in United States ports.

2. The definition of "ocean waters" may give some problems. The meaning of "territorial waters" is not clear, although the term is probably intended to be limited to offshore territorial waters, since inclusion of internal territorial waters would conflict with the generic "ocean waters." The scope of "high seas adjacent to the territorial waters" is also not clear.

3. EPA has reservations about the provision which would prohibit the issuance of permits for the disposal of wastes in the United States territorial sea. The provision is unnecessary since under H.R. 4723 and similar bills the Administrator would have authority to prohibit dumping in such waters where appropriate, and very little dumping is carried out in such waters in any event. Furthermore, some carefully planned and controlled disposal of waste materials in these waters may be desirable, *e.g.*, the sinking of car bodies or other similar material to serve as a shelter for fish.

H.R. 1383; H.R. 805 (also 807, 808, 1329, 2581, and 5705)

Under H.R. 1383, the Secretary of the Interior, acting through the Fish and Wildlife Service, would be required to establish standards applicable to the deposit or discharge into the "coastal waters" of the United States of all industrial wastes, sludge, and spoil, and all other materials that might be harmful to the wildlife or ecology of these waters. These standards would require any person, before discharging such materials into such waters, to present sufficient evidence to sustain a burden of proof that such materials will not endanger the natural environment and ecology of such waters. These standards would be required to be adopted and enforced by any agency of Federal or State government that issues licenses for disposal of materials in coastal waters. The States would be authorized to establish more stringent standards provided they contain adequate procedures for enforcement. District courts would have jurisdiction to restrain violations. Violators of standards would be liable to civil penalties of not more than $10,000 or less than $5,000 per day of violation. Outstanding Federal permits would be terminated as of the effective date of the proposal.

H.R. 805 is essentially the same as H.R. 1383, except (1) the standards would be established jointly by the Secretary of the Interior and the Administrator of EPA; (2) the standards would be applicable to "ocean, coastal, and other waters" rather than simply to "coastal water," and (3) EPA rather than the Interior Department would be the agency charged with administrative responsibilities. In H.R. 805, "ocean, coastal, and other waters" are defined in the same way as these words are defined in H.R. 4723, except that the bill's application to ocean waters would appear to be limited to the territorial sea and the contiguous zone. The term "coastal waters" as used in H.R. 1383 is not defined. The words "deposit or discharge" as used in both bills would appear to embrace continuous discharges as well as intermittent dumping.

EPA is opposed to the enactment of these bills because they overlap existing [illegible]. Water quality standards have already been established under the Federal Water Pollution Control Act for all of the waters to which these bills relate except the waters of the contiguous zone, a gap which will be closed if H.R. 5966, an Administration proposal to amend the Federal Water Pollution Control Act, is enacted. H.R. 5966 would also make these standards enforceable by civil penalty and injunction. Under H.R. 4723, the Administration's ocean dumping proposal, the Administrator of EPA would be precluded from issuing permits which violate water quality standards, and under the Refuse Act Permit Program, the Corps of Engineers will not issue permits which violate or permit a violation of these standards. Moreover, H.R. 1383 and 805, by calling for Federal standards which shall govern unless the States adopt more stringent standards, are inconsistent with the established policy of the Federal Water Pollution Control Act, which places the primary responsibility for the establishment of water quality standards on the States.

H.R. 285 and H.R. 983

H.R. 285 would require the Secretary of the Interior, acting through the Fish and Wildlife Service, after a two-year study, to designate those portions of the navigable waters of the United States and of the waters above the Outer Continental Shelf into which he determines that sewage, sludge, spoil and other waste can be safely discharged (in terms of ecological and environmental values). After making such designations, the Secretary of the Interior would be required to establish standards applicable to the discharge of material within

such designated areas. The purpose of the standards would be to insure that no damage to wildlife, or pollution of United States navigagle waters, results from such discharges. States would be authorized to establish standards of equal or greater stringency provided they contain adequate procedures for enforcement. Discharges of sewage, sludge, spoil or other waste into any waters within the jurisdiction of the United States which are not within a designated discharge area would invite civil penalties of up to $10,000 per offense. Violators of discharge standards applicable to discharge areas would be subject to comparable civil penalties. District courts would have jurisdiction to restrain violations. Outstanding Federal discharge permits would be nullified on the effective date of the proposal. Thereafter, no Federal permits could be issued which would authorize any activity prohibited by this bill.

H.R. 983 is the same as H.R. 285 except that (1) designation of discharge areas would be carried out jointly by Interior and EPA; (2) standard setting and enforcement would be carried out by EPA rather than by Interior; and (3) the maximum authorized civil penalty per violation would be $40,000 rather than $10,000. Both bills define covered "discharges" to include "any spilling, leaking, pumping, pouring, emitting, emptying, or dumping."

H.R. 285 and 983 are similar to H.R. 1383 and 805, discussed above, except that they would be applicable to *all* United States navigable waters, and would call for the designation of safe discharge areas as well as for the establishment of discharge standards. EPA is opposed to the enactment of these bills for the same reasons it is opposed to enactment of H.R. 1363 and 805: basically, the fact that they are designed to accomplish, in a somewhat different way, what is already being accomplished under the Federal Water Pollution Control Act. The overlap is even greater than in the case of H.R. 1383 and 805, in view of the broad application to all "navigable" waters. Interstate navigable waters are already subject to the standard-setting provisions of the Federal Water Pollution Control Act, and intrastate navigable waters will be brought within the coverage of that Act if the Administration's H.R. 5966 is enacted.

H.R. 1095

H.R. 1095 would require the Secretary of the Interior, acting through the Fish and Wildlife Service, after a one-year study, to designate those portions of the navigable waters of the United States and those portions of the waters above the Outer Continental Shelf into which he determines that sewage, sludge, spoil, landfill, heated effluents, or other wastes or substances *cannot* be safely discharged, such areas to be known as "marine sanctuaries." Persons who discharge (defined to include spilling, leaking, pouring, etc.) any wastes or substances into such designated waters would be subject to fines of up to $10,000 per offense. All Federal permits would be terminated to the extent that they authorize any discharges into such areas, and no new Federal permits authorizing such dumping could be issued.

The Secretary of the Interior would be required to establish standards applicable to the discharge of all wastes and substances into areas not so designated as marine sanctuaries. Such standards would be for the purpose of insuring against damage to marine life or wildlife, or pollution of United States navigable waters. The standards would be required to provide that no sewage or industrial waste may be discharged: (1) after January 1, 1973, unless it has received at least primary treatment or its equivalent; (2) after January 1, 1975, unless it has received at least secondary treatment or its equivalent; and (3) after January 1, 1977, unless it has received at least tertiary treatment or its equivalent. States would be authorized to establish standards of equal or greater stringency provided they contain adequate provisions for enforcement. Dischargers of any waste or substance in violation of the established standards would be subject to a civil penalty of not more than $10,000 per day of violation. All Federal permits would be terminated to the extent they authorize discharges which violate such standards. District courts would have authority to restrain violations.

The Secretary of Defense would be required to make a complete inventory of all existing munitions, chemical, biological, and radiological warfare agents, and other military materials, the disposition of which may present a danger to man, the environment, or to fish and wildlife, and to determine the date beyond which each such item cannot be safely retained. He would also be required to prepare a plan for the demilitarization, detoxification or decontamination of such military materials. After the date of enactment of the bill, he would be required to

determine such disposition dates and to prepare such disposition plans for any new military materials prior to acquiring them. After the date of enactment of the bill, all disposal of such military materials into any navigable or coastal waters of the United States, or into any international waters, would be prohibited.

EPA has the following comments with respect to this bill:

1. The establishment of "marine sanctuaries" has been discussed above in connection with H.R. 4359.

2. The establishment of discharge standards has been discussed above in connection with H.R. 1383, 805, 285, and 983.

3. The prohibition against the discharge of sewage or industrial wastes which have received less than a specified level of treatment has been discussed above in connection with H.R. 3662.

4. EPA does not believe that a legislated ban on the dumping of military materials is necessary. Recent policy declarations by the Department of Defense indicate that an effective ban is already in effect or is being implemented. Furthermore, as already discussed in connection with H.R. 3662, there may be the rare exceptional case in which ocean disposal will present a lesser threat to human health, welfare or the environment than land-based disposal.

H.R. 337 (also 549, 1381); H.R. 4584; H.R. 4217 (also 4218, 4719)

H.R. 337 would prohibit any person from discharging, into any of the navigable waters of the United States or into international waters, any munition, or any chemical, biological, or radiological warfare agent, or any other military material, except in accordance with a certificate issued by the Council on Environmental Quality establishing the terms, conditions and limitations of such disposal. H.R. 4584 is the same as H.R. 337, except that the certificate would be issued jointly by EPA and NOAA rather than by CEQ. H.R. 4217 is the same as H.R. 4584, except that the certifying authority would be EPA exclusively, and the bill's requirements would apply not only to military materials but also to "any other refuse matter of any kind or description whatsoever."

EPA has the following comments on these bills:

1. All of them, applying to discharges by any person into international waters, without regard to citizenship or point of origin of the discharged material, may raise problems under international law.

2. EPA prefers the comprehensive approach taken in H.R. 4723, which would apply a dumping permit requirement to a broad range of materials, including military materials, to the *ad hoc* approach of H.R. 337 and H.R. 4584.

3. CEQ serves an advisory rather than a regulatory function and should not be the certifying authority as provided in H.R. 337. CEQ supports H.R. 4723, under which such regulatory authority would be vested in EPA.

4. With respect to discharges into navigable waters, H.R. 4217 duplicates the requirements of the Refuse Act of 1899, which requires a permit from the Corps of Engineers for the discharge of any refuse matter into navigable waters other than refuse flowing from streets and sewers in a liquid state. Discharges not covered by the Refuse Act are subject to control under the Federal Water Pollution Control Act, and proposed amendments thereto.

H.R. 336 (also 548, 1382, 1674)

This bill requires the CEQ to make an investigation and study of all aspects of existing national policy with respect to the discharge of materials into the Atlantic and Pacific Oceans, the Gulf of Mexico, and other waters within the territorial sea or contiguous zone of the United States, and to report to the President and Congress the results thereof, and its recommendations for a national ocean dumping policy, including any treaties, agreements or legislation necessary in connection therewith. EPA is of the opinion that CEQ has already performed this task, as evidenced by its report entitled "Ocean Dumping—A National Policy" submitted to the President in October, 1970. The Administration's ocean dumping bill, H.R. 4723, is based on the recommendations contained in that report.

The Office of Management and Budget has advised that there is no objection to the presentation of this report and that enactment of H.R. 4723 would be in accord with the program of the President.

Sincerely yours,

William D. Ruckelshaus,
Administrator.

FEDERAL POWER COMMISSION,
Washington, D.C., April 16, 1971.

Combined report on H.R. 285, H.R. 805, H.R. 983, and H.R. 1095, 92d Congress, related bills to amend the Fish and Wildlife Coordination Act

Hon. EDWARD A. GARMATZ,
Chairman, Committee on Merchant Marine and Fisheries, House of Representatives, Longworth House Office Building, Washington, D.C.

DEAR MR. CHAIRMAN: In response to your requests of February 9 and February 17, 1971, we enclose 20 copies of the report of the Federal Power Commission on the subject bills.

The Office of Management and Budget advises there is no objection to the presentation of this report and, that enactment of H.R. 4723 would be in accord with the program of the President.

Sincerely,

JOHN N. NASSIKAS,
Chairman.

FEDERAL POWER COMMISSION

REPORT ON RELATED BILLS, H.R. 285, H.R. 805, H.R. 983, AND H.R. 1095—92D CONGRESS

H.R. 285, A bill, "To amend the Fish and Wildlife Coordination Act to provide additional protection to marine and wildlife ecology by requiring the designation of certain water and submerged lands areas where the depositing of certain waste materials will be permitted, to authorize the establishment of standards with respect to such deposits, and for other purposes."

H.R. 805, A bill, "To amend the Fish and Wildlife Coordination Act to provide additional protection to marine and wildlife ecology by providing for orderly regulation of dumping in the ocean, coastal, and other waters of the United States."

H.R. 983. A bill, "To amend the Fish and Wildlife Coordination Act to provide additional protection to marine and wildlife ecology by requiring the designation of certain water and submerged lands areas where the depositing of certain waste materials will be permitted, to authorize the establishment of standards with respect to such deposits, and for other purposes."

H.R. 1095, A bill, "To amend the Fish and Wildlife Coordination Act to provide additional protection to marine and wildlife ecology by requiring the designation of certain water and submerged land areas where the depositing of certain waste materials is prohibited, to require the establishment of standards with respect to such deposits in all other areas, and for other purposes."

H.R. 285 would amend the Fish and Wildlife Coordination Act to provide additional protection to the ecology of the Nation's marine and fresh waters by authorizing the Secretary of the Interior acting through the Fish and Wildlife Service to designate those portions of the navigable waters of the United States, of the waters above the Outer Continental Shelf, and of the submerged lands relating to those waters, on which sewage, sludge, spoil or other waste can be safely discharged. H.R. 285 would direct the Secretary of the Interior to establish standards applicable to the discharge of material within designated discharge areas "for the purpose of insuring that no damage to, or loss of, any wildlife or wildlife resources or pollution of the navigable waters of the United States will result from such activity." The bill would also permit the States to establish more stringent discharge standards. Initial designation of discharge areas would be delayed for two years after enactment of the bill pending completion of an investigation and study of potential discharge areas by the Secretary of the Interior in cooperation with the Secretary of the Army acting through the Chief of Engineers. H.R. 285 contains enforcement provisions (subsections (g) and (k) and provides civil penalties for discharge of waste in undesignated areas and for violation of applicable discharge standards (subsection (i)). Subsection (j) provides that:

"(j) Upon the designation of waters or submerged lands under subsection (a) of this section, all licenses, permits, or authorizations which have been issued by any officer or employee of the United States under authority of any other provision of law shall be terminated and of no effect to the extent

they authorize any activity prohibited by subsection (i) of this section. Thereafter no license, permit, or authority shall be issued by any officer or employee of the United States which would authorize any activity prohibited by subsection (i) of this section."

H.R. 805 would require the Administrator of the Environmental Protection Agency and the Secretary of the Interior (acting through the United States Fish and Wildlife Service) in consultation with the Secretary of the Army (acting through the Chief of Engineers), to establish standards for the discharge of waste:

" . . for the purpose of insuring that no damage to the natural environment and ecology including but not limited to marine and wildlife ecology of the ocean, coastal, and other waters of the United States, will result from any such activity. . . ."

H.R. 805 would also permit the imposition of more stringent state standards.

H.R. 805 does not provide for the designation of areas within which waste may be safely deposited. Instead, the bill would require any person, before depositing or discharging industrial wastes, sludge, spoil or other materials into the ocean, coastal, or other waters of the United States, to "present sufficient evidence to sustain a burden of proof that such materials in the location in which they are to be deposited will not endanger the natural environment and ecology of these waters and to meet such additional requirements as the Administrator may deem necessary for the orderly regulation of such activity." The bill further provides in subsection (d) that the standards established "shall be applicable to all of the departments, agencies, and instrumentalities of the Federal Government, to the States and their agencies, including any person having any license, permit, or other authorization from such State or agency for any such activity with respect to any such ocean, coastal, and other waters." The civil penalties set forth under H.R. 805 are less stringent than those contained in H.R. 285 and apply only to violations of discharge standards. Subsection (i) of H.R. 805 is much more stringent than the parallel subsection (j) of H.R. 285 *supra* in that it provides:

"(i) Upon the effective date of this section all licenses, permits, or authorizations which have been issued by any officer or employee of the United States under authority of any other provision of law shall be terminated."

Unlike the parallel provisions[1] in H.R. 285, H.R. 983 and H.R. 1095, subsection (f) of H.R. 805, which relates to recordkeeping and reporting, does not provide for confidential treatment of information relating to trade secrets.

H.R. 983 is substantially the same as H.R. 285, except for the following differences. Under H.R. 983, the Secretary of the Interior, acting through the Fish and Wildlife Service, and the Administrator of the Environmental Protection Agency would have joint responsibility for designating discharge areas. However, H.R. 983 would give the Administrator of the Environmental Protection Agency, instead of the Secretary of the Interior, sole responsibility for the determination of applicable federal discharge standards. The civil penalties which H.R. 983 would establish are the most stringent of those provided in any of the bills included in this report.

H.R. 1095 is similar to both H.R. 285 and H.R. 805, but is drafted in a converse form. Under H.R. 1095, the Secretary of the Interior, acting through the Fish and Wildlife Service, would be authorized to designate those areas into and onto which he determines certain waste materials *cannot* be safely discharged. Such areas then would be known as "marine sanctuaries." Persons discharging waste[2] in "marine sanctuaries" would be subject to heavy fines (Sec. 5B(e)). Initial designation of these areas would be delayed for one year after enactment of the bill pending completion of an investigation and study of potential "marine sanctuaries" by the Secretary of the Interior in cooperation with the Secretary of the Army acting through the Chief of Engineers.

Section 5B(d) of H.R. 1095 would provide that once such areas were designated as "marine sanctuaries".

". . . all licenses, permits, or authorizations which have been issued by any officer or employee of the United States under authority of any other provision of law shall be terminated and of no effect to the extent they authorize any

[1] H.R. 285, subsection (h); H.R. 983, subsection (h); H.R. 1095, section 5C(b).

[2] In describing the wastes affected by the bill, H.R. 1095, unlike H.R. 285, H.R. 805 and H.R. 983, refers specifically to heated effluents and to solid, liquid or gas wastes (§§ 5B(e), 5C(a)).

activity prohibited by subsection (e) of this section. Thereafter no license, permit, or authority shall be issued by any officer or employee of the United States which would authorize any activity prohibited by subsection (e) of this section."

Section 5C(a) of H.R. 1095 would require the Secretary of the Interior, within one hundred and eighty days after the designation of areas as "marine sanctuaries", to establish standards for the discharge of waste materials[2] in all other areas. The standard contained in this section is again a federal "no damage" standard.[3] The standard also includes requirements for the treatment of wastes and like H.R. 805 would require persons before discharging wastes to "present sufficient evidence that discharging materials in the location in which they are to be deposited will not endanger the natural environment and ecology" of the navigable and coastal waters of the United States and international waters. Subject to certain exceptions which would allow the States to establish more stringent standards, these standards would be binding on the States and state agencies as well as the Federal Government and all federal agencies. Section 5C(b) would allow the Secretary of the Interior to appoint officers to enter and inspect property, plants and facilities in order to determine whether there has been compliance with this section.

Section 5C(f), of H.R. 1095 would provide that:

"(f) Upon the issuance of standards under subsection (a) of this section applicable to any area, all licenses, permits, or authorizations which have been issued by any officer or employee of the United States under authority of any other provision of law with respect to discharges in an area shall be terminated and of no effect to the extent they authorize any activity prohibited by subsection (g) of this section."[4]

Unlike H.R. 285, H.R. 805, and H.R. 983, H.R. 1095 contains specific requirements for disposal of military materials including chemical, biological, and radiological warfare agents.

It is not entirely clear from the language of the bills, what impact H.R. 285, H.R. 805, H.R. 983 and H.R. 1095 would have on the Commission's responsibilities for licensing non-federal hydroelectric projects under Part I of the Federal Power Act (16 U.S.C. 792–823), and for issuing certificates of public convenience and necessity for the construction and operation of natural gas pipeline facilities under Section 7 of the Natural Gas Act (15 U.S.C. 717f). It could well be argued that the definitions of wastes used in the bills are not intended to encompass discharges from non-federal hydroelectric power plants or from natural gas pipeline facilities. H.R. 805 could have a similarly limited impact by virtue of its narrower definition of "ocean, coastal, and other waters".

The Commission opposes enactment of H.R. 805 in its present form because subsection (i) would terminate all FPC licenses, permits and certificates on the date H.R. 805 becomes effective. We believe that enactment of H.R. 805 would seriously impair the attainment of an adequate supply of electric energy throughout the United States. The proposed bill is contrary to the national policy of comprehensive development of the Nation's water resources articulated in Part I of the Federal Power Act. (*First Iowa Hydro-Electric Cooperative* v. *F.P.C.*, 328 U.S. 152, 180–181 (1946).

While the Commission supports their basic intent, we question whether the provisions in H.R. 285, H.R. 983 and H.R. 1095 represent the best or most orderly means of achieving the general objectives of these bills. We believe that the comprehensive approach embodied in H.R. 4723, the Administration's proposed "Marine Protection Act of 1971" offers a significantly better solution to the growing problem of unregulated ocean dumping. Under that proposal the Administrator of the Environmental Protection Agency would be authorized to issue permits for the dumping in the oceans, coastal and other waters of materials which he determines "will not unreasonably degrade or unreasonably endanger human health, welfare or amenities of the marine environment, ecological systems or economic potentialities". In reviewing and evaluating individual permit applications the Administrator would apply criteria which extend to both (1) the likely impact of the proposed dumping on human health and welfare and the

---

[2] See footnote on p. 104.

[3] "Such standards shall be for the purpose of insuring that no damage to, or loss of, any marine life or wildlife or other resources necessary for the ecological balance of the area or pollution of the navigable waters of the United States will result from any such activity . . ." § 5C(a).

[4] Subsection (g) would subject persons discharging wastes in violation of established standards to heavy fines.

62-513 O—71——8

marine environment and (2) alternative disposal locations, the probable impact of requiring the use of such alternative locations and the public interest considerations associated with issuing or denying permits. In establishing or revising such criteria the Administrator would have the benefit of the comments and suggestions of various Federal agencies, including those of the Federal Power Commission.

The Commission also questions the practicality of the absolute "no damage" standard contained in the bills. In practice, this standard would have the effect of prohibiting any discharge of waste material into navigable or coastal waters. The federal "no damage" standard and the more stringent state standards which could be imposed under H.R. 285, H.R. 805, H.R. 983 and H.R. 1095, could well, if pressed too far, impair or defeat the attainment of other national objects, including the development of adequate utility services and the production of needed supplies of industrial goods. The Commission is cognizant of the importance of protecting marine and wildlife resources. However, the Commission believes the more flexible case-by-case approach utilized in H.R. 4723, the Administration bill, would be preferable.

The Commission has no comments to offer on the provisions of H.R. 1095 which relate to the disposal of military wastes.

The Office of Management and Budget advises there is no objection to the presentation of this report and, that enactment of H.R. 4723 would be in accord with the program of the President.

FEDERAL POWER COMMISSION,
JOHN N. NASSIKAS, *Chairman.*

DEPARTMENT OF HEALTH, EDUCATION, AND WELFARE,
May 21, 1971.

Hon. EDWARD A. GARMATZ,
Chairman, Committee on Merchant Marine and Fisheries, House of Representatives, Washington, D.C.

DEAR MR. CHAIRMAN: This letter is in response to your request of February 19, 1971, for a report on H.R. 336 and H.R. 548, bills "To require the Council on Environmental Quality to make a full and complete investigation and study of national policy with respect to the discharging of material into the oceans."

These bills would provide that the Council on Environmental Quality make a full and complete investigation and study of all aspects of existing national policy with respect to the discharge of any material of any kind into the waters of the Atlantic and Pacific Oceans, the Gulf of Mexico, and any other waters within the territorial sea and the contiguous zone of the United States. The bill provides that upon completion of such investigation and study the Council would report to the President and Congress its recommendations for a national policy with respect to discharge into such waters. Such recommendations would include treaties, agreements, and legislation necessary in connection therewith.

The Administrator of the Environmental Protection Agency transmitted to the Congress on February 10, 1971, the Administration's proposal, which is embodied in H.R. 4247 and H.R. 4723, to regulate the dumping of waste material into the oceans, coastal, and other waters of the United States. The need for such regulation is made clear in the President's message of February 8, 1971, transmitting a program to save and enhance the environment. This Department strongly supports the Administration's proposal.

While this Department would defer to the views of the Council on Environmental Quality and the Environmental Protection Agency with respect to whether the study contemplated by H.R. 336 and H.R. 548 is necessary, it would appear that the bills' basic objectives have already been achieved. In October 1970, the President made public a Council on Environmental Quality report entitled "Ocean Dumping: A National Policy," which included recommendations for a comprehensive national policy in the area of ocean dumping and which was the basis for the Administration's proposed legislation.

We are advised by the Office of Management and Budget that there is no objection to the submission of this report, and enactment of H.R. 4247 or H.R. 4723 would be in accord with the Administration's program.

Sincerely,

(S) ELLIOTT L. RICHARDSON,
Secretary.

DEPARTMENT OF HEALTH, EDUCATION, AND WELFARE,
May 10, 1971.

Hon. EDWARD A. GARMATZ,
Chairman, Committee on Merchant Marine and Fisheries,
House of Representatives, Washington, D.C.

DEAR MR. CHAIRMAN: This letter is in response to your request of February 26, 1971, for reports on H.R. 4247 and H.R. 4723, bills "To regulate the dumping of material in the oceans, coastal, and other waters and for other purposes."

These identical bills embody an Administration proposal transmitted to the Congress by the Administrator of the Environmental Protection Agency on February 10, 1971. They would prohibit, except as authorized by the Administrator of the Environmental Protection Agency, the transportation of material from the United States for the purpose of dumping it into the "oceans, coastal, and other waters," and the dumping of material into the "oceans, coastal, and other waters" of the United States. Nevertheless, the proposal would authorize the Administrator of the Environmental Protection Agency to issue permits for such purposes where, in his judgment, such transportation or dumping will not unreasonably degrade or endanger human health, welfare, or amenities, or the marine environment, ecological systems, or economic potentialities. It would require the Administrator to develop criteria for reviewing and evaluating the issuance of such permits, after consultation with the Secretaries of Commerce, Interior, State, Defense, Agriculture, Health, Education, and Welfare, and Transportation, the Atomic Energy Commission, and other appropriate Federal, State, and local officials.

In addition, the proposal would authorize the Administrator to designate recommended sites for the dumping of specified materials. Provision would be made for penalties for violation of the Act. The proposal would also direct the Secretary of State, in consultation with the Administrator of the Environmental Protection Agency, to seek effective international action and cooperation to ensure protection of the marine environment and would authorize him to formulate, present, or support specific proposals in the United Nations and other competent international organizations for such purposes.

The need for this new program is made clear in the President's message of February 8, 1971, "Program for a Better Environment". We urge its enactment.

We are advised by the Office of Management and Budget that enactment of this proposal would be in accord with the Administration's program.

Sincerely,

(S) ELLIOT L. RICHARDSON,
Secretary.

U.S. DEPARTMENT OF THE INTERIOR,
OFFICE OF THE SECRETARY,
Washington, D.C., April 5, 1971.

Hon. EDWARD A. GARMATZ.
Chairman, Committee on Merchant Marine and Fisheries,
House of Representatives, Washington, D.C.

DEAR MR. CHAIRMAN: We respond to your recent requests for comment on H.R. 285. H.R. 805, H.R. 983, H.R. 1095. H.R. 1383, H.R. 1661, H.R. 3662, H.R. 4359, and H.R. 5050, bills which have as their common objective the regulation of ocean dumping to retard degradation of the marine environment.

While these bills are similar in terms of the problem addressed. they can be distinguished with respect to the mechanism or procedure proposed as a solution to that problem. H.R. 285. H.R. 983. and H.R. 1095 would amend the Fish and Wildlife Coordination Act to vest in the Secretary of the Interior and the Administrator of the Environmental Protection Agency responsibility for the designation of ocean sites into and onto which waste material could be safely dumped. H.R. 1095 would require, further, that the Secretary establish environmental standards for waste disposal in areas not so designated, and would establish a schedule for minimum treatment of sewage and industrial waste discharged into areas subject to such standards. Each of these bills also provides for the State acceptance of disposal standards comparable to those promulgated by Federal authority.

enactment will help to curtail the use of our coastal waters for waste disposal and contribute to the development of feasible land-based alternatives.

The Office of Management and Budget has advised that this report is in accord with the program of the President.

Sincerely yours,

HARRISON LOESCH,
Assistant Secretary of the Interior.

U.S. DEPARTMENT OF THE INTERIOR,
OFFICE OF THE SECRETARY,
Washington, D.C., April 5, 1971.

Hon. EDWARD A. GARMATZ,
Chairman, Committee on Merchant Marine and Fisheries,
House of Representatives, Washington, D.C.

DEAR MR. CHAIRMAN: We respond to your request of February 19 for comment on H.R. 336 and H.R. 548, identical bills "To require the Council on Environmental Quality to make a full and complete investigation and study of national policy with respect to the discharging of material into the oceans."

H.R. 336 and H.R. 548 would require that the Council on Environmental Quality make an investigation and study of national policy with respect to the discharge of all materials into the territorial sea and contiguous zone of the United States. Upon completion of its study, the Council would be required further to report its findings, both to the President and the Congress, together with its recommendations for a national policy concerning such discharges.

Commenting last year on similar legislation pending before the 91st Congress, we noted that President Nixon had already directed the Council to conduct such a comprehensive study of ocean dumping, and to recommend such action as may be appropriate to the establishment of a national policy on ocean dumping. Those recommendations of the Council, "Ocean Dumping—A National Policy," were endorsed by the President and transmitted to the Congress on October 7, 1970 (*Congressional Record*, Oct. 7, 1970, p. H9780; H. Doc. 91-399). Further, a legislative proposal to implement the Council's recommendations, the "Marine Protection Act of 1971," has been submitted to the 92nd Congress by the Environmental Protection Agency as part of the President's environmental program.

Thus, while we feel there is no longer a need for enactment of H.R. 336 or H.R. 548, we urge that your Committee give prompt and favorable consideration to the "Marine Protection Act of 1971." We agree with EPA Administrator Ruckelshaus that "this legislation would provide a comprehensive framework for regulating the transportation and dumping of materials and forestalling pressures to dispose of a vast new influx of wastes in the oceans, coastal waters and the Great Lakes."

The Office of Management and Budget has advised that there is no objection to the presentation of this report from the standpoint of the Administration's program.

Sincerely yours,

HARRISON LOESCH,
Assistant Secretary of the Interior.

U.S. DEPARTMENT OF THE INTERIOR,
OFFICE OF THE SECRETARY,
Washington, D.C., April 5, 1971.

Hon. EDWARD A. GARMATZ,
Chairman, Committee on Merchant Marine and Fisheries, House of Representatives, Washington, D.C.

DEAR MR. CHAIRMAN: We respond to your recent requests for comment on H.R. 337, H.R. 549, H.R. 4217 and H.R. 4584, similar bills that would require certification either by the Council on Environmental Quality, the Environmental Protection Agency, or the National Oceanic and Atmospheric Administration prior to ocean disposal of military waste material.

We recommend the enactment of H.R. 4247 or H.R. 4723, this Administration's proposal to regulate all ocean dumping and the transportation of material to be dumped, in lieu of H.R. 337, H.R. 549, H.R. 4217, and H.R. 4584.

As the sponsors of these bills recognize, the unregulated ocean disposal of military material, including obsolete munitions and chemical, biological or radiological warfare agents, constitutes a grave threat to the marine environment. As we noted during hearings held last summer on the Army's "Operation Chase" by your Subcommittee on Oceanography, the disposal of wastes at sea has been poorly monitored, making it difficult to measure the extent of damage already done. We do know, however, that degradation of water quality and physical alteration of marine habitat will take its toll from among species of sport fish and other aquatic wildlife.

H.R. 4247 and H.R. 4723 would vest in the Administrator of the Environmental Protection Agency responsibility for the issuance and enforcement of permits to regulate all kinds of ocean dumping. We think it appropriate that such authority be given to an operating agency broadly charged with protection of the environment, and that its Administrator be required to establish environmental standards for the transportation and disposal of all waste material, whatever its source. It should be noted, too, that the Administrator would be empowered to prohibit absolutely the dumping of a specified material when he finds that such material cannot be dumped without harmful impact upon the marine environment.

Thus, while we support the objectives of H.R. 337, H.R. 549, H.R. 4217, and H.R. 4584, we believe that they can be best attained by the enactment of more comprehensive legislation pending before your Committee as H.R. 4247 and H.R. 4273.

The Office of Management and Budget has advised that there is no objection to the presentation of this report from the standpoint of the Administration's program.

Sincerely yours,

HARRISON LOESCH,
Assistant Secretary of the Interior.

U.S. DEPARTMENT OF THE INTERIOR,
OFFICE OF THE SECRETARY,
Washington, D.C., April 5, 1971.

Hon. EDWARD A. GARMATZ,
Chairman, Committee on Merchant Marine and Fisheries,
House of Representatives,
Washington, D.C.

DEAR MR. CHAIRMAN: We respond to your request of February 26 for comment on H.R. 4247 and H.R. 4723, identical bills "To regulate the dumping of material in the oceans, coastal, and other waters and for other purposes", the "Marine Protection Act of 1971".

The Department of the Interior strongly recommends enactment of this Administration proposal to provide long sought regulation of waste disposal in ocean, coastal, and Great Lakes waters of the United States.

H.R. 4247 and H.R. 4723 would vest in the Administrator of the Environmental Protection Agency authority to control ocean dumping of waste materials through issuance of permits and enforcement of a prohibition against the unauthorized transport or dumping of such material. In determining whether or not to approve a permit application, the Administrator would be required to consider (1) the impact of dumping on the marine environment and human welfare and (2) other possible locations and methods of disposal, including land-based alternatives, but in no event would a permit be issued for a dumping in violation of applicable water quality standards. Section 5 provides authority to designate recommended sites for the dumping of specified materials, and would allow the Administrator to deny, alter or revoke a permit for the disposal of any material that could threaten human health or the marine environment.

Jurisdiction would extend to all persons, including Federal, State, and foreign governmental organizations, who seek to dispose in territorial waters of the United States or the adjacent contiguous zone, to the extent that such disposal

in the contiguous zone may affect the territorial sea or territory of the United States. Section 6 provides a civil penalty of not more than $50,000 for each violation of the prohibition against unauthorized transport or disposal and criminal sanctions for knowing and willful violations. Surveillance would be conducted by the Coast Guard, and legal action taken by the Attorney General upon request of the Administrator. A thorough analysis of its draft bill was transmitted to the Congress on February 10 by the Environmental Portection Agency.

As your Committee is aware this Department has frequently expressed its opposition to the use of ocean waters for waste disposal. Implicit in our opposition to *all* ocean dumping, however, has been the recognition that feasible alternatives are not always available. Our concern for the environmental effects of uncontrolled dumping led to recent studies of the New York Bight and participation in the review of ocean dumping generally which preceded the issuance on October 7, 1970 of "Ocean Dumping—A National Policy", a report prepared by the Council on Environmental Quality.

We participated, too, in the preparation and review of legislation to implement the Council's recommendations. The bills now pending before your Committee, H.R. 4247 and H.R. 4723, are the end result of close cooperation among those several Federal agencies with responsibility for the protection, conservation and management of our Nation's natural resources. The Department of the Interior will provide whatever assistance it can to the Administrator of the Environmental Protection Agency under section 5(a) of the Marine Protection Act of 1971.

President Nixon noted in his environmental message of February 8 that ocean disposal has a number of harmful effects, including destruction of marine life, decreased abundance of fish and other economic resources, modification of marine ecosystems, and impairment of aethetic values. We urge prompt enactment of H.R. 4247 or H.R. 4723, as the President suggested, "to assure that our oceans do not suffer the fate of so many of our inland waters, and to provide the authority needed to protect our coastal waters, beaches, and estuaries".

The Office of Management and Budget has advised that this report is in accord with the program of the President.

Sincerely yours,

HARRISON LOESCH,
Assistant Secretary of the Interior.

DEPARTMENT OF THE NAVY,
OFFICE OF LEGISLATIVE AFFAIRS,
Washington, D.C., April 21, 1971.

Hon. EDWARD A. GARMATZ,
Chairman, Committee on Merchant Marine and Fisheries,
House of Representatives,
Washington, D.C.

DEAR MR. CHAIRMAN: Your request for comment on H.R. 4359, a bill "To amend the Act of August 3, 1968 (82 Stat. 625), to protect the ecology of estuarine areas by regulating dumping of waste materials, to authorize the establishment of a system of marine sanctuaries, and for other purposes," has been assigned to this Department by the Secretary of Defense for the preparation of a report expressing the views of the Department of Defense.

The purpose of the bill is to amend the Act of August 3, 1968 (82 Stat. 625), to provide for the protection of the ecology of estuarine areas by regulating the dumping of waste materials, the authorization of the establishment of a system of marine sanctuaries, and the implementation of these general goals.

The Department of the Navy, on behalf of the Department of Defense, is deeply concerned about the adverse ecological and environmental effects associated with the discharge of wastes and other materials into the oceans, coastal and other waters. We are also concerned, however, that certain features of H.R. 4359 could unnecessarily prohibit some important activities not necessarily harmful to the marine environment. We are especially concerned that the proposed new section 7(c)(1) to the Act of August 3, 1968, as set forth in section 3 of

H.R. 4359, could be construed to preclude operation of U.S. nuclear powered warships, including the strategic deterrent Fleet Ballistic Missile Submarine force. Such a result would be untenable to the security of the United States.

We are also concerned that the bill could be construed to apply to areas over which the United States does not have jurisdiction. Under international law a state has complete jurisdiction over its territorial seas, subject only to the right of innocent passage. The United States' territorial waters extend three miles seaward from the mean low-water line. Beyond this territorial sea the United States has sovereign rights for the purpose of exploring and exploiting the natural resources of its continental shelf and also has the right to enforce its customs, fiscal, immigration or sanitary regulations within a zone of the high seas contiguous to its territorial sea. (Article 2, 1958 Geneva Convention on the Continental Shelf, TIAS 5578; Article 24, 1958 Geneva Convention on the Territorial Sea and the Contiguous Zone, TIAS 5639). Under the 1958 Geneva Convention on the Territorial Sea and Contiguous Zone the extent of the contiguous zone must be no more than 12 nautical miles. In addition, customary international practice presently recognizes the coastal state's right to control fishing within 12 miles of its coast. In consonance with the recognized international practice, a 9-mile fisheries zone contiguous to the United States 3-mile territorial sea was established by the United States in 1966 (Public Law 89-658; 16 U.S.C. 1091-1094).

As presently formulated, H.R. 4359 would provide for unilaterial United States regulation and control of activities well beyond these specialized jurisdictional rights recognized under international law. Such unilateral claims which go beyond the confines of recognized international law, although couched in terms of domestic legislation, can and frequently are used as a basis for exaggerated offshore jurisdictional claims by other nations. Such unwarranted extensions of offshore jurisdiction erode the principle of freedom of the high seas which is essential for naval mobility.

H.R. 4359 would authorize the Secretary of Commerce to designate as marine sanctuaries those areas which the Secretary determines should be preserved or restored. The exercise of this authority conceivably could restrict or prohibit research, development, testing, survey work, or training exercises conducted by, or under the sponsorship of, the Department of Defense, without prior coordination with the Department of Defense.

The Department of the Navy, on behalf of the Department of Defense, believes that the Administration's well drafted, comprehensive bill, H.R. 4723, introduced by you on February 22, 1971, to the 92nd Congress, realistically provides for the intent expressed in H.R. 4359 with respect to preventing harmful, unregulated dumping into the oceans, coastal, and other waters. The Department of the Navy, on behalf of the Department of Defense, therefore favors H.R. 4723, in lieu of H.R. 4359.

This report has been coordinated within the Department of Defense in accordance with procedures prescribed by the Secretary of Defense.

The Office of Management and Budget advises that, from the standpoint of the the Administration's program, there is no objection to the presentation of this report for the consideration of the Committee.

For the Secretary of the Navy.

Sincerely yours,

LANDO W. ZECH, Jr.,
Captain, U.S. Navy,
Deputy Chief.

DEPARTMENT OF STATE,
Washington, D.C., April 22, 1971

Hon. EDWARD A. GARMATZ,
Chairman, Committee on Merchant Marine and Fisheries,
House of Representatives.

DEAR MR. CHAIRMAN: Thank you for your letter of February 17 giving this Department the opportunity to comment on H.R. 1095, a bill to amend the fish and wildlife coordination act to provide additional protection to marine and wildlife ecology by requiring the designation of certain water and submerged land areas where the depositing of certain waste materials is prohibited, to

require the establishment of standards with respect to such deposits in all other areas and for other purposes.

The Department of State in agreement with the general intent of Section 5 B(a) of this bill to establish marine sanctuaries—but only insofar as those sanctuaries would be within the territorial sea limits of the United States. The United States may, of course, restrict dumping within its territorial sea. Beyond that, the Geneva Convention on the Territorial Sea and the Contiguous Zone provides that a coastal state may, in a zone of high seas contiguous to its territorial waters, prevent infringement of its customs, fiscal, immigration or sanitary regulations within its territory or territorial sea. The United States may, therefore, restrict dumping in the contiguous zone which would contravene the sanitary regulatoins of its territory or territorial sea. The high seas beyond the 12-mile limit of the contiguous zone are, however, entirely beyond U.S. jurisdiction.

The Department notes that the establishment of sanctuaries in or under international waters would require international action and thus could not be accomplished unilaterally.

The Department is also in favor of the establishment of general dumping standards in areas other than marine sanctuaries as provided in Section 5C—but only insofar as they apply to United States nationals or to areas under the jurisdiction of the United States. Again, the establishment of dumping standards for foreign nationals in international waters would require international action and could not be accomplished unilaterally. The Department would propose that the general problem of ocean dumping as discussed in this Section of the bill be dealt with by a comprehensive regulatory measure such as that proposed in H.R. 4247 which would prohibit the transport from the United States by any person of material to be dumped in the ocean without a permit.

The Department sees no objection to the enactment of Section 5 D as far as the foreign policy interests of the United States are concerned.

The Office of Management and Budget advises that from the standpoint of the Administration's program, there is no objection to the submission of this report.

Sincerely yours,

David M. Abshire,
Assistant Secretary for Congressional Relations.

Department of State,
Washington, D.C., April 21, 1971.

Hon. Edward A. Garmatz,
Chairman, Committee on Merchant Marine and Fisheries, House of Representatives.

Dear Mr. Chairman: Thank you for your letter of February 26, 1971, requesting the views of the Department of State on H. R. 1661, a bill to regulate the discharge of wastes in territorial and international waters.

The Department of State agrees that there is a need to regulate dumping in order to protect the marine environment in the oceans. Also, it is clear that there is growing international concern over the effects of indiscriminate ocean dumping.

From the viewpoint of foreign policy and international law, the Department of State has no objection to the enactment of this bill. However, the Department favors the adoption of H. R. 4247, the Marine Protection Act of 1971, which is before your Committee for consideration.

The Office of Management and Budget advises that from the standpoint of the Administration's program there is no objection to the submission of this report.

Sincerely yours,

David M. Abshire,
Assistant Secretary for Congressional Relations

DEPARTMENT OF STATE,
Washington, D.C., April 21, 1971.

Hon. EDWARD A. GARMATZ,
Chairman, Committee on Merchant Marine and Fisheries, House of Representatives, Washington, D.C.

DEAR MR. CHAIRMAN: Thank you for your letter of March 9, 1971, requesting the views of the Department of State on HR 3662, a bill to amend the Fish and Wildlife Coordination Act in order to protect the marine environment by regulating the dumping of wastes in the coastal and ocean waters of the United States.

The Department of State is in agreement with the need to protect the marine environment by regulating dumping. However, we wish to point out two problems of international law raised by the present language in HR 3662.

Section 5B (a) provides that "no person may dump waste material into the ocean waters of the United States *or transport such material through such waters* unless he has first obtained a permit from the Administrator of the Environmental Protection Agency authorizing such dumping" (emphasis added). The Geneva Convention on the Territorial Sea and the Contiguous Zone, to which the United States is a party, provides the right of innocent passage through territorial waters for all vessels. Article 14 (4) of that Convention states that passage is innocent so long as it does not prejudice the peace, good order or security of the coastal State. This provision places on the United States an international legal obligation not to restrict, prevent or regulate passage through its territorial waters except on the above grounds. The simple transport of waste material through United States territorial waters would not be considered prejudicial to the peace, good order or security of the United States. Of course, the United States does have jurisdiction to control the transport *from* its territory of materials to be ocean dumped. Therefore, the Department of State recommends that the phrase on lines seven and eight of Section 5B (a) "transport such material through such waters" be deleted.

In addition, Article 24 (1) of the Convention provides that a coastal State may, in a zone of high seas contiguous to its territorial sea, prevent infringement of its customs, fiscal, immigration or sanitary regulations *within its territory or territorial sea.* The United States may, therefore, restrict dumping in the contiguous zone which would contravene the sanitary regulations of its territorial sea or territory. However, the high seas beyond the 12-mile limit of the contiguous zone are entirely beyond United States jurisdiction.

The Department of State strongly favors enactment of comprehensive legislation to regulate ocean dumping. It is quite clear that indiscriminate ocean dumping of waste products from the United States in the territorial waters and contiguous zone and on the high seas is a matter of great international concern. In order to regulate such activities the Department of State favors the adoption of HR 4247, the Marine Protection Act of 1971, which is before your committee for consideration.

The Office of Management and Budget advises that from the standpoint of the Administration's program there is no objection to the submission of this report.

Sincerely,

DAVID M. ABSHIRE,
Assistant Secretary for Congressional Relations.

DEPARTMENT OF STATE,
Washington, D.C., April 7, 1971.

Hon. EDWARD A. GARMATZ,
Chairman, Committee on Merchant Marine and Fisheries, House of Representatives, Washington, D.C.

DEAR MR. CHAIRMAN: The Secretary has asked me to respond to your letter of February 11, 1971 on H.R. 549, and your letter of February 25, 1971 on H.R. 337 and H.R. 4217, bills concerning the discharging of material into any of the navigable waters of the United States.

The proposed bills would regulate discharges in international waters by any person of certain materials. There is no basis in international law for an exercise of jurisdiction over the conduct of foreign nationals on the high seas. We would propose that this problem be dealt with in the context of a comprehensive regulatory scheme such as that proposed in H.R. 4247, the Administration's pro-

posed "Marine Protection Act of 1971." That proposal would meet the essential objectives of the subject bills by prohibiting the transport from the United States by any person of material to be dumped in the ocean without a permit.

The Office of Management and Budget advises that from the standpoint of the Administration's program there is no objection to the submission of these reports.

Sincerely yours,

DAVID M. ABSHIRE,
Assistant Secretary for Congressional Relations.

DEPARTMENT OF STATE,
Washington, D.C., April 21, 1971.

Hon. EDWARD A. GARMATZ,
Chairman, Committee on Merchant Marine and Fisheries, House of Representatives.

DEAR MR. CHAIRMAN: Thank you for your letter of March 9, 1971 requesting the Department of State to comment on H.R. 1383, a bill to amend the Fish and Wildlife Coordination Act to provide additional protection to marine and wildlife ecology by providing for the orderly regulation of dumping in the coastal waters of the United States.

The Department of State shares the concern regarding the protection of the marine environment to which H.R. 1383 is directed and has no objection to its enactment from a foreign policy viewpoint. However, the Department would prefer a more comprehensive proposal to regulate the dumping of materials in the oceans as contained in the Marine Protection Act of 1971 (H.R. 4247 or H.R. 4723).

The Office of Management and Budget advises that from the standpoint of the Administration's program there is no objection to the submission of this report.

Sincerely yours,

DAVID M. ABSHIRE,
Assistant Secretary for Congressional Relations.

DEPARTMENT OF STATE,
Washington, D.C., April 7, 1971.

Hon. EDWARD A. GARMATZ,
Chairman, Committee on Merchant Marine and Fisheries, House of Representatives, Washington, D.C.

DEAR MR. CHAIRMAN: The Secretary has asked me to reply to your letter of February 26, 1971, enclosing for the Department's comments copies of H.R. 4247 and H.R. 4723, bills cited as the "Marine Protection Act of 1971".

The Department's views on this legislation, which we fully support, are set forth in the prepared statement delivered to your Committee in advance of the hearings today at which the Department's Legal Adviser, John R. Stevenson, is testifying on this general subject.

The Department recommends favorable action on this legislation which the Office of Management and Budget advises is in accord with the program of the President.

Sincerely yours,

DAVID M. ABSHIRE,
Assistant Secretary for Congressional Relations.

(The position paper referred to follows:)

ANNEX I.—POSITION PAPER ON OCEAN PROTECTION

THE NEED

The oceans, the common heritage of mankind and essential to his survival, have always been regarded as too vast and productive to be damaged by man. But today we know that existing threats to the health and productivity of the oceans are both real and grave. We can no longer afford the myth that the oceans are an unlimited cornucopia for exploitation by mankind.

Man poses two direct threats: overexploitation and pollution. Through overfishing some species, such as the Blue Whale, the largest animal ever to exist on the Earth, have been virtually exterminated. Pollution in the form of oil spills, pesticides, radioactive material and other toxic substances, is increasing rapidly.

Man's impact on the marine environment also occurs through the simplification of a complex ecosystem through arbitrary selection in species and fish population. The elimination of competing predators to increase production from a fishery or the elimination of an anadromous fish environment in order to extend irrigation agriculture, can be guaranteed to produce adverse environmental consequences.

Existing institutions have not yet been able to prevent pollution or overfishing. A few efforts, now under way, show some small signs of success in handling parts of the problem but new and innovative efforts are urgently required. The urgency stems from the fact that the time scale within which we must respond may be measured in years, not decades.

Additionally, the growing pressures upon the resources of the sea will make the solution of these problems even more difficult.

Oceans, atmosphere and land are interdependent: a disturbance in one is quickly reflected in the others. A particularly critical area is the coastal zone, where the land meets the sea—and much of the seafood used by man depends directly or indirectly upon this zone. Exploitation and pollution in the estuaries and continental shelf area thus pose significant threats to the health of a great portion of the world fishery.

A decline in the productivity of the oceans, combined with rising population levels, would cause serious problems for any nation. *To the underdeveloped countries, this represents imminent disaster.*

In the past the developed countries, owing to their advanced technology, have taken the greater share of marine resources without proper measures to induce rational utilization. If this trend continues the developing nations will never receive their equal share of this finite resource. The present catch from the world's oceans might, under ideal conditions be increased by a factor of 50% to 200%. Increasing pollution of the oceans however must and will decrease their productivity, and the nevitable losses will be suffered by those who can least afford them.

Some believe, almost as an article of faith, that when the problems of the oceans become sufficiently acute, technology will somehow produce a miraculous cure. The history of the deteriorating aquatic environment does not support this faith. Until better evidence is available that irreversible changes are *not* taking place, reason demands that we proceed more carefully, with greater concern for the health of the seas. Mankind cannot assume the risk of precipitating irreversible changes.

THE POTENTIAL THREAT

Without positive protection of the oceans, we can anticipate with varying degrees of certainty and severity:

Diminution or destruction of coastal and oceanic fishery resources through physical, chemical and/or biological disturbances in the ecosystem.

Inadvertent modification of weather and climate, inadequately monitored by existing or planned weather systems, in time producing major adverse changes in ecosystems.

Disturbance of the diversity, stability and productivity of the oceans.

Accelerating reduction in the recreational value of the oceans.

THE PROPOSALS

1. We recognize the need for the conservation and rational use of the ocean environment consonant with the foregoing discussion, organized to assure the protection and rational use of the oceans for the benefit of all nations, particularly the undeveloped nations.

2. To work toward this goal, we urge governments jointly to strengthen existing international organizations concerned with the ocean-atmosphere environment and we stress the value and importance of non-governmental public service groups to support this concern.

3. We recognize the need for the development of an international network of monitoring stations for the ocean-atmosphere environment coupled with adequate facilities for data collection and distribution to nations and organizations desiring them.

4. We further recommend that the following functions be considered as integral to the establishment of any organization purporting to act for the protection of the ocean-atmosphere environment:

(*a*) long-range forecasting and evaluation of data, including (where appropriate) simulation studies.

(*b*) continuing surveillance of potential long and short-range problems, specifically incorporating means for providing public information and recommendations for action.

5. We express the strong hope that preparations for the United Nations Conference on the Human Environment will lead to carefully prepared international conventions covering the primary threats to the ocean ecosystems (see attached Appendix).

6. We propose that an interdisciplinary interim committee be established to refine and develop recommendations 1-3 above, including the preparation of progress reports to the participants in this convocation.

7. Finally, we express gratitude to the Center for the Study of Democratic Institutions, with particular reference to the work of Mrs. Elisabeth Mann Borgese, for its leadership in preparing for and conducting the present convocation and we hope that its work on behalf of an effective Ocean Regime will continue undiminished.

APPENDIX

1. Effective international regulations are necessary to prevent irreversible changes in the ocean environment. Areas in which such regulation may be appropriate include:

(*a*) Prohibition of emission of oil into the oceans.

(*b*) Prohibition or reduction of the manufacture and use of persistent environmental poisons, such as certain pesticides, and heavy-metal compounds.

(*c*) Rules for the transportation of toxic substances harmful to the environment.

(*d*) Rules for the storage and final disposal of environmental poisons and radioactive materials.

(*e*) Rules to prevent overfishing of certain stocks.

2. There is a need for education and training in both developed and especially underdeveloped and developing countries with respect to the ecological requirements of international planning and development. We recommend the setting up of training centers and the publishing of supporting information to this end, as well as the dissemination of relevant information produced by existing organizations and those organizations established pursuant to the Malta recommendations of July 1970.

3. We realize that an increase in the number of organizations working on behalf of the world's oceans adds to the difficulty of coordinating the work of the new with that of the old, and also adds to the probability of duplication. However, we also believe that the increase in organizational complexity is a concomitant of expanding population and technology. Moreover, accelerating threats to the ocean ecosystems and the need for innovative and rapid corrective action require adding to the diversity and consequent strength of the forces available to mankind to protect the global environment.

Mr. LENNON. Our first witness is a very distinguished and able member of our full committee, the Honorable John M. Murphy.

Let me see if the distinguished member from Florida, Mr. Fascell, is here. I don't know whether Congressman Whitehurst, Congressman Pepper, and the chairman of the full committee are here or not, but we will, gentleman, with your permission, hear the several Members of Congress in the order that I have called them and with your

permission we will defer questioning of the Members of Congress until they have finished, if there is no objection to that, because we have to be ready at 11 o'clock to hear Chairman Train.

Now, if you gentlemen have something that you would like to have go on the record, you can appeal to the Chair to rescind that decision I have just made.

We welcome you, Mr. Murphy.

STATEMENT OF HON. JOHN M. MURPHY, A REPRESENTATIVE IN CONGRESS FROM THE STATE OF NEW YORK

Mr. MURPHY. Thank you, Mr. Chairman.

Since I last testified before this committee, the administration has conducted a massive study of ocean dumping. In October of 1970, the Council on Environmental Quality issued a complex report of its findings and recommendations. I find myself in almost complete agreement with them, at least as far as they go.

This is not unusual as they follow the precepts that I outlined in the bill I introduced 1 year ago and in my testimony before the committee on July 27, 1970. In the meantime, nothing much has been done by the administration in the way of substantive action, and an additional 620 billion gallons of garbage have been dumped into New York Harbor.

During that year, another 16,400 billion gallons of industrial waste were poured into our rivers, lakes, and coastal waters; 7,300 billion gallons of waste water were pumped into our sewers.

Lake Erie, one of our many "dead seas," received its annual 3 million tons of pollutants.

Almost 40 million tons of dredge spoils were dumped on our coastal waters, 14 million tons of which were polluted.

Five million tons of industrial wastes polluted our seas.

Four and a half million tons of sewage sludge were dumped at sea, 4 million tons off New York Harbor alone.

I will agree with the conclusion of the report that "the volume of wastes dumped in the ocean is increasing rapidly, that many are harmful or toxic to marine life, hazardous to human health, and esthetically unattractive."

However, I cannot and will not accept the conclusion of the report that the volume of ocean-dumped wastes will "increase greatly" in the future. We cannot let this happen. This committee has the power to stop it. The solution, however, will not be found by creating more pollution farther out to sea; the solution will come from our scientists and other ecology experts who must be given a firm mandate to find new answers to the disposal of our cosmic trash problem. Otherwise, we are all going to end up, those of us left alive, living on one gigantic garbage pile.

The issues are clear and simple. We are throwing too much of society's excrement into our water and it is coming back to haunt us. If I cannot appeal to you on esthetic or ecological grounds let me do it on a gastronomical level. Your very lives may depend on it.

How many would enjoy a lobster salad at lunch knowing that shellfish have been found containing hepatitis virus? How many would

enjoy a lobster dinner not knowing if it came from those polluted areas where shellfish contain concentrated polio virus 60 times that of the fish in surrounding waters? Pollution has already closed 20 percent of our commercial shellfish beds and of the large clam industry areas, particularly in my area.

Oil in the water is a real killer of our marine life, but it may come to the time where it has a second strike capacity—it may kill human beings. Direct contact with the breathing apparatus of undersea life kills many and weakens others. Cancer in fishes is a likely result of oil pollution with cancers, growths, and concentrated cancer producing agents being found in a variety of marine life exposed to those parts of the ocean polluted by oil refineries. Oysters and mussels from polluted areas have been found to contain concentrations of hydrocarbons known to cause cancer in man. And don't think because your oysters are fried you're safe. These potentially lethal hydrocarbons, odorless and invisible, are still locked into seafood tissues even after frying.

Food and Drug Administration scientists say it is possible that these cancerous fish could cause cancer in humans, although they have not had medical evidence of this yet. I, for one, do not want to take that chance and I don't think the American people want to take that chance, either.

For those of you who are clam lovers, I would remind you that clams harvested from the New York Bight contained coliform bacteria 50 to 80 times above acceptable levels set by the Food and Drug Administration. And when you consider the poor little shrimp, I call attention to the ironic case in Florida where uncontaminated shrimp were contaminated by being cleaned on land with polluted water taken from the harbor at Key West.

Of course, the recent mercury pollution flap is receding from our minds, but let us not forget that mercury contamination is still with us; it will be with us for a long time, and its dangers are still very real.

You may remember my statement last July when I described the greatest cesspool of our seas, the New York Bight. I carefully outlined the "plight of the bight" in my remarks then. Nothing has changed, but the fact is that pollution in our harbor is getting worse.

The importance of all this is that it is not only happening in the New York area but in all coastal areas of the United States. There are 121 other ocean-dumping sites on the Atlantic coast, 56 on the gulf coast, and 68 on the Pacific coast, where we are dumping upward of 50 million tons of trash from tin cans to cannons and poisonous isotopes to poisoned gas.

New York and its own "dead sea" is being emulated by a string of fledging dead seas from Maine to Washington State, and we must not forget our polluted inland waterways and lakes that are fast turning into a massive national disgrace.

I have an explanation of my bill that I have submitted in two Congresses now, H.R. 285. H.R. 285 offers a total program for the solution of the water pollution problem not only in New York Harbor but throughout America wherever wastes are disposed of in our waters. In a nation where 85 percent of the population lives in the coastal environment, and in which 100 percent of the people depend on that

environment, the problem is nationwide in scope and needs a comprehesive national solution.

The bill amends the Fish and Wildlife Coordination Act to provide additional protection to marine and wildlife ecology by requiring the designation and regulation of certain water and submerged-land areas where the depositing of any waste material will be permitted. T[illegible] bill established a mechanism for developing effective disposal standards within these areas and provides that all other marine areas will be maintained in a "no dumping" status and preserved and protected as marine sanctuaries.

The guiding principle is to require the Secretary of Interior or the new Environmental Protection Agency to identify and designate those areas in which certain dumping can be safely accomplished. For example, some quantities of cellar dirt may be safely dumped on the Continental Shelf without damaging the ecology of the marine environment if carefully controlled and regulated. Elsewhere the bottom configuration and other factors may permit disposal of certain chemicals or other wastes that are absorbed into the water without causing imbalance.

There has never been a comprehensive program to determine what kinds can be safely disposed of in which waters. Previously, factors such as effects on navigation and distance from population centers were considered but specific ecological effects were generally ignored.

My bill tasks the Secretary of Interior—or EPA—with studying the national marine environment with a view to identifying each river, harbor, and coastal area and designating which of these areas can accept certain types of waste disposal. Standards for the types and amount of dumping would follow in cooperation with the States and the vast majority of our marine environment would be maintained as disposal-free marine sanctuaries where wildlife and fish could exist without the threat of foreign introduction of harmful materials.

The bill includes stiff penalties which I am convinced are justified for dumping in nondesignated areas and for illegal dumping in designated areas: $10,000 per day, per violation, with each day of violation constituting a separate offense. Two years are permitted for completion of the study and identification and designation of disposal areas, and the Secretary of Interior—or EPA—is required to cooperate with the Secretary of the Army in the execution of the study of potential water and submerged-land areas.

Following formal designation by Interior—or EPA—all existing licenses will be revoked and suspended and the Army Corps of Engineers will receive new applications for controlled disposal in designated areas. Enforcement of dumping standards—standards based on the capacity of a specific marine area to absorb wastes harmlessly—shall be undertaken by the Coast Guard.

The foreging represents an innovative approach to the problem of waste disposal in our harbor, river, and coastal waters, and has application to every type of waste disposal throughout the Nation. I strongly urge your prompt approval of this approach and hope that we may see House action on this proposal before the close of the current session.

So I implore the committee, let us take the first step. I have a great deal of pride of authorship in this legislation but I am perfectly will-

ing to cooperate ... the committee in reporting out an administration bill which contains the provisions needed to get the job done. But I do urge cautious speed.

I urge this committee to act quickly to report out a bill that contains those provisions needed to halt the destruction of our marine and wildlife ecology. My only qualification is that the committee consider these sections of the administration bill that fall short of the standards contained in my bill, H.R. 285.

In that respect I point out that the administration bill is lacking a major provision of my original legislation, the establishment of "no dumping" sanctuaries for marine life. I insist that proposals which simply move dumping grounds from one area to another are myopic and only increase the danger of prolonged pollution and international complications growing out of contaminating the world's oceans.

I ask the members of this committee to carefully consider the incorporation of the concept of no-dumping sanctuaries for marine life into any bill they report.

I ask that a physical description of the New York Bight and a map showing the location of ocean disposal sites in the area be printed at the conclusion of my remarks today.

Mr. Chairman, there were proposals made last year that ocean dumping be mandated at 100 miles off the coast and other people said 25 miles off the coast. In effect they were trying to move the dumping areas off the Continental Shelf. What they would do is make it impossible to dispose of wastes that have to be disposed of.

In the New York area I think there were three or four oceangoing barges that would be permitted by the Coast Guard to go those distances at sea. In effect what that type of legislation or approach would do would be simply to cut off dumping that could not be cut off. Those wastes were formerly dumped in New York Harbor and now they are dumped in a controlled area. H.R. 285 goes into the problem of controlled dumping areas without affecting marine sanctuaries and protecting the ecology.

I appreciate the opportunity to appear before this committee and particularly its chairmen, Chairman Dingell and Chairman Lennon, who worked so long in the ecology field and are so identified with the progress we have made.

Mr. LENNON. Without objection, at the gentleman's request, following his remarks a description of the New York Bight and a map showing disposal sites in the area should be printed in the record.

(The description and map follow:)

THE NEW YORK BIGHT

The New York Bight is a slight indentation of the Atlantic coast, extending northeasterly from Cape May inlet, New Jersey, for some 200 miles to the eastern end of Long Island, New York, at Montauk Point. Its coastline is generally a moderately sloping sand beach shore, broken by indentations of the sea into the land. Among these are a number of small inlets along the New Jersey coast, Lower Bay of New York Harbor, East Rockaway Inlet, Jones Inlet, Fire Island Inlet, Moriches Inlet, and Shinnecock Inlet.

Depths in the Bight generally exceed 100 feet about 50 miles off shore but are substantially less than that in most inshore areas. The bottom is mostly sandy and is subject to shifts due to tidal actions or storm surges. Consequently, channels have been dredged and maintained by the U.S. Engineers to accom-

62-513 O—71——9

modate the large volume of sea commerce into the industrial and commercial complex of Greater New York. Sandy Hook Channel leads into Sandy Hook Bay and Raritan Channel branches off into Raritan Bay. Ambrose Channel is the principal entrance into New York Harbor leading to Upper Bay and New City. The inlets to the east (East Rockaway, Jones, and Fire Island) are also subject to shifting sands from time to time.

The New York Bight is a contract in extremes. It contains the only remaining strip of virgin barrier beach between Cape Cod and Cape Hatteras (Island Beach State Park, New Jersey) and supports the most heavily populated and industrialized complex in the country—between Sandy Hook, New Jersey, and Jamaica Bay, New York. The Bight supports some of the most heavily utilized and valuable recreation areas in the country. For example, New Jersey's four-county coastal waterway supports a two-billion-dollar recreation industry

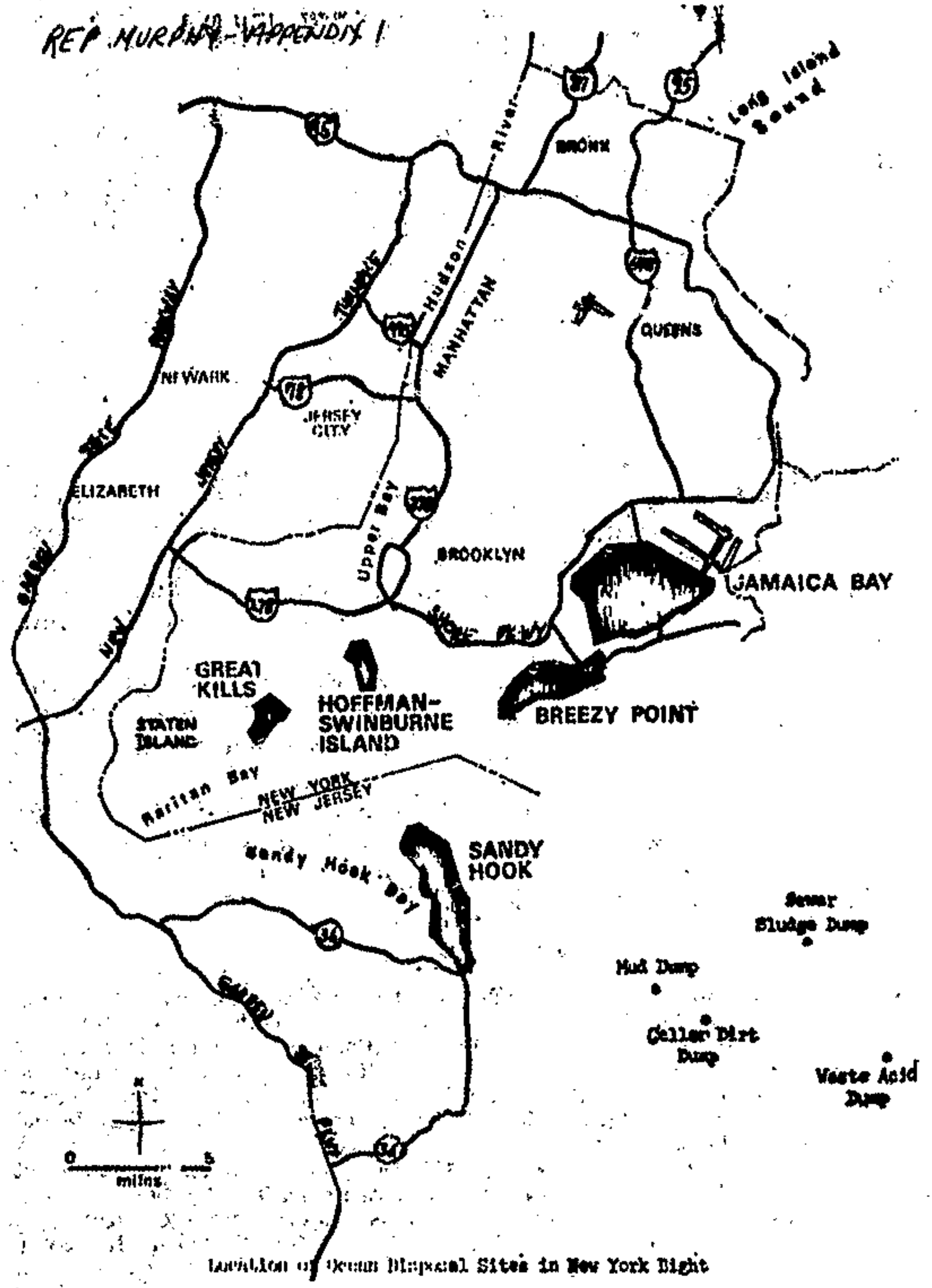

Location of Ocean Disposal Sites in New York Bight

annually and New York's Coney Island beach recorded 22 million visitors in 1968. The Bight area also supports excellent sport and commercial fishing resources. Some of the finest oyster grounds are found in this area; approved shellfish harvesting operations for inshore and offshore clams continue within sight of the New York skyline. Both New York and New Jersey contemplate removal of inadequately treated sewage effluent from condemned inshore shellfish waters that will assure even greater shellfish production in this area.

Mr. LENNON. I would like to ask you also, please, Congressman, if you can furnish us for the record, following the introduction in the record of what we have just agreed to, the 121 ocean-dumping sites on the Atlantic coast, and the 56 on the gulf coast and the 68 on the Pacific coast that you have referred to in the third paragraph of your statement on page 3. Do you have those sites by identification?

Mr. MURPHY. Yes, Mr. Chairman, I have those sites and I would be happy to furnish them.

Mr. LENNON. Without objection, then, they shall be inserted in the record at this point.

(The information referred to was included in chapter I of the Report of the Council of Environmental Quality on Ocean Dumping and is as follows:)

House Document No. 91–399

REPORT OF THE COUNCIL ON ENVIRONMENTAL QUALITY ON OCEAN DUMPING

MESSAGE

FROM

THE PRESIDENT OF THE UNITED STATES

TRANSMITTING

A REPORT OF THE COUNCIL ON ENVIRONMENTAL QUALITY ON OCEAN DUMPING

CHAPTER I

Ocean Dumping: Location, Quantities, Composition, and Trends

ABOUT 48 million tons of wastes were dumped at sea in 1968. These wastes included dredge spoils, industrial wastes, sewage sludge, construction and demolition debris, solid waste, explosives, chemical munitions, radioactive wastes, and miscellaneous materials. This chapter indicates rapid increases in ocean dumping activity over the last two decades and the potential for great increases in the future. At the same time, ocean dumping of wastes from other sources should decrease through implementation of water quality standards and new Federal laws dealing with control of sewage from vessels and with oil pollution.

DISPOSAL SITE LOCATIONS

Data on disposal sites are still incomplete, with little definitive information on sites off Alaska and Hawaii and outside the U.S. contiguous zone (more than 12 miles offshore). There are almost 250 disposal sites off U.S. coasts. Fifty percent are located off the Atlantic Coast, 28 percent off the Pacific Coast, and 22 percent in the Gulf of Mexico. Table 1 summarizes the number of sites for each major area and the number of permits issued for their use. The locations of the disposal sites are indicated in Figure 1.

TABLE 1.—*Ocean Dumping: Site Location Summary (22, 66)*

Coastal area	Number of sites	Active Corps disposal permits
Atlantic Coast	122	136
Gulf Coast	56	50
Pacific Coast	68	71
Total	246	257

Not included in Table 1 are some 100 artificial reefs constructed by private concerns under permits issued by the U.S. Army Corps of Engineers. (66) These reefs, sometimes formed of old car hulks or tires, are intended to provide artificial shelters for fish.

QUANTITIES AND TYPES OF WASTES

The categories of wastes covered in this report are used because of the large quantities of materials currently dumped, their potential for increase, or their special characteristics, such as toxicity. The quantities for each category are summarized by coastal region in Table 2. Radioactive wastes and chemical munitions are not included in the table because weight is not a meaningful descriptor. Each, however, will be discussed later.

The Bureau of Solid Waste Management estimates that the data in Table 2 represent about 90 percent of ocean dumping. However, the data undoubtedly underestimate the size and scope of the problem because of the time lapse and the possibility of many small community operations or illicit operations by private firms. Also not included in the table are those wastes that are piped to sea.

Each major category of ocean dumping sources is now discussed and the possible chemical composition of the wastes delineated as an aid in evaluating their present and potential effects on the marine environment.

Dredge Spoils

A large percentage of dredging is done directly by the Corps. The remainder is done by private contractor under Corps permit. Spoils are generally disposed of in open coastal waters less than 100 feet deep.

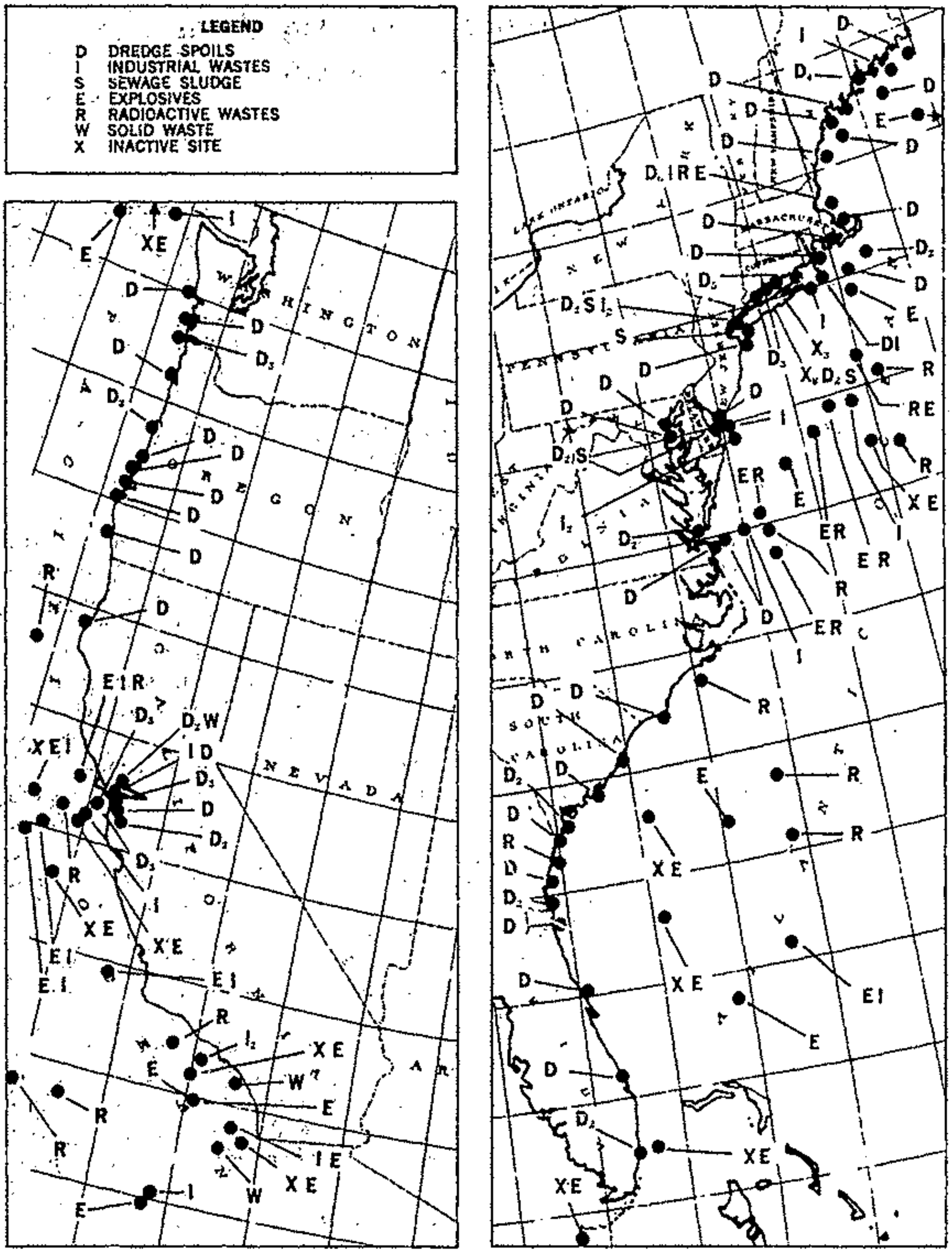
LEGEND
D DREDGE SPOILS
I INDUSTRIAL WASTES
S SEWAGE SLUDGE
E EXPLOSIVES
R RADIOACTIVE WASTES
W SOLID WASTE
X INACTIVE SITE
NEVADA
SOUTH CAROLINA

Figure 1.—Known Dumping Sites Off U.S. Coasts (22, 66)

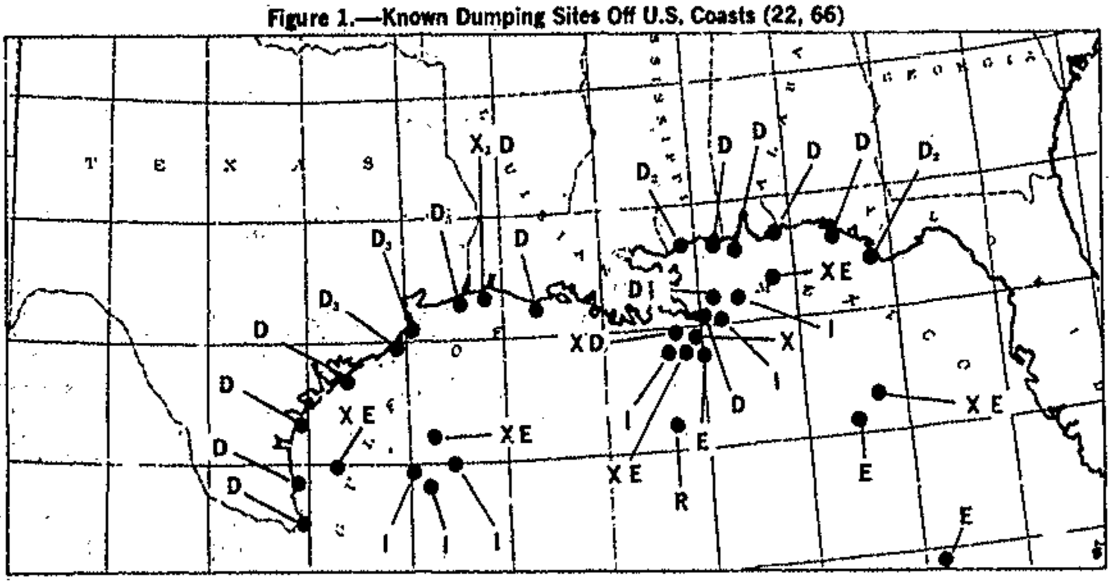

TABLE 2.—*Ocean Dumping: Types and Amounts, 1968* (66)

(In tons)

Waste type	Atlantic	Gulf	Pacific	Total	Percent of total
Dredge spoils	15,808,000	15,300,000	7,320,000	38,428,000	80
Industrial wastes	3,013,200	696,000	981,300	4,690,500	10
Sewage sludge	4,477,000	0	0	4,477,000	9
Construction and demolition debris	574,000	0	0	574,000	<1
Solid waste	0	0	26,000	26,000	<1
Explosives	15,200	0	0	15,200	<1
Total	23,887,400	15,996,000	8,327,300	48,210,700	100

Dredge spoils account for 80 percent by weight of all ocean dumping The Corps of Engineers estimates that about 34 percent (13 million tons) of this material is polluted. Contamination occurs from deposition of pollutants from industrial, municipal, agricultural, and other sources on the bottom of water bodies. The quantities of polluted dredge spoils are shown in Table 3.

Polluted dredge spoils vary at every location according to the land-based sources of pollution. Detailed quantitative analyses of the pollutants in dredge spoils in the coastal

TABLE 3.—*Estimated Polluted Dredge Spoils* (22)

Coastal area	Total spoils (in tons)	Estimated percent of total polluted spoils [1]	Total polluted spoils (in tons)
Atlantic Coast	15,808,000	45	7,120,000
Gulf Coast	15,300,000	31	4,740,000
Pacific Coast	7,320,000	19	1,390,000
Total	38,428,000	34	13,250,000

[1] Estimates of polluted dredge spoils consider chlorine demand; BOD; COD; volatile solids; oil and grease; concentrations of phosphorous, nitrogen, and iron; silica content; and color and odor of the spoils.

areas are not available. An analysis by the Federal Water Quality Administration (FWQA) of polluted spoils from Lake Erie indicates that a total of 82,091 tons of spoils created 10,500 tons of chemical oxygen demand (COD). (23) These large quantities oxygen-demanding materials can reduce the oxygen in the receiving waters to levels at which certain fish and other aquatic populations cannot survive. Also present were toxic heavy metals. Even with substantial dilution, the levels of heavy metals in the spoils may deleteriously affect marine life, as shown in Table 4.

TABLE 4.—*Heavy Metals Concentrations in Dredge Spoils* (23, 36)

(In parts per million)

Metal	Concentrations in dredge spoils	Natural concentrations in sea water	Concentrations toxic to marine life
Cadmium	130	.08	.01–10.0
Chromium	160	.00005	1.0
Lead	310	.00003	.1
Nickel	610	.0054	.1

Industrial Wastes

Industrial wastes were the second largest category of pollutants dumped at sea in 1968 (4.7 million tons, or 10 percent of the total). (66)

Most industrial wastes are commonly transported to sea in 1,000- to 5,000-ton-capacity barges. Sites are 4 to 125 miles off the Atlantic Coast, from 25 to 125 miles off the coast of the Gulf of Mexico, and from 5 to 75 miles off the Pacific Coast. Most of the sites are at the nearshore end of the range.

Highly toxic industrial wastes are sometimes contained in 55-gallon drums and are je[illegible]isoned from either merchant ships or disposal vessels at least 300 miles from shore. The containers are sometimes weighted and sunk. More frequently, they are ruptured at the surface, either manually with axes or by small arms or rifle fire. (66)

The breakdown for disposal methods by geographic area is shown below.

TABLE 5.—*Industrial Wastes by Method of Disposal* (66)

(In tons)

Coastal area	Number of sites	Bulk wastes	Containerized wastes	Total
Atlantic Coast	10	3,011,000	2,200	3,013,200
Gulf Coast	6	690,000	6,000	696,000
Pacific Coast	7	981,000	300	981,300
Total	23	4,682,000	8,500	4,690,500

Table 6 shows the relative quantities of major industrial wastes found in a survey of 50 producers in 20 cities.

TABLE 6.—*Industrial Wastes by Manufacturing Process* (66)

Type of waste	Estimated tonnage	Percent
Waste acids	2,720,500	58
Refinery wastes	562,900	12
Pesticide wastes	328,300	7
Paper mill wastes	140,700	3
Other wastes	938,100	20

The types of contaminants in industrial wastes dumped at sea vary greatly because of the diversity of industries and production processes involved. Many of the wastes are toxic—some highly toxic. For example, refinery wastes, which are 12 percent of the total ocean-disposed industrial wastes, can include cyanides, heavy metals, mercaptides, and chlorinated hydrocarbons. Pulp and paper mill wastes may contain "black liquor" and various organic constituents which are toxic to the marine environment. Chemical manufacturing and laboratory wastes that are dumped include arsenical and mercuric compounds and other toxic chemicals. (66)

Sewage Sludge

Sewage sludge is the waste solid byproduct of municipal waste water treatment processes. These solids can be further treated by digestion, a process which allows accelerated decomposition of the sludge to control odors and pathogens. Most sewage sludge is disposed of on land or is incinerated. Relatively small amounts (4.5 million tons on a wet basis) are currently dumped at sea, of which almost 4.0 million tons are dumped off New York harbor. (66) As of 1968, there were no similar operations on either the Gulf or Pacific Coasts, although sludge is being discharged from Los Angeles by pipeline.

Sewage sludge in digested or undigested form contains significant quantities of heavy metals. A study by the FWQA indicated that copper, zinc, barium, manganese, and molybdenum are present in sewage sludge. (9) The concentrations and types of toxic materials vary because sludge is the residual of waste water treatment and contains whatever domestic and industrial contaminants have entered the system. Table 7 shows the minimum, average, and maximum values for three heavy metals found in one analysis of sewage sludge.

TABLE 7.—*Heavy Metals Concentrations in Sewage Sludge (8, 9, 36)*

(In parts per million)

Metal	Concentrations in sewage sludge			Natural concentrations in sea water	Concentrations toxic to marine life
	Min.	Avg.	Max.		
Copper	315	[illegible]	1,960	.003	.1
Zinc	1,350	2,459	3,700	.01	10.0
Manganese	30	262	790	.002	....

Sewage sludge also contains significant amounts of oxygen demanding materials. In 1969, sludge dumped in the New York Bight, encompassing the New York harbor and some adjacent coastal areas, had an oxygen demand of about 70,000 tons. (15) These wastes also include some bacteria that cause diseases in man.

Construction and Demolition Debris

Only New York City disposes of debris at sea in significant quantities because of the lack of nearby available landfill. Sea disposal is conducted with 3,000- to 5,000-ton capacity barges that are towed some 9 miles offshore. These materials are generally inert and nontoxic.

Solid Waste

Solid waste, the byproducts and discards of our society, amounts to approximately 5.5 pounds per capita per day collected by municipal and private agencies. (28) Although these wastes total approximately 190 million tons per year, ocean disposal accounted for only about 26,000 tons. (66) Ocean dumping of solid waste occurred exclusively on the Pacific Coast, where they were generated by cannery operations and commercial and naval shipping operations. Other sources no doubt exist, but the overall magnitude of the current problem is minor.

The composition of solid waste, ascertained by sampling, is shown in Table 8. It is presented here to indicate the materials that would be introduced into the marine environment if ocean dumping of solid waste becomes a common practice.)

Solid waste disposed of in the ocean interacts with the water, but the resultant chemical products are difficult to determine. Studies have been done on the interaction between solid waste and fresh water in sanitary landfills as the water percolates through the waste materials. (The resultant mixture of water and chemicals is called leachate.)

TABLE 8.—*Composition of Solid Waste* (28)

Type of waste	Average (percent)
Paper products	43.8
Food wastes	18.2
Metals	9.1
Glass and ceramics	9.0
Garden wastes	7.9
Rock, dirt, and ash	3.7
Plastics, rubber, and leather	3.1
Textiles	2.7
Wood	2.5
Total	100.0

The percentage of pollutants in solid waste is not nearly as high as in sewage sludge or dredge spoils, but it does contain nutrients, oxygen-demanding materials, and heavy metals. Laboratory studies of water contaminated by solid waste have shown significant quantities of heavy metals, with zinc, nickel, and magnesium present in concentrations of 13, .27, and 378 parts per million respectively. (29) These concentrations are well above toxic levels for marine life.

Up to 50 percent of solid waste is usually paper, wood, plastics, and rubber, all of which can float to the surface. Particularly significant are the plastics which will not become water soaked and will not degrade for many, perhaps even hundreds, of years. Even if baled before ocean disposal, it is almost certain that over time the bales will disintegrate and the floatables will rise to the surface. The potential esthetic problems of large quantities of solid wastes floating to the surface and then being carried to shore are staggering.

Explosives and Chemical Munitions

Unserviceable or obsolete shells, mines, solid rocket fuels, and chemical warfare agents have been disposed of in deep water for many years. In 1963, the Navy initiated Operation "CHASE," in which munitions were disposed of by sinking them in obsolete hulks. Since then, 19 gutted World War II Liberty ships containing munitions have been scuttled. In the last six operations, the weapons were to detonate, but the S.S. ROBERT LOUIS STEVENSON failed to do so as planned and is located on the continental shelf near Alaska in 2,200 feet of water.

Since 1964 at least 18,342 tons of ammunition and explosives have been dumped in this manner. Additional cargoes of approximately 35,000 tons containing an unknown proportion of net explosives were also scuttled. A detailed listing of the ships scuttled, their cargoes, and disposition are shown in Table 9.

Detonation of explosives can result in trace amoun [illegible] of lead, nickel, bronze, and other metals [illegible] the water, depending on corrosion processes and the materials used in the munitions.

Radioactive Wastes

Most nuclear waste products are liquid and of low radioactivity. They consist mostly of decontaminated process and cooling waters from reactors, fuel processing, and other operations. Small amounts of liquid wastes are highly radioactive; they result from the reprocessing of reactor fuel elements.

Solid radioactive wastes are produced by contamination of equipment and other materials during nuclear power plant operations, from medical use, and by research and development activities.

Solid radioactive wastes have been buried in carefully controlled landfill sites. Low-level liquid nuclear wastes are treated and/or stored to reduce radioactivity before disposal. High-level liquid wastes are stored exclusively in tanks at land-based sites.

TABLE 9.—*Explosives and Chemical Munitions, 1964–1970 (30)*

Year	Name	Total cargo (in tons)	Nature of cargo	Net explosives (in tons)	Disposition
1964	S.S. John F. Shafroth	9,799	A&E	Unknown	SDW
	S.S. Village	7,535	A&E	Unknown	SDW
1965	M.V. Coastal Mariner	4,040	A&E	512	D at 1,000′
	S.S. Santiago Iglesia	8,715	A&E	406	D at 1,000′
1966	S.S. Isaac Van Zandt	7,600	A&E	1,625	D at 4,000′
	S.S. Horace Greely	6,033	A&E	442	D at 4,000′
1967	S.S. Robt. L. Stevenson	6,600	A&E	2,327	S
	S.S. Corporal Eric G. Gibson	9,005	Chem.	None	SDW
	S.S. Monahan	333	A&E	Unknown	SDW
1968	S.S. Mormactern	7,763	Chem.	N.A.	SDW
	S.S. Richardson	7,437	A&C	128	SDW
1969	S.S. Cape Tryon	7,626	A&E	1,145	DU
	S.S. Cape Catoche	6,342	A&E	1,359	DU
	S.S. Cardinal O'Connell	6,431	A&E	2,144	DU
1970	S.S. Frederick E. Williamson	5,245	A&E	478	DU
	S.S. Cape Comfort	6,200	A&E	N.A.	DU
	S.S. Walker D. Hines	6,500	A&E	N.A.	DU
	S.S. David Hughes	5,000	A&E	N.A.	DU
	S.S. LeBaron Russell Briggs	2,664	Chem.	N.A.	SDW

Definitions: A&E=ammunition and explosives; N.A.=not available; DU=Detonated unintentionally; SDW=sunk in deep water; D=detonated; S=sunk at less than 4,000 feet and did not detonate as planned; A&C=ammunition and cylinders contaminated with residues of GB nerve gas.

Liquid and solid radioactive wastes which have been dumped in the ocean are usually in concrete-filled metal drums or containers. Table 10 summarizes the amounts of these wastes disposed of at sea.

The quantities of radioactive materials disposed of at sea have decreased dramatically for several reasons. First, in 1960 the Atomic Energy Commission placed a moratorium on new licenses for disposal of radioactive wastes in the ocean. Only one commercial organization (which has never conducted any sea disposal), two Government agencies, and one university are still authorized to dispose of radioactive wastes in the ocean. Second, the major contractors of the AEC have not disposed of any wastes at sea since 1962. And for economic reasons, those firms with licenses are phasing out sea disposal of radioactive wastes in favor of land disposal.

TABLE 10.—*Radioactive Wastes: Historical Trends, 1946–1970 (70)*

Year	Number of containers	Estimated activity at time of disposal (in curies)
1945-1960	76,201	93,690
1961	4,087	275
1962	6,120	478
1963	129	9
1964	114	20
1965	24	5
1966	43	105
1967	12	62
1968	0	0
1969	26	26
1970	2	3
Total	86,758	94,673

Two sites have been used for disposal of most of the wastes in the Pacific Ocean. These sites are approximately 48 nautical miles west of the Golden Gate Bridge. One commercial firm has disposed of wastes in the Pacific Ocean farther than 150 miles from the U.S. coast; these disposals, 11 in number, were at depths greater than 6,000 feet. In the Atlantic Ocean, the major sites for disposal were in the area of Massachusetts Bay, approximately 12 to 15 miles from the coast; approximately 150 miles southeast of Sandy Hook, N.J.; and approximately 105 miles from Cape Henry, Va. With the exception of the Massachusetts Bay site, disposal was at depths greater than 6,000 feet. The Massachusetts Bay site was in 300 feet of water.

PAST TRENDS

Figure 2 shows significant increases in ocean dumping activities during the years 1951–1968. These data do not include dredge spoils or explosives because historical data could not be readily reconstructed. Radioactive wastes are also excluded because of their negligible weight contribution.

Table 11, on which Figure 2 is based, shows a fourfold increase in tonnage dumped at sea from 1949 to 1968. The 28 percent increase between the 1959–1963 period and the 1964–1968 period is largely attributable to dramatic increases in industrial wastes and sewage sludge disposal. In 1959, industrial wastes disposed of at sea approximated 2.2 million tons. By 1968, the amount had increased to over 4.7 million tons, a 114 percent increase in 9 years. The amount of sewage sludge disposed of at sea increased by 61 percent in the same period, from 2.8 million tons to 4.5 million tons. (66)

FUTURE TRENDS

Assessing future trends in ocean dumping requires analysis of basic population trends. Population growth is accompanied not only by increased amounts of wastes but also by decreased space available for their disposal.

Between 1930 and 1960 the coastal population increased by 78 percent, compared with a 48 percent increase nationwide. (36) The figures below (25) indicate the population growth in the coastal region projected through the year 2000:

1960	57,946,000
1970	68,397,000
1980	76,607,000
1990	92,940,000
2000	106,900,000

TABLE 11.—*Ocean Dumping: Historical Trends, 1949–1968*[1] *(66)*

Coastal area	1949-1953		1954-1958		1959-1963		1964-1968	
	Total	Avg./Yr.	Total	Avg./Yr.	Total	Avg./Yr.	Total	Avg./Yr.
Atlantic Coast	8,000,000	1,600,000	[2] 16,000,000	3,200,000	27,270,000	5,454,000	31,100,000	6,200,000
Gulf Coast	[3] 40,000	8,000	283,000	56,000	860,000	172,000	2,600,000	520,000
Pacific Coast	487,000	97,000	850,000	170,000	940,000	188,000	3,410,000	682,000
Total	8,527,000	1,705,000	17,133,000	3,426,000	29,070,000	5,814,000	37,110,000	7,422,000

[1] Figures do not include dredge spoils, radioactive wastes, and military explosives.

[2] Estimated by fitting a linear trend line between data for preceding period and data for succeeding period.

[3] Disposal operations in the Gulf of Mexico began in 1952.

Figure 2.—Average Annual Tonnage Dumped at Sea—by Coastal Area (66)

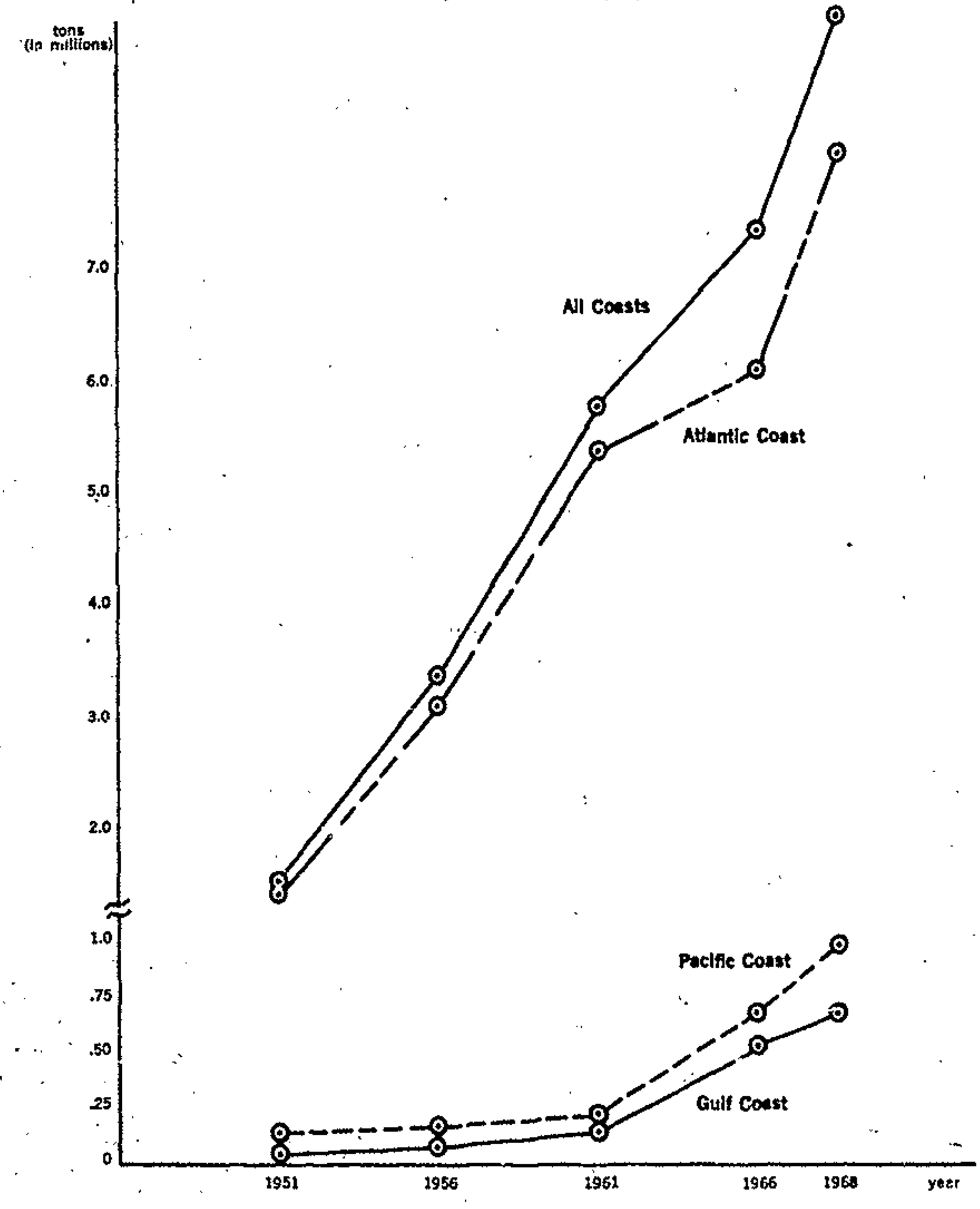

Solid Waste

About 65 million tons of solid waste are generated annually in the coastal region. Based on a conservative estimate of 8 pounds of waste generated per person per day in the year 2000—the generation rate which will be reached by 1980—over 150 million tons will need to be disposed of for that one year. (28) If 10 pounds per person per day are generated, total wastes in the coastal area will be close to 200 million tons, more than triple current levels. The pressure to use the ocean for waste disposal will increase as land disposal sites become more scarce, costs increase, and metropolitan areas face political problems in obtaining new land disposal sites. Several cities are currently exploring the use of the ocean as a solid waste disposal site, and this interest is expected to increase. In some cases operations may begin within a year. If even a small percentage of the solid waste annually generated in the coastal area were disposed of at sea, the quantities entering the marine environment would be many orders of magnitude greater than all solid waste disposed of at sea to date.

Sewage Sludge

Based on an average of .119 pounds of sludge generated per person per day, potential sludge disposal quantities for the coastal region can be roughly estimated. (37) In 1970, approximately 1.4 million tons of sludge will be disposed of in the coastal areas, and in the year 2000, approximately 2.1 million tons will be generated, an increase of 50 percent in 30 years. If anything, these figures may underestimate future quantities of sludge. For example, between 1960 and 1980, 20-year period, the sludge generated by the Baltimore-Washington area is expected to increase from 70,000 tons to 166,000 tons, or about a 140 percent increase. New York City's sludge barged to sea is expected to increase from 99,000 tons in 1960 to about 220,000 tons in 1980, a 120 percent increase in 20 years. (66)

Industrial Wastes

The volume of industrial production, which gives rise to waste production, is increasing at a rate of 4.5 percent annually, or three times the population growth rate. Additionally, the FWQA estimates that the manufacturing industry is responsible for three times as much waste as that produced by the Nation's population. And about 40 percent of the Nation's industrial activity is concentrated in the estuarine economic region. (36) Given increasingly stringent water quality standards and the ever expanding level of industrial waste generation in the coastal zone, the potential for increased industrial waste dumping at sea is great.

Radioactive Wastes

The amount of liquid and solid radioactive wastes will rise with projected increases in nuclear power generation. The amount of high-level liquid radioactive wastes will increase from 100,000 gallons in 1970 to 6,000,000 gallons by the year 2000 and radioactive solid wastes, from approximately 1 million cubic feet in 1970 to 3 million cubic feet by 1980. (70) As mentioned earlier, however, ocean dumping has been virtually nonexistent since the early 1960's because of the AEC moratorium and the economic advantage of land disposal.

Large radioactive structures, an additional source of radiation, are not yet a significant problem. In the past, the few that became obsolete have been decontaminated, dismantled, and kept under surveillance on land—with the exception of parts of one nuclear sub-

marine, which were disposed of in the ocean. Currently, however, there are 16 nuclear power plants in operation, 55 under construction, and 25 for which construction permit applications are pending with the Atomic Energy Commission. (70) If current forecasts are realized, by the year 2000, the equivalent of up to 1,000 nuclear power units, each with a capacity of some 1,000 megawatts, may be operating. In addition, the Navy has about 90 nuclear-powered submarines and surface ships, and many more may be built in the next 30 years as a large portion of the current naval fleet is replaced. Commercial nuclear ships—currently the N.S. SAVANNAH is the only one—may become economically feasible in the future.

A lifetime of 10 to 30 years for the power plants' and ships' reactor vessels is reasonable in terms of physical or technological obsolescense. Their radiation levels vary considerably, up to 50,000 curies of induced radiation in each structure. (70)

Individually none of these sources adds significant amounts of radioactivity to the ocean. Taken together, however, the increases could be of significant concern.

Dredge Spoils

In the long run, the reduction of polluted discharge from municipal and industrial sources, brought about by water quality standards, will lessen the problem from dredge spoils. However, they will remain a problem for at least the next 5 to 10 years. During this period, there will be pressures for more dredging to deal with increasing marine commerce, to meet the desire of cities for new deep-water harbors, and to provide draft for larger vessels (including the supertankers used to transport oil). These needs will all increase total dredging and hence dredge spoils.

Explosives and Chemical Munitions

The following are Department of Defense estimates of conventional munitions planned for disposal: in 1970, 103,777 tons; in 1971, 88,835 tons; and in 1972, 80,000 tons. (26) These quantities are several times larger than the total volume of these wastes disposed of at sea in the last two decades. They indicate the quantities which would enter the marine environment if no other disposal technique were employed.

Chemical munitions have also been disposed of at sea in three deep-water disposal operations, but actual quantities involved are not known. No future ocean disposal operations are planned. Biological agents have not previously been disposed of at sea, and no future disposal is projected.

SUMMARY

The data indicate that the volume of wastes dumped in the ocean is increasing rapidly. Many are harmful or toxic to marine life, hazardous to human health, and esthetically unattractive. In all likelihood, the volume of ocean-dumped wastes will increase greatly due to decreasing capacity of existing disposal facilities, lack of nearby land sites, higher costs, and political problems in acquiring new sites.

Mr. LENNON. Gentlemen, without objection we will follow the rule that I established, and we would be delighted if you could find the time to stay here in case the members wanted to question you after we have heard the other Members scheduled to be heard.

Mr. MURPHY. Thank you, Mr. Chairman.

Mr. LENNON. Would you submit a list of the definitive amendments to the so-called administration bill which would implement specifically your philosophy that you enunciated in your statement, which had to be done in this point of time but which the administration bill did not do?

Mr. MURPHY. I will be happy to provide that also, Mr. Chairman.

Mr. LENNON. Thank you.

(The list follows:)

AMENDMENTS TO H.R. 4723, AS INTRODUCED, OFFERED BY MR. MURPHY OF NEW YORK

Page 2, lines 17 and 18, strike out "and the Great Lakes." and insert "the Great Lakes, and the navigable waters of the United States."

Page 8, between lines 5 and 6 insert the following:

Designation of Dumping Areas and Application of State Dumping Standards Thereto in Certain Cases

SEC. 6. (a) During the two-year period after the date of the enactment of this Act, the Administrator, in cooperation with the Federal officers listed in section 5(a)(1), shall make a full and complete investigation with respect to the oceans, coastal, and other waters for the purpose of designating specific areas therein where material can be safely discharged. Before designating any such area, the Administrator shall take into account (1) those factors specified in section 5(a) relating to the protection of the general welfare and the environment, and (2) the criteria developed in the administration of such section 5. After designating any such area, the Administrator shall prescribe such standards relating to the discharge of material within such area as may be necessary and appropriate. Such standards (hereafter referred to in this section as the "Federal standards") shall apply with respect to permits issued under section 5, but nothing in this subsection shall be deemed to prohibit the Administrator from appropriately modifying any Federal standard prescribed under this subsection in order to take care of any exceptional circumstance which may be raised in connection with the issuance of any permit.

(b)(1) If within one year after the Administrator prescribes Federal standards with respect to any area designated by him pursuant to subsection (a) of this section, a State establishes standards with respect to dumping within such area, such State standards shall apply with respect to any dumping in such area if such area is within the jurisdiction of such State if, within such one year period, the Secretary, after public hearings—

(A) determines that such State standards are equal to or more stringent than the Federal standards prescribed by him pursuant to subsection (a) of this section, and

(B) that there are adequate procedures by which the State can enforce such standards.

If the Administrator determines that such State standards are not as stringent as the Federal standards then the Federal standards shall apply within such area.

(2) Any State standards found to be applicable within an area, designated by the Administrator pursuant to subsection (a) of this section shall continue to apply until such time as the Administrator, after public hearing, determines that such standards are not equal to or more stringent than the comparable Federal standards. The Administrator shall review all such standards of each State at least once each calendar year for purposes of comparing them with the applicable Federal standards.

(c) The Administrator is authorized to issue new Federal standards and to amend existing Federal standards from time to time as he determines necessary

with respect to any area designated by him under subsection (a) of this section, and such new or amended standards shall be considered to be the initial Federal standards issued pursuant to such subsection (a) for the purposes of this section.

And redesignate the succeeding sections of the bill accordingly.

Page 12, line 15, after the period insert the following: "No license, permit or authorization issued by any officer or employee of the United States shall be of any effect to the extent that it authorizes or regulates any activity regulated by a State pursuant to State standards which apply to any area designated by the Administrator under section 6(a) of this Act. For purposes of the preceding sentence, an 'activity regulated by a State pursuant to State standards' includes transportation of material to the designated area concerned."

Mr. LENNON. Now we are delighted to recognize the distinguished gentleman from Florida, Mr. Dante Fascell.

Mr. FASCELL. Thank you, Mr. Chairman.

Mr. ROGERS. Mr. Chairman, would you yield?

Mr. LENNON. Delighted.

Mr. ROGERS. I am particularly pleased to see my colleague from Florida here. He has taken a big interest in this problem and has been a leader in the field and we welcome him to the committee.

Mr. DINGELL. I would like to take this opportunity to welcome our good friend this morning and if we might take just a moment I think we should take note of some well-deserving facts about the gentleman from Florida.

Congressman Dante B. Fascell is serving his ninth term in the House of Representatives. He is a ranking member of the Committee on Foreign Affairs and chairs its Subcommittee on Inter-American Affairs.

I want the record to show that Congressman Fascell was appointed by President Nixon to be a U.S. delegate to the 24th General Assembly of the United Nations, and he recently represented the United States at the inauguration of Mexico's President, Luis Echeverria.

An acknowledged leader in the fight to protect our beleaguered environment, I want the record to further show that Congressman Fascell is presently a member of the Conference Committee of the International Conference on Scientific Aspects of the Global Environment, which will be held in November in Miami, Fla. In addition, while he was chairman of the Foreign Affairs Subcommittee on International Organizations and Movements in 1968, Mr. Fascell conducted extensive hearings which were reported under this title: "The Oceans: A Challenging New Frontier."

STATEMENT OF HON. DANTE B. FASCELL, A REPRESENTATIVE IN CONGRESS FROM THE STATE OF FLORIDA

Mr. FASCELL. Thank you.

Mr. Chairman and gentlemen, I appreciate the opportunity to appear once again before this committee on this very important problem. I commend the committee for its prompt action initially in pursuing this most urgent and difficult problem.

Mr. Chairman, before I begin my formal statement I think that I would like to make this comment. The chairman of the Conservation and Natural Resources Subcommittee of the Committee on Government Operations has by letter, just recently, asked several agencies of Government to do that which ought to be done under current law, and that is one of the things we all have to consider here. Chairman

62-513 O—71——10

Reuss stated to the Corps of Engineers and to the Coast Guard that the corps can control under supervisory law where wastes are transported from New York, Norfolk, and Baltimore Harbors. The Coast Guard controls all hazardous materials; for example, poisons, and so forth. So one of the problems, in other words, that we face is that we have several agencies in the act and one of the things we want to consider is whether or not we want to change that; and it is an important consideration.

The other, gentlemen, it seems to me is the question of whether or not any kind of dumping ought to be permitted unless we have a sound scientific base for it. I cannot get too excited by saying that dumping is bad inside of 3 miles but it might be all right a hundred miles out. I know that the committee will have to struggle with that problem also. The whole question of designated dumps and supervised dumps and authorized dumps is not one easily resolved.

We have reached the point in the crisis of the pollution of our oceans where anything less than comprehensive legislation to strictly control ocean dumping internationally would mean that the cancer we face is terminal.

Not too long ago, 1966 as a matter of fact, the philosophy which the chairman has enunciated in his opening statement was rather strongly urged. A delegate at the First International Conference on Waste Disposal in the Sea in 1966 said this:

> The great economy inherent in the discharge of urban sewage and industrial wastes into near-shore waters for final disposal is apparent to all who will investigate. It is doubly apparent to those charged with the responsibility of disposing of such wastes without excessive cost to the public or menace to public health. If the ocean, or one of its arms, can be reached with a sewer outfall within the bounds of economy, the grim spectre of an expensive complete treatment plant grows dimmer and dimmer until it fades entirely and, to the great satisfaction of those who have to gather funds for the public budget, as well as they (you and I) who have to pay the bill, the good old ocean does the job free.
>
> And small wonder that we look to the sea for this assist. Its vast area and volume, its oxygen-laden waters, its lack of potability or usefulness for domestic and most industrial purposes, present an unlimited and most attractive reservoir for waste assimilation.

Now, that was in 1966. I guess we have come a long way. I think we have come a considerable way, and we have got a long way to go. We in this Government have already taken strong steps to protect water quality within the 3-mile limit, where most of this type of dumping takes place. But the increase in the problem of dumping in waters beyond the 3-mile limit has been exponential.

The concept of the sea as an infinite source of waste absorption must be laid to rest before the disruption in the life cycle of this planet lays the human race to rest.

Evidence? There is plenty of evidence, gentlemen; some of which we touch upon here.

Thor Heyerdahl, in his attempt to cross the ocean in a reed ship, found that he could not fill tooth mugs in the middle of the ocean because of the filthy condition of the water.

Dr. Max Blumer of the Woods Hole Oceanographic Institute has declared that the amount of tar on the surface of the sea already equals the amount of its surface sea life.

Two officials of the Federal Water Pollution Control Agency recently rode a dredge-clammer 14 miles out into the Atlantic from the

Delaware Bay and watched as the ship dredged 200 pounds of sewage sludge containing many dead clams.

The presence of mercury, lead, DDT, and pesticides is increasing dangerously in sea life. It has been shown that pesticides inhibit the ability of diatoms in the ocean to produce oxygen. The world's supply of oxygen comes mainly from the photosynthetic activity of these tiny diatoms.

Dr. Jacques Cousteau, famed oceanologist who has traveled nearly 155,000 miles in the last 3½ years exploring the oceans of the world, recently concluded: "The oceans are in danger of dying. The pollution is general."

Mr. Chairman, we are all familiar with inside the United States and the waters close by, but down our way we have the specter of the Gulf of Mexico becoming a dead gulf in a very short time and it is a very real danger. It is going to take a tremendous effort in terms of technology to restore the Gulf of Mexico to what it ought to be in terms of clear water and as an asset for mankind.

The hearings I am sure will be replete with evidence, Mr. Chairman, that the need is urgent and that the time is short.

At the present time there is neither legal provisions for the control of ocean dumping nor enforcement beyond the 3-mile limit. Furthermore, there is a regulatory vacuum in international waters which lie outside even the 12-mile outer limit of the territorial seas of a coastal nation.

Recently the United Nation's Intergovernmental Maritime Consultative Organization adopted in London a U.S. resolution calling for an end to willful ocean dumping and accidental spills by 1975 if possible, but certainly by the end of the decade. This is a positive step, but I fear we cannot afford the luxury of waiting until 1980 or even 1975.

The various legislative proposals before this distinguished committee offer unilateral restraints on the part of the United States. My bill is not an exception. I have got a repeat of the package put in here last year. H.R. 4719 is part of a legislative package which includes, among other things, a concurrent resolution calling for an international agreement, under the auspices of the 1972 United Nations Conference on the Human Environment, to prohibit dumping in the waters of the world and provide the necessary framework for review and enforcement.

In the final analysis, Mr. Chairman, this is a problem affecting all the nations of the world, and we must have international cooperation in order to meet it successfully. House Concurrent Resolution 146 on that point is now pending before the House Committee on Foreign Affairs.

The part of my legislative package which is before this committee is, of course, H.R. 4719. Simply and comprehensively it prohibits the discharge into the waters of the world of any military or waste material without a certification by the Administrator of the Environmental Protection Agency approving such discharge.

As the operational agency charged with the responsibility of enforcing and administering air and water quality standards, the EPA is the logical watchdog against pollution of our oceans.

Another encouraging aspect of our attempts to control ocean dumping is the recent announcement by Department of Defense Secretary Laird that no more military chemicals or munitions would be dumped into the ocean and that alternative, ecologically safe methods would be developed for future disposal of such agents.

We have come a long way in the period since last year's dumping of obsolete nerve gas by the Department of the Army in the Atlantic Ocean off the coast of Florida. The volatile condition of the material left us with no choice in the matter.

Still, these are voluntary restraints. The third part of my package of legislative proposals, Mr. Chairman, which could be before this committee and I think it will be, now pending before the House Armed Services Committee, would require that before any new munition of a chemical or biological nature is added to the U.S. arsenal there must first be submitted to EPA and approved a date by which the material must be disposed and the means of disposal. In addition, the Department of Defense would be required to inventory all such munitions on hand, the future disposal of which might present a potential harm to mankind or the environment. Now, the thrust of that is so obviously logical, Mr. Chairman, that I wonder that we have not done it before but maybe it is like the safety pin.

Legislation of this kind will prevent us from again finding ourselves in the untenable position of having tons of a lethal agent in a volatile condition, with the ocean as the least objectionable place to get rid of it.

One of the deficiencies in the administration's bill, it seems to me, Mr. Chairman, is its provision that:

> Nothing in this act shall prohibit any transportation for dumping or dumping of material where such transportation or dumping is necessary, in an emergency, to safeguard human life.

Now, that sounds like an easy, necessary provision, but on the other hand it might be an out. It seems to me that if we could take the cautions in advance for the inventory of those dangerous agents, decide on the methods of disposal, the time of disposal scientifically, that we would eliminate if not all at least a substantial part of the emergency.

As you can see, H.R. 4719 is an integral part of a three-pronged legislative attack which in its entirety forms an effective control and regulation of the various and harmful practices of ocean dumping. I am pleased that more than 65 of our colleagues have joined me in the sponsorship of these proposals.

I am also cosponsoring a bill which combines the provisions of the bill before this committee and the bill which I have referred to earlier about the defense inventory which got before the Armed Services Committee, and I hope this committee can consider that joint bill.

In the face of a problem as potentially dangerous as ocean dumping, however, the important thing is not who sponsors the legislative remedy but how effective the legislation is in protecting the environment and the citizens of this Nation. I am confident that this committee will report out an effective bill.

Mr. Chairman, let me just remind you by quoting from the summary of the findings of the Council on Environmental Quality's report on ocean dumping in which they say:

> The Nation has an opportunity unique in history—the opportunity to act to prevent an environmental problem which would otherwise grow to a great mag-

nitude. In the past, we have failed to recognize problems and to take corrective action before they became serious. The resulting signs of environmental degradation are all around us, and remedial actions heavily tax our resources. This is clearly the time for a conscious national decision to control ocean dumping.

I am delighted that these committees which are presently here today have that challenge, have that opportunity, and I am confident that they will take the necessary action to close the gaps at least legislatively.

Thank you very much.

Mr. LENNON. Thank you very much, Congressman.

Now we would like to make the same request of you, sir, if you would submit for the consideration of the committee and the counsel of the committee the specific amendments to the so-called administration bill that would accomplish the purpose you seek.

Mr. FASCELL. Be very happy to do that, Mr. Chairman.

Mr. LENNON. Now our next witness is a very distinguished Member of our body in Congress who served on both sides of the Capitol. We are delighted to have you, Senator Pepper, or Congressman Pepper, whichever you prefer. The gentlemen is again from Florida.

Mr. ROGERS. Thank you, Mr. Chairman.

I want to welcome to the committee again another colleague from Florida who has been vitally interested in this question. You can see, because of the location of our State and the problems we have, I think that is one reason that so much interest is centered in Florida. So I join in welcoming Congressman Pepper.

STATEMENT OF HON. CLAUDE PEPPER, A REPRESENTATIVE IN CONGRESS FROM THE STATE OF FLORIDA

Mr. PEPPER. Thank you very much, Mr. Chairman and members of the committee. I particularly thank my distinguished colleague from Florida for his kind words.

My remarks will be brief. I appear here in support of H.R. 808 which I am one of the cointroducers of and which I think means a great deal to our State as it does to many other parts of the country.

I have not heard any technical definition of it but I know we have one. If you take in all of the indentations of our coast, I suppose that we have probably the longest water coastline in the United States.

Would you not think that might be true, Mr. Rogers? Or one of the longest at least.

I think the definition of this bill which defines oceans, coastal and other waters would include oceans, gulfs, bays, salt-water lagoons, salt-water harbors, other coastal waters where the tide ebbs and flows, the Great Lakes and so on is a very appropriate description to which this proposed legislation should apply basically.

Mr. Chairman and members of the committee, what is proposed in this legislation is that anybody who puts anything of a deleterious character or may be proved to be polluting in its confluence into one of these waterways as so defined shall be required before he is authorized to do so to get the approval of the appropriate agency which in this case is the administration of the Environmental Protection Agency acting through the U.S. Fish and Wildlife Services and in consultation with the Army Chief of Engineers.

Nobody, it seems to me, has a right to pollute the waters of our cast—the waters, for that matter, of the interior, but that is another matter. The presumption is that any material capable of polluting does pollute unless unless one of these bodies determines after an appropriate application and license is applied for that it does not have any polluting effect upon the water to which it can be discharged.

I think it is extremely necessary that we enact legislation of this sort because along many of our bays—Biscayne Bay, for example, in my area and Mr. Fascell's area is generally referred to as being polluted to the degree that it is not even suitable for bathing. It certainly is not desirable that a great beautiful body of water like that has any contamination or any pollution, and that is due to the fact that over the years we have simply poured sewerage and poured these polluting and contaminating properties into this beautiful bay without any regard for the public interest at all. We have also done the same thing for the ocean.

I don't think anybody particularly is to blame because up until recently we thought it was all right to build an outfall into the ocean with only preliminary treatment of the material that goes into the outfall and therefore is distributed into the ocean. We thought that was just a public dumping ground where you could put anything you wanted to and it was not going to hurt anything or anybody; it was so big and so much water and generally so deep that you could dump anything into the ocean with impunity.

It has been many months since I have had the honor to appear here before the committee to talk about the dropping of these weapons into the ocean. Even 2 or 3 years ago nobody would ever have thought of questioning that because our awareness of this ecology problem and contamination and pollution problem had not come to be so acute as it has in the recent past. So I think it perfectly proper to put the burden of proof that it will not harm a public waterway upon whoever disposes materials into such a waterway that would have some deleterious effect.

Let me comment on this. In the county of Dade, of which I have the honor to represent a part here, Mr. Fascell a part, Mr. Burke a part, many of our municipalities have these outfall lines out into the ocean where they dump their sewage and those outfall lines have been built at quite a lot of expense—in fact, they have been wrestling with the financial problem of building those outfalls. My understanding is that generally speaking they have only given preliminary treatment to the sewage that has gone through those outfalls and dumped into the ocean.

I don't know just how our public bodies would be affected by this legislation. While I think it is right in principle, on the other hand, if acting under permissible conceptions and permissible authority in the past, public bodies have done things that have burdened them with debt and they should be required to set up other facilities before they should be permitted to such dumping, why I think the Federal Government ought to help them to make the necessary adjustments to accommodate themselves to the criteria of the present in respect to the pollution of waterways.

I know we have had sewerage money available. To my regret the administration has reduced the request for such money, perhaps held

up quite a bit of it, but we are going to encounter collateral problems probably. What private industry should have to do to make adjustments to this is also a matter that should give us concern. It may well be that we should have loan programs that would permit private industry which has been set up in the past to dispose of its waste in this way to make the necessary adjustment. It might bankrupt some of them if they did not get such assistance.

I would not propose giving money to private industry but that we might well set up a system of loans to enable private industry to convert its functioning so that it would not in any way be in conflict with the public interest. Although this principle is sound, Mr. Chairman.

Now I want to say this. I guess it is not politic to say. I think some of our people who are dedicated and devoted to the cause of conservation and the ecology—one of the Cracker politicians calls it "ec'o log'ogy" because everybody has not got accustomed to this new phrase or new wording yet. I think some of those good people are going to the extreme in some of their demands that they are making, and they are going to run into and provoke a reaction, I think, if they do insist upon going to the extreme.

I think they therefore ought to contemplate that they are now where you cannot talk about anything but what they say that it pollutes something. I wonder sometimes whether when you walk across the grass you might step on the bugs that might have a right to live in the luxurious green foliage of many of our grassy areas.

But this kind of thing, this is sound and this should be insisted upon and this should have priority. Any other pollution to any of our streams should have priority. Pollution of the air, nobody has a right to pollute the air which we breathe. I think the pollution of the water, the pollution of the air should have priority and those programs should go strongly ahead, but it gets to the point now you cannot build an airport or you cannot fly a plane without somebody telling you it is going to damage the ecology.

There is going to be eventually, I am afraid, a reaction. I don't want to see any reaction toward the very desirable ends of protecting the environment of our people but we are on solid ground when we are talking about preserving our waterways and preserving the air of our people, and that is what this bill primarily relates to.

Thank you very much, Mr. Chairman.

Mr. Lennon. We thank you very much for your excellent presentation and recognition that there must be a balance somewhere.

Mr. Pepper. Right.

Mr. Lennon. Now we will hear from the distinguished chairman of the full committee who wants to address the two subcommittees this morning on this subject.

Mr. Garmatz.

STATEMENT OF HON. EDWARD A. GARMATZ, A REPRESENTATIVE IN CONGRESS FROM THE STATE OF MARYLAND

Mr. Garmatz. Thank you, Mr. Chairman.

I introduced H.R. 4723, which is an administration bill, because I think it is essential for this Nation to protect its marine resources

from pollution by the indiscriminate and uncontrolled dumping of harmful waste material into America's coastal and offshore waters.

I scheduled hearings on this issue because I think this is a critical problem. On March 16, 1971, when I announced these hearings, I said they would probably comprise the most important consideration of environmental legislation to be held in this session of Congress. And I explained that statement by saying there is an urgent need to establish a national policy on ocean dumping now—before this Nation resorts to an irreversible pattern of wholesale dumping into the oceans.

I might add to that by saying that I think the legislation which ultimately evolves from these hearings will become landmark legislation.

But I want to emphasize at this point that, even though I introduced H.R. 4723, I am concerned over some of the proposals it contains.

Approximately 40 other ocean-dumping bills have been referred to this committee, and some of them also contain disturbing proposals. Original bills, of course, are not expected to be perfect, they merely provide a vehicle for formulating a more effective approach to any problem. That is the reason for these hearings.

My concern can best be explained, I think, by pointing to the broad scope of responsibility of the full House Committee on Merchant Marine and Fisheries. Among its many other functions, the full committee plays a rather unique dual role. The Subcommittee on Merchant Marine is responsible for the protection and promotion of a healthy maritime industry, including shipping and port facilities and port development. On the other hand, the Subcommittee on Fisheries and Wildlife Conservation is responsible, as its title implies, for the conservation of our fish and wildlife resources and for the protection and enhancement of our environment, including our estuaries, our fresh waters, and our oceans, and all the precious living resources in those waters. The Subcommittee of Oceanography is also concerned over any activity which might adversely affect the ecology of the oceans and of America's coastal waters.

Because of the joint concern of the latter two subcommittees, I decided hearings on these ocean-dumping bills should be held jointly, with both subcommittees participating. I might add that Congressman John Dingell, chairman of our Subcommittee on Fisheries and Wildlife Conservation, and Congressman Alton Lennon, chairman of our Subcommittee on Oceanography, both held extensive hearings on ocean dumping in the last session of Congress. Both of these distinguished chairmen are equally concerned over this serious problem, and I think it appropriate that they share the responsibilities of the chair during these proceedings.

I am here today to speak both as chairman of the full House Committee on Merchant Marine and Fisheries and as chairman of the Subcommittee on Merchant Marine. In the latter capacity, I want to reiterate my concern over certain proposals contained in many of the bills we will be considering during these hearings.

My primary concern is that some of these bills, if enacted as now drafted, could seriously impede future port development and, therefore, adversely affect the entire maritime industry.

For instance, in the case of the administration bill, H.R. 4723, no dumping of waste materials in our coastal or Great Lakes waters would be allowed without a permit from the Environmental Protection Agency, known as EPA. Many representatives of various port authorities have personally come to me to express their fear over this provision. They are convinced that, if this provision is enacted, it would seriously delay the completion of many dredging projects—such as port and harbor channels—which are vitally needed if these ports are to survive in the fiercely competitive fight for cargo.

In the past, dredging permits have been obtained from the Corps of Engineers. The administration bill, however, would require the corps to obtain a permit for any dredging in port and harbor areas from the EPA. It is sincerely felt by many that such a requirement would introduce a significant element of delay in processing applications, in gaining firm approval, and in actually completing the channel project. Such delays could be economically disastrous to many American ports. It is also sincerely felt by many that the corps has the expertise, the staff, and the total capability to justify that agency's retention of authority for approving channel-dredging projects, and that the EPA, a newly created agency, has neither the staff, the experience, nor the capability, to do the necessary research and administrative work needed for rapid approvals.

We all realize the disposal of dredging spoil is a practice which can be harmful to any marine ecology if the spoil is discharged indiscriminately. There have been occasions in the past when the Corps of Engineers has shown a lack of interest or indifference toward the conservation of our great natural resources. But I have recently been impressed with the fact that the corps has been converted to the cause of conservation, and that it now seems dedicated as much to that cause as it is to industrial development.

I think that Chairman Dingell, who is a staunch conservationist, has done more than his share to convert the Corps of Engineers and I would be happy to hear his expert views upon what I considered to be the corps' healthy and sensible approach to this problem by attempting to establish a balance between the need to promote industrial and economic development and the need to protect and preserve our environment.

Achieving that kind of balance may be difficult, but that is the kind of balance I would like to see personified in the bill that ultimately comes from this committee. I might say there is a precedent for such an approach to this seeming dilemma. Public Law 90–454, which was enacted August 3, 1968, and which calls for the protection of our estuarine areas, says in part, and I quote:

> It is therefore the purpose of this Act to provide a means for considering the need to protect, conserve and restore these estuaries in a manner that adequately and reasonably maintains a *balance* between the the national need for such protection in the interest of conserving the natural resources and natural beauty of the nation and the need to develop these estuaries to further the growth and development of the nation.

Mr. Chairman, I am proud to say that this act and this language emanated from our Subcommittee on Fisheries and Wildlife Conservation, which held hearings on the original estuarine legislation and

was responsible, on the House side, for its passage and ultimate enactment.

It should also be noted that the first annual report of the Council on Environmental Quality, in its declaration of national environmental policy, said, and again I quote:

> . . . It is the continuing policy of the Federal Government, in cooperation with State and local governments, and other concerned public and private organizations, to use all practical means and measures, including financial and technical assistance in a manner calculated to foster and promote the general welfare, to create and maintain conditions under which men and nature can exist in productive harmony . . .

I am also proud to say that the Environmental Quality Council was established by a law which originated in legislation introduced by our distinguished chairman, Congressman Dingell, and that it was his subcommittee which held hearings on the enabling legislation.

It is natural that I am concerned about the impact of y legislation on port deveolpment, since the port facilities are vital to our national economy and to a healthy American maritime industry. In my own State of Maryland, the Maryland Port Authority plans to spend $90 million in the next 10 years to improve its port facilities. Other ports around the country are making similar plans for future development.

Mr. Chairman, I am not here to plead for any select interest but I am here to speak out on behalf of our national interest and for the general welfare.

America has been blessed with a wonderful abundance of natural resources, which have helped to make our country great. But these resources have been abused, and now that they are threatened, there is a justifiable move to protect them. What we must guard against is overreaction, against an anti-industry movement which could contain the seeds of destruction. After all, industry and technology have also helped to make this a great nation.

I know that Chairman Dingell and Chairman Lennon are aware of the essential nature of industry, just as they are aware of the need for strong environmental legislation; and I know they will, therefore, give all concerned parties a chance to present their views on this important and controversial subject.

Mr. Chairman, I am sorry that my statement has been so lengthy. But there was no brief way for me to get my thoughts on such a complex matter on the record.

In the beginning of my opening statement, I said that the legislation which is ultimately reported out by this committee, as a result of these extensive hearings, will become landmark legislation.

I say this because I sincerely feel that the final bill our full committee reports out will reflect the balanced interest of the entire committee, and will, therefore, be designed to promote our Nation's total welfare, by protecting our environment and at the same time assuring continued economic and industrial growth, not only for the immediate generations but for all future Americans.

Thank you very much, Mr. Chairman.

Mr. Lennon. Thank you, Mr. Chairman, for the fine statement that will certainly be considered in depth by the joint subcommittees.

I would now like to call on our good friend from Texas, an ex-member of this committee, the Honorable Bob Casey.

STATEMENT OF HON. BOB CASEY, A REPRESENTATIVE IN CONGRESS FROM THE STATE OF TEXAS

Mr. CASEY. Mr. Chairman, and distinguished colleagues of these two important subcommittees, permit me to express my personal thanks to you for the expeditious hearings on the grave problem of waste discharge in our coastal waters. I am especially pleased that the bill which I have had the pleasure of cosponsoring with my friend, Representative John M. Murphy of New York, is included in legislation being considered by these hearings.

I think the gravity of the problem is well recognized, and the fact that two important subcommittees are holding joint hearings on this issue certainly testifies to that fact. Having been privileged to serve on the House Merchant Marine and Fisheries Committee for several terms, I am well aware of the leadership it has taken through the years in protecting our natural resources under its legislative jurisdiction.

We, along the Texas gulf coast, know only too well that there is much to do in conserving and protecting our great coastal areas. We know that it cannot be done by the county, or the State of Texas, and we must have broad Federal legislation. For decades, we have callously disregarded the vital resource provided by our seas and coastal zones, and have looked upon them as vast dumping grounds for every type of refuse—from municipal garbage to obsolete munitions and explosives.

Along the Gulf of Mexico, few people realize the enormity of the problem we face. Our great gulf is the dumping ground for pollution of all types carried by river drainage from 31 of our States. Our five coastal States—Alabama, Florida, Louisiana, Mississippi and Texas—have 17,141 miles of tidal shore, 18 percent of the U.S. total. In our own State of Texas, almost three-fourths of our population live within 50 miles of the gulf, and I am sure the same fact is true in many of our sister States. This tremendous rate of growth is continuing to accelerate as new heavy industries seek the many advantages offered by the gulf's shoreline.

As I am sure my colleagues know, the gulf is a vast reservoir of natural resources, mostly undeveloped, for our Nation. Petroleum is but one of the great assets to be found there. It is, for example, the production area for 80 percent of our country's oil and gas—and it is estimated that 60 percent of the enormous Continental Shelf petroleum reserve lies under the warm waters of the gulf. This vast area of the gulf holds 33 separate bay systems, averaging each about 550 miles, which are the spawning area for our seafood resources—and the principal drainage pits for waterborne pollution. The importance of protecting these areas becomes more evident when you realize that about one-third of the country's commercial fish crop comes from the gulf.

We, along the gulf, are fortunate to one extent. As I advised the Subcommittee on Fisheries and Wildlife Conservation in hearings on the similar bill we introduced in the 91st Congress, there are invaluable studies underway which will provide the basis for intelligent and concerted action in years to come. We have underway now a major study of Galveston Bay, a joint State-Federal project. And we have in the initial stages a massive 10-year study of the gulf environment by the Gulf Universities Research Corp., a consortium of 17 major uni-

versities in our five coastal States, and including the University of Mexico, and many major industries. It is estimated the total cost of this mammoth project will total $150 million, and it holds exciting promise for the future. It is, I might add, the only study focused in its entirety on a single oceanic system adjacent to our coastline, and on the common but vital problems of marine preservation, conservation, and development. This study will utilize the great wealth of talent of these institutions—some 1,400 of our Nation's top scientists.

We cannot, of course, wait 10 years to begin work in cleaning up—and preventing further degradation of our ocean systems. Some scientists even now point out that the Gulf of Mexico is a prime candidate to be another Lake Erie, unless immediate remedial action is taken to protect it from pollution. A great deal of effort is underway, and it is not my intention to fault the effort by our county, State, and Federal agencies concerned with pollution, for they are moving against those who are causing the problem. But I believe that H.R. 285, or legislation similar to it, will give us the long-range answer to this problem and would be an incentive to the States to take the lead in resolving this problem.

Many of you, I know, are familiar with my own home of Houston and Harris County. Although 50 miles inland, we alternate between being second- or third-ranking port in tonnage in the country. To reach the port of Houston, ocean-going vessels traverse the Houston ship channel, a 40-foot-depth channel dredged the length of Buffalo Bayou. This dead body of water has often been termed the most polluted in our country. Along the banks of this great channel stand massive petroleum and petrochemical complexes, steel mills, and foundries. The waste product of one is often piped into the plant next to it as its raw product. These great industrial complexes have brought dynamic growth and economic prosperity to our area. They also brought major problems of air and water pollution, still unresolved. Much is being done at the local and State level to control emissions, and most of these plants are fully cooperating in a responsible manner. But the Houston ship channel is the main drainage system for a highly devloped urban area of nearly 2 million people, and it empties into Galveston Bay all of the accumulated wastes from sewage-treatment plants, the industrial complex, and the unbelievable residential runoff from our 6-, 8-, or 10-inch tropical rainstorms. Adding to this are the spills from oil and chemical tankers, from chemical plants, or from those obtuse industrialists who in spite of warnings from man and nature continue to view any body of water as their own private industrial sewer.

Unlike the problems of the eastern seaboard, municipalities in Texas have not as yet viewed the Gulf of Mexico as its private garbage dump for municipal solid wastes. But I need not tell you here today that it is but a matter of time before this will be viewed as the easiest and cheapest solution to the urban problem of garbage disposal. The cost of land, and the vigorous objections of those neighboring counties to being a dump for nearby city, are forcing city officials to seek this method. This is why action is needed urgently to enact this legislation, to chart those areas where dumping will not be allowed, and to set guidelines to control it.

Mr. Chairman and members of the subcommittee, on behalf of the people of my district and my State, I wish to say that I know you share our concern for protection of our environment and our great natural resources. We cannot delay too long in moving to protect our estuarine areas, and I believe our bill, H.R. 285, provides a solid basis for remedy of this difficult and complex problem. I commend you highly for your prompt consideration of legislation to control this problem.

Mr. LENNON. Thank you for an excellent statement, Mr. Casey.

Our next witness will be the gentleman from Connecticut, our colleague Bob Giaimo.

STATEMENT OF HON. ROBERT N. GIAIMO, A REPRESENTATIVE IN CONGRESS FROM THE STATE OF CONNECTICUT

Mr. GIAIMO. Mr. Chairman and members of the committee, I am pleased to offer my support for legislation designed to curtail unregulated discharge of industrial waste into coastal waters, oceans, and related bodies of water. In particular, my testimony is in support of H.R. 807, of which I am a cosponsor.

The time has long passed when America believed that large bodies of water and large masses of air could absorb the byproducts and aftereffects of industry and remain unaffected, or that the many and complex forms of life living in oceans, bays, harbors, and marshes could survive our assaults on their environment.

Certain of our industries are indiscriminately turning coastal waters in particular into repositories for toxic materials, and all industries must belatedly concern themselves with the unintended but devastating consequences of their prosperity.

The coastal waters of Connecticut, and in particular the shellfish and fishing industries dependent on those waters, have suffered considerable long-term damage from unregulated dumping of waste, and the economic consequences have spelled disaster for the men who work those waters.

While the Marine Science Council has estimated that 8 percent of all shellfish that could potentially be harvested are unsafe now for human consumption, that figure in Connecticut is surely much higher since major harbors and shoreline areas, for example New Haven Harbor, no longer can yield edible shellfish.

H.R. 807 is a measure with several features of particular importance in regulating this coastal carnage. For example, by placing the burden of proof—not absolute proof, of course, but a "preponderance of evidence" proof—on those seeking permits to dump wastes, that such wastes will meet standards to be set by the Environmental Protection Agency, industry will have to take a larger role in protecting oceanic environments.

In addition, provision for stronger State regulations, which would supersede Federal regulations if States could also demonstrate enforcement capacity, would allow those areas particularly damaged by waste discharge additional protection from stricter State standards.

Finally, the standards adopted by the EPA under this measure would supersede standards promulgated by any other agency of the

Federal Government and would help to both clarify and to strengthen the Federal role in enforcement.

It is inexcusable that in the midst of concern for other areas of our environment no standards have ever been adopted by the Federal Government which would effectively regulate the discharge of industrial or other waste into ocean or coastal waters, that no industry wishing to dump in coastal waters has been required to demonstrate the effect of that dumping, and that, as a result, some 48 million tons of materials are yearly deposited in what is a vanishing but hopefully salvageable natural resource.

Mr. LENNON. The subcommittee appreciates your testimony here this morning, Mr. Giaimo.

Our friend from New York, the able Seymour Halpern, will now be our next witness.

STATEMENT OF HON. SEYMOUR HALPERN, A REPRESENTATIVE IN CONGRESS FROM THE STATE OF NEW YORK

Mr. HALPERN. Thank you for this opportunity to speak in favor of legislation designed to halt the promiscuous dumping of waste materials into the ocean and to develop orderly arrangements for regulation of the practice.

I am told that over 44 bills aimed at regulating ocean dumping have been introduced in Congress so far this session. Some of these bills call for designating specific dumping sites and for standards to apply. Others specify areas of sanctuary wherein nothing can be dumped. There are bills dealing solely with oil and other hazardous substances while others are concerned with disposal of munitions and poisonous gases. Obviously all of these bills have merit and serve to demonstrate how broad the problem is.

Perhaps the outstandingly bad example of environmental abuse by ocean dumping is in the New York Bight into which the City of New York dumps its sewage and sludge along with all sorts of garbage euphemistically called solid wastes. Studies by the Corps of Engineers, the State University of New York at Stony Brook, the Fish and Wildlife Service have recently been completed. Their report has been released and is contained within hearings before this committee held July 27, 28, September 30, 1970, entitled "Dumping of Waste Material," Serial No. 91-39. I would like to quote for the committee certain of the summary conclusions drawn by this report:

1. The New York Harbor Complex must rank as one of the largest grossly polluted areas in the United States.

2. The major sources of pollution in the New York Bight are (1) sewer and industrial outfalls, (2) ocean disposal of sewage sludge and dredge spoil, (3) river discharge and land runoff, (4) wastes from vessels, (5) accidental spills, and (6) harbor debris.

3. No significant improvement in the water quality in the New York Bight can be expected until the mid-70's. Complete secondary treatment is not scheduled for New York City and Passaic Valley Sanitation Commission until 1976. Additional pollution treatment facilities in up-river and shoreline communities will not be completed until the mid-70's. Vessel pollution should be significantly reduced under the provisions of the Water Quality Improvement Act of 1970.

4. Even with completion of all currently proposed pollution abatement programs, conditions in the New York Bight will fall short of what must be the ultimate goal of protecting coastal ocean environments from serious degradation.

5. There will be increased pressure for more ocean disposal of sewage sludge and dredge materials in the New York Bight. This will raise to a potentially critical level the threat of pollution to land and surrounding ocean.

6. The projected increase in pollution from ocean disposal practices calls for stricter control of future ocean disposal practices in the New York Bight.

7. The major threat to full enjoyment of the proposed Gateway National Recreation Area and other beaches in the New York Bight is pollution. To date, however, there has not been demonstrated any connection between present ocean dumping practices and water pollution at any of the proposed Gateway sites.

8. The present ocean disposal of sewage sludge and dredge fill may be a serious threat to the sanitary quality of local populations of ocean quahogs and surf clams (4–10 mile radius).

9. Accumulation by fish and shellfish of heavy metals and other persistent toxic compounds is another potential health hazard in the New York Bight. This threat appears to be most serious from the sludge disposal areas.

10. Ocean disposal of sludge and dredge spoil materials, along with pollution from other sources, offer a potential threat to local fish populations.

No amount of rhetoric will present a more eloquent argument for the need to develop orderly regulation of needless destruction of the marine environment due to dumping wastes into the ocean and coastal waters, especially since the situation in the New York Bight is by no means unique. This same report and others such as the report of the Council on Environmental Quality demonstrate that similar problems exist elsewhere. For example, the report of the CEQ identifies 246 sites off the coasts of the United States into which 48 million tons of dredge spoil, sewage sludge, building debris, industrial wastes, radioactive wastes, and outdated munitions are dumped every year. A later report by a responsible Federal agency states that there are 281 dumping sites receiving 62 million tons of wastes of all sorts.

All indications are that, as serious a problem as this is now, it is becoming more so at an alarmingly rapid rate. Time is no longer available to luxuriate in detailed studies and deliberations. We must move expeditiously to enact legislation which will halt this senseless destruction of our coastal waters. I vigorously support the legislation before this committee as a sound approach to prevent further destruction of the marine environment.

Mr. LENNON. You gave a very enlightening statement, Congressman. We appreciate your time.

Our next witness will be the gentleman from Florida, the Honorable Charles E. Bennett.

STATEMENT OF HON. CHARLES E. BENNETT, A REPRESENTATIVE IN CONGRESS FROM THE STATE OF FLORIDA

Mr. BENNETT. Mr. Chairman, I appreciate the opportunity to testify in support of legislation which I have pending to end ocean dumping without a certificate from the Council on Environmental Quality. This legislation is section 2 of my bill, H.R. 1214, which is a comprehensive environmental protection bill, which was referred to the House Public Works Committee.

For some time now, I have been extremely concerned about deterioration of our oceans by pollutants. Oceans cover 140 million square miles of water surface and over 70 percent of the area of the earth. The world environment and our very existence are dependent on the

oceans, and we must stop using them as huge open sewers which can absorb pollutants on an unrestricted and indefinite basis.

Reports on ocean contaminants from leading scientists and engineers are truly alarming. Thor Teyderdahl, in his attempt to cross the ocean in a reed ship, found that he could not fill containers for desalting for drinking water in the middle of the ocean because of the filthy condition of the water. Dr. Jacques Cousteau, famed oceanologist who has traveled nearly 155,000 miles in the last 3½ years exploring the oceans of the world, recently concluded: "The oceans are in danger of dying. The pollution is general."

I am pleased that the President has taken affirmative action in seeking greatly reduced ocean dumping and I am glad that the thrust of the administration bill and my bill are similar. My bill requires a certification from the Council on Environmental Quality before any ocean dumping would be allowed, while the administration bill requires a certificate from the Environmental Protection Agency. In light of the increased responsibility of the Environmental Protection Agency for monitoring various aspects of environment pollution, I favor amending my bill to place jurisdiction with the Environmental Protection Agency.

It has been estimated that in 1968 slightly over 48 million tons of waste were dumped at sea off the shores of the United States. Many of these wastes are oxygen-demanding materials, which have the potential to reduce oxygen in ocean waters to levels in which the aquatic life cannot live. The volume of waste dumping is growing rapidly, and with many major cities running out of landfill areas, they will be looking toward the oceans to get rid of their wastes.

As the most prosperous industrialized nation in the world, we must set an example for other nations to follow in cleaning up and keeping clean our oceans.

I hope that the committee will give careful consideration to section 2 of my bill, H.R. 1214, to control ocean dumping, as I believe it directly and firmly attacks the problem and will aid in preserving our life-giving oceans.

I thank the committee for the opportunity to present this statement.

Mr. LENNON. Thank you for an excellent statement, Congressman.

I would now like to call the gentleman from New York, our friend Jack Kemp.

STATEMENT OF HON. JACK F. KEMP, A REPRESENTATIVE IN CONGRESS FROM THE STATE OF NEW YORK

Mr. KEMP. The problem of water pollution has, unfortunately, been ignored for too long. The results of such apathy are now being paid for, and they have been and will be paid in far larger amounts than would have been expended upon an enlightened and effective policy of preventing such pollution.

In the last 7 years, for example, commercial fishing off the New York-New Jersey coast has dropped from 673 million pounds to 133 million, a decline of 80 percent. Oyster production for the same period is off by 43 percent. As the New York Times recently noted, "A major factor in these staggering losses is the sludge dumped offshore

daily, as though the Atlantic were an infinite catch-basin for the wastes of man."

In January 1971 the Governments of Canada and the United States released a report by the International Joint Commission on Pollution of Lake Erie, Lake Ontario, and the international section of the St. Lawrence River.

The report concluded that water pollution extends throughout the lakes; the principal causes are wastes discharged to the boundary waters and tributaries by municipalities and industries; pollution is taking place in all jurisdictions sharing the boundary waters.

The Commission expressed the view that urgent remedial measures are required, including adoption and adherence to the recommended water quality objectives; immediate reduction of the phosphorus content in detergents; and the prompt implementation of a vigorous program to treat municipal and industrial waste and to reduce phosphorus inputs into these waters.

In 1969 the IJC was asked to extend its inquiry to the adequacy of existing requirements for the prevention of oil leakage into Lake Erie as well as of existing measures for cleaning up any major oil spill.

The IJC concluded, in an interim report submitted to both Governments and confirmed in its present report, that safety requirements and procedures applicable to drilling and production operations were adequate if effectively supervised and properly enforced. With respect to cleaning up a major oil spill, the IJC found current methods to be primitive and inadequate. In addition, an urgent need now exists for a formal plan of international cooperation on oil spills.

In the present report on pollution in the Lower Lakes, the IJC finds that Lake Erie, particularly its western basin, is in an advanced state of eutrophication, or aging, and that accelerated eutrophication is occurring in Lake Ontario.

As a result, the IJC recommends that both Canada and the United States adopt the water quality objectives set out in the report and enter into agreement on programs, measures, and schedules to achieve them.

Just last month Secretary of Transportation John A. Volpe announced that a committee of the Intergovernmental Maritime Consultative Organization meeting in London had adopted a U.S. resolution calling for the end of willful marine pollution and minimization of accidental spills by 1975.

The magnitude of our water pollution has been made evident to Americans in at least one dramatic respect. Last December, Prof. Bruce McDuffie, a chemist at the State University of New York, tested a can of tuna fish and the question of the concentration of mercury in fish erupted into worldwide proportions.

The tuna contained a mercury concentration well above the U.S. Food and Drug Administration limit. So did frozen swordfish steaks also tested by McDuffie. The FDA confirmed his findings and took a million cans of tuna, and most swordfish, off the market.

For many years no one worried about dumping insoluble, inorganic mercury into water. In 1967, however, scientists discovered that bacteria could convert inorganic mercury into a highly toxic, soluble, organic form called methylmercury. The bacteria are eaten by tiny

62-513—71——11

forms of aquatic life, which are eaten by small fish; the small fish are eaten by bigger fish, and as the mercury moves up the food chain, it becomes more and more concentrated. In a body of water containing .00001 parts of mercury per million, the food-fish might contain 10 parts per million. Tuna and swordfish have large amounts, in part because both are wide-ranging predators, at the top of long food-chains.

We should have been alerted before this to the danger of mercury. In Japan, between 1953 and 1960, at least 43 people died and scores were permanently disabled—suffering blindness, deafness, convulsions, coma, mental retardation—from eating fish caught in a bay where a plastics factory had been dumping mercury.

In the 1950's, Sweden discovered that mercury was responsible for her dwindling bird population, and that her fresh-water fish were contaminated. The Government there has banned the use of mercury-treated seed, and recommends that people eat no more than one fish meal a week.

Pollution has many sources. This applies to the disposal of industrial wastes and sewage from urban communities, insecticides, and fertilizers from land runoff, seepage of petroleum from offshore drilling, as well as the pollutants that accumulate in the marine food chain since many species of fish and other marine biota tend to inhabit the relatively shallow areas of the ocean.

This bill, which I am pleased to cosponsor, states clearly that it is the policy of the United States "to regulate the dumping of all types of material in the oceans, coastal and other waters and to prevent or vigorously limit the dumping into oceans, coastal, and other waters of any material which could adversely affect human health, welfare, or amenities, or the marine environment, ecological systems, or economic potentialities."

In addition, the bill provides for the means of making this policy a reality.

It is also my hope that our private industries will voluntarily do everything possible to limit pollution. We have already seen many instances of such action. In one instance, for example, the Dow Chemical Co. in Midland, Mich., has prevented waste through such steps as recycling raw materials. It has made each man—"right down to the janitor"—accountable for pollution, using the rule: "If you mess it up, you clean it up." Dow has attached the same emergency-type importance to a pollution incident as to a fire, explosion, or personal injury.

Two years ago when the program started, Dow's monitors turned up 1,100 potential pollution problems. Three hundred of them serious enough to require immediate action—such as the fact that contaminants sometimes get into cooling water. Last year Dow spent $800,000 to put in a system with devices that can sense contaminants in the cooling water and immediately divert the water into a 50-million gallon pond. There it is treated before it is allowed to get back into the Tittabawassee River.

There are many other instances of such private initiative. It is my hope that a bill such as the one we are discussing today, and private efforts by business and industry, can together help us to ease this major problem of water pollution.

For too long we have ignored this question. It is now essential that we act as quickly as possible to gain some of the valuable time we have lost.

Mr. LENNON. That was an excellent statement, Mr. Kemp. We certainly appreciate your efforts.

Now I would like to call the gentleman from Massachusetts, the very able Silvio Conte.

STATEMENT OF HON. SILVIO O. CONTE, A REPRESENTATIVE IN CONGRESS FROM THE STATE OF MASSACHUSETTS

Mr. CONTE. I wish to thank the Committee on Merchant Marine and Fisheries for the opportunity to express my support for H.R. 805 and H.R. 3662, two bills which I cosponsored, that deal with the serious problems of dumping waste materials into the oceans.

The dangers of indiscriminate dumping are very grave as I am sure this committee is aware. Thousands of square miles of ocean have been reduced to lifelessness, with a resulting threat not only to the economy of the fishing industry, but to health and even life itself.

We know now that the depositing of waste materials in certain of the Great Lakes was responsible for their present lifelessness and, indeed, poisonous condition. If we continue to pollute our oceans at the present rate, it is only a matter of time before they, too, cease to support life.

Scientists may disagree about the extent to which the oceans are now polluted, and they may disagree as to how long the oceans can absorb an increasing quantity of waste without serious and perhaps permanent harm. There is substantial agreement, however, on two facts. First, there is a limit to the amount of waste that the oceans can absorb over a given period of time. Second, even the waste now being dumped in the oceans is producing effects which are not evident and visible, but which are real, nonetheless, and will have consequences which cannot now be foreseen.

Mr. Chairman, your committee has a number of bills before it dealing with this matter, and eventually you will draw the conclusions as to which of these bills will best serve the interest of our Nation.

I wish at this time to urge you to support a strong bill that will reflect what I feel is a newer and tougher sentiment in Congress regarding pollution. I assure you that there exists in this country a vast constituency which would not only support, but which wholeheartedly desires, such legislation.

I believe that both H.R. 805 and H.R. 3662 are strong, responsible bills. There are differences between them which I shall not discuss at this time. These differences are matters of degree since both bills have substantially the same thrust.

Both H.R. 805 and H.R. 3662 would have the Federal Government set standards with regard to dumping, require permits or licenses to be obtained before dumping is allowed, and provide for penalties should violations occur.

These two bills deserve your careful consideration. I recommend them to you and urge you to either give one of them your approval, or else to devise a measure which in your view embodies the best elements of both of these proposals.

Thank you.

Mr. LENNON. We also thank you for an excellent presentation.

I understand the gentleman from Arizona, the Honorable John J. Rhodes, would like to give a very brief statement at this time.

STATEMENT OF HON. JOHN J. RHODES, A REPRESENTATIVE IN CONGRESS FROM THE STATE OF ARIZONA

Mr. RHODES. Mr. Chairman, as a cosponsor of the legislation before you today I appreciate the opportunity to speak on behalf of these bills proposing a limit on the dumping of hazardous materials into our coastal waters.

I do not believe that pollution must be the end product of our Nation's industry. I believe this country can conquer the menace of environmental pollution as it has other problems in the past. However, in order to do this there will have to be legislation enacted by Congress.

Presently there are no effective standards to regulate the dumping of waste products in our coastal waters. No one wishing to dump waste products is required to demonstrate that the material is harmless.

We must have effective standards now. I hope that this committee will act favorably on this legislation as soon as possible.

Mr. LENNON. Thank you very much, Mr. Rhodes.

I note two other Members of Congress would like to give statements. Would Congressman Mikva please take the witness chair?

STATEMENT OF HON. ABNER J. MIKVA, A REPRESENTATIVE IN CONGRESS FROM THE STATE OF ILLINOIS

Mr. MIKVA. Thank you very much, Mr. Chairman.

The sea stirs the spirit of man. In many respects, it is the last unexplored and untouched region of the earth. Its vastness defies the imagination; its savage independence frustrates our feeble attempts to tame it. Yet man is slowly killing the majestic oceans by quietly and relentlessly dumping his garbage into the sea.

It is not fair to say that the problem is only beginning. Many areas of our coastal waters are already irrevocably contaminated. The New York Bight is a prime example. A study conducted by the U.S. Marine Laboratory at Sandy Hook, N.J., and completed last year indicates that 40 years of dumping has destroyed the marine ecosystem and rendered most of the area uninhabitable for any sea life. The bight is appropriately referred to as "The Dead Sea."

The prevalence of disease and contamination, which not only threatens the Atlantic coastal fisheries, but also gravely endangers public health, was indicated by studies conducted in this area. More than a dozen species of fish captured in the befouled area of the bight were suffering from a disease known as fin rot. Lobsters and crabs exposed under laboratory conditions to the same pollutants as are pouring daily into the bight developed a fouling of their bronchial chambers and gills. The test animals all perished in 3 to 4 days. A report recently prepared by M. Grant Gross, Research Oceanographer at the Marine Sciences Center, State University of New York at Stony Brook, warns of high concentrations of a number of toxic and cancer-

causing elements. If these elements enter or have entered the food chain, we are faced with a serious hazard to public health. Studies conducted by a group of scientists under the direction of the Smithsonian Institute substantiate these terrible conclusions.

Unfortunately, the situation in the New York Bight is not an isolated phenomenon. Commercial fisheries have collapsed all along the Atlantic shore because of the deterioration of previously abundant fishing grounds. There are some 49 dumps off the populous East Coast and all of them pose a continuing threat to the health of the Atlantic and to the livelihood of those who depend on it. If left alone, things are only going to get worse. As sanitary landfill sights become more crowded and less practical, the ocean is fast becoming the cheapest and most convenient garbage dump for many coastal cities.

If our oceans are to remain a source of food, esthetic pleasure, and sheer wonder for future generations, then we must stop treating them like a gigantic open sewer. It is for this reason that I vigorously endorse H.R. 2581, a bill introduced by my colleague from Massachusetts, Mr. Harrington. This bill represents an important initial step toward reducing ocean pollution. The proposal authorizes the Administrator of the Environmental Protection Agency, in conjunction with the Secretary of the Interior and the Chief of the Army Corps of Engineers, to promulgate standards designed to protect the delicate marine ecology of our coastal waters. It then requires any potential dumper within a 12-mile limit of U.S. snores to demonstrate that such dumping will not violate these standards. In short, this legislation puts the burden where it should be—on the dumper—to show that the dumping of his garbage is not detrimental to marine life.

While H.R. 2581 is vital to the attack on ocean pollution, it will not eliminate the problem. It unquestionably makes it more difficult for polluters, but it does not preclude further pollution. Cities could simply take their garbage past the 12-mile limit and dump it or they may attempt to evade the law through nighttime or clandestine dumpings. I personally favor an outright ban against ocean dumping. The United States should prohibit American citizens or vessels from dumping any deleterious matter into any ocean. Such a unilateral declaration could prompt other nations to follow suit. The International Oceanographic Commission established by UNESCO in 1961 is a prime example of international cooperation to stop the contamination of international waters. The organization is now sponsoring a program of research on the Mediterranean Sea, involving some 20 nations including the U.S.S.R., Israel, Syria, and the United States. The mutual cooperation of these normally antagonistic nations demonstrates the pragmatism of international policing of our seas.

Ultimately, however, the problem is one of solid waste disposal. We in America have developed a throwaway society of historic proportions. With sheer arrogance born of obsession with convenience, we use things once and throw them away, not caring or realizing that all that garbage piling up has to go somewhere—whether to an incinerator, a landfill site, or some body of water. All three alternatives are unsatisfactory. The final solution is recycling, and we must begin to implement ways of reutilizing our resources before we are inundated with our own waste.

The sea has played a great spiritual role in the history of man, and I would hate to see it die of our own neglect. But that is what is happening. No event dramatizes this fact better than the voyage of Thor Heyderdahl who traversed the Atlantic in a small papyrus boat. This brave man, like Leif Erickson, Columbus, and Magellan before him challenged the sea and, by enduring the pain and brutality of the Atlantic, conquered it. And yet thousands of lonely miles at sea the water was sometimes so full of oil and other junk that bathing was impossible. What a sad commentary on the imprint man has made on his earth.

Mr. LENNON. Thank you for a very interesting and informative statement.

Our next witness will be the Honorable Lawrence Coughlin, a very able Member of Congress from the State of Pennsylvania.

STATEMENT OF HON. LAWRENCE COUGHLIN, A REPRESENTATIVE IN CONGRESS FROM THE STATE OF PENNSYLVANIA

Mr. COUGHLIN. I am pleased to testify today on a bill, H.R. 805, that provides specific Federal authority to enforce waste disposal regulations and curb ocean pollution within a 12-mile limit of the shoreline of the United States. The ultimate goal of this legislation is to contribute to the improvement of the ocean environment by allowing only that matter into the oceans that is essentially inert or which could be assimilated without adverse effects.

As cosponsor of this bill, I recognize that we cannot undo with one law what has been allowed to happen over the many decades. I feel, however, that we as Federal legislators must initiate action that will enable the United States to make meaningful contributions toward ending rampant pollution of the very basis of our life on earth—the oceans.

The President's Council on Environmental Quality in its October 1970 report indicates to what degree the cavalier dumping of wastes off our shores has affected our environment and our economy. Closing of beaches and bays has become so commonplace that it is accepted almost as a fact of life in the United States today. Many of us in the Congress have supported legislation to protect and increase the use of recreation areas in and around the coastal waters of the country. Yet, our efforts in this field cannot succeed in any reasonable measure until we abate the wholesale polluting of these coastal waters.

According to the Council, about 48 million tons of waste were dumped at sea in 1968. Dredge spoils accounted for 80 percent by weight of all our ocean dumping. The Army Corps of Engineers estimated about 34 percent, or 13 million tons, of the material was polluted.

In 1969, sewage sludge dumped in the New York Bight, an area encompassing the New York Harbor and adjacent coastal areas, had an oxygen demand of approximately 70,000 tons. Tests conducted on wastes taken from this area contained bacteria that cause hepatitis in man.

All of us are aware of the reports of mercury and DDT which are discharged from industrial plants or run off from our lands, flow into our streams and rivers, and eventually find their way into the ocean.

Sludge generated by the Baltimore-Washington area is expected to increase between 1960 and 1980 from 70,000 to 160,000 tons, a rise of about 140 percent. For the New York area during the same period, the increase is expected to be from 99,000 to 220,000 tons for a 120-percent increase.

Industrial wastes are growing at a rate of 4.5 percent annually or three times the population growth. Many of these wastes are harmful or toxic to marine life, hazardous to human health, have gravely damaged the shellfish industry, and are destroying the esthetic beauty and use of recreational areas.

The volume of wastes can only increase as existing facilities decrease. Landfill sites are becoming scarce or are being outlawed. Escalating costs from land-based methods of disposal are further encouraging dumping in our oceans.

The destruction to our shellfish industry by dumping of wastes is appalling. The Council stated that pollution from these dumpings had closed at least 20 percent of the Nation's shellfish beds. Affected shellfish had been found to contain hepatitis, polio virus and other pathogens hazardous to human health.

The economic effect, aside from obvious and dangerous health hazards, has been devastating. The National Estuarine Pollution Study, conducted by the Federal Water Quality Administration in 1970, noted that the annual commercial harvest of soft-shelled clams was between 100,000 to 300,000 pounds before 1935. This clam digging is virtually nonexistent because of pollution.

The annual commercial landings of the shrimp fishery before 1936 were as high as 6.5 million pounds. Landings in 1965 were only 10,000 pounds.

The potential value of the U.S. shellfish catch in 1969 was $320 million. As a result of increased pollution in ocean and coastal waters, the actual value in 1969 was $257 million, a $63 million loss in this specific industry alone from the effects of ocean-dumping wastes.

Commercial fisheries, of course, are on the decline with the resultant loss in business and jobs. Pollution caused by dumping has wiped out many fishing grounds and sent United States commercial fleets to distant waters.

A side effect of this situation has been the overfishing of prime grounds for such species as tuna and salmon. The supply of salmon particularly is in peril from huge foreign fishing fleets that have found their feeding grounds in the North Atlantic.

In assessing the damage to fishing, we should not overlook sports-fishing.

Millions of sports fishermen can attest to the dwindling supplies of ocean fish. At a time when we are attempting to increase recreational activities, the sport of fishing is being hit hard by the pollution of rivers, bays, and coastal waters.

Specifically, in the Pennsylvania-New Jersey-Delaware area I cite the virtual disappearance of the croaker which once roamed the Delaware Bay and even the lower Delaware River in vast quantities. Once the prime fish for anglers in those waters, the croaker no longer is available for sports fishing or commercial fishing in the Delaware Bay. It is a rare occasion when a fisherman catches a croaker in these waters.

It is clear to me that the authority to establish standards should rest with the EPA. Problems have arisen in the past with current authorities, such as the Army Corps of Engineers, which are mainly concerned with the navigability of our waterways and not the ecology.

Section 5B(a) defines "ocean, coastal, and other waters" as "oceans, gulfs, bays, salt-water lagoons, salt-water harbors, other coastal waters where the tide ebbs and flows, the Great Lakes, and all waters in a zone contiguous to the United States extending to a line 12 nautical miles seaward from the baseline of the territorial sea as provided in article 24 of the Convention on the Territorial Sea and the Contiguous Zone." This 12-mile limit would prevent those infractions which occur today outside of the current territorial sea limit of 3 miles which has left the Army Corps of Engineers helpless to act.

Section 5B(a) also requires that the person wishing to dump sustain the burden of proof that the materials that are dumped will not endanger the natural environment of those waters and will meet any additional requirements as the Administrator of the EPA deems necessary for the orderly regulation of such activity. Certainly, the time has come for those who persist in dumping harmful wastes in the ocean to be held accountable for their action and, in fact, through this legislation, begin to terminate the amount of ocean dumping entirely.

The legislation provides that the standards established be adopted and applied to all parts of the Federal and State authorities which have the right to issue authorizations to discharge or deposit material into these waters.

Furthermore, States may establish their own standards with respect to the activity covered by the Federal standard with the condition that the State standard would have to be more stringent than the Federal standard and provide adequate procedures for enforcement. This allows the States not to be hampered by past Federal uniform minimum standards which serve to hamper rather than to effect the causes of the activity.

Section 5B(f) provides that every State and Federal instrumentality and every person applying for authorization to discharge or otherwise dispose of any material into these waters maintain records, make reports, and provide whatever additional information the Administrator of the EPA needs to determine that there is compliance with the standards.

Section 5B(g) provides that the district courts of the United States have jurisdiction to restrain violations of this act.

Section 5B(h) provides that each violation of these standards shall be punishable by a fine of not more than $10,000 nor less than $5,000.

This section would make each dumping in violation of the standards punishable by fine.

It is clear to me that we must take those steps which are necessary to turn this spiraling rate of pollution spoilage around, so that we begin to protect not only human lives but the use of the sea, both economically and from a recreational purpose. The dumping of any waste materials which could create hazardous conditions, toxic or otherwise, in ocean and coastal waters, must stop. Ocean disposal of polluted dredge spoil, undigested sludge, and improperly treated sewage effluent must be terminated. Disposal of unpolluted dredge spill,

rubble, and similar wastes, which have been demonstrated to be inert and nontoxic, should be evaluated on a case-by-case basis. Municipal or industrial refuse, such as garbage, should not be dumped into the sea. Finally, ocean dumping of digested or other stabilized sludge should be discontinued as soon as feasible.

I believe the time to act is now or we may find ourselves in a position where we cannot change the biochemical reactions which are occurring in the ocean at this very minute.

The state of the oceans is rapidly approaching the crisis stage. This bill would provide the means for effective and prudent restraints on ocean dumping within a 12-mile limit from our shores and would be an instrumental step in seeking international cooperation so indispensable if we are to save our oceans, and life itself, from death by pollution.

Passage of legislation of this type is especially important in view of the attitude of foreign countries. For instance, American initiatives for an absolute ban on ocean dumping have been rejected by our NATO allies.

The United States alone cannot end the killing of our seas, but we can provide the leadership necessary. The spread of dead and dying sections of our oceans must be stopped.

I hope that the committee will review all testimony and act favorably on measures to curb ocean dumping.

In support of this testimony on H.R. 805, I submit as exhibit A a letter from the person most expert on the oceans of the world—Jacques-Yves Cousteau.

I feel that his testimony, based on 30 years of exploration, is the best available to alert us to the damages we have done to our oceans and to the dangers we face if we do not act quickly and constructively. I am grateful for his support of H.R. 805 and offer, as exhibit B, biographical information on him that attests to his knowledge and expertise.

I thank you for the privilege of presenting this testimony and Jacques-Yves Cousteau's letter on behalf of H.R. 805.

Mr. LENNON. Congressman, we are grateful to you for an excellent statement. If there is no objection, the exhibits you mentioned will appear at this point in the record.

Mr. COUGHLIN. Thank you for your time, Mr. Chairman.

(The exhibits referred to follow:)

EXHIBIT A

LIVING SEA CORP.,
Los Angeles, Calif., April 8, 1971.

Congressman LAWRENCE COUGHLIN,
336 Cannon Office Building,
Washington, D.C.

DEAR CONGRESSMAN COUGHLIN: It is gratifying to learn of the introduction of bill HR-805 to prevent the dumping of pollutants into the oceans.

For nearly thirty years my companions and I have been studying the waters of our unique planet. Our observations have been made in stations all over the world—in the Mediterranean, the Red Sea, in the northern and sounthern Indian Ocean, the southern Atlantic, the Caribbean and the Pacific—places we have visited not just once, but often. These observations lead us to an assessment, true everywhere, that the intensity of life has diminished by more than thirty percent over the past twenty years. This reduction applies to fixed fauna, to vege-

tation, plankton, shellfish, edible and non-edible fish, coral, and, in fact, all marine life.

There are reasons other than pollution for the diminution of life in the seas: overfishing is one, and the destruction of breeding and living areas by alteration of underwater environments is another. But the primary reason is pollution, for every pollutant on land and in the air eventually finds its way to the sea. Cleansing rains run through streams and rivers and pipelines directly or indirectly to the ocean.

Because 96 percent of the water on earth is in the ocean, we have deluded ourselves into thinking of the seas as enormous and indestructible. We have not considered that earth is a closed system. Once destroyed, the oceans can never be replaced. We are obliged now to face the fact that by using it as a universal sewer, we are severely over-taxing the ocean's powers of self-purification.

The sea is the source of all life. If the sea did not exist, man would not exist. The sea is fragile and in danger. We must love and protect it if we hope to continue to exist ourselves.

Men of all nations must join together in an effort to save our seas. I am sure that by such measures as are called for in HR-805, we will succeed.

Sincerely yours,

JACQUES-YVES COUSTEAU.

EXHIBIT B

BIOGRAPHY OF JACQUES-YVES COUSTEAU

For centuries man has dreamed of unlocking the secrets of the mysterious world beneath the sea. Through his inventions, books, films and television specials, undersea explorer Jacques-Yves Cousteau has taken man into this inner space—both vicariously and personally.

Since the day Cousteau donned a pair of goggles more than thirty years ago and looked into the sea, his goal has been to go deeper, stay longer and learn more. His dissatisfaction with existing breathing devices led him to design the compressed-air Aqualung in 1943 in collaboration with the French engineer Emile Gagnan. Throughout World War II he dived and made underwater films with companions Frederic Dumas and Philippe Tailliez as a cover for his underground intelligence work.

Cousteau was born in 1910 in St. André de Cubzac, France. He is a graduate of the French Naval Academy of Brest and served in the French Navy as a gunnery officer. For his wartime services he was made a Knight of the Legion of Honor and awarded the Croix de Guerre. Subsequently he was made Officer of the Legion of Honor in recognition of his contribution to Science. After the liberation, Cousteau with Dumas and Tailliez founded the Group for Undersea Research in the French Navy. The Group participated in many underwater activities including the clearing of German mines from Mediterranean harbors, testing the effects of compressed air divi[illegible]g and explosions underwater on the human body, exploring the romantic Fountain of Vaucluse, excavating a Roman ship sunk off Tunisia in 80 B.C. and aiding with the first dive of a bathyscaphe in 1948.

Fearing that he would be rotated away from the sea to a desk job, Cousteau took leave from the Navy in 1950 to create the non-profit Compagnes Océanographiques Françaises, through which the American-built *Calypso*, a former minesweeper, is now operated as an oceanographic research vessel.

Calypso made her maiden voyage as a research vessel in 1951 to the Red Sea where, for the first time, an underwater television system developed by Cousteau and French engineer Andre Laban, was put in use. In 1953, Cousteau in collaboration with Frederic Dumas published his first book, The Silent World, a classic account of exploration and adventure and an immediate best-seller. In the years between the book's publication and release of the film of the same name in 1956, Calypso Expeditions was involved in an archeological dig near Grand Congloue, off Marseilles, of a Greek wine ship dating from 205 B.C. To trace the route of the ship, *Calypso* visited the island of Delos where evidence of the ship's owner was found in the ruins of his villa. In 1954, *Calypso* and divers explored for oil deposits in the Persian Gulf before making two long cruises to the Seychelles Islands, the Indian Ocean and Red Sea to film The Silent World, winner of the

"Palme d'Or" at Cannes in 1956, and the first of three Cousteau films to be awarded the Motion Picture Academy Award "Oscar".

In 1957, Cousteau resigned from the Navy and was elected Director of the Musée Océanographique of Monaco. His engineering organization in Marseilles, now known as CEMA (for Centre d'Etudes Marines Avancés), began, under the direction of engineer Jean Mollard, the design and construction of the Diving Saucer (DS-2) Denise, a revolutionary two-man research and observation submarine of circular design, propelled by water jets capable of going to 1,000 feet of depth.

On her voyage to New York to participate in the International Geophysical Year in 1959, *Calypso* towed a deep-sea camera sled or "Troika", built by CEMA, through the depths of the Atlantic's Rift Valley, making the first continuous photographic record of that bottom. The Diving Saucer was successfully tested during that same year and became a major tool for exploration of the continental shelf.

In 1962, Cousteau and his group establishhed the world's first underwater station, Conshelf I, in which two divers lived continuously for one week in thirty-five feet of water. The following year a more amibitious underwater community was established in the Red Sea, and the feature film, World Without Sun, was made to record the experience. In Coneshelf II five men lived in Starfish House, submergea in thirty-five feet of water for a month, while further down at 85 feet, two men lived for one week in Deep Cabin. A hangar for the Diving Saucer and a tool shed completed the underwater station.

During the period from *Calypso's* acquisition through the Conshelf III experiment, Calypso expeditions and many Cousteau group projects were funded by the French Ministry of Education and by the National Geographic Society. Accounts of his experiences were made periodically by Cousteau and appeared in the National Geographic Magazine. In 1963, in collaboration with James Dugan, *The Living Sea*, which enlarged and expanded on these accounts, was published.

In the following year the construction of *Deepstar-4000* for the Westinghouse Corporation was completed and the three-man submarine was delivered from CEMA's manufacturing facility in Marseilles, France. That year also saw another unique Cousteau project—the world's first anchored open-sea oceanographic buoy, Mysterious Island—put into operation in the Mediterranean. Currently, CEMA is constructing an advance version of the Diving Saucer (the S.P. 3000) capable of operating at 10,000 feet of depth and the "Argyronete", a ten-man submarine designed to operate at 2,000 feet and from which four oceanauts will emerge to carry on assigned tasks on the ocean floor.

Conshelf III in 1965 was a major advance in underwater habitats which housed six oceanauts at a depth of 328 feet for three weeks. Their experiences were filmed as a television special entitled *The World of Jacques-Yves Cousteau* for the National Geographic-CBS-TV. The popularity of this program triggered a contract for twelve television specials with Metromedia Producers Corp. and ABC called *The Undersea World of Jacques Cousteau*. The series of specials has since received numerous awards throughout the United States and Europe.

In 1967, carrying special new equipment including two one-man minisubs, (the "S.P. 500"), *Calypso* left Monaco for an extended voyage of underwater exploration and filming. The long cruise took the ship and its crew to the Red Sea, Indian Ocean, around the Cape of Good Hope, to the South Atlantic, the Caribbean, Pacific, Peru, Alaska, the Galapagos Islands, the British Honduras and the Bahamas before she returned to France in September of 1970. The enthusiastic response from critics and viewers to the first twelve programs resulted in a contract for a new television series.

On his return with the *Calypso*, Cousteau voiced his growing concern over pollution of the seas. "The sea is the universal sewer", he said. "All pollutants on land eventually reach the sea". In 1960, Cousteau had lead a successful campaign to prevent the French Atomic Energy Commission's dumping of radioactive wastes into the Mediterranean. At that time he remarked, "We risk poisoning the sea forever just when we are learning her science, art and philosophy and how to live in her embrace."

Cousteau is also Chairman of the Board of U.S. Divers Co., a diving equipment manufacturing firm; Les Requins Associés, a French film production company; Living Sea Corporation, a marine structural and design firm in charge of the design of the Museum of the Sea Aboard the *Queen Mary*; and Thalassa,

Incorporated, which specializes in feature and television films. He is President of the World Underwater Federation, representing free divers in thirty countries, a member of the U.S. National Academy of Science and he holds degrees from the University of California at Berkeley and Brandeis University as an honorary Doctor of Science. In October 1970, Cousteau was again honored. He received the *Potts Medal* from the Franklin Institute and the *Spirit of St. Louis* award from Saint Louis University.

More recently, Cousteau is the founder and Chairman of the Board of EUROCEAN, a new European organization set up to study and implement the exploitation of the ocean.

Mr. LENNON. We have at this time the distinguished chairman of the Council on Environmental Quality, Mr. Russell E. Train. If you have anyone with you, Mr. Train, you may bring them forward and have them sit at the table with you.

Mr. TRAIN. I have another member of the staff in the audience but I don't believe I will bring him up to the table except in case of need perhaps to answer questions, but at this point I will deal with it myself.

Mr. LENNON. All right.

STATEMENT OF RUSSELL E. TRAIN, CHAIRMAN, COUNCIL ON ENVIRONMENTAL QUALITY

Mr. TRAIN. Mr. Chairmen—and I have used that word in the plural here as I gather we have cochairmen; I was not sure whether to say Mr. Co-Chairmen—Congressman Pelly and Congressman Mosher, I appreciate the opportunity to meet with your subcommittees and to testify in support of the President's proposals for the control of ocean dumping. Protection of the marine environment has been and continues to be a high priority concern of this administration.

The Council on Environmental Quality has been deeply concerned about and involved with the problems of ocean dumping from its inception slightly over 1 year ago. In his message to Congress of April 15, 1970, on the subject of Great Lakes and other dumping, the President directed the Council to make a study and report on the ocean disposal of wastes. Through the summer of last year the Council worked to prepare a report to the President on the subject.

On October 7, 1970, the President transmitted the completed report to Congress, endorsing the Council's recommendations and stating that specific legislative proposals in the form of a bill would be presented to the 92d Congress. The bill was transmitted to Congress as a part of the President's recent environmental message on February 8, This bill has been introduced by Congressman Pelly as H.R. 4247 on February 10 and also has been introduced as H.R. 4723 by Congressman Garmatz, your distinguished chairman; as H.R. 5239 by Congressman Kemp; as H.R. 5268 by Congressman Ruppe; as H.R. 5477 by Congressman Pelly; and as H.R. 6771 by Congressman Gerald Ford.

During our formal study we became convinced that there is a critical need for Federal legislation to implement a national policy on ocean dumping. I would like briefly to present our reasons for reaching this conclusion and for adhering to it in the light of our subsequent work. Then I would like briefly to describe our legislative proposal and comment on several of the other proposals now pending before the House and this committee.

We often do not take adequate account of the fact that oceans—140 million square miles of water surface—cover over 70 percent of the earth. They are critical to maintaining the world's environment, contributing to the oxygen-carbon dioxide balance in the atmosphere, affecting global climate, and providing the base for the world's hydrologic system. Oceans are economically valuable to man, providing, among other necessities, food and minerals.

The coastlines of the United States are long and diverse, ranging from the tropical waters of Florida to the Arctic coast of Alaska. These areas, as biologically productive as any in the world, are the habitat for much of our fish and wildlife. They also provide transportation, recreation, and a pleasant setting for more than 60 percent of the Nation's population.

These waters are also the final receptacle for many of our wastes. Sewage, chemicals, garbage, and other wastes are carried to sea through the watercourses of the Nation from municipal, industrial, and agricultural sources or directly by barges, ships, and pipelines.

The amount of wastes actually transported and dumped in the ocean is small in terms of the total volume of pollutants reaching the oceans. But even so, the Council estimated that in 1968 slightly over 48 million tons of waste were dumped at sea off the shores of the United States. Of this total, the main source of ocean dumping were:

(1) Dredge spoils—the solid materials removed from the bottom of water bodies, generally for the purpose of improving navigation (80 percent of the total by weight);

(2) Industrial wastes—acids, refinery, pesticide and paper mill wastes, and assorted liquid wastes (10 percent);

(3) Sewage sludge—the solid material remaining after municipal waste water treatment (9 percent by weight);

(4) Construction and demolition debris—masonry, tile, stone, excavation dirt, and similar materials (about 1 percent);

(5) Solid waste—the common refuse, garbage, or trash generated by residences, commercial, agricultural, and industrial establishments (less than 1 percent).

And, as we all know, small but potential tonnages of other materials, such as explosive munitions and chemical warfare agents, have been dumped.

Tonnages are not a good indicator of the effect of the dumped material. Dredge spoils, for example, can be contaminated with pollutants from industrial, municipal, agricultural, and other sources on the bottom of water bodies. If these contaminants are oxygen-demanding materials, they can reduce the oxygen in the receiving waters to levels at which certain acquatic life cannot survive. Heavy metal contamination can also create water concentrations toxic to marine life. Sewage sludge, whether or not digested to control odors and pathogens, can also contain significant concentrations of heavy metals and of oxygen-demanding materials.

Most of the dumping takes place in designated sites for the disposal of certain types of wastes. Disposal sites for dredge spoils are scattered off the Atlantic, gulf, and Pacific coasts, but most of the ocean disposal of other wastes is concentrated in Atlantic sites off the heavily polluted Northeastern States. The effects of dumping in a designated

area can be disastrous, as studies of the New York Bight and the areas off Rehoboth Beach indicate.

The problem that faces us is not limited to the effects of materials presently being dumped. The volume of waste dumping is growing rapidly, and the future impact of dumping could increase significantly relative to other sources of pollution in the ocean. Because the capacity of land-based disposal sites is becoming exhausted in some coastal cities, some communities are increasingly looking to the ocean for disposal. And, higher water-quality standards could lead industries to also look to the ocean for disposal.

A number of alternatives are presently available for wastes now being dumped at sea. Our report discusses these alternatives in detail and also evaluates present efforts to develop other disposal options, some of which such as land reclamation and recycling can be environmentally beneficial. After an evaluation of the effect of specific types of wastes currently being dumped and of the alternatives to dumping available, the Council recommended adopting certain policies respecting the ocean disposal of given types of materials.

Mr. Chairman, the next three pages are essentially summaries from the ocean dumping report. I would just as soon skip over these with the request that they be inserted in the record, or if you would prefer I will be happy to read through them.

Mr. LENNON. If you have the time, I would like you to read through them; I think it might be better.

Mr. TRAIN. All right, sir. I would be happy to do so.

OCEAN-DUMPING POLICY

Ocean dumping of undigested sewage sludge should be stopped as soon as possible and no new sources allowed.

Ocean dumping of digested or other stabilized sludge should be phased out and no new sources allowed. In cases in which substantial facilities, and/or significant commitments exist, continued ocean dumping may be necessary until alternatives can be developed and implemented. But continued dumping should be considered an interim measure.

Ocean dumping of existing sources of solid waste, other than sewage sludge, should be stopped as soon as possible. No new sources should be allowed; that is, no dumping by any municipality that currently does not do so, nor any increase in the volume by existing municipalities.

Ocean dumping of polluted dredge spoils should be phased out as soon as alternatives can be employed. In the interim, dumping should minimize ecological damage. The current policy of the Corps of Engineers on dredging highly polluted areas only when absolutely necessary should be continued, and even then navigational benefits should be weighed carefully against environmental costs.

The current policy of prohibiting ocean dumping of high-level radioactive wastes should be continued. Low-level liquid discharges to the ocean from vessels and land-based nuclear facilities are, and should continue to be, controlled by Federal regulations and international standards. The adequacy of such standards should be continually reviewed. Ocean dumping of other radioactive wastes should

be prohibited. In a very few cases, there may be no alternative offering less harm to man or the environment. In these cases ocean disposal should be allowed only when the lack of alternatives has been demonstrated. Planning of activities which will result in production of radioactive wastes should include provisions to avoid ocean disposal.

No ocean dumping of chemical warfare materials should be permitted. Biological warfare materials have not been disposed of at sea and should not be in the future. Ocean disposal of explosive munitions should be terminated as soon as possible.

Ocean dumping of industrial wastes should be stopped as soon as possible. Ocean dumping of toxic industrial wastes should be terminated immediately, except in those cases in which no alternative offers less harm to man or the environment.

Ocean dumping of unpolluted dredge spoils, construction and demolition debris, and similar waste which are inert and nontoxic should be regulated to prevent damage to estaurine and coastal areas.

Use of waste materials to rehabilitate or enhance the marine environment, as opposed to activities primarily aimed at waste disposal, should be conducted under controlled conditions. Such operations should be regulated, requiring proof by the applicant of no adverse effects on the marine environment, human health, safety welfare, and amenities.

That concludes the summary of the policy recommendations included in our report.

Current regulatory activities and authorities are not adequate to carry out such a policy. The States, the Army Corps of Engineers, the Coast Guard, the Atomic Energy Commission and the Environmental Protection Agency each exercise some control, but the dispersion of authority along with an accompanying inadequate [illegible]risdictional basis and lack of statutory standard-setting guidance prevent an effective governmental response to ocean dumping problems.

Government witnesses who will appear before you in the coming days will describe our bill in detail. But, in a nutshell, to control ocean dumping adequately, the administration bill would provide a ban on the unregulated dumping of all materials into the oceans, estuaries, and Great Lakes and would provide authority to limit strictly ocean disposal of any materials harmful to the marine environment. It would require a permit from the Administrator of the Environmental Protection Agency, for the transportation for dumping in estuaries, the Great Lakes, and the oceans anywhere in the world of wastes which originate in the United States and for dumping by United States and foreign nationals in our territorial waters and in the contiguous zone when the dumping would affect our territory or territorial sea.

The Administrator would be empowered to ban ocean dumping of certain materials and to designate safe disposal sites for others. Transportation for dumping or dumping without a permit, or dumping in violation of a permit would be subject to civil and criminal penalties. The Coast Guard would perform surveillance and other appropriate enforcement activities.

Specific considerations are set out for use by EPA in developing criteria for ocean dumping. EPA could refine and modify the criteria as additional knowledge on the effects of ocean dumping is gained.

In no case could dumping violate Federal-State water quality standards in the United States territorial sea or contiguous zone. The proposal would encourage Federal research on the effects of materials dumped or spilled into the oceans and the development of means of monitoring and controlling such disposal. In developing the criteria and the enforcement programs, EPA would work with the Coast Guard and the National Oceanic and Atmospheric Administration.

Our premise is that action is necessary now to avoid a serious national problem from ocean dumping. Yet, the proposed action is not all preventive. Adequate regulation could contribute to the restoration of many of the presently damaged areas.

Congress now has before it a number of other legislative proposals which also seek to control ocean dumping. I would like to comment on these proposals briefly, discussing these aspects which involve principles essential to effective control over ocean dumping. The committee is very fortunate to have the benefit of a number of fine proposals, and I commend not only the interest which you have demonstrated in this subject but also the cooperative and bipartisan spirit in which you have approached the matter. We would hope that you will consider our comments as constructive suggestions and that we could work together in arriving at a solution.

H.R. 3662 would amend the Fish and Wildlife Coordination Act by providing for a new section 5B which would prohibit dumping waste material into or transportation of such material through estuarine areas, the territorial sea, the Great Lakes, and the waters above the Outer Continental Shelf, except where a permit has been obtained from the Administrator of EPA. We obviously appreciate the recitation of factors such as land-based alternatives and the effect of the dumping on human health and welfare, fisheries resources, and marine ecosystems which the Administrator would consider before acting on a permit application.

Nonetheless, the State Department has advised us that the jurisdictional provisions of this bill are unsound as a matter of international law. Mr. Stevenson, the State Department's Legal Adviser, will be discussing the jurisdictional problems with you on Wednesday. Notwithstanding the jurisdictional issues, we are concerned with the bill's failure to regulate transportation for dumping beyond the 200-meter depth contour for the Outer Continental Shelf.

We suggest that the United States should control the transport of material from the United States for dumping in the outlying areas of the high seas. The need for such control is demonstrated by the recently disclosed practice of disposing of solid arsenic wastes originating in Pennsylvania by dumping it in steel drums 1,000 miles out to sea.

We also suggest that effective and efficient implementation of the regulatory concept would be aided by placing primary emphasis on control of the transportation for dumping of material which originates within the United States. Very nearly all of our problems arise from such dumping and accordingly our concerns must be with regulating this dumping along with developing environmentally sound domestic alternatives to the ocean dumping means of disposal of our wastes.

Nonetheless, direct control seems desirable for all dumping in our territorial sea and in our contiguous zone where it affects our territory

or our territorial sea. Further direct controls over dumping in the waters above the Outer Continental Shelf of wastes not originating in the United States do not seem necessary. In light of the experience gained through our study such dumping does not present a practical problem. As far as we know, no such dumping takes place.

Paragraph (b)(4) of H.R. 3662 would prohibit the Administrator from issuing permits to dump radioactive wastes, toxic industrial wastes, and chemical and biological warfare materials. Subsection 5(f) of our bill would give the Administrator more flexibility in dealing with the problems caused by particular pollutants, providing, where and when appropriate, for a complete ban on the dumping of particular materials.

Satisfactory alternatives to ocean dumping may not be available in all cases. We would not favor foreclosing the Administrator from considering a disposal option which in a given case may be environmentally the most desirable or, put another way, the least undesirable. Some have said that the specific ban on dumping particular materials actually only carries out the policy as expressed in our report and which I have described earlier. This is not the case.

For example, we recommended continuing the present prohibition on the dumping of high-level radioactive wastes. We recommended maintaining careful controls on dumping certain low-level wastes. And, where careful advance planning could not avert occurrence of a situation where the ocean dumping alternative was demonstrably the least harmful, we recognized that it should be chosen. The upshot of our whole approach is that the Administrator will be in the best position to assemble, not only from his agency but other agencies, the scientific and technical data necessary to choose between an immediate ban, a gradual phasing out, or other regulatory control as warranted by the facts of each case.

A further bill, H.R. 1661, would regulate the discharge from vessels of wastes originating in the United States, requiring that a permit be obtained from the Administrator of EPA prior to such loading. This bill would also bar the discharge of wastes in waters between the seaward edge of the Continental Shelf and the coast of the United States. Again, we agree with much that is contained in H.R. 1661, and particularly with its focus on regulating the transport of wastes from the United States.

We do recommend extending regulation to transport other than by vessel, since much dumping of material such as dredge spoils does not take place from vessels but rather from special conveyer systems or pipelines which are not considered outfalls. And, we do not advocate an absolute ban on dumping for any area which is selected only by geography and not by its ecological characteristics. Relationship of the area to alternatives to ocean dumping also is important because many materials such as unpolluted dredge spoil can be dumped in the particular general sea area from which they originated, and returning them to a carefully selected nearby site may be the action most in accord with maintaining and preserving the existing land and marine environments.

Another bill, H.R. 4359, would combine many of the elements of the bills previously mentioned, but would also direct the Secretary of

62-513—71——12

Commerce, acting through NOAA, to establish marine sanctuaries where, among other things, dumping would not be permitted. We wholeheartedly agree that dumping should not be permitted in given areas and have provided authority for the Administrator to take effective action to achieve such an end by simply not issuing any permits for dumping in such areas. As he desired, the Administrator could designate recommended sites for dumping and thereafter only grant permits relating to such sites.

I would not wish to neglect research needs in my comments. As our report pointed out, serious information deficiencies exist, and research is required in such broad and diverse areas as the pathways of waste materials in marine ecosystems and the recycling of wastes and the development of alternatives to ocean dumping. Agencies such as EPA, NOAA, and the Coast Guard have authority and have presently operating programs to gain such information. Implementing an ocean-dumping policy by enactment of appropriate legislation would provide a focus for a cooperative research effort. The Council is presently entering upon a study of recycling to evaluate the policy options available in that area. Other such efforts will be stepped up.

My remarks have been extensive and I would not wish to prolong them further except to comment briefly on our international efforts and prospects in this area. Through domestic legislation such as that which we have proposed, in my judgment very effective action can be taken to curb the present and potentially harmful effects of ocean dumping. Further, such action can be taken consistent with accepted principles of international law. Very nearly all of our problems in the United States with ocean dumping arise as a result of disposing of waste material which originates within the United States.

We can and should through domestic legislation control the transport for dumping of such material. We can and should also control all dumping in our territorial sea and dumping in our contiguous zone which affects our territory or territorial sea. And, as the President has stat[illegible], we will urge other nations to adopt similar measures and enforce them. But, a completely effective system for the control of ocean dumping would involve regulation of at least all harmful materials, wherever they may be generated, and wherever and by whomever they may be dumped.

The administration bill contains a section requiring the Secretary of State to "seek effective international action and cooperation to insure protection of the marine environment. . . ." State, in conjunction with the Council and other concerned agencies, is taking steps to assure accomplishment of this objective. We anticipate that the 1972 United Nations Stockholm Conference on the Environment will be a useful forum in this respect.

If the United States is in fact to exercise leadership in this critical area, if it is to persuade other nations to control their ocean disposal of wastes, then it is essential that the United States first put its own house in order. In my opinion, prompt and favorable action by Congress to establish effective regulation of ocean dumping is a prerequisite to action by other nations.

Thank you, Mr. Chairman. That concludes my prepared statement.

Mr. LENNON. Thank you, Mr. Train. I personally do not think that

your remarks have been too extensive; they were very interesting, informative, and certainly will be most helpful.

Mr. Rogers.

Mr. ROGERS. Thank you, sir.

Mr. Train, I notice that you take pretty much the position the bills have taken generally that certain elements should be permitted all together. My understanding is that the President said there would be no more ocean dumping for Federal agencies. Am I correct or incorrect on that?

Mr. TRAIN. It is the President's policy that Federal agencies prior to the enactment of legislation endeavor, to the extent practicable, to conform to the policy which he has recommended for legislation. Certainly the Federal agencies would be covered by our proposed legislation.

Mr. ROGERS. What I am concerned with—we had a problem with the Army where we got into all this nerve gas thing, as you recall, and then we had problems with the Navy in my own State where they dumped acids and oil. Now I find, and I have predicted this would happen, we would have to go service by service to get anything done. Now I find the Air Force is out dumping in California and I am sure from other bases in an attempt that I think will soon kill off part of the water in the ocean there off southern California and that is the Norton Air Force Base.

They have been dumping chemicals into the ocean via barge. Now they are supposed to dump them 5 to 10 miles off, but if it is good and foggy they say they do it quickly and they even had a problem there where they could not get them to sink so they used rifles to sink the containers and even had one blow up and blow part of a ship out, too, I understand. Now they have stopped the dumping by barges since the President's direction came out, but do you know what they are now doing? They are now taking it in trucks and just dumping it into the sewerage system and it goes right into the bay.

Now I think that is a perversion of what the President intended and I would hope that there is some action that could be taken to prevent that. That is probably the largest film depository where they develop a lot of film in Hollywood, so all of these chemicals that are most deadly are dumped right into the sea. In fact, there are some that directly effect cancer.

Are you aware of this situation?

Mr. TRAIN. No; I am not, Mr. Rogers. That is new information to me and I am certainly very glad to have it. I assure you that I will be in touch with the Air Force as soon as this hearing is over to discover what is going on.

Mr. ROGERS. Should they not be filing an impact statement with you if they dump 1.2 million gallons of chemicals, including cyanides, sulfates, hexa chromium, and 94 other chemicals? The truck leaves the base each morning by 7:30 to 8:30 and they simply dump into the sewer lines into the bay, and the truck is marked, incidentally, corrosives.

Mr. TRAIN. In specific answer to your question, Mr. Rogers, from what you describe I would consider these actions to be subject to the National Environmental Policy Act and the subject of environmental impact statements under section 102; yes.

Mr. ROGERS. I understand they have not filed such a statement.

Mr. TRAIN. To my knowledge, no.

Mr. ROGERS. I think you are correct, we have also checked with EPA and have not found that to be the fact.

Also, I would just like to recall while we are going into this just a minute the fact that when the Navy ordered the dumping into the St. Johns River of the acids we went into the problem of who is responsible, what happens. The Corps of Engineers under the law is supposed to recommend an action by the Attorney General. Well, they are afraid to do it against the Navy. We have talked to the counsel over there; "Oh, we don't think we can do that."

We said; "What about the individual who gives the command?"

"Well, there may be some authority there, we better let the Navy do it."

Now the Navy told us they were going to investigate. It has been over 6 months and still no action taken against the person who gave that order. Now if this continues, the perversion of what the President has ordered has gone astray, the intent of the law is not being carried out. We will never stop dumping unless we begin to center responsibility and take some action or at least a reprimand, and I have not even seen that done. I don't see as much point in us passing a lot of laws if our own establishments are going to ignore the law and ignore the intent of Presidential directives.

Do you suppose we can get any action on that?

Mr. TRAIN. Well, as I say, having this information I will certainly take it up with the Air Force right away. We are very much dependent on the council on information to provide a basis for identifying problems.

Mr. ROGERS. I understand.

Mr. TRAIN. As you understand, this kind of information is not generally volunteered to you by either a government agency or a private source if that is what is involved. So it is helpful to us to have this kind of information and we can certainly proceed to look into the matter and see what is going on.

Mr. ROGERS. Would you, and let us know?

Mr. TRAIN. I certainly will.

Mr. ROGERS. I think it is very vital.

Mr. TRAIN. And I do. At the same time, with all of the continued actions which are clearly inconsistent with the policy which the President has recommended, I do note that the Defense Department has been moving to put its house into order. The Secretary has banned, as you know, all dumping of chemical and biological warfare agents. This has been completely stopped. Of course no biological warfare agents have ever been dumped at sea, but chemical warfare agents, as this committee well knows, were dumped and this has been now completely banned. There are no explosive dumpings presently underway. There was one that was scheduled but that has been held up pending an extensive Navy study of alternative methods of disposal.

The Navy has also to my knowledge moved, I hope effectively since the dumping of the oily wastes off the Florida coast some months back, to prohibit any reoccurrence of that kind of action from any of its other bases around the coastline. Now on the other hand, the kinds of

cases to which you referred do arise and we have to keep working on them.

Mr. ROGERS. I think some action needs to be taken, and also on mercury. I think we could have prohibited the dumping of mercury but the agencies still have not done this. We know it takes action, we know the deadliness of it. We have had a few suits brought where they still permit them to continue, and I would hope you could encourage some action, too.

I won't continue now because I know the other members have questions. There are many questions I would like to go into, Mr. Chairman, with Mr. Train at a later time.

Mr. LENNON. Would you like to submit the specific questions and let him include them in the policy review?

Mr. ROGERS. Some of them. I think it would be actually helpful to have a little rapport because you don't always get the answer you need.

Mr. LENNON. Thank you.

Mr. Pelly.

Mr. PELLY. Thank you, Mr. Chairman.

Mr. Train, before you came into the hearing room one of the witnesses testifying in behalf of his own bill said that the administration bill is lacking in the major provision of his own original legislation—the establishment of no dumping sanctuaries for marine life. His comment was that "Proposals which simply move dumping grounds from one area to another are myopic and only increase the danger of prolonged pollution and international complications growing out of contaminating the world's oceans."

I think you referred to the subject of sanctuaries in your discussion. Would you care to comment on this witness's statement?

Mr. TRAIN. I suspect I was commenting upon the same bill, although I am not certain. I was simply noting for the information of the committee that under the administration bill—while it does not have a marine sanctuary section in it, that was not really the purpose of the legislation—it does have authority for the Administrator of EPA to prevent any form of dumping in certain fixed areas at his discretion.

Mr. PELLY. In other words, you don't agree that the administration bill would result in just moving a contaminated area from one place to another?

Mr. TRAIN. Oh, no; certainly not. The administration bill, that is, the purpose of the bill, is to either stop or to phase out as rapidly as possible all harmful dumping.

Mr. PELLY. In the administration bill there would be a requirement for a permit for transportation of material in the United States to be dumped anywhere at sea and then also it would require a permit for the actual dumping by any person. Is that duplication or is there any reason for the distinction?

Mr. TRAIN. We are not talking about two permits. It is the transportation from the United States which is the jurisdictional hook, if you will, upon which we hang the authority of the United States to regulate dumping anywhere in the world. So if a ship carries wastes from a U.S. port to dump anywhere in the world, it will have to have a permit from the Administrator of the EPA.

Mr. PELLY. In other words, there is a legal power to control carrying pollutant materials through the the territorial sea and on that basis you propose to prevent it from being dumped outside.

Mr. TRAIN. It is actually the taking from the United States proper, not the passing through the territorial sea.

Mr. PELLY. In other words, it is an export permit. What legal authority would there be for taking material from a port as against going through our sovereign territorial waters?

Mr. TRAIN. Well, of course, you could not leave a port without going into the waters but it would be under the commerce power, I presume. We do not by that authority seek to govern the case, for example, where wastes are loaded in a foreign port and for some reason carried through the U.S. territorial waters and then back out to the high seas somewhere and dumped. We would not seek to assert U.S. jurisdiction over that dumping by this legislation.

Mr. PELLY. Does the International Convention on the Law of the Sea give us authority to prevent dumping over the Continental Shelf?

Mr. TRAIN. If we get into the complexities of international law in this area, you might be better advised to rely on Mr. Stevenson of the State Department. But, I would say this: that our jurisdiction insofar as territorial seas are concerned extends only 3 miles, and 9 miles beyond that to the contiguous ozone.

Mr. PELLY. Over the bottom, but we don't actually cover the free-swimming fish, for example.

Mr. TRAIN. Under the Geneva Convention with respect to the deep seabed we have jurisdiction over the resources of the seabed out to the 200-meter isobath or so much further out as we have the ability to exploit it. I hope I am close to the language.

Mr. PELLY. You are almost word for word but I just thought perhaps there would be some limit on our power to prevent anybody dumping over our seabed to the extent of 12 miles, the Continental Shelf.

Mr. TRAIN. We believe that we have complete authority to regulate the dumping by anyone within our territorial waters, no matter from where those wastes come or whether there is any touching at a U.S. port. We likewise believe that we have authority completely to regulate any such dumping in the contiguous zone to the extent that it could affect the territorial waters or our shores. In effect, we say this gives us authority to regulate ocean dumping within the 12 miles. To the extent that a dumping takes place outside of the 12-mile limit, whether on the Continental Shelf or beyond the Continental Shelf, we do not believe that the United States has unilateral sovereign authority to regulate that except where the wastes originate in the United States.

Mr. PELLY. I think some of the bills that are before the committee actually do seek to control outside the 12-mile limit.

Mr. TRAIN. They do, sir; and this is one of the problems to which I have referred in my statement. To the extent that they do seek to regulate dumping on the Outer Continental Shelf beyond the contiguous zone where the wastes do not orignate from the United States. we believe there is raised a very serious question under international law. At the same time I go to great pains to point out there is no evidence of such dumping so it is not at the present time a real problem.

Mr. PELLY. Mr. Train, I just have two quick questions, and answers to which I think should be in the record. One has to do with the matter of violations. The administration's bill provides that each day of the continuous offense shall be counted as a separate violation. With respect to the dumping of material from barges, however, would it not be better to consider each incident a separate violation since more than one barge load of material could be transported within a given day?

Mr. TRAIN. I think I better give you a response for the record on that.

Mr. PELLY. Yes.

(The response follows:)

Subsection 6(c) of the bill provides that "each day of a continuing violation shall constitute a separate offense." Dumping from a single waste-carrying barge would be a separate, identifiable incident and not a "continuing violation", even if the same barge were to make a second dumping trip later in the same day. Accordingly, each such barge dumping would be considered a separate violation under the present language of the bill.

Mr. PELLY. Then the other question I have has to do with the rights that are given to the States to establish their own standards. Well, I don't see any provision in the administration bill that requires that the State standards should have greater strength than actually the standards provided in this bill.

Mr. TRAIN. Where are you reading from in the bill, Mr. Pelly?

Mr. PELLY. Well, I have some notes here and I will read them. I don't have the actual bill before me.

The bill does not state that the State requirements must be more stringent than the Federal law.

It is section 7(e) I am told.

In other words, should we imply or should the bill be amended to assure that any State requirements would be more stringent than the Federal requirement?

Mr. TRAIN. The Federal Government is taking over—well, no, that is not entirely correct.

What the bill provides in section 7(e) is that the fact of Federal regulation as provided by this bill will not preempt the States from exercising regulatory authority if they wish. Now in effect what this means is that if a State wishes to set more stringent rules than the Federal within the 3-mile limit of the territorial waters, it will be free to do so. On the other side of the coin, a State could not set less vigorous standards within the 3-mile limit, or if it did the Federal regulations would override them because the Federal permit would be needed and would rest on the Federal criteria.

Mr. PELLY. So I will not take too much of the other Members' time, I will ask you to supply for the record the answer to my question with regard to the jurisdiction in connection with oil.

Mr. TRAIN. Yes, sir.

(The information follows:)

Discharges of oil are strictly regulated by other Federal laws. For example, section 11 of the Federal Water Pollution Control Act bars making discharges of oil determined to be harmful into the territorial sea or the waters of the contiguous zone. By regulation, any discharge creating a visible sheen has been determined to be harmful. Further restrictions, particularly on the high seas, are imposed under the International Convention for the Prevention of Pollu-

tion of the Sea by Oil, 1954, as amended. Pertinent further amendments to this Convention were adopted by a Conference of Contracting Governments in 1969, and the President has transmitted these Amendments to the Senate for its advice and consent.

Mr. PELLY. Thank you, Mr. Chairman.

Mr. LENNON. Thank you, Mr. Pelly.

Mr. Karth.

Mr. KARTH. Mr. Chairman, I know time is fleeting. I merely would like to make a request, if I may. Pursuing what our distinguished colleague from Florida started, if he is correct about the Air Force dumping, I would like to have you, Mr. Chairman, submit for the record the name or names of those who are responsible for having given the order to dump and the name or names of those responsible for filing with you or the EPA an application to dump. Would you do that for the record?

Mr. TRAIN. Certainly. I will endeavor to get the information—that should be my correct answer.

Mr. KARTH. I am sure you can.

(The material follows:)

We have been informed that the Air Force has transmitted to you a factual statement describing the circumstances involved in Norton Air Force Base's handling of liquid wastes. To our present knowledge, the treatment given the Norton wastes is consistent with applicable water quality standards. We have asked the Environmental Protection Agency to review the present Norton handling practices for such wastes and to assess the adequacy of the applicable treatment standards. At your request, we have also asked the Air Force to identify those officials who are responsible for the Norton practices and to send you their names in a separate, subsequent letter.

Mr. KEITH. Would the gentleman yield?

Mr. KARTH. Yes.

Mr. KEITH. I hope you would add the corrective action that is necessary to overcome this condition and to make certain it does not occur again.

Mr. KARTH. If you would, Mr. Chairman, the results of your investigation and your recommended action.

Mr. ROGERS. Would the gentleman yield?

And also the point I mentioned about the Navy doing nothing about the dumping at the St. Johns, I think we need some information on what action is taken there, what investigations, what action has been taken against the officer who gave the dumping order. I intend to pursue it but I think it might be helpful if you could request it also.

Mr. TRAIN. Which exact case was this?

Mr. ROGERS. Where they dumped the acid in the St. Johns River. The command was given from the Naval District Office, not the little Lieutenant who actually translated the order but the order was given from up the line. We need to know who did that, and some action should be taken against those people.

Mr. TRAIN. I will to the best of my ability get together these answers for you. As you may know, there will be a witness here from the Navy, I believe, later this week.

Mr. ROGERS. Maybe you can ask if he has the answer when he comes.

Mr. TRAIN. I certainly will mention this to him.

Mr. LENNON. We are talking about the Mayberry incident, in which the contractor for the Navy dumped, with the permission of the Navy,

first of all sulfuric acid, and then subsequently caustic soda, which the Administration on Oceanography became involved in and had the assurance of the Navy there would be an investigation, and a report and an assurance, too, that they would take it up with you.

We would like to know, and we think we are entitled to know, if they did carry out that directive which obviously they have not because you have no recollection of it.

I thank the gentleman for yielding. Mr. Mosher.

Mr. MOSHER. Judge Train, at the bottom of page 10 you commend the committee for its interest in the subject and you speak of the cooperative and bipartisan spirit. I judge you are implying there that the administration has considerable flexibility in its attitude on this legislation, and you are prepared for some give and take as the committee considers legislation, and you anticipate that the administration bill will be changed to include provisions in those bills introduced by members of our committee. I hope that is the case.

Mr. TRAIN. That is absolutely correct, Mr. Mosher. The administration's purpose is to achieve strong ocean-dumping control legislation as soon as possible.

Mr. MOSHER. So now the issue between the administration bill and the bills that have been generated on the Hill seems to be the degree of discretion which the Administrator of the Environmental Protection Agency would have. The administration bill gives him almost whole discretion—at least it is very wide discretion—whereas most of the other bills restrict his options in one way or another.

I think that inevitably will be taken into consideration as we ponder this legislation. Do you have a certain degree of flexibility at that point? Do you anticipate that perhaps we should sharpen and strengthen, and perhaps limit that discretiionary power to a greater degree than in the administration bill?

Mr. TRAIN. I think it would be my view that while the policy should be very clearly announced, and while the criteria which are developed by the Administrator of the Environmental Protection Agency should be made a matter of public record, and settled only after public comment, that the Congress should permit great flexibility in the administration of this program rather than trying to legislate fixed rules concerning what can and what cannot be dumped, and fix time tables or specific references to geographic areas.

I believe that we are dealing with a highly complex problem. I do not believe that you can pretend to, or seek to, regulate the marine environment in some sort of separation from the land environment. We are talking about one total interrelated problem. This is the reason why, for example, the administration's legislation contains the requirement that the Administrator of the Environmental Protection Agency in setting standards must take into account the effect on the marine environment, and also the availability of alternative disposal methods. I think that it would be both unrealistic, and I think incorrect, to require ocean-dumping decisions to be made irrespective of the effect of other alternatives.

Mr. MOSHER. Well, the complexity of the job is very apparent. I agree that there is a need for considerable discretionary flexibility, but at the same time I think that we are going to have to have this

very serious and thorough consideration as to certain limits, certain standards, certain requirements as guidance. I think Congress cannot relinquish its responsibility just by shoving off to the administrative agency complete discretionary authority. This is something that we will all be discussing, of course, in the days ahead.

Mr. ROGERS. Would the gentleman yield?

I think it will be well to have your comment, Mr. Train, about the provisions that those of us who introduced H.R. 3662 provided in this proposed legislation, my bill, to begin to set deadlines as to treatment of wastes before they can be dumped into the water; for instance, primary, secondary, and tertiary treatment to set specific dates just like you did in the air pollution bill.

Now, unless we have goals and dates set, I am afraid all of this discretion in the administration bill will end up in no decisions and no real progress being made. I think it is essential for us to have deadlines as to when we must have primary, secondary, and tertiary treatment before they can be dumped. I think it would be helpful if we could have your opinion in the record on this.

Mr. MOSHER. Of course, the Air Quality Act was unique, because Congress for the first time did establish quantitative standards and deadlines, and I think this will be almost the prime question before us as to whether we want to follow that example or not.

Mr. ROGERS. I think the gentleman is correct. If you could let us have your comment on that?

(The comment follows:)

Requirements for primary, secondary, and tertiary treatment of wastes would generally apply to those liquid wastes which are discharged through outfalls. Discharges of effluent from outfalls are excluded from regulation under H.R. 4723, with the expectation that such discharges will be addressed under the Federal Water Pollution Control Act and the Refuse Act. The Administration has proposed amendments to the Federal Water Pollution Control Act which are now being considered by other Committees; Mr. Ruckelshaus, the Administrator of the Environmental Protection Agency, could and to my knowledge will discuss the details of these water quality proposals with you.

Mr. LENNON. The gentleman from California, Mr. Anderson.

Mr. ANDERSON. Yes. Thank you, Mr. Chairman.

Mr. Train, to follow up Mr. Pelly's question a moment ago concerning the establishment of no-dumping sanctuaries for marine life, would you be opposed to an amendment to your bill that would incorporate the concept of the no-dumping sanctuaries?

Mr. TRAIN. No, I am not opposed to it. I am personally rather in favor of the establishment of marine sanctuaries and some system of that sort. As you know, the administration bill proposed legislation last year for the acquisition of Santa Barbara oil leases and the establishment of a marine sanctuary in that particular area off the Santa Barbara Channel. So this is a concept which, I think, speaking very generally, I would strongly favor.

There can be, obviously, differences in detail. Whether this is the proper vehicle or not is something else again. I think that the authority for the establishment of marine sanctuaries is probably more appropriately placed in the Secretary of the Interior, which I think the bill in question does do, rather than in the Administrator of the Environmental Protection Agency, for example.

Mr. ANDERSON. Thank you. I have another question.

Mr. TRAIN. Let me also comment beyond that. There is a great deal involved, obviously, in the establishment of a marine sanctuary that goes far beyond questions of dumping which define the scope of this particular legislation. There are questions of resource development, and, I suppose, other questions of international law, a whole range of concerns which we have not gone into, either in our report or in this legislation, and I would think that perhaps ought to be dealt with separately.

Mr. ANDERSON. I believe Congressman Murphy's idea is to have regulated dumping in these areas but, in addition to the dumping areas, to have no-dumping sanctuaries, areas free from dumping concessions, along our coasts, where no dumping whatsoever is allowed. This appeals to me, particularly on sections of the California coast, where I would like to see areas set up wherein no dumping of any kind occurs. Furthermore, in those areas where dumping is allowed, some regulation should be required. I think that concept probably should be included in your bill.

Mr. TRAIN. Well, it is included in the bill to the extent that the Administrator has the authority to ban all dumping in given areas. That is, as I pointed out, part of the authority in the bill.

Mr. ANDERSON. Such authority could almost be construed as the no-dumping sanctuary which he is recommending in his bill.

Mr. TRAIN. Yes, I think it is; but also, as I repeat, a lot other than dumping is involved in the marine sanctuary. There is oil development, for example; deep sea mining. I would imagine that is involved. I think these are very complex questions that should be gone into before you put the label of marine sanctuary on a given area. That would be my only suggestion, that all of those concerns in fact should be taken into account in a marine sanctuary proposal.

Mr. ANDERSON. I have another question. In your bill your definition of the word "dumping" does not include the disposition of any effluent from any outfall structure. I interpret this to mean the exclusion of any industrial-waste outfall. Am I right on that?

Mr. TRAIN. Well, yes and no. The outfall at the present time that is within the 3-mile territorial sea is governed by the Water Pollution Control Act and by the water quality standards. The President recommended last year, and again in his environment message this year, that water quality standards be extended to include the contiguous zone also; so that would then cover all outfalls out to the 12-mile limit. To the extent that you have an outfall that is beyond the 12-mile limit, I would question whether the water quality approach is an effective one, and would think that, if it were desired to control such outfalls, such control be accomplished through a permit system under this legislation. The relationship of the Water Quality Act to this permit authority is one I think you will want to discuss very closely with the Administrator of the Environment Protection Agency; it is a complex relationship.

Mr. ANDERSON. In your remarks this morning on page 14, you said you recommended "extending regulations to transport other than by vessel, since much dumping of material such as dredge spoils does not take place from vessels but rather from special conveyor systems or pipelines which are not considered outfalls."

I would like to determine in my own mind when you consider a pipeline an outfall and when you do not. Would a pipeline built for the purpose of conveying sludge and ground-up refuse be considered an outfall and would your proposed legislation apply? Municipal and industrial sewer lines are considered outfalls and would not be covered in your bill, according to your definition.

Mr. TRAIN. I am not sure that is correct, Mr. Congressman. I would like to direct myself to that in a written answer if I may.

Mr. ANDERSON. That is all I have.

(The answer follows:)

The first proviso to subsection 3(f) excepts from "dumping" covered by the bill, "a disposition of any effluent from any outfall structure." We note that "outfall structure" is a term of art used as a matter of practice in such related areas as the regulations promulgated by the Corps of Engineers for administration of the Refuse Act. See 33 C.F.R. § 209.131(f)(2), published at 36 Fed. Reg. 6567. We consider an "outfall structure" to be an identifiable artificial or artificially-adapted-natural discharge point for effluents which are transmitted either from facilities located on shore or from artificial islands or other fixed structures located off shore.

To our knowledge, the three primary means of dredging used in the United States would not involve outfall structures. A "pipeline" dredge uses a cutter head and suction to remove material from the bottom of a water body. The removed material is then pumped through a pipe to the designated disposal location. A "sidecast" dredge disposes of the removed material by casting it to one side of the dredging implement. It is mainly used in intercoastal or inland waterways where the removed material is unpolluted; e.g., where it is clean sand. A "hopper" dredge is often used in seagoing operations and involves taking up the removed material, transporting it to the designated disposal area, and then dumping it.

Mr. LENNON. Mr. Keith.

Mr. KEITH. Thank you, Mr. Chairman.

I was glad to have some introduction on the subject of marine sanctuaries. As you know, that is a pet of mine. Massachusetts has legislation establishing a sanctuary within the 3-mile limit off the cape of our national seashore along the shorelines of Massachusetts and they now have legislation establishing a marine sanctuary for many more areas.

Does the administration have the authority for creating marine sanctuaries within the area known as the fishing area—3 to 10 miles?

Mr. TRAIN. This is an international law question. Twelve miles could well go beyond the Continental Shelf in some areas. I would say that to the extent that the jurisdiction over deep sea dead resources extends the United States would have jurisdiction to establish a marine sanctuary related to the exploitation of those resources.

Mr. KEITH. Pertinent to the shelf but not the water column?

Mr. TRAIN. That falls under another body of law with which I am less familiar. I just don't know the answer to that.

Mr. KEITH. From the State Department I suspect that is really a very rough one and that is perhaps one of the reasons that we have not moved more readily in this area. I am concerned of course about the fishing grounds and the possible conflict as we continue our search for oil, the exploration phase followed by the exploitation phase. The State of Massachusetts has moved forward and I would hope that we could.

You referred to the State Department. Would the concept of zoning of the Continental Shelf be the kind of a vehicle that we would utilize

for improving the environment of the shelf? Could we as a nation abutting the Continental Shelf be able to use that as the authority for unilateral action to zone C?

Mr. TRAIN. Well, I don't think that by a process of zoning we can do anything which we are not otherwise entitled to under international law. In other words, I don't think any unilateral action can give us greater jurisdiction than we already have under international law.

Mr. KEITH. So if we wanted to proceed with legislation protecting the water column, we would have to go to IMCO and the international treaty group.

Mr. TRAIN. Within the 3-mile limit you can do anything you want with the water. Within the contiguous zone I am uncertain what you can do. I just don't know, there are a number of rules. You can control dumping to an extent but only if it affects your territorial waters and your shore.

Mr. KEITH. Does your concept, or your role, have a positive nature as well as a negative one? Can you take steps to——

Mr. TRAIN. I very definitely hope so, Mr. Keith.

Mr. KEITH. I have particular reference to the fishing zone.

Mr. TRAIN. Can I answer very quickly before you proceed because I think the previous question and maybe my answer will look a little strange here on the record. I would say that the record of our Council in terms of positive proposals, including the ocean dumping policy sent to the Congress by the President last October, is a positive one. I think most of our work is of a positive nature. The legislative package which the President sent to this Congress, some 15 to 20 bills, is a very positive program which we have developed.

Mr. KEITH. Unfortunately, we have to undo a lot that has been done and we must police the area before we can improve it. Most of the legislation which is in effect police action and we want to improve the environment by positive roles and it can't be done until we establish our authority on these resources and further establish the authority on the Continental Shelf and in the water above it.

We have to try to clear the area before we can improve it, and with the shortage of protein I would hope that we could spend some time and thought as to what we might do to make it more rewarding from a resource point of view, particularly as relates to fisheries. Because we have to police that area in a positive way; we have to have conservation measures and we have to have some way of making a better habitat for the fish and the shellfish on the ocean floor. And it is going to require an imaginative course of action if we are going to get the resources that will be required to take care of the population.

I have no further questions at this time.

Mr. LENNON. Thank you, Mr. Keith.

Mr. Kyros.

Mr. KYROS. Thank you, Mr. Chairman.

Mr. Train, while oil and radioactive wastes are glamorous issues at present, you point out that 80 percent of what is removed from the bottom is dredge spoils, and 34 percent is polluted. And every day, while we sit here the State of Maine people are dredging off the Atlantic coast. I don't see in all of your materials any real solution of the problem. Possibly taking dredge spoils out a little further, instead of 3 miles from the coast line, possibly taking them out 10 miles; and

then, perhaps, some long-range efforts. But what are we going to do in the interim with the dredge spoils? Is there some crash program that we are going to have, something more practical in solving the problem?

Mr. TRAIN. Of course in the Great Lakes the administration has instituted a program for shifting from the dumping of dredge spoils in the lakes themselves to a system of diked disposal areas along the shoreline which in itself is not completely satisfactory but is certainly a major improvement over what was done before.

I don't think that that is probably a feasible solution over much of our exterior coastline because there we run into the problem of destruction of wetlands whose protection we are equally concerned with. I have indicated that the Corps of Engineers has instituted a policy of taking the water quality effects of the disposal of spoils very much into account as part of the process of deciding whether to dredge a given area or not. This is a new development and I think a significant improvement.

This is being weighed in the process along with the navigational and economic benefits of dredging. I can't hold out to the committee any real hope that the dumping of spoils is going to come to a very rapid end because the alternative of disposal on land is not a very appealing alternative. As I mentioned a moment ago it probably would involve wetland destruction and this may well be a far more environ[illegible]lly harmful alternative than a continued disposal at sea.

[illegible]king well down the road, as water quality standards generally become increasingly effective, the polluted nature of our river bottoms and port bottoms should steadily improve so that hopefully, again looking somewhat down into the future, these dredge spoils will not be as polluted and environmentally harmful as they are at the present time.

Mr. KYROS. Do we have a cutoff date for municipal sewage dumping? Where the dredge is going to happen, is there a set date State-by-State, port-by-port?

Mr. TRAIN. There are implementation schedules under the Water Pollution Control Act, yes.

Mr. KYROS. So you could look forward to those dates. In the interim, I notice that under 7(c) of the act the Army and the Corps of Engineers retain authority as to where to dump. In other words, they still don't have to come to you or to the EPA, for a permit do they?

Mr. TRAIN. You are referring to their dredging and filling authority under the Rivers and Harbors Act. We do not supersede that by this legislation, but the legislation does require that the Corps of Engineers must get certification by EPA that a given dredge and fill operation is acceptable from the standpoint of the criteria of this legislation, so they are meshed together.

Mr. KYROS. Do you feel that this is sufficient authority in order to have your own agencies exercise complete and uniform control over the dumping of dredge spoils, as well as over the other materials which you will be watching?

Mr. TRAIN. Yes, I believe so. It is as broad as we think you can provide. I would also suggest that this is a brand new program and

I think that this and other committees of the Congress would want to keep a very close eye on its administration. At the present time we really have no control over ocean disposal in any effective sense. We are recommending total regulation of all disposal offshore originating from the United States. So it is a very broad authority and a very basic shift, almost a revolutionary shift in the extent of our existing authority. How it is in fact administered is exceedingly important. We believe that this is a matter of urgency, that harmful dumping should either be stopped or phased out as rapidly as possible as a matter of urgency.

Now if it turns out in effect that this is not being administered as vigorously as you would desire, then I think that it would be entirely appropriate for the Congres to set specific deadlines if it wished along with other specific rules, but I do believe that in the meantime it is very important to have a strong element of flexibility in the way this is done.

Mr. KYROS. But on section 7(c) there will be this kind of conflict. As I see it, people want their harbors dredged, and Congressmen want their own particular districts to get the dredging because of their boating and fishing and commercial fishing. Then, on the other hand, we have budgetary restraints which will prevent us from taking the dredged spoils out so far to sea as we would like to in the optimum situation. There will be a conflict.

I am just wondering if you are going to have sufficient power, within these restraints, to see that the dredging is done properly.

Mr. TRAIN. As you will note in section 7(c)(2), as I indicated the Corps must in all of these cases go to the administrator of EPA and get a certification, and I quote here from the bill, "that the activity proposed to be conducted is in conformity with the provisions of this act and with the regulations issued hereunder."

That concludes the quote.

Now I would say that is a requirement that would be enforcible in the courts by injunctive proceedings and otherwise. So I think there are very strong tools here.

Mr. KYROS. Thank you, Mr. Train.

Thank you Mr. Chairman.

Mr. LENNON. Mr. McCloskey.

Mr. McCLOSKEY. Thank you, Mr. Chairman.

Mr. Train, on line 10 of page 3 I note that the—

Mr. TRAIN. Let me just add one point here. I am reminded that under the bill before the Corps could in fact dump as a result of its own dredging activities, they would have to go to EPA to get a permit. So to that extent dredging is covered even more strongly than I have indicated by speaking of the certification process. The certification process primarily goes to the granting by the Corps of a permit to some private party or municipality to conduct dredging.

Mr. LENNON. Would the gentleman yield.

Do I infer from what you said under existing regulations now the Corps of Engineers does not not have to go to the Department of the Interior or the Secretary or the Regional Director of the Department of Interior to get a permit for dredging?

Mr. TRAIN. No, sir. Under the Fish and Wildlife Coordination Act they get comment.

Mr. LENNON. This is in addition?

Mr. TRAIN. Yes.

Mr. LENNON. I thank the gentleman for yielding.

Mr. McCLOSKEY. In the administration bill that the chairman has introduced, Mr. Train, on page 3 at line 10 I note that this bill applies to any person, including employees of the Federal Government, with the exception that Department employees are exempt from civil or criminal penalties. Can you tell me why a Federal employee should not be subject to penalty if he violates this law?

Mr. TRAIN. I don't know what the answer to that is, Mr. McCloskey, offhand.

Mr. McCLOSKEY. This may be a legal matter.

Mr. TRAIN. It definitely is, and I see it refers to section 6.

Mr. McCLOSKEY. That is the penalty section. They can violate this law but not be subject to criminal or civil prosecution.

Mr. TRAIN. That is correct.

Mr. McCLOSKEY. I wonder if perhaps at this point in the record we can afford the Council the opportunity to give us the legal explanation as to why they seek to exclude Federal employees from civil and criminal penalties.

Mr. LENNON. Does Mr. Train understand the question?

Mr. TRAIN. Yes, and I would be happy to provide an answer for the record.

Mr. LENNON. Would you provide a definitive answer because one of the things that concerns me is the image of the American Government particularly in relation to what its agencies and departments can do with immunity and what industry and municipalities across the country cannot do.

So definitively answer that question for the record.

(The answer follows:)

The exception from the penalty provisions is that contained in section 3(e), which provides that the penalty and enforcement provisions of section 6 do not apply to "any employee, agent, department, agency, or instrumentality of the Federal Government." The exception should be retained for Federal departments and agencies since it would be pointless for one Federal agency to sue another Federal agency to collect a penalty. The exception for Federal employees and agents could be removed by rephrasing section 3(e) as follows:

> "(e) 'Person' means any private person or entity, any employee, agent, department, agency, or instrumentality of any State or local unit of government, or foreign government, any employee or agent of the Federal Government, and except as to the provisions of section 6, any department, agency, or instrumentality of Federal Government."

Elimination of the exception for Federal employees and agents is not recommended, however, since the Federal Government has traditionally used internal disciplinary measures, as authorized and sanctioned by Congress and the courts, to secure compliance by Federal employees with Federal recommendations or requirements.

Mr. McCLOSKEY. Thank you, sir.

The second question goes to a policy question, Mr. Train. I note that in the bill the administration excludes sewer outfall structures under the provisions of this act, and to my understanding the administration is submitting separate legislation to apply to sewer outfalls going into the oceans and the estuaries. I wonder if you could comment briefly on the administrative structure which would result be-

cause, as I understand it, the quality of ocean waters will then be regulated in the following manner:

First, for industrial waste the 1899 Refuse Act applies to any industry water or pollution into the navigable waters of the United States.

Second, the sewage which is excluded from the operation of the Refuse Act is also excluded from this act. It will come under water quality. Third, the Environmental Protection Agency will regulate ocean dumping and fill.

Thus, out of the four areas, sewage is apparently the one excluded. It will be regulated by some other agency under some other standards. Could you tell us why?

Mr. TRAIN. Well, the Water Pollution Control Act which is administered by the Environmental Protection Agency.

Mr. MCCLOSKEY. Does the Environmental Protection Agency set water standards for the ocean adjacent to the United States?

Mr. TRAIN. Well, they are set under the Water Pollution Control Act; they are part of the standards adopted by the States and reviewed and approved by the Federal Government.

Mr. DINGELL. Would the gentleman yield?

But those are to extend only 3 miles.

Mr. TRAIN. That is correct, and the President has recommended that this be extended to the 12-mile limit.

Mr. DINGELL. You are exempting outfall structures so that today if somebody runs an outfall 3 miles and a quarter off shore he is exempt from water quality standards and he also would be exempt from the ocean-dumping prohibition. Now assuming that this committee and the Congress enacts this bill, you will find yourself in a situation where you will be able to control dumping but not from outfalls.

Now if the Congress does not enact legislation extending water quality standards out to 12 miles, then you will be in the situation where the addition of perhaps a quarter of a mile of pipe to existing outfall will completely exempt it from any Federal regulation whatsoever.

Mr. TRAIN. That is correct, Mr. Chairman. It is important that all of these legislative proposals be considered as part of an inter-related mechanism and that the Congress be aware that it is at the present time considering water quality legislation which would extend the water quality standards to the contiguous zone. Now as I indicated earlier, there is no legislation before the Congress which would extend beyond the contiguous zone, so to that extent at least you would have to rely upon this permit system under the ocean dumping authority if you wish to control that.

Mr. DINGELL. What gives you reason to believe that the States have the right to regulate from 3 to 12 miles insofar as water quality standards? It is an awful big area and it is a goodly distance beyond the shore.

Mr. TRAIN. I am not aware that this would be a State regulatory activity at all but I believe Federal standards——

Mr. DINGELL. If you extend water quality standards to 12 miles, you are making this a possibility.

62-513—71——13

Mr. TRAIN. No, sir; I believe these would be standards set by the Federal Government which would apply to the contiguous zone.

Mr. DINGELL. You are not going to go through the mechanism then that you have with water quality standards?

Mr. TRAIN. We have mechanisms under the existing law for the Federal Government to set standards in cases where States refuse to set standards.

Mr. DINGELL. I am going unduly into my colleague's time but the State sets standards, the Federal Government approves them and the State enforces them. Now are you going to use a different mechanism with regard to the distance between 3 and 4 miles offshore?

Mr. TRAIN. That is the legislation that has been before Congress since last year; yes. The States have no regulatory authority over the contiguous zone, it is entirely a matter of Federal regulation and it always has been.

Mr. DINGELL. Do you propose to set up specific Federal water quality standards of the contiguous zone between the 3 and 12 mile limit?

Mr. TRAIN. That is my understanding of the proposal.

Mr. DINGELL. As opposed to the State mechanism?

Mr. TRAIN. Yes, sir.

Mr. DINGELL. I see.

Mr. McCLOSKEY. I have a question that I think also justifies consideration here in view of your last answer, Mr. Train. In the San Francisco Bay area one of the proposals is the so-called Kaiser plan which would take sewage an undetermined number of miles out into the Pacific Ocean for disposal. I gather from your testimony under this Act that if dumping occurs 12 miles out, those dumpings would be immune to Federal regulation.

We have the danger here that, unless this legislation goes precisely to this question, the entire local government-State government operation, involving hundreds of millions of dollars, would not know what to expect from the Federal Government on the question of the right to dump sewage 12 miles out. It seems to me that the question of the long 15-mile sewer outfall ought to receive concern, for example.

The other question, though, is with our separate act.

Mr. LENNON. Would the gentleman yield?

Let the gentleman comment on that question.

Mr. TRAIN. I agree with you. I think the legislation should address itself to that problem.

Mr. LENNON. Thank you.

Mr. DINGELL. Would the gentleman just yield?

Would you want to give us some language that might be helpful to the committee in coming to some proper judgments as to how we could meet the approval of the legislation and carry out the matter that our good friend from California just suggested?

Mr. TRAIN. I think what I would suggest is that we will work with the Environmental Protection Agency on this together with your staff and see what we can suggest along those lones.

Mr. KYROS. Would the chairman yield?

Mr. LENNON. We are really cutting in on Mr. McCloskey's time.

Mr. KYROS. Don't sections 4 and 5 already cover that, Mr. Train? It says here you can issue permits for transportation, and then, under

prohibited acts, 4(b), no one can dump material in a zone contiguous to the 12-mile zone that would affect our territorial waters. So, if someone had a pipeline that transported the materials 12½ miles out, but you concluded that this would affect our territorial waters within 12 miles, you could take action?

Mr. TRAIN. No; that is outside the contiguous zone.

Mr. KYROS. It says in 4(b):

No person shall dump material * * * in a zone contiguous to the territorial sea of the United States, extending to a line 12 nautical miles seaward from the base line of the territorial sea * * * to the extent that it may affect the territorial sea or the territory of the United States.

So if that dumping affected water within 12 miles because of currency——

Mr. TRAIN. No, sir.

Mr. KYROS. No?

Mr. TRAIN. It is confusing, I will certainly agree with that. What this is saying is that from the 3-mile limit to the 12-mile limit of the contiguous zone any dumping that occurs within that zone is subject to the control of the act if it has an effect or if it may affect the territorial sea which is within the 3-mile limit or the territory of the United States itself.

Mr. KYROS. I see. But does the term "transportation" include within it the definition by pipeline as well as by ship?

Mr. TRAIN. I am not sure of the answer to that.

Let me say for the record this is Mr. Charles Lettow, an attorney with the staff of the Council.

This apparently falls in the definition of dumping rather than in any definition of transportation, so that if there is a gap here in the coverage of the act this is probably where the language would be operated on.

Mr. LENNON. Would the gentleman from California yield further to the gentleman from Maine?

Mr. MCCLOSKEY. Yes, sir.

Mr. KYROS. Thank you. I am all through, sir.

Mr. TRAIN. It excludes the disposition of an effluent from the outfall in the definition of dumping so that your normal pipe would not be covered by this act. Now, excuse me.

Mr. MCCLOSKEY. Thank you, Mr. Chairman. I have no further questions.

Mr. LENNON. The gentleman from Delaware.

Mr. DUPONT. Thank you, Mr. Chairman.

Mr. Train, I would like to raise some questions in what I think is an area that maybe we ought to make a direct about face from the previous Federal practice and that is in the question of permitting overlapping State jurisdiction. I have recently had some contacts with the Environmental Protection Agency in regard to the enforcement of the Air Quality Act and frankly it is an administrative disaster over there and I will take that up with those people when they come.

Looking for a minute at ocean dumping and the fact that you might permit States to set up standards of their own, it seems to me that there are four questions raised that suggest very strongly that we

BEST COPY AVAILABLE

ought to completely free up this area and get the States out of it. I would like your comments.

First of all, in an area such as the Delaware River where you have two States bounding either side of the river you have the problem of allowing a dumper to choose his jurisdiction. New Jersey sets up one standard and Delaware another one and they are different. The dumping company or the dumping concern could choose between the two of those if both of them had standards that were more stringent than the Federal.

Second, as soon as you permit a State to set up its own law that is more stringent than the Federal law and preempt the Federal law thereby, then you throw out the window all the Federal enforcement procedures. If you take away the Federal law, you take away Federal enforcement, you take away the Federal court system, any citizen suits that you permit, anything of that kind.

Mr. LENNON. Would the gentleman let Mr. Train comment on both of these various good questions?

Mr. DUPONT. All right. I have two more.

Mr. LENNON. He may not get a chance to answer any of them.

Mr. DUPONT. Perhaps we could start with those two and get your comment generally.

Mr. TRAIN. Let me be sure I understand the question. Your concern with the case within the 3 mile limit is where a State would have more stringent standards for disposal than would Federal Government and what that would do to the system. Now as I understand the act, Mr. DuPont, all dumping will require a permit from EPA, all of it in every case. Under the Water Quality Improvement Act of last year, Section 21(b) wherever the Federal Government grants a license for an activity which by a discharge could affect State and water quality standards it must get a certification from the State of the effect of the discharge in light of the standards and that I think is what would happen in this case. The State would say, "We have stronger water quality standards with respect to this 3 mile area and we would not approve a dumping tested only by the Federal standards, and this is what you would have to do for us to go along with it.

Mr. DUPONT. So the State would have the final authority.

Mr. TRAIN. No, the Federal Government would give the permit, as I understand it, but only if it met the State water quality standards.

Mr. DUPONT. In other words——

Mr. TRAIN. You better permit me to send you an answer for the record on that.

Mr. DUPONT. I would appreciate it.

Mr. TRAIN. I am really not familiar enough with the interrelationship of these more than to just muddy the record for you.

Mr. LENNON. Mr. Train, I think the two questions, you have two more yet to go, are very vital to the ultimate decision this committee would have to make in the point of time that it has to report out the bill. You can see, a permit issued by the State of South Carolina within the 3 mile limit but who is going to hear that? You said you cannot just move the length of the ship off the coast of North Carolina and dump or off the coast of Georgia and dump, move out of Port Charleston, Wilmington, or Morehead, N.C. I am just illustrating those because I do know them. Do go into that in depth.

BEST COPY AVAILABLE

(The answer follows):

The interelationship of Federal and State controls over ocean dumping involves consideration of both water quality standards and ocean dumping criteria. H.R. 4723 saves from preemption State rules of either type.

Moreover, state-adopted water quality standards would be a consideration in the evaluation of a Federal ocean dumping permit application, if the application were for a dumping to take place within the three mile territorial sea. Section 21(b)(1) of the Federal Water Pollution Control Act provides in part that: "Any applicant for a Federal license or permit to conduct any activity . . . which may result in any discharge into the navigable waters of the United States, shall provide the licensing or permitting agency a certification from the State in which the discharge originates . . . that there is reasonable assurance . . . that such activity will be conducted in a manner which will not violate applicable water quality standards. . . .No license or permit shall be granted until the certification required by this section has been obtained or has been waived . . ." Section 21(b)(2) goes on to provide for a means of dealing with the situation where a discharge would affect more than one State's waters.

Mr. duPont. The third question and fourth question are kind of wrapped together on the same subject. If you are going to have EPA issuing permits on the basis of State law, then I think you have a very difficult jurisdictional question and that is whether a Federal agency has the power to interpret and enforce State law. I think you will find those who want to dump will take advantage of this and bring innumerable suits involving due process and a lot of things to set the whole thing.

Perhaps if you are going to make some comments in this area you would let us have the benefit of your thoughts on taking the States out of this field entirely. We are generally dealing with non-State bodies of water, we are dealing with really interstate and international water almost exclusively. If you would give us your comments on eliminating the States completely, and let the Federal Government preempt entirely.

Mr. Train. Be happy to do that.

Mr. Du Pont. Thank you.

(The answer follows:)

States could be involved in controlling ocean dumping only where they have jurisdiction, *i.e.*, ordinarily in their territorial sea, which normally extends three miles from shore. Since States would necessarily become involved through establishing their own permit system alongside a Federal permit system, and since both permits would be required, the State standards would necessarily have to be stricter than the Federal criteria to be meaningful. In consideration of a State's close interest in the waters near its shore, and in light of the fact that ordinarily is exercise of jurisdiction is restricted to these ocean waters, we did not believe that preemption of State regulatory efforts was necessary.

Mr. duPont. No further questions, Mr. Chairman.

Mr. Lennon. The gentleman from Michigan, Mr. Ruppe.

Mr. Ruppe. Thank you very much.

Is there anything in the existing legislation, Mr. Train, or in this legislation here before us that would actually reduce the pollution in our territorial seas to the extent that perhaps in 5 years or 10 years the dredge material will be removed therefrom? This is a major problem in the Great Lakes and I am sure in the Atlantic Ocean. Is there a chance then of alleviating the basic pollution problem in this dredge material?

Mr. Train. I think that the impact of the dumping of these materials will lessen, as I pointed out in response to an earlier question. It

BEST COPY AVAILABLE

is the policy of the port at the present time to take the impact of disposal into account in making a decision as to whether to dredge in the first place, and I understand this is reducing the amount of dredging and dumping situations in the case of polluted spoils.

As I also pointed out, the increasing impact of water quality standards over the years will certainly reduce the pollution of bottom materials in harbors and river bottoms.

Mr. RUPPE. You cannot do much to eliminate the pollution that is already there but you feel it can immeasurably reduce the pollution that would take place in the future.

Mr. TRAIN. Yes; over a long period of time and then also the impact of the dumping of these spoils can be minimized by careful selection of site, shifting of sites, and so forth.

Mr. RUPPE. Do you know if the program in the Great Lakes of not dumping back any polluted dredge spoils has actually taken effect yet? Is there anything to demonstrate concrete results?

Mr. TRAIN. I believe this is underway. It is something to be done over a period of years. It is not a complete answer at the present time.

Mr. RUPPE. Have you had anything to do in your agency with the dumping of mine tailings or taconite sands in Lake Superior?

Mr. TRAIN. I am generally familiar with the problem. No; we have not had any specific activity.

Mr. RUPPE. Would they fall into one of the classifications you consider should be eliminated or terminated under the identifications you have listed in this testimony?

Mr. TRAIN. Well, I think it quite clear that they would be either under this dumping control or they would be under the permit system set up under the Refuse Act which the President ordered in December.

Mr. RUPPE. You say they would be effective?

Mr. TRAIN. Yes. They certainly would be controlled one way or the other.

Mr. RUPPE. But the determination would have to be made, either controlled or eliminated. This bill would not specifically eliminate them as such, is that correct?

Mr. TRAIN. That is correct. This bill provides the authority for regulating.

Mr. RUPPE. Thank you.

Thank you, Mr. Chairman.

Mr. LENNON. Thank you.

The subcommittees will stand in recess until 2:15.

Whereupon, at 12:45 p.m., the subcommittee recessed, to reconvene at 2:15 p.m.)

AFTERNOON SESSION

Mr. DINGELL. The subcommittee will come to order.

This is a continuation hearing on ocean dumping begun this morning.

At the time the subcommittee adjourned we were hearing from our good friend, Russell E. Train, Chairman of the Council on Environmental Quality.

The Chair will recognize for purpose of questioning our counsel, Mr. Everett, for any questions he wishes to ask.

Mr. EVERETT. Thank you, Mr. Chairman.

[illegible]

Mr. TRAIN. Before Mr. Everett begins, Mr. Chairman, there is one point we were talking about this morning where I think I may have misspoken, and that is with respect to the sewer outfalls that extend or coextend beyond the limits of the contiguous zone.

It is my understanding that the present version of the administration's proposals now before the Congress would extend water quality standards to outfalls that reached the high seas, and that is beyond the contiguous zone, if they originate in the United States. So, I was incorrect to the extent I suggested that neither under the pending legislation in the water quality field nor under this legislation as presently written would the Federal Government have a hand.

That was not correct.

Mr. DINGELL. Thank you.

Mr. EVERETT. Mr. Train, in connection with that same title, on page 3 of the bill, section (f), goes on to read, and says:

And provided further, That it does not mean the intentional placement of any device in the oceans, coastal, or other waters or on the submerged land beneath such waters, for the purpose of using such device there to produce an effect attributable to other than its mere physicial presence.

I was wondering if you could elaborate on that particular provision of the bill, as to what type of device we are talking about.

Is that part of the national defense feature, or just what?

Mr. TRAIN. I am sure it covers all of those things. It would cover oil well drilling and production platforms, military installations, I presume, of various kinds, and oceanographic surveillance, and monitoring systems.

None of these things would normally be thought of as examples of dumping, but for purposes of certainty, the bill seeks to insure that the definition of dumping does not include them.

Mr. EVERETT. But some of these devices could result in a discharge of an effluent that could be constituted pollution, could it not?

Mr. TRAIN. Yes.

Mr. EVERETT. But based on the way this section is written, it would exclude those from being covered under the act, things like pipelines, drilling structures, things of that sort?

Mr. TRAIN. Drilling structures, and I think pipelines also are covered by regulations of the Department of the Interior, which include very stringent environmental protection elements.

Pipelines are to some extent, I believe, regulated by the Department of Transportation, and also, I believe, by the Coast Guard.

Mr. EVERETT. How about nuclear reactors?

I understand there is a program on the way to investigate the desirability of placing nuclear reactors in these offshore waters.

They would also be excluded?

Mr. TRAIN. I do not think we are saying quite the same thing when we say they are excluded.

We are excluding the placing of the structure from the definition of dumping.

This does not go to discharges, and things of that sort, from the structure itself.

Mr. DUPONT. Mr. Train, are you saying if an oil structure, or nuclear powerplant, or something is placed a mile offshore, will or will not this bill cover the effluent from that structure?

Mr. TRAIN. It would not cover any oil discharges, as that is specifically excluded by the bill, as it is governed by other legislation.

Mr. duPONT. If you had people living on it, how about simply sewage from the structure?

Mr. DINGELL. I believe that is excluded, too, am I correct, Mr. Train?

Mr. TRAIN. No, I am not positive. I am not sure whether it is covered. I would think by this legislation, or o her water quality standards, and the Refuse Act permit authority, but I cannot answer your question specifically.

Mr. DINGELL. Would you get us an answer to that?

That is a very interesting point.

Mr. duPONT. If the chairman would yield, also, and perhaps a little more relevant is the question of heated water discharge from the nuclear powerplant in the Delaware River, and I don't know if this bill covers that, or if it is covered under the previous Water Quality Act.

Mr. TRAIN. Well, that is being dealt with under the permit authority, under the Refuse Act.

This has been construed to include thermal discharges now by the administration, so that I would be pretty sure would not be covered by the dumping legislation.

Mr. DINGELL. Mr. Train, counsel has observed to me the relevant section of the bill to which we are addressing ourselves appears to be on page 3, at the top of the page, and the question I think concerns outfall, and since we are not talking about a vessel, does this fall under the meaning of dumping, or just what does this matter of the runoff and effluent fall under?

Mr. TRAIN. I think it could fall under either one, depending on how the different acts are administered, and I think this could probably be clarified as to which of the intentions it is.

I do not know the specific answer to the question.

Mr. duPONT. Let me focus a minute on procedural questions from another point of view.

How do you envision the permit issuing process as working?

An application is made from some firm. Is there a hearing on each permit, or once the EPA has generally classified areas, can they issue permits with no hearing?

Mr. TRAIN. EPA clearly has the authority to hold hearings, and they doubtless will have hearings in a number of cases.

There is no requirement in the statute that they hold a hearing in every single case of an application for a permit under this authority, and I would not think that Congress would require a permit and hearing in every such case.

This is true, for example, with the Corps of Engineers dredging and filling permit authority under the Rivers and Harbors Act.

They do have hearings in many cases, but they decide this of their own discretion, such as in cases of great public interest, new questions, or questions of importance and so forth.

They issue some 6,000 such permits a year, and it would be an administrative impracticality to have hearings in each case.

Mr. duPONT. As the environment groups get more and more strength, I wonder that each time a permit is sought, that some group, either

continuing or ad hoc, will not spring up and fight the dumping and request a hearing.

I wonder if we ought to have anything in our legislation to deal with a situation of that type.

Mr. TRAIN. I think it would be perfectly appropriate to require public notice of an application for a permit.

This has been done, for example, under the Refuse Act permit program.

The final regulations being issued by the Army, I think later this week, on Wednesday, as a matter of fact, will spell out this in great detail.

I happen to have a copy of the Refuse Act regulations with me. The requirement of publication extends to all of the information and data pertaining to the application, and I would imagine that administratively this will be required in the case of the dumping permits.

Mr. DUPONT. I would think that would be a sensible approach, and then if you had to have a public notice, you could have some further mechanism.

Mr. TRAIN. And, of course, there is as in all such cases, opportunity for judicial review.

Mr. DUPONT. All right.

Thank you. I have no further questions.

Mr. EVERETT. I have some further questions.

Mr. DINGELL. I am just curious about this.

In regard to language in a piece of legislation of this type such as "After notice and opportunity for hearing," would you have any objection to that kind of language with regard to proceeding with the issuance of a dumping permit?

Mr. TRAIN. I do not know that there is any problem in principle, Mr. Dingell.

There is no such statutory language pertaining to the dredge and fill permit authority.

Having said that, I would like to have the opportunity to expand on that for the record.

Mr. DINGELL. We are running into a constitutional problem that you deny the applicant a hearing.

Anybody could have a constitutional question, and I suspect probably the Administrative Procedures Act requires it anyway, so it occurs to me there probably would not be too much objection.

Mr. TRAIN. No. It may be in some cases superfluous, and in some cases may raise a question because of rights under other legislation or the constitution.

Mr. DINGELL. Let me ask this question.

There is no intent anywhere in the legislation to repeal or modify the National Environmental Policy Act in section 102(2)(C) thereof, is there?

Mr. TRAIN. No, sir.

Mr. DINGELL. As I understand, 102(2)(C) requires the issuance at this time, am I correct?

Mr. TRAIN. Not in the way the National Environmental Policy Act has been administered to date. I think, as you know, we have construed the legislative history of the act as expressing an intent to exclude

environmental regulatory activities from the environmental impact statement requirement.

Mr. DINGELL. It never has referred to the Corps of Engineers' permits.

The Corps of Engineers has always required the 102(2)(C) statements?

Mr. TRAIN. Yes, sir; but we do not consider their functions as one of the environmental regulatory activities.

Mr. DINGELL. You have a very clear question on this point, wherein the agreement between the corps and the Department of Interior was sanctioned by the courts, stating therein that the corps could disapprove of permits of this kind on environmental grounds.

This is permit to deposit material in the waters, and I am curious to understand just what the legislative history is.

My personal interpretation is an absence of expression would mean 102(2)(C) would apply.

I would like to hear your testimony. We have to have the matter very clearly nailed down here.

Mr. TRAIN. Well, obviously it could be administered either way, and I see no objection as a matter of principle to including an environmental impact statement requirement.

Certainly the criteria announced by EPA will be subject to very careful review, and it will be a matter of review by our Counsel under both the enabling legislation of the Environmental Policy Act and——

Mr. DINGELL. Let me go to another question.

Mr. TRAIN. I am trying to think what the policy considerations would be which would suggest that a dumping permit either should or should not be the subject of the environmental impact statement.

Mr. DINGELL. Let me ask this question, if I may.

Who carries the burden of proof on whether dumping will unreasonably degrade or endanger human health?

Mr. TRAIN. Well, mainly it is the applicant.

Mr. DINGELL. The applicant bears that burden?

Mr. TRAIN. Yes.

Mr. DINGELL. Now, tell me, must there be specific findings, must the Administrator make specific findings in each case where the permit is issued subject to public review?

This relates to the question that I raised before.

Should he file the 102(2)(C) statement under the Environmental Policy Statement Act?

Mr. TRAIN. It is my understanding the Administrator would make such findings.

Mr. DINGELL. And would the materials submitted by the applicant for permit be open to public inspection?

Mr. TRAIN. As I indicated in response to Mr. DuPont's question, following the same procedures which have been or are being announced for the permit program under the Refuse Act, yes, but this is not set forth in the legislation, and I am just assuming this would be the practice followed by the Administrator.

Of course, the Freedom of Information Act has some generally applicable requirements that would extend to this entire procedure.

Mr. DINGELL. I am sure you are familiar with the Fish and Wildlife Coordination Act which requires consultation with sister agencies,

particularly with the Interior Department and with other similar conservation oriented organizations.

I note a reading of the Administration's bill permits the Administrator of the EPA to consult, but does not require him to do so.

Is it the intention of the Administration by this particular language to change or to amend the Fish and Wildlife Coordination Act?

Mr. TRAIN. No, it is not my opinion that it does.

Mr. DINGELL. The question would be does this amend the Fish and Wildlife Coordination Act?

Mr. TRAIN. Mr. Chairman, rather than give an off-the-cuff answer, I would like to look at the exact language of the Fish and Wildlife Coordination Act in this language and render you a legal opinion on that.

Mr. DINGELL. Very well. It would be helpful to me if you did, and I also would like you to indicate to us what is your intention, if it is to alter the Fish and Wildlife Coordination Act as it applied traditionally to the Corps of Engineers in its dumping permits.

As a matter of fact, they are specifically included in that statute, and in their dredging and fill permits, and I would be curious to know why a rather similar permit issued by the Environmental Protection Agency would not be under the Fish and Wildlife Coordination Act, so in your response, would you indicate to me any difference in treatment, if you propose to have a difference in treatment.

Mr. TRAIN. I would be glad to do that.

(The information follows:)

The Fish and Wildlife Coordination Act does not contain a statement defining the territorial extent of its requirements, including the requirement of consultation found in Section 2 (16 U.S.C. § 662). Normally, however, a statute of the United States is presumed to be applicable only within the territorial jurisdiction of the United States, *i.e.*, includling, but not beyond, the waters of the territorial sea. *American Banana Co. v. United Fruit Co.*, 213 U.S. 347 (1909) (Holmes, J.). The special authorization for consultation contained in Subsection 5(a) of H.R. 4723 is designed to allow the Administrator to accomplish consultations such as those described in the Fish and Wildlife Coordination Act, even if the terms of that Act might not otherwise apply because the dumping itself is to take place within the contiguous zone or on the high seas. The fact that the material is transported from a U.S. port may not be a factor bringing the Fish and Wildlife Coordination Act's provisions into play. In any event, there is no intention to amend or limit in any way the terms of the Fish and Wildlife Coordination Act.

Mr. DINGELL. Mr. Train, now, this does concern the Chair, because there is no requirement, as I read the bill, that the other agencies provide comments. For example, the Department of Interior would not be compelled to give information as to dumping permits, or give comments, and it occurs to me philosophically, there should be direction that there be consultation to protect the fish and wildlife values. This would tend to indicate to me there might be some reason to be very apprehensive of a bill of this kind, until and unless there is a requirement for clear consultation and the views from relating agencies to be put in writing.

This is in direct confrontation to the Fish and Wildlife Coordination Act.

Would you like to comment on that also, sir?

Mr. TRAIN. There is certainly ample authority in the legislation to permit consultation, and I would certainly suggest strongly that the Administrator consult widely in the granting of these permits.

Mr. DINGELL. As I read it, Mr. Train, there is no requirement to do so.

It says he may. There is a very wide difference between may and shall, as I am sure you would agree.

It only says he may consult. It does not say he should receive the comments of the sister agencies. I am sure you can understand there is a very distinct difference there.

Mr. TRAIN. There is a very definite difference. I think it is a question as to what extent such a formal requirement of written request and written comment in each of what may be many thousands of applications is really serving the purpose of environmental protection; such a thing could hold down the administrative machinery in paper work.

Mr. DINGELL. I am glad you mentioned that, because as you will notice, the statute that the administration has presented us with, in its criteria, that the Administrator of the Environmental Protection Agency must consider, no mention is made to fish and wildlife values, or protection of fish and wildlife from hazards, and, of course, you can understand the importance of the living resources, and I am curious to know why that should be omitted if EPA is not being required to consult with other related agencies.

Mr. TRAIN. The language of the bill very clearly covers fish and wildlife through its reference to marine environmental and ecological systems.

This covers fish, wildlife, and all other elements of the ecosystem.

Mr. DINGELL. But I am referring now to the latter section dealing with the specific matters he is considering.

For example, on page 5, line 9, he is considering "the likely impact of the proposed dumping on human health, welfare, and amenities, and on the marine environment, ecological systems, and economic potentialities, including an assessment of—(*a*) the possible persistence or permanence of the effects of the proposed dumping; (*b*) the volume and concentration of materials involved; and (*c*) the location proposed for the dumping."

You are saying ecological system, ecological potentialities including fish and wildlife values?

Mr. TRAIN. Marine environment and ecological systems certainly includes fish and wildlife.

Mr. DINGELL. Now, would you please, Mr. Train, comment on this particular point.

The report of the Council on Environmental Quality recommended to the President that ocean dumping be immediately halted on chemical biological warfare materials, such as high level radioactive wastes and toxic industrial wastes.

While the Administrator would have the power to do this he would not. Can you tell us why the difference in the drafting of the bill and the recommendation of your Agency?

Mr. TRAIN. First, the recommendation as to chemical warfare and biological warfare weapons has already been implemented by the Department of Defense by order of the Secretary, so legislation is not necessary in that case.

Mr. DINGELL. Of course, the administrative regulation can be changed by administrative move.

BEST COPY AVAILABLE

Mr. TRAIN. I believe as I pointed out in my statement, that before you ban something, you had better be sure that there is no better way, or that there is a better way of disposal available.

Mr. DINGELL. I am simply taking your recommendations, and contrasting with the bill, and I am asking you for your comment.

I would assume the Agency was dead serious when it made that recommendation.

Mr. TRAIN. You will also find in our report, Mr. Chairman, a full recognition of the need for the development of an alternative disposal site and methods; this runs through the entire report.

It is in our view unrealistic just to ban things, when there is no suggestion as to any alternative method of disposition.

Mr. DINGELL. You are bringing up another matter which I think is important.

Your report indicated the necessity for substantial research in the area of ocean dumping.

The bill provides neither authority nor funding for such research.

Can you comment to us as to the reason for that, or whether or not some consideration by this committee should be given to the establishment and funding of a research program in this area?

Mr. TRAIN. There is, as I pointed out in my statement, we believe ample authority at the present time for the various agencies interested in the marine environment for research, and that has a bearing on this legislation.

That is true for the Environmental Protection Agency. It is true for the Coast Guard. It is true for the National Oceanic and Atmospheric Administration.

Mr. DINGELL. This question I am certain will be asked of this committee when the matter comes to the floor.

Can you outline to us, not at this particular time, but at your convenience, when you have more opportunity for an understanding, precisely what kind of research is going on, and the kind of research that the administration contemplates will be needed to receive the answers that are necessary in the field of ocean dumping.

I would like this so we can understand precisely what you have in mind with regard to the research program.

I am sure our colleagues will ask this question when the matter comes before us on the House floor.

Mr. TRAIN. I can certainly try to break down the prevalent research programs as they exist, and as they have been proposed by the President in his 1972 budget.

It is just a bit difficult to speculate what will be proposed subsequent to that.

I would note that it is not easy to break down the parts of the research programs, as, for example, in NOAA, to define exactly those portions which you would say ocean dumping related research, or simply marine environment in some other, but related matter.

You could say that practically all of the marine biological research being done by the US. Government in any agency is of importance, and real significant to this legislation.

Mr. DINGELL. Well, I am curious to find out the limit of it.

I have the feeling we are always told the research is related to something, but on more careful study, oftentimes one finds this is really not so.

The bill refers to emergencies where permits will not be required.

What would these emergencies constitute, and what would be the handling of emergency procedures?

Mr. TRAIN. It has been suggested to me that this is dealing with, in part, the case where a vessel may be in distress on the seas, and dumps its cargo, or some part of it as a matter of saving lives, or something of that sort.

Mr. DINGELL. Is that the only emergency to which that section alludes?

Mr. TRAIN. It is the only one I have any knowledge of myself.

I will be glad to explore this further.

Mr. DINGELL. I think the committee would be grateful for your help.

I have a distinct feeling that if the emergency is alluded to, the bill should be more precise.

Do you contemplate any emergency disposal matter from land sources, like carrying radioactive materials, rockets, nerve gas, or something for dumping on an emergency basis?

Mr. TRAIN. Certainly nothing of that sort is contemplated.

I suppose it is perfectly possible, if for some reason something will blow up inside of a half hour, you will try to get rid of it.

I do not see anything of that sort here, and it is not the reason for this provision as far as I know.

Mr. DINGELL. Would it not be possible in some kind of land origination to ocean disposal, that if that was necessary, you could authorize the Administrator of EPA to set up some kind of emergency procedures to handle this kind of thing on a quick basis, rather than say they could proceed without a permit?

Would you want to give the committee your views on that, Mr. Train?

Mr. TRAIN. Well, I do not think I can really add to what I have already said, Mr. Chairman, without a little more information as to just the kind of situation that this covers.

It is in fact a vessel in distress sort of thing, I do not think there are any procedures that could be set up that would not be too slow.

Mr. DINGELL. Now, the Chair would like to consider very carefully the relationship of permits called for under the legislation to other permits authorized under present laws.

You have, first of all, the permit under the Refuse Act.

That would not be impaired; am I correct?

Mr. TRAIN. That is correct.

Mr. DINGELL. You have the 1888 act, am I correct, which dealt with the harbor in Philadelphia, and so forth, that would not be impaired?

Mr. TRAIN. This would be superseded by this legislation.

Mr. DINGELL. All right.

You have the requirement of the Fish and Wildlife Coordination Act.

Mr. TRAIN. No.

Mr. DINGELL. Then I am rather curious, we seem to be getting ourselves in a situation where there is a potentiality for two permits, possibly three permits in these matters.

Is that really desirable?

Mr. TRAIN. Well, I do not suppose it is desirable, although there are many cases wherein both State and Federal permits are required, but insofar as multiple Federal permits, that certainly is not desirable.

Mr. DINGELL. Of course, it is clear the Refuse Act permit system now under the corps is beginning to work rather well; is that correct?

Mr. TRAIN. It is a little early to say. It has just gotten underway, and the regulation will be issued this week, but I am hopeful it will be working well.

Mr. DINGELL. The indications are that it will be working well, and it includes requirements of the Fish and Wildlife Coordination Act, and an agreement between the Interior and the corps.

Would the legislation before us be subject to the same limitation, that is, the requirement of the Fish and Wildlife Coordination Act, and also the requirement of the agreement between Interior and the corps, with regard to fish and wildlife values and dumping?

Mr. TRAIN. As I said, this legislation requires consideration of fish and wildlife values.

It requires this consideration not only within the territorial limits with regard to the Fish and Wildlife Coordination Act, but also to the high seas, where it does not extend.

Mr. DINGELL. Now, on page 6, lines 6 to 9—and I would hope that you would refer to those sections, Mr. Train—the statute refers to the following language.

It says, and I quote:

In reviewing applications for permits, the Administrator shall make such provision for consultation with interested Federal and State agencies as he deems useful or necessary.

I know we have gone over this ground a couple of times, but would you tell us again, does this amend the Fish and Wildlife Coordination Act in any fashion?

Mr. TRAIN. Not to my knowledge.

Mr. DINGELL. Now, this does trouble me greatly.

There are no provisions in the bill for public participation, or in consideration of permit applications, such as notice in public hearings.

Can you tell us why that requirement was excluded from the bill?

Mr. TRAIN. I suppose we thought it was not necessary. I have no doubt that permit applications and all of the data that is part of the application will be made available to the public as a matter of course under the regulations of the Administrator.

That is done under the permit authority.

Mr. DINGELL. Let's suppose the Administrator of EPA was determined to set up a dumping ground right in the midst of the best oyster beds off the U.S. coast.

Under this bill, there is no requirement in the legislation before we hold hearings.

What would a citizen do if he were a shell fisherman, or something of that kind, to protest if he felt under the particular circumstances that the bill provides no relief to him?

Mr. TRAIN. I presume one thing he might do, if he is well advised, to go into court to get an injunction for violation of the National Environment Act.

Mr. DINGELL. With regard to 102(2) (C) statement?

Mr. TRAIN. Not in violation of the 102(2) (C) statement, but there is the general violation of the congressional policy that environmental conditions and circumstances must be taken into account in making decisions.

Mr. DINGELL. I am delighted to hear you say that.

I want you to know that. I think that would be excellent, but I have no recollection of the courts ever extending the breath and width of the National Environmental Policy Act so wide as that.

Now, they have enjoined governmental actions where there was a failure to care for the requirement of 102(2) (C), but never do I recall them enjoining actions of policy statements.

If there is some new change in the law, it would help the committee to know.

Mr. TRAIN. Well, I am not changing the law, Mr. Dingell.

Mr. DINGELL. I am not accusing you of that.

I know of no changes in the law which dealt with this particular point.

Well, you suggested, I gather, if the Administrator of EPA violated his proper discretion, and issued a dumping permit for an area where it is clearly harmful, in your opinion, under this act, and under the National Environmental Policy Act, a citizen, or a citizens' organization, would have a pretty good chance of going into court and enjoining such an act.

I would like as wide as possible an interpretation of a statute in this direction.

I enthusiastically support your interpretation, but even I in my enthusiasm have never interpreted it quite so broadly as the point you alluded to.

I do not think the courts have joined either one of us on this point.

How would a citizen get a hearing under the statute before us?

He might have a redress under some other statute, but I see no redress under the statute you have here.

Mr. TRAIN. All I can say relates to the way it works under the dredge and fill permit authority, for example, of the Corps of Engineers. Under the Rivers and Harbors Act, there is no statutory provision for public hearing.

The corps provides for public hearing at its own discretion.

Whenever there is any interest in public hearings, the corps has them.

While there has been a lot of interest with some of the final permit actions on the part of the public, very little dissatisfaction has come about with the hearing procedure, to my knowledge.

So far as I know, we expect this to also be true under the administration of the permit program under the Refuse Act, and as I indicated there is nothing in the Refuse Act which requires public hearing in connection with those permits.

However, the Department of the Army's regulations are being issued this week, and they do provide for public notice, and I believe

hearing in cases where this is held to be desirable, and I would expect this will work equally efficiently.

Mr. DINGELL. This being so, then there would be no strong objection to require notice of hearing in connection with the bill before us?

Mr. TRAIN. This could well be. You certainly do not want to require a hearing as a matter of course in all of these permit applications, I would think.

Mr. DINGELL. Now, you are going to have some cases where you are going to have dual permit authority.

Who will issue the permit for ocean dumping, will it be done by both agencies, or will it be done through some joint permit issuing process?

Mr. TRAIN. The issuing authority is the Environmental Protection Agency.

Mr. DINGELL. You will find certain instances where ocean dumping will be covered by the permit requirements of the statute, that are proposed before us today, and also through the 1899 act.

Mr. TRAIN. I think I may have misspoken before. This supersedes the Refuse Act.

Mr. DINGELL. It does supersede the Refuse Act in total?

Mr. TRAIN. In connection with dumping covered by this act.

Mr. DINGELL. Well, thank you very much, Mr. Train.

Mr. Everett.

Mr. EVERETT. Thank you, Mr. Chairman.

In connection with the question you just answered, you did state a moment ago this act did not supersede the 1899 Refuse Act, and I note now you say that it does.

Mr. TRAIN. Yes. You will find that on page 14.

Mr. EVERETT. Yes, sir.

Mr. TRAIN. Subparagraph (e).

Mr. EVERETT. It is not clear to me what the net effect would be with respect to those portions of the 1899 act that are superseded, and I was wondering if you could summarize just what the net effect of subsection (e) is on section 13 of the Rivers and Harbors Act of 1899.

Mr. TRAIN. Frankly, Mr. Chairman, it sounds like I am ducking the question. I do not mean to. This is a highly technical complicated area of the interrelations of three or four different statutes.

One program is really not even in effect yet. One is the legislation before you, much of the application of which is going to turn upon the kinds of regulations which will be issued by the Administrator.

I would be happy to address myself in writing to the committee as best I can, in talking with other agencies on these questions, but I think I probably will be doing a disservice to this committee if I should try to speak off the cuff to that particular question.

I will be most happy to answer that in writing.

Mr. DINGELL. In perfect fairness to you, it is a highly technical question.

I will direct this question to be answered in writing.

(Answer follows:)

Section 7 of H.R. 4723 deals with the relationship of this legislation to other laws. Generally, except as provided in subsections 7(b) and 7(c), it provides that after the Act's effective date, existing licenses, permits, or authorization would be terminated to the extent they authorize activity covered by this proposal, and

62-513—71——14

that further licenses, permits, or authorizations of a similar nature could not be issued.

Subsection 7(b) maintains present responsibility and authority contained in the Atomic Energy Act of 1954, and provides that the provisions of Sections 4 and 7(a) of the bill do not apply to actions taken under that Act. However, the AEC must consult with the Administrator before issuing a permit to conduct any activity otherwise regulated by this proposal. Moreover, the AEC must comply with the radioactive-material standards set by the Administrator, and the Administrator is directed to consider the policy expressed in subsection 2(b) of this proposal along with the factors stated in subsections 5(a)(1) and 5(a)(2) in setting such standards for the waters covered by this proposal.

Subsection 7(c) relates to authorities contained in the Rivers and Harbors Act of 1899, respecting dredging, filling, harbor works, and maintenance of navigability. The powers are exercised for the most part by the Secretary of the Army and the Chief of Engineers. Except for the limited supersession found in subsection 11(e), the Rivers and Harbors Act authorities are not negated or abrogated, nor are existing licenses or permits issued under the Act terminated. Rather, in situations where this bill and the Act of 1899 both would apply to dumping of material in connection with a dredge, fill or other permit issued by the Corps of Engineers, issuance of the permit requires a certification by the Administrator of EPA that the activity is in conformity with this proposal and any regulations issued under it. The Administrator will not issue separate permits in such cases.

Subsection 11(e)'s limited supersession of the Rivers and Harbors Act pertains only to Section 13 (the "Refuse Act"). Nonetheless, after this Act becomes effective, the Department of the Army's permit program under the Refuse Act, which is administered in close cooperation with EPA on all water quality matters, will continue to regulate the disposition of any effluent covered by the Refuse Act from any outfall structure regardless of the waters into which this disposition occurs. In addition, the Refuse Act will continue to apply to all depositing of material into those navigable waters of the United States or their tributaries which are not covered by subsection 4(b) of this Act.

The objective of the limited supersession is to remove a double permit requirement in the area of overlap between H.R. 4723 and the Refuse Act. To achieve this objective, subsection 11(e) supersedes the Refuse Act only insofar as it applies to dumping as defined in subsection 3(f), of material in the waters covered by subsection 4(b). One further consideration deserves mention. Simple supersession of part of the Refuse Act's coverage would leave an accompanying gap in protection of navigation. Accordingly, subsection 7(d) provides for consultation by the Administrator of EPA with the Secretary of the Army in cases where the Administrator finds that proposed activity regulated by the ocean dumping system may affect navigation or create an artificial island on the Outer Continental Shelf.

Besides the provision relating to Refuse Act, Section 11 contains a number of other repeals or supersessions. Subsections 11(a) and 11(b) repeal the Supervisory Harbors Act of 1888, as amended (33 U.S.C. §§ 441–451b), and the provision of the Rivers and Harbors Act of 1899 (33 U.S.C. § 418) which preserved the Supervisory Harbors Act from supersession by the 1899 Act. The Supervisory Harbors Act provides a special authority to control transit in and from the harbors of New York, Baltimore, and Hampton Roads, Virginia. This authority has been used to regulate ocean dumping. The proposed Act would replace that authority. Subsection (e) repeals Section 2 of the Act of August 5, 1886 (33 U.S.C. § 407a), which pertains to deposits of debris from mines and stamp works. These deposits are covered by this bill or the Refuse Act. Lastly Section 4 of the Rivers and Harbors Act of 1905 (33 U.S.C. § 419), which has been used to buttress the Corps of Engineers' authority to regulate ocean dumping, is superseded, insofar as it authorizes action that would be regulated by this proposal.

Section 4 of the 1905 Act has been used to set aside areas for oyster cultivation. If the oysters and other material placed in the oyster beds were "devices", they would be exempt from the coverage of this bill under the second proviso to subsection 3(f). A "device", however, ordinarily connotes a mechanism or a piece of equipment, and accordingly oysters and thus oyster beds are not exempted from coverage. As a further result, section 4 of the 1905 Act would be superseded in the area.

As an alternative to the foregoing approach, the Committee could delete the supersession of section 4 of the 1905 Act. The Corps could then continue to use

this section to designate areas for oyster cultivation. While H.R. 4723 would also apply, the Administrator could use the authority of subsection 5(e) to issue general permits for "dumping" oysters and supporting material in the areas so designated.

Mr. EVERETT. Mr. Train, if you would provide for the record, particularly at the bottom of page 13, just what section 11(a), which relates to a portion of the 1899 act, and subsection (e) relate to, it would be most helpful, and that I would appreciate.

Now, with respect to the 1905 act, I am concerned about the areas that are set aside for oyster cultivation, under section 4 of the 1905 act, and I am wondering what this particular language in the bill would have on that public law. Also with respect to the N.Y. Harbor, Hampton Roads Harbor law I note that one of the provisions that law has a finders fee in it.

I am going to ask you what your thinking is on how effective that finders fee provision has been?

Mr. TRAIN. I do not really know how well that has been working.

Of course, for the finders fee under the 1899 Refuse Act, I cannot answer your question.

I do not know whether that has proven to be an effective device or not.

Mr. EVERETT. After you have had a chance to reflect on that also, I wish you would give the committee the benefit of your thoughts.

Could you do that for us?

Mr. TRAIN. I would be most happy to.

(Answer follows:)

We do not advocate adapting to this bill an informer's fee similar to that found in Section 16 of the Rivers and Harbors Act of 1899. Problems have arisen with administration of Section 16, see *United States v. Transit Mix Concrete Corp.*, 2 E.R.C. 1074 (S.D. N.Y. 1970), and we would prefer to rely on other avenues for citizen participation in administration of an ocean dumping regulatory statute. For example, we believe that citizens and citizen groups are most likely to be helpful in reviewing criteria and in commenting on individual applications for a dumping permit. We have also gone on record as favoring citizen suit provisions analogous to Section 304 of the Clean Air Act as "an important complement to Federal enforcement efforts where Federal environmental standards and procedures have been established."

Mr. EVERETT. Also, would you provide, somebody has to do it for us, the estimated cost of this legislation?

If you would also submit that for the record, I would greatly appreciate it.

I think one of the requirements ought to be what you anticipate for the next 5 years if the legislation were enacted, what would be the cost to the Government?

Mr. TRAIN. We do have those estimates. We will be glad to provide those.

Mr. DUPONT. I would like that also to include enforcement from the Coast Guard point of view.

Mr. TRAIN. Yes; that will be included.

Mr. DINGELL. That is an admirable suggestion, because I am apprehensive with the limited resources the Coast Guard has at this time to carry out its responsibilities, whether it would take on any additional chores like chasing ocean dumpers.

(Answer follows:)

Our estimate of the cost both of administering the permit system and of developing and using alternate disposal means to ocean dumping were made at or

BEST COPY AVAILABLE

near the time we issued our ocean dumping report. See, for example, the table on page 20 of the report comparing estimated solid waste disposal costs. The operating agencies, particularly the Environmental Protection Agency, the Coast Guard, and the National Oceanic and Atmospheric Administration have more recently developed estimates of the cost of administration. We understand that these agencies can provide the desired information for you.

Mr. EVERETT. On page 13, under subsection (c) of section 8, it says:

The Secretary of the department in which the Coast Guard is operating shall conduct surveillance and other appropriate enforcement activity to prevent unlawful transportation of material for dumping or dumping.

Does this give the Coast Guard sufficient authority to enforce deviation of the permit?

Mr. TRAIN. What do you mean by that?

Mr. EVERETT. It looks like the prohibition goes to the transporting or the dumping, where there has been no permit issued.

Now, where a permit has been issued, and the dumping is in a different locality, this does not meet the requirements or conditions attached to the permit, and who has the authority to enforce this?

Does subsection (c) give the Coast Guard the authority, or should the Coast Guard have that authority?

Mr. TRAIN. I think clearly——

Mr. EVERETT. The language seems to be written narrowly to prevent transportation or dumping.

Mr. TRAIN. Certainly an unlawful transportation would in my view include any transportation followed by a dumping in violation of a permit, and I would include as a violation of a permit a substantial divergence from its terms.

Mr. EVERETT. I notice under the provisions under section 6, you say:

A person who violates section 4 of this Act, or regulations promulgated under this Act, or a permit issued under this Act by the Administrator shall be liable to a civil penalty of not more than $50,000 for each violation to be assessed by the Administrator.

But I am just wondering if the language is broad enough in subsection (c) on page 13 to include violations of a permit issued under the act.

You might reflect on that.

Mr. TRAIN. I think if there is any uncertainty there, it could well be clarified.

We will be glad to take a look, another look at that to see if that could be strengthened.

Mr. EVERETT. You have the authority to designate areas where safe sites can be located for purposes of dumping.

Do you have any indication as to how long it would take to study these areas with respect to identifying them for this purpose?

Mr. TRAIN. No, Mr. Everett; I do not have any information I could give you on that.

The act takes effect, as you know, 6 months after enactment, but that does not give any indication as to when that study could be concluded.

Mr. EVERETT. One other thought with respect to the Coast Guard.

It is not spelled out in the bill, but do you think, either in the bill or the regulations, that there should be some requirement that the vessel operator, or the vessel itself, should have a permit displayed on the vessel in some appropriate place so that it could be readily seen and checked by the Coast Guard?

BEST COPY AVAILABLE

Mr. TRAIN. I would certainly assume that the regulations would cover this.

I would rather doubt that it is necessary to put it into the statute.

Mr. EVERETT. One last question with respect to the Atomic Energy Act of 1954.

I am not sure as to the effect of this legislation on that act. Would you exclude all radioactive waste from the provisions of the Atomic Energy Act?

I wonder if you could enlighten us on that some?

Mr. TRAIN. There is not any exclusion. It is just a matter of how it is handled.

First, as you know, none of these things are being dumped at the present time.

The Atomic Energy Commission has stopped all of these disposals; however, what we have done under the legislation is to continue the Atomic Energy Commission's existing permit authority, rather than try to shift it to another agency, yet at the same time requiring with the language beginning on line 21 of page 10, that prior to issuing any permit for dumping radioactive materials the Atomic Energy Commission would be required to consult with the Administrator, and that in issuing any such permit, the Atomic Energy Commission would be required to comply with standards set by the EPA Administrator, respecting limits on radioactive exposure levels, or concentrations or quantities of radioactive materials.

Mr. EVERETT. I am wondering, why you would go to all of the trouble to require conditions to be established by EPA and to be met in consultations with AEC, why not just require the Atomic Energy Commission to comply with the legislation like all other Federal agencies?

What justifies their exclusion, if I may ask, or different treatment from the other Federal agencies?

You say they are not dumping now.

I am at a loss to understand the rationale with respect to the other agencies.

Mr. TRAIN. The Atomic Energy Commission traditionally has had regulatory authority throughout an entire process that in any way relates to radioactive materials, transport, and so on.

Mr. EVERETT. The whole range of activities related to radioactive materials?

Mr. TRAIN. That is correct.

Mr. DINGELL. Is the Atomic Energy Commission permitted to dump radioactive matter into the waters is defiance of Federal policy standards?

Mr. TRAIN. At the present time——

Mr. DINGELL. Are they permitted to violate those policies?

Mr. TRAIN. They are not doing any disposal offshore.

Mr. DINGELL. The statutes, the Air and Water Quality Acts apply to the Atomic Energy Commission; do they not?

Mr. TRAIN. I do not know what the relation is between those acts, Mr. Chairman.

Mr. DINGELL. I am curious why the Atomic Energy Commission would be different from all other agencies, and why it would be given this rather special treatment under the proposed statute.

Mr. TRAIN. It had the authority ever since the beginning, and all of the handling of radioactive materials is under regulation by the

Atomic Energy Commission throughout every step of the process and it always has been, and this legislation would simply continue that legal arrangement, but it does say that no permit can be granted by the Atomic Energy Commission for the disposal of radioactive wastes without the agreement of the Environmental Protection Agency.

It says:

In issuing any such permit, the Atomic Energy Commission shall comply with standards set by the Administrator respecting limits on radiation exposures or levels, or concentrations or quantities of radioactive material.

It says:

In issuing any such permit, the Atomic Energy Commission shall comply with standards set by the Administrator * * *.

Mr. DINGELL. Go ahead, Mr. Everett.

Mr. EVERETT. The previous sentence that concerned me, it says "shall consult" and then you say, "in issuing any such permit, and so on," but I am wondering where is the language that requires the Environmental Protection Agency or directs them to set the standards.

It says AEC shall abide by standards set by the Administrator. Where is the authority directing him to set the standards?

Mr. TRAIN. The Environmental Protection Agency has that authority in my opinion under existing law through Reorganization Plan No. 3 of last year.

Mr. DINGELL. Is not this going to give us one set of standards administered by two different agencies?

Mr. TRAIN. Well, in one sense, I suppose; yes.

Mr. DINGELL. Now, the Atomic Energy Commission usually does not in its disposal of substances use the oceans, and it will be giving permits to other persons who will in turn engage in practice of ocean dumping; am I correct, Mr. Train?

Mr. TRAIN. I believe that would be the case, as a rule.

It would be an Atomic Energy Commission contractor or something of that sort.

Mr. DINGELL. I find myself hard put. I might be able to swallow the idea that the Atomic Energy Commission could do its own dumping, but I find myself hard put to see why the Atomic Energy Commission should become the permitting agency.

Mr. TRAIN. Well, it is.

Mr. DINGELL. For dumping.

Is not the reason the Environmental Protection Agency set up to have one place for all environmental matters, where all of those matters will be handled?

Was not that the basis on why Reorganization Plan No. 3 was issued?

Mr. TRAIN. I do not see any great objection in principle either way the Congress would prefer to handle it.

Mr. DINGELL. I really think we ought to keep it all in one place, to having one set of standards administered by two different agencies, I am rather curious as to why, and as I read the wind, the Atomic Energy Commission will be rather anxious to get rid of this stuff, and that being so, they might tend to view the environmental protection standards rather differently than would the Environmental Protection Agency, and it occurs to me, where you allow the Atomic

Energy Commission to engage in permits under the circumstances, it is not good.

Mr. TRAIN. Let's remember the Atomic Energy Commission has taken the lead worldwide in stopping ocean disposal of radioactive waste.

Mr. DINGELL. I can remember the day when they were loading the rivers with just hundreds of tons of curious radioactive materials, and this was the water source of many communities out there in New Mexico, Arizona and Colorado.

Now, it may be the Atomic Energy Commission has arrived in the 20th century, but I am prepared to see that more clearly than I have so far.

Well, thank you.

Mr. EVERETT. That is all I have.

Mr. duPONT. One final question.

I have looked at your data in your policy report concerning tonnage dumping, and so forth, but nowhere in here do I see an estimate of, if you had a permit issuing authority in Environmental Protection Agency, how many permits a year you think you would be dealing with.

Are you talking of 10,000, or a thousand?

Have you any feel for that at all?

Mr. TRAIN. No, Mr. duPont.

Now, maybe we do have that information. I am sure that in arriving at estimates of expenditure requirements for the administration of this permit system, that there are some estimates that have been developed.

How accurate they are, I don't know, but I will certainly see to it we develop something before you.

Mr. duPONT. I think the primary responsibility of EPA is in trying to pull its air quality standards together. If you take 10,000 permits, and require a hearing, and put this in addition, they will have to have a substantial increase of manpower, and we appreciate that.

Mr. TRAIN. Yes, sir.

(Material follows:)

The Environmental Protection Agency has estimated that at least 3,000 permit applications per year will be received under this bill if enacted. We generally concur with this estimate. The number of total regulatory actions required by EPA may be higher if requests for EPA certification of the dumping aspects of corps dredge and fill permit applications under the Rivers and Harbors Act of 1899 are added to the number of direct applications for an ocean dumping permit.

Mr. DINGELL. Mr. Anderson.

Mr. ANDERSON. Mr. Train, in circumstances where degrees of statutory stringency exist among various regulatory bodies, I can see the possibility of serious complication and much confusion arising.

For example, if a State or local government has its own regulations and the Federal Government its regulations, would the applicant be required to obtain a permit from each of the local governments as well as the Federal?

Mr. TRAIN. It is the intention that EPA would regulate all dumping offshore, including within the 3-mile limit, but that in cases where

[illegible]

the State in question had stricter rules than those prescribed by the Administrator, that the Administrator in granting the permit would take those more strict rules into account.

Mr. ANDERSON. Who would be the one to determine which is more strict?

I could see where a small jurisdiction could have more stringent requirements in a particular area while a larger State jurisdiction, for example, could have strict requirements overall. Who determines which requirements apply?

Mr. TRAIN. Well, of course, the Administrator will be referring his permit application for comment to the applicable State and the local water quality agency where there is a case of dumping within the 3-mile limit, and the State would make representations at least as to whether or not the proposed dumping would contravene State water quality standards.

I suppose in the final analysis, the Administrator of EPA would have to make that determination.

Mr. ANDERSON. So there would then be one permit, but the applicant would in effect have to comply with the requirements of both?

Mr. TRAIN. Really it would be the requirements laid down by the Environmental Protection Agency, but they could in that case be stricter standards than EPA would be applying perhaps in some other coastal area.

Mr. ANDERSON. That is all, Mr. Chairman.

Mr. DINGELL. Mr. Pelly.

Mr. PELLY. No questions.

Mr. DINGELL. Mr. Train, it is always a privilege to have you before this committee, and you deserve congratulations with respect to your very fine work.

It is a long time since we have had you before this committee, and everytime it has always been a pleasure.

Mr. TRAIN. Thank you, Mr. Chairman. It is always a pleasure to be here.

Mr. DINGELL. We also express our thanks to your associate.

Our next witness is Mr. Paul A. Amundsen, executive director of the American Association of Port Authorities.

Mr. AMUNDSEN. Mr. Chairman, I understood we were to testify tomorrow afternoon.

Mr. DINGELL. The witness list indicates you were to be heard today.

If you wish to be heard tomorrow afternoon, we will make an opportunity for you to be heard tomorrow.

Mr. AMUNDSEN. I would be glad to be heard, but I do not have my statement with me.

Mr. DINGELL. We will try to hear you tomorrow afternoon.

I suspect we have a rather full schedule tomorrow, but we will try to give you an opportunity to get your statement before this committee.

Mr. AMUNDSEN. Mr. Langlois is our witness tomorrow of the Port of Portland, Maine.

Mr. DINGELL. Perhaps it would be helpful for the Chair to read off the list of witnesses.

Tomorrow morning we have several of our colleagues scheduled to testify.

BEST COPY AVAILABLE

We have representatives of the Atomic Energy Commission. We have Mr. Harrison Loesch of the Department of the Interior.

In the afternoon we have our colleague, Congressman Williams, and we have Dr. Ketchum, associate director of Woods Hole Oceanographic Institute.

We then have Mr. Langlois of Port of Portland, Maine, and he will be heard at 3:15, and he is your associate, is that correct?

Mr. AMUNDSEN. That is correct.

Mr. DINGELL. Is there anyone else here who is on the witness list?

Well, perhaps, it would assist the committee to read the list of witnesses for Wednesday, but to finish for tomorrow first.

We have at 4 o'clock Mr. James Beggs, and then at 4:30 we have Mr. James J. Reynolds.

On Wednesday we have Congressman Harrington, Congressman Gallagher.

Also Mr. William D. Ruckelshaus, Mr. John R. Stevenson, Mr. Henry Douglas, Mr. James Wakelin, Brig. Gen. George Hayes, Dr. Richard Barber, Louis Clapper, and then the Sierra Club.

Mr. PELLY. It appears to me we have some important legislation on Wednesday, Mr. Chairman.

There may be a controversial issue, and now that we have a recorded teller vote, I doubt we will be able to hear all of the witnesses.

Mr. DINGELL. You may be correct.

Mr. PELLY. That may present a problem for Wednesday, Mr. Chairman.

Mr. DINGELL. The gentleman is probably correct as always.

The subcommittee will stand adjourned until 10 o'clock tomorrow morning.

(Whereupon, the committee meeting was adjourned at 3:30 p.m., scheduled to reconvene at 10 o'clock, April 6, 1971.)

THIS PAGE INTENTIONALLY LEFT BLANK

OCEAN DUMPING OF WASTE MATERIALS

TUESDAY, APRIL 6, 1971

HOUSE OF REPRESENTATIVES,
JOINT SUBCOMMITTEES ON OCEANOGRAPHY AND
FISHERIES AND WILDLIFE CONSERVATION,
Washington, D.C.

The joint subcommittees met, pursuant to notice, at 10 a.m. in room 1334, Longworth House Office Building, Hon. Alton Lennon (chairman of the Subcommittee on Oceanography) and Hon. John D. Dingell (chairman of the Subcommittee on Fisheries and Wildlife Conservation) presiding.

Mr. DINGELL. The subcommittees will come to order.

This is a continuation of the hearings begun yesterday on the series of bills regarding the matter of ocean dumping.

Our first witness this morning will be our good friend and colleague, the Honorable Charles W. Sandman, Jr.

Mr. Sandman, we are glad to welcome you.

Will you identify yourself fully, for the record.

STATEMENT OF HON. CHARLES W. SANDMAN, A REPRESENTATIVE IN CONGRESS FROM THE STATE OF NEW JERSEY

Congressman SANDMAN. Thank you, Mr. Chairman, and the distinguished members of the Subcommittees on Fisheries and Wildlife Conservation and Oceanography. These hearings are most timely.

I say timely because starting this Easter weekend and continuing through late fall, millions of American tourists and sportsmen will flock to the seashore resorts such as Atlantic City, the Wildwoods, and Cape May in the congressional district I represent.

They are attracted primarily by the Atlantic Ocean, its clean white beaches, its surf, the cool fresh air it brings to the shore and the bathing, fishing, boating and other recreational opportunities it provides. They will spend an estimated $3 billion this summer alone, an income upon which a large number of the people I represent are dependent for their livelihood. Yet the seashore as a vacationland may soon be a thing of the past if we do not act now to stop ocean pollution.

This committee's deliberations are also timely because one of the Nation's most historic, basic and important industries may well be threatened with near extinction because of ocean pollution. Commercial fishing hauls continue to go down in quantity and quality; the fleets are dwindling in size and recently, over 80 percent of the inshore waters in my district and large portions of offshore waters have been declared off limits for the taking of shellfish. All because of pollution.

Therefore on opening day of this session of Congress, I introduced H.R. 1661 (H.R. 5049 and 5050 for cosponsors), a bill to regulate and eventually eliminate indiscriminate ocean dumping.

The premise of this legislation is simply that the oceans and other waters are not the proper places to dispose of man's wastes: human, industrial or any other kind. Our marine resources are too valuable as a source of food and other natural resources and valuable as a source of recreation to spoil.

Therefore, my intention and hope is that this committee and Congress will, through unequivocal legislation, declare the oceans unconditionally off-limits as dumping grounds.

Now I realize, Mr. Chairman, that ocean dumping has been going on for some time and that elimination of this practice in some cases cannot be expected to happen overnight. But this must not divert our attention from the real need: to phase out ocean dumping altogether.

I had the privilege of testifying before the U.S. Senate Public Works Committee hearing on ocean pollution on March 26 at Rehoboth Beach, Del. I spoke, I listened to and I later read the remarks of all who attended the hearings, conducted by the Subcommittee on Air and Water Pollution. I was the only Member of the House present.

Also, I personally initiated the two, already historic and precedent setting the U.S. district court cases on ocean dumping of industrial wastes. The research and legal strategy and negotiations on these cases was most extensive.

I mention these items of background, Mr. Chairman, not so much to establish my credentials, but rather to spare the distinguished members of these subcommittees a lengthy blow-by-blow account of events upon which I base the recommendations I will make to you here today. I am prepared to justify any of these points to you in detail.

There are eight points I want to bring to your attention briefly.

(1) There is no excuse for dumping wastes of any kind in the oceans. I am not impressed with any of the arguments advanced by those who try to defend or justify ocean dumping and I've heard them all. Other than the fact that the oceans contain vast amounts of water in which some materials can be diluted easier and perhaps cheaper than on land, ocean dumping exists for only one reason, best identified with the cliché: "Out of sight, out of mind." Ocean polluters operate with the very same intent as housewives who sweep dirt under the carpet.

(2) Safe and economical land-based waste disposal sites and facilities can be developed. The technology now exists and given a congressional ultimatum to cease ocean dumping, the creative genius of American enterprise will develop new, possibly cheaper and more effective methods of disposing of wastes on land.

In this connection, I do want to note in all fairness that waste disposal site selection and acquisition is a serious problem. Nobody, as the old saying goes, wants to live next to a dump. As a result of this, local zoning laws and sanitary landfill and other disposal site licensing requirements in most communities of the Nation tend to "freeze out" disposal facilities. It is possible there is a need for Federal legislation to cope with this very real problem.

As further testimony that ocean dumping is not necessary, I point with pride at the fact that the city of Bridgeton—the only municipal-

ity in my district that barged sewage sludge to the ocean to be dumped—has now ceased this practice. In January, Bridgeton stopped ocean dumping and is now allowing private nurserys to pick up its treated sewage sludge for use as fertilizer, one of the best available.

(3) Until all dumping can be phased out, the practice should and can be regulated effectively without undue hardship. I have proposed a simple permit system in my bill and am pleased to note this approach is included in other legislation now before these subcommittees. Simply, no dumping could take place unless a permit is first obtained from the Environmental Protection Agency (EPA). This permit would only be issued if the EPA Administrator is satisfied that the proposed dumping will not damage the marine environment.

Additionally, H.R. 1561 establishes an immediate ban against dumping anything between the Continental Shelf and the coast of the United States. This is a reasonable requirement in my opinion, for it is inside the first 75 to 100 miles that the current damage from ocean pollution has been done. Congressional support for this minimum distance for dumping, effective immediately, will accomplish two very important things. First, the immediate threat to fishing and recreation will have been alleviated. In time, the nearshore ocean floor will be cleansed by the flush of tidal action and therefore shellfishing and commercial and sport fishing will improve. Next, the increased distance a load of waste would have to be transported will tend to discourage dumping and encourage development of land-based disposal sites and facilities.

I want to point out briefly that I have seen detailed proposals by a private contractor showing that sewage sludge could be transported 100 miles offshore in the interim for approximately the same cost and in approximately the same time it now takes to transport the sludge only a few miles offshore. The idea is simple and sensible. It is to use fast, high-capacity ships instead of slow, low-capacity barges to transport the material. Based on this, I am confident such a dumping minimum as I propose will not pose an unsolvable problem to those who, in fact, will be unable to stop dumping immediately altogether.

(4) International regulations on ocean pollution are essential. In the long run, even assuming the United States is eventually able to phase out all dumping of pollutants in the oceans, cooperation from the other nations of the world must be sought immediately. I urge this committee, perhaps by a separate joint resolution, to urge adoption of pollution abatement programs such as ours by the other countries and through the various international organizations.

(5) No arbitrary time limits for ending dumping or allowing it to continue should be included in this legislation. There are a variety of proposals, Mr. Chairman, before Congress now to establish a specific time by which dumping will no longer be allowed. One bill asks 5 years; another says 2. My view is that more can be accomplished faster without any arbitrary time limits. If the committee reports a proposal that dumping shall be illegal after 5 years, for example, I am certain many of the municipal and industrial polluters will take the full limit to cease operations. I urge that scheduling be left up to the EPA which will act on each case individually to establish phasing-out of mean dumping.

(6) The traditional jurisdictional limits of the United States should not be tampered within order to regulate ocean pollution. We simply

don't have the time. The business of rewriting international treaties is a time-consuming one and in my opinion, this approach is cumbersome and unnecessary in order to establish the necessary jurisdiction to control ocean pollution.

(7) Adequate research and development funds should be available to help establish land-based waste disposal technology and facilities. I urge this committee to investigate whether existing Federal grant and loan programs are adequate to meet the needs for the transition from ocean disposal to land-based disposal of wastes. If it is determined that exising programs are inadequate, I will support new or increased appropriations for this purpose.

(8) New jurisdiction is needed to deal with ocean dumping. This is my final point, Mr. Chairman. It is one with which I am completely familiar because the matter of U.S. jurisdiction was the crux of my U.S. district court suits on ocean pollution.

Academically, I feel the Federal Government does now have a form of jurisdiction over ocean pollution. It is based on the concept that in our Federal Union, every State is expected to exercise reasonable regard for the health and welfare of the people of every other State. I have maintained in court that the loading, transporting, and dumping of certain pollutants constitutes a threat to the health and welfare of certain of our citizens.

The fact is, however, this case has not yet been argued to a decision in the courts. In both civil actions I initiated this year on ocean pollution, the defendants consented to the restraints I requested rather than to contest the jurisdiction upon which I based my cases. But I know from years of courtroom experience what raised eyebrows on the part of the presiding judge means. At best, Mr. Chairman, our current jurisdiction to control dumping beyond 3 miles is highly questionable. At worst, it doesn't exist.

In my bill (H.R. 1661) therefore, I propose that Congress establish controls where we now have jurisdiction without question; namely at the loading docks and ports of the Nation. To load any vessel with waste material intended for ocean dumping, the permit I have previously mentioned must be obtained.

Armed with this new jurisdiction, Mr. Chairman, and with the increased surveillance and minimum dumping distance required under the permit system I have proposed, this Nation can finally proceed to clean up and strive to protect forever our precious marine and coastal environment.

Mr. SANDMAN. Very basically, I think the most important thing we have to accomplish, Mr. Chairman, and the biggest obstacle we have is time.

We do not have time to lose in establishing a law that is going to give us some teeth by which we can control the promiscuous dumping in the ocean outside the 3-mile limit.

I feel that the only kind of measure that we can pass, and pass with dispatch, will be a bill such as the one I have introduced. Other members have introduced the same kind of bill in both the House and the Senate.

My bill is known as H.R. 1661, and it requires any person or company who is moving any kind of substance, any kind of waste substance,

BEST COPY AVAILABLE

that is going to be dumped in the territorial waters of the United States, to first get a permit from the Environmental Protection Agency.

That permit has in it a condition precedent that each applicant must show exactly how the wastes are going to be loaded, where it is going to be loaded, how it is going to be transported, and most of all, where it is going to be disposed of, and by what method.

This permit will give the U.S. district court jurisdiction, and it can give it a supervisory ability which we do not have now in the present law, and which we sorely need.

It will require that the U.S. Coast Guard be given at least 4 hours notice before any ship is leaving any port in the United States, and, secondly, broad powers are given to the Environmental Protection Agency on a regulation of the entire bill.

I have suggested in this bill that very heavy penalties be inflicted, because we are dealing with something highly dangerous to our environment.

I have recommended a first offense penalty of $50,000, a second offense penalty of a hundred thousand dollars, or confiscation of the vessel or both, and the liability should be joint and several with everybody involved.

The other measures that have been introduced basically have only few deviations from what I have proposed.

Some bills would like to set a cutoff time. So that we understand each other, it is my view that the ocean is not the proper place to dump anything; any kind of waste, whether it be toxic or otherwise. I would like to see this ended as quickly as possible, but I think it is wrong to put a time limit in any legislation, Mr. Chairman. You know, and I know, if you give any industry 5 years, or 2 years to do something, that is how long they will take to do it.

I would rather see the Environmental Protection Agency have the authority, because of experience with the various people and industries, as to when dumping must end.

Now, here is a good example. Of the three cities that have dumped only 10 miles from where I live, off the coast of Cape May, N.J., one of those cities, only 3 months ago developed a new method—new to them—on how they can use this waste for a fertilizer, and this is what they are doing today.

I can see that the city of Philadelphia, because of its tremendous problem, and its great volume, perhaps cannot stop disposing of the sludge immediately.

This might be too much of a hardship, and I can understand that. But as for the city of Bridgeton, which has already reached a point through its own exploration where it does not have to dump any of its waste, the cutoff time has already arrived as far as the city of Bridgeton is concerned, and they should not be permitted at any time to dump sludge into the Atlantic Ocean.

When the day arrives that the other two cities have reached that point, whether it be next month or next year, that is when their permits should expire.

There are many other things that I could elaborate upon; however, I know you have many other witnesses, and I have touched on those things I think are the most important, and, again, gentlemen, this is a very dangerous situation.

The Federal courts, in most legal minds today, does not have the jurisdiction that it should have to handle this kind of a problem adequately, and I am hopeful that your committee will be working, with dispatch to pass a measure that will regulate the dumping in the ocean.

Mr. DINGELL. Mr. Sandman, the committee is grateful to you. We are aware of your long assistance to this committee, during the last session of the Congress, and we are glad to say that this time we think we are able to move forward on legislation.

Mr. PELLY. Mr. Chairman, Mr. Sandman has made an unusual contribution, because he has had an active fight against the dumping, and in his experience I think it will be very helpful to this committee.

Mr. DINGELL. I thoroughly agree.

Mr. SANDMAN. Thank you, gentlemen.

Mr. DINGELL. The Chair is happy to welcome as our next witness, and a former member of this committee, Congressman Louis Frey, Jr.

We are sorry he is not here with us on the committee this year.

Mr. Frey, we are certainly happy to welcome you.

STATEMENT BY HON. LOUIS FREY, JR., A REPRESENTATIVE IN CONGRESS FROM THE STATE OF FLORIDA

Mr. FREY. Thank you, Mr. Chairman. These bills, H.R. 4359, H.R. 4360, and H.R. 4361, were cosponsored by 52 members from both sides of the aisle, including the distinguished chairman of the oceanography subcommittee, Mr. Lennon. Other cosponsors who are members of the Merchant Marine and Fisheries Committee include Mr. Grover, Mr. McCloskey, Mr. Hathaway, and Mr. Keith. In addition, the bill introduced by Mr. Rogers—H.R. 662—and cosponsored by Messrs. Dingell, Pelly, McCloskey, Keith, Moss, and Conte differ in only one major respect from my bill—it does not provide for the establishment of a system of marine sanctuaries.

Mr. Chairman, we are all well aware of the seriousness of the present situation. Marine pollution has seriously damaged the environment and in some areas it is posing a great threat to human life. There have been heavy kills of fish and at least one-fifth of the Nation's commercial shellfish beds have been lost due to pollution.

Shellfish have been found to contain hepatitis, polio virus, and other pathogens. In the lagoons and estuaries in Brevard County, Fla., for example, heavy fresh water runoffs from agricultural areas have resulted in the banning of shellfish harvesting, which was a major industry in the area. Lifeless zones in the marine environment have actually been created.

Man has also been seriously affected. Not only has there been a loss of recreational opportunities and adverse esthetic effects, but there have been instances of lethal and sublethal effects. By 1965, over 41 persons living on Minamata Bay, Japan, had died due to mercury poisoning in fish and shellfish. Recent tests off Long Island indicate the presence of mercury in fish that is within the range reported for the first that caused the Minamata Bay disease in Japan.

According to the Council on Environmental Quality, 48 million tons of waste were dumped at sea in 1968. These wastes included dredge spoils, industrial wastes, sewage, construction and demolition

BEST COPY AVAILABLE

debris, solid waste, explosive chemical munitions, radioactive wastes, and miscellaneous materials.

There are at least 250 known official and unofficial disposal sites off U.S. coasts. Half of the ocean dumping grounds are located off the Atlantic coast while the other half is evenly divided between the gulf and Pacific coasts. A large area of the Atlantic Ocean off New York Harbor has become an ecological desert as a result of the dumping of sewage sludge in that location for 40 years.

Aside from areas such as that off New York Harbor which have been used continuously for the dumping of deposition by outfall of sewage and other toxic material, there have been hundreds of other incidents of spillage of hazardous material. A very thorough study recently compiled by the Coast Guard listed 157 separate instances of reported spillages of dangerous or hazardous materials in the past 2 years.

Decisions made by municipalities and industries in the next few years could lead to dramatic increases in the level of dumping. Because the capacity of land-based disposal sites is rapidly being exhausted in some coastal cities, communities are looking to the ocean as a dumping ground for their wastes.

Forced with higher water quality standards, the industries may also look to the ocean for disposal. The result could be a massive increase in the already growing level of ocean dumping.

It is largely the responsibility of the Federal Government to halt and reverse the environmental deterioration taking place along coastal areas, and particularly those adjacent to large urban centers. However, the Federal Government presently lacks sufficient authority and some of the Federal roles are overlapping with no clear-cut idea as to the lead agency responsibility. It was this tangled bureaucratic web that impelled me to search for a more comprehensive approach that includes specific guidelines.

Let's look at the present system. Ocean dumping in territorial waters is not "regulated" under the Refuse Act of 1899, the 1888 Supervisory Act, the 1905 River and Harbors Act, and the Federal Water Pollution Control Act of 1965, as amended. The first three acts are administered by the Corps of Engineers and the Water Quality Act by FWQA which is now a part of the Environmental Protection Agency.

Beyond the territorial sea, authority to enforce pollution laws applicable to U.S. nationals is assigned to the Coast Guard. Apparently, the authority applies only to vessels carrying oil. No Federal agency has authority to completely regulate or control dumping beyond the territorial sea. Applications to the Corps of Engineers for disposal of wastes at sea are hardly ever denied beyond the 3-mile limit even when public health groups object, because of a lack of explicit regulations and guidelines.

A recent study for the Bureau of Solid Waste Management concluded:

> Although there are many Federal, State, and local agencies involved in one way or another with the disposal of wastes from barges and ships in any one city, rarely did more than one of these agencies have a comprehensive picture of the total activities of this city. This lack of effective data management appears to be due primarily to both a lack of communication between agencies

62-513—71——15 ""ST COPY AVAILABLE

involved and the concentration of interest in a given agency in only specific types of wastes.

Also, the expertise in one agency often isn't made available to another. There is also breakdown in obtaining and processing environmental data to assess future waste disposal activities. At present, there isn't a continuous monitoring and surveillance of disposal activities.

Furthermore, there is no regulation of dumping by her nationals within the contiguous zone. Legislation has not been passed to implement article 24 of the Convention of the Territorial Sea and Contiguous Zone of 1958.

Mr. Chairman, the legislation which I have introduced not only clears up this administrative mess, but also creates the authority necessary to effectively regulate clean dumping.

In my opinion, this bill offers several important and desirable features which are lacking in the bill proposed by the administration. In addition, by bill, unlike that of the administration, conforms closely to the recommendations of the Council of Environmental Quality as to what provisions ocean dumping legislation should contain. These recommendations, it should be remembered, were the result of a comprehensive study initiated by President Nixon on April 15, 1970.

It is important that we in the Congress decide the kinds and places of dumping that should be proscribed and not leave it simply to the discretion of the Administrator. After all, it is the lack of specific guidelines and direction which has caused indecisiveness on the part of the administering agencies. Furthermore, this is an area of such importance that strong legislation is required. Delegating full authority to the Administrator of EPA could easily result in a so-called balancing of interests which would result in less stringent regulations and enforcement. The Congress should establish a national policy embodying specific guidelines to halt the practice of using our coastal areas and Great Lakes as a dumping ground simply because it is the "cheapest method of disposal." The fact is that the dollar cost of ocean dumping coupled with the ecological costs make it the most costly method.

The legislation I have introduced has a threefold approach. First, instead of designating areas where dumping may be conducted safely, my bill concentrates on determining which areas of our marine environment are most valuable and setting them aside as sanctuaries. Second, similar to the administration's proposal, my bill also prohibits the dumping of waste material into the oceans, coastal waters, and estuarine areas, except under a permit signed by the Administrator of the EPA. Third, the bill proscribes absolutely the dumping of toxic, radioactive, and chemical biological warfare material.

I have chosen as a vehicle for this legislation the act of August 3, 1969, which declared as a national policy the concept that the estuarine areas of the United States are of great value to America and must be protected and conserved for the future of this Nation. This act was chosen, Mr. Chairman, because of the need to relate the problem of ocean dumping to the broader problem of preserving certain ecosystems within the coastal zone area. This need exists because the dumping of dredge spoil constitutes the largest single element in the growing volume of refuse being dumped into the ocean.

An[illegible] st dredge spoil is dumped relatively inshore, where it may contam[illegible]ate the valuable shellfish and fish species generally.

The report of the Council on Environmental Quality recommended:

> High priority be given to protecting those portions of the marine environment which are biologically most active, namely, the estuaries and the shallow, near-shore areas in which many marine organisms breed or spawn. These biologically critical areas should be delineated and protected.

Both the act of August 3, 1969, and the report of the Council would seem to mandate that any ocean-dumping legislation would have to include a provision for setting aside as sanctuaries those areas of our marine environment which are determined most valuable.

Section 3 of the bill authorizes the Secretary of Commerce acting through the newly established National Oceanic and Atmospheric Administration, in conjunction with the Secretary of the Interior, the Administrator of EPA, and the Council on Environmental Quality, to designate as marine sanctuaries those areas which the Secretary determines should be preserved or restored for their recreation conservation, ecological or esthetic value. This section directs the Secretary to make an initial designation of marine sanctuaries within 2 years following the date of enactment of the legislation, and require him to submit an annual report to the President and Congress reviewing the activities under this act. Adequate funds are authorized for the conduct of studies leading to the designation of marine sanctuaries. The Secretary of Interior may not renew any license or permit for marine mining activity within an area under study for designation as a marine sanctuary, nor may the Administrator of EPA issue or renew any permit for dumping in any areas under study.

Section 2 of the bill amends section 6 of the 1968 act to prohibit disposal of waste materials without a permit issued by the Administrator of EPA, under such terms and conditions as he determines necessary to insure that the dumping or disposal will not damage the ecology of the marine environment.

The minimum guidelines which this bill sets forth for the issuance or permits corresponds with those suggested by the Council on Environmental Quality and differs markedly from those contained in the administration's proposed bill.

My bill calls for a gradual phasing out of municipal sewage and industrial waste outfalls. These outfalls constitute one of the major sources of marine pollution. Sewer outfall is the primary pollutant in the New York Bight. 130 municipal waste outfalls discharge 2 billion gallons per day.

Waste material is defined so as to include all solid and liquid industrial byproducts, chemicals, sewage, sludge, dredging spoils, and debris. Disposal is defined as the placing, releasing or discharging by any means whatsoever.

The administration's proposal, on the other hand, specifically excludes municipal sewage outfalls or industrial waste outfalls from the definition of dumping.

Following the recommendations of the Council on Environmental Quality, my bill proposes a phasing out of the dumping, or disposal of municipal sewage, or industrial wastes. After January 1, 1972, no disposal could take place unless such sewage or industrial waste has

received primary treatment in accordance with standards established by the Administrator.

After January 1, 1974, no permit may be issued unless such sewage or industrial waste has received primary and secondary treatment, and after January 1, 1976, no permit can be issued unless primary, secondar[illegible] and tertiary treatment has been received.

This gradual strengthening of standards will allow the companies and municipalities involved leadtime to develop new processes for treatment and also eradicate a major source of ocean pollution.

The dumping of radioactive wastes, toxic industrial wastes, and chemical and biological warfare material are completely prohibited. The serious adverse effects which the dumping of these materials could and do have, coupled with interim and long-term alternatives to their dumping in the oceans leads me to believe that no rational balance of interests requires the use of our oceans and coastal waters for their dumping.

In this regard, the Council on Environment Quality concluded that "no ocean dumping of chemical warfare materials should be permitted," and "ocean dumping of industrial wastes should be stopped as soon as possible." They also called for more stringent standards regulating the dumping of radioactive materials.

It should be noted that other studies, including a recent study of the New York Bight for the Department of [illegible]r and a study by the Coast Guard, also recommend that the dumping of these categories of material should cease entirely.

Alternatives exist to outfalls of sewage sludge, industrial wastes, and the dumping of other toxic, chemical-biological and radioactive materials. In some cases these alternatives actually cost less. And when you add in the ecological costs imposed on the marine environment by dumping at sea, in almost every instance it would be less expensive, in both economic and social terms, to revert to land-based disposal systems.

Sewage sludge can be disposed of in sanitary landfills or used as a soil conditioner. Industrial wastes can be treated and disposed of on land, or they can be incinerated. Radioactive materials can be entombed in salt mines, and dismantled chemical and biological warfare material can be neutralized, incinerated, or buried. Of course, longer-term alternatives such as recycling can and should be explored.

Finally, the bill recognizes the fact that stringent enforcement of these regulations is required in the purpose and spirit of the act is to be accomplished. Fines for unauthorized use of dumping range from a minimum of $2,000 up to $10,000 for the first offense, and from $10,000 to $25,000 for each succeeding offense.

It further provides that any vessel or barge engaged in dumping in violation of the act shall be forfeited. The Administrator of EPA, the Coast Guard, and the Corps of Engineers, acting jointly, shall enforce the act. This legislation also empowers the Coast Guard to stop and search ve[illegible]els in our territorial waters and in the contiguous zone to determine whether they are engaged in unauthorized dumping activities or related transportation activities.

I guess the problem of ocean dumping really hit home to me during the hearings on the dumping off the cape, when we found the laws we had just were not adequate, and did not do the job.

There is no reason to relate the problems to the committee of the great growth in pollution, and the fact that the ocean is becoming a garbage dump.

I think we recognize this, and we also recognize we have to do something about it.

The question in front of the committee is what are we going to do about it. Are we going to just make a pass at it, or are we going to put it under one roof and make it a tough law that has teeth in it.

Very briefly, my bill has a somewhat different approach from the administration's bill that has been introduced.

To begin with, the initial thrust of the bill is to set up sanctuaries, areas, where as a matter of national policy, there can be no dumping of any kind under any circumstances.

Second, it prohibits the dumping of waste materials in the ocean, the coastal waters, and the estuaries, except under a permit signed by the Administrator of the Environmental Protection Agency.

Third, we allow, or we prohibit absolutely the dumping of any toxic, radioactive, or chemical biological warfare material in any place.

The bill that I propose follows very closely the report to the President, prepared by the Council on Environmental Quality on ocean dumping which was filed in October, 1970.

The act also provides that the Coast Guard be given the funds to carry out surveillance of any dumping by the shipping that may be using these waters.

It also calls for Federal preemption and uniform regulation. I think we can see it in the Delaware Bay area. If we allow the States themselves to act, and Delaware, for instance, does not have as strict a standard as New Jersey, you will find people going over the line and dumping their garbage.

I think this is a national question. I do not think it should be one that the States themselves should decide.

The act I propose is somewhat different from the administration's act, in that it is broader. It prohibits, not only our flagships from dumping in our territorial and contiguous waters, but any foreign national from doing it.

It addresses itself to one other problem, and that is the question of waste disposal.

This is extremely important. The administration's bill does not say anything about waste disposal. It just ignores it. Part of the rational as I understand it, is that this will be handled by the Public Works Committee under the Water Quality Control Act. But even under that act, only the question of effluent standards is considered and not other factors, such as where the dumping takes place.

My bill calls for a gradual phaseout beginning in 1972 and ending in 1976. And, of course, there is precedence for this under the Air Control Act.

It, in essence, brings everything under one head, and it gives one agency, the power to carry this out.

I am somewhat puzzled at the reluctance to follow the recommendations of the ocean-dumping report of Council on Environmental Quality, which is I think of importance in drawing this act.

BEST COPY AVAILABLE

The penalties themselves are on a graduated basis for a first offense, being between $2,000 and $10,000, and more for the second offense.

I am not tied in my bill to any particular approach. I am not tied necessarily to the penalties, if they could be increased, but I feel very deeply the problem must be met.

It cannot be done piecemeal, and I am not for just delegating broad discretion to an agency to make up the rules.

I think this is of such importance to our Nation that the legislation should restrict to a good deal the ability to trade off, or to balance off the various interests, because I think the interests in preserving our ocea are greater than any other interests that might come into conflict.

I would be glad to answer any questions on this that you may have, but I think my statement is fairly complete in laying out this matter.

One last point, I do want to emphasize the difference between this approach, and the approach I think that the administration has taken. This bill, or a bill like it is a complete, not a piecemeal, approach to the problem.

It just does not take one particular part of it, and try and isolate it. It just does not work. It has not worked in the past, and I think we need to do a lot more in this area.

Mr. Dingell. Mr. Frey, you have given the committee an excellent statement, and we are indeed grateful to you.

The Chair would appreciate receiving, at your convenience, such amendments to the administration's bill you might deem appropriate in addition to the comments you have made this morning.

Mr. Frey. Thank you very much, Mr. Chairman.

Mr. Dingell. Our next witness is our good friend and colleague, Hon. Sam Gibbons, Congressman.

Mr. Gibbons, we are glad to welcome you to present such statement as you wish to give at this time.

STATEMENT OF HON. SAM GIBBONS, A REPRESENTATIVE IN CONGRESS FROM THE STATE OF FLORIDA

Mr. Gibbons. Thank you, Mr. Chairman.

I am a cointroducer of a couple of the bills that are before you this morning, and I want to state I am not wedded to the specific language of any of the bills I have introduced.

I respect the ability of this committee.

I would urge you to bring out as early as possible a strong tough law on the matter of dumping, and the matter of the pollution of our coastal waters and estuaries.

I came here this morning to tell you from the point of view of a person who has lived very close to the sea all of his life, that I see what is developing, and I think something needs to be really done.

Let me describe my background. I am a native of Florida. I have lived near the gulf all of my life. I live on one of the most highly polluted estuary waters in the United States. That is not something to brag about, but it is a fact.

The port of Tampa is one of the largest, probably the eighth or ninth largest port in the United States.

The Bay of Tampa has been badly treated by the merchant marine and by the people of the Tampa area.

Last month when I was home, I took a tour of the bay, and it was the filthiest body of water I have seen. It has gotten progressively worse. It is a combination of things of sanitary sewage dumping, run-off from the streets, and also dumping from plants in the area, as well as the merchant marine ships in that area.

As you recall, we had one of the largest oil spills in my bay last year, and we have had very serious repercussions from that.

On the gulf coast of Florida, as far as the committee jurisdiction is concerned, you will find the bay scallops, and the small fish that used to grow in the bays around there no longer exist.

For years I have fished, and retrieved scallops and things of that sort from this area, and they no longer exist.

The whole opportunity to catch fish and use the estuary qualities of the bay has practically ceased to exist in my part of Florida.

It is a very hardy fish that can survive in the kind of pollution that has been created there.

The gulf beaches of Florida have become littered with broken bottles, with trash of all kinds, with all kinds of floating things from ships, and from people who are careless in their use of the beaches, and from people who use fishing piers in that area.

I think that in drawing a law, you must make sure it touches all of these things.

At Christmas time, I was using my boat, and I landed on a little island in the mouth of one of the passes, and there had been quite a bit of erosion.

Frankly, it was almost impossible to use the beach in a barefooted condition, because there were so many broken bottles on it.

I realize that is not completely within the jurisdiction of this committee.

It is not something the Merchant Marine and Fisheries Committee ordinarily would be taking up, but I think someone in the Congress must do so.

Some method must be found, either by putting a tax on bottles, requiring them to be returned, or to require them to be made from material that does not shatter, so that the beaches won't be a complete boobytrap for people.

I know of these hazards personally. A few years ago, I went in swimming in front of my house on the Gulf of Mexico, and I tripped, fell down, and put my hand down on the bottom, and cut myself on a very jagged piece of glass.

This is the kind of thing that is happening today. I say today because it is something that is happening more frequently.

For years I have fished in the gulf waters, and I find that there is more and more trash in these waters, beer cans, bottles, everything you can think of.

Fishing fleets have been putting out for years, and I have watched nets retrieved on my shore, and frankly now, you catch a few fish, and an awful lot of bottles and can. These are coming from not only people but from boats on the sea, from fishing piers, and from party boats.

Although the beaches in the Miami area, are some distance from my area, I have gone there many times to fish.

After the war, they said because so many subs, and so many merchant marine ships were sunk, there was a lot of oil on the beach.

Here 25 years or a quarter of a century since that time, there is still fresh oil floating up on Miami Beach, and you cannot go swimming at any of the most expensive hotels, or any of the hotels, down there, without having to come out and practically take a bath in some kind of solvent to get the oil off your feet.

It gets on everything.

Of course, I think most of this is coming from ships that just dump the material out, helter-skelter all over the ocean.

I think the time is now, and the time is very critical, and I hope that you gentlemen will use all of the skill and all of the determination you have on this committee to do something about it. It needs to be done.

Mr. DINGELL. Thank you.

This committee is grateful to you for your very helpful testimony.

The committee would appreciate any suggestions you might give us with regard to amendatory language to the administration's bill.

We thank you very much for your fine statement, Mr. Gibbons, and we appreciate your help.

Mr. GIBBONS. Mr. Chairman, I have a prepared statement I would like inserted into the record.

Mr. DINGELL. Without objection, let the prepared statement of the gentleman appear at this point in the record.

(The statement follows:)

STATEMENT OF HON. SAM GIBBONS, A REPRESENTATIVE IN CONGRESS FROM THE STATE OF FLORIDA

Mr. Chairman: Thank you for this opportunity to present testimony to this Committee concerning the need to regulate the dumping of waste materials into the ocean. I had the privilege of introducing H.R. 4218, a bill to prohibit the discharge into any of the navigable waters of the United States or into international waters of any military material or other refuse without a certification by the Environmental Protection Agency approving such discharge. I am also a co-sponsor of H.R. 807, a bill to amend the Fish and Wildlife Coordination Act to provide additional protection to marine and wildlife ecology by providing for the orderly regulation of dumping in the ocean, coastal, and other waters of the United States.

Recent reports have left no doubt that our current practice of haphazard disposal of wastes by ocean dumping has seriously damaged certain areas of our coastal zone and is on the verge of causing worldwide, irreversible environmental effects. In order to alleviate the existing problem and to forestall it from becoming worse, many members of the Congress have introduced, either as individuals or in conjunctions with one or more of their colleagues, legislation regulating ocean dumping. I am told that the number of bills dealing solely and directly with ocean dumping exceeds 44, and that many other measures concerned with water pollution or with coastal zone management have sections regulating ocean dumping. Because there have been so many measures introduced, I would like to briefly review the major provisions of HR 4218 and HR 807.

HR 4218 is a very short bill, yet quite inclusive. It states that after the date of enactment, no person shall discharge, either directly or indirectly, into any of the navigable waters of the United States or into international waters, any munitions, or any chemical, biological, or radiological warfare agent, or any other military material except in accordance with a certificate issued by the Administrator of the Environmental Protection Agency. Further, this bill would prohibit the dumping of any other kind of refuse material of any kind or de-

BEST COPY AVAILABLE

scription whatever except as approved by the Administrator of the Environmental Protection Agency.

HR 807 is far more specific in its approach. It identifies the waters to be protected including the oceans, gulfs, bays, salt-water lagoons, and other coastal waters where the tide ebbs and flows, the Great Lakes, and all waters in a zone contiguous to the United States extending twelve miles seaward from the baseline of the territorial seas as provided for in the Convention of the Territorial Sea and Contiguous Zone.

Like HR 4218, this bill declares that the Administrator of the Environmental Protection Agency shall be responsible for administering this act. The Administrator will, in conjunction with the Secretary of the Interior, and in consultation with the Secretary of the Army, establish standards for the deposit or discharge of waste materials into the coastal waters of the United States. The kinds of materials which will be regulated include dredge spoil, sewage sludge, industrial wastes, building rubble, and all other materials which might be harmful to the quality of the receiving waters or to its inhabitants. The purpose, of course, of the standards program is to guarantee that disposal of waste materials will cause no damage to the natural environment.

In the administration of this act, the person seeking to discharge waste will bear the burden of proof that his action will not violate the standards established, and he must present evidence to this effect before any permit can be granted. The Administrator may, at his discretion, include additional requirements that he feels are necessary for the orderly regulation of ocean dumping.

These standards will be adopted and enforced by any arm of the Federal and State Governments issuing any license, permit, or any other authorization which regulates dumping into coastal waters. Further, these standards will be applicable to all the departments, agencies and other instrumentalities of the Federal Government, the various State governments involved, and to any person operating under any kind of license or permit from any of these authorities.

Unless the State standard is more stringent than the one established by the Administrator of the Environmental Protection Agency, the Federal standards will apply in all coastal waters. The decision as to which standard applies in the various State jurisdictions will be made by the Administrator. The actual legal jurisdiction shall fall to the District courts of the United States. Violation of the standards will make a person liable to a civil penalty of not more than $10,000 nor less than $5,000. In the case of a continuing violation, each day counts as a separate offense.

Upon the effective date of this Act, all licenses, permits, and authorities which have been issued under any other provision of law shall be terminated.

Mr. Chairman, I feel that there is real need for the Congress of the United States to enact strong legislation establishing a national policy on ocean dumping and establishing an effective mechanism for regulating this common and widespread method of waste disposal. There is no doubt of the destructiveness of promiscuous ocean dumping. Within the last year, several excellent reports have been released documenting the extent of environmental damage being wrought. An example of the extent of this abuse may be inferred from just one statistic taken from the report submitted to the Secretary of Interior on June 24, 1970, entitled: "Evaluation of Influence of Dumping in the New York Bight." On page 26 of this report, the following statement is made: "During fiscal year 1968 disposal of materials in dumping grounds amounted to 17,110,144 cubic yards . . ." For purpose of comparison, this volume is almost exactly four times that of Hoover Dam on the Lower Colorado River. Bearing in mind that this is the amount of material dumped into the New York Bight alone, it is disheartening to note that the report on Ocean Dumping prepared by the Council on Environmental Quality identified 246 dumping sites off the coasts of the United States. And even at the present vast levels of dumping, the Council felt that ocean dumping is not a serious nationwide problem now but could become one within the next few years because of the rapid rate of increase in ocean dumping.

I urge this Committee to move quickly in the direction of effective legislation which will control this growing threat to the marine ecosystems of the United States. It is imperative that legislation be enacted promptly which will provide for strong and effective regulation of ocean dumping.

Mr. DINGELL. Our next witness will be the gentleman from California, Hon. Don Edwards.

STATEMENT OF HON. DON EDWARDS, A REPRESENTATIVE IN CONGRESS FROM THE STATE OF CALIFORNIA

Mr. EDWARDS. Mr. Chairman, to say that effective legislation establishing a national policy on ocean dumping is needed is to greatly understate the crisis this country is likely to observe in the near future. The relative and absolute increase of pollution in the face of disastrous health, economic and environmental consequences is shocking. The President's Council on Environmental Quality has reported that between 1964–68 an average of about 7.5 million tons of waste was dumped at sea. Without proper controls, this can be expected to climb to over 150 million tons by 1980. If such activity continues, the only and inevitable consequences we can expect is the unleashing of a Pandora's box producing a mixed bag of interrelated and almost insoluable problems.

What is astonishing is to discover that the Federal Government is the Janus-headed offender in the field of water pollution. By virtue of the Government's tardiness in handling the problem of ocean dumping, toxic pollutants have been deposited in the ocean, creating harmful and unattractive waters. By its laxity in enforcing statutes already on the books, the Government has been a conspirator to the pollution of our coastal and inland waterways. But the blame cannot end here.

Above all, what is most horrifying is that the U.S. Government has been traditionally the major offender in the area of ocean dumping. To begin with, it is estimated that the Department of Defense plans to dump 88,835 tons of munitions alone this year; exploding large quantities of explosives results in pollution and destroys marine life. The Defense Department believes that a detonation of 1,000 tons of explosives—not an unusual amount to be disposed—will be sufficiently strong to kill marine organisms from 1 to 4 miles—depending on the species.

Yet, this activity is miniscule in scope when compared with the extent of other offenses. Empowered with the right to dump sludge into waters, the Army Corps of Engineers is responsible for most if not all of dredge dumping, which experts calculate constitutes 80 percent of the weight of all ocean dumping. The Army has admitted that at least one-third of these wastes are polluted. This type of dumping is responsible for adding oxygen-demanding materials and heavy metals which are detrimental to whole communities of marine organisms.

The administration's bill is but a slight improvement over what we have on the books now. Due to the application of legislative cosmetics, H.R. 4723 would make it difficult to halt Government dumping operations. I refer specifically to the construction of section 3(e) which does not place "any employee, agent, department, agency, or instrumentality of the Federal Government" under the sanctions of section 6. Then too, because the bill fails to clearly delineate the jurisdiction of the courts, it is unlikely that private citizens will have the opportunity for a forum in which to seek redress from Government dumping operations. Moreover, section 11 of the administration's bill discourages public involvement in reporting illegal dumping activities by repealing the finder's fee provisions of the Rivers and Harbors Act of 1899.

It is for these reasons, Mr. Chairman, that I suggest the committee report out H.R. 805 as a substitute for H.R. 4723.

Mr. DINGELL. Thank you for a fine statement, Congressman.

We will now hear from our colleague from New York, Hon. Lester Wolff.

STATEMENT OF HON. LESTER L. WOLFF, A REPRESENTATIVE IN CONGRESS FROM THE STATE OF NEW YORK

Mr. WOLFF. I very much appreciate this opportunity to express my strong support for legislation to control ocean dumping. I am a cosponsor of two of the bills under consideration by the subcommittee—H.R. 808, to provide for the orderly regulation of dumping in the ocean, coastal, and other waters of the United States; and H.R. 4359, to regulate dumping of waste materials and authorize the establishment of a system of marine sanctuaries.

For several years, I have been deeply involved in the fight to restore and protect Long Island Sound, which is used for recreational purposes by residents of New York, Connecticut, and Rhode Island. Eleven million residents of those States live within 15 miles of the sound. Unfortunately, however, the sound's waters are becoming less and less appealing for recreational activities.

One of the chief causative factors in the sound's rapid deterioration has been man's dumping of his wastes—sewage, sludge, or, worse yet, untreated sewage; dredge spoils; industrial wastes. Strong Federal legislation to control such dumping is urgently needed if we are to prevent further destruction of the sound and other bodies of water, and eventually reclaim and preserve them.

Both of the bills I have cosponsored would help halt indiscriminate dumping of man's waste products in our coastal waters. H.R. 808, of which Congressman Harrington is the chief sponsor, would place the burden of proof on the person who wishes to dump to demonstrate that the waste materials would not endanger the natural environment and ecology of the area in which he plans to dump. The Administrator of the Environmental Protection Agency and the Secretary of the Interior would be authorized to establish standards to govern ocean dumping—standards to insure that no damage to the natural environment and ecology of the ocean, coastal, and other waters of the United States would result from any discharge or dumping activity. Failure to comply with the established standards would result in a fine of $5,000 to $10,000.

H.R. 4359, of which Congressman Frey is the principal sponsor, carries these principles a step further by authorizing the Secretary of Commerce to designate as marine sanctuaries those areas of the Nation's tidelands which should be protected for their recreation, conservation, ecologic, or esthetic values. These marine sanctuaries, which would be analogous to the wilderness areas in our national parks system, would be out of bounds for mining activities, industrial development, and dumping or disposal of waste material.

In addition, H.R. 4359 would prohibit the dumping of waste material into the oceans, coastal waters, and estuarine areas, except under a permit issued by the Administrator of the Environmental Protection Agency after he determines the dumping will not damage the ecology

of the marine environment. The dumping of radioactive wastes, toxic industrial wastes, and chemical and biological warfare materials would be flatly prohibited, and standards would be established for treatment of sewage and industrial wastes disposed of under a permit. Violation of the permit requirement would be punishable by a fine of $2,000 to $10,000 for a first offense, and $10,000 to $25,000 for each succeeding offense.

Recent news reports about the contamination of marine life, including the fish we eat, by mercury, DDT, and NTA, and the destruction of the waters of the New York Bight through excessive sludge dumping, prove the danger of indiscriminate dumping. Remarkable as the marine ecosystem may be, it cannot continue to cleanse itself indefinitely while man dumps infinite amounts of waste into the ocean.

Action to protect our marine environment is urgently needed, and I urge adoption of a strong bill by these subcommittees and by your full committee.

Mr. DINGELL. The subcommittee appreciates your time for an excellent and informative statement.

Next we wish to have our good friend and very able minority leader of the House, Hon. Gerald R. Ford.

STATEMENT OF HON. GERALD R. FORD, A REPRESENTATIVE IN CONGRESS FROM THE STATE OF MICHIGAN

Mr. FORD. Thank you Mr. Chairman. The horrendous problems created by our pollution of the environment have finally come to the forefront of the national conscience and are receiving the attention they deserve.

Cleanup has become an everyday word. But how much better it would have been had we forestalled the pollution which is necessitating cleanups throughout the Nation and throughout the world.

There are environmental problems that are far more critical than ocean dumping. But there is no time better than the present for acknowledging that the current level of ocean dumping is creating serious environmental damage in some areas.

We should recognize now that the volume of wastes dumped in the ocean is increasing rapidly.

We should warn ourselves now that a vast new influx of wastes is likely to occur as municipalities and industries look to the oceans as a convenient spot to dump their wastes.

We should view with alarm now the trends indicating that ocean dumping could become a major, nationwide environmental problem.

The oceans cover nearly three-fourths of the world's surface. They are critical to maintaining our environment. They contribute to the basic oxygen-carbon dioxide balance upon which human and animal life depends.

We must act now to safeguard our basic environmental balance by banning unregulated dumping of any materials into the oceans and by strictly limiting the ocean disposal of any materials harmful to the environment.

I have introduced a bill, H.R. 6771, which would accomplish these objectives. Needless to say, I strongly endorse the identical bills now

being considered by the subcommittee. I urge favorable committee action on this legislation.

Mr. DINGELL. Thank you Gerry, we are very grateful for your thoughts.

The gentleman from Pennsylvania, the Hon. Lawrence G. Williams, will be our next witness.

STATEMENT OF HON. LAWRENCE G. WILLIAMS, A REPRESENTATIVE IN CONGRESS FROM THE STATE OF PENNSYLVANIA

Mr. WILLIAMS. Mr. Chairman and members of the committee, I am here this morning to testify in behalf of H.R. 4723, introduced by Mr. Garmatz, and H.R. 5050, which was introduced by Mr. Charles Sandman of New Jersey for himself and for a number of other members, including myself.

I believe that it is long past the time when we must stringently control the discharge of waste into our oceans in territorial and international waters. We know that there are methods in which to dispose of waste that will not contaminate our oceans or other bodies of water. These methods may be somewhat more expensive but this increased cost is a small price to pay to avoid pollution of our oceans.

I can remember as a young teenager seeing parts of Lake Erie posted for "no swimming" due to pollution. This pollution was ignored and during the 91st Congress, I heard a number of Members refer to Lake Erie as being "dead." Of course, back in the 1920's, everyone thought that Lake Erie could take everything that was put into it. This, of course, was a complete fallacy. During the intervening years, raw sanitary sewage and industrial wastes were dumped into Lake Erie until, today, Lake Erie has almost no marine life worthy of mention and the lake is entirely polluted.

Even during the 91st Congress when Members were deploring the sad condition of Lake Erie and called it "dead," raw sewage and industrial waste continued to flow into Lake Erie.

Privately, I asked some of these same Members how long it had taken the States abutting Lake Erie to kill it and why they were surprised that the Lake "died."

Precisely the same thing can happen in our oceans. In many cases, raw sanitary sewage sludge from sanitary sewage treatment plants is being taken by barge down our rivers and dumped into our oceans. I have heard some comments to the effect that this has no detrimental effect on our oceans. This is just plain nonsense.

If the solids from sanitary sewage have no effect on marine life, why has it killed all of the fish that used to be in our rivers on which metropolitan areas are located? Why has the shad disappeared from the Delaware River?

Further, what sense does it make to build sanitary sewage treatment plants costing millions of dollars in order to remove the solids from the sanitary sewage and then dump these same solids in the form of sludge back into the ocean?

We must make certain that all sanitary sewage is processed by sewage treatment plants that contain primary, secondary and tertiary facilites. This type of plant provides a 90–94 percent efficiency in treat-

BEST COPY AVAILABLE

ment when measured by the biological oxygen demand (BOD). These sewage treatment facilities must include on-site sludge incineration facilities.

The fact is that sludge incineration facilities can be built with the ultimate in air pollution controls. A proper sludge incineration plant can reduce sludge to 3 percent of its original volume and produce an inorganic ash that can be used as fill without fear of contamination.

Modern incinerators, with adequate air pollution controls, have been developed that will do almost the same thing as the disposal of trash, garbage and other solid waste materials.

Industrial waste which is now being dumped in our oceans must be treated by the industry that is producing such waste. The cost of properly treating industrial waste to avoid pollution must be part of the cost of doing business and this is just one reason why national standards must be established to control air and water pollution.

As far as dumping dangerous materials into the ocean, I am confident that proper scientific research will produce means of treating these dangerous materials chemically so that they can be rendered harmless. Of course, this chemical treatment of such things as nerve gases and other types of gases could be a slow and rather costly program. However, again, the cost would be justified by keeping these dangerous materials out of our oceans.

It is a matter of public record that thousands, and perhaps millions, of pounds of seafood have had to be taken off the market due to mercury contamination and other forms of contaminants. Unless we stop dumping waste materials of any kind in our oceans, this problem will continue to become more acute and have an adverse effect on our entire population.

I want to commend these subcommittees for devoting the time to consider these matters and I want to express my appreciation for having the opportunity to appear before you.

Mr. DINGELL. Thank you Congressman, that was an excellent statement.

Our next witnesses are from the Atomic Energy Commission. Welcome to the committee.

STATEMENT OF HON. JAMES T. RAMEY, MEMBER, JOSEPH F. HENNESSEY, GENERAL COUNSEL, AND HAROLD L. PRICE, DIRECTOR OF REGULATIONS, ATOMIC ENERGY COMMISSION

Mr. RAMEY. Thank you, Mr. Chairman.

I am James T. Ramey, member, of the Atomic Energy Commission.

I have with me Mr. Joseph F. Hennessey, General Counsel of the Atomic Energy Commission.

On my right is Mr. Harold L. Price, Director of Regulations of the Atomic Energy Commission.

Mr. DINGELL. Gentlemen, we are happy to welcome all of you to the committee.

Mr. RAMEY. Mr. Chairman, members of the committee, I have a prepared statement which I will read.

We are pleased to accept the subcommittee's invitation to appear before you today to testify concerning legislation dealing with the

problem of ocean dumping. The committee counsel has asked that we address our remarks to section 7(b) of H.R. 4723, "a bill to regulate the dumping of materials in the oceans, coastal, and other waters, and for other purposes."

We strongly s pport effective measures to protect and preserve our environment. The problem of ocean dumping has been extensively examined by the Council of Environmental Quality in its report, "Ocean Dumping; a National Policy" October 1970. In implementation of the recommendation in this report, a proposal for legislation dealing with ocean dumping was included in the President's message to the Congress on pollution control, dated February 8, 1971, and has been introduced in the House as H.R. 4723.

Since the committee is fully familiar with the provisions of H.R. 4723, rather than present a summary of the bill, I shall address myself directly to the caption "Relationship to Other Laws," in the middle of page 10 of the bill. Under that caption the bill provides in section 7 (b) that:

> Nothing in this Act shall abrogate or negate any existing responsibility or authority contained in the Atomic Energy Act of 1954, as amended, and section 4 and subsection 7(a) of this Act shall not apply to any activity regulated by that Act: Provided, the Atomic Energy Commission shall consult with the Administrator prior to issuing a permit to conduct any activity which would otherwise be regulated by this Act. In issuing any such permit, the Atomic Energy Commission shall comply with standards set by the Administrator respecting limits on radiation exposures or levels, or concentrations or quantities of radioactive material. In setting such standards for application to the oceans, coastal, and other waters, the Administrator shall consider the policy expressed in subsection 2(b) of this Act and the factors stated in subsections 5(a)(1) and 5(a) (2) of this Act.

Under the Atomic Energy Act of 1954, as amended, the Atomic Energy Commission regulates the receipt, use and disposal of source, special nuclear and byproduct material to assure the common defense and security and public health and safety. AEC regulations governing the disposal of radioactive materials have been established in 10 CRF parts 2, 20, 30, 40, and 70.

Because of the nature of radioactivity, the AEC has vigorously exercised its authority in regulating the marine disposal of radioactive wastes and materials. No new licenses authorizing radioactive waste disposal at sea have been issued in the past 10 years. Only one commercial organization (which has never conducted any sea disposal), two Government agencies, and one university are still authorized to dispose of radioactive wastes in the ocean. The major contractors of the AEC have not disposed of any wastes at sea since 1962.

We believe that the AEC has exercised and is exercising effective regulation over the ocean disposal of radioactive substances to the extent that this type of operation poses no threat to the marine environment now or in the foreseeable future. In this regard, the AEC has as one of its primary responsibilities the protection of the ocean and its ecosystem from any harmful effects of radioactivity.

Thus, we believe that the policy and purpose of the proposed bill, that is, "to regulate the dumping of materials in the oceans, coastal, and other waters and for other purposes," already is being effectively carried out with respect to radioactive substances regulated by the AEC. Section 7(b) recognizes this fact and continues, in effect, the

full licensing and enforcement authority of the AEC over ocean disposal of radioactive material. This provision eliminates the possibility of dual regulation over these materials, yet it provides for full participation by the Environmental Protection Agency in the program. The provision in section 7(b) of the bill requiring consultation and the setting of standards is a useful one in that it will assure an "across-the-board" approach to the problems associated with ocean dumping. Also, it will assure that the relationship between EPA and the AEC will be consistent with the relationship set forth in Reorganization Plan No. 3.

In continuing to regulate the disposal of radioactive materials at sea under the provisions of section 7(b), the AEC would plan to continue its practice of prohibiting the disposal of high-level radioactive wastes in the ocean. We believe that ocean disposal of other solid radioactive wastes should be prohibited to the extent that practicable alternatives are available which provide less risk to man and his environment.

I will be glad to answer any questions you may have.

Mr. DINGELL. We are very grateful to you for your very helpful testimony.

Mr. Lennon.

Mr. LENNON. Thank you, Mr. Chairman.

I want to be as specific for the record as I can. Your representation here this morning that you gentlemen are making is on behalf of the Atomic Energy Commission, and you are the representatives of the Atomic Energy Commission, is that correct?

Mr. RAMEY. Yes, sir.

Mr. LENNON. Are either one of you three gentlemen members of the Commission?

Mr. RAMEY. Yes, sir; I am.

Mr. LENNON. Well, I am delighted to see this spirit of cooperation and recognition on the part of the Atomic Energy Commission, and for the sake of the new members, I am going to just quickly recapitulate my experience with the Atomic Energy Commission last year.

When the Congress learned after the fact the decision had been made to dump off Cape Kennedy some 416, as I recall, coffins of nerve gas, which was, and I recall that the Secretary of the Army, and the recognition that the fuses on the rockets, and the propellers were deteriorating, and, therefore, there was some reason to believe there was imminent danger, so that the Secretary of the Army called on the National Scientific Foundation to recommend an ad hoc committee, of scientists to determine how best we could dispose of the caskets, or coffins of the nerve gas. He convened this committee of scientists, and they came up with recommendation No. 1, that they could be disposed of by the Atomic Energy Commission, and the Atomic Energy Commission was asked to look into the matter, which they did. This committee of scientists said we do not have experience in ordnances, munitions, or explosives, and they suggested to convene an ad hoc committee of these gentlemen who have experience, and they came up with the same specific and definitive recommendation, that the Atomic Energy Commission be called on to dispose of these 416 plus coffins of nerve gas, and the Atomic Energy Commission in turn went to its, I believe you called the Lawrence Radiation Laboratory, and they came back, and said yes, we can dispose of it.

BEST COPY AVAILABLE

They said, we have a place, where it can be done, but it will interfere with—if you gentlemen want to read the record, I will not misstate anything, but it will interfere with our on-going projects.

We suggest you ship it out here. This is a recommendation of your laboratory, ship it out here, and we will store it in a safe isolated spot, and in time we will dispose of it.

I recall in those hearings, some of them were held in the evenings, nighttime, and we confirmed this, and the Commission made a decision that this was not politically the smart thing to do for the Atomic Energy Commission, instead of writing to the Army, and putting it in writing, saying, we do not want to do this, because of the problems we are having around the country with atomic energy reactor power, we suggest you move it from Alabama, out of Blue Grass Kentucky, down to a point in North Carolina, and ship it to Cape Kennedy, and dump it in the ocean, but you did not put it in writing.

You called the Army late one night, and you told them you just could not get involved in it, and the Army did not have the intestinal fortitude——

We call it guts in my part of the country, to take that decision to the President, and let him resolve it, and I lost most of the respect I ever had for the Atomic Energy Commission, because of your unwillingness, or gutless attitude in that respect, and as much as I love the services, and I am on the Armed Services Committee, I lost a great deal of respect for them, that they did not have the courage to take that decision to the President.

I am sure he would have followed the recommendation of your laboratory, the Lawrence Laboratory.

Now, you have moved right far, and I am delighted to see it.

If you gentlemen do not believe what I said is the truth, all I ask you is to do is to take the record and read it.

It is all in there.

That is the reason I am delighted that you have finally accepted the fact that Government agencies should be as responsible for ocean dumping as private enterprise.

I am pleased to see the change in attitude, but having lived with this thing between the Federal district court, and North Carolina was a trying experience. We brought the Surgeon General here before the committee, and he having just made a public statement that they were giving every hospital in the entire area some sort of antidote to be injected in case anybody inhaled this gas. We asked him, how long it took him to park his car, and get to his office, and he then said you would take this injection of antidote within 2 minutes, from the time you inhaled it, or within 10 minutes, or it was lethal. When he came here, I asked him the question if he drove to work occasionally. He said he did. I said how long does it take you to get from your car where you park it in the office, and he said about 10 or 12 minutes.

I said then the only way this antidote would avoid a lethal dose of this gas would be to be fortunate enough, unfortunate enough to be sitting in the emergency room of a hospital, but not for that purpose, and after you inhaled the gas that came through the window, you might avoid it by getting an immediate injection. I want to make that crystal clear to you gentlemen, it was your decision which caused those coffins to be dumped off the Florida coast.

62-513—71——16

You told the Army no, we do not want to do this, our political interests would be hurt, and you did not have the guts to put it in writing.

You did it in a late telephone call one night.

Thank you.

Mr. DINGELL. Thank you very much.

Mr. RAMEY. Mr. Chairman——

Mr. DINGELL. I think you are entitled to at least say something at this point.

Go ahead.

Mr. LENNON. And then that will permit me to say something. (Laughter.)

Mr. RAMEY. Mr. Chairman, I would be glad to provide for the record a little statement on this.

This sort of starts us out in the hole, you might say. Of course I was familiar with this problem when it occurred. However, I do not recollect that, and I would doubt that, the Commission would recommend any type of disposal of some other agency's materials.

One of the things the Commission gets accused of a lot is of not being very aggressive in any of its recommendations.

I would also point out that the method of disposal of these gases so far as the possibilities of it being done by the Atomic Energy Commission through nuclear means, was a type of disintegration using atomic explosives underground. This was a method that some of the laboratory people thought could be effective, but it was one that was essentially untried, and in the required time sequence this would have been a rather difficult thing to have been accomplished. The Commission did not believe that it could undertake this—certainly not at that time.

That is about all I have to say, Mr. Chairman.

Mr. DINGELL. We thank you very much, Commissioner.

Mr. Pelly.

Mr. PELLY. I have just one question, Mr. Chairman.

I would like to ask the Commissioner why is it possible for the Commission to revoke the authority that he referred to in the statement?

How can he revoke that authority of other agencies when his own agency is disposing of the same radioactive waste of one university and two agencies that I know of?

Mr. RAMEY. These are essentially licensees involved in research activities, and the licenses are in the process of being terminated.

Mr. PELLY. I think for the record, we would like you to name the university and the two agencies.

Mr. RAMEY. One of the agencies is the NIH, and the other is the National Oceanographic and Atmospheric Administration. The university is the University of Hawaii.

Mr. PELLY. And then there is one commercial organization, is there not?

Mr. RAMEY. There is one commercial organization, yes, sir. The California Salvage Co. of San Pedro, Calif.

As I mentioned, they have not engaged in the actual disposal of radioactive waste.

Mr. PELLY. It might be well before they do to revoke it, because that word "salvage" kind of scares me.

It sounds like they want to get rid of anything they have.

Anyway, I raised the point, and you have indicated that the Agency has taken its course. I would indicate that you implement that action.

Mr. DINGELL. Thank you.

Mr. Rogers.

Mr. ROGERS. I would like to question on this.

How much has been dumped in the ocean?

Mr. RAMEY. Mr. Price, do you have the figures?

Mr. PRICE. We have some figures, Mr. Rogers, going all the way back to 1946.

Mr. ROGERS. Why don't you just give us the last two nearest.

Mr. PRICE. 1970?

Mr. ROGERS. Can you submit that?

Mr. DINGELL. Without objection, the full list will be inserted in the record at this point, subject, gentlemen, to appropriate discussion of your staff and our staff with regard to the actual security questions.

(The subject list follows:)

SEA DISPOSAL OF RADIOACTIVE WASTES—HISTORICAL TRENDS, 1946-70

Year	Number of containers	Estimated activity at time of disposal (in curies)
1946 through 1960	76,201	93,690
1961	4,087	275
1962	6,120	478
1963	129	9
1964	114	20
1965	24	5
1966	43	105
1967	12	62
1968	0	0
1969[1]	36	26
1970[1]	22	3
Total	86,758	94,673

[1] The number of containers differs from the number contained in ocean dumping—A national policy, A report to the President prepared by the Council on Environmental Quality, October 1970 to reflect information recently received on disposals made by the University of Hawaii in 1969 and 1970. The quantity of radioactive material disposed by the University of Hawaii in 1969 was approximately 7 millicuries and in 1970 approximately 25 millicuries which does not significantly change the quantity of radioactive material disposed in 1969 and 1970.

Mr. PRICE. In the last 2 years: 1970, two containers, 3 curies; 1969, 26 containers, 26 curies.

Mr. ROGERS. What is a curie?

Mr. PRICE. It is a measure of radioactivity.

Mr. ROGERS. How could you explain it to a layman?

Mr. PRICE. I will have to get some technical help, please.

Dick Cunningham, could you say something?

This is Richard Cunningham.

Mr. CUNNINGHAM. A curie is a measure of radioactivity in terms of a rate of decay. It is 3.7×10^{10} disintegrations per second.

It is a measure of rate of decay of radioactive material.

Now, as an example, an average wristwatch being manufactured today has 5 millicuries on it, and a millicure is one-thousandth of a curie.

Mr. ROGERS. In other words, the curie is a thousand times more than you have in your wristwatch?

Mr. CUNNINGHAM. Yes, approximately.

Mr. ROGERS. And you say it is the rate of disintegration?

Mr. CUNNINGHAM. Yes.

Mr. ROGERS. How long does it take to disintegrate?

Mr. CUNNINGHAM. That depends on the type of radioactive material.

Mr. ROGERS. Give us this example of what you are dumping in the ocean.

Mr. CUNNINGHAM. Sir, I do not know what radioisotopes were involved on the list of disposals I have here.

The rate of decay, depends on the type of radioisotope.

It may go to half its activity in seconds or it may take hundreds of thousands of years, depending on the radioisotopes.

Mr. ROGERS. Could we estimate?

Mr. CUNNINGHAM. For any specific radioisotope, yes, sir; we do know.

Mr. ROGERS. Do you have those figures on the dumpings?

Mr. CUNNINGHAM. I do not have them available here.

Mr. ROGERS. Does the Commission have them?

Mr. RAMEY. Yes, sir; we do.

Mr. CUNNINGHAM. Could you let us know what those are for the record?

Mr. PRICE. We have those; yes, sir.

Mr. CUNNINGHAM. We have the coordinates on the dump locations.

Mr. ROGERS. Where have these been dumped?

Mr. PRICE. In the Atlantic Ocean, beyond the Continental Shelf, east of Norfolk.

Now, all of these places are beyond the Continental Shelf, and we could furnish for the record the exact locations.

Mr. ROGERS. I would appreciate that.

(The information follows:)

The radioactive material disposed of at sea contained types and quantities of radioactive material associated with research and development activities of the Atomic Energy Commission, use of radioactive materials by hospitals, universities, industrial firms, and other places where radioisotopes are used for various purposes. Such wastes were most often in the form of contamination on equipment such as test tubes, bottles, rubber gloves, paper wipes, etc. Most of the radioactivity involved radioisotopes with atomic numbers 1 through 83. It also includes some uranium and thorium and to a very much lesser extent, transuranium elements.

Mr. ROGERS. Now, is the dumping supervised?

Mr. PRICE. Well, the packaging is supervised, but we do not send somebody out.

I cannot tell you for sure whether we send somebody out on each ship.

I think we have in cooperation with the Coast Guard and the NIH in connection with their dumping, but I am not sure.

Mr. CUNNINGHAM. The Coast Guard has taken the NIH materials out to sea.

On other dumpings, we have had an inspector on board ship.

Mr. ROGERS. In all instances?

Mr. CUNNINGHAM. No, sir.

Mr. PRICE. Not in all.

Mr. ROGERS. I think this would be a rather important point.

You leave it to a contractor or some agency. I am not sure they go all the way out, as you say, or put it in the proper place.

How do you know they do?

Mr. PRICE. Well, if we are not there, we cannot be sure.

Mr. ROGERS. That is correct.

Mr. PRICE. But these quantities, they are so small, that it really is not very important.

Mr. ROGERS. Even though you are dumping them all at the same spot, maybe, there is no buildup?

Mr. PRICE. No, sir, we do not think there is.

Mr. ROGERS. You do not think so?

Mr. PRICE. Not for these quantities, that is right, sir.

Mr. RAMEY. Last year there were only two containers.

Mr. ROGERS. But there were 26 in the year before.

What is the highest you had?

Mr. PRICE. In 1961, 4,000 containers, approximately 275 curies; 1962, 6,000 containers, approximately 478 curies.

Now, they were not all at one place. Some of them were off Norfolk. Some of them were off New Jersey.

All of them were out at about 150 miles.

Mr. ROGERS. What is the area that you had been concerned with recently?

Mr. PRICE. We have stopped ocean dumping effectively.

Mr. ROGERS. What is the level that you are really concerned with?

Mr. PRICE. I do not think we know of a level, Mr. Rogers.

Mr. ROGERS. You could dump any amount, is that right?

Mr. RAMEY. No, sir. The permissible level of radioactivity on the surface of the cask effectively limits the quantity of radioactive material which can be handled and dumped. But back in the late fifties, and early sixties, there was concern expressed, particularly on the problem of a few casks which were allegedly washed up. But these were low-level radioisotopes being disposed of; and with the means of land disposal the Commission has adopted, it is no longer a problem.

Mr. ROGERS. I do not think you have quite gotten to what I asked.

I asked at what level do you consider a dangerous amount, and you said some of them even washed up on shore.

Mr. RAMEY. A very few, yes, sir.

Mr. ROGERS. When did that happen?

Mr. RAMEY. Back in around 1959, 1960, 1961.

Mr. ROGERS. Have we had any other examples of that washing ashore?

Mr. RAMEY. Not in recent years.

Mr. PRICE. There have been none in recent years.

I think, Mr. Rogers, if we would be permitted, we would like to supplement the record on this quantity safety situation that you have raised, because back in the early sixties, the National Academy of Sciences published a report on ocean disposal, in which they concluded that there were about 25 sites along the Atlantic coast, I think it is about 25, close in, as close as 3 or 4 miles, where it would be perfectly all right to dump *x* quantities per year.

We never did that, but it was in the furor over that report that people thought we were dumping in those close-in areas, in those quantitie-, that we in effect stopped all ocean dumping for all practical purposes, except for these few little amounts that are now being cleaned up, so I think we would like to supplement the record in the light of that report, because I do not think we today can give you a number that would say up to here it is all right, and beyond that it is not, but that report would help to put this in perspective.

Mr. ROGERS. I think we should have that, and I think we should have a summary on where the heavy dumpings have been.

(The information follows:)

DUMPING SITES OF RADIOACTIVE WASTES

The major areas used for dumping of radioactive wastes in the ocean were at the following locations:

ATLANTIC OCEAN

1. 30°38′N, 72°06′W approximately 150 miles southeast of Sandy Hook.
2. 37°50′N, 70°35′W approximately 230 miles southeast of Sandy Hook.
3. 36°56′N, 74°23′W approximately 105 miles east of Cape Henry, Virginia.
4. 42°25.5′N, 70°35′W approximately 12 to 15 miles from the coast in Massachusetts Bay.

PACIFIC OCEAN

1. Within an area bounded by points designated as
37°38′N, 123°18′W; 37°38′N, 123°30′W;
37°43′N, 123°24′W; and 37°43′N, 123°30′ W
approximately 48 miles west of the Golden Gate.
2. 33°39′N, 119°28′W approximately 53 miles west of Point Vicente, California.

NOTE: All sites listed above are beyond the continental shelf where the depth exceeds 1000 fathoms except No. 4 for the Atlantic Ocean which was a toxic chemical dumping area designated by the Corps of Engineers. The last disposal at this site was in August 1959.

Mr. ROGERS. Do you monitor where you dump this material?

Mr. PRICE. We did where we were authorizing the dumping.

There was a periodic monitoring of these dumping areas.

Mr. ROGERS. How often?

Mr. PRICE. We would have to check the record.

Mr. ROGERS. But there is no more monitoring?

Mr. RAMEY. There is very little dumping.

Mr. ROGERS. It is already down there. We are not monitoring what may be happening then?

Mr. RAMEY. I think it is not being monitored now.

Mr. ROGERS. I think we ought to know that for the record.

Mr. PRICE. We would be glad to clear the record.

(The information follows:)

MONITORING OF RADIOACTIVE DUMPING SITES

In October, 1957, a survey of the Atlantic Ocean disposal area located approximately 150 miles southeast of Sandy Hook was conducted by the Chesapeake Bay Institute in cooperation with the U.S. Coast and Geodetic Survey. The survey consisted of taking a series of samples of ocean bottom in and near the disposal site. Radiological analyses of these samples indicated no radioactivity detectable above background levels. A similar survey by the Scripps Institute of Oceanography of Pacific Ocean disposal areas located off the Farallon Islands and in the Santa Cruz Basin indicated comparable results.

In June, 1959, a site in Massachusetts Bay was surveyed by the U.S. Coast and Geodetic Survey. Core samples, sediment samples, and water samples were

analyzed. There was no indication that there had been any change in the level of radioactivity at the site as compared with levels of radioactivity found at other ocean sites where no wastes were disposed.

In March, April, and November of 1960, studies were made by the Pneumo Dynamics,Corporations, El Segundo, California, of the two Pacific Ocean sites noted above. Assays of samples of bottom sediment, organisms, and bottom-caught fish revealed no evidence of radioactivity above natural background levels.

The sea disposal of radioactive waste diminished considerably in the 1960's because of the opening of land burial facilities for disposal of waste. In view of the 1957–1960 survey results and the diminution in the quantities of radioactive wastes dumped in the ocean since the studies were made, there has not appeared to be a need for further monitoring studies of the type conducted. Accordingly, no further surveys of waste disposal sites have been made.

Mr. ROGERS. Would 275 curies injure anybody in this room?

Mr. PRICE. It certainly could, but not on the bottom of the ocean.

Mr. ROGERS. If it washed ashore?

Mr. RAMEY. 275 curies would not be injurious in a cask. Not in the way it would be shielded.

Mr. ROGERS. If it is shielded, what is the point of dumping it out in the ocean?

Mr. RAMEY. It was a means of providing for permanent disposal.

Mr. PRICE. Please understand, we are not arguing now for ocean dumping.

We have effectively stopped it, but in the time when waste was being dumped it was mostly in solid concrete mixtures in 55 gallon drums designed to sink to the bottom in whatever the depth is beyond the Continental Shelf.

Mr. ROGERS. But evidently some did not.

Mr. PRICE. I think a few drums did wash up on the shore, and there never was——

I would have to go back to the record.

This is a long time ago.

Mr. ROGERS. I would be concerned about this, because if you have dumped 6,000 drums 1 year, 4,000 drums another, it seems to me without any monitoring, I do not know how many would be out there, and I do not think this committee would, and I think we should at least have some monitoring.

Mr. PRICE. There was a followthrough during those years.

I do not believe it has been continued in recent years, but I would have to supplement the record on that.

Mr. ROGERS. I understand, but I think it ought to be done.

I should think in quantities of that amount, they are dumped off the shore, and where we have had examples where they have come into the shore, we certainly ought to keep track of this.

Mr. PRICE. I will submit some information on that.

(The information follows:)

RECOVERY OF ITEMS DUMPED IN THE OCEANS

The following summaries identify the 13 occasions on which drums were found either washed ashore or picked up at sea. Of the 13 cited items, only 3, 11, and 12 appeared to involve radioactive materials. None of these three occasions involving radioactive materials appeared to have resulted from any violations of Atomic Energy Commission regulations and, consequently, no action was taken by the Atomic Energy Commission.

1. *July 1959.*—A 55-gallon drum washed ashore at Coos Bay, Oregon, was thought to contain radioactive material but analysis determined the contents to be lubricating oil and sea water. No radioactive materials were associated with either the drum or its contents.

2. *September 1959.*—A 55-gallon drum was found on a beach at Tacoma, Washington, on September 27, 1959. The drum bore a radiation symbol and the words, "Do not open." Investigation showed that neither the drum nor its fluid contents were radioactive. The fluid contents were a type of mineral oil used in refrigerator compressors. No information could be obtained as to the source of the drum.

3. *September 1960.*—A 30-gallon drum was netted on September 29, 1960, in about 275 feet of water about 12 miles east of Marblehead, Massachusetts. The drum was examined by the Coast Guard, Boston, Massachusetts, on October 3, 1960, and no breaks were found in the drum. The radiation levels at the surface of the drum were found to be well below the radiation levels permitted for normal transportation of radioactive materials as specified in Interstate Commerce Commission regulations. Crossroads Marine Disposal Corporation, an AEC license, identified the drum as one dropped by it. Crossroads took possession of the drum and shipped it to Oak Ridge National Laboratory for disposal.

4. *October 1960.*—On October 22, 1960, Mr. Eisendrath, Daily Herald, Biloxi, Mississippi, informed Oak Ridge Operations Office that some picnickers had found a plastic container with a wooden top and bottom on the beach at Biloxi. The container had stenciled on it, "Danger—Radiation. Use Equipment Within 10 Feet. AEC 19637 MISS." The container was not seaworn and appeared to have been recently placed in the water. The police were informed and they notified Kessler Air Force Base. A representative from the Air Force Base surveyed the container with a radiation survey meter and found no evidence of radiation. The container was disassembled and the inside of a vacuum bottle was found therein. Inside the vacuum bottle insert were flashlight batteries connected to a buzzer which was activated when the container was moved. It was concluded that this was a hoax.

5. *March–April 1961.*—Seven commercial fishing trawlers netted about 40 steel drums about 80 miles southeast of Manaquan, New Jersey. All the drums were dumped back into the sea by the fishermen shortly after they were netted. No evidence was found by AEC that any radioactive waste had been disposed of in that area, nor was there any evidence that the drums contained any radioactive materials.

6. *January 1962.*—Three steel drums, two of 55-gallon size and one of 30-gallon size were netted by a fishing trawler off the New Jersey coast. Two of the drums contained a plastic-like material and the third drum was empty. A sample of the material was analyzed and found to be organic material and non-radioactive. The Bureau of Explosives concluded that the material was a residue from a plastic manufacturing process.

7. *June 1962.*—Ten drums were found along the Florida coast which were determined to contain metallic sodium and were not radioactive. They were taken out to sea and destroyed by a Naval Demolition Team. The drums were from a shipment aboard the motor vessel "Heedless" which sank in the Gulf of Mexico on January 29, 1962.

8. *July 1962.*—A 2-inch by 2½-inch cork wrapped in lead foil was found on the beach at Oceanside, Oregon. There were no AEC markings but there was a radiation symbol, apparently attached as a tag. It did not appear to have been in the water very long. It was taken to the Coast Guard Station at Garibaldi, Oregon. The cork was surveyed at Hanford and no radiation above background was found.

9. *March 1965.*—A refrigerator door was found on the beach at Long Branch, New Jersey, on March 28, 1965. There was a 1-inch by 6-inch tape on the door with the radiation symbol and the words, "Caution—Radioactive Material." The Safety Director from Fort Monmouth, New Jersey, surveyed the door and the beach in the immediate vicinity and found no radiation above background.

10. *April 1967.*—Four drums and three smaller containers washed ashore near Gloucester, Massachusetts. Investigation by the Coast Guard revealed that the contents were petroleum derivative wastes and were nonradioactive. They had been dumped 10 miles out by a Massachusetts firm.

11. *May 1967.*—A fishing boat netted a concrete container about 5 miles off the coast at Scituate, Massachusetts. Markings that were noted on the container before it was dumped back overboard identified it as a container that had been delivered to Crossroads Marine Disposal Corporation in August 1957 for disposal

by the Naval Research Laboratory, Washington, D.C. It reportedly contained radium dials and thorium oxide but was dumped back overboard before authorities could check it. Surveys of the vessel showed no evidence of contamination.

12. *September 1968.*—The fishing trawler "Resolve" picked up three drums while trawling about 60 miles off Atlantic City. Two of the drums were returned to the ocean by the "Resolve." A Coast Guard Cutter rendezvoused at sea with the "Resolve" and monitored the third drum and found a maximum radiation level of 0.7 milliroentgens per hour. The drum was returned to the ocean. The drums appeared to have been in the area for several years and had started to deteriorate so that it was not possible to identify them. A survey of the trawler by the Virginia State Health Department upon return of the trawler to Hampton, Virginia, revealed no contamination.

13. *July 1970.*—The Coast Guard at Galveston, Texas, reported on July 30, 1970, to the Manned Spacecraft Center at Houston, Texas, that a shrimp boat had retrieved a 55-gallon drum, which was labeled "Atomic Waste," from the Gulf of Mexico on July 29, 1970, and had docked at Freeport, Texas, with the drum aboard. Investigation was made by a Coast Guard monitoring team from Freeport and the investigation revealed that the drum was labeled "Resin Paint Thinner," bore no "Atomic Waste" wording or caution labels, and was not radioactive.

Mr. ROGERS. Now, I would like to know something about the relationship between the Environmental Protection Agency and the Atomic Energy Commission.

I notice now in all of these pollution matters, there is always a little clause that nobody can tell the Atomic Energy Commission what to do.

Now, you have the final determination on what standards will be, or does the Environmental Protection Agency?

Mr. RAMEY. The Environmental Protection Agency establishes the standards.

Under Reorganizational Plan No. 3 of the Federal Radiation Council, which had been responsible for establishing radiation standards, and its functions were transferred to the Environmental Protection Agency. The plan provided that EPA would exercise the standard setting, but that the Atomic Energy Commission would continue to license, and to implement the standards that the Environmental Protection Agency sets. This proposed legislation, concerning ocean disposals provides that the Environmental Protection Agency will provide the standards, and that the Atomic Energy Commission consult with them before any type of radioactive waste disposal could be made.

Mr. ROGERS. It says nothing in this act shall abrogate or negate any existing responsibility or authority contained in the Atomic Energy Act.

What does that mean?

Mr. RAMEY. That means that the Atomic Energy Commission has the licensing authority.

Mr. ROGERS. That is all?

Mr. RAMEY. Essentially, I believe so, yes, sir; and then at the bottom here, it provides, that:

In issuing any such permit, the Atomic Energy Commissiion shall comply with standards set by the Administrator respecting limits on radiation exposures or levels, or concentrations or quantities of radioactive material.

Just before that, it says:

The Atomic Energy Commission shall consult with the Administrator prior to issuing a permit to conduct any activity which would otherwise be regulated by this act.

Mr. ROGERS. Let me ask you a final question here.

Do you see any reason why you should continue to permit radioactive wastes to be dumped in the ocean?

Mr. RAMEY. Essentially, no; except in very minor situations.

Mr. ROGERS. Would you have the Commission review this, and let this committee know whether you are going to continue these existing permits?

Mr. RAMEY. As I indicated, we are not going to do any dumping.

Mr. ROGERS. When are you going to take action?

Mr. RAMEY. Existing licenses are in the process of being phased out now.

Mr. ROGERS. What is the time element?

Mr. RAMEY. I would say 6 months, a year.

They are essentially research activities, you understand, Mr. Rogers, by these agencies.

Mr. ROGERS. To dump wastes?

Mr. RAMEY. They are using radioactive materials for their research, as I understand it.

Mr. ROGERS. But you plan to phase all of this out?

Mr. RAMEY. Yes, sir. Now, the report by the Environmental Quality Council did indicate that there may be in the future situations where in balancing the risk and benefit, that some type of disposal might be necessary, but again——

Mr. ROGERS. Would you file an impact statement in that instance?

Mr. RAMEY. Yes, sir; we would be required to.

Mr. ROGERS. That is clear

Mr. RAMEY. Yes, sir.

Mr. ROGERS. Thank you very much.

Mr. DINGELL. Mr. Keith.

Mr. KEITH. Thank you, Mr. Chairman.

Were any of you gentlemen on the Commission in 1960?

Mr. RAMEY. Mr. Price was Director of Regulations, and Mr. Hennessey was Associate General Counsel at that time.

I happened to have been the Staff Director of the Joint Congressional Committee of the Atomic Energy Commission at that time, watching this situation.

Mr. KEITH. Well, good.

Were the casts identifiable? Were they marked in case one of them washed ashore so you would know the contractor that put them there and where they came from?

Mr. PRICE. They were required to be, as far as I know.

Mr. KEITH. Do you happen to know what action was taken to get damages for failure to comply with the terms of the contract?

Mr. PRICE. Well, sir; I will have to go back to the record.

You mean on those few that washed up on the shore?

Mr. KEITH. Yes.

Mr. PRICE. My recollection is they were never positively identified as containing radioactive material.

Some of them had some labels on them, but I would have to check back.

Mr. KEITH. You are hung by your own statement.

You just said they were identified.

Mr. PRICE. I said that disposal containers are supposed to have identification marks on them, and I would like to check the record.

Mr. KEITH. I did not realize you said they were supposed to have been marked.

I thought you said they were identifiable.

Mr. PRICE. Here again, if somebody does not comply with the requirements, and we do not know about it, I am just recognizing that that could happen.

Mr. KEITH. It seems to me, as you said, you did know about it.

I do not want to pursue it, but it does not look very good on the record.

Mr. PRICE. Sir, I am trying to clear the record. I think you are talking about the cans that washed up on the shore.

I do not think we were ever able to trace them to any particular person, but I would have to check the record.

That was a long time ago.

Mr. KEITH. My question was certainly leading up to whether or not they were identifiable, and, therefore, corrective action could be taken.

I would have thought all of you as witnesses would have anticipated what I had in mind.

Mr. RAMEY. I might say, Mr. Chairman, we were requested to testify yesterday, to come in this morning.

Mr. KEITH. Excuse me.

Mr. PRICE. I would like to submit something on that to clear the record.

Mr. KEITH. Fine.

(The information follows:)

RADIOACTIVE WASTE LABELING REQUIREMENTS

Each container of radioactive waste dumped at sea was required to be labeled with the following information:

1. Total activity in millicuries, or in the case of source and special nuclear material, the total weight of the radioactive material;
2. Principal radioisotopes;
3. Radiation level at the surface of the container and at one meter; and
4. The name and address of the licensee.

In 1970, two disposals at sea were made. One disposal, by Chevron Research Company, Richmond, California, consisted of two drums containing about 25 millicuries of cobalt 60 in sealed sources, 6 millicuries of cobalt 60 metal, 35 millicuries of strontium 90 in sealed sources, 22 millicuries of cesium 137 fixed on catalyst beads, and 3 curies of tritium (hydrogen 3). The other disposal, by the University of Hawaii, consisted of 20 packages containing about 5 millicuries of carbon 14, 10 millicuries of calcium 45, and 10 millicuries of tritium (hydrogen 3).

Mr. KEITH. Now, the Atomic Energy Commission in accordance with this bill will be excluded from complying with the terms of the act, as though they had sufficient capability to police their own actions.

Mr. RAMEY. It is not just our own actions, Mr. Keith. The Commission is a regulatory authority. The Commission is regulating nuclear powerplants, utilities, and others, and under its authority, as we indicated, we have cutback on ocean disposal, and are in the process of phasing it out.

Mr. KEITH. I think what we are concerned about is what the people are concerned about.

The reliability of the Government agencies, is to conform to the statutes, and the intent of the statute, and you said you were responsible for the regulation of nuclear atomic powerplants.

Mr. RAMEY. Yes.

Mr. KEITH. I have in my constituency a nuclear powerplant, and I am naturally concerned about how thoroughly it is being monitored and regulated and how much statutory authority there is, to make certain that the plant does not get constructed in an area where it could be a contaminant, either with atomic or other radioactive fallout, and also in terms of thermal pollution.

Is it your agency that is responsible for monitoring, first anticipating any possible hazard, and then monitoring the operation?

Mr. RAMEY. Of the nuclear powerplant?

Mr. KEITH. Yes.

Mr. RAMEY. Yes, sir.

Mr. KEITH. Would you briefly describe how the public is protected in the authorizing of a site, and the design, ad operation of the plant?

Mr. RAMEY. That is a——

Mr. KEITH. That is a big thing.

I would like you to give us a quick appraisal that will reassure the members of this committee and our constituencies.

Mr. RAMEY. The Commission under the Atomic Energy Commission Act is responsible for issuing rules and regulations concerning the use of radioactive materials and it has issued quite a number to regulate the safety and site selection of nuclear powerplants.

It is also responsible for listening to individual complaints.

We have a separate regulatory organization under Mr. Price here as Director of Regulation through which the Commission regulates the site selection, the design, the construction, and the operation of these plants.

In establishing its regulations, the Commission is implementing radiation standards which have been issued by the Federal Radiation Council, and which will now be issued by the Environmental Protection Agency.

The Commission has issued regulations that essentially cover both the design and the site for nuclear powerplants. The regulations, governing the suitability of sites, from a radiological standpoint, also factor in population density, meteorological conditions, and so forth.

When a utility, called an applicant, under the Commission's regulations files an application to construct a nuclear powerplant, the application has to be submitted with a great deal of factual material, and it is usually about 12 inches thick. Copies of these applications have in the past, and are at present, sent to Federal and State agencies that have an interest in them, and their comments are solicited. Included are agencies such as the Fish and Wildlife Services, and also the Geological Survey, both of which are in the Department of Interior. The Commission gets their comments on the radiological aspects of the proposed plant, and its relationship to the environment.

More recently, under the National Environmental Policy Act, we required the utilities to submit, not only this application and safety analysis, but also an environmental report.

Copies of that report are also sent to all interested agencies of the Federal and State Governments, and comments are obtained on it. I previously testified before this committee concerning the Commission's regulations on this. Based on the comments, and on the original report, the regulatory staff then puts out a final environmental statement, analyzing the environmental impact of a proposed nuclear powerplant.

The regulatory staff also, as I mentioned, reviews the safety analysis report which the utility has submitted on radiological safety aspects.

The applicant's safety analysis report and these environmental statements go through sort of a four-part process in the Comission's regulatory consideration of the license application.

First, the safety analysis report is reviewed by the Commission's regulatory staff. Then there is revie[illegible] [illegible] our Advisory Committee on Reactor Safeguards, which is a——

Mr. KEITH. May I interrupt?

It sounds quite impressive—and may in fact be. The thing that concerns me a little bit and I have seen it in the advisory commissions statute, it says that the Atomic Energy Commission shall consult.

Now, in these procedu[illegible]s you have outlined, is there any agency of Federal or State Government that can say stop, or are you the final authority?

Mr. RAMEY. Well, this licensing authority that the Commission is exercising is essentially a quasi-judicial function. I had not gotten quite to that, but the Commission has Atomic Safety and Licensing Boards. We have a mandatory hearing process on each nuclear powerplant before this Board. It is an independent board, independent of our regulatory staff, and its decisions are reviewed by an appeal board, under limited conditions.

It can be reviewed by the Commission, and then can ultimately go to the courts.

Mr. KEITH. Someb[illegible]y over here asked if you would kindly say, "Yes" or "No."

Mr. RAMEY. Unfortunately it is not a question that one can give a yes or no answer to, because it is a quasi-judicial process, similar to any other regulatory proceeding.

Mr. KEITH. May I interrupt?

If a State agency, or another agency of the Federal Government disagreed with your decision to put a plant in a particular place, would that agency then have to take it to the administrative procedure in order to adjudicate the difference of opinion?

Mr. RAMEY. On the question——

Mr. KEITH. Yes or no; is that correct?

Is the Administrative Procedures Act the recourse that the other agencies of Government have for appealing a decision you have made?

Mr. RAMEY. Generally, where a Government agency appears as a party before the Licensing Board, on the question of the radiological safety of the plant, and the adequacy of the site from the radiological safety standpoint——

Mr. KEITH. Is "Yes" the answer in those areas?

Mr. RAMEY. In those areas; yes, sir.

Mr. KEITH. I appreciate your generosity with your time.

Mr. RAMEY. I would point out, Mr. Chairman, if I might, just to have a complete record here, that we have been working with interdepartmental agencies and groups, including the Environmental Protection Agency, on the President's legislative proposal on the siting of powerplants, and in this context, the role of the States. As the traditional organization that worries about land use, and other aspects of the siting of industrial facilities including powerplants, the States could exercise a fairly large role if they chose.

Mr. DINGELL. Thank you.

Mr. PELLY. I just want to recall the fact that we have had witnesses before this committee from the Federal Power Commission. Their ecological adviser was an engineer, formerly consultant to private power companies, and he has no background as far as I could tell as an expert in either radiation or thermal pollution, or anything else as far as the environment is concerned, so I assume in that case, you perform that function; is that right?

Mr. RAMEY. Well, the Federal Power Commission does not have any jurisdiction over the licensing of nuclear powerplants.

They have some authority over hydroelectric plants, and they also regulate rates of plants from an economic standpoint in interstate commerce, as I understand it.

Mr. PELLY. All right. I feel better.

Mr. RAMEY. As I indicated, we have people technically trained on our regulatory staff.

We also, as I indicated, go to the expert agencies in the Federal and State Governments for their advice, such as the Fish and Wildlife Service, the Geological Survey, and we have worked with these and other agencies for many years.

Mr. DINGELL. We must move rapidly along here. I want to give you all of the time you need to respond to questions, and the Chair is trying to give every member a full opportunity to ask questions.

I hope that you will limit yourself, if you please, to the point of the question, and also, if you please, as briefly as possible, so that the members can get the questions that they desire to ask into the record.

Mr. Downing.

Mr. DOWNING. How do you dispose of atomic waste now?

Mr. RAMEY. The high level waste is stored at the present time on an interim basis, at Atomic Energy Commission sites.

These wastes are in tanks at Hanford and Savannah River, and at one commercial facility south of Buffalo, N.Y.

Low-level waste is buried in AEC or State-regulated land-based disposal facilities.

Mr. DOWNING. What is done with it?

Are they still in containers somewhere?

Mr. RAMEY. Yes, sir.

Mr. DOWNING. So you have not really disposed of them?

Mr. PRICE. The low-level waste, Mr. Downing, of the kind that used to be disposed of at sea is packaged and buried at land burial sites, and those sites of course are monitored, but it is buried, covered up.

This is low-level waste, and as Mr. Ramey said, the high-level waste is kept in tanks at Commission installations.

Mr. Downing. Does this constitute a danger?

Mr. Price. Yes.

Mr. Ramey. The high level?

Mr. Downing. Yes.

Mr. Ramey. The program and plan the Commission has established is to require that these high-level wastes which come from licensed nuclear powerplants, must be solidified, and permanently stored far below the ground, such as in a salt mine.

Mr. Downing. When are you going to do this with the high-level waste?

Mr. Ramey. We are in the process of this now. We have plans and a program for a demonstration facility to be prepared in the next few years.

We have done many experiments on storing there high-level wastes in dry salt mines, and we believe this is the best technological form for this permanent storage.

Mr. Downing. When a submarine carrier is recalled, what do you do with that?

Is that radioactive?

Mr. Ramey. The reactor is, of course, but there is essentially no disposal of waste in a harbor.

Mr. Price. The "spent" fuel cores, from U.S. Navy vessels are sent to an AEC chemical reprocessing plant, and it is the waste from that operation which is the so-called high-level waste. It is stored in tanks at a Commission installation, and then solidified and put in long-term storage.

Mr. Downing. You have not pursued any further with the idea of atomic disposal of this material?

Mr. Ramey. This has not been an alternative method that has been seriously considered.

Mr. Rogers. I am somewhat concerned about these plants that have been built, and the thermal pollution.

Did you previously allow any thermal pollution to exist?

Mr. Ramey. Mr. Congressman, this is again a pretty broad subject. I have to remember the chairman's prescription here.

Mr. Rogers. Just yes or no.

Mr. Ramey. Until NEPA was passed, and the Muskie act was passed, the Commission did not have authority to consider the thermal effects of nuclear powerplants.

Since that time, as I testified before this committee, the Commission does apply the requirements of NEPA and the Muskie act.

Mr. Rogers. Do you still permit mixing areas?

Mr. Ramey. We essentially apply the requirements of the State agencies, and we get a certificate from the State water pollution people as a condition of our granting a construction permit, so it would depend on what the State water pollution people did in consultation with the Environmental Protection Agency.

Mr. Rogers. As I understand it then, your standards are set by the States, not by you as far as thermal pollution is concerned?

Mr. Ramey. That is right.

Mr. Rogers. Should you set the standards?

Mr. Ramey. This has been discussed a great deal. We believe that under the current Federal-State pattern, with the States in con-

sultation with, and subject to arrangements and regulations that they have with the Environmental Protection Agency, the States are the appropriate group.

Mr. ROGERS. You mean the Environmental Protection Agency now sets the standards?

Mr. RAMEY. Yes, sir. They have to approve the State standards.

Mr. ROGERS. Do you advise the Environmental Protection Agency on the standards?

Mr. RAMEY. Only in the sense that we do have authority to conduct research and development.

Mr. ROGERS. I thought you said they would consult with you.

Mr. RAMEY. Under this proposed bill.

Mr. ROGERS. They do not presently?

Mr. RAMEY. No, sir.

Mr. ROGERS. They do not presently consult with you as to what the standards should be?

Mr. RAMEY. We discuss in a more general sense the results of our research and development.

Mr. ROGERS. But not standards specifically?

Mr. RAMEY. It is fairly early at this stage, they have only been in existence for less than a year, and we are——

Mr. ROGERS. We have had the Water Pollution Control Agency for some time, the quality of standards of water, but you have not consulted that?

Mr. RAMEY. Not as to specific standards.

Mr. ROGERS. That is shocking. I am surprised.

Thank you.

Mr. DINGELL. Mr. McCloskey.

Mr. MCCLOSKEY. Thank you, Mr. Chairman.

Mr. Commissioner, as I understand your testimony, within 6 months you expect to phase out all ocean dumping of radioactive materials?

Mr. RAMEY. Yes.

Mr. MCCLOSKEY. If that is true, and this act does not become effective for 6 months, under section 12, why is there any need in the act for section 7(b)?

Since you are going to do it in 6 months anyway, why not allow this act to absolutely prohibit any waste disposal of radioactive waste in the ocean?

Mr. RAMEY. I think the only possible future use is, as far as it was brought out in the CEQ report on ocean dumping, I think it is on page 27, in which they recommended that the door sort of be left open on a benefit risk basis.

For example——

Mr. MCCLOSKEY. Let me refer you to Chairman Train's testimony. He said ocean dumping of other radioactive waste should be prohibited.

That was as I understand the administration's testimony. He says it should be prohibited.

Do you have any objection to that?

Mr. RAMEY. I think there was a sentence before that.

Mr. MCCLOSKEY. It says that low-level liquid discharge to the ocean from vessels and land-based nuclear facilities are and should continue to be controlled by Federal regulations and international standards.

Do you have any objection to this bill prohibiting ocean dumping of radioactive solid wastes, yes or no?

Mr. RAMEY. I would just refer you to this report as stated.

Mr. MCCLOSKEY. Mr. Ramey, you are one of the most intelligent lawyers I know. We have had a good deal of experience in the courts together. You know what a yes or no answer would be.

Does the Commission object to the prohibiting of dumping of radioactive solid wastes in this bill?

Mr. RAMEY. I would request we be given an opportunity to answer that question.

Mr. MCCLOSKEY. You cannot answer it with a yes or no?

Mr. RAMEY. I can.

Mr. MCCLOSKEY. What is your answer?

Mr. RAMEY. I would say that——

Mr. MCCLOSKEY. Yes or no.

Mr. RAMEY. I would have to speak from my own standpoint on this, because the Commission's position has been essentially what was the CEQ position in its report.

Mr. MCCLOSKEY. The answer to the question is yes, you do not feel we should prohibit solid radioactive solid waste disposal?

You feel you should have freedom to dispose of some radioactive solid waste?

Mr. RAMEY. I think it has to be that the door should possibly be left open in a very minor way.

Let me give you a kind of example.

Mr. MCCLOSKEY. I want to make sure I understand your testimony.

You do not want this committee to prohibit solid radioactive waste disposal by the Atomic Energy Commission, do you?

Mr. RAMEY. What I would like would be an opportunity for the Commission to take up this specific question that you are raising, and to supply you with our position on it.

. Mr. MCCLOSKEY. The answer to my former question is at this time you cannot answer the question yes or no because the Commission has not deliberated on this question.

Is that your answer to my question?

Mr. RAMEY. Yes, sir; that is what I was trying to say.

Mr. MCCLOSKEY. Well, that is fine. That is a perfectly legitimate answer. If you do not want to answer the question at this time, because the Commission has not deliberated on it, that is fine.

Now, Chairman Train has testified ocean dumping of radioactive waste should be prohibited, including solid radioactive waste.

You will take up that question?.

Mr. RAMEY. Yes.

Mr. MCCLOSKEY. How soon?

Mr. RAMEY. Within the next week or 10 days.

Mr. MCCLOSKEY. Then we will await that answer.

(The information follows:)

U.S. ATOMIC ENERGY COMMISSION,
Washington, D.C., April 26, 1971.

Hon. PAUL N. MCCLOSKEY, Jr.,
House of Representatives.

DEAR MR. MCCLOSKEY: I am pleased to submit for the record the position of the Atomic Energy Commission with respect to three questions which you raised during my appearance before the Subcommittees on Oceanography and Fisheries and Wildlife Conservation on April 6, 1971.

Question 1. Does the Commission object to a prohibition of the dumping of radioactive solid waste in H.R. 4723?

62-513 O—71——17

Answer. The Commission participated in the writing of the report of the Council on Environmental Quality entitled "Ocean Dumping: A National Policy", dated October 1970. At page 28, the report stated:

> Because of the need to keep all sources of radioactivity at the lowest possible level, ocean disposal of the wastes should be avoided except when no alternative offers less harm to man or the environment. These cases should be carefully examined to assure that no safe and practical alternatives do exist. If ocean disposal is necessary, it should be carefully controlled.

As I indicated in my testimony, the AEC has not permitted ocean disposal of high level radioactive waste and is phasing out licenses to dump waste materials containing low levels of radioactivity into the ocean and has no intention of dumping any such material from its own operations. However, it does not follow that a complete prohibition of such dumping is indicated. We are in the early stages of a great deal of national research into the effects of various actions on the environment. We believe that until more is known about the environmental effects of other types of waste disposal, it is not in the national interest to make a decision at this time which would foreclose a particular disposal, in the future, of radioactive waste under proper conditions and controls in some part of the ocean. A possible candidate for such disposal could be a reactor pressure vessel from the dismantling of a civilian power plant located at an ocean-side site or from the dismantling of a propulsion reactor in a military vessel. Until scientific bases are sufficiently advanced for enlightened decisions with respect to alternate methods of waste dispose' we believe it would be premature to foreclose all options.

Question 2. Does the Commission object if it is brought under the Ocean Dumping Act and required to secure a permit from EPA in connection with the disposal of radioactive waste generated by its own operations?

Answer. We do not believe that it is necessary, in order to assure that AEC waste disposal actions are consistent with the Act, for the AEC to secure a permit from EPA. Under the bill as presently drawn, if the Commission should wish to make an ocean disposal of its own radioactive waste under some unusual circumstances, as described in the CEQ report, it would first consult with the Administrator, and comply with the standards set by the Administrator respecting limits on radiation exposures or levels, or concentrations or quantities of radioactive materials, in the same manner as under section 7b. of the bill required for AEC licenses. If the Congress were to amend section 7b. to require an EPA permit for all Government agencies proposing ocean disposal of radioactive waste, we foresee no resulting undue interference with Commission operations. Other Government agencies, however, planning ocean disposal of radioactive waste would still be required, in addition to securing an EPA permit, to secure an AEC license.

Quesion 3. Is the Commission agreeable to the applicability of civil and criminal penalties to AEC employees for violation of laws on ocean dumping?

Answer. As a Government-wide matter, it is not desirable to impose civil and criminal penalties on Federal employees arising in the course of the substantive performance of agency missions. To do so can seriously inhibit the performance of necessary Government functions. Thus, the Commission opposes applicability of civil or criminal penalties to Federal employees for violation of laws on ocean dumping. To charge each Federal employee with knowledge of laws pertinent to the discharge of his official duties, pursuant to direction of his supervisors, and with the requirement that he pre-assess at his peril compliance with such laws, is, in our view, unrealistic, unduly burdensome, and not likely to result in the prompt and efficient conduct of official business. We doubt that, in the private sector, subordinates would, in fact, be charged with violation of ocean dumping laws—rather, the violation would be charged to the head of the organization. Thus, the responsibility for violations of ocean dumping laws should likewise be that of the head of the Government agency conducting the operations. No useful purpose would be served by subjecting higher echelon Government officials to such penalties. (Agencies are, in appropriate cases, answerable civilly under the Tort Claims Act.) In any event, we think the responsibility for assuring compliance by Federal departments and agencies with such laws in the operation of their facilities should rest with the President of the United States.

Submissions for the record in connection with other matters discussed at the hearing will be transmitted to you separately.

Sincerely,

JAMES T. RAMEY, *Commissioner.*

Mr. McCLOSKEY. Now we come to the question that Chairman Train testified to about low liquid waste discharges, of vessels and land-based.

I take it you want the Commission to have the right to continue to regulate those nuclear facilities?

Mr. RAMEY. Yes.

Mr. McCLOSKEY. Section 7(b) of this act reads as follows:

> *Provided*, The Atomic Energy Commission shall consult with the Administrator prior to issuing a permit to conduct any activity which would otherwise be regulated by this act. In issuing any such permit, the Atomic Energy Commission shall comply with standards set by the Administrator respecting limits on radiation exposures or levels, or concentrations or quantities of radioactive material.

That refers to a permit. Presumably a private party of governmental agency is included in that.

Do you intend that the Atomic Energy Commission should have the ability without permit to continue this kind of liquid low-level waste from vessels and land-based facilities?

Mr. RAMEY. That would have to be under a permit.

Mr. McCLOSKEY. The language of the administration's bill would leave you free to do this without a permit.

Mr. RAMEY. Under the Atomic Energy Commission Act, it has to be by means of a license or permit.

Mr. McCLOSKEY. Even of your own agency when you are referring to yourself?

Mr. RAMEY. You mean the operational activities of the Atomic Energy Commission?

Mr. McCLOSKEY. Yes.

Mr. RAMEY. I would prefer to have Mr. Price answer that question. Mr. Hennessey can also if he wishes.

Mr. HENNESSEY. I interpret this section 7(b) would have the effect of exempting the Atomic Energy Commission's own activities from any requirement for a permit.

Mr. McCLOSKEY. Do you have any objection if the Atomic Energy Commission is brought under the act and required to comply with the act as an agency itself?

You would have to get a permit from the Environmental Protection Agency before you could do any of this dumping yourselves. Would you object to that?

Mr. RAMEY. Again, I would like to provide you with a written answer.

This is again something which we have not specifically taken up in connection with this testimony.

As I pointed out, we were informed that we were being requested to testify yesterday, and we have not had a Commission meeting in that period.

Mr. McCLOSKEY. You will examine that second question?

Mr. RAMEY. Yes, sir.

(See section 2 of the letter dated April 26, 1971, which may be found on p. 249.)

Mr. McCLOSKEY. Could you also examine a third question? That is whether or not you are willing to be bound by an administration permit, like any other agency of government, and to respond as to whether or not civil or criminal penalties should be or could be assessed against your employees if they violate the law?

Mr. RAMEY. We will examine that. This is more of an across-the-board policy for the Federal Government.

Mr. McCLOSKEY. That is exactly correct.

(See paragraph 3 of letter on p. 249.)

Mr. McCLOSKEY. Do you recall, that the President, shortly after taking office, created an Environmental Policy Council, and it was stated by Dr. DuBridge that we need not pass an environmental protection act because environmental protection was already being handled by the Federal agencies?

The Department of Defense and the Atomic Energy Commission were specifically excluded from that Environmental Policy Council as it was first set up, and I think it is extremely important we have your reaction whether your agency should be excluded from criminal and civil penalties, and whether you are willing to submit to Environmental Protection Agency proceedings.

Mr. DINGELL. I would just like to have Mr. Ramey tell us why the Atomic Energy Commission is the only agency in government which is exempt from the Environmental Protection Agency's license requirement.

What I am curious to know is if there is any logic behind that.

Mr. RAMEY. Do you mean under——

Mr. DINGELL. Under the administration's bill, why is the Atomic Energy Commission exempt from licensing requirements?

Why is it and its contractors not compelled to go to the Environmental Protection Agency?

I am very curious as to that very quaint situation.

Mr. RAMEY. Well, I believe that one of the main reasons is that under the reorganization plan, it was determined by the Congress and the administration that in the field of radiation, and radiological matters, affecting nuclear powerplants, and the whole nuclear cycle, the Government had set up a pattern with the Atomic Energy Commission continuing to exercise a fairly broad licensing authority. The standards making authority had always been in the Federal Radiation Council, so this was following the same pattern in transferring the standards making to the Environmental Protection Agency, and retaining the licensing authority in the Atomic Energy Commission.

Mr. DINGELL. We find ourselves then in passing strange conditions, where the Atomic Energy Commission will issue the permit, but according to the language on page 10, line 24, under 7(b), this lanuague appears as follows:

> In issuing any such permit, the Atomic Energy Commission shall comply with standards set by the Administrator respecting limits on radiation exposures or levels, or concentrations or quantities of radioactive material.

So as I read this particular statute, the Atomic Energy Commission will be issuing the permit, out to comply with the standards and qualifications set out by the Environmental Protection Agency.

Now, is that not a very, very strange thing?

Why do we need to have two different permit issuing agencies, when effectively they will meet the qualifications and standards which will be laid down by the Environmental Protection Agency.

It appears that the Atomic Energy Commission will simply be serving as a ministerial clerk to process some of the Environmental Protection Agency's papers.

Now, why should that legislation go through?

Mr. RAMEY. Well, my understanding of the Environmental Protection Agency's role, generally, is that in their standard-setting in the radiological area, they have taken over what the Federal Radiation Council has accomplished for the last 10 or 12 years. This language in the pending administration bill would carry forward that general pattern, of an agency setting overall standards, and another agency, as the licensing agency, implementing them.

Now, I recognize in the case of ocean dumping we are talking about something that from a practical standpoint is of no very great consequence. I just think the general pattern as set forth in the bill is consistent with the reorganization plan, and the way the Commission will be operating in the general radiological area.

Mr. DINGELL. We keep coming back to the same point: that the Atomic Energy Commission will be doing what the Environmental Protection Agency is supposed to be doing.

Mr. RAMEY. Well, as I said, the Environmental Protection Agency, presumably will be issuing relatively broad scale types of standards.

Mr. DINGELL. I am curious as to why we have to have an Atomic Energy Commission doing this work though, and regardless of your organization plan, and the wisdom of it, I do not think we should be debating.

I happen to be one who opposes the particular organizational plan, so I would like to challenge its validity, but regardless of its validity, why should the Atomic Energy Commission be doing the Environmental Protection Agency's work, if you are not going to have any discretion, you will simply be stamping these things approved or disapproved, according to the standards the Environmental Protection Agency has laid down.

It seems to me we have a very curious situation before us.

Mr. RAMEY. As I mentioned, Mr. Chairman, in this field of radiological regulation, the Commission has by far the greater amount of experience and expertise, particularly in relation to the whole sequence of the fuel cycle.

Mr. DINGELL. Excepting that here you shall be issuing permits using the Environmental Protection Agency's standards. That does not indicate that whoever drafted this bill had much greater faith in your capacity to issue permits than set standards, does it? I am still curious to find out why you folks will be doing essentially what they will be doing.

Why should you folks be excepted from the act at all?

Mr. RAMEY. As I indicated, Mr. Chairman, in the whole radiological field, the Environmental Protection Agency has taken over the responsibility from the Federal Radiation Council to issue standards, so in the future, the Atomic Energy Commission will be implementing EPA general standards.

They will be reviewed from time to time by the Environmental Protection Agency. I do not think we will be exercising anything unusual in this whole subsidiary regulation area, and in the specific licensing of powerplants, and so on, we will not be exercising a ministerial or clerical role.

I do think in relation to ocean disposal, in view of the fact that we are phasing out even the very small amounts going on, and that any

future uses of the ocean would be very minimal, from a practical standpoint, we are not arguing about a lot.

Mr. DINGELL. Commissioner, I want to thank you, and I just want to observe you play a bad hand rather well.

The Chair recognizes Mr. Tiernan.

Mr. TIERNAN. Thank you, Mr. Chairman.

With regard to your first answer to Mr. McCloskey's questions, you indicated the Commission had not taken any position, and you want to supply that for the record.

Mr. RAMEY. Yes. We have supported the language in the CEQ report.

Mr. TIERNAN. Mr. McCloskey quoted Mr. Train's statement before this committee yesterday, and that report was filed in October.

I assume you have had chance to review the language in that report, and also the administration's bill, and it seems you are in contradiction to the Administrator who testified here with regard to the objectives.

Mr. RAMEY. I would not make it quite that degree of disagreement.

Mr. TIERNAN. Well, what degree would you make it?

Mr. RAMEY. The bill of course does not contemplate, it does not have authority for——

Mr. TIERNAN. But the Administrator saw no other reason for that authority, except to liquefy radioactive materials, is that correct?

Mr. RAMEY. I am not sure that that was it.

Mr. TIERNAN. You question the quote of Mr. Train's statement?

Mr. RAMEY. I would take it that he was saying under this authority he would expect that it be prohibited.

Mr. TIERNAN. And you do not agree with that?

Mr. RAMEY. I have stated that I would like to review this with my fellow commissioners.

Mr. TIERNAN. You mean since October, and since the bill was introduced in February, that the Commission has not considered this at all?

Mr. RAMEY. No. I say we have not considered it since we saw Mr. Train's statement.

Mr. TIERNAN. When did you see Mr. Train's statement?

Mr. RAMEY. This morning.

Mr. TIERNAN. You saw the bill the administration placed before the committee?

Mr. RAMEY. Yes, sir.

Mr. TIERNAN. And in view of that, you think that Mr. Train's statement is of further restriction than the language in the bill?

Mr. RAMEY. It is not a restriction in the language of the bill. It indicated——

Mr. TIERNAN. You think his statement indicates a further restriction in the application of the language of the bill?

Mr. RAMEY. It indicated how he would expect the bill to be administered, and it is a further restriction on what is stated in the report of the CEQ, of which he was chairman.

Mr. TIERNAN. And if we were to act on his recommendation, you say the Commission has not taken a position yet?

Mr. RAMEY. No, sir.

As I indicated, from a practical standpoint, I do not think it is a big problem.

Mr. TIERNAN. Now, with regard to the issuing of permits and sites of construction of nuclear powerplants, have you ever denied a permit for construction?

Mr. RAMEY. Yes.

Mr. TIERNAN. When?

Mr. RAMEY. In the case of the Malibu Atomic Power Plant, the city of Los Angeles, this must have been about 4 or 5 years ago, the Commission approved the denial of a construction permit by a Licensing Board for building of that powerplant.

Mr. TIERNAN. At that particular site?

Mr. RAMEY. Yes.

Mr. TIERNAN. Was it built at another site?

Mr. RAMEY. No.

Mr. TIERNAN. How many applications have been filed for construction before the Commission, how many applications have been filed?

Mr. RAMEY. I would like Mr. Price to answer that.

Mr. PRICE. I think probably close to a hundred. I don't know exactly. That is for powerplants.

Mr. TIERNAN. That is powerplants, generating plants.

How many have been denied?

Mr. PRICE. I would have to check that.

Mr. RAMEY. There have been other examples, whereby in the process of review, the applicant—the utility has withdrawn its application.

For example, Con Edison proopsed to build a plant across the East River in Queens. That did not get through the Commission's Advisory Committee on Reactor Safeguards, before they asked enough questions that the utility decided to withdraw the application.

Mr. TIERNAN. Mr. Price, how many of those hundred applications have been refused?

Mr. PRICE. Let me just give my best guess, and I will provide the specifics for the record, but I would say that either by the staff, my staff, or the ACRS telling the applicant that the site was not good enough, or that the case had too many problems in it, that about six to 10.

Mr. TIERNAN. Six to 10?

Mr. PRICE. Yes.

Mr. TIERNAN. Could you tell me whether or not there has been——

Well, when was the first application filed with your agency?

Mr. PRICE. Back in 1955 or 1956.

Mr. TIERNAN. 1955 or 1956?

Mr. PRICE. Yes.

Mr. TIERNAN. Those six or seven applications. Were they recent denials, or were they over the period of 1955 to 1956?

Mr. PRICE. They are spread out, because sometimes we will have the applicant come in and talk about a site, and we will say we do not think that is a very good site.

Mr. TIERNAN. Is that Con Edison application included in those six or seven?

Mr. PRICE. That is right.

Mr. TIERNAN. Do you feel that your Commission has an objective viewpoint on the applications?

Mr. PRICE. We try to, Mr. Tiernan.

Mr. TIERNAN. Do you have any outside advisory group?

Mr. PRICE. We sure do. We have not only the technical staff of the Commission, but we have an Advisory Committee on Reactor Safeguards that are independent, and then when we get through, and they get through, there is a public hearing before a licensing board, and that is reviewed by the appeals board, and sometimes the Commissioners themselves.

In addition, in working through these cases, we consult, like on earthquakes, with the Geological Survey group.

Mr. TIERNAN. Mr. Chairman, I think that was pretty well gone over step by step as to that procedure.

I would like to request the Commission supply for the record the actual step-by-step procedures.

Mr. DINGELL. I think that would be helpful, if you would do so, and without objection, that will be inserted in the record.

(The information follows:)

STEP BY STEP PROCESS FOR LICENSING OF POWER REACTORS BY THE ATOMIC ENERGY COMMISSION

Introductory Note

As was pointed out by the Commission's responses to Mr. Tiernan's questions on April 6, before a formal application is made by a utility for a construction permit, the prospective applicant is encouraged to meet with AEC regulatory staff to discuss the overall suitability of the plant site. Such discussions have in the past led to applicants deciding not to build specific plants at specific locations.

Once an application is submitted, the Commission regulatory staff and ACRS spend from 12-20 months reviewing the utility's application and environmental report and discussing the site and the plant design with him. There frequently are revisions made in the applicant's plans, including the design of the plant, as a result of these discussions.

The numbers of withdrawals by applicants and denials and suspensions of construction permit or site approval applications have to be considered within the context of the above two features of the licensing process.

The role of nuclear reactors in the production of electricity is growing rapidly. With this increased use of nuclear power reactors has come greater public interest in, and awareness of, the safeguards to public health and safety which are provided for these nuclear power plants.

When the atom is split, or "fissioned," in a reactor, it produces energy—heat energy and radiation energy. Positive control of this energy is achieved in a reactor through the design of the facility and by careful operation. By these means, constant care is taken to prevent injury to employees or to the general public.

Comprehensive safeguards to protect public health and safety are engineered into power reactor plants. These include: a system of controls to regulate the reactivity and rate of energy release in the reactor core where the nuclear fuel elements are placed; a heat extraction system to convert the thermal energy from the reactor to the generation of electric power; containment systems designed to prevent the escape of harmful amounts of radioactivity in the event, however remote, of an accident; and a waste handling system to control the release to the environment of the low-level radioactive effluents produced during normal operation. These systems are checked periodically to assure that they are working properly.

Protection of health and safety is the primary goal of the Atomic Energy Commission's regulatory program. Under this program, the licensed uses of radioactive materials and the construction and operation of licensed nuclear facilities, of which reactors are one type, are regulated by AEC.[1] State and local officials are kept informed of AEC licensing actions taken in connection with a power reactor project.

---

[1] Commission owned power reactors located at non-AEC sites and operated as part of conventional utility systems are not licensed. However, procedures which are parallel to those discussed here are used in the issuance of authorizations for construction and operation of these reactors.

SELECTION OF A REACTO SITE

The selection of a reactor site is the responsibi ty of the company proposing to build the reactor. To assist prospective applica s, the Atomic Energy Commission has published criteria which it uses as guides in the safety evaluation of proposed sites for stationary licensed power and test reactors.

Factors considered by the Commission in judging the safety of proposed sites for power reactors include dimensions and characteristics of the site under the operator's control; population density in the area surrounding the proposed site, and the uses which are made of this area, such as industrial, farming or residential; and the seismology, meteorology, geology and hydrology of the area. Other factors considered are the characteristics of the proposed reactor, including maximum power level, and the particular safeguards to be engineered into the plant either to prevent accidents or to limit their consequences; and the extent to which the design of the reactor incorporates unique or unusual features that may have a significant bearing on the probability or consequences of an accident.

Before formally filing an application for construction and operation of a power reactor a prospective applicant is encouraged to discuss informally the possible sites for the reactor with the Commission's regulatory staff. In this way an applicant can receive additional guidance as to the acceptability of a site and the information which must be included in the application for a license to construct and operate a reactor.

APPLICATION FOR A CONSTRUCTION PERMIT

Before a company can begin to build a power reactor at a particular site, it must obtain a construction permit from the Commission. Such things as site exploration, site excavation, procurement or manufacture of components and construction of non-nuclear facilities may be done before an AEC construction permit is received.

As a major part of the application, the company files a preliminary safety analysis report. This report presents the preliminary design and safety features of the proposed reactor, as well as comprehensive data on the proposed site. The report discusses various accident situations and the safeguards which will be provided to prevent accidents or, if they should occur, to prevent overexposure of the public and employees to radiation.

Copies of the application are sent to the Commission's Advisory Committee on Reactor Safeguards (ACRS). The ACRS is a committee established by law to advise the Commission on safety aspects of reactors. It is composed of scientists and engineers who are eminently qualified in the various fields related to reactor technology. Copies also are sent to the state and local officials, and are placed in the AEC's Public Document Room. A public announcement of the receipt of the application is issued by AEC and a notice is published in the Federal Register and in trade and news publications which will give reasonable notice to municipalities, public bodies, private utilities and corporations which might have a potential interest in the facility. Copies of all correspondence and filings relating to the application are placed in the public records of the Commission, which are available to any member of the public at the Commission's Washington office.

REVIEW AND SAFETY EVALUATION

The application is reviewed by technical experts of the Commission's regulatory staff. This staff is headed by the Director of Regulation, who reports directly to the Commission. There are seven divisions under the Director of Regulation. They are the Divisions of Reactor Licensing, Reactor Standards, Materials Licensing, Radiological & Environmental Protection, Compliance, State and Licensee Relations and Nuclear Materials Safeguards.[2] The review includes

[2] The Division of Reactor Licensing handles AEC staff review of applications to construct and operate nuclear reactors. The Division of Reactor Standards develops standards, criteria and guides for location, design, construction and operation of reactors. The Division of Materials Licensing issues licenses for the use of radioactive materials and reviews applications to build and operate reactor fuel reprocessing plants. The Division of Radiological and Environmental Protection develops and recommends to the Commission regulations to limit exposures of persons to radiation from licensed activities; and develops, as required by the National Environmental Policy Act, statements which evaluate the environ-

consideration of all the radiation safety aspects of the proposed reactor, as well as the applicant's technical and financial qualifications. The Division of Reactor Licensing supplements this study of the safety analysis report with conferences with the technical staff of the applicant, and may ask for further information if required.

The data submitted should provide the necessary information to permit evaluation of the adequacy of the proposed site for a reactor of the power level and type planned. Even though final design details are usually not available at the time of the application for a construction permit, the data submitted should provide reasonable assurance that the proposed reactor can be constructed and operated at the selected site without endangering the health and safety of the public, including plant employees.

ACRS SAFETY EVALUATION

The Division of Reactor Licensing prepares an evaluation of the safety aspects of the proposed power reactor for the Advisory Committee on Reactor Safeguards, which has already received the applicant's preliminary safeguards report. Particular problems which may exist are identified for consideration by the ACRS. The Advisory Committee considers the applicant's preliminary safety analysis report, together with the evaluation prepared by the Division of Reactor Licensing. Representatives of the applicant and members of the technical staff of the Division of Reactor Licensing meet with the ACRS to deal with questions that arise during the Committee's review of the reactor. Usually a subcommittee meeting is held, often at the proposed site, before full Committee review. When it has reached a conclusion as to the safety aspects of the proposed reactor, the ACRS reports its views to the Commission. This letter report is made public.

HEARING ON CONSTRUCTION PERMIT

The Commission has begun the practice of giving earlier notice in nuclear power plant licensing proceedings which is expected to facilitate participation by the public in licensing hearings and at the same time minimize licensing delays. In addition, a public announcement is issued by AEC and sent to the news media in the vicinity of the site.

The Commission's rules of practice permit persons whose interests may be affected by the proceeding to intervene as parties in accordance with the requirements of the regulations. Person who wish only to make a statement of their views concerning the project may be permitted to make "limited appearances."

In advance of the public hearing, an evaluation of the safety aspects of the proposed reactor, prepared by the Division of Reactor Licensing, is made available to the public. This evaluation takes into account the recommendations and advice of the ACRS. Copies of the evaluation are also furnished to state and local officials and to newspapers in the area which surrounds the proposed site of the reactor.

An Environmental Report is submitted by the applicant in accordance with AEC regulations (10 CFR 50, Appendix D). As required by the AEC's policy statement implementing the National Environmental Policy Act of 1969, the regulatory staff prepares a draft detailed environmental statement and forwards it along with the Applicant's report to the appropriate Federal, State, and local agencies for comment. At the conclusion of the comment period, the regulatory staff prepares a final detailed environmental statement which includes a discussion of any problems or objections raised and the disposition thereof. The detailed environmental statement is then made available to the public and will accompany the application through the Commission's review process.

---

mental impact of nuclear facilities proposed for license by the AEC. The Division of State and Licensee Relations administers the AEC program for transferring part of the Commission's regulatory authority to the states, conducts the licensee indemnity program, and export licensing. The Division of Compliance conducts inspections of licensees and initiates enforcement actions to assure that the provisions of licensees and AEC regulations are being met. The Division of Nuclear Materials Safeguards reviews safeguards programs of those licensees authorized to possess and use more than 5,000 grams of contained U-235, U-233 and/or plutonium in a form other than sealed sources and issues safeguards amendments to licensees to incorporate nuclear material controls as licensee conditions. Safeguards inspections are conducted at licensed facilities to determine compliance with regulations.

The public hearing usually is conducted by a three-member atomic safety and licensing board appointed by the Commission. The board is composed of two technical experts and one lawyer, who acts as chairman of the board for the hearing.

The application, any amendments to the application which may have been filed, and any other pertinent documents are submitted for the record. The proposed findings by the regulatory staff and the proposed construction permit are presented. Testimony is given both by the applicant and by the AEC regulatory staff on the safety aspects of the reactor and on the applicant's technical and financial qualifications to construct the facility.

The board considers matters of radiological safety involved in the application for this proposed reactor at the selected site. In addition, any party to the proceeding may raise as an issue whether the issuance of the permit would be likely to result in a significant, adverse effect on the environment. If such a result were indicated, in accordance with the declaration of national policy expressed in the National Environmental Policy Act of 1969, consideration will be given to the need for the imposition of requirements for the preservation of environmental values consistent with other essential considerations of national policy, including the need to meet on a timely basis the growing national requirements for electric power.

With respect to those aspects of environmental quality for which environmental quality standards and requirements have been established, proof that the applicant is equipped to observe and agrees to observe such standards and requirements will be considered a satisfactory showing that there will not be a significant, adverse effect on the environment. Certification by the appropriate agency that there is reasonable assurance that the applicant for the permit will observe such standards and requirements will be considered dispositive for this purpose. In any event, there will be incorporated in construction permits a condition to the effect that the licensee shall observe such standards and requirements for the protection of the environment as are validly imposed pursuant to authority established under Federal and State law and as are determined by the Commission to be applicable to the facility that is subject to the licensing action involved.

In a hearing on an uncontested application for a construction permit, the licensing board will determine (1) whether the application and the record of the proceeding contain sufficient information and (2) whether review of the application by the AEC regulatory staff has been adequate, to support the findings proposed to be made by the Director of Regulation and to support issuance of a construction permit also proposed by the Director of Regulation. If an application is contested—that is, if there is a controversy between the AEC regulatory staff and the applicant concerning the issuance of a permit or any of its terms or conditions, or if the application is opposed by an intervening party to the proceeding—then the licensing board will consider any matters in controversy.

The proceedings are conducted informally, consistent with legal requirements and fairness to all parties.

FINAL ACTION ON CONSTRUCTION PERMIT

The licensing board considers the evidence which has been presented, together with any briefs which may have been filed, and issues a decision. The decision is effective immediately, unless the board finds that good cause has been shown by a party why it should not be. If authorized by the decision, a construction permit is issued. The decision and the permit are subject to review by the Atomic Safety and Licensing Appeal Board or by the full Commission upon filing of exceptions or on its own motion. The Commission may, on its own motion, review certain appeal board decisions on certain specified grounds.

The permit includes a finding, however, that AEC is satisfied it has enough information to provide reasonable assurance that the proposed facility can be constructed and operated safely at the proposed location.

OPERATING LICENSE

As construction proceeds on the reactor, it is inspected periodically by representatives of the Commission's Division of Compliance to assure that the requirements of the construction permit are met. Amendments to the application and reports may be submitted from time to time for review by the Division of Reactor Licensing.

When final design is completed, and plans for operation are ready, the applicant submits the final safety analysis report in support of an application for an operating license. The information includes plans for operation, procedures for coping with emergency situations, and pertinent details on the final design of the reactor itself—such as containment design, design of the nuclear core, and waste handling systems. Once again the Division of Reactor Licensing makes a detailed review of the information on the reactor and presents an evaluation of it to the Advisory Committee on Reactor Safeguards. The ACRS again makes an independent evaluation and reports its opinion to the Commission. This is made public.

Applicants for operating licenses are required to submit an Environmental Report discussing the same environmental considerations, to the extent that they differ significantly from those discussed in the Report submitted at the construction permit stage. The same procedure is then followed as during the construction stage review.

A public hearing is not required by law on every application for an operating license. Under the new practice of early noticing the 30-day notice to the public, that the Commission is considering issuance of an operating license, will be issued while the technical reviews by the AEC staff and the ACRS are in the later stages. The regulatory staff's evaluation of the safety aspects is prepared and made available to the public after technical reviews have been completed. Normally the Commission will not direct that a hearing be held at this stage unless there is a difficult safety problem of unusual importance, or substantial public interest which would warrant that course. In the event no hearing is scheduled initially, the published notice states that in the absence of a timely petition to intervene and a request for a hearing, the license will be issued.

If a public hearing is held, the decision of the licensing board is subject to Commission or appeal board review.

Each license for operation of a nuclear reactor contains specific license conditions called technical specifications which set forth the particular safety characteristics of the facility and the conditions of its operation which are to be met in order to assure protection of the health and safety of the public.

Persons who operate the controls of a power reactor are required to be individually licensed by the Commission. The AEC conducts examinations of individuals which include an operating test and a written examination on knowledge of specific details of the facility and the procedures used in its operation.

CONTINUING REVIEW

AEC inspection and review of power reactors does not stop when the operating license is issued. The reactors are inspected periodically by representatives of the Commission's Division of Compliance to make certain operations are being conducted in accordance with terms of the license.

Thus, reactors are subjected to detailed review by technical experts before construction is permitted, before operation is permitted and during the entire period of their operation. In the event an unsafe condition is discovered after operation begins, the Commission has authority to order the licensee to shut down the reactor and take any safety measures which may be necessary. It should be emphasized, however, that the outstanding safety record of the atomic energy industry has been achieved because persons who deal with atomic energy respect the potential hazards and exercise great care in the handling and use of atomic materials.

Since April 1955, 85 applications have been filed requesting construction of 110 central station nuclear power plants.

Construction Permit or Site Approval Applications Withdrawn, Denied or Suspended

1. Ravenswood—Consolidated Edison Co.

Application Filed—12/10/62
Withdrawn—1/17/64

(Opposition from citizens of New York City due to high population density. Alleged availability of alternative cheaper sources of power from Canada and other areas. Note AEC regulatory staff and ACRS wer asking a series of penetrating questions on siting of th[illegible] plant.)

2. Bolsa Island—Depa[illegible]ent of Water & Power of the City of Los Angeles—Southern California Edison Co.—San Diego Gas & Electric Co.

Applications Filed—9/5/67
Withdrawn—11/4 & 12/30/68

(The dual nuclear power and desalting plant project was postponed due to economic and organizational problems.)

3. Florida West Coast Nuclear Power Group, Inc.

Application Filed—12/10/59
Withdrawn—6/26/61

(The 50 MWe gas-cooled heavy water reactor project was terminated in mid-1961 because of technical and economic uncertainties.)

4. Easton Station—Niagara Mohawk Power Corp.

Application Filed—8/1/67
Withdrawn—8/22/68

(Application had difficulties obtaining site approval from other governmental bodies dealing with matters not related to radiation safety.)

5. Bodega Bay—Pacific Gas & Electric Co.

Application Filed—12/31/62
Withdrawn—11/4/64

(Application withdrawn due to opposition of the AEC regulatory staff based on a need for a design against positive ground displacement, because of the proximity to the San Andreas Fault.)

6. Malibu—Los Angeles Department of Water & Power

Application Filed—11/23/63

(The Atomic Energy Commission's decision, issued 3/27/67, upheld an Atomic Safety and Licensing Board's determination that the probability of permanent ground displacement at the proposed site was sufficiently high to require that the design criteria for the plant be modified and supplemented to include provision for ground displacement from earthquake activity before a construction permit could be issued. Although the application was not formally withdrawn, the applicant's contract with reactor supplier was terminated.)

7. Burlington—Public Service Electric & Gas Co.

(The AEC regulatory staff and the Commission's Advisory Committee on Reactor Safeguards (ACRS) told the company informally that they found no way in which Burlington could be approved. The applicant amended its application changing the site to Salem County, New Jersey.)

8. Bell Station—New York State Electric and Gas Corp.

(The applicant announced indefinite postponement of its plans to build a nuclear plant at Cayuga Lake, New York, in order to provide more time for additional research of cooling systems for thermal discharge from the plant and for consideration of the economic effect of such systems. Although this application has not been formally withdrawn, it is considered to be inactive by the AEC.)

9. Seabrook—Public Service of New Hampshire

(In November 1969 the applicant announced deferrment of plans for the projected Seabrook Nuclear Station as a result of a decision by one of the participants not to contribute to the funding. Although this application has not been formally withdrawn, it is considered to be inactive by the AEC.)

Examples of Results of Informal Site Reviews by AEC

10. Jamestown Site, New York

(Proposed for the Small Size Pressurized Water Reactor Project.) First site (35 acres of city-owned land located in the northwest corner of the city approximately 1.75 miles from the center) was disapproved by the ACRS (letters 3/14/60 and 6/30/60) due to smallness of site, proximity of City of Jamestown with its high population density, unfavorable meteorlogy, and consequent adverse effects on liquid waste disposal.

Two additional sites (located east of Jamestown) were found suitable by the Committee (letter 11/7/60), but project was abandoned.

11. Point Loam Site, California

(Proposed for the Experimental Low-Temperature Process-Heat Reactor Project) The ACRS (letter 3/14/60) considered the site unsuitable due to unfavorable meteorology and high population density.

12. Cayucos, Oxnard, Sycamore Canyon and Tehachapi Sites, California (City of Los Angeles)

The ACRS (letter 7/22/66) found the Cayucos site acceptable provided additional geological studies confirmed expectations and proper attention was given to seismic design.

The Oxnard and Teha[illegible]pi sites had specific problems requiring considerable effort and additional safeguards. Insufficient geological information was available concerning the Sycamore Canyon site.

Mr. TIERNAN. Thank you. I have no further questions.

Mr. DINGELL. Mr. DuPont.

Mr. DUPONT. Thank you, Mr. Chairman.

Mr. Ramey, under the 1954 act, did the Atomic Energy Commission have authority to issue permits for dumping of anything other than radioactive material?

Mr. RAMEY. Not in the sense that you are talking about, no, sir.

Mr. DUPONT. How can the Atomic Energy Commission get involved in the question that Mr. Lennon was asking about, the nerve gas?

Mr. RAMEY. Well, as I indicated, I did not believe that the Commission made any recommendations as to disposal of something in the jurisdiction of the Department of Defense.

Where the Commission got involved was in the question as to whether it might be possible to disintegrate these nerve gases by means of an underground atomic explosion. In any such an arrangement, the Commission would be essentially providing a service, if you want to call it that, as a fellow Government agency on the disintegration of this material.

As I indicated, this had never been done on an experimental basis, and it was something that needed a great amount of experiment and study in our judgment before we made a decision on it.

Mr. DUPONT. But you have no authority to dump anything, or to issue permits for the dumping of anything other than radioactive material?

Mr. PRICE. Radioactive material is the only material that we have regulatory authority over to control the dumping by others.

Mr. DUPONT. No, that is not quite the question.

Does the Atomic Energy Commission have the permission, if I came to you with a sack, and said I want a permit to dump this in the ocean, and it does not contain radioactive material, do you have statutory authority to give me a permit?

Mr. PRICE. No, sir.

Mr. DUPONT. Do you have exclusive authority over the dumping permits of radioactive material?

That is, could a State enact a radioactive dumping law that could in any way impinge on your jurisdiction?

Mr. PRICE. No; we have exclusive authority under section 274 of the Atomic Energy Act.

The Act permits some limited delegation of the States to regulate radioactive material, but it expressly excludes ocean disposal.

Mr. DUPONT. And, finally, would you object to an amendment to eliminate section 7(b) of the administration's bill?

Mr. RAMEY. This is one of these points that I would like to consult my fellow Commissioners on.

Mr. DUPONT. Well, I understand that.

Maybe the question is not quite a fair one.

I am sure the Atomic Energy Commission will have a position, but do you see, from a personal point of view, any particular problem, do you feel you would be in any kind of a bind if you did not have this authority and if you had to go to the Environmental Protection Agency for a permit?

Mr. RAMEY. You mean with respect to the Atomic Energy Commission's own proprietary activities in disposing of waste?

Mr. DUPONT. Yes.

Mr. RAMEY. Again, this is something that I personally cannot answer.

I would have to consult the Commissioners on that.

Mr. DUPONT. I have no further questions.

Mr. DINGELL. Mr. Kyros.

Mr. KYROS. Thank you, Mr. Chairman.

I would like to ask you about nuclear plants situated on the coastline, where a discharge of heated water goes into the ocean. That causes some problems, does it not, such as thermal pollution?

Mr. RAMEY. That has raised some questions in certain cases.

Mr. KYROS. Now, within this act, under the definition of dumping, is the discharge of this heated water included? I am referring to section 3, subsection F.

Mr. RAMEY. I would like to have Mr. Hennessey comment on that.

Mr. HENNESSEY. Our understanding is that it is not covered as dumping under this bill.

Mr. KYROS. Yes. Is the dumping of refuse into navigable waters, under the 1899 Rivers and Harbors Act, is that covered here?

Mr. RAMEY. That is the position that has been taken by the Environmental Protection Agency, and the Justice Department, and being contested in the courts now.

Mr. KYROS. Under the exemption of section 7(b), in which your agency becomes exempted, is nothing contemplated as to the discharge of such thermal pollution? Is nothing contained in there which refers to that?

Mr. RAMEY. This is already being regulated, of course, by the Federal Water Quality Act of 1970, and by Executive order under the 1899 Refuse Act.

Mr. KYROS. But the Atomic Energy Commission has no jurisdiction over that part of the discharge from a nuclear plant. Is that correct?

Mr. RAMEY. We come in in connection with our licensing authority.

We do serve as the enforcement agency for these other Federal and State requirements.

Mr. KYROS. For example, a power company putting in an atomic plant along the coast at Wiscasset, Maine when they come to you to get a license, is this one of the issues you have to consider? That is, whether they will thermally pollute the bay?

Mr. RAMEY. We require they will provide a certificate from the appropriate state water pollution agency that they have met the State water quality standard that has been approved by the Environmental Protection Agency.

Mr. KYROS. Now, do you automatically accept that, if certificate is so provided? Or do you examine the matter initially yourself?

Mr. RAMEY. We accept that as being adequate.

Mr. KYROS. Because, you know, one of the problems we hear about is the fact that numerous plants are planning to set themselves up along the coastlines, because of the available cooling sea water. And I just wanted to know, apart from the radioactive waste you have already discussed, what would happen with the problem of thermal pollution, if it is as great as we believe it to be?

Mr. RAMEY. This would come under the States and the Environmental Protection Agency, under their water pollution standards.

Mr. KYROS. This would not be within the jurisdiction of your agency in other words?

Mr. RAMEY. Only as I say, where the Commission, under the NEPA and the Muskie act, conditions its license on meeting the State and Federal requirements.

Mr. KYROS. Thank you very much.

Mr. DINGELL. Mr. Everett.

Mr. EVERETT. One question, Mr. Chairman.

Mr. Ramey, will you supply for the record the Atomic Energy Commission's regulations mentioned on page 2 of your statements?

Mr. RAMEY. Yes, I will be glad to.

(The information follows:)

The attached copies of AEC regulations—10 CFR Parts 2, 20, 30, 40 and 70—were referred to by Commissioner Ramey as those which related to the receipt, use and disposal of source, special nuclear and byproduct material. It should be noted that there are no specific references in any of these regulations to the *ocean* dumping of radioactive waste. (The regulations were placed in the files of the committee.)

Mr. EVERETT. One final question. I was wondering, with respect to 7(b), if you give us any indication as to the number of permits for ocean dumping of material we might be talking about in the future?

Mr. RAMEY. We will be glad to do this. I would say there will be very few.

Mr. EVERETT. But you will supply that for the record?

Mr. RAMEY. Certainly.

(The information follows:)

All routine dumping of radioactive wastes into the ocean was, for all practical purposes, discontinued in 1962, when two commercial land burial facilities commenced operations. The few remaining licenses, referred to previously, are being phased out. We do not have plans for issuing licenses for ocean disposal of radioactive material in the foreseeable future and we have no intention of dumping any such material from our own operations. Thus, the number of permits for ocean disposal of radioactive material will be zero.

We believe, however, it does not follow that a complete prohibition of ocean disposal is indicated. Until more is known about the environmental effects of other types of waste disposal, it is not in the national interest to make a decision at this time which would foreclose a particular disposal, in the future, of radioactive waste under proper conditions and controls in some part of the ocean. We have no way of estimating at this time the frequency which such proposals would be made and, if made, favorably considered.

Mr. EVERETT. That is all, Mr. Chairman.

Mr. DINGELL. Mr. Commissioner, could you tell us, in matters like ocean dumping, what has been the practice of the Atomic Energy Commission?

Have you given public notice, and had hearings, or have you simply issued permits to dump?

Mr. RAMEY. Mr. Price, do you want to comment on this?

Mr. PRICE. Mr. Chairman, we have not issued any licenses since 1960, when we stopped that part of the program.

I think the rules still provide that in order to issue a license, for waste disposal, commercial waste disposal, we have to publish a notice, and offer a public hearing.

I do not remember what cases ended up in a hearing, if any.

It was 10 years ago. I would have to check the record.

Mr. DINGELL. Would you check that, and inform us if you please, first, what were the requirements with regard to public notice and hearing, and second, whether in those days, a public hearing was held on any of the permits which were ultimately issued.

I remember I was a member of this committee, and we had some very heated discussions with the Atomic Energy Commission on some dumping off the coast of California. It may be that you remember those discussions, and there was great displeasure expressed by the oceanographic subcommittee at that time over the practice of dumping, and I think that may have contributed at that time to the halting of dumping off the California coast, and I remember, the California Salvage were the people doing it at that time, am I correct about that?

Mr. PRICE. I think it was probably another company, Mr. Chairman, but I would have to check.

Mr. DINGELL. Would you get us the information on those?

Mr. PRICE. I would be happy to.

(The information follows:)

RULES OF PRACTICE AUTHORIZING OCEAN DUMPING

The Atomic Energy Commission's "Rules of Practice," 10 CFR Part 2, became effective on March 6, 1956, and provided for notice to others of the filing of the application as required by Atomic Energy Commission regulations and such additional notice as it deems appropriate. Although not formally required at the time, notices of receipt of applications for license [illegible]thorizing sea disposal of radioactive waste were filed in the *Federal Regis[illegible]*. Notices of proposed issuances of such licenses were also published in the *Federal Register*. The notices offered the opportunity for public hearings.

The Commission's "Rules of Practice" have been amended so that the regulation now provides that an applicant for a license authorizing commercial disposal of waste radioactive material shall serve a copy of the application on the chief executive of the municipality in which the facility is to be located or the activity is to be conducted or, if the facility is not to be located or the activity conducted within a municipality, on the chief executive of the county. The Governor or other appropriate official of the State is sent a copy of the application by the AEC and a notice of receipt of the application is published in the *Federal Register*.

A hearing on an application for commercial disposal of radioactive waste at sea is not mandatory. A notice of proposed action is required to be published in the *Federal Register* which provides the opportunity for any person whose interest may be affected by the proceeding to file a petition for leave to intervene within 15 days from the date of publication in the *Federal Register*. At the time the notice of proposed action is published in the *Federal Register*, State and local officials are notified of the proposed action and a public announcement concerning the proposed action is issued by the AEC.

If no requests for leave to intervene are received, a notice of issuance of the license is published in the *Federal Register* and State and local officials are notified of the license issuance.

There have been two hearings relating to applications for licenses authorizing sea disposal of radioactive wastes.

1. A hearing was held on November 19, 1958, on the application filed by the Walker Trucking Company, which requested authority to establish a w[illegible]ste storage facility in Portland, Connecticut, and to dispose of radioactive waste at

62-513 O—71——18

sea. Subsequent to the hearing, the license was granted on March 18, 1959. Walker Trucking Company never disposed of any radioactive waste at sea. On April 9, 1964, Walker Trucking Company withdrew its request for authority to dispose of radioactive waste at sea; a license amendment issued November 27, 1964, deleted sea disposal.

2. A hearing was held on January 22 and 23, 1959, in Houston, Texas, on an application filed by Industrial Waste Disposal Corporation, Houston, Texas, which requested authorization to dispose of radioactive waste in the Gulf of Mexico. The Initial Decision by the Hearing Examiner on May 29, 1959, granted the license. After review by the Commission, the matter was remanded to the Hearing Examiner to take testimony concerning the integrity of low-level waste containers after their disposal in the Gulf of Mexico. On April 20, 1962, the Commission denied the application for disposal of radioactive waste in the Gulf of Mexico because of objections by the Government of Mexico.

The following licensees have disposed of radioactive waste off the coast of California:

1. Chevron Research Company, Richmond, California.
2. Coastwise Marine Disposal Corp., Long Beach, California.
3. Isotopes Specialties Company, Burbank, California.
4. U.S. Naval Radiation Development Laboratory, San Francisco.
5. Nuclear Engineering Company, Walnut Creek, California.
6. Ocean Transport Company, Richmond, California.

Mr. DINGELL. Gentlemen, the committee wishes to thank you for your statement, and for your being here on short notice.

We are very grateful to you. You have been very helpful. We have had a very vigorous and enlightened discussion.

We will take our next witness this afternoon at 1:45, and the staff will notify the members of the committee as to the hour.

The committee stands in recess.

(Whereupon, the subcommittee recessed at 12:30 o'clock p.m.)

AFTERNOON SESSION

Mr. DINGELL. The subcommittee will come to order.

This is a continuation of the hearings begun yesterday on the subject of dumping of waste materials of different kinds in the ocean.

Our first witnesses this afternoon are witnesses on behalf of the Interior Department, Mr. Harrison Loesch, Assistant Secretary for Public Lands Management.

Mr. Loesch, we are happy to welcome you to the committee. The Chair notes that you have with you an old friend of the present occupant of the Chair, Mr. Linduska. If you will identify yourself and Mr. Linduska for the purposes of the record, we will be most happy to listen to any statement you choose to give.

STATEMENT OF HON. HARRISON LOESCH, ASSISTANT SECRETARY FOR PUBLIC LANDS, OF THE DEPARTMENT OF THE INTERIOR, ACCOMPANIED BY DR. JOSEPH P. LINDUSKA, SPECIAL DIRECTOR, BUREAU OF SPORT FISHERIES AND WILDLIFE, DEPARTMENT OF THE INTERIOR

Mr. LOESCH. Thank you very much, Mr. Chairman. I appreciate very much being before the committee.

I am Harrison Loesch, and I have with me Dr. Joseph P. Linduska, Associate Director, Bureau of Sport Fisheries and Wildlife.

I am sure, Mr. Chairman, that Dr. Linduska will be able to give me the expertise here which I personally lack.

Mr. DINGELL. I have always found you a capable witness.

Mr. LOESCH. Thank you, sir.

Mr. Chairman, my statement, as you may have noticed, is a rather general one, and unless the Chair prefers otherwise, I would just as soon have it placed in the record, and highlight it very briefly.

Mr. DINGELL. Without objection, that is ordered and while we are discussing matters of that sort, Mr. Loesch, I will observe that the Interior Department has submitted to this committee a series of reports on legislation before us. I will tell you that you are the first, and the only department which has done so, and I must confess it is with a measure of pleasure that I compliment you and regretfully observe that other agencies have not carried out their duties in the same fashion.

Mr. LOESCH. I must say, Mr. Chairman, that that is a compliment I am not really used to receiving. Before certain other committees of this House, I am quite often criticized because the reports didn't get there until I did. I appreciate it.

Well, Mr. Chairman, my report, of course, adverts to the dangers and damages of dumping in the ocean, which I think this committee is especially very well acquainted with already. I think we need not go into the general nature of the damage and pollution which the legislation before this committee is designed to correct.

We especially are commenting on H.R. 4247 and H.R. 4723, now pending before the full committee, which is the result of cooperation between a number of the Federal agencies, including our own.

And those bill vest, of course, in the Administrator of the EPA the authority to control the ocean dumping through permits and enforcement of prohibitions against unauthorized transport or dumping of waste materials.

In determining whether or not to approve the permit applications provided for by the legislation, the Administrator would have to consider the impact of dumping on the marine environment and human welfare, and other possible locations and methods of disposal, including land based alternatives.

To my mind, it is quite similar to the situation under the National Environmental Policy Act, which requires us in performing any or in studying any proposal to consider all possible alternatives which might have a less adverse effect or no effect at all upon the environment.

I haven't any doubt, though I am not acquainted with what has gone on before in this hearing before this committee, that the Environmental Protection Agency has presented the specific of this matter, and I think we should note that the proposal by that agency incorporates several provisions of the other bills, of which I believe, if I am not mistaken, there are about 20, altogether.

Mr. DINGELL. At least.

Mr. LOESCH. The comprehensive framework provided by these two bills, we wish to support. We believe that the consultation provided for in the act between the Administrator and the Department of the Interior and others, would establish the proper criteria, and would afford our Department an opportunity to contribute the knowledge and expertise of the marine environment that we have, and to seek the protection of wildlife, minerals, recreation resources, for which we have the primary responsibility.

In this connection, we agree with the Council on Environmental Quality that regulatory authority should be vested in an agency whose chief role is the enforcement of environmental standards.

Amendment of the Fish and Wildlife Coordination Act, as several bills, propose, would tend to disperse the regulatory authority and to discourage effective coordination with air and water quality programs already administered by EPA, and, of course, since the loss by the Interior Department of the Bureau of Commercial Fisheries and FWQA, an amendment of the Fish and Wildlife Coordination Act would not at this time appear to be very productive, especially in view of the possibility of further reorganization of the executive branch of the Government.

That concludes a summary of my formal statement, Mr. Chairman, and I am prepared to answer questions if I can.

Mr. DINGELL. The Chair thanks you for a very helpful statement, Mr. Loesch.

Mr. DINGELL. The Chair notes also that we have the reports from your agency, which the Chair without objection directs the staff to insert into the record at a place appropriate, together with such other reports as may be received from the agencies of the executive branch.

There is also a letter from the Attorney General relative to this matter, which the Chair without objection directs the staff to insert in the record at the appropriate point.

Mr. Pelly?

Mr. PELLY. Mr. Secretary, you support this bill without equivocation, and I am going to quit while I am ahead.

I don't want to invite any questions which might in any way cause you to weaken as far as your support is concerned. I think you made a fine statement, and we are very happy to have it.

Mr. LOESCH. I hope, Mr. Pelly, that any questions you might ask would not have that effect.

Mr. PELLY. Thank you.

Mr. DINGELL. Thank you very much, Mr. Pelly.

Mr. Loesch, the Chair wishes you and your able associate there, Dr. Linduska, to direct your attention very carefully to the Fish and Wildlife Coordination Act, and to the agreement that was executed by and between your agency and the Department of the Army relative to dredging, filling and dumping.

Would it be too much to ask that you submit to us a copy of that agreement which I am satisfied is in the files of your agency for purposes of inclusion in the record at this particular point?

Mr. LOESCH. I am sure we can do so.

(Document referred to follows:)

MEMORANDUM OF UNDERSTANDING BETWEEN THE SECRETARY OF THE INTERIOR AND THE SECRETARY OF THE ARMY

In recognition of the responsibilities of the Secretary of the Army under sections 10 and 13 of the Act of March 3, 1899 (33 U.S.C. 403 and 407), relating to the control of dredging, filling, and excavation in the navigable waters of the United States, and the control of refuse in such waters, and the interrelationship of those responsiblities with the responsibilities of the Secretary of the Interior under the Federal Water Pollution Control Act, as amended (33 U.S.C. 466 *et seq.*) the Fish and Wildlife Coordination Act, as amended (16 U.S.C. 661–666c), and the Fish and Wildlife Act of 1956, as amended (16 U.S.C. 742a

et seq.) relating to the control and prevention of water pollution in such waters and the conservation of the Nation's natural resources and related environment, including fish and wildlife and recreational values therein; in recognition of our joint responsibilities under Executive Order No. 11288 to improve water quality through the prevention, control, and abatement of water pollution from Federal and federally licensed activities; and in recognition of other provisions of law and policy, we, the two Secretaries, adopt the following policies and procedures:

POLICIES

1. It is the policy of the two Secretaries that there shall be full coordination and cooperation between their respective Departments on the above responsibilities at all organizational levels, and it is their view that maximum efforts in the discharge of those responsibilities, including the resolution of differing views, must be undertaken at the earliest practicable time and at the field organizational unit most directly concerned. Accordingly, District Engineers of the U.S. Army Corps of Engineers shall coordinate with the Regional Directors of the Secretary of the Interior on fish and wildlife, recreation, and pollution problems associated with dredging, filling, and excavation operations to be conducted under permits issued under the 1899 Act in the navigable waters of the United States, and they shall avail themselves of the technical advice and assistance which such Directors may provide.

2. The Secretary of the Army will seek the advice and counsel of the Secretary of the Interior on difficult cases. If the Secretary of the Interior advises that proposed operations will unreasonably impair natural resource or the related environment, including the fish and wildlife and recreational values thereof, or will reduce the quality of such waters in violation of applicable water quality standards, the Secretary of the Army in acting on the request for a permit will carefully evaluate the advantages and benefits of the operations in relation to the resultant loss or damage, including all data presented by the Secretary of the Interior, and will either deny the permit or include such conditions in the permit as he determines to be in the public interest, including provisions that will assure compliance with water quality standards established in accordance with law.

PROCEDURES FOR CARRYING OUT THESE POLICIES

1. Upon receipt of an application for a permit for dredging, filling, excavation, or other related work in navigable waters of the United States, the District Engineers shall send notices to all interested parties, including the appropriate Regional Directors of the Federal Water Pollution Control Administration, the United States Fish and Wildlife Service, and the National Park Service of the Department of the Interior, and the appropriate State conservation, resources, and water pollution agencies.

2. Such Regional Directors of the Secretary of the Interior shall immediately make such studies and investigations as they deem necessary or desirable, consult with the appropriate States agencies, and advise the District Engineers whether the work proposed by the permit applicant, including the deposit of any material in or near the navigable waters of the United States, will reduce the quality of such waters in violation of applicable water quality standards or unreasonably impair natural resources or the related environment.

3. The District Engineer will hold public hearings on permit applications whenever response to a public notice indicates that hearings are desirable to afford all interested parties full opportunity to be heard on objections raised.

4. The District Engineer, in deciding whether a permit should be issued, shall weigh all relevant factors in reaching his decision. In any case where Directors of the Secretary of the Interior advise the District Engineers that proposed work will impair the water quality in violation of applicable water quality standards or unreasonably impair the natural resources or the related environment, he shall, within the limits of his responsibility, encourage the applicant to take steps that will resolve the objections to the work. Failing in this respect, the District Engineer shall forward the case for the consideration of the Chief of Engineers and the appropriate Regional Director of the Secretary of the Interior shall submit his views and recommendations to his agency's Washington Headquarters.

5. The Chief of Engineers shall refer to the Under Secretary of the Interior all those cases referred to him containing unresolved substantive differences of

views and he shall include his analysis thereof, for the purpose of obtaining the Department of Interior's comments prior to final determination of the issues.

6. In those cases where the Chief of Engineers and the Under Secretary are unable to resolve the remaining issues, the cases will be referred to the Secretary of the Army for decision in consultation with the Secretary of the Interior.

7. If in the course of operations within this understanding, either Secretary finds its terms in need of modification, he may notify the other of the nature of the desired changes. In that event the Secretaries shall within 90 days negotiate such amendment as is considered desirable or may agree upon termination of this understanding at the end of the period.

(S) Stewart L. Udall,
Secretary of the Interior.

JULY 13, 1967.

(S) Stanley Resor,
Secretary of the Army.

JULY 13, 1967.

Mr. DINGELL. The Chair regards that as very important, because the Chair is very interested in observing with some care, one, whether the Fish and Wildlife Coordination Act is in any way amended, altered, or repealed by the legislation before us, and two, whether or not the memorandum of agreement would in any way be affected, altered, superseded, or changed by the legislation that is before us.

Are you able to give us your interpretation of whether or not the legislation before us, specifically the administration's bill, or identical measures, would in any fashion alter or amend or change, first of all, the Fish and Wildlife Coordination Act?

Mr. LOESCH. The legislative counsel of the Department advises me, Mr. Chairman, that it will not.

Mr. DINGELL. In no fashion whatsoever?

Mr. LOESCH. In no way. Am I correct?

Dr. LINDUSKA. That is correct.

Mr. DINGELL. Now can you give us your understanding of the impact of this legislation on the agreement between the Fish and Wildlife and the Interior Department on the one side, and the Department of the Army Corps of Engineers on the other regarding dumping, dredging, and filling?

Or is it your impression that that legislation as construed by the *Boca Ciega* case, carried forward, unimpeded, unimpaired, and unaffected under the legislation before us, under the new kind of permit which would be issued by EPA for similar ocean dumpings?

Mr. LOESCH. Yes, it is my impression, Mr. Chairman, that the old agreement, the agreement we have, would remain in full force and effect without alteration.

Dr. Linduska, do you have a comment?

Dr. LINDUSKA. We have had no reason to believe it would be changed in any way, up to this point, Mr. Chairman.

Mr. DINGELL. Very well. The Chair is going to direct the staff to see to it that the Supreme Court case, it finally deciding the *Boca Ciega* case, be inserted in the record at this point, so as to make very clear precisely the case, the agreement, and the statute to which you and I have been allowed in our colloquy at this time, for purposes of establishing a firm and a hard and very clear legislative record.

(The document follows:)

IN THE

United States Court of Appeals

FOR THE FIFTH CIRCUIT

No. 27555

ALFRED G. ZABEL and DAVID H. RUSSELL,
Plaintiffs-Appellees,

versus

R. P. TABB, COLONEL, CORPS OF ENGINEERS, DISTRICT ENGINEER, DEPARTMENT OF THE ARMY, JACKSONVILLE, FLORIDA, DISTRICT; STANLEY R. RESOR, SECRETARY OF THE ARMY; AND UNITED STATES OF AMERICA,
Defendants-Appellants.

Appeal from the United States District Court for the Middle District of Florida

---

(July 16, 1970)

Before BROWN, Chief Judge, TUTTLE and MORGAN, Circuit Judges.

BROWN, Chief Judge: It is the destiny of the Fifth Circuit to be in the middle of great, oftentimes explosive issues of spectacular public importance. So it is here as we enter in depth the contemporary interest

in the preservation of our environment. By an injunction requiring the issuance of a permit to fill in eleven acres of tidelands in the beautiful Boca Ciega Bay in the St. Petersburg-Tampa, Florida area for use as a commercial mobile trailer park, the District Judge held that the Secretary of the Army and his functionary, the Chief of Engineers, had no power to consider anything except interference with navigation. There being no such obstruction to navigation, they were ordered to issue a permit even though the permittees acknowledge that "there was evidence before the Corps of Engineers sufficient to justify an administrative agency finding that [the] fill would do damage to the ecology or marine life on the bottom." We hold that nothing in the statutory structure compels the Secretary to close his eyes to all that others see or think they see. The establishment was entitled, if not required, to consider ecological factors and, being persuaded by them, to deny that which might have been granted routinely five, ten, or fifteen years ago before man's explosive increase made all, including Congress, aware of civilization's potential destruction from breathing its own polluted air and drinking its own infected water and the immeasurable loss from a silent-spring-like disturbance of nature's economy. We reverse.

I

Genesis: The Beginning

In setting the stage we draw freely on the Government's brief. This suit was instituted by Landholders,

Zabel and Russell, on May 10, 1967, to compel the Secretary of the Army to issue a permit to dredge and fill in the navigable waters of Boca Ciega Bay, in Pinellas County near St. Petersburg, Florida. On August 15, 1967, the United States and its officers, Defendants-Appellants, filed a motion to dismiss the suit for lack of jurisdiction which was denied. The United States and other defendants then answered the complaint alleging lack of jurisdiction and that the Court lacks power to compel a discretionary act by the Secretary of the Army. The United States and other defendants moved for summary judgment. Landholders, Zabel and Russell, also moved for summary judgment. After a hearing, the District Court, on February 17, 1969, granted summary judgment for Landholders and directed the Secretary of the Army to issue the permit. It granted a stay of execution of the judgment until this appeal could be heard and decided. We invert the summary judgments, reversing Appellees and rendering judgment for the United States.

Landholders own land riparian to Boca Ciega Bay and adjacent land underlying the Bay. It is navigable water of the United States, being an arm of Tampa Bay which opens into the Gulf of Mexico. The Zabel and Russell property is located about one mile from the Intracoastal Waterway.

Landholders desire to dredge and fill on their property in the Bay for a trailer park, with a bridge or culvert to their adjoining upland. To this purpose they first applied to the state and local authorities for permission to perform the work and obtained the consent

or approval of all such agencies having jurisdiction to prohibit the work, namely Pinellas County Water and Navigation Control Authority (which originally rejected permission, but ultimately issued a permit pursuant to state Court order),[1] Trustees of the Internal Improvement Fund of the State of Florida, Central and South Florida Flood Control District, and Board of Pilot Commissioners for the Port of St. Petersburg.

Landholders then applied to the Corps of Engineers for a federal permit to perform the dredging and filling. The Pinellas County Water and Navigation Control Authority (which originally rejected permission, but ultimately issued a permit pursuant to state Court order) continued to oppose the work as did the Board of County Commissioners of Pinellas County, who also comprise the Pinellas County Water and Navigation Control Authority, the County Health Board of Pinellas County, the Florida Board of Conservation, and about 700 in-

[1]The Authority's denial of a permit was affirmed by the Florida District Court of Appeal in Zabel v. Pinellas County Water & Navigation Control Authority, Fla. Ct. App., 1963 154 So.2d 181. The Supreme Court of Florida reversed that decision because Zabel had been required by the Authority to show that there would be no adverse effect on the public interest, rather than the burden of adverse effect being placed on the Authority. It held that on this record there was insufficient showing of adverse effect, so that denial of a permit would be a taking of property without compensation. It said (p. 381). "In view of the foregoing, the decision appealed from is quashed and the cause remanded for disposition consistent herewith." Zabel v. Pinellas County Water & Nav. Con. Auth., Fla., 1965, 171 So.2d 376. Against the Authority's contention that this ruling intended further proceedings on the application, to accord it a chance to establish adverse effect, the District Court of Appeal directed issuance of a permit. Pinellas County Water & Nav. Con. Auth. v. Zabel, Fla. Ct. App., 1965, 179 So.2d 370.

dividuals who filed protests. The United States Fish and Wildlife Service, Department of the Interior, also opposed the dredging and filling because it "would have a distinctly harmful effect on the fish and wildlife resources of Boca Ciega Bay."

A public hearing was held in St. Petersburg in November, 1966, and on December 30, 1966, the District Engineer at Jacksonville, Florida, Colonel Tabb, recommended to his superiors that the application be denied. He said that "The proposed work would have no material adverse effect on navigation"[2] but that:

> "Careful consideration has been given to the general public interest in this case. The virtually unanimous opposition to the proposed work as expressed in the protests which were received and as exhaustively presented at the public hearing have convinced me that approval of the application would not be in the public interest. The continued opposition of the U.S. Fish & Wildlife Service despite efforts on the part of the applicants to reduce the extent of damage leads me to the conclusion that approval of the work would not be consistent with the intent of Congress as expressed in the Fish & Wildlife Coordination Act, as amended, 12 August 1958. Further, the opposition of the State of Florida and of county authorities as

---

[2] There was evidence both that it would aid navigation and that it would obstruct navigation. There was similar evidence on pollution.

described in paragraph 5 above gives additional support to my conclusion that the work should not be authorized."

The Division Engineer, South Atlantic Division, Atlanta, Georgia, concurred in that recommendation stating: "In view of the wide spread opposition to the proposed work, it is apparent that approval of the application would not be in the public interest." The Chief of Engineers concurred for the same reasons. Finally, the Secretary of the Army denied the application on February 28, 1967, because issuance of the requested permit:

1. Would result in a distinctly harmful effect on the fish and wildlife resources in Boca Ciega Bay,

2. Would be inconsistent with the purposes of the Fish and Wildlife Coordination Act of 1958, as amended (16 U.S.C. 662),

3. Is opposed by the Florida Board of Conservation on behalf of the State of Florida, and by the County Health Board of Pinellas County and the Board of County Commissioners of Pinellas County, and

4. Would be contrary to the public interest.

Landholders then instituted this suit to review the Secretary's determination and for an order compelling him to issue a permit. They urged that the proposed

work would not hinder navigation and that the Secretary had no authority to refuse the permit on other grounds. They acknowledged that "there was evidence before the Corps of Engineers sufficient to justify an administrative agency finding that our fill would do damage to the ecology or marine life on the bottom." The Government urged lack of jurisdiction and supported the denial of the permit on authority of §10 of the Rivers and Harbors Act of March 3, 1899, 30 Stat. 1121, 1151, 33 U.S.C.A. § 403, giving the Secretary discretion to issue permits and on the Fish and Wildlife Coordination Act of March 10, 1934, 48 Stat. 401, as amended, 16 U.S.C.A. §§ 661 and 662 (a), requiring the Secretary to consult with the Fish and Wildlife Service and state conservation agencies before issuing a permit to dredge and fill.

The District Court held that it had jurisdiction, that the Fish and Wildlife Coordination Act was not authority for denying the permit, and that:

> "The taking, control or limitation in the use of private property interests by an exercise of the police power of the government or the public interest or general welfare should be authorized by legislation which clearly outlines procedure which comports to all constitutional standards. This is not the case here.
>
> As this opinion is being prepared the Congress is in session. Advocates of conservation are both able and effective. The way is open to obtain a remedy for future situations like this

one if one is needed and can be legally granted by the Congress."

The Court granted summary judgment for Landholders and directed the Secretary of the Army to issue the permit. This appeal followed.

The question presented to us is whether the Secretary of the Army can refuse to authorize a dredge and fill project in navigable waters for factually substantial ecological reasons even though the project would not interfere with navigation, flood control, or the production of power. To answer this question in the affirmative, we must answer two intermediate questions affirmatively. (1) Does Congress for ecological reasons have the power to prohibit a project on private riparian submerged land in navigable waters? (2) If it does, has Congress committed the power to prohibit to the Secretary of the Army?

II

Constitutional Power

The starting point here is the Commerce Clause[3] and its expansive reach. The test for determining whether Congress has the power to protect wildlife in navigable waters and thereby to regulate the use of private property for this reason is whether there

[3] "The Congress shall have power to regulate Commerce with foreign nations, and among the several states, and with the Indian Tribes." U.S. Const. Art. I, § 8, Cl. 3.

BEST COPY AVAILABLE

is a basis for the Congressional judgment that the activity regulated has a substantial effect on interstate commerce. *Wickard v. Filburn*, 1942, 317 U.S. 111, 125, 63 S.Ct. 82, ——, 87 L.Ed. 122, 135. That this activity meets this test is hardly questioned.[4] In this time of awakening to the reality that we cannot continue to despoil our environment and yet exist,[5] the nation knows, if Courts do not, that the destruction of fish and wildlife in our estuarine waters does have a substantial, and in some areas a devastating, effect on interstate commerce. Landholders do not contend otherwise. Nor is it challenged that dredge and fill projects

---

4Landholders cite Weber v. State Harbor Comm'rs, 1873, 85 U.S. (18 Wall.) 65, 21 L.Ed. 798 and United States v. River Rouge Improvement Co., 1926, 269 U.S. 411, 46 S.Ct. 144, 70 L.Ed. 339 as limiting the power of the Federal Government over navigable waters to control for navigational purposes. Not surprisingly, the narrow view these cases take of the commerce clause is pre-United States v. Darby, 1941, 312 U.S. 100, 61 S.Ct. 451, 85 L.Ed. 609.

5Complete documentation of the concern over environmental problems would surely be voluminous, but it is indirectly evidenced by the amount of very recent legal activity. See National Environmental Policy Act of 1969. Pub. Law 91-190 (Jan. 1, 1970), *infra* note 24; *Our Waters and Wetlands: How the Corps of Engineers Can Help Prevent Their Destruction and Pollution*, H. Rep. 91-917, 91st Cong., 2d Sess, March 18, 1970, *infra* text at note 26; Executive Order 11507, Feb. 4, 1970, 38 L.W. 2436; United States v. Ray, 5 Cir., 1970, —— F.2d —— [No. 27888, Jan. 22, 1970]; E.B. Elliott Advertising Co. v. Hill, 5 Cir., 1970, —— F.2d —— [No. 27589, April 3, 1970]; Citizens Committee for the Hudson Valley v. Volpe, S.D.N.Y., 1969, 302 F. Supp. 1083, aff'd, 2 Cir., 1970, —— F.2d —— [No. 428-33, April 16, 1970]; National Advertising Co. v. Monterey, Calif., 1970, —— Calif. Rptr. —— [38 L.W. 2433, Jan. 30, 1970; MacGibbon v. Duxbury Board of Appeals, Mass., 1970, —— N.E.2d —— [38 L.W. 2429, Jan. 29, 1970]; California v. SS Bournemouth, C.D. Cal., 1969, 307 F. Supp. 922; Creation of ABA Special Committee on Environmental Quality, 15 Am. Bar News No. 3, March 1970.

BEST COPY AVAILABLE

are activities which may tend to destroy the ecological balance and thereby affect commerce substantially. Because of these potential effects Congress has the power to regulate such projects.

III

Relinquishment of the Power

Landholders do not challenge the existence of power. They argue that Congress in the historic compromise over the oil rich tidelands controversy abandoned its power over other natural resources by the relinquishment to the states in the Submerged Lands Act.[6] By it they urge the Government stripped itself of the power to regulate tidelands property except for purposes relating to (i) navigation, (ii) flood control, and (iii) hydroelectric power. This rests on the expressed Congressional reservation of control for these three purposes over the submerged lands, title to and power over which Congress relinquished to the states.[7]

---

6 43 U.S.C.A. § 1301, et seq. See Continental Oil Co. v. London Steamship Owners' Mut. Ins. Ass'n., 5 Cir., 1969, 417 F.2d 1030, —— A.M.C. ——, *cert. denied*, 1970, —— U.S. ——, —— S.Ct. ——, 25 L.Ed.2d 92, —— A.M.C. ——; Atlantis Development Corp. v. United States, 5 Cir., 1967, 379 F.2d 818.

7 The relinquishing provision states, 43 U.S.C.A. § 1311 (a) and (b):

> "(a) It is determined and declared to be in the public interest that (1) title to and ownership of the lands beneath navigable waters within the boundaries of the respective States, and the natural resources within such lands and waters, and (2) the right and power to manage, administer, lease, develop, and use the said lands and natural resources all in accordance with applicable State law be, and they are, subject to the provisions hereof, recognized, confirmed,

BEST COPY AVAILABLE

The argument assumes that when Congress relinquished title to the land and the right and power to manage and use the land, it relinquished its power under the commerce clause except in particulars (i), (ii), and (iii). It also assumes that reservation of these three enumerated aspects of the commerce power implied that Congress gave up its plenary power over the myriad other aspects of commerce. See, e.g., *Heart of Atlanta Motel, Inc. v. United States*, 1964, 379 U.S.

> established, and vested in and assigned to the respective States or the persons who were on June 5, 1950, entitled thereto under the law of the respective States in which the land is located, and the respective grantees, lessees, or successors in interest thereof;
>
> (b) (1) The United States releases and relinquishes unto said States and persons aforesaid, except as otherwise reserved herein, all right, title, and interest of the United States, if any it has, in and to all said lands, improvements, and natural resources * * *."

The reservation provision referred to states, 43 U.S.C.A. § 1311 (d):

> "(d) Nothing in this chapter shall affect the use, development, improvement, or control by or under the constitutional authority of the United States of said lands and waters for the purposes of navigation or flood control of the production of power, or be construed as the release or relinquishment of any rights of the United States arising under the constitutional authority of Congress to regulate or improve navigation, or to provide for flood control, or the production of power * * *."

The term "natural resources" is broadly defined to include both the animate and inanimate:

> "The term 'natural resources' includes, without limiting the generality thereof, oil, gas, and all other minerals, and fish, shrimp, oysters, clams, crabs, lobsters, sponges, kelp, and other marine animal and plant life but does not include water power, or the use of water for the production of power;"

43 U.S.C.A. § 1301 (e)

BEST COPY AVAILABLE

62-513 O - 71 - 19

241, 58 S.Ct. 348, 13 L.Ed.2d. 258; *Katzenbach v. McClung*, 1964, 379 U.S. 294, 85 S.Ct. 377, 13 L.Ed.2d 290.

A nice argument can be contrived that the net effect of these provisions was to vest in the adjacent states [1] title in these tidelands and their natural resources and [2] [a] the exclusive power to use, exploit and manage these lands [b] only subject to the reserved power of the Federal government regarding (i) navigation, (ii) flood control, and (iii) production of power. Certainly, this brief synopsis of (1) and (2) (a) is the literal import of §1311 (a) (1) (2). Likewise, the reservation summarized as (2) (b) is literally specified in § 1311 (d). On this approach, the Federal Government turned over to adjacent states the full management and use of the tidelands reserving only those limited powers over commerce comprehended within the three particulars.

But this argument ignores both language found elsewhere and the legislative purpose of the Act. The controversy, often pressed with emotional overtones, was over oil and gas and whether the states were to reap the economic benefits of development royalties and to regulate the exploration and development or whether these benefits and these controls were to be exercised by the Federal Government as an adjunct of then newly declared "paramount' rights", *United States v. California*, 1947, 332 U.S. 804, 805, 68 S.Ct. 20, ——, 92 L.Ed. 382, 383. The Act and this relinquishment reflect the legislative compromise found in the combination of the Submerged Lands Act and the Outer

BEST COPY AVAILABLE

Continental Shelf Act.[8] The adjacent states were to be the "owner" of the resources and reap exclusively the economic benefits of resources in the tidelands and have full control over management and exploitation. The Federal government, on the other hand, was given exclusive ownership and control *vis-a-vis* the states in the Outer Continental Shelf.

Although it was easy to make this division, the nature of the physical area of the controversy presented immediate operational problems growing out of the water. The Federal government's traditional concern with navigation, especially on the high seas, its later but then quite extensive concern in flood control, hydroelectric power production, and the frequent combination of both under grandiose projects of a Corps of Engineers, raised specific problems calling for accommodation of the (i) sweeping Federal divestiture and (ii) the continued fulfillment of the Federal government's role in these activities. Thus, for example, the states' exclusive right to grant exploration privileges, determine the location and spacing of development wells or drilling platforms posed prospects of maritime hazards. Without imposing its own notions of how development ought to be conducted, restricted, expanded, or controlled, the Federal government had to have, and reserved expressly this power even to prohibit a drilling rig platform at a particular location These specific reservations eliminated these frequent and extensive activities as a source of further state versus national controversy.

---

[8] 43 U.S.C.A. § 1331, et. seq.

BEST COPY AVAILABLE

Whatever remaining doubt there might be on this reading was expressly eliminated by language in § 1314 (a) which specifically retains in the Federal government "all of its * * * rights in and powers of regulation and control of said lands and * * * waters for the constitutional purposes of commerce * * *" 43 U.S.C.A. § 1314 (a).[9] This section, which encompasses and pervades the entire Act, makes it clear that Congress intended to and did retain all its constitutional powers over commerce and did not relinquish certain portions of the power by specifically reserving others.[10]

---

[9] "The United States retains all its navigational servitude and rights in and powers of regulation and control of said lands and navigable waters for the constitutional purposes of commerce, navigation, national defense, and international affairs, all of which shall be paramount to, but shall not be deemed to include, proprietary rights of ownership, or the rights of management, administration, leasing, use, and development of the lands and natural resources which are specifically recognized, confirmed, established, and vested in and assigned to the respective States and others by section 1311 of this title."

43 U.S.C.A. § 1314 (a).

[10] It is argued that the retention in § 1314(a) is limited to the three aspects enumerated in § 1311(d) by the words "[the commerce power] shall be paramount to, but shall not be deemed to include [relinquished rights]." But we have already shown that the enumeration of these three, which are explicitly stated because they are particularly relevant to the regulation of land lying under navigable waters, does not imply that Congressional power over other types of commerce was among the rights relinquished. Because Congress did not give up any of its power over all of interstate commerce in § 1311 (see note 7, *supra*), they are not "[relinquished rights]" and the limitation portion of § 1314(a) is inapplicable.

To hold otherwise would render the reservation of constitutional commerce power in § 1314(a) a useless reiteration of

BEST COPY AVAILABLE

All of this is additionally borne out by the legislative history[11] and *United States v. Rands*, 1967, 389 U.S. 121, 127, 88 S.Ct. 265, ___, 19 L.Ed.2d 329, 335:

> "Finally, respondents urge that the Government's position subverts the policy of the Submerged Lands Act, which confirmed and vested in the States title to the lands beneath navigable waters within their boundaries and to natural resources within such lands and waters, together with the right and power to manage, develop, and use such lands and natural resources. However, reliance on that Act is misplaced, for it expressly recognized that the United States retained all its navigational servitude and rights in and powers of regulation and control of said lands and navigable waters for the constitutional purposes of commerce, navigation, national defense, and international affairs, all of which shall be paramount to, but shall not be deemed to include,

---

the impliedly retained powers in § 1311(d). But to hold that it is an explicit reservation of all commerce powers gives the section meaning. The section may be unneeded and overly cautious in that it reserves a constitutional power that has never been relinquished, but it should not be read in such a way as to render it otherwise useless.

> "This title does not affect any of the Federal constitutional powers of regulation and control over these areas within State boundaries. Such powers, as those over navigation, commerce, national defense, international affairs, flood control, and power production where the United States owns or acquires the water power."

H. R. Rep. No. 215, 83d Cong., 1st Sess. (March 27, 1953), 1953 U.S.C.C. & A.N. 1385, 1389.

BEST COPY AVAILABLE

> proprietary rights of ownership .... Nothing in the Act was to be construed as the release or relinquishment of any rights of the United States arising under the constitutional authority of Congress to regulate or improve navigation, or to provide for flood control, or the production of power. The Act left congressional power over commerce and the dominant navigational servitude of the United States precisely where it found them."

Congress clearly has the power under the Commerce Clause to regulate the use of Landholders' submerged riparian property for conservation purposes and has not given up this power in the Submerged Lands Act.

IV

Prohibiting Obstructions to Navigation

The action of the Chief of Engineers and the Secretary of the Army under attack rests immediately on the Rivers and Harbors Act, 33 U.S.C.A. § 403, which declares that "the creation of any obstruction * * * to the navigable capacity of any of the waters of the United States is prohibited."[12] The Act covers both

12 "The creation of any obstruction not affirmatively authorized by Congress, to the navigable capacity of any of the waters of the United States is prohibited; and it shall not be lawful to build or commence the building of any wharf, pier, dolphin, boom, weir, breakwater, bulkhead, jetty, or other structures in any port, roadstead, haven, harbor, canal, navigable river, or other water of the United States, outside established

BEST COPY AVAILABLE

building of structures and the excavating and filling in navigable waters. It is structured as a flat prohibition *unless* — the unless being the issuance of approval by the Secretary after recommendation of the Chief of Engineers.[13] The Act itself does not put any restrictions on denial of a permit or the reasons why the Secretary may refuse to grant a permit to one seeking to build structures on or dredge and fill his own property. Although the Act has always been read as tempering the outright prohibition by the rule of reason against arbitrary action, the Act does flatly forbid the obstruction. The administrator may grant permission on conditions and conversely deny permission when the situation does not allow for those conditions.

But the statute does not prescribe either generally or specifically what those conditions may be. The ques-

---

harbor lines, or where no harbor lines have been established, except on plans recommended by the Chief of Engineers and authorized by the Secretary of the Army; and it shall not be lawful to excavate or fill, or in any manner to alter or modify the course, location, condition, or capacity of, any port, roadstead, haven, harbor, canal, lake, harbor of refuge, or inclosure within the limits of any breakwater, or of the channel of any navigable water of the United States, unless the work has been recommended by the Chief of Engineers and authorized by the Secretary of the Army prior to beginning the same."

33 U.S.C.A. § 403.

13 This Court recently held that under this same section together with the Outer Continental Shelf Lands Act, 43 U.S.C.A. § 1333(f), a permit must be obtained before a project can be begun on the Outer Continental Shelf. United States v. Ray, *supra*, note 5, which followed the remand and trial on the merits in Atlantis Development Corp. v. United States, 5 Cir., 1967, 379 F.2d 818.

BEST COPY AVAILABLE

tion for us is whether under the Act the Secretary may include conservation considerations as conditions to be met to make the proposed project acceptable. Until now there has been no absolute answer to this question. In fact, in most cases under the Rivers and Harbors Act the Courts have been faced only with navigation problems.[14] See, e.g., *Sanitary Dist. v.*

---

[14]Landholders cite authority holding that the Secretary is empowered to deny a permit only for navigational reasons, United States Attorney General's opinion of February 13, 1925, 30 U.S. Atty. Gen. Ops. 410 at 412, 415, 416; Miami Beach Jockey Club, Inc. v. Dern, D.C. Cir., 1936, 86 F.2d 135, 136 (on petition for rehearing). These determinations, by no means inexorable under the wording of the statute, see Greathouse v. Dern, *infra*, pre-date the changes wrought by the Fish and Wildlife Coordination Act, *infra*.

And they are out of step with the sweeping declaration of power over commerce in United States v. Appalachian Electric Power Co., 1940, 311 U.S. 377, 423-27, 61 S.Ct. 291, ——, 85 L.Ed. 243, 261-63:

> "The state and respondent, alike, however, hold the waters and the lands under them subject to the power of Congress to control the waters for the purpose of commerce. The power flows from the grant to regulate, i.e., to 'prescribe the rule by which commerce is to be governed.' This includes the protection of navigable waters in capacity as well as use. This power of Congress to regulate commerce is so unfettered that its judgment as to whether a structure is or is not a hindrance is conclusive. Its determination is legislative in character. The Federal Government has domination over the water power inherent in the flowing stream. It is liable to no one for its use or non-use. The flow of a navigable stream is in no sense private property; 'that the running water in a great navigable stream is capable of private ownership is inconceivable.' Exclusion of riparian owners from its benefits without compensation is entirely within the Government's discretion."
>
> * * * *
>
> "In our view, it cannot properly be said that the constitutional power of the United States over its

BEST COPY AVAILABLE

United States, 1925, 266 U.S. 405, 45 S.Ct. 176, 69 L.Ed. 352; *Wisconsin v. Illinois*, 1929, 278 U.S. 367, 49 S.Ct. 163, 73 L.Ed. 426; *United States v. Republic Steel Corp.*, 1960, 362 U.S. 482, 80 S.Ct. 884, 4 L.Ed.2d 903.

One very big exception is *United States ex rel. Greathouse v. Dern*, 1933, 289 U.S. 352, 53 S.Ct. 614, 77 L.Ed. 1250. There petitioners sought a writ of mandamus to compel the Secretary of War and the Chief of Engineers to issue a permit to build a wharf in navigable waters. The Secretary, specifically finding that it would not interfere with navigation, denied the permit. The Supreme Court held that mandamus would not issue because the allowance of mandamus "is controlled by equitable principles * * * and it may be refused for reasons comparable to those which would lead a Court of equity, in the exercise of a sound discretion, to withhold its protection of an undoubted legal

---

waters is limited to control for navigation. By navigation respondent means no more than operation of boats and improvement of the waterway itself. In truth the authority of the United States is the regulation of commerce on its waters. Navigability, in the sense just stated, is but a part of this whole. Flood protection, watershed development, recovery of the cost of improvements through utilization of power are likewise parts of commerce control. * * * That authority is as broad as the needs of commerce. * * * The point is that navigable waters are subject to national planning and control in the broad regulation of commerce granted the Federal Government. The license conditions to which objection is made have an obvious relationship to the exercise of the commerce power. Even if there were no such relationship the plenary power of Congress over navigable waters would empower it to deny the privilege of constructing an obstruction in those waters."

BEST COPY AVAILABLE

right." The reason was that the United States had plans to condemn petitioners' land for use as a means of access to a proposed parkway. Allowing a wharf to be built would increase the expense to the government since it would increase the market value of the land and would require the government to pay for tearing down the wharf. The importance of *Greathouse* is that it recognized that the Corps of Engineers does not have to wear navigational blinders when it considers a permit request. That there must be a reason does not mean that the reason has to be navigability.

Another case holds that the Corps has a duty to consider factors other than navigational. *Citizens Committee for the Hudson Valley v. Volpe*, S.D.N.Y., 1969, 302 F.Supp. 1083, aff'd., 2 Cir., 1970 ____ F.2d ____ [No. 428-33, April 16, 1970]. There the District Court held that the Corps must consider a fill project in the context of the entire expressway project of which it was a part rather than just considering the fill and its effect on navigation. The reasoning was that the approval of the Secretary of Transportation was necessary before a proposed causeway could be constructed. The causeway, along with the fill, was an integral part of the expressway project. However, if the Corps and Secretary of the Army approved the fill and the State completed it, the Secretary of Transportation, considering the enormous expense of the fill, would have no choice, other than approving the causeway. The Army thus had exceeded its authority in approving the fill on only navigational considerations since

approval of the fill was effectually approval of the causeway.[15]

But such circuity is not necessary. Governmental agencies in executing a particular statutory responsibility ordinarily are required to take heed of, sometimes effectuate and other times not thwart other valid statutory governmental policies. And here the government-wide policy of environmental conservation is spectacularly revealed in at least two statutes, The Fish and Wildlife Coordination Act[16] and the National Environmental Policy Act of 1969.[17]

The Fish and Wildlife Coordination Act[18] clearly re-

---

[15]The Court essentially held that the Corps, where approval of Transportation is also required, cannot be oblivious to the effect of fill projects on the beauty and conservation of natural resources. This inference arises from the fact that the Secretary of Transportation is statutorily required to consider conservation before granting a permit. But if the fill on which the causeway was to be built were completed at the time the permit for the causeway was requested, there would be no conservation factors for Transportation to consider. The Court held that the Corps could not blind itself to this fact and thereby cut off considerations of conservation by granting a fill permit without Transportation's approval of the causeway.

[16] 16 U.S.C.A. §§661-666.

[17]Public Law 91-190, 42 U.S.C.A. §§4331-47.

[18]The Fish and Wildlife Coordination Act states:

> "Except as hereafter stated in subsection (h) of this section [not applicable], whenever the waters of any stream or other body of water are proposed or authorized to be impounded, diverted, the channel deepened, or the stream or other body of water otherwise controlled or modified for any purpose whatever, including navigation and drainage, by any department or agency of the United States, or by any public or private agency under Federal permit or license, such department or agency first shall consult with the

quires the dredging and filling agency (under a governmental permit), whether public or private, to consult with the Fish and Wildlife Service,[19] with a view of conservation of wildlife resources. If there be any question as to whether the statute directs the licensing agency (the Corps) to so consult it can quickly be dispelled. Common sense and reason dictate that it would be incongruous for Congress, in light of the fact that it intends conservation to be considered in private dredge and fill operations (as evidenced by the clear wording of the statute), not to direct the only federal agency concerned with licensing such projects both to consult and to take such factors into account.

The second proof that the Secretary is directed and authorized by the Fish and Wildlife Coordination Act to consider conservation is found in the legislative history. The Senate Report on the Fish and Wildlife Coordination Act states:

> "Finally, the nursery and feeding grounds of valuable crustaceans, such as shrimp, as well

---

> United States Fish and Wildlife Service, Department of the Interior, and with the head of the agency exercising administration over the wildlife resources of the particular State wherein the impoundment, diversion, or other control facility is to be constructed, with a view to the conservation of wildlife resources by preventing loss of and damage to such resources as well as providing for the development and improvement thereof in connection with such water-resource development."

16 U.S.C.A. § 662(a).

[19] Presumably Landholders must first obtain the Corps of Engineers permit before becoming a "private agency under Federal permit or license."

as the young of valuable marine fishes, may be affected by dredging, filling, and diking operations often carried out to improve navigation and provide new industrial or residential land.

* * * *

Existing law has questionable application to projects of the Corps of Engineers for the dredging of bays and estuaries for navigation and filling purposes. More seriously, existing law has no application whatsoever to the dredging and filling of bays and estuaries by private interests or other non-Federal entities in navigable waters under permit from the Corps of Engineers. This is a particularly serious deficiency from the standpoint of commercial fishing interests. The dredging of these bays and estuaries along the coastlines to aid navigation and also to provide land fills for real estate and similar developments, both by Federal agencies or other agencies under permit from the Corps of Engineers, has increased tremendously in the last 5 years. Obviously, dredging activity of this sort has a profound disturbing effect on aquatic life, including shrimp and other species of tremendous significance to the commercial fishing industry. The bays, estuaries, and related marsh areas are highly important as spawning and nursery

BEST COPY AVAILABLE

grounds for many commercial species of fish and shellfish."[20]

S. Rep. No. 1981, 85th Cong. 2d Sess. (July 28, 1958). 1958 U.S.C.C. & A.N. 3446, 3448, 3450. This Report clearly shows that Congress intended the Chief of Engineers and Secretary of the Army to consult with the Fish and Wildlife Service before issuing a permit for a private dredge and fill operation.

This interpretation was judicially accepted in *Udall v. FPC:*

"Section 2(a), 16 USC § 662(a), provides that an agency evaluating a license under which 'the waters of any stream or other body of water are proposed ... to be impounded first shall consult with the United States Fish and Wildlife Service, Department of the Interior ... with a view to the conservation of wild-

[20]The Senate Report also shows how the exercise of the commerce power in the conservation arena ties in with its exercise in other areas:

"The amendments proposed by this bill would remedy these deficiencies and have several other important advantages. The amendments, would provide that wildlife conservation shall receive equal consideration with other features in the planning of Federal water resource development programs. This would have the effect of putting fish and wildlife on the basis of equality with flood control, irrigation, navigation, and hydroelectric power in our water resource programs, which is highly desirable and proper, and represents an objective long sought by conservationists of the Nation."

1958 U.S.C.C. & A.N. at 3450.

BEST COPY AVAILABLE

life resources by preventing loss of and damage to such resources .... Certainly the wildlife conservation aspect of the project must be explored and evaluated."

1967, 387 U.S. 428, 443-44, 87 S.Ct. 1712, ——, 18 L.Ed.2d 869, 879.

The meaning and application of the Act are also reflected by the actions of the Executive that show the statute authorizes and directs the Secretary to consult with the Fish and Wildlife Service in deciding whether to grant a dredge and fill permit.

In a Memorandum of Understanding[21] between the

---

[21] "POLICIES

1. It is the policy of the two Secretaries that there shall be full coordination and cooperation between their respective Departments on the above responsibilities at all organizational levels, and it is their view that maximum efforts in the discharge of those responsibilities, including the resolution of differing views, must be undertaken at the earliest practicable time and at the field organizational unit most directly concerned. Accordingly, District Engineers of the U.S. Army Corps of Engineers shall coordinate with the Regional Directors of the Secretary of the Interior on fish and wildlife, recreation, and pollution problems associated with dredging, filling, and excavation operations to be conducted under permits issued under the 1899 Act in the navigable waters of the United States, and they shall avail themselves of the technical advice and assistance which such Directors may provide.

2. The Secretary of the Army will seek the advice and counsel of the Secretary of the Interior on difficult cases. If the Secretary of the Interior advises that proposed operations will unreasonably impair natural

BEST COPY AVAILABLE

Secretary of the Army and the Secretary of the Interior, it is provided that, upon receipt of an application for a permit to dredge or fill in navigable waters, the District Engineer of the Corps of Engineers concerned is required to send notices to all interested parties, including the appropriate Regional Directors of the Federal Water Pollution Control Administration, the Fish and Wildlife Service, the National Park Service and the appropriate state conservation, resources, and water pollution agencies. The District Engineer is given the initial responsibility of evaluating all relevant factors in reaching a decision as to whether the particular permit involved should be granted or denied. The Memorandum also provides that in case of conflicting views the ultimate decision shall be made by the Secretary of the Army after consultation with the Secretary of the Interior.

This Executive action has almost a virtual legislative imprimatur from the November 1967 Report of the House Committee on Merchant Marine and Fish-

---

resources or the related environment, including the fish and wildlife and recreational values thereof, or will reduce the quality of such waters in violation of applicable water quality standards, the Secretary of the Army in acting on the request for a permit will carefully evaluate the advantages and benefits of the operations in relation to the resultant loss or damage, including all data presented by the Secretary of the Interior, and will either deny the permit or include such conditions in the permit as he determines to be in the public interest, including provisions that will assure compliance with water quality standards established in accordance with law. * * *."

BEST COPY AVAILABLE

eries, in reporting favorably on a bill[22] to protect estuarine areas which was later enacted into law.[23] As a result of the effective operation of the Interdepartmental Memorandum of Understanding, the Interior Department and the Committee concluded that it was not necessary to provide for dual permits from Interior and Army.

The intent of the three branches has been unequivocally expressed: The Secretary must weigh the effect a dredge and fill project will have on conservation before he issues a permit lifting the Congressional ban.

---

[22] H. Rept. 989, 90th Cong., 1st sess., to accompany H.R. 25, pp. 4-5. See also S. Rept. No. 1419, July 17, 1968, 90th Cong., 2d sess., Senate Committee on Commerce, reporting on S. 695 and H.R. 25, pp. 13-14. H.R. 25 with revisions became the Act of August 3, 1968, 82 Stat. 625 (Pub. L. 90-454).

[23] "As a result of the hearings and the discussions which ensued from the circularized draft proposal — particularly with respect to the permit provision for dredging, filling, and excavation — a memorandum of understanding was entered into between the Secretary of the Interior and the Secretary of the Army. This agreement set forth the policies and procedures to be followed regarding the control of dredging, filling, and excavation in the navigable waters of the United States, which would include many of our Nation's estuarine areas.

On August 2, the Department of the Interior filed a supplemental report on the bill. In its report to the committee, the Department stated that we believe that this memorandum of understanding provides an effective administrative solution to the problem of preventing unreasonable impairment of the natural resources of the Nation's waterways and related environment, and preventing the pollution of the waters. In our opinion, the agreement makes the legislative approach set forth in H.R. 25 . . . for control for dredging, et cetera, unnecessary . . . (Omissions by the Committee.)"

BEST COPY AVAILABLE

The parallel of momentum* as the three branches shape a national policy gets added impetus from the National Environmental Policy Act of 1969, Public Law 91-190, 42 U.S.C.A. §§4331-47. This Act essentially states that every federal agency shall consider ecological factors when dealing with activities which may have an impact on man's environment.[24]

---

[24]Its newness, relevance and significance warrants reproduction in full.

"This Act may be cited as the 'National Environmental Policy Act of 1969'.

PURPOSE

Sec. 2 The purposes of this Act are: To declare a national policy which will encourage productive and enjoyable harmony between man and his environment; to promote efforts which will prevent or eliminate damage to the environment and biosphere and stimulate the health and welfare of man; to enrich the understanding of the ecological systems and natural resources important to the Nation; and to establish a Council on Environmental Quality.

TITLE I

DECLARATION OF NATIONAL ENVIRONMENTAL POLICY

Sec. 101 (a) The Congress, recognizing the profound impact of man's activity on the interrelations of all components of the natural environment, particularly the profound influences of population growth, high-density urbanization, industrial expansion, resource exploitation, and new and expanding techological advances and recognizing further the critical importance of restoring and maintaining environmental quality to the overall welfare and development of man, declares that it is the continuing policy of the Federal Government, in cooperation with State and local governments, and other concerned public and private organizations, to use all practicable means and measures, including financial and technical assistance, in a manner calculated to foster and promote the general welfare, to create and maintain conditions under which man and nature can exist in productive harmony, and fulfill the social, economic, and other

BEST COPY AVAILABLE

requirements of present and future generations of Americans.

(b) In order to carry out the policy set forth in this Act, it is the continuing responsibility of the Federal Government to use all practicable means, consistent with other essential considerations of national policy, to improve and coordinate Federal plans, functions, programs, and resources to the end that the Nation may —

(1) fulfill the responsibilities of each generation as trustee of the environment for succeeding generations;

(2) assure for all Americans safe, healthful, productive, and esthetically and culturally pleasing surroundings;

(3) attain the widest range of beneficial uses of the environment without degradation, risk to health or safety, or other undesirable and unintended consequences;

(4) preserve important historic, cultural, and natural aspects of our national heritage, and maintain, wherever possible, an environment which supports diversity and variety of individual choice;

(5) achieve a balance between population and resource use which will permit high standards of living and a wide sharing of life's amenities; and

(6) enhance the quality of renewable resources and approach the maximum attainable recycling of depletable resources.

(c) The Congress recognizes that each person should enjoy a healthful environment and that each person has a responsibility to contribute to the preservation and enhancement of the environment.

Sec. 102. The Congress authorizes and directs that, to the fullest extent possible: (1) the policies, regulations, and public laws of the United States shall be interpreted and administered in accordance with the policies set forth in this Act, and (2) all agencies of the Federal Government shall —

(A) utilize a systematic, interdisciplinary approach which will insure the integrated use of the natural and social sciences and the environmental design arts in planning and in decision-

BEST COPY AVAILABLE

making which may have an impact on man's environment;

(B) identify and develop methods and procedures, in consolidation with the Council on Environmental Quality established by title II of this Act, which will insure that presently unquantified environmental amenities and values may be given appropriate consideration in decisionmaking along with economic and technical considerations;

(C) include in every recommendation or report on proposals for legislation and order major Federal actions significantly affecting the quality of the human environment, a detailed statement by the responsible official on —

(i) the environmental impact of the proposed action,

(ii) any adverse environmental effects which cannot be avoided should the proposal be implemented,

(iii) alternatives to the proposed action,

(iv) the relationship between local short-term uses of man's environment and the maintenance and enhancement of long-term productivity, and

(v) any irreversible and irretrievable commitments of resources which would be involved in the proposed action should it be implemented.

Prior to making any detailed statement, the responsible Federal official shall consult with and obtain the comments of any Federal agency which has jurisdiction by law or special expertise with respect to any environmental impact involved. Copies of such statement and the comments and views of the appropriate Federal, State, and local agencies, which are authorized to develop and enforce environmental standards, shall be made available to the President, the Council on Environmental Quality and to the public as provided by section 552 of title 5, United States Code, and shall accompany the proposal through the existing agency review processes;

(D) study, develop, and describe appropriate alternatives to recommended courses of action in any proposal which involves unresolved conflicts

BEST COPY AVAILABLE

concerning alternative use of available resources;

(E) recognize the worldwide and long-range character of environmental problems and, where consistent with the foreign policy of the United States, lend appropriate support to initiatives, resolutions, and programs designed to maximize international cooperation in anticipating and preventing a decline in the quality of mankind's world environment;

(F) make available to States, counties, municipalities, institutions, and individuals, advice and information useful in restoring, maintaining, and enhancing the quality of the environment;

(G) initiate and utilize ecological information in the planning and development of resource-oriented projects; and

(H) assist the Council on Environmental Quality established by title II of this Act.

Sec. 103. All agencies of the Federal Government shall review their present statutory authority, administrative regulations, and current policies and procedures for the purpose of determining whether there are any deficiencies or inconsistencies therein which prohibit full compliance with the purposes and provisions of this Act and shall propose to the President not later than July 1, 1971, such measures as may be necessary to bring their authority and policies into conformity with the intent, purposes, and procedures set forth in this Act.

Sec. 104. Nothing in Section 102 or 103 shall in any way affect the specific statutory obligations of any Federal agency (1) to comply with criteria or standards of environmental quality, (2) to coordinate or consult with any other Federal or State agency, or (3) to act, or refrain from acting contingent upon the recommendations or certification of any other Federal or State agency.

Sec. 105. The policies and goals set forth in this Act are supplementary to those set forth in existing authorizations of Federal agencies."

Public Law 91-190, Title I, 83 Stat. 852.
42 U.S.C.A. §§4331-47.

BEST COPY AVAILABLE

Although this Congressional command was not in existence at the time the permit in question was denied, the correctness of that decision must be determined by the applicable standards of today. The national policy is set forth in plain terms in § 101 and the disclaimer of § 104(3) neither affects it nor the duty of all departments to consider, consult, collaborate and conclude. For we hold that while it is still the action of the Secretary of the Army on the recommendation of the Chief of Engineers, the Army must consult with, consider and receive, and then evaluate the recommendations of all of these other agencies articulately on all these environmental factors. In rejecting a permit on non-navigational grounds, the Secretary of the Army does not abdicate his sole ultimate responsibility and authority. Rather in weighing the application, the Secretary of the Army is acting under a Congressional mandate to collaborate and consider all of these factors.[25]

To judge the ebb and flow of the national tide, he can look to the Report of the House Committee on

[25]For like reasons the following disclaimer in the Fish and Wildlife Act of 1956, 70 Stat. 119, 16 U.S.C.A. §§741-754, specifically 70 Stat. 1124, 16 U.S.C.A. §742i is not decisive:

> "The rights of States. - Nothing in this Act (subsection 742a and note - 742d, 742e - 742j of this title; 15 subsection 713c - 3 and note) shall be construed (1) to interfere in any manner with the rights of any State under the Submerged Lands Act (Public Law 31, Eighty-third Congress) (43 subsection 1301 and notes - 1303, 1311-1315) or otherwise provided by law, or to supersede any regulatory authority over fisheries exercised by the States either individually or under interstate compacts;"

BEST COPY AVAILABLE

Government Operations. Although this perhaps lacks traditional standing of legislative history, it certainly has relevance somewhat comparable to an Executive Commission Report. On March 17, 1970, it approved and adopted a Report,[26] based on a study made by its Conservation and Natural Resources Subcommittee, entitled *Our Waters and Wetlands: How the Corps of Engineers Can Help Prevent Their Destruction and Pollution.* (H. Rep. No. 91-917, 91st Cong. 2d Sess. (1970)) The first section stifles any doubt as to how this part of Congress construes the Corps' duty under the Rivers and Harbors Act. The section traces the historical interpretation of the Corps' power under the Rivers and Harbors Act. It commends the Corps for recognizing ecological considerations under the Act to protect against unnecessary fills and cites the instant case.[27] But following the temper of the times, the re-

---

26The heading of the Report reads:

"The Corps of Engineers, which is charged by Congress with the duty to protect the nation's navigable waters, should, when considering whether to approve applications for landfills, dredging and other work in navigable waters, increase its consideration of the effects which the proposed work will have, not only on navigation, but also on conservation of natural resources, fish and wildlife, air and water quality, esthetics, scenic view, historic sites, ecology, and other public interest aspects of the waterway."

27 "In 1968, the Corps revised its regulations to state that the Corps, in considering an application for a permit to fill, dredge, discharge or deposit materials, or conduct other activities affecting navigable waters, will evaluate "all relevant factors, including the effect of the proposed work on navigation, fish and wildlife, conservation, pollution, esthetics, ecology, and the general public interest." 33 CFR 209.120(d) (1).[4] The Corps applied this policy when it recently rejected the

BEST COPY AVAILABLE

port by bold face black type cautions against any easy overconfidence and charges the Corps with ever-increasing vigilance.[28]

When the House Report and the National Environmental Policy Act of 1969 are considered together with the Fish and Wildlife Coordination Act and its inter-

---

> efforts of land developers to fill in a major part of Boca Ciega Bay, near St. Petersburg, Fla. See *Zabel v. Tabb*, 296 F. Supp. 764 (D.C. M.D. Fla., Tampa Div., Feb. 17, 1969), now on appeal to the U.S. Court of Appeals, Fifth Circuit, No. 27555.
>
> The committee commends the Corps for recognizing its broader responsibilities to protect against unnecessary fills and other alteration of water bodies. * * *"

H. Rep. No. 91-917, p. 5.

28
> "The Corps of Engineers should instruct its district engineers and other personnel involved in considering applications for fills, dredging, or other work in estuaries, rivers, and other bodies of navigable water to increase their emphasis on how the work will affect all aspects of the public interest, including not only navigation but also conservation of natural resources, fish and wildlife, air and water quality, esthetics, scenic view, historic sites, ecology, and other public interest aspects of the waterway."

H. Rep. No. 91-917.

As the Committee views it, not only should the Corps consider conservation, but it should consider conservation to be endangered by every dredge and fill project and place the burden of proving otherwise on the applicant. See, e.g., the conclusion of the first section of the Report and its bold face type recommendation:

> "The Corps of Engineers should permit no further landfills or other work in the Nation's estuaries, rivers and other waterways except in those cases where the applicant affirmatively proves that the proposed work is in accord with the public interest, including the need to avoid the piecemeal destruction of these water areas."

H. Rep. No. 91-917, p. 6.

BEST COPY AVAILABLE

pretations, there is no doubt that the Secretary can refuse on conservation grounds to grant a permit under the Rivers and Harbors Act.

V

Due Process

Landholders next contend that the denial of a permit without a hearing before the Fish and Wildlife Service is a deprivation of property without due process of law. Administrative law requires that before an agency can regulate a party, it must allow that party to be heard. Here, Landholders were given such a hearing before the Corps of Engineers, the body empowered to grant or deny a permit. They were not entitled to a hearing before the Fish and Wildlife Service because it is not "the one who decides." *Morgan v. United States*, 1935, 289 U.S. 468, 481, 56 S.Ct. 906, 912, 80 L.Ed. 1288, 1295. They were allowed to rebut the findings and conclusions of the Fish and Wildlife Service before the deciding body and thus were not denied due process for lack of a hearing.

VI

Taking Without Compensation

Landholders' last contention is that their private submerged property was taken for public use without just compensation. They proceed this way: (i) the denial of a permit constitutes a taking since this is the only use to which the property could be put; (ii) the public

BEST COPY AVAILABLE

use is as a breeding ground for wildlife; and (iii) for that use just compensation is due.

Our discussion of this contention begins and ends with the idea that there is no taking. The waters and underlying land are subject to the paramount servitude in the Federal government which the Submerged Lands Act expressly reserved as an incident of power incident to the Commerce Clause. (See Part II *supra*).

VII

Conclusion

Landholders' contentions fail on all grounds. The case is reversed and since there are no questions remaining to be resolved by the District Court, judgment is rendered for the Government and the associated agent-defendants.

REVERSED and RENDERED.

Adm. Office, U.S. Courts—Scofields' Quality Printers, Inc., N. O., La.

BEST COPY AVAILABLE

Mr. DINGELL. Now, Mr. Loesch, in fairness to you, I would like to have you refer to the bill, and I am going to have to ask counsel here to help me. The administration bill, does not provide that the Environmental Protection Agency must consult with the Interior Department.

It occurs to me that in many of the statutes which we have had before this body, it has been a clear requirement of the statute, for example, as in the Fish and Wildlife Coordination Act, that the agency issuing the permit must consult with your agency.

Mr. LOESCH. Mr. Chairman, if I am not mistaken also, the bill provides for the consultation, as adverted to in my formal statement, with the Department of the Interior, in creating or developing the standards on which the permit applications will be measured, but does not provide for consultation as to the issuance or refusal of individual permits.

Mr. DINGELL. That is the precise point.

Now we have situations where they must consult, in establishing or revising criteria.

Mr. LOESCH. Exactly.

Mr. DINGELL. But we have a situation where they may issue permits, without consultation with the Interior Department. Now, I am wondering, what are your views on that particular point? Would you have any objection to having them consult with your agency on matters of this sort?

Mr. LOESCH. No, I don't think we would have any objection, Mr. Chairman.

I bring to your attention the fact that if we are fully consulted, as we certainly expect to be, in the development of the necessary criteria, that it would then seem a little supererogatory, perhaps, to require consultation by EPA on every single permit, and could, I sense, slow down the process, perhaps.

But we wouldn't have any objection, and, of course, we would have the expertise to judge a permit application.

Mr. DINGELL. Right.

Now the Fish and Wildlife Coordination Act says that:

> Whenever the waters of any stream or body of water are proposed or authorized to be impounded, diverted, the channel deepened, or the stream or other body of water otherwise controlled or modified for any purpose whatever, including navigation and drainage, by any department or agency of the United States, or by any public or private agency under federal permit or license, such department or agency first shall consult with the United States Fish and Wildlife Service, Department of the Interior, and with the head of the agency exercising the administration over the wildlife resources of the particular State wherein the impoundment, diversion or other control facility is to be constructed, with a view to the consideration of wildlife resources by preventing loss of and damage to such resources as well as providing for the development and improvement thereof in connection with such water-resource development.

Now that is the requirement of the Fish and Wildlife Coordination Act. There is no such requirement here.

Mr. LOESCH. No; but the Coordination Act is still going to be in full force and effect, so that any agency who is presently required under that act to consult with us will have to consult with us as well under the new act.

Mr. DINGELL. Well, of course, here we have the peculiar situation where the language of a later statute says they "may", and the earlier statute, the Coordination Act says they "shall".

Now in aiding this committee in arriving at a judgment as to what is the legislative history, I personally feel that the Fish and Wildlife Coordination Act is still in effect. But I am apprehensive here because we see possibly a situation where we might be amending or repealing or altering Fish and Wildlife Coordination Act, and I seek your counsel and guidance on this particular point.

Mr. LOESCH. Well, of course out of my own head, Mr. Chairman, I am probably not competent to say. But I did take the precaution before coming before you, to discuss this matter with our legislative counsel, and legislative counsel advises me that it is his opinion that this act in no way impinges on the other act, the Coordination Act, and that in effect what this act will do is to require a permit from EPA for any dumping at all, even if it were dumping that wouldn't have been within our purview under the Coordination Act.

And they still will have to get the permits for us, under the Coordination Act. Isn't that correct?

Mr. LINDUSKA. Yes.

Mr. DINGELL. This is a matter to which the committee is going to have to address its very careful attention, and we will have to clarify this matter with some care, either in the report or, I suspect, in the fundamental legislation, by indicating that the section shall not be construed as amending, altering, and so forth, the requirements of the Fish and Wildlife Coordination Act.

The Chair is going to recognize our able counsel, Mr. Everett.

Mr. Everett?

Mr. LOESCH. May I comment, Mr. Chairman.

Mr. DINGELL. Certainly.

Mr. LOESCH. That certainly for my part, and Dr. Linduska concurs, I think we would have no possible objection to the addition of such a section.

Mr. DINGELL. I think that is wise counsel, and I suspect that most of the Government agencies who are terribly attuned to the public interest would view it the same way you do.

Mr. Everett?

Dr. LINDUSKA. Mr. Chairman, if I may make an observation.

Mr. DINGELL. Certainly.

Dr. LINDUSKA. I think there is one difference between the problem of ocean dumping as compared with our work with the Corps of Engineers on dredge-fill permits. There is no question that the Coordination Act is a tight document, and we have substantial powers under that act to veto projects in the field, and they are subject to appeal at departmental level, if they can't be resolved in the field.

But I would remind you that this type of activity represents an individual case in each instance, each one is different. There is a need to judge and rule on the basis of individual applications. But I think with the establishment of criteria, in connection with ocean dumping, we will have satisfied that need in large measure and from there on, the passing on individual permits will be almost of a perfunctory nature, there will be guidelines to follow.

I think there is a substantial difference in the type of problem that we are dealing with here.

Mr. DINGELL. Dr. Linduska, my old Daddy said, "Son, trust everyone but cut the cards." And in dealing with these permit issues, I have always adhered to that practice, and been much better served for it.

Mr. Everett?

Mr. EVERETT. Mr. Loesch, it is not clear in my mind, and I don't think it has been made clear at the hearings, as to how this system is going to work with respect to the Corps of Engineers' present authority in regard to the changes that will take place.

I wish you would have your counsel study these acts, with respect to the protection of fish and wildlife resources, to see that this protection is not diminished.

I understand that the Corps will continue to issue permits, in the inland areas, that is, up to a certain point, where the Environmental Protection Agency takes over, and then the Corps authority extends seaward to the high seas; the Environmental Protection Agency will issue permits for ocean dumping, or dumping of any sort in those offshore areas.

Also, I wish your counsel would take a look at the last two pages of the bill, section 11 (A), (B), (C), (D), and (E), pages 13 and 14 of the bill, and give us an opinion as to what the effects of these provisions on present law would be.

For instance, section 20 of the 1899 act is being repealed. Section 4 of the 1905 act is being superseded. Section 13 of the 1899 act is being superseded, insofar as it is inconsistent with this act.

We haven't anything on the record that explains the effects of these sections on these public laws with respect to fish and wildlife resources.

I don't know whether you gentlemen have had a chance to reflect on these issues, but it certainly would be helpful to have your comments.

Mr. LOESCH. No, I haven't had a chance to reflect on that, and as a matter of fact, quite honestly, I don't know, for instance what section 11(B) means. I don't know what it is really repealing. And besides, I think it would probably be more—be better if counsel responded on that, so I will try to get a response from our legislative counsel.

Mr. EVERETT. One of the acts, the 1905 act, has to do with the protection of oyster cultivation areas in some of the harbors, particularly New York Harbor, and the way this amendment is written, I am not sure as to what the effect would be with respect to those oyster cultivation areas.

This is something that concerns me.

Under section 8 of the act, the Administrator has the authority to transfer his responsibility with respect to issuing permits to other Federal agencies, of which the Department of Interior might be one.

Do you have an opinion as to whether Interior will be used to implement some of these permits from time to time? Has any indication been given to your Department that you might be given this responsibility?

Mr. LOESCH. We haven't any indication on that, but I would suppose that if a dumping proposal which would directly affect fish and wildlife, and that alone, perhaps, in an area of special expertise

for the Bureau of Sport Fisheries and Wildlife, that it would be referred to our Department, and consequently, that Bureau, for evaluation.

Mr. EVERETT. Well, I have mixed emotions with respect to giving this authority to EPA, and then allow EPA the authority to transfer the responsibility to other agencies. I don't know whether this is good or bad. I would like to have your comments on that idea.

Mr. LOESCH. Well, of course all through the Government, there is a substantial amount of cooperation, and many times, you would prevent by such a delegation the necessity of duplicating a staff, for instance.

And I think perhaps it might be appropriate, in the interests of the taxpayers' dollar, to occasionally delegate responsibility back to an agency with special expertise in a particular field, rather than gearing up to handle the entire matter in the initiating agency.

And it may be that that is what is in mind here.

For instance, I think one illustration within my own shop in this regard, we have a rather substantial fire protection program, in the Bureau of Indian Affairs, but we delegate that entirely to the Bureau of Land Management and the Forest Service, under their Interbureau Fire-Fighting Agency, on a reimbursement basis. They do the work, and research, and fight the fires, and we pay them back for it.

Mr. EVERETT. Well, one section of the bill exempts the AEC from the provisions of this act, and there is some consideration of eliminating that provision, or to modifying it considerably so when it comes to a disposal of high radioactive waste, the Environmental Protection Agency would have the authority under the legislation to transfer this responsibility back to the AEC, which in effect would allow the AEC to dispose of its own waste.

And this is the part under the authority that gives me concern.

Mr. LOESCH. Yes, I see why you have that concern, all right, and I may say I heard some of the testimony this morning. But frankly, on that, I am just not knowledgeable enough on that aspect of the matter to make any cogent comment.

Certainly I think we should all be concerned with the disposition of atomic wastes. What is the best way to have that concern paid attention to and proper steps taken, I don't know.

Mr. EVERETT. Mr. Chairman, that's all the questions I have, thank you.

Mr. DINGELL. Mr. Loesch, it isn't often we have the privilege of your being here; can you answer me a question on another matter?

I am very much distressed about the slowness in which Interior is reconstituting the vacancies that exist down there. Can you tell us when a director for the Fish and Wildlife Service is going to be appointed, and when the other vacancies down there are going to be filled?

I must tell you, I am very much distressed about this. I have not had a chance to complain lately to the Secretary of Interior, but now that I have got you here, I might as well let you know of my distress in this matter, and hope that those positions will be filled at an early time. I am sure you are aware of the fact you have a major morale problem inside that agency, by reason of the failure to fill these positions.

In fact, we have, by reason of a number of events of recent dates, a whole sequence of morale problems that are plaguing your agency. I am not sure you want to comment as when——

Mr. LOESCH. I can comment on it to this extent, Mr. Chairman.

I can't say when we will have a new director. I can say that we look forward with great confidence to having a new Assistant Secretary very shortly.

Mr. DINGELL. I hoped that would be so, but I certainly hope that the Director's position will be filled. You have got major morale problems down there, that if you don't do something, the whole agency, I am afraid, insofar as fish and wildlife, is liable to just fall apart on you.

I hope something will be done at a very near time.

Mr. LOESCH. I hope so, too. Frankly, I don't know anything about that. I can say, Mr. Chairman, that while I fully recognize your concern, I think that you will find that the Bureau of Sport Fisheries and Wildlife is much tougher than you think. I don't think it is going to fall apart just because it hasn't a director for a period.

Mr. DINGELL. It isn't just the absence of a director. I have great respect for the agency, as you know, and these are friendly questions, but Mr. Secretary, I express to you an honest and friendly concern.

Mr. LOESCH. Yes, I understand.

Mr. DINGELL. And I hope that this message will be carried back by you to the Interior Department.

Mr. LOESCH. I will carry that message.

Mr. DINGELL. And of the apprehensions I had and concern I feel as to the failures of that agency to reconstitute itself more rapidly.

Now I have great respect for Secretary Morton. I think he is a distinguished Secretary of the Interior, who was a fine and valuable Member of this Congress, and I have great affection and respect for him as a person, but time is fleeting, and it is a long time since the openings occurred, I think that the filling of them is long overdue, and it is my hope something will happen down there at an early time to correct this. Frankly, it has taken too long, Mr. Secretary.

Mr. LOESCH. I have been, just by way of an aside, Mr. Chairman, attempting to fill the post of Associate Director of the Bureau of Outdoor Recreation since last July.

Mr. DINGELL. I guess that sort of answers my question.

Mr. Secretary, we are grateful you could be with us. We thank you for your presence. It is always a pleasure to have you here, and we thank our old friend, Dr. Linduska, for his kindness in being here.

(The following letter was received in reference to the foregoing:)

U.S. DEPARTMENT OF THE INTERIOR,
OFFICE OF THE SECRETARY,
Washington, D.C., May 19, 1971.

Hon. EDWARD A. GARMATZ,
Chairman, Committee on Merchant Marine and Fisheries, House of Representatives, Washington, D.C.

DEAR MR. CHAIRMAN: We return herewith the transcript of testimony presented by Assistant Secretary Loesch to your Subcommittee on Fisheries and Wildlife Conservation concerning the general subject of ocean dumping.

A question has been raised concerning the effect of H.R. 4247 and H.R. 4723 upon our responsibilities under the Fish and Wildlife Coordination Act. While it is our opinion, as stated to the Subcommittee by Assistant Secretary Loesch, that

the enactment of this legislation will not impair the exercise of those responsibilities, we intend to provide a more detailed statement on this subject at a later date.

Sincerely yours,

FRANK A. BRACKEN,
Legislative Counsel.

STATEMENT OF HARRISON LOESCH, ASSISTANT SECRETARY OF THE INTERIOR

I think the Chairman of both Subcommittees and the members for this opportunity to discuss briefly the subject of ocean dumping and the need for its regulation. As others have already testified, the threat to oceans and our coastal waters is a real one that grows worse with their continued utilization for waste disposal. The value of these waters for commerce, recreation, transportation and as the habitat for a vast variety of marine life was recognized early in the life of our country. Centers of population grew along the coasts, and came to regard the coastal waters not only as a source of bounty and as a trade link to the world, but as a convenient medium for the disposal of urban waste. Suddenly, it seems, we have come to realize that the oceans, too, have limits and that urban society is to be held accountable for its misuse of a great natural resource. We talk in terms of "dead seas," while counting numbers of fish killed and beaches despoiled. We are awed by accounts of debris afloat on the high seas, thousands of miles from shore.

The number of bills under consideration today bespeaks a conviction that the time has come for regulation of ocean dumping. Most of the sponsors recognize, I am sure, that their bills are but a first step. The problem is world-wide, and will require a solution of comparable scope. I am equally sure that most would prefer an absolute prohibition to regulation, however strict. We, too, look forward to the day when it will not be necessary to use our lakes, coastal waters and oceans for the disposal of any waste material. Until technology provides the alternatives, however, we must seek to assure ourselves that such disposal as is allowed will be accomplished with a minimum risk of injury to the marine environment.

In announcing a program for the control of pollution in our Great Lakes, President Nixon last year asked the Council on Environmental Quality to study the threat posed by ocean dumping, and to propose a national policy to meet that threat. The Council assembled a panel of experts to conduct such a study, and made its recommendations in the report, "Ocean Dumping—A National Policy," adopted by the President and released on October 7, 1970. The Department's concern for the environmental effects of uncontrolled dumping led to studies of waste disposal in the New York Bight and participation in the Council's review of ocean dumping generally. We participated, too, in the preparation and review of legislation to implement the Council's recommendations.

That legislation is now pending before the full Committee as H.R. 4247 and H.R. 4723. It is the result of close cooperation among those several Federal agencies, and others, with responsibility for and interest in the protection, conservation and management of our Nation's natural resources. As you are aware, the bill would vest in the Administrator of the Environmental Protection Agency authority to control ocean dumping through issuance of permits and enforcement of a prohibition against the unauthorized transport or dumping of waste material. In determining whether or not to approve a permit application, the Administrator would be required to consider (1) the impact of dumping on the marine environment and human welfare and (2) other possible locations and methods of disposal, including land-based alternatives. The Administrator would be authorized to designate recommended sites for the dumping of specified materials, and would be permitted to prohibit absolutely the disposal of any material that could threaten human health or the marine environment.

Additional species of this, the proposed "Marine Protection Act of 1971", have no doubt been presented by the Environmental Protection Agency. It should be noted, I think, that its proposal combines several provisions of other bills also pending before the Committee. The result, we believe is a comprehensive framework for regulating the transportation and dumping of wastes in the oceans, coastal waters, and the Great Lakes. The Department of the Interior and others would be consulted by the Administrator in establishing criteria against which to measure permit applications. We believe that such consultation will afford an

opportunity to contribute our knowledge of the marine environment, and to seek protection of the wildlife, mineral, and recreation resources for which we have primary responsibility. In this connection, we agree with the Council on Environmental Quality that regulatory authority should be vested in an agency whose chief role is enforcement of environmental standards. Amendment of the Fish and Wildlife Coordination Act, as several bills propose, would tend to disperse regulatory authority and to discourage effective coordination with air and water quality programs already administered by EPA.

We urge prompt enactment of H.R. 4247 or H.R. 4723, as the President asked in his environmental message of February 8, "to assure that our oceans do not suffer the fate of so many of our inland waters, and to provide the authority needed to protect our coastal waters, beaches, and estuaries." Dr. Linduska and I would be pleased to answer your questions.

Mr. DINGELL. The Chair is going to recess this hearing very briefly to allow the members of the subcommittee an opportunity to go over and answer their names.

We will be back within 10 or 15 minutes, so the subcommittee will stand in recess for that period of time.

(Recess taken.)

Mr. DINGELL. The subcommittee will come to order for continuation of the hearings conducted on the subject of ocean dumping and bills relating to that matter.

I would like to insert in the record a statement the subcommittee received from the Maryland Port Authority.

(The statement follows:)

STATEMENT OF MARYLAND PORT AUTHORITY

The Maryland Port Authority is an agency of the State of Maryland charged with the responsibility for promoting the waterborne commerce of the State. This is centered primarily in the port of Baltimore which is the third ranking port in the country.

We are fully in accord with the intent of the Bill to prevent or limit dumping into ocean, coastal or Great Lakes waters of hazardous, noxious, or environmentally detrimental substances.

However, we think that it would be a mistake to include dredging spoil in the same category as "solid waste, garbage, sewage sludge, munitions, chemical, biological and radiological warfare agents, radioactive materials", etc. Also, we do not think that waters for which the States have been authorized to establish water quality standards by the Water Quality Act of 1965 should be included with ocean and coastal waters. And finally, we believe it would be desirable to leave the permit authority for disposal of dredging spoil within the U.S. Army Corps of Engineers.

Consequently, we urge that the Bill be amended to exclude from its coverage the deposit of dredging spoil in waters to which State or Federal-State water quality standards apply.

The reasons for our position are that we believe that:

1. Dredging spoil disposal is already adequately regulated by the States and the Army Corps of Engineers.

2. Transferring the Federal permit authority for dredging spoil disposal from the Corps of Engineers to the Environmental Protection Agency will increase the time involved in processing applications for such permits, and thereby impede navigation channel projects.

With respect to the adequacy of the preesnt regulatory setup:

1. At the State level, deposit of dredging spoil requires compliance with the water quality standards which have been established by the State, or where a State has not established such standards, compliance with standards established by the Environmental Protection Agency.

2. At the Federal level, dumping of dredging spoil requires a permit from the Corps of Engineers which, under current Federal statutes and regulations, requires:

(a) Certification that State water quality standards are complied with.

62-513—71——21

(b) Compliance with the Corps of Engineers "Section 403" criteria regarding environmental and ecological effects as required by:

The Fish and Wildlife Coordination Acts (16 USC 661 and 16 USC 742-A)

The National Environmental Policy Act (P.L. 91-90)

The Water Quality Improvement Act. (P.L. 91-224)

The reason we fear delay in the processing of applications for permits as a result of shifting the permit authority from the Corps of Engineers to the Environmental Protection Agency is that the Corps of Engineers is equipped for the job with personnel experienced in this field and 40 district offices, whereas the Environmental Protection Agency does not have a comparable staff and, as we understand it, envisions only 10 field offices. We believe there is a significant advantage in the more decentralized organization of the Corps of Engineers which brings the application and permit process much closer to the applicant.

Since we are recommending changes in the Bill involving dredging spoil and the Corps of Engineers, we would like to offer some comments on these two subjects.

First, as to dredging spoil. This is not necessarily the ogre that it is frequently considered to be, in spite of the unpleasant connotation of the word "spoil". There is "good" spoil and "bad" spoil. Typical of the former is natural uncontaminated bay or river bottom. Moving in from one location on the bottom to another nearby location on the bottom can hardly be considered as polluting the body of water involved. "Bad" spoil is typified by bottom material which has been subjected to industrial or municipal wastes and become contaminated as a consequence. Such "bad" spoil can be a pollutant and should be disposed of so that it does not degrade water quality. To this end, Maryland is constructing at its own expense a $13 million disposal area to receive and confine such "bad" spoil.

In Maryland we are confronted with the problem of simultaneously advancing our most important economic asset, the port of Baltimore, with its port-oriented heavy industry, and also preserving the environmental and ecological quality of our highly cherished Chesapeake Bay. This has caused us to give a great deal of attention to reconciling the requirements of the two assets, with particular attention to the handling of dredging spoil, and as a consequence we have learned some interesting things:

1. Since 1924 a deep, natural trough in the bottom of the Bay, known as "The Dumping Ground" has received most of the dredging spoil from Baltimore harbor and channels. However, this same "Dumping Ground" is the most popular sport fishing location on the Bay, particularly for striped bass; and on any summer weekend, hundreds of sport fishing boats can be seen there.

2. Last year's oyster harvest from the Chesapeake Bay was the largest on record. The "Dumping Ground" lies near the center of the relatively small area of the Bay which was the most productive.

3. A $268,000 study of an actual case of overboard disposal of dredging spoil conducted by the Natural Resources Institute of the University of Maryland in 1966 concluded that there were no observable detrimental effects from such spoil disposal. (See Exhibit A)

4. In the opinion of knowledgeable people concerned with natural resources conservation, it is recognized that not all dredging spoil is harmful, and that uncontaminated spoil need not be kept out of the Bay. (See Exhibit B)

With respect to the Corps of Engineers. We would like to call attention to the changes in their permit criteria resulting from the passage of the National Environmental Policy Act of 1969 (P.L. 91-190, January 1, 1970), and the Water Quality Improvement Act of 1970 (P.L. 91-224, April 3, 1970), and promulgated by the Secretary of the Army. These are succinctly expressed in Press Release 70-8 of May 15, 1970, by the Baltimore District, Corps of Engineers, concerning evaluation of permit applications, to the effect that,

> '"The decision . . . will be based . . . on an evaluation of the proposed work on the public interest." "Public interest" is described as including factors such as: "navigation, fish and wildlife, water quality, economics, conservation, aesthetics, recreation, water supply, flood damage prevention, ecosystems and, in general, the needs and welfare of the people." This change clarifies the standard against which permit applications are to be judged and re-emphasizes that the Corps is no longer concerned only with the impact which a proposed project may have on navigation.'

We respectfully request that the Committees give due consideration to the above points in their deliberations on H.R. 4723.

Sincerely,

HENRY T. DOUGLAS,
Chief of Planning,
Maryland Port Authority.

Mr. DINGELL. The Chair notes that we have our good friend and colleague, Mr. Howard W. Robison.

STATEMENT OF HON. HOWARD W. ROBISON, CONGRESSMAN FROM THE STATE OF NEW YORK

Mr. ROBISON. Mr. Chairman, I was privileged to address members of the Merchant Marine and Fisheries Committee last September on the subject of the dumping of military weapons and refuse, and I thank you for allowing me to present this statement today on the same subject.

When I last appeared before you, I was able to invoke recent instances of the disposal of military equipment in the ocean which were not only shortsighted in terms of environmental effect, but also terrifyingly dangerous, as in the cases involving nerve and mustard gases.

Since that time, intense public reaction to these events has abated, and it would be easy to evade the same sense of urgency. Certainly, we must count ourselves fortunate that we are not again meeting in the aftermath of a potential disaster.

My testimony may lack a bit of the dramatic quality it held last September, yet, Mr. Chairman, I hope it has not lost its note of urgency. Neither this committee nor the House of Representatives, for that matter, can afford a leisurely approach to this problem, at the expense of our citizens and our oceanic resources.

We are all aware of the recent efforts by the Secretary of Defense to halt the dumping of obsolete weapons into the oceans. There can be no doubt of his sincerity in this matter. He has taken noteworthy steps to protect our ocean environment from the hazards of indiscriminate dumping, and he rightfully deserves the praise of all citizens.

Yet, at the same time, we must remain aware that it is our duty as members of Congress to provide Americans with legislation which will secure them and our oceanic resources from the dangers of indiscriminate dumping.

Many of us spent a great deal of time last year answering correspondence on this question and making statements on the floor and to this committee. Several of us introduced or cosponsored legislation.

Yet that session has passed. The possibility of new—even fatal—incidents remains, and Congress has no significant statement of policy to show for its efforts.

Mr. Chairman, we have all been shaken out of our lethargy by the death-dealing potentials of past incidents. We know why we need this legislation. The dangers are evident. The magnitude of the problem is evident.

Indeed, many of us have seen the oceans shrink before our eyes. Those seemingly infinite bodies which could once swallow all of the refuse from our society like a small pill are now disgorging this trash

BEST COPY AVAILABLE

on our beaches and strangling ocean life. These oceans and bays, together with our country's lakes and navigable rivers, are increasingly rejecting animal and human life. We cannot swim, or fish, or sail, or even stand the smell in larger and l[illegible]ger stretches of water.

A reasonable policy for disposal of obsolete military equipment, one regulated by the Environmental Protection Agency, can greatly ease the mind of our citizens. We must not forget that the conditions we now seek to correct are not solely a problem created by the military. Both Congress and the Executive, in their haste to provide new and growing quantities of weapons have also helped to provide a legacy of obsolete equipment which no longer defends but only contaminates when disposed of improperly. We share with the military the responsibility for ending these practices, and we owe it to our citizens to do so as soon as possible.

To these ends, Mr. Chairman, I have introduced a proposal, H.R. 6884, which is now before you. By introducing this legislation, I do not mean to question the advisability or soundness of our weapons acquisition policies. Our defense requirements are not the point of argument.

However, in striving to provide for the security of our Nation, we should not tread insensitively on that which we are attempting to defend. If, in the course of maintaining weapons systems and rendering obsolete arsenals harmless, we contaminate our oceans and render our rivers and lakes lifeless, our defense must be considered our most aggressive enemy.

My proposal attempts to insure that in any plan to acquire military hardware, proper consideration is given to the disposal process. By requiring a disposal scheme for all present and future military weapons, we can insure that the legacy of our Nation's defense is not pollution.

Mr. Chairman, I thank you again for allowing me the opportunity to present my statement to you. I hope that your committee will accept the spirit of my remarks as it considers my proposal and those of my colleagues.

Mr. Dingell. Our next witness is Dr. Bostwick H. Ketchum, associate director of Woods Hole Oceanographic Institution.

Dr. Ketchum, we are greatly privileged to have you with us, and we thank you for your presence, and your very helpful testimony. We look forward to hearing your words.

STATEMENT OF DR. BOSTWICK H. KETCHUM, ASSOCIATE DIRECTOR OF THE WOODS HOLE OCEANOGRAPHIC INSTITUTION, WOODS HOLE, MASS.

Dr. Ketchum. Thank you, Mr. Chairman. It is indeed a pleasure for me to have this opportunity to speak to you about this important problem.

My name is Bostwick Ketchum, and I am Senior Scientist and Associate Director of the Woods Hole Oceanographic Institution.

This committee knows our institution well, since I and many of my colleagues have testified previously before this committee.

Mr. Dingell. It is always in a very helpful fashion, may I say, Doctor. We are certainly privileged to have you with us.

Dr. KETCHUM. Thank you.

I would like to emphasize, in speaking on the three bills that I have had the opportunity to read, some of the scientific problems which must be considered, in evaluating any proposed disposal of waste material at sea.

I am speaking mainly concerning H.R. 4723, which was introduced by Mr. Garmatz, and appears to be more comprehensive than either of the other two bills I have studied, which are, H.R. 2581 and H.R. 3662.

The former bill proposes a separate act, to be cited as the Marine Protection Act of 1971, whereas the latter two propose amendment of the Fish and Wildlife Coordination Act. I am not qualified to express a preference concerning the two proposed mechanisms.

It is a pleasure to endorse heartly the objective of the three bills, each of which would require regulation of the disposal of any waste material at sea. The Administrator of the Environmental Protection Agency would be given the authority to issue permits of the disposal of waste materials at sea or for the transportation of waste which is to be dumped at sea beyond the area of U.S. jurisdiction.

The administrator is instructed to take into consideration the likely impact of the proposed dumping, not only on human health, welfare and amenities, but also on the marine environment and ecological systems.

The Administrator could deny a permit in cases where ecological or environmental damage would be produced. Protection of the marine environment is certainly essential but unfortunately in only a few cases do we have the information which will be needed to make a wise judgment concerning the possible impact of dumping operations.

In my testimony, I would like to emphasize the scientific problems which have to be approached in order to evaluate and decide wisely how how we should operate.

I would like to emphasize that in my opinion, sea disposal of waste materials should be considered an interim or temporary solution to our waste disposal problems.

Mankind has been changing his environment for hundreds of years, but it is only during recent generations that we have been faced with one pollution disaster after another.

In large part, this is due to the rapid growth of the human population, which reached a billion people in 1830, increased to 2 billion people in the next century, and to 3 billion people in the next 30 years.

Each individual in this expanding population is demanding more and more material things. Our average daily per capita production of solid wastes today is now about 5 pounds, and this is expected to increase to about 7½ pounds per capita per day in the next decade.

The problems are greatly magnified by the concentration of people in metropolitan areas where tremendous tonnages of waste materials are continuously produced.

The technology which has satisfied the increasing demands for material things must produce a solution to the question of what to do with them once they are discarded.

More than half of the U.S. population lives close to the seacoast and it appears inevitable that pressures will increase for the use of

BEST COPY AVAILABLE

the estuaries and coastal waters for the disposal of various kinds of waste materials of our population and our expanding technology.

These waters are of tremendous importance to mankind. There are multiple demands for the use of these waters, and some uses are compatible with others and some exclude others.

Transportation and fishing are classical nonexclusive uses of estuarine and coastal waters.

The need for animal protein which is provided by our marine fisheries steadily increases, as our population expands.

Most fisheries products are harvested from these coastal waters and estuaries and the success of the fisheries depends upon these waters for breeding grounds.

The survival of these fisheries must be assured. Our increasing affluence makes it inevitable that we must also protect the quality of these waters for recreational and esthetic purposes.

Waste disposal is one use of these waters which carries the ever-present threat of degrading the quality and of prohibiting other uses.

Probably any disposal operation at sea would have some impact upon the marine ecological system.

The effects may range from negligible to disastrous. I believe that the disposal of toxic industrial wastes, of chemical and biological warfare materials and of high-level radioactive wastes at sea should be prohibited.

As this committee knows from previous hearings the disposal of sewage sludge and dredging spoils in the New York Bight has had disastrous effects on the bottom populations there.

Dr. Pearce testified before this committee last July that the affected area appears to be expanding with continued dumping. In cases like these, the evidence is already clear that disposal of waste materials at sea can be seriously detrimental.

I urge the committee to consider including specific instructions to the Administrator when the damaging effects of disposal operations are already well known. Some operations should be prohibited; others should be phased out as rapidly as alternative methods of disposal can be developed.

Instructions to the Administrator are included in subsection (4) of H.R. 3662 which was introduced by Mr. Rogers but is not in the administration bill.

Very few disposal operations have been adequately studied. I know of only one case where continuing scientific studies have failed to produce evidence of a detrimental effect of a waste disposal operation at sea.

And this is the National Lead Co. discharge of acid-iron wastes in which I was personally involved in some of the early studies.

We have looked at this operation over the last 20 years and the most recent studies by members of the staff of the Woods Hole Oceanographic Institution, were made last summer, when an effort was made to discover why the so-called "acid-grounds" are now good areas for blue fishing. We were unable to find an explanation for the good fishing, but neither were our scientists able to find any evidence of ecological damage from this operation, even though it has been carried on now for a period of over 20 years.

[illegible]

Some disposal operations may even be beneficial. Ground fish tend to concentrate near natural reefs and artificial reefs have been created in several coastal areas by dumping old car bodies and rubble. The fishing over these artificial reefs is said to be better than that over flat, level bottoms, presumably because the fish congregate on these reefs for protection from their natural enemies.

Municipal solid wastes present a constantly increasing problem. Areas suitable for sanitary landfill are becoming evermore scarce near our major cities. It has been proposed to compress and bale these wastes for disposal at sea.

There are obvious problems to be solved, such as insuring that the density of the bale is greater than that of sea water so that it will sink and that floatable materials will not be released from the bale. These are comparatively easy.

However, there have been no tests of the long-term effects of such a disposal on the marine environment and ecological systems. With our present knowledge on the marine environment and ecological systems, it is impossible to predict with any degree of certainty what the effect of such a disposal operation would be.

Would such disposal act as an artificial reef and improve the environment or would it have detrimental effects. Will materials gradually leach out of the bales and have a delayed effect on the ecology of the area? How fast will the organic materials decompose?

Recent experiments have shown that this process is very slow in the deep sea, and disposal of waste material there may merely preserve it for posterity.

These examples illustrate the fact that the impact of waste disposal at sea can vary widely from severe damage to potential benefits. For many of the waste materials which are being dumped at sea today there have been no scientific studies or monitoring of the environmental effects.

It appears obvious that a great deal of basic ecological research in the marine environment is needed before the Administrator of the Environmental Protection Agency can make wise decisions concerning the acceptability of many proposed waste disposal operations.

It is apparent to those of us who have studied these problems that the ocean has a considerable capacity to assimilate waste materials and to recover from abuse.

Once this capacity is exceeded, however, the quality of the environment may deteriorate rapidly. Some of the effects may be subtle and may develop over a long period of time so that monitoring of the effects of each disposal operation will be essential to observe the impact of the operation and to give advance warning when the capacity of the marine system to recover is being reached.

Several research needs were given in the report to the President prepared by the Council on Environmental Quality entitled "Ocean Dumping—a National Policy," October 1970.

I would be pleased if authorization could be included in this bill for funds to support the needed marine ecological research. Equally important would be funds for research on alternative methods, including recycling and reuse of the materials.

The studies could be carried out by the Environmental Protection Agency, or by grants and contracts to organizations capable of undertaking the research.

I like one provision in H.R. 2581, which was introduced by Mr. Harrington. This provision would force the producer or the dumper of the waste material to bear some of the research costs by requiring that he "must present sufficient evidence to sustain a burden of proof that such materials in the location in which they are to be deposited will not endanger the natural environment and ecology of these waters."

It seems appropriate to me that this information might properly be required by the Administrator of EPA as a part of any application for a permit for waste disposal at sea. Even if this were required of each applicant, the Administrator would need, within his organization, the capability to interpret and evaluate whatever predictions were made.

For several reasons, however, as I mentioned before, I believe that sea disposal of waste materials should be looked upon as an interim and temporary solution.

In part this is because chronic detrimental effects may develop slowly and may not be anticipated. In part this is because our uses of the seabed may expand and change greatly during the next generation.

Most importantly, however, the resources of this planet are limited and mankind cannot continue indefinitely to discard large quantities of our nonrenewable resources.

We must adopt a policy of recycling and reuse of as much of our waste materials as is humanly possible. Only in this way can we be sure that coming generations will have the natural resources that they need and will have an environment of quality so that the amenities of life can be enjoyed.

I thank you very much for the opportunity to appear before you and to make this statement, Mr. Chairman.

Mr. DINGELL. Doctor, it is the committee's privilege that you can be with us. I wish to commend you for a helpful statement. The Chair notes you have appended to your statement biographical information which indicates a most distinguished career.

Dr. KETCHUM. Thank you sir.

Mr. DINGELL. The Chair would like, if you have no objection, by unanimous consent, to insert that at this point in the record.

Dr. KETCHUM. You are welcome to use it, sir.

(Biographical information follows:)

BIOGRAPHICAL INFORMATION, BOSTWICK H. KETCHUM

Dr. Bostwick H. Ketchum is Senior Scientist and Associate Director of the Woods Hole Oceanographic Institution. He was born in Cleveland, Ohio, and received his undergraduate education at St. Stephen's College, Columbia University (A.B. 1934) and did his graduate studies at Harvard University (Ph.D. 1938). He has been awarded honorary Doctorate of Science degrees by Bard College (1964) and by Clarkson College of Technology (1970).

After working for a year as a Research Assistant at Harvard University (1938–39) and teaching at Long Island University (1939–40), he returned to the Woods Hole Oceanographic Institution where he hed held graduate student fellowships during the summers of 1935 to 1937. At the Woods Hole Oceano-

BEST COPY AVAILABLE

graphic Institution, he has held positions as Associate Marine Biologist (1940–45), Marine Microbiologist (1945–53), Senior Biologist (1953–54), Senior Oceanographer (1954–63), Senior Scientist (1963–). He has been Associate Director since 1962. He has been Lecturer in Biological Oceanography at Harvard University (1960–68) and is at present Associate Member of the Department of Biology. During 1968–69, he was on leave of absence and served as Head of the Section on Ecology and Systematic Biology of the National Science Foundation, Washington, D.C.

He is a Fellow of the American Association for the Advancement of Science and of the New York Academy of Sciences and a member of Phi Beta Kappa, Sigma Xi, Ecological Society of America (Vice President, 1961–62; President, 1965–66), American Society of Limnology and Oceanography (Secretary-Treasurer, 1952–58; Vice President, 1958–59; President, 1959–60), American Institute of Biological Sciences, American Geophysical Union, Marine Technology Society, and of the Corporation of the Marine Biological Laboratory. He is a Trustee of the Bermuda Biological Station, of the Falmouth Hospital, and of the Inter-American Institute of Ecology. He was co-editor of the book "Marine Fouling and Its Prevention" (1952), associate editor of the journal "Limnology and Oceanography" (1966–69) and is co-editor of "Coastal Marine Science" (1971–).

Dr. Ketchum's research interests have included the production of organic material by the marine planktonic algae, the cycling of nutrients in the sea, and the circulation of coastal and estuarine waters. He has long been interested in pollution of the sea and the effects of pollution on marine organisms. He has published over 50 scientific papers on these subjects.

Mr. DINGELL. The Chair recognizes Mr. Griffin.

Mr. GRIFFIN. No questions.

Mr. DINGELL. Mr. LaGarza?

Mr. LAGARZA. This does not relate specifically to the marine aspect of it, Doctor, but a man of your experience I can readily see might shed some light on this for me because it is related in part to this.

I had occasion to visit in Africa last fall. In the center part of the old French Equatorial Africa where there are no disposal problems and no residual pesticide or insecticide problems, the rivers are all polluted nonetheless.

You can't drink the waters, swim in the river. Do you have any explanation environmentalwise or ecologywise why this happens?

Dr. KETCHUM. I am not acquainted with Africa at all, having set foot in it only once at Dakar.

It is true, however, that natural rivers will vary from clear sparkling mountain streams to the rather sluggish rivers that carry tremendous loads of terrestrial sediments and of organic materials which has fallen from the trees and bushes that line the stream and these, as they rot and decay, can be pollutants just as can the things that we are introducing in our human waste.

They are not in the same category as things like DDT and other exotic chemicals that the normal bacteria flora of the world has never been exposed to before and evolution has never produced organisms capable of decomposing some of these materials.

Mr. LAGARZA. Thank you, Doctor.

For this reason I was most happy and I heartily agree with your statement that there should be some funds dedicated to research in order that we might continue the research and probably get to the point where we might know what exactly it is that causes some of the problems and in order to control what we know now we must continue our research.

I most heartily concur with your statement.

BEST COPY AVAILABLE

Thank you, Mr. Chairman.

Dr. KETCHUM. I thank you. I think it is very important to understand the system that we are working with.

Mr. DINGELL. Thank you, Mr. LaGarza.

Mr. Pelly?

Mr. PELLY. Dr. Ketchum, your testimony is always of great interest to this committee. You have a regular representative down here from Woods Hole, my colleague on my left. He looks after your interest even more than I do.

Dr. KETCHUM. To our pleasure, sir.

Mr. PELLY. There are some questions I would like to ask. One has to do with reference to your statement that additional funding was needed for research. I certainly agree with that.

In the Roger's bill you referred to, H.R. 3662, there is an authorization for $1 million. I don't think the administration has any provision for additional funding as I recall.

Dr. KETCHUM. That is correct.

Mr. PELLY. I take it what you are referring to is that the committee would do well to add a provision such as in the Roger's bill if they amend the administration's bill, the chairman's bill, and bring it out. That is correct?

Dr. KETCHUM. I would be pleased to see that done if it can be done.

Mr. PELLY. On page 8 of your statement you indicated marine studies could be done by EPA. How about the National Science Foundation or NOAA? Wouldn't they be agencies that would provide for additional research work?

Dr. KETCHUM. They certainly would, sir.

I mentioned EPA specifically because it is the Administrator of EPA that the bill provides will have the authority to issue permits.

In order to understand the system he must have within his organization a group of knowledgable people on the subject. I meant the next phase "grants and contracts to organizations capable of undertaking the research" to be comprehensive and to include both private institutions, universities, other government agencies. I did not spell it out in detail but used the word "organization" as being all inclusive.

Mr. PELLY. I do not think that I know of any environmentalist or ecologist that is presently of a high level on the NOAA roster. So I think in asking that question I probably touched on a rather weak spot in the present organization, although they may have added some. I did know of many biologists or good people that they have but not marine scientists.

Dr. KETCHUM. They do have what was the Bureau of Commercial Fisheries, now the National Marine Fisheries Service I believe they are calling it, with several good people in that organization.

Mr. PELLY. I may underrate them because they have never had adequate funding to do the kind of job they should have done.

Dr. KETCHUM. That is true.

Mr. PELLY. Which material should be flatly prohibited and which should be phased out? You might not only answer that question off the top of your head but if you would supply it for the record I think it would be helpful.

Dr. KETCHUM. I listed specifically toxic industrial wastes, biological and chemical warfare materials, and high level radioactive wastes.

Mr. PELLY. In other words, the three that are spelled out in the bill that you referred to as the Roger's bill?

Dr. KETCHUM. Yes. These are the ones that I consider the most hazardous and generally in not tremendous quantities. Some of the nontoxic wastes are in large weights at this time but the toxic wastes are in comparably small quantities.

The things that I noticed that I thought needed phasing out are problems such as the sluge disposal of the city of New York. It is simple to say, let us stop this immediately but this will cover Manhattan Island to a depth of 6 inches in a year and something must be done about it.

So that here I believe that alternative methods must be vigorously pursued and as soon as these are developed, phase out this type of activity which is damaging.

Mr. PELLY. I think the administration has indicated that they do not understand exactly what the proper definition of toxic waste is. Do you have a clear understanding of what it is? Would you be willing to submit it for the record? If they have any doubt I think maybe you could clear it up.

Dr. KETCHUM. The problem is this, there are all grades of toxicities from a few parts per million to much higher concentrations. So that you have a very wide spectrum. In terms of being toxic in the environment, it depends on the concentration that it reaches in the environment. I can see that there are difficulties.

However, there are wastes that I think all scientists agree are toxic, the mercury compounds that are being disposed of in various industrial operations and have had already an impact on the environment, the chromium which results from chrome plating operations, a highly toxic chemical. Silver in the fresh water environment more than the marine because it preciptates a silver chloride in the marine environment, is a toxic chemical and should be removed from the waste before it is introduced into the environment or even into a waste disposal system.

Mr. PELLY. I would assume that if we were to abandon the toxic materials there might be some lesser strength of toxity or whatever the word is which might be permitted, is that correct?

Dr. KETCHUM. That is correct.

Mr. PELLY. On page five of your testimony you indicated that sewage should not be covered in this legislation. What specific reasons do you have for eliminating sewage?

Dr. KETCHUM. I think because it opens a pandora's box, that it extends into every city and town in this Nation. Here I am talking about the sewer effluents.

Mr. PELLY. In other words, that is the effluent that comes from a sewage treatment plant?

Dr. KETCHUM. I would be glad to make a few observations.

Of course in brief testimony one tries to use examples which seem the most pertinent to the question at hand.

In terms of domestic waste, if they do not include toxic industrial wastes they do inevitably include vertilizing elements which can be beneficial if they are not added to natural waters in excess.

Just as one fertilizes his garden to make it grow, one can fertilize the sea with these materials and make it more fertile.

If you add too much fertilizer to your garden you kill everything. This is indeed what we are doing in many of our estuaries, we are just overloading the system so that it is incapable of recovering from the amount that we are putting in.

I think on ecological grounds for example it is possible to compute that the H[illegible]son River estuary is capable of taking care of the sewage from 1.2 m[illegible]lion people on an annual basis and at the lowest flow of the Hudson River it is capable of taking care of 400,000 people and we are expecting it to recover from sewage of 12 million people.

The system cannot handle it.

Likewise another example of beneficial uses would be the possibility that a waste heat from power plants, whether they be nuclear reactors or fossil fuel plants, can be utilized. As you well know there are many places along the coast of Maine where the swimming could be improved. It is quite possible that the growth of many of the natural fauna there could be improved and extended throughout the year by lagooning and preserving some of this heated seawater.

It is even possible that you could introduce exotic species into warmed water who could survive that would not be able to survive otherwise.

One can find the possibilities of use of our waste materials. It is this use or recycling of these materials that I think is the only long-term solution to our problems of pollution, whether it be marine, terrestrial, air or any other part of our environment.

Mr. DINGELL. Would the gentleman yield for a question?

Mr. KEITH. Certainly.

Mr. DINGELL. Doctor, you and Mr. Keith are getting into a point that I hoped to be able to discuss with you at a little time later: this problem of nutrients. One of the reasons that great fisheries resources exist in certain parts of the world is that you have these upwellings of nutrient-rich waters. There has been discussion of the creation of synthetic upwellings to pump the cold bottom waters up.

Dr. KETCHUM. Yes. These are problems but these may be introduced well up stream in a river and reach the sea after along transport down the river stream and to try to control these throughout the country adequately in this bill did not seem to me reasonable.

Mr. PELLY. In my own city of Seattle we had a very deep and long lake, Lake Washington, where all the little individual towns and cities sewage treatment plants emptied their effluent into the lake and it became completely polluted.

Then they joined together under one great disposal program and all emptied into the deep waters of the Puget Sound where the tide carried it out and we have no such problem any more.

Dr. KETCHUM. That is correct. I know Dr. Edmondson very well and I think he has done a fine service not only to Seattle but to science and the who[illegible] problem of pollution abatement.

Mr. PELLY. That was done before Federal Government aid was received. The people did it on their own and they are the greatest salesmen for any antipollution program you have ever seen because they have seen it with their own eyes.

The water had turned green and suddenly it was restored crystal clear now and there are fish in the lake. It is a great example of what can be done.

Thank you.

BEST COPY AVAILABLE

Dr. KETCHUM. Very good.

Mr DINGELL. Thank you, Mr. Pelly.

Doctor, I will recognize our good friend and distinguished member of this body—as Mr. Pelly says we constantly hear about Woods Hole—our good friend from Massachusetts, Mr. Keith.

Dr. KETCHUM. It is a pleasure to see Mr. Keith to.

Mr. KEITH. I can hardly resist smiling at this most recent exchange, because what Puget Sound did, Falmouth didn't do. I suspect that the Doctor would not want to hang Falmouth linen on this line at this time.

Dr. KETCHUM. I don't think it will serve any useful purpose, Mr. Keith.

Mr. KEITH. I was particularly interested in that you are the first one I believe who has talked about some of the positive things that you can do. I asked former Secretary Train yesterday, if his testimony could not be a little bit more positive. It seemed to me to be quite negative. He was constantly talking about the restrictions that we must apply.

He blushed a bit. But what I had in mind is what you mentioned here, the positive utilization of techniques that would make the sea a better place in which to practice aquaculture. Perhaps we could use the sea as a receptacle for old automobiles to create reefs that would provide breeding grounds for fish.

I wonder if you can think of any other techniques that might improve the problem.

Do you have any observations to make in that respect?

It occurs to me that sewage, at an appropriate point in treatment, could conceivably provide the phosphates and nitrates to provide a flow of nutrients in the ocean that would be advantageous for fish.

Am I correct in this impression?

Dr. KETCHUM. I think you are entirely correct. We have one member of our staff, a very distinguished member, Dr. John Ryther, whom I know Mr. Keith knows, who is now on a laboratory scale studying just exactly this possibility of growing phytoplankton on secondary treated sewage, feeding this phytoplankton to oysters, growing the oysters on the plant produced in the seawater and ultimately he will add scavengers in the system so that in essence one can conceive of having the tertiary treatment not only remove these materials but produce the product that would be beneficial to mankind.

Mr. DINGELL. You have answered precisely my point.

It is possible also to utilize this just by releasing the effluent in a certain fashion in certain places and times in the ocean to perhaps create an increase in the plankton growth and the fish life in a given area through this particular device?

Dr. KETCHUM. There are many parts of the ocean which are limited by a lack of nutrients. The problem here is in maintaining the right balance of these elements—they are not balanced just the way you would like them in sewage—and of keeping them at the proper concentration so that you don't get these excessive growths of eutrophication.

Mr. DINGELL. Thank you, Doctor. I thank the gentleman.

Mr. KEITH. I think you have lent a balanced philosophy to this discussion that is badly needed. I think Japan has done a great deal

BEST COPY AVAILABLE

with aquaculture even as they have with agriculture in the use of human waste.

I hope that it will keep us from overreacting.

Thank you, Mr. Chairman.

Mr. DINGELL. Thank you, Mr. Keith.

Mr. Kyros?

Mr. KYROS. I thank the gentleman.

I was interested in what you said about Maine, of course. Did you mean, for example, that if discharge from one of these nuclear plants was going into Sheepscot Bay near Wiscasset, you could dam that discharge somehow, and utilize it?

Dr. KETCHUM. Yes. As I remember that part of the coast there are long lagoons which you could utilize either for growing lobsters or other forms in the water where they now grow only about 4 months of the year because of the cold water. You might indeed get other things to grow there which are no longer able to grow because of the cold winter.

Mr. KEITH. Will t[illegible] gentleman yield?

I think you hinte[illegible] at that before when you said we could warm the waters of Maine so that some of us could swim there.

Dr. KETCHUM. That is one of the advantages.

Mr. KYROS. Doctor, the other day I had occasion to climb Mt. Katahdin on snowshoes. At about the 4,000 foot level I met someone by the name of Charles Hollister. He said he was an oceanographer testing for mercury and nitrates in the snow.

Dr. KETCHUM. Is he? I didn't know what he was doing up there.

Mr. KYROS. We were standing on a sterile pond up there where, I must admit, it was kind of incongruous to see him. He said he was going to make a trip to the Himalayas next year.

Dr. KETCHUM. He is also a famous mountain climber. He has climbed some of the mountains in the Antarctic.

Mr. KYROS. I went last summer to New Meadows, where they are growing oysters. Do you think that could become a profitable business up there?

Dr. KETCHUM. It certainly is in Japan. At present state of our demands for food it is profitable when and only when a high quality product is produced. So that in Japan the profitable aquaculture consist of oysters, shrimp, other delicacies. There are a few parts in the Far East where mullet is grown in aquaculture, and they may consider it a delicacy, I am not sure that I would. Most of the aquaculture that is profitable today is high quality, high priced material.

Mr. KYROS. Thank you very much, Doctor.

Thank you, Mr. Chairman.

Mr. DINGELL. Doctor, the committee is grateful to you.

The Chair is going to recognize a good friend and counsel, Mr. Everett.

Mr. EVERETT. Thank you, Mr. Chairman.

Dr. Ketchum, on two occasions you referred to the expression that the ocean should be an interim or temporary solution to our waste disposal problem.

I was wondering if you had formulated in your mind the time when there should be a complete ban on ocean dumping of these waste materials?

BEST COPY AVAILABLE

Dr. KETCHUM. I am afraid that I can't give you a time limit on this. There are engineering problems that have to be solved for the reuse of some of these materials. There is the natural lethargy of spending money to get rid of something that you don't really want.

I would find it very difficult to say how quickly this could be done. I think if Congress pushes the country a little it will get done more quickly than if we relax and say maybe it will happen some day.

Mr. EVERETT. Mr. Pelly discussed with you a few moments ago the funding of the legislation and a specific authorization for funding.

The Rogers bill I think indicated a $1 million a year authorization for the purpose of this act for each year in the future.

Do you think $1 million is sufficient to do the job that would be required to implement this legislation? Or should it be larger?

Dr. KETCHUM. I think it will have to be larger in the long run. There are just so many problems in the marine environment that are not now being adequately studied that I think this would be merely a start on the problem.

Mr. DINGELL. Mr. Everett, would you yield at this point?

Doctor, Mr. Everett is touching on a question which has bothered me rather considerably. That is, I assume that a good research program authorized by law has to have certain parameters and directions so as to achieve the desired legislative goal. The requirement in the Rogers bill of which Mr. Pelly and Mr. Keith and I are cosponsors does not include specific boundaries and parameters.

I have been groping for what boundaries and parameters the research program should have.

Would you be able to give us some assistance in laying out and in outlining what should be the boundaries and the directions and the goals of a research program of that sort together with the amount which should be expended to achieve that kind of goal?

Dr. KETCHUM. To do that just quickly here would be a difficult thing because I am sure that if I attempted to do it I would leave out many of the things which would be important.

Mr. DINGELL. Doctor, you mentioned this.

Your guidance on an off-the-cuff basis at this time would be highly desirable and helpful. But your thought and assistance when you have had more opportunity to reflect and come up with a more complete judgment, perhaps after consulting with some of your colleagues up at Woods Hole would be also eminently useful.

In fact, that is really what I am asking for.

Dr. KETCHUM. Very well. May I say that I have prepared a proposal to the National Science Foundation—it is still in a preliminary form—which outlines a very small part of the coastal ecosystem study with orientation toward the understanding of man's impact upon this environment.

I would be glad to send the part of that proposal which describes the proposed research program if this would be helpful.

Mr. DINGELL. It would be immensely helpful. Could you fashion it in legislative terms for guidance which will be necessary for this research program?

Dr. KETCHUM. I will try to do so. I think I might need some assistance in this regard.

Mr. DINGELL. Your reputation satisfies me that you have the competence to do that.

Dr. KETCHUM. To give a concept of the magnitude of the job, as I say this proposal that I have prepared would only do a small part of the overall job. I would include 17 scientists and would have an annual budget, for just one operation and just one small part of the problem, a cost of $500,000 a year.

I think you need for the Nation as a whole a minimum of 10 or 20 of these programs. The sea grants program that this committee is cognizant of has now a budget of something like $15 million a year and much of the work that they are supporting would feed information into this type of evaluation of the coastal zone.

Mr. DINGELL. Thank you, Doctor.

(The information follows:)

WOODS HOLE OCEANOGRAPHIC INSTITUTION,
Woods Hole, Mass., April 16, 1971.

MEMORANDUM

To: The Honorable John D. Dingell.
From: Dr. Bostwick H. Ketchum.
Subject: Ocean Dumping Hearings, April 6, 1971, P. 318, Line 22.

The following information is submitted for the record by Bostwick H. Ketchum. It was requested by Mr. Dingell at the Hearings on Ocean Dumping. It describes a research program proposed for a Center for Environmental Quality at the Woods Hole Oceanographic Institution.

I would like to preface this supplementary material by stating that the research proposed by the Council of Environmental Quality which is quoted below constitutes an excellent brief summary of what needs to be done. The research areas given are directly pertinent to ocean dumping. I hope that this summary will be helpful to the Committee in its efforts to cast an appropriate research program in legislative terms.

The balance of the attached statement is more broadly applicable to all forms of pollution of the coastal zone. The entire program needs to be done, but parts of it, such as disposal of waste heat and sewage, may not be appropriate for legislation concerned with ocean dumping.

BOSTWICK H. KETCHUM.

PROPOSED RESEARCH PROGRAM

In its report entitled "Ocean Dumping, A National Policy," the Council on Environmental Quality listed a number of research needs which are quoted below:

"*Research needs*

In the long term, additional information is required in the implementation of this policy. Serious information deficiencies exist, and research is required in the following major areas:

Broad-based ecological research is needed to understand the pathways of waste materials in marine ecosystems. Such studies should be directed to a better understanding of the food chain from microscopic plants and animals to high predators; how pollutants concentrate in the food chain; the origin and ultimate fate of pollutants in the oceans; and the effects of concentration on the marine environment and eventually man.

Marine research preserves should be established to protect representative marine ecosystems for research and to serve as ecological reference points—baselines by which man-induced changes may be evaluated.

Oceanographic studies of basic physical and chemical processes should be directed toward gaining a thorough understanding of the marine environment, with special emphasis on estuaries and coastal areas.

Toxic materials should be identified and their lethal, sub-lethal and chronic long-term effects on marine life investigated. Information is needed on the

persistence of toxic substances; how pollutants are degraded chemically and biologically; the effects of radioactivity on the marine environment and man; and the capacity of waters to assimilate waste materials.

More information is needed about public health risks from ocean pollution. Studies should determine what pathogens are transported in marine ecosystems and how. Better methods of measuring public health dangers are also needed.

Research is needed on the recycling of wastes and the development of alternatives to ocean dumping. Technical problems must be solved, but there is also a great need to study the social, institutional, and economic aspects of waste management.

Effective national and international monitoring systems need to be developed. Research is necessary to develop improved methods and technology so that alterations in the marine environment may be detected. But there is also a need for data coordination so that data gathering and analysis efforts are not duplicated."

These research needs emphasize the fact that a coordinated, interdisciplinary approach to the problems of the pollution of our inshore waters is essential. The unique capabilities of the Woods Hole Oceanographic Institution in marine studies can be well employed in addressing some of these critical problems.

The continuing research program of the proposed Woods Hole Oceanographic Center for Environmental Quality should focus upon major problems which are of general applicability to marine pollution problems, rather than on evaluation of local and specific problems of waste disposal in the sea. Studies of local problems would be undertaken when the results of the investigations might be expected to have general applicability. It is hoped, however, that the study of specific cases may be undertaken by other marine stations along the coastline which possess special capabilities for local work with the cooperation of or consultation with will be made with many of these marine stations to discuss with them our general program and to determine whether they would be interested in studying the pertinent details of their particular local environment. For these specific case studies it is anticipated that funds will be available from sources other than NSF. The following discussion defines the type of research program which the Center should undertake actively in a continuing way. Research proposals for several of the subject areas would be prepared for inclusion in a formal proposal.

a. Coastal circulation, mixing and effects on the biota

Fundamental to any evaluation of waste disposal in the coastal waters is an understanding of the circulation and mixing of these waters. It is proposed that the Center for Environmental Quality undertake a general analysis of the circulation and mixing of the coastal waters extending from Cape Cod to Cape Hatteras. About 20% of the population of the United States live along watersheds emptying into this coastal zone, and examples of many pollution problems of general interest can be found in this area.

In addition to describing the circulation and mixing of this specified area of coastal waters, one of the prime objectives of the program would be to develop standards which could be applied in similar investigations elsewhere. It is obviously important that observations be arranged, both in space and time, so that important fluctuations in environmental conditions will not be missed or overlooked. This spacing may be different for different parts of the program and may, indeed, be different for different geographical regions. Criteria for the determination of the proper spacing of observations is a necessary step in the development of adequate environmental monitoring programs both for the description of existing baselines and for the prediction of future effects of modifications.

The principal investigator and several other members of the staff have already conducted a number of investigations in this general area and some specific questions which still require a definitive answer for a better understanding can already be identified. For a complete understanding of the system, the study must include the entire continental shelf from the beach to the slope water offshore since only in this way can the exchanges between the coastal zone and the deep sea be evaluated.

There are several critical areas where direct current measurements are needed in order to reach definitive conclusions about the rates of circulation and exchange. We still do not know the mechanisms for the exchanges between slope water and coastal water, but it seems probable that tidal action in the numerous

submarine canyons along this stretch of the continental shelf may play an important role in the mixing processes. Direct current measurements in the canyons are needed to evaluate this hypothesis.

While the observations necessary for an evaluation of the physical oceanography are being made, water samples will also be collected for chemical analyses. Various heavy metals, pesticides and their residues, petroleum products and detergents analyzed in samples throughout this part of the continental shelf should provide a present-day baseline for evaluation of further changes (see Section c. below).

Biological samples for both plankton and benthos would also be necessary to define the present distribution of the organisms in this marine ecosystem. In some cases, particularly for oil and pesticide residues which accumulate in the lipid pool, an analysis of the organism may be more informative than an analysis of the water. It is known that the biological characteristics of this stretch of the continental shelf are quite variable and it is important to know existing conditions so that future changes can be evaluated. The biological sampling plan of operations would be to select pairs of "standard" stations at various locations along the coast. One station of each pair would be selected as a potential indicator of pollution effects, while the other station would be selected for similar environmental characteristics (type of bottom, water flow, etc.) in an area more remote from pollution. Periodic sampling of these pairs of stations will establish the present-day baselines and detect biotic changes as a result of additional environmental modifications.

In this field program, cooperation of other marine laboratories to evaluate local conditions would be particularly valuable. Each of the major estuaries discharging into this stretch of the Atlantic coastline will require intensive study and evaluation. All of them are definitely polluted at present. Studies of the local distribution and the seaward extension of selected pollutants will give us an indication of how far the effects extend from shore and the pollutants may also act as tracers and help to evaluate the rate of exchanges between the coastal water and the deep sea.

b. Domestic pollution

There are many questions concerning the advisability of adding domestic pollution directly into the sea, and the rate at which this can be done without deleterious effects. Our ongoing program concerning the use of domestic pollution as a possible fertilizer for sea water has already been mentioned. This program may answer some questions, such as the rate of addition of domestic pollution that can be tolerated by the ocean without over fertilization and eutrophication, the advantages of secondary treatment prior to discharge of the domestic pollution, the potential advantages of tertiary treatment in which the fertilizing elements, such as phosphorus and nitrogen, are removed from the pollution, and the survival of various viruses in the sea water with the possible danger of transmittal of disease by discharging sewage at sea. We have some approximations to some of these questions, and it is easy to see, for example, that the estuary of the Hudson River is over fertilized by the pollution discharged by the Metropolitan New York area. It seems clear that secondary treatment alone will not cure the problems of the pollution of the Hudson River estuary but it is uncertain how much more could be achieved by more refined treatment methods.

The sewage slude resulting from secondary treatment plants presents another kind of problem. For forty years, sewage sludge has been dumped at sea off New York and it has had seriously damaging effects on the bottom populations in the dumping area. There is evidence for the accumulation of petrochemicals and of heavy metals in the sludge deposits. The presence of these toxic materials may have inhibited the bacterial breakdown of the organic materials in the sludge. The rate of addition of organic sludges to the coastal zone should not exceed the rate at which these materials can decay so that the marine environment can recover from the addition. Unfortunately, we do not know the rate of decay nor whether this is inhibited by toxic components of the sludge. Precise answers are not yet available as to an acceptable rate of disposal of this type of material.

There are several proposals for the disposal of sewage in the deep water off the edge of the continental shelf, either by a direct pipeline or by barging material out to sea. At first glance this seems to have the advantage that the best fishing areas can be avoided. The deep sea, however, is a very constant environ-

ment and the biota living there have not been exposed to the stresses which are characteristic of the coastal zone environment. It is possible that disposal in the deep sea may have much more drastic effects on this biota than has been observed with the hardier forms living under continual stress. As mentioned above, evidence is accumulating that the rate of decomposition of organic materials under the low temperature and high pressure conditions of the deep sea is much slower than it is at normal pressure even at the same low temperature. Disposal in the deep sea may, therefore, be merely a mechanism for preserving our waste materials for posterity. We certainly need to know a great deal more about the sensitivity of the biota of the deep sea to this type of pollution before millions of dollars are spent on pipelines or barges to contaminate the area.

c. Toxic materials

Toxic materials are becoming common in the coastal zone environment but very few studies have been made concerning the resistance of marine organisms and populations to these toxins. A continuing study is needed to evaluate the toxic effects of heavy metals and of the wide variety of organic compounds. Long-term effects, especially on breeding and behavior of organisms may be particularly important but are almost unknown. These problems are intensified because we are producing new organic compounds at the rate of several hundreds per year and many of these are reaching the environment with unknown consequences.

There is adequate evidence that unsuitable levels of mercury contamination are present in all of our aquatic environments. Lakes, rivers, streams and estuaries have been extensively closed to the harvesting of fish and shellfish because the mercury levels in the flesh exceeds the amounts recommended by the Food and Drug Administration. The Minimata disease in Japan demonstrated clearly that the organic compounds of mercury are particularly hazardous.

Extensive studies have also been made of the distribution of lead in the oceans. As a result of the burning of tetraethyl lead in gasoline, the lead content of the surface water of the world ocean has been increased substantially in recent decades. Less adequate information is available about the many other toxic heavy metals which are produced and discharged in large amounts by our industrial technology.

The organic compounds are particularly troublecome. The effects of DDT on the breeding failure of oceanic bird populations is amply documented. There is also evidence that DDT can effect the rate of photosynthesis of several species of marine phytoplankton. The distribution of DDT and its derivatives in the marine ecosystem is, however, very poorly known so that one cannot even estimate the proportion of the total DDT produced which has reached the ocean reservoir. Even if we knew how much is there, we do not have any evidence concerning its persistence in the marine environment. Even less is known about the distribution of the highly toxic polychlorinated biphenyls, but we now know how to identify and measure these and the evidence so far available indicates that they may be as broadly distributed in the environment as DDT and its derivatives.

d. Petrochemicals

Although oil contains several toxic materials, and could have been discussed with the other toxic organic materials, it deserves a special mention because of the magnitude of sea transport of oil. In an energy-based civilization, transportation of oil by tankers is certain to increase in coming years. Larger and larger tankers are being designed and built and an accident to one of the huge super tankers would have much broader effects than the accidental releases of the past. Although there are laws and international compacts concerning the discharge of oil at sea, they are more frequently ignored than obeyed.

There are many aspects of oil spills which require more intensive investigation. There is considerable need for more intensive studies of containment and recovery of the oil following an accident, an aspect of the problem which might well be considered by our Department of Ocean Engineering in cooperation with some of the engineering groups at M.I.T. From an ecological point of view the use of detergents to disperse the oil merely makes it more available to the marine biota, and some of the detergents which have been used are more toxic than the oil itself. Also efforts at sinking the oil to the bottom merely moves the problem to another location where the toxic effects may have just as drastic and serious results. Intensive studies on all of these problems are needed.

[illegible]

Modern methods of chemical analysis make it possible to "fingerprint" the source of a specific oil spill and thus make it easier to assess the blame for an accidental spill. Chemical studies to improve the fingerprinting potential and biological studies to assess the ecological effects of oil in the marine environment are greatly needed.

e. Solid wastes

One of the very serious problems co[illegible]ng our civilization is the disposal of solid waste materials. At the same tim[illegible]at our major cities are running out of waste land suitable for land fill operations, our population is using and discarding materials at an accelerating pace. Many proposals are pending for the disposal of this solid waste material at sea, both in the coastal zone and off the edge of the continental shelf. We have very little information about the possible effects of this type of waste disposal on the marine environment.

One of the many problems is the varied character of the waste material to be thrown away. Organic compounds, such as garbage, sewage sludge and paper, will certainly have very different rates of decomposition in the marine environment. Other solid waste materials, such as the non-returnable bottles, and various plastics, may have an indefinite life in sea water. Parts of old cars and other metallic objects may rust away and disappear in time but the rubber tires may be expected to persist almost indefinitely. Mixed household wastes can be compacted and bailed and tossed in the ocean where they are "out-of-sight, out-of-mind."

It seems clear that the only long-term solution to the solid waste problem is the recycling of as much of the material as can be made technologically possible. The Council on Envorenmental Quality in its publication on ocean dumping emphasized the need for recycling and also suggested as an interim alternative to ocean disposal the use of the approximately 2 million acres of unreclaimed surface mine lands which are available in the middle Atlantic States. They again re-emphasize the fact that if the United States population is to continue along its present pathway of development, the recycling of solid waste materials is the only ultimate solution to the problem.

As an interim solution, some types of solid wastes may be dumped at sea without apparent damage to the environment. There are several studies of the potential of developing improved fishing reefs by the dumping of building rubble or old cars on the continental shelf. There is evidence that the fishing over these artificial reefs is better than over a smooth bottom, but it is not yet clear whether the improved fishing represents an accumulation of existing organisms or an actual improvement in the productivity of new organisms.

Numerous questions concerning the impact of dumping of solid material on the ocean floor cannot be adequately answered with our present knowledge and understanding. It seems worthwhile to initiate some studies which might provide the answers which are required.

f. Heated effluents

The effluent cooling water of power plants may be 25° F warmer than the ambient temperature of the area. Because of the large demand for cooling water, particularly in the nuclear plants which are less efficient than the fossil fuel plants, the power industry is increasingly planning to site plants on coastal waters, where large volumes of cooling water are available. This raises a host of problems, ranging from the detrimental effects of the heated effluents to the possibility of beneficial uses of low grade waste heat.

As the water passes through the cooling system of the power plant, there may be direct effects on the biota, the effects of entrainment. The direct effects may not only be the result of the increased temperature of the water but may also include the effect of excessive turbulence on the organisms, the effect of pressure drops within the circulating system and the effects of chlorination when this is used to keep the cooling tubes from becoming fouled by marine organisms. If the amount of water used is a small proportion of the amount of water available because of the circulation, the entrainment effects themselves may also be a small proportion of the effect on biota. Thus, it is important to have a good understanding of the circulation in the neighborhood of the site which may be used for the dilution of the heated effluents.

The general environmental effects will also be very different depending on the location of the plant. In northern waters, the problems are quite different than they would be in sub-tropical regions where the summer temperatures

BEST COPY AVAILABLE

are near the upper limit which can be tolerated by the biota. In either case, knowledge of the vertical temperature distribution in the sea would be valuable in deciding where to place the intake and the outfall. If the intake could be placed in cold deeper water, it is possible that the effluent would not have a greatly different temperature than the nearby surface waters. By discharging the effluent at some depth rather than at the surface, it might be possible to provide a great deal of mixing as the warmer, less dense water rises to the surface.

In the colder parts of the ocean, it is comparatively easy to conceive of various ways in which the heated effluent could be utilized. Throughout much of the year in the Gulf of Maine, for example, the surface waters are too cold for the optimum growth rate of lobsters, clams and other marine products. It is possible that lagooning the heated effluents could provide a more favorable environment of the growth of these organisms. It is also possible that transplanting organisms which would not be able to survive the rigors of New England winters might produce useful products in such heated lagoons. A cost benefit analysis of such processes might indicate the economic feasibility of the procedure if the detrimental effects of such discharges of the effluent to the environment were taken into account and properly assessed.

Another possible use of heated effluents would be to place them at some depth in the ocean where they would mix with the nutrient rich deep water, rise to the surface and produce an artificial upwelling. As mentioned previously, productivity of the marine ecosystem is frequently limited by nutrient deficiencies. The artificial upwelling would both mix the heated effluent with colder deep water and bring up nutrients from the deeper waters. In each case a careful assessment of the magnitude of the possible effects would have to be made.

Another possible use of heated effluents is to speed up the biological processes in a sewage treatment plant by providing an environment of optimum temperature. Again, the winter effects may be quite different from the effects in the summer so that a careful evaluation would be neded. It is also possible that some industries which spend a great deal of fossil fuel energy in heating materials could make use of this low grade heat if the total system were designed as a unit rather than having each part designed separately.

Various other possible uses of heated effluents in the marine environment could be postulated. The present difficulty is that we are looking at only one aspect of these problems and reasonable solutions cannot be achieved without looking at the problem as a system in which the benefits of the use of the heated effluent are carefully weighed against the possible detrimental effects of its release to the environment.

g. Task Forces for Environmental Crises

The personnel of the Center should also be capable of a quick response to an environmental crisis such as an oil spill or a fish kill. It is very important that these phenomena be looked at quickly if the optimum evaluation is to be achieved. It is not proposed, of course, that this activity be undertaken for distant events but phenomena which occur close to home can frequently give clues as to the general environmental effects of man's activities. The Smithsonian Institution Center for Short-Lived Phenomena provides a good source of information. It would be possible to have the Woods Hole Oceanographic Center notified by phone when an event of interest occurs within reasonable distance of Woods Hole.

a. Monitoring

It is not proposed that the Center undertake any extensive system of monitoring except insofar as the activity would provide suitable baselines for evaluation of future changes in the environment or the biota. Samples could be taken, for example, in specific areas such as the dumping grounds off New York City during the studies of circulation and mixing. If progressive changes are identified during any part of the research program, it is apparent that monitoring for specific rates of change could be of value in the further development of the program.

The techniques of monitoring coastal environments have been given inadequate attention. The Center should accept the responsibility for evaluating and developing such techniques so that monitoring programs would give useful information. Such a study would consider not only what types of measurements should be made, but also evaluate the importance of the frequency and spatial distribution of

BEST COPY AVAILABLE

the observations. The various main sources of pollution—i.e. rivers, atmosphere and direct discharges at sea—will certainly influence the design of proper monitoring systems for pollution of the coastal zone.

Mr. DINGELL. Mr. Everett?

Mr. EVERETT. Doctor, with respect to identifying areas where the dumping could take place safely, do you have an opinion as to how long it would take EPA to make a study to identify these areas?

Dr. KETCHUM. This is a question of identifying areas where dumping could be safely carried on?

Mr. EVERETT. Yes, sir.

Dr. KETCHUM. With the qualification that this will be different areas for different types of material I think that the characteristics of the Atlantic shelf waters at any rate are already pretty well known.

The geological characteristics of the bottom have been studied over a period of 10 years in a joint program between Woods Hole and the U.S. Geological Survey and they have a vast fund of information on this type of thing.

In the specific case one would probably have to have a minimum of a few months predumping study and then a periodic reevaluation of the site after the dumping had occurred to detect any progressive changes in the course of time.

Mr. DINGELL. Are you referring to essentially a monitoring program?

Dr. KETCHUM. Yes.

Mr. DINGELL. Monitoring and assessment.

Dr. KETCHUM. And assessment of the effects.

Mr. EVERETT. That brings me to another question.

With respect to the monitoring, I also wanted to question you on this. There is no provision in the bill that provides for monitoring. What department or agency do you think should carry out the monitoring if such a provision were put in the bill? Should it be the Coast Guard, NOAA, or should it be EPA?

Dr. KETCHUM. I debated including a statement on monitoring and enforcement and decided that this was not my area of competence. I found myself vacillating between the Coast Guard and NOAA and EPA as to which would be the appropriate agency.

I really could not reach a firm conclusion.

Mr. EVERETT. Those are all the questions I have, Mr. Chairman.

Mr. DINGELL. Dr. Ketchum, you stated at page 4, at the bottom of your statement:

I urge the committee to consider including specific instructions to the Administrator when damaging effects of disposal operations are already known. Some operations should be prohibited, others should be phased out as rapidly as alternative methods of disposal can be developed.

Would you like to give this committee your assistance with regard to those two sentences?

First of all what specific instructions do you believe should be directed toward the administration of EPA. I think that would be of use. I am not asking you to give us your answer now. Perhaps you want to give it to us after more deliberate thought.

Then secor [illegible]ly, you have indicated some operations should be prohibited, others should be phased out as soon as alternative methods of disposal can be developed.

Do you want to address yourself to that question, sir?

Dr. KETCHUM. Yes. sir. I believe we discussed this indirectly before in that I have specifically identified toxic industrial waste, chemical and biological warfare material and high level radioactive waste disposal at sea should be prohibited.

I was asked to define toxic industrial waste and I believe that I sidestepped the question and failed to give a definition which would have any legal continuity, any legal reality at all.

In terms of the phased-out operations I was thinking of the operations which are already known to be damaging such as the disposal of sewage sludge and dredging spoils off New York Harbor and it is happening in other parts of our coastal zone where we know that these are damaging.

We need to have alternate methods of disposing of this material because it is being produced on a continuous basis. These I think should be phased out as rapidly as the alternatives could be developed.

Mr. DINGELL. Earlier you mentioned specific instructions to the Administrator when damaging effects of disposal operations are already well known.

What specific instructions would you have in mind? Again I indicated to you the possibility of your giving some comments on these points after you have had an opportunity to reflect on them.

Dr. KETCHUM. I think I would almost be willing to quote directly from the report of the Council on Environmental Quality which had some rather specific statement that this type of material should be prohibited, other types should be phased out in the course of time.

Mr. DINGELL. Are there any further questions of Dr. Ketchum?

Doctor, the committee is grateful to you. We thank you. You have been most kind and most helpful to the committee. We appreciate your assistance.

Dr. KETCHUM. Thank you for the opportunity of appearing before you, sir.

Mr. DINGELL. Our next witness is Mr. Edward Langlois, of Portland, Maine. Mr. Langlois.

Mr. Langlois, I notice you have an associate with you. If you will identify yourself and your associate for the purpose of the record by name and address, we will be happy to recognize you for your statement.

STATEMENT OF EDWARD LANGLOIS, CHAIRMAN, COMMITTEE ON ENVIRONMENTAL AFFAIRS, THE AMERICAN ASSOCIATION OF PORT AUTHORITIES; ACCOMPANIED BY PAUL AMUNDSEN, WASHINGTON, D.C., EXECUTIVE DIRECTOR OF THE AMERICAN ASSOCIATION OF PORT AUTHORITIES

Mr. LANGLOIS. Mr. Chairman, and gentlemen, may I introduce Mr. Paul Amundsen, Washington, D.C., Executive Director of the American Association of Port Authorities.

Mr. DINGELL. You may proceed.

Mr. LANGLOIS. Mr. Chairman, and gentlemen.

My name is Edward Langlois and I am appearing before this committee in my capacity as chairman of the Committee on Environmental

[illegible]

Affairs of the American Association of Port Authorities. I am regularly employed as general manager of the Maine Port Authority whose principal office is at Portland, Maine.

The AAPA is a corporate body whose membership includes all of the public port agencies, boards, commissions or authorities responsible for the planning, development, operation and maintenance of the seaports and seaport facilities along the coasts, bays, rivers and Great Lakes of the United States and its insular possessions. Our U.S. voting members are variously formed as State, city or district bodies responsible to the public for the development of commerce and navigation.

In 1970 the Nation's seaports handled 559 million tons of foreign trade (as versus 417 million in 1969) plus heavy volumes of coastal and insular trade and defense shipments. To help do so efficiently and economically, over 3 billion in non-Federal funds have been invested by local port interests in terminal and cargo handling facilities since the end of World War II.

AAPA interest in H.R. 4723 is based largely on the fact that seaport facilities are totally dependent on Federal and private channel and pierside dredging, which, in turn, would be affected by the new spoils disposal permitting requirement contained in the subject legislation. The port industry unquestionably supports the goal of improving, to appropriate standards, the quality of the water of the Nation's harbors, and would approve of strict regulation of dumping of materials such as garbage, sewage, munitions, chemical and various other deleterious commodities and agents into the waters, navigable or otherwise, of the country. We do wish to question, however, the inclusion of dredged spoil in this category and oppose the transfer of dredged spoil disposal permitting from the Corps of Engineers, U.S. Army, wherein it was reaffirmed as recently as Friday, December 25, 1970, with the President's Executive Order 11574.

The handling and disposition of dredged spoil is an engineering matter and should continue to reside with the Engineers, for the better protection of the Nation's environmental well being. There are two basic considerations: (1) where the material is to be placed and (2) how it is to be handled to placement.

Location of disposal is primarily a planning problem and increasingly a long range planning problem, for the community. Large land areas such as are needed for the receipt of spoil, particularly along harbor waterfronts, are both exceedingly scarce and costly in many of the Nation's older, highly urbanized and heavily populated areas. Some available areas are wetlands which are prohibited for disposal. Some areas are earmarked for recreational or residential use which lend themselves to spoil disposal under carefully managed fill conditions.

In port areas faced with problems such as these, progress toward locating, obtaining and condemning or helping to finance the construction of land containment areas must be measured against very patient long-range standards. Such problems cannot be "ordered" to be solved according to the regulations of a policing-oriented body focused on regulating the outfall of new material and effluents into the waters.

The handling of spoil material from the dredging site to the containment or disposal area, like planning, is an engineering function.

BEST COPY AVAILABLE

Local conditions and the distance the material is to be transported must be weighed on the basis of economics. This is a thoroughly integrated decision having a strong bearing on the overall cost of the project. We believe this function should remain with the U.S. Army Corps of Engineers as it has historically. The environmental safeguards are built into the corps procedure, including a requirement for local approval, and we see no useful purpose in retaining "dredged spoil" under the definition of "material" in this proposed legislation.

Procedures are adequately and effectively regulated now from the standpoint of the environment and of marine transportation. We further respectfully suggest that this distinguished committee look with great care at the growth of world dependence on merchant shipping, as it views this legislation.

It has been estimated that back in the year 1900, comparing total world population with total tonnage of merchant shipping, there were approximately 200 pounds of shipping for each person. Today that figure has grown to 600 pounds for each individual, reflecting a threefold dependence, and this continues to grow. World population, meanwhile, is growing also. The world fleet could reach a billion gross tons of shipping by the year 2000.

Reflecting the demands of world shipping on our Nation's harbors, total local public investment in marine terminals had reached $861 million by 1941 and adding the previously cited investments post-World War II, amounts to almost $4 billion today.

This nationwide harbor development has been done in partnership with a Federal investment in ports, mainly in the form of deepwater channels, the U.S. Army Corps of Engineers being responsible for the Nation's navigable waterways.

So that we may visualize this partnership, the Federal investment in channels since 1824 totals almost 1.5 billion including maintenance. Comparing this to the historic local public investment in marine terminals means that competing local port authorities have invested more than $2 for every Federal dollar.

The resulting plant, a product of the forces of competition, is considered to be the finest port system in the world. We estimate that our current seaport waterfronts occupy 1,650 miles in the aggregate, or 2 percent of the national shoreline which, measured point to point on a 100-foot unit basis, totals 93,653 miles. This may have to be increased to as much as about 5 percent over the next three decades, to meet the demands of world interchange of goods. This leaves 55 percent of the shoreline as the national playground or for perpetuation of the ecocycle, or for other healthful uses. We suggest to the committee, and the Congress, that the above ratio offers a certain sense of balance and proportion in the area of dredge spoil disposal as in many other areas requiring balanced considerations.

This entire port structure has been developed, of course, with the movement and redeposit of countless tons of dredged spoil. We ought now to take a look at the material itself.

Mud, clay, rock, and sand are reportedly not major, lasting or widespread "pollutants", even under the most severe definition of that flexible word, when stirred up by dredging activities. Only when man has added a coating of his own ingredients do they become carriers of pollutants. For that type of dredged spoil, at issue is one long-standing

BEST COPY AVAILABLE

practice of dredging harbors, either for maintenance or improvement, and disposing of the silt by hauling it to designated dumping grounds in deep open waters.

This long-standing practice has been challenged, on the grounds that silt from centers of population tends to be highly polluted and that its transfer to open waters would present a new source of pollution. In most cases the actual risk is debatable, since the handling process may very well tend to minimize the pollutants and transfer from one point to another may not really affect the total situation. Nevertheless, the environmental viewpoint has prevailed and the placement of this kind of spoil into designated containment areas has become widespread practice in the last several years. A growing number of approaches have been carefully worked out as between the Corps of Engineers and local authorities on a planned basis. These approaches involve designated containment areas tied in as before stated with area development planning and soils management techniques.

It seems obvious to us who are directly dealing with the problem that contaminated spoil, where it exists, is a result rather than a cause. The approach, therefore, should be, and is, a careful technological cooperation employing Federal and local expertise on an economic base. Current regulatory safeguards and permitting procedures are more than adequate, and we do not approve of the intrusion of yet another permitting agency in the spoil disposal program.

We would hope that such an agency would be directed to concentrate its efforts upon causative effects, such as sewage outfall and industrial effluents. Development of adequate treatment or recycling, in which the Federal Government should play a positive role, would greatly simplify spoil disposal, eventually, in heavily populated areas. Man's overlay would be eliminated, leaving us to deal only with mud, clay, rock and sand.

Mr. Chairman, if I may, I would like to read a short supplement.

SUPPLEMENTARY AAPA STATEMENT

We would like to compliment Chairman Edward Garmatz of the House Committee on Merchant Marine and Fisheries for his very perceptive plea, as these hearings opened, for balanced consideration of all of the factors. We share the Chairman's concern that bills such as H.R. 4723 as now drafted could seriously impede future port development and therefore adversely affect the entire maritime industry and, indeed, the Nation.

We join wholeheartedly in the Chairman's recognition of the corps' role in environmental protection, and, even further, would suggest that the Nation's environmental interests would be better served in retaining spoil disposal permitting authority with the Corps of Engineers. Our statement presented here points to the complexity of spoil disposal as an engineering and planning function requiring careful coordination at Federal and local levels.

The Congress has recognized that complexity as recently as December 31, 1970 in Public Law 91-611, River and Harbor Act of 1970, which states (sec. 123(i) A):

The Chief of Engineers, under the direction of the Secretary of the Army, is hereby authorized to extend to all navigable waters, connecting channels, tribu-

tary streams, other waters of the United States and waters contiguous to the United States, a comprehensive program of research, study, and experimentation relating to dredged spoil. This program shall be carried out in cooperation with other Federal and State agencies, and shall include but not be limited, to investigations on the characteristics of dredged spoil, and alternative methods of its disposal. To the extent that such study shall include the effects of such dredge spoil on water quality, the facilities and personnel of the Environmental Protection Agency shall be utilized.

We greatly appreciate the chairman's call for balance and wish to express our thanks to the chairman and members of the respective subcommittees for this opportunity to present our views.

Mr. DINGELL. Mr. Langlois, the committee is very grateful to you for a very helpful statement.

The Chair recognizes Mr. Kyros. As I understand, he is your representative, a very able one, and we have a great deal of respect for him.

Mr. KYROS. Thank you, Mr. Chairman.

I would like to welcome Mr. Langlois here. I have known him for many years as general manager for the Portland, Maine, authority, and am well aware of what he is trying to do for our city of Portland. Now, in Portland Harbor we dump, I believe, 16 million gallons of raw municipal sewage every day from the out-falls of the city of Portland, and other out-falls. Is that correct?

Mr. LANGLOIS. Mr. Kyros, we dump a great deal, and I don't have the numbers. We are aware of it, and I know the community is and they are taking steps to redirect it.

Mr. KYROS. Of course, we conduct dredging regularly in Portland Harbor, using it, for the moment, as an example of a typical port?

Mr. LANGLOIS. That is correct.

Mr. KYROS. I note from your statement your feelings with regard to contaminated spoils, dredging spoils; where they should be located after they are dredged and how this matter should be left to the Corps of Engineers.

Mr. LANGLOIS. That is correct.

Mr. KYROS. Also, I understand from your statement that you feel that the real problem lies with the contaminated factor, things like sewage. And that if we just started out with the clay, silt, or soil that is on the ocean bottom, we would not have as much trouble relocating this dredged material.

Mr. LANGLOIS. That is correct.

Mr. KYROS. But don't you feel that someone like the Environmental Protection Agency should have some uniform voice or idea as to what the corps is doing? The act as proposed here so provides, that the corps will seek to obtain permits from the Environmental Protection Agency.

Mr. LANGLOIS. We are concerned, Congressman Kyros, with excessive delays at this time in granting permits from the corps for projects that are basic and important to the development of ports throughout the country. We understand it and we accept it. We are concerned however that another agency participating in the lengthy debates of the granting of the permit would tie us up to such an extent that the cost-benefit ratio that has been established could well be jeopardized and the project could then die. And this is very serious.

Mr. KYROS. What about the fact that the EPA would be concerned with other agencies regarding the quality of pollutants that are

dumped into a harbor, and from that vantage point they would have some rational perspectives in dealing with the corps about contaminated dredging material. In other words, would it not be better to have them all operating and cooperating together than to have the Corps of Engineers operating independently of the EPA?

Mr. LANGLOIS. I say no, and I might say, if I may, the case that involved the dredging of Portland Harbor in the middle 1960's from 35 feet to 48 feet which is the basis of our survival as a major port because, without it, we would have lost perhaps the major shipping.

We felt—and certainly no one took issue in 1963, when the permit was granted to dump off the coast of Portland, no one took issue that we were going to spoil the fishing grounds, we are going to contaminate all the areas which were richly blessed with fish. We are satisfied that no one has raised a hand since 1965 when we finished the job to come forward to say that because we deposited spoils off the Portland lightship that it in any way affected the fishing habitat.

So, we are satisfied that in our own backyard the corps acted prudently in behalf of their responsibilities and our own, and we think they can continue to do so.

Mr. KYROS. But your primary concern, as indicated by your statement, is that it would be a time-consuming process, if the corps had to consult and obtain a permit from the EPA.

Mr. LANGLOIS. Time consuming would be one factor. The second would be that for decades the Army corps has accepted this responsibility and we feel have the expertise. If you transfer that to another department, it could well be that these two factors alone could jeopardize our future dredging projects and the disposal of the spoils.

Mr. KYROS. Up and down the coast of Maine, do you know how many areas we have had shut off from shellfish because of pollution?

Mr. LANGLOIS. Right.

Mr. KYROS. Dredged spoils, inevitably from harbors where municipal sewage has been dumped for many years, carriers the effects of this pollution. Would it not be logical and rational to have this agency, the EPA, the agency that uniformly oversees all forms of pollution, at the same time act to expedite the dredging of harbors that might be silted up, that can't be used? Why can't they live together? I can't quite see that.

Mr. LANGLOIS. Perhaps Mr. Amundsen would like to comment a little further on this.

Mr. AMUNDSEN. I think in order to do that logically you would have to bring EPA into the basic considerations of each and every one of these projects, which normally now take nine years to evolve. So that you would have, I think, a duplication of function. When you get into the economics of the project, you have to transport the material and so on, and choosing a disposal site, each one of these problems is different. It all wraps up closely with the local considerations and the corps has within the last year insisted on a local permit as well as its own. So that the system is pretty well stafeguarded now.

I might say with these additional local restrictions it is taking 6 months to a year to get even a simple dredging maintenance permit where we are already experiencing vessel groundings on this basis. So there is a lot to be taken into consideration on this question, sir.

BEST COPY AVAILABLE

Mr. KYROS. I certainly sympathize with the last point you made, about the need to expedite the dredging. But the facts presented to this committee indicate that dredge spoils account for 60 percent of the waste of all ocean dumping, and that 34 percent of all the dredged spoils are already contaminated.

So, somewhere we have to work a line between the need to get the dredging done, to get the projects started, and the need to assure everyone that the dredge spoils that are located somewhere else will not pollute. I think that is the problem that this committee is going to face.

Thank you very much.

Thank you, Mr. Chairman.

Mr. DINGELL. Thank you.

Mr. Pelly? Thank you.

Mr. PELLY. Thank you, Mr. Chairman.

Mr. Langlois, I have the port of Seattle in my district. I don't have the problems obviously of those ports that have to dredge, to keep their channels open, so that the large ships with greater draft can continue to utilize the ports and port facilities. However, I have been thinking as you have testified and I wonder if you have done any research as to the changes which have taken place in our merchant marine.

In other words, we are going to have a vastly different type of ship now, one that loads and unloads very quickly with containers and moves out, but further than that the new Lash type of ship in which a whole barge is floated downstream or taken from industrial areas where it does not require deep draft, and then loaded entirely onto a ship, one after the other. This might mean that many areas that have had to be channeled in the past will not have to be channeled under this new type of operation. The barge will go in shallow waters and will be floated right up on board the new type of ship.

Mr. LANGLOIS. You have asked if we have put any time and study on it; and my answer is yes, we certainly have. I am sure you have seen the vessels themselves or at least the plans. These vessels are deep-draft vessels. That is, they are down to 35, close to 40 feet. The primary purpose of these vessels is to reduce in-port time and also to bring cargo that was upriver down to the point of loading. So that even the smaller conventional steamers never went up the river for most of the cargo that the Sea Bees and Lash vessels are going to pick up.

So I strongly feel if we are going to protect our ports for these vessels and these vessels are here and I think they are here to stay, in great numbers—then we must make certain that we have existing channels and that another very important issue is that we have the proper maintenance of these channels. That provides for maintenance dredging. Again, when you do maintenance dredging, you have to have your permit. In some of our port areas vessels are grounded because of silting, and therefore the dredging must be accomplished in order to continue the traffic. With the Lash and the Sea Bee ships, they are drawing more water than the steamers you and I knew about in World War II.

Mr. PELLY. Yes; but there will be fewer ships coming into Portland with the Lash type of vessel, because they are moving faster and

they will, as I see it, dock in the channel, the deep channel, and then the barges will be brought. So it will reduce the number of channels tha [illegible]ave to be built because now the ships have to go right into the industrial areas to load and unload, and it is going to simplify the operation. I would think it would mean less in the way of dredging and change the entire port operation.

Mr. LANGLOIS. Without a doubt, there will be changes. I see because of the Lash and the Sea Bee, however, an increase in the movement of cargoes into overseas markets because it opens up new areas for the exporter and importer.

Mr. PELLY. Fewer ships?

Mr. LANGLOIS. Fewer in number of ships, but not fewer in number of sailings. I think the cargo is going to increase enough so that the larger vessel is going to operate even more today than it has in the past. But I do agree with you regarding the fact that perhaps they could go into a channel if that [illegible]annel is self-protected enough so that this intricate movement can take place.

Mr. PELLY. Isn't it true that many ports today never were intended by the Almighty to be ports? They channel rivers. So that those of us who were blessed with deep water lost our advantage, and the big ships have been going upriver. In the future that operation would seem to me to be one in which the barges come down the river and meet the ship in deeper water, at the mouth. But I am glad you have studied it, and I want to say that I am glad to see you recognize that we are going to have more trade.

Thank God, in my community there is more cargo going over our dock than ever before. It is the only bright spot we have, but I hope it will continue, and under the new *Lash* type ship and the President's new maritime program, I would think that our trade will expand, as you say.

Thank you.

Mr. DINGELL. Thank you, Mr. Pelly.

Mr. Griffin?

Mr. GRIFFIN. No questions.

Mr. DINGELL. Gentlemen, I am curious as to the precise thrust of your statement. I would like your assistance, if you please. You are, I assume, taking the position that you don't want to have two permits to meet in connection with dredging and fill operations; is that correct?

Mr. LANGLOIS. That is correct.

Mr. DINGELL. Are you taking the position that the ocean dumping will be entirely within the purview, as I understand the bill, of the Environmental Protection Administration, the dredging would remain under the Corps of Engineers? Is that your complaint, gentlemen?

Mr. LANGLOIS. We understand from the bill that the permitting to dump the spoils into the ocean would be granted by the EPA.

Mr. DINGELL. That is correct.

Mr. LANGLOIS. We think it should remain with the Army Corps of Engineers.

Mr. DINGELL. I am curious to have you explain to me how you would be differently treated by the Environmental Protection Agency than you have been treated by the Corps of Engineers. You have

had an increasing conspicuous pattern of rules and regulations with regard to ocean dumping and with regard to dumping in the Great Lakes under the administration of the Corps. I have been reading the bills before us as you gentlemen were testifying to try to see how you would be differently treated by EPA on ocean dumping than you have been treated by the Corps under existing Executive order under the various other statutes relevant to ocean dumping. Fish and Wildlife Coordination Act, and other matters.

Now can you address yourselves to that? This poses a difficult question to the committee.

Mr. LANGLOIS. Mr. Chairman, as we stated, we feel that the Corps' experience in granting permits in the past—they have the expertise, they are engineers, they are involved in the planning. As it is an engineering function to dredge, as it is to dump, that here lies the expertise. This is used and is available.

Mr. DINGELL. Let me simplify this. Are you telling us that you want this regarded simply as an engineering problem and not as an environmental problem?

Mr. LANGLOIS. Not at all.

Mr. DINGELL. Is it your position that you want us to ignore, or the agencies granting these permits for dumping to totally ignore the environmental questions involved?

Mr. LANGLOIS. Not in any way. I wanted to add that we understand and attempt to assist the Corps in its role today, which is more prominent now than it has been in the past regarding the environment. The granting of permits in the past, as I mentioned, in Portland Harbor in 1963 was a routine matter. It did not even have a public hearing. We have appeared at public hearings before the Corps and we certainly appreprriate and understand. As we mentioned, we are ecology minded [illegible] the ports, we have a program in great scope regarding all phases [illegible] pollutants: oil spillage, debris, deterioration, disposal of spoils. So we understand and we appreciate. We are only putting forth our thoughts that we have a responsibility to commerce and industry and we must maintain these channels in order that the bridge between our industry and world industry does not break down.

Mr. DINGELL. Let me make you a very simple and very clear statement. I can understand your apprehension. By this legislation, we are not going to hold up the ocean dumping or other dumpings entirely. We are not, by this legislation before us, going to halt, let us say, dredging and filling in the harbors. It is not our intention to do that. It is the intention I think, and the intention of the administration, to handle it in careful fashion and in an environmentally sound fashion that would consider fish, wildlife, and other things. That, very strongly, is the intention of the Chair.

Now I must tell you that it is my opinion that the administration will not settle for less than something of the order of the bill before us. But I would be interested, as one of the two subcommittee chairmen considering this matter, in having specific amendments and suggestions from you folk as to precisely what you want.

Now I could tell you, just sitting here, that it is my personal judgment that EPA is going to issue the ocean-dumping permits. I will tell you, speaking here as a subcommittee chairman, that I have much doubt that you will be radically differently treated by EPA than you would by the Corps. I must tell you that I have observed the Corps

in recent years and I am well satisfied that they have been behaving both from an engineering standpoint and from the standpoint of preservation and care of the environment. They have been doing some mighty good things.

I have a feeling you will not find yourselves afflicted with radically different situations if EPA does it than you will if the corps does it. I will be glad to have your suggestions, but I also would like to have your suggestions raised in the cold light of reality.

Mr. LANGLOIS. I might comment and tell you that I share your remarks regarding the corps' performance to this country and to our ports and keeping in mind its responsibility as far as ecology is concerned and dredging is concerned. We understand it and we appreciate it, and we work with them very closely.

Mr. DINGELL. I am satisfied that you want them to behave in a responsible fashion so far as environment is concerned, and I know you will not do anything clearly outrageous or anything of that sort. If you will give us your suggestions as to amendments to the administration's bill, we will be happy to consider them very sympathetically.

Mr. LANGLOIS. Thank you.

(The following letter was received in reference to the above:)

THE AMERICAN ASSOCIATION OF PORT AUTHORITIES,
Washington, D.C., April 8, 1971.

Chairman JOHN D. DINGELL,
Subcommittee on Fisheries and Wildlife Conservation, House Committee on Merchant Marine and Fisheries, U.S. House of Representatives, Washington, D.C.

DEAR MR. CHAIRMAN: Thank you for the courtesies afforded in hearing the statement of The American Association of Port Authorities on April 6th. Supplementary to our testimony, the following would apply:

You asked if the ports would not fare just as well under EPA as they now do under the Corps, in the disposition of dredge spoil, in view of the recent stringent environmental activity of the Corps. We would note that the Corps has both a navigational and an environmental approach. We suggest that the alternative to planned permitting under the Corps could often be the navigational emergency, resulting in turn in emergency permitting and chaotic disposal.

In connection with specific amendatory language, we appreciate your offer that we send our suggestions, which are as follows:

(1) Strike the words "dredge spoil" from line 20, page 2.

(2) Exclude the civil functions of the Corps of Engineers from this act, specifically those functions involving the Corps responsibility for the navigable waters. This exclusion should replace the language of Section (d) which begins on page 12, line 3.

We would re-emphasize in this connection that ocean dumping of dredge spoil is not a national problem. Rather it is a series of specific localized problems the complexities of which have been alluded to in our statement and which are more than adequately dealt with by the sections of the Rivers and Harbors Act of 1970 previously cited.

We would like to see this material in its various forms much more definitely characterized, as the result of the Corps study authorized in that Act, before any further national legislation affecting spoil disposition is considered by the Congress.

Respectfully submitted.

EDWARD LANGLOIS,
Chairman, Committee on Environmental Affairs.

SUPPLEMENTARY STATEMENT OF THE AMERICAN ASSOCIATION OF PORT AUTHORITIES

We would like to compliment Chairman Edward Garmatz of the House Committee on Merchant Marine and Fisheries for his very perceptive plea, as these hearings opened, for balanced consideration of all of the factors. We share the Chairman's concern that bills such as H.R. 4723 as now drafted could seriously

impede future port development and therefore adversely affect the entire maritime industry and, indeed, the nation.

We join wholeheartedly in the Chairman's recognition of the Corps role in environmental protection, and, even further, would suggest that the nation's environmental interests would be better served in retaining spoil disposal permitting authority with the Corps of Engineers. Our statement presented here points to the complexity of spoil disposal as an engineering and planning function requiring careful coordination at Federal and local levels.

The Congress has recognized that complexity as recently as December 31, 1970 in Public Law 91-611, River and Harbor Act of 1970, which states (Sec. 123(i)):

"The Chief of Engineers, under the direction of the Secretary of the Army, is hereby authorized to extend to all navigable waters, connecting channels, tributary streams, other waters of the United States and waters contiguous to the United States, a comprehensive program of research, study, and experimentation relating to dredged spoil. This program shall be carried out in cooperation with other Federal and State agencies, and shall include, but not be limited to investigations on the characteristics of dredged spoil, and alternative methods of its disposal. To the extent that such study shall include the effects of such dredge spoil on water quality, the facilities and personnel of the Environmental Protection Agency shall be utilized."

We greatly appreciate the Chairman's call for balance and wish to express our thanks to the Chairman and members of the respective Subcommittees for this opportunity to present our views.

Mr. DINGELL. Gentlemen, if there are no further questions, thank you very much for your presence and your very helpful testimony. The Chair notes that we have a vote on the floor. Probably if the members leave to go to vote, we will be back here within a matter of 20 minutes, at which time our next witnesses are Mr. James M. Beggs, Under Secretary of the Department of Transportation, accompanied by an old friend of this committee, Rear Adm. Robert Hammond, Chief, Office of Operations, Coast Guard.

Mr. Secretary, are those conditions unduly onerous?

Mr. BEGGS. No, sir.

Mr. DINGELL. Is that all right?

Mr. BEGGS. Yes, sir; that will be fine.

Mr. DINGELL. We will be back, then, within just a matter of minutes. After that we will hear another old friend of this committee, Mr. James J. Reynolds, assistant secretary of the American Institute of Merchant Shipping.

If there is no further business to come before this committee at this particular time, the subcommittee will stand in recess.

(Recess.)

Mr. DINGELL. The subcommittee will come to order. This is continuation of the hearings on ocean dumping and legislation relating to that matter.

Our next witness is Hon. James M. Beggs, Under Secretary of the Department of Transportation, accompanied by Rear Adm. Robert Hammond, Chief, Office of Operations, Coast Guard.

You are most welcome, gentlemen. You may give whatever statement you wish to give.

STATEMENT OF HON. JAMES M. BEGGS, UNDER SECRETARY, DEPARTMENT OF TRANSPORTATION; ACCOMPANIED BY REAR ADM. ROBERT HAMMOND, CHIEF, OFFICE OF OPERATIONS, COAST GUARD

Mr. BEGGS. Thank you, Mr. Chairman, and members of the committee.

62-513—71——23 BEST COPY AVAILABLE

I appreciate the opportunity to testify today on H.R. 4723 and a number of other bills, all directed at the serious problem of the contamination of our oceans. To my right is Rear Adm. Robert E. Hammond, Chief of the Office of Operations of the Coast Guard.

Secretary Volpe has said often that at the Department of Transportation environmental quality is a goal, not a constraint. I am pleased to be here today to explore with the subcommittee how the Department, through the Coast Guard, can play a positive and effective role in regulating ocean dumping.

I graduated from the Naval Academy in 1947. At that time, no one questioned the capacity of the oceans to absorb our waste. We now realize, however, the peril of ocean dumping on a major scale. We now understand that we cannot continue to poison our oceans merely because they seem large enough to dilute the poison.

Administrator Ruckelshaus of the Environmental Protection Administration and Chairman Train of the Council on Environmental Quality will testify during this hearing. Both of those gentlemen will address themselves to the environmental concerns involved. I need not reiterate their statement of the problem or their commitment to solving it. I would like, however, to assure this subcommittee that my Department, through the Coast Guard, stands ready to cooperate immediately with the Environmental Protection Agency to carry out those portions of the program which they choose to delegate to us. I can assure the subcommittee that the Coast Guard can, today, fulfill the responsibilities for enforcement under section 8(c) of the proposed legislation.

In this regard, let me review for you the capabilities of the Coast Guard in this area. I have attached to my statement, as appendix A, a more thorough analysis of what is available in the way of support personnel, materials, and technical expertise.

The Coast Guard is already active in the area of ocean dumping as an adjunct to its marine environmental protection program. On a day-to-day basis, they actively seek information on planned dumping and record the location, identity of materials, and persons involved in all observed ocean dumping activities. This information is routinely furnished to interested agencies such as the Council on Environmental Quality and the Environmental Protection Agency. They also monitor many dumping operations upon request.

Effective regulation of ocean waste disposal requires three elements: a permit issuing authority, an available surveillance and enforcement capability, and effective monitoring activity. Based on the Coast Guard's considerable past experience in the field of maritime law enforcement, we believe that the agency vested with the responsibility for enforcement should have some involvement at each stage of the regulation scheme, particularly the issuance of permits. It is self-evident that effective surveillance and enforcement calls for knowledge of any permits issued and the terms of those permits. H.R. 4723 makes provision for this in section 8(b), and we have already begun to discuss with the Environmental Protection Agency how we can be most useful to them and to the program.

The Coast Guard engages in a variety of mission areas which would support the role of the Environmental Protection Agency in the reg-

ulation of ocean dumping. They have long been established as the Federal maritime law enforcement agency. Their officers and men are trained and experienced in matters of law enforcement just as they are trained and experienced in seamanship. The Coast Guard provides the bulk of the Federal force for enforcement of the Refuse Act and the various other oil pollution laws. They also have a long history of involvement with the marine community in such areas as the handling of dangerous cargo and the issuance of permits for various regulated marine activities. In addition, they have the facilities to carry out these functions, and with little augmentation, can utilize these same facilities to undertake a program of ocean dumping regulation.

Appendix A outlines in detail the Coast Guard units available for ocean dumping regulatory activity. I would only say here that they cover the east, west and gulf coasts of the continental United States, as well as Hawaii, Alaska, the Virgin Islands and Puerto Rico, and, of course, the Great Lakes.

The Coast Guard can aid the Environmental Protection Agency in the issuance of permits, and I have listed in appendix A the locations of the already-established Coast Guard captains of the port and marine inspection offices. I wish to emphasize that these offices are well known to all members of the marine industry and are presently involved in many tasks closely related to ocean waste disposal regulation. Captains of the port are responsible for the inspection of port structures housing hazardous materials and for the supervision of explosives loading. Marine inspectors are responsible for the inspection and certification of merchant vessels. These two types of facilities represent, if the Environmental Protection Agency choose to utilize them, an already available administrative force.

In addition, our experience and the data we have collected, in the regulation of hazardous material transport, would be readily available to assist the Environmental Protection Agency in making the necessary determinations as to whether or not particular substance should be disposed of at sea. The Coast Guard also collects oceanographic data in support of other missions which, while presently limited in scope, could be utilized in baseline determination.

Captains of the port and port safety officers have small boats, ranging from 31 to 44 feet, at their disposal for a variety of tasks including the supervision of loading of material to be dumped at sea and other calm water surveillance and monitoring. Eighty-two and 95-foot patrol boats are also available for the same tasks. Medium and high endurance cutters can be utilized too, and these vessels are capable of long-range operations of extended duration. Finally, Coast Guard helicopters and fixed wing aircraft can respond in less than 1 hour for surveillance and other duties.

If requested, we could also aid the Environmental Protection Agency in oceanographic monitoring of dump sites and adjacent waters. The Coast Guard has, for several years, been a major participant in the U.S. oceanography program. This participation takes the form of daily observations from lightships and offshore light stations as well as from a variety of ships. Seasonal oceanographic cruises are also carried out in specially equipped vessels. In addition, the Coast Guard Office of Research and Development and the Office

of Engineering are developing monitoring technology and instrumentation to support our present operational programs. Of particular importance will be the development of sensors specifically for pollution control purposes. Finally, our oceanographic unit and our marine scientists are skilled and experienced in the evaluation of environmental data as a result of our efforts in oceanography survey work including the international ice patrol.

In short, the Department of Transportation stands ready and able to aid the Environmental Protection Agency in any way they desire.

This concludes my prepared statement, gentlemen. I would now be willing to answer any questions you might have.

Mr. DINGELL. Mr. Secretary, the chairman wants to thank you for your presence and for your patience and for a very helpful statement.

The Chair is going to recognize at this time Mr. Everett, the counsel for the committee.

Mr. EVERETT. Thank you, Mr. Chairman.

Mr. Secretary, on page 23 of the bill, subsection (c), it reads as follows: "The Secretary of the Department in which the Coast Guard is operating shall conduct surveillance and other appropriate enforcement activity to prevent unlawful transportation of material for dumping or dumping."

Does this give you sufficient authority, in your opinion, to also enforce deviations from any permit that may be issued, such as dumping in a no-dumping area or dumping in violation of the permit or dumping closer inshore than the permit would allow?

Mr. BEGGS. I believe that section, in conjunction with the already existing statutory authority of the Coast Guard for enforcement, would be sufficient. In 14 U.S. Code 89, Coast Guard is given authority to "make inquiries, examinations and inspections and searches, seizures and arrests upon the high seas and waters over which the United States has jurisdiction for the prevention and detection of suppression of violation of laws of the United States." This would provide satisfactory authority for surveillance, yes.

Mr. EVERETT. Would you not think it would be advisable to make a reference in subsection (c) to that particular law that would be utilized for purposes of enforcing the Act and deviations?

Mr. BEGGS. Yes, I think that would be appropriate.

Mr. EVERETT. Another matter that gives me concern is how are you going to keep abreast of the permits that are issued in order to carry out your responsibilities with respect to the present language of the bill? How do you know when a permit has been issued or is there any requirement that the permit be displayed on the vessel at all times so that when you board a vessel it will be readily available for investigation?

Mr. BEGGS. I do think that there will have to be a procedure worked out with the Environmental Protection Agency so the Coast Guard is informed by copy of each permit or by appropriate notification of what has been involved in the issuance of the permit. We have talked already with EPA about procedures of this type. These have been preliminary discussions, but I believe that a satisfactory procedure can be worked out with them so that the Coast Guard is kept informed at all times.

Mr. DINGELL. Would it be helpful to you if you have specific directions in the statute from EPA to inform the Coast Guard?

Mr. BEGGS. Again, I think that would be appropriate.

Mr. DINGELL. That would make your job of enforcement rather easier?

Mr. BEGGS. Yes, it would. Although, again, I think that the motivation here will be in the direction of both parties keeping each other informed.

Mr. DINGELL. Do you want something said about having the permit displayed at some appropriate place on the vessel carrying cargo?

Mr. BEGGS. Perhaps I should have Admiral Hammond comment on that, since he is more familiar with the operational details.

Admiral HAMMOND. Mr. Chairman, I think it would be difficult to have a permit displayed on a vessel so that it could be seen and identified closely enough to be of real value.

Mr. DINGELL. I am not thinking of your standing off a thousand yards and reading the permit on the bridge, but I am thinking it should be readily available for inspection.

Admiral HAMMOND. It should be available so that the barge and tug operators could show it to the Coast Guard or other law enforcement agency upon demand.

Mr. DINGELL. Does the bill, in your opinion, definitely require amendments to afford that direction to permit holders?

Admiral HAMMOND. I would say that this again would be something that we could certainly work out very easily with EPA. It would be included in their regulations implementing the legislation.

Mr. DINGELL. Would you take a look at this particular matter that we are discussing at this time and when you return give us your views as to whether or not you need statutory authority with regard to the display of permits, et cetera?

Mr. BEGGS. Yes, sir; we certainly will.

(The information follows:)

The display of the permit can be required by regulation, and we do not recommend that a statutory requirement for its display be included in the Bill.

Mr. EVERETT. Mr. Secretary, I notice that you mentioned monitoring several times in your statement. Do you find anything in the bill that would require a monitoring program to be carried out?

Mr. BEGGS. No, not specifically. However, to accomplish the intent of the bill would require monitoring and the establishment of baseline data on the dumping grounds and the waters in general. The Coast Guard is doing a certain amount of this already. In pursuit of the responsibility they would have under this bill for surveillance they will have to develop, or assist in developing, probably with NOAA and EPA, a satisfactory baseline and continuous monitoring activity to note any adverse changes that were developing.

Mr. EVERETT. I get the impression from reading your statement that the Coast Guard is ready and willing and would be delighted to accept this responsibility. I was wondering if you were in a position to suggest an amendment to this effect that we put the monitoring responsibility on the Coast Guard.

Mr. BEGGS. I would suggest that this requires some discussion with NOAA, which has a broad responsibility in this area of developing

baseline data on contiguous waters. The division of responsibility between NOAA and the Coast Guard, I think, is something that will have to be carefully worked out. I do not think that we are prepared to offer an amendment at this time which would delegate this responsibility to the Coast Guard.

There is a developing relationship, I should say, between the Coast Guard and NOAA as to what assistance NOAA requires from the Coast Guard. Clearly, the NOAA organization will have to rely to a large extent on the data gathering facilities of the Coast Guard, both data gathering in the harbor areas and in the contiguous waters. So, here again I think we would have to consult with NOAA to find out what an appropriate division of responsibilities would be there.

Mr. EVERETT. Under section 8(b) of the bill, pages 12 and 13, the administrator has the authirty to delegate responsibility to other Federal agencies. I note you refer in your statement to the issuance of permits. Is there any contemplation that EPA will transfer this responsibility or delegate this responsibility to the Coast Guard?

Mr. BEGGS. No. As a matter of fact, I think EPA has in mind developing the capability to issue the permits directly themselves. What I had reference to was the existing organization and facilities of the Coast Guard and the captiains of the port, the marine inspection offices, and so forth, which are in place and are available to EPA to assist them in judging the background and details of any request for a permit. I am sure that EPA will take advantage of that.

Mr. EVERETT. Those are all the questions I have.

Mr. DINGELL. Mr. Pelly?

Mr. PELLY. I want to apologize to you both for being unavoidably detained.

I have had a chance, Mr. Secretary, to look through your statement. I am curious as to what is involved in monitoring. Have you the present equipment to take on such a responsibilitiy?

Mr. BEGGS. To a large extent; yes, sir. Although we will need some augmentation, I am sure, as the program develops. But in the Coast Guard's program of gathering oceanographic data in pursuit of their normal mission, they can indeed gather data that will be needed to establish the baseline as well as continue to gather further data as the dumping grounds are observed from time to time, after each dump.

So I think that the facilities and capabilities exist now, although I would expect that as time goes on there will be need of some augmentation, depending on how large a program we get into. I suspect it will eventually get quite large.

Mr. PELLY. You have quite a worldwide responsibility. I must say that I would hope that you would get additional up-to-date equipment. I think the authorization bill here moves in that direction. But you can't monitor from the air, can you?

Mr. BEGGS. Only to a modest extent. My understanding of the technology is that there is some capability, although modest, to do a certain amount of monitoring from the air. But very basically the information you need has to be taken through sampling and soundings by ships.

Mr. PELLY. I would be hopeful that this monitoring responsibility would not interfere with your duty to patrol our waters, because we

have had ever increasing encroachment by foreign vessels. They have damaged our crab pots recently. They have intruded into our waters which are an exclusive fishing zone where they are not permitted, and I think you need more equipment for that responsibility. That is why I asked the question.

Mr. BEGGS. Yes, sir, we are very conscious of our responsibility in this area. As you pointed out, we are getting an increasing frequency of foreign vessels intruding and violating our fishing laws.

Mr. PELLY. Meanwhile, we loan our vessels, including the Coast Guard's, to Latin American countries and they use them to patrol international waters and to seize our fishing fleet. So, if we could get some of those back maybe we could use them to protect the fishing zone and the contiguous zone and so forth without really requiring modern, new equipment.

Mr. BEGGS. In answer to your previous question, it is this administration's intent, as this program proceeds, to request additional facilities and equipment from the Congress as the need develops. We think we have adequate equipment to start the program and to get some feel for what additional equipment will be necessary.

Mr. PELLY. I would like one particular vessel that would be assigned off the coast of Latin America to cruise and add some sense of security to our fishing fleet down there, although I am not advocating that we go to war.

Thank you, Mr. Chairman.

Mr. DINGELL. Thank you, Mr. Pelly.

Gentlemen, I will recognize Captain Heyward, who is the Oceanographic Subcommittee counsel.

Mr. HEYWARD. Mr. Beggs, this is my maiden attempt on the subcommittee staff to ask you a few questions.

In connection with the discussion on surveillance and enforcement activity, would you care to comment as to whether or not there should be a provision in the bill which might authorize the Secretary to schedule the permitted dumping so that you might be able to have a better grasp of the timing of the dumping and therefore utilize the facilities in a more reasonable fashion?

Mr. BEGGS. My current feeling on this is that scheduling is not necessary. I might ask Admiral Hammond to comment on this further, but I feel we are in a position to adequately handle the situation without scheduling. If it becomes necessary, I think we would be back up here asking for that authority.

Mr. HEYWARD. I have another question in connection with section 5(c) in connection with permits. This question might be better addressed to the Administrator of EPA. I wonder whether you would have any comment on whether the word "may" in subsection (c) under (2) should be "shall". I am addressing myself to the question of what these permits would show that you are expected to enforce when you go aboard a ship to determine what they are doing. Shouldn't the legislation perhaps require each permit to contain certain information which would give you the knowledge upon which you could respond?

Mr. BEGGS. Perhaps I should ask my counsel to tell me what the implication of that might be in terms of the Administrator's responsibilities.

BEST COPY AVAILABLE

Mr. DINGELL. Excuse me, Mr. Secretary. Counsel is perfectly welcome to sit at the table.

Mr. BEGGS. This is Frederic Schwartz, from my General Counsel's Office.

Mr. HEYWARD. In connection with section 8 and the assessment of the penalties, do you have any reaction as to whether or not the process of violation of permits should in some way authorize you to act rather than to enforce this through the Administrator in connection with violation of permits which you discover?

Mr. BEGGS. I believe, in light of our general statutory authority, we would have the power to enforce the act.

Mr. DINGELL. Captain, if you will yield just briefly, for purposes of laying out the legislative history on this matter.

Then what you are saying is that this bill would not affect your existing statutory authority in these areas, and that you have statutory authority in these areas to handle violations, for example, of the kind you meet in improper dumping or illegal dumping or dumping in violation of the administration's statute now before us?

Mr. BEGGS. Yes, sir; that is my understanding as well. While the assignment of the actual penalty under this act does lie with the Administrator of EPA, there are general enforcement powers in the Coast Guard under the existing statute.

Mr. DINGELL. Would you have authority sufficient to assess civil penalties or criminal penalties under your existing authority?

Mr. BEGGS. No, I think again that would have to go to the Administrator.

Mr. DINGELL. I would appreciate if you would review those and give us more deliberate thoughts, after you have had an opportunity to reflect on it more fully.

Mr. BEGGS. Yes, sir.

(The information follows:)

Section 6 of H.R. 4723 provides for both civil and criminal penalties. These penalties would be assessed by the Administrator of the Environmental Protection Agency, unless he chose to delegate all or part of that function.

Mr. HEYWARD. I have one question which I think is important, Mr. Chairman. That is in connection with the facilities for this surveillance and enforcement that Mr. Pelly alluded to.

I recognize that you may be able to do some part of the job in connection with other missions, provided you can schedule the dumps so that they can be surveyed. But I wonder whether or not you are not really saying that you are going to take on another job without additional resources.

Could you furnish the committee some idea, depending on the number of permits involved, the distance offshore, the frequency of the dumping that may be permitted, the hourly cost of plane surveillance to ascertain the adherence to the dump site, the vessel cost per day to escort vessels out to the dump site, so that the committee might have some idea of the amount of money they are talking about in connection with the total enforcement problem?

Mr. BEGGS. Yes. We will certainly do that.

I might make this comment, though. We don't envision that in every case there would have to be surveillance on the site. We believe that in

many cases administrative surveillance, that is, review of log books and navigation records, and so forth, would be sufficient to determine whether the dumping had taken place in the proper spot and under proper conditions.

So I do not believe that we are in a position where we will be unduly taxed at this time, but we certainly will furnish for the record the data you ask for.

(The information follows:)

We assume from 3,000 to 6,000 permits will be issued during the first year. Assuming we will conduct surveillance of all the toxic loads, but only a low percentage of all other loads, we believe this will require about 600 aircraft hours per year at a cost of $500 per hour and 350 ship days per year at a cost of about $3,500 per day. This comes to a total of approximately $1,350,000 per year for enforcement operations.

Mr. HEYWARD. May I ask whether or not, in connection with the inspection program of the Coast Guard, is there a possibility under the inspection program to take a look at the license of the vessel to actually carry out these dumps, such as the dump barges that operate out of New York, Philadelphia, and other ports?

Mr. BEGGS. Admiral Hammond, do you wish to comment on that?

Admiral HAMMOND. I am not sure I understand your question exactly.

Do you mean could we determine if the barge or dump vessel is actually licensed for this business?

Mr. HEYWARD. Yes.

Admiral HAMMOND. Certainly this is true concerning many barges and vessels. I am sure, however, that some small contractors would be engaging in dumping operations using vessels not so licensed.

Mr. HEYWARD. I was wondering how far the present inspection program might be extended, or whether under the present inspection program there might be a way to regulate the particular vessels that would be utilized in carrying out the dumping.

Perhaps if you could inquire and have a comment further for the committee.

Admiral HAMMOND. Fine.

(The information follows:)

The existing marine inspection program can be partially utilized to police the permit requirements, however, it is anticipated that a complete policing program would require the cooperative efforts of the Officer in Charge, Marine Inspection at the Captain of the Port of a given port. Another way to regulate particular vessels, such as dump barges, et cetera, would be to place a restriction on their Certificate of Inspection that would preclude operations without a permit.

Mr. HEYWARD. One final question. I am not sure that you are able to answer it, but I will take a chance.

In 6(e), the bill talks about forfeiture of vessels, and it says, "A vessel, except a public vessel . . . or other public property of a similar nature. . . ."

Do you have any idea of what the "other public property of a similar nature," which would have an in rem action against it, other than a vessel?

Admiral HAMMOND. The term "other public property of a similar nature" would, under the general definition, refer to federal, state,

county or municipally-owned facilities not used for commercial purposes. In this case, examples might be navy yards, army port facilities, NOAA shore facilities, government owned fuel farms, etc."

The only thing that comes to mind is a dredge or pipeline, something of that sort. Perhaps we can amplify that again for you.

Mr. DINGELL. Gentlemen, I have looked with interest on the Coast Guard facilities that you have listed to us, and it brings to mind several things.

First of all, the Reorganization Act requires this committee to submit with our bill a statement of the 5-year expenses anticipated under the legislation.

Since your statement does not indicate what your estimate for the 5-year cost is, I would appreciate it if you will submit to us a statement indicating the 5-year cost for this particular program.

(The statement follows:)

The estimated five year cost of the program would be $6,500,000. This includes personnel augmentation and aircraft and vessel operating costs. The first year is estimated to be $1,610,000, tapering off in the fourth and fifth years if dumping is curtailed as CEQ desires. This does not include costs of acquiring vessels or monitoring instruments.

I would secondly like to make a statement on which I would like your comment.

I think the Coast Guard is a fine service, one of the finest I have ever had the privilege of observing, but they are perhaps in terms of their total mission the most undermanned and underequipped that it has ever been my misfortune to look upon.

I was in Alaska, for example, an area where you have a coastline of 33,000 miles, longer than the coastline, I understand, of the rest of the United States. At the time you had, and, Admiral Hammond, you remember, because you were the officer up there, you had three aircraft, two of which were down, one for routine maintenance and one as a result of gusty landing. You had two buoy tenders, one high endurance cutter, and two low endurance cutters.

Admiral HAMMOND. We had a few more buoy tenders than that.

Mr. DINGELL. That was substantially your capacity to police the fishing problem, which is your major problem.

Perhaps, Mr. Secretary, you and the Admiral can give us some appreciation of how you are going to add this to your search and rescue responsibilities, the fish protection responsibility that you have, and how you are going to add this to the hospitalization chores that you undertake to get people to the hospital when it is indicated, so as to properly protect the ocean areas from illegal dumping in an area of that size, with that kind of resource.

I must tell you I don't see how in the heck you will be able to do it, but I am curious to hear your explanation on that matter.

Mr. BEGGS. Certainly the Coast Guard, as you point out, has very broad responsibilities in the area of search and rescue and navigation.

But it is our view that as this program starts up, we can handle the additional chores, where physical surveillance is necessary, within the current resources.

It was recognized that as the program developed in forthcoming years, we would very likely have to request additional resources. But

we do not have a clear idea at the present time of our total responsibilities under the Act or of what the Environmental Protection Agency might assign us in addition to that.

So we feel that we can start up on our current resource base and build from there. The monitoring activities, of course, are going forward at present under our general responsibility to gather oceanographic data.

Mr. DINGELL. Are you saying, then, that you are not going to ask for additional resources initially in regard to this particular program?

Mr. BEGGS. Not this year. That is correct.

Mr. DINGELL. Well, let us take Alaska, because that is the area, because of my study up there, I am perhaps the most familiar with.

I am of the flat opinion that your resources up there are grossly inadequate to the mission that is assigned to the Coast Guard at this particular time. I am curious as to how you are going to take on any additional responsibilities in that area.

I am satisfied that you are no better off in the rest of the United States.

If I were to take the trouble to go through on an item-by-item basis, discussing different Coast Guard facilities as listed in your statement, which the Chair will at this time insert by unanimous consent request at the appropriate point in the record, I am satisfied that each and every one of those would be hopelessly inadequate to the missions presently assigned to them, let alone discussing the additional responsibilities which this statute is going to impose.

I say this without criticism, because it is very clear in my mind that the Coast Guard is doing an outstanding effort under almost hopeless circumstances, almost hopelessly limited in terms of equipment, to carry out their existing responsibilities.

Yet this legislation would overlay a broad additional responsibility.

You are indicating to the committee that with this understaffed, undermaned, very fine agency you propose to take on additional very broad responsibilities.

I cannot look with much comfort on your statement that you think you are going to be able to do it initially. They will certainly try, but I am not satisfied that they will be able to do it.

I would like your comments. It would help the committee and the record.

Mr. BEGGS. I agree with you, Mr. Chairman. We are very proud of the Coast Guard and the way they have handled all of the challenges thrown at them. They have done a magnificent job.

Mr. DINGELL. The Bureau of the Budget does not seem to appreciate their work. I am not sure whether the Department of Transportation does.

I have not observed whether you good folks are at fault in this matter or not. I know where the major part of the responsibilities lies. That is in the laps of the Bureau of the Budget.

We have not gotten around the fact that you are hopelessly underequipped to carry out your responsibilities.

Mr. BEGGS. We have augmented the Coast Guard budget to some extent over the past 2 years for surveillance of oil pollution. There is additional money in the 1972 budget. We have added to the Coast Guard budget each of the last 2 years for additional equipment.

As I say, we are going to have to closely observe how far those resources stretch. If they do not stretch far enough, we will have to come back for more.

Mr. DINGELL. Just to be perfectly fair and I am not critical with you, because I understand your problems with the Bureau of the Budget, but you don't want me to walk away from here with the impression that the Coast Guard has resources to do really an adequate job of superintending this ocean dumping problem, any more than they have full capability to superintend any of the responsibilities that the Administration has given them by law and regulation; do you?

Mr. BEGGS. Again, I do not have right now an assessment of just how much this is going to entail in additional workload for the Coast Guard.

It may well be that when we size up everything required by the act, we will require more funds. But I don't think I can say that at the present time.

Mr. DINGELL. Do you have any idea of the number of dumping permits which will be issued under this, or the number of dumpings which take place? It is on the order of hundreds of thousands of tons.

Mr. BEGGS. I don't have any idea of the number of specific dumping permits that are involved, or, the number of surveillance missions that the Coast Guard will have to carry out.

Mr. DINGELL. I have less trouble about the rascals that are going to be dumping perhaps outside of their assigned areas than I have about the rascals that will be dumping without permits at all, or dumping substances for which they have no permit.

I am curious how many boardings are you going to be able to carry out to ascertain whether these people are actually dumping what they purport to dump.

Mr. BEGGS. Generally speaking, and I will ask the admiral to comment on this, our experience has been that if a statute requires that something be done in accordance with certain rules and regulations, the maritime industry and the folks who are associated with the operation of barges along the coast will comply. I think the more serious problem will be those who dump without permit.

Mr. DINGELL. That is right.

Let us take another for instance. You have another problem that is almost as discouraging, and that is the very obvious one of bilge pumping. One of our major problems is people pumping bilges up and down the coast. Nobody knows who does it. Nobody knows how you survey it. All of a sudden, you have oil on the beach.

Mr. BEGGS. We think the surveillance activity there can be carried out with aircraft.

As I stated earlier, we have requested additional resources to do that. I think the 1972 appropriations request contains an amount for six additional aircraft for the specific purpose of surveillance in the coastal contiguous zones.

Mr. DINGELL. Let me add this. I am not quarreling with you. I just want you to understand at this time that I am fully aware of the magnitude of the problem that you are taking on. I think you are. I hope you are.

I am not satisfied that your resources, despite your optimistic statements today, are going to be adequate even to the initial responsibilities, let alone the long-range responsibilities which you are undertaking in this particular piece of legislation.

I think you had better give careful thought to keeping this committee advised as to, one, adequacy of resources, and, two, changes in the law that will enable the Coast Guard to take on the responsibilities here.

Mr. BEGGS. We will do that, Mr. Chairman.

Mr. DINGELL. Gentlemen, I hope you don't take unkindly my remarks. Thank you very much for your presence. It is always a pleasure to see you here, Mr. Secretary, and, Admiral Hammond, who I regard as a conscientious and able public servant, and an old friend, it is particularly nice to see you.

Mr. BEGGS. Thank you, gentlemen.

Mr. DINGELL. Our last witness is James J. Reynolds, appearing here on behalf of the American Institute of Merchant Shipping.

Mr. Reynolds, we are happy to welcome you.

STATEMENT OF JAMES J. REYNOLDS, PRESIDENT, JOHN PROKOP, LEGISLATIVE ATTORNEY, AND O. LINCOLN CONE, STAFF MEMBER, AMERICAN INSTITUTE OF MERCHANT SHIPPING

Mr. REYNOLDS. On my left is John Prokop, legislative attorney with the American Institute of Merchant Shipping. On my right is O. Lincoln Cone, member of my staff, who is particularly conversant and expert in the field of navigation improvements, channels, et cetera.

Mr. Chairman, I will attempt to make this statement a little briefer in my presentation than it reads. I would like permission to submit the full statement, Mr. Chairman. I will attempt to shorten it out of forbearance for your patience and that of your colleagues.

Mr. DINGELL. Without objection, your full statement will appear in the record at this point, and we are happy to recognize you.

(Statement follows:)

STATEMENT OF JAMES J. REYNOLDS, PRESIDENT, AMERICAN INSTITUTE OF MERCHANT SHIPPING

My name is James J. Reynolds. I am President of the American Institute of Merchant Shipping. AIMS is a national trade association composed of 34 United States companies which own and operate about 500 oceangoing vessels of all types registered under the U.S. flag. These vessels aggregate approximately 8,300,000 deadweight tons and are engaged in the foreign and domestic trades of the United States.

We are very grateful for the opportunity afforded us of appearing before the above Subcommittees and presenting this statement of our views and recommendations relative to H.R. 4723, cited as the "Marine Protection Act of 1971."

Section 2 of H.R. 4723 states that "it is the policy of the United States to regulate the dumping of all types of material in the oceans, coastal, and other waters and to prevent or vigorously limit the dumping into the oceans, coastal, or other waters of any material which could adversely affect human health, welfare, or amenities, or the marine environment, ecological systems, or economic potentialities." Section 2 further states that to carry out the foregoing policy "it is the purpose of this Act to regulate the transportation of material from the United States for dumping into the oceans, coastal, or other waters, and the dumping of

material by any person from any source if the dumping occurs in waters over which the United States has jurisdiction."

At the outset I wish to state that the AIMS and its members companies wholeheartedly support the above policy and purpose of H.R. 4723 and it is our desire to cooperate in the accomplishment of this policy and purpose. In order to accomplish this policy and purpose, H.R. 4723 would transfer from the Secretary of the Army and Chief of Engineers of the Department of the Army to the Administrator of the Environmental Protection Agency the authority to issue permits for the (1) transportation of material from the United States for dumping in the oceans, coastal and other waters and (2) dumping of material in ocean, coastal and other waters which are within the territorial jurisdiction of the United States i.e. within the three mile limit, except as provided in 33 USC 441. The term "material" is defined in section 3(c) of the bill to mean among other things dredged spoil, rock and sand which result from deepening and widening the nation's waterways.

For about 147 years the U.S. Army Corps of Engineers has, since 1824, studied, investigated, recommended, constructed and maintained waterway improvement projects authorized by Congress and the President. Needless to say, the Corps of Engineers has acquired a great amount of knowledge, understanding and experience during this period of time in respect to determining suitable, economical and safe areas for the disposal of dredged material resulting from waterway improvements. Under the terms of H.R. 4723, however, the Secretary of the Army and Chief of Engineers could no longer make the foregoing determination with respect to disposal of dredged material. This determination would be made by the Administrator of the EPA and the Secretary of the Army and Chief of Engineers would be required to obtain a permit from the EPA Administrator for disposal of such dredged material at locations to be determined by the EPA Administrator.

The American Institute of Merchant Shipping is unalterably opposed to the transfer of the permit authority from the Secretary of the Army and Chief of Engineers to the Administrator of the EPA for the above purpose. We do not believe this transfer of the permit authority is necessary to achieve the policy and purpose set forth in H.R. 4723.

As you probably know, one of the major activities of AIMS is the initiation and accomplishment of navigation improvements in Federal channels in U.S. ports and waterways to more adequately accommodate large vessels, particualrly by tankers, dry bulk carriers, container ships, LASH (Lighter Aboard Ship) and Seabee ships. The deepening of channels enable these vessels to increase their cargo-carrying capacity and revenue earning capability, thereby achieving reductions in transportation costs. It is a well known fact that the cost of transportation is an important factor in determining the price of goods to consumers. Therefore, an increase or decrease in the transportation cost has a corresponding effect upon the consumer price structure.

The planning, construction and maintenance of the extensive and excellent system of waterways serving the transportation requirements of our country is a result of the expert and dedicated work which the Corps of Engineers has performed since 1824. The entire nation owes a great debt of gratitude for the fine job they have done and are still doing. For this reason. AIMS urges that no action be taken which would interfere with or delay the continued progress of the waterway improvement program under the direction of the Army Engineers.

Generally speaking, under existing laws and regulations all dredging, filling, erection of structures and depositing of refuse in the navigable waters of the United States is permitted only when recommended by the Chief of Engineers and authorized by the Secretary of the Army through issuance of a permit. The delegation of this permit authority to the Chief of Engineers and Secretary of the Army had its origin in the Act of June 29, 1888. The permit authority was considerably enlarged and etxended by the Act of March 3, 1899. The Corps of Engineers has therefore been exercising this permit authority for a period of 83 years and has acquired extensive experience and expertise in this area which is indispensible to the administration of the permit authority.

For many years the Corps of Engineers administered its authority to issue permits taking into consideration only the effect of the proposed work on navigation. However, following the enactment of the Fish and Wildlife Coordination Act of 1956, consideration given by the Corps of Engineers in connection with permit applications was extended to include the impact of proposed waterway improvements and deposits on fish and wildlife.

As the problems of water pollution and maintenance of water quality became of increasing concern, they have become significant factors in the evaluation of permit applications. The regulations of the Chief of Engineers governing issuance of permits now include requirements for evaluation of effects of the proposed Federal and non-Federal works, including disposal of dredged material, not only on navigation but also on fish and wildlife, water quality, pollution, conservation, aesthetics, ecology and other environmental factors. We wish to point out that the policy and practice of the Corps of Engineers of evaluating many of the foregoing factors were initiated *prior* to the enactment of the National Environmental Policy Act of 1969, approved by the President January 1, 1970 (Public Law 91-190). This Act has served to confirm the policy and practices of the Corps of Engineers of giving due weight to the preservation and enhancement of the quality of the environment in connection with the consideration of applications for permits for dredging, filling, erection of structures or depositing of refuse in navigable waters. Accordingly, the enactment of the National Environmental Policy Act is regarded as a strong affirmation of the administrative policy of the Chief of Engineers.

We wish to point out that section 123(a) of the River and Harbor Act of 1970 (Public Law 91-611) approved by the President December 31, 1970 provides that with respect to the Great Lakes and their connecting channels "the Secretary of the Army, acting through the Chief of Engineers, is authorized to construct, operate, and maintain * * * contained spoil disposal facilities of sufficient capacity for a period not to exceed ten years," and that "before establishing each such facility, the Secretary of the Army shall obtain the concurrence of appropriate local governments and shall consider the views and recommendations of the Administrator of the Environmental Protection Agency and shall comply with the requirements of section 21 of the Federal Water Pollution Control Act, and of the National Environmental Policy Act of 1969." Subsection (b) provides that the "Secretary of the Army, acting through the Chief of Engineers, shall establish the contained spoil disposal facilities authorized in subsection (a) at the earliest practicable date, taking into consideration the views and recommendations of the Administrator of the Environmental Protection Agency as to those areas which, in the Administrator's judgment, are most urgently in need of such facilities and pursuant to the requirements of the National Environmental Policy Act of 1969 and the Federal Water Pollution Control Act."

In addition, subsection (i) of section 123 of the River and Harbor Act of 1970 stipulates that "the Chief of Engineers, under the direction of the Secretary of the Army, is hereby authorized to extend to all navigable waters, co[illegible]ecting channels, tributary streams, other waters of the United States and [illegible]waters contiguous to the United States, a comprehensive program of research, study, and experimentation relating to dredged spoil. This program shall be carried out in cooperation with other Federal and State agencies, and shall include, but not be limited to, investigations on the characteristics of dredged spoil, and alternative methods of its disposal. To the extent that such study shall include the effects of such dredge spoil on water quality, the facilities and personnel of the Environmental Protection Agency shall be utilized."

Accordingly, in view of the above provisions of section 123(i) of the River and Harbor Act of 1970, and for reasons set forth in this statement, the American Institute of Merchant Shipping strongly urges that H.R. 4723 be amended to provide that the authority to issue permits for the transportation and disposal of dredged material resulting from waterway improvement projects shall be retained by the Secretary of the Army, acting through the Chief of Engineers, taking into consideration the views and recommendations of the Administrator of the Environmental Protection Agency in respect to criteria and guidelines to be followed in the seltcion of disposal areas.

Legislation similiar to that contained in section 123(a) and (b) for the Great Lakes should also be enacted to authorize the Secretary of the Army, acting through the Chief of Engineers, to construct, operate and maintain contained land spoil disposal facilities on other U.S. waterways, taking into consideration the views and recommendations of the EPA Administrator.

Our proposal for retention of the permit authority in the Secretary of the Army and Chief of Engineers is in accord with the delegation of authority made by the President himself to the Secretary of the Army in Executive Order 11574 issued under date of December 23, and published in the Federal Register of December 25, 1970. Under the terms of this Executive Order, the President specifically delegated to the Secretary of the Army the authority to administer the permit pro-

gram under section 13 of the Act of March 3, 1899, commonly known as the Refuse [illegible] (33 USC 407). Section 2(a)(1) of the President's Executive Order stipulates that the Secretary of the Army "after consultation with the Administrator [of the EPA] respecting water quality matters, issue and amend, as appropriate, regulations, procedures, and instructions for receiving, processing, and evaluating applications for permits pursuant to the authority of the Act." Paragraph (2) provides that the Secretary of the Army "shall be responsible for granting, denying, conditioning, revoking, or suspending Refuse Act permits." It is the position of AIMS that the foregoing procedure prescribed by the President in his Executive Order 11574 should be followed as the most practicable and expeditious method of considering and acting on dumping permit applications.

To transfer from the Secretary of the Army and Chief of Engineers to the Administrator of the EPA complete authority to issue permits for the disposal of dredged material resulting from waterway improvement projects, including dredging of access channels and berths to private facilities, would in our opinion seriously jeopardize the economic justification and progress of essential waterway improvement projects now under study or recommended by the Army Engineers and those which have been authorized by Congress, including perhaps those projects for which funds have already been appropriated.

While the Corps of Engineers has consistently endeavored throughout the years to develop and maintain a balanced evaluation of the effects of a waterway improvement project on navigation, industrial and economic growth, fish and wildlife, water quality, pollution, conservation, aesthetics, ecology and other environmental factors, we are of the opinion that because it is the primary function and concern of EPA to preserve the environment, no matter how laudable it may be, EPA will not be in a position to evaluate on an impartial and equitable basis all the foregoing factors related to a waterway improvement project. It is logical to conclude that from the standpoint of EPA environmental considerations will outweight all others by far and influence the EPA Administrator to require that dredged material be transported for disposal far at sea or to inland locations. In either case, the effect of such a requirement on projects under study or recommended by the Corps of Engineers or authorized by Congress would be to greatly increase the cost of such projects and thereby jeopardize their economic justification by adversely affecting the benefit-cost ratio. We have been reliably informed that for each 30 miles the dredged material is transported the cost of spoil disposal is doubled, thereby substantially increasing the cost of the waterway improvement project. If the material is ordered to be disposed at sea, it would be necessary to use oceangoing barges. Most of the barges now in use for transporting spoil disposal are not constructed for oceangoing operation. The cost of constructing oceangoing barges for spoil disposal at sea would be very substantial and would of course be added to the cost of the project.

Should the EPA Administrator take the above action with respect to disposal of dredged material, this would also seriously affect the continued maintenance of channels at their authorized project depths since the cost of such channel maintenance would be greatly increased. If equivalent appropriation increases are not provided annually in the President's budget and by Congress in Public Works Appropriation Acts, the maintenance of channels at their authorized project depths will not be possible and the estimated return to the Federal Government on its original investment in deepening of the channels, based primarily on savings in transportation costs, will not be realized. It is axiomatic that if channels are not maintained, the cargo-carrying capacity of vessels will be reduced due to reductions in draft occasioned by lack of channel maintenance, thereby causing an increase in transportation cost per ton of cargo. Such increased transportation costs are usually reflected in increases in the prices of goods and services to consumers. Thus it is the general public that will ultimately bear the burden of higher costs involved in the construction and maintenance of waterway improvements that may be caused by requirements imposed by the EPA Administrator for disposal of dredged material far at sea or at inland locations, rather than at for more economical waterway or shore locations adjacent to or in the vicinity of the river and harbor construction or maintenance projects, which for the most part is the present practice. The Corps of Engineers has endeavored to follow the latter practice in the interest of maintaining the cost of waterway improvement and maintenance projects at a minimum, thus helping to achieve a favorable benefit-cost ratio so as to establish the economic justification of improvement projects. We could not be sure that the Administrator of the EPA

would give appropriate consideration to project cost factors in the event the authority to issue dumping permits is transferred from the Secretary of the Army and Chief of Engineers to the EPA Administrator.

A case in point, which is typical of other projects, is the Baltimore Harbor and Channels, Maryland and Virginia, navigation improvement project which would primarily provide for the deepening of the channels through Chesapeake Bay into Baltimore Harbor from 42 to 50 feet mean low water. As you are aware, this project was authorized by the River and Harbor Act of 1970 with the stipulation, however, "that construction shall not be initiated until approved by the Secretary of the Army and the President." This project has not yet been cleared by the Office of Management and Budget, including the Bureau of the Budget. Until it is, the Secretary of the Army and the President cannot be expected to approve the project for construction.

We wish to call attention to the fact that one of the conditions of local cooperation stipulated by the Chief of Engineers, which has been accepted by the states of Virginia and Maryland, is that these States will "provide without cost to the United States * * * suitable areas *determined by the Chief of Engineers* to be required in the general public interest for initial and subsequent disposal of spoil, and also necessary retention dikes, bulkheads, and embankments therefor, or the costs of such retaining works" (emphasis supplied).

The Baltimore District Engineer in his report (page 53) on the Baltimore Harbor and Channels project states as follows: "Disposal in deep water in the Atlantic Ocean is planned for the material dredged from the Cape Henry Channel while disposal in deep water in Chesapeake Bay is planned for the York Spit and Rappahannock Shoal Channels." The Board of Public Works of the State of Maryland "has given assurance, * * * * * that disposal areas will be provided in the waters of Chesapeake Bay opposite Kent Island, or in overboard or diked areas near Baltimore Harbor, or in combinations of the two areas. *The cost estimates for the plans of improvement in Baltimore Harbor are based on disposal of the dredged material in Chesapeake Bay opposite Kent Island below the William Preston Lane, Jr. Memorial Bridge.* This area is considered to be economically equal to and representative of all the potential disposal areas, both diked and overboard. A final determination will be made at the time of preparation. The effect of such action would be to substantially increase the cost of the method of dredging" (Emphasis supplied).

According to the report of the District Engineer, the above plans for disposal of dredged spoil have been coordinated with the proper Federal agencies and concerned agencies of Virginia and Maryland.

The total estimated cost of the Baltimore Harbor and Channels improvement project is about $100,000,000 resulting in a benefit-cost ratio of 2 to 1. I would like to point out, however, that if H.R. 4723 is enacted in its present form, the Administrator of the EPA would then have the authority and power to revise the above spoil disposal plans outlined in the report of the Baltimore District Engineer as approved by the Chief of Engineers, and require that some or all of the dredged spoil be transported for disposal at sea or some other more costly location. The effect of such action would be to substantially increase the cost of the project. This would adversely affect the benefit-cost ratio and might endanger the economic justification of the project. This could create a problem with respect to clearance of the project by the Office of Management and Budget for approval by the President and Secretary of the Army for construction.

As I have previously stated, the U.S. Army Corps of Engineers is making every effort in consultation with the Environmental Protection Agency, Department of the Interior, Maritime Administration and other Federal, State and local government agencies to determine suitable locations for the disposal of dredged material resulting from waterway improvement projects. In our opinion, there is nothing to be gained and much to be risked from the standpoint of the formulation and progress of the waterway improvement program if the authority to determine spoil disposal sites is transferred from the Secretary of the Army and Chief of Engineers to the Administrator of the EPA. We therefore strongly reaffirm our recommendation that H.R. 4723 be amended to provide that the authority to issue permits for transportation and disposal of dredged material resulting from waterway improvement projects shall be retained by the Secretary of the Army and Chief of Engineers.

The Lake Carriers Association, representing companies operating U.S. flag ships on the Great Lakes, has endorsed our position and recommendation with respect to H.R. 4723.

62-513—71——24

BEST COPY AVAILABLE

The favorable consideration of our views and recommendations will be most helpful and appreciated.

Mr. REYNOLDS. Thank you very much, Mr. Chairman.

The association I represent represents some 34 major merchant marine companies owning and operating about 500 oceangoing vessels of all types registered under the U.S. flag. These vessels aggregate approximately 8,300,000 deadweight tons and are engaged in the foreign and domestic trades of the United States.

We are very grateful for the opportunity afforded us of appearing before your subcommittee and presenting this statement of our views and recommendations relative to H.R. 4723, cited as the "Marine Protection Act of 1971."

I am privileged, Mr. Chairman, to inform you that the Lake Carriers Association, which is the association representing U.S. flag carriers on the Great Lakes, endorses our observation and recommendations with respect to this bill today.

Mr. Chairman, I would find it difficult to contemplate any responsible American who would not applaud the basic objectives of this piece of legislation; namely, to limit the dumping into the oceans, coastal, and other waters of any material that could adversely affect human health, welfare, or the amenities, or the marine environment, ecological systems, or economic potentialities.

The objectives of the bill are superb, we support them completely. Our concern focuses on only one feature of the bill.

H.R. 4723 would transfer from the Secretary of the Army and Chief of Engineers of the Department of the Army to the Administrator of the Environmental Protection Agency the authority to issue permits for (1) transportation of material from the United States for dumping in he oceans, coastal, and other waters, and (2) dumping of material in ocean, coastal, and other waters which are within the territorial jurisdiction of the United States. That is, within the 3-mile limit except as provided in 33 U.S.C. 441.

The term "material," we note, is defined as meaning among other things dredged spoil, rock and sand which result from deepening and widening of the Nation's waterways.

Parenthetically, Mr. Chairman, of course, when we speak of the issuance of permits, we speak of the authority of the Corps of Engineers to recommend to the Secretary of the Army to issue permits not only to private parties, under contract to the corps or otherwise, but indeed to the Corps of Engineers, itself.

For some 147 years, the U.S. Army Corps of Engineers has studied, investigated, and recommended, constructed, et cetera, waterway improvements authorized by Congress and the President.

Needless to say, it acquired a great deal of knowledge, understanding, and experience during this period of time in respect to determining suitability, economical, and safe areas for the disposal of dredged material resulting from waterway improvement.

The planning, construction, and maintenance of the extensive and excellent system of waterways serving the transportation requirements of our country is the result of the expert and dedicated work which the corps has performed since 1824.

Indeed, I think you would agree with me the entire Nation owes a great debt of gratitude for the fine job they have done, and they are still doing.

BEST COPY AVAILABLE

Generally speaking, under existing laws and regulations, all dredging, filling, erection of structures, depositing of refuse in the navigable waters of the United States is permitted only when recommended by the Chief of Engineers and authorized by the Secretary of the Army through issuance of a permit.

This delegation of this permit authority to the Chief of Engineers and the Secretary of the Army had its origin in the act of June 29, 1888. The permit authority was considerably enlarged and extended with the act of March 3, 1899.

Therefore, the corps has been exercising this permit authority for a period of 83 years, and has certainly acquired extensive experience and expertise in this area.

For many years the Corps of Engineers administered its authority to issue permits taking into consideration only the effect of the proposed work on navigation. However, following the enactment of the Fish and Wildlife Coordination Act of 1956, consideration given by the Corps of Engineers in connection with permit applications was extended to include the impact of proposed waterway improvements and deposits on fish and wildlife.

As the problems of water pollution and maintenance of water quality became of increasing concern, they have become significant factors in the evaluation of permit applications. The regulations of the Chief of Engineers governing issuance of permits now include requirements for evaluation of effects of the proposed Federal and non-Federal works, including disposal of dredged material, not only on navigation but also on fish and wildlife, water quality, pollution, conservation, aesthetics, ecology, and other environmental factors.

We wish to point out that the policy and practice of the Corps of Engineers of evaluating many of the foregoing factors were initiated prior to the enactment of the National Environmental Policy Act of 1969, approved by the President January 1, 1970, Public Law 91-190.

This act has served to confirm the policy and practices of the Corps of Engineers of giving due weight to the preservation and enhancement of the quality of the environment in connection with the consideration of applications for permits for dredging, filling, erection of structures, or depositing of refuse in navigable waters.

Accordingly, the enactment of the National Environmental Policy Act is regarded as a strong affirmation of the administrative policy of the Chief of Engineers.

We wish to point out that section 123(a) of the River and Harbor Act of 1970 (Public Law 91-611) approved by the President December 31, 1970, provides that with respect to the Great Lakes and their connecting channels:

> The Secretary of the Army, acting through the Chief of Engineers, is authorized to construct, operate, and maintain * * * contained spoil disposal facilities of sufficient capacity for a period not to exceed ten years * * *

and that:

> Before establishing each such facility, the Secretary of the Army shall obtain the concurrence of appropriate local governments and shall consider the views and recommendations of the Administrator of the Environmental Protection Agency and shall comply with the requirements of section 21 of the Federal Water Pollution Control Act, and of the National Environmental Policy Act of 1969.

BEST COPY AVAILABLE

Subsection (b) provides that the:

Secretary of the Army, acting through the Chief of Engineers, shall establish the contained spoil disposal facilities authorized in subsection (a) at the earliest practicable date, taking into consideration the views and recommendations of the Administrator of the Environmental Protection Agency as to those areas which, in the Administrator's judgment, are most urgently in need of such facilities and pursuant to the requirements of the National Environmental Policy Act of 1969 and the Federal Water Pollution Control Act.

In addition, subsection (i) of section 123 of the River and Harbor Act of 1970 stipulates that:

The Chief of Engineers, under the direction of the Secretary of the Army, is hereby authorized to extend to all navigable waters, connecting channels, tributary streams, other waters of the United States and waters contiguous to the United States, a comprehensive program of research, study, and experimentation relating to dredged spoil. This program shall be carried out in cooperation with other Federal and State agencies, and shall include, but not be limited to, investigations on the characteristics of dredged spoil, and alternative methods of its disposal. To the extent that such study shall include the effects of such dredge spoil on water quality, the facilities and personnel of the Environmental Protection Agency shall be utilized.

Accordingly, in view of the above provisions of section 123(i) of the River and Harbor Act of 1970, and for reasons set forth in this statement, the American Institute of Merchant Shipping strongly urges that H.R. 4723 be amended to provide that the authority to issue permits for the transportation and disposal of dredged material resulting from waterway improvement projects shall be retained by the Secretary of the Army, acting through the Chief of Engineers, taking into consideration the views and recommendations of the Administrator of the Environmental Protection Agency in respect to criteria and guidelines to be followed in the selection of disposal areas.

Legislation similar to that contained in section 123 (a) and (b) for the Great Lakes should also be enacted to authorize the Secretary of the Army, acting through the Chief of Engineers, to construct, operate, and maintain contained land spoil disposal facilities on other U.S. waterways, taking into consideration the views and recommendations of the EPA Administrator.

Our proposal for retention of the permit authority in the Secretary of the Army and Chief of Engineers is in accord with the delegation of authority made by the President himself to the Secretary of the Army in Executive Order 11574 issued under date of December 23, and published in the Federal Register of December 25, 1970.

Under the terms of this Executive order, the President specifically delegated to the Secretary of the Army the authority to administer the permit program under section 13 of the act of March 3, 1899, commonly known as the Refuse Act, 33 U.S.C. 407. Section 2(a)(1) of the President's Executive order stipulates that the Secretary of the Army:

After consultation with the Administrator [of the EPA] respecting water quality matters, issue and amend, as appropriate, regulations, procedures, and instructions for receiving, processing, and evaluating applications for permits pursuant to the authority of the Act.

Paragraph (2) provides that the Secretary of the Army "shall be responsible for granting, denying, conditioning, revoking, or suspending Refuse Act permits."

[illegible]

It is the position of AIMS that the foregoing procedure prescribed by the President in his Executive Order 11574 should be followed as the most practicable and expeditious method of considering and acting on dumping permit applications.

To transfer from the Secretary of the Army and Chief of Engineers to the Administrator of the EPA complete authority to issue permits for the disposal of dredged material resulting from waterway improvement projects, including dredging of access channels and berths to private facilities, would in our opinion seriously jeopardize the economic justification and progress of essential waterway improvement projects now under study or recommended by the Army Engineers and those which have been authorized by Congress, including perhaps those projects for which funds have already been appropriated.

While the Corps of Engineers has consistently endeavored throughout the years to develop and maintain a balanced evaluation of the effects of a waterway improvement project on navigation, industrial and economic growth, fish and wildlife, water quality, pollution, conservation, esthetics, ecology, and other environmental factors, we are of the opinion that because it is the primary function and concern of EPA to preserve the environment, no matter how laudable it may be, EPA will not be in a position to evaluate on an impartial and equitable basis all the foregoing factors related to a waterway improvement project.

It is logical to conclude that from the standpoint of EPA, environmental considerations will outweigh all others by far, and influence the EPA Administrator to require that dredged material be transported for disposal far at sea or to inland locations.

In either case, the effect of such a requirement on projects under study or recommended by the Corps of Engineers or authorized by Congress would be to greatly increase the cost of such projects, and thereby jeopardize their economic justification by adversely affecting the benefit-cost ratio.

We have been reliably informed that for each 30 miles the dredged material is transported, the cost of spoil disposal is doubled, thereby substantially increasing the cost of the waterway improvement project.

If the material is ordered to be disposed at sea, it would be necessary to use oceangoing barges. Most of the barges now in use for transporting spoil disposal are not constructed for oceangoing operation. The cost of constructing oceangoing barges for spoil disposal at sea would be very substantial and would, of course, be added to the cost of the project.

Should the EPA Administrator take the above action with respect to disposal of dredged material, this would also seriously affect the continued maintenance of channels at their authorized project depths, since the cost of such channel maintenance would be greatly increased.

If equivalent appropriation increases are not provided annually in the President's budget and by Congress in Public Works Appropriations Acts, the maintenance of channels at their authorized project depths will not be possible, and the estimated return to the Federal Government on its original investment in deepening of the channels, based primarily on savings in transportation costs, will not be realized. It is axiomatic that if channels are not maintained, the cargo-carrying

BEST COPY AVAILABLE

capacity of vessels will be reduced, due to reductions in draft occasioned by lack of channel maintenance, thereby causing an increase in transportation cost per ton of cargo.

Such increased transportation costs are usually reflected in increases in the prices of goods and services to consumers. Thus, it is the general public that will ultimately bear the burden of higher costs involved in the construction and maintenance of waterway improvements that may be caused by requirements imposed by the EPA Administrator for disposal of dredged material far at sea or at inland locations, rather than at far more economical waterway or shore locations adjacent to or in the vicinity of the river and harbor construction or maintenance projects, which for the most part is the present practice.

The Corps of Engineers has endeavored to follow the latter practice in the interest of maintaining the cost of waterway improvement and maintenance projects at a minimum, thus helping to achieve a favorable benefit-cost ratio so as to establish the economic justification of improvement projects.

We could not be sure that the Administrator of the EPA would give appropriate consideration to project cost factors in the event the authority to issue dumping permits is transferred from the Secretary of the Army and Chief of Engineers to the EPA Administrator.

A case in point, which is typical of other projects, is the Baltimore Harbor and channels, Md. and Va., navigation improvement project, which would primarily provide for the deepening of the channels through Chesapeake Bay into Baltimore Harbor from 42 to 50 feet mean low water.

As you are aware, this project was authorized by the River and Harbor Act of 1970, with the stipulation, however, "that construction shall not be initiated until approved by the Secretary of the Army and the President." This project has not yet been cleared by the Office of Management and Budget, including the Bureau of the Budget. Until it is, the Secretary of the Army and the President cannot be expected to approve the project for construction.

We wish to call attention to the fact that one of the conditions of local cooperation stipulated by the Chief of Engineers, which has been accepted by the States of Virginia and Maryland, is that these States will:

> Provide without cost to the United States * * * suitable areas determined by the Chief of Engineers to be required in the general public interest for initial and subsequent disposal of spoil, and also necessary retention dikes, bulkheads, and embankments therefor, or the costs of such retaining works.

The Baltimore district engineer in his report (p. 53) on the Baltimore Harbor and channels project states as follows:

> Disposal in deep water in the Atlantic Ocean is planned for the material dredged from the Cape Henry Channel while disposal in deep water in Chesapeake Bay is planned for the York Spit and Rappahannock Shoal Channels.

The Board of Public Works of the State of Maryland:

> Has given assurance, * * * that disposal areas will be provided in the waters of Chesapeake Bay opposite Kent Island, or in overboard or diked areas near Baltimore Harbor, or in combinations of the two areas. * * * *The cost estimates for the plans of improvement in Baltimore Harbor are based on disposal of the dredged material in Chesapeake Bay opposite Kent Island below the William Preston Lane, Jr. Memorial Bridge.* This area is considered to be economically equal to and representative of all the potential disposal areas, both diked and overboard. A final determination will be made at the time of preparation of

plans for the dredging, both as to the disposal areas to be used and the method of dredging. (Emphasis supplied.)

According to the report of the district engineer, the above plans for disposal of dredged spoil have been coordinated with the proper Federal agencies and concerned agencies of Virginia and Maryland.

The total estimated cost of the Baltimore Harbor and channels improvement project is about $100 million, resulting in a benefit-cost ratio of 2 to 1.

I would like to point out, however, that if H.R. 4723 is enacted in its present form, the Administrator of the EPA would then have the authority and power to revise the above spoil disposal plans outlined in the report of the Baltimore district engineer, as approved by the Chief of Engineers, and require that some or all of the dredged spoil be transported for disposal at sea or some other more costly location.

The effect of such action would be to substantially increase the cost of the project. This would adversely affect the benefit-cost ratio, and might endanger the economic justification of the project. This could create a problem with respect to clearance of the project by the Office of Management and Budget for approval by the President and Secretary of the Army for construction.

As I have previously stated, the U.S. Army Corps of Engineers is making every effort, in consultation with the Environmental Protection Agency, Department of the Interior, Maritime Administration, and other Federal, State, and local government agencies to determine suitable locations for the disposal of dredged material resulting from waterway improvement projects.

In our opinion, there is nothing to be gained, and much to be risked, from the standpoint of the formulation and progress of the waterway improvement program if the authority to determine spoil disposal sites is transferred from the Secretary of the Army and Chief of Engineers to the Administrator of the EPA.

We therefore strongly reaffirm our recommendation that H.R. 4722 be amended to provide that the authority to issue permits for transportation and disproval of dredged material resulting from waterway improvement projects shall be retained by the Secretary of the Army and Chief of Engineers.

In conclusion, I would say I believe the important thing is that it is the Corps of Engineers which has the basic expertise with respect to the needs of the waterways of this Nation, but what is of equal importance, on the basis of the policy and the objectives of this bill, is that before they decide on any disposal area, they consult with and get the advice of the Director of the Environmental Protection Administration.

I believe it is a matter of Government teamwork and consultation, but the utlimate decision should reside where it has been for some 84 years, and where it has been exercised responsibly.

I know full well the problems that people bring up, that spoils taken from harbors that have been despoiled by the outrages of our human irresponsibility and have been dropped in the seas, and have visited injury to marine ecology.

I believe that is a thing of the past. I don't believe that the Corps of Engineers, or any other agency at this moment in history, or in the future, is going to do anything which is going to be inconsistent with the basic objectives of this statute, Mr. Chairman.

Thank you.

Mr. DINGELL. Thank you very much, Mr. Reynolds.

Mr. Everett.

Mr. EVERETT. I have one question, Mr. Chairman.

Mr. Reynolds, is it your opinion that if this legislation passes, it is going to prohibit the dumping of dredged spoils back into some of the areas where they are presently being dumped?

Mr. REYNOLDS. Mr. Everett, I think that it would only depend on whether or not the Administrator of EPA determines that it was a suitable place and site for their disposition.

If he so decided after due deliberation, then the spoil could be deposited exactly as it is being placed today. And, hopefully, that may be what will happen, because if my thesis is correct, that the Corps of Engineers is acting responsibly, and is using a whole list of criteria, and not just the economics of the situation, then I am sure there will be an agreement.

Mr. EVERETT. That brings up another point.

The National Environmental Policy Act, as well as the Fish and Wildlife Coordination Act, in essence requires the corps to meet the same requirements that the Environmental Protection Agency will have to meet under the bill.

So, in effect, these areas that you are probably dumping in now are probably already spoiled, and of no further use for fish and wildlife resources, or for sound ecological programs. It might be that after the study is made, many of these areas over which you are concerned will still be available for dumping.

Mr. REYNOLDS. This may very well be, Mr. Everett, but no one can be certain of that.

Mr. DINGELL. Could I ask at this point: How would you be differently served by having EPA issue the permit? With the corps doing essentially what this statute is going to impose on it, how would you be in any worse state if EPA issi ed the permit, using the same general criteria?

Mr. REYNOLDS. I think that the application of the criteria by an organization which for a generation has been engaged in the improvement of waterways of this Nation might conceivably, bluntly, come to a different result than if a group, very laudably and commendably given only the mission of preserving the ecology, were to apply the same criteria.

I think it is as blunt as that, Mr. Chairman.

I believe that the delay, the waiting for the permits, the evaluation of permits by individuals who are not attuned to the engineering methods, or the waterway needs, or the broad concepts of the need of the merchant marine and need of commerce, but only ecology, would add to cost and delay.

Maybe that is what is desired. I will be very frank about it. If the Nation is prepared to say that it is the ecology which shall be paramount, and all other considerations take second and third place, then one would say let us put the permit-issuing authority in the hands of the Administrator.

I think, frankly, it would be a mistake. I don't think there is that much of a monopoly on concern for the ecology.

I believe the Corps of Engineers share that view. I believe they have the same devotion to the basic objectives of this bill as the director of EPA.

Mr. DINGELL. Are there any further questions?

Captain Heyward.

Mr. HEYWARD. I would like to ask a couple of questions on section 11, Mr. Reynolds.

I am sure the Army Engineers tomorrow will probably be able to address themselves to the same problem. However, if I can straighten my own thinking out in connection with section 11, I don't think that we are talking about subsection (a) here in connection with your statement.

Is that correct?

Mr. REYNOLDS. No, we do not believe subsection (a) should be repealed.

Mr. HEYWARD. In connection with subsection (b), does your statement address itself to the repeal of subsection (b), which has to do with the New York Harbor refuse control?

Mr. REYNOLDS. We do not believe it should be repealed.

Mr. HEYWARD. I am merely asking whether or not the Engineers issue any dredging deposit permits presently under subsection (b), which is being repealed by this act.

It is my impression that they do not, but I may be mistaken.

Mr. REYNOLDS. Let me ask Mr. Cone.

Do you know whether any permits are presently issued under this statute of EPA?

Mr. CONE. One of the big points here is that the Corps of Engineers determines where the channel shall be dredged, and where the spoils shall be deposited at the present time, after taking into consideration the views of all the other Government agencies.

Mr. HEYWARD. I understand that, Mr. Cone, but I am really trying to focus our attention on what specific act we are talking about. As you are perfectly well aware, the Rivers and Harbors Act contains so many different features, as well as these other acts that we are talking about.

For instance, I call your attention to subsection (c), which repeals 407(a). We are not concerned with that. That is mine tailings, et cetera.

When you get down to subsection (e), this act supersedes the Refuse Act, this section 13, to the extent that it applies to dumping.

If you look at 407 in the act, you will find it does not prohibit operations in connection with the improvement of navigable waters or construction of public works. So, to the extent, it seems to me, that 407 is superseded, you are not superseding anything about public works.

Am I wrong in my interpretation?

Mr. CONE. You can not only look at this section 11. I think you have to turn back to section 7(c), subsection (c), paragraph 2 on page 11.

Mr. HEYWARD. I understand that. I was trying to narrow down the effect of these repealers first, before I get back over to the cooperation.

Mr. CONE. I realize what you are driving at.

I would like to call your attention to paragraph 2 on page 11, the proviso clause.

Mr. HEYWARD. I was coming to that next.

Mr. CONE. Which states that:

After the effective date of this Act, no Federal license or permit shall be issued under the authority of the Rivers and Harbors Act of 1899 to conduct any activity otherwise regulated by Section 4 of this Act and the regulations issued hereunder—

and so forth and so on.

Mr. HEYWARD. This says, "No Federal license or permit shall be issued under the authority of the Rivers and Harbors Act of 1899."

I am trying to get to what section of the Rivers and Harbors Act of 1899 are we talking about.

It is my impression that what we are talking about is section 419 of title 33. If we can narrow our attention to that, I think we could come up with——

Mr. CONE. You say 419?

Mr. HEYWARD. It is subsection (d) of section 11.

Mr. CONE. That is true. 419 of title 33 is applicable.

Mr. HEYWARD. That is the one under which I believe the Secretary of the Army is authorized to prescribe regulations concerning the dumping of dredging.

Mr. CONE. That is right.

Mr. HEYWARD. Now, if that is so, would you take a look at the second part of that, "whenever in his judgment such regulations are required in the interest of navigation."

Does that mean he does not have authority to issue regulations unless he finds it necessary in the interest of navigation?

I am not challenging your thesis, Mr. Reynolds, but I am trying to clarify what this bill is doing to the present law.

Mr. REYNOLDS. I appreciate that thoroughly.

Mr. HEYWARD. Maybe in pursuing your thesis, this bill needs some positive authority. If the committee accepted your thesis, to give the Engineers certain definite authority, but I am not sure in what you are saying, in merely not repealing some of these things, that you are really doing what you want to do, because I point out that under this act, depending on what you mean by "waters adjacent thereto," or what Congress meant, the Engineers do not now have general authority in the ocean dumping field beyond the navigable waters, except to the extent that their protection of waters on the Outer Continental Shelf gives them the authority in connection with the navigational protection and permit for construction.

But in some of those cases do I see authority in the dumping field, and this is what concerns me.

Mr. REYNOLDS. I see your point, because 419 only refers to the navigable waters or waters adjacent thereto. It does not get into contiguous waters.

Mr. HEYWARD. In connection with the EPA authority under this act, which requires the administrator to consult with the Secretary of the Army, and no permit shall be issued if the Secretary of the Army determines that navigation will be unreasonably impaired, does that not protect what 419 authorized the Secretary of the Army to do, to protect navigation?

Mr. REYNOLDS. I question that that would do it.

Indeed, he has the responsibility to consult with the Secretary of the Army to determine that there will be no impairment of the stream for navigation, but I think that is quite another matter, is it not?

Mr. HEYWARD. I agree with you, but I am suggesting that perhaps confining your testimony here today to authorize the Secretary of the Army to issue certain permits, that perhaps you might be ready to recommend some specific language other than these general repealers, and accept some whereases that we are faced with in section 7 and section 11.

I don't know about the committee, but it is very confusing to me.

Mr. CONE. This section 419 that you just mentioned, under that section the Army issues permits to applicants who want to dredge a private channel. They are all required to make applications for a permit.

Mr. HEYWARD. I think his authority under excavations you will find over in section 10 of the Rivers and Harbors Act is not disturbed by this legislation. That is section 403 of title 33.

Mr. CONE. Do you mean to imply that this bill does not withdraw from the Secretary of the Army the right to determine where channels shall be dredged, or the dredged spoil shall be put?

Mr. HEYWARD. I mean that this bill, in my opinion, does not affect the Secretary of the Army's authority to regulate any tpe of construction, dredging, or any other public works in the navigable waters of the United States.

The question that comes to my mind is whether or not it is superseding something that the Army presently has in connection with deposit of the results of the dredging, which is a different matter, which should be retained by the Army.

I am not implying anything, Mr. Cone. I am asking for your expert testimony to straighten out some of my confusion.

Mr. CONE. The Army Engineers at the present time determines where the dredge spoil from the channel improvement which they are dredging is to be put.

Mr. DINGELL. Yes, but this is subject to the fact they only have it in navigable waters of the United States. This is subject to the fact that it is highly doubtful whether the corps has jurisdiction over dumping or transporting for the purpose of dumping on the high seas outside the 3-mile limit, or outside the 12-mile limit, depending on how far one may interpret the navigable waters of the United States to extend.

Mr. CONE. They do have authority to determine dumping outside the 3-mile limit in three areas only. That is New York, Baltimore, and Norfolk.

Mr. DINGELL. And that is all?

Mr. CONE. That is all. In those cases, I forget the section number, but the supervisor of the harbor can extend the jurisdiction out into the ocean.

Mr. DINGELL. But as Captain Heyward has indicated, they have never issued a permit in those areas for dumping of——

Mr. CONE. They don't have to issue the permits to themselves, because they are doing the dredging, the Army Engineers are doing the dredging.

Mr. DINGELL. The Army Engineers do not do the dredging. The Army Engineers do it through contractors, in most instances. Sometimes they do their own dredging, but rarely do they do so.

Mr. CONE. On a Federal project, the Army Engineers award the dredging contract to a contractor. That is true.

Mr. DINGELL. Very little maintenance dredging is done by the corps.

Mr. CONE. That is true.

Actually, as a practical matter, there is no permit, as I understand it, involved, because the Army Engineers will award the contract to a dredging contractor, and that is that.

Mr. REYNOLDS. I think the point you have raised is very well taken, sir. I most respectfully suggest it might be well to explore with the Corps of Engineers people and find out exactly what their authority is, and what it is not.

I have been assuming, on the basis of my reading of the enabling statute, that they had all this authority. It has been my approach that they should retain it, and possibly maybe something more is needed, if indeed it is a valid assumption that they should have the authority vis-a-vis the Administrator in this one particular area.

Mr. HEYWARD. That is the point I was bringing up.

Mr. REYNOLDS. It is a good point.

The real measure, it seems to me, is the problem of controlling permits for the disposal of industrial waste, the human waste and municipal waste.

All we are concerned about is this one specific area, Mr. Chairman. I appreciate your patience in listening to us.

Mr. DINGELL. We are very happy to have had you here.

The chairman wants to compliment Captain Heyward for a very useful contribution to the hearings.

The chairman will appreciate, gentlemen, that after we have had an opportunity to discuss these matters in more detail with the corps, as we shall do, as I understand it, tomorrow, it will probably be very helpful if you will give us your further thoughts in writing in regard to this.

I don't want you to take my comments today as commitment that I will do anything one way or the other.

I think it would be helpful to the committee if you would give us your judgment in the light of such additional testimony and information we receive not only as a result of this afternoon's deliberations, but also after we have heard tomorrow the corps' responsibilities, and so forth.

Mr. REYNOLDS. Thank you very much, Mr. Chairman.

Mr. DINGELL. Gentlemen, we are grateful to you.

The Chair has received two papers on the subject of the risks involved to the oceans from radioactive pollution, and without objection, these papers will be placed in the record at this point. The author of these papers, Dr. Jerold Lowenstein, is a well-known expert on the subject, being simultaneously a qualified physician and nuclear physicist. Also, and particularly significant, in the light of the scope of these hearings, I should add that Dr. Lowenstein is a director of the Oceanic Society, a group providing an important voice in the dialog on the protection of the oceans.

If there is no further business to come before the committee, the committee will stand adjourned until 10 o'clock tomorrow morning.

(The documents mentioned follows:)

RISKS OF RADIOACTIVE POLLUTION OF THE OCEANS—JEROLD M. LOWENSTEIN, ASSOCIATE CLINICAL PROFESSOR OF MEDICINE, RADIOACTIVITY RESEARCH CENTER, UNIVERSITY OF CALIFORNIA, SAN FRANCISCO, CALIFORNIA, U.S.A., MEMBER, BOARD OF DIRECTORS OCEANIC SOCIETY—PRESENTED AT INTERNATIONAL COLLOQUIUM ON THE EXPLORATION OF THE OCEANS, BORDEAUX, FRANCE, MARCH 9–12, 1971.

At a recent meeting of the United Nations Food and Agriculture Organization in Rome, it was reported that all types of pollutants in the ocean are increasing except for radioactivity. The report was widely disseminated in the news media and probably reassured millions of people who are concerned about the dangers of radioactivity in the environment. This happy situation can last only a short time. The major source of radioactive pollution of the oceans now is nuclear fallout due to atomic weapons testing in the atmosphere, which has been decreasing for the past ten years, since the U.S. and the U.S.S.R. stopped atmospheric testing. Very soon, however, this trend will be reversed because of resumed atmospheric testing of nuclear weapons by other nations, and because of the increasing numbers of nuclear power plants and nuclear ships in operation. Within a generation, we may be seeing serious radiation effects on ocean ecology and human health.

Due to fallout that will continue for another generation from nuclear weapons already tested, the world's oceans have already been contaminated with approximately twenty million curies of strontium-90 and cesium-137, isotopes with half-lives of thirty years, which enter the metabolic cycles of all living organisms (1). There are, at present, measurable amounts of these two radioactive isotopes in all living creatures, including man. There is considerable scientific controversy as to the "safe" concentration of these materials, or whether there is a safe concentration. But it is important to realize that if at some point we should decide that the "safe" concentration has been exceeded, we must then wait at least 30 years for that amount to be reduced by fifty percent.

Present levels, whether safe or not, are low indeed compared with those that may be projected to the end of the century. Until now, nuclear power has been largely experimental, but by 1980 there are expected to be about 100 plants of 1000 megawatt capacity in operation. Under present U.S. regulations, the allowable release of radioactive materials into coolant water will be 22,000 curies per year. The nuclear industry claims it is only releasing 2.5% of the allowed amount, but it violently opposes any downward change in the regulations. The direct discharge, however, accounts for less than one hundred millionth of the total radioactive wastes, which are either stored in tanks as corrosive liquids that will boil for more than a hundred years, or incorporated into glassy materials and stored in abandoned salt mines. By 1980, it is estimated that ten trillion curies of accumulated wastes will be stored, of which one trillion will be strontium-90 (2). Although precautions are taken to prevent these lethal and long-lived radioactive poisons from entering the environment, a number of storage tanks have already developed leaks, and the heat from wastes stored in salt mines have been observed to deform the walls of the mines and raise the ground temperature at the surface by several degrees. Inevitably some of these radioisotopes will find their way into the world's waters and into the hydrobiosphere.

What I have said so far takes the most optimistic view of future radioactive pollution, for it assumes that present U.S. standards will be adhered to, and that there will be no major accidents. But some other nations already have less rigorous controls of nuclear wastes, and it cannot be expected that developing nations, which are viewed as possible customers for nuclear power plants exported by the advanced nations, will adhere to waste disposal techniques which are expensive and require a high level of technology. With regard to accidents, even at the present minimum stage of nuclear activity, there have been several major blow-ups (at Windscale, England; Chalk River, Canada; and Colorado, U.S.A., for examples) which spilled vast quantities of radioisotopes into the surrounding areas. With the extreme safety-consciousness of the nuclear industry, I think it fair to predict that major accidents will be rare—but serious, for unlike other industrial explosions, the radioactive hazard may persist for months

or years. As most nuclear plants are being built along rivers or the sea, the aquatic environment is the most vulnerable.

Nuclear shipping presents an even more direct threat to the aquatic environment. Not only does it discharge fission products into the water, especially during warmup, but a nuclear vessel carries all its radioactive power source and radioactive wastes with it, and in case of accident, the entire amount event[illegible]lly may go into the ocean. Two U.S. and one Soviet nuclear submarine have al[illegible]ady been lost, with millions of curies of fission products on board. Although the reactors of these vessels are strongly contained so as to prevent accidental release, it seems likely that over many years these corrosive radioactive wastes, with half-lives of thirty, a hundred, or a thousand years, will escape into the sea. Collisions in closed harbors, where most such accidents occur, could endanger large population centers and result in closure of a harbor to commercial activities for months or years.

From these sources—continued fallout, effluents and wastes from nuclear power and nuclear shipping—we see the prospect of steadily rising radioactive pollution of the ocean for several decades. During the next ten years, there will be a tenfold increase in the production of radioactive wastes and, as yet, there are no international agreements limiting the disposal of these wastes into the oceans.

So violent are the disagreements among scientists regarding the biological hazards of radioactivity, that the general public has become quite confused. Citizens' groups in the U.S. have succeeded in blocking the construction of several nuclear power plants and are fighting legal battles against several others. The power companies have counterattacked by a massive advertising campaign to persuade the public that nuclear power is safer and cleaner than conventional power.

What are the facts?

As usual, they are complex enough to provide argum[illegible]nts for both sides.

Many aquatic organisms concentrate radioactive elements. Oysters, for example, have been observed to concentrate zinc-65, a common fission product, by a factor of 250,000 over its level in the surrounding water, and cobalt-60 by a factor of a million. Certain edible seaweeds concentrate iodine-131. Other typical radionuclides which may be avidly incorporated by aquatic organisms are tritium, chromium-51, iron-59, manganese-54, as well as the familiar cesium-137 and strontium-90. Some investigators point out that though the concentration factors may be high, the absolute amounts of the radionuclides in sea animals and plants are still small, and that one would have to eat very large amounts of any species in order to exceed the "allowable limit" for a particular isotope. A scientist studying zinc-65 concentration in oysters near the Humboldt Bay nuclear reactor in California claimed that one would have to eat nothing but oysters in order to exceed the "allowable limit" of zinc-65.

Other scientists insist that many of the present limits are set too high, that they are based on ignorance of the detailed, or long-term effects of the radionuclide. For example, more recent studies of zinc-65 in rabbits have revealed that though the animals appeared well after small daily doses for several months, more sensitive studies showed deleterious effects on a number of proteins, on blood clotting and on immunity from disease (3).

In humans, the allowable limits of radiation have been reduced progessively, as effects have been observed at lower and lower levels. For example, the permitted total body dose to radiation workers was set at 2500 rem/yr in 1902 (this is about three times the mean lethal dose, if given all at once); it was reduced to 100 in 1925, to 25 in 1936, to 5 in 1955; and Gofman and Tamplin believe it should be reduced now to 0.5, which is just the value that the previous downhill slope would predict for 1970! (4)

Gofman and Tamplin, vocal critics of the present radiation standards, have compiled voluminous evidence that there are increased rates of cancer and leukemia at currently permitted radiation levels (5). Other experts have denied this and supported the concept of a "threshold" radiation dose below which no ill effects occur. Against the "threshold" concept and suporting the Gofman and Tamplin view, is a recent study by Stewart and Kneale, in England, showing that children whose mothers had x-rays taken while pregnant are more likely than other children to develop cancer, and that the probability increases with the number of x-ray pictures taken (6). The radiation dose in these cases is extremely small and, until the time of this study, were considered completely safe for humans at any age. It appears now that unborn babies and infants may be

a hundred times more sensitive than adults to the carcinogenic effects of radiation.

As with DDT, the direct effects on man may prove to be less important than the indirect ecological impact due to eradication of vulnerable species. Among the disastrous effects of DDT, discovered many years after it was pronounced a safe insecticide, are the fragility it induces in some birds' eggs, such as the pelican's which in certain areas are now threatened with extinction. The Soviet biologist Polikarpov has observed that extremely low concentrations of strontium-90 cause abnormalities, especially in the spinal cords, of developing fish eggs. Almost all fish eggs float in the upper five centimeters of sea water, where they are vulnerable to fallout and industrial discharge (1). Polikarpov predicts that one result of radioactive pollution will be to shift the ecological balance from more radiosensitive species like fish to less sensitive species like plants. Whether or not some commercial species of fish will be wiped out by radiation, as this line of research suggests, the possibility itself illustrates, in analogy with DDT, that trace amounts of pollutants can cause unexpected catastrophes by breaking a weak link of the ecological chain.

Radiation does literally break a link in the helical chain that transmits genetic information, causing abnormalities and depth in descendants. These effects, while they occur at the lowest levels of radiation, may not become apparent for several generations. Therefore some of the most serious delayed consequences of radioactive pollution may not appear for ten to fifty years in affected species, which includes all species on earth. It can be argued that some mutations are useful, that improved strains of food plants have been produced by deliberate irradiation of seeds, that the process of evolution may ultimately depend on radiation-induced mutations (7). But the ratio of harmful tc useful mutations is at least a million to one, so radioactive pollution constitutes genetic experimentation on a global scale, with unpredictable consequences to all life on earth.

I am very much disturbed by the massive advertising campaign which has been launched by power companies in the United States, aimed at convincing the public that nuclear power is clean, virtually free of radiation, good for the environment, and necessary to meet the power demands which their advertising has helped to create. The parallel with the cigarette companies, which for years made unsupported health claims for their products, and have persisted in their promotional efforts despite the proved carcinogenic and other disease-inducing results of smoking, are only too striking. It seems to me grossly irresponsible to substitute the techniques of mass persuasion for the scientific investigation and careful search for answers which only many years of experience and observation will assure. In the meantime, restraint and careful planning in nuclear exploitation of the oceans, and worldwide agreements limiting radioactive pollution, are urgently needed.

REFERENCES

(1) Polikarpov, GG, "Radioecology of Aquatic Organisms," Reinhold, 1966.
(2) Mawson, CA, "Management of Radioactive Wastes," Van Nostrand, 1965.
(3) Letavet, AA and Kurlandskaya, EB, Eds., "The Toxicology of Radioactive Substances, vol. 5, Zinc-65," Pergamon Press, 1970.
(4) Schubert, J and Lapp, RE, "Global Radiation Limits," Bull. Atomic Scientists 14-23, January 1958.
(5) Gofman, JW and Tamplin, AR, "Radiation, Cancer and Environmental Health," Hospital Practice, p. 71, October 1970.
(6) Stewart, Alice and Kneale, GW, "Radiation dose effects in relation to obstetric x-rays and childhood cancers." The Lancet 1: 1185, June 6. 1970.
(7) Schultz, V et al., Eds., "Radioecology," Reinhold, 1961.

RADIOACTIVE POLLUTION OF THE SEAS—Jerold M. Lowenstein, Associate Clinical Professor of Medicine, Radioactivity Research Center, Univerisity of California, San Francisco, California, U.S.A. Member, Board of Directors, Oceanic Society—Presented on July 1, 1970, at the International Convocation, Bacem in Maribus, Malta

The first decade of intense exploitation of nuclear power, during which there will be a ten-fold increase in production of radioactive wastes, begins with no international agreements regarding the disposal of these wastes into the seas, wth inadequate but ominous data regarding their effects on aquatic organisms, and with mounting evidence that the current permissible radiation doses in man

are too high and would, if given to large segments of the population, result in thousands of cases [illegible] cancer and leukemia, and inestimable numbers of genetic defects being passed on to future generations.

In fact, the major impact of radioactive pollution will be felt by future generations, both because the projected production of radioactive wastes ascends steeply to and beyond the end of this century, and because the genetic and ecological effects of low level radiation emerge slowly. When they do emerge, it will be too late to do anything about it: the most troublesome waste, strontium-90, has a half-life of 30 years, and others go into the hundreds and thousands of years.

Strontium-90 levels in the Irish Sea, due to the nuclear power station at Windscale, are already about a hundred times as high as in most other waters, in a concentration range where the Soviet biologist Polikarpov (1) observed abnormalities, especially in the spinal cords, of developing fish eggs. He concluded that one effect of radioactive pollution will be to shift the ecological balance from more radiosensitive species like fish to less sensitive species like plants. As in terrestrial ecology, the more highly developed organisms are the first eliminated by radiation (2).

Aside from nuclear power plants, the principle sources of radioactive pollution will be radioactive fallout from nuclear weapons tests in the atmosphere, and nuclear shipping. As a result of nuclear weapons tests more than a decade ago by the U.S.A. and the U.S.S.R., the world's oceans were contaminated with approximately twenty million curies of strontium-90 and cesium-137 (1), and the fallout will continue for another generation, supplemented by the current nuclear weapons tests of France and China and those of other nations when they are ready. When one considers that Polikarpov observed deleterious effects on fish eggs at ^{90}Sr concentrations of 10^{-12} curies/liter, and that most fish eggs are in the upper five centimeters of seawater, the most vulnerable to fallout, the potential for ecological disaster may be appreciated.

Nuclear shipping represents hazards of yet another magnitude. Radioactive wastes discharged directly into the water by nuclear plants and nuclear ships are only about one-millionth of the total fission product produced in these reactors. The remainder are buried or stored in tanks as corrosive liquids that will boil for more than a hundred years (3). Two serious "blow-ups" have occured in stationary nuclear reactors—at Chalk River, Canada, and Windscale, England—that resulted in thousands of curies of isotopes being released to the environment. With ships, accidents are inevitable, and can be expected to become increasingly frequent as more and more vessels are nuclear powered. The loss of the U.S. Nuclear Submarine Thresher in 1963 may have released a million curies of fission products · enough, according to Polikarpov, to contaminate a volume of water as great as both the Black and the Irish Seas. Such a catastrophe within a closed harbor, where most shipping accidents ocur, could result in closure of the harbor to all commercial activities for years: In the open sea, the effects will not be as immediate but merely add to the increasing radiation burden of all sea life.

The proponents of nuclear power consistently minimize the risks, though their reassuring statements often have the opposite effect; as, for instance this one, from Mawson, in his book on Management of Radioactive Wastes (3), writing in 1965 regarding nuclear ships: "Liberation of radioactive material into the confined waters of harbors would appear very hazardous, and liberation at sea would contaminate an international resource, with no control other than that of the captain of the ship. However, similar problems concerning the discharge of fuel oil have been faced with considerable success . . ."

The nuclear power advocates, which include the U.S. Atomic Energy Commission, point out that effluents from reactors do not exceed the maximum permissible concentrations set as safe by the Federal Radiation Council. But aquatic organisms may concentrate trace amounts of radioactive materials a thousandfold or more. An example occurred in a worker at the Hanford reactor, which discharges large amounts of zinc-65 into the Columbia River. The worker was found somehow to have taken in excessive amounts of ^{65}Zn. Finally the source was traced not to industrial carelessness, but to some oysters he had eaten which came from the Pacific Ocean 250 miles away, and which contained 200,000 times as much radioactive zinc as the surrounding sea (1). We do not know how many such cases are occurring in people who are not routinely checked for absorption of fission products.

Another objection to the "safe limits" argument is the questionable adequacy of present radiation standards. Recently Gofman and Tamplin (4,5), and others,

have adduced impressive evidence that there are increased rates of cancer and leukemia at the present allowable total body dose for radiation workers of five rem per year. The permitted dose has been progressively reduced since the beginning of this century, as radiation effects were observed at lower and lower levels (6) : from 2500 rem/yr in 1902, to 100 in 1925, to 25 in 1936, to 5 in 1955. If one extrapolates this downslope to 1970, one predicts a reduction to 0.5, which is about what Gofman and Tamplin are suggesting! More and more it appears that there is no really safe dose of radiation, but the risk is proportional to the dose.

What, then, is an acceptable risk? It seems to me that there is a critical difference between a risk which one chooses, in order to gain a larger benefit, such as having an x-ray taken, or working professionally with radioactive materials, as I do, and the situation with which we are now confronted, in which every living thing on the land, in the air, and on and under the sea, is being poisoned with radioactive wastes because some decision-makers, in some countries, have decided that their people must have atomic bombs and atomic power, regardless of the consequences to the environment.

Polikarpov concluded his book, Radioecology of Aquatic Organisms: "It has been shown that further radiocative contamination of the seas and oceans is inadmissible, because it entails great risk of a) producing irreversible changes in the hydrobiosphere, b) disrupting the resources upon which the fisheries depend and c) producing dangerous levels of contamination in the marine organisms consumed by man. To avoid these radiation consequences, it is essential to end all nuclear weapons tests and the dumping of liquid and solid radioactive waste into the seas and oceans."

What can we do to save this great "common heritage of mankind" from further contamination?

The most hopeful signs I see in a generally discouraging world trend are conferences such as this one, exploring the possibility of an Ocean Regime, that would involve agreement among all nations not to turn the seas into a radioactive sump (7,8,9). Just as international fishing agreements are necessary in order to save desirable species from extinction, limitations on waste disposal will benefit all nations by preserving healthy aquatic species (10). The frustrated individual, confronted with reckless industrial and military interests (11) and their enormous money and power, can join conservation organizations, such as the Oceanic Society, which I represent here, and make their voices heard. Collective action has proved to be effective at times, as in blocking the building of a reactor at Bodega Bay, California, on the San Andreas Fault. At stake is the health of the oceans, on which all life depends.

References

1. Polikarpov, GG, Radioecology of Aquatic Organisms, Reinhold, 1966.
2. Woodwell, GM, The ecological effects of radiation, Scientific American, 221: issue of Sept. 1969.
3. Mawson, CA, Management of Radioactive Wastes, Van Nostrand, 1965.
4. Gofman, JW, and Tamplin, AR, A proposal for at least a Ten-fold reduction in the FRC guidelines for radiation exposure to the population-at-large. Testimony at hearings of the Committee on Atomic Energy 91st Congress, Jan. 28, 1970.
5. Lawson, HG, Nuclear Split, an account of the Gofman-Tamplin AEC controversy, The Wall Street Journal, May 20, 1970.
6. Schubert, J, and Lapp, RE, Global Radiation Limits, Bull. Atomic Scientists 14: 23, Jan. 1958.
7. Brynielsson, H, Radioactive Waste Disposal into the Sea, International Atomic Energy Agency Safety Series No. 5, Vienna, 1961.
8. Olson, TA, and Burgess, FJ, eds., Pollution and Marine Ecology, Interscience Publishers, 1967.
9. Hedgpeth, JW, The Oceans: World Sump, Environment 12: 40, 1970.
10. The Ocean, Scientific American 221; entire issue of Sept. 1969.
11. Inglis, DR, The Hazardous Industrial Atom (Book Review) Bull. Atomic Scientists 26: 50, Feb. 1970.

(Whereupon, at 6 p.m., the committee adjourned, to reconvene at 10 a.m., Wednesday, April 7, 1971.)

62-513—71——25

THIS PAGE INTENTIONALLY LEFT BLANK

OCEAN DUMPING OF WASTE MATERIALS

WEDNESDAY, APRIL 7, 1971

HOUSE OF REPRESENTATIVES,
JOINT SUBCOMMITTEES ON OCEANOGRAPHY
AND FISHERIES AND WILDLIFE CONSERVATION,
Washington, D.C.

The joint subcommittees met, pursuant to adjournment, at 10 a.m., in room 1334, Longworth House Office Building, Hon. John D. Dingell (chairman of the Subcommittee on Fisheries and Wildlife Conservation) presiding.

Mr. DINGELL. The subcommittees will come to order.

This is a continuation of the hearings scheduled on the subject of ocean dumping and on legislation related thereto.

The Chair observes that we have a statement presented to the committee by our colleague, Michael Harrington of Massachusetts. Without objection, that document will be inserted in the record in its entirety.

STATEMENT OF HON. MICHAEL J. HARRINGTON, A REPRESENTATIVE IN CONGRESS FROM THE STATE OF MASSACHUSETTS

Mr. HARRINGTON. Mr. Chairman, it is my pleasure to appear before these subcommittees for the second time on this most important subject of ocean dumping.

When I testified last July 28 before the Fisheries and Wildlife Conservation Subcommittee, I had hoped that some form of strong anti-dumping bill would be reported out of committee. That did not happen, but the hearings were valuable in bringing to public attention the danger in which we find ourselves—of losing our oceans to the pollution which is consuming our air and inland waters.

I am confident that a bill will come out of committee this year. Chairman Garmatz has introduced his own bill, as subcommittee Chairman Dingell, and there are several others, aside from my own, pending before the committee. All of these bills have merit, and all recognize the obvious need for legislation in this field.

We cannot wait any longer for firm and binding prohibitions against dumping ecologically harmful materials into our oceans. We are in the position of being able now to save our last remaining natural resource from certain destruction by pollution.

We are hearing more and more about the incredible value of our oceans. We hear that our food supply may eventually come in greater proportion from the ocean than from the land. Untapped mineral resources lie within these waters. As a source of oxygen and through

its interaction with the terrestrial ecosystem, a healthy ocean may well have critical importance for the survival of the human species.

Distinguished marine biologists are warning that unless we act, the current accelerating pace of ocean pollution could destroy significant life in the sea in 50 years or less. This would be catastrophic.

To halt this devastation, Congress will have to act immediately to legislate major new policies backed by a massive aid and action program.

If we are to legislate effectively to save our oceans, we need a bill which—at a minimum—includes the following four provisions:

1. The broadest definition of the waters to be covered under the act.
2. The strongest prohibitions against the dumping or discharge of ecologically harmful wastes made in conjunction with the establishment of strong standards before permission to dump is granted.
3. Stringent enforcement of those standards with all necessary authority provided.
4. Strong penalties—including fines and jail sentences for those who fail to comply with those standards.

I am testifying before you today in support of a bill which I believe contains the provisions which I have outlined. H.R. 805, 807, 808, 1329, and 2581 (all identical) will, if enacted, go a long way toward keeping our oceans clean. I am gratified by the fact that more than 65 of my colleagues have joined me in cosponsoring this legislation.

The committee is also considering legislation introduced by Congressman Garmatz, H.R. 4723. I endorse the basic concept of Chairman Garmatz' bill, but there are several changes which I would like to see incorporated in the bill before I could fully support it. I believe the changes are essential, and I urge the committee to enact them.

In some respects, my bill is similar or identical to Mr. Garmatz' bill. In other areas, we differ greatly. I would like to take the four requirements I believe are essential and compare my bill with that of Chairman Garmatz in those areas.

1. DEFINITION OF THE WATERS TO BE COVERED

Dumping of harmful substances should be prohibited not only in what we normally think of as ocean, but in all coastal waters. Salt marshes and other such "inland" tidal features, because they nourish and shelter many marine organisms, are as important to the marine ecosystem as open water areas.

Section 5B(a) of my bill defines:

> Oceans, coastal and othe rwaters as "oceans, gulf, bays, saltwater lagoons; saltwater harbors, other coastal waters where the tide ebbs and flows, the Great Lakes, and all waters in a zone contiguous to the United States extending to a line 12 nautical miles seaward from the baseline of the territorial sea as provided in article 24 of the Convention on the Territorial Sea and the Contiguous Zone."

This wording is identical to that found in Congressman Garmatz' bill.

2. STANDARDS AND PROHIBITIONS

Section 5B(b) of my bill would require the Administrator of the Environmental Protection Administration acting through the Fish

and Wildlife Service, and in consultation with the Army Chief of Engineers, to establish standards—

which apply to the deposit or discharge into the ocean, coastal and other waters of the United States of all industrial wastes, sludge, spoil and all other materials that might be harmful to the wildlife or wildlife resources or to the ecology of these waters.

This section gives the Administrator jurisdiction over all wastes entering the coastal waters.

I have included the U.S. Army Corps ot Engineers in the standard making, because they have some experience with ocean dumping, and the mechanism is already established for issuance of dumping permits by them under the provisions of the 1899 Refuse Act.

The Corps has not distinguished itself in its enforcement of the Refuse Act, but with the Presidential order of last December and the new regulations presently being reviewed—and most importantly with new legislation, which this committee is considering, mandating protection of our oceans—the Corps will have the mechanism ready to go to enforce antidumping regulations.

Congressman Garmatz' bill would have the Administrator of the EPA establish or revise criteria (section 5(a)) in consultation with the Secretaries of Commerce, Interior, State, Defense, Agriculture, Health, Education, and Welfare, and Transportation, the Atomic Energy Commission, and other appropriate Federal, State, and local officials.

I find this long list a little bewildering. The problem with the Government now is red tape and vested interests. Is it not time to allow the person in charge of protecting the environment some latitude in doing his job? Will not consultations with so many different agencies merely delay the setting of criteria? No one has done any kind of job in this area to date. We need less consultation and more action.

Congressman Garmatz' bill exempts "effluent from any outfall structure," and oil. I see no point in relying on the existing fragmented regulatory structure dealing with these types of waste. Oil spillage and effluent from sewer outfalls contribute heavily to the pollution of our most valuable recreational and commercial coastal waters. The problem of ocean pollution, from whatever source, deserves treatment with coherent, comprehensive legislation.

I consider the exemptions of oil and effluents from outfall structures to be one of the most serious deficiencies of the Garmatz bill. The exemption of these two pollutants is totally unacceptable.

The disposal of domestic wastes of all kinds into our coastal waters has introduced toxic heavy metals and organics into these waters. The result has been to lower the available oxygen content of the bottom water.

Our new technology has also created new kinds and larger amounts of material which must be disposed of. During the past 30 years we have disposed of many synthetic chemicals heretofore unknown.

These chemicals are foreign to organisms, and natural pathways of biodegradation are lacking or inefficient. Thus, many chemicals now dumped into our coastal waters enter the marine food chain and increase in density as they move through the chain until they become harmful to both marine and human life.

The problem is particularly acute when we look into the amount of municipal and industrial wastes being dumped.

The estuaries of this country are being fed approximately 30 billion gallons of sewage and industrial waste every day. In the New York City area, five of the 18 municipal systems are still pumping raw sewage into the heavily polluted waters. And, in Boston, five municipal systems dump 400 million gallons a day of only primary-treated sewage into the harbor.

The story is the same on the West Coast. In San Francisco Bay, for exam[illegible], about 700 million gallons a day of effluents are being poured in—[illegible]ith about half of these wastes being treated at the primary level.

The situation is intolerable now. But the October 1970, Council on Environmental Quality report stated that we can expect an increase of 50 percent by the year 2000 in sewage sludge generated in the coastal zone.

With tougher water quality standards and coastal area industrialization, we can expect a massive increase in pressure for industrial dumping at sea. Forty percent of the Nation's industry is in coastal regions right now.

We cannot duck this issue by omitting these effluents from the legislation before us, as does the Garmatz bill. My bill covers all materials harmful to the ecology of the waters.

But we also cannot expect the cities and towns of this country to build waste treatment facilities without additional Federal help. I would, therefore, urge the committee to recommend greater Federal aid to municipalities and industries for waste treatment.

We cannot legislate standards that are impossible to attain, nor can we be so remiss as to ignore the fact that these wastes are a major source of our ocean's pollution.

I therefore have two recommendations. First, it is my intention shortly to introduce legislation which will provide for 90 percent Federal funding of municipal waste treatment plants, and which will also provide advance funding for waste treatment planning grants.

Many of our cities are on the verge of bankruptcy. They cannot afford to pay 70 or 60, or even 50 percent of the cost of these treatment facilities. They must receive our help.

We supply a greater Federal share for urban renewal, mass transit, solid waste disposal, and law enforcement assistance than we do for municipal sewage treatment.

When this Nation decided that it required coast-to-coast highways, it did not expect the States or localities to bear the great portion of the cost. Instead, we established a Federal Highway Trust Fund, with the Federal Government putting up 90 percent of the cost.

We are faced now with a need as great or greater than our need for highways. Without clean water—and I include all inland waters as well as our oceans—we cannot survive. It is time we squarely faced that need and provided the funds required to build these municipal waste treatment plants. Ninety percent is what is needed.

Second, on January 22, 1971, I introduced the Industrial Water Pollution Abatement Loan Act, H.R. 806. This bill, presently pending before the Public Works Committee, would provide low-cost loans to marginal industries to build waste treatment facilities beyond their financial abilities. The cost of building a treatment plant in compli-

BEST COPY AVAILABLE

ance with State water quality standards would force some companies to go out of business.

My bill would allow the Administrator of EPA to provide loans to these marginal industries where he finds that:

(1) such business firm could not continue to operate competitively if it were required to bear the burden of the cost of such construction without such financial assistance, or (2) that other financing credit is not reasonably available to such business firm for the cost of such construction.

Of course, all loans made under this act would require that the treatment facility be in compliance with a comprehensive plan approved by the administrator for the abatement of water pollution in the city, town, or water pollution abatement district where the business form applying for the loan is located.

This coordinated plan of 90 percent Federal grants to municipalities and loans to marginal industries for waste treatment facilities, in conjunction with strong antidumping bills, would save our oceans.

H.R. 805 states that the standards established "shall be for the purpose of insuring that no damage to the natural environment and ecology including but not limited to marine and wildlife ecology of the ocean, coastal, and other waters of the United States will result from any such activity."

This language is considerably more stringent and explicit than section 5(a) of Congressman Garmatz' bill, which permits such dumping as "will not unreasonably degrade or unreasonably endanger human health, welfare, or amenities or the marine environment, ecological systems, or economic potentialities."

I would like to know, Mr. Chairman, what constitutes an unreasonable degradation of human health? We must realize that after we are through temporizing and equivocating on this issue, we are faced with a matter of human survival. There is only one standard that can be applied to the establishment of standards or the issuance of dumping permits—there must be no damage to the environment.

If we persist, as provided by section 5(a) (2) of Congressman Garmatz' bill, in shortsighted assessment of "the probable impact of issuing or denying permits on considerations affecting the public interest," our oceans will never be clean.

I am deeply disturbed by the lack of clarity of this language. Before I could support such legislation, I would have to know what is meant by not unreasonably degrade or unreasonably endanger human health. The language in H.R. 805 is, I believe, far more precise.

Another imprecise definition may be found in section 5(e) of Mr. Garmatz' bill which states that the administrator "may issue general permits for the transportation of dumping or dumping, or both of classes of materials which he determines will have a minimal impact considering the factors cited in subsection (a)." How do we define minimal? Certainly, mercury pollution was considered minimal until last year.

Can we afford to take the chance with other substances? Once again, H.R. 805 states that there shall be no environmental damage.

Because the environmental effects of many wastes are not yet perfectly understood it is important that no material be assumed safe for dumping without decisive scientific evidence.

BEST COPY AVAILABLE

As Dr. Max Blumer, senior scientist at Wood's Hole, notes:

> The marine food web is so involved and the biochemical processes necessary for the survival of every species are so complex that it is virtually impossible to foresee which species might be damaged by a certain persistent chemical. The award of the Nobel Prize to the discoverer of the insecticide DDT illustrates our ignorance in this area. Lacking sufficient foresight we need to be much more cautious in the use of persistent chemicals lest we disrupt inadvertently processes in the sea upon which our survival may depend.

Accordingly, section 5B(a) of my bill requires that the person wishing to dump sustain a burden of proof that the materials that are dumped will not endanger the natural environment of these waters and will meet any additional requirements as the Administrator of the EPA deems necessary for the orderly regulation of such authority.

I feel that placing the burden of proof on the dumper is an important part of this legislation. It is time that those who wish to dispose of refuse material be required to consider the ecological consequences of their actions.

The public must not be asked to assume the risk of environmental damage because there has been insufficient time to study the problem thoroughly.

The Federal Government should not have to do all the work in this one area. Dumping is a privilege—not a right. The right involved here is the right of every American to a decent environment. This is neither a new nor particularly startling concept.

I have introduced a constitutional amendment (H.J. Res. 522) to accomplish this end. The right to a decent environment is as basic as the right to life and liberty, for without a decent environment we can have neither.

Therefore, it is the obligation of the people and the Government to protect the environment as the Government.

Burden of proof does not require the person wishing to dump to prove beyond a shadow of a doubt that the materials will be harmless. Rather, burden of proof requires a preponderance of evidence which demonstrates that the dumper can abide by the standards.

In addition, this legislation takes into account the fact that in some locations materials can be dumped without harm to the waters, whereas the same materials would be harmful to other areas.

I have always felt that a unilateral prohibition against dumping was both unjust and unrealistic. Ocean currents in some areas will disperse most refuse material to the point where it does no harm. In other locations, however, the materials may stagnate.

H.R. 805 also provides that different amounts of the same type of refuse may be dumped in different locations. Each dumping site and material has its own particular characteristics and these must be taken into account, as they will have to be by the person wishing to dump. There are, of course, certain materials, such as mercury, which would not be dumped at all.

The standards set by the Administrator of the EPA and the burden of proof required by the dumper would effectively prohibit any dumping of such materials. Therefore, this section provides a flexible approach to the problem of dumping into the coastal waters.

BEST COPY AVAILABLE

Section 5B(c) of H.R. 805 provides that the standard established by the Administrator of the EPA shall be adopted and applied to all Federal and State authorities which have the right to issue authorizations to discharge or deposit material into these waters.

In his role as standardmaker, the Administrator would find it possible to establish longer term goals for reducing ocean pollution, as well as issuing standards for the immediate guidance of regulatory bodies.

I should point out that these provisions do not require every permit application to cross the Administrator's desk. My bill allows the retention of the present regulatory structure, subject to new standards established by the administator. Since these standards will be designated to prevent all environmental damage, it is not essential that the Administrator consult formally with the Secretary of every department, and with every State board.

3. Stringent enforcement:

Section 5B(d) requires that the standards apply to all parts of the Federal and State governments and all persons who have authorization from the State or its agency to deposit or discharge such materials into these waters.

Section 5B(e) permits the States to establish and enforce standards covering these activities within their jurisdiction only on the condition that the State standards are stricter than the Federal standards and that the States provide adequate procedures for enforcement. I believe this section is important because, as we have seen in the case of automobile pollution, many States have wished to enact stricter regulations than the Federal ones but have been unable to do so because Federal laws require that the Federal standards apply.

The Garmatz bill sets no minimum requirements for the States or territories. Although section 7(e) states that Federal standards could not preempt the requirements or liabilities imposed by States or territories, the State or territory could set a much less strict standard than the Federal Government's.

The oceans are interstate bodies of water. The currents carry pollution from one State seacoast to another. It is not fair to require strong standards of one State when another would be allowed to set weak ones. I therefore. recommend that the wording of sections 5B(d) and 5B(e) of H.R. 805 be incorporated into this bill.

Section 5B(f) of H.R. 805 provides that every State and Federal instrumentality and every person applying for authorization to discharge or otherwise dispose of any material into these waters maintain records, make reports. and provide whatever additional information the Administrator of the EPA needs to determine that the standards are being complied with. The Administrator may also, upon request, have access to these records.

Thus, we require that dumpers continually sustain a burden of proof that their activities are harmless. The Administrator may revoke a permit if the dumper fails to do so. Due process of law requires that a person whose permit is revoked is subsequently entitled to a hearing, but I feel that section 5(f) of Congressman Garmatz's bill. providing for formal hearings. before a permit may be revoked is unwise. No one has an inalienable right to dump wastes into the oceans.

Denying the Administrator decisive power over permits forces the public to bear much of the risks of non-compliance by dumpers.

Section 5B(g) of H.R. 805 provides that the Distric courts of the United States have jurisdiction to restrain violations of this act. The courts have subpena power and failure to obey the subpena may be punishable by a charge of contempt of court.

I endorse Section 6 (a), (b), and (d) of the Garmatz bill which provides for both criminal and civil penalties with the District courts having jurisdiction to grant relief as the equities of the case require. I would like to see the District courts having subpena power.

4. Punishment:

Section 5B (h) of H.R. 805 provides that each violation of these standards shall be punishable by a fine of not more than $10,000 nor less than $5,000. This means that each time refuse is dumped in violation of these standards, the violator is liable for this fine. In many cases, several dumpings or discharges occur per day and each instance is a violation punishable by the fine.

Mr. Garmatz's bill would provide for a civil penalty of $50,000 for each violation and a criminal penalty of "not more than $50,000 or imprisonment for not more than one year, or both."

I heartily endorse these higher fines and would amend my bill to include them. In my amendment, however, I would set a minimum fine in criminal penalties of $25,000, retaining the maximum $50,000. I would also increase the jail sentence to 5 years for repeated convictions. The minimum fine is a stiff one—but the destruction of our environment is a deadly serious matter. Stiff minimum fines would be a deterrent from the beginning.

I would also like to see amended the language of section 6(c) of H.R. 4723 which states, "For the purpose of imposing civil penalties and criminal fines under this section, each day of a continuing violation shall constitute a separate offense."

I realize that the differences between Chairman Garmatz's bill and mine are very great. This testimony has emphasized those differences but I want to say that there are many portions of H.R. 4723 which I applaud and support. We basically agree on the need for an end to ocean pollution, and this fundamental agreement is what is required for effective legislation.

I would hope that the committee will seriously consider making the change I have suggested in my testimony.

All forms of ocean pollution must be covered—including municipal and industrial effluents and oil. Without a coordinated and comprehensive program, we are never going to be able to regulate cleaner oceans.

The Federal Government must make standards which are applicable to the States—otherwise chaotic enforcement will ensue, and we could continue to have serious pollution problems throughout the country.

Most importantly, Mr. Speaker, our standard must be no pollution. We have already done irreparable damage to our waters through ignorance and laziness. We must develop alternate means of waste disposal and we need a far greater Federal investment in municipal and industrial waste treatment.

BEST COPY AVAILABLE

Everyone has the responsibility for saving our oceans and coastal waters. The dumper has an obligation to all of us to demonstrate that his material is not harmful. The Government has an equal responsibility to set and enforce standards for dumping.

We need a strong law—a clear law—a comprehensive law —to halt the degradation of our oceans and coastal environment. I urge the committee to report out a bill of this nature. We in Congress bear a primary responsibility for a decent environment. We have failed thus far to act to save our oceans and coastal waters.

We must act now.

Mr. DINGELL. The same unanimous request is made with regard to the statement of our colleague, Cornelius Gallagher.

STATEMENT OF HON. CORNELIUS E. GALLAGHER, A REPRESENTATIVE IN CONGRESS FROM THE STATE OF NEW JERSEY

Mr. GALLAGHER. Mr. Chairman, I am very pleased to have the opportunity to testify this afternoon before your distinguished commiteee on legislation to control the dumping of waste materials into our oceans. For far too long, we have looked to the oceans as an inexhaustible source for the disposal of side effects of man's technological abundance and urbanization. I contend, as have the many Members of the Congress who have appeared before you, that it is no longer possible.

The problem of ocean dumping is one in which I am particularly interested for two specific reasons: (1), in May 1969, the Subcommittee on International Organizations and Movements of the House Committee on Foreign Affairs, which I chaired at the time, held what is widely regarded as the first hearing on the dumping of any substance into the ocean; and (2), there are 250 major dumping areas nationwide; 15 of these areas are located off the coast of my home State of New Jersey. Two of these 15 areas have been closed to shellfish harvesting due to the impact of the dumpings.

The major feature of the bills I have cosponsored is that before any discharge can take place in any ocean an approval must be obtained from the Administrator of the Environmental Protection Agency. This may seem like a somewhat small step to take when so many in our land are speaking the language of doomsdaymanship and are freely predicting ecocatastrophies, but I think this is vital.

Let me illustrate the essential nature of fixing responsibility in the Federal Government for assessing the dangers of each ocean dump by describing what we learned during our Foreign Affairs Subcommittee consideration of plans to dump obsolete poison gas into the oceans.

This plan vitally affected the mandated responsibilities of the Department of Defense, the Department of State, the Department of Transportation, and the Department of the Interior. Each of these Federal Agencies had a particular responsibility in the transportation of the trainloads of poison gas across our Nation and in the disposition of the poison gas in the oceans.

Yet, the only time representatives of these Agencies ever gathered in the same room to discuss their mutual problem was in the hearing

room of the Subcommittee on International Organizations and Movements.

This was after 12 similar shipments had already taken place. Not only was the Congress in the dark, but the affected Federal Agencies had not been informed, either.

It may be of special interest to subcommittees of the Merchant Marine and Fisheries Committee that the only citizens who were informed of the movement of this lethal gas were recreational boaters who were warned to stay clear as the disposal ships were towed to sea.

Residents of the crowded urban areas which trains moved through were not informed, and this includes a city in my 13th District of New Jersey, Elizabeth. Fortunately, many changes have been made in national policy since our subcommittee took its pioneering actions, but I do believe that a significant weakness in Federal structure was disclosed.

That is why it is absolutely essential that one man and one agency be firmly fixed with the ultimate responsibility in this area. One staff should evaluate competing interests and competing proposals, and one decisionmaking apparatus should be the focus of evaluation and review, and when necessary, of the wrath of those who disagree with the final decision.

I, therefore, feel that no matter what the final form of the many bills you are debating during these hearings may take, retention of centralized control is essential. In this way we can prevent what we discovered in May 1969; policy by default and pollution by indifference.

I appreciate the opportunity to make this brief statement, Mr. Chairman, and I would close by stating that the environmental issue is now closing in on each of us.

It is closing in a symbolic sense, as the operational arm of man's concern with the welfare of his fellow man. It is also closing in a physical sense, by forcing us to realize that no man is an environmental island, complete unto himself. The dangers of ocean dumping must be dealt with before the accumulated sludge fills up the oceans and there are no islands and probably no men, at all.

Mr. DINGELL. The Chair observes that the Sierra Club has called advising the committee that it will not be possible for the scheduled witnesses on behalf of the Sierra Club to be present. For that reason, the same order is made with regard to that and a like order from the Chair with regard to a statement from distinguished Mr. Louis Clapper, Conservation Director of National Wildlife Federation, and you will see to it that is also inserted in the record.

(The statements referred to follow:)

STATEMENT OF EUGENE V. COAN, THE SIERRA CLUB

We appreciate this opportunity to present our views on the question of dumping into the ocean and the Great Lakes, for this has become a matter of growing concern to us. Of all our many environmental problems, this one appears to hold some especially grave dangers.

We have reached the time when we can no longer consider the ocean and the Great Lakes to be a dumping grounds of last resort. We can no longer indiscriminately place our waste materials in the sea and assume that they will not return to haunt us again. For as large and powerful is the sea, it is surprisingly fragile. It is also of growing importance to us. Given time, the fresh water of the world can cleanse itself, but the sea cannot. Once we put our chemicals and trash into

it, they may be there for a very long time. We are already familiar with the tragic story of DDT, the first recognized and best understood of a growing list of materials we have come to call "environmentally hazardous substances." It is probable that other chemicals will prove to be as long-lived and as harmful, for instance the heavy metals, other chlorinated hydrocarbons, the petrochemicals lost in oil spills, and radioactive plutonium.

When we commit environmental injustice on land, it is often within our power to undo the damage, given enough time and money. If we do further-harm to the seas, it will be beyond our power to undo that harm.

There are three broad, interrelated areas of marine conservation. First, water quality. Second, conservation of marine organisms, including fisheries and other wildlife. Third, shore conservation and planning. Some of our fisheries have already declined. In some cases, it is very difficult to determine the reasons for the declines, whether poor management of the fishery or the result of coastal contamination. In some ways, the use of our shoreline is dictated by the degree of water pollution. So, maintain water quality is a key factor in marine conservation.

Obviously, the answer is that we must stop all pollution of the sea. The sea is just as insufficient an answer to the problem of waste disposal as food from the sea is the solution to the problem of hunger and over-population.

Moreover, we cannot allow the deep sea to be the ultimate carpet under which to sweep our trash. We know less about the life of the deep sea than any other area of our planet. Recent work at Woods Hole has begun to show that the creatures of the deep sea are more diverse and abundant than we had previously supposed. We have no more right to contaminate and kill the organisms of the deep sea than those of shallow water simply because they are less well known and more distant from us.

We must move now to extract ourselves from this potential dangerous situation by establishing two basic goals. First, we must set in motion the machinery which will enable us to find out what is happening to the sea and what we can do about it. Then, we must take action now where we can.

To an extent, the necessary studies have already been initiated by the Environmental Protection Agency, by the National Science Foundation, and by the Sea Grant Program, now under the National Oceanic and Atmospheric Administration. What is required is the proper funding of these programs, an overall, clear set of goals, and coordination among these granting programs.

These research goals must be (1) to established baselines for the existing levels of contamination or natural levels for as many substances as possible over as wide an area as possible, (2) to establish water quality criteria based on extensive testing of a wide array of substances on a wide array of marine organisms. We must look not only for immediate toxicity of the substances but their subtle effects on the health and reproductive ability of marine organisms for more than one generation. We need to have this information about as many organisms as possible, but certainly about the most important ones both commercially and ecologically. Until we have such exhaustive information, we cannot possibly set water quality standards. In this regard, the Environmental Protection Agency has a very long way to go. (3) Finally, we need to put as much money as possible into finding ways to reclaim and recycle so-called wastes. It will only be after we have extensive information in all of these three fields that we can deal adequately and knowledgeably with the problem of dumping.

Needless to say, it will be a considerable time before we have most of the required answers. We must act now upon the most serious problems and give ourselves deadlines for eliminating marine pollution.

The legislation which should be enacted this Congress should, at a minimum, do the following things:

(1) Establish a national policy and goal of eliminating environmentally harmful dumping.

(2) Establish a permit system under the Environmental Protection Agency for all forms of ocean dumping, including sewage discharge.

(3) Place the burden of proof on the parties wishing to dump materials into the sea or Great Lakes to prove that no environmental harm would be done.

(4) Establish broad criteria for the use of the Environmental Protection Agency in evaluating dumping permit applications.

(5) Allow public hearings when a dumping permit is contested.

(6) Completely prevent dumping into the sea of environmentally hazardous substances, being defined as substances which persist in the marine environment
[illegible]

because of their physical, chemical, and biological properties, and/or which become widespread in the marine environment because of their physical and chemical properties, and/or which tend to become more concentrated in living organisms than in the surrounding environment, *and* which may present a danger to living organisms by their direct toxicity or their influence on the health or reproductive ability of living organisms or the health of man.

(7) Establish fines for parties violating the regulations and necessary enforcement procedures.

(8) Establish dates after which sewage to be placed in the sea or Great Lakes must be upgraded. We would expect that increased Federal assistance will make it possible to meet these deadlines.

(9) Enable substantial areas to be set aside in which no dumping would be allowed. These would be especially fragile areas and a substantial number of areas to serve as baselines for biological study.

(10) Finally, it would establish a national goal for the Department of State to seek international cooperation with regard to preserving the oceans.

STATEMENT OF LOUIS S. CLAPPER ON BEHALF OF THE NATIONAL WILDLIFE FEDERATION

Mr. Chairman, I am Louis S. Clapper, Conservation Director of the National Wildlife Federation which has its national headquarters at 1412 Sixteenth Street, N.W., here in Washington, D.C.

The National Wildlife Federation has affiliates in all 50 States and the Virgin Islands. These affiliates, in turn, are made up of local groups and individuals who, when combined with associate members and other supporters of the Federation, number an estimated 3 million persons.

Mr. Chairman, we want to congratulate the Subcommittees for holding these hearings on ocean dumping. In our opinion, this is a major problem—one which merits immediate action and we are pleased by the interest of members of these Subcommittees and by other members of the Congress who have introduced appropriate legislation on the subject.

Basically, the Federation does not believe that the oceans or the Great Lakes or other areas of the U.S. shorelines should be used for dumping or waste disposal purposes. Ed Chaney, one of our Staff members, is outlining this attitude in a forthcoming article for our NATIONAL WILDLIFE Magazine and he points out quite appropriately that the earth is a closed system—that nothing actually can be thrown away. Unwanted wastes must be reclaimed or recycled back into the overall ecological system.

We note that H.R. 4723 defines "material" as "matter of any kind or description, including, but not limited to, dredge spoil, solid waste, garbage, sewage sludge, munitions, chemical, biological and radiological warfare agents, radioactive materials, wrecked or discarded equipment, rock, sand, cellar dirt, and industrial wastes." H.R. 3662 and H.R. 4359 defines "waste materials" as "all solid and liquid products or byproducts of the industrial process (including tailings, sediment, and like materials resulting from marine mining or dredging activities), industrial waste acids, chemicals, sewage, sludge, garbage, dredge spoils, radioactive materials, construction and demolition debris, military ordinance, explosives, and any other form of discarded material or equipment." After reviewing these definitions, Mr. Chairman, *we simply cannot see any valid reason for using the Nation's offshore water areas for waste disposal.* For far too long, this has been another indication of the "out-of-sight," "out-of-mind" attitude toward waste disposal and we no longer can afford this type of degradation in our environment.

If dumping is to be allowed, however, then we generally are in agreement with the below listed principles which are expressed by one or more of the bills under consideration:

1. For the reasons already expressed, we note with exceptional interest that some bills "phase out" ocean dumping. In the Senate, S. 192 would terminate all dumping by June 30, 1975. H.R. 3662 and H.R. 4359 would phase out ocean dumping of municipal and industrial wastes with primary treatment by 1972, by secondary treatment by 1974, and tertiary treatment by 1976. In lieu of outright prohibition, we would be in accord with that schedule.

BEST COPY AVAILABLE

2. It is our firm belief that any regulation of dumping should apply to all U.S. waters and the oceans outside this Nation's territorial waters and the contiguous zone. In this connection, we like the definition contained in Section 3(b) of H.R. 4723 which specifies the coverage as "oceans, gulfs, bays, salt-water lagoons, salt-water harbors, other coastal waters where the tide ebbs and flows, and the Great Lakes." However, definitions in H.R. 3662 and H.R. 4359 are essentially the same.

3. We think it is appropriate for the Environmental Protection Agency to be empowered to issue permits, if any dumping is to be allowed, if the action will not degrade the environment or ecological systems or endanger human health, welfare, or the amenities. H.R. 4723 appears to give the EPA Administrator suitable discretion in issuing permits and we like the provision in this proposal which burdens the applicant with providing information to justify a permit.

4. We agree that the EPA Administrator should be authorized to designate by a permit the type and amount of materials to be transported and/or dumped and the location, as well as the period of time that the permit is valid. This is outlined in H.R. 4723. And, we also are in accord with that proposal's requirement that a permit shall not violate applicable water quality standards.

5. We concur with the principle expressed in H.R. 4723, whereby EPA will establish and apply criteria for evaluating permit applications. We prefer this discretionary process on this criteria more than formal regulations as in some other bills before the Committee.

6. We do not disagree with the provision which names the Department of Justice responsible for conducting any legal actions which may be necessary, or with surveillance by the Coast Guard. However, we do note a wide variance in the amounts of maximum fines to be applied to violators for each offense: $2,000 in H.R. 4359, $5,000 in H.R. 808, $50,000 in both H.R. 4723 and S. 1238, and $25,000 in S. 1286. Penalties should serve as deterrents and we question whether $2,000 is enough in some instances. On the other hand, penalties can be so severe that many courts would be reluctant to impose them. However, in our judgment, we do not feel that $50,000 is so severe as to impede such sentencing when this is a maximum.

7. We are in concurrence with provisions in H.R. 3662 and H.R. 4359 which would immediately suspend the dumping or disposal of radioactive wastes, toxic industrial wastes, and chemical or biological warfare materials.

8. We note with particular interest the provisions in H.R. 3662 which would authorize and direct the Administrator of the EPA to conduct research and investigation on the marine ecology. We believe that the authorized appropriation of $1 million per year for this purpose is both reasonable and appropriate.

9. We think favorably of the provision in H.R.4359, proposed new Section 7(g) to the Act of August 3, 1968, wherein: "The Administrator of the Environmental Protection Agency may by regulation prohibit the disposal or dumping of any waste material which he determines may damage the ecology of the marine environment, and in making such determination he may rely upon whatever indicators are currently available to him, regardless of the fact that such indicators may not be conclusive."

10. We recommend that the Subcommittees give consideration to proposed Section 7 in S.1082. This proposal would provide for determining means of recovering useful materials from wastes. Certainly, in our opinion, if a deleterious waste can be transferred into a positive value, the entire environmental movement will have been strengthened.

To conclude, Mr. Chairman, we have welcomed this opportunity of making these remarks and we certainly hope that these Committees can take joint action to report a bill to the full Committee at the earliest possible time.

Mr. DINGELL. The Chair is very pleased to welcome for our first witness the very able Administrator of the Environmental Protection Agency, Mr. William D. Ruckelshaus.

We are happy to welcome you to the committee. The Chair observes that you probably have members of your staff present in the room, and if you would like to have any of them present with you at the table, you may feel free to do so, if you will only identify them for the record by name and by responsibility.

BEST COPY AVAILABLE

STATEMENT OF WILLIAM D. RUCKELSHAUS, ADMINISTRATOR, ENVIRONMENTAL PROTECTION AGENCY, ACCOMPANIED BY DAVID DOMINICK, ACTING COMMISSIONER OF THE WATER QUALITY OFFICE, EPA

Mr. RUCKELSHAUS. Thank you, Mr. Chairman.

I have with me today Mr. David Dominick, Acting Commissioner of Water Quality Office of EPA, who will participate in the answering of any questions that the committee or chairman may have.

Mr. DINGELL. Mr. Dominick, we are happy to welcome you.

You may proceed, sir.

Mr. RUCKELSHAUS. Mr. Chairman and members of the committee, I appreciate the opportunity to meet with you today to discuss the Administration's proposal for the control of ocean dumping entitled, "The Marine Protection Act of 1971." This bill, H.R. 4723, has been introduced in recognition of the critical need for a national ocean dumping policy.

Our proposal is the product of an intensive and comprehensive study of the problems of ocean dumping. That study and the recommendations for a strong policy of preventive and remedial measures were reported to the Congress in the Report on Ocean Dumping prepared by the Council on Environmental Quality. I understand that Chairman Train has discussed the study and recommendations with you.

I believe we are all in agreement as to the need for a strong bill to control ocean dumping. We endeavored in the drafting of our proposal to translate the recommendations of the Ocean Dumping Report into law. Our purpose here is to recommend to the committee and to the Congress the creation of the farthest reaching and strongest authority that law and technology will allow.

Members of this committee and other members of Congress have introduced bills which in many cases are similar to our own proposal. Other proposals take somewhat different approaches.

Mr. Chairman, we wish to work with you and the committee to develop the most effective legislation possible.

Our proposal, H.R. 4723, would invest regulatory authority over ocean dumping in the Administrator of EPA. As that Administrator, I propose to administer H.R. 4723, if it is enacted, in a way that will fully implement the recommendation of the Council on Environmental Quality as set forth in its Ocean Dumping Report.

I would like to describe briefly the principal provisions of our bill and our thinking about it.

The purpose of H.R. 4723 is to regulate the dumping of all types of material in the oceans, estuaries, and the Great Lakes, and to prevent or strictly control the dumping into such waters of any material which could adversely affect human health or welfare or the marine environment. These objectives would be carried out by means of a permit system established and administered by the EPA.

An important feature of the bill is that it would require a permit for two different kinds of activity. In the first place, persons desiring to transport materials from this country for dumping into ocean or coastal waters, anywhere, whether or not within our territorial jurisdiction, would be required to obtain a permit.

This requirement is based on the authority of the United States to control the disposition of materials transported from U.S. territory.

Secondly, a permit would be required for the dumping of materials—whether transported from this country or not—in waters covered by the bill which are within our territorial jurisdiction, including the 3-mile territorial sea, or in waters of the 9-mile contingous zone beyond the territorial sea where the dumping may affect our territory or territorial sea.

Both requirements would apply to foreign nationals and foreign governments, as well as to United States citizens and to all agencies and instrumentalities of Federal, State and local government.

Thus, the bill would utilize the regulatory authority of the United States to its fullest extent consistent with the established principles of international law.

The bill would apply to any disposition of material with several exceptions, the most important of which is the disposition of effluents from outfall structures. The bill is aimed at intermittent dumping as opposed to continuous discharges from fixed sources. This is an important distinction.

Continuous discharges from outfall structures into territorial waters covered by the Act are already subject to regulation under the Federal Water Pollution Control Act.

Amendments to that Act proposed by the Administration would extend its coverage to outfalls in the contiguous zone and also to outfalls in the high seas beyond the continguous zone which discharge matter originating within U.S. territory.

The Administrator, in issuing permits to dump materials or to transport them for dumping would be required to determine that such activity will not unreasonably degrade or endanger human health, welfare or amenities, or the marine environment, ecological systems, or economic potentialities.

He would be required to establish criteria for evaluating permit applications, to include—

The likely present and future impact of the dumping on human health and welfare and the marine environment.

The possible persistence or permanence of the effects of the dumping.

The volume and concentration of the materials involved.

Alternative locations and methods of disposal, including land-based alternatives.

The probable impact on the public interest of either issuing or denying a permit or of requiring alternative locations or methods of disposal.

These criteria would be refined as additional knowledge is gained about the environmental impact of ocean dumping and about the acceptability and feasibility of various land-based alternatives.

The Administrator would be permitted to impose restrictions in permits relating to the type and amount of materials to be dumped, the place of dumping, and the period of validity of the permit.

He would be authorized to deny the issuance of a permit where he finds that the materials in question cannot be dumped consistently

62-513—71——26

with the criteria established for the issuance of permits, as well as to alter or revoke permits upon such finding.

The Administrator would be authorized to require applicants for permits to provide such information as he considers necessary to evaluate the application. Information required by the Administrator might include detailed plans for conversion to land-based disposal. The Administrator would also be authorized to prescribe reporting requirements for actions taken pursuant to permits.

Any person who violates the act or the provisions of any regulations or permit issued thereunder would be liable to a civil penalty of up to $50,000 per day, imprisonment of up to one year, or both. The Attorney General would be authorized to bring actions for equitable relief to redress any such violations, and the Administrator would be authorized to revoke or suspend a violator's permit. The bill would require the Coast Guard to conduct surveillance and other enforcement activity.

No permit would be denied, suspended, or revoked, or a civil penalty assessed, without notice and opportunity for a hearing.

An important aspect of the bill is the clear definition of its relationship with other Federal laws related to ocean dumping and water pollution control. As I have already indicated, the bill would be inapplicable to internal navigable waters, except for estuarine areas and the Great Lakes, and would be inapplicable to effluents discharged from outfall structures.

Overlap with the Federal Water Pollution Control Act and the Refuse Act of 1899, which between them deal with discharges of all types into navigable waters, is avoided by specific provisions which would prevent duplication or conflict with the provisions of these other laws.

The Refuse Act requires a permit issued by the Army Corps of Engineers for the discharge of wastes other than municipal sewage into navigable waters. Duplicate permit requirements for the disposal of wastes into waters covered by both Acts would be avoided, since H.R. 4723 would expressly supersede the Refuse Act in areas in which both apply.

With respect to the Federal Water Pollution Control Act, under which water quality standards are established and enforced, H.R. 4723 provides that no permit may be issued for dumping of material which would violate such standards.

Under another Administration proposal relating to standards and enforcement, the Administrator of EPA would be given authority to establish water quality standards for the contiguous zone with respect to the discharge of matter originating within U.S. territory.

Such standards, as well as the standards already established by joint Federal-State action for coastal waters out to the 3-mile limit, will be of great assistance in implementing H.R. 4723 if it is enacted.

Except as I have just indicated with respect to the Refuse Act, all existing authorities and actions taken under the Rivers and Harbors Act of 1899 would be preserved. The authority of the Atomic Energy Commission to regulate the disposal of radioactive materials would be affected.

In implementing H.R. 4723, EPA would rely on assistance provided by other Federal agencies. In establishing or revising criteria for the issuance of permits, the Administrator would consult with

the Secretaries of Commerce, Interior, State, Defense, Agriculture, Transportation, and Health, Education and Welfare, and with the Atomic Energy Commission.

He would consult with interested Federal and State agencies in reviewing individual permit applications, and would be precluded from issuing a permit where the Secretary of the Army determines that it would cause an unreasonable impairment of navigation.

In administering the Act, EPA would be guided by the ultimate objective of terminating all ocean dumping which is damaging to the marine environment.

We would adopt a precautionary, preventive approach, aimed at terminating all dumping not clearly demonstrated to be safe. Ocean dumping of materials clearly identified as harmful would be stopped as soon as possible. Where existing information on the effects of ocean dumping of particular materials is inconclusive, yet the best indications are that such materials may create adverse conditions when dumped, the dumping of these materials would be phased out.

If further information conclusively proves that such dim dumping does not damage the environment, it could be allowed to continue under regulation.

The dumping of some materials, such as chemical warfare materials and toxic industrial wastes, would be stopped immediately. The dumping of other materials, such as sewage sludge and solid waste, would be discontinued as soon as possible, and no new sources of such dumping would be allowed.

It might prove unnecessary to discontinue the dumping of some inert, nontoxic materials, such as unpolluted dredge spoil and construction and demolition debris, although the dumping of such materials would be strictly regulated to prevent damage to estuarine and coastal areas.

As one example of how H.R. 4723 might be implemented, consider the case of ocean disposal of sewage sludge. Some communities have substantial financial investment in facilities and equipment for the barging of digested sewage sludge to sea. To impose an immediate ban on ocean dumping by these communities would be uneconomic and possibly self-defeating where acceptabl land-based disposal methods are not immediately available.

In such cases, EPA would temporarily allow the dumping to be continued but would require it to be phased out entirely within a reasonable period of time. No new sources of ocean disposal of sewage sludge would be permitted.

This would mean that communities already dumping at sea would not be allowed to increase the volume of such dumping over current levels or what the existing barging facilities will accommodate. In the case of municipalities which do not currently dump sewage sludge at sea, they would not be allowed to start.

H.R. 4723 would not place an absolute ban on the dumping of specified classes of materials, nor would it ban the dumping of materials in specified waters within the coverage of the bill. Instead, the Administrator would be authorized, based on criteria developed by him in consultation with other agencies, to permit, limit, or ban the dumping of particular materials, in all or portions of the waters covered by the bill, depending on all the circumstances of a particular case.

BEST COPY AVAILABLE

It would not be feasible to ban all ocean dumping at once. In some instances, waste disposal methods which are less damaging to the environment than ocean dumping are not immediately available. Research is needed on the recycling of wastes and the development of other alternatives to ocean dumping.

Conversion to land-based disposal methods will require a substantial reallocation of resources by municipalities and others presently disposing of wastes at sea.

EPA is making every effort to develop solutions to the often very complex problems of recycling and alternate disposal of wastes which otherwise would find their way into inland and ocean wastes.

Some of the projects now underway include an examination and demonstration of the recycling of solid wastes, an examination of the feasibility of mixing municipal sewage sludge and solid waste into a composting material, the location of national land disposal sites for the disposition of hazardous and toxic industrial wastes, the incineration of solid wastes as a fuel for power production, and the use of sewage sludge for soil enrichment or as landfill—especially in strip mined areas.

We are also making an intensive effort through our grant and contract authority to develop and demonstrate practical industrial waste water recycling and by-product recovery as well as industrial methods which minimize the production of pollutants.

In addition to the technological problems, we face an array of social, legal, and economic problems when we seek answers to the puzzle of waste disposal sites and waste transportation.

A great deal of effort and investment is necessary.

Some of the bills pending before these subcommittees would ban the dumping of all materials into the territorial sea or into waters over the Continental Shelf. EPA is not favorable to a ban of this nature which would be applicable to all classes of materials since it might lead to undesirable results in some instances.

For example, again using the case of sewage sludge, it might be necessary for some communities which presently barge sludge for dumping in near-shore ocean waters over the Continental Shelf to invest substantial amounts in new equipment to barge a farther distance from shore. It might be preferable to allow such communities to continue near-shore dumping on an interim basis and to invest their money instead in developing a capability for land-based disposal.

However, EPA does agree that special protection should be accorded to those portions of the marine environment which are biologically the most productive and the most sensitive, that is, estuaries and shallow, near-shore areas in which many marine organisms breed or spawn. In many cases, a complete ban of dumping in such areas might be appropriate.

Several of the bills pending before this committee would require designated levels of treatment for municipal sewage and industrial wastes by specified dates. This approach does not take into account variations in water use designations, the quality or characteristics of the receiving waters, or other factors which bear on the appropriate level of treatment in a given instance.

[illegible]

We believe that water quality standards established under the Federal Water Pollution Control Act provide more flexible and responsive vehicle for the determination of base levels of treatment for these continuous discharges.

Mr. Chairman, I wish to emphasize again our intention to cooperate with the committee to the fullest extent.

We will be pleased to provide you with more detailed information on any of the matters I have dealt with here today, and to make such information a part of the record of these hearings.

We will endeavor to answer now any questions you may have.

Thank you.

Mr. DINGELL. The committee thanks you for a very helpful statement.

The Chair has an observation I would like to bring to the attention of my colleagues on the committee. I am informed that the House will meet at 11 o'clock. This being the case, the Chair observes that this will leave a very limited amount of time for the members and it has been the practice, the Chair observes, of this occupant of the Chair not to impose the 5-minute rule. So the Chair will request that the members do voluntarily restrict themselves to that period of time so that everyone here present can have a full opportunity to question Mr. Ruckelshaus on matters of interest and concern.

On the conclusion of that, the Chair will then afford members additional time on appropriate sequence to ask questions of Mr. Ruckelshaus.

Mr. Pelly?

Mr. PELLY. Thank you, Mr. Chairman.

I will be very limited in my questions and then, if I may, Mr. Ruckelshaus, refer questions to you for written reply for the record.

Mr. RUCKELSHAUS. Certainly.

Mr. PELLY. I am concerned about the fact the Administration bill doesn't have any provision for authorizing additional funds. EPA would do the research work in recycling, for example, would it?

Mr. RUCKELSHAUS. Yes, Mr. Pelly, we have under the Resources Recovery Act of 1970, the specific responsibility for a great deal of research in recycling of solid waste. In section 212 of that act, we have a mandate from Congress to institute a 2-year study on national disposal sites for solid waste. The results of that study are to be made available to Congress. It would now be a year from this October when the bill passed in October 1970.

Mr. PELLY. Would you have adequate funds for carrying on the necessary research?

Mr. RUCKELSHAUS. In our supplemental request for appropriation to Congress, and also in our fiscal year 1972 request, we have allocated funds for this study and for implementation of the Resource and Recovery Act, and we believe those funds are adequate, Mr. Pelly.

Mr. PELLY. If we could have some additional material placed in the record with regard to the present state of recycling, I think it would be very interesting and helpful to us, and I am not going to ask you any questions on that.

There are one or two matters that have come up and that have been brought up by other witnesses that I think I would like to ask you to

comment on now. One is the port authorities, who are greatly concerned over the time that would be consumed in their channeling and dumping of what I would say would be non-toxic materials. They sought to have the bill provide that they could get permission from the Coast Guard, as I recall, without having to go to two different agencies of Government. Do you think that in connection with the necessary channeling of ports to provide for the adequate depth for ships that come into the port, that this procedure could be simplified?

Mr. RUCKELSHAUS. I think it could, Mr. Pelly. Under the provisions of the present law, any dredging of a channel of that nature in a port would be handled under the dredge and fill permits of the Coprs of Engineers. There is a specific provision in H.R. 4723 providing that where a permit is issued under section 403 of the Rivers and Harbors Act of 1899 there is no necessity for two permits to be used. But there is a requirement for consultation with the Environmental Protection Agency by the Corps in the instances in which they issue such permits.

Mr. PELLY. I should think that would be desirable and general for EPA to keep surveillance over this commercial activity that is not confined to just the channeling into port, but goes up rivers and has a vast effect on the environment, I would think.

Mr. RUCKELSHAUS. Yes, I think it is under section 7(c)(3), Mr. Pelly, where it provides that there is no need for duplication of permit under this type of situation that you describe.

Mr. PELLY. I know the chairman of this committee testified as to his concern as to some of the effects of this legislation and his interest, of course, was the Port Authority of Baltimore. And I know there are others of us who have various ports and we do want to make it possible to conduct this necessary work as expeditiously as possible. But I am sure none of us want to violate the principles of good environment.

Mr. RUCKELSHAUS. Yes, sir; that is precisely our position, too, Mr. Pelly.

Mr. PELLY. You would have no objection, would you, if we added to the bill the provision which is in Mr. Rogers' bill for authorization of—I think Mr. Rogers had a million dollars. Some seemed to think it should be more than that.

Mr. RUCKELSHAUS. We have some preliminary figures which I can give to the committee now as to implementation of our bill, and this does not include any of the other provisions which have been suggested in other bills or in previous hearings.

Mr. PELLY. Would you feel that there is reason to have an authorization clause in the bill, so that we can fund?

Mr. RUCKELSHAUS. There is a general authorization clause in section 13 of the bill, as I understood the committee was interested in how much we estimated the implementation of the act or the bill would cost.

Mr. PELLY. Chairman Dingell reminded another witness of the fact that there is a new provision which is required that any legislation now has to give the 5-year cost, and I presume you have that?

Mr. RUCKELSHAUS. Yes, we have the 6-year cost, Mr. Pelly, so we will be glad to provide that.

Mr. PELLY. Will you put that in the record?

Mr. RUCKELSHAUS. Yes, we will.

(The information follows:)

SIX-YEAR COST ESTIMATE ON IMPLEMENTATION OF H.R. 4247

WATER QUALITY (PROJECTED PROGRAM) MARINE PROTECTION ACT OF 1971

[In millions of dollars]

	Fiscal year—					
	1972	1973	1974	1975	1976	1977
**Budget authority:**						
Permit program	0.5	0.5	0.5	0.3	0.2	0.2
Disposal research:						
In-house	.1	.4	.3	.2	.2	.2
Contracts	1.0	1.1	.7	.5	.5	.5
Subtotal	1.1	1.5	1.0	.7	.7	.7
Technical studies and monitoring:						
In-house	.1	.4	.6	.6	.6	.6
Contracts	.3	.6	2.4	2.4	2.4	2.4
Subtotal	.4	2.0	3.0	3.0	3.0	3.0
Total	2.0	4.0	4.5	4.0	3.9	3.9
**Obligations:**						
Permit program	.5	.5	.5	.3	.2	.2
Disposal research:						
In-house	.1	.4	.3	.2	.2	.2
Contracts	1.0	1.1	.7	.5	.5	.5
Subtotal	1.1	1.5	1.0	.7	.7	.7
Technical studies and monitoring:						
In-house	.1	.4	.6	.6	.6	.6
Contracts	.3	.6	2.4	2.4	2.4	2.4
Subtotal	.4	2.0	3.0	3.0	3.0	3.0
Total	2.0	4.0	4.5	4.0	3.9	3.9
**Outlays:**						
Permit program	.25	.40	.45	.3	.2	.2
Disposal research:						
In-house	.05	.32	.27	.2	.2	.2
Contracts	.65	.88	.43	.4	.7	.6
Subtotal	.7	1.20	.70	.6	.9	.8
Technical studies and monitoring:						
In-house	.05	.32	.55	.6	.6	.6
Contracts	.2	.128	1.6	2.2	2.7	2.6
Subtotal	.25	1.60	2.15	2.8	3.3	3.2
Total	1.2	3.2	3.3	3.7	4.4	4.2
Positions	70	100	110	90	79	79

COST OF IMPLEMENTING THE MARINE PROTECTION ACT OF 1971

INTRODUCTION

In a message to the Congress on April 15, 1970, the President directed the Council on Environmental Quality to work with other Federal Agencies and with State and local governments on a comprehensive study of ocean disposal that would result in research, legislative, and administrative recommendations. Their report was issued in October 1970 and a legislative proposal has been prepared by the Administration for submission to the Congress.

If the proposal is adopted, the transportation and dumping of all materials in the oceans, estuaries, and the Great Lakes will be regulated by the issuance of permits. The Administrator would be authorized to establish criteria which would consider the possible detrimental effects of ocean disposal and the impact of the use of alternative locations and methods; to ban ocean dumbing of specific

BEST COPY AVAILABLE

materials and to designate safe sites; and to issue permits where the applicant presents information indicating that proposed transportation and/or dumping will not unreasonably degrade or unreasonably endanger human health, welfare, or amenities, or the marine environment, ecological systems, or economic potentialities.

Implementation of this legislation will require the development of criteria and regulations for the granting of permits. This development must recognize both the characteristics of the materials to be dumped and their potential impact on receiving waters. To accomplish this will require not only an augmented research effort and understanding on the effects of different waste materials on the marine environment but also a series of baseline technical investigations of existing water quality conditions in present and potential dumping areas.

As a continuing effort it will be necessary to continuously evaluate and update the impact of ocean dumping on the marine environment. While the act specifically assigns surveillance and enforcement functions to the Coast Guard, it will be necessary for EPA to work closely with the Coast Guard in carrying out these functions and in modifying permit requirements and regulations as environmental requirements or conditions change.

EPA already has available staff expertise on marine pollution problems and has laboratory capabilities of running a wide variety of laboratory tests. It must be recognized, however, that EPA does not presently have any ocean-going ship capabilities; it is therefore anticipated that EPA will make full use of the Coast Guard, National Ocean Survey, and Corps of Engineers' ocean-going equipment and personnel to carry out environmental investigations of marine pollution problems. It may also be anticipated that the services of private contractors will be utilized for this purpose under the supervision of EPA personnel. (A contract for establishing the organizational framework of an overall coastal monitoring network, which will include monitoring of ocean dumping zones, will be negotiated during FY 1971. The report from this study should be available about the middle of FY 1972.)

It should also be recognized that extension of water quality standards to the contiguous zone will require the same type of technical investigation and monitoring as will be required by the Marine Protection Act specifically for ocean dumping problems. The program outlined here is directed specifically toward implementation of the Marine Protection Act; however, it should be recognized as part of an overall attack on coastal and marine pollution problems.

STAFFING

A supervisory headquarters staff will be required to initiate the program, to supervise its operations, coordinate efforts within EPA and all other Federal and State agencies concerned with the program. The staff would control final granting of permits. After development of criteria, regulations, and guidelines for the granting of permits had been developed, some of the authorities for the granting of permits will be delegated to the regions.

Headquarters staff will also be required to coordinate research efforts on a continuing basis and to assist in the continuing review and up-dating of criteria and regulations. It is anticipated that much of the research necessary will be carried out by grants or contracts or integrated into the surveillance and monitoring programs and baseline technical investigations of environmental conditions.

Development of a broadly based technical staff to supervise the technical investigations necessary to provide a viable program with the ultimate goal of stopping ocean dumping completely is a necessary part of the overall research and monitoring effort. A broadly based headquarters technical staff is needed to work with the Coast Guard, NOAA, Corps of Engineers, and other Federal and State agencies whose facilities will be used to carry out monitoring and surveillance functions and to make full use of the facilities of other [illegible]ncies in carrying out ocean disposal research programs. It is expected that e[illegible]h coastal region will develop its own specialized technical expertise to deal with the overall problem as well as problems unique to the region.

OCEAN DISPOSAL PERMIT PROGRAM

Fiscal year 1972: Positions: 48; Budget: $500,000

The law as proposed will become effective 6 months after passage. Within this time interim criteria and regulations for the granting of ocean disposal permits

must be established and guidelines for permit evaluation must be developed and promulgated.

The headquarters staff will convene an advisory committee and set up a task force of in-house personnel to develop the necessary criteria, regulations, and guidelines. The work of this group should be closely coordinated with the setting of water quality standards for the contiguous zone if such laws are enacted. An approximate $100,000 is required for the operation of this advisory committee, and $100,000 for the salaries of eight headquarters personnel for an average of 6 months during FY 1972. A total of 40 personnel will be placed in the eight EPA coastal regions: Boston, New York, Philadelphia, Atlanta, Dallas, San Francisco, Seattle, and Chicago. The tasks of these personnel will be to: coordinate input from all EPA offices; develop working relationships with all Federal and State agencies concerned; receive and review applications for permits; issue permits where applications fall within established criteria or send to the headquarters control group those applications having unusual problems that cannot be resolved locally; coordinate permit information with the enforcement and surveillance agency (Coast Guard); establish a time schedule with other existing or proposed regulations; coordinate and select suitable interim disposal sites with NOAA and the fisheries agencies; and coordinate with State and Federal agencies selection of alternative methods of disposal. Approximately $300,000 will be required for salaries of regional personnel during FY 1972.

Fiscal year 1973: Positions: 48; Budget: $500,000

During this fiscal year, the permit regulations and guidelines will be re-evaluated in terms of actual practice and a stronger set of criteria will be developed to discourage ocean disposal except of the most innocuous materials. It is anticipated that during this fiscal year, the initial results of research and technical studies will become available and consideration can be given to the elimination of some ocean disposal areas and relocation of others.

Fiscal year 1974: Positions: 48; Budget: $500,000

During this fiscal year, a solid environmental data base on the impact of ocean dumping on the environment should begin being available and the thrust of the ocean disposal permit program will be toward the elimination of ocean disposal where possible and the relocation of dumping grounds to areas of small ecological significance.

Fiscal year 1975: Positions: 35; Budget: $300,000

By fiscal year 1975, research and technological development should have advanced to the point where ocean dumping as a disposal technique can begin to be phased out. The reduced staff allocation to this part of the program during this year assumes this condition.

Fiscal years 1976 and 1977: Positions: 24; Budget: $200,000

By this time, ocean disposal should be eliminated except for demonstratively innocuous materials and the criteria and guidelines should be developed to the point where only the most routine permit granting efforts are required. It is expected that a regional staff of twenty people will be maintained in this part of the program with a supervisory staff of four in headquarters.

OCEAN DISPOSAL RESEARCH

Fiscal year 1972: Positions: 11; Total Budget: $1,100,000; Contracts: $1,000,000

During this fiscal year, a major effort will be initiated to evaluate the impact of ocean disposal on the marine environment and alternative methods of disposal of material presently being dumped. A combination of 11 new personnel and $100,000 for their salaries during fiscal year 1972 and grants, contracts, and interagency agreements totaling $1,000,000 in fiscal year 1972 will be required. Much of the work will be integrated into technical studies, monitoring, and baseline studies for surveillance; the bulk of this work will be done in cooperation with the Coast Guard, NOAA, the Corps of Engineers, and various fisheries agencies. Specific research projects to be initiated during this fiscal year are:

(1) Alternative Methods of Disposal—$400,000.
(2) Environmental Impact Studies—$300,000.
(3) Beneficial Use of Wastes in the Marine Environment—$300,000.

Fiscal year 1973: Positions: 22; Total Budget: $1,500,000; Contracts: $1,100,000

[illegible]

During this fiscal year, $1,100,000 will be used in contracts, grants, and reimbursable agreements for ocean disposal research. This money will be split between developing technical and alternative methods of disposal to eliminate ocean dumping and toward determining environmental impacts of continuing ocean disposal in the past. Research activities during this fiscal year will begin being channeled into the beneficial uses of wastes in enhancing the marine environment. Some funds will also be allocated toward the solution of ocean disposal problems of particular concern in certain regions and an increase of regional staff is indicated to support this action.

Fiscal year 1974: Positions: 22; Total Budget: $1,000,000; Contracts: $700,000

This fiscal year research efforts specifically directed toward ocean dumping should decrease as problems are solved. It is not anticipated that additional efforts in new directions will be initiated, but that funds will be directed toward the more difficult problems as found during the preceding two years.

Fiscal years 1975, 1976, and 1977: Positions: 15; Total Budget: $700,000; Contracts: $500,000

During these years, a continuing research effort will be maintained directed toward eliminating the environmental impact of past dumping and toward the solution of specific regional problems.

TECHNICAL STUDIES AND MONITORING

Fiscal year 1972: Positions: 11; Total Budget: $400,000; Contracts: $300,000

Contractual or reimbursable agreements will be negotiated with Federal agencies, such as the Coast Guard, or with private contractors for carrying out studies of specific dumping areas as an interim guide for the granting of permits. In carrying out such studies, it is anticipated that EPA personnel will be very closely associated with the survey efforts, either as on-board observers, as project officers or both. EPA laboratories will be required to provide some laboratory support, particularly in the more difficult types of determination. Therefore studies of the dumping grounds of the New York Bight and the New Jersey coast will be in the first order of priority.

During this year, the mechanism needed for continued surveillance of ocean disposal sites and the continued laboratory support of surveillance operations will be developed. The ocean disposal surveillance program will be integrated into the overall EPA coastal and ocean monitoring network.

Fiscal year 1973: Positions: 30; Total Budget: $2,000,000; Contracts: $1,600,000

During this fiscal year, an intensive series of studies of ocean dumping grounds will be carried out, with particular emphasis being given to the development of disposal sites with small ecological significance. Part of the work carried on will be of a research nature and will absorb funds from the research part of the program. Much of the contract money will go for vessel support and laboratory facilities. Sixteen personnel will be distributed among the regions and will provide technical expertise on specific regional problems. It is anticipated that these personnel will participate in surveys and will assist the Coast Guard in its surveillance activities.

Fiscal year 1974: Positions: 40; Total Budget: $3,000,000; Contracts: $2,400,000

By fiscal year 1974, the Coast Guard should be maintaining a full scale surveillance of all dumping activities with close support from EPA personnel and laboratories. Additional personnel will be needed in the regions to deal with specific local problems.

Fiscal year 1975: Positions: 40; Total Budget: $3,000,000; Contracts: $2,400,000

During this fiscal year, a major effort will be made on the overall impact of ocean dumping on the coastal and marine environment. The bulk of the contract money will go to reimbursable agreements or contracts or ship support to assist in these activities. By the end of this fiscal year, all the important areas of the coastal environment subject to dumping impact will have been evaluated in terms of their ecological significance and usability for continued ocean disposal.

Fiscal years 1976 and 1977: Positions: 40; Total Budget: $3,000,000; Contracts: $2,400,000

By this time, a continuing program of surveillance can be carried out by contract or reimbursable agreements with the major EPA effort going into laboratory support and studies of particular local or regional programs. The

BEST COPY AVAILABLE

personnel and budget indicated are those which it now appears necessary to commit to have a sustained surveillance program through the Coast Guard to meet EPA objectives. It should be noted, however, that this surveillance program and the studies associated with ocean disposal will probably be integrated into an overall coastal and oceanic monitoring network and that the personnel and budget indicated specifically for the ocean dumping programs will also continue toward this overall effort.

COST OF IMPLEMENTING THE MARINE PROTECTION ACT

[In millions of dollars]

	1971	1972	1973	1974	1975	1976	1977
Budget authority	0	2.0	4.0	4.5	4.0	3.9	3.9
Obligations	0	2.0	4.0	4.5	4.0	3.9	3.9
Outlays	0	1.2	3.2	3.3	3.7	4.4	4.2
Man-years	0	70.0	100.0	110.0	100.0	79.0	79.0

Mr. DINGELL. Mr. Rogers?

Mr. ROGERS. Mr. Ruckelshaus? It is good to see you.

Just to pursue for a moment the question on solid wastes, what is the Administration's request for funds? What are your requests for funds?

Mr. RUCKELSHAUS. I don't have those figures before me, Mr. Rogers. I can provide them for you. We have requested in our Solid Waste Office as we inherited it, a budget of approximately $17 million. We have requested in our supplemental request which is still in the process of approval more money for the purposes of new and innovative demonstration projects under the Resource Recovery Act. And in addition to that, we have requested additional funds for the fiscal year 1972 submission for this same purpose, for new and innovative demonstration projects, and also for all of the studies which we were required to make under the Resource Recovery Act.

Mr. ROGERS. What is your 1972 request? It is only about $17 million, isn't it?

Mr. RUCKELSHAUS. No, it is more than that.

Mr. ROGERS. What is your figure?

Mr. RUCKELSHAUS. I can't give you the figure now, because there was a separate request in the budget that was sent to Congress of $85 million.

Mr. ROGERS. For how much?

Mr. RUCKELSHAUS. Eighty-five million dollars. This did not just cover the Solid Waste Office of the Environmental Protection Agency. This was because our agency came into existence so late in the budget cycle that the Office of Management and Budget gave us this extra $85 million to meet the additional needs that we would have as an agency. This budget information will be submitted as soon as we have allocated the funds.

Mr. ROGERS. This hasn't come up to the Congress yet?

Mr. RUCKELSHAUS. Eighty-five million dollars has come up, but there has been no designation as to what the request represents.

Mr. ROGERS. You don't know whether you will get any of it for Solid Waste or not?

Mr. RUCKELSHAUS. We have requested some of it for that purpose. I can't tell you the exact figure at this time, because I can't recall.

Mr. ROGERS. What is your 1972 budget request for solid waste, which

BEST COPY AVAILABLE

was already submitted? In the President's budget, what figure is that for solid waste?

Mr. RUCKELSHAUS. I think it is between $17 and $19 million.

Mr. ROGERS. As I recall, it is about $17 million, maybe it is $19 million, which is far under the authorization, isn't it?

Mr. RUCKELSHAUS. Yes, that is right.

Mr. ROGERS. About how much under?

Mr. RUCKELSHAUS. Well again I would have to look at that authorization figure in the act.

Mr. ROGERS. Are any of your people here who could help us? I realize you may not have every figure in your mind. Don't you have some Solid Waste people here who can help us?

Mr. RUCKELSHAUS. No, we don't have any Solid Waste people here with that figure.

Mr. ROGERS. Well, I won't pursue that at this time, if you will furnish that for the record.

(The following was submitted in response to the above:)

This information has not yet been cleared by the Office of Management and Budget. It will be provided as soon as such clearance has been obtained.

Mr. ROGERS. Don't you think it is a good idea to have specific deadlines as to when we should say everything must have primary, secondary, tertiary treatment before you dump it in the waters? Would you support that principle?

Mr. RUCKELSHAUS. I think it is a good idea to have deadlines. I think it is a question of whether it is to have deadlines in the act or whether to have deadlines administratively proposed.

Mr. ROGERS. Have you set any deadlines?

Mr. RUCKELSHAUS. No, because we don't have authorization to set deadlines at all.

Mr. ROGERS. Then we ought to either give you deadlines or give you the authorization to do it?

Mr. RUCKELSHAUS. Yes, I think that is right.

Mr. ROGERS. In principle, you are for that, as I recall, and you were for that in the air pollution bill.

Mr. RUCKELSHAUS. Yes.

Mr. ROGERS. We have that in the proposal and I am hopeful we can do it, and I see no reason why it could not be done legislatively.

Mr. RUCKELSHAUS. It is my belief that, where you have a problem that may be complex, as where different kinds of dumpings that are involved, that there ought to be legislative authorization to have administrative agency set the deadlines.

Mr. ROGERS. Perhaps if we set the goal, we may have some slippage—we hope not—but in principle you agree to the deadline idea?

Mr. RUCKELSHAUS. Yes.

Mr. ROGERS. Let me ask you this. Do you think municipal sewage and discharge of waste should be exempt from any requirement of permits?

Mr. RUCKELSHAUS. That is covered under the Water Pollution Control Act, Mr. Rogers. We think it is better to control it under that act than to try to control under the provisions of this act. We don't want to exempt them from regulation, but we think the regulation under the Water Pollution Control Act is a more effective way of doing it than through ocean dumping control legislation.

Mr. ROGERS. I thought you said the Refuse Act requires a permit issued by Army Corps of Engineers for discharge of waste other than municipal sewage.

Mr. RUCKELSHAUS. That is right. But we control the discharge of municipal sewage into the territorial sea under present law, the Federal Water Pollution Control Act, not under the Refuse Act. We are asking in our proposed amendment to the Federal Water Pollution Control Act to extend that coverage to the contiguous zone, and even beyond, when it originates here in our own country.

Mr. ROGERS. In other words, you do believe municipal systems should be covered?

Mr. RUCKELSHAUS. They should be controlled, not necessarily by permits.

Mr. ROGERS. Well, standards that they would have to meet, and so forth?

Mr. RUCKELSHAUS. Yes.

Mr. ROGERS. Now you also say dumping of some materials, such as chemical warfare materials and toxic industrial wastes, would be stopped immediately. Should this be stopped immediately?

Mr. RUCKELSHAUS. We think it should be, Mr. Chairman. That is why we suggested that power should be given to the agency to stop it, Mr. Rogers.

Mr. ROGERS. Is there any reason now why Federal installations couldn't immediately be stopped in line with the Presidential intent?

Mr. RUCKELSHAUS. I think that the Federal agencies, in compliance with Presidential Executive Order 11507, should stop the discharge of any of these materials.

Mr. ROGERS. Are you aware of the dumping that the Norton Air Force Base has been carrying on?

Mr. RUCKELSHAUS. I understand, Mr. Rogers, you brought that up yesterday and that Chairman Train was going to get you an immediate answer to it. I have just gotten back late last night and will work with him in getting that answer for you.

Mr. ROGERS. There was some discussion by the Norton Air Force Base that they were dumping, but they said it was treated by the public body into which they have arrangement to make their dumpings into the waste system. When we talked to the body out there, they said. "Oh, no, it must be treated by the people who dump before we accept it".

So, we have a chicken and an egg, and nobody does it. And do you recall any impact statement having been filed by them?

Mr. RUCKELSHAUS. No, I do not.

Mr. ROGERS. As a matter of fact, has any military installation filed an impact statement with your office?

Mr. RUCKELSHAUS. I think some of them have, but I can't give you any specific examples. But I would be glad to supply them for you.

Mr. ROGERS. I presume they would file it with counsel, but they would ask for your comment, wouldn't they?

Mr. RUCKELSHAUS. That is right. Under the Security Order we are the agency which gives them technical assistance in treating their waste of every kind.

Mr. ROGERS. Well, we checked with EPA yesterday and we find that no impact statements have been filed for military bases. Don't you think that is unusual?

Mr. RUCKELSHAUS. I not only think it is unusual, I am not sure it is even so.

Mr. ROGERS. That is what your people are telling us. I don't know whether I can believe them or not.

Mr. RUCKELSHAUS. Mr. Dominick says he knows of one or two.

Mr. DOMINICK. We have had impact statements filed with the agency.

Mr. ROGERS. For military bases?

Mr. DOMINICK. From military bases. I believe we had one filed from Fort Dietrick the other day with respect to certain chemicals that they were proposing to discharge into a sewage system.

Mr. ROGERS. Would you let us know for the record how many?

Mr. DOMINICK. We certainly will.

Mr. ROGERS. Because the information we got from your information people yesterday was that none had been filed.

(The information follows:)

ENVIRONMENTAL IMPACT STATEMENTS RECEIVED BY EPA AND ITS PROGRAM UNITS FROM THE DEPARTMENT OF DEFENSE—MAY 15, 1970–APRIL 15, 1971

DEPARTMENT OF THE ARMY, CORPS OF ENGINEERS

Anclote River, Fla.
Anacostia River and tributaries, Md. and D.C.
Atlantic Intracoastal Waterway bridges
Alpine Lake project, Texas
Arcadia Reservoir, Okla.
Arkansas-Red River Basins water quality control study, Texas, Oklahoma, and Kansas
Alpine, Pecos River, Tex.
Baker Brook flood protections project, Massachusetts
Baltimore Harbor and Channels, Md. and Va.
Brazos Island Harbor, Tex.
Brush Bayou, La.
Beals Creek at Big Springs, Tex.
Beaver Brook Reservoir, N.H.
Big Creek Watershed, Kans.
Black River Harbor, Alcona County, Mich.
Big Creek, Lower White River, Ark.
Baldwin and Hannon Sloughs, Ala.
Blue Marsh Lake project, Pennsylvania
Bucks Harbor project, Maine
Bristol Harbor navigation project, Rhode Island
Blue River, Mo. and Kans.
Bodega Bay dredging, California
Brookfield Lake, Mo.
Blue Springs Lake, Mo.
Brownsville Washington navigation improvement
Buchanan Reservoir project, Chowchilla River Basin, Calif.
Butler Valley Reservoir project, California
Cascadia Dam and Reservoir, Wash.
Cottonwood Creek, Stuart Gulch, Wash.
Calcasieu River, Devil's Elbow, La.
Cedar Bayou, Tex., navigation
Choptank River, Md.
Central and southern Florida, small boat navigation
Champlin Slough Watershed, Tehama County, Calif.
Chino Canyon improvement project, Polin Springs, Calif.
Citadel, santitary landfill permit request, Charleston, S.C.

Calcasieu River at Coon Island. La.
Charles River Dam, Mass.
Columbia River and tributaries, canyons 1 and 2, Wenatchee, Wash.
Clear Creek, Tex., flood control project
Cliff Walk, scienic restoration project, Rhode Island
Coos Bay, Oreg.
Corpus Christi ship channel—45-foot project, Texas
Corpus Christi ship channel, Port Aranas, Tex.
Corpus Christi Beach, Tex.
Corte Madera Creek, channel improvements, Marin County, Calif.
Candy Dam and Reservoir, Okla.
Cottonwood Creek, Calif.
City of Munday, Tex.
Crescent City Harbor Dredging and breakwater extension, California
Cucamonga Creek, San Bernardino, and Riverside Counties, Calif.
Clayton Dam and Reservoir, Jack Ford Creek, Okla.
Crutcho Creek, channel improvements
Delaware Bay-Chesapeake Bay Waterway
Delaware coast protection, Delaware
Des Moines River at Ottumwa, Iowa
Dewitt, Ark., flood control project
Dickey-Lincoln School Reservoir, Maine
Dunkirk Harbor, N.Y.
Dog and Fowl River, Mobile Bay, Ala.
Dodge City, Las Animas, Arkansas River and tributaries, Great Bend, local protection, New Mexico
East River and Steinway Creek, N.Y.
Duck Creek, channel improvement, Garland, Tex.
Buffalo Bayou and tributaries, Texas
Birch Dam, Birch Creek, Okla.
Cow Creek, channel improvement
Big Hill Dam and Reservoir, Big Hill Creek, Kans.
Copan Dam, Little Chaney River, Okla. and Kans.
Clinton Lake, Kans.
Chariton and Little Chariton Rivers, Mo.
Davis Creek Lake, Iowa
Entiat River, Seattle, Wash.
Elk Creek, Wash.
East River, N.Y. (spur channel to Astoria waterfront)
El Paso, local protection project and central area, Texas
Eastern Rapids and South-Central Avoyelles Parishes
Ellicott Creek, N.Y.
Boone, Colo., flood protection project
Bayou Des Gloises Division, channel and tributaries, Louisiana
Bayou Coden, Ala.
Bayous Rapids, Boeuf, and Cocodria and outlets, Louisiana
Edgartown Harbor, Edgartown, Mass.
Eldorado Dam and Reservoir, Walnut Creek, Kans.
Ellicott Creek project, New York
Fall River Harbor, channel project, Massachusetts
Fall River Harbor, navigation project, Massachusetts
Flood control on Merced County streams, California
Flood control project, Bennington, Vt.
Flood protection project, Danbury, Conn.
Fort Charles and Ivy Landing, drainage, district No. 5; and Stringtown Drainage and Levee District No. 4, Illinois
Fisheating Creek Area, Fla.
Fort Meyers Beach, Fla.
Four Mile Run, Va.
Flat Rock Creek, Tulsa, Okla.
Freeport Harbor, Tex.
Gilham Reservoir, Ark.
Gulf Intracoastal Waterway, Texas section
Frenchboro Harbor, Maine
Gulf Intracoastal Waterway, Offatts Bayou, Galveston, Tex.
Highland Bayou, Tex.

Frio River in the vicinity of Three Rivers, Tex.
Gulf Intracoastal Waterway, Chocolate Bayou, Tex.. navigation project
Gulf Intracoastal Waterway, mouth of Colorado River, Tex.
Galveston Harbor and Channel, Tex.
Giww, Port Isabel side, channels, Texas
Genesee River Basin study, New York
Geneva-on-the-Lake, Ohio
Gila River, below Painted Rock Dam, Ariz.
Gila River Canal, improvements, Arizona
Goleta, Calif., and vicinity, Santa Barbara County
Gordons Creek, Miss.
Intracoastal Waterway to vicinity of Boute, La.
Great Lakes and St. Lawrence Seaway, navigation season extension
Grand Lagoon, Fla.
Gypsum, Kans.
Hidden Reservoir, Madera County, Calif.
Hoonah Harbor, Alaska, harbor improvements
Hogtown Creek, Fla.
Jacksonville Harbor, section 2, Florida
Humboldt Harbor at Sand Point, Alaska
Ipswich River, navigation project, Massachusetts
Ico Harbor Dam, Wash.
Indian Bend Wash project, Arizona
Jack and Simmerly Sloughs area, Calif.
John F. Baldwin Ship Canal project, California
Kaimu Beach, Hawaii
Kaneohs-Kailua Area, Oahu, Hawaii
Lahaina small boat harbor project, Hawaii
Lake Port Reservoir, Lake County, Calif.
Lee County, Fla., beach erosion control
Jacksonville Harbor, section 1, Florida
Lavon Dam and Reservoir, modification; and East Fork Channel, improvement, Texas
Lido Key, beach erosion control, Sarasota County, Fla.
Las Cruces, local protection project, Las Cruces, N. Mex.
Lake View Dam and Reservoir project
Ludington Harbor, Mich., commercial harbor improvement
Lake Wichita, Holiday Careek, Tex.
Los Esteros, Pecos, Espanola, N. Mex.
Manteo (Shallow-Bay) Bay, N.C., navigation
Logan Airport, Mass.
Lagoon Pond, navigation project, Massachusetts
Local protection project, Marion, Kans.
Long Sands-Beach project, Maine
Lower San Joaquin River and tributaries, Sanislaus County, Calif.
Merrimack River, N.H., Hooksett project No. 1913
Marysville Reservoir, Yuba River, Calif.
Massachusetts 1 ort Authority, airport expansion
Merimentau River, La.
Mountain Creek, Trinity River Basin, Tex.
Laneport, North Fork and South Fork Lakes, San Gabriel River, Tex.
Lufkata Reservoir, Okla.
Little Rock Levee (Eastend-Fourche Bayou) Ark.
Kaw Dam and Reservoir, Arkansas River, Okla.
Little Blue, channel improvement, Missouri
Loup River, Nebr.
Lower Granite, Walla Walla, Wash.
Lake Stevens, Wash., section 205, Seattle, Wash.
Lapwai Creek, Idaho
Metlakatla Harbor, Metlakatla, Alaska
Mill Creek, interior survey report, southwest Ohio
Quachita, Monroe, Bayou Bartholemew, and Yazoo River, La.
Mississippi River between Columbus and Huhman, Ky.
Mississippi River, east bank, Warren to Wiikinson, La.
Mojave Forks Reservoir, recreational facilities, California
Mississippi River, flood control, Winona, Minn.

St. Bernard Parish, La.
Scayaquada Creek, N.Y.
San Diego Harbor, navigation channel improvements, California
Sacramento River, bank protection, California
Selma and Selmont, Ala.
Singlaw River, navigation channel, Washington
Shidler Dam and Reservoir, Salt Creek, Okla.
Sheyenne River N. Dak.
Short Sands Beach project, Maine
Smithfield Lake, Mo.
Snohomish River and tributaries, Washington
Slaughters Bar, Wash.
Scappoose drainage district, flood protection
Souris River, N. Dak.
South shore of Lake Ontario, Fort Niagara State Park, N.Y.
Streams in vicinity of Fairfield, Calif.
Tahquitz Creek, debris basin and channel, Palm Springs. Calif.
Tampa Harbor, Fla., navigation
Taylor Bayou, Tex., flood control and major drainage project
Texas City Channel, Tex., industrial canal
Tocks Island Reservoir project
Tybee Island, Ga., beach erosion control and hurricane protection Tyler Island, Ga.
Verona (Staunton) Dam and Reservoir project, Virginia.
Great South Bay and Patchoque River, N.Y.
Pascagoula River Basin, Miss. and Ala.
Pajaro River levies, Santa Cruz and Monterey Counties, Calif.
Potomac River Basin, Sixes Bridge, Dam, and Reservoir project
Panama City Harbor, Fla.
Port Huen[illegible]me Harbor project, Ventura County, Calif.
Protective sandfill, Key Biscayne Beach, Fla.
San Diego River, Mission Valley, San Diego, Calif.
Spring River and tributaries, Mo., Kans. and Okla.
Mississippi River from Cassville, Wisc. to mile 300
Mississippi River at Moline, Ill. and Davenport, Iowa
Mississippi River, Gulf outlet, Michoud Canal, La.
Mobile Harbor, Ala.
Monterey Harbor, breakwater project, California
Missouri River, N. Dak., S. Dak., and Nebr.
Murrells Inlet, Georgetown County, S.C.
Napa River flood control project, California
Nawiliwili, deep-draft harbor, Kauai, Hawaii
New London Harbor project, Connecticut.
New London hurricane barrier, Connecticut.
New Jersey coastal inlets and beaches, Great Egg Harbor Inlet to Stone Harbor
Nookagee Reservoir project, Massachusetts
North Nashua flood protection project, Massachusetts
North Nashua River, channel project, Massachusetts
Okeechobee Waterway, Fla.
New Madrid, Mo.
Osceola, Ark.
Jefferson River, Mont.
North Shore of Long Island, Suffolk County, N.Y.
North Harbor, Wisc. (Dorr County)
Lytle and Warm Creeks, San Bernardino, Calif.
Oachita and Black Rivers, navigation project, Arkansas and Louisiana
Ottawa River Harbor, Mich. and Ohio
Oakland, interharbor dredging, Alameda County, Calif.
Ohio River locks and dams, annual list of repair and maintenance projects
Pamlico River and Morehead City Harbor, N.C., navigation
Park River flood protection project, Connecticut
Pax Creek, snagging and clearing project, West Virginia
Peyton Creek, Tex.
Pattonsburg Lake, Mo.
Peripheral Canal, Central Valley project, California
Placer Creek at Wallace, Idaho

62-513—71——27

Phillips Reservoir, Mass.
Piogahammond Reservoir, Pa.
Pleasant Bay, Cape Cod, Mass.
Oak Bluff Beach project, Massachusetts
Pisquatua River project, New Hampshire.
Point Place, flood control, Toledo, Ohio.
Platte River, channel, Missouri
Port San Luis project, California
Port Sutton, Fla. navigation
Pine Bluff, Bayou Bartholomew, flood study
Portugues and Bucance Rivers. Puerto Rico
Posten Bayou, proposed flood control study
Red Brook Harbor project, Massachusetts
Red Run Drain, Clinton River, Mich.
Roseau River, Minn.
Reservoir operations
Reedy River, S.C., Salvda River Basin
Red River of the North, flood control project, Pembina, N. Dak.
Revene and Nantasket Beaches, Mass.
Skiatook Dam and Reservoir, Okla.
Ridgecraft Wash., channel, Kern County, Calif.
Stillwater Creek and tributaries, Stillwater, Okla.
San Antonio, improvement, San Antonio River and tributaries, Texas
Running Water Draw Watershed, Plainview, Tex.
Running Water Draw, Brazos River, Tex.
Spring Creek, Springdale, Ark.
Sabine River and tributaries, Texas and Louisiana
San Antonio, channel improvement project, Texas
Sand Bar, Milton, Vt.
San Leandro Marine, Alameda County, Calif.
Stratford hurricane barrier, Connecticut
Saxonville flood protection project, Massachusetts
San Luis Rey River, San Diego County, Calif.
Sanoma Creek, channel improvement, California
Small beach erosion project, Broadkill Beach, Del.

DEPARTMENT OF THE ARMY, OFFICE OF THE SECRETARY

Project Eagle, phase I—Disposal of mustard gas at Rocky Mountain Arsenal, Denver, Colo.
Plan to dispose of biological agents and toxins at Fort Detrick, Md.; Pine Bluff Arsenal, Ark.; Beale Air Force Base, Calif.; and Rocky Mountain Arsenal, Colo.

DEPARTMENT OF THE AIR FORCE

Luke Air Force Base, runway improvements

DEPARTMENT OF THE NAVY

Underwater demolition of dud ordnance near the island of Culebra
Santa Rosa Wash project, Pinal County, Ariz.
Santa Paula Creek project, Ventura County, Calif.
Sunset Cliff, segment B, shore protection improvement, Osprey Street to Ladera Street, city of San Diego, Calif.
Edwards Underground Reservoir, Guadalupe, San Antonio, and tributaries, Texas
Tennessee, Tombigbee Waterway project
Theodore Ship Channel, Mobile Harbor, Ala.
Corps of Engineers Circular No. 1120-2-69, guidelines
Tangipahoa River and tributaries, Louisiana
Vicksburg, Yazoo areas, Miss.
Project No. 1194, Union River, Maine
Whiteriver to Augusta, Ark.
Survey of Gulf Intercoastal Waterway to vicinity of Boutte, La.
Ventura, Marina project, California.
Trumbull Pond Reservoir, Conn.
Tahquitz Creek, debris basin and channel improvement, Palm Springs, Calif.

Walnut Creek, Contra Costa County, Calif.
Trexler Lake, Lehigh County, Pa.
Westerly, R.I., hurricane barrier
Whitmanville Reservoir, Mass.
West Tennessee tributaries projects
Wallisville Lake, Trinity River, Tex.
Turtle Creek, Yukon, Okla.
Whitney Lake, Brazos River, Tex.
Waurika Dam and Reservoir, Beaver Creek, Okla.
Water quality control projects, study, Arkansas, Red River Basins, Tex., Okla., and Kans., part II
Woodbine Lake, Kans.

Mr. ROGERS. Do you know of any others besides the one you mentioned?

Mr. DOMINICK. We have had a number of statements filed with respect to the dumping of munitions and other matters. Many of those, of course, have been thoroughly reviewed by the Congress and by others. But impact statements were filed in the more celebrated cases.

Mr. ROGERS. The nerve gas dumping?

Mr. DOMINICK. That is correct.

Mr. ROGERS. I was speaking of other than those that the Congress has gone into. Well, if you will submit that.

I think it is important, if we are going into this whole question, to set up some organization with some toxicologists in it. Do you have any toxicologists operating in your Agency?

Mr. RUCKELSHAUS. Yes, we do.

Mr. ROGERS. What is your setup there?

Mr. RUCKELSHAUS. We are attempting to increase the number of toxicologists that we have in the Agency.

Mr. ROGERS. How many do you have?

Mr. RUCKELSHAUS. I can't give you the specific number, because it changes from day to day. We are trying to hire toxicologists, who are in short supply in our Agency and, indeed, in the country. Particularly in our pesticides office we have a program to hire immediately approximately 50 additional toxicologists, in order to comply with the provisions of the Pesticides Act.

Mr. ROGERS. But you don't know how many you have presently?

Mr. RUCKELSHAUS. I can't give that exact figure. I can supply it for the record.

Mr. ROGERS. Do any of your people here know?

Mr. RUCKELSHAUS. Not that I know of.

Mr. ROGERS. Nobody knows? I won't pursue it, if no one knows.

Mr. RUCKELSHAUS. We can supply that information for the record.

Mr. ROGERS. I think it would be helpful. I realize you can't keep everything in your head, but I think it would be helpful to have supporting people here who could give us some of these answers that we need to pursue.

(The information follows:)

TOXICOLOGISTS EMPLOYED BY THE ENVIRONMENTAL PROTECTION AGENCY

At the present time the Environmental Protection Agency employs 45 toxicologists, all of them in the Pesticides Office. There are plans for the hiring of 13 additional people in this field in fiscal year 1971.

In the Pesticides Office toxicologists review products, labels, and laboratory reports for determination of compliance with label requirements. Toxicologists

also review petition toxicology data for evaluation of sufficiency, and participate in the establishment of tolerances for pesticides residues on or in food and feedstuffs, They conduct (a) studies on the toxic action of pesticides in small animals from low and high level exposure, (b) research on the toxicological effects of pesticides administered to subhuman primates to determine potential risks from long-term low level exposure, and (c) toxicological studies of pesticides residues in estuarine and marine life.

Mr. ROGERS. Let me ask you this, Mr. Ruckelshaus. You have the authority to ban mercury being discharged into the waters?

Mr. RUCKELSHAUS. We would have the authority, Mr. Chairman. In any instances where a toxic substance is discharged into a waterway it is in violation of water quality standards. And in the case of mercury—we have, in all of the industries and installations where we have found discharge of mercury to be occurring, stated that it was our goal to eliminate any continued discharge of mercury from manmade sources into the environment.

Mr. ROGERS. Is there any reason why it shouldn't be banned? Really, we know the results of mercury in the water, don't we?

Mr. RUCKELSHAUS. That is right.

Mr. ROGERS. We know how to remove it and to neutralize it. It is very simple. Is there any reason why it should not be banned?

Mr. RUCKELSHAUS. In discharging into the water, no: not that I know of.

Mr. ROGERS. Would you consider doing that?

Mr. RUCKELSHAUS. That is what we have stated.

Mr. ROGERS. You say you want to phase it out. I am saying a ban, a deadline; no more mercury in the water.

Mr. RUCKELSHAUS. That is essentially what we have done, Mr. Rogers.

Mr. ROGERS. I don't think it is at all, Mr. Ruckelshaus. I know we can talk about this, but it has not been banned. Mercury is still being put into the waters of the United States. You know it and I know it. In fact, even some of the cases that were settled authorized them to do it. Why shouldn't it be banned, if you have that authority? We know it is bad, there is no question about it. Shouldn't it be banned?

Mr. RUCKELSHAUS. I believe that what we are working toward is the ban of mercury.

Mr. ROGERS. I am not saying working toward, I said ban now, a deadline saying "No more after next week." Is there any reason this shouldn't be done?

Mr. RUCKELSHAUS. There is no reason.

Mr. ROGERS. Would you consider doing it?

Mr. RUCKELSHAUS. Yes; I would.

Mr. ROGERS. Would you let me know what your decision finally is?

Mr. RUCKELSHAUS. Yes: I will.

(The information follows:)

EPA POSITION ON DISCHARGES OF MERCURY

The position of the Environmental Protection Agency regarding the discharge of mercury to public waters is to eliminate all man-made discharges. EPA's approach to this goal is to seek from all known industrial mercury dischargers an immediate and substantial reduction. Experience shows this to be possible and practical in most cases with a nominal effort on the part of industry. In

a few cases substanital effort was required by industry, but significant results have been achieved nevertheless. Given additional short periods, a matter of a few months, more sophisticated process, product, or raw material changes plus special treatment techniques have resulted in further reductions in mercury discharges. The search for additional measures which will eliminate the addition of mercury to the aquatic environment then continues, and complete elimination has been achieved in some cases. This effort to identify and eliminate major and specific point sources of industrial mercury discharge has resulted in measurably improved conditions in waters formerly affected.

In addition to the identifiable industrial sources of mercury entering the nation's waters, there are ubiquitous sources from man's activity, which at the source may be undectectable but when collected constitute measurable amounts. A proposal to immediately ban the discharge of all mercury into navigable waters would not recognize the practical limitations imposed by the fact of innumerable sources. Hospitals, dental clinics, university laboratories, medical and scientific research facilities and many varieties of small businesses may all discharge quantities of materials containing mercury. Most often these are collected in municipal systems, where after treatment mercury may still be found in the treatment plant discharge. The methods and alternatives available to these small but numerous sources are far more limited than to a large industrial source. Additionally, the capabilities of municipal waste treatment plants are limited for purposes of completely eliminating any particular waste constituent, including mercury.

The Environment Protection Agency along with other governmental agencies and private concerns, having recognized the potential threat from increasing mercury discharges before any known damage to man has occurred in the United States, have reversed that trend. With continued progress in the reduction of man-made mercury discharges and continued enforcement of appropriate limitations on water and food and the use of mercury in materials such as pesticides and fungicides, it is believed that public health is adequately protected.

Mr. Rogers. Then one last question. How many enforcement actions have been taken by your agency under the Air Pollution Act? I know when you first came in you said you were concerned because only one of any significance had been taken. Have you instituted any enforcement actions?

Mr. Ruckelshaus. One of the problems we have under the Air Pollution Act is that we have two essential enforcement responsibilities. One is to enforce the implementation plans as adopted by the States now. Under the Clean Air Act of 1970, those implementation plans are not adopted for 17 to 19 months after the last day of last year. We have an emergency power under the act where there is a substantial endangerment to human health.

Mr. Rogers. Has that authority been used at all?

Mr. Ruckelshaus. That authority has not been used, because we have not as of this time found any circumstances where this occurred where we were not able to get the particular discharger into compliance without going to court. But we would certainly use that power if we found it necessary.

Mr. Rogers. How many actions have we had? The only ones I recall were in West Virginia, the chicken case; and the ones in West Virginia were initiated by the Governor.

Mr. Ruckelshaus. That is because of the cumbersome administrative hearing procedures that were superceded by the Clean Air Act of 1970. Once we get implementation plans adopted, we don't have to go through those cumbersome procedures of having enforcement conferences and hearings before we get into court.

Mr. Rogers. We passed the act last year and I want to know if any actions have been instituted this year.

Mr. RUCKELSHAUS. There is no difference in the emergency provisions under the old act and under the new act, but all of our enforcement procedures under the new act are tied into implementation plans which will not be adopted for 17 or 19 months.

Mr. ROGERS. I would like to pursue this more, but the chairman tells me I have exceeded my time. I am very much concerned that no enforcement action has been taken by the agency.

Mr. DINGELL. Mr. Goodling?

Mr. GOODLING. Thank you, Mr. Chairman. I shall be very brief.

Mr. Ruckelshaus, has your agency been in existence long enough to have given thought to ocean dumping of automobiles?

Mr. RUCKELSHAUS. No. The Federal Water Quality Administration, which has been doing some research into certain problems involving ocean dumping, prior to coming into EPA.

Mr. GOODLING. Have you come up with any answers whether it is good or not? Is any harm being done by dumping of junked automobiles?

Mr. RUCKELSHAUS. I don't know the answer to that question.

Mr. DOMINICK. No, we don't have specific answers to that question, Mr. Goodling. I believe that there may be some areas in the oceans where the placing of junk automobiles would be beneficial to fish life, to provide a proper habitat. We can supply answers to you on what studies have been done by the Bureau of Sport Fisheries and Wildlife on that aspect.

(The information follows:)

STUDIES ON FISH HABITATS OF JUNKED AUTOMOBIES

Such information is contained in the attached reports at the pages indicated:
Progress in Sport Fishery Research, 1966, p. 12.
Progress in Sport Fishery Research, 1967, p. 176.
Progress in Sport Fishery Research, 1968, p. 17.
Progress in Sport Fishery Research, 1969, p. 185.

[Progress in Sport Fishery Research, 1966, p. 12]

ARTIFICIAL FISHING REEFS

Automobile body reef study

We made monthly SCUBA observations on the laboratory's pilot artificial reef, two miles off Monmouth Beach, New Jersey. Construction material consists of 16 junk automobile bodies sunk in a depth of 55 feet. The cars are supporting a moderately heavy growth of encrusting organisms—primarily barnacles and hydrozoans. The initial fouling rate during the last summer was very rapid, but with decreasing temperatures the accumulation of encrusting organisms has been reduced. A differential setting of organisms occurred on the car surfaces. The heaviest fouling was on the painted and chrome surfaces of the bodies. The unpainted and rusted surfaces of the engine and chassis have few marine organisms attached.

The fish attracting quality of this test reef became evident soon after it was established. The fish observed in or about the reef were: tautog, cunner, black sea bass, scup, summer flounder, Atlantic mackerel, pollock, longhorn sculpin, puffer, searobin, and ocean pout. Although most of the fish observed were adults, there were also juvenile pollock, puffers, searobins, and hake. If the reef can function as a nursery habitat, it will not only attract angling size fish from other areas, but may enable fish to survive past the larval stage.

We noted a seasonal change in the reef fauna in December. Water temperatures dropped and longhorn sculpins, a boreal species, had moved to the reef.

The two most common demersal invertebrates encountered in the reef were starfish and sand dollars. We found both species densely packed under and

around the car bodies. The reason for the aggregation is not understood, but it might be a response to eddy currents that are set up by the car bodies. The starfish were frequently aggregated into "balls," made up of several individuals.

The Bureau of Economic Research, Rutgers University, in cooperation with our Laboratory, has submitted a research proposal to the Ford Foundation for an economic feasibility study on the utilization of metropolitan wastes for the construction of artificial fishing reefs.

A cooperative artificial fishing reef program was arranged with the Bureau of Marine Fisheries, New York State Department of Conservation. We plan to construct a pilot-reef off Rockaway Beach utilizing a permit issued to the State of New York by the Corps of Engineers. Other areas along the Long Island littoral zone and some old artificial reef sites will be made available to us to study. This joint effort with New York will extend our research activity into an area of heavy recreational angling.

LARRY OGREN.

[Progress in Sport Fishery Research, 1967, p. 176]

ARTIFICIAL REEF ECOLOGY

Our artificial reef program which began last year with the construction of a pilot study reef off Monmouth Beach, New Jersey, is designed to determine the effect of artificial fishing reefs on the distribution and abundance of marine game fish. From the data that we have gathered this year, and information available from other sources, there is no doubt that artificial reefs attract fish. However, we believe these reefs may not only congregate available fish, but also serve to increase the size of some populations by providing additional spawning sites for adults and protected areas and food for the young.

We selected five sites between New York and Miami in addition to our Monmouth Beach site, to compare species composition of fish and invertebrates attracted to reefs in different latitudes and environments. In choosing these sites, we considered depth of water, bottom type, ease of access by our survey team and distance to coastal population centers. After selecting sites off Atlantic Beach, Long Island, New York; Kiawah Island, South Carolina (south of the Charleston Harbor entrance); Jacksonville Beach, Florida; Palm Beach, Florida, and in Biscayne Bay, Miami, Florida, we surveyed each area and assembled a list of the marine plants and animals present before placement of the reef materials.

The initial construction phase is now complete on five of our sites and a sixth, off Palm Beach, Florida is in the planning stage.

Part of our study is to determine (1) what type of reef material is best for attracting fish and encrusting organisms, (2) life expectancy of the material and (3) cost, both of material and handling. We are using a number of test materials including scrap metal, mostly in the form of junk car bodies, concrete culvert and old tires. Tires were arranged in units of twelve, spaced on reinforcing rods and weighted with concrete.

We plan to complete construction of our Palm Beach reef and add a number of different materials at the other reef sites. Our direct observational techniques using SCUBA to study the distribution of game fish on the reef sites will be complemented with the use of fish traps for mark and recapture studies and hook and line methods by project personnel and cooperating sport fishermen. Our plan includes close and continual cooperative research efforts with State conservation departments. We are compiling a checklist of artificial and natural reefs along the Atlantic coast from Maine to Florida to aid in future reef site selections, for possible comparisons of new and old artificial reef habitats with natural reefs and for dissemination to interested sport fishermen.

RICHARD B. STONE.

Life history and behavior of reef fishes

We expanded our research diving activities to include the newly constructed artificial reefs in waters from New York to Jacksonville, Florida. On the older, established reefs we saw new and changing faunas. Reef materials have not been scattered or buried by storms or currents.

In New Jersey, the year-old car body reef still afforded considerable relief above the flat surrounding area. Currents scoured the sands out from under chas-

sis, which now rest on bed rock. Early in the year the reef took on a different appearance; the thin barnacle growth that covered the reef the first year was almost completely replaced by a settlement of small mussels. Tautog, cunner and black sea bass replaced the winter-spring fish fauna of ling and ocean pout. We observed breeding behavior and collected ripe specimens of the cunner population occupying the reef. Later in the summer, juvenile cunner appeared on the reef, darting in among the hydroid growth. Such evidence suggests that artificial reefs can increase fish production as well as afford temporary haven and feeding sites to adult fishes.

Preliminary trapping efforts to obtain animals for tagging and recapture data were successful. A non-baited trap (modified lobster pot design) caught tautog, cunner and black sea bass. The range of tautog appeared to be confined to the immediate reef site since only traps placed within the reef caught tautog.

In August we constructed and placed a twenty unit tire reef (12 tires per unit) about a mile south of the existing Monmouth Beach car reef. Black sea bass immediately occupied this reef.

In November, off Jacksonville, Florida, divers observed a large assemblage of fish on the three-month-old-reef. Over twenty species were counted, many prime sport fish. Several hundred large amberjack schooled over the top of the reef. Grouper, snapper, sheepshead, and black sea bass occurred in and around the culverts and cars. Groups of car bodies, cabled together, were completely hidden from view by dense schools of small porgies and grunts. Of the organisms encrusting the reef, barnacles dominated, with lesser numbers of hydroids and tube-building worms present. Although we saw many species of fishes in this area before construction of the reef, such dense concentration had not been observed before. This reef is successfully attracting large numbers of adult animals from other areas.

In Charleston, South Carolina, a similar but much more dramatic change in the local fauna was effected by construction of a car reef on the sand flats off shore. Pre-construction dives revealed a fish fauna of scattered sea robins, razorfish and jawfish. In December, twelve species of fishes were observed on the first inspection dive on the reef. Principal forms were bluefish, black sea bass, filefish and longspine porgies, with several large sheepshead and black drum. Barnacles, an important food item, were the most numerous encruster on the car bodies. The sediments around car bodies in contact with the sand bottom had been scoured out about one foot, exposing a fossil oyster reef, a stratum which should prevent the reef from settling any deeper.

We completed necessary plans for a January survey of our artificial reef in Biscayne Bay, Miami, Florida.

LARRY OGREN.

[Progress in Sport Fishery Research, 1968, p. 17]

ARTIFICIAL REEF DEVELOPMENT AND MANAGEMENT

In 1968, we added a sixth unit to our series of experimental artificial reefs. This one off Palm Bleach, Fla., is made up of three sunken ships—the 185-foot vessel Mizpah, a 165-foot steel Navy patrol craft, and a 485-foot section of the Greek freighter *Amaryllis*. It is in 85 feet of water, about a mile offshore just north of Lake Worth Inlet. Observations on the reef have shown a good population of fishes around the vessels.

During 1968, we also added to existing reefs. In August we put a small tire reef down on the Monmouth Beach, N.J. site. To minimize preparation costs we tried the simple method of stringing tires on scrap anchor chain and put 1,100 tires into a 1,250 square foot area. Our reef off Rockaway Inlet, N.Y. was increased in October when the New York State Conservation Department deposited 200 tons of concrete culvert and approximately 25 three-tire units.

Our experience shows scrap tires make the best reef material in many areas. Tires are easy to obtain because they are a nuisance to dispose of on land but are easy to handle and can be formed into units of almost any size. Techniques for handling the masses of tires necessary to cover acres of bottom are under study with funds supplied by the U.S. Public Health Service. The development of an effective disposal technique will have many benefits: it will produce more favorable habitat for coastal fish, help solve a critical disposal problem, and increase the esthetics of the landscape.

We received reports of excellent catches made by our Miami and Jacksonville test reef sites. Fishermen in the Charleston, S.C. area also reported successful fishing on our small test reef about 9 miles off Kiawah Island. To develop a more stringent test of the utility of artificial reefs, we began planning a quantitative study of ang'er use and fishing success. Our preliminary studies will incorporate various techniques to determine the most reliable and efficient method of executing a major creel survey. We will expand the study to obtain comparative estimates of total harvest, fishing pressure and catch per unit of angling effort for each reef site and adjacent natural fishing areas.

Our survey of artificial fishing reefs for the Atlantic and Gulf coasts is near completion. When finished it will show the location, history, composition, and fish life for all major Atlantic and Gulf coasts artificial reefs.

RICHARD B. STONE AND CHESTER BUCHANAN.

Monitoring and fish population studies

During 1968, we inspected all the test reef localities except Jacksonville in underwater surveys. We also made a pre-construction survey for the Palm Beach, Fla., site that showed the ocean nearly barren of fish before the reef was built—only a few individual grunts, porgies, and snappers had been observed foraging over the bottom. We also were able to inspect an artificial reef in the Virgin Islands, built 8 years ago from 800 concrete building blocks. Fishes were abundant and diverse, but the low biomass of encrusting organisms was the principal ecological difference between tropical and temperate reefs.

We conducted trapping and tagging experiments at the New Jersey and South Carolina automobile reefs during spring and summer, and estimated catch rates at the New York reef using multiple hook sampling rigs. We received one unusual tag return. A tautog marked and released on our New Jersey car reef in November, 1967, was recaptured one year later in eastern Long Island Sound, about 100 miles from the release point.

Fish stomachs and gonads were collected and preserved since analysis of the prey and forage items can help us describe the degree of reef dependency of each species.

Population estimates of the larger black sea bass on the South Carolina automobile reef showed that 200 adults occupied the 0.1 acre reef in April and May. A related species, the rock sea bass, showed a different behavior which should reduce interspecific competition. Rock sea bass were confined to the lower reaches of the reef and always in contact with the substrate. Black sea bass, the dominant form, were widely distributed, occasionally resting on parts of the reef and bottom but usually swimming in and around the reef. They do not appear to exclude rock sea bass from occupying their restricted lower portion of the reef.

Our Monmouth Beach, N.J., scrap tire reef (.03 acre) was observed to have a five to ten foot profile. Initial occupants included tube-building polychaete worms, small lobsters, and cunner. Our New York reef was inhabitated by hundreds of 2-3 pound squirrel hake last summer. In December we caught codfish as well as tautog, cunner and squirrel hake.

A variety of bottom animals died in considerable numbers along the northern New Jersey coast in September. First reports were received from SCUBA divers visiting wrecks. We investigated and found dead and dying lobsters, cancrid crabs, ocean pout, and cunner on both wrecks and natural reefs. In addition to these species, sport divers reported seeing dead surf clams, sea stars, black sea bass, and flatfish. We recorded such low levels of dissolved oxygen (0.34–0.72 ml/liter) at one wreck where we found distressed fish and dead lobsters that the cause of death here was obviously suffocation. We are not sure what caused the oxygen deficiency. The coastal area affected was extensive, and apparently restricted to depths of less than 100 feet. Die-off of the red tide blooms are implicated.

LARRY OGREN AND JAMES CHESS.

Distribution and ecology of attached marine organisms

We continued investigation of attached marine organisms using the Multiple Disc Sampling Apparatus (MDSA). The third MDSA was placed off Cow and

BEST COPY AVAILABLE

Calf Reef, St. Thomas, V. I., in February, and a fourth unit was installed at Gloucester, Mass. in May. Cooperating scientists subsequently developed MDSA sites at Mattituck, L.I., N.Y. and Key West, Fla.

Discs from the site near the New Jersey car reef have been collected monthly and analyzed. Attached organisms fluctuated seasonally (fig. 21) with general low levels of reproduction and colonization during winter months. Unattached, motile organisms such as gammarid and caprellid amphipods tend to occur when habitat or cover is provided by hydroids or tube-building polychaete worms.

Severe competition for settling space develops between those epibenthic invertebrate species fed upon by finfish and forms not generally used as food. Several large invertebrate species have been noted to compete directly with finfish for available food species. The seastar, *Asterias forbesii*, is an important predator on mussels and barnacles at the New Jersey site. The sea urchin, *Lytechinus sp.*, is the dominant invertebrate predator on the Charleston, S. C. site and competes with game fish for barnacles, a dominant encrusting food species at this site. A small flatworm, *Stylochus sp.*, is an important predator on barnacles at both the New Jersey and Charleston, S. C. site.

Gut content of fishes associated with car reefs and MDSA sites were analyzed to determine game fish use of epibenthic resources. The diet of many fishes indicates they feed exclusively upon attached and motile epifauna and are in direct competition with each other and invertebrate predators. Other species appear to forage on invertebrate species which live in bottom sediments adjacent to car reefs. These infaunal species are therefore important in the ecology of certain reef dwelling fishes.

During the first 10 months of the study, rubber appeared to be the most desirable substratum for colonization by most epibenthic organisms. After 18 months, however, concrete appeared to be an equally effective or superior substratum. Certain chemical components of raw concrete may have leached out which had inhibited normal settlement or attachment of invertebrate larvae on the concrete discs during the early months of submergence. Steel, the poorest substratum in terms of colonization by epibenthic invertebrates, undergoes rapid corrosion which prevents formation of well developed communities.

Discs from the Virgin Island MDSA site indicated the development of epibenthic associations on artificial habitats proceeds slowly in this tropical environment. Evidently, finfishes grazing removed the fauna as rapidly as it became established.

JACK B. PEARCE AND JAMES R. CHESS.

[Progress in Sport Fishery Research, 1969, p. 185]

ARTIFICIAL REEF DEVELOPMENT AND MANAGEMENT

During the first four years of our artificial reef program we found answers for many of the questions we posed at the inception of this study. Some of the information we can now provide includes: 1) the cost and methods of building reefs with several different materials, 2) life expectancy of car body reefs, 3) techniques to use in building effective tire reefs, 4) which substrate appears to be most effective for colonization by epibenthic organisms, and 5) feeding habits of various fish on artificial habitats.

There are still many questions we are trying to answer. One of the problems that has confronted us throughout our study is highly restricted visibility on our artificial reefs in the New York Bight because of turbid water conditions. We had hoped to obtain quantitative data on fishes and study their behavior on artificial habitats through the use of SCUBA. With poor visibility, however, this has proved impractical.

With the addition of two reefs, one off Sea Girt, N.J., and the other off the coast of southern Georgia, we now have 8 experimental reefs under study. We gave technical assistance to groups creating 8 more reefs along the east coast, two off the coast of New York, one in Chesapeake Bay, three off the coast of South Carolina, and one each in Georgia and Florida. We completed a preconstruction survey and site selection off Chincoteague, Va., in a cooperative experimental reef effort between the Chincoteague National Wildlife Refuge and the Sandy Hook Marine Laboratory.

Our cooperative study with the Environmental Control Administration's Bureau of Solid Waste Management investigating the use of scrap tires as arti-

[illegible]

ficial reefs was highlighted by the installation of 35,000 tires on two reef sites in the New Jersey-New York area. We tested different techniques of incorporating scrap tires as reef-building material in configurations that provided necessary relief, ease of handling, and low cost. These are necessary criteria if the material is to be practical for use by sport fishing groups and conservation agencies. After selecting a combination of rod units and single tire units, we deposited 30,000 tires between June and October on the Atlantic Beach artificial reef off southern Long Island. We then deposited 5,000 tires in November on our new experimental reef site off Sea Girt, N.J.

Our inspection dives on the Jacksonville and Palm Beach, Fla., reefs revealed numerous game fishes of many species and a thick growth of encrusting organisms on the materials at both reefs. The car bodies on the two-year-old Jacksonville reef showed appreciable deterioration. The car frames remained intact and supported a considerable growth of invertebrates but the thin metal of the roof and sides of many cars had disappeared.

To compare the biomass of encrusting organisms on artificial reefs with populations on natural bottom around the reef, we resumed and refined the tabulation of data collected on a benthic survey off southeastern Long Island from February 1966 to January 1967. Two polychaetes were tentatively identified as new to this area. We found three types of invertebrate distribution present in this area, two specific and one ubiquitous.

RICHARD STONE AND CHESTER BUCHANAN.

Creel survey technique

We developed and tested several creel survey methods for estimating fishing pressure, catch per angler hour, and anglers' total harvest around artifical reefs. We defined the angling population in our study area as all sport fishermen fishing beyond the surf zone between Manasquan Inlet, N.J., and Jones Inlet, N.Y. To sample this population, we divided the anglers into two groups: 1) party and charter boat anglers and 2) private boat anglers.

In our first attempts to gather information from party boat anglers, we distributed a limited number of log books to the captains and attempted to interview the anglers when they returned to the docks. The dockside interviews proved impractical. However, we are getting encouraging results from the log book returns.

We designed a mail survey which proved to be the best sampling method for private boat anglers. We identified the owner of a particular boat by recording his registration number as he passed an observation point and then checking with the State Marine Police to see who owned the boat. Then we mailed questionnaires to 196 boat owners. We received completed questionnaires from over 80 percent of the boat owners sampled. Errors introduced from non-response were minimal—a follow-up survey differed by only 0.07 fish per hour in the estimate of fish per angler hour and 4 percent in the number of unsuccessful anglers. We are using aerial surveys to estimate total angling pressure in the test area.

CHESTER BUCHANAN AND RICHARD STONE.

Mr. GOODLING. Are you going to continue to attempt to rid the countryside of these unsightly automobiles? To my way of thinking, they are a pollutant just as much as many other things.

Mr. RUCKELSHAUS. Yes, we are, Mr. Goodling. Under the Resource Recovery Act of 1970, we are specifically authorized and told to look into this problem and come up with some solutions for it.

Mr. GOODLING. You are working on that problem?

Mr. RUCKELSHAUS. Yes, we are.

Mr. GOODLING. That is all, Mr. Chairman.

Mr. DINGELL. Thank you, Mr. Goodling.

Mr. Downing?

Mr. DOWNING. Thank you, Mr. Chairman.

Mr. Ruckelshaus, this bill excludes rivers and harbors, does it not?

Mr. RUCKELSHAUS. It does not exclude harbors from its provisions.

Mr. DOWNING. It excludes rivers?

Mr. RUCKELSHAUS. It would exclude, yes, it excludes rivers. These are covered under Federal Water Pollution Control Act.

Mr. DINGELL. If the gentleman would yield, it doesn't include rivermouths.

Mr. RUCKELSHAUS. No, that is right. Estuaries and harbors and tidal waters are specifically included.

Mr. DOWNING. The Federal Water Pollution Act would cover the rivers, then?

Mr. RUCKELSHAUS. That is right, insofar as they are tidal.

Mr. DOWNING. But this act does include harbors?

Mr. RUCKELSHAUS. That is right.

Mr. DOWNING. What about dumping of ships, dumping into harbors, does it cover that?

Mr. RUCKELSHAUS. You are talking about oil dumping?

Mr. DOWNING. Oil or sewage, either.

Mr. RUCKELSHAUS. Oil is specifically included under section 11 of the Water Pollution Control Act. The vessel regulation for the discharge of sewage is covered under section 12 of the Water Pollution Control Act.

Mr. DOWNING. Does this include ships?

Mr. RUCKELSHAUS. Yes, it does. All vessels. We are in the process of adopting regulations now for the control of discharge of sewage from vessels.

Mr. DOWNING. The Navy discharges sewage while in the harbors?

Mr. RUCKELSHAUS. That is right.

Mr. DOWNING. Would that be in violation of the law?

Mr. RUCKELSHAUS. It is not now in violation of the Federal water pollution control law. We are in the process of adopting standards for regulations to control the discharge of sewage from any vessels.

Mr. DOWNING. A ship won't have to file an impact statement to discharge sewage, would it?

Mr. RUCKELSHAUS. Not that I know of.

Mr. DOWNING. How many people do you have in your office, Mr. Ruckelshaus?

Mr. RUCKELSHAUS. We have 6,000 employees in the agency.

Mr. DOWNING. Do you think that is adequate to handle the new duties under this legislation?

Mr. RUCKELSHAUS. No, we have requested in our fiscal year 1972 submission to Congress a 41-percent increase in our personnel in order to meet the responsibilities, not only of this act, but under other acts that we are requesting Congress pass.

Mr. DOWNING. Thank you very much.

Thank you, Mr. Chairman.

Mr. DINGELL. Mr. du Pont?

Mr. DU PONT. Thank you, Mr. Chairman.

Mr. Ruckelshaus, following up on Mr. Rogers' line of thinking, one of the reasons in my view that we haven't had any effective enforcement yet of the Air Pollution Act of 1970 is because we have a tremendous tangle of jurisdictions. There have been three air pollution standards approved by your agency at different times. We have State jurisdiction. We have Federal jurisdiction. I am not convinced that we are ever going to get out of that bog, but isn't this kind of legislation an opportunity to avoid that bog the second time around? Isn't

BEST COPY AVAILABLE

this an ideal place to take jurisdiction away from the States? Let's give it all to the Federal Government, let's take AEC out of the business. Let's get one comprehensive statute which you can enforce that won't have multiplicity of jurisdictions, that won't have a lot of different agencies passing paper back and forth, and get the job done a little bit faster.

I wondered if you could comment on the jurisdictional questions.

Mr. RUCKELSHAUS. I think you are asking a number of questions here. No. 1, I do not agree that the reason we haven't enforced the Air Pollution Act of 1970 is because of conflicting standards or because of the fact that there are standards at local, State and Federal level. In the 1970 Clean Air Act, all of our enforcement responsibilities are tied into the adoption of an implementation plan. Implementation plans cannot be adopted until we have published national standards, which we will do at the end of this month. And then every air quality region in the country has to adopt emission control within their region in order to achieve a national ambient air quality standard.

Once these implementation plans are submitted and approved by the EPA, which again under the act is a 17- to 19-month proposition, then that is when the act permits us to start enforcing these implementation plans. We just don't have any power to enforce them at this point. We can utilize emergency provisions of the act, but under that provision you have to find an imminent and substantial endangerment to human health.

Mr. DU PONT. Mr. Ruckelshaus, to avoid 17 to 19 months delay type problem in ocean dumping, can't we get legislation noww hich will give all of the power to you? Why do we need AEC? The AEC man was here yesterday, and I could see nothing that he said that convinced me that you couldn't do his job better than he could do it.

Mr. RUCKELSHAUS. I read part of the testimony yesterday by the representative of the AEC, and I must say I was somewhat confused myself as to the precise reasons for that exclusion in the act, given what he said yesterday. What I think we are going to have to get is a clear understanding of the reasons for the exclusions that were given in the testimony yesterday. Otherwise I must agree with you I don't see why we can't do it as well as they.

Mr. DU PONT. Do you see any reason that we can't take State jurisdictions out of this? There is a clause in here that permits a State to enact a statute that might be stricter than your standards.

Mr. RUCKELSHAUS. This is so throughout the pollution control laws. It presents tremendous problems for us, but the air and water pollution laws both are joint Federal-State statutes. They contain Federal-State enforcement provisions in which the State, being the primary enforcement arm, is called upon to act first and the Federal Government coming in when the State refuses to act. States are given authority to adopt stricter standards, if they so desire. It is my own feeling that, as a general rule, the Federal Government should set a base line of treatment that is necessary across the country. If a State decides in its own wisdom that it would prefer to have a much stricter standard in order to have cleaner air or cleaner water than provided under Federal standards, that State ought to be allowed to do that as long as it knows what its doing.

BEST COPY AVAILABLE

Its the same in the area of ocean dumping; if we set standards for ocean dumping—and there is bound to be conflict of opinion about these standards—there are going to be people who disagree. If States want to have strict standards so the beaches are absolutely pristine in their purity, it again seems to me within their jurisdictional right they ought to have the power to do that. And it is for that reason that we have not provided for preemption in this bill.

Mr. du Pont. I am not sure that I agree with you. It seems to me that there is an opportunity here for dumpers to select their jurisdiction. If a barge of sludge and sewage is coming down the Delaware River from Philadelphia, and Delaware has one kind of a law and New Jersey has another one, that guy has an option. You are dealing with navigable waters of the United States and it seems to me here is a clear opportunity to get rid of a whole layer of redtape. I have a lot of confidence that you can set some good standards and enforce them, and I would appreciate if we could get for the record your considered thoughts on whether you really think it would be not in the best interests of preserving our oceans to eliminate the State clause from the bills.

Mr. Ruckelshaus. Certainly the State would have no power to be any more lenient than we should be. They can only be stricter and it is not our intention to make these standards lenient. We intend to protect the oceans in the sense that I think the ocean dumping report of the Council on Environmental Quality implies. It is a preventive measure in those cases where we haven't gotten in trouble in the ocean. Although off of Delaware we already have problems, and in many other areas of the country, and we don't want to see that proliferate. It is our intention to set standards that are strict in their application to the prevention of any degredation of the marine environment.

Mr. du Pont. If we could have some detailed comments from you for the record.

(The information follows:)

Federal Preemption of State Control of Ocean Dumping

We have long opposed complete Federal preemption of water quality control. Section 1(b) of the Federal Water Pollution Control Act expresses a Congressional policy in favor of State action to control pollution, and recognizes the primary responsibilities and rights of the States in this regard.

We recognize that there are areas, such as the control of wastes from watercraft, in which an unnecessary burden on interstate commerce or upon Federal-State relationships would result if the States all exercised independent regulatory powers. However, we believe that the existing partnership between the Federal Government and the States is the most fruitful approach to dealing with most environmental problems.

Under H.R. 4247, for example, the Administrator would be precluded from issuing permits for disposal which would violate water quality standards, which are set by the States. In the event that the Congress enacts S. 1014, the Administration's proposed amendments to section 10 of the Federal Water Pollution Control Act, the States would be required, pursuant to Federal regulations, to develop effluent limitations for discharges into all waters, including ocean waters. These limitations, as well as the other components of water quality standards, would be subject to Federal approval.

As the Administrator testified before this Committee on April 7, 1971:

"It is my own feeling that, as a general rule, the Federal Government should set a base line of treatment that is necessary across the country. If a State decides in its own wisdom that it would prefer to have a much stricter standard in order to have cleaner air or cleaner water than provided under Federal stand-

[illegible]

ards, that State ought to be allowed to do that as long as it knows what it is doing.

"It is the same in the area of ocean dumping; if we set standards for ocean dumping—and there is bound to be conflict of opinion about these standards—there are going to be people who disagree. If States want to have strict standards so the beaches are absolutely pristine in their purity, it again seems to me within their jurisdictional right they ought to have the power to do that. And it is for that reason that we have not provided for preemption in this bill."

Mr. du Pont. Mr. Chairman, I am over my time. Let me ask one more question.

Do you have an estimate of the number of ocean dumping permits that you think you would have to handle every year under this legislation, and do you think you have adequate provisions for staff to take care of that?

Mr. Ruckelshaus. Our present estimate is approximately 3,000 permits would be issued. We have requested, and we will submit for the record our request for increased funding that will be necessary to take care of this permit program. We don't have adequate staff now. We will need more people to handle this program, and we think the funding we have requested will provide us with the additional staff necessary.

Mr. du Pont. Thank you.

Mr. Dingell. Thank you, Mr. du Pont.

Mr. Karth?

Mr. Karth. Thank you, Mr. Chairman.

Mr. Administrator, getting back to deadlines, one of our colleagues testified in opposition to deadlines. His reasoning was, if you set a 5-year limit, everyone affected by the deadline will wait 4 years, 11 months, and 29 days, to comply and they would probably make a greater effort and spend more money to run out the clock than to comply with the act.

Mr. Ruckelshaus. I think you are right. That is why I am against the legislatively imposed deadlines. In the case you mention we are talking about one industry, the automotive industry, and the problem is common to everyone in the industry. To set a deadline legislatively makes some sense in that case, but to try to set a deadline legislatively for all dumping in the ocean when we have so many kinds of problems involved in dumping does not make as much sense to me. Because, wherever you set it, there are some people who even though they could stop immediately, would be inhibited from doing this, or at least not encouraged, because of this 12-month deadline.

Mr. Karth. What do you think is a reasonable deadline so long as you suggest that this committee allow you that authority? What do you think is fair, 6 months or a year?

Mr. Ruckelshaus. As I say, the authorization would be for deadlines to be set administratively and you might put it within a certain framework. You might set an upper limit.

Mr. Karth. That is right, but I think this committee ought to know what you have in mind.

Mr. Ruckelshaus. It would depend on the particular dumper. If we are talking about municipal sludge, there may be an available alternative site for them to take care of the sludge problem. There should be no deadline. They should simply put the sludge in a different place.

For another community, there may be incredible problems trying to find an alternative site. It seems to me we, as an agency, through the Solid Waste Office, have to cooperate with them in attempting to find an alternative way to get rid of the sludge, and set a deadline in a reasonable way so they can develop this alternative site. And at the same time we can phase out their ocean dumping.

Mr. KARTH. On page 11 you talk about "Toxic industrial waste should be stopped immediately upon effective date of this act." Is that direct toxic waste disposal into the waterways of the country that would be affected by the bill, or is that also toxic waste that would go through channels of municipal disposal?

Mr. RUCKELSHAUS. No; this has to do with dumping. This doesn't have to do with continuous outfall. We have under Federal-State standards presently in existence regulations against discharge of toxics.

Mr. KARTH. Would it make any difference if they dump it by pipeline in the ocean?

Mr. RUCKELSHAUS. No; it doesn't make any difference.

Mr. K[illegible]TH. What about offshore oil, what do you think is a reasonable tim[illegible] to eliminate any pollution from that particular source? Maybe you don't call that dumping, but I do.

Mr. RUCKELSHAUS. That is also covered under regulations issued by the Interior Department and under Section 11 of the Federal Water Pollution Control Act for the control of oil as a pollutant.

Mr. KARTH. Every one of the offshore oils, I assume, has some toxic materials that every minute goes into the water. What are your regulations with respect to offshore oil?

Mr. RUCKELSHAUS. With respect to the dumping of oil from——

Mr. KARTH. No; I am talking about offshore oil wells, the actual drilling operations, and the leaks once the oil wells have been established.

Mr. RUCKELSHAUS. The regulations deal in terms of a visible sheen as being a violation of the regulations any time a dumping or discharge occurs.

Mr. KARTH. Subject to the penalties of this act for a $50,000 fine?

Mr. RUCKELSHAUS. No; they would be subject to penalties under Federal Water Pollution Control Act.

Mr. KARTH. What are those penalties?

Mr. RUCKELSHAUS. Mr. Dominick might be able to give you an answer.

Mr. KARTH. Mr. Dominick said $5,000. Is that sufficient, in your judgment, Mr. Administrator?

Mr. RUCKELSHAUS. I don't believe it is. That is an act we inherited.

Mr. DOMINICK. That is, for failure to report, I believe it is up to $50,000.

Mr. KARTH. How many of these oil rigs or drilling companies have been fined under that act? Do you know? What is their track record?

Mr. DOMINICK. There are none under the act passed last spring. We are in the process of developing regulations under section 11(j) to impose equipment requirements on the offshore facilities in the territorial waters.

BEST COPY AVAILABLE

Mr. KARTH. Was it because there has been no visible sheen or because they are nice fellows? Which one is it?

Mr. RUCKELSHAUS. I don't think it is an either-or situation. We are trying to develop regulations which can be met in a very short time period by the industry on the many facilities which are located in territorial waters.

Mr. KARTH. But you are constantly pursuing that, are you, Mr. Ruckelshaus?

Mr. RUCKELSHAUS. That is right. Don't think for a minute that where we have had these repeated instances of accidents that we can permit this to continue.

Mr. KARTH. I am glad to hear you say that.

Mr. Chairman, one more question.

I thought yesterday I might be the only one confused by AEC's testimony and apparently both of us, and other members of the committee, have been confused. What is your position on that? Do you feel they really ought to have exclusionary clause in this 4723?

Mr. RUCKELSHAUS. It is my understanding from testimony yesterday that the present permits would run out before this act actually came into existence, and the question in my mind is as to whether there should be a distinction between AEC issuing a permit and any other agency a permit.

Mr. KARTH. That is the same question in my mind. What is your judgment on that?

Mr. RUCKELSHAUS. It depends on the degree of consultation that the act provides and the veto power that we would have over any permit they issued. And if the testimony as I understand it yesterday was correct, I really don't see any reason for the exclusion.

Mr. KARTH. Well, I agree with you that I don't really understand it; but as I understand it, probably that is the conclusion I would have to come to. Basically, you do agree that there should be no exclusion for AEC, irrespective of whether we understand their testimony or not? Do you agree?

Mr. RUCKELSHAUS. I will have to qualify the statement by saying, based on what I understand the testimony to be yesterday, I don't see. There may be some reasons that I am not aware of that I will have to inform myself on.

Mr. KARTH. Whatever the reasons might be, and whatever their testimony said, isn't it fair to treat them the same as all other agencies of the Government?

Mr. RUCKELSHAUS. Certainly. No one can quarrel with that. I don't know that it is a question of equal treatment or fair treatment. If that is the only question, then——

Mr. KARTH. Well, let's say the same treatment, afford them the same treatment.

Mr. RUCKELSHAUS. That is right.

Mr. KARTH. Nothing better and nothing worse.

Mr. RUCKELSHAUS. I can't quarrel with that.

Mr. KARTH. Thank you, Mr. Chairman.

Thank you Mr. Ruckelshaus.

Mr. DINGELL. Mr. Ruppe?

Mr. RUPPE. Thank you, Mr. Chairman.

BEST COPY AVAILABLE

62-513—71——28

Mr. Ruckelshaus, does your agency have any kind of policy or general attitude toward the dumping today, continued dumping or future dumping of mine tailings or mine waste material in the Great Lakes and particularly Lake Superior?

Mr. RUCKELSHAUS. We have had at least three enforcement conferences regarding the Reserve Mining Co. which I assume you are alluding to in Lake Superior. We have presently the report from a committee that was assigned the responsibility under the last enforcement conference to develop an on-land disposal site for the taconite tailings involved in that situation. We don't find that that report is adequate and we have, and will shortly be announcing, the recommendation of the Environmental Protection Agency as to what further action should be taken relating to that particular incident.

Mr. RUPPE. Without going into that specific case, what are the ranges of recommendations or directives that can come out of your agency in a situation of that kind? What are the possibilities?

Mr. RUCKELSHAUS. There are a number of possibilities. One of the problems we have is that the enforcement conference it is a rather cumbersome technique. In our water pollution control amendments that are presently pending before Congress we have asked for the authority to move much more quickly than we can under these enforcement conferences. We can make recommendations and the recommendations are ignored as they have been in many instances. We then go to an administrative hearing and make findings and further recommendations and then from that hearing we have to go to court. That is a very cumbersome administrative procedure. What we are asking for in the water bill is precisely the same thing that was in the air bill, that is, that the Federal presence come right up behind the States in terms of their enforcement authority. In that instance, if we go to court of equity as opposed to attempting to get a fine, the range of options as to what is done is as broad as the equity power of that court, which is limitless in terms of providing equitable solutions to the problem.

Mr. RUPPE. Thank you.

Thank you, Mr. Chairman.

Mr. DINGELL. Thank you, Mr. Ruppe. Mr. Anderson?

Mr. ANDERSON. Thank you, Mr. Chairman.

Mr. Ruckelshaus, who asked for the exclusion of AEC? Was this asked for by the Commission itself?

Mr. RUCKELSHAUS. I can't give you the specific answer to that, Mr. Anderson. This bill was prepared before our agency really came into existence. We have reviewed the bill and this is the way the bill was drafted in its final form. It was my understanding there were extenuating circumstances as to reasons for the specific exclusion of AEC, and that is why I say that my understanding again of what was testified to yesterday is somewhat different from my understanding again of the reason. For that reason, I will have to consult with AEC to find out.

Mr. ANDERSON. I had understood this was an administration bill, so I assumed it would come from your office.

Mr. RUCKELSHAUS. It, as most of the environmental bills presently pending in Congress, was drafted under sponsorship of CEQ. We didn't come into existence until December 2. We are supporting these

bills. But in answer to your specific question, I can't give you the history and genesis of all of these bills because we weren't there at the time this happened.

Mr. ANDERSON. Municipal and industrial sewage is excluded in this act. Yesterday when we were discussing the dumping of rubbish, it was brought out that the act would apply in cases where rubbish was being transported by trucks, barges and conveyor belt. In my district, we are experimenting with a rubbish disposal project, in which rubbish is ground up and delivered in pipes. If rubbish were delivered in pipes, would this law be applicable?

Mr. RUCKELSHAUS. It would be under the Federal Water Pollution Control law. It would be under the same agency.

Mr. ANDERSON. The fact that it would be conveyed in pipes rather than on a belt would be the difference?

Mr. RUCKELSHAUS. Well, the question is, the distinction is between the continuous discharge and intermittent dumping. The continuous discharge, which is normally through a pipe, is controlled under Federal Water Pollution Control Act. What we need to do is to get control over intermittent dumping, which is what this bill is aimed at.

That doesn't mean that the standards would be any different in terms of what we would do as far as water quality control is concerned as far as what we would permit to be discharged from a pipe into any ocean or river or lake under the Water Pollution Control Act.

Mr. DINGELL. If you would yield, Mr. Anderson.

I am curious, isn't that going to mean that a fellow who puts the same amount of stuff in the water through an outfall or through barge dumping or through a conveyor belt or through a ground-up dry discharge through a pipe is going to get different treatment insofar as the amount that he can put in and insofar as the deadlines that he is going to have to meet? For example, let's say a fellow would be banned absolutely by an order that you would issue with regard to putting toxic industrial wastes or substances of this kind in from a barge, but he would be able to continue, if he were to alter his operation. He would then be able to continue by simply piping it in from an outfall as liquid waste, so long as it didn't impair the water quality standards; or if he were to divert it by a conveyor belt or through a pipe, ground up in dry from, he might be able to get still different treatment.

Now it occurs to me that probably to treat it as dumping he is going to get more stringent treatment than he is going to get under the Water Quality Act, because the Water Quality Act is not going to prohibit against the positive dumping in the waterways; but it is going to deal with the end result of what the deposit has to be.

In other words, you would then be fixing a tolerance as opposed to quite possibly an absolute prohibition. Would you want to comment on that, Mr. Ruckelshaus.

Mr. RUCKELSHAUS. Yes, Mr. Chairman. No. 1, if we are talking about toxic substances, and we were prohibiting the dumping of a toxic substance under the ocean dumping authority, we would likewise prohibit it from being discharged by an industry through the municipal system or directly into a stream.

Mr. DINGELL. This is not so, Mr. Ruckelshaus, and you well know it. The law allows, and you do, I know—for example, up in our coun-

[illegible]

try you are allowing deposit of chemicals under water quality standards. These are toxic substances. You would never allow that under the water quality standards bill. You are allowing mercury to go into water. You have reduced it significantly, and I applaud you, but you are still allowing mercury to go in water. You are allowing cadmium waste to go into water by fixing the amount which may go. But under dumping provisions you would probably ban totally the deposit of those substances into the waters.

What I am asking you is, aren't we going to treat the fellow who runs it in, through outfall or through the kind of device Mr. Anderson is alluding to, differently than we treat the fellow who would casually barge it out and dump it?

Mr. RUCKELSHAUS. To the extent that the discharge now of any substance labeled "toxic" would not violate water quality standards as they are presently established, they are permitted to continue to discharge them.

Mr. DINGELL. If he is dumping in a big waterway, the Mississippi River or Detroit River or Hudson River that has a large flow, that is going into an outflow like the Atlantic that has a heavy stream flow, the probability is he is going to be able to put an awful lot in terms of waste and volume of a particular waste which he would not be able to do were he in a situation where he would try to barge it out or put it in by conveyor belt?

Mr. RUCKELSHAUS. That again depends in part on whether the effluent standard which we have attempted to get Congress to adopt in the bill that we have presently submitted, whether we could set effluent standards to limit very carefully how many of these substances go into water as opposed to setting water quality standards in dealing with similar capacity of the waters that receive them.

If we get this additional authority, I think we can obviate much of the problem you are discussing. That does not mean there may not be some minimal amount of discharge to be permitted of a substance that may normally be called toxic, even under effluent standards, which could not be dumped. But the problem of dumping, it seems to me, is concentration of that material into a certain area where there is no flow going through or no way of dispering it into the waterway.

Mr. DINGELL. My question was not critical of you. I am trespassing on my good friend's time, but I wanted us to have a clear understanding of the anomaly we have here. I hope that you will consider this particular problem. I want to mention I was probably the first fellow around here to come up with the idea of effluent standards on water, and I support your endeavor in that particular direction.

Thank you, Mr. Anderson.

Mr. RUCKELSHAUS. I might say, Mr. Chairman, I did not mention this problem before, but if somebody were prohibited from dumping a particular substance by any prohibition that we issue and they simply put a pipe on and dumped it through the pipe, it seems to me this would be a clear violation of the spirit of this statute, if not in substance.

Mr. DINGELL. I understand the violation of the spirit, but I would like you to carefully address yourself to a violation of the substance so we can understand whether or not we aren't setting up an anomaly

BEST COPY AVAILABLE

which might justify some kind of rascality that neither this committee or yourself wants to sanctify at this time. I would suggest that you give us suggestions as to how we can meet this particular challenge, and hope that you might be able to give us some legislative suggestions for amendment to this particular bill to prevent that kind of situation.

Mr. RUCKELSHAUS. We will certainly do that.

Mr. DINGELL. Thank you.

Thank you, Mr. Anderson.

Mr. ANDERSON. A last brief question to follow up on Mr. Karth's discussion on the oil well spillage or dumping: You cited the $5,000 penalty. Isn't there a difference on the 3-mile limit application? What happens beyond the 3 miles? Is that area under the Department of Interior's supervision with a different set of penalties, or are the penalties the same?

Mr. RUCKELSHAUS. I think they are, Mr. Anderson.

Mr. DOMINICK. Mr. Anderson, the application of regulations under section 11 to offshore facilities would only apply to territorial waters, and the regulation of offshore facilities in the contiguous zone is by regulation of the Department of the Interior, U.S. Geological Survey. I think the answer to your question is basically "Yes": as to offshore oil facilities, there is a difference between application of Federal Water Pollution Control Act and application——

Mr. ANDERSON. Are the penalties the same?

Mr. DOMINICK. I am not aware of what penalties are now being employed by the Department of Interior on the contiguous zone.

Mr. ANDERSON. That is the area beyond the 3 miles?

Mr. DOMINICK. That is correct.

Mr. ANDERSON. Thank you.

Mr. DINGELL. Mr. Kyros?

Mr. KYROS. Thank you, Mr. Chairman. I will yield my time to Mr. Rogers.

Mr. ROGERS. Thank you, Mr. Kyros.

Thank you, Mr. Chairman.

Mr. Ruckelshaus, to pursue this question just a minute on setting a deadline, you say you believe it should be done administratively although where there is one industry like oil you had no objection to that. You say it might make some wait until the deadline. Suppose we say that you could ban it on or before a certain date. Would you have any objection to that?

Mr. RUCKELSHAUS. No, I think that would obviate some of the problem, but still when you have an outside date, there is still a tendency for anybody who is discharging to look to that outside date as to time.

Mr. ROGERS. And when there is no date at all, they think they may never have to do it unless you catch up with them?

Mr. RUCKELSHAUS. If we don't set a deadline, that is right.

Mr. ROGERS. Sure. So I think it is better for us to try to set deadlines.

Now, I think it would be helpful to the committee to have some suggested dates from you in the agency and you may not be able to give them now, I realize, but I think we should have them very shortly. For instance, why shouldn't everybody have primary treatment, which is practically nothing, by 1972, this coming year, the end of 1972?

Mr. RUCKELSHAUS. Our goal under the Water Pollution Control Act is secondary treatment for every municipality, and it is for that

BEST COPY AVAILABLE

reason that we discussed or we introduced the $6 billion, 3-year matching funds program for $12 billion total.

Mr. ROGERS. Give us a date for secondary, where we can skip primary if you don't think we should. Secondary or equivalent.

Mr. RUCKELSHAUS. We anticipate that in the $6 billion figure that we will have—this is a 3-year program of $12 billion—that we will have all of the projects underway and approved in 3 years, to be completed within 5 years. This is our goal and again we have to constantly reassess the amount of money that is involved, because it changes almost monthly as to how much is needed.

Mr. ROGERS. What I am saying is, should it be tertiary by 1976, 1978, or should it be secondary by 1974, 1975? What should be the goal?

Mr. RUCKELSHAUS. Again it differs widely depending on what area you are in and just exactly what the standards themselves provide, if we have water use designation, which we do have in most of the State-Federal standards.

Mr. ROGERS. Of course, you know what this does, it changes the theory of saying you are licensing a certain amount of pollution in every river, which the Water Pollution Act does. It authorizes and licenses pollution. To set a deadline change the whole philosophy and says, "We don't want any waters polluted after a certain date to this degree of treatment." Right?

Mr. RUCKELSHAUS. That is right.

Mr. ROGERS. Is that a good philosophy or isn't it?

Mr. RUCKELSHAUS. Our bill still provides ——

Mr. ROGERS. I know what your bill provides. What I am asking is, is that a good philosophy? We are going to write the bill. We want to consider yours, but we are going to write the bill in the committee. I want to know your thinking. is it an equitable philosophy not to pollute the waters or to license pollution?

Mr. RUCKELSHAUS. If you put it between those two choices, obviously not to pollute is the better philosophy.

Mr. ROGERS. Of course, it is. And I knew that you would agree with that and I think that is what the committee will do to try to be helpful to you, to give you some tools to work with. Because his Water Pollution Act just says you can continue polluting and in certain rivers you let them pollute tremendous amounts, as the chairman brought out, even deadly chemicals.

Now, my concern about allowing administrative decision as to deadlines, when certain things should be done now, is proved by your lack of banning mercury when we know the effects. Yet they are making administrative decisions and saying, "We will let a little go in here and there", and so it is still being done. The Air Force base, that we brought out the other day, putting cyanide into public dumping systems without being treated, and each one thinks that the other is treating it. And so what is happening, they are having a dead sea between Catalina Island and the mainland and Westinghouse has been out there with their submarines to take pictures to show what has happened. So I think it would be helpful if you could furnish to the committee suggested dates or deadlines on or before which primary, secondary, tertiary, or equivalent treatment, or any other designation

BEST COPY AVAILABLE

that you think would be helpful would be required. Could you furnish that?

Mr. RUCKELSHAUS. I would be glad to furnish you with a statement of my philosophy as to how we go about——

Mr. ROGERS. I say I would like to have from the agency for the committee the suggested dates.

Mr. RUCKELSHAUS. As to when primary, secondary, and tertiary could be achieved?

Mr. ROGERS. That is right.

(The information follows:)

QUESTION: DEADLINE DATES FOR MUNICIPAL AND INDUSTRIAL WASTES

ANSWER

The postulation of a specific date for the attainment of a particular level of waste treatment by all dischargers has the appeal of simplicity and enforceability. However, it is not advantageous to have such a proposal expressed in the general terms of primary, secondary, and tertiary treatment. These terms relate generally to types of treatment processes which have been historically associated with the clarification of domestic sewage. They are not easily defined or related to the treatment of industrial wastes. As an example of problems associated with their definition, a treatment plant containing a series of "primary" treatment processes (principally settling) with the addition of a phospate removal process designed to control eutrophication in the wastes' receiving waters would be essentially a tertiary treatment plant from the standpoint of nutrient removal and a primary treatment plant from the standpoint of the removal of oxygen demanding wastes.

In addition, these terms relate to the percent removal of pollutants and do not account for the absolute amounts of pollutants being added to the receiving waters.

The Federal Water Pollution Control Act, as amended, and the Administration's proposed legislation, S. 1014, do not relate to the attainment of primary, secondary, or tertiary treatment but instead refer to the treatment required to meet the imposed water quality criteria to insure the full and designated uses of the Nation's navigable waters. It is imperative that all municipal and industrial waste discharges be in compliance with water quality standards.

All wastes both municipal and industrial should be required to provide on a national basis a standard effluent level. Specific treatment requirements should be imposed on those municipalities or industries above the standard effluent level as necessary to insure the meeting and compliance with water quality standards. This approach permits the maximum utilization of available financial resources being applied in the most advantageous and cost effective manner, to insure that the major effort be made in those areas where it is most urgently required.

The Federal Water Pollution Control Act, as amended, and the Administration's proposal, S. 1014, required that each State adopt as part of their water quality standards effluent criteria for its navigable waterways. The achievement of these effluent criteria as discussed in the above paragraph will insure the orderly attainment of water quality standards throughout the Nation. We therefore endorse the concept of effluent guidelines, as called for in S. 1014, and recommend that the approach not be based on simply primary, secondary, or tertiary treatment but on the concept of applying initially a base level of treatment reflecting the best level of technology.

The WQO/EPA is currently developing effluent criteria guidelines, in effect standard effluent limitations, for major industrial categories as well as determining the current state of the art for treatment and control within these industries.

In dealing on an industry-by-industry basis we can specify effluent requirements consistent with available technology and for industries where technology is presently not proven we allow for the scheduling of the necessary research and development.

It must be remembered that municipal-sewered wastes and industrial wastes do not constitute all of the national water quality problem areas. In addition,

there are such national problem areas as agricultural wastes, other urban wastes (including urban runoff), power plant wastes (thermal), mining wastes (including acid mine drainage), and oil and hazardous material discharges. All of these problem areas, not just municipal and industrial wastes, must be corrected in order to meet water quality goals. Consequently, the WQO/EPA is addressing effort in each of these directions to achieve national goals.

Mr. ROGERS. Now, let me ask you this. You have 6,000 employees you say?

Mr. RUCKELSHAUS. That is right.

Mr. ROGERS. How many do you plan to hire in addition to the 6,000?

Mr. RUCKELSHAUS. We have requested an increase of 41 percent so we will go to 9,000 at this time next year, if the budget passes.

Mr. ROGERS. Where will these people be used mainly?

Mr. RUCKELSHAUS. Primarily in the Water Quality Office and in the Air Pollution Control Office, although we are in the process again of reorganizing the Agency so that to strictly say they will be in one or two of these offices may not be true after the reorganization, because it may be we will functionalize in area of standard setting and enforcement so that more people would be in those areas that might be specifically dealing with these problems, but not in those offices.

Mr. ROGERS. What is the number of personnel you have for monitoring in the field what is happening?

Mr. RUCKELSHAUS. We presently have, as best we can determine because some people perform more than just one function, in the neighborhood of 250 to 300 people involved in monitoring. We have an assistant Administrator for research and monitoring. One of his prime functions is monitoring. There are a number of Federal agencies which also do monitoring, NEAA and Interior Department and Corps of Engineers that are involved in this, along with State agencies which we finance and which we match funds for State pollution control agencies which do a lot of monitoring. We do not have, in all honesty, a very good fix on just how much monitoring is going on, how much we need, and we need to get a lot more information about this.

Mr. ROGERS. I agree. I think we are very deficient on efforts in monitoring. I would think one of the primary areas you would look at immediately is the monitoring of outfalls. Is there any program for that specifically?

Mr. RUCKELSHAUS. Under the permit program, there is a provision for monitoring by the industry itself which is discharging into any stream. There has to be a sworn statement by the industry what is in their effluent, and we will in turn monitor those statements to insure that they have told us the truth, and that there is a provision in the permit program itself for self-monitoring by industry.

Mr. ROGERS. When is the effective date of your permit program?

Mr. RUCKELSHAUS. The application has to be in by July 1.

Mr. ROGERS. Is there a cutoff date, when they cannot discharge unless they have a permit?

Mr. RUCKELSHAUS. We don't have any strict cutoff date as of this time. We are going to process these permits as fast as we possibly can.

Mr. ROGERS. I believe you estimated it would be 40,000.

Mr. RUCKELSHAUS. That is right.

Mr. ROGERS. What is the time element you have estimated that you can work through those?

Mr. RUCKELSHAUS. One of the things that we do know is that in traveling around to our regions in the country, we basically know who the people are that are giving us the most problems, giving us the most trouble, so what we want to do is select the permits and get at the permits where we really are having the problems first, and then gradually get down to eliminate all of them, and we had set a deadline approximately 12 months to process all of the permits. Whether we achieve that or not, that is in itself imposed as an internal deadline.

Mr. ROGERS. How many people have you asked to do that specific work?

Mr. RUCKELSHAUS. We have requested in a supplemental budget to Congress 400 people in our agency to do that, and it is my understanding that the Corps of Engineers has something like 300 that they have requested.

Mr. ROGERS. So it will be a joint effort. Is that it?

Mr. RUCKELSHAUS. That is right.

Mr. ROGERS. Do you review all of the permits?

Mr. RUCKELSHAUS. We review them as to whether they meet water quality standards.

Mr. ROGERS. Water quality standards are set by whom?

Mr. RUCKELSHAUS. Joint Federal-State standards in interstate waters, and State in intrastate waters.

Mr. ROGERS. How do you find out what those standards are in intrastate waters?

Mr. RUCKELSHAUS. As far as intrastate standards, they are supplied to us by the State pollution control agency.

Mr. ROGERS. You are going to check to see that they meet those standards intrastate, too?

Mr. RUCKELSHAUS. We are going to do more than that. In some instances they don't have standards, in which case we will see that they are in compliance with purposes of the Federal Water Pollution Control Act, which will be essentially the standards we have set in other areas of the country.

Where standards are so weak that they might as well not be any standards at all, we are also not going to simply rubberstamp those certifications from the States as they come through for our approval.

Mr. ROGERS. Well, what authority do you have to set your standards in intrastate waters? The State does not set any?

Mr. RUCKELSHAUS. To be perfectly frank, the authority is not as clear as we would like to have it.

Mr. ROGERS. Did you ask for such authority?

Mr. RUCKELSHAUS. We certainly have. We have asked in our amendments to the Federal Water Pollution Control Act last year, and this year, for authority to set standards in instrastate waters.

Mr. ROGERS. Has it been granted?

Mr. RUCKELSHAUS. It has not. It did not pass last year, and it is pending before Congress this year.

Mr. ROGERS. Maybe we should include it in here.

Mr. RUCKELSHAUS. The ocean-dumping bill has been included in the Federal Water Pollution Control Act. We had hearings in the Senate Public Works Committee. The problems in determining who is

responsible to do what probably can be cleared up if these bills are treated together.

Mr. ROGERS. What concerns me, Mr. Ruckelshaus, and I know you have not had an opportunity to be in this long, for instance, you tell me we are going to do this, and then I find out that you don't even have authority to do it.

Mr. RUCKELSHAUS. I have not said we don't have authority to do it. Under the Refuse Act of 1899, when read in conjunction with 21(b) of the Water Pollution Control Act, we have arguable authority to do it.

So we believe under that arguable authority we can look at these certifications by the States and pass on those, weher clearly they are deficient in any kind of water pollution control program at all.

Mr. ROGERS. Do you have the authority, or does the Corps of Engineers have authority under the Refuse Act?

Mr. RUCKELSHAUS. Under the Refuse Act, they have the authority to issue permits, and read in conjunction with section 21(b), we believe the authority to pass on water quality is given to the Environmental Protection Agency, and that we advise the Corps on the exercise of that authority.

I admit this is not without some dispute, and that there are those who say we don't have that authority.

Mr. ROGERS. If you had deadlines, you could get at it quickly, could you not?

Mr. RUCKELSHAUS. If we had the amendments that we suggested to Congress, we could get at it quickly.

Mr. ROGERS. Well, if this committee decided, that could really get you going on this problem.

Mr. RUCKELSHAUS. We would hope all of the committees would do that.

Mr. ROGERS. Regarding solid wastes, I just checked, we authorized for fiscal 1972 $152 million. The committee thought the problem was that significant. I notice the budget request was $19 million. Do you think this is a proper request to meet the needs in this area?

Mr. RUCKELSHAUS. This is not the final, total request that we will make, as I explained.

Mr. ROGERS. You said there is $85 million, but you are not sure whether any of that goes to solid waste.

Mr. RUCKELSHAUS. We are requesting that some of it go to solid waste.

Mr. ROGERS. What is the major request for?

Mr. RUCKELSHAUS. For new and innovative demonstration projects.

Mr. ROGERS. Of the $85 million, I mean.

Mr. RUCKELSHAUS. Probably to implement the Clean Air Act of 1970.

Mr. ROGERS. What are the total requests for air pollution?

Mr. RUCKELSHAUS. Something in the neighborhood of $41 million.

No, that is not the total request. That is of the $85 million.

Mr. ROGERS. Then for water pollution it is what?

Mr. RUCKELSHAUS. Very little of the $85 million goes to water pollution.

The reason I am a litte fuzzy on these figures is because we are still in the process of trying to get a final answer from OMB as to just

where these will be allocated, and there is no final answer that I can give you right now.

Mr. ROGERS. I wondered what you had requested. I realized OMB often does not agree with your request.

Mr. RUCKELSHAUS. We requested allocation of $85 million between the problems of air and water pollution, and pesticides, radiation, and solid wastes, and obviously we don't come up with the authorization in the bill, if it is even a portion of the $85 million.

Mr. ROGERS. Very, very short?

Mr. RUCKELSHAUS. That is right.

Mr. ROGERS. Do you agree with that approach?

Mr. RUCKELSHAUS. I believe that in looking at the new and innovative projects which the bill authorizes us to fund as they come in, there are not that many new and innovative projects. We have already been criticized by GAO for funding projects that were not new and innovative at all. In fact, they were redundant as to other projects.

I think we have to be careful to perform what Congress has said we are supposed to perform, which is to put this money into new and innovative demonstration projects.

Mr. ROGERS. No; it is not just demonstration projects. There is a section for demonstration projects. There is also a section to help communities bring about new methods, but it is not just demonstration, which does not mean it has got to be new over what they have out in Seattle, Wash., the very latest there, but it means it is new in that area, an improvement.

Mr. RUCKELSHAUS. I suppose this is an argument that in looking at legislative history was an argument that went on through Congress.

It is our interpretation of the act that it does mean new in terms of the Nation, because if the purpose of the act is for this Federal agency to provide means of the treatment of solid wastes, that can be adopted in communities around the country when they see they work.

Mr. ROGERS. Well, may I say, then, you probably will end up with only one project in the United States a year, or a month.

Mr. RUCKELSHAUS. No; I don't think that is right.

Mr. ROGERS. I thought you said there were not that many new and innovative projects.

Mr. RUCKELSHAUS. Well, there are not, but there are more than one.

Mr. ROGERS. If you will analyze, and I happen to be the author of that section, I would tell you that the intent is that it does not have to be the same in Florida, on the same level as it does in Washington, but it should be new and innovative in that area, an improvement for that grant section for the demonstrations, yes.

I think if you will check that, maybe that will help in getting more funds.

Then finally, let me conclude by saying I believe you do have primary responsibility for enforcement in the Air Pollution Act for new stationary sources.

Mr. RUCKELSHAUS. That is right.

Mr. ROGERS. You don't have to wait for the State.

Mr. RUCKELSHAUS. That is right.

Mr. ROGERS. For airplane pollution?

Mr. RUCKELHAUS. We have a year before we have any authority for airplane.

Mr. ROGERS. You can set standards.

Mr. RUCKELSHAUS. In a year.

Mr. ROGERS. Well, I think that is true all over the act.

Mr. RUCKELSHAUS. You asked why we don't have enforcement standards. We don't have authority to set standards for a year.

Mr. ROGERS. If you are going to get into that, which I was not going to get into because of the time element, but there are a number of procedures that already have taken place as to regional establishments under the prior act.

Mr. RUCKELSHAUS. That is right. We have no implementation plans approved as of this time.

Mr. ROGERS. I don't know why not, because some have been submitted.

Mr. RUCKELSHAUS. That is right. Nineteen have been submitted.

Mr. ROGERS. Sure. They told us we were going to have 52.

Mr. RUCKELSHAUS. They are not in compliance. The implementation will not implement.

Mr. ROGERS. Under the old act, there are areas where you can proceed with enforcements, no question about it. You can have conferences even under the old act.

Mr. RUCKELSHAUS. I agree with that. Under the conference procedure, we could, but under the new act, we have new procedures.

Mr. ROGERS. But we did not stop those procedures under the old act in the new act. If they were on-going, they are still in existence. Now, you know that.

Mr. RUCKELSHAUS. And we are going on with those.

Mr. ROGERS. But not much is going on. That is the point I was making. I will not pursue that with you.

Thank you, Mr. Ruckelshaus.

Thank you, Mr. Chairman.

Mr. DINGELL. Mr. Du Pont.

Mr. DUPONT. I have an additional question, Mr. Chairman.

Mr. Ruckelshaus, you said that you thought there would be about 3,000 permit applications.

Mr. RUCKELSHAUS. That is the best estimate we can come up with. That is a ball park estimate.

Mr. DUPONT. Do you envisage for each of these 3,000 applications a public hearing, or at least an opportunity for public hearing?

Mr. RUCKELSHAUS. I think there should be very similar to the Corps of Engineers dredge and fill permits provisions for public hearing, when a hearing is requested by affected citizens.

Mr. DU PONT. I agree with that. There ought to be an opportunity, but do you foresee another bogging down of procedures here?

It seems to me on every application that comes up that the more militant citizens and conservation groups are going to want to have a hearing; they will want to testify; and they are going to want to string out procedures as long as they can to prevent the dumping.

Do you think if you go through all of this procedure that you are going to issue any permits?

If you request enough permits, you will never get a permit, because it will go on and on.

Mr. RUCKELSHAUS. A lot of hearings go on in the Corps of Engineers dredge and fill permits, and there is sometimes vigorous advo-

cacy on both sides. Sometimes the permits are issued, and sometimes denied.

I don't see that the hearing procedure itself would keep us from issuing any permits. I think if you get the hearing procedure too familiarized, and make it automatic for even a rather minor issuance of a permit, you might well be bogging the agency down.

Mr. DUPONT. You might be better off to provide for notification, and not fit the hearing clause into the law.

Mr. RUCKELSHAUS. That is my present thinking on it. It is not a question without some difficulty, because we are dealing with due process and the right of people to be heard.

I would think when the application was filed, and with all of the information in it that is provided in the act, that application would be made public immediately, and that anybody would have within a certain number of days to comment on the application and request a hearing on the application, if they so desire, and then, if it seemed that the subject was of enough significance, and there was enough controversy about it, we should hold a hearing.

That is essentially what the Corps does under dredge and fill permits, and this procedure has worked very well under their regulation.

In that way, we could provide due process, and people the right to be heard, and at the same time not get the program and issuance of permits bogged down in a lot of endless procedure.

Mr. DUPONT. I would like to ask one more question, Mr. Chairman.

Mr. Ruckelshaus, I wonder if you would consult the legal arm of your organization and get a legal opinion on the question of substantive due process when there are multiple standards approved by the Federal Government or by overlapping Federal and State jurisdictions.

What I am getting at here is under the Air Quality Act, for example, in Delaware, the Federal Government has independently approved three standards. HEW set one under the Federal criterion. Delaware submitted one that your agency approved, and now you are about to issue a third one on your new standards.

There are three. All of the numbers happen to be different in one of these cases. If I were an attorney on the other side, I would argue that this is totally lacking in substantive due process here, because they are irrelevant criteria. There are three criteria, all approved by the Federal Government.

Mr. RUCKELSHAUS. I think clearly the national standard we are about to approve would preempt the other two.

Mr. DUPONT. The reason this is important is if we allow the States to legislate in the area, and you end up with two conflicting criteria on ocean dumping, I suggest that maybe the whole thing could be brought down by one lawsuit.

Mr. RUCKELSHAUS. We will look into it. I am sure the criteria is conflicting. One is more strict, and that one would apply.

Mr. DUPONT. Well, they are overlapping, if you will. But I think this is a chink in the armor that we should not perpetuate in this law.

Mr. RUCKELSHAUS. I agree with you, and I also think that I don't agree that we should not have stricter standards. I am not sure I agree with that.

But one of the problems we do have are sometimes conflicting standards between air and water pollution agencies, and then agencies at various levels of Government, and standards that change very quickly, so it allows two things to happen.

One is an industry that sincerely wants to do something about it does not know what the standard is, and they don't do anything. It also gives the industry which does not want to do anything an excuse to drag their feet and say they will wait until they find out what the standard is going to be.

I see it as a function of our agency to try and make some sense out of these conflicting standards, and try to give some clear signals to people as to what is expected of them.

We have not achieved it yet, but we are still trying.

Mr. duPont. Mr. Ruckelshaus, you put it far better than I could have put it, and that is exactly the problem. I think when we get to ocean dumping, maybe we can avoid making a mistake made in the Air Quality Act by removing the State jurisdictions to enact standards at all.

Thank you.

Mr. Dingell. Mr. Sharood.

Mr. Sharood. Mr. Ruckelshaus, I wonder if we could clarify one problem I have with the State preemption requirement.

If a State establishes requirements or standards under the act, will you continue in the role of issuing or denying permits for dumping, or will the total burden be transferred to the State?

Mr. Ruckelshaus. Not under the present provision of the act. Anybody who is going to dump any material in the ocean will have to have a permit from, or any of the other areas of water covered under the act, a permit from the Administrator of the EPA.

The fact that a State requires another permit is a burden on the dumper, but that is not a burden that is not duplicated in other areas of our law.

Mr. Sharood. Let's assume that the State did not require a permit, but simply established a higher standard of some sort, and I have some difficulty envisioning the kind of conditions the State might impose with respect to ocean dumping.

Would you then be guided by those State standards and deny or approve a permit based upon the higher State standards?

Mr. Ruckelshaus. No, we would not have any authority, other than what were provided in our own standards.

If we are going to treat people equally across the country, it seems to me as far as our agency is concerned, you are entitled to a permit if you comply with our standards.

If States want to prohibit you from dumping within their jurisdictions, then that is a problem between the State and the person who wants to dump.

Mr. Sharood. If the State adopts a higher standard, the party wishing to dump would simply have to go into a contiguous zone in order to avoid the State requirement?

Mr. Ruckelshaus. There is a question whether a State can, from a ship originating in its ports, control beyond the territorial seas, as being one of their citizens of that State.

There is a California State law that apparently calls that into question, but that is not without some ambiquity at this point.

But that is true. If the State did not have any authority outside of the territorial sea, then that is where they would end up going.

Mr. SHAROOD. You would not look to those State standards, if the party came to you with a permit to go out 6 miles, or 4 miles from shore, you would not take into account those higher State standards. Is that true?

Mr. RUCKELSHAUS. Well, I think what our agency has to do a lot more of, and it is difficult for me to promise in the context of this bill that we ought to do it, is a lot closer coordination and cooperation with the States in adopting standards as such.

There is a certain impetus, when a Federal agency adopts a standard, and makes it clear what that standard is, and makes clear the reasons behind the standard, there is a certain preemptive force in this kind of standard setting itself in that States then say, "Well, they are handling it, and therefore there is no reason for me to do it."

This has happened in some areas, particularly with HEW.

Mr. SHAROOD. It seems pointless to provide in this legislation for a provision such as this, if you are then going to ignore the State standards and are not going to take them into account, but issue permits solely on the basis of the Federal standards. It does not seem to serve any useful purpose.

Mr. RUCKELSHAUS. I don't think we can ignore them, but by the same token, I don't think we should be in the business of enforcing State regulations. This could get to be a very confusing problem, if we tried it, and we have not done it in the past.

Mr. SHAROOD. My second question is perhaps a bit far out, but I want to clarify another point for the record.

Let's say the Army decided to transport surplus munitions of some type from Vietnam to the central Pacific and dump them. That would not come under the coverage of this act. Is that correct?

Mr. RUCKELSHAUS. I think it would. I think they would have to have a permit to do so.

Mr. SHAROOD. As I read your permit system, you have to have a permit to transport from the United States out into the ocean, and you have to have a permit to dump within our territorial sea.

I don't read anything in there that requires a permit for the U.S. citizen or entity to transport material from a foreign country out into the ocean for dumping.

Mr. RUCKELSHAUS. I may not be in the act.

Mr. SHAROOD. I am not suggesting that they would do this, but I am posing the question.

Mr. RUCKELSHAUS. We are asking for the authority under the Federal Water Pollution Control Act to set standards beyond the contiguous zone.

Mr. SHAROOD. That relates to material coming out from the United States.

Mr. RUCKELSHAUS. And also affecting the territorial sea of the United States.

It also, I think, might run into a clear problem under the Executive order, or under the National Environmental Policy Act, but arguably, this might be outside of the coverage of any present law.

Mr. SHAROOD. That is the way I read it, and I wanted to get your view on that, to make sure I was not misinterpreting it.

Do you think we should?

Mr. RUCKELSHAUS. I think it ought to be made clear as to the coverage of the act in this kind of situation.

Mr. SHAROOD. Will you submit some language?

Mr. RUCKELSHAUS. Yes, we will.

Mr. SHAROOD. Fine.

(The information follows:)

DUMPING MATERIAL FROM FOREIGN TERRITORY IN WATERS SEAWARD OF THE UNITED STATES

A paragraph could be added after subsection 4(b) as follows:

"This Act does not apply (1) to transportation for dumping carried out by United States citizens when such transportation originates in territory other than United States territ[illegible]y, or (2) to dumping of material by United States citizens when such mate[illegible] is not transported from the United States for the purpose of dumping it an[illegible] the dumping takes place in ocean, coastal, or other waters other than those described in subsection (b)."

The definitional section could be amended to define "United States citizen" to include natural persons who are citizens of the United States as well as United States corporations and agencies of the Federal or of any State or local government of the United States.

The amendment is not recommended because it is believed that section 4 is already very clear that the activities in question would not be regulated by the bill. Furthermore, without a careful reading, the additional language might be misconstrued to mean that United States citizens would be given more favorable treatment than foreign nationals, clearly an undesirable result.

Mr. SHAROOD. I have another question with regard to the overlap or tie-in between the Dumping Act and the proposed amendments to the Water Quality Act.

As I read the proposal under section 10 of the Water Quality Act, you will be establishing standards for the contiguous zone of the high seas with respect to effluent which flows from the United States into those waters, but until you have in fact established standards, there is no effective prohibition or limitation upon a municipality or private industry to run an effluent pipe, let us say, out into the contiguous zone or the high seas.

Mr. RUCKELSHAUS. There is not now.

Mr. SHAROOD. But even after this act is presumably in force you must first establish standards?

Mr. RUCKELSHAUS. Well, the way we will get at it is through effluent standards, which makes much more sense than water quality standards that distance from the shore.

Mr. SHAROOD. The next question is: viewing this from a nationwide standpoint, effectively how long is it going to take you to establish effluent standards which will encompass all of the seacoasts of the United States, let us say?

Mr. RUCKELSHAUS. We are in the process now of establishing effluent standards for 21 basic classes of industries in the country, and we expect to have the preliminary reports on those standards by the first part of next month, and hopefully, we will have an ability to establish effluent standards prior to July 1 for these 21 basic industries.

As far as effluent standards for municipalities are concerned, where we have pipes outside of the 3-mile zone, they might well be estab-

lished on the basis of secondary treatment, which has a meaning in terms of municipalities that is not nearly as clear in the case of industry.

Mr. SHAROOD. Would your water quality standards say for outfalls that extend far beyond the contiguous zone, differ from your standards for outfalls that extend only 2 or 3 miles out from shore?

Mr. RUCKELSHAUS. I think again it depends on the nature of the ocean, and where the outfall is. I don't know of any outfall that extends beyond the 12-mile zone.

Mr. SHAROOD. Mr. McCloskey yesterday said San Francisco is contemplating building one a 100 miles out in the Pacific. I don't know if this is true or not.

Mr. RUCKELSHAUS. This is why we have asked for rather flexible authority, granted a great deal of authority, into the ocean dumping bill, because there is a lot we don't know, and it may well be that there are places in the ocean in which it would be the most desirable environmental place to either dump or discharge wastes, and as far as examples are concerned, they don't readily come to mind, but I think we need to keep this power and have this ability built into the statute.

Mr. SHAROOD. Do you see any utility in this legislation requiring that after a year your agency will report back to Congress on the implementation and follow-through.

Mr. RUCKELSHAUS. No, we are doing that in all of the bills that we now have. We report up here about once a month.

Mr. SHAROOD. I have two other short questions.

One is on the penalties. You have an exemption there for Federal employees. This is probably more traditional, I suppose, than anything else, from the criminal penalties, and yet it would cover State employees, municipal employees, and other governmental types. Why carve out an exemption for a Federal employee who might willfully violate this act, on a personal liability basis?

Mr. RUCKELSHAUS. I think in stating your question, you stated the reason, that it is a traditional reason for the Government suing itself, and in this case——

Mr. SHAROOD. No, we are talking about a person, an employee, an official of the Government.

Mr. RUCKELSHAUS. Of course, he would be acting outside of the scope of his authority as governmental authority, if he was doing it.

I have some difficulty, myself, in saying exactly why there should be an exemption, except that there are administrative procedures for handling employees of the Federal Government who violate any rules or regulations of their employment, and traditionally it has been thought that these regulations and procedures were sufficient to handle the situation in the case where an employee was involved in some part of civil violation.

Now, governmental employees are subject to criminal penalties, just as are any other citizens. To the extent that this offense is similar to a criminal offense, it becomes more difficult to exclude the Federal employee.

It is a difficult question.

Mr. SHAROOD. Is it logical to exempt a Federal employee and not the State or local employee?

62-513—71——29

Mr. RUCKELSHAUS. It is more logical, because it is a Federal program, and the Federal program can be administered more carefully by the Federal Government, and there is more authority over the employees of our level of government than there is over the State level.

For instance, if any employee of the Federal Government violated provisions of this act, he could be discharged, and there would be means or ways of forcing his discharge. In the case of the State employee, if the State wanted to completely ignore the fact that he violated it, there would be very little we could do.

Mr. SHAROOD. On the question of emergencies, you have a provision here for the safety of human life, or words to that effect, that requires a report, I believe.

Who determines whether or not there is a situation that endangers human life, the private party who decides to go out and dump? Is it totally their judgment, or must they consult with you, or will you have any handle on this at all?

Mr. RUCKELSHAUS. Clearly, if there was a dumping, it would be a dumping without a permit, so on the basis of the flat basis of the statute itself, it would be a violation of the statute.

Their claim would be that they did this to safeguard human life, as the statute gives them an exception from having a permit.

They make a report to me, the Administrator of this agency, indicating that while they did violate the act on its face, they were exempted from the act because of the need to safeguard human life, and there would be a need for them to prove to me this is what happened.

Mr. SHAROOD. What you are saying, in effect, is that this is a defense.

Mr. RUCKELSHAUS. That is right.

Mr. SHAROOD. But the way it is written in the bill, it seems to me it is more of an exemption than a defense.

I wonder if you will consider rewriting that section to spell out a little more clearly that this is a defense to a civil or criminal penalty, and not an exemption, as it appears to be.

Mr. DINGELL. Would you yield?

Mr. Ruckelshaus, this is a matter that does concern me, too. I have the distinct feeling that, as the bill is presently constituted, anyone would be able to come forward and say, "Well, it was an emergency, and we had to run out and dump the stuff to get rid of it."

And I am not sure precisely what you would do in that event, and I think some great care should be devoted to preventing this exemption, or whatever you want to call it, from becoming virtually a license to evade the bill.

Mr. RUCKELSHAUS. I agree with you, Mr. Chairman. If that is the import of the section, I think that the mere fact that they raised that as an excuse for dumping without a permit would be pretty transparent, unless they had some really good evidence as to why they actually needed to engage in this particular dump in order to safeguard human life.

It seems to me it would be a pretty extreme situation in which this defense would come up.

Mr. DINGELL. Would you take a careful look at that and see whether or not some amendatory language is necessary, and try to assist the committee with that particular provision?

Mr. RUCKELSHAUS. We will.

(The information follows:)

EMERGENCY DUMPING WITHOUT A PERMIT TO SAFEGUARD HUMAN LIFE

To accomplish this purpose we would recommend that subsection 5(h) be renumbered as subsection 6(g) and revised to read as follows:

"No person shall be subject to a civil penalty or to a criminal fine or imprisonment for transportation or dumping without a permit or in violation of a permit if such person can prove that such transportation or dumping was necessary, in an emergency, to safeguard human life. Any such transportation or dumping shall be reported to the Administrator within such times and under such conditions as he may prescribe by regulation."

Mr. SHAROOD. One last question on your recommended dumping sites.

As I read the bill, you can recommend dumping sites, but is there anything in the bill which enables you to require that the dumping of the given material take place in a given site? Or will this be discretionary with the permittee?

Mr. RUCKELSHAUS. I don't think so.

If we found, for instance, a particular site was preferable for that permittee, I think we would simply stipulate in the permit that that is where the dumping was to take place, and that they could not do so otherwise, and that in that manner be able to control very carefully where the material is dumped.

Mr. DINGELL. Would you want to give us your counsel, Mr. Ruckelshaus, as to whether or not you would be able under this to require a time for dumping? For example, when the current might be flowing strongly, or the tide might be moving in a particular direction, or the wind might be moving in a certain way, or the currents might be moving in a particularly desirable direction, or when water temperature might be at a particular level? Do you have the authority to control those particular events with regard to dumping?

Mr. RUCKELSHAUS. I think we clearly do, under the provisions of the act, have the discretion to, in the permit itself, limit very carefully the location and time and manner in which any dumping would occur.

Mr. DINGELL. I don't read the bill as being entirely clear on that point.

Mr. RUCKELSHAUS. We would certainly be glad to agree to any language that would make it more clear, or suggest language.

Mr. DINGELL. I think it would be helpful to the committee if you would suggest language which would enable you to give rather clear direction as to all of the conditions of the permit.

(Suggested language to be furnished follows:)

CONDITIONS OF A DUMPING PERMIT

Section 5(c) of the bill as presently drawn provides that—

"Permits issued under subsection (a) may designate and include (1) the type of material authorized to be transported for dumping or to be dumped; (2) the amount of material authorized to be transported for dumping or to be dumped; (3) the location where such transport for dumping will be terminated or where such dumping will occur; (4) the length of time for which the permits are valid and their expiration date; and (5) *such other matters as the Administrator deems appropriate.*" (Emphasis added)

In view of the underlined language, further specificity as to the matters which may be dealt with in a permit is not considered necessary. Such addi-

tional matters could deal with a great variety of things, such as the route to the dumping site, safety precautions to ensure that the transportation and dumping are carried out safely, the precise times when dumping will be allowed. monitoring and reporting requirements, and the like. Greater specification of the allowable permit provisions might lend support to the argument that the listing is intended to be exclusive. EPA prefers the language of the bill as presently drawn, which enumerates only the *basic* provisions of the typical permit, namely, those relating to type and amount of material, location of dumping, and expiration date.

Mr. DINGELL. Now, it occurs to me, Mr. Ruckelshaus, that you are going to be dealing under this section with persons who will constitute a category of ocean dumpers. Are you going to want licensing authority for those people, as opposed to permit?

Mr. RUCKELSHAUS. You mean a more general license?

Mr. DINGELL. I am talking about contractors who will engage in this business.

That is the way it is done today. Are you going to need or want that, or will that be desirable for you to have?

Mr. RUCKELSHAUS. I really——

Mr. DINGELL. Would you like to reflect on that and give us your guidance on that point, please?

(The information follows:)

EPA AUTHORITY TO LICENSE TRANSPORT CONTRACTORS FOR OCEAN DUMPING

We favor the minimum amount of regulation necessary to control ocean dumping. The provisions of H.R. 4247 requiring a permit for each incident of dumping or transportation for dumping provide all the control that is necessary.

Mr. DINGELL. Mr. Everett.

Mr. EVERETT. Thank you, Mr. Chairman.

Mr. Ruckelshaus, also, when you reflect on the emergency provision, please give some thoughts to a way of expediting a procedure with respect to these type dumpings, and still require a permit or some notification to you or the Department prior to the dumping of such material.

Mr. RUCKELSHAUS. All right.

Mr. DINGELL. If you will yield, the Chair comes to the thought that perhaps we ought at least to require that they do give you notice, in the event of these so-called emergencies, so that if they are going to do something that is hazardous, and it is not a bona fide emergency, then you could bring into play whatever powers you felt necessary, including resort to the courts for equity for appropriate injunctive relief.

Mr. RUCKELSHAUS. It is my understanding, Mr. Chairman, the purpose of that provision is primarily for a distressed ship at sea, where it is necessary to dump over a number of ballasts, or whatever might preserve the ship.

Mr. DINGELL. This was the Coast Guard's interpretation, but I am sure you recognize that the particular section is rather more broadly drawn than that.

Mr. RUCKELSHAUS. Yes, it is.

Again, I would think that other than that specific application, even if you are talking about toxic materials in the question as to whether to dump them on land or in the ocean is safeguarding human life, I cannot conceive of such a sitaution, but it is possible, and there is no reason that could not be worked out as the general permit procedure

itself, with prior notice and a hearing, if necessary, and all of the provisions of the granting of any permit.

Mr. DINGELL. It would be possible, if somebody wanted a very fine device to evade the probability of a hearing over a very unpopular subject.

Mr. RUCKELSHAUS. I suppose that is right.

Mr. DINGELL. Mr. Everett.

Mr. EVERETT. Mr. Ruckelshaus, I am still not clear in my mind as to how this would work with respect to the Corps of Engineers.

The chairman of the full committee is concerned about the plans for the Baltimore harbor project. In a statement issued by Mr. Reynolds yesterday, on behalf of the American Institute of Merchant Shipping, he indicated that the project had already been approved, and that the State of Maryland, the board of public works and State of Maryland have given assurance that disposal areas will be provided in the Chesapeake Bay opposite Kent Island and/or in diked areas off Baltimore Harbor.

If this bill is passed in its present form, what will take place with respect to a permit application that would have to be obtained under this legislation, so far as the dredge and fill permit is concerned?

Mr. RUCKELSHAUS. Well, as it would ordinarily work, if we are talking about a dredge and fill permit from the Corps of Engineers, where under their authority under the Rivers and Harbors Act, there would only be one permit issued, and if the permit is requested of them, any dumping provisions relating to the issuance of that permit would come under the purview of our responsibilities, under this act, and they would have, under the act, as I read it, there is no requirement for a separate permit.

Mr. EVERETT. The State of Maryland said they would like to have——

Mr. RUCKELSHAUS. Excuse me. There has to be certification under section 7(b)(2) to the Corps of Engineers by our agency that there is no violation of the criteria.

Mr. DINGELL. Mr. Everett, if you yield, what you as saying here, if you please, Mr. Ruckelshaus, is that under this particular proposal, you would still have the corps continue to issue dredge-fill permits as they do under existing law, but that the dredge-fill permits would require an additional activity by your agency under lines 18, 19, 20, 21, and 22 of the bill appearing on page 11, which, for the purpose of the record, says:

> And regulations issued hereunder, unless the Administrator has certified that the activity proposed to be conducted is in conformity with provisions of this Act and with regulations issued hereunder.

Am I correct in that?

Mr. RUCKELSHAUS. That is right.

Mr. DINGELL. So essentially, the corps would have to defer to you on environmental matters, even though they would actually issue the permit in that particular instance. Am I correct?

Mr. RUCKELSHAUS. I think the theory of this is one agency ought to have responsibility for assuring that there is uniform treatment for dumping whatever the material is in the ocean, and if you divide this

BEST COPY AVAILABLE

responsibility up, it would be more difficult to get consistent treatment for dumping of material in the ocean.

Mr. DINGELL. This would refer also to estuaries, bays, salt marshes, and also to the Great Lakes, which is a matter we have not discussed, which I hope we will be able to address ourselves to a little later.

Thank you, Mr. Everett.

Mr. EVERETT. Does the certification requirement in effect give you veto authority over this type of permit that would be issued by the corps?

Mr. RUCKELSHAUS. That is right. They could not issue a permit without the certification from us.

Mr. DINGELL. Mr. Ruckelshaus, for the purposes at this point, I think it would be useful for you to give us, for the record, a statement.

Does this bill in any way amend or alter the Fish and Wildlife Coordination Act?

Mr. RUCKELSHAUS. No, it does not.

Mr. DINGELL. Does the bill in any way amend the requirements of the National Environmental Policy Act, particularly section 102?

Mr. RUCKELSHAUS. No, it does not.

Mr. DINGELL. Does it in any fashion at all change or amend the agreement and understanding between the Corps of Engineers and and the Interior Department, which has just been reviewed by the Supreme Court in *Zabze* v. *Tabb* where they said they would consider questions regarding fish and wildlife in issuing permits of this kind?

Mr. RUCKELSHAUS. I am not familiar with that case. I would have to defer that answer.

Mr. DINGELL. I will see to it that the precise title of the case is made available to you, and you may submit the additional information to us.

Briefly, the Corps and Interior Department made an agreement which said that the corps would condition dredge and fill permits on consideration of 50-mile values.

This was challenged in the courts and was reviewed up in the Supreme Court, and the agreement was upheld, it was held that the corps agreement was proper, rather than Interior Department, and that in light of the Coordination Act and in light of the National Environmental Policy Act, these matters could be appropriate conditioning devices to dredge and fill permits issued by the corps.

After you have had a chance to review that more carefully, we would like your guidance on that.

(The information follows:)

H.R. 4247 EFFECT ON MEMORANDUM OF UNDERSTANDING

This Memorandum of Understanding deals with consultations between the Department of the Interior and the Department of the Army on fish and wildlife, recreation, and water pollution problems associated with dredging, filling and excavation operations conducted under permits issued under the Rivers and Harbors Act of 1899. H.R. 4247 would have no effect on this Memorandum of Understanding (although it might be pointed out that Reorganization Plan No. 3 of 1970, by transferring responsibility for water pollution control from the Department of the Interior to EPA, has narrowed the scope of application of the

BEST COPY AVAILABLE

Memorandum). Subsection 7(c)(2) of H.R. 4247 would require consultations between the Department of the Army and EPA *in addition to* the consultations between the Department of the Army and the Department of the Interior required by the Memorandum of Understanding.

Mr. EVERETT. Mr. Ruckelshaus, how will this act affect applications and permits that have already been approved?

I notice the act takes effect 6 months after its effective date. How will this affect projects underway, or applications that have been approved by previous agencies?

Mr. RUCKELSHAUS. It would not affect them at all, unless there were some dumping that was going to occur after the effective date of the act. So if you had a dredge and fill permit that had been issued, unless there was some violation of the act itself, it would not affect that particular permit, although it is conceivable to me that there may have been a dredge and fill permit authorized in which we were dumping polluted dredges in some sections of the ocean which seemed to be inadvisable, where we would ask them to get a permit to do that, or ask them to go through this procedure.

I think once the act took effect, that it would impact any dumping that occurred after that, and, if a permit were issued for a dredge and fill that covered several months, or even years, then at that point, when the act took effect, there would have to be some control over that continued dumping.

Mr. EVERETT. Mr. Ruckelshaus, there has been some concern expressed by port authorities that maybe industrial and economic development considerations would not be taken into account as much so as they would under the National Estuarine Act and the National Environmental Policy Act.

I don't find too much language in the bill that relates to that. Are you going to take all of these considerations in mind when you consider where a dumping should take place, or if it should take place at all?

Mr. RUCKELSHAUS. What specific kinds of considerations?

Mr. EVERETT. Under the National Environmental Policy Act, all Federal agencies shall carry out their responsibilities in such way as to achieve a balance between population growth, urbanization, industrialization, and resource use, in order to attain the widest range of beneficial uses of the environment without degradation to health, safety, or other consequences.

Mr. RUCKELSHAUS. Well, of course, we will carry out the provisions of the National Environmental Policy Act, and any other act of Congress.

Mr. EVERETT. And to create or maintain conditions under which man can exist in harmony and fulfill social and economic requirements of present and future generations.

Under the National Estuarine Act, it likewise requires similar consideration, that the estuaries will be utilized in such a manner that balance will be maintained between the national need to protect such areas in the interest of conservation and the need to develop these estuaries to further growth and development.

Now, the concern expressed is that these concepts and requirements might not be kept in mind under the administration of this act as they would be under the administration of the National Environmental Policy Act and National Estuarine Act.

Mr. RUCKELSHAUS. I think we are clearly mandated to keep those considerations in mind, and I think, given the present state of the law, we have no ability to keep those considerations in mind in our ocean policy, and we certainly would keep them in mind in the administration of this act.

Mr. DINGELL. Mr. Ruckelshaus, in the Great Lakes area you have generally a freeze on open water dumping of polluted dredge spoil. These spoils are deposited now, and must be deposited in closed, diked areas.

Is it your intention to continue that practice with regard to the Great Lakes area?

Mr. RUCKELSHAUS. Mr. Dominick may be able to give you an answer. He has been involved in this directly, and he may be able to give you a better answer.

Mr. DINGELL. I would like your comments, if you please, Mr. Dominick.

Mr. DOMINICK. Mr. Chairman, I think the important legislation that has directed activity in this area is the Rivers and Harbors Act of 1970, section 123 of that act which refers to alternate disposal sites in the Great Lakes for the dumping of polluted dredge spoils.

We are in the process of identifying now, with the Corps of Engineers, those harbors where polluted dredge spoils are a significant problem, and we have identified up to 40 or more at the present time.

Mr. DINGELL. In fact, you have not got a single Great Lakes harbor that does not have polluted dredge spoil in it. Is that not a fact?

Mr. DOMINICK. I think most of them very definitely will have some degree of pollution in them. We also want to evaluate whether additional dredging is going to be required in order to keep navigational channels open.

As that evaluation is completed, we will be making determinations and referring these determinations to the Administrator on the availability of on-land disposal sites, and on the Federal and State or local funding required to implement those alternative sites.

Mr. DINGELL. Mr. Ruckelshaus, on page 11, you mentioned toxic industrial wastes. You may not wish to do so at this particular time, but would you give this committee a definition of what those are, not necessarily the exclusive definition, but one which would give us an understanding of how you interpret that particular word?

Mr. RUCKELSHAUS. Yes, we will.

(The definition follows:)

"TOXIC INDUSTRIAL WASTES" DEFINITION

"Toxic industrial waste" is a general term used to describe various materials having the ability to produce harmful effects in living organisms. These materials are dumped by industry either because there are no straight forward or economi-

cal treatment methods or because the materials cannot be technically or economical recovered. These materials may be in liquid, solid or semi-solid form. When discharged into the marine environment, the materials may be (1) deposited on the ocean floor, (2) dissolved and transported by ocean diffusion, dispersion and currents, (3) suspended in colloidal form and transported by currents, (4) float and be transported by currents. The position that the discharged materials occupy in the ocean determine what life forms may be damaged. The toxicity of the wastes is dependent upon several ocean factors, such as pH, salinity, turbidity, temperature and mixing.

Examples of the types of industries that can produce toxic wastes are petrochemical facilities, steel mills, paper mills, metal plating plants and others. Very simply, much of these toxic industrial wastes are the residual materials from "still bottoms", settling basins, lagoon storage systems or concentrated precipitates and sludges from solid-liquid industrial processing streams. Elements and compounds that may be regarded as hazardous substances are common in toxic industrial wastes. These materials would include elements in their elemental form or as ions, compounds, or in any combination or mixture such as antimony, arsenic, beryllium, boron, cadmium, chromium, lead, mercury, nickel, selenium, silver, thallium and zinc. In addition, organic materials, includuing certain pesticides and residues that have been classified by Department of Transportation as Class B poisons in 46 CFR 146.25 are also toxic. A major threat to the marine environment from toxic industrial wastes is the complexity of these mixtures and the high potential for synergism once discharged.

Mr. DINGELL. Are you satisfied that oil may be safely excluded from the purview of this act because it is adequately covered elsewhere in the statutes?

Mr. RUCKELSHAUS. I think under section 11 of the Federal Water Pollution Control Act, Mr. Chairman, there has been a very recent effort by this Congress to cover comprehensively the problems of oil pollution, and we are in the process of attempting to implement that act, and maybe after a little more experience with our efforts to implement it, we can give you a clearer answer, but it is a comprehensive effort to treat the problem of oil, and certainly we have by no means got it completely under control, but we are in the process of trying.

Mr. DINGELL. You have here a question of existing legislation and the question is whether bilge and hold pumping anywhere by American ships is adequately handled, out of the 20-mile limit, or possibly 50 miles out.

Could you give us a comment on that particular problem?

Mr. DOMINICK. We feel that the role of the United States in handling the question of ballast dumping and bilge pumping beyond our territorial waters or the contiguous zone must be undertaken in the international convention areas, and, as you know, Secretary Volpe has taken a very strong role with the NATO countries in seeking international agreements to totally ban the discharge of ballast waters on the high seas. I believe the date was by 1975 or 1976.

Mr. DINGELL. This brings to mind the question regarding matters of some importance to the Chair.

Legislation pending elsewhere in the Congress would instruct the administration, if it is the wish of Congress, that they should at an early time enter into endeavors to achieve international controls on ocean dumping through treaties and similar devices.

Would you have any feeling as to the relative usefulness of a direc-

tion of this kind from Congress, and whether or not it would be necessary, or would it conflict with any administration policy?

Mr. RUCKELSHAUS. Well, it does not conflict with any policy, because we are attempting to do this right now. I am going to Brussels next week for a meeting of the Committee on Challenges to Modern Society, in an effort to get some international cooperation with this problem.

There is in section 10 of this bill, of course, a provision relating to international cooperation, and I think that a direction like this, indicating Congress is concerned that we make these international efforts, is very beneficial.

Mr. DINGELL. Now, there are, if you please, gentlemen, in the last two pages of the bill, pages 13 and 14, section 11, entitled "Repeal and Supersession," there are a whole series of statutes, sections, and so forth, which are referred to at that point.

Would you gentlemen please inform the committee, would you, please, gentlemen, give the committee at your convenience some statement of precisely what the meaning of this particular section happens to be?

Mr. RUCKELSHAUS. Yes; we will.

Mr. DINGELL. It will be very helpful to the committee.

(The statement follows:)

"REPEAL AND SUPERSESSION" DEFINITION

We understand that this statement will be provided by the Council on Environmental Quality.

Mr. DINGELL. Now, in your statement, Mr. Ruckelshaus, page 5, you state the Administrator in issuing permits to dump materials, to transport them for dumping, will be required to determine that such activity will not, and so forth.

On whom would be the burden of proof in that matter? Would it be upon you, or would it be on the would-be dumper?

Mr. RUCKELSHAUS. I think it would be clearly on the dumper.

Mr. DINGELL. Is there amendatory language needed to make that crystal clear?

Mr. RUCKELSHAUS. I think it is clear from the present statute, but I certainly would have no objection to making it clearer if the committee felt it was necessary.

Mr. DINGELL. I must tell you that I am not satisfied that it is as clear as I personally would like.

Your words at this time have great impact on me, and I am satisfied under the legislative history, but it would be appreciated if you would review that point and let the committee have the benefit of your views.

(The information follows:)

BURDEN OF PROOF FOR PROPOSED DUMPING PERMIT

In our view subsection 5(a) is sufficiently clear that such burden of proof is upon the permit applicant.

Of course the Administrator must exercise his judgment as to whether such

burden has been met in determining whether, and on what conditions, a permit will be issued.

It is the applicant, however, who must provide all the necessary information upon which the Administrator makes his determination.

Mr. DINGELL. Now, at page 6 you mention that the Administrator will be permitted to impose restrictions, and a little later you say he would be authorized to deny the issuance of a permit, and then at the last paragraph, he would be authorized to require applicants with permits to provide information. And then, on down, you say he would be authorized to prescribe reporting requirements.

I wonder if we ought not simply require you to do that, Mr. Ruckelshaus. Do you have any feelings on that matter?

Mr. RUCKELSHAUS. I think the whole question involved is a question as to whether it is better to permit Administrator flexibility in the administration of a statute of this nature, or to put more mandatory language in from the legislative branch, and the one reason I think we have opted for the more flexible administrative approach in this statute is that there are so many unknowables in the area of ocean dumping.

While we may feel today that one particular kind of activity is terribly harmful to the environment, further research may show us this is the most beneficial we could be doing.

And to attempt to lock too many specifics into the statute in an area where there are so many unknowns may have some hazards.

Where we are dealing with specific problems that everybody agrees are known in terms of, for instance, high degree of radioactive waste, I see no reason not to get more specific in the statute, but I also can see in trying to implement this kind of statute, in reading the report of the Council on Environmental Quality, that there are a number of areas that we simply don't have the knowledge we should have, and for that reason there is a need for flexibility.

Mr. DINGELL. You are coming to a matter that is of considerable concern.

H.R. 6332, which is authored by Mr. Rogers and the present occupant of the Chair, and a number of our colleagues, generally sets out a research program at a level of $1 million per year. You and every other witness have indicated the great plausibility of knowledge, and great need for research.

I am satisfied that you are carrying out research down there at your agency, but I am troubled, since we really do not have any program which has been here in a unified form, which the Congress may scrutinize and understand precisely what you folks in the administration propose to do in this area, and I am wondering, would it be possible for you to list for this committee the research requirements which you would need to conduct a good research program in this area, so that we could have before us, when we go into this statute, a very clear understanding of what you are doing, and how it conforms with the requirement of law to carry out a carefully thought out and integrated program of research?

Mr. RUCKELSHAUS. We will certainly supply that to the committee.

Mr. DINGELL. Would you do that for us, please?

(The information follows:)

FISCAL YEAR 1972 PROGRAM PLAN FOR OCEAN DISPOSAL RESEARCH

[Dollar amounts in thousands]

Project	Program office	Positions (12K/year, at lapsed rate)	Grants	Contracts
Objective No. 1—Determine the size and nature of the ocean and Great Lake disposal problem:				
(1) Identify sources and amounts of municipal and industrial sludges, dredge spoils, and other wastes.[1]	SWO/WQO			
(2) Characterize disposed materials for each disposal site (physical, chemical, biological, and bacteriological characteristics).	WQO:			
	1607	1	$100	
	6112	1	75	
Objective No. 2—Establish scientific criteria for disposal site selection:				
(A) Determine natural ecology:[2]				
(1) Establish natural marine preserves and sanctuaries.	NOAA, Interior, Smithsonian Institution, WQO			
(2) Determine natural ecology-food web systems.	WQO, NSF, AEC, Smithsonian Institution.			
(3) Delineate biologically critical areas	WQO, Interior, NOAA			
(B) Determine effects in existing disposal sites:				
(1) Study the physical and geochemical character and extent of deposits (continue studies of New York Bight, initiate studies in Pacific, Guld of Mexico, and Great Lakes.	WQO:			
	1607	1		$200
	1612	1		75
(2) Characterize water circulation at disposal sites (initiate multilayer circulation model of coastal waters and plan field measurements).	WQO:			
	1607	1	150	
	1612			50
(3) Characterize and chemically analyze the benthic biota in disposal sites.	WQO:			
	1602	2		
	1607	1	182	
	1805	1		
	1808	1		
	1612		75	
(4) Determine the time/space distribution of bacteria and viruses.	WQO:			
	1602	1		
	1607		50	
Objective No. 3—Establish marine water quality criteria for disposal sites:				
(1) Determine toxicity effects of sludges and their constituents—organic and inorganic (marine and freshwater).	WQO:			
	1607			
	1805	2	75	
	1808	3	75	
(2) Develop testing protocol for use by waste disposers in permit applications.	WQO:			
	1607	1		50
	1 1808	1		
Objective No. 4—Establish monitoring systems for ocean disposal:[2]				
(1) Develop site monitoring techniques	WQO, USCG, NASA, NOAA, NOIC&NODC			
(2) Develop water quality sampling procedures and sensors for estuaries, coastal waters, open ocean, and Great Lakes				
(3) Establish and maintain ocean pollution data management system.				
Objective No. 5—Establish environmentally optimum dumping methods:				
(A) Characterize methods:				
(1) Survey and study existing operational technology and effects.	WQO-1607		25	
(2) Scale model proposed methods				
(3) Conduct full-scale demonstrations				
Objective No. 6—Establish optimum waste management procedures relative to ocean dumping				
(1) Develop and demonstrate alternatives to ocean dumping.	WQO, SWO			
	WQO-1707	1		100
(2) Study beneficial uses of waste[3]	WQO-1607			
(3) Develop marine restoration techniques	WQO:			
	1607			
	1707			
Objective No. 7—Program coordination and management: Coordinate, Plan and Manage Ocean Disposal R. & D.[4]	WQO Headquarters	3		

[1] This may have already been done.
[2] During the 1st year, headquarters will plan the program with other agencies.
[3] During the 1st year, a program plan will be developed by headquarters.
[4] Includes 1 secretary.

Mr. DINGELL. One of the reasons I ask this is I happen to have read some articles in the paper; for example, one mentioned that it is possible perhaps to properly encase and package these substances and put them into places between the plates in the earth crust, where there is a downward movement into the crust, and dispose of waste that way with minimum hazard to the environment.

Then I observed another comment about utilization of pumps to bring cold water up to create nutrient, and I wonder if we ought not perhaps consider giving you the authority to engage in a program of converting municipal sewage into useful nutrients. We have done that in the Great Lakes with great success.

It occurs we might not have the putrification problem in the ocean, if we treat sewage in the proper way and deposit it in the right place to create a significant benefit.

Mr. RUCKELSHAUS. I agree, Mr. Chairman, there are those who believe that this can happen, and I think it may well be right. I think we need more research to determine whether this would be a proper way to dispose of sewage sludge.

Mr. DINGELL. Perhaps a device of this kind might be highly desirable to include in the research authorization in the direction we are discussing at this time.

Mr. RUCKELSHAUS. We have had this same problem come up, the same question, in virtually every bill which we presently have before Congress, and in our general appropriations bill we have included a considerable sum of money for research across the agency, and we have attempted to deal with the problem of research money in our general appropriation bill rather than trying to deal with it in each individual bill that comes across.

We are trying to in the agency centralize the general research overview, and the effort to coordinate all of our research into areas where it would seem to bear the most fruit for the environment.

Mr. DINGELL. I can see the desirability from your viewpoint, but from the standpoint of congressional review, we have the other problem, and that is comparing what you are doing as opposed to congressional direction, and that is the reason I seek your assistance in this.

Mr. Ruckelshaus, the Chair is a little bit concerned here about the language at page 12. You mentioned that no new sources of ocean disposal of sewage sludge would be permitted, and then you went on to say you would not allow an increase in volume of dumping over current levels, that is a very strong statement, for what existing barging facilities can accommodate.

How do you propose to carry out that particular policy statement?

Mr. RUCKELSHAUS. How do I propose to carry out that statement?

Mr. DINGDELL. To carry out that statement.

I happen to be in entire agreement with it. I want to be sure we have before us your proposal.

Mr. RUCKELSHAUS. There may be some specific problems with this statement in sections of the country, in particular New York City, but with the 6-month period in which we have to implement the act, I think we can develop alternatives sites that will allow us to carry out this pledge, at least not to have any increase.

Mr. DINGELL. You have indicated here again at pages 13 and 14 that a great deal of effort and investment is necessary, and research is needed in recycling wastes.

BEST COPY AVAILABLE

Could you give us and idea of the dollar amount of research that you actually feel is needed in this particular area, in connection with the questions that I asked earlier that you said you would furnish?

Mr. RUCKELSHAUS. We have a submission in our 1972 budget of $1,100,000 for ocean disposal research.

Mr. DINGELL. That level will rise to what figure over a 5-year period?

Mr. RUCKELSHAUS. The level for the bill itself, for the implementation of the act, which would not necessarily include research, because we don't have that built into these figures in fiscal year 1972, starts at $2 million, and then by 1977 we are down to 3.9, because I believe the phasing out of much of ocean dumping that we now know, it might not necessitate as grave an expenditure of money.

Mr. DINGELL. You have mentioned—at the bottom of page 15, top of page 16, you said as follows:

> Several of the bills pending before this committee would require designated levels of treatment for municipal sewage and industrial wastes by specified dates. This approach does not take into account variations in water use designations, the quality or characteristics of the receiving waters, or other factors which bear on the appropriate level of treatment in a given instance.

That statement does not change or alter or indicate an intention by your agency to change the no degradation requirement, over which a monumental fight took place some time back, does it?

Mr. RUCKELSHAUS. No, it does not.

It might imply a certain qualification of the statement by Mr. Rogers a little earlier, when we discussed deadlines in terms of secondary and tertiary treatment, as to how we would go about insuring clean water in the country.

Mr. DINGELL. Now, the reason I asked that is, I am apprehensive in looking over my shoulder at some of the industrial and municipal folks who have ideas we should utilize the streams and lakes and oceans up to their assimilative capacity. The understanding I have had with your agency previous to your taking office was that was not going to be the policy of the Public Health Service, and then the Interior Department, and I think that is still not the policy, to allow the utilization of these waters up to that capacity.

Mr. RUCKELSHAUS. No, that is not the policy.

Mr. DINGELL. There is a requirement that you consult with the Department of the Interior with regard to achievement with the levels, or, rather with regard to criteria and so forth with regard to ocean dumping, but there is no similar requirement with regard to the issuance of permits.

Can you explain the difference there?

Mr. RUCKELSHAUS. I think the difference is mainly a difference between substance and procedure, and that on the substantive matters, which we would hope to cover in the criteria, upon which the issuance of the permits would be based, we would want to insure that we had complete concurrence in these other affected agencies with substantive requests that we were going to be making from the permittees, but as far as actual administrative process of issuing the permits themselves, there did not seem to be as great a need for coordination between the agencies.

BEST COPY AVAILABLE

That does not mean that in given instances, where clearly another agency is involved, and policy and statutory duty of another agency is involved, we would not consult with them even on the issuance of a permit.

Mr. DINGELL. Would you have any objection to requiring that you should consult with them on the issuance of these permits? It is done under the Coordination Act in every instance.

Mr. RUCKELSHAUS. The thing that disturbs me, as apparently disturbed Mr. duPont and some of the witnesses yesterday, is administrative difficulty of issuing permits where you have multiagency responsibility, and the time lag between the request and ultimate issuance of the permit can bog the program and destroy its effectiveness.

Certainly we have no objection to inclusion of language that we should consult in every case where a problem arises, or where responsibility with another agency occurs.

Mr. DINGELL. Perhaps maybe to assist you and assist this committee, you can submit the language that you would deem appropriate to carry that particular purpose out.

(The proposed language follows:)

CONSULTATION PROVISIONS PRIOR TO ISSUING PERMITS

This matter could be dealt with by amending subsection 5(a) to include immediately prior to the last sentence of that subsection, the following:

"Such provision shall require consultation with Federal agencies with respect to factors specified in paragraphs (1) and (2) of this subsection for which such agencies have responsibilities."

Mr. DINGELL. With regard to the question of notice of hearing on these matters, there is no clear statutory requirements for notice of hearings.

You mentioned in response to questions from others of our colleagues that there are both constitutional and due process, and also Administrative Procedures Act requirements would come into play.

As I recall it, and I don't want you to give me a specific answer at this particular moment, but I would like to have you reflect and give us your answer after you have had a chance to consider the matter soberly, it is more or less standard legislative boilerplate that says that after notice and opportunity for hearing.

Would this be objectionable? Obviously, some permit requests are going to be highly controversial, and it occurs to me the courts are going to probably require you to give both notice and hearing in any event, and probably statutory language of this kind would not do too much mischief.

Mr. RUCKELSHAUS. Let me, as you stated, upon sober reflection give you a detailed answer on that, because notice of hearing can mean so many different things, and I want to be sure that our position is crystal clear on how we can provide due process and at the same time provide an expedited administrative process, if possible.

(The information follows:)

NOTICES AND/OR HEARINGS PRIOR TO THE ISSUING OF DENIAL PERMITS

We would not be opposed to the addition of the following paragraph as a new subsection of section 5 of the bill:

"The Administrator shall issue a public notice of each application for a permit, which shall provide a reasonable period of time within which inter-

ested persons may express their views concerning the application. The Administrator shall hold a public hearing on an application whenever he determines that there is substantial public interest in the application or that a hearing will be of assistance to him in reaching a determination as to whether a permit should be issued, and if so, the terms of the permit."

Mr. DINGELL. It is also my intention to be fair, and give you the opportunity to have you give us your best judgment, because we intend to lean heavily on this matter.

Mr. Everett.

Mr. EVERETT. Mr. Ruckelshaus, when you have an opportunity, also, section 5(f) and 6(f) of the bill are not clear to me, and I wondered if they are duplicative. If not, would you provide a brief explanation of what you are trying to get at with these two different sections?

Mr. RUCKELSHAUS. Yes, we will.

(The information follows:)

INTENTIONS OF SECTIONS 5(f) AND 6(f)

Section 5(f) authorizes the Administrator to establish, by regulation, categories of dumping or transportation for dumping for which permits will not be granted because of inability to comply with the criteria established under section 5(a). Section 5(f) also authorizes the Administrator to alter or revoke permits where he determines that the materials in question cannot be dumped consistently with such criteria. Section 6(f) authorizes the Administrator to revoke or suspend a permit which has been violated.

The sections are not duplicative. They both deal with revocation of permits, but in different circumstances: 5(f) applies where the Administrator determines that the material cannot be dumped consistently with the criteria (even though there has been no violation of the permit), whereas 6(f) applies where there has been a violation (even though the dumping authorized by the permit could be carried out consistently with the criteria).

Mr. EVERETT. Section 12 of the bill pertains to delegation of your responsibility to other agencies, including the issuance of permits. Do you contemplate that you will transfer or delegate this responsibility to some other agency?

Mr. RUCKELSHAUS. We have no present contemplation of doing that. It just may be that in the administration of the act itself there would be some area where dual permits or dual administration of the act would not seem to be wise, that we would want to make such a delegation.

We have no present intention, nor do I foresee any in the near future, of making such a delegation

Mr. EVERETT. As Mr. Dingell mentioned a while ago, it is broad language, and it opens the door.

One problem we have with respect to AEC is that they already are exempted; but if this provision should be dropped from the legislation, you could still turn around and delegate the same responsibility to AEC with respect to dumping of their own waste materials.

This is something that gives the committee some concern.

Mr. DINGELL. Mr. Everett, if you will yield, as a matter of fact it has been interpreted by some of the members of the committee as affording a complete exemption to AEC from EPA regulation in certain instances of ocean dumping, and I would like it very much if you would please to direct your attention to the responsibility as to whether or not some amendatory language is not necessary for the bill, so that

AEC will not be able to escape entirely your permit and regulatory requirements.

Mr. RUCKELSHAUS. That is not the intention, and so we will direct our attention to amendatory language of that nature.

Mr. DINGELL. I think it would be very helpful.

(The proposed language follows:)

AEC PERMIT AND REGULATORY REQUIREMENTS

One way of accomplishing this would be to delete section 7(b) of the bill altogether, so that the AEC would be subject to the requirements of the Act just like other Federal agencies.

As section 7(b) is now written, the AEC would be required to consult with the Administrator prior to issuing permits for dumping, but would not, however, be required to consult with the Administrator prior to carrying out the dumping itself. Thus, another way of tightening regulatory control with respect to the AEC would be to require consultation with the Administrator in the latter case as well as in the former. The first two sentences of the proviso in section 7(b) could be amended to read as follows:

> "*Provided*, The Atomic Energy Commission shall consult with the Administrator prior to conducting any activity or issuing a permit to conduct any activity which would otherwise be regulated by this Act. In conducting any such activity or in issuing any such permit, the Atomic Energy Commission shall comply with standards set by the Administrator respecting limits on radiation exposures or levels, or concentrations or quantities of radioactive material."

Mr. EVERETT. One final question, Mr. Ruckelshaus, with respect to finder's fee. I wondered what your thinking would be on including a provision in the bill that would provide for an informer's fee.

Mr. RUCKELSHAUS. The informer's fee has worked in some instances, and has not in others.

The question I suppose really is whether you want to proceed with the fine or through injunctive process. An informer's fee is of no particular benefit through the injunctive process, and here, where you have a specific act that is violated, maybe an informer's fee would be a good way to insure that the statute was complied w[illegible].

Mr. EVERETT. Would you have any particular obje[illegible]ion to including a provision of this sort?

Mr. RUCKELSHAUS. I have not thought about it, but offhand I cannot think of any.

Mr. EVERETT. Thank you very much, Mr. Chairman.

Mr. DINGELL. Mr. Sharood had a question he would like to direct at you, please, Mr. Ruckelshaus.

Mr. SHAROOD. In the penalty provision, dealing with actions against the vessel, you have an exception for public vessels, and then it goes on to say "as defined in the Water Quality Act or Water Pollution Control Act or other public property of a similar nature."

Could you tell us what you are referring to there, "other public property of a similar nature"?

Mr. RUCKELSHAUS. I am not sure what they do have in mind, although an action against a public vessel would again be taking out of one pocket and putting it into another.

Mr. SHAROOD. I want to know how far this exemption goes. There may be some optional aspect to this. I cannot visualize what you mean by "other public property."

62-513—71——30

Mr. RUCKELSHAUS. It apparently refers to channels and pipelines and things of that nature.

We will supply for the committee the specific thing we have in mind.

(The information follows:)

Upon further consideration, we believe that this phrase is unnecessary and may appropriately be deleted from the bill.

Mr. DINGELL. Mr. Ruckelshaus, this is probably a question that should have been asked earlier. It relates to just what authority and which particular statute will apply where.

Dealing with the different kinds of permits, the Water Pollution Control Office will apply out to 3 miles, or 12 miles, under existing statute?

Mr. RUCKELSHAUS. Under existing statute, they apply out to the edge of the territorial sea, 3 miles.

Mr. DINGELL. From 3 miles out, if somebody puts a sewage outfall or something of that kind, they are exempt from the water pollution control law?

Mr. RUCKELSHAUS. We have no authority now, except possibly under the dredge and fill permit, the navigational permit of the Corps of Engineers, if they put an outfall out that far, they have to get a permit to do so from the corps, and at the beginning of that process we may be able to insure under section 21(b) of the Water Pollution Control Act that they meet pollution standards as well, water quality standards as well, but once it is there, we cannot do anything about it.

Mr. DINGELL. The corps' permitting authority goes to 3 miles, as I understand it, or do they go out?

Mr. RUCKELSHAUS. It is a navigational permit, and I think under the control of the navigational aspects of anything in the water, we could control what goes out at the end of that pipe.

Mr. DINGELL. Providing it had an impact on navigation, or would you go unde. the guidelines of the Executive order that has been issued by the President?

Mr. RUCKELSHAUS. We cannot go any further, under the permit program, than the 3-mile limit, unless we can try and get hold of it prior to construction of any outfall through the corps' other permit authority under the dredge and fill.

It is not at all clear that we can. I don't want to put too much emphasis on this, because I am not sure we can.

Mr. DINGELL. This bill exempts outfalls, which would mean that it would exempt sewage emissions between 3 and 12 miles. Am I correct?

Mr. RUCKELSHAUS. That is right.

Mr. DINGELL. If we were to strike outfalls, what problems would that create for you or the administration with regard to the projected legislation which you are going to submit, or which has been submitted, to cover underwater quality standards the areas between 3 and 12 miles?

Mr. RUCKELSHAUS. It would present problems, because we would have two separate permit programs going on for discharges into waters, and in the case of the Great Lakes, or in the case of estuarine areas, or the ocean.

We have one permit program under ocean dumping provisions, and another program under the Water Pollution Control Act.

Mr. DINGELL. You don't have permits under the Water Pollution Control Act, and the new legislation does not apply to permits, does it?

Mr. RUCKELSHAUS. That is right, except we do have for the discharge of industrial wastes not going through municipal systems. We have a permit program including that.

Mr. DINGELL. All right. Now, would it be possible for you through administrative action to control, or rather to merge the two programs? For example, Secretary Volpe has merged the reporting requirements under section 4(f) of the Transportation Act and 102(2)(C) of the National Environmental Policy Act. Could you merge the two without any difficulties?

Mr. RUCKELSHAUS. Assuming we had the statutory framework to do so, I am sure we could, and I think that is what we have got to be very careful about, and I think the passage of this act has got to be looked at carefully in terms of passage of the Water Pollution Control Act, so we don't have an overlap and so that the two programs can clearly be merged under the two statutes.

Mr. DINGELL. We would be giving the same agency the authority to issue two permits. That should not create too much problem, if you would administer it wisely and well, would it?

Mr. RUCHELSHAUS. It would not, except we would be issuing one permit under the Refuse Act, if industry was involved, and then another permit under the Ocean Dumping Act, so they would have to get two permits for doing essentially the same thing.

Mr. DINGELL. It would not create problems with water pollution activity, would it?

Mr. RUCKELSHAUS. Other than the dual permit, which is not a very administratively neat way to do it, I don't see that it would affect our effort to clean up the water.

Mr. DINGELL. Actually, you are effectively, in some areas, doing away with the Refuse Act permit, anyway, in other parts of the bill, are you not?

Mr. RUCKELSHAUS. Yes, but that is by distinguishing between intermittent dumping and continuous outfall.

If you put outfalls into this act, we would be treating outfalls differently, depending on where they are located.

Mr. DINGELL. I am talking outside of the 3 miles. Does the corps' Refuse Act apply to the running of these outfalls beyond the 3-mile limit?

Mr. RUCKELSHAUS. No.

Mr. DINGELL. That would not be a major problem, except insofar as construction is concerned?

Mr. RUCKELSHAUS. As far as we know now, it is no problem because I don't know of any industrial outfall that goes out that far.

Mr. DINGELL. There are some long lines that are contemplated in some parts of the country very shortly, and is it your view that these are being done in part to avoid the requirements of the water pollution control laws?

Mr. RUCKELSHAUS. I don't think that they are being done to avoid the requirements as much as they are to maybe take advantage of what

BEST COPY AVAILABLE

their engineers say is a better mechanism for dispersing the waste in the environment.

I am not prepared to say they are trying to avoid the law. If they are, I think they are going to be included very quickly.

Mr. DINGELL. That is one of the things I propose to try to do.

Let me ask you this other question. Outside 12 miles, what would be the requirements for permits, under this legislation, under the other proposed legislation that you have been addressing yourself to, and under existing law, if you please?

Mr. RUCKELSHAUS. Outside of the 12 miles, under existing law we have no authority to do anything about either outfalls or dumping.

Under our proposed legislation, we would control the dumping of any material from ships originating in our ports outside the 12-mile limit. We would attempt to control the dumping from ships not orginating on our shores only in the contiguous zone as it affected the territorial sea, and not outside the contiguous zone.

In terms of the Water Pollution Control Act, which we have submitted to Congress under the other provisions, we would control outfalls originating in the United States, wherever they ended up in the ocean, whether it was in the contiguous zone or in the high seas.

Mr. DINGELL. I see.

Mr. Ruckelshaus, you and Mr. Dominick have been very patient with the committee, and very helpful. We thank you for your kindness and great assistance for the committee this morning. It has been a privilege to have you before us.

If there is no further business to come before us at this time, the committee will stand adjourned until the hour of 2 o'clock.

(Whereupon, at 12:50 p.m. the joint subcommittees recessed, to reconvene at 2 p.m., the same day.)

AFTERNOON SESSION

Mr. DINGELL. The subcommittee will come to order.

This is a continuation of the hearings commenced this morning on the general subject of ocean dumping, and a number of pieces of legislation related to that matter.

Our first witness is Mr. John R. Stevenson, legal advisor, Department of State.

Mr. Stevenson, we are pleased that you could be with us, and we will be happy to recognize you for such statement as you choose to give.

Do you have any members of your staff or associates you would like to have with you at the witness table?

Mr. STEVENSON. I have several with me, Mr. Chairman, but I don't think it is necessary for them to come forward.

Mr. DINGELL. It is entirely appropriate if you want to have them at your side.

Mr. STEVENSON. It may be that on some of the questions, I will need to ask them to join me.

Mr. DINGELL. We will accede to your wishes.

If you will identify yourself fully for purpose of the record, the chair will recognize you for your statement, Mr. Stevenson.

BEST COPY AVAILABLE

STATEMENT OF JOHN R. STEVENSON, LEGAL ADVISER, DEPARTMENT OF STATE

Mr. STEVENSON. Thank you very much, Mr. Chairman. I am John Stevenson, the legal adviser of the State Department.

Mr. Chairman, committee members: I appreciate this opportunity to meet with you to testify in support of the President's proposal to control ocean dumping. I would like to discuss with the committee the jurisdictional aspects of the Marine Protection Act of 1971, H.R. 4247 and H.R. 4723, and the international efforts of the administration to protect the marine environment.

The proposed Marine Protection Act of 1971 has been carefully drafted to maximize U.S. control over ocean dumping activities consistent with accepted principles of international law. In this connection, I wish to note the fact that to our knowledge all dumping off our coasts at present originates from the United States and that we have no reason to believe the situation will change. I would like to briefly discuss the relevant principles of international law on which the President's ocean dumping proposal is based.

Traditionally, the law of the sea has been faced with two fundamental problems—defining the extent of coastal state jurisdiction over the ocean and accommodating conflicting uses of the high seas. Although we continue to work on several aspects of these problems, great advances were made in 1958 with the adoption of the four Geneva Law of the Sea Conventions. These conventions codified the existing international law of the sea and established several important new international legal principles. These conventions, to which we and many other nations are parties, establish the present legal basis for coastal state control of c[illegible]n activities.

The Convention on the Territorial Sea and the Contiguous Zone provides in its first article that the sovereignty of a coastal state extends beyond its land territory to its territorial sea. With the exception of the right of innocent passage through the territorial sea, the United States under this convention and customary international law enjoys complete control over all activities in our 3-mile territorial sea.

Furthermore, this convention provides that a coastal state in a zone of the high seas contiguous to its territorial sea may exercise control necessary to prevent the infringement of its customs, fiscal, immigration, or sanitary regulations within its territory or territorial sea. The convention specifies that this contiguous zone may not extend beyond 12 miles from the coast. Thus, within the contiguous zone, the United States can enact measures to prevent unlawful pollution of its territory or territorial sea. It is important to bear in mind that the U.S. authority under the convention does not derive from a right to prevent pollution of the contiguous zone as such, but from a right to prevent pollution of our territory or territorial sea.

A State, of course, has jurisdiction over vessels flying its flag on the high seas irrespective of their location. A state may also determine the conditions under which materials may be removed from its territory, and specifically has the power to prohibit such removal by its own or foreign nationals and vessels. But, beyond 12 miles, a state has

BEST COPY AVAILABLE

no rights under international law to regulate the activities of foreign vessels on the high seas in the absence of an international agreement. The convention on the high seas is most explicit on this point; article 2 provides in relevant part that:

"The high seas being open to all nations, no state may validly purport to subject any part of them to its sovereignty."

Article 6, in part, provides:

Ships shall sail under the flag of one state only and, save in exceptional cases expressly provided for in international treaties or in these articles, shall be subject to its exclusive jurisdiction on the high seas.

The Administration's proposed Marine Protection Act of 1971 establishes control over the transportation of material by any person from the United States for dumping on the high seas. It also establishes control over the dumping of material by any person from any source in the 3-mile territorial sea of the United States and in the additional 9-mile contiguous zone adjacent to its territorial sea. Both provisions would apply to American and foreign nationals and vessels. We believe this is the proper exercise of our jurisdiction under international law, and that it fully meets all cases of dumping arising now or likely to arise.

As there is legislation before this committee to control ocean dumping on the basis of our jurisdiction over the continental shelf, I would like to briefly discuss the legal problems inherent in this approach. Under the convention on the continental shelf, the United States has exclusive sovereign right for the purpose of exploring the continental shelf and exploiting its natural resources. The convention does not give the United States sovereignty over the continental shelf for all purposes, and it explicitly preserves the status of the superjacent waters as high seas. The drafters of the convention carefully considered what rights and obligations necessarily flowed from the general right of the coastal state over exploration and exploitation, and they were quite explicit. The coastal state, for example, has a right to erect installations and exercise jurisdiction over them for the exploration or exploitation of natural resources, as well as to establish safety zones around the installations. It has certain rights over research undertaken on the shelf and has an obligation to prevent unjustifiable interference with other uses of the sea. Nowhere does the convention authorize the coastal state to regulate dumping. Indeed, it is the convention on the high seas, not the Continental Shelf Convention, which specifically refers to the dumping of radioactive wastes. It is clear that the Geneva conventions consider that dumping should be treated under the high seas regume, that is by regulation of one's nationals and by international agreement.

In this connection, we must also consider the question of enforcement. The basic principle regarding vessels on the high seas is that they are subject to the exclusive jurisdiction of the flag state except as otherwise agreed. There is no treaty giving the United States authority to arrest a foreign vessel on the high seas for dumping.

Thus, legislation regarding dumping activities on the high seas above the continental shelf would amount to a unilateral assertion of jurisdiction by the United States without a firm basis in international

law, and in detogation of certain general principles of international law. Such an assertion would be contrary to our established policy of opposing unilateral claims as a means of solving high seas problems, could result in protests by other states, is unnecessary in the light of the fact that the prospects for effective international action under this country's leadership are quite good.

The accommodation of various uses of the high seas, as I mentioned earlier, has been a fundamental issue in the development of the law of the sea. Article 2 of the Tigh Seas Convention provides *inter alia:*

"The freedoms that is navigation, fishing, laying submarine cables and pipelines, flying over the high seas, and others which are recognized by the general principles of international law, shall be exercised by all states with reasonable regard to the interests of other states in their exercise of the freedom of the high seas."

It is thus clear that dumping must be conducted with reasonable for the interests of other states in their exercise of the various freedoms of the high seas. This obviously includes the protection of the high seas environment and its fisheries and living resources. Moreover, it can be anticipated that a future international treaty on dumping would be likely to place very heavy emphasis on the need to protect the common interests states have in preservation of the marine environment.

What I would like to emphasize is that we cannot unilaterally resolve these marine pollution problems by extending our jurisdiction in violation of accepted principles of international law. We must resolve these problems multilaterally and we are presently working to do so in several different forums.

The Preparatory Committee for the 1973 Law of the Sea Conference has charged one of its three subcommittees to work on the problem of marine pollution. I expect this committee to produce treaty provisions for the 1973 Law of the Sea Conference establishing international protection for the marine environment. In this connection, I had the honor to serve as U.S. representative to the Preparatory Committee for the Law of the Sea Conference which met in Geneva last month, and I there specifically suggested ocean dumping as one aspect of the marine pollution problem that required international action. I am certain the committee will be pleased to note that the United States was the first country to present such proposals to the preparatory committee for specific action to combat marine pollution. With your permission, I would like to make copies of our recent statement in the preparatory committee available to this committee.

Mr. DINGELL. Without objection, they will be received and inserted in the record at this point.

(The document follows:)

[Press release from Enlarged United Nations Committee on the Peaceful Uses of the Seabed and the Ocean Floor Beyond the Limits of National Jurisdiction, Subcommittee III, Mar. 25, 1971]

STATEMENT BY THE HONORABLE JOHN R. STEVENSON, U.S. REPRESENTATIVE

Mr. CHAIRMAN: The two principal subjects assigned to Subcommittee III are "preservation of marine environment (including, inter alia, the prevention of pollution) and scientific research."

The United States believes strongly that preservation of the marine environment and marine pollution are appropriate subjects for international action, and has supported various activities in this area for many years.

On May 23, 1970, President Nixon announced a new U.S. Oceans Policy in which he pointed out that States are becoming apprehensive about the ecological hazards of unregulated use of the oceans and seabeds. He urged that a new international seabed regime should, inter alia, protect the ocean from pollution. He stated that if new international agreements with respect to Law of the Sea can be obtained, over two-thirds of the earth's surface can be saved from national conflict and rivalry be protected from pollution, and put to use for the benefit of all.

The problems of pollution are no longer restricted to our cities nor to the streams, rivers and estuaries of our land masses. Residuals of some pollutants can now be found in all the seas and oceans of the world. Such pollutants have their origin in both maritime and land-based activities. Pollution from the latter is carried to the oceans by air currents, by fresh water run-off or is the result from deliberate disposal of materials into the oceans.

Growth in the world population and advances in technology have produced rapid increase in waste products and the long held view that the oceans have infinite ability to accommodate the consequences of human activity has been proven false. We now recognize that pollutants reaching the seas and ocean are a threat to the health and general welfare of mankind, as well as to the productivity of living resources of the oceans.

Pollution affects the maritime environment, its living resources and ultimately human beings in a variety of ways. Toxic substances can kill animals or plants which come in contact with them. Still other pollutants may alter the oceans' environment making it unsuitable for animal life. Finally, pollutants can be concentrated into living resources of the ocean and as consequence these resources may become unsuitable as human food.

The impact of some pollutants on the ocean and its inhabitants and its consequence to human beings is not yet clear. It is, however, obvious that increased understanding of the amount, distribution and effects of pollution in the marine environment will be required to implement effective measures for pollution control and that a variety of actions will be required depending on the nature and origin of the pollutant.

The complexity of the problem is further evidenced by the growing number of international organizations dealing with some aspect of the marine pollution problem. At the same time we have to recognize that the marine pollution problem and the need to preserve the marine environment are an important part, but only a part, of the global environment problems to be discussed at the 1972 Stockholm Conference on the Human Environment. The measures we decide to take here in the marine area must take into account the wider problems of human environment and should in no way conflict with measures which might be taken in that wider area.

To be effective. action must be taken in concert among states to prevent pollution and they must be prepared to implement agreed actions. If only a few states should take the needed anti-pollution measures, any resultant improvement might prove to be temporary only. In the absence of cooperative international action, competitive economic pressures will severely limit national abilities to take or require the costly measures needed to protect the marine environment. Only a broad international approach can provide sufficient incentives for all states concerned to do their part.

The Seabed Committee, of course, has been assigned the responsibility for dealing with seabed pollution. The Declaration of Principles regarding the seabed adopted by the General Assembly in December 1970 contemplates that the international seabed regime will include such provision. Accordingly, this aspect of the marine pollution problem must necessarily have an important relationship to the work of Subcommittee I on the international seabed regime and machinery as well as to the work of this Subcommittee. It is our view that the regime should provide that all activities in the international seabed area shall be conducted with strict and adequate safeguards for the protection of human life and safety and of the marine environment. Moreover, the safe development of seabed resources necessarily requires appropriate provisions in the regime treaty itself as well as a major environmental protection role for the international machinery to be established under the regime.

In his second Foreign Policy Report, issued on February 25, 1971, the President of the United States suggested that the following essential measures be taken by the international community in the near future:

Identification of pollutants and other ecological hazards which are dangerous on a global scale.

Establishment of an effective world monitoring network to keep track of these environmental dangers.

Initiation of a global information system to facilitate exchange of experience and knowledge about environment problems.

Establishment of internationally accepted air and water quality criteria and standards.

Development of international guidelines for the protection of the environment.

Achievement of comprehensive international action programs to prevent further environmetal deterioration and to repair the damage already done.

Development and improvement of training and education programs to provide the skilled capability to meet the environmental challenge.

Almost all of these suggestions apply with special urgency to the marine environment. In particular we need to focus our attention on drafting articles on major problems relating to marine pollution.

In identifying such problems, we should acknowledge work accomplished by IMCO concerning oil spills from ships, FAO and its technical conference of December 1970 dealing with issues of marine pollution on living resources of seas and expected contributions of the 1972 Stockholm Conference. Examples of major areas of concern might include the following:

A. Such international machinery as may be required for determining marine pollution research priorities, for coordinating research efforts, and for collecting research infromation and arranging for its exchange.

B. Regulation of deliberate disposal of materials into the ocean.

We recommend that drafting of articles begin promptly. In the preparation of draft articles we should seek assistance as required from the appropriate specialized agencies and other public and private international organizations active in the field. Experts from these organizations should participate in a consultative capacity. Similarly, we believe that our preparatory work should be closely coordinated with the related work for the Stockholm Conference. We should avoid duplication. In particular, should some parallel working group be established by the committees engaged in the preparatory work for the two conferences, a member of the bureau of each group should be invited to participate in the other group.

I would like to emphasize again the complexity of the issues before us, the need to take into account other efforts in this area, and the importance of tailoring each solution to the special requirements of each particular problem.

I turn now to the second of the two subjects assigned to Subcommittee III, scientific research.

The United States has long identified itself with the need to expand world efforts in scientific research of the oceans. Our initiative calling for an International Decade of Ocean Exploration exemplifies that posture. We consider that scientific research should not be interferred with and should be conducted with the view to open publication for the benefit of all. We whole-heartedly support the applicable principle stated in the Declaration of Principles (Resolution 2749) which states in relevant part: "States shall promote international cooperation in scientific research exclusively for peaceful purposes . . . through effective publication of research programs and dissemination of the results of research through international channels". I have discussed already some forms of cooperation we favor in connection with preservation of the marine environment and marine pollution. In general, it is our belief that cooperation in scientific research in the marine environment will help ensure that the oceans will be developed and used in ways which will benefit mankind. Through increased knowledge we can all better understand the oceans and make optimum use of their resources.

We recognize the particular interest of developing countries in learning how the seas may help solve such problems as chronic shortfalls of protein for their populations; how fresh water may be obtained from the sea; how weather may be modified to improve crop porduction and to avoid such catastrophes as rampaging hurricanes; and how new, inexpensive energy sources might be tapped. Answers to such questions will require further scientific activity and cooperation in the period ahead.

Scientists have in fact a long tradition of sharing information, although the vast quantities of data accumulated and the limitations on their processing and interpretations often delay their dissemination and use. The best means of insuring that there is a flow of scientific information is actual participation in scientific projects and continued support for existing scientific mechanisms for the exchange of data, such as the World Data Center System and the World Weather Watch. New means of data acquisition, such as Earth Resource Survey Satellites, may offer new opportunities for international cooperation and sharing of benefits as they fulfill their promise.

In our view this Subcommittee should draw upon the experience and knowledge of other bodies, such as the specialized agencies and intergovernmental organizations, in performing its work. Resolution 2750 (C) invites inter alia, the IOC to cooperate fully with the Seabed Committee, in particular by preparing such scientific and technical documentation as the Committee may request. We favor taking full advantage of this suggestion. Similarly, the Committee may well wish to draw upon the FAO, IMCO, and the Human Environmental Secretariats for support.

In this connection, it would be most helpful to our work if the Secretariat would provide each of the members of this Committee with copies of treaties and other basic documents produced by other international and intergovernmental organizations concerned with marine pollution and scientific research.

Thank you, Mr. Chairman.

Mr. STEVENSON. Thank you, Mr. Chairman.

President Nixon's proposals regarding the seabeds beyond the limits of national jurisdiction are also relevant. The Draft Convention on the International Seabed Area submitted by the United States as a working paper last August to the U.N. Seabeds Committee provides that "all activities in the International Seabed Area shall be conducted with strict and adequate safeguards for the protection of human life and safety and of the marine environment." The draft contains regulatory provisions to further these ends and contains provisions for compulsory settlement of disputes. Accordingly, international means would be available to insure that all seabed activities, including dumping, are conducted in agreement with the requirement that there be strict and adequate safeguards for the protection of the marine environment.

An International Working Group on Marine Pollution has been established by the Preparatory Committee for the 1972 United Nations Conference on the Human Environment. The Working Group will prepare a marine pollution agenda submission for the Conference. This will probably include proposals that nations ban the dumping of certain harmful substances in the ocean and adopt systems for the regulation of ocean dumping.

Work is also under way in the NATO Committee on the Challenges of Modern Society and the Intergovernmental Maritime Consultative Organization. The latter is preparing for a 1973 Conference to ban all intentional discharges into the seas by ships of oil, oily wastes, and other noxious substances.

Accordingly, I am confident that in the next few years we will see major international developments banning the ocean disposal of toxic industrial wastes, highly radioactive materials, heavy metals, chemical warfare agents, as well as the setting of international standards to prevent damage to the marine environment from exploration and exploitation of the seabed.

I strongly urge the adoption of this comprehensive ocean dumping bill—the Marine Protection Act of 1971—as an important domestic

first step which should lead to international control of the universal problem of marine pollution.

Thank you very much, Mr. Chairman.

I would be pleased to answer any questions that you or other members of the committee might have.

Mr. DINGELL. Thank you very much, Mr. Stevenson. Your testimony is most helpful to the committee, and the committee is grateful to you.

Mr. DuPont?

Mr. DUPONT. Just one question, I think, Mr. Chairman.

On page 3 of your testimony, Mr. Stevenson, you commented that our authority to regulate the 9-mile contiguous zone, as far as antipollution activities are concerned, really derives only from our right to protect the 3-mile coastal zone. Does that imply any jurisdictional weakness, as far as the United States is concerned?

Mr. STEVENSON. No, sir; this just reflects the provisions of the 1958 Geneva Convention with respect to the contiguous zone. The concept of the contiguous zone is that you are able to do certain additional things in that zone of the high seas, in order to protect our territorial sea or our own territory. I think for present purposes, that this is entirely adequate, because most of the dumping in the contiquous zone we are talking about, would have an effect in the waters within 3 miles of our coast, so we could take the necessary action between 3 and 12 miles, to prevent that sort of activity.

Mr. DUPONT. I have been proceeding on the assumption that we could pass a piece of legislation that would have full and complete jurisdiction now for 12 miles. There is nothing, in your view, nothing in international law that prevents us from passing antidumping legislation of the type being considered, that would be effective fully out to the 12-mile limit?

Mr. STEVENSON. The actual wording of the Geneva Convention gives you the authority to exercise the control within the zone between 3 and 12 miles necessary to implement certain national policies with respect to sanitation, among other things. It seems to me that the power provided to control certain actions in the contiguous zone would probably justify most of the type of regulatory activity that is involved here. This is not the same thing as saying we have a 12-mile territorial sea. Our position still is that the United States has a 3-mile territorial sea, with an additional contiguous zone between 3 and 12 miles. While we have agreed that we would be willing to accept a 12-mile territorial sea, by international agreement, until there is international agreement, our position remains at a 3-mile territorial sea. You could not equate the legal situation in the contiguous zone with the situation in the territorial sea. There are differences.

Mr. DUPONT. Can you give me a practical example of something we could do within the 3-mile limit in regard to limiting dumping that we could not do in the 3- to 12-mile zone?

Mr. STEVENSON. Well, basically, with respect to the sort of dumping that we are talking about here, the only problems we have had have been with the ocean dumping of materials originating on the U.S. mainland. So that basically, everything that we need to control is dealt with satisfactorily by the provision which controls the transporting of materials from U.S. territority for ocean dumping purposes.

BEST COPY AVAILABLE

Now the main difference between the territorial sea and the contiguous zone would have to do with regulation of vessels. As far as I can see, we contemplate taking the most effective type of action that the contiguous zone provision enables us to take.

Now the only possible difficulty that I could see would be where there was some dumping of materials that clearly would not affect the area within 3 miles. Something that clearly wouldn't move shoreward. Perhaps some type of nonliquid type of dumping. There might be a problem with respect to that. I don't think as a practical matter we are talking about anything that is a serious problem.

Mr. duPont. I was with you until you said that last phrase, but now you have got me concerned about dumping construction debris. Are you saying if we have a prohibition against dumping cinder blocks, broken cinder blocks, outside of 3 miles, that under international law we are perhaps not able to prohibit that?

Mr. Stevenson. Well, I am not an ocean pollution specialist, so I am not sure that there wouldn't be a problem even with materials such as cinder blocks, even though dumped within the contiguous zone. It might very well come landward, and interfere with our territorial sea. So that I think even in that area, I am not sure that we couldn't act if we wanted to.

Mr. Dingell. Would the gentleman yield?

We have got several concepts involved here. First of all, there is a question of the relationship of the United States to other nations and citizens of other nations, and the concept of the United States with regard to its relationship with its own citizens. Then you have the question of the area in the territorial sea which is 3 miles from our shoreline. Then you have the question of the contiguous zone, which is from 3 to 12 miles out.

We can clearly control the activity of anybody within 3 miles—our own citizens or citizens of other nations. As between the 3 and the 12, or even 12 all over the high seas, we can control the actions of our own citizens.

Between the 3 and the 12 mile limits, we may only engage in certain acts which would control the activities, rather than we can control certain activities of citizens of other nations. Is that a kind of simplification of the question which you are discussing with Mr. duPont at this time?

Mr. Stevenson. Yes, I think that is accurate.

Mr. Dingell. And so as to the dumping of cinder blocks, there would be no problem of dumping cinder blocks out anywhere out to 12 miles, if it was an American citizen; if it was between 3 and 12 miles, as to the nationals of other nations, we might have a problem, you are saying, if we couldn't demonstrate clearly that this would involve some effect which would adversely affect the U.S. territorial sea or the shoreline or its nationals. Is that correct?

Mr. Stevenson. That is correct. I think the most important point here, and I think it is the same point that Judge Train made before, is that at the present time, there is no dumping off our shores that

BEST COPY AVAILABLE

doesn't originate from our territory. So that the provision in this act that regulates transporting of material for dumping covers at the present time all dumping that is involved, wherever that dumping takes place. It is regulated by the provision that affects transportation from our shores.

Now as far as we know, there is no foreign dumping at all involved off our shores. So that the question of what you do about foreign dumping has not arisen as a practical problem. Though I think, basically, as you pointed out, with respect to regulating our own nationals there is no problem in any event. We can do what we wish. In the contiguous zone or beyond would only be a problem with respect to foreign nationals, and that just isn't a problem at the present time.

Mr. duPont. No further questions.

Mr. Dingell. Mr. Everett.

Mr. Everett. Thank you, Mr. Chairman.

At the bottom of page 3, Mr. Stevenson, you said that a state, of course, has jurisdiction over vessels flying its flag on the high seas, irrespective of their location. The question arose this morning as to whether the language in the bill would cover a situation where an American-flag vessel brought material in from a foreign country to be dumped, beyond the 12-mile zone, shall we say. Would we have the authority under your statement to regulate an American-flag vessel bringing this material back to the U.S. waters and dumping it beyond 12 miles from our shores?

Mr. Stevenson. As far as U.S. vessels are concerned, there is no international law problem. The only problem is the practical problem of Coast Guard or other enforcement action against U.S. vessels.

Mr. Everett. The bill itself just says, "No person can transport material from the United States", and it doesn't say about American-flag vessels bringing material into these waters.

Mr. Stevenson. I think that is more a policy than an international law question. Because once you do start to regulate the U.S. vessels generally, you have the problem of effectively administering those provisions. The other consideration is that since we are dealing not only with U.S.-flag vessels, but with others, it would be better to handle this worldwide problem by international agreement, so you have uniform standards and people know what to expect, in terms of regulation.

Mr. Everett. At the top of page 4 of your statement you say:

"A state may also determine the conditions under which materials may be removed from its territory, and specifically has the power to prohibit such removal by its own or foreign nations and vessels."

Now, this does not extend to the dumping of it, does it?

Mr. Stevenson. No. This is just the initial act of removing the material.

Mr. Everett. Well, under this bill, though, if a foreign-flag vessel picks up material at a U.S. port then EPA can control the dumping anywhere in the world, supposedly. Is that correct?

Mr. Stevenson. I am sorry?

Mr. EVERETT. Under the legislation you could control the dumping by a foreign-flag vessel that picks up material in a U.S. port, as far as a hundred miles off the coast of the United States. That is what I have been led to believe. And I was wondering if this statement you have at the top of page 4 covers that?

Mr. STEVENSON. Well, it was intended to cover that.

Mr. EVERETT. So, actually, you can control the removal as well as the dumping, if it is picked up at a U.S. port—even though it is a foreign-flag vessel.

Mr. STEVENSON. Yes.

When you are talking about the question of transporting material from the United States for the purpose of dumping it, we could regulate that—it wouldn't matter where they were intending to dump, in terms of the regulation that we imposed, in terms of not letting them do it or accepting whatever conditions we were talking about—but that does not mean that we would have enforcement jurisdiction, or could, on the basis of this, set forth some sort of a general code regulating dumping beyond our jurisdiction. What we are regulating is the removal of the materials from our shores for the purpose of dumping.

Mr. EVERETT. Now with respect to these international conventions you mentioned, they are only binding as to those nations that are signatory to the Convention. Is that correct?

Mr. STEVENSON. In all cases we would only be talking about countries that became parties to the Convention.

Mr. EVERETT. Now, with respect to the Continental Shelf Convention, you indicated in the statement that, normally, controls over the exercise of jurisdiction over the resources of the Continental Shelf were primarily for the purpose of exploitability, I believe, and exploration. One of the bills before us today has a provision that would try to control the dumping of materials out to the 200-meter depth contour by foreign-flag vessels irrespective of whether the material was received on board the vessel at a U.S. port or foreign port.

Do you think a coastal nation has the right to protect its resources of the Continental Shelf from pollution as it has in its 3- to 9-mile zone?

Mr. STEVENSON. Well, the Continental Shelf Convention does not deal with the question of dumping. It deals with the coastal states' rights to explore and exploit the Continental Shelf's resources. The clear intention of the Geneva Conventions is to make dumping beyond the territorial sea and the contiguous zone governed by the High Seas Convention. Therefore, the rules for the area beyond the territorial sea and contiguous zone are based on the high-seas conception of regulating our own flag vessels, or reaching international agreement as to the appropriate regulatory rules. There is no right on the basis of your jurisdiction over the Continental Shelf to regulate what happens on the seas above with respect to dumping.

Now, this does not mean that you have no rights. Even under existing law, in the absence of international agreement with respect to dumping, the high-seas regime requires that you exercise the freedoms

of the high seas with reasonable regard for the interests of other States in their exercising the freedoms of the sea. So that if another country's flag vessel were to dump in an area of the high seas, which adversely affected the rights of our Nation—for example, to carry on fishing or another of the high-seas rights—we would have a right to complain to that the country, and have a valid international claim under the High Seas Convention, because they would be unduly interfering with our interests.

Mr. EVERETT. Mr. Chairman, that is all the questions I have, thank you.

Mr. DINGELL. Thank you very much, sir. The committee is grateful to you for your very helpful testimony. We appreciate your kindness.

Our next witness is Mr. Henry Douglas, chief of planning, Maryland Port Authority.

Mr. Douglas, we are happy to welcome you for such statement as you choose to give, and if you will identify yourself in full for purpose of the record, identifying the associates with you at the main table, you may proceed to give your statement.

STATEMENT OF HENRY T. DOUGLAS, CHIEF OF PLANNING, MARYLAND PORT AUTHORITY

Mr. DOUGLAS. Thank you, Mr. Chairman.

My name is Henry Douglas, chief of planning for the Maryland Port Authority.

The Maryland Port Authority is an agency of the State of Maryland, charged with the responsibility for promoting the waterborne commerce of that State.

I am here to address myself to House bill 4723, the Marine Protection Act of 1971, and I have given Mr. Everett copies of my prepared statement. However, I would like to follow your wishes, Mr. Chairman, as to whether I simply give a brief summary of the position set forth in that statement, or read the entire statement.

Mr. DINGELL. As far as the Chair is concerned, it is a matter of choice to you. If you were asking the counsel of the Chair in this matter, I would make the statement to you we will be happy to have your entire statement in the record; and you may then choose to highlight such sections as you may wish.

Mr. DOUGLAS. Mr. Chairman, we are fully in accord with the intent of the bill to prevent or limit dumping into the ocean, coastal or Great Lakes waters, of hazardous, noxious, or environmentally detrimental substances.

However, we think that it would be a mistake to include dredging spoil in the same category as "solid waste, garbage, sewage sludge, munitions, chemical, biological and radiological warfare agents, radioactive materials, etc.". Also, we do not think that waters for which the States have been authorized to establish water quality standards by the Water Quality Act of 1965 should be included with ocean and

coastal waters. And finally, we believe it would be desirable to leave the permit authority for disposal of dredging spoil within the U.S. Army Corps of Engineers.

Consequently, we urge that the bill be amended to exclude from its coverage the deposit of dredging spoil in waters to which State or Federal-State water quality standards apply.

The reasons for our position are that we believe that:

(1) Dredging spoil disposal is already adequately regulated by the States and the Army Corps of Engineers.

(2) Transferring the Federal permit authority for dredging spoil disposal from the Corps of Engineers to the Environmental Protection Agency will increase the time involved in processing applications for such permits, and thereby impede navigation channel projects.

With respect to the adequacy of the present regulatory setup:

(1) At the State level, deposit of dredging spoil requires compliance with the water quality standards which have been established by the States, or where a State has not established such standards, compliance with standards established by the Environmental Protection Agency.

(2) At the Federal level, dumping of dredging spoil requires a permit from the Corps of Engineers which, under current Federal statutes and regulations, requires:

(a) Certification that State water quality standards are complied with.

(b) Compliance with the Corps of Engineers "section 403" criteria regarding environmental and ecological effects as required by:

The Fish and Wildlife Coordination Acts, 16 U.S.C. 661 and 16 U.S.C. 742-A; The National Environmental Policy Act, Public Law 91-90; The Water Quality Improvement Act, Public Law 91-224.

The reason we fear delay in the processing of applications for permits as a result of shifting the permit authority from the Corps of Engineers to the Environmental Protection Agency is that the Corps of Engineers is equipped for the job with personnel experienced in this field and 40 district offices, whereas the Environmental Protection Agency does not have a comparable staff and, as we understand it, envisions only 10 field offices. We believe there is a significant advantage in the more decentralized organization of the Corps of Engineers which brings the application and permit process much closer to the applicant.

Since we are recommending changes in the bill involving dredging spoil and the Corps of Engineers, we would like to offer some comments on these two subjects.

First, as to dredging spoil. This is not necessarily the ogre that it is frequently considered to be, in spite of the unpleasant connotation of the word "spoil." There is "good" spoil and "bad" spoil. Typical of the former is natural uncontaminated bay or river bottom. Moving it from one location on the bottom to another nearby location on the bottom can hardly be considered as polluting the body of water involved.

BEST COPY AVAILABLE

"Bad" spoil is typified by bottom material which has been subjected to industrial or municipal wastes and become contaminated as a consequence. Such "bad" spoil can be a pollutant and should be disposed of so that it does not degrade water quality. To this end, Maryland is constructing at its own expense a $13 million disposal area to receive and confine such "bad" spoil.

In Maryland we are confronted with the problem of simultaneously advancing our most important economic asset, the port of Baltimore, with its port-oriented heavy industry, and also preserving the environmental and ecological quality of our highly cherished Chesapeake Bay. This has caused us to give a great deal of attention to reconciling the requirements of the two assets, with particular attention to the handling of dredging spoil, and as a consequence we have learned some interesting things:

(1) Since 1924 a deep, natural trough in the bottom of the Bay, known as the dumping ground has received most of the dredging spoil from Baltimore Harbor and channels. However, this same "Dumping Ground" is the most popular sport fishing location on the bay, particularly for striped bass; and on any summer weekend, hundreds of sport fishing boats can be seen there.

(2) Last year's oyster harvest from the Chesapeake Bay was the largest on record. The dumping ground lies near the center of the relatively small area of the bay which was the most productive.

(3) A $268,000 study of an actual case of overboard disposal of dredging spoil conducted by the Natural Resources Institute of the University of Maryland in 1966 concluded that there were no observable detrimental effects from such spoil disposal. See exhibit A.

(4) In the opinion of knowledgeable people concerned with natural resources conservation, it is recognized that not all dredging spoil is harmful, and that uncontaminated spoil need not be kept out of the bay. See exhibit B.

With respect to the Corps of Engineers, we would like to call attention to the changes in their permit criteria resulting from the passage of the National Environmental Policy Act of 1969 (Public Law 91-190, Jan. 1, 1970), and the Water Quality Improvement Act of 1970 (Public Law 91-224, Apr. 3, 1970), and promulgated by the Secretary of the Army. These are succinctly expressed in press release 70-8 of May 15, 1970, by the Baltimore District, Corps of Engineers, concerning evaluation of permit applications, to the effect that:

"The decision . . . will be based . . . on an evaluation of the proposed work on the public interest." "Public interest" is described as including factors such as: "navigation, fish and wildlife, water quality, economics, conservation, aesthetics, recreation, water supply, flood damage prevention, ecosystems, and, in general, the needs and welfare of the people." This change clarifies the standard against which permit applications are to be judged and reemphasizes that the Corps is no longer concerned only with the impact which a proposed project may have on navigation."

We respectfully request that the committees give due consideration to the above points in their deliberations on H.R. 4723.

BEST COPY AVAILABLE

62-513—71——31

(Attachments to Mr. Douglas' statement follow:)

NRI
SPECIAL
REPORT
No. 3
JULY 1970

GROSS PHYSICAL AND BIOLOGICAL EFFECTS OF OVERBOARD SPOIL DISPOSAL IN UPPER CHESAPEAKE BAY

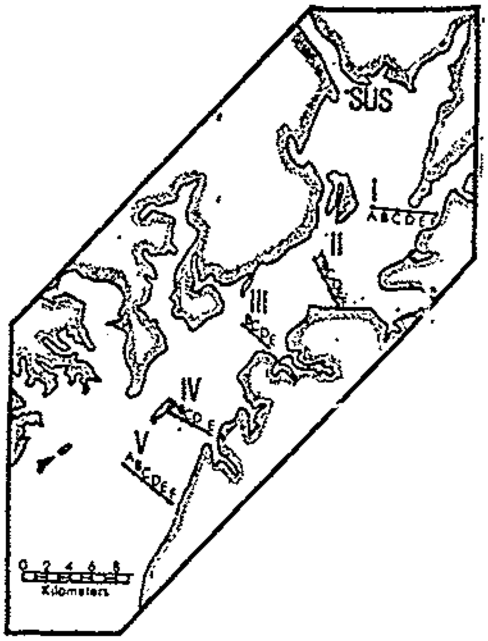

FINAL REPORT
to the
Bureau of Sport Fisheries and Wildlife
United States Department of the Interior
Under Contract 14-16-0005-2096

NATURAL
RESOURCES
INSTITUTE

UNIVERSITY OF MARYLAND

From: Gross Physical and Biological Effects of Overboard Spoil Disposal in Upper Chesapeake Bay

Fish and Wildlife Ser ice, Maryland Department of Natural Resources, Maryland Board of Public Works and other agencies concerned with optimal management of estuarine areas.

These papers also make a major contribution to understanding of an important estuarine area which is becoming subject to growing and sometimes conflicting demands and uses. It is highly probable that the results will serve many uses in the solution of important practical and fundamental questions.

The publications and reports are listed in the Appendix.

Effects of Dredging and Spoil Disposal in 1966

The following summaries of effects draw freely from data and conclusions in subsequent detailed final reports by Biggs, Flemer, Pfitzenmeyer, Goodwyn, Dovel and Ritchie. Supporting evidence and discussion appear in those reports. Suggestions of guidelines for dredging projects and of certain recommendations have involved all of the scientists of the program.

1. Fine sediments from the channel were released in shoal water over similar sediments, as a semi-liquid mixture.
2. Turbidity increased over an area of 1.5-1.9 square miles (4-5 square kilometers) around the disposal site (Fig. S-3). Over most of the area, the suspended sediment load was within the range of natural variation observed, but at a different season from observed natural maxima (see Biggs).

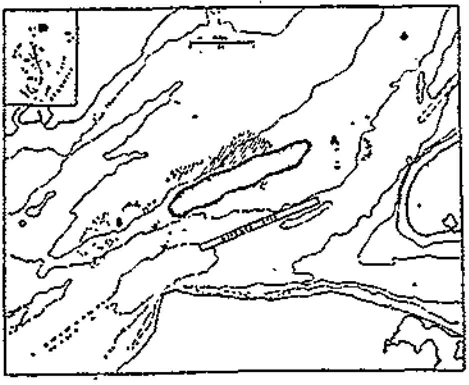

Fig. S-3. The area studied during the 1966 hydraulic dredging from the DREDGED AREA and pipe-line disposal into the DISPOSAL AREA. "A" illustrates the spread of water-borne sediments observed on flood tide, and "B" is the spread seen on ebb tide. The dark hatched area of bottom was covered by at least 1 foot of sediment.

3. Suspended sediments (in the top of 10 feet of water) were carried in a tide-related plume to a maximum distance of about 3.1 miles (5000 meters), and virtually disappeared within two hours when pumping ceased (see Biggs).
4. Total phosphate and nitrogen were increased in the immediate vicinity of the discharge by factors of about 50 and 1000, respectively, over ambient levels (see Biggs), but limited field experiments did not show any detectable effects on photosynthesis by phytoplankton (see Flemer).
5. The spoil material deposited on the bottom covered to at least 1 foot (.3 meter) an area at least 5 times as large as that of the defined disposal site (see Biggs and Fig. S-3).
6. Approximately 12% of the deposited sediment disappeared from the spoil "pile" in 150 days after deposition (see Biggs).
7. No gross effect of dredging or spoil disposal was observed on phytoplankton primary productivity, zooplankton, fish eggs and larvae, or fish (see Flemer, Goodwyn, Dovel and Ritchie).
8. There was a reduction of about 70% in the average number of benthic individuals per square yard and of about 65% in the benthic biomass in the spoil disposal area, accompanied by a marked reduction in the number of species present. After one and a half years, numerical abundance, biomass, and species diversity had recovered to approximately the pre-disposal levels. Individual species varied greatly in susceptibility to damage and in recovery patterns (see Pfitzenmeyer).
9. At the site of dredging in the channel, an erratic series of species fluctuations occurred. After one year, the channel had about the same number of individuals as during the pre-dredging period, but not as many species were present (see Pfitzenmeyer).

Guidelines for Dredging and Spoil Disposal

The environment of this project must be considered in applying the results to other dredging and disposal sites. This is the variable low salinity area of a large estuary. A sediment trap is in effect, natural turbidities are often high, and wind and wave effects on sediments are considerable. The sediments involved do not, so far as we know, contain any highly toxic metals, oils, or other deleterious materials. The sediments of the channel and the disposal area are both of fine grain size, similar to the sediments of many upper estuarine areas. Wherever comparable conditions exist, the following guidelines are probably useful.

1. In estuarine areas like the Upper Chesapeake, which is of high value as a fish nursery and supports populations of plankton, benthic animals and useful fish, proposed large-scale environmental modifications should be thoroughly analyzed, with special concern to avoid or rigorously minimize damage to aquatic resources.
2. Disposal of fine sediments on flat bottom areas from hydraulic pumping will affect a wide area because of the spread of semi-liquid spoil and movement of sediment after original deposition (see Biggs). Since the

BEST COPY AVAILABLE

DEPARTMENT OF NATURAL RESOURCES.
Annapolis, Md., February 2, 1971.

Mr. ANDREW HEUBECK, Jr.,
Secretary, Board of Public Works,
State Office Building, Annapolis, Md.

DEAR MR. HEUBECK: By letter dated November 17, 1970, I sent you comments of the Department of Natural Resources concerning the proposed dredging project in the approach area to the C. & D. Canal as outlined in the Corps proposal dated September 11, 1970.

In my letter of November 17th I stated that this department recommends that overboard disposal not be allowed. I went on to point out that "Since the Confined Disposal Area for the Upper Bay should be in operation by the time this dredging project is to be initiated (winter of 71–72), the Department recommends that the spoil be placed in this disposal area."

Since writing to you, I have determined that it is quite unlikely that the Confined Disposal Area will be completed in time to accommodate the proposed maintenance dredging in the approach to the C. & D. Canal. Furthermore, it now seems prudent to reserve, during the initial years at least, the capacity of the Confined Disposal Area for the contaminated dredging that will be removed from Baltimore Harbor. Thus, it might be unwise to use the high cost Confined Disposal Area for the type of spoil that will result from the C. & D. maintenance dredging.

I have corresponded with Dr. Cronin of the Natural Resources Institute and he confirms the conclusion of their special report number 3, July, 1970, which indicated that there was no gross effect of the overboard disposal in Upper Chesapeake Bay from the deepening of the C & D approach channel in 1965–67.

In view of these considerations, the Department of Natural Resources with Corps of Engineers proposal dated September 11, 1970 provided that:

(1) a safety zone of 500 feet for each foot of expected disposition be provided between the receiving area and any shellfish beds or other areas of special significance;

(2) to protect fish eggs and larvae as well as other biological activities in the Bay, the dredging should be conducted during the months of February-March or September-October; and

(3) that the Corps obtain and provide to the State, accurate information on the deposition and movement of the spoil.

If the aforementioned three conditions are acceptable to the Corps of Engineers, the Department of Natural Resources withdraws its opposition to the overboard disposal of spoil in the Upper Chesapeake Bay near the site of the C & D Canal approach channel.

Please let me know if we can be of further assistance in this matter.

Sincerely yours,

JAMES B. COULTER,
Deputy Secretary.

Mr. DINGELL. Perhaps we could address ourselves to one particular point. The comments I have heard from port authorities, so far, would tend to indicate the particular areas of your apprehension. Yesterday, we heard from the American Institute of Merchant Shipping, and also from the American Association of Port Authorities, who indicated their apprehension was the transfer projected by the legislation before this committee of the authority over dumping from the Corps of Engineers to the——

Mr. DOUGLAS. Environmental Protection Agency, sir.

Mr. DINGELL. Yes, to the Environmental Protection Agency.

The chair notes, in the bill before us that their objection may well have been met, and I think the greatest help you could afford this committee at this particular time would be to comment with regard to that particular point.

The chair notes that under the bill before us, as interpreted this morning, the Corps of Engineers will continue to issue the permits, as they have in times past, but will do so in conformity with the

provisions of the act and regulations issued thereunder by the Environmental Protection Agency.

I would refer you to page 11, to subsection 7(c)(2), in suggesting that you give particular attention to line 15 of page 11, down through line 22. The language to which I refer is as follows: That after the effective date of this Act—

No Federal license or permit shall be issued under the authority of the Rivers and Harbors Act of 1899 to conduct any activity otherwise regulated by section 4 [illegible] this Act, and regulations issued thereunder, unless the Administrator has certified that the activity proposed to be conducted is in conformity with the provisions of this Act, and with regulations issued hereunder.

Now Mr. Ruckelshaus this morning testified that that language indicated that the Corps would continue to issue regulations for dredging, filling, and dumping, as they have in the past, in conformity with the statutes that you have cited in your statement. And that those actions would be simply done in conformity with regulations and so forth, and subject to certification by the Environmental Protection Agency. I think this may change somewhat the understanding that the port authorities have with regard to the impact of this particular legislation. Am I correct?

Mr. DOUGLAS. Yes. I would say that you are correct. We were concerned at the prospect of the permit issuance procedure being transferred from the Corps of Engineers to the EPA, because we felt——

Mr. DINGELL. Excuse me, Mr. Douglas. This is a new problem that we have here. I apologize to you. The Chair notes that we have at this moment a teller vote with clerks on the floor of the House, which gives us exactly 12 minutes to get over there and cast our vote on a very important question, and for that reason, with due apologies to you, I must recess the committee briefly. We should be back within the next 15 minutes.

Mr. DOUGLAS. Certainly, sir.

Mr. DINGELL. If there is no further business at this time, the subcommittee will stand in recess for a period of approximately 15 minutes.

(Brief recess.)

Mr. DINGELL. The subcommittee will come to order.

At the time when the subcommittee was constrained to recess, we were hearing from Mr. Henry Douglas, Chief of Planning of the Maryland Port Authority.

Mr. Douglas, I am sure you recall my question. Perhaps you would want to comment on the point raised.

Mr. DOUGLAS. Yes, Mr. Chairman. I have had an opportunity now to consider the implications of the paragraph you referred me to. And frankly, sir, as I understand it, with all due respect to Mr. Ruckelshaus, whose possible interpretation is to the contrary, I would read this as meaning that although the Corps of Engineers would issue the permit, it would have to obtain certification from the EPA that it was in order so to do. So that for all practical purposes, we would have imposed an additional layer of regulatory procedure on top of that already existing. And in all candor, this is one of the things that we are concerned about, the addition of additional layers of permanent processing procedure, which in this particular instance with which we are concerning ourselves, we think is superfluous.

Please understand that we have no disagreement whatsoever with the major thrust of the bill, but that we believe that it might very well, and should, exclude from its coverage the deposit of dredging spoil in waters to which State or Federal-State water quality standards apply.

This doesn't mean offshore waters. This is not ocean waters. There are no State or Federal-State water quality standards applicable there. We are referring to disposal in waters which come under the jurisdiction of the State, if they have issued water quality standards, and of course, if they have not, why, then, the EPA has the authority to issue water quality standards.

So perhaps I have been unduly roundabout, but as an answer to your question, we would deplore the addition of another step in the process of obtaining a permit, after the applicant has complied with the requirements of the water quality standards of his State, and has complied with the requirements of the Corps of Engineers, as their new criteria have been established, since the passage of the acts of January 1 and last April, which have made the Corps of Engineers very much more sensitive to environmental and ecological standards.

Mr. DINGELL. Thank you very much, sir.

Mr. Everett.

Mr. EVERETT. Thank you, Mr. Chairman. No questions.

Mr. DINGELL. Mr. Rogers?

Mr. ROGERS. I have no questions, Mr. Chairman. Thank you very much.

Mr. DINGELL. Mr. Douglas, the committee is grateful to you for your presence and for your very helpful statement. It is a pleasure indeed to have you with us and we appreciate it very much.

Mr. DOUGLAS. Thank you, Mr. Chairman.

Mr. DINGELL. Our next witness is Mr. James Wakelin, Assistant Secretary for Science and Technology of the Department of Commerce, accompanied by an old friend, Mr. Howard Pollock, Deputy Administrator of National Oceanic and Atmospheric Administration.

Gentlemen, we are happy to welcome you both to the committee for such statements as you choose to give, and if you will see to it that the other gentlemen at the table with you are identified for the record, we will be more than happy to recognize you for such statements as you may wish to give.

You may proceed.

STATEMENT OF JAMES H. WAKELIN, JR., ASSISTANT SECRETARY FOR SCIENCE AND TECHNOLOGY, ACCOMPANIED BY HOWARD POLLOCK, DEPUTY ADMINISTRATOR FOR NATIONAL OCEANIC AND ATMOSPHERIC ADMINISTRATION AND JAMES BRENNAN, ACTING ASSISTANT GENERAL COUNSEL, DEPARTMENT OF COMMERCE

Mr. WAKELIN. Thank you, Mr. Chairman.

Before I engage in the statement, with your permission I would like to again reintroduce an old colleague of ours, Dr. Howard Pollock, who is my associate in the National Oceanic and Atmospheric Admin-

istration, a member of these two committees for the past 4 years; Mr. James Brennan, who is Acting Assistant General Counsel for the Department of Commerce. Mr. Howard Eckles of NOAA, Dr. Donald Martineau of NOAA, and Dr. Robert Hanks, also of NOAA, helping us to support answers, possibly, to questions of your interest.

Mr. DINGELL. Thank you very much, sir.

Mr. Wakelin, you are certainly welcome. This committee recalls your presence before us on other days, and it is always a pleasure to welcome you back, as it is our old friend, Mr. Pollock, for whom I have great personal affection.

Mr. WAKELIN. Thank you, Mr. Chairman. I am delighted to be here. It is a privilege to be before this distinguished committee to testify in support of H.R. 4723, and also H.R. 4247. These identical bills would enable the Administration to proceed with its plan to regulate all types of ocean dumping and to prevent or vigorously limit dumping of any material which could adversely affect human health, welfare, or amenities, the marine ecology, or economic potential.

In April 1970 President Nixon indicated his concern about the dumping of waste into the ocean. The Chairman of the Council on Environmental Quality was asked to work with other Federal agencies and with State and local governments on a comprehensive study of ocean dumping that would result in appropriate recommendations for research, legislation, and policy. A report of the Council released in October of last year stated that serious information deficiencies exist and that research is required to understand the pathway of waste material in marine ecosystems.

While we can point to examples where continued unregulated dumping over a long period of time have led to ecological damage to waters adjoining our coastlines, we still have insufficient detailed knowledge to state accurately what is happening to our ocean environment or to set fully adequate regulations.

To more accurately predict the present and future impact of ocean dumping on the marine environment and set the most desirable standards for regulating it, we must identify materials potentially harmful to the marine ecosystem and conduct oceanographic surveys to establish baseline conditions and variations as a consequence of dumping.

I am not suggesting that efforts to establish regulations or standards in the environmental field must await our complete mastery of the ecological sciences. The time to act is now. Regulation of ocean dumping should be based upon evaluation of the criteria stated in section 5(a) of H.R. 4723.

However, recognizing that the ecological knowledge available upon which to develop such regulations is far from complete we believe that regulations governing ocean dumping will be refined and adjusted as our knowledge grows.

H.R. 4723 provides the essential criteria for developing appropriate regulations but does not specify the regulations in detail. This, we believe, is appropriate in view of the current state of our knowledge.

You have under your consideration several bills which would control ocean dumping in a variety of ways and would place responsibility for the control of ocean dumping in one of several departments. The

Department of Commerce believes that the approach of H.R. 4723 which places the regulatory authority in the Environmental Protection Agency is most appropriate since EPA is an agency which has as its chief purpose the protection of the environment and which possesses the necessary regulatory capability to develop and carry out a comprehensive ocean dumping policy.

We see the Department of Commerce as a logical agency to support EPA in the development of appropriate regulations.

This conforms with the intention of the President expressed in his message of July 9, 1970, where, in speaking of the role of NOAA, he stated:

> I expect it to maintain continuing and close liaison with the new Environmental Protection Agency and the Council on Environmental Quality as part of an effort to insure that environmental questions are dealt with in their totality and that they benefit from the full range of the Government's technical and human resources.

I see NOAA as playing an important role in support of the Environmental Protection Agency especially in the areas of marine research and monitoring.

NOAA and other organizations in the Department can contribute in the following areas:

1. Ecological research and determination of the processes controlling the dispersion and concentration of pollutants in the marine environment;
2. Engineering and economic analysis of alternatives to ocean dumping as presently practiced involving both field and laboratory tests and analysis;
3. Development of more reliable and adequate instrumentation as well as precise measurement techniques; and, finally,
4. Assisting with improved information exchange to shorten the timelag between acquisition of information and application for pollution control.

I would like to discuss the special capabilities of the Department of Commerce as they relate to the ocean-dumping problem.

NOAA has already begun research in the following problem areas:

1. Broad-based ecological research to understand the pathways of wastes in marine ecosystems.
2. Oceanographic studies of basic physical and chemical processes, with special emphasis on estuaries and coastal areas.
3. Identification of toxic materials and their lethal, sublethal, and chronic effects on marine life.
4. Development of effective monitoring systems.

NOAA's 1972 budget request provides for expansion in these four areas.

NOAA resources that could be used in ocean dumping research include some 43 research vessels and 25 laboratories. At the present time we are also working on the development of ocean monitoring buoys which would contribute to our understanding of the marine environment and which may provide information useful in dealing with ocean-dumping problems.

In addition, as part of the Environmental Data Service, NOAA has the National Oceanographic Data Center, and I should also add, for the record, the National Oceanographic Instrumentation Center,

which now have extensive data, records, and information which can be used to help predict the effects of ocean dumping.

We have available in the Department hundreds of scientists, engineers, and economists who working together can provide knowledge and understanding of the processes of the marine environment which can assist EPA in the formulation of policies and development of effective regulations on ocean dumping.

We feel it is the role of the Department, particularly through NOAA, to perform ocean monitoring and to provide information which will be useful to EPA in evaluating requests for permits.

Section 5(a) of H.R. 4723 identifies the two primary considerations that would be considered in reviewing and evaluating permit applications. These are (1) consideration of the likely ecosystem, human health, and economic impact of the proposed dumping and (2) alternative locations and methods of disposal including land-based alternatives.

These criteria provide a proper basis for issuance of permits. A rational investigation of the benefits and costs associated with alternatives, including land-based alternatives, will help identify least-cost solutions within overall environmental protection objectives.

The Department of Commerce has a wide range of resources capable of participating in the assessment of alternatives to ocean dumping.

The National Bureau of Standards, for example, has for many years maintained programs of research on corrosion of metals in soils and in marine environment, and on degradation of a wide variety of materials, including plastics, under unfavorable conditions of temperature and pressure.

The capabilities of NOAA which I mentioned a moment ago would also be valuable in assisting to design and to carry out suitable experiments.

It is widely recognized that there is a need to improve measurement techniques and instrumentation which are used today to measure concentrations of pollutants in our waters. Especially needed are reliable and cheap field tests which can be rapidly performed to give precise results.

The NBS has the capability to respond to the need for better measurement techniques and calibration standards. There is now work underway at NBS on new methods of detection and measurement of low-level pollutants in water, and a survey of needs is being made to determine how NBS research can best contribute to the solution of the more pressing measurement problems that other agencies such as EPA now face.

The capability of EPA to effectively apply the criteria of dumping impacts and consideration of alternatives as required under section 5(a) of H.R. 4723 can be enhanced by full utilization of existing information exchange capabilities within the Department of Commerce.

The National Technical Information Service (NTIS) of the Department is a potential vehicle for the needed information exchange. NTIS now handles about 45,000 new technical reports each year. The reports cover mainly research and engineering work done with Federal funds. They are cataloged, abstracted, indexed, placed on microfiche, and sold to the public.

The public learns about the reports on a biweekly basis. NTIS is already publishing special bulletins announcing new reports and other documents in the air pollution and water resources fields. This type of information exchange could be extended to include reports on pollution control devices and techniques.

The capabilities of the Department of Commerce in assisting in the implementation of ocean dumping legislation are impressive. Of particular significance is the initiative of Secretary Stans in placing departmentwide responsibility for environmental quality matters, including implementation of the Environmental Policy Act of 1969, in my office.

I have been directed to provide coordination and guidance in bringing about maximum contribution by Commerce programs to the solution of national problems of environmental quality of concern to the Department as a whole; and to serve as the Department's principal point of contact with the Council on Environmental Quality, and with organizations, both public and private, on environmental matters of concern to the Department as a whole.

Already, an environmental work group has been established under my Deputy for Environmental Affairs. The Department is in a position to function as a member of the administration's team in protecting and preserving our environment.

I will do my best to assure that Commerce is an effective member.

Following this, sir, I would be happy to attempt to answer questions of your interest.

Thank you, sir.

Mr. DINGELL. Thank you very much, Mr. Wakelin.

Mr. Rogers?

Mr. ROGERS. Thank you, Mr. Chairman.

First of all, I want to join with the chairman in welcoming you distinguished witnesses and old friends. Jim Wakelin, who has done an outstanding job of service to the Nation, and is known to this committee, and to others, for his work in the Navy as Assistant Secretary for Research and Development, an outstanding job, and his preeminence in the field of the environment, particularly as it affects the oceans.

And of course our colleague and good friend, Mr. Howard Pollock, who is now contributing in a significant way to the executive branch in the same manner, I am sure, as he did here, so we welcome you.

Mr. WAKELIN. Thank you, Mr. Rogers.

Mr. ROGERS. I am interested just a little bit in the detail of your research programs, your budget, and personnel involved in these programs that you have discussed with us. Perhaps you could just give us a quick rundown, and I don't want to take too long. Perhaps you could furnish some for the record.

Mr. WAKELIN. Yes, Mr. Rogers.

Mr. ROGERS. Just give us a summary of the main things.

Mr. WAKELIN. I would think that Mr. Pollock could talk to those research efforts and programs that are going on within our principal activity in the oceans, and that is in NOAA, if I might defer to him, sir.

Mr. ROGERS. That would be fine, and the budget that you have allocated, and foresee as being applied to those.

Mr. Pollock. Well, in the particular areas that Dr. Wakelin talked about, we have already begun research, they are stated on page 4 and page 5 at the top of our testimony. I think I could state very briefly, Mr. Rogers, that No. 1 is an area that is accomplished by our National Marine Fisheries Service. The current fiscal year figure is $3,170,000, and for the next fiscal year it is $4,170,000.

In No. 2, the oceanographic studies of basic physical and chemical processes, our Environmental Research Laboratories for the current fiscal year are spending $1,471,000, and for the next fiscal year $2,437,000. And the National Marine Fisheries Service is also engaged in this work at a level of $100,000.

In paragraph 3, the identification of toxic materials and their lethal, sublethal, and chronic effects on marine life and No. 4 being the development of effective monitoring systems, our National Marine Fisheries Service is spending $500,000 in the current fiscal year, and we have budgeted $1,060,000 for the next fiscal year.

Now you will note that on the middle of page 5 of our testimony we talk about NOAA's National Oceanographic Data Center, and Dr. Wakelin included also the National Oceanographic Instrumentation Center. The figures for the current fiscal year, I believe, are $1.8 million for the Instrumentation Center, and I believe it is $2.015 million for the Data Center.

Mr. Wakelin. If I might just go on to complete, Mr. Rogers, the work at the Bureau of Standards on the pollution problem of detection and measurement, their particular budget in NBS is $613 million for fiscal year 1971, and $884 million for fiscal year 1972. That is, the request for 1972.

Our National Technical Information Service has a budget, but not an appropriation—a budget of $5 million, of which I believe the budget request is $1.477 million for fiscal year 1972.

Other funds coming into NTIS come either from the Department of Defense or from public sale of our technical reports.

Mr. Rogers. I see. I was particularly concerned about the little amount budgeted for toxic materials, lethal, sublethal and chronic effects on marine life and human life, I would presume, too, that you get into. Don't you think this figure is rather insufficient, with the state of the knowledge?

Mr. Pollock. Well, I think the answer to the question is that we would certainly like to go a lot further than we have. As we have indicated, we have more than doubled the budget for next year, over what we presently have. I think this is an extremely important subject. Most of the work here, as you might imagine, is being done in the area of heavy metals in fish, and we are moving along with it. There is a lot more we could do, but we have to live at the present time within the budget we have for doing the job. And I think we are doing an effective job.

Mr. Rogers. Also, we are somewhat concerned about the budget limitations on the sea grant college programs. It is my understanding that they are not able to really go into any new programs, but simply to fund those that are ongoing, really because of the inflationary factor. Is that about correct?

Mr. Pollock. Yes, sir. We have a problem; I think all of us that are concerned with the whole marine environment would certainly

like to move further than we have now, but obviously, this is just one of many, many problems in the national economy, and we are trying to live within that framework. But your statement is correct, that we are fairly well limited to the existing programs, because we are in the position of having certain studies and programs started at certain universities, and we don't want to curtail those, in order to start new ones.

Mr. ROGERS. We are going to try to get some more money this year.

Mr. POLLOCK. We would be very happy.

Mr. ROGERS. Thank you very much.

Thank you, Mr. Chairman.

Mr. DINGELL. Thank you, Mr. Rogers.

Mr. Everett?

Mr. EVERETT. Thank you, Mr. Chairman.

Mr. Wakelin, several times throughout your statement, you mentioned monitoring, and I noticed the Coast Guard under the bill has the responsibility of surveillance and enforcement. And I was wondering if there has been any thought given to extending this monitoring requirement to the Coast Guard, or to NOAA, or just to whom?

It is not clear under the bill as to who would do the monitoring.

Mr. WAKELIN. I would suggest that the word "surveillance" there refers to the surveillance of surface activities and surface vessels. Our point here, I think, in the NOAA capabilities, presents a fairly strong case for the functions, at least at first, being done by NOAA, in the monitoring operations.

These are pretty sophisticated programs, and while I am sure the Coast Guard has the capability, I don't think it is a broad as NOAA. We are talking about monitoring in the physical, chemical, and biological areas.

Mr. EVERETT. I see, sir.

Now on page 5, Mr. Wakelin, you mention that you have 43 research vessels and 25 laboratories available. Do these numbers include the four vessels that were tied up last year by the BCF and also the four research laboratories that were either closed or phased down considerably in their activities?

Mr. WAKELIN. Well, let me answer first the question about the vessels, and then I think our supporting people can answer about the laboratories.

There are 43 vessels in active service.

Now with respect to the laboratories, I would like to call on Howard Eckles, if I may.

Mr. EVERETT. Yes, sir.

Mr. POLLOCK. I believe the answer to your question is that all of the vessels that are listed here are the number of operational vessels and we would be happy to provide for the committee, if they would like, a list of those vessels and indeed, a list of the laboratories and facilities that we have which are capable of working in the general area of dumping as distinct from other aspects of our responsibility.

Mr. EVERETT. If you would, provide for the record a list of those research services, as well as laboratories, in total, and indicate those which are not operational at this time.

BEST COPY AVAILABLE

Mr. POLLOCK. We will be pleased to do that.
(The information follows:)

NOAA RESEARCH VESSELS

I. Vessels Equipped for Oceanographic Research

National Marine Fisheries Service	
Albatross IV	Woods Hole, Mass.
David Star Jordon	San Diego, Calif.
Charles H. Gilbert	Honolulu, Hawaii
Delaware II	Woods Hole, Mass.
George B. Kelez	Seattle, Wash.
John N. Cobb	Do.
Miller Freeman	Do.
Oregon	Juneau, Alaska
Oregon II	Pascagoula, Miss.
Townsend Cromwell	Honolulu, Hawaii
Undaunted	Wilmington, N.C.
National Ocean Survey	
Discoverer	Miami, Fla.
Oceanographer	Seattle, Wash.
Researcher	Miami, Fla.
Surveyor	Seattle, Wash.

II. Vessels Equipped for Limited Oceanographic Research

National Ocean Survey (Hydrographic vessels)	
Fairweather	Seattle, Wash.
Mt. Mitchell	Norfolk, Va.
Pathfinder	Seattle, Wash.
Rainer	Do.

III. Vessels Equipped for Fisheries Research

National Marine Fisheries Service	
Alosa	Oxford, Md.
Blueback	Woods Hole, Mass.
Challenger	Highlands, N.J.
Dolphin	Do.
George M. Bowers	Panama City, Fla.
Kingfish	St. Petersburg, Fla.
Martha E	Highlands, N.J.
Murre II	Juneau, Alaska
Phalarope II	Booth Bay Harbor, Me.
Point of Marsh	Beaufort, N.C.
Rorqual	Booth Bay Harbor, Me.
Sablefish	Juneau, Alaska
Shang Wheeler	Milford, Conn.
Sockeye	King Salmon, Alaska
Tommy Box	Galveston, Tex.
Cripple Creek	Juneau, Alaska

IV. Vessels Utilized in Nautical Charting that Have Secondary Oceanographic Capabilities

National Ocean Survey	
Davidson	Seattle, Wash.
Heck	Norfolk, Va.
McArthur	Seattle, Wash.
Pierce	Norfolk, Va.
Rude	Do.
Whiting	Do.
Launch #1255	Do.
Launch #1257	Do.
Schenahon	Detroit, Mich. (going to Fla.)

V. Vessels Equipped for Unique Estuarine Water Circulation Studies

National Ocean Survey
Ferrel — Norfolk, Va.

NOAA LABORATORIES CAPABLE OF ASSISTING WITH OCEAN DUMPING PROBLEMS

	Budget fiscal year 1971 (K)
National Marine Fisheries Service	
Eastern Gulf Marine Laboratory—Panama City, Fla	75
Tiburon Marine Laboratory—Tiburon, Calif	225
Sandy Hook Marine Laboratory—Highlands, N.J	631
Narragansett Marine Gamefish Research Laboratory—Narragansett, R.I	190
Biological Laboratory—Seattle, Wash	3285
Biological Laboratory—St. Petersburg Beach, Fla	228
Biological Laboratory—Galveston, Tex	1013
Tropical Atlantic Biological Laboratory—Miami, Fla	752
Center for Estuarine and Menhaden Research—Beaufort, N.C	989
Biological Laboratory—West Booth Bay Harbor, Maine	528
Biological Laboratory—Woods Hole, Mass	1023
Biological Laboratory—Milford, Conn	392
Biological Laboratory—Oxford, Md	497
Fishery Oceanography Center—La Jolla, Calif	1416
Hawaii Area Fisheries Research Center—Honolulu, Hawaii	995
Biological Laboratory—Auke Bay, Alaska	1647
Environmental Research Laboratories	
Geophysical Fluid Dynamics Laboratory—Princeton, N.J	2192
Atlantic Oceanographic and Meteorological Laboratories—Miami, Fla	2127
Pacific Oceanographic Laboratories—Seattle, Wash	423
Marine Mineral Technology Center	
Marine Mineral Technology Center—Tiburon, Calif	1000
National Oceanographic Instrumentation Center	
National Oceanographic Instrumentation Center—Washington, D.C	1800
NOAA Facilities	
Research Flight Facility—Miami, Fla	1664
Pacific Marine Center—Seattle, Wash	974
Atlantic Marine Center—Norfolk, Va	914
Mississippi Test Facility (Aerospace Remote Sensing Program)—Bay St. Louis, Miss	

Mr. EVERETT. I might ask if any of your staff would know, with respect to the Ann Arbor and Milford Conn. laboratories if there are any requests in your 1972 budget for these laboratories?

Mr. ECKLES. Howard Eckles, Mr. Chairman.

Concerning the Ann Arbor Laboratory, I think you may know that that laboratory rests now with the Department of the Interior, underneath the Reorganization Plan No. 4. There were funds for the operation of that laboratory requested in the budget. Funds have also been requested for the Milford, Conn. Laboratory. We do have plans to continue the operation of this laboratory, and to concentrate there work on ocean contamination.

There are some personnel being adjusted between Ann Arbor and Milford, to concentrate on this problem. So the answer to your question is that both of them are operating and there have been funds requested for both of them. The dollar figure for the Milford Laboratory is $392,000.

Mr. EVERETT. I was going to ask. I think that there is around $400,000 for fiscal 1971.

That is all the questions I have, Mr. Chairman.

Mr. DINGELL. Gentlemen, the committee is grateful to you for your presence today, and for your very helpful statement.

We thank you very much.

Mr. WAKELIN. Thank you, Mr. Chairman.

Mr. DINGELL. Our next witness is Brig. Gen. George Hayes, Deputy Assistant Secretary of Defense for Health and Environment, accompanied by Brig. Gen. Richard H. Groves, and Rear Adm. E. R. Crawford, U.S. Navy.

Gentlemen, you are certainly welcome here to the committee. If you will identify yourselves by name and duty, for our reporter for the purpose of the record, we will be happy to recognize you for such statements as you may give.

Is there anyone else among your staff associates you would like to have at the witness table with you?

General HAYES. Not at this time.

Mr. DINGELL. All right, you may feel free to call on such members of your staff as you wish, and if you will identify yourselves for our reporter here, we would be most happy to recognize you.

STATEMENT OF BRIG. GEN. GEORGE J. HAYES, M.C., U.S. ARMY PRINCIPAL DEPUTY ASSISTANT SECRETARY OF DEFENSE FOR HEALTH AND ENVIRONMENT; REAR ADM. E. R. CRAWFORD, U.S. NAVY ASSISTANT DEPUTY CHIEF OF NAVAL OPERATIONS (LOGISTICS), AND BRIG. GEN. RICHARD H. GROVES, DEPUTY DIRECTOR OF CIVIL WORKS, OFFICE, CHIEF OF ENGINEERS, DEPARTMENT OF THE ARMY

General HAYES. Mr. Chairman, members of the committee, I am Brig. Gen. George J. Hayes, Principal Deputy Assistant Secretary of Defense, Health and Environment.

With me are Rear Adm. Earl R. Crawford, Assistant Deputy Chief of Naval Operations, and Brig. Gen. Richard H. Groves, Deputy Director, Civil Works, Office of the Chief of Engineers.

I welcome the opportunity to appear before you today to discuss the views of the Department of Defense on H.R. 4723, a bill to regulate the dumping of materials in the oceans, coastal, and other waters and for other purposes. I propose to limit my testimony to the environmental issues of the bill. Admiral Crawford will subsequently provide testimony concerning military operational issues and General Groves will testify on civil works functions of the Corps of Engineers.

The Department of Defense is deeply concerned about the adverse ecological and environmental effects associated with the unregulated discharge of wastes and other materials into the navigable, coastal, and ocean waters of the United States. We believe that legislation to regulate such activities is desirable.

The Department of Defense has already taken action to initiate and implement comparable policy guidance which was first promulgated in the Report of the Council on Environmental Quality on Ocean Dumping—a National Policy and transmitted to Congress by President Nixon on October 7, 1970.

It is clear that some of the provisions of the proposed legislation will have a significant influence on some of the functions of the Department of Defense but actions are already underway by the military departments to exert leadership in cleaning up the oceans.

On February 24, 1971, Secretary Laird approved the U.S. Navy order to suspend deepwater dumping of obsolete, unserviceable munitions until all alternative methods of disposal have been completely studied. An intensive research and development program has also been directed with the aim of seeking alternative methods of disposal which will have minimal impact on the environment. In effect, Secretary Laird's announcement put a freeze on ocean dumping of all military munitions by the United States, since the U.S. Navy provides deepwater dump services of obsolete munitions for all the armed services. All chemical munitions disposal at sea has been terminated, and biological warfare agents or munitions have never been disposed of at sea.

The Department of the Army has assessed in detail its proposed biological warfare agents and munitions demilitarization program and its currently staffing and coordinating with other Federal, State, and local authorities, its environmental statement in accordance with Public Law 91-190 "The National Environmental Policy Act of 1969." The Department of the Army also has draft environmental statements under preparation which address the disposal of chemical munitions by demilitarization on land. These programs are intended to protect the environment during disposition of these materials.

The Department of Defense believes that H.R. 4723 embodies a realistic approach by establishing a regulatory authority rather than by prohibiting all ocean disposal or establishing unreasonable deadlines for such termination. It is possible that future research and study may reveal that some waste materials would contribute to the rehabilitation or enhancement of the marine environment. Absolute prohibition of all ocean disposal could result in disposal techniques which pose greater hazards to man and his environment than those which currently exist. In some cases there may be no current technologically acceptable alternative. These facts weigh heavily against any proposal which would prohibit all such ocean disposal operations. H.R. 4723 avoids these difficulties by providing for a balancing of interests that would integrate technological or scientific knowledge with operational requirements. It also envisions and accepts current standards which have proven to be effective in protecting human health and the marine environment. It does so by excluding the regulation of routine discharge of effluents from facilities, discharges which are already effectively regulated by existing laws.

In summary, the Department of Defense supports H.R. 4723 in lieu of the other bills related to marine protection which we have reviewed. This proposal takes a giant stride forward in the ever expanding effort to enhance the quality of our environment, a goal with which the Department of Defense is vitally concerned in our everyday actions, wherever we may be located.

If enacted, we believe that the implementing regulations of the Department of Defense would be a valuable addition to the more general rules that are currently in effect under the National Environmental Policy Act.

Mr. Chairman, this concludes my testimony on behalf of the Department of Defense. If there are questions related to the environmental aspects of the Department of Defense program, I would be pleased to answer them. Should you have questions concerning the operational aspects of the legislation, the military department witnesses to follow will be pleased to answer those questions.

Mr. DINGELL. General, the committee thanks you for a very helpful statement.

Which of your associates would you like to follow?

General HAYES. Admiral Crawford.

Mr. DINGELL. Admiral?

Admiral CRAWFORD. I will follow, Mr. Chairman.

I am happy to have the opportunity to appear before this committee to present the Navy's views on the proposed Marine Protection Act of 1971.

At the outset I want to point out, to the committee, the overall concern the Navy has in regard to all of the environmental aspects of our operations. The need to improve and enhance the quality of life in all of its forms is receiving a great deal of attention within the Navy—from the Secretary's office down through the commands to the local activities.

We are making every effort to cooperate with the President's Council of Environmental Quality, the Environmental Protection Agency and the National Oceanic and Atmospheric Administration. Also, our pollution abatement programs and projects are prepared to meet evolving local, State and Federal standards and regulations.

When it comes to the environment, the Navy is confronted with a many-faceted problem. Our efforts to date in such areas as smoke elimination at powerplants, incinerators and firefighting schools, our program to convert ships to cleaner burning distillate fuel together with the expanded effort in the construction of sewage and industrial waste treatment plants are positive examples of our desire to seek a cleaner and more healthful environment. We are hard at work on developing acceptable methods to handle sewage, trash, garbage, oil, and other wastes from our ships. Smoke and noise abatement from our planes are also areas that we are concentrating on. These problems are receiving appropriate priority within the Navy and we are increasing our effort to develop the technology and the equipment required to do the job.

In recent months, Secretary Chafee has taken a number of initiatives with regard to ocean dumping. In December 1970, the Secretary prohibited the transporting and dumping, at sea, of wastes generated in-port, or ashore, such as trash, refuse, oily wastes, and industrial sludges. In February of this year, Secretary Laird approved Secretary Chafee's proposal to suspend the deepwater dumping of obsolete and unserviceable conventional ammunition until the Navy is able to determine the precise environmental effects of these dumps, as well as possible environmentally acceptably alternative methods of disposal. The deepwater disposal of chemical and biological munitions has been specifically prohibited by Secretary Laird.

We have been asked, by your committee's counsel, to focus in our statements today, on several areas of the ocean dumping question that are of particular interest to members of your committee.

62-513—71——32

MUNITIONS DISPOSAL

Each year the Navy must dispose of a significant amount of Conventional ammunition that becomes unserviceable, unsafe, or obsolete. In recent years we have accomplished this through a number of means which have included demilitarization (that is, by taking the ammunition apart), by controlled burning, by exploding, and by ocean dumping.

For many years, the Navy has used deepwater dumping extensively for ammunition disposal and we have experienced no major safety problems with this method. Since 1964, 19 deepwater dump operations have taken place in which shiploads of ammunition were scuttled. As I mentioned, in February we suspended all deepwater dumping of ammunition. While the present evidence indicates that the environmental impact of deepwater dumping is minimal, some environmental questions remain unanswered. Consequently, plans for ocean dumping have been postponed while a reassessment of all alternative methods of disposal is carried out.

The Assistant Secretary of the Navy for Research and Development has established a select high level working group from the Navy laboratories and systems commands to examine the disposal problem and to develop a plan which will minimize or, hopefully, eliminate the need for ocean dumping. This is being done in consultation with the Army and the Air Force. In addition, the Navy is investigating the redesign of ammunition with the objective of easier, safer demilitarization.

NAVY CONTRACTOR OPERATIONS

There are few requirements for the Navy or Navy contractors to dispose of Navy generated materials into the ocean.

Perhaps the most significant situation would involve the disposal, at sea, of dredge material resulting from maintenance dredging of naval port facilities to insure adequate draft for ships. These dredging operations are now conducted under the direction and approval of the U.S. Army Corps of Engineers.

Under the proposed bill, a permit issued by the Environmental Protection Agency would be required for the regulated disposal of Navy dredgings, whether performed by Navy in-house capability or by civilian contractor. Certainly, all such disposal will be performed in accordance with the standards and criteria to be developed by the Environmental Protection Agency. In any event, obtaining an EPA permit for the relatively small amount of dredging in Navy ports should not impose a hardship on the Navy or on Navy contractors.

CONCLUSION

It is the Navy's contention that an ocean dumping act should provide a rational means of controlling ocean dumping which is based on the effect of the material to be dumped on the ocean environment—present and future—as well as the effect of alternative means of disposal of the materials on man's environment. In other words, discussion of legislation regarding regulating ocean dumping should include consideration for man's total environment, not only the ocean en-

vironment. We feel that the proposed act, calling for a permit system administered by the Environmental Protection Agency, which is charged with overall protection of the environment, meets this criteria. We also feel that a good ocean dumping act should clearly exclude waste waters discharged from shore facilities through sewers into coastal waters, because the mechanisms for controlling these discharges are included in the Clean Waters Act, as amended. The proposed act is clear in this regard.

Finally, a good ocean dumping act should exclude the day to day operational discharges from ships, such as sewage, and oily bilge, which are properly subject to regulation by other laws. In this connection, it is our understanding that "dumping" as defined in the proposed act would not include the incidental discharge of some debris or other material in the water from an activity provided that disposition is not the primary objective of the activity. For example, wastes incidental to the operation of ships, the material and debris from missiles, spent bombs, and other projectiles would be excluded from this act.

In summation, the Navy supports the proposed act as a rational, effective means of protecting the ocean environment while, at the same time, providing for consideration of man's total environment.

Mr. Chairman, this concludes my statement.

Mr. DINGELL. Thank you very much, Admiral Crawford.

General Groves.

General GROVES. Mr. Chairman and members of the committee, I am Brig. Gen. Richard H. Groves, Deputy Director of Civil Works, Office, Chief of Engineers, Department of the Army.

I am accompanied by members of the staff of that office, Mr. Mark S. Gurnce chief of our operations division, and Mr. Errol Tyler from the general counsel's office.

I appreciate having this opportunity to testify on the numerous bills before your committee concerned with the subject of ocean dumping.

Mr. Chairman, the bills before your committee embody a variety of approaches to the problems associated with the dumping of waste materials in the oceans. Rather than discuss each of them in detail, I shall confine myself to discussing the administration bill and attempting to answer any questions you may have concerning the other bills.

Last year, at the request of the President, the Council on Environmental Quality investigated the problem of ocean pollution and concluded that there is a critical need for a national policy on ocean dumping. The recommendations of the Council were incorporated in the proposed Marine Protection Act of 1971, submitted to the Congress by the President and introduced as H.R. 4247, 4723, 5239, 5268, and 5477.

The administration bill provides that, except as may be authorized in a permit issued by the Administrator of EPA, no person may transport material from the United States for the purpose of dumping it into ocean or other waters covered by the bill, or dump any materials into any such waters which are within the territorial jurisdiction of the United States, or dump any materials into the waters of the con-

tiguous zone to the extent that such dumping may affect the territorial sea or the territory of the United States. The bill would apply to all Federal, State and foreign governmental organizations, employees and agents, as well as to private persons and entities. The waters to which the bill applies would include the Great Lakes. It would apply to all kinds of matter including dredge spoil, solid waste, sewage sludge, industrial wastes, radioactive materials, munitions, and chemical, biological and radiological warfare agents.

The Administrator would be authorized to issue permits to dump materials or to transport them for dumping where in his judgment such activity will not unreasonably degrade or endanger human health, welfare or amenities, or the marine environment, ecological systems or economic potentialities. He would be required to establish criteria for evaluating permit applications, taking into account the likely environmental effects of the proposed dumping, as well as alternative locations and methods of disposal and the impact on the public interest of issuing or denying permits or of requiring such alternative disposal. The Administrator would be authorized to impose restrictions relating to the type and amount of materials to be dumped, and the time and place of dumping. He would be authorized to limit, deny, alter, or revoke permits where he finds that materials cannot be dumped consistently with the criteria established for the issuance of permits. Civil and criminal penalties of up to $50,000 per violation would be provided for violations of the act or of any regulations or permit issued thereunder. The Attorney General would be authorized to bring actions for equitable relief to redress such violations. Surveillance and enforcement authority would be given to the Coast Guard.

This legislation would provide a means of regulating the dumping of material in the oceans so as to prevent or seriously limit the dumping of material which could adversely affect human health or welfare or the environment. We urge its enactment.

Mr. Chairman, that concludes my statement. I will be pleased to answer any questions that the committee may have.

Mr. Dingell. General, thank you very much.

Mr. Rogers?

Mr. Rogers. Thank you, Mr. Chairman.

Gentlemen, thank you for these statements.

Let me ask General Groves: Now I presume, you just announced, I think, today, haven't you, a program for issuing permits?

General Groves. A program for issuing industrial effluent permits under the Refuse Act was announced April 7, sir.

Mr. Rogers. UPI——

General Groves. Which is today, yes, sir.

Mr. Rogers (continuing). Said you announced something else today.

General Groves. T[illegible] is correct.

Mr. Rogers. It is in the Federal Register today.

General Groves. Yes.

Mr. Rogers. Now who issues a permit to the Corps of Engineers, when you do the dredging?

General Groves. No one, sir. We issue it to ourselves, in effect.

Mr. Rogers. Is that a good system, do you think?

General GROVES. We think, sir, all things considered, it probably is.

Mr. ROGERS. Do you have to have any approval? Do you file any impact statements?

General GROVES. Yes, sir, we do, under certain conditions.

Mr. ROGERS. To do your projects?

General GROVES. Oh, yes.

Mr. ROGERS. So that Environmental Council and EPA would comment on it?

General GROVES. Yes, sir.

Mr. ROGERS. And you would take their comments into consideration before you issued yourself a permit?

General GROVES. We would have to, yes, sir.

Mr. ROGERS. But you wouldn't have to necessarily abide by what they suggest. The final authority to issue it is yourself, I believe. Is that correct?

General GROVES. Yes, sir, our understanding of the section 102(c) of the Environmental Policy Act is that it provides us with the information, those of us who have to make the decision, the information we need in order to reach a good and valid decision.

Mr. ROGERS. And you are going to have, I believe it is estimated, between 40,000 and 100,000 permits?

General GROVES. That is our present estimate, yes, sir.

Mr. ROGERS. What is going to be the time limit? How are you going to handle it? Have you got sufficient manpower?

General GROVES. We are talking, sir, now, in this matter about the Refuse Act, the section 13 permits.

Mr. ROGERS. Yes.

General GROVES. This is going to require a considerable effort, of an administrative nature, of course.

Mr. ROGERS. Yes.

General GROVES. The appropriation request this year, the budget request this year, provides the necessary funding for it. As a matter of fact, we have some 1971 funds for it.

Mr. ROGERS. How much?

General GROVES. $1 million in 1971, sir. $4 million in 1972.

Mr. ROGERS. How many people will this provide?

General GROVES. We have asked for 200 spaces. However, when you consider other losses, it comes out about even. There will be about 200 spaces earmarked for this.

Mr. ROGERS. But you say you have lost other personnel, so it would even up?

General GROVES. Our total strength doesn't change, particularly, sir.

Mr. ROGERS. So you are really not getting any increase in manpower, as I understand it.

General GROVES. No, sir, but we are in one way of looking at it, because we are getting the spaces required to do this job, which we otherwise would have lost.

Mr. DINGELL. Mr. Rogers, would you yield? Where are these other spaces coming from, and what were the functions of the personnel who are being removed from the corps' roster engaged in at the time of their departure from service?

BEST COPY AVAILABLE

General GROVES. Sir, essentially, we have had a steady drain or a steady decrease in our overall strength, due primarily to productivity increases, increased efficiency of our operations, and planning.

Mr. DINGELL. Is it due to that, or to costs in appropriations?

General GROVES. No, sir; our appropriations are running fairly constant.

Mr. DINGELL. Are they running up to the increase in the costs of labor and salaries and wages and so forth?

General GROVES. Not entirely; no, sir.

Mr. DINGELL. So actually, this is just a cutback forced in part by budgetary strings. Am I correct?

General GROVES. No, sir; I wouldn't really say so.

Mr. DINGELL. Well, in any event, what I am trying to find out is what were the functions of these people who have vanished from the corps' rolls.

In other words, what activities were they engaged in? Were they engaged, for example, in the issuance of permits? Were they engaged in writing 102(2)(c) statements? What were they doing? I am curious to know whether or not maybe this gain that appears is not really a gain, but a shifting of personnel from one place to another.

General GROVES. Essentially, sir, it is shifting around to meet our changing missions. If you care to, I could elaborate for the record and be very specific. I am unable to right now.

Mr. DINGELL. I think that would be fine.

General GROVES. We are prepared to do so.

(The information follows:)

ESTABLISHMENT OF THE ISSUANCE OF PERMITS PROGRAM

The President's budget for FY 1971 contained an increase of 365 civilian positions to meet the requirements for the FY 1971 program. In view of the uncertainties of the FY 1971 program pending a resolution by the President of the FY 1972 budget, the increased spaces were not allocated to our field installations as early as they normally would be. It was against this increase personnel hire ceiling that the Corps temporarily borrowed the 200 positions required to implement the permit program in view of the high priority of that program. The 200 positions are included in the personnel ceiling in our FY 1972 budget now being considered by the Congress. Since the necessary permit spaces (200) are included in the FY 1972 figure, the borrowed spaces will be reallocated for program support.

Mr. ROGERS Well, it is interesting you can handle this in 200, and EPA, I think, said they added 300 or 400. I wonder why.

General GROVES. I am unable to comment, sir. We have evaluated our position very carefully, and——

Mr. ROGERS. Have you estimated the time it will take to approve these permits?

General GROVES. Yes, sir.

Mr. ROGERS. What is that time?

General GROVES. It depends on how controversial it becomes, but I would guess that on the average, one that is not controversial could be processed within 6 months.

Mr. ROGERS. It will take 6 months?

General GROVES. I would think so, yes, sir. By the time it is fully coordinated that is so. And in an extreme, where there is a total lack of controversy, it should move much faster. For the ones that are more controversial, it will take considerably longer.

Mr. ROGERS. Would you have hearings on these permits, is that it?

General GROVES. Where they become contentious, yes, sir.

Mr. ROGERS. Public hearings or written comments?

General GROVES. The first step will be to issue a public notice. If the public notice or the coordination——

Mr. ROGERS. Basically the way you handle it now?

General GROVES. Yes, sir; the same way we handle it now, except that in some conditions the hearings could become adversary hearings.

Mr. ROGERS. Yes, under the Administrative Procedures Act.

General GROVES. Yes, sir.

Mr. ROGERS. I think it might be well for you to get this for the record, on your——

General GROVES. On the section 13 permits?

Mr. ROGERS. Yes.

General GROVES. All right, sir.

Mr. ROGERS. All right, sir.

(The information follows:)

REFUSE ACT PERMIT PROGRAM

The Refuse Act permit program is specified in regulations appearing in the 7 April edition of the Federal Register. The program involves coordination among Corps, State, and officials from other Federal agencies as well as applicants. I shall briefly outline the usual procedure for processing applications for Department of the Army permits.

Processing of a permit application begins with the State in which the proposed activity is to occur certifying that it has reasonable assurance that the activity will not result in violation of applicable water quality Standards. Once the certified application is in order, the District Engineer issues pubic notices announcng the proposal. Public notices are distributed to the EPA, other appropriate Federal and State agencies, media and other interested parties. Based on the response to the public notice and on the requirements of Sections 21(b)(2) and 21(b)(4) of the Federal Water Pollution Control Act, as amended, the District Engineer decides whether a public hearing is necessary. At this time, a decision is also made regarding the necessity for an Environmental Impact Statement (EIS) in accordance with Section 102 (2)(c) of the National Environmental Policy Act of 1969. If a hearing is deemed necessary, notices are sent out to all known interested parties announcing a hearing to be scheduled at least 30 days hence.

After the hearing, if EPA objects for water quality reasons, the application must be denied unless the District Engineer disagrees with the EPA's evaluation. In this case, the application is forwarded through channels to the Secretary of the Army. The Secretary may then coordinate the matter with the Administrator of the EPA, but must, in the end abide by the Administrator's recommendations. of the EPA does not object, but the Regional Director of the Bureau of Fish and Wildlife or NOAA object, the District Engineer may deny the permit. If the District Engineer disagrees with these objections and intends to issue the permit, he must inform NOAA and Interior. These agencies then have 30 days in which to contact their superiors in Washington who in turn contact Secretary of the Army. If the Secretary of the Army requests a review, the District Engineer will forward the case through channels to Washington. Otherwise the application will be approved. If the District Engineer believes the proposed activity would be detrimental to anchorage or navigation interests, the application may be denied immediately.

Mr. ROGERS. General Hayes, I recall your testimony before. How many impact statements have been filed by the Department of Defense?

General HAYES. We have at the present time seven environmental statements that have been submitted to CEQ. We have, including those seven, a total of 26, which are in the process of internal review within the DOD.

Mr. ROGERS. They have not been forwarded?

General HAYES. They have not been forwarded to CEQ.

Mr. ROGERS. I see. These are being worked on within the Department?

General HAYES. Or in coordination with other agencies.

Mr. ROGERS. They have not gone to CEQ yet?

General HAYES. Not gone to CEQ yet.

Mr. ROGERS. So you have five to go?

General HAYES. That'rs right.

Mr. ROGERS. What are the actions on those seven? Have they been acted on?

General HAYES. We have three environmental statements that are final. We have not had any adverse response to these as yet. Four are in process, in CEQ. We have no report on these yet.

Mr. ROGERS. Yes. Would you list for us those—for the record, not now—but those impact statements and the significant projects?

General HAYES. We will be glad to, sir.

Mr. ROGERS. I think that would be helpful.

(Supplemental report follows:)

DOD ENVIRONMENTAL STATEMENTS

FINAL

1. Operation Chase
2. Luke Training Mission Change
3. Operation Red Hat

DRAFT TO CEQ

1. Demilitarization of Biological Weapons
2. Underwater Demolition of Ordnance near the Island of Culebra
3. Project Eagle
4. SAFEGUARD

DRAFT—INTERNAL DOD PROCESSING

1. Western Med. Institute of Research Phase II
2. Airfield Complex—Fort Campbell
3. Relocation of Harry Diamond Laboratory
4. New Walter Reed Hospital
5. Land Acquisition, Lemoore, Calif.
6. Mark 48 Torpedo Shop, Keyport, Washington
7. Land Acquisition Naval Sub Base-New London, Connecticut
8. Land Acquisition, Norfolk, Va.
9. DoD Building, Bowling AFB
10. Exercises using Target—Ship Hulls
11. Land Acquisition, Homestead, Fla.
12. SANGUINE
13. Culebra
14. B-1
15. F-15
16. Laser Program
17. AF Decontamination Study
18. Projectile Test
19. Fate on Soil and Vegetation

Mr. ROGERS. Now I notice you don't think it is a very good idea to put any deadlines. Am I correct in my understanding of your statements?

General HAYES. Where is that?

Mr. ROGERS. I may not have observed it.

General HAYES. Is it page 2, the bottom paragraph?

Mr. ROGERS. Yes, I believe that is right.

BEST COPY AVAILABLE

General HAYES. The statement is realistic by establishing a regulatory authority rather than by prohibiting all ocean disposal.

Mr. ROGERS. That——

General HAYES. Or establishing unreasonable deadlines for such determination.

Mr. ROGERS. Yes.

General HAYES. Well, the key word is "reasonable," Congressman Rogers.

Mr. ROGERS. Yes.

General HAYES. Because technologically we can't meet some things at certain deadlines too close in the future.

Mr. ROGERS. Yes. Would you let the committee have your thinking on deadlines? Now we have incorporated one bill, deadlines for sewage and industrial wastes.

General HAYES. Right.

Mr. ROGERS. Saying they should have primary, secondary, and tertiary treatment by certain dates. I believe that is the only proposal on dates in the legislation.

General HAYES. That is my understanding.

Mr. ROGERS. And I presume you would have no objection to that.

General HAYES. Well, again, I think a certain latitude, not an unreasonable latitude, but a certain latitude of industry and the technological capability to accomplish the end point, on the concept of priorities to each of these, and the effort put forward, we have no argument with that.

Mr. ROGERS. Well, would you let us have your thinking on these dates, for the record?

General HAYES. We will be glad to, sir.

(The information follows:)

Several of the bills on Marine protection would establish a rigid timetable for the installation of primary, secondary and tertiary waste treatment. The DoD has identified all requirements to meet existing water quality standards as we know them and intends to be in full compliance with Executive Order 11507, "Prevention, Control and Abatement of Air and Water Pollution at Federal Facilities," by 31 December 1972. To impose a deadline where neither standards have been developed nor technological know how exists does not seem prudent. In any case the degree of treatment provided should be based upon that required for the protection and enhancement of the receiving body of water.

Mr. ROGERS. Now also, you say absolute prohibition of all ocean disposal could result in techniques which pose greater hazards, and also you thought some dumping might even help the ocean. Is that my understanding?

General HAYES. This is thought of some people in the technological area. There are things which might be either indifferent or helpful. Some of them are rather technical in this aspect. If you would like that explored a little further, I can do it for the record.

Mr. ROGERS. I think you should.

General HAYES. Fine.

(The information follows:)

Existing information on the environmental effects of a variety of materials which find their way to the ocean are at present inconclusive. Current and future research may conclusively prove that ocean dumping of some materials does not damage the marine environment. The placement of discarded auto-

BEST COPY AVAILABLE

mobiles in certain waters has seemed to create artificial reefs and provided shelter and protection for spawning and young marine life. Some forms of construction of demolition debris composed of dense and inert materials may be similarly used without threat to the ocean.

Mr. ROGERS. As I recall, the only prohibition goes to about three items, don't they? I think they would prohibit radioactive wastes, toxic industrial wastes, and chemical and biological warfare materials. Have you seen in any provisions of the bill any other absolute prohibitions?

General HAYES. Well, that is the point. We feel that there aren't really absolute prohibition provisions in H.R. 4723, and that does allow some latitude for accommodation to what further knowledge may indicate to us.

Mr. ROGERS. Well, I thought the Secretary had already ruled out most of these, hasn't he?

General HAYES. We have done that. That is correct.

Mr. ROGERS. But you said an absolute prohibition.

General HAYES. For the time being.

Mr. ROGERS. You mean the Secretary is now going to change his mind?

General HAYES. It may be found that technologically, and this is a supposition, that some of the old ammunition, and I am thinking primarily of conventional explosive ammunition, that we may not be able to demilitarize it safely on land. We may have to, after due consideration and effort, request an ocean dump. But, we don't know at the present time. Now our goal is not to dump.

Mr. ROGERS. Well, it seems to me the Secretary has already made a determination that this is not a wise procedure, and I think it might be well to strengthen his hand in termination in the law. Then if you need a specific exemption, you could come to ask for a specific act, you know, if it got to that point.

General HAYES. I can assure you Mr. Rogers, that the Secretary and his advisers do not intend to act in any capricious manner.

Mr. ROGERS. Well, it is difficult sometimes even to get all the commands to carry out some of the directives. For instance, the Air Force, we just have an example where they have been dumping, as you are probably aware, toxic materials into the California water.

General HAYES. Well, we have had a preliminary investigation into that already, and from our current information, it appears that the Air Force is complying with the applicable laws and regulations. So I would like to—again for the record—give you a detailed report on that.

Mr. ROGERS. Well, we have checked pretty thoroughly, and we don't agree with your preliminary finding, because the Air Force says they are paying local agencies to do the detoxification, and the local agencies say nobody can dump in there until they do the detoxification. But they are not doing it.

So somebody is not pretty well squared away.

General HAYES. As I said, our preliminary indication is such, but we will have a report for you for the record.

[illegible]

(The information follows:)

AIR FORCE DISPOSAL OF PHOTOGRAPHIC WASTES

The Department of the Air Force began operation of the Aerospace Audio Visual Service at Norton Air Force Base, California, in May 1969. The processing of film at the facility results in liquid wastes which contain a number of chemical materials.

The Air Force determined that, for environmental and economic reasons, it would be better to contract with a responsible party for the disposal of the photographic wastes rather than to alter existing industrial waste processing facilities at the Air Force Base to accommodate these wastes. Prior to beginning operations, the Air Force entered into a contract with the Chino Basin Municipal Water District for the disposal of photographic wastes. The contract cites the significant components of the wastes as well as certain maximum concentrations and the pH at the time of delivery to the contractor. It provides that the contractor shall furnish ". . . an acceptance and disposal service as required by the Government, and shall receive, carry, treat, and dispose of all chemical waste originating at the reception locations in such amounts as the Government desires to release into contractor's system, and in a manner and by such means as will constitute no hazard to the public health. Contractor shall operate its disposal and treatment facilities in conformity with applicable laws, rules, and regulations promulgated by the State and Federal Government authorities." The contract specifically states that "Contractor acceptance and disposal service shall include all necessary treatment."

Since May 1969 the Air Force has disposed of approximately 1.86 million gallons of photographic wastes by transporting them to the Chino Basin Collection Point in Fontana, California. The Chino Basin Municipal Water District pipes the wastes from the collection point to the Los Angeles Sewage Treatment Plant located in Carson City, California, where the wastes are treated and the resulting effluent piped from that plant to a point in the ocean two and one-half miles from shore. The outflow at that point is diffused two hundred feet below the surface.

The wastes delivered by the Air Force to the Chino Basin Collection Point have met the standards specied in the contract, and this fact was confirmed in writing by the General Manager of the Chino Basin Municipal Water District on April 8, 1971. All operations from the time of delivery of the wastes by the Air Force to the final disposition in the ocean are monitored by the California State Water Resources Control Board.

In an effort to reduce operational costs, the Air Force changed its processing procedures in September 1970 so that a large portion of the photographic wastes are recycled through an oxidation process, thereby permitting reuse of some of the liquids prior to disposal. As a result of these changes the concentration of certain chemicals in the resulting wastes has been greatly reduced.

Mr. ROGERS. Now while you are getting us that report, could you also get us a report on the Navy dumping, or maybe Admiral Crawford could, in Jacksonville. I have still not seen a report after our hearings on that, of what happened there and who actually gave the order of dumping.

Admiral CRAWFORD. Mr. Rogers, before I answer that question, I would like to clarify a point.

Mr. ROGERS. Surely.

Admiral CRAWFORD. You said that the Secretary prohibited dumping. Were you speaking of the deepwater dumps of conventional munitions?

Mr. ROGERS. Well, I thought I read in the statement——

Admiral CRAWFORD. He suspended them, while this study goes on seeking alternative methods. There is no positive prohibition.

Mr. ROGERS. Oh, all chemical munition disposal at sea has been terminated?

Admiral CRAWFORD. Yes, sir.

Mr. ROGERS. That's what General Hayes said on biological warfare agents or munitions have never been disposed.

Admiral CRAWFORD. But not conventional munitions, is my point. Those are the ones we are investigating alternatives at this time.

Mr. ROGERS. Yes. I understand that category, but there is an absolute prohibition against chemical munitions disposal at sea. Isn't it?

Admiral CRAWFORD. Yes, sir. I just wanted to make sure that you weren't including conventional munitions in that.

Mr. ROGERS. No. I understood this has been held up, but you know final determination has been made, whether you would come back to ask that that be done. I presume that there would be an impact statement filed if you decide.

Admiral CRAWFORD. Yes, sir.

Mr. ROGERS. Yes. Well, maybe you could go now to the question of Jacksonville, where they dumped the acid into St. Johns. I understood, when General Hayes testified before, I think, that they were going to—the Navy was doing an investigation, and was going to report and going to let us know.

I have never seen that. Maybe you filed it with the committee, but I don't recall it.

Admiral CRAWFORD. I think I can satisfy you very shortly.

Mr. ROGERS. Yes, all right.

Admiral CRAWFORD. A member of your staff had a conversation with Rear Adm. Means Johnston, Chief of Legislative Affairs, on April 6, and your continued concern on this matter was made known to the Chief of Naval Operations, who has directed that this matter be further investigated. We will expect to hear from that investigation shortly.

Mr. ROGERS. Yes. And you will advise us as soon as you get that complete?

Admiral CRAWFORD. Yes, sir.

Mr. ROGERS. Thank you.

Now what about—you say biological warfare agents and munitions have never been disposed of at sea. Could you give for the record how this is done, General?

General HAYES. We can do that. It really will be both plans for some chemical demilitarization and biological disposal also, so it will be actual as well as proposed.

Mr. ROGERS. Well, if you would differentiate, which is actual and which is proposed?

General HAYES. Yes.

Mr. ROGERS. And the extent of it, if you could.

(The information follows:)

DISPOSING OF BIOLOGICAL WARFARE AGENTS

Biological warfare agents by virtue of their disease producing capability have always been treated with the most rigid and absolute controls known. Detoxification and sterilization to render any such agents nonpathogenic have always involved exposure of the organisms to extremely high temperatures, chemical sterilization or both with subsequent test and retest to absolutely determine that no live or virulent organisms remained. Following such an elaborate procedure the inert residue was either incinerated in pathological destruction facilities or treated in other waste disposal installations.

BEST COPY AVAILABLE

The Department of the Army's Draft Environmental Statement on Biological Demilitarization provides a detailed description of the elaborate program prepared for biological agents and munitions disposal. Currently this statement has been reviewed by other Federal Agencies and appropriate state organizations. The final E.I.S. is being filed with the CEQ in the immediate future.

Mr. ROGERS. Now, Admiral, let me ask you a question or two that I have on your statement. I believe you said that a good ocean dumping act should clearly exclude waste waters discharged from shore facilities through sewers into coastal waters, because the mechanism for controling these discharges are included in the Clean Waters Act.

Admiral CRAWFORD. Yes, sir.

Mr. ROGERS. I thought there was some question as to whether this bill goes into actual coastal waters. Isn't there some question as to whether that actually would apply? I am not sure they set standards, as far as all of the coastal waters.

Admiral CRAWFORD. This is Commander D'Emido.

Commander D'EMIDO. Mr. Chairman, section 10 of the Federal Water Pollution Control Act, as amended, provides for the States to develop their effluent standards. Once these standards are approved by EPA, these become the enforcement documents in each particular State. The mechanism for developing these standards for effluents eminating from industrial plants and from sewage treatment plants, provides adequate discharge controls to protect the receiving waters.

Mr. ROGERS. Suppose the State has not established.

Commander D'EMIDO. They must by law. They had to establish them by a certain date in consultation with the EPA, otherwise the Government would establish the standards for the States.

Mr. ROGERS. Essentially it might happen, if they get to it. Well, would you have any objection to deadlines being set as we stated before, Admiral, on industrial and sewage wastes, having primary and secondary treatment by certain dates? What would be——

Admiral CRAWFORD. I would refer back to General Hayes' comment. It would depend upon what those dates were, because anything that we are required to do in changing sewage treatment plant facilities requires money. This comes out of our appropriation and it is a constrained appropriation. Based on current standards, we hope within a few years to have every deficiency corrected ashore, but we can't do it all at once.

So I would say that if it is reasonable, yes, because we are laying out a program to complete all these things as far as we can.

Mr. ROGERS. Would you let us have your thinking on the time limits that would seem to you to be reasonable?

Admiral CRAWFORD. Yes, sir.

(Supplemental statement follows:)

DEFENSE DEPARTMENT SEWAGE TREATMENT TIMETABLE

H.R. 4359 referred to by Congressman P. Rogers would establish a rigid timetable for the installation of primary, secondary, and tertiary treatment. To meet these standards, especially tertiary treatment, under the present DOD Planning, Programming and Budgeting System, would be impractical to achieve by 1976. Also tertiary treatment, which is a very expensive process, should be provided only if it is determined, based on site surveys and analysis, that this level of treatment is required and essential to protect the marine life and marine habitat. In most cases the quality of the effluent from secondary treatment facilities is adequate to prevent degradation of the ocean, coastal, and other waters.

BEST COPY AVAILABLE

Mr. Rogers. Now I want to commend the Navy on some of the actions you have taken. For instance, I think you put out some clarifying orders on setting up personnel to be responsible in every command for impact statements and looking at this. Also I think you clarified some of the orders to your fleets on their dumping policies, which I think is an improvement.

I wonder if you have given any thoughts, and I notice you comment some on that problem. I am not sure. I think you say we should not do—make certain requirements. I am not sure that I can find that right off.

Can you recall where you say we should not require certain actions to be taken by the ships?

Admiral Crawford. I think this is in my next to last paragraph, Mr. Rogers.

Mr. Rogers. All right.

Should exclude the day-to-day operation and discharge from ships, such as sewerage or oily bilge, which is subject to regulation by other laws.

Now what other laws are those that you are referring to?

Admiral Crawford. The Oil Pollution Act of 1961 takes care of regulations within the 50-mile limit, the prohibited zone. The Water Quality Improvement Act of 1970 takes care of standards within the contiguous zone.

Mr. Rogers. Well, is there any reason that you see why a ship that is coming into port shouldn't maintain all of its wastes, if it is, say, within a day, unless there is some emergency, 2 days, or 3 days?

Admiral Crawford. These are their instructions, sir; namely, to make maximum use of shore facilities. But to put a blanket restriction on our ships, I don't think, is very practicable.

Mr. Rogers. Well, I think you have to have some operational judgment.

Admiral Crawford. Yes, sir.

Mr. Rogers. But I read over the regulations, and I did not get the thrust that you have stated that the ships, if say they are within 2 or 3 days of port, should not. I thought you equated it to the 50 miles out.

Admiral Crawford. Well, we have said that maximum use will be made of shore facilities, where they exist.

Mr. Rogers. Well, if they are in port, I understand.

Admiral Crawford. Well, this is also ships returning to port.

Mr. Rogers. Now also you say the Oil Pollution Act would apply, but I just am trying to think, the public vessels really are not subject to that; are they?

Admiral Crawford. They are not subject to the law, but the Navy has stated that we will abide by it. This has been a matter of record for many years. We have never claimed exemption under this law as a matter of policy.

Mr. Rogers. Now did you have a clarifying statement?

Admiral Crawford. I would like to read you a paragraph. A paragraph from an OPNAV instruction which I think we furnished you, Mr. Rogers.

Mr. Rogers. Yes.

Admiral Crawford. OPNAV Instruction 6240.3 of March 2, 1971.

Mr. Rogers. Yes, you may have furnished that. I didn't recall it.

BEST COPY AVAILABLE
[illegible]

Admiral CRAWFORD. It says:

In the routine operation of Naval vessels maximum use is to be made of all available port disposal facilities for all wastes prior to getting underway, and upon returning.

And then it goes on to put the prohibitions for the 50-mile and the 12-mile limits.

Mr. ROGERS. Well, I understand that, but I don't think it is still quite clear. If I understand, you are saying that you make it if you are returning to port. But I don't think it is quite clear, the point I was trying to make, that if they are coming into port, say, within 2 days or a reasonable time, they shouldn't be dumping out there, even though it is 50 miles out.

If they are going to come into port, where there is no operational problem, when they could not dump outside the 50 miles, and just deposit that in the facility at port. That's the point I was making.

Admiral CRAWFORD. Yes, sir.

Mr. ROGERS. And I wonder if it is possible to perhaps clarify that. I think possibly that is what you are getting at, but I don't think it is quite clear. Would you be willing to consider looking at that?

Admiral CRAWFORD. Modifying our instructions?

Mr. ROGERS. Yes.

Admiral CRAWFORD. Yes, sir, we will.

Mr. ROGERS. Now let me conclude. General Hayes, have you issued any instructions for the Department of Defense in all of the services to check on filing statements, to have that responsibility? I think we went into this before, and you were going to consider it, and I wondered if you had ever taken any action.

General HAYES. We didn't have to take the actions because the services were so responsive.

Mr. ROGERS. I have only seen one response, and that was the Navy.

General HAYES. No, the Air Force and the Army both.

Mr. ROGERS. Would you submit those?

General HAYES. We will, sir.

(The documents follow:)

DEPARTMENT OF THE AIR FORCE,
HEADQUARTERS U.S. AIR FORCE,
Washington, D.C., May 6, 1970.

Reply to attention of: AFOCE
Subject: Pollution Abatement (Our letter, 7 April 1970)
To: ACIC, ADC, AFCS, AFLC, AFRES, AFSC, ATC, AU, AAC, MAC, OAR, SAC, TAC, USAFA, USAFSS, HQ COMD USAF, CINCPACAF (Commander).

1. The referenced letter furnished copies of the recent Executive Orders, legislation and a summary of Air Force actions in environmental enhancement.

2. In order to carry out the intent of the President's Executive Orders we must view environmental pollution on a much broader scale than in the past. Previously, the Civil Engineer and the Surgeon have been tasked with assuring that our bases had potable water, adequate sewage treatment and solid waste disposal and, along with our maintenance personnel, tried to control aircraft noise to within safe levels. We have been successful within this limited sphere but now must address the broader aspects of environmental enhancement and this involves virtually all functional elements of the Air Force. The Environmental Policy Act of 1969 (copy provided with referenced correspondence) requires that we examine the environmental impact of all our actions.

3. In order to carry out the expanded Air Force responsibilities and to insure increased emphasis in support of announced national objectives, the Directorate of Civil Engineering has been designated the OPR at Headquarters USAF for

environmental pollution matters regardless of the source of funds or program authority. The Deputy Director of Civil Engineering is personally responsible for coordinating all staff actions bearing on the subject. In addition, a HQ USAF Environmental Pollution Control Committee has been established, chaired by Civil Engineering, with members from all staff offices having collateral interests and responsibilities. Attached for your information is a listing of personnel and offices. Representatives from Civil Engineering, Maintenance Engineering and the Surgeon General's office constitute the nucleus of the HQ, USAF committee.

4. In order to effectively carry out our mutual responsibilities and provide for rapid communications and coordination at all levels, it is requested that an OPR and working committee, along the lines just described, be established at each major command headquarters having jurisdiction over installations in the U.S., Guam and Puerto Rico. We further suggest that similar organizational arrangements be made at each principal installation.

5. Request you advise HQ USAF (AFOCE) of the name of your designated command pollution abatement coordinator on or before 25 May 1970. A HQ USAF Conference on pollution abatement and environmental enhancement will be scheduled for early summer and invitations tendered to each command designee.

6. Commands having jurisdiction over bases in foreign countries will be receiving separate instructions relative to pollution abatement responsibilities.

For the Chief of Staff:

GUY H. GODDARD,
Major General, HQ Air Force,
Director of Civil Engineering.

Pollution abatement HQ USAF contacts

Office Symbol and Name:	*Telephone extension*
AFAAC, Mr. Stan Woodard	72632
AFIGO, Col. Augie T. Ong	70066
AFJAG, Maj. D. C. Reid	35648
AFMSG, Lt. Col. Herbert Bell	35775
AFOCE, Brig. Gen. M. R. Reilly	77366
Lt. Col. S. A. Jacobs	79257
AFPDC, Lt. Col. C. E. Waggoner	79977
AFRDC, Mr. C. Kurrle	74958
AFSDC, Col. John Hileman	56233
AFTOR, Capt. H. L. Stricklen	55057
AFXDC, Col. R. A. Jameson	77124/52735
SAFOI, Capt. J. Malloy	71128

DEPARTMENT OF THE AIR FORCE
Headquarters US Air Force
Washington DC 20330

AF REGULATION 161-22

23 September 1970

Aerospace Medicine

ENVIRONMENTAL POLLUTION CONTROL

This regulation establishes policies, assigns responsibilities, and provides criteria and standards for an environmental pollution abatement program. It requires HQ USAF Air Staff offices and MAJCOMs to establish and implement procedures to insure that their activities are conducted in a way to minimize environmental pollution. It implements DOD Directives 5100.50, June 23, 1970, and 5030.41, May 23, 1969. It applies to all Air Force installations and facilities, including Air Force Reserve and contractor activities located on real property under Air Force jurisdiction.

	Paragraph
Environmental Pollution Explained	1
Air Force Policy	2
HQ USAF Responsibilities	3
Major Command Responsibilities	4
Criteria and Standards	5
Submitting Reports and Comments	6

Attachment	Page
1. References	7

1. Environmental Pollution Explained. As used in this regulation, environmental pollution is the presence of physical, chemical, and biological elements or agents that adversely affect human health or welfare, unfavorably alter ecological balances of importance to human life, affect species of animal or plant life that are of importance to man, or degrade the utility of the environment for aesthetic and recreation purposes. Control of environmental pollution requires consideration of air, water, and land, and must extend to noise, improper solid waste management, and electromagnetic energy, as well as things conventionally thought of as pollutants.

2. Air Force Policy

a. **Air Force commanders will:**

(1) Eliminate or control environmental pollutants generated by or resulting from Air Force operations or from contractor operations on real property owned or leased by the Air Force.

(2) Lead in preventing, controlling, and abating environmental pollution by accelerating corrective measures at Air Force installations and by actively seeking the cooperation of local communities in developing area-pollution abatement programs.

(3) Provide for environmental pollution control measures in designs for new buildings, facilities, weapon systems, operations, tests, exercises, procedures, and projects for rehabilitation or modification of structures.

(4) Comply not only with Air Force directives relating to pollution criteria and standards but also with related directives published by the Department of Health, Education, and Welfare (HEW); the Federal Water Quality Administration (FWQA), Department of the Interior; and state and local pollution abatement agencies in the area. HEW or FWQA standards and criteria apply when state or local agencies prescribe less stringent one or none at all. Refer to the Surgeon General, HQ USAF/SGP for resolution of the problem with the appropriate agency, if:

(a) A conflict arises on the applicability of any criteria or standard, or

(b) Applying a prescribed criteria or

Supersedes AFR 161-22, 21 April 1966. (For summary of revised, deleted, or added material, see signature page.)

OPR: SGPAAP

DISTRIBUTION: S

standard is considered not in the national interest.

(5) Provide preventive pollution control by:

(a) Reducing or eliminating waste at the point of generation.

(b) Considering potential environmental pollution control problems when selecting chemical compounds and materials to be used in Air Force operations.

(c) Including pollution abatement as an element in specifications.

(d) Urging manufacturers of products used by the Air Force to make their products from materials and compounds that have the least pollution potential.

(6) When resources to accomplish pollution control are limited, give priority in this order:

(a) Situations that constitute a hazard to the health or safety of man.

(b) Situations that are cost effective.

(c) Situations that affect the recreational and esthetic value of our natural resources.

(7) Insure, to the extent practicable, that oversea installations conform to the same pollution abatement policies that are prescribed for installations located in the United States, particularly policies pertaining to cooperating with community programs.

(8) Fully coordinate environmental pollution matters with all agencies concerned to avoid duplication and insure timely solutions to mutual problems.

b. **HQ USAF Environmental Pollution Control Committee.** An environmental pollution control committee will obtain coordination of environmental pollution matters at HQ USAF.

(1) The committee will include, as a minimum, representatives of the following Air Staff offices:

(a) The Director of Civil Engineering (Office of the Deputy Chief of Staff, Programs and Resources).

(b) The Deputy Chief of Staff, Systems and Logistics.

(c) The Surgeon General.

(d) The Deputy Chief of Staff, Research and Development.

(2) Other staff agencies that have strong collateral interest and responsibility (such as the Comptroller; the Inspector General; the Assistant for Weather; the Deputy Chief of Staff, Personnel; the Deputy Chief of Staff, Plans and Operations; the National Guard Bureau; and the Secretary of the Air Force Office of Information) will support the committee.

(3) The Director of Civil Engineering, or his designee, will chair the committee, which will meet at the chairman's call.

3. HQ USAF Responsibilities:

a. All Air Staff offices will develop procedures to insure that their activities are conducted in a manner consistent with the spirit and intent of this regulation and of Executive Orders 11507, 5 February 1970, and, 11514, 7 March 1970.

b. **The Director of Civil Engineering (PRE):**

(1) Is the Air Staff office of primary responsibility (OPR) for pollution abatement, regardless of the source of funds or program authority.

(a) All Air Staff actions on pollution abatement that may alter existing policy or reflect on the stated policies of the Air Force must be coordinated through PRE.

(b) When PRE requests it, an organizational coordinator will be designated in an Air Staff activity that has a strong collateral interest and responsibility. That organizational coordinator must be fully cognizant of all environmental pollution control actions within the designating staff agency.

(2) Establishes engineering design criteria and standards to meet health and welfare requirements established by the Surgeon General for facilities provided under the Military Construction Program and consistent with those of civil authority.

(3) Develops plans and programs to meet the intent of the various statutes on air and water pollution control.

(4) Supervises utility and waste disposal practices and operations to insure they meet required standards.

(5) Includes in the Operation and Maintenance (O&M) and the Military Construction (MC) Programs estimates for funding waste disposal and abatement measures.

(6) Establishes requirements for development efforts involving environmental pollution control.

c. **The Surgeon General, USAF,** is the office of primary responsibility for establishing standards and criteria to protect the health and welfare of US Air Force personnel and for coordinating with other Federal agencies and health authorities on contami-

nation matters involving environmental pollutants. (See AFM 161-2 and AFR 161-18.)

d. **The Director of Maintenance Engineering** establishes policy and criteria for use of sound suppressors during maintenance testing of jet engines, and develops plans and programs to insure that adequate sound suppression equipment is available where needed.

e. **The Director of Procurement Policy:**

(1) Develops policies and procedures for:

(a) Contract administration of contractual provisions for environmental quality.

(b) Preaward surveys that include determining whether the contractor supports good environmental quality practices.

(2) Monitors development of new or the revision of existing military specifications and standards to insure that they are not in conflict with environmental pollution controls.

(3) Assures Armed Service Procurement Regulation clause coverage that is adequate to insure compliance with applicable public laws.

f. **The Director of the Industrial Resources Division (RDPI),** consistent with the provisions of SAFSO 715.1, 15 August 1969, when contractors are performing on real property owned or leased by the Air Force:

(1) Initiates new funding required to insure continued contractor compliance with pollution abatement standards.

(2) Coordinates pollution abatement actions with PRE, SGP, and the Federal Water Pollution Control Administration

g. **The Deputy Chief Of Staff For Research and Development** plans, programs, and budgets for environmental pollution control research and development which is of singular pertinence to the Air Force Mission or which comprises a DOD responsibility and can be conducted most expeditiously by the Air Force.

4. Major Command Responsibilities:

a. **Each major command** will:

(1) Appoint an organizational coordinator for pollution control matters to assure full coordination and avoid duplication. The organizational coordinator must be fully cognizant of all command environmental pollution control actions. Notify HQ USAF/PRE Wash DC 20330 when the appointment is made.

(2) In budget estimates and funded programs, provide for environmental pollution control consistent with this regulation. The cost of environmental pollution control must be accomplished within funding available to the Air Force.

(3) Identify and control or eliminate existing environmental pollution sources at all installations; maintain records of monitoring results; assure that in all new plans, programs, and specifications any potential environmental pollution problems have been recognized and their abatement or control provided for; and identify requirements for research and exploratory and advanced development for environmental pollution control consistant with mission requirements.

(4) Coordinate each new water pollution control project with the regional administrator of the Federal Water Quality Administration during its early planning stages. The Air Force installation develops the initial concepts of the project. It then obtains the advice of FWQA regional administrator about applicable state and local criteria and requirements and the various alternative corrective measures that are available. If any differences of opinion arise that cannot be resolved at regional level, advise HQ USAF.

(5) Assure that:

(a) Officials who conduct operational and training programs that result in emission of pollutants into the atmosphere (such as use of open fires for fire-fighting training) exercise proper constraint, consistent with meteorological conditions and operational training requirements.

(b) The Office of Information is given appropriate help in preparing background information on local environmental pollution in response to queries from the public or news media.

b. **HQ AFLC, HQ USAFE, HQ AFSC, and HQ MAC** fulfill the following additional responsibilities:

(1) *HQ AFLC:*

(a) Develops procedures to minimize the amount of air and water pollution generated by normal base industrial-type operations (corrosion control, engine repair, paint removal, etc.) and assures that they are included in appropriate Air Force technical orders.

(b) Programs and procures pollution control equipment to support Air Force weapon systems, aerospace ground equip-

ment, motor vehicles, and other industrial equipment on Air Force bases that is not covered by military construction.

(2) *HQ AFLC and HQ USAFE—USAF Radiological and Environmental Health Laboratories and USAFE Environmental Health Laboratory (AFR 161-17).* These laboratories:

(a) Give Air Force installations and organizations advice and guidance on sampling equipment, instruments, methods, and frequency of sample collection appropriate to establishing an installation environmental pollution abatement and monitoring program.

(b) Recommend methods of sanitary and industrial waste treatment and air pollution control.

(c) Test and evaluate instruments and equipment used, or proposed for use, in USAF pollution programs.

(d) Maintain a repository of analytical results and of information on methods and techniques for measuring, evaluating, and controlling environmental pollutants of concern to the Air Force.

(e) Send HQ USAF/SGP, Wash DC 20330 information copies of reports, studies, and other information relating to environmental pollution abatement and control that are developed by the laboratories.

(f) When requested by a major command, conduct periodic environmental pollution surveys at military installations to determine the need for, effectiveness of, and degree and type of treatment or control required to comply with this regulation and related statutory requirements.

(g) Respond to requests for support from the Departments of the Army or Navy, or from other DOD components or Federal agencies.

(h) Collaborate with the Air Force Weapons Laboratory (AFWL) and, when directed by HQ USAF or when otherwise appropriate, evaluate jointly with the AFWL any unique or unusual environmental pollution problems.

(3) *HQ MAC—Air Weather Service (AWS):*

(a) Provides guidance and helps Air Force facilities and organizations obtain and interpret meteorological data on the diffusion of gaseous and particulate pollutants in the atmosphere. AWS detachment commanders and staff meteorologists will provide staff weather services to commanders confronted with pollution problems.

(b) As required, provides specialized fixed and mobile meteorological observations to support hazardous operations and transportation of hazardous materials and to help bioenvironmental and medical personnel in problem areas.

(c) As directed by HQ USAF (XOOTR), provides aerial sampling or other aerial reconnaissance data for air pollution control agencies.

(d) Develops and maintains procedures to interpret and tailor for USAF installations the available air pollution potential forecasts of the National Meteorological Center. Issues forecasts and warnings as required to support Air Force global operations.

(e) Provides, or arranges for, predictions of atmospheric pollutant diffusion under various meterological conditions to help USAF environmental pollution abatement and control personnel determine the requirement for, establish, and enforce pollution abatement and control programs.

(f) Through staff meteorologists, helps AFSC insure that air pollution control is considered as an integral part of all RDT&E activities.

(g) Participates in studies and research projects concerned with the diffusion and transport or propagation of pollutants in the atmosphere.

(h) Develops and maintains a capability to provide the meteorological support necessary to interpret the environmental effects of noise and to help monitor and assess hazardous aircraft-operating noise levels for compliance with FAA regulations and local laws.

(4) HQ AFSC:

(a) Assures that environmental pollution control:

1. Is an element in RDT&E projects and programs and in specifications for materials that support them (see AFR 161-18).

2. Is an integral part of all weapon system development.

(b) Monitors the following procedure for industrial facilities that support production and RDT&E programs:

1. The contractor or his A&E secures the necessary approvals of facilities procurement contracting officer.

2. The contracting officer obtains civil engineering technical review of plans and specifications and, at the earliest stage of planning, asks the Federal Water Quality Administration's advice on state and local criteria for pollution abatement.

(c) Through the Air Force Weapons Laboratory, conducts research and develops exploratory and advanced environmental pollution controls. The laboratory develops methods and techniques for detecting, controlling, and abating environmental pollution and continuously exchanges information with HQ AFLC. Joint evaluation and collaboration with the USAF may be necessary to solve, or determine approaches to, particularly difficult problems.

(d) Conducts environmental pollution toxicology studies to help develop realistic standards and criteria for chemical compounds of special interest or peculiar to the Air Force.

(e) Prepares, for the Surgeon General, HQ USAF, manuals, regulations, pamphlets, and other documents on environmental pollution abatement and control in Air Force systems operation and systems support functions.

5. Criteria and Standards. Normally, Air Force activities must comply with the quality standards and related implementing plans, including emission standards, adopted and approved by Federal regulatory agencies. Issued criteria and standards apply to all facilities (buildings, installations, structures, public works, equipment, aircraft, vessels, and other vehicles and property) owned by, or constructed or manufactured for lease to, the Federal government.

a. If no air or water quality standards are in force for a particular geographic area or if more stringent requirements are considered advisable for Federal facilities, use Federal standards issued by regulatory authority under section 4b, Executive Order 11507, 5 Feb 70.

b. From time to time facilities may be identified that should, in the interest of national security or the nation, be exempted from complying with approved environmental control standards. To exempt the facility, the installation must apply for a waiver through command channels to HQ USAF/SGP, Wash DC 20330. Send an information copy of the request to HQ USAF/PRE or HQ USAF/RD, as appropriate. The application must explain why the waiver is requested, justify the waiver, and include the comments or recommendations of the State or local pollution control agency or the regional office of the Department of Interior.

c. Standards in the Code of Federal Regulations, title 42, chapter I, subchapter F, part 76, "Prevention, Control, and Abatement of Air Pollution from Federal Government Activities, Performance Standards, and Techniques of Measurement" (published in the Federal Register, volume 13, No. 107, June 3, 1966) apply to Air Force installations and facilities.

d. Do not discharge into waters or the atmosphere any waste that contains any substance in concentrations that are hazardous to the health of people; that can result in substantial harm to domestic animals, fish, shellfish, or wild life; or that may cause economic loss through damage to plants or crops.

e. Do install facilities or establish procedures to prevent cooling water from increasing stream temperatures above acceptable limits.

f. Store and handle gasoline, jet fuels, and other volatile petroleum distillates or organic liquids in a way to minimize vapor emission. For storage, use pressure tanks or reservoirs or containers equipped with floating roofs or vapor recovery, vapor emission control devices or systems to control loss of vapor to the atmosphere.

g. Avoid or minimize the creation of wastes throughout the complete cycle of operations of each facility.

h. Preferably, use municipal or regional waste collection or disposal systems to dispose of wastes from Air Force facilities. When use of such a system is not feasible or appropriate, do whatever is necessary to satisfactorily dispose of such wastes, including the following:

(1) When appropriate, install and operate waste treatment and disposal facilities.

(2) Provide trained manpower, laboratories, and other supporting facilities, as appropriate, to meet the requirements of issued standards.

(3) Require operators of Air Force pollution control facilities to meet levels of proficiency consistent with the operator certification requirements of the State in which the facility is located. If the State has no requirements, use the guidelines on operator qualifications and performance issued by the Secretary of HEW or Interior.

i. Insure that all materials (including solid fuels, ashes, petroleum products, and other chemical and biological agents) are used, stored, and handled to avoid or minimize the possibilities of water and air pollution.

j. Provide the engineering safeguards (such as dikes, catchment areas, relief ves-

sels) that are necessary to prevent pollution of water by acc'dental discharge of stored fuels, solvents, oils, and other chemicals.

k. Do not dispose of or discharge waste in a way that could cause ground water pollution that would endanger the health or welfare of the public.

l. Assure that discharges of radioactivity conform with the applicable rules, regulations, and requirements of the Atomic Energy Commission and with the policies and guidance of the Federal Radiation Council, as published in the Federal Register.

NOTE: Attachment 1 lists Air Force documents that give guidance on various aspects of pollution abatement and control programs. If questions about criteria and standards arise, direct them to HQ USAF/SGPAA.

6. Submitting Reports and Comments. HQ USAF requires copies of reports and comments by representatives of State or local pollution abatement agencies, HEW, or Department of Interior about pollution caused by Federal installations. Send documents by letter of transmittal and indorsement to HQ USAF/PRE. Include comments on proposed actions. Send information copies of reports and comments to HQ USAF/SGPAA.

BY ORDER OF THE SECRETARY OF THE AIR FORCE

OFFICIAL

JO'IN D. RYAN, *General, USA?*
Chief of Staff

JOHN F. RASH, *Colonel, USAF*
Director of Administration

Summary of Revised, Deleted, or Added Material

This revision expands explanation of "environmental pollution (para 1); requires control of environmental pollutants generated by contractor operations on real property owned or leased by the Air Force (para 2a(1)); establishes an Air Force Environmental Pollution Control Committee, chaired by the PRE or his designee (para 2a(8)); requires all Air Staff agencies to insure that their activities are conducted in a manner consistent with this regulation and Ex Os 11597 and 11514 (para 3a); designates HQ USAF/PRE the Air Staff OPR for pollution abatement and authorizes a single point of contact in each Air Staff activity (para 3b(1)). Adds to HQ USAF responsibilities—PRE to establish requirements for funds and direct necessary mission-related research and development to establish new and improve existing environmental pollution control methods (para 3b (2)-(6)); SME to be responsible for jet engine sound suppressors (para 3d); Dir of Industrial resources to insure that contractors on real property owned or leased by AF comply with pollution abatement standards, and initiate funding to insure continued compliance (para 3f). It requires MAJCOMs to appoint a single point of contact for environmental pollution matters (para 4a(1)), exercise caution to minimize pollution when conducting firefighting training (para 4a(5)(a)); help the Office of Information respond to public or news media queries (para 4a(5)(b)), directs HQ AFLC to program and procure control equipment to support weapon systems, AGE, motor vehicles, and other industrial equipment not covered by military construction (para 4b(1)), USAF Environmental and Radiological Health Laboratories to collaborate with Air Force Weapons Laboratory (para 4b(2)(h)), and HQ AFSC to require industrial facility contractors and A&E to obtain the procurement contracting officer's approval and the PCO to obtain civil engineering technical review (para 4(b)(4)(b). Revises Air Weather Service responsibilities (para 4b(3)), and adds Air Force Weapons Laboratory responsibilities (para 4b(3)(c)). It defines criteria and standards for facilities (para 5); specifies procedures for areas without established air or water quality standards (para 5); adds requirements of ExO 11507 (para 5g, h, i, k, and l); deletes reporting requirement for environmental contamination problems and aligns reporting procedures with new Air Force policy (para 6); and updates references and spells out new requirements of recent public laws and executive orders (throughout regulation).

REFERENCES

(Each publication listed in this attachment contains references that apply to some aspect of an environmental pollution abatement and control program.)

1. Statutory and Executive Office Requirements. (Do not requisition these publications; they are not available through distribution channels.)

a. The Federal Water Pollution Control Act, Act of June 30, 1948 (62 Stat. 1155), as amended by the Act of October 2, 1965 (79 Stat. 903; 33 USC 466 et. seq.).

b. The Clean Air Act, Act of July 14, 1955, as amended (42 USC 1857 et. seq.).

c. Code of Federal Regulations, Title 42, Subchapter F, Pa[illegible] 76, as amended, 23 March 1967 and 10 July 1969.

d. Executive Order 10779, "Directing Federal Agencies to Cooperate with State and Local Authorities in Preventing Pollution of the Atmosphere," August 20, 1958.

e. Executive Order 11507, "Prevention, Control, and Abatement of Air and Water Pollution at Federal Facilities," February 5, 1970, Federal Register, Vol 35, No. 25.

f. Excutive Order 11514, "Protection and Enhancement of Environmental Quality," March 7, 1970, Federal Register, Vol 35, No. 46.

g. Message of the President to Congress, February 10, 1970 (H.R. Doc 91-225).

h. National Environmental Policy Act of 1969, Act of January 1, 1970 (83 Stat. 852).

2. DOD Directives. (Do not requisition these publications; they are not available through distribution channels.)

a. DOD Directive 6015.5, "Joint Utilization of Military Health and Medical Facilities and Services," December 5, 1955. (Implemented by AFR 160-62; AFMs 162-1 and 168-4)

b. DOD Directive 4000.19, "Basic Policies and Principles for Interservice Support," March 26, 1960. (Implemented by AFR 400-27)

c. DOD Directive 5410.18, "Community Relations," April 21, 1965. (Implemented by AFR 190-20)

d. DOD Directive 5500.5, "Natural Resources—Conservation and Management," May 24, 1965. (Implemented by AFRs 91-36 and the 126 series)

e. DOD Directive 5230.9, "Clearance of DOD Public Information," December 24, 1966. (Implemented by AFR 190-12)

f. DOD Directive 5030.41, "Implementation of National Multi-Agency Oil and Hazardous Materials Pollution Contingency Plan," May 23, 1969. (Implemented by AFRs 161-18; 190-27; 355-9, -11; and AFM 355-1)

g. DOD Directive 5100.50, "Environmental Pollution Control," June 23, 1970.

3. Air Force Regulations:

85-5	Operation and Maintenance of Installations Facilities
85-6	Real Property Maintenance, Repair and Construction
85-18	Trailer Parks for Privately-Owned and Government-Owned Trailers
86-4	Master Planning
88-2	Evaluation of New Materials and Methods
88-3	AF Contract Construction
88-9	Transfer and Acceptance of Facilities Constructed for the Air Force
88-15	Engineering Manuals and Guide Specifications for Military Construction
88-16	Surveys of Installations in CONUS, Alaska, and Hawaii
88-18	Air Force Regional Civil Engineers
89-2	AF Form 378, "USAF Construction Program"
89-11	Air Force Responsibilities for the Military Construction Program
91-2	Custodial Services
91-5	Utility Services
91-7	Heating
91-8	Refrigeration, Air Conditioning, Mechanical Ventilation Evaporative Cooling, Dehumidifying, Ice Manufacturing and Cold Storage
91-9	Sewage and Industrial Waste Works
91-10	Water Works
91-11	Refuse Collection and Disposal
91-12	Utilities Management and Conservation Program
91-13	Permanently Installed Storage and Dis-

pensing Facilities for Petroleum and Unconventional Fuels

91-21 Pest Control

91-22 Aerial Dispersal of Pesticides

126-1 Conservation and Management of Natural Resources

126-2 Management and Conservation Programs for Fish and Wildlife

126-3 Presentation of Awards for the Outstanding Management and Conservation of Natural Resources

126-4 Soil and Water Management

161-1[illegible] Environmental Health, Forensic Toxicology, and Radiological Health Professional Support Functions

161-18 Use of Potentially Toxic Agents and Hazardous Materials

4. Air Force Manuals:

85-1 Resources and Work Force Management

85-6 Land Management and Grounds Maintenance

85-7 Military Entomology Operational Handbook

85-10 Custodial Services

85-11 Refuse Collection and Disposal

85-12 Vol I—Operation and Maintenance of Central Heating Plants and Distribution Systems

85-12 Vol II—Operation and Maintenance of Space Heating Equipment and Systems, and Process Heat Utilization

85-13 Maintenance and Operation of Water Plants and Systems

85-14 Maintenance and Operation of Sewage and Industrial Waste Plants and Systems

85-15 Coal Handling

85-16 Maintenance of Permanently Installed Storage and Dispensing Systems for Petroleum and Unconventional Fuels

85-1[illegible] Maintenance and Operation of Electric Plants and Systems

85-18 Maintenance and Operation of Refrigeration, Air Conditioning, Evaporative Cooling, and Mechanical Ventilation Systems

85-19 Maintenance and Operation of Electric Power Generating Plants

85-20 Plumbing

85-25 Standard Guide Specifications for Family Housing Construction

85-26 Military Construction Programming

85-28 Policies, Procedures, and Criteria for the Management and Conservation of Utilities
Vol I: Policies, Procedures and Criteria
Vol II: Utility Measurement Systems

85-31 Industrial Waste Management

86-1 Programming Civil Engineer Resources

86-4 Standard Facility Requirements

86-6 Air Base Master Planning Manual

88-2 Definitive Designs of Air Force Structures

88-5 Soils, Site Preparation, Grading and Drainage

88-8 Mechanical Design

88-10 Water Supply

88-11 Sewerage, Refuse and Industrial Waste

88-12 Fuel Storage and Distribution

88-15 Standard Outline Specifications for Air Force Facilities

88-17 Planting (Chapters 3 and 4)

88-19 Arctic and Sub-Arctic Construction (Chapters 1, 5, and 7)

88-20 Chapter 1—Heating and Air Conditioning Underground Installations

88-29 Air Force Design Manual

88-40 Emergency Construction (Chapter 5, Water Supply; Chapter 19, Garbage and Rubbish Disposal; Chapter 26, Dust Control)

88-54 Air Force Civil Engineer Handbook

160-39 The Handling and Storage of Liquid Propellants

161-2 Conducting the Aerospace Medicine Program

5. Air Force Pamphlets:

AFP 161-19 Environmental Health Engineering Handbook—Air Pollution

AFP 161-20 Environmental Health Engineering Handbook—Water Pollution

DEPARTMENT OF THE ARMY
OFFICE OF THE ADJUTANT GENERAL
WASHINGTON, D.C. 20310

IN REPLY REFER TO
AGDA-A (M) (24 Mar 71) LOG-C-PDBB | 29 March 1971

SUBJECT: Environmental Protection and Preservation

SEE DISTRIBUTION

1. References:

a. Public Law 91-190, 1 January 1970

b. AR 11-21

c. TAGO Ltr AGDA(M) (10 Sept 1970) LOG-C-PDBB-8316B, dtd 11 Sept 1970, subject: Interim Guidelines on Environmental Statements.

2. Since the passage of the National Environmental Policy Act in January 1970, there have been a number of Executive Orders and a great variety of new laws all designed to curtail pollution and preserve the national environment. In addition to the efforts at the national level, almost every state and community has taken up the ecological banner, so that today control of pollution has become a national theme and to a certain extent a major political issue. To those in the Army who are a witness to all of this activity there can be little doubt that preserving the environment is an effort in which we must play a part.

3. In response to a Presidential edict directing all Federal Agencies to demonstrate leadership, both DA and subordinate commands and agencies have initiated measures to demonstrate a genuine concern toward protecting our environment. Actions taken to date include such measures as programing for new construction to abate air and water pollution emanating from Army installations, issuing guidelines on the preparation of Environmental Impact Statements for those actions and plans which could have adverse impact on the environment, supporting the use of low-lead gasoline in military vehicles, encouraging greater efforts toward expanding the conservation and fish and wildlife protection programs on our posts, and stimulating a greater awareness of the environmental considerations as part of normal DA staff activity.

4. For the most part there has been good response by subordinate commands and agencies to comply with specific pollution abatement requirements. In a number of instances most noteworthy initiative has

SUBJECT: Environmental Protection and Preservation

been displayed in the development of dynamic local programs in the absence of specific guidance or direction from DA. In one instance, the efforts at one post received laudatory Congressional recognition as a direct result of local popular support of the actions taken. Unfortunately, this type of recognition has been the exception rather than the rule.

5. At this time, when a great deal remains to be done to bolster the image of the Army, direct advantage can be taken of the public interest in the environment to aid this effort - particularly if there are accomplishments or activities that reflect that the Army is supporting the fight against pollution. Activities which should be capitalized upon through public information media are the construction, regardless of funding source, which has been accomplished to abate air and water pollution, local cooperative agreements involving regional sewage treatment facilities, local efforts to promote the purchase of beverages at Post Exchanges and commissaries in returnable bottles and the use of low phosphate detergent soaps, salvage of or recycling of waste materials (glass containers and waste papers), and local forestry, fish and wildlife conservation programs. Particularly significant and newsworthy activities of the types listed which should be publicized beyond the local area should be forwarded to DA (ATTN: CINFO).

6. The pervasive nature of and the public sympathy for environmental issues make it essential that staff responsibilities in this matter be clearly defined and assigned at each level of command. Consideration should even be given to the establishment of an Environmental Quality Control Committee to assist the commander in the formulation of local policies and the planning and coordination of programs which have environmental implications. A particular responsibility of such a committee should be the preparation, review and reduction of the all important impact statements which are now required for any program which could have a significant adverse impact on the environment. Very careful attention must be given to the timely preparation and submission of environmental impact statements for failure to do so could cause Congress to deny funds for a program until the requirements of the National Environmental Policy Act are fully observed.

7. In addition to the foregoing suggestions which primarily focus on the management aspects, there is also a clear need to gain the active support of all command elements, down to the individual soldier himself. During recent Congressional hearings on federal agency implementation of the National Environmental Policy Act, it was clearly demonstrated that there was a failure on the part of the Services in disseminating environmental policies down to the operating level. It was the consensus that the effectiveness of this program is heavily dependent on the wholehearted support of all members of a command but that individuals will only contribute assistance if they clearly understand the basic policies and objectives involved. Obviously, to achieve this end there must be

AGDA-A (M) (24 Mar 71) LOG-C-PDBB 29 March 1971
SUBJECT: Environmental Protection and Preservation

increased publicity on what is being done to protect the environment, an opportunity for those within a command to contribute support to local programs and appropriate recognition and publicity of the efforts to assure productive and enjoyable harmony between us and our environment.

BY ORDER OF THE SECRETARY OF THE ARMY:

Kenneth G. Wickham
KENNETH G. WICKHAM
Major General, USA
The Adjutant General

DISTRIBUTION:
Commander in Chief
  US Army, Pacific
Commanding Generals
  US Continental Army Command
  US Army Materiel Command
  US Army Combat Developments Command
  US Army Strategic Communications Command
  US Army Security Agency
  US Army Intelligence Command
  US Army Air Defense Command
  US Army, Alaska
  US Army SAFEGUARD System Command
Commanders
  US Army Forces Southern Command
  Military Traffic Management and Terminal Service
Superintendent
  US Military Academy
CF:
Office, Secretary of the Army
Office, Chief of Staff, US Army
Assistant Secretary of the Army (FM)
Assistant Secretary of the Army (I&L)
Chief of Legislative Liaison
Deputy Chiefs of Staff
Comptroller of the Army
Chief of Research and Development
Chief, Office of Reserve Components
Assistant Chiefs of Staff
The Adjutant General
Chief of Engineers
The Surgeon General
Chief, National Guard Bureau
Chief of Information
Chief, Army Reserve
The Provost Marshal General
Chief of Personnel Operations
Chief of Support Services
Commanders in Chief
  US Army, Europe
Commandants
  US Army Command and General Staff College
  US Army War College

Memorandum
U. S. ARMY

DISTR A EXPIRES 31 August 1971

CSM 70-323

DATE 14 September 1970

FILE CS 723.1 (14 Sept 70)

ACTION OFFICER EXT LTC Thomason/78641/sez

SUBJECT: Protection and Enhancement of Environmental Quality

MEMORANDUM FOR: HEADS OF ARMY STAFF AGENCIES

1. REFERENCES.

a. The National Environmental Act of 1969, P.L. 91-90.

b. Executive Order 11507, 5 February 1970, 35 F.R. 2573, "Prevention, Control and Abatement of Air and Water Pollution at Federal Facilities."

c. Executive Order 11514, 5 March 1970, "Protection and Enhancement of Environmental Quality."

d. DOD Directive 5100.50, 23 June 1970, Environmental Pollution Control.

e. DOD Instruction 4120.9, 30 December 1966, Air Pollution Control.

f. DOD Instruction 4120.10, 5 June 1967, Water Pollution Control.

g. AR 11-21, 3 November 1967, Army Programs, Environmental Pollution Abatement.

2. PURPOSE. This CSM:

a. Applies to all Army Staff agencies and programs except the Civil Works Program.

b. Identifies Army Staff interests and responsibilities in the protection and enhancement of environmental quality.

c. Directs the review, and revision as appropriate, of all Department of the Army policies and directives which affect environmental quality.

d. Assigns Army General Staff responsibility for environmental pollution control and abatement programs to the DCSLOG.

3. DEFINITIONS.

a. Environmental Pollution: That condition which results from the presence of chemical, physical, or biological agents in the air, water, or soil which so alter the natural environment that an adverse effect is created on human health or comfort, fish and wildlife, other aquatic resources and plant life, structures and equipment to the extent of producing economic loss, impairing recreational opportunity or marring natural beauty.

DA3 FORM 62, 1 MAR 68

BEST COPY AVAILABLE

SUBJECT: Protection and Enhancement of Environmental Quality.

b. Environmental Quality Enhancement: Environmental preservation and improvement activities of the Army, including all actions to curtail pollution of the environment by installations, facilities, buildings, structures, equipment, aircraft, vehicles, vessels, and any other property owned, leased, and/or operated by the Army.

4. DISCUSSION.

a. While significant public interest in environmental factors is relatively new, elements of the Army have been actively concerned with ecological problems and projects for many years. It is noteworthy that in the present era of austere budgets, Army fiscal planning includes provisions for projects relating to protection and enhancement of environmental quality.

(1) In planning for operations and in designing materiel and facilities, individual elements of the Army Staff have long recognized an interest and responsibility in protecting environmental resources. However, the Army Staff as a whole has not given deliberate and coordinated consideration to the requirement to avoid contamination and destruction of land, water, and air resources.

(2) The increasing severity and pervasiveness of the contamination and depletion of environmental resources require a universal awareness and a thoroughly coordinated response by the Army. The inherent nature of military operations and the professional and technical resources of the Army place it in a unique position - to contribute both to the problem and to its solution.

(3) The increasing attention being given the national pollution abatement effort requires that Army planning consider the impact of Federal and local environmental controls on the accomplishment of the Army's primary mission and on the availability of resources.

b. Environmental conservation issues have two common characteristics which must be recognized in both Army planning and execution:

(1) Environmental considerations are pervasive and impact on almost every aspect of Army activity. Each Army Staff agency must acquire and sustain a knowledge of current developments in this field, outside as well as within the Army. Planners and action officers must develop a sufficient understanding of environmental pollution control to be able to give it due consideration in their planning.

(2) Both the cause of deterioration and programs for environmental protection and enhancement operate slowly and over a period of many years. Army Staff planning for environmental pollution control must be constant and continuous in nature, rather than oriented only to immediate problems, if the Army's long-range responsibilities are to be met.

BEST COPY AVAILABLE

62-513 O - 71 - 34

SUBJECT: Protection and Enhancement of Environmental Quality

5. POLICY.

a. The Army will comply fully with both the requirements and the spirit of Executive Order 11507, subject: Prevention, Control and Abatement of Air and Water Pollution at Federal Facilities, and with the National Environmental Policy Act of 1969 as implemented by Executive Order 11514, subject: Protection and Enhancement of Environmental Quality.

b. Pollution of the environment by installations, facilities, equipment, vehicles, and other property owned and/or operated by the Army will be curtailed. Continued efforts will be made to conserve or protect the natural environment and to improve or enhance the changed environment when it is altered by Army activities.

c. Resources for the control and abatement of environmental pollution will be allocated first to those programs which provide the most significant, tangible results with the earliest payoff. Toward this end, cost and effectiveness measures will be developed for each resource programing proposal, and alternatives will be considered to insure priority treatment for only the most cost-effective proposals. Effort will be allocated in the following order of priority:

(1) To correct situations which constitute a direct hazard to the health of man.

(2) To conserve economic resources.

(3) To improve the recreational and aesthetic value of the environment.

d. Maximum effort will be given to incorporating environmental pollution preventive measures in the basic design for weapon systems, military materiel, tests and exercises, and projects for rehabilitation or modification of existing structures and new construction.

6. RESPONSIBILITIES.

a. DCSLOG:

(1) Exercise primary Army Staff responsibility for coordinating environmental preservation and improvement activities within the Army.

(2) Establish a central point of contact for the coordination of environmental control and abatement actions (Suspense: 18 September 1970).

(3) Conduct, on a continuing basis and in coordination with appropriate Staff agencies, a comprehensive review of Department of the Army statutory authority, administrative regulations, policies, and procedures - including those relating to loans, grants, contracts, leases, licenses, or permits -

BEST COPY AVAILABLE

SUBJECT: Protection and Enhancement of Environmental Quality

to identify deficiencies or inconsistencies which prohibit or limit full compliance with the provisions of the National Environmental Policy Act of 1969 and Executive Orders 11507 and 11514. DCSLOG will task other Staff agencies, as appropriate, to accomplish portions of this review.

(a) Propose actions to be accomplished by the Department of the Army to correct identified deficiencies and inconsistencies to assure compliance with the National Environmental Policy Act of 1969 and Executive Orders 11507 and 11514.

(b) With the concurrence of interested Staff agencies, implement those actions developed in response to subparagraph (3)(a) above which are within the scope of DCSLOG's authority.

(c) Prepare and submit necessary action documents to the Chief of Staff to implement actions developed in response to subparagraph (3)(a) above which require approval by the Chief of Staff or the Secretary of the Army.

(d) As necessary, recommend actions to be accomplished by OSD or higher authority to provide DA the capability of conforming its policies to the intent, purposes and procedures of the National Environmental Policy Act of 1969.

b. COA: Assist DCSLOG and other Army Staff agencies in the development of cost and effectiveness measures to insure that the programing of resources to accomplish pollution control and abatement is consistent with the policy established by paragraph 5c, above.

c. CRD: Review Army Research and Development activities which contribute to the control and abatement of environmental pollution. Develop recommendations to insure that Research and Development programs fully support the goals established by the National Environmental Policy Act of 1969 and Executive Orders 11507 and 11504.

d. ACSFOR: Review current procedures for establishing Qualitative Materiel Development Requirements and recommend changes necessary to insure that future Army materiel meets Federal pollution control standards (unless exception thereto has been granted).

e. TJAG: Review existing pollution control and abatement laws and identify legal requirements not currently satisfied by Department of the Army regulations, programs, and procedures.

f. CofEngrs:

(1) Assist DCSLOG in the review directed by paragraph 6a(3) above, with special emphasis on the engineering and construction aspects of pollution abatement and control.

BEST COPY AVAILABLE

SUBJECT: Protection and Enhancement of Environmental
Quality

(2) When appropriate advise DCSLOG of Civil Works activities and programs which are related to military functions.

g. TSG: Assist DCSLOG in the review directed by paragraph 6a(3) above, with special emphasis on the health and welfare aspects of pollution, health and medical policy guidance, and pollution monitoring.

h. CINFO: Prepare, in coordination with DCSLOG and other appropriate Staff agencies, national news releases which focus public attention on Army programs for the protection and enhancement of environmental quality.

i. SMD, OCofSA: In coordination with each Staff agency, insure that appropriate responsibilities are included in the functional statements of Chief of Staff Regulations.

j. Each Army Staff agency:

(1) Designate a point of contact for environmental protection and enhancement matters (Suspense to DCSLOG: 24 September 1970).

(2) Establish a planning capability for environmental pollution control and assure its consideration in the discharge of agency functional responsibilities.

(3) Coordinate planning and actions which impact on environmental quality control with DCSLOG.

(4) Review, in coordination with DCSLOG and SMD, OCofSA, current Mission and Function Statements and recommend revisions necessary to insure that responsibilities for protection and enhancement of environmental quality are included in appropriate Chief of Staff Regulations.

BY DIRECTION OF THE CHIEF OF STAFF:

Warren K. Bennett

SUSPENSE:
DCSLOG-18 Sep 70--Central Point of Contact
Other Addresses-24 Sep 70--Points of Contact to DCSLOG

WARREN K. BENNETT
Major General, GS
Secretary of the General Staff

BEST COPY AVAILABLE

Mr. ROGERS. That is fine if they have. Each one has done that?

General HAYES. That is correct.

Mr. ROGERS. Said that they should have a person in each command specifically responsible?

General HAYES. That's right.

Mr. ROGERS. That it be done. That is fine, and I commend you.

Thank you very much.

Thank you, Mr. Chairman.

Mr. DINGELL. Thank you, Mr. Rogers.

Mr. EVERETT. Thank you, Mr. Chairman.

Mr. ROGERS. Let me ask this before you proceed.

You will let us have this report on the Air Force dumping?

General HAYES. Air Force dumping?

The Air Force base in California, yes, sir.

Mr. ROGERS. Thank you.

(The information may be found on p. 541.)

Mr. DINGELL. Mr. Everett.

Mr. EVERETT. Admiral Crawford, in conjunction with Mr. Rogers' question with respect to the operation of vessels, in the next to the last paragraph of your statement, it says as follows.

> Finally, a good ocean dumping act should exclude the day-to-day operational discharges from ships, such as sewage and oily bilge, which are properly subject to regulation by other laws. In this connection it is our understanding that 'dumping' as defined in the proposed act would not include the incidental discharge of some debris or other material the water from an activity provided that disposition is not the primary objective of the activity. For example, waste incidental to the operation of ships, the material and debris from missiles, spent bombs and other projectiles would be excluded from this act.

Mr. Chairman, I would like to introduce into the record a letter from Mr. Barry Sullivan, Washington representative of the River and Harbor contractors, addressed to Congressman Lennon, who raises this same point.

Mr. DINGELL. Without objection, the document referred to will be inserted in the record at this particular point.

(Letter follows:)

THE NATIONAL ASSOCIATION OF RIVER AND HARBOR CONTRACTORS,
Washington, D.C., March 31, 1971.

HON. ALTON LENNON,
Chairman, Subcommittee on Oceanography, Committee on Merchant Marine and Fisheries, Rayburn House Office Building, Washington, D.C.

DEAR MR. CHAIRMAN: With reference to H.R. 4723, a bill to regulate the dumping of material in the oceans, coastal, and other waters and for other purposes, may we suggest that consideration be given to amendment of Sec. 3. (f) definitions. As amended, the provision would read as follows:

"Dumping" means a disposition of material: Provided, That it does not mean a disposition of any effluent from any outfall structure, or a routine discharge of effluent incidental to the propulsion *or operation* of vessels. And provided further, That it does not mean the intentional placement of any devise in the oceans, coastal, or other waters, or on the submerged land beneath such waters, for the purpose of using such devise there to produce an effect attributable to other than its mere physical presence. (Proposed amendment italic above.)

In the operation of vessels, cooling water is required for equipment, such as winches and derricks, auxiliary machinery, etc., and as effluent this cooling water is the same as the effluent from propulsion.

Accordingly, we request such effluent receive the same statutory treatment as "effluent incidental to propulsion of vessels."

Sincerely,

BARRY SULLIVAN,
Washington Representative.

Mr. Everett. And he is concerned that operation of the vessel and equipment are not covered under the exclusion. And he suggests language that would include language similar to what you did, that would exclude the operation of the vessel from the coverage of dumping.

And so I take it that if the interpretation is that the discharges from regular operation of vessels are not covered, then it is your suggestion that these items be covered under the bill as an exclusion to dumping?

Admiral Crawford. Well, we would like to make it clear that it is certainly the intent, if not specifically in the bill, that routine operations of ships are to be excluded.

Mr. Everett. He suggested an amendment more or less to this effect, in the operation of vessels, cooling waters required for equipment, such as winches and derricks, auxiliary machinery and so forth, as effluent, is the same as the effluent from propulsion, and the bill only speaks of propulsion rather than operation.

Do you agree with Mr. Sullivan's suggestion with respect to operation?

Admiral Crawford. I would agree to that, and if you think that it is not sufficient to ha[illegible] the intent clarified on this, I would welcome the opportunity to subr[illegible]t a statement as to how a portion of the bill should read to satisfy these considerations.

Mr. Everett. If you would, we would appreciate it, Admiral, if you would submit suggested language to accomplish the purpose you seek.

(The amendment follows:)

Amendatory Language Re Vessel "Dumping"

S. 1238—Sec. 3(f)—Recommend change in definition for "Dumping" to read as follows:

f. "Dumping" means a disposition of material: provided that it does not mean a disposition of any effluent from any outfall, or a routine discharge of *wastes and* effluent incidental to the *operation and* propulsion of vessels: and . . .

Mr. Everett. General Groves, one of the definitions of the section 3(b) of the bill is that oceans, coastal, and other waters, means oceans, gulfs, bays, salt water lagoons, salt water harbors, and other coastal waters where the tide ebbs and flows, and the Great Lakes. I was wondering if you could give us some indication as to how far inland the coverage of this bill would be with respect to the ebbing and flowing of tides.

General Groves. I can give you a couple of very specific examples. In the case of the Hudson, it would be up to about Troy, N.Y.

Mr. Everett. How far is that, sir? Roughly.

General Groves. About 150 miles in. And on the Mississippi it goes up to, oh, slightly above Mile 350.

Mr. Everett. 350 miles?

General Groves. Yes. And I would be happy to give you some others for the record, if you want.

Mr. Everett. If you would, Mr. Chairman, I would like to ask that this information be supplied as to the effects of this definition concerning the ebbs and flows of tides, with respect to these coastal areas and the rivers and tributaries extending therefrom.

Mr. Dingell. General, I think that kind of counsel would be helpful, if you could give it to us.

General Groves. All right, sir, we will try to work up something along that line.

(The information follows:)

LIMITS OF TIDAL INFLUENCE ALONG CERTAIN RIVERS

River and location:	*Approximate miles*
Connecticut, Windsor Locks, Conn	64
Hudson, Troy, N.Y	134
Delaware, Trenton, N.J	127
Susquehanna, Conowingo, Md	9
Potomac, Little Falls, Washington, D.C	114
Rappahannock, Fredricksburg, Va	107
James, Richmond, Va	91
Mississipi, Red River Landing, La	(AHP) 302
Klamath, Klamath, Calif	[1]
Columbia, Bonneville Dam	140

[1] 2 miles inside bar.

Mr. EVERETT. General, we have had some discussions about the harbor at Baltimore, and I was wondering if you could give me some indication as to the answer to this question. What would be the effect on the cost and economic justification of waterway improvement projects, such as that which the Port of Baltimore has, in the event that the Administrator of EPA should revise the dredge material disposal plans of the corps, to require such material to be transported for disposal at sea or other locations remote from the improvement project?

General GROVES. In the case of Baltimore the present project is based upon disposal of the spoil near Kent Island in the Chesapeake Bay.

Mr. DINGELL. Would you yield? Is that open or dike-enclosed?

General GROVES. It is open disposal.

Mr. DINGELL. Is that polluted dredging? Or is this clean dredging?

General GROVES. I am sure it would be classed as polluted, yes, sir.

Mr. DINGELL. It is polluted?

General GROVES. I would expect so, yes, sir.

Mr. DINGELL. There is no question in your mind on that point?

General GROVES. I wouldn't think so, no, sir.

Mr. DINGELL. Very good.

General GROVES. I would say within whatever jurisdiction we might have we would look to EPA for the establishment of water quality standards. They would govern anything else that we do just as they do govern in the case of the section 13 permits now.

Mr. DINGELL. So that when we referred to the provisions here that we have, section 7(c) (2), and I hope if you don't have a copy of it, I will see that the staff makes one available to you, 7(c) (2), referring down here:

> Provided, That after the effective date of this act, no Federal license or permit shall be issued under the authority of the Rivers and Harbors Act of 1899 to conduct any activity otherwise regulated by section 4 of this act and the regulations issued hereunder, unless the Administrator has certified that the activity proposed to be conducted is in conformity with the provision of this act and with the regulations issued hereunder.

Would you behave strikingly differently if this statue were in effect with regard to the dredging and depositing of spoil in the Baltimore Harbor than you would if it were not in effect under the water pollution contract, national environmental policy acts and your agreement with the Interior Department?

General GROVES. I believe the essential difference would be this. This particular act has a 6-month effective date on it. As I read this, we would have to have applicable standards in order to proceed.

Under present conditions we would proceed using the best means available to us.

In other words, if the EPA standards were not fully developed we would proceed as at present. If they developed further we would modify our work.

Mr. DINGELL. Would you have any reason to believe tha' you would apply any lesser environmental safety standards before the enactment of this legislation than you would after?

General GROVES. I don't really think, sir, it makes much difference. We would use the best that we have. EPA is the source of those standards and that is where we would look to get them.

Mr. DINGELL. You would in any event get the standards on water pollution and so forth from EPA?

General GROVES. Yes, sir. This is inherent in the five point environmental statement if nothing else. We would have to coordinate it with the various governmental agencies.

We would solicit and I am sure we would obtain EPA's comments. We would certainly abide by them.

Mr. DINGELL. What you are saying is that really your behavior, either before or after the enactment of H.R. 4723 insofar as environmental protection, will be very little different.

General GROVES. I see no basic difference, except in the time necessary to adopt alternate methods.

General GROVES. Now if the law required that the dredged spoil be disposed of at some more distant location, perhaps in an enclosed location on land, say, in any case the cost of the project would undoubtedly go up. This can lead you to a number of possibilities

If we were to follow the one course that might be taken when you run a five-point environmental statement analysis through section 102(c) of the act and you arrive at the point where you have certain irreconcilable conflicts and if at that point you decided you would invoke the provisions of section 102(d) which says, in effect, you reformulate and start over and you come up with a project that does have an adverse environment effect which, to eliminate would result in higher cost, the first question you have to answer is to whom do you charge these higher costs?

If you could charge it to mitigation of the environmental damage under our present understanding of the system, it would have no effect on the project economics. If, on the other hand, you had to pull them into the project cost and, in effect, charge them to navigation the cost of the project would go up, the benefits would remain constant.

So, it is conceivable that the B-C ratio could become unacceptable. This is one possible outcome.

Another possible outcome, almost a certain outcome, is that if you had to go to sea and if you were going to have to do this on a large scale, we have only a limited hopper dredge capacity, which is already fully employed. It would take us about 5 years to build new dredges. If you went to the industry, using a scow and dipper, it would take them at least 2 years probably to react. So if this hits you throughout the Nation, the effect on Baltimore Harbor might be that the work would stop while we got a new capability.

This is one of the possible outcomes. It might become economically unfeasible. In any case, it might be delayed or stopped.

Mr. DINGELL. You have been referring to what would happen under existing law, General, or under a transfer to EPA or a compliance with section 102(c) and (d)?

General GROVES. My understanding of the question was, sir, that it involved the assumption that we would have some requirement to haul the dredged spoil to some more distant location than we now put it.

Mr. DINGELL. Let me ask you a hard question here. Is it your assumption that the Environmental Protection Agency would behave strikingly different under the legislation in H.R. 4723 than you would under the existing law, your agreement with the Interior Department, and under the Environmental Protection Act? I am talking about issuing permits generally for the dumping of polluted dredgings.

Mr. DINGELL. Do you see any basic difference in terms of the cost of the project before and after the enactment by reason of the fact that you are going to have to get formal certification from EPA instead of having informal certification as you do at this particular time?

General GROVES. Here I think, sir, the main difference is one of timing. I think that if we were to apply the rule that we are applying in section 13 which is that EPA governs water quality standards, they are the same.

Mr. DINGELL. They are the same?

General GROVES. They would be the same. If on the other hand we were to consider the total public interest it might be slightly different.

Mr. DINGELL. Thank you.

Mr. Everett?

Mr. EVERETT. General Groves, would the Corps be immediately capable of maintaining the harbors and channels at their authorized project depth if alternate disposal methods should become necessary as a result of this legislation?

General GROVES. Would you repeat the beginning of that, please, sir.

Mr. EVERETT. Would the Corps be immediately capable of maintaining the harbors and channels at their authorized project depth if alternate disposal methods should become necessary as a result of this legislation?

General GROVES. I think the answer to that is that we would be unable to maintain our total level of effort.

In other words, we would have to maintain a lesser amount initially for the reasons that I mentioned, that the available plant, both corps and contractor and especially the corps hopper dredge are fully employed.

If we went to diked land disposal, our experience leads us to believe that it would be several years before we would enter into the agreements necessary and get the funds necessary and to build the enclosures.

It would be quite a while, maybe a period of 5 years. If we went to scow and dipper type operation it would take industry or we or both several years to get the funds to build these things and put them in operation.

62-513 O - 71 - 35

So, to answer your question, you are imposing a greater workload on a fixed capability and less is going to get done overall for a while.

Mr. EVERETT. General Groves, there is still some ambiguity as to what the intent of section 11 is.

I wonder if you could provide us with a memo with respect to section 11(a), 11(b), 11(d) and 11(e), on the effects of these reactions on existing law if the bill is enacted as recommended in H.R. 4723.

General GROVES. Do you want me to supply that for the record?

Mr. EVERETT. Yes.

General GROVES. I will be glad to.

Mr. EVERETT. Does the bill in any way change your method of operation or procedure under the Fish and Wildlife Coordination Act?

General GROVES. No, indeed.

Mr. EVERETT. How about under the national Environmental Policy Act?

General GROVES. No.

Mr. EVERETT. That is all I have, Mr. Chairman.

Mr. DINGELL. Captain Heyward.

Mr. HEYWARD. General Groves, in connection with section 11 there were some discussions with other witnesses as to the effect of the EPA procedures for issuing permits.

There has been disagreement on the interpretation of exactly what supersession of corps permit authority is involved in this act.

May I ask you, can you tell me whether a particular section is used by the corps as authority for issuing permits to dispose of dredge spoil?

Is that under 33 U.S.C. 403 or are you aware of the specific number?

General GROVES. I am not aware of the code numbers. Essentially they derive from the supervisory act and from the 1905 act, section 4.

Mr. HEYWARD. Section 4 of the 1905 act?

General GROVES. Yes, sir.

Mr. HEYWARD. That is not being touched in any of this repealing material, is that correct?

Yes, that is 33 U.S.C. 419, whch is being superseded. This is the question I was really getting to, whether or not the authority under section 10 of the Rivers and Harbors Act is utilized by the corps in connection with its permit for excavation and filling or whether you rely on section 4 of the act of 1905 which is being superseded here.

General GROVES. Now we are talking about ocean dumping, are we not?

Mr. HEYWARD. We are talking about dumping in all these defined waters, yes, sir.

General GROVES. Section 10 of course applies to the territorial waters. It is used primarily for structures and dredge and fill type operations.

The subject of today's hearing, ocean dumping, is covered under the section 4 of the 1905 act.

Mr. HEYWARD. This is the problem that we face in discussing this because in section 7(c)(2) it says except as provided in subsection 11(e) nothing in the Rivers and Harbors Act is going to be superseded.

Now, if section 11 then turns around and superseded those elements of the Rivers and Harbors Act that you rely on there is nothing left for you to do in issuing the dredge spoil disposal permit.

This is the point I am sure Mr. Everett was getting to.

It would be very helpful for the committee to have a complete analysis of what you need to retain your permit authority consistent with the scheme envisioned here in 7(c)(2) which would not be adversely affected by any of the repealers in section 11.

General GROVES. All right, sir, we will be very happy to supply that for the record.

(The information requested follows:)

Subsections 11(a) and 11(b) repeal the Supervisory Harbors Act of 1888, as amended (33 U.S.C. §§ 441–451b), and the provision of the Rivers and Harbors Act of 1899 (33 U.S.C. § 418) which preserved the Supervisory Harbors Act from supersession by the 1899 Act. The Supervisory Harbors Act provides a special authority to control transit in and from the harbors of New York, Baltimore, and Hampton Roads, Virginia. This authority has been used to regulate ocean dumping. The proposed Marine Protection Act would replace that authority. A portion of the Act of August 5, 1888 (33 U.S.C. § 407a), which pertains to deposits of debris from mines and stamp works, and which is covered by the proposed Act or the Refuse Act, is also repealed. A provision contained in the Rivers and Harbors Act of 1905 (33 U.S.C. § 419), which has been used to buttress the Corps of Engineers' authority to regulate ocean dumping, is superseded, insofar as it authorizes action that would be regulated by the proposed Act. Lastly, Section 13 of the Rivers and Harbors Act of 1899 (33 U.S.C. § 407), commonly known as the Refuse Act, is superseded, but only insofar as it applies to dumping of material in the waters covered by subsection 4(b) of the proposed Act. Except for the limited supersession found in subsection 11(e), the Rivers and Harbors Act authorities are not negated or abrogated, nor are existing licenses or permits issued under that Act terminated. Rather, in situations where the proposed Act and the Act of 1899 both apply to dumping of material in connection with a dredge, fill or other permit issued by the Corps of Engineers, issuance of the permit requires a certification by the Administrator of EPA that the activity is in conformity with this proposal and any regulations issued under it. The Administrator will not issue separate permits in such cases.

After the proposed Act becomes effective, the Department of the Army's permit program under the Refuse Act, which is administered in close cooperation with EPA on all water quality matters, will continue to regulate the disposition of any effluent covered by the Refuse Act from any outfall structure regardless of the waters into which this disposition occurs, in addition to regulating all depositing of material into other navigable waters of the United States not covered by subsection 4(b) of this Act.

Mr. HEYWARD. There is one other question.

In 7(b)(2) there is provisional authority as the EPA certification comes only on permits issued after the effective date of the act, that is to the extent that it does not repeal something, in effect, any permits which you issued before the acts:

Would the Corps of Engineers in those cases where permits have already been issued but where disposal of the dredging had not been undertaken confer with the EPA consistent with the policy required in section 7(c)(2) even if the statute does not specifically require it?

General GROVES. I would expect so, sir. In any case, these permits are for finite durations and when they came up for renewal, they would have to be considered again.

Mr. HEYWARD. Thank you, General.

Mr. DINGELL. Gentlemen, can you tell us about that impact of enactment of this bill on existing practices with regard to dredge and fill operations in the Great Lakes?

General GROVES. The dredge and fill operations in the Great Lakes, sir, if this were enacted, would, of course, have to be conducted under license from EPA, under permit from EPA.

Mr. DINGELL. As I read the requirements of section 7 they require that the permit be issued by the corps and that you carry out the requirements of EPA.

As I read section 7(c)(2) you would still continue to issue permits but you would have the added requirement that it would have to be certified that the activity to be conducted is in conformity with the provisions of the act and the regulations issued thereunder.

Captain Heyward and Mr. Everett were both inquiring as to this particular point.

General GROVES. Sir, I believe this is one of the points that probably requires some clarification. It would depend in part as to whether we were doing the dredging or one of our contractors.

Mr. DINGELL. If you were doing the dredging and your contractors were doing the dredging you would have the same situation.

EPA would have to certify that your permit was issued in conformity with the requirements of the law and in conformity with the regulations?

General GROVES. My question really dealt more with specifics.

Mr. DINGELL. I assume that in the Great Lakes you anticipate you will probably continue to be obligated to utilize dike disposal areas for pollutant spoil.

Am I correct on this?

General GROVES. Yes, sir.

The Rivers and Harbors Act of 1970 provided a means by which that can be accomplished.

We are certainly headed in that direction. I should point out though that the proviso that the local interests be excused from their 25 percent contribution if EPA certifies that they have an on-going acceptable program is making it rather difficult for us to come to grips with them so far.

Mr. DINGELL. Would you amplify that, please?

General GROVES. Yes, sir.

The initial reaction of any of these local interests that we go to is that they will go to EPA first and see if they can't be excused from their contribution.

Mr. DINGELL. What is the effect of that on your program?

Does it jump your cost?

General GROVES. It means that we are not making much progress toward getting these dike enclosures underway.

Mr. DINGELL. Are you telling us that EPA is excusing them from the requirement of using dike enclosures or simply paying the cost and shifting the cost to you?

General GROVES. Sir, a pattern has not really emerged yet.

Mr. DINGELL. Obviously, when they go to EPA to be excused from the 25 percent they either try to get out of the construction of the dike area in its entirety or they are expecting to shift the burden to someone else.

General GROVES. I think it is the latter.

Mr. DINGELL. Which is it, shift the burden to you?

General GROVES. We get that impression.

Mr. DINGELL. You then have to rush out and get more appropriations?

General GROVES. Yes, sir.

Mr. DINGELL. That is what you referred to when you said it will make it a little harder?

General GROVES. Yes, sir.

Mr. DINGELL. I think you have answered all of my questions at this time.

Gentlemen, if there are further questions the committee wishes to have answered or further assistance, I assume we can come back to you for guidance and counsel.

General HAYES. We will be glad to assist you in any way.

Mr. DINGELL. Gentlemen, the Chair wishes to commend you for a very able and very helpful presentation.

The Chair, as I am sure you know, has long been an admirer of the efficiency of the corps. Of late I have come to be an admirer of the corps for its environmental concerns. So, I wish to express that to each of you for your presence today.

Mr. DINGELL. Our next witness is Dr. Richard Barber, Duke Marine Laboratory, Beaufort, N.C.

Dr. Barber, I don't want you to feel that we have downgraded your appearance. As a matter of fact, it might be said that we have saved the best for last.

We are grateful for your attendance.

Will you identify yourself in full by name and address.

We will be happy to recognize you.

STATEMENT OF DR. RICHARD BARBER, DIRECTOR, OCEANOGRAPHIC PROGRAM, DUKE MARINE LABORATORY, BEAUFORT, N.C.

Dr. BARBER. I am Richard Barber, director of the oceanographic program at Duke University Marine Laboratory in Beaufort, N.C.

My experience with the ocean dumping is as an oceanographer studying certain parts of the biological system in the waters over the New York Bight dump sites.

The proposed bill, H.R. 4723 seems to me to be a very good first step in improving relations between our way of life and the ocean.

The fact that the bill regulates the transportation, as well as the dumping (sec. 4) of materials is wise and necessary in my opinion.

I favor passage of the bill, but I feel it could be strengthened.

This bill will perhaps be adequate in the future when we have good water treatment facilities, solid waste recycling, and other environmental amenities, but I question whether this bill will enable the Administrator to deal in a reasonable manner with our current ocean dumping problems.

The acutely dangerous, small volume dumping practices, such as chemical warfare agents and explosives, can be halted, but the large volume practices such as the New York Bight dumps cannot be halted.

We have lived with and rely on this kind of ocean disposal. How will the Administrator deal with this problem?

One means would be to establish procedures such as the air pollution abatement schedules which generate solutions in a realistic manner.

Given: (1) authority and responsibility over the Continental Shelf to a depth of 200 meters;

(2) public dissemination of information on the condition of the environment; and

(3) the establishment of grades of environment—sanctuaries, moderately protected areas and interim lower quality sites—the administrator could effectively protect the ocean system at present.

On the basis of my New York Bight experience, I feel that involved Government units are devoting almost no effort to solving the problems of ocean dumping.

The very small studies to define the problem were good. Now that the problem is defined it is time to start on the more difficult and risky work of decreasing the degradation caused by this dumping.

I hope this bill will provide an interim mechanism for controlling existing dumping practices as well as an ultimate means for regulating future dumping practices.

That is the end of my prepared statement. I will provide my resume for the record later.

Mr. DINGELL. Without objection, that will appear at this point in the record.

We will be happy to hear any other comments you have.

(Information to be furnished follows:)

RÉSUMÉ OF BIOGRAPHICAL INFORMATION

RICHARD T. BARBER

Dr. Richard T. Barber is Director of the Oceanographic Program at Duke University Marine Laboratory and Associate Professor of Zoology and Botany of Duke University, Durham, North Carolina. He was born in Bridgeton, New Jersey and received his undergraduate education at Brown University, Utah State University (B.S., 1962), and did his graduate studies at Stanford University (Ph.D., 1967).

After completion of the Ph.D. degree at Stanford University, he was a Postdoctoral Fellow in Biological Oceanography at the Woods Hole Oceanographic Institution, Woods Hole, Massachusetts. During tenure of the fellowship he served as Chief Scientist for three months on a Stanford University Oceanographic Expedition to the Galapagos Islands. In 1968 he joined the staff of Woods Hole Oceanographic Institution as Assistant Scientist in the Department of Biology. In 1970 he assume his present position at Duke University.

He is a member of the National Academy of Sciences Committee on Water Quality, Panel on Marine Aquatic Life and Wildlife; the National Science Foundation Panel on Research Needs for the Council on Environmental Qualities Report on Ocean Dumping; and the National Advisory Board of the *R/V HELIX*.

Dr. Barber's work on the inhibition of plankton growth in the New York Bight dumping area was selected by the National Science Foundation for inclusion in the 1970 Annual Report of the President to the Congress on Marine Resources and Engineering Development. Other research interests include studies on the growth and decay of organic matter in the sea and the effect of metals on the growth of plankton. Specific work has examined the speed with which organic matter decays and is recycled in the ocean and the effect of organic matter on metal uptake by plankton. He is the author of ten publications on these subjects.

Dr. BARBER. My statement that no effort is made in solving the current problems of ocean dumping is based on the fact that when you are out over the New York Bight dumps you don't see anyone else out there doing research.

It is not being done.

The amount of effort and the dollars spent on work in this area is vanishingly small and some of the work that I am familiar with is

supported by the National Science Foundation and supported by the Army Corps of Engineers, but they are very small projects.

The other Government agencies such as State and city authorities and other Federal authorities just seem to be missing from the scene.

There is very currently concern among the people that I know working there this bill has a potential effect of decreasing work on the problem.

Mr. DINGELL. You say this bill has the potential effect of decreasing work?

Dr. BARBER. The idea that dumping is going to be ended means that there is no longer a need to study it.

That, of course, is the thrust of my statement.

I know and anyone who looks at the economics of the coastal population centers knows that dumping is not going to be ended so that the question is to work out some way to cope with it.

This different message seems to be going out that it is going to be ended so that it is no longer necessary to work in the area.

I refer specifically, though my information may be incorrect, that the Army Corps of Engineers has a 3-year plan for working on the New York Bight, which in my opinion is an excellent one.

I hear from the Army Corps of Engineers they will only carry out one year of that. After that year it will presumably be EPA's responsibility, but EPA has not announced that this is their plan.

The Bureau of Solid Waste Management, again by verbal exchange, has informed us that it is no longer funding any work on dumping since dumping will be ended.

Mr. DINGELL. Your counsel in these matters is useful.

We may find it necessary to communicate with the agency involved regarding the points you raised.

Your assistance to this committee in framing the appropriate questions to the agencies to which you have alluded for inquiry by the committee will be much appreciated.

Dr. BARBER. Thank you.

Are there any questions?

Mr. EVERETT. I have no questions, Mr. Chairman.

Mr. DINGELL. Doctor, we thank you very much for your presence. You have come a considerable distance to be of assistance to the committee.

We thank you for your very generous and kind assistance.

We are most grateful for your presentation.

Dr. BARBER. Thank you.

Mr. DINGELL. If there are no further witnesses to be heard, the subcommittee will stand adjourned pending the call of the Chair.

(The following information was submitted for inclusion in the printed record:)

DUKE UNIVERSITY MARINE LABORATORY,
Beaufort, N.C., April 19, 1971.

MR. NED P. EVERETT,
Counsel, Committee on Merchant Marine and Fisheries, Longworth House Office Building, Washington, D.C.

DEAR MR. EVERETT: When I testified on the ocean dumping bills on April 7 I expressed my concern that the proposed regulatory acts appeared to be having the unexpected and unintentional effect of placing in jeopardy some of the ongoing research programs on the environmental consequences of ocean dumping

in the New York Bight. The basis for my concern is described in the following letter. This material may be included in my statement for the record on H.R. 4723, the Marine Protection Act of 1971, if you desire to use this information.

I support the intent of the Marine Protection Act of 1971 because I think there is a very real need for regulating the various ocean dumping practices that are going on at present. The acutely dangerous and small volume dumps of explosives, chemical warfare agents, highly radioactive wastes, and very toxic industrial wastes can be halted. The relatively small volumes involved in this kind of dumping make it economically feasible for this waste material to be disposed of in alternative ways.

The large volume dumps of dredge spoils and sewage sludge will have to be brought under "control" by a slow and complex process which will require greatly improved land-based waste treatment facilities. The dredge spoil and sewage sludge are in themselves not acutely dangerous materials but the large areas of environmental degradation at the site of the New York dumps testify to the fact that the present methods of managing this ocean disposal are no good. Whether the recycling capacity of the system has recently been exceeded or whether the dredge spoils and sewage sludge have become more acutely toxic in the last five years is not known.

What is known is that an area of the sea floor is devoid of animals and the bottom sediment smells of petrochemicals. I feel that we have defined the problem clearly and we are ready to start research on an interim solution for managing this kind of dumping while we work towards the ultimate goal of no harmful dumping.

I am familiar with the studies supported by the Army Corps of Engineers and carried out by Dr. Jack Pearce and Dr. Grant Gross. When the Coastal Engineering Research Center of the Corps of Engineers was developing its second phase research program on the New York Bight they contacted Dr. Dirk Frankenberg of the National Science Foundation to discuss the Army's proposed research program.

Dr. Frankenberg is the Biological Oceanography Program Director at the National Science Foundation; he contacted me and asked if I had suggestions for a man to administer the research contract program for the Army. He outlined the Army's program as being a three-year program budgeted at $300,000 the first year, $2,000,000 the second year, and $2,000,000 the third year. After discussion with Dr Frankenberg I said I would be glad to serve as an advisor to the Army on this work because I feel that problems associated with the New York Bight dumping are important and I feel that they can be solved, but I am dissatisfied with the amount of effort that government agencies, other than the Corps of Engineers and National Science Foundation, have devoted to this important problem. Dr. Frankenberg knew that I was interested in this problem because my NSF supported research studied some of the biological consequences of the dumping. Some of the results of my work in the New York Bight were selected by National Science Foundation for mention in the 1970 Annual Report of the President to the Congress on Marine Resources and Engineering Development. This report is entitled "Marine Science Affairs—Selecting Priority Programs" and was communicated to the Congress in April 1970. The relevant section of page 156 of the Report is given below:

> "It has been demonstrated that phytoplankton growth is prohibited or prevented in waters over New York City's sewage dumping area.[13] The toxicity is increased by the addition of small amounts of trace metals and is partially decreased by the addition of metal chelators indicating the toxicity results from high concentration of toxic metals in the sewage sludge."

On March 31, 1971 an advisory group met at the Army Corps of Engineers' Coastal Research Center, 5201 Little Falls Road, Washington, D.C., to review the specifications of their proposed research program. At that time, Lieutenant Colonel Edward M. Willis of the Center informed the advisory group that the Army's participation in the study had been reduced from the original three-year program to a one-year program. The staff at the Army's Coastal Research Center mentioned that the future of the research program has been discussed with individuals from EPA but as late as 6 April 1971 EPA had not announced that it was going to adopt and carry out the Army's proposed program of research.

I think that the Army's proposed program is a good first step towards further understanding of the New York dumping problems. It would take several

---

[13] Barber, Richard T., Unpublished results from progress report on NSF Grant GB–13663 to Woods Hole Oceanographic Institution 1969.

months, perhaps even a year, for another agency to develop a similar program from scratch; therefore I would regret seeing the three-year program discarded until a better and more comprehensive plan of attack is in hand. Everyone involved in these studies recognizes that the Army Corps of Engineers may not be the best agency for administering the research program, because their responsibility for maintaining the harbor depths is one of the sources of the problem. However, the Corps has acted in the public interest in supporting research on the problem up to now and they are well acquainted with the nature and mechanics of the dumping activities.

In addition to the Army's familiarity with the problem I feel that other agencies of the government that are charged with protecting the environment have not demonstrated a willingness to become involved in work on problems related to the actual dumping activities. Our present knowledge of the problem is based on the excellent, but small, Army supported programs of Dr. Jack Pearce and Dr. Grant Gross. A Smithsonian advisory group has contributed its skills and the National Science Foundation and Atomic Energy Commission have supported research work done at sea by Woods Hole Oceanographic Institution and Duke University groups. If there are other major research efforts the Army's advisory group and the major researchers are not aware of them.

It is disappointing that NOAA and what is now EPA have not been active in this research. In view of the lack of major involvement of these agencies at present I feel that a clear *charge* of research responsibility to some agency is needed in the Marine Protection Act of 1971 to insure that the necessary work is carried out.

One example of necessary work is high precision depth and seismic mapping of the dump sites. To "manage" the dumping we need to know whether the material dumped in the New York Bight has been accumulating over the past forty years and has produced a series of low hills or hummocks or whether the dredge spoil and sewage sludge is dispersed by currents and winter storms over a broad area. The vessels of the Environmental Science Services Administration, under the direction of NOAA in the Department of Commerce, are specifically equipped for carrying out a precision depth and seismic survey of the sea floor. The question of dispersal versus accumulation would perhaps be answered by such a survey, and in addition a sensitive seismic survey (such as are used in oil prospecting on the continental shelf) might tell us what kind of material is accumulating on the bottom in dump sites. Since the New York Bight dumps have been receiving publicity for over three years I am puzzled as to why ESSA has not assisted the Corps by making such a survey. This work would be a small and perhaps even trivial effort for NOAA to undertake but until work of this sort is started I do not feel that we are making a reasonable effort to solve the problems caused by ocean dumping.

If the Marine Protection Act of 1971 is going to result in the transfer of research responsibility out of the Army Corps of Engineers then the responsibility should be assigned to another agency and that agency should carry on the ongoing programs until it has a better research program.

The present research effort of my lab is NSF supported; I do not intend to seek support from the above described Army research program. My concern is that an environmental crisis resulting from ocean dumping is gradually occurring with the full knowledge of political and scientific authorities. This low level crisis deserves as much attention as the acute hazards of transporting and dumping of chemical warfare agents.

RICHARD T. BARBER,
Director, Oceanographic Program.

Santa Ana, California, April 7, 1971.

HON. ALTON LENNON,
Chairman, Subcommittee on Oceanography,
U.S. House of Representatives, Washington, D.C.

DEAR SIR: Please place this on the record for the hearings of April 5–7.

I ask that you by-pass H.R. 4723, which only meets *some* of the recommendations of the Council on Environmental Quality in banning the dumping of waste material.

I ask your support for the bill co-sponsored by Dingell and Rogers which meets all *CEQ* recommendations.

Sincerely,

FERN ZIMMERMAN.

THE PORT OF NEW YORK AUTHORITY
New York, N.Y. April 13, 1971.

Hon. EDWARD A. GARMATZ,
Chairman, Committee on Merchant Marine and Fisheries,
U.S. House of Representatives, Washington, D.C.

DEAR CHAIRMAN GARMATZ: The Port of New York Authority is vitally interested in H.R. 4723, titled the "Marine Protection Act of 1971", which was considered jointly at public hearings held by the Subcommittee on Fisheries and Wildlife Conservation and Subcommittee on Oceanography in Washington on April 5, 6 and 7, 1971. In general, we agree with the statement you made at the hearing on April 5, with particular regard to provisions for dredge spoils in the proposed bill.

Under the Port Compact of 1921, the Port Authority is responsible for promoting and protecting the commerce of the Port of New York, as well as for the development of transportation and terminal facilities in the Port District. Development of these facilities often entails the creation of land along the waterfront by fill, the dredging and maintenance of ocean vessel berths, and the provision of safe, efficient and economic Federal navigation channels to serve the Port of New York. Such activities involve moving dredged spoils, rock and sand from one area to another in and around the waters of the Harbor under permits granted by the U.S. Army Corps of Engineers, since land areas in the Port of New York have been deemed unsuitable for this purpose in various Corp navigation improvement reports. It is the sections of the proposed legislation dealing with spoils disposal on which we wish to comment.

The Port Authority believes that H.R. 4723, sensibly interpreted, would provide a reasonable approach to balancing development needs with environmental needs in the matters of spoils disposal in the Port of New York. The proposed Act obviously recognizes that while disposal in ocean and adjacent waters must be intelligently regulated, it cannot be capriciously or arbitrarily prohibited. All uses of the world's oceans—for transportation as well as for food and as a source of natural resources—must be accommodated.

We feel, however, that the bill may be negating some of its general spirit of reasonableness and unbiased regulation by expressing a policy "to prevent or vigorously limit the dumping into the oceans, coastal and other, which could adversely affect human health, welfare, or amenities, or the marine environment, ecological systems, or economic potentialities" as prescribed in Section (2)(b). It is suggested that the world "could" be changed to "will", or the phrase "prevent or vigorously limit" revised to read "control", thus avoiding a situation in which a harsh curb might be levied against materials which have not been proved to be harmful.

Section 3(e) would appear to apply the proposed Act with equality to both Federal and non-Federal dumping activities. We trust this means that non-Federal requirements will be made no more restrictive than Federal requirements of compliance. We are also assuming that the wording in Section 3(f) excludes fill operations from application of the term "dumping", and thus, from inclusion in the proposed Act.

In Section 5, we commend the inclusion of such criteria as "unreasonable degrade", "unreasonable endanger", "likely impact" and "probable impact", since these terms evidence an intent to attain not only desirable but practical goals in a field which still lacks considerable scientific knowledge about cause and effect. The Federal, State and local consultative obligation is likewise desirable, as is the establishment and issuance of various categories of permits commensurate with the nature, volume, dumping location and impact period of the material to be dumped. Particularly commendable is the option of issuing general permits for materials which will have a minimal adverse impact. The designation of dumping sites is also reflective of the constructive approach to the dumping problem displayed throughout much of the proposed Act.

There is another section which gives us some cause for concern, namely, Section 7(a), which rescinds all outstanding permits "after the effective date of this Act," which is established as "six months after its enactment". We recommend an additional six month grace period after enactment, to soften hardships during the period for conversion to new permits, and to permit processing of applications with minimum delay to ongoing dumping needs.

Dredged spoils in their natural state, unlike other materials such as sewage sludge and certain industrial wastes which are obvious pollutants, are no more

detrimental to the environment than littoral drift or fluvial deposits. Areas which are to be dredged sometimes have been polluted by indiscriminate sewage and sludge outfalls which, for the lack of proper treatment and disposal, deposit polluted material on nearby harbor bottoms. In such cases, dredged spoils are the victim, rather than cause of pollution. It would be grossly unfair in view of these facts to place an economic burden on navigation improvements in the form of excessive transportation costs with what, in effect, is a form of interim pollution abatement. Just as pollution control facilities are funded specially for this purpose, any extra costs for disposal of polluted dredged spoils should not be borne by navigation improvements, but by pollution abatement programs. We respectfully submit that these factors must be carefully considered in this legislation.

Sincerely,

HAYDEN B. JOHNSON,
Coordinator, Office for Environmental Programs.

STATEMENT OF THE MANUFACTURING CHEMISTS ASSOCIATION

The vital importance of the ocean environment to our total ecological well being is an undisputed scientific fact. It is important to recognize, however, that the oceans are the ultimate sink in the hydrologic cycle. An inquisitive child might ask the question, "Why are the oceans salty?" The answer is not so apparent. The oceans receive, concentrate, and to a degree provide natural treatment to water-borne substances. Natural and man-made water-borne substances eventually find their way to the oceans where the water evaporates. Rains then fall over the land and fresh water resources are replenished.

We are faced with a dichotomy. On the one hand, oceans are the source of life, vital to ecological well being, and a significant economic factor in many parts of the world. On the other hand, oceans are the natural and ultimate depository for water-borne residues of man and nature.

Decisive action must be taken to regulate and control the practice of dumping deleterious wastes into oceans and coastal waters. In some instances serious problems have arisen from irresponsible dumping practices—primarily dredging spoils and municipal sewage sludges in coastal waters where the contaminating materials have washed shoreward or affected commercial and sport fishing. Such practices should be prohibited or strictly controlled. We submit, however, that there are instances where ocean disposal, if responsibly and conscientiously performed is justified and that this disposal method should not be categorically prohibited.

We would like to put forth for consideration some basic regulatory concepts and then comment specifically on the proposed "Marine Protection Act of 1971".

The hydrologic cycle is an element in a complex ecological system which must be balanced to serve mankind's needs. How we go about maintaining this balance is the central issue. It is our view that control of ocean disposal practices should not be totally divorced from protection of coastal and estuarine waters or inland fresh water lakes, streams, and ground waters. The interplay between all aspects of water pollution control dictates that a flexible grant of authority be given to the regulatory agency. The agency should have latitude to determine within reasonable bounds the control strategy and alternatives most appropriate in a particular instance.

It should be recognized that there are technical limitations on what can be accomplished in wastewater treatment and control. It is not always possible, with today's state of the art, to adequately treat and control all wastewaters to a degree which would allow the safe discharge to surface waters. Some wastewaters are presently untreatable; others may be treatable, but where treatment does not result in complete destruction, residuals may have adverse environmental consequences.

The diversity and scope of the chemical industry present a wide range of waste residuals that must be disposed of. The industry has been forced to seek various disposal alternatives when such wastes cannot be safely or economically recycled. These alternatives are deep well injection, land application, incineration, and ocean dumping. Each has its place if properly selected and conscientiously performed. The central question is which disposal alternative poses the least risk

of environmental harm. In certain instances, ocean disposal may be the only responsible alternative.

We reiterate that indiscriminate ocean dumping should be outlawed, but maintain that ocean disposal should be allowed under strict regulation. It is appropriate that a Federal agency, specifically the Environmental Protection Agency, be given regulatory authority in this matter. EPA is the only agency at either the Federal or State governmental level with broad authority for waste-water control. Other Federal agencies, such as NOAA, Corps of Engineers, and the Coast Guard, along with State agencies, may very well play an active role in the regulatory process or surveillance, but we recommend that ultimate responsibility be centered at the Federal level in EPA.

Regulatory control should take into account the quantity as well as type of material to be disposed of, the disposal site, and method of disposal. The Environmental risks of various alternative disposal means should be weighed, taking into consideration technically feasible control methods and the possible effect of onshore disposal. We envision that certain potentially toxic materials which can be practically treated should not be allowed to be discharged into the ocean, whereas other potentially toxic substances which might create greater hazards of land, air or surface water pollution be disposed of at sea under strictly controlled conditions. Disposal areas should be carefully selected and monitored for any adverse effect. Marine sanctuaries should be maintained, and fishing and recreational areas protected for present as well as future generations.

The issue is not simply one of ocean disposal, since wastes eventually find their way to the oceans, if not by direct disposal then by conveyance in surface streams and subsurface waters. Rather it is a matter of farsighted wastewater control management, soundly and effectively administered. The controlling agency must have a broad grant of authority, alternative choices, flexibility of action, and the resources to fully implement its program.

We have reviewed the various legislative proposals presently pending and feel that the "Marine Protection Act of 1971" offers the more complete and appropriate approach to regulatory control. Our recommendations in reference to it follow.

Recommendation 1—Incorporate regulation of ocean disposal as a separate Title of the Federal Water Pollution Control Act.

Control of ocean disposal should be regarded as simply one element of water quality management. Accordingly, we suggest that control of ocean disposal would most appropriately be provided for as a separate Title of the Federal Water Pollution Control Act.

Recommendation 2—Subject agencies of the Federal Government to injunctive proceedings for violations.

As the proposed Act now reads, departments and agencies of the Federal Government are exempt from the penalty provisions of Section 6. Ocean dumping, significantly the dumping of dredging spoils, has been practiced by a number of Federal agencies with alleged detriment to the environment. We believe Federal agencies should be subject to injunctive proceedings brought by the Administrator, and recommend the exception provided under Section 3(e) be limited specifically to those penalties provided in Subsections 6(a) and (b), i.e., fines and imprisonment.

Recommendation 3—Criteria establishment under Section 5 should afford interested persons an opportunity for written comment.

The development of ocean disposal criteria will affect a large number of interested parties, including other agencies of the Federal Government, State control officials, conservation and economic interest groups, permit applicants, etc. On matters as important and complex as this, criteria should be published as a proposed regulation with reasonable time given for interested persons to submit written comments thereon.

Valuable assistance and added expertise can be made available when government fosters a common spirit of cooperation and coordination in the resolution of environmental problems.

Recommendation 4—Clearly delineate between responsibility for (a) the nature of the material to be disposed of and (b) the proper deposition of such material at the permitted site.

Many barging activties are conducted by independent waste haulers who are under contract to the waste-generating party. A barge may contain wastes from a number of different sources and a party turning his wastes over to the inde-

pendent hauler may not have control over other types of wastes included with the load, the exact disposal location, or the actual disposal technique practiced.

We feel that the various responsibilities of the waste generator and the waste hauler should be specifically delineated. A possible approach would be to issue permits for various types of waste materials specifying the zone or area in which the material could lawfully be discharged. The waste generator would certify the quantity, nature of the waste material, and the permitted disposal area when the waste material was loaded upon the barge. It would then be the responsibility of the waste hauler to dispose of the material in the permitted disposal area. This could be checked by requiring reports of the log of the barge's activities including copies of the waste certificates of the barge load. This system is in accordance with the realities of most ocean disposal activities, and would provide a workable and enforceable system of control.

Recommendation 5—Include a provision to allow continued ocean disposal pending implementation of the permit system.

The reasons for not categorically banning all ocean disposal of waste material were stated earlier. Similarly, a precipitous moratorium on ocean disposal pending the implementation of the permit system would be unwise and could result in adverse environmental and economic consequences. We suggest two years as an appropriate and realistic lead time for obtaining the requisite permits. Interim measures such as prohibiting the discharge of waste materials within a 30-mile limit could be initiated if thought necessary or desirable.

Recommendation 6—Provide funding to initiate and support fundamental scientific and social research related to ocean disposal practices.

Existing knowledge of effects of ocean disposal on the actual physical, chemical and biological properties of the oceans is sadly lacking. Active research in this area should be sponsored by the Federal Government. We recommend that a system of Federal grants be established to initiate and support fundamental scientific and social research related to ocean disposal practices.

In conclusion, the chemical manufacturing industry shares the national and international concern over indiscriminate ocean dumping practices. We urge recognition that ocean disposal has its place in a sound and comprehensive water management program We support strict regulatory control of ocean disposal of waste materials.

STATEMENT OF RICHARD T. BARBER, DIRECTOR, OCEANOGRAPHIC PROGRAM, DUKE UNIVERSITY MARINE LABORATORY, BEAUFORT, NORTH CAROLINA

My experience with ocean dumping is as an oceanographer studying certain parts of the biological system in the waters over the New York Bight dump sites.

The proposed Bill H.R. 4723 seems to me to be a very good first step in improving relations between our way of life and the ocean.

The fact that the Bill regulates, the transportation, as well as dumping, (Sec. 4) of materials is wise and necessary in my opinion. I favor passage of the Bill, but I feel it could be strengthened. This bill will perhaps be adequate in the future when we have good water treatment facilities, solid waste recycling, and other environmental amenities, but I question whether this Bill will enable the Administrator to deal in a reasonable manner with our current ocean dumping problems. The acutely dangerous, small volume dumping practices, such as chemical warfare agents and explosives, can be halted, but the large volume practicer such as the New York Bight dumps, can not be halted. We have lived with and rely on this kind of ocean disposal. How will the Administrator deal with this problem?

One means would be to establish procedures such as the air pollution abatement schedules which generate solutions in a realistic manner. Given 1) authority and responsibility over the continental shelf to a depth of 200 meters; 2) public dissemination of the condition of the environment; and 3) the establishment of grades of environment (sanctuaries, moderately protected areas, and interim lower quality sites); the Administrator could effectively protect the ocean system.

On the basis of my New York Bight experience I feel that involved government units are devoting almost no effort to solving the problems of ocean dumping. The very small studies to define the problem were good. Now that the problem is defined it is time to start on the more difficult and risky work of decreasing the degradation caused by this dumping.

I hope this Bill will provide an interim mechanism for controlling existing dumping practices as well as an ultimate means for regulating future dumping practices.

STATEMENT OF HAROLD L. PERRY, WILDLIFE REPRESENTATIVE, THE HUMANE SOCIETY OF THE UNITED STATES

Mr. Chairman and Members of the Subcommittee: My name is Harold L. Perry. I am a wildlife representative for The Humane Society of the United States, with headquarters in Washington, D.C. I am stationed in Phoenix, Arizona, where I have worked on wildlife problems for the past twenty years.

The Humane Society strongly endorses the provisions of H.R. 5060 to amend the Fish and Wildlife Act of 1956 to provide a criminal penalty for shooting at certain birds, fish, and other animals from aircraft. We are totally and vehemently opposed to shooting any species of wildlife from any type of aircraft. Such activity is the worst form of distortion of the true meaning of the word "sport." More than one species is being depleted in this absurd and inhumane method of hunting. Some of them are threatened with total extinction.

We take exception to one provision of the bill, namely (b)(1), exempting employees or agents of federal or state governments. My personal observation in Arizona has proved to me that coyotes and their dens can be spotted and easily shot from aircraft. I see no need to leave an open door for government representatives to use this unfair method of killing.

We also urge that this Subcommittee consider extending H.R. 5060 to cover snowmobiles. This is another cruel and unsportsmanlike method of hunting that has grown enormously in popularity in recent years. The hunted animal has no chance as it is run down by the snowmobile and killed. Laws controlling this activity have been passed by some states but federal legislation is badly needed to stop snowmobile hunting on public lands and to declare it is against the policy of the United States Government to hunt animals in this manner.

Snowmobiles can be included in H.R. 5060 with minor changes in wording. The Humane Society urges that you do so.

STATEMENT OF ANDREWS M. LANG, PRESIDENT, WASTE MANAGEMENT AND COMPACTION SYSTEMS, INC., CHESTNUT HILL, MASS.

I would like to draw the Committee's attention to the experience which Japan has had with ocean dumping. They have, in fact, been disposing of solid wastes for some time both in shallow water situations and in deep water—i.e. Tokyo Bay which is several thousand feet deep.

In all cases, the Japanese have treated their solid wastes prior to disposal in water. The treatment has been through the process known as Intensive Compaction—which is similar to baling but at much higher compression levels and with special equipment so that the compacted wastes are consistently heavier-than-water. Also, the compacted wastes are wrapped with wire mesh and dipped in asphalt in order to maximize stability and minimize sanitation problems. The equipment used comes from the Tezuka Kosan Company of Tokyo.

There have been no significant negative results from this Japanese exercise in ocean dumping. I would, therefore, urge that the pending legislation permit ocean diposal of solid wastes so long as the wastes are given a pre-disposal treatment sufficient to guard against any adverse effects to the marine ecology.

I would also urge that the Committee, the Congress and the appropriate government agencies carry out more intensive investigation of the Japanese activities in the field so as to permit this greater application in the U.S.A.

STATEMENT OF THE AMERICAN PETROLEUM INSTITUTE

The American Petroleum Institute would like to comment on currently pending federal legislation concerning the disposal of wastes in the oceans. The Institute is in agreement with the general thrust of the various measures now being considered—namely, to bring an end to uncontrolled use of the oceans as a dumping ground for the wastes of our civilization.

Without really effective control, the problem of ocean disposal may become critical within a few years. The amount of waste to be disposed of is growing rapidly. Some of the proposed alternatives to ocean disposal—such as recycling—are still in the developmental stage and are not yet technically or economically feasible. At the same time, the responsibilities of the various jurisdictions involved—local, state, national, and international—are poorly defined.

Clearly, it is time for an effective federal program to deal with the problem. For as the Council on Environmental Quality stated in its *Report to the President* last October: "The Nation has an opportunity unique in history—the opportunity to prevent an environmental problem which will otherwise grow to a great magnitude." It may be added that an effective legislative response to the challenge laid down in the Council's report is of great importance not only nationally, but also internationally, particulary at this time. Positive action in this area now would contribute toward U.S. leadership in pollution control in the family of nations, whose representatives will be meeting at the United Nations Conference on the Human Environment in Stockholm next year. Ocean pollution is expected to be a major topic at this conference.

We believe that in order to be effective, federal legislation should embody a system for issuing permits for ocean disposal. Further, the issuance of such permits should be based upon consideration of such elements as what types of wastes may be safely disposed of in the oceans, in what quantities, and at what sites and times. Effective criteria need to be developed, along with adequate enforcement procedures and appropriate penalties for violations.

Some of the legislation now pending before Congress proposes absolute deadlines and absolute bans against all ocean disposal. Although research may one day demonstrate that some kinds of waste should not be disposed of at any site, at any depth, in any ocean, research may also demonstrate that some kinds of waste can be so disposed of without damage to the marine ecosystem. The Institute therefore would support legislation authorizing the issuance of federal permits, in accordance with carefully developed criteria.

The logical agency to coordinate such a program would seem to be the Environmental Protection Agency (EPA). We believe that EPA's Administrator should develop the criteria on which permits are based. In this task he should develop be required to consult with the National Oceanic and Atmospheric Administration (NOAA), which has or is developing the relevant expertise. We suggest further that the job of serveillance, enforcement, and general administration be delegated to the Coast Guard, which already has the necessary experienced manpower, shore installations, communications network, and ships.

In summary, the American Petroleum Institute believes:

1. That the ocean disposal of wastes should be controlled
2. That the federal level is the only level of government that can achieve effective control
3. That a system of permits based on carefully developed criteria is far preferable to absolute bans on all ocean disposal.
4. That in developing such criteria, EPA should be required to consult with NOAA
5. That the responsibility for enforcement and day-to-day administration should be delegated to the Coast Guard.

[Telegram]

WESTERN OIL AND GAS ASSOCIATION,
Los Angeles, Calif., May 11, 1971.

R. J. McELROY,
Chief Clerk, House Committee on Merchant Marine and Fisheries,
House of Representatives, Washington, D.C.:

Regret inability to participate in recent hearings H.R. 4723. Request following comments be added to hearing record which understand still open.

We are concerned that definition "material" in section 3(c) will prohibit present practice of depositing cleaned drill cuttings (rock chips) to ocean from offshore drilling operations. Also, that present practice approved by California water quality boards of depositing excess drilling mud which is an oil free and nontoxic clay and water combination into ocean will be prohibited. "Offshore

oil drilling its effect upon the marine environment" conducted by California Department of Fish and Game, January 31, 1962, shows conclusively that depositing washed drill cuttings on ocean bottom not deleterious to marine life in adjacent area.

If such ocean depositing prohibited by strict construction of section 3(c) decision would add approximately 7–9 percent to cost of offshore drilling operations. Request opportunity to discuss matter with appropriate committee personnel and staff in greater detail. Copy of report cited earlier in mail to you for attachment to this wire.

Appreciate your consideration of these belated remarks.

HENRY W. WRIGHT,
Manager, Land and Water Department.

[Telegram]

PORT OF NEW ORLEANS,
New Orleans, La., April 5, 1971.

Hon. EDWARD A. GARMATZ,
Chairman, House Merchant Marine and Fisheries Committee
House of Representatives, Washington, D.C.:

Please accept this as the position of board of commissioners of the Port of New Orleans an agency of the State of Louisiana with respect to H.R. 4723 entitled Marine Protection Act of 1971 and on which bill joint hearings are being currently held. This board administers the second largest port in the United States and is charged with the legal duty to develop and improve the port and harbor in the interest of maritime activities and the development of the Foreign Commerce of the Port and of the United States.

While this Board supports the objectives of H.R. 4723, it is its firm and considered opinion that the provisions of this bill which would relieve the Army Corps of Engineers of its authority to issue permits for the disposition of dredge spoil and place such authority with the Administrator of the new Environmental Protection Agency (EPA), will be seriously detrimental to the day-to-day operations and to the long-range development and improvement of ports and harbors and navigation channels by reason of the fact that the Corps of Engineers is eminently qualified and experienced by virtue of its vast professional knowledge and long established familiarity with and understanding of the engineering requirements in connection with dredging and other related activities required for the successful development, maintenance, and operation of ports, harbors, and navigation channels, and the like, and historically is qualified and experienced in the economic and environmental aspects and, therefore, to transfer such authority to another agency or Federal Government such as the EPA, is unnecessary, and will, in our said firm opinion, result only in delaying urgently needed projects, and further in discouraging future developments of kind in the public interests, particularly since no other Federal agency is as qualified as the Corps of Engineers to weigh the balance between economic and national defense necessities and the environmental quality considerations.

It must be remembered that the Corps of Engineers is controlled not only by the congressional processes, but itself must seek the comments and approval of environmental agencies.

EDWARD S. REED,
Executive Director, Board of Commissioners.

SIERRA CLUB, NEW ENGLAND CHAPTER,
Somerville, Mass., April 29, 1971.

Chairman EDWARD A. GARMATZ,
Committee on Merchant Marine and Fisheries,
Longworth Building, Washington, D.C.

DEAR CHAIRMAN GARMATZ: The Sierra Club, New England Chapter, strongly supports certain environmental policies of H.R. 895 as a significant first step in protecting the oceans against the reckless and excessive dumping of industrial wastes. In your consideration of this and other bills on ocean dumping, we hope you will adopt the sound policies that Mr. Harrington's bill stresses.

First, a glaring defect of the present Water Pollution Control Act, as amended, and other proposed legislation on ocean dumping is the assumption that those chemicals and wastes that are toxic can be enumerated and thus barred from the oceans. It is safe to state that experts in the field would not be able to compose such a list. The reason is that our knowledge is much too primitive; substances we once thought to be much too insoluble to be harmful turn out to be solublized by micro-organisms or by reacting with other pollutants and consequently are capable of being amplified in the food-web to toxic levels. Further, toxicity is always a function of concentration—low levels of fluoride are beneficial but higher concentrations are deadly metabolic poisons—and it is impossible to predict at present, what concentrations of industrial wastes become toxic to marine life. H.R. 805 does not contain this defect. Instead, the burden of proof of non-toxicity is on the dumper. This may appear, at first, too controversial, too unjust to business but remember a dying or dead ocean may have unfathomable effects on all mankind. In reality, this requirement will limit dumping and tend to stimulate recycling of wastes. Our knowledge and technology can make recycling a reality. If ecological considerations are paramount then suddenly recycling wastes will become much cheaper than dumping.

Secondly, H.R. 805 includes thermal pollution which the present federal legislation does not. The proliferation of nuclear and fossil power plants using ocean or estuary water for cooling purposes demands immediate Federal regulation. The rejection of excess heat into estuaries can do profound damage to these all important ocean nursery grounds.

It is these two substantive aspects of this bill, not covered by present Federal legislation, that are essential if this nation is to move towards sound ecological solutions to our very serious waste disposal problems.

Don Comb, and
Jeannette Hargroves,
Ocean Studies Group,
Sierra Club—New England Chapter.

(Committee Note.—The following information is a follow-up on material supplied for the record in the committee's hearing entitled "Administration of the National Environmental Policy Act—Part 1," Serial 91–41, on page 1264:)

Department of the Navy,
Office of Legislative Affairs,
Washington, D.C., June 23, 1971.

Hon. John D. Dingell,
Chairman, Subcommittee on Fisheries and Wildlife Conservation, Committee on Merchant Marine and Fisheries, House of Representatives, Washington, D.C.

Dear Mr. Chairman: In response to the requests made by the Subcommittee on Fisheries and Wildlife Conservation, House Committee on Merchant Marine and Fisheries, during the hearing held on December 11, 1970, I am enclosing a summary embodying the contents of a final report released by the Secretary of the Navy, of the results of the investigation of the oil dumping incident which occurred off Mayport, Florida on November 30, 1970.

Sincerely yours,

Lando W. Zech, Jr.,
Captain, U.S. Navy,
Deputy Chief.

Enclosure.

Final Report of results of Navy investigation to inquire into the circumstances surrounding the discharge of waste material which occurred off the coast of Jacksonville, Fla., on November 30, 1970

1. The review of the investigation has now been completed within the Department and the intermediate and final reviewing authorities have concurred in the findings of fact, opinions and recommendations of the investigation as they were prevously reported.

2. Review of the investigation revealed that the directive of the Chief of Naval Operations, dated October 30, 1970, regarding the implementation of the National Environmental Policy Act of 1969 was in the process of being prepared for dissemi-

nation to individual commands on November 30, 1970. By virtue of Secretary Chafee's message on this subject to all ships and stations on December 3, 1970, it became unnecessary for such implementing directives to be published by intermediate commands.

3. In paragraph III.A. of the interim summary, it was stated that the failure at Naval Station, Mayport, to insure distribution of the Chief of Naval Operations' message of September 1970, enjoining a review of procedures to prevent incidents such as this, was a breakdown in internal communications. A command inspection was conducted of the Naval Station at Mayport, with particular attention to the message distribution practices used. It was the conclusion of the inspecting officer that messages were being handled and routed in accordance with standardized methods under the supervision of knowledgeable and trained personnel, and that a recurrence of the unfortunate communications breakdown that occurred in this instance was extremely unlikely.

4. The method of disposal of oily waste waters outlined in paragraph IV of the interim summary continues in effect. This procedure has been approved, on an interim basis, by the Department of Air and Water Pollution Control of the State of Florida. The Navy is developing a water separation installation as a pilot project and, upon satisfactory testing, will prepare an emergency Military Construction item for installation at the Naval Station, Mayport, to replace the interim system.

5. The Navy has initiated a number of positive steps to reduce to an absolute minimum any future actions which could have an adverse impact on the environment. Deep water disposal of munitions has been halted while alternative methods of disposal are being reassessed. Instructions have been issued to the Fleet stressing the requirement for maximum use of in-port facilities for the disposal of shipboard wastes. The Navy is in the process of investigating various methods of recycling waste products to attain maximum recovery of usable products. A man at each major command will be designated to act as the focal point for all matters relating to the environment.

6. The Navy is most concerned with all aspects of environmental pollution and is determined to continue aggressive and, hopefully, farsighted and imaginative programs, aimed at not only eliminating degradation of the environment, but enhancing it as well.

Ecology, Law, and the 'Marine Revolution'

CARLETON RAY, Ph.D. (Columbia)

Departments of Pathobiology and Biology, The Johns Hopkins University, 615 North Wolfe Street, Baltimore, Maryland 21205, USA

ABSTRACT

The attainment of a world-wide high standard of living depends upon vastly increased resource exploitation, including the seas. Man's exploitive activities heretofore have simplified environments, reducing their stability and leading to ecosystem collapse (Fig. 1). Ecosystem ecology is rarely definitive; however, laws by which Man regulates his activities should be subject to change according to ecosystem reality. Many historically-developed modes of human activity make little sense ecologically. What we may call the 'Marine Revolution' follows the Agricultural and Industrial Revolutions as a significant change in Man's relationship with his environment. Advanced technology for ocean research and development are becoming available, but cultural and legal frameworks for regulation have not matured. A major problem in the development of a marine tradition whereby Man will not destroy marine ecosystems lies in the application of ecological 'laws' to our activities at sea. The uses of the seas are for fisheries, minerals, and mining, for furthering military interests, and for recreation. Science and technology are advanced for all of these, while conservation attempts to integrate them wisely. Legal régimes for regulation stem mainly from the four Geneva Conventions which formalize a three-and-a-half-centuries-old history of marine law. These Conventions themselves emphasize that marine law needs modification along new lines. Debate intensifies over régimes of res nullius *(belonging to no one) versus* res communis *(property of the community) for ocean exploitation. This paper concludes that exploitive 'conquest' can no longer serve as a guide for Man's use of the sea. An emphasis must be given to marine ecosystems and to the rôle of the marine ecologist in the oceanological debate. In the past, provincialism and tradition have stood in the way of international control of ocean-resource use. Should an over-riding consideration be given to ecology and to internationalism, the Marine Revolution will affect Man's future life far more beneficially than a mere evaluation of resources alone would indicate.*

INTRODUCTION

Man has not yet solved the age-old paradox upon which his civilizations have many times foundered; namely, that high population numbers with high cultural levels demand high environmental productivity, yet exploitation of Nature produces environmental destruction and ecological collapse. When the numbers of humans will come to exceed the total carrying capacity on Earth, as is already the case in many nations, no one can say; but if Man does not learn the lessons of history, there is no doubt that this catastrophic situation will occur relatively soon. The survival of Man, or anyway of civilization as we now know it, will surely depend upon how he handles this challenge.

There are two dominant features of the marine part of this challenge: first, the development of international law with enforcement for exploitation of the sea, and, second, the development of ecosystem-based conservation practices. The latter includes the cessation of

existing destructive practices, the assessment of marine environments relative to the carrying capacity of Earth for Man, and the creation of marine parks, sanctuaries, and control areas for research. These ecological aspects have to date been attacked in a piecemeal fashion. Ultimately, the answers will depend upon value judgements about what sort of a world we choose to live in. The late Fairfield Osborn (1953) asked: 'Is the purpose of our civilization really to see how much the earth and human spirit can sustain?'

This paper considers biology and law as they reflect upon what we may call the Marine Revolution. Biology and law require different approaches. The body of law by which we exercise control and responsibility is of Man's cr[illegible]on. It should reflect common sense and be capable [illegible] rational alteration. Natural phenomena may make no 'sense' at all, and their complexities are infinite. It has been stated that the ecosystem is not only more complex than we think it is; it is also more complex than we can think. The ecologist can rarely be definitive. He often experience[illegible] great difficulty in explaining, even to some fellow scientists and especially to engineers and technicians, the real nature of the ecological crisis. Ehrlich's (1969) 'Eco-catastrophe' sounds to many like alarmist stuff. yet it has a fundamental basis of perception.

To a great extent we are slaves of our own history. The *laissez faire* spirit of exploitation, the goal of economic growth, Man's socio-religious beliefs which separates him from Nature, and the conflict and case-history methods of law make little sense when applied to the environment. The emerging 'Marine Revolution' poses to those concepts a challenge which magnifies the importance of the sea far beyond its resource value. The wide recognition that this is so is reflected by the numbers of recent symposia and reports on the exploration, use, and legal régimes of the sea. Unfortunately, meetings of the American Bar Association and the Marine Technology Society, among others, have been composed almost entirely of industry representatives, lawyers, and a scattering of government officials, naval personnel, and fisheries biologists—the last mostly representing mission-oriented governmental agencies or industry. Marine ecologists have been virtually absent!

In spite of this, the intensifying debate has produced the beginnings of workable ideas. The ecosystem approach may be just over the horizon, the greatest present need being for marine ecologists to make their voices heard. If consideration for the ecosystem be added to the debate, it is possible that non-destructive and cooperative exploitation on an international basis will result, and perhaps then marine ecosystems will not suffer further.

THE MARINE REVOLUTION

Man's massive entry into 'inner space' initiates what we are calling the Marine Revolution. It is resulting in increased resource utilization and new régimes for law, politics, and socio-economics, as Man investigates, uses, and hopefully will conserve, that three-quarters of the world's surface which has been mostly foreign to him.

Agricultural and Industrial Revolutions. Some thousands of years ago, Man began to grow his own food. This change from the hunter-gatherer to the agriculturalist comprized the Agricultural Revolution It led to the diversity of occupations which marks present urban culture. The Agricultural Revolution produced more food in a more accessible form than was available to the hunter-gatherer. Food, which presumably had been a limiting factor, was limiting no more. The carrying capacity of land for humans rose and the population grew accordingly.

The Industrial Revolution has been going on for the last two centuries or more. It has been marked by the growth of science and technology, by increased resource-use, and by expanded diversity and efficiency of human skills. It has meant a turning away from the agricultural way of life to an increasingly urbanized and 'artificial' one. It once again increased the carrying capacity of the land for human beings and led to a spectacular decrease in death control without concomitant birth control. Most significantly of all, the Industrial Revolution, in its greed for resources, has produced environmental destruction at an astounding and dangerous pace. Forests have been cut, land has been eroded and stripped, bays have been polluted and filled, and the result of all of these and other activities has been to lower the long-term carrying capacity of land for future human populations, notwithstanding the temporary increase which technology has made possible. Such environmental wastage makes our wish to provide a better life for our children seem to be sheer hypocracy.

The Marine Revolution. Thus does Man turn to the seas which become increasingly vital for his resources. However, the Marine Revolution is not totally a consequence of the exhaustion of the land. Man also turns to the sea as it lies before him in the form of a challenge which he is now becoming technologically able to accept. 'Products are sold on an open world market that cares nothing about the origin of the material; one competes only against price' (Bascom, 1966).

Thus, we accept the challenge of the sea, being not a little starry-eyed over our technology. But we must remind ourselves that Man remains a hunter-gatherer in the oceans; in only an insignificant few places does

he farm the sea. This contrast between developing technology and the inadequacies of cultural and legal frameworks for regulation is a characteristic of 'revolution'.

The Marine Revolution is, to my mind, quite as important a development as the previous Agricultural and Industrial Revolutions. It is no more obvious on a day-to-day basis than the Agricultural and Industrial Revolutions were in their time. Future Man will clearly see this Revolution as his inner-space logistics and utilization increase.

ECOSYSTEMS AND HOMEOSTASIS

The ecosystem is the fundamental functional unit of the natural world. It is comprised of all the living and non-living components of an environment and the totality of their interrelationships. An ecosystem has properties of self-sustainment. Solar energy must be added, but nutrients and other materials are recycled. Examples are a lake, a forest, an estuary, and a coral reef.

Carrying Capacity, Limiting Factors, and Synergisms. Carrying capacity may be defined as the number of individuals of a species within a particular ecosystem beyond which no major increase in numbers may occur. It fluctuates about an equilibrium level and may change seasonally or even daily. It is regulated according to Liebig's 'law' of the minimum and Shelford's 'law' of tolerance, which together state that the presence or abundance of an organism locally is determined by the amounts of critical materials available or by the local levels of environmental factors such as salinity or temperature.

It is typical of ecology that 'laws' are easy to state but difficult to prove. A major reason for this is *synergism*; that is, environmental factors often act together to produce effects which are different quantitatively or qualitatively from the effects expected separately or additively. Carrying capacity and limiting factors apply to all living things. The foolish assumption is that technology may negate them for Man. Technology cannot alter ecological laws, though it can redirect utilization in limited ways.

Productivity. Productivity is determined by turnover rate. The standing crop or biomass is a poor indicator of this, as it tells little about how often materials are recycled. Plants absorb about one per cent of solar energy for photosynthesis. An examination of trophic levels from these producers to primary, secondary, or tertiary consumers, reveals that each step involves about a 90 per cent loss of energy. Thus, food-chains are usually short and each trophic level shows much lower productivity than its predecessor.

Nutrients, unlike energy, are recycled. The biogeochemical cycles of gases, salts, and minerals, are most efficient in complex ecosystems. Man can occasionally increase productivity through the addition of substances which once were limiting. More often, his 'making the desert bloom' fails in the long run through failure to recognize the interrelationships of these cycles.

Primary productivity varies widely. Deserts and the waters of the deep oceans, which together cover most of the Earth, produce less than one gram of dry organic matter per square metre per day. Grasslands, waters over the continental shelf, and marginal agriculture produce 0·5 to 3 gm; moist forests and agriculture produce 3 to 10 gm; estuaries, inshore seas, and intensive agriculture produce 10 to 25 gm (Odum, 1959).

Owing to their large total productive area and volume, the seas contain more living material than the land supports. However, Man's utilization is at a higher trophic level in the sea: land = sun → grass → cow; sea = sun → phytoplankton → zooplankton → primary carnivore (e.g. herring) → secondary carnivore (e.g. tunny). The seas contain a much greater total diversity of life in terms of classes of animals than does the land, but owing to the lower oxygen content of water than air, the seas are dominated by animals of lower metabolic rate, but higher ecological efficiency than birds and mammals. Lastly, the sea provides a more stable environment than the land; in it, the 'weather' is mild and the productive season is long. For all these reasons, marine productivity is not equivalent to that of land.

Homeostasis, Simplification, and Pollution. Homeostatis defines the 'balance of nature'. All ecosystems depend upon recycling for sustainment and upon complexity for stability. These involve intricate mechanisms analogous to (but more complex than) the heat-producing, dissipating, and conserving mechanisms which regulate human body temperature. Ecosystems are never perfectly balanced, but homeostatic mechanisms give them recuperative power which, when exceeded, leads to breakdown, the eutrophication of Lake Erie is a classic example of such excess.

A major part of homeostasis lies in complexity which insures both productivity and stability, and also has aesthetic value for Man (Elton, 1958; Dasmann, 1968). Man is a simplifier of ecosystems and thus reduces their recuperative power. The many forms of pollution are the most serious stresses in this regard. Historically, Man has depended upon maximum homeostatic capacities of the environment to endure pollution; but in simplifying and polluting at the same time, he attacks with a two-edged sword.

Is the ocean too large to disrupt? I think not. According to the Task Force on Environmental Health and Related Problems (1967), the American people and their environment are being exposed to half-a-million different alien substances with 20,000 new ones being added each year. Some of these go to sea. For instance, pesticides have been distributed throughout the world's oceans through the vectors of air and precipitation (Frost, 1969). Polikarpov (1966) suggests that radionuclide pollution of the seas may already be at a dangerous level for some organisms Hedgpeth (in press) remarks than our standards for waste disposal are anthropocentric and that laboratory tests on pollutants are 'interesting, but possibly academic as far as the real world is concerned'—in other words, waste-level standards set for Man are not necessarily those which ecosystems will tolerate.

MAN'S USE OF THE SEA

Only recently has Man begun to explore the sea throughout its three dimensions. The first extensive exploration of the deep sea was in 1873–76 by *H.M.S. Challenger*. Not quite a century later, Man has visited the ocean's deepest place in a research submarine and knows that all marine waters are capable of supporting life.

The Marine Revolution consists of five major aspects, which are related to, but by no means coincidental with, its dominating challenges mentioned in the Introduction. These aspects are: fisheries, minerals and mining, military interests, science and technology, and conservation and recreation Emery (1966) gives world values of marine resources in 1964 as follows: biological—US $\$6{\cdot}4 \times 10^9$; geological—US $\$3{\cdot}6 \times 10^9$; and chemical—US $\$1{\cdot}3 \times 10^9$. Biological resources will always be the most valuable, even if surpassed economically, for Man cannot exist without them, and they are largely renewable.

Fisheries. Fisheries remain the most difficult aspect of international law of the sea. This is due mainly to the fact that most commercially important marine animals move and cannot be claimed. It is ludicrous to discover that certain benthic organisms are, in fact, classified as 'minerals' under the Convention of the Continental Shelf. In some cases it is of advantage to the exploiter that they should be so classified, an instance being the Alaska King Crab (Oda, 1968); in other cases the reverse is true, instances including some shrimps (Neblett, 1966). Fisheries resources include various Algae, plankton, shellfish, fishes, turtles, and mammals (Walford, 1958); but, as has been pointed out above, Man's utilization represents only a fraction of total marine productivity.

Over-utilization continues to dominate fisheries, especially off-shore ones. Clark (1967) states that Japanese long-lining accounted for almost a million billfishes in 1965. Even larger quantities of tunny were taken. Evidence is accumulating that such utilization cannot be sustained. Perhaps even more serious than overfishing is inshore habitat destruction. Over two-thirds of all commercial and sport fishes of the eastern United States depend upon inshore environments at some critical time of their life-cycle. The most effective way to extirpate a species is by environmental disruption, and this is being done inshore at a rapid pace.

Consideration of energetics lead many to propose exploitation at lower trophic levels. Complex size/metabolic factors and fishing efficiency strongly indicate, however, that higher-order consumers are more effective fishermen and converters of energy than Man is. A total 'plankton' fishery should be considered as a last, and none too satisfactory, resort. Those who have taste-tested swordfish and plankton might agree! The choice, however, should not be between swordfish and plankton; given proper management, we could have both.

The concept of 'yield' is vital biologically and legally. Fisheries biologists have emphasized the asymptotic attainment of maximum biomass through controlled utilization. Such a yield may or may not conform to economic efficiency or to local market value—hence the preference of 'optimum' over 'maximum' yield (Crutchfield, 1968).

W. M. Chapman (1966) states an exploitive point of view: 'When the fishing effort has increased beyond the point of maximum sustainable yield, the fishing can ordinarily be permitted to expand without serious damage to the resource'. He ignores Allee's principle (Odum, 1959), which is that density is in itself a limiting factor for population growth and survival. Relative abundance of the species in a community is a contributor to homeostasis. Thus, it is biologically most sound to change population size as little as possible in natural systems.

Christy (1966) considers broader aspects of utilization: '. . . somehow or other it will be necessary to limit the number of fishermen that can participate in a fishery. Such limitations can be achieved only by further restricting the "freedom of the seas"; and this clearly raises questions about the meaning of this freedom and about the distribution of wealth.' This approach appears to me more susceptible to ecological application than Chapman's more narrowly-stated views.

Aquaculture presents different sorts of problems from hunter-gathering, and may be the dominant provider of the future. Aquaculture is a major concern of the US Sea Grant Program (Abel, 1968). Ryther & Bardach (1968) and Bardach & Ryther (1968) review aquaculture and make the point that it will be carried out largely along coasts—exactly the areas currently most stressed at the hand of Man. To reconstitute coastal environments, or to fertilize them artifically, is difficult or impossible. The key to aquaculture is clearly the maintenance of natural productivity.

Minerals and Mining. Reading in this field often leaves one impressed with the viewpoint that somehow we are slaves to 'economic growth'. Close (1968) speaks of 'the care and feeding of a gigantic industrial complex'. One hopes that only a segment of industry would speak so carelessly, but it does appear true that an awareness of ecology and a willingness to exploit the non-living resources at little or no expense to the living are indeed rare. If mineral exploitation continues by sea as it has by land, the predictable results are frightening to contemplate. Strip mining is one parallel example.

Mero (1966, 1968), Luce (1968), and Young (1968), review the diversity of mineral resources in the sea. Inshore mineral exploitation is already heavy, but a consensus exists that only a few minerals, such as oil and gas, are currently feasible of exploitation. This is evidently based upon the lack of a favourable legal and economic climate, not upon the lack of technological capability. Further, it is not true that exploitation will progress from shallower to deeper water, any such progress being a function of the resource sought (Wilkey, 1969).

Off-shore mineral production in 1968 was 6 per cent of the world total and of it oil and gas accounted for 84 per cent (Economic Associates, 1968). In 1965, 16 per cent of the free world's oil was produced off-shore, the result of the work of 325 rigs which have drilled many thousands of wells (Dozier, 1966); oil has been produced from wells in as much as 104 m of water (Wilkey, 1969), and exploratory drilling was carried out in 1968 in the Gulf of Mexico in over 3600 m. At any one time, about 30,000,000 tons of oil are at sea in tankers. From US off-shore wells alone the production of oil has so far been 2×10^9 barrels,* and of gas $5{\cdot}5 \times 10^{12}$ ft^3,* at an investment of US $6 thousand million, and with the ultimate potential of 15-35 thousand million barrels of oil and $90–170 \times 10^{12}$ ft^3 of gas (Nelson & Burk, 1966). The massive pollution potential of the oil industry has been previewed by the tragic *Torrey Canyon* and Santa Barbara disasters. We can be certain that these episodes are not the last of their kind, and probably there will be far bigger ones.

* 1 barrel = *ca* 200 litres, 1 cubic metre = *ca* 30 cubic feet.

Military Interests. Military activities in the oceans are shrouded in secrecy. It would, for instance, be interesting to know what the degree of radio-nuclide pollution is from Soviet and US nuclear-powered submarines. Both Harlow (1966) and Hearn (1968) give as the US Navy's viewpoint the contention that maximum freedom to use all dimensions of the sea must be maintained in order to exploit naval strength to the fullest in the best national interest. I think it fair to state that such a position is shared by the military of other major powers. The effect is to raise a serious obstacle to internationalization, to expanded territorial jurisdiction, and to peaceful use of the sea-floor.

It is difficult for me to understand why putting the sea-bed under a 'peaceful purposes only' treaty, as has already been done for outer space and Antarctica, is not in the 'best national interest'. Evidently, military influence was a major factor in preventing that principle from being accepted at the 1967 United Nations debate on the subject (Eichelberger, 1968). As yet the sea-bed is not much utilized militarily, though the waters over the floor of the sea certainly are. Thus, it is particularly disturbing to read that 'military strategists . . . have been looking for better ways to put the sea to use for the purposes of national defense' (New York Times, 1969).

It must be pointed out that military interests are not necessarily contrary to fishing or mineral exploitation. In any case, international progress on these last should not be held up by conflicts with the military authorities.

Science and Technology. The United States, among other nations, is heavily committed to marine exploration, science, development, and conservation. Reports on the highest level are numerous, including. Interagency Committee on Oceanography (1963, 1967); National Academy of Sciences (1964, 1967, 1969); Panel on Oceanography, President's Science Advisory Committee (1966); National Council on Marine Resources and Engineering Development (1967, 1968*a*, 1968*b*); and the Commission on Marine Science, Engineering and Resources (1969).

The last-mentioned, the so-called Stratton Commission Report, departs courageously from—while also building upon—the baseline established by its predecessors and is no doubt the most significant of them all. It is broadly ecological and international in nature, and recommends a US National Oceanographic and Atmospheric Agency for centralization of US research, exploration, data collection, and education. Further, it proposes an International Registry Authority for

ocean claims—with régimes for ocean bottoms, a delineated continental shelf, and an intermediate zone. The Commission also stresses optimal use of coastlines on a long-term basis in which industry, water quality, and aquaculture would be regulated under Federal law to guard against deterioration of the inshore marine environment. A useful review of this Report, including both the pros and the cons, is provided by the Program of Policy Studies in Science and Technology (1969).

Looking not at reports, but at budgets, produces some dismay. Ocean Science News (1969) states the current US Federal commitment to marine matters to be $528 million per annum, of which only $150·6 million is in basic and applied research, $143 million being in national security—and this in the very year of Man's travel to the moon and continued development of supersonic transport! The overall oceanic budget has grown 22 per cent since 1968, when Economic Associates, Inc. (1968) remarked: 'what remains to be pointed out is the very low level of Federal expenditure on . . . resources and their environment, compared with Federal oceanologic programs in general and, decidedly so, with the Federal effort in such a field as outer space'.

The International Biological Programme's Marine Productivity section deserves mention. The IBP theme of 'The biological basis of productivity and human welfare' is ideally suited to the needs of Man during the initial period of the Marine Revolution. However, at the current level of funding (only US $7 million for all US IBP sections in fiscal year 1970), it is certain that IBP cannot fulfil its goals.

Conservation and Recreation. To many, conservation and recreation involve *inter alia* the establishment of parks, sanctuaries, and control areas for research (Ray, 1961, 1965, 1966, 1968; V. J. Chapman, 1968; Randall, 1969). However, conservation and recreation must not be confined to protected areas. Both must principally be concerned with the maintenance of ecosystem homeostasis on a world-wide basis, and this is a large order indeed.

The concepts of conservation have been developed for terrestrial environments and are only vaguely applicable to the sea. The oceans together occupy a vastly larger part of the biosphere than does the land, and they are more continuous. The sea's rate of change, its biotic complexity, and our ignorance of its three-dimensional hydrosphere, are of a different order of magnitude from their counterparts on the more familiar land. For both land and sea, modern conservationists have become less concerned with the placing of 'fences' about sea or landscape, valuable as protective measures are, than with an ecological concept of the total ecosystem of which Man forms a part. A good basis of conservation policy exists for land and, in part, for inshore seas. For the high seas, this is not the case.

LEGAL RÉGIME OF THE SEA

Ultimately, Man's marine activities of all kinds must be legally regulated. Griffin (1967) states: 'To a large extent, a period of legal conjecture is ending.' The problem is '. . . to evolve policies and a legal régime which will maximize all beneficial uses of ocean space. . . . Under no circumstances, we believe, must we ever allow the prospects of rich harvest and mineral wealth to create a new form of colonial competition among the maritime nations.' A contrary view is that of Ely (1967*a*): 'Above all, we should not now cede to any international agency whatsoever the power to veto American exploration of areas of the deep sea which are presently open to American initiative. We can give away later what we now keep, but the converse is sadly false.' Ely (1967*b*, 1968) later extended these views.

Basically, the argument concerns whether the sea and sea-floor are *res nullius* (belonging to no one but subject to claim) or *res communis* (property of the world community).

Eichelberger (1968) puts the matter another way when he says: 'Either [the sea] opens up another threat of conflict or another area of cooperation.' Of course, the argument is not so simple. As Friedham (1966) and Belman (1968) point out, traditional law of the sea is imperfect, but there is legitimate hesitancy towards creating new modes when our experience with the sea and our ignorance of its resources are both still great.

Historical Background. In 1609, Grotius wrote *Mare Liberum* as a challenge to national jurisdiction of areas of ocean. This brief for the Dutch Government was directed towards breaking the Portuguese monopoly of the East Indies spice trade. Gradually, and in partial response to struggles for supremacy between Britain and Spain, the principle of 'freedom of the high seas' was accepted.

The concept of a territorial sea was born when Bijnkershoek wrote *De Domino Maris* in 1702. A territorial width of three nautical miles (*ca* 6 km) has been attributed to the distance of a cannon-ball shot, but the range of cannon at the time was only a single nautical mile. Probably the three-mile limit began with a British instruction to her Ambassadors, in 1672, that control should be exercised one marine league (= 3 nautical miles) from shore (Weber, 1966). Three

nautical miles was never adopted as a limit universally; claims of up to 12 such miles (*ca* 24 km) have always been valid.

A Convention of 1884 sustained all states' rights to lay cable on the deep sea-floor; but it was not until the Treaty of Paria, between Britain and Venezuela in 1942, and the Truman Proclamation of 1945, that any state claimed jurisdiction and control over any part of the sea-floor. By its important action, the United States effectively laid claim to an area of shelf larger than Alaska and Texas combined.

Three-and-a-half centuries of precedent thus led to recognition of the following zones: (1) internal waters and bays within the control of the coastal state; (2) territorial sea under the control of the coastal state; (3) continental shelf over which the coastal state might claim control; (4) contiguous zones for special purposes; (5) the high seas, held to be *res communis*; and (6) the deep sea-floor, held to be *res nullius*. New technology for ocean research and exploitation after World War II indicated obvious conflict under this system.

The International Law Commission had been created in 1947 under the United Nations. It proposed in 1956 that a Conference on Law of the Sea be held. This occurred in 1958 at Geneva and adopted four Conventions as follows:

(1) *Territorial Sea and the Contiguous Zone*: ratified 10 September 1964. This Convention confirmed the control of the coastal state over all resources within a territorial sea. In addition, the coastal state might declare control over a contiguous zone for security, customs, fiscal, immigration, or sanitary purposes, but not to interfere with the right of innocent passage. The width of the territorial sea is still undecided. Of 91 coastal states, 50 declare 12 nautical miles, 17 declare more than 12, 10 declare between 3 and 12, and 14 declare 3 such miles (Oda, 1968). A narrow territorial sea is favoured by military interests and by states with international fishing fleets; Japan is the only major fishing nation which adheres to three miles. A wide territorial sea is favoured by states wishing to protect a coastal fishery. Obviously, the US has been in a delicate position and only recently declared 12 nautical miles to be the width of its territorial waters.

(2) *High Seas*: ratified 30 September 1962. This includes all waters outside territorial ones and declares freedoms of navigation, overflight, fishing, and the laying of submarine cables and pipelines. Also included are regulations on piracy and pollution.

(3) *Continental Shelf*: ratified 10 June 1964. This Convention is mainly concerned with the sea-floor and does not include the water lying above. It has already been pointed out that certain living resources are included. The most serious contention concerns the extent of the shelf, which is defined in the Convention as extending: '. . . to the sea-bed and subsoil of the submarine area adjacent to the coast, but outside the area of the territorial sea, to a depth of 200 metres or, beyond that limit, to where the depth of the superjacent waters admit of the exploitation of the natural resources of the said areas.' Two schools of thought prevail here. One contends that as this Convention is entitled 'Contenental Shelf', the sea bottom beyond its geographic limits of about 200 m depth is not included. The other contends that the exploitability provision defines a 'juridical shelf' which could include the slope or even the whole ocean bottom. It should be kept in mind that the shelf area is a huge one; without the slope it comprises 10×10^6 mi^2 (about 28×10^6 km^2), which is equal to 20 per cent of the total land area on Earth (Mero, 1966, 1968). An excellent review of the problem is that of Tubman (1966).

(4) *Fishing and Conservation of Living Resources of the High Seas*: ratified 20 March 1966. This remains the most controversial of the Conventions, being the only one which did not more or less standardize a body of existing custom but which contained genuine innovation. The problem that one non-cooperating state could vitiate fishery conservation efforts was a major reason for calling the Geneva Conference. This Convention 'virtually forces consideration of the need for conservation of a fish stock by all participating nations if only one (or an adjacent coastal state) insists on it,' but 'it says nothing about the principles to be followed, nor, more fundamentally, about the objectives sought' (Crutchfield, 1968). It does not treat allocations or provide more than case-by-case consideration of conservation.

Prognosis. Christy (1968) outlines four approaches to the developing law of the sea. The 'wait and see' approach leaves exploitation to chance. Support for wait-and-see comes in part from proponents of case law who heed the dictum of Oliver Wendell Holmes: 'The life of the law is not logic, but experience.' Additional support accrues from those who note our lack of knowledge and experience in the sea.

The second approach is that of the 'national lake'. The obstacle here is that the division of the sea would be highly inequitable. The USSR would get little, whereas tiny oceanic islands would gain title to huge territories.

The 'flag' approach is the third. It is supported mainly by mineral and military interests of powerful nations. Burke (1966*a*, 1966*b*, 1968, 1969), McDougal (1968), and Wilkey (1969), all defend this point of

view, emphasizing traditional processes of mineral claim on and under a sea-bed held to be *res nullius*. Some are willing to make concessions on an international registry or towards cooperation in pollution and security. On the other hand, Young (1968), Krueger (1968), and Eichelberger (1968), hasten to point out that the flag approach is but a form of neo-colonialism which would rapidly lead to a gold-rush. Nor does the flag approach, with its unavoidable competitive nature, make much sense ecologically.

The last alternative is 'international'. Kruger (1968) and Eichelberger (1968) lucidly point out the obsolescence of nationalism and the fact that most small nations will view internationalism as the only legitimate approach to the sea. Furthermore, mineral resources required by the industrial nations are spread throughout the international market, necessitating international trade.

The United Nations has shown its resolve by a series of resolutions. One of 31 December 1968, designated Resolu.ion 2467A–2467D (XXIII), includes the following points: (1) promotion of international co-operation; (2) exploitation for the benefit of mankind; (3) prevention of pollution; (4) desirability of peaceful use of the sea-bed; and (5) endorsement of an International Decade of Ocean Exploration.

I find it impossible to argue against any of these goals, and equally impossible to see an alternative to internationalism in achieving any of them. Precedents of treaties on Antarctica and outer space exist though both Young (1968) and Eichelberger (1968) point out that the ocean floor is not *tabula rasa* (i.e. a 'blank slate') as were in some senses both Antarctica and outer space. However, they do not point out that virtually all of Antarctica was under territorial claim, and that nuclear testing and exploration had been carried on in outer space before those treaties were signed. Both treaties involved a yielding of claims and nullifications of military interests. It is difficult to see why such yielding could not also take place for the sea-floor, the superjacent waters, and even some sections of shelf. One thing is certain; under no reasonable circumstances would the exploiter lose by international control. All that might ensue would be more efficient utilization and a cleaner sea.

Gargantuan problems exist with regard to internationalism. Burke (1966*a*, 1966*b*, 1968, 1969), Alexander (1966), and Griffin (1967), review the problems of disarmament, bilateral and multilateral agreements, the extent of off-shore claims, scientific freedom in research, and many others. Burke (1969), particularly, examines difficulties in applying the Stratton Commission Report. However, one should not be deterred from a path simply because it is stony.

CONCLUSION

The sea lies today like a huge plum which Man is ready to pluck but towards which he gropes in quandary. This paper emphasizes the application of ecology to this Marine Revolution. We see that historically we have grown to treat the sea as the land—exploitively and as a 'frontier' to be conquered. There is no longer room for doubt that this is a collision course and that the 'conquest' of Nature threatens Man's existence as a species with high 'culture'.

Much as we might wish it so, the sea is not a placebo for our destruction of the land. The very existence of Conventions on the sea are cause for optimism and proof of awareness of the need for change To the international lawyers belongs most of the credit. However, there persist such items as the 'house' lawyer's fear of loss of proprietary rights, the industrialist's fear of loss of claim, and the fisherman's fear of loss of *laissez faire* exploitation. Many maintain that we do not yet know enough about the sea, nor do we have sufficient experience with it, to change our *modus operandi*. Nevertheless, one must agree with Belman (1968): 'If law awaits developments, it loses the ability to shape them.'

The ecosystem principle must serve as the overriding guide for shaping our future resource use. We simply do not dare exceed limits of homeostasis in the sea. Ripley (1966) states: 'The basic problem therefore is to acquire sufficient knowledge about our ecosystems to provide feedback controls essential to homeostasis.' It is true that we do not as yet have all the knowledge we might desire, but it is also true that we know enough now to be able intelligently to monitor our actions. We *can* assume that every one of our actions puts some stress on the environment. We *can* put aside expediency, tradition, and false economic idols. We *can* negate flimsy and obsolescent national boundaries. We *can* shift the burden of proof for ecological damage from the plaintiff-community to the defendent-exploiter. The problem is not the ability to change; it is the desire and necessary understanding.

A new brand of environmental biologist must become increasingly involved in the Marine Revolution; without him, no purely political or legal solution will suffice. Non-biologists, even lay conservationists, have too rarely shown comprehension of the complexities of the living world and they are not equipped to deal with the sophistication of ecosystem ecology. However, the biologists have been largely unwilling to commit themselves. Darling (1967) has pinpointed part of the problem: '. . . public policy has to be ahead of public consensus. . . ecology and conservation

can move surely into the hurly-burly without losing scholarly integrity, a course most of us must be prepared to follow . . .'. Biology must to a new degree achieve interaction with politics and the law. Scientific integrity must be defended and this is not in conflict with a willingness to 'stick one's neck out'.

There is apparently no end in sight either to Man's reproductive potential or to his infinite conceit that he shall inherit the (still productive?) Earth. Yet there is a limit to the sea as to the land. The uniqueness of the Marine Revolution lies in part in the fact that Man is recognizing the limits of the Earth as he is developing exploitation of its most remote and unknown region—the oceans and seas. It also lies in the fact that the oceans' and seas' uncertain ownership forces Man at last to consider alternatives to provincialism and nationalism. Indeed it may be said that the Marine Revolution, for the first time in Man's history, ties survival with international cooperation.

Acknowledgements

To the following I owe thanks for helpful comments on this paper: Eugenie Clark, University of Maryland; Raymond F. Dasmann, Conservation Foundation; Sidney R. Galler, Smithsonian Institution; Roger M. Herriott, The Johns Hopkins University; A. Starker Leopold, University of California (Berkeley); Nicholas Polunin, *Biological Conservation*; John E. Randall, Bishop Museum, Honolulu, Hawaii; George W. Ray, jr, private lawyer; Frank M. Potter, jr., Environmental Clearinghouse; Charles H. Southwick, The Johns Hopkins University; Richard Young, private lawyer. I am indebted to all these persons, for their very constructive criticism.

References

ABEL, Robert B. (1968). A history of federal involvement in marine sciences: emergence of the National Sea Grant Program. *Natural Resources Lawyer*, 1, 105–14.

ALEXANDER, Lewis M. (1966). Offshore claims of the world. Mimeo. for *Law of the Sea Institute*. University of Rhode Island, June–July, 8 pp.

BARDACH, John E. & RYTHER, John H. (1968). *The Status and Potential of Aquaculture. Vol. II: Particularly Fish Culture*. Amer. Inst. Biol. Sci., Clearinghouse for Fed. Sci. & Tech. Information, Springfield, Virginia, 225 pp., illustr.

BASCOM, Willard (1966). Mining in the sea. Mimeo. for *Law of the Sea Institute*, University of Rhode Island, June–July, 1 p.

BELMAN, Murray J. (1968). The role of the State Department in formulating federal policy regarding marine resources. *Natural Resources Lawyer*, 1, 14–22.

BURKE, William T. (1966*a*). Technological development and the law of the sea. Mimeo. for *Law of the Sea Institute*, University of Rhode Island, June–July, 18 pp.

BURKE, William T. (1966*b*). Legal aspects of ocean exploitation—status and outlook. Pp. 1–23 in *Exploiting the Ocean. Trans. Second Annual Mar. Tech. Soc. Conf. & Exhibit.*, Mar. Tech. Soc., Washington D.C.

BURKE, William T. (1968). A negative view of a proposal for United Nations ownership of ocean mineral resources. *Natural Resources Lawyer*, 1, 42–62.

BURKE, William T. (1969). Law, science and the ocean. Mimeo. for *Law of the Sea Institute*, University of Rhode Island, August, 34 pp.

CHAPMAN, V. J. (1968). Underwater reserves and parks. *Biol. Conserv.*, 1, 53.

CHAPMAN, Wilbert M. (1966). Fishery resources in offshore waters. Mimeo. for *Law of the Sea Institute*, University of Rhode Island, June–July, 18 pp.

CHRISTY, Francis T., Jr. (1966). The distribution of the seas' fisheries wealth. Mimeo. for *Law of the Sea Institute*, University of Rhode Island, June–July, 14 pp.

CHRISTY, Francis T., Jr. (1968). Alternative régimes for the marine resources underlying the high seas. *Natural Resources Lawyer*, 1, 63–77.

CLARK, Eugenie (1967). The need for conservation in the sea. *Oryx*, 9, 151–3.

CLOSE, Frederick, J. (1968). *The Sleeping Giant. An Industrial Viewpoint on the Potential of Oceanography*. Mar. Tech. Soc., Washington, D.C. Reprinted by Alcoa, Pittsburgh, 12 pp.

COMMISSION ON MARINE SCIENCE, ENGINEERING AND RESOURCES (1969). *Our Nation and the Sea*. US Government Printing Office, Washington D.C., xi + 305 pp.

CRUTCHFIELD, James A. (1968). The convention on fishing and living resources of the high seas. *Natural Resources Lawyer*, 1, 114–24.

DARLING, F. Fraser (1967). A wider environment of ecology and conservation. *Daedalus*, 96, 1003–19.

DASMANN, Raymond F. (1968). *A Different Kind of Country*. Macmillan, New York, and Collier-Macmillan, London, viii + 276 pp., illustr.

DOZIER, J. R. (1966). Offshore oil and gas operations present and future. Mimeo. for *Law of the Sea Institute*, University of Rhode Island, June–July, 11 pp.

ECONOMIC ASSOCIATES, INC. (1968). *The Economic Potential of the Mineral and Botanical Resources of the US Continental Shelf and Slope*. A Study Prepared for the National Council on Marine Resources and Engineering Development, Clearinghouse for Fed. Sci. & Tech. Information, Springfield, Virginia, 520 pp.

EHRLICH, Paul (1969). Eco-catastrophe. Reprinted from *Ramparts*, Int. Planned Parenthood Fed., New York, September, 5 pp.

EICHELBERGER, Clark M. (1968). A case for the administration of mineral resources underlying the high seas by the United Nations. *Natural Resources Lawyer*, 1, 85–94.

ELTON, Charles S. (1958). *The Ecology of Invasions by Animals and Plants.* Methuen, London, and John Wiley, New York, 181 pp., illustr.

ELY, Northcutt (1967*a*). American policy options in the development of undersea mineral resources. Mimeo. for *90th Ann. Meeting Amer. Bar Assn*, Honolulu, August, 10 pp.

ELY, Northcutt (1967*b*). The administration of mineral resources underlying the high seas. Mimeo. for *Amer. Bar Assn National Institute on Marine Resources*, Long Beach, California, June, 12 pp.

ELY, Northcutt (1968). A case for the administration of mineral resources underlying the high seas by national interests. *Natural Resources Lawyer*, 1, 78-84.

EMERY, K. O. (1966). Geological methods for locating mineral deposits on the ocean floor. Pp. 24-43 in *Exploiting the Ocean. Trans. Second Ann. Marine Tech. Soc. Conf. & Exhibit.*, Mar. Tech. Soc., Washington D.C.

FRIEDHAM, Robert L. (1966). Conflict over law: voting behavior at the United Nations Law of the Sea Conference. Mimeo. for *Law of the Sea Institute*, University of Rhode Island, June-July, 16 pp.

FROST, Justin (1969). Earth, air, water. *Environment*, 11, 14-33.

GRIFFIN, William L. (1967). The emerging law of ocean space. *Int. Lawyer*, 1, 548-87.

HARLOW, Bruce A. (1966). Territorial sea concept. Mimeo. for *Law of the Sea Institute*, University of Rhode Island, 1 p.

HEARN, Wilfred A. (1968). The role of the United States Navy in the formulation of federal policy regarding the sea. *Natural Resources Lawyer*, 1, 23-31.

HEDGPETH, Joel (in press). Atomic waste disposal in the sea: an ecological dilemma. In *The Careless Technology* (Ed. M. Taghi Farvar & John Milton). Natural History Press, Doubleday, New York.

INTERAGENCY COMMITTEE ON OCEANOGRAPHY (1963). *Oceanography, the Ten Years Ahead.* ICO Pamphlet No. 10, Federal Council for Science and Technology, Washington D.C., 54 pp.

INTERAGENCY COMMITTEE ON OCEANOGRAPHY (1967). *National Oceanographic Program.* ICO Pamphlet No. 24, Federal Council on Science and Technology, iv + 107 pp.

KRUEGER, Robert B. (1968). The Convention on the Continental Shelf and the need for its revision and some comments regarding the régime for the lands beyond. *Natural Resources Lawyer*, 1, 1-18.

LUCE, Charles F. (1968). The development of ocean minerals and law of the sea. *Natural Resources Lawyer*, 1, 29-35.

McDOUGAL, Myers S. (1968). Revision on the Geneva Convention on the law of the sea (Comments). *Natural Resources Lawyer*, 1, 19-28.

MERO, John L. (1966). Review of mineral values on and under the ocean floor. Pp. 61-78 in *Exploiting the Ocean. Trans. Second Ann. Mar. Tech. Soc. Conf. & Exhibit.*, Mar. Tech. Soc., Washington D.C.

MERO, John L. (1968). Mineral deposits in the sea. *Natural Resources Lawyer*, 1, 130-7.

NATIONAL ACADEMY OF SCIENCES (1964). *Economic Benefits from Oceanographic Research.* NAS Publ. 1228, 50 pp.

NATIONAL ACADEMY OF SCIENCES (1967). *Oceanography 1966: Achievements and Opportunities.* NAS Publ. 1492, 183 pp.

NATIONAL ACADEMY OF SCIENCES (1969). *An Oceanic Quest: The International Decade of Ocean Exploration.* NAS Publ. 1709, 115 pp.

NATIONAL COUNCIL ON MARINE RESOURCES AND ENGINEERING DEVELOPMENT (1967). *Marine Science Affairs—A Year of Transition.* US Govt. Printing Office, Washington D.C., v + 157 pp.

NATIONAL COUNCIL ON MARINE RESOURCES AND ENGINEERING DEVELOPMENT (1968*a*). *International Decade of Ocean Exploration.* US Govt. Printing Office, Washington D.C., i + 7 pp.

NATIONAL COUNCIL ON MARINE RESOURCES AND ENGINEERING DEVELOPMENT (1968*b*). *Marine Science Affairs—A Year of Plans and Progress.* US Govt. Printing Office, Washington D.C., xiii + 228 pp.

NEBLETT, William R. (1966). A fishery view of recent law of the sea conferences. Mimeo. for *Law of the Sea Institute*, University of Rhode Island, June-July, 15 pp.

NELSON, T. W. & BURK, C. A. (1966). Petroleum resources of the continental margins of the United States. Pp. 116-33 in *Exploiting the Ocean. Trans. Second Ann. Mar. Tech. Soc. Conf. & Exhibit.*, Mar. Tech. Soc., Washington D.C.

NEW YORK TIMES (1969). Seabed potential for arms studied, 8 Oct.

OCEAN SCIENCE NEWS (1969). 1970 federal ocean market. *Ocean Science News*, 11, 2-3.

ODA, Shigeru (1968). The Geneva Conventions on law of the sea: some suggestions for their revision. *Natural Resources Lawyer*, 1, 103-13.

ODUM, Eugene P. (1959). *Fundamentals of Ecology*, second edn. W. B. Saunders, Philadelphia & London, xvii + 546 pp., illustr.

OSBORN, Fairfield (1953). *The Limits of the Earth* Little, Brown, Boston, x + 238 pp.

PANEL ON OCEANOGRAPHY, PRESIDENT'S SCIENCE ADVISORY COMMITTEE (1966). *Effective Use of the Sea.* US Govt Printing Office, Washington D.C., xv + 144 pp.

POLIKARPOV, G. G. (1966). *Radioecology of Aquatic Organisms.* Reinhold, New York, xxviii + 314 pp.

PROGRAM OF POLICY STUDIES IN SCIENCE AND TECHNOLOGY (1969). A critical review of the Marine Science Commission Report. The George Washington Univ. and the Mar. Tech. Soc. Law Committee, Washington D.C., 114 pp.

RANDALL, John E. (1969). Conservation in the sea. *Oryx*, 10, 31-38.

RAY, Carleton (1961). Marine Preserves for ecological research. Pp. 323-8 in *Trans. 26th No. Amer. Wildlife & Nat. Res. Conf.*, Wildlife Man. Inst., Washington D.C.

RAY, Carleton (1965). The scientific need for shallow-water marine sanctuaries. Pp. 83–98 in *Symp. on Sci. Use of Natural Areas*, XVI Int. Cong. Zool. Reprinted by *Field Research Projects, Natural Area Studies*, No. 2, Coconut Grove, Fla, x + 103 pp.

RAY, Carleton (1966). Inshore marine conservation. Pp. 77–87 in *First World Conference on National Parks*. US Govt Printing Office, Washington D.C., xxxiv + 471 pp.

RAY, Carleton (1968). *Marine Parks for Tanzania*. Conservation Foundation, Washington D.C., 47 pp., illustr.

RIPLEY, S. Dillon (1966). The future of environmental improvement. Reprinted by *The Graduate School Press*, from *Environmental Improvement: Air, Water and Soil*, US Dept Agriculture, Washington D.C., pp. 85–93.

RYTHER, John H. & BARDACH, John E. (1968). *The Status and Potential of Aquaculture. Vol. 1: Particularly Invertebrates and Algal Culture*. Amer. Inst. Biol. Sci., Clearinghouse for Fed. Sci. & Tech. Infor., Springfield, Virginia, 261 pp.

TASK FORCE ON ENVIRONMENTAL HEALTH AND RELATED PROBLEMS (1967). *A Strategy for a Liveable Environment*. Rept to the Sec. of Health, Education & Welfare, US Govt Printing Office, Washington D.C., xxi + 90 pp.

TUBMAN, William C. (1966). The legal status of minerals located on or beneath the sea floor beyond the continental shelf. Pp. 379–404 in *Exploiting the Ocean. Trans. Second Ann. Mar. Tech. Soc. Conf. & Exhibit.*, Mar. Tech. Soc., Washington D.C.

WALFORD, Lionel A. (1958). *Living Resources of the Sea*. Ronald Press, New York, xi + 321 pp.

WEBER, Alban (1966). Our newest frontier: the sea bottom. Some legal aspects of the continental shelf status. Pp. 405–11 in *Exploiting the Ocean. Trans. Second Ann. Mar. Tech. Soc. Conf. & Exhibit.*, Mar. Tech. Soc., Washington D.C.

WILKEY, Malcolm R. (1969). The role of private industry in the deep ocean. *Symp. on Private Industry Abroad*. Southwestern Legal Foundation, Dallas, Texas, June. Reprinted by Matthew Bender, Washington D.C., 40 pp.

YOUNG, Richard (1968). The legal régime of the deep sea floor. *Amer. Jour. Int. Law*, 62, 641–53.

(Whereupon, at 5 p.m., the subcommittee hearing was adjourned, subject to call.)

STANDARD FORM NO. 64

Office Memorandum • UNITED STATES GOVERNMENT

TO : James R. Mason, Chief, Isotopes Branch
Division of Licensing and Regulation

DATE: February 28, 1961

FROM : W. S. Cool, Isotopes Branch
Division of Licensing and Regulation

SUBJECT: MATTERS TO BE DISCUSSED WITH A. GOLDSTEIN DURING OFFICE VISIT

L&R:IB:WSC

1. U. S. Nuclear Corporation application August 24, 1959, amended by letter of January 13, 1961 to request land burial only. Notice of Proposed Issuance (15 day) sent to H. L. Price on February 17, 1961.

2. January 13, 1961 request for transfer of Isotopes Specialties Co., waste disposal License No. 4-580-6 to Isotopes Specialties Company, Division of U. S. Nuclear Corporation. The following items of information are required:

   A. Clear statement of the facilities to be available to this program at 170 West Providencia, Burbank, California.

   B. Indication that all administrative instructions, radiation safety procedures and packaging techniques previously submitted by Isotopes Specialties Co., Division of Nuclear Corporation of America, Inc., will apply to Isotopes Specialties Co., Division of U. S. Nuclear Corporation, or a detailed statement of those which will apply.

   C. Indication that equipment such as instrumentation and heavy handling equipment previously described will be available.

   D. Reply to our letter of December 2, 1960 (copy attached)/

3. California Salvage Company application for amendment, with particular reference to pressure relief tube location outside of metal inside container; the size of reinforcing rods or bars and wire and how they are attached to each other. (Note, arrangements are being made to test their type of drum in the laboratory phase of the Reactor Development container testing program.)

A/128

9701270014 970110
PDR FOIA
SIEFERT96-448 PDR

DEC 2 1960

L&R:LS:WSC
Docket No. 27-7

DOCKETED
2 [illegible]

Isotopes Specialties Company
Division of Nuclear Corporation
of America, Inc.
170 West Providencia
Burbank, California

Attention: Mr. Alfred J. Moses, Chairman
Isotope Committee

Gentlemen:

Receipt is acknowledged of your application dated July 25, 1960.

Pursuant to Section 30.35(b) of 10 CFR 30, since your application for renewal was received more than thirty (30) days prior to expiration, the license shall not expire until the application has been finally determined by the Commission.

In reviewing this application we find that there are several items which require clarification or further information, before licensing action can be taken.

Supplement A of the application states that waste material received under License No. 4-530-6 may be reclaimed and used under License No. 4-533-7. You are requested to provide a detailed description of the types of anticipated operations indicating the nature of the isotopes and/or equipment to be reclaimed and the maximum levels of contamination which will be allowed on reclaimed items. The radiation safety procedures for such operations should also be submitted. You should also indicate whether this authorization is desired to provide a decontamination service to customers. Such use may be considered for coverage under a separate license.

Supplement B, III, "Storage of Radioactive Waste" should be expanded to indicate how waste material will be stored prior to encasement in concrete or impervious containers, to protect it from damage or dispersion due to weather.

9701230321

SPP

Supplement B, II.A.4 indicates the information to be logged on radioactive waste received from Isotopes Specialties Company's customers. Upon amendment of License No. [illegible], we propose to add a condition specifying the records which shall be kept regarding each incoming container and each container as finally prepared for disposal, substantially in the following form:

The licensee shall maintain:

A. Records of the following items of information regarding each container of waste received from customers:

(1) Name and address of customer.
(2) Principal radioactive isotopes.
(3) Amount of radioactivity.
(4) Radiation level at the surface of the container and at 1 meter.
(5) Level of removable contamination on the container surface.
(6) Date received.

B. Records of the following items of information regarding each container of waste packaged for disposal:

(1) Amount of radioactivity.
(2) Radiation level at the surface of the container and at 1 meter.
(3) Level of removable contamination on the container surface.
(4) Most hazardous radioisotope.
(5) Date of packaging.
(6) Weight of final container.
(7) Disposal location and date.

Therefore, any record system which will include this information may be used. Indication of the acceptability of this condition would be appreciated.

Supplement [illegible] indicates that wastes may be processed by chemical dissolution, filtration, extraction, ion exchange, chemical polymerization and physical absorption. You are requested to provide detailed information regarding the types of waste material to be treated by each technique, the facilities available for such processing, and the procedures to be followed.

DEC 2 1960

Supplement B, VI.B.1.b. indicates the information to be provided on all radioactive waste containers. You are requested to indicate how this information will be semipermanently attached to the final sea disposal containers.

Supplement B, VI.B.1.d. states that solid waste will be put into the drum in increments of [illegible] to [illegible] and a slurry of cement will be carefully added to each incremental layer of waste to produce a solid mass. We are concerned that this technique may result in laminations of waste in which significant voids may exist, which may be compressible such that voids may be produced when the drum is subjected to pressure, or which may separate and allow buoyant items to rise to the surface of the ocean. If the technique proposed is such as to result in an integral mixing of the waste and concrete, please so verify. If laminations may result you are requested to describe the provision to be made for pressure relief, including the size and type of tube to be used and the method of installation; reinforcement to be provided to assure adherance of the multiple layers of waste and the securing of the 3 to [illegible] concrete cap to the drum contents to provide assurance that it will not come off during handling, transportation and disposal operations.

Supplement B, VI.B.1.e. states that other special containers may be used dependent upon the shape, toxicity and intensity of radiation of the waste. Drawings [illegible] and [illegible] showing an I beam and a [illegible] encased in [illegible] concrete have previously been approved. If it is proposed to construct forms and encase irregularly shaped items in concrete blocks, it will be necessary to submit a complete description of each type of such container to be used including such information as the [illegible] wall thickness, the reinforcement to be provided and the provision to be made for pressure relief if significant voids may exist in the package.

Review of your application will be continued upon receipt of the requested information.

Sincerely yours,

James [illegible]. Mason, Chief
Isotopes Branch
Division of Licensing and Regulation

OFFICE ▶	L&R:IB	L&R:[illegible]	L&R:IB			
SURNAME ▶	Coolidge	Nussbaumer	Mason			
DATE ▶	12/[illegible]	12/[illegible]	12/[illegible]			

Form AEC-318 [illegible]

UNITED STATES DISTRICT COURT

IN AND FOR THE STATE OF CALIFORNIA

STATE DEPT. OF HEALTH SERVICES MAR 15 2000 SACRAMENTO

CERTIFIED COPY

JOSEPH A. THOMSON and VIRGINIA)
THOMSON,)
Plaintiffs,)
vs.) No. CV-97-5220
ICN PHARMACEUTICALS, INC., a) RAP (AJWx)
Delaware Corporation; NUCOR) VOLUME I
CORPORATION, a Delaware)
Corporation; RHONE-POULENC,)
INC., a New York Corporation,)
Defendants.)

Deposition of RICHARD DONELSON, at 221 North Figueroa Street, Los Angeles, California, commencing at 11:49 A.M., Monday, September 27, 1999, before Marceline F. Noble, CSR No. 3024.

PAGES 1-138

BEN HYATT
CERTIFIED DEPOSITION REPORTERS
(888) 272-0022
18226 Ventura Boulevard, Suite 103 • Tarzana, California 91356
323.225.2040 310.273.5040 818.343.7040
Toll-Free 888.272.0022 FAX 818.343.7119

APPEARANCES OF COUNSEL:

FOR THE PLAINTIFFS:

LOEB & LOEB
BY: MALISSA HATHAWAY McKEITH, ESQ.
1000 Wilshire Boulevard
Suite 1800
Los Angeles, California 90017-2475
(213) 688-3400

FOR THE DEFENDANT, COUNTER-DEFENDANT AND COUNTER-CLAIMANT NUCOR CORPORATION:

LEWIS, D'AMATO, BRISBOIS & BISGAARD LLP
BY: CHRISTOPHER P. BISGAARD, ESQ.
JOHN H. SHIMADA, ESQ.
221 North Figueroa Street
Suite 1200
Los Angeles, California 90012-2601
(213) 250-1800

BEN HYATT
CERTIFIED DEPOSITION REPORTERS
(888) 272-0022

APPEARANCES (CONTINUED):

FOR THE DEFENDANT ICN PHARMACEUTICALS, INC.:

PROSKAUER ROSE LLP
BY: GREGORY J. PATTERSON, ESQ.
2049 Century Park East
Suite 3200
Los Angeles, California 90067-3206
(310) 284-5676

FOR THE DEFENDANT RHONE-POULENC, INC.:

McCUTCHEN, DOYLE, BROWN & ENERSEN LLP
BY: BRYAN K. BROWN, ESQ.
355 South Grand Avenue
Suite 4400
Los Angeles, California 90071
(213) 680-6400

ALSO PRESENT:

DUDLEY FETZER, VIDEOGRAPHER

THE VIDEOGRAPHER: Good morning.
We're on the record at 11:49 a.m. on Monday, September 27th, 1999, for the videotaped deposition of Richard Donelson.
We're taking this deposition at 221 North Figueroa Street in Los Angeles, California, on behalf of the defendants, counter-defendants and counter-claimants in the action entitled Joseph Thomson and Virginia Thomson versus ICN Pharmaceuticals, Inc., et al., case No. CV-97-5220 RAP, parens, (AJWx), closed parens.
My name is Dudley Fetzer. I'm the video production specialist from Ben Hyatt Certified Deposition Services located at 18226 Ventura Boulevard, Tarzana, California.
This is tape No. 1 of Volume 1.
Would counsel and all present please identify themselves for the record.
MR. BISGAARD: My name is Christopher P. Bisgaard of Lewis, D'Amato, Brisbois & Bisgaard, attorneys for Nucor Corporation.
MR. BROWN: I am Bryan K. Brown with McCutchen, Doyle, Brown & Enersen for defendant Rhone-Poulenc, Inc.

MR. PATTERSON: Gregory Patterson from the law firm of Proskauer Rose on behalf of INC Pharmaceuticals, Inc.

MS. HATHAWAY McKEITH: Malissa McKeith, law firm of Loeb & Loeb, on behalf of Virginia and Joseph Thomson.

THE VIDEOGRAPHER: The court reporter may swear the witness.

RICHARD DONELSON,

having been first duly sworn, testified as follows:

EXAMINATION

BY MR. BISGAARD:

Q. Good morning, Mr. Donelson.

Again, let me introduce myself. My name is Chris Bisgaard. I represent Nucor Corporation --

A. I did not understand your name.

Do you have a card that I could look at?

Q. Yes, I do. Let me hand that to you. It's Chris Bisgaard.

A. Bisgaard.

Q. Bisgaard, right.
I represent Nucor Corporation, which is a party to this litigation. And we have noticed your deposition and served you with a subpoena to attend these proceedings today.
Before we get to the formal question-and-answer portion of the deposition, let me take a moment or two to provide some background, explanation as to the game -- the ground rules that we follow in taking a deposition.
First off, let me ask, have you ever been deposed before today?
A. Yes.
Q. On how many occasions?
A. I don't recall. I recall one, possibly two -- twice.
Q. Have you ever given testimony at a trial?
A. Yes.
Q. Very briefly, tell us the matters in which you have previously been deposed.
A. One of the groups I worked for, specifically it was called Isotopes Incorporated, who at one point purchased the controlling

interest in U.S. Nuclear. And there was a -- under a government contract, there was a facility that U.S. Nuclear was responsible for, and the cost overrun meant that it would -- Isotopes Incorporated was trying to get an increase in -- in the contract price.

So I was deposed in regard to that.

Q. Do you recall approximately when you gave that deposition?

A. Oh, probably in the 1950's somewhere.

Q. Okay.

A. Is that good enough?

Q. It certainly is. All we can ask, Mr. Donelson, is for you to provide us with your best recollection.

We understand that we -- we'll be talking about things that took place literally decades ago.

A. Yes.

Q. And it's difficult to recall things that took place that long ago.

So what your -- your obligation is is to search your memory to give us your -- your best, your most complete answer but if you don't

recall, that's perfectly appropriate.

But if you have any recollection, no matter how slight it may be, we would like to -- to know that.

And I appreciate you're -- you're giving us your best estimate.

A. I left the company in 1957, and it must have been a couple of years before that.

Q. Okay. And what company was that that you left, Mr. Donelson?

A. It was U.S. Nuclear, I think that was still the name that was used, although the controlling interest was -- well, it was purchased by ICN somewhere in that area. It was before that.

So let me search my memory again now. I think -- I think I'm mistaken on the timing. I left U.S. Nuclear in 1967. Is that right?

Yeah.

Q. I believe so.

A. So it would have been somewhere a year or two before that.

Q. Sometime in the mid-1960's?

A. Yeah.

Q. Okay. Do you recall who was suing whom in that matter?

A. Well, I -- I'm not sure it was a real lawsuit. It was a government -- ended with a government contract. The contractor was trying to get an increase in the price.

Q. Okay.

A. And it was conducted like a courtroom, but I'm not sure it really was.

Q. Okay. You mentioned that you gave one or possibly two depositions. You've mentioned one proceeding.

Do you recall any other proceedings in which you gave a deposition?

A. Not precisely, no, I don't.

Q. In any case, it's been many years since you have been deposed; correct?

A. Yes. A good amount.

Q. The ground rules are pretty much as follows: I will begin asking questions and the other lawyers around the table will have their opportunity after I complete my examination.

It's very important that you listen to each one of our questions. Make sure that you understand the question. If for any reason you

don't understand the question, the lawyers here would be more than happy to restate or rephrase the questions so that you do understand it.

It's important that you understand the question and it is important to allow the lawyer who is asking the question to complete his or her question before you begin responding.

On occasion, the last couple of words in a question can change the entire meaning of the question. And if you begin answering before the lawyer has finished the question, you may be answering a different question than what you think you're answering.

So please allow us to complete our -- our questions. It is possible that there may be objections raised to questions posed to you. Objections such as "the question is vague and ambiguous" or something else.

You are not represented by counsel today; is that correct?

A. Pardon me?

Q. You don't have an attorney with you --

A. No.

Q. -- today?

Okay. If there are objections raised, there may be discussion between the lawyers about the objection, but it really isn't of concern to you unless someone asks you do you understand the question, and then it will be up to you to tell us if you do, and if you do understand the question, then we can proceed.

But if at any time you don't understand the question, please let us know, whether an objection has been made or not.

It is important that you answer by verbally responding to the question in contrast to a shake of the head, that type of thing.

Also, if the question calls for an affirmative answer, it's better to say yes versus "uh-huh"; and if the question calls for a no response to say no versus "huh-uh," because on the record it's sometimes difficult to differentiate between "uh-huh" and "huh-uh."

If at any time you feel like you need to take a break because you are becoming tired or for any other reason, please let us know, and we will certainly be happy to accommodate you in that regard.

At the conclusion of your deposition,

it will be transcribed and will be put into a booklet form and that will become your deposition. You'll be given an opportunity to read your deposition and at the conclusion of that to sign the deposition under penalty of perjury, making notes of any corrections or changes that you may make to the transcript.
I should note that the oath that has been administered to you has the same force and effect as if we were sitting in a courtroom with a judge and jury present and your obligation to tell the truth is the same as if we were in that courtroom setting.
You understand that; correct?
A. Yes.
Q. Okay. If at any time as the proceedings go forth, you remember that a question or -- I'm sorry, an answer that you gave earlier perhaps wasn't the best answer, please feel free to say that you'd like to correct a response, that you've given further thought to it and you'd like to amend or change. That's perfectly appropriate.
So you'll have that opportunity to think about that as the day goes -- goes forward,

and also when you get your deposition transcript, you'll have an opportunity to read it and you may -- having seen it now in black and white -- say, "well actually that response wasn't my best response," and you can cross out what is incorrect and write above that what is correct.

However, it's important that you do your very best today to give us your best responses because if you make changes in your deposition transcript when it's presented to you for reading, counsel will be free to comment on those changes at the time of trial, and it could prove embarrassing, and it's just better to be as accurate as we can be today.

Do you have any questions about what we've been talking about so far?

A. No.

Q. Okay. All right. Mr. Donelson, what is your date of birth?

A. December 20th, 1921.

Q. That makes you pretty close to 78 years old?

A. Will be 78 in December, yes.

Q. What's your health?

A. So-so.

Q. Okay.
A. I have been treated for prostate cancer, and I'm under medication for hypertension all the time.
Q. What medication are you currently taking at this time?
A. Atacand.
Q. Can you spell that?
A. A-t-a-c-a-n-d, I think.
Q. Okay.
A. Two others for my hypertension, and I take a -- I can't recall really.
Q. How frequently do you take your hypertension medication?
A. Daily.
Q. Okay. You took medication this morning?
A. Yes.
Q. Okay. Besides suffering from hypertension and having been treated for prostate cancer, are there any other illnesses or physical maladies that you suffer from at this time?
A. I have allergies.
Q. Okay. All right. As you sit here today, Mr. Donelson, in this conference room, you

BEN HYATT
CERTIFIED DEPOSITION REPORTERS
(888) 272-0022

believe that you're able to give us your best recollection of events that took place some years ago, even though you are taking some medication?

A. Yes.

Q. Okay. Do you think that the medication that you're under in any way will impact your ability to give your best and most accurate testimony?

A. No.

Q. Okay. All right. Let's begin, Mr. Donelson, with your educational background.

Could you tell us, beginning with your graduation from high school, what educational institutions you attended.

A. I attended what was then the College of Puget Sound, now it's a university, in Tacoma, Washington; I attended the University of Washington, Seattle.

While I was in the army, I attended Alabama Polytechnic Institute, which is better known as Auburn, Alabama, and I finally finished my education back at the University of Washington after World War II.

Q. Okay. What degree did you obtain?

A. Bachelor of Science in chemical

engineering.

Q. And do you recall the year in which that degree was granted to you?

A. 1948, I believe.

Q. And that was from the University of Washington at Seattle?

A. Yes.

Q. Okay. With respect to any of the institutions you'd attended prior to that, did you receive any degrees from them?

A. No.

Q. No?

A. No.

Q. You have --

A. No.

Q. Okay. Thank you.

A. I'm a little hoarse.

Q. I understand.

Do you have water or something to drink there?

A. Yes.

Q. Okay. Approximately what period of time did you attend the college of Puget Sound?

A. 1940, '41; '41, '42, I believe.

Q. What was your course of study there?

A. Oh, pre-engineering. They didn't have an engineering degree.

Q. Okay. And when did you begin your study for the first time at the University of Washington, Seattle?

A. 1942.

Q. Okay. And how long did you attend the University of Washington on that first occasion?

A. Less than a year. I don't recall whether it was one quarter or two.

Q. Okay. And you joined the army after that?

A. Yes.

Q. That's what caused you to interrupt your education was to join the military?

A. Actually, I joined before but I was called to duty at that time, yes. And that's what --

Q. And you served in the U.S. Army?

A. Yes.

Q. What was the highest rank that you attained?

A. Corporal.

Q. Okay. And you served in

World War II?

A. Yes.

Q. What theater of actions?

A. I had one battle star in the European Campaign; I also wound up in the Philippines in the Asiatic-Pacific Campaign.

Q. Okay. While you were the University of Washington, what was your course of study?

A. Chemical engineering.

Q. Okay. And what were the years that you were in the service, Mr. Donelson?

A. 1943 through, I guess, '45, I guess the war was over.

Q. And after being discharged honorably from the army, you then began attending University of Washington for a second time; is that correct?

A. Yes.

Q. And you attended the University of Washington on the second occasion during what period of time?

A. Well, it would have been from 1945 or '46, I guess, until 1948.

Q. Okay. And when you went back to the University of Washington, you continued your study in the area of chemical engineering?

A. Yes.

Q. Okay. Since your graduation from the University of Washington in 1948, have you taken any other classes or courses offered by any educational institution?

A. Yes.

Q. Please tell us what that additional education has been.

A. I -- I'm not sure I'm getting ahead or not. But I took some courses in Richland, Washington, that were put on by the -- extension courses, I guess, put on by the University of Washington.

I also attended -- not a formal college -- but attended a course in health physics while I was working at Richland.

Q. While working where?

A. Richland, Washington.

Q. Any other formal educational courses that you've taken since your graduation that you can recall at this time?

A. I don't recall any.

Q. Okay. Do you recall what the course of subject -- what the course of study was in connection with the extension courses you took in

the University of Washington?

A. It was a mathematics course, I believe advanced math.

Q. And how about the health physics course? Was that one course or more than one course?

A. It was a course given by General Electric Company, whom I worked for, and in the subject of radiation safety, health physics.

Q. Over what period of time was that course taught?

A. It was probably half a year in 1948.

Q. How -- was this a full-time class work or what was it?

A. A little of both. Both full time and on the job.

Q. Who taught the class?

A. A number of people.

Q. Okay. Were these people that were employed by General Electric?

A. Yes.

Q. Okay. Did you get any sort of certificate for completing that course?

A. I don't recall that I did, no.

Q. Okay. Can you describe generally what the course consisted of in terms of radiation safety?
A. Well, it started out with basic physics, I would think. We do basic atomic physics. It had to do then with the biological effects of radiation, the procedures that were enforced by General Electric Company at the Hanford Works.
I guess I should have mentioned that.
Q. Anything else that you recall?
A. Oh, the radiation limits, which were more or less standard at the time.
Q. When you say "radiation limits," what do you mean by that?
A. Exposure to atomic radiation in terms of the amount that is permissible to receive.
Q. Do you recall what those standards were at the time?
A. 100 millirem.
Q. How do you spell that word?
A. M-i-l-l-i-r-e-m.
Q. "M" as in Mary --
A. Yes.

Q. -- or "N" as Nancy?
A. Mary.
Q. Millirem?
A. Yes.
Q. Okay.
A. Per week.
Q. And who established that standard, do you know?
A. Atomic Energy Commission, I believe.
Q. I'm really going to stretch your memory now, Mr. Donelson.
Do you recall if you received any written material in connection with your course work in health physics?
A. Yes.
Q. Okay. What written materials did you receive?
A. It was a handbook put out by General Electric Company.
Q. By any stretch of the imagination, do you still that handbook?
A. No, I do not.
Q. Do you recall the title of that handbook?

A. No.
Q. Okay. Do you have any recollection as the year -- as to the year it was published?
A. Sometime in the 1940's. I wouldn't -- I don't know.
Q. Was this a hardback set of materials or loose-leaf?
What was its form?
A. It was loose-leaf.
Q. Okay. In the course that you took while you were employed by General Electric at the Hanford facility, was there any discussion about decommissioning atomic facilities?
A. No.
Q. In any of your -- your course work that you took that you described for us this morning, have you had any course work that would be relevant to the subject of decommissioning a facility where radioactive materials had been used?
A. No.
Q. Mr. Donelson, in your career, did you ever receive any licenses that may have been relevant to your profession?
A. I received a certificate from the

American Board of Health Physics.
They call it Certified Health Physicist.
Q. The American Board of Health Physicists?
A. American Board of Health Physics.
Q. Health physics.
A. Yes.
Q. And when did you receive that certificate?
A. Oh, let me guess around 1960.
MR. PATTERSON: I'm sorry, could you repeat that?
THE WITNESS: 1960.
MR. PATTERSON: '60?
BY MR. BISGAARD:
Q. You used the word "guess," and that prompted another thought that I wanted to share with you, Mr. Donelson.
As we ask you questions today -- and it may go into tomorrow -- especially as we go back many years, you may not have a clear recollection of the -- the area we're asking about. And you may be inclined to say, "Well, I guess this."

For example: "What color was the car?" "Well, I guess it was black."

If you have any recollection that the car, in fact, was black, you're required to give us that. If you don't and it's just based on, perhaps, supposition or just pure guess, we don't want that.

So when you give us your response, if you don't recall specifically, you can certainly tell us, "I'm not sure, but I think it was around 1960," by way of example, but we don't want you to guess or to speculate.

Understand?

A. (No audible response.)

Q. You have to answer that question yes or no.

A. Yes.

Q. Okay. All right. When you received the certificate from the American Board of Health Physics, was this based on taking some class work or taking some test?

How was it you came to receive the certificate?

A. May I use the word "grandfather clause"?

Q. Certainly.
A. There were a number of us in the business, in the health physics business that started the certification process, and those of us were considered grandfathered did not go through the rigid course present-day certified health physicists do have to.
Q. Have you ever held any position with the American Board of Health Physics?
A. No.
Q. Are -- other than having a certificate --
A. Let me correct that.
Q. Certainly.
A. There is a society called the Health Physics Society, which is separate from the Board of Health Physics. And I have had -- been off to the Health Physics Society. It's just a professional society. In the local chapter of an office.
Q. So you are a certified health physicist; is that correct?
A. Yes, sir.
Q. And you have held that certificate since around 1960?

A. Yes.
Q. Any other certificates or licenses that you can recall?
A. When I -- some years later, I -- when I went into business for myself, and I had a license from the State of California, a radioactive material license.
Q. What is a radioactive materials license?
A. Pardon me?
Q. What is a radioactive materials license?
A. It is a license issued by -- at the present time -- the state, and in some cases the Atomic Energy still issues those licenses. It allows the named person, company, institution, whatever, to possess and use radioactive material.
Q. Any other licenses or certificates that you have held?
A. Not that I have held, per se. I've been on other institutions' licenses, my name has been on.
Q. What other institutions?
A. There have been many.

Q. Give us your best recollection of what institutions that you've been on their licenses.
A. Isotope Products Company, J.L. Shepherd & Associates.
Q. T.L.?
A. J.L.
Q. J.L.
A. Standard Oil Company of California, Aerospace Corporation, Hughes Aircraft.
There have been many.
Q. In addition to the ones you've just mentioned.
A. Yes.
Q. What does it mean to be on a license?
A. Well, each license is individual. I can't answer that question --
Q. Okay.
A. -- in words.
Q. We'll come back to that.
Have you received any recognition from any institution or professional society for your professional activities?
A. Not that I recall.

Q. Any awards?
A. Not that I recall.
Q. Have you ever published anything?
A. I can't recall.
MR. BISGAARD: Off the record.
(Off record discussion.)
MR. BISGAARD: Back on the record.
Q. Mr. Donelson, could you please provide us with your education -- I'm sorry, your employment background from the point in time that you graduated from the University of Washington in 1948.
A. I was employed by General Electric Company at Richland, Washington, between 1948 and 1957.
Q. Okay. What was your position at General Electric, sir?
A. There were several.
Q. Okay. Starting from the first, if you could go chronologically, that would be great.
A. Engineer, supervisor, area supervisor, superintendent; that was about it.
Q. Okay. What were your duties and responsibilities as an engineer at

General Electric?

A. Supervising the radiation protection program in the facility.

Q. This was at the Hanford atomic facility?

A. Hanford Atomic Works I believe it was called at that time.

Q. Just generally, what was the Hanford Atomic -- Hanford Atomic Works?

A. I don't believe it's a secret anymore. At one time it was.

Producing plutonium was the main purpose.

Q. Okay. And as an engineer at that facility, you were supervising the radiation protection program; is that correct?

A. Yes.

Q. Okay. What were your duties and responsibilities as a supervisor for General Electric?

A. Pretty much the same.

Q. Okay. How about your duties and responsibilities as an area supervisor?

A. Well, that had to do with a certain location. Supervising the same program and --

Q. Within the Hanford facility itself?
A. Yes.
Q. I see.
And then you became a superintendent, and what were your duties and responsibilities as a superintendent?
A. The same. They just changed titles. Every -- periodically.
Q. Okay.
MS. HATHAWAY McKEITH: Just for the record, what time period were you at Hanford?
THE WITNESS: Pardon me?
MS. HATHAWAY McKEITH: What were the years that you were at Hanford?
THE WITNESS: 1948 through 1957.
MS. HATHAWAY McKEITH: Thanks.
THE WITNESS: Is that what I said before?
BY MR. BISGAARD:
Q. Yes.
Mr. Donelson, prior to going to work for General Electric, had you worked for anyone where radioactive materials were used?
A. No.
Q. Your -- your time in the service had nothing to do with radioactive materials?

A. No.
Q. You weren't on the Manhattan Project?
A. I didn't know it at the time.
Q. Okay. After leaving General Electric in 1957, what was your next employment?
A. Isotopes Specialties Company.
Q. And when you first began working for Isotopes Specialties Company, where were they located?
A. Burbank, California.
Q. Okay. How long did you work for Isotopes Specialties Company?
A. In the order of a year. I'm not sure that's a good answer, but --
Q. If we could all agree on this -- I think it would save us a lot of syllables -- if we refer to Isotopes Specialties Company as ISC. Would that be okay?
A. Yes.
Q. Okay. Are you familiar with Isotopes Specialties Company being referred to as ISC?
A. Excuse me.
Q. Sure.
When you were working for Isotopes Specialties Company, did people in the company

refer to it as ISC?
A. I don't recall that they did.
Q. Okay. Well, let's -- we'll refer to it as that.
Okay?
A. All right.
Q. What was your position or what were your positions at ISC?
A. Chief engineer.
Q. And what were your duties and responsibilities as chief engineer?
A. Designing equipment, radioactive sources.
Q. Reactive what?
A. Radioactive sources.
Q. What type of equipment did you design?
A. Radioactive sources.
Q. Okay. What are radioactive sources?
A. Radioactivity.
Do I need to define that?
Q. No. I think we're okay on that one.
A. All right. Radioactive sources, object that contains the radioactivity.
Q. Any other duties and responsibilities

at ISC besides designing equipment for radioactive sources?

A. I was on an isotope committee, which is something I suppose I should define.

Q. Please, if you would.

A. A committee that sets down the radiation protection program in the facility.

Q. How long did you serve on the isotope committee for ISC?

A. As long as I was there.

Q. How many members were on the isotope committee?

A. You want me to give a precise answer, and I can't.

Q. I understand that.

Give me your best recollection if it's more than a guess.

A. Eight.

Q. Okay. Do you recall the names of any of the people who served on the isotope committee with you?

A. Yes.

Q. What are their names?

A. Allen Goldstein, Philip Shepherd, Joseph Shepherd.

Q. I'm sorry?
A. Joseph Shepherd.
Q. Joseph.
MS. HATHAWAY McKEITH: What was the first -- were there two different Shepherds?
MR. BISGAARD: Philip and Joseph.
MS. HATHAWAY McKEITH: Okay.
THE WITNESS: I'm sorry. Did I say Philip Shepherd?
MS. HATHAWAY McKEITH: You said Philip Shepherd.
THE WITNESS: Philip Gill.
BY MR. BISGAARD:
Q. Okay. I've got Allen Goldstein, Philip Gill, Joseph Shepherd.
Do you recall the names of anybody else?
A. Karl Amlauer.
Q. Is that A-m-l-a-u-e-r?
A. Yes.
Q. Okay. Anybody else that you can recall?
A. Richard Dickey. I don't recall any more.
Q. Okay. Were these individuals whom

you have just identified members of the isotope committee for the entire period that you were a member of the isotope committee?

A. I don't recall.

Q. Okay. I'm sure they'll be further questions about the isotope committee as the deposition proceeds.

Any other duties and responsibilities at ISC in your capacity as the chief engineer over and above designing equipment for radioactive sources and serving as a member of the isotope committee?

A. We all did many -- we wore many hats.

It's hard to answer that question.

Q. Can you tell me anything you can recall what you did at ISC over and above what you've already told us.

A. I can't -- I can't recall.

Q. Okay. That's fine.

Did you have an office at ISC?

A. Not as such.

Q. Okay. Where was ISC located in terms of street address in Burbank?

Do you recall?

A. Main Street.
Q. Did you ever work for ISC at any other facility other than its Main Street facility?
A. Yes.
Q. Okay. What other facility?
A. On Providencia Street in Burbank.
Q. While you were working for ISC, did you work at both the Main Street facility and the Providencia Street facility?
A. Yes.
Q. Okay. Any other facility besides those two?
A. For ISC?
No.
Q. Yes, sir.
A. No.
Q. Okay. How -- what were the circumstances of your coming to work for Isotopes Specialties Company in about 1957?
A. I and my whole family was having allergic problems in eastern Washington, so I sent out some applications.
Q. And you sent an application to, among others, Isotopes Specialties Company?

A. Yeah.
Q. How do you first hear of Isotopes Specialties Company?
A. I don't recall.
Q. What business was Isotopes Specialties Company in at the time that you made your application for employment?
A. Producing materials, radioactive materials, sources and equipment.
Q. Who hired you at ISC?
A. Allen Goldstein.
Q. And what was his position at the time?
A. President.
Q. I believe you earlier testified that you worked for Isotopes Specialties Company for approximately one year; is that correct?
A. Yes.
Q. After Isotopes Specialties Company, for whom did you work?
A. U.S. Nuclear Corporation.
Q. And you recall the year that you went to work for U.S. Nuclear Corporation?
A. When it was formed.
Is that a good enough answer?

Q. I'm sorry?
A. When it was formed.
Q. Okay. You testified earlier you went to work for Isotopes Specialties Company in around 1957; you worked for them about a year.
So we're talking about sometime in the late 50's --
A. Yes.
Q. -- when you went to work for U.S. Nuclear?
A. Yes.
Q. Okay. What were the circumstances of you going to work for U.S. Nuclear Corporation?
A. Everybody I was associated left U.S. Nuclear and started -- left ISC and started U.S. Nuclear.
Q. Who was it who started U.S. Nuclear?
A. The same people that I mentioned before.
Q. Okay. Let's just quickly go through that.
Mr. Goldstein?
A. Yes.
Q. Was he the president of U.S. Nuclear --

A. Yes.
Q. -- when it was formed?
And Philip Gill also transferred over?
A. Yes.
Q. As did Joseph Shepherd?
A. Yes.
Q. And Karl Amlauer and Richard Dickey?
A. Yes.
Q. Okay. Any other names you can recall that began at U.S. Nuclear when it was first formed?
A. Ray Ballard, I believe.
Q. What, to your knowledge, was the purpose of forming U.S. Nuclear Corporation?
A. To produce radioactive materials, sources and equipment.
Q. Did some people stay at ISC that were formerly with the company?
A. I believe so.
Q. Tell us, generally, what you can recall about the split between ISC and U.S. Nuclear.
A. I don't really recall except that the people that I knew all left for some reason.

Q. Okay. Were you ever a shareholder of either Isotopes Specialties Company or U.S. Nuclear?

A. U.S. Nuclear, yes.

Q. Okay. When you -- when U.S. Nuclear was formed, were you one of the original shareholders?

A. Yes.

Q. Okay. Do you recall what percentage of the company stock you held?

A. Maybe one percent.

Q. Okay. So it was certainly a minority interest?

A. Yes.

Q. Okay. Do you recall the names of anyone who stayed at ISC?

A. No, I don't.

Q. Do you know what business ISC was in after U.S. Nuclear was formed?

A. No, I don't.

Q. Okay. When U.S. Nuclear was formed and you went to work for it, where were you located?

A. At home or --

Q. Bad question. Let me -- let me

strike that question.
A. Thank you.
Q. What was the business address of U.S. Nuclear Corporation when it was formed?
A. I don't recall right at the moment. I will later perhaps.
Q. Okay. Do you know what town it was in?
A. It was in Burbank.
Q. Okay. Was it at the Providencia Street or --
A. No. It was on Lake Street as I recall now.
Q. So when U.S. Nuclear Corporation was formed, it did not continue to be involved with the Providencia Street property; is that correct?
A. Difficult question.
A few years later, this facility was to be decommissioned, as I understand it, and U.S. Nuclear was hired as an agent to help decontaminate.
Q. This was the Providencia Street facility?
A. Yes.
Q. Was U.S. Nuclear in any way involved

in the decommissioning of the Main Street ISC facility?

A. U.S. Nuclear Corporation?

No.

Q. Okay. Do you know if the Main Street facility was at some point decommissioned?

A. I don't know first hand.

I assume it was, but that's not a good answer, is it?

Q. Do you have any personal knowledge, you, yourself, independent of any other sources of -- as to whether the Main Street facility of Isotopes Specialties Company was in fact decommissioned?

A. I have no recollection.

Q. Have you seen any documents on that subject?

A. No.

Q. Okay. Do you have any personal knowledge regarding the decommissioning of the Providencia Street facility?

A. Yes.

I was involved in it.

Q. Okay. We'll come back to that subject in a little while, Mr. Donelson.

How long were you with U.S. Nuclear Corporation?
A. Until 1967.
Q. Okay.
A. Is that right?
Excuse me.
Q. Mr. Donelson, where did you go to work at that time?
A. I started my own company.
Q. And what was the name of that company?
A. Richard Donelson and Associates.
Q. And what was the business of Richard Donelson and Associates?
A. Providing health physic services.
Q. To whom?
A. Anybody that would require -- would request it.
Q. Would this be companies that were dealing with radioactive materials?
A. Yes.
Q. Okay. Did you also provide professional services to companies that did not handle radioactive materials while you were with Richard Donelson and Associates?

A. I provided services for facilities that used X rays, which are not radioactive materials per se.

Q. All right. How long were you with Richard Donelson and Associates?

A. Until I retired in 19- -- now I have to think.

It was approximately 20 years.

Did you say how long?

Q. Approximately when it was that you retired.

A. Approximately. Start again. Sorry.

Q. Okay.

A. 1987, would that be?

Q. Ten or 12 years ago?

A. Yes.

Q. Approximately?

Okay. And what form -- strike that.

Was Richard Donelson and Associates a sole proprietorship, a corporation?

A. Corporation.

Q. Corporation.

And were you the sole shareholder?

A. Yes.

Q. And you were the president of the

company?

A. Yes.

Q. Okay. How many employees did Richard Donelson and Associates have at its peak, so-to-speak?

A. My wife, my dog.

Q. Okay. So this was pretty much a --

A. My daughter.

Q. Oh, your daughter?

A. Yeah.

Q. So this was pretty much a family business?

A. Yes.

Q. Okay. Have you provided any consulting services since your retirement in approximately 1987?

A. Not that I remember -- recall.

Q. Okay. Is there any other employment that we have not talked about since your graduation from the University of Washington?

A. No.

Q. Okay. All right. Good.

Let's take a lunch break at this time.

And just for the record,

Mr. Donelson, I have to go to a court hearing and so Mr. Shimada, my partner, will continue your examination.

THE VIDEOGRAPHER: The time is approximately 12:46.

We're off the record.

(Off record discussion.)

(The luncheon recess was taken at 12:46 P.M.)

(The deposition of RICHARD DONELSON was reconvened at 1:35 P.M.)

RICHARD DONELSON,

having been previously duly sworn, testified further as follows:

MR. SHIMADA: All right.

THE VIDEOGRAPHER: The time is approximately 1:35.

We're on the record.

EXAMINATION (CONTINUING)

BY MR. SHIMADA:

Q. Good afternoon, Mr. Donelson.

As you know my name is John Shimada, and I'm another attorney for Nucor. I have a couple of preliminary questions for you.

Prior to today, have you had any communications with anyone about this lawsuit? And apart from our conversations about scheduling this deposition.

A. I believe Mr. --

MR. PATTERSON: Patterson.

THE WITNESS: -- Patterson came to see me

BEN HYATT
CERTIFIED DEPOSITION REPORTERS
(888) 272-0022

about it one day, and your colleague and you were going to show up and didn't.

And I expect I talked to her, Martha.

MR. PATTERSON: Martha Sharp.

THE WITNESS: On the phone.

BY MR. SHIMADA:

Q. So you met with -- actually met with Mr. Patterson?

A. Not --

MR. PATTERSON: That's me.

THE WITNESS: Yes, I met him before.

I'm a little bit hard of hearing.

BY MR. SHIMADA:

Q. That's okay. I'll speak up.

A. Okay.

Q. Do you recall when this meeting with Mr. Patterson took place?

A. A few months ago.

Q. Where did the two of you meet?

A. At my house. At my home.

Q. Was it just the two of you that were there at the meeting?

A. Yes.

Q. And this was a meeting arranged by Mr. Patterson?

A. Yes.
Q. What did Mr. Patterson tell you about who he represented?
A. He gave me his card. He represented ICN, I believe.
Q. And did Mr. Patterson ask you questions at this meeting?
A. Yes.
Q. And what was the nature of those questions?
A. Well, considering the property at Providencia Street in Burbank.
Q. Were there specific areas about the Providencia property that he was inquiring about? Specific subjects?
A. I don't recall.
Q. During this meeting, did Mr. Patterson show you any documents?
A. I don't recall that he did.
Q. Do you recall how long this meeting lasted?
A. Maybe an hour.
Q. Did Mr. Patterson ask you about your recollections of the nature of the operations at the Providencia facility?

A. I believe so.
Q. What specifically did he ask you about, if you can recall?
A. I don't recall.
Q. Did Mr. Patterson also ask you questions about any possible decommissioning of the Providencia property?
A. I believe he asked me that type of question.
Q. Do you recall what the nature of your answers were?
A. I recall that I discussed the fact that the Atomic Energy Commission had released the property and that I had been involved in the decontamination of it.
Q. Are there any other subjects that you recall discussing with Mr. Patterson?
A. We discussed personal people we knew, personally. Nothing -- nothing -- nothing about the case.
Q. People at ICN?
A. I think we might have mentioned somebody at the ICN.
Q. Do you recall who those individuals were?

A. Probably Matt -- mentioned Mr. Panic.
Q. Have you met Mr. Panic before?
A. Yes.
Q. Do you recall when your telephone conversation with Ms. Sharp took place?
A. I have some notes I might bring back a little bit of memory.
Q. Okay.
A. All right.
MR. SHIMADA: Let's go off the record.
THE VIDEOGRAPHER: The time is approximately 1:40.
We're off record.
(Off record discussion.)
THE WITNESS: I have two letters here.
MR. SHIMADA: All right. Let's go back on the record.
THE VIDEOGRAPHER: The time is approximately 1;41.
We're on the record.
BY MR. SHIMADA:
Q. Mr. Donelson, immediately before our break, you indicated that you had some notes regarding a conversation that you had with Ms. Sharp.

You have in front of you the folder that you were going to make reference to. Having had a chance to look another that folder, do you, in fact, have any notes of a conversation with Ms. --

A. No.

Q. -- Sharp?

Your folder did contain two letters from Ms. Sharp; correct?

A. Yes.

Q. And these discussed the scheduling and then the cancellation of your deposition?

A. Yes.

Q. Apart from discussing the mechanics of the scheduling of your deposition with Ms. Sharp, did you have any other conversations with Ms. Sharp?

A. I don't recall that I did.

Q. Do you recall discussing at any time with Ms. Sharp the -- your recollections concerning the Providencia facility?

A. I may have.

I don't recall.

Q. Is the only subject that you can recall discussing with Ms. Sharp presently is the

mechanics of the scheduling of your deposition?
A. Yes.
Q. Was that a yes?
A. Yes.
Q. That's okay. I drop my voice too, sometimes.
Do you recall having any communications prior to this deposition with anyone from the McCutchen law firm?
A. No.
Q. Have you had discussions with David Bernhardt regarding the Providencia facility?
A. I don't recall that name.
Q. Do you recall speaking with anyone from a firm called Rodgers & Associates regarding the ISC facility at Providencia?
A. I have spoke to somebody on the telephone one time, I don't recall the name or the company.
He was calling from Providencia Street.
Q. Do you recall when this conversation took place?
A. Sometime this year.

Q. And this individual told you he was calling from the Providencia facility?
A. Yes.
Q. And do you recall the name of this person?
A. No, I don't.
Q. Do you recall what company he was calling --
A. No, I don't.
Q. -- from?
Did he tell you why he was calling you?
A. Yes.
Q. And what did he tell you?
A. He invited me to come down to Providencia Street; he asked me about a few things at the property, some of which I had forgotten.
Other than that, I don't recall anything.
Q. What was the nature of the questions this individual asked you regarding the Providencia property?
A. The one I recall specifically was he mentioned some holes in the floor that I had forgotten about, and he asked me what they had

been used for, as I recall.

Q. Where were these holes in the floor located?

A. How do I answer that question?

In the building.

Q. Did you -- what did you tell him that you recalled about these holes in the floor?

A. I told him that I thought they had been used for storing radioactive materials and that they had been installed with liners which could be removed when the decontamination came along.

Q. Did this individual tell you where at the Providencia property these holes were located?

A. In a way.

Q. What did he tell you?

A. I don't recall.

Q. Apart from these holes in the floor, do you recall anything else he asked you about?

A. Not specifically.

Q. Did you take this individual up on his invitation and go to the Providencia Street facility?

A. No, I did not.

Q. When this individual asked you a

question regarding holes in the floor, that brought back memories to you of holes you recall being present at the Providencia property?

A. Yes.

Q. And these were holes that you recall were used for storing radioactive materials?

A. Yes.

Q. Do you recall the nature of the materials that were stored in these holes that you have a memory of?

A. No.

Q. Is it your recollection that these were holes that were in the concrete floors of the property?

A. Yes.

Q. Do you recall how many holes there were of this type?

A. No.

Q. Do you recall the diameter of the holes?

A. No.

Q. It's your recollection that there -- that these holes that you had in mind had liners?

A. I recall that they did, yes.

Q. What is your recollection -- to your

recollection, what was the -- what was these
liners made of?
A. Metal of some sort. I -- I don't
recall which -- whether it was iron or aluminum or
the other, probably.
Q. During your time at the -- working at
the Providencia property, did you have an
opportunity to personally see these liners in
these holes?
A. I imagine.
I don't recall.
Q. So you don't recall presently whether
these are -- these liners are something that you
were told about or whether these were liners that
you actually saw?
A. I don't recall.
Q. Previously you mentioned in
describing these liners that they were designed so
that they could be removed for purposes of
decontamination.
Do you recall that testimony?
A. I believe so, yes.
Q. Now, is that an understanding -- let
me rephrase that.
Do you recall how you came to have

that understanding as to why the liners were removable?

A. It was just good practice.

Q. And what do you mean by that?

A. One has to consider decontaminating at some future time, and it maximizes your -- the ability to do so.

Q. Did you mention these liners to this individual on the telephone --

A. I don't.

Q. -- who was calling from the property?

A. I don't recall.

Q. Prior to beginning work at -- for ISC, had you ever had any training in the decommissioning of facilities at which radioactive materials were handled?

A. Yes.

Q. What was the nature of that training that you received?

A. We went through this already once today.

Well, when I left -- worked at Hanford Works for General Electric Company, I attended a training course in the use and safety handling of radioactive material.

Q. And did -- as part of that training in the handling of the use of radioactive materials, did that training also cover the subject of decommissioning facilities?

A. I don't believe so.

Q. Prior to working at ISC, did you ever receive any training in the decommissioning of radioactive facilities?

A. Not complete facilities, no. Decontaminating areas that were in facilities, yes.

Q. From whom did you receive the training in the decontamination of areas?

A. Any number of people. My supervisors.

Q. So this was not formal training by way of classroom work --

A. Yes, it was.

Q. -- this was --

This was?

A. Yes.

Q. Where did you receive this training at?

A. At Richland, Washington.

Q. While you were working for

General Electric?
A. Yes.
Q. And what type of activities were you -- were you trained concerning? What -- what type of activities --
A. To --
Q. What type of procedures were you trained --
A. General radiation safety procedures.
Q. And did these general radiation safety procedures include decontamination of discrete areas?
A. Of what?
Q. Of discrete areas?
A. Yes.
Q. What were you taught about that, about how to do that?
A. Hard work.
Q. What do you mean by that?
A. It's hard work. Scrubbing. Sweeping.
Q. What was the nature of the surfaces that you were -- that would be scrubbed and swept?
A. Anything you can think of.

Q. So this is training that you received at General Electric?
A. Yes.
Q. What would the scrubbing be done with?
What would you use to scrub?
A. Brooms, mops, sandpaper.
Q. Did you actually have an occasion to perform actual decontamination work while you were at General Electric?
A. Yes.
Q. Apart from scrubbing and sweeping, what other types of procedures were you -- did you receive training in the subject of decommissioning and decontamination?
A. Oh, probably use of various chemical agents in decontaminating.
Q. What kind of chemical agents? Do you recall what they were?
A. No.
Q. How long were these classes at General Electric, the classes that you had concerning general radiation safety procedures?
A. Eight hours at a time.
Q. Do you recall how many days of

training did you receive?
A. Less than a year. I don't recall a number of days.
Q. Do you recall what portion of that time was spent in learning about decontaminating procedures?
A. No, I don't.
Q. During this training at General Electric, did you receive training in the cleanup levels that you had to have achieve during the process of this decontaminating work?
A. Yes.
Q. Do you recall whether those were levels set by the Atomic Energy Commission?
A. No.
Q. Were -- were these levels sent -- do you recall who set those levels?
A. G -- General Electric personnel.
Q. General Electric had their own set of cleanup criteria at that time?
A. Yes.
Q. Do you recall whether at that time the Atomic Energy Commission had issued its own cleanup criteria for decontamination?
A. I don't recall.

BEN HYATT
CERTIFIED DEPOSITION REPORTERS
(888) 272-0022

Q. Do you recall what the cleanup levels were that were established by G.E. at that time?
A. No, I don't.
Q. Would you describe the nature of your training at General Electric in decontamination as being various forms of cleaning the surface of objects and buildings?
A. I don't understand the question.
Q. Let me -- let me rephrase that.
The training that you received at General Electric in decontamination concerned various procedures for cleaning the surface of objects; correct?
A. Yes.
Q. Did any of that training also include going down deeper, say, digging up concrete and the procedures that should be followed for doing that type of work?
A. I don't recall.
Q. Did any of that training involve the taking of radioactive assays of soil, for example?
A. Of soil?
Q. Soil.
A. I don't recall.

Q. I'm going to show you a document and ask you just a couple of brief questions about it, primarily to see if it refreshes your recollection.

It's a document dated August 20, 1958, and it's entitled "Isotopes Specialties Company, Inc., Administrative Procedures." And specifically I'll direct your attention to page 8 of the document.

THE WITNESS: Is this the same?

MS. HATHAWAY McKEITH: Uh-huh.

THE WITNESS: Yes.

BY MR. SHIMADA:

Q. You have page 8 in front of you?

A. Yes.

Q. There's a section 8 on page 8 entitled "Isotope Committee."

Do you see that?

A. Yes.

Q. And it recites that the various individuals were members of the isotopes committee: Karl Amlauer, Richard Dickey, Allen Goldstein, Philip Gill, Richard Donelson and Charles Miller.

Do you recall each of those

individuals being members of the Isotopes Committee at ISC?
A. Yes.
Q. This document is dated August 20, 1958. I believe you previously testified that you began working at ISC in 1957.
Having seen this document, do you recall working at ISC through at least August of 1958?
A. I don't recall the date specifically.
MR. SHIMADA: I'll ask the reporter to mark that as our first exhibit.
Have we been using --
MS. HATHAWAY McKEITH: We haven't marked any exhibits yet -- oh, I don't know.
MR. PATTERSON: I didn't think to look it up.
MR. SHIMADA: Let's go off the record.
THE VIDEOGRAPHER: The time is approximately 2:03.
We're off the record.
(Off record discussion.)
MR. SHIMADA: Back on the record.
THE VIDEOGRAPHER: One second.
The time is approximately 2:04.

We're on the record.

MR. SHIMADA: We'll mark the August 20, 1958, "Administrative Procedures" as Exhibit 1 to this deposition.

For the record it also bears the production Nos. JT0148 through JT0156.

(Deposition Exhibit 1
was marked for identification and is
annexed hereto.)

BY MR. SHIMADA:

Q. Mr. Donelson, I'm going to place a second document in front of you and ask you to take a moment to look at it.

For the record, it is a document dated January 13, 1961, and appears to be a letter from Allen M. Goldstein to the Atomic Energy Commission.

MS. HATHAWAY McKEITH: Thank you.

BY MR. SHIMADA:

Q. Specifically I'd ask you, Mr. Donelson, to look at paragraph 4 on the first page of the document, which states that the "Isotopes Specialties Company Isotope Committee will be altered as of January 16, 1961, to include the following personnel, resumes of whom are

already in your possession: Philip Gill, R.N. Donelson, Charles G. Miller, Allen M. Goldstein and Karl Amlauer."

Do you see that?

A. Yep.

Q. Do you recall each of those individuals as being members of the isotope committee for the Isotopes Specialty Company after the sale to U.S. Nuclear?

Let me do that again.

Do you recall each of those individuals being members of the isotopes committee for U.S. Nuclear?

A. I recall the individuals.

The committee membership changed from time to time, so I can't say specifically.

Q. Do you recall being on the isotopes committee for U.S. Nuclear?

A. Yes.

Q. Okay. And that was shortly after the formation of the company?

A. Yes.

Q. Now, this letter is dated 1961. The -- as you will recall, the prior document I showed you was dated 1958.

A. Uh-huh.

Q. With these two documents in mind, does it refresh your recollection that you were employed by ISC for -- for several years?

A. I don't recall much about these letters at all.

Q. Do you recall working for ISC for several years?

A. I don't recall how long it was.

MR. SHIMADA: We'll mark this document as Exhibit No. 2 to this deposition.

For the record, it bears production Nos. JT0400 through 401.

(Deposition Exhibit 2 was marked for identification and is annexed hereto.)

BY MR. SHIMADA:

Q. Do you -- while you were at ISC, did you receive any training in the decommissioning of facilities?

A. No.

Q. Do you recall for how long you worked for U.S. Nuclear?

A. It was totally with U.S. Nuclear and Isotopes Specialties, it was about 10 years, I

believe.

Q. So you believe that your total time of employment by ISC and then U.S. Nuclear was approximately 10 years, that total period of time?

A. Yes.

Q. While you were employed by U.S. Nuclear, do you recall receiving any training in the decommissioning of facilities?

A. No training.

Q. After your employment with U.S. Nuclear, you formed your own company; correct?

A. Yes.

Q. And did you during that time period have any involvement with the decommissioning of facilities?

A. I'm sure I must have, but I don't recall any specifics.

Q. While you were with General Electric during the time you received the training that you described in decontamination procedures, were there specific types of measuring devices that were used to determine the level of radioactivity in surfaces that you -- that were being cleaned?

A. Yes.

Q. What was the nature of those devices?

A. Radiation detectors of several kinds are Geiger counters or chambers or scintillation chambers. Ion chambers. I've forgotten the question.

Q. The question was what was the nature of the devices that were used during your training in decontamination procedures at General Electric for the purpose of determining how high or low the level of radioactivity was and the surfaces that were being cleaned?

A. Did I answer the question?

Q. We'll you've listed ion chambers, scintillation chambers and Geiger counters?

A. Yes.

Q. Were there any other devices?

A. So difficult.

There are different types of radiation and you use different detectors to determine different types of radiation.

Q. Do you recall any other specific devices apart from those three you already mentioned?

A. Well, those are categorically -- I

remember probably dozens of different devices, but those are the -- the overall general description of them, I guess.

Q. So those are three categories of devices.

A. Yes.

Q. This morning you testified that a few years after the formation of U.S. Nuclear the Providencia facility was to be -- was to be decommissioned.

Do you recall that testimony?

A. (No audible response.)

Q. What do you mean by "was to be decomissioned"?

Why did you phrase it that way?

A. Did I phrase it that way?

Q. Yes.

A. I don't think I ever used the word "decomissioned."

Q. Is it your understanding that appearing -- is it your recollection that something was to be done with the Providencia facility at some time after the formation of U.S. Nuclear?

A. "Something was to be done," I don't

understand the question.
Q. Is it your understanding that U.S. Nuclear was supposed to decontaminate the Providencia property?
A. If it was contaminated, yes.
Q. And how did you come to have that understanding?
A. I imagine my supervisor told me.
That's not a good answer, I know.
Q. When you first started working with U.S. Nuclear, did you have a supervisor?
A. Yes.
Q. And do you recall who that was?
A. Allen Goldstein.
Q. Do you recall being advised at some time following the start of your employment with U.S. Nuclear that U.S. Nuclear was going to decontaminate the Providencia property?
A. No.
Q. Is that something that you recall being told?
A. Yes.
Q. Do you recall who told you that?
A. No.
Q. Do you recall when you were told

that?

A. No.

Q. Do you recall the circumstances in which you came to have this understanding?

A. No.

Q. Were you told that U.S. Nuclear was going to decontaminate the property -- strike that.

Earlier you testified that U.S. Nuclear was going to be the agent for decontaminating the property.

What did you mean by "the agent"?

A. Is that a bad word? "Agent"?

Q. No. I'm just trying to understand what you were trying to say.

A. I'm trying to understand your question and I can't.

Q. Okay. Was it your understanding at the time this conversation with this person who told you that U.S. Nuclear was going to decontaminate the property, that U.S. Nuclear had been hired by someone to decontaminate the property?

A. Yes.

Q. Is that something you were

specifically told, that U.S. Nuclear had been hired?

A. Would you rephrase that question, please.

Q. To the best of your recollection, what were you told about why U.S. Nuclear was going to decontaminate the Providencia facility?

A. So it could be used for other purposes, I assume.

Q. Well, now, is that something that you recall or is that just something you're assuming right now?

A. I'm assuming.

Q. Do you have any recollection as to what you were specifically told about why U.S. Nuclear was going to -- or intended to decontaminate the property?

A. No.

Q. Now, do you have any personal knowledge as to whether any steps were taken by U.S. Nuclear to decontaminate the property?

A. Yes.

Q. And what do you know about that subject of your own personal knowledge?

A. You lost me.

Q. Okay. All right.
MS. HATHAWAY McKEITH: He wants you to tell him about the decommissioning.
BY MR. SHIMADA:
Q. What do you recall about decommissioning -- the decommissioning of the Providencia property?
A. I recall --
Q. That you witnessed.
A. I recall being there off and on. I recall taking many measurements, many instrument readings, many samples. I recall --
Well, that's enough for now.
Q. You say you recall being there off and on.
A. Yes.
Q. Do you recall when you first went to the Providencia facility for the purpose of participating in these activities?
A. No.
Q. Do you recall for how long a period of time you went to the property off and on to perform the various activities you described?
A. A matter of a few months.
Q. Do you recall who else was present at

the Providencia facility during the time that you went to the property to conduct these activities? Do you recall any names?

A. The -- half a building was used by another corporation, the Research Chemicals company, and I believe they were all still there and the names I can remember Nathanson. I can't remember -- the boss's name.

Q. Mr. Kleiber?

A. Kleiber. Gene -- Cleaver? Cleaver, I think it is, yeah.

Q. Anyone else at research chemical you can recall being at the property during this period of time?

A. I don't recall any names.

MS. HATHAWAY McKEITH: Nathanson's first name?

BY MR. SHIMADA:

Q. Do you recall Mr. Nathanson's first name?

A. Fred.

Q. I'm sorry. Was that Fred?

A. Fred.

Q. Do you recall the names of any of the individuals that were present on the ISC portion

of the property during these activities?

A. I don't recall.

Q. Do you recall there being an individual who was in charge of these activities at ISC at the Providencia property?

A. What activities?

MR. PATTERSON: Vague as to activities.

BY MR. SHIMADA:

Q. During this period of time when you were going to the Providencia facility on -- on an off-and-on basis to take readings and to do other activities, do you recall was there someone supervising -- conducting overall supervision of the work at the Providencia facility?

A. I was.

Q. Okay. Who did you report to?

A. Allen Goldstein.

Q. Do you recall who else besides yourself was -- was performing the work?

A. I don't recall names. I recall faces. But --

Q. Do you recall how many individuals were actually involved in performing the work at the site?

A. Well, less than three.

Q. Counting or -- counting or not counting yourself?

A. Yes.

Q. So there's three other persons besides yourself?

A. I don't remember how many. I can remember two faces.

I can't remember names.

Q. Were these two individuals persons who were U.S. Nuclear employees?

A. Yes.

Q. Had they also been ISC employees during the time you were an ISC employee?

A. No.

Q. Do you recall whether these two individuals were hired specifically to perform the work at the Providencia facility?

A. No. I don't recall.

Q. Do you recall ever seeing more than two persons at the Providencia facility performing this work?

A. I don't recall.

Q. What was the nature of the work that these two persons were doing?

A. Mopping, scrubbing. Probably a

BEN HYATT
CERTIFIED DEPOSITION REPORTERS
(888) 272-0022

little jackhammering.

Q. Do you specifically recall seeing them mopping and scrubbing?

A. No, I don't.

Q. Do you specifically recall seeing them doing a little jackhammering?

A. No, I don't.

Q. Do you have any recollection as to exactly what type of work was being done by these two individuals?

A. I'm sorry, I -- your voice did --

Q. Right.

A. I'm a little bit hard of hearing.

Q. My voice is dropping.

Do you have any recollection as to exactly what type of activities these two individuals were performing at the Providencia facility during this process?

A. I don't recall.

Q. Do you recall where at the facility they were performing this work?

A. On the isotopes half of the building. Other than that, memory is rather vague.

Q. Do you recall seeing these

individuals perform work on the outside of the buildings?

A. I don't recall.

Q. Do you recall seeing these two individuals doing any -- doing any work on the asphalt surrounding the buildings?

A. I don't recall.

Q. Okay.

MS. HATHAWAY McKEITH: John, it's about 2:30.

Can we take a break?

MR. SHIMADA: That's fine.

Let's go off the record.

THE VIDEOGRAPHER: It's approximately 2:29. This is the end of tape No. 1. We're off the record.

(Short recess.)

MR. SHIMADA: Back on the record.

THE VIDEOGRAPHER: One second.

The time is approximately 2:50. This is the start of tape No. 2.

We're on the record.

BY MR. SHIMADA:

Q. Mr. Donelson, before we go on, as part of your deposition, we had served you with a

subpoena that had asked you to bring certain categories of documents.

A. Uh-huh.

Q. Were you able to find anything in response to that subpoena?

A. Not very much.

MR. SHIMADA: Let's go off the record.

THE VIDEOGRAPHER: Approximately 2:50. We're off record.

(Off record discussion.)

MR. SHIMADA: Let's go back on the record.

THE VIDEOGRAPHER: The time is approximately 2:53.

We're on the record.

BY MR. SHIMADA:

Q. We've had a chance to -- or I've had a chance to review the folder that Mr. Donelson brought with him.

We'll mark copies of those documents at a later time. Mr. Donelson's been kind enough to consent to leaving the documents here so that we can photocopy them and return the originals to him on Wednesday.

Were those the only documents that you were able to find in response to the subpoena?

A. Yes.
Q. Would you say that those documents could be described as being sales brochures from U.S. Nuclear and from ISC?
A. Yes.
Q. Now, you've testified that as part of the decontamination activities you were taking instrument readings and samples.
A. Yes.
Q. What kind of instrument readings were you taking?
A. Geiger counter readings, ion chamber readings, scintillation counter readings, all detecting radiation.
Q. Now, what type of radiation is detected by a Geiger counter?
A. There are several types of Geiger counters.
Q. Do you --
A. Basically gamma and beta are detected by practically all Geiger counters and alpha contamination does not.
But some Geiger counters will detect alpha also.
Q. Do you recall whether the Geiger

counters that you were using as part of these activities were capable of detecting alpha radiation also?

A. No, they were not.

Q. Were they limited to detecting gamma and beta radiation?

A. Yes.

Q. Do you recall the names of the specific Geiger counters you were using?

A. The manufacturer you mean?

Q. Yes.

A. I don't recall specifically.

Q. What was the purpose for the ion chamber detectors that you were using?

A. Well, measuring radiation levels, external, mostly gamma ray radiation.

Q. Do you recall the manufacturer or manufacturers of the ion chambers that you were using?

A. I don't recall.

Q. What was the purpose for the scintillation chambers that you were using?

A. May I digress here a little bit and say there are two types of measurements to take one -- direct measurements where you actually

measure a surface of something, and the other is to take what we call wipe samples, which you then take to a laboratory and -- and -- and measure the amount of contamination picked up by the wipe sample.

And I believe the alpha scintillator would have been one of the measurements to detect alpha contamination.

Q. Now, the Geiger counter is a direct-reading instrument; correct?

A. Yes, but it can be also used in a laboratory for measuring samples.

Q. Did -- when -- did you use Geiger counters as a direct-reading instrument during this activity at the Providencia facility?

A. I -- as I recall, yes.

Q. The ion and scintillation chambers, those are all devices used to analyze wipe samples?

A. What was the first word now?

Q. Ion chamber?

A. No. No. Ion chamber generally is not used for wipe samples.

Q. What type of -- is the ion chamber also a direct-reading instrument?

BEN HYATT
CERTIFIED DEPOSITION REPORTERS
(888) 272-0022

A. Yes.

Q. And you recall using that at the Providencia property?

A. Not specifically, but I would guess I did.

That's not a good answer, I know.

Q. As has been explained to you before, recognizing that this -- we're asking about things that took place sometime ago, it's still important that you testify based on what you recall versus what you suppose happened.

A. Yes.

Q. Now, do you specifically recall using Geiger counters or a Geiger counter at the property?

A. No, I don't specifically recall.

Q. Do you recall using an ion chamber at the property?

A. That's what you just asked me.

Q. No. I asked you about Geiger counter first.

A. Yes.

Q. Do you recall using a Geiger counter at the property?

A. Yes.

Q. Okay. Do you recall using an ion chamber --
A. No.
Q. -- at the property?
Now, the scintillation chamber, is that also direct reading or is that also used for wipe samples?
A. It can be both.
Q. Do you recall using a scintillation chamber at the property?
A. No, I don't.
Q. As you sit here today, do you have any actual recollection as to the type of instruments you used at the property?
A. No, I do not.
Q. Now, you've also testified that you took samples at the property.
A. Yes.
Q. What type of samples do you recall taking?
A. Would you explain what you mean by "type"?
Q. Okay. When -- these samples, what were you taking samples of?
A. Floor, walls, equipment.

Q. And in taking these samples, were you wiping the surface of the floors, the walls and the equipment?
A. Yes.
Q. And what were you wiping the surface of the floors, wall and equipment with?
A. Probably some sort of a paper sample type.
Q. Do you recall using some sort of sample paper to take --
A. Not specifically.
Q. -- to take specific wipe samples?
A. No.
Q. Do you recall wiping the floors, the walls and the equipment with some type of material?
A. Yes.
Q. And was that material treated with anything prior to wiping the walls or surface?
A. I don't recall.
Q. Do you recall whether there was any type of solvent used on the material before it was wiped across the surface of the walls, floors and equipment?
A. I don't recall.

Q. Do you recall how you decided where at the property to take these wipe samples?

A. I don't understand your question.

Q. Let -- let's talk about the wipe samples that you took of the floors.

Okay?

A. (No audible response.)

Q. How did you decide what parts of the floor to wipe to take the sample?

A. How did I decide? Basically I decided to wipe everything, at least a portion of everything.

In some areas, I would have taken it more frequently, because I knew to some extent where the radioactive material had been.

Q. Now, was it -- is that also true for where on the walls you wiped?

A. Yes.

Q. Some areas were wiped more frequently than others?

A. Yes.

Q. Did you, prior to taking these wipe samples, prepare some sort of sampling plan, a plan ahead of time as to where you would take the samples and how frequently --

A. I don't recall specifically.

Q. -- those areas?

Who decided where the wipe samples would be taken?

A. I did.

Q. In deciding where the wipe samples were to be taken, did you consult with anyone else for their input?

A. I don't recall specifically.

Q. Do you recall anyone else in addition to yourself having some sort of supervisory role over this work that was done at the Providencia property?

A. I don't believe so.

Q. After you took these wipe samples, was there a type of container that you put the material into after taking that wipe sample?

A. Only ones I can recall are paper envelopes like little -- what do they use those for? Something.

They are just little envelope -- little envelope like this with flap on it.

Q. And you just put the piece of paper in there?

A. Yes.

Q. Or whatever it was?
A. Yes.
Q. Were these samples -- were the wipe samples analyzed in the laboratory, offsite laboratory?
A. Well, they were probably taken to U.S. Nuclear Corporation and analyzed there.
I know that's not a good answer, but --
MS. HATHAWAY McKEITH: Why do you --
Could we go off the record a second?
MR. SHIMADA: Sure.
(Off record discussion.)
MR. SHIMADA: Go back on the record.
MS. HATHAWAY McKEITH: Okay.
BY MR. SHIMADA:
Q. Do you recall exactly where those wipe samples were analyzed?
A. No.
Q. Were some of the samples analyzed at the property itself with a Geiger counter --
A. Yes.
Q. -- or something?
Other than wipe samples, was there any other type of sample that you took at the

property?

A. Not that I recall.

Q. Did you take any -- do you recall taking any samples of the -- any soil at the property?

A. No, I don't.

Q. Do you recall any -- taking any samples of concrete at the property?

For instance, taking some chips out of the concrete?

A. No, I don't recall.

Q. In taking these wipe samples, were you following some sort of established sampling protocol?

A. Yes.

Q. And what was that protocol that you were following?

A. My own.

Q. Okay.

A. No. There are certain methods in the business that are standard methods. I -- I don't recall what they -- any specifics, but that -- that's not -- say it was done according to the standard methods.

Q. Do you recall making reference to

certain standards before deciding --
A. No.
Q. -- how to conduct the wipe samples?
A. No.
Q. Prior to this activity at the Providencia property, had you ever taken wipe samples before as part of the decontamination process?
A. Yes.
Q. And was that at General Electric?
A. Yes.
Q. And while you were doing this at General Electric, was it part of actual decontamination work or was it simply being trained in how to do it?
A. It was actually decontamination work.
Q. And at that time doing that work for General Electric, did General Electric have its own company protocols as to how to conduct that wipe sampling work?
A. I don't recall.
Q. Do you recall approximately how many wipe samples you took --
A. No.
Q. -- at the Providencia property?

A. No.

Q. As part of this work at the Providencia property, do you recall any materials being removed from the property?

MR. PATTERSON: Objection; vague.

Maybe you can --

MR. SHIMADA: Sure.

Q. Do you -- as part of this work at the property, do you recall any portions of the structures of the building being removed and taken offsite?

A. I don't recall.

Q. As part of this work at the Providencia property, do you recall any portions of the structures being torn down?

A. I don't recall any.

Q. At the time this work was performed, do you recall making reference to any guidance or regulations issued by the Atomic Energy Commission?

A. I don't recall.

Q. Was there -- let me rephrase that.

The purpose in taking the wipe samples was to determine the level of radioactivity on the surface of the particular

BEN HYATT
CERTIFIED DEPOSITION REPORTERS
(888) 272-0022

item that you were sampling; correct?
A. That portion of it that is removable, yes.
Q. And were there -- was there a cleanup level that you were looking for to see if -- if -- the radiation --
A. I'm sure there was.
Q. -- was at an acceptable level?
A. I'm sure there was.
Q. Do you recall what that --
A. No.
Q. -- numeric level was?
A. No.
Q. Do you recall where you -- how you came to choose that particular standard, whatever it was?
A. I would -- to the best of my memory, it was agreed with between the company and the Atomic Energy Commission as to what the levels were to be to be released.
Q. Now, is that something that you were -- that you have personal knowledge of?
A. Yes.
Q. Do you recall being present during discussions between representatives of

U.S. Nuclear and the Atomic Energy Commission about a cleanup of the property?

A. Yes.

Q. Do you recall when the first of these discussions took place?

A. No, I don't.

Q. Do you recall where the first discussion that you participated in between U.S. Nuclear and the Atomic Energy Commission --

A. No.

Q. -- took place?

Do you recall who was present during this discussion?

A. I recall one name, has passed away, Herbert Book.

Q. And was Mr. Book with U.S. Nuclear or the Atomic Energy Commission?

A. Atomic Energy Commission.

Q. So you have a recollection of being present during a discussion at which Mr. Book was also present?

A. Yes.

Q. Do you recall who else, if anyone, was present at this discussion?

A. No.

BEN HYATT
CERTIFIED DEPOSITION REPORTERS
(888) 272-0022

Q. Do you recall whether there were, in fact, other people there?
A. No, I don't.
Q. Do you recall having a discussion with Mr. Book at this meeting, you personally?
A. I don't remember any specifics, but I'm sure I was.
Q. Do you have any specific recollection of what was discussed at this discussion with Mr. Book?
A. No.
Q. Did this discussion with Mr. Book take place before the start of the activities at the Providencia facility that you've described?
A. I don't recall.
Q. So you don't recall when in the timeline of the work at the facility this discussion took place?
A. I would imagine -- no, that's a poor answer.
I don't recall specifically --
Q. It's okay.
A. -- but I would think that --
That's not a good answer either.
Q. If you don't --

A. There would have been several meetings.
Q. And you recall several meetings with Mr. Book?
A. Not specifically.
Q. You recall at least one?
A. I don't even recall that.
Q. Do you have some recollection of Mr. Book having some involvement with the Providencia facility?
A. Yes.
Q. And you don't -- do you -- do you have a recollection now as to whether that involvement with the Providencia facility by Mr. Book specifically concerned any decontamination or decommissioning of the facility?
A. I don't recall.
Q. Do you recall any specific discussions between U.S. Nuclear and the Atomic Energy Commission concerning the decontamination or decommissioning of the Providencia property?
A. No, I don't.
Q. Do you recall any modifications being made to the structures at the Providencia property

during this work?

A. Define what you mean by "the structure."

Q. Okay. The building, the floors, the -- any asphalt surrounding the property.

A. What was the question again?

Q. Do you recall any changes being made to that, the structures for the property itself?

A. I don't recall.

Q. Now, it's your recollection from working at the Providencia property that there were various holes or sumps at the property --

A. Uh-huh.

Q. -- correct?

A. Yes.

Q. Do you recall any of those holes or sumps being filled in or otherwise removed during the course of this activity at the site?

A. I recall one facility, which we called pool, which was essentially a -- a pool, but deep to provide water shielding for radioactive materials down at the bottom of the pool.

And I recall that that was decontaminated and the area filled in.

Q. And do you recall -- let me rephrase that.
That -- that work that you recall with respect to the pool was performed during this time frame that we've talking about that you were supervising the work --
A. Yes.
Q. -- at the property?
Do you recall what was done to decontaminate the pool prior to it, filling it in?
A. There were certain chemical agents that are known to collect certain elements which may be radioactive and it involved an application of solution to the walls of the pool and then removal of that material.
Q. So you recall those solutions being applied to the walls of the pool?
A. Uh-huh. Yes.
Q. Was that work performed by yourself or was that work performed by one or both of these individuals that you've mentioned previously?
A. All -- all three I would say.
Q. Do you recall actually doing some of that work yourself?
A. Yes.

Q. And do you recall the nature of the chemical agents that you were using?
A. No, I don't.
Q. The wall --
A. I -- I recall another name that came to my mind.
There was a Mr. Newman that came into the picture somewhere, I'm not sure what he was, but he also was the president of a company called Turco, I believe.
And that company did a lot of work with chemicals, particularly in the area of removing radioactive contamination.
Q. Do you recall Mr. Newman's first name?
A. You don't want me to guess.
I believe it was Ken. But that's a bad answer, I know.
Q. Do you recall how Mr. Newman spelled his name?
A. I think the standard way. N-e-w-m-a-n.
Q. And he was president of the company called Turco?
A. Turco, yeah.

Q. How do you spell Turco?
A. T-u-r-c-o.
Q. And one of their business activities was removing radioactive contamination?
A. Yes.
Q. Do you recall where --
A. I'm sorry. Let me say providing chemicals to do so.
Q. And where was that -- where was Turco based?
Where was its place of business?
A. I don't know.
Q. Is it your recollection that during the course of this work Turco supplied some chemicals for use in these activities at the -- at the property?
A. Using them? The -- the decontaminating --
Q. Yes.
A. -- materials?
Yes.
Q. Getting back to the pool and your work on the pool, do you recall what the walls of the pool were made out of?
A. Concrete.

Q. And the walls of the pool were -- were wiped with some sort of chemical?
A. More sprayed than wiped.
Q. So the chemicals are sprayed on the walls with what?
A. I don't recall.
Q. With a hand sprayer or with something more powerful?
A. I don't recall.
Q. And then the rinse was collected at the bottom of the pool; correct?
A. Uh-huh.
Q. Yes?
A. Yes.
Q. And was that pumped out somehow?
A. Yes.
Q. What was done with the water or the solution?
A. It was probably -- it was analyzed, and if considered to be radioactive, it was probably taken to a radioactive waste site.
Q. Do you recall at the conclusion of this spraying of liquid on the swimming pool walls taking some sort of radioactive reading from the walls with a Geiger counter --

A. Yes.
Q. -- some other direct reading instrument?
A. Yes.
Q. And do you recall there being detectable levels of radiation being present in the walls of the swimming pool following this spraying of the solvents?
A. In this business, you never say there's none.
Radioactivity is everywhere.
Q. Do you recall there being --
Do you recall detecting levels of radioactivity in the concrete walls that you would consider to be something other than background radiation?
A. Before the decontamination or after?
Q. After spraying this liquid on the walls.
A. I don't recall specifically.
I can only guess, which isn't any good.
Q. Do you recall whether you looked for cracks in the concrete walls of the pool?
A. I don't recall.

Q. Do you recall taking any steps to check whether or not there had been cracks in the walls?
A. I don't recall. I can say there was never any sizable cracks, because the pool held water.
Q. Do you -- do you recall what that pool was filled in with?
A. Dirt.
Q. Was some sort of concrete cap placed on the dirt?
A. As I recall about it, yes.
Q. Do you recall taking any samples of the chip samples or actual physical samples of the concrete walls of the pool and -- and testing those samples for radiation?
A. I don't recall.
Q. Apart from the pool, do you recall any other holes being filled in at the facility?
A. No.
Q. Previously you testified that you recalled there being some holes at the facility --
A. Yes.
Q. -- a recollection that was prompted by this telephone conversation you received from

someone at --

A. Yes.

Q. -- the property recently.

A. Yes.

Q. Do you recall what, if anything, was done to those holes?

A. I don't recall specifically.

Q. Do you recall preparing a written report of the sample results of your wipe samples at the property?

A. I don't recall specifically, but I'm almost positive it was done.

Q. Why are you almost positive that it was done?

A. It's standard practice.

Q. Do you recall there being any representatives of the Atomic Energy Commission or any other governmental agency present at the property during any portion of the work that you've been describing this afternoon at the property?

A. Yes.

Q. Were the -- was this -- was it just one person you recall or more than one?

A. Only one I recall is Herbert Book.

Q. Now, is it your recollection that Mr. Book -- there was at least one occasion during which Mr. Book was at the property during the time this decontamination work was being conducted?
A. Would you repeat that?
Q. Do you recall Mr. Book being present during the decontamination work at any time?
A. Yes.
Q. Do you recall what office Mr. -- do you recall where Mr. Book's office was? What city?
A. I believe it was in Walnut Creek, although their offices were in San Francisco at one time.
I'm not sure the date that they moved.
Q. How many times do you recall seeing Mr. Book at the property during the decontamination process?
A. I don't recall how many.
Q. But you recall at least one time?
A. Yes.
Q. Do you recall speaking with Mr. Book on this occasion that he was at the property?
A. Yes.

Q. What -- do you recall what the two of you discussed?
A. I don't recall.
Q. Is Mr. Book the only representative of the Atomic Energy Commission that you recall ever being present at the property during the decontamination work?
A. None that I recall.
Q. How many --
A. I believe Mr. Patterson spoke of a Mr. Fish, whose name I remember, but I cannot place him, and he may or may not have been present.
He was also with the Atomic Energy Commission.
Q. So you recall a Mr. Fish as having worked with the Atomic Energy Commission?
A. Yes.
Q. And you recall Mr. Patterson at some point in time asking you if you recalled speaking with Mr. Fish?
A. I recalled that he said he spoke to Mr. Fish.
Q. Do you recall ever personally speaking with Mr. Fish?

A. Not specifically.
Q. Do you recall how many man-hours were spent on this work that we've been talking about?
A. No, I don't.
Q. Prior to this work, had you ever supervised the decontamination of a facility?
A. A facility?
Q. Yes.
A. No.
Q. Mr. Donelson, I'm going to ask you to take a look at a document. It's just a single page.
A. Can I look, too?
Q. Yes. It's coming around the table.
MR. SHIMADA: For the record, it's a document that's entitled "Figure 1-2, Thomson property facility map."
It's taken from the September 1999 report by Rodgers & Associates concerning the property.
Q. Mr. Donelson, when is the last time you've been to the Providencia property?
A. Probably when I was decontaminating.
Q. Do you recall when -- when that was?
A. We've already gone over that.

Q. Okay. Does this diagram correspond with your recollection of the configuration of the property and the various structures of the property?

A. Well, I don't -- where it says, "former hot cells," that's what I remember -- I remember what we called the hot lab to be. It doesn't show it like there, it's not there anymore.

Q. Do you refer to something you recall being a hot lab being in the area where it indicates here hot cells, former hot cells?

A. Former hot cells, yes.

Q. Other than that, does this diagram correspond with your recollection of the layout of the property?

A. Yes.

The half occupied by Richards Chemicals is not shown at all.

Q. What type of activities were conducted in the hot labs that you recall?

A. Probably fabrication of radioactive sources primarily.

Q. Was that work performed in sealed boxes?

A. Some.
Q. They had what I refer to at least as glove boxes?
A. They what?
Q. They had glove boxes?
A. Yes.
Q. In the hot lab area?
A. Yes.
Q. This diagram shows a feature in the upper right-hand corner of the main building where it says, "CO-60 pool."
Do you see that?
A. Yes.
Q. Is that where you recall the pool being located?
A. Yes.
Q. And was that pool used to store cobalt?
A. Yes.
Q. And CO-60 a form of cobalt?
A. Yes.
Q. And this -- where it shows CO-60 pool, that's the location of the pool that you've described as having been cleaned and then filled in with dirt?

A. Yes.

Q. Now, that built -- this diagram also shows a feature that on this diagram is described as sump with CS-137.

Do you see that?

A. Yes.

Q. Do you recall there being a sump there at the property in that location?

A. No, I don't.

Q. At the top of the figure where it says, "Bay B," do you see that?

"Bay B"?

A. Yes.

Q. And again, there's a feature being described as "floor sump with thorium material" --

A. Yes.

Q. -- "was cleaned out"?

Do you recall a sump being there?

A. I don't recall that bay at all.

Q. Do you recall there being a back set of buildings at the property?

A. Yes.

Q. Do you recall what -- what's your recollection as to the nature of the activities that was conducted in those back buildings?

MR. PATTERSON: By ISC?

BY MR. SHIMADA:

Q. During --

MS. HATHAWAY McKEITH: By anybody.

MR. SHIMADA: By anybody.

THE WITNESS: I recall at one time we had a, what we called a rat facility. When we were irradiating rats.

That isn't even shown. I'm sure that was removed by the time this went -- this survey --

BY MR. SHIMADA:

Q. There's no rat building.

A. The other thing I notice there is thorium in Bay B, I don't know -- I don't recall a Bay B, but thorium happens to be one of the materials that goes along with the Research Chemical operations.

Thorium is a natural radioactive material.

Q. How did you become familiar -- or how did you come to have an understanding that thorium was used in Research Chemicals operations?

A. It -- Research Chemicals are very rare and they are found in nature along with

thorium ore.

Q. Did you ever have a chance to observe the operations that were going on at Research Chemicals?

A. No.

Q. Did you ever have a chance to talk to anyone at Research Chemicals as to the type of radioactive materials they were handling?

A. No.

MS. HATHAWAY McKEITH: Can I, just for clarification, when you were saying "rat," r-a-t --

THE WITNESS: R-a-t, rat.

MS. HATHAWAY McKEITH: -- are you talking about the rodent rat?

THE WITNESS: Yes.

MS. HATHAWAY McKEITH: Yes. I see.

BY MR. SHIMADA:

Q. So they were irradiating laboratory animals?

A. Yes. We were.

Q. You were?

A. Yes. That was over to the left there in one of those bays.

Other than that, all I recall is

storage back there.

Q. Now, the area between the -- what's described as being storage bays and the main building containing the shop area --

A. Uh-huh.

Q. -- that area right there, was that an asphalt parking surface?

A. I believe it was asphalt.

I don't know that there was much parking went on there.

Q. Was it an asphalt-covered --

A. Yes.

Q. -- surface?

Okay. During the course of your -- the work at the property to decontaminate various portions of it, do you recall taking any samples, wipe samples or otherwise of the part -- that asphalt area?

A. I don't recall.

Q. Do you recall using a Geiger counter or other direct reading instrument in that area behind the shop area?

A. I don't recall.

Q. Now, to the best of your recollection, what was the -- that asphalt area

used for?

A. One corner is not shown in here, Isotopes Specialties had a storage, waste storage area. It was partly underground and partly above ground and, apparently, it's been removed.

The rest of it, as far as I can remember, except for that rat facility, was used by Research Chemicals.

Q. Where in this asphalt area was this waste storage area that you've made reference to?

A. Right behind the building in the corner where it says, "Cobalt 60," in that area right there.

Q. So within the southwest portion of the asphalt area?

A. I can't see what -- I can't see the north arrow on this thing.

MS. HATHAWAY McKEITH: Oh, north is to --

BY MR. SHIMADA:

Q. This map is unusual. North is not to the top.

It's to the left.

A. North is to the left.

So that would be in the south --

Q. -west corner?

A. -west corner, yes.

Q. And you say that this waste storage area was partly underground?

A. Yes.

Q. Could you describe the configuration for me?

A. It was probably about three feet deep, oh, I can't guess at the lateral dimensions. It had a wall of dirt surrounding it about two feet thick.

Q. So this was a three-foot deep hole in the asphalt?

A. Yeah, concrete lined, yes.

Q. So it was a -- it was three feet deep and lined with concrete?

A. Yes.

Q. And then around the rim of this hole, there was a two-foot thick dirt wall?

A. Uh-huh.

Q. Yes?

A. Yes.

Q. How high was this dirt wall?

A. Five feet. In the order of five feet, approximately.

Q. What type of materials were -- were

stored, to your recollection, in this area?
A. Waste.
Q. Do you recall what was done with the -- do you recall if anything was done with this hole during the decontamination activities you've described?
A. Well, I recall a lot of waste was removed and was surveyed. But that's -- that's about it.
Q. Is it your recollection that when the -- your activities at the property started there was still some waste containers in that area?
A. Yes. In fact, we made more in the decontamination process.
Q. The waste that you generated from the decontamination process, did that include liquid that had rinsed off from surfaces like the pool that you washed?
A. Any liquid at that time had to be absorbed in some material so we had no liquid waste as such.
So I don't recall how it was disposed of, but I think it must have been taken up in some form.

Q. Do you have a recollection of some sort of an absorbent material being used?
A. I don't recall exactly what it is right now.
Q. Do you recall what type of materials were -- what type of waste materials were generated as part of the decontamination?
A. Oh, floor scrapings, in addition to the pool water I'm talking about, sweepings. There may have been some concrete chipping, I don't recall.
Q. Do you recall in this waste storage area that the con- -- that this concrete-lined hole was filled in in some manner?
A. I don't recall that. It certainly doesn't show it here.
Q. You said that the waste area was assayed?
How was it assayed?
A. The same as the rest of the building.
Q. With?
A. With wipe samples and direct readings.
As I recall, I started to say there was not much contamination in that area. Ever.

Q. The sweeping and scrubbing process that you've described, did that also -- did that include removal of some layer of the -- of concrete also?
A. Not specifically a layer. There may have been spots in small areas.
Q. The spots being loose concrete that might have --
A. No.
Loose concrete that we generated by removing it.
Q. Do you recall there being areas --
Do you have a specific recollection of some areas of concrete having a layer of it removed --
A. No.
Q. -- during this process?
A. I don't.
Q. Do you recall how many -- the waste that was generated from this process, was it put in 55-gallon size drums?
A. Yes.
Q. Do you recall how many drums were generated as part of this process?
A. No.

THE WITNESS: I'm going to need a break.

MS. HATHAWAY McKEITH: Thank you.

I didn't want to be the one this time who insisted.

THE VIDEOGRAPHER: The time is approximately 3:52.

We're off the record.

(Off record.)

THE VIDEOGRAPHER: Okay. One moment.

The time is approximately 4:08. We're on the record.

BY MR. SHIMADA:

Q. Mr. Donelson, do you recall where the drums containing the waste from the decontamination process was sent?

A. The best I recall they were sent to a waste disposal company.

I believe their name was California Salvage Company.

Q. Do you recall where this company was located?

A. In -- I know. San Pedro.

Q. Do you recall what was done --

Do you have an understanding as to what was done with this waste material by

California Salvage?

A. I believe at that time it was legal and permissible to take it out in the ocean and dump it.

Now, the time frame somewhere along there that disposal method was stopped, but at that time I believe that's where it went.

Q. Apart from the two individuals whose names you can't recall and yourself, were there -- did you ever see any other persons employed by U.S. Nuclear at the Providencia property during this decontamination activity?

A. I don't recall.

Q. Do you recall, for example, Mr. Goldstein ever being present during this process?

A. I don't recall specifically.

MR. SHIMADA: Before I forget, I'll ask the reporter to mark this Figure 1-2 as Exhibit 3 to the deposition.

(Deposition Exhibit 3 was marked for identification and is annexed hereto.)

BY MR. SHIMADA:

Q. At the time this work was being

performed at the Providencia facility, do you recall whether U.S. Nuclear had its own internal company guidelines for the decontamination or decommissioning of facilities?

A. I don't recall.

Q. The Figure 1-2, Exhibit 3, shows an area called "Tritium & C-14 Labs."

Do you see that?

A. Yes.

Q. Do you recall work involving Tritium and Carbon 14 being performed in that area?

A. Yes, I recall it was done.

I had very little to do with it.

Q. Do you recall what type of sampling, if any, that you performed in that Tritium & C-14 Lab area during this decontamination process?

A. The same as in the rest of the building.

Q. And that is wipe samples of some sort?

A. Wipe samples, direct measurements, yes.

Might explain the Tritium and Carbon are very low level in their radiation output so that they don't require any shielding or anything

like that.

Q. And as you sit here today, you don't have any specific recollection of the number of wipe samples you took in the Tritium --

A. No, I do not.

Q. -- and C-14 areas?

And as you sit here today, you don't have any specific recollection as to where in those labs you actually took those samples?

A. No, I do not.

Q. Do you recall the nature of any sweeping and scrubbing that was done in the Tritium & C-14 Labs?

A. Not specifically.

Q. Do you recall removing -- having any equipment removed from those labs and taken offsite?

A. Not that I recall.

Q. The -- Exhibit 3 shows an area entitled "QA Lab" next to the Tritium & C-14 Labs.

Do you see that?

A. Do I have that?

Q. Yes. It's kind of small. It's immediately to the left.

A. Excuse me. Would you ask the question again?

Q. I wanted to direct your attention to where it says, "QA Lab." To the left.

A. QA Lab. Okay. Yes.

Q. Do you recall there being a quality assurance laboratory in that area?

A. I recall I've been in an area there. What was in it, I don't recall.

Q. And, again, do you have any recollection of the number of samples or the location of the samples that, if any, that you took in the QA Lab area?

A. No, I do not.

Q. And likewise, is it true that you have no recollection of what type of cleaning activity was performed, if any, in that laboratory?

A. That's right. No, I don't.

Q. Immediately to the east or just directly above on this figure of the Tritium & C-14 Labs, there's an area called "Cooler."

Do you see that?

A. I see it.

Q. What was that area used for, to your

recollection?

A. I don't recall.

Q. You recall -- and again, is it true that you don't recall the number of samples or the location of any sampling that you may have done in that area?

A. That's correct.

Q. And is it also true that you don't recall any -- the nature of any cleanup that may have been performed in that area?

A. I don't recall.

Q. Now, the area described in this figure as being the "Shop Area," do you see that?

A. Yes.

Q. What kind of operations were conducted in that area?

A. Lathes, mill work, probably some welding, some shop type work.

Q. That was work that did not involve the handling of radioactive materials?

A. That's true of many cases, the items were being constructed to house radioactive material but it was not handled in there.

Q. You don't recall -- isn't it true that you don't recall whether you took any samples

in that area and, if so, the number or the location?

A. I don't recall.

Q. So that's a true statement?

A. Yes.

Q. And -- and likewise, you don't recall what type of cleaning, if any, was done in that area, is that true?

A. That is correct.

Q. The area that's described here as being "Former Hot Cells" but you recall as everything to the hot labs --

A. Yes.

Q. -- is it accurate to say that, again, you do not recall the number of samples or the location of the samples that you took in that area?

A. That's correct.

Q. And likewise, you do not recall the nature of whatever cleaning was done in that area?

A. That's correct. I recall a little bit.

Q. What do you recall?

A. When we started operations in the hot lab, we covered the walls with plastic. All the

walls and the ceiling.

And that was removed, of course, during the decontamination work.

Q. Do you recall the nature of the plastic that was removed?

A. Polyethylene.

Q. Do you recall how many -- how thick it was?

A. No.

Q. Was this a -- a coating that was applied or were they actual sheets of --

A. Sheets.

Q. -- polyethylene?

How were the sheets attached to the walls?

A. I don't recall.

Stapled.

Q. And it's your recollection that those sheets were removed during the course of this work?

A. Yes.

Q. And what was done with those sheets?

A. Went to the waste.

Q. Apart from the removal of those sheets, do you recall the nature of any other

cleanup that might have been done in those hot labs or hot cells?

A. Scrubbing and mopping is all I recall.

Q. Was a particular type of scrubbing material you used during the process of scrubbing the facility?

A. General household cleaners, I would think. Bon Ami.

I don't really recall specifically, though.

Q. So you recall some sort of cleaning product being used --

A. Yes.

Q. -- in addition to whatever was being used to scrub?

A. I'm sure the material they used to, when they sweep large areas, whatever it is, oil impregnated --

What do you call that? Do you know what I'm talking about?

Q. A sweeping compound?

A. Yes, a sweeping compound.

Q. So do you recall a sweeping compound being used?

A. Yes.
Q. Do you recall the nature of that compound?
A. No. No.
Q. Do you recall --
Do you recall what was actually used to perform the scrubbing? Was it a brush, a sponge?
What was used to do to the scrubbing that you recall?
A. Brush, mop, broom.
Q. All of the above?
A. Yes.
MS. HATHAWAY McKEITH: High tech. High tech.
THE WITNESS: Real.
BY MR. SHIMADA:
Q. The workers that were doing this work, the scrubbing and the sweeping, were they wearing any kind of protective clothing?
A. As I recall they were, yes.
Q. What were they wearing?
A. Coveralls. It was considered dust -- dusted -- to be dusty, we wore masks of some sort. Probably some sort of shoe covering, I

don't remember what.

Q. Did they wear any kind of dosimeters?

A. Oh, yes.

Q. And was it your response -- one of your responsibilities to review those dosimeters on a regular basis?

A. Yes. Focimeter.

Q. Focimeter.

There is an area on Exhibit 3 entitled "former thorium operations area."

Do you see that?

A. Yes.

Q. What is your recollection as to the nature of the activities that were conducted in that area?

A. I believe that is a mistake.

Q. What --

A. I can't remember any thorium operations going on.

Q. What do you recall taking place in that area of the facility?

A. I can't really recall.

I almost seem to remember that the hot lab, as I called it, where it says hot cells,

went all the way to the wall.
Q. Which wall?
A. The left in the picture.
Q. Across the area denominated on this as being the shop area?
A. Yes.
Q. Was the asphalt area behind the shop -- between the bays and the main building swept, to your recollection?
A. Sorry, I don't recall.
Q. Do you recall any kind of cleaning being done in that area?
A. I recall making surveys. We may have found a few spots and took up some asphalt, I don't recall specifically.
That would be the standard way of doing it.
Q. But you don't recall whether, in fact, you --
A. I don't.
Q. -- actually dug up any asphalt?
A. No, I don't.
Q. Did you, as part of your activities at the property, take wipe samples of the exterior walls of the building?

A. I don't recall if we did.
Q. Do you recall using a direct reading instrument to conduct a survey of the exterior walls of the building?
A. I don't recall.
Q. The area that is described as storage bays, do you recall whether you took samples in that area?
A. Which area?
Q. The storage -- what's depicted here as storage bays, those back buildings.
Do you recall whether you took any --
A. I don't --
Q. -- samples of that?
A. I don't recall.
I don't recall that there was ever any loose radioactive materials out there from the Isotopes Specialties part.
I can't vouch for Research Chemicals.
Q. Now, there was -- there were labs being irradiated --
A. Yes.
Q. -- back there?
A. A short-term project.
Q. So it's possible that you didn't take

samples back there?
Is that what you're saying?
A. I don't recall.
Q. And likewise, you don't recall, what, if any, cleaning activities were performed in those back buildings?
A. I don't recall.
MR. SHIMADA: Let's go off the record.
THE VIDEOGRAPHER: It's approximately 4:26.
We're off the record.
(Off record discussion.)
THE VIDEOGRAPHER: Okay. One moment.
The time is approximately 4:29. We're on the record.
MR. SHIMADA: It's been decided that the deposition will resume on Wednesday at 10 o'clock, and that at the conclusion of that session we'll enter into the appropriate stipulation concerning preparation of the transcript and the like.
But today's session is concluded.
THE VIDEOGRAPHER: Approximately 4:29.
It's the conclusion of tape 2 of 2 in Volume 1 in the deposition of Richard Donelson.
We're off the record.
(TIME NOTED: 4:29 P.M.)

I declare under penalty of perjury under the laws of the State of California that the foregoing is true and correct.

Executed on ____________________, 1998, at__________________, California.

____

SIGNATURE OF THE WITNESS

STATE OF CALIFORNIA)
) ss:
COUNTY OF LOS ANGELES)

I, MARCELINE F. NOBLE, C.S.R. No. 3024, do hereby certify:

That the foregoing deposition of Richard Donelson was taken before me at the time and place therein set forth, at which time the witness was put under oath by me;

That the testimony of the witness and all objections made at the time of the examination were recorded stenographically by me, were thereafter transcribed under my direction and supervision and that the foregoing is a true record of same.

I further certify that I am neither counsel for nor related to any party to said action, nor in any way interested in the outcome thereof.

IN WITNESS WHEREOF, I have subscribed my name this 8th day of October, 1999.

Marceline F. Noble, CSR

MARCELINE F. NOBLE, C.S.R. No. 3024

INDEX

VOLUME I

MONDAY, SEPTEMBER 27, 1999

WITNESS		EXAMINATION
RICHARD DONELSON		
	(By Mr. Bisgaard, a.m.)	5
	(By Mr. Shimada, p.m.)	48

DEPOSITION EXHIBITS

RICHARD DONELSON

NUMBER	DESCRIPTION	IDENTIFIED
1	Nine-page document entitled, "Isotopes Specialties Company, Inc., Administrative Procedures," dated August 20, 1958.	67
2	Two-page letter dated January 13, 1961, to James Mason from Allen Goldstein.	69
3	One-page document entitled, "Figure 1-2 Thomson property facility map."	122

ERRATA SHEET

Pursuant to the Federal Rules of Procedure and the California Code of Civil Procedure, any changes in form or substance you wish to make to your deposition testimony shall be included in the deposition transcript. Should you have any corrections, please enter them below.

PAGE LINE

____ ____ Change: ________________________________

Reason: ________________________________

____ ____ Change: ________________________________

Reason: ________________________________

____ ____ Change: ________________________________

Reason: ________________________________

____ ____ Change: ________________________________

Reason: ________________________________

____ ____ Change: ________________________________

Reason: ________________________________

____ ____ Change: ________________________________

Reason: ________________________________

____ ____ Change: ________________________________

Reason: ________________________________

____ ____ Change: ________________________________

Reason: ________________________________

____ ____ Change: ________________________________

Reason: ________________________________

NOTES

Page No.	Line No.	Notes

PDR advance cy per J. Rathye
PART 50

44 FR 61372

THE NUCLEAR INDUSTRY

1966

0/1
DF02

☆U.S. ATOMIC ENERGY COMMISSION☆

DIVISION OF INDUSTRIAL PARTICIPATION

Ernest B. Tremmel, Director

9903160021 661231
PDR PR
50 44FR61372 PDR

Prepared for delivery to
Atomic Industrial Forum's Annual Conference
Pittsburgh, Pennsylvania
November 3, 1966

FOREWORD

Section 1, the Declaration, of the Atomic Energy Act states that it shall be the policy of the United States to develop, use, and control atomic energy "so as to......... strengthen free competition in private enterprise." As part of its fulfillment of this mandate, this booklet, Nuclear Industry, is published annually to present the AEC's assessment of the state of the nuclear industry and make available facts believed to be of interest and in accordance with the President's program to improve service to the public.

It is intended that the booklet serve both as introductory reading for persons who desire to become better acquainted with the nuclear industry as well as a handy reference for important items of interest to the industry. It is prepared yearly by the AEC's Division of Industrial Participation (in September of each year) and released at the Atomic Industrial Forum and American Nuclear Society joint meeting held in the Fall of each year.

The Division of Industrial Participation acknowledges the assistance given by other organizational units of the Atomic Energy Commission in contributing and reviewing information presented in the Report. The assistance of industrial firms has been particularly helpful and should not be overlooked in any [illegible]knowledgment.

It is sincerely hoped that Nuclear Industry - 1966 will [illegible] value to the many readers of past year's reports as well [illegible] future [illegible]aders and that it will provide information which [illegible] serve as one basis for guidance to industry in determining their future role in the nuclear field.

For sale by the Superintendent of Documents, U.S. Government Printing Office
Washington, D.C. [illegible] - Price [illegible]

REPORT ON THE NUCLEAR INDUSTRY
1966

Contents

Page

SUMMARY 1

A. Shipments of Products 1
B. Nuclear Power 4
C. Investment 6
D. Exports 6
E. Employment 7

HIGHLIGHTS 9

A. Civilian Nuclear Power 9
B. Desalting 12
C. Maritime 12
D. Radioisotopes 13
E. Meetings with Industry 14
F. Encouraging Competition 14
G. Operating Contractors 14
H. Nuclear Safety 14
I. Regulation 15
J. NUCLEX '66 16
K. Appointment of New Commissioners 16

PRIVATE OWNERSHIP -- TOLL ENRICHING 18

A. Legislative History 18
B. Background on Private Ownership 19
C. Time-Table for Private Ownership 20
D. Implementation Required 21
E. Commission Actions to Implement 22
F. Safeguarding of Special Nuclear Materials . . 23
G. Uranium Sale and Lease Policies 24
H. Toll Enriching Criteria and Contracts 25
I. Future Areas of Action 25
J. Other Highlights of JCAE Hearings 26

Page

CATEGORY I -- RAW FUEL MATERIALS 28

A. Uranium 28
B. Thorium 37

CATEGORY II -- MATERIALS PROCESSING AND FABRICATION 41

A. Uranium 41
B. Plutonium 49
C. Thorium 55
D. Uranium-233 56
E. Other Materials 57

CATEGORY III -- NUCLEAR COMPONENTS AND EQUIPMENT 62

A. Nuclear Instruments 62
B. Components for Sodium Cooled Reactor 67
C. Other Components 68

CATEGORY IV -- REACTORS 69

A. Expenditures for Reactor Projects 69
B. Civilian Power 74
C. Civilian Maritime Reactors 96
D. Military Reactors 97
E. Research and Teaching Reactors 100
F. Space Reactors 101

CATEGORY V -- RADIOISOTOPES 105

A. Producers 105
B. Processors 111
C. Source Fabrication 113
D. Equipment Manufacturers 113
E. AEC Advisory Committee on Isotopes
and Radiation Development 125

CATEGORY VI -- MATERIALS REPROCESSING 127

A. Irradiated Fuel 127
B. Unirradiated Fuel 135

Page

CATEGORY VII -- PLOWSHARE -- PEACEFUL USES OF NUCLEAR EXPLOSIVES . . 139

A. Introduction 139
B. Budget 139
C. Types 140
D. Potential Applications 140
E. Proposed Projects 141
F. Charges 145

CATEGORY VIII -- SERVICES 147

A. Radioactive Waste Management 147
B. Film Badge Processing 154
C. Decontamination Laundry Services 158
D. Transportation 161
E. Whole Body Counting Service 169
F. Reactor Servicing 170
G. Decontamination 171
H. Consultants and Engineers 172

ASSISTANCE FOR INDUSTRY AT AEC FIELD OFFICES 173

List of Charts iv

List of Tables v

List of Charts

Page

SUMMARY

Chart A -- Shipments of Atomic Energy Products 1
Chart B -- Value of Net Orders Received 2
Chart C -- Nuclear Power Growth 5

RAW FUEL MATERIALS

Chart I-A -- Effect of Stretch-Out Program 30
Chart I-B -- Procurement of Uranium 32
Chart I-C -- Industry Drilling Plans 38

MATERIALS PROCESSING AND FABRICATION

Chart II-A -- Plutonium Processing and Fabrication 54

NUCLEAR COMPONENTS AND EQUIPMENT

Chart III-A -- Growth of Nuclear Instrument Industry 66
Chart III-B -- Nuclear Instrument Sales 66
Chart III-C -- Reactor Components 67

REACTORS

Chart IV-A -- Domestic Reactor Projects 73
Chart IV-B -- Nuclear Power Reactors in the U. S. 75
Chart IV-C -- Estimated Nuclear Capacity 81
Chart IV-D -- Energy Requirements for Electric Generation 83
Chart IV-E -- Estimated Increases in Steam Plant Capacity 84

MATERIALS REPROCESSING

Chart VI-A -- Domestic Central Station Spent Fuel Forecast 132

Page

PLOWSHARE -- PEACEFUL USES OF NUCLEAR EXPLOSIVES

Chart VII-A -- Transisthmian Canal 141
Chart VII-B -- Applications of Deeply Buried Explosions 142
Chart VII-C -- Charges for Thermonuclear Explosives 146

SERVICES

Chart VIII-A -- Transfer of Licensing Functions 149
Chart VIII-B -- Commercial Burial Sites 150
Chart VIII-C -- Film Badge Operations 155
Chart VIII-D -- Contaminated Laundry Services 161

List of Tables

Page

SUMMARY

Table 1 -- Orders Received for Atomic Energy Products 2
Table 2 -- Shipments of Atomic Energy Products 3
Table 3 -- United States Exports (Bureau of the Census) 7
Table 4 -- Employment 7
Table 5 -- Number of Establishments 8

PRIVATE OWNERSHIP -- TOLL ENRICHING

Table 6 -- Time-Table for Private Ownership 21

RAW FUEL MATERIALS

Table I-1)
Table I-2) -- Domestic Uranium Processing Mills 29
Table I-3 -- Approved Barter Requests 33
Table I-4 -- Uranium Requirements 34
Table - -- Uranium Ore Reserves by States 35
Table I-5 -- U. S. Uranium Reserves 36
Table I-6 -- World Uranium Reserves 36

Page

MATERIALS PROCESSING AND FABRICATION

Table II-1 -- Principal Producers of Uranium Fuels 44
Table II-2 -- Enriched Uranium Furnished to Industry 45
Table II-3 -- Principal Fabricators of Uranium Fuel 47
Table II-4 -- AEC Orders for Fabrication of Uranium Fuel 48
Table II-5 -- Projected Plutonium Requirements 52
Table II-6 -- Anticipated Plutonium Recovery from Commercial Power Reactor Fuels 53
Table II-7 -- Industrial Interest in Plutonium Work 55
Table II-8 -- Principal Firms Processing Thorium 56
Table II-9 -- Principal Commercial Suppliers of Heavy Water 59
Table - -- Companies in Beryllium Metal Industry 61

NUCLEAR COMPONENTS AND EQUIPMENT

Table III-1 -- Companies with 1965 Nuclear Instrument Sales Exceeding $0.5 Million 63

REACTORS

Table IV-1 --) Reactors Under Design or Construction,) 69
Table IV-2 --) showing Costs Incurred, Total Estimated) 70
Table IV-3 --) Costs, Source of Funds, etc.) 71
Table IV-4 -- Domestic Reactor Projects Under Design or Construction 72
Table IV-5 -- Nuclear Plant Announcements Through 1965 76
Table IV-6 -- Nuclear Plant Announcements - 1966 77
Table IV-7 -- Announcements of New Steam-Electric Plant Additions - 1965 78
Table IV-8 -- Announcements of New Steam-Electric Plant Additions - 1966 79
Table IV-9 -- Fuel Cycle Steps by Suppliers 88
Table IV-10 -- Power Reactors for Export 93
Table IV-11 -- Army Reactors 99
Table IV-12 -- Producers of Research and Teaching Reactors 101

Page

RADIOISOTOPES

Table V-1 -- Principal Industrial Suppliers of Cyclotron Radioisotopes 106
Table V-2 -- AEC Sales of Radioisotopes 108
Table - -- AEC Revenue from Isotope Sales 111
Table V-3 -- Principal Processors of Radiochemicals and Radiopharmaceuticals 112
Table V-4 -- Principal Suppliers of Radiographic Units 113
Table V-5 -- Principal Suppliers of Radioisotope Teletherapy Units . 114
Table - -- Sales of Tritium 115
Table V-6 -- Principal Suppliers of Self-Illuminating Materials and Devices 116
Table V-7 -- Principal Firms Supplying Accelerator Equipment and Services 121
Table V-8 -- Principal Suppliers of Isotopic Irradiators 122
Table V-9 -- Regulations and Petitions on Radiation Preservation of Foods 123

MATERIALS REPROCESSING

Table VI-1 -- Principal Firms Recovering Uranium Scrap 136
Table VI-2 -- AEC Orders for Scrap Recovery 137
Table VI-3 -- Scrap Recovery Awards in FY-1966 137

PLOWSHARE -- PEACEFUL USES OF NUCLEAR EXPLOSIVES

Table - -- Plowshare Budget, 1965-1967 139

SERVICES

Table VIII-1 -- Private Waste Burial 150
Table VIII-2 -- AEC Solid Waste Burial 152
Table VIII-3 -- Commercial Firms Licensed to Receive and Dispose of Radioactive Wastes 153
Table VIII-4 -- Film Badge Processors 155
Table VIII-5 -- Commercial Contaminated Laundry Processors 159
Table VIII-6 -- AEC Sites Using Commercial Laundries / AEC Sites Not Using Commercial Laundries 160
Table VIII-7 -- U. S. Ports for Irradiated Fuels 165
Table VIII-8 -- Fuel Shipments to Foreign Countries 166
Table - -- Firms Supplying Special Transportation Services . . 169
Table - -- Firms Offering Whole-Body Counting Services 169
Table - -- Reactor Servicing Firms 170
Table - -- Firms Offering Decontamination Services 171

Page

ASSISTANCE FOR INDUSTRY AT AEC FIELD OFFICES

Table - -- Industrial Participation Representatives at AEC Field Offices 173

STATUS OF THE NUCLEAR INDUSTRY

SUMMARY

Central station nuclear power plants continued to receive the most attention of all the applications of nuclear energy during 1966. Expanding radiation applications in areas such as medicine, industry and space; the decision to move ahead with the world's largest desalting plant as a nuclear-powered, dual-purpose facility; and increasing interest in commercial applications of peaceful nuclear explosives; all added to the bright promise for the nuclear industry's future. All segments of the industry should feel the impact of the expansion that lies ahead.

A. Shipments of Products

One of the principal indicators of the growth of the industry is the Census Bureau report on Selected Atomic Energy Products (Series MA-38Q) which gives the dollar value for shipments of certain products and services and the value of orders for a more limited group of products. Charts A and B show these shipments and orders for the years for which the data have been collected. Tables 1 and 2 show the detailed information collected for 1965.

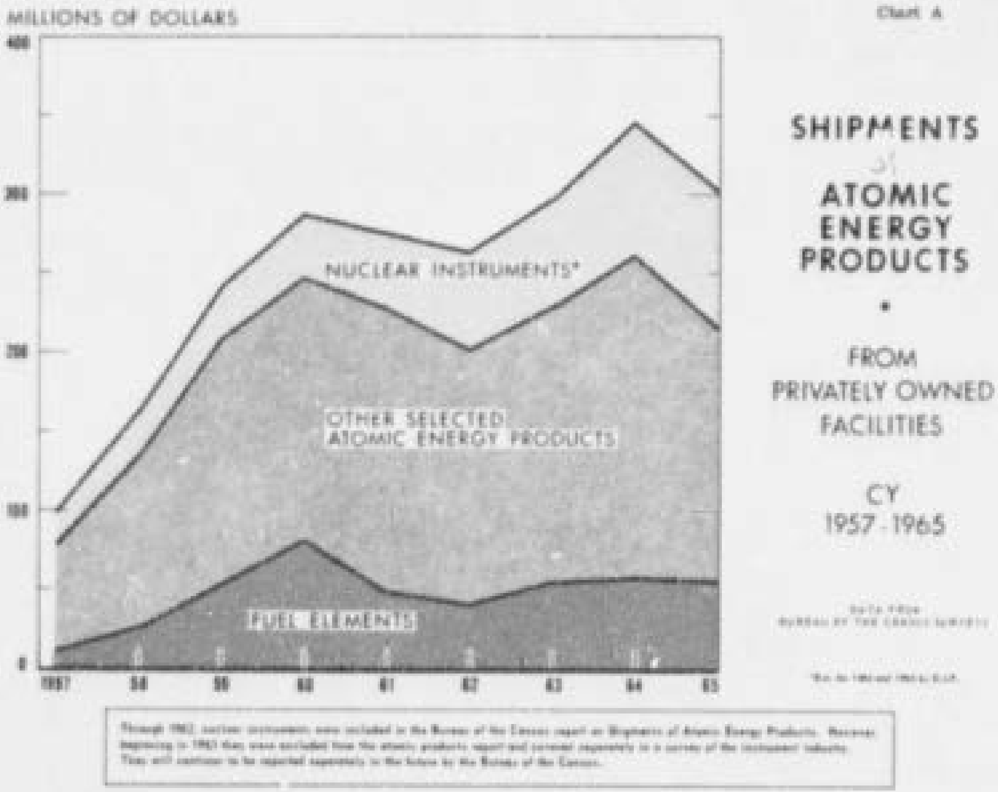

Chart B

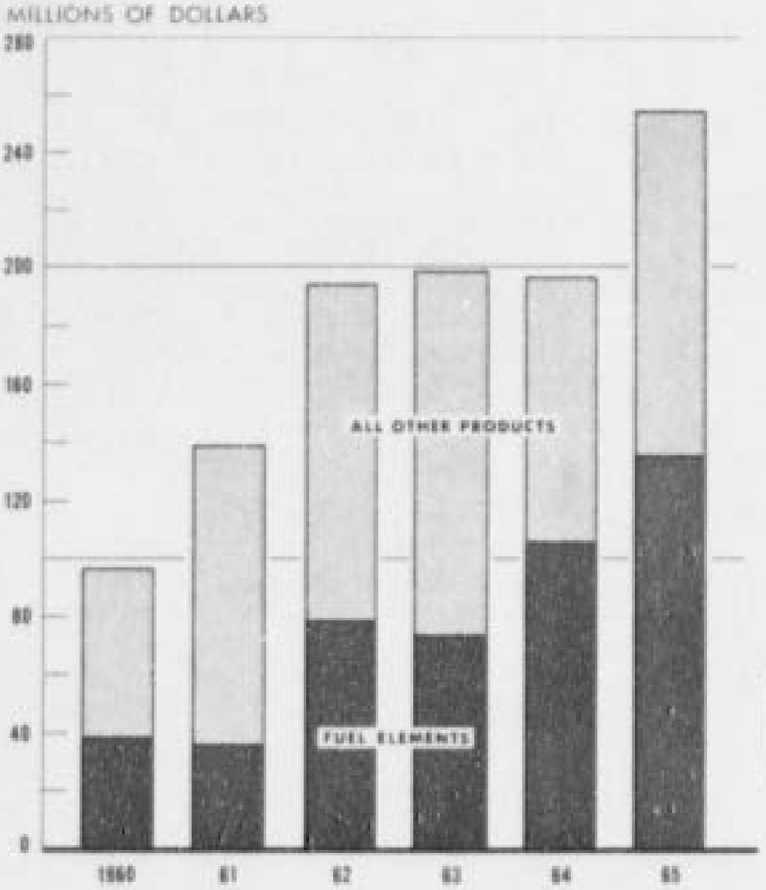

Value of NET ORDERS RECEIVED *for* SELECTED ATOMIC ENERGY PRODUCTS

The orders reported do not include orders for nuclear instruments or certain of the other categories included in Chart A, Shipments of Atomic Energy Products. See Tables 1 and 2 for details of these differences.

TABLE 1

Value of Net Orders Received for Selected Atomic Energy Products

1965

(In thousands of dollars)

Product	Net orders received
Nuclear reactors*	2,302
Reactor components and equipment:	
Primary vessels and tanks	15,742
Control rod drive mechanisms and components for:	
Power plant	18,[illegible]
Propulsion	732
Other	-
Heat exchangers and condensers	49,568
Pressurizers, components, and auxiliary equipment	16,294
Valves	7,793
Fuel handling equipment	2,567
Complete reactor fuel elements and control rods shipped directly for installation or use in a reactor for:	
Power plant	57,509
Propulsion	69,455
Other	7,871
Partially fabricated reactor fuel element materials and control rods not shipped directly for installation or use in a reactor	4,536

* Includes only reactors shipped fully assembled from the place of manufacture, such as AGN-201 and other similar types.

Table 2.--VALUE OF SHIPMENTS OF SELECTED ATOMIC ENERGY PRODUCTS: 1963 AND 1964

[In thousands of dollars]

Product	1963 Total	1963 To U.S. Government: Atomic Energy Commission	1963 To U.S. Government: Other Agencies	1963 Export	1963 Other	1964 Total	1964 To U.S. Government: Atomic Energy Commission	1964 To U.S. Government: Other Agencies	1964 Export	1964 Other
TOTAL (excluding radiation detection and monitoring devices and control and measuring devices containing radioactive isotopes)..	216,563	[1]83,387	[1]37,512	[1]40,111	[1]35,553	238,282	[1]113,481	[1]68,521	[1]36,902	[1]29,388
Nuclear reactors*	2,185	(D)	(D)	(D)	(D)	7,392	(D)	(D)	(D)	(D)
Reactor components and equipment:										
Primary vessels and tanks	12,033	3,513	8,320	200	([2])	16,230	2,378	13,573	289	([2])
Control rod drive mechanisms and components for:										
Power plant	17,058	11,655	([3])	5,403	([2])	15,360	11,374	([3])	3,986	([2])
Propulsion	} 949	{ (D)	(D)	-	-	} 9,956	{ (D)	(D)	-	(D)
Other		(D)	(D)	-	-		(D)	(D)	(D)	-
Accessory instrumentation for reactor control	9,676	2,438	6,122	270	846	18,050	5,368	9,651	154	2,877
Heat exchangers and condensers	26,297	1,344	7,158	17,793	([2])	30,619	3,488	14,950	12,181	([2])
Pressurizers, components, and auxiliary equipment	4,881	254	1,704	2,923	([2])	8,460	340	4,848	3,272	([2])
Pumps	22,169	1,141	15,526	5,502	([2])	22,727	1,347	17,116	4,264	([2])
Valves	9,590	981	6,406	90	2,113	9,052	876	6,297	117	1,762
Fuel handling equipment	792	588	([3])	204	([2])	562	492	([3])	70	([2])
Complete reactor fuel elements and control rods shipped directly for installation or use in a reactor for:										
Power plant	15,276	4,712	([3])	2,941	7,622	22,212	17,823	([3])	1,701	2,688
Propulsions	23,689	23,689	-	-	-	30,337	30,337	-	-	-
Other	15,040	14,229	([3])	811	([2])	3,954	2,635	([3])	1,319	([2])
Partially fabricated reactor fuel element materials and control rods not shipped directly for installation or use in a reactor	7,688	2,244	2,263	600	2,581	13,634	7,813	1,489	356	3,976
Core structurals (barrels, cans, boxes, plates, etc., not included above)	3,719	3,065	(D)	(D)	(D)	5,182	3,116	(D)	(D)	(D)
Reactor moderators, coolants, and reflectors	4,043	3,034	([3])	1,009	([2])	3,023	905	([3])	2,118	([2])
Specialized reactor components and equipment not included elsewhere in this report	8,463	2,013	1,644	236	4,565	13,300	2,105	2,441	5,856	2,898
Hot laboratory equipment	3,620	2,539	([3])	610	484	5,726	5,156	([3])	274	296
Shielding	5,941	1,080	2,247	44	2,570	6,692	1,998	2,354	128	2,220
Radioactive isotopes shipped from non-AEC plants producing isotopes	4,437	326	([3])	321	3,790	1,284	121	([3])	116	1,037
Radiation sources and other radioactive materials produced from purchased isotopes	10,043	47	1,366	866	7,764	8,051	18	1,325	801	5,907
Conversion of enriched uranium metal and compounds (excluding production or conversion of normal and depleted uranium)	4,371	} 3,909	1,851	{ (X)	2,167	5,374	} 4,851	1,086	{ (X)	3,376
Commercial irradiation service	4,550			(X)	977	4,690			(X)	717

- Represents zero.
* Includes only reactors shipped fully assembled from the place of manufacture, such as AGN-201 and other similar types.
(D) Withheld to avoid disclosing figures of individual companies.
(X) Not applicable.
[1] Includes the value of those products for which separate figures are not shown to avoid disclosing figures of individual companies. Also see footnotes 2 and 3.
[2] Included in export figure.
[3] Included in shipments to A.E.C.

The effects of the rapid advances during the year in the uses of nuclear energy are not yet reflected in the Census Bureau reports. This is due partly to the time required to collect and summarize the data. It is also due to the fact that orders for nuclear power plants are not reflected directly in the Census Bureau reports, but indirectly through values reported for nuclear power plant components. Shipments of these components, of course, lag orders for complete plants by from two to four years. Orders for some of the principal components, such as pressure vessels, on the other hand, should begin to be reflected in reports for 1966 and 1967. Thus, although shipments for 1965 are down from a year earlier, in future years they may be expected to show a substantial rise as components are shipped to fulfill power plant orders placed by electric utilities over the past two years.

The report on orders received, however, better reflects the general health of the nuclear industry, the total dollars being about 30% above the amount for orders reported for any prior year. However, even these figures do not yet reflect the full impact of recent utility orders for nuclear plants.

It now seems probable that reports for 1966 and later years will show a substantial upturn. However, the actual effect of the recent upturn in nuclear-electric plant orders will become apparent only as reports for later years become available.

Today, we have a broadly based competitive industry with future prospects finally beginning to appear promising and current optimism especially in certain areas such as nuclear power and radioisotopes well justified.

B. Nuclear Power

Any summary of the nuclear industry would be incomplete without mentioning the expanding market for energy to produce electricity and the important position nuclear energy is rapidly attaining in this market. The upsurge in orders for nuclear power plants, which began in 1965, not only continued but accelerated in 1966. During the first nine months of the year, orders for nuclear electric plants exceeded orders for fossil fueled plants, an event unforeseen by even the most optimistic forecasters a year ago.

Chart C

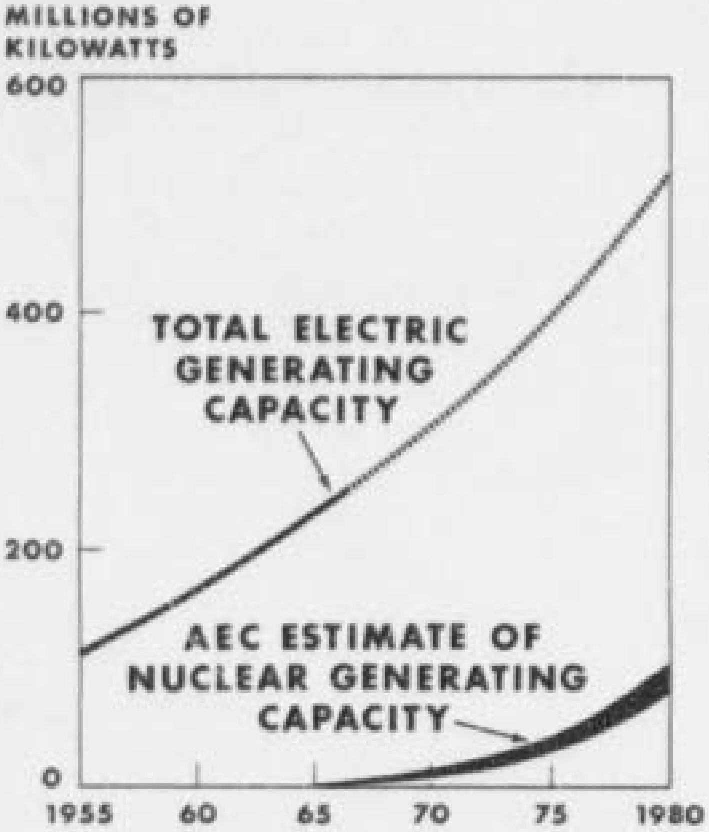

U. S. ELECTRIC GENERATING CAPACITY and NUCLEAR POWER GROWTH

The year 1966 will no doubt be remembered as the year that nuclear energy was fully recognized as competitive with fossil fuel for producing electric power in many areas of the United States and in the world. The portion of this report on reactors gives the details of this large increase in orders and shows the AEC estimate which was revised in 1966, for installed nuclear-electric capacity - 80,000 kw to 110,000 kw by 1980. This is approximately one-third greater than the AEC 1964 estimate and more than double that made only four years ago. The current AEC estimate, together with the projected estimated increase in total electric generating capacity through 1980, is shown in Chart C. The rapid expansion of the nuclear power market promises a bright future for the nuclear industry.

C. Investment

Investment by private industry in nuclear energy activities continued to increase as new central station power plants were announced or came into operation. Examples were the opening of new plants for forming reactor vessels and for reprocessing spent reactor fuel.[1/] New reactor power plants ordered by the electric utility industry during the first nine months of 1966 are estimated to cost more than $1 billion. By the end of the year, the total cost of plants ordered in 1966 could even equal the $1.5 billion estimate of the cost of all nuclear power plants ordered since the first nuclear plant at Shippingport, Pennsylvania, was announced in 1953.

Although little information is publicly available on private industry's investment in nuclear energy, the available indicators -- sales of products, employment, number of establishments and orders for nuclear-electric generating plants -- all point to a rapidly increasing impact on our country's economy by this newest primary energy source.

D. Exports

The Bureau of the Census collects information on export shipments of a number of products peculiar to the nuclear industry. A summary of these data is given in Table 3 on the next page. It is interesting to note that Special Nuclear Materials, Radioactive and Stable Isotopes and Nuclear Radiation Instruments have shown a continuing increase, while Uranium and Thorium Concentrates and Nuclear Reactors and Parts show a decrease. The over-all upward trend over the period shown is encouraging and also gives promise of future growth as applications for peaceful uses of nuclear energy expand overseas.

1/ See Highlights section of this report.

TABLE 3

United States Exports

Commodity Description	Total Value - Thousands of Dollars 1963	1964	1965
Uranium Ores and Concentrates	$ 659	$ 20	$ -0-
Thorium Ores and Concentrates	322	353	79
Uranium and Thorium Compounds	144	182	265
Special Nuclear Material	23,560	15,339	35,559
Radioactive and Stable Isotopes, Their Compounds & Mixtures & Radioactive Elements	2,554	2,921	6,647
Nuclear Reactors and Parts	798	27,618	13,209
Nuclear Radiation Detecting and Measuring Instruments	11,804	15,338	18,108
Total for Items Reported	$39,841	$61,771	$73,867

E. Employment

Reports on employment in private establishments in the atomic energy field are prepared for the Atomic Energy Commission by the Bureau of Labor Statistics. Total employment in atomic energy work was reported as 132,000 in March 1966. Of this total, about 101,000 were employed in AEC-owned facilities and approximately 31,000 were employed in privately-owned facilities. Of the latter number, over two-thirds were employed in private industrial establishments on work now primarily associated with peaceful uses of atomic energy. These are shown in Table 4 together with comparable figures for the five preceding years. Table 5 shows the number of establishments covered by each of the annual surveys.

TABLE 4

Employment
Selected Economic Segments

Industry Segment	Jan. 1961	Jan. 1962	Jan. 1963	Jan. 1964	Jan. 1965	Mar. 1966
Milling	4,793	3,381	2,579	2,255	2,079	2,054
Production of Feed	522	538	620	643	352	343
Special Materials	1,970	1,890	1,507	1,757	1,228	1,503
Fuel Fabrication	506	786	1,819	2,236	1,658	1,693
Reactors & Components	7,992	7,966	10,091	12,245	14,094	12,068
Waste Disposal	51	44	86	79	67	58
Instruments	3,717	4,237	4,745	3,853	4,979	4,068
Isotopes	215	291	365	348	407	642

TABLE 5

Number of Establishments
Selected Economic Segments

Industry Segment	Jan.* 1961	Jan. 1962	Jan. 1963	Jan. 1964	Jan. 1965	Mar. 1966
Milling	23	23	23	26	24	23
Production of Feed	6	6	5	8	6	4
Special Materials	15	15	19	33	31	28
Fuel Fabrication	6	6	11	14	13	13
Reactors & Components	38	38	50	58	61	48
Waste Disposal	4	4	8	10	9	8
Instruments	106	106	94	115	106	82
Isotopes	21	21	25	22	22	33

It is interesting to note in analyzing the data on employment and number of establishments that certain industry segments have increased while others have decreased. It is also interesting to note that these increases and decreases in the employment figures are consistent with those occurring in the number of establishments. Significant decreases occurred in the reactors and components and instrument segments of the industry between 1965 and 1966. Detailed data in order to analyze why these decreases occurred was not available at the writing of this report. In the reactor and components field the decrease may reflect the low period in orders for nuclear reactors by utilities in the 1960-1964 period. In the case of instruments the decrease may well reflect consolidations in the industry even though the market is growing.

Despite some year-to-year declines, a long-range comparison between 1961 and 1966 indicates a substantial advancement in the nuclear industry as a whole. Also, if milling and the production of feed are excluded, better than a one-third increase over the five-year span of the reports is indicated. As the effects of the rapidly expanding market for nuclear power are felt at the production level, further increases in employment should result.

---

* Employment data for January 1961 came from the January 1962 survey and covered the same establishments. Comparable surveys were not made in 1961 and earlier years.

NUCLEAR INDUSTRY HIGHLIGHTS - 1966

Listed below are the noteworthy events of the year relating to the commercial nuclear energy industry. Detailed information on these activities can be found in the respective categories of this report. Last year this highlights section was divided between "industrial actions" and "Commission actions". This year the two have been consolidated so that all actions in individual subject areas may be covered in one place.

A. Civilian Nuclear Power

1. New Orders

By far the outstanding event of the year was the dramatic number of orders for nuclear power plants placed by the utility industry.1/ The total electric generating capacity of nuclear power plants sold in 1963-64 was at the two thousand Mwe level and in 1965 at the five thousand Mwe level. The capacity of plants sold in 1966 had reached twelve thousand Mwe by the end of September, indicating a good possibility of fifteen thousand Mwe for the year.

2. Competitive Power

1966 will be remembered as the year in which the atom became economically competitive with fossil fuel in many parts of the United States and, possibly, throughout the world. This was demonstrated most forcefully with the announcement of the selection of nuclear power by the Tennessee Valley Authority for a plant in the southeastern United States, which is near a location of major coal fields.

3. Private Ownership

The Commission took a number of major actions required to implement the Congressional Act providing for the transfer of ownership of nuclear fuels from the Government to private industry, and the JCAE held extensive hearings on this legislation in August of this year. This subject is considered of such far reaching importance that a special section of this year's report is devoted to it immediately following these Highlights. At these hearings, industry spokesmen emphasized the need to plan now for an industry-owned enriching operation.

1/ For a fuller discussion, see remarks by Dr. Glenn T. Seaborg, Chairman of the Atomic Energy Commission, at the National Association of Manufacturers' Conference on Industrial Science and Technology, Washington, D. C. June 7, 1966, available as AEC Public Release S-20-66.

4. Uranium Supply

Large purchases of uranium by utilities and manufacturers began to materialize during the year. These purchases tended to alleviate the earlier concern over problems the uranium mining and milling industry would have in making the transition from a Government market to a private market.

5. Fuel Reprocessing

The first commercial fuel reprocessing plant, built by Nuclear Fuel Services, Inc., near Buffalo, New York, commenced operation and shipped its first processed material in 1966. The plant began operation close to the estimated schedule and without major difficulties. Its operation marked another significant milestone in the transfer of nuclear activities to the commercial sector of the economy.

6. Turnkey Bidding

A trend toward turnkey arrangements in the construction of nuclear power plants was reversed during the year. The trend initially stemmed from the relative unfamiliarty of utilities with nuclear power. The change reflects the growing confidence of utilities in this new energy source. The announcement by General Electric that it would no longer offer nuclear power plants on a turnkey basis helped to bring nuclear power plants under the more normal utility-supplier relationship which has prevailed in the past in the construction of fossil fueled plants. Helping to change the trend was the fact that all four of the major power reactor suppliers sold plants during 1966 on a non-turnkey basis.

7. Reactor Suppliers

The commercial power reactors ordered on a competitive basis in the earlier years were sold by either General Electric or Westinghouse. This year additional reactor manufacturers were successful in selling nuclear plants on the commercial market. In the beginning of the year, Consumers Power Company of Michigan ordered a nuclear power plant from Combustion Engineering, and at mid-year the Duke Power Company ordered two large units from Babcock & Wilcox. This expansion in the number of commercial suppliers of large power reactors promises keen competition for utility orders in the future.

8. Nuclear Fuel Market

The first utility order for a reactor fuel core was placed with a supplier other than a reactor builder. This was the first indicator of the development of a reactor supply market independent of the major reactor suppliers. The contracting parties in this pioneering arrangement were Commonwealth Edison and the United Nuclear Corporation.

9. Reactor Vessels

At the start of the year, Babcock & Wilcox and Combustion Engineering had emerged as the primary sources of large pressure vessels. B&W expanded its capability to produce large pressure vessels of the type used as primary tanks for very large light water reactors by opening a new plant at Mt. Vernon, Indiana. A third firm entered this field, Chicago Bridge & Iron, which received a contract to fabricate a reactor primary tank at the reactor site of Northern States Power. This not only represented a broadening of the competitive base for reactor primary tanks, but also established a precedent for field fabrication of reactor primary tanks. Both B&W and Combustion Engineering are prepared to offer similar services.

10. Plutonium

Interest in the use of plutonium once again accelerated as the Atomic Industrial Forum, The American Nuclear Society and the Edison Electric Institute joined the AEC in sponsoring a Commercial Plutonium Fuels Conference in April 1966.2/ The meeting brought together representatives of industry, electric utilities and Government for an exchange of views and information on this potentially useful energy source for electric generation for both light water reactors and breeders.3/ Of particular significance, was the extent of private support for development work looking toward the use of plutonium in thermal reactors. The Edison Electric Institute is now sponsoring a particularly worthwhile effort in this area.

2/ The proceedings of the conference are available as AEC report CONF-660308 from the Clearinghouse for Federal Scientific and Technical Information, National Bureau of Standards, U. S. Department of Commerce, Springfield, Virginia, 22151.

3/ Dr. Glenn T. Seaborg, Chairman of the AEC, addressed the conference on "Plutonium, Past, Present, Future", available as AEC Public Release S-7-66.

11. Fast Reactors

Breeder reactors were the subject of greatly accelerated interest during the year by both the AEC and the industry. This interest was demonstrated in detailed discussions by the AEC's Division of Reactor Development & Technology at hearings before the Congressional Joint Committee on Atomic Energy in February and March. At these hearings, both the plans of the AEC and industry were heard. General Electric and Westinghouse also indicated an interest in cooperative ventures with utilities in prototype fast reactor plants.

B. Desalting

A major step toward construction and operation of a large, dual-purpose desalting and electric power plant was taken when agreement on its construction and operation was reached by the AEC, the Department of the Interior, the Metropolitan Water District of Southern California, the Los Angeles Department of Water and Power and two investor-owned utilities -- Southern California Edison Company and San Diego Gas & Electric Company. The plant will be designed to produce 150 million gallons of water daily (enough to meet the needs of a city of 750 thousand), and 1,800 megawatts of electric power. The planned capacity for the plant is more than the total capacity of all desalting plants in the world today.[4]

C. Maritime

1966 was another controversial year for the maritime nuclear program. American Export Isbrandtsen Lines proposed construction and operation of three "high speed, super vessels." With nuclear propulsion plants, these ships were characterized as being able to do the work of six conventional ships. The Commission has continued to advocate a two-pronged program: (1) cooperation with the Maritime Administration in that agency's shipbuilding program, and (2) R&D by AEC at a land-based test facility directed toward an advanced maritime plant. During the year, MarAd continued its broader studies of the over-all problems associated with United States merchant shipping. The AEC and the DOD coolaborated with MarAd in a comprehensive report on the U. S. maritime program prepared at the request of the Senate Commerce Committee.

4/ For a full discussion of the nuclear desalting program see testimony by AEC Commissioner James T. Ramey at Hearings before the Joint Committee on Atomic Energy on AEC Authorizing Legislation for Fiscal Year 1967.

D. Radioisotopes

1. Space Applications

Radioisotopic systems for a variety of auxiliary power uses came into prominence as their value for space missions, requiring up to 10 kilowatts, was recognized by agencies developing space vehicles. The prospective demand for fuel for these systems stimulated a variety of plans for production or separation of isotopes useful for these purposes.5/

2. AEC Withdrawal from Production

In keeping with its established policy of withdrawing from the production of radioisotopes as private industry attains competence in this activity, the AEC withdrew from the production of 19 radioisotopes in early 1966. This brought to 34 the number of isotopes now available commercially.

3. Production in Power Plants

The first large-scale production in nuclear power plants of radioisotopes for commercial sale began at the Big Rock Point Plant of Consumers Power Company at Charlevoix, Michigan. This is the first evidence of utility participation in by-product uses for power reactors. Production of Cobalt-60 at Big Rock Point Plant began in May and is expected to reach 2 million curies annually by late 1967.

4. Commercial SNAP6/

The first commercial offering of compact, electric power systems powered by radioisotopes was made by the Martin-Marietta Corporation. The units provide 25 watts of power over guaranteed periods of up to five years. The first sale was to the Department of the Navy.

5/ For a fuller discussion see remarks by AEC Commissioner Gerald F. Tape on "Plutonium and Other Radioisotope Sources -- Discovery to Demand" before the American Nuclear Society, Atlanta, Georgia, March 22, 1966, available as AEC Public Release S-11-66.

6/ SNAP stands for Systems for Nuclear Auxiliary Power.

5. Wood-Plastic Alloys

The AEC deferred for a year construction of a demonstration plant for the production of wood-plastic alloys when private industry indicated that it could move ahead commercially in this field without further Government assistance. The AEC's position will be reviewed in 1967 in the light of commercial accomplishments in this area at that time. Should the use of this new material not be developing rapidly enough, the AEC will consider additional steps to accelerate its commercial exploitation.

E. Meetings with Industry

The Commission continued its series of meetings with the nuclear industry. Meetings were held with a number of industrial concerns and utilities to discuss current problems and future plans.

F. Encouraging Competition

The development of the nuclear industry continued to be followed closely by the AEC. During the year, ways of stimulating the growth of a competitive industry were discussed with both utilities and their suppliers. Discussions were also initiated with the Department of Justice on ways in which the nuclear industry might be strengthened without loss of competition.

G. Operating Contractors

The AEC continued to implement its policy of changing operating contractors to provide broader opportunities to industrial concerns, particularly those with a substantial investment in the commercial nuclear energy field. At the National Reactor Testing Station in Idaho, proposals were invited on a portion of the work performed by Phillips Petroleum Company. Idaho Nuclear Corporation, a company formed for this purpose by Aerojet-General and Allied Chemical, was awarded an operating contract for this work. So far, the transfer of this work to a new contractor at the Idaho site and the changes in contractors at the Richland, Washington, facilities have proceeded satisfactorily.

H. Nuclear Safety

The AEC's Steering Committee on Reactor Safety Research, established in 1965, continued to provide an important means of

keying the Commission's extensive program of reactor safety research to the needs of continuing development of the nuclear industry and to the requirements of the Commission's regulatory program. The Committee was active in R&D planning, in augmenting channels of information exchange and in reviewing plans for the development of criteria, standards and codes. Additional industry participation came with the appointment by the Atomic Industrial Forum of a task force to review the AEC's safety program. Comments, together with recommendations for specific types of research and development, were expected late in 1966.

I. Regulation

1. Improved Procedures

The AEC essentially completed its implementation of recommendations by an independent review panel chaired by attorney William E. Mitchell for improvements to its regulatory procedures. The review panel was the first established by the Commission to review and report on the regulatory system. A second panel was subsequently established to review procedures followed in contested reactor licensing cases. A report by this second panel is expected in late 1966.

2. Reactor Standards

In late 1965, the AEC released for comment general design criteria for reactors. Supplemental criteria for components, systems and processes are under development. These steps could be taken at this time because of the trend toward standardization in the design of light water reactors.

3. Non-Proliferation

During the year, the Commission announced that its planning to require reports from licensees on receipts, transfers and inventories of privately owned special nuclear (fissionable) material.[7/] The Commission also announced that it is proposing amendments to its regulations concerning

7/ See AEC Public Release J-75, dated March 28, 1966.

the responsibilities of licensees who posess special nuclear material to control and account for the material in the interest of national security.[8/]

An independent advisory panel was established by the Commission to make a comprehensive review and appraisal of AEC's policies and procedures for safeguarding SNM. In advancing the further development of international controls, the first commercial plant for processing spent reactor fuels, the Nuclear Fuel Services plant near Buffalo, New York, was made available by the AEC to the IAEA for the purpose of developing and testing safeguard techniques.

J. NUCLEX '66

The AEC, the Department of Commerce and representatives of American industry cooperated in planning U. S. participation in NUCLEX '66, an international commercial exhibit and technical symposium in Basel, Switzerland, in September 1966. Commission activities in connection with this exhibit consisted of preparing an AEC Exhibit, helping to assure international sales opportunities for United States producers of nuclear equipment and services, and in coordinating the technical program. About 60 U. S. firms presented exhibits, and about 50 technical papers were presented by U. S. participants.[9/]

K. Appointment of New Commissioners

During the year several changes occurred at the Commission level.

Commissioner John Palfrey left the Commission on June 30, 1966, to become a Fellow for a year at Professor Neustadt's Institute of Politics at Harvard in the field of science and public policy. Mr. Wilfred E. Johnson was appointed on August 1, 1966, to fill the vacancy left by Commissioner Palfrey. Mr. Johnson brings to the Commission a long background in engineering in American industry.

8/ See AEC Public Release J-136, dated May 26, 1966.
9/ Former AEC Commissioner John G. Palfrey was the official AEC representative and delivered the opening address.

On August 1, 1966, Dr. Samuel M. Nabrit was appointed to fill the vacancy on the Commission left when Commissioner Mary Bunting returned on June 30, 1965, to the presidency of Radcliffe College. Dr. Nabrit brings to the Commission a background in both biology and education.

PRIVATE OWNERSHIP - TOLL ENRICHING

The private ownership of special nuclear material and toll enriching are reported in a separate section of this report this year because of their far-reaching importance. The hearings held in August of this year by the JCAE represented the most important and comprehensive review of the nuclear power industry made available publicly since the 1964 hearings on the same subject.

A. Legislative History

The importance of the private ownership legislation is still very appropriately summarized in a statement by Representative Holifield of the Joint Committee on Atomic Energy when he opened the hearings on private ownership in 1963. He stated, "This legislation, representing as it does the most sweeping amendment to the Atomic Energy Act since 1954, will vitally affect the future legal and economic structure of the entire atomic energy industry."

No discussion of this legislation would be complete without mentioning the untiring efforts of former AEC Commissioner Robert E. Wilson whose vision and foresight played an important role in the development of this far-reaching amendment to the Atomic Energy Act. The farsightedness of Dr. Wilson and his fellow Commissioners in working on this legislation, with the support of the Joint Committee on Atomic Energy, is now evident as the dramatic growth of nuclear power on an economic and commercial basis during the year became a reality. The legislation opened the door to the toll enriching of privately owned uranium and to the eventual transfer of this remaining activity in the fuel cycle from Government to industry. By permitting the private ownership of special nuclear materials within the United States, the development of the civilian nuclear power program can proceed as a normal commercial program.

On March 15, 1963, the Atomic Energy Commission transmitted a proposed bill to the Congress, based on studies initiated by Dr. Wilson and his fellow Commissioners. On March 21, 1963, Senator Pastore, Chairman of the Joint Committee on Atomic Energy, and Vice-Chairman Holifield introduced bills on private ownership to the Senate and the House. Extensive public hearings were held in 1963 and 1964 which permitted a full discussion of the viewpoints of the different industry interests, including the uranium

mining industry, nuclear fuel fabricators, nuclear suppliers, electric utilities and even the coal industry. The 1964 hearings, in particular, made a public record of the extensive amount of study that was given to this subject by the AEC, the industry and the Joint Committee on Atomic Energy and demonstrated the effectiveness of the Government and industry working together in developing appropriate legislation to transfer activities in the nuclear field to our private system in the United States. Revised bills were reported to the Congress early in August 1964, and, as a result of the aggressive action of the Joint Committee on Atomic Energy and its staff with the cooperation of the AEC, the Private Ownership of Special Nuclear Materials Act was approved on August 26, 1964.

B. Background on Private Ownership

The Atomic Energy Act of 1946 created a Government monopoly because of the military character and newness of atomic energy. After some ten years time, the Atomic Energy Act of 1954 was passed which permitted, among other things, private industry to participate in the development of nuclear energy and private persons to possess and use special nuclear materials under license from the AEC.

Prior to the private ownership amendment, private industry could own all components of a nuclear power plant except the enriched uranium fuel and plutonium produced therein by irradiation. The law had stipulated that:

> "...All rights, title and interest in or to any special nuclear material within or under the jurisdiction of the United States, now or hereafter produced, shall be the property of the United States and shall be administered and controlled by the Commission as agent of and on behalf of the United States by virtue of this Act...."

Enactment of the amendment for private ownership in 1964, eliminated this requirement. The decline in recent years in military requirements for enriched uranium will eventually completely reverse the situation which has prevailed in the past -- that peaceful requirements were insignificant compared to military requirements and that material could be diverted to meet them with no significant change in enriched uranium production operations -- and the major demand for enriching services in the future will be

-

for requirements for civilian power.

One of the most important concepts in the private ownership legislation is the provision of toll enrichment of privately owned uranium. Because private ownership of enriched uranium was made possible, with passage of the amendment (August 1964), the delivery of privately owned uranium can be made to the AEC for enrichment in the isotope U-235 and the subsequent return of a lesser amount of uranium containing a greater concentration of that isotope to the customer upon his payment of an enrichment services charge. Providing for toll enrichment is consistent with the AEC's policy of withdrawing from areas of potential commercial activity through the encouragement of direct relationships between ore or concentrate suppliers and users of enriched uranium with minimum Government involvement.

C. Time-Table for Private Ownership Legislation

The Private Ownership Act provides guidance to the AEC and industry and establishes a time-table for its implementation. With its enactment the Commission can distribute special nuclear material by sale domestically. (Distribution to Foreign government by sale had been AEC policy for the past ten years.) After December 31, 1970, the AEC is prohibited from distributing special nuclear materials to power reactor operators, for use in power reactors, except by sale. The Commission can continue leasing to fuel fabricators and converters, although the material will have to be acquired by the reactor operator by sale. Distribution for use in research reactors by lease or grant is not affected. In addition, the AEC is authorized to offer uranium enrichment services, commonly referred to as toll enriching, beginning on January 1, 1969. The Commission's guaranteed purchase prices for plutonium delivered to the AEC which is produced in reactors by persons discussed under section 104 through the use of uranium leased or sold by the AEC will expire on January 1, [illegible]. Guaranteed prices for U-233 can continue past that date for [illegible] up to ten years. Finally, all Government owned special nuclear materials previously leased for use in section 103 or 104b facilities to operators of such facilities must be returned or converted to private ownership by July 1, 1973. On the following page there is a timetable for these key actions.

TABLE 6

Time-Table

Private Ownership Permitted	August 26, 1964
Toll Enriching of Privately Owned Uranium Can Begin	January 1, 1969
AEC Prohibited From Entering Into New Lease Agreements For Power Reactor Fuel	January 1, 1971
AEC Guaranteed Purchase Price For Plutonium Will Terminate	January 1, 1971
Private Ownership of Special Nuclear Material Mandatory For Commercial Utilitization Facilities (section 103 or 104b facilities)	July 1, 1973

Thus, the JCAE and the Congress wisely provided for a period of transition for the nuclear industry to adjust to this new concept allowing a ten-year period from mid-1963, when the first hearings were held, to July 1, 1973, for the principal fuel, enriched uranium, to become a part of the private nuclear power industry.

D. Implementation Required of the Commission for Private Ownership and Toll Enriching

Under the authorization of the amendment of 1964, the Commission was committed to undertake a number of steps to implement private ownership and toll enriching services. These are:

1. To expand its procedures to include the licensing of imports and exports of special nuclear material by private parties. (Exports would be in accordance with agreements for cooperation).1/

2. To maintain and establish regulatory controls for privately owned SNM by the licensing process, or otherwise, concerning protection of public health and safety and common defense and security and the possession, use, accountability and safeguarding of special nuclear material within the United States.

1/ See AEC Public Release IN-716, dated September 21, 1966.

3. To establish guaranteed purchase prices (not to exceed AEC's estimated value of the material as fuel in a nuclear reactor) for special nuclear material produced by licensees through the use of AEC sold or leased uranium and delivered to the Commission prior to January 1, 1971, for plutonium and for periods up to ten years for U-233.

4. To establish reasonable sale prices for special nuclear material distributed by sale (by the AEC).

5. To establish criteria for the determination of how and whether special nuclear material will be distributed by grant.

6. Pursuant to the authorization to enter into contracts to produce special nuclear material from privately owned uranium, the Commission is directed to establish criteria setting forth the terms and conditions under which such toll enriching service would be provided and the general features of standard contracts for this service.

Special provision is made in the legislation prohibiting AEC from enriching uranium of foreign origin intended to be used in the United States until such time as the viability of the domestic uranium industry is established. In this regard, the Commission is to periodically review the condition of the domestic uranium industry.

E. Commission Actions to Implement Private Ownership and Toll Enriching

In response to the steps required under the authorization, the Commission published guaranteed purchase prices for plutonium and U-233 on March 25, 1965.2/ The prices established are:

> For plutonium in the forms of nitrate, dioxide or metal and meeting AEC specifications, $10 per gram of the contained isotopes plutonium-239 and plutonium-241.
>
> For uranium enriched in uranium-233 in the forms of nitrate, dioxide or metal and meeting AEC specifications, $14 per gram of the contained isotope uranium-233, subject to adjustment for the presence of other uranium isotopes and deductions for handling costs.

2/ See AEC Public Release H-65, dated March 25, 1965, and Federal Register 3886, dated March 25, 1965.

F. Control, Protection and Safeguarding of Special Nuclear Materials

During the year, the Commission announced that it is planning to require reports from licensees on receipts, transfers and inventories of privately owned special nuclear (fissionable) material. The Commission also announced that it is proposing amendments to its regulations concerning the responsibilities of licensees who process special nuclear material to control and account for the material in the interest of national security. These proposed changes would provide the Government with necessary information in the interest of national security comparable to that which is required for special nuclear material leased from the Commission.3/

In connection with the evaluation of its policies and procedures on material control, in order to assure that special nuclear material is properly accounted for, the Commission established an Ad Hoc advisory panel to conduct a comprehensive review and appraisal of the AEC's policies and procedures for safeguarding special nuclear materials.4/

The panel is composed of the following representatives from Government and industry.

Dr. Ralph F. Lumb, Chairman, Director
Western New York Nuclear Research Center, Buffalo, New York

Francis P. Cotter, Vice President, D.C. Office
Westinghouse Corporation

Gerald Charnoff, Attorney
Shaw, Pittman, Potts, Trowbridge & Madden, Washington, D. C.

Paul Grady, Ex-Partner (Retired)
Price, Waterhouse and Company, New York, N. Y.

Ashton J. O'Donnell, Manager of Development
Bechtel Corporation, San Francisco, California

Louis Roddis, President
Pennsylvania Electric Company

Fred H. Tingey, Head, Operations Analysis Branch
Idaho Nuclear Corporation, Idaho Falls, Idaho

Vincent Vespe, Director, Division of Operational Safety
USAEC, Albuquerque Operations

Ralph G. Page, Chief, Enforcement Branch
Division of State and Licensee Relations, USAEC Hqtrs.

3/ See AEC Public Releases J-75, March 28, 1966, and J-136, May 26, 1966.
4/ See AEC Public Release J-179, dated July 22, 1966.

[illegible]

G. Uranium Sale and Lease Policies

To provide policy guidance and to assist industry in planning for the transition from Government to private ownership of nuclear fuels, the Commission announced in July 1966,[5] its proposed post-1968 policies covering the supply of uranium. The principal features of these policies are summarized below:

1. The natural uranium component of the AEC schedule of charges for lease and sale of enriched uranium will continue to be based upon a price of $8 per pound of U_3O_8 at least through June 30, 1973.

2. With certain exceptions not involving commercial U. S. power projects, any AEC sales of enriched uranium will be on the basis of single transactions.

3. Beginn[illegible] January 1, 1971, enriched uranium on lease may be converted to private ownership by a mechanism, called "in situ" toll enriching, under which the lessee would furnish to the AE[illegible]ecified amounts of uranium feed and dollars and would thereby acquire ownership of the leased material.

4. The transition to private ownership of all material held or being procured by licensees for power reactor use will be accomplished by AEC's terminating distributions of such material by lease on December 31, 1970, and terminating outstanding leases for such material on June 30, 1973. However, AEC will, on a case-by-case basis, consider requests by fabricators for deferred payment of enrichment service charges on toll enriched material -- at an interest charge on the unpaid balance equal to the then-current use charge rate -- during a fabrication period of up to one year.

5. AEC was unable to specify quantative criteria on the disposition of AEC's available uranium feed. However, the AEC announced that it does not plan to sell its available uranium until this can be done in a manner that will not damage the domestic uranium industry. It is expected that, once the industry attains viability, AEC distributions will be based on an appropriate sharing of the market growth beyond the point of viability.

5/ See AEC Public Release IN-704, "AEC Announced Proposed Policies on Supply of Uranium," dated July 25, 1966.

H. Toll Enriching Criteria and Contracts

In October 1965, the Commission published proposed criteria and supply contracts for toll enriching services after first obtaining the comments of an AIF Study Committee which provided a wide range of imput for AEC to consider in developing another draft. A second review by the AIF Committee in late spring 1966 was helpful to the Commission in developing its final draft of the criteria and contracts that were sent to the Joint Committee on Atomic Energy, July 1, 1966.6/

The toll enriching criteria provide:7/ (a) for AEC entering enrichment service contracts for up to 30 years; (b) customer option to take tails if desired; (c) a ceiling on the cost of separative work of $30/kg subject to escalation based on power and labor; (d) a prohibition against enrichment of foreign uranium intended for use in domestic reactors; (e) two standard types of contracts for enriching services covering "firm quantities" and "requirements" and (f) that quantities of feed and charges will be determined by a standard table of enriching services.8/

I. Future Areas for Action

In August of this year, the JCAE again held extensive hearings on the actions proposed by the Commission. Based on these public hearings, it appeared that industry was generally satisfied with the criteria and the enrichment service contracts.

The hearings focused on several areas that will require examination and future action by the Commission. These were: (1) the possible declassification of capacity and economics of one or more of the Commission's gaseous diffusion plants; (2) of the feasibility, methods and economics involved in transfer of one or more of these plants to private industry; (3) greater customer flexibility in the enriching service by being able to specify the assay of tails to be used as the basis of the enrichment of this material and (4) AEC publication of its standard table of enriching services at the earliest possible date.

6/ See AIF Report on Ad Hoc Committee on Toll Enrichment, "An Analysis of AEC's Uranium Enrichment Service Contracts".

7/ See AEC Public Release S-28-66, Remarks by Dr. Gerald F. Tape, USAEC Commissioner at the Annual Meeting of Pennsylvania Electric Association, Pittsburgh, Pennsylvania, September 21, 1966.

8/ The AEC standard table of enriching services is expected to be available early in 1967.

At the hearings it was also recognized, however, that additional action by both the Government and industry is still required to bring into existence commercial services and practices throughout the entire fuel cycle of sufficient capacity in order to keep pace with the growing industry needs. In particular, there will be need for establishing or strengthening the development of industry contracting approaches for uranium procurement; to provide for industrial sampling and umpire services; to provide conversion services for both natural and irradiated material; for industrial procurement of equipment and containers for transporting UF_6, plutonium and other materials; to develop practices for financing material under private ownership; and finally, utilization of our nuclear resources so as to achieve a full realization of the peaceful potential of nuclear energy.

J. Other Highlights of JCAE Hearings

The Joint Committee on Atomic Energy Hearings in August of this year brought out views on the following additional related areas to private ownership which are of considerable importance to the nuclear power industry.

1. The Embargo on the Import of Foreign Uranium for Domestic Use

During the Hearings, a number of witnesses for utilities raised questions on how and when the embargo on the import of foreign uranium for domestic use could be lifted. They suggested that the condition of the domestic uranium industry be reviewed at an early date in view of the recent surge of orders for nuclear power plants and the resulting purchases that are occurring from the reactor and utility industry for deliveries of uranium.

2. The AEC's Natural Uranium Stockpile

Questions were raised by a number of witnesses on when and how the AEC's stockpile would be released to the market. Several of the witnesses urged that, if possible, some indication of the magnitude of the Government stockpile be made available to industry.

3. Competition in the Nuclear Fuel Cycle

In response to questions from the JCAE, a number of industry representatives gave their views on competition in

the nuclear industry. In general, the utilities pointed out that competition and lower fuel costs can be accomplished by purchasing replacement cores through the use of competitive bidding. There seemed to be general agreement that competition will be governed to a large extent by the utilities themselves.

4. The Uranium Supply Situation

There was considerable testimony on the outlook for uranium supply, and the witnesses from the mining industry seemed, in general, to be confident that the private uranium industry can supply at reasonable prices the raw material requirements of the growing nuclear power industry.

5. Private Enriching Operations

The industry witnesses urged the AEC to open the way for planning a non-Government uranium enrichment operation. They emphasized the importance of their having access to capacity and economics of the Government's enriching plants in order to make future management decisions.

CATEGORY I
RAW FUEL MATERIALS

As reported in past years, private industry supplies all of the materials reported under this category.

This category will review the status of uranium and thorium, the two source materials.1/

A. Uranium

1. Domestic Uranium Processing

At present, the uranium ore processing mills own or control an estimated 90% of the uranium reserves. The remaining 10%, which are independently owned mines, sell ore to the larger firms having processing mills. Table I-1, lists the companies which currently sell uranium concentrates to the AEC. No attempt has been made to list the independent mines.

In Fiscal Year 1966, AEC purchased $161 million worth of uranium concentrates from domestic producers.

Domestic uranium processing mills which are not participating in the stretch-out program are listed in Table I-2.

1/ The term "source material" means (1) uranium, thorium or any other material which is determined by the Commission pursuant to the provisions of Section 61 of the Atomic Energy Act to be source material; or (2) ores containing one or more of the foregoing materials, in such concentration as the Commission may by regulation determine from time to time.

TABLE I-1

Domestic Uranium Processing Mills Participating in Stretch-Out Program	Estimated Deliveries to AEC Under the Stretch-Out Program, CY 1967-1970	
	Tons U_3O_8	% of Total
Anaconda Co., Bluewater, N.M.	3,000	9.2
Atlas Corp., Moab, Utah	3,500	10.8
Federal-Radorock-Gas Hills, Fremont Co., Wyo.	1,500	4.6
Homestake-Sapin, Grants, N.M.	4,300	13.2
Kerr-McGee Corp., Grants, N.M.	6,100	18.8
Union Carbide Corp., Uravan & Rifle, Colo.	3,800	11.7
Union Carbide Corp., Globe, Wyo.	750	2.3
United Nuclear, Grants, N.M.	3,850	11.9
Utah Construction, Fremont Co., Wyo.	2,150	6.6
Vanadium Corp. of America, Shiprock, N.M.	1,400	4.3
Western Nuclear, Jeffrey City, Wyo.	2,150	6.6
	32,500	100.0

TABLE I-2

Domestic Uranium Processing Mills Not Participating in Stretch-Out Program

Climax Uranium, Grand Junction, Colo.
Cotter Corp., Canon City, Colo.
Dawn Mining Co., Ford, Washington
El Paso Natural Gas Co., Tuba City, Ariz.
Mines Development, Inc., Edgemont, S. Dak.
Petrotomics Co., Shirley Basin, Wyo.
Susquehanna-Western, Inc., Falls City, Texas

2. AEC Uranium Procurement Stretch-Out Program

In order to bring purchases more nearly in balance with requirements and to assist in maintaining a viable uranium producing industry before a commercial market materializes, the AEC announced in November 1962 a program under which mills could

elect to stretch-out base contract deliveries into 1967 and 1968. The AEC would then purchase in 1969 - 1970 an additional quantity of U_3O_8 in concentrates equal to the amount deferred for delivery after December 31, 1966. The firms participating in this stretch-out program are shown also in Table I-1.

The total deferral will amount to approximately 15,350 tons U_3O_8. About $600 million worth of uranium (or 42 thousand tons) will be bought by the AEC from domestic producers between January 1, 1966, and the end of 1970. Chart I-A illustrates the effects of the stretch-out program.

Chart I-A

RAW FUEL MATERIALS

ESTIMATED EFFECT OF 'STRETCH OUT' PROGRAM

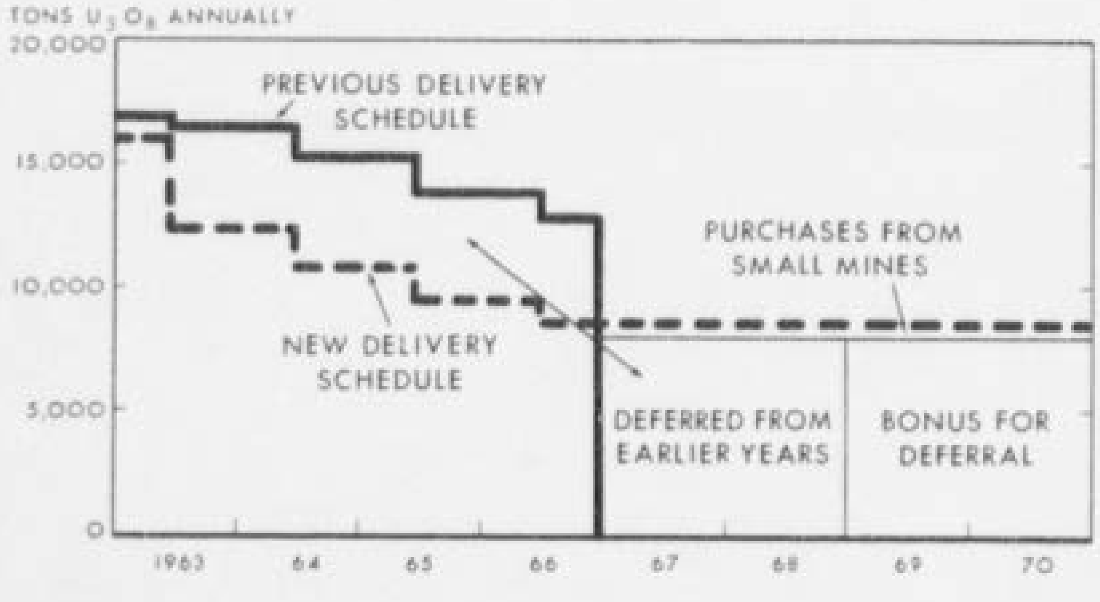

There have been no significant changes in the domestic uranium industry. The Cotter Corporation, Canon City, Colorado, announced plans to reactivate their mill to produce uranium for sale to private firms.

During the year, interest in the nuclear field continued to be shown by the oil industry. The City Service Company held discussions with the United Nuclear Corporation in regard to a possible acquisition. Interest continued to be expressed by other oil companies, especially in the uranium mining field. Also, there was some interest by the coal industry in ways that they might enter the nuclear field.

In August 1966, the Commission requested comments on a proposed modification of the stretch-out program to permit ore produced in 1967 and 1968 from eligible small properties to be processed in mills not participating in the stretch-out program in those instances where there is no participating mill serving the area.[2] Several non-stretch-out mills have announced their intention to continue operations, after completion of their AEC contracts, to produce uranium for private sales.

In the Spring of 1966, the Atomic Industrial Forum Mining and Milling Committee suggested that the Commission consider the possibility of announcing, at an early date, policies on the leasing and sale of enriched uranium; and that it consider some additional assurance to the industry on the disposition of surplus uranium inventories.

The AEC's post-1968 policies on supply of uranium are reviewed in the section of this report on private ownership and toll enriching.

3. Forecasts of Uranium Requirements and Reserves

The Growing Uranium Market

The overall procurement of uranium concentrates by the AEC is shown in Chart I-B. In the past, it appeared that there would be a period after 1970 when the uranium industry would see

---

2/ See AEC Public Release No. J-201, dated August 26, 1966.

a declining market. The dramatic number of announcements by utilities in 1966 to install large nuclear power plants has resulted in a much brighter outlook for the uranium industry for the period immediately following the termination of uranium concentrate procurement by the AEC in 1970. During the year, several private industry orders for the supply of uranium concentrates were announced. A large order for uranium concentrates was placed by Westinghouse ($70 million from Anaconda) for delivery over a period of eight years. The General Electric Company announced orders

Chart I-B

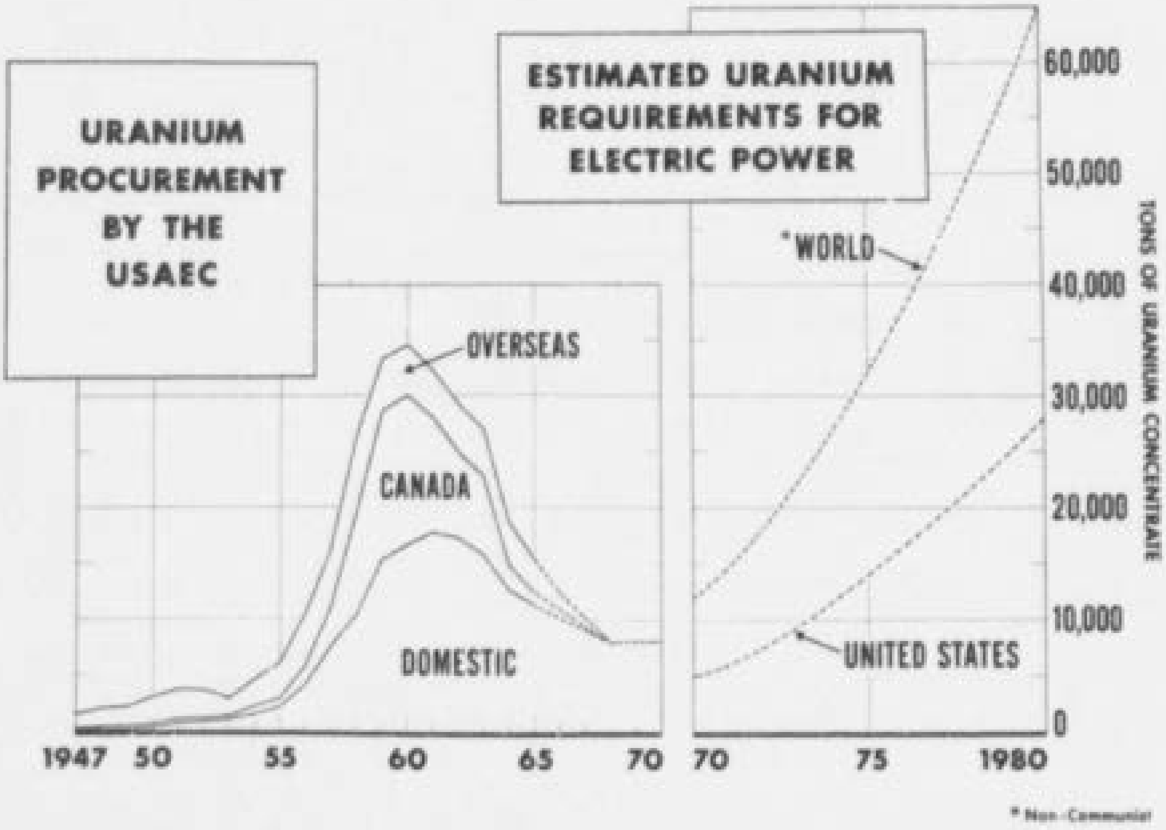

with Climax Uranium Company, Cotter Corporation and Utah Construction Company for near-term requirements for TVA and other reactors. In addition, Commonwealth Edison placed an order for $15-20 million worth of U_3O_8 for delivery in 1968-1970 from Kerr-McGee. Therefore, it now appears that no substantial drop in sales in the post stretch-out program period may occur as the market for this segment of the industry changes from the Government to a commercial one.

4. Ad Hoc Barter Transactions

Five barter transactions with foreign countries were permitted prior to the beginning of toll enriching services in 1969 as shown in Table I-3. The legislative history leading to private ownership and toll enriching services, in general, required that the AEC consider the effect of any barter arrangements on the international trade position of the United States, including the ability of domestic companies to furnish nuclear reactors to foreign nations and the value at which the foreign natural uranium to be received should be credited. The AEC announced in March 1966 that no further barter arrangements will be considered.3/

TABLE I-3

Approved Barter Requests

Country	Project	Megawatts Electrical	Tons U_3O_8
Germany	VEW	160	220
Germany	KRB	237	435
Spain	U.E.M.	154	147
Spain	NUCLENOR	440	430
Switzerland	NOK	350	370
TOTALS		1,341	1,602

3/ See AEC Public Release J-60, dated March 11, 1966.

Of the 1,600 tons of U_3O_8 estimated to be required, it is anticipated that about 1,000 tons will be purchased from domestic producers.

Because of the Commission's policy not to provide services that are available from private industry, uranium must be furnished to Government enriching plants in the form of UF_6, thus requiring conversion to the UF_6 form by private firms (See Materials Processing and Fabrication Category.)

Uranium Requirements and Reserves[4/]

The following summary reviews the status of uranium requirements and reserves.

As a result of the growth in the nuclear power industry, the near-term domestic commercial uranium requirements have been revised upward from those given in 1965. Sales to AEC in 1969, when toll enrichment becomes available, and in 1970, will be about 8 thousand tons of U_3O_8 per year under the stretch-out program. Private requirements are projected to be about 3,700 tons for 1969 and 5000 tons for 1970. It is also possible that domestic producers may make some foreign sales. The projected foreign requirement for enriched uranium reactors is 2,400 tons in 1969 and 3,500 tons in 1970.

Table I-4 summarizes the estimated uranium requirements for the non-Communist world through 1980.

TABLE I-4

URANIUM REQUIREMENTS FOR NON-COMMUNIST WORLD

(Short Tons U_3O_8)

	1970		1975		1980	
	Annual	Cumultv.*	Annual	Cumultv.	Annual	Cumultv.
U. S.	5,000	13,000	14,000	65,000	28,000	172,000
Other Non-Communist						
Enriched	3,500	10,000	13,000	53,000	27,000	159,000
Natural	3,500	15,000	5,000	38,000	10,000	78,000
Totals	12,000	38,000	32,000	156,000	65,000	409,000

* Beginning 1966

4/ Also see remarks by Rafford L. Faulkner, Director, Division of Raw Materials, U.S.A.E.C., at International Nuclear Industries Fair and Technical Meetings, Basel, Switzerland, September 8, 1966.

This table shows that annual requirements by 1975 on a non-Communist world basis will be in the neighborhood of 32 thousand tons per year, rising to 65 thousand by 1980. In terms of annual supply, this means that the present annual production rate of the non-Communist world - about 18 thousand tons - will have to be nearly doubled by 1975 and more than tripled by 1980. The cumulative requirement through 1980 is about 400 thousand tons of U_3O_8. Requirements for enriched uranium are expected to be about 80 per cent of the total. Neither the introduction of breeder reactors nor the use of thorium is considered likely to be sufficiently significant before 1980 to affect any of the uranium requirement estimates given.

The current estimates of uranium ore reserves for the U. S. and the Free World, recoverable at prices up to $30/lb. U_3O_8, are given in Tables I-5 and I-6,[5/] on the following page.

The low cost ($8/lb. U_3O_8 or less) uranium ore reserve estimates in the United States, as of January 1, 1966, are given below:

State	Tons of Ore	% U_3O_8	Tons U_3O_8
Arizona	220,000	0.32	710
Colorado	3,850,000	0.25	9,590
New Mexico	29,700,000	0.24	70,400
North & South Dakota	480,000	0.29	1,400
Utah	1,750,000	0.24	4,300
Wyoming	22,700,000	0.23	53,100
Others: California, Idaho, Montana, Nevada, Oregon, Texas, Alaska, Washington	2,900,000	0.18	5,500
Total (rounded)	61,600,000	0.235	145,000

5/ Obtained from AEC's Division of Raw Materials.

TABLE I-5

U. S. URANIUM RESERVES
(in tons U_3O_8 x 1000)

Price Per Lb. U_3O_8	Reasonably Assured	Possible Additional*	Total
$8 or less	145	--	145
$8 - $10	45	325	370
$10 - $15	150	200	350
$15 - $30	170	440	610
Total	510	965	1,475

TABLE I-6

WORLD URANIUM RESERVES**

Price Per Lb. U_3O_8	Reasonably Assured	Possible Additional*	Total
$10 or less	690	680	1,370
$10 - $15	715	500	1,215
$15 - $30	570	1,100	1,670
Total	1,975	2,280	4,255

* The figures for possible additional resources refer to geologic estimates of future discoveries for those regions in which important efforts have been made in the field of prospecting or evaluation.

** Including United States, but excluding USSR, China and Eastern Europe.

- -

As a result of the dramatic surge of orders for nuclear power plants in 1966, the availability of adequate long-term reserves was questioned during the year by coal intersts. Estimates of uranium requirements and of the availability of reserves have been reviewed in considerable detail in the JCAE Hearings last August and in speeches by AEC officials and others.

In his statement to the JCAE on August 2, 1966, concerning AEC's steps to implement the Private Ownership of Special Nuclear Materials Act, Dr. Seaborg discussed the subject of uranium reserves and the prospects of a future supply of low-cost uranium. He pointed out that the Commission regards the "reasonably assured" reserves merely as a working inventory which can be drawn upon with minimum lead time. He also indicated that the "possible additional" reserves are not intended to represent all that will be found and that the Commission expects uranium reserves will respond to exploration and market pressures as have other commodities.

Representatives of the uranium industry, testifying in August 1966 at the JCAE hearing on implementation of private ownership and the toll enriching criteria, were all optimistic that adequate low cost reserves would be developed as exploration increased. They also believed that the AEC's post 1968 policies, recently announced, would provide an incentive to encourage uranium exploration.

It can be noted in Chart I-C, on the following page, that annual drilling rates reached a low in 1965 and are just beginning to increase.

B. Thorium 6/

There have been no significant changes in the status of thorium as a nuclear fuel since our last report.

Development of advanced converter reactors for commercial applications (and of breeder reactors for subsequent applications) is expected to lead eventually to the expanded use of thorium as a source of nuclear fuel. The use of thorium, of course, will further expand our nuclear fuel resource base.

6/ See also: Some Recent Developments of Interest to the Nuclear Fuel Industry, by Commissioner James T. Ramey at the National Western Mining Conference, Denver, Colorado, February 5, 1965, AEC Public Release No. IN-561.

U.S. URANIUM RESOURCES

Chart I-C

ANNUAL DRILLING RATES AND RESERVE ADDITIONS

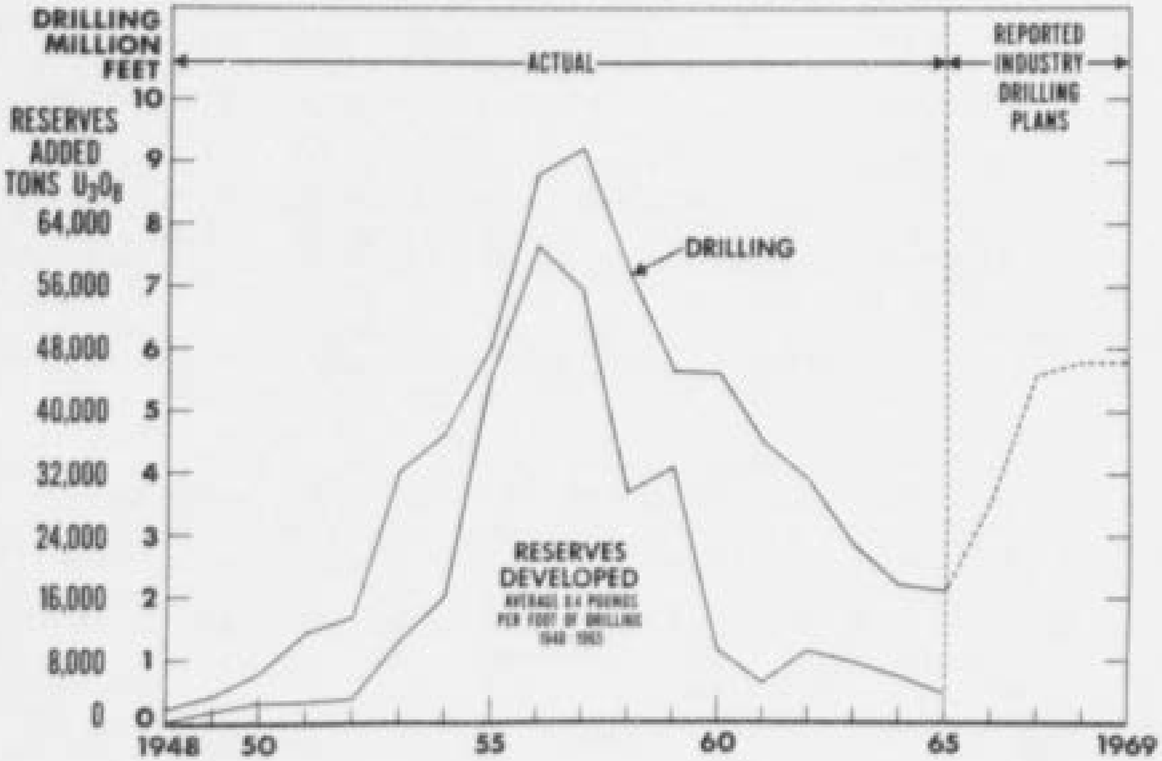

1. <u>Use of Thorium in Reactors</u>

Thorium can be used in power reactors as a fertile material· that is, a material that can be converted into a fissionable material -- uranium-233. In this respect, it is similar to uranium-238 which can be converted into the fissionable material plutonium-239. The fissionable material uranium-233 is of interest because in a thermal reactor it releases more neutrons than either uranium-235 or plutonium-239. Hence, reactor designers can get a greater efficiency on conversion of fertile to fiss[illegible]able materials in thermal reactors using the thorium uranium-233 cycle than they can get in the uranium-plutonium cycle.

2. Supply of Thorium

Although there has been little demand for thorium to date for both non-nuclear and nuclear purposes, and, therefore, little prospecting activity, the United States has large thorium resources. They are located in Lemhi County, Idaho, and in neighboring Montana, where preliminary investigation indicates deposits containing more than 100 thousand tons ThO_2 from which thorium can be produced at prices comparable to those for uranium, and there is a possibility of several times that amount.

The present lack of market is not conducive to exploration. As increased requirements for thorium develop, increased exploration activity will undoubtedly result. The geologic potential for new discoveries would appear to be promising.

3. Future Thorium Requirements

Thorium reactor technology is at a much earlier stage than uranium, and therefore, it is only possible to make general observations.

The principal conclusion is that for the foreseeable future, uranium requirements for power reactors will be much larger than those for thorium for several reasons. The number of power reactors using thorium will be limited for some time. More importantly, however, large amounts of natural uranium will be required as feed material in our gaseous diffusion plants to produce the fissionable isotope U-235 needed to fuel initially all our reactors. The demand for thorium will be based only on the need for it as a fertile material.

Furthermore, even a thorium-based reactor will require large amounts of natural uranium to produce its initial fuel charge of fissionable material, whether it be U-235, U-233 or plutonium. To illustrate, one recently designed reactor of approximately 500 megawatts electrical will require 1,250 tons of natural uranium to supply its enriched uranium inventory, but only 77 tons of thorium.

[illegible]

Total thorium requirements in the next 15 years probably will be measured only in hundreds of tons, with annual requirements during most of that period of less than 100 tons. After about 1980, requirements may gradually increase, and as we approach the end of the century may amount to several thousand tons per year.

CATEGORY II
MATERIALS PROCESSING AND FABRICATION

A. Uranium

1. Feed Preparation

Feed (UF_6) for AEC diffusion cascades is obtained primarily from uranium concentrates, from uranium scrap recovery operation and from unspent reactor fuels after chemical reprocessing.

a. Conversion of Concentrates to UF_6 -- Both the AEC's Paducah and the privately owned Allied Chemical Corporation uranium hexaflouride plants ceased operations on June 30, 1964. Prior to ceasing operations, Allied Chemical placed in inventory some privately owned UF_6 to meet possible private requirements. The Allied Plant, with a capacity of about 6 thousand tons U_3O_8 per year, continues to be the only private capability available to convert uranium concentrates to UF_6 and their capacity will, no doubt, be greater than the market for conversion for a number of years. The Commission looks to the industry to take over every step possible in the fuel cycle and hence, to supply UF_6 under barter arrangements and toll enriching. Accordingly, as noted in the AEC proposed criteria for uranium enriching services (published on July 1, 1966.) feed delivered to the AEC will be required to be in the form of UF_6. Reactivation of the Allied plant and/or establishment of new private capability, therefore, will eventually be required to meet the future needs for UF_6 conversion services.

The Commission has had no requirements for UF_6 conversion in the past few years, but sometime in the next few years should again require UF_6. There are, as noted in the Raw Fuel Materials Category, foreign requirements for this service for about 1,600 tons U_3O_8 between now and January 1969 through ad hoc barter arrangements. There is also developing a potential volume of uranium conversion business, for initial fuel requirements, for the recently announced nuclear power reactors scheduled

to be in operation in the early 1970's. The Commission's announcement at the JCAE Hearings in August of this year of its policy concerning post-1968 uranium leasing and sales is intended to favor toll enriching over leasing from AEC and this is expected to create a requirement for UF_6 conversion services earlier than forecast previously. In anticipation of the toll enriching market, Allied Chemical Corporation announced in July 1966, a reduction in its charge for conversion of uranium concentrates to UF_6 from $1.25 to $1.04 per pound of contained uranium. The AEC's factor for conversion of U_3O_8 to UF_6 has been approximately $1.21/lb. Allied Chemical anticipates reopening its plant sometime during 1967 or 1968 at the latest.

As indicated in the Raw Fuel Materials Category and based on the estimated uranium requirements for power reactors being built, it appears that there will begin to be a market for the production of UF_6 in 1968 and 1969. When the market develops sufficiently, other companies will, no doubt, provide this service.

b. Conversion of Enriched Uranium to UF_6

Adequate industrial capacity exists to recover uranium from unirradiated scrap. In 1966, the new Nuclear Fuel Services plant at West Valley, New York, began recovery of uranium from irradiated fuel elements. Industrial capacity does not exist, however, to convert the resulting uranium, such as the nitrate, from either unirradiated scrap or irradiated fuels to UF_6.

A number of firms, such as Allied Chemical, Kerr-McGee, Nuclear Fuel Services and United Nuclear, have expressed interest in providing this service to both the industry and the Government. The main factor presently delaying entrance by the industry into this area is the uncertainty in the market forecast. Up to now, only enriched uranium derived from the scrap recovery operations has been potentially available for conversion to UF_6. Also, the quantities and batch sizes have been relatively small; however, with all the nuclear power plants announced in the last quarter of 1965 and in 1966 and with the advent of uranium enriching services and operation of the Nuclear Fuels Services reprocessing

plant in 1966, substantial quantities of recovered uranium from both unirradiated scrap and irradiated fuel recovery operations should begin to become available for conversion to UF_6 by 1968. This market may not grow as rapidly, however, if reactor operators decide to recycle their uranium from the spent fuel operations. This would require adding only enough new fuel to blend with the recycled uranium. The selection of such a mode of operation would depend on the relative economics involved.

2. Enriching

This activity continues to be the only major area in the fuel cycle not in private industry. A separate section of this report reviews the status of private ownership -- toll enriching, and discusses the background leading to toll enriching as well as the implementation of private ownership and toll enriching.

During the JCAE Hearings on private ownership and toll enriching in 1964, the transfer of this activity to the private sector was the subject of considerable testimony. In testimony the industry witnesses were unanimous in suggesting that the AEC start studying the feasibility of operation one of the AEC's diffusion plants as a private enterprise as soon as possible. Dr. Seaborg stated at these hearings that "the Commission is willing to cooperate with industry representatives in exploratory studies of the feasibility and timing of private participation in uranium enrichment activities".

Many industry representatives, testifying during the JCAE Hearings on toll enrichment in August 1966, urged that a review of the AEC gaseous diffusion plant classification policies be made to attempt to reduce or eliminate the classification of gaseous diffusion plant economics and capacity in order to permit business managements to make realistic evaluations and projections of fuel cycle costs and of providing enrichment services.

3. Processing

Adequate industrial capability and competition exists today to convert depleted, normal or enriched UF_6 to any of

the required forms for commercial use.

The principal producers of depleted, normal and enriched uranium metal, oxides, compounds and coated particles are listed in Table II-1.

TABLE II-1

Principal Producers of Uranium Fuels

	Metals, Oxides & Compounds	Coated Particles*
Combustion Engineering	X	
General Electric Company, San Jose, Cal.	X	
General Dynamics Corp., San Diego, Cal.		X
Kerr-McGee Oil Industries, Inc., Oklahoma City, Oklahoma	X	
National Lead Company, Albany, New York	X	
National Carbon Company, Lawrenceburg, Tenn.		X
Nuclear Fuel Services, Inc., Erwin, Tenn.		X
Nuclear Materials and Equipment Corp. Apollo, Pennsylvania	X	
United Nuclear Corp., Hematite, Missouri	X	X

* Used in certain AEC fuels such as NERVA but, as yet, not being used in commercial fuels.

- -

In January 1966, Diamond Alkali Company ceased manufacturing of coated particles.

The quantities of enriched uranium distributed by the AEC over the years are reflected in Table II-2 on the following page.

As predicted, the total quantity of enriched uranium distributed by the AEC in Fiscal 1966 increased significantly as compared to Fiscal 1965. The increase in total uranium requirements in Fiscal Year 1966 was due to increased requirements in both the domestic and foreign programs which more than offset sharply reduced AEC requirements.

TABLE II-2

Enriched Uranium Furnished to Industry

(Kilograms of Uranium)

Fiscal Year	Furnished as UF_6	Furnished in Forms Other Than UF_6	Total
1956	0	2,400	2,400
1957	6,000	4,000	10,000
1958	24,000	21,000	45,000
1959	110,300	6,300	116,600
1960	86,200	3,400	89,600
1961	119,400	6,900	126,300
1962	125,600	3,000	128,600
1963	100,273	3,918	104,191
1964	116,401	2,037	118,438
1965	152,786	1,441	154,227
1966	285,773	5	285,778

As can be noted in Table II-2, requirements for uranium furnished in forms other than UF_6 during FY-1966 dropped greatly from FY-1965. Also as predicted, the AEC requirements for uranium oxides and compounds increased in FY-1966 as compared to FY-1965.

In FY-1967 the total uranium requirements are expected to again increase significantlv. Some of this increase will result from foreign projects, but the main increases will be principally from requirements for domestic power reactors. This segment of the industry should, therefore, experience a substantial increase in volume of sales in FY-1967.

4. Fabrication

A complete industry capability exists to produce various types of fuels from various compounds.

a. Metallic Fuels -- During the year, M&C Nuclear announced that they intend to phase out all nuclear fuel work, including naval reactor fuels

and possibly HIFIR (High Flux Isotope Reactor) fuel elements. The phase out of M&C Nuclear (Westinghouse and Combustion Engineering withdrew last year), leaves United Nuclear and Babcock & Wilcox as the remaining suppliers of naval fuels. With the probable withdrawal of M&C Nuclear from fabrication of HIFIR fuel elements, the AEC took steps during the year to develop more commercial suppliers, so that AEC would be assured of continuity of a fuel supply for this reactor and for metallic fuel for all the research and testing reactors. The High Flux Isotope Reactor is designed to produce research quantities of the transuranium elements and is the first of its kind in the United States.[1/]

At present, the potential suppliers of research and test reactor fuels are United Nuclear Corp., National Lead Co., NUMEC and Atomics International. The market for research and test reactor fuels, including MTR, ETR, ATR, ORR, HFBR, HIFIR, Plumbrook, GETR and university reactors, is estimated at about $7 million annually.

During the year, the National Lead Company acquired the nuclear fuel fabrication facilities of the Sylcor Division of Sylvania Electric Products, Inc. Many of Sylcor's management, engineering and supervisory personnel also joined the National Lead staff. Atomics International entered this field and obtained their first contract from Idaho to produce ETR fuel elements.

b. Oxide Fuels -- This segment of the industry is highly competitive and, as a result, adjustments in the firms active in this area may be anticipated in the years ahead. At the present time, the independent fabricators are still concerned about obtaining a portion of the fuel business, because power fuels have, in general, been purchased by utiliteis from the companies supplying their reactor.

1/ See AEC Public [illegible] J-210, dated September 2, 1966.

This year, Commonwealth Edison placed an order with United Nuclear Corporation for a replacement core for its Dresden #1 plant. This was a major milestone in establishing the first instance of a utility obtaining competitive bids for their fuel requirements and selecting a supplier other than a reactor manufacturer. There is a trend for independent fabricators to become core suppliers in order to assure themselves of a market.

The principal fabricators of oxide uranium fuels are listed in Table II-3. For convenience those also fabricating metallic fuels are shown.

TABLE II-3

Principal Fabricators of Uranium Fuel

	Oxide	Metal
Atomics International, Canoga Park, Cal.	X	X
Babcock & Wilcox Co., Lynchburg, Va.	X	
Combustion Engineering, Inc., Windsor, Conn.	X	
General Dynamics Corp., San Diego, Cal.	X	
General Electric Company, San Jose, Cal.	X	
National Lead Company, Albany, N. Y.	X	X
Nuclear Fuel Services, Inc., Erwin, Tenn.	X	X
Nuclear Materials & Equipment Corp., Apollo, Pa.	X	X
United Nuclear Corp., New Haven, Conn.	X	X
Westinghouse Electric Corp., Pittsburgh, Pa.	X	

- -

Combustion Engineering will build a fuel fabrication facility addition to their Windsor, Connecticut, plant. The new facility, expected to be completed by mid-1967, will be used to fabricate the first core for the Consumers Power Company Palisades Reactor.

The following firms have some fabrication capacity and/or perform R&D on fuels:

Aerojet-General Corp., San Ramon, Cal.
The Carborundum Co., Niagara Falls, N.Y.
Battelle Memorial Institute, Columbus, Ohio
Martin-Marietta Co., Baltimore, Md.
Metals & Controls, Inc., Attleboro, Mass.
(Are completing current order for HIFIR fuel elements and plan to phase out of fuel business early in 1968)
Nuclear Metals, Inc., Division of Whittaker Corp., Concord, Mass.

AEC orders placed with commercial suppliers for the fabrication of fuels are given in Table II-4.

TABLE II-4

AEC Orders for Fabrication of Uranium Fuel
From Commercial Suppliers
(Millions of Dollars)

Fiscal Year	Naval Purposes	All Other Purposes	Total
1959	38.6	2.8	41.4
1960	48.6	3.8	52.4
1961	29.2	4.0	33.2
1962	60.9	4.5	65.4
1963	36.8	2.2	39.0
1964	28.5	8.7	37.2
1965	52.3	5.4	57.7
1966	63.4	2.0	65.4

Table II-4 indicates a substantially higher volume of orders was placed by AEC in FY-1966 over FY-1965. In FY-1966, the naval orders increased about 20% over FY-1965.

This segment of the industry should experience a substantial increase in sales in FY-1967 as a result of increased requirements for AEC, domestic and foreign programs. U. S. industry continues to be successful in the foreign market; however, as anticipated, substantial portions of

the processing and fabrication work for foreign reactors are being conducted overseas. In FY-1966, approximately 9% of the total enriched uranium destined for foreign reactor projects was shipped overseas as UF_6 and processed and fabricated overseas compared to approximately 7.5% in FY-1964 and 11% in FY-1965.

B. Plutonium

1966 marked the 25th anniversary of the discovery of plutonium by Dr. Joseph W. Kennedy, Dr. Arthur C. Wahl, Dr. Edwin M. McMillan and Dr. Glenn T. Seaborg. Within a week of the date of this anniversary, some 300 persons were attending a commercial plutonium fuels conference in Washington, D. C. This conference, held March 1 and 2, 1966, was sponsored by the Atomic Industrial Forum, the Edison Electric Institute and the American Nuclear Society. The conference featured papers and panel discussions on plutonium fuels and requirements, availability and industry processing capability, technical aspects of plutonium processing and plutonium as a reactor fuel. The conference proceedings are contained in AEC report CONF-660308.2/

While the word plutonium has historically referred mainly to the isotope Pu-239, which results from converting uranium-238, after irradiation in thermal reactors, there are also other very important isotopes of plutonium. One cannot help being intrigued by the existence of four important isotopes of plutonium which are indistinguishable chemically, but due to different nuclear properties may have values differing from a few cents to $1 thousand per gram.3/ For example, Pu-240 may be used as the means to provide future fuels with almost constant nuclear reactivity. Pu-241, which is a fissile isotope, and Pu-242, a parasitic neutron absorber, are also produced upon irradiation of Pu-239. There is every reason to believe that Pu-238, another isotope, because of its desirable properties, may become one of the most important heat sources for many applications.

2/ "Commercial Plutonium Fuels Conference," March 1-2, 1966, available for $3.00 from Clearinghouse for Federal Scientific and Technical Information, National Bureau of Standards, U. S. Department of Commerce, Springfield, Virginia 22151

3/ Introductory Remarks by E. B. Tremmel before the Conference on Commercial Plutonium Fuels, Washington, D. C., March 1, 1966.

Dr. Seaborg in his address to the plutonium fuels conference placed the economic significance of plutonium in perspective when he stated: "This impending growth in the production rate of plutonium poses some very meaningful economic issues . . . .this may be simply viewed by considering that plants installed through 1980 would be producing throughout the world about one-half billion dollars worth of plutonium per year, calculated at a cost of $10 per gram of fissionable isotopes. In other words, plutonium will be produced at a rate of more than a million dollars worth every day, by about 1980!"4/

Plutonium has been of interest to those concerned with the Government's research and reactor programs for many years, but the dramatic developments of the past two or three years in the growth of nuclear power has greatly increased the interest of reactor suppliers and electric utilities in this element. This is due to several reasons; the principal ones being:

1. The value of plutonium as a fuel to recycle in thermal reactors and the impact on the fuel cycle and power cost estimates for reactor systems.

2. The potential of using plutonium to extend the available supply of fissionable material through breeding.

3. Fissionable plutonium can be used in a reactor instead of U-235, thereby providing greater flexibility of independence from an enriched uranium supply.

4. The termination of January 1, 1971, of the Commission's guaranteed purchase price for plutonium produced in domestic or foreign reactors fueled with AEC supplied enriched uranium as required under the private ownership amendment of 1965.

The Commission's work on plutonium fuel recycle in thermal reactors will be gradually phased out over the next few years, because the basic questions of feasibility have been answered and a large body of experience, data and design information has been developed. It is expected that the information, which is

4/ Remarks by Chairman Glenn T. Seaborg at the Confernece on Commercial Plutonium Fuels, Washington, D. C., March 1, 1966, "Plutonium - Past, Present, Future."

being obtained by the AEC in its programs and that which is also being obtained through the efforts of privately sponsored plutonium fuel development work, will be the basis for large-scale plutonium loadings in commercial reactors in the early 1970's. The economic pressure to use this valuable fuel and the increasing quantities which will become available in the early 1970's, coupled with the termination of AEC's guaranteed purchase price should establish the time-table for the beginning of plutonium recycle. This should occur in the early to mid-1970's for power reactors which will be producing plutonium and discharging spent fuel during the next five to ten years.

The U. S. utilities, through the Edison Electric Institute and its Committee on Nuclear Fuels, have carefully studied the status and future potential of plutonium fuels technology and its application to both thermal and fast breeder reactors. Their "Plutonium" survey reports for 1963 and 1964 were released to inform EEI members and the nuclear power industry of the status of plutonium as a power reactor fuel and its economic importance to the industry. The EEI Committee on Nuclear Fuels recommended in its 1964 report that EEI make an independent forecast of the nuclear power growth rate, sponsor programs which will lead to plutonium loadings in existing commercial reactors at an early date; and that the electric power industry participate more extensively in plutonium-fueled fast reactor development. These recommendations were accepted by the EEI Policy Committee on Atomic Power.

A program for research and development, leading to commercial loadings of plutonium bearing fuels in large BWR's and PWR's, has been negotiated with General Electric Company and Westinghouse Corporation. This program would generally complement the AEC's program in this area. The Phase I part of this program is estimated to cost about $690 thousand. A Phase II program will be developed on the basis of the results obtained from Phase I.

In support of fast reactors fuel development, the EEI has entered into a first phase contract with Atomic Power Development Associates ($1.13 million) based upon contributions from EEI members, Detroit Edison and Belgo-Nucleaire. The entire EEI fast reactors plutonium fuel program will extend over five years and is expected to cost about $4.8 million.

It is directed toward demonstration of PuO_2-UO_2 fuel in a fast breeder reactor and would also complement the AEC's fast reactor fuel development programs.

As reported last year, the Commission adopted the recommendation of a committee representing several AEC Divisions which had studied the potential commercial work which could be done in private plutonium facilities. One of the principal results of this committee's work was that the Commission took additional steps to encourage the growth of a competitive commercial capability in the field of plutonium processing and fabrication. The Commission issued a Public Release No. H-63 on March 19, 1965, stating that the Commission will procure commercial services for most plutonium processing in non-weapons programs and encourage private efforts in plutonium technology development. This policy was emphasized further by AEC officials at the Commercial Plutonium Fuels Conference mentioned earlier.

The plutonium supply-demand situation has been examined from time to time by AEC, the EEI and by private firms interested in the processing or use of plutonium. Program projections of requirements generally favor the more optimistic side and, thus, create forecasts that are excessive in quantity and advanced in timing. An estimate of the potential plutonium requirements for the next five years for AEC and licensee programs are listed in Table II-5 below.

TABLE II-5

Projected Plutonium Requirements
(In Kilograms)

	FY-68	69	70	71	72
AEC R&D	150	100	100	100	100
AEC Critical Experiments	1700	1900	2000	2200	1700
AEC Reactors	50	25	2700	770	1600
Licensed Uses	70	500	400	300	--
Total	1970	2525	2770	3370	3400

There seems to be general agreement that over-all plutonium requirements will exceed the available supply from power reactors until the early or mid-1970's.

In a paper entitled "Commercial Plutonium - Its Availability and Composition," given at the Commercial Plutonium Fuels Conference mentioned above, R. E. L. Stanford and C. R. Moore of Nuclear Fuel Services presented the following estimates of anticipated plutonium recovery from commercial power reactor fuels.

TABLE II-6

Anticipated Plutonium Recovery
From
Commercial Power Reactor Fuels

Year	Pu Discharged Kgs	Pu Recovered This Year Kgs	Pu Recovered Cumulative Kgs
Thru 1966	700	499	499
1967	181	59	558
1968	366	337	895
1969	844	499	1394
1970	870	625	2019
1971	1841	628	2647
1972	3564	1830	4477
1973	5034	3564	8041
1974	6377	5034	13075
1975	8073	6358	19433

Based on the projected requirements in Table II-6 shown above, it is expected that commercial fabricators may process and fabricate at least 80% of these quantities. At an estimated fabrication cost of $5,000 per kg of contained plutonium, the potential fabrication market is shown in Chart II-A on the following page.

As stated previously these forecasts usually tend to be optimistic. They, therefore, should be used only as rough indications of future requirements.

PLUTONIUM PROCESSING AND FABRICATION

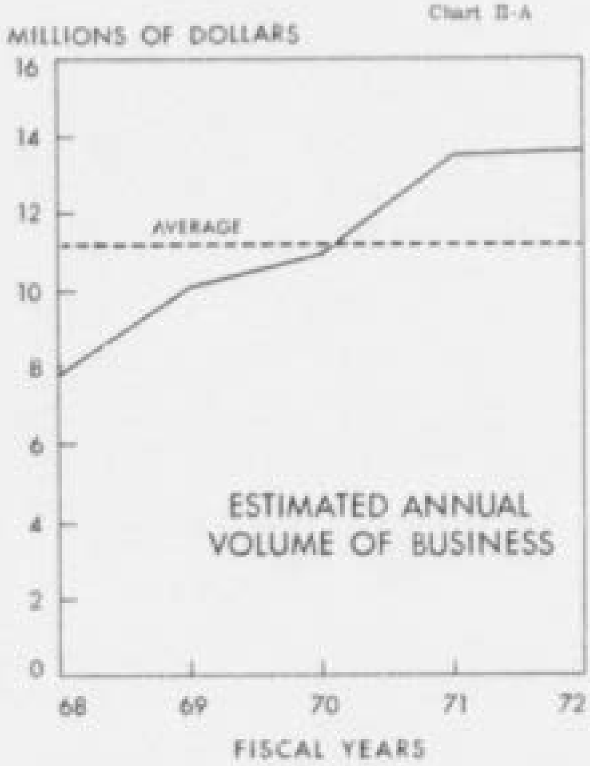

A substantial amount of the above fabrication will be the ZPPR (Zero Power Plutonium Reactor) fuel amounting to about 3 thousand kg plutonium to be fabricated through 1970.

Plutonium bearing fuels are now being or have been irradiated in programs using the EBR-II, PRTR, EBWR and the Saxton reactors. Plutonium fuels will be irradiated soon in SEFOR and later on in the FFTR. During the year, Nuclear Fuel Services was awarded a large order for fabrication of the mixed uranium and plutonium oxide fuel for SEFOR.

During the year, Atomics International announced that they had awarded a contract for the construction of a new plutonium fabrication facility for their company. The new facility and equipment are expected to cost about $1.2 million and are scheduled to be completed in early 1967.

Table II-7 lists private firms which have various degrees of capability in plutonium processing.

TABLE II-7

Industrial Interest in Plutonium Work

	Fuel Processing	Fuel Fabrication	Scrap Recovery	Source Fabrication	R&D
Atomics International	X	X			X
Babcock & Wilcox					X
Battelle Memorial Inst. (Columbus, Ohio)					X
Carborundum Co.					X
General Electric Co.	X	X			X
Monsanto Research Corp.				X	X
Nuclear Fuel Services	X	X	X		X
Nuclear Materials & Equipment Corp.	X	X	X	X	X
United Nuclear Corp.					X

C. Thorium

The present need for thorium as a reactor fuel is limited and is used primarily for research and development purposes. Orders for thorium metal powder and ingots in the past year remained the same as for 1965. As noted in the Raw Fuel Materials Category, the development of certain advanced converter reactors for commercial applications would expand the use of thorium as a source of nuclear fuel.

No changes have occurred in the industry in the processing and fabrication of thorium. The firms that fabricate thorium are identical to those listed in Table II-3. The principal firms processing thorium are shown in Table II-8 on the following page.

TABLE II-8

Principal Firms Processing Thorium

American Potash & Cehmical Corp., Chicago, Illinois
Babcock & Wilcox Company, Lynchburg, Virginia
The Dow Chemical Company, Midland, Michigan
General Dynamics Corporation, San Diego, California
Kerr-McGee Oil Industries, Inc., Cushing, Oklahoma
Metal Hydrides, Inc., Beverly, Massachusetts
National Lead Company, Albany, New York
Nuclear Fuel Services, Inc., Erwin, Tennessee
Nuclear Materials & Equipment Corp., Apollo, Pennsylvania
United Nuclear Corporation, Hematite, Missouri

D. Uranium-233

The present need for uranium-233 is limited and is essentially of a developmental nature. Due to the interest in the thorium - uranium-233 fuel cycle, development work in this area may increase in the future.

In addition to the capability at Babcock & Wilcox's Nuclear Development Center at Lynchburg, Virginia, Nuclear Fuel Services, Nuclear Materials and Equipment Corporation and Atomics International have some processing and fabrication capabilities.

The Private Ownership Act of 1964 directed the Commission to establish guaranteed purchase prices for U-233 not to exceed 10 years with no fixed termination date on the Commission's authority. Accordingly, the AEC established prices for uranium enriched in uranium-233 in forms of nitrate, dioxide, or metal and meeting AEC specifications of $14 per gram of the contained isotope uranium-233, subject to adjustment for the presence of other uranium isotopes and deductions for handling costs. The U-233 purchase prices continue until the Commission has made a determination that U-233 technology has reached the point where the Government should no longer provide a guaranteed market for this material.

E. Other Materials

1. Heavy Water

Heavy water plants were originally built by the Atomic Energy Commission in the early 1950's at Dana, Indiana, and at Savannah River to furnish heavy water for the production reactors at Savannah River. The Dana plant was shut down in 1957 and the Savannah River plants were gradually reduced in operation to about one-third their total capacity during 1957 and 1958. As of today, the AEC continues to operate the Savannah River heavy water plant at approximately one-third its capacity in order to meet AEC production requirements.

Since 1955, the AEC has sold heavy water in quantities of 125 pounds or more to commercial users. Smaller quantities are available from distributors who buy large quantities from the AEC for resale. The principal commercial suppliers are given in Table II-9. Since heavy water use frequently involves degradation of the heavy water, the AEC also provides a service to rework degraded heavy water. The Bio-Rad Company of California has established an operation for upgrading small quantities of heavy water. The Deuterium Corporation of New York is now offering a broad range of services, including design, engineering and construction of heavy water plants. The AEC will continue to offer purification services for larger quantities, until this service is reasonably available from industry for all quantities.

Growing heavy water inventory requirements for natural uranium fueled heavy water moderated reactors created considerable interest in heavy water production during the past few years. It is estimated that Canadian, Swedish, UK, French and Indian reactor programs for heavy water reactors will require initial inventories and make-up ranging from 200-500 tons annually in the next few years, to about 1,000 tons in 1970. As mentioned above, present AEC production is about 190 tons per year, utilizing about one-third of the capacity of the Savannah River plant.[5]

5/ Total capacity of AEC's Savannah River plant is approximately 500 tons per year.

Canadian, European and Asian capacity, outside the Soviet Bloc, in operation is about 35-40 tons, with another 1,100 tons under construction or planned, for a total of about 1,150 tons. Most of the planned capacity will be in Canada, with about 900 tons planned, but none yet in production. The Deuterium of Canada, Ltd. plant, presently under construction at Glace Bay, Nova Scotia, was designed for 200 tons per year, and the company has indicated that they are planning to double the capacity of this plant. The Glace Bay Plant expansion was announced September 10, 1966, by the Honorable Jean Pepin, Canadian Minister of Energy, Resources and Mines, and The Honorable Allan MacEachen, member of the Federal Cabinet from Nova Scotia, following an agreement between Deuterium of Canada and Atomic Energy of Canada Limited, under which AECL would underwrite the purchase of about 5,800 tons of heavy water over a 15 year period. The sales price to AECL will be $20.50 per pound (Canadian) for the initial 1,830 tons of heavy water, decreasing to $19.25 and then, to $16.00 per pound at the end of the contract term, for an average sales price of $18.50 per pound.

The Canadian General Electric Company is planning a plant to produce 500 tons a year, with operation scheduled to begin about 1969. India is planning a 200 ton a year plant. France, India, Norway and Switzerland have limited capacity to produce heavy water (estimated at about 35-40 tons total annual capacity.)

Because of the increasing interest in heavy water for natural uranium reactors, an Atomic Industrial Forum Ad Hoc Committee was appointed in 1965 at the request of the AEC to review the feasibility and interest of industry in taking over the operation of the Savannah River heavy water facilities on a commercial basis. This Committee made a careful study of the heavy water production market and projected requirements and forwarded their study to the AEC in the Spring of 1966. The Committee concluded, that because of the limited domestic market and competition from Government-assisted foreign production plants, it would be unlikely that a private company would be willing to assume the risk of building new heavy water production facilities in the United States at the present time. They pointed out, however, that about 1969 the future national and international supply and demand situation should be much clearer.

In view of the AIF Committee's study report, the AEC decided to continue operation of the Government plant at Savannah River at about one-third its capacity for Fiscal Years 1967 and 1968 and to continue to closely follow the heavy water market.

Domestic heavy water requirements will, of course, increase if the heavy water organic cooled reactor (HWOCR) is developed successfully.

TABLE II-9

Principal Commercial Suppliers of Heavy Water
(In Quantities Less Than 125 Lbs.)

Bio Rad Labs, Inc., Richmond, California
Columbia Organic Chemicals - Columbia, S.C.
Deuterium Corporation, New York, New York
Diaprep, Inc., Atlanta, Georgia
International Chemical Nuclear Corp.,
City of Industry, California
Isomet, Corporation, Palisades Park, N. J.
Liquid Carbonic, Division of General Dynamics
Corporation, San Carlos, California
Matheson, Coleman & Bell, East Rutherford, N. J.
Merck & Company, Rahway, New Jersey
New England Nuclear Corporation, Boston, Mass.

2. Beryllium

In early 1965, the beryllium industry began expressing considerable concern over an AEC cost-type contractor's attempts to attract beryllium work from other Government agencies and their cost-type contractors. Industry spokesmen indicated that their companies had capabilities equal to that of the Commission's facilities, as well as excess capacity of their own.

To provide an objective review of the several interrelated questions concerning AEC beryllium capability and requirements, a study group was established in the Spring of 1965. This group subsequently was instrumental in resolving the United Kingdom proprietary information problems which involved certain areas of the wrought process technology; the adoption

of internal procedures at Oak Ridge and Rocky Flats to screen work to determine whether or not it can be performed by industry; improving lines of communication between representatives of the industry and the Commission's laboratories and production facilities; and providing prompt publication and dissemination of the AEC-developed technology.

Later in 1965, the industry became concerned with developments at Rocky Flats, when that AEC-owned facility began producing beryllium parts to meet its weapons schedule requirements.

In response to industry's concern with this in-house fabrication, the AEC invited the four beryllium metal companies to attend a meeting at Rocky Flats on June 16-17, at which time the AEC and the operating contractors discussed in detail the technical aspects of the process and the basis for the procurement decision. The beryllium industry also met with the Commissioners concerning this matter.

As a result of the subsequent review by the Commission, the AEC reiterated the Government's interest in maintaining a strong domestic beryllium industry and that it was the AEC's desire to procure these parts on a normal commercial basis if the industry can offer these parts at acceptable prices. To assist the companies in achieving production economics through the wrought process or some other equally effective process, the AEC intends to issue an invitation for proposals from industry for the manufacture of small quantity lots to enable industry to obtain the required production experience.

In recognition of the industry's general interest in the continuation of AEC sale of its scrap holdings, the companies were also informed that the AEC will continue to offer for for sale a portion of the scrap generated in AEC operations. Offerings will be in small lots in accordance with existing practice. In the last two years, the AEC has sold 77 thousand pounds of scrap.

The four companies constituting the basic beryllium metal industry are listed on the following page.

The Brush Beryllium Company
17876 St. Clair Avenue
Cleveland, Ohio 44110

Beryllium Corporation
P. O. Box 1462
Reading, Pennsylvania 19603

General Astrometals Corporation
320 Yonkers Avenue
Yonkers, New York 10701

Beryllium Metals, Inc.
500 Fifth Avenue
New York, New York 10036

CATEGORY III
NUCLEAR COMPONENTS AND EQUIPMENT

As reported previously, all nuclear components and equipment are available commercially. Also, there does not appear to be any substantial competition between the Government and private industry in furnishing these items.

This category continues to be characterized by companies not now dependent on nuclear energy activities for their growth and well-being. Within this category, however, there are also some smaller producers of specialty items whose origin and growth have been entirely dependent on nuclear energy. The manufacturers of radiation detection and measuring instruments, to a considerable degree, fall into this group. They are among the first, and most interesting, independent groups to form after the Government's atomic energy program encouraged commercial procurement of certain components. During the past year, additional information has been obtained about this segment of the nuclear industry.

A. Nuclear Instruments

There are now about 170 firms in the U. S. manufacturing nuclear instruments in one form or another, or equipment which employs some type of radiation detecting element and some form of read-out system to achieve the desired measurement or control. Companies making primarily nuclear instruments, whose 1965 sales reportedly have exceeded $0.5 million, are shown in Table III-1 on the following page.

Companies manufacturing nuclear industrial gauges and process control equipment are listed in Category V, Radioisotopes, Section D-3. A separation of industrial gauges from the remainder of the equipment broadly referred to as nuclear radiation detection

TABLE III-1

Companies With 1965 Nuclear Instrument Sales Exceeding $0.5 Million

Baird-Atomic	Nuclear Data, Inc.
Beckman Instruments, Inc.	Nuclear Measurement Corp.
Bendix Corporation	Oak Ridge Technical Enterprises Corporation
Chronetics, Inc.	Ohio Nuclear
Controls for Radiation, Inc.	Packard Instrument Company, Inc.
Cosmic Radiation Labs	Picker X-Ray Corporation
Curtis Nuclear Corporation	Reuter-Stokes Electronic Components, Inc.
Dero Research & Development Corp.	Solid State Radiations, Inc.
Eberline Instrument Corporation	Technical Associates
Edgerton, Germeshausen & Grier, Inc.	Technical Measurement Corp.
EON Corporation	Tennelec Instrument Co., Inc.
Hamner Electronics Company	Tracerlab, A Division of LFE, Inc.
Landsverk Electrometer Company	Transistor Specialties, Inc.
Miles Laboratory	The Victoreen Instrument Co.
Northern Scientific	
Nuclear-Chicago Corporation*	

* The G. D. Searle Company and Nuclear-Chicago Corporation merged during the year.

and monitoring instruments has been made this year. The other categories within the radiation detection and monitoring instruments section of the Bureau of the Census Current Industrial Reports series MA-38B "Selected Instruments and Related Products" are as follows:

- Radiation detecting elements, including ion chambers.
- Nuclear Monitoring Instruments-portable hand held.
- Nuclear Monitoring Instruments-Environmental (not hand held)
- Medical monitors - whole body scanners
- Scalers
- Special amplifiers for nuclear applications
- Pulse analyzers - single channel and multi-channel
- Sample changers
- Count rate meters
- Miscellaneous

This separation was made because the requirement for radioisotopes as sources of radiation in nuclear industrial gauging equipment provides

a convenient and practical division between the somewhat overlapping areas of nuclear instruments and industrial nuclear gauges. Also, a committee for the formation of an Association of Nuclear Instrument Manufacturers (which is discussed later) does not include industrial nuclear gauges, or reactor instrumentation, as falling within their sphere of interest.

Sales of nuclear instruments are expected to increase around 15% annually and should approach $180-$200 million by 1970. (See Chart III-A).

As reported last year, data on shipments of radiation detection and measuring instruments were formerly reported along with shipments of other "atomic products" in the Census Bureau Current Industrial Reports series "Selected Atomic Energy Products". For 1963 and subsequent years, data for nuclear instruments have been published annually in the Census Bureau CIR series M-38B "Selected Instruments and Related Products". Chart III-B shows annual sales of nuclear instruments and associated equipment through 1964 and estimated sales for 1965. This chart was compiled by the Division of Industrial Participation.

A report was issued in 1965 by the Atomic Industrial Forum regarding the achievements and capabilities of the nuclear instrument industry.

The Division of Industrial Participation continued to work closely with industry groups to explore potential areas of cooperation between the Government and industry. These instrument manufacturers provide an excellent example of one of the ways in which the transfer of technology and production operations from Government laboratories to private industry can assist in the general expansion of the country's economy. Nuclear instruments were originally developed and built in AEC laboratories to meet specific needs of the nuclear energy program. As information on these instruments became available to private industry, private companies entered this field and became capable of meeting the needs of AEC programs and also developed commercial markets.

Nuclear instrument makers are spending from 10% to 20% of sales for private R&D leading to new or improved products. This is somewhat more than instrument manufacturers in general commit to R&D, but is necessary in this highly competitive field where the market can demand and obtain design and performance improvements

on short notice. The private R&D investment for 1965 should amount to approximately $8 million for this segment of the industry.

Exports of nuclear instruments have been steadily growing (see Chart III-B) having increased from $4.6 million in 1959, when data was first collected, to $18.1 million in 1965[2]. It is expected that exports in 1966 may exceed $22 million. The export of nuclear instruments is currently about 20% of the total shipments reported, or roughly five times as large as exports for other manufactured atomic energy products shipped.

A committee for the formation of an Association of Nuclear Instrument Manufacturers (mentioned previously) has been working as a temporary small group of 13 companies during the year to scope its program and basis of operation. The organization presently limits its sphere of interest to single and multi-channel analyzers; nuclear power supplies, scalers, countrate meters, monitoring instruments and detecting heads; solid-state detectors; sample and flow counting systems; medical and biological counting systems. Several committees have been named within the group to keep appropriately informed about areas of special interest and also of potential problem areas including organization, membership, standards, statistics, exhibits, marketing and finance.

Twelve manufacturers of nuclear instruments exhibited products at the NUCLEX-66 exhibition held in Basel, Switzerland, September 8-14, 1966. (Eight of these firms are included in Table III-1.) The results of the NUCLEX-66 commercial promotion of nuclear energy products will not be known for some time.

Reactor control instrumentation is not considered within the nuclear instrument area reported above although there are similar elements in each type of equipment. This is due to the unique requirements for these components and the fact that reactor instrumentation is usually supplied by the reactor manufacturers. This equipment is reported as reactor components (see Section C and Chart III-C).

2/ "Analysis o U. S. Exports of Scientific, Industrial and Technical Instruments, 1959-1965" Available from U. S. Department of Commerce, Washington, D. C., 20230, for $.10.

Chart III-A

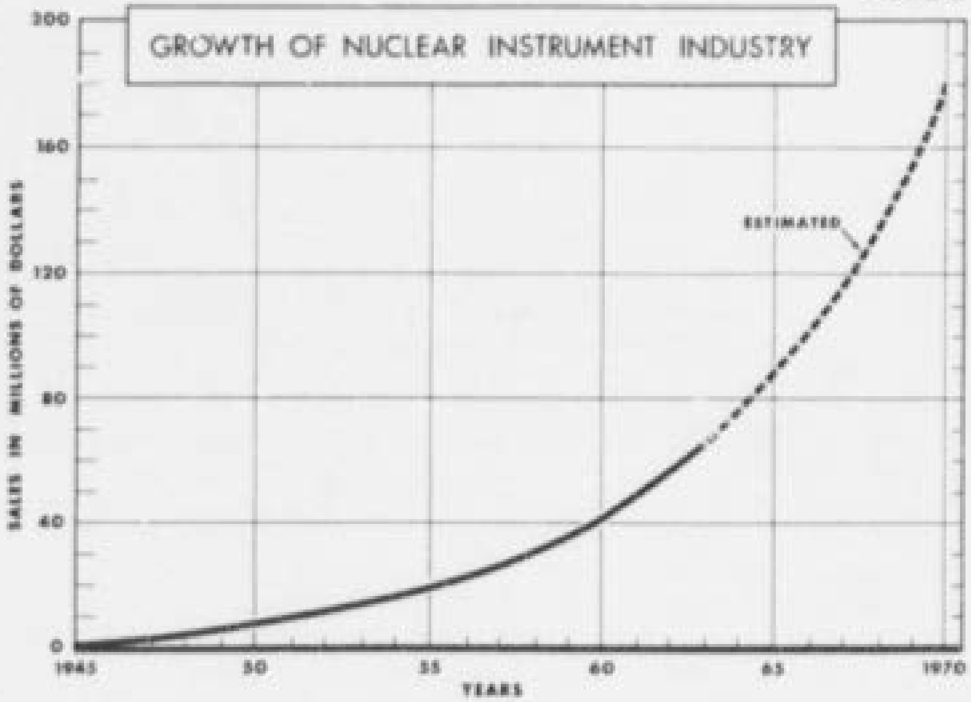

Chart III-B

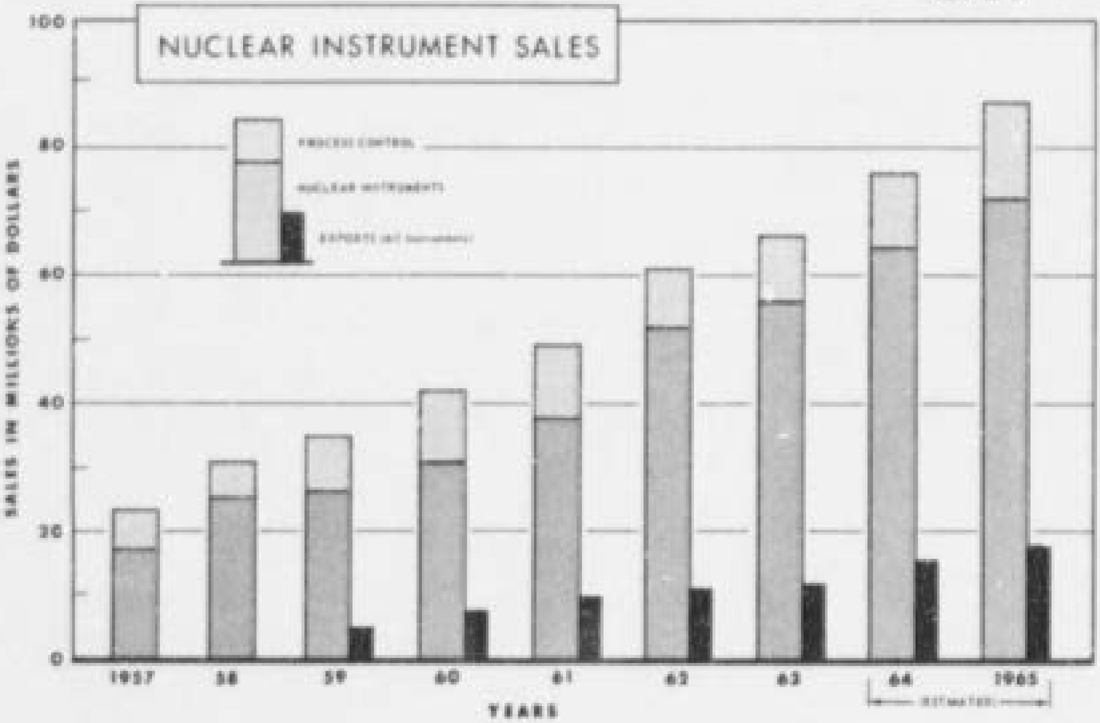

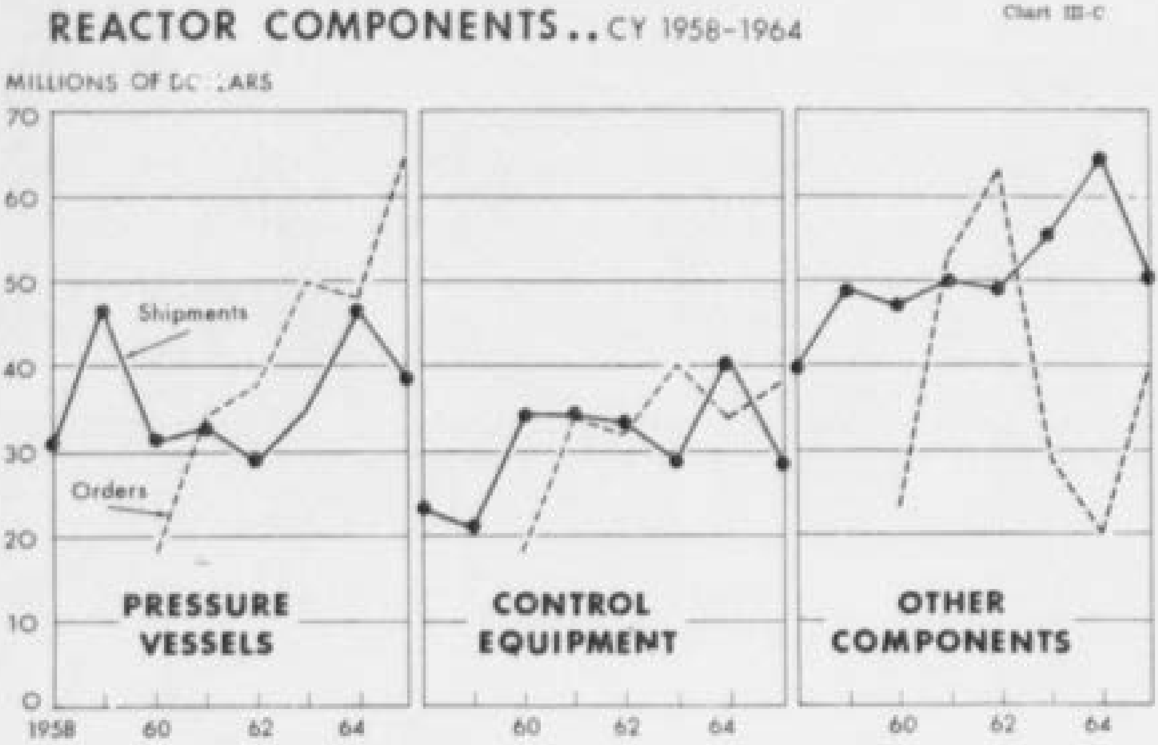

B. Components for Sodium Cooled Reactor Systems

Liquid metal pumps, valves and related components will be required for liquid metal cooled fast breeder reactors. During the year, the Commission decided to locate a sodium pump test facility at the Atomics International sodium components test facility at Santa Susana, California. The existing facilities have a value of about $4.3 million and the additional project will cost about $6.8 million. The total facilities will be known as the Liquid Metal Engineering Center and will proof test major sodium components such as: heat exchangers, steam generators, instrumentation, valves, reactor control rod assemblies and small and large liquid metal pumps up to 120 thousand gpm at temperatures up to 1200° F.[3/]

Atomics International will also operate the Liquid Metals Information Center for the AEC at Canoga Park, California. The Center will maintain data on current research and development on liquid metal technology, disseminate information and answer technical inquiries. It plans to produce a revised and expanded edition of the AEC "Liquid Metals Handbook".

C. Other Components

Shipments of reactor components other than fuel elements and nuclear instruments as reported by the Bureau of the Census are shown in Chart No. III-C. The components reported here are only those designed specifically for nuclear applications.

The data shown for "pressure vessels" include shipments reported to Census for primary vessels and tanks and heat exchangers and condensers. Data for "control equipment" include shipments of control rod drive mechanisms and components and accessory instrumentation for reactor control. "Other components" include shipments of pressurizers, components, and auxiliary equipment, pumps, valves, fuel handling equipment, core structurals and other specialized reactor components and equipment.

3/ See Public Release J-121, dated May 13, 1966.

CATEGORY IV
REACTORS

As mentioned in the Highlights, commercial sales of nuclear reactors are expanding more rapidly than anticipated several years ago, as electric utilities in many sections of the country find nuclear heat sources more economical than fossil fuels. Future prospects are promising, and current optimism for the application of nuclear reactors for a number of purposes appears well justified. The various markets for reactors are discussed in the following parts of this category.

A. Expenditures for Reactor Projects

Estimated expenditures for reactors under construction in the United States for the year ending June 30, 1966, were nearly a third higher than the previous year's level, as shown in Table IV-1.

TABLE IV-1

Comparative Costs Incurred For Domestic Reactor Projects

	FY-1965	FY-1966
	(in millions)	
All Projects	$167.0	$218.9
Federal Government	85.6	49.9
Atomic Energy Commission	76.3	42.8
Department of Defense	6.5	6.6
Other Federal Agencies	2.8	.5
Industry and Others	81.4	169.0
Privately Owned Utilities	74.0	165.5
Publicly Owned Utilities	5.1	.2
Universities and Manufacturers	2.3	3.3

Expenditures by utilities exceeded those by the AEC for the first time in FY-1965. For FY-1966, they were at nearly four times the AEC level. In future years, expenditures by utilities are expected to rise to levels that will greatly exceed expenditures for reactors for other purposes.

The number of reactors under construction and their total estimated cost are shown in Table IV-2. Table IV-3 shows cumulative cost incurred for these reactors and estimated costs yet to be incurred. This is also shown graphically on Chart IV-A. Electric utility central station power plants are not included in these summaries until applications for construction permits are received by the AEC. Therefore, since the cut-off date for the information included here was June 30, 1966, most of the large plants which have been announced this year are not included in these tables or Chart IV-A.

TABLE IV-2

Domestic Reactor Projects
Under Design or Construction

	At June 30, 1965		At June 30, 1966	
	Number of Projects	Total Estimated Cost (millions)	Number of Projects	Total Estimated Cost (millions)
All Projects	34	$1,322.8	31	$1,804.2
Civilian Reactor Projects	30	1,175.8	29	1,782.1
Civilian Power*	17	1,019.4	18	1,623.3
Civilian Testing	1	63.6	1	64.5
Civilian Research	12	92.8	10	94.3
Military Reactor Projects	4	147.0	2	22.1

* Projects are not included in this tabulation until license applications are received. For listing of projects included see Table IV-4.

TABLE IV-3

Costs and Estimates by Source of Funds
(Projects Active as of June 30, 1966)

	Costs Incurred FY-1966	Total Estimated Costs of Projects	Cumulative Costs Incurred Through June 30, 1966	Estimated Costs to be Incurred After June 30, 1966
	(in millions)			
All Projects	$218.9	$1,804.2	$585.4	$1,218.8
Federal Government	49.9	303.0	191.8	111.2
Atomic Energy Commission	42.8	271.9	162.2	109.7
Department of Defense	6.6	22.1	21.0	1.1
Other Federal Agencies	.5	9.0	8.6	.4
Industry and Others	169.0	1,501.2	393.6	1,107.6
Privately Owned Utilities	165.5	1,398.0	379.8	1,018.2
Publicly Owned Utilities	.2	92.7	8.2	84.5
Universities and Manufacturers	3.3	10.5	5.6	4.9

The estimates in all tables and the chart are based on surveys conducted by the AEC which cover all civilian and military land-based reactors and reactors in the maritime program. A listing of reactors included in these surveys appears in Table IV-4, together with the criteria followed in adding projects to, and dropping projects from, this tabulation.

TABLE IV-4

List of Domestic Reactor Projects
Under Design or Construction
At June 30, 1966 and June 30, 1965

Reactor Projects	June 1965	June 1966
**Civilian Power**		
Carolinas-Virginia Tube Reactor	X	-
Pathfinder (Northern States)	X	-
Experimental Gas Cooled Reactor (TVA)	X	-
BONUS (Puerto Rico Water Res. Auth.)	X	-
PWR Modifications	X	-
*EBOR - Exper. Beryllium Oxide Reactor	X	-
*UHTREX - Ultra High Temp. Reactor Exper.	X	-
FARET - Fast Reactor Test Facility	X	-
Enrico Fermi (PRDC)	X	X
Peach Bottom (Philadelphia Electric)	X	X
LaCrosse Boiling Water Reactor (Dairyland)	X	X
San Onofre (So. Cal. Ed./San Diego G. & E.)	X	X
Malibu Nuclear Plant (Los Angeles)	X	X
Connecticut Yankee	X	X
Nine Mile Point (Niagara Mohawk)	X	X
Oyster Creek (Jersey Central Pwr. & Lt.)	X	X
Dresden #2 (Commonwealth Edison)	X	X
Dresden #3 (Commonwealth Edison)	-	X
Public Service Company of Colorado	-	X
Quad Cities Station (Commonwealth Edison)	-	X
Robert E. Ginna #1 (Rochester Gas & Electric)	-	X
Indian Point #2 (Consolidated Edison)	-	X
Millstone Pt. (Northeast Utilities)	-	X
Turkey Point #3 (Florida Power & Light)	-	X
Turkey Point #4 (Florida Power & Light)	-	X
Palisades Nuclear Station (Consumers Pwr. Co.)	-	X
	17	18
**Civilian Testing**		
Advanced Test Reactor	1	1
**Civilian Research**		
Aerojet General	X	-
General Dynamics	X	-
HFBR - High Flux Beam Research Reactor	X	-
HFIR - High Flux Isotope Reactor	X	-
University of Missouri	X	X
Columbia University	X	X
University of California	X	X
Lowell Technological Institute	X	X
Oregon State University	X	X
National Bureau of Standards	X	X
HTLTR - High Temperature Lattice Test Reactor	X	X
LOFT - Loss of Flow Test Facility	X	X
*EBOR - Exper. Beryllium Oxide Reactor	-	X
*UHTREX - Ultra High Temp. Reactor Exper.	-	X
	12	10
**Military Reactors**		
MH-1A - Army Barge Mounted Reactor	X	X
Army Pulsed Reactor Facility - Aberdeen	X	X
NETR - Air Force Nuclear Engr. Test Fac.	X	-
S5G - Navy Natural Circulation Test Plant	X	-
	4	2

Chart IV-A

REACTORS

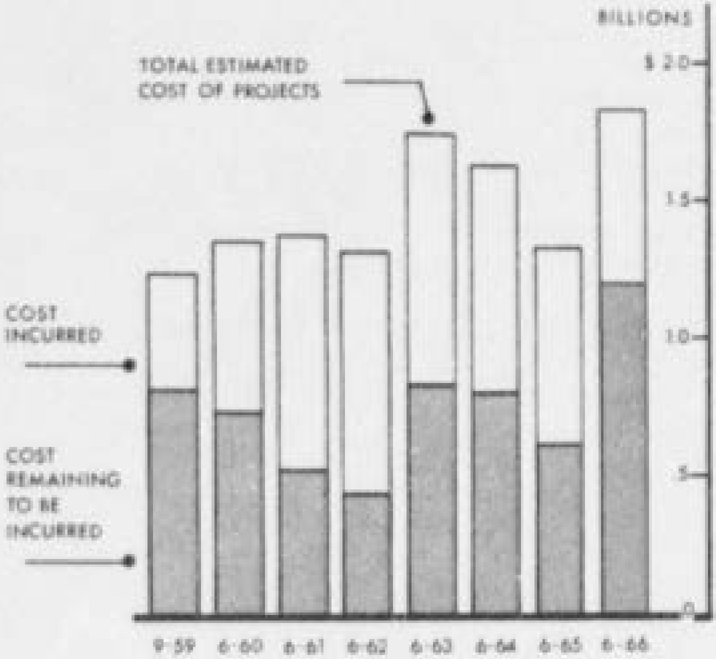

DOMESTIC REACTOR PROJECTS UNDER DESIGN OR CONSTRUCTION

•

THIS SERIES EXCLUDES REACTORS FOR AIRCRAFT AND SPACE APPLICATIONS AND FOR THE PROPULSION OF NAVAL VESSELS (but land-based Naval prototypes are included).

NOTES FOR TABLE IV-4

X - indicates project included in tabulation.
* - reclassified from Civilian Power to Civilian Research.

Projects are added to this tabulation:

(1) During the calendar quarter in which license applications are received.

(2) For projects under the cooperative demonstration program, during the quarter in which a basis for arrangements is submitted to the JCAE.

(3) For Government projects not subject to licensing and not subject to the submission of a basis for arrangements to the JCAE, during the quarter in which Title I work begins. (However, Government projects are not added prior to a decision to proceed with construction at a specific location even though design work may be under way.)

Projects are dropped from this tabulation in the quarter following the calendar quarter in which they first operate at design power.

Modifications to existing projects are included only when a substantial increase in the rated power output will result.

B. Civilian Power

1. General

The number of utilities which announced the signing of contracts or intentions to construct nuclear power plants in 1966 was particularly rewarding to those who have pioneered in the nuclear industry. The volume of orders has resulted in most forecasters revising their estimates of nuclear growth upward. Because of the volume of announced installations, nuclear fuel suppliers are now in a better position to make plans to meet the forthcoming markets.

As in 1965, utilities continued to make their decisions on the basis of economics. Of particular significance during the year was the TVA's evaluation of coal and nuclear plants, followed by its decision to construct two 1,065 Mwe nuclear units at Browns Ferry, Tennessee. The TVA published the results of its evaluation[1] which showed the nuclear plants fuel cost approximately seven cents per million Btu less expensive and the bus bar power cost 0.46 mills per kilowatt hour less expensive than the competitive coal fired plant. Many utilities which announced nuclear plants during the year, on the basis of internal evaluations, did not obtain fossil fuel bids and many ordered repeat plants. Commonwealth Edison ordered an additional plant, the 715 Mwe Dresden #3 unit, early in 1966 and then announced the signing of contracts for two additional 715 Mwe plants to be constructed in conjunction with its neighboring utility, the Iowa-Illinois Gas & Electric Company.

The TVA's award of the two unit 2,130 Mwe Browns Ferry station to General Electric marked not only one of the largest awards as of September 1966, but also the entry of nuclear power in another low cost fossil fuel area. TVA's acceptance of GE's twelve year fuel contract also marked the first instance where a utility accepted such an arrangement, although many other utilities have been considering similar arrangements offered by other reactor suppliers. The fuel proposals, however, give TVA the right to cancel the fuel contract effective in 1975 or later, and presumably TVA could take advantage of such a circumstance should it find other qualified suppliers with lower cost fuel.

1/ Office of Power, Tennessee Valley Authority, "Comparison of Coal-Fired and Nuclear Power Plants for the TVA System", Chattanooga, Tennessee, June, 1966.

While Jersey Central's decision in 1963 to construct Oyster Creek may be considered the first major nuclear power plant anticipated to become competitive in high-cost areas of the United States, TVA's award during 1966 is a similar milestone in that it indicated that large nuclear plants can be competitive in low-cost fossil fuel areas of the United States. Areas of the country having mine-mouth plant sites and areas of the country having direct access to natural gas continue to remain fossil fuel supply areas.

Nuclear plant announcements through 1965 and for the first nine months of 1966 are shown in Tables IV-5 and IV-6. Chart IV-B shows the locations of these plants. The tables show that over three times as much nuclear capacity was announced in the first nine months of 1966 than in the entire year of 1965. This includes utilities that announced decisions to order nuclear plants, but have not yet selected suppliers.

Chart IV-B

CENTRAL STATION NUCLEAR-ELECTRIC PLANTS

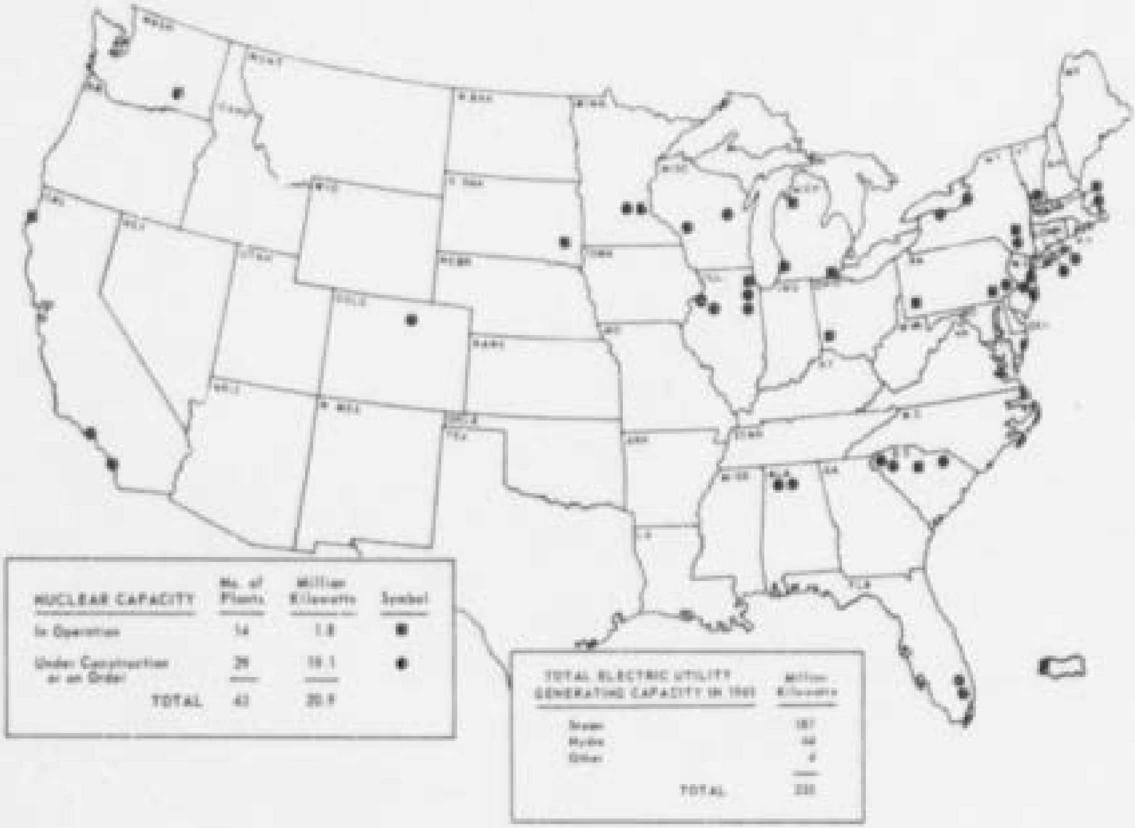

TABLE IV-5

Central Station Nuclear Power Plants

Operating, Under Construction, or on Order

Announced	Utility & Plant	State	Mfgr.	Size (Mwe-Net)	Est. Cost* (Millions)	Year of Initial Operation
1953	Duquesne (Shippingport)	Pa.	W	135[a/]	$ 69	1957
1955	Commonwealth Ed. (Dresden #1)	Ill.	GE	200	51	1959
	Consolidated Ed. (Indian Pt. #1)	N.Y.	B&W	151[b/]	107	1962
	Pwr. React. Dev. Co. (Fermi)	Mich.	PRDC	61	66	1963
				412	224	
1956	Yank. At. El. Co. (Yankee)	Mass.	W	175	39	1960
1957	Northern States (Pathfinder)	S.D.	AC	58	26	1964
1958	Car's-Va. Nuc. Pwr. Assoc. (CVTR)	S.C.	W	14[b/]	24	1963
	Phila. El. (Peach Bottom)	Pa.	GA	40	28	1966
	City of Piqua, Ohio (Piqua)	O.	AI	11	16	1963
	Puerto Rico Wtr. Res. Auth. (BONUS)	P.R.	CE	16	19	1964
	Rur. Coop. Pwr. Assoc. (Elk River)	Minn.	AC	16[b/]	14	1962
	Washington Pub. Pwr. Sup. Sys. (NPR)	Wash.	GE	786	315	1966
				883	416	
1959	Consumer Power Co. (Big Rock Pt.)	Mich.	GE	70	28	1962
	Pac. Gas & Electric (Humboldt Bay)	Cal.	GE	68	24	1963
				138	52	
1960	SCE and SDG&E (San Onofre)	Cal.	W	375	97	1966
1961	Dairyland Power Coop. (LaCrosse)	Wisc.	AC	50	19	1966
1963	Conn. Yank. A. P. Co. (Conn. Yankee)	Conn.	W	462	87	1967
	Los Angeles D. W. & P. (Malibu)	Cal.	W	462	83	1970
	Jersey Central (Oyster Creek)	N.J.	GE	515	66	1967
	Niagara Mohawk (Nine Mile Pt.)	N.Y.	GE	500	87	1967
				1,939	323	
1965	Boston Edison (Unnamed)	Mass.	GE	540	65	1971
	Commonwealth Ed. (Dresden #2)	Ill.	GE	715	79	1968
	Northeast Utilities (Millstone Pt.)	Conn.	GE	549	81	1969
	Consolidated Ed. (Indian Pt. #2)	N.Y.	W	873	106	1969
	Fla. Power & Lt. (Turkey Pt. #3)	Fla.	W	722	70	1970
	Fla. Power & Lt. (Turkey Pt. #4)	Fla.	W	722	63	1971
	Pub. Ser. Co. of Colo. (Fort St. Vrain)	Colo.	GA	330	69	1971
	Rochester Gas & Elect. (Robt. E. Ginna)	N.Y.	W	420	63	1969
				4,871	596	

a/ Turbine-Generator capacity currently limited to 90 Mw.

b/ Does not include capacity added by fossil fueled superheater. Net electrical output with superheater is: Indian Point -- 270 Mw; CVTR -- 17 Mw; Elk River -- 22 Mw.

* Total construction cost. Land, fuel and transmission plant excluded.

TABLE IV-6

Central Station Nuclear Power Plants

Contracts Awarded in 1966<br>Utility & Plant	State	Mfgr.	Size (Mwe-Net)	Est. Cost* (millions)	Year of Initial Operation
Commonwealth Ed. (Dresden #3)	Ill.	GE	715	$ 81	1969
Carolina Power & Lt. Co. (Robinson #2)	S.C.	W	663	76	1970
Consumers Power Co. (Palisades)	Mich.	CE	710	75	1970
Wisc. Mich. Power Co. (Point Beach)	Wisc.	W	450	60	1970
Common. Ed./Iowa-Ill. (Quad Cities #1)	Ill.	GE	715	90	1970
Common. Ed./Iowa-Ill. (Quad Cities #2)	Ill.	GE	715	80	1971
Northern States (Monticello)	Minn.	GE	472	74	1970
T. V. A. (Browns Ferry #1)	Ala.	GE	1,065	124	1970
T. V. A. (Browns Ferry #2)	Ala.	GE	1,065	123	1971
Duke Power Company (Lake Keowee #1)	S.C.	B&W	822	78	1971
Duke Power Company (Lake Keowee #2)	S.C.	B&W	822	79	1972
Vermont Yankee N. P. Corp. (Vt. Yankee)	Vt.	GE	540	88	1970
PSEG/PE/ACE/DPL** (PSEG Area)	N.J.	W	993	125	1971
PSEG/PE/ACE/DPL** (Peach Bottom)	N.J.	GE	1,064	125	1971
PSEG/PE/ACE/DPL** (Unnamed)	N.J.	GE	1,064	125	1973
			11,875	1,403	

Announced as Planned for Nuclear Power - (Contracts Not Awarded)

	Size (Mwe-Net)	Year of Initial Operation
Maine Yankee A. P. Co. (Bailey Pt.)	700	1972
Long Island Lighting Co. (Shoreham)	500	1973
PSEG/PE/ACE/DPL**	993	1974
SCE and SDG&E (Bolsa Chica)	900	1971
Los Angeles D. W. & P. (Bolsa Chica)	900	1972
Virginia Electric & Power	750	1971
Omaha Public Power Dist.	400	1971
Consumers Publ. Power Dist./Iowa Power & Lt.	800	1972
Pacific Gas & Electric	800/1050	1971

* These are mostly unofficial figures taken from various published sources and are not necessarily on comparable bases.

** Public Service Electric & Gas, Philadelphia Electric, Atlantic City Electric and Delmarva Power & Light.

TABLE IV-7

Announcements of New Steam-Electric Plant Additions
As Reported by "Electrical World"

1 9 6 5

Electrical World Issue Date	Utility - Plant	Capacity, Mwe Fossil	Nuclear
1/18	Pub. Ser. Electric & Gas (Hudson #2)	600 C	
2/1	Five Owners (Conemaugh)	1,800 C	
2/8	New Orleans Pub. Ser. (Michaud)	587 G	
2/15	Commonwealth Ed. (Dresden #2)		714
3/1	Cleveland El. Illum./Ohio Ed. (Sammis)	1,200 C	
3/22	Pub. Ser. Co. of Colo. (Fort St. Vrain)		330
4/5	Cincinnati G. & E. and Others (Beckjord)	434 C	
4/26	Detroit Ed./Consumers Pwr. (St. Clair)	500 C	
"	Jacksonville, Florida (Northside)	276 G/O	
"	Carolina Power & Lt. Co. (Robinson)	650 C	
5/3	Iowa Southern Utilities (Burlington)	200 C	
5/10	Rochester G. & E. (Robert E. Ginna)		400
"	Puerto Rico (San Juan)	220 O	
5/17	San Antonio, Texas	256 G	
5/24	Penna. Power & Lt. (Brunner Island)	765 C	
5/31	T. V. A. (Browns Ferry)	2,260 C	
7/26	Central Power & Lt. Company	238 G	
8/2	Fla. Power Corp. (Crystal River #2)	421 C	
8/9	NYSE&G/Pa. El. Power (Homer City)	1,280 C	
8/16	Boston Edison		600
"	Pub. Ser. Co. of N. H. (Merrimack)	350 C	
8/23	Detroit Edison (Harbor Beach)	121 C	
"	Virginia El. & Power (Chesterfield)	690 C	
"	Detroit Edison (St. Clair)	500 C	
9/13	Northeast Utilities (Millstone Pt.)		600
11/1	Louisiana Power & Lt. (Little Gypsy)	560 G	
"	New England Electric (Brayton Point)	640 C	
11/22	Fla. Power & Lt. (Turkey Pt.)		1,520
"	Ohio Power Co. (Muskingum River)	615 C	
11/29	Consolidated Ed. (Indian Pt.)		873
12/13	Oklahoma G. & E. (Horseshoe Lake)	415 G	
"	Dairyland Power (Genoa)	350 C	
12/20	Vermont Yankee N. P. Corp.		450
	Total Each	15,928	5,487
	Total - %	74%	26%

1965 TOTAL FOSSIL & NUCLEAR- 21,415

C - Coal G - Gas O - Oil

TABLE IV-8

Announ:cements of New Steam-Electric Plant Additions
As Reported by the Trade Press

1 9 6 6

Utility - Plant	Capacity, Mwe Fossil	Nuclear
Maine Yankee A. P. Co. (Bailey Pt.)		700
Consolidated Ed. (Arthur Kill)	500 C/O	
Commonwealth Ed. (Dresden #3)		715
Consumers Power Co. (Palisades)		710
Carolina Power & Lt. Co. (Robinson)		652
Cincinnati Gas & Electric and Others (Stuart)	2,000 C	
American Electric Power (3 units)	2,400 C	
Long Island Lighting Co. (Shoreham)		500
Wisc. Mich. Power Co. (Point Beach)		454
Georgia Power Co. (Harlee Branch)	490 C	
Cleveland El. Illum. (Avon Lake)	600 C	
Kansas City Power & Lt. (Hawthorne)	500 C/G	
Duquesne Light	500 C	
Allegheny Power (Hatfield Ferry)	1,080 C	
Common. Ed./Iowa-Ill. (Quad Cities)		1,430
Northern States (Monticello)		472
WEST (Four Corners)	1,510 C	
WEST (Kaiparowits)	1,500 C	
WEST (Mohave)	1,500 C	
PSEG/FE/ACE/DPL		1,800
Utah Power & Lt. (Naughton #2)	220 C	
T. V. A. (Browns Ferry)		2,200
Virginia Electric & Power		750
Omaha Public Power Dist.		400
Union Electric Company (Labadie)	1,200 C	
Minnkota Power Cooperative	200 C	
Consumers Pub. Power Dist./Iowa Power & Lt.		800
Duke Power Company (Lake Keowee)		1,644
Associated Electric Co-op (Mo.)	250 C	
South Mississippi Electric & Power Assoc. (4 units)	64 G	
Arkansas Electric Co-op	125 G	
PSEG/PE/ACE/DPL		2,000
Illinois Power Company (Baldwin)	550 C	
SCE and SDG&E/LADWP (Bolsa Chica)		1,800
Pub. Serv. Co. of Okla. (Northeastern #2)	430 C	
Southwestern El. Power Co. (Wilkes)	350 G	
Wisc. Power & Lt. (Edgewater)	300 C	
Western Power & Gas (Ft. Dodge)	157 O/G	
Tampa Electric (Big Bend #1)	425 C	
Pacific Power & Lt.	250 C	
Total Each	17,101	17,742
Total - %	49%	51%

1966 TOTAL FOSSIL & NUCLEAR - 34, 843

A comparison, based on published information, of fossil fuel versus nuclear announcements is also interesting and is shown as Tables IV-7 and IV-8. In 1965 nuclear plants apparently captured about one-fourth of the market for new power plants, while in the first nine months of 1966, nuclear plants appear to have captured approximately one-half of the market.

This rapid growth rate probably can be attributed to several factors. The economic competitiveness of nuclear power, coupled with the general utility management recognition of this fact, was probably the most important factor. Prior to 1965, some utilities were relectant to commit nuclear plants in borderline economic cases. However, announcements early in 1965 by utilities which had experience with nuclear power plants no doubt served to bolster utility confidence.

It appears that once utilities realized that those with first-hand experience with nuclear plants were adopting them with confidence, many accelerated their planning in order to allow for the longer lead time currently required to bring a nuclear plant into operation. Increased demands for electricity probably also helped to make utilities more conscious of their expansion plans. In addition, many utilities had concluded a round of hard negotiating with coal suppliers and had clear indications of fossil fuel prices. Increased emphasis on air pollution and attractiveness of plant also may have contributed to the nuclear decisions.

All of the above factors combined to make the climate for nuclear plant orders very favorable and orders were at an all time high. Should some of these favorable factors not continue, this dramatic pace of orders could slow in 1967.

2. Estimates of Growth

The increase in nuclear plant orders resulted in most forecasters revising their estimates upward. Dr. Glenn T. Seaborg, Chairman of the AEC, traced the history of AEC forecasts and released revised estimates for the AEC in June.2/ In the

---

2/ Remarks by Dr. Glenn T. Seaborg, "Nuclear Energy and the Generation of Power", at National Association of Manufacturers' Conference on Industrial Science and Technology, Washington, D. C., June 7, 1966, AEC Public Release S-20-66.

1962 Civilian Power Report to the President, the AEC predicted that there would be 40 thousand megawatts of installed nuclear capacity by 1980. In 1964, only two years after the 1962 forecast, the AEC predicted that by 1980 there would be between 60 thousand and 90 thousand megawatts of installed nuclear capacity. The AEC now estimates that by 1970-71 there will be more than 10 thousand Mwe of nuclear electric generating capacity in the United States, and by 1980 there will be between 80 thousand and 110 thousand Mwe. The new and old estimates are shown in Chart IV-C. The General Electric Company also revised its estimates during the year, increasing them to 105 thousand installed Mwe in 1980.

Chart IV-C

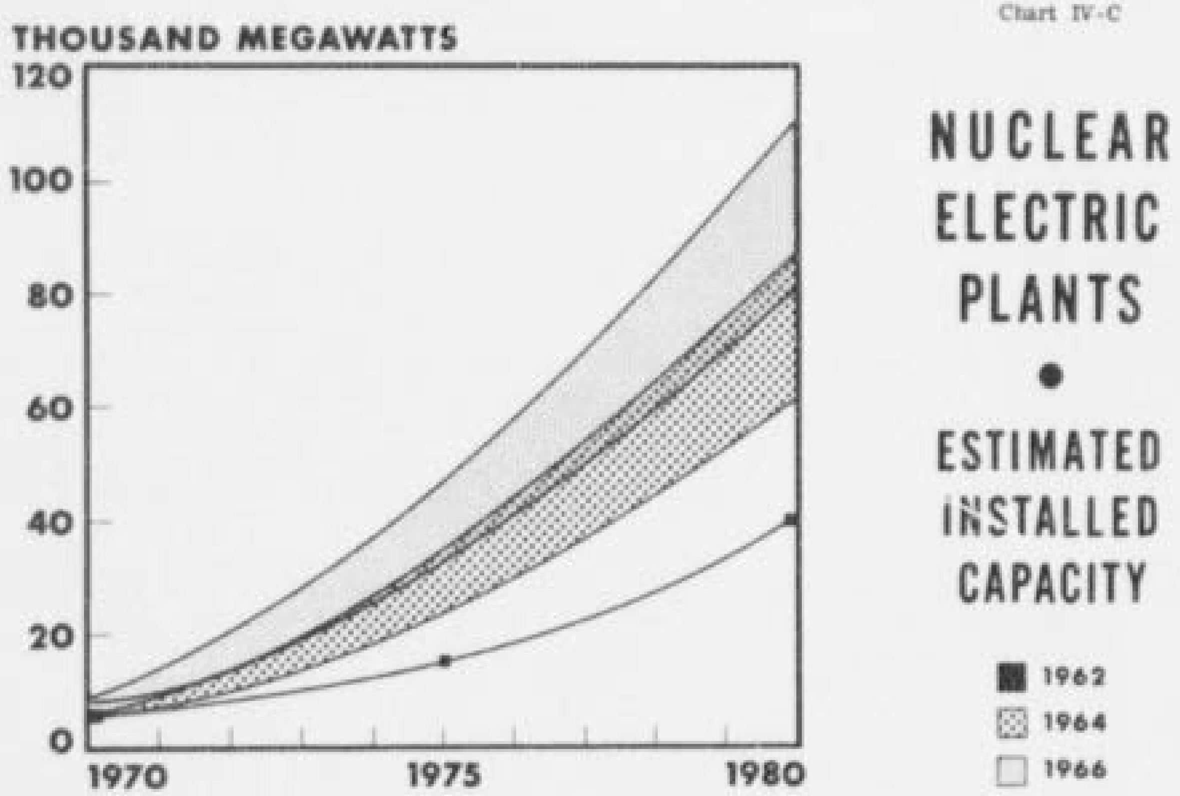

One factor which may have been partly overlooked in the earlier estimates and which may have contributed to the fact that they were low has been the trend to larger plant sizes, pooling of loads and the construction of plants jointly owned by several utilities (undivided interest plants). The success of this type of arrangement was particularly illustrated in New England where the construction of plants continued with the announcement of Maine Yankee early in the year and Vermont Yankee in August; and in the Middle Atlantic States where Public Service of New Jersey, Philadelphia Electric, Delmarva Power and Light and Atlantic City Electric announced their joint interest in two 993 Mwe and two 1064 Mwe nuclear units. This trend is expected to continue and will enable the smaller utilities to take advantage of large plant sizes.

Chart IV-D shows estimated annual energy requirements for electric generation. Chart IV-E shows the estimated increases in steam electric capacity. These charts illustrate the magnitude of future energy and capacity demands.

Because of the above developments, the Commission is now updating the 1962 Civilian Power Report to the President. This should be completed next year.

3. Industrial Structure

In accordance with Section 1.b. of the Atomic Energy Act, the Commission has continued to encourage an active viable and competitive nuclear industry. The present backlog of orders for nuclear power plants is enabling many firms to make future plans based upon reliable predictions of future business. While some segments of the industry still lack enough business to be self-sustaining, future prospects are considerably brighter and companies are now in a better position to make more positive plans for their future role in the nuclear field.

During the year the only major withdrawal in the industry in the area of civilian power was that of Allis-Chalmers as a reactor manufacturer.

Chart IV-D

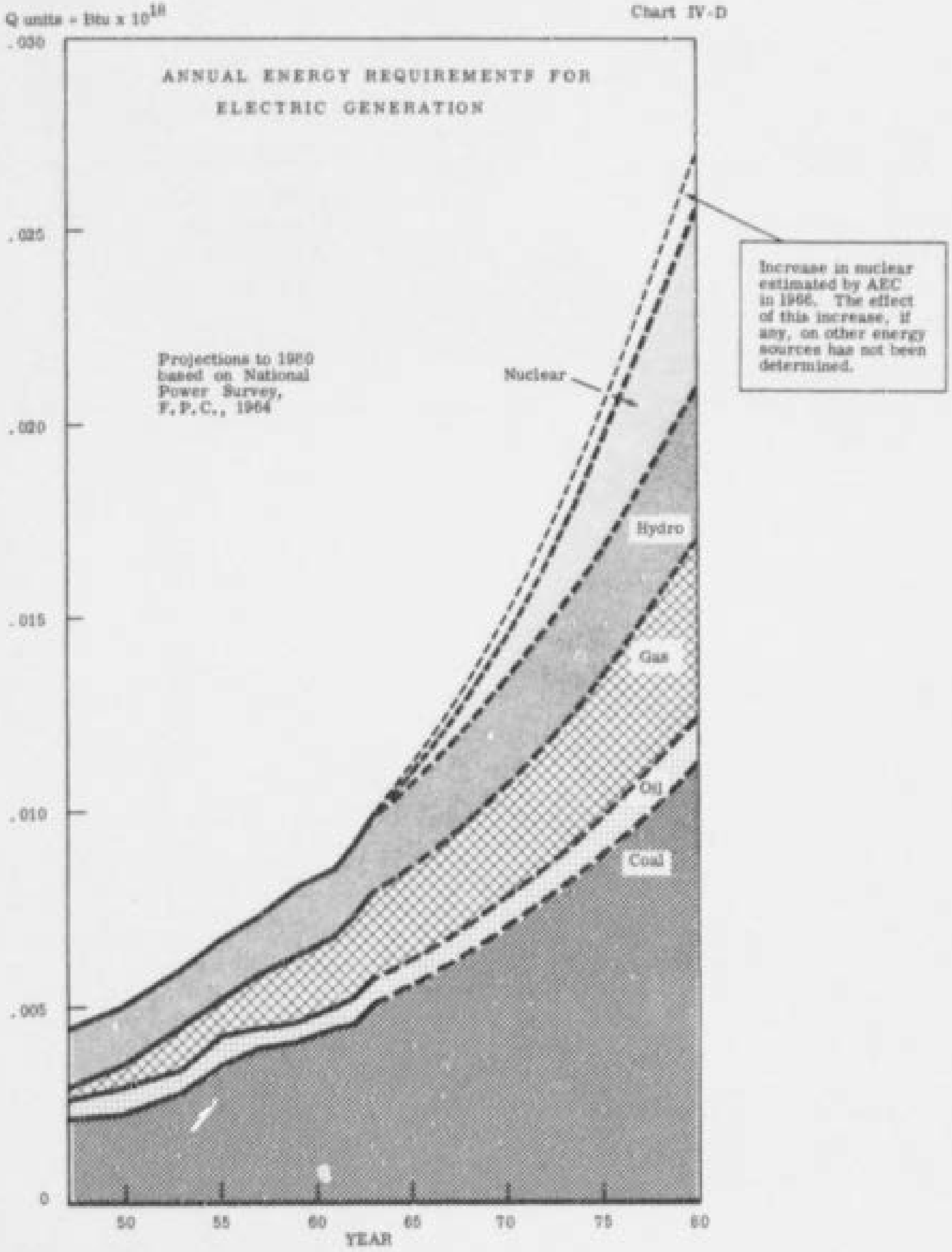
Q units = Btu x 10^18
.030
.025
.020
.015
.010
.005
0
ANNUAL ENERGY REQUIREMENTS FOR ELECTRIC GENERATION
Projections to 1980 based on National Power Survey, F.P.C., 1964
Nuclear
Hydro
Gas
Oil
Coal
Increase in nuclear estimated by AEC in 1966. The effect of this increase, if any, on other energy sources has not been determined.
50
55
60
65
70
75
80
YEAR

Chart IV-E

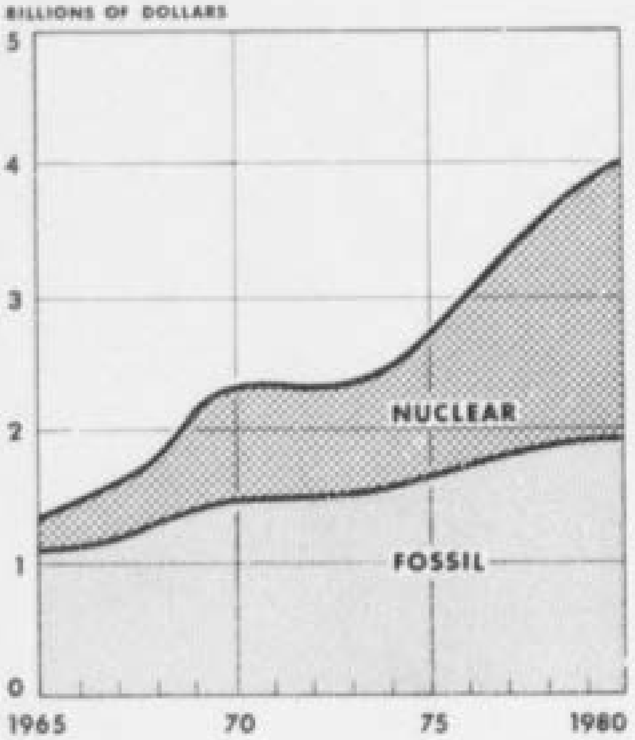

ESTIMATED INCREASES IN STEAM ELECTRIC PLANT CAPACITY

•

PROJECTED ANNUAL EXPENDITURES

a. Commission Actions

The Commission has taken many steps over the years and in cooperation with industry to help define and achieve a competitive industry.3/ The AEC stretch-out program for purchase of uranium ore has served to sustain the uranium industry, while the transition from military to civilian power purchase of uranium takes

3/ Statement of Commissioner James T. Ramey to the JCAE at Hearings on the Development, Growth and State of the Atomic Energy Industry, September 8, 1965.

place. The AEC made a base load spent fuel processing guarantee available to private industry to encourage a reprocessing industry. The AEC is taking into account the interest and investment which members of industry have made in the civilian nuclear industry when awarding operating contracts. Informal discussions are continuing to be held with industrial organizations to learn of their plans and problems. The Advisory Committee on Isotopes and Radiation Development in the last few years was expanded to bring in representatives from appropriate segments of the industry.

The AEC has always encouraged close cooperation and good working relationships between the National Laboratories and the nuclear industry, and is aware of the need for insuring that industry becomes involved in, and assumes the responsibility for, many of the tasks that are parts of major programs. This support will continue, and be augmented, as the need exists to assist industry in alleviating problems which occur in the developing nuclear industry.

b. Important Events

Several noteworthy events occurred during the year that helped to expand the competitive base of the industry.

Contracts were awarded for construction of nuclear power plants to Combustion Engineering and Babcock & Wilcox. These two manufacturers are thus assured of the experience of constructing large nuclear power stations, and, thereby, having the opportunity to become established competitors of General Electric and Westinghouse. General Atomic continues to be a competitor in the reactor field, but, of course, its position depends on a considerable extent on the success of the gas-cooled reactor concept. The gas-cooled Peach Bottom reactor is in the process of being started up, and the Public Service Company of Colorado has announced its intention to construct a 330 Mwe gas-cooled power reactor if the Peach Bottom reactor produces favorable results. Thus, there are at lease five potential suppliers of nuclear steam supply systems. In addition, Atomics International is

participating with Combustion Engineering in the development of the organic cooled, heavy water moderated concept and recently announced its intention to devote a major effort to fast reactors.

Another important development was the announcement that the contract for the primary pressure vessel of the Northern States, 472 Mwe, Monticello Power Plant had been awarded to Chicago Bridge & Iron and that it would be assembled by field fabrication techniques. The demonstration of field fabrication techniques to assemble primary pressure vessels will permit the construction of large nuclear power plants at locations not accessible to deep water. It seems probable that Babcock & Wilcox is offering, and Combustion Engineering is prepared to offer, similar services.

The trend away from turnkey arrangements for the construction of nuclear power plants was also evidenced during the year. Several utilities purchased plants on a non-turnkey basis, and the General Electric Company announced that it would no longer bid on a turnkey basis. Helping to change the trend were the positions of Babcock & Wilcox, Combustion Engineering and Westinghouse, all of whom sold plants during 1966 on a non-turnkey basis. Thus, the construction of nuclear power plants seems to be returning to the normal utility, architect-engineer, constructor, supplier relationship which existed before the advent of nuclear power plants.

There are several companies either prepared or preparing themselves to enter the fuel supply field, but this will not be possible until utilities are willing to purchase replacement cores from other than the reactor manufacturers. Commonwealth Edison's award of a replacement core for the General Electric Dresden #1 reactor to United Nuclear was the first real indicator of this possible trend. The full significance of this will probably not be felt by the industry for several years. Commonwealth Edison's decision to buy this core from other than a reactor supplier should encourage other utilities to consider this same approach to replacement cores, thus broadening the industry.

c. Fuel Cycle Arrangements

It is interesting to note that during the year utilities began to develop patterns for requesting fuel cycle proposals. As examples, three approaches are described below:

(1) Complete fuel cycle service. Under this arrangement the core supplier takes responsibility for all fuel cycle steps except ownership of core while in the utility's plant. Utility pays core supplier for energy extracted from core.

(2) Initial fuel supply. The core supplier delivers a fabricated core to the utility and guarantees the performance of the core. The utility is responsible for handling all steps of spent core recovery.

(3) Fuel fabrication service. The utility handles purchasing of uranium ore, conversion to UF_6 and enrichment, and delivers enriched UF_6 to core supplier. The core supplier purchases zirconium tubing and fabricates the core. The utility pays the core supplier for zirconium tubing and fabrication of the core. Core supplier guarantees performance of core and utility handles all steps of the recovery of the spent core.

It should be realized that there are many variations of the above pattern and that each utility uses the method best suited to its system.

d. Status of Competition in Nuclear Fuel Cycle

The status of competition in supplying the various steps of the nuclear fuel cycle is shown in Table IV-9, on the following page, which lists the major steps of the nuclear fuel cycle and the reactor manufacturing and fuel cycle suppliers capabilities to perform these steps.

Only two companies in the mining and milling operation are shown, because these are the only two which have carried their operation beyond the milling of ore to U_3O_8 (yellow cake). However, there are sixteen domestic uranium processing mills with about 90% of the uranium reserves owned by companies engaged in both mining and milling and about 10% by independent mines.

[illegible]

NUCLEAR FUEL CYCLE STEPS BY SUPPLIERS

TABLE IV-4

Approximate Distribution of Costs %		Reactor Suppliers: Atomics International	Babcock & Wilcox	Combustion Engineering	General Dynamics*	General Electric	Westinghouse	Fuel Cycle Suppliers: Allied Chemical	Kerr-McGee	[illegible]	National Lead	Nuclear Fuel Services	NUMEC	United Nuclear	AEC
100%	FABRICATION OF CORE														
20	Exploring, mining & milling ore to U_3O_8								X					X	
3	Conversion of U_3O_8 to UF_6							X							X
31	Enrichment of UF_6					A		A				A			X
	Manufacture of Zr sponge and ingot														
	Fabrication of Zr ingot into tubing													O	
26	Conversion of UF_6 to UO_2					X			X		X	X	X	X	
	Pelletizing of UO_2	X	X			X	X		X			X	X	X	
	Fabrication of fuel elements	X	X	X	X	X	X				X		X	X	
	Fuel Warranty		X	X		X	X					O	O	X	
	MANAGEMENT OF FUEL IN REACTOR														
	Design of fuel management schemes		X	X	X	X	X				X		X	X	
	RECOVERY OF SPENT CORE														
1	Spent fuel shipping					X	X				X	X			X
12	Reprocessing fuel elements and waste disposal					O		O				X			X
2	Conversion of $UO_2(NO_3)_2$ to UF_6 for re-enrichment														X
unknown	Conversion of $Pu(NO_3)_4$ into PuO_2		X			X						X	X	X	X
unknown	Fabrication of Pu fuel elements		X			X						X	X	X	X
unknown	Fission product recovery and encapsulation									X					X

*General Dynamics is selling a gas-cooled reactor in which some of these steps are not the same.

X - Company now capable of performing these steps.

O - Company planning additional step.

A - Study teams from GE/Allied Chemical and Electro-Nucleonics/W. R. Grace (majority owner of NFS) are working on centrifuge enrichment methods.

The companies engaged in the remaining steps of the nuclear fuel supply cycle are of three types; reactor manufacturers (all of which also manufacture fuel for their reactors), firms that manufacture either fuel cores or perform certain steps of the nuclear fuel cycle and firms which perform certain individual services which support the nuclear fuel cycle.

The conversion of U_3O_8 to UF_6 is presently performed by only one firm, the Allied Chemical Corporation. However, there is no doubt that other companies will be entering this field as soon as the market materializes.

The manufacture of zirconium sponge and ingot is presently performed by Wah Chang and Carborundum Metals Climax. The fabrication of zirconium ingot into tubing is performed by three competitors: Harvey Aluminum,

Wolverine Tube and Superior Tube. In addition, a Swedish firm, The Sandvik Steel Works Company, announced in Stockholm that its wholly owned U. S. subsidiary, Sandvik Special Metals Corporation, would build a zirconium tubing plant in the United States.

Zirconium tubing is purchased by fabricators of commercial reactor cores to specifications which the core fabricator specifies to meet the guarantees he provides to the utility. Conversion of UF_6 to UO_2, pelletizing of UO_2 and fabrication of fuel elements are all steps also presently performed or carefully controlled by the core fabricator in order to meet his warranty.

Reactor manufacturers and core suppliers are presently able to undertake the management of fuel in a reactor, and it is probable that consulting firms and the utilities themselves will undertake this step as the nuclear power industry matures.

Spent fuel shipping services are available from reactor suppliers and fuel cycle suppliers, as well as from commercial transport firms. The reprocessing of fuel elements and handling and storage of the residual radioactive material is presently being performed by only one commercial firm, Nuclear Fuel Services. While the AEC will no longer receive reactor fuel for processing if the fuel can be processed commercially at reasonable charges, it will, if requested by a reactor operator, review reprocessing charges and other terms and conditions offered by NFS and determine if they are reasonable. If they are determined not to be reasonable, the AEC will negotiate, pursuant to its published Federal Register Notices on this subject, with the reactor operator for acceptance of the material for a financial settlement.

Both the General Electric Company and Allied Chemical Corporation have indicated intentions to build private reprocessing plants. At present, there is insufficient market to fully load the NFS plant at West Valley, New York, and the timing of the entry of a second reprocessor could be important.

The state of competition in the remaining steps in the recovery of a spent core, such as conversion of uranyl nitrate to UF_6 for re-enrichment, conversion of plutonium nitrate into plutonium oxide, fabrication of plutonium fuel elements and fission product recovery and encapsulation, is not yet too well defined. The AEC expects that industrial interest in these steps will become firm as power reactor fuels begin to be processed in greater volume.

e. Industry Study

The AEC has been very interested in stimulating the growth of a competitive industry. In this connection, there has been some concern in regard to the vertical integration of suppliers, as well as the inability of others to survive because they perform insufficient steps in the fuel cycle. The Atomic Energy Commission and the Department of Justice are presently working on initiation of a joint study to make a review of the nuclear industry, with the objective of possibly developing guidelines which could serve to assist industry in its future operations.

4. Desalting

Desalting attracted major interest in 1966 and was highlighted by a cooperative agreement for construction of the world's largest nuclear fueled desalting and electric power plant in the Los Angeles area. The significance of this agreement was emphasized by President Johnson's statement that the agreement is a dynamic step forward in the development of processes for economical conversion of saline water for beneficial consumption and that the proposal was of paramount importance in the effort to find new ways and means of conserving and increasing the water resources of the nation.[4/]

4/ AEC Public Release J-190, dated August 10, 1966.

The plant will produce 150 million gallons of fresh water per day, along with 1,800 megawatts of electric power. The Metropolitan Water District of Southern California, the Federal Government, represented by the Department of the Interior and the Atomic Energy Commission, and the electric utilities of the Los Angeles and San Diego area will finance the construction of the plant. Plans presently call for locating the plant on a 40-acre man-made island 2,800 feet offshore between Huntington Beach and Seal Beach, California.

The program of studies and evaluations of the application of nuclear energy to desalting and other processes, initiated at ORNL, are continuing. Promising techniques for dual purpose plant coupling and operating flexibility are being investigated and detailed design and economic data are being developed.5/

Studies of large plant application of interest to foreign countries are also continuing. The Joint U. S.-Mexico Study Team was appointed late in 1965 to study the water and power needs of the Mexico states of Sonora and Baja California and the States of California and Arizona in the U. S. The study is being conducted under the auspices of the International Atomic Energy Agency. Technical representatives of the U. S.-Mexico are currently studying water needs of the area and the feasibility of using a large dual purpose plant located on the northern part of the Gulf to meet these needs.

Kaiser Engineers, in association with Catalytic Construction Company, completed a detailed engineering study of a dual purpose plant to serve the water and power needs of Israel. The study was undertaken jointly by the Governments of the U. S. and Israel. The report6/ issued in January recommended a plant using a light water reactor to produce 200 megawatts salable electric power and 100 MGD of fresh water.

5/ For more information, see statement by Commissioner James T. Ramey at AEC Authorization Hearings for Fiscal Year 1967. AEC Authorizing Legislation for FY-1967, Part 2, pages 610-632.

6/ Engineering Feasibility and Economic Study for Dual-Purpose Electric Power-Water Desalting Plant for Israel, January, 1966, - Report No. -6-1-RE. Available from Clearinghouse for Federal Scientific and Technical Information of the U. S. Department of Commerce, Springfield, Virginia, 22151.

An interdepartmental task force investigated the potential use of large nuclear power desalting facilities to help "drought proof" the northeast metropolitan area. The task force concluded that while large nuclear desalting plants are technically feasible and have reasonable economics, utilization of the Hudson River appeared to be a more attractive immediate solution to augment the area's water supply. The report further concluded, however, that in consideration of the potential advancement both in desalting and nuclear technology, large nuclear desalting plants might be attractive alternatives in the next 10-20 years for this application.

Plans are being initiated to convene an "Water for Peace" conference in Washington, D. C., in May 1967. The conference is being organized by the Department of the Interior with the assistance of several other Government agencies. A portion of the conference will be devoted to desalting activities and will provide an opportunity for industry to participate.

In September, the AEC participated with the Department of the Interior in a joint U. S.-Greek study to investigate the feasibility of desalting as a means for supplying fresh water for the Athens area. Economic attractiveness of combined power generation with desalting will be analyzed. The study will consider both nuclear and fossil fuel heat sources. Studies of alternative sources of fresh water, currently being conducted by the Greek government, will be reviewed in order that desalting economic data will be presented on a comparable basis. A report of this study is expected to be available next year.

The AEC has held discussions with representatives from Tunesia, Italy, Lebanon and other nations, including the IAEA and other international organizations interested in nuclear desalting, and has continued to demonstrate willingness to share its technology with its friends abroad.

5. Small Reactors

While large power reactors continue to receive the bulk of the attention, progress has also been made in smaller reactors for power generation. The increasing volume of nuclear business should assist in broadening the base of the nuclear industry and lowering unit costs. This should help to lower the costs of small plants and decrease the size at

which nuclear plants can compete with fossil fuels. The Commission also has a study of small reactors under way at the Chicago Operations Office which is presently nearing completion. It is expected that there will be continuing interest in small reactors, because many utilities and countries around the world do not have sufficient loads to utilize large power plants on their system. The potential market for small reactors could be significant in many regions of the world.

6. Export Sales

Table IV-10 shows a list of power reactors which have been sold by U. S. firms to foreign utilities. During the first nine months of 1966, only one power reactor was ordered from a U. S. manufacturer for use abroad. This was an order on GE for a boiling water reactor for a 300 Mwe electric power plant to serve Bern, Switzerland.

TABLE IV-10

Power Reactors for Export

Owner	Country	Mfgr.	Mwe.	Start-up
RWE	Germany	GE	16	1960
SENN	Italy	GE	150	1963
JAERI	Japan	GE	12	1963
SELNI	Italy	W	240	1964
SENA	France	W	210	1966
Gov't-owned	India	GE	380	1967
KRB	Germany	GE	237	1966
Zorita	Spain	W	153	1968
Gov't-owned	Pakistan	W	70	1969
NOK	Switzerland	W	350	1969
Nuclenor	Spain	GE	440	1970
JAPCO	Japan	GE	310	1969
BKW	Switzerland	GE	300	1971

7. Advanced Converters

In order to extend uranium reserves, assure better utilization of uranium and minimize the effect of higher cost ore, the Commission is continuing its development of advanced reactors. The three advance systems being pursued with this objective in mind are the high temperature gas cooled reactor, the heavy water organic cooled reactor and the large seed blanket reactor.

8. Breeders[7/]

The primary objective of the civilian power reactor program is to achieve widespread commercial use of nuclear energy while fully exploiting the energy available in our resources of uranium and thorium. The meeting of this objectives will require the development of breeder reactors and their successful introduction into commercial use. With the utility industry's adoption of slightly enriched water reactors for commercial use, the Commission considers the fast breeder program one of its highest priority missions. Based on an assessment of the various breeder concepts and the over-all development of the liquid metal technology, the Commission has decided to focus on the sodium cooled fast breeder reactor concept. However, the Commission is also looking at other concepts such as steam and gas cooled fast reactors and molten salt thermal reactors.

Reviews are being made in each technical area of the Liquid Metal Fast Breeder Reactor (LMFBR) program by AEC's Division of Reactor Development and Technology. Industry

---

7/ Testimony by Milton Shaw, Director of Division of Reactor Development and Technology, AEC, before the Congressional Joint Committee on Atomic Energy at Hearings on AEC Authorizing Legislation for Fiscal Year 1967, page 719. Also see a talk given by Mr. Shaw, "Fast Breeder Reactor Program in the United States," British Nuclear Energy Society Conference on Fast Breeder Reactors, London, England, May 17, 1966, AEC Public Release No. IN-688.

is contributing to these reviews. The initial reviews on fuels and materials, physics and associated facilities have been completed and were published in early 1966.[8] Reviews are continuing in the areas of fuel reprocessing and recycling, safety, components, instrumentation and control, sodium technology, fuel handling, plant design and in each case, the related facilities. Though development work is continuing in each of the other areas, and has in part been substantially augmented, the results of each formal review are required to bring into proper perspective the parts requiring strengthening and/or augmentation, including interactions with other program elements which may be affected by the review.

In these reviews, considerable emphasis is being placed on assessing the specific status of the program, defining the program needs and initiating those actions required to strengthen the fast breeder reactor program.

In its earlier reviews, the Commission determined that there was a significant lack of realistic irradiation environment test space. In recognition of this, the AEC has placed a high priority on the design and construction of a Fast Flux Test Facility (FFTF). The major objective of this facility is to provide an adequate controlled and instrumented environment, in a representative LMFBR fast flux, for test instrumented fuel specimens, fuel rods, fuel subassemblies and clad materials.

The results of 1,000 Mwe reactor studies performed by industrial firms within the last few years will be factored into these follow-on studies initiated by the Commission. The results of these studies will assist in making determinations regarding the application of resources to those areas most likely to result in the greatest over-all benefit to the program.[9]

8/ WASH-1065, "Liquid Sodium Metal Cooled Fast Breeder Reactor (LMFBR) Program", WASH-1066, "Reactor Physics Efforts Required in Support of the Fast Breeder Development Program", available from the Clearinghouse for Federal Scientific and Technical Information, National Bureau of Standards, U. S. Department of Commerce, Springfield, Virginia, 22151, for $1.00 and $3.00, respectively.

9/ A discussion of the types of reactors mentioned here and their differences will be found in a talk by Ernest B. Tremmel to the National Coal Association, June 14, 1965.

C. Civilian Maritime Reactors

Over the past year, the Commission has continued to study the application of nuclear power to the civilian maritime industry. The Commission has continued to advocate a two-pronged approach: (1) cooperation with the Maritime Administration in that agency's nuclear shipbuilding program; and (2) research and development by the AEC directed toward an advanced maritime reactor plant, utilizing a land-based test facility. The objective of this approach is to develop an economical, reliable, and safe maritime plant that will be competitive with fossil-fueled plants. A major difficulty has been the absence of a clear definition of short and long range civilian maritime shipbuilding needs, an understanding that would permit establishing specific objectives and goals for the R&D work.

A major effort this year has been directed toward a joint study of the civilian nuclear merchant marine program by the AEC, the Maritime Administration and the Department of Defense. At the time this report was written, the study group had completed its considerations, but its report had not been released.

The only specific interest from ship operators continued to come from American Export Isbrandtsen Lines (AEIL). Early in 1966, AEIL submitted a revised application for a construction differential subsidy covering three "high speed super vessels". As this report went to press, this application was still pending.

Meanwhile, the Savannah completed an annual overhaul on schedule (June 2 - July 1) and is continuing in operation on her original fuel loading. This original loading is expected to last through 1968, providing power for more than a quarter million miles of operation. The Savannah is also providing training for the crew of the Otto Hahn, a new nuclear merchant ship under construction in West Germany. The Savannah's power plant was built by Babcock & Wilcox, and the plant for the Otto Hahn is being built by a B&W affiliate in West Germany.

In addition to B&W, Combustion Engineering, United Nuclear and Westinghouse have maintained active interest in the application of nuclear plants for the civilian maritime program.

It may well be that the construction of advanced merchant ships in this country will have to await a resolution of the broader problems facing our maritime program. However, there are indications that propulsion plants for the next generation of American merchantmen may be nuclear powered.

Nuclear Powered Icebreaker. The feasibility of using nuclear power for icebreakers is being studied by NUS Corporation (in association with New York Shipbuilding and Hydronautics), under a Coast Guard contract. The AEC has met with the Coast Guard for discussions on the prospects for a nuclear icebreaker, and AEC information applicable to the Coast Guard's studies has been provided. First results from the studies should become available toward the end of the year.

D. Military Reactors

1. Army Reactors

Over the past 14 years, a joint AEC-Department of the Army program has developed reactors for the production of power to meet defense requirements, other than for naval propulsion, air and space applications. Over the past four year, five such plants have provided power for military operations in locations spread throughout the world. Today, four of these continue to provide experimental and operating data necessary for the design of more advanced plants. In addition, a fifth plant (barge mounted) is undergoing initial testing this year at Ft. Belvoir, Virginia.

Originally designed to provide the military services with nuclear power plants which could further the mission of the Armed Forces operationally, logistically or economically, this program at one time was at a $13 million annual cost for reactor development alone. Approximately $80 million has been spent on Army reactor work over the past 12 years. The plants developed under this program are small, compact and readily transportable. In common with reactor plants for civilian power purposes, they cannot yet compete on an economic basis with conventional plants. Until advanced, more economical designs become available, plants of this type probably will not come into widespread use.

Because the technology of these plants in general has been demonstrated, the Army reactor program, as such, has been reduced to a level which mainly provides support for the operating field plants. The total budget for Fiscal Year 1967 is less than a million dollars.

Table IV-11 lists the operating Army reactor plants with their manufacturers. Nuclear support services in connection with the operation of these plants is provided by NUS Corporation. Aerojet-General has participated in this program in the past in the design and operation of the ML-1, a gas cooled experimental reactor. This project was terminated in October 1965, following advice from the Army that ML-1 type field plants would be too costly to procure. General Motors' Allison Division and United Nuclear also did preliminary work on a military compact reactor (MCR).

TABLE IV-11

Plant Designation	Location	Manufacturer
MH-1A	Barge mounted<br>Ft. Belvoir, Va.	Martin-Marietta<br>" "
PM-1	Sundance, Wyo.	" "
PM-3A	McMurdo Sound, Antartica	" "
SM-1	Ft. Belvoir, Va.	*
SM-1A	Ft. Greely, Alaska	*

*These plants were built by Alco Products, Inc., whose nuclear activities were taken over by Allis-Chalmers. Allis-Chalmers has since left the reactor business.

Although the Army reactor program now represents a specifically limited level of effort, work is beginning at a low level under another AEC program which could provide developments of interest to the Armed Services. This is the terrestrial low power reactor systems program which is directed toward the development of technology for systems from 100 Kwe to 2 Mwe applicable to a wide variety of potential uses. This effort could lead to advanced designs for compact systems for military use. The AEC's program is just getting under way as a part of the Satellite and Small Power Systems Program. Other work along this line is being done by Martin-Marietta under contract to the Department of Defense. Martin calls its approach TURPS for Terrestrial Unattended Reactor Power System. There may be a number of potential uses for reactor plants in this size range.

2. Naval Reactors

At the end of 1966, the United States will have 70 nuclear powered submarines and four nuclear powered surface ships in operation. The four surface vessels constitute a carrier task group. The last of these surface vessels, the frigate Truxtun, which was authorized in 1961, will be completed this year.

This year, advocates of nuclear surface vessels were encouraged by the statement from the Secretary of Defense that three new nuclear powered carriers will be called for over the next several years. Additional nuclear vessels, to make up nuclear-powered task groups, will be needed. Two of these, frigates, have already been authorized (one last year, one this year), and there is considerable reason to believe that increasing percentages of the fleet will have nuclear power from year to year in the future.

In addition to the 70 submarines referred to above, 34 others have been authorized. Most of these are in various stages of construction, and continue to provide a significant market for reactor components.

Westinghouse, General Electric and Combustion Engineering are the reactor designers in the naval program. For a number of years there were four principal fabricators of fuel for Navy reactors. Partly because of substantial increases in core life and a consequent reduction in fuel loadings needed, and partly because other areas appeared more promising for future growth, two of these fabricators withdrew from this field during the past two years. The fuel fabricators remaining in Naval work are Babcock & Wilcox and United Nuclear.

Oceanographic Applications. A seminar on Ocean Needs and Nuclear Capabilities was held in June by the Atomic Industrial Forum. The objective was an exchange of views between potential users and suppliers of nuclear devices. Areas discussed in which atomic energy could prove valuable included deep-sea well drilling operations, the Navy's "Man in the Sea" project, studies of sea farming and underseas mining.

Although small, compact reactor systems for terrestrial use, mentioned elsewhere in this report, are expected to have ultimate applications as power sources for underwater work, the major reactor development effort aimed at ocean bottom exploration is the NR-1 Project. This is a deep submergence submarine for a crew of five, plus two scientists. Capability will be substantially greater than that of any other vehicle developed or planned to date, because of the greatly increased endurance made possible by nuclear power. It will be relatively independent of surface support ships and of relatively unlimited power for propulsion and auxiliary uses.

The NR-1, sometimes referred to as the DSRV or Deep Submergence Research Vehicle, is being designed and built by the Electric Boat Company. Operation is expected in about a year. It is being jointly developed by the AEC and the Department of the Navy, and is a part of the Naval Reactors Program under Admiral Hyman G. Rickover.

E. Research and Teaching Reactors

During the year, there was a resurgence of interest in reactors for research and teaching purposes, with orders for five new facilities being placed over the past year. These were: an AMF Pulstar for North Carolina State University, a General Atomic TRIGA-Mk I for Reed College, an Atomics International C-77 for Brigham Young, a TRIGA-Mk I for Dow Chemical at Midland, Michigan, and a TRIGA-Mk I for the Denver Laboratory of the U.S. Geological Survey.

The principal producers of reactors in this category are listed in Table IV-12, on the following page.

The potential market for these reactors, both at home and abroad, however, still appears to be nearly saturated. Most institutions that can make effective use of these facilities have been able to build and bring them into operation. There are undoubtedly a few institutions which will order new reactors from time to time as their facilities become obsolete. In this regard, the market for expansion and upgrading of existing facilities is expected to provide a limited market in the future.

TABLE IV-12

Producers of Research and Teaching Reactors

Firm	Number of Reactors Domestic	Number of Reactors Foreign
Advanced Technology Labs	4	4
Aerojet-General	17	3
AMF Atomics	9	14
Atomics International	10	6
Babcock & Wilcox	4	3
Combustion Engineering	2	0
General Atomic	18	14
General Electric	8	4
Lockheed	8	0
United Nuclear	1	0
Internuclear Company	1	1
	82	53

In Fiscal 1966, the AEC and the National Science Foundation jointly provided funding which permitted Pennsylvania State University to raise the power level of its reactor from 200 to 1,000 Kwt by installing a TRIGA Core with pulsing capabilities.

In Fiscal 1967, four universities received AEC grants for upgrading reactors in somewhat the same way as Penn State. These were Texas A&M, Utah State, Illinois and Wisconsin. Of 43 schools with reactors, some seven (in addition to those mentioned above) are know to be interested in upgrading their facilities.

F. Space Reactors

The importance which the Commission attaches to the space reactor programs is reflected in the level of support for this work - an amount nearly equal to that spent for research and development on naval reactors or on civilian power.

The past year has seen further advances in the development of nuclear power for space uses. Operational tests of the reactor system, under development for rocket propulsion, have been very successful and indicate the potential for a high degree of reliability. A 500 watt space reactor power system completed a 10,000 hour endurance test - longer continuous operation than any other known reactor.

Although our space program is young and its future difficult to predict, "there is a general agreement that nuclear reactor energy systems for propulsion and also for power will be needed ...(for)... missions beyond the Apollo manned lunar landing mission, involving delivery of very large payloads at the very high velocities that are required in order to achieve distant space objectives."10/ The reason lies in the compactness of atomic energy sources in comparison with the great bulk of chemical fuels that would provide equivalent amounts of energy.

The missions referred to are a number of years in the future (the mid-1970's and beyond) making it difficult to predict when and to what extent industrial opportunities may materialize.

The propulsion and auxiliary power reactors are discussed separately below.

1. Propulsion

The rocket propulsion program is in the stage of developing a sound technology base for future development of specific systems. Successful tests of graphite reactors over the past several years paved the way for tests of a complete engine system in breadboard layout early this year. Next year a complete engine system with components assembled in a configuration more closely approaching a flight configuration will be tested. This program is being carried out under joint sponsorship with NASA by Los Alamos Scientific Laboratory, Aerojet-General and Westinghouse. Work on other more advanced concepts is also under way. The needs of the program should provide opportunities in the future for specialty firms in the fabrication of prototypes and test equipment. For example, advanced technology in the fabrication of Hastelloy-X has resulted in the forging for this program of the largest ingot of this material ever produced in this country. The forging was done by Wyman-Gordon in Worcester, Massachusetts.

10/ Harold B. Finger, Director of AEC's Space Nuclear Systems Division, at hearings before the Congressional Joint Committee on Atomic Energy on AEC Authorizing Legislation for 1967, March 8, 1966.

2. Auxiliary Power

The need for electric power in space and other remote, unattended locations has led to the SNAP (Systems for Nuclear Auxiliary Power) program. This program includes units powered by the heat of decaying radioisotopes, as well as heat from reactors. The radioisotope units are discussed under the Radioisotopes Category following this Reactor Category.

Reactor powered SNAP units are expected to play their greatest role in applications requiring tens of kilowatts or more of power. At low powers, isotope powered units are preferred because of better power-to-weight ratios. For example, in the 500-watt range an isotope system would weigh less than half as much as a reactor system. Three SNAP reactor systems are under development. The experimental reactor of the SNAP 8 system has been tested on land, running continuously for 200 days and producing over 5.1 megawatt hours of thermal power. In 1965, a SNAP 10 500-watt system performed satisfactorily in space for 43 days, before shutting down because of a series of failures in the spacecraft's electrical system. A duplicate system has operated satisfactorily on land for 10,000 hours, longer than any other known reactor test. The SNAP 8 reactor and the SNAP 10 system development contractor is Atomics International.

Work looking toward advanced high temperature, fast reactors has been assigned to the Lawrence Radiation Laboratory. The objective of this program is development of technology for SNAP systems ranging up to ten megawatts in size, intended to meet needs which will become more clearly defined in the mid-1970's. A comprehensive program to develop advanced power conversion technology is being conducted in coordination with NASA and DOD. Other advanced reactor work is being carried out by General Electric (Vallecitos, California) and General Atomic (LaJolla, California) on fuel elements for thermionic reactors and by General Electric (Evandale, Ohio) on high temperature gas cooled reactor technology applicable to space power generation.

3. Industry and Space

The opportunities for firms with highly specialized skills has been referred to above. Any substantial expansion of opportunities over the next few years seems unlikely. Nonetheless, as AEC Commissioner Ramey has indicated, as the development of these devices reach their final testing stages, new uses will probably be seen which had not been visualized earlier. Such uses, of course, would provide increased opportunities in future years.

[illegible]

Aircraft Nuclear Propulsion. Although the AEC's program directed toward nuclear propulsion plants for aircraft was cancelled in 1961, some interest in the concept has continued. The use of nuclear power in heavier-than-air craft continues to be studied by NASA and the Air Force. United Nuclear, Westinghouse, GM's Allison Division and NUS Corporation are participating in this work through study contracts. A dirigible with nuclear power has been proposed by Henry Irwin & Associates of Oklahoma and by Professor Frances Morse of Boston University.

The likelihood of industrial opportunities for work beyond preliminary feasibility studies on reactors for aircraft use appears distinctly limited for at least the next several years.

CATEGORY V
RADIOISOTOPES

During the year, radioisotope-fueled systems for auxiliary power for a variety of uses came into prominence as their value for space missions requiring up to ten kilowatts was recognized by agencies developing space vehicles. The prospective demand for fuel for these systems stimulated a variety of plans for production or separation of isotopes useful for these purposes. It has now been approximately 20 years since the first commercial shipment of radioisotopes from Oak Ridge National Laboratory, and the possibilities for growth in the radioisotopes segment of the nuclear industry continue to appear extremely favorable. In fact, in such major areas as industry and medicine, it may well be that the period of greatest growth is still in the future. Another example, developmental work on a radioisotope-powered cardiac pacemaker is well under way; and studies indicate that a completely implantable artificial heart powered with a radioisotopic source could become a reality.

Although the packaging and distribution of radioisotopes are performed almost entirely by industry, the AEC is still a principal domestic producer and distributor of radioisotopes. However, in the past year industry has continued to add capability and is showing an ever-increasing interest in the production, distribution and use of radioisotopes.

For the purpose of our report we have divided the radioisotope industry into four categories: producers, processors, source fabricators and equipment manufacturers.

A. Producers

A producer is defined as one who can perform target irradiation and may also perform target preparations and separation of the desired radioisotope for ultimate distribution. Radioisotopes are obtained from cyclotron bombardments, reactor irradiations and from high-level waste streams.

1. Cyclotron Radioisotopes

In 1955, the AEC discontinued processing and distributing cyclotron-produced radioisotopes. Thus, except for industry's dependence upon the University of Pittsburgh cyclotron, which

became available this year, or the AEC's 86-inch cyclotron and upon Oak Ridge National Laboratory for preparation of targets, the industry supplies cyclotron radioisotopes. Cyclotron radioisotopes such as Na-22, Co-57 and Rb-84 are sold to radiopharmaceutical suppliers for use in medical, industrial and fundamental research programs. Among the most promising gamma or positron emitters which are not available, but which could be produced by cyclotron techniques, are Carbon-11, Nitrogen-13, Oxygen-15, Iron-55, Mercury-199, Chromium-49, Copper-61, Zinc-62 and Selenium-73.

The growth in cyclotron-produced radioisotopes may be estimated from the AEC revenue for special cyclotron irradiations which amounted to $126,508 in FY-1964 and $258,665 in FY-65.

As a result of work conducted by AEC and industry, a sizeable market (estimated at $0.5 to $1 million per year) has been developed for cyclotron radioisotopes. Up to now, the United States has been the dominant supplier of cyclotron radioisotopes; however, the UK, South Africa and USSR also compete in the world market for these radioisotopes.

The principal industrial suppliers of cyclotron radioisotopes are listed in Table V-1.

TABLE V-1

Principal Industrial Suppliers of Cyclotron Radioisotopes

Abbott Laboratories

Cambridge Nuclear Corporation

New England Nuclear Corporation

Nuclear Science & Engineering Corporation

U. S. Nuclear Corporation (Subsidiary of Isotopes, Inc.)

Nuclear Consultants, Division of Mallinckrodt Chemical Company

2. Reactor Radioisotopes

The AEC is still a principal domestic producer and distributor of reactor-produced radioisotopes. However, the industry continues to show increasing interest in and capability to produce and distribute these radioisotopes. There has been an increasing emphasis on the part of industry to produce higher specific activity material, especially for radiopharmaceutical applications.

A list of companies offering radioisotope target irradiation services is as follows: (1) General Electric (GETR), (2) Union Carbide (Tuxedo, New York), (3) Babcock & Wilcox (Lynchburg, Virginia) and (4) Western New York Nuclear Research Center (Buffalo, New York). In the future, however, additional private reactors, such as power reactors, should become available for large volume and low specific activity radioisotope production. A first approach to the use of power reactors for isotope production was announced this year after AEC approved the production of Co-60 in the Big Rock Point nuclear power reactor. Consumers Power Company is carrying out the production, in cooperation with Neutron Products, Inc., subsidiary of Nuclear Utility Services, Inc., of Washington, D. C.1/

During the year, the AEC formally opened an Isotope Information Center at Oak Ridge National Laboratory to collect, evaluate and disseminate world-wide information on production of radioisotopes and their use in industry and research.2/

Up to 1965, the Commission had withdrawn, on an ad hoc basis, from producing and distributing selected radioisotopes. In March 1965, the AEC adopted and published in the Federal Register, formal policies and procedures for the transfer of commercial AEC radioisotope production and distribution activities to private industry. These policies and procedures

---

1/ See AEC Public Release J-99, dated April 18, 1966.
2/ See AEC Public Release IN-676, dated March 18, 1966.

provide for the orderly withdrawal from this area of activity. During 1965, the Commission withdrew from the routine distribution of six radioisotopes and on March 31, 1966, withdrew from the production and sale of an additional 19. Since 1963, the AEC has withdrawn from routine production of 34 reactor-produced radioisotopes.[3/]

Industrial interest in radioisotopes has increased steadily in the last few years. Although industry is producing and distributing more radioisotopes each year, AEC sales (distributions outside the AEC) have also increased as noted in Table V-2. This increase is primarily due to the distribution of large quantities of Cobalt-60 for irradiators.

TABLE V-2

AEC Sales of Radioisotopes

Fiscal Years	Million Dollars
1963	$1.2
1964	1.3
1965	1.6
1966	2.0

Statistics are not currently available on industrial sales; however, indications are that commercial sales are rapidly increasing.

Effective May 1, 1966, the AEC increased prices for 13 radioisotopes in order to recover full costs of production and distribution.[4/]

In 1961, the AEC withdrew from the routine production and distribution of cobalt-60 with specific activity greater than 30 curies per gram (increased to 45 curies per gram in 1965). Effective December 18, 1965, the AEC

3/ See AEC Public Releases: J-79, dated March 13, 1966; H-14, dated January 19, 1965; G-170, dated July 14, 1964; G-96, dated April 30, 1964; and F-184, dated September 20, 1963.
4/ See AEC Public Release J-80, dated April 1, 1966.

established revisions in its Co-60 price schedule to encourage development of large-scale industrial applications and to assist American firms in competing more effectively in the world market for radiation systems.5/

The AEC's Advisory Committee on Isotope and Radiation Development had reviewed the AEC Cobalt-60 price revisions prior to its acceptance by the Commission and agreed that this was an appropriate action to take to encourage industrial uses and stimulate further development of new applications.

In 1961, both Westinghouse and General Electric started producing limited quantities of high-specific activity Cobalt-60 in their test reactors. The Westinghouse Test Reactor was subsequently shut down and today, General Electric is the only commercial firm engaged in the production of substantial quantities of predominately teletherapy and radiography grade high-specific activity Cobalt-60. As previously mentioned, Neutron Products, Inc., recently initiated a joint program with Consumers Power Company to produce mega-curie quantities of Cobalt-60 in the Big Rock Point power reactor. This Co-60 will range from 15-60 curies per gram specific activity and will be used primarily for radiation processing source requirements. With the gradual entry of commercial Cobalt-60 production in power reactors, the demands for large-volume material should be able to be met in terms of both quantity and price.

The AEC has, therefore, been supplying the lower-specific activity material at costs designed to stimulate research and development and use for industrial purposes, while leaving the higher-specific activity, higher priced medical and radiographic material, to be supplied by the private producers. We expect that power reactor produced Co-60 will gradually serve a portion of the market now being supplied by the Commission as radiation processing source requirements continue to grow.

The problem of foreign competition in the Cobalt-60 market has been and continues to be a problem for American firms. In addition to price competition, the Canadian and UK firms also provide a more complete package of services than is yet available from individual U. S. companies.

5/ See AEC Public Release H-249, dated November 17, 1965.

Reactor produced radioisotopes are used in a large number of applications; to name only a few, medicine, agriculture, crime detection, industrial gauges and space. An estimate of the future potential dollar market for radioisotopes is difficult to make, but it is growing at a very rapid pace as indicated in several sections of this category.

3. Fission Product Radioisotopes

In addition to cyclotron and reactor produced radioisotopes, fission product radioisotopes are presently being separated from AEC high-level wastes. Isochem, Inc., (jointly owned by Martin-Marietta and U. S. Rubber) has agreed to construct and operate an $8 million fission product conversion and encapsulation plant at Richland, Washington, completely on a commercial basis and has received a contract to operate the AEC chemical processing complex at Richland. Present plans are for the Isochem plant to be operational in the fall of 1968. The radioisotopes from fission products of principal interest are Sr-90, Ce-144, Cs-137 and Pm-147. Similar fission products can also be made available in large quantities, in the future, when their production is warranted by market demands, from commercial firms operating chemical processing plants such as Nuclear Fuel Services, Inc. Thus, the expanding uses of these isotopes should eventually create a source of revenue for utilities from these by-products resulting from reprocessing of fuel from reactor operation.

In 1965, the AEC announced sharp price reductions on the four radioisotopes mentioned above.[6] These lower prices were set at levels approaching the prices which were expected to be charged by Isochem, in order to further stimulate the market for these materials in the period before Isochem can begin operation. However, as noted in a paper given by E. T. O'Sullivan, of Isochem, Inc., at the NUCLEX technical meeting in Basel, Switzerland, this September, the prices expected to be charged by Isochem will probably be significantly lower than the AEC's prices.[7] The AEC will withdraw from supplying these four radioisotopes when the Isochem plant commences commercial operation in 1968.[8]

6/ See AEC Public Release H-196, dated August 27, 1965.

7/ Reference NUCLEX Technical Meeting #9 Paper by E. T. O'Sullivan, "Applications of Fission Products for Large Production Sources".

8/ See AEC Public Release H-222, dated October 11, 1965.

Fission product radioisotopes are used in a number of industrial applications, as well as for space, power and heat purposes. Promethium-147 and Strontium-90 are used as luminous sources and atomic "batteries". Strontium-90 is also employed in industrial thickness gauges and in the treatment of certain eye disorders. Cerium-144 is used for various research purposes and has good potential for use as a heat source. Cesium-137 is used as a radiation source for both industrial and medical uses and is a competitor of Cobalt-60 in certain areas.

AEC revenue for the distribution of the above four radioisotopes is given below for FY-65 and FY-66.

	Revenue in $	
	1965	1966
Cesium-137	192,000	27,000
Promethium-147	28,000	12,000
Strontium-90	3,000	65,000
Cerium-144	negl.	negl.
TOTAL	$223,000	$104,000

B. Processors

A processor is defined as one who can perform target preparation and separation of the desired radioisotopes, radiochemicals and/or radiopharmaceuticals for ultimate distribution. Industry continues to assure more and more of these activities.

The principal commercial firms in this area are listed in Table V-3, on the following page.

Union Carbide Corporation entered the radiopharmaceutical field this year through their recently acquired Neisler Labs. Mallinckrodt Chemical Company acquired Nuclear Consultants Corporation. The G. D. Searle Company and Nuclear-Chicago Corporation merged during the year, thereby giving Nuclear-Chicago an expansion opportunity into nuclear medicine. As shown in the table, Isotopes, Inc., has acruired Hazleton Nuclear, Volk Radiochemical

TABLE V-3

Principal Processors of
Radiochemicals & Radiopharmaceuticals

	Radiochemicals	Radiopharmaceuticals
Abbott Laboratories	X	X
Ames Lab - Tex, Inc.		X
Atomic Corporation of America	X	
Bio-Rad Laboratories	X	
Calbiochem	X	
Cambridge Nuclear Corporation	X	X
General Nuclear, Inc.	X	X
General Radioisotope Processing Co.	X	
Hastings Radiochemical Works	X	
International Chemical & Nuclear Corporation	X	
Isotopes, Inc., Subsidiaries: Hazleton Nuclear Science Corp. U. S. Nuclear Corporation Volk Radiochemical Company	X	
New England Nuclear Corporation	X	
Nuclear-Chicago Corporation (G. D. Searle Company)	X	
Nuclear Consultants Corporation (Division of Mallinckrodt Chemical Works)	X	X
Nuclear Research Chemical, Inc.	X	
Nuclear Science & Engineering Co.	X	
Nucleonic Corporation of America	X	
Schwartz Bio-Research, Inc.	X	
E. R. Squibb & Sons		X
Tracerlab, Inc. (Division of L.F.E., Inc.)	X	

Company and U. S. Nuclear. U. S. Nuclear had previously acquired Isotopes Specialties, Inc. International Chemical and Nuclear Corporation purchased Baird-Atomic's radiochemical production facilities during the year. Nuclear Science and Engineering Corporation set up a wholely owned subsidiary, Radioactive Materials Corporation, in Buffalo, New York, to produce and distribute short-lived reactor produced isotopes.

It is estimated that the total annual sales of radiochemicals ($6 million) and radiopharmaceuticals ($7 million) will be approximately $13 million. Commercial sales of radiopharmaceuticals are increasing at a rate of about 20-30% per year.

C. Source Fabrication

There are presently approximately 50 commercial firms producing alpha, beta, gamma and neutron sources. Although the alpha and neutron source business is small (about $500 thousand per year) and may continue to increase slightly over the years, the beta and gamma source business can be expected to increase substantially in the future as a result of increased interest in heat and process radiation applications. A number of firms that own large hot cells such as Battelle Memorial Institute, Babcock & Wilcox, General Electric, Gamma Process Corporation (through Westinghouse) and Lockheed-Georgia have bid on large Cobalt-60 source encapsulation work.

Nuclear Materials and Equipment Corporation, Apollo, Pennsylvania, and Monsanto Research Corporation, Dayton, Ohio, are the principal commercial suppliers of plutonium and americium neutron sources.

D. Equipment Manufacturers

There are six principal areas of interest in equipment: namely, radiographic and teletherapy units, gauges, irradiators, isotopic power devices and self-illuminating devices.

1. Radiographic Units

Radiographic units are manufactured by industry and not by the AEC. The commercial firms active in this field are listed in Table V-4.

TABLE V-4

Principal Suppliers of Radiographic Units

The Budd Company, Phoenixville, Pennsylvania
Gamma Industries, Inc., Baton Rouge, Louisiana
Radionics Corporation, Norristown, Pennsylvania
Picker X-Ray, Technical Operations, Inc., Burlington, Mass.
U. S. Nuclear Corporation, Burbank, California (A subsidiary of Isotopes, Inc.)

The annual volume of sales in this field is approximately $1 million and is expected to continue to increase. The radioisotope units, utilizing such isotopes as Iridium-192, Cobalt-60, Cesium-137 and Thulium-170 compete with machines.

2. Teletherapy Units

There are eight commercial suppliers of teletherapy units. Four of the eight suppliers (General Electric, North American, Philips Company, Siemens New York and Schick X-Ray Company) are distributors of foreign-made teletherapy units. The firms active in this field are listed in Table V-5.

TABLE V-5

Principal Suppliers of Radioisotope Teletherapy Units

W. F. and John Barnes Company, Automated Machine Division of Babcock & Wilcox, Rockford, Illinois

General Electric Company, Milwaukee, Wisconsin

Laboratories for Electronics, Waltham, Massachusetts (Tracerlab)

North American Philips Company, Inc., New York, New York

Picker X-Ray Company, Cleveland, Ohio

Schick X-Ray Company, Chicago, Illinois

Siemens New York, Inc., New York, New York

Westinghouse Electric Corporation, Baltimore, Maryland

There are approximately 17 hundred teletherapy units in the world -- 7 hundred of which are in North and South America. The annual volume of sales for these units is approximately $2 million. The average new unit being installed in the U.S. contains 3 thousand to 4 thousand curies of high-specific activity Cobalt-60 with some containing up to 8 thousand to 10 thousand curies. The International Atomic Energy Agency plans to up-date their 1963 survey of installations throughout the world.

3. Gauges

There are between 20 and 30 firms active in the nuclear gauge field. The industrial uses of nuclear gauging for measurement and control of various processes include gauges for flow rate, thickness, level, density and also activation analysis systems. Estimated 1966 sales of industrial nuclear gauges is between $13-16 million, including exports of about $3 million, but excluding rental or lease revenue which is estimated to be approximately $4 million.

The principal suppliers of industrial nuclear gauges and gauging systems are listed below:

Foxboro Corporation
Industrial Nucleonics
Nuclear-Chicago Corporation, Subsidiary of
  G. D. Searle
Ohmart Corporation
Tracerlab, Division of L. F. E., Inc.
Robertshaw Controls Company

Sales in this field are expected to continue growing at about the same rate as the remainder of the nuclear instrument industry or about 15% per year and could reach $40 to $50 million by 1976.

4. Self-Illuminating Materials and Devices

The use of self-illuminating materials on watches, signs, etc., continues to increase. The increased usage and interest is reflected by the fact that tritium sales by the AEC have almost tripled in the past three years. The quantities and total dollar sales are given below.

Fiscal Year	Curies	AEC Sales
1963	60,000	$ 95,000
1964	112,000	$125,000
1965	118,000	$128,000
1966	171,000	$196,000

The firms active in this field are listed in Table V-6.

TABLE V-6

Principal Commercial Suppliers of Self-Illuminating Materials and Devices

American Atomics, Tucson, Arizona
Canrad Precision Industries, Inc., New York, New York
Laboratories for Electronics, Inc., Waltham, Massachusetts
Luminous Products Corporation, Boston, Massachusetts
Minnesota Mining & Manufacturing Company, St. Paul, Minnesota
Radium Chemical Company, New York, New York
U. S. Radium Corporation, Morristown, New Jersey

The AEC has received numerous petitions to exempt from licensing the use of small amounts of Tritium, Promethium-147 and Krypton-85, for various applications in the field of self-luminous materials and devices.

Last year Canrad Precision Industries petitioned AEC to exempt the use of Tritium contained in luminous thermostat dials and pointers, radio dials and pointers, automobile shift quadrants and marine compasses. All except the use in radio dials and pointers have been approved by the AEC. A petition also was received from Canrad Precision Industries requesting an exemption from licensing of luminous-level vials each containing up to 300 millicuries of Tritium gas.

This year General Electric Company filed a petition requesting exemption from licensing of glow lamps containing up to 10 microcuries of Tritium per lamp. This petition was considered by the Commission and the proposed exemption has been published for public comment.

A similar petition this year from Brookes and Gatehouse, Inc., to exempt the possession and use of Tritium in marine navigational instruments was considered by the AEC and the proposed exemption has been published for public comment.

The Luminous Products Corporation recently requested exemption of Tritium in self-luminous screws containing the

Tritium in the form of a self-luminous injection molded plastic. If such a petition were approved, it would permit a wide range of applications for small self-luminous sources.

U. S. Radium Corporation has recently petitioned for an increase from four curies of Tritium per device (now approved for general license) to ten curies of Tritium per device to be used in luminous safety markers for aircraft.

American Atomics has petitioned the AEC this year for exemptions from licensing for small quantities of Krypton-85 for use in light sources for highway signs and airport runway markers and commercial and industrial switching equipment and control panels.

Previously the AEC exempted from licensing the use of Tritium or Promethium-147 to provide self-illuminating automobile locks and Tritium for self-luminous dial timepieces.

In March 1965, the AEC published criteria which it will use in considering applications to use radioactive materials in products intended for distribution to the general public.[9/] In June 1965, the AEC simplified the format of its regulations for the use of radioisotopes retaining common requirements applicable to all radioisotopes licensing in Part 30 and recodified new parts applicable to certain classes or categories of uses or users as follows:[10/]

Part 31 -- "General Licenses for Certain Quantities of By-Product Material and By-Product Material Contained in Certain Items."

Part 32 -- "Specific Licenses to Manufacture, Distribute, or Import Exempted and Generally Licensed Items Containing By-Product Material."

Part 33 -- "Specific Licenses of Broad Scope for By-Product Material."

9/ See AEC Public Release IN-568, dated March 15, 1965.
10/ See AEC Public Release IN-601, dated June 29, 1965.

Part 34 -- "Licenses for Radiography and Radiation Safety Requirements for Radiographic Operations."

Part 35 -- "Human Uses of By-Product Material."

Part 36 -- "Export and Import of By-Product Material."

5. Radioisotopic Power

Taking advantage of the heat released when isotopes decay, electric power systems for several classes of specialized applications are being developed. Several units at power levels up to 60 electrical watts have been used successfully in remote terrestrial sites, and five units have been launched in the DOD Navigation Satellite Program.

For terrestrial applications, power systems have been used in remote weather stations, navigation buoys and as beacons on off-shore oil rigs. Additional systems are under development for use in the following:

(1) Unattended sea surface and land surface systems (light houses, navigational buoys, automatic weather stations) for one to five year life in the power range of 10-200 watts.

(2) Extremely small, long-life systems such as the cardiac pacemaker, and the military Prescribed Action Link (PAL) systems, in the power range of one microwatt to one watt.

(3) Deep-sea generators for use in various undersea applications in the ten watt to the low kilowatt range.

a. Space

For space applications, four units, two of 2.7 watts and two of 25 watts, have been successfully launched in the TRANSIT 4 and subsequent DOD novigation satellites. The first, SNAP 3A, launched in 1961, now has been operating longer than any other orbiting space power system. Four of these units supplied their full assigned

power during the whole life of the parent satellite system. A fifth unit at 25 watts was aboard a navigation satellite which failed to go into orbit. Evidence from fallout studies indicates the unit burned up safely in the upper atmosphere on re-entry, as designed. Units are now being developed for the following:

(1) Nimbus "B" satellite at 60 watts (two 30 watt modules on each satellite) to be launched in 1967.

(2) The Apollo Lunar Surface Experiment Package (ALSEP to be left on the lunar surface by the first Apollo astronauts - 50 watts).

(3) A family of DOD and NASA 90-day missions, at 500 watts, to be available for earliest possible flight in 1971, probably as part of a qualification test of a space satellite system.

A thorough development program in advanced heat source and power conversion technology is also being carried out. There is also under way a program of heat source technology development directed toward low-thrust, high-specific impulse isotopic thermal rockets for specialized space applications.

b. Oceanographic

The AEC began developing an oceanographic nuclear power program in 1966 which includes both isotopic and reactor power sources. It is expected that isotopic power sources would range from milliwatts to multi-kilowatts and reactor power sources would range to 2 Mwe or above depending on the intended use. This program would be integrated into the national oceanographic effort to assure that the AEC's program is oriented to meet the demands of the national effort.

c. Markets

At present, the Federal Government is the principal customer for these devices, although commercial markets, especially in the marine and terrestrial areas, and in commercial space applications such as communications satellites are also promising. Radioisotopes such as Plutonium-238, Strontium-90 and Polonium-210 are being used in these devices.

d. Cardiac Pacemaker

One of the most exciting and important new areas for the use of isotopic power devices is the development of a cardiac heart pacer. During the year, the AEC selected NUMEC to develop and test a highly compact unit with a power source capable of operating up to ten years without refueling. The power source will be fueled with Pu-238. The potential market for these units has been estimated at about 10 thousand per year with a Plutonium-238 fuel requirement of about three to four kg per year.

e. Industry

Isotopic power generation is a large and actively growing field. There are approximately 30 firms active in this area. Isochem, Inc., will process and supply heat source materials for sale to this segment of the industry. Undoubtedly other firms will enter the heat source material supply picture as markets develop.

The formation of a new company to develop isotopic power generation and heat sources, Sanders Nuclear Corporation, was announced this year by Cambridge Nuclear Corporation and Sanders Associates, Inc.

During the year NUMEC received an order for two Sr-90 powered thermoelectric generators from the U. S. Naval Facilities Engineering Command.

The Martin-Marietta Corporation announced fixed-price isotopic powered generators for sale. The 25 watt Sr-90 powered generators are intended for use in terrestrial applications. A deep-sea version is also available. The first Martin unit was sold to the Naval Electronics Lab for installation on an island off the Alaskan coast for powering an oceanographic measuring station.

6. The Radiation Processing Industry

During the past year, industry has evidenced a rapidly growing interest in commercial radiation processing applications. This field is divided into machine or accelerator

produced radiation and isotope irradiation, each possessing some advantages and disadvantages over the other. Radiation processing applications using one or the other method as sources include upgrading of plastics, curing of paints, upgrading of solid state devices, sterilization of medical supplies, food preservation, production of wood plastic combinations, production of shrinkable plastics, sterilizing parts for the space programs, bio-degradable detergents and synthesis of chemical compounds.

The following table lists the principal firms supplying accelerators equipment and/or services.

TABLE V-7

Company	Supply Accelerators	Supply Services
Cyclotron Corporation	X	
General Electric Company	X	
Hughes Aircraft Company	X	
Applied Radiation Corporation	X	X
Electra Process Industries, Inc.		X
General Atomics, Division of General Dynamics	X	X
High Voltage Engineering Corp.	X	
Electronized Chemicals Corp., Subsidiary of High Voltage Engineering Corporation		X
Midwest Irradiation Center		X
Raycon		X
Radiation Dynamics, Inc.	X	X
Rad-Mat, Inc.		X
Varian Associates	X	X
High Energy Process Company		X
Radiation Polymer Corporation		X

Rad-Mat, Inc., Gaithersburg, Maryland, was formed this year to provide radiation processing services to the medical, plastics, food, electronics and other industries. The company will use a 3 million volt Dynamitron accelerator which will be the highest powered source in industrial use.

The current commercial accelerator market including Van de Graaffs, linacs, linacs, insulating core transformers and Dynamitrons is approximately $17 million annually. About three fourths of this market is for Van de Graaffs for research.

Large scale (tonnage) private radioisotope process service irradiation facilities in the U. S. are practically non-existent at the present. While firms such as Lockheed-Georgia and American Novawood Corporation have substantial capacity for production of special products, no one firm offers a large-scale processing service.

TABLE V-8

Principal Suppliers of Isotopic Irradiators

Company	Medical	Process	Research
Ameray Corporation			X
American Nuclear Corporation	X	X	X
Budd Company	X		X
Gamma Process Company, Inc.		X	X
Iso Nuclear Corporation		X	
Knapp Mills, Inc.	X	X	X
Martin-Marietta Corporation			X
Radiation Facilities, Inc.	X	X	X
Radionics, Inc.			X
Isotopes, Inc. (and subsidiaries)	X	X	X
Lockheed-Georgia		X	X
Babcock & Wilcox		X	X
Nuclear Materials & Equipment Corporation		X	X
Burns & Roe		X	
Stone & Webster		X	
American Novawood		X	
General Electric		X	
Westinghouse		X	

Irradiation, Inc., a subsidiary of Isotopes, Inc., was formed to introduce radiation processing to private industry and to aid businesses in bringing radiation processing into their production lines. The company has been awarded a contract to operate AEC's Portable Cesium Irradiator for the Commission's program to demonstrate to food processors on a small-scale basis the potential advantages provided by radiation processing.

TABLE V-3

Regulations and Petitions on Radiation Preservation of Foods

Product	Petitioner	Source	Dose	FDA Petition			FDA Regulation		
					Ref. F.R.			Ref. F.R.	
				Filing Date	Vol.	Page	Issue Date	Vol.	Page
Bacon	US Army	Cobalt 60	4.5-5.6 megarads	[illegible]	27	[illegible]	2-8-63	28	1469
Bacon	Gen. Electric	5 Mev. electron	4.5-5.6 megarads	6-5-63	28	5537	8-30-63	28	9526
Bacon	US Army	10 Mev. electron	4.5-5.6 megarads	8-23-63	28	9325	4-21-65	30	5631
Bacon	AEC	Cesium 137	4.5-5.6 megarads	12-10-63	28	13707	4-1-64	29	467
Bacon	Radiation Dynamics, Inc.	X-rays from 5 Mev. elec.	4.5-5.6 megarads	7-23-64	29	9910	12-15-64	29	18056
Wheat, Wheat Products	Brownell et al.	Gamma 22 Mev.	20,000-50,000 rads	11-4-62	28	1465	8-21-63	28	9259
Wheat, Wheat Products	High Voltage Engr. Corp.	5 Mev. electron	20,000-50,000 rads	12-18-63	28	13797	2-26-64	29	3186
Wheat, Wheat Products	AEC	Cesium 137	20,000-50,000 rads	8-1-64	29	12801	10-13-64	29	14027
White Potato	AEC	Cesium 137	5,000-10,000 rads	8-1-64	29	12801	10-13-64	29	14027
White Potato	US Army	Cobalt 60 Cesium 137	5,000-10,000 rads	1-30-65	30	1113	10-16-64	29	14027
White Potato	US Army	Cobalt 60	5,000-10,000 rads	8-6-63	28	5580	7-8-64	29	9329
White Potato	US Army	Cobalt 60 Cesium 137	15,000 rads	1-30-65	30	1013	11-5-65	30	11851
Strawberries	AEC	Cobalt 60 Cesium 137	100,000-250,000 rads	5-18-66	31	7256	(Pending)		
Citrus (oranges, lemons, grapefruit)	AEC/US Army	Cobalt 60 Cesium 137	75,000-500,000 rads	12-26-63	28	13797	(Pending)		
Citrus (oranges)	AEC/US Army	Cobalt 60 Cesium 137	75,000-300,000 rads	-	-	-	(Pending)		
Marine Products (haddock, pollock, ocean perch, cod, flounder, and sole)	AEC/US Army	Cobalt 60 Cesium 137 10 Mev. elec. 5 Mev. x-rays	100,000-250,000 rads	9-15-65	30	11803	(Pending)		
Packaging Materials 9 classes of materials	AEC	Gamma	1 megarad	2-6-[illegible]	29	2232	8-14-64	29	11651
Packaging Materials Amendments:									
Cellophane (Saran-coated)	AEC	Gamma	1 megarad	2-15-65	30	8134	6-11-65	30	7590
Nylon	AEC	Gamma	1 megarad	9-5-65	30	11406	-	-	-
Polyester (Saran-coated)	AEC	Gamma	1 megarad	7-30-65	30	9553	(Pending)		
Polypropylene (Saran-coated)	AEC	Gamma	1 megarad	7-30-65	30	9553	(Pending)		
[illegible] Films	US Army	Gamma	6 megarads	7-21-65	30	8719	(Pending)		
Parchment Paper	US Army	Gamma	6 megarads	1-15-65	30	587	3-12-65	30	3354

An indication of the over-all market for radiation processing and its growth may be obtained from the value of products processed (1963 - $75 million, 1964 - $150 million and 1965 - estimated over $400 million).

The irradiation processing of food is developing into one of the most important areas of applications of radiation. The AEC and U. S. Army programs for irradiation processing of food have been under way for many years, but only in the past three years have specific clearances for food been issued by the Food and Drug Administration. The current status of petitions on radiation processing of foods is given in Table V-9, taken from the statement of Commissioner James T. Ramey, USAEC, at the Joint Committee on Atomic Energy Hearings on the Food Irradiation Program, September 12, 1966.

One of the major areas of interest which began in 1961 is the development of irradiated wood-plastic materials. The AEC has sponsored a substantial amount of development work at West Virginia University, the Air Reduction Company and Lockheed-Georgia Company. The Arthur D. Little, Inc., the Southern Interstate Nuclear Board, Battelle-Northwest Laboratories and Western New York Nuclear Research Center have conducted market surveys to determine the steps necessary to achieve the earliest possible commercialization.

The Atomic Industrial Forum and AEC sponsored an information meeting on irradiated wood-plastics materials in September 1965 for persons interested in the production, marketing or use of these materials. In August 1966, the AEC released a report, KLX-1876, "Engineering and Evaluation Study for the Manufacture of Wood-Plastic Composites", prepared by Vitro Engineering Company.[11]

Under a demonstration program with Lockheed-Georgia, 80 companies cooperated in end product testing programs to evaluate specific wood-plastics product applications.

11/ AEC Public Release IN-710, dated August 24, 1966, "AEC Reports on Wood-Plastic Research".

During 1966, the AEC solicited from industry expressions of interest in a cooperative program for a wood-plastic materials demonstration facility and in polyethylene production. The industry responses generally indicated that the private capability now existing and planned might be adequate for development and production of these materials. In view of this, the AEC concluded that an AEC-industry cooperative program was not warranted at the present time but that AEC would review the situation after approximately a year to determine the extent of industrial progress.

During 1966, commercial prices for significant quantities of a variety of wood-plastic materials were announced by American Novawood Corporation, Lynchburg, Virginia, and Lockheed-Georgia Company, Marietta, Georgia. These wood-plastic composites are being routinely manufactured in their radiation facilities. American Novawood is using Cobalt-60 and Lockheed is using both their reactor and Cobalt-60. Louisiana-Radiant, Inc., Baton Rouge, Louisiana, has installed a Cobalt-60 radiation source at the Louisiana State University's Nuclear Science Center for the manufacture of wood-plastic samples, doing additional product development work, plus training of company personnel. In addition, two new companies, Gen Irradiated Wood, Inc., Dothan, Alabama, and Iso Nuclear Corporation, New York City, announced plans to build plants for the commercial production of various wood-plastic composites. Both groups will be using Cobalt-60 for their radiation sources.

One private firm has plans to install a pilot plant for polyethylene production using ionizing radiation to initiate the reaction.

E. AEC Advisory Committee on Isotopes and Radiation Development

The present AEC Advisory Committee on Isotopes and Radiation Development was established in 1958. This committee grew out of the Advisory Committee on Isotope Production and Distribution, which was formed in 1946 and terminated in 1958 when the Commission's regulatory and licensing activities were separated from the operating activities.

The Advisory Committee on Isotopes and Radiation Development reviews AEC policies and the potential development of new applications of isotopes and radiation and makes appropriate recommendations for the Commission's consideration.

The composition of this committee has been gradually changed to bring in additional representatives from various segments of the isotope industry. At the committee's suggestion, the membership was reduced from 23 to 18 during the year. The present membership is given below:

Chairman - John L. Kuranz, Nuclear-Chicago Corporation
Vice Chairman - John W. Landis, Babcock & Wilcox Company
AEC Member - Dr. George M. Kavanagh, Assistant General Manager for Reactors

<u>Subcommittee on Commercial Activities</u>
Chairman - Howard K. Nason, Monsanto Research Corp.
Dr. John C. Brantley, Nuclear Science & Engineering Corp.
David E. Trumbull, General Electric Company
Dr. Charles Artandi, Ethicon, Inc.

<u>Subcommittee on Process Radiation</u>
Chairman - Dr. Willard P. Conner, Hercules Research Center
Dr. Vivian T. Stannett, Research Triangle Institute
Dr. David E. Harmer, Dow Chemical Company
E. Robert Kinney, Gortons of Gloucester, Inc.

<u>Subcommittee on Radioisotope Power</u>
Chairman - Dr. Leonard Reiffel, IIT Research Institute
Dr. Nathan W. Snyder, Georgia Institute of Technology
Dr. Thomas E. Hicks, University of California at Los Angeles
Dr. Ernst Stuhlinger, Department of the Army, Marshall Space Flight Center

<u>Subcommittee on Radiation Systems</u>
Chairman - Dr. Rodman A. Sharp, Beckman Instruments, Inc.
Henry R. Chope, Industrial Nucleonics Corporation
Dr. Paul M. Stier, Union Carbide Corporation
Dr. Donald W. Pritchard, Johns Hopkins University

In 1965 a study was conducted for the AEC by Dr. Lauchlin M. Currie, then Chairman of the AEC Advisory Committee on Isotopes and Radiation Development, to determine the extent of industrial development of isotopes and radiation, to identify factors that limited their use and obtain recommendations on how AEC might better help this portion of the atomic energy program. Recommendations were submitted to the Commission and all have been acted upon or are in the process of being handled.

CATEGORY VI

MATERIALS REPROCESSING

A. Irradiated Fuel

Fuel elements must be removed from the reactor core for reprocessing, before all the fissile atoms have been consumed, for two primary reasons. The first is the accumulation of fission product poisons that are produced during the fissioning process. These products have a high affinity for the absorption of neutrons necessary to sustain the chain reaction. The second is that the damaging effects of the radiation environment in the reactor necessitates removal of the fuel element prior to warping or rupture. The discharged elements still contain significant amounts of the valuable fissionable materials uranium-233, uranium-235 and plutonium, in addition to certain fission products which can be economically recovered by chemical reprocessing.

The recovery of the fissionable material remaining in the spent fuel involves a series of operations performed at a chemical separations facility. The process currently being used is the aqueous process, developed and used in AEC separation facilities for over 20 years. An updated version of the aqueous process is also being used at the commercial reprocessing plant of Nuclear Fuel Services, Inc. (NFS), West Valley, New York. Other, more advanced methods of separation are under development in both the AEC and private industry and will be discussed further in this section.

For purposes of this report the reprocessing of spent fuels is divided into the following three areas:

1. Production Reactor Fuels
2. Power Reactor Fuels
3. Highly Enriched Fuels

1. Production Reactor Fuels

The AEC's production reactors were built specifically for the purpose of providing fissionable material for our defense needs. The Government's production reactor fuel loads are processed at the large reprocessing facilities at its Hanford Plant at Richland, Washington, and at the Savannah River Plant near Aiken, South Carolina.

The President, in his January 8, 1964, State of the Union message, which was further amplified in April of 1964, announced the decision to curtail the production of special nuclear materials. In response to this determination the AEC has shut down four production reactors; one at Savannah River, South Carolina, on June 15, 1964, and three reactors at the Hanford Works within the first six months of 1965. Because the demand for processing production fuels has decreased, the Redox chemical separations facility at Hanford is scheduled for shutdown at the end of 1966.

Another production reactor, the AEC's $200 million New Production Reactor (NPR) at Richland, Washington, achieved designed power of 4000 Mwt in 1965 and became operational for plutonium production during the same year. In the near future this plant will serve a dual purpose role in that it will also provide steam which will be sold to the Washington Public Power Supply System for use in their $125 million, 800 Mwe electric generating plant located adjacent to the reactor. Because of the similarity of its fuel to power reactor fuel, part of the spent fuel discharged from this reactor has been designated for commercial reprocessing.

As of January 1, 1966, Isochem, Inc., (jointly owned by Martin-Marietta Corporation and the U. S. Rubber Company) became the operating contractor for the chemical reprocessing complex at Richland, Washington.

2. Power Reactor Fuels

In order to encourage the development of a commercial reprocessing industry, the Commission, in 1957, announced that it would enter into contracts with private companies for the chemical reprocessing of irradiated fuel. In order to further encourage a commercial capability in this area, the

Commission also announced prices based on a conceptual plant design for guidance to the industry.[1] While industry has for some time been interested in establishing a reprocessing service, part of the problem has been the lack of a sufficient commercial reprocessing load.

a. Commercial Chemical Reprocessing

In 1959 the Industrial Reprocessing Group, IRG, was formed for the purpose of establishing the technical and economical feasibility of the design, construction and operation of a privately owned facility for the chemical re[illegible]ocessing of spent nuclear fuels. The group consisted c[illegible]. R. Grace and Company and five utility companies: Consolidated Edison Company of New York; Commonwealth Edison Company of Chicago; Yankee Atomic Electric Company of Boston; Northern States Power Company of Minneapolis; and Power Reactor Development Corporation of Detroit.

After an expenditure of more than $450 thousand during 1960 and 1961, a conclusion was reached late in 1961 whereby W. R. Grace and Company decided to pursue this project at its own expense. In the spring of 1962 American Machine & Foundry Company joined with W. R. Grace in support of this effort, and a separate corporation was formed called Nuclear Fuel Services, Inc. (NFS). In early 1963, NFS contracted with the Bechtel Corporation for the design and construction of a spent fuel reprocessing and waste disposal facility on land leased from the State of New York. Plant construction began in June 1963. The same five utility companies who cooperated with W. R. Grace and Company in the early study supported this plant by contracting for a certain amount of reprocessing services.

In order to assist in the development of private reprocessing capability the AEC has contracted with NFS for the reprocessing of a base load of Government irradiated fuels equivalent to about 100 metric tons of uranium per year for the five-year period 1966 to 1971.

1/ AEC Public Release F-201, dated October 14, 1963.

On April 20, 1966, Chairman Glenn T. Seaborg announced that the AEC had issued a license (chemical separations facility operating license #1) for operation of the first privately owned nuclear fuel reprocessing plant in the U. S. The license was issued to NFS and the New York State Atomic and Space Development Authority for operation of the NFS plant located at the Western New York Nuclear Service Center, West Valley, New York, about 30 miles south of Buffalo.[2/] The first spent fuel was delivered to the mechanical cell of their facility on April 19, 1966, and the first product shipment (3-1/2 tons of uranyl nitrate) was delivered on May 2, 1966, to the AEC's feed materials plant at Fernald, Ohio.

The capacity of the plant is 300 metric tons of spent nuclear fuel per year. The facility employs a mechanical (chop-leach) head end with Purex solvent extraction. Fuel is chopped into pieces before entering the dissolver and the uranium content is leached from the cladding. The solid cladding is then inspected and buried. The products uranium and plutonium are recovered as nitrate solution.

b. Other Company Programs

General Electric in September of 1964 announced its intention to build a reprocessing plant and on February 19, 1965, gave public notice of the award of a construction contract to the Fluor Corporation. Under the terms of the contract, Fluor will provide engineering, procurement and construction services. In November of 1965 GE made available to the nuclear industry firm prices for complete recovery services which would be based on a plant utilizing the GE patented Aquafluor process.[3/] In a public release on September 15, 1966, General Electric announced that it would apply to the AEC for a permit to construct a fuel recovery plant to be located near Morris, Illinois. General Electric's announcement pointed out

2/ AEC Public Release J-101, dated April 20, 1966.
3/ "Aquafluor" - Trademark of General Electric Company, Patent No. 3,222,124, December 7, 1965.

that depending upon continued favorable evaluation of technical and economic factors, as well as approval from the AEC, state and local regulatory bodies, their Board of Directors was expected to consider the project in 1967. If approved by the Board, construction probably would start that same year.

Their present plans anticipate operation of the reprocessing plant to begin in 1970. The plant capacity is designed for 300 metric tons of irradiated nuclear fuel per year.

It was previously reported that the Allied Chemical Corporation had formally approved and committed funds for a nuclear fuels reprocessing development program. During 1966 Allied affirmed their interst in eventually building a commercial reprocessing plant, but stated they had abandoned their initial plans for the use of the combination aqueous fluoride-volatility process which they had been studying since January 1963. Following this decision Allied curtailed construction of the full-scale cold pilot plant it was building at Metropolis, Illinois. They are presently concentrating their research and engineering efforts on several processes within their advanced fluorine research group. A process selection is expected to be made in 1967 for use in its commercial plant. Although Allied Chemical has made no decision as to site location, present indications favor the Eastern United States.

c. Spent Fuel Forecast

Chart VI-A, on the following page, presents an estimate of the low-enriched domestic, power reactor spent fuel market through 1980.

Although 1966 has been a very encouraging year for nuclear electric plant announcements, the spent fuel discharges available for reprocessing from these forthcoming reactors will not be available in significant quantities until the early 1970's. Another factor which must be considered when viewing the spent fuel market is the highly successful development of increased fuel life in reactors. The success of this development in the last few years has resulted in reduced requirements for chemical reprocessing services in the near future.

DOMESTIC CENTRAL STATION SPENT FUEL FORECAST
QUANTITIES AVAILABLE ANNUALLY

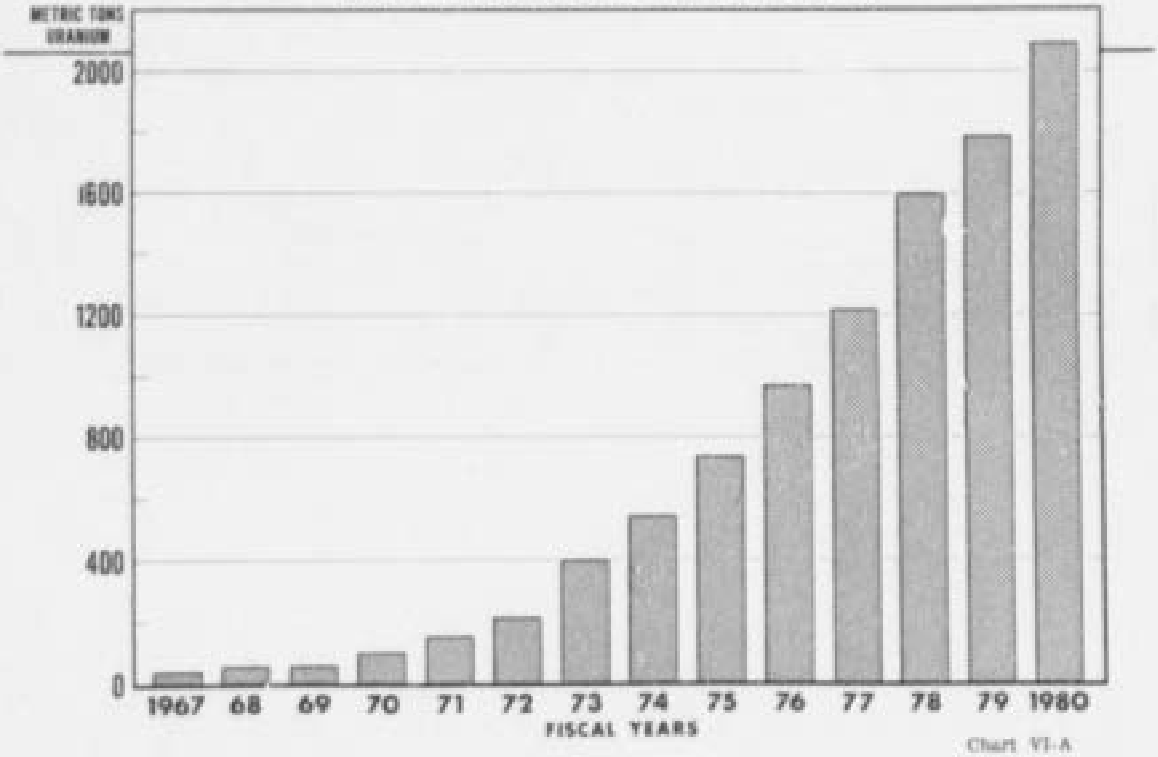

Chart VI-A

d. AEC Actions with Industry

In order to determine if any additional steps were necessary in connection with the development of a competitive processing industry, the AEC on March 10, 1965, issued Public Release H-57, soliciting information from private companies concerning their plans to provide chemical processing service. As a result of this release, the AEC held detailed discussions with representatives of potential chemical processors, reactor manufacturers and electric utilities concerning their plans and suggestions. On the basis of these discussions and the future plans to construct new chemical processing plants by companies such as Allied Chemical and GE, the Commission announced

in Public Release IN-692 dated May 23, 1966, that it had concluded that support in the form of a second base load of fuels, such as was furnished NFS, is not required to encourage the development of a competitive reprocessing industry. The Commission intends to follow the progress of industry in this area and will reconsider its offer of a second base load of Government fuels if it should prove to be necessary at some future date.

As stated earlier, the AEC established a conceptual plant in 1957 to provide, on an interim basis, firm charges for spent fuel reprocessing service for a period from March 12, 1957, until June 30, 1967.4/ This plant has been used as a guideline for establishing prices for commercial reprocessing.5/ The use of the conceptual plant charge schedule and the necessity of extending this policy beyond June 30, 1967, is presently being evaluated in order to determine whether there is sufficient competition in reprocessing to eliminate this kind of control.

e. AEC Research Programs

At the same time that the Commission held discussions with industry representatives on the plans and viewpoints in regard to the construction of additional reprocessing plants, the Commission also reviewed the research program being carried on within the Commission on advanced technology. In AEC Public Release IN-692, mentioned above, the Commission also announced that as a result of its review with industry, it would continue its development program on the fluoride volatility process. The Commission also stated that it would continue to evaluate the merits and need for a future cooperative program for the industrial demonstration of the fluoride volatility process. The AEC, through prompt dissemination of technical information and through its work experience program, will continue to encourage the active interest of all potential processors in following its research and development program.

4/ 22 F.R. 1591, March 12, 1957, as amended. Also 28 F.R. 11462, October 25, 1963.
5/ WASH-743, "AEC Reference Fuel-Processing Plant", October, 1957.

In the early years of power fuel process development, it was natural to choose as a first approach the extension of the aqueous technology well proven in the AEC production program. It was fairly obvious that a commercial venture would probably utilize the vast experience gained in Government programs with aqueous technology. Development programs have now reached a point where the fluoride volatility technology appears to be the only known practical high decontamination alternate to the aqueous technology. Two major fluoride volatility alternates have been studied in varying degrees; the fused salt process and fluid bed process. Both alternates were initially developed for application to high-enrichment fuels. Subsequent development indicated the superiority of the fluid bed process for this application, and the fused salt process development was deferred. At the present time the development of volatility processing technology for high-enrichment fuels is considered essentially complete. Investigations of the fluid bed head end processes for high-enrichment aluminum, zirconium and graphite base fuels have been carried to a stage where plant design can be initiated. Further work will be limited to confirming the compatibility of high-enrichment fuel processing. Subject to continued success in the development program, it is felt that volatility technology may in due course be capable of processing the low-enriched civilian power reactor fuels, and thus make a significant contribution to the competitive private chemical processing industry. Several companies are actively supporting their own R&D programs emphasizing fluoride volatility technology.

Closed cycle processes utilizing close coupled reprocessing and refabrication concepts are being evaluated for possible application in the processing of breeder reactor fuels. The reactor and Fuel Cycle Facility (FCF) of the Second Experimental Breeder Reactor (EBR-II) represents the first demonstration of the integrated operation of a nuclear reactor and a reprocessing and refabrication plant. All of the operations in the FCF are conducted by remote control in an argon filled processing cell through five foot thick high-density concrete shielding. Irradiated fuel was first introduced to the cell of this facility for processing in September of 1964.

3. Highly Enriched Fuels

The highly enriched spent fuel loads include those fuels used in the Space Propulsion Program (Rover), Naval reactors, test reactors such as MTR/ETR/ATR and research reactors. The capability to reprocess most of these highly enriched fuel types and the actual processing of irradiated fuel elements from Japan, Sweden, France and Germany is currently available at the AEC Idaho and Savannah River Plants.[6/] In addition, NFS has the capability of handling selected types of the above fuels. Graphite fuels are one source of high enriched fuels for which suitable AEC or private processing facilities are not now available.

B. Unirradiated Fuel

The principal source of radioactive material in the commercial fuel cycle occurs when fuel undergoes fissioning in a nuclear reactor. The radioactive materials generated are the so-called fission products. The part of the fuel cycle which includes the operating reactor, spent fuel reprocessing and radioactive waste disposal activities is known as the "hot" side. The fuel which has not undergone fissioning makes up the "cold" side of the nuclear fuel cycle; the unirradiated fuel side.

1. Enriched Uranium Scrap Recovery

The major source of enriched uranium scrap comes from the fuel fabrication activity. Since the nuclear material involved in this scrap is quite expensive its recovery is economically very desirable.

Adequate private capability exists to recover enriched uranium from all types and forms of scrap generated within both private and AEC programs.

The principal commercial firms having capability in this area are listed in Table VI-1. Not all of the firms listed have capability to recover all types of fuel forms.

6/ AEC Public Release J-194, dated August 15, 1966.

[illegible]

For example, Babcock & Wilcox has the capability to recover selected internally generated scrap but is not bidding on the recovery of scrap from outside sources. General Dynamics has the capability to recover carbide scrap only. The remaining firms (Kerr-McGee Oil Industries, Inc., Nuclear Fuel Services, Inc., Nuclear Materials and Equipment Corporation, and United Nuclear Corporation) have the capability of recovering all types of uranium scrap generated internally as well as from outside sources, with the exception of carbide scrap.

TABLE VI-1

Principal Firms Recovering Uranium Scrap

Babcock & Wilcox Company, Lynchburg, Virginia
General Dynamics Corporation, San Diego, California
Kerr-McGee Oil Industries, Inc., Oklahoma City, Oklahoma
Nuclear Fuel Services, Inc., Erwin, Tennessee
Nuclear Materials and Equipment Corporation, Apollo, Pennsylvania
United Nuclear Corporation, Westerly, Rhode Island

The National Lead Company, who was previously listed under this activity, has discontinued this service for the present. They do, however, intend to provide this service at some time in the future depending on the evaluation of technical and economic factors.

AEC scrap is recovered commercially either as part of a fixed-price fabrication contract, such as for MTR-ETR fuels, or through the Scrap Recovery Program administered by the Oak Ridge Operations Office. Since 1959 contracts totaling $5.9 million have been awarded through the Scrap Recovery Program. As noted in Table VI-2, on the following page, the dollar value of contracts awarded through the AEC Scrap Recovery Program in 1966 decreased by about 31% over the awards made in 1965.

In FY-1967, it is anticipated that the value of the contract awards through the AEC Scrap Recovery Program may decline even further due to the emphasis on having the fabricator handle its own scrap either in-house, if he has the capability, or by sub-contract, if he does not have the capability. In FY-1967 the total over-all anticipated

quantities of scrap are expected to increase with the addition of work stemming from new domestic and foreign reactors. FY-1967 should result in a progressively larger volume of work for this segment of the industry.

TABLE VI-2

Orders Placed with Commercial Firms Through The Scrap Recovery Program

Fiscal Years	Value of Awards
1959	$ 115,757
1960	275,223
1961	1,438,379
1962	927,501
1963	554,240
1964	592,463
1965	1,168,526
1966	802,833

The following table lists the total contract awards (including modifications) during FY-1966.

TABLE VI-3

Total Uranium Scrap Reprocessing Contracts Awarded in FY-1966

Contractor	No. of Contracts	Value of Contracts	Est. U Content of Scrap		
			Kgs.	% U-235	$ Value
Nuclear Fuel Services	9	$265,618	521.239	79.48	$ 4,953,512
Nuclear Materials & Equipment Corp.	2	82,894	1,345.600	6.18	845,405
United Nuclear Corp.	2	141,110	619.420	35.44	2,588,831
Kerr-McGee Corp.	4	313,211	831.818	72.20	7,213,957
TOTAL	17	$802,833	3,318.077		$15,601,705

2. Plutonium Scrap Recovery

Nuclear Materials & Equipment Corporation and Nuclear Fuel Services, Inc., have private capability to recover plutonium from scrap.

With the increased interest in the use of plutonium as a reactor fuel, it can be anticipated that in the future additional private scrap recovery capability will be established, probably by the plutonium fuel fabricators.

CATEGORY VII

PLOWSHARE - PEACEFUL USES OF NUCLEAR EXPLOSIVES

Plowshare is the name given to AEC's program to study and develop peaceful uses for nuclear explosives in science and industry. In the last few years, industrial interest in this unique utilization of one of man's most powerful energy sources has increased and consideration is being given, or preparations being made, for several joint Government-industry experiments.

A. Introduction

The Plowshare Program was formally established by the Atomic Energy Commission in 1957. The program has many potential applications in such areas as: deeply buried explosions for gas and oil recovery, gas storage, waste disposal, mining, and water management; excavation of harbors, canals and mountain passes; production of transplutonium isotopes; and neutron physics research. The program consists of laboratory and field experiments to develop basic technology and the exploration and evaluation of proposed applications.

B. Budget

The following tabulation lists the AEC operating budget for the Plowshare Program for FY-1965 through FY-1967:

	FY-1965 (Actual)	FY-1966 (Actual)	FY-1967 (Estimate)
Total Operating Program	$10,677,714	$14,605,444	$16,200,000
Research and Development	$ 5,373,012	$ 6,323,906	$ 6,185,000
Excavation Experiments	3,025,722	6,597,014	8,165,000
Device Testing	602,795	3,954,188	3,000,000
Cratering Technology	2,422,927	2,642,826	5,165,000
Scientific Experiments	2,278,980	1,684,524	350,000
Natural Resources Experiments (Gasbuggy)	- 0 -	- 0 -	1,500,000

C. Types

There are two general types of peaceful nuclear explosions: contained and cratering. In a contained explosion, emplacement depth is such that the earth's surface remains essentially intact. Thus, essentially all radioactive effects are contained deep underground. A cratering explosion, on the other hand, is designed to make a crater in the earth's surface. One of the objectives of a cratering explosion, however, is to also contain most of the radioactivity deep underground. Success is being achieved in accomplishing this goal in this type of explosion by device development and emplacement techniques.

D. Potential Applications

Generally, the peaceful applications of nuclear explosives are divided into three classes of applications, known as Scientific, Excavation, and Underground Engineering.

1. In the Scientific application, nuclear explosives are used to produce the very high neutron flux needed to produce heavy elements and for other research requiring large neutron generation. In addition, the resultant seismic signals have been used to obtain a more accurate understanding of the earth's interior and have produced new information on earthquakes.

2. For the Excavation application, the objective is to develop the technology required to use nuclear explosives safely in large earth moving projects -- which have heretofore been either economically infeasible or conventionally impossible. Such projects include building harbors, cutting mountain passes for highways and railroads and in blasting for canals such as the interoceanic sea-level canal presently being investigated by the Atlantic-Pacific Interoceanic Canal Study Commission. Chart VII-A illustrates comparative cost of one possible route based on a previous study made in 1960, on the following page.

3. In the Underground Engineering application, both Government and private industry are interested in the development of the completely contained explosion technology because of the probability that underground chimneys of broken rock and fractured areas can be used in the recovery of natural

TRANSISTHMIAN CANAL

Chart VII-A

CONVERT EXISTING CANAL TO SEA LEVEL ROUTE	← EACH → ABOUT 50 MILES	SASARDI · MORTI ROUTE
**CONVENTIONAL EXCAVATION**		**NUCLEAR EXCAVATION**
**$ 2.3 BILLION**	**ESTIMATED COST**	**$ 0.8 BILLION**
**10 YEARS**	**EXCAVATION TIME**	**6 YEARS**
**600' WIDE 60' DEEP**	**CHANNEL SIZE**	**1000' WIDE 250' DEEP**
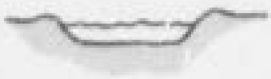	**PROFILE**	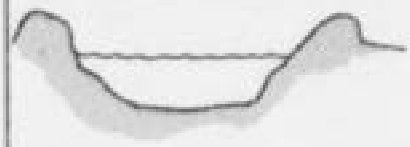

resources such as oil, gas, and mineral ores and storage of water, gases and wastes. Here, the Commission[1] has indicated that it wishes to be guided by the interests of industry in undertaking joint demonstration projects to test the practical utilization of nuclear explosives for industry. Chart VII-B, on the following page, illustrates applications of Deeply Buried Nuclear Explosions.

E. Proposed Projects

The following projects are at various stages of consideration as a result of expressions of industry interest.

1/ Statement of Chairman Glenn T. Seaborg, on January 5, 1965, during hearings on the Plowshare Program before the Joint Committee on Atomic Energy.

Chart VII-D

APPLICATIONS OF DEEPLY BURIED NUCLEAR EXPLOSIONS

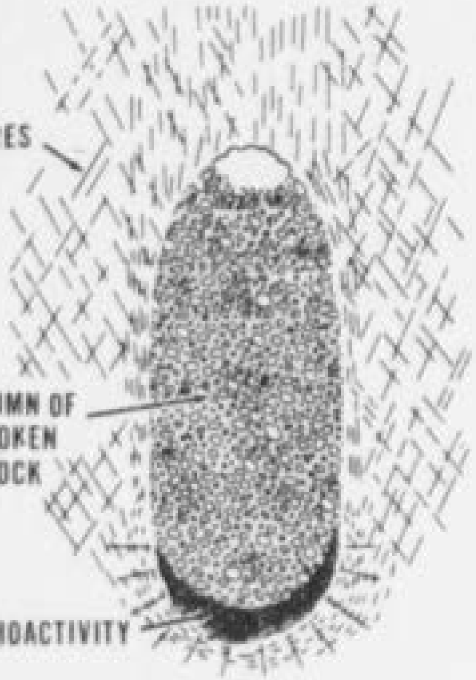

- TERMINAL GAS STORAGE
- WASTE DISPOSAL
- RECOVERY OF GEOTHERMAL HEAT
- GAS RESERVOIR STIMULATION
- LEACHING OF MINERAL ORE
- BREAKAGE OF ORE FOR MINING
- WATER CONSERVATION

1. Project Gasbuggy

Gasbuggy is the name given a proposed joint Government-industry experiment involving the AEC, U. S. Bureau of Mines and the El Paso Natural Gas Company to test the capability of nuclear explosives to stimulate the production of natural gas from "tight" underground formations. The project, proposed for location in Rio Arriba County of northwestern New Mexico, received considerable attention by the Congress during the Joint Committee on Atomic Energy authorization hearings for AEC for Fiscal Year 1967. Gasbuggy was included in AEC's 1967 authorization law. Plans are being made for the conduct of the experiment during 1967, when fuels become available.

The U. S. Bureau of Mines has estimated that if the Gasbuggy technology can be demonstrated, as much as 317 trillion cubic feet of natural gas would become available from Rocky Mountain reserves alone. This would have the effect of more than doubling the Nation's proven reserves.

In Gasbuggy, as in all other nuclear detonations, the AEC's Nevada Operations Office is responsible for conducting all nuclear explosive operations, both at the Nevada Test Site and at selected off-site locations. The Nevada Operations Office is specifically responsible for preparing for and conducting the detonation and attendant safety aspects of all nuclear explosive operations.

2. Projects Dragontrail and Rulison

Two follow-on projects to Gasbuggy, which would also be joint experiments in gas stimulation by nuclear explosives, have recently been proposed to AEC. Project Dragontrail has been proposed by the Continental Oil Company, Houston, Texas, and CER Geonuclear, Inc., Last Vegas, Nevada. Project Rulison was proposed by the Austral Oil Company of Houston, Texas, and CER Geonuclear, Inc. Both projects would be located in the Piceance Creek area of western Colorado.

3. Project Ketch

The Columbia Gas System Service Corporation and AEC are now completing a feasibility study concerning the application of nuclear explosives to create underground gas storage facilities. In this application the chimney and permeable zone created by the underground explosion is expected to provide economic gas storage space geographically near large market areas to satisfy peak load demands at a cost below that of using a depleted gas field remote from the market.

Mr. Charles Atkinson of the U. S. Bureau of Mines, Bartlesville (Oklahoma) Research Station, writing in the Oil and Gas Journal for August 22, 1966, indicates that the importance of natural gas storage to the growth of the gas industry is increasing each year. The article points out that with the major supply of natural gas located in the Southwest, adequate storage capacity must be provided in the major market areas of the East and West, or alternatively long and expensive transmission lines must be constructed.

4. Project Sloop

A joint study with the Kennecott Copper Company for investigating the feasibility of in-situ leaching of copper-bearing ore following fracturing of the ore body by nuclear explosives is being completed. Consideration is now being given to the possibility of a joint project by Kennecott, AEC, and the U. S. Bureau of Mines.

5. Oil Shale

Following a July 20, 1966, meeting in Washington, D. C., CER Geonuclear Corporation, in conjunction with approximately 25 American Oil companies, is continuing with plans and studies to investigate the feasibility of utilizing nuclear explosives in the recovery of oil from shale rock. The recovery procedure would involve an in-situ retorting of the shale after fracturing of the oil bearing strata with a nuclear explosion. The U. S. Bureau of Mines is continuing their extensive long-term research program in oil shale technology including the use of nuclear explosives.

Dr. Walter R. Hibbard, Jr., Director of the U. S. Bureau of Mines, has indicated that nuclear fracturing may turn out to be the key to making the 2 trillion barrels of oil locked up in Western shale deposits available for production. In an interview in the Oil and Gas Journal, dated August 15, 1966, he indicated that nuclear explosives may be the key for accomplishing the badly needed expansion of this Nation's oil and gas reserves.

The Commission will be happy to receive other industry suggestions for specific projects which can be jointly examined to determine their nuclear and industrial feasibility. More detailed information concerning the Plowshare Program may be found in:

1. "Plowshare Program During 1965", for sale by the Superintendent of Documents, U. S. Government Printing Office, Washington, D. C., 20402, at $0.15, and

2. "Plowshare", a booklet in AEC's "Understanding the Atom" series. Single copies are available free from the U. S. Atomic Energy Commission, P. O. Box 62, Oak Ridge, Tennessee, 37830.

Two recently produced films describing the program are also available through AEC film libraries. They are entitled, "Plowshare" and "Safety in the Plowshare Program".

Companies interested in discussing such joint projects may direct their inquiries to the Division of Industrial Participation or to Mr. John S. Kelly, Director, Division of Peaceful Nuclear Explosives, U. S. Atomic Energy Commission, Washington, D. C., 20545.

F. Charges

The AEC is not authorized at present to supply nuclear explosive services on a commercial basis; however, as noted above, it can enter into research and development arrangements with respect to peaceful uses of such explosives, including demonstration of a particular application. Potential users may plan on a charge of about $350 thousand per nuclear explosive with a yield equivalent to 10 kilotons (10 thousand tons) of TNT, gradually increasing to a charge of about $600 thousand for a nuclear explosive with a yield equivalent to about two megatons (2 million tons) of TNT. The tentative charge would cover arming and firing services, as well as the explosive itself. These charges represent a projection based on current and future developments and production in quantity and should be used only for planning purposes. Nevertheless, the charges are believed to be sufficiently appropriate to warrant use in feasibility studies. A projection of the future charge for nuclear explosives, for use in studies, is shown in Chart VII-C.

Chart VII-C

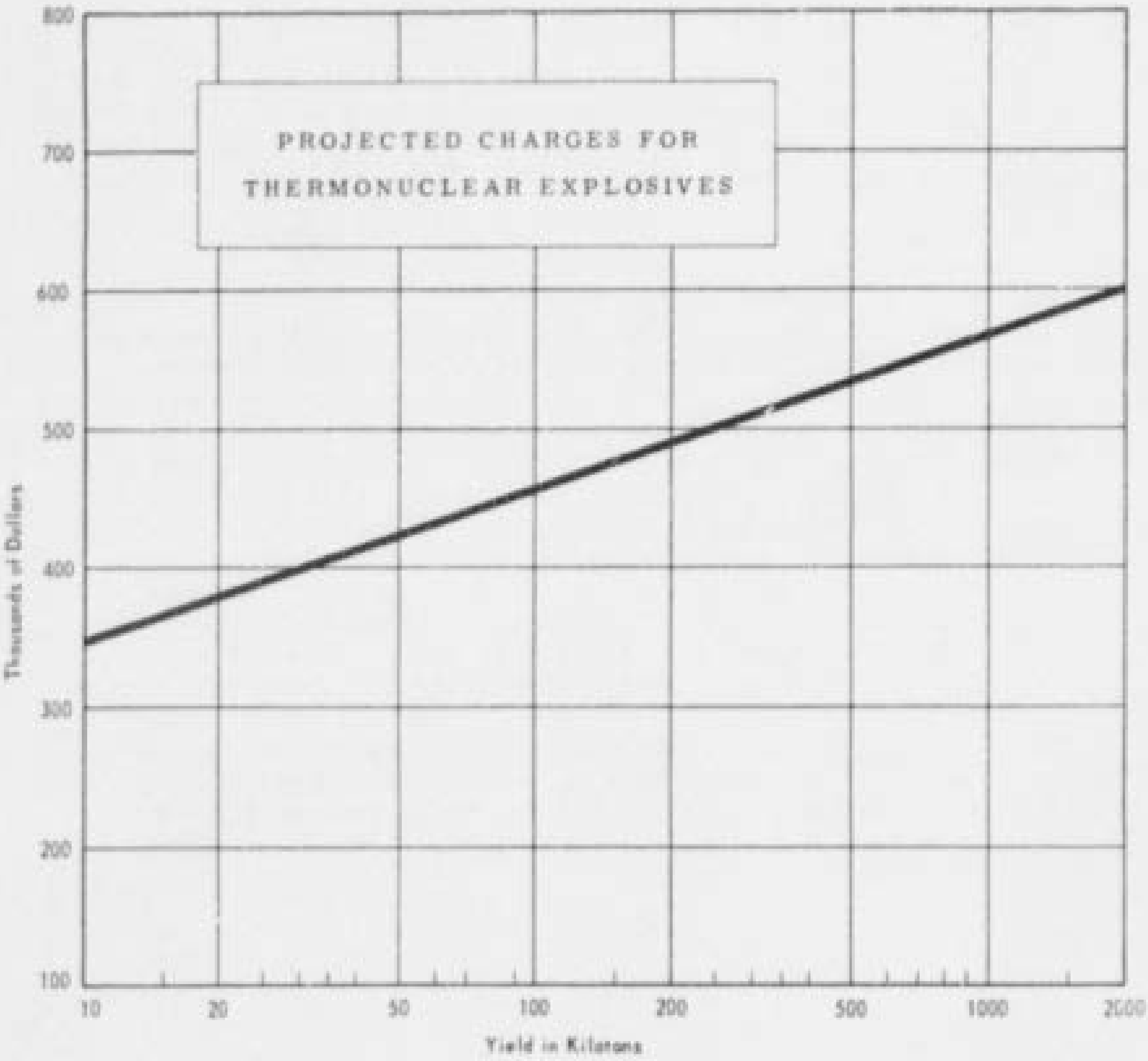
PROJECTED CHARGES FOR
THERMONUCLEAR EXPLOSIVES
800
700
600
500
400
300
200
100
Thousands of Dollars
10
20
50
100
200
500
1000
2000
Yield in Kilotons

CATEGORY VIII
SERVICES

This category includes a wide range of nuclear service type activities, many of which are performed by organizations with interests outside the nuclear energy field.

Last year we reported on five specific service areas: waste management, film badge processing, decontaminated laundry, transportation and private nuclear consulting firms. This year three new areas, portable whole body counting, reactor servicing and decontamination, have been included in this category.

A. Radioactive Waste Management

Radioactive wastes are generated in various forms (liquids, solids and gases) and radiation levels in the nuclear fuel cycle. Wastes, which are generated in the cold or pre-irradiation phase of the fuel cycle (from the mine to the reactor), as well as wastes resulting from laboratory and medical research and in industrial applications of radioisotopes, are generally considered as low level or low hazard potential wastes. In terms of radioactivity concentration, the liquid wastes from these sources are normally in the range of millionths of a curie per gallon. The emphasis in the disposal of low level solid radioactive wastes has been increasingly shifting toward land burial.

High level wastes are almost entirely generated as a result of fuel irradiation and the subsequent chemical reprocessing which produces wastes of high radioactivity concentration. These wastes are normally stored in large underground tanks. An interesting comparison on the amount of high level liquid waste that will be generated in the reprocessing of fuel from commercial power reactors is that the total estimated accumulated volume to the year 2000 will not add up to the total volume existing today at AEC sites. At the present time the AEC has about 70 million gallons of high level liquid waste in storage. It is estimated that the waste resulting from chemical reprocessing commercial power reactor fuels (based on 734,000 Mwe installed nuclear electric capacity at the year 2000) will be approximately 31 millions gallons by the year 2000, if it all remains in the liquid form.1/ With the improvements expected from advanced methods, particularly solidification, and the removal of some fission products

---

1/ R.E. Blanc, ORNL, "Waste By Product Production," Presented at the Nuclear Power Briefing for the Coal Industry, September 29-30, 1966, Oak Ridge, Tennessee.

for useful industrial applications, the quantity of waste to be stored and the problems of storage should continue to decrease. Considerable research and development work has been concentrated in the high level waste area in order to improve the technology utilized in its treatment and storage. High level wastes are stored near the plants where irradiated fuel is processed. There are five Government plants and one private plant now in operation for reprocessing fuel.

The terms "low level" and "high level" are relative terms and are subject to varying interpretations at different nuclear installations. For purposes of this report, low level radioactive wastes are those of such penetrating radiation level and contamination potential that they may be shipped by simple modification of conventional packaging. Conversely, high level radioactive wastes are those of such penetrating radiation level or contamination potential that they require complex or costly handling and packaging techniques.

The Atomic Energy Commission directly, and by delegation of authority to agreement states, is charged with the responsibility of controlling the handling, transporting and disposal of this material. The general requirement for establishment of a land burial site is that the land be owned by the Federal Government or a State Government. The AEC's Rules and Regulations, 10 CFR Part 20, spell out in detail the standards established by the Commission in the control of waste disposal.

Under subsection 274b of the Atomic Energy Act of 1954, as amended, the Commission has the authority to enter into agreements with individual states for the discontinuance and transfer of certain of the Commission's regulatory responsibilities. Among these responsibilities are those relating to the disposal of low and high level wastes within a state. The Commission has, thus far, entered into agreements with fourteen states (Chart VIII-A). A map showing the location of commercial radioactive waste burial sites is presented in Chart VIII-B.

1. <u>Low-Level Waste Disposal</u>

On May 14, 1963, the Commission approved withdrawal of the AEC from its interim low level waste burial operations provided to licensees, based upon the availability of commercial service from Nuclear Engineering Company (NECO) at its burial sites at Beatty, Nevada, and Morehead, Kentucky.

NECO actually began burial operations at its Nevada site in October 1962 and its Kentucky site in May 1963. A second, competitive burial activity was established in the East, with the entrance in November 1963 of Nuclear Fuel Services' (NFS) burial site at West Valley, New York. A third burial service company, California Nuclear, Inc. (CNI) obtained a license on September 10, 1965, for operation of its disposal site near Richland, Washington. The land being used by CNI is being leased to the State of Washington by the AEC for a 99-year period. Table VIII-1 gives a summary of commercial waste burial operations of the above companies since January 1963. Several other companies, in cooperation with various states, are currently studying the possibility of establishing this service.

TRANSFER OF MATERIALS LICENSING FUNCTIONS TO STATES

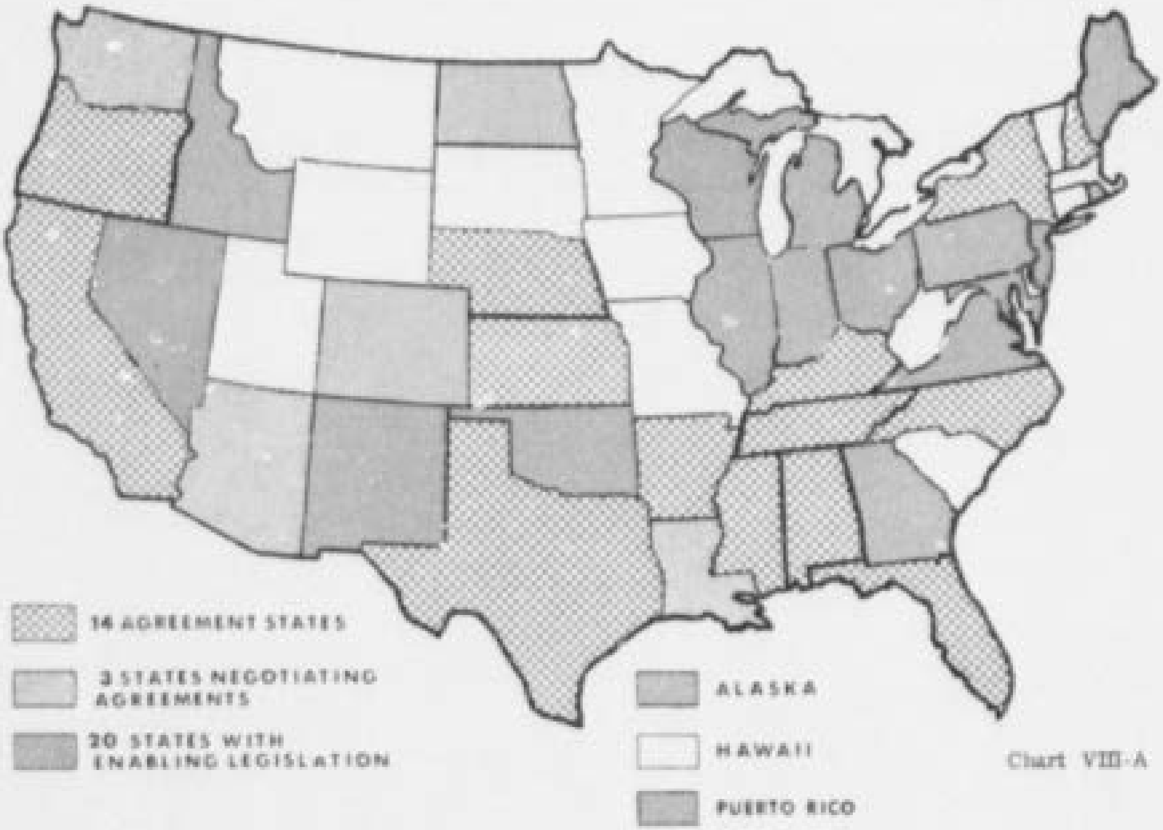

Chart VIII-A

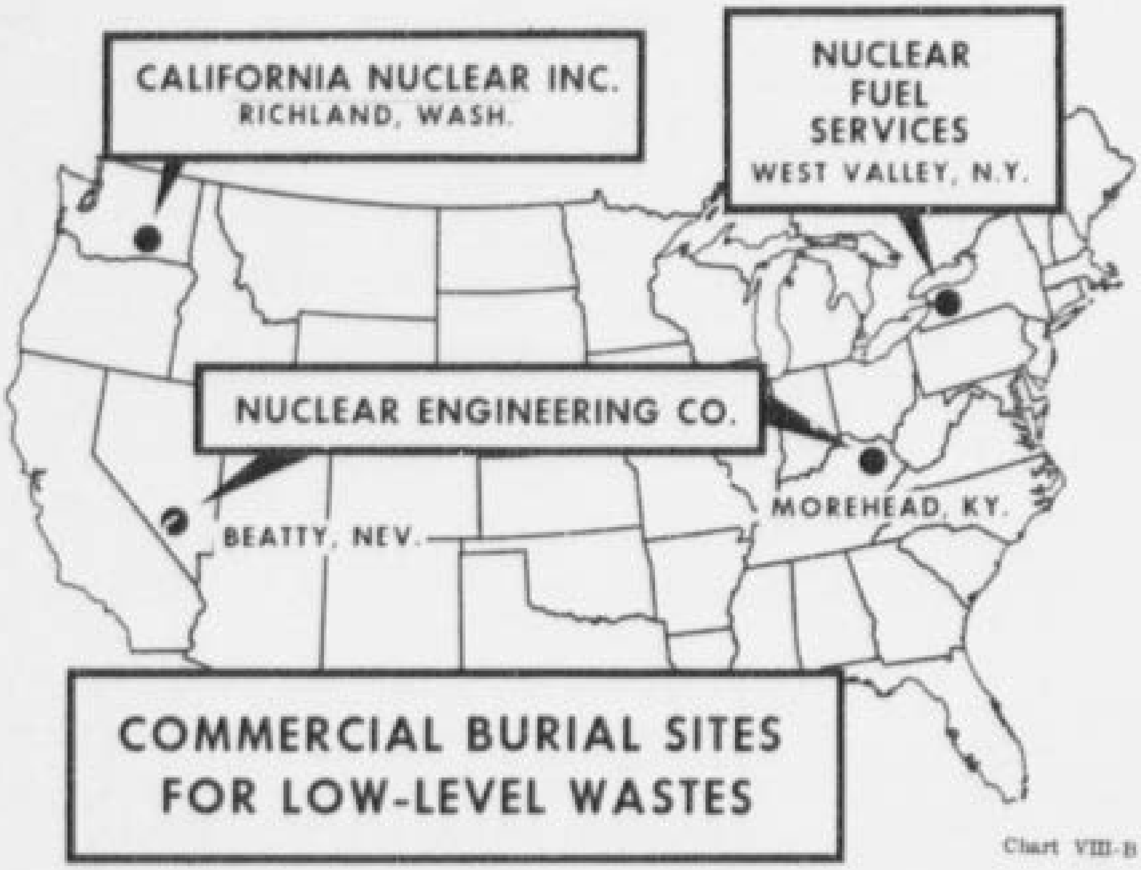

Chart VIII-B

TABLE VIII-1

Private Waste Burial (Cubic Feet)

	Jan.-June	July-Dec.	Annual Total
1963	120,069	95,821	215,890
1964	241,660	205,434	447,094
1965	258,997	234,480	493,477
1966	259,419	-	-

Another facet of the radioactive waste business, in addition to burial itself, is the operation of collection, transportation and storage services. Companies licensed by the AEC in the handling of radioactive wastes are listed in Table VIII-3. As of August 1966, Allied Crossroads Nuclear Corporation ceased operations in their radioactive waste handling and decontamination service. A new addition during the year is ATCOR, Inc., of Hawthorne, New York. This waste disposal firm is specializing in on-site decontamination and material-handling techniques, as well as providing A-E services for the design of integrated waste systems.

Six AEC Operations Offices maintain one or more on-site waste disposal facilities. Their use is limited to waste generated on-site, off-site classified waste and, in some minor exceptions, to licensees. A breakdown of disposal activity at these AEC sites is given in Table VIII-2.

2. High-Level Waste Disposal

At present, only a very small percentage of the total volume of radioactive materials generated throughout the commercial nuclear industry falls into the high level category, although large quantities have been generated over the years from the reprocessing of government reactor fuel elements. Spent fuel elements, after removal from the production reactor, are shipped intact to a fuel reprocessing plant for recovery of the uranium and plutonium. It is in this part of the fuel cycle that essentially all of the high level wastes originate. Nuclear Fuel Services, Inc., in addition to its low level waste disposal activity, is now generating and storing high level waste from its chemical spent fuel reprocessing plant which began operations on April 19, 1966. (See Category VI-A).

3. Waste Research and Development Program

The AEC is supporting a number of research activities aimed at developing new and improved methods for the storage and disposal of high level wastes. In the last few years, the AEC has made significant progress in the development of means of solidifying high level wastes. The conversion of large volume high level liquid wastes to more compact and easier handling inert solid forms is one process which has received R&D emphasis. Several liquid/solid conversion approaches are under study, including such processes as fluidized bed, heat

[illegible]

pots, radiant heat spray columns and glass forming additives. In December 1963, the Waste Calcination Facility (WCF) in Idaho became the world's first plant-scale facility for converting high level waste to solid form. The Waste Solidification Engineering Prototype Plant (WSEP) at Pacific Northwest Laboratory is another facility which is designed to demonstrate, on an engineering scale, the conversion of power reactor fuel reprocessing waste to a solid form. This plant started operations in the fall of 1966.

TABLE VIII-2

AEC Solid Waste Burial
(Cubic Feet)

SITE	July - Dec. 1963	Jan. - June 1964	July - Dec. 1964	Jan. - June 1965	July - Dec. 1965
Savannah River	236,000	201,000	234,000	218,000	286,000
Richland	98,000	143,000	173,000	164,000	140,000
Idaho	131,000	116,000	105,000	142,000	117,000
Oak Ridge Nat. Lab	199,000	122,000	72,000	116,000	94,000
Los Alamos Sc. Lab	110,000	31,000	45,000	32,000	63,000
Fernald	141,000	116,000	50,000	29,000	15,000
Y-12 (Oak Ridge)	20,000	30,000	27,000	18,000	15,000
Others*	10,000	8,000	7,000	10,000	24,000
TOTAL	945,000	767,000	713,000	729,000	754,000

* Sandia, Pantex, NTS, ORGDP (K-25), Paducah, Portsmouth.

TABLE VIII-3

Commercial Firms Licensed by the U.S. Atomic Energy Commission to Receive and Dispose of Radioactive Wastes

New England Tank Cleaning Company
135 First Street
Cambridge, Massachusetts 02141

California Salvage Company
700-745 N. Pacific Avenue
San Pedro, California 90731

ATCOR, Inc.
360 Bradhurst Avenue
Hawthorne, New York 10532

Nuclear Engineering Company, Inc.
Box 594
Walnut Creek, California 93002

California Nuclear, Inc.
2323 South Ninth Street
Lafayette, Indiana 47905

Laboratory for Electronics, Inc.
Tracerlab Division
1601 Trapelo Road
Waltham, Massachusetts 02154

The Walker Trucking Company
1283-1285 East Street
New Britain, Connecticut 06053

Long Island Nuclear Service Corp.
Station Road
Bellport, New York 11713

Radiological Service Company, Inc.
811 West Merrick Road
Valley Stream
Long Island, New York 11101

Atomic Disposal Company, Inc.
Box 141
Matteson, Illinois 60443

These firms should be contacted directly for information regarding services which they can provide.

NOTE: This list does not include commercial firms licensed entirely by "Agreement States." An "Agreement State" is defined as any State with which the Commission has entered into an effective agreement under subsection 274b of the Atomic Energy Act of 1954.

The Oak Ridge National Laboratory has recently adopted the hydraulic fracturing technique as its routine means for the disposing of intermediate level liquid wastes. The method involves blending the waste with cement and other additives and pumping the mix, under high pressure, into underground shale fractures 700 to 1000 feet underground.

Byproducts that we now call waste are emerging as valuable sources of useful products and one can question whether they should be referred to as "waste". In this regard, the Commission is supporting a program whereby long life fission products, such as strontium, cesium, curium and promethium will be removed before the remaining wastes are stored. Some of these fission products are now becoming valuable for use as radiation or heat sources in a number of applications. In fact, Isochem, Inc. (a company formed by Martin-Marietta and U.S. Rubber) is investing approximately $8 million for the construction of a Fission Products Conversion and Encapsulation Plant (FPCE) on the basis that they will be able to find a market for the long life fission products from high level waste generated at the AEC's Hanford Works. These fission products are being recovered for use by industry, as well as to assist in the waste management program. The nuclear utilities that are having their fuels reprocessed at NFS, along with NFS, are looking at the possibility of extracting fission products from their high level waste.

D. Film Badge Processing

In 1963, we reported that there were from 2-1/2 to 3 million film badges processed commercially at a dollar volume of about $1-1/4 million. In the same year, about 1-3/4 million badges were processed at AEC sites representing a dollar volume of about $1 million. No new survey has been made since 1963. As a result of the general increase in nuclear activities since 1963, however, the volume of film badges being processed is no doubt increasing. Any increase is probably offset to some extent by the changes in badge use and control, since badge utilization time has been continuously increasing.

Private companies are well represented in this phase of the nuclear industry with approximately 18 companies offering this service (Table VIII-4). Chart VIII-C shows the principal film badge operations in the United States. One company, AND, Inc., of Chicago,

TABLE VIII-4

Film Badge Processors

Applied Health Physics, Inc.
Bethel Park, Pennsylvania

Atomic Energy Industrial Laboratories
Houston, Texas

Controls for Radiation, Inc.
Cambridge, Massachusetts

Eberline Instrument Corporation
Santa Fe, New Mexico

T. M. Gaines Company
Berkeley, California

Gard-Ray Film Badge Service
Burlington, Massachusetts

Health Physics Services, Inc.
Baltimore, Maryland

R. S. Landauer, Jr., and Company
Matteson, Illinois

Nuclear-Chicago Corporation
Des Plaines, Illinois

Nuclear Consultants Corporation
St. Louis, Missouri

Nucleonic Corporation of America
Brooklyn, New York

Nuclear Services Laboratory
Knoxville, Tennessee

Radiation Detection Company
Mountain View, California

Tracerlab
Waltham, Massachusetts

St. John X-Ray Laboratory
Califon, New Jersey

Technical Associates
Burbank, California

United States Testing Company
Hoboken, New Jersey

PRINCIPAL FILM BADGE OPERATIONS IN THE UNITED STATES

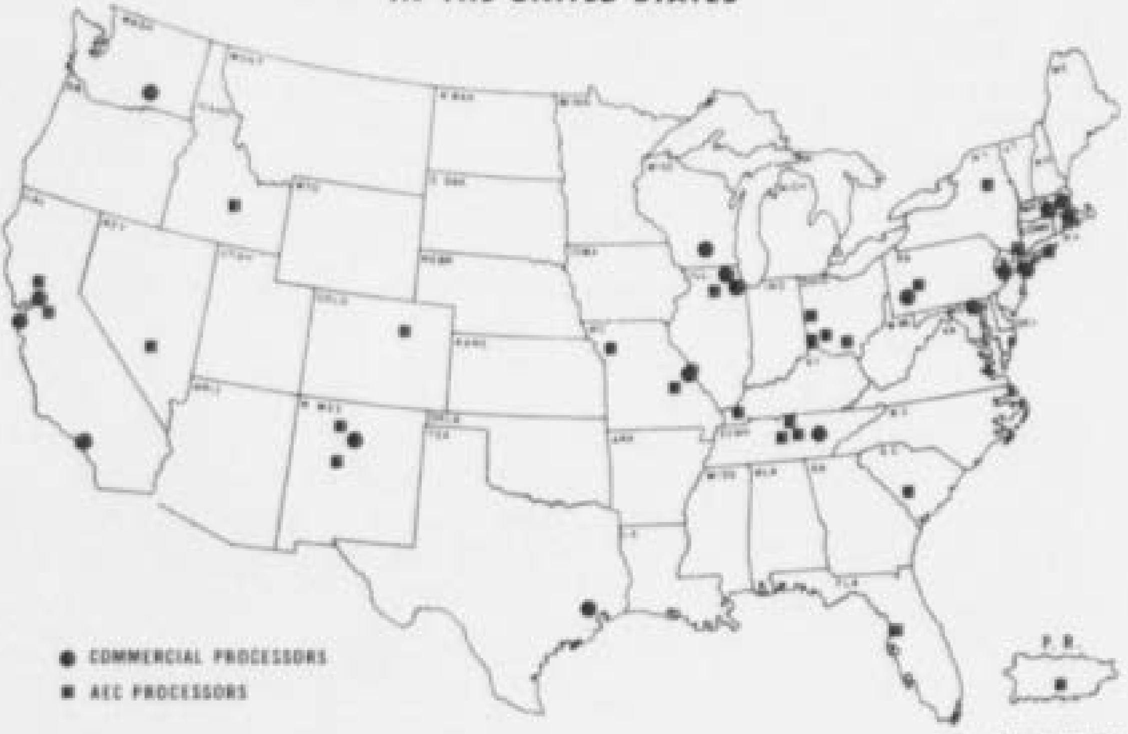

Chart VIII-C

Illinois, has suspended operations in this service during the past year.

The film badge industry is currently providing film badge service for 16 of 39 AEC sites and contractors. For the most part, however, these are small order contracts, with the exception of the U.S. Testing contract at Richland, Washington. In general, the AEC sites with large volumes are not using commercial services.

To date, the film badge industry in general, has been unsuccessful in providing film badge service for AEC sites and contractors. Some of the problems associated with industry providing this service are: (1) the investment already existing at Government sites, (2) the potential labor problems in shifting work from Government facilities and contractors to private industry, (3) the use of incremental costing under BOB Circular A-76 for calculating existing AEC operating contractor costs versus private industry bids, (4) the difficulty in establishing performance specifications rather than emphasizing detailed technical specifications, and (5) the setting up of administrative specifications which are acceptable to both the AEC operating contractors and the film badge processors.2/

It has been recognized for some time that a dosimetry standards laboratory would be desirable from the standpoint of the users of dosimetry equipment, including AEC, the suppliers and regulatory groups. Some of the factors which must be considered in establishing standards are: (1) the AEC's responsibility to the public in regard to health and safety matters and (2) the long-term maintainence of all personnel film dosimetry records.3/ The Commission has been giving considerable attention to the development of a program leading to appropriate standards and testing procedures whereby personnel dosimetry can be evaluated. The need for criteria to measure the adequacy of dosimetry services, including AEC's interest in the establishment of a film dosimetry calibration laboratory was discussed in a Federal Register Notice in September 1963.4/

2/ See remarks given by Ernest B. Tremmel before the AEC and Contractor Health Protection Meeting in Los Angeles, October 14, 1964.

3/ Remarks by Commissioner James T. Ramey at the International Association of Machinists, August 25, 1966, AEC Public Release IN-714. Also see AEC Public Release IN-715, dated September 14, 1966.

4/ 22 FR 9678.

Possible industrial sponsorship of a laboratory to establish standards was also the subject of a meeting with representatives of the nuclear industry, the Atomic Energy Commission, National Bureau of Standards and the U.S. Public Health Service during July 1964, under the auspices of the Atomic Industrial Forum. After continuing study of this problem, the Commission established a program at its Pacific Northwest Laboratory for the further development of performance criteria and procedures for the evaluation of film dosimetry services (see AEC Public Release IN-616 issued September 9, 1965). In addition, this study will consider ways and means to appraise any evaluation laboratory that might be established. The final program report by Battelle is scheduled for completion shortly after July 1, 1967. During the study, coordination will be maintained with appropriate industrial, private and Government groups. The results of the study will be made available to interested persons. Information on this program can be obtained from Dr. S. Allan Lough, Division of Biology and Medicine, U.S. Atomic Energy Commission, Washington, D. C. 20545.

A non-profit group, the National Sanitation Foundation (NSF), has embarked on the establishment of a film dosimetry calibration and certification laboratory. They announced on May 17, 1966, that their Board of Trustees had adopted a performance standard for film badge services on May 4, 1966, and formal evaluations of film badge services began on May 16, 1966. Those participating companies who demonstrate the ability to meet the requirements of the NSF standard will be eligible to apply for use of the Foundation's Seal of Approval beginning in the early summer of 1967. The copyrighted seal will certify that a service has satisfied the requirements of the Standard.

This is the first program of periodic evaluation of film badge services to be announced in this country. The Standard which serves as the basis for the evaluations has taken three years to develop. The Standard, as developed, was supported with technical and financial contributions from many different organizations interested in radiation dosimetry with film badges. A research program which provided basic data utilized in the development of the Standard was supported by the U.S. Public Health Service. Information in regard to this program can be obtained from Mr. Charles A. Farish, Executive Director, National Sanitation Foundation, University of Michigan, School of Public Health, Ann Arbor, Michigan 48103.

On September 8, 1966, a group of companies, representing about 85% of the current film badge reprocessing industry, met with representatives of the AEC. The purpose of their visit was to discuss various phases of the film badge processing service with particular emphasis on how private industry can provide this service for AEC sites and contractors not now utilizing commercial services. It was concluded from this meeting that the AEC sites would be informed of the situation and encouraged to give consideration to using private reprocessing services.

C. Decontamination Laundry Services

On December 22, 1964, the Commission approved a policy of encouraging the use of commercial services for processing AEC-generated contaminated laundry. As part of AEC's effort to increase industrial participation in the atomic energy program, most locations where AEC now conducts in-house laundry operations have established specifications for their laundry needs. During the year, several AEC locations solicited proposals for this service, but problems were encountered in attempting to place this work commercially.

Among the problems in transferring this activity to industry are those of assuring continuity of jobs for workers at the AEC sites, the investment already existing at Government sites and assuring comparable wage rates when a commercial laundry is established at or in the vicinity of the AEC plant. In addition, during the year BOB Circular A-76 ("Policies for Acquiring Commercial or Industrial Products and Services for Government Use") was issued, which allows the use of incremental costing of existing Government activities in the cost analysis when comparing on-site versus commercial bids.

Eight private companies are actively engaged in the nuclear laundry business (Table VIII-5). Collectively, they service such nuclear clients as the public utilities, hospitals, various segments of our private nuclear industry and 14 AEC sites and contractors. The AEC load processed by industry is estimated at about 2,500,000 pounds.

There are currently 18 AEC contractor sites processing contaminated laundry on-site (Table VIII-6). The total annual load of these on-site operations is estimated at about 17 million pounds.

Chart VIII-D indicates the location of the decontamination laundry operations in the United States.

TABLE VIII-5

Commercial Licensees
Companies Actively Processing Contaminated Laundry

1. Interstate Industrial Laundry Service, Inc.
   Santa Fe, New Mexico
2. Interstate Industrial Laundry Service, Inc.
   Pleasanton, California
3. Interstate Industrial Uniform Service, Inc.
   Indian Orchard, Massachusetts
4. Nuclear Laundry Rental Services, Inc.
   Jeanette, Pennsylvania
5. Nuclear Laundry Service, Inc.
   Waterbury, Connecticut
6. Nuclear Materials and Equipment Corp.
   Apollo, Pennsylvania
7. Penn Overall Supply Company
   Pittsburgh, Pennsylvania
8. Interstate Industrial Uniform Rental Service, Inc.
   N. Charleston, South Carolina

Companies Licensed
But Not Currently Processing Contaminated Laundry

1. Santa Fe Electric
   Santa Fe, N. M.
2. Carter Industrial Laundry
   Washington, D. C.
3. Idaho Nuclear Laundry Corp.
   Idaho Falls, Idaho
4. Consolidated American Services, Inc.
   Hawthorne, California
5. Sanitary Overall Supply Co.
   Tarantum, Pennsylvania
6. Wilfred S. Kearse
   Charleston, South Carolina

TABLE VIII-6

AEC Sites and Contractors Using
Commercial Decontamination Laundering Facilities

Los Alamos Scientific Laboratory	-	Los Alamos, New Mexico
Atomics International	-	Canoga Park, California
Argonne National Laboratory	-	Argonne, Illinois
Knolls Atomic Pwr. Lab.	-	Schenectady, New York
Lawrence Radiation Lab.	-	Livermore, California
Lawrence Radiation Lab.	-	Berkeley, California
Bettis Laboratory	-	Pittsburgh, Pennsylvania
Sandia Laboratory	-	Albuquerque, New Mexico
Ames Laboratory	-	Ames, Iowa
Argonne Cancer Hospital	-	Chicago, Illinois
Laboratory of Nuclear Medicine	-	Los Angeles, California
Univ. of California Medical Ctr.	-	San Francisco, California
Univ. of Rochester Atomic Energy Project	-	Rochester, New York
Notre Dame Radiation Laboratory	-	Notre Dame, Indiana

Estimated volume: 2,500,000 lbs/year
$500,000/year

AEC Sites and Contractors Not Using
Commercial Decontamination Laundering Facilities

National Lead Company	-	Fernald, Ohio	OR
Union Carbide - Y-12	-	Oak Ridge, Tennessee	OR
Union Carbide - ORNL	-	Oak Ridge, Tennessee	OR
Union Carbide - K-25	-	Oak Ridge, Tennessee	OR
General Electric	-	Hanford, Washington	RL
Du Pont - Savannah River	-	Aiken, South Carolina	SR
Dow Chemical Company	-	Rocky Flats, Colorado	AL
Mason & Hanger	-	Burlington, Iowa	AL
Mason & Hanger - Pantex	-	Amarillo, Texas	AL
Brookhaven National Lab.	-	Upton, New York	BH
Phillips - NRTS	-	Idaho Falls, Idaho	ID
Mallinckrodt Chem. Lab.	-	Weldon Springs, Missouri	OR
Goodyear	-	Portsmouth, Ohio	OR
Union Carbide		Paducah, Kentucky	OR
Reynolds Electric	-	Mercury, Nevada	NEV
Monsanto Chemical- Mound	-	Miamisburg, Ohio	AL
Bendix	-	Kansas City, Missouri	AL
Lucius Pitkin	-	Grand Junction, Colorado	GJ

Estimated Volume: 17,000,000 lbs/year
$3,000,000/year

CONTAMINATED LAUNDRY OPERATIONS IN THE UNITED STATES

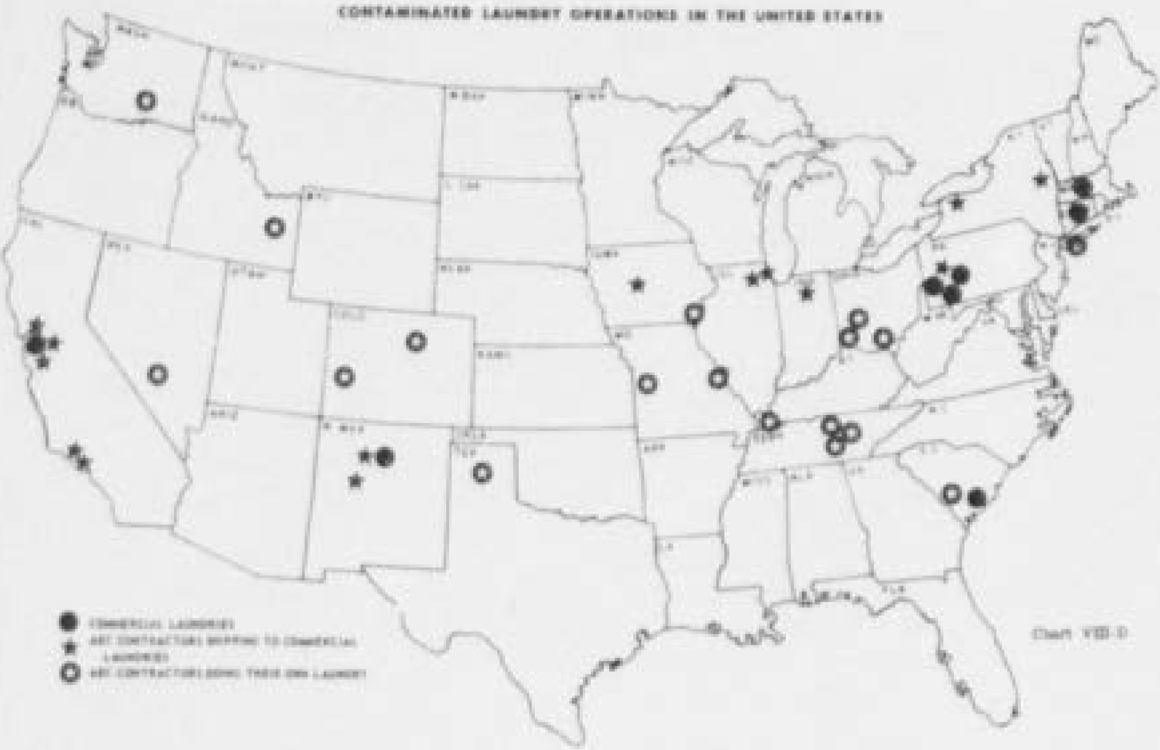

D. Transportation

Shippers of radioactive materials during the past 15-20 years have been principally concerned with the transportation of radioisotopes, weapons, radioactive wastes, uranium and thorium ores and concentrates, unirradiated nuclear fuel materials, fabricated fuel elements and irradiated fuels.

All modes of transportation -- air, surface and water -- are used to ship radioactive materials. Rail and steamship or barge transportation is used for large power reactor spent fuel shipments due to the heavy shielding of the shipping casks required for this type of material. Trucks are also used within their weight carrying limitations. It has been estimated that the annual transportation charges associated with nuclear power plants alone may reach $50-80 million in a nuclear economy of 80 thousand Mwe installed nuclear capacity.

There are no insurmountable technical problems hindering the movement of radioactive materials. However, there are some problems in the area of insurance and indemnity which can adversely affect their shipment. There is a lack of appropriate insurance coverage to protect against the risks of transporting radioactive materials not subject to indemnification under the Price-Anderson Act. Upon request of a carrier, the AEC will issue a certification regarding the Price-Anderson Act indemnification coverage applicable to any particular shipment within the United States.

Air -- Air transportation of radioactive materials has frequently been the only solution to shipping cold nuclear fuels and radioactive materials overseas because of the difficulty in obtaining indemnification for steamship service.

Truck -- Motor carriers are willing to accept shipments of irradiated fuels to and from all parts of the country within the weight limitations permitted.

Rail -- The eastern railroads do not consider themselves to be common carriers for spent fuels and have not published rates for transporting spent fuel. To date, acceptance of private power reactor spent fuel for transportation by eastern railroads has been contingent upon a private contract basis with the shipper providing the railroad written assurances on protection should an accident occur. Continued efforts will need to be made by individual owners of spent fuel to work out long-range solutions with the eastern railroads. Railroads in other areas of the country have published rates, and there has been no difficulty in shipping irradiated fuels in other areas.

Water -- Transportation of radioactive materials, especially irradiated fuels, and also cold nuclear fuels on the high seas is difficult and frequently impossible to arrange due to lack of satisfactory nuclear indemnification. Transportation of these materials by water domestically has not been a problem.

1. Studies

A number of studies concerning transportation of radioactive materials have been made in the past. The most recent is the Southern Interstate Nuclear Board (SINB) study completed this year under contract to the AEC on "International Traffic of Radioactive Materials". This report covers a broad range of subjects relating to the transportation of radioactive materials in domestic and foreign commerce, including detailed study of the following general areas: land, water and air transportation; bridge, tunnel and turnpike matters; insurance and indemnity; legal factors; casks and containers; economics of spent fuel transportation; health and safety; physical and human capabilities and present and future markets. The report concludes that three major obstacles presently affecting the transportation of radioactive materials are: (1) the refusal of the eastern railroads to transport fissile materials as common carriers; (2) the lack of satisfactory nuclear indemnification for movements on the high seas and by means of air transport and (3) the lack of indemnification which, in the view of bridge, tunnel and turnpike authorities, would enable them to offer the use of their facilities as applied to other types of cargo.

2. Costs

An estimate of the cost of transporting irradiated fuels to the United States is contained in the SINB study mentioned previously. This estimate is based on the most up-to-date information available and was made by individuals directly concerned with the shipment of irradiated fuels and radioactive materials.

For a single round trip using a 100 ton power reactor cask carrying ten fuel elements or 10 thousand pounds uranium (4540 kg), the estimated cost of transportation from Europe to a chemical reprocessing facility such as the NFS facility near Buffalo would range from $45 thousand to $50 thousand including insurance and indemnification. The cost of uranium shipped would range from $9.90 to $11.00 per kilogram. The reprocessing charge for this quantity of power reactor fuel would be approximately $160,000.

As noted in our report last year, the transportation costs to the NFS reprocessing facilitity at West Valley, New York, including casks rental amounted to approximately $2.50 per kilogram of uranium shipped for the Yankee fuel and $3.00 for the Dresden fuel. Insurance was not required in these instances.

The costs of insurance and indemnification for the transportation of radioactive materials depends upon many factors including type of material, mode of transportation, distance, value of material, country of origin of shipment and destination.

3. Transportation Regulations

Shipments of radioactive material are covered by the regulations of several Federal agencies, including the AEC, the Interstate Commerce Commission, the Coast Guard, the Federal Aviation Agency and the Post Office Department. The AEC is coordinating its regulatory control in this area with these other agencies.

In a Memorandum of Understanding between AEC and ICC, signed on March 21, 1966,5/ the agencies agreed that AEC will adopt regulations applicable to preparation for shipment of fissile material and large quantities of radioactive material, and, as necessary, for radioactive material when its shipment is outside the jurisdiction of ICC. ICC will adopt regulations consistent with AEC standards, on transportation of all radioactive material and on preparation for shipment of radioactive material other than fissile material and large quantities of radioactive material.

The ICC issued a revision of its regulations (ICC Order #70) with an effective date of July 12, 1966, which, for the first time, provides rules for shipping fissile material as a routine commodity in transportation.

The AEC's regulations govern the packaging of radioactive materials for transport and are contained in the U.S. Code of Federal Regulation TITLE 10 PART 71. A revision to 10 CFR Part 71 became effective on August 21, 1966.6/ The revision incorporated performance standards for shipping containers and for packing and shipping the fissile materials, uranium-233, uranium-235 and plutonium-238, 239 and 241. The standards include a minimum of detail on required packaging specifications. The emphasis has been placed on performance standards to provide the needed flexibility to develop improved shipping methods. The scope of the regulation has been extended to apply these same standards to large quantities of other licensed radioactive material, such as radioisotopes, in addition to fissile material. The performance standards are compatible with those developed by the International Atomic Energy Agency during the past years.

5/ See AEC Public Release J-64, dated March 18, 1966.
6/ See AEC Public Release J-178, dated July 21, 1966.

4. U.S. Ports Cleared for Receipt of Irradiated Fuels -- Shipments to Foreign Countries

Table VIII-7 lists the U. S. ports cleared for receipt of irradiated fuels. Port clearance involves the filing of a joint resolution by port authority and community officials, with the U. S. Coast Guard and the AEC, certifying their acceptance of irradiated fuels. Table VIII-8 shows shipments to foreign countries. These figures include enriched uranium and plutonium fuel materials, fabricated fuel elements for research and power reactors, research materials, National Bureau of Standards samples and Pu-Be neutron sources. The amount of material in individual shipments varies from milligrams to tons.

TABLE VIII-7

U.S. Ports Cleared for Receipt of Irradiated Fuels

Charleston, South Carolina	Portland Maine
Savannah, Georgia	Searsport, Maine
Norfolk, Virginia	Los Angeles, California
Philadelphia, Pennsylvania	Gulfport, Mississippi
Seattle, Washington	Albany, New York
Houston, Texas	Buffalo, New York
Port Everglades, Florida	Corpus Christi, Texas
Wilmington, North Carolina	Redwood City, California
Morehead City, North Carolina	Portland, Oregon
New Orleans, Louisiana	Tampa, Florida
New York, New York	Jacksonville, Florida
Baton Rouge, Louisiana	Port St. Joe, Florida
Benton, Washington	Bellingham, Washington
Baltimore, Maryland	Olympia, Washington
Willapa Harbor, Washington	Anacortes, Washington
Grays Harbor, Washington	Mattawa, Washington
Everett, Washington	Longview, Washington
Tacoma, Washington	San Francisco, California
Lake Charles, Louisiana	Hilo, Hawaii
Kawaihae, Hawaii	Kahului, Hawaii
Honolulu, Hawaii	Nawiliwili, Hawaii
Port Allen, Hawaii	Kennewick, Washington

TABLE VIII-8

Shipments to Foreign Countries Pursuant To Agreements for Cooperation

Year	Approximate Number of Shipments
1962	250
1963	400
1964	350
1965	270
1966	250

To date AEC has shipped abroad under bilateral agreements approximately 10 thousand kg of U-235 (10 metric tons) representing a value of about $100 million.

AEC expects to ship abroad nuclear fuel at the rate of 2 thousand to 5 thousand kg U-235 per year, primarily for power reactors.

Beginning in 1964, the AEC has received 34 shipments of irradiated research reactor fuel for chemical reprocessing in Commission facilities at Savannah River and Idaho. The material returned represents only about 2% of the enriched uranium transferred abroad. The reprocessing of spent fuel will become an important economic activity as more and more power reactors come into use. There is every reason to believe that U.S. industry can obtain a large share of the foreign reprocessing business provided the various elements in the industry are willing to cooperate.

In the area of irradiated research reactor fuels, the AEC's Division of International Affairs estimates shipments will level off at about 34 shipments per year which may contain a total of about 300 kg of U-235 and could represent some 1300 tons of shipping. A single power reactor could utilize the service of a 100 ton shipping cask for about one year (six round trips) to transport its spent fuel to a chemical reprocessing plant. With the foreign nuclear power growth expected to about equal the U.S. domestic growth, U. S. industry will no doubt want to obtain the greatest possible share of this business.

5. Shipping Containers

a. Efforts to Obtain Standardized Snipping Containers

Efforts need to be made by the shippers of radioactive and fissile material so as to facilitate handling and reduce transportation costs in the area of shipping containers. Shipping casks need to be standardized so that greater interchangability is possible and cask utilization may be increased.

With the increased traffic of nuclear material, particularly that of enriched unirradiated uranium for power reactor fuels, the AEC. in cooperation with the nuclear fuels industry, recognized the need for a universally acceptable off-the-shelf type shipping container.

Special emphasis, therefore, is being directed toward the development of ICC specification containers for the transportation of radioactive and fissile materials, and efforts are currently in process in cooperation with the ICC and the AIF Committee on the Shipment of Nuclear Materials to develop a variety of ICC specification containers. One container for unirradiated fissile material has already been adopted in the ICC regulations as a specification container. In further promoting this program, the AIF is cooperating with the AEC in coordinating industry's review of specification container design proposals. As a part of its role in encouraging the development of appropriate specification containers, the AEC's Division of Construction is developing a guide for the preparation of container design proposals for guidance in submitting proposals to the ICC.

It is expected that many AEC and industry shipping containers which have been approved by AEC for specific uses will be proposed as ICC specification containers. For example, the UF_6 cylinders are presently being proposed as specification containers along with a suitable protective structural package designed and tested by Union Carbide Corporation at Oak Ridge, Tennessee. The thermal shield and the structural package developed for

[illegible]

the 5", 8", 12" and 30" diameter UF_6 cylinders should prove useful for packaging other materials using different inner containers as appropriate.

The AEC has also licensed for use, improved lightweight, low cost conventional type containers and unique transportation container designs submitted by private fuel processors which permit the shipment of greater amounts of material than those used previously. One of these was the NFS 3600 gallon tank trailer for shipping low concentration uranium product solutions.

b. Plutonium Shipping Containers

As noted last year, a series of plutonium nitrate solution and oxide shipping containers have been developed and tested by Dow Rocky Flats, under a contract with the AEC's Division of Nuclear Materials Management. A substantial part of this contract involved the study of shipper-receiver differences in AEC plutonium shipments and the container development was a necessary part of this study. Both the Class I and II containers resulting from this work are satisfactory for licensing and have been approved for plutonium nitrate solution and U-233 solution under specified conditions. A plutonium nitrate solution shipping container designed by NUMEC has also been licensed during the year.

While the three and ten liter Pu shipping containers are adequate for the level of plutonium shipments expected during the next few years, there is a growing need to begin development of larger capacity shipping containers which would permit greater economy in transport and reduce the investment in equipment.

Private firms supplying special services and/or products for the transport of radioactive materials are listed below:

ATCOR, Inc.	Hawthorne, New York
Edlow and Isbrandtsen Assoc.	Columbus Ohio, and New York, N.Y.
Long Island Nuclear Services	Bellport, New York
National Lead Company	New York, New York
Nuclear Engineering Co.	Walnut Creek, California
Nuclear Fuel Services	West Valley, New York
Tri-State Motor Transit Co.	Joplin, Missouri
International Transport, Inc.	Rochester, Minnesota

E. Whole Body Counting Service

A number of commercial organizations are now offering on-site whole body counting services using portable trailer-mounted counters. These services may be useful to hospitals, research laboratories, nuclear materials production plants, nuclear power plants and radioisotope users that must rely on bio-assay to monitor personnel for internal deposition of radioactive material.

The whole body counter measures the gamma and x-ray emitting radioactive nuclei in humans and in animals, while, at the same time, indicating their disposition and, by repeated use, their retention in the body. Accurate measurements of the natural potassium content of the body and inhaled or ingested radioactive isotopes are readily made.

Further information concerning portable whole-body counting services may be obtained by contacting the following commercial firms offering this service:

California Nuclear, Inc.
Mrs. Lugene Hungerford
2323 South Ninth Street
Lafayette, Indiana 47905

Helgeson Nuclear Services
Mr. George Lewis Helgeson
872 Abbie Street
Pleasanton, California 94566

Permian Corporation
Mr. Charles R. Keck
Rte. 2, Box 50
Okmilger, Oklahoma

F. Reactor Servicing

Newport News Shipbuilding and Dry Dock Company, announced on September 14, 1965, the establishment of a wholly-owned subsidiary, Nuclear Engineering and Construction Company, Inc. (now known as Nuclear Service and Construction Company, Inc.), primarily for the purpose of performing specialized work such as refueling and periodic overhaul of land-based nuclear plants for both Government and utility reactor facilities. NSC plans to broaden this service in the coming years to include facility construction.

The New York Shipbuilding and Dry Dock Company of Camden, New Jersey, is now offering a similar type of service. They are prepared to provide any of the reactor servicing activities except refueling.

Todd Shipyards Corporation of Galveston, Texas, has already established this service with the construction and operation of its fuel handling barge and associated equipment and personnel for servicing the N. S. Savannah at the ship's overhaul and maintenance dock at Galveston. Although primarily set up for nuclear ship service, Todd is also interested and capable of offering a similar type service for land-based nuclear facilities.

Additional information concerning this service may be obtained from the following firms:

Nuclear Service and Construction Company
S. A. Foster, Vice President
c/o Newport News Shipbuilding and Drydock Company
Newport News, Virginia

New York Shipbuilding and Dry Dock Company
John R. Thorpe, Manager, Nuclear Services
Camden, New Jersey

Todd Shipyards Corporation
Charles Hathway, Manager, Nuclear Division
Galveston, Texas

G. Decontamination

Several companies now offer the service of removing radioactive contaminants from plant and equipment items. This activity normally includes such routine work as decontamination of reactor parts and equipment, hot cell parts and equipment, glove boxes and the tools and equipment of the nuclear industry which become radioactively contaminated through use. Washing, vacuum cleaning and paint removing are some of the methods used in reducing or eliminating the contaminants.

The companies listed below are offering this service. In every case, they provide this capability in addition to other services such as waste handling, storage, transportation, burial and radiation safety services.

Applied Health Physics, Inc.
2986 Industrial Boulevard
Bethel Park - P.O. Box 197
Pennsylvania 15102

ATCOR, Inc.
360 Bradhurst Avenue
Hawthorne, New York 10532

California Nuclear, Inc.
2323 South Ninth Street
Lafayette, Indiana 47905

Long Island Nuclear Service Corp.
Station Road
Bellport, New York 11713

Nuclear Engineering Company, Inc.
Box 594
Walnut Creek, California 93002

Radiological Service Co., Inc.
35 Urban Street
Westbury, New York 11590

H. Consultants and Engineers

Because of the continuing increase in the number of firms specializing in the nuclear field, the growth of this segment of the industry has been viewed with considerable interest. In past years, this report has contained a listing of consultants in nuclear work. However, the increasing number of firms specializing in nuclear work makes it impractical to continue such a listing.

The Division of Industrial Participation maintains brochure files of consultants and engineers and will be glad to make this information available to the public. The Buyers' Guide issue of Nucleonics magazine (the August issue each year) also contains a very detailed and complete directory of services and consultants. References to these sources should be helpful to those seeking assistance for specific purposes.

ASSISTANCE FOR INDUSTRY AT AEC FIELD OFFICES

Managers of each of the AEC Field Offices have designated representatives from their staffs as primary points of contact on Industrial Participation activities. These representatives serve as focal points within their respective offices for activities for which the Division of Industrial Participation is responsible.

These representatives are:

Mr. Charles C. Campbell
Deputy Assistant Manager
for Administration
Albuquerque Operations Office
U. S. Atomic Energy Commission
P. O. Box 5400
Albuquerque, New Mexico 87115
(505) - 667-5105

Mr. Alfred K. Huse
Chief of Technical Projects
Operations Division
Brookhaven Office
U. S. Atomic Energy Commission
Upton, New York 11973
(516)-924-2360

Mr. D. P. Rudolph, Director
Technical Services Division
Chicago Operations Office
U. S. Atomic Energy Commission
9800 South Cass Avenue
Argonne, Illinois 60439
(312) - 739-2091

Mr. J. C. Westbrook
Assistant Manager for Administration
Grand Junction Operations Office
U. S. Atomic Energy Commission
Grand Junction, Colorado 81502
(303) - 242-8201

Mr. V. V. Hendrix, Chief
Technical Services Branch
Contracts and Support Division
Idaho Operations Office
U. S. Atomic Energy Commission
P. O. Box 2108
Idaho Falls, Idaho 83401
(208) - 526-2521

Mr. William R. Cooper, Director
Plans Division
Nevada Operations Office
U. S. Atomic Energy Commission
P. O. Box 1676
Las Vegas, Nevada 89101
(702) - 734-3791

Mr. Lewis C. Cooper
Classification & Technical
Information Staff
New York Operations Office
U. S. Atomic Energy Commission
376 Hudson Street
New York, New York 10014
(212) - 989-1260

Mr. Ray C. Armstrong
Assistant Manager for Operations
Oak Ridge Operations Office
U. S. Atomic Energy Commission
P. O. Box E
Oak Ridge, Tennessee 37831
(615) - 483-4462

LCDR Gerald J. Thompson, Chief
Contract & Procurement Branch
Pittsburgh Naval Reactors Office
U. S. Atomic Energy Commission
P. O. Box 109
West Mifflin, Pennsylvania 15122
(412) - 462-0240

Mr. Leonard F. Perkins, Director
Division of Contracts & Procurement
Richland Operations Office
U. S. Atomic Energy Commission
P. O. Box 550
Richland, Washington 99352
(509) - 942-1111, X-6-4138

Mr. Robert Hughey, Director
Technical Services Division
San Francisco Operations Office
U. S. Atomic Energy Commission
2111 Bancroft Way
Berkeley, California 94704
(415) - 841-4311

Mr. Harry D. Rahner
Special Asst. to the Deputy Manager
Savannah River Operations Office
U. S. Atomic Energy Commission
P. O. Box A
Aiken, South Carolina 29802
(803) - 842-2678

Mr. Stanley W. Hitzman, Manager
Schenectady Naval Reactors Office
U. S. Atomic Energy Commission
P. O. Box 1069
Schenectady, New York 12301
(518) - 393-4441

U.S. GOVERNMENT PRINTING OFFICE [illegible]

PDR

PART 50

44 FR 61372

THE NUCLEAR INDUSTRY

1967

0/1

DF

☆ U.S. ATOMIC ENERGY COMMISSION ☆

9905130088 671231
PDR PR
50 44FR61372 PDR

ACKNOWLEDGEMENT

The Division of Industrial Participation wishes to acknowledge the assistance by other Divisions and staff of the Atomic Energy Commission in the preparation of this report. Without their help and the assistance of industrial firms, associations, and state groups, this report could not have been prepared.

Ernest B. Tremmel

Ernest B. Tremmel, Director
Division of Industrial
Participation

Prepared for delivery to
Atomic Industrial Forum's Annual Conference
Chicago, Illinois
November 6, 1967

FOREWORD

Section 1, the Declaration of the Atomic Energy Act states that it shall be the policy of the United States to develop, use, and control atomic energy "so as to........strengthen free competition in private enterprise." As part of its fulfillment of this mandate, this booklet, Nuclear Industry, is published annually to present the AEC's assessment of the state of the nuclear industry and make available facts believed to be of interest and in accordance with the President's program to improve service to the public.

It is intended that the booklet serve both as introductory reading for persons who desire to become better acquainted with the nuclear industry as well as a handy reference for important items of interest to the industry. It is prepared yearly by the AEC's Division of Industrial Participation (in September of each year) and released at the Atomic Industrial Forum and American Nuclear Society joint meeting held in the Fall of each year.

It is sincerely hoped that Nuclear Industry - 1967 will be of value to the many readers of past year's reports as well as future readers and that it will provide information which will serve as one basis for guidance to industry in determining their future role in the nuclear field.

For sale by the Superintendent of Documents, U.S. Government Printing Office
Washington, D.C., 20402 - Price $1

REPORT ON THE NUCLEAR INDUSTRY

1 9 6 7

Contents

		Page
SUMMARY		1
A. Shipments of Products	1	
B. Nuclear-Electric Power	1	
C. Investment	6	
D. Exports	6	
E. Employment	7	
NUCLEAR INDUSTRY HIGHLIGHTS - 1967		9
A. Civilian Nuclear Power	9	
B. Private Ownership & Enriching	11	
C. Safeguards	11	
D. Desalting	12	
E. adioisotopes	12	
F. mpetition	12	
G. Plowshare	13	
H. Spinoff	13	
I. Interstate Agreements	13	
J. Meetings with Industry	13	
K. Operating Contractors	14	
L. Nuclear Safety	14	
M. Licensing and Regulation	14	
N. International Confernece	15	
O. Foreign Activities	15	
PRIVATE OWNERSHIP - TOLL ENRICHING	16	
A. Actions Taken During 1965 and 1966	17	
B. Actions Taken During 1967	19	
C. Areas That Still Require Attention	21	
CATEGORY I -- RAW FUEL MATERIALS		22
A. Uranium	22	
B. Thorium	30	

		Page
CATEGORY II -- MATERIALS PROCESSING AND FABRICATION		31
A.	Uranium Fuel Preparation - Conversion of Feed Materials to UF_6 31	
B.	Uranium Enriching 40	
C.	Uranium Processing and Fabrication 44	
D.	Plutonium Processing and Fabrication 52	
E.	Thorium 57	
F.	Uranium-233 58	
G.	Other Materials 59	
CATEGORY III -- NUCLEAR COMPONENTS AND EQUIPMENT		64
A.	Radiation Detection and Monitoring Instruments 64	
B.	Reactor Components 68	
CATEGORY IV -- REACTORS		71
A.	Expenditures for Reactor Projects 71	
B.	Civilian Power 76	
C.	Civilian Maritime Reactors 101	
D.	Military Reactors 101	
E.	Research and Teaching Peactors 104	
F.	Space Systems 105	
CATEGORY V -- RADIOISOTOPES		112
A.	Producers 113	
B.	Processors 118	
C.	Source Fabrication 119	
D.	Equipment Manufacturers 119	
CATEGORY VI -- MATERIALS REPROCESSING		133
A.	Irradiated Fuel 133	
B.	Unirradiated Fuel 144	
CATEGORY VII -- PLOWSHARE -- PEACEFUL USES OF NUCLEAR EXPLOSIVES		147
A.	Introduction 147	
B.	Budget 147	
C.	Industrial Participation 149	
D.	Natural Resources Projects 149	
E.	Excavation 153	

Page

F. Scientific Research 154
G. Charges for Nuclear Explosives 154
H. Program Information 154

CATEGORY VIII -- SERVICES 156

A. Radioactive Waste Management 156
B. Film Badge Processing 165
C. Contaminated Laundry 168
D. Transportation 172
E. Whole Body Counting Service 178
F. Reactor Servicing 180
G. Decontamination 181

ASSISTANCE FOR INDUSTRY AT AEC FIELD OFFICES 182

A. At AEC Field Offices 182
B. At AEC Laboratories 183
C. From AEC Publications 183
D. From Congressional Publications 184
E. Through the Access Permit Program 184
F. Through the Work Experience Program 184

List of Charts iv

List of Tables v

List of Charts

Page

SUMMARY

Chart A -- Shipments of Nuclear Products 2
Chart B -- Net Orders Received for Selected Atomic Energy Products 2
Chart C -- Growth Expected in Electric Utility Operating Capacity 5
Chart D -- Economic Impact of Nuclear Plants 5
Chart E -- Export Shipments of Selected Nuclear Products . . 7

RAW FUEL MATERIALS

Chart I-A -- Estimated Annual Uranium Requirements 23
Chart I-B -- World Uranium Resources 25
Chart I-C -- Annual Exploratory Drilling Rates and Reserve Additions 26
Chart I-D -- Uranium Procurement by the AEC 28

MATERIALS PROCESSING AND FABRICATION

Chart II-A -- Estimated Requirements for Conversion of Ore Concentrates to UF_6 34
Chart II-B -- Irradiated Scrap Estimates 38
Chart II-C -- Estimated Separative Work Requirements 43
Chart II-D} -- Estimated Requirements for Processing}
Chart II-E} and Fabricating Fuel} 50
Chart II-F -- Potential Plututonium Fabrication Market . . . 56

NUCLEAR COMPONENTS AND EQUIPMENT

Chart III-A -- Annual Sales of Radiation Detection Instruments 69
Chart III-B -- Shipments of Reactor Components and Equipment 70

REACTORS

Chart IV-A -- Costs for Reactors Under Design or Construction 75
Chart IV-B -- Locations of U.S. Nuclear-Electric Plants . . 81
Chart IV-C -- Estimates for Generating Capacity in the U.S. by 1980 83
Chart IV-D -- Estimated Energy Sources for Electric Generation 84

Page

MATERIALS REPROCESSING

Chart VI-A -- Domestic Central Station Spent Fuel Forecast . 138

PLOWSHARE -- PEACEFUL USES OF NUCLEAR EXPLOSIVES

Chart VII-A -- Applications of Deeply Buried Nuclear Explosions 151
Chart VII-B -- Transisthmian Canal 153

SERVICES

Chart VIII-A -- Transfer of Commission Regulatory Responsibilities to States 157
Chart VIII-B -- Location of Commercial Radioactive Waste Burial Sites 158
Chart VIII-C -- Schedule of Rates and Charges for Low Level Waste Burial Services 160
Chart VIII-D -- Principal Film Badge Operations in the U.S.. 166
Chart VIII-E -- Principal Contaminated Laundry Operations in the U.S. 172

List of Tables

Page

SUMMARY

Table 1 -- Orders Received for Atomic Energy Products . . . 3
Table 2 -- Shipments of Atomic Energy Products 4
Table 3 -- United States Exports 6
Table 4 -- Employment 8
Table 5 -- Number of Establishments 8

PRIVATE OWNERSHIP -- TOLL ENRICHING

Table 6 -- Time-Table for Private Ownership 17

Page

RAW FUEL MATERIALS

Table - -- Market Prediction for Uranium 22
Table - -- Uranium Ore Reserves by States 24
Table I-1 -- Domestic Uranium Companies Supplying AEC and Commercial Market 27
Table I-2 -- Domestic Uranium Processing Mills Operating Primarily or Entirely for Private Sales . . . 27
Table - -- Estimated Sales of Uranium to the Government . 28
Table I-3 -- Domestic Sales of Uranium 29

MATERIALS PROCESSING AND FABRICATION

Table - -- Market Predictions - Uranium Fuel Preparation 31
Table - -- Summary of Present and Future Industry Capability 39
Table - -- Market Predictions - Uranium Enriching 40
Table II-1 -- Enrichment Capacity of AEC's Gaseous Diffusion Complex 42
Table - -- Market Predictions - Uranium Processing and Fabrication 44
Table II-2 -- Principal Producers of Uranium Fuels 45
Table II-3 -- Principal Fabricators of Uranium Fuels 47
Table II-4 -- Companies Having Capability to Process and Fabricate Fuel Materials 48
Table II-5 -- Enriched Uranium Furnished to Industry 49
Table II-6 -- Foreign Reactor Fuel Fabricated Abroad 51
Table II-7 -- AEC Orders for Fabrication of Uranium Fuel . . 52
Table II-8 -- Projected Plutonium Recovered from Domestic Commercial Power Reactor Fuels 53
Table II-9 -- Annual Net Requirements for Plutonium 54
Table II-10 -- Industrial Interest in Plutonium Work 57
Table II-11 -- Principal Firms Processing Thorium 58
Table II-12 -- Principal Commercial Suppliers of Heavy Water 59
Table - -- Companies in Beryllium Metal Industry 62

NUCLEAR COMPONENTS AND EQUIPMENT

Table - -- Market Predictions 64
Table III-1 -- Companies Manufacturing Radiation and Monitoring Instruments with 1966 Sales Exceeding $0.5 Million 65
Table III-2 -- Exports of Nuclear Instruments 67

Page

REACTORS

Table IV-1 -- Reactors Under Contract, Design or 71
Table IV-2 -- Construction, showing Costs Incurred, 72
Table IV-3 -- Total Estimated Costs, Source of Funds, etc. 73
Table IV-4 -- Domestic Reactor Projects Under Design or Construction 74
Table IV-5 -- Nuclear Plant Announcements through 1966 . . . 78
Table IV-6 -- Nuclear Plant Announcements through 1967 . . . 79
Table IV-7 -- Announcements of New Steam-Electric Plant Additions 80
Table IV-8 -- Central Station Nuclear Power 86
Table IV-9 -- Fuel Cycle Steps by Suppliers 89
Table IV-10 -- Estimated Future Requirements for Zirconium Tubing for Commercial Power Reactors . . . 92
Table IV-11 -- Power Reactors for Export 97
Table IV-12 -- Army Reactors 102
Table IV-13 -- Producers of Research and Teaching Reactors . 105
Table IV-14 -- Reactor Space Power Technology Growth 108
Table IV-15 -- Isotopic Space Power Technology Growth . . . 108
Table IV-16 -- Space Electric Power Systems 109
Table IV-17 -- Space Electric Power Systems 111

RADIOISOTOPES

Table - -- 1967 Market Prediction 112
Table V-1 -- Principal Industrial Suppliers of Cyclotron Radioisotopes 114
Table V-2 -- AEC Sales of Radioisotopes 115
Table - -- AEC Revenue from Isotope Sales 117
Table V-3 -- Principal Processors of Radiochemicals and Radiopharmaceuticals 118
Table V-4 -- Principal Suppliers of Radiographic Units . . . 119
Table V-5 -- Principal Suppliers of Radioisotope Teletherapy Units 120
Table V-6 -- Sales of Radioisotope Gauges 121
Table - -- Sales of Tritium 122
Table V-7 -- Principal Commercial Suppliers of Self-Illuminating Materials and Devices 122
Table V-8 -- Principal Firms Supplying Accelerator Equipment and/or Services or Finished Products 127
Table V-9 -- Principal Suppliers of Isotopic Irradiators . . 128

Page

MATERIALS REPROCESSING

Table - -- Market Predictions 133
Table VI-1 -- Fuel Reprocessing Load Forecasts 139
Table VI-2 -- Status of the Chemical Reprocessing Plants . . 141
Table VI-3 -- Principal Firms Recovering Uranium Scrap . . . 144
Table VI-4 -- Dollar Value of the AEC's Commercial Uranium Scrap Recovery Program 145
Table VI-5 -- Uranium Scrap Reprocessing Contracts Awarded in FY-1967 146

PLOWSHARE -- PEACEFUL USES OF NUCLEAR EXPLOSIVES

Table VII-1 -- AEC Operating Budget for Plowshare Program, FY 1966 to FY 1968 148
Table VII-2 -- FY 68 Budget Estimate 148
Table VII-3 -- Project Gasbuggy - Total Estimated Cost . . . 150

SERVICES

Table - -- Market Predictions 156
Table VIII-1 -- Private Waste Burial 159
Table VIII-2 -- Commercial Firms Licensed by the U.S. Atomic Energy Commission to Receive Radioactive Wastes (As of April 12, 1967) 161
Table VIII-3 -- Commercial Firms Licensed by Agreement States to Receive Radioactive Wastes (As of June 6, 1967) 161
Table VIII-4 -- AEC Solid Radioactive Waste Burials 162
Table VIII-5 -- Reactor Resin Waste Handling and Burial Forecast 163
Table VIII-6 -- Film Badge Processors 166
Table VIII-7 -- National Sanitation Foundation Testing Laboratory, Inc. 1967 Approved Listing for Film Badge Services 168
Table VIII-8 -- Commercial Licensees 169
Table VIII-9 -- AEC Sites Using Commercial Launderies AEC Sites Not Using Commercial Launderies . 171
Table VIII-10 -- U.S. Ports for Irradiated Fuels 177
Table VIII-11 -- Fuel Shipments to Foreign Countries 178
Table - -- Firms Supplying Special Transportation Services 178
Table - -- Firms Offering Whole-Body Counting Services 179
Table - -- Reactor Servicing Firms 180
Table - -- Firms Offering Decontamination Services . . 181

Page

ASSISTANCE FOR I[illegible]TRY

Table - -- Industrial Participation Representatives to AEC Field Offices 182

STATUS OF THE NUCLEAR INDUSTRY

SUMMARY

Of the many peaceful applications of nuclear energy, the one that continues to have the most impact on our country's economy is the use of nuclear reactors to produce steam for the generation of electric power. Orders for nuclear-electric generating units had reached 19 million kilowatts for the first nine months of 1967, compared with 17 million kilowatts for all of 1966. For each of these years, these figures represent more than half the total new steam-electric generating capacity ordered. Part B below indicates the impact the growth of nuclear power is expected to have between now and 1980.

Other applications of nuclear energy -- for desalting water, providing electric power for space and other remote locations such as the ocean bottom, the many promising commercial uses of nuclear explosives, preserving food, and producing new wood-plastic products -- all continued their advance through developmental stages in 1967 and offer a bright outlook for continuing growth of the nuclear industry in the years ahead.

A. Shipments of Products

The Bureau of the Census has collected data on shipments of a selected group of nuclear products for the past ten years. In 1966 shipments of these specialized items reached an all-time high -- up by more than a third from a year ago -- as the impact of orders for nuclear power plants began to be reflected in the shipment of components used in these plants. Data for both 1965 and 1966 are shown in Table 2. Chart A shows a comparison of shipments for the past ten years.

Table 1 and Chart B, showing net orders received for selected atomic energy products, reflect the impact of new orders even more dramatically, with the total for 1966 being nearly three times the 1965 total. Orders for control rod drive mechanisms and components and pumps specially designed for nuclear use were included in the 1965 total but were not included in the Census Bureau report for 1966. Had they been, the 1966 total would be substantially higher.

B. Nuclear-Electric Power

1967 was a year in which the Commission again raised its estimates of the rate of installation of nuclear power units by U.S. electric utilities. Chart C indicates the growth expected in electric utility generating capacity in the next 13 years as estimated by the Federal Power Commission. The portion of the total now expected to be nuclear is shown as estimated by the AEC in 1967.1/

1/ The official Commission estimate has been given as "from 120,000 to 170,000 Mwe." The median of this range has been used for Chart C and other charts in this report. Chart IV-C shows earlier Commission estimates in comparison with this 1967 estimate.

Chart A

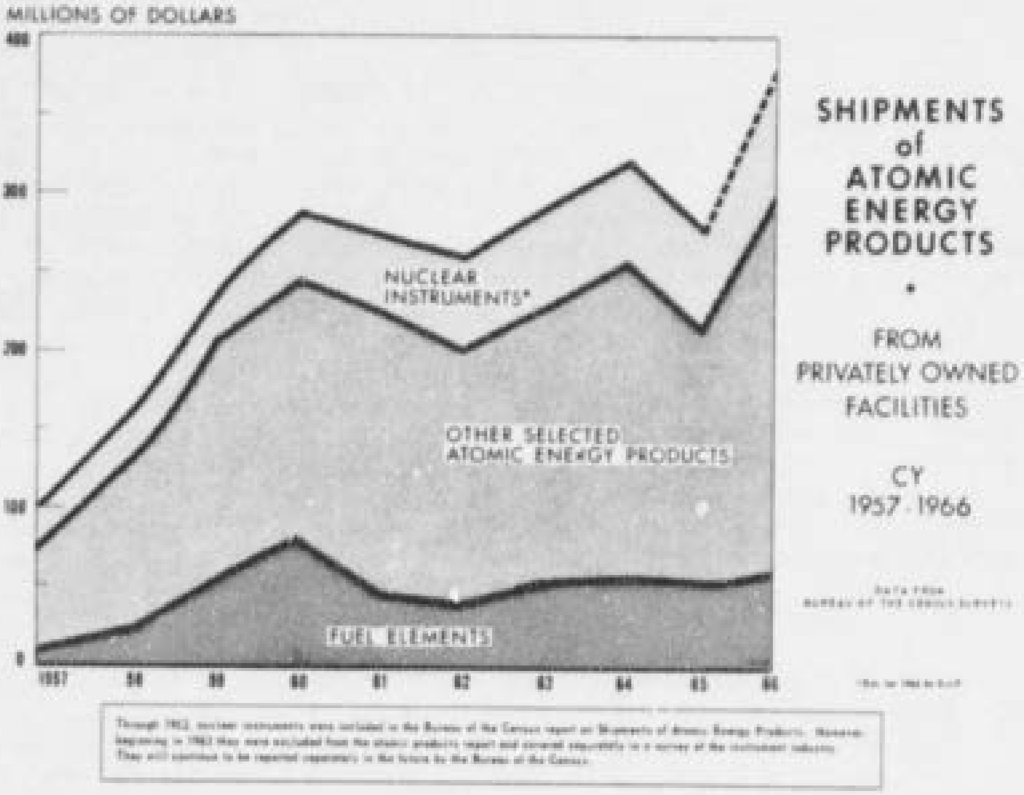

Chart B

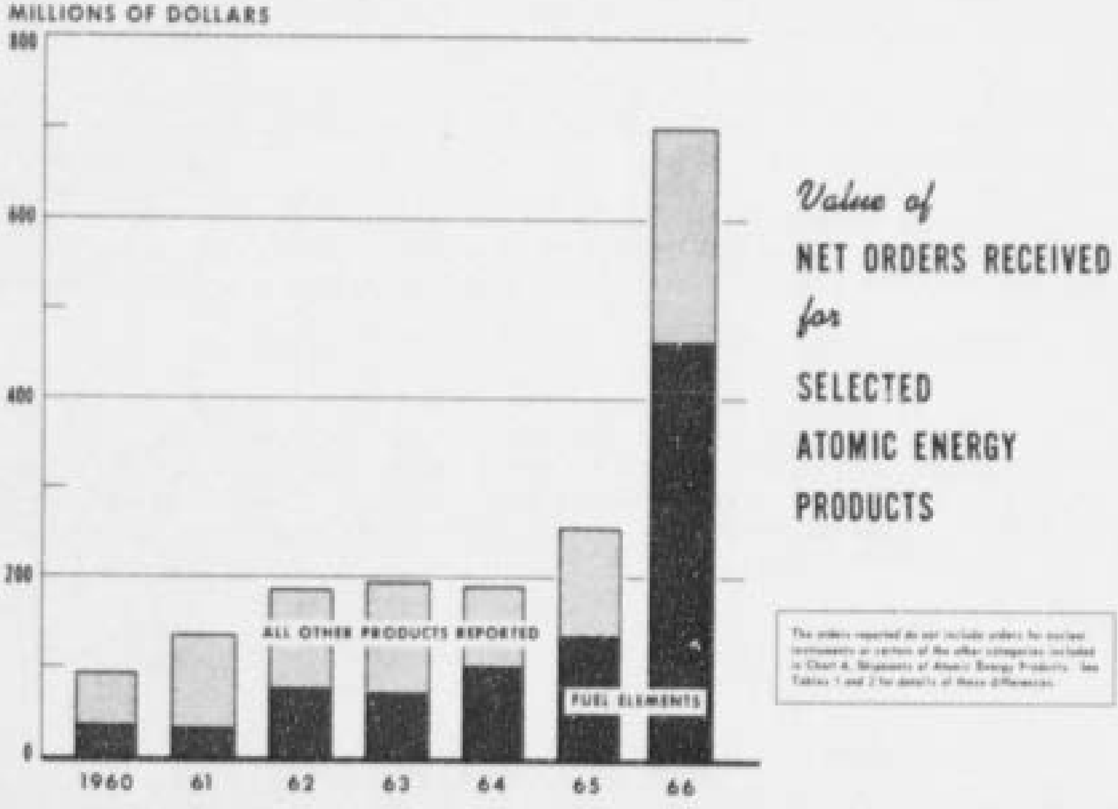

Chart D shows the cumulative economic impact of the construction and operation of nuclear plants at the rate now predicted -- a total for nuclear power plants plus fuel cycle costs well in excess of $30 billion over the next 12 to 13 years.

TABLE 1

Value of Net Orders Received for Selected Atomic Energy Products

1 9 6 6 and 1 9 6 5

(In thousands of dollars)

Product	Net orders received 1966	1965
Nuclear reactors*	780	2,302
Reactor components and equipment:		
Primary vessels and tanks	105,553	15,748
Control rod drive mechanisms and components for:		
Power plant	(D)	18,932
Propulsion	(D)	133
Other	-	-
Heat exchangers and condensers	92,128	49,568
Pressurizees, components, and auxiliary equipment	7,864	16,294
Pumps	(D)	X
Valves	8,694	7,193
Fuel handling equipment	2,399	2,587
Complete reactor fuel elements and control rods shipped directly for installation or use in a reactor for:		
Power plant	444,892	57,509
Propulsion	11,880	69,455
Other	4,109	7,871
Partially fabricated reactor fuel element materials and control rods not shipped directly for installation or use in a reactor	21,790	6,536

(L) Withheld to avoid disclosing figures of individual companies.

* Includes only reactors shipped fully assembled from the place of manufacture, such as AGN-201 and other similar types.

X Not reported in 1965.

Table 2.--VALUE OF SHIPMENTS OF SELECTED ATOMIC ENERGY PRODUCTS: 1966 AND 1967

(Thousands of dollars)

Product code	Product	1966 Total	1966 To U.S. Government: Atomic Energy Commission	1966 To U.S. Government: Other Agencies	1966 Export	1966 Other	1967 Total	1967 To U.S. Government: Atomic Energy Commission	1967 To U.S. Government: Other Agencies	1967 Export	1967 Other
	TOTAL (including radiation detection and monitoring devices and control and measuring devices containing radioactive isotopes)[1]	[illegible]	[illegible]	[illegible]	[illegible]	[illegible]	[illegible]	[illegible]	[illegible]	[illegible]	[illegible]
[illegible]	Nuclear reactors*	[illegible]	[illegible]	[illegible]	[illegible]	[illegible]	[illegible]	[illegible]	[illegible]	[illegible]	[illegible]
	Reactor components and equipment:										
[illegible]	Primary vessels and tanks	[illegible]	[illegible]	[illegible]	[illegible]	[illegible]	[illegible]	[illegible]	[illegible]	[illegible]	[illegible]
	Control rod drive mechanisms and components for--										
[illegible]	Power plant	[illegible]	[illegible]	[illegible]	[illegible]	[illegible]	[illegible]	[illegible]	[illegible]	[illegible]	[illegible]
[illegible]	Propulsion						[illegible]	[illegible]	[illegible]	[illegible]	[illegible]
[illegible]	Other							[illegible]	[illegible]	[illegible]	[illegible]
[illegible]	Auxiliary instrumentation for reactor control						[illegible]	[illegible]	[illegible]	[illegible]	[illegible]
[illegible]	Heat exchangers and condensers	[illegible]	[illegible]	[illegible]	[illegible]	[illegible]	[illegible]	[illegible]	[illegible]	[illegible]	[illegible]
[illegible]	Pressurizers, components, and auxiliary equipment	[illegible]	[illegible]	[illegible]	[illegible]	[illegible]	[illegible]	[illegible]	[illegible]	[illegible]	[illegible]
[illegible]	Pumps	[illegible]	[illegible]	[illegible]	[illegible]	[illegible]	[illegible]	[illegible]	[illegible]	[illegible]	[illegible]
[illegible]	Valves	[illegible]	[illegible]	[illegible]	[illegible]	[illegible]	[illegible]	[illegible]	[illegible]	[illegible]	[illegible]
[illegible]	Fuel handling equipment	[illegible]	[illegible]	[illegible]	[illegible]	[illegible]	[illegible]	[illegible]	[illegible]	[illegible]	[illegible]
	Complete new-core fuel elements and control rods shipped directly for installation or use in a reactor for--										
[illegible]	Power plant	[illegible]	[illegible]	[illegible]	[illegible]	[illegible]	[illegible]	[illegible]	[illegible]	[illegible]	[illegible]
[illegible]	Propulsion	[illegible]	[illegible]	[illegible]	[illegible]	[illegible]	[illegible]	[illegible]	[illegible]	[illegible]	[illegible]
[illegible]	Other	[illegible]	[illegible]	[illegible]	[illegible]	[illegible]	[illegible]	[illegible]	[illegible]	[illegible]	[illegible]
[illegible]	Partially fabricated reactor fuel element materials and control rods not shipped directly for installation or use in a reactor	[illegible]	[illegible]	[illegible]	[illegible]	[illegible]	[illegible]	[illegible]	[illegible]	[illegible]	[illegible]
[illegible]	Core structurals (barrels, cans, tubes, plates, etc., not included above)	[illegible]	[illegible]	[illegible]	[illegible]	[illegible]	[illegible]	[illegible]	[illegible]	[illegible]	[illegible]
[illegible]	Reactor moderators, coolants, and reflectors	[illegible]	[illegible]	[illegible]	[illegible]	[illegible]	[illegible]	[illegible]	[illegible]	[illegible]	[illegible]
	Specialized reactor components and equipment not included elsewhere in this report	[illegible]	[illegible]	[illegible]	[illegible]	[illegible]	[illegible]	[illegible]	[illegible]	[illegible]	[illegible]
[illegible]	Hot laboratory equipment	[illegible]	[illegible]	[illegible]	[illegible]	[illegible]	[illegible]	[illegible]	[illegible]	[illegible]	[illegible]
	Shielding	[illegible]	[illegible]	[illegible]	[illegible]	[illegible]	[illegible]	[illegible]	[illegible]	[illegible]	[illegible]
[illegible]	Radioactive isotopes shipped from non-AEC plants producing isotopes	[illegible]	[illegible]	[illegible]	[illegible]	[illegible]	[illegible]	[illegible]	[illegible]	[illegible]	[illegible]
[illegible]	Radiation sources and other radioactive materials produced from purchased isotopes	[illegible]	[illegible]	[illegible]	[illegible]	[illegible]	[illegible]	[illegible]	[illegible]	[illegible]	[illegible]
	Receipts for the processing of source and special nuclear materials for--										
[illegible]	Conversion to [illegible]	[illegible]	[illegible]	[illegible]	[illegible]	[illegible]	[illegible]	[illegible]	[illegible]	[illegible]	[illegible]
[illegible]	Reprocessing of irradiated reactor fuels										
[illegible]	Receipts for commercial irradiation service	[illegible]			[illegible]	[illegible]	[illegible]			[illegible]	[illegible]
[illegible]	Isotopic power devices	[illegible]	[illegible]	[illegible]	[illegible]	[illegible]	[illegible]	[illegible]	[illegible]	[illegible]	[illegible]

[illegible]

*Includes only reactors shipped fully assembled from the place of manufacture, such as AGN-201 and other similar types.

Chart C

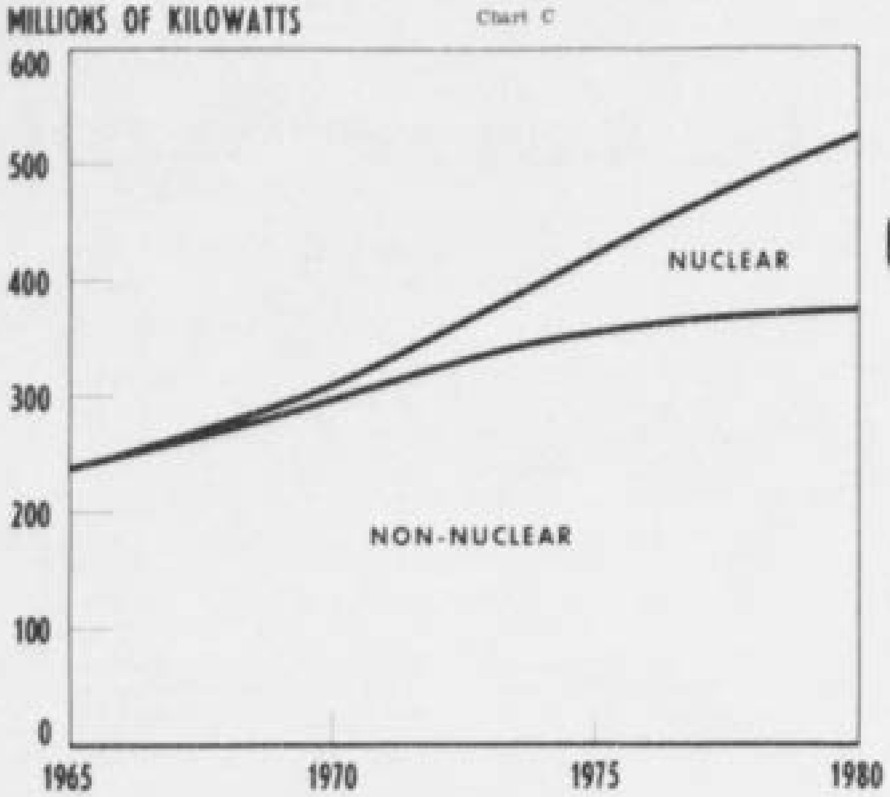

U. S. ELECTRIC GENERATING CAPACITY *and* NUCLEAR POWER GROWTH

Chart D

NUCLEAR POWER PLANT

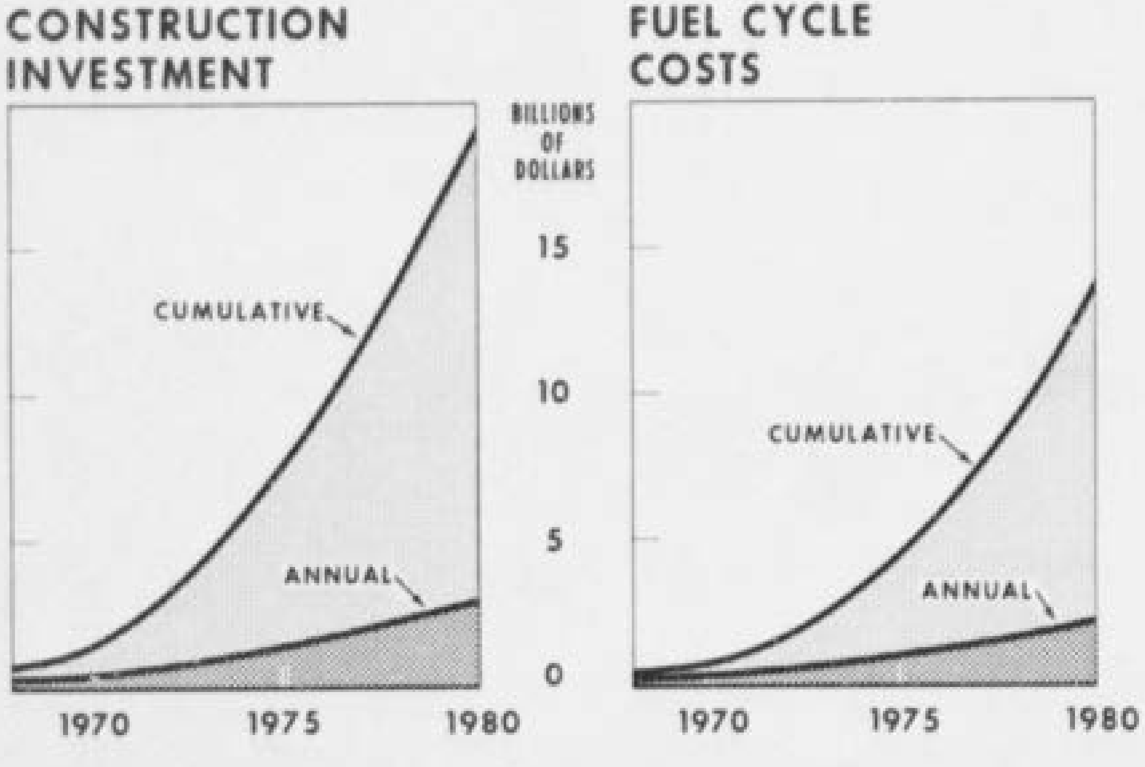

C. Investment

Investment by private industry in nuclear energy continued to increase and at an accelerated pace. Nuclear steam supply systems ordered for electric generation during the 9 months ended in September 1967 totaled around $2.6 billion. New facilities for the production of reactor components and associated equipment and services were announced by all the major organizations in reactor and reactor fuel activities.

Although complete information is not available publicly on private industry's investment in nuclear energy, the available indicators -- shipments of products, employment, and orders for nuclear-electric generating plants and their components -- all point to a rapidly increasing impact on our country's economy by this newest primary energy source.

D. Exports

The Bureau of the Census collects information on export shipments of a number of products peculiar to the nuclear industry. These data are summarized in Table 3 and Chart E. The over-all upward trend over the period shown, and particularly the more than 60% increase in 1966 over 1965, reflects the growth in applications for peaceful uses of nuclear energy overseas and the competitive position of the U.S. nuclear industry. Data reported on exports during the first four months of 1967 indicate the probability of a continuation of the uptrend this year.

TABLE 3

United States Exports

Commodity Description	Total Value - Thousands of Dollars 1963	1964	1965	1966
Uranium Ores and Concentrates	$ 659	$ 20	$ -0-	$ 161
Thorium Ores and Concentrates	322	353	79	3
Uranium and Thorium Compounds	144	182	265	296
Special Nuclear Material	23,560	15,339	35,559	41,906
Radioactive and Stable Isotopes Their Compounds & Mixtures	2,554	2,921	6,647	15,641
Nuclear Reactors and Parts	798	27,618	13,209	43,363
Nuclear Radiation Detecting And Measuring Instruments	11,804	15,338	18,108	19,194
Total for Items Reported	$39,841	$61,771	$73,867	$120,564

Chart E

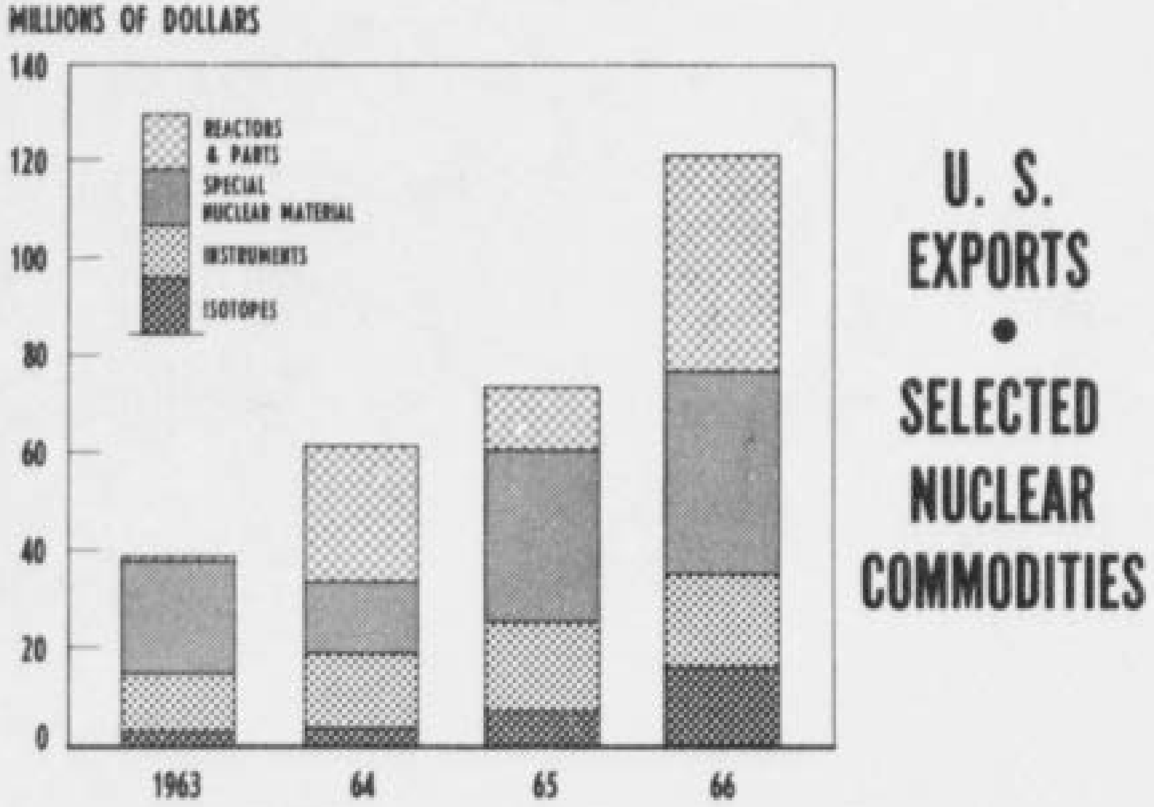

E. Employment

Reports on employment in private establishments in the atomic energy field are prepared for the Atomic Energy Commission by the Bureau of Labor Statistics. The Report on employment in 1967 was not available at the time this report went to the printer. Total employment in atomic energy work was reported as 132,000 in March 1966. Of this total, about 101,000 were employed in AEC-owned facilities and approximately 31,000 were employed in privately owned facilities. Of the latter number, over two-thirds were employed in private industrial establishments on work now primarily associated with peaceful uses of atomic energy. These are shown in Table 4 together with comparable figures for the five preceding years. Table 5 shows the number of establishments covered by each of the annual surveys.

Despite some year-to-year declines, a long-range comparison between 1961 and 1966 indicates a substantial advancement in the nuclear industry as a whole. Also, if milling and the production of feed are excluded, better than a one-third increase over the five-year span of the reports is indicated. As the effects of the rapidly expanding market for nuclear power are felt at the production level, further increases in employment should result.

TABLE 4

Employment
Selected Economic Segments

Industry Segment	Jan. 1961	Jan. 1962	Jan. 1963	Jan. 1964	Jan. 1965	Mar. 1966
Milling	4,793	3,381	2,579	2,255	2,079	2,054
Production of Feed	522	538	620	643	352	343
Special Materials	1,970	1,890	1,507	1,757	1,228	1 503
Fuel Fabrication	506	786	1,819	2,236	1,658	1,693
Reactors & Components	7,992	7,966	10,091	12,245	14,094	12,068
Waste Disposal	51	44	65	79	67	58
Instruments	3,717	4,237	4,745	3,853	4,979	4,068
Isotopes	215	291	365	348	407	642

TABLE 5

Number of Establishments
Selected Economic Segments

Industry Segment	Jan.* 1961	Jan. 1962	Jan. 1963	Jan. 1964	Jan. 1965	Mar. 1966
Milling	23	23	23	26	24	23
Production of Feed	6	6	5	8	6	4
Special Materials	15	15	19	33	31	28
Fuel Fabrication	6	6	11	14	13	13
Reactors & Components	38	38	50	58	61	48
Waste Disposal	4	4	8	10	9	8
Instruments	106	106	94	115	106	82
Isotopes	21	21	25	22	22	33

* Employment data for January 1961 came from the January 1962 survey and covered the same establishments. Comparable surveys were not made in 1961 and earlier years.

NUCLEAR INDUSTRY HIGHLIGHTS - 1967

1967 has been a year of exciting progress for the nuclear industry! It is especially notable because of the continued growth in peaceful applications of nuclear energy. The more noteworthy events are summarized below. Detailed information on them can be found in the following sections of this report.

A. Civilian Nuclear Power

1. New Orders

This year the outstanding event has been the continued upward trend in orders for nuclear steam supply systems placed by U.S. electric utilities.[1] By the end of the first nine months of 1967, orders for these systems for the generation of electricity stood at one-and-a-quarter times the nuclear electric generating capacity ordered during all of 1966.

Repeating the trend of 1966, nuclear orders in the first nine months of 1967 represented more than half of all generating capacity ordered.

2. Uranium Supply

Only two years ago there was considerable concern about the ability of the uranium mining and milling industry to make a healthy transition from a guaranteed Government market of approximately 8,000 tons per year in 1969 and 1970 to a market dependent entirely on commercial orders. This no longer appears to be of the same concern since the domestic requirements for uranium concentrate in 1971 are estimated to be at least 9,000 tons increasing to nearly 40,000 tons in 1980. The present AEC policy of restriction on import of foreign uranium for domestic use and the associated question of viability of the U.S. uranium industry are being studied by the Commission.

3. Uranium Exploration

During 1967 plans for further exploration for new deposits expanded so rapidly that by September exploratory drilling activities were scheduled at a level nearly twice that of any previous year.

4. Fuel Reprocessing

In May, the Southern Interstate Nuclear Board and the AEC sponsored a symposium on fuel reprocessing at Augusta, Georgia,

1/ Remarks by Dr. Glenn T. Seaborg, "Fast Breeder Power Reactors - A World Outlook," at Annual Meeting of the Canadian Nuclear Association, Montreal, Quebec, Canada, May 31, 1967, AEC Public Release S-24-67.

which emphasized the importance and the future of fuel reprocessing with respect to the entire nuclear power cycle.

In November of 1966, General Electric Company applied to the AEC for a construction permit to build a commercial fuel reprocessing plant. Allied Chemical Corporation continued to affirm its interest in eventually building a commercial fuel reprocessing plant. National Lead Company announced plans to finance further engineering work directed toward a commercial fuel reprocessing plant. Atlantic Richfield Company announced that it expects to have a fuel reprocessing plant operating on the West Coast some time in the 1970's. The Nuclear Fuel Services, Inc., facility, which started operating at West Valley, New York, in 1966, continued to pioneer in the processing of commercial fuels.

5. Reactor Competition

The reactor orders awarded to The Babcock & Wilcox Company and Combustion Engineering, Inc., increased during the year thereby broadening the competitive base of the industry. In addition, General Atomic Division of General Dynamics Corporation and Atomics International Division of North American Aviation, Inc., continued to be prospective suppliers of advanced converter and breeder types of reactors.

6. New Plant Investment

All four producers of nuclear steam supply systems who have been successful in selling to electric utilities on a competitive basis announced plans for substantial expansions of their nuclear production facilities. In addition, the independent fuel suppliers also announced plans for expansion.

7. Nuclear Fuel

Utilities exhibited an increasing independence from reactor suppliers in making arrangements for reactor fuel loadings beyond those contracted for at the time of placing orders for initial cores. For example, American Electric Power Company, Inc., placed a $75 million order with an independent fuel supplier for second cores for the two 1,000 Mw units at the proposed plant to be built by its subsidiary, Indiana & Michigan Electric Company, near St. Joseph, Michigan.

8. Fast Reactors

Industry recognition of the importance of developing breeder reactors was reflected in arrangements between utilities

and reactor suppliers to develop plans for prototype fast reactor electric generating units. The Edison Electric Institute (EEI) undertook a major study of the role they should play in developing breeder reactors. This study is to be completed by the end of the year.

9. Plutonium Recycle

While a number of joint programs are under way between the Edison Electric Institute and some of the reactor manufacturers to develop a plutonium recycle technology, the AEC is increasingly concerned about the amount of effort in this area and is encouraging the reactor core manufacturers and the electric utilities to accelerate work in this area.

B. Private Ownership & Enriching

Considerable attention was focused during the year on enriching. This is the only segment of the nuclear industry that is still under the control of the Government.

1. The Atomic Industrial Forum, Inc. (AIF), with the cooperation of the AEC, initiated a study of the feasibility and desirability of transferring to private industry one or more of the Commission's gaseous diffusion plants. The Commission declassified quantitive information on the capacity of its gaseous diffusion plants to produce enriched uranium. The Commission is undertaking a parallel study, which is also concerned with matters of national security, and has retained four consultants to provide expert advice on questions relating to the transfer of plants.

2. The AEC announced on September 21, 1967,[2/] that its current charge for the toll enrichment of privately owned uranium in the U-235 isotope is $26 per unit of work. This charge was announced to assist the U.S. and foreign nuclear industry in determining future costs of reactor fuel and future uranium requirements.

C. Safeguards

The AEC domestic program for safeguarding special nuclear materials (SNM) was strengthened with organizational changes within the Commission and the designation of resident inspectors, on a trial basis, at four private facilities processing significant quantities of SNM. Industry has been very cooperative in volunteering its plants for safeguards inspection.

2/ See AEC Public Release K-228, dated September 21, 1967.

D. Desalting

Plans for a large, dual-purpose, desalting and electric power plant moved decisively forward with passage of legislation providing for construction of the Bolsa Chica installation on a man-made island off the coast of southern California. Initially producing 50 million gallons of water daily, with an ultimate capacity of 150 million gallons daily, and generating 1,600 megawatts of electric power, this plant will have a larger capacity than the total of all other desalting plants in the world today.

E. Radioisotopes

1. Meat Irradiator

Irradiation-pasteurization of food advanced to a commercial basis with the announcement that Irradiated Foods, Inc. would build and operate a pilot plant with a capacity of 1,000,000 pounds of meat annually.

2. Heart Pacer

A prototype nuclear powered heart pacer produced by Nuclear Materials and Equipment Corporation (NUMEC) was completed and is now under test.

3. Fission Product Recovery

Isochem, Inc. (jointly owned by Martin-Marietta and Uniroyal) withdrew from its agreement to construct and operate a fission product conversion and encapsulation plant at Richland, Washington, because projected demands for the isotopes of particular interest (Sr-90, Ce-144, Cs-137, and Pm-147) appeared insufficient to support the proposed plant for a number of years. Nevertheless, there was increasing attention during the year to the use of fission products for many applications.

F. Competition

1. Study of Industry

A broad economic study[3/] of the nuclear supply industry was initiated by Arthur D. Little, Inc. under contract to AEC. It is hoped that the study will provide a basis for preparation and consideration of guidelines for healthy competitive development of the emerging nuclear industry.

3/ Remarks by James T. Ramey, "Reminiscences on the Atomic Energy Program - A Look Backward with Some Forward Implications," at Briefing Conference on Nuclear Power sponsored by FBA, AIF, ANS, and BNA, Washington, D. C. January 27, 1967, AEC Public Release IN-756.

2. Conversion of U_3O_8 to UF_6

Kerr-McGee Oil Industries, Inc. plans to bring a new 5-10,000 tons/day conversion facility into operation in 1970. The announcement, together with indications of serious interest in this work on the part of other firms, should assure competition in this area. Allied Chemical plans to open their plant at Metropolis, Illinois, in 1968.

3. Corporate Changes

The quickening pace of nuclear activities generated increased interest in mergers and consolidations to enhance competitive positions as the industry moved from a primarily developmental phase to a phase of strong competition for production orders.

G. Plowshare

The first experiment to study the feasibility of using nuclear explosives for peaceful underground engineering applications was planned for mid-November with the underground detonation of a 26-kiloton nuclear explosive near Farmington, New Mexico. This experiment, undertaken by the AEC in cooperation with the El Paso Natural Gas Company and the U.S. Department of the Interior, was planned to study the use of nuclear explosions in freeing natural gas from "tight" rock formations from which it could not otherwise be recovered.

H. Spinoff

Use of the Science Information Exchange for compiling data on privately funded nuclear research was initiated with the registration of privately funded R&D projects of about 50 industrial concerns in the radioisotope field. Under the SIE system, R&D is registered and cataloged at the time it is initiated rather than one or more years later in journals, libraries, and documentation centers.

I. Interstate Agreements

The Commission continued to work closely with the Southern Interstate Nuclear Board (SINB) and jointly sponsored conferences on several subjects.

The Western Interstate Nuclear Compact moved nearer reality, with approval of the compact in principle by the Western Governors' Conference in June.

J. Meetings with Industry

The Commission continued its series of meetings with the nuclear industry. Meetings were held with a number of industry

associations, industrial concerns, and utilities to discuss current problems and future plans.

K. Operating Contractors

Implementation of the AEC policy of changing operating contractors to provide broader opportunities for participation in AEC programs continued with the issuance of a request for proposals for the operation and maintenance of test site facilities in Nevada.

L. Nuclear Safety

The safety record of the nuclear industry has been so outstanding that the insurance pools, set up a decade ago to provide protection to the private industry, refunded during 1967 most of the premiums charged in 1957. The refunds are made under special rating plans which recognize the fact that there have been virtually no losses since the insurance pools were organized ten years ago.

M. Licensing and Regulation

1. Design Criteria

Further evidence of approaching maturity in the private reactor industry was the issuance by the AEC of a revised set of General Design Criteria. Development of these criteria is part of a longer-range Commission program to develop criteria, standards, and codes for nuclear reactor plants. This includes codes and standards that industry is developing with AEC participation. The ultimate goal is the evolution of industry codes and standards based on accumulated knowledge and experience as has occurred in various fields of engineering and construction.

2. Review Panel Recommendations

Valuable recommendations on procedures for handling contested cases were received from a three-member panel from outside the Government. The Commission also received a report with recommendations on the licensing and regulation of radioisotopes from an 11-member panel from outside the Government. Because the principal responsibility of the AEC regulatory program is the protection of the health and safety of the public, the AEC licensing process is unique in its framework and purpose. Both studies are helpful in the continuing development of the AEC regulatory program to better accommodate the needs of licensees and license applicants consistent with the AEC responsibility for the protection of the public health and safety.

3. JCAE Hearings

The Joint Committee on Atomic Energy held hearings on the licensing and regulation of nuclear reactors. AEC witnesses were heard in April and May, followed by witnesses from the nuclear industry in September.

4. Exposure Limits for Uranium Miners

In 1967 exposure levels for underground uranium miners came under prolonged and vigorous examination. As this report went to press, an exposure limit had been fixed by the Secretary of Labor (with a temporary 18-month exemption for mines meeting certain conditions), but final recommendations had not yet been sent to the President by the Federal Radiation Council.

N. International Conference

Initial plans were made for the Atomic Industrial Forum International Conference and Exposition to be held in Washington, D.C. in 1968.

O. Foreign Activities

The Commission continued its program of exhibits in foreign countries such as "Atoms in Action" which opened at Panama City, Panama,4/ May 4, 1967. The Commission also participated in a number of foreign conferences such as the Canadian Nuclear Associations Annual Conference5/ at Quebec, Canada, May 1967.

4/ Remarks by Dr. Gerald F. Tape, at Ceremonies Opening the "Atoms in Action" Nuclear Science Demonstration Center, Panama City, Panama, May 4, 1967, AEC Public Release S-19-67.

5/ Remarks by Ernest B. Tremmel, "The Role of Industrial Participation in Assisting Industry and Promoting Nuclear Exports," at Canadian Nuclear Association's Annual Conference, Montreal, Quebec, Canada, May 31, 1967, AEC Public Release IN-794.

PRIVATE OWNERSHIP - TOLL ENRICHING

Details regarding the private ownership of special nuclear material and toll enriching were reported in a special section of this report last year because of their far-reaching importance. This year again, because of its importance, we are repeating some of the basic information on the legislation and summarizing actions taken since our last report.

Background on Private Ownership

As we reviewed in last year's report, the Atomic Energy Act of 1946 placed complete control of atomic energy in the Government because of the military character and the lack of general knowledge about this new form of energy. Almost 10 years later, the Atomic Energy Act of 1954 was passed which permitted, among other things, private industry to participate in the development of nuclear energy and private persons to possess and use special nuclear materials under license from the AEC.

The Private Ownership of Special Nuclear Materials Act was approved on August 26, 1964.

Prior to the private ownership amendment, private industry could own all components of a nuclear power plant except the enriched uranium fuel and plutonium produced therein by irradiation.

Enactment of the amendment for private ownership in 1964 eliminated this exclusion.

The provision of toll enrichment of privately owned uranium was one of the most important concepts in the private ownership legislation. Under this concept, the AEC will receive privately owned uranium and perform U-235 enrichment services on a toll basis. This results in the return of a lesser amount of uranium, containing a greater concentration of U-235, to the customer.

Time-Table for Private Ownership Legislation

In order to assist the reader of this report, we are again reporting the time-table for implementation of private ownership.

As can be seen from the time-table, after December 31, 1970, the AEC is prohibited from distributing special nuclear materials for use in power reactors, except by sale. The Commission can continue leasing to fuel fabricators and converters, although the material will have to be acquired by the reactor operator by sale. Distribution for use in research reactors by lease or grant is not affected. In addition, the

AEC is authorized to offer uranium enrichment services, commonly referred to as toll enriching, beginning on January 1, 1969. The Commission's guaranteed purchase prices for plutonium delivered to the AEC, which is produced in reactors by persons licensed under section 104 through the use of uranium leased or sold by the AEC, will expire on January 1, 1971. Guaranteed prices for U-233 can continue past that date for periods up to 10 years. Finally, all Government-owned special nuclear materials, previously leased for use in section 103 or 104b facilities to operators of such facilities, must be returned or converted to private ownership by July 1, 1973.

TABLE 6

Time-Table

Private Ownership Permitted	August 26, 1964
Toll Enriching of Privately Owned Uranium Can Begin	January 1, 1969
AEC Prohibited From Entering Into New Lease Agreements For Power Reactor Fuel	January 1, 1971
AEC Guaranteed Purchase Price For Plutonium Will Terminate	December 31, 1970
Private Ownership of Special Nuclear Material Mandatory For Commercial Utilization Facilities (section 103 or 104b facilities) All lease arrangements must terminate	July 1, 1973

Status of Implementing Private Ownership and Toll Enriching

Under the authorization of the Private Ownership of Special Nuclear Materials Act of 1964, a number of steps to implement private ownership and toll enriching were to be undertaken by the Commission. The status of those implementing actions is given below.

A. Actions Taken During 1965 and 1966

1. Control, Protection and Safeguarding of Special Nuclear Material

In the area of maintaining and establishing regulatory controls, the Commission announced on September 21, 1965, that it was planning to amend its regulations concerning its licensing authority over privately owned special nuclear material and the import and export of special nuclear material. (On February 7, 1967, the Commission published amendments

to Part 50, "Licensing of Production and Utilization Facilities"; Part 70, "Special Nuclear Material"; Part 115, "Procedures for Review of Certain Nuclear Reactors Exempted from Licensing Requirements"; and Part 140, "Financial Protection Requirements and Indemnity Agreements". The amendments became effective on March 9, 1967.)

Of particular importance is the amendment to Part 70 requiring licensees who possess special nuclear material to control and account for the material in the interest of national security. This control has been exercised over SNM by the Commission through contractual relationships in the lease of Government-owned material and a new regulation was required for privately owned SNM.

During 1966 the Commission established an advisory panel[1/] to provide an independent review and assist in appraising AEC's policies and procedures for accounting for and protecting special nuclear materials domestically and internationally. This panel transmitted a report to the Commission in March 1967 presenting a number of detailed recommendations for consideration by the Commission. The panel concluded that safeguards against diversion of special nuclear material are very important and warrant the most careful attention of the AEC, other Government agencies, and the industry.

2. Guaranteed Purchase Prices for Pu and U-233

Another implementing step taken by AEC was the establishment of guaranteed purchase prices (not to exceed AEC's estimated value of the material as fuel in a nuclear reactor) for special nuclear material produced by licensees through the use of AEC sold or leased uranium and delivered to the Commission prior to January 1, 1971, for plutonium and for periods up to 10 years for U-233. The prices established are:[2/]

> For plutonium in the forms of nitrate, dioxide or metal and meeting AEC specifications, $10 per gram of the contained isotopes plutonium-239 and plutonium-241.

1/ See AEC Public Release J-179, dated July 22, 1966; and Remarks by Dr. Samuel M. Nabrit, "Atom and Evil - The Problem of Nuclear Proliferation", at American Nuclear Society Annual Meeting, San Diego, California, June 14, 1967, AEC Public Release S-28-67.

2/ See AEC Public Release H-65, dated March 25, 1965, and Federal Register 3886, dated March 25, 1965.

For uranium enriched in uranium-233 in the forms of nitrate, dioxide, or metal and meeting AEC specifications, $14 per gram of the contained isotope uranium-233, subject to adjustment for the presence of other uranium isotopes and deductions for handling costs.

3. Uranium Sale and Lease Policies

A further implementing step required AEC to establish reasonable sale prices for special nuclear material distributed by sale (by the AEC). In July 1966[3/] the Commission announced its proposed post-1968 policies concerning the supply of uranium.

4. Toll Enriching Criteria and Contracts

The AEC published in the Federal Register effective December 23, 1966,[4/] the final criteria under which it will perform toll enrichment of privately owned uranium. These criteria provide: (a) for AEC entering enrichment service contracts for up to 30 years; (b) customer option to take tails if desired; (c) a ceiling on the cost of separative work of $30/kg subject to escalation based on power and labor; (d) a prohibition against enrichment of foreign uranium intended for use in domestic reactors; (e) two standard types of contracts for enriching services covering "firm quantities" and "requirements"; and (f) that quantities of feed and charges will be determined by a standard table of enriching services.

Copies of the standard contracts may be obtained from the U.S. Atomic Energy Commission, Oak Ridge Operations Office, Oak Ridge, Tennessee 37831.

B. Actions Taken During 1967

1. Declassification of Gaseous Diffusion Plant Information

Declassification of capacity and economics of one or more of AEC's gaseous diffusion plants was suggested by many witnesses during the 1966 JCAE hearings on private ownership and toll enriching. This action was taken in 1967[5/] and is discussed further under Category II of this report.

3/ See AEC Public Release IN-704, dated July 25, 1966.
4/ See AEC Public Release IN-750, dated December 22, 1966.
5/ See AEC Public Release K-145, dated June 14, 1967.

2. Charge for Uranium Enrichment Services

The AEC announced[6/] on September 21, 1967, its current charge for the toll enrichment of privately owned uranium of $26 per kilogram unit separative work. It was announced simultaneously that the "tails" assay (U-235 content of tails material) on which this charge is based is 0.2 percent U-235. This subject is discussed further under Category II of this report.

3. Toll Enriching Contracts

The first toll enriching contract was signed in June 1967 with the Swedish utility OKG to provide about 19,000 kg of U-235 for the Oskarshamn 400 Mwe boiling water reactor over a 30-year period. There were no domestic toll enriching contracts under negotiation as of September 1967.

4. Control, Protection and Safeguarding of Special Nuclear Material

In May 1967, the AEC announced the establishment of two new safeguards organizations: an Office of Safeguards and Materials Management, reporting to the General Manager; and a Division of Nuclear Materials Safeguards, reporting to the Director of Regulation. Delmar L. Crowson is Director, Office of Safeguards and Materials Management, and Dr. Russell P. Wischow is Director, Division of Nuclear Materials Safeguards.

Also in May 1967, the AEC developed plans to station resident safeguards inspectors at five private facilities. These are: Nuclear Fuel Services, Inc., West Valley, New York, and Erwin, Tennessee, facilities; Nuclear Materials and Equipment Corporation facilities, Apollo and Leechburg, Pennsylvania; and United Nuclear Corporation, Hematite, Missouri.[7/]

In August 1967, the AEC announced the appointment of an advisory committee[8/] on nuclear materials safeguards. The committee is composed of the following members: John Palfrey, Chairman; Delmar L. Crowson and Dr. Russell P. Wischow, Vice Chairmen; Roger E. Batzel; Francis P. Cotter; Paul Grady; Dr. Jane Hall; Ralph F. Lumb; Horace W. Norton III; Clement J. Rodden; Louis H. Roddis, Jr.; Walton A. Rodger; Lombard Squires; Charles D. W. Thornton; Fred H. Tingey; Francis O. Wilcox; and J. Ernest Wilkins.

6/ See AEC Public Release K-228, dated September 21, 1967.
7/ See AEC Public Release K-121, dated May 12, 1967.
8/ See AEC Public Release K-212, dated August 29, 1967.

C. Areas That Still Require Attention

There are a number of areas relating to the implementation of toll enriching and private ownership that still require attention by the AEC. As of September 1967, the principal items are as follows:

The publication of a new schedule of charges for toll enriching, based on the $26 per kilogram unit of separative work and 0.2 percent "tails" assay, is necessary to enable determination of charges and feed requirements for various enrichments of enriched uranium product. This schedule will also apply to AEC's sale and leasing of enriched uranium for establishing the separative work component.[9] The uranium feed component will be $8.00/lb. U_3O_8 through June 30, 1973.

The AEC is reviewing its current rate of use charge for leasing enriched uranium and heavy water.

Clarification of AEC policy relating to sale of enriched uranium on the basis of single transactions, the question of removal of the restriction on import of foreign uranium for domestic use, and the viability of the U.S. uranium industry are under review. The Commission will probably issue statements regarding these matters in the near future.

Under the existing toll enriching policy, the customer is not given a choice in the isotopic content (% U-235) of tails material resulting from enrichment of his uranium. Numerous industry spokesmen have urged that greater customer flexibility should be permitted in this aspect of AEC's enriching service. This suggestion will continue to be considered by the AEC.

9/ The establishing of a $26 charge will result in a lowering of the guaranteed purchase prices for Pu and U-233 inasmuch as these prices are based on the value of Pu and U-233 as fuel material. Revised Pu and U-233 purchase prices will probably be published at the same time as a schedule of charges for toll enriching.

[illegible]

CATEGORY I
RAW FUEL MATERIALS

This category reviews the status of uranium supply and requirements.

Market Prediction for Uranium
(Annual Sales)

	Domestic	Foreign
1970	$120 million	$ 80 million
1975	$340 million	$260 million
1980	$600 million	$600 million

The area of nuclear raw materials has probably attracted as much public attention during 1967 as any other segment of the nuclear industry. Whereas a year or two ago there was considerable concern regarding the ability of the uranium industry to survive several lean years following the termination of AEC's procurement program, because of the rapid surge to nuclear power this concern has now diminished. In fact, concern is now being expressed as to whether an adequate supply of uranium will be found in time to meet the expected requirements. An extensive program in uranium exploration is under way, however, which is expected to increase the production capability of the U.S. uranium industry substantially.

A. Uranium

1. Uranium Requirements

As noted in the introduction, the status of uranium requirements has changed significantly during the past year. Chart I-A shows the estimated annual uranium requirements to fuel projected nuclear power plants through 1980.

The Commission's forecast of nuclear power growth was up-dated in May 1967 and predicts a range of 120,000 to 170,000 Mwe with a median of 150,000 Mwe installed by 1980. The nuclear growth potential in the non-Communist world (excluding U.S.) was reviewed for AEC in 1966 by A. D. Little, Inc.[1/] Their forecasts indicate that by 1980, nuclear installations in the non-Communist world would reach approximately 130,000 megawatts electrical installed.

1/ TID-22973 - "The Growth of Foreign Nuclear Power", April 1966.

Chart I-A

ESTIMATED ANNUAL URANIUM REQUIREMENTS

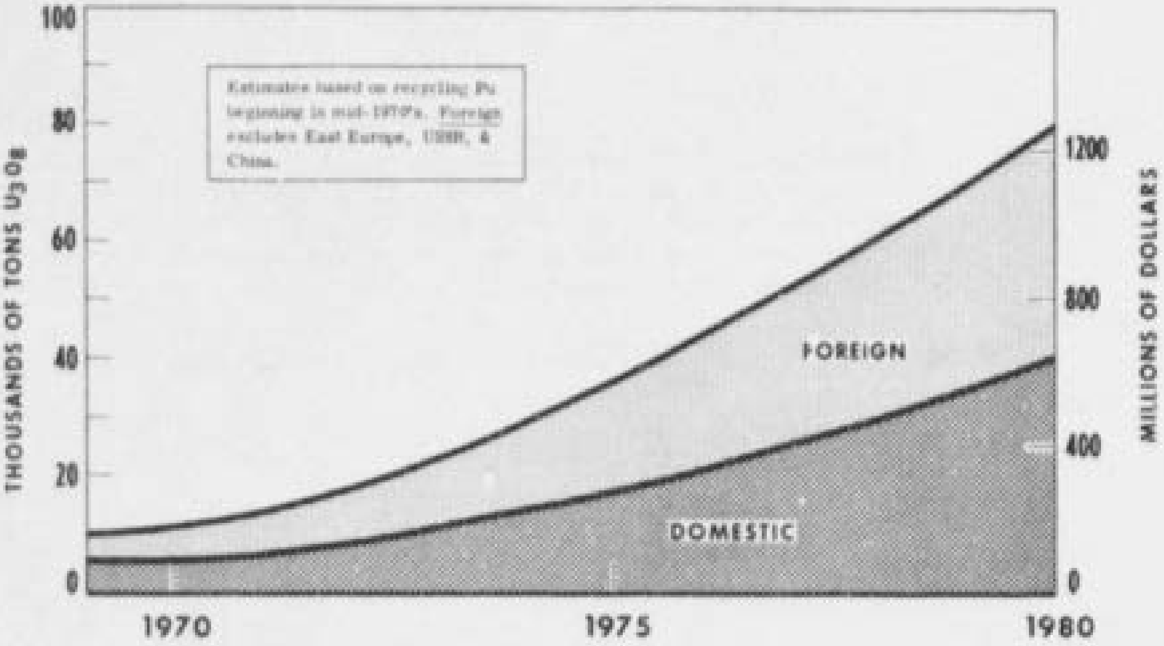

The requirements shown in Chart I-A, above, are based on the 150,000 Mwe domestic and 130,000 Mwe foreign power growth forecasts and assume that plutonium recycle will be adopted beginning in the mid-70's. If plutonium is not recycled, the projected uranium requirements would be about 10% higher by 1980. The dollar figures in Chart I-A are arbitrarily based on $8.00 per pound U_3O_8 although this figure could change considerably by 1980. On the basis of this forecast, the cumulative uranium requirements through 1980 amount to about 250,000 tons U_3O_8 for the U.S. and about 240,000 tons for foreign needs or nearly 500,000 tons for the non-Communist world.

If priced at $8.00 per pound U_3O_8, the combined foreign and domestic estimated uranium requirement of about 500,000 tons through 1980 would represent a value of about $8 billion.

Investment in new mines and mills to meet the needed capacity through 1980 may require between $20,000 and $30,000 per ton of annual output of U_3O_8 or a total investment of around $1 billion.

2. Uranium Reserves

The uranium reserves picture is based on information available as of January 1967. The next annual updating of reserves information will be January 1968. Estimates of domestic uranium ore reserves by states available at a price of $8.00/pound U_3O_8 are given below:

State	Tons of Ore (1000's)	% U_3O_8	Tons U_3O_8
New Mexico	28,300	0.24	67,200
Wyoming	23,400	0.22	53,000
Colorado	4,600	0.21	9,800
Utah	1,700	0.24	4,100
Others*	3,000	0.21	6,900
TOTALS (Rounded)	61,000	0.23	141,000

*Alaska, Arizona, California, Idaho, Montana, Nevada, North Dakota, Oregon, South Dakota, Texas, and Washington.

It is anticipated that uranium will be found in areas of the country other than those states identified previously with ore reserves.

U.S. and world uranium reserves estimates are shown in Chart I-8.

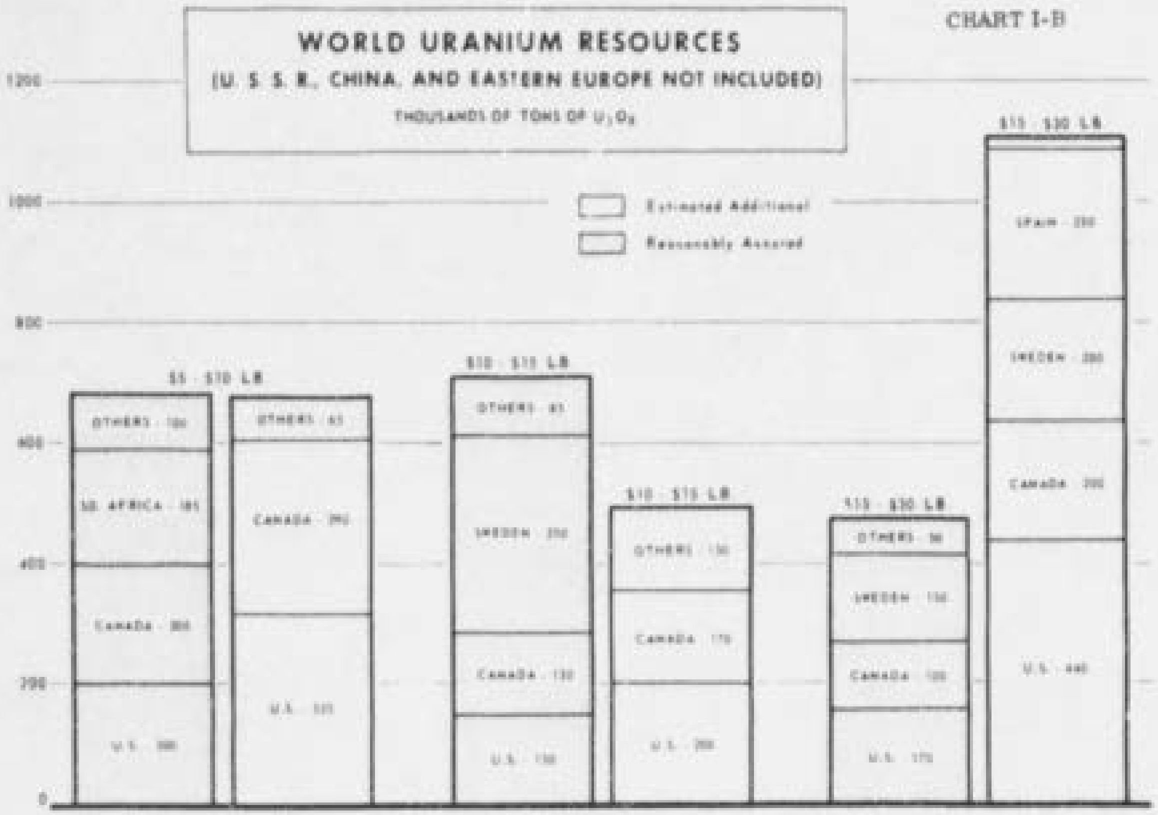

3. <u>Uranium Explorations</u>

During 1967 plans for further exploration for new deposits expanded so rapidly that by September exploratory drilling activities were scheduled at a level nearly twice that of any previous year. Private exploration effort has increased to about double that of 1966. In the past, the rate of discovery and development of new reserves has been directly related to the amount of drilling carried out. Chart I-C shows the current and planned drilling for discovery and development of new uranium reserves. For 1967 through 1970 it is estimated that drilling will amount to 53.8 million feet, costing some $77 million. The exploration investment required to meet U.S. uranium requirements of about 250,000 tons U_3O_8 through 1980, and also have an eight-years ore reserve of some 400,000 tons at that time, has been estimated at over $250 million.

Chart I - C

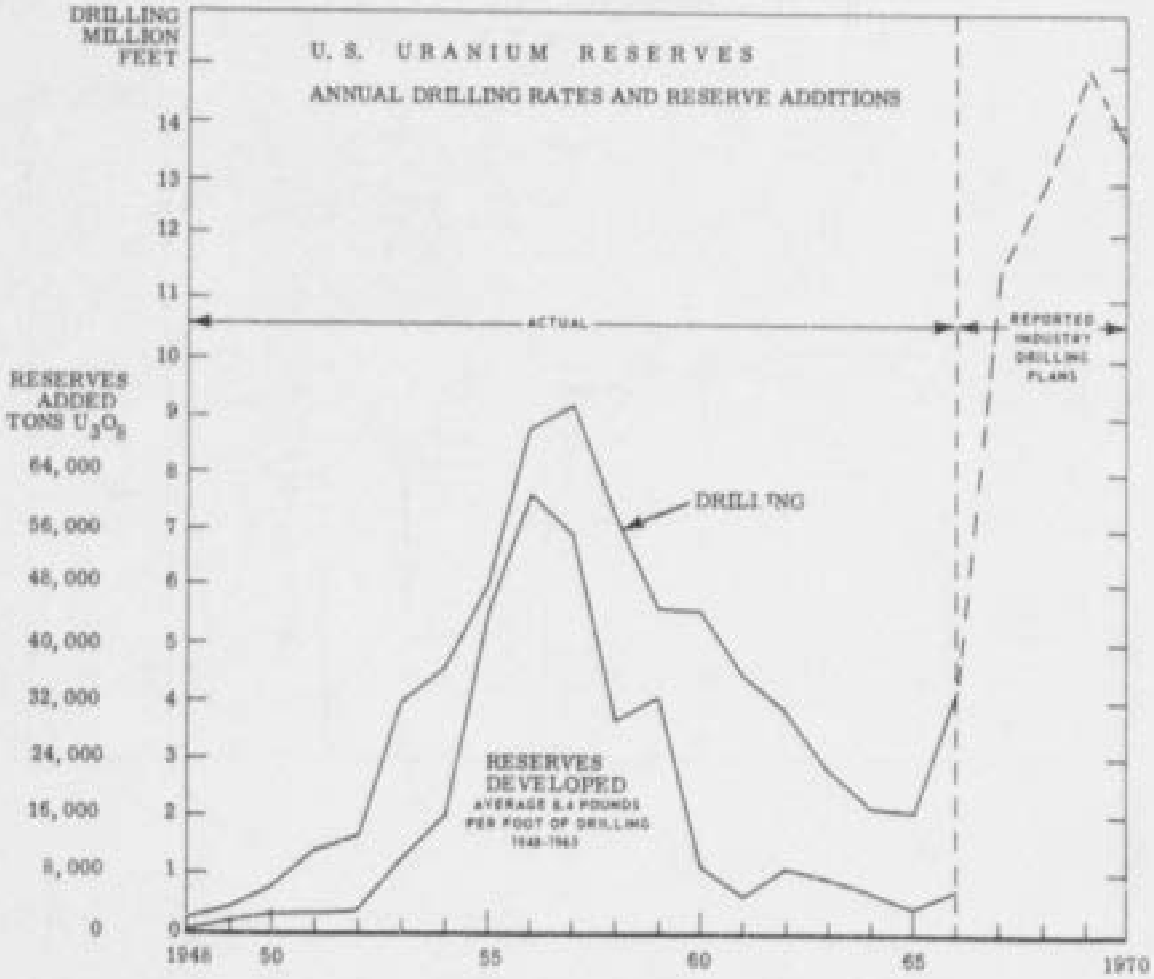

4. Domestic Uranium Processing Industry

At present, the uranium ore processing mills own or control an estimated 90% of the uranium reserves. The reamining 10% is in the hands of independently owned mines which sell ore to the larger firms having processing mills. Tables I-1 and I-2, on the following page, list the companies which currently sell uranium concentrates to the AEC and to the commercial market. No attempt has been made to list the independent mines.

Present world production capability is about 20,000 tons U_3O_8 per year of which 13,000-14,000 tons represents current U.S. capability.

In addition to the mining companies previously engaged in the uranium business, there are a growing number of major petroleum companies and other firms which have indicated plans for substantial expenditures in uranium exploration.

TABLE I-1

Domestic Uranium Companies
Supplying AEC and Commercial Market

Anaconda Company, Bluewater, New Mexico
Atlas Corporation, Moab, Utah
Federal-Radorock-Gas Hills, Fremont Co., Wyoming
Homestake-Sapin, Grants, New Mexico
Kerr-McGee Corporation, Grants, New Mexico
Union Carbide Corporation, Uravan & Rifle, Colorado
Union Carbide Corporation, Globe, Wyoming
United Nuclear Corporation, Grants, New Mexico
Utah Mining and Construction, Fremont Co., Wyoming
Vanadium Corp. of America, Shiprock, New Mexico
Western Nuclear, Jeffrey City, Wyoming

TABLE I-2

Domestic Uranium Processing Mills Operating
Primarily or Entirely for Private Sales

Climax Uranium, Grand Junction, Colorado
Cotter Corporation, Canon City, Colorado
Mines Development, Inc., Edgemont, South Dakota
Petrotomics Company, Shirley Basin, Wyoming
Susquehanna-Western, Inc., Falls City, Texas

Five barter transactions with foreign countries were permitted prior to the beginning of toll enriching in 1969. Approximately 1600 tons U_3O_8 is involved to fuel two power reactors in Germany, two in Spain, and one in Switzerland. The AEC announced in March 1966 that no further barter arrangements would be considered.

5. AEC Procurement Program

In order to bring purchases more nearly in balance with requirements and to assist in maintaining a viable uranium producing industry before a commercial market materialized, the AEC announced in November 1962 a program under which mills could elect to stretch-out base contract deliveries into 1967 and 1968. The AEC would then purchase in 1969 - 1970 an additional quantity of U_3O_8 in concentrates equal to the amount deferred for delivery after December 31, 1966. All of

the companies listed in Table I-1 participated in this program. Sales of uranium to the Government during 1967 - 1970, shown below, are the annual quantities remaining to be delivered to AEC under the "stretch out" commitments.

Estimated Sales of Uranium to the Government

Fiscal Year	Tons U_3O_8	$ Millions
1967	10,000	$165
1968	8,000	129
1969	8,000	112
1970	8,000	95
1971	4,000	47

Chart I-D, below, shows the over-all procurement of uranium concentrate by the AEC.

Chart I-D

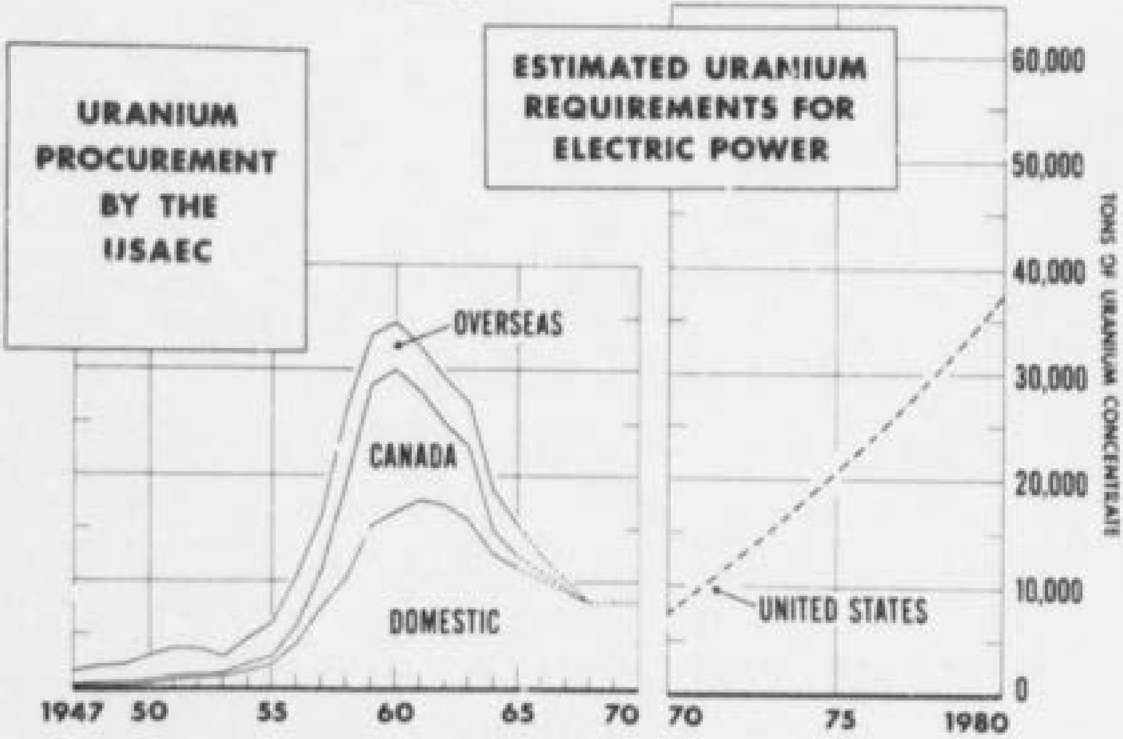

6. Commercial Purchases of Uranium

The commercial sales of uranium that have been reported to date for private nuclear power plants are given in Table I-3.

TABLE I-3

Domestic Sales of Uranium
(in short tons U_3O_8)

Delivery Year	Sales to Private Industry Total Including Options
1966	100
1967	600
1968	4,900
1969	5,000
1970	6,000
1971	7,200
1972	7,900
1973	6,200
1974	4,400
1975	3,300
1976 - 1980	940
	46,540

7. Viability

The question of viability of the domestic industry and the present AEC policy of restriction on import of foreign uranium for domestic use are matters of great interest to all segments of the nuclear power industry. The resolutions of these questions, now under study by the Commission, involves several complex and conflicting variables including: (1) The stimulation of foreign exploration and development of reserves

that would be in the long-term interest of the U.S., (2) uncertainty about the U.S. domestic uranium market in 1971 and 1972, (3) the risk of discouraging or delaying U.S. exploration by importing foreign uranium too early, (4) the U.S. balance of payments effects, and (5) protection of consumers interests.

8. Sampling of Uranium Concentrates

An opportunity for a limited number of specialized analytical firms to enter the uranium concentrates sampling business to provide independent commercial sampling services should develop over the next several years. It appears that sampling plants will probably be established at UF_6 conversion facilities. The volume of concentrates that would be subject to sampling may be estimated from the uranium requirements shown in Chart I-A. A very rough estimate of the annual business is $250,000 - $350,000 in the early 1970's and $750,000 - $1 million in the mid-70's.

Uranium concentrates purchases by the Government had been sampled at both the Weldon Springs and the Grand Junction AEC facilities. At the present, only the Grand Junction sampling facility is in operation. As an AEC service to industry, the AEC's Grand Junction facility presently performs sampling of commercial purchases of U_3O_8 concentrates until these services become available commercially. Lucius Pitkin, operator of the AEC's Grand Junction facility, plans to operate an independent commercial sampling plant at the Allied Chemical Corporation's Metropolis, Illinois, UF_6 conversion facility. It is understood that this sampling facility will begin operation in the spring of 1968.

B. Thorium

There have been no significant changes in the status of thorium as a nuclear fuel since our last report.

CATEGORY II
MATERIALS PROCESSING AND FABRICATION

A. Uranium Fuel Preparation - Conversion of Feed Materials to UF_6

Market Predictions

	Normal U	Slightly Enriched Uranium (<5.0% U-235)	Highly Enriched Uranium (>5.0% U-235)
In 1970	$ 16,500,000	$ 225,000	$160,000
In 1975	$ 55,000,000	$1,100,000	$160,000
In 1980	$115,000,000	$4,250,000	$160,000

Feed (UF_6) for AEC's gaseous diffusion plants is being obtained, at present, largely by the re-feeding of depleted uranium from AEC's stockpile of former gaseous diffusion tails material. Future feed will consist primarily of the following:

Normal UF_6
Produced from uranium having normal, or naturally occurring, U-235 content (0.711 wt. % U-235).

Enriched UF_6
Produced from uranium having higher than normal U-235 content, both Government-owned and privately owned, obtained by:

(a) recovery of unirradiated uranium1/ from scrap generated during fuel conversion and fabrication, and

(b) recovery of uranium from irradiated fuel elements.2/ (The amount of burn-up to be achieved in commercial light water power reactors is expected to result in uranium residues assaying 0.75 to 0.95 wt. % U-235.)

1/ Unirradiated Uranium - Refers to uranium that has not been subjected to radiation in a nuclear reactor following its withdrawal from an enriching plant.

2/ Irradiated Fuel Elements - Fuel elements that have been subjected to radiation in a nuclear reactor resulting in a fissioning or "burn-up" of a portion of their U-235 content.

<u>Production Reactor Tails UF_6</u>
Produced from uranium whose U-235 content has been partially consumed through burn-up in AEC's plutonium production reactors. (Since decisions have not yet been reached as to when and how this material will be converted to UF_6, it is not treated further in this section.)

Since commercial capability exists to convert ore concentrates to UF_6, AEC requires that all normal-assay uranium feed furnished for toll enriching be in the form of UF_6.

Commercial capability does not exist, at present, for conversion to UF_6 of the enriched uranium described as (a) and (b) above. AEC has been accepting the return of recovered leased uranium in these categories in forms other than UF_6, but with a charge to cover conversion to UF_6, and will probably continue a similar policy under private ownership arrangements until such time as private capability is established to convert these materials to UF_6.

1. <u>Conversion of Normal Uranium to UF_6</u>

a. <u>Industry Plans</u>

As reported last year, Allied Chemical Corporation's plant at Metropolis, Illinois, is the only commercial facility capable of converting ore concentrates to UF_6. This plant, which has a capacity to convert about 5,000 tons U per year from ore concentrates to UF_6, has been shut down since mid-1964, but is scheduled to be reopened early in 1968. In April 1967, Allied announced a $10 million, two-stage, expansion program which will increase the plant's capacity to about 7,000 tons U early in 1968 and to about 10,000 tons U some time in 1969.

Allied announced in August 1966, that its future charge for converting ore concentrates to UF_6 will be reduced from $1.25 per pound U to $1.04 per pound U. The reduced price will apply to UF_6 to be delivered to AEC for toll enrichment, but the $1.25 charge will continue to apply for conversion accomplished in conjunction with ad hoc barter arrangements between U.S. and foreign countries.

Allied, at present, is negotiating for a six-months processing period for the orders it has been accepting. It is believed that, as more experience is gained with a variety of ore concentrates, and, as other plants come into being, the industry will achieve a three-months processing period.

In July 1967, the Kerr-McGee Corporation announced plans to build a $25 million UF_6 conversion plant, with a capacity of 5-10,000 tons U per year at a site yet to be announced. The plant is expected to go on stream early in 1970.

b. AEC UF_6 Conversion Facilities

In the past, AEC has met most of its needs for converting ore concentrates to UF_6 by the following sequences:

(1) By solvent extraction refining of ore concentrates at its Weldon Spring, Missouri, and its Fernald, Ohio, feed processing plants, yielding product as either UO_3 or UF_4; and

(2) By converting UO_3 to UF_6 at its Oak Ridge, Tennessee, or its Paducah, Kentucky, gaseous diffusion sites; or

(3) As an alternate to (2), by converting UF_4 to UF_6 at its Portsmouth, Ohio, site.

The last of the three UF_6 producing plants to discontinue operations (the Paducah Feed Plant) was shut down in mid-1964.

c. Future Commercial Requirements

Commercial requirements for conversion of ore concentrates to UF_6 in 1968 will be those associated with ad hoc foreign barter arrangements, and will amount to about 1,000 short tons U, plus a portion of the domestic privately owned uranium that will be toll enriched starting in 1969. After 1968 the requirements for conversion of ore concentrates to UF_6 will be principally those of U.S. nuclear power plants. Annual power reactor requirements for conversion of ore concentrates to UF_6 from 1969 through 1980 are projected in Chart II-A, along with dollar value of the conversion service.

Chart II-A

CONVERSION OF ORE CONCENTRATES TO UF_6

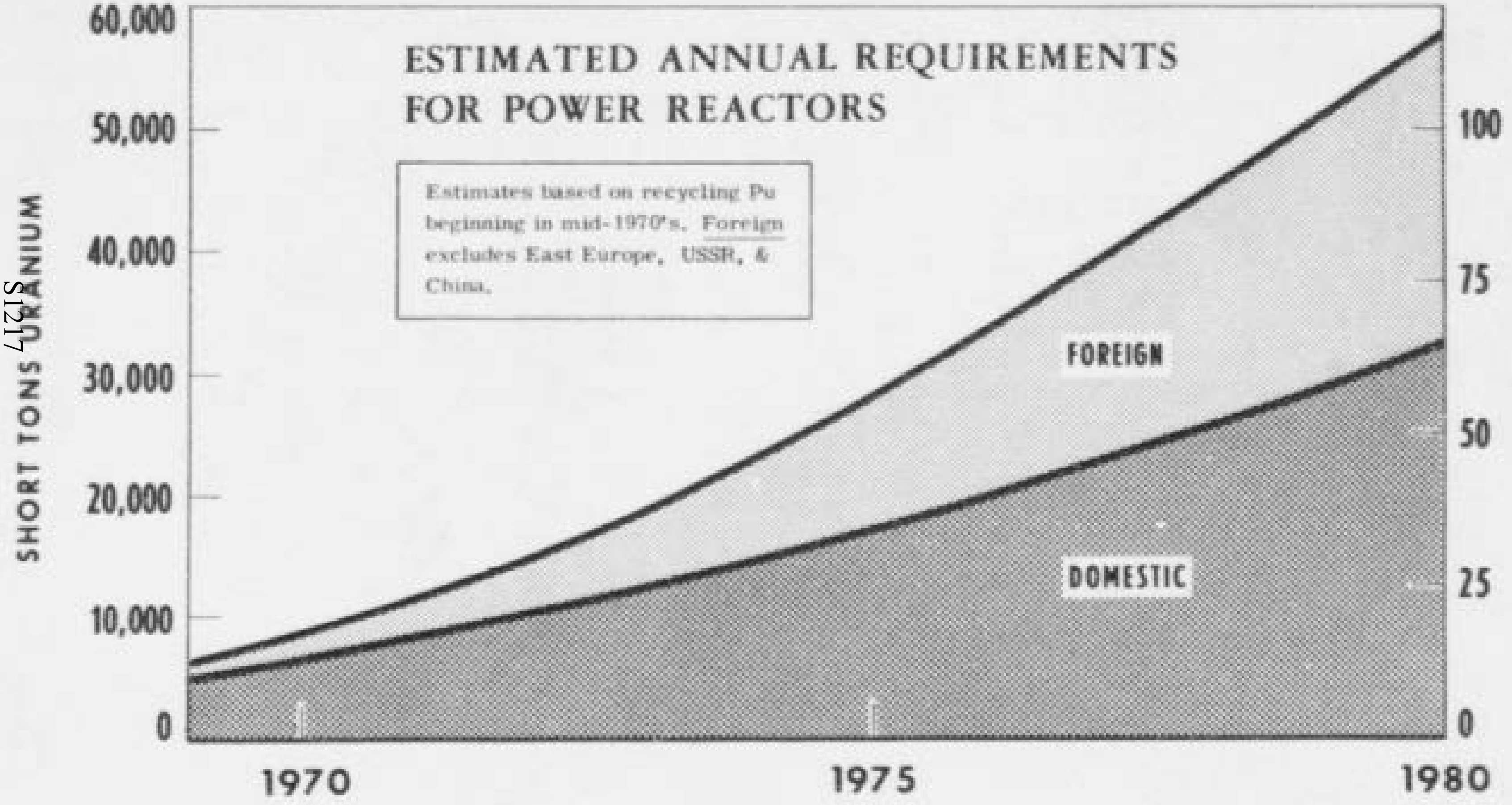

2. Conversion of Enriched Uranium to UF_6

The quantities and batch sizes of enriched uranium recovered from (a) unirradiated scrap, and (b) irradiated fuels, that have been returned to AEC's gaseous diffusion plants for re-feeding to date have been relatively small, and no industrial capability exists for conversion of these recovered materials to UF_6. The criticality limitations and health protection aspects of a facility designed to convert such materials to UF_6 would necessitate a sizable capital expenditure; however, private companies will no doubt enter this field at such time as a private market develops. A private facility capable of converting slightly enriched uranium to UF_6 can probably be expected within the next two or three years with a UF_6 conversion facility for highly enriched uranium following some time later.[3/] Several companies, such as Allied Chemical, Kerr-McGee, Nuclear Fuel Services, and United Nuclear have shown interest in considering such facilities.

It is probable that, as soon as private capability is achieved to convert enriched materials to UF_6, the AEC will require that these materials, when returned to AEC as feed to the gaseous diffusion plants, must be in the form of UF_6. At present, however, the Code of Federal Regulations permits the return of leased enriched uranium to AEC in the form of uranyl nitrate, upon payment of charges calculated to cover AEC's cost of converting to UF_6. The conversion charge is \$5.60 per kilogram of contained uranium for low U-235 enrichments (<5.0% U-235) and \$32.00 per kilogram of contained uranium for high U-235 enrichments (>5.0% U-235), plus allowances for losses and conversion time.

a. Recycling and Blending of Enriched Uranium

The extent to which recovered, enriched uranium will need to be converted to UF_6 for re-feeding to the gaseous diffusion plants is dependent on how much recycling and blending of these materials can be expected.

(1) Unirradiated Uranium

It is normal practice to recover some of the unirradiated enriched scrap generated during a fuel processing or fuel fabricating campaign and recycle

---

3/ It would not be practical from the standpoint of economics to use the same equipment for conversion of slightly enriched uranium to UF_6 that is used for conversion of highly enriched uranium. The equipment for highly enriched uranium must be severely limited in size to prevent accidental nuclear criticality. The limited throughput of this equipment would be much too costly for processing of slightly enriched uranium.

it back before completion of the campaign. To the extent that this can be done without introducing contaminants to the fuel or altering its isotopic enrichments, considerable savings can be achieved through higher conversion efficiency. It is usually inevitable, however, that some small portion of the scrap cannot be recycled back into the campaign or into later campaigns producing identical fuel material. The portion that is not directly recycled back to processing or fabrication must be converted to UF_6 and re-fed to the gaseous diffusion plants. The percentage of enriched UF_6 starting material that ends up in this portion varies greatly for different types and enrichments of fuels. For light water power reactor fuels, being fabricated during the next year or two, it is estimated that about 1.5% of the uranium withdrawn from the gaseous diffusion plants for this purpose will be returned to them as recovered unirradiated scrap for re-feeding. It is expected to be less than 1.5% under private ownership when more and more recycling and blending will probably take place.

(2) Irradiated Uranium

The recycling of recovered, irradiated fuel back to fuel processing and fabrication involves much more complex problems than are encountered with unirradiated material. The first, and probably the most difficult, obstacle is that the isotopic enrichment will have been greatly reduced during irradiation, thereby requiring blending back to the required enrichment by combining it with uranium of considerably higher enrichment. Although blending has been successfully accomplished in AEC plants over a period of years, in such forms as UF_6, UF_4, and uranium metal, it requires a high degree of precision in isotopic analysis. It is further complicated by the fact that some economic disadvantage is inherent as a result of the blending, (i.e., the total market value of the resultant material can be expected to be less than the total value of the two portions that were blended), and the fact that irradiated uranium acquires a build-up of U-232; U-233, and U-236, which may be objectionable from the standpoint of fuel homogeneity and safety.

b. Future Market for Conversion of Enriched Uranium to UF_6

It can be seen from the above that considerable uncertainty exists concerning the amount of enriched fuel recycling that can be expected without re-introduction to the gaseous diffusion plants. This uncertainty, in turn, greatly affects accuracy of estimates of the future requirements for conversion of enriched uranium to UF_6.

If irradiated fuel reprocessing plants in the future employ a fluoride volatility process or an aqueous process followed by a volatility process, their recovered uranium will be in the form of purified UF_6. Although UF_6 is an ideal chemical form in which to blend two different isotopic enrichments, its owners would probably find it more economical to return the recovered material to the gaseous diffusion plants for re-enriching, than would be the case if it were recovered as uranyl nitrate hexahydrate.

Future requirements for conversion to UF_6 of recovered enriched uranium of isotopic enrichment less than 5% U-235 are forecast in Chart II-B. The estimates are separated into unirradiated material and irradiated material and were based on the assumptions described below:

Unirradiated Material - It was assumed that, prior to 1969, 1-1/2% of the uranium withdrawn at less than 5% enrichment will end up as unirradiated scrap to be returned to the gaseous diffusion plants. Starting in 1969, a figure of 1% is used; however, even this percentage might prove to be too high if recycling is utilized as extensively as possible.

Irradiated Material - The irradiated scrap estimates shown in Chart II-B are based primarily on commercial power reactor spent fuel. It was arbitrarily assumed that, starting sometime in the 1970's, 50% of the spent fuel will be reprocessed in facilities using the fluoride volatility or modified volatility process and, as a result, will already be in the form of UF_6 when it is recovered. Of the remaining material (recovered as UNH), it was assumed that about half will be blended and recycled without being returned to the gaseous diffusion plants. Consequently, the irradiated scrap estimates in Chart II-B, represent about 25% of recovered power reactor spent fuel.

[illegible]

Chart II - B

CONVERSION OF RECOVERED, SLIGHTLY-ENRICHED (< 5.0% U-235) URANIUM TO UF_6

ESTIMATED ANNUAL REQUIREMENTS

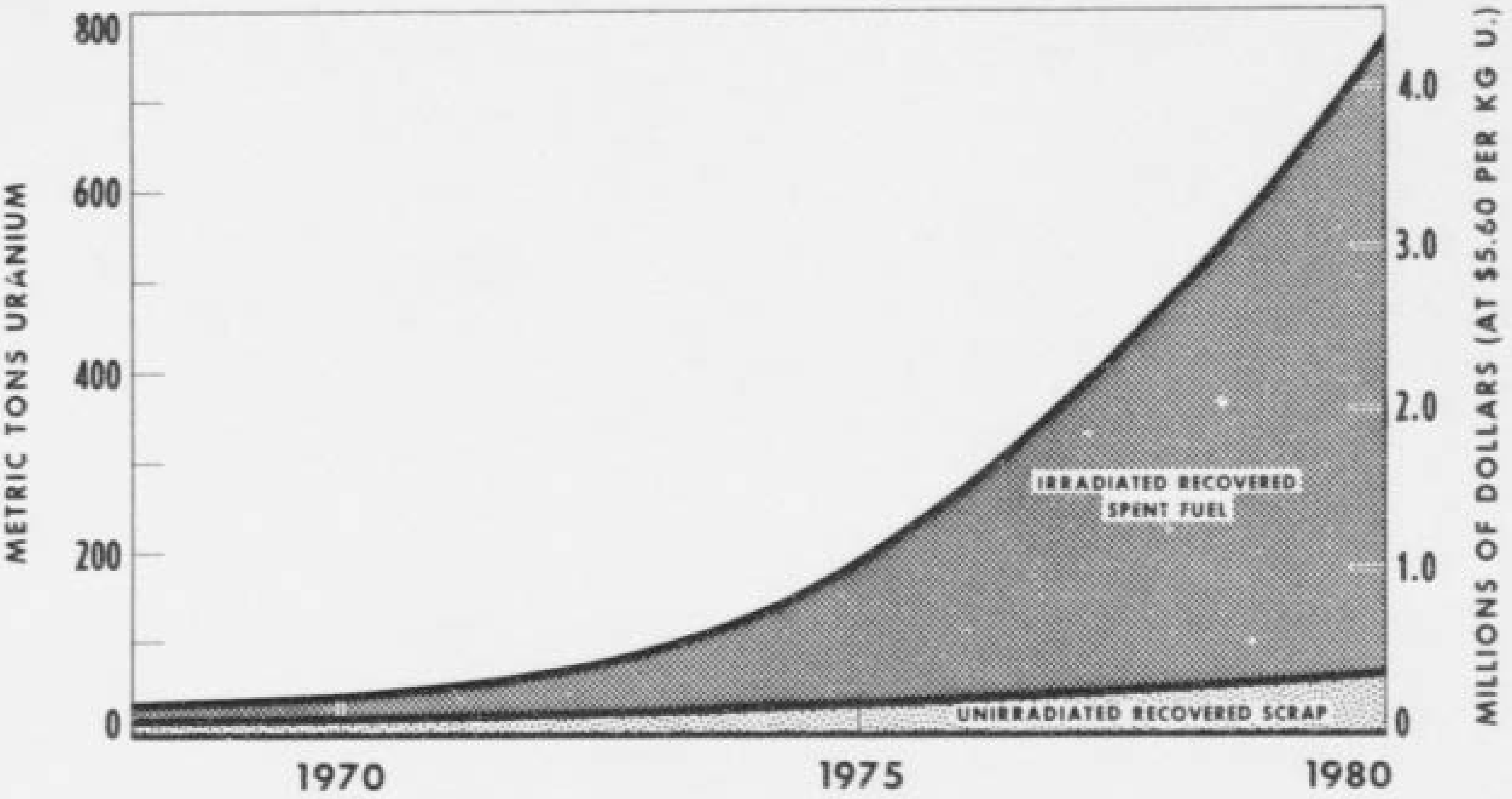

The annual quantities of enriched uranium containing greater than 5% U-235, both irradiated and unirradiated, that will require recovery, conversion to UF_6, and return to the gaseous diffusion plants are expected to remain at approximately the present level: therefore, they have not been plotted in chart form. These quantities have been averaging in the range of 5,000 kgs U per year, which would represent an annual UF_6 conversion cost of $160,000 based on $32 per kg U.

3. Summary of Present and Future Industry Capability

Facility	Annual Capability	Calendar Year
**Conversion of Ore Concentrates to UF_6**		
Allied Chemical Corp. Plant at Metropolis, Illinois	5-7,000 STU	early 1968
	10,000 STU	1969
Kerr-McGee Plant (site not yet announced)	5-10,000 STU	1970

Additional capacity or additional plants needed about 1974

Conversion of Enriched Recovered Uranium to UF_6

None in existence at present

Probable Future Plants	Needed Capacity	
Facility Capable of Converting U Containing <5.0% U-235 Expected in Several Years.	40 MTU	1970
	200 MTU	1975
	800 MTU	1980
Facility Capable of Converting U Containing >5.0% U-235 Expected in 5 or 6 years	Approx. 5 MTU	

B. <u>Uranium Enriching</u>

Market Predictions

	Value of Sep. Wk.
1970	$ 190,000,000
1975	$ 495,000,000
1980	$1,050,000,000

Of the total cost of a fuel core for a power reactor, about one-third represents the cost of uranium enriching.

Since the isotopic enrichment of uranium is the only major step in the nuclear fuel cycle in which private industrial capability does not exist, it is not surprising that a great deal of attention is now being focused on possible transfer of the gaseous diffusion plants to private industry. The Government's three gaseous diffusion plants, which were built for national defense purposes at a cost of $2.3 billion, are available to produce fuel for civilian reactors, now that Government requirements for enriched uranium have greatly decreased.

AEC announced in March 1967, that it had agreed to cooperate with the Atomic Industrial Forum in a special study that AIF proposed to undertake to evaluate the feasibility and desirability of transferring one or more of AEC's gaseous diffusion plants to private industry.4/

The Forum estimated that its study would require six to nine months. It is directed to answer the following questions:

Is it feasible and desirable for AEC to lease or sell some or all of its gaseous diffusion plants to industry?

If the feasibility and desirability can be established, when and how can this transfer best be accomplished?

The Forum established a seven-member study team to carry out the detailed study. The study team has been authorized by the AEC to have access to certain classified information pertinent to its study. The Forum report on its study, however, will be unclassified and available to the public.

4/ See AEC Public Release K-62, dated March 10, 1967.

The study team is assisted by an AIF [illegible]visory Committee made up of approximately 41 representatives of a broad segment of utilities and industry.

AEC is also conducting an internal classified study of the feasibility and desirability of transferring diffusion plants. Some of the factors to be considered are national security, weapons proliferation, competition in uranium enriching, assurances of supply at reasonable costs, and national financial interests. AEC has retained the following consultants to assist in its study:

James K. Carr, General Manager of Public Utilities for the City and County of San Francisco
Dr. J. Elton Cole, a chemist who retired from DuPont Company in 1965
Dr. Edward W. More[illegible], a consultant in public utility economics
Joseph C. Swidler, senior member of the law firm of Swidler and Freeman in Washington, D. C.

In addition, AEC will use the services of Dr. Manson Benedict, one of its consultants and a member of its General Advisory Committee.

In order to permit the nuclear power industry, both in this country and abroad, to make more reliable judgments concerning future enriching capacities, AEC declassified and released in June 1967, quantitative information on the total capacity of its three gaseous diffusion plants to produce enriched uranium.5/ The separative capability of the plants varies with the amount of electric power utilized, although not necessarily in direct proportion; consequently, AEC's initial release expressed the total capacity for three different power levels. Similar information is presented in Table II-1.

AEC announced on September 21, 1967,6/ that its current charge for the toll enrichment of privately owned uranium is $26 per kilogram separative work unit. This charge is within the $30 per unit ceiling price specified in the uranium enrichment criteria established in December 1966. The $30 ceiling continues in effect, subject to upward escalation for the cost of electric power and labor.

5/ See AEC Public Release K-145, dated June 14, 1967.
6/ See AEC Public Release K-228, dated September 21, 1967.

TABLE II-1

Enrichment Capacity of AEC's Gaseous Diffusion Complex

Total Power Level, Megawatts	Annual Production Capability in Millions Of Kilogram Separative Work Units*	Examples of Use of Separative Capacity, Approximate Amount of Enriched Product That Could Be Produced by Feeding Normal (0.711% U-235) Uranium and Withdrawing Tails at 0.2% U-235.	
		Product At 2.0% U-235	Product At 3.0% U-235
2000 (Maximum projected level, to be reached September 31, 1968)	6.9	3.15 million kgs U product from 11.1 million kgs U of normal feed	1.60 million kgs U product from 8.74 million kgs U of normal feed
3000 (Approximate Present Level)	9.8	4.51 million kgs U product from 15.[illegible] million kgs U of normal feed	2.30 million kgs U product from 12.6 million kgs U of normal feed
4000 (Approximate maximum level reached in the past)	17.0	7.75 million kgs U product from 27.3 million kgs U of normal feed	3.96 million kgs U product from 21.5 million kgs U of normal feed

* Separative Work Units - The AEC expresses capacity and product rate of its gaseous diffusion plants in terms of separative work units. A separative work unit is not a quantity of material, but is a measure of the effort expended in the plants to separate a quantity of uranium of a given assay into two components, one having a higher percentage of uranium-235 and one having a lower percentage. Separative work is generally expressed in kilogram units to give it the same dimensions as material quantities, i.e., kilograms or metric tons of uranium.

The total enriched uranium shipped from AEC's gaseous diffusion plants during fiscal year 1967, to meet domestic commercial and foreign requirements, represents approximately 1.3 million kilogram separative work units when calculated on the basis of using normal uranium feed and operating at a tails assay of 0.20% U-235. This amounts to only about 13% of the total production capability of the three plants when operated at the present total power level of about 3 million electrical kilowatts (see Tab'e II-1). Estimated annual separative work requirements through 198U, along wich the corresponding dollar value based on $26 per kilogram separative work unit are shown in Chart II-C.

Chart II - C

URANIUM ENRICHING

ESTIMATED TREND IN FUTURE REQUIREMENTS FOR SEPARATIVE WORK

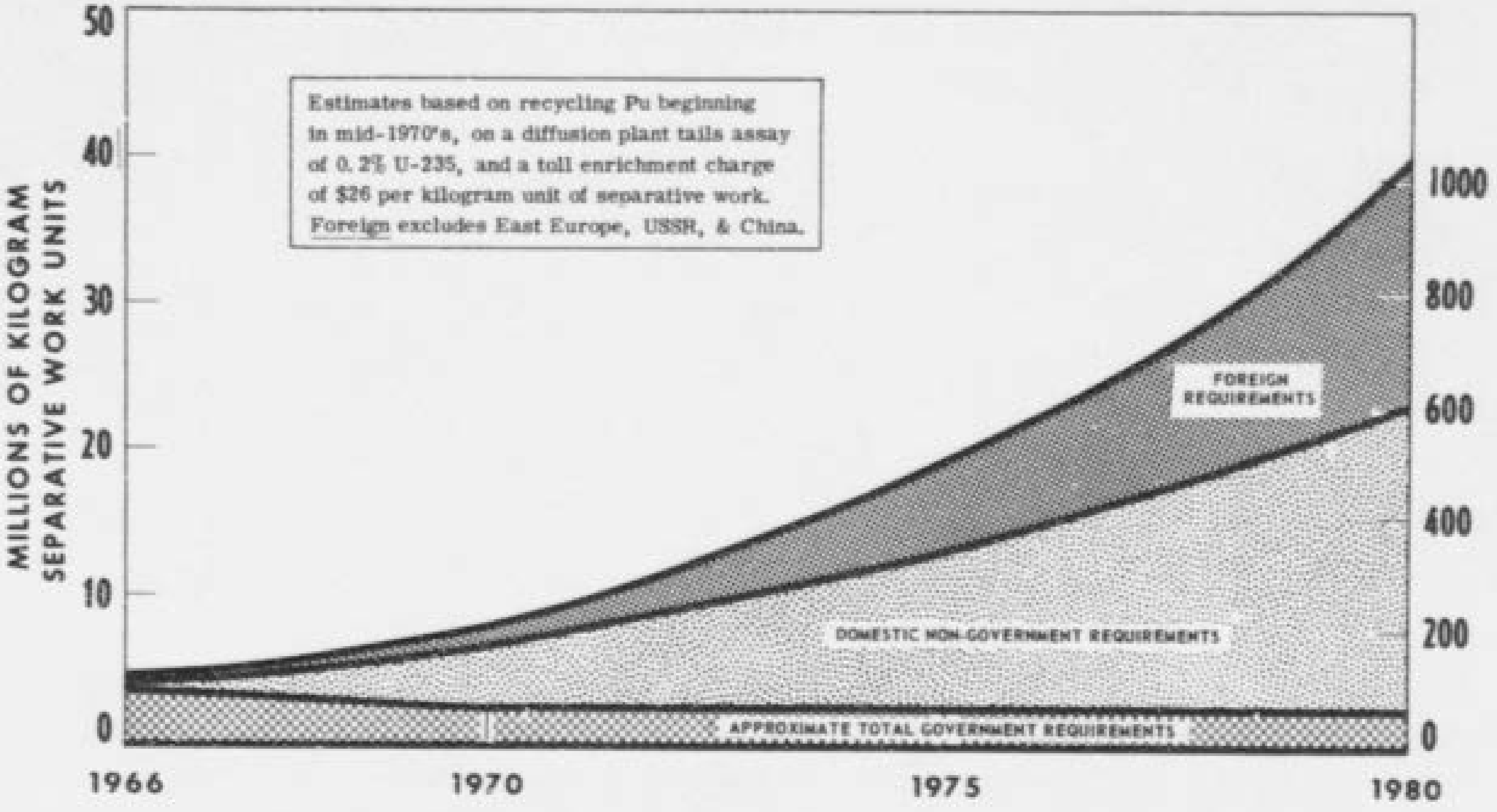

Many factors must be considered in determining the optimum power level for operating the gaseous diffusion plant complex. Among these are cost of power, cost of feed, future requirements for product, capability of plants to meet future requirements, desirability of building up advance inventory of product in order to optimize the production schedule on economic grounds and with the additional effect of deferring the date by which additional plants will be needed, etc. The planning is further complicated by the uncertainties associated with predicting future requirements and the need for five or six years advance lead time in starting construction of new plants and securing under contract the large blocks of power needed to operate them. When military and other Government requirements began to decrease, it became necessary to decide between (a) building up a huge inventory of enriched uranium to meet future needs, or (b) cutting down on the operating power level of the gaseous diffusion plants. Even though it was clearly recognized at that time that requirements for enriched uranium would eventually exceed total production capability of the existing plants, continued operation of the plants at full capacity was determined to be unjustified from an economic standpoint. Consequently, Presidential announcements in January 1964, April 1964, and February 1965 resulted in a series of power reduction steps which, when completed late in 1968, will bring the total power level of AEC's three gaseous diffusion plants down to 2 million electrical kilowatts. It is apparent, however, when the total separative capability of the existing plants (Table II-1) is compared with estimated requirements (Chart II-C), that the power level of the plants will again have to be increased. Although a direct comparison between Table II-1 and Chart II-C indicates that additional production capacity will be necessary early in 1975, the advance inventory that will be available at that time, and the additional production capacity that can be obtained by improvement of existing plants should make it possible to postpone need for an additional plant until about 1980.[7]

C. Uranium Processing and Fabrication

Market Predictions

	Initial Fuel	Replacement Fuel
In 1970	$ 70,000,000	Relatively Small
In 1975	$113,000,000	$119,000,000
In 1980	$144,000,000	$276,000,000

7/ See Chairman Seaborg's testimony on Uranium Enrichment Services Criteria before the JCAE in August 1966.

Adequate industrial capability and competition exist today to process and fabricate depleted, normal, and enriched uranium to the various fuel forms required in research, test, and power reactors.

1. Processing

The principal producers of depleted, normal, and enriched uranium metal, oxides, compounds, and coated particles are listed in Table II-2.

TABLE II-2

Principal Producers of Uranium Fuels

	Metals, Oxides & Compounds	Coated Particles*
General Electric Company, San Jose, Cal.	X	
General Dynamics Corp., San Diego, Cal.		X
Kerr-McGee Oil Industries, Inc., Oklahoma City, Oklahoma	X	
National Lead Company, Albany, New York	X	
National Carbon Company, Lawrenceburg, Tenn.		X
Nuclear Fuel Services, Inc., Erwin, Tenn.	X	X
Nuclear Materials and Equipment Corp., Apollo, Pennsylvania	X	X
United Nuclear Corp., Hematite, Missouri	X	X

* Used in certain AEC fuels such as NERVA and in HTGR fuel elements.

No major changes have taken place during the past year in the capabilities of private industry to produce uranium fuel. Several announcements were made concerning future plants that will convert and fabricate uranium fuels. These announcements are mentioned in the following section under Oxide Fuels.

2. Fabrication

A complete industry capability exists to produce various types of fuels from various compounds; however, the number of capable suppliers is very limited for several highly specialized types of fuels.

a. Metallic Fuels -- It was mentioned in last year's report that M&C Nuclear might possibly withdraw from fabrication of HIFIR (High Flux Isotope Reactor) fuel elements; however, they have since decided to continue in this field and are, at present, the only fabricators of HIFIR fuel elements. The AEC is interested in developing more commercial suppliers of this type of fuel in order to assure continuity of fuel supply. The High Flux Isotope Reactor is designed to produce research quantities of the transuranium elements and is the first of its kind in the United States.

A stronger industrial base for the fabrication of research and test reactor fuels of all kinds is needed and the AEC has been working to encourage wider participation. The present and potential suppliers of research and test reactor fuels of the uranium-aluminum type are United Nuclear Corporation, National Lead Company, M&C Nuclear, Atomics International, and Aerojet-General Corporation. The market for research and test reactor fuels, including MTR, ETR, ATR, ORR, HFBR, HIFIR, Plumbrook, GETR, and university reactors, is estimated at about $7 million annually.

Fuel for the EBR-II differs from other test reactor fuels in that it consists of a uranium-fissium alloy that is sodium-bonded with stainless steel cladding. Aerojet-General is producing this fuel.

b. Oxide Fuels -- This is the type of fuel used for all the light water reactors being presently built or ordered. This segment of fuel element fabrication continues to be highly competitive.

The four major reactor manufacturers have announced decisions to add to their fuel fabrication capabilities, as follows:

It was announced in last year's report that Combustion Engineering was building a fuel fabrication facility addition to their Windsor, Connecticut, plant.

In December 1966, General Electric announced plans to build a plant near Wilmington, North Carolina, to produce fuel for nuclear power plants as well as equipment components. It was announced that the plant was scheduled to go into operation in early 1969.

In August 1967, Babcock & Wilcox Company announced plans to build a commercial reactor fuel fabrication plant at its Campbell County site in Virginia at a first-phase cost in excess of $2 million. The plant is expected to be ready for production by 1970.

In September 1967, Westinghouse announced plans to build a $20 million manufacturing plant near Columbia, South Carolina, to fabricate nuclear fuel elements for commercial nuclear power plants. Initial operations are scheduled for the third quarter of 1969 with full production to be reached early in 1970.

The principal fabricators of oxide uranium fuels are listed in Table II-3. For convenience those also fabricating metallic fuels are shown.

TABLE II-3

Principal Fabricators of Uranium Fuel

	Oxide	Metal
Aerojet-General Corp., San Ramon, Cal.		X
Atomics International, Canoga Park, Cal.	X	X
Babcock & Wilcox Co., Lynchburg, Va.	X	
Combustion Engineering, Inc., Windsor, Conn.	X	
General Dynamics Corp., San Diego, Cal.	X	
General Electric Company, San Jose, Cal.	X	
National Lead Company, Albany, N. Y.	X	X
Nuclear Fuel Services, Inc., Erwin, Tenn.	X	X
Nuclear Materials & Equipment Corp., Apollo, Pa.	X	X
United Nuclear Corp., New Haven, Conn.	X	X
Westinghouse Electric Corp., Pittsburgh, Pa.	X	

3. Comprehensive Tabulation of Industry Capabilities

A comprehensive tabulation of capabilities that exist at commercial facilities for the processing and fabrication of various types of fuel elements, uranium and plutonium fuel research and development, and uranium and plutonium unirradiated scrap recovery is presented as Table II-4. The AEC Division of Industrial Participation will appreciate hearing of any changes or additions that should be made to this table.

TABLE II-4 [illegible]

X DENOTES PRESENT CAPABILITY
F DENOTES FUTURE CAPABILITY

COMPANY AND FACILITY LOCATION	[illegible]
[illegible]	[illegible]

4. Present and Future Requirements for Processing and Fabrication Services

During Fiscal Year 1967, the amount of enriched uranium withdrawn from the gaseous diffusion plants and shipped to domestic processors and fabricators totaled 169,983 kgs U. (See Table II-5). This quantity includes requirements for all types of privately sponsored reactors requirements for Government reactors exclusive of production and military types, and fuel for foreign reactors processed and/or fabricated in the United States. The corresponding quantity that was withdrawn and shipped in FY-1966 amounted to 285,773 kgs U. Although the quantity decreased from FY-1966 to FY-1967, the reason is not readily apparent. It could be due to the fact that no orders were placed for nuclear power plants in 1964. A part of the explanation lies in the fact that a smaller portion of enriched uranium destined for foreign reactors is now being

fabricated in this country (see section that follows). The quantity shipped to domestic fabricators is expected to increase again rapidly as the orders for commercial nuclear power plants materialize.

TABLE II-5

Enriched Uranium Furnished to Industry
(Kilograms of Uranium)

Fiscal Year	Furnished as UF_6	Furnished in Forms Other Than UF_6	Total
1956	0	2,400	2,400
1957	6,000	4,000	10,000
1958	24,000	21,000	45,000
1959	110,300	6,300	116,600
1960	86,200	3,400	89,600
1961	119,400	6,900	126,300
1962	125,600	3,000	128,600
1963	100,273	3,918	104,191
1964	116,401	2,037	118,438
1965	152,786	1,441	154,227
1966	285,773	5	285,778
1967	169,983	96	170,079

Annual domestic requirements for processing and fabrication of commercial power fuel are projected in Chart II-D, and corresponding dollar values for the service are estimated in Chart II-E. Since the total quantity of fuel will not be significantly affected, depending on whether plutonium is or is not recycled, Chart II-D may be used for either assumption. The annual dollar values projected in Chart II-E are based on an assumption that unit costs for processing and fabrication will decrease from a present cost of about $100 per kilogram U to about $60 per kilogram U in 1980 for uranium fuel. If plutonium recycle is adopted, the annual dollar totals can be expected to increase slightly above the non-recycle estimates in the late 1970's, but by something less than 10% in 1980.

Chart II - D & E

PROCESSING & FABRICATING POWER REACTOR FUELS

ANNUAL DOMESTIC REQUIREMENTS

Chart II - D

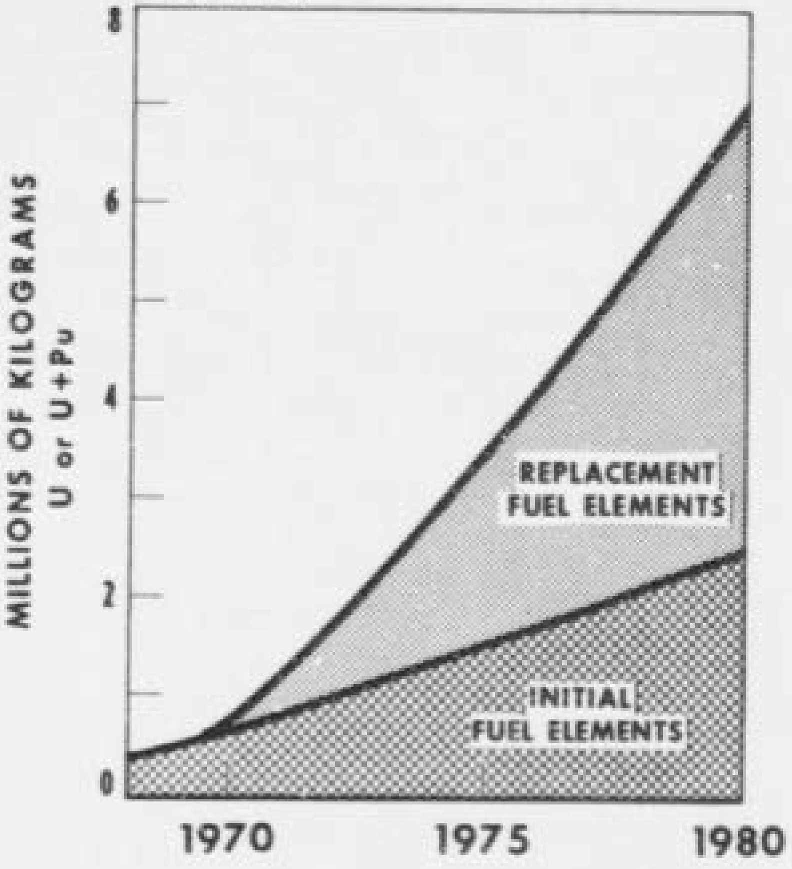

Chart II - E

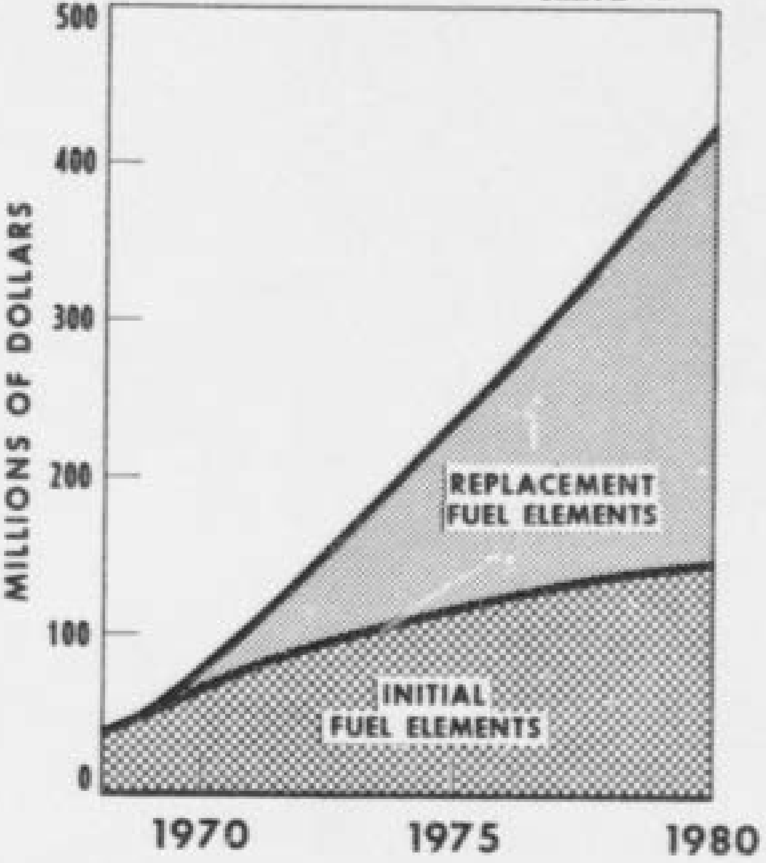

5. Processing and Fabrication of Foreign Reactor Fuel

In addition to the quantity shown in Table II-5 of UF_6 shipped to domestic processors and fabricators in FY-1967, 80,925 kilograms of enriched uranium were withdrawn and shipped to foreign countries in the form of UF_6 during FY-1967 as compared to 9,524 kgs U in FY-1966.

Also in FY-1967, domestic processors and fabricators furnished 79,085 kilograms of enriched uranium to foreign customers, which when compared with the 80,925 kilograms shipped as UF_6, indicates that about 50% of the enriched uranium fuel being produced for foreign reactors is being processed and fabricated in foreign countries. By way of comparison, in FY-1966, only about 9% was processed and fabricated in foreign countries. See Table II-6. It is probable that this trend will continue and that, in a few years, essentially all enriched uranium used in foreign reactors, will be fabricated abroad. For this reason, foreign fuel was not taken into account in the preparation of Charts II-D and II-E.

TABLE II-6

	FY-1966	FY-1967
A. Enriched Uranium Shipped Abroad in Form of UF_6 for Foreign Reactors Kgs U	9,524	80,924
B. Enriched Uranium Shipped Abroad in Processed or Fabricated Form for Foreign Reactors Kgs U	93,626	79,085
C. Approximate Percentage of Foreign Reactor Fuel Enriched in USA That is Fabricated in Foreign Countries, $(\frac{A}{A\&B} \times 100\%)$	9.2%	50.6%

6. Commercial Fabrication of AEC Fuel

The magnitude of orders placed each year by AEC for commercial processing and fabrication of enriched uranium fuels is indicated in Table II-7.

It can be seen from Table II-7 that the commercial fabrication of naval fuels has been running at a fairly constant level, and that the commercial fabrication of other fuels for AEC increased appreciably in FY-1967.

TABLE II-7

AEC Orders for Fabrication of Uranium Fuel
From Commercial Suppliers
(Millions of Dollars)

Fiscal Year	Naval Purposes	All Other Purposes	Total
1959	38.6	2.8	41.4
1960	48.6	3.8	52.4
1961	29.2	4.0	33.2
1962	60.9	4.5	65.4
1963	36.8	2.2	39.0
1964	28.5	8.7	37.2
1965	52.3	5.4	57.7
1966	63.4	2.0	65.4
1967	55.1	13.0	68.1

7. Fuel Cycle Arrangements

Various different fuel cycle arrangements, including fabrication and reprocessing, are being offered to utilities by the fuel suppliers. These are discussed under Category IV of this report.

D. Plutonium Processing and Fabrication

Our 1966 report reviewed some of the significant background information on plutonium that was presented at a commercial plutonium fuels conference sponsored by the AIF, EEI, and ANS in March 1966. One of the most significant statements from that conference was contained in Dr. Seaborg's address in which he forecast that plutonium generation in nuclear power plants would be at a rate of more than one million dollars per day in 1980.

A great amount of interest has also been expressed by nuclear utilities and fuel cycle suppliers in the value of plutonium as a fuel to recycle in thermal reactors and the impact on the fuel cycle and power cost estimates for reactor systems. In addition to its effect on thermal reactor system economics, plutonium used in a reactor as a fuel in place of U-235 provides greater flexibility of independence from an enriched uranium supply and also permits extending the available supply of fissionable material through breeding.

In view of the termination on December 31, 1970, of AEC's guaranteed purchase price for plutonium produced in domestic or foreign reactors fueled with AEC-supplied enriched uranium, utilities having operating nuclear power plants and faced with increasing supplies of plutonium must eventually plan to recycle this valuable fuel or hold it in inventory until a breeder market develops.

1. Availability of Plutonium

The projected quantities of plutonium expected to be recovered annually from domestic power fuels are shown in Table II-8. This table is based on the AEC's estimates of power reactor spent fuel discharges and eight kilograms of plutonium recovered per metric ton of uranium processed.

TABLE II-8

Projected Plutonium Recovered From Domestic Commercial Power Reactor Fuels
(KG Total Pu as the Nitrate)

Year	Annual Kg Pu Recovered
1968	200
1969	240
1970	320
1971	800
1972	1300
1973	2200
1974	3600
1975	5400

The availability of plutonium is based on the quantity expected to be recovered from reprocessed spent fuels rather than plutonium generated in power reactor operation during any given year.

2. Requirements for Plutonium

AEC, private industry, and foreign programs will require substantial quantities of plutonium for research and development, critical assemblies, experimental reactors, and demonstration reactors. The annual plutonium requirements for foreign, AEC, and private domestic programs are presented in Table II-9.

Comparison of annual requirements versus availability indicates that the demand exceeds supply until around 1973. The remainder would have to be obtained from AEC production reactor operations.

TABLE II-9

Annual Net Requirements for Plutonium in Kgs.

	FY-68	FY-69	FY-70	FY-71	FY-72	FY-73	FY-74	FY-75
Research & Development	400	320	81	85	50	60	51	74
Critical Assemblies	1145	1769	495	-122*	1075	-99*	-120*	-11*
Experimental Reactors	106	8	413	-102*	140	-575*		
Fast Flux Test Prog.	390	365	1105	570	300	900	800	440
Fast Reactor Demonstrators	0	0	0	0	0	850	780	690
Foreign	97	430	550	600	190	70	50	30
EEI Thermal Recycle Test Prog.	30	40	0	400	300			
ROUNDED TOTALS	2200	2900	2600	1400	2000	1000	1600	1200

* The negative sign indicates a return of Plutonium from a project to the Government.

3. Transition of Plutonium Recycle From AEC to Industry

As noted in our 1966 report, the Commission's work on plutonium fuel recycle in thermal reactors will be gradually phased out over the next few years, because the basic questions of feasibility have been answered and a large body of experience, data, and design information has been developed. The AEC expects the nuclear power utilities and the major reactor suppliers to assume a substantial amount of plutonium fuel development work. There is presently some concern by the AEC that these efforts may not be adequate to assure the efficient utilization of plutonium during the mid-1970's when the supply is expected to begin to rapidly exceed the demands.

Many electric utilities which own or have ordered nuclear power plants have been studying the utilization of plutonium as a fuel to recycle in their plants. In 1966 the Edison Electric Institute initiated a plutonium recycle R&D program which is being conducted in two phases, the first involving parallel recycle technology and economic studies with Westinghouse and General Electric and totaling about $750,000 in contributions from EEI members. The second phase includes fabricating and irradiation testing of plutonium recycle fuels in power reactors. The first plutonium fuel to be placed in a commercial power reactor under this program was the insertion in June 1967 of four plutonium fuel rods in fuel assemblies in Dresden #1. The plutonium used was recovered from reprocessed Dresden #1 fuel elements.

The AEC program of plutonium utilization in light-water reactors has plutonium dioxide - uranium dioxide fuel mixtures in fabricated fuel elements under irradiation in the Plutonium Recycle Test Reactor at Richland, Washington, the Experimental Boiling Water Reactor at Argonne, and the Saxton Nuclear Experimental Reactor at Saxton, Pennsylvania.

The EEI also has a program in support of fast reactor fuel development. The first phase of this program is with Atomic Power Development Associates at a level of $1.1 million. The entire EEI fast reactor plutonium fuel program will extend over five years and is expected to cost about $5 million.

4. Commercial Fabrication

Based on the projected annual plutonium requirements in Table II-9 on page 54, it is expected that commercial fabrication may process and fabricate about 80% of these quantities excluding foreign requirements. An estimated fabrication cost of $5,000 per kilogram of contained plutonium was used to develop the potential fabrication market shown in Chart II-F.

Chart II-F

PLUTONIUM PROCESSING AND FABRICATION

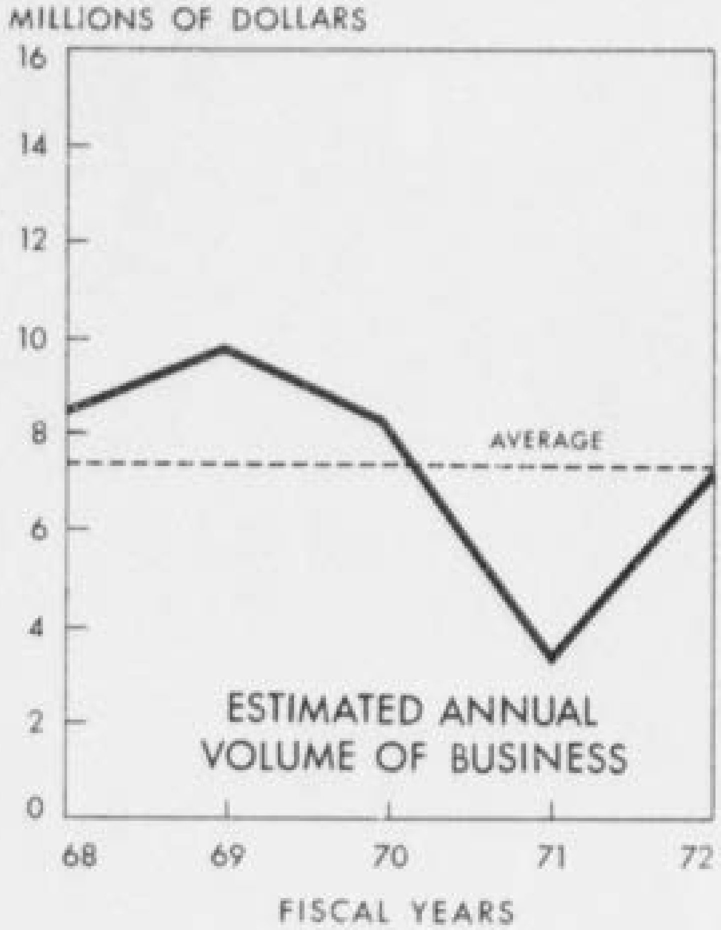

5. Industry Capability

Table II-10 lists private firms which have various degrees of capability in plutonium processing.

In September 1967, Douglas United Nuclear and AEC announced that revisions had been negotiated to the DUN commitments with the AEC which would expand DUN's commercial operations in the Richland, Washington, area in lieu of announced plans to construct a plutonium fuel fabrication facility there.[8/]

8/ See AEC Public Release IN-821, dated September 7, 1967.

TABLE II-10

Industrial Interest in Plutonium Work

	Fuel Processing	Fuel Fabrication	Scrap Recovery	Source Fabrication	R&D
Atomics International	X	X			X
Babcock & Wilcox					X
Battelle Memorial Inst. (Columbus, Ohio)					X
Carborundum Co.					X
General Electric Co.	X	X			X
Monsanto Research Corp.				X	X
Nuclear Fuel Services	X	X			X
Nuclear Materials & Equipment Corp.	X	X	X	X	X
United Nuclear Corp.					X

Nuclear Materials and Equipment Corporation was awarded an order for about 1,000 kg and options for an additional 1,200 kg of ZPPR (Zero Power Plutonium Reactor) plutonium fuel. Atomics International has also submitted proposals to supply a portion of this fuel.[9/]

Babcock & Wilcox was awarded a contract for $3.2 million to develop the gel-addition method for production of uranium-plutonium oxide fuel for the fast breeder program.

In August 1967, the New York State Atomic and Space Development Authority announced that they had retained the Ralph M. Parsons Company to provide engineering assistance to the Authority in the siting and conceptual design of a plutonium fuel manufacturing facility to be capable of fabricating fuel for breeder power reactors.

E. Thorium

The present need for thorium as a reactor fuel continues to be limited primarily to research and development purposes. AEC orders for thorium metal powder and ingots for fuel R&D use during the past year amounted to only a few hundred dollars. The development of certain advanced converter and breeder reactors for commercial applications could eventually expand the use of thorium as a source of nuclear fuel.

9/ A certain portion of the ZPPR fuel is being fabricated in Government facilities at Rocky Flats.

No changes have occurred in the industry in the processing and fabrication of thorium. The firms that fabricate thorium are identical to those listed in Table II-2. The principal firms processing thorium are shown in Table II-11.

TABLE II-11

Principal Firms Processing Thorium

American Potash & Chemical Corp., Chicago, Illinois
Babcock & Wilcox Company, Lynchburg, Virginia
The Dow Chemical Company, Midland, Michigan
General Dynamics Corporation, San Diego, California
Kerr-McGee Oil Industries, Inc., Cushing, Oklahoma
Metal Hydrides, Inc., Beverly, Massachusetts
National Lead Company, Albany, New York
Nuclear Fuel Services, Inc., Erwin, Tennessee
Nuclear Materials & Equipment Corp., Apollo, Pennsylvania
United Nuclear Corporation, Hematite, Missouri

F. Uranium-233

The present need for uranium-233 continues to be limited and is essentially of a developmental nature. Due to the interest in the thorium - uranium-233 fuel cycle, development work in this area will no doubt increase in the future.

The principal companies having U-233 processing and fabrication capabilities are Nuclear Fuel Services, Nuclear Materials and Equipment Corporation, and Babcock & Wilcox Company.

The Private Ownership Act of 1964 directed the Commission to establish guaranteed purchase prices for U-233 not to exceed 10 years with no fixed termination date on the Commission's authority. Accordingly, the AEC established prices for uranium enriched in uranium-233 in forms of nitrate, dioxide, or metal and meeting AEC specifications,of $14 per gram of the contained isotope uranium-233, subject to adjustment for the presence of other uranium isotopes and deductions for handling costs. The U-233 purchase prices continue until the Commission has made a determination that U-233 technology has reached the point where the Government should no longer provide a guaranteed market for this material.

G. Other Materials

1. Heavy Water

There has been no change in the status of heavy water since last year's report.

Heavy water plants were originally built by the Atomic Energy Commission in the early 1950's at Dana, Indiana, and at Savannah River to furnish heavy water for the production reactors at Savannah River. The Dana plant was shut down in 1957 and the Savannah River plants were gradually reduced in operation to about one-third their total capacity during 1957 and 1958. As of today, the AEC continues to operate the Savannah River heavy water plant at approximately one-third its capacity in order to meet AEC production requirements.

Since 1955, the AEC has sold heavy water in quantities of 125 pounds or more to commercial users. Smaller quantities are available from distributors who buy large quantities from the AEC for resale. The principal commercial suppliers are given in Table II-12. Since heavy water use frequently involves degradation of the heavy water, the AEC also provides a service to rework degraded heavy water. The Bio-Rad Company of California has established an operation for upgrading small quantities of heavy water. The Deuterium Corporation of New York is now offering a broad range of services, including design, engineering, and construction of heavy water plants. The AEC will continue to offer purification services for larger quantities, until this service is reasonably available from industry for all quantities.

TABLE II-12

Principal Commercial Suppliers of Heavy Water
(In Quantities Less Than 125 Lbs.)

Bio Rad Labs, Inc., Richmond, California
Columbia Organic Chemicals, Columbia, S. C.
Deuterium Corporation, New York, New York
Diaprep, Inc., Atlanta, Georgia
International Chemical Nuclear Corp., City of Industry, California
Isomet Corporation, Palisades Park, N. J.
Liquid Carbonic, Division of General Dynamics Corporation, San Carlos, California
Matheson, Coleman & Bell, East Rutherford, N. J.
Merck & Company, Rahway, New Jersey
New England Nuclear Corporation, Boston, Mass.

Growing heavy water inventory requirements for natural uranium fueled heavy water moderated reactors created considerable interest in heavy water production during the past few years. It is estimated that Canadian, Swedish, UK, French, and Indian reactor programs for heavy water reactors will require initial inventories and make-up ranging from 200-500 tons annually in the next few years, to about 1,000 tons in 1970. As mentioned above, present AEC production is about 190 tons per year, utilizing about one-third of the capacity of the Savannah River plant.[10]

Canadian, European, and Asian capacity, outside the Soviet Bloc, in operation is about 35-40 tons, with another 1,100 tons under construction or planned, for a total of about 1,150 tons. Most of the planned capacity will be in Canada, with about 900 tons planned, but none yet in production. The Deuterium of Canada, Ltd. plant is presently in the start-up operations of the first phase (200 tons per year) which is expected to begin production early in 1968. The second phase of 200 additional tons per year capacity is expected to be in operation by the end of 1968.

The Canadian General Electric Company is planning a plant to produce 500 tons a year, with operation scheduled to begin about 1969. India is planning a 200 ton a year plant. France, India, Norway, and Switzerland have limited capacity to produce heavy water (estimated at about 35-40 tons total annual capacity.)

As reported last year an Atomic Industrial Forum Ad Hoc Committee made a careful study of the heavy water production market and projected requirements and concluded, that because of the limited domestic market and competition from Government-assisted foreign production plants, it would be unlikely that a private company would be willing to assume the risk of building new heavy water production facilities in the United States at the present time. They pointed out, however, that about 1969 the future national and international supply and demand situation should be much clearer.

In view of the AIF Committee's study report, the AEC decided to continue operation of the Government plant at Savannah River at about one-third its capacity for Fiscal Years 1967 and 1968 and to continue to closely follow the heavy water market.

Domestic heavy water requirements will, of course, increase if the heavy water organic cooled reactor (HWOCR) is developed successfully.

10/ Total capacity of AEC's Savannah River plant is approximately 500 tons per year.

2. Beryllium

In line with AEC's desire to support, develop, and utilize the services of a strong domestic beryllium industry, steps were taken in 1966 to improve AEC's communications with the private beryllium industry and to utilize private industry to satisfy a portion of AEC's production needs. As a continuation of this effort, AEC issued both production and development type orders to private industry during 1967.

a. Production Orders

As a follow-up on AEC's meeting with the four major beryllium metal companies at Rocky Flats in June 1966 (see last year's report) AEC invited competitive bids late in 1966 for a portion of its beryllium production requirements and awarded a sizable production order to a private company. An additional order was awarded based on competitive bids in mid-1967, and it is planned that AEC will determine similar requirements semiannually in the future.

Supply-type contracts with three beryllium companies were in effect during 1967.

b. Dissemination of Information

It became apparent during last year's discussions with the beryllium company representatives that there was room for improvement in making information available between Government and industry. In order to facilitate communications, and early notice of AEC technical requirements for beryllium, arrangements were made to designate company liaison men who would contact counterpart AEC, and AEC laboratory and production personnel.

c. Development Orders

To assist the companies in achieving production economies and experience through the wrought beryllium process, or some other equally effective process, the AEC has negotiated development order contracts with three different companies.

d. Beryllium Scrap Program

In response to industry's indicated interest in bidding on AEC beryllium scrap holdings, a small lot sale of about 15,000 pounds was completed during the year. The AEC has sold about 100,000 pounds of scrap since the beginning of this program three years ago.

e. Beryllium Industry

The four companies constituting the basic beryllium metal industry are listed below.

The Brush Beryllium Company
17876 St. Clair Avenue
Cleveland, Ohio 44110

Beryllium Corporation
P. O. Box 1462
Reading, Pennsylvania 19603

General Astrometals Corporation
320 Yonkers Avenue
Yonkers, New York 10701

Beryllium Metals, Inc.
500 Fifth Avenue
New York, New York 10036

3. Boron-10

The Eagle-Picher Company has expressed to the Commission its interest in building a boron-10 facility to meet the needs of the AEC and any developing commercial market. The proposed plant would be capable of producing a high purity boron-10 product.

In consideration of the Eagle-Picher proposal, the AEC is conducting an extensive study of its long-range requirements for this isotope. A Commission review of this matter is expected to be completed before the end of this calendar year.

The Government's total requirements for boron-10 are now being met by the AEC-owned Model City Plant (MCP) in New York State, operated by the Nuclear Materials and Equipment

Corporation (NUMEC). A small portion of this plant's output is upgraded under contract to the Eagle-Picher Company. The MCP is currently operating at a level of about 400 kilograms per year with expectations to reach annual design capacity of 500 kilograms in the near future.

CATEGORY III
NUCLEAR COMPONENTS AND EQUIPMENT

All nuclear components and equipment are available commercially, usually from companies not wholly depenoent on nuclear energy activities for profitable markets. Establishments manufacturing pumps or valves for a wide variety of industrial applications, for example, are usually the suppliers of pumps and valves for specialized nuclear applications. However, as orders for nuclear power reactors have accelerated, demands for reactor components have also expanded rapidly, and it seems probable that some establishments will eventually be devoted exclusively to the supply of reactor parts. Part B of this category discusses reactor components.

Other components within this category are, in some cases, produced by smaller firms whose origin and growth has been more dependent on nuclear energy applications. Nuclear instruments largely fall into this group, and, because of their particular interest and importance to practically all phases of nuclear work, are reported on separately.

The term "nuclear instruments" has been generally used in the past to describe a broad area of application. Their use has now developed to a point where they can be divided into several general areas. These are defined below and are covered under several categories in this report as indicated.

Reactor Control Instruments - Instrumentation built into reactor facilities for control and operation purposes. This use is covered under Part B of this category, "Reactor Control Equipment".

Gauges and Process Control Equipment Utilizing Radioactive Materials
Gauges and process control equipment utilizing radioisotopes in their design. These are covered in Category V, Radioisotopes.

Radiation Detection and Monitoring Instruments - Instruments designed to detect, measure, and monitor radiation, as well as associated equipment. These are covered in Part A which follows.

A. Radiation Detection and Monitoring Instruments

Market Predictions

In 1967	$75-80 millions
In 1970	$ 120 million

This area covers the following types of instruments which are reported by the Census Bureau under their current Industrial Report series, MA-38B, "Selected Instruments and Related Products".

Radiation detecting elements, including ion chambers	Scalers
Nuclear Monitoring Instruments portable hand held	Special amplifiers for nuclear applications
Nuclear Monitoring Instruments Environmental (not hand held)	Pulse analyzers - single channel and multi-channel
Medical monitors - whole body scanners	Sample changers
	Count rate meters
	Miscellaneous

1. Principal Manufacturers

Companies concerned with the manufacture of radiation detection and monitoring instruments in the above categories and whose 1966 sales reportedly have exceeded $0.5 million are shown in Table III-1.

TABLE III-1

Companies Manufacturing Radiation and Monitoring Instruments with 1966 Sales Exceeding $0.5 Million

Baird-Atomic
Beckman Instruments, Inc.
Bendix Corporation
Chronetics, Inc.
Cosmic Radiation Labs
Curtis Nuclear Corporation
Dero Research & Development Corp.
Eberline Instrument Corporation
E. G. & G. Inc.
  ORTEC, Div. of E. G. & G.
EON Corporation
Hamner Electronics Company
Landsverk Electrometer Company
Miles Laboratory
Northern Scientific
Nuclear-Chicago Corporation
Nuclear Data, Inc.
Nuclear Measurement Corp.
Ohio Nuclear
Packard Instrument Company, Inc.
Picker X-Ray Corporation
Reuter-Stokes Electronics Components, Inc.
Solid State Radiations, Inc.
Technical Associates
Technical Measurement Corp.
Teledyne, Inc.
  Isotopes, Inc.
  Controls for Radiation, Inc.
Tennelec Instrument Co., Inc.
Tracerlab, A Division of LFE, Inc.
Transistor Specialties, Inc.
The Victoreen Instrument Co.

Nuclear instrument companies in this category now number[1/] about 170 firms and were among the first types of private firms to form as a result of the Government's encouragement of private industry in the atomic energy program. The large number of firms and complexity of the industry structure, the question of its definition, and the variations in Government purchases and exports have led to a rather wide range of estimates by various groups attempting to forecast future sales. The Association of Nuclear Instrument Manufacturers (discussed later in this category), is attempting to assess the scope of the industry. The efforts of this association to define the industry and the change to more detailed reporting of sales of radiation detection and monitoring instruments to the Census Bureau, beginning in 1964, should help achieve a better understanding of this industry.

2. Competition

As reported last year, the radiation detection and monitoring instrument field is highly competitive, requiring a sizeable expenditure for private research and development in order to keep pace with the market. It is estimated that the private R&D for 1967 may amount to $8-$10 million for this segment of the industry, ranging from 10 per cent to 20 per cent of sales in individual companies.

All radiation detection and monitoring instruments are available commercially and there does not appear to be any substantial competition between the Government and private industry in furnishing these items.

3. Markets

Chart III-A shows annual sales of radiation detection instruments and related equipment through 1965 and estimated sales for 1966 and 1967.

The market for radiation detection and monitoring instruments is intimately associated with the remarkable growth of all nuclear applications and can be expected in general to grow with an expanding nuclear power economy.

The market for radiation detection and monitoring instruments has increased from $17 million in 1957 to $55 million in 1965. Estimates for 1966 and 1967 sales are $65 and $75-$80 million respectively. If this level is achieved, growth over the past 10 years will have averaged about 15 per cent annually, although as Chart III-A indicates, there has been a leveling off of manufacturers' shipments during the past three years. It

1/ Remarks by Dr. Glenn T. Seaborg, "Two Partnerships for Nuclear Progress", at the dedication of the New Facilities for the Nuclear-Chicago Corporation, Des Plaines, Illinois, April 25, 1967, AEC Public Release S-14-67.

is expected that growth in this industry will continue over the next several years as a result of increasing uses for nuclear instrumentation in medical applications, uranium mining exploration and safety, research and development and analytical areas, and may reach $120 million in 1970.[2]

4. Exports

Exports of nuclear instruments amount to about 25 per cent of total shipments. For 1966, exports were reported as $19.2 million, $1.1 million over 1965. Exports are shown below:

TABLE III-2

Exports of Nuclear Instruments

Year	Exports (In Millions $)
1959	4.6
1960	7.1
1961	9.4
1962	11.0
1963	11.8
1964	15.3
1965	18.1
1966	19.2
1967	(est.)23.0

5. Association of Nuclear Instrument Manufacturers (ANIM)

The Association of Nuclear Instrument Manufacturers, (ANIM), mentioned previously, became formally established as a trade association in January 1967. The Association was preceded by a committee which had been working for over a year on the formation of this association.

6. Corporate Changes

In 1967, the Packard Instrument Company was acquired by American Bosch Arma Corporation; Controls for Radiation, Inc.

2/ This estimate is lower than the estimate shown in our 1966 report for growth of the "nuclear instrument industry" ($180-200 million) as a result of dividing the use of nuclear instrumentation into segments.

was acquired by Teledyne, which already owns Isotopes, Inc.; Victoreen Instrument Company announced plans to expand or acquire firms that would complement their capability; Tennelec Instrument Company acquired Princeton Gamma Tech; E G & G acquired ORTEC; and EON Corporation purchased Par-Metal Products Corporation.

B. Reactor Components

1. Orders and Shipments

Shipments of reactor components and equipment over the past nine years, as reported by the Bureau of the Census, are shown in Chart III-B. For pressure vessels[2/] net orders received are also shown for the years for which they have been reported by the Bureau of the Census.

The effect of the upsurge in orders for nuclear power plants is reflected most significantly in the orders reported for pressure vessels in 1966. Orders for other components[3/] are not available for 1966 on the same basis as shipments -- orders for control rod drive mechanisms and pumps were not reported for 1966 -- and hence are not shown on Chart III-B. Were these figures available, it is believed that orders for other components would show an upturn in 1966 comparable to that shown for pressure vessels.

2. Components for Sodium Cooled Reactors

Liquid metal pumps, valves, and other components which will be needed for liquid metal cooled fast breeder reactors will be tested and standards and specifications established through a program to be carried out in the Liquid Metals Engineering Center, now under construction at the Santa Susanna, California, site of Atomics International.

3. Reactor Control Equipment

The reactor control instrumentation and control rod drive mechanisms are of potential interest to various nuclear and non-nuclear firms who are not suppliers of complete power reactors. These components are usually supplied by the reactor vendors but there may be areas in the future that companies specializing in reactor control instrumentation could share in their market.

2/ primary vessels and tanks, [illegible] heat exchangers and condensers

3/ all other reactor components listed in Table 2 in the summary section of this report, except fuel elements, moderators, coolants, and reflectors

A typical 1,000 Mwe PWR would include an estimated $3.7 million out of $134 million for reactor control instrumentation, control rod drive mechanism and area monitors adjacent to the reactor. A typical 1,000 Mwe BWR would include $5.08 million out of about $130 million for comparable equipment. BWR's require more control rod drives than PWR's.

Chart III - A

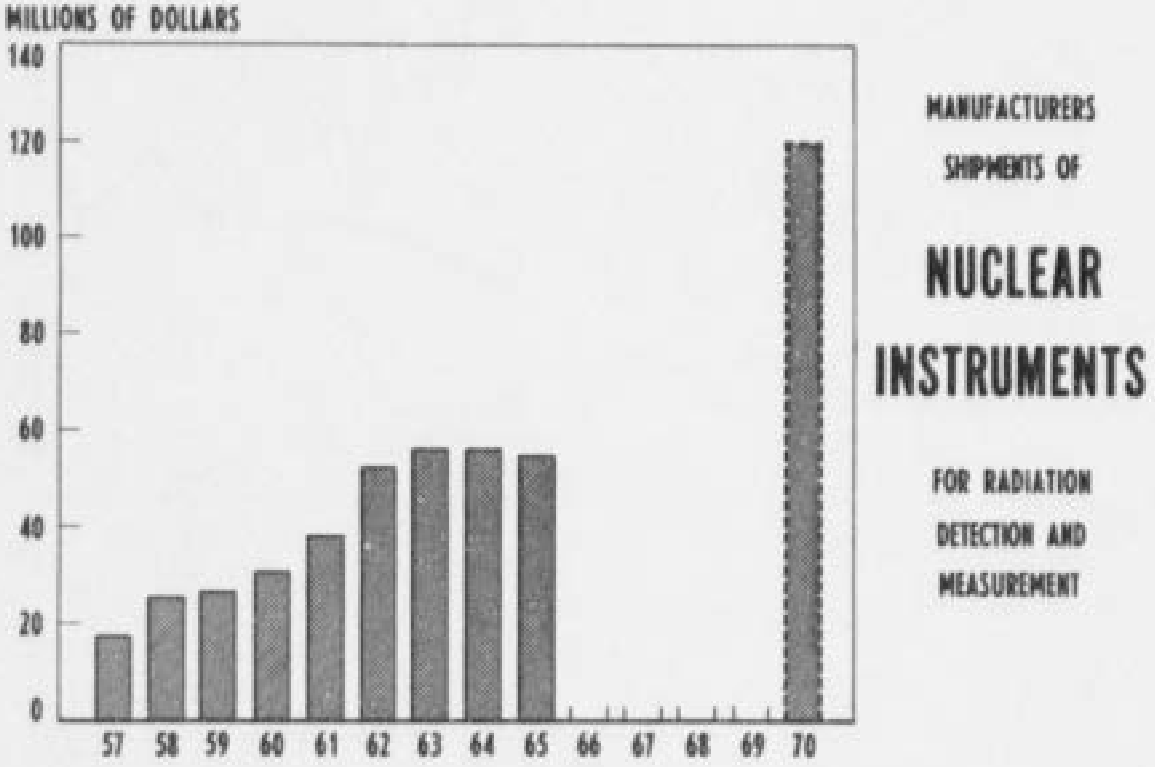

Chart III - B

REACTOR COMPONENTS

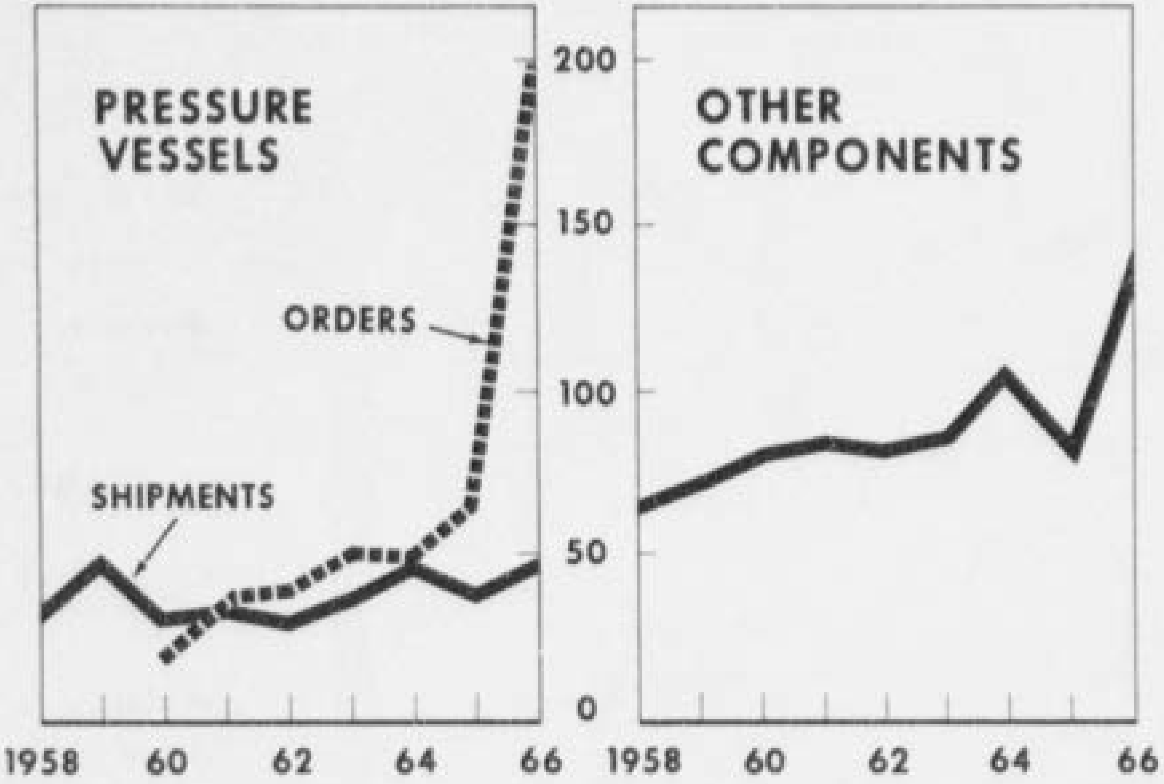

CATEGORY IV

REACTORS

During 1967, a continuing surge to nuclear power occurred at a pace that again surprised even those closest to the industry. In the first nine months, as much nuclear capacity was ordered as in the entire year of 1966. Electrical utilities over a much broader section of the country considered nuclear heat sources more economical than fossil fuels. In some cases, a decision to go nuclear was made even on borderline economic evaluations because of the advantages of nuclear power in solving air pollution problems. There is continuing optimism for the application of nuclear reactors for several purposes. The various markets for reactors are discussed in this section.

A. Expenditures for Reactor Projects

Estimated expenditures for reactors under construction in the United States for the year ending June 30, 1967, were nearly 25% higher than the previous year's level as shown in Table IV-1.

TABLE IV-1

Comparative Costs Incurred For Domestic Reactor Projects

	FY-1966	FY-1967
	(in millions)	
All Projects	$190.8	$241.3
Federal Government	25.1	25.1
Atomic Energy Commission	24.6	14.6
TVA-Browns Ferry	.5	10.5
Utilities	165.7	216.2
Privately Owned Utilities	165.5	215.9
Publicly Owned Utilities	.2	.3

Expenditures by utilities exceeded those by the AEC for the first time in FY-1965. For FY-1966, they were at nearly four times the AEC level. For FY-1967, they were about eight times the AEC level. As the nuclear power market grows in future years expenditures by utilities will dwarf expenditures for reactors for other purposes.

The number of reactors under construction and their total estimated cost are shown in Table IV-2. Table IV-3 shows cumulative costs incurred for these reactors and estimated costs yet to be incurred. This is also shown graphically on Chart IV-A. Electric utility central station power plants are not included in these summaries until contracts have been awarded to reactor vendors Since the cut-off date for the information reported here was June 30, 1967, the plants which have been placed under contract since then are not included in these tables or Chart IV-A.

TABLE IV-2

Domestic Reactor Projects
Under Contract, Design or Construction

	At June 30, 1966		At June 30, 1967	
	Number of Projects	Total Estimated Costs (millions)	Number of Projects	Total Estimated Costs (millions)
All Projects	34	$2,028.8	60	$5,956.7
Civilian Power*	24	1,934.5	54	5,806.8
Civilian Research	10	94.3	6	149.9

* Projects are not included in this tabulation until contracts have been awarded to Reactor Vendors. For listing of projects included see Table IV-4.

TABLE IV-3

Costs and Estimates by Source of Funds
(Projects Active as of June 30, 1967)

	Costs Incurred FY-1967	Total Estimated Costs of Projects	Cumulative Costs Incurred Through June 30, 1967	Estimated Costs to be Incurred After June 30, 1967
	(in millions)			
All Projects	$241.3	$5,956.7	$537.4	$5,419.3
Federal Government	25.1	696.6	70.0	626.6
Atomic Energy Commission	14.6	255.2	59.6	195.6
TVA - Browns Ferry	10.5	441.4	10.4	431.0
Utilities	216.2	5,260.1	467.4	4,792.7
Privately Owned Utilities	215.9	4,998.7	458.9	4,539.8
Publicly Owned Utilities	.3	261.4	8.5	252.9

A listing of reactors included in these Tables appears in Table IV-4, together with the criteria followed in adding projects to and dropping projects from this tabulation.

TABLE IV-4

List of Domestic Reactor Projects
Under Design or Construction
At June 30, 1967 and June 30, 1966

Reactor Projects	June 1966	June 1967
**Civilian Power**		
Peach Bottom #1 (Philadelphia Electric)	X	-
Enrico Fermi (PRDC)	X	-
San Onofre (SCE and SDG&E)	X	X
LaCrosse Boiling Water Reactor (Dairyland)	X	X
Malibu (Los Angeles D. W. & P.)	X	X
Connecticut Yankee (Conn. Yankee A. P. Co.)	X	X
Fort St. Vrain (Pub. Ser. Co. of Colo.)	X	X
Oyster Creek #1 (Jersey Central Pwr. & Lt. Co.)	X	X
Nine Mile Point (Niagara Mohawk Pwr. Corp.)	X	X
Dresden #2 (Commonwealth Ed. Co.)	X	X
Dresden #3 (Commonwealth Ed. Co.)	X	X
Millstone Pt. (Northeast Utilities)	X	X
Pilgrim (Boston Edison Co.)	X	X
Robt. E. Ginna (Rochester G.&.E.)	X	X
Turkey Point #3 (Florida Pwr. & Lt. Co.)	X	X
Turkey Point #4 (Florida Pwr. & Lt. Co.)	X	X
Indian Point #2 (Consolidated Edison Co.)	X	X
Robinson #2 (Carolina Pwr. & Lt. Co.)	X	X
Palisades (Consumer Pwr. Co. of Michigan)	X	X
Point Beach #1 (Wisconsin Mich. Pwr. Co.)	X	X
Quad-Cities #1 (Commonwealth Ed./Iowa-Ill.)	X	X
Monticello (Northern States Pwr. Co.)	X	X
Browns Ferry #1 (Tennessee Valley Auth.)	X	X
Browns Ferry #2 (Tennessee Valley Auth.)	X	X
Indian Point #3 (Consolidated Edison Co.)	-	X
Vermont Yankee (Vermont Yankee Nuclear Pwr. Co.)	-	X
Quad-Cities #2 (Commonwealth Ed./Iowa-Ill.)	-	X
Peach Bottom #2 (Phila. Elec. & Others)	-	X
Peach Bottom #3 (Phila. Elec. & Others)	-	X
Burlington #1 (Pub. Ser. E.&.G. & Others)	-	X
Surry #1 (Virginia Elec. & Pwr. Co.)	-	X
Surry #2 (Virginia Elec. & Pwr. Co.)	-	X
Fort Calhoun (Omaha Pub. Pwr. Dist.)	-	X
Oconee #1 (Duke Pwr. Co.)	-	X
Oconee #2 (Duke Pwr. Co.)	-	X
Oconee #3 (Duke Pwr. Co.)	-	X
Diablo Canyon (Pacific G.&.E.)	-	X
Three Mile Island (Metrop. Ed. Co.)	-	X
Prairie Is. #1 (Northern States Pwr.Co.)	-	X
	24	37
**Civilian Research**		
EBOR - Exper. Beryllium Oxide Reactor (AEC)	X	-
University of Missouri	X	-
Columbia University	X	-
University of California	X	-
Lowell Technological Inst.	X	-
Oregon State University	X	-
National Bureau of Standards	X	-
HTLTR - High Temp. Lattice Test Reactor (AEC)	X	X
LOFT - Loss of Flow Test Facility (AEC)	X	X
UHTREX - Ultra High Temp. Reactor Exper. (AEC)	X	X
PBF - Pwr. Burst Facility (AEC)	-	X
AARR - Argonne Advanced Research Reactor (AEC)	-	X
FFTF - Fast Flux Test Facility (AEC)	-	X
	10	6

NOTES FOR TABLE IV-4

X - indicates project included in tabulation.

Projects are added to this tabulation:

(1) During the calendar quarter in which contracts are awarded to reactor vendors.

(2) For Government projects not subject to licensing and not subject to the submission of a basis for arrangements to the JCAE, during the quarter in which Title I work begins. (However, Government projects are not added prior to a decision to proceed with construction at a specific location even though design work may be under way.)

Projects are dropped from this tabulation in the quarter following the calendar quarter in which they first operate at design power.

Modifications to existing projects are included only when a substantial increase in the rated power output will result.

REACTORS

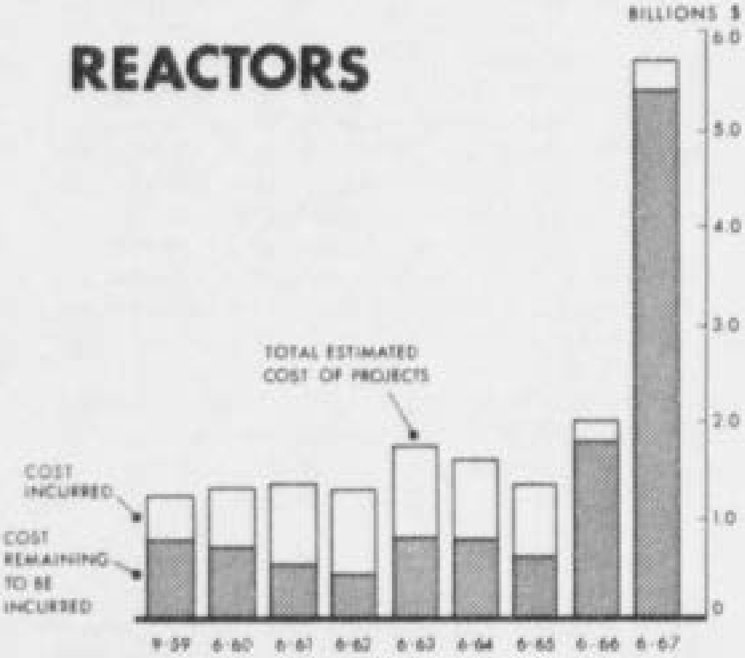

Chart IV-A

DOMESTIC REACTOR PROJECTS UNDER DESIGN OR CONSTRUCTION

●

FOR FY 1964 AND PRIOR YEARS DATA WAS BASED ON THE DATE LICENSE APPLICATIONS WERE RECEIVED.

FOR FY 1966 & 67 DATA IS BASED ON THE DATE CONTRACTS WERE AWARDED TO REACTOR VENDORS.

B. Civilian Power

1. General

In 1967, the electrical utilities again ordered more nuclear power plants or announced intentions to construct nuclear power plants at a greater rate than had been anticipated. This increased volume of business has stimulated the interest of additional industrial participation and resulted in the announcement of plans for facility expansion by a number of companies. The dramatic increase in orders also focused attention on the requirements for additional exploration in the mining industry, the need for accelerated development in plutonium recycle and emphasized the need to develop breeder reactors. This substantial growth in nuclear systems also emphasizes the need for utilities to develop in-house nuclear capabilities on a timely and thorough basis.

Of particular significance during the year was a decision made by Middle South Utilities, Inc. to build an 800 Mwe plant for operation on the Arkansas Power and Light system because the power generation on this system is primarily by gas-fired boilers.

TVA's award of an additional 1,065 Mwe power unit to General Electric for its Browns Ferry station and the award of a 1300 Mwe coal-fired power unit, both to be placed in service in 1972, reflected that the relative cost of nuclear and coal-fired units is about a stand-off on the TVA system. Power from these two units will cost approximately 2.75 mills per kwh. A year ago nuclear power had an appreciable advantage in the calculated cost per kwh generated compared to conventional units. Calculations at that time showed a cost of 2.39 mills per kwh for nuclear generation.[1/]

The announcement of the American Electric Power award of two 1100 Mwe units to Westinghouse, to be located at Bridgman, Michigan, marks the entry of nuclear power on that large electrical power system.

The announcements by Commonwealth Edison Company and Northern States Power Company of the purchase of power units from Westinghouse is a significant milestone in that these are the first utilities to order both pressurized water and boiling water reactor power plants for operation on their systems.

1/ TVA's News Release dated June 22, 1967.

The electrical utilities continue to order their reactor power plants with the provision that the first reactor core in all cases will be provided by the reactor manufacturer, and in most cases, that the replacement fuel for a certain period of time would be provided by the reactor manufacturer. Although nuclear power has only recently emerged, utilities are exploring alternative fuel element suppliers and in some cases replacement fuel awards have been made to independent fabricators. (See pages 90 and 91.)

The nuclear power plant manufacturing base was broadened significantly during the year through the award of an increasing number of plants to Babcock & Wilcox and Combustion Engineering, which indicates that the electrical utilities are becoming more confident and desirable of wider industrial participation to provide the competition that has been characteristic of American industry.

It is interesting to note that areas of the country having mine mouth plant sites and areas of the country having direct access to natural gas, continue to remain primarily conventional fuel supply areas. The demand for coal for electric power generation also continued to increase and it is estimated that the 1980 utility coal market could range from 400 million to 500 million tons[2/] as compared to 243 million tons in 1965.

Nuclear plants placed under contract through 1966 and for the first nine months of 1967 are shown in Tables IV-5 and IV-6. Chart IV-B shows the locations of these plants. The tables show that about 15% more nuclear capacity was placed under contract in the first nine months of 1967 than in the entire year of 1966.

A comparison, based on published information, of fossil fuel versus nuclear announcements in 1967 is also interesting and is shown as Table IV-7. The table shows that announcements for nuclear plants during the first nine months of 1967 were at a slightly higher level than the announcements for fossil fuel plants.

This continued phenomenal growth can be attributed to some extent to the trend to large size power generating units on large scale power systems and to the fact that the utility industry has accepted nuclear power as safe, reliable, and the most economic means of meeting a sizeable proportion of its new requirements.

2/ Secretary Stewart L. Udall - Conference of Coal Industry Executives at the Department of Interior - reported in Coal News, August 18, 1967.

TABLE IV-1

Central Station Nuclear Power Plants

Operating, Under Construction or on Order

Contract Awarded	Utility & Plant	State	Reactor Supplier	Size (Mwe-Net)	Est. Cost* (Millions)	Commercial Operation
1953	Duquesne (Shippingport #1)	Pa.	W	90a/	$ 69	1957
1955	Commonwealth Ed. (Dresden #1)	Ill.	GE	200	51	1960
	Consolidated Ed. (Indian Pt. #1)	N.Y.	B&W	265b/	107	1963
				465	$ 158	
1956	Yank. At. El. Co. (Yankee)	Mass.	W	175	$ 39	1961
1957	Pwr. React. Dev. Co. (Fermi)	Mich.	PRDC	61	$ 66	--
	Northern States (Pathfinder)	S.D.	AC	59	26	1967
				120	$ 92	
1958	Phila. El. (Peach Bottom #1)	Pa.	GA	40	$ 28	1967
	Rur. Coop. Pwr. Assoc. (Elk River)	Minn.	AC	22b/	14	1964
	Pacific Gas & El. (Humboldt Bay)	Cal.	GE	69	24	1963
				131	$ 66	
1959	City of Piqua, Ohio (Piqua)	O.	AI	11	$ 16	1964
	Consumers Power Co. (Big Rock Pt.)	Mich.	GE	70	28	1963
				81	44	
1960	Puerto Rico Wtr. Res. Auth. (BONUS)	P.R.	CE	17	$ 19	1965
1962	Dairyland Power Coop. (LaCrosse)	Wisc.	AC	50	$ 19	1968
	Conn. Yank. A. P. Co. (Conn. Yankee)	Conn.	W	462	87	1968
	Washington Pub. Pwr. Sup. Sys. (NPR)	Wash.	GE	790	315	1966
				1,302	$ 421	
1963	SCE and SDG&E (San Onofre)	Cal.	W	430	$ 97	1967
	Los Angeles D. W. & P. (Malibu)	Cal.	W	462	83	1972
	Jersey Central (Oyster Creek)	N.J.	GE	515	67	1968
	Niagara Mohawk (Nine Mile Pt.)	N.Y.	GE	500	89	1968
				1,907	$ 336	
1965	Boston Edison (Pilgrim)	Mass.	GE	625	$ 65	1971
	Commonwealth Ed. (Dresden #2)	Ill.	GE	715	79	1969
	Northeast Utilities (Millstone)	Conn.	GE	549	85	1969
	Consolidated Ed. (Indian Pt. #2)	N.Y.	W	873	108	1969
	Fla. Power & Lt. (Turkey Pt. #3)	Fla.	W	722	66c/	1970
	Pub. Svc. Co. of Colo. (Fort St. Vrain)	Colo.	GA	330	69	1973
	Rochester Gas & Elect. (Robert E. Ginna)	N.Y.	W	420	65	1969
				4,234	$ 537	
1966	Commonwealth Ed. (Dresden #3)	Ill.	GE	715	$ 81	1970
	Carolina Power & Lt. Co. (Robinson #2)	S.C.	W	663	76	1970
	Consumers Power Co. (Palisades)	Mich.	CE	700	75	1970
	Wisc. Mich. Power Co. (Point Beach #1)	Wisc.	W	455	61	1970
	Commw. Ed./Iowa-Ill. (Quad Cities #1)	Ill.	GE	715	90	1970
	Commw. Ed./Iowa-Ill. (Quad Cities #2)	Ill.	GE	715	77	1971
	Northern States (Monticello)	Minn.	GE	472	76	1970
	T. V. A. (Browns Ferry #1)	Ala.	GE	1,065	} 235	1970
	T. V. A. (Browns Ferry #2)	Ala.	GE	1,065	}	1971
	Duke Power Co. (Oconee #1)	S.C.	B&W	874	86	1971
	Duke Power Co. (Oconee #2)	S.C.	B&W	874	86	1972
	Vermont Yankee N.P. Corp. (Vt. Yankee)	Vt.	GE	514	88	1971
	P. S. El. & Gas & Others (Burlington #1)	N.J.	W	999	139	1971
	Phila. El. & Others (Peach Bottom #2)	Pa.	GE	1,065	138	1971
	Phila. Electric (Peach Bottom #3)	Pa.	GE	1,065	125	1973
	Va. El. & Power (Surry #1)	Va.	W	783	130	1971
	Va. El. & Power (Surry #2)	Va.	W	783	108	1972
	Niagara Mohawk (Easton)	N.Y.	GE	755	100	1971
	Omaha Pub. Pwr. Dist. (Fort Calhoun)	Nebr.	CE	450	70	1971
	Pacific Gas & Elect. (El Diablo)	Cal.	W	1,060	154	1971
	Metropolitan Edison (Three Mile Island)	Pa.	B&W	831	116	1971
				16,618	$2,109	
Total through 1966 (47 units)				25,160	$3,890	

a/ Limit of T-G capacity. Reactor power 135 Mwe.
b/ Includes capacity of fossil fueled superheater. Electric power from reactor only: Indian Point - 151 Mw; CVTR - 14 Mw; Elk River - 16 Mw.
c/ ½ option price on Units #3 & #4, option exercised in 1967.
* Total construction cost. Land, fuel and transmission plant excluded.

TABLE IV-6

Central Station Nuclear Plants

Utility & Plant	State	Reactor Supplier	Size (Mwe-Net)	Est. Cost* (Millions)	Commercial Operation
NUCLEAR STEAM SUPPLY SYSTEM ORDERS PLACED IN 1967					
Florida Pwr. & Lt. (Turkey Point #4)	Fla.	W	722	$ 66 a/	1971
Northern Indiana Pub. Ser. Co. (Bailly)	Ind.	GE	515	91	1973
Jersey Central Pwr. & Lt.	N.J.	B&W	800	100	1973
Northern States Pwr. Co. (Prairie Is. #1)	Minn.	W	550	100	1972
Wisconsin Pub. Ser. & Others (Kewaunee)	Wisc.	W	527	85	1972
Commonwealth Ed. (Zion #1)	Ill.	W	1,050	164	1972
Commonwealth Ed. (Zion #2)	Ill.	W	1,050	153	1972
Florida Pwr. Corp. (Crystal River #3)	Fla.	B&W	825	110	1972
Wisc.-Mich. Pwr. Co. (Point Beach #2)	Wisc.	W	455	57	1971
Maine Yankee A.P. Co. (Maine Yankee)	Me.	CE	800	100	1972
Long Island Lighting Co. (Shoreham)	N.Y.	GE	540	105	1973
Ark. Pwr. & Lt. Co.	Ark.	B&W	800	140	1973
Consolidated Ed. (Indian Pt. #3)	N.Y.	W	965	159	1971
Duke Power Co. (Oconee #3)	S.C.	B&W	874	92	1973
Consumers Pub. Pwr. Dist. (Cooper)	Nebr.	GE	778	125	1973
Baltimore G.&.E. Co. (Calvert Cliffs #1)	Md.	CE	848	118	1973
Baltimore G.&.E. Co. (Calvert Cliffs #2)	Md.	CE	848	105	1975
P.S. El. & Gas (Burlington #2)	N.J.	W	993	121	1973
N.Y. State El. & Gas Corp. (Milliken)	N.Y.	GE	829	130	1973
TVA (Browns Ferry #3)	Ala.	GE	1,065	115	1972
Northern States Pwr. Co. (Prairie Is. #2)	Minn.	W	550	98	1974
Indiana & Mich. Elect. Co. (Bridgman #1)	Mich.	W	1,100	} 300	1972
Indiana & Mich. Elect. Co. (Bridgman #2)	Mich.	W	1,100		1973
Duquesne (Shippingport #2)	Pa.	W	800	-- b/	1973
Sacramento Mun. Util. Dist. (Rancho Seco)	Cal.	B&W	800	-- b/	1973
1967 total through 9/30/67	(25 units)		20,184	2,634	
ANNOUNCED AS PLANNED FOR NUCLEAR POWER BUT CONTRACTS NOT AWARDED					
SCE and SDG&E (Bolsa Island)			800		1972
Los Angeles D.W. & P. (Bolsa Island)			800		1972
New England Elect. System			800		1973
Portland General Elect. Co. (Trojan)			1,000		1975
Pub. Ser. Co. of N. Hampshire			800		1975
Wash. Pub. Pwr. Sup. Sys.			1,000		1973
Penna. P. & L. (Susquehanna)			800		1974
Northeast Utilities (Millstone Pt. #2)			800		1974
So. Cal. Ed. (Cojo Canyon)			1,000		1975
Georgia Pwr. Co.			500*		1974
Atlantic City El. Co. (Bayside)			1,000		1973
Florida Pwr. Corp.			825		1974
			10,125		

* These are mostly unofficial figures taken from various published sources and are not necessarily on comparable bases.

a/ 1/2 option price on Units #3 and #4.
b/ Cost estimate not available.

TABLE IV-7

Announcements of New Steam - Electric Plant Additions
As Reported by The Trade Press
Through September 30, 1967

Utility & Plant	Capacity, Mwe Fossil	Capacity, Mwe Nuclear
Detroit Edison	1,500 C	
Potomac El. Power Co. (Morgantown)	1,146 C	
Pac. Pwr. & Lt./Wash. Wtr. Pwr. Co.(Centralia)	1,400 C	
Northern Indiana Pub. Ser. Co.		500
Jersey Central Power & Lt.		800
Northern States Power Co. (Prairie Is.)		550
Wisconsin Pub. Service		547
Commonwealth Edison		2,200
Florida Power Corp.		850
Wisc.-Mich. Power		455
So. Car. El. & Gas	400 C	
Wisc. El. Power (Valley #2)	140 C	
Delmarva Pwr. & Lt. (Indian River #3)	167 C	
Georgia Pwr. Co. (Hammond #4)	500 C	
Gulf Pwr. Co. (Crist #6)	320 C	
Utah Pwr. & Lt.	300 C	
Portland Gen. Elect.		1,000
Washington Pub. Pwr. Sup. Sys.		1,000
N. Y. State El. & Gas		750
Pub. Ser. Co. of N. Hampshire		800
Middle South (Ark. P. & L.)		800
West Penn Pwr. (Hatfield Ferry #3)	540 C	
Savannah El. & Pwr.	125 C/G	
So. Indiana G. & E./Alcoa	300 C	
Central P. & L. (Corpus Christi)	240 G	
Central P. & L.	150 G	
Central P. & L.	235 G	
Dallas P. & L. (Lake Hubbard #1)	375 G	
Gulf States Utils. (Conroe #1 & #2)	500 G	
Houston Ltg. & Pwr.	725 G	
Kansas P. & L. (Lawrence #5)	420 C/O	
La. P. & L. (Nine Mile Pt. #4)	750 G	
Okla. G. & E. (Konawa #1)	550 G/O	
Springfield, Mo. (James River #5)	112 G/C	
Texas P. & L. (Valley #3)	375 G	
Central La. El. (Teche #3)	350 G	
Southern Co.	505 C	
Jacksonville, Fla. (Northside #2)	268 O	
Penna. P. & L. (Washingtonville)	765 C	
Tampa Elect. (Big Bend #2)	434 C	
Consolidated Ed. (Indian Pt. #3)		965
Duke Power Co. (Oconee #3)		874
Penna. P. & L. (Susquehanna)		800
Northern Ind. P. S.	100 C/G	
T.V.A.	1,300 C	
New England El. Sys. (Salem Harbor)	400 C/O	
Central La. El.	350 G	
Kentucky Utilities (E. W. Brown #3)	420 C	
Northeast Utilities (Millstone Pt. #2)		800
So. Cal. Ed. (Cojo Canyon)		1,000
T.V.A. (Browns Ferry #3)		1,152
Atlantic City El. Co. (Bayside)		500/1,065
Baltimore G. & E. (Calvert Cliffs)		1,600
Middle South (Vicksburg)	550 G	
Florida P. & L.	700 G/O	
Northern States (Prairie Is. #2)		550
Georgia Power		500/800
Indiana & Mich. El. Co. (Bridgman #2)		1,100
Pacific Pwr. & Lt. (Wyoming)	330 C	
Duquesne Light Co. (Shippingport)		800
Ohio Edison Co. (W. H. Sammis)	625 C	
Cleveland Elec. Illum. Co.	625 C	
Oklahoma G. & E. (Seminole)	550 G	
Kansas City P. & L.	400 C/G	
Georgia Pwr. (Cartersville)	700 C	
Total Each	20,642	20,893
Total - %	50	50

Sept. 30, 1967 Total Fossil & Nuclear 41,535

CENTRAL STATION NUCLEAR - ELECTRIC PLANTS

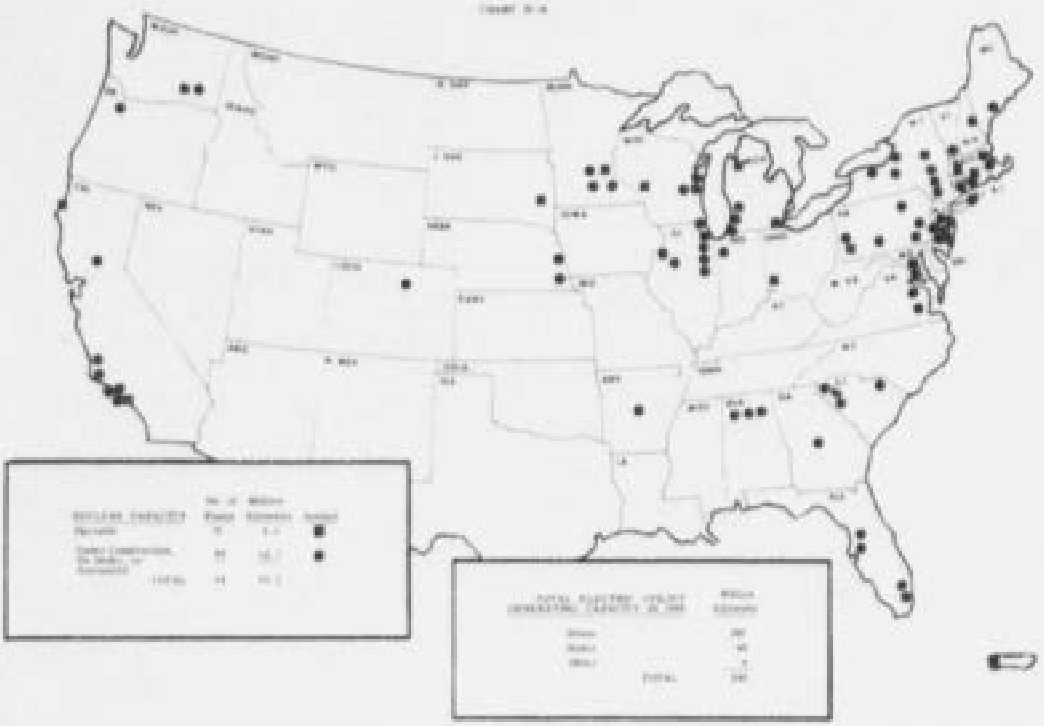

Prior to 1965, some utilities were reluctant to commit nuclear plants in borderline economic cases. However, announcements early in 1965 by utilities which had experience with nuclear power plants no doubt served to bolster utility confidence. This confidence continued into 1967 and is supported by the accumulation of operating experience by four plants. These are in the size range of 200 Mwe and they include Shippingport, Dresden I, Yankee, and Indian Point I. Of these four plants, Shippingport and Dresden I in 1966 had plant availability factors in excess of 90% and the Dresden I and Yankee plants both had operating factors in excess of 80%. Moreover, Dresden I and Yankee have had a plant availability of about 80% over a five-year period from 1962 to 1967, although they were operated at only 64% and 71% of rated capacity respectively.3/

It appears that once utilities realized that those with firsthand experience with nuclear plants were adopting them with confidence, many accelerated their planning in order to allow for the longer lead time currently required to bring a nuclear plant into operation. Thus, the utilities rushed to get their plants on order while they could. It would,

---

3/ Remarks by Wilfrid E. Johnson, U.S. Atomic Energy Commission, "Nuclear Energy for Electric Power", at the Society of American Military Engineers, Portland, Oregon, April 14, 1967, AEC Public Release S-13-67.

therefore, not be surprising if the rate of orders for nuclear plants decreased in 1968.

2. Estimates of Growth

The continuing announcements of plans for new nuclear power plants prompted the AEC to revise its forecast for nuclear power in the United States. It is now estimated that by 1980 the U.S. will have between 120 million and 170 million Kwe of electricity generated by nuclear power. It is believed that somewhere near the mid-range of this projection -- about 150,000 Kwe -- is probably the best single estimate that can be made at this time for 1980.[4/] The difficulty in making these forecasts is indicated by tracing the history of the AEC forecasts to date. In the 1962 Civilian Power Report to the President, the AEC predicted that there would be 40,000 megawatts of installed nuclear capacity by 1980. In 1964, only two years after the 1962 forecast, the AEC predicted that by 1980 there would be between 60,000 and 90,000 megawatts of installed nuclear capacity. In June 1966, the AEC estimated that by 1970-71 there would be more than 10,000 Mwe of nuclear-electric generating capacity in the United States, and by 1980 there would be between 80,000 and 110,000 Mwe. The new and old estimates are shown in Chart IV-C.

The 1967 Supplement to the 1962 Report to the President on Civilian Nuclear Power examines the civilian power program prospects; notes the changes that have taken place since 1962 in the technical, economic and resources picture; and considers the present AEC program in relation to the recommendations of the earlier report. The report is intended to provide an updated description of circumstances and programs as of January 1967 to serve as background for extensive studies currently under way. These studies are considering in depth the tebhnical prospects of the individual reactor concepts, will relate these prospects to resource projections and to economic considerations, and will make available detailed and continuing assessments of program content and direction. It is expected that the individual studies will be completed and published during the latter part of 1967 or early 1968.

Chart IV-D shows estimated energy requirements and sources for electric generation.

4/ Remarks by Dr. Glenn T. Seaborg, "Fast Breeder Power Reactors - A World Outlook", at the Annual Meeting of the Canadian Nuclear Association, Montreal, Quebec, Canada, May 31, 1967, AEC Public Release No. S-24-67.

3. Industrial Structure and Competition

The contin[illegible] pace of nuclear power plant orders in 1967 enabled many firms to announce future plans based upon assured projections of future business. While some segments of the industry still lack enough business to show a reasonable return on investment, long-range prospects look bright and companies are now making positive plans for properly timing their participation in the nuclear field. As a result of the many orders placed by utilities, renewed interest was shown during the year by additional companies entering the nuclear business. There was special interest on the part of the oil industry in uranium exploration and mining.

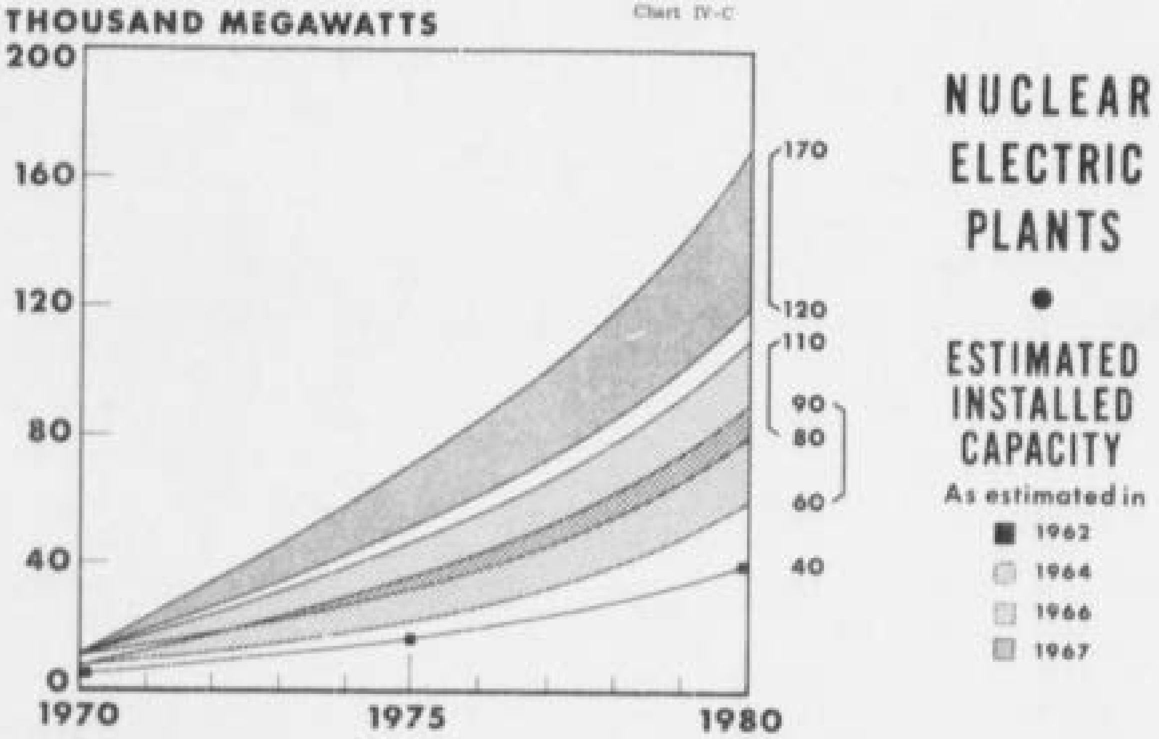

Chart IV-D

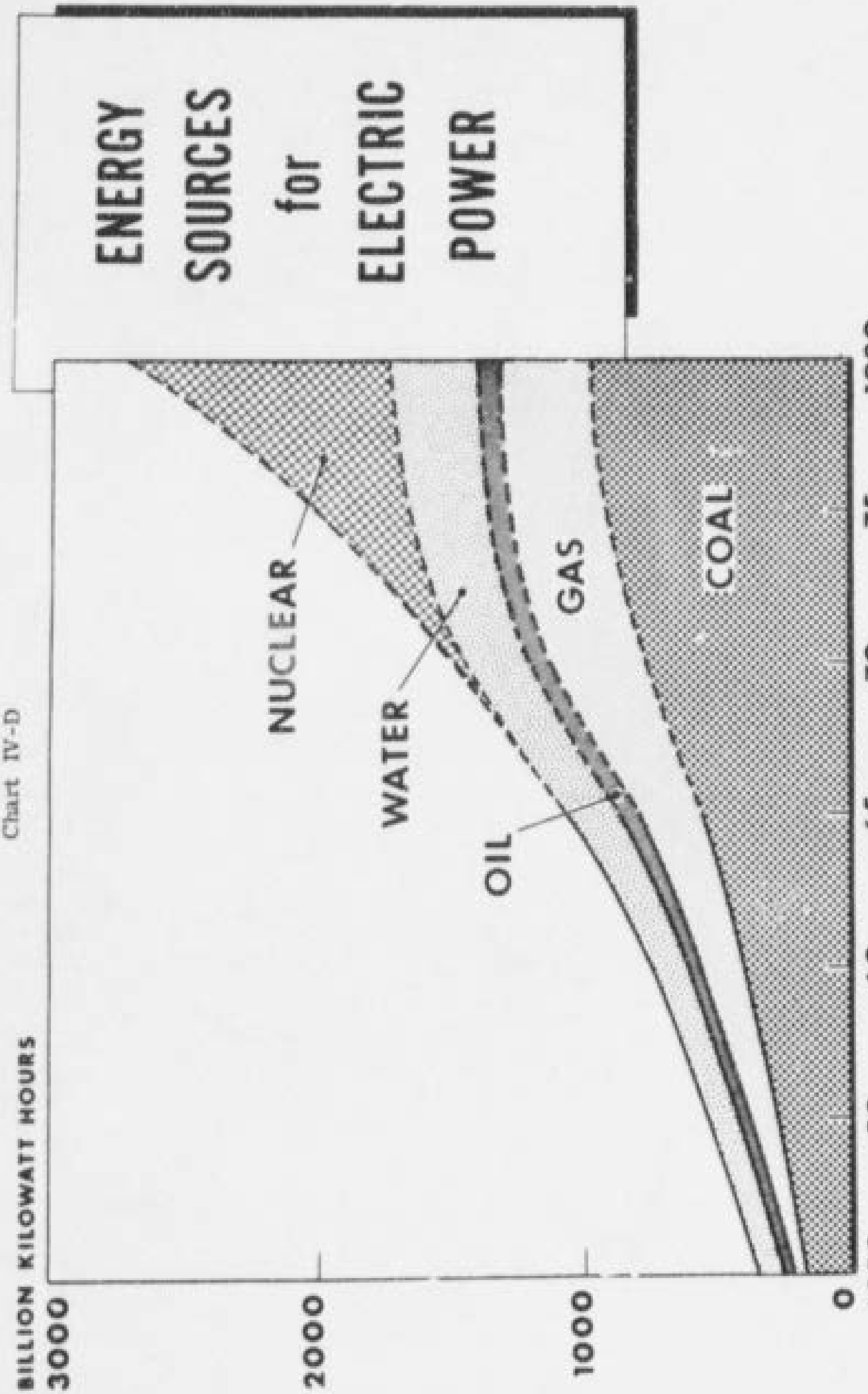
ENERGY
SOURCES
for
ELECTRIC
POWER
BILLION KILOWATT HOURS
3000
2000
1000
0
1950
55
60
65
70
75
1980
NUCLEAR
WATER
OIL
GAS
COAL

a. Commission Actions

The Commission has taken many steps over the years and in cooperation with industry to help define and achieve a competitive industry.5/ These were outlined in detail in last year's report.

In continuing this policy of stimulating competition, during 1967 the AEC and the Department of Justice engaged Arthur D. Little, Inc., to perform a study of competition in the nuclear industry. This study is part of the Department of Justice-AEC joint project of examining all aspects of competition in the relatively new and developing nuclear industry to assist the two agencies in fulfilling their responsibilities under the Atomic Energy Act and antitrust legislation.

The nuclear power supply industry for the purposes of this study consists of firms engaged in the business of supplying reactors, reactor components, nuclear fuel, or other products and services associated with the design, construction, and operation of nuclear electric plants, including their entire fuel cycle.

It is hoped that the study will provide the basis for preparation and consideration of guidelines for the healthy competitive development of the emerging nuclear industry.6/

Informal discussions were again held throughout the year with industrial organizations to learn of their plans and problems. Members of the Advisory Committee on Isotopes and Radiation Development were rotated during the year to bring in representatives from different segments of the industry. The AEC continued to consult with the Atomic Industrial Forum and the Edison Electric Institute to obtain their valuable assistance in formulating its policies and programs by obtaining views of a cross-section of the industry through the use of their members. The Division of Industrial Participation maintained close contact with the nuclear industry and with the Commission's programs to determine and advise on what activities might be accomplished more appropriately in industry.

5/ Statement of Commissioner James T. Ramey to the JCAE at Hearings on the Development, Growth and State of the Atomic Energy Industry, September 8, 1965.

6/ AEC Public Release K-106, dated May 3, 1967.

The AEC has always encouraged close cooperation and good working relationships between the National Laboratories and the nuclear industry, and through the years has been constantly aware of the need for insuring that there is a smooth transition from the laboratories' role of technology development to industry's role of specific engineering development and fabrication.

b. Important Events

Several noteworthy events occurred during the year that helped to expand the competitive base of the industry, and improve nuclear power technology. 7/

Additional contracts were awarded to Combustion Engineering and Babcock & Wilcox for construction of a significant percentage of nuclear power plants. Through these awards these two manufacturers improved their positions of becoming established competitors in the reactor manufacturing area as indicated in Table IV-8.

TABLE IV-8

Central Station Nuclear Power

Total Sales

	No. of Units	Mwe	%
Babcock & Wilcox	9	6,943	16
Combustion Engineering	6	3,566	8
General Dynamics	2	370	1
General Electric	24	15,905	36
Westinghouse	26	17,450	39
Total	67	44,234	100

7/ Remarks by Ernest B. Tremmel, Director, Division of Industrial Participation, USAEC, "Challenges in the Electric Power Field", before the Southeastern Electric Exchange, Boca Raton, Florida, March 27, 1967, AEC Public Release IN-774.

The Peach Bottom reactor, developed by General Atomic, began full power operation in June 1967. Operation of this reactor marked the first time a U.S. nuclear reactor system has achieved high steam temperatures and pressures comparable to those of a coal-fired system. GA is designing a 330 Mwe gas-cooled reactor for the Public Service Company of Colorado (PSC). This cooperative project is to continue into the construction phase in 1968 subject to issuance of a construction permit and a review and determination by General Atomic, Public Service of Colorado and the Commission, which will include a review of Peach Bottom operating experience.

The Connecticut Yankee atomic power plant achieved criticality on July 24, 1967, and had operated up to 50 Mwe by September. This 462 Mwe reactor is the largest commercial plant in the country to achieve criticality and begin power production. The 430 Mwe San Onofre plant of Southern California Edison and San Diego Gas & Electric had achieved operations up to 135 Mwe by September.

A new technological development that shows promise of enhancing the safety and reducing the capital costs of nuclear power plants was announced by Westinghouse Electric Corporation. This concept uses an ice condenser unit, and Westinghouse claims that it could swiftly and safely absorb any heat that might be released to the interior of any reactor's container. It is proposed for use in the atomic power plant announced by Indiana and Michigan Electric Company of the American Electric Power System and Westinghouse has indicated that it will be incorporated in any Westinghouse reactor plant designs to follow.

An interesting event during the year was the announcement that the English Electric Company, as low bidder, was awarded a $13.5 million contract by the Department of Water and Power of the City of Los Angeles for the most powerful single shaft steam turbine ever scheduled to be imported into the U.S. The 800 Mwe turbo-generator will be one of two large condensing units used at the Bolsa Island nuclear generating and desalinization plant at Los Angeles. This is the first time that a turbo-generator has been ordered for importation for a nuclear plant in the U.S. Later, Southern California Edison and San Diego Gas and

Electric ordered a similar unit from the English Electric Company for their generating portion of the Bolsa Island plant.

The TVA ordered a 1300 Mwe two-shaft turbo-generator from Brown-Boveri during the year for a coal-fired plant.

The trend away from turnkey bids continued and all of the reactor manufacturers sold plants during 1967 on a non-turnkey basis. The construction of nuclear power plants continues to return to the normal utility, architect-engineer, constructors supply relationship which existed before the advent of nuclear power plants.

c. Fuel Cycle Arrangements

The nuclear utilities can select from a multitude of fuel cycle arrangements being offered by both the reactor manufacturer, as well as independent fuel cycle suppliers. The range varies from that of the reactor manufacturers full fuel cycle service to that of the utility controlling many of the major steps in the fuel cycle. Typical arrangements in order of increasing utility participation are as follows:

1. Initial Core Sale. The core supplier sells a fabricated core to the utility and guarantees the performance of the core. The utility owns the core and is responsible for handling spent fuel recovery. Normally, several follow-on cores are ordered; however, in some cases, particularly the larger nuclear staffed utilities, only the first core and several spare elements are ordered.

2. Complete Fuel Cycle Service. Under this arrangement the core supplier takes responsibility for all fuel cycle steps. Utility pays the core supplier for energy as it is extracted from the core. The utility takes title ownership of the core while in the utility's possession.

3. Fuel Fabrication Service. The utility handles purchasing of uranium ore, conversion to UF_6 and enrichment, and delivers enriched UF_6 to core supplier. The core supplier purchases zirconium

tubing and fabricates the core. The utility pays the core supplier for zirconium tubing and fabrication of the core. Core supplier guarantees performance of core and utility handles all steps of the recovery of the spent core. A recent survey of nuclear power utilities concerning their fuel cycle arrangements, indicated that the majority intend to control as many of the separate steps in the fuel cycle as they consider practical, once they have obtained the necessary experience from their first round reactor orders.

d. Status of Competition in Nuclear Fuel Cycle 8/

During the year, most of the companies involved in the nuclear fuel cycle announced plans for expansion and several new companies announced intentions to enter the nuclear field. Those companies that are presently participating in the various steps of the fuel cycle are listed in Table IV-9. Only two companies in the mining and milling operation are shown, because these are the only two which have carried their operations beyond the milling of ore to U_3O_8 (yellow cake).

NUCLEAR FUEL CYCLE STEPS BY SUPPLIERS

TABLE IV-9

Approximate Distribution of Costs %		Reactor Suppliers: Atomics International	Babcock & Wilcox	Combustion Engineering	General Dynamics*	General Electric	Westinghouse	Fuel Cycle Suppliers: Allied Chemical	Kerr-McGee	National Lead	Nuclear Fuel Services	NUMEC	United Nuclear	AEC
100	FABRICATION OF CORE													
20	Exploring, mining & milling ore to U_3O_8								X				X	
3	Conversion of U_3O_8 to UF_6							X	O					X
31	Enrichment of UF_6													X
38	Manufacture of Zr sponge and ingot						O							
	Fabrication of Zr ingot into tubing					O	O						O	
	Conversion of UF_6 to UO_2					X	O		X	X	X	X	X	X
	Pelletizing of UO_2	X	X	X		X	X		X		X	X	X	X
	Fabrication of fuel elements	X	X	X	X	X	X			X	X	X	X	
	Fuel Warranty		X	X	X	X	X			O	O	O	X	
	MANAGEMENT OF FUEL IN REACTOR													
	Design of fuel management scheme		X	X	X	X	X			X		X	X	
	RECOVERY OF SPENT CORE													
1	Spent fuel shipping					X	X			X	X			X
12	Reprocessing fuel elements and waste disposal					O		O		O	X	O		X
8	Conversion of $UO_2(NO_3)_2$ to UF_6 for re-enrichment					O		O						X
unknown	Conversion of $Pu(NO_3)_4$ into PuO_2	X				X				O	X	X	X	X
unknown	Fabrication of Pu fuel elements	X				X				O	X	X	X	X
unknown	Isotope recovery from spent fuel					O		O		O	O			X

* General Dynamics produces the HTGR which uses coated U-Th carbide or oxide particles in an all-graphite matrix fuel element.

X - Company now capable of performing these steps

O - Company planning additional step.

8/ Remarks by Ernest B. Tremmel, Director, Division of Industrial Participation, USAEC, "The Surge to Nuclear Power", before the Southern Electric Exchange, Atlanta, Georgia, October 13, 1966.

The companies engaged in the remaining steps of the nuclear fuel supply cycle are of three types; reactor manufacturers (all of which also manufacture fuel for their reactors), firms that manufacture either fuel cores or perform certain steps of the nuclear fuel cycle, and firms which perform certain individual services which support the nuclear fuel cycle.

Reactor Suppliers

During the year, the General Electric Company announced several general expansions of its facilities. Their fuel production includes the conversion of UF_6 to UO_2 powder, making UO_2 pellets, and fabrication of the complete reactor core. They have also announced intentions of proceeding with the manufacture of zirconium tubing and the construction of a chemical fuel reprocessing plant.

At the present time, Westinghouse purchases UO_2 powder and fabricates UO_2 pellets into the complete reactor core. Its plans for facility expansion include installing a capability for producing UO_2 powder and pellets and for manufacturing zirconium tubing. Babcock & Wilcox plans to produce UO_2 powder and pellets and Combustion Engineering presently purchases UO_2 pellets. They both do most of the remaining fabrication in the manufacture of a reactor core, but have not indicated plans to manufacture zirconium tubing.

Fuel Core Manufacture

United Nuclear Corporation is the only independent producer so far that makes UO_2 powder and pellets, and fabricates the complete reactor core for commercial reactors that has bid and received orders for replacement cores.

Commonwealth Edison's award of a replacement core for the GE Dresden I reactor to United Nuclear during 1966 was the first breakthrough of the independent supplier in the replacement core business. This trend was re-enforced during 1967 with the award of the contract to United Nuclear to provide demonstration assemblies for the Westinghouse Yankee reactor. The General Electric Company is also providing demonstration assemblies for the Yankee pressurized water

reactor. American Electric Power Company awarded replacement cores II and III to United Nuclear for AEP's first nuclear plant that they purchased from Westinghouse. The first segment of this replacement core would be manufactured in 1972-1973. This is the first time that a replacement core was purchased from other than a reactor manufacturer at the same time the nuclear plant was purchased. The decision by Commonwealth Edison and American Electric Power Company to buy fuel from other than a reactor supplier will no doubt encourage other utilities to consider this same approach to procurement of replacement cores, thus broadening the industry and achieving competition possibly at an earlier date.

Suppliers of Segments of the Fuel Cycle

The potential market in the nuclear fuel cycle is sufficiently attractive that several companies are making plans to enter all phases of the fuel cycle even though the magnitude of business may not be adequate to assure a reasonable return on an investment until the mid-1970's. It appears, however, that there will be some delay in accelerated activities in this area until utilities are in a position to purchase replacement cores on a larger scale from other than the reactor manufacturers.

At the present time, Kerr-McGee, Nuclear Fuel Services, and Atlantic Richfield (NUMEC) produce UO_2 powder and pellets for sale to the reactor manufacturer. The Gulf Oil Corporation has indicated plans to enter the total fuel cycle business, but has not announced at the present time which phase of the fuel cycle they will undertake first. The National Lead Company has indicated plans to enter the replacement core business and other steps in the fuel cycle such as fuel reprocessing. Aerojet-General states it could have an automated fuel production facility in operation during 1972-1973. It would convert UF_6 to UO_2, as well as fabricate fuel elements. Atomics International is involved in reactor development with a major effort in fast breeder reactors and plans for replacement fuel business, including plutonium recycle.

The conversion of U_3O_8 to UF_6 is presently performed by only one firm -- the Allied Chemical

Corporation, who announced plans during the year for expansion of their capacity. The Kerr-McGee Corporation announced plans to build a conversion facility at a site yet to be announced.

The manufacture of zirconium sponge and ingot is presently performed by Wah Chang Corporation, recently acquired by Teledyne, Inc., and by AMAX Specialty Metals, Inc., formerly Carborundum Metals Climax. Zirconium tubing is fabricated by five competitors - Harvey Aluminum, Wolverine Tube, Superior Tube, Reactive Metals, and AMAX. In addition, A Swedish firm, the Sandvik Steel Works Company is building a zirconium tube plant, in partnership with United Nuclear Corporation, at Richland, Washington. The company is known as the Sandvik Special Metals Corporation. In addition, General Electric and Westinghouse have announced plans to construct zirconium tubing fabrication plants. The total existing zirconium tube fabrication capacity in the U.S. is 4.7 millions of feet per year. Plans for expansion will increase this capacity to 9.8 million feet by 1969. The zirconium tubing requirement for industrial power reactors is shown in Table IV-10.

TABLE IV-10

Estimated Future Requirements for Zirconium Tubing For Commercial Power Reactors

Calendar Year	Millions of Linear Feet as Tubing
1967	1.7
1968	3.7
1969	6.4
1970	7.5
1971	8.6
1972	10.7
1973	13.5
1974	17.0
1975	20.4
1976	23.5
1977	27.6
1978	32.1

Reactor manufacturers and core suppliers are presently able to undertake the management of fuel in a reactor, and it is probable that consulting firms and the utilities themselves will undertake this step as the nuclear power industry matures. Spent fuel shipping services are available from reactor suppliers and fuel cycle suppliers, as well as from commercial transportation firms. The reprocessing of fuel elements and handling and storage of the residual radioactive material is presently being performed by only one commercial firm, Nuclear Fuel Services.

The General Electric Company has applied for a construction permit for a chemical reprocessing plant and Allied Chemical Corporation has previously indicated intentions to build a private reprocessing plant. During the year, National Lead announced studies for a chemical reprocessing project. Also Atlantic Richfield, who acquired NUMEC and is operating the chemical reprocessing facilities for the AEC at Richland, indicated that they would eventually consider building a chemical reprocessing plant for commercial purposes at Richland, Washington. At present, there is insufficient market to fully load the NFS plant at West Valley, New York, and a sufficient load to support a second plant probably will not be available until about 1973.

The state of competition in the remaining steps in the recovery of a spent core is not yet too well-defined. Further attention must be given to such things as fission product recovery and encapsulation, the conversion of uranyl nitrate to UF_6 for re-enrichment, or blending, conversion of plutonium nitrate into plutonium oxide, and especially the fabrication of plutonium fuel.

There are joint programs between the Edison Electric Institute and some of the reactor manufacturers to develop plutonium recycle technology. However, the AEC is becoming increasingly concerned that not enough effort is being devoted to the demonstration of the recycling of plutonium on a timely basis. Therefore, the AEC is encouraging the reactor core manufacturers and the electric utilities to accelerate work in this area. Reactor and independent fuel suppliers will no doubt eventually have to achieve a plutonium recycle capability to maintain a competitive position in the replacement core business.

4. Desalting

General

The importance of desalting was emphasized during the year at the International Conference on Water for Peace, which was held in Washington, D. C. At the conference, Commissioner James T. Ramey outlined the present status and future possibilities of desalting and pointed out that few technical developments in history have attracted the attention and raised the hopes of so many as has the promise of desalting of sea and brackish water.[9/]

The AEC's program for desalting and other process applications is directed toward analyzing, developing, and demonstrating nuclear reactor systems for desalting and other process applications.

Since the program's inception, ORNL has been conducting a broad-based program of technical studies, evaluations, and research and development to support the near-term and long-range application of nuclear power for desalting.

A significant activity under the ORNL program has been the investigation of the extrapolation of several reactor concepts to 10,000 Mwt single-unit sizes. The reactor concepts studies included the boiling water reactor, the high temperature gas-cooled reactor, th[illegible] heavy water moderated organic cooled reactor, and the liquid metal cooled fast breeder reactor.

United States

The most prominent of the current desalting program activities in the U.S. is the Bolsa Island Nuclear Power and Desalting Project, a cooperative project involving the Department of the Interior, the Atomic Energy Commission, the Metropolitan Water District of Southern California (MWD), the Department of Water and Power of the City of Los Angeles, Southern California Edison, and San Diego Gas and Electric.

As presently planned, the Bolsa Island Project would use two light water reactors of approximately 3,000 megawatts each and would have an initial capacity of about 1,800 gross megawatts of electricity and 50 million gallons per day (MGD) of fresh water. Following

---

9/ Remarks by James T. Ramey, Commissioner, USAEC, "Policy Considerations in Desalting and Energy Development and Utilization," at the International Conference on Water for Peace, Washington, D.C., May 25, 1967, AEC Public Release S-23-67.

the initial operation, the desalting portions would be expanded to have a capacity of 150 million gallons per day.

The AEC, working with the Bureau of Reclamation, is participating in a reconnaisance-level study of the potential for nuclear desalting in the Santa Barbara Channel area of California. The plant being considered would use a light water reactor to produce about 100 MGD and 850 Mwe (gross) for application sometime in the mid-1970's.

International

Studies of large plant applications of interest to foreign countries are continuing. An engineering and economic study of a dual purpose desalting plant for Israel was jointly supported by the Governments of Israel and the United States.

A cooperative study under the auspices of the International Atomic Energy Agency (IAEA) is being undertaken to assess the potential of desalting to meet the power and water needs of the southwestern United States and northwestern Mexico. Preliminary estimates indicate that near term water requirements in this region might be on the order of one billion gallons per day (BGD) and that long-range water needs could be as high as 50 BGD, depending on the cost of water and the extent of regional development.

The AEC participated in a preliminary study of the engineering and economic feasibility of desalting to meet the water and power needs of Athens, Greece. The study was conducted by a team of Department of the Interior and AEC specialists working with a similar Greek group.

In addition to specific site studies, other international activities have continued to be an important aspect of the desalting program. The IAEA has been kept informed regarding U.S. developments in the nuclear desalting field; and the AEC has participated in IAEA panel meetings, two of which were held during the past year.

At the initiative of the Russians, the agreement for cooperation on desalting, which was to expire in November 1966, was extended for two more years.10/

10/ See AEC Authorizing Legislation, Fiscal Year 1968, Hearings Before the Joint Committee on Atomic Energy, Congress of the United States, Ninetieth Congress, First Session on Reactor Development Program, Part 2, dated March 14 and 15, 1967.

5. Small Reactors

The anticipated economics of large power reactors in competition with conventional power plants is evidenced by the amazing number of orders placed this year. The future of small reactors as economically competitive power sources in the U.S. is not encouraging. Utility systems that need relatively less power might best take advantage of nuclear power by the cooperative use of large reactors. The smallest reactor units ordered for commercial power production since 1962 are in the range of 400-455 Mwe.

The economics of reactor units at lower power levels of course, depends on the particular location and availability and cost of other sources of power. In certain situations, one may want to consider nuclear units for other than economic reasons. In high cost fuel areas, where transportation and other problems exist, one should make careful economic comparisons even at power level requirements lower than 400 Mwe.

Small reactors are finding a place, however, as sources of power in remote areas. The Navy for almost five years now has been using a 1500 Kwe reactor, the Pm-3A nuclear plant at McMurdo Sound in the Antarctic, to supply power to a National Science Foundation-sponsored scientific community there. During the past year this reactor has also been effectively used to desalt seawater for the community, although it was not originally designed for this purpose.11/

Because of the interest which has been expressed in small reactors (around 150 Mwe), the AEC initiated a study of small nuclear power plants at the Chicago Operations Office. This study was completed and published during the Year.12/ Volume I of the small nuclear power plant study pertains to the design, construction, and operating experience. Volumes II and III deal with the economics of these plants. Interesting information provided by the study is the cost reduction which might be possible through joint action by several utilities in the areas, such as procurement of a standardized plant, licensing and operating services.

It is expected that there will be continuing interest in small reactors because many utilities and countries around the world do not have sufficient loads to utilize large power plants on their system.

11/ Remarks by Dr. Glenn T. Seaborg, Chairman, USAEC, "The Proliferation of the Peaceful Atom", at the 24th Annual Conference of the American Public Power Association, Denver, Colorado, May 11, 1967, AEC Public Release S-20-67.

12/ COO-284, Small Nuclear Power Plants.

6. Export Sales

Previous reports on the nuclear industry have summarized sales of power reactors abroad. Over the past two years, it has become apparent that power reactors built abroad in the future will tend more and more to be constructed of locally fabricated components, with participation by U.S. reactor builders largely through licensing or joint venture arrangements and the supply of specialized components. For example, a second pressurized water nuclear unit for Japan's Kansai Electric Power Company was announced in August. About 70% of this unit is expected to be manufactured in Japan, but with Westinghouse providing design information, fuel, and certain components such as cooling water pumps. At the present time, 18 enriched uranium power reactors of the United States type, totaling more than 4-1/2 million kilowatts of electricity and representing an investment of at least $1.0 billion, are in operation, under construction, or on order in nine countries outside of the United States.

The increasing impact of nuclear power plant construction abroad is reflected in the rising exports of special nuclear materials (reactor fuel) and reactor components shown in the report on exports in the Summary section of this report. Exports in these two categories were reported for 1966 at $85 million, 75% above the 1965 level.

Table IV-11 shows a list of U.S. type power reactors for operation abroad.

Table IV-11

Power Reactors for Export

Plant	Country	Mfg. or Licensor	Mwe	Start-up
RWE	Germany	GE	16	1960
SENN	Italy	GE	150	1963
JAERI	Japan	GE	12	1963
SELNI	Italy	W	240	1964
SENA	France	W	248	1966
TARAPUR	India	GE	380	1967
KRB	Germany	GE	237	1966
Zorita	Spain	W	153	1968
GKN	Netherlands	GE	48	1968
KWL	Germany	GE	160	1968
KWO	Germany	W	200	1968
NOK	Switzerland	W	350	1969
Nuclenor	Spain	GE	440	1970
JAPCO	Japan	GE	342	1969
BKW	Switzerland	GE	306	1971
OKG	Sweden	GE	400	1970
Tokyo El. Pwr.	Japan	GE	460	1970
Kansai	Japan	W	340	1970

7. Advanced Converters

The Commission's effort to develop advanced converters has been narrowed to three approaches, heavy water moderated reactors, the high temperature gas-cooled concept, and the seed-blanket light water breeder concept. Each of these concepts has promise of higher conversion ratios than current light water reactors and can greatly extend the usefulness of our uranium resources.13/

Development of the heavy water organic cooled reactor (HWOCR) concept was discontinued during the year.14/ This was the major effort in the U.S. program to develop heavy water moderated reactors.

The continuing heavy water reactor base research and development program will take advantage of the heavy and light water reactor experience in this country and the HWR experience in Canada and Euratom. It will permit the U.S. to maintain the option, in the future, to exploit heavy water reactors for power production and for the extremely large size needed for dual purpose and desalting applications. The need to maintain such an option has been confirmed. Such a base HWR program can be executived without imposing an additional burden on the limited available resources, reactor design and engineering personnel, equipment vendors, and facilities currently committed to the heavy backlog of orders for light water reactors, as well as on the high priority commitments to develop and construct breeder reactors.

Continuation of the Public Service of Colorado Project for a high temperature gas-cooled reactor into the construction phase, as cited previously, is scheduled to lead to completion and power operation of this 330 Mwe high temperature gas-cooled reactor nuclear power plant in early 1972.

Work on the seed-blanket light water breeder, which shows promise of breeding in the light water medium, is directed toward the possibility of demonstrating the breeding potential in the existing light water reactor, such as the Shippingport reactor.

The extent to which these reactor concepts will be accepted for industrial power plants will depend on the comparative economics with other systems, availability of low-cost ore, and the relative schedule for the development of breeder reactors.

13/ Remarks by James T. Ramey, Commissioner, USAEC, "The U.S. Atomic Power Program", at the International Conference on Reactors, Mexico City, May 2, 1967, AEC Public Release S-18-67.

14/ AEC Public Release IN-769, dated March 9, 1967.

8. Breeders

The development of breeder reactors to provide a long term source of low cost electric power while using the nation's nuclear fuel resources most efficiently is receiving the highest priority and support in the AEC's reactor development program. Of the concepts considered for breeders, the highest priority has been assigned to the development of the liquid metal-cooled fast breeder reactor (LMFBR) which at the present time has the best potential for most economically achieving the most efficient fuel utilization.

The development of the breeder reactor will increase fuel utilization from 1-2% now available to over 50%. The high gain breeders will be capable of producing significantly more fissionable fuel material than they consume, thereby providing an excess of burnable plutonium which will be used to fuel new fast breeders in an expanding power system.[15/]

The fast breeder program in the United States has an extensive background dating from 1945. Present programs are aimed toward the development of satisfactory fast reactor fuels and cladding materials, components, instrumentation and control, sodium technology, core design, fuel recycle, physics, safety and plant design. During 1967, initial versions of detailed program plans were completed for seven of these nine technical areas and were forwarded to industry and the national laboratories for review and comment.

Current fuels and materials irradiation programs center on the operation of the sodium-cooled Experimental Breeder Reactor II (EBR-II). Another important facility is the sodium-cooled Fermi reactor; however, future utilization of the Fermi reactor will depend on the determination of the cause of the fuel failure of October 1966, the extent of the damage and the success of the recovery program. The new 400 Mwt Fast Flux Test Facility (FFTF) will serve as the major facility for development of fuels and materials for the demonstration plants and commercial sodium-cooled fast breeder reactor power plants. The SEFOR reactor is expected to provide valuable information on fast reactor safety. It is anticipated that the decision to build the first LMFBR demonstration plant in the United States will not be made before about 1970. It is expected that the first demonstration plants will have a capacity of 300-500 Mwe and that they will be

---

15/ Remarks by Milton Shaw, Director, Division of Reactor Development & Technology, USAEC, "Current AEC Program in Fuel Reprocessing Research and Development", at the SINB/AEC Conference on "Nuclear Power Fuel Reprocessing -- Technology and Economics", Augusta, Georgia, May 11, 1967, AEC Public Release IN-792.

designed to provide as much information as possible on all features of the 1,000 Mwe or larger plants that will be built subsequently.

The AEC awarded $3.2 million in contracts for fast breeder reactor plant design studies on 1,000 Mwe liquid metal fast breeder reactor (LMFBR) plant concepts. Designs will be based on estimates of the state of technology by 1980. The contracts were awarded to Atomics International, Babcock & Wilcox, Combustion Engineering, General Electric, and Westinghouse.

An example of the increasing utility interest in breeder reactor development was evidenced by the award by the Empire State Atomic Development Associates, Inc. (ESADA), an organization of seven New York utility systems, of a $5 million three-year contract to the General Electric Company for the development of some key components for a liquid sodium-cooled fast breeder reactor plant. It is expected that ESADA members will decide about mid 1970 whether to build a 300 Mwe breeder-type atomic power plant to demonstrate the concept and components. Previously, GE began conducting a $750,000 preliminary design study of a 300 Mwe prototype sodium-cooled fast breeder jointly with Pacific Gas & Electric Co. (PG&E), Commonwealth Edison Co., Duke Power Co., and the Southwest Atomic Energy Associates (SAEA). In addition, Atomics International is doing a conceptual design study of a 1,000 Mwe sodium-cooled fast breeder for ESADA.

Commonwealth Edison and Westinghouse joined in a design study of a sodium-cooled fast breeder reactor plant with a capacity of 200-400 Mwe. Participating also are Duke Power Co., Detroit Edison Co. and Commonwealth Associates.

The design and feasibility of a 50 Mwe experimental steam-cooled breeder is being investigated by General Electric and the East Central Nuclear Group (ECNG) with American Electric Power Service Corp. performing the architect-engineering role.

The leading proponent of the gas-cooled fast breeder reactor has been General Atomic. They have been working on a 1,000 Mwe breeder study with the East Central Nuclear Group which includes further design work on a 100 Mwt experimental plant.

C. Civilian Maritime Reactors

The Nuclear Ship Savannah, the first nuclear-powered merchant ship, has been in regular experimental commercial service for over two years. During this period it has traveled more than 158,000 miles to the world's leading shipping ports. It traveled an additional 90,000 miles during its public port visits. The Savannah is operating on its original nuclear core installed November 1961. It has consumed a total of about 91 pounds of uranium-235, which is the equivalent of 23 million gallons of fuel oil. During the Savannah's five years at sea, it has demonstrated the feasibility of nuclear power for the propulsion of merchant ships. In the ship's two years of experimental commercial operation, the reactor was at power 81% and 82% of the time, respectively.

At the present time, the NS Savannah is in the third year of commercial operation, has completed one round trip to the Far East and has departed on the second trip.

The construction of advanced nuclear merchant ships in this country and a definitive program of development is presently awaiting the resolution of definitive program needs of the maritime industry. While awaiting an administrative decision on this matter, AEC efforts during fiscal year 1967 have been limited to assessments of specific reports on nuclear fuel costs, capital costs, cost trends, and nuclear power plant designs for selected maritime applications. It is planned to continue development of the applicable reference technology necessary to enable proceeding with AEC support of the civilian nuclear maritime program.

D. Military Reactors

1. Army Reactors

During the past year, valuable operating experience was gained with the reactor power plants that were developed under the joint AEC-Department of the Army program. As reported previously under Small Reactors, the Navy, for almost five years, has been using the PM-3A nuclear plant at McMurdo Sound in the Antarctic in support of a scientific community there.

Another portable medium power nuclear plant, the PM-1, has been in use since 1962 in a remote section of Wyoming supplying power for a small isolated Air Force station. Both the PM-3A plant in the Antarctic and the PM-1 in Wyoming have made record power runs of over 3,600 hours of continuous operation.

[illegible]

Still another interesting power plant is the MH-1A reactor which has been installed on the barge Sturgis. This 10,000 kilowatt plant (to be operated by the Army) can be towed to offshore anchorages where emergency power is needed and can supply enough electricity to meet the needs of a community of from 10,000 to 20,000 people.14/ This is undergoing testing at Fort Belvoir, Virginia.

The reactor power plants were developed under this program to provide military services with power sources that could further the mission of the Armed Forces.

Table IV-12 lists the operating Army reactor plants with their manufacturers.

Although the Army reactor program currently represents a reduced level of effort, work is beginning at a low level under another AEC program which could provide developments of interest to the Armed Services. This is the terrestrial low power reactor systems program which is directed toward the development of technology for systems from 100 Kwe to 2 Mwe applicable to a wide variety of potential uses.

TABLE IV-12

Plant Designation	Location	Manufacturer
MH-1A	Barge mounted	Martin-Marietta
	Ft. Belvoir, Va.	" "
PM-1	Sundance, Wyo.	" "
PM-3A	McMurdo Sound, Antartica	" "
SM-1	Ft. Belvoir, Va.	*
SM-1A	Ft. Greely, Alaska	*

*These plants were built by Alco Products, Inc., whose nuclear activities were taken over by Allis-Chalmers. Allis-Chalmers has since left the reactor business.

2. Naval Reactors

During 1967 the advantages of nuclear power for surface ships continued to be demonstrated under actual military operations associated with the Vietnam conflict. The Joint Committee on Atomic Energy and the Atomic Energy Commission continued to vigorously support Admiral Rickover's Naval Nuclear Propulsion Program. The U.S. has a formidable fleet of nuclear ships - 75 submarines will be in service by the end of the year with 32 more authorized or under construction, and four nuclear surface ships are in service.

With a continuing demonstration of the advantages of nuclear power for naval propulsion, it is anticipated that additional nuclear surface vessels will be authorized in the future.

Westinghouse, through the operation of the AEC's Bettis Laboratory, and General Electric, through the operation of the AEC's Knolls Atomic Power Laboratory (KAPL), are the reactor designers in the naval program. The reactor core manufacturers are The Babcock & Wilcox Co. at its Lynchburg, Virginia, plant, and the United Nuclear Corporation, at its New Haven, Connecticut, plant.

a. Oceanographic Applications

One of the most significant activities applicable to oceanography, in which the AEC is participating, is the development of the Deep Submergence Research Vehicle (NR-1) which is being built by the Navy and the AEC. It is believed that this vehicle will help greatly to open up this new frontier of "inner space." The NR-1 will be able to cruise along the bottom of the ocean for weeks at a time, supplying all the necessities of life and tools for undersea investigation to its crew of five and two scientists. Other nuclear-powered undersea vessels will no doubt follow, not only for scientific and technical use, but perhaps someday as pleasure craft revealing to us a new world of natural wonders.16/ The reactor for NR-1 is being designed by KAPL and the submarine is being designed and built by the Electric Boat Division of General Dynamics. This work is part of the Naval Reactors Program under Vice-Admiral Hyman G. Rickover.

16/ Remarks by Dr. Glenn T. Seaborg, Chairman, U.S. Atomic Energy Commission, "The Proliferation of the Peaceful Atom", at the 24th Annual National Conference of the American Public Power Association, Denver, Colorado, May 11, 1967, AEC Public Release S-20-67.

In addition to the NR-1, it is expected that nuclear power plants will play an important role in the provision of auxiliary power and power for use in the exploration and recovery of minerals and other materials from the ocean's floor.

It has been conservatively estimated that the world's oceans contain some 50 quadrillion metric tons of minerals, including two quadrillion tons of magnesium, 100 trillion tons of bromine, 20 billion tons of uranium, 15 billion tons of copper, and -- don't all rush to sea -- 10 billion tons of gold! Other materials down there include boron, manganese, silver, nickel phosphorous, platinum, and a variety of rare earths. When it comes to extracting and processing these and the many more valuable materials in the sea and on the ocean floor, the extensive use of nuclear power will probably become essential.17/

The many uses of atomic energy in oceanography are described in a recent pamphlet entitled, "The Atom and the Ocean" which can be obtained from the Division of Technical Information Extension; USAEC; P.O. Box 62; Oak Ridge, Tennessee 37830.

E. Research and Teaching Reactors

The potential market for these reactors, both at home and abroad, appears to be nearly saturated. Most institutions that can make effective use of these facilities have been able to build and bring them into operation. However, the need for expansion and upgrading of existing facilities should provide a continuing limited market in the future.

One factor contributing to the absence of orders for new reactors of this type has been the difficulty experienced by many owners in finding work to help finance operating costs. This has been alleviated to some degree recently by arrangements with electric utilities under which reactor owners have been training utility personnel. Electric utilities should take advantage of these facilities for this purpose.

The principal producers of reactors in this category are listed in Table IV-13.

17/ Remarks by Dr. Glenn T. Seaborg, Chairman, U.S. Atomic Energy Commission, "The Proliferation of the Peaceful Atom", at the 24th Annual National Conference of the American Public Power Association, Denver, Colorado, May 11, 1967, AEC Public Release S-20-67.

TABLE IV-13

Producers of Research and Teaching Reactors

Firm	Number of Reactors	
	Domestic	Foreign
Advanced Technology Labs	4	4
Aerojet-General	17	3
AMF Atomics	9	14
Atomics International	10	6
Babcock & Wilcox	4	3
Combustion Engineering	2	0
General Atomic	18	14
General Electric	8	4
Lockheed	8	0
United Nuclear	1	0
Internuclear Company	1	1
	82	53

F. Space Systems

The objective of the AEC space system program is to develop nuclear power sources that are more economical than other means of producing power for space applications and in some cases, the provision of power for missions that could not be achieved by any other means. Mission analyses have shown significant mission payload advantages and very significant economic advantages by the use of nuclear power.

The importance which the Commission attaches to the space reactor programs is reflected in the level of support for this work -- an amount nearly equal to that spent for research and development on naval reactors or on civilian power.

The future of the nuclear space program is tied to the military and civilian space efforts. As the space program expands into advanced missions, the requirements for developed nuclear systems for space should materialize. Because nuclear systems require a long time for their development, the Commission has undertaken a broad-ranging program of nuclear space systems technology so as to be in a position to provide developed systems when the requirements are firmly established. In working to advance the nation's capability in space power and propulsion, the AEC's space systems program concentrates on the two vital elements

of space technology -- the development of reactors and radioisotopes as heat sources for propulsion, and the production of auxiliary electrical power. A recent staff study of the Committee on Science and Astronautics, U.S. House of Representatives, stated:

> "The future progress of this Nation in achieving its constantly expanding objectives in space exploration is primarily dependent upon significant accomplishment in the development of (1) propulsion power and (2) on-board electrical energy. These two sources of energy provide the fundamental basis by which all other space research and technology is made feasible and practical. Given an abundance of these two energy sources, all other factors can be overcome."

There have been significant advances in the development of technology in these areas as discussed below:

1. Propulsion

During the past year, the objective of the joint NASA-AEC nuclear rocket program has been established as the development of an advanced rocket engine, using nuclear energy as its heat source, which will provide a thrust of between 200,000 and 250,000 pounds (a reactor power level of 4,000-5,000 megawatts). The goal is to establish a chamber temperature of 4,500° Rankine to give a specific impulse of 825 seconds. This is a factor of two better than specific impulses that can be achieved with the best chemical systems. This system would substantially increase mission payload capability for such missions as earth orbital operations, direct lunar landing missions, unmanned deep space missions, and manned planetary operations. For certain missions, such as the Mars landing, the use of this propulsion system rather than a chemical propulsion means we could save as much as $1 billion or more per mission, depending on the mission requirements and the year in which it is conducted. It is estimated that it would take 10 years and $1 billion to develop this propulsion system, therefore, this development cost could be saved in one mission.

Up to the present time, eight reactor tests have been completed, providing significant information on the reliability of this type of system. During the past year, a

reactor test was conducted at nearly 1,500 megawatts with 30 minutes at more than 1,250 megawatts. The long term objective is to develop the high power system that would provide the flexibility at minimum time and cost to do all the missions that the country may want to consider.

The contractors that are carrying our program under joint AEC-NASA sponsorship are Los Alamos Scientific Laboratory, Aerojet-General, and Westinghouse.18/

2. Auxiliary Power

Nuclear auxiliary power sources have successfully operated in orbit generating electricity through the use of radioisotopes and small compact reactors. These have ranged from the SNAP 10-A reactor system, which operated in orbit for 43 days generating 500-watts of electricity, to the SNAP-3 isotopic system, which has been supplying several watts of power to a Navy navigational satellite in orbit for the past six years.

There are three choices for long-lived power in space; solar, isotopic, and reactor systems. The assessment of these three for any given mission must be mainly on the basis of weight, cost, availability, size, reliability, lifetime, and any peculiarities of the mission that would favor one over the other. Reactor power SNAP units are expected to play their greatest role in applications requiring tens of kilowatts or more of power. The anticipated reactor space power technology growth is indicated in Table IV-14. At low power, isotope powered units are preferred because of better power-to-weight ratios. The projected growth of isotopic space power technology is depicted in Table IV-15.

The approach that is being taken in this program is to provide the technology for these systems and perform development of specific systems as needed by using agencies. As new requirements appear for specific systems that are designed to use the best technology that is available. The systems that have been under development for space electric power, including the costs and contractors, are listed in Table IV-16. The development status of the various numbered SNAP systems is tabulated in Table IV-17.

18/ Milton Klein, Director, USAEC Division of Space Nuclear Systems, AEC Authorizing Legislation, Fiscal Year 1968, Part 3.

TABLE IV-14

Reactor Space Power Technology Growth

	Fuel Surface Temperature Degrees Fahrenheit	Power Electrical KW	Weight Objective Lbs. Per KW
SNAP 10A - 8	1300	Up to 50	300 - 1,000
Gas-Cooled Reactor w/Brayton	2200 - 2700	100 - 200	100 - 200
Liquid Metal Reactor w/Potassium Turbogenerator	2000	100 - 10,000	20 - 100
In-Core Thermionic Reactor	2800 - 3400	100 - 10,000	20 - 100

TABLE IV-15

Isotopic Space Power Technology Growth

	Power per Unit Electric Watts	Efficiency Per cent	Specific Power Pounds/Watt	Available
SNAP 9A, SNAP 19	3 - 30	4 - 5	1.0	Today
SNAP 27	50	4 - 5	0.6	1968 (Apollo)
SNAP 29	500	4 - 5	1.0	1970 (Ground Test)
Isotope - Improved Thermoelectric	-	7 - 9	0.3-1.0	1970 (Ground Test Capability)
Isotope - Organic or Mercury Rankine	2,000-10,000	10- 20	0.3-0.5	Early 1970's (Ground Test Capability)
Isotope - Brayton	5,500	20+	0.15-0.25	Early 1970's (Ground Test)

Table IV-16

[In millions]

Operating costs	Through fiscal year 1966	Fiscal year 1967	Fiscal year 1968	Location
1. Radioisotope:				
a. Thermoelectric	27.2	25.5	19.5	
SNAP 9-A	1.3			Martin Co.
SNAP 11	2.0	.2		Do.
SNAP 19-A and B Nimbus	4.0	3.0	.9	Do.
DoD/GE-M		.1	1.0	Not under contract.
SNAP 27 (ALSEP)	7.6	12.0	4.6	General Electric (MSD).
250-watt generator	.1	.4	1.0	Development not under contract.
NANS (formerly NAVSAT)	0	.5	2.5	Not under contract.
SNAP 17 (Sr[90])	1.8			Martin Co.
Sr[90] ground handling equipment	.3	.2	.4	Development not under contract.
SNAP 29 (Po[210] space generator)	.7	7.5	8.3	Martin Co.
SNAP 1 and 1-A	7.7			Do.
Lightweight converter	.7	.3	.5	General Atomic.
Modules and materials	1.0	.3	.3	RCA and TEECO.
b. Thermionic	2.2	.2	0	
SNAP 13	1.7	0	0	Martin Co.
Thermionic space generator	0	.2	0	General Electric (MSD).
Studies and investigations	.5	0	0	Martin Co.
c. Dynamic	.5	.9	.7	
Organic cycle fundamental investigations	.1	.5	.2	Philco-Sundstrand
Large heat source (kilowatt) (for Brayton cycle being supported by NASA)	.4	.1	.5	Proposals requested.
Operating costs exclude space nuclear safety costs.	(42.4)	(5.0)	(5.6)	
d. Supporting technology (radioisotopes)	3.1	2.2	1.9	Sandia. Johns Manville. TEECO. Linde.
e. Isotope fuel (space)	8.1	8.4	10.5	Mound-ORNL. Dupont. PNL (Battelle).
Total radioisotope	40.1	36.2	32.6	
2. Thermal reactor	120.6	10.1	9.7	
a. Mercury rankine:				
SNAP 2 system[1]	59.9	0	0	A.I. and TRW.
SNAP 8 reactor[1]	45.6	5.0	4.5	A.I.
Zr-hydride reactor technology	5.5	1.9	2.4	Do.
Mercury rankine cycle (CRU)	16.3	2.0	1.8	A.I. and TRW.
Basic studies and investigations	9.3	1.2	1.5	A.I. and ORNL.
b&c. Brayton cycle	0	0	0	(Technology being supported by NASA).
d. Thermoelectric	85.2	3.4	2.3	
SNAP 10-A system development	78.8	0	0	A.I. and RCA.
Direct radiating converter	4.5	1.4	.3	Do.
Compact converter (also applicable to isotopes)	1.9	2.0	2.0	RCA and Westinghouse.
e. Thermionic	6.5	.5	.5	
Plasma thermocouple[2]	6.5	.5	.5	LASL.
Total thermal reactor	232.3	14.0	12.5	
3. Fast reactor:				
a. Potassium rankine cycle	113.9	6.1	5.8	
Liquid metal cooled reactor	3.2	5.2	5.8	LRL.
SNAP 50 and LCRE	86.4	.1	0	Pratt & Whitney.
MPRE	12.9	.3	0	ORNL.
Potassium rankine cycle development	11.3	.5	0	AiResearch-ORNL, Pratt & Whitney.
b. Gas cooled reactor (710)	9.8	2.9	2.7	
c. Thermoelectric	0	0	0	
d. Thermionic, in-pile	7.0	3.4	4.0	
Thermionic R. & D.[3]	7.0	3.4	1.5	General Atomic—TEECO, RCA, G.E.
Thermionic reactor development	0	0	2.5	Not under contract.
Total fast reactor	130.7	12.8	12.0	

[1] Includes $[illegible] for mercury rankine development.
[2] Conversion system development conducted by NASA.
[3] Budgeted under "Advanced Systems R. & D."

Work looking toward advanced high temperature, fast reactors has been assigned to the Lawrence Radiation Laboratory. The objective of this program is development of technology for SNAP systems ranging up to ten megawatts in size, intended to meet missions which will become more clearly defined in the mid-1970's. A comprehensive program to develop advanced power conversion technology is being conducted in coordination with NASA and DOD. Other advanced reactor work is being carried out by General Electric (Vallecitos, California) and General Atomic (LaJolla, California) on fuel elements for thermionic reactors and by General Electric (Evendale, Ohio) on high temperature gas-cooled reactor technology applicable to space power generation.

3. Industry and Space

Because of the broad range of disciplines involved in the nuclear space program, a great deal of scientific and technological information has evolved which is of value not only to aerospace, but to the Nation's consumer economy as well. A cooperative AEC-NASA effort has been in operation for over a year, aimed at announcing these developments to the public through a Technology Utilization (TU) Program.

A small sampling of recent disclosures includes:

....New High Temperature Thermocouples
....A number of highly practical Computer Codes useful in the teaching of nuclear physics and in the design of commercial reactors
....Materials Properties Handbooks which fill-in serious gaps in the literature
....Electronic circuits with fail-safe, high reliability features of significant value to commercial processing, medicine, etc.
....New machine and hand tools
....Workmanship Standards for Welding
....Radioactive techniques for measuring surface areas

As measured by the number of inquiries received from sources throughout the United States and overseas, requesting amplification of the disclosures already in print, the public response is highly encouraging.

In recent months, a retrospective search of advancements made in the nuclear rocket program over a ten-year span has resulted in the cataloguing of a large number of additional candidates for disclosure through the TU system. These items

vary from basic hardware, to advanced new materials such as carbide coatings, to fundamental mathematical theories. Coupled with the ideas being reported from the on-going nuclear rocket program, this added backlog of useful technology is certain to find its way to the market place.

1. Aircraft Nuclear Propulsion

The decision to build a massive aircraft such as the C-5A type seems to have stimulated renewed interest in nuclear propulsion for aircraft. An in-house study is under way at Lockheed Aircraft Corporation, the C-5A contractor, on the system effectiveness and the performance capabilities of a nuclear powered aircraft. The use of nuclear power for aircraft continues to be studied by NASA and the Air Force. United Nuclear Corporation; Westinghouse; General Motors; Allison Division; and Nuclear Utility Services Corporation have been participating in this work through study contracts.

Table IV-17

Radioisotope power system and prime contractor [1]	Development period: Started (fiscal year)	Development period: Completed (fiscal year)	Design space useful life	Generator power (electrical watts)	Fuel	Total development cost (millions)	Application	Development status
SNAP 1 and 1-A (Martin)	[illegible]	[illegible]	[illegible]	[illegible]	[illegible]	[illegible]	DOD space missions	[illegible]
SNAP 3 (Martin)	[illegible]	[illegible]	90 days	[illegible]	Polonium 210	[illegible]	[illegible]	Completed.
SNAP 3-A (Martin)	[illegible]	[illegible]	5 years	[illegible]	Plutonium 238	[illegible]	[illegible]	[illegible]
SNAP 9-A (Martin)	[illegible]	[illegible]	do.	[illegible]	do.	[illegible]	[illegible]	[illegible]
SNAP 11 (Martin)	[illegible]	[illegible]	90 days	[illegible]	Curium 242	[illegible]	[illegible]	[illegible]
SNAP 13 (Thermionic unit) (Martin)	[illegible]	[illegible]	do.	[illegible]	do.	[illegible]	[illegible]	[illegible]
SNAP [illegible] (Martin/[illegible])	[illegible]	[illegible]	[illegible]	[illegible]	[illegible]	[illegible]	[illegible]	[illegible]
SNAP [illegible] (Martin)	[illegible]	[illegible]	5 years	[illegible]	Plutonium 238	[illegible]	[illegible]	[illegible]
SNAP [illegible] (Martin)	[illegible]	[illegible]	do.	[illegible]	do.	[illegible]	[illegible]	[illegible]
SNAP [illegible] (Lockheed)			do.	[illegible]	do.		[illegible]	[illegible]
SNAP 27 (General Electric)	[illegible]	[illegible]	do.	[illegible]	do.	[illegible]	[illegible]	[illegible]
SNAP [illegible] (Martin)	[illegible]	[illegible]	90 days	[illegible]	[illegible]	[illegible]	[illegible]	[illegible]

Reactor power systems [1] and prime contractors	Development period: Started (fiscal year)	Development period: Completed (fiscal year)	System power (electrical)	Reactor power (thermal kilowatts)	Reactor type	Total development cost (millions)	Application	Development status
SNAP 2 system (AI)	[illegible]	[illegible]	3 kilowatts	[illegible]	Thermal	[illegible]	DOD space missions	[illegible]
SNAP 8 reactor (AI)	[illegible]	[illegible]	[illegible] kilowatts	[illegible]	do.	[illegible]	NASA space missions	[illegible]
SNAP 10 system (AI)	[illegible]	[illegible]	[illegible] watts	[illegible]	do.	[illegible]	DOD space missions	[illegible]
SNAP 50 (including [illegible]) (Pratt & Whitney)	[illegible]	[illegible]	[illegible] kilowatts	[illegible]	Fast	[illegible]	High power space missions	[illegible]

[1] [illegible]
[2] [illegible]
[3] [illegible]
[4] [illegible]

CATEGORY V
RADIOISOTOPES

The total U.S. market for radioisotopes and radiopharmaceuticals is estimated at about $18-22 million.

1967 Market Prediction

Radiochemicals	$ 8-10 million
Radiopharmaceuticals	$10-12 million
TOTAL	$18-22 million

The estimated private sales of radiochemicals include the value of AEC supplied radioisotopes purchased by private suppliers for further processing or repackaring and distribution.

Radioisotopes of commercial interest fall into four classes: those found in nature, such as radium-226; those produced in nuclear reactors, such as cobalt-60 and carbon-14; those produced in accelerators, such as sodium-22 and iodine-123; and those resulting from the fission of other elements, such as strontium-90 and cesium-137. A radiochemical is a chemical compound prepared with a radioactive element. A radiopharmaceutical is a radiochemical which has been prepare[illegible] [illegible]o meet requirements for use in the human body.

The trend in radioisotopes uses indicates the market will continue to expand, especially in the fields of radioisotope-fueled power systems for space, medical, and terrestrial applications and in radiopharmaceuticals. It is our belief that in areas such as medicine, isotopic-fueled power systems, and radiation processing the growth period has only started.

As discussed in our previous reports, although the packaging and distribution of radioisotopes is performed almost entirely by industry, th[illegible] AEC is still a principal domestic producer and distributor of radioisotopes. Industry has continued to show increa[illegible]g interest in enlarging processing capacity and in the production, distribution, and use of radioisotopes.

As in our last report, we have divided the radioisotope industry into four categories: producers, processors, source fabricators, and equipment manufacturers.

A. Producers

A producer is defined as one who can perform target irradiation and may also perform target preparations and separation of the desired radioisotope for ultimate distribution. Radioisotopes are obtained from cyclotron bombardments, reactor irradiations, and from high-level waste streams.

1. Cyclotron Radioisotopes

In 1955, the AEC discontinued processing and distributing cyclotron-produced radioisotopes. Except for target preparation and irradiation services provided by Oak Ridge National Laboratory or by the University of Pittsburgh, the industry supplies cyclotron radioisotopes. The interest in cyclotron-produced radioisotopes may be estimated from the AEC revenue for special cyclotron irradiations listed below:

FY-1964 - $126,508
FY-1965 - $258,665
FY-1966 - $277,713
FY-1967 - $258,236

Some of the cyclotron-produced radioisotopes of interest include sodium-22, cobalt-57, rubidium-84, iodine-123, carbon-11, nitrogen-13, oxygen-15, iron-55, mercury-199, chromium-49, copper-61, zinc-62, and selenium-73. Of particular interest is I-123 with a short half life of 13 hours and which can reduce radiation exposures in patients to a small fraction of that with I-131 for thyroid work. It is also very promising for brain scans and cancer research. Because of the special interest in cyclotron-produced I-123 by the medical profession and with the encouragement of the private processors, the Commission has agreed to make it available on a special order basis[1/] as a processed target similar to Rb-84 and Co-57.

Because of the very short half life of the radioisotopes desired for medical applications and the difficulty in transporting useable quantities over great distances from the cyclotron, a growing need is developing for commercial cyclotron facilities near large population centers to provide medical researchers and nuclear medicine departments with the necessary isotopes on a timely basis.

1/ See AEC Public Release K-67, dated March 21, 1967.

The first commercial installation of a medical cyclotron facility was completed in the fall of 1967 with acceptance by the Sloan-Kettering Institute of the accelerator supplied by The Cyclotron Corporation of Berkeley, California. This facility was supplied under a $272,000 grant from the Atomic Energy Commission. Other facilities are planned for the Argonne Cancer Research Hospital and by the National Institutes of Health (NIH) as funding permits. The Cyclotron Corporation hopes to have off-the-shelf sales of their compact machine in the future. It has been estimated that a market for 20 or more such facilities may develop for medical isotope production by 1975.

The present market for cyclotron-produced radioisotopes is estimated at about $0.5 to $1 million per year. While the U.S. has been the principal supplier, cyclotron radioisotopes are also marketed by the United Kingdom, South Africa, and the USSR.

The principal industrial suppliers of cyclotron radioisotopes are listed in Table V-1.

TABLE V-1

Principal Industrial Suppliers of Cyclotron Radioisotopes

Abbott Laboratories, North Chicago, Illinois
Cambridge Nuclear Corporation, Cambridge, Mass.
Hastings Radiochemical Works, Houston, Texas
International Chemical and Nuclear Corporation, Cal.
*Nuclear Science and Engineering Corporation, Pittsburgh, Pennsylvania
*U.S. Nuclear Corporation, Burbank, California
New England Nuclear Corporation, Boston, Mass.
Nuclear Consultants, Division of Mallinckrodt Chemical Company, St. Louis, Missouri

---

* Wholly owned subsidiary of International Chemical and Nuclear Corporation.

2. Reactor Radioisotopes

As indicated previously, the AEC is still a principal domestic producer and distributor of radioisotopes, including those produced in reactors. However, the industry is offering an increasing number of products and higher specific activity material than in past years.

Reactor irradiation services for production of radioisotopes in targets supplied by customers is offered by the following companies: (1) General Electric (GETR), (2) Union Carbide (Tuxedo, New York), (3) Babcock & Wilcox (Lynchburg, Virginia), and (4) Western New York Nuclear Research Center (Buffalo, New York). In the future it is expected that additional private reactors, especially power reactors, will become available for large volume, low specific activity radioisotope production. Neutron Products Inc. has been producing Co-60 in the Big Rock Point nuclear power reactor owned by Consumers Power Company and in May 1967 published a price schedule for this material. The General Electric Company announced it has initiated irradiation of Np-237 to produce Pu-238 in research quantities for sale commercially.

Up to 1965, the Commission had withdrawn, on an ad hoc basis, from producing and distributing selected radioisotopes. In March 1965, the AEC adopted and published in the Federal Register formal policies and procedures for the transfer of commercial AEC radioisotope production and distribution activities to private industry. These policies and procedures provide for the orderly withdrawal from this area of activity. In 1967 AEC withdrew from production of one isotope, Sr-87m. Since 1961, the AEC has withdrawn from routine production of a total of 37 reactor-produced radioisotopes.[2/]

AEC sales of reactor-produced radioisotopes declined slightly in Fiscal Year 1967 primarily due to reductions in sales of cobalt-60 and carbon-14. Table V-2 lists AEC sales of radioisotopes.

TABLE V-2

AEC Sales of Radioisotopes

Fiscal Years	Million Dollars
1963	$1.2
1964	1.3
1965	1.6
1966	2.0
1967	1.5

2/ See AEC Public Releases: J-79, dated March 13, 1966; H-14, dated January 19, 1965; G-170, dated July 14, 1964; G-96, dated April 30, 1964; F-184, dated September 20, 1963; and IN-777, dated March 31, 1967.

Consistent with the March 1965 policies published in the Federal Register, the AEC gave notice on March 21, 1967, of an increase in prices of 15 radioisotopes in order to recover the full costs of production and distribution.[3/]

Reactor-produced radioisotopes are used in a large number of applications; to name only a few, medicine, agriculture, crime detection, industrial gauges, and space. An estimate of the future potential dollar market for radioisotopes is difficult to make, but it is believed to be growing at about 10% annually.

There are currently more than 10,000 doctors and medical[4/] organizations in the United States alone routinely using some 30 different radioisotopes in the diagnosis and treatment of many diseases. Some of the isotopes and applications include: cobalt-60 as a source of radiation for treating cancer; iodine-131 is used in blood volume and cardiac studies, to locate brain tumors and diagnose kidney and liver disorders, but primarily it is used for diagnosing and treating thyroid disorders (over one-half million thyroid cases annually); carbon-14 is used to study metabolic diseases; sodium-22 and 24 are used to measure blood volume and diagnose circulatory disorders; gallium-68 and strontium-85 are used in bone scanning; technicium-99m is used to make radio-cardiograms; phosphorous-32 in locating eye tumors; and iron-59 in hemoglobin studies. It is estimated that between three and four million patients each year are treated with procedures using isotopes. About 98% of these procedures are diagnostic.

Foreign competition in the radioisotope market is increasing and will continue to be a problem for American firms. This is especially so in the cobalt-60 market where Canada, the United Kingdom, and Australia are major suppliers. The Canadian and UK firms also provide a more complete package of services than is yet available from individual U.S. companies.

3. Fission Product Radioisotopes

In addition to cyclotron and reactor-produced radioisotopes, fission product radioisotopes are separated from high-level waste streams from AEC production facilities. The radioisotopes from fission products of principal interest are Sr-90, Ce-144, Cs-137, and Pm-147. These fission products will also be available in large quantities in the future,

3/ See AEC Public Release K-67, dated March 21, 1967.
4/ Remarks by Dr. Glenn T. Seaborg, "The Proliferation of the Peaceful Atom", at 24th Annual National Conference of the American Public Power Association, Denver, Colorado, May 11, 1967, AEC Public Release S-20-67.

when their production is warranted by market demands from commercial firms operating the chemical processing plants, such as Nuclear Fuel Services, Inc., Allied Chemical Company, and General Electric Company. It is expected that the expanding uses of these isotopes may eventually create a source of revenue for utilities from these by-products resulting from reprocessing of fuel from reactor operation.

Isochem, Inc. (which was jointly owned by Martin-Marietta and Uniroyal) in 1965 had agreed to construct and operate a fission product conversion and encapsulation plant at Richland, Washington on a commercial basis, along with a contract to operate the AEC chemical processing complex at Richland. In early 1967, the firm decided to terminate this contract because of increasing costs of the commercial plant, in addition to the lagging development of a commercial market for these isotopes. Atlantic Richfield was chosen to replace Isochem as the AEC's contractor at Richland; and for the present, there are no plans to recover fission product radioisotopes on a commercial basis at Richland. At present fission product isotopes are obtained from waste streams in the Richland chemical separations plant and are distributed through the Oak Ridge National Laboratory, Isotopes Sales Department.

Fission product radioisotopes are used in a number of industrial applications, as well as for space, power, and heat purposes. In this regard during 1967 both Atomics International and Donald W. Douglas Laboratories announced that they were sponsoring significant R&D programs using Pm-147 for heat sources. Promethium-147 and strontium-90 are used to prepare and activate luminous sources and atomic "batteries". Strontium-90 is also employed in industrial thickness gauges and in the treatment [illegible] certain eye disorders. Cerium-144 is used for various resea[illegible]h purposes and has good potential for use as a heat source. [illegible]esium-137 is used as a radiation source for both industrial and medical uses and is a competitor of cobalt-60 in certain areas.

AEC revenue for the distribution of the above four radioisotopes is given below for FY-65, FY-66, and FY-67.

Revenue in $

	1965	1966	1967
Cesium-137	192,000	27,000	60,000
Promethium-147	28,000	12,000	55,000
Strontium-90	3,000	65,000	59,000
Cerium-144	negl.	negl.	negl.
TOTAL	$223,000	$104,000	$174,[illegible]00

[illegible]

B. Processors

A processor is defined as one who can perform target preparation and separation of the desired radioisotopes, radiochemicals, and/or radiopharmaceuticals for ultimate distribution. Industry continues to assume more and more of these activities.

The principal commercial firms in this area are listed in Table V-3.

TABLE V-3

Principal Processors of
Radiochemicals and Radiopharmaceuticals

	Radiochemicals	Radiopharmaceuticals
Abbott Laboratories		X
Atomic Corp. of America	X	
Bio-Rad Laboratories	X	
Calbiochem	X	
Cambridge Nuclear Corp.	X	X
General Nuclear, Inc.	X	
General Radioisotope Processing Co.	X	X
Hastings Radiochemical Works	X	X
International Chemical & Nuclear Corp.	X	
Nuclear Science & Engrg. Corp.	X	
U.S. Nuclear Corp.	X	
Volk Radiochemical Co.	X	
Isotopes Inc. (sub. of Teledyne)	X	
Isotope Development Laboratories	X	
New England Nuclear Corp.	X	X
Nuclear-Chicago Corp. (G. D. Searle Company)	X	
Nuclear Consultants Corp. (Div. of Mallinckrodt Chem. Works)	X	X
Schwartz Bio-Research Inc. (sub. of Becton, Dickinson & Co.)	X	
E. R. Squibb & Sons		X
Tracerlab (Div. of L.F.E. Inc.)	X	
Union Carbide Corp.	X	X

Since our last report, there have been additional changes in the industry structure. The following radioisotopes firms were involved: Mallinckrodt Chemical Works purchased Nuclear Research Chemicals Inc. of Orlando, Florida; Isotopes Inc. and Controls for Radiation were acquired by Teledyne, Inc.; International Chemical and Nuclear Corporation acquired Nuclear Science and Engineering Corporation (they previously had purchased U. S. Nuclear Corporation, Volk Radiochemical Company, and Baird Atomic's radiochemical interests in 1966).

C. Source Fabrication

There are presently approximately 50 commercial firms producing alpha, beta, gamma, and neutron sources. The alpha and neutron source business is small (about $500 thousand per year) but may continue to increase slightly over the years. The beta and gamma source business can be expected to increase substantially in the future, as a result of increased interest in heat and process radiation applications. A number of firms that own large hot cells, such as Battelle Memorial Institute, The Babcock & Wilcox Company, General Electric, Gamma Process Corporation, and Lockheed-Georgia, have bid on large cobalt-60 source encapsulation work.

Nuclear Materials and Equipment Corporation, Apollo, Pennsylvania; Monsanto Research Corporation, Dayton, Ohio; and General Nuclear Inc., Houston, Texas, are the principal commercial suppliers of plutonium and americium neutron sources.

D. Equipment Manufacturers

There are six principal areas of interest in equipment: these are radiographic and teletherapy units, gauges, irradiators, isotopic power devices, and self-illuminating devices.

1. Radiographic Units

Radiographic units are manufactured by industry and not by the AEC. The commercial firms active in this field are listed in Table V-4.

TABLE V-4

Principal Suppliers of Radiographic Units

The Budd Company, Phoenixville, Pennsylvania
Gamma Industries, Inc., Baton Rouge, Louisiana
Radionics Corporation, Norristown, Pennsylvania
Picker X-Ray, Technical Operations, Inc., Burlington, Mass.
U. S. Nuclear Corporation, Burbank, California (a subsidiary of International Chemical & Nuclear Corporation)

The annual volume of sales in this field is approximately $1 million and is expected to continue to increase. The radioisotope units, utilizing such isotopes as iridium-192, cobalt-60, cesium-137, and thulium-170 compete with machines.

2. Teletherapy Units

There are eight commercial suppliers of teletherapy units. Four of the eight suppliers (General Electric, North American Philips Company, Siemens New York, and Schick X-Ray Company) are distributors of foreign-made teletherapy units. The firms active in this field are listed in Table V-5.

TABLE V-5

Principal Suppliers of Radioisotope Teletherapy Units

W. F. and John Barnes Company, Automated Machine Division of Babcock & Wilcox, Rockford, Illinois

General Electric Company, Milwaukee, Wisconsin

Laboratories for Electronics, Waltham, Massachusetts (Tracerlab)

North American Philips Company, Inc., New York, New York

Picker X-Ray Company, Cleveland, Ohio

Schick X-Ray Company, Chicago, Illinois

Siemens America Inc., New York, New York

Westinghouse Electric Corporation, Baltimore, Maryland

There are approximately 1700 teletherapy units in the world -- 7 hundred of which are in North and South America. The annual volume of sales for these units is approximately $2 million. The average new unit being installed in the U.S. contains 3 thousand to 4 thousand curies of high-specific activity cobalt-60 with some containing up to 8 thousand to 10 thousand curies.

3. Radioisotope Gauges

There are between 20 and 30 firms active in the radioisotope gauge field. This type of gauge combines a radioisotope (alpha, beta, gamma, or neutron) source of radiation and appropriate radiation detection elements and control equipment which permit measurement and control of various processes. These include flow rate, thickness, level, density, and activation analysis systems. The current market for radioisotope gauges is estimated between $20-$25 million and is expected to grow at about 10-15% annually.

The principal suppliers of industrial radioisotope gauges and gauging systems are listed below:

Brun Corporation
Foxboro Corporation
Industrial Nucleonics
Instruments, Inc.
Nuclear-Chicago Corp. (subsidiary of G. D. Searle)
Ohmart Corporation
Robertshaw Controls Company
Tracerlab, Division of L.F.E. Inc.
Troxler Corporation

Table V-6 gives sales of radioisotope gauges as reported by the Bureau of the Census.

TABLE V-6

Sales of Radioisotope Gauges

Year	$ in Millions
1957	$ 6
1958	6
1959	9
1960	10
1961	11
1962	9
1963	9.9
1964	9.7
1965	13.1
1966	est. 20 - 25

Some examples of radioisotope gauge applications include measuring and automatically adjusting the thickness of steel sheet, paper, tire cords, textiles, plastics, and other materials; controlling sugar, fat, and meat content in food packaging; measure soil density and moisture content; measure snow density for water run-off forecasting; detect gold; and measuring the density of ocean bottom sediments.

4. Self-Illuminating Materials and Devices

Beta emitting radioisotopes, such as krypton-85, tritium, and promethium-147, are used in the preparation of self-illuminating materials and devices. Tritium is the most frequently used radioisotope. The growing use and interest in self-illuminating materials is reflected in the sales of tritium by the AEC given below.

Fiscal Year	Curies	AEC Sales
1963	60,000	$ 95,000
1964	112,000	$125,000
1965	118,000	$128,000
1966	171,000	$196,000
1967	178,874	$200,000

The firms active in this field are listed in Table V-7.

TABLE V-7

Principal Commercial Suppliers of
Self-Illuminating Materials and Devices

American Atomics, Tucson, Arizona
Canrad Precision Industries, Inc., New York, New York
Laboratories for Electronics, Inc., Waltham, Mass.
Luminous Products Corporation, Boston, Mass.
Minnesota Mining & Manufacturing Company, St. Paul, Minn.
Radium Chemical Company, New York, New York
U.S. Radium Corporation, Morristown, New Jersey

5. Commission Licensing Actions in Radioisotopes Area

During 1967, the AEC acted to amend several of its regulations concerning radioactive materials. The most significant items are given below.

The 3M Company requested AEC to increase the maximum quantity of Pm-147 in any single aircraft luminous safety device used under general license from 100 to 300 millicuries. This was approved by the AEC in October 1967.

The U.S. Radium Corporation petitioned AEC to increase the quantity of tritium in any one aircraft luminous safety device from four to ten curies. This was approved by the AEC in May 1967.

The Chevron Research Company petitioned AEC to exempt from licensing the possession and use of radioactive scandium-46 in a synthetic plastic used for consolidating loose sand in oil wells to permit greater production. Location of the sand would be determined by detection of the radioactivity with an oil well logging tool. This request was approved by AEC in March 1967.

Siemens America Inc. petitioned the AEC to exempt from licensing the use of small amounts of Pm-147 in surge voltage protectors. This was approved in April 1967.

In June 1967, the AEC amended Part 36 of its regulations, "Export and Import of Byproduct Material" to permit licensees to export under general license radioisotopes having an atomic number from 3 to 83 inclusive in quantities not to exceed one curie per shipment and tritium at specific activities not to exceed 10 curies per gram of hydrogen in quantities not to exceed 10 curies per shipment.

In May 1967, the AEC amended Part 40 of its regulations and its licensing practices to permit a five-year effective period in licenses for radioactive materials. Licenses had generally been issued for three years or more, depending on the form of the material and its intended use.

6. Radioisotopic Power

During 1967, the AEC reviewed its current programs and future plans for the development of the large-scale use of isotopes in electrical power, thermal energy, and process radiation applications. AEC policy and objectives in each of the several major programs have also been set forth and the result of this review is a comprehensive white paper, "Radioisotopes Production and Development of Large Scale Uses," which was published in 1967.

The utilization of the heat released when isotopes decay for various specialized applications requiring power is now beginning to approach large-scale quantities. The growth of isotopic power applications has been limited by availability of several of the isotopes which in turn affects the demand of potential large-scale users such as the DOD and NASA. Also, space and military requirements for isotopic powered systems have not developed as fast as expected. Detailed forecasts of requirements for Pu-238, Sr-90, for the intermediate lifetime isotopes Co-60 and Cs-137, and for short-lived isotopes, such as Po-210, Tm-170, and Pm-147, are given in the white paper referenced above for various applications.

It is expected that the expanding nuclear power economy should contribute significantly to the production of isotopes, such as Pu-238, Cm-244, and Sr-90, as a by-product of reactor operation and chemical reprocessing operations. As mentioned previously, some isotopes may be produced in power reactors through the irradiation of target materials, i.e., Co-60 and Tm-170.

Radioisotopic power applications fall into the following general categories:

(a) Space Electric Power - These applications include weather, communications, and navigation satellites; space exploratory power systems, such as that for the Apollo Lunar Surface Experiment; and space mission requirements.

(b) Terrestrial Power - Applications in this area include remote weather stations, navigation buoys and beacons, for power on off-shore oil rigs, cardiac pacemaker, and undersea power systems.

(c) Thermal Applications - These applications include spacecraft and swimsuit heaters, waste water reprocessing systems for manned space missions, and energy source for a heart pump and small undersea propulsion systems.

(d) Isotopic Propulsion Systems - are primarily for auxiliary spacecraft propulsion.

Markets for radioisotope power - The Federal Government is the principal market for these devices at the present time. While forecasts of isotope requirements are given in the AEC white paper, the dollar value of power producing devices cannot really be estimated yet. Substantial commercial markets for applications, such as direct broadcast satellites, marine and

remote land power systems, could develop over the next several years. The cardiac pacemaker and the artificial heart appear to be among the most promising future markets. It is estimated that there could be a need for about 10,000 cardiac pacemakers per year in the future. These units produce 150-200 electrical microwatts of power and do not require large quantities of Pu-238. The need for artificial heart implantation operations is estimated as high as 100,000 patients or more per year and could use all foreseen supplies of Pu-238 if this device is successfully developed.

Industry - There are some 30 companies active in the isotopic power generation area and it is expected that other firms will enter this field as the market develops. A number of the most important activities by commercial firms are described below.

NUMEC delivered the first model of an isotopically powered cardiac pacemaker to AEC for testing under the first phase of a two phase design and development contract with AEC.

In May 1967, the AEC selected four firms to conduct design studies of a radioisotope powered heart pump engine, which is being co-funded by the AEC and the National Heart Institute of the NIH. The companies are Aerojet-General Corporation, San Ramon, California; Donald W. Douglas Laboratories, Richland, Washington; Thermo Electron Engineering Corporation, Waltham, Massachusetts; and Westinghouse Astronuclear Laboratory, Pittsburgh, Pennsylvania.

Lockheed Missiles and Space Division was awarded an AEC contract for a design study of an integrated isotope powered life support system for manned space missions.

Atomics International was selected by AEC to develop the technology of a high-temperature radioisotope fuel capsule that would operate at 2000°F for five years and would be used to power the Brayton cycle power conversion system being developed by NASA for potential space missions.

Battelle Memorial Institute has been selected by AEC to work on Pu-238 isotopic fuel form development.

Sanders Nuclear Corporation fueled a milliwatt radioisotope generator with thulium-170 oxide encapsulated fuel in August 1967. This is the first such generator to use Tm-170 oxide and was developed entirely with private funding.

The Navy purchased a 25-watt radioisotope thermoelectric generator from the Martin-Marietta Company for use in an undersea oceanographic data collection system. Martin had previously sold a 3-watt unit to the Navy. Martin also has a Navy order for 3 more generators for use in NOMAD weather buoys. NUMEC has been awarded a Navy contract for a 25-watt submersible generator unit. Atomics International has a Navy order for a 3-watt unit. Aerojet-General sold the Navy a 1-watt URIPS unit and NUMEC sold two 100 milliwatt generators for Navy testing.

7. The Radiation Processing Industry

Industry continues to show a growing interest in commercial radiation processing applications. This interest was demonstrated by the number and varied backgrounds of industrial groups which submitted proposals to AEC on the Pilot Plant Meat Irradiator Project and also by the large attendance at the SINB-AEC Food Irradiation Briefing held at Oak Ridge in February 1967.

The radiation processing field is divided into machine or accelerator-produced radiation and isotope radiation, each of which possesses some advantages and disadvantages over the other. For example, machines can be operated at variable energy output levels to accomodate the work to be processed and can be turned off. Isotope radiation cannot be turned off, but is suitable for bulk processing of chemicals and products requiring great penetration where machine radiation is inadequate. At the present time, machine irradiation facilities account for over 90% of all the radiation facilities in the U.S. Radiation processing applications include sterilization of medical supplies, food preservation, production of wood plastic combinations, production of shrinkable plastics, sterilizing parts for the space programs, preparation of bio-degradable detergents, synthesis of chemical compounds, upgrading of solid state devices and plastics, and curing of paints and special coatings.

The radiation processing market has been estimated in terms of the value of products processed during the past few years (1960 - $20 million, 1964 - $75 million, 1965 - $150 million, and 1966 estimated at $250 million).

Table V-8, on the following page, lists the principal firms supplying accelerator equipment and/or services or finished products.

TABLE V-8

Company	Supply Accelerators	Supply Services	Supply Finished Products
Boise Cascade Corp.		X	X
Cyclotron Corporation	X		
General Electric Company	X		
Hughes Aircraft Company	X		
Applied Radiation Corporation	X	X	
Electra Process Industries, Inc.		X	
General Atomic, Division of General Dynamics	X	X	
High Voltage Engineering Corp.	X		
Electronized Chemicals Corp., Subsidiary of High Voltage Engineering Corporation		X	
Midwest Irradiation Center		X	
Raycon		X	
Radiation Dynamics, Inc.	X	X	
Rad-Mat, Inc.		X	
Varian Associates	X	X	
High Energy Process Company		X	
Radiation Polymer Corporation		X	X

The market for commercial accelerators is currently estimated between $20-$25 million and is divided about three fourths for Van de Graaffs for research and the remainder for linacs, insulating core transformers, and Dynamitrons.

Table V-9, on the following page, lists the principal firms supplying isotopic irradiators.

The Lockheed-Georgia and American Novawood companies have substantial capacity for the production of special products and there is some capacity available for small to moderate-scale irradiation processing service in facilities at Atomics International, Louisiana State University, NUMEC, Iso Nuclear Corporation, High Energy Processing Corporation, and General Electric Company.

Irradiation preservation of foods, as noted earlier, has generated a great amount of interest in the food processing industry, the food producers, and a newly developing radiation

TABLE V-9

Principal Suppliers of Isotopic Irradiators

Company	Medical	Process	Research
Atomchem Corp.		X	X
Ameray Corporation			X
American Nuclear Corporation	X	X	X
Budd Company	X		X
Gamma Process Company, Inc.		X	X
National Lead Company	X	X	X
Martin-Marietta Corporation			X
Radiation Facilities, Inc.	X	X	X
Radionics, Inc.			X
Isotopes, Inc., Irradiations Systems Division	X	X	X
Lockheed-Georgia		X	X
Babcock & Wilcox		X	X
Nuclear Materials & Equipment Corp.		X	X
Burns & Roe		X	
Stone & Webster		X	
American Novawood		X	
General Electric		X	
Westinghouse		X	

processing industry. To assist in familiarizing these different industry groups with this subject, the Southern Interstate Nuclear Board and the AEC co-sponsored a briefing on radiation preservation of food in February 1967. The briefing was attended by some 250 representatives of interested private firms, Government and state agencies, and university groups. It covered the current program of the Army and the AEC; technology of preservation of fruit, fish, grain and potatoes, meat and dehydrated vegetables; the Food and Drug Administration responsibilities; and commercialization aspects of food preservation.

While only a few foods (bacon, wheat and wheat products, and white potatoes) have been cleared by FDA for processing to date, there is a great amount of testing under way by Government agencies and private firms on additional foods such as strawberries, citrus fruits, and marine products. There has been no change in the status of FDA approvals since our last report.

In May 1967 the U.S. Department of Agriculture and the AEC dedicated the world's first pilot plant grain irradiator at Savannah, Georgia. The irradiator will be used to treat insect

infested grains in a five-year program that will treat packaged commodities and bulk grain.5/

In August 1967, AEC announced the start of a cross-country tour of its truck-mounted irradiator to visit private firms for demonstration of the benefits and commercial potential of food preservation by irradiation. The irradiator uses 170,000 curies of cesium-137 for the radiation source and will test-demonstrate various food products including wheat and wheat flour, potatoes, tomatoes, onions, oranges, mushrooms, mink feed (fish), and seeds.6/

The AEC entered into an agreement with Irradiated Foods, Inc. on the Pilot Plant Meat Irradiator Project under which AEC furnishes $185,000 for the Co-60 course and $370,000 for design and construction and the Department of Defense agrees to procure some 300,000 pounds of meat for a three-year period. The pilot plant will process about 1 million pounds annually and is a joint venture of Allen Products, Isotopes Inc., Martin-Marietta, and Uniroyal.

Dr. Samuel M. Nabrit, Commissioner of the AEC, participated in the dedication of a Hawaii Development Irradiator on July 3, 1967. This facility, designed by NUMEC, was optimized for semi-commercial scale development work in the radiation processing of fresh fruits, especially tropical fruits, such as papayas and mangoes. In his remarks at the dedication cermony, Dr. Nabrit indicated that the effects of radiation energy on various foods encompass a fairly wide range of end uses. One possibility that might be applied to increasing food supplies is the knowledne that very, very low radiation doses applied to items such as grain or seed potatoes actually stimulate earlier plant germination and growth. Planting irradiated seed potatoes could enable farmers to grow two potato crops, where only one is now possible.7/

The development of irradiated wood-plastic combination materials (WPC) is another major area of interest in radiation processing. The following summarizes the status of current and planned commercial activity in this new field.

5/ See AEC Public Release K-116, dated May 10, 1967.
6/ See AEC Public Release K-214, dated August 31, 1967.
7/ Remarks by Dr. Samuel M. Nabrit, "The Peaceful Atom Comes to Hawaii", at the Dedication of the Hawaii Development Irradiator, Honolulu, Hawaii, July 3, 1967, AEC Public Release S-30-67.

American Novawood Corporation, Lynchburg, Virginia, began operation in 1966 and since then has been offering wood plastic products on a commercial basis. They are now producing items such as flooring and building products and also specialty items for musical instruments, sporting goods, textiles, and furniture.

Lockheed-Georgia Company, Marietta, Goergia, has been producing development quantities of their trade name wood plastic combination, Lockwood, for testing by private firms, under an AEC contract and also as a private development. They have an irradiator with a capacity of about 250,000 board feet per year.

Louisiana Radiant Inc., Baton Rouge, Louisiana, is considering the construction of a large-scale WPC facility which would specialize in producing building materials.

Gem Irradiated Wood Company, Dathan, Alabama, is also considering construction of a WPC facility with a capacity of about 3 million board feet per year, which would produce building materials, flooring, marine products, and novelty items.

Significant Industry Changes and Announcements - Union Carbide announced plans to use Co-60 in its large-scale commercial production of polyethylene oxide, used as an intermediate agent in chemical manufacturing.

NUMEC announced plans to enter the food irradiation business using facilities at Quehanna, Pennsylvania. The first items to be market tested are expected to be bacon and potatoes.

Gamma Process Company acquired Iso Nuclear Corporation as a wholly owned subsidiary and Iso Nuclear has an agreement with the New York State Atomic and Space Development Authority to develop a meat preservation plant at the Authority's Center at West Valley, New York.

8. AEC Advisory Committee on Isotopes and Radiation Development

The present AEC Advisory Committee on Isotopes and Radiation Development was established in 1958. This committee grew out of the Advisory Committee on Isotope Production and Distribution, which was formed in 1946 and terminated in 1958 when the Commission's regulatory and licensing activities were separated from the operating activities.

The Advisory Committee on Isotopes and Radiation Development reviews AEC policies and the potential development of new applications of isotopes and radiation and makes appropriate recommendations for the Commission's consideration.

The composition of this committee has been gradually changed to bring in additional representatives from various segments of the isotope industry. The present membership is given below:

Chairman - John L. Kuranz, Nuclear-Chicago Corporation
Vice Chairman - John W. Landis, The Babcock & Wilcox Co.
AEC Member - Dr. George M. Kavanagh, Assistant General Manager for Reactors
Dr. Howard K. Nason, Monsanto Research Corporation
Dr. John C. Brantley, Nuclear Science & Engineering Corp.
Dr. Charles Artandi, Ethicon, Inc.
Dr. Vivian T. Stannett, Research Triangle Institute
Dr. David E. Harmer, The Dow Chemical Company
Dr. Thomas E. Hicks, University of California at Los Angeles
Dr. Rodman A. Sharp, Beckman Instruments, Inc.
Henry R. Chope, Industrial Nucleonics Corporation
Dr. Paul M. Stier, Union Carbide Corporation
Dr. Donald W. Pritchard, Johns Hopkins University
Milton Burton, Director of Radiation Laboratory, University of Notre Dame
Cdr. M. Scott Carpenter, Department of Navy
Dr. Bernard A. Fries, Research Chemist, California Research Corporation
Dr. Wilfred R. Konneker, General Manager, Nuclear Consultants Division of Mallinckrodt Chemical Works
Dr. James [illegible]. Maxfield, Maxfield Clinic Hospital

9. Science Information Exchange Program

A cooperative program to register private R&D in isotopes and radiation was initiated by the AEC and the Science Information Exchange of the Smithsonian Institute in April 1967.[8]

This program should be mutually beneficial to industry and Government in that the SIE system registers and catalogs research work at the time it is initiated, rather than after it appears in reports and journals. The reporting system, which

8/ See AEC Public Release IN-784, dated April 28, 1967.

is entirely voluntary, is expected to be useful and informative without at the same time revealing proprietary information concerning work in specific companies. The purpose of this program is to develop an over-all isotope and radiation program with a minimum of overlapping between private industry-sponsored and Government-sponsored work.

The Science Information Exchange registers over 95,000 basic and applied research projects annually from Federal Agencies, private foundations, universities, and private industry. SIE answers, without charge, some 50,000 questions each year from scientists, investigators and research administrators.

CATEGORY VI

MATERIALS REPROCESSING

A. Irradiated Fuel

Market Predictions

1970	-	$ 3,000,000
1975	-	$ 30,000,000
1980	-	$115,000,000

Fuel elements must be removed from the reactor core for reprocessing and recovery of the remaining reusable fissile atoms before they have been completely consumed, for two principal reasons. One is that the fission product poisons that build up in the reactor fuel have a high affinity to absorb the neutrons necessary to sustain the critical reaction. The other is that the damaging effects of radiation in the reactor necessitate removal of fuel elements prior to warping or rupture.

The reprocessing and recovery of valuable fissionable materials (U-233, U-235, and plutonium) remaining in spent fuel discharged from the reactor involve a series of operations in a chemical separations facility. The technology currently being used commercially is an updated version of the aqueous process that was developed and used initially in AEC separations facilities.

To be somewhat consistent with the format of last year's report, the remainder of this section will be divided and discussed under the following three headings:

1. Production Reactor Fuels
2. Power Reactor Fuels
3. Highly Enriched Fuels

1. Production Reactor Fuels

The AEC's production reactors were built specifically for the purpose of providing fissionable material for our defense needs. The production reactor fuel loads are processed at the Hanford Plant at Richland, Washington, and the Savannah River Plant near Aiken, South Carolina. Because the production fuel load has decreased, the Redox chemical reprocessing facility at the Hanford Plant was shut down at the end of 1966. The Purex reprocessing plant is now handling all the production reactor fuel loads at Richland.

The AEC's New Production Reactor (NPR) at Richland, Washington, began dual purpose operation during the year. In addition to plutonium production, it is providing steam to the

Washington Public Power Supply System for use in the utility's 800 Mwe generating plant located adjacent to the reactor. In order to assist in the development of a private reprocessing capability for central station power reactor fuels, the Commission is making available part of the spent fuel load discharged from this reactor for reprocessing under an AEC base load contract to the NFS, West Valley reprocessing facility which is the first private plant in operation in the United States.

2. Power Reactor Fuels

a. Policy for Reprocessing Commercial Fuels

In order to encourage the development of a commercial reprocessing industry, the Commission, in 1957, announced that it would enter into contracts to receive and make financial settlement for irradiated non-production fuels, if commercial reprocessing services were not available at reasonable terms.

This policy, which was to expire on June 30, 1967, but was extended through December of 1967, is described in two Federal Register notices, 22 F.R. 1591, March 12, 1967, as amended, and 28 F.R. 11462, October 25, 1963.

(1) The 1957 notice provides that, in the absence of private processing services at reasonable prices, AEC will undertake under individually negotiated contracts to receive irradiated reactor fuel and blanket materials at AEC designated facilities and to make a financial settlement therefor. The financial settlement will take into account the charges for chemical processing and conversion of the returned materials to the standard forms for which specifications and prices have been established by AEC. The processing charge is based upon the costs associated with processing certain specified types of irradiated materials in an assumed chemical processing plant (conceptual plant) described in AEC's report WASH-743, "Summary Report AEC Reference Fuel Processing Plant". There is one exception in that the charge for chemical processing of enriched uranium-aluminum alloy fuels is $145 per kilogram of the total weight of uranium and aluminum metal where the fuel batch delivered to AEC is less than 400 kilograms of metal.

(2) The 1963 notice advised of the planned Nuclear Fuel Services, Inc. (NFS), private processing plant and defined that after services are available from NFS the AEC will execute processing contracts only when AEC has determined, on a request by a reactor operator including operators of foreign reactors, that chemical processing services are not available to the reactor operator from NFS at reasonable terms and charges.

The Commission during the year reviewed the need for AEC to continue a policy beyond December 31, 1967, and asked for and received comments from the Atomic Industrial Forum regarding a proposed extension. The Commission study and AIF comments take into consideration such factors as existing capability, competition, fuel types, fuel loads, charges, etc. The Commission's renewal of its policy for receipt and settlement of private spent fuel, when established, will be published in the Federal Register for public comment.

b. Private Capability

(1) Nuclear Fuel Services, Inc. (NFS). The first private commercial reprocessing plant in the United States was completed and began operations on April 20, 1966. The plant is owned and operated by NFS, a company formed in 1962 as a joint venture by W. R. Grace & Co. (80% ownership), and American Machine & Foundry Co. (20%). About 125 metric tons of uranium (MTU) have been reprocessed by the plant during the first year of operation.

The following information summarizes pertinent facts covering this facility.

Site: West Valley, N. Y.	Technology: Aqueous
Construction: 1963-1966	Cost: $32 Million
Capacity: 300 Tons/Year	Operation: 20 Apr.66
Capability: Various Fuels	A/E: Bechtel

The process used at the NFS plant employs a mechanical (chop-leach) head end with Purex solvent extraction. The final products from the plant are uranium and plutonium in the form of nitrate solutions. To date the recovered uranium has been returned to the AEC's Feed Materials Production Center at Fernald, Ohio.

Fuels reprocessed to date at the West Valley, New York, facility consisted of: (1) New Production Reactor (NPR) fuels under the AEC/NFS base load contract, (2) Dresden 1 reactor fuel from Commonwealth Edison Company, and (3) fuel from the Rowe, Massachusetts, power reactor of the Yankee Atomic Power Company.

This first plant has so far played a key role in the national and international safeguards inspection programs. Under an AEC contract, a safeguards manual was completed by NFS which was used as guidance for the International Atomic Energy Agency's safeguards inspection of spent fuel elements from the Yankee reactor reprocessed at NFS during 1967[1]. The plant will also be one of several private nuclear facilities where AEC resident inspectors will be stationed as part of this country's safeguards program to prevent diversion of special nuclear material.

c. Other Company Programs

(1) General Electric

In November of 1966, the General Electric Company submitted an application to the AEC for a permit to construct a 300 MTU per year chemical reprocessing plant near Morris, Illinois.[2] A review of the application has been essentially completed by the AEC Regulatory staff and the Advisory Committee on Reactor Safeguards. The AEC has scheduled a public hearing for November 7, 1967, at Morris, Illinois, to consider the GE application. (See AEC Public Release K-232, dated October 2, 1967.)

Some pertinent information on the proposed plant follows:

Midwest Fuel Recovery Plant

Site: Morris, Illinois	Technology: Aquafluor
Construction: 1967-1969	Operation: 1970
Capacity: 300 Tons/Year	A/E: Fluor
Capability: Low Enriched UO_2	

The plant is expected to recover uranium, plutonium and neptunium, with the final product uranium being in the hexafluoride form. GE's proposed "Aquafluor" process makes use of aqueous and fluoride volatility fuel recovery technologies. The process uses fuel recovery technologies including fuel shearing and leaching, anion exchange, calcination, as well as the newer techniques of fluorination and uranium hexafluoride distillation.

1/ See AEC Press Release K-188, dated July 28, 1967.
2/ See AEC Public Release J-269, dated November 29, 1966.

(2) Allied Chemical Corporation

After making detailed comparisons of alternate processes and plant sizes, Allied Chemical decided last spring to design and construct a multiton reprocessing plant basically utilizing the aqueous process. Since then, work has proceeded on staffing, process optimization, and siting. Discussions on contract provisions are being held with utilities. They are directing their efforts toward a plant on-stream approximately in 1973 but are willing to consider contracts for processing loads earlier. Allied Chemical has said it hopes to announce its plant site by year-end.

(3) National Lead Company

NL has publicly announced its intentions to be a part of the commercial reprocessing industry, probably commencing in the 1973 time period. A contract was signed during the year with Saint-Gobain Techniques Nouvelles (SGN) of Paris, France, calling for preliminary process and plant design, and cost estimating studies.

(4) Atlantic Richfield

AR has expressed its intention to be a leader in commercial irradiated fuel reprocessing on the West Coast. Details concerning the process to be used and its timetable for construction and operation have not yet been decided. They have indicated that they are planning to have a plant operating in the 1970's, costing in the range of $20 million or more. Details will probably be provided after a comprehensive study of the subject has been completed, estimated to take about one and one-half years.

d. Spent Fuel Forecast

Chart I presents AEC estimates of power reactor spent fuel discharges through 1980,3/ along with the dollar market. The dollar market estimate is based on the AEC/NFS base load contract formula, which amounts to about $32,000 per metric ton of uranium for typical, low-enriched, UO_2 spent reactor fuels. All estimates are based on the AEC's

3/ See AEC Public Release IN-818, dated August 31, 1967.

recently revised estimate for nuclear power growth of approximately 150,000 Mwe of installed nuclear power capacity by 1980.

As can be seen from Chart VI-A, sufficient load does not materialize to support more than one reprocessing plant until about 1972. Although the projected reprocessing requirements of domestic nuclear power plants appeared adequate to support the West Valley plant when it was designed and built, slippages in nuclear plant schedules, cancellation of certain reactor projects and improvements in fuels technology resulting in longer life have reduced the load that would have been otherwise available during this time period.

Chart VI - A

DOMESTIC CENTRAL STATION SPENT FUEL DISCHARGE FORECAST

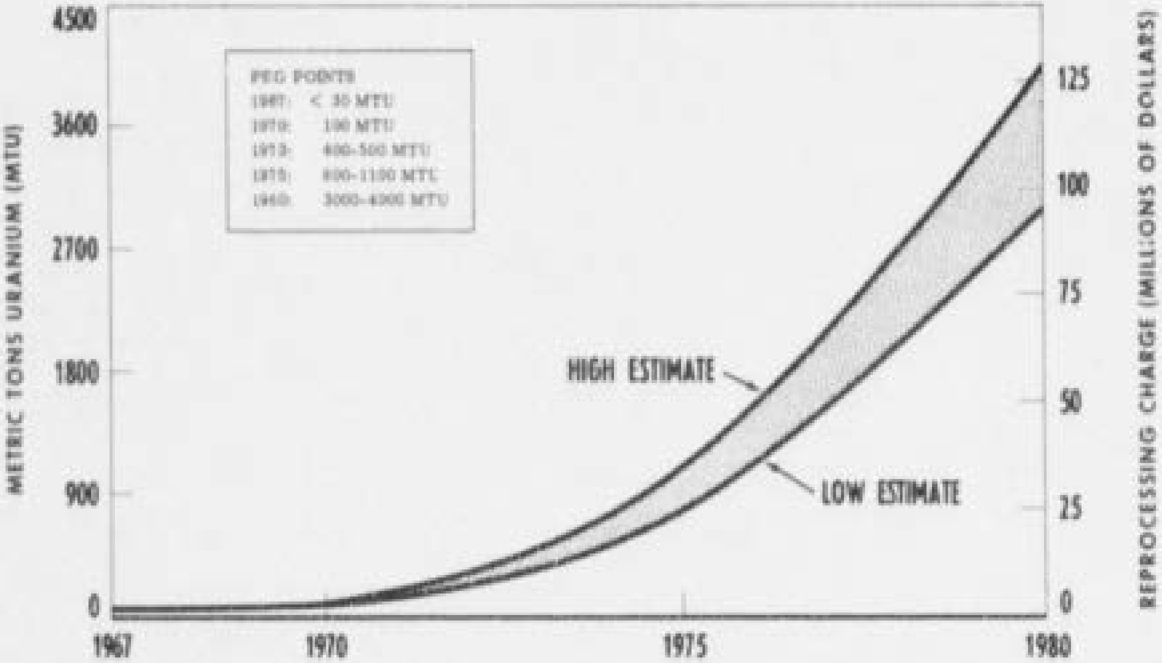

Several independent forecasts on the growth of reprocessing load were released during the year. Table VI-1 presents these projections along with the AEC forecast.

Table VI-1

Fuel Reprocessing Load Forecasts
(In metric tons uranium)

Calendar Year	AEC 8/67	NFS 5/67	GE 7/67	DUKE 5/67
1967	30	32		
1968		14		37
1969		69		78
1970	100	130	114	143
1971		214	163	320
1972		268	330	590
1973	400-500	600	676	910
1974		900	1188	1260
1975	800-1100	1200	1527	1660
1976		1350	2050	2110
1977		1600	2500	2570
1978		2000	3100	3080
1979		2300	3800	3610
1980	3000-4000	2650	4600	4190

NOTE: 1. The AEC figures are reactor discharges.
2. The NFS, GE, and Duke figures represent fuel available for reprocessing.

e. AEC Research Programs[4/]

In line with the Commission's programs to provide a technological base to afford possible reductions in the costs of nuclear power, a research and development program utilizing fluoride volatility technology for spent fuel reprocessing has been underway for some years. In this process uranium, plutonium, and fission products are separated by taking advantage of the difference in volatilities or boiling points of the fluorides of the various components of the irradiated fuel. The AEC program has included laboratory work at the Argonne National Laboratory, a hot-pilot plant program at the Oak Ridge National Laboratory, and a scale-up and evaluating program at the Oak Ridge Gaseous Diffusion Plant.

4/ AEC Public Release IN-792, dated May 26, 1967.

It appears now that the near-term commercial reprocessing plants for the fast breeder fuels will be based on aqueous technology, and the AEC is actively engaged in programs aimed at adapting aqueous processes to the more complex process of recovery and recycle of fast reactor fuels. Fast reactor fuels, if they are cooled long enough, chopped or otherwise declad in the head end, and sufficiently diluted, can be processed using more or less conventional aqueous techniques.

The work on the adaptation of advanced processes to fast breeder fuels includes head end treatments such as sodium removal, mechanical chopping, and dissolution of highly irradiated, short cooled fuels. Control of I-131 and Xe-133 is being investigated in the dissolution step and elsewhere throughout the process. Within the separations part of the process, investigations are under way on radiation damage to solvents, plutonium valence control, and solubility of fission products, to cite a few examples. High efficiency centrifugal contactors have been developed and are in use at Savannah River, and stacked cyclone contactors are under development at Oak Ridge. These contactors permit very short residence times and very low inventory of the process solutions, about 1/50 that of conventional contactors, because the mixed organic-aqueous phases are rapidly separated from each other in centrifugal fields of several hundred g's.

Also, for the longer term, there is a program on pyrochemical processing under way at Argonne National Laboratory. In this process, uranium, plutonium, and fission products are separated from each other by a preferential distribution between molten salt (magnesium chloride) phases and molten metal (magnesium-copper alloy) phases at elevated temperatures of about 1500 F. Such reprocessing systems could be extremely resistant to radiation damage. This process is in the very early stages of development and, while it appears technically feasible from a chemistry standpoint, considerable additional work is required to demonstrate its feasibility from an engineering and economic standpoint. A much simplified version of a "pyro" process has been used successfully in the recovery and refabrication of a metal alloy driver fuel from the EBR-II in Idaho.

f. <u>Chemical Reprocessing Plants</u>

Table VI-2 presents the current status of existing and announced reprocessing plants throughout the world.

Table [illegible]

Status of Chemical Rep[illegible]ng Plants

Country	Location	Operator	Size	Operation
United States	West Valley, N.Y.	NFS	300 T/Y	1966
United States	Morris, Illinois	GE	300 T/Y	1970
England	Windscale	UKEA	1500 T/Y-Nat.U<br>300 T/Y-Oxide	1966<br>1968
France	Cap de la Hague	CEA	1 T/D-Nat.U	1966
Belgium	Mol	OECD	350 Kg/D	1966
Scotland	Dounreay	UKAEA	1 Kg/D-UAL<br>1200 Kg/Y-U+Pu	1958<br>1960
Germany	Karlsruhe	GWK	35 T/Y	1969
Italy	Saluggia	EUREX	.75 Kg/D-UAL	1967
Italy	Rotondella	PCUT	15 Kg/D-UAL	1967
India	Trombay	AEET	350 Kg/D	1964
Japan	Tokai-Mura	J.A.P.C.	700 Kg/D	1971
Norway	Kjeller	Nor./Swed./Neth.	12 Kg/D	1961
Spain	Moncloa	LaJunta	6.3 Kg/Y-UAL	1966

g. Conference on Chemical Reprocessing

The Souchern Interstate Nuclear Board and the AEC Savannah River Operations Office jointly sponsored a conference on spent fuel reprocessing at Augusta, Georgia, on May 11-12, 1967. A speakers list, along with the papers presented at this conference is given below. It is suggested that interested persons contact the speakers directly for copies of the papers presented.

Conference on "Nuclear Power Fuel Reprocessing - Technology and Economics"
Augusta, Georgia
May 11 - 12, 1967

Speakers	Title
Robert H. Gifford<br>Executive Director, SINB	"Opening Remarks"
Nathaniel Stetson, Manager<br>Savannah River Operations Office	"Welcome"
Representative W. J. Bryan Dorn<br>U.S. Congress	"Introduction Address"
Representative Chet Holifield<br>Vice Chairman, JCAE<br>U.S. Congress	"Nuclear Fuel Reprocessing: Crux of National and International Policy"
Governor Robert E. McNair<br>South Carolina	"Introduction Address"
James T. Ramey, Commissioner<br>U.S. Atomic Energy Commission	"Future role of the AEC in Reprocessing" (AEC IN-787, 5/11/67)
William S. Lee, Vice President<br>Duke Power Company	"Importance to the Utilities"
Ernest B. Tremmel, Director<br>Division of Industrial Participation<br>U.S. Atomic Energy Commission	"The History of Spent Fuel Reprocessing"
George F. Quinn<br>Assistant General Manager for Plans and Production<br>U.S. Atomic Energy Commission	"AEC Conceptual Plant Policy"
Milton Shaw, Director<br>Division of Reactor Development and Technology<br>U.S. Atomic Energy Commission	"Current AEC Program"(AEC IN-792, 5/26/67)
I. A. Hobbs, Director<br>Technical & Production Division<br>Savannah River Operations Office<br>U.S. Atomic Energy Commission	"Description of Atomic Energy Commission Chemical Processing Facilities"

Speakers	Title
T. C. Runion<br>President<br>Nuclear Fuel Services, Inc.	"Current Considerations of Chemical Reprocessing"
L. R. Braun<br>Executive Vice President<br>Nuclear Fuel Services, Inc.	"Description of NFS' Facilities for the Reprocessing of Nuclear Power Fuel"
Paul H. Reinker<br>General Manager<br>Fuel Recovery Operation<br>Nuclear Energy Division<br>General Electric Company	"General Electric Commercial Reprocessing Plans"
Kenneth R. Osborn<br>Director, Nuclear Fuels Department<br>Industrial Chemicals Division<br>Allied Chemical Division<br>Allied Chemical Corporation	"Allied Chemical Corporation Nuclear Power Fuel Reprocessing Facilities and Plans"
Wallace B. Behnke, Jr.<br>Assistant to the President<br>Chairman of Nuclear Fuel Committee<br>Commonwealth Edison Company	"Nuclear Fuel Reprocessing Experience and Costs"
Eugene T. O'Sullivan<br>Vice President-Marketing<br>Isochem Incorporated<br>Richland, Washington	"Fission Products, By-Products or Waste"
W. P. Overbeck, Director<br>Savannah River Laboratory<br>E. I. du Pont de Nemours & Co.<br>Aiken, South Carolina	"Transuranium Elements as By-Products of Nuclear Fuel Reprocessing"
Robert E. Kettner<br>Assistant to the President<br>Consumers Power Company<br>Jackson, Michigan	"By-Products or Waste - A Utility Viewpoint"

3. Highly Enriched Fuels

The highly enriched spent fuel loads include those fuels used in the Space Propulsion Program (Rover), high temperature graphite reactors, naval reactors, test reactors such as

MTR/ETR/ATR, and research reactors. Both the AEC and NFS have the capability to handle selected types of these fuels. The graphite fuels (Rover and HTGR) are one type of highly enriched fuel for which no suitable processing capability now exists.

B. Unirradiated Fuel

The part of the nuclear fuel cycle starting at the mine and ending at the reactor is known as the "cold" side or unirradiated side, of the cycle. Since this material has not undergone fissioning, it can be easily handled compared to the highly radioactive material discharged from the reactor or "hot" side of the cycle.

1. Enriched Uranium Scrap Recovery

a. Commercial

The major source of enriched uranium scrap comes from the fuel fabrication activity. Since the nuclear material involved in this scrap is quite valuable its recovery is economically very desirable. Normally a large part of the scrap generated during the fabrication operation is recycled back into the system at the site. It is estimated that only a small percentage (about 1.0 to 1.5%) of the scrap cannot be recycled back into the fabrication campaign and must be treated separately by converting it to UF_6 and refeeding it to the enrichment plant (see Category II, Materials Processing and Fabrication, for additional information).

Adequate private capability exists to recover enriched uranium from all types and forms of scrap generated within both private and AEC programs. Table VI-3 indicates those firms capable of recovering uranium scrap, in addition to the principal reactor manufacturers.

TABLE VI-3

Principal Firms Recovering Uranium Scrap

General Dynamics Corporation, San Diego, California
Kerr-McGee Oil Industries, Inc., Oklahoma City, Oklahoma
Nuclear Fuel Services, Inc., Erwin, Tennessee
Nuclear Materials and Equipment Corporation, Apollo, Pennsylvania
United Nuclear Corporation, Westerly, Rhode Island

b. AEC-Industrial Program

AEC generated uranium scrap is recovered commercially as part of the fuels fabrication contract (such as the prime contract procurement for MTR, ETR, and ATR fuels), or through the Scrap Recovery Program administered by the AEC's Oak Ridge Operations Office. Since 1959 contracts totaling about $6.3 million have been awarded through the Scrap Recovery Program. Table VI-4 indicates the dollar value of scrap recovery contracts awarded to private industry since 1959.

TABLE VI-4

Dollar Value of the AEC's Commercial Uranium Scrap Recovery Program

Fiscal Years	Values
1959	$ 115,757
1960	275,223
1961	1,438,379
1962	927,501
1963	596,409
1964	566,141
1965	1,171,066
1966	802,833
1967	579,425

Table VI-5 presents information on contracts awarded to commercial firms during FY-1967 for the recovery of about 1780 kgs of uranium scrap generated in Commission programs.

Table VI-5

Uranium Scrap Reprocessing Contracts
Awarded in FY-1967

Contractor	No. Awards	Dollar Award	Scrap Value	Kgs, U
Kerr-McGee Corp.	1	$ 64,888	$ 622,000	-
Nuclear Fuel Services	5	226,995	6,356,000	-
United Nuclear	7*	287,542	8,367,000	-
Total	13	$579,425	$15,345,000	1780.4

* Includes 1 contract at no-cost to allow UNC to test its procedure for processing NERVA scrap.

As can be seen from Table VI-5, above, the value of contract awards have been steadily decreasing since 1965. It is anticipated that the value of contracts awarded through the AEC Scrap Recovery Program will tend to level off in future years because of the emphasis on having the Commission contracted fuel fabricator handle scrap recovery within its own organization or under subcontract.

CATEGORY VII
PLOWSHARE -- PEACEFUL USES OF NUCLEAR EXPLOSIVES

Probably there is no more fitting introduction to Plowshare than the following quotation from remarks by Dr. Glenn T. Seaborg, Chairman of the Atomic Energy Commission.

> "Explosives have always been used for constructive purposes as well as for military purposes and there is good evidence that the powerful force of nuclear explosions can be put to constructive use... The potential benefits of many of these applications are well worth the experimental effort which is going on now to develop them."1/

A. Introduction

Plowshare is the name given to the AEC's research and development effort to find ways to adapt and control the effects of underground nuclear explosions for peaceful purposes in such areas as excavation, natural resources development, and scientific research.

The program is currently developing the technology to apply two types of underground explosions: contained explosions which create conditions suitable for a number of natural resources and scientific applications and cratering explosions for excavation projects.

B. Budget

Table VII-1, on the following page, shows the AEC operating budget for the Plowshare Program for FY 1966 through 1968.

Table VII-2, on the following page, identifies the organizations responsible for the various areas of work included in the FY 68 Budget.

1/ Remarks by Dr. Glenn T. Seaborg, "Nuclear Energy -- A Generation of Progress", at Buenos Aires, Argentina, July 5, 1967, AEC Public Release S-32-67.

TABLE VII-1

AEC Operating Budget for Plowshare Program

	FY 1966 (Actual)	FY 1967 (Actual)	FY 1968 (Estimate)
Total Operating Program	$14,605,444	$12,852,846	$20,715,000
Research and Development	$ 6,323,906	$ 6,403,337	$ 6,625,000
Excavation Experiments	$ 6,597,014	$ 5,601,142	$11,445,000
Device Testing	3,954,188	4,030,775	5,205,000
Cratering Technology	2,642,826	1,570,367	6,240,000
Scientific Experiments	$ 1,684,524	$ 468,817	$ 350,000
Underground Engineering Experiments	- 0 -	$ 379,550	$ 2,295,000

TABLE VII-2

FY 68 Budget Estimate
(In Millions)

R&D		$6.6	Principally at LRL*
Excavation Experiments			
Cratering Equipment	6.2		
Device Testing	5.2		
Total Excavation Experiments		11.4	By LRL at NTS* - In Support of Inter-oceanic Canal Studies
Scientific Experiments		0.4	Principally at LASL*
Underground Engineering Experiments		1.5	Gas, Oil & Mineral Applications by LRL
TOTAL		$20.7	

*LRL - Lawrence Radiation Laboratory
NTS - Nevada Test Site
LASL- Los Alamos Scientific Laboratory

C. Industrial Participation

Project Gasbuggy -- the first Government-industry joint experiment to investigate the use of a nuclear explosion for an industrial application -- the stimulating of natural gas fields of low productivity is scheduled for the end of 1967. In addition, three Government-industry feasibility studies on gas storage, in-situ copper recovery, and oil shale development were completed and published. These and previous studies resulted in proposals to the AEC from industrial organizations for joint experiments to follow Project Gasbuggy.

A Plowshare committee was formed during the year by the Atomic Industrial Forum to keep that organization abreast of current developments in the Plowshare Program. In addition, a number of insurance companies[2/] have been looking into the Plowshare Program from the standpoint of potential insurance considerations in future Plowshare projects.

D. Natural Resources Projects

It is extremely interesting to note the high degree of industry interest in the various Plowshare projects. The Commission is currently cooperating with industry in the following projects:

1. Project Gasbuggy

The Gasbuggy experiment grew out of an 18-month feasibility study of the gas stimulation application conducted by the El Paso Natural Gas Company (EPNG), the U.S. Bureau of Mines (Bumines), the Lawrence Radiation Laboratory at Livermore, California (LRL-L), and the AEC's San Francisco Operations Office (SAN). A contract to conduct a joint experiment was signed by EPNG, the Department of the Interior, and the AEC on January 31, 1967. Areas of responsibility were assigned by the contract on a functional basis: no exchange of funds between the parties was involved.

Project Gasbuggy will involve the detonation of a 26-kiloton nuclear explosive 4,200'deep in the Lewis Shale Formation of the San Juan Basin of New Mexico, approximately 55 air miles east of Farmington, New Mexico. There are four primary objectives of the Gasbuggy experiment:

2/ Nuclear Energy Liability Insurance Association and Mutual Atomic Energy Liability Underwriters.

[illegible]

1. to measure the gas production changes caused by nuclear explosions and to determine the mechanisms responsible for these changes,

2. to determine the gas quality in regard to radioactivity,

3. to develop the technical engineering knowledge to use nuclear explosions to stimulate natural gas fields, and

4. to refine existing predictive capability for seismic effects on gas field equipment and other structures.

Dr. Walter R. Hibbard, Director, U.S. Bureau of Mines, in commenting on the gas stimulation application in a letter to the Atomic Energy Commission, dated December 22, 1965, said:

> "If nuclear explosives can be used to stimulate production by fracturing rock in * * * deep, thick, low-productivity reservoirs, as our engineers are convinced, significant increases in recovery can be effected. We estimate that, in Rocky Mountain reservoirs alone, the total increase in production may be as great as 317 trillion cubic feet. This is a greater quantity of natural gas than our present proved reserves of 281 trillion cubic feet"

TABLE VII-3

Project Gasbuggy
Tota. Estimated Cost (In Thousands)

	AEC	EPNG	TOTAL
Field Construction	$1,135	$ 960	$2,095
Technical Support	630	50	680
Logistical Support	385	250	635
Maintenance and Operations	125	90	215
Technical Program	500	575	1,075
Total	$2,775	$1,925	$4,700

The State of New Mexico also contributed to this project by providing an all-weather road from State Highway 17 to the Gasbuggy site.

Chart VII - A

APPLICATIONS OF DEEPLY BURIED NUCLEAR EXPLOSIONS

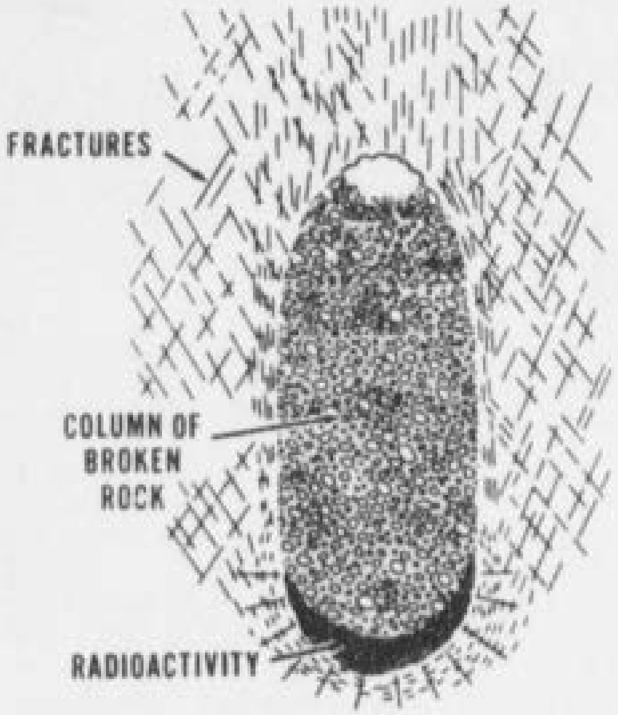

- TERMINAL GAS STORAGE
- WASTE DISPOSAL
- RECOVERY OF GEOTHERMAL HEAT
- GAS RESERVOIR STIMULATION
- LEACHING OF MINERAL ORE
- BREAKAGE OF ORE FOR MINING
- WATER CONSERVATION

2. Follow-on Gas Stimulation Experiments

Two follow-on experiments to Gasbuggy, Projects Dragon Trail and Rulison, have been proposed to the AEC.

Project Dragon Trail was proposed jointly by the Continental Oil Company and CER Geonuclear Corporation. The proposed experiment would extend data received from the Gasbuggy experiment to a gas-bearing formation with different geological characteristics.

Project Rulison was proposed by the Austral Oil Company in conjunction with CER Geonuclear Corporation. The experiment would extend data to gas-bearing formations at deeper depths.

Both the Dragon Trail and Rulison proposed sites are located near the Piceance Creek area of western Colorado.

3. Project Ketch

The Columbia Gas System Service Corporation, Bumines, LRL-L, and SAN completed and published the Ketch feasibility study (PNE-1200) in August of this year. The study concluded that:

> "Nuclear explosives offer the potential for creating underground reservoirs for storing natural gas. This potential if realized could lead to the expansion of the gas industry in areas in which inexpensive underground storage is not now available and continued growth in areas where natural storage is available, but growing scarce."

On August 28, the AEC received a proposal from Columbia Gas to conduct jointly an experiment to assess the technical and economic feasibility of this proposed application. A site located in the Sproul State Forest in north central Pennsylvania is currently being evaluated for the proposed Ketch experiment.

4. Project Sloop

In October of this year, Kennecott Copper Corporation, Bumines, LRL-L, and SAN completed and published the Sloop study which investigated the feasibility of using nuclear explosives to fracture a low-grade copper ore body preparatory to recovery of the copper by an in-situ leaching process. The study concluded that:

> "The development of an operable process using this low cost recovery method would allow the immediate exploitation of large, marginal low-grade deposits at production cost that would be completely competitive with the copper now being produced conventionally from higher-grade ores."

Upon the completion of the feasibility study, Kennecott submitted a proposal to the AEC to conduct a joint experiment to test this application. The AEC is currently evaluating this proposal.

5. Project Bronco

On January 27, 1967, Secretary of Interior Stewart L. Udall announced a Five Point Oil Shale Development Program, point four of which was to cooperate with the AEC in an investigation of the

use of underground nuclear explosions to fracture deep deposits of oil shale for subsequent recovery of the oil content of the shale by an in-situ retorting process. In accord with this policy, the Department of Interior's Bureau of Mines participated in the Bronco feasibility study with CER Geonuclear Corporation (representing 20 oil companies), LRL-L, and SAN. The study was completed in the fall of 1967 and an experiment to further assess the technical and economic feasibility of this application has been recommended.

E. Excavation

The excavation program is currently directed at providing the Atlantic-Pacific Interoceanic Canal Study Commission with the necessary technical data to reach a decision regarding the feasibility of using nuclear explosives to construct a sea-level canal across the American Isthmus. Project Cabriolet, a 2.5 kiloton nuclear cratering experiment in hard, dry rhyolite at the Nevada Test Site, is scheduled for the fall of 1967 as one of a series of nuclear cratering experiments to develop this technology. The objectives of the test are twofold:

1. to obtain data on cratering mechanisms and final crater geometry necessary to refine and verify existing predicative capability, and

2. to study radioactivity patterns in order to refine existing fallout models and atmospheric diffusion predictions of airborne concentration.

Chart VII-B

TRANSISTHMIAN CANAL

CONVERT EXISTING CANAL TO SEA LEVEL ROUTE	← EACH → ABOUT 50 MILES	SASARDI - MORTI ROUTE
CONVENTIONAL EXCAVATION		NUCLEAR EXCAVATION
$ 2.3 BILLION	ESTIMATED COST	$ 0.8 BILLION
10 YEARS	EXCAVATION TIME	6 YEARS
600' WIDE 60' DEEP	CHANNEL SIZE	1000' WIDE 250' DEEP
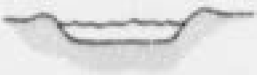	PROFILE	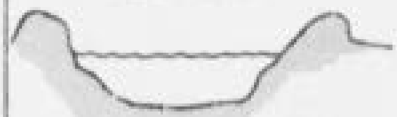

F. Scientific Research

For several years, the Los Alamos Scientific Laboratory has been developing techniques and equipment to conduct neutron-physics experiments, particularly neutron-fission and cross-section measurements, using nuclear explosions as the source of neutrons. A number of such measurements pertinent to reactor development were carried out on February 27, 1967, in connection with the "Persimmon" event, an underground nuclear device test at the Nevada Test Site. The Plowshare Program funded two of these experiments, one involved a highly radioactive sample and the other a very small sample. The former involved a cross-section measurement of promethium-147, a reactor-produced isotope which acts as a reactor poison by absorbing the neutrons which propogate the chain reaction. The Pm-147 measurement marked the first time that the nuclear characteristics of a short-lived, intensely radioactive substance were measured.

G. Charges for Nuclear Explosives

In order to aid industry in developing cost estimates for proposed projects, the AEC published in May of 1964 projected charges for thermonuclear explosives, which are expected to be particularly relevant for excavation applications. The announced charges range from a charge of about $350,000 for a 10 kiloton explosive to $600,000 for a 2 megaton explosive. Arming and firing services are included in these charges, as well as the cost of the explosive. Charges for related services and safety studies are not included.

Estimates of charges for explosives suitable for contained applications will be issued as soon as the technical information on which to base these estimates is developed. In fact, one of the objectives of the Gasbuggy experiment is to determine some of the explosive design constraints in the gas stimulation application relative to radioactivity.

H. Program Information

The AEC is not authorized, at present, to supply nuclear explosive services on a commercial basis; however, it can enter into research and development arrangements with respect to peaceful uses of such explosions, including demonstration of a particular application.

Companies interested in discussing such joint projects may direct their inquiries to the Division of Industrial Participation or to Mr. John S. Kelly, Director, Division of Peaceful Nuclear Explosives, U.S. Atomic Energy Commission, Washington, D. C. 20545.

More detailed information concerning the Plowshare Program may be found in:

1. "Plowshare Program During 1966", for sale by the Superintendent of Documents, U.S. Government Printing Office, Washington, D. C. 20402, at $0.15;

2. "Plowshare," a booklet in AEC's "Understanding the Atom" series. Single copies are available free from the U.S. Atomic Energy Commission, P. O. Box 62, Oak Ridge, Tennessee 37830; and

3. Plowshare Biography, TID-3522 (8th Revision), available from the Clearinghouse for Federal, Scientific and Technical Information, National Bureau of Standards, U.S. Department of Commerce, Springfield, Virginia 22151, price $1.25.

In addition, a series of reports (listed below) on the economics of Plowshare may be obtained from the Federal Clearinghouse:

1. Morgenstern, P., Heiss, K. P., "General Report on the Economics of the Peaceful Uses of Underground Nuclear Explosives," August 31, 1967;

2. Heiss, K. P., "Technical and Economic Potential of Gas Stimulation by Nuclear Explosives," August 31, 1967;

3. Heiss, K. P., "Technical and Economic Potentials of Shale Oil Production by Nuclear Explosives," August 31, 1967;

4. Whiten, T., Heiss, K. P., and Cohen, G. D., "The Economic Feasibility of Mining Operations Using Nuclear Explosives with Particular Reference to Copper," August 31, 1967; and

5. Sand, F., Cohen, G. D., "Three Studies of the Use of Nuclear Explosives in the Areas of Water Resource Development, Storage and Waste Disposal," August 31, 1967.

Two films describing the program are also available through AEC film libraries. They are entitled "Plowshare" and "Safety in the Plowshare Program."

CATEGORY VIII
SERVICES

This category includes a wide range of nuclear service type activities, many of which are performed by organizations with interests outside the nuclear energy field.

Included in this category this year are seven major nuclear service areas: waste management, film badge processing, contaminated laundry, transportation, whole body counting, reactor servicing, and decontamination.

A. Radioactive Waste Management

Market Predictions

1970	$1,250,000
1975	$3,500,000
1980	$6,600,000

Radioactive wastes are generated at various different steps in the nuclear fuel cycle and accumulate as liquids, solids, and gases at varying radiation levels. Wastes generated in the cold or pre-irradiation phase of the fuel cycle (from the mine to the reactor), as well as wastes resulting from research laboratories and from medical and industrial applications of radioisotopes, are generally considered as low level, low hazard potential wastes. In terms of radioactivity concentration, the liquid wastes from these sources are normally in the range of millionths of a curie per gallon. Low level solid wastes are normally defined as those wastes reading less than 10 curies per cubic foot.

High level wastes (wastes having high radioactivity concentration) are generated almost entirely in connection with the chemical reprocessing of irradiated fuels. These wastes are presently stored as liquids in large underground tanks; however, there has been increasing emphasis on research and development programs aimed at converting and reducing these high level liquids to solid form. High level wastes are normally defined as wastes exceeding 1000 curies per cubic foot, but, in any case, they are of such penetrating radiation level or have such contamination potential that they require complex handling and packaging techniques.

The Atomic Energy Commission has the responsibility for controlling the handling, transporting, and disposal of this radioactive waste material. The Commission has the authority, under subsection 274b of the Atomic Energy Act of 1954, as amended, to enter into agreements with individual states for the transfer of certain of the Commission's regulatory responsibilities. Among these responsibilities are those relating to the disposal of low and high level wastes within a state. The Commission has, thus far, entered into agreements with 17 states (Chart VIII-A). A map showing the location of commercial radioactive waste burial sites is presented in Chart VIII-B, on the following page.

A general requirement for establishment of a land burial site is that the land be owned by the Federal or state Government. The AEC's Rules and Regulations, 10 CFR Part 20, spell out in detail the standards established by the Commission in the control of waste disposal.

TRANSFER OF MATERIALS LICENSING FUNCTIONS TO STATES

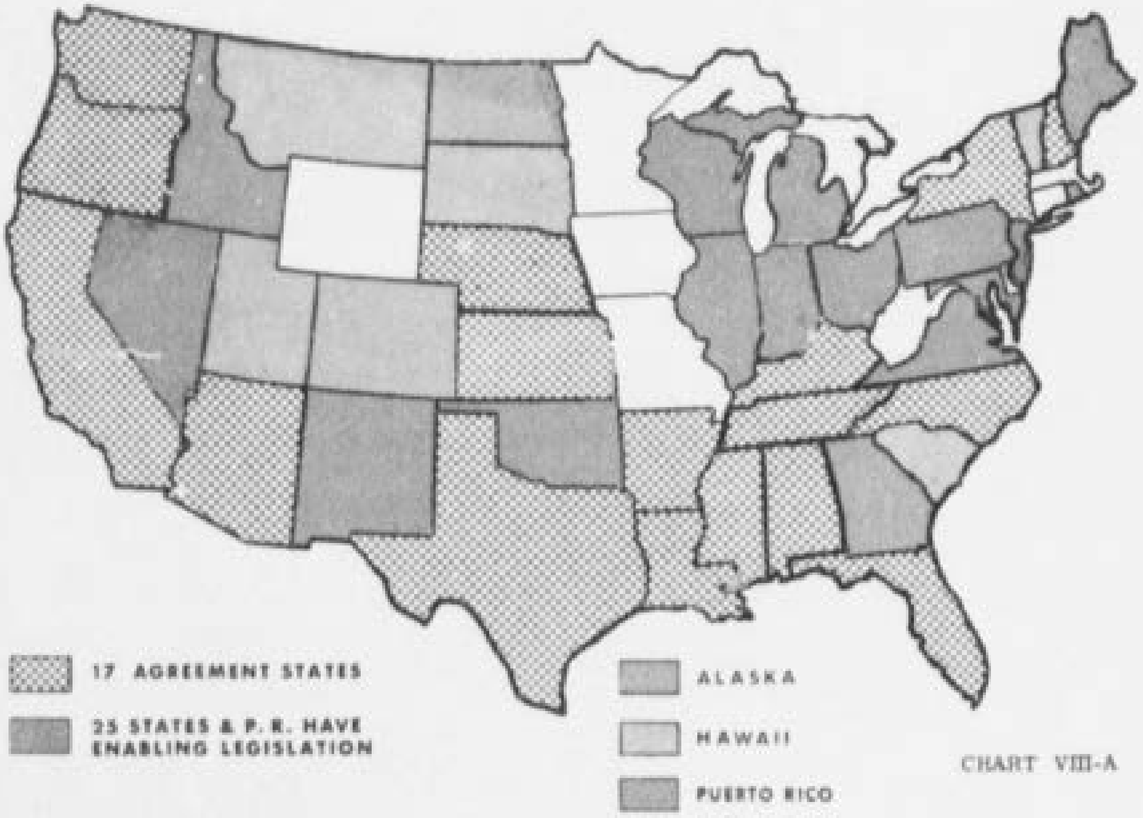

CHART VIII-A

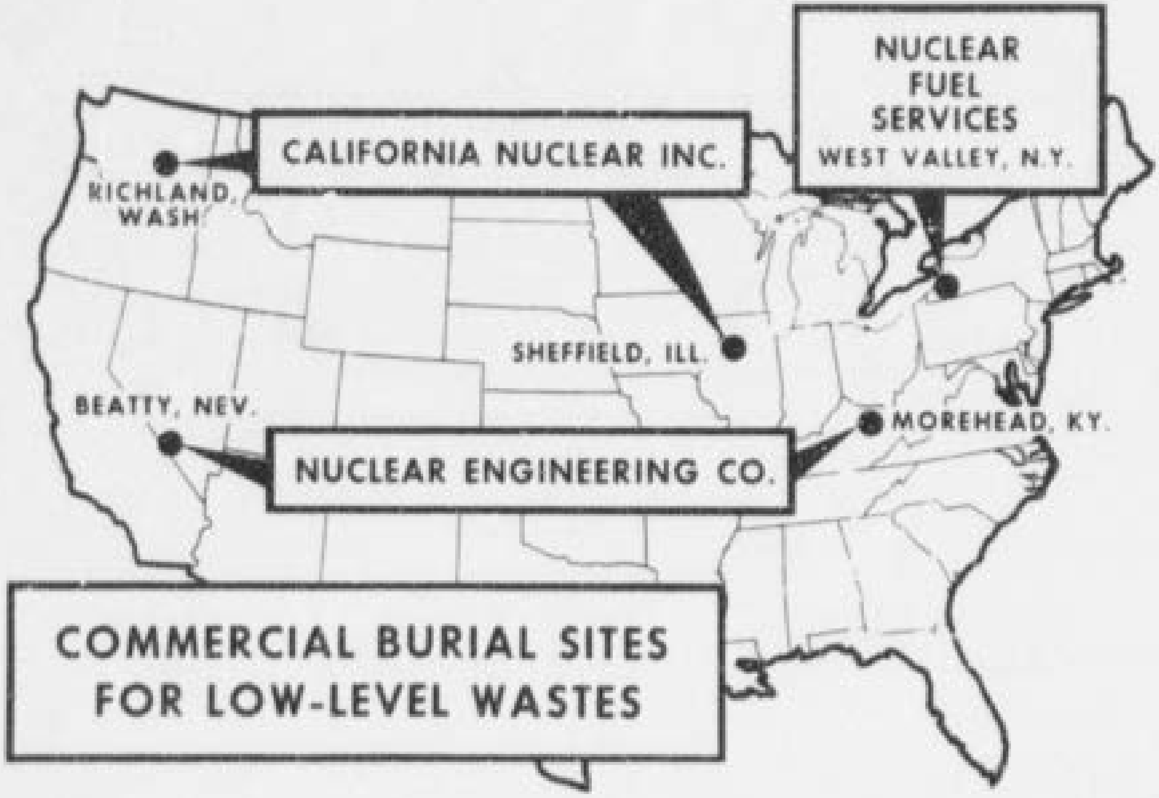

Chart VIII-B

1. Low Level Waste Disposal

On May 14, 1963, the Commission approved withdrawal of the AEC from its interim low level waste burial operations provided to licensees, based upon the availability of commercial service from Nuclear Engineering Company (NECO) burial sites at Beatty, Nevada, and Morehead, Kentucky. In November of 1963, Nuclear Fuel Services, Inc., (NFS) established a low level waste disposal facility near West Valley, New York.

A new waste burial site was recently established by California Nuclear, Inc. (CNI) on state-owned land near Sheffield, Illinois. The AEC issued an amendment to an existing CNI waste handling license, permitting them to begin operations on August 1, 1967.[1/] CNI also operates a waste disposal site at Richland, Washington.

1/ See AEC Public Release K-176, dated July 14, 1967.

Chart VIII-B indicates the three commercial radioactive waste disposal companies in operation along with the location of the five burial sites in the United States.

Table VIII-1 shows, by semi-annual breakdown, the quantities of low level commercial waste buried since October 1962.

TABLE VIII-1

Private Waste Burial (Cubic Feet)

	Jan.-June	July-Dec.	Annual Total
1962		36,281	36,281
1963	120,069	95,821	215,890
1964	241,660	205,434	447,094
1965	258,997	234,480	493,477
1966	259,419	238,172	497,591
1967	380,584		

Burial charges have varied over the last few years from a high of $1.50 to a low of about $.70 per cubic foot of waste buried. Based on an estimated current average disposal charge of about $1.00 per cubic foot, Table VIII-1 can be used as a reasonable indication of the dollar market for this service by substitution of dollars for cubic feet. Chart VIII-C presents a typical breakdown of rates and charges offered by a commercial waste disposal company.

Another facet of the radioactive waste business, in addition to burial itself, is the operation of collection, transportation, and storage services. Companies licensed by the AEC in the handling of radioactive wastes are listed in Table VIII-2, on the following page.

As mentioned earlier in this section, the AEC has transferred many of its regulatory responsibilities to "Agreement States". Table VIII-3 lists those private firms licensed by agreement states to receive radioactive waste.

The burial of AEC solid radioactive waste at AEC burial sites has been proceeding at a fairly constant rate of about 1.5 million cubic feet per year since the Commission's withdrawal of waste burial services to licensees in 1963. This volume of wastes requires about 20 to 25 acres of land per year. Table VIII-4 gives a fiscal year breakdown of solid waste burial at AEC facilities.

CHART VIII-C

SCHEDULE OF RATES AND CHARGES

The following is a published list of rates and fees Company "X" will charge users F. O. B. the "X" Burial Facility. Waste will be received from all sources on a prior booking arrangement.

All shipments must be in accordance with AEC, Bureau of Explosives, Government Bill of Lading and/or ICC regulations. All disposable containers must be self-supporting and capable of remaining intact during handling under normal weather conditions prior to burial. All containers that do not comply with the packaging and labeling requirements of Company "X", must be negotiated separately. Shipper must furnish statement to Company "X" disclaiming any right to title of wastes after the acceptance by Company "X". The following statement has been found acceptable by the Federal Government:

> "Title to all materials, which were previously Government-owned, contained in waste furnished for disposal under this contract (except material which is required by Section 52 of the Atomic Energy Act of 1954, as amended, to be owned by the Government) shall be deemed to be vested in Company "X" immediately upon acceptance of such material by Company "X" from the shipper or from the common carrier. The Government shall have no right to the recovery of any materials contained in such waste nor any credit for their potential value."

Waste shipments will be accepted at the "X" Burial Facility during the hours of 0830 to 1600 on week days, legal holidays excepted. Emergency shipments will be received at other times, however extra charges for such receipt may be added. Each truck or railroad car will normally be unloaded before the end of the next succeeding normal business day. However, an effort will be made to unload all trucks on the day of arrival. Company "X" will not take responsibility for demurrage on unscheduled shipments.

All shipments must be accompanied by completed "Radioactive Shipment Record Form". These forms are available from Company "X". Shipments received without these forms may be delayed as the information must be available prior to acceptance of the wastes at the "X" Burial Facility.

Company "X" will make the following charges for operation of the "X" Burial Facility:

> Unload and bury containers approved by the AEC, ICC, or Bureau of Explosives, or containers shipped under special ICC permit, which are less than 15 tons total weight per container -- $0.70 per cubic foot plus state charge (which presently is) -- $0.05 per cubic foot.

Surcharges as follows will be added for containers in excess of fifteen tons:

Weight, Tons	Surcharges Per Shipment	Per Container
0 - 15	0.00 plus	0.00
15 - 30	130.00 plus	200.00
30 - 50	260.00 plus	330.00
50 - 60	520.00 plus	475.00
60 - 80	1,600.00 plus	1,200.00
80 - 130	3,200.00 plus	2,500.00

Surcharges will be made for inner packaging from shielded containers as follows:

> Density over 200 pounds per cubic foot -- $0.005 per pound for total weight -- over 200 pounds per cubic foot.

Lower charges will be made for very large volumes delivered to the facility by a single organization in any single calendar year as follows:

Cubic Feet	Cost per Cubic Foot*
1 to 100,000	$0.700
100,000 to 200,000	0.675
200,000 to 300,000	0.650
Over 300,000	0.625

*Plus state charges

Surcharges as follows will be added for special handling of containers consisting of two or more parts. This is for removing and burying inner containers which have been shipped inside a shielded coffin, cask or container:

Primary Containers with Surface Dose Rates	$ Per Shipment		$ Per Hour**
0.2 to 10 r/hr	$ 25.00	plus	$26.00
10 to 50 r/hr	50.00	plus	26.00
50 to 100 r/hr	100.00	plus	26.00
100 to 500 r/hr	250.00	plus	26.00

**The $26.00 per hour referred to includes consulting and preparation of proper procedures as well as the additional equipment, time and labor used to handle this equipment.

The minimum charge for any shipment will be $20.00.

TABLE VIII-2

Commercial Firms Licensed by the U.S. Atomic Energy Commission to Receive Radioactive Wastes (As of April 12, 1967)

ATCOR, Inc.
360 Bradhurst Avenue
Hawthorne, New York 10532

Laboratory for Electronics, Inc.
Tracerlab Division
1601 Trapelo Road
Waltham, Massachusetts 02154

Nuclear Diagnostic Laboratories, Inc.
1000 Lower South Street
Peekskill, New York 10566

The Walker Trucking Company
1283-1285 East Street
New Britain, Connecticut 06053

California Salvage Company
700-745 North Pacific Avenue
San Pedro, California 90731

Long Island Nuclear Service Corp.
Station Road
Bellport, New York 11713

Nuclear Engineering Company, Inc.
Box 594
Walnut Creek, California 93002

Radiological Service Company, Inc.
35 Urban Avenue
Westbury, New York 11590

California Nuclear, Inc.
2323 South Ninth Street
Lafayette, Indiana 47905

Atomic Disposal Company, Inc.
14532 South Kedzie Avenue
Midlothian, Illinois 60445

These firms should be contacted directly for information regarding services they can provide.

TABLE VIII-3

Commercial Firms Licensed by Agreement States To Receive Radioactive Wastes (As of June 6, 1967)

Atomic Energy Industrial Laboratories of the Southwest
6413 South Main Street
Houston, Texas 77025

Laboratory for Electronics
Tracerlab Division
2030 Wright Avenue
Richmond, California 94804

California Nuclear, Inc.
P. O. Box 59
Cornell, California 91315

U.S. Nuclear Corporation
801 North Lake Street
Burbank, California 91502

General Nuclear, Inc.
6740 Telean
Houston, Texas 77034

William Wayne Electronics, Inc.
Hastings Radiochemical Works
P. O. Box 60448
Houston, Texas 77060

McCormack's Highway Transportation, Inc.
151 Erie Boulevard
Schenectady, New York 12305

Nuclear Engineering Company, Inc.
Box 146
Barns Building
Morehead, Kentucky 40351

Nuclear Fuel Services, Inc.
P. O. Box 124
West Valley, New York 14171

These firms should be contacted directly for information regarding services they can provide.

TABLE VIII-4

AEC Solid Radioactive Waste Burials
(Cubic Feet)

Fiscal Year*	At AEC Burial Grounds: From AEC and AEC Contractor Operations	At AEC Burial Grounds: From Other Government Agencies**	From Licensees**	Total
1961	2,892,600	20,600	74,400	2,987,600
1962	2,268,200	21,800	68,900	2,358,900
1963	1,698,900	24,500	77,700	1,801,100
1964	1.697,400	2,700***	15,300***	1,715,400
1965	1,454,300			1,454,300
1966	1,413,000			1,413,000
July-Dec. 1966	775,000			775,000

* Fiscal Year is from July 1 to June 30.
** Buried at Oak Ridge and National Reactor Testing Station (Idaho) under AEC Interim Burial Program.
***Buried during period of July/August, 1963.

The volumes of low level solid wastes available for commercial burial are estimated to reach about 1 million cubic feet by 1970; 2 million cubic feet by 1975; and 3 million cubic feet by 1980.

2. Reactor Resin Waste

In a typical water-cooled power reactor, traces of impurities are carried along with the coolant and irradiated as they pass through the core of the reactor. These radioactive products, such as iron, nickel, and other corrosion products, are removed from the system in ion-exchange columns. The radioactive resins from these columns are periodically removed for burial at commercial waste disposal sites. Table VIII-5, on the following page, is an estimate of the future reactor

resin waste market, based on the AEC's recent "most likely" forecast of about 145,000 Mwe of installed nuclear capacity in the year 1980 and a factor of 500[2/] cubic feet of reactor resins discharged per year per 1000 Mwe. The cost column assumes handling and burial charges of about $50.00 per cubic foot.

TABLE VIII-5

Reactor Resin Waste Handling and Burial Forecast

Year	Cubic Feet	Dollars
1970	5,000	$ 250,000
1975	30,000	$1,500,000
1980	73,000	$3,600,000

3. High Level Waste Disposal

a. Commercial

Radioactive liquid waste effluents from chemical reprocessing plants contain essentially all of the radionuclides generated within the nuclear cycle not recovered as by-products. The commercial reprocessing operations started last year at West Valley, and the operation of additional reprocessing plants in the near future, will generate wastes in ever-increasing quantities. Present estimates indicate an accumulated volume of wastes from the reprocessing of nuclear power fuels of less than 4 million gallons by 1980, and 40 million gallons (equivalent to a cube 180 feet on back side) by the year 2000. There is no reason to believe, however, that these quantities cannot be handled and controlled safely.

b. Method of Handling

Principally, there are two types of high level wastes stored: "Self-boiling" and "Non-boiling". The difference depends on the relative fission product concentration of the

2/ Remarks by Dr. Stuart McLain, Purdue University, "Disposal of Low Level Radioactive Wastes", at Argonne National Laboratory to the Fuel Management Seminar, August 30, 1967.

waste solution. In either case, the primary concern is to assure the containment and control of the material involved. Within Government operations, these wastes are being stored in special underground tanks which range in size from about 300,000 to 1,330,000 gallons of capacity. At West Valley, high level wastes are stored in large, concrete-enclosed, carbon steel tanks (600,000 gallons design capacity) located in close proximity to the separations plant.

c. AEC Experience in Waste Handling

Significant amounts of radioactive waste solutions are generated and stored at the AEC's Hanford, Idaho Falls, and Savannah River sites. Over 65 million gallons of high level waste have been satisfactorily handled to date, mainly as a result of chemical processing operations on production reactor fuels.[3/] It can be concluded, based on AEC experience, that: (1) radioactive waste management practices have not resulted in any harmful effects on the public, its environment, or its resources; and (2) the general problem of radioactive wastes need not retard the future development of the nuclear energy industry.

4. Research and Development

The AEC has had a research and development program in connection with reprocessing operations at its production and test sites ever since the first fuel from production reactors was processed. This research and development program has provided considerable information that is applicable to the handling of high level wastes. The conversion of large-volume, high level, liquid wastes to more compact and easier handled inert solid forms has received considerable R&D emphasis. Several liquid/solid conversion approaches are under study, including such processes as fluidized bed, heated pots, spray solidification, and phosphate glass fixation. It is hoped that techniques can be developed by the year 2000 such that the total accumulated volumes can be reduced from the 180 foot cube previously mentioned to the equivalent of a 60 foot cube. This would amount to a reduction factor of 27, as compared to the volume required for total liquid storage.

3/ "The 1967 Supplement To The 1962 Report To The President on Civilian Nuclear Power."

Storage of high level liquid wastes in geological formations, such as salt mines, has also been under investigation. A two-year demonstration program with radioactive material in a salt mine near Lyons, Kansas, will be completed in 1968. To date this demonstration program has not revealed any major obstacles to ultimate disposal of highly radioactive wastes in salt.

In December 1963, the Waste Calcination Facility (WCF) in Idaho became the world's first plant-scale facility for converting high level waste to solid form. The Waste Solidification Engineering Prototype Plant (WSEP) at Pacific Northwest Laboratory is another waste management facility. One of its major objectives is to demonstrate, on an engineering scale, the conversion of power reactor reprocessing waste to solid form. This demonstration plant started operations in the fall of 1966.

B. Film Badge Processing

In order to detect and control exposure to ionizing radiation by personnel working in or around potential source areas, external dosimetry devices are utilized. The most common external personnel monitoring device is the film badge. There are increasing indications, however, that Thermoluminescent Dosimetry (TLD) will be a major competitor to the film badge in the near future.

Anyone working with x-ray equipment or radioactive materials is a potential customer for this service. Research laboratories, nuclear industrial plants, hospitals, physicians' and dentists' offices, and institutes of higher education are some of the more common users of this service.

In 1963, we reported that there were from 2-1/2 to 3 million film badges processed commercially at a dollar volume of about $1-1/4 million. In the same year, about 1-3/4 million badges were processed at AEC sites representing a dollar volume of about $1 million. No new survey has been made since 1963; however, we expect the volume of business from private sources to increase in step with the growth of nuclear power.

Private companies are well represented in this phase of the nuclear industry with approximately 18 companies offering this service (Table VIII-6). Chart VIII-D shows the principal film badge operations in the United States.

TABLE VIII-6

Film Badge Processors

Applied Health Physics, Inc.
Bethel Park, Pennsylvania

Atomic Energy Industrial Laboratories
Houston, Texas

Controls for Radiation, Inc.
Cambridge, Massachusetts

Eberline Instrument Corporation
Santa Fe, New Mexico

T. M. Gaines Company
Berkeley, California

Gard-Ray Film Badge Service
Burlington, Massachusetts

Health Physics Services, Inc.
Baltimore, Maryland

R. S. Landauer, Jr., and Company
Matteson, Illinois

Nuclear-Chicago Corporation
Des Plaines, Illinois

Nuclear Consultants Corporation
St. Louis, Missouri

Nucleonic Corporation of America
Brooklyn, New York

Nuclear Services Laboratory
Knoxville, Tennessee

Radiation Detection Company
Mountain View, California

Tracerlab
Waltham, Massachusetts

St. John X-Ray Laboratory
Califon, New Jersey

Technical Associates
Burbank, California

United States Testing Company
Hoboken, New Jersey

PRINCIPAL FILM BADGE OPERATIONS IN THE UNITED STATES

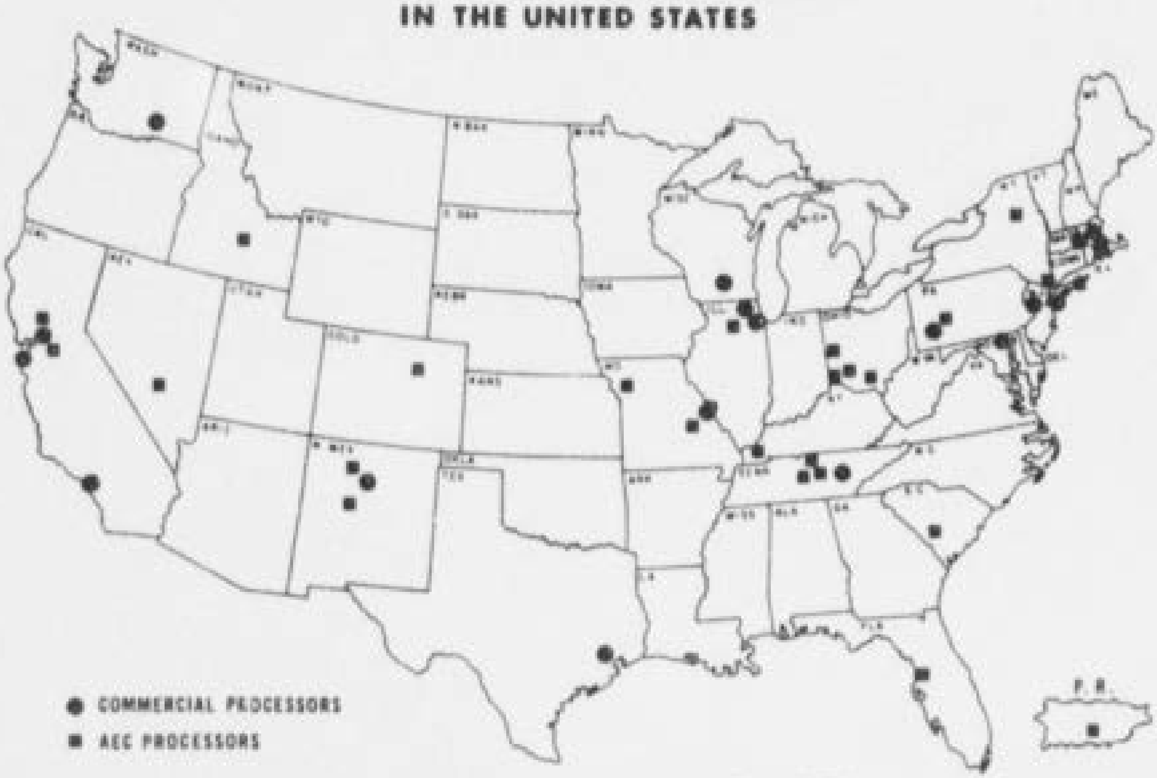

Chart VIII-D

Most of the companies noted in Table VIII-6 offer a complete line of service including film and film holder supply, exchange badges, processing and interpretation, and reporting. The processing procedures employed by various companies range from those consisting of many individually controlled steps to those of a highly automated nature.

The price charged for this service depends on the type of badge (beta-gamma or neutron), the size of order, and the exchange period (weekly, bi-weekly, monthly, etc.).

As mentioned earlier, there has been a growing use of, and interest in, thermoluminescent dosimetry devices (TLD) in both Government and private installations. Many of the film badge processing companies are offering the sale and service of these re-usable dosimeters and the reading instrument. In operation, the working material becomes excited to a higher energy state when exposed in an ionizing radiation environment. The reading instrument heats the TLD which then releases its excess energy in the form of light. The intensity of the emitted light is proportional to the cumulative dose, so that an exposure measurement can be determined.

As reported last year, the Commission established a program at its Pacific Northwest Laboratory aimed at the development of performance criteria and procedures for the evaluation of film dosimetry services.4/ This study was to also consider ways and means by which any evaluation laboratory might be appraised. The program was essentially completed by mid-summer, and final reports should be available to interested persons prior to the end of this year. Information on this program can be obtained from Dr. S. Allan Lough, Division of Biology and Medicine, U.S. Atomic Energy Commission, Washington, D. C. 20545.

The National Sanitation Foundation's (NSF) film dosimetry and calibration laboratory has been in official operation for over a year. NSF has extended the right to use its "Seal of Approval" to seven film badge processing companies and two national laboratories, effective July 1, 1967 (see Table VIII-7). The NSF Seal of Approval indicates certification that a film badge service has satisfactorily passed the requirements of Standard No. 16 established by the Foundation.

The NSF program is the first periodic evaluation of film badge services to be announced in this country. The Standard took three years to develop, and was supported with technical and financial

4/ See AEC Public Release IN-616, dated September 9, 1965.

contributions from many different organizations interested in radiation dosimetry with film badges. A research program which provided basic data utilized in the development of the Standard was supported by the U.S. Public Health Service. Information regarding this program can be obtained from Mr. Charles A. Farish, Executive Director, National Sanitation Foundation, University of Michigan, School of Public Health, Ann Arbor, Michigan 48103.

TABLE VIII-7

National Sanitation Foundation Testing Laboratory, Inc.
1967
Approved - Listing
for
Film Badge Services
NSF Standard No. 16

Eberline Instrument Corporation
P. O. Box 2108
Santa Fe, New Mexico 87501

Gard-RayFilm Badge Service
P. O. Box 117
Burlington, Massachusetts 01803

R. S. Landauer Jr., & Company
3920 - 216th Street
Matteson, Illinois 60443

Nuclear-Chicago Corporation
333 East Howard Avenue
Des Plaines, Illinois 60018

Nucleonic Corporation of America
196 Degraw Street
Brooklyn, New York 11231

Radiation Detection Company
385 Logue Avenue
Mountain View, California 94042

Tracerlab
Film Badge Department
1601 Trapelo Road
Waltham, Massachusetts 02154

U.S. Air Force Radiological
Health Laboratory
Wright Patterson Air Force Base, Ohio 45433

U.S. Atomic Energy Commission
Idaho Operations Office
P.O. Box 2108
Idaho Falls, Idaho 83401

C. Contaminated Laundry

Nine companies are actively engaged in the nuclear laundry business (see Table VIII-8) and, with the exception of NUMEC, are operated as part of a larger industrial non-nuclear laundering complex. In many cases, both the contaminated and the industrial operations are housed under the same roof, separated by partition and by independent system arrangement.

TABLE VIII-8

Commercial Licensees

Companies Actively Processing Contaminated Laundry

1. Interstate Industrial Laundry Service, Inc.
   Santa Fe, New Mexico
2. Interstate Industrial Laundry Service, Inc.
   Pleasanton, California
3. Interstate Industrial Uniform Service, Inc.
   Indian Orchard, Massachusetts
4. Nuclear Laundry Rental Services, Inc.
   Jeanette, Pennsylvania
5. Nuclear Laundry Service, Inc.
   Waterbury, Connecticut
6. Nuclear Materials and Equipment Corporation
   Apollo, Pennsylvania
7. Penn Overall Supply Company
   Pittsburgh, Pennsylvania
8. Interstate Industrial Uniform Rental Service, Inc.
   N. Charleston, South Carolina
9. Southern Space, Inc.
   Macon, Georgia

Companies Licensed But Not Currently Processing Contaminated Laundry

1. Santa Fe Electric
   Santa Fe, New Mexico
2. Carter Industrial Laundry
   Washington, D. C.
3. Idaho Nuclear Laundry Corporation
   Idaho Falls, Idaho
4. Consolidated American Services, Inc.
   Hawthorne, California
5. Sanitary Overall Supply Company
   Tarantum, Pennsylvania
6. U.S. Services and Production Company, Inc.
   Charleston, South Carolina

[illegible]

Contaminated laundry facilities are unique in that they cannot economically be used for other laundry use. They require additional equipment (holdup tanks, monitoring equipment, etc.) and personnel training over and above those needed in conventional laundries.

The commercial contaminated laundry companies service such nuclear clients as the nuclear power utilities, hospitals, research and development laboratories, various companies in the private nuclear industry, and 14 AEC sites and contractors. The estimated annual volume from these 14 AEC sites totals about 2,500,000 pounds and is expected to remain fairly constant around this figure for the near term. This volume represents about $500,000 of business to the contaminated laundry market. The laundered items consist of coveralls, laboratory coats, boots, undergarments, socks, towels, and caps.

The possession of by-product materials, source material, and special nuclear material is subject to the licensing authority of the AEC, or in some cases, an "Agreement State".[5/] Title 10, Code of Federal Regulations, Parts 20, 30, 40, and 70 are pertinent to this licensing. In this context, possession includes contamination associated with garments and equipment, and the waste solutions or sludges that accumulate.

Chart VIII-E indicates the location of AEC and private contaminated laundry operations in the United States. The total annual load of the AEC on-site operation is estimated at about 17 million pounds.

On December 22, 1964, the Commission approved a policy of encouraging the use of commercial services for processing AEC-generated laundry and the development of specifications and requests for bids. Table VIII-9 lists the AEC sites both "doing and procuring" this service. Also indicated in the table are the estimates of the volumes and dollars involved.

5/ See page 157 for further information on "Agreement States".

TABLE VIII-9

AEC Sites and Contractors Using
Commercial Decontamination Laundering Facilities

Los Alamos Scientific Laboratory	-	Los Alamos, New Mexico
Atomics International	-	Canoga Park, California
Argonne National Laboratory	-	Argonne, Illinois
Knolls Atomic Pwr. Lab.	-	Schenectady, New York
Lawrence Radiation Lab.	-	Livermore, California
Lawrence Radiation Lab.	-	Berkeley, California
Bettis Laboratory	-	Pittsburgh, Pennsylvania
Sandia Laboratory	-	Albuquerque, New Mexico
Ames Laboratory	-	Ames, Iowa
Argonne Cancer Hospital	-	Chicago, Illinois
Laboratory of Nuclear Medicine	-	Los Angeles, California
Univ. of California Medical Ctr.	-	San Francisco, California
Univ. of Rochester Atomic Energy Project	-	Rochester, New York
Notre Dame Radiation Laboratory	-	Notre Dame, Indiana

Estimated volume: 2,500,000 lbs/year
$500,000/year

AEC Sites and Contractors Not Using
Commercial Decontamination Laundering Facilities

National Lead Company	-	Fernald, Ohio	OR
Union Carbide - Y-12	-	Oak Ridge, Tennessee	OR
Union Carbide - ORNL	-	Oak Ridge, Tennessee	OR
Union Carbide - K-25	-	Oak Ridge, Tennessee	OR
General Electric	-	Hanford, Washington	RL
Du Pont - Savannah River	-	Aiken, South Carolina	SR
Dow Chemical Company	-	Rocky Flats, Colorado	AL
Mason & Hanger	-	Burlington, Iowa	AL
Mason & Hanger - Pantex	-	Amarillo, Texas	AL
Brookhaven National Lab.	-	Upton, New York	BH
Phillips - NRTS	-	Idaho Falls, Idaho	ID
Mallinckrodt Chem. Lab.	-	Weldon Springs, Missouri	OR
Goodyear	-	Portsmouth, Ohio	OR
Union Carbide	-	Paducah, Kentucky	OR
Reynolds Electric	-	Mercury, Nevada	NEV
Monsanto Chemical- Mound	-	Miamisburg, Ohio	AL
Bendix	-	Kansas City, Missouri	AL
Lucius Pitkin	-	Grand Junction, Colorado	GJ

Estimated Volume: 17,000,000 lbs/year
$3,000,000/year

CONTAMINATED LAUNDRY OPERATIONS IN THE UNITED STATES

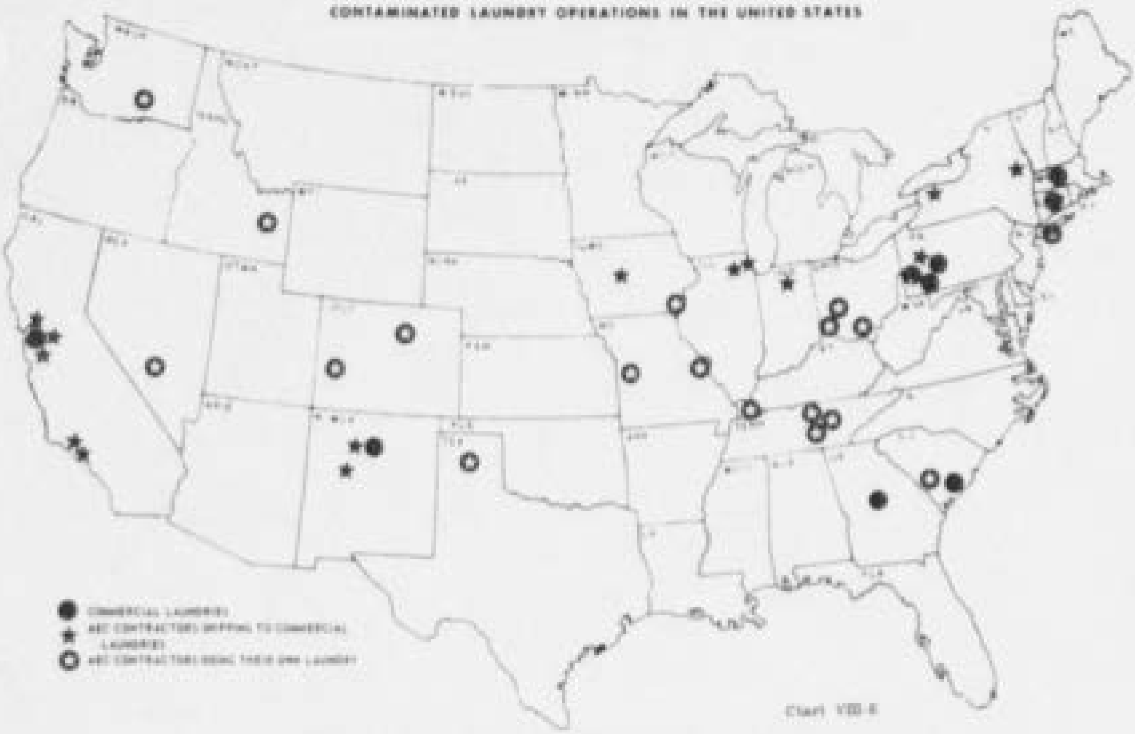

Chart VIII-6

D. <u>Transportation</u>

This service, provided to various users or producers of nuclear materials, is principally concerned with the packaging and transport of radioactive materials, including nuclear radioisotopes, radioactive wastes, weapons, ores and concentrates, unirradiated nuclear fuel materials, fabricated fuel elements, and irradiated spent fuel elements.

All modes of transportation, air, surface, and water, are used in the performance of this service. Large power reactor spent fuel shipments normally use rail, steamship, or barge due to the heavy shielding of the shipping casks required for this type of material. Piggyback transport of unirradiated power reactor fuel elements is also used extensively. Trucks are also used within their weight carrying limitations.

Some idea of the packaging requirements and movements of radioactive materials can be gained from the total flow of materials in the nuclear fuel cycle starting at the uranium ore mill and ending with the return of uranyl nitrate and plutonium nitrate to the Government or the owners for further use. The principal packaging and material movements in the nuclear fuel cycle are shown below.

Commodity	Packaging	Point to Point Movements
Uranium Ore Concentrates	Drums	Uranium mill to Feed Preparation Plant
Normal UF_6	Pressurized cylinders	Feed Preparation Plant to enriching (gaseous diffusion) plant
Enriched UF_6	Pressurized cylinders and protective packaging	Enriching plant to materials processing and/or fuel fabrication plant
Enriched uranium oxide power or pellets	Drums and birdcages	Materials processing plant to fuel fabrication plant
Cold new fabricated fuel assemblies	Protective packages and birdcages	Fuel fabrication plant to nuclear power reactors
Irradiated fuel	Shielded casks	Nuclear power reactor to chemical reprocessing plant
Uranyl nitrate	Tank trucks, tank cars and protective packages	Chemical reprocessing plant to fuel fabrication plant or to UF_6 feed preparation plant
Plutonium nitrate	Protective packages and plutonium containers	Chemical reprocessing plant to fuel fabrication plant
Fission product radioisotopes	Shielded casks	Chemical reprocessing plant to government or industry users.

Using the projected median figure of installed nuclear power capacity, (150,000 Mwe through 1980), and the associated estimates of U_3O_8 requirements, estimates have been made of the tonnage shipments for the domestic nuclear power industry. These are given below.

Commodity	1970	1975	1980
U_3O_8	7,500 tons	20,000 tons	40,000 tons
Normal UF_6	9,400 tons	25,000 tons	50,000 tons
Enriched UF_6	1,800 tons	5,000 tons	10,000 tons
UO_2	1,400 tons	3,800 tons	7,600 tons
New Fuel Elements	4,500 tons	12,000 tons	24,000 tons
Irradiated Fuel	4,500 tons	12,000 tons	24,000 tons
Total, Rounded	29,000 tons	78,000 tons	156,000 tons
Add Weight of Containers	56,000 tons	150,000 tons	300,000 tons
Total, Rounded	85,000 tons	228,000 tons	456,000 tons
Plus return of re-usable containers	56,000 tons	150,000 tons	300,000 tons
Grand Total	141,000 tons	378,000 tons	756,000 tons

Foreign nuclear power programs are expected to require tonnage shipments of the same order of magnitude as those in the U.S.

1. Studies

The AEC is currently working on a study program for a light weight spent fuel shipping cask using laminated depleted uranium. The final report on the demonstration laminated uranium cask project will be available in early CY-1968.

2. Costs

In a paper presented at the June 1967 annual meeting of the Institute for Nuclear Materials Management, Mr. Samuel Edlow, a traffic management consultant, gave the following costs for shipping nuclear materials abroad, based on a dollar

value per kg of material or per kg of uranium including the cost of freight, insurance, and interest charges on the material value:

- Uranium hexafluoride in 16,000 lb. lots (total value of material amounting to $750,000.00) from Oak Ridge to Germany. Total cost $1,250.00.

- Same shipment above by air. Total cost $1,975.00.

- Plutonium oxide in 5 kg lots, by air, from Richland Washington, to inland airports in Europe. Total cost $500.00 (not including insurance costs).

In a paper presented at the 1966 AIF Conference the National Lead Company indicated that total costs for shipping irradiated fuel are in the neighborhood of $5.00 per kilogram of uranium (includes loading and unloading, shipping equipment and shipping costs, and fuel lease charges and insurance). In an effort to reduce costs, National Lead has under study a cask design which shows promise in reducing the turn-around time in the loading and unloading pools. The design employs lead, steel, and uranium.

National Lead also has under preliminary study shipments by barge using water for shielding and heat transfer. Nuclear power projections indicate that in about ten years there would be adequate volume for such a system to function economically giving shipping costs below $1/kg of fuel for 1000-mile distances.

A recent conference on chemical reprocessing held at Augusta, Georgia, brought forth the following information concerning spent fuel shipping:

- Nuclear Fuel Services, Inc., cited total shipping costs in the range of $4,000 to $10,000 per metric ton of uranium fuel, as a factor favoring regional reprocessing plants.

- Commonwealth Edison Company gave the following breakdown of costs for shipping Dresden #1 spent fuel assemblies to NFS during 1966-67 for reprocessing:

Assemblies	Mode	Freight Cost/Assembly	$/Kg U
319	Rail	$176.00	$1.45
165	Truck	$245.00	$2.00

3. Transportation Regulations

Shipments of radioactive material are covered by the regulations of several Federal Agencies, including the AEC, the Interstate Commerce Commission, the Coast Guard, the Federal Aviation Agency, and the Post Office Department. The AEC is coordinating its regulatory control in this area with these other agencies.

In a Memorandum of Understanding between AEC and ICC, signed on March 21, 1966,[6/] the agencies[7/] agreed that AEC will adopt regulations applicable to preparation for shipment of fissile material and large quantities of radioactive material, and, as necessary, for radioactive material when its shipment is outside the jurisdiction of ICC. ICC will adopt regulations consistent with AEC standards, on transportation of all radioactive material and on preparation for shipment of radioactive material other than fissile material and large quantities of radioactive material.

The ICC issued a revision of its regulations (ICC Order #70) with an effective date of July 12, 1966, which, for the first time, provides rules for shipping fissile material as a routine commodity in transportation.

The AEC's regulations govern the packaging of radioactive materials for transport and are contained in the U.S. Code of Federal Regulation, Title 10, Part 71. A revision to 10 CFR Part 71 became effective on August 21, 1966.[8/] At the same time an AEC manual chapter (AECM-0529) was issued that placed AEC contractors under identical packaging requirements, thus establishing a common standard for all shippers of fissile and large quantities of radioactive material to use in designing shipping packages. AEC contractors shipping packages must be in compliance with AECM-0529 by July 1, 1968.

4. U.S. Ports Cleared for Receipt of Irradiated Fuels - Shipments to Foreign Countries

Table VIII-10 lists the U.S. ports cleared for receipt of irradiated fuels.

6/ See AEC Public Release J-64, dated March 18, 1966.

7/ On April 1, 1967, the DOT assumed the responsibilities of the CG, FAA, and the safety activities of the ICC.

8/ See AEC Public Release J-178, dated July 21, 1966.

TABLE VIII-10

U.S. Ports Cleared for Receipt of Irradiated Fuels

Charleston, South Carolina	Portland, Maine
Savannah, Georgia	Searsport, Maine
Norfolk, Virginia	Los Angeles, California
Philadelphia, Pennsylvania	Gulfport, Mississippi
Seattle, Washington	Albany, New York
Houston, Texas	Buffalo, New York
Port Everglades, Florida	Corpus Christi, Texas
Wilmington, North Carolina	Redwood City, California
Morehead City, North Carolina	Portland, Oregon
New Orleans, Louisiana	Tampa, Florida
New York, New York	Jacksonville, Florida
Baton Rouge, Louisiana	Port St. Joe, Florida
Benton, Washington	Bellingham, Washington
Baltimore, Maryland	Olympia, Washington
Willapa Harbor, Washington	Anacortes, Washington
Grays Harbor, Washington	Mattawa, Washington
Everett, Washington	Longview, Washington
Tacoma, Washington	San Francisco, California
Lake Charles, Louisiana	Hilo, Hawaii
Honolulu, Hawaii	Kahului, Hawaii
Kawaihae, Hawaii	Nawiliwili, Hawaii
Port Allen, Hawaii	Kennewick, Washington
Walla Walla, Washington	Pasco, Washington

To date AEC has shipped abroad under bilateral agreements approximately 20 thousand kg of U-235 (20 metric tons) representing a value of about $200 million. (See Table VIII-11)

AEC expects to ship abroad nuclear fuel at the rate of 10 to 15 thousand kg U-235 per year over the next several years, primarily for power reactors.

Since 1963 the AEC has received 79 shipments of irradiated research reactor fuel for chemical reprocessing in Commission facilities at Savannah River and Idaho.

TABLE VIII-11

Shipments to Foreign Countries Pursuant To Agreements for Cooperation

Year	Approximate Number of Shipments
1962	250
1963	400
1964	350
1965	270
1966	250
1967	225

5. Efforts to Obtain Standardized Shipping Containers

Following extensive development work at Oak Ridge Gaseous Diffusion Plant,(ORGDP), four sizes (5-inch, 8-inch, 12-inch, and 30-inch) of protective shipping containers have been designed, drop tested, and fire tested to the extent that they satisfy AEC Part 71 and IAEA requirements. OR plans to hold an information meeting in the near future aimed at disseminating information on the ORGDP protective container designs for the benefit of companies that might be considering fabrication of these containers commercially.

6. Commercial Services

Private firms supplying special services and/or products for the transport of radioactive materials are listed below:

ATCOR, Inc., Hawthorne, New York
Samuel Edlow, Columbus, Ohio
Long Island Nuclear Services, Bellport, New York
National Lead Company, New York, New York
Nuclear Engineering Company, Walnut Creek, California
Nuclear Fuel Services, Inc., West Valley, New York
Tri-State Motor Transit Company, Joplin, Missouri
International Transport, Inc., Rochester, Minnesota
Transnuclear, Inc., New York, New York

E. Whole Body Counting Service

Whole body counters are sensitive radiation detection instruments that provide information on the internal deposition of certain radionuclides by measuring the radiation emanating from the human

or animal body. Their proven value lies in their ability to reveal the kinds, amounts, and location of gamma emitting radioactive nuclei that have accumulated in various parts of the body from natural sources, fallout, or from medical tracer isotopes. From this information, it is possible to calculate the radiation absorbed by tissues and organs in which radioactive materials have accumulated.[9]

The most common whole body counter employs a sodium iodide crystal as the radiation detector. In another type the subject is surrounded by a tank filled with a scintillation fluid that detects gamma rays.

A number of commercial organizations are offering whole body counting services at the customer's site using portable trailer-mounted counters. In the last year this service has been provided to several nuclear power reactors, pharmaceutical companies, research laboratories, nuclear shipyards, radioactive waste disposal companies, and a fuel reprocessing facility. In addition, one company (Permian) has leased its counter over the last few years to several midwestern universities for use in animal research.

It is estimated that about 3,000 counts were performed during the past year by the commercial firms offering this service. The price charged per whole body count is dependent on the number of people to be counted per contract and ranges from about $20 to $35 per count.

Further information concerning portable whole body counting services may be obtained by contacting the following commercial firms offering this service:

California Nuclear, Inc.
Mrs. Lugene Hungerford
2323 South Ninth Street
Lafayette, Indiana 47905

Helgeson Nuclear Services
Mr. George Lewis Helgeson
872 Abbie Street
Pleasanton, California 94566

Permian Corporation
Mr. Charles R. Keck
Rte. 2, Box 50
Okmulgee, Oklahoma 74447

9/ See "Whole Body Counters", a booklet available from USAEC, Post Office Box 62, Oak Ridge, Tennessee 37830.

F. Reactor Servicing

A number of private companies are now established which offer specialized service in the area of reactor refueling and periodic overhauling of land-based nuclear power plants.

For example, Todd Shipyards Corporation of Galveston, Texas, has the capability of shipboard nuclear servicing at its overhaul and maintenance dock at Galveston.

During the year the AEC invited proposals from private industry to provide technical support services for the Elk River, BONUS, and LaCrosse reactors. The services required include an assessment of the performance of each plant; and evaluation and recommendations for reactor modifications: and participation in the incorporation and testing of modifications.[10/] The AEC is currently reviewing the proposals. Companies offering this service are listed below:

ATCOR, Inc.
360 Bradhurst Avenue
Hawthorne, New York

Atomics International
Division of North American
Rockwell Corporation
P. O. Box 309
Canoga Park, California 91305

Burns & Roe, Inc.
700 Kinderkamack Road
Oradell, New Jersey 07649

Hittman Associates, Inc.
P. O. Box 2685
Baltimore, Maryland 21215

Jackson & Moreland, Inc.
600 Park Square Building
Boston, Massachusetts 02116

Martin-Marietta Corporation
Baltimore, Maryland 21203

New York Shipbuilding Corp.
2430 Broadway
Camden, New Jersey 08101

Nuclear Associates
International Corp.
12601 Twinbrook Parkway
Rockville, Maryland

Nuclear Service and
Construction Company
c/o Newport News Shipbuilding
and Drydock Company
Newport News, Virginia

NUS Corporation
1730 M Street, N. W.
Washington, D. C. 20036

Todd Shipyards Corporation
P. O. Box 1600
Galveston, Texas

United Nuclear Corporation
1735 Eye Street, N. W.
Washington, D. C. 20006

10/ See AEC Public Release K-97, dated April 26, 1967.

G. Decontamination

The removal of radioactive contaminants from plant and equipment items is a nuclear service offered by several private companies specializing in this area.

This activity usually involves washing surfaces with chemicals, vacuum cleaning, or paint removing, along with frequent monitoring to determine when the area is suitable for use. Types of items frequently decontaminated include reactor parts and equipment, hot cell parts and equipment, glove boxes, shipping casks and all other tools and equipment of the nuclear industry which become radioactively contaminated through use or exposure.

The companies listed below offer this service in addition to other nuclear services such as waste handling, storage, disposal, transportation, and radiation safety services.

Applied Health Physics, Inc.
2986 Industrial Boulevard
Bethel Park - P. O. Box 197
Pennsylvania 15102

ATCOR, Inc.
360 Bradhurst Avenue
Hawthorne, New York 10532

California Nuclear, Inc.
2323 South Ninth Street
Lafayette, Indiana 47905

Isotopes, Inc.
123 Woodland Avenue
Westwood, New Jersey

Laboratory for Electronics
230 Wright Avenue
Richmond, California 94802

Long Island Nuclear
Service Corporation
Station Road
Bellport, New York 11713

Nuclear Diagnostic Labs, Inc.
755 Park Avenue
New York, New York

Nuclear Engineering Co., Inc.
Box 594
Walnut Creek, California 93002

Radiological Service Co., Inc.
35 Urban Street
Westbury, New York 11590

H. Consultants and Engineers

The Division of Industrial Participation maintains brochure files of consultants and engineers and will be glad to make information on these firms available to the public. Also, consultants and engineers with a particular interest in nuclear work are usually members of the Atomic Industrial Forum. The Forum would be glad to make the names of these members available on request.11/

11/ Atomic Industrial Forum; 850 Third Avenue, New York, New York 10022.

ASSISTANCE FOR INDUSTRY

A. At AEC Field Offices

Managers of each of the AEC Field Offices have designated representatives from their staffs as primary points of contact on Industrial Participation activities. These representatives serve as focal points within their respective offices for activities for which the Division of Industrial Participation is responsible.

These representatives are:

Mr. Charles C. Campbell
Assistant Manager for Administration
Albuquerque Operations Office
U.S. Atomic Energy Commission
P.O. Box 5400
Albuquerque, New Mexico 87115
(505) - 667-5105

Mr. Alfred K. Huse
Chief of Technical Projects
Operations Division
Brookhaven Office
U.S. Atomic Energy Commission
Upton, New York 11973
(516) - 924-2368

Mr. D.P. Rudolph, Director
Technical Services Division
Chicago Operations Office
U.S. Atomic Energy Commission
9800 South Cass Avenue
Argonne, Illinois 60439
(312) - 739-2091

Mr. J.C. Westbrook
Assistant Manager for Administration
Grand Junction Operations Office
U.S. Atomic Energy Commission
Grand Junction, Colorado 81502
(303) - 242-8201

Mr. John F. Kaufmann
Assistant Manager
Technical Services
Idaho Operations Office
U.S. Atomic Energy Commission
P.O. Box 2108
Idaho Falls, Idaho 83401
(208) - 526-1524

Mr. Jack H. Hooker
Administrative Assistant
Program & Budget Division
Nevada Operations Office
U.S. Atomic Energy Commission
P.O. Box 1676
Las Vegas, Nevada 89101
(702) - 734-3743

Mr. Lewis C. Cooper
Classification Officer
Classification & Technical
Information Staff
U.S. Atomic Energy Commission
376 Hudson Street
New York, New York 10014
(212) - 989-1260

Mr. John W. Ruch, Deputy Assistant
Manager for Operations
Oak Ridge Operations Office
U.S. Atomic Energy Commission
P.O. Box E
Oak Ridge, Tennessee 37831
(615) - 483-4468

Lieutenant James A. Schroeder
Senior Contract Administrator
Contract & Procurement Branch
Pittsburgh Naval Reactors Office
U.S. Atomic Energy Commission
P.O. Box 109
West Mifflin, Pennsylvania 15122
(412) - 462-0248

Mr. Leonard F. Perkins, Director
Division of Contracts & Procurement
Richland Operations Office
U.S. Atomic Energy Commission
P.O. Box 550
Richland, Washington 99352
(509) - 942-1111, X-6-4138

Mr. Robert Hughey, Director
Technical Services Division
San Francisco Operations Office
.S. Atomic Energy Commission
2111 Bancroft Way
Berkeley, California 94704
(415) - 841-4311

Mr. Harry B. Rahner, Special
Assistant to the Deputy Manager
Savannah River Operations Office
U.S. Atomic Energy Commission
P.O. Box A
Aiken, South Carolina 29802
(803) - 642-2678

Mr. Stanley W. Nitzman, Manager
Schenectady Naval Reactors Office
U.S. Atomic Energy Commission
P.O. Box 1069
Schenectady, New York 12301
(518) - 393-4441

B. At AEC Laboratories

Offices of Industrial Cooperation have been established at three of the major AEC laboratories. These offices operate principally to promote and maintain the types of individual laboratory-industry contacts that can result in "spinoff" of AEC-developed technology into commercial use. Occasionally the laboratories also use the offices for contacting industrial groups. Thus, the OIC at Argonne National Laboratory has hosted briefings for industry by members of the laboratory staff to explain the Federal Government's technology transfer programs. Such briefings may be conducted in cooperation with other Federal agencies and state and local industrial development groups. Further information on these offices and assistance which they might provide for specific purposes or companies may be obtained directly from the following:

Office of Industrial Cooperation
Oak Ridge National Laboratory
P.O. Box X
Oak Ridge, Tennessee 37831
(615) 483-6883

Office of Industrial Cooperation
Argonne National Laboratory
9700 South Cass Avenue
Argonne, Illinois 60440
(312) 739-4762

Office of Industrial Cooperation
Sandia Corporation
P.O. Box 5800
Albuquerque, New Mexico 87115
(505) 264-3904

C. From AEC Publications

The annual report which the Commission prepares for submission to the Congress in January each year contains summary descriptions of all the AEC programs. This is available to the public as Major Activities in the Atomic Energy Programs -- January-December (each year) from the U.S. Government Printing Office, Washington, D.C. 20402. Also available from the same source is an annual supplemental report, Fundamental Nuclear Energy Research (each year). This report summarizes the noteworthy advances resulting each year from AEC basic research studies in the physical, biological, medical, and environmental areas.

During the past two decades, a body of literature describing the science, technology, and application of atomic energy has been

developed. This literature appears in many forms. It is available from numerous sources and is announced and indexed in a variety of publications. A description of the types of material available and how to obtain them is contained in a booklet, What's Available in the Atomic Energy Literature, available on request to the USAEC Division of Technical Information Extension; P.O. Box 62; Oak Ridge, Tennessee 37830.

A good source of current information on AEC activities are the AEC Public Releases. To get on the mailing list for these, write to the Division of Public Information; USAEC; Washington, D.C. 20545.

D. From Congressional Publications

The Joint Committee on Atomic Energy publishes transcripts of its hearings on atomic energy legislation and programs and issues reports on a wide range of nuclear subjects. Those interested in these publications should write to the Joint Committee on Atomic Energy; Congress of the United States; Washington, D.C. 20510, asking to be placed on the Committee's mailing list.

E. Through the Access Permit Program

Certain classified technical information may be obtained for private use in developing peaceful applications of nuclear energy through the Commission's Access Permit Program. Information on this program is contained in the booklet Questions and Answers on USAEC Access Permits, available from the Division of Industrial Participation; USAEC; Washington, D.C. 20545.

F. Through the Work Experience Program

Employees of private firms engaged in civilian applications of nuclear energy are eligible for training through work experience in AEC plants and laboratories. Further information on this program may be obtained from the Division of Industrial Participation; USAEC; Washington, D.C. 20545.

U.S. GOVERNMENT PRINTING OFFICE : 1967 O - [illegible]

The Division of Industrial Participation was established in 1961 to strengthen industry's participation in the United States atomic energy program. Within this over-all objective of bolstering the Commission's cooperation with U.S. industrial organizations, the Division of Industrial Participation has the following objectives:

1. Provide a focal point for industry within the Commission.

2. Identify activities performed in Government facilities that can be performed by private industry.

3. Appraise and report on the effectiveness of Commission policies for broadening industry participation in the U.S. nuclear program.

4. Serve as a contact point for state and local governments on non-regulatory matters.

5. Encourage non-nuclear applications of AEC-developed innovations.

THE NUCLEAR INDUSTRY

1968

☆U.S. ATOMIC ENERGY COMMISSION☆

9903250387 681231
PDR MISC
9903250387 PDR

THE NUCLEAR INDUSTRY

19[illegible]

050063

9903250387 681231
PDR MISC
9903250387 PDR

S1370

ACKNOWLEDGEMENT

The Division of Industrial Participation wishes to acknowledge the assistance by other Divisions and staff of the Atomic Energy Commission in the preparation of this report. Without their help and the assistance of industrial firms, associations, and state groups, this report could not have been prepared.

Ernest B. Tremmel

Ernest B. Tremmel, Director
Division of Industrial
Participation

Prepared for delivery to
Atomic Industrial Forum's International Conference
Washington, D. C.
November 14, 1968

FOREWORD

Section 1, the Declaration of the Atomic Energy Act states that it shall be the policy of the United States to develop, use, and control atomic energy "so as to........strengthen free competition in private enterprise." As part of its fulfillment of this mandate, this booklet, Nuclear Industry, is published annually to present the AEC's assessment of the state of the nuclear industry and make available facts believed to be of interest and in accordance with the President's program to improve service to the public.

It is intended that the booklet serve both as introductory reading for persons who desire to become better acquainted with the nuclear industry as well as a handy reference for important items of interest to the industry. It is prepared yearly by the AEC's Division of Industrial Participation (in September of each year) and released at the Atomic Industrial Forum and American Nuclear Society joint meeting held in the fall of each year.

It is sincerely hoped that Nuclear Industry - 1968 will be of value to the many readers of past years' reports as well as future readers and that it will provide information which will serve as one basis for guidance to industry in determining their future role in the nuclear field.

For sale by the Superintendent of Documents, U.S. Government Printing Office
Washington, D.C. 20402 - Price [illegible]

REPORT ON THE NUCLEAR INDUSTRY
1968
Contents

Page

SUMMARY. 1

A. Shipments of Products 1
B. Investment 6
C. Exports 6
D. Employment 7
E. Nuclear-Electric Power 9

NUCLEAR INDUSTRY HIGHLIGHTS - 1968 13

A. Civilian Nuclear Power 13
B. Private Ownership 17
C. Enriching 18
D. Safeguards 18
E. Radioisotopes 19
F. Plowshare 19
G. Spin-Off 19
H. Licensing, Regulation and Legislation 20
I. Interstate Agreements 21
J. Federal-State Relations 21
K. Meetings with Industry 21
L. International Activities 21

PRIVATE OWNERSHIP - TOLL ENRICHING 22

A. Actions Taken During 1965, 1966 and 1967 23
B. Status of Toll Enriching Contracts 26
C. Actions Taken in 1968 26
D. Areas That Still Require Attention 27

CATEGORY I -- RAW FUEL MATERIALS 28

A. Uranium 29
B. Thorium 36

Page

CATEGORY II -- MATERIALS PROCESSING AND FABRICATION 37

A. Uranium Fuel Preparation - Conversion of Feed Materials to UF_6 37
B. Uranium Enriching 46
C. Uranium Processing and Fabrication 54
D. Plutonium Processing and Fabrication 64
E. Thorium 69
F. Uranium-233 70
G. Zirconium 70
H. Heavy Water 73
I. Beryllium 75
J. Boron-10 76

CATEGORY III -- NUCLEAR COMPONENTS AND EQUIPMENT 78

A. Reactor Components 79
B. Radiation Detection and Monitoring Instruments 84

CATEGORY IV -- REACTORS 89

A. Expenditures for Reactor Projects 89
B. Civilian Power 94
C. Civilian Maritime Reactors 125
D. Military Reactors. 126
E. Research and Teaching Reactors 129
F. Space Systems 130
G. Aircraft Nuclear Propulsion 138

CATEGORY V -- RADIOISOTOPES AND RADIATION APPLICATIONS 139

A. Radiochemicals and Radiopharmaceuticals 140
B. Equipment Manufacturers 148
C. Commission Licensing Actions in Radioisotope Area 152
D. Radioisotopic Power 154
E. The Radiation Processing Industry 156
F. Significant Industry Changes and Announcements 161
G. AEC Advisory Committee on Isotopes and Radiation Development 162
H. Science Information Exchange Program 163

CATEGORY VI -- MATERIALS REPROCESSING 164

A. Irradiated Fuel 164
B. Unirradiated Fuel Scrap 182

Page

CATEGORY VII -- PLOWSHARE -- PEACEFUL USES OF NUCLEAR EXPLOSIVES 185

A. Introduction 185
B. Budget 187
C. AEC Authority to Provide Plowshare Services 187
D. Industrial Participation 187
E. Natural Resources Projects 187
F. Arizona Water Study 193
G. Charges for Nuclear Explosives 193
H. Miscellany 193
I. Program Information 194

CATEGORY VIII -- SERVICES 196

A. Radioactive Waste Management 196
B. Personnel Dosimeter Services 209
C. Contaminated Laundry 213
D. Transportation 217
E. Whole Body Counting Service 223
F. Reactor Servicing .224
G. Decontamination 225

LICENSING AND REGULATION .226

A. Facility Licensing 226
B. Control of Materials. 227
C. Nuclear Safety 229
D. AEC License Fees Established 229

PROPOSED LEGISLATION 230

A. S. 1934 - H.R. 15340 230
B. S. 2564 - H.R. 13838 - H.R. 15273 230
C. S. 2889 - H.R. 14971 231
D. S. 3851 - H.R. 18667 231
E. S. 3960 231
F. S. 3330 232

ASSISTANCE FOR INDUSTRY 233

A. At AEC Field Offices 233
B. At AEC Laboratories 234
C. From AEC Publications 234
D. From Congressional Publications 235
E. Through the Access Permit Program 235
F. Through the Work Experience Program 235

Page

PRIVATE ORGANIZATION INDEX. 236

List of Charts v

List of Tables vi

List of Charts

Page

SUMMARY

Chart A -- Flow Chart of the Nuclear Industry 2
Chart B -- Shipments of Nuclear Products 2
Chart C -- Orders for Selected Nuclear Products 5
Chart D -- Exports - Selected Nuclear Commodities 7
Chart E -- Electric Utility Generating Capability and Nuclear Power Growth 9
Chart F -- Nuclear Power Plant Construction Investment and Fuel Cycle Costs 10
Chart G -- Nuclear Fuel Cycle - Predicted Annual Market . . . 11
Chart H -- U.S. Utility Orders for Electric Generating Equipment 12

RAW FUEL MATERIALS

Chart I-A -- Estimated Annual Uranium Requirements 29
Chart I-B - World Uranium Resources 31
Chart I-C -- Uranium Reserves and Cumulative Requirements . 31
Chart I-D -- Uranium Exploration 33
Chart I-E -- Uranium Procurement by the USAEC and Estimated Requirements 35

MATERIALS PROCESSING AND FABRICATION

Chart II-A -- Conversion of Ore Concentrates to UF_6 44
Chart II-B -- Conversion of Recovered, Slightly Enriched U to UF_6 44
Chart II-C -- Uranium Enriching 51
Chart II-D -- Fuel Fabrication 61
Chart II-E -- Plutonium Processing and Fabrication 67

NUCLEAR COMPONENTS AND EQUIPMENT

Chart III-A -- Shipments of Reactor Components 79
Chart III-B -- Manufacturers Shipments of Nuclear Instruments 87

REACTORS

Chart IV-A -- Domestic Reactor Projects on Order or Under Design or Construction 93
Chart IV-B -- U.S. Map - Nuclear Power Plants 100
Chart IV-C -- Nuclear Electric Plants - Estimates of Installed Capacity 102
Chart IV-D -- Energy Sources for Electric Power Generation .104

Page

MATERIALS REPROCESSING

Chart VI-A -- Nuclear Fuel Services, Inc., Spent Fuel Process. 168
Chart VI-B -- Proposed GE Process 170
Chart VI-C -- Domestic Central Station Spent Fuel Discharge Forecast 173

PLOWSHARE -- PEACEFUL USES OF NUCLEAR EXPLOSIVES

Chart VII-A -- Gas Reservoir Stimulation 190
Chart VII-B -- Fallout Patterns. 192
Chart VII-C -- Projected Charges for Thermonuclear Explosives 194

SERVICES

Chart VIII-A -- Materials Licensing - U.S. Map 197
Chart VIII-B -- Commercial Burial Sites for Low Level Wastes . 199
Chart VIII-C -- Schedule of Rates & Charges for Low Level Waste Disposal 200
Chart VIII-D -- Principal Film Badge Operations in the United States 211
Chart VIII-E -- Contaminated Laundry Operations in the United States 215

List of Tables

Page

SUMMARY

Table 1 -- Value of Shipments of Selected Atomic Energy Products 3
Table 2 -- Value of Net Orders Received for Selected Atomic Energy Products 4
Table 3 -- United States Exports 6
Table 4 -- Employment in Private Establishments (Selected Economic Segments) 8
Table 5 -- Number of Private Establishments (Selected Economic Segments) 8

(List of Tables - Continued) Page

PRIVATE OWNERSHIP - TOLL ENRICHING

Table 6 -- Time-Table 23

RAW FUEL MATERIALS

Table I-1 -- Domestic Uranium Companies Supplying AEC and Commercial Market 34
Table I-2 -- Domestic Uranium Processing Mills Operating Primarily or Entirely for Private Sales. 34
Table I-3 -- Domestic Sales of Uranium (in short tons U_3O_8) . . 35

MATERIALS PROCESSING AND FABRICATION

Table II-1 -- Enrichment Capacity of AEC's Gaseous Diffusion Complex 48
Table II-2 -- Producers of Uranium Fuels 55
Table II-3 -- Fabricators of Uranium Fuel 57
Table II-4 -- Commercial Capability to Process and Fabricate Nuclear Fuels 60
Table II-5 -- Enriched Uranium Furnished to Industry (Kilograms of Uranium) 59
Table II-6 -- Enriched Uranium Shipped Abroad 62
Table II-7 -- AEC Orders for Fabrication of Uranium Fuel From Commercial Suppliers (Millions of Dollars). . . 63
Table II-8 -- Projected Plutonium Recovered from Domestic Commercial Power Reactor Fuels (Kg Total Pu as the Nitrate) 64
Table II-9 -- Major Programs Requiring Plutonium (Annual Net Requirements in Kgs.) 65
Table II-10 - Industrial Interest in Plutonium Work 68
Table II-11 - Principal Firms Processing Thorium 69
Table II-12 - The Principal U.S. Companies Having Capability to Produce Zirconium and Zirconium Products 71
Table II-13 - Estimated Annual Requirements for Zirconium in U.S. Nuclear Reactors 73
Table II-14 - Principal Commercial Suppliers of Heavy Water (In Quantities Less Than 125 Lbs.) 74

NUCLEAR COMPONENTS AND EQUIPMENT

Table III-1 - Companies Manufacturing Radiation and Monitoring Instruments with 1966 Sales Exceeding $0.5 Million 85
Table III-2 - Exports of Nuclear Instruments 87

(List of Tables - Continued)

REACTORS Page

Table IV-1 -- Estimated Comparative Costs Incurred for Domestic Reactor Projects 90
Table IV-2 -- Domestic Reactor Projects Under Contract, Design, or Construction . . . . [illegible]0
Table IV-3 -- Costs and Estimates by Source of Funds Projects Under Contract, Design, or Construction. 91
Table IV-4 -- List of Domestic Reactor Projects Under Contract, Design, or Construction at June 30, 1967, and June 30, 1968 92-93
Table IV-5a - Central Station Nuclear Plants Operable, Under Construction, or On Order 97a
Table IV-5b - Central Station Nuclear Plants Operable, Under Construction, or On Order (Continued) 97b
Table IV-6 -- Central Station Nuclear Plants Announced as Planned for Nuclear Power but Contracts not Awarded. . . . 98
Table IV-7 -- Announcements of New Steam-Electric Plant Additions. 99
Table IV-8 -- Central Station Nuclear Power Reactor Sales Through 9/30/68 106
Table IV-9 -- Commercial Power Reactor Fuel Cycle Steps by Suppliers 110
Table IV-10 - Foreign Installations of U.S.-Type Power Reactors ..119
Table IV-11 - Army Reactor Plants 127
Table IV-12 - Categories of Nuclear Systems and Technology 133
Table IV-13 - Radioisotope Power Systems 136
Table IV-14 - Space Electric Power Development Operating Costs FY-1968 and FY-1969. 137

RADIOISOTOPES AND RADIATION APPLICATIONS

Table V-1 -- Principal Industrial Suppliers of Cyclotron Radioisotopes 142
Table V-2 -- AEC Sales of Radioisotopes 143
Table V-3 -- Principal Processors of Radiochemicals and Radiopharmaceuticals 147
Table V-4 -- Principal Suppliers of Radioactive Sources 148
Table V-5 -- Principal Suppliers of Radiographic Units 148
Table V-6 -- Principal Suppliers of Radioisotope Teletherapy Units 149
Table V-7 -- Sales of Radioisotope Gauges 151
Table V-8 -- Principal Commercial Suppliers of Self-Illuminating Materials and Devices 152
Table V-9 -- Irradiators Supporting the Food-Irradiation Effort .161

(List of Tables - Continued)

Page

MATERIALS REPROCESSING

Table VI-1 -- Fuel Reprocessing Load Forecasts (In Metric Tons Uranium). 173
Table VI-2 -- Status of Chemical Reprocessing Plants 177
Table VI-3 -- Principal Firms Recovering Uranium Scrap 182
Table VI-4 -- Dollar Value of the AEC's Commercial Uranium Scrap Recovery Program 183
Table VI-5 -- Uranium Scrap Reprocessing Contract Awarded In FY-1968 183

PLOWSHARE - PEACEFUL USES OF NUCLEAR EXPLOSIVES

Table VII-1 -- Operating Budget for Plowshare 186
Table VII-2 -- FY-69 Budget Estimate (In Millions) 186

SERVICES

Table VIII-1 - Private Waste Burial (Cubic Feet) 199
Table VIII-2 - Commercial Firms Licensed by the U.S. Atomic Energy Commission to Receive Radioactive Wastes (As of August 21, 1968) 201
Table VIII-3 - Commercial Firms Licensed by Agreement States to Receive Radioactive Wastes (As of August 21, 1968). 202
Table VIII-4 - AEC Solid Radioactive Waste Burials 203
Table VIII-5 - Reactor Resin Waste Handling and Burial Forecast 204
Table VIII-6 - Film Badge Processors 211
Table VIII-7 - National Sanitation Foundation Testing Laboratory, Inc. 1968 Approved Listing for Film Badge Services (NSF Standard No. 16) . .213
Table VIII-8 - Commercial Licensees - Companies Actively Processing Contaminated Laundry. 214
Table VIII-9 - AEC Sites and Contractors Using Commercial Decontamination Laundering Facilities 216

AEC Sites and Contractors Having Their Own Commercial Decontamination Laundering Facilities 216

Table VIII-10 - U.S. Ports Cleared for Receipt of Irradiated Fuel 220
Table VIII-11 - Shipments to Foreign Countries Pursuant to Agreements for Cooperation 221

STATUS OF THE NUCLEAR INDUSTRY

SUMMARY

Of all the peaceful uses of nuclear energy, the use of nuclear reactors to produce steam for the generation of electric power continued to attract the most public attention and have the largest impact on the country's economy. Orders for all electric generating equipment, however, were down considerably from the 1967 peak (as had been expected). Orders for nuclear plants were down roughly in proportion to the overall total. Because of the broad interest in this facet of the nuclear industry, it is discussed in more detail in Section E of this summary.

Nuclear reactors for other applications continued to receive attention, particularly dual-purpose plants which would desalt water or produce process steam, as well as produce electric power (see Category IV of this report).

Overall uses of nuclear energy, such as the extensive applications of radioisotopes and radiation and the many promising uses of peaceful nuclear explosives in the Plowshare program, made further advances in 1968. The bright outlook for the future in all applications of nuclear energy offers assurance of a continuing expansion of the nuclear industry in the years ahead.1/

The overall inter-relationships for the various applications of nuclear energy are shown graphically on the flow diagram included as Chart A on the following page.

A. Shipments of Products

The Bureau of the Census has collected data on shipments of selected groups of nuclear products each year beginning in 1957. In 1967, shipments of these products again reached an all-time high, more than 20 percent above 1966, as reflected in Chart B and Table 1.

Several changes were made in the report for 1967 (Table 1), as compared to 1966, in groupings of products to avoid disclosure of information on individual companies. It is interesting to note that significant increases were reflected in Table 1 in almost every product code. For fuel elements there was a decrease in shipments for propulsion applications but a substantial increase in shipments for power plants and other uses.

1/ See remarks by Ernest B. Tremmel, Director, Division of Industrial Participation, USAEC, before the Financial Analysts Federation 21st Annual Conference, Boston, Mass., May 1968.

CHART A

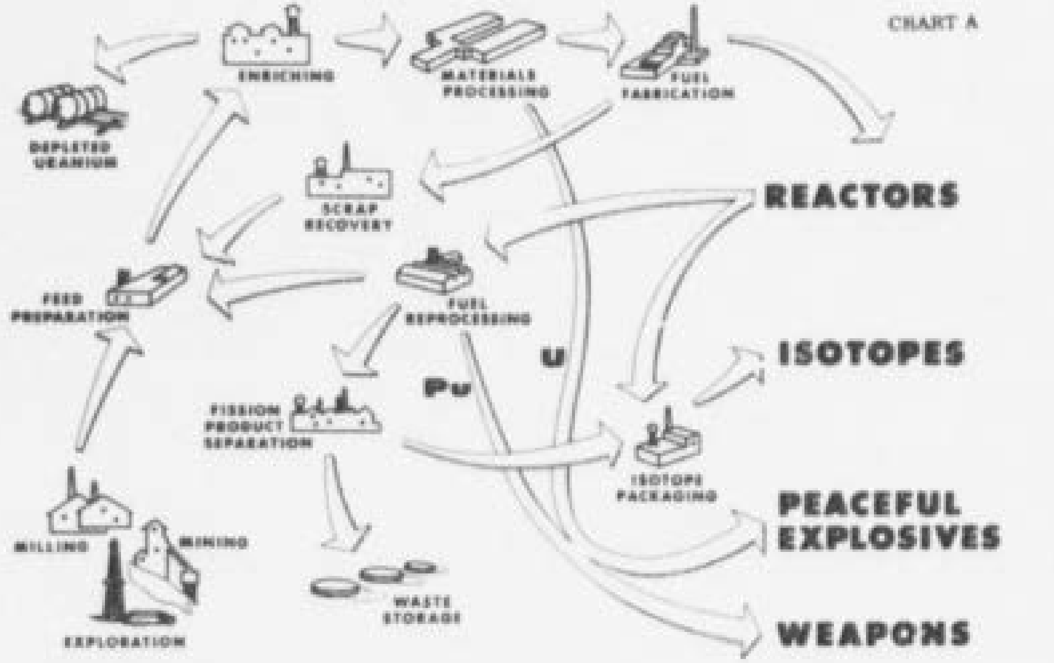

FLOW CHART *of the* NUCLEAR INDUSTRY

SHIPMENTS OF NUCLEAR PRODUCTS

CHART B

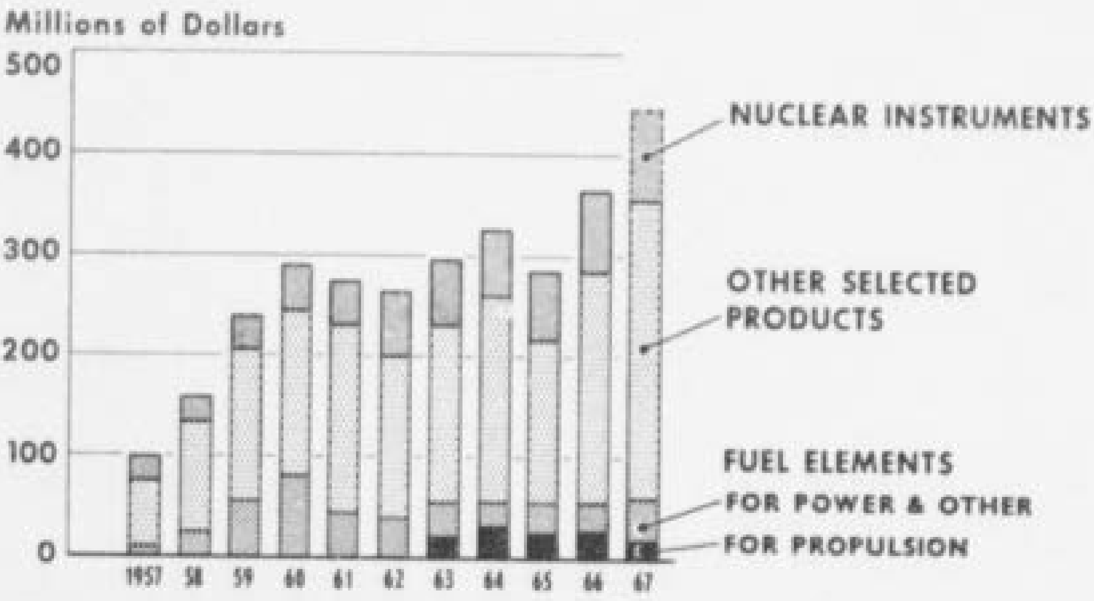

TABLE 1. —VALUE OF SHIPMENTS OF SELECTED ATOMIC ENERGY PRODUCTS: 1967 AND 1966

(Thousands of dollars)

Product code	Product	1967 Total	1967 To U.S. Government: Atomic Energy Commission	1967 To U.S. Government: Other agencies	1967 Export	1967 Other	1966 Total	1966 To U.S. Government: Atomic Energy Commission	1966 To U.S. Government: Other agencies	1966 Export	1966 Other
	TOTAL (excluding radiation detection and monitoring devices and control and measuring devices containing radioactive isotopes)[1]	[illegible]	[illegible]	[illegible]	[illegible]	[illegible]	[illegible]	[illegible]	[illegible]	[illegible]	[illegible]
[illegible]	Nuclear reactors[6]	[illegible]	(D)	(D)	(D)	(D)	[illegible]	(D)	(D)	(D)	(D)
	Reactor components and equipment:										
[illegible]	Primary vessels and tanks						[illegible]	[illegible]	[illegible]	[illegible]	[illegible]
	Control rod drive mechanisms and components for—										
[illegible]	Power plant										
[illegible]	Propulsion										
[illegible]	Other	[illegible]	[illegible]	[illegible]	[illegible]	[illegible]	[illegible]	[illegible]	[illegible]	[illegible]	[illegible]
[illegible]	Core structurals (barrels, rods, boxes, plates, etc., not included above)										
[illegible]	Accessory instrumentation for reactor control										
[illegible]	Heat exchangers and condensers	[illegible]	[illegible]	[illegible]	[illegible]	[illegible]	[illegible]	[illegible]	[illegible]	[illegible]	[illegible]
[illegible]	Pressurizers, components, and auxiliary equipment	[illegible]	(D)	(D)	-	[illegible]	[illegible]	[illegible]	[illegible]	[illegible]	[illegible]
[illegible]	Pumps	[illegible]	[illegible]	[illegible]	[illegible]	[illegible]	[illegible]	[illegible]	[illegible]	[illegible]	[illegible]
[illegible]	Valves	[illegible]	[illegible]	[illegible]	[illegible]	[illegible]	[illegible]	[illegible]	[illegible]	[illegible]	[illegible]
[illegible]	Fuel handling equipment	[illegible]	[illegible]	[illegible]	[illegible]	[illegible]	[illegible]	[illegible]	[illegible]	[illegible]	[illegible]
	Complete reactor fuel elements and control rods shipped directly for installation or use in a reactor for—										
[illegible]	Propulsion	17,950	17,950	-	-	-	[illegible]	[illegible]	-	-	-
[illegible]	Power plant	[illegible]	(D)	(D)	(D)	(D)	[illegible]	[illegible]	[illegible]	[illegible]	[illegible]
[illegible]	Other						[illegible]	[illegible]	[illegible]	[illegible]	[illegible]
[illegible]	Partially fabricated reactor fuel element materials and control rods not shipped directly for installation or use in a reactor	[illegible]	[illegible]	[illegible]	[illegible]	[illegible]	[illegible]	[illegible]	[illegible]	[illegible]	[illegible]
[illegible]	Reactor moderators, coolants, and reflectors	[illegible]	[illegible]	[illegible]	[illegible]	[illegible]	[illegible]	[illegible]	[illegible]	[illegible]	[illegible]
	Specialized reactor components and equipment not included elsewhere in this report	[illegible]	[illegible]	[illegible]	[illegible]	[illegible]	[illegible]	[illegible]	[illegible]	[illegible]	[illegible]
[illegible]	Hot laboratory equipment	[illegible]	[illegible]	[illegible]	[illegible]	[illegible]	[illegible]	[illegible]	[illegible]	[illegible]	[illegible]
	Shieldings	[illegible]	[illegible]	[illegible]	[illegible]	[illegible]	[illegible]	[illegible]	[illegible]	[illegible]	[illegible]
[illegible]	Radioactive isotopes shipped from non-AEC plants producing isotopes	[illegible]	[illegible]	[illegible]	[illegible]	[illegible]	[illegible]	[illegible]	[illegible]	[illegible]	[illegible]
[illegible]	Radiation sources and other radioactive materials produced from purchased isotopes	[illegible]	[illegible]	[illegible]	[illegible]	[illegible]	[illegible]	[illegible]	[illegible]	[illegible]	[illegible]
	Receipts for processing of source and special nuclear materials for—										
[illegible]	Conversion to uranium						[illegible]			(X)	[illegible]
[illegible]	Reprocessing of irradiated reactor fuels	[illegible]	[illegible]	[illegible]	[illegible]	[illegible]		[illegible]	[illegible]	-	-
[illegible]	Receipts for commercial irradiation service						[illegible]			(X)	[illegible]
[illegible]	Isotopic power devices	(D)	(D)	(D)	(D)	(D)	(D)	(D)	(D)	(D)	(D)

-Represents zero. (D) Withheld to avoid disclosing figures of individual companies. (NA) Not available. [r]Revised. (X) Not applicable.
[1]Includes the value of those products (except isotopic power devices) for which separate figures are not shown to avoid disclosing figures of individual companies.
[2]"Export" shipments included with "other."
[3]Shipments to other U.S. Government agencies included with AEC shipments.
[4]Fuel handling equipment is included in control rod drive mechanisms and components, etc.

[6]Includes only reactors shipped fully assembled from the place of manufacture, such as AGN-201 and other similar types.

Current Industrial Reports Series MA-38(67)-1

- 3 -

TABLE 2

Value of Net Orders Received
for Selected Atomic Energy Products
1967 and 1966
(In thousands of dollars)

Product	Net orders received 1967	1966
Total	1,687,728	NR
Nuclear reactors*	(D)	780
Reactor components and equipment:		
Primary vessels and tanks	(D)	105,553
Control rod drive mechanisms and components for:		
Power plant	(D)	(D)
Propulsion	-	(D)
Other	(D)	-
Heat exchangers and condensers	260,539	92,128
Pressurizers, components, and auxiliary equipment	(D)	7,864
Pumps	(D)	(D)
Valves	18,440	8,694
Fuel handling equipment	(D)	2,399
Complete reactor fuel elements and control rods shipped directly for installation or use in a reactor for:		
Power plant	885,865	444,892
Propulsion	(D)	11,880
Other	(D)	4,109
Partially fabricated reactor fuel element materials and control rods not shipped directly for installation or use in a reactor	34,213	21,790

(D) Withheld to avoid disclosing figures of individual companies.

* Includes only reactors shipped fully assembled from the place of manufacture, such as AGN-201 and other similar types.

NR Not reported.

Chart B includes shipments of radiation detection and monitoring instruments which, beginning in 1963, have been shown separately by the Bureau of the Census in a report on shipments of "Selected Instruments and Related Products." Since that report for 1967 was not available at the end of September, the nuclear instrument shipments shown on Chart B are as estimated by the Division of Industrial Participation, USAEC.

Net orders received for a selected group of nuclear products, as reported by the Census Bureau, exceeded the $1-1/2 billion level in 1967, as shown in Table 2 and Chart C. Although data reported for 1966 and 1967 are not on exactly comparable bases, there is no doubt that 1967 orders in total were at a rate more than twice that reported for the prior year. This, of course, reflects the tremendous impact of orders for nuclear power plants over the past year. The increase in orders for nuclear products reported for 1967 are expected to be reflected in significant increases in shipments of nuclear products in future years.

ORDERS FOR SELECTED NUCLEAR PRODUCTS

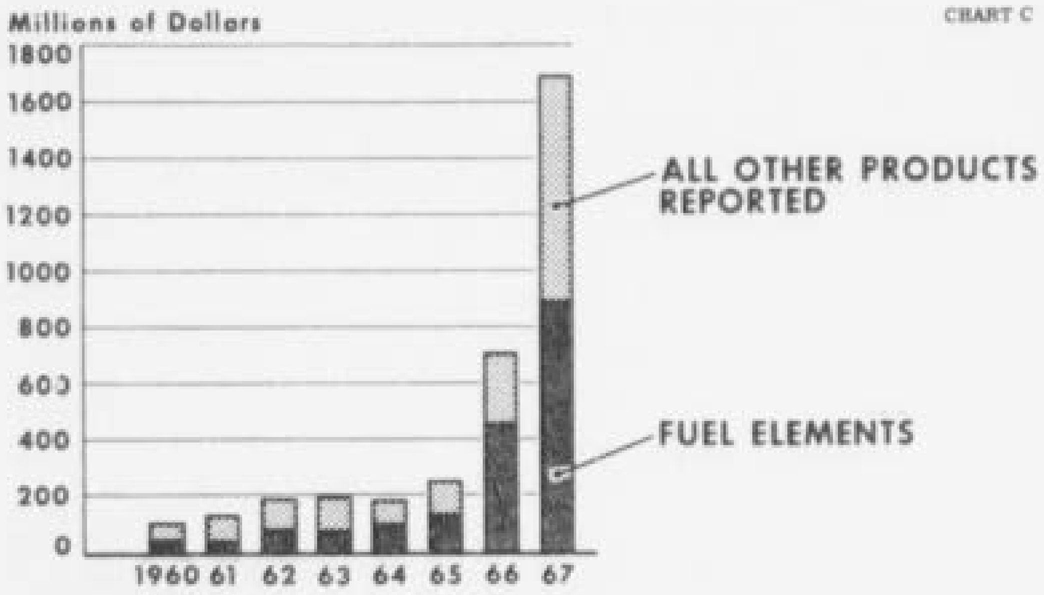

[illegible]

B. Investment

The estimated cost of nuclear-electric power plants announced by electric utilities during the first nine months of 1968 totaled about $2 billion. This brings the estimated cost of nuclear-electric plants operating, under construction, or planned, by electric utilities to about $12 billion.

New facilities for the production of reactor components and associated equipment and services now underway or planned by private industrial suppliers is estimated at $300 million or more.

C. Exports

Export shipments for a number of nuclear products, as reported by the Bureau of the Census, are summarized in Table 3 and Chart D. The reduction in 1967 from the 1966 high came from decreases in exports of isotopes, reactors, and reactor parts. The unusually high figures for isotope exports in 1966 reflected a shipment to France of about $6 million worth of heavy water. Exports of nuclear instruments continued to increase at an encouraging rate.

Shipments through the first half of 1968 indicate exports for the current year at about the 1967 level.

TABLE 3

United States Exports

Commodity Description	Total Value - Thousands of Dollars 1963	1964	1965	1966	1967
Uranium Ore and Concentrates	$ 659	20	-0-	161	-0-
Thorium Ores and Concentrates	322	353	79	3	-0-
Uranium and Thorium Compounds	144	182	[illegible]	296	203
Special Nuclear Material	23,560	15,339	35,[illegible]	41,906	44,015
Radioactive and Stable Isotopes Their Compounds & Mixtures	2,554	2,921	6,647	15,641	6,422
Nuclear Reactors and Parts	798	27,618	13,209	43,363	23,955
Nuclear Radiation Detecting and Measuring Instruments	11,804	15,338	18,108	19,194	22,346
Total for Items Reported	$39,841	$61,771	$73,867	$120,564	$96,941

EXPORTS-SELECTED NUCLEAR COMMODITIES

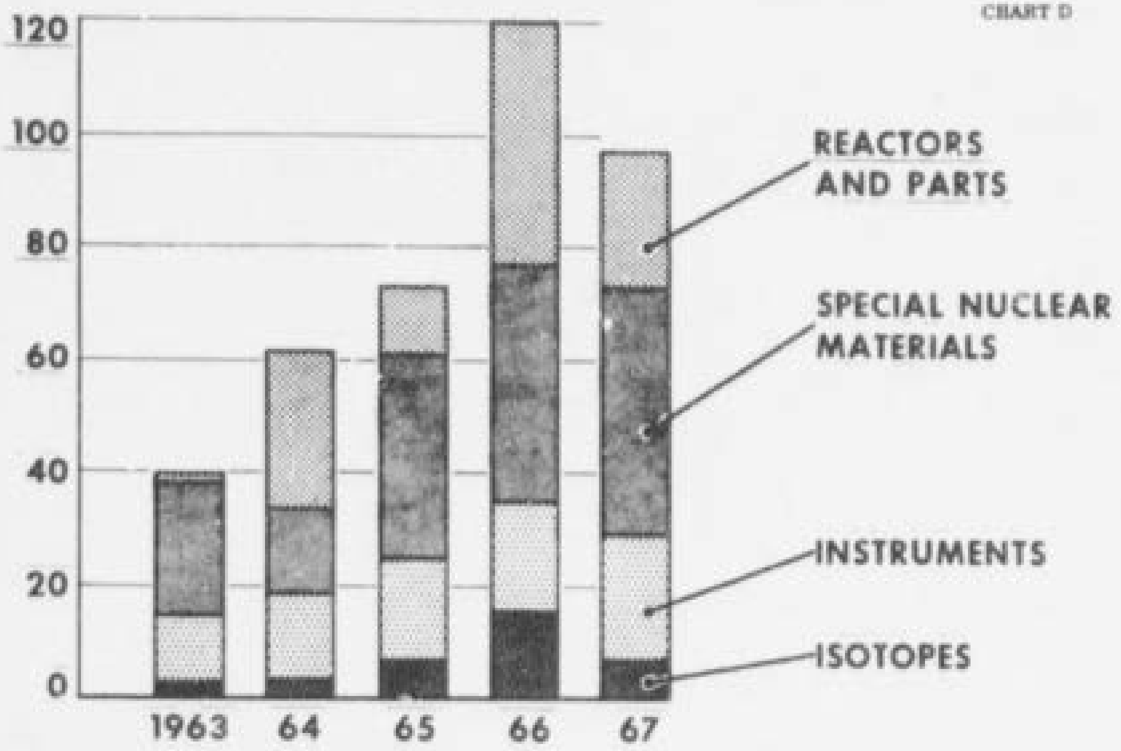

D. Employment

Reports on employment in atomic energy work are prepared for the AEC by the Bureau of Labor Statistics. The most recent report, covering employment in March 1967, gives a total industrial employment at that time of 138,000 in nuclear work. This is about a six percent increase over employment as reported for March 1966. Of the total employed in March 1967, 35,000 were employed in privately-owned facilities, and 103,000 in Government-owned facilities. Tables 4 and 5 show somewhat comparable year-to-year employment figures for eight private industry segments.

As new facilities now under construction come into operation, and as the effects of a rapidly expanding market for nuclear power are reflected in employment figures, increases over the next few years should be significant.

TABLE 4

Employment in Private Establishments
Selected Economic Segments

Industry Segment	Jan. 1961	Jan. 1962	Jan. 1963	Jan. 1964	Jan. 1965	Jan. 1966	Jan. 1967
Mill,ng	4,793	3,381	2,579	2,255	2,079	2,054	1,962
Production of Feed	522	538	620	643	352	343	1.154
Special Materials	1,970	1,890	1,507	1,757	1,228	1,503	1,350
Fuel Fabrication	506	786	1,819	2,236	1,658	1,693	1,934
Reactors & Components	7,992	7,966	10,091	12,245	14,094	12,068	12,725
Waste Dispos?l	51	44	86	79	67	58	34
Instruments	3,717	4,237	4,745	3,853	4,979	4,068	4,027
Isotopes	215	291	365	348	407	642	824

TABLE 5

Number of Private Establishments
Selected Economic Segments

Industry Segment	Jan.* 1961	Jan. 1962	Jan. 1963	Jan. 1964	Jan. 1965	Jan. 1966	Jan. 1967
Milling	23	23	23	26	24	23	15
Production of Feed	6	6	5	8	6	4	6
Sperial Materials	15	15	19	33	31	28	20
Fuel Fabrication	6	6	11	14	13	13	13
Reactors & Components	38	38	50	58	61	48	41
Waste Disposal	4	4	8	10	9	8	7
Instruments	106	106	94	115	106	82	70
Isotopes	21	21	25	22	22	33	30

* Employment data for January 1961 came from the January 1962 survey and covered the same establishments. Comparable surveys were not made in 1961 and earlier years.

E. Nuclear-Electric Power

The AEC continues to expect nuclear generating capacity in 1980 to total about 150 million kilowatts, as indicated in Chart E. Other data in Chart E are based on estimates by the electrical industry's weekly magazine, "Electrical World," in the September 16, 1968, issue. This is a current estimate which reflects a generally anticipated overall electric utility generating capacity some 20 percent above the estimate used in a similar chart in this report for 1967. The estimate used in last year's report was made by the Federal Power Commission in 1964 and is presently in the process of being updated.

CHART E

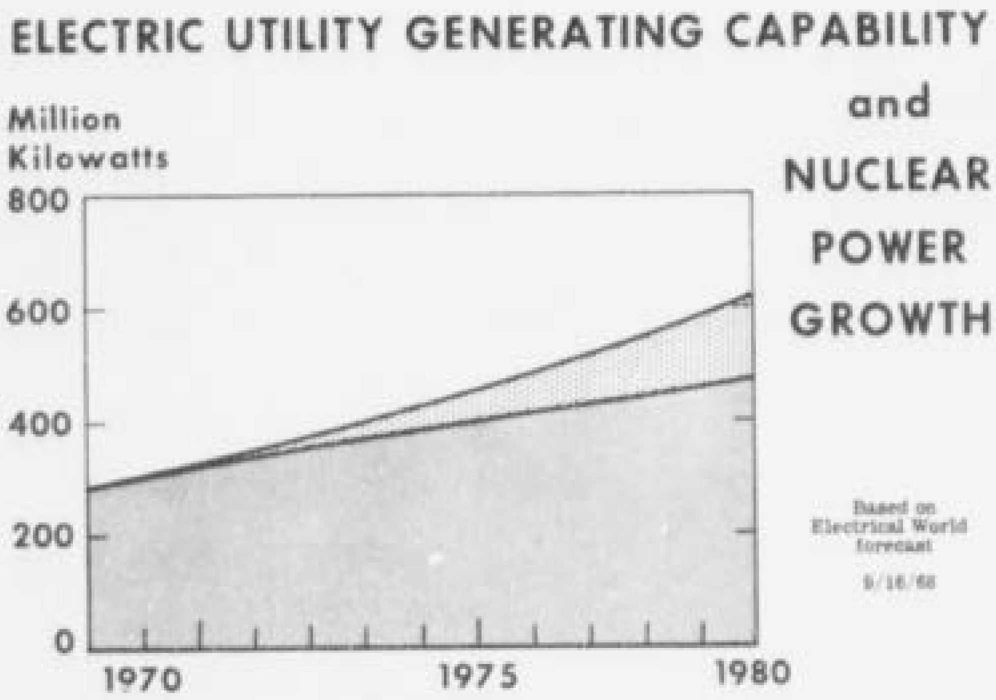

The year-by-year economic impact of nuclear power plant construction[2] and operation is shown on Chart F, together with estimated cumulative market totals for the 13-year period 1968-1980. The cumulative total for plant construction and fuel costs are well in excess of $40 billion. This is a substantial increase from the estimate included in this report for last year. The increase comes primarily from a change in the estimated cost of constructing nuclear plants based on additional information received during the year. A year ago this was estimated at $130/kilowatt of electrical capacity. The estimate shown in Chart F is based on an average estimated construction cost of $150/kilowatt.

Chart F shows the predicted market for the individual steps in the nuclear fuel cycle.

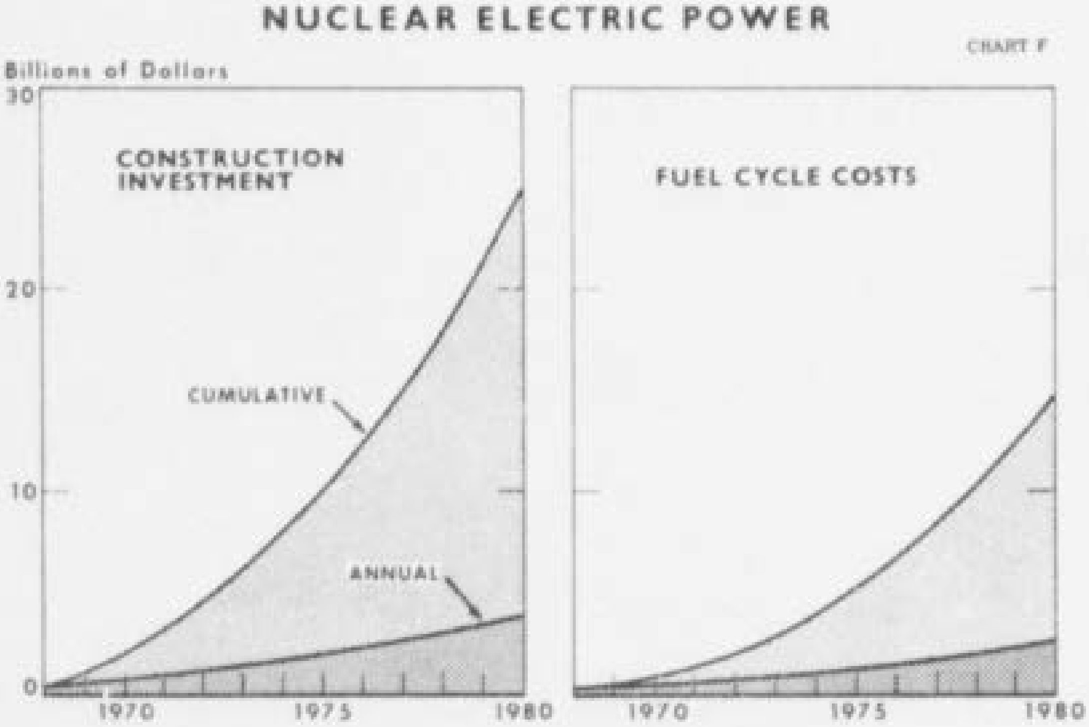

2/ See 5/23/68 remarks by Ernest B. Tremmel, Director, Division of Industrial Participation, USAEC, at the Mechanical Contractors Association Convention, San Juan, Puerto Rico.

Chart G shows the predicted market for the individual steps in the nuclear fuel cycle. Each of these steps is discussed in detail in the following parts of this report.

NUCLEAR FUEL CYCLE - PREDICTED ANNUAL MARKET

CHART G

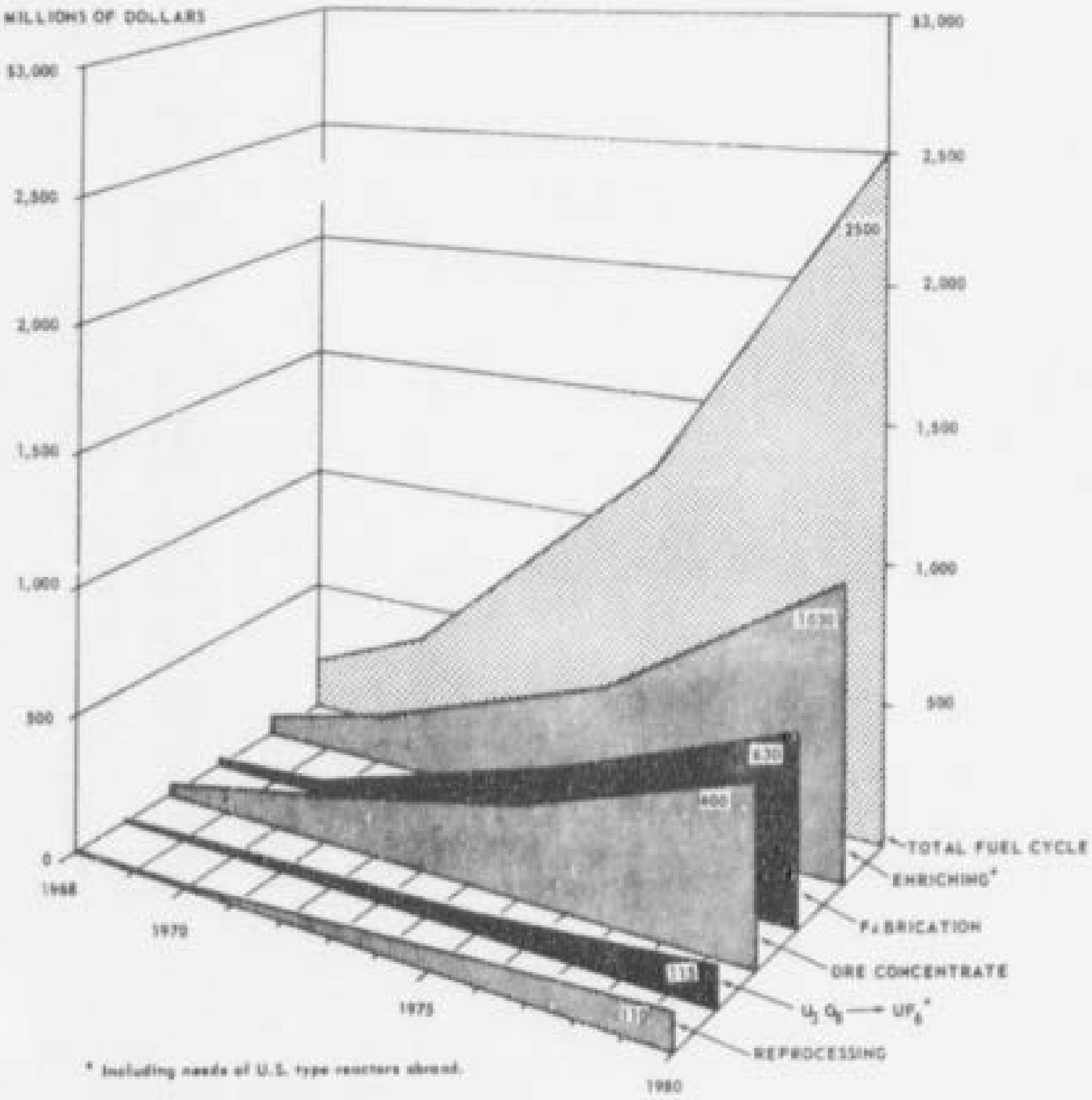

Chart H shows orders by U.S. electric utilities for electric generating equipment f.r the past eleven years as reported by the Edison Electric Institute. The cyclical nature of orders in past years shows clearly in the 1958-62 period. This cyclical trend is expected to be evident again as orders are expected to be lower in 1968 than in the peak year of 1967.

CHART H

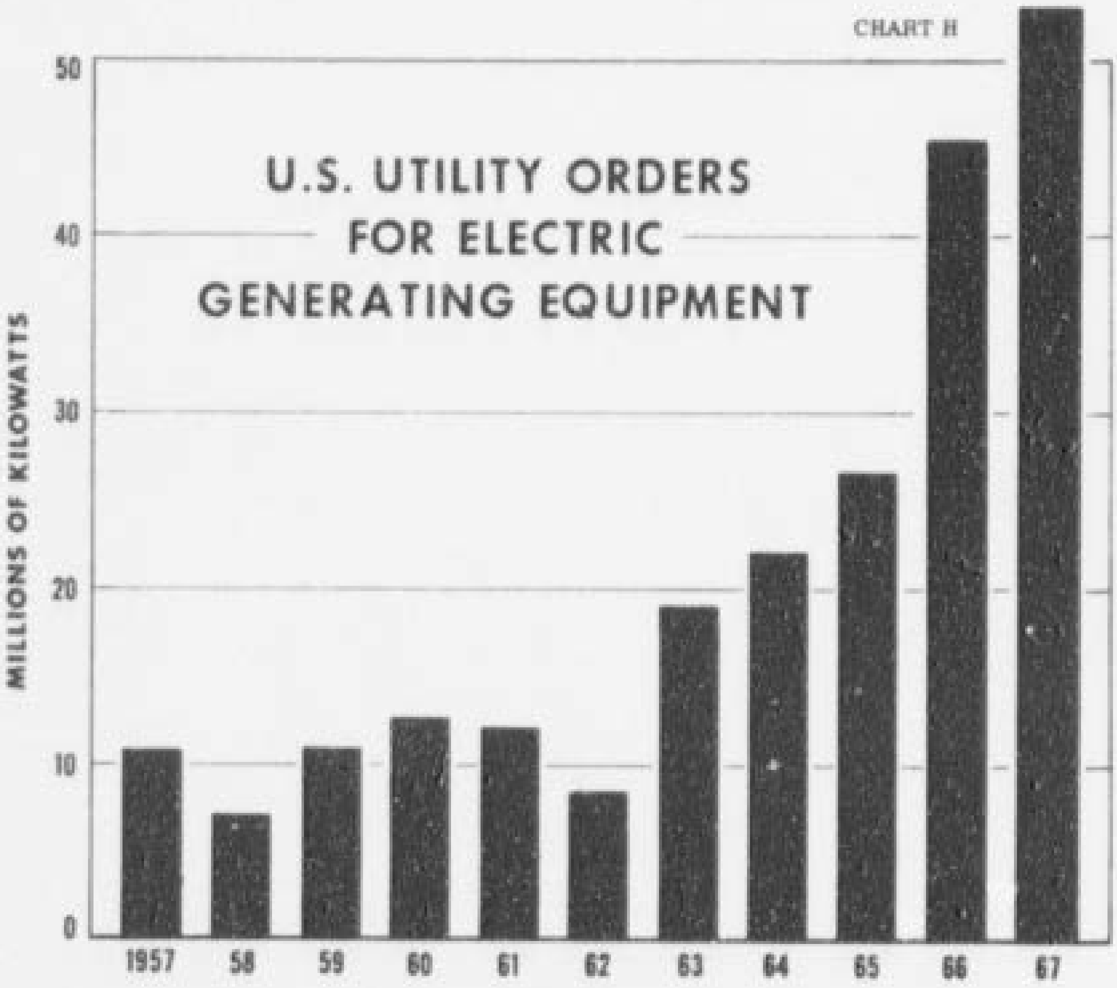

NUCLEAR INDUSTRY HIGHLIGHTS - 1968

1968 was another year of growth for the nuclear industry, with the peaceful applications of nuclear energy showing continuing progress in many areas. The following are the highlights of the year. Detailed information on the various subjects may be found in later sections of the report.

A. Civilian Nuclear Power

1. New Orders

Utility orders for nuclear steam supply systems continued to attract considerable attention and although at a high level, were below the record-breaking number of orders in 1967. By fall, it became apparent that the rate of orders for central station power plants was beginning to slow down. This trend represents a long predicted leveling off that had been anticipated for some time. Toward the end of the year the long lead time for ordering, licensing and constructing nuclear plants in comparision with fossil fuel plants began to be reflected in a hedge by some utilities to purchase fossil plants.

The long predicted leveling off of orders was also reflected in the decline of new plant announcements. For both fossil and nuclear plants announcements totaled approximately 35,000 megawatts electrical (MWE) for the first nine months of 1968 as compared to approximately 42,000 MWE for the first nine months of 1967, reflecting a decrease of about 16 percent.

Orders for nuclear steam supply systems (as distinguished from new plant announcements) were about 13,000 MWE for the first nine months of 1968 compared with about 20,000 MWE in the same period in 1967, a decrease of close to 33 percent.

2. Uranium Supply and Exploration

Uranium supply and exploration continue to receive considerable public attention and the uranium industry was reviewed in considerable detail at a Nuclear Fuels Conference held in Oklahoma City in May 1968.1/ Exploration activity during 1968 reached an all-time high, and again, as in 1967, more than doubled the rate of activity for the previous year.

1/ Conference on "Nuclear Fuel - Exploration to Power Reactors," Oklahoma City, Oklahoma, May 23-24, 1968, presented by SINB and AIF in cooperation with USAEC.

The transition from a Government to a private market seemed well on its way and by September 1968, private domestic sales and contract commitments extending through 1977 were nearly 60,000 tons of U_3O_8 in uranium concentrate reflecting nearly two-thirds of the total uranium that will be required by the commercial market through 1975. In comparison, under AEC contracts which end December 31, 1970, purchase commitments for 1969-70 total about 15,000 tons.

The Commission during the year again reviewed its uranium supply policies and on September 6, 1968, issued a revised policy statement. The details of this statement are reviewed under Category I in this report. While no date is set for removal of the restriction on enrichment of foreign uranium for domestic use, the revised policy states that it appears that it may be possible to remove it by July 1, 1973, or earlier.[2/]

3. Fuel Reprocessing

During 1968 this area of the nuclear fuel cycle attracted more attention from private industry than in any previous year. The NFS plant continued in operation, its main problem continuing to be the lack of an adequate market in the near future. The General Electric Company started construction on its plant nerr Morris, Illinois. Allied Chemical, Atlantic Richfield, National Lead, and Gulf General Atomic continued their activities in regard to offering reprocessinn services.

During the year several utilities and at least one reactor manufacturer were inviting proposals for their requirements for reprocessing from potential candidates in this area.

In contrast to the great interest by commercial companies in this area, the near term loads to support this part of the industry are quite small and if recent announcements of plant slippages and longer burn-ups are taken into consideration, indications are that there will not be any significant market for this service until well into the 1970's.

On January 3, 1968, the AEC announced in the Federal Register 33FR30 under Miscellaneous Notices, revisions to its policy to receive and process commercial reactor fuels.

2/ See AEC Public Release L-210, dated 9/6/68.

4. Nuclear Steam Supply System Competition

Considerable attention was focused during the year on competition in the nuclear steam supply industry. Highlighting this area was the completion of a report by Arthur D. Little, Inc., under the sponsorship of the Commission and the Department of Justice entitled "Competition in the Nuclear Power Supply Industry." This report was made public near the end of the year and provides a very comprehensive study of the growing nuclear industry.

While all four manufacturers of light water reactors continued to actively submit proposals, the larger suppliers, G. E. and Westinghouse, together, continued to receive a large percentage of all new orders.3/

Attention was directed to this area by the purchase by Combustion Engineering of approximately 21 percent of the stock of the United Nuclear Corporation and the filing of a suit by United Nuclear Corporation against Combustion Engineering. This was the first time an antitrust question of a serious nature had been raised in Court by the nuclear supply industry.

Additional competition from Gulf General Atomic continued to be in the offing by approval of all parties concerned for the construction of the Fort St. Vrain project for the Public Service Company of Colorado, and their proposal to a utility for a large high temperature gas-cooled reactor, thus bringing into possible competition a third technology in addition to the boiling water and pressurized water types.

5. New Plant Investment

Expansion of manufacturing facilities for the supply of nuclear-electric plant components continued. Either new projects or expansion of existing facilities were underway at some fifteen locations in the United States at an estimated cost of a quarter of a billion dollars. These included the plans by the four light water reactor suppliers to expand their manufacturing facilities for the fabrication of reactor fuel, the manufacture of turbine generator sets for nuclear plants and the fabrication of pressure vessels.

Several new companies were organized in the past year in the service industry and radiation applications fields. Industry in these and related fields also announced plans for significant new plant investment.

3/ See Commissioner Ramey's article, "Competitive Vigor in Nuclear Power," in July-August 1968 issue of Harvard Business Review.

6. Nuclear Fuel

During 1968, the utilities continued to purchase the first fuel loads for reactors from the reactor manufacturers but continued to show their desire to be independent of reactor suppliers in making arrangements for subsequent fuel loadings. United Nuclear, the only independent supplier of fuel core loadings for nuclear-electric plants, received one of its largest orders since its entry into this field, namely, an order for replacement fuel from the Consolidated Edison Company of New York.

Other companies continued to indicate an interest in entering the market for reload cores.

7. Dual-Purpose Plants

The year was one both of encouragement and disappointment in progress on dual-purpose plants. In the areas of desalting, the originally planned Bolsa Island project was reevaluated for a number of reasons, and studies of alternative measures to achieve the significant objectives of the project originally planned are underway.[4/] The world's first privately owned dual-purpose nuclear plant (process heat and electrical) was announced by Consumers Power Company and Dow Chemical Company. This plant is to be located near Midland, Michigan, and will guarantee 1,300 megawatts of electricity and also four million pounds of steam per hour for industrial use by the Dow Chemical Company.

A joint United States/Mexico study, in cooperation with the International Atomic Energy Agency, for a dual-purpose desalting plant in the arid area adjoining the two countries was scheduled for completion by the end of the year.

Studies of a very preliminary nature are also underway regarding dual-purpose desalting plants in New York, Utah, and Puerto Rico.

8. Fast Reactors

The AEC's program for the development of fast breeder reactors was brought into sharper focus during the year with the issuance of a ten-volume comprehensive plan for the development of a liquid metal fast breeder reactor by the AEC. The AEC reports which were prepared with participation by industry, define objectives, identify necessary R&D work, establish schedules

---

4/ Remarks by James T. Ramey, Commissioner, USAEC, "Nuclear Desalting -- A Look to the Future," at Conference on "Seawater Conversion -- State of the Art," Sacramento, California, June 18, 1968, AEC Public Release S-30-68.

and milestones and estimate the cost required in order to meet the program objective of producing commercial power with breeder reactors in the 1980's.

Work on the steam cooled fast breeder reactor by General Electric and the East Central Nuclear Group was discontinued during the year.

The Edison Electric Institute also completed and made available to the public a report reviewing existing breeder programs. The re ort recommended an acceleration of utility participation in breeder programs with a goal of construction of demonstration plants. The report was consistent with the AEC's program in that it focused primary attention on the liquid metal breeder, although it recommended accelerated effort on other breeder concepts such as the gas-cooled.

9. Plutonium Recycle

The AEC continued to encourage the utilities and nuclear supply industry to devote more attention to the demonstration of plutonium recycle for use in thermal reactors. Additional programs in this area were undertaken during the year by the industry with the engagement of United Nuclear by Commonwealth Edison to fabricate plutonium recycle fuel assemblies for operation in the Dresden I nuclear power plant. In addition, the Edison Electric Institute continued with its plans and negotiations with General Electric and Westinghouse for [illegible] plutonium recycle fuel element demonstration program. They [illegible]so placed a contract with Gulf General Atomic for a feasib[illegible]ity study of plutonium recycle in gas-cooled reactors.

B. Private Ownership

The transition to private ownership continued in an orderly fashion attributing to the workability of the private ownership legislation and the careful and thorough attention given to its drafting by the AEC and the Joint Committee on Atomic Energy.

1. In November the AEC amended Part 70 of its regulations covering special nuclear material to provide a general license for the ownership of special nuclear material.5/ The new general license will permit an individual or firm to own special nuclear material, but not physically possess or use it. This arrangement will permit private companies to finance fuel inventories for nuclear power plants and still allow the Commission to retain controls over possession, use, and physical

---

5/ See AEC Public Release L-158, dated July 8, 1968.

transfer of special nuclear materials as required for national security purposes.

2. Interest in providing a commercial fuel leasing service expanded with four or more organizations actively discussing such arrangements with utilities.

C. Enriching

This area continued to receive increasing attention and in the next few years will no doubt be an area of far reaching interest since it represents approximately one-third the cost of a fuel core for a light water power reactor and is the only major step in the nuclear fuel cycle not performed by industry.

1. The Atomic Industrial Forum completed a study of the feasibility and desirability of transferring to private industry one or more of the Commission's gaseous diffusion plants. The Forum committee recommends transferring the plants to private industry. Its report suggests beginning preliminary action at once and completing the change-over by 1972.

2. A Commission parallel study, which is also concerned with matters of national security, has not yet been completed. The question of future methods of operation of these plants represents one of the most challenging and difficult problems facing the Commission in the coming years.

3. A schedule of charges[6/] for toll enrichment of privately owned uranium was established by the Commission on November 29, 1967. Charges are based on $26 per kilogram unit of a separative work, a tails assay of 0.2 percent U-235, and a uranium feed component of $8.00 per pound of U_3O_8.

4. The first toll enriching agreement with a U.S. customer was signed with Kerr-McGee. It covered fuel for the Omaha Public Power District, Fort Calhoun plant at an estimated cost of $9.4 million for the enriching services.

D. Safeguards

Considerable attention was given to this area during the year. The Commission's program developed in cooperation with industry to station resident inspectors at five privately owned facilities was completed on June 30, 1968. A report on this program is being prepared and a study will be made to determine what role, if any, resident inspectors could play in the safeguards program.

6/ See AEC Public Release K-277, dated November 28, 1967.

The first safeguards training course[7/] opened at Argonne National Laboratory on September 16, 1968. Sixteen students were enrolled, about evenly divided between foreign, U.S. domestic industry, and AEC personnel. A second course and a management seminar will be offered in the Spring of 1969.

E. Radioisotopes

1. The AEC's Division of Isotopes Development completed a study of the radiation processing industry and predicts a probable growth rate of 25 percent per year. Curing of surface coatings, production of wood-plastics products, sterilization of medical supplies, and the production and synthesis of special copolymers has reached a total volume of radiation processing estimated by private industry at about $250 million in 1967.

2. A new report, "Radioisotopes - Production and Development of Large Scale Uses," summarizing major uses of isotopes and outlining related Atomic Energy Commission development programs, was published by the AEC.[8/]

3. A setback occurred for the AEC-Army food irradiation program this year when the Food and Drug Administration rejected the Army's petition to permit use of irradiated ham to feed soldiers. FDA also withdrew its approval of bacon.

F. Plowshare

Significant progress was made with the successful detonation of a nuclear explosive on December 10, 1967, in the Project GASBUGGY experiment. Preliminary results from gas flow tests to date appear promising. The industrial community has expressed considerable interest and submitted a number of proposals for other commercial applications for various purposes.

Fourteen companies have either established organizations or otherwise indicated their interest in providing partial or complete design, advisory, and operational services to user firms contemplating industrial applications of nuclear explosions.

G. Spin-Off

Keen industry interest was shown in the commercial development of two non-nuclear inventions originating in the course of AEC research

---

7/ See AEC Public Release L-31, dated February 15, 1968.

8/ See AEC Public Release L-156, dated July 5, 1968. Available from the Superintendent of Documents, U.S. Government Printing Office, Washington, D. C., 20402, for $0.50 per copy.

and development.

A private company was formed to assist industry in applying "rolamite" technology. "Rolamite" is an elementary mechanism, like the wheel or the lever, that was developed at the AEC's Sandia Laboratory in an advanced development area.

A symposium was held in AEC Headquarters to discuss possible applications of, and the potential market for, TRIP Steels. These are a new series of alloy steels combining high strength with high ductility with the name TRIP coming from Transformation Induced Plasticity, the steels being of a wrought austenitic type which gradually transforms to the martensitic condition when stresses exceeding the yield strength are applied. These steels were invented under AEC-supported basic research work at the University of California.

H. Licensing, Regulation and Legislation

1968 has been the AEC's most active year of civilian nuclear power licensing to date. Prospects are that by year's end 44 nuclear power units will be under construction in 19 states, nearly all of which are scheduled to begin commercial operations over the next five years.

To help assure that licensing procedures keep pace with rapid expansion of the nuclear industry, the Commission appointed a new internal study group to review the licensing process as a follow-up to several previous studies by special panels and the Joint Committee on Atomic Energy.

A number of bills were introduced in the 90th Congress which would broaden the areas of consideration in the licensing of nuclear reactors. Proposed legislation was pending in Congress which would give the AEC, or in some bills, other agencies such as the Federal Power Commission, authority to consider thermal effects and antitrust implications in reactor licensing proceedings.

A survey of injury frequency and severity rates again showed the work-injury experience of the private sector of the atomic energy industry to be better than the average for all manufacturing industries as compiled by the Bureau of Labor Statistics. Reflecting the excellent safety record of the nuclear industry, the insurance pools which were created 11 years ago to provide private nuclear energy liability insurance refunded the major portion of premiums paid by policy holders in 1958.

I. Interstate Agreements

The Commission continued to work closely with the Southern Interstate Nuclear Board (SINB) in sponsoring projects of mutual interest. The Western Interstate Nuclear Compact moved nearer reality with legislation being considered by state legislatures and the U.S. Congress. Both the Midwest states and the New England states are actively considering establishing compacts.

J. Federal-State Relations

Colorado and Idaho became the 18th and 19th states to sign agreements with the AEC under which certain of the AEC's regulatory authority over byproduct, source, and small quantities of special nuclear materials are transferred to the states.

K. Meetings with Industry

The Commission continued to meet with representatives of the nuclear industry. Meetings were held with a number of industry associations, industrial concerns, and utilities to discuss current problems and future plans.

L. International Activities

Discussions were initiated between Brown-Boveri and North American Rockwell looking toward an agreement under which heavy electrical equipment produced by Brown-Boveri in Switzerland, including large turbine-generator sets for nuclear-electric plants, would be marketed and manufactured in the U.S. by North American Rockwell.

A further trend toward competition in the U.S. by European manufacturers was indicated by the placement of orders by Westinghouse for two large reactor primary tanks with Rotterdam Dockyard Company.

The Atomic Industrial Forum and the American Nuclear Society joined in sponsoring an International Conference and Exposition in Washington, D. C., November 10-15.

The Commission continued its program of exhibits in foreign countries with exhibits at Taipei, Taiwan; Caracas, Venezuela; Seoul, Korea; Cordoba, Argentina; and Mexico City, Mexico.

[illegible]

PRIVATE OWNERSHIP - TOLL ENRICHING

A discussion of private ownership of special nuclear material (SNM) and toll enriching was presented in a special section of this report in 1966 and 1967. We are repeating some of the basic information again this year together with a summary of actions taken since our last report.

Background Information on Private Ownership and Toll Enriching

As reviewed in past reports, the Atomic Energy Act of 1946 placed complete control of atomic energy in the Government because of its military character and the lack of general knowledge about this new form of energy. Almost 10 years lat r, the Atomic Energy Act of 1954 was passed permitting private industry to participate in the development of nuclear energy and private persons to possess and use special nuclear materials under license from the AEC.

The Private Ownership of Special Nuclear Materials Act was approved on August 26, 1964.

Before the private ownership amendment, private industry could own all components of a nuclear power plant except the enriched uranium fuel and the plutonium produced in the reactor. Enactment of the amendment for private ownership in 1964 eliminated this exclusion.

The provision to permit enrichment of privately owned uranium for a charge or "toll" (called toll enrichment) was one of the most important developments in the private ownership legislation. Under this concept, the AEC will receive privately owned uranium and perform U-235 enrichment services (that is, the enrichment of the uranium in the isotope U-235) on a toll basis. This results in the return of a lesser amount of uranium, containing a greater concentration of U-235, to the customer.

Time-Table for Private Ownership Legislation

In order to assist the reader of this report, we are again including the time-table for implementation of private ownership.

As can be seen from the time-table, January 1, 1971, is a significant turning point in the AEC's furnishing of special nuclear material on a lease basis. Starting at that time, the AEC is prohibited from distributing special nuclear materials for use in power reactors, except by sale. Distribution for use in research reactors by lease or grant is not affected. In addition, the AEC is authorized to offer uranium enrichment services, commonly referred to as toll enriching, beginning on January 1, 1969.

TABLE 6

Time-Table

Private Ownership Permitted	August 26, 1964
Toll Enriching of Privately Owned Uranium May Begin	January 1, 1969
AEC Prohibited From Entering Into New Lease Agreements For Power Reactor Fuel	January 1, 1971
AEC Guaranteed Purchase Price For Plutonium Will Terminate	December 31, 1970
Private Ownership of Special Nuclear Material Mandatory For Commercial Utilization Facilities (section 103 or 104b facilities). All Lease arrangements must terminate	July 1, 1973

December 31, 1970, is also the expiration date of the Commission's guaranteed purchase prices for plutonium (produced in reactors from the use of uranium leased or sold by the AEC) delivered to the AEC by persons licensed under section 104 of the Atomic Energy Act of 1954, as amended. Guaranteed prices for U-233 can continue past that date for periods up to 10 years. Finally, all Government-owned special nuclear materials, previously leased for use in facilities licensed under section 103 or 104b of the Act, must be returned or converted to private ownership by July 1, 1973.

Status of Implementing Private Ownership and Toll Enriching

Under the authorization of the Private Ownership of Special Nuclear Materials Act of 1964, a number of steps to implement private ownership and toll enriching were to be undertaken by the Commission. The status of these implementing actions is given below.

A. Actions Taken During 1965, 1966, and 1967

1. Control, Protection, and Safeguarding of Special Nuclear Material

In the area of maintaining and establishing regulatory controls, the Commission announced on September 21, 1965, that it was planning to amend its regulations concerning its

licensing authority over privately owned special nuclear material and the import and export of special nuclear material. On February 7, 1967, the Commission published amendments to Part 50, "Licensi[illegible] of Production and Utilization Facilities"; Part 70, "Special Nuclear Material"; Part 115, "Procedures for Review of Certain Nuclear Reactors Exempted from Licensing Requirements"; and Part 140, "Financial Protection Requirements and Indemnity Agreements". The amendments became effective on March 9, 1967.

Of particular importance is the amendment to Part 70 requiring licensees who possess special nuclear material to control and account for the material in the interest of national security. This control had been exercised over SNM by the Commission through contractual relationships in the lease of Government-owned material, but a new regulation was required for privately owned SNM.

In May 1967, the AEC announced the establishment of two new safeguards organizations: an Office of Safeguards and Materials Management, reporting to the General Manager; and a Division of Nuclear Materials Safeguards, reporting to the Director of Regulation.

Also in May 1967, the AEC developed plans to station resident safeguards inspectors at five private facilities. These are: Nuclear Fuel Services, Inc., West Valley, New York, and Erwin, Tennessee, facilities; Nuclear Materials and Equipment Corporation facilities, Apollo and Leechburg, Pennsylvania; and United Nuclear Corporation, Hematite, Missouri.[1]

In August 1967, the AEC announced the appointment of an advisory committee[2] on nuclear materials safeguards. The committee is composed of the following members: John Palfrey, Chairman; Delmar L. Crowson and Dr. Russell P. Wischow, Vice Chairmen; Roger E. Batzel; Francis P. Cotter; Paul Grady; Dr. Jane Hall; Ralph F. Lumb; Horace W. Norton III; Clement J. Rodden; Louis H. Roddis, Jr.; Walton A. Rodger; Lombard Squires; Charles D. W. Thornton; Fred H. Tingey; Francis O. Wilcox; and J. Ernest Wilkins.

1/ See AEC Public Release K-121, dated May 12, 1967.
2/ See AEC Public Release K-212, dated August 29, 1967.

2. Guaranteed Purchase Prices for Pu and U-233

Another implementing step taken by AEC was the establishment of guaranteed purchase prices (not to exceed AEC's estimated value of the material as fuel in a nuclear reactor) for special nuclear material produced by licensees through the use of AEC sold or leased uranium and delivered to the Commission prior to January 1, 1971, for plutonium, and for U-233 for periods up to 10 years. The prices established are:3/

For plutonium in the forms of nitrate, dioxide, or metal and meeting AEC specifications, $9.28 per gram of the contained isotopes plutonium-239 and plutonium-241.

For uranium enriched in uranium-233 in the forms of nitrate, dioxide, or metal and meeting AEC specifications, $13 per gram of the contained isotope uranium-233, subject to adjustment for the presence of other uranium isotopes and deductions for handling costs.

3. Charge for Uranium Enrichment Services

The AEC published on November 29, 1967, a new schedule of charges for the toll enrichment of privately owned uranium based on $26 per kilogram unit of separative work. The "tails" assay (U-235 content of tails material) on which this charge is based is 0.2 percent U-235. In the schedule of charges for lease or sale of enriched uranium, the charge for the natural uranium feed component would continue to be based on $8 per pound of U_3O_8 through June 30, 1973.

4. Uranium Sale and Lease Policies

A further implementing step required AEC to establish reasonable sale prices for special nuclear material distributed through sale (by the AEC). In July 1966 4/ the Commission announced its proposed post-1968 policies concerning the supply of uranium and in November 1967 and September 1968 issued more detailed statements on its policies and proposed procedures for their implementation.5/6/7/

3/ See AEC Public Release H-65, dated March 25, 1965, and Federal Register 3886, dated March 25, 1965.

4/ See AEC Public Release IN-704, dated July 25, 1966.

5/ See AEC Public Release K-263, dated November 6, 1967.

6/ Remarks by Wilfred E. Johnson, Commissioner, U.S. Atomic Energy Commission, at the Atomic Industrial Forum 1967 Annual Conference, Chicago, Illinois, November 6, 1967, AEC Public Release S-48-67.

7/ See AEC Public Release L-210, dated September 6, 1968.

5. Toll Enriching Criteria and Contracts

The AEC published in the Federal Register effective December 23, 1966,[8] the final criteria under which it will perform toll enrichment of privately owned uranium. These criteria provide: (a) for AEC entering into enrichment service contracts for up to 30 years, (b) customer option to take tails if desired, (c) a ceiling on the cost of separative work of $30/kg. subject to escalation based on costs of power and labor, (d) a prohibition against enrichment of foreign uranium intended for use in domestic reactors, (e) two standard types of contracts for enriching services covering "firm quantities" and "requirements", and (f) that quantities of feed and charges will be determined by a standard table of enriching services.

Copies of the standard contracts may be obtained from the U.S. Atomic Energy Commission, Oak Ridge Operations Office, Oak Ridge, Tennessee 37830.

6. Declassification of Gaseous Diffusion Plant Information

Declassification of capacity and economics of one or more of AEC's gaseous diffusion plants was suggested by many witnesses during the 1966 JCAE hearings on private ownership and toll enriching. This action was taken in 1967. See Category II of this report for further discussion of this subject.

B. Status of Toll Enriching Contracts

The first toll enriching contract was signed by the AEC in June 1967 with the Swedish utility OKG to provide about 19,000 kg. of U-235 for the Oskarshamn 400 MWE boiling water reactor over a 30-year period. The first domestic contract was executed on September 14, 1968, between AEC and Kerr-McGee Corporation for fuel for the Omaha Public Power District's Fort Calhoun Unit #1.

C. Actions Taken in 1968

1. AEC Uranium Policy

Further clarification of AEC's uranium policies was provided the nuclear fuel industry at a symposium sponsored by the Southern Interstate Nuclear Board in May 1968 when AEC Commissioner Wilfred Johnson in his address "The Uranium Fuel Industry in Transition" reviewed the Commission's policies.[9]

8/ See AEC Public Release IN-750, dated December 22, 1966.

9/ Remarks by Wilfred E. Johnson, Commissioner, U.S. Atomic Energy Commission, at Southern Interstate Nuclear Board Nuclear Fuel Symposium, Oklahoma City, Oklahoma, May 23, 1968, AEC Public Release S-25-68.

2. Regulatory Action

The AEC amended Part 70 of its regulations covering special nuclear material to provide a general license for the ownership of special nuclear material.[10/] The new general license will permit an individual or firm to own special nuclear material, but not physically possess or use it. This arrangement will permit private companies to finance fuel inventories for nuclear power plants and still allow the Commission to retain controls over possession, use, and physical transfer of special nuclear materials as required for national security purposes.

D. Areas That Still Require Attention

While essentially all of the implementing actions required of the AEC to get private ownership and toll enriching underway have been taken, there does exist a question raised by industry spokesmen on the need for flexibility in AEC toll enriching policy for the customer to choose the isotopic content of tails material resulting from the enriching of his uranium.

The major step of transferring the Government enriching plants to private industry is under study by AEC and by private industry. This subject is treated in more detail in Category II of this report.

10/ See AEC Public Release L-158, dated July 8, 1968.

CATEGORY I
RAW FUEL MATERIALS

This category again received considerable public attention during the year and the uranium industry was reviewed in considerable detail at a nuclear fuels conference held in Oklahoma City in May 1968.1/ 2/

Market Prediction for Uranium (U_3O_8)
(Annual Sales)

	Domestic	Foreign
1970	$120 million	$ 80 million
1975	$340 million	$260 million
1980	$600 million	$600 million

While some concern was still being expressed about the ability of the uranium mining and milling industry to meet the long-term demands for uranium, exploration activity during the year reached an all-time high and again, as in 1967, more than doubled the rate of activity for the previous year. Plans for 1968 indicated that there would be nearly 25 million feet of drilling, compared with approximately 11 million feet in 1967.

The transition from a Government to a private market seemed well on its way and by September 1968, private domestic sales and contract commitments, extending through 1977, were nearly 60,000 tons of U_3O_8 in uranium concentrates, reflecting nearly two-thirds of the total uranium that will be required by the commercial market through 1975. In comparison, under AEC contracts which end December 31, 1970, purchase commitments for 1969-1970 total about 15,000 tons.

The Commission again during the year reviewed its uranium supply policies and on September 6, 1968, issued a revised policy statement. In this statement, the Commission reaffirmed its preference for toll enrichment of privately owned uranium in AEC facilities over sales from Government stocks. The statement points out, however, that the AEC is willing, in certain circumstances, to sell Government-owned uranium on a single transaction basis, and notes that preference will be placed on the sale of natural uranium over enriched uranium if circumstances permit. While no date is set for removal of the restriction on enrichment of foreign uranium for domestic use, the policy states that it appears that it may be possible to remove it by July 1, 1973, or earlier.3/

1/ Remarks by Wilfred E. Johnson, Commissioner, USAEC, "The Uranium Fuel Industry in Transition," at the Southern Interstate Nuclear Board Symposium, Oklahoma City, Oklahoma, May 23, 1968, AEC Public Release S-25-68.

2/ Remarks by Rafford L. Faulkner, Director, Division of Raw Materials, USAEC, "Reserves," at the Conference on Nuclear Fuels, Oklahoma City, Oklahoma, May 23, 1968, AEC Public Release S-24-68.

3/ See AEC Public Release L-210, dated 9/6/68.

A. Uranium

1. Uranium Requirements

The Commission's May 1967 forecast of nuclear power growth predicts a range of 120,000 to 170,000 MWE, with a median of 150,000 MWE installed by 1980. New plant orders for operation in the early and mid-70's have kept pace with AEC's forecast for that period. The nuclear growth potential in the non-Communist world (excluding U.S.), reviewed for AEC in 1966 by A. D. Little, Inc.4/ indicates that by 1980, nuclear installations in the non-Communist world would reach approximately 130,000 megawatts electrical installed.

The requirements shown in Chart I-A are based on the 150,000 MWE domestic power growth forecasts and assume that plutonium recycle will be adopted beginning in the mid-70's. If plutonium is not recycled, the projected uranium requirements would be about 10 percent higher by 1980. The dollar figures in Chart I-A are arbitrarily based on $8.00 per pound U_3O_8, although this figure could change considerably by 1980. On the basis of this forecast, the cumulative uranium requirements through 1980 amount to about 250,000 tons U_3O_8 for the U.S. About 240,000 tons for foreign needs have also been forecast, making nearly 500,000 tons for the non-Communist world through 1980.

ESTIMATED
ANNUAL URANIUM REQUIREMENTS

CHART I-A

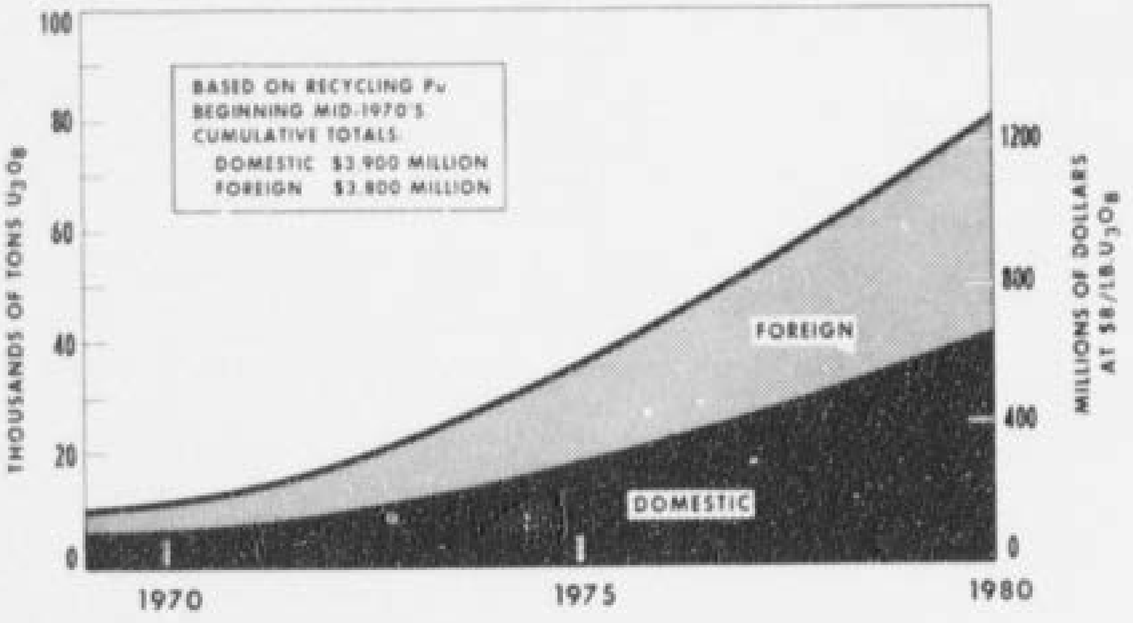

4/ TID-22973 - "The Growth of Foreign Nuclear Power," April 1966.

If priced at \$8.00 per pound U_3O_8, the combined foreign and domestic estimated uranium requirements of about 500,000 tons through 1980 would represent a value of about \$8 billion.

Investment in exploration and production facilities may require between \$20,000 and \$30,000 per ton of annual output of U_3O_8. Therefore, the total investment to meet the needed production capability of nearly 40,000 tons yearly by 1980 is estimated in excess of \$1 billion.

2. Uranium Reserves

The AEC updates the uranium reserves information each January. The estimates of domestic uranium ore reserves by states available at a price of \$8.00/pound U_3O_8 are as follows:

Area	Reserves as of January 1, 1968 (Tons U_3O_8)	% of Total	Previous Years 1966 (Tons U_3O_8)	Previous Years 1967 (Tons U_3O_8)
Colorado	9,400	6	9,600	9,800
New Mexico	70,000	47	70,400	67,200
Wyoming	53,000	36	53,100	53,000
Others	15,600	11	11,900	11,000
	148,000		145,000	141,000

As exploration proceeds, it is anticipated that reserves of uranium will be found in other areas of the country, as well as in those states identified previously with ore reserves.

U.S. and world uranium resource estimates, as compiled by the European Nuclear Energy Agency in cooperation with the International Atomic Energy Agency5/, are shown in Chart I-B. The U.S. AEC participated in this effort.

Chart I-C compares reasonably assured uranium reserves at \$10 or less per pound with the cumulative requirements 1968 through 1980. Physical and economic limitations on mining rates of the reserves shown on I-C would permit only about two-thirds of the uranium to be mined and made available to the consumer by 1980.

5/ Joint Report by the ENEA and IAEA, "Uranium Resources Revised Estimates," December 1967.

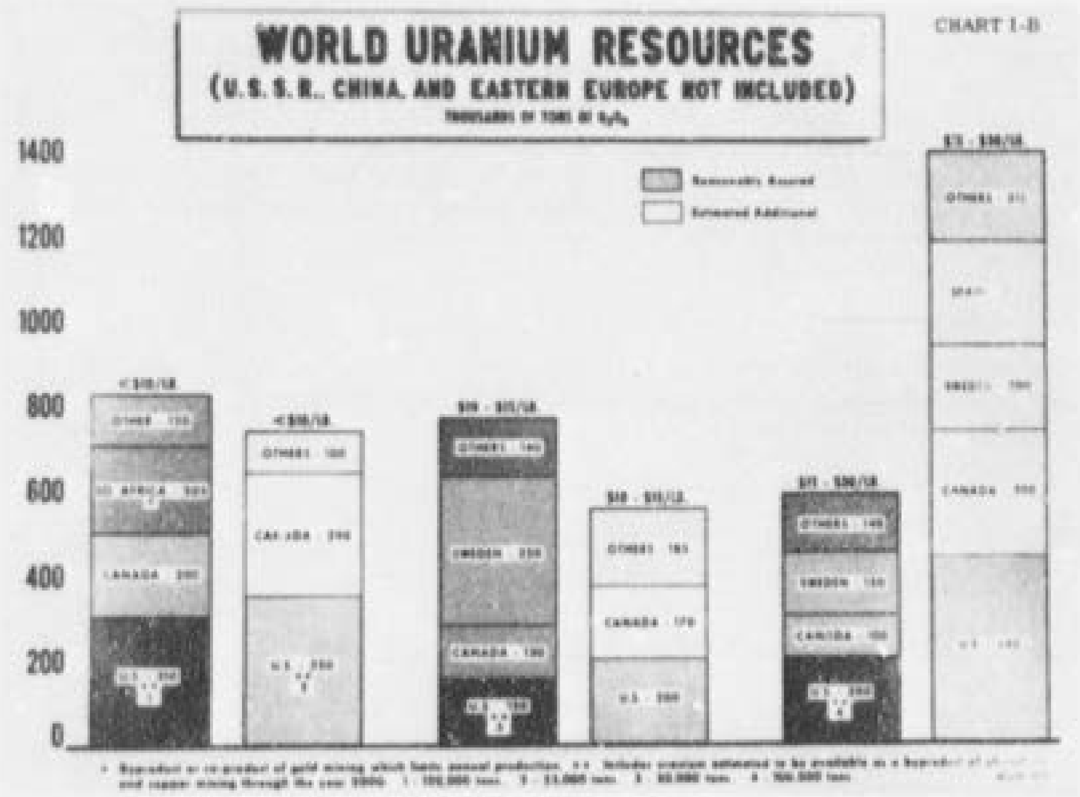
WORLD URANIUM RESOURCES
(U.S.S.R., CHINA, AND EASTERN EUROPE NOT INCLUDED)
CHART I-B
1400
1200
1000
800
600
400
200
0

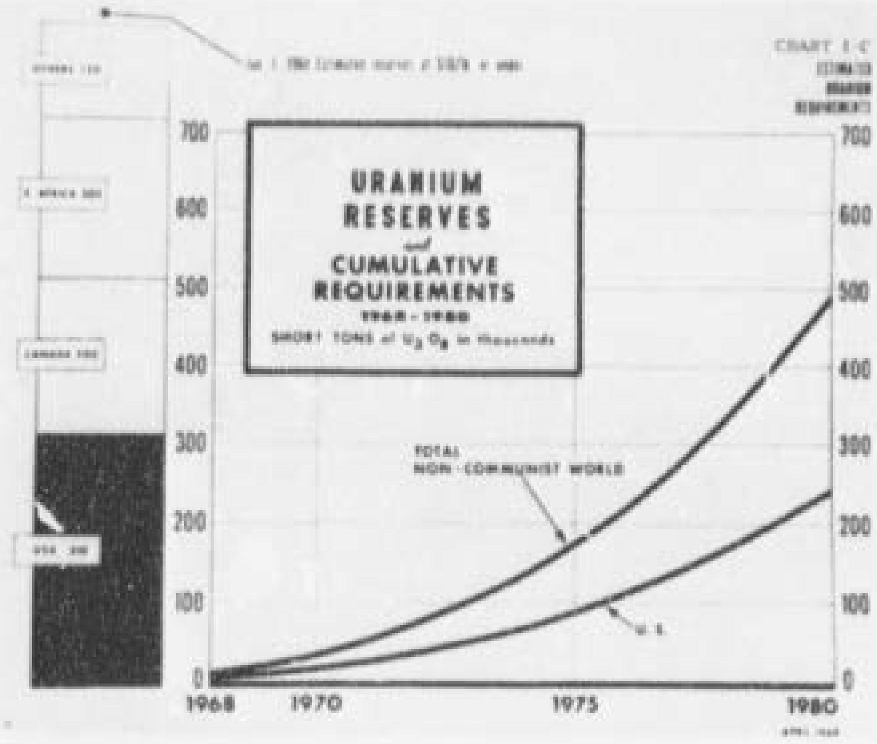
URANIUM
RESERVES
and
CUMULATIVE
REQUIREMENTS
CHART I-C
700
600
500
400
300
200
100
0
TOTAL
NON-COMMUNIST WORLD
U.S.
1968
1970
1975
1980

To meet the anticipated requirements, production rates must be backed up by adequate reserves to provide a reasonable production base. The AEC has suggested an eight-year forward reserve -- that is, sufficient to meet projected requirements for the next eight years. Thus, for domestic nuclear power, during the period through 1980, production plus reserves should be about 650,000 tons of U_3O_8, which will require new discoveries exceeding 500,000 tons (presently known reserves at prices up to $8 per pound are about 150,000 tons).

3. Uranium Exploration

Domestic uranium exploration activities increased rapidly in 1967 when 10.7 million feet of surface drilling was reported. This activity exceeded the previous peak year of 1957 with 9.2 million feet of drilling. Exploration activity continued to accelerate in 1968 and it is expected that the industry will drill some 25 million feet in 1968.

The uranium industry reported its four year forward plans for drilling in 1967 and again in 1968. The projected drilling rates increased from 53.8 million feet for the 1967-1970 four-year period to 98.8 million feet for the 1968-1971 period, about equally divided between exploratory and development drilling. In all, some 65 companies reported their drilling plans to the AEC.

The 1968 industry estimates of planned drilling activity are given below:

Calendar Year	Est. Feet of Drilling
1968	24,854,000
1969	26,340,000
1970	25,740,000
1971	21,916,000
	98,850,000

The estimated cost of this drilling is $135 million.

The historical and planned drilling and the reserves added are shown in Chart I-D.

The 7,000 tons U_3O_8 increase in U.S. reserves, reported as of January 1, 1968, over 1967 (see page 30) is probably not indicative of future rates of ore development, because the companies conducting uranium exploration activities have indicated that it requires several years subsequent to initial exploration to develop firm reserves information.

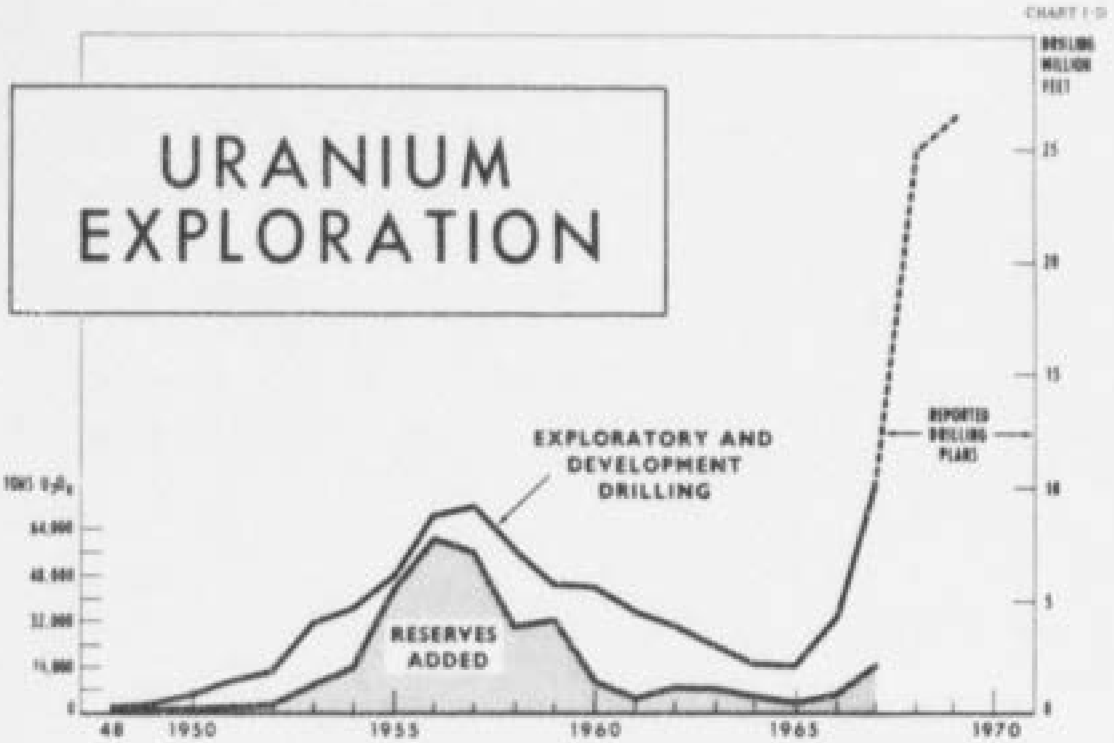

4. Domestic Uranium Processing Industry

The domestic uranium industry processing capability is approximately 15,000 tons U_3O_8 annually.

Fourteen uranium ore processing mills are currently in operation in the U.S. These milling companies produce about 90 percent of the ore from mines they own or control. Indepently-owned mines produce about 10 percent of the ore and deliver it to the operating mills for processing into concentrates. Tables I-1 and I-2 list the companies which currently sell uranium concentrates to the AEC and to the commercial market.

Many new companies, not originally part of the uranium mining and milling industry, have been pressing uranium exploration. These include railroads, oil companies, specialized minerals exploration firms, and other mining companies.

TABLE I-1

Domestic Uranium Companies
Supplying AEC and Commercial Market

Anaconda Company, Bluewater, New Mexico
Atlas Corporation, Moab, Utah
Federal-Americam Partners, Fremont Co., Wyoming
Kerr-McGee Corporation, Grants, New Mexico
Union Carbide Corporation, Uravan & Rifle, Colo., and Globe, Wyoming
United Nuclear Corporation - Homestake Partners Grants, New Mexico
Utah Mining and Construction, Fremont Co., Wyoming
Western Nuclear, Jeffrey City, Wyoming

TABLE I-2

Domestic Uranium Processing Mills Operating
Primarily or Entirely for Private Sales

Climax Uranium, Grand Junction, Colorado
Cotter Corporation, Canon City, Colorado
Mines Development, Inc., Edgemont, South Dakota
Petrotomics Company, Shirley Basin, Wyoming
Susquehanna-Western, Inc., Falls City, Texas

5. AEC Procurement Program

Sales of uranium to the Government during 1968-1970, shown below, are the annual quantities remaining to be delivered to AEC under existing commitments.

Estimated Sales of Uranium to the Government

Fiscal Year	Tons U_3O_8	$ Millions
1969	7,300	104
1970	7,300	87
1971	3,600	44

Chart I-E on the next page shows the overall procurement of uranium concentrate by the AEC.

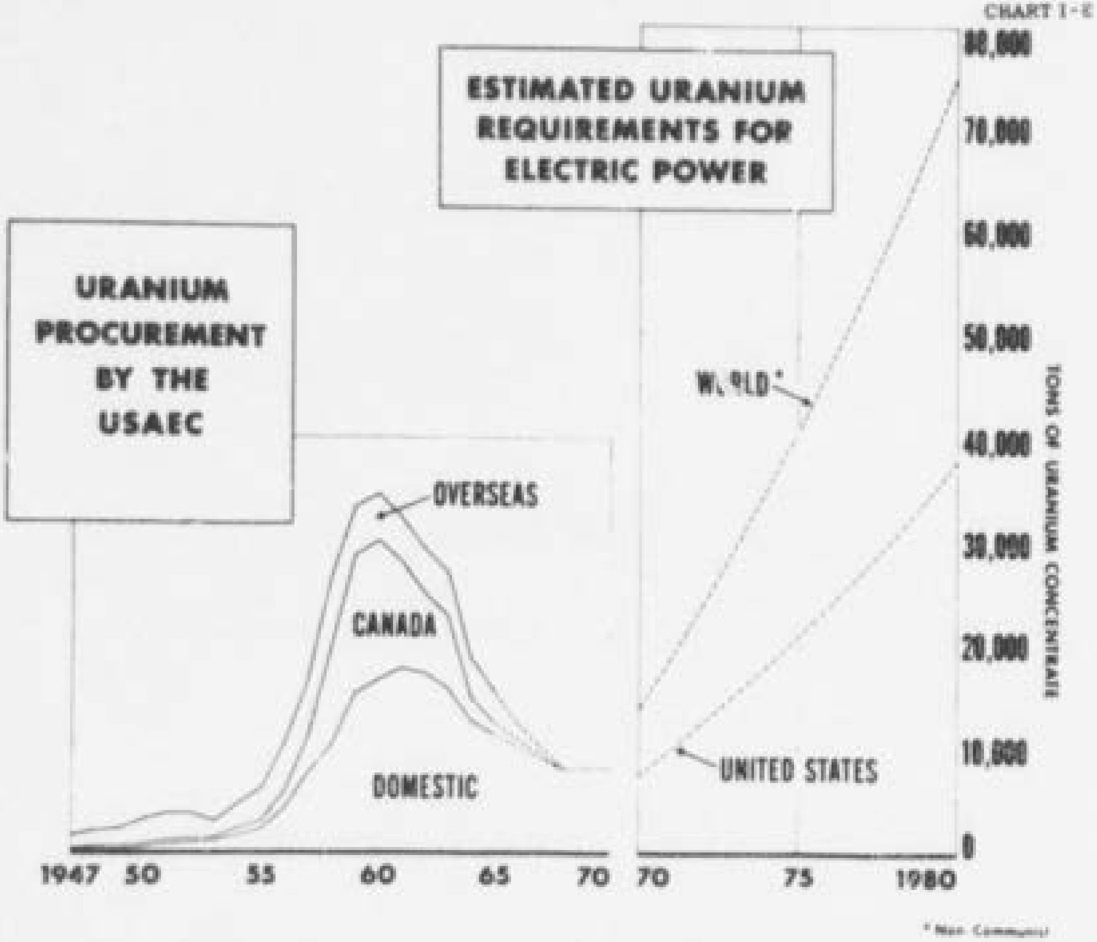

6. Commercial Purchases of Uranium

The commercial sales of uranium for private nuclear power plants, reported in a survey completed in April 1968, are listed in Table I-3.

TABLE I-3

Domestic Sales of Uranium
(in short tons U_3O_8)

Year of U_3O_8 Delivery	Tons of U_3O_8	Cumulative Quantity Tons of U_3O_8
Pre-1968	900	900
1968	5,300	6,200
1969	4,500	10,700
1970	7,500	18,200
1971	9,800	28,000
1972	10,500	38,500
1973	7,900	46,400
1974	6,000	52,400
1975-77	6,200	58,600

In contrast to sales to the Government, which total about 15,000 tons U_3O_8 for calendar years 1969-1970, the private domestic sales and contract commitments as of September 1968 were about 60,000 tons U_3O_8 or nearly two-thirds of the cumulative domestic uranium requirements for nuclear power through 1975.

In addition to these sales to domestic buyers, some 3,100 tons of U_3O_8 have been delivered or committed as of April 1, 1968, to foreign buyers by domestic producers.

7. Viability

This question has received a considerable amount of attention in trade journals and in meetings and speeches by Government and industry representatives. Under the Private Ownership Act, the Commission may not offer toll enriching services for uranium of foreign origin intended for domestic use, until there is reasonable assurance of the viability of the domestic mining and milling industry. The Commission is continuing to study this situation and has indicated that it may be possible to remove this restriction by July 1, 1973, or earlier. A discussion of the Commission's uranium supply policies is contained in AEC's revised statement of policies released September 6, 1968, and published in the Federal Register of September 7, 1968.[6/]

8. Sampling of Uranium Concentrates

It appears that sampling plants will probably be established at UF_6 conversion facilities. The volume of concentrates that would be subject to sampling may be estimated from the uranium requirements shown in Chart I-A. A very rough estimate of the annual business is \$250,000-\$350,000 in the early 1970's and \$750,000-\$1,000,000 in the mid-70's.

As an AEC service to industry, the AEC's Grand Junction facility performed sampling of commercial purchases of U_3O_8 concentrates, until these services became available commercially. Lucius Pitkin, operator of the AEC's Grand Junction facility, now operates an independent commercial sampling plant at the Allied Chemical Corporation's Metropolis, Illinois, UF_6 conversion facility.

B. Thorium

There have been no significant changes in the status of thorium as a nuclear fuel since our 1966 report.

6/ See AEC Public Release L-210, dated 9/6/68.

CATEGORY II
MATERIALS PROCESSING & FABRICATION

A. Uranium Fuel Preparation - Conversion of Feed Materials to UF_6

Market Predictions

	Normal U	Slightly Enriched Uranium (<5.0% U-235)	Highly Enriched Uranium (>5.0% U-235)
In 1970	$ 16,500,000	$ 225,000	$160,000
In 1975	$ 55,000,000	$1,100,000	$160,000
In 1980	$115,000,000	$4,250,000	$160,000

Our market predictions remain unchanged from those furnished in last year's report.

The feed to AEC's gaseous diffusion plants in 1967 consisted largely of depleted UF_6 1/ contained in AEC's stockpile of former gaseous diffusion tails material. Gaseous diffusion economics favored the stripping of additional U-235 content from the stockpile material.

Feed to the plants at present consists of depleted and normal UF_6 2/. It will be supplemented to some extent, however, by uranium recovered from unirradiated 3/ fuel fabrication scrap and irradiated 4/ reactor fuel. The unirradiated uranium recovered from scrap ranges in isotopic content all the way from depleted (<0.711 wt. % U-235) to very highly enriched 5/ uranium. The uranium recovered from irradiated reactor fuel varies widely in its U-235 content depending

---

1/ Depleted UF_6 - Refers to uranium that has been depleted of a portion of its U-235 content and, therefore, has a U-235 content less than that contained in normal, or naturally occurring, uranium.

2/ Normal UF_6 - Refers to uranium having normal, or naturally occurring, U-235 content (0.711 wt. % U-235).

3/ Unirradiated Uranium - Refers to uranium that has not been subjected to radiation in a nuclear reactor following its withdrawal from an enriching plant.

4/ Irradiated Fuel - Fuel that has been subjected to radiation in a nuclear reactor resulting in a fissioning or "burn-up" of a portion of its U-235 content.

5/ Enriched - Refers to uranium that has had its U-235 content enriched, or increased above that occurring in normal, or naturally occurring, uranium.

[illegible]

on the type of reactor. For example, the irradiated fuel discharged from commercial light water power reactors will ordinarily contain uranium having less than 1.0 wt. % U-235 content, whereas most research and test reactors will discharge irradiated fuel containing highly enriched uranium.

The AEC requires that all normal-assay uranium feed furnished for toll enriching be in the form of UF_6, and commercial capability exists to convert ore concentrates to UF_6.

Commercial capability does not exist, at present, for conversion to UF_6 of the enriched uranium recovered from unirradiated scrap or irradiated fuel elements. AEC has been accepting the return of recovered leased uranium in these categories in forms other than UF_6, but with a charge to cover conversion to UF_6, and will probably continue a similar policy under private ownership arrangements, until such time as private capability is established to convert these materials to UF_6.

1. Conversion of Normal Uranium to UF_6

a. Industry Plans

As reported last year, Allied Chemical Corporation's plant at Metropolis, Illinois, is the only commercial facility that has the capability, at present, to convert ore concentrates to UF_6. This plant, which has a capacity to convert about 7,000 tons U per year from ore concentrates to UF_6, was reopened early in 1968 following a 3-1/2 year shutdown. Immediately prior to restarting the plant, Allied increased its annual capacity from 5,000 tons U to about 7,000 tons U and plans to further increase its capacity from 7,000 tons to 10,000 tons U late in 1969.

Allied's base charge for converting ore concentrates to UF_6 for use in toll enrichment is $1.04 per pound U, subject to escalation, with provision for a 0.5 percent process loss. An additional processing charge is applied if the ore concentrate has a sodium content of greater than 0.5 percent.

On orders it accepts for UF_6 delivery during 1968 and 1969, Allied is requiring that the ore concentrates be furnished six months in advance of the UF_6 delivery date. The specified processing period will be reduced from six months to three months starting in 1970.

The Kerr-McGee Corporation is proceeding with construction of a $25 million UF_6 conversion plant at Sequoyah, Oklahoma. The plant is scheduled for completion in January 1970 and is expected to be at its full initial operating capacity of 5,000 tons U per year by July 1970. Plans call for an increase in the plant's capacity to 10,000 tons U per year at a later date, dependent on the growth of the market.

Kerr-McGee is quoting a toll conversion base charge of $2.52 per kg U, subject to escalation, which covers sampling, conversion, and shipping of product UF_6 to the gaseous diffusion plants. This compares very closely with Allied's charge of $1.04 per pound U. Like Allied, Kerr-McGee sells its conversion service on the basis of guaranteeing a yield of 99.5 percent.

Eldorado Nuclear Limited, Port Hope, Ontario, Canada, which has had a capability for a number of years to convert ore concentrates to purified UO_3, is now going ahead with plans to add a capability to convert UO_3 to UF_6. The UF_6 conversion facility is scheduled for full operation at an initial capacity of 2,500 tons UF_6 per year in 1970. Design of the plant will permit ready expansion to a capacity of 5,000 tons UF_6 per year at a later date. Other countries, besides the U.S. and Canada, to be considered in the future UF_6 industry are the United Kingdom and France, who have UF_6 conversion capability at Springfields and Pierrelatte, respectively. There have been indications that Springfields has been offering UF_6 conversion services to other foreign countries.

Other U.S. companies have indicated in the past that they were seriously considering future entry into the UF_6 conversion business; however, no firm decisions have been announced.

b. AEC UF_6 Conversion Facilities

In the past, AEC has met most of its needs for converting ore concentrates to UF_6 by the following sequences:

(1) By solvent extraction refining of ore concentrates at its Weldon Spring, Missouri, and its Fernald, Ohio, feed processing plants, yielding product as either UO_3 or UF_4; and

(2) By converting UO_3 to UF_6 at its Oak Ridge, Tennessee, or its Paducah, Kentucky, gaseous diffusion sites; or

(3) As an alternate to (2), by converting UF_4 to UF_6 at its Portsmouth, Ohio, site.

The entire Weldon Spring Feed Processing Plant was shut down in 1966 and is no longer under the jurisdiction of AEC. The Portsmouth and Oak Ridge UF_6 facilities were shut down in February and October 1962, respectively, and the Paducah UF_6 facility was shut down in mid-1964. Numerous items of equipment from the Oak Ridge and Portsmouth Feed Plants have since been sold or converted to other uses. A decision was announced by AEC in February 1968,[6/] however, that the Paducah Feed Plant will be restarted and used to convert a quantity of AEC's plutonium production reactor tails UO_3 to UF_6 in FY-1969. AEC explained that the primary factors in deciding to start up the plant were economics and limited commercial capabilities at the time of AEC's needs.

c. Future Commercial Requirements

Commercial requirements for conversion of ore concentrates to UF_6 in 1968 consisted of those associated with ad hoc foreign barter arrangements, amounting to about 1,000 short tons U, plus a portion of the domestic privately owned uranium to be delivered to the gaseous diffusion plants early in 1969 for toll enrichment. After 1968 the requirements for conversion of ore concentrates to UF_6 in domestic conversion plants will be largely those from U.S. nuclear power plants. Annual power reactor requirements for conversion of ore concentrates to UF_6 from 1969 through 1980 are projected in Chart II-A, along with dollar value of the conversion service. The estimated domestic requirements are based on AEC's estimate of installed nuclear power capacity of about 150,000 MWE by 1980.

2. Conversion of Enriched Uranium to UF_6

The quantities and batch sizes of enriched uranium recovered from (a) unirradiated scrap, and (b) irradiated fuels, that have been returned to AEC's gaseous diffusion plants for

---

6/ In an unclassified statement before the JCAE during the AEC FY-1969 authorization hearings in February 1968.

refeeding to date have been relatively small, and no industrial capability exists for conversion of these recovered materials to UF_6. The criticality limitations and health protection aspects of a facility designed to convert such materials to UF_6 necessitate a sizable capital expenditure.

In their plans to build spent fuel reprocessing plants, both Allied Chemical and Atlantic Richfield have indicated plans to include facilities capable of converting recovered, slightly enriched, uranium nitrate solutions to UF_6, and to start operations in 1973 and 1974, respectively. Also, NUMEC has announced that, starting in December 1968, it will be able to handle some conversion to UF_6 of uranium recovered from cold scrap.

There have been no firm plans announced on the part of private industry concerning future construction of a facility capable of converting highly enriched uranium to UF_6.7/

It is probable that, as soon as private capability is achieved to convert enriched materials to UF_6, the AEC will require that these materials, when returned to AEC as feed to the gaseous diffusion plants, must be in the form of UF_6. At present, however, the Code of Federal Regulations permits the return of leased enriched uranium to AEC in the form of uranyl nitrate, upon payment of charges calculated to cover AEC's cost of converting to UF_6. The conversion charge is $5.60 per kilogram of contained uranium for low U-235 enrichments ($<$5.0% U-235) and $32.00 per kilogram of contained uranium for high U-235 enrichments ($>$5.0% U-235), plus allowances for losses and conversion time. (These charges are presently under review.)

Quantities of recovered slightly enriched uranium that have been returned to AEC from commercial programs in recent years are as follows:

Fiscal Year	Metric Tons U
1962	2.4
1963	7.2
1964	10.9
1965	7.8
1966	18.3
1967	22.3
1968	26.7

7/ It would not be practical from the standpoint of economics to use the same equipment for conversion of slightly enriched uranium to UF_6 that is used for conversion of highly enriched uranium. The equipment for highly enriched uranium must be severely limited in size to prevent accidental nuclear criticality. The limited throughput of this equipment would be much too costly for processing of slightly enriched uranium.

a. Recycling and Blending of Enriched Uranium

The extent to which recovered, enriched uranium will need to be converted to UF_6 for refeeding to the gaseous diffusion plants is dependent on how much recycling and blending of these materials can be expected.

(1) Unirradiated Uranium

It is normal practice to recover some of the unirradiated enriched scrap generated during a fuel processing or fuel fabricating campaign and recycle it back before completion of the campaign. To the extent that this can be done without introducing contaminants to the fuel or altering its isotopic enrichments, considerable savings can be achieved through higher conversion efficiency. It is usually inevitable, however, that some small portion of the scrap cannot be recycled back into the campaign or into later campaigns producing identical fuel material. The portion that is not directly recycled back to processing or fabrication must be converted to UF_6 and refed to the gaseous diffusion plants. The percentage of enriched UF_6 starting material that ends up in this portion varies greatly for different types and enrichments of fuels. For light water power reactor fuels being fabricated during the next year or two, it is estimated that about 1.5 percent of the uranium withdrawn from the gaseous diffusion plants for this purpose will be returned to them as recovered unirradiated scrap for refeeding. It is expected to be less than 1.5 percent under private ownership when more and more recycling and blending will probably take place.

(2) Irradiated Uranium

The recycling of recovered, irradiated fuel back to fuel processing and fabrication involves much more complex problems than are encountered with unirradiated material. The first, and probably the most difficult, obstacle is that the isotopic enrichment will have been greatly reduced during irradiation, thereby requiring blending back to the required enrichment by combining the recovered uranium with uranium of higher enrichment. Although blending has been successfully accomplished in AEC plants over a period of years, in such forms as UF_6, UF_4, and uranium metal, it requires a high degree

of precision in isotopic analysis. It is further complicated by the fact that some economic disadvantage is inherent as a result of the blending, (i.e., the total market value of the resultant material can be expected to be less than the total value of the two portions that were blended), and the fact that irradiated uranium acquires a build-up of U-232, U-233, and U-236, which may be objectionable from the standpoint of fuel homogeneity, fuel efficiency, and safety.

b. Future Market for Conversion of Enriched Uranium to UF_6

It can be seen from the above that considerable uncertainty exists concerning the amount of enriched fuel recycling that can be expected without reintroduction to the gaseous diffusion plants. This uncertainty, in turn, greatly affects accuracy of estimates of the future requirements for conversion of enriched uranium to UF_6.

If irradiated fuel reprocessing plants in the future employ a fluoride volatility process or an aqueous process followed by a volatility process, their recovered uranium will be in the form of purified UF_6. Although UF_6 is an ideal chemical form in which to blend two different isotopic enrichments, its owners would probably find it more economical to return the recovered material to the gaseous diffusion plants for reenriching, than would be the case if the uranium were recovered as uranyl nitrate hexahydrate (UNH).

Future requirements for conversion to UF_6 of recovered enriched uranium of isotopic enrichment less than 5 percent U-235 are forecast in Chart II-B. The estimates are separated into unirradiated material and irradiated material and were based on the assumptions described below:

Unirradiated Material - It was assumed that, prior to 1969, 1-1/2 percent of the uranium withdrawn at less than 5 percent enrichment will end up as unirradiated scrap to be returned to the gaseous diffusion plants. Starting in 1969, a figure of 1 percent is used; however, even this percentage might prove to be too high if recycling is utilized as extensively as possible.

CHART II-A

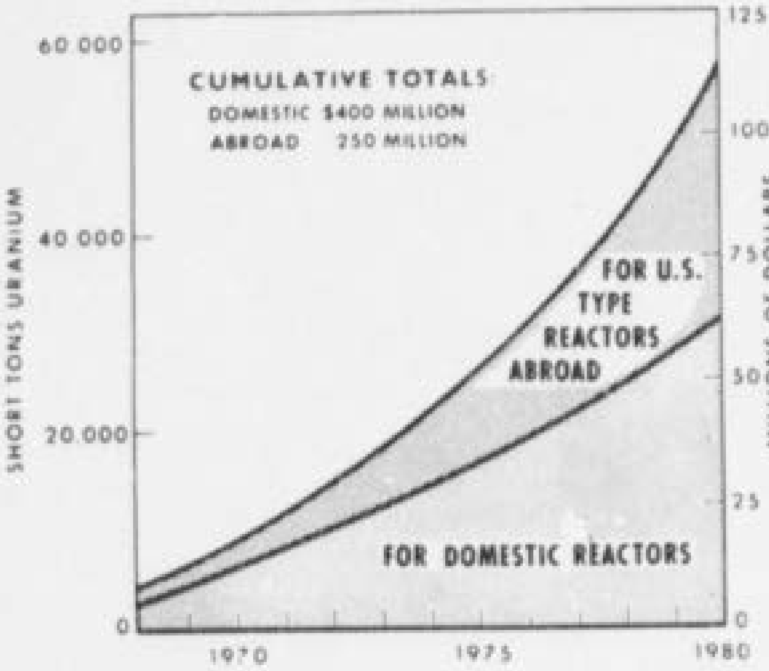

CONVERSION OF ORE CONCENTRATES TO UF_6

CHART II-B

CONVERSION OF RECOVERED, SLIGHTLY-ENRICHED (< 5.0% U-235) URANIUM TO UF_6

ESTIMATED ANNUAL REQUIREMENTS

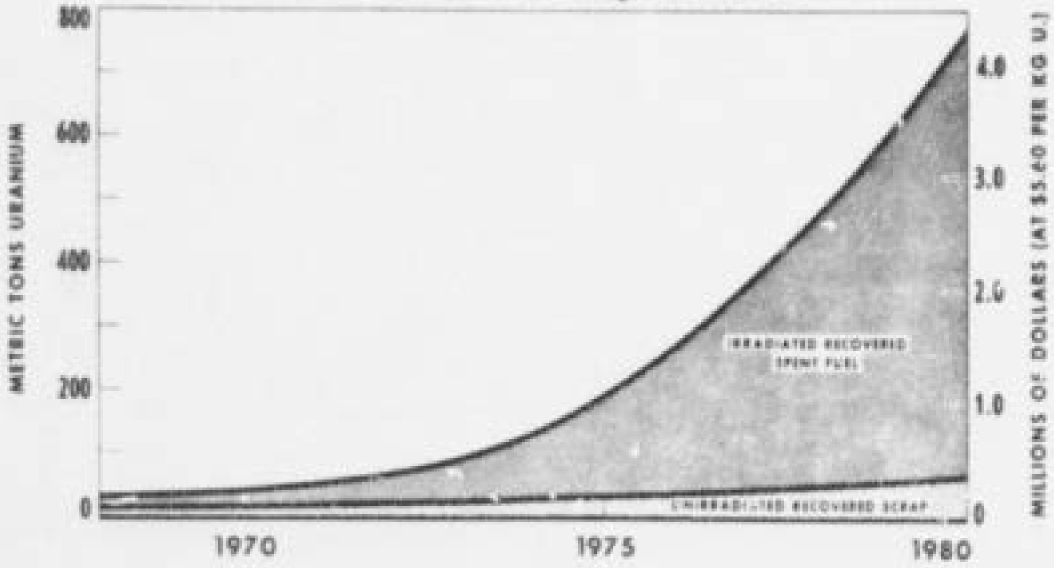

Irradiated Material - The irradiated scrap estimates shown in Chart II-B are based primarily on commercial power reactor spent fuel. It was arbitrarily assumed that, starting sometime in the 1970's, 50 percent of the spent fuel will be reprocessed in facilities using the fluoride volatility, or modified volatility, process and, as a result, will already be in the form of UF_6 when it is recovered. Of the remaining material (recovered as UNH), it was assumed that about half will be blended and recycled without being returned to the gaseous diffusion plants. Consequently, the irradiated scrap estimates in Chart II-B, represent about 25 percent of recovered power reactor spent fuel.

The annual quantities of enriched uranium containing greater than 5 percent U-235, both irradiated and unirradiated, that will require recovery, conversion to UF_6, and return to the gaseous diffusion plants are expected to remain at approximately the present level; therefore, they have not been plotted in chart form. These quantities have been averaging in the range of 5,000 kgs U per year, which would represent an annual UF_6 conversion cost of $160,000 based on $32 per kg U.

3. Summary of Present and Future Industry Capability

Facility	Annual Capability	Calendar Year
Conversion of Ore Concentrates to UF_6		
Allied Chemical Corp. Plant at Metropolis, Illinois	7,000 STU<br>10,000 STU	early 1968<br>late 1969
Kerr-McGee Plant Sequoyah, Oklahoma	5,000 STU<br>10,000 STU	mid 1970<br>at later date not yet announced
Eldorado Nuclear Limited Port Hope, Ontario, Canada	2,500 ST UF_6<br>5,000 ST UF_6	mid 1970<br>at later date not yet announced

Based on Chart II-A, it appears that total conversion capability of 20,000 STU/year will be needed by about 1974 and that about 25,000 STU/year will be needed about one year later.

Conversion of Slightly Enriched (<5.0% U-235)
Recovered Uranium to UF_6

Facility	Estimated Start-Up
Nuclear Materials & Equipment Corp.	December 1968
Allied Chemical Corp. Spent Fuel Reprocessing Plant	1973
Atlantic Richfield Corp. Spent Fuel Reprocessing Plant	1974

The future capacity that will be required is estimated at 40 MTU in 1970, 200 MTU in 1975, and 800 MTU in 1980.

Conversion of Highly Enriched (>5.0% U-235)
Recovered Uranium to UF_6

No firm plans have been announced.

The future capacity that will be required is estimated to be relatively constant at about 5 MTU/year.

B. Uranium Enriching

Enriching Requirements

	Value of Sep. Wk.
1970	$ 150,000,000
1975	$ 484,000,000
1980	$1,035,000,000

The above market predictions have been changed only slightly from last year's predictions. These changes result from slight changes in estimated Government requirements.

Of the total cost of a fuel core for a power reactor, about one-third represents the cost of uranium enriching. The isotopic enrichment of uranium is the only major step in the nuclear fuel cycle for which private industrial capability does not exist.

Although a number of nuclear reactors have been designed, constructed, and operated using normal uranium (0.711 wt. % U-235)

as a fuel,[8/] the overwhelming choice of fuel for light water type, commercial power reactors in this country has been slightly enriched uranium, usually having an enrichment between 2 and 4 wt % U-235. On the other hand, high temperature, gas-cooled advanced converters, and probably some breeder reactors, will use highly enriched uranium during their early years of operation. Also, many specialized reactors, such as test, research, and military power reactors, use highly enriched uranium as their fuel.

The Government's three gaseous diffusion plants, which were built for national defense purposes at a cost of $2.3 billion, are available to produce fuel for civilian reactors, now that Government requirements for enriched uranium have greatly decreased. Since Government requirements are expected to remain very low while commercial requirements continue to grow, it is expected that Government requirements will amount to less than 10 percent of total requirements by the mid-1970's.

Based on authority granted by the Atomic Energy Act of 1954, procedures were established for private parties to lease enriched uranium (as well as other special nuclear materials) from the Government for approved purposes under AEC licenses. The lease charge is computed by applying an established use charge rate to the values of enriched uranium published in AEC's Table of Base Charges, with provisions for reimbursing the Government for approved losses and consumption. All of the early commercial reactors are fueled with leased material.

As a result of the Private Ownership of Special Nuclear Materials Act approved in August 1964, the AEC will provide enriching of privately owned uranium on a toll basis starting January 1, 1969 (see section entitled "Private Ownership - Toll Enriching," starting on page 22 of this report).

1. Increased Availability of Gaseous Diffusion Information

AEC announced on September 21, 1967,[9/] that its current charge for the toll enrichment of private owned uranium is $26 per kilogram separative work unit. This charge is within the $30 per unit ceiling price specified in the uranium enrichment criteria established in December 1966. The $30 ceiling continues in effect, subject to upward escalation for the cost of electric power and labor. On November 28, 1967,[10/] AEC released a Table for Enriching Services, based on the $26 charge.

8/ Some of the U.S. plutonium production reactors and a number of foreign power reactors, particularly those built in Canada and the United Kingdom, use normal uranium as their fuel.
9/ See AEC Public Release K-228, dated September 21, 1967.
10/ See AEC Public Release K-277, dated November 28, 1967.

In order to permit the nuclear power industry, both in this country and abroad, to make more reliable judgments concerning future enriching capacities, AEC declassified and released in June 1967, quantitative information on the total capacity of its three gaseou" diffusion plants to produce enriched uranium.11/ The separati.. capability of the plants varies with the amount o. .lectric power utilized, although not necessarily in direct proportion; consequently, AEC's initial release expressed the total capacity for three different power levels. Similar information is presented in Table II-1.

TABLE II-1

Enrichment Capacity of AEC's Gaseous Diffusion Complex

Total Power Level, Megawatts	Annual Production Capability in Millions Of Kilogram Separative Work Units*	Examples of Use of Separative Capacity, Approximate Amount of Enriched Product That Could Be Produced by Feeding Normal (0.711% U-235) Uranium and Withdrawing Tails at 0.2% U-235	
		Product At 2.0% U-235	Product At 3.0% U-235
2000 (Minimum projected level, to be [illegible]d June 30, 1969)	6.9	3.15 million kgs U product from 11.1 million kgs U of normal feed	1.60 million kgs U product from 8.76 million kgs U of normal feed
3000 (Approximate level in 1967)	9.9	4.51 million kgs U product from 15.9 million kgs U of normal feed	2.30 million kgs U product from 12.6 million kgs U of normal feed
6000 (Approximate maximum level reached in the past)	17.0	7.75 million kgs U product from 27.3 million kgs U of normal feed	3.95 million kgs U product from 21.5 million kgs U of normal feed

* Separative Work Units - The AEC expressed capacity and product rate of its gaseous diffusion plants in terms of separative work units. A separative work unit is not a quantity of material, but is a measure of the effort expended in the plants to separate a quantity of uranium of a given assay into two components, one having a higher percentage of uranium-235 and one having a lower percentage. Separative work is generally expressed in kilogram units to give it the same dimensions as material quantities, i.e., kilograms or metric tons of uranium.

In February 1968,12/ the AEC released a 45-page report dealing in considerable detail with the AEC gaseous diffusion plants, their mode of operation, plant and operating cost experience, future cost projections, future plant improvements,

11/ See AEC Public Release K-145, dated June 14, 1967.
12/ See ORO 658, entitled "AEC Gaseous Diffusion Plant Operations," copies may be requested from: U.S. Atomic Energy Commission, Division of Technical Information Extension, P. O. Box 62, Oak Ridge, Tennessee 37830.

estimates concerning future plants, and information concerning enrichment services. Information on gaseous diffusion was also presented at a meeting of the Eastern Idaho Nuclear Council in March 1968, by Charles Keller, USAEC, Oak Ridge.

2. Possible Transfer of Gaseous Diffusion Plants to Industry

A great deal of attention has been focused on possible transfer of the gaseous diffusion plants to private industry. Two studies have been underway during the past year on this subject. One was conducted by an AIF Study Committee and another is still underway by the AEC staff.

a. AIF Committee Study

An AIF Study Committee was appointed by the Atomic Industrial Forum in early 1967 to study private ownership and operation of uranium enrichment facilities. The committee completed its study and published a report of its conclusions and recommendations in June 1968.[13/]

The committee concluded that it is both desirable and feasible for the AEC to transfer its three enrichment plants promptly to the private sector. Their conclusion that such a transfer is desirable was based largely on their opinion that existence of a profit incentive would enhance the advancement of technology, improve efficiency, and reduce costs and that private industry could provide maximum flexibility in meeting requirements of an expanding nuclear industry.

Two members of the Study Committee expressed differing views on some of the committee's conclusions and recommendations. They expressed more concern than the other committee members over the question of whether an enriching industry consisting of only three private operators would provide adequate competition. They expressed support for an alternate approach that would delay transfer of the three existing plants until after a fourth plant has been committed or constructed. Another alternate plan that received only minority committee support was one whereby the Government would retain one of the existing plants for an interim period of perhaps five to 10 years, in order to facilitate continued Government-sponsored development and better transfer of technology to future participants in the industry.

13/ "Private Ownership and Operation of Uranium Enrichment Facilities - Report of a Forum Study Committee," available from Atomic Industrial Forum, 850 Third Avenue, New York, New York 10022, price to members, $5.00, to nonmembers, $10.00.

b. AEC Staff Study

The Atomic Energy Commission continues to study the various alternatives that appear possible for future operation of the enriching plants.[14/]

Before any decision can be reached, numerous complex and substantive matters must be carefully weighed in the following broad categories: (1) national security aspects of private participation in uranium enriching, (2) potential problems of establishing a competitive or adequately regulated industry, and (3) national economic and Government financial interests.

3. FY-1968 Operations

The total enriched uranium shipped from AEC's gaseous diffusion plants during fiscal year 1968, to meet domestic commercial and foreign requirements, represents approximately 1.63 million kilogram separative work units when calculated on the basis of using normal uranium feed and operating at a tails assay of 0.20 percent U-235. This represents an increase of about 25 percent over the separative work required to meet similar requirements in FY-1967. The FY-1968 requirement, however, represents only about 22 percent of the current production capability of the three gaseous diffusion plants operating at the present total power level of about 2215 electrical megawatts. (The total power level of the three plants was reduced to about 2,215 MWE June 30, 1968, and is expected to remain at that level until June 30, 1969, when it is to be further reduced to about 2,000 MWE. Approximately one year later, however, it is expected that the total operating power level will commence a step-wise series of upratings until the total operating level reaches 6,000 MWE by the mid-1970's.)

4. Future Requirements for Separative Capacity

Estimated annual separative work requirements through 1980, along with the corresponding dollar value, based on $26 per kilogram separative work unit, are shown in Chart II-C.

5. Additional Capacity from Plant Improvements

A direct comparison between Table II-1 and Chart II-C suggests that additional production capacity will be necessary early in 1975; however, the advance inventory that will be available at that time, and the additional production capacity that can be obtained by improvement of existing plants should

---

14/ See page 41, "The Nuclear Industry - 1967."

CHART II-C

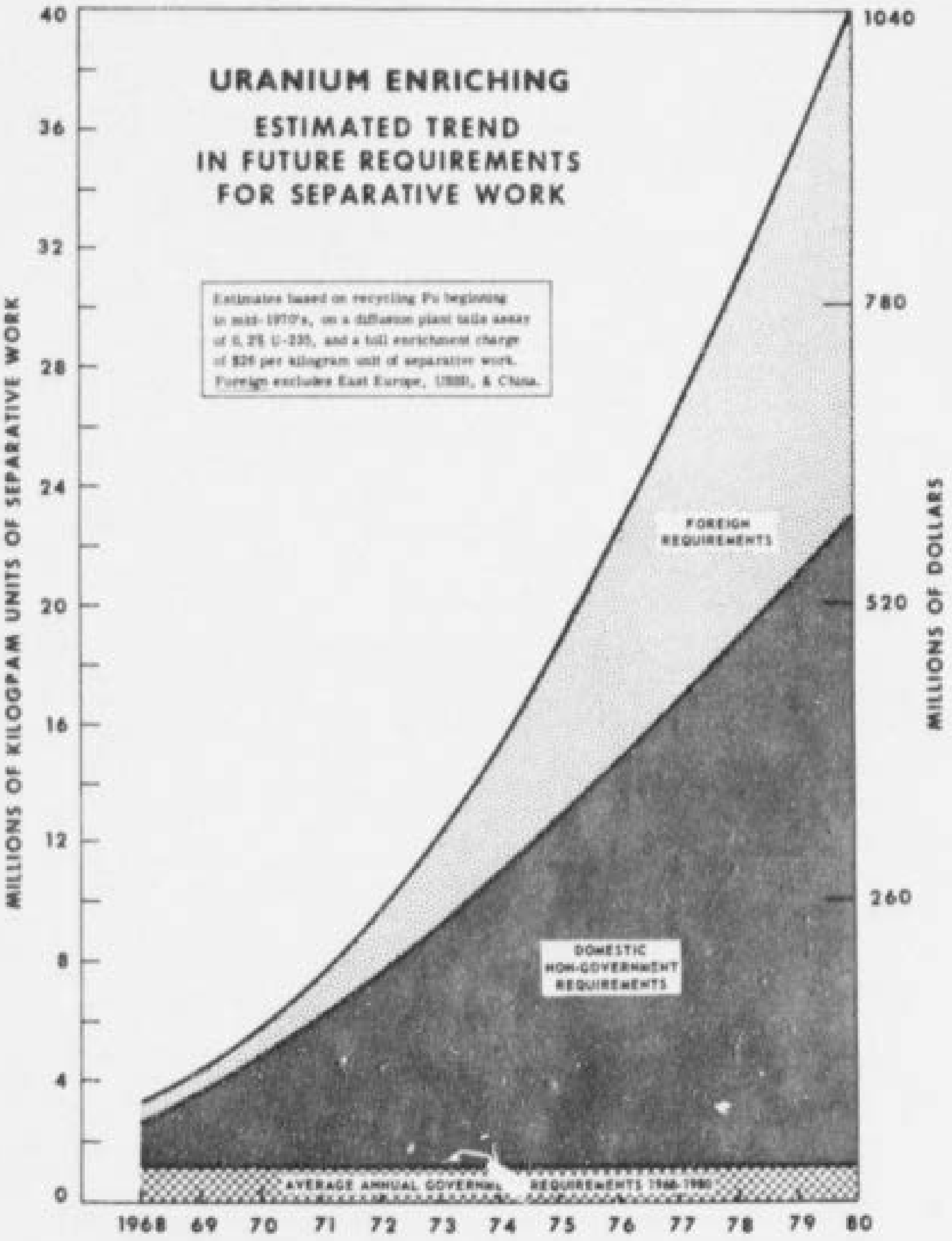
URANIUM ENRICHING
ESTIMATED TREND
IN FUTURE REQUIREMENTS
FOR SEPARATIVE WORK
Estimates based on recycling Pu beginning
in mid-1970's, on a diffusion plant tails assay
Foreign excludes East Europe, USSR, & China.
FOREIGN REQUIREMENTS
DOMESTIC NON-GOVERNMENT REQUIREMENTS
MILLIONS OF KILOGRAM UNITS OF SEPARATIVE WORK
MILLIONS OF DOLLARS
0
4
8
12
16
20
24
28
32
36
40
260
520
780
1040
1968
69
70
71
72
73
74
75
76
77
78
79
80

make it possible to postpone need for an additional plant until about 1980.[15/] It has been estimated[16/] that the total separative capacity of the existing three plants can be increased from 17.0 million kilogram separative work units per year to about 21.7 million kilogram separative work units per year (both figures based on a total power level of 6,000 megawatts) by carrying out an improvement program at a cost of several hundred million dollars using technology that will be available by the early 1970's, as a result of the five-year development program now in progress. In addition, it is estimated that 1970 technology would permit total separative capacity to be further increased to about 25.8 million kilogram separative work units per year by making improvements that would permit the three existing plants to operate at a higher total power level. The capital cost of the uprating is estimated over $100 million for changes that would enable increasing the total power level from 6,000 to 7,300 megawatts. Operation at the higher power level would, of course, require increased operating costs. This would make the increment of additional capacity obtained by power uprating less attractive than that obtained without power uprating, but still probably more attractive than that to be obtained by building new plants (ORO-658).

6. Additional Capacity from New Plants

Although it is impossible to extrapolate requirements for enriching capacity beyond 1980 with a high degree of confidence and accuracy, it would appear that the total free world requirements will be increasing each year by about 5 million kilogram separative work units per year or greater. This projection, combined with the assumption that the total annual capacity of the existing three plants will be about 14 million kilogram separative work units less than annual requirements when the advance inventory of enriched uranium runs out in 1979 or 1980, indicates that, starting perhaps a year prior to that time, new enriching plants should go into operation at the rate of about one per year for about five consecutive years. This should enable the installed enriching capacity to catch up with enriching requirements, following which, new plant additions will probably be required less frequently than one per year. These rough predictions are based on the assumption that new plants will have initial annual separative capacities of about 8 million kilogram separative work units and will stay at that level. This is purely an arbitrary assumption, however, and should not be interpreted as a prediction of the trend that will prevail when new plants are added.

15/ See Chairman Seaborg's testimony on Uranium Enrichment Services Criteria before the JCAE in August 1966.

16/ ORO 658, entitled "AEC Gaseous Diffusion Plant Operations," copies may be requested from: U.S. Atomic Energy Commission, Division of Technical Information Extension, P. O. Box 62, Oak Ridge, Tennessee 37830.

It has been estimated (see ORO 658) that, based on the level of gaseous diffusion technology expected to be available in the early 1970's, new plants can be constructed at the capital costs indicated below:

Size of Plant		Capital Cost
Kg S.W. Units/Year	Operating Power Level	
5 million	1,280 MWE	$ 506 million
8 million	2,080 MWE	$ 743 million
17.5 million	4,600 MWE	$1,446 million

These numbers are preliminary estimates only and are not based on detailed engineering studies.

If it is assumed that construction of a new plant must be initiated four years prior to the time it is to go fully into operation, and if it is assumed that commitments for the plant's operating power must be made at least five years prior to its operation, it becomes apparent that some firm plans for new plants must be made before the mid-1970's.

A logical question at this point is, "How long will requirements for enriching capacity continue to increase?" The answer appears to depend entirely on the rate at which commercially competitive breeder reactors come into being in significant numbers, reach their doubling times, and become self-sustaining. On this basis, it does not appear that enriching requirements will begin to level off much before the year 2000.

7. Foreign Enriching Capability

The only gaseous diffusion plants in the Free World outside of the United States are the Capenhurst Plant in Great Britain and the Pierrelatte Plant in France. It has been stated 17/ that the Capenhurst Plant will have an annual separative capacity of 400,000 kilogram separative work units after modifications to the plant have been completed in 1970 and that subsequent modifications should enable the plant to meet UK requirements after 1973 with some spare capacity for other European requirements. The Pierrelatte Plant is said to be about the same size as the Capenhurst Plant. 17/

17/ Nuclear Engineering, April 1968.

Although the AEC has offered its toll enrichment services to other countries of the Free World without discrimination between domestic and foreign customers, there is a feeling on the part of various European groups that Europe should have its own enriching capability above and beyond the spare capability that might be made available at Capenhurst and Pierrelatte. Such groups as Euratom, the European Nuclear Energy Agency, and Foratom have conducted studies or talked about the possibility of a new enrichment plant in Europe. Most of these groups, however, have acknowledged that an enrichment plant in Europe would require a very sizable capital investment and would have an extremely difficult time matching, or even approaching, the toll enrichment charges offered by this country. It does not appear likely that a decision to build such a plant will be reached in the next few years.

The prospects for a Canadian enrichment plant were discussed by a special panel at the Canadian Nuclear Association Conference in June 1968. Although some opinions were expressed favoring such a plant, financing appears to be the biggest obstacle. An opinion was expressed that at least 50 percent of the financing should be non-government. The CNA Uranium Enrichment Study Committee has concluded that the time is not yet ripe for embarking on a costly enrichment program.

The Japanese are said to be devoting considerable effort to ultracentrifuge development, but have not indicated any definite intention to build an enriching plant.

C. Uranium Processing and Fabrication

Market Predictions

	Initial Fuel	Replacement Fuel
In 1970	$ 67,500,000	Relatively Small
In 1975	$144,000,000	$166,000,000
In 1980	$216,000,000	$414,000,000

Although the above market predictions reflect sizable changes when compared with last year's predictions, the quantities on which they are based remain essentially the same. The changes result from the use this year of a constant $90 per kg U fabrication price, whereas last year's predictions were based on a gradual reduction of fabrication price from $100 down to $60 per kg U over the period 1968 through 1980.

Adequate industrial capability and competition exist today to process and fabricate depleted, normal, and enriched uranium to the various fuel forms required in research, test, and power reactors.

1. Processing

The principal producers of depleted, normal, and enriched uranium metal, oxides, compounds, and coated particles are listed in Table II-2.

TABLE II-2

Producers of Uranium Fuels

	Metals, Oxides Or Compounds	Coated Particles*
General Electric Co., San Jose, Calif.	X	
Gulf General Atomic, San Diego, Calif.		X
Kerr-McGee Corp., Oklahoma City, Okla.	X	
National Lead Co., Albany, N. Y.	X	
Nuclear Fuel Services, Inc., Erwin, Tenn.	X	X
Nuclear Materials and Equipment Corp., Apollo, Pa.	X	X
United Nuclear Corp., Hematite, Mo.	X	X

*Used in certain AEC fuels such as NERVA and in HTGR fuel elements.

No major changes have taken place during the past year in the capabilities of private industry to produce uranium fuel, although several companies have expanded their capacities to process fuel materials. Expansions are mentioned further in [illegible] following section under Fabrication.

2. [illegible]ication

A complete industry capability exists to produce essentially all types of fuel elements that have been used or are being considered; [illegible]owever, the number of capable suppliers is very limited for several highly specialized types of fuel elements.

a. Oxide Fuels

This is the type of fuel used for all the light water reactors being presently built or ordered. This segment of fuel element fabrication continues to be highly competitive.

As was stated in last year's report, all four of the manufacturers of commercial light water power reactors are expanding or have recently expanded their fuel fabrication capabilities as follows:

Combustion Engineering has essentially completed a fuel fabrication facility addition to its Windsor, Connecticut, plant.

A new General Electric fuel facility near Wilmington, North Carolina, is almost completed.

Babcock & Wilcox is building a fuel fabrication plant at its Campbell County site in Virginia.

Westinghouse is building a $20 million manufacturing plant near Columbia, South Carolina, to fabricate nuclear fuel elements.

In addition to the above, expansions of fuel fabricating capability have taken place or been announced by other companies as follows:

Aerojet-General Corporation has added capability to produce highly specialized ur[illegible]nium-plutonium-fissium metallic fuel elements for the EBR-II and plans to continue expanding its production capability on various other fuels. It has been reported to be considering entry into the complete commercial core replacement market in the early 1970's.

Kerr-McGee Corporation is greatly increasing its capacity for production of UO_2 pellets at Cimarron, Oklahoma, in addition to the expansions it is undertaking in the fields of UF_6 conversion and research and development on reactor fuel. Kerr-McGee has been reported to be considering eventual participation in the complete commercial core replacement market.

Nuclear Materials and Equipment Corporation plans a major expansion of its fuel fabrication capability; however, this is largely directed toward plutonium-type fuels at present.

United Nuclear substantially increased both its UF_6-to-UO_2 capacity and its UO_2 pelletizing capacity at Hematite, Missouri, during the past year.

The principal fabricators of oxide uranium fuels are listed in Table II-3. For convenience, those fabricating metallic fuels are also shown.

TABLE II-3

Fabricators of Uranium Fuel

	Oxide	Metal
Aerojet-General Corp., San Ramon, Calif.		X
Atomics International, Canoga Park, Calif.	X	X
Babcock & Wilcox Co., Lynchburg, Va.	X	
Combustion Engineering, Inc., Windsor, Conn.	X	
General Electric Co., San Jose, Calif.	X	
Gulf General Atomic, Inc., San Diego, Calif.		X
M&C Nuclear, Inc., Attleboro, Mass.		X
National Lead Co., Albany, N. Y.		X
Nuclear Fuel Services, Inc., Erwin, Tenn.	X	
Nuclear Materials and Equipment Corp., Apoll[illegible], Pa.	X	X
Nuclear Metals Div., Whittaker Corp., West Concord, Mass.		X
United Nuclear Corp., New Haven, Conn.	X	X
Westinghouse Electric Corp., Chesw[illegible]k, [illegible]a.	X	

b. Metallic Fuels

A stronger industrial base for the fabrication of research and test reactor fuels of all kinds is needed and the AEC has been working to encourage wider participation. The present and potential suppliers of research and test reactor fuels of the uranium-aluminum type are United Nuclear Corporation, National Lead Company, M&C Nuclear, Atomics International, Aerojet-General Corporation, Nuclear Materials and Equipment Corporation, and the Nuclear Metals Division of Whittaker Corporation.

In a number of cases there are limited commercial suppliers for the highly specialized fuel required in specific reactors. Some examples are:

HFIR - [illegible] H[illegible]gh Flux Isotope Reactor is designed to produc[illegible] [illegible]search quantities of the transuranium elements [illegible] is the first of its kind in the United States. Its fuel assemblies consist of concentric,

cylindrical fuel elements made up of vertically positioned, curved fuel plates. The fuel plates are made from a mixture of enriched U_3O_8 and aluminum with aluminum cladding. M&C Nuclear, Inc., is the present commercial fabricator of HFIR fuel assemblies.

EBR-II - This experimental breeder reactor serves as an irradiation test reactor for experiments on the priority fast reactor fuels. Its fuel elements are made up of fuel pins containing injection cast uranium-plutonium-fissium alloy, sodium bonded, and clad in stainless steel. The present commercial supplier is the Aerojet-General Corporation.

CP-5 - The reactor, also called the Argonne Research Reactor, is a heavy-water moderated reactor designed for irradiation of materials and equipment. Its fuel elements are in the form of hollow tubes that permit experimental material to be placed inside them for irradiation purposes. The fuel material is enriched uranium and aluminum with aluminum cladding. The Nuclear Metals Division of Whittaker Corporation is the present commercial supplier.

ZPPR - The Zero Power Plutonium Reactor is to be used to perform reactor physics experiments. Its fuel will consist primarily of a Pu-U-Mo alloy clad in stainless steel to form fuel plates; however, it will also utilize some fuel produced from mixed oxides. Nuclear Materials and Equipment Corporation is the present commercial producer of this fuel. The same type of fuel plates will be used in ZPR reactors.

Although a few of the metal fuel fabricators have increased their capabilities during the past year, there have been no major expansions.

The market for research and test reactor fuels, including MTR, ETR, ATR, ORR, HFBR, HFIR, Plumbrook, GETR, and university reactors, is estimated at about $5 million annually. This market is small relative to the future market for commercial power reactor fuels.

3. Comprehensive Tabulation of Industrial Capabilities

A comprehensive tabulation of capabilities that exist at commercial facilities for the processing and fabrication of various types of fuel materials, research and development on

uranium and plutonium fuels, and chemical recovery of uranium and plutonium is presented as Table II-4, on the following page. The AEC Division of Industrial Participation will appreciate hearing of any changes or additions that should be made to this table. Table IV-9, page 110 of this report, also indicates industrial capabilities in the fuel cycle. It shows capabilities as they exist or are planned for commercial power reactor fuels only.

4. Present and Future Requirements for Processing and Fabrication Services

During fiscal year 1968, the amount of enriched ura[illegible] [illegible] withdrawn from the gaseous diffusion plants and shipped [illegible] to domestic processors and fabricators totaled 387,382 kg (See Table II-5). This quantity includes requirements for all types of privately sponsored reactors, requirements for Government reactors exclusive of plutonium production types, and fuel for foreign reactors processed and/or fabricated in the United States. The corresponding quantity that was withdrawn and shipped in FY-1967 amounted to 169,983 kgs U. This represents a very significant, but not unexpected, increase over previous years. The requirements are expected to continue increasing in future years in line with the increased rate at which commercial nuclear power stations are constructed since they lag by some time the ordering of nuclear steam supply systems.

TABLE II-5

Enriched Uranium Furnished to Industry
(Kilograms of Uranium)

Fiscal Year	Furnished as UF_6	Furnished in Forms Other Than UF_6	Total
1956	0	2,400	2,400
1957	6,000	4,000	10,000
1958	24,000	21,000	45,000
1959	110,300	6,300	116,600
1960	86,200	3,400	89,600
1961	119,400	6,900	126,300
1962	125,600	3,000	128,600
1963	100,273	3,918	104,191
1964	116,401	2,037	118,438
1965	152,786	1,441	154,227
1966	285,773	5	285,778
1967	169,983	96	170,079
1968	387,382	63	387,445

COMMERCIAL CAPABILITY TO PROCESS AND FABRICATE NUCLEAR FUELS

TABLE II-4

X DENOTES PRESENT CAPABILITY F DENOTES FUTURE CAPABILITY

CAPABILITY \ NAME OF COMPANY	A-G	ALLIED	A.I.	B&W	B.M.I.	C.E.	G.E.	GRACE	G.G.A.	K-Mc	M&C	N.L.C.	NUMEC	N.M.	N.F.S.	U.N.C.	W
(company name)	[illegible]	[illegible]	[illegible]	[illegible]	[illegible]	[illegible]	[illegible]	[illegible]	[illegible]	[illegible]	[illegible]	[illegible]	[illegible]	[illegible]	[illegible]	[illegible]	[illegible]
$U_3O_8 \rightarrow UF_6$ [illegible]		X								F							
$UF_6 \rightarrow UO_2$ [illegible]	F		F	F		F	X X			X			X		X	X	F
UO_2 PELLETS [illegible]	F		F	F		F	X X			X		X	X		X	X X	X F
UO_2 FUEL FABR. [illegible]	F		X	X		X	X X						X		X	X	X F
CARBIDE FUELS [illegible]			X						X	X			X		X	X X	X
METAL FUELS [illegible]	X		X						X	X	X	X	X	X		X X	
U-233 FUELS [illegible]													X		X		
THORIUM [illegible]									X	X		X	X		X	X X	
Pu FUELS [illegible]	X		X	F		F	X						X		X	X	X
U FUEL R&D [illegible]	X		X	X	X	X	X	X	X			X	X	X	X	X X	X
Pu FUEL R&D [illegible]	X		X	X	X	F	X	F					X		X	X	X
DEPL. U-METAL [illegible]	X		X							X		X	X		X	X	
DEPL. U-CPDS [illegible]										X		X	X		X	X	
COLD U SCRAP [illegible]				F			X X			X			X		X	X	F
COLD Pu SCRAP [illegible]				F			F						X		X	F	X
SPENT FUEL [illegible]		F					F								X		
LOCATION OF FACILITY	[illegible]	[illegible]	[illegible]	[illegible]	[illegible]	[illegible]	[illegible]	[illegible]	[illegible]	[illegible]	[illegible]	[illegible]	[illegible]	[illegible]	[illegible]	[illegible]	[illegible]

Division Of Industrial Participation [illegible]

Annual domestic requirements for processing and fabrication of commercial power fuel, along with corresponding dollar values for the service, are projected in Chart II-D. Since the total quantity of fuel will not be significantly affected, depending on whether plutonium is or is not recycled, the quantities shown in Chart II-D may be used for either assumption. The annual dollar values projected in Chart II-D are based on a present average price of about $90 per kilogram U for going from enriched UF_6 to the complete fuel elements. It is quite possible, of course, that this unit price will decrease by 1980. If plutonium recycle is adopted, the annual dollar totals can be expected to increase slightly above the non-recycle estimates in the late 1970's, but by something less than 10 percent in 1980.

CHART II-D

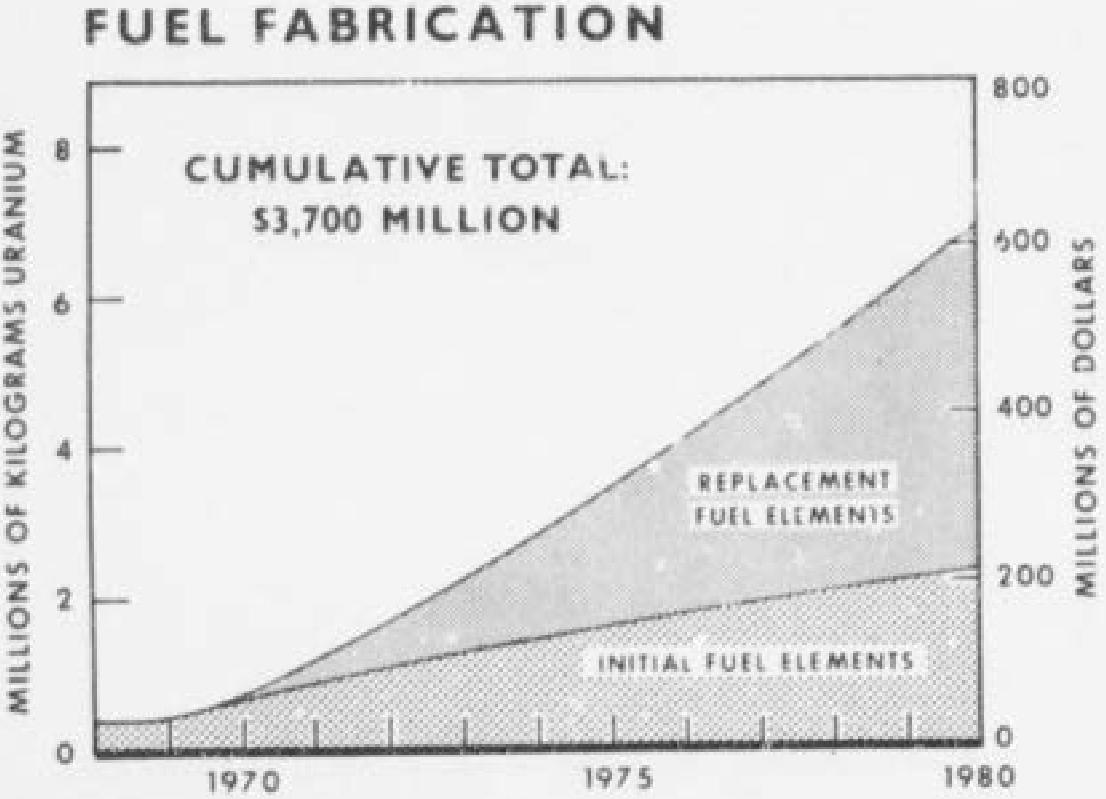

5. Processing and Fabrication of Foreign Reactor Fuel

In addition to the quantity shown in Table II-5 of UF_6 shipped to domestic processors and fabricators in FY-1968, 38,619 kilograms of enriched uranium were withdrawn and shipped to foreign countries in the form of UF_6 during FY-1968 as compared to 80,925 kgs U in FY-1967.

Also in FY-1968, domestic processors and fabricators furnished 74,180 kilograms of enriched uranium to foreign customers which, when compared with the 38,619 kilograms shipped as UF_6, indicates that about 34 percent of the enriched uranium fuel produced for foreign reactors was processed and fabricated in foreign countries. The corresponding percentages in FY-1967 and FY-1966, respectively, were 50 percent and 9 percent processed and fabricated in foreign countries. See Table II-6. It appeared last year, in comparing FY-1967 with FY-1966, that the fabrication of fuel for foreign reactors was shifting rapidly to foreign countries. Although the trend slowed down in FY-1968, it is expected to pick up again until, in a few years, essentially all enriched uranium used in foreign reactors will probably be fabricated abroad. For this reason, foreign fuel was not taken into account in the preparation of Chart II-D.

TABLE II-6

Enriched Uranium Shipped Abroad

	FY-1966	FY-1967	FY-1968
A. Enriched Uranium Shipped Abroad in Form of UF_6 for Foreign Reactors Kgs U	9,524	80,924	38,619
B. Enriched Uranium Shipped Abroad in Processed or Fabricated Form for Foreign Reactors Kgs U	93,626	79,085	74,180
C. Approximate Percentage of Foreign Reactor Fuel Enriched in USA That is Fabricated in Foreign Countries. $(\frac{A}{A\&B} \times 100\%)$	9.2%	50.6%	34.2%

6. Commercial Fabrication of AEC Fuel

The magnitude of orders placed each year by AEC for commercial processing and fabrication of enriched uranium fuels is indicated in Table II-7.

TABLE II-7

AEC Orders for Fabrication of Uranium Fuel
From Commercial Suppliers
(Millions of Dollars)

Fiscal Year	Naval Purposes	All Other Purposes	Total
1959	38.6	2.8	41.4
1960	48.6	3.8	52.4
1961	29.2	4.0	33.2
1962	60.9	4.5	65.4
1963	36.8	2.2	39.0
1964	28.5	8.7	37.2
1965	52.3	5.4	57.7
1966	63.4	2.0	65.4
1967	55.1	13.0	68.1
1968	107.4	5.3	112.7

It can be seen from Table II-7 that the commercial fabrication of naval fuels increased significantly in FY-1968 over previous years. The commercial fabrication of other AEC fuels, however, dropped much lower in FY-1968 than its level in FY-1967, although it compared well with years prior to FY-1967. This seems to indicate that the level was abnormally high in FY-1967. The FY-1967 total, for example, included one single item that amounted to greater than $8 million for fabrication of about a five-year supply of HFIR fuel elements.

7. Fuel Cycle Arrangements

Various different fuel cycle arrangements, including fabrication and reprocessing, are being offered to utilities by the fuel suppliers. These are discussed under Category IV of this report.

D. Plutonium Processing and Fabrication

There is increasing interest on the part of utilities operating nuclear power plants in planning the utilization of their plutonium in recycle fuel or its sale or storage until early breeder reactor requirements develop.

This concern over the management of such a valuable source of energy will increase during the next two years in view of the termination, on December 31, 1970, of AEC's guaranteed purchase price for plutonium that was produced in reactors fueled with AEC-supplied enriched uranium.

It has been estimated that plutonium generation in nuclear power reactors may exceed $1 million per day in 1980. One private utility expert recently estimated that 700 metric tons of total plutonium will have been recovered by 1990. This quantity of plutonium would be worth about $4.5 billion at the current value of $9.28 per gram. About 200 grams of fissile plutonium per megawatt per operating year would be produced, assuming 80 percent capacity factor and a 70 percent fissile composition.

With its greater neutron yield in fast reactors, the value of plutonium is expected to be substantially higher than in thermal reactors, as much as 50 percent greater in efficient types.

1. Availability of Plutonium

The projected quantities of plutonium expected to be recovered annually from domestic power fuels are shown in Table II-8. This table is based on the AEC's estimates of power reactor spent fuel discharges and about eight kilograms of plutonium recovered per metric ton of uranium processed.

TABLE II-8

Projected Plutonium Recovered From Domestic Commercial Power Reactor Fuels
(Kg Total Pu as the Nitrate)

Calendar Year	Annual Kg Pu Recovered
1969	280
1970	330
1971	640
1972	1000
1973	2100
1974	3900
1975	6600

The availability of plutonium is based on the quantity expected to be recovered from reprocessing spent fuels, rather than plutonium generated in power reactor operation during any given year.

2. Requirements for Plutonium

Over the next four to five years, AEC, private industry, and foreign programs will require substantial quantities of plutonium for research and development, critical assemblies, experimental reactors, and demonstration reactors. The annual plutonium requirements for foreign, AEC, and private domestic programs are estimated in Table II-9.

TABLE II-9

Major Programs Requiring Plutonium
(Annual Net Requirements in Kgs.)

Program or Project	FY-69	FY-70	FY-71	FY-72	FY-73	FY-74	FY-75
ZPR's	1900	370	600	600	360	0	0
Fast Flux Text Project	470	[illegible]50	850	1200	1100	900	890
Fast Reactor Demonstration	0	0	0	700	700	940	1400
Foreign	700	500	200				
EEI Thermal Recycle Test Program	100	500	400	300			
Rounded Totals	3200	1800	2100	2800	2200	1800	2300

Comparison of annual requirements versus availability indicates that the demand exceeds supply until around 1973. The difference probably will be obtained from AEC production reactor operations.

Fuels containing plutonium for the ZPR family of reactors have been primarily in the metallic form and are interchangeable to permit the greatest flexibility in conducting experiments.

Plutonium will be recycled in light water reactors in the oxide form, mixed with uranium oxide. The first fast breeders probably will use mixed oxides. Later, as experience permits, carbides and nitrides of plutonium will probably be used in fast breeders to achieve higher specific power and fuel rod sizes that are larger and easier to fabricate.

In addition to the requirements shown in Table II-9, above, research and development requirements should average about 100 kg annually.

3. <u>Transition of Plutonium Recycle Effort From AEC to Industry</u>

As noted in our 1966 and 1967 reports, the Commission's work on plutonium fuel recycle in thermal reactors will be gradually phased out over the next few years, because the basic questions of feasibility have been answered and a large body of experience, data, and design information has been developed. The nuclear power utilities and the major reactor suppliers have already assumed a substantial amount of plutonium recycle development work. In 1966 the electric power industry, through the Edison Electric Institute, sponsored a two-phase program aimed at the use of plutonium-bearing fuel loadings in light water power reactors. General Electric and Westinghouse are bearing part of the cost of the initial phase of this program which is to evaluate economic and technical aspects and specify the optimum method of plutonium utilization. A follow-on phase will include design and manufacture of test fuel assemblies for the demonstration of Pu recycle in large water reactors. EEI also contracted with Gulf General Atomic for a feasibility study of plutonium recycle in gas cooled reactors.

The first commercial power reactor to irradiate plutonium in a recycle test program is Dresden #1. Four plutonium fuel rods in fuel assemblies were installed in June 1967, using plutonium recovered from reprocessed Dresden #1 fuel elements.

A larger scale irradiation test of plutonium fuel in Dresden was initiated during the year with a joint Commonwealth Edison-United Nuclear program to fabricate fuel rods for assembly into 11 fuel assemblies. Some 12 kg of plutonium will be involved in this program.

The AEC program of plutonium utilization in light water reactors has plutonium dioxide - uranium dioxide fuel mixtures in fabricated fuel elements under irradiation in the Plutonium Recycle Test Reactor at Richland, Washington, and the Saxton Nuclear Experimental Reactor at Saxton, Pennsylvania.

The Edison Electric Institute also has a program in support of fast reactor fuel development. The first phase of this program is with Atomic Power Development Associates at a level of $1.1 million. The entire EEI fast reactor plutonium fuel program will extend over five years and is expected to cost about $5 million.

4. Commercial Fabrication

Although the major plutonium requirements have been forecast by the using organizations, as indicated in Table VII-9, there is expected to be some slippage in awarding fabrication contracts and reductions in the quantity of plutonium to be fabricated. Because of AEC budget restrictions, shortages of plutonium of the required composition and the need to qualify some of the new commercial suppliers, it is expected that commercial fabricators may process and fabricate from 30-50 percent of the quantities forecast in Table II-9 during the next year or two (foreign requirements excluded). An estimated fabrication cost of $9000 per kilogram of contained plutonium in mixed oxides and $5000 per kilogram of contained uranium in metal fuels was used to develop the potential fabrication market shown in Chart II-E.

CHART II-E

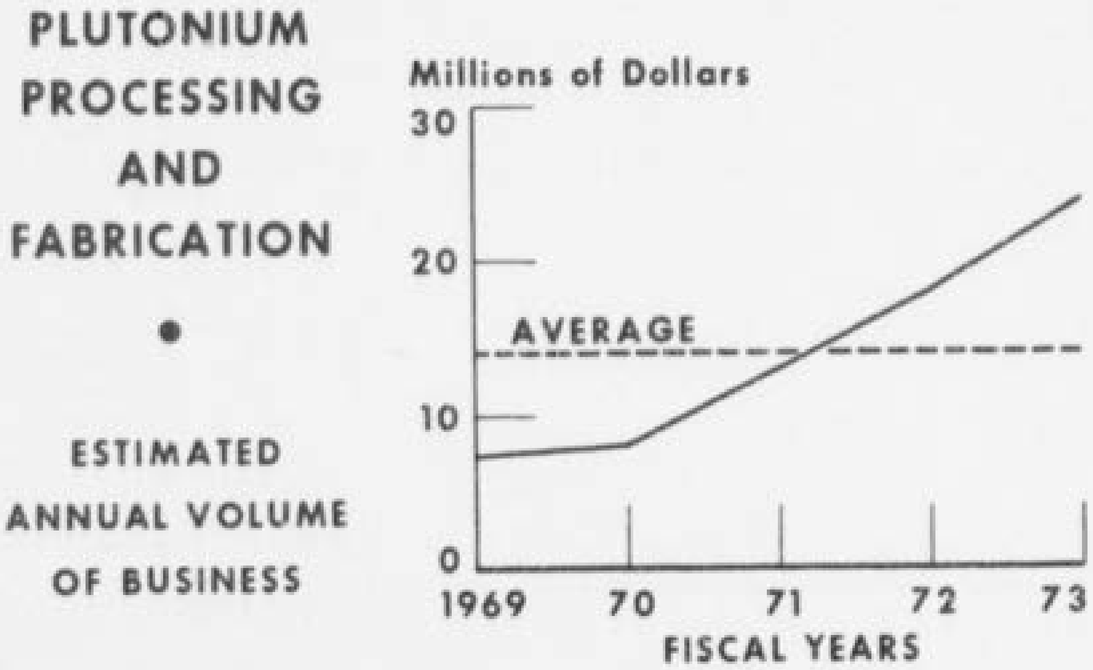

5. Industry Capability

Table II-10 lists private firms which have various degrees of capability in plutonium processing.

TABLE II-10

Industrial Interest in Plutonium Work

	Fuel Processing	Fuel Fabrication	Scrap Recovery	R&D
Aerojet-General Corp. San Ramon, Calif.	X	L		X
Atomics International Canoga Park, Calif.	X	X	X	
Babcock & Wilcox Lynchburg, Va.	X	L		X
Battelle Memorial Inst. Columbus, Ohio	X	L		
General Electric Co. San Jose, Calif.	X	X		X
Nuclear Fuel Services Erwin, Tenn.	X	X	X	X
Nuclear Materials & Equipment Corp. Leechburg, Pa.	X	X	X	X
United Nuclear Corp. Pawling, N. Y.	X	L		X
Westinghouse Cheswick, Pa.	X	X	X	X

NOTE: "L" - Limited Fabrication Capability

NUMEC has planned a major expansion of its facilities in Leechburg, Pennsylvania, including a $3 million fuel facilities program, enlarging its plutonium processing plant, and adding research and development facilities.

Aerojet-General Corporation announced an expansion program at its fuel research facilities at San Ramon, California. This expansion will include a plutonium processing facility for process development work on mixed oxide fuels and limited production of these fuels.

E. Thorium

Requirements for thorium as a reactor material continue to be limited primarily to research and development purposes. AEC orders for thorium metal powder and ingots for fuel R&D use, during the past year, amounted to only a few hundred dollars. Further development and construction of reactors employing the thorium - U-233 cycle for commercial applications could eventually expand the use of thorium as a source of nuclear fuel. Examples of the application of the thorium - U-233 fuel cycle are the HTGR, the Molten Salt Breeder Reactor, the Elk River Reactor, seed-blanket loadings for the Shippingport Pressurized Light Water Reactors, and the initial loading in Indian Point No. 1. The future market for thorium will depend, in the near term, upon the success of Gulf General Atomic in marketing the high temperature gas-cooled reactor and, in the long range future, on the successful development of breeder reactors that utilize thorium, such as the molten salt concept.

Industry capabilities to process and fabricate thorium have changed very little. The firms that have capability to fabricate thorium are identical to those listed in Table II-2. The principal firms processing thorium are shown in Table II-11.

TABLE II-11

Principal Firms Processing Thorium

American Potash & Chemical Corp., Chicago, Ill.
Gulf General Atomic, San Diego, Calif.
Kerr-McGee Corp., Oklahoma City, Okla.
Metal Hydrides, Inc., Beverly, Mass.
National Lead Co., Albany, N. Y.
Nuclear Fuel Services, Inc., Erwin, Tenn.
Nuclear Materials & Equipment Corp., Apollo, Pa.
United Nuclear Corp., Hematite, Mo.

[illegible]

F. Uranium-233

The present need for uranium-233 continues to be limited and is essentially of a developmental nature. Due to the interest in the thorium - uranium-233 fuel cycle, development work in this area should increase in the future.

The principal companies having U-233 processing and fabrication capabilities are Nuclear Fuel Services and the Nuclear Materials and Equipment Corporation.

The Private Ownership Act of 1964 directed the Commission to establish guaranteed purchase prices for U-233, not to exceed 10 years, with no fixed terminatic[illegible] date on the Commission's authority. Accordingly, the AEC establishec prices for uranium enriched in uranium-233 in forms of nitrate, dioxide, or metal and meeting AEC specifications, of $13 per gram of the contained isotope uranium-233, subject to adjustment for the presence of other uranium isotopes and deductions for handling costs.[18/] The U-233 purchase prices continue until the Commission has made a determination that U-233 technology has reached the point where the Government should no longer provide a guaranteed market for this material.

G. Zirconium

The metal used for fuel cladding or as a core structural material in nuclear reactors must meet difficult requirements as to strength at operating temperatures, resistance to corrosion by the coolant, resistance to irradiation damage, and neutron absorption cross section. The remarkable ability of zirconium to meet all of these requirements under the operating conditions of light water reactors has made it the universal choice in fuel cladding and fuel element fabrication for commercial light water reactors.

Since zirconium occurs in nature in combination with hafnium, a metal which, unlike zirconium, has a high neutron absorption cross section, it is absolutely essential that the hafnium be removed before zirconium can qualify as nuclear grade zirconium. This separation is normally accomplished by solvent extraction.

Zirconium metal is derived from two principal minerals - zircon ($ZrSiO_4$) and baddeleyite (ZrO_2), with zircon being by far the more important source. Zircon is found in beach sands throughout the world and most American zircon comes from Florida beaches. A sizable portion of the zircon used in this country is imported, principally from Australia. Zirconium metal requirements, however, account for only a small portion (about 5 percent in 1967) of the zircon consumed in this country. Zircon is used in much greater quantities in foundry sands, blasting grains, refractories, and ceramics.

18/ See AEC Public Release K-277, dated November 28, 1967.

Although it is common practice to refer simply to "zirconium" cladding or "zirconium" tubing, the material actually used contains small amounts of alloying agents. Zircaloy-2, usually used in boiling water reactors (BWR's), contains tin, iron, chromium, and nickel, while zircaloy-4, used by all three manufacturers of commercial pressurized water reactors (PWR's), contains little or no nickel but an increased amount of iron, in addition to tin and chromium. The tubing used for commercial fuel cladding measures about 0.570" o.d. by about 0.0355" wall thickness for BWR's and about 0.425" o.d. by about 0.025" wall thickness for PWR's.

Wah Chang Albany Corporation of Albany, Oregon, and AMAX Specialty Metals, Inc., of Akron, New York, are the only two companies in this country that are currently processing zircon ore to zirconium metal (frequently called zirconium sponge, when produced by the method used by these companies). Both companies have expanded their capacity to produce zirconium metal during the past two years, and both have been reported to be considering further expansion of their capacity.

In addition to metal production, both Wah Chang and AMAX also melt sponge to ingot and produce rolled mill products. AMAX Specialty Metals also produces zirconium tubing, but Wah Chang does this only on an experimental basis.

The principal companies that have capability to produce zirconium products are shown in Table II-12.

TABLE II-12

The Principal U.S. Companies Having Capability to Produce Zirconium and Zirconium Products

Company	Zr Sponge	Zr Ingots	Zr Plates & Strips	Zr Tubing
Wah Chang Albany Corp.	X	X	X	Experimental Only
AMAX Specialty Metals, Inc.	X	X	X	X
Superior Tube Company	---	---	---	X
Wolverine Tube Division Calumet & Hecla, Inc.	---	---	---	X
Harvey Aluminum	---	X	---	X
Sandvik Special Metals Corporation	---	---	---	X
Westinghouse Electric Corporation	---	X	X	X

In addition to the companies shown in Table II-12, General Electric Company plans to install zirconium tubing capability at the fuel fabrication plant it is building near Wilmington, North Carolina. Both GE and Westinghouse will probably produce only a portion of their tubing and continue to purchase the remaining requirements from other tubing fabricators.

It was recently announced that a new company by the name of Zirconium Technology Corporation will be constructed in or near Albany, Oregon, and begin producing tubing late in 1969. Its initial zirconium plans call only for the production of tubing and the rod stock required in fuel rod end plugs; however, it will probably also produce tubing from other materials.

Other organizations not listed in Table II-12 that have very specialized zirconium production capabilities are: Carpenter Steel Company, which makes large diameter tubing for use in heavy water reactors or in the fabrication of BWR fuel channels; Chase Brass and Copper Company, which produces large diameter, relatively heavy-walled, tubing for specialized uses; and Oregon Metallurgical Corporation, which concentrates primarily on non-nuclear zirconium products, such as corrosion resistant, chemical processing equipment. There are also a number of additional companies that are converters of zirconium metal, including some not indicated in Table II-12 that have rolling capability.

The present total domestic capability to produce zirconium sponge runs at about four million pounds per year and to produce tubing for fuel cladding use, at about 8.5 million linear feet per year.

Eldorado Nuclear Limited of Canada has been constructing a zirconium plant. It is to produce about 600,000 pounds per year of zirconium metal, using a process that by-passes zirconium sponge and produces the metal directly as ingots. The plant has been sized primarily to meet Canadian requirements, which have been estimated at an average of about 400,000 pounds per year for the next five years and in excess of 600,000 pounds per year by 1976. The Eldorado plant is designed to accommodate ready expansion. Most of Canada's zirconium requirements have been produced in the United States in the past.

Estimated annual reactor requirements are shown in Table II-13 in terms of both the linear feet of tubing required for fuel cladding in commercial light water reactors and the total pounds of zirconium required for U.S. nuclear reactors of all types (including AEC and Navy). It should be noted, however, that not all types of reactors use zirconium, for example, the HTGR and fast breeder reactors.

TABLE II-13

Estimated Annual Requirements for
Zirconium in U.S. Nuclear Reactors

Calendar Year	Zr Fuel-Clad Tubing Required for Commercial Reactors (Millions of Feet)	Total Pounds of Zr Sponge Required for U.S. Nuclear Reactors (Millions of Pounds)
1968	1.5	1.3
1969	3.1	1.8
1970	4.8	2.1
1971	6.8	2.5
1972	8.9	2.8
1973	11.0	3.2
1974	13.4	3.7
1975	15.8	4.1
1976	18.2	4.6
1977	20.7	5.1
1978	23.4	5.6
Cumulative Totals	127.6	36.8

If one compares the above-indicated market figures with the existing sponge production capacity of four million pounds, it appears that existing capacity is adequate to meet requirements through about 1974. Since both of the present producers have indicated they are considering further expansion, there appears to be no reason for concern about adequate capacity for a number of years.

A comparison of the tubing requirements indicated in Table II-13 with the existing production capability of about 8.5 million feet indicates that capacity should meet requirements through 1971. The anticipated additional plants and expansions of present plants should provide adequate tubing capability indefinitely.

H. Heavy Water

Heavy water plants were originally built by the Atomic Energy Commission in the early 1950's at Dana, Indiana, and at Savannah River to furnish heavy water for the production reactors at Savannah River. The Dana plant was shut down in 1957 and the Savannah River plants were gradually reduced in operation to about one-third their total capacity during 1957 and 1958. As of today, the AEC continues

to operate the Savannah River heavy water plant at approximately one-third its capacity. This level of operation meets AEC production requirements and provides some excess material.

Since 1955, the AEC has sold heavy water in quantities of 125 pounds or more to commercial users. Smaller quantities are available from distributors. The principal commercial suppliers are given in Table II-14. Since heavy water use frequently involves degradation of the heavy water, the AEC also provides a service to rework degraded heavy water on a large-quantity basis. The Bio-Rad Company of California has established an operation for upgrading small quantities of heavy water. The AEC will probably continue to offer purification services for larger quantities, until this service is reasonably available from industry for all quantities.

TABLE II-14

Principal Commercial Suppliers of Heavy Water
(In Quantities Less Than 125 lbs.)

Bio Rad Labs, Inc., Richmond, California
Columbia Organic Chemicals, Columbia, S.C.
Deuterium Corporation, New York, New York
Diaprep, Inc., Atlanta, Georgia
International Chemical & Nuclear Corporation
City of Industry, California
Isomet Corporation, Palisades Park, N.J.
Liquid Carbonic, Division of General Dynamics
Corporation, San Carlos, California
Matheson, Coleman & Bell, East Rutherford, N.J.
Merck & Company, Rahway, New Jersey
New England Nuclear Corporation, Boston, Mass.

The Deuterium Corporation of New York and the Lummus Company of Bloomfield, New Jersey, offer a broad range of services, including design, engineering, and construction of heavy water plants.

The worldwide heavy water inventory requirements for natural-uranium fueled, heavy-water moderated, reactors have received attention during the past few years. The countries principally involved in the heavy water reactor concept are: Argentina, Canada, France, Germany, India, Japan, Sweden, and the United Kingdom.

Canadian, European, and Asian capacity, outside the Soviet Bloc, in operation at present is about 55-60 tons, with another 820 tons under construction or planned. Most of the planned capacity will be in Canada.

The Deuterium of Canada, Ltd. plant is scheduled for start-up operations of the first phase in early 1969 and the second phase is expected to be in operation by the end of 1969. The plant is to have an ultimate capacity of 400 tons per year.

The Canadian General Electric Company is building a plant that is designed to produce 420 tons a year, with operation scheduled to begin in 1969. Also, AECL of Canada is considering an additional 400 ton plant. The Siemens Company of West Germany is considering a 200 ton a year plant. France, India, Norway, and Switzerland each have limited capacity to produce heavy water that adds up to make the previously mentioned total of 55-60 tons/year.

As was mentioned above, present AEC production is based on operation of one-third of the Savannah River heavy water plant. Current production runs about 200 tons per year whereas the rated capacity of the total plant is about 500 tons per year.

The AEC announced in March 1968[19/] that it was increasing its sale price for heavy water from $24.50 to $28.50 per pound, effective May 11, 1968, and that its lease charge would also be based on the higher figure. In view of the limited U.S. production capacity for heavy water, the AEC has not attempted to meet worldwide long-term requirements for this material as it has for enriched uranium. To the extent, however, that heavy water is available in excess of the needs of Commission-sponsored programs, the AEC contracts to supply heavy water for peaceful purposes to domestic and foreign customers on a first-come, first-served basis.

I. Beryllium

Although beryllium is an excellent moderator and reflector material in test reactors, the total amount used in nuclear reactors of all kinds is very small since light water is used as the moderator in the current commercial reactors. The discussion that follows therefore primarily concerns highly specialized, non-reactor, Governmental needs for beryllium parts.

In line with AEC's desire to support, develop, and utilize the services of a strong, domestic beryllium industry, AEC held meetings with representatives of the four major beryllium metal companies in 1966 to plan methods of better accomplishing these goals. As a result, steps were initiated to (a) improve communications and exchange

---

19/ See AEC Public Release L-39, dated March 11, 1968.

of technical information between Government and industry, (b) provide development order contracts with industry to enable them to gain experience and prove capability in meeting specialized Government needs, and (c) provide for periodic solicitations of bids from industry to meet at least part of AEC's beryllium needs.

As a result of AEC's increased efforts to meet more of its production requirements for beryllium parts from commercial sources, a sizable order for beryllium parts was placed with industry in November 1966. Additional orders for the same type of part were placed with industry, following receipt of competitive bids, in August 1967, in December 1967, and in April 1968. In July 1968, AEC placed its first order with industry for a quantity of a second type of beryllium part. Efforts are continuing to find additional cases where AEC's specialized beryllium requirements can be met satisfactorily from commercial sources.

The four companies constituting the basic beryllium metal industry as as follows:

Beryllium Metals and Chemicals Corporation
500 Fifth Avenue
New York, New York 10036

The Beryllium Corporation
P. O. Box 1462
Reading, Pennsylvania 19603

The Brush Beryllium Company
17876 St. Clair Avenue
Cleveland, Ohio 44110

General Astrometals Corporation
320 Yonkers Avenue
Yonkers, New York 10701

J. Boron-10

The Government's total requirements for boron-10 are now being met by the AEC-owned Model City Plant (MCP) in New York State, operated by the Nuclear Materials and Equipment Corporation (NUMEC). A small portion of this plant's output is upgraded under contract to the Eagle-Picher Company. In the past, the MCP operated at a level of about 400 kilograms per year and, at present, is operating very close to the annual design capacity of 500 kilograms.

It was noted in last year's report that the Eagle-Picher Company had expressed to the Commission its interest in building a boron-10 facility to meet the needs of the AEC and any developing commercial market. The proposed plant was to be capable of producing a high purity boron-10 product.

A number of discussions took place during the past year between AEC staff members and Eagle-Picher representatives in regard to their possible interest in building such a facility. In these discussions, the AEC pointed out that it had concluded that AEC requirements were not sufficiently firm to provide a sound and attractive basis for seeking proposals from industry to meet these requirements.

CATEGORY III
NUCLEAR COMPONENTS AND EQUIPMENT

Nuclear components and equipment are generally available commercially, usually from companies not wholly dependent on nuclear energy activities for profitable markets. Establishments manufacturing pumps or valves for a wide variety of industrial applications, for example, are usually the suppliers of pumps and valves for specialized nuclear applications. As orders for nuclear power reactors have accelerated, however, demands for reactor components have also expanded rapidly, and it seems probable that some establishments will eventually be devoted exclusively to the supply of reactor parts. This seems particularly true of the suppliers of light water reactors who, as orders have expanded, have tended to integrate vertically to bring control over production of many principal components within their own facilities. Part A of this category discusses reactor components and lists producers of the principal components.

Other components within this category are, in some cases, produced by smaller firms whose origin and growth has been more dependent on nuclear energy applications. Nuclear instruments largely fall into this group, and, because of their particular interest and importance to practically all phases of nuclear work, are reported on separately in Part B of this category.

The term "nuclear instruments" has been generally used in the past to describe a broad area of application. Their use has now developed to a point where they can be divided into several general areas. These are defined below and are covered under several categories in this report, as indicated.

Reactor Control Instruments

Instrumentation built into reactor facilities for control and operation purposes. This use is covered under Part A of this category, "Reactor Control Equipment."

Radiation Detection and Monitoring Instruments

Instruments designed to detect, measure, and monitor radiation, as well as associated equipment. These are covered in Part B of this category.

Gauges and Process Control Equipment Utilizing Radioactive Materials

Gauges and process control equipment utilizing radioisotopes in their design. These are covered in Category V, Radioisotopes.

A. Reactor Components

Because of increasing attention and interest in the sale of components for nuclear power plants, a more detailed review of this area is included in this section in the following paragraphs.

1. Orders and Shipments

Shipments of reactor components over the past eight years as reported by the Bureau of the Census are shown in Chart III-A with pressure vessels[1/] shown separately from other components.[2/] Both groups were up substantially in 1967 over 1966 -- pressure vessels by nearly 60 percent and other components by 26 percent.

In past years new orders for pressure vessels have also been reported by the Bureau of the Census. For 1967, however, orders for primary vessels and tanks were not reported to avoid disclosing figures for individual companies. Orders for a number of products in "other components" were also withheld to avoid disclosing figures for individual companies. Based on the upsurge in orders reported in total (as shown in Chart B in the Summary part of this report) shipments of reactor components are expected to rise very sharply in 1968 and 1969 as the impact of orders for nuclear power plants is felt at the component level.

CHART III-A

SHIPMENTS OF REACTOR COMPONENTS

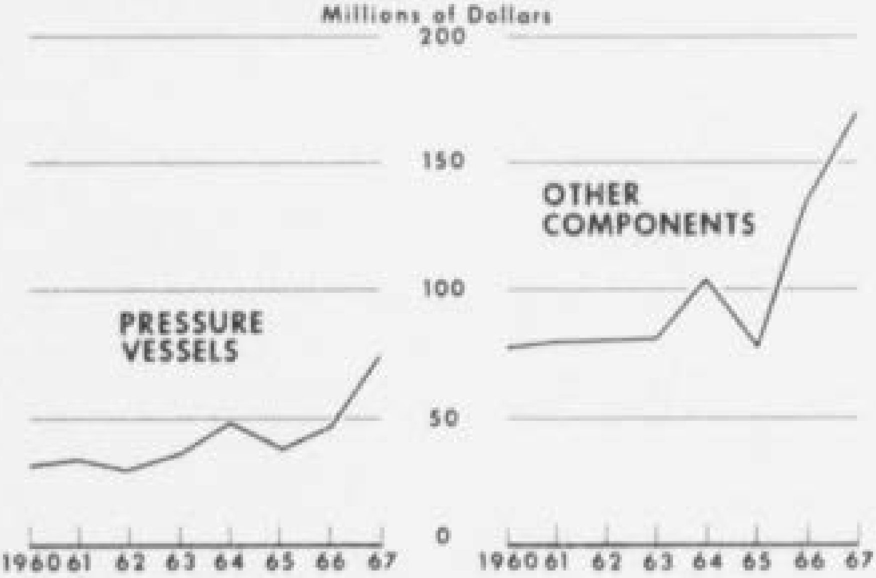

1/ primary vessels and tanks, and heat exchangers and condensers
2/ all other reactor components and equipment listed in Table 2 in the summary portion of this report, except fuel elements, moderators, coolants, and reflectors.

2. Components for Light Water Cooled Systems

Components are available on a competitive basis for the types of light water reactors now under construction or planned in the United States. The following listing shows the principal components of these systems and the current suppliers competing for U.S. orders.

a. Pressure Vessels

Babcock & Wilcox
Chicago Bridge & Iron
Combustion Engineering
Rotterdam Dockyard Co.
(foreign company)

b. Steam Generators

Babcock & Wilcox
Combustion Engineering
Westinghouse

c. Pressurizers

Babcock & Wilcox
Chicago Bridge & Iron
Combustion Engineering
Westinghouse

d. Primary Pumps

Bingham Pumps
(G. F. Atkinson Co.)
Byron Jackson (Borg-Warner)
Westinghouse

e. Valves

Anchor
Atwood & Morrill
Black-Sivalls-Bryson
Crane
Darling
Hoke
Wm. Powell
Rockwell Manufacturing
Velan
Walworth

f. Core Structurals

Babcock & Wilcox
Carpenter Steel
Combustion Engineering
General Electric
Mesta Machine
NUCOM Div., Sealol
Rotterdam Dockyard
Westinghouse
Williamette Steel Div.,
G. F. Atkinson Co.

g. Control Rod Drives

Babcock & Wilcox
Combustion Engineering
General Electric
Marvel-Schebler Div.,
Borg-Warner
Reactor Controls
Westinghouse

h. Fuel Handling and Service Equipment

AMF
Babcock & Wilcox
Combustion Engineering
Gifford-Wood
National Annealing Box
Pittsburgh-Des Moines Steel
Programmed & Remote Systems
Schutte & Koerting
Stearns-Roger
Westinghouse

i. Turbine - Generators

Associated Electric Industries (foreign company)
Brown-Boveri (foreign company)
English Electric (foreign company)
General Electric
Westinghouse

j. Condensers

Foster Wheeler
Ingersoll-Rand
Maryland Dry Dock
Southwestern Engineering
Westinghouse
Worthington Div., Studebaker
Yuba

k. Feed Water Heaters

BLH
Foster Wheeler
Southwestern Engineering
Struthers Wells
Westinghouse
Yuba

l. Containment Structures

Steel

Chicago Bridge & Iron
GATX
Pittsburgh-Des Moines Steel
Union Tank

Concrete

Engineer-Constructor Firms

3. Reactor Control Equipment

Reactor control instrumentation and control rod drive mechanisms are of potential interest to various nuclear and non-nuclear firms in addition to the suppliers of complete power reactors. These components are usually supplied by the reactor vendors but there could be areas in the future where companies specializing in control instrumentation could compete.

A typical 1,000 MWE PWR would include nearly $4 million worth of control instrumentation, control rod drive mechanisms and area monitors adjacent to the reactor. A typical 1,000 MWE BWR would include about $5 million for comparable equipment. (BWR's require more control rod drives than PWR's.)

4. Foreign Competition for Components

In 1967, two California utilities placed orders for 800 MW turbine-generators for the Bolsa Island project with English Electric. TVA ordered a 1,300 MW turbine-generator for a coal plant from Brown-Boveri & Co. of Switzerland, and American Electric Power placed an order with Brown-Boveri for two 1,100 MW turbine-generators and obtained options for two more. In January 1968, Consolidated Edison ordered a turbine-generator for its 1,115 MW Nuclear Four plant from Associated Electric Industries, Inc. of England and obtained an option on a second unit. In placing these orders with foreign suppliers, utilities have generally noted a need for more competition than is provided by the two U.S. firms now offering large turbine-generators.

At the end of 1967, Brown-Boveri and North American Rockwell announced that they were studying the possibility of building Brown-Boveri generating equipment in this country and considerable progress was made in discussions which continued during 1968.

Competition from abroad was also reflected in an order placed by Westinghouse with the Rotterdam Drydock Company in Holland for two large pressure vessels (primary tanks) for reactors. This marked the first order placed overseas for an important nuclear reactor component.

As a result of these activities, utility executives are looking forward to a stronger competitive situation in these important power plant components in the future.

5. Components for Breeder Reactors

a. Sodium Cooled Reactors

Both the private nuclear industry and the AEC are pursuing very active programs looking toward future operation of breeder reactors.3/ Important parts of these programs are concerned with the development of components for sodium cooled systems.

3/ These programs are discussed in Part IV of this report dealing with reactors.

The AEC program has been detailed in a ten-volume report, Volume 3 of which covers the plan for components.[4/] The technology to be developed under this program includes design practices, engineering standards, analytical methods, fabrication methods, quality-assurance practices, installation, and operation and maintenance procedures.

The components covered by Volume 3 of the plan are: the reactor primary vessel with its cover, guard vessel, support, and surveillance systems; the core-components handling equipment for moving fuel and other removable core components to and from reactor vessel and storage, cleaning, and canning stations; and reactivity control drives up to, but not including, the control members (covered in the LMFBR Plan as part of the core). Also included are the components of the primary and secondary coolant-transport systems, i.e., valves and piping (fittings, supports, heaters, expansion and drain tankage, surveillance systems, etc.), pumps, intermediate heat exchangers, and the steam generators with associated vent-relief systems. (Technology for other LMFBR components is covered by other volumes of the Plan, for example, under Volume 4, Instrumentation and Control, or Volume 7, Fuels and Materials.)

The AEC plan for component development specifically states: "The participation of industry is a vital feature of the program. The technology to be developed ultimately will be used by industry in the design and construction of LMFBR central-station power plants. Therefore, the plan stipulates that development activities described be performed principally by industrial firms. To foster a competitive and viable industry, the utilities, reactor designer-suppliers, architect-engineers, equipment vendors, and construction firms are to be encouraged by contracts with AEC to participate in development activities and to acquire the necessary capabilities to meet exacting requirements for LMFBR components. AEC laboratories are to participate principally in supporting roles, particularly in fundamental investigations, testing, and evaluating or coordinating data."

In the industry-supported programs, electric utilities and industrial organizations are cooperating in R&D programs which are coordinated with the AEC plan. These include the development of components. For example, the Empire State Atomic Power Development Associates (an organization formed and financed by seven New York State electric utilities) is providing $5 million to the General Electric Company for the

---

4/ The Liquid Metal Fast Breeder Reactor Program Plan, consisting of ten volumes, is available from the Clearinghouse for Federal Scientific and Technical Information, U.S. Department of Commerce, Springfield, Virginia 22151, for $3.00 each.

development of key components for a 300 MWE prototype sodium cooled plant; GE, in turn, has entered into a three-year program with Foster Wheeler to establish the basis on which to build steam generators for such plants. Other R&D programs which include studies of sodium components by North American Rockwell and Westinghouse are also being supported by electric utilities.

b. Liquid Metal Engineering Center

An important step in the LMFBR component program has been the establishment of the Liquid Metal Engineering Center, an AEC-owned facility at Santa Susana, California, operated by the Atomics International Division of North American Rockwell. This Center is discussed in Category IV of this report under Breeders.

c. Components for Gas Cooled Systems

Gulf General Atomic has underway a $3.5 million two-year program for a fast reactor system which includes component development and which is supported by 21 electric utilities.

B. Radiation Detection and Monitoring Instruments

Market Predictions

In 1968	$100-$110 million
In 1970	$120-$130 million

This area covers the following types of instruments which are reported by the Census Bureau under their current Industrial Report series, MA-38B, "Selected Instruments and Related Products."

- Radiation detecting elements, including ion chambers
- Nuclear Monitoring Instruments portable hand held
- Nuclear Monitoring Instruments Environmental (not hand held)
- Medical Monitors - whole body scanners
- Scalers
- Special amplifiers for nuclear applications
- Pulse analyzers - single channel and multi-channel
- Sample changers
- Count rate meters
- Miscellaneous

1. Principal Manufacturers

Companies concerned with the manufacture of radiation detection and monitoring instruments in the above category and whose 1966 sales reportedly have exceeded $0.5 million are shown in Table III-1.

TABLE III-1

Companies Manufacturing Radiation and Monitoring Instruments with 1966 Sales Exceeding $0.5 Million

Baird-Atomic, Inc.
  Cambridge, Massachusetts
Beckman Instrument Company
  La Jolla, California
Bendix Corporation
  Cincinnati, Ohio
Canberra Industries, Inc.
  Middletown, Connecticut
Chronetics, Inc.
  Yonkers, New York
Cosmic Radiation Labs
  Bellport, Long Island, N.Y.
Curtis Nuclear Corporation
  Los Angeles, California
Dero Research & Development Corp.
  Huntington, New York
Eberline Instrument Corporation
  Santa Fe, New Mexico
E. G. & G., Inc., (incl. ORTEC Div.)
  Salem, Massachusetts
EON Corporation
  Brooklyn, New York
Hamner/Harshaw
  Cleveland, Ohio
Hewlett Packard
  Palo Alto, California
Kaman Nuclear
  Colorado Springs, Colorado
Landsverk Electrometer Company
  Glendale, California
LeCroy Research Systems Corp.
  Elmsford, New York
Miles Laboratory
  Elkhard, Indiana
Northern Scientific
  Madison, Wisconsin
Nuclear Associates, Inc.
  Westbury, New York
Nuclear-Chicago Corporation
  Des Plaines, Illinois
Nuclear Data, Inc.
  Palatine, Illinois
Nuclear Diodes, Inc.
  Prairie View, Illinois
Nuclear Measurement Corp.
  Indianapolis, Indiana
Nucleonic Corp. of America
  Brooklyn, New York
Ohio Nuclear
  Cleveland, Ohio
Packard Instrument Co., Inc.
  Downers Grove, Illinois
Picker Nuclear Corporation
  White Plains, New York
Princeton Gamma Tech
  Princeton, New Jersey
Reuter-Stokes Electrical
  Components, Inc., Cleveland, Ohio
Solid State Radiations, Inc.
  Los Angeles, California
Technical Associates
  Burbank, California
Isotopes Div. of Teledyne, Inc.
  Westwood, New Jersey
Tennelec Instrument Co., Inc.
  Oak Ridge, Tennessee
Tracerlab, A Div. of LFE, Inc.
  Waltham, Massachusetts
Transistor Specialities, Inc.
  Plainview, Long Island, N. Y.
Victoreen Instrument Company
  Cleveland, Ohio

[illegible]

Nuclear instrument companies in this category now number about 170 firms and were among the first type of private firms to form as a result of the Government's encouragement of private industry in the atomic energy program.

2. Competition

As reported in our previous reports, the radiation detection and monitoring instrument field is highly competitive, requiring a sizable expenditure for private research and development in order to keep pace with the market. It is estimated that the private R&D for 1967 may amount to $10-12 million for this segment of the industry, ranging from 10 percent to 20 percent of sales in individual companies.

All radiation detection and monitoring instruments are available commercially and there does not appear to be any substantial competition between the Government and private industry in furnishing these items.

3. Markets

Chart III-B shows annual sales of radiation detection instruments and related equipment through 1966 and estimated sales for 1967 and 1968, with a projection through 1970.

The market for radiation detection and monitoring instruments is intimately associated with the remarkable growth of all nuclear applications and can be expected, in general, to grow with an expanding nuclear power economy.

The market for radiation detection and monitoring instruments has increased from $17 million in 1957 to $80 million in 1966. Estimates for 1967 and 1968 sales are $90 and $100-110 million, respectively. It is expected that growth in this industry will continue over the next several years, as a result of increasing uses for nuclear instrumentation in medical applications, uranium mining exploration and safety, research and development and analytical areas, and may reach $120-130 million in 1970.

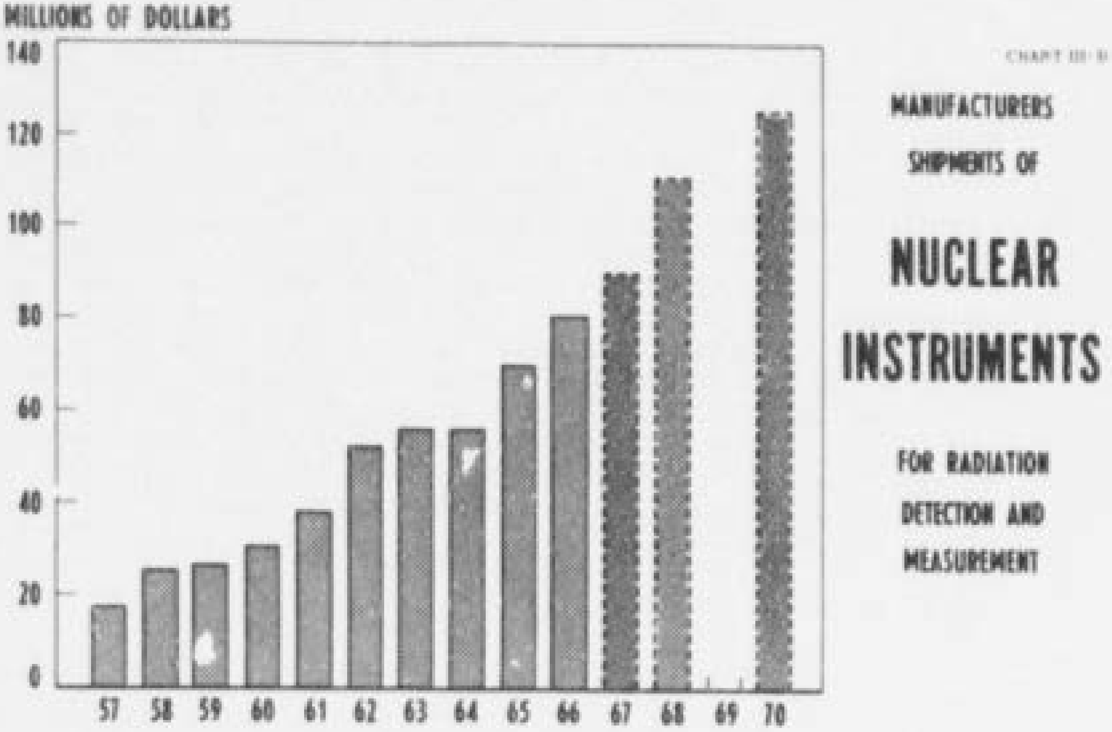

4. Exports

Exports of nuclear instruments amount to about 25 percent of total shipments. For 1967, exports were reported as $24.0 million, $4.8 million over 1966. Export figures are shown below.

TABLE III-2

Exports of Nuclear Instruments

Year	Exports (In Millions $)
1959	4.6
1960	7.1
1961	9.4
1962	11.0
1963	11.8
1964	15.3
1965	18.1
1966	19.2
1967	24.0
1968	est. 30.0

5. Association of Nuclear Instrument Manufacturers (ANIM)

The Association of Nuclear Instrument Manufacturers (ANIM) became formally established as a trade association in January 1967. The Association's basic objective is to foster the development of a healthy and more vigorous nuclear instrument industry. Its activities include the development of industry statistics and standards, cooperation with Government agencies in the evolution of regulations and laws that affect the industry, promoting exports, and up-grading trade show exhibits. Its officers and member companies met with the AEC Commissioners early this year to brief the Commission on the Association, the products of its members, and some of the problems facing the industry. The current membership of about 25 firms represents 75 percent to 80 percent of the sales volume of commercial catalog-type nuclear radiation detection and monitoring instruments (exclusive of industrial process control equipment, reactor instruments, and radiation sources). The Association has reported 1967 sales of $88 million for 12 of its member firms for the fiscal year, ending March 31, 1968.

6. Corporate Changes

Due to financial difficulties resulting largely from acquisitions outside the nuclear instrument field, the Technical Measurement Corporation of North Haven, Connecticut, terminated operations in January 1968. Three new companies have been formed by former TMC employees during the year. These are: Technical Instruments Corporation, North Haven, Connecticut; Kevax Corp., Burlingame, California; and Geoscience Nuclear Division of Geoscience Instruments Corporation, Mount Vernon, New York.

CATEGORY IV
REACTORS

During the past four years, the growth of nuclear power in the United States has been remarkable and orders for new plants have been placed at a pace that surprised even the most optimistic forecasters. Many electrical utilities, over a broad section of the country during this period, considered nuclear heat sources more economical than fossil fuels, and in some cases, a decision to go nuclear was made even on borderline economic evaluations, because of certain inherent advantages of nuclear power.

During 1968, there was a significant reduction in the orders for all types of electrical power plants, including nuclear power plants, and a reduction in the number of announcements for the construction of future power plants - both nuclear and fossil fueled. This is a trend that was anticipated and that may continue for several years before orders again increase.

Overall, there is a continuing optimism for the application of nuclear reactors for a number of other purposes, even though the budgets for some of these development programs, such as the space reactor program, were recently reduced, due to national budget policies. The various markets for all types of reactors are reviewed in this section.

A. Expenditures for Reactor Projects

Estimated expenditures for reactors under construction in the United States, for the year ending June 30, 1968, were more than 2-1/2 times the previous year's level and more than three times the FY-1966 level, as shown in Table IV-1. These figures reflect the high level of orders for nuclear power plants which began in 1965.

Expenditures for reactors by utilities exceeded those by the AEC for the first time in FY-1965. For FY-1968, utility expenditures were more than 20 times the AEC level. As the nuclear power market grows in future years, expenditures by utilities are expected to greatly exceed expenditures for reactors for other purposes.

The number of civilian power and AEC research reactors under construction and their total estimated cost are shown in Table IV-2. Table IV-3 shows cumulative costs incurred for these reactors and estimated costs yet to be incurred. This is also shown graphically on Chart IV-A. Electric utility central station power plants are not included in these summaries until contracts have been awarded to reactor vendors. Since the cut-off date for the information reported here was June 30, 1968, the plants which have been placed under contract since then are not included in these tables or Chart IV-A.

TABLE IV-1

Estimated Comparative Costs Incurred For Domestic Reactor Projects

	FY-1966	FY-1967 (in millions)	FY-1968
All Projects	$190.8	$241.3	$638.6
Federal Government			
Atomic Energy Commission	24.6	14.6	28.0
Utilities	166.2	226.7	610.6
Investor-Owned Utilities	165.5	215.9	545.3
Publicly Owned Utilities*	.7	10.8	65.3

*Including Tennessee Valley Authority.

TABLE IV-2

Domestic Reactor Projects Under Contract, Design, or Construction

	At June 30, 1966		At June 30, 1967		At June 30, 1968	
	Number of Projects	Total Estimated Costs (millions)	Number of Projects	Total Estimated Costs (millions)	Number of Projects	Total Estimated Costs (millions)
All Projects	34	$1,756.5	60	$5,540.6	77	$8,490.5
Civilian Power*	24	1,662.2	54	5,390.7	73	8,399.9
Civilian Research	10	94.3	6	149.9	4	90.6

* Projects are not included in this tabulation until contracts have been awarded to Reactor Vendors. For listing of projects included see Table IV-4.

TABLE IV-3

Costs and Estimates by Source of Funds
Projects Under Contract, Design, or Construction

	Costs Incurred FY-1968	Total Estimated Costs of Projects	Cumulative Costs Incurred Through June 30, 1968	Estimated Costs To be Incurred After June 30, 1968
	(in millions)			
All Projects	$638.6	$8,490.5	$928.4	$7,562.1
Federal Government				
Atomic Energy Commission	28.0	122.2	48.0	74.2
Utilities	610.6	8,368.3	880.4	7,487.9
Investor-Owned Utilities	545.4	7,279.3	804.7	6,474.6
Publicly Owned Utilities	65.2	1,089.0	75.7	1,013.3

Data on cumulative costs and FY-1968 costs have been submitted on a voluntary basis by utilities. Where information was incomplete, estimates were made and incurred costs assumed. Approximately 12 percent of FY-1968 costs were estimated.

A listing of reactors included in these Tables appears in Table IV-4, together with the criteria followed in adding projects to and dropping projects from this tabulation.

TABLE IX-4

List of Domestic Reactor Projects
Under Design or Construction
At June 30, 1967 and June 30, 1968

Reactor Projects	June 1967	June 1968
**Civilian Power**		
San Onofre (SCE and SDG&E)	X	-
LaCrosse Boiling Water Reactor (Dairyland)	X	-
Malibu (Los Angeles D. W. & P.)	X	X
Connecticut Yankee (Conn. Yankee A. P. Co.)	X	-
Fort St. Vrain (Pub. Ser. Co. of Colo.)	X	X
Oyster Creek #1 (Jersey Central Pwr. & Lt. Co.)	X	X
Nine Mile Point (Niagara Mohawk Pwr. Corp.)	X	X
Dresden #2 (Commonwealth Ed. Co.)	X	X
Dresden #3 (Commonwealth Ed. Co.)	X	X
Millstone #1 (Northeast Utilities)	X	X
Pilgrim (Boston Edison Co.)	X	X
Robt. E. Ginna (Rochester G. & E.)	X	X
Turkey Point #3 (Florida Pwr. & Lt. Co.)	X	X
Turkey Point #4 (Florida Pwr. & Lt. Co.)	X	X
Indian Point #2 (Consolidated Ed. Co. of N. Y.)	X	X
Robinson #2 (Carolina Pwr. & Lt. Co.)	X	X
Palisades (Consumers Pwr. Co.)	X	X
Point Beach #1 (Wisconsin Mich. Pwr. Co.)	X	X
Quad Cities #1 (Commonwealth Ed./Iowa-Ill.)	X	X
Monticello (Northern States Pwr. Co.)	X	X
Browns Ferry #1 (Tennessee Valley Auth.)	X	X
Browns Ferry #2 (Tennessee Valley Auth.)	X	X
Indian Point #3 (Consolidated Edison Co.)	X	X
Vermont Yankee (Vermont Yankee Nuclear Pwr. Co.)	X	X
Quad Cities #2 (Commonwealth Ed./Iowa-Ill.)	X	X
Peach Bottom #2 (Phila. Electric)	X	X
Peach Bottom #3 (Phila. Electric)	X	X
Salem #1 (Public Service E. & G.)	X	X
Surry #1 (Virginia Elec. & Pwr. Co.)	X	X
Surry #2 (Virginia Elec. & Pwr. Co.)	X	X
Fort Calhoun (Omaha Pub. Pwr. Dist.)	X	X
Oconee #1 (Duke Pwr. Co.)	X	X
Oconee #2 (Duke Pwr. Co.)	X	X
Oconee #3 (Duke Pwr. Co.)	X	X
Diablo Canyon (Pacific G. & E.)	X	X
Three Mile Island (Metrop. Ed. Co.)	X	X
Prairie Is. #1 (Northern States Pwr. Co.)	X	X
Easton (Niagara Mohawk)	X	X
Bailly (Northern Indiana Pub. Service)	X	X
Oyster Creek #2 (Jersey Central P. & L.)	X	X
Prairie Island #2 (Northern States Pwr.)	X	X
Kewaunee (Wisconsin Public Service)	X	X
Zion #1 (Commonwealth Ed.)	X	X
Zion #2 (Commonwealth Ed.)	-	X
Crystal River #3 (Florida Pwr. Corp.)	X	X
Point Beach #2 (Wisconsin Mich. Pwr. Co.)	X	X
Maine Yankee (Maine Yankee A. P. Co.)	X	X
Shoreham (Long Island Lighting Co.)	X	X
Russellville (Arkansas P. & L. Co.)	X	X
Cooper (Consumers Pub. Pwr. Dist.)	X	X
Calvert Cliffs #1 (Baltimore G. & E.)	X	X
Calvert Cliffs #2 (Baltimore G. & E.)	X	X
Salem #2 (Public Service E. & G.)	X	X
Bell (N. Y. State E. & G.)	X	X
Browns Ferry #3 (Tennessee Valley Authority)	X	X
D. C. Cook #1 (Indiana & Mich. Electric Co.)	-	X
D. C. Cook #2 (Indiana & Mich. Electric Co.)	-	X
Beaver Valley #1 (Duquesne)	-	X
Rancho Seco (Sacramento Municipal Utility Dist.)	-	X
Philadelphia Electric	-	X
Philadelphia Electric	-	X
No. Anna #1 (Virginia Electric & Pwr. Co.)	-	X
Millstone #2 (Northeast Utilities)	-	X
E. I. Hatch (Georgia Pwr. Co.)	-	X
Fort Slocum (Consolidated Edison)	-	X
Brunswick #1 (Carolina P. & L.)	-	X
Brunswick #2 (Carolina P. & L.)	-	X
Unit 3 (Carolina P. & L.)	-	X
D. Arnold (Iowa Electric Light & Pwr.)	-	X
Hutchinson Island #1 (Florida Power & Light)	-	X
Sequoyah #1 (Tennessee Valley Authority)	-	X
Sequoyah #2 (Tennessee Valley Authority)	-	X
Unit #1 (Pennsylvania Pwr. & Light)	-	X
Unit #2 (Pennsylvania Pwr. & Light)	-	X
Midland #1 (Consumers Pwr. Co.)	-	X
Midland #2 (Consumers Pwr. Co.)	-	X
TOTAL CIVILIAN POWER	54	72

(Cont.)

TABLE IV-4
(Cont.)

Reactor Projects	June 1967	June 1968
Civilian Research		
HTLTR - High Temp. Lattice Test Reactor (AEC)	X	-
LOFT - Loss of Flow Test Facility (AEC)	X	X
UHTREX - Ultra High Temp. Reactor Experiment (AEC)	X	-
PBF - Power Burst Facility (AEC)	X	X
*AARR - Argonne Advanced Research Reactor (AEC)	X	X
FFTF - Fast Flux Test Facility (AEC)	X	X

* Cancelled 7/68

NOTES FOR TABLE IV-4

X - Indicates project included in tabulation.

Projects are added to this tabulation:
(1) During the calendar quarter in which contracts are awarded to reactor vendors. For FY-1965 and prior years, projects were added during the calendar quarter in which an application for a construction permit was received.

(2) For Government projects not subject to licensing and not subject to the submission of a basis for arrangements to the JCAE, during the quarter in which Title I work begins. (However, Government projects are not added prior to a decision to proceed with construction at a specific location even though design work may be underway.)

Projects are dropped from this tabulation in the quarter following the calendar quarter in which they first operate at design power.

Modifications to existing projects are included only when a substantial increase in the rated power output will result.

CHART IV-A

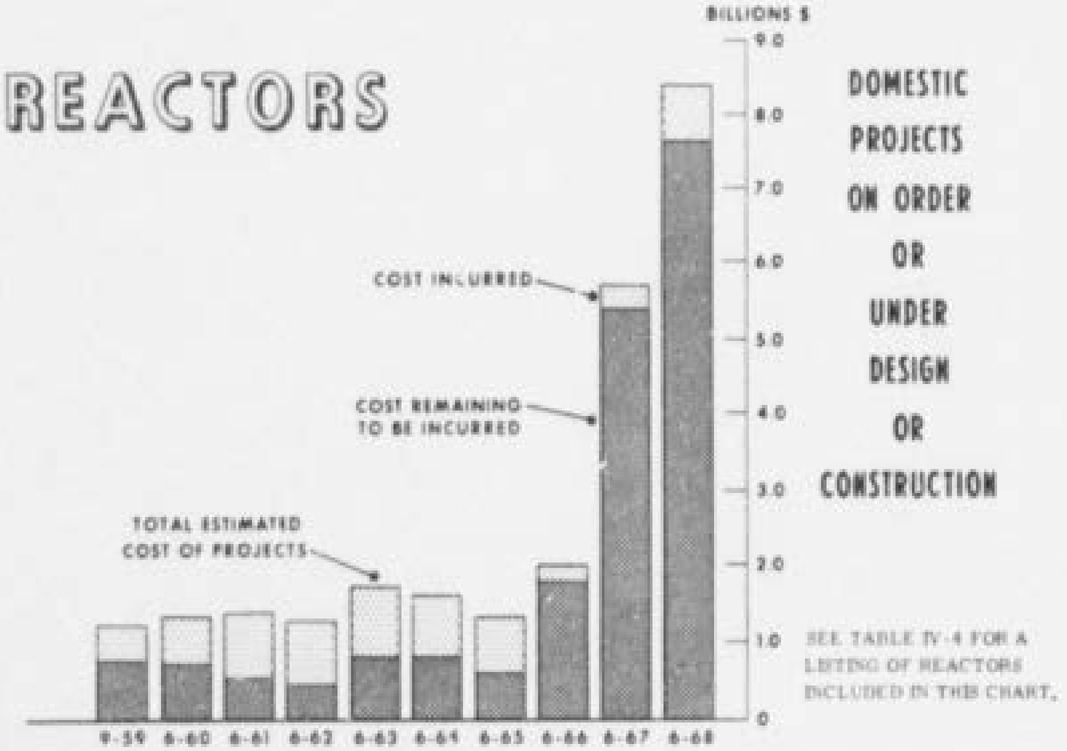

B. Civilian Power

1. General

During the past four years, the rapidly increasing volume of orders by the utilities resulted in either the construction of new facilities or the expansion of manufacturing facilities by a number of companies who supply reactors, their components, or parts of the fuel cycle. Many of the new plants or expansions are taking place in the southern and southeastern part of the country[1/] and construction was well underway during 1968 on most of the new plants that were announced last year. It is estimated that several hundreds of millions of dollars are being invested in these new facilities.

The dramatic increase in orders over this four-year period also focused attention on the requirements for the development of additional uranium reserves by the mining industry, the need for accelerated work in the demonstration of plutonium recycle fuel elements, and the need to develop breeder reactors. This growth also emphasized the additional requirements for training personnel for the operation and management of these plants.[2/] The AEC completed a study of this subject and will soon publish a booklet entitled "Guide to Nuclear Power Plant Education & Training Programs," which is intended to assist utilities considering the purchase of nuclear power plants.

By the fall of 1968, it became apparent that orders for electrical generating capacity from all sources of power would be considerably less than orders during 1967. As previously stated, this is a trend that was anticipated and that may continue for a few years before orders again increase. Orders for nuclear steam supply systems were about 13,000 MWE for the first nine months of 1968 as compared with about 20,000 MWE for the same period in 1967.

It is estimated that there will also be a reduction in 1968 of about 16 percent in the announcements of new electrical generating capacity from fossil and nuclear sources combined. For both fossil and nuclear, announcements totaled approximately 35,000 megawatts electrical (MWE) for the first nine months of 1968 as compared to approximately 42,000 MWE for

---

1/ Annual Report to the President, 1968, from the Federal Representative to the Southern Interstate Nuclear Board.

2/ Remarks by A. Giambusso at the American Public Power Association's 12th Annual Engineering and Operations Workshop, Sacramento, California, February 14, 1968, AEC Public Release S-8-68, dated February 27, 1968.

the same period in 1967. It is estimated that announcements for new nuclear plants will be down by about 33 percent for 1968. For the first nine months of this year, the orders for nuclear plants were also running about one-third less than orders for nuclear plants for the same period.

During the past five years, the utility orders for central station electrical plants have doubled about every two years. Since the doubling time on projected requirements of installed electrical power is about 10 years, it seemed apparent that a reduction in orders would occur. In connection with nuclear plants, it appears that this gap between the rate of orders and electrical power requirements is closing and, hopefully, will result in a reduction of capital plant costs in the near future to offset recent substantial increases. If it were possible for utilities to order plants at a more uniform rate, any price increases occurring as a result of manufacturer's plant being saturated with orders might be alleviated.

The average of capital costs of nuclear plants ordered from 1965 through 1968 is about $150/KWE. These plant costs have varied during this period from about $90/KWE to about $200/KWE. Some reactor manufacturers now state that their estimate of such average capital plant costs is about $170/KWE. However, larger sized plants of 1,200 to 1,300 MWE, which are now being offered for sale, may show lower capital plant costs of around $145 to $150/KWE. The National Coal Association reports that capital costs for fossil plants at the present time are around $140/KWE.[3/] The reduction in capital costs, referred to above, may occur in the future when overcommitment of the reactor manufacturers' plant capacity is alleviated and as improvement in manufacturing processes continues. Caution must be used in making comparisons of capital costs because of the differences in accounting practices followed by the various electrical utilities. Regardless of the recent increases in capital costs for nuclear plants, during the year there again were purchases of nuclear plants in sections where coal was highly competitive. For example, the Detroit Edison Company and Iowa Electric Light and Power Company ordered their first nuclear power plants during 1968.

The electrical utilities continue to order their reactor power plants with the first reactor core in all cases being provided by the reactor manufacturer, and in many cases, for

---

3/ Memorandum to Participants in "Conference on Abundant Nuclear Energy," Gatlinburg, Tennessee - August 26-29, 1968, dated August 26, 1968, from Brice O'Brien, General Counsel, National Coal Association.

the replacement fuel for a certain period of time as well. Many of the orders for replacement fuel, however, are of an optional nature so the utility has some freedom to purchase from other suppliers. A recent survey indicates that the electrical utilities have ordered or have options for approximately 50 percent of the replacement fuel that will be needed through 1980.[4/] Although the use of nuclear power by utilities is relatively new, they are already exploring the possibility of using alternative fuel element suppliers and, in some cases, replacement fuel awards have been made to independent fabricators.

The nuclear power plant manufacturing base was broadened significantly during 1967 through the award of a number of plants to Babcock & Wilcox and Combustion Engineering. During 1968, all four manufacturers of light water reactors continued to actively submit proposals, with the two larger companies continuing to receive a large percentage of all new orders. This situation may change as the electrical utilities become more experienced with nuclear plants and as their interest in wider industrial participation develops the competition that has been characteristic of American industry.

Additional competition from Gulf General Atomic continued in the offing by approval of all parties concerned for the construction of the Fort St. Vrain project for the Public Service Company of Colorado and by a proposal from Gulf General Atomic to a utility for a large high temperature gas-cooled reactor, thus bringing into possible competition a third technology in addition to the boiling water and the pressurized water types.

Nuclear plants, placed under contract through 1967 and for the first nine months of 1968, are shown in Tables IV-5 and IV-6. Chart IV-B shows the locations of these plants. The tables show that about 33 percent less nuclear capacity was placed under contract in the first nine months of 1968 than in the first nine months of 1967.

A comparison based on published information of announced fossil fuel versus nuclear plants in 1968 is also interesting and is shown as Table IV-7. The table shows that announcements of nuclear plants during the first nine months of 1968 were at

---

4/ Remarks by Glenn T. Seaborg, Chairman, USAEC, "Nuclear Industry - 1968 and Beyond," at a Financial Forum on Nuclear Energy, New York City, October 30, 1968, AEC Public Release S-39-68.

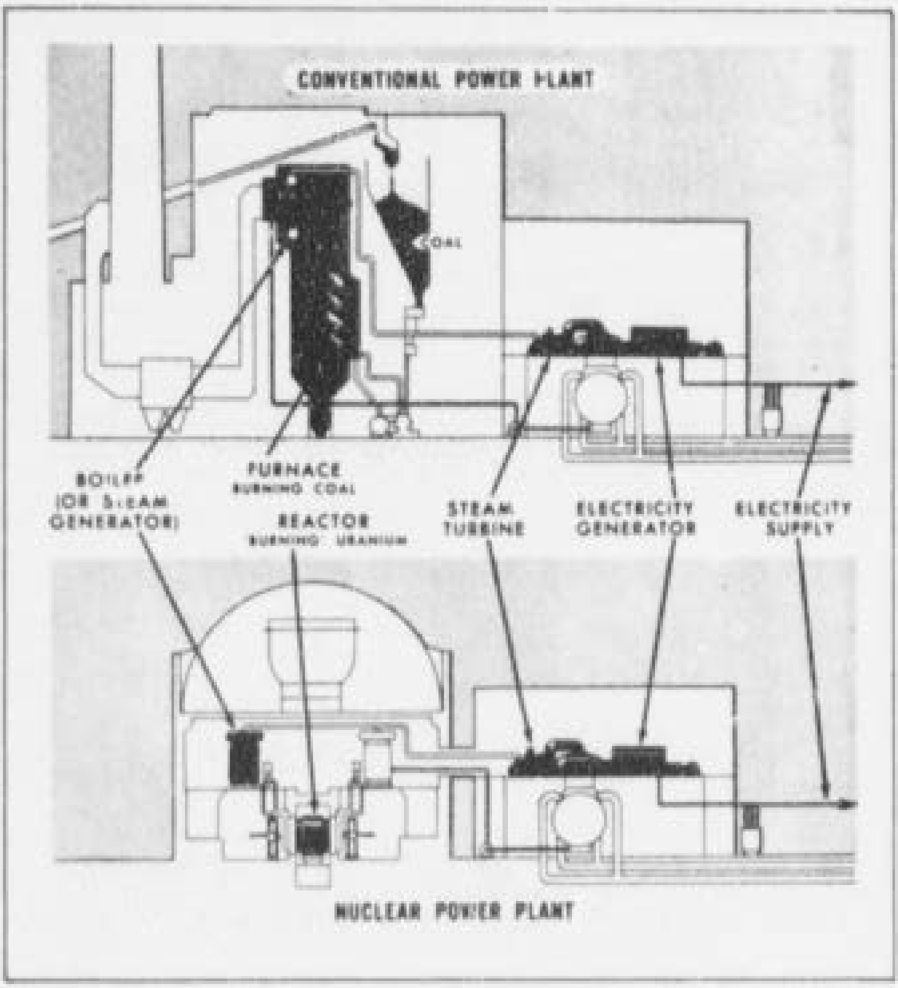
CONVENTIONAL POWER PLANT
COAL
BOILER
(OR STEAM
GENERATOR)
FURNACE
BURNING COAL
REACTOR
BURNING URANIUM
STEAM
TURBINE
ELECTRICITY
GENERATOR
ELECTRICITY
SUPPLY
NUCLEAR POWER PLANT

TABLE IV-5a

CENTRAL STATION NUCLEAR PLANTS

Operable, Under Construction, or on Order

Contract Awarded	Operating Utility & Plant	State	Reactor Supplier	Size (Mwe-Net)	Est. Cost* (Millions)	Commercial Operation	Regulatory Status**
1953	Duquesne (Shippingport #1)	Pa.	W	90 a/	$ 69	1957	c/
1955	Commonwealth Ed. (Dresden #1)	Ill.	GE	200	51	1960	O
	Consolidated Ed. (Indian Pt. #1)	N.Y.	B&W	265 b/	124	1963	O
				465	$ 175		
1956	Yank. At. El. Co. (Yankee)	Mass.	W	175	39	1961	O
1957	Pwr. React. Dev. Co. (Fermi)	Mich.	PRDC	61	68	--	O
	Northern States (Pathfinder)	S.D.	AC	59	29	1967	O
				120	$ 97		
1958	Phila. El. (Peach Bottom #1)	Pa.	GA	40	28	1967	O
	Rur. Coop. Pwr. Assoc. (Elk River)	Minn.	AC	22 b/	14	1964	O
	Pacific Gas & El. (Humboldt Bay)	Cal.	GE	68	24	1963	O
				130	$ 66		
1959	Consumers Pwr. Co. (Big Rock Pt.)	Mich.	GE	70	25	1963	O
1962	Dairyland Power Coop. (LaCrosse)	Wisc.	AC	50	18	1968	O
	Conn. Yankee A.P. Co. (Conn. Yankee)	Conn.	W	462	95	1968	O
	Washington Pub. Pwr. Sup. Sys. (NPR)	Wash.	GE	790	315	1966	c/
				1,302	$ 428		
1963	Southern Cal. Edison (San Onofre)	Cal.	W	430	98	1968	O
	Los Angeles D.W. & P. (Malibu)	Cal.	W	462	92	1972	C/P
	Jersey Central (Oyster Creek #1)	N.J.	GE	515	83	1969	O/P
	Niagara Mohawk (Nine Mile Pt.)	N.Y.	GE	500	134	1969	O/P
				1,907	$ 407		
1965	Boston Edison (Pilgrim)	Mass.	GE	625	85	1971	C
	Commonwealth Ed. (Dresden #2)	Ill.	GE	715	[illegible]	1969	O/P
	Northeast Utilities (Millstone #1)	Conn.	GE	652	86	1969	C
	Consolidated Ed. (Indian Pt. #2)	N.Y.	W	877	108	1969	C
	Fla. Pwr. & Lt. (Turkey Pt. #3)	Fla.	W	652	66	1970	C
	Pub. Ser. Co. of Colo. (Ft. St. Vrain)	Colo.	GA	330	68	1973	C
	Rochester Gas & Elec. (Robert E. Ginna)	N.Y.	W	420	85	1969	O/P
	(7 Units)			4,287	$ 558		
1966	Commonwealth Ed. (Dresden #3)	Ill.	GE	715	81	1970	O/P
	Carolina Pwr. & Lt. Co. (Robinson #2)	S.C.	W	663	76	1970	C
	Consumers Pwr. Co. (Palisades)	Mich.	CE	700	89	1970	C
	Wisc. Mich. Pwr. Co. (Pt. Beach #1)	Wisc.	W	455	61	1970	C
	Common. Ed./Iowa-Ill. (Quad Cities #1)	Ill.	GE	715	88	1970	C
	Common. Ed./Iowa-Ill. (Quad Cities #2)	Ill.	GE	715	82	1971	C
	Northern States (Monticello)	Minn.	GE	472	74	1970	C
	T. V. A. (Browns Ferry #1)	Ala.	GE	1,064	} 249	1970	C
	T. V. A. (Browns Ferry #2)	Ala.	GE	1,064		1971	C
	Duke Pwr. Co. (Oconee #1)	S.C.	B&W	841	85	1971	C
	Duke Pwr. Co. (Oconee #2)	S.C.	B&W	841	86	1972	C
	Vermont Yankee N.P. Corp. (Vt. Yankee)	Vt.	GE	514	88	1971	C
	Pub. Ser. Elec. & Gas (Salem #1)	N.J.	W	1,050	152	1972	C
	Phila. Elec. (Peach Bottom #2)	Pa.	GE	1,065	163	1971	C
	Phila. Elec. (Peach Bottom #3)	Pa.	GE	1,065	145	1973	C
	Va. Elec. & Pwr. (Surry #1)	Va.	W	783	144	1971	C
	Va. Elec. & Pwr. (Surry #2)	Va.	W	783	112	1972	C
	Omaha Pub. Pwr. Dist. (Ft. Calhoun)	Nebr.	CE	457	70	1971	C
	Pacific Gas & Elec. (Diablo Canyon #1)	Cal.	W	1,060	154	1972	C
	Metropolitan Edison (Three Mile Is.)	Pa.	B&W	831	124	1971	C
	(20 units)			[illegible]	$ [illegible]		
	Total through 1966 (44 units)			24,379	$ [illegible]		

* Total construction cost, including land and land rights. Transmission plant, fuel, training and R&D excluded.

** O - Operating License, O/P - Operating License Pending, C - Construction Permit, C/P - Construction Permit Pending.

a/ Limit of T-G capacity. Reactor power 135 Mwe.

b/ Includes capacity of fossil fueled superheater. Electric power from reactor only: Indian Point - 151 Mw; Elk River - 16 Mw.

c/ Operable - License not required.

TABLE IV-[illegible]

CENTRAL STATION NUCLEAR PLANTS

Operable, Under Construction, or on Order

Contract Awarded	Operating Utility & Plant	State	Reactor Supplier	Size (Mwe-Net)	Est. Cost* (Millions)	Commercial Operation	Regulatory Status**
	Total through 1966 (44 units)			24,379	$ 3,985		
1967	Florida Pwr. & Lt. (Turkey Pt. #4)	Fla.	W	652	$ 66	1971	C
	Northern Indiana Pub. Ser. Co. (Bailly)	Ind.	GE	515	91	(no date)	
	Jersey Central Pwr. & Lt. (Oyster Cr. #2)	N.J.	B&W	810	150	1975	C/P
	Northern States Pwr. Co. (Prairie Is. #1)	Minn.	W	530	97	1972	C
	Northern States Pwr. Co. (Prairie Is. #2)	Minn.	W	530	95	1974	C
	Wisconsin Pub. Ser. (Kewaunee)	Wisc.	W	527	111	1972	C
	Commonwealth Ed. (Zion #1)	Ill.	W	1,050	166	1972	C/P
	Commonwealth Ed. (Zion #2)	Ill.	W	1,050	153	1973	C/P
	Florida Pwr. Corp. (Crystal River #3)	Fla.	B&W	825	113	1972	C
	Wisc.-Mich. Pwr. Co. (Point Beach #2)	Wisc.	W	455	54	1971	C
	Maine Yankee A.P. Co. (Maine Yankee)	Me.	CE	790	131	1972	C/P
	Long Island Lighting Co. (Shoreham)	N.Y.	GE	523	127	1973	C/P
	Ark. Pwr. & Lt. Co. (Russellville)	Ark.	B&W	850	132	1972	C/P
	Consolidated Ed. (Indian Pt. #3)	N.Y.	W	965	156	1971	C/P
	Duke Power Co. (Oconee #3)	S.C.	B&W	841	93	1973	C
	Consumers Pub. Pwr. Dist. (Cooper)	Nebr.	GE	778	127	1972	C
	Baltimore G.& E. Co. (Calvert Cliffs #1)	Md.	CE	800	125	1973	C/P
	Baltimore G.& E. Co. (Calvert Cliffs #2)	Md.	CE	800	106	1974	C/P
	Pub. Ser. El. & Gas (Salem #2)	N.J.	W	1,050	127	1973	C
	N.Y. State El. & Gas Corp. (Bell)	N.Y.	GE	829	141	1973	C/P
	T. V. A. (Browns Ferry #3)	Ala.	GE	1,064	124	1972	C
	Indiana & Mich. El. Co. (D. C. Cook #1)	Mich.	W	1,054)	235	1972	C/P
	Indiana & Mich. El. Co. (D. C. Cook #2)	Mich.	W	1,060)		1973	C/P
	Duquesne (Beaver Valley #1)	Pa.	W	785	150	1973	
	Sacramento Mun. Util. Dist. (Rancho Seco)	Cal.	B&W	800	134	1973	C/P
	Phila. Electric	Pa.	GE	1,065	150	1973	
	Phila. Electric	Pa.	GE	1,065	150	1977	
	Virginia Elec. & Pwr. Co. (No. Anna #1)	Va.	W	800	135	1974	
	Northeast Utilities (Millstone #2)	Conn.	CE	828	148	1974	
	Georgia Pwr. Co. (E. I. Hatch)	Ga.	GE	786	160	1973	C/P
	1967 Total (30 units)			24,475	$ 3,711		
	Total through 1967 (74 units)			48,854	$ 7,696		
1968	Consolidated Edison (Nuclear Four)	N.Y.	GE	1,115	$ 210	1973	
	Carolina Pwr. & Lt. Co. (Brunswick #1)	N.C.	GE	821	130	1974	C/P
	Carolina Pwr. & Lt. Co. (Brunswick #2)	N.C.	GE	821	130	1973	C/P
	Carolina Pwr. & Lt. Co.	N.C.	GE	821	130	1975	
	Iowa Elec. Lt. & Pwr. Co. (D. Arnold)	Iowa	GE	538	103	1973	
	Florida Pwr. & Lt. Co. (Hutchinson Is. #1)	Fla.	CE	800	112	1973	
	T. V. A. (Sequoyah #1)	Tenn.	W	1,125	150	1973	
	T. V. A. (Sequoyah #2)	Tenn.	W	1,125	150	1974	
	Penna. Pwr. & Lt.	Pa.	GE	1,052	150	1975	
	Penna. Pwr. & Lt.	Pa.	GE	1,033	150	1977	
	Consumers Pwr. Co. (Midland #1)	Mich.	B&W	685	134	1974	
	Consumers Pwr. Co. (Midland #2)	Mich.	B&W	685	134	1975	
	Detroit Edison (Fermi #2)	Mich.	GE	1,100	150	1974	
	Pub. Serv. Co. of New Hamp. (Newington)	N.H.	W	800	150	1974	
	Pac. G. & E. (Diablo Canyon #2)	Cal.	W	1,060	160	1974	C/P
	1968 Orders Through 9/30/68 (15 units)			13,601	$ 2,143		
	Total Orders Through 9/30/68 (89 units)			62,454	$ 9,839		

* These are mostly unofficial figures taken from various published sources and are not necessarily on comparable bases.

** O - Operating License, O/P - Operating License Pending, C - Construction Permit, C/P - Construction Permit Pending.

TABLE IV-6

CENTRAL STATION NUCLEAR PLANTS

Announced, Planned for Nuclear Power but Contracts Not Awarded

Year Announced	Utility & Plant	Size (Mwe-Net)	Commercial Operation
1966	New England Electric System	800	1973
1967	Portland General Electric Co. (Trojan)	1,000	1974
	Washington Pub. Pwr. Sup. System	1,000	1973
	So.California Ed. (Cojo Canyon)	1,000	1975
	Atlantic City Electric Co. (Bayside)	1,000	No date
	Virginia Electric & Pwr. Co. (No. Anna #2)	800	1974
1968	Toledo Edison Co.	800	1974
	Cincinnati Gas & Electric	800	1975
	Pacific Power & Light	2 x 1,000	1975/77
	Power Auth. of State of N. Y.	750	1973
	Total through 9/30/68 (11 units)	9,950	

Total: Operable, Under Construction, on Order and Announced

Through 9/30/68	(100 units)	72,404	

Operable, Under Construction, or On Order

Commercial Operation	No. of Units Annual	No. of Units Cumulative	Capacity - Mwe Annual	Capacity - Mwe Cumulative
Thru 1965		8		951
1966	1	9	790	1,741
1967	2	11	99	1,840
1968	3	14	942	2,782
1969	6	20	[illegible]	6,457
1970	8	28	[illegible],436	11,893
1971	12	40	8,967	20,860
1972	14	54	11,664	32,524
1973	18	72	15,126	47,650
1974	10	82	8,549	56,199
1975	4	86	3,623	59,822
1976	-	86	-	59,822
1977	2	88	2,117	61,939
No Completion Date	1	89	515	62,454

TABLE IV-7

Announcements of New Steam-Electric Plant Additions

1 9 6 8

Utility & Plant	Generating Capacity - Mwe Fossil	Nuclear
Allegheny Power System, Inc.	2 x 650 C	
Appalachian Pwr. Co. (Amos #1, #2)	2 x 800 C	
Carolina Power & Light Co.		3 x 821
Central Ill. P.S. Co. (Cofeen #2, size increase)	200 C	
Cincinnati Gas & Electric		800
Columbus & Southern Ohio Electric (Conesville #4)	580 C	
Commonwealth Edison	800 C	
Connecticut Light & Power (Montville)	400 O	
Consolidated Edison (Nuclear Four)		1,115
Consolidated Edison (Newburg)	1,000 O	
Detroit Edison Co. (Monroe #3)	800 C	
Detroit Edison Co. (Fermi #2)		1,100
El Paso Electric Co.	150 G	
Florida Power & Light (Hutchinson Island)		800
Georgia Power Co. (Etowah #1, #2)	2 x 712 C	
Iowa Electric Light & Pwr. (Arnold)		538
Iowa Public Service (Neal #2)	325 C	
Kansas City Bd. of Pub. Utils. (Quindaro)	145 C	
Kansas City Power & Light	400 C/G	
Kansas G. & E.	840 C	
LADWP	450 O/G	
Louisville Gas & Electric (Kosmos #1)	330 C	
Louisville G. & E. (Mill Creek #1)	330 C	
Middle South	750 G	
Mississippi Power & Light Co. (Baxter Wilson)	750 G	
Oklahoma Gas & Electric (Seminole #2)	550 G	
Pacific Gas & Electric (Pittsburg #7)	735 O/G	
Pacific Gas & Electric (Diablo Canyon #2)		1,060
Pacific Power & Light		2 x 1,000
Pennsylvania Power & Light		1,052
Pennsylvania Power & Light (increase 800 to 1,100)		300
Public Service Co. of Indiana (Cayuga #2)	500 C	
Public Service of Oklahoma	600 G	
Salt River Power District (Navajo #1, #2)	2 x 750 C	
Sierra Pacific Power (Ft. Churchill #2)	110 G	
Southern Indiana G. & E. (Culley #3)	200 C	
Southwestern Public Service Co.	2 x 200 G	
Tennessee Valley Authority		2 x 1,125
Texas Electric Service Co. (Eagle Mt. #3)	375 G	
Texas Power & Light (Tuco Big Brown #1, #2)	2 x 575 G	
Texas Power & Light (Tradinghouse Creek)	775 G	
Toledo Edison Company		800
Union Electric Company	600 C	
West Texas Utilities Company (Paint Creek #4)	107 G	
Total Each	20,196	14,278
Total %	59%	41%
Total Fossil & Nuclear September 30, 1968	34,474	

	FOSSIL Mwe	FOSSIL %	NUCLEAR Mwe	NUCLEAR %	TOTAL
Announcements in					
1967	32,320	55	26,460	45	58,780
1966	20,096	47	22,477	53	42,573
1965	15,928	73	6,009	27	21,937

[illegible]

NUCLEAR POWER PLANTS *in the* UNITED STATES

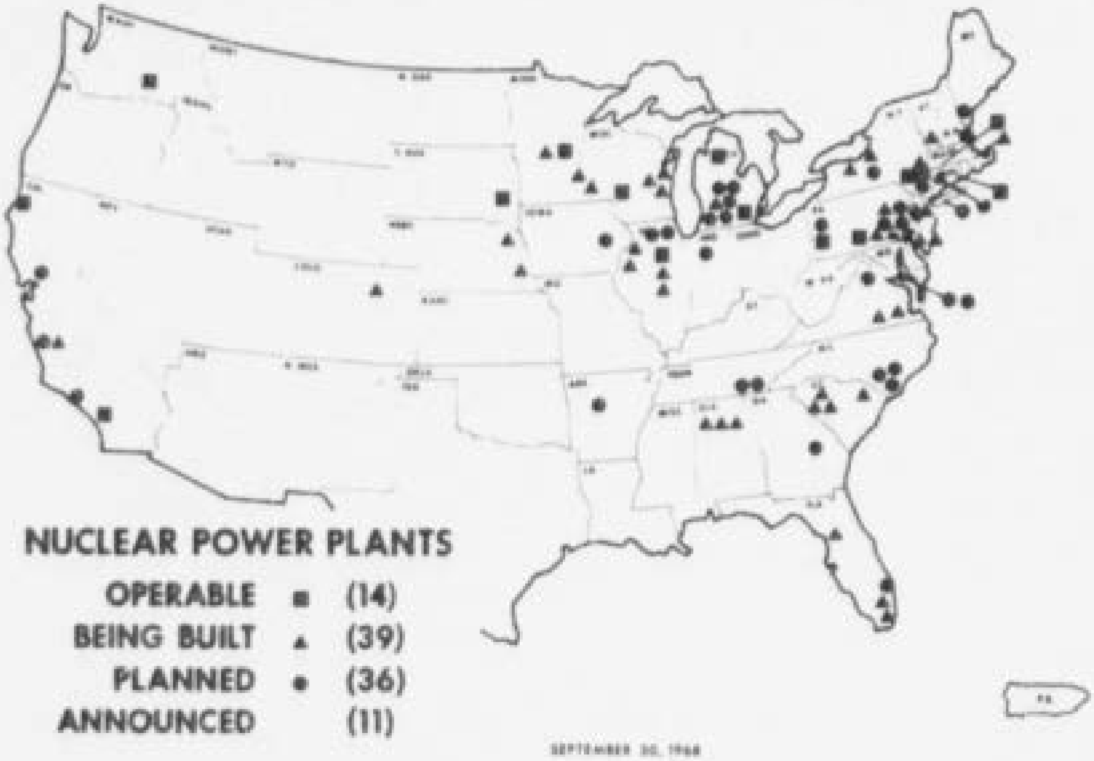

a lower level than those for fossil fuel plants. The average size of units ordered continued to increase from approximately 790 MWE for the year ending September 30, 1967, to an average of about 835 MWE in the current year for the same period. On orders for large plants, nuclear units continued to dominate. For example, for plants 800 MWE and larger, nuclear units were selected for 14 of a total of 19 in the first nine months of 1968.

The phenomenal growth of nuclear power over the past four years can be attributed to a considerable extent to the trend toward large size power generating units on large scale power systems and to the fact that the utility industry has accepted nuclear power as safe, reliable, and as the most economic means of meeting a sizable proportion of its new requirements.

The electrical utilities continued to accumulate experience through the operation of a number of nuclear power plants. Of the plants of significant size range, Shippingport, Dresden I,

and Yankee had plant availability factors of 95.5 percent, 53.9 percent, and 92.1 percent, respectively, for 1967. Moreover, Dresden I and Yankee had a plant availability of about 80 percent over a five-year period, although they were operating at only 64 percent and 71 percent of rated capacity, respectively, during that period. Indian Point I had a plant capacity factor of 68.3 percent for 1967. Dresden I was down for refueling during the year, and this reduced its availability factor.

The 462 MWE Connecticut Yankee atomic power plant operated at full power for the first time during this year with a plant capacity factor of 66 percent. It achieved criticality on July 4, 1967, and began commercial operation at significant levels during September 1967. There have been no problems with the nuclear systems, but trouble did develop in the steam throttling valves, and they were replaced. The 430 San Onofre plant of Southern California Edison and San Diego Gas and Electric also began commercial operation during the latter part of 1967. During 1968, the plant was shut down for six months to repair damage that resulted from a fire in the electrical wiring system. It resumed operation in September 1968.

The plant availability factors and plant capacity factors for all of these plants should not be considered as representative of commercial nuclear power plants. These plants should be considered as experimental and/or demonstration plants. In many cases, plants are shut down normally as part of the demonstration program. The availability factors for commercial nuclear plants, after their initial shakedown period, are expected to be very high and most nuclear plants are expected to be operated as baseload plants.

The Oyster Creek plant of Jersey Central Power and Light is expected to become operational in 1969. This plant encountered serious difficulties with its pressure vessel, in addition to construction delays that have emphasized the importance of careful supervision, rigid inspection, and quality assurance for all types of components. No serious problems of a nuclear nature have, however, been encountered in any of the commercial nuclear plants to date.

Five large nuclear plants, in addition to Oyster Creek, are scheduled to become operational in 1969.

2. Estimates of Growth

During 1968, the AEC reviewed its 1967 forecast for nuclear power in the United States. This review indicated no basis, at the present time, for changing the AEC's previous estimate that by 1980 the U.S. will have between 120 million and 170 million KWE of nuclear electric generating capacity. It is believed that somewhere near the mid-range of this projection - about 150 million KWE - is probably the best single estimate for 1980 for planning purposes.[5/] The difficulty in making such forecasts is indicated by tracing the history of the AEC forecasts to date. In the 1962 Civilian Power Report to the President, the AEC predicted that there would be 40,000 megawatts of installed nuclear capacity by 1980. In 1964, only two years after the 1962 forecast, the AEC predicted that by 1980 there would be between 60,000 and 90,000 megawatts of installed nuclear capacity. In June 1966, the AEC estimated that by 1970-71 there would be more than 10,000 MWE of nuclear-electric generating capacity in the United States, and by 1980 there would be between 80,000 and 110,000 MWE. The new and old estimates are shown in Chart IV-C.

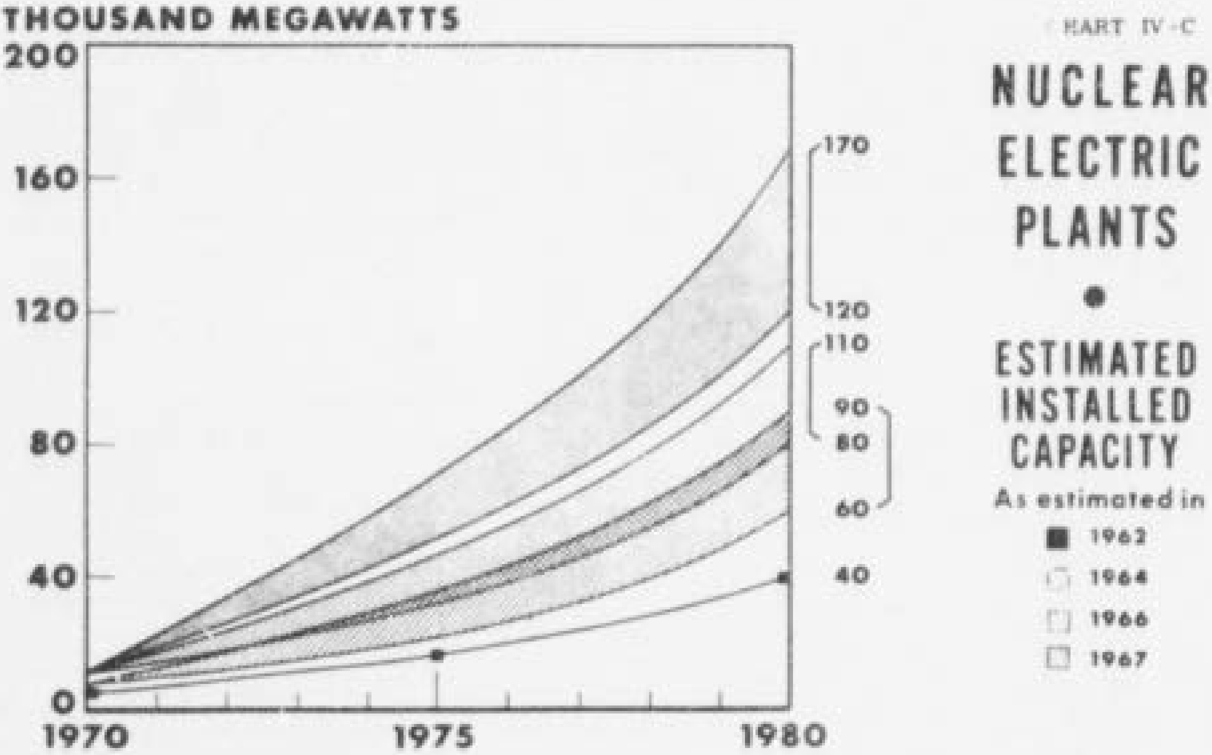

5/ Remarks by Glenn T. Seaborg, Chairman, USAEC, "Nuclear Industry - 1968 and Beyond," at a Financial Forum on Nuclear Energy, New York City, October 30, 1968, AEC Public Release S-39-68.

Some estimates of the projected growth of nuclear power have been made by the industry, by electrical utilities, and by manufacturers that are higher than the AEC estimate. An estimate contained in the "Fast Breeder Reactor Report," published by the Edison Electric Institute in April, indicates a total installed nuclear capacity in the range of 160,000 to 195,000 MWE by 1980 and 1 million MWE by the year 2000.

The Commission continued to review its overall program for civilian nuclear power. The "1967 Supplement to the 1962 Report to the President on Civilian Nuclear Power" examined the civilian power program prospects; noted the changes that had taken place since 1962 in the technical, economic, and resources picture; and considered the AEC program in relation to the recommendations of the earlier report. The 1967 Supplement to the report, therefore, provided a description of circumstances and programs as of January 1967 to serve as background for further extensive studies. Such studies have been underway since the Supplement was issued and are considering in depth the technical prospects of the individual reactor concepts, and are relating these prospects to resources projections and to economic considerations.

During 1968, the following individual studies were completed and published: "An Evaluation of Heavy Water Moderated-Organic Cooled Reactors," (WASH-1083) and "Current Status and Future Technical and Economic Potential of Light Water Reactors," (WASH-1082). It is expected that the remaining studies, in regard to other reactor concepts and related programs, will be completed and published during 1969.

Chart IV-D shows estimated energy requirements and sources for electric generation.

3. Industrial Structure and Competition

While i general there continued to be healthy competition among the nuclear steam system suppliers, some segments of the nuclear fuel cycle lack sufficient market, such as replacement fuel fabrication and reprocessing, to allow present or prospective suppliers a reasonable return on investment for some time into the future, thus delaying further competition. Long-range prospects, however, look bright for healthy competition in all segments of the business and many companies are studying plans for properly timing their participation in the nuclear field.[6/]

6/ Article by James T. Ramey, Commissioner, U.S. Atomic Energy Commission, Harvard Business Review, July-August 1968, "Competitive Vigor in Nuclear Power."

There was again considerable interest on the part of the oil industry in uranium exploration and additional oil companies are making extensive studies of the possibility of participating in other segments of the industry.

During the year, the purchase by Combustion Engineering of 21 percent of the United Nuclear Corporation's stock, which was previously owned by Olin Mathieson Chemical Corporation, attracted considerable attention. UNC, the only independent core supplier, filed suit for an injunction against Combustion Engineering contending that Combustion Engineering should not be allowed to exercise their rights through the voting of this block of stock in matters concerning the management of this company. One of the reasons for their objection was that they considered Combustion Engineering a competitor in the replacement core business. Hearings were started in Philadelphia in the United States District Court for the Eastern District of Pennsylvania on September 16, 1968. At the hearing, Combustion Engineering indicated that they needed to acquire United Nuclear Corporation in order to enhance their ability to compete with the two large reactor suppliers. As of the completion of this report, no decision had been reached by the Court on this case. This was the first time an antitrust question of a serious nature had been raised in Court by the nuclear supply industry. The case focused attention on how many reactor suppliers the utility market can support, especially during the low periods of the ordering cycle.

CHART IV-D

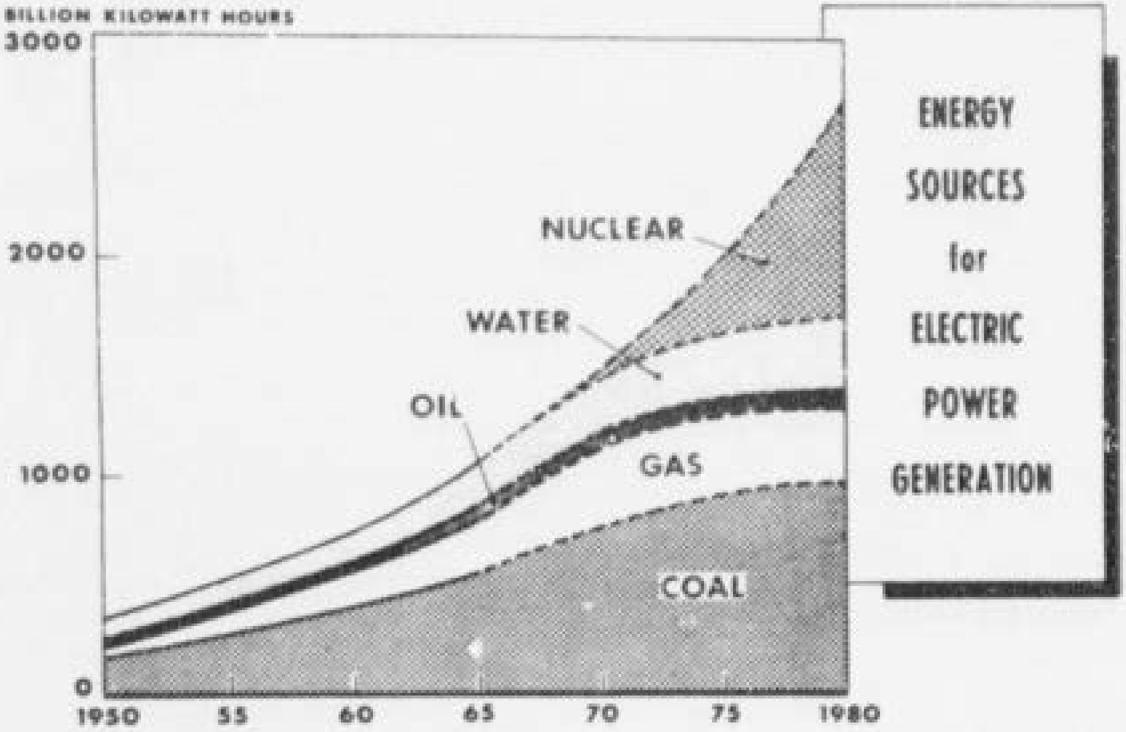

a. Commission Actions

During 1968, A. D. Little, Inc., completed its study of competition in the nuclear industry. This study is part of the Department of Justice/AEC joint project of examining all aspects of competition in the relatively new and developing nuclear industry, to assist the two agencies in fulfilling their responsibilities under the Atomic Energy Act and antitrust legislation.

The nuclear power supply industry, for the purpose of this study, consists of firms engaged in the business of supplying reactors, reactor components, nuclear fuel, or other products and services associated with the design, construction, and operation of nuclear-electric plants, including their entire fuel cycle.

One of the reasons for this study is to provide the Department of Justice and the AEC with detailed information for use in possibly developing guidelines to assure the healthy competitive development of the emerging nuclear industry.7/

Informal discussions were held throughout the year with industrial organizations to learn of their plans and problems. The Commission met with the Policy Committee of the Edison Electric Institute to discuss subjects such as licensing and regulation (currently proposed legislation that may affect the nuclear power industry), the Edison Electric Institute's report on breeder reactors, and other matters of mutual interest. The Commission also met with representatives of the American Public Power Association to discuss matters of mutual interest. The Commission usually meets semiannually with the officers of the Atomic Industrial Forum to exchange views in regard to the development of peaceful applications of nuclear energy. All of these meetings have proven very valuable in providing for an exchange of information between the Government and the industry.

The Division of Industrial Participation continued to maintain close contact with the nuclear industry, in order to determine and advise the Commission on what activities might be accomplished in industry, and in

---

7/ See AEC Public Release K-106, dated May 3, 1967, and Remarks by Glenn T. Seaborg, Chairman, USAEC, "Nuclear Industry - 1968 and Beyond," at a Financial Forum on Nuclear Energy, New York City, October 30, 1968, AEC Public Release S-39-68.

regard to the transfer of activities from the Government to the private sector of the economy. Meetings were held with the management of many electrical utilities, reactor manufacturers, and suppliers participating in fuel cycle activities, to exchange views and to assist industry in obtaining information and solving problems of mutual interest.[8/]

The AEC has always encouraged close cooperation and good working relationships between the national laboratories and the nuclear industry, and through the years has been constantly aware of the need for insuring that there is a smooth transition from the laboratories' role of technology development to industry's role of specific engineering development, fabrication, and marketing.

b. Important Events

Although during the past year there was much discussion of increased capital costs of nuclear plants, the electrical utilities continued to place orders for new plants in areas where other sources of energy were very competitive, such as the orders by Detroit Edison and Iowa Electric Light and Power Company previously mentioned. Even though the orders for new nuclear plants were considerably less than the previous year, the total backlog, including new purchases, provides an impressive workload for the reactor manufacturers. A tabulation of total sales is contained in Table IV-8.

TABLE IV-8

Central Station Nuclear Power
Reactor Sales Through 9/30/68

Reactor Suppliers	No. of Units	Capacity MWE	Capacity %
Babcock & Wilcox	11	8,274	13
Combustion Engineering	7	5,175	8
General Electric	33	24,679	40
Gulf General Atomic	2	370	1
Westinghouse	31	22,974	37
Other*	5	982	1
TOTAL	89	62,454	100

* Fermi, Pathfinder, Elk River, LaCrosse, NPR.

8/ Remarks by Ernest B. Tremmel, Director, Division of Industrial Participation, USAEC, "Peaceful Applications of Nuclear Energy," Before the Financial Analysts Federation 21st Annual Conference, Boston, Mass., May 5-9, 1968.

The Public Service Company of Colorado, Gulf General Atomic, and the Commission reviewed the operating experience of Peach Bottom and decided to proceed with the construction of a 330 MWE gas-cooled nuclear plant on the Public Service system near Platteville, Colorado, for operation in 1973. The AEC issued a construction permit for this plant in September 1968. The total cost of construction, exclusive of fuel, is estimated to be $75.6 million, of which $52 million will be borne by the Public Service Company.[9/] The Peach Bottom reactor continued to operate satisfactorily after beginning full power operation in June 1967. Operation of this reactor demonstrates the use of a nuclear power plant for achieving high steam temperatures and pressures comparable to those of the coal-fired plant.

Some purchases of turbo-generators from foreign firms continued during 1968. The purchase by American Electric Power of turbo-generating equipment from Brown Boveri, for installation at its first nuclear power plant, caused a considerable amount of interest in industrial circles relative to the impact these foreign purchases would have on the sales policy of the two United States manufacturers of turbo-generating equipment. Additional information on foreign purchase of steam turbo-generators and other components of central station power plants is discussed in Category III, "Nuclear Components and Equipment."

Of considerable interest was the announcement early in the year that North American Rockwell and Brown Boveri were studying the possibility of a joint venture in the manufacture of turbo-generating equipment in the United States and, by fall, indications were that the venture appeared promising. There is no doubt but that utilities in the United States would greatly welcome the possibility of a third supplier to improve competition.

In regard to dual-purpose plants, the first nuclear power plant, having a dual capability of producing electricity and process steam, was purchased by the Consumers Power Company from the Babcock & Wilcox Company during May 1968. Each of the two Babcock & Wilcox pressurized-water type units included in this plant will have a nominal rated capacity of 2,452 megawatts thermal. They will generate 1,300,000 electrical kilowatts to be fed into

---

9/ See AEC Public Release L-221, dated September 18, 1968.

the Michigan power pool. At the same time, they will provide a minimum of four million pounds per hour of steam for process use by the Dow Chemical Company.

With reference to dual-purpose plants for desalting, many in the industry were disappointed with the announcement in 1968 that the Bolsa Island project was to be re-studied. The original plan called for construction on a man-made island of two nuclear power reactors capable of generating 1,800,000 kilowatts of electricity and a desalting plant ultimately capable of producing 150,000,000 gallons of fresh water a day. All the participants in this project reiterated their interest in the construction of nuclear power producing and water desalting plants, including the use of island sites to meet the growing need of Southern California for these essential resources.[10/]

The 462 MWE Connecticut Yankee Atomic Power plant operated at full power for the first time during this year and the 430 MWE San Onofre nuclear power plant of Southern California Edison and San Diego Gas and Electric resumed operation after repairing damage resulting from an electrical fire as previously discussed in more detail under this category. The operation of these plants represents an important benchmark since they represent an increase in size of double the first Dresden and Yankee plants and an intermediate step toward the operation of the next larger plants of 700 to 1,000 MWE scheduled for operation in 1969 and 1970.

The design and marketing by reactor suppliers of a 1200-1300 MWE power plant was a milestone in the trend to go to higher power levels. These larger systems should be attractive because of achieving significant reductions in the capital costs of nuclear power plants.

A new technological development was accomplished which verifies the potential for improved economics of power reactors now on order, when the Saxton Nuclear Station's reactor attained a record power density level. It reached a peak linear power density of 18.5 kilowatts per foot which is approximately 25 percent higher than that in presently operating pressurized water reactors. This power density work was conducted by Westinghouse and the Saxton Nuclear Experimental Corporation which is a subsidiary of the General Public Utilities Corporation.

10/ See AEC Public Release L-171, dated July 25, 1968.

c. Fuel Cycle Arrangements

The nuclear utilities can select from a multitude of fuel cycle arrangements being offered by the reactor manufacturers, as well as independent fuel cycle suppliers. The range varies from that of the reactor manufacturer's offer of a full fuel cycle service to the opposite extreme, wherein the utility controls most of the major steps in the fuel cycle. Typical arrangements are as follows:

(1) Comprehensive Fuel Cycle Service

Under this arrangement, the core supplier takes responsibility for all fuel cycle steps. The utility pays the core supplier for energy as it is extracted from the core. The utility usually takes title to ownership of the core while it is in the utility's possession.

(2) Initial Core Sale

The core supplier sells a fabricated core to the utility and guarantees the performance of the core. The utility owns the core and is responsible for spent fuel recovery. Usually, replacement fuel for several years operation is ordered at the time the initial core is ordered.

(3) Fuel Fabrication Service

The utility handles purchasing of uranium ore, conversion to UF_6 and enrichment, and delivers enriched UF_6 to core supplier. The core supplier furnishes zirconium tubing and fabricates the core. The supplier guarantees performance of the core and the utility handles all steps involved in reprocessing the spent core.

A survey in regard to the plans of nuclear power utilities for fuel cycle arrangements indicated that the majority ultimately intend to control as many of the separate steps in the fuel cycle as possible, once they have obtained the necessary operating experience with their first reactor plants.

A new approach introduced during the year was the establishment of a uranium bank, so to speak, in which an owner of uranium can, in effect, deposit his uranium in the bank to accumulate interest while it is on loan to

others. He may withdraw this uranium later on when he needs it. This would be a valuable service by reducing the burden of carrying charges on a large inventory of very expensive material. Another prospect being considered by the banking institutions and others is to purchase uranium and lease it to the utilities after it is fabricated into fuel for use in the nuclear plant.

One new company, called National Assurance Corporation, has been formed to serve the utilities by having fuel in various forms from UO_2 powder to finished fuel assemblies in stock so as to provide the necessary item of fuel on short notice whenever it is needed by the utility. In addition, they may stock other vital components, such as pumps, valves, etc.

d. <u>Status of Competition in Nuclear Fuel Cycle</u>

The companies that are presently participating in the various steps of the fuel cycle are listed in Table IV-9.

TABLE IV-9

COMMERCIAL POWER REACTOR FUEL CYCLE STEPS BY SUPPLIERS

X - Company now capable of performing these steps.
O - Company planning additional step.

	Reactor Suppliers						Fuel Cycle Suppliers							
	Atomics International	Babcock & Wilcox	Combustion Engineering	General Electric	Gulf GGA (Gulf General Atomic*)	Westinghouse	Aerojet-General	Allied Chemical	Atlantic Richfield (Nuclear Materials)	Kerr-McGee	National Lead	Nuclear Fuel Services	United Nuclear	AEC
**FABRICATION OF CORE**														
Exploring, mining & milling ore to U_3O_8					X	X			X	X		**	X	
Conversion of U_3O_8 to UF_6								X		O				X
Enrichment of UF_6														X
Fabrication of Zr ingot into tubing				O		X							X	
Conversion of UF_6 to UO_2	O	O	O	X	*	O	O		X	X		X	X	X
Pelletizing of UO_2	O	O	O	X	*	X	O		X	X	X	X	X	X
Fabrication of fuel elements	X	X	X	X	X*	X	O		X			X	X	
Fuel Warranty	X	X	X	X	X	X			O			O	X	
**MANAGEMENT OF FUEL IN REACTOR**														
Design of fuel management scheme	X	X	X	X	X	X			X				X	
**RECOVERY OF SPENT CORE**														
Spent fuel shipping				X	O	X		O	O		X	X		X
Reprocessing fuel elements and waste disposal				O				O	O		O	X		X
Conversion of $UO_2(NO_3)_2$ to UF_6 for re-enrichment				O				O	O					X
Conversion of $Pu(NO_3)_4$ into PuO_2	O	O		X		O	O		X			X	X	X
Fabrication of Pu fuel elements	X	O	O	X		X	O		X			X	X	X
Isotope recovery from spent fuel				O				O			O	O		X

*Gulf General Atomic produces the HTGR which uses coated U-Th carbide or oxide particles in an all-graphite matrix fuel element.
** Nuclear Fuel Services is partially owned by Getty Oil and Skelly Oil, who are involved in uranium exploration.

November 1968

The companies engaged in the remaining steps of the nuclear fuel supply cycle are of three types: reactor manufacturers (all of which also manufacture fuel for their reactors), firms that manufacture either fuel cores or perform certain steps of the nuclear fuel cycle, and firms which perform certain individual services which support the nuclear fuel cycle but do not manufacture the complete fuel core.

(1) Reactor Suppliers

Earlier in this category the competition in the reactor industry was reviewed. The parts of the fuel cycle being undertaken by each of the reactor suppliers are discussed in this section.

The General Electric Company continued with the general expansion of its facilities. Their fuel production includes the conversion of UF_6 and UO_2 powder, making UO_2 pellets, and fabrication of the complete reactor core. They also intend to manufacture zirconium tubing and are proceeding with the construction of a chemical fuel reprocessing plant.

At the present time, Westinghouse purchases UO_2 powder and fabricates UO_2 pellets for a complete reactor core. Its new facilities under construction include installing a capability for producing UO_2 powder, in addition to pellets, and for manufacturing zirconium tubing.

Babcock & Wilcox plans to produce UO_2 powder and pellets. Combustion Engineering presently purchases UO_2 pellets, but is considering developing a capability to produce both UO_2 powder and pellets. Both Babcock & Wilcox and Combustion Engineering do most of the remaining fabrication in the manufacture of a reactor core, but have not indicated plans to manufacture zirconium tubing.

Gulf General Atomic is performing most of the steps in the fuel cycle for the gas-cooled reactor, will fabricate the complete reactor core, and is considering entering fuel reprocessing.

(2) Fuel Core Manufacture

As stated earlier, United Nuclear Corporation is the only independent producer so far that has bid and received orders for replacement cores. They produce UO_2 powder and pellets, fabricate the complete reactor core, and warrant its performance for commercial reactors.

Commonwealth Edison's award of a replacement core for the CE Dresden I reacto· to United Nuclear during 1966 was the first breakthrough of an independent supplier in the replacement core business. This trend was continued during 1967 with the award of the contract to United Nuclear to provide demonstration assemblies for the Westinghouse Yankee reactor. The General Electric Company is also providing demonstration assemblies for the Yankee pressurized water reactor. American Electric Power Company awarded replacement cores II and III to United Nuclear for AEP's first nuclear plant that they purchased from Westinghouse. The first segment of this replacement core would be manufactured in 1972-1973. This is the first time that a replacement core was purchased from other than a reactor manufacturer at the same time the nuclear plant was purchased. The decision by Commonwealth Edison and American Electric Power Company to buy fuel from an independent fuel supplier has encouraged other utilities to take this same approach to procurement of replacement cores, thus broadening the industry and achieving competition possibly at an earlier date. For example, during 1968, Consolidated Edison placed a large order for a replacement core from United Nuclear for Consolidated's fourth nuclear power plant. This nuclear unit will be manufactured by General Electric and they will fabricate the initial core. United Nuclear is also fabricating the pellets for Consumers Power Company's Palisades plant under subcontract to Combustion Engineering.

(3) Suppliers of Segments of the Fuel Cycle

The potential market in the nuclear fuel cycle is sufficiently attractive that companies have entered or are considering the possibility of entering all phases of the fuel cycle, even though the magnitude of business may not be adequate to assure a reasonable return on investment until the mid-1970's.

(a) Conversion of U_3O_8 to UF_6

The only industrial capability in existence for the conversion of U_3O_8 to UF_6 is the Allied Chemical plant at Metropolis, Illinois. Allied has announced plans for expanding their annual capacity to 10,000 tons of uranium by 1970. In addition, Kerr-McGee is building a conversion facility in Oklahoma, with an initial capacity of 5,000 tons uranium per year, to be operational in 1970. Other companies have indicated an interest in going into this phase of the fuel cycle. (See Category II).

(b) UO_2 Powder and Pellets

At the present time, Kerr-McGee, Nuclear Fuel Services, and Atlantic Richfield (NUMEC) produce UO_2 powder and pellets for sale to the reactor manufacturer. The Gulf Oil Corporation has indicated plans to enter the total fuel cycle business for high temperature gas-cooled reactors. The National Lead Company continued to indicate plans to possibly enter the replacement core business and other steps in the fuel cycle, such as fuel reprocessing. Aerojet-General states it could have an automated fuel production facility in operation during 1972-1973. It would convert UF_6 to UO_2, as well as fabricate fuel elements. Atomics International is involved in reactor development with a major effort in fast breeder reactors and may ultimately enter the replacement fuel business, including plutonium recycle. Indications are that there will be extensive competition in this area, when the market becomes significant, and as utilities gain experience with the fuel cycle. (This is further discussed in Category II.)

(c) Fuel Reprocessing

The reprocessing of fuel elements and handling and storage of the residual high level radioactive material is presently being performed by only one commercial firm, Nuclear Fuel Services. Other companies are entering this field as discussed under Category VI. Spent fuel shipping services are available from reactor suppliers and fuel cycle suppliers, as well as from commercial transportation firms.

(d) Management of Fuel

All four reactor manufacturers and the replacement fuel supplier are presently able to undertake the management of fuel in a reactor, and it is probable that consulting firms and the utilities themselves will undertake this step as the nuclear power industry matures. As the utilities become more experienced and knowledgeable in nucleonics and fuel cycle technology, it is expected that they will not attach as much importance to fuel warranty contracts as they have in the past. The utilities may find that without a fuel warranty they may have more flexibility in the manner in which their fuel may be "shuffled" and a better opportunity to achieve additional savings by purchasing from broader competitive sources. Each of these features may be conducive in reducing the cost of power production.

It appears that there will be some delay in accelerated activities for independent purchases of replacement fuel until more utilities are in a position to purchase replacement cores on a large scale from other than the reactor manufacturers. As indicated earlier, the electrical utilities have already ordered, or have options for, more than 50 percent of the replacement fuel fabrication that will be needed through 1980. Under their option, however, they should have the opportunity to solicit alternate proposals from the independent suppliers.

The state of competition for some of the steps related to the recovery of a spent core are not yet too well-defined. For example, further attention must be given to such activities as fission product recovery and encapsulation, the conversion of uranyl nitrate to UF_6 for reenrichment, or blending, recovery of neptunium-237, conversion of plutonium nitrate into plutonium oxide, and especially the fabrication of plutonium fuel.

Reactor and independent fuel suppliers will, no doubt, eventually have to achieve a plutonium recycle capability to maintain a competitive position in the replacement core business. Beginning about 1975, large quantities of plutonium will become available for recycling in the nuclear power plants. The estimated value of plutonium that will be generated through 1980 is around a billion dollars. More detail on this subject is included in Category II, D.

4. Desalting

a. General

During the past year, the role of nuclear energy for desalting and other industrial process applications continued to receive considerable attention. The AEC's program for desalting and other process applications is directed toward analyzing, developing, and demonstrating nuclear reactor systems for desalting and other industrial processes. The AEC's efforts are closely coordinated with those of the Office of Saline Water, U.S. Department of the Interior, which has the responsibility within the Federal Government for desalting process development.

For the AEC's desalting program, the Oak Ridge National Laboratory acts as the principal technical center in the conduct of a broad-based program of technical studies, evaluations, and research and development for the near-term and long-range application of nuclear energy.

b. United States

During the past few years, considerable effort was devoted to the initiation of a large dual-purpose (power and desalting) nuclear project in southern California. This project, the Bolsa Island Nuclear Power and Desalting Project, has been a cooperative effort involving the Department of the Interior, the Atomic Energy Commission, the Metropolitan Water District of Southern California (MWD), the Department of Water and Power of the City of Los Angeles, Southern California Edison, and San Diego Gas and Electric.

The plans for the Bolsa Island Project considered the use of two light water reactors of approximately 3,000 megawatts thermal capacity each, which would generate about 1,800 megawatts of electricity, and produce 50 million gallons per day of fresh water from the sea. The project also considered the expansion of the desalting portions, following initial operation, to a total capacity of 150 million gallons per day. On July 25, 1968, increases in the estimated capital cost of the project caused the participants to investigate other alternatives which would be less costly, but still achieve the significant objectives of the project originally planned. These investigations are currently underway.

The AEC, in cooperation with the Office of Saline Water and various domestic groups, conducted studies of the potential of large scale desalting. Included in such studies were the possible role of nuclear desalting plants in the long-range and resource development in the State of Utah, and the role that large nuclear desalting plants could play in providing fresh water from the sea in the New York metropolitan area.

A cooperative study was initiated with the Commonwealth of Puerto Rico to assess the potential of multi-purpose nuclear plants in the economical industrial development of the island.

c. International

Studies of nuclear desalting plants of interest to foreign countries were conducted, and support of the IAEA as the focal point for large scale nuclear desalting in the international field was continued. The results of a U.S./Mexico/IAEA team study were prepared and submitted to the Governments of Mexico and the United States and to the International Atomic Energy Agency. This international study assessed the potential of very large dual-purpose plants to serve the power and water needs of the region consisting of the Mexican states of Sonora and Baja California and Arizona and southern California in the United States. Reference plants in this study have outputs of approximately 2,000 megawatts electric and one billion gallons per day of fresh water. The results of this study are currently being reviewed by the participating Governments.

AEC representatives participated in an International Symposium on Nuclear Desalting, which was sponsored by the IAEA, and held in Madrid, Spain, in November.

d. Energy Center Studies

The results of an energy center study, which was conducted by the Oak Ridge National Laboratory during the past year, were released during the latter half of 1968. This ORNL study investigated the use of large nuclear "energy centers" as the nuclei for industrial and agro-industrial complexes. The study results indicated that such complexes, based on predicted low cost nuclear energy, complimentary industrial processes, and intensified agriculture (food factories) might be expected to provide attractive returns on investment and assist in the development of many areas around the world.

In response to a Senate Resolution, and based in large measure on the favorable results of the ORNL energy center study, a detailed investigation of the potential of agro-industrial complexes for the Middle East was initiated during the summer of 1968. One of the objectives of this Middle East study will be to determine the extent to which the energy center concept might convert the arid waste lands of the Middle East into productive agro-industrial centers, drawing on nuclear and desalting technology and the mineral resources available to these areas.

5. Small Reactors

Because of the interest that has been expressed in the past in small reactors (around 150 MWE), the AEC's Chicago Operations Office initiated a study in 1966 of small nuclear power plants. This study was completed and published during 1967.11/ Volume I of the small nuclear power plant study pertains to the design, construction, and operating experience. Volumes II and III deal with the economics of these plants. Interesting information provided by the study is the cost reduction which might be possible through joint action by several utilities in areas, such as procurement of a standardized plant, licensing, and operating services. However, this study indicated that the future of small reactors as economically competitive sources of power in the United States is not very encouraging.

The economics of reactor units at lower power levels, of course, depends on the particular location and availability and cost of other sources of power. In certain situations, one may want to consider nuclear units for other than economic reasons. In high cost fuel areas, where transportation and other problems exist, one should make careful economic comparisons even at power level requirements lower than 400 MWE.

Small reactors are finding a place as sources of power in remote areas. The Navy for several years now has been using a 1,500 KWE reactor, the PM-3A nuclear plant at McMurdo Sound in the Antarctic, to supply power to a National Science Foundation-sponsored scientific community there. This reactor has also been effectively used to desalt seawater for the community, although it was not originally designed for this purpose.12/

11/ COO-284, "Small Nuclear Power Plants."
12/ Remarks by Dr. Glenn T. Seaborg, Chairman, USAEC, "The Proliferation of the Peaceful Atom," at the 24th Annual Conference of the American Public Power Association, Denver, Colorado, May 11, 1967, AEC Public Release S-20-67.

Utility systems that need relatively less power might best take advantage of nuclear power by the cooperative use of large reactors. The smallest reactor units ordered for commercial power production, since 1962, are in the range of 400-455 MWE.

It is expected that there will be continuing interest in small reactors, because many utilities in the United States and countries around the world do not have sufficient loads to utilize large power plants on their system.

The National Nuclear Corporation, a new company that was formed during the past year, is studying new concepts and reevaluating the economics of small plants.

6. Export Sales

Previous reports on the nuclear industry have summarized sales of power reactors abroad. In recent years, it has become apparent that power reactors installed will increasingly tend to be constructed of locally fabricated components, with participation by U.S. reactor builders largely through licensing or joint venture arrangements and the supply of specialized components. For example, about 70 percent of a second pressurized water nuclear unit for Japan's Kansai Electric Power Company is expected to be manufactured in Japan, but with Westinghouse providing design information, fuel, and certain components such as cooling water pumps.

At the present time, 26 enriched uranium power reactors of the United States type, totaling about 10 million kilowatts and representing an investment of about two billion dollars are in operation, under construction, or on order in 10 countries outside of the United States. Additional plants have been announced but not contracted for.

The impact of nuclear power plant construction abroad is expected to be reflected in the future in rising exports of special nuclear materials shown in the report on exports in the Summary section of this report. Special nuclear material exports were reported in 1967 as $44,015 as compared to $41,906 in 1966.

U.S. commercial reactors in general have demonstrated excellent performance. While difficulties have been experienced with only three overseas reactors, these are being corrected, and design improvements will be incorporated in future reactors.

Table IV-10 shows a list of U.S.-type power reactors for operation abro^d.

TABLE IV-10

Foreign Installations of U.S.-Type Power Reactors

Plant	Country	Mfr. or Licansor	MWE	Start-Up
Tihange	Belgium	W	740	1973
SENA	France	W	248	1966
RWE	Germany	GE	16	1960
KRB	Germany	GE	237	1966
KWL	Germany	GE	160	1968
KWO	Germany	W	280	1968
NWK-HEW	Germany	W	630	1972
Wuergassen	Germany	GL	612	1972
TARAPUR	India	GE	380	1967
SENN	Italy	GE	150	1963
SELNI	Italy	W	240	1964
JAERI	Japan	GE	12	1963
JAPCO #2	Japan	GE	342	1969
Tokyo #1	Japan	GE	460	1970
Kansai #1	Japan	W	340	1970
Tokyo #2	Japan	GE	784	1973
Chubu #1	Japan	GE	500	1973
Kansai #2	Japan	W	500	1974
Chugohu #1	Japan	GE	460	1975
GKN	Netherlands	GE	48	1968
Zorita	Spain	W	153	1968
Nuclenor	Spain	GE	440	1970
OKG	Sweden	GE	400	1970
NOK	Switzerland	W	350	1969
BKW	Switzerland	GE	306	1971
NOK	Switzerland	W	350	1972

7. Advanced Converters

In the United States, work on advanced converters is centered on the high temperature gas-cooled reactor concept (HTGR) and the seed-blanket light water breeder concept (LWBR).

As was discussed last year, other nations have placed emphasis on other approaches. In this regard, we have a cooperative program on heavy water reactors with the Canadians. Whatever the approach, however, the benefits to be realized are essentially similar. The advanced converters and near breeders represent a step forward from present reactors and provide a means of alleviation of fuel resource requirements during a period before high gain breeders are fully developed.13/

The major effort in the U.S. program to develop the heavy water moderated-organic cooled reactor under contract with Atomics International and Combustion Engineering was discontinued during 1967.14/

The AEC is continuing a heavy water reactor base research and development program that will take advantage of the heavy and light water reactor experience in this country and the HWR experience in Canada and Euratom. This program will permit the U.S. to maintain the option, in the future, to pursue heavy water reactors for power production and for the extremely large size if needed for dual-purpose and desalting applications.

During the year, a review of the operating experience of the Peach Bottom High Temperature Gas-Cooled reactor by the Atomic Energy Commission, Gulf General Atomic, and the Public Service of Colorado led to a decision to proceed with the construction of a 330 MWE high temperature gas-cooled reactor nuclear power plant. This plant will be located near Platteville, Colorado, and is scheduled for operation by the Public Service of Colorado in 1972. Gulf General Atomic also was asked to submit a proposal to an electrical utility in the Northwest for a large gas-cooled nuclear plant.

Work on the seed-blanket light water breeder, which shows promise of breeding in the light water medium, is directed toward the possibility of demonstrating the breeding potential in an existing light water reactor, such as the Shippingport reactor.

13/ Remarks by Gerald F. Tape, Commissioner, USAEC, "The Increasing Importance of the Breeder Program," at the American Power Conference Symposium, April 23, 1968, Chicago, Illinois, AEC Public Release S-17-68.

14/ See AEC Public Release IN-769, dated March 9, 1967.

The extent to which these reactor concepts will be accepted for industrial power plants will depend on the comparative economics with other systems, availability of low-cost ore, and the relative schedule for the development of breeder reactors.

B. Breeders

The development of the breeder reactor will increase fuel utilization from 1-2 percent now available to over 50 percent. The high gain breeders will be capable of producing significantly more fissionable fuel material than they consume, thereby providing an excess of burnable plutonium which will be used to fuel new fast breeders in an expanding power system.15/

A top priority program in the AEC's fast breeder work is the Liquid Metal Cooled Fast Breeder Reactor (LMFBR). The objective of this program is the development of commercially competitive, central station power plants with capacities of 1,000 MWE and greater. Achievement of this goal will require development of technology and the design, construction, and operation on utility grids of two or more "demonstration plants" as intermediate objectives.

With respect to the demonstration plant program on the liquid metal breeders, the kind of work and how it will be performed will be a matter for future negotiations between industry and the AEC. The plans now provide for financial assistance for a total of at least three demonstration plants, each constructed at about two year intervals. Each of these first demonstration plants, which may have a capacity of 300 to 500 MWE, could be based on a different plant concept as proposed by different proposer groups. Each plant could be owned and operated by a utility as a part of a utility grid.16/

The Liquid Metal Cooled Fast Breeder Reactor's position of priority in the AEC's advanced reactor program was based on potential economics, industrial and worldwide interest, technological experience, and its capability to utilize efficiently the uranium resources. Sodium has been chosen as the coolant for the LMFBR: It is an excellent heat transfer agent; boils at 1618° which allows for high steam temperature and high steam pressure conditions and easily allows for natural circulation

---

15/ Remarks by Milton Shaw, Director, Division of Reactor Development & Technology, USAEC, "Current AEC Program in Fuel Reprocessing Research and Development," at the SINB/AEC Conference on Nuclear Power Fuel Reprocessing - Technology and Economics, Augusta, Georgia, May 11, 1967, AEC Public Release IN-792.

16/ Remarks by James T. Ramey, Commissioner, USAEC, "Utility Participation in the AEC Fast Breeder Reactor Program," before a Panel at the American Power Conference, Chicago, Illinois, April 23, 1968, AEC Public Release S-20-68.

during shutdown emergency conditions; is neutronically suitable since it is a good scatterer, but a poor moderator; and has a low viscosity coefficient, low density, and low specific heat. These last three properties allow for large temperature differentials across the reactor, and low pumping powers. Low pumping power requirements during emergency cooling allow for small pump units easily supplied from small standby power units. Sodium is a relatively low corrosion agent. It has an established technological base, and in conjunction with plutonium fuel and fast flux should provide a low plutonium doubling time of less than 10 years.17/

The present programs are aimed toward the development of satisfactory fast reactor fuels and cladding materials, components, instrumentation and control, sodium technology, core design, fuel recycle, physics, safety, and plant design. Current fuels and materials irradiation programs center on the operation of the sodium-cooled Experimental Breeder Reactor II (EBR-II), and experimental work to be carried out in Fermi. A new 400 Mwt Fast Flux Test Facility (FFTF) will serve as the major facility for development of fuels and materials for the demonstration plants and commercial sodium-cooled fast breeder reactor power plants. The FFTF should become operational in 1973-74.

Another important facility in the fast breeder development program is the SEFOR reactor located near Fayetteville, Arkansas. This experimental facility is operated as part of a cooperative program involving the Southwest Atomic Energy Associates, (a group of electrical utilities), the General Electric Company, West Germany, Euratom, and the AEC. It is expected to provide valuable information on fast breeder reactor safety.

An important step in the LMFBR component and systems program was the establishment of the Liquid Metal Engineering Center (LMEC), an AEC-owned facility located at Santa Susana, California, and operated by Atomics International. The activities of the LMEC include: operation and administration of LMFBR component proof testing facilities; compilation and dissemination of information through the Liquid Metal Information Center (LMIC); development and coordination of the identification of LMFBR codes, standards, specifications, and practices; conduct of training programs; technical surveillance of component development; and participation

---

17/ Remarks by Milton Shaw, Director, Division of Reactor Development & Technology, USAEC, "The U.S. Fast Breeder Reactor Program," at the American Power Conference, Chicago, Illinois, April 23, 1968, AEC Public Release S-18-68.

in special studies. The AEC is encouraging industry, including the utilities, to make maximum use of LMEC by assigning personnel to these facilities and coordinating their efforts with the programs to be conducted in the facility.17/

In April 1968, the Edison Electric Institute published a Fast Breeder Reactor report which was completed by their subcommittee for fast breeder reactor development. The conclusions and recommendations of this report are essentially in consonance with the program that has been delineated by the AEC.18/

Cooperative programs continued during the year between electrical utilities and the reactor manufacturers. The General Electric Company is working with the Southwest Atomic Energy Associates (SAEA) and four other utilities in the design and study of a 300 MWE prototype sodium-cooled plant. GE is also working with the Empire State Atomic Development Associates (ESADA) on the development of key components for a sodium-cooled fast breeder nuclear plant, the objective of the work being to lead to the construction of a prototype breeder reactor. According to an ESADA announcement in August 1967, one of the provisions of the ESADA contract with GE is that a member company will have the right of first refusal to proceed with the project by June 30, 1970, by which time a decision regarding construction of the demonstration plant will have been reached.

Westinghouse Electric Corporation is working with about 21 utilities and an architect-engineering firm on a four-year program concerning the development of a sodium-cooled fast breeder nuclear plant. It is expected that they will make a decision in 1970 on the construction of a 200 to 400 MWE prototype unit.

In addition to General Electric and Westinghouse who are working with utilities toward fast breeder liquid metal demonstration programs, the potential of another company entering this field was enhanced during the year. The Atomics International Division of North American Rockwell announced a three-year R&D study, supported by General Public Utilities and three other electric companies, directed toward a decision in 1970 on the construction of a prototype liquid metal fast breeder plant - probably on the GPU system.

There are other approaches being considered for the development of breeder reactors in addition to the liquid metal fast breeder as previously discussed. These are the gas-cooled fast breeder reactor, the steam-cooled fast breeder reactor, and the

---

18/ "Fast Breeder Reactor Report," published by Edison Electric Institute, April 1968.

molten salt breeder. During the past year, work which had been conducted by General Electric and the East Central Nuclear Group on the steam-cooled fast breeder reactor was discontinued. The leading proponent of the gas-cooled fast breeder has been Gulf General Atomic. In June this company announced a two-year study to be undertaken with 21 utilities regarding a gas-cooled fast breeder reactor.

The Molten Salt Breeder Reactor, as envisioned by the Oak Ridge National Laboratory, is a thermal reactor, based on the thorium breeding cycle. It would represent a reactor concept dependent on availability of thorium rather than uranium as a source of fertile material.

Through continual reprocessing at the reactor site, removal of fission products and protactinium would be achieved. This would contribute to good neutron economy and to a low total fuel inventory. It is believed that the low inventory of fissile material that is required, coupled with a potential breeding ratio of about 1.05 could result in a relatively short doubling time. In addition, a molten salt reactor might be started up economically on U-235, U-233, or plutonium and hence could use whichever fissile material would be available.

Present work at ORNL on this concept is centered on operation of the Molten Salt Reactor Experiment. This incorporates a single region, non-breeding circulating molten fluoride salt fuel, graphite moderator, and an air-cooled radiator for heat dissipation. Its operation is directed to the development of technology for a large breeder reactor. Present research and development efforts include development of improved moderator graphite, detailed evaluation of improved nickel base alloys for use as structural material, and study of optimum on-site processing techniques for separating the uranium, protactinium, and fission products from the salt.19/

Some electrical utilities have expressed an interest in the AEC continuing the molten salt reactor development program because of its possible long-range potential for conservation of resources and the achievement of cheap power.

19/ Remarks by Gerald F. Tape, Commissioner, USAEC, before the American Power Conference Symposium on The Increasing Importance of the Breeder Program, Chicago, Illinois, April 23, 1968, AEC Public Release S-17-68.

It is expected that an investment of $1,500 million or more will be required in the development of the breeder before a safe, reliable, and economic machine can be brought to the market place and that the date of substantial market penetration is, in all likelihood, some 15 years away.

Indications are that by the year 2000 savings in electric power costs from the introduction of the breeder will be about $2 billion every year, which would be a valuable return on the Government's investment. Because this return matures so far in the future and will tend then to accrue to the public at large rather than to any individual company, it is not an attractive risk venture for private industry at this time. Consequently, the breeder is one area where investment by the Government is desirable and necessary - both to reduce the cost of electricity and to conserve our resources of thermal energy.[20]

C. Civilian Maritime Reactors

In August 1968, the first nuclear-powered merchant ship, the Nuclear Ship Savannah, completed its third year of experimental commercial service. During these three years and the period of public port visits, it traveled about 330,000 miles. It has operated on its original nuclear core installed in November 1961 and has now consumed a total of about 120 pounds of uranium-235. This is equivalent to about 96,000 tons of fuel oil. In the three years of commercial use the reactor was at power 81 percent, 82 percent, and 87 percent of the time respectively.

Since the ship began its public port visits and its commercial voyages, it has entered 32 domestic ports in 20 states and 44 foreign ports in 25 countries, including 2 trips to the Far East and 17 trips to the North Atlantic and Mediterranean areas.

At present the Savannah has returned to its service port in Galveston, Texas, for its first fuel addition. Four new elements will be added and the remaining 28 will be "shuffled". It is planned that an additional two years of continued operation can be expected before a complete core is replaced.

The schedule for the N. S. Savannah to resume service is October 1968.

20/ Remarks by Wilfrid E. Johnson, Commissioner, USAEC, "Perspectives in Nuclear Energy," before the Royal Society of Engineers, Brussels, Belgium, April 22, 1968, AEC Public Release S-15-68.

There are no funds in the AEC budget for continuing work on Merchant Ship Reactors at the present time.

On the foreign scene, West Germany's nuclear merchant ship, the Otto Hahn, began sea trials under nuclear power during October 1968. The Japanese began construction of their first nuclear merchant ship and the Russians announced that they would begin construction of two additional nuclear powered icebreakers that would serve as prototypes for a nuclear merchant marine fleet.

D. Military Reactors

1. Army Reactors

Valuable operating experience has been gained with the reactor power plants that were developed under the joint AEC/Department of the Army program. As reported previously under Small Reactors, the Navy, for several years, has been using the PM-3A nuclear plant at McMurdo Sound in the Antarctic in support of a scientific community there.

Another portable medium power nuclear plant, the PM-1, has been in use since 1962 in a remote section of Wyoming, supplying power for a small isolated Air Force station. Both the PM-3A plant in the Antarctic and the PM-1 in Wyoming have made record power runs of over 3,600 hours of continuous operation. However, in 1968, the Air Force PM-1 was shutdown along with the radar station to which it supplied power.

Another operational power plant is the MH-1A reactor which has been installed on the barge Sturgis. This 10,000 kilowatt plant (operated by the Army) is towed to offshore anchorages where emergency power is needed and supplies enough electricity to meet the needs of a community of from 10,000 to 20,000 people. During 1968, the Sturgis was used for the first time outside the United States to provide power to the Panama Canal Zone to meet the present power shortage there.

The reactor power plants were developed under this program to provide military services with power sources that could further the mission of the Armed Forces.

Table IV-11 lists the operating Army reactor plants with their manufacturers.

TABLE IV-11

Plant Designation	Location	Manufacturer
MH-1A	Barge mounted Panama Canal Zone	Martin-Marietta Martin-Marietta
PM-1	Sundance, Wyoming	Martin-Marietta
PM-3A	McMurdo Sound, Antarctica	Martin-Marietta
SM-1	Ft. Belvoir, Virginia	*
SM-1A	Ft. Greely, Alaska	*

*These plants were built by Alco Products, Inc., whose nuclear activities were taken over by Allis-Chalmers. Allis-Chalmers has since left the reactor business.

During 1968 work that could lead to plants of interest to the Armed Services was conducted under the terrestrial low power reactor program. This program provides for development of technology for improved reactor systems covering the 100 KWE to 2,000 KWE range and applicable to a wide variety of uses, such as oceanographic (non-mobile) and terrestrial power plants, both attended and unattended.21/

2. Naval Reactors

The Naval Propulsion Reactors Program is a joint program of the AEC and the Department of the Navy which has as its objective the design and development of improved nuclear propulsion plants and reactor cores for installation in Navy ships ranging in size from small submarines to large combatant surface ships.

Design, development, testing, and evaluation of these improved nuclear propulsion plants and cores is carried out principally by the AEC's Bettis Atomic Power Laboratory, Pittsburgh, Pennsylvania, and Knolls Atomic Power Laboratory, Schenectady, New York. These two Government-owned research and development laboratories are operated for the AEC by the Westinghouse Electric Corporation (Bettis) and the General Electric Company (Knolls). The principal reactor core

21/ See AEC Authorizing Legislation, Fiscal Year 1969, Hearings Before the Joint Committee on Atomic Energy, Congress of the United States, Ninetieth Congress, Second Session on General and Reactor Development Programs, Part 1, dated January 30, 31 and February 5 and 6, 1968.

manufacturers are the Babcock and Wilcox Company at its Lynchburg, Virginia, plant, and the United Nuclear Corporation at its New Haven, Connecticut, plant.

Congress has authorized 108 nuclear-powered submarines, including 41 of the Polaris missile-launching type and one deep submergence research vehicle, as well as seven nuclear-powered surface ships. It is planned that 81 nuclear-powered submarines will be in operation by the end of the year. Four nuclear-powered surface ships -- the aircraft carrier ENTERPRISE, the guided-missile cruiser LONG BEACH, and the guided-missile frigates BAINBRIDGE AND TRUXTUN -- are now in operation. During 1968 the TRUXTUN completed her first Vietnam combat deployment; the LONG BEACH completed her second; and the ENTERPRISE completed her third. The operation of these nuclear-powered surface ships continues to demonstrate, under actual combat conditions, the significant advantages of nuclear propulsion for surface warships.

A high level of effort continued during 1968 on the development of a two-reactor nuclear propulsion plant for the Navy's second nuclear-powered aircraft carrier, the NIMITZ, CVAN 68. The keel of the NIMITZ was laid June 22, 1968. Her two reactors are the highest-powered reactors under development in the Naval Program, each producing about as much power as four of the ENTERPRISE reactors.

a. Oceanographic Applications

A nuclear-powered deep submergence research vehicle, the NR-1, is also presently under development. The capability of this submarine -- to be manned by a crew of five and two scientists -- will be significantly greater than any other oceanographic research vessel developed or planned to date because of the vastly increased endurance afforded by nuclear power. NR-1 is designed to operate on and near the ocean bottom for periods of time limited only by the provisions carried on board. Nuclear propulsion in a vessel of this nature provides greater independence from surface support ships and essentially unlimited endurance in propulsion for exploration of the ocean. NR-1 will provide the capability of exploring an area of the ocean bottom several times as great as that of the United States and can be used to perform studies and mapping of the ocean bottom, temperature, and currents for military, scientific, and commercial uses.

In addition to the NR-1, it is expected that nuclear power plants will play an important role in the provision of auxiliary power and power for use in the exploration and recovery of minerals and other materials from the ocean's floor.

It has been conservatively estimated that the world's oceans contain some 50 quadrillion metric tons of minerals, including two quadrillion tons of magnesium, 100 trillion tons of bromine, 20 billion tons of uranium, 15 billion tons of copper, and 10 billion tons of gold! Other materials down there include boron, manganese, silver, nickel, phosphorous, platinum, and a variety of rare earths. When it comes to extracting and processing these and the many more valuable materials in the sea and on the ocean floor, the extensive use of nuclear power will probably become essential.22/

The many uses of atomic energy in oceanography are described in a pamphlet entitled, "The Atom and the Ocean" which can be obtained from the Division of Technical Information Extension, U.S. Atomic Energy Commission, P. O. Box 62, Oak Ridge, Tennessee 37830.

E. Research and Teaching Reactors

The potential market for these reactors, both at home and abroad, appears to be nearly saturated. Most institutions that can make effective use of these facilities have been able to build and bring them into operation. However, the need for expansion and upgrading of existing facilities provides a continuing limited market.

In the year ended September 30, 1968, an operating license was issued for a new TRIGA-Mk I (Gulf General Atomic) reactor at Reed College, Portland, Oregon. Two construction permits were issued: to the U.S. Geological Survey at Denver, Colorado, for a TRIGA-Mk I and to Michigan State University at East Lansing for a TRIGA-Mk II. A third construction permit was expected to be issued (probably in October 1968) for an AMF Pulstar reactor at North Carolina State University at Raleigh. Four other educational institutions were planning new reactors. For one of these, the University of California at Irvine, an application for a construction permit for a TRIGA-Mk I had been received. Another, the Mississippi State College at State College, Mississippi, had received a permit to store reactor parts

22/ Remarks by Dr. Glenn T. Seaborg, Chairman, USAEC, "The Proliferation of the Peaceful Atom," at the 24th Annual National Conference of the American Public Power Association, Denver, Colorado, May 11, 1967, AEC Public Release S-20-67.

but had not started assembling the reactor. (This reactor had originally been located at North Carolina State University where it was first operated in 1953. It was dismantled by N. C. State in 1963 and transferred to Mississippi State in 1966.)

One factor tending to limit orders for new reactors of this type has been the difficulty experienced by many owners in finding work to help finance operating costs. This has been alleviated to some degree, in some instances, by arrangements with electric utilities under which reactor owners have been training utility personnel. Electric utilities may want to take advantage of these facilities for this purpose.

In upgrading existing facilities, interest has centered primarily on going to higher operational power levels to achieve higher fluxes and broaden the usefulness of the reactors in activation analysis work. In the reactors most often under consideration, the TRIGAs and PULSTARs, pulsing capability is an additional feature.

Although more than ten companies have been interested in this field and have built more than 80 of these small research and training reactors in the past, only two firms appear to be actively looking for this business at this time: Gulf General Atomic (TRIGAs), San Diego, California, and Hittman Associates (PULSTARs), Columbia, Maryland.23/

Three university reactors are now being upgraded, and modifications to nine others are under consideration.

F. Space Systems24/

The objective of the AEC space program is to develop nuclear energy devices to produce electrical power, and a nuclear rocket for propulsion applications, for missions of the National Space Program. The AEC program is guided largely by the results of studies of energy requirements conducted by or in coordination with missions planners in the user agencies. These studies lead to performance objectives which define needed technology capabilities. In addition to these technology needs, actual space hardware systems are developed at the request of the NASA and DOD agencies to meet needs of specific space missions. Several nuclear power generators have been operated in space, one unit, SNAP-3A, aboard the Transit IV Satellite, is still operating after seven years in orbit.

23/ Final negotiations are underway to assign all rights to the PULSTAR, originally developed by AMF Atomics, to Hittman Associates.

24/ Milton Klein, Director, Division of Space Nuclear Systems, USAEC, AEC Authorization Legislation, Fiscal Year 1969, Part 2.

1. Propulsion

During the past year, the major emphasis in the joint NASA-AEC Nuclear Engine for Rocket Vehicle Application (NERVA) program was on the completion of the technology phase and NERVA engine development. The objective of the NERVA engine development effort is to provide an advanced nuclear rocket engine of approximately 75,000 pounds thrust, with a specific impulse of approximately 825 seconds, an endurance capability of approximately one hour, the capability for multiple restarts in space and the highest reliability possible. Apart from NERVA, work also continued under the program in support of advanced research and technology activities, e.g., advanced reactor technology, advanced non-reactor component technology, and stage technology. The objective of this work is to advance the technologies pertinent to nuclear rocketry beyond the initial NERVA performance objectives.

The NERVA technology effort is directed toward the accumulation of data and experience on which to base the development of the NERVA engine. Since 1964, twelve major test programs have been successfully completed at the Nuclear Rocket Development Station (NRDS) in Nevada in support of this objective. Ten were tests of nuclear rocket reactors and two were tests of experimental ground-based nuclear rocket engines. Two of these test programs were conducted during the past year. These were the Phoebus-2A reactor test program and XECF (ground-experimental engine/cold-flow) test program.

The Phoebus-2A reactor was the most powerful nuclear rocket reactor ever to be tested at NRDS. Originally, it was planned to demonstrate high-power reactor performance for direct application to a 200,000 pound thrust NERVA-2 engine. When the proposed NERVA engine thrust level was reduced to 75,000 pounds, the Phoebus-2A reactor became a test article for demonstrating high-power-density operation, for obtaining data on reactor design concepts being considered for use in the 75,000 pound thrust engine, and for studying new modes of reactor control. In two power cycles, the Phoebus-2A was operated for a total test time of approximately 62 minutes, about 12 minutes of which were at a power level above 4,000 megawatts (4,200 megawatts, peak).

The Experimental Engine Cold Flow (XECF) tests were the first to be conducted under simulated altitude conditions in the new down-firing test stand at NRDS, Engine Test Stand No. 1. The major objective of the engine experiments was to verify that Engine Test Stand No. 1 was ready for hot engine operation. All systems operated as intended and all primary test objectives were met.

[illegible]

A reactor of approximately 1,500 megawatts, based on the current Phoebus and NERVA reactor technology, will be used to heat the liquid hydrogen propellant, flowing through the engine at 90 pounds per second, to the 4,500-degree Rankine chamber temperature required to achieve this performance. It has been established that a single engine of this size can be used to perform all of the missions for which nuclear rockets are currently being considered.

During the past year, preliminary planning in preparation for the development of the 75,000 pound thrust engine was completed, and a development schedule was defined which indicated preliminary flight tests could be accomplished in seven to eight years. In addition, work proceeded on the preliminary design of the NERVA reactor and critical engine hardware, i.e., the nozzle, reactor pressure vessel, and turbopump, and on modifications required to test the NERVA engine in Engine Test Stand No. 1.

The major contractors that are involved in the joint AEC-NASA nuclear rocket program are Los Alamos Scientific Laboratory, Aerojet-General, and Westinghouse.

2. Auxiliary Power

Nuclear auxiliary power sources have successfully operated in orbit generating electricity using the heat of radioisotopes and a compact fission reactor.

The development program is designed to provide technology for those categories of future missions wherein nuclear systems promise advantages over solar cells, their main competitor for missions of longer than a few weeks duration. These consist mainly of missions on the moon (where nights last 350 hours), missions in which spacecraft must go to distant planets, where the invulnerability of nuclear devices to space radiation belts is at a premium, or for spacecraft requiring tens of kilowatts or higher.

Consideration of these factors and the likely future missions reveals several categories of space nuclear power needs, by power level, as shown in Table IV-12.

The dividing lines between these categories are not exact and inflexible; however, there are special programmatic and technical problems which define each category. At power levels up to about 10 kw, isotope systems are attractive; at power levels above a few kilowatts, reactors are advantageous. There is some overlap between these two energy sources in the range of 1-10 kilowatts.

TABLE IV-12

CATEGORIES OF NUCLEAR SYSTEMS AND TECHNOLOGY

CATEGORY	SYSTEM CHARACTERISTICS	ESTABLISHED SYSTEMS	PRINCIPAL TECHNOLOGY GOALS
0-100 W	Self-Contained Isotope - Thermo Electric (TE)	SNAP 3A, 9A, 19, 27	
100-1000 W<br>a. Short Life (2-5 Mos.)<br>b. Long-Life	Modular Isotope TE<br>Short Half-Life<br>Reusable Fuel and or Higher Efficiency Power Conversion	--	Increase Fuel and Capsule Temperature<br>Increase Power Conversion Efficiency<br>Increase TE Life
1-10 KW<br>a. Recoverable Fuel	Modular, Reusable Isotopes and High-Efficiency Power Conversion	--	Reduce Power Conversion System Weight
b. Unrecovered Fuel, Unmanned	Partly Shielded Reactor	SNAP 10A	Develop Higher Temperature to Reduce Specific Weight and Radiator Area
10-100 KW	Reactor-TE (10-25 KW)<br>Reactor-Rankine (25-100 KW)	--	Develop More Efficient Power Conversion
100 KW AND ABOVE	Reactor-Thermionic or Reactor Rankine	--	Increase Power Conversion System Life

In the lowest power ranges, the spacecraft tend to be small, so that the small, self-contained radioisotope-thermoelectric generator (RTG) is used. Within this low-power region, fuel cost is a small enough part of the overall mission cost that the current 5 percent thermoelectric efficiency seems to be acceptable. Also, long lifetime is important; for example, if the useful lifetime of a satellite can be doubled by use of an isotopic power system, launch costs required to keep the satellite system operating can be halved. In this 100-1000 watt category, certain short-lived (3-5 months) earth orbit missions have been identified by the Department of Defense. The use of the cheaper fuel, Po-210 (half-life 138 days), for these missions is possible because of the short mission life. For longer-lived missions (years) at these power levels, or above these power levels, a more economical approach is required than the Pu-238 (half-life 87 years) one-stage thermoelectric concept. At 5 percent efficiency, fuel costs are large; and, in the general case, the reference power concept in this region will require technology that promises

either cheaper, more readily available fuel, the capability of fuel reuse, or improved efficiency which will allow better utilization of available fuels.

In the 1-10 kw region, two types of systems are of interest. For manned missions (or any missions that require radiation-sensitive payloads), Pu-238 fuel will be acceptable if the fuel can be recovered and reused and if high-efficiency power conversion is available, such as the Brayton or organic Rankine cycle. For unmanned missions, permitting low payload shielding, the SNAP-10A class of zirconium hydride reactors at temperatures of 1000-1300°F with thermoelectric conversion is available.

In the 10-100 kw region, the reactor-thermoelectric and reactor-Rankine systems can provide the power plants for a lunar base, TV broadcast satellites, manned orbiting stations, and manned and unmanned interplanetary spacecraft.

Finally, at power levels of more than 100 kw, the performance expected from the advanced reactors -- the in-core thermionic or the advanced liquid metal-cooled reactor with potassium Rankine power conversion -- is most attractive.

The third column of Table IV-12 shows the nuclear power systems which are now available, as they fit these power categories. Only in the 0-100 watt range and the 1-10 kw range are there established systems, the SNAP-3A, 9A, 19, and 27, fitting the low-watt range, and the SNAP-10A available in the low-kilowatt range. The AEC program is aimed at providing the technology which will permit the development of systems to fill the voids in this list, as well as lead to improvements as necessary in the existing systems.

To do so, the program includes the technology goals listed in the final column of Table IV-12. In the isotope area, higher heat source temperature and higher power conversion efficiency are needed. These factors become more important as power level requirements increase. Similarly for reactors, at the lower power levels a relatively low-temperature reactor and thermoelectric power conversion technology are acceptable. But as power levels go up, advanced, high-temperature reactors and longer-lived, more efficient power conversion will be needed. The program is directed to the achievement of these basic goals.

These categories are wide enough that a single, integrated design will not be attractive over the whole range. But sub-sized modules can be designed which can serve as building blocks to create attractive power systems over each range of interest.

Development of these modules will then bring the concept to a state of readiness for adoption by a spacecraft designer. Such an approach will also allow demonstration of lifetime capability of these modules, avoiding or reducing the need to add long-life demonstration time to the usual 2-3 year lead time from time of mission approval to launch. The AEC program is directed at acquiring the technology needed for each of these categories, and conducting the work needed to bring these concepts to a state of readiness. The program is coordinated with a NASA program in the area of high-power, dynamic power conversion technology.

Table IV-13 indicates the isotopic and reactor power systems which are or have been under development. In addition to these, at the request of the Department of Defense, AEC is starting development of a 20-watt RTG for the improved Navigation Satellite (NAVSAT).

Table IV-14 is a summary of existing space electric power development contracts, including the FY-1968 budget level and the expected FY-1969 levels.

3. Industry and Space

Because of the broad range of disciplines involved in the nuclear space program, a great deal of scientific and technological information has evolved which is of value not only to aerospace, but to the Nation's consumer economy as well. A cooperative AEC-NASA effort has been in operation for over a year, aimed at announcing these developments to the public through a Technology Utilization (TU) Program.

A small sampling of recent disclosures includes:

....New High Temperature Thermocouples
....A number of highly practical Computer Codes useful in the teaching of nuclear physics and in the design of commercial reactors
....Materials Properties Handbooks which fill-in serious gaps in the literature
....Electronic circuits with fail-safe, high reliability features of significant value to commercial processing, medicine, etc.
....New machine and hand tools
....Workmanship Standards for Welding
....Radioactive techniques for measuring surface areas

TABLE IV-13

Radioisotope Power Systems

Radioisotope power system & prime contractor*	Development Period: Started (Fiscal year)	Development Period: Completed (Fiscal year)	Design Operational Life	Generator power level (electrical watts)	Fuel	Total Development cost (millions)	Application	Development Status
SNAP-1 and 1A (Martin)	1956	1960	60 days/1 yr.	250-500	Cerium-144	$ 7.7	DOD space missions	Terminated in favor of reactor heat source
SNAP-3 (Martin)	1959	1961	90 days	3	Polonium-210	.7	AEC demonstration	Completed
SNAP-3A (Martin)	1961	1962	5 years	2.7	Plutonium-238		Transit 4A and B	Launched June 1961 and November 1961
SNAP-9A (Martin)	1961	1963	5 years	25	Plutonium-238	1.3	Navigation satellite (DOD)	Launched June 1963, December 1963, and April 1964
SNAP-11 (Martin)	1962	1967	90 days	25	Curium-242	2.3	Surveyor (NASA)	Experimental unit fueled and tested
SNAP-13 (thermionic unit) (Martin)	1962	1967	90 days	12.5	Curium-242	1.7	AEC demonstration unit	Experimental unit fueled and tested
SNAP-17A & 17B (Martin/GE)	1964	1965	3 to 5 years	30	Strontium-90	1.6	Communications satellite (DOD)	Terminated after preliminary design and fabrication test of T/E components
SNAP-19A (Martin)	1963	1964	5 years	20	Plutonium-238	.4	Imp satellite (NASA)	Terminated after completion of a design and component test
SNAP-19B (Isotopes, Inc.**)***	1964	1968	5 years	30	Plutonium-238	9.1	Nimbus B (NASA)	Nimbus launch aborted due to booster failure, May 68. Replacement unit being assembled for early 1969 launch
SNAP-2[illegible] (Lockheed)			5 years	75	Plutonium-238		Various satellites	Development not initiated
SNAP-27 (General Electric)	1966	1969	5 years	50	Plutonium-238	15.0	ALSEP (NASA)	5 flight units, including [illegible] follow-on units, delivered 1969
SNAP-29 (Isotopes, Inc.**)	1966	1970	90 days	400	Polonium-210	34.0	Short lifetime space missions (DOD/NASA)	Preliminary design phase completed; component development underway; fueled ground demonstration planned for FY 1970

Reactor Power Systems

Reactor power system**** and prime contractor*	Development Period: Started (Fiscal year)	Development Period: Completed (Fiscal year)	System power (electrical)	Reactor power (thermal) kilowatts	Reactor type	Total development cost (millions)	Application	Development Status
SNAP-2 system (AI)	1956	1966	3 kilowatts	60	Thermal	$39.9	DOD space missions	System development terminated after withdrawal of DOD support. Technology continued under other space electric power programs
SNAP-8 reactor (AI)*****	1960	1971	35 kilowatts	600	Thermal	63.4	NASA space missions	Plan to test the S8DR reactor with flight-type control system in fiscal year 1969-70
SNAP-10 system (AI)	1960	1966	500 watts	40	Thermal	78.8	DOD space missions	System successfully launched in April 1965; operated 43 days prior to non-reactor oriented shutdown
PWAR-20 (including LCRE) (Pratt & Whitney)	1962	1965	300 kilowatts	2,200	Fast	84.4	High power space missions	Reoriented and assigned to LRL as liquid metal-cooled reactor technology program; terminated due to budget priorities, 1968

* Systems designated SNAP 5, 7, 15, 21, 23, are terrestrial power systems
** Martin Nuclear Division sold to Isotopes, Inc., August 1968
*** Includes $1,400,000 for expanded safety
**** System designated SNAP-4 was a terrestrial system; SNAP-6 was conceptual study only of a [illegible] electrical kilowatts terrestrial generator
***** R&D development funded by NASA; total shown is estimated to completed S8DR testing

TABLE IV-14

Space Electric Power Development
Operating Costs - FY.1968 and FY-1969
(In Millions)

	Fiscal Yr. 1968	Estimate Fiscal Yr. 1969	Major Contractors
Total Space Power Development Program	$ 53.6	$ 42.2	
Radioisotope Power System	17.8	13.7	
SNAP-19B (Nimbus)	2.6	.5	Martin Co.
SNAP-27 (ALSEP)	4.7	1.5	General Electric (MSD)
NAVSAT	.5	2.5	(Not under contract for system)GA
SNAP-29 (Po-210 Space Generator Dev.)	6.7	6.0	Martin Co.
Thermionic Space Generator Dev.	0	.3	General Electric (MSD)
Supporting Technology (Incl. Capsule Tech.)	3.3	2.9	Sandia, Hittman, AI, Johns Manville, TEECO, Union Carbide
Reactor Power Development	18.0	12.0	
Zirconium-Hydride Reactor	6.1	5.9	AI
Liquid Metal Cooled Reactor	4.6	.1	LRL
Gas Cooled Reactor	1.6	0	General Electric
Thermionic Reactor	5.0	5.5	TEECO, RCA, LASL, ORNL, General Atomic, GE
Supporting Technology	.7	.5	ORNL
Power Conversion Development	3.9	4.2	
Direct Radiating Converter	.2	.2	AI and RCA
Compact Converter	1.7	1.8	Westinghouse, ICA
Lightweight Converter	.3	.3	General Atomic
Modules and Materials	.7	1.4	RCA, 3M, GE
Mercury Rankine Cycle	.1	0	AI and TRW
Potassium Rankine Cycle	.2	0	Westinghouse
Organic Rankine Cycle	.6	.5	Philco - Sundstrand - AOC
Space Nuclear Safety	4.0	2.9	Sandia, AI, LASL, NRDL
Isotope Fuels (Space)	9.9	9.4	Mound, ORNL, BMI, LASL, ANL, Sandia, PNL (Battelle), NRDL

A large number of additional items for disclosure through the technology utilization system have been catalogued. These items vary from basic hardware, to advanced new materials such as carbide coatings, to fundamental mathematical theories. Coupled with the ideas being reported from the on-going nuclear rocket program, this added backlog of useful technology is certain to find its way to the market place.

G. Aircraft Nuclear Propulsion

Lockheed Aircraft Corporation's C-5A aircraft achieved its first test flight during the year. Lockheed has indicated that it is studying the possibility of using nuclear power for propulsion of the aircraft to extend its range capabilities. The use of nuclear power for aircraft continues to be studied by NASA and the Air Force. Two articles on the subject of nuclear power for aircraft were published in the March issue of Astronautics and Aeronautics magazine. There is no activity within the AEC at present directed toward nuclear aircraft propulsion.

CATEGORY V RADIOISOTOPES AND RADIATION APPLICATIONS

Because of the many facets of industry's participation in this category, we have divided our discussion into the following sections:

A. Radiochemicals and Radiopharmaceuticals

1. Producers

a. Cyclotron
b. Reactor
c. Fission Product

2. Processors

3. Source Fabrication

B. Radiation Equipment Suppliers

1. Radiographic Units

2. Teletherapy Units

3. Radioisotope Gauges

4. Self-Illuminating Materials and Devices

C. Commission Licensing Actions in Radioisotopes Area

D. Radioisotopic Power

E. Radiation Processing

1. Review of Radiation Processing Applications and Industrial Interest

2. Firms Associated with Radiation Processing

a. Principal Firms Supplying Electron Accelerator Equipment and/or Services or Finished Products

b. Commercial Irradiation Processing Facilities

c. Architectural Design Engineering and Construction

d. Irradiation Equipment, Design, and Fabrication

e. Radiation Processing Source Suppliers and Encapsulators

3. Irradiation Processing of Food

F. Industry Changes

G. AEC Advisory Committee on Isotopes and Radiation Development

H. Science Information Exchange Program

A. Radiochemicals and Radiopharmaceuticals

The domestic market for radiochemicals and radiopharmaceuticals has increased during the past year by about 25 percent over 1967. It is estimated that the radioisotope industry's growth will continue at this rate for the next several years.

1968 Market Prediction

Radiochemicals	$10-$13 million
Radiopharmaceuticals	$12-$15 million
TOTAL	$22-$28 million

The estimated private sales of radiochemicals include the value of AEC-supplied radioisotopes purchased by private suppliers for further processing or repackaging and distribution.

Radioisotopes of commercial interest fall into four classes: those found in nature, such as radium-226; those deliberately produced in nuclear reactors, such as cobalt-60 and carbon-14; those unavoidably produced in nuclear reactors through the fission process, such as strontium-90 and cesium-137; and those produced in accelerators, such as sodium-22 and iodine-123. A radiochemical is a chemical compound prepared with a radioactive element. A radiopharmaceutical is a radiochemical which has been prepared to meet requirements for use in the human body.

We expect the market for radioisotopes will continue to expand at the rate of 25 percent annually, as a result of new developments and increasing requirements for applications in areas such as radiation processing, medicine, and isotopic-fueled power systems. Industry

continues to make significant increases to its capacity to produce, process, and distribute isotopes. While the packaging and distribution of radioisotopes is performed to a great extent by private industry, the AEC is still one of the principal producers and distributors.

As in our two previous reports, we have divided the radioisotope industry into four categories: producers, processors, source fabricators, and equipment manufacturers.

1. Producers

A producer is defined as one who can perform target irradiation and may also perform target preparations and separation of the desired radioisotope for ultimate distribution. Radioisotopes are obtained from cyclotron bombardments, reactor irradiations, and from high-level waste streams.

a. Cyclotron Radioisotopes

In 1955, the AEC discontinued processing and distributing cyclotron-produced radioisotopes. Except for target preparation and irradiation services provided by Oak Ridge National Laboratory or by the University of Pittsburgh, the industry supplies cyclotron radioisotopes. An indication of the interest in cyclotron-produced radioisotopes may be obtained from the AEC revenue for special cyclotron irradiations listed below:

FY-1964	-	$126,508
FY-1965	-	$258,665
FY-1966	-	$277,713
FY-1967	-	$258,236
FY-1968	-	$163,670

Cyclotrons can generate a wide variety of radioisotopes which cannot be produced in reactors. Many have unique properties which make them especially useful or necessary for certain applications, in spite of their relatively high cost. For example, the use of iodine-123, with a half life of 13 hours, instead of iodine-131 for in-vivo thyroid uptake studies, reduces radiation exposure to patients substantially because it emits no beta radiation. It is also very promising for brain scans and cancer research. Because of the special interest in cyclotron-produced I-123 by the medical profession and with the encouragement of the private processors, the Commission makes it available on a special order basis as a processed target, similar to Rb-84 and Co-57. Other cyclotron-produced radioisotopes of interest include: sodium-22, cobalt-57, rubidium-84, carbon-11, nitrogen-13, oxygen-15, iron-55, mercury-199, chromium-49, copper-61,

zinc-62, and selenium-73.

Because of the very short half life of the radioisotopes desired for medical applications and the difficulty in transporting useable quantities over great distances from the cyclotron, there is a need for commercial cyclotron facilities near large population centers to provide medical researchers and nuclear medicine departments with the necessary isotopes on a timely basis.

Small cyclotrons, designed for medical research and isotope production, are now in use and planned at the following locations:

Washington University Medical Center, St. Louis, Missouri (first commercially installed unit)
Massachusetts General Hospital, Boston, Massachusetts
Sloan-Kettering Institute, New York, New York
Argonne Cancer Research Hospital, Chicago, Illinois
New England Nuclear Corporation, Boston, Massachusetts

The Cyclotron Corporation, Berkeley, California, supplied or has on order the last three units listed above and Allis-Chalmers supplied the Washington University Medical Center and the Massachusetts General Hospital machines. Other facilities are planned in the future as the National Institutes of Health (NIH) and AEC funding permits. It has been estimated that a market for some 20 such facilities may develop for medical radioisotope production by 1975.

The present market for cyclotron-produced radioisotopes is estimated at about $1.5 to $2 million per year. While the U.S. has been the principal supplier, cyclotron radioisotopes are also marketed by the United Kingdom, South Africa, and the USSR.

The principal industrial suppliers of cyclotron radioisotopes are listed in Table V-1.

TABLE V-1

Principal Industrial Suppliers of Cyclotron Radioisotopes

Abbott Laboratories, North Chicago, Illinois
Cambridge Nuclear Corporation, Cambridge, Mass.
Hastings Radiochemical Works, Houston, Texas
International Chemical and Nuclear Corporation City of Industry, California
Isotope Products Lab, Los Angeles, California
New England Nuclear Corporation, Boston, Mass.
Nuclear Consultants, Division of Mallinckrodt Chemical Company, St. Louis, Missouri

b. Reactor Radioisotopes

As indicated previously, the AEC is still a principal domestic producer and distr'butor of radioisotopes, including those produced in reactors. However, the industry is offering an increasing number of products and higher specific activity material than in past years.

Commercial reactor irradiation services, for production of radioisotopes in targets supplied by customers, are offered by the following facilities: (1) General Electric (Pleasanton, California), (2) Union Carbide (Tuxedo, New York), and (3) Babcock & Wilcox (Lynchburg, Virginia). In the future, it is expected that additional private reactors, especially power reactors, will become available for large volume radioisotope production. In a first such venture, Neutron Products, Inc. (Dickerson, Maryland) has been producing Co-60 in the Big Rock Point nuclear power reactor owned by Consumers Power Company.

Up to 1965, the Commission had withdrawn, on an ad hoc basis, from producing and distributing selected radioisotopes. In March 1965, the AEC adopted and published in the Federal Register formal policies and procedures for the transfer of commercial AEC radioisotope production and distribution activities to private industry. These policies and procedures provide for orderly AEC withdrawal from this area of activity.

Since our last report, the AEC has withdrawn from production of low specific activity cobalt-60,[1/] making a total of 38 reactor-produced isotopes that have been withdrawn from routine production. AEC earlier had withdrawn from furnishing high specific activity Co-60.

Table V-2 lists AEC sales of radioisotopes.

TABLE V-2

AEC Sales of Radioisotopes

Fiscal Years	Million Dollars
1963	$ 1.2
1964	1.3
1965	1.9
1966	2.3
1967	1.8
1968	1.8

1/ See AEC Public Release K-290, dated December 27, 1967.

In September 1968, the AEC reduced prices for large quantity orders of tritium and polonium-210.[2] The AEC is also considering reducing its price for carbon-14 to assist in meeting price competition from foreign suppliers. The full cost of production and distribution would still be recovered for these isotopes at the reduced prices.

The AEC made available for sale limited quantities of plutonium-238,[3] californium-252, americium-243, and curium-244 for use in private research and development. Pu-238 is useful for isotopic heat sources and alpha and neutron sources.

Cf-252 is the most concentrated source of neutrons known and will enable significant improvements and the miniaturization of such analytical tools as activation analysis and gauging applications.

Reactor-produced radioisotopes are used in a large number of applications: to name a few, medicine, agriculture, crime detection, industrial gauges, and space. As indicated in the introduction, the market is estimated to grow at about 25 percent annually for the next several years.

There are currently more than 5,000 doctors and medical organizations in the United States, alone, routinely using some 30 different radioisotopes in the diagnosis and treatment of many diseases. Some of the isotopes and applications include: cobalt-60 as a source of radiation for treating cancer; iodine-131 is used in blood volume and cardiac studies, to locate brain tumors and diagnose kidney and liver disorders, but primarily it is used for diagnosing and treating thyroid disorders (over one-half million thyroid cases annually); carbon-14 is used to study metabolic diseases; sodium-22 and 24 are used to measure blood volume and diagnose circulatory disorders; gallium-68 and strontium-85 are used in bone scanning; technicium-99m is used to make radio-cardiograms; phosphorus-32 in locating eye tumors; and iron-59 in hemoglobin studies. It is estimated that between three and four million patients each year are benefiting from medical procedures using isotopes. About 98 percent of these procedures are diagnostic.

Foreign competition in the radioisotope market is increasing and will continue to be a problem for American

---

2/ See AEC Public Release L-232, dated September 27, 1968.

3/ See AEC Public Release L-71, dated April 19, 1968.

firms. This is especially so in the cobalt-60 market where Canada, the United Kingdom, and Australia are major suppliers.

c. Fission Product Radioisotopes

In addition to cyclotron and reactor-produced radioisotopes, fission product radioisotopes are separated from high-level waste streams from AEC production facilities. The radioisotopes from fission products of principal interest are Sr-90, Cs-137, and Pm-147. At present, fission product isotopes are obtained from waste streams in the Richland chemical separations plant and are distributed through the Oak Ridge National Laboratory, Isotopes Sales Department. These fission products will also be available in large quantities in the future, when their production is warranted by market demands, from commercial firms operating chemical processing plants, such as Nuclear Fuel Services, Inc., Allied Chemical Company, and General Electric Company. It is expected that the expanding uses of these isotopes may eventually create a source of revenue for utilities from these by-products resulting from reprocessing of fuel from reactor operation.

Fission product radioisotopes are used in a number of industrial applications, as well as for space, power, and heat purposes. Promethium-147 and strontium-90 are used in radioisotope power generators. Strontium-90 is also employed in industrial thickness gauges and in the treatment of certain eye disorders. Promethium-147 is also used to prepare and activate luminous sources. Cesium-137 is used as a radiation source for both industrial and medical uses.

AEC revenue for the distribution of the above three radioisotopes is given below for FY-65, FY-66, FY-67, and FY-68.

Revenue in $

	1965	1966	1967	1968
Cesium-137	192,000	27,000	60,000	83,000
Promethium-147	28,000	12,000	55,000	56,000
Strontium-90	3,000	65,000	59,000	188,000
TOTAL	$223,000	$104,000	$174,000	$327,000

During the year, the AEC reviewed its price schedules for the fission product isotopes. In June 1968, the Commission announced its intention to increase the price of cesium-137 to recover full costs of production and distribution[4/] and in September 1968, decided to retain the existing price schedules for strontium-90 and promethium-147.[5/] The Commission had reduced the prices of the above three fissionproduct radioisotopes in November 1965 to assist in stimulation of a market, primarily to assist the then-planned private fission products conversion and encapsulation plant to be operated by Isochem, Inc. As reported previously (1967 report) plans for this plant were canceled in early 1967.

2. Processors

A processor is defined as one who can prepare irradiation targets and (subsequent to irradiation in an accelerator or nuclear reactor) can separate and prepare the desired radiochemicals or radiopharmaceuticals for ultimate distribution.

A number of changes in the radioisotope industry have taken place since our last report. The following companies were involved: General Nuclear, Inc. (Houston, Texas) purchased General Radioisotope Processing Company of San Ramon, California; Teledyne Inc. acquired the Nuclear Division of Martin-Marietta Corporation; Cambridge Nuclear Corporation acquired Nuclear Components, Inc.; G. D. Searle, Inc., formed a joint company with the UKAEA Radiochemical Center Amersham group known as Amersham/Searle Corporation.

The principal commercial firms in this area are listed in Table V-3.

3. Source Fabrication

There are approximately 30 commercial firms producing alpha, beta, gamma, and neutron sources. The current volume of business in radioactive sources is estimated at $2.5-$3 million per year, significantly higher than our previous estimate, and is based on information furnished by several of the principal suppliers.

The principal commercial firms supplying radioactive sources are listed in Table V-4.

4/ See AEC Public Release L-118, dated June 6, 1968.

5/ See AEC Public Release L-209, dated September 6, 1968.

TABLE V-3

Principal Processors of
Radiochemicals and Radiopharmaceuticals

	Radio-Chemicals	Radio-Pharmaceuticals
Abbott Laboratories, North Chicago, Ill.		X
Atomic Corp. of America, Sun Valley, Calif.	X	
Bio-Nuclear, Houston, Tex. (Div. of Bio-Assay Labs)	X	
Bio-Rad Laboratories, Richmond, Calif.	X	
Calbiochem, Los Angeles, Calif.	X	
Cambridge Nuclear Corp., Cambridge, Mass.	X	X
General Electric Co., Pleasanton, Calif.	X	
General Nuclear, Inc., Houston, Tex.	X	
Hastings Radiochemical Works, Houston, Tex.	X	X
International Chemical & Nuclear Corp., Burbank, Calif.		
INC Division, City of Industry, Calif. (includes Volk Radiochemical)	X	
Nuclear Science Div., Pittsburgh, Pa.	X	
U.S. Nuclear Corp., Burbank, Calif.	X	
Isotopes Inc., Westwood, N. J. and Baltimore, Md. (Subsidiary of Teledyne)	X	
New England Nuclear Corp., Boston, Mass.	X	X
Nuclear-Chicago Corp., Des Plaines, Ill. (G. D. Searle Company)	X	
Nuclear Consultants Corp., St. Louis, Mo. (Div. of Mallinckrodt Chemical Works)	X	X
Nuclear Equipment Chemical Corp., Farmingdale, N. Y.	X	X
Schwartz Bio-Research Inc., Orangeburg, N. Y. (Sub. of Becton, Dickinson & Co.)	X	
E. R. Squibb & Sons, New Brunswick, N. J.		X
Tracerlab, Waltham, Mass. (Div. of L.F.E. Inc.)	X	
Union Carbide Corp., Tuxedo, N. Y.	X	X

[illegible]

TABLE V-4

Principal Suppliers of Radioactive Sources

Atlantic Research Corp., Alexandria, Virginia
Bio-Nuclear, Div. of Bio-Assay Labs, Houston, Texas
The Budd Co., Phoenixville, Pa.
Gamma Industries, Inc., Baton Rouge, La.
General Nuclear, Inc., Houston, Texas
Hastings Radiochemical Works, Houston, Texas
Minnesota Mining and Manufacturing Co., St. Paul, Minn.
Monsanto Research Corp., Dayton, Ohio
Nuclear-Chicago Corp., Des Plaines, Ill.
Nuclear Materials & Equipment Corp., Apollo, Pa.
Picker X-Ray, Technical Operations, Inc., Burlington, Mass.
Radionics Corp., Norristown, Pa.
Tracerlab, Div. of L.F.E., Waltham, Mass.
U. S. Nuclear Div. of ICN, Burbank, Calif.

B. Equipment Manufacturers

There are six principal areas of interest in equipment: these are radiographic and teletherapy units, gauges, irradiators, isotopic power devices, and self-illuminating devices.

1. Radiographic Units

Radiographic units are manufactured by industry and not by the AEC. The commercial firms active in this field are listed in Table V-5.

TABLE V-5

Principal Suppliers of Radiographic Units

The Budd Company, Phoenixville, Pennsylvania
Gamma Industries, Inc., Baton Rouge, Louisiana
Picker X-Ray, Technical Operations, Inc., Burlington, Mass.
U.S. Nuclear Corp., Burbank, Calif., (a subsidiary of International Chemical & Nuclear Corpor[illegible]ion)

The annual volume of sales in this field is approximately $1.5 million and is expected to continue to increase. The radioisotope units, utilizing such isotopes as iridium-192, cobalt-60, cesium-137, and thulium-170, compete with X-ray machines.

2. Teletherapy Units

There are eight commercial suppliers of teletherapy units. Four of the eight suppliers (General Electric, North American Philips Company, Siemens New York, and Schick X-Ray Company) are distributors of foreign-made teletherapy units. The firms active in this field are listed in Table V-6.

TABLE V-6

Principal Suppliers of
Radioisotope Teletherapy Units

W. F. and John Barnes Company, Automated Machine Division of Babcock & Wilcox, Rockford, Illinois

General Electric Company, Milwaukee, Wisconsin

Laboratories for Electronics, Waltham, Massachusetts (Tracerlab)

North American Philips Company, Inc., New York, New York

Picker X-Ray Company, Cleveland, Ohio

Schick X-Ray Company, Chicago, Illinois

Siemens America, Inc., New York, New York

Westinghouse Electric Corporation, Baltimore, Maryland

Since our last report the IAEA has published a new "Directory of High Energy Radiotherapy Centres"6/ which lists radioisotope and other high energy teletherapy installations throughout the world. Some 1,578 radiotherapy centers in 62 countries are listed with details on 2,074 treatment units.

The world total of 2,074 units includes 1,676 cobalt-60 units and 140 cesium-137 units. Since 1959, the annual increase in cobalt units has been nearly constant at about 160-170 per

6/ Published May 1968, available from IAEA Sales Agents - In USA from National Agency International Publications Inc., 317 East 34th Street, New York, New York 10016, for $3.50.

year. The average new unit being installed in the U.S. contains 3 thousand to 4 thousand curies of high-specific activity cobalt-60, with some containing up to 8 thousand to 10 thousand curies. There are some 258 non-isotope teletherapy units reported including betatrons, linear accelerators, Van de Graaff machines, and high-energy resonant transformer x-ray generators.

3. Radioisotope Gauges

There are between 15 and 20 firms active in the radioisotope gauge field. This type of gauge combines a radioisotope (alpha, beta, gamma, or neutron) source of radiation and appropriate radiation detection elements and control equipment, which permit measurement and control of various processes. These include flow rate, thickness, level, density, and activation analysis systems. The current market for radioisotope gauges is estimated at about $25 million and is expected to grow at about 10-15 percent annually.

The principal suppliers of industrial radioisotope gauges and gauging systems are listed below:

Avionics Corporation, Succasunna, New Jersey
Brun Corporation, Columbus, Ohio
Densitronics, Inc., Los Angeles, California
Electronic Automation Systems, Grand Island, N.Y.
Foxboro Corporation, Foxboro, Massachusetts
General Nucleonics Corporation, Claremont, Calif.
Industrial Nucleonics, Columbus, Ohio
Instruments, Inc., Tulsa, Oklahoma
Gene Lowey, Inc., Hamilton, Ohio
Ludlum Measurements, Inc., Sweetwater, Texas
Nuclear-Chicago Corporation, Des Plaines, Illinois (subsidiary of G. D. Searle)
Ohmart Corporation, Cincinnati, Ohio
Robertshaw Controls Company, Anaheim, Calif.
Tracerlab, Waltham, Massachusetts (Division of L. F. E., Inc.)
Troxler Corporation, Raleigh, North Carolina

Table V-7, on the following page, gives sales of radioisotope gauges, as reported by the Bureau of the Census.

Some examples of radioisotope gauge applications include: measuring and automatically adjusting the thickness of steel sheet, paper, tire cords, textiles, plastics, and other materials; controlling sugar, fat, and meat content in food packaging; measuring soil density and moisture content; measuring snow density for water run-off forecasting; detecting gold; and measuring the denrity of ocean bottom sediments.

TABLE V-7

Sales of Radioisotope Gauges

Year	$ in Millions
1957	$ 6
1958	6
1959	9
1960	10
1961	11
1962	9
1963	9.9
1964	9.7
1965	13.1
1966	14.5
1967	est. 20
1968	est. 25

4. Self-Illuminating Materials and Devices

Beta-emitting radioisotopes, such as krypton-85, tritium, and promethium-147, are used in the preparation of self-illuminating materials and devices. Tritium is the most frequently used radioisotope. The growing use and interest in self-illuminating materials is reflected in the sales of tritium by the AEC given below.

Fiscal Year	Curies	AEC Sales
1963	60,000	$ 95,000
1964	112,000	125,000
1965	118,000	128,000
1966	171,000	196,000
1967	178,874	200,000
1968	178,549	199,000

The firms active in this field are listed in Table V-8.

TABLE V-8

Principal Commercial Suppliers of
Self-Illuminating Materials and Devices

American Atomics, Tucson, Arizona
Canrad Precision Industries, Inc., New York, New York
Laboratories for Electronics, Inc., Waltham, Mass.
Luminous Products Corporation, Boston, Mass.
(Subsidiary of New England Nuclear Corp.)
Minnesota Mining & Manufacturing Company, St. Paul, Minn.
Radium Chemical Company, New York, New York
U.S. Radium Corporation, Morristown, New Jersey

C. Commission Licensing Actions in Radioisotope Area

Since our last report, the AEC has taken a number of significant steps in its regulatory program and has amended, or proposed amendments of, several of its regulations concerning radioactive materials. The most important items are summarized below.

The Division of Materials Licensing issued a booklet, "[illegible] to Get a License to Use Radioisotopes",[7] which explains in n[illegible]echnical terms the objectives of the regulatory program, how it functions, and the safety responsibilities of persons who use radioisotopes.

Effective in November 1967, Part 35 of the AEC's Regulations was amended to simplify the licensing process for the medical use of radioisotopes in diagnostic procedures.[8]

At the request of Abbott Laboratories and Jones Medical Instrument Company of Chicago, the AEC published a proposed amendment of its regulations to provide a general license to physicians, hospitals, and clinical laboratories for the use of iodine-125 and 131 in tests not involving administration of radioactive material to human beings.[9]

In response to numerous petitions received for exemption of by-product materials in self-luminous products, the Commission has revised 10 CFR Parts 30 and 32 of its Regulations to exempt

---

7/ For sale by Superintendent of Documents, U.S. Government Printing Office, Washington, D. C. 20402, for 30 cents.
8/ See AEC Public Release K-243, dated October 13, 1967.
9/ See AEC Public Release IN-865, dated April 26, 1968.

self-luminous products containing tritium, krypton-85, and promethium-147 meeting specific safety standards and for which the end uses can reasonably be foreseen.10/

The AEC amended Part 33 of its Regulations to provide for more flexibility to users, who have small to intermediate sized radio-isotope programs, by establishing new types of broad licenses and expanding the scope of the uses authorized in all broad licenses.11/

In July 1968, the AEC adopted licensing fees, effective October 1, 1968, for facilities and for certain materials licenses. Fees will be charged for licenses for by-product material of 100,000 curies or more in sealed sources used for irradiation of materials; for licenses for special nuclear material in quantities sufficient to form a critical mass (except plutonium-beryllium neutron sources), and for waste disposal licenses specifically authorizing the receipt of waste materials from other persons for the purpose of commercial disposal by the waste disposal licensee.12/

In February, the AEC proposed amendment of its regulations which would redefine the basis of continued regulatory authority in agreement states over the manufacturer's transfer of products whose subsequent transfer is exempt from regulatory controls.

The AEC amended its regulations in April to increase the quantity limit of generally licensed promethium-147 in any single aircraft safety device from 100 millicuries to 300 millicuries.13/

In June 1968, the Commission proposed an amendment to revise the present labeling requirements for generally licensed luminous aircraft safety devices, so that the label could be appropriately used on such devices within either agreement states or non-agreement states.

In August 1968, the Commission proposed to exempt from licensing the use of small quantities of certain isotopes now under general license and to increase the number of radioisotopes to which the exemption would apply from 65 to 171.14/ The proposed amendments to Part 31 are responsive to recommendations made by the Commission's Advisory Committee on Isotopes and Radiation Development and the Commission's ad hoc Radioisotopes Licensing Review Panel.

10/ See AEC Public Release L-138, dated June 21, 1968.
11/ See AEC Public Release L-228, dated September 27, 1968.
12/ See AEC Public Release L-177, dated July 31, 1968.
13/ See AEC Public Release IN-864, dated April 26, 1968.
14/ See AEC Public Release L-187, dated August 9, 1968.

D. Radioisotopic Power

Publication of AEC's report WASH-1095, "Radioisotopes - Production and Development of Large Scale Uses," (which was referenced in Nuclear Industry - 1967) was delayed until May 1968.[15/] This report reviews AEC's current programs and future plans for the development of large-scale uses of radioisotopes in electrical power, thermal energy, and process radiation applications.

The utilization of the heat released, when isotopes decay, for various specialized applications requiring power is now beginning to approach large-scale quantities. The growth of isotopic power applications has been limited by availability of several of the isotopes which, in turn, affects the demands of potential large-scale users such as the DOD and NASA. Also, space and military requirements for isotopic-powered systems have not developed as fast as expected. Detailed forecasts of requirements for Pu-238; Sr-90, for the intermediate lifetime isotopes, Co-60 and Cs-137; and for short-lived isotopes, such as Po-210, Tm-170, and Pm-147; are given in WASH-1095, referenced above, for various applications.

It is expected that the expanding nuclear power economy should contribute significantly to the production of isotopes, such as Pu-238, Cm-244, and Sr-90, as a by-product of reactor operation and chemical reprocessing operations. As mentioned previously, some isotopes may be produced in power reactors through the irradiation of target materials, i.e., Co-60 and Tm-170.

Radioisotopic power applications fall into the following general categories:

1. Space Electric Power

These applications include weather, communications, and navigation satellites; space exploratory power systems, such as that for the Apollo Lunar Surface Experiment; and space mission requirements.

2. Terrestrial Power

Applications in this area include remote weather stations, navigation buoys and beacons, for power on off-shore oil rigs, cardiac pacemakers, and undersea power systems.

15/ Available from the Superintendent of Documents, U.S. Government Printing Office, Washington, D. C. 20402, for 50 cents.

3. Thermal Applications

These applications include spacecraft and swimsuit heaters, waste water reprocessing systems for manned space missions, and as energy sources for a heart pump and small undersea propulsion systems.

4. Isotopic Propulsion Systems

These are primarily for auxiliary spacecraft propulsion.

Markets for Radioisotope Power

The Federal Government is the principal market for these devices at the present time. While forecasts of isotope requirements are given in WASH-1095, the dollar value of power-producing devices cannot really be estimated yet. Substantial commercial markets for applications, such as direct broadcast satellites, marine and remote land power systems, could develop eventually. The cardiac pacemaker and the artificial heart appear to be among the most promising future markets. It is estimated that there could be a need for about 10,000 cardiac pacemakers per year in the future. These units produce 150-200 electrical microwatts of power and do not require large quantities of Pu-238. The need for artificial heart implantation operations has been estimated as high as 100,000 patients or more per year and could use all foreseen supplies of Pu-238, if this device is eventually successfully developed.

Industry

The following companies are active in the isotopic power generation field:

Aerojet-General Corporation, San Ramon, California
Atomics International, Canoga Park, California
Douglas Laboratories, Richland, Washington
Minnesota Mining & Manufacturing Company, St. Paul, Minn.
(NUMEC) Nuclear Materials & Equipment Corp., Apollo, Penna.
Sanders Nuclear Corporation, Nashua, New Hampshire
Teledyne (Isotopes Division), Westwood, N.J. and Baltimore, Maryland

It is expected that other firms will enter this field as the market develops. Significant activities by commercial firms are described on the next page.

NUMEC has shipped one 50 milliwatt generator for Navy testing and sold two 2-1/2 watt units to a foreign customer.

Martin-Marietta Corporation sold its nuclear activities to Teledyne, whose Isotopes (Isotopes, Inc.) subsidiary will take over the Martin contracts and programs. Martin-Marietta now has supplied ten units, nine of which are still operating. Georgia Institute of Technology, the Navy, and Sincl[illegible]r Oil are the principal users. A new company, formed by Martin-Marietta, Offshore Systems, Inc., plans to market an underwater wellhead control system powered by two, three-watt radioisotope generators and a blowout preventer system for underwater wells, also powered by a radioisotope generator.

E. The Radiation Processing Industry

1. Review of Radiation Processing Applications and Industrial Interest

During the year, the AEC's Division of Isotopes Development completed a study of radiation processing applicable to chemical systems. This study was conducted to determine the status of industrial interest and investment in radiation processing, to determine the present attitude of industrial companies regarding radiation processing, and to assess the AEC's programs of research and development in radiation processing.[16/]

While the study has been reviewed in at least one trade journal, a number of the more significant observations and statistics resulting from this study are presented in this section.

The radiation processing market has been estimated, in terms of the value of sales of products processed, at about $100 million in 1967. Industrial radiation processing could possibly grow at a rate of 50 percent annually for the next two years and is expected to grow at 25 percent annually.

AEC has spent some $8 million on radiation processing R&D and industry, an estimated $50 to $75 million over the past 2[illegible] years. The current annual level of support by AEC is $1.4 million and by industry, $10-15 million.

The radiation processing field is divided into machine or accelerator-produced radiation and isotope radiation, each of which possesses some advantages and disadvantages over the other. For example, machines can be operated at variable energy output

---

16/ Copies of this report may be obtained from AEC's Division of Isotopes Development.

levels to accommodate the work to be processed and can be turned off. Isotope radiation cannot be turned off, but is suitable for bulk processing of chemicals and products requiring great penetration where machine radiation is inadequate. In applications where a low dose rate is a factor, such as the polymerization of a monomer in a fibrous substance (i.e. wood plastics), isotopes are most suitable. At the present time, of the total energy output from radiation sources in irradiation facilities, 90 percent is delivered by machines.

The most promising radiation processing applications in use or under development are listed below.

Curing of surface coatings by machine irradiation of special lacquers and paints. The Boise Cascade Corporation using the Electrocure process under license to the Ford Motor Company, and the Radiation Dynamics, Inc., Dynacote Process, are the principal firms. PPG Industries is also doing development work in this area.

Irradiation of wood plastic combinations to produce superior qualities in strength, appearance, and surface properties has been underway for some time by American Novawood Corporation and Lockheed-Georgia Company. Atlantic Richfield Company, Gamma Process Company, and Radiation Machinery Corporation have recently entered this field. All of the above firms use cobalt-60.

Crosslinking of polyethylene films to produce special wire coatings, insulating materials, heat shrinkable packaging film, and bags of improved physical properties. The major companies in this area are Raychem, W. R. Grace (Cryovac Division), High Energy Processing Corporation, the Electronized Chemical subsidiary of High Voltage Engineering Corporation, and International Telephone & Telegraph.

Preparation of specialty copolymers for use as battery separators for Department of Defense requirements. Radiation Applications, Inc., and High Energy Processing Corporation are active in this area.

Synthesis of new graft copolymer fibers with improved "crease resistance" and "soil resistance" properties by Deering-Milliken Corporation.

Synthesis of ethyl bromide by Dow Chemical Company.

The controlled degradation of polyox, a polyethylene oxide polymer, by Union Carbide Corporation.

2. Firms Associated with Radiation Processing

In January 1968, the Business and Defense Services Administration of the Department of Commerce released a new "Guide to Services, Suppliers, and Research on Food Irradiation."17/ The guide lists commercial firms in all areas of radiation processing, including design, engineering, and construction of facilities; design and fabrication of irradiation equipment, radiation source suppliers; encapsulators; and electron machine manufacturers. This year, because of the additional companies that have been identified in the above categories, we are listing firms associated with radiation processing in all categories.

a. Principal Firms Supplying Electron Accelerator Equipment and/or Services or Finished Products

Company	Supply Accelerators	Supply Services	Supply Finished Product
Accelerators, Inc., Austin, Texas	X		
Applied Radiation Corp., Walnut Creek, California	X	X	
Boise Cascade Corp., Boise, Idaho		X	X
Columbia Research Corp., Gaithersburg Maryland		X	
Electra Process Industries, Inc., Spokane, Washington		X	
Electronized Chemicals Corp., Burlington, Massachusetts (Subsidiary of High Voltage Engrg.)		X	X
Ford Motor Co./Texas Nuclear	X		X
General Electric Co., Milwaukee, Wis.	X		
High Energy Processing Corp., New Bedford, Mass.		X	X
High Voltage Engineering Corp., Burlington, Mass.	X		
Radiation Dynamics, Inc., Westbury, New York	X	X	
Radiation Polymer Corp., Plainfield, Illinois		X	
Raychem, Redwood City, California			X
Raycon, Inc., South Windsor, Conn.		X	
Varian Associates, Livingston, N. J.	X	X	
Westinghouse Electric Corp., Baltimore, Maryland	X		

17/ Available from U.S. Department of Commerce, Sales and Distribution Branch, Washington, D. C. 20230, price 50 cents.

b. Commercial Irradiation Processing Facilities

The American Novawood Corp., Lynchburg, Va.
American Nuclear Corp., Oak Ridge, Tenn.
Electra-Process Industries, Inc., Spokane, Wash.
Electronized Chemicals Corp., Burlington, Mass.
Gamma Process Co., New York, New York
High Energy Processing Corp., New Bedford, Mass.
Isotopes, Inc., Westwood, N. J.
Lockheed-Georgia Co., Dawsonville, Ga.
Neutron Products, Inc., Dickerson, Maryland
Nuclear Materials & Equipment Corp., Apollo, Pa.
Radiation Dynamics, Inc., Westbury, L. I., N. Y.
Radiation Machinery Corp., Parsippany, New Jersey
Raycon, Inc., South Windsor, Conn.
Raychem, Redwood City, California

c. Architectural Design, Engineering and Construction

The American Novawood Corp., Lynchburg, Va.
American Nuclear Corp., Oak Ridge, Tenn.
Atomchem Corp., Long Island, New York
Burns & Roe, Inc., Oradell, N. J.
Commonwealth Associates, Inc., Jackson, Mich.
A. Epstein and Sons, Inc., Chicago, Ill.
Food Industries Research & Engineering, Yakima, Wash.
Gamma Process Company, New York, New York
General Electric Co., Pleasanton, California
Gibbs and Hill, Inc., New York, New York
Holmes & Narver, Inc., Los Angeles, California
Isotopes, Inc., Westwood, New Jersey
Lockheed-Georgia Co., Dawsonville, Georgia
Chas. T. Main, Inc., Boston, Massachusetts
Nuclear Materials & Equipment Corp., Apollo, Pa.
Nuclear Technology Corp., White Plains, New York
Neutron Products, Inc., Dickerson, Maryland
Parson-Jurden Corp., New York, New York
(Subsidiary of Ralph M. Parsons Co.)
Radiation Dynamics, Inc., Westbury, L. I., N. Y.
Radiation Facilities, Inc., Lodi, New Jersey
Stearns-Roger Corp., Denver, Colorado
Stone & Webster Engineering Corp., Garden City, Long Island, New York

d. Irradiation Equipment, Design and Fabrication

The American Novawood Corp., Lynchburg, Va.
American Nuclear Corp., Oak Ridge, Tenn.
Applied Radiation Corp., Walnut Creek, Calif.
Atomchem Corp., Long Island, New York
Gamma Process Co., New York, New York
General Electric Co., Pleasanton, Calif.
Isotopes, Inc., Westwood, N. J.
Lockheed-Georgia Co., Dawsonville, Ga.
National Lead Co., Wilmington, Del.
Nuclear Materials & Equipment Corp., Apollo, Pa.
Nuclear Technology Corp., White Plains, N. Y.
Neutron Products, Inc., Dickerson, Md.
Radiation Facilities, Inc., Lodi, N. J.
Radiation Machinery Corp., Parsippany, N. J.
Stearns-Roger Corp., Denver, Colorado

e. Radiation Processing Source Suppliers and Encapsulators

American Nuclear Corp., Oak Ridge, Tenn.
Atomchem Corp., Long Island, New York
The Babcock & Wilcox Co., Lynchburg, Va.
Gamma Process Co., New York, New York
General Electric Co., Pleasanton, Calif.
Industrial Reactor Lab, Albinsville, N. J.
Lockheed-Georgia Co., Dawsonville, Ga.
Minnesota Mining & Manufacturing Co., St. Paul, Minn.
Nuclear Materials & Equipment Corp., Apollo, Pa.
Neutron Products, Inc., Dickerson, Maryland
Radiation Machinery Corp., Parsippany, N. J.

3. Irradiation Processing of Foods

Irradiation processing of foods attracted a great amount of attention this year, as a result of the Food and Drug Administration's refusal to approve ham, and its withdrawal of approval of bacon. Both products are in the Army Program and the petitions were submitted by the Army. This action delayed the food programs of the Army, the AEC, and commercial firms for at least the four to five year period required to conduct new animal feeding tests.

At present, wheat and wheat products and white potatoes have been cleared by FDA for irradiation processing. A great amount of testing is underway by Government agencies and private firms on additional foods such as strawberries, citrus fruits, and marine products. Other than withdrawal of bacon, there has

been no change in the status of FDA approvals since our 1966 report.

The principal irradiators supporting the food irradiation effort are listed in Table V-9.

TABLE V-9

Irradiators Supporting the Food-Irradiation Effort

Irradiator	Description	Location	Purpose
	Research		
Research	30,000-40,000 curies of ^{60}Co, pool type	Massachusetts Institute of Technology<br>University of California (Davis)<br>University of Washington<br>University of Florida	On-site research
On-Ship No. 3	36,000 curies of ^{60}Co, 18-ton portable unit	University of Nebraska	On-site research
Portable Cesium No. 1	170,000 curies of ^{137}Cs, 18-ton portable unit	Operated for AEC by Isotopes, Inc., Westwood, N. J.	Demonstration to and use by industry
	Pilot Plant		
Marine Products Development Irradiator (MPDI)	250,000 curies of ^{60}Co, 1,000 lb/hr. at pasteurizing dose	Gloucester, Massachusetts	Seafood irradiation
Mobile Gamma Irradiator (MGI)	100,000 curies of ^{60}Co, 1,000 lb/hr. at pasteurizing dose	Davis, California	Fruit irradiation
Grain Products Irradiator (GPI)	25,000 curies of ^{60}Co, 5,000 lb/hr. bulk or 2,800 lb/hr. packaged	Savannah, Georgia	Grain products disinfestation
Hawaii Development Irradiator (HDI)	225,000 curies of ^{60}Co, 4,000 lb/hr. at pasteurizing dose	Honolulu, Hawaii	Tropical fruits

F. Significant Industry Changes and Announcements

Columbia Research Corporation took over operation of the 3 MEV Dynamitron electron accelerator, previously operated by Rad Mat Inc. in Gaithersburg, Maryland.

Gamma Process Company and the New York Atomic and Space Development Authority contracted for the construction and operation of ASDA's Malta Test Station north of Schenectady, New York. This will be the nation's first full-scale radiation processing facility. Gamma Process also acquired the Rose Alloy Industrial Corporation, a heavy-metals fabricator, based in Uniontown, Pennsylvania.

As noted previously, Martin-Marietta sold its nuclear interests to Teledyne. The Isotopes Division of Teledyne will take over the Martin-Marietta activities.

Radiation Machinery Corporation, Parsippany, New Jersey, was formed as a new company in the radiation processing field.

G. AEC Advisory Committee on Isotopes and Radiation Development

The present AEC Advisory Committee on Isotopes and Radiation Development was established in 1958. This committee grew out of the Advisory Committee on Isotope Production and Distribution, which was formed in 1946 and terminated in 1958 when the Commission's regulatory and licensing activities were separated from the operating activities.

The Advisory Committee on Isotopes and Radiation Development reviews AEC policies and the potential development of new applications of isotopes and radiation and makes appropriate recommendations for the Commission's consideration.

The composition of this committee has been gradually changed to bring in additional representatives from various segments of the isotope industry. The present membership is given below:

Chairman - John W. Landis, Gulf General Atomic, Inc.
AEC Member - Dr. Nathanial F. Barr, Technical Advisor to the Assistant General Manager for Research and Development
Mr. Robert E. Kettner, private consultant
Dr. Milton Burton, University of Notre Dame
Commander Scott Carpenter, U. S. Navy, Deep Submergence System Project
Dr. Bernard Fries, Chevron Research Company
Dr. David E. Harmer, Dow Chemical Company
Dr. Wilfred R. Konneker, Mallinckrodt/Nuclear
Dr. James R. Maxfield, Jr., Maxfield Clinic Hospital
Dr. Donald W. Pritchard, Johns Hopkins University
Dr. Vivian T. Stannett, North Carolina State University
Dr. Paul M. Stier, Union Carbide Corporation

H. Science Information Exchange Program

A cooperative model program, to register private R&D in isotopes and radiation, was initiated by the AEC and the Science Information Exchange of the Smithsonian Institute in April 1967.

This model program with isotopes has been completed and is regarded as moderately successful. This type of program is mutually beneficial to industry and Government, in that the SIE system registers and catalogs research work at the time it is initiated, rather than after it appears in reports and journals. The reporting system, which is entirely voluntary, is expected to be useful and informative without, at the same time, revealing proprietary information concerning work in specific companies. The purpose of this SIE program is to develop an overall isotope and radiation program, with a minimum of overlapping between private industry-sponsored and Government-sponsored work.

The Science Information Exchange registers over 95,000 basic and applied research projects annually from Federal Agencies, private foundations, universities, and private industry. SIE answers, without charge, some 50,000 questions each year from scientists, investigators, and research administrators.

[illegible]

CATEGORY VI MATERIALS REPROCESSING

A. Irradiated Fuel

Domestic Market Predictions

	Metric Tons Uranium (MTU) Discharged[1/]	Chemical Reprocessing Dollars at $32,000/MTU
1970	110	3,500,000
1975	1,100	35,000,000
1980	3,600	115,000,000

Some minor revisions were made in the above market predictions from last year's report in order to reflect certain refinements in these estimates.

Fuel elements must be removed from the reactor core before they have been completely consumed, for two principal reasons. One is that the fission product poisons that build up in the reactor fuel have a high affinity to absorb the neutrons necessary to sustain the critical reaction. The other is that the damaging effects of radiation in the reactor necessitate removal of fuel elements prior to warping or rupture.

The reprocessing and recovery of valuable fissionable materials (U-233, U-235, and plutonium) remaining in spent fuel discharged from the reactor, as well as any by-product or fission product material (neptunium, americium, strontium, etc.) involve a series of operations in a chemical separations facility. The technology currently being used commercially is a modified version of the aqueous process that was developed and used initially in AEC separations facilities.

As in last year's report, this section will be divided and discussed under the following three headings:

1. Production Reactor Fuels
2. Power Reactor Fuels
3. Highly Enriched Fuels

1/ WASH-1084, "Forecast of Growth of Nuclear Power," USAEC.

In addition, a special section will highlight some of the major events which have occurred in the foreign reprocessing field over the last several years.

1. Production Reactor Fuels

The AEC's production reactors were built specifically for the purpose of providing special nuclear materials for our defense needs. The production reactor fuel loads are processed at the Hanford Plant at Richland, Washington, and the Savannah River Plant near Aiken, South Carolina. In line with the reduced requirements for special nuclear materials, particularly for military programs, the AEC's Hanford Works at Richland, Washington, shut down the Redox chemical reprocessing facilities toward the end of 1966. The Commission has under consideration the possibility of offering this facility to private industry for use as a commercial reprocessing plant. The Purex reprocessing plant at Richland is now handling all the Richland production reactor fuel loads.

The Atlantic Richfield Hanford Company (ARHCO) was selected by the AEC last year to operate the chemical reprocessing complex at the Hanford plant. In addition to its operations under the AEC contract, the company plans to invest in a private diversification program.

The AEC's New Production Reactor (NPR) at Richland, Washington, began dual purpose operations during 1967. In addition to plutonium production, it is providing steam to the Washington Public Power Supply System for use in the utility's 800 MWE generating plant located adjacent to the reactor. In order to assist in the development of a private reprocessing capability for central station power reactor fuels, the Commission is making available part of the spent fuel load discharged from this reactor for reprocessing under an AEC base load contract with Nuclear Fuel Services at the West Valley facility.

The base load contract with NFS provides that AEC will supply about 95 metric tons of spent fuel per year over the period 1966-1971. Under the contract, about 100 MTU of power reactor spent fuels delivered to the Commission for financial settlement prior to January 1, 1966, by Yankee-Rowe and Commonwealth Edison have also been supplied to NFS and already have been processed.

2. Power Reactor Fuels

a. AEC Policy for Reprocessing Commercial Fuels

To encourage the development of a commercial reprocessing industry, the Commission, in 1957, announced

that it would enter into contracts to receive and make financial settlement for irradiated non-production fuels, if commercial reprocessing services were not available at reasonable terms.

This policy, which was to expire on June 30, 1967, was revised by notice in the Federal Register 33FR30 dated January 3, 1968, to become effective January 1, 1968. The history and details concerning this policy are described in the three following Federal Register notices: (1) 22 FR 1591, March 12, 1967, as amended, (2) 28 FR 11462, October 25, 1963, and (3) 33 FR 30, January 1, 1968.[2]

(1) The 1957 notice provided that, in the absence of private processing services at reasonable prices, the AEC will undertake, under individually negotiated contracts, to receive irradiated reactor fuel and blanket materials at AEC designated facilities and to make a financial settlement therefor. Any financial settlement is to take into a count the charges for chemical processing and conversion of the returned materials to the standard forms under specifications and prices established by AEC. The processing charge is based upon the costs associated with processing certain specified types of irradiated materials in an assumed chemical processing plant (conceptual plant) described in AEC's report WASH-743, "Summary Report AEC Reference Fuel Processing Plant."

(2) The 1963 notice advised of the planned Nuclear Fuel Services, Inc. (NFS), private processing plant and defined that after services are available from NFS the AEC will execute processing contracts only when AEC has determined, on a request by a reactor operator, including operators of foreign reactors, that chemical processing services are not available to the reactor operator from NFS at reasonable terms and charges.

(3) The notice of extension of policy, 33 FR 30, supersedes the above-mentioned notices, and, in general, extends the AEC policy to receive light water and research reactor fuels through December 30, 1970, and through December 31, 1977, for all other power reactor fuels (such as graphite matrix fuels) for cases where the AEC has determined that reprocessing services are not available to the reactor operator from private industry at reasonable terms and charges.

2/ See AEC Public Release IN-830, dated October 31, 1967.

b. Private Capability

(1) Nuclear Fuel Services, Inc. (NFS)

The first private commercial reprocessing plant in the United States began operations on April 20, 1966. Since that time the fuels reprocessed at the West Valley, New York, facility include: (1) New Production Reactor (NPR) fuel under the AEC/NFS base load contract, (2) Dresden I reactor fuel from Commonwealth Edison Company, and (3) fuel from the Rowe, Massachusetts, power reactor of the Yankee Atomic Power Company.

The following information summarizes pertinent facts covering this facility.

Site: West Valley, N. Y.	Technology: Aqueous
Construction: 1963-1966	Cost: $33 Million (approx.)
Capacity: 300 Tons/Year	Operation: 20 Apr. 66
Capability: Various Fuels	A/E: Bechtel
	Employees: Approx. 170

On April 29, 1968, it was announced that Getty Oil Company and Skelly Oil had agreed to purchase American Machine and Foundry's 20 percent stock interest in NFS. The other major partner in the company, formed in 1962, is W. R. Grace, which currently holds an 80 percent interest. Getty Oil would acquire two-thirds and Skelly one-third of the AMF interest. Getty and Skelly also agreed to purchase additional stock issued by NFS so as to acquire a 49 percent ownership over the next four years.

The process used at the NFS plant employs a mechanical (chop-leach) head end with Purex solvent extraction. The final products from the plant are uranium and plutonium in the form of nitrate solutions. A simplified flow diagram and description of the NFS process is presented in Chart VI-A.

The NFS plant continues to play a key role in the national and international safeguards inspection programs. Under an AEC contract, a safeguards manual was completed by NFS, which was provided to the International Atomic Energy Agency for their use in formulating procedures during the safeguards inspection of Yankee-Rowe spent fuel elements reprocessed

CHART VI-A

NUCLEAR FUEL SERVICES, INC., SPENT FUEL PROCESS

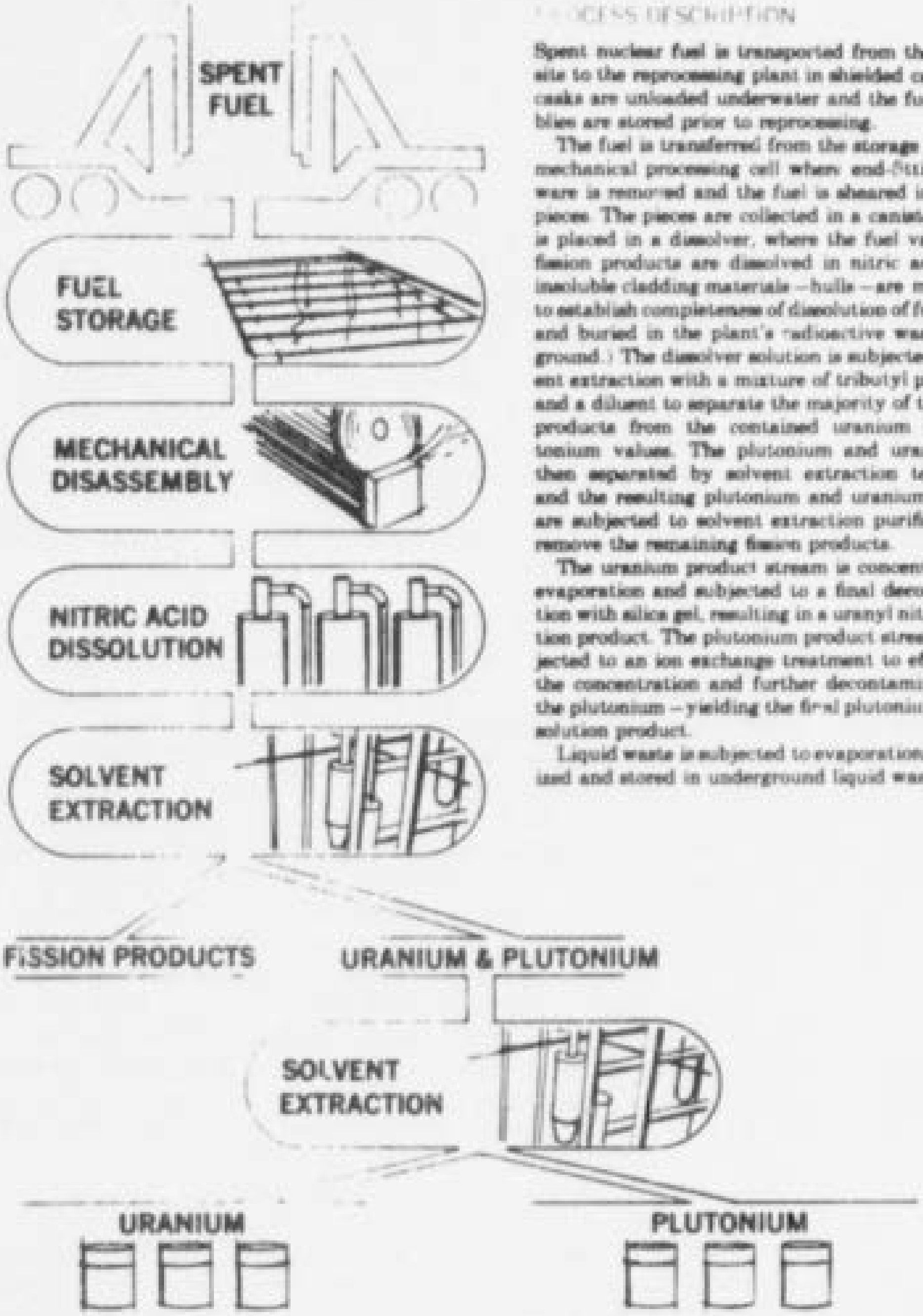

[illegible]OCESS DESCRIPTION

Spent nuclear fuel is transported from the reactor site to the reprocessing plant in shielded casks. The casks are unloaded underwater and the fuel assemblies are stored prior to reprocessing.

The fuel is transferred from the storage pool to a mechanical processing cell where end-fitting hardware is removed and the fuel is sheared into small pieces. The pieces are collected in a canister, which is placed in a dissolver, where the fuel values and fission products are dissolved in nitric acid. (The insoluble cladding materials—hulls—are monitored to establish completeness of dissolution of fuel values and buried in the plant's radioactive waste burial ground.) The dissolver solution is subjected to solvent extraction with a mixture of tributyl phosphate and a diluent to separate the majority of the fission products from the contained uranium and plutonium values. The plutonium and uranium are then separated by solvent extraction techniques and the resulting plutonium and uranium streams are subjected to solvent extraction purification to remove the remaining fission products.

The uranium product stream is concentrated by evaporation and subjected to a final decontamination with silica gel, resulting in a uranyl nitrate solution product. The plutonium product stream is subjected to an ion exchange treatment to effect both the concentration and further decontamination of the plutonium—yielding the final plutonium nitrate solution product.

Liquid waste is subjected to evaporation, neutralized and stored in underground liquid waste tanks.

at NFS during 1967.[3/] The plant is also one of several private nuclear facilities where use of AEC resident inspectors will be evaluated in connection with development of a domestic safeguards program to prevent diversion of special nuclear material.

c. Other Company Programs

(1) General Electric

The General Electric Company received a permit from the AEC in late December 1967 to construct a 300 MTU per year chemical reprocessing plant near Morris, Illinois. A public hearing on the construction plan was held in Morris, Illinois, in November 1967. Construction was initiated in August 1968 and plans call for completion in 1970 with production initiated before the end of that year.

Some pertinent information on the proposed plant follows:

Midwest Fuel Recovery Plant

Site: Morris, Illinois	Technology: Aquafluor
Construction: 1968-1970	Operation: 1970
Capacity: 300 Tons/Year	A/E: Fluor Corp.
Capability: Low Enriched UO_2	

The plant will recover uranium, plutonium, and neptunium, with the final product uranium being in the hexafluoride form. GE will use its Aquafluor process which makes use of aqueous and fluoride volatility fuel recovery technologies. The process uses fuel recovery technologies including fuel shearing and leaching, anion exchange, calcination, as well as the newer techniques of fluorination and uranium hexafluoride distillation.

The facility is planned to include means to convert all liquid waste to a solid form.

Chart VI-B presents a simplified block diagram of the proposed GE process.

3/ See AEC Public Release K-188, dated July 28, 1967.

CHART VI-B

PROPOSED GE PROCESS

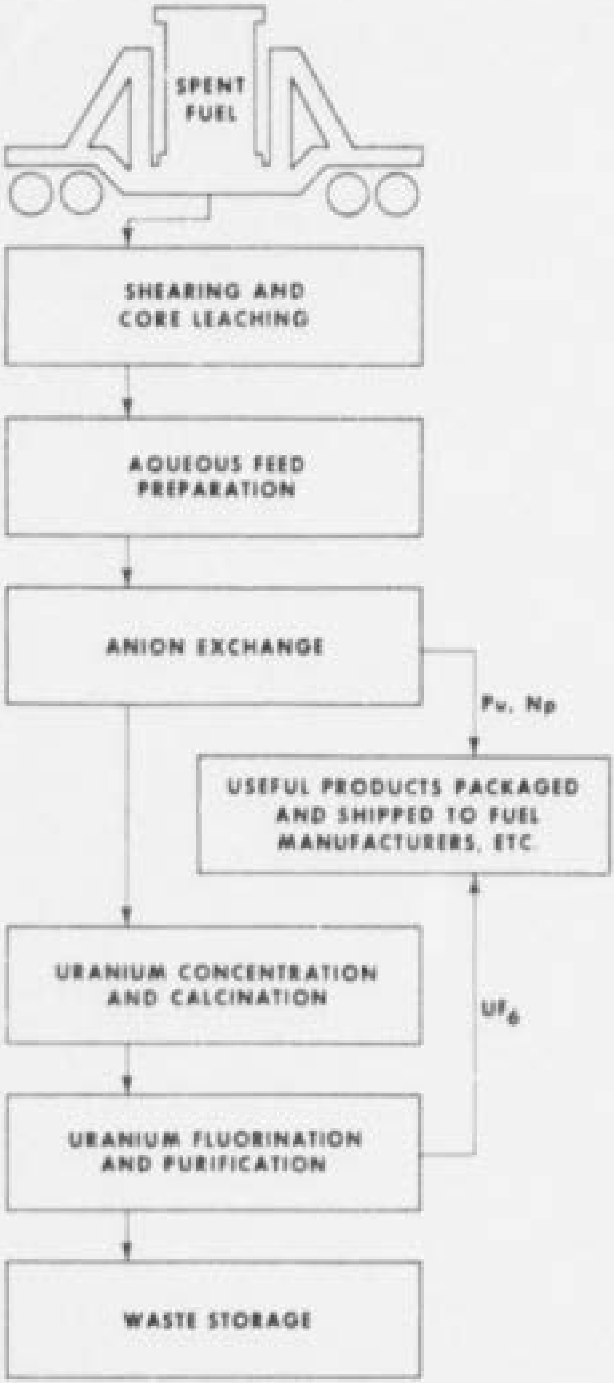

General Electric's proposed Aquaflour process makes use of aqueous and fluoride volatility fuel recovery technologies. The process uses well-demonstrated fuel recovery techniques including fuel shearing and leaching, solvent extraction, anion exchange, calcination, fluorination, and uranium hexafluoride distillation. Anion exchange is used in the recovery and purification of plutonium and neptunium. Uranium will be converted to the volatile uranium hexafluoride (UF_6) and purified to make it suitable for toll enrichment. The high level liquid wastes will be converted to solid form and stored temporarily on-site in a waterfilled concrete storage basin.

(2) Allied Chemical Corporation

Allied Chemical continued its activities in regard to the design and construction of a multi-ton reprocessing plant using an aqueous process.

During the year, Allied Chemical announced that it would file a construction permit application, probably in the fall, for a five metric ton per day reprocessing plant with "primary consideration" being given to a site adjacent to the AEC's Savannah River Plant in South Carolina.

Commercial operations at the plant are anticipated to begin in 1973. The cost of the plant is estimated to be in the range of $50 million.

Allied has submitted reprocessing proposals to a number of utilities, and both the marketing and technical staffs of the company have been expanded in conjunction with the announced reprocessing project.

Allied Nuclear Fuels Plant

Site: Undetermined	Technology: Modified Aqueous
Construction: 1968 (Start)	Operation: 1973
Capacity: 5 MTU/Day	Employees: Approx. 200
Capability: Low Enriched UO_2	
Cost: $50 Million	

(3) National Lead

The National Lead Company continued its studies in regard to their possibly building a chemical reprocessing plant. A contract with Saint Gobain Techniques Nouvelles (SGN) of Paris, France, for preliminary process and plant design and cost estimating studies was completed during the year. The company is continuing to evaluate the economics of entering the reprocessing market.

(4) Atlantic Richfield

ARCO continued its activities in regard to entering the reprocessing area. They plan to construct a five metric ton U per day plant near Leeds, South Carolina, which will provide for recovery of uranium, plutonium, and neptunium from irradiated light water fuel elements. Services to be offered will include: shipping of spent fuels and products, on-site burial of wastes, storage facilities for plutonium products, and conversion facilities for uranyl nitrate to UF_6. Work is continuing on process evaluation, plant design, and site investigations necessary for the preliminary safety analysis report (PSAR) which is planned for 1969.

Site: Near Leeds, S. C. Capacity: 1,500 MTU/year
Construction: 1972-73 Technology: Aqueous
Operation: 1974

They are also continuing to develop plans for a West Coast facility for late 1970 operation.

(5) Gulf General Atomic

Gulf General Atomic is continuing to study the reprocessing of high temperature gas reactor graphite fuels. They have, both independently and in cooperation with others, completed several studies involving the technical and economical aspects of graphite spent fuel recovery.

d. Spent Fuel Forecast

Chart VI-C presents AEC estimates of power reactor spent fuel discharges through 1980, along with the dollar market. The dollar market estimate is based on the AEC/NFS base load contract formula, which amounts to about $32,000 per metric ton of uranium for typical, low-enriched UO_2 spent reactor fuels. All estimates are based on the AEC's 1967 figures for nuclear power growth of approximately 150,000 MWE of installed nuclear power capacity by 1980.[4/]

Several independent forecasts of reprocessing load are presented in Table VI-1 along with the latest AEC estimate.[5/] The AEC estimate has been further refined since last year's report to forecast loads for each year between 1968 and 1980.

4/ See AEC Public Release IN-818, dated August 31, 1967, and S-43-67, dated November 2, 1967.
5/ See WASH-1084, "Forecast of Growth of Nuclear Power," December 1967.

CHART VI-C

SPENT FUEL DISCHARGE FORECAST

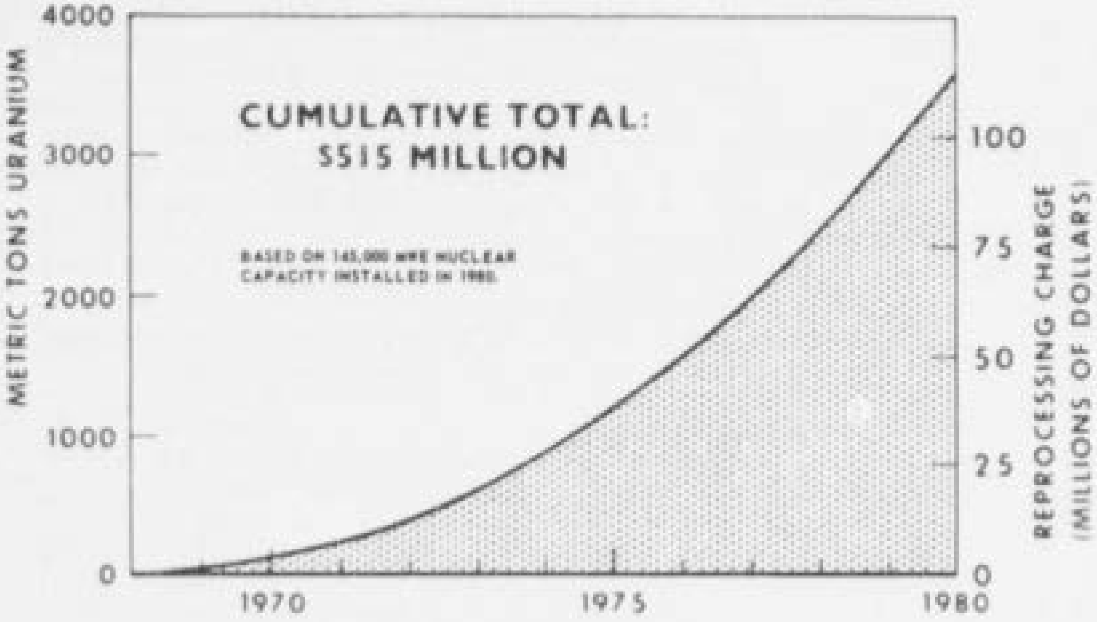

TABLE VI-1

Fuel Reprocessing Load Forecasts
(In metric tons uranium)

Calendar Year	AEC - 12/67	NFS - 9/68	GE - 7/67	DUKE - 5/67
1968	43	10		37
1969	58	32		78
1970	110	109	114	143
1971	210	69	163	320
1972	350	194	330	590
1973	580	338	676	910
1974	870	843	1188	1260
1975	1100	1288	1527	1660
1976	1600	1852	2050	2110
1977	2100	2068	2500	2570
1978	2500	2446	3100	3080
1979	3000	3013	3800	3610
1980	3600	3551	4600	4190

NOTE: The AEC figures are reactor discharges. The company figures represent fuel available for reprocessing. In order to compare the AEC figures with those of the companies noted, the AEC forecast would need to be slipped somewhat to account for spent fuel cooling and shipping. A one-year slip would be more than ample to cover the time required to have the AEC figures represent fuel available for reprocessing.

e. AEC Research Programs

Currently AEC research programs are directed toward recovery of fuels from advanced reactor concepts, particularly the Liquid Metal Fast Breeder (LMFBR), the High Temperature Gas Cooled Reactors (HTGR), the Molten Salt Breeder Reactor (MSBR), and the like.

A continuing program of development is required to assure achievement of the economic objectives and technical requirements for a fuel-recovery process for fuels from advanced commercial reactors.

Because aqueous technology has been extensively used in Government processing plants to extract plutonium bred in uranium and a commercial plant to recover LWR fuels, development of this technology for LMFBR fuels will be emphasized in the near term. The near-term efforts are to determine whether the aqueous technology will meet satisfactorily the needs of a commercial LMFBR, and will provide an adequate technology for processing early LMFBR fuels. If the aqueous technology is judged to be satisfactory for long-term use, its development will be continued to complete process developments and optimize process conditions.

Until there is sufficient assurance that the aqueous technology will be successful, it will be necessary to continue -- on a more modest scale -- the development of the fluoride-volatility and pyrochemical processes. If major inadequacies become apparent in the aqueous technology, one of the alternate process technologies will be developed intensively as a substitute.

f. Southern Interstate Nuclear Board

In October 1967, SINB released a report which presented the results of a survey of projected nuclear power growth and related fuel service requirements in the 17 state region served by the Board. The fuel reprocessing load forecast for the SINB region indicated a sharp increase from 84 MTU in 1972, to 1,286 MTU in 1980. Further details on this survey as well as a copy of the report can be obtained from Mr. Robert H. Gifford, Executive Director, Southern Interstate Nuclear Board, 800 Peachtree Street, N. E., Atlanta, Georgia 30308.

g. Utility Actions

During the year, Duke Power invited proposals from industry for the reprocessing of all of its spent fuel

scheduled for discharge over the next ten years. Several other utilities and one reactor manufacturer were informally inviting proposals for their near-term requirements. This was the first time utilities had invited proposals for reprocessing services covering more than one reactor.

h. Competition

Inherent with the expanded interest in chemical reprocessing on the part of private industry during the past year, is the problem of establishing and developing this service on a healthy, sound basis.

NFS will experience a load short situation for some time to come, mainly because of the continuing development of long-burn-up UO_2 power reactor fuels and the recent announcements concerning slippages in various nuclear plants. Other developments which could further delay the growth of the reprocessing market are the fuel-sharing and reinsertion fuel management schemes offered by several of the reactor manufacturers. Taking all these aspects into consideration, it appears that a spent fuel market sufficient to support NFS operations will not appear until at least 1973. Comparing the available market with the 300 MTU per year GE plant schedule for operation in 1970, the Allied Chemical announcement of a five MTU per day plant scheduled for commercial operation in 1973, and the plans of Atlantic Richfield and National Lead to be part of the commercial reprocessing industry in the 1970's, a further over supply of reprocessing capability seems to be developing.

In recognition of the load-short reprocessing market that will continue to exist for some time to come, the Commission advised the UKAEA last year that the AEC believes it would not be in the U.S. interest to amend the bilateral agreement with the U. K. to permit reprocessing of U.S. fuels abroad during the critical transition period required for private industry to reach a level of economic viability. While the Commission could not forecast precisely when the domestic reprocessing industry would reach the level of economic viability that would permit export of U.S. fuel for reprocessing abroad, it did indicate that this would most likely take place during the first part of the 1970's. The U. K. Winscale facility has been actively competing throughout the world for spent fuel reprocessing. The Eurochemic Plant in Belgium is also actively competing for spent fuel reprocessing primarily in Europe. (Foreign reprocessing capability is discussed in detail later in this category).

3. Highly Enriched Fuels

The highly enriched spent fuel loads include those fuels used in the Space Propulsion Program (Rover), high temperature gas cooled reactors (HTGR), naval reactors, research reactors, and test reactors such as the MTR/ETR/ATR reactors at the AEC's National Reactor Testing Station, Idaho Falls, Idaho.

Both the AEC and NFS have the capability to handle selected types of these fuels; however, for the graphite fuels (Rover and HTGR), TRIGA (UZrH) fuel, and several other special fuels, there exists no suitable processing capability. Gulf General Atomic, the AEC, and NFS have conducted studies associated with the reprocessing of HTGR fuels. GGA naturally has a serious interest in this service in connection with the commercial sale of its gas cooled reactor. The AEC is currently undertaking studies of several alternative bases upon which to develop specific processing. charges, rather than on a case-by-case basis which now exists, for the graphite matrix power fuels. The alternatives are to include evaluations of appropriate single-purpose conceptual plants, and modifications to existing commercial and AEC facilities. Further information in this regard is covered in the extension of the Commission's policy on accepting commercial fuels for reprocessing which was released in the Federal Register on January 3, 1968.

4. Conference on Spent Fuel Reprocessing

The Southern Interstate Nuclear Board and the AEC Savannah River Operations Office jointly sponsored a conference on spent fuel reprocessing at Augusta, Georgia, on May 11-12, 1967. Information on this conference was presented in last year's report in "The Nuclear Industry - 1967" on page 142.

5. Foreign Reprocessing Plants and Programs

a. Foreign Plants

Table VI-2 presents a listing of foreign spent fuel processing plants along with several significant facts about each plant.

TABLE VI-2

Status of Chemical Reprocessing Plants

Country	Location	Operator	Size	Operation
England	Windscale	UKEA	1500 T/Y-Nat. U	1966
			300 T/Y-Oxide	1968
France	Cap de la Hague	CEA	1 T/D-Nat. U	1966
Belgium	Mol	OECD	350 Kg/D Nat. U	1966
			200 Kg/D <5% enr.	
Scotland	Dounreay	UKEA	1 Kg/D-UAL	1958
			1200 Kg/Y-U+Pu	1960
Germany	Karlsruhe	GWK	35 T/Y <3% enr.	1969
Italy	Saluggia	EUREX	.75 Kg/D-UAL	1968
Italy	Rotondella	PCUT	15 Kg/D-Th+U	1968
India	Trombay	AEET	350 Kg/D Nat. U	1964
India	Tarapur	AEET	500 Kg/D Nat. & Low enr.	1971
Japan	Tokai-Mura	PNC	700 Kg/D	1972
Norway	Kjeller	Nor./Swed./Neth.	12/D <5% enr.	1961
Spain	Moncloa	JEN	6.3 Kg/Y-UAL	1966
Argentina	Buenos Aires	CNEA	200 Kg/Y	1967

b. Programs

(1) United Kingdom

The Windscale plant in England was built primarily for reprocessing fuels from the British Nuclear Power Program of magnox reactors, as well as the Calder Hall and Chapelcross reactors. Although designed for a capacity of 1500 metric tons of natural uranium per year, it is reported to be capable of a somewhat higher capacity.

A "head-end" section for handling enriched oxide fuel elements is being added to the plant, and is expected to be operational in 1968. The modification is designed for 300 MTU per year and is estimated to cost about $4.2 million.

The UK-Windscale facility has been very active in competing for spent fuel loads throughout the world, and to date, they have been successful in contracting reprocessing services in Italy, Japan, Canada, and Switzerland.

The Dounrey plant in the North of Scotland is used for the reprocessing of enriched fuels from the Prototype Fast Reactor, and from materials test reactors both in the UK and overseas.

(2) France

In the La Hague reprocessing plant, both the final purification and concentration steps of plutonium are carried out in a single extraction - stripping cycle using trilaurylamine. This solvent is claimed to have a very high distribution ratio for plutonium, as well as a selectivity comparable to tributyl phosphate. Its radiation stability is reported to be high enough if temperature increases are avoided and if nitrous acid is eliminated. Industrial use of this solvent has confirmed laboratory performances.

In order to improve the operation conditions and decrease the operating cost of the Marcoule weapons oriented processing plant, a second plutonium purification cycle, also used as a concentration cycle, has been developed. This cycle includ s a plutonium extraction step with tributyle phosphate followed by a reducing - stripping extraction step. The reducer which is used is uranous nitrate stabilized with hydrazine. A reflux extraction provides a constant concentration solution of plutonium. A new facility equipped with pulsed columns and based on this flowsheet came into operation in August 1967.

The treatment of irradiated fuels by non-aqueous methods is being studied for two kinds of fuel: highly enriched uranium zirconium or uranium-aluminum alloys, and mixed UO_2-PUO_2 stainless steel clad oxides. Experimentation with the processing of irradiated alloys is to be undertaken in the "ATTILA" facility. "ATTILA" is a hot cell facility, 11.6 meters x 5.89 meters in dimension, located in Fontenay-aux-Roses.

Two good references on France's reprocessing program, as well as other nuclear interests are adequately described in (1) Bulletin d'Informations Scientifiques et Techniques," July 1968, and (2) "Energie Nucleaire," May 1968.

(3) Belgium

The European Company for the Chemical Reprocessing of Irradiated Fuels (Eurochemic) is a joint effort of 13 European nations formed to construct a multi-fuels reprocessing plant primarily to serve the needs of the

13 member nations. The company operates under the auspices of the European Nuclear Energy Agency, an offshoot of the Organization for Economic Cooperation and Development.

The Eurochemic plant at Mol, Belgium, some 50 miles northeast of Brussels started operations in July 1966. Since that time, and until mid-1968, the plant has recovered approximately 46 tons of natural or slightly enriched uranium, four tons of uranium-aluminum containing 160 Kg of highly enriched uranium and around 40 Kg of plutonium.

The following presents pertinent details on the plant.

<u>Eurochemic</u>

<u>Site</u>: Mol, Belgium	<u>Technology</u>: Purex
<u>Construction</u>: 1961-1966	<u>Operation</u>: July 66
<u>Capacity</u>: 350 Kg/Day (<5% U-235)<br>10 Kg/Day (highly enriched)	<u>A/E</u>: Saint Gobain<br><u>Project Cost</u>: $38 Million<br><u>Employees</u>: 350
<u>Capability</u>: Multiple Fuels	

(4) <u>Germany</u>

Construction began last year on the German Reprocessing Plant Karlsruhe (WAK). The $15 million project, capable of handling 35 metric tons per year of low enriched uranium, is scheduled for completion in late 1969. Operator of the plant will be the Gesellschaft zur Wiederaufarbeitung Bestrahlter Kernbrennstaffe Gmbh (GWK), a joint subsidiary of the chemical firm Farbwerke Hoechst Ag, the coal-oil company Gelsenkirchener Bergwerke AG, and the nuclear-fuel manufacturer NUKEM.

(5) <u>Japan</u>

The Saint Gobain Company of France has contracted with Japanese Power Reactor and Nuclear Fuel Development Corporation (PNC) to design a chemical reprocessing plant at Tokai-mura, Japan. The Japanese Atomic Energy Commission's budget submittal of $830,000 for design and site preparation work has been approved.

[illegible]

PNS plans to start site preparation work this Fall with construction starting in 1969. The schedule calls for the plant to be completed in 1972. The plant will initially reprocess about .7 MTU per day (200 MTU per year) of both Calder Hall type fuel and light water reactor fuel. Construction cost is estimated to total about $45 million. The plant is expected to ultimately employ 300 people. PNC has submitted its application for safety evaluation of the plant to the Prime Minister's office.

The recent Japan Atomic Power Company (JAPCO) and UKAEA contract for reprocessing Tokai Power Plant fuel specified a charge of $41,600 per ton, including transportation and insurance.

(6) Italy

The PCUT Processing Plant was conceived as a pilot plant with two major functions: to test reprocessing methods for thorium-uranium 233 fuel cycles, and to test the feasibility and economics of remote fabrication of reactor fuel elements. The plant was designed by a U.S. firm (Allis Chalmers) for the CNEN, and construction of the plant began in late 1962 at a site near Rotondella in extreme southern Italy. In mid-1963 the project ran into budgetary problems. Construction has been reduced to a very low rate of progress and the whole PCUT and thorium fuel program was carefully reevaluated by CNEN. As a result, the project was again approved and construction was resumed at a normal rate in July 1964. The approved schedule called for cold start-up in August 1967 and hot start-up about January; however, these dates have slipped somewhat, and plant operations are not expected until late 1968. The plant is estimated to cost $9.3 million.

The plant is designed to process at a rate of 0.015 tons of uranium-thorium mixture per day. If operated at this design rate, the plant could achieve and annual throughput of approximately 140 effective kilograms.

The U.S. has contracted with the CNEN to process certain fuels from the Elk River Reactor at PCUT.

A contract was awarded several years ago to the Societa Bomprini Parodi Delfino of Rome for the construction of a chemical processing plant near Saluggia. The plant is designed to handle three MTR type elements daily, and is estimated to cost in the range of $6-10 million. The plant, which is now scheduled for completion by the end of 1968, will use the Eurex aqueous process. A feed of 3 MTR elements per day of 93 percent uranium equals 0.18 ton per year (300 days).

(7) India

The Indian Chemical Processing Plant at Trombay began operation in June 1964. The plant reportedly cost $7.7 million and uses the Purex aqueous process. The plant was reported at the 1964 Geneva Conference to be operating at .12-.24 tons uranium per day with the expressed belief that the rate could be doubled. The plant was reportedly designed to have a cpaacity of 10 tons total metal processing per day.

(8) Norway, Sweden, Netherlands

A pilot reprocessing plant jointly sponsored by the three countries, built at Kjeller, Norway, has been operating since June 1961. The plant which uses the Purex aqueous process and which operates on a batch basis can process approximately 0.012 ton of uranium per day.

(9) Spain

A small pilot reprocessing plant is in operation at Moncloa, Spain. The plant processes highly enriched uranium at the rate of approximately two MTR fuel plates per day. The plant, completed in April 1966, uses the aqueous solvent extraction process.

(10) Argentina

A chemical reprocessing plant proposed at Buenos Aires is designed to process approximately 200 grams of U-235 (10 RA-1 fuel elements) at a time. The plant would use the modified Dounreay method, based on extraction with tributyl phosphates.

B. Unirradiated Fuel Scrap

The part of the nuclear fuel cycle starting from the mine to the reactor is known as the "cold" side or unirradiated side, of the cycle. Since this material has not undergone fissioning, it can be easily handled compared to the highly radioactive material discharged from the reactor or "hot" side of the cycle.

1. Enriched Uranium Scrap Recovery

a. Commercial

The major source of enriched uranium scrap comes from the fuel fabrication activity. Since the nuclear material involved in this scrap is quite valuable, its recovery is economically very desirable. Normally a large part of the scrap generated during the fabrication operation is recycled back into the system at the site. It is estimated that only a small percentage (about 1.0 to 1.5%) of the scrap cannot be recycled back into the fabrication campaign and must be treated separately by converting it to UF_6 and refeeding it to the enrichment plant (see Category II, Material Processing and Fabrication, for additional information).

Adequate private capability exists to recover enriched uranium from all types and forms of scrap generated within both private and AEC programs. Table VI-3 indicates those firms capable of recovering uranium scrap, in addition to the principal reactor manufacturers.

TABLE VI-3

Principal Firms Recovering Uranium Scrap

Gulf General Atomic Inc., San Diego, California
Kerr-McGee Corporation, Oklahoma City, Oklahoma
Nuclear Fuel Services, Inc., Erwin, Tennessee
Nuclear Materials and Equipment Corporation, Apollo, Penna.
United Nuclear Corporation, Westerly, Rhode Island

b. AEC-Industrial Program

AEC generated uranium scrap is recovered commercially as part of the fuels fabrication contract (such as the prime contract procurement for MTR, ETR, and ATR fuels), or through the Scrap Recovery Program administered by the AEC's Oak Ridge Operations Office. Since 1959 contracts

totaling about $6.8 million have been awarded through the Scrap Recovery Program. Table VI-4 indicates the dollar value of scrap recovery contracts awarded to private industry since 1959.

TABLE VI-4

Dollar Value of the AEC's Commercial Uranium Scrap Recovery Program

Fiscal Years	Value
1959	$ 115,757
1960	275,223
1961	1,438,379
1962	927,501
1963	597,744
1964	568,499
1965	1 [illegible]71,066
1966	789,678
1967	576,975
1968	355,230

Table VI-5 presents information on contracts awarded to commercial firms during FY-1968 for the recovery of about 1690 kgs of uranium scrap generated in Commission programs.

TABLE VI-5

Uranium Scrap Reprocessing Contracts Awarded in FY-1968

Contractor	No. Awards	Dollar Award	Scrap Value	Kgs. U
Nuclear Fuel Services	6	$283,138	$4,689,000	1530
United Nuclear	2	72,092	1,757,000	160
Total	8	$355,230	$6,446,000	1690

As can be seen from Table VI-5, the value of contract awards has been steadily decreasing since 1965. However, this trend is expected to be reversed during the next three years as a result of program changes which should release large quantities of unirradiated scrap for reprocessing. There is some question, however, as to whether AEC will be able to obtain funds in the immediate future to contract for this service because of the critical national budget situation. The Commission will continue to require that its fixed price contractors handle scrap recovery within their own organizations or under contract.

CATEGORY VII
PLOWSHARE - PEACEFUL USES OF NUCLEA[illegible] [illegible]XPLOSIVES

In July 1968, Dr. Glenn T. Seaborg, Chairman, Atomic Energy Comm[illegible]ssion, made the following statement in testifying before the U.S. Senate Committee on Foreign Relations:

> "I am confident that the technology of using nuclear explosions for peaceful purposes will produce enormous civil dividends not only for ourselves, but also for the non-nuclear weapon countries with whom we will cooperate...."1/

This statement summarizes the challenging potential of the use of this technology in the future.

A. Introduction

The Plowshare Program, formally established by the United States Atomic Energy Commission in 1957, has the task of investigating and developing peaceful uses for nuclear explosives. As a result of the nuclear experiments conducted by the AEC, a significant body of information has been accumulated on the physical effects of both contained explosions (underground engineering) and cratering explosions (for excavation projects). The contained explosions create conditions suitable for a number of natural resources and scientific applications.

The information being accumulated concerning contained and cratering explosions will, it is hoped, in the near future enable industry to utilize this new technology effectively in the recovery of natural resources, in large scale engineering projects, and in other useful applications for the good of mankind.

B. Budget

Table VII-1 and VII-2, on the following page, give a dollar breakdown of the Plowshare budget. The former shows the AEC operating budget for the Plowshare program for FY-1967 through FY-1968, with an estimate for FY-1969, while the latter identifies the various areas of work included in the FY-1969 budget.

Since industry and Government share the cost of the various joint underground engineering projects, the level of the AEC budget does not accurately reflect the true breadth of the Plowshare program.

1/ Statement by Dr. Glenn T. Seaborg, Non-Proliferation Treaty Hearings before the U.S. Senate Committee on Foreign Relations, July 10, 1968, page 105.

TABLE VII-1

Operating Budget for Plowshare

	FY-1967 Actual	FY-1968 Actual	FY-1969 Estimate
Total Operating Program	$12,852,846	$17,826,[illegible]	$14,450,000
Research and Development....	$ 6,403,337	$ 6,849,[illegible]	$ 5,500,000
Excavation Experiments......	5,601,142	7,841,790	6,950,000
Device Testing...........	4,030,775	1,869,726	3,050,000
Cratering Technology Exp.	1,570,367	5,972,064	3,900,000
Scientific Experiments......	468,817	346,268	500,000
Underground Engineering Exp.	379,550	2,788,767	1,500,000

TABLE VII-2

FY-69 Budget Estimate
(In Millions)

Research and Development		$5.5	- Principally at LRL*
Excavation Experiments			
Device Testing	3.0		
Cratering Tech. Experiments	3.9		
		6.9	
Scientific Experiments		0.5	
Underground Engineering Experiments		1.5	
	TOTAL	14.4	

*LRL - Lawrence Radiation Laboratory, Livermore

C. AEC Authority to Provide Plowshare Services

The Non-Proliferation Treaty, which was signed by the United States on July 1, 1968, and currently awaits ratification, specifies in Article V that the nuclear weapon states shall make available the potential benefits of peaceful nuclear explosives to the non-nuclear weapon states party to the treaty on a nondiscriminatory basis. Legislation has been introduced in the Congress which would establish and extend the AEC's statuatory authority to fulfill that obligation for the United States. This legislation, if enacted, would have the added effect of authorizing the AEC to engage in Plowshare projects with industry for other than research purposes which, under the present Act, is not permitted. This would be a necessary and significant step toward the goal of providing a useful and economic service to users of the Plowshare technology.

D. Industrial Participation

A Plowshare Committee was formed in 1967 by the Atomic Industrial Forum to keep that organization abreast of current developments in the Plowshare program. The Committee has now organized into five subcommittees: (a) procedures for industrial participation, (b) legislative matters, (c) economics, (d) safety, and (e) public acceptance.

The growing Plowshare service industry has been stimulated by the interest of a number of companies in this new field. The following companies have either established organizations or otherwise indicated their interest in providing partial or complete design, advisory, and operational services to user firms contemplating industrial applications of nuclear explosions.

Applied Nuclear Company, LaJolla, California
ATCOR, Inc., Hawthorne, New York
The Bechtel Corporation, San Francisco, Calif.
El Paso Natural Gas Company, El Paso, Texas
Fenix & Scisson, Inc., Tulsa, Oklahoma
Geonuclear Nobel-Paso, Inc., Paris, France
Gulf General Atomic, Washington, D. C.
Holmes & Narver, Inc., Los Angeles, Calif.
Kaman Sciences Corporation, Bethesda, Maryland
Lockheed Missiles and Space Company, Sunneyvale, Calif.
The Ralph M. Parsons Company, Washington, D. C.
Teledyne Geonuclear, Garland, Texas
Texas Instruments, Dallas, Texas

E. Natural Resources Projects

During the year, there was a continuing high degree of interest in the various Plowshare projects. The Commission is currently

cooperating with industry in the following projects.

1. Project Gasbuggy[2/]

Project Gasbuggy is the first Government-industry joint experiment to investigate the use of a nuclear explosive in stimulating natural gas fields of low productivity.

On December 10, 1967, a nuclear explosive with a yield of about 20 to 30 kilotons was detonated 4,240 feet beneath the surface in the San Juan Basin, New Mexico. It was jointly sponsored by the AEC, the El Paso Natural Gas Company, and the Department of the Interior.

Flow tests began June 28, 1968, and consisted of producing the well at a rate of five million cubic feet per day for six days. The well was then shut down for one day to measure bottom-hole pressure. Testing was then resumed at the five million cubic feet per day flow rate for five additional days.

Preliminary calculations indicate that the chimney created by the explosion has a void volume of at least 2 million cubic feet which was about as predicted. Additional test data indicate that only a trace of radioactive iodine was encountered and that this was absorbed in the rock. The amount of tritium was much less than expected and the flow test data showed very encouraging indications that surface wellhead pressure was 954 psia and the bottomhole pressure was about 1,100 psia. However, the degree of success of the experiment whose purpose was to increase the ultimate recovery of gas, will not be known for some time.

2. Other Gas Stimulation Experiments

Other follow-on experiments to Gasbuggy that have been proposed or brought to the attention of the AEC are as follows.

a. Project Dragon Trail

This was proposed jointly by the Continental Oil Company and the CER Geonuclear Corporation and calls for the detonation of a 26 kiloton nuclear explosive, 2,700 feet underground, at a site about 50 miles north of Grand Junction, Colorado. The experiment would extend knowledge to a gas-bearing formation with different geological characteristics than the Gasbuggy site. At present Technical and Operational Safety Planning are being carried

---

2/ Remarks by Glenn T. Seaborg, Chairman, USAEC, "Nuclear Industry - 1968 and Beyond," Financial Forum on Nuclear Energy, New York City, October 30, 1968, AEC Public Release S-39-68.

out in preparation for contract negotiations.

b. Project Rulison

This was proposed by the Austral Oil Company in conjunction with the CER Geonuclear Corporation and is now being technically defined prior to contract negotiations. The experiment, which is planned for late 1969, with a yield of about 40 kilotons, would extend data to gas-bearing formations at depths of eight thousand to ten thousand feet. The Los Alamos Scientific Laboratory is providing the technical direction for the nuclear portion of this experiment.

Both the Dragon Trail and Rulison proposed sites are located near the Piceance Creek area of Western Colorado.

c. Additional Gas Stimulation Plans

Two suggestions have been submitted for gas stimulation projects in the Pinedale area of Wyoming. The International Nuclear Corporation has proposed project WASP (Wyoming Atomic Stimulation Project) and the El Paso Natural Gas Company also has submitted a recommendation for a joint study in the Pinedale area. At present, discussions concerning feasibility studies for these proposed projects are underway.

3. Project Ketch

The Ketch Project is intended to explore the use of nuclear explosives for creating underground reservoirs for storing natural gas.

The Columbia Gas System Service Corporation, U.S. Bureau of Mines, LRL-Livermore, and SAN completed and published the Ketch feasibility study (PNE-1200) in August 1967. The study concluded that:

> "Nuclear explosives offer the potential for creating underground reservoirs for storing natural gas. This potential, if realized, could lead to expansion of the gas industry in areas in which inexpensive underground storage is not now available and continued growth in areas where natural storage is available but growing scarce."

Columbia Gas System has now withdrawn its request to utilize Pennsylvania State Forest land and is looking for a new site in Pennsylvania or elsewhere in the Appalachian area. In this connection, the Southern Interstate Nuclear Board has offered its services to the Government and Columbia in locating

an appropriate site. When a site location is agreed upon, project planning will be undertaken.

4. Project Sloop

Project Sloop is intended to study the feasibility of fracturing copper ore bodies with nuclear explosives, and the extraction of copper by in-situ leaching methods. A Sloop feasibility study was completed by the Kennecott Copper Corporation, BUMines, LRL-Livermore, and SAN in October of 1967. In this report the U.S. Bureau of Mines indicated that an additional 58 million tons of copper probably exist in potential ores, averaging about 9.4 pounds of copper per ton of ore. It is hoped that these copper ore bodies, which at present are not ec[illegible]ically mineable, can be fractured with nuclear explosives, and followed up with the extraction of the copper by in-situ leaching methods.

Following this study, Kennecott submitted a proposal to the AEC and the Department of the Interior to conduct a joint experiment to test this application in a copper ore body nine miles northeast of Safford, Arizona. The Government and Kennecott are currently engaged in technical definition activities prior to contract negotiations.

CHART VII-A

GAS RESERVOIR STIMULATION

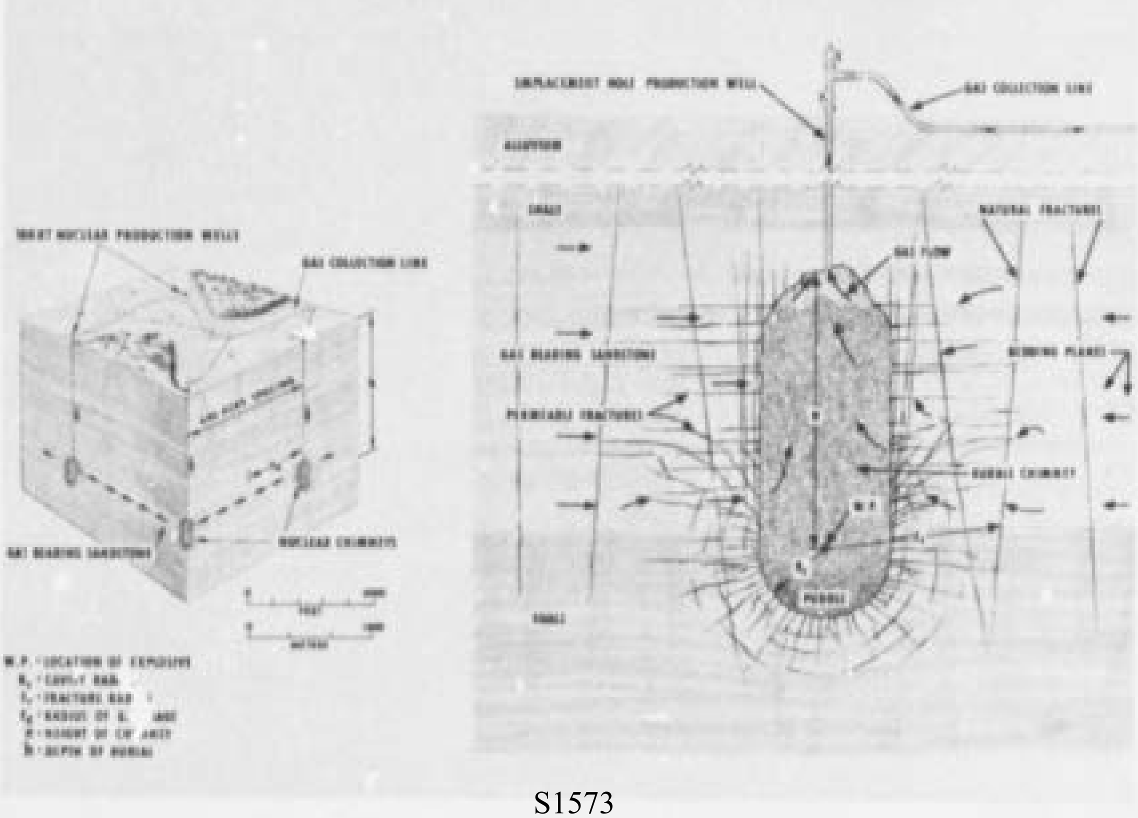

5. Project Bronco

The Bronco project would involve the fracturing of oil shale with nuclear explosions and the extraction of oil by an in-place retorting process.

On October 25, 1967, CER Geonuclear Corporation (CER), on behalf of about eighteen oil and related companies, formally proposed to conduct the Bronco experiment jointly with the AEC and the Department of the Interior.

The negotiations between the Government and CER were essentially completed during July 1968. Following expected contract execution, work will commence at the project site located 23 miles west of Rangely, Colorado.

The U.S. Bureau of Mines estimates that if in-place retorting of domestic oil shale in nuclear chimneys proves feasible, 160 billion barrels of oil might be recovered. Secretary of the Interior Stewart L. Udall, in an article written for the July 1967 issue of Western Oil Reporter, indicates that the Green River oil shale deposits of Colorado, Wyoming, and Utah, alone, are estimated to contain the equivalent of about 70 times the nation's proved reserves of crude petroleum.

6. Excavation

The Nuclear excavation program is currently planned to develop a technical feasibility determination for nuclear excavation in time to be considered by the Atlantic-Pacific Interoceanic Canal Study Commission in its final report to the President. In its report to the President, due before December 1, 1970, the Commission needs to make a determination regarding the feasibility of nuclear excavation to construct a sea-level canal in the region of the American Isthmus. Project Cabriolet, the first in a series of nuclear cratering experiments designed to develop a nuclear excavation technology, was detonated January 26, 1968, at the Nevada Test Site. The experiment involved the detonation of a two and one half kiloton nuclear explosive buried 170 feet in hard, dry rhyolite. A crater was formed having a diameter of about 360 feet and a depth of about 120 feet. Useful data was obtained on cratering mechanisms and cratering geometry to verify existing predictive capability, to refine existing fallout models, and to study atmospheric diffusion of airborne radioactivity.

Project Buggy, the first nuclear row-charge cratering experiment and second in the series mentioned above, was detonated March 12, 1968, at the Nevada Test Site. This experiment consisted of the simultaneous detonation of five nuclear explosives

each having a yield of about one kiloton (equivalent to 1,000 tons of TNT), buried at a depth of 135 feet and spaced 150 feet apart in a dry, complex basalt formation. The explosion created a huge ditch 250 feet wide, 850 feet long, and 65 feet deep. It is considered a major step toward the development of an economic and practical nuclear excavation technology for use in digging harbors, canals, and highway passes through mountains.

An aggregate production experiment has been p oposed by the U.S. Army Corps of Engineers, with the objective of developing a nuclear technology for producing large amounts of broken rock for use in rockfill dams. The Corps of Engineers' feasibility study estimates that approximately seven million cubic yards of rockfill material would be produced by detonation of a deeply buried 40 kiloton nuclear explosive. Before any decision can be reached on proceeding with such a nuclear detonation, a thorough study must be made of the proposed experiment to evaluate all of the considerations related to public safety.

CHART VII-B

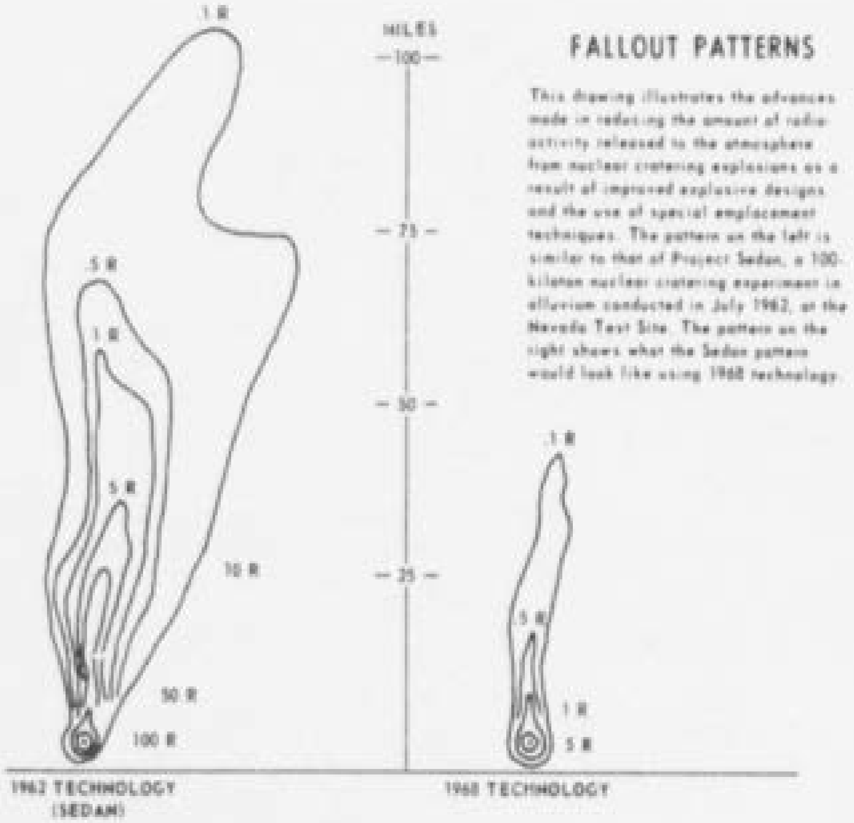

R - INFINITE DOSE FROM ARRIVAL IN ROENTGENS

F. Arizona Water Study

In response to a proposal by Governor Williams of Arizona, the AEC and the Department of the Interior are cooperating in a study of the feasibility of the application of nuclear explosives to water management in Arizona. The AEC is participating in the study principally through the Lawrence Radiation Laboratory of the University of California at Livermore, and the Department of the Interior is participating through the U.S. Geological Survey and the Bureau of Reclamation. The Arizona AEC is contributing to the study through the University of Arizona, Bechtel Corporation, and CER Geonuclear Corporation. AEC's San Francisco Operations Office has been named the Government coordinator for the study.

Work on this study began July 1, 1968, and should be completed in 12-15 months.

G. Charges for Nuclear Explosives

The AEC is currently conducting studies regarding the economics of nuclear explosions. These studies will provide the basis for a comprehensive program to examine the related costs in providing a commercial nuclear explosion service. Upon completion of the study, it is anticipated that a projected schedule of charges will be made available to industry for contained and cratering explosions, as well as estimated charges for simplified fielding systems, safety, and security activities.

In May of 1964, the AEC published projected charges for thermonuclear explosives, which are expected to be particularly relevant for excavation applications. The announced charges range from about $350,000 for a 10 kiloton explosive to $600,000 for a 2 megaton explosive. Arming and firing services are included in these charges, as well as the cost of the explosive. Charges for related services and safety studies are not included.

H. Miscellany

Included in the agenda of the International Conference on the Constructive Uses of Atomic Energy, sponsored by the Atomic Industrial Forum and the American Nuclear Society, was a discussion of the Plowshare program. Considerable interest is also being shown by the American Nuclear Society which featured the Plowshare program in the March 1968 issue of its publication entitled "Nuclear News."

CHART VII-C

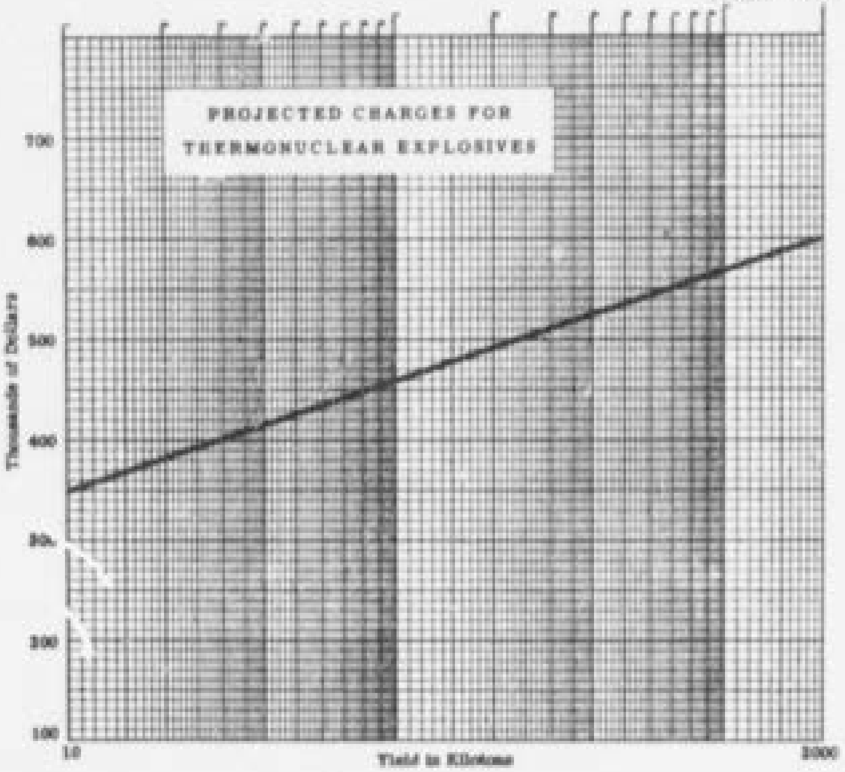

I. Program Information

Companies interested in discussing Plowshare applications may address their inquiries to the Division of Industrial Participation or to Mr. John S. Kelly, Director, Division of Peaceful Nuclear Explosives, U.S. Atomic Energy Commission, Washington, D. C. 20545.

More detailed information concerning the Plowshare program may be found in the following publications:

1. "Plowshare Program During 1967," for sale by the Superintendent of Documents, U.S. Government Printing Office, Washington, D. C. 20402, at $0.15.

2. "Plowshare," a booklet in AEC's "Understanding the Atom" series. Single copies are available free from the U.S. Atomic Energy Commission, P. O. Box 62, Oak Ridge, Tennessee 37830.

3. Plowshare Bibliography, TID-3522 (8th Revision), available from the Clearinghouse for Federal, Scientific and Technical Information, National Bureau of Standards, U.S. Department of Commerce, Springfield, Virginia 22151, price $1.25.

In addition, the following reports on the economics of Plowshare may be obtained from the Federal Clearinghouse:

1. Dagum, E., "A Cost-Benefit Model for Nuclear Explosive Stimulation of Natural Gas Reservoirs," June 30, 1968.

2. Dagum, E., Heiss, K. P., "An Econometric Study of Intermediate and Small Diameter Drilling Costs for the United States," June 30, 1968.

3. Whiten, T., "Primer on Pricing Principles Relevant to Plowshare Explosives," June 30, 1968.

Three films describing the program are also available through AEC film libraries. They are entitled "Plowshare," "Safety in the Plowshare Program" and "Gasbuggy."

[illegible]

CATEGORY VIII SERVICES

A wide range of nuclear service type activities are performed by organizations with interests outside the nuclear field. These activities are reviewed in this Category.

Included are seven major nuclear service areas: waste management, radiation dosimeter services, contaminated laundry, transportation, whole body counting, reactor servicing, and decontamination.

A. Radioactive Waste Management

Market Predictions
(Low Level Waste: Handling & Burial)

1970	$ 1,500,000
1975	$ 4,500,000
1980	$10,000,000

The above estimates have been increased slightly over those presented last year, mainly because of the fairly significant increase in the quantity of low level waste buried in 1967 as compared to the three previous years. If this trend continues, even at a minimum rate as compared to the forecasted growth of nuclear power, the levels of business indicated in the table should be reached.

Radioactive wastes are generated in practically all areas of the nuclear fuel cycle and accumulate as liquids, solids, and gases at varying radiation levels. Wastes generated in the cold or pre-irradiation phase of the fuel cycle (from the mine to the reactor), as well as wastes resulting from research laboratories, and from medical and industrial applications of radioisotopes, are generally considered as low level, low hazard potential wastes.

Most workers in the nuclear industry use the terms low level, intermediate level, and high level to characterize, in a general way, radioactive wastes. On the basis of gross activity concentration, low level wastes are defined as wastes with a radioactivity concentration of less than 10 curies per cubic foot, intermediate level wastes in the range of 10 to 1000 curies per cubic foot, and readings exceeding 1000 curies per cubic foot as high level wastes.

The chemical reprocessing of irradiated fuels is the primary source of all high level wastes. These wastes are presently stored as liquids in large underground tanks; however, increasing emphasis is being focused on research and development programs aimed at converting and reducing these high level liquids to solid form. While high level wastes are normally defined as wastes exceeding 1000 curies per cubic foot, they are, in any case, of such penetrating radiation level or have such contamination potential that they require complex handling and packaging techniques.

The Atomic Energy Commission has the responsibility for controlling the handling and disposal of this radioactive waste material. A general requirement for establishment of a land burial site is that the land be owned by the Federal or a state Government.

The Commission has the authority, under subsection 274b of the Atomic Energy Act of 1954, as amended, to enter into agreements with individual states for the transfer of certain of the Commission's regulatory responsibilities. Among these responsibilities are those relating to the disposal of radioactive wastes within a state. The Commission has, thus far, entered into agreements with 19 states. (Chart VIII-A).

MATERIALS LICENSING

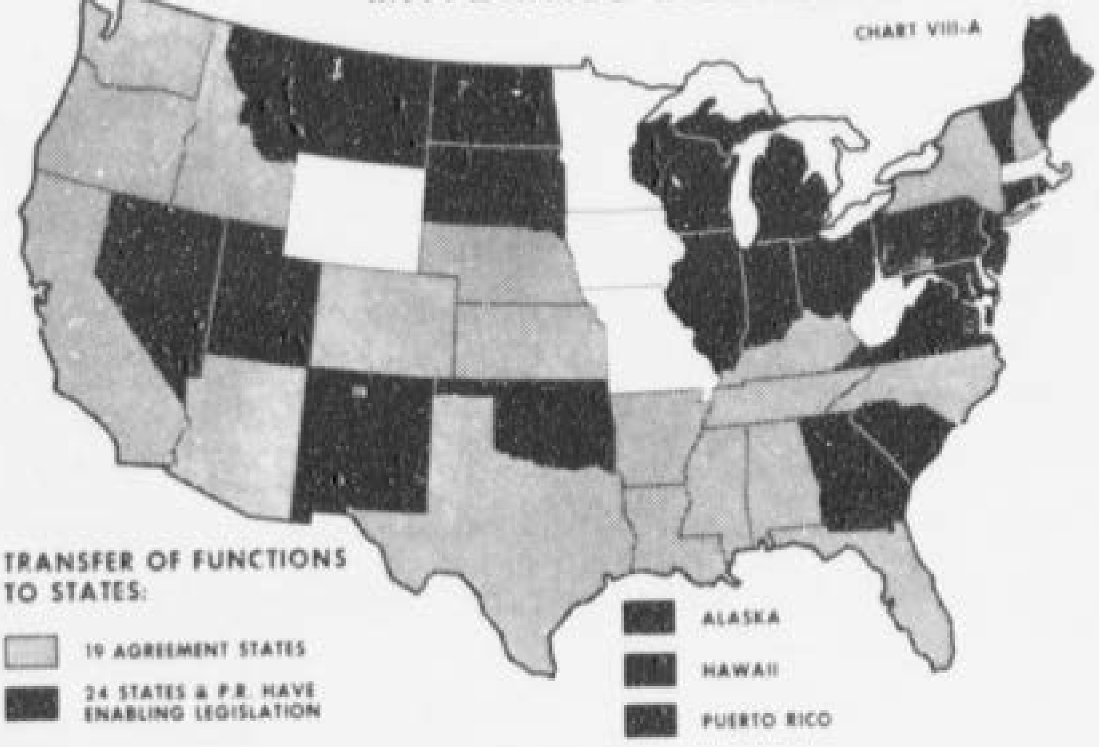

The Atomic Energy Commission and the National Academy of Sciences recently announced the establishment of a Committee on Radioactive Waste Disposal to review and advise the AEC concerning long-range radioactive waste management plans and programs for the rapidly expanding nuclear energy industry. The primary task of this new Academy committee includes the assessment of the adequacy of the present and projected technology to meet long-range health, safety, and other environmental requirements and the identification of new research and development needs.1/

The AEC's Rules and Regulations, 10 CFR Part 20, spell out in detail the standards established by the Commission in the control of waste disposal.

1. Low Level Waste Disposal

On May 14, 1963, the Commission approved withdrawal of the AEC from its interim low level waste burial operations provided to licensees, based upon the availability of commercial service from Nuclear Engineering Company (NECO) burial sites at Beatty, Nevada, and Morehead, Kentucky. In November of 1963, Nuclear Fuel Services, Inc., (NFS) established a low level waste disposal facility near West Valley, New York.

In 1965, California Nuclear, Inc. (CNI) began operation of its first waste burial site at Richland, Washington. On August 1, 1967, CNI was granted a license to operate its second burial site on state-owned land near Sheffield, Illinois. CNI merged with NECO in August 1968, with Nuclear Engineering becoming the surviving company.

Chart VIII-B, on the following page, indicates the two commercial radioactive waste disposal companies in operation, along with the location of the five burial sites in the United States.

Table VIII-1 shows the semiannual quantities of low level commercial waste buried since October 1962.

Burial charges have varied over the last few years from a high of $1.50 to a low of about $0.70 per cubic foot of waste buried. Based on an estimated current average disposal charge of about $1.00 per cubic foot, Table VIII-1 can be used as a reasonable indication of the dollar market for this service by

---

1/ AEC Public Release L-184, dated August 6, 1968.

substitution of dollars for cubic feet. Chart VIII-C, on the following page, presents a typical breakdown of rates and charges offered by a commercial waste disposal company.

TABLE VIII-1

Private Waste Burial (Cubic Feet)

	Jan.-June	July-Dec.	Annual Total
1962		36,281	36,281
1963	119,069	95,821	214,890
1964	241,660	205,434	447,094
1965	258,997	230,982	489,979
1966	264,800	238,172	502,972
1967	380,584	393,266	773,850
1968	324,940		

CHART VIII-B

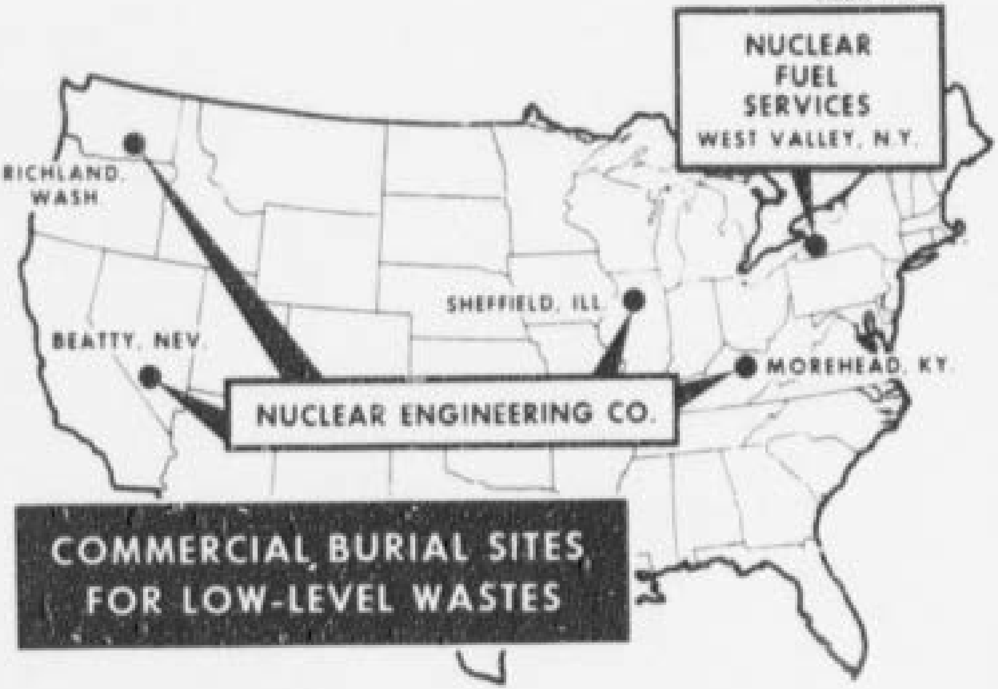

CHART VIII-C

SCHEDULE OF RATES AND CHARGES

The following is a published list of rates and fees Company "X" will charge users F. O. B. the "X" Burial Facility. Waste will be received from all sources on a prior booking arrangement.

All shipments must be in accordance with AEC, Bureau of Explosives, Government Bill of Lading and/or ICC regulations. All disposable containers must be self-supporting and capable of remaining intact during handling under normal weather conditions prior to burial. All containers that do not comply with the packaging and labeling requirements of Company "X", must be negotiated separately. Shipper must furnish statement to Company "X" disclaiming any right to title of wastes after the acceptance by Company "X". The following statement has been found acceptable by the Federal Government:

> "Title to all materials, which were previously Government-owned, contained in waste furnished for disposal under this contract (except material which is required by Section 52 of the Atomic Energy Act of 1954, as amended, to be owned by the Government) shall be deemed to be vested in Company "X" immediately upon acceptance of such material by Company "X" from the shipper or from the common carrier. The Government shall have no right to the recovery of any materials contained in such waste nor any credit for their potential value."

Waste shipments will be accepted at the "X" Burial Facility during the hours of 0830 to 1600 on week days, legal holidays excepted. Emergency shipments will be received at other times, however extra charges for such receipt may be added. Each truck or railroad car will normally be unloaded before the end of the next succeeding normal business day. However, an effort will be made to unload all trucks on the day of arrival. Company "X" will not take responsibility for demurrage on unscheduled shipments.

All shipments must be accompanied by completed "Radioactive Shipment Record Form". These forms are available from Company "X". Shipments received without these forms may be delayed as the information must be available prior to acceptance of the wastes at the "X" Burial Facility.

Company "X" will make the following charges for operation of the "X" Burial Facility:

> Unload and bury containers approved by the AEC, ICC, or Bureau of Explosives, or containers shipped under special ICC permit, which are less than 15 tons total weight per container -- $0.70 per cubic foot plus state charge (which presently is) -- $0.05 per cubic foot.

Surcharges as follows will be added for containers in excess of fifteen tons:

	Surcharges	
Weight, Tons	Per Shipment	Per Container
0 - 15	0.00 plus	0.00
15 - 30	130.00 plus	200.00
30 - 50	260.00 plus	330.00
50 - 60	520.00 plus	475.70
60 - 80	1,600.00 plus	1,200.00
80 - 130	3,200.00 plus	2,500.00

Surcharges will be made for inner packaging from shielded containers as follows:

> Density over 200 pounds per cubic foot -- $0.005 per pound for total weight -- over 200 pounds per cubic foot.

Lower charges will be made for very large volumes delivered to the facility by a single organization in any single calendar year as follows:

Cubic Feet	Cost per Cubic Foot*
1 to 100,000	$0.700
100,000 to 200,000	0.675
200,000 to 300,000	0.650
Over 300,000	0.625

*Plus state charges

Surcharges as follows will be added for special handling of containers consisting of two or more parts. This is for removing and burying inner containers which have been shipped inside a shielded coffin, cask or container:

Primary Containers with Surface Dose Rates	$ Per Shipment		$ Per Hour**
0.2 to 10 r/hr	$ 25.00	plus	$26.00
10 to 50 r/hr	50.00	plus	26.00
50 to 100 r/hr	100.00	plus	26.00
100 to 500 r/hr	250.00	plus	26.00

**The $26.00 per hour referred to includes consulting and preparation of proper procedures as well as the additional equipment, time and labor used to handle this equipment.

The minimum charge for any shipment will be $20.00.

Another facet of the radioactive waste business in addition to burial itself, is the operation of collection, transportation, and storage services. Companies licensed by the AEC in the handling of radioactive wastes are listed in Table VIII-2.

TABLE VIII-2

Commercial Firms Licensed by the U.S. Atomic Energy Commission to Receive Radioactive Wastes
(As of August 21, 1968)

ATCOR, Inc.
360 Bradhurst Avenue
Hawthorne, New York 10532

Atomic Disposal Company, Inc.
14532 South Kedzie Avenue
Midlothian, Illinois 60445

California Salvage Company
700-745 North Pacific Avenue
San Pedro, California 90731

Laboratory for Electronics, Inc.
Tracerlab Division
1601 Trapelo Road
Waltham, Massachusetts 02154

Long Island Nuclear Service Corp.
Station Road
Bellport, New York 11713

Nuclear Diagnostic Laboratories, Inc.
19 Hubbard Drive
White Plains, New York 10605

Nuclear Engineering Company, Inc.
Box 594
Walnut Creek, California 93002

Radiological Service Company, Inc.
35 Urban Avenue
Westbury, New York 11590

The Walker Trucking Company
1283-1285 East Street
New Britain, Connecticut 06053

These firms should be contacted directly for information regarding services they can provide.

As mentioned earlier in this section, the AEC has transferred many of its regulatory responsibilities to "Agreement States". Table VIII-3, on the following page, lists those private firms licensed by agreement states to receive radioactive waste.

TABLE VIII-3

Commercial Firms Licensed by Agreement States
To Receive Radioactive Wastes
(As of August 21, 1968)

ATCOR, Inc.
360 Bradhurst Avenue
Hawthorne, New York 10532

Atomic Energy Industrial
Laboratories of the Southwest
6413 South Main Street
Houston, Texas 77025

California Salvage Company
700-745 North Pacific Avenue
San Pedro, California 90731

General Nuclear, Inc.
6740 Telean
Houston, Texas 77034

Laboratory for Electronics, Inc.
Tracerlab Division
2030 Wright Avenue
Richmond, California 94804

Long Island Nuclear
Service Corp.
Station Road
Bellport, New York 11713

McCormack's Highway
Transportation, Inc.
151 Erie Boulevard
Schenectady, New York 12305

Nuclear Diagnostic Laboratories, Inc.
19 Hubbard Drive
White Plains, New York 10605

Nuclear Engineering Company, Inc.
Box 146
Barns Building
Morehead, Kentucky 40351

Nuclear Engineering Company, Inc.
243 Symons
Richland, Washington 99352

Nuclear Engineering Company, Inc.
P. O. Box 594
Walnut Creek, California 94597

Nuclear Fuel Services, Inc
P. O. Box 124
West Valley, New York 14171

Radiological Service Company, Inc.
Waste Disposal Department
35 Urban Avenue
Westbury, New York 11590

U.S. Nuclear Corporation
801 North Lake Street
Burbank, California 91502

William Wayne Electronics, Inc.
Hastings Radiochemical Works
P. O. Box 60448
Houston, Texas 77060

These firms should be contacted directly for information regarding services they can provide.

The burial of AEC solid radioactive waste generated at AEC burial sites has been proceeding at a fairly constant rate of about 1.5 million cubic feet per year since 1963. Table VIII-4 gives a fiscal year breakdown of solid waste burial at AEC facilities.

TABLE VIII-4

AEC Solid Radioactive Waste Burials

Fiscal Year*	At AEC Burial Grounds: From AEC and AEC Contractor Operations	From Other Government Agencies**	From Licensees**	Total
1961	2,892,600	20,600	74,400	2,987,600
1962	2,268,200	21,800	68,900	2,358,900
1963	1,698,900	24,500	77,700	1,801,100
1964	1,697,400	2,700***	15,300***	1,715,400
1965	1,454,300			1,454,300
1966	1,413,000			1,413,000
July-Dec. CY-1966	775,000			775,000

* Fiscal Year is from July 1 to June 30.
** Buried at Oak Ridge and National Reactor Testing Station (Idaho) under AEC Interim Burial Program.
***Buried during period of July/August, 1963.

The volumes of low level solid wastes available annually for commercial burial are estimated to reach over 1 million cubic feet by 1970, 3 million cubic feet by 1975, and 6 million cubic feet by 1980.

2. Reactor Resin Waste

In a typical water-cooled power reactor, traces of impurities are carried along with the coolant and irradiated as they pass through the core of the reactor. These radioactive products, such as iron, nickel, and other corrosion products, are removed from the system in ion-exchange columns. The radioactive resins from these columns are periodically removed for burial at commercial waste disposal sites. Table VIII-5 is an estimate of the future reactor resin waste market, based on the AEC's recent "most likely" forecast of about 150,000 MWE of installed nuclear capacity in the year 1980, and a factor of 500 cubic feet[2/] of reactor resins discharged per year per 1000 MWE. The cost column assumes handling and burial charges of about $50.00 per cubic foot.

TABLE VIII-5

Reactor Resin Waste Handling and Burial Forecast

Year	Cubic Feet	Dollars
1970	5,000	$ 250,000
1975	30,000	$1,500,000
1980	73,000	$3,600,000

3. Intermediate Level Waste Disposal

Intermediate level wastes, like high level wastes, are practically all produced during the processing of spent fuel. At the present time, they are generated at AEC production sites, several AEC national laboratories, and at the NFS commercial fuel reprocessing plant.

The AEC's Oak Ridge National Laboratory has developed, and is now routinely operating, a "hydraulic fracturing" technique by which intermediate level wastes are disposed of by injection into the ground. The radioactive waste is first concentrated

2/ Remarks by Dr. Stuart McLain, Purdue University, "Disposal of Low Level Radioactive Wastes," at Argonne National Laboratory to the Fuel Management Seminar, Argonne, Illinois, August 30, 1967.

by evaporation, then mixed in storage tanks with cement and other additives, and then pumped under high pressure into suitably oriented fractures in impermeable shale formations 700-1,000 feet deep. The slurry establishes itself in a thin horizontal sheet configuration several hundred feet across which permanently sets in this position.

4. High Level Waste Management

a. Commercial

Radioactive liquid waste effluents from chemical reprocessing plants contain essentially all of the radionuclides generated within the nuclear cycle not recovered as by-products. The first commercial reprocessing plant which began operations in 1966 at West Valley, New York, and the operation of additional reprocessing plants, in the near future, will generate high level wastes in ever-increasing quantities. Present estimates indicate an accumulated volume of wastes from the reprocessing of nuclear power fuels of approximately 6 million gallons by 1980, and 75 million gallons by the year 2000, assuming all the wastes are stored as liquid. These figures were increased over those presented in last year's report to reflect the latest AEC forecast of nuclear power growth, which reaches a "medium" of about 150,000 MWE by 1980. As conversion-to-solids technology, being developed by the AEC, becomes operational, these volumes could be expected to be reduced by about a factor of 10.

b. Method of Handling

Principally, there are two types of high level wastes stored: "Self-boiling" and "Non-boiling." The difference depends on the relative fission product concentration of the waste solution. In either case, the primary concern is to assure the containment and control of the material involved. At the Nuclear Fuel Services reprocessing plant at West Valley, New York, the high level liquid wastes are stored in large, concrete enclosed, carbon steel tanks (600,000 gallons design capacity) located in close proximity to the separations plant. The NFS operation results in a generation rate of 400 gallons of high level liquid waste per metric ton of uranium processed. GE, in its plant to be constructed at Morris, Illinois, plans to convert to solid form all process waste streams, except the gaseous effluents. In the GE process the high level

wastes will first be concentrated in an evaporator, then fluid-bed calcined to a solid form, and the resulting calcined solids placed in a corrosion-resistant steel container. The packaged solids will then be submerged in a water-filled basin to provide cooling and shielding, pending shipment to a permanent storage site. GE has indicated that about one cubic foot of high level waste will be generated for each metric ton of uranium processed.

c. AEC Experience in Waste Handling

Significant amounts of radioactive waste solutions are generated and stored at the AEC's Hanford, Idaho Falls, and Savannah River sites. Over 75 million gallons of high level waste have been satisfactorily handled to date, mainly as a result of chemical processing operations on production reactor fuels. These wastes are being stored in special underground tanks, which range in size from about 300,000 to 1,330,000 gallons of capacity. It can be concluded, based on AEC experience, that: (1) radioactive waste management practices have not resulted in any harmful effects on the public, its environment, or its resources; and (2) the general problem of radioactive wastes need not retard the future development of the nuclear energy industry.

5. Research and Development[3/]

The AEC has had a research and development program in connection with nuclear fuel reprocessing operations at its production and test sites ever since the first fuel from a production reactor was processed. This research and development program has provided considerable information that is applicable to the handling of high level wastes. The conversion of large-volume, high level, liquid wastes to more compact and easier handled inert solid forms has received extensive R&D emphasis. Several liquid/solid conversion approaches are under study, including such processes as fluidized bed, heated pots, spray solidification, and phosphate glass fixation. With the techniques under development, wastes can be made immobile while at the same time achieving volume reductions by a factor of about 10, as compared to the volume required for total liquid storage.

3/ See "Waste Management and Environmental Aspects of Nuclear Power," J. A. Lieberman and W. G. Belter, Environmental Science & Technology, June 1967, pages 466-475.

Solidification and disposal technology for high-activity waste has reached the pilot plant and field demonstration phase. In December 1963, the Waste Calcining Facility at the National Reactor Testing Station became the world's first plant-scale facility for converting actual high level radioactive wastes to solid form. The plant was satisfactorily operated during its initial run, which ended in late 1964. The plant then remained down for about 1-1/2 years, while additional solids storage bins were constructed. The facility's second campaign began on April 1, 1966, and ended on March 24, 1968. Pertinent operational data follows:

Waste Calcining Facility

	Run No. 1	Run No. 2	Total Feed (Gal.)	Total Product (Ft.^{3})	Volume Reduction
Start	12-8-63	4-1-66			
End	10-15-64	3-24-68			
Gal. Feed					
Al. Type	511,350	879,310	1,390,660	17,856	10.4
Zr. Type	--	109,370	109,370	1,667	8.8
TOTAL	511,350	988,680			

At the AEC's Hanford Plant near Richland, Washington, a program is underway to demonstrate, on an engineering scale, the technical economics and safety aspects of various solidification processes for converting high level liquid wastes to solid form. Three processes have been selected for testing in the Waste Solidification Engineering Prototype Plant (WSEP), which began operation in the fall of 1966.4/

Storage of high level liquid wastes in geological formations, such as salt mines, has also been under investigation. A two-year demonstration program with radioactive material in a salt mine, near Lyons, Kansas, was completed in 1968. Irradiated fuel assemblies from the Engineering Test Reactor at NRTS, Idaho, served as the source of irradiation in place of actual solidified wastes. The program has not revealed any major obstacles to the ultimate disposal of highly radioactive wastes in salt. Final reports on the operation are expected to be released in the near future.

4/ See "Progress in Waste-Disposal Research & Development," Phillip Fineman, Reactor and Fuel-Processing Technology, Spring 1968, Vol. 11, No. 2, pages 107-116.

6. Foreign Plans and Programs

The International Atomic Energy Agency held a symposium on the disposal of radioactive wastes into the ground. The meeting was held in Vienna from May 29 to June 2, 1967. The meeting was broken down into the following three sessions: (1) operational experiences, (2) hydrology and geochemistry, and (3) research and development on mines and cavities for waste disposal. The proceedings of this meeting have been published.5/

a. Low Level Waste Disposal

The European Nuclear Energy Agency has been sponsoring an experimental project involving sea disposal of radioactive waste. In the summer of 1967, 11 thousand tons of solid radioactive waste were dumped into the Atlantic Ocean. Waste from nuclear reactors in Britain, France, West Germany, Belgium, and The Netherlands was packaged in containers and dumped at a depth of 16,400 feet. The Agency's annual report concludes that the dumping will represent no risk either to man or marine organisms.

The French have under construction at Marcoule, a pilot facility for the vitrification of low level wastes. Different kinds of glass, aluminosilicate, or alumino phosphate have been used in experimentally developing this waste disposal technique. Glass blocks, weighing several kilograms and having a specific radioactivity of 2 curies per milliliter, have been prepared on the laboratory scale. Two industrial scale processes are under study (Service de Genie Chimique Radioactif, Groupe des Produits de fission). The first is a continuous process, using a preliminary calcination in a rotary kiln; the other, of more advanced design, is a semicontinuous method involving vitrification in a metallic pot. It is this latter method that will be used in the pilot plant under construction at Marcoule. (Bulletin d'Informations Scientifiques et Techniques, July 1968)

5/ "Disposal of Radioactive Wastes Into The Ground," International Atomic Energy Agency, Vienna, 1967.

b. High Level Waste Management

(1) Windscale, UK

In the past highly active wastes from the solvent extraction plant have been concentrated in a number of small evaporators and stored in intermediate-sized tanks. These are still in use, but in the course of expanding the reprocessing plant, new larger evaporators and storage tanks are being installed.

(2) Eurochemic

The high level wastes generated at the Eurochemic reprocessing plant at Mol, Belgium, are concentrated in an evaporator and stored in heavily shielded tanks.

7. Useful By-Products From Reactor Wastes

Fission products, such as strontium, cesium, and promethium, recovered from irradiated fuel processing are already finding useful commercial applications. Others, such as xenon, krypton, rhodium, and palladium, are being considered for recovery because of potential uses. Possible markets for the expanded use of these materials in the near future offers many challenging opportunities.

Of particular interest in the by-product category is neptunium, which is used as the target material in the production of plutonium-238. It is possible that at some future date there will be a very large demand for this by-product in the space program. General Electric is offering to recover neptunium (as well as uranium and plutonium) from irradiated nuclear fuel at its chemical reprocessing plant being constructed at Morris, Illinois. Nuclear Fuel Services, Inc., New York State Atomic and Space Development Authority, and all the other companies with interests in the chemical reprocessing business, are giving serious consideration to this and other isotopes for which a market and economic conditions justify recovery.

B. Personnel Dosimeter Services

In order to detect and control exposure to ionizing radiation by personnel working in or around potential source areas, external dosimetry devices are utilized. The most common external personnel monitoring device is the film badge. There are indications, however, that Thermoluminescent Dosimetry (TLD) may become a major competitor to the film badge in the future. Though the current usage of TLD's

is only a few percent of the total market, several groups are using them on a limited trial basis with a view to eventual change-over. In recognition of this and other potential developments, the title and content of this section have been broadened from "Film Badge Processing," which appeared in previous reports, to include all "Personnel Dosimeter Services" of commercial interest.

Anyone working with x-ray equipment, electronic equipment which may produce x-rays, or radioactive materials, is a potential customer for this service. Research laboratories, nuclear industrial plants, hospitals, physicians' and dentists' offices, and institutes of higher education are some of the more common users of this service.

In 1963, we reported that there were from 2-1/2 to 3 million film badges processed commercially at a dollar volume of about $1-1/4 million. In the same year, about 1-3/4 million badges were processed at AEC sites representing a dollar volume of about $1 million. No new survey has been made since 1963; however, a recent spot check with several major commercial and Commission processors indicates that commercial business has doubled in the five intervening years. In the same period, the number of units processed at AEC sites has decreased about 50 percent. This decrease is due largely to longer film use before processing and, to a lesser extent, the change-over to TLD's. A significant portion of the recent commercial increase has come from non-nuclear electronic firms who work with electronic equipment which may produce x-rays (such as TV tubes).

Private companies are well represented in this phase of the nuclear industry with approximately 16 companies offering this service (Table VIII-6, on the following page). Chart VIII-D, on the following page, shows the principal commercial film badge operations in the United States.

Most of the companies noted in Table VIII-6 offer a complete line of service including film and film holder supply, exchange badges, processing and interpretation, and reporting. The processing procedures employed by various companies range from those consisting of many individually controlled steps to those of a highly automated nature.

The price charged for this service depends on the type of badge (beta, gamma, or neutron), the size of order, and the exchange period (weekly, bi-weekly, monthly, etc.).

TABLE VIII-6

Film Badge Processors

Applied Health Physics, Inc.
Bethel Park, Pennsylvania

Atomic Energy Industrial Laboratories
Houston, Texas

Eberline Instrument Corporation
Santa Fe, New Mexico

Gaines Nuclnar Health Services, Inc.
Berkeley, California

Gard-Ray Film Badge Service
Burlington, Massachusetts

Health Physics Services, Inc.
Baltimore, Maryland

Isotopes, Inc., A Division of Teledyne Company
Westwood, New Jersey

R. S. Landauer, Jr., and Company
Glenwood, Illinois

Nuclear-Chicago Corporation
Des Plaines, Illinois

Nuclear Services Laboratory
Knoxville, Tennessee

Nucleonic Corporation of America
Brooklyn, New York

Radiation Detection Company
Mountain View, California

St. John X-Ray Laboratory
Califon, New Jersey

Technical Associates
Burbank, California

Tracerlab, Div. of L. F. E.
Waltham, Massachusetts

United States Testing Company
Hoboken, New Jersey

PRINCIPAL FILM BADGE OPERATIONS IN THE UNITED STATES

CHART VIII-D

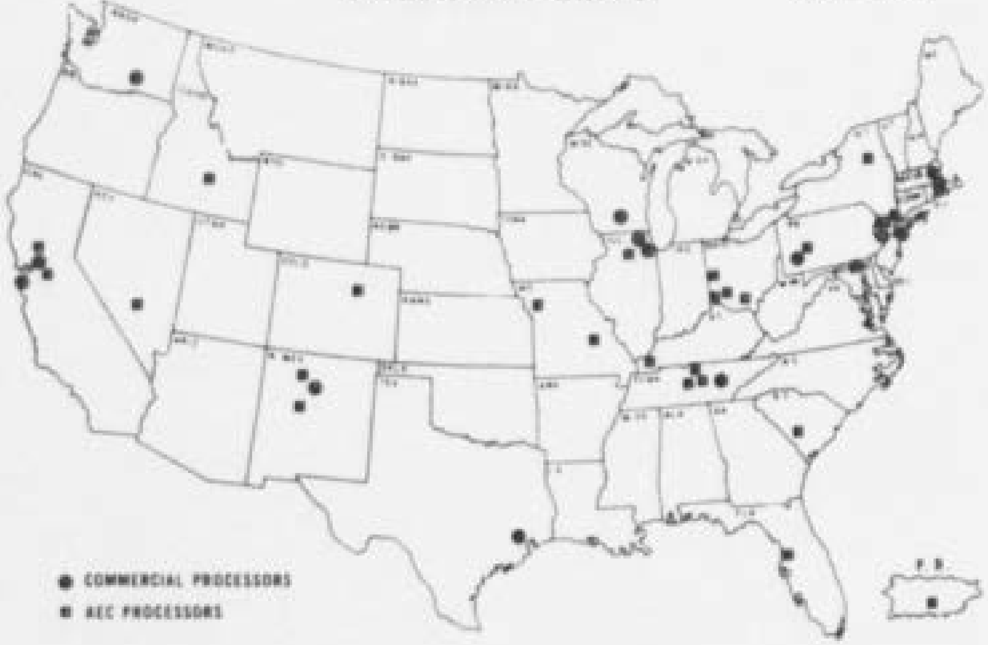

[illegible]

As mentioned earlier, there has been a growing use of, and interest in, thermoluminescent dosimetry devices (TLD) in both Government and private installations. Many of the film badge processing companies are offering the sale and service of these re-usable dosimeters and the reading instrument. In operation, the working material becomes excited to a higher energy state when exposed in an ionizing radiation environment. The reading instrument heats the TLD which then releases its excess energy in the form of light. The intensity of the emitted light is proportional to the cumulative dose, so that an exposure measurement can be determined.

The radiation-sensitive thermoluminescent material is a crystaline powder such as calcium fluoride. It can be supplied in a variety of configurations, the most common one being as a coating on a piece of teflon which fits into the familiar cassette of a film badge. Because of the light insensitivity of thermoluminescent materials, their use is very flexible; e.g., they can be taped to the body or incorporated in a pen-type holder. Because of the tenfold sensitivity advantage of TLD's over film, they tend to be preferred for use by non-radiation workers.

The Commission published broad guidelines for film badge processing standardization in the Federal Register in 1963.6/ In 1967, the Battelle Northwest Laboratory developed, under a Commission contract, performance criteria and procedures for the evaluation of film dosimetry services.7/ One of their conclusions was that "a program that would provide an assessment of the quality of film processing services appears to be desirable." The Commission is considering what official action, if any, should be taken in regard to the mandatory use of these performance criteria and procedures. Information about this program can be obtained from the Division of Biology and Medicine, U.S. Atomic Energy Commission, Washington, D. C. 20545.

The National Sanitation Foundation (NSF) has had a film dosimetry and calibration laboratory in operation for over two years now. NSF has developed a standard test procedure (NSF Standard No. 16) for evaluation of film badge services on a periodic basis. The Foundation has established a "Seal of Approval" and extended the right to use this Seal to any requesting processor who can pass the periodic tests established in the Standard. To date, seven film badge processing companies and two Government facilities have qualified to use the Seal, as shown in Table VIII-7.

6/ U.S. Atomic Energy Commission, "Notice Regarding Need for Establishment of Film Dosimetry Calibration Laboratory," Federal Register 63, p. 9411, September 1963.

7/ BNWL-542, "The Establishment and Utilization of Film Dosimeter Performance Criteria," September 1967.

TABLE VIII-7

National Sanitation Foundation Testing Laboratory, Inc.
1968
Approved - Listing
for
Film Badge Services
NSF Standard No. 16

Eberline Instrument Corporation
P. O. Box 2108
Santa Fe, New Mexico 87501

Gard-Ray Film Badge Service
P. O. Box 117
Burlington, Massachusetts 01803

R. S. Landauer, Jr., & Company
Science Road
Glenwood, Illinois 60425

Nuclear-Chicago Corporation
333 East Howard Avenue
Des Plaines, Illinois 60018

Nucleonic Corporation of America
196 Degraw Street
Brooklyn, New York 11231

Radiation Detection Company
385 Logue Avenue
Mountain View, California 94042

Tracerlab, Div. of L. F. E.
Film Badge Department
1601 Trapelo Road
Waltham, Massachusetts 02154

U.S. Air Force Radiological
Health Laboratory
Wright Patterson Air Force Base,
Ohio 45433

U.S. Atomic Energy Commission
Idaho Operations Office
P. O. Box 2108
Idaho Falls, Idaho 83401

The NSF Standard No. 16 took three years to develop, and was supported with technical and financial contributions from many different organizations interested in radiation dosimetry with film badges. A research program, which provided basic data utilized in the development of the standard, was supported by the U.S. Public Health Service. The NSF program to evaluate film badge services is the only one to be announced in this country to date. Information regarding this program can be obtained from National Sanitation Foundation, University of Michigan, School of Public Health, Ann Arbor, Michigan 48103.

C. Contaminated Laundry

Ten companies are actively engaged in the nuclear laundry business (see Table VIII-8, on the following page) and, with the exception of the Nuclear Decontamination Corporation, are operated

as part of a larger industrial non-nuclear laundering complex. In many cases, both the contaminated and the industrial operations are housed under the same roof, separated by partition and by an independent system arrangement.

TABLE VIII-8

Commercial Licensees
Companies Actively Processing Contaminated Laundry

1. Interstate Industrial Laundry and Decontamination Service, Inc.
   Santa Fe, New Mexico

2. Interstate Industrial Laundry Service, Inc.
   Pleasanton, California

3. Interstate Industrial Uniform Rental Service, Inc.
   Indian Orchard, Massachusetts

4. Nuclear Laundry Rental Services, Inc.
   Jeannette, Pennsylvania

5. Nuclear Laundry Service, Inc.
   Waterbury, Connecticut

6. Nuclear Decontamination Corporation
   Apollo, Pennsylvania

7. Penn Overall Supply Company
   Pittsburgh, Pennsylvania

8. Interstate Industrial Uniform Rental Service, Inc.
   N. Charleston, South Carolina

9. Southern Space, Inc.
   Macon, Georgia, and Portsmouth, Virginia

10. Sanitary Overall Supply Company
   Tarantum, Pennsylvania

Contaminated laundry facilities are unique in that they cannot economically be used for other laundry use. They require additional equipment (holdup tanks, monitoring equipment, etc.) and personnel training over and above those needed in conventional laundries.

The commercial contaminated laundry companies service such nuclear clients as the nuclear power utilities, hospitals, research and development laboratories, various companies in the private nuclear industry, and 14 AEC sites and contractors. Two years ago, the annual volume from these 14 AEC sites totaled about 2,500,000 pounds, representing about $500,000 of business to the contaminated laundry market. While no new survey has been made, because of the large increase in central station nuclear power plants and in fuel fabrication and reprocessing facilities, there is no doubt that the contaminated laundry business has increased substantially in the two years since [illegible] last survey. The laundered items consist of coveralls, laboratory [illegible]oats, boots, undergarments, sock[illegible], towels, and caps.

The possession of by-product materials, source material, and special nuclear material is subject to the licensing authority of the AEC, or in some cases, an "Agreement State."8/ Title 10, Code of Federal Regulatiuns, Parts 20, 30, 40, and 70 are pertinent to this licensing. In this context, possession includes contamination associated with garments and equipment, and the waste solutions or sludges that accumulate.

It is the Commission's policy to encourage the use of commercial services for processing AEC-generated laundry on a competitive basis. Chart VIII-E indicates the location of AEC and private contaminated laundry operations in the United States. Table VIII-9, on the following page, lists the AEC sites both "doing and procuring" this service.

CONTAMINATED LAUNDRY OPERATIONS IN THE UNITED STATES

CHART VIII-E

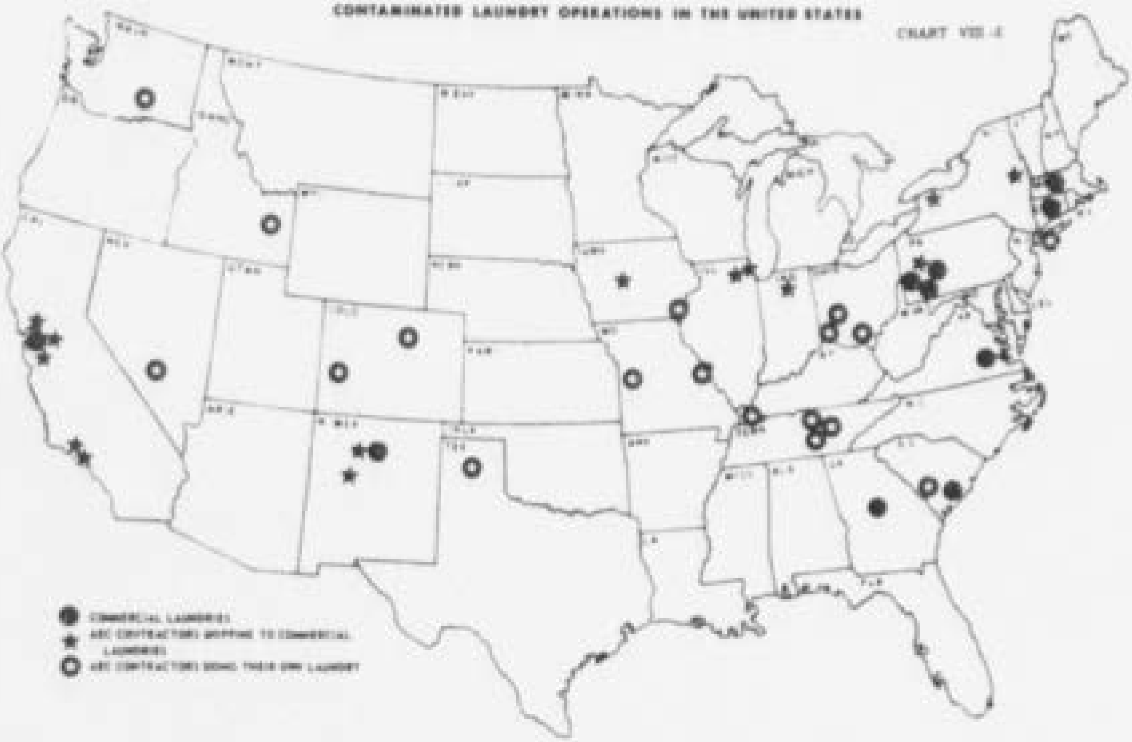

8/ See page for further information on "Agreement States."

TABLE VIII-9

AEC Sites and Contractors Using
Commercial Decontamination Laundering Facilities

Los Alamos Scientific Laboratory	- Los Alamos, New Mexico
Atomics International	- Canoga Park, California
Argonne National Laboratory	- Argonne, Illinois
Knolls Atomic Power Laboratory	- Schenectady, New York
Lawrence Radiation Laboratory	- Berkeley, California
Bettis Laboratory	- Pittsburgh, Pennsylvania
Sandia Laboratory	- Albuquerque, New Mexico
Ames Laboratory	- Ames, Iowa
Argonne Cancer Hospital	- Chicago, Illirois
Laboratory of Nuclear Medicine	- Los Anoeles, California
Univ. of California Medical Ctr.	- San Francisco, California
Univ. of Rochester Atomic Energy Project	- Rochester, New York
Notre Dame Radiation Laboratory	- Notre Dame, Indiana

AEC Sites and Contractors Having Their Own
Commercial Decontamination Laundering Facilities

National Lead Company	- Fernald, Ohio	OR
Union Carbide - Y-12	- Oak Ridge, Tennessee	OR
Union Carbide - ORNL	- Oak Ridge, Tennessee	OR
Union Carbide - K-25	- Oak Ridge, Tennessee	OR
International Tel./FSS	- Hanford. Washington	RL
Du Pont - Savannah River	- Aiken, South Carolina	SR
Dow Chemical Company	- Rocky Flats, Colorado	AL
Mason & Hanger	- Burlington, Iowa	AL
Manson & Hanger - Pantex	- Amarillo, Texas	AL
Brookhaven National Lab.	- Upton, New York	BH
Idaho Nuclear - NRTS	- Idaho Falls, Idaho	ID
Goodyear	- Portsmouth, Ohio	OR
Union Carbide	- Paducah, Kentucky	OR
Reynolds Electric	- Mercury, Nevada	NEV
Monsanto Chemical - Mound	- Miamisburg, Ohio	AL
Bendix	- Kansas City, Missouri	AL
Lucius Pitkin	- Grand Junction, Colorado	GJ

D. Transportation

This service includes the packaging and transportation of radioactive materials such as radioisotopes, radioactive wastes, weapons, ores and concentrates, unirradiated nuclear fuel materials, fabricated fuel elements, and irradiated spent fuel elements.

In performing this service, all modes of transportation - air, surface, and water are used. Large power reactor spent fuel shipments normally use rail or water due to the heavy shielding of the shipping casks required for this type of material. Piggyback transport of unirradiated power reactor fuel elements is also employed extensively. Trucks are also used within their weight carrying limitations.

An understanding of the packaging requirements and movements of radioactive materials can be obtained by reviewing the total flow of materials in the nuclear fuel cycle starting at the uranium ore mill and ending with the return of uranyl nitrate and plutonium nitrate to the Government or the owners for further use. The principal types of packaging and the usual flow patterns for these materials are shown below.

Commodity	Packaging	Point to Point Movements
Uranium Ore Concentrates	Drums	Uranium mill to feed preparation plant
Normal UF_6	Pressurized cylinders	Feed preparation plant to enriching (gaseous diffusion) plant
Enriched UF_6	Pressurized cylinders and protective packaging	Enriching plant to materials processing and/or fuel fabrication plant
Enriched uranium oxide powder or pellets	Drums and birdcages	Materials processing plant to fuel fabrication plant
Cold new fabricated fuel assemblies	Protective packages and birdcages	Fuel fabrication plant to nuclear power reactors
Irradiated fuel	Shielded casks	Nuclear power reactor to chemical reprocessing plant
Uranyl nitrate	Tank trucks, tank cars and protective packages	Chemical reprocessing plant to fuel fabrication plant or to UF_6 feed preparation plant
Plutonium nitrate	Protective packages and plutonium containers	Chemical reprocessing plant to fuel fabrication plant
Fission product radioisotopes	Shielded casks	Chemical reprocessing plant to government or industry users

Based on the projected median figure of installed nuclear power capacity (150,000 MWE through 1980), and the associated estimates of U_3O_8 requirements, estimates have been made of the tonnage of shipments for the domestic nuclear power industry which are presented below.

Commodity	1970	1975	1980
U_3O_8	7,500 tons	23,000 tons	43,000 tons
Normal UF_6	9,000 tons	29,000 tons	54,000 tons
Enriched UF_6	1,800 tons	5,800 tons	11,000 tons
UO_2	1,400 tons	4,500 tons	8,400 tons
New Fuel Elements	1,900 tons	6,000 tons	11,000 tons
Irradiated Fuel	200 tons	1,700 tons	6,000 tons
Total, Rounded	22,000 tons	70,000 tons	133,000 tons
Add Weight of Containers	40,000 tons	130,000 tons	250,000 tons
Total, Rounded	66,000 tons	200,000 tons	383,000 tons
Plus return of re-usable containers	40,000 tons	130,000 tons	250,000 tons
Grand Total	106,000 tons	330,000 tons	633,000 tons

Foreign nuclear programs are expected to require tonnage shipments of the same order of magnitude as those in the U.S.

1. Transportation and Packaging Symposium

In October 1968 a second international symposium on packaging and transportation of radioactive materials was held in Gatlinburg, Tennessee. Papers presented at this symposium covered a very broad range of subjects within the field of packaging and transportation, including regulatory matters, shipping economics, container research and development, design and testing of containers for processed fissionable fuel materials such as UO_2 and PuO_2, cold fuel assemblies and irradiated spent fuels. Three papers were presented reporting on the AEC's demonstration fuel element shipping cask fabricated from

laminated depleted uranium metal that was referred to in our 1967 report. Copies of the symposium proceedings may be purchased from the Federal Clearinghouse, Springfield, Virginia.

2. Costs

The largest single item of shipping cost in the nuclear fuel cycle is for irradiated fuel. This year additional information on spent fuel shipping costs became available with the publication of the AEC's Civilian Nuclear Power report "Current Status & Future Technical & Economic Potential of Light Water Reactors" - WASH 1082. In projecting fuel cycle costs, the shipping cost component of fuel cycle recovery (shipping spent fuel, reprocessing it, and reconverting the recovered uranium to UF_6) has been projected from 1970 through 2000. This cost, composed of two major components - amortization of the shipping cask and the ton-mile cost of transportation - is estimated to be $5.50 per kg uranium in 1970; $4.50 in 1975; $4.00 in 1980 and remains at $4.00 through 2000. The costs are based on rail transportation.

The shipping costs projected in WASH-1082 are of the same order of magnitude as the spent fuel shipping costs estimated by the National Lead Company at about $5.00 per kg U and by Nuclear Fuel Services at $4.00 to $10.00 per kg U in Nuclear Industry - 1967.

3. Transportation Regulations

The AEC's regulations govern the packaging of radioactive materials for transport and are contained in the U.S. Code of Federal Regulations, Title 10, Part 71. A revision to 10 CFR Part 71 became effective on August 21, 1966. At the same time an AEC Manual Chapter (AECM-0529) was issued that placed AEC contractors under identical packaging requirements, thus establishing a common standard for all shippers of fissile and large quantities of radioactive material to use in designing shipping packages.

On April 1, 1967, the Department of Transportation (DOT) assumed the overall regulatory responsibilities of the Coast Guard, Federal Aviation Agency, and the safety activities of the Interstate Commerce Commission. The AEC coordinates its regulatory control of radioactive materials with the DOT and the Post Office Department.

The DOT published 49 CFR Part 170 in the Federal Register Volume 33 - 108 on June 4, 1968. This regulation covers the rule-making procedures of the Hazardous Materials Regulations

Board of the DOT and formalizes that agency's procedures for the application and its handling of special permits.

On October 4, 1968, the DOT published in the Federal Register its revised transportation regulations applicable to radioactive aterials, 49 CFR Parts 171 through 190. These regulations had previously been published in the Federal Register on January 20, 1968, for public comment.

TABLE VIII-10

U.S. Ports Cleared for Receipt of Irradiated Fuel

Charleston, South Carolina	Portland, Maine
Savannah, Georgia	Searsport, Maine
Norfolk, Virginia	Los Angeles, California
Philadelphia, Pennsylvania	Gulfport, Mississippi
Seattle, Washington	Albany, New York
Houston, Texas	Buffalo, New York
Port Everglades, Florida	Corpus Christi, Texas
Wilmington, North Carolina	Redwood City, California
Morehead City, North Carolina	Portland, Oregon
New Orleans, Louisiana	Tampa, Florida
New York, New York	Jacksonville, Florida
Baton Rouge, Louisiana	Port St. Joe, Florida
Benton, Washington	Bellingham, Washington
Baltimore, Maryland	Olympia, Washington
Willapa Harbor, Washington	Anacortes, Washington
Grays Harbor, Washington	Mattawa, Washington
Everett, Washington	Longview, Washington
Tacoma, Washington	San Francisco, California
Lake Charles, Louisiana	Hilo, Hawaii
Honolulu, Hawaii	Kahului, Hawaii
Kawaihae, Hawaii	Nawiliwili, Hawaii
Port Allen, Hawaii	Kennewick, Washington
Walla Walla, Washington	Pasco, Washington

To date AEC has shipped abroad under bilateral agreements slightly more than 20 thousand kg of U-235 (20 metric tons) representing a value of about $250 million. (See Table VIII-11).

AEC expects to ship abroad nuclear fuel at the rate of 7 to 10 thousand kg U-235 per year over the next several years, primarily for power reactors.

Since 1963 the AEC has received 92 shipments of irradiated research reactor fuel from foreign users for chemical reprocessing in Commission facilities at Savannah River and Idaho.

TABLE VIII-11

Shipments to Foreign Countries Pursuant To Agreements for Cooperation

Year	Approximate No. of Shipments
1962	250
1963	400
1964	350
1965	270
1966	250
1967	250
1968	225

5. Commercial Services

Private firms supplying special services and/or products for the transport of radioactive materials are listed below:

ATCOR, Inc., Hawthorne, New York
Samuel Edlow, Columbus, Ohio
Long Island Nuclear Services, Bellport, New York
National Lead Company, New York, New York
Nuclear Engineering Company, Walnut Creek, California
Nuclear Fuel Services, Inc., West Valley, New York
Tri-State Motor Transit Company, Joplin, Missouri
International Transport, Inc., Rochester, Minnesota
Transnuclear, Inc., New York, New York
Whitehead & Kales Corp., Detroit, Michigan

6. AEC Shipping Container Program

In recognition of a need for coordination of container research and development, standardization of shipping containers, and the effective utilization of shipping containers once they are built, the AEC's Division of Construction, Transportation Management Branch, has as a first step prepared the following publications:

a. Draft Directory of Shipping Containers for Radioactive Materials

This directory provides data on approximately 250 different types of AEC-owned shipping containers. It includes an inventory of approximately 26,000 containers having an estimated value of $30 million for loan or leasing.

The directory includes information on shipping containers such as permit or DOT specification number, description, dimensions, types of shielding, authorized contents, special conditions of transport and availability for lease or loan. The draft directory is being updated in order to maximize its usefulness to both AEC and industry.

b. Compendium of AEC Container Testing Facilities

This listing was prepared to assist AEC contractors and private industry in identifying potential testing facilities and, thus, possibly avoid the costly duplication of such facilities. Descriptions of vertical, horizontal, thermal, and other testing facilities are given, as well as supporting services related to testing containers. AEC policy regarding the use of AEC facilities is given in AEC Manual Chapter 7603.

c. Information Brochure on Department of Transportation Specification Shipping Containers and Guidelines for the Preparation of Proposals for DOT Specifications for Radioactive and Fissile Material Shipping Containers

This brochure presents information to assist those preparing specification (standardized) container designs for DOT approval and publication under 49 CFR Parts 178 and 179. The Division of Construction, Transportation Management Branch, in conducting reviews of shipping containers to maximize the utilization of container design cooperates with the Atomic Industrial Forum to obtain input from industry. A long-range objective is to achieve the development of a broad spectrum of off-the-shelf type delivery of containers.

E. <u>Whole Body Counting Service</u>

Whole body counters are sensitive radiation detection instruments that provide information on the internal deposition of certain radionuclides by measuring the radiation emanating from the human or animal body. Their proven value lies in their ability to reveal the kinds, amounts, and location of gamma emitting radioactive nuclei that have accumulated in various parts of the body, from either artificial or natural sources, such as fallout, accidental ingestion, or from medical tracer isotopes. From this information, it is possible to calculate the amount of radioactive materials which have accumulated in various body tissues and organs.9/

The most common whole body counter employs a sodium iodide crystal as the radiation detector. In another type, the subject is surrounded by a tank filled with a scintillation fluid that detects gamma rays.

This service is provided by industry to several nuclear reactors operators, pharmaceutical companies, research laboratories, nuclear shipyards, radioactive waste disposal companies, and fuel fabrication and reprocessing facilities. For example, the measurement of U-235 and Pu-239 in the lungs is of interest to fuel fabrication companies. One company (Nuclear Electronics Counter Corporation) has leased its counter over the last few years to several midwestern universities for use in animal research. There are two companies presently in this business and both offer their counting services at the customer's site, using trailer-mounted counters.

It is estimated that about 3,000 counts were performed during the past year by the commercial firms offering this service. The price charged per whole body count is dependent on the number of people to be counted per contract and ranges from about $20 to $45 per measurement.

Further information concerning portable whole body counting services may be obtained by contacting the following commercial firms offering this servic[illegible]

Mr. Ge[illegible]ge Lewis Helgeson
Helges[illegible] Nuclear Services
872 Ab[illegible]ie Street
Pleasanton, California 94566

Mr. Charles R. Keck
Nuclear Electronics Counter Corporation
McCulloch Building, Box 604
Okmulgee, Oklahoma 74447

9/ See "Whole Body Counters," a booklet available from USAEC, P. O. Box 62, Oak Ridge, Tennessee 37830.

F. Reactor Servicing

A number of private companies are now established which offer specialized service in the area of reactor refueling and periodic overhauling of land-based nuclear power plants. For example, Todd Shipyards Corporation of Galveston, Texas, has the capability of shipboard nuclear servicing at its overhaul and maintenance dock at Galveston.

Reactor engineering services which are required include: assessment of plant performance, evaluation and recommendation for reactor modifications, and installation and testing of modifications. For example, during the last year an AEC contract to provide these services for the Elk River, BONUS, and LaCrosse reactors was awarded to the United Nuclear Corporation, on a competitive basis.

Companies offering these services are listed below:

ATCOR, Inc.
360 Bradhurst Avenue
Hawthorne, New York 10532

Atomics International
Division of North American
Rockwell Corporation
P. O. Box 309
Canoga Park, California 91305

Burns & Roe, Inc.
700 Kinderkamack Road
Oradell, New Jersey 07649

Hittman Associates, Inc.
9190 Red Branch Road
Columbia, Maryland 21043

Jackson & Moreland, Inc.
600 Park Square Building
Boston, Massachusetts 02116

Isotopes, A Division of
Teledyne Company
50 Van Buren Avenue
Westwood, New Jersey 07675

New York Shipbuilding Corp.
2430 Broadway
Camden, New Jersey 08101

Nuclear Associates
International Corporation
12601 Twinbrook Parkway
Rockville, Maryland 20850

Nuclear Service and
Construction Company
c/o Newport News Shipbuilding
and Drydock Company
Newport News, Virginia 23600

NUS Corporation
1730 M Street, N. W.
Washington, D. C. 20036

Todd Shipyards Corporation
P. O. Box 1600
Galveston, Texas 77550

United Nuclear Corporation
1735 Eye Street, N. W.
Washington, D. C. 20006

G. Decontamination

The removal of radioactive contaminants from plant and equipment items is offered by several private companies specializing in this area.

This activity usually involves washing surfaces with chemicals, vacuum cleaning, or paint removing, along with frequent monitoring to determine when the area is suitable for use. Types of items frequently decontaminated include reactor parts and equipment, hot cell parts and equipment, glove boxes, shipping casks, and all other tools and equipment of the nuclear industry which become radioactively contaminated through use or exposure.

The companies listed below offer this service, in addition to other nuclear services, such as waste handling, storage, disposal, transportation, and radiation safety services.

Applied Health Physics, Inc.
2986 Industrial Boulevard
P. O. Box 197
Bethel Park, Penna. 15102

ATCOR, Inc.
360 Bradhurst Avenue
Hawthorne, New York 10532

Isotopes, A Division of Teledyne Company
50 Van Buren Avenue
Westwood, New Jersey 07675

also

Radiological Service Co., Inc.
Division of Isotopes
50 Van Buren Avenue
Westwood, New Jersey 07675

Laboratory for Electronics
230 Wright Avenue
Richmond
California 94802

Long Island Nuclear Service Corp.
Station Road
Bellport, New York 11713

Nuclear Diagnostic Labs, Inc.
755 Park Avenue
New York
New York 10021

Nuclear Engineering Co., Inc.
Box 594
Walnut Creek
California 93002

LICENSING AND REGULATION

Because of their importance and the attention given to these areas during the year, we have included the following special summary of the Commission's licensing and regulatory activities.

A. Facility Licensing

1968 has been the AEC's most active year of civilian nuclear power licensing to date. By the end of September, 18 nuclear power units had been authorized for construction, and public hearings before atomic safety and licensing boards had been either completed or scheduled on applications for five additional units. These 23 power reactors represent a total of more than 18,000 net MWE of design capacity -- seven times the combined capacity of all nuclear power plants in operation to date. Prospects are that by year's end 44 nuclear power units will be under construction in 19 states, nearly all of which are scheduled to begin commercial operations over the next five years.

1. New Regulatory Study

To help assure that licensing procedures keep pace with rapid expansion of the nuclear industry, the Commission appointed a new internal study group to review the licensing process as a follow[illegible] to several previous studies by special panels and the Jo[illegible]ommittee on Atomic Energy. Primary areas of the tech[illegible]ly oriented study are timing and coordination in the decision-making and review processes and whether a degree of standardization has been reached in reactor design that could affect regulatory procedures.

2. Criteria, Standards, and Codes

As the number of reactor applications from electric utilities tapered off from the peak of 1967, attention was focused on quality in design, fabrication, and construction of plants underway, and on early operating experience of the large power reactors being placed in operation. Substantial progress was made, with increasing cooperation of industry and professional code groups, in developing more detailed regulatory criteria for light water reactors.

An AEC review group continued to establish detailed safety criteria for important components of reactor coolant systems. The ASME strengthened its Nuclear Pressure Vessel

Code, published a new Code for Nuclear Power Piping for trial use, and was engaged in developing standards for in-service inspection. Work progressed on codes and standards for instrumentation, control, and electrical systems for nuclear plants under direction of the Institute of Electrical and Electronic Engineers and the United States of America Standards Institute. The cooperative efforts in the standards field have helped assure that the ideas and experience of industry are incorporated in regulatory criteria, and that regulatory needs are reflected in appropriate industry codes.

Development by the AEC of criteria for design and siting of reactors in earthquake zones reached an advanced stage.

3. Nonradiological Factors in Licensing

Increasing attention was placed during the year on nonradiological issues over which the AEC has no jurisdiction, and which were raised in AEC licensing proceedings and elsewhere. In one instance, a utility withdrew its application for authority to construct a nuclear power plant because of difficulties in obtaining site approvals from other Federal and state bodies dealing with nonradiological matters.

A number of bills were introduced in the 90th Congress which would broaden the areas of consideration in the licensing of nuclear reactors. Primary issues of concern are the "thermal effects" of heated condenser water discharges from power plants on the ecology of rivers and other bodies of water, and efforts of smaller utilities -- mostly publicly or cooperatively owned -- to participate in ownership of large-scale electric generating plants and the benefits of their anticipated low-cost power. As this report went to press, proposed legislation was pending in Congress which would give the AEC, or in some bills, other agencies such as the Federal Power Commission, authority to consider thermal effects and antitrust implications in reactor licensing proceedings.

A brief analysis of pending legislation affecting the nuclear industry appears later.

B. Control of Materials

As expansion and diversification in uses of atomic energy materials continued, new actions were taken to simplify the licensing process. Interest of the states in assuming regulatory authority remained high, and the program for safeguarding special nuclear materials in the hands of licensees was strengthened. The

[illegible]

AEC's Federal-state program, authorized by Sec. 274 of the Atomic Energy Act, provides for agreements for the transfer of certain of AEC's regulatory authority over by-product (radioisotopes), source, and small quantities of special nuclear materials. The licensing and regulation of reactors and other nuclear facilities is reserved to the AEC.

1. State Programs

Colorado and Idaho assumed regulatory authority over some 250 former AEC licenses, bringing the number of Agreement States to 19. About 41 percent of the estimated 15,800 materials licenses in effect in the United States are under regulatory authority of Agreement States.1/

2. Improved AEC Licensing Procedures

Several actions were concluded or initiated to simplify AEC licensing procedures, several of which reflected recommendations of the ad hoc Radioisotopes Licensing Review Panel which conducted a study in 1967. Wider use of broad licenses for research and development is now permitted, giving greater flexibility to qualified licensees and reducing administrative effort. A program was started to exempt from licensing certain classes of products containing radioisotopes, with safety criteria established for authorizing their distribution. A comprehensive schedule was proposed designating small quantities of radioisotopes that can be used under an exemption, with safety controlled by requirements on suppliers concerning such matters as packaging and labeling. The generally licensed distribution was authorized in 1968 of seven new devices using radioisotopes, ranging from determination of zinc coating thickness of a galvanizing process to measuring underwater currents at ocean depths exceeding 15,000 feet. A general license also was provided for ownership of special nuclear materials.

3. Nuclear Materials Safeguards

AEC regulations were changed to extend reporting requirements applicable to special nuclear material (SNM) to all privately owned material, regardless of origin, except for specified small quantities. Three district offices were established under the Director of Regulation to conduct safeguards inspections of licensees' SNM, and a guide was prepared for licensees and applicants which describes the elements of material control and accounting procedures for safeguarding SNM against unlawful diversion.

1/ See Chart VIII-A on page 197.

C. Nuclear Safety

A survey of injury frequency and severity rates again showed the work-injury experience of the private sector of the atomic energy industry to be better than the average for all manufacturing industries as compiled by the Bureau of Labor Statistics.

1. Insurance Premium Refunds

Reflecting the excellent safety record of the nuclear industry, the insurance pools which were created 11 years ago to provide private nuclear energy liability insurance refunded the major portion of premiums paid by policy holders in 1958. It was the second year in a row that refunds were paid out under a retrospective rating plan based on loss experience over a 10-year period.

2. Government Indemnity

The AEC has revised its regulations to reflect 1966 amendments to the Price-Anderson indemnification legislation which provides for waivers of defense in the event of an "extraordinary nuclear occurence." The waivers of defense would enable persons affected by a nuclear incident meeting the criteria for an "extraordinary nuclear occurrence" to obtain prompt compensation for damage resulting from hazardous properties of radioactive material or radiation. All current indemnity agreements with AEC licensees and financial protection insurance policies have been amended to provide for waivers of defenses.

D. AEC License Fees Established

Effective October 1, 1968, the AEC adopted a schedule of fees to be charged for: (a) licenses to construct and to operate nuclear reactors and other production or utilization facilities, (b) licenses for by-product material (radioisotopes) of 100,000 curies or more in sealed sources, (c) licenses for special nuclear material in quantities sufficient to form a critical mass (except plutonium/beryllium neutron sources), and (d) waste disposal licenses specifically authorizing the receipt of radioactive materials for commercial disposal. The adopted schedule differs from the proposed schedule published for public comment in March 1967. Licenses generally exempt are those for materials or facilities (other than power reactors) issued to nonprofit educational institutions for training, teaching, or medical purposes, and Government agency and export licenses. The AEC is studying the subject of fees for material licenses not covered in the schedule.

PROPOSED LEGISLATION

An area of potential impact this year on central station nuclear power has been the legislative actions pending and proposed. Bills before Congress now concern subjects such as electric power reliability, exemption from antitrust suits, opportunity for ownership participation by all interested persons in proposed nuclear generating units, elimination of the statutory requirement for a finding of "practical value," and the present distinction between "commercial" and "developmental" licenses, jurisdiction over water quality and thermal effects, as well as power plant siting. The effect of the passage of any one of the relevant bills could be to alter the planning and regulatory procedures for all subsequent central station nuclear power plants. A brief summary of some of the significant legislative proposals follows:

A. <u>S. 1934 (1967) - H.R. 15340 (1968)</u>

The Administration-sponsored "Electric Power Reliability Act," introduced by Senator Muskie in the Senate, would create new mechanisms for regional coordination and planning to insure that plans for bulk power facilities are adequate. It would authorize the Federal Power Commission to establish planning and operating standards to enhance reliability. Immunity from private antitrust suits would be granted if the FPC makes certain determinations.

The Hearings, held by both the Senate and House Committees, have not been completed.

B. <u>S. 2564 - H.R. 13838 - H.R. 15273</u>

The "Aiken-Kennedy Bill," introduced by Senator Aiken and the late Senator Robert Kennedy, describes itself as a bill "to insure a reasonable opportunity for all electrical utilities to participate in the benefits of nuclear power." This bill would amend the Atomic Energy Act of 1954 to require a showing, by both "research and development" and "commercial" license applicants, that they had offered all other electric utilities and other interested persons in the affected areas an opportunity to purchase a portion of the proposed nuclear power plant, and to sell power from the particular nuclear power plant involved to all persons engaged in the distribution, transmission, or sale of electrical energy. In addition, the bill proposes AEC regulatory jurisdiction to include such matters as antitrust, thermal effects, and aesthetics.

Hearings were held by the JCAE on April 30 through May 3 and June 11-13. Comments have been received by the Committee from the Agriculture, Interior, and Justice Departments; the Atomic Energy Commission; the Securities and Exchange Commission; the Federal Power Commission; and the Water Resources Council. One Federal Power Commissioner submitted a separate statement.

C. S. 2889 - H.R. 14971

The "Kennedy-Ottinger Bill" or "Electric Power Reliability Act of 1968" was introduced by Senators Robert and Edward Kennedy in the Senate and Congressman Ottinger in the House. In essence, this bill is a revision and refinement of S. 1934 and would vest in the FPC broad new regulatory powers over electric utilities. It would treat nuclear and non-nuclear facilities alike.

D. S. 3851 - H.R. 18667 (introduced 7/16/68)

The JCAE-sponsored "Practical Value Bill" would amend the Atomic Energy Act of 1954 to eliminate the statutory requirement that the Commission make a finding of "practical value" before it issues a facility license under section 103 of the Act. The Commission would be authorized to issue licenses under section 104b. only after a determination that the principal purpose of the facility is the performance of substantial research and development activities for the demonstration of significant advances in the commercial or industrial application of atomic energy. Under an amended section 105c., the Attorney General would be required to render antitrust advice to the Commission within 180 days of receipt of an application under section 103 to construct a facility. Other changes in present section 105c. would also be made. A new section 111 is proposed, requiring obtaining advice from the Secretary of the Interior, within 90 days of receipt of a license application under sections 103 or 104b., on whether the applicant is equipped to observe applicable Federal and state standards for the control of thermal effects of the release of liquid effluents from the facility. A license could not be issued unless the applicant is so equipped and agrees to observe the standards. (There are other bills before other Congressional committees relating to thermal effects.)

E. S. 3960 (introduced 8/1/68)

AEC's proposed "Practical Value Bill" differs from the JCAE bill S. 3851 in that it would abolish the distinction between licenses granted under sections 103 and 104b. under the Act. [illegible] would also delete the requirements for a finding of "practical value" and provide for antitrust review by the Department of Justice in all power reactor proceedings. Certain of the changes which would be made in section 105c. differ from those proposed under S. 3851. There are no provisions relating to thermal effects.

Hearings on both "Practical Value Bills" S. 3851 and S. 3960 have no date set but are expected to be held during the next session of Congress. AEC comments on S. 3851 supported the elimination of the "practical value" concept, but expressed a preference for the approach in S. 3960 which would also eliminate the distinction between classes of licenses. The comments indicated that the provision in S. 3851 for a new type of determination might cause unnecessary delay and litigation in licensing. Under either approach, Congress would retain authority to authorize research and development assistance on a case-by-case basis. The comments urged that the "thermal effects" provisions of S. 3851 not be enacted on the ground that they would have an unwarranted, discriminatory effect on nuclear plants, since there is no comparable licensing restriction applicable to thermal discharges from non-nuclear plants. The Commission indicated, however, that if the Congress believed such federal licensing control was necessary, it favored the approach to the problem proposed in the bill. Moreover, the Water Quality Act of 1965 provides a comprehensive program through which water quality standards are developed by the states and approved by the Secretary of the Interior.

F. S. 3330

The bill was introduced by Senator Edward Kennedy "to amend the Federal Power Act to provide for a national powerplant siting study and a national powerplant siting plan." No hearings have been held.

The Energy Policy staff of the White House Office of Science and Technology, is coordinating an interdepartmental examination of siting problems.

ASSISTANCE FOR INDUSTRY

A. At AEC Field Offices

Managers of each of the AEC Field Offices have designated representatives from their staffs as primary points of contact on Industrial Participation activities. These representatives serve as focal points within their respective offices for activities for which the Division of Industrial Participation is responsible.

These representatives are:

Mr. Charles C. Campbell
Assistant Manager for Administration
Albuquerque Operations Office
U.S. Atomic Energy Commission
P. O. Box 5400
Albuquerque, New Mexico 87115
(505) - 264-7241 (FTS and Commercial)

Mr. Alfred K. Huse
Chief of Technical Projects
Operations Division
Brookhaven Office
U.S. Atomic Energy Commission
Upton, New York 11973
(516) - 924-2368 (FTS)
(516) - 924-6262, x-2368 (Commercial)

Mr. Thomas P. Heckman
Safety and Technical Services Division
Chicago Operations Office
U.S. Atomic Energy Commission
9800 South Cass Avenue
Argonne, Illinois 60439
(312) - 739-2091 (FTS)
(312) - 739-7711, x-2091 (Commercial)

Mr. J. C. Westbrook
Assistant Manager for Administration
Grand Junction Operations Office
U.S. Atomic Energy Commission
Grand Junction, Colorado 81502
(303) - 242-8201 (FTS)
(303) - 242-8621, x-8201 (Commercial)

Mr. John F. Kaufmann
Assistant Manager
Technical Services
Idaho Operations Office
U.S. Atomic Energy Commission
P. O. Box 2108
Idaho Falls, Idaho 83401
(208) - 526-1301 (FTS)
(208) - 522-6640, x-1301 (Commercial)

Mr. Jack H. Hooker
Administrative Assistant
Office of Assistant Manager
for Administration
Nevada Operations Office
U.S. Atomic Energy Commission
P. O. Box 14100
Las Vegas, Nevada 89114
(702) - 734-3148 (FTS)
(702) - 734-3011, x-3148 (Commercial)

Mr. J. Allentuck, Director
Engineering & Technical Services Division
New York Operations Office
U.S. Atomic Energy Commission
376 Hudson Street
New York, New York 10014
(212) - 989-1353 (FTS)
(212) - 989-1000, x-1353 (Commercial)

Mr. John W. Ruch, Deputy Assistant
Manager for Operations
Oak Ridge Operations Office
U.S. Atomic Energy Commission
P. O. Box E
Oak Ridge, Tennessee 37830
(615) - 483-4469 (FTS)
(615) - 483-8611, x-4468 (Commercial)

Lieutenant James A. Schroeder
Senior Contract Administrator
Contract & Procurement Branch
Pittsburgh Naval Reactors Office
U.S. Atomic Energy Commission
P. O. Box 109
West Mifflin, Pennsylvania 15122
(412) - 462-0248 (FTS)
(412) - 462-5000, x-0248 (Commercial)

Mr. Leonard F. Perkins, Director
Division of Contracts and Procurement
Richland Operations Office
U.S. Atomic Energy Commission
P. O. Box 550
Richland, Washington 99352
(509) - 942-1111, x-6-4138 (FTS & Commercial)

Mr. Milton F. Moore
Technical Services Division
San Francisco Operations Office
U.S. Atomic Energy Commission
2111 Bancroft Way
Berkeley, California 94704
(415) - 841-3667 (FTS & Commercial)

Mr. Harry B. Rahner, Special
Assistant to the Deputy Manager
Savannah River Operations Office
U.S. Atomic Energy Commission
P. O. Box A
Aiken, Sout[illegible]arolina 29802
(803) - [illegible]678 (FTS)
(803) - 824-6331, x-2678 (Commercial)

Mr. Stanley W. Witzman, Manager
Schenectady Naval Reactors Office
U.S. Atomic Energy Commission
P. O. Box 1069
Schenectady, New York 12301
(518) - 393-4441 (FTS)
(518) - 393-6611, x-4441 (Commercial)

B. At AEC Laboratories

Offices of Industrial Cooperation have been established at three of the major AEC laboratories. These offices operate principally to promote and maintain the types of individual laboratory-industry contacts that can result in "spinoff" of AEC-developed technology into commercial use. Occasionally the laboratories also use the offices for contacting industrial groups. Thus, the OIC at Argonne National Laboratory has hosted briefings for industry by members of the laboratory staff to explain the Federal Government's technology transfer programs. Such briefings may be conducted in cooperation with other Federal agencies and state and local industrial development groups. Further information on these offices and assistance which they might provide for specific purposes or companies may be obtained directly from the following:

Office of Industrial Cooperation
Oak Ridge National Laboratory
P. O. Box X
Oak Ridge, Tennessee 37831
(615) - 483-8611, x-6883 (Commercial)

Office of Industrial Cooperation
Argonne National Laboratory
9700 South Cass Avenue
Argonne, Illinois 60439
(312) - 739-7711, x-4762 (Commercial)

Office of Industrial Cooperation
Sandia Corporation
P. O. Box 5800
Albuquerque, New Mexico 87115
(505) - 264-3904 (Commercial)

C. From AEC Publications

The annual report which the Commission prepares for submission to the Congress in January each year contains summary descriptions of all the AEC programs. This is available to the public as Major Activities in the Atomic Energy Programs -- January-December (each year) from the U.S. Government Printing Office, Washington, D. C. 20402. Also available from the same source is an annual supplemental report, Fundamental Nuclear Energy Research (each year). This report summarizes the noteworthy advances resulting each year from AEC basic research studies in the physical, biological, medical, and environmental areas.

During the past two decades, a body of literature describing the science, technology, and application of atomic energy has been developed. This literature appears in many forms. It is available from numerous sources and is announced and indexed in a variety of publications. A description of the types of material available and how to obtain them is contained in a booklet, What's Available in the Atomic Energy Literature, available on request to the USAEC Division of Technical Information Extension, P. O. Box 62, Oak Ridge, Tennessee 37830.

A good source of current information on AEC activities are the AEC Public Releases. To get on the mailing list for these, write to the Division of Public Information, USAEC, Washington, D. C. 20545.

D. From Congressional Publications

The Joint Committee on Atomic Energy publishes transcripts of its hearings on atomic energy legislation and programs and issues reports on a wide range of nuclear subjects. Those interested in these publications should write to the Joint Committee on Atomic Energy, Congress of the United States, Washington, D. C. 20510, asking to be placed on the Committee's mailing list.

E. Through the Access Permit Program

Certain classified technical information may be obtained for private use in developing peaceful applications of nuclear energy through the Commission's Access Permit Program. The usefulness of and the continuing need for this program is presently being evaluated. Information on this program is contained in the booklet Questions and Answers on USAEC Access Permits, available from the Division of Industrial Participation, USAEC, Washington, D. C. 20545.

F. Through the Work Experience Program

Employees of private firms engaged in civilian applications of nuclear energy are eligible for training through work experience in AEC plants and laboratories. Certain of these facilities throughout the country will be available to supplement industrial facilities in meeting the nuclear orientation requirements of the electrical utility industry. Further information on this program may be obtained from the Division of Industrial Participation, USAEC, Washington, D. C. 20545.1/

1/ See AEC Public Release L-207, dated 9/4/68.

PRIVATE ORGANIZATION INDEX

NAME	PAGE NO.
Abbott Laboratories	142, 147, 152
Accelerators, Inc.	158
Aerojet-General Corporation	56, 57, 58, 60, 68, 69, 113, 132, 137, 155
Alco Products, Inc.	127
Allegheny Power Systems, Inc.	99
Allied Chemical Corporation	14, 36, 38, 39, 41, 45, 46, 60, 113, 145, 171, 175
Allis-Chalmers Manufacturing Company	127, 142, 180
AMAX Specialty Metals, Inc.	71
American Atomics Corporation	152
American Electric Power Company, Inc.	82, 107, 112
American Machine and Foundry Company	80, 167
The American Novawood Corporation	157
American Nuclear Corporation	159
American Nuclear Society	21, 193
American Potash & Chemical Corporation	69
American Public Power Association	94, 105, 117, 129
American Society of Mechanical Engineers	226
Amersham/Searle Corporation	146
Anaconda Company	31
Anchor Alloys, Inc.	80

NAME	PAGE NO.
Appalachian Power Company	99
Applied Health Physics, Inc.	211, 225
Applied Nuclear Company	187
Applied Radiation Corporation	158, 160
Argonne Cancer Research Hospital	142
Arizona Atomic Energy Commission	193
University of Arizona	193
Arkansas Power & Light Company	90, 97b
Associated Electric Industries, Inc.	81, 82
Association of Nuclear Instrument Manufacturers, Inc.	88
ATCOR, Inc.	187, 201, 202, 221, 224, 225
Atlantic City Electric Company	98
Atlantic Research Corporation	148
Atlantic Richfield Company	14, 41, 46, 113, 157, 172, 175
Atlantic Richfield Hanford Company	165
Atlas Corporation	31
Atomchem Corporation	159
Atomic Corporation of America	147
Atomic Disposal Company, Inc.	201
Atomic Energy Industrial Laboratories of the Southwest	202, 211
Atomic Industrial Forum	13, 18, 21, 25, 49, 105, 193

NAME	PAGE NO.
Atomic Power Development Associates, Inc.	67
Atomics International, Division of North American Rockwell Corporation	57, 60, 68, 84, 113, 123, 136, 137, 155, 224
Atwood & Morrill Company	80
Austral Oil Company	189
Avionics Corporation	150
The Babcock and Wilcox Company	56, 57, 60, 68, 80, 96, 106, 107, 111, 128, 143, 160
Paird-Atomic, Inc.	85
Baldwin-Lima-Hamilton Corporation	81
Baltimore Gas & Electric Company	92, 97b
W. F. and John Barnes Company	149
Battelle Memorial Institute	60, 68, 137
Bechtel Corporation	187, 193
Beckman Instrument Company	85
Bendix Corporation	85
The Beryllium Corporation	76
Beryllium Metals and Chemicals Corporation	76
Bingham Pump Company (G. F. Atkinson Company)	80
Bio-Nuclear, Division of Bio-Assay Labs	147, 148
Bio-Rad Labs, Inc.	74, 147

NAME	PAGE NO.
Black-Sivalls-Bryson	80
Boise Cascade Corporation	157, 158
Boston Edison Company	92, 97a
Brown Boveri Corporation	21, 81, 82, 107
Brun Corporation	150
The Brush Beryllium Company	76
The Budd Company	148
Burns & Roe, Inc.	159, 224
Byron Jackson Pumps, Inc. (Borg Warner)	80
Calbiochem, Inc.	147
California Nuclear, Inc.	198
California Salvage Company	201, 202
University of California	20, 129, 161,193
Cambridge Nuclear Corporation	142, 146, 147
Canadian General Electric Company	75
Canberra Industries, Inc.	85
Canrad Precision Industries, Inc.	152
Carolina Power & Light Company	92, 97a, 97b, 99
Carpenter Steel Company	72, 80
Central Illinois Public Service Company	99
CER Geonuclear Corporation	188, 189, 191, 193
Cha[illegible] Brass and Copper Company	72

NAME	PAGE NO.
Chevron Research Company	162
Chicago Bridge & Iron Company	80, 81
Chronetics, Inc.	85
Cincinnati Gas & Electric Company	98, 99
Climax Uranium	34
Columbia Gas System Service Corporation	189
Columbia Organic Chemicals	74
Columbia Research Corporation	158, 161
Columbus & Southern Ohio Electric Company	99
Combustion Engineering, Inc.	15, 56, 57, 60, 80, 96, 104, 106, 111, 112
Commonwealth Associates, Inc.	159
Commonwealth Edison Company	17, 66, 92, 97a, 97b, 99, 112, 165, 167
The Connecticut Light & Power Company	99
Connecticut Yankee Atomic Power Company	92, 97a
Consolidated Edison Company of New York, Inc.	16, 82, 92, 97a, 97b, 99, 112
Consumers Power Company	16, 92, 97a, 97b, 107, 112, 143
Consumers Public Power District	92, 97b
Continental Oil Company	188
Cosmic Radiation Labs	85
Cotter Corporation	34
Crane Company	80

NAME PAGE NO.

Curtis Nuclear Corporation 85

Cyclotron Corporation 142

Dairyland Power Cooperative 92, 97a

Darling Valve & Manufacturing Company 80

Deering-Milliken 157

Densitronics, Inc. 150

Dero Research & Development Corporation 85

Detroit Edison Company 95, 97b, 99, 106

Deuterium of Canada, Ltd. 75

Deuterium Corporation 74

Diaprep, Inc. 74

Donald W. Douglas Laboratories 155

Dow Chemical Company 16, 108, 157, 162

Duke Power Company 92, 97a, 97b, 174

Duquesne Light Company 92, 97a, 97b

Eagle-Picher Company 76, 77

East Central Nuclear Group 17, 124

Eberline Instrument Corporation 85, 211, 213

Edgerton, Germeshausen, & Grier, Inc. (E. G. & G.). 85

Edison Electric Institute 12, 17, 66, 67, 103, 105, 123

Samuel Edlow 221

NAME PAGE NO.

Eldorado Nuclear Limited 39, 45, 72

Electra Process Industries, Inc. 158, 159

Electronic Automation Systems 150

Electronized Chemical Corporation 157, 158, 159

El Paso Electric Company 99

El Paso Natural Gas Company 187, 188, 189

Empire State Atomic Power
Development Associates 83, 123

The English Electric Company, Ltd. 81

EON Corporation 85

A. Epstein and Sons, Inc. 159

Federal-Americam Partners 34

Fenix & Scisson, Inc. 187

Financial Analysts Federation 1, 106

Florida Power Corporation 92, 97b

Florida Power & Light Company 92, 97a, 97b, 99

University of Florida 161

Food Industries Research & Engineering 159

Ford Motor Company 157, 158

Ford Philco 137

Foster Wheeler Corporation 81, 84

Foxboro Corporation 150

NAME	PAGE NO.
Gaines Nuclear Health Services, Inc.	2'1
Gamma Industries, Inc.	148
Gamma Process Company	157, 159, 160, 162
Gard-Ray Film Badge Service	211, 213
General Astrometals Corporation	76
General Electric Company	14, 15, 17, 55, 56, 57, 60, 66, 68, 72, 80, 81, 83, 84, 106, 111, 112, 119, 122, 123, 124, 127, 136, 137, 143, 145, 147, 149, 158, 159, 160, 169, 175, 205, 206, 209
General Nuclear, Inc.	146, 147, 148, 202
General Nucleonics Corporation	150
General Public Utilities Corporation	108, 123
General Radioisotope Processing Company	146
Geonuclear Nobel-Paso, Inc.	187
Georgia Institute of Technology	156
Georgia Power Company	92, 97b, 99
Geoscience Nuclear, Division of Geoscience Instruments Corporation	88
Getty Oil Company	167
Gibbs and Hill, Inc.	159
Gifford-Wood, Inc.	80
W. R. Grace & Company	60, 157, 167
Great American Transportation Company	81

NAME PAGE NO.

Gulf General Atomic, Inc. 14, 15, 17, 55, 57, 60, 66, 69, 84, 96, 106, 107, 111, 120, 124, 129, 130, 137, 162, 172, 176, 182, 187

Gulf Oil Corporation 113

Hamner/Harshaw 85

Harvey Aluminum 71

Hastings Radiochemical Works 142, 147, 148

Health Physics Services, Inc. 211

Helgeson Nuclear Services, Inc. 223

Hewlett-Packard Company 85

High Energy Processing Corporation 157, 158, 159

High Voltage Engineering Corporation 158
(See also Electronized Chemical)

Hittman Associates, Inc. 130, 137, 224

Hoke, Inc. 80

Holmes & Narver, Inc. 187

Indiana & Michigan Electric Company 92, 97b

Industrial Nucleonics Corporation 150

Industrial Reactor Laboratories, Inc. 160

Ingersoll-Rand Company 81

Institute of Electrical and Electronic Engineers 227

NAME PAGE NO.

Instruments, Inc. 150

International Chemical & Nuclear Corporation . . . 74, 142, 147

International Nuclear Corporation 189

International Telephone & Telegraph Corporation . . 157

International Transport, Inc. 221

Interstate Industrial Laundry and
Decontamination Service, Inc. 214

Interstate Industrial Laundry Service, Inc. 214

Interstate Industrial Uniform
Rental Service, Inc. 214

Iowa Electric Light and Power Company 92, 95, 97b, 99, 106

Iowa-Illinois Gas & Electric Company 92

Iowa Public Service Company 99

Isochem, Inc. 146

Isomet Corporation 74

Isotope Products Laboratory 142

Isotopes, Division of Teledyne, Inc. 85, 136, 147, 156, 159, 160, 161, 211, 224, 225

Jackson & Moreland, Inc. 224

Jersey Central Power and Light Company 92, 97a, 97b, 101

Johns Hopkins University 162

Johns Manville Corporation 137

Jones Medical Instrument Company 152

NAME	PAGE NO.
Kaman Nuclear	85
Kaman Sciences Corporation	187
Kansas Electric Power Company	118
Kansas City Board of Public Utilities	99
Kansas City Power & Light Company	99
Kansas Gas & Electric Company	99
Kennecott Copper Corporation	190
Kerr-McGee Corporation	18, 26, 34, 39, 45, 55, 56, 60, 69, 113, 182
Kevax Corporation	88
Laboratory for Electronics, Inc.	149, 152, 201, 202, 225
R. S. Landauer, Jr., and Company	211, 213
Landsverk Electrometer Company	85
LeCroy Research Systems Corporation	85
Liquid Carbonic, Division of General Dynamics Corporation	74
Arthur D. Little, Inc.	15, 29, 105
Lockheed Aircraft Corporation	136, 138
Lockheed-Georgia Company	157, 159, 160
Lockheed Missiles and Space Company	187
Long Island Lighting Company	92, 97b
Long Island Nuclear Service Corporation	201, 202, 221, 225

NAME PAGE NO.

Department of Water and Power of the City of Los Angeles 92, 97a, 99, 115

Louisville Gas & Electric Company 99

Gene Lowey, Inc. 150

Lucius Pitkin, Inc. 36

Ludlum Measurements, Inc. 150

Luminous Products Corporation 152

Lummus Company 74

McCormack's Highway Transportation, Inc. 202

Chas. T. Main, Inc. 159

Maine Yankee Atomic Power Company 92, 97b

Mallinckrodt/Nuclear 162

Martin-Marietta Corporation 127, 136, 137, 146, 156, 162

Marvel-Schebler Division, Borg-Warner 80

Maryland Shipbuilding & Dry Dock Company 81

Massachusetts General Hospital 142

Massachusetts Institute of Technology 161

Matheson, Coleman & Bell 74

Maxfield Clinic Hospital 162

M&C Nuclear, Inc. 57, 58, 60

Merck & Company 74

NAME	PAGE NO.
Mesta Machine Company	80
Metal Hydrides, Inc.	69
Metropolitan Edison Company	92, 97a
Michigan State University	129
Middle South Utilities, Inc.	99
Miles Laboratory	85
Mines Development, Inc.	34
Minnesota Mining and Manufacturing Company	137, 148, 152, 155
Mississippi Power & Light Company	99
Mississippi State College	129, 130
Monsanto Research Corporation	148
National Annealing Box Company	80
National Assurance Corporation	110
National Coal Association	95
National Lead Company	14, 55, 57, 60, 69, 113, 171, 175, 219, 221
National Nuclear Corporation	118
National Sanitation Foundation	212, 213
University of Nebraska	161
Neutron Products, Inc.	143, 159, 160
New England Electric System	98
New England Nuclear Corporation	74, 142, 147

NAME	PAGE NO.
New York Atomic and Space Development Authority	162, 209
New York Shipbuilding Corporation	224
New York State Electric & Gas Corporation	92, 97b
Power Authority of the State of New York	98
Niagara Mohawk Power Corporation	92, 97a
North American Philips Company	149
North American Rockwell Corporation (See also Atomics International)	21, 82, 84, 107
North Carolina State University	129, 130, 162
Northeast Utilities Service Company	92, 97a, 97b
Northern Indiana Public Service Company	92, 97b
Northern Scientific, Inc.	85
Northern States Power Company	92, 97a, 97b
University of Notre Dame	162
Nuclear Associates, Inc.	85
Nuclear Associates International Corporation	224
Nuclear-Chicago Corporation	85, 147, 148, 150, 211, 213
Nuclear Components, Inc.	146
Nuclear Consultants, Division of Mallinckrodt Chemical Company	142, 147
Nuclear Data, Inc.	85
Nuclear Decontamination Corporation	213, 214
Nuclear Diagnostic Laboratories, Inc.	201, 202, 225
Nuclear Diodes, Inc.	85

NAME	PAGE NO.
Nuclear Electronics Counter Corporation	223
Nuclear Engineering Company, Inc.	198, 199, 201, 202, 221, 225
Nuclear Equipment Chemical Corporation	147
Nuclear Fuel Services, Inc.	14, 24, 55, 57, 60, 68, 69, 70, 113, 145, 165, 166, 167, 169, 172, 175, 176, 182, 183, 198, 199, 202, 204, 205, 209, 219, 221
Nuclear Laundry Rental Services, Inc.	214
Nuclear Laundry Service, Inc.	214
Nuclear Materials and Equipment Corporation	24, 41, 46, 55, 56, 57, 58, 60, 68, 69, 70, 76, 148, 155, 156, 159, 160, 182
Nuclear Measurement Corporation	85
Nuclear Metals Division, Whittaker Corporation	57, 58, 60
Nuclear Service and Construction Company	224
Nuclear Services Laboratory	211
Nuclear Technology Corporation	159, 160
Nucleonic Corporation of America	85, 211, 213
NUCOM Division, Sealol, Inc.	80
NUS Corporation	224
Offshore Systems, Inc.	156
Ohio-Nuclear, Inc.	85
Ohmart Corporation	150

NAME PAGE NO.

Oklahoma Gas & Electric Company 99

Olin Mathieson Chemical Corporation 104

Omaha Public Power District 18, 26, 92, 97a

Oregon Metallurgical Corporation 72

Pacific Gas & Electric Company 92, 97a, 97b, 99

Pacific Power & Light Company 98, 99

Packard Instrument Company, Inc. 85

Parson-Jurden Corporation 159

The Ralph M. Parsons Company 187

Penn Overall Supply Company 214

Pennsylvania Power & Light Company 92, 97b, 99

Petrotomics Company 34

Philadelphia Electric Company 92, 97a, 97b

Picker Nuclear Corporation 85

Picker X-Ray Company 148, 149

Pittsburgh - Des Moines Steel Company 80, 81

Portland General Electric Company 98

The Wm. Powell Company 80

Power Reactor Development Company 97a

PPG Industries, Inc. 157

Pratt & Whitney Aircraft 136

Princeton Gamma-Tech, Inc. 85

NAME	PAGE NO.
Programmed & Remote Systems Corporation	80
Public Service Company of Colorado	15, 92, 96, 97a, 107, 120
Public Service Company of Indiana, Inc.	99
Public Service Company of New Hampshire	97b
Public Service Company of Oklahoma	99
Public Service Electric & Gas Company	92, 97a, 97b
Purdue University	204
Rad Mat Inc.	161
Radiation Applications, Inc.	157
Radiation Detection Company	211, 213
Radiation Dynamics, Inc.	157, 158, 159
Radiation Facilities, Inc.	159, 160
Radiation Machinery Corporation	157, 159, 160, 162
Radiation Polymer Corporation	158
Radio Corporation of America	137
Radiological Service Company, Inc.	201, 202
Radionics Corporation	148
Radium Chemical Company	152
Raychem Corporation	157, 158, 159
Raycon, Inc.	158, 159
Reactor Controls, Inc.	80
Reed College	129

NAME PAGE NO.

Reuter-Stokes Electrical Components, Inc. 85

Robertshaw Controls Company 150

Rochester Gas and Electric Corporation 92, 97a

Rockwell Manufacturing 80

Rose Alloy Industrial Corporation 162

Rotterdam Dockyard Company 21, 80, 82

Rural Cooperative Power Association 97a

Sacramento Municipal Utility District 92, 97b

St. John X-Ray Laboratory 211

Salt River Power District 99

Sanders Nuclear Corporation 155

San Diego Gas and Electric Company 92, 101, 108, 115

Sandvik Special Metals Corporation 71

Sanitary Overall Supply Company 214

Schick X-Ray Company 149

Schutte & Koerting Company 80

Schwartz Bio-Research, Inc. 147

G. D. Searle, Inc. 146
(See also Nuclear-Chicago)

Siemens America, Inc. 75, 149

Sierra Pacific Power Company 99

Sinclair Oil Company 156

Skelly Oil Company 167

NAME PAGE NO.

Sloan-Kettering Institute 142

Solid State Radiations, Inc. 85

Southern California Edison Company 92, 97a, 98, 101, 108, 115

Metropolitan Water District of Southern California. 115

Southern Indiana Gas & Electric Company 99

Southern Interstate Nuclear Board 13, 21, 26, 28, 94, 121, 174, 176, 189

Southern Space, Inc. 214

Southwest Atomic Energy Associates 122, 123

Southwestern Engineering Company 81

Southwestern Public Service Company 99

E. R. Squibb & Sons 147

Stearns-Roger Corporation 80, 159, 160

Stone & Webster Engineering Corporation 159

Struthers Wells Corporation 81

Sundstrand Corporation 137

Superior Tube Company 71

Susquehanna-Western, Inc. 34

Technical Associates 85, 211

Technical Instruments Corporatic 88

Technical Measurement Corporation 88

Teledyne Geonuclear 187

NAME	PAGE NO.
Teledyne, Inc.	146, 155, 162
Tennelec Instrument Company, Inc.	85
Texas Electric Service Company	99
Texas Power & Light Company	99
Texas Instruments Inc.	187
Thermo Electron Corporation	137
Todd Shipyards Corporation	224
Toledo Edison Company	98, 99
Tracerlab, a Division of L.F.E., Inc.	85, 147, 148, 150, 211, 213
Transistor Specialties, Inc.	85
Transnuclear, Inc.	221
Tri-State Motor Transit Company	221
Troxler Corporation	150
TRW, Inc.	137
Union Carbide Corporation	34, 137, 143, 147, 157, 162
Union Electric Company	99
Union Tank Car Company	81
United Nuclear Corporation	15, 24, 34, 55, 56, 57, 60, 66, 68, 69, 104, 112, 128, 182, 183, 224
United States of America Standards Institute	227
U. S. Nuclear Corporation	147, 148, 202

NAME	PAGE NO.
U. S. Radium Corporation	152
United States Testing Company	211
Utah Mining and Construction	34
Varian Associates	158
Velan Engineering C[illegible]any	80
Vermont Yankee Nuclear Power Corporation	92, 97a
Victoreen Instrument Company	85
Virginia Electric & Power Company	92, 97a, 97b, 98
Wah Chang Albany Corporation	71
The Walker Trucking Company	201
Walworth Company	80
Washington Public Power Supply System	97a, 98, 165
University of Washington	161
Washington University Medical Center	142
William Wayne Electronics, Inc.	202
West Texas Utilities Company	99
Western Interstate Nuclear Compact	21
Western Nuclear Corporation	34
Westinghouse Electric Corporation	15, 17, 56, 57, 60, 66, 68, 71, 72, 80, 81, 82, 84, 106, 108, 111, 112, 118, 119, 123, 127, 132, 137, 149, 158

NAME PAGE NO.

Whitehead & Kales Corporation 221

Williamette Steel Division,
G. F. Atkinson Company 80

Wisconsin-Michigan Power Company 92, 97a, 97b

Wisconsin Public Service Company 92, 97b

Wolverine Tube Division,
Calumet & Hecla, Inc. 71

Worthington Division,
Studebaker 81

Yankee Atomic Elect ic Company 97a, 167

Yuba Industries, Inc. 81

Zirconium Technology Corporation 72

[illegible]

The Division of Industrial Participation was established in 1961 to strengthen industry's participation in the United States atomic energy program. Within this overall objective of bolstering the Commission's cooperation with U.S. industrial organizations, the Division of Industrial Participation has the following objectives:

1. Provide a focal point for industry within the Commission.

2. Identify activities performed in Government facilities that can be performed by private industry.

3. Appraise and report on the effectiveness of Commission policies for broadening industry participation in the U.S. nuclear program.

4. Serve as a contact point for state and local governments on non-regulatory matters.

5. Encourage non-nuclear applications of AEC-developed innovations.

The principal staff of the Division of Industrial Participation are:

Ernest B. Tremmel, Director
James H. Hill, Assistant Director
Clifford E. McColley, Assistant Director
Walter M. Babb
Robert J. Berte
Beatrice S. Braitman
Richard L. Egli
Jane T. Karlson
Walter Koester, Jr.

Form AEC-313
8-64
10 CFR 30

UNITED STATES ATOMIC ENERGY COMMISSION

APPLICA[illegible]ON FOR BYPRODUCT MATERIAL LICENSE

Form approved
Budget Bureau No. 38-R027

INSTRUCTIONS. – Complete Items 1 through 16 if this is an initial application or an application for renewal of a license. Information contained in previous applications filed with the Commission with respect to Items 8 through 15 may be incorporated by reference provided references are clear and specific. Use supplemental sheets where necessary. Item 16 must be completed on all applications. Mail two copies to: U.S. Atomic Energy Commission, Washington, D.C., 20545, Attention: Isotopes Branch, Division of Materials Licensing. Upon approval of this application, the applicant will receive an AEC Byproduct Material License. An AEC Byproduct Material License is issued in accordance with the general requirements contained in Title 10, Code of Federal Regulations, Part 30, and the Licensee is subject to Title 10, Code of Federal Regulations, Part 30.

1. (a) NAME AND STREET ADDRESS OF APPLICANT (Institution, firm, hospital, person, etc. Include ZIP Code.)

Philco-Ford Corporation
Aeronutronic Division & Space and
Re-entry Systems Division
Ford Road
Newport Beach, California 92663

(b) STREET ADDRESS(ES) AT WHICH BYPRODUCT MATERIAL WILL BE USED (If different from 1 (a). Include ZIP Code.)

Support and Launch Facilities and Land Flight Corridors on Vandenberg AFB, and Pacific Ocean areas of the Air Force Western Test Range (AFWTR).

2. DEPARTMENT TO USE BYPRODUCT MATERIAL

Field Test Operations
Vandenberg Air Force Base

3. PREVIOUS LICENSE NUMBER(S) (If this is an application for renewal of a license, please indicate and give number.)

21-4114-7 Expired 31 March 1963
4-11694-1 Expired 30 November 1966

4. INDIVIDUAL USER(S) (Name and title of individual(s) who will use or directly supervise use of byproduct material. Give training and experience in Items 8 and 9.)

S. E. Golian
R. E. Kay
T. F. Morrissey
R. K. Murray
E. R. Walwick

5. RADIATION PROTECTION OFFICER (Name of person designated as radiation protection officer if other than individual user. Attach resume of his training and experience as in Items 8 and 9.)

T. F. Morrissey

6. (a) BYPRODUCT MATERIAL (Elements and mass number of each.)	(b) CHEMICAL AND/OR PHYSICAL FORM AND MAXIMUM NUMBER OF MILLICURIES OF EACH CHEMICAL AND/OR PHYSICAL FORM THAT YOU WILL POSSESS AT ANY ONE TIME (If sealed source(s), also state name of manufacturer, model number, number of sources and maximum activity per source.)	
A. Any by-product material	A. Irradiated Phenolic Plugs (1/4" diameter, 1-1/2" long)	A. No single plug to exceed 10 millicuries
B. Iridium 192	B. Sealed Metallic Wire in Graphite Phenolic Plugs (1/4" diameter, 1" long)	B. No single plug to exceed 10 millicuries
C. Tantalum 182	C. Sealed Metallic Wire in Graphite Phenolic Plugs (1/4" diameter, 1" long)	C. No single plug to exceed 10 millicuries
D. Tungsten 181<br>183<br>184<br>187	D. Sealed Metallic Wire in Graphite Phenolic Plugs (1/4"diameter, 1" long)	D. No single plug to exceed 10 millicuries<br>NOTE: Maximum supply at any one time, not to exceed ten (10) Plugs.

7. DESCRIBE PURPOSE FOR WHICH BYPRODUCT MATERIAL WILL BE USED (If byproduct material is for "human use," supplement A (Form AEC-313a) must be completed in lieu of this item. If byproduct material is in the form of a sealed source, include the make and model number of the storage container and/or device in which the source will be stored and/or used.)

A, B, C and D to be assembled by Philco-Ford personnel in research re-entry vehicles for launch flights by assigned USAF Test Wings. The responsibility of Philco-Ford Corporation, Aeronutronic Division and Space and Re-entry Systems Division for the care and handling of the radioactive materials hereunder shall continue until, but not beyond the lift off (or attempt to lift off in the event of a failure) of the missile from Vandenberg Air Force Base. Responsibility for launch pad and post launch operations is to be assumed by the cognizant Air Force Agency and/or booster range contractors.

Information in this record was deleted in accordance with the Freedom of Information Act, exemptions outside scope
FOIA 95-105 9509130114

RECEIVED FEB 13 1967

91975 A/31

(Continued on reverse side)

9509130114 950425
PDR FOIA
PARKS95-105 PDR

TRAINING AND EXPERIENCE [illegible] EACH INDIVIDUAL NAMED IN ITEM 4 (Use supplemental sheets if necessary)

8. TYPE OF TRAINING	WHERE TRAINED	DURATION OF TRAINING	ON THE JOB (Circle answer)	FORMAL COURSE (Circle answer)
a. Principles and practices of radiation protection	See resumes Attachment #1		Yes No	Yes No
b. Radioactivity measurement standardization and monitoring techniques and instruments			Yes No	Yes No
c. Mathematics and calculations basic to the use and measurement of radioactivity			Yes No	Yes No
d. Biological effects of radiation			Yes No	Yes No

9. EXPERIENCE WITH RADIATION (Actual use of radioisotopes or equivalent experience.)

ISOTOPE	MAXIMUM AMOUNT	WHERE EXPERIENCE WAS GAINED	DURATION OF EXPERIENCE	TYPE OF USE
		See resumes Attachment#1		

10. RADIATION DETECTION INSTRUMENTS (Use supplemental sheets if necessary.)

TYPE OF INSTRUMENTS (Include make and model number of each)	NUMBER AVAILABLE	RADIATION DETECTED	SENSITIVITY RANGE (mr/hr)	WINDOW THICKNESS (mg/cm²)	USE (Monitoring, surveying, measuring)
	See Attachment #2				

11. METHOD, FREQUENCY, AND STANDARDS USED IN CALIBRATING INSTRUMENTS LISTED ABOVE

Survey instruments at least semi-annually, scintillation spectrometer, twice/week standard sources used as references, calibrated over entire detection range.

12. FILM BADGES, DOSIMETERS, AND BIO-ASSAY PROCEDURES USED (For film badges, specify method of calibrating and processing, or name of supplier.)

Film badges - renewed monthly
R. S. Landauer, Jr. & Company, Matteson, Illinois

INFORMATION TO BE SUBMITTED ON ADDITIONAL SHEETS IN DUPLICATE

13. FACILITIES AND EQUIPMENT. Describe laboratory facilities and remote handling equipment, storage containers, shielding, fume hoods, etc. Explanatory sketch of facility is attached. (Circle answer) Yes No See Attachment #3

14. RADIATION PROTECTION PROGRAM. Describe the radiation protection program including control measures. If application covers sealed sources, submit leak testing procedures where applicable, name, training, and experience of person to perform leak tests, and arrangements for performing initial radiation survey, servicing, maintenance and repair of the source.

See Attachment #4

15. WASTE DISPOSAL. If a commercial waste disposal service is employed, specify name of company. Otherwise, submit detailed description of methods which will be used for disposing of radioactive wastes and estimates of the type and amount of activity involved. California Salvage Company, San Pedro, California

CERTIFICATE (This item must be completed by applicant)

16. THE APPLICANT AND ANY OFFICIAL EXECUTING THIS CERTIFICATE ON BEHALF OF THE APPLICANT NAMED IN ITEM 1, CERTIFY THAT THIS APPLICATION IS PREPARED IN CONFORMITY WITH TITLE 10, CODE OF FEDERAL REGULATIONS, PART 30, AND THAT ALL INFORMATION CONTAINED HEREIN, INCLUDING ANY SUPPLEMENTS ATTACHED HERETO, IS TRUE AND CORRECT TO THE BEST OF OUR KNOWLEDGE AND BELIEF.

Aeronutronic Division, Philco-Ford Corporation
Applicant named in Item 1

By [signature]

J. B. Lawson, Vice-President and General Manager
Title of certifying official

Date ____________

RECEIVED JAN 31 1967 [illegible]

WARNING. — 18 U.S.C., Section 1001; Act of June 25, 1948; 62 Stat. 749; makes it a criminal offense to make a willfully false statement or representation to any department or agency of the United States as to any matter within its jurisdiction.

Department of the Air Force
Headquarters Air Force Logistic Command
Wright-Patterson Air Force Base, Ohio

MCDPE

30 January 1967

Application for Byproduct Material License, Philco-Ford Corporation, Newport Beach, California

Chief, Isotopes Branch
Division of Materials Licensing
U. S. Atomic Energy Commission
Washington, D. C. 20545

1. The USAF Radioisotope Committee has reviewed the attached application for a Byproduct Material License submitted by Philco-Ford Corporation, Aeronutronic Division and Space and Re-Entry Systems Division, Newport Beach, California. The Committee has determined that the use of the material does not present a hazard to Air Force personnel or degradation of Air Force property; therefore, we offer no objection to the issuance of this license.

2. It is requested that the license issued pursuant to your review of this application be transmitted through the USAF Radioisotope Committee.

FOR THE COMMANDER.

LAWRENCE T. ODLAND, LtCol, USAF, MC
Chairman, USAF Radioisotope Committee

1 Atch
Application

9509130111

ATTACHMENT NO. 1

ITEMS 8 and 9

RECEIVED
JAN 31 1967

RADIATION TRAINING AND EXPERIENCE SERGE E. GOLIAN

1.	Type of Training	Where Trained	Duration of Training	On Job	Formal Course
(a)	Principles of radiation protection	Naval Research Lab.	3 yrs.	3 yrs.	
		Various Nuclear Weapons Test Sites	1-1/2 yrs.	1-1/2 yrs.	
		Brookhaven National Lab.	1 yrs.	1 yr.	
(b)	Radioactivity measurement standardization and monitoring techniques	Naval Research Lab	3 yrs.	3 yrs.	
		Various Nuclear Weapons Test Sites	1-1/2 yrs.	1-1/2 yrs.	
		Brookhaven National Lab.	1 yr.	1 yr.	
(c)	Mathematics and calculations regarding radioactivity	Geo. Washington Univ.	1 yr.	1	1 yr.
		University of Chicago	4 yrs.		4 yrs.
(d)	Biological effects of radiation	Naval Research Lab.	3 yrs.	3 yrs.	
		Various Nuclear Weapons Test Sites	1-1/2 yrs.	1-1/2 yrs.	
		Brookhaven National Lab.	1 yr.	1 yr.	

2. Experience with Radiation - Type of isotope, amount, where handled, duration of experience, etc.

I. Director of Naval Research Laboratory, Research Reactor Facility - 2 yrs.
   1. Directed operation of 100 KW pool type research reactor.
   2. Directed operation of hot cells involving used fuel elements with activity up to 1,000 curies.
   3. Directed design, construction, and safety studies for reactor safeguards.
   4. Directed presentations to Reactor Safeguards Committee.

II. Head of Detector Branch, Radiation Division, Naval Research Laboratory - one year.
   1. Directed calibration of detection systems for nuclear weapon diagnostic test at Naval Research Laboratory, Nevada Proving Grounds, and Pacific Proving Grounds. Worked with sources of approximately 1,000 curies of cobalt-60.
   2. Designed and operated high voltage accelerators and performed radiation monitoring work involving neutrons.

III. Brookhaven National Laboratory - one year.
   Performed work on experimental problems of nuclear interactions with the Brookhaven Graphite Reactor.

IV. Author of paper analyzing "Safety Features of Water Moderated Parallel Plane Fuel Elements."

91375

RADIATION TRAINING AND EXPERIENCE R. E. KAY

1.	Type of Training	Where Trained	Duration of Training	On Job	Formal Course
(a)	Principles of radiation protection	U.S. Naval Radiological Defense Laboratory, San Francisco 24, California	8 yrs., & 8 mo.	X	X
(b)	Radioactivity measurement standardization and monitoring techniques	U.S. Naval Radiological Defense Laboratory, San Francisco 24, California	8 yrs., & 8 mo.	X	
(c)	Mathematics and calculations regarding radioactivity	U.S. Naval Radiological Defense Laboratory, San Francisco 24, California	8 yrs., & 8 mo.	X	
(d)	Biological effects of radiation	U.S. Naval Radiological Defense Laboratory, San Francisco 24, California	8 yrs., & 8 mo.	X	

2. Experience with Radiation - Type of isotope, amount, where handled, duration of experience, etc.
From 1952 to 1961, a period of 8 years, 8 months, was employed as a biochemist (radiological) by the U.S. Naval Radiological Defense Laboratory, San Francisco, California. During the period used for the investigation of the intermediary metabolism of irradiated mammals, mc amounts of the following radioactive isotopes: C^{14}, S^{35}, H^{3}, P^{32}, Na^{22}, Fe^{59}, and I^{131}. High voltage x-rays were employed as the radiation source, and studies were carried out at the whole animal and cellular levels. During this period, was the senior author of 15 scientific publications and 14 technical reports dealing with the biological effects of ionizing radiations. From 1958 to 1961, a period of 3 years, was the instructor in atomic, biological, and chemical warfare for Naval Reserve Officer's School 12-1. Approximately one-half of the course was concerned with the effects of atomic warfare. This included instruction in the basic physics of nuclear radiation, radioactivity measurements, fallout hazards, shielding, biological effects, and decontamination.

RADIATION TRAINING AND EXPERIENCE | T. F. MORRISSEY

1. Type of Training	Where Trained	Duration of Training	On Job	Formal Course
(a) Principles of radiation protection	Illinois Institute of Technology	1/2 yrs.		1/2 yr.
	Harvard University	1 yr.		
(b) Radioactivity measurement standardization and monitoring	Harvard University	1 yr.		1 yr.
	Aeronutronic Div. Ford Motor Co.	1-1/2 yrs.	1-1/2 yrs.	
(c) Mathematics and calculations regarding radioactivity	Harvard University	1 yr.		
	Aeronutronic Div. Ford Motor Co.	1-1/2 yrs.	1-1/2 yrs.	
(d) Biological effects of radiation	Harvard University	1 yr.		1 yr.

2. Experience with Radiation - Type of isotope, amount, where handled, duration of experience, etc. Worked with small quantities of isotopes representing all types of emitters at Harvard University for one year. Administered health physics aspects of operations at Aeronutronic involving millicurie quantities of a variety of isotopes and radiation generating machines for past seven years.

RADIATION TRAINING AND EXPERIENCE R. K. MURRAY

1.	Type of Training	Where Trained	Duration of Training	On Job	Formal Course
(a)	Principles of radiation protection	Univ. of Colorado	1/2 yr.		1/2 yr.
		Univ. of California	1 yr.		1 yr.
		AEC Formal Course	16 weeks		16 weeks
		State of California	3 years	3 yrs.	
(b)	Radioactivity measurement standardization and monitoring techniques	Univ. of Colorado	1/2 yr.		1/2 yr.
		Univ. of California	1 yr.		1 yr.
		Lawrence Radiation Laboratory	1/2 yr.	1/2 yr.	
		State of California	3 yrs.	3 yrs.	
(c)	Mathematics and calculations regarding radioactivity	Univ. of Denver	2 yrs.		2 yrs.
		Univ. of California	1 yr.		1 yr.
		Univ. of Colorado	1/2 yr.		1/2 yr.
(d)	Biological effects of radiation	Lawrence Radiation Laboratory	1/2 yr.	1/2 yr.	
		Univ. of California	1 yr.		1 yr.
		State of California	3 yrs.	3 yrs.	

2. Experience with Radiation - Type of isotope, amount, where handled, duration of experiences, etc.

I. Worked with small quantities of isotopes representing all type of emitters at University of California at Berkeley.

II. Conducted applied research in Radiation Physiology at Lawrence Radiation Lab for one-half year. Bio assay, radio chemical studies, and accelerator operation comprised the major effort in this period. Sr^{90}, Y^{89}, Co^{60}, H^{3}, I^{131}, Ra^{226} and P^{32}, up to 250 millicuries was used during this period.

III. Administered and conducted Health Physics responsibilities with the Division of Industrial Safety, State of California. Made preliminary surveys and inspections of radiation users, conducted studies of potential radiation hazards in industries or processes, or where specific radionuclides were used, prepared reports of findings and made recommendations for the elimination or control of hazardous conditions, assisted in design and construction of special field survey - bio assay techniques, collected air and biological samples to evaluate contamination, assisted in design and construction of special radionuclide facilities, enforced radiation standard, calibrated instrumentation, and conducted radiation protection training.

RADIATION TRAINING AND EXPERIENCE E. R. WALWICK

1.	Type of Training	Where Trained	Duration of Training	On Job	Formal Course
(a)	Principles of radiation protection	U.S. Naval Radiological Defense Lab.	3 yrs.	3 yrs.	
(b)	Radioactivity Measurement standardization and monitoring techniques	Univ. of Calif. Berkeley	1/2 Semester Course of 4 units.		4-1/2 mo.
		U.S. Naval Radiological Defense Lab. San Francisco 24, California	3 yrs.	3 yrs.	
(c)	Mathematics and calculations regarding radioactivity	Univ. of Calif. Berkeley	1/2 Semester Course of 4 units.		4-1/2 mo.
		U.S. Naval Radiological Defense Lab.	3 yrs.	3 yrs.	
(d)	Biological effects of radiation	U.S. Naval Radiological Defense Lab.	3 yrs.	3 yrs.	

2. Experience with Radiation - Type of isotope, amount, where handled, duration of experience, etc.
Tritium labeled thymidine, 1-5 mc - U.S. Naval Radiological Defense Laboratory, San Francisco, 3 years.

Tritiated water, 3 c - U.S. Naval Radiological Defense Laboratory.

C^{14}, C amounts, University of California, 2 months. This was a formal course in the use of radioactive tracers in biochemical research.

Work at USNRDL resulted in a paper on tritium assay. R. K. Main and E. R. Walwick, Biochem., Biophys., Res. Comm., 4-52 (1961). "A Simplified Quantitative Assay of Tritiated Thymidine Incorporated into Deoxyribonucleic Acid."

While at the Naval Radiological Defense Laboratory, I handled 5 millicurie amounts of tritiated thymidine routinely. During this period of time, in collaboration with Dr. R. K. Main, I developed a procedure for the "Assay of Tritiated Dioxyribonucleic Acid by Liquid Scintillation Counting." We employed this procedure over this period of 3 years for investigation of nucleic acid synthesis. One particular group of experiments involved the effect of x-ray (250 KVP) on the synthesis system. For a period of six months, I studied the tritiation of thymidylic acid by tritiated water and tritiated acetic acid (formed from tritiated water). This required the removal of exchangeable tritium and isolation of the labeled material free of the water or acetic acid. The area of work was monitored for vaporized material.

(See attached supplement)

RADIATION TRAINING AND EXPERIENCE - (Continued) **E. R. Walwick**

The Naval Radiological Defense Laboratory is directly involved in research concerned with the biological effect of radiation and problems of protection against radiation. Consequently, knowledge was obtained of radiation effects in systems and animals other than the specific problem on which work was performed.

ATTACHMENT NO. 2

ITEM 19

RECEIVED
JAN 31 1967
U.S. ATOMIC ENERGY COMMISSION

S1375

ATTACHMENT NO. 2

ITEM 10

RADIATION DETECTION INSTRUMENTS

The following instrumentation is available at the Newport Beach Facility. The proper instruments to monitor the specific isotope will be transported by the Radiation Safety Officer to the Philco-Ford Vandenberg Facility. Monitoring will be continuous by the Radiation Safety Officer during receipt, storage and handling of the radioactive material.

Type	No. Avail.	Radiation Detected	Sensitivity Range	Window Thickns.	Use
eiger-Muller Survey Meter<br>uclear Chicago<br>odel 2612	1	Beta Gamma	0-0.2mr/hr<br>0-20 mr/hr<br>0-20 mr/hr	1.2mg/cm^2	monitor<br>survey
eiger-Muller Survey Meter<br>.S. Nuclear Corp.<br>odel 121	1	Beta Gamma	0-0.2mr/hr<br>0-2 mr/hr<br>0-20 mr/hr	1.3mg/cm^2	survey
eiger-Muller Survey Meter<br>racer Lab. Inc.<br>odel SU-14	1	Alpha<br>Beta<br>Gamma	0-50 cpm<br>0-500 cpm<br>0-5000 cpm<br>0-0.25 mr/hr<br>0-25 mr/hr<br>0-2.5 mr/hr	1 mg/cm^2	survey
cintillation<br>pectrometer<br>.S. Nuclear Corp.	1	Beta Gamma	Wide	5 mil Be<br>1 mil AL	measur
utie Pie Radiation<br>urvey Meter<br>racer Lab. Inc.<br>odel 1542	1	Alpha<br>Beta<br>Gamma	0-15 mr/hr<br>0-150 mr/hr<br>0-1500 mr/hr	1 mg/cm^2	survey
atie Pie Survey Meter<br>echnical Associates<br>odel CP-3	1	Alpha<br>Beta<br>Gamma	0-50 mr/hr<br>0-150 mr/hr<br>0-1500 mr/hr	1 mg/cm^2	survey
nizudon Chamber<br>ctoreen<br>odel 440	1	Gamma	0-3 mr/hr<br>0-10 hr/hr<br>0-30 mr/hr<br>0-100 mr/hr<br>0-300 mr/hr	1 mg/cm^2	survey
ondenser R-Meter<br>ictoreen<br>odel 570	1 set	X-Ray<br>Gamma	0-25 R<br>0-100R<br>0-250R		measur

ATTACHMENT NO. 3

ITEM 13

ATTACHMENT NO. 4

ITEM 14

RECEIVED
MAR 3 1 1967

91975

RADIATION PROTECTION AND CONTROL (VANDENBERG AFB)

1. SCOPE

This standard outlines the radiation protection requirements and instructions, which will be followed by all personnel at Philco-Ford's Vandenberg Air Force Base facility. This standard is applicable when working with, or around, radioactive material and/or in radiation fields. Supplemental appendices will be issued as required for specific operations.

2. PURPOSE

The fundamental purpose of this standard instruction is:

2.1 To prevent ingestion, inhalation, or other modes of entry of radioactive particulates into the body.

2.2 To reduce the amount of external irradiation exposure to below maximum permissible levels.

2.3 To prevent contamination of the environment.

3. RESPONSIBILITY

The responsibility of Philco-Ford Corporation, Aeronutronic Division and Space and Re-entry Systems Division for the care and handling of the radioactive material shall continue until, but not beyond the lift off (or attempt to lift off in the event of a failure) of the missile from Vandenberg AFB. Air Force personnel are not involved in the handling of any radioisotope material, except as in an advisory capacity.

3.1 The Philco-Ford Radiation Safety Officer will:

3.1.1 Administer, and provide health physics services to assure adequacy of an adherence to this radiation safety standard.

3.1.2 Notify and coordinate in advance with the 392nd Aerospace Medical Group (SAC) all planned operations involving sources of radiation in accordance with the General Range Safety Plan.

3.1.3 Provide copies of Test Procedures, Operational Procedures, accepted operating procedures, Test Memoranda, Program Documents, AEC licenses and work schedules relating to the storage or use of sources of radiation to responsible Test Wing

and the 392nd Aerospace Medical Group (SAC).

3.1.4 Provide notice of intent to deviate significantly from planned procedures or schedules to responsible Test Wing and 392nd Aerospace Medical Group (SAC).

3.1.5 Provide advance notification of the arrival, use, conditions, and departure of sources of ionizing radiation to 392nd Aerospace Medical Group (SAC).

3.1.6 Notify Range Support at least two hours prior to any test operation involving use of radioactive materials or radiation producing equipment.

[illegible].1.7 Ensure that Philco-Ford personnel are properly trained in radiological safety prior to working in radiation areas.

3.1.8 Maintain appropriate radiological monitoring instrumentation.

3.1.9 Monitor safe receipt, transportation, handling and storage of radioactive material on the Vandenberg AFB. If this involves launch complexes, spin test facilities, or areas where explosive ordnance hazards exists, actions will be coordinated with Pad Safety and the 392nd Aerospace Medical Group (SAC).

3.1.10 Review all design or modification of all facilities to be used for nuclear purposes by Philco-Ford and advise the 392nd Aerospace Medical Group (SAC) and AFWTR of any hazard potential which may exist.

3.1.11 Maintain the following records:

3.1.2.1 Radiation exposures of all individuals for whom personnel monitoring is required.

3.1.2.2 Results of required surveys and radioactive waste disposals.

3.1.2.3 A current record of all radioactive isotopes possessed by Aeronutronic and intended use.

3.1.2.4 A record of radioactive material receipts, disposal and transfer.

3.1.2.5 Current official regulatory licenses and registrations with required communications.

3.1.2.6 A record of occupational medical examination dates for personnel at intervals not exceeding six (6) months.

4. RADIOACTIVE MATERIAL PROCUREMENT

4.1 Required amendments to existing licenses for radioactive material including by-product and special nuclear materials, shall be secured through the Radiation Safety officer.

4.2 Plans for facilities and equipment, procedures for handling materials, and information pertaining to the radioactive material involved, shall be submitted to the Radiation Safety Officer for review and approval.

4.3 Only the radioactive material listed on Philco-Ford's current licenses, and not exceeding the total amount specified at any time, may be procured for use.

4.4 All radioactive material requires Hazardous Material Form ARD 7001 to be submitted to the Safety Engineering Section, Newport Beach, prior to purchase in accordance with Procedure No. 5 and Purchasing Procedure No. 2.

5. RADIATION GENERATING MACHINE REGISTRATION

5.1 Plans for facilities and equipment, procedures and specifications shall be submitted to the Radiation Safety Officer prior to purchase of any radiation generating machine for use at Vandenberg AFB.

6. TRAINING

Supervisors responsible for operations involving radioactive materials and workers who enter radiation areas for any reason must be oriented in standards for protection, radiological safety work practices, decontamination, and contamination control. Training time for both supervisors and workers will normally vary depending upon the particular needs of the group. A general program of instruction which may be tailored to fit the needs of various groups is given in the following table:

RECEIVED
JAN 31 1967
U.S. ATOMIC ENERGY COMMISSION
Regulatory Mail Section

PROGRAM OF INSTRUCTION
RADIOLOGICAL HEALTH AND SAFETY

Subject	Scope of Instruction
Structure of Matter	Sources of radiation and modern concepts of matter; Discussion of building blocks of matter and the structure of the atom to enable a general understanding of the mechanics of radioactive emission.
Radioactivity	Nature and types of radioactivity; Radioactive decay; Ionization; Units of measurement; Dose and dose rate.
Biological Effects of Radiation	Biological response to radiation exposure; Radiosensitivity of cells; Acute and chronic exposures; Radiation syndrome.
Basis of Radiation Detection and Dosimeters	Principles of radiation detection; Comparison of dose and dose-rate measuring, film badges and pocket dosimeters.
IC Meters, Alpha Meters	Operation of ionization chamber type radiac meters and alpha survey meters.
Shielding Properites of Materials	Absorption of alpha, beta, X and gamma radiations; Shielding materials for X and gamma radiations; Half-thickness concept.
Radiological Safety Work Practices	Personnel dosage control (External and Internal); Dose and dose rate estimates; Exposure from a point source; Safe handling, Protective clothing.

91975

7.0 CONTROLLED AREA REQUIREMENTS

7.1 Each area in which a potential or actual exposure to radioactivity in an average concentration in excess of two millirems in any one hour from radiation generating machines and in excess of the limites specified by the General License Material Standards shall be declared a CONTROLLED AREA.

7.2 No person under 18 years of age shall be assigned to a CONTROLLED AREA.

7.3 Radiation CONTROLLED AREAS shall be clearly defined and designated by a standard magenta on yellow sign containing the radiation symbol and the wording to denote the level of hazard in the area.

7.4 Access to CONTROLLED AREAS shall be restricted to authorized persons. CONTROLLED AREAS entrances shall be of a minimum number, well posted in sufficient number to assure adequate warning.

7.5 A written operating procedure shall be prepared by the responsible supervisor for each CONTROLLED AREA. This procedure shall be posted at the CONTROLLED AREA and include the following:

7.5.1 The handling and use of radiographic exposure devices and the manner of employment to insure that no individual is likely to be exposred to radiation doses in excess of the limits of this standard.

7.5.2 Methods and occasions for conducting radiation surveys.

7.5.3 Methods and occasions for controlling access to radiographic areas.

7.5.4 Methods and occasions for locking and securing radiographic exposure devices and storage containers.

7.5.5 Personnel monitoring and the use of personnel monitoring devices and protective clothing.

7.5.6 Minimizing exposure of individuals in the event of an accident.

7.5.7 The procedure for notifying the Radiation Safety Officer and Medical Section in the event of an accident.

7.5.8 Maintenance of accountability records.

7.6 CONTROLLED AREAS should not include unnecessary area, equipment, materials, people, etc. They should be limited to areas actually requiring controls for radiation safety.

7.7 No person shall enter CONTROLLED AREAS except under the conditions of the written procedure for the area in question or emergency conditions.

7.8 Protective apparel and personnel monitors will be worn by all personnel in CONTROLLED AREAS.

7.9 CONTROLLED AREA housekeeping, including the regulation of used protective clothing, shall be sufficient to minimize exposure of personnel.

8. PERSONNEL MONITORING AND APPAREL FOR CONTROLLED AREAS

8.1 Each person who is assigned to a CONTROLLED AREA shall have his exposure monitored with approved personnel dosimeters. Responsible supervisors will submit the individuals name, age, birth date, sex and social security number to the Radiation Safety Officer.

8.2 The minimum personnel monitoring device for closed radiographic room operation shall be a film badge worn on the outside of the protective clothing above waist height. For field radiographic work both a film badge and pocket dosimeter are required.

8.3 For exposures which represent significant variations, pocket dosimeter and/or other dosimeter devices may be required at the determination of the Radiation Safety Officer.

8.4 For those operations with potential extremity exposure, ring badges and/or wrist film packets will be worn in addition to body film badge.

8.5 Supervisors shall be responsible for any mistreatment or contamination of any monitoring device, or if any unit is lost, and shall arrange for replacement equipment before the person is permitted further access to a CONTROLLED AREA.

8.6 Film badges, ring badges, wrist film packets and any other monitoring device will be controlled by the Radiation Safety Officer. Personnel monitoring devices will be evaluated on a monthly basis, unless excess exposure is suspected.

8.7 Detailed protective clothing standards will be issued by the Radiation Safety Officer when the scope of radioisotope operations indicate this is necessary.

9. CONTROLS FOR SEALED SOURCES

9.1 Sealed sources, including calibration source shall be used only under the conditions of a written procedure approved by the Radiation Safety Officer. This procedure shall be posted at, or near the source or source container.

9.2 Sealed sources shall be leak tested on receipt and semi-annually. The Radiation Safety Officer will be responsible for this function.

9.3 The responsible supervisor will be charged with accountability of the source.

9.4 No radiographic operation using a sealed source shall be conducted unless a calibrated and operable radiation survey instrumentation is available at each site.

9.5 A physical radiation survey shall be made after each radiographic exposure during a radiographic operation in which a sealed source is utilized has been returned to its shielded condition.

9.6 A physical radiation survey shall be made to determine that each sealed source is in its shielded condition prior to locking the radiographic exposure device or storage container.

9.7 Records shall be kept of required surveys by the responsible supervisor and submitted to the Radiation Safety Officer.

9.8 Any sealed source which is not fastened to or contained in a radiographic exposure device shall have permanently attached to it a durable tag at least one inch square bearing the radiation symbol as prescribed by the Radiation Safety Officer.

10. RADIATION GENERATING MACHINES

10.1 Radiation generating machines shall be operated only under the conditions of a written procedure approved by the Radiation Safety Officer. This procedure shall be posted at or near the machine.

10.2 All radiation generating machines must be under the supervision of a qualified supervisor as determined by the Radiation Safety Officer.

10.3 Periodic radiation surveys of radiation producing devices shall be conducted by the responsible supervisor. Results shall be submitted to the Radiation Safety Officer on a prescribed frequency.

10.4 In any application in which the operator and any other individuals are required to be in the room during exposure and are not located behind a protective barrier, clothing consisting of a protective apron having a lead equivalent of not less than 0.25 millimeter shall be worn.

10.5 During each radiographic operation, the supervisor or operator shall maintain a direct surveillance of the operation to protect against unauthorized entry into the CONTROLLED AREA.

11. DEVICES CONTAINING RADIOACTIVE MATERIALS (General License Material

11.1 Devices containing radioactive material shall be plainly labeled to indicate radioactive material content, activity, radiation characteristics and date of containment.

11.2 The location of such devices shall be reported to the Radiation Safety Officer.

11.3 Such devices shall be used only under the conditions of a procedure approved by the Radiation Safety Officer. The procedure shall provide for adequate control of contamination from broken or leaking devices. The procedure shall be posted at or near the area of use.

11.4 Environmental surveys and leak testing shall be completed by the responsible user at the direction of the Radiation Safety Officer.

12. EMERGENCY PROCEDURES AND INSTRUCTIONS FOR RADIATION INCIDENTS

12.1 Emergency procedures shall be established for each CONTROLLED AREA by the responsible supervisor and approved by the Radiation Safety Officer.

12.2 General Instructions for Area Contamination Accident

12.2.1 Notify the supervisor and the Radiation Safety Officer. (If this can be done without excessive spread of contamination). The Radiation Safety Officer will then notify AFWTR, Pad Safety and the 392nd Aerospace Medical Group (SAC) as appropriate.

12.2.2 Limit spread of contamination.

12.2.2.1 Do not allow newcomers to enter the contaminated area unless satisfactory precautions can be taken to prevent spreading the contamination.

12.2.2.2 Permit no one to leave the contaminated area without clean footwear. Exceptions may be made to avoid overexposure. Shoe covers should be worn while in the contaminated area.

12.2.2.3 Prevent spread of activity by liquid carriers, apply absorbers, raise barriers (putty, etc.), seal cracks in floors, desk tops, etc. (putty, quick drying plastics, etc.).

12.2.2.4 If contamination is airborne, hold breath, hold wet handkerchief or immediately put on respiratory equipment over nose and mouth, close windows and doors, shut off fans or air conditioners and leave room as quickly as possible. Seal it in.

12.3 General instructions for Personnel Contamination Accidents

12.3.1 Notify the supervisor and Radiation Safety Officer immediately.

12.3.2 Remove any contaminated clothing and isolate until properly disposed of.

12.3.3 Procedure for contaminated unbroken skin: Wash area thoroughly (preferably in a shower) for two to three minutes, repeatedly "soaping" and rinsing. Consideration should be given to the chemistry of the contaminate and an attempt made to find a suitable agent for dissolving it.

12.3.4 Instructions for contaminated wounds: Flush the wound with water immediately, preferably under running water from a tap. If much area around the wound is contaminated the surrounding skin should be covered with a layer of adhesive tape to prevent washing more contaminate into the wound. After the wound is cleansed, it may be covered while the surrounding skin is decontaminated.

12.4 General Instructions for External Radiation

12.4.1 If any individual suspects that he has received an over-dose of external radiation from any source, he should immediately inform the Radiation Safety Officer.

12.4.2 He should be removed from areas in which he might conceivably receive more radiation, and will not be allowed to return to work in such areas until approved by the Radiation Safety Officer.

12.5 All radiation incidents shall be documented by the responsible supervisor to the extent necessary to describe the equipment contamination and evaluation of personnel exposures.

12.6 All radiation incidents shall be investigated and documentated by the Radiation Safety Officer. The Radiation Safety Officer shall be responsible for notifying regulatory agencies, as necessary, of personnel exposures exceeding exposure limits or release of radioactive materials in excess of permissible concentrations.

13. PERMISSIBLE DOSES AND LIMITS

13.1 No individual shall possess, use, receive or transfer sources of radiation in such a manner as to cause in any period of one calendar quarter excess of the limits specified.

13.1.1 Whole body, head and trunk; active blood forming organs, lens of eyes or gonads - - - 1-1/4 rem per calendar quarter.

13.1.2 Hands and forearms, feet and ankles - - - 18-3/4 rem per calendar quarter.

13.1.3 Skin of whole body - - - 7-1/2 rem per calendar quarter.

13.2 Emergency exposure limits

13.2.1 Exposure of the whole body of any individual over 18 years of age to 25 rems.

13.2.2 Exposure of the skin of the whole body of an individual over 18 years of age to 75 rems.

13.2.3 Exposure of the feet, ankles, hands or forearms of any individual over 18 years of age to 375 rems.

13.3 Each person assigned to work in a CONTROLLED AREA shall have his previous exposure to external and internal radiation evaluated prior to beginning work.

14. RECORDS AND REPORTS

14.1 The Radiation Safety Officer shall maintain the following records:

14.1.1 Radiation exposures of all individuals for whom personnel monitoring is required.

14.1.2 Results of required surveys and radioactive waste disposals.

14.1.3 A current record of all radioactive isotopes possessed by Philco-Ford and location of intended use.

14.1.4 A record of radioactive material receipts, disposal and transfer.

14.1.5 Current official AEC licenses and registrations with required communications.

14.2 The responsible supervisor for each CONTROLLED AREA shall maintain the following records:

14.2.1 Results of required CONTROLLED AREA surveys and disposal.

14.2.2 A current record of all radioactive material and amount within the designed CONTROLLED AREA.

14.2.3 A record of radioactive material receipts and transfers.

14.2.4 Authorized personnel assigned to the CONTROLLED AREA.

14.2.5 A record of occupational medical examination dates for personnel at intervals not exceeding six months.

14.3 The Medical Section will maintain the following records:

14.3.1 Occupation exposures to radiation for each individual.

14.3.2 Records of his bio assay tests and examination.

14.3.3 Prescribed medical physical examination findings.

15. SHIPPING AND RECEIVING - RADIOACTIVE MATERIALS

15.1 Receipt of radioactive materials or shipment suspected to contain radioactive materials shall be held in an approved location.

15.2 Shipments of radioactive material leaving Philco-Ford - Vandenberg AFB shall be coordinated with the 392nd Aerospace Medical Group (SAC).

16. STORAGE OF RADIOACTIVE MATERIALS

16.1 Ordinarily, only enough radioactive materials to meet current needs will be kept on hand. Large stocks shall not be maintained. There may be some lapse of time between receipt of the materials and actual use. During this period, the material shall be kept in containers that will prevent breakage or spillage. Storage duration will normally not exceed three (3) working days.

16.2 Radioactive material containers should be kept locked and stored in the designated Radioactive Area.

16.3 Movement of radioactive materials shall be only in properly shielded carriers protected against accident (e.g., by double containers when liquid)

16.4 All containers of radioactive substances, as well as all contamination supplies or equipment shall be clearly labeled.

16.5 Radioactive materials shall be housed in fireproof containers and preferably placed in a fireproof room or enclosure for storage.

16.6 Storage areas shall be denoted by a standard magenta on yellow sign bearing the standard radiation symbol with appropriate wording.

17. RADIOACTIVE WASTE DISPOSAL

17.1 The possibility of radioactive wastes at Philco-Ford's Vandenberg Facility is not expected, however, in the event of an accident which requires decontamination procedures, as outlined in Section 12.0 of this standard, the waste material shall be removed from Philco-Ford (Vandenberg AFB) premises by an approved waste disposal contractor. He shall be licensed by the State of California and the AEC to receive and dispose of radioactive waste.

17.2 Prior to radioactive waste removal from Philco-Ford's Vandenberg Facility, coordination with the 392nd Aerospace Medical Group (SAC) will be completed.

18. MEDICAL RADIATION SURVEILLANCE

18.1 Every individual for whom personnel monitoring is required shall be placed under medical surveillance when the potential acute or chronic exposure to radiation is such that somatic radiation effects are capable of being detected by medical examination.

18.2 Medical examination as specified by the Medical Section, Newport Beach, shall occur prior to the potential radiation exposure, at subsequent intervals not to exceed one year and at termination of program.

18.3 Where necessary or desirable in order to aid in determining the extent of an individuals exposure to radioactive material, the Medical Section, Newport Beach, may require bio-assay examination and tests.

18.4 The Medical Section, Newport Beach, shall record all radiation exposures on the individuals medical records and maintain a complete file pertinent to radiation medical surveillance.

UNITED STATES
ATOMIC ENERGY COMMISSION
DIVISION OF COMPLIANCE
REGION V
2111 BANCROFT WAY
BERKELEY, CALIFORNIA 94704

TELEPHONE: 841-5121
EXT. 651

October 3, 1968

H. E. Book, Senior Radiation Specialist
Region V, Division of Compliance

INSPECTOR'S EVALUATION AS PER MM 900-15
VETERANS ADMINISTRATION CENTER, WADSWORTH HOSPITAL
LOS ANGELES, CALIFORNIA - LICENSE NO. 4-181-4

The licensee's program (inspected September 20, 1968) remains essentially unchanged since the last previous inspection. The most significant changes consist of the transfer of the 1.15-curie strontium-90 extracorporeal blood irradiator to UCLA and the acquisition of an Anger camera. The licensee is also utilizing fluorine-18 (with a half life of 111 minutes) in conjunction with the Anger camera in the performance of bone scans. Fluorine-18 is produced by irradiation in the UCLA reactor. The material is analyzed for assay, sterility and pyrogenicity.

It is the inspector's opinion that the licensee's activities do not constitute a threat to the health and safety of the licensee's patients, employees, or the general public.

H. S. North
Radiation Specialist

8204150581 820216
PDR FOIA
NELSON82-11 PDR

591 Notes
North/msb
10/3/68

[signature] Inspector [initials]
[signature] Reviewer 10-11-68

Veterans Administration Center
Wadsworth Hospital
Los Angeles, California

License No. 4-181-4

Summary

1. An announced reinspection of the subject licensed program was conducted on September 20, 1968, by H. S. North, Radiation Specialist, Region V, Division of Compliance. The inspector was unaccompanied.

Inspection Findings and Exit Interview

2. As a result of the inspection, no items of noncompliance were noted and a Form AEC-591 reflecting this finding was issued at the conclusion of the inspection. The results of the inspection were discussed ~~were discussed~~ with Dr. William H. Blahd, M.D., Chairman of the Radioisotope Committee, and Chief of the Radioisotope Service; Mr. L. W. Wetterau, Physicist and Radiation Safety Officer.

Persons Interviewed

3. During the course of the inspection, the following persons were either interviewed or met by the inspector. Mr. L. W. Wetterau, Physicist and Radiation Safety Officer, whose responsibilities are Center-wide in connection with the licensed programs with the exclusion of the Radiology Department. He is responsible for the control of receipt and disposal of radioactive materials, the use of licensed materials as authorized by individual user authorizations approved by the committee, and radiation safety training. Dr. W. H. Blahd, M.D., Chief of the Radioisotope Service and Radioisotope Committee Chairman.

His responsibilities include use and supervision of use of isotopes in humans. Dr. A. Yuwiler, Head of the Neurobiochemistry Research Section. Mr. K. Shoaf, Research Technician, Wadsworth Hospital, is responsible for operation of the Scanning Laboratory. Mary Ann Lederer, Technician, David Maryn, Technician, operate the whole body counter facility at the Veterans Administration Center.

Organization

4. The VA Center is divided into two organizations - research and hospital. Dr. Blahd holds positions in both organizations as the Chief of the Radioisotope Service under the hospital and the Chief of the Radioisotope Research Service under the research organization. Mr. Wetterau is the RSO for the VA Center. The licensee's administrative and operating organization is as shown in paragraph 4 of the notes of the last previous inspection with exception of the following changes. The Assistant Hospital Medical Director, B. Mandelstam, has been added to the staff. He reports to the Hospital Medical Director. Dr. Seymor Dayton has replaced Dr. L. Fred as Head of Hospital Medical Services. Dr. E. Weiss is now Dr. Blahd's assistant. Dr. J. M. Koplowitz, M.D., is now a consultant to the Radioisotope Service. Other portions of the organization remain unchanged.

5. The licensee's isotopes and research committees include approximately 12 members, among them the Chiefs of all services and medical and research investigators. The Research Committee meets weekly as a committee and on occasion Isotope Committee business is included in the Research Committee meetings. Formal Isotope Committee meetings are rare in that

they revolve primarily around policy and there have been few policy changes. Most normal committee business (i.e., review and approval of research protocols) is conducted by mail. A description of the procedure and samples of the forms used by the committee are a part of paragraph 5 of the notes of the last previous inspection.

Radiation Safety

6. Mr. Wetterau's status as the RSO remains unchanged from that described in paragraph 6 of the previous inspection notes.

Instruction of Personnel

7. Written procedures are required by the license. Copies of the procedures are provided to all persons who require them in the performance of their work. Mr. Wetterau provided a copy of the licensee's Radiation Safety Program Manual, revised July 1968. Mr. Wetterau stated that this revision was a republication of the existing document with minor changes to correct grammatical and typing errors. This manual is attached to the inspection notes as Appendix A.

Training

8. Mr. Wetterau conducts a training course on radiation protection and isotope handling. A description of the training program is included in paragraph 8 of the notes of the last previous inspection. Mr. Wetterau stated that since the last inspection, approximately 25 technicians and 25 physicians have attended the training course. Training consists of lectures, with no examination. No certificates are issued. Since the last inspection, the training course has been presented a total of four times.

Records of Receipt and Transfer

9. The licensee's methods of procurement and receipt are the same as those described in paragraphs 9 and 10 of the notes of the last previous inspection. Wetterau stated that an estimate of present procurement would be approximately 50 curies per year at the maximum. The licensee is maintaining current and effective records showing the receipt of materials. Occasional transfers of materials occur between the VA Center and other Veterans Hospitals or UCLA; however, such transfers are very infrequent. Transfers are well documented. Since the last inspection, the licensee returned to UCLA an extracorporeal blood irradiation unit containing 1150 millicuries of strontium-90. This was returned to UCLA on June 19, 1967.

10. The major significant change in the licensee's procurement program is the use of fluorine-18 (half life 111 minutes). This material is produced in the UCLA reactor by the irradiation of lithium-6 carbonate. The fluorine-18 is used in the performance of bone scans and results in a lower bone and whole body dose than similar scans performed with strontium-85.

11. The production of fluorine-18 involves the irradiation of approximately 10 grams of lithium-6 carbonate sealed in a Vicor tube contained in a screw-top teflon irradiation container. The material is irradiated for approximately four hours at a thermal flux of 5×10^{12} or 5×10^{13}. When the container is removed from the reactor, the surface of the teflon container reads between 600-700 mr/hr. The irradiation produces approximately four millicuries of fluorine-18. Irradiated

material is transferred to the VA Center, where the capsule is opened in a glove box, with the resulting whole body dose of approximately 25 millirad. After the capsule has been opened, the irradiated lithium carbonate is distilled with sulfuric acid into sodium hydroxide. The sodium hydroxide - sodium fluoride mixture is titrated with hydrochloric acid to produce a physiological saline solution. The distillation is performed in a closed system in a hood. Following preparation, which is designed to produce a sterile hydrogen-free material, Wetterau determines the assay of fluorine-18, M. Tubis, Ph.D. (formerly with the USDA) checks sterility, and pyrogenicity is evaluated by Bioscience Laboratories. The short half life of the material, pyrogenicity and sterility findings are not avilable until after the material has been used. Based on calculations made by the staff of the VA Center, it appears that a one-millicurie dose of fluorine-18 results in a 250 millirad bone exposure and approximately 25 millirad whole body exposure. This may be compared to 100 microcuries strontium-85 dose which would produce approximately 1.2 rad bone exposure and 625 millirad whole body exposure.

Inventory

12. Attached as Appendix B is a copy of the licensee's inventory prepared by Mr. Wetterau.

Use of Licensed Materials

13. The licensee operates under a broad license authorizing medical research diagnosis and therapy as well as tracer studies on laboratory animals. The license also authorizes possession of certain specific materials

in greater quantities for certain specified uses. On the basis of the review of the licensee's activities and facilities, it did not appear that materials were used in any unauthorized activities. The licensee stated that materials were used only as authorized by the license.

[illegible]

<u>Facilities and Equipment</u>

14. The licensee's definition of a restricted area and his facilities and equipment are essentially as described in paragraphs 15, 16 and 17 of the notes of the last previous inspection.

15. Since the last inspection, the only new portable survey instrumentation acquired by the licensee is a Technical Associates PUG-1A alpha survey instrument.

16. Paragraph 17 of the notes of the last previous inspection noted that funds had been alloted for the purchase of an Anger camera. This device has now been acquired and installed in Wadsworth Hospital in a room adjoining the uptake room. The licensee also has installed at Wadsworth a Picker Magnascanner II with a three-inch crystal and a Picker Magnascanner III with a five-inch crystal. Small amounts of licensed material are stored at Wadsworth for use in conjunction with the diagnostic work performed at this facility.

17. A review of the facilities at Building 114, Wadsworth Hospital, and the Biochemistry Laboratories revealed that all areas were posted as required by the regulations. Materials were secured against unauthorized removal or were stored in areas where access to the facility was controlled. Individual containers were appropriately

labelled and Forms AEC-3 were posted in restricted areas.

Survey Program

18. The licensee's survey program remains essentially unchanged from the last previous inspection. A new record log of surveys was begun in May 1968. This record records surveys on a laboratory-by-laboratory basis so that all records associated with any one laboratory are found in a particular portion of the logbook. premises of the surveys are stated the date, identity of the surveyor, dose rates, and maximum contamination in counts per minute. One section of the record identifies whether wipes were taken or not and provides space for remarks. In the event that wipes are collected, records of the results of counting are retained in another logbook. In all cases where anything above technical contamination in laboratory areas was observed, immediate contamination was performed. The effectiveness of the decontamination was confirmed by additional wipes. The licensee collects and analyzes room air and stack samples from the isotope laboratory. Samples are collected at any time that I-125 or I-131 are used to iodinate ACTH which is used for in vitro immunio assay work in connection with the experimental program. Stack samples were observed to run over the MPC; however, the samples were collected on a spot basis only during the iodination work. The licensee had made no effort to average the results. Averaging on a weekly or yearly basis would produce results substantially below the MPC.

Personnel Monitoring

19. The licensee uses Landauer badges which are supplied monthly. A total of approximately 52 persons are subject to personnel monitoring. Complete Forms AEC-4 and 5 are maintained. The licensee has pocket dosimeters available; however, these are not routinely used. Summary of high quarterly exposures is noted below.

	1st Quarter	2nd Quarter	3rd Quarter	4th Quarter
1967	-	280 mr	410 mr	120 mr<br>170 mrem
1968	220 mr<br>*1240 mrem	130 mr		

*This particular badge was issued to Dr. Yuji, who is responsible for handling the I-131 in the ACTH iodination work. The results of this exposure were recorded on Dr. Yuji's record; however, a notation was made that the beta exposure was due to direct badge contamination with iodine. A Form AEC-4 had been completed for Dr. Yuji showing that he had received 0 prior exposure. It is the inspector's opinion that the licensee adequately documented that this was a badge only exposure. It is noted that, in spite of the fact that this was a badge only exposure, the licensee removed Dr. Yuji from further isotope work for that quarter.

20. A review of all exposures generally noted that most exposures are minimal ~~at an~~ average ranging from 30-100 mr/quarter. It is noted that the licensee conducts routine whole body counts of personnel involved in the use of radioisotopes. Wetterau stated that there had been no significant results from the whole body counting program.

Waste Disposal

21. The licensee disposes of no waste by sewer on an intentional basis. All wastes are accumulated. Higher level wastes are stored for later transfer to California Salvage Company. Records are maintained of all such transfers of waste. The licensee also disposes of significant quantities of waste by burial on VA property. The following is specific information relating to two disposals, with general information relating to several others which have occurred since the last previous inspection. Waste was disposed by burial on the following dates: May 4, June 23, August 23, November 27, 1967; January 26, March 28, June 20 and August 15, 1968. On June 20, 1968, the following materials and quantities were disposed: tritium, 33.92 millicuries; carbon-14, 1.928 millicuries; sodium-22, 0.002 millicuries; sulfur-35, 0.2 millicuries; iron-59, 0.148 millicuries; chromium-51, 0.05 millicuries; iodine-125, 0.42 millicuries (not decay corrected); iodine-131, 0.606 millicuries; contained in 30 cubic feet plus 162 gallons of dry and liquid waste. The August 15, 1968 disposal consisted of: tritium, 5.7 millicuries; carbon-14, 2.16 millicuries; sodium-22, 0.025 millicuries; iron-59, 1.06 millicuries; iodine-125, 0.45 millicuries; iodine-131, 1.5 millicuries; molybdenum-99, technetium-99m, one millicurie; contained in 41 cubic feet plus 175 gallons of dry and liquid waste. It is noted that all liquid waste is packaged in breakage-resistent containers which are packaged inside drums with dry waste.

Leak Tests

22. The licensee is authorized to perform tests for leakage and/or contamination. Sealed sources possessed by the licensee were leak tested

at the following times:

Material	Leak Test Dates		
Cobalt-60, 5 millicuries Model # R-30	4/27/67	10/2/67	4/2/68
Strontium-90 medical applicator, Model # RA-1	4/27/67	10/2/67	4/2/68
Strontium-90 blood irradiator	4/27/67	Returned to UCLA by transfer.	

The results of the tests for leakage were recorded in units of microcuries. In all cases, removable contamination was less than 0.005 microcuries.

Incidents

23. The licensee stated that there had been no unreported incidents. In connection with unusual occurrences, the licensee stated that there had been several occasions when leaking packages (i.e., the individual container leaked into the packing material, which was contained in a sealed metal can). These occurrences involved shipments of iodine-125 from Isoserve in Cambridge, Massachusetts. In these cases, the VA Hospital notified Isoserve, calling attention to the problem and suggesting corrective action. The licensee received a prompt response from Isoserve stating that corrective action would be taken. It is noted that in connection with this type of activity, the licensee has maintained nonroutine surveys which are well documented.

24. The licensee has no AEC contracts.

UNITED STATES
ATOMIC ENERGY COMMISSION
DIVISION OF COMPLIANCE
REGION V
2111 BANCROFT WAY
BERKELEY, CALIFORNIA 94704

TELEPHONE: 841-5121
EXT. 651

August 22, 1969

H. E. Book, Senior Radiation Specialist
Region V, Division of Compliance

INSPECTOR'S EVALUATION AS PER MM 900-15
VETERANS ADMINISTRATION CENTER, WADSWORTH HOSPITAL
LOS ANGELES, CALIFORNIA - LICENSE NO. 4-181-4

The subject broad licensed medical program includes the diagnostic and therapeutic use of radioisotopes in the clinical program, as well as an active research program. The hospital is affiliated with UCLA and is currently beginning an extensive residency program. The use of licensed materials ranges from laboratory tracer work with carbon and tritium containing compounds through substantial quantities of I-125 and 131 used in the preparation of labelled materials for the research program and the production of Tc-99m from a commercially supplied milker for diagnostic purposes.

During the inspection, the licensee advised the inspector that property presently owned by the VA Center might possibly be transferred to the City of Los Angeles for development as a park. The licensee was concerned with this possible transfer of property since the land involved had been used for a number of years as a land burial site for low-level radioactive waste, as authorized by 10 CFR 20. Mr. Wetterau, the RSO, was advised that although there appears to be no statuatory restrictions concerning transfers or use of land used for low-level waste disposal in accordance with 10 CFR 20, it would appear advisable to fully inform DML in the event plans for the transfer appeared to be approaching consumation. The inspector also commented that it appeared that good public relations concerning the release of information regarding the former use of this property could well be extremely important to both the hospital and the City.

It is the inspector's opinion that the licensee's activities have not resulted in a threat to the health and safety of the licensee's patients, employees or the general public.

H. S. North
Radiation Specialist

8204150399 820316
PDR FOIA
NELSON82-11 PDR

591 Notes
North/msb
8/22/69

[signature] Inspector [date]
[signature] Reviewer [date]

Veterans Administration Center
Wadsworth Hospital
Wilshire and Sawtelle Boulevards
Los Angeles, California

License No. 4-181-4

Introduction

1. An announced reinspection of the subject licensed program was conducted on August 11, 1969, by H. S. North, Radiation Specialist, Region V. The inspector was unaccompanied. As a result of the inspection, no items of noncompliance were noted and a Form AEC-591 reflecting these findings was issued at the conclusion of the inspection.

Exit Interview

2. Since Dr. William H. Blahd, Chairman, Radioisotopes Committee, was not present, the inspector conducted the exit interview with Mr. L. W. Wetterau, Physicist and Radiation Safety Officer for the hospital. This discussion involved two items: (1) the fact that no items of noncompliance had been noted in connection with the inspection (therefore a Form AEC-591 was issued at the conclusion) and (2) the possible transfer of government-owned VA-administered lands adjoining the hospital to the City of Los Angeles. Mr. Wetterau pointed out that certain areas on this land had been used for the disposal of low-level radioactive waste by means of burial, as authorized in 10 CFR 20. The inspector commented to Mr. Wetterau that it appeared there were no statuatory restrictions which would prohibit such a transfer, but the inspector was certain that DML and CO:HQ would be extremely interested in any such transfer of control. The inspector also

commented that, because of the unusual sensitivity associated with radioactive waste, it would probably be an excellent idea to have expert guidance in connection with news releases relating to the proposed transfer.

Inspection History

3. The subject licensed program was last inspected on September 20, 1968. At a result of that inspection, no items of noncompliance were noted and Form AEC-591 was issued confirming those findings.

Administration

4. During the course of the inspection, the inspector interviewed Mr. L. W. Wetterau, Physicist and Radiation Safety Officer, who has Center-wide responsibility in connection with the licensed programs with the exception of the teletherapy unit. The inspector also met Dr. A. Euwiler, Head of the Neurobiochemistry Research Section; Dr. J. Gambino (Ph.D.), Educational Director in charge of the resident training program; and Dr. Melvin Golden, one of the Residents in training at the hospital.

Organization

5. Mr. Wetterau stated that the licensee's organization is as follows:

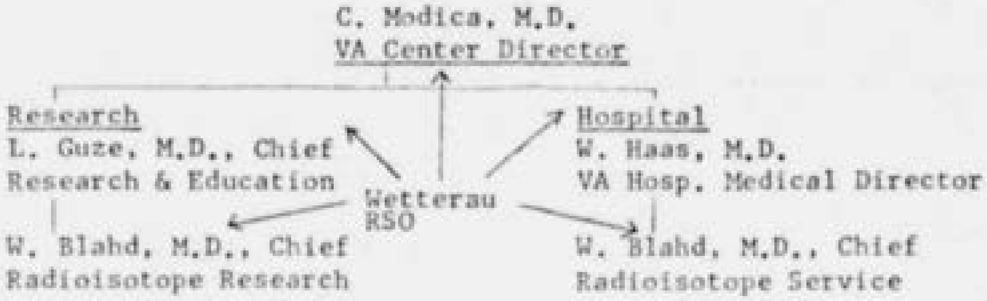

6. Mr. Wetterau stated that the licensee has an Isotopes Committee which meets on call at least twice a year. Most of the committee business is carried out by telephone and mail. Membership on the committee is as follows: W. Blahd, M.D., Chief, Radioisotope Service, and Chief, Radioisotope Research, responsible for both research and clinical applications at the center; S. Dayton, M.D., Internist and Chief of Medicine at the hospital; B. Fishkin, M.D., Pathologist; L. Fred, M.D., Special Assistant to the Director for Outside Affiliations and Associate Dean for VA -UCLA Affairs, UCLA School of Medicine, acting as a training liaison officer between the University Med School and the hosp'tal; O. True, M.D., Radiologist, Head of the Diagnostic Radiology Program, and license-identified user on the teletherapy license 4-181-10; and L. Wetterau, Physicist and Radiation Safety Officer. Mr. Wetterau commented that the following persons are ex-officio members of the committee: L. Guze, M.D., Internist Chief of Research and Education; W. Haas, M.D., VA Hospital Medical Director; C. Modica, M.D., VA Center Director.

Radiation Safety, Instruction of Personnel

7. Mr. Wetterau stated that as of July, a formal training program at the M.D. postgraduate level had been started. A total of four physicians were enrolled; however, it is planned that this enrollment will climb to between five and eight in the near future. This program is totally involved in the clinical use of radioisotopes. Mr. Wetterau stated that his in-hospital radiation safety and safe handling course continues, with an enrollment ranging from 12-14 persons/quarter. This course is primarily familiarization with good health physics

practices, the rules, regulations and the licensee's procedures. The licensee maintains a record of individuals having completed this training. Condition 21 of the license identifies statements, representations and procedures contained in applications dated January 21 and February 8, 1965. Mr. Wetterau stated that copies of these procedures are given to individuals who need them in the performance of their work as a part of the safe handling training course. Mr. Wetterau stated that there have been no changes in the procedures without the prior approval of DML except for some minor organizational and language changes. Mr. Wetterau commented that with the gradually expanding program at the hospital, it may be appropriate to consider amendment of these procedures in the near future.

Receipt and Transfer

8. Mr. Wetterau stated that there had been no exports of licensed materials. Procurement of radioisotopes requires prior approval by the Radioisotopes Committee which, for new or unusual uses, will require submission of a protocol which must be reviewed by the committee. Materials are delivered to Wetterau. Mr. Wetterau is responsible for assuring that materials ordered are received and for transferring the materials to the appropriate recipient. Stock materials, such as I-131 used in the clinical program, are acquired on the basis of standing orders. Occasional purchases are made from a GSA price list on a government purchase order. In any case, all licensed materials are delivered to Wetterau on receipt. Mr. Wetterau stated that there have been occasional small transfers of materials to hospitals in the Los Angeles area. He stated that these customarily are cases where a physician on the staff of the VA Hospital also holds an appointment

with a non-VA hospital in the area. If this individual is conducting VA-sponsored radioisotope research, the materials may be transferred to the non-VA hospital for the use of the physician provided the physician is also authorized to receive, possess and use materials under the State of California license issued to the non-VA hospital. Mr. Wetterau had records showing that materials had been delivered to Mt. Sinai Hospital under such circumstances. The records revealed the isotope, quantity, physician, hospital, hospital license number, and date of the transfer. The licensee makes from six to eight transfers per year, with total quantities ranging from 1 uCi to 10 mCi per year.

Inventory

9. The licensee had performed a physical inventory immediately prior to the inspection. A copy of the inventory is attached as Appendix A.

Use of Licensed Material

10. The subject licensed program, operating under a broad license, allows diagnostic and therapeutic aspects of clinical medicine as well as medical research on small animals and in vitro systems. In association with the clinical program, the licensee elutes Tc-99m from a Mo-Tc generator. All material eluted is checked for pyrogenicity, sterility and break-through of Mo-99. At the time of the last inspection, the licensee was doing some experimental work involving fluorine-18 for bone scans. This material was produced in the UCLA reactor; however, this reactor is currently shut down and no irradiations are being performed. On the basis of statements made by the licensee's representatives and the inspector's observations, it appears that the

licensee is using materials as authorized by the license.

Facilities and Equipment

11. The locations of use of licensed materials are as described in paragraphs 15 and 16 of the notes of the inspection conducted May 23, 1967. In the case of the rooms associated with the hot laboratory and preparation room, a new suite of offices is being built in Room 214. These facilities will provide a combination of office space and preparation and assay laboratory areas. The licensee's preparation lab and storage facilities remain unchanged from the last previous inspection. At the time of the last inspection, the licensee defined the hot laboratory, Rooms 212 and 212A in Building 114, as the restricted area. Access to these rooms is controlled by means of lock and key. The licensee's main clinical counting and scanning areas in Wadsworth Hospital have been somewhat expanded, and additional expansions are planned. The licensee has acquired a new Picker dual-probe Magnascanner with color readout. The licensee has also acquired an Ampex vidio tape recorder for use with the Pho-Gamma III Anger camera. Mr. Wetterau stated that generally the licensee's instrumentation has remained substantially the same.

Posting and Labelling

12. All areas in which licensed materials were used or stored were observed to be posted with a conventional symbol and the words "Caution or Danger - Radioactive Materials". All containers of licensed materials display the conventional symbol and the words "Caution or Danger - Radioactive Materials", identify the isotope, quantity and date of measurement of

the quantity. The licensee had posted Forms AEC-3 in all areas where individuals work with licensed materials.

Survey Program

13. The licensee performs monthly surveys of all laboratories, which include measurements of dose rates and, where applicable, wipes or surveys for removable contamination. Records of these surveys show the date, the maximum exposure in mr/hr, whether wipes were collected, with space for remarks. A separate record shows the results of the analysis of smear test samples. A review of the results of these surveys showed that the radioisotope clinic surveys range from 0.1 to 1.5 mr/hr at the storage refrigerator. This cl[illegible]c is located in Room 4748 of Wadsworth Hospital. The radioisotope research area ranged from 0.2 to 0.8 mr/hr and wipes were generally taken throughout this area. The licensee's program of iodination of ACTH continues, with substantially no changes in the procedures or equipment in use. The inspector discussed with Mr. Wetterau the possible use of the uCi hr/cc concept of evaluating exposure. Mr. Wetterau said that this had been considered. A review of the records revealed the following room and stack air concentrations of I-125 and 131:

Isotope	Air Concentrations uCi/cc Room Air	Stack Air	Date	Duration of Exposure or Release
I-125	7.4×10^{-9}	8.6×10^{-8}	5/20/69	3 hrs. 20 min.
I-131	4.6×10^{-9}	9.0×10^{-10}	5/20/69	3 hrs. 20 min.
I-131	4.0×10^{-8}	4.7×10^{-8}	6/9/69	45 min.
I-131	7.0×10^{-10}	1.0×10^{-10}	6/16/69	2 hrs. --

The entry for June 9, 1969 was the highest recorded concentration shown in the licensee's records. The review of these concentration levels revealed that they were substantially below the MPC's of soluble materials permitted by Columns I of Tables I and II of Appendix B, 10 CFR 20.

14. Mr. Wetterau also maintained, as a part of his survey records, special surveys of unusual occurrences. A summary of these unusual surveys revealed the following items of special interest.

October 23, 1968	A 200-uCi shipment of calcium-47 fluoride leaked into packing material. Contamination ranged from 300-700 cpm maximum. Some hand contamination resulted; however, the leakage was caught and cleaned without spread. All contamination was limited to the inside of the container.
January 14, 1969	Post-mortem on cadaver containing 8 mCi P-32 in plural cavity. Record and copy of notes to Funeral Director advising for appropriate handling of deceased.
February 12, 1969	3 mCi I-125 contained in a centrifuge tube fell on the floor. Approximately 75% of the material was recovered. The laboratory technician's shoes were contaminated and held for decay. The floor and centrifuge were decontaminated to background.

Personnel Monitoring

15. The licensee uses monthly film badges supplied by Radiation Detection Company. Approximately 45-50 persons are monitored on a routine basis. Badges are issued to individuals using gamma or hard beta emitting radioisotopes but not to individuals using tritium or C-14. The

licensee possesses pocket dosimeters, but they are not in routine use. The review of the results of personnel monitoring provided the following information. During second quarter 1969, the average exposure was less than 50 mr. The high exposures were received by Endow at 350 mrem and Thomas at 120 mrem. The first quarter 1969 exposures averaged less than 50 mr, with a high to Thomas of 120 mrem. Fourth quarter 1968 exposures again showed average exposures of less than 50 mr, with a high to Ashcraft of 320 mrem. Third quarter 1968 showed an average exposure of less than 75 mr, with a high to Thomas of 220 mrem. During second quarter 1969, the licensee did some extremity monitoring in the high potential exposure population. The results indicated the following quarterly exposures. Those operating the technetium milking operation showed extremity exposure of 130 and two at 230 mrem. The nurse and technicians working in the clinic showed exposures of 240, 390 and 430 mrem. Individuals carrying out the ACTH iodination showed exposures of 110 and 140 mrem.

Waste Disposal

16. Mr. Wetterau stated there is no intentional waste disposed to the sanitary sewage system. All waste is presently transferred to California Salvage Company. Historically, this licensee has disposed by burial on VA property since 1952, and approximately 50 disposals have occurred. The total material disposed by burial is approximately 1.2 Ci and was principally composed of C-14 and tritium. The last burial occurred October 28, 1968 and consisted of the following materials.

Isotope	Millicuries	Isotope	Millicuries
Tritium	5.445	Sodium-22	0.050
Carbon-14	3.350	Iron-59	0.020
Iodine-131	6.265	Molybdenum-99	2.000
Iodine-125	1.175		

These wastes were contained in the following forms and volumes: 3 gallons of liquid, 8 cubic feet of animals, two 30-gallon containers of solids, one 20-gallon container of solids, three 10-gallon containers of solids, 4 cubic feet scintillation vials, 4 cubic feet miscellaneous solids. The licensee's most recent disposals to California Salvage were as follows: one 55-gallon drum containing 80 mCi of C-14 and tritium on September 18, 1968; eight 55-gallon drums containing 13.94 mCi on February 12, 1968; and seven 55-gallon drums containing 52.3 mCi on May 22, 1969.

Leak Tests

17. The licensee performs leak tests of sealed sources. Sources leak tested include the Tracerlab Model R-30 cobalt-60 sealed source and the Tracerlab Model R-1A strontium-90 medical applicator. These sources were tested for leakage on October 2, 1968 and April 1, 1969. No removable contamination was detected.

Miscellaneous

18. Mr. Wetterau stated that there have been no unusual occurrences, no MIS administrations of radioisotopes, and that there have been no unreported incidents.

OPTIONAL FORM NO. 10
MAY 1962 EDITION
GSA FPMR (41 CFR) 101-11.6

UNITED STATES GOVERNMENT

Memorandum

TO : Mr. Harry North
USAEC

DATE: Aug. 11, 1969

FROM : Radiation Safety Officer
V.A. Center, Los Angeles

SUBJECT: Radioisotopes on Hand as of This Date

^{3}H	as labeled compounds	897.175 mCi
^{14}C	" " "	33.276
^{22}Na	" " "	0.200
^{32}P	" " "	1.000
^{45}Ca	" " "	0.400
^{51}Cr	" " "	2.800
^{57}Co	" " "	0.110
^{75}Se	" " "	0.800
^{85}Sr	" " "	0.680
^{99}Mo-^{99m}Tc	Generator (8/12/69 precalibrated)	200.000
^{125}I	as labeled compounds	44.260
^{131}I	" " "	56.000
^{133}Xe	in saline	0.100
^{137}Cs	in solution	0.005
^{203}Hg		0.400
^{90}Sr	sealed eye therapy source (Tr. Lab RA-1A)	31.000 mCi
^{60}Co	sealed calibration source (Tr. Lab R-30)	0.100
^{3}H	Foils - Barber Coleman model 4168 for gas Chromat. - 4 ea @ 100 mCi =	400.000 mCi
	TOTAL =	1,668.306 mCi

L. W. Wetterau Jr.
L.W. Wetterau, Jr.

Buy U.S. Savings Bonds Regularly on the Payroll Savings Plan

UNITED STATES
ATOMIC ENERGY COMMISSION
DIVISION OF COMPLIANCE
REGION V
2111 BANCROFT WAY
BERKELEY, CALIFORNIA 94704

TELEPHONE: 841-5121
EXT. 651

August 6, 1970

H. E. Book, Senior Radiation Specialist
Region V, Division of Compliance

INSPECTOR'S EVALUATION AS PER MM900/15
VETERANS ADMINISTRATION CENTER - WADSWORTH HOSPITAL
WILSHIRE & SAWTELLE BOULEVARDS - LOS ANGELES, CALIF.
LICENSE NO. 4-181-4

There appeared to be no health or safety problems associated with activities under the license. Facilities at the clinic (Building 114) had not changed since the previous inspection; however, additional facilities have been added at Wadsworth Hospital for routine diagnostic and scanning procedures. Additional rooms at Wadsworth were being modified for radioisotope work at the time of this inspection. Since the previous inspection there has been a moderate reduction in the area of research at the center due to a cutback in government spending. Clinical uses of radioisotopes remains about the same.

It was found during the course of the inspection that the licensee has held no formal radioisotope committee meetings since the previous inspection. Approvals for new protocols had been done on an informal basis, through telephone discussions and individual review of protocols. During an inspection of another license (-10 Teletherapy) at Wadsworth Hospital, the day following the present inspection, it was learned that the principle user under the -10 license had not been contacted for any approvals although he is a member of the Radioisotopes Committee under the subject license. For these reasons the next inspection should include an in depth review of the Isotope Committee functions and review policies. It may be that Dr. Blahd, Chief of Nuclear Medicine, is assuming the greater share of the Radioisotopes Committee responsibilities while other members give only token approvals of new uses of radioisotopes.

Nothwithstanding, the licensee appears to maintain an adequate program to insure health and safety with sufficient authority invested in the Radiation Safety Officer. The RSO appeared to have a complete understanding of all

8204150418 820216
PDR FOIA
NELSON82-11 PDR

activities for which he has health and safety responsibilities; records appeared to be well maintained and adequate surveys performed.

At the conclusion of the inspection the licensee was issued a Form AEC-591 showing no items of noncompliance.

J. R. Metzger
J. R. Metzger
Radiation Specialist

591 Notes
Metzger:ejn
8-4-70

J R Metzger Inspector 8-6-70
H E Book Reviewer [illegible]

Veterans Administration Center
Wadsworth Hospital
Wilshire and Sawtelle Boulevards
Los Angeles, California 90073

License No. 4-181-4

General Information

1. On July 23, 1970, an announced reinspection of the subject broad medical license was conducted at the above address. There were no representatives from the State of California present. The last inspection was conducted on August 11, 1969, at which time no items of noncompliance were found. The present inspection revealed no items of noncompliance and a Form AEC-591 reflecting these findings was issued at the conclusion of the inspection.

Exit Interview

2. At the conclusion of the inspection a summary of the inspection findings was held in the presence of Dr. W. J. Blahd, Chief of Nuclear Medicine and Mr. L. W. Wetterau, Radiation Safety Officer for the Hospital. At that time those present were told that there appeared to be no items of noncompliance or any apparent health or safety problems. Due to the cutback in research activities there followed some reduction in the frequency of bioassays on employees and in wipe surveys for hydrogen-3 and carbon 14 compounds in the laboratories. It was emphasized to those present to consider carefully reviewing the research activities and determine whether or not the bioassay and survey frequencies were adequate for the activities being performed. It was emphasized that there appeared to be no problems in these areas but due to the infrequencies of bioassays

and surveys in recent months a reevaluation may be in order.

3. It was also brought out that the procedures referenced by the license had been revised as of April 1970, and that the Division of Materials Licensing should be notified of the revision. Again it was brought out that there was no significant difference between the old and new procedures but that the AEC wished to be kept current on the procedures used.

Organization and Persons Contacted

4. An organizational diagram as it relates to licensed activities is attached as exhibit A. The organization is essentially the same as found during the previous inspection with the exception that now Dr. W. Blahd is Chief of Nuclear Medicine reporting directly to the Center Director, whereas before he reported to the Hospital Medical Director. Also there is a new Head of Principle Research, Dr. N. Baker who reports to Dr. Blahd and conducts in vitro procedures and animal research with radioactive materials. Dr. Blahd is head of all activities involving human research, however. Also, the use of radioisotopes has been divided into three categroies under Dr. Blahd, which includes the Radioisotope Research group, the Nuclear Medicine Training group and the Clinical group.

5. Mr. Wetterau stated that he had the authority for insuring the health and safety of personnel using radioisotopes which includes responsibility for the conduct of isotope use, supervising disposal, approvable on procurement of radioactive materials, dosimetry, health physics training,

research activities (health and safety) and protocol approvals for radiation safety considerations. He stated that administratively he reports to Dr. Blahd but for health and safety policy answers to the center director, Dr. C. Modica.

6. Persons contacted during the course of the inspection were as follows:

NAME	FUNCTION
L. W. Wetterau	Radiation Safety Officer for the Hospital
W. Blahd	M. D., Chief, Nuclear Medicine, Head, Human Research and Chairman, Radioisotope Committee
M. Tubis	M. D., Chief, Research Biochemistry and Radiopharmacology
Dr. Erwiler	M. D., Neurobiochemist
T. Braun	Research Biologist
L. Wilson	Research Chemist
J. J. Gambino	PhD., Head Nuclear Medicine Training Department
C. Walsh	Nuclear X-Ray Technologist (Registered)
B. J. Kinney	Radioisotope Technician
Panchita Thomas	Radioisotope Technician & Biologist

Radioisitope Committee

7. Although the radioisotopes committee in the past has met once or twice a year on a formal basis there have been no formal meetings since the previous inspection. Mr. Wetterau explained that the meetings between concerned individuals have been informal involving telephone conversations

and individual review of protocols and papers submitted. The committee membership consists of the following people:

W. Blahd, M.D., Chairman

C. Modica, M.D., Ex-officio member (Center Director)

J. F. Rouse, M.D., Chief, Hemotology

L. Fred, M.D., Special Assistant Director for Outside Affiliations

L. Guze, M.D., Chief of Staff for Research and Education

S. Dayton, M.D., Chief Medical Services, Wadsworth Hospital

E. G. Fishkin, M.D., Chief Laboratory Services, Wadsworth Hospital

J. E. Thornhill, M.D., Chief Radiation Therapy, Wadsworth Hospital

L. W. Wetterau, Physicist and Radiation Safety Officer

8. Since the previous inspection seven new protocols for the use of radioactive materials have been submitted and approved by the Isotopes Committee.

9. The new protocols were examined and contained essentially the following information.

   a. Approval was granted for the use of Chromium-51 as labelled $CrCl_3$ in 40 human subjects to be used as a dilution marker for acid in gastric ulcers (work not yet begun).

   b. Ytterbium-169 in DTPA to be used in the measurement of Glomeruler filtration rate (work not yet begun).

   c. Calcium-45 in $CaCl_2$ to be used in animals in studies of calcium deprivation depending on vitamin deficiency in conjunction with Hydrogen-3 in vitamin D3.

   d. Selenium-75 as selenomethionine for brain uptake studies involving amino acid uptake from the blood system.

   e. Technetium 99m in sulfur colloid for studies in patients suspected of pulmonary ailments.

f. [illegible]echnetium 99m as pertechnetate with iron and asorbic acid complexes f[illegible]r application in kidney scans.

g. Iodine-131 as labled fibrogen for the detection for venous thrombosis.

Instruction of Personnel

10. Written procedures are required by the license. The procedures attached to the application for the license are entitled "Radiation Safety Progra[illegible] Manual, Veterans Administration Center, Los Angeles, Calif. Revised December 1964." Mr. Wetterau stated that the procedures had been revised in a revision dated April 19, 1970, a copy of which is attached as exhibit B. A comparision of the two revisions show that the only change in the new procedures is the disposal of animal carcases quarterly rather than monthly as stated in the old procedures. All other procedures remain the same.

11. Mr. Wetterau stated that all investigators have received copies of the new revised procedures and that the procedures were being followed.

12. A rotational training program for physicians is in effect at the Center. At the time of this inspection Mr. Wetterau stated that 4 physicians were currently undergoing nuclear medicine training with lectures provided by himself and Dr. Blahd and other members of the staff. During the facility tour the lecture room for training of physicians was observed
used in the training program. Mr. Wetterau stated that most of the new equipment
to have desks and various types of equipment that they had procured for use in clinical programs had been purchased with funds allocated for training. Mr. Wetterau also stated that the procedures used in both clinical and research activities were strictly enforced by himself with backing from his superiors.

Procurement - Records of Receipt, Transfer and Export

13. Radioactive material procurement has been described in previous inspection notes. The RSO stated that standing orders included only two radionuclides which were 200 millicuries of Technetium-99m in generators biweekly and 50 millicuries of I-131 per week. He said that all other materials were ordered on a demand basis.

14. Several log books were maintained for the various radioisotopes received which showed the labels removed from the shipping containers together with the isotope, chemical form, volume, date of assay, quantity in millicuries, total activity, and from whom received. Also included in the same books were the name of the patient to whom doses were administered, the physician prescribing the dose, the total activity given to the patient, and the date of assay at the time of the dose administration. All receipts for compounds of carbon-14 and hydrogen-3 were maintained
in a second log book and all other isotopes in a third log book. [illegible] transfers of radioact[illegible]
in one log book (research), iodine 125, 131 compounds had been made since the pervious inspection - all at UCLA. The records of transfer showed that 14 transfers involving a total of 345 microcuries of carbon-14, calcium 45, 47 had taken place. Mr. Wetterau explained that prior to any transfers he telephonically confirmed with the UCLA RSO, whether or not the institution was licensed to receive the material. The records of transfer also showed the date of transfer, the user to whom transferred at UCLA and the appropriate UCLA license number together with the amount in microcuries.

16. Mr. Wetterau stated that there had been no exports of radioactive material

Inventory of Licensed Material

Attached as Exhibit C is a recent inventory of materials held under the license as of June 21, 1970. Mr. Wetterau stated that such inventories

are conducted quarterly or semi-annually and usually prior to the AEC annual inspection visit. An examination of the inventory sheet showed that the institution possessed amounts within those authorized by the license.

Use of Licensed Material

18. Some of the nonroutine uses of licensed material were described under the heading "Radioisotopes Committee." Other uses of radioactive material involved routine clinical work and areas of research involving small animals and in vitro systems. Of particular interest was the production of various new compounds used in human research as indicated earlier. Dr. Tubis, Head of Pharmacology, is in charge of proper preparation of the materials with respect to purity and Pyrogen testing. Dr. Tubis is also responsible for a quality assurance program with respect to the production of various isotopic compounds.

19. The VA Center publishes for internal distribution a yearly summary of clinical procedures performed on patients. An examination of the report for fiscal year 1970 was examined. The report showed that a total of 5,457 diagnoses were performed on 4,982 patient for the year. The report showed that more than 2,000 of the diagnoses involved iodine-131 125, for routine uses such as thyroid function studies and scans, determination of blood and blood plasma volume, liver function studies and scans, lung scans, fat absorbtion studies, kidney functions studies and scans and cardiacs scans. Iodine-131 was also used in 33 patients for treatment of hyperthyroidism.

20. Technetium-99m was used formore than 1,000 brain scans during the period but was also used in kidney transplants, cerebral blood flow studies, liver and heart scans. Other radioactive materials used included Xenon-133 for pulmonary studies, selenium-75 for pancreas scans, chromium-51 for spleen scans, iron-59 in iron turnover studies, hydrogen-3 as tritiated water for water turnover studies, bromine-82 for extraceluar water studies, sodium 24 for exchangable sodium studies and fluorine-18 for bone scans. Phosphorous-32 was used in therapy for polycythemia vera. Also used was mercury 203 in brain scans and strontium-95 in bone scans.

Facilities and Equipement

21. The facilities at the clinic (building 114) were found to be the same as described in previous inspection notes. Therefore, only new equipment and facilities will be described. New equipment for the clinic included two Nuclear Chicago Mediac assay analyzers and dose calibrators, a Packard TriCarb Scintillation device which was used in training and for bioassay analysis, and a Nuclear Supplies single channel analyser. Also new was a Nuclear Chicago portable ionization instrument, Cutie Pie Model 2588, which had low range and high range ionization chambers with ranges of respectively, 2.5 r/hr and 250 r/hr. A tour of the laboratory areas and hot storage room (room 212) revealed that they were properly posted in accordance with the regulations and the containers in storage were properly labeled as required.

22. Room 228 which is used for iodine-131 uptake studies and brain scans contained an adjacent foom used for education and training of physicians in nuclear medicine. Noted in the room were various pieces of equipment used in training such as projectors, X ray readers, desks and the usual

class room materials.

23. The tour was made of the Neurobiochemistry laboratory (Building T-85) which had been used in carbon-14 and hydrogen-3 research studies involving about 3 millicuries per month of the compounds, total. The building and equipment had not changed since the previous inspection. Also, the building was posted and most containers labelled in accordance with the regulations. For those containers which were not labeled there was a readily available log book showing the amounts, date, and the isotope in the containers which had been made up from stock solutions for research.

24. A new waste handling facility has been added and was located adjacent to Building 114. The building was observed to be built with concrete blocks with a 9-inch thick concrete roof. Mr. Wetterau stated that since the on-site burial had created some problems with respect to disposing of the real estate that they had decided that all of their waste would be transferred it to a licensed waste handling firm. As a result, Mr. Wetterau stated that they were allocated funds to construct the waste storage building. The door to the building was observed to be locked and posted "Caution Radiactive Materials" with the words "Restricted Area" with the usual symbol and colors. Two freezers were stored in the room, one for carbon-14 and hydrogen-3 compounds in animal carcasses, and the second freezer for all other isotopes in animal carcasses. There were sign sheets located on each freezer for the investigators to show the amount of isotope and date that the animal carcass was put into the freezer. Near the entrance door there was a Nuclear Chicago count rate

meter used for surveying personnel after loading waste. The room was divided up into two sections, one used for low level storage of radioactive materials and another used for high level materials and liquid waste. Several waste drums were stored near the freezers for beta emitters which also contained sign sheets to indicate the amounts put in the barrels on a given day. The second half of the room contained three liquid waste jugs and two concrete caves, one used for gamma emmitters with dose rates less than 5 mr/hr and the second highly shielded cave for gamma emitters with dose rates of greater than 5 mr/hr. Mr. Wetterau stated that these were called decay pits where higher level wastes were left for decay for ease in handling later on.

25. Other rooms visited in Building 114 included room 118 used in carbon-14 research which contained a Barbara Colman Gas chromatography unit with two H-3 100 millicurie detector cells which were noted to be properly labeled. There was also a temperature indicating device which was set at 160° centigrade on the Barbara Coleman unit. Room 135 also contained another Barbara Coleman unit which had a temperature control mechanism.

26. Room 317 contained a Varian Aerograph gas chromatography unit with 250 millicuries of hydrogen 3 which had not been used.

27. Since the previous inspection new rooms have been modified in the Wadsworth Hospital for additional radiosotope clinic space. Room B-1 on the main floor was being used as a thyroid uptake and thyroid scanning room and contained a new nuclear Chicago thyroad scanner. There was also a new Picker Renogram scanner in the room. In Room B-48 there was a Picker

Magna Scanner-500 with a 5 inch sodium iodide crystal and two lead caves for storage of compounds of iodine-131 (uptake capsules). The caves were posted in accordance with the regulations.

28. Room B-47 contained two new Picker Magna Scanners, Models II and 500, respectively, and space was allocated for radiopharmaceutical preparations. Mr. Wetterau stated that part of the hot lab in building 114 would be relocated to Wadsworth Hospital. Room B-47 was not quite completed and a new fume hood was being installed. In room B-245 there was new Nuclear Chicago Pho/Gamma III scanner and a new Picker scanner.

29. One more area used in research was a laboratory in a quonset hut near Wadsworth (Building T-22) which had been used in carbon-14 and hydrogen-3 studies in animals. At the time of the inspection there was no work being performed in the building and the compounds were stored in a freezer which was properly posted and the containers properly labelled. There was also an Indium generator stored in the room. The principle user in laboratory T-22 was Mr. T. Braun who stated that at any one time no more than ½ microcuries of carbon-14 or hydrogen-3 labeled amino acids were ever used. Other new instrumentation purchased since the previous inspection included a Picker Magna Scanner III with a 3-inch sodium iodide crystal and a Nuclear Chicago model 8725 uptake scanner with a two inch and a three inch sodium iodide crystal.

30. Restricted areas are defined to be those laboratories in all the buildings where radioactive materials are used or stored, according to Mr. Wetterau. He stated that in addition to the VA Center being patroled

at night that all rooms and building where radioisotopes were stored were kept locked during nonworking hours.

31. All other facilities and equipment have been fully described in previous inspection notes.

32. A number of AEC Form 3's were posted in each facility where radioactive materials were either being used or stored.

Survey Program

33. Surveys of the laboratories are performed monthly and include a direct survey of equipment, room bkgd. radiation, and wipes of surfaces as needed. Records of surveys were examined and showed the date the surveys were made, the highest dose rates on the containers or areas surveyed, and the highest contamination found on wipes. Results of the surveys indicated radiation levels equivalent to background in the rooms and up to 2 mr/hr on storage caves; all wipes taken showed less than 100 d/m per 100 cm^2. Mr. Wetterau stated that although routine surveys of the labs are done monthly that surveys are made by informal request as well.

34. Air sampling is performed as needed during iodine-125 iodination and samples are taken of stack air exhaust when synthesizing ACTH with iodine-125 or iodine-131. Results of the air samples recorded in a logbook showed that the average concentration during iodination was 10^{-9} μCi/cc and for stack air the maximum concentration was shown to be 5 x 10^{-9} μCi/cc. Iodination lasts about 3 hours at any one time. Mr. Wetterau explained that air sampling is not routine but done each time these activities are performed, principally for radioiodine compounds since these posed the greatest potential for airborne radioactivity problems.

35. Bioassays on personnel and whole body counting are performed as needed in accordance with the levels of radioactivity used. Mr. Wetterau explained that bioassays are performed for personnel using greater than 10 milli

curies of H-3 per week by internal procedure and he stated that there had been only one case where anyone used greater than 100 millicuries in one week. He stated that due to the cutback in research there was no bioassays performed for hydrogen-3 at the present since very little was being used. Some urine samples were provided by personnel who used calcium-45 and carbon-14 compounds. It was noted that urine samples have not been provided for the past several months because of the reduction in the use of these compounds. For those samples submitted within the last year there had been no significant internal exposure from the radioisotopes used.

36. The results of the whole body counting which was last performed in December of 1969 were examined. These records showed that 12 people were given whole body counts with only one showing 1/100th of a microcurie of iodine-131 and no detectable activity in other personnel or for calcium-45 and technetium 99m. The limit for iodine-131 is 0.7 microcuries with the thyroid taken as the principle organ.

Personnel Monitoring

37. Film badges are provided for the employees and exchanged monthly. The film badges are supplied and analyzed by the Radiation Detection Company of Mt. View, California. The records supplied by the Radiation Detection Co
of 1969 was 140 mr; for the 4th quarter 1969 270 mr; for 1970 first quarter
were examined and showed that the highest exposure for the third quarter ^ the maximum exposure was 165 mr and for the second quarter 195 mr.

38. The Radiation Detection Company reports showed no unusually high doses received as reported to this office by the Radiation Detection Company on May 28, 1970 (Memo Route Slip - H. E. Book to File 5/28/70). Mr.

Wetterau was asked if there had been any unexplainable or unusually high exposures to badges in the past year; he said there had not been any.

39. Pocket dosimeters are available but generally not used according to Mr. Wetterau. The types available are self reading Landauer model No. L-49 with the range of 0 - 200 mr.

Waste Disposal

40. Prior to the last inspection of August 11, 1969 the licensee had buried low level waste on VA Center land and at that time had considered releasing the land to the City of Los Angeles for a park. (Reference: Memo to File December 22, 1969 signed by H. S. North). Mr. Wetterau stated that there had been nothing new with respect to the land disposal but that at present he felt that it was not going to be released. He stated that in any event they had not buried any additional waste on the land in question and had decided to make it a policy to transfer all wastes to a disposal firm.

41. According to Mr. Wetterau there have been no disposals by sanitary sewage and this had been discouraged as a policy. He stated that all wastes were being saved, both liquids and solids and transferred once per quarter to California Salvage Company waste disposal firm. As a result of the new changes in waste handling policy a new waste building was constructed which has been described earlier in these notes. Records of waste transfer to California Salvage were maintained. An examination of these records showed that four transfers had taken place since the previous inspection as follows:

Date	Quantity	Principle Isotopes
8-18-69	59.6 millicuries	Iodine 131, Carbon-14, Hydrogen-3, Iodine-12

Date	Quantity	Principle Isotopes
11-5-69	35 millicuries	Iodine-131, Iodine-125, Hydrogen-3
2-20-70	1,847.6 millicuries	Iodine-131, Iodine-125, Carbon-14, hydrogen-3, Radium-226. (included 1 hydrogen-3 gas cromatography foil)
6-25-70	30 millicuries	Iodine-131, Iodine-125, Carbon-14, Hydrogen-3, rubidium-86, and Molybdenum 99

42. As stated earlier both solids and liquids were transfered to California Salvage Company. Such methods of disposal are the only ones currently in use at the VA Center.

Leak Tests

43. Leak tests are required by conditions of the license at 6 months intervals for gamma emmitting sources. Currently, the licensee possesses only two sources requiring leak tests, one cobalt-60, 100 microcuries - Tracerlab Model R-30 source and one strontium-90 eye applicator model R-1-A of 50 millicuries. Records showed that both sources were leak tested on October 1, 1969, and again on April 1, 1970 with the results of less than 0.005 microcuries of removable contamination. The leak tests are performed and analyzed by the licensee.

Miscelianeous

44. According to Mr. Wetterau there had been no unusual occurrences or incidents since the previous inspection. Mr. Wetterau maintains a special log book with a summary of occurrences the last of which occurred on October 23, 1968 involving a radioisotope leak inside a shipping container. Mr. Wetterau also stated that they had no AEC contracts.

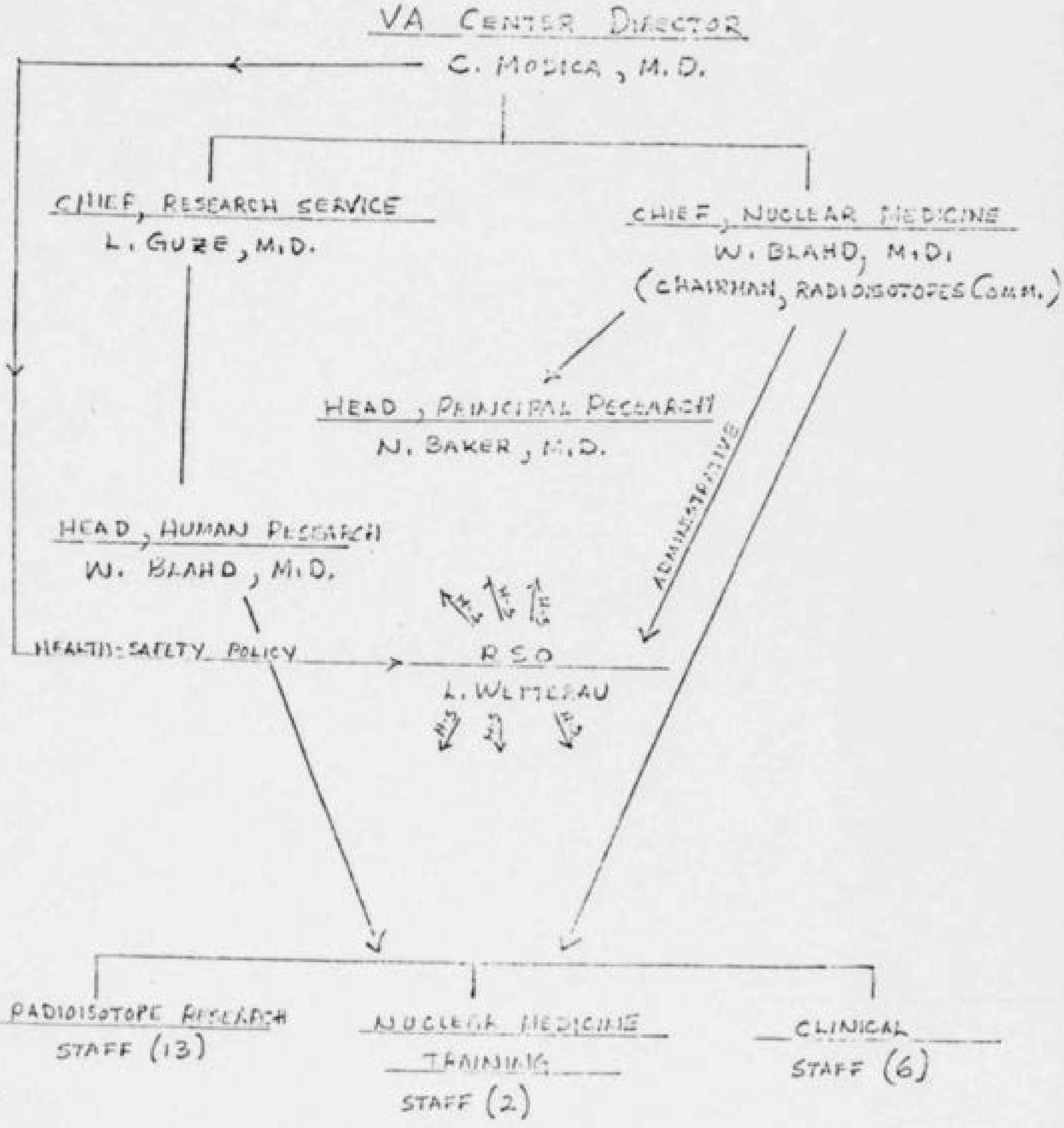

ORGANIZATION - VA CENTER

[illegible]

DRAFT INSPECTION REPORT FORM

1. Name and address of licensee
Veterans Administration Hospital
Wilshire and Sawtelle Boulevards
Los Angeles, California 90073

2. Date of Inspection 9/28/73

3. Type of Inspection Announced Reinspection

4. License number(s), docket number (s), number and date of last amendment for each license. Category and Priority of each licensee.

04-00181-04, Amendment No. 61, March 20, 1973, G(1), II

5. Date of previous inspection February 3, 1972

6. Proprietary information None

7. Scope of inspection if other than routine Routine

8. Participants (Licensee representatives and titles, State representatives, etc.)

Mr. L. Wettereau, Physicist, Nuclear Medicine Service, RSO

9. Management Interview (Information required for N/C cases)

W. H. Blahd, M.D.; Chief, Nuclear Medicine Service
Mr. L. Wettereau, RSO

10. Action and Date: Letter to Licensee ______

AEC-591 Clear September 28, 1973

AEC-591 n/c ______

11. Recommend reinspection date September 28, 1974

12. H. S. North [signature] — 10/[illegible]/73
Inspector — Date of Report

H. E. Book — 10/16/73
Reviewer — Date of Review

Attachment to Section 1010

8204150467 820216
PDR FOIA
NELSON82-11 PDR

Veterans Administration Hospital
Los Angeles, California 90073
License No. 04-00181-04

13. Inspection Summary

The inspection of September 28, 1973 disclosed no items of noncompliance or health and safety significance. The inspector did observe that the licensee is operating under difficult conditions due to space limitations resulting from the closure of Wadsworth Hospital. At the time of the inspection, Wadsworth Hospital had been razed, and excavations are in progress for a new 800-bed hospital. The inspection was concluded with the issuance of a Form AEC-591 reflecting no items of noncompliance. The previous inspection disclosed no items of noncompliance and resulted in the issuance of a clear AEC-591.

14. Summary of Licensed Program

The licensee operates, under a broad license, a program of nuclear medicine diagnosis and therapy with major activities in various fields of research. A nuclear pharmacy is presently active. Summaries of the licensee's Nuclear Medicine Service are contained in the Nuclear Medicine Service Report for Fiscal Year 1973, which is attached to this draft report as Appendix A. This report notes that a total of 7,434 procedures were performed which included approximately 1,800 in-vitro tests. The licensee also treated

ten cases of hyperthyroidism and one case of thyroid CA using iodine-131 and made therapeutic use of phosphorus-32 in five cases. The personnel associated with the use of licensed materials in the Nuclear Medicine Service are described on Page 7 of Appendix A of this report. In addition, the Research Service has staffs that operate in various laboratories under the direct supervision of specifically authorized supervising individuals. The licensee estimated that procurement of licensed material by the hospital is in the range of approximately 25 curies per year, mostly technetium-99m. The licensee is currently using no generators and is buying all technetium in precalibrated form. On the basis of discussion with the licensee and as a result of the examination of records, it appeared that the use of materials was as authorized by the license.

15. Organization and Administration

The current hospital Director is Mr. John Cox, who at the time of the inspection was a patient in the hospital. The Assistant Director, Joseph Burmingham, was acting for Mr. Cox. [illegible] to the Director as the [illegible] Administrative Service is Doctor Ed Wright, Chief of Staff. Reporting to Dr. Wright are various medical services, plus the Research Service, who use radioactive material in medical and biological research. Also reporting to Dr. Wright

is Dr. Blahd as the Chief of the Nuclear Medicine Service. Mr. Wettereau, RSO, reports to Dr. Blahd. Mr. Wettereau of the Nuclear Medicine Service is identified on Page 7 of Appendix A. The VA Hospital is continuing a program of residency and technician training, and this program is described on Page 11 of Appendix A. Mr. Wettereau, as RSO, provides training for users of radioactive materials on a continuing basis. This activity requires approximately 5% of his time. The training course repeats quarterly, and attendance may be a prerequisite to ~~these~~ licensed materials based on the decision of the Isotopes Committee. Attendance at the course is open to users of licensed materials ______________ for refresher training. In addition to the training program described, Mr. Wettereau devotes approximately 10% of his time to the training of resident physicians and nuclear medicine technologists. It is noted that the principal user of licensed material at this facility, based on curies or quantity of materials handled, is the Nuclear Medicine Service. The Research Service, which encompasses a much larger number of individuals, actually uses relatively small quantities of materials in the form of tracer activity. Mr. Wettereau's duties include supervision of receipt, possession and use of licensed materials and has authority to c~~heck on~~ activities in the case of radiation safety problems. The licensee has a Radioisotopes Committee that consists of the individuals named in an attachment to the licensee's application of January 21, 1965, with the exception of Dr. James Davis who has been replaced by Dr. Otis H. True, Chief,

Radiotherapy-Radiology Service. Mr. Wettereau is not a member of the Committee but is in an advisory capacity through Dr. Blahd. The licensee has in fact two separate Committees, the Radioisotope Committee and the Human Use Committee, both of which must evaluate proposals for use of materials in humans. The Radioisotope Committee also approves use of materials in connection with research activities, which do not involve human use. The licensee maintains well documented files reviewing Committee actions on proposed uses and users. It appeared that the Committee is actively carrying out its responsibilities as described in the Radioisotope Committee Administrative Procedures attachment to [illegible] application of January 21, 1965.

16. <u>Facilities</u>

The licensee's clinical facilities consist of two clinical areas on the hospital grounds which provide space for the administration [illegible] and patient counting facility, a nuclear pharmacy and the old hot lab in Building 114, which is used to handle iodine-131. In addition, the licensee maintains one thyroid clinic as a part of the Nuclear Medicine Service. The licensee has approximately 24 laboratories using small quantities of materials in in-vitro and small animal research. These activities are located in Buildings 114, 115 and T85. During the inspection, the inspector toured the Radioisotope

Research Laboratories in Building 114, where the licensee was using tri[illegible] liquid scintillation counting systems for analysis of low level samples, ~~and~~ labeled organics and biological materials were stored in two different refrigerators or freezers. The whole body counter is in a small building adjacent to Building 114, ~~which~~ is presently equipped with 5 x 8 inch NaI detector and a Nuclear Data 512 Channel Analyzer. The Nuclear Medicine Service facilities in Building 213 include, dual probe and ~~electro~~linear scanner and uptake equipment in addition to the scintillation camera and Clinicon Computer. The facility also has a dual opposed three-inch NaI scintillation detector, TOBOR counter scaler and apparatus to perform xenon-133 pulmonary studies. The licensee maintains control of access to locations where licensed materials are stored or used by means of personal surveillance or lock and key.

17. Equipment

A listing of the equipment available to the Nuclear Medicine Service is contained in Appendix A, Page 8, under "Equipment, Present Inventory." In addition, the licensee has a number of freezers and refrigerators used for storage of licensed materials in the research programs, as well as liquid scintillation counting equipment. At the time of the inspection, the licensee's inventory was stated to be as reported in the document dated September 28, 1973, which is attached as Appendix B.

18. Radiological Safety Procedures

The licensee's procedures remain as identified in Paragraphs 21 and 23 of the license. The procedures had not been changed without prior approval of DOL. Mr. Wettereau stated that when an individual first receives approval to use licensed materials they are required to complete a Form AEC-4, at which time they are provided with a copy of the procedures and invited to attend the Health Physics Radiation Safety Training Course. Attendance at this course may be a prerequisite to receiving permission to utilize materials unless the Radioisotopes Committee is satisfied concerning the level of training and proficiency of the individual. It was noted that Forms AEC-3 were posted at all facilities visited during the inspection. The licensee had attached to each AEC-3 a small type-written notice informing the readers that copies of the AEC license and the licensing procedures were available for review as were the AEC's regulations in compliance with 10 CFR 19 at a specified location. The location was Mr. Wettereau's office, and his name and office location were provided.

19. Personnel Monitoring and Exposure to External Radiation

The licensee uses monthly film badges supplied by Radiation Detection Company. The licensee maintains records of exposure in terms of dose per month and per quarter. Information

equivalent to Forms AEC-5 are maintained. Finger rings are used by individuals who are likely to receive high extremity exposures, principally due to handling technetium. Pocket dosimeters are available but are not routinely used. During 1972, the high quarterly whole body exposure was 480 millirem, and the finger ring measured high exposure was 1560 millirem. During 1973 through August, the maximum quarterly whole body exposure was 225 millirem. The maximum finger ring measured exposure was 1850 millirem. The average exposure ranges from approximately 25 to 50 millirem per month.

The licensee has a requirement placed by Condition 18 which requires the performance of bioassays on individuals using at one time more than 100 millicuries of tritium in an uncontained form. The licensee stated that no such use of tritium had occurred, and no bioassays had been performed for tritium. The licensee does perform spot check bioassays on radioisotope technicians, however, the results of the spot checks have been negative.

20. Exposure of Employees to Airborne Radioactive Materials

The only significant potential for exposure to airborne activities would be in the case where patients are administered xenon-133 for pulmonary studies. Mr. Wettereau stated that a gas is administered

in a saline solution by injection and that the patient's exhaled breath is collected in a Douglass bag and vented through a chemical fume hood in one of the counting facility laboratories.

21. Effluents to Unrestricted Areas

This licensee makes no intentional sewer disposals of radioactive materials. Some small disposal probably occurs during washing of glassware. This licensee has in the past buried waste at their facility. There have been no recent burials. Waste is disposed by transfer to California Salvage Company. Since the last inspection, the licensee has disposed of a total of 53, 55-gallon barrels of waste which contained 629.8 millicuries of activity. The licensee maintains records of disposal in the form of disposal company supply records.

22. Miscellaneous Surveys, Evaluations and Records

Mr. Wettereau stated that he performs surveys for record of activity using licensed materials at this facility. He stated that he also instructs and advises users of materials to contact him in the event of any problems. Mr. Wettereau maintains a log-book which records the results of surveys and problems. These records include the date, dose rates or measured maximum contamination, the results of sewer tests, with space for remarks. The

frequency of visits to the laboratories varies from weekly to approximately every few month depending upon the use of materials at that facility. Most of the surveys indicate no significant levels. Wettereau makes a particular point of conducting thorough surveys of vacated facilities including plumbing and records the results for future reference. The survey records indicated that four surveys had been performed and were well documented.

23. Special License Conditions

None.

24. Posting and Labeling

A tour of the licensee's facilities disclosed that locations of use and storage were posted in accordance with Regulatory requirements. A spot check of materials in storage disclosed that containers were labeled in accordance with Regulatory requirements. Forms AEC-3 and notices relating to Part 19 requirements were posted in each facility or laboratory where licensed materials were used.

25. Independent Measurements

None.

26. Operations Observed

None.

27. Incidents, Overexposures, Theft or Loss, Equipment Malfunction

Mr. Wettereau maintains a log of problems and unusual occurrences. From this record, the following items of interest were noted:

A shipment from Cambridge Nuclear Pharmaceutical Corporation, P. O. Box 528, Princeton, New Jersey 3540 -- A packing slip from this supplier dated March 21, 1972, identified the shipment as sodium chloride-24, NaCl-24, at 2.2 millicuries per milliliter containing two milliliters with the activity shipped as 0.4 millicuries. The licensee's receipt analysis showed that the shipment contained 22 millicuries of sodium-24. A telephone call by Wettereau to Cambridge Nuclear did not result in an appropriate response according to Wettereau.

In a second case, a shipment from Isomed was stated to contain 50 millicuries technetium-99 and was labeled as of April 5, 1972; however, the material was actually shipped April 17, 1972 and on that date contained 47.9 millicuries.

Mr. Wettereau noted one occurrence on April 19, 1973, in which a resident physician preparing to do a bolus IV injection of 15 millicuries technetium-99 pertechnetate inserted the needle in the patient's arm after having applied a tourniquet above the injection site, verified the presence of veinous blood, removed the tourniquet, removed the hypodermic from the patient's arm and ejected the contents of the hypodermic on the patient rather than injecting the material into the vein as intended. Mr. Wettereau stated that the particular resident physician in question was a pathologist who he stated apparently had had insufficient contact with living patients. Wettereau was notified and, with the assistance of the physician and the nursing staff, the patient was decontaminated. The resident physician terminated his training program without completion.

On September 10, 1973, a sink overflowed in a carbon-14 lab resulting in localized flooding. Wettereau participated in the cleanup of the facility and surveyed the floor and made certain that it was decontaminated to background levels.

Mr. Wettereau stated that there had been no unreported incidents or unusual occurrences which he felt were necessary to report to the Commission.

28. Other Information or Continuation From Previous Paragraphs

Addition to Paragraph 23 -

The licensee has two sealed sources, a Model R-30 cobalt-60 source and a Model RA-1A strontium-90 Medical Applicator. The licensee performs leak tests at regular intervals. Records of leak tests are maintained. Leak tests have been performed at the required intervals, and the records disclosed no evidence of leakage.

Attachments:
1. Appendix A
2. Appendix B

RCS 10-108

NUCLEAR MEDICINE SERVICE REPORT

VA WADSWORTH HOSPITAL CENTER, Los Angeles, Calif. 90073

CHIEF William H. Blahd, M. D.

FISCAL YEAR 1973

DUE 10 working days after receipt of this form

I. DIAGNOSIS

	FORM	PURPOSE	DOSAGE RANGE	NO. OF PTS. VISITS (TOTAL 7,446)	NO. OF PROCEDURES OR SCANS (TOTAL 7,434)
		**By-product Material**			
		**131-Iodine**			
1.1	Iodide	Thyroid function (uptake)	2-10 µCi	625	206
1.2	Iodide	Thyroid scan	100-500 µCi	101	101
1.3	IHSA	Plasma volume	10-15 µCi	392	196
1.4	IHSA	Cardiac scan-output			
1.5	IHSA	Cisternography	100 µCi	111	37
1.6	MAA	Lung scan			
1.7	MAA	Liver scan			
1.8	Rose Bengal	Liver function	250 µCi	1	1
1.9	Hippuran	Kidney function	250 µCi	74	74
1.10	Hippuran	Kidney scan	250 µCi	73	73
1.11	Fats/Fatty Acids	GI absorption			
1.12	T-3	In vitro			
1.13	T-4	In vitro			
1.14	Iodide	Thyroid function ½-life	2-5 µCi	108	18
1.15	Iodide	Thyroid & chest scan	5 mCi	38	19
1.16					
1.17					

	FORM	PURPOSE	DOSAGE RANGE	NO. OF PTS. VISITS	NO. OF PROCEDURES OR SCANS
		**125-Iodine**			
2.1	Iodide	Thyroid function (uptake)			
2.2	Iodide	Thyroid scan			
2.3	IHSA	Plasma volume			
2.4	T-3	In vitro	In vitro	-----	1451
2.5	T-4	In vitro	In vitro	-----	241
2.6	Digoxin	In vitro	In vitro	------	118
2.7	Insulin	In vitro			
2.8	Renin	In vitro			
2.9	Australian Antigen	In vitro			
2.10	RISA	Blood Volume	5 μCi	15	15
2.11					
2.12					
		**^{99m} Technetium**			
3.1	Pertechnetate	Brain scan	15-20 mCi	1454	1454
3.2	Pertechnetate	Thyroid scan	1-6 mCi	6	6
3.3	Pertechnetate	Thyroid uptake			
3.4	Pertechnetate	Cerebral blood flow	15-20 mCi	953	953
3.5	Pertechnetate	Renal blood flow			
3.6	Pertechnetate	Joint scan			
3.7	Pertechnetate	Cardiac scan			
3.8	Pertechnetate	Arterial flood flow			
3.9	Pertechnetate	Cardiac blood flow			
3.10	Pertechnetate	Brain blood flow			
3.11	Pertechnetate	Parotid scan	10 mCi	1	1
3.12	Sulphur Colloid	Liver scan	500 μCi-2.5 mCi	862	862
3.13	Sulphur Colloid	Spleen scan	500 μCi-2.5 mCi	50	50
3.14	Sulphur Colloid	Bone marrow scan	10 mCi	10	10
3.15	Sulphur Colloid	Cardiac scan	8 mCi	4	4
3.16	Sulphur Colloid	Lymphangiogram			
3.17	Sulphur Colloid	Stomach & Duodeum			
3.18	Albumin	Cardiac scan			
3.19	Albumin	Cysternogram			
3.20	Human Serum Albumin Microspheres	Lung scan	3 mCi	424	424
3.21	Antimony-sulphur Colloid	Aerosol lung scan			
3.22	Sulfur Colloid	Vena Cava	2-4 mCi	42	42
3.23	Tc DTPA	Kidney Scan	1-3 mCi	63	63
3.24	Sulfur Colloid MA	Venogram	2 mCi	1	1
3.25	TcO_4 Meckle's Diverticulum		10 mCi	2	2
	Tc-PYP	Bone	15 mCi	279	27[illegible]
	Tc-DPP	Bone	15 mCi	10	10

	FORM	PURPOSE	DOSAGE RANGE	NO. OF PTS. VISITS	NO. OF PROCEDU[illegible] OR SCAN[illegible]
		**51-Chromium**			
4.1	Sodium Chromate	RBC Survival	75-150 µCi	117	18
4.2	Sodium Chromate	Spleen scan			
4.3	Sodium Chromate	RBC volume	50-75 µCi	72	24
4.4	Sodium Chromate	Spleen sequestration	75-150 µCi	150	15
4.5	Sodium Chromate	G.I. bleeding			
4.6	Sodium Chromate	Liver/Spleen ratio			
4.7	Sodium Chromate	Platelet survival			
4.8	Albumin	GI protein loss			
4.9					
4.10					
4.11					
		**59-Iron**			
5.1	Chloride	Iron turnover			
5.2	Chloride	Body distribution, external counts			
5.3	Citrate	Iron turnover	<20 µCi	90	6
5.4	Citrate	Plasma clearance	<20 µCi	90	6
5.5	Sulphate	Iron absorption			
5.6	Amomonium Citrate In vitro	Iron binding capacity			
5.7					
5.8					
5.9					
5.10					
		**197 or 203-Mercury**			
6.1	Chlormerodrin	Kidney			
6.2	Chlormerodrin	Brain scan			
6.3					
6.4					
		**198-Gold**			
7.1	Colloid	Liver scan			
7.2	Colloid	Lymph node scan			
7.3					
7.4					
7.5					

	FORM	PURPOSE	DOSAGE RANGE	NO. OF PTS. VISITS	NO. OF PROCEDU[illegible] OR SCAN[illegible]
		**111-Indium**			
8.1	Transferin	Lymph node scans			
8.2	DTPA	Cisternogram	500 µCi	204	68
8.3					
8.4					
		**113^m-Indium**			
9.1	Colloid	Liver scan			
9.2	Colloid	Lung scan			
9.3	Colloid	Cardiac scan-output			
9.4					
9.5					
		**57-Cobalt**			
10.1	Vit. B_{12}	Absorption (P.A.)	0.5-1 µCi	114	57
10.2	Vit. B_{12}	Absorption (G.I.)			
10.3	Vit. B_{12}	Serum binding capacity			
10.4					
10.5					
		**58 or 60-Cobalt**			
11.1	Vit. B_{12}	Absorption (P.A.)			
11.2	Vit. B_{12}	Absorption (G.I.)			
11.3					
11.4					
11.5					
		**85-Strontium**			
12.1	Nitrate	Bone scan			
12.2	Chloride	Bone scan			
12.3					
12.4					
12.5					

	FORM	PURPOSE	DOSAGE RANGE	NO. OF PTS. VISITS	NO. OF PROCEDURES OR SCANS
		**18-Fluorine**			
13.1	Sodium fluoride	Bone scan	1-5 mCi	39	39
13.2					
13.3					
		**133-Xenon**			
14.1	Gas	Cardiac studies			
14.2	Gas	Pulmonary studies	5-10 mCi	2	2
14.3	Saline	Muscle blood flow	2-10 mCi	72	72
14.4	Saline	Cerebral blood flow			
14.5					
14.6					
14.7					
		**3-H Tritium**			
15.1	Digoxin	In vitro			
15.2					
15.3					
		**Other**			
16.1	Methionine Se 75	Pancreas Scan	250 µCi	30	30
16.2	Hydrogen 3 (Tritium)	Total Body Waters	150-200 µCi	28	14
17.1	Bromine 82	Extracellular Water	25 µCi	28	14
17.2	Sodium 24	Exchangeable Sodium	150-200 µCi	28	14
17.3	K 40	Total Body Potassium	-----	31	31
17.4	Gallium 67	Gallium	4-5 mCi	342	114
	-----	Ultrasound	----	168	168
18.1	-----	TSH studies	10 units	45	15
18.2		Thyroid Followup		61	
18.3					
19.1					
19.2					
19.3					

II. THERAPY

	FORM	PURPOSE	DOSAGE RANGE	NO. OF PTS. VISITS	NO. OF PROCED[illegible] OR SCA[illegible]
		**131-Iodine**			
20.1	Iodide	Hyperthyroidism	2-20 mCi	10	10
20.2	Iodide	Thyroid CA	50-150 mCi	1	1
20.3	Iodide	Heart Disease			
		**198-Gold**			
21.1	Colloid	Intercavitary CA			
21.2	Colloid	Interstitial CA			
		**32-Phosphorous**			
22.1	Phosphate	Polycythemia Vera			
22.2	Phosphate	Leukemia			
22.3	Phosphate	Bone CA			
22.4	Colloidal chromic	Intercavitary CA			
22.5	Phosphate	Interstitial CA	10-50 mCi	1	1
	Phosphate	Interstitial (Arthritis)	500 μCi	24	4

III. Scope of Services

1. Coverage: 40 hrs per week, plus emergency callback capability

2. Sites: There are 38 locations within our hospital where radioactive materials were used for any purpose.

IV. Cost

1. Personnel (Name, Position Title, Grade, State Licensure, Certification or Registration)

W. H. Blahd, M. D.
Chief, Nuclear Medicine Service (DM&S 15)
California license
Certified in Nuclear Medicine, and Internal Medicine
Fellow, American College of Physicians

M. A. Winston, M. D.
Assistant Chief, Nuclear Medicine Service (DM&S 14)
California license
Certified in Nuclear Medicine, and Internal Medicine

G. T. Krishnamurthy, M. D.
Staff Physician, Nuclear Medicine Service (DM&S 14)
Licensed in California and Washington
Certified in Nuclear Medicine
Fellow, American College of Physicians

J. H. Pritchard, M. D.
Staff Physician, Nuclear Medicine Service (DM&S 14)
California license
Certified in Nuclear Medicine

L. W. Wetterau, B. A., Physicist, GS-12

Manuel Tubis, Ph. D., Clinical Radiobiochemist, GS-15

Panchita B. Thomas, B. S., Biologist, GS-11

Robert J. Huebotter, B. A., Biologist, GS-11

Jerome J. Gambino, Ph. D., Biologist (Instructor), GS-13

Carol F. Walsh, Health Technician (Instructor), GS-9, ARRT(RT Nucl Med)

Billie Jean Kinney, Health Technician, GS-9

Shirley N. Stichler, Health Technician GS-7, ARRT (RT Nucl Med)

IV. Cost

1. Personnel (continued)

Vacancy to be filled - Health Technician, GS-7

Kazuko Endow, Secretary-Steno, GS-6

Ruth V. Adams, Dictation Machine Transcriber, GS-4

TOTAL SALARY COST: $ 237,112

2. Supplies: $ 60,000

3. Equipment

3.1. Purchase cost: $ 59,998

3.2. Repair cost: 8,300

V. Special Items

1. Personnel needs: GS-4 Clerk-typist (new position)

2. Equipment

2.1. Present inventory

2.1.1. Imaging equipment

Nuclear-Chicago, Pho/Gamma Scintillation Camera System, Model HP, 1972

Nuclear-Chicago, Pho/Gamma III Scintillation Camera System, Model 6403, 1969

Searle Nuclear-Chicago, Clincom, Model PDS-3, 1973

Radx, 70 mm camera, Model M-600, 1972

2.1.2. Scanners

Picker Nuclear, Magnascanner II, 3" crystal, Model 6184-D, 1964

Picker Nuclear, Magnascanner 500, 3" crystal, Model 2806-K, 1970

Picker Nuclear, Magnascanner III, 5" crystal, Model 2852, 1972

V. Special items

2. Equipment

2.1.2. Scanners (continued)

Picker Nuclear Magnascanner 500, 5" crystal, Model 500, 1969

Nuclear-Chicago, Pho/Dot V, 5" crystal, Model 1775, 1973

2.1.3. Probes

Nuclear-Chicago Probe-Scaler Assembly, Model 820582, 1970

Nuclear-Chicago Probe-Scaler Assembly, Model 8725, 1971

2.1.4. Other equipment

Picker Renogram Assembly Dual Analyzer/Ratemeter, Model 620-085, 1966

Nuclear-Chicago Auto/Gamma Spectrometer, Model 1085, 1971

Packard Tri/Carb Liquid Scintillation Spectrometer, Model 3320, 1971

Kodak X-Omat Dry Film Processor, Model M6A-N, 1970

Kodak X-Omat Dry Film Processor, Model M6A-N, 1972

Picker Nuclear Ultrasonoscope, Model 1020-2, 1972

Olivetti Desk Programmer, Model 101, 1969

Whole-Body Counter (constructed in 1959)

5" steel chamber

4" x 8" crystal

Nuclear Data 512-Channel Analyzer System, Series 2200, 1971

V. Special Items

2. Equipment (continued)

2.2. Needs

2.2.1. <u>Initial equipment</u>

Nuclear Chicago 2-channel, 3" Automatic Gamma Counter with eye-level control, Model 1185D	$ 14,500
Nuclear Chicago Model 09009 Dual Isotope Analysis Display for H.P. Camera	4,500
Nuclear Chicago Whole-body Scanning Table	22,000
Refrigerated Centrifuge - International PR-J with 8-place head	2,544
Van Waters & Rogers, Sterilizer, Steri Quick, #58626-007	650
Van Waters & Rogers, Waterbath, Shaker, #13300-007 with Tray, #13301-065	770
Total Cost	$ 44,964

2.2.2. <u>Replacement equipment</u>

04-6493 Scanner, Ser. #196 - replacement requested is Ohio Nuclear Dual [illegible]" Scanner, M.C. price	$ 33,000
18-0614 Camera, Osc. Trace - replacement requested is Riverside Bio Engineering 70 mm camera M-800	1,795
04-2214 Counter Prop Gas Flow Ser. #569 - replacement requested is Nuclear Chicago Model 8725	3,000
04-2240 Counter, Well Scintillation - replacement requested is Baird Atomic M-98810C	1,450
18-0470 Camera and Video Monitor - replacement requested is Shibaden HV-14 & Shiba VM-903	738
Total Cost	$ 39,983

V. Special Items

2. Equipment (continued)

2.3. Repairs

2.3.1. 24 repair service calls

2.3.2. Down time, inpatient-hours/week: 1 hr/week

2.3.3. Service is readily available

2.3.4. Service is not by VA personnel.

3. No untoward reactions to any clinical procedures.

4. Space used for clinical activities:

4.1. Total space in square feet: 4,000 sq ft

4.2. Number of rooms: 20

4.3. Service is not situated in one geographic area

5. Training and Education Activities

The Nuclear Medicine Service offers training at three levels: physician, radiopharmacist, and technologist. Physicians seeking eligibility for certification by the American Board of Nuclear Medicine take a two-year program. During this time, resident physicians receive instruction in the theoretical and practical aspects of diagnostic and therapeutic nuclear medicine. Didactic and laboratory instruction is provided in relevant basic sciences, including medical nuclear physics, radiation biology, radiation protection, instrumentation, radiopharmaceuticals, and statistics. In the second year of training, emphasis is on independent investigation, and the residents' responsibility in the nuclear medicine clinics is progressively increased. The program is structured so as to provide balanced training in the clinical and research applications of radionuclides.

The radiopharmacist training program provides one year of training to qualified licensed pharmacists. Trainees attend the University of Southern California for two academic semesters, receiving didactic and laboratory training. Upon completion of the academic portion of the program, trainees intern for a three-month period at VA Wadsworth Hospital Center. Their internship provides practical experience in the Nuclear Medicine Service Radiopharmacy Laboratory where they participate in the preparation of radiopharmaceuticals used in routine clinic procedures, observe the performance of clinical procedures, and also participate in developmental research in radiopharmaceuticals. During this period, the trainees attend many of the same lectures and seminars attended by physicians in training.

V. Special Items

5. Training and Education Activities (continued)

The Technologist Training Program offers one year of training, including both theory and practice. Trainees attend and participate in many of the same lectures, laboratories, conferences, and seminars that the physicians in training attend. In addition, the technologists receive ample practical experience under the supervision of the training staff in the Nuclear Medicine Clinics and in vitro laboratories.

The Training Program in Nuclear Medicine at Wadsworth Hospital Center was implemented in May of 1969. During the period of time the Program has been in effect, a total of 10 physicians, 10 technologists, and 8 radiopharmacists have been or are being trained.

6. Research Activities - title of research project and investigator(s)

Treatment of Thyrotoxicosis in Graves' Disease
(W.H. Blahd and M.A. Winston)

A Comparison of Radioiron and Indium 111 Chloride
(W.H. Blahd, H.G. Berger)

Radiolabeled Anti-CEA for External Detection of GI Tumors
(W.H. Blahd, J.H. Pritchard, M. Tubis, and J.J. Gambino)

Body Composition Changes in Radiation Therapy of Malignancy
(W.H. Blahd and J.J. Gambino)

Urecholine, Glucagon, to Aid Pancreatic Scans
(M.A. Winston)

^{32}P Chromic Phosphate for Recurring Synovial Effusions
(M.A. Winston and R. Bluestone)

Detection of Increased Iron Absorption Using ^{57}CO
(M.A. Winston and P. Guth)

Technetium 99m-Penicillamine for Cholescintigraphy
(G.T. Krishnamurthy, M. Tubis, W.H. Blahd, and J.S. Endow)

Kinetics of Technetium-Labeled Skeletal Radionuclides
(G.T. Krishnamurthy, W.H. Blahd, and M. Tubis)

Gamma Camera Study of Venacaval Obstruction without Syndrome
(G.T. Krishnamurthy, M.D. Kehr, Z. Piotrkowicz, and W.H. Blahd)

Tc 99m and I 131-Labeled Metronidazole for Liver Imaging
(M. Tubis, W.H. Blahd, G.T. Krishnamurthy, and R. Suwanik)

V. Special Items

6. Research Activities (continued)

Indium 113m Sulfide Macroaggregate for Lung Scanning
(M. Tubis, M. B. Cohen, and C. D. Gilliam)

Technetium 99m-Penicillamine-Acetazolamide for Scanning
(M. Tubis and W. H. Blahd)

Indium 111 Colloid for Lymph Node Imaging
(H. G. Berger and W. H. Blahd)

7. Notable advance made in Nuclear Medicine Service within the past year

7.1. The Nuclear Medicine Service has recently acquired a Clincom system that will become an integral part of one of our scintillation cameras. It is anticipated that this dedicated computer system will provide improved diagnostic services and new avenues for clinical investigation.

7.2. Ultrasound B mode scanning has been used extensively during the past year as a complementary modality to nuclear medicine imaging procedures. It has greatly augmented our diagnostic capabilities and it is hoped that this technique will have even greater application during the coming year.

7.3. Two new radiopharmaceuticals have been devised during the past year. One of these, I 131 labeled-metronidazole, will be used for the detection of amebic abscesses of the liver. A second radiopharmaceutical, Tc 99m penicillamine, has been developed for imaging of the gallbladder.

7.4. A technique has been developed to improve imaging of the pancreas, involving the use of urecholine and pancreazymin. Early results using this technique have been encouraging.

7.5. The metabolism and distribution of a new radioisotope tracer, Indium 111, has been investigated. Preliminary studies suggest that this tracer may be extremely useful for bone marrow imaging, and for the evaluation of patients with hematologic disease associated with disordered bone marrow function.

VI. Significant Administrative Problems and Future Plans

1. Administrative Problems

1.1. One important problem has developed as a result of the earthquake of 1971 necessitating the relocation of the Nuclear Medicine Service into two clinical areas. It has been impossible to obtain through Administration, clerical help in one of these clinical areas. As a result, the efficiency of the Service operation in this area has been greatly hampered since Nuclear Medicine technicians have been required to perform clerical and secretarial work.

1.2. As a result of the increased workload and expanding activities of the Nuclear Medicine Service during the past several years requiring the handling and preparation of numerous and large quantities of radiopharmaceuticals, there has developed an urgent need for a radiopharmacist. Despite numerous appeals, it has not been feasible to obtain the services of such an individual despite the fact that he would play a major role in increasing the efficiency and productivity of Service operations.

1.3. The Service operates a scintillation camera in each of its two clinical areas. In one area an older camera is in use that urgently requires overhauling and upgrading, such that its performance will be comparable to the current standards of scintillation camera operation. The cost to upgrade the above instrument is approximately $12,000. Funds have been requested for this project but have not been forthcoming.

1.4. The Nuclear Medicine Service has need of replacement equipment funding as indicated above, and in particular, the need of a dual probe system to replace an existing rectilinear scanning instrument that would also improve and extend the efficiency and productivity of Service operations.

2. Future Plans

The Nuclear Medicine Service is implementing an In Vitro Laboratory for the performance of radioimmunoassay procedures. The Laboratory was constructed largely from surplus laboratory furniture and equipment and is nearing completion. It is anticipated that the Laboratory will be functional within the next 30 days. According to present plans, a number of procedures will be immediately available, including T3, T4, and Digoxin analyses. Future plans include performance of other techniques, such as HAA, Vitamin B-12, TSH, and angiotensin. A major problem regarding this activity is the lack of adequate personnel. At the present time the Service has one high-level person who is assigned to this area on a part-time basis, who has available primarily the assistance of one or two technicians in training. Further personnel support for this Laboratory is urgently needed.

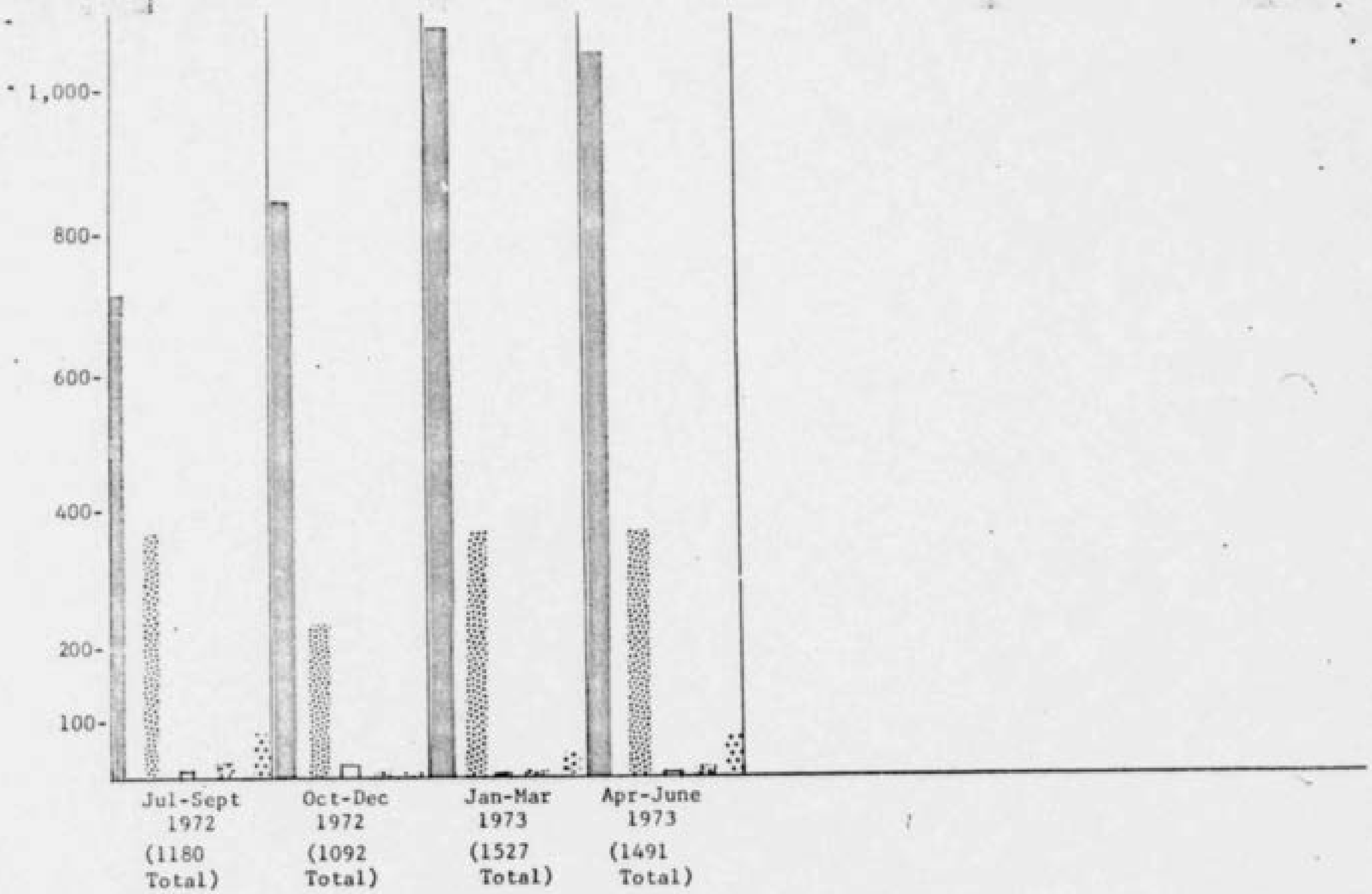

Key:
In-patients =
Out-patients =
Intermediate Care =
Patient-Member Svc. =
Brentwood Hospital =

FY 1973

FY 1974

TOTAL PATIENT VISITS

NUCLEAR MEDICINE SERVICE

OPERATIONS PROFILE

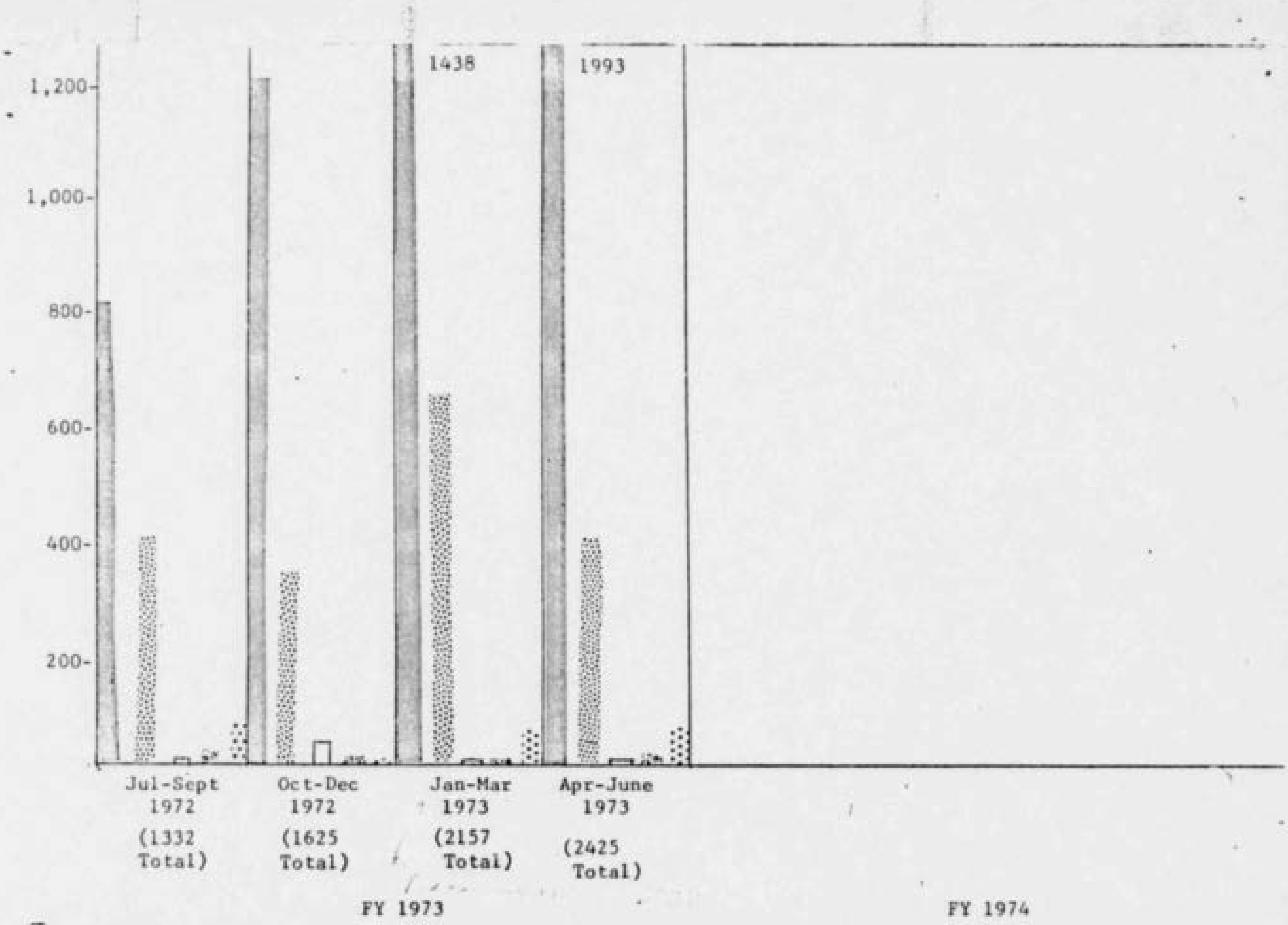

Key:
In-patients =
Out-patients =
Intermediate Care =
Patient-Member Svc. =
Brentwood Hospital =

TOTAL NUMBER OF TESTS PERFORMED

NUCLEAR MEDICINE SERVICE

OPERATIONS PROFILE

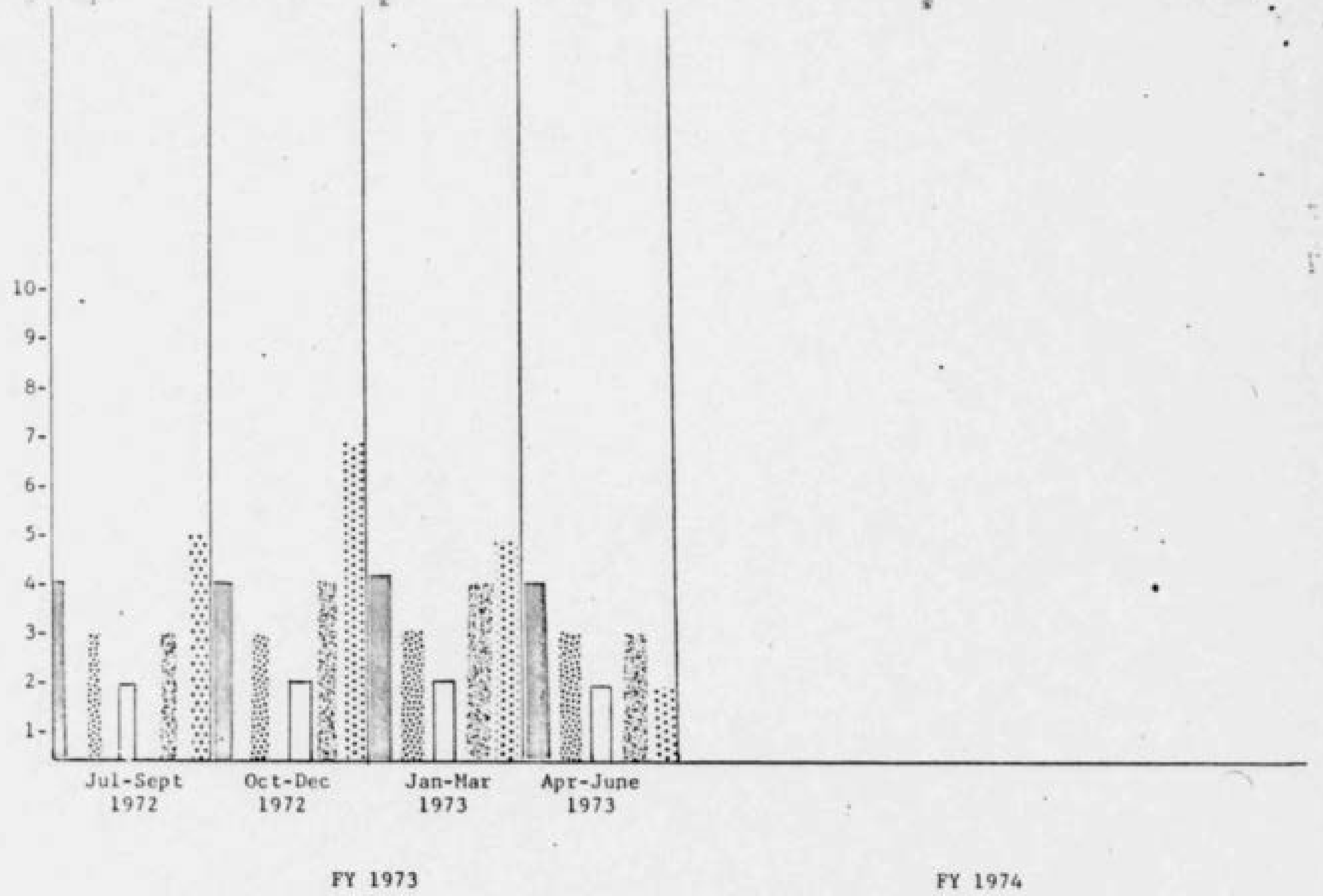

Key:
Physicians =
Radiopharmacists =
Technologists =
Residents (Other Services) =
Other [illegible] Personnel =

NUMBER OF PERSONNEL IN TRAINING PROGRAM

NUCLEAR MEDICINE SERVICE

OPERATIONS PROFILE

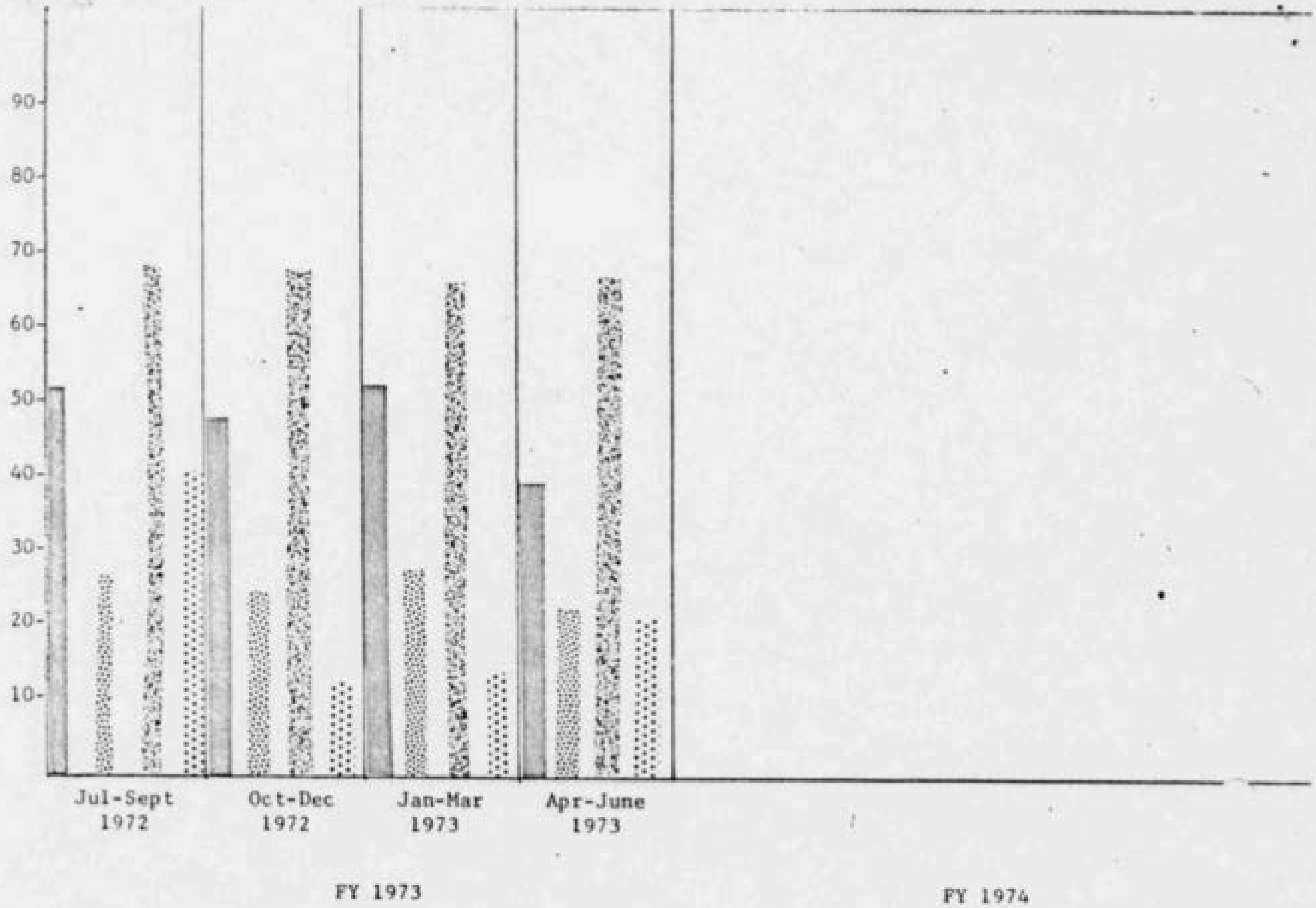

Key:
Lectures =
Seminars =
Conferences =
Laboratory Exercises =

TRAINING ACTIVITIES IN HOURS

NUCLEAR MEDICINE SERVICE

S1739

[illegible]ERATIONS PROFILE

VETERANS ADMINISTRATION
WADSWORTH HOSPITAL CENTER
WILSHIRE AND SAWTELLE BOULEVARDS
LOS ANGELES, CALIFORNIA 90073

September 28, 1973

IN REPLY REFER TO: 691/172A

Mr. Harry North
U. S. Atomic Energy Commission

SUBJ: Radioisotope inventory as of 9-28-73 - License 04-00181-04

		Millicuries
^{3}H	as Labeled Compounds	158.520
^{14}C	as Labeled Compounds	18.127
^{35}S	as Labeled Compounds	0.675
^{45}Ca	as Labeled Compounds	0.495
^{51}Cr	as Labeled Compounds	2.100
^{59}Fe	as Labeled Compounds	0.138
^{57}Co	as Labeled Compounds	0.040
^{63}Ni	as Labeled Compounds	9.800
^{67}Ga	as Labeled Compounds	6.000
^{75}Se	as Labeled Compounds	1.500
^{99m}Tc	as Pertechnetate	400.000
^{99m}Tc	as Labeled Compounds	20.000
^{111}In	as Labeled Compounds	4.500
^{114}In	as Labeled Compounds	0.030
^{125}I	as Labeled Compounds	26.500
^{131}I	as NaI	27.970
^{131}I	as Labeled Compounds	5.200
^{133}Xe	as Gas in Saline	25.000
^{137}Cs	as Labeled Compounds	0.138

Show veteran's full name, VA file number, and social security number on all correspondence.

Appendix B

Mr. Harry North
U. S. Atomic Energy Commission

Sept. 28, 1973

		Millicuries
^{3}H	as Sealed Source-Varian Gas Chromatograph Model 02-0645 detector cell	199.000
^{60}C	as Sealed Source - Tracerlab Model R-30	0.060
^{90}Sr	as Sealed Source - eye therapy - Tracerlab Model RA-1A	24.900
	TOTAL	923.693 millicuries

L. W. Wetterau

L. W. WETTERAU
Radiation Safety Officer
